# Supplementary material for: A novel Drosophila injury model reveals severed axons are cleared through a Draper/MMP-1 signaling cascade
Source: eLife. 2017 Aug 21;6:e23611. doi: 10.7554/eLife.23611 (PMC5565368; doi:10.7554/eLife.23611)
Supplement: Table 1—source code 1. — DOI: http://dx.doi.org/10.7554/eLife.23611.013 [file elife-23611-table1-code1.zip › Table 1 - Source Data 7-drosophila_annokey_report.html]

Annokey Search Report


# Annokey Search Report

- **annokey version:** 1.0.2
- **annokey homepage:** http://bjpop.github.io/annokey/
- **hostname:** barcoo
- **directory:** /vlsci/VLSCI/bjpop/scratch/annokey\_sean\_speese/drosophila
- **command:** annokey --email bjpope@unimelb.edu.au --terms ../search\_terms.csv --genes drosophila\_genes\_symbols.csv --log log --pubmed --organism drosophila
- **date:** 30 Apr 2017

## DptA

- NCBI Gene
- GeneRIF
- Pubmed

| Search Term | Rank | Fields |
| --- | --- | --- |
| [Mm]igration | 6 | PubMed(1) |
| [Ee]xtracellular [Mm]atrix | 8 | PubMed(1) |
| [Ii]nvasive | 10 | PubMed(1) |

  
hide/show details for DptA

### [Mm]igration

#### PubMed

1. The unconventional myosin CRINKLED and its mammalian orthologue MYO7A regulate caspases in their signalling roles. Caspases provide vital links in non-apoptotic regulatory networks controlling inflammation, compensatory proliferation, morphology and cell **migration**. How caspases are activated under non-apoptotic conditions and process a selective set of substrates without killing the cell remain enigmatic. Here we find that the Drosophila unconventional myosin CRINKLED (CK) selectively interacts with the initiator caspase DRONC and regulates some of its non-apoptotic functions. Loss of CK in the arista, border cells or proneural clusters of the wing imaginal discs affects DRONC-dependent patterning. Our data indicate that CK acts as substrate adaptor, recruiting SHAGGY46/GSK3-β to DRONC, thereby facilitating caspase-mediated cleavage and localized modulation of kinase activity. Similarly, the mammalian CK counterpart, MYO7A, binds to and impinges on CASPASE-8, revealing a new regulatory axis affecting receptor interacting protein kinase-1 (RIPK1)>CASPASE-8 signalling. Together, our results expose a conserved role for unconventional myosins in transducing caspase-dependent regulation of kinases, allowing them to take part in specific signalling events.

### [Ee]xtracellular [Mm]atrix

#### PubMed

1. Drosophila melanogaster as an animal model for the study of Pseudomonas aeruginosa biofilm infections in vivo. Pseudomonas aeruginosa is an opportunistic pathogen capable of causing both acute and chronic infections in susceptible hosts. Chronic P. aeruginosa infections are thought to be caused by bacterial biofilms. Biofilms are highly structured, multicellular, microbial communities encased in an **extracellular matrix** that enable long-term survival in the host. The aim of this research was to develop an animal model that would allow an in vivo study of P. aeruginosa biofilm infections in a Drosophila melanogaster host. At 24 h post oral infection of Drosophila, P. aeruginosa biofilms localized to and were visualized in dissected Drosophila crops. These biofilms had a characteristic aggregate structure and an **extracellular matrix** composed of DNA and exopolysaccharide. P. aeruginosa cells recovered from in vivo grown biofilms had increased antibiotic resistance relative to planktonically grown cells. In vivo, biofilm formation was dependent on expression of the pel exopolysaccharide genes, as a pelB::lux mutant failed to form biofilms. The pelB::lux mutant was significantly more virulent than PAO1, while a hyperbiofilm strain (PAZHI3) demonstrated significantly less virulence than PAO1, as indicated by survival of infected flies at day 14 postinfection. Biofilm formation, by strains PAO1 and PAZHI3, in the crop was associated with induction of diptericin, cecropin A1 and drosomycin antimicrobial peptide gene expression 24 h postinfection. In contrast, infection with the non-biofilm forming strain pelB::lux resulted in decreased AMP gene expression in the fly. In summary, these results provide novel insights into host-pathogen interactions during P. aeruginosa oral infection of Drosophila and highlight the use of Drosophila as an infection model that permits the study of P. aeruginosa biofilms in vivo.

### [Ii]nvasive

#### PubMed

1. **Invasive** and indigenous microbiota impact intestinal stem cell activity through multiple pathways in Drosophila. Gut homeostasis is controlled by both immune and developmental mechanisms, and its disruption can lead to inflammatory disorders or cancerous lesions of the intestine. While the impact of bacteria on the mucosal immune system is beginning to be precisely understood, little is known about the effects of bacteria on gut epithelium renewal. Here, we addressed how both infectious and indigenous bacteria modulate stem cell activity in Drosophila. We show that the increased epithelium renewal observed upon some bacterial infections is a consequence of the oxidative burst, a major defense of the Drosophila gut. Additionally, we provide evidence that the JAK-STAT (Janus kinase-signal transducers and activators of transcription) and JNK (c-Jun NH(2) terminal kinase) pathways are both required for bacteria-induced stem cell proliferation. Similarly, we demonstrate that indigenous gut microbiota activate the same, albeit reduced, program at basal levels. Altered control of gut microbiota in immune-deficient or aged flies correlates with increased epithelium renewal. Finally, we show that epithelium renewal is an essential component of Drosophila defense against oral bacterial infection. Altogether, these results indicate that gut homeostasis is achieved by a complex interregulation of the immune response, gut microbiota, and stem cell activity.

---

## CecA1

- NCBI Gene
- GeneRIF
- Pubmed

| Search Term | Rank | Fields |
| --- | --- | --- |
| [Ee]xtracellular [Mm]atrix | 8 | PubMed(1) |

  
hide/show details for CecA1

### [Ee]xtracellular [Mm]atrix

#### PubMed

1. Drosophila melanogaster as an animal model for the study of Pseudomonas aeruginosa biofilm infections in vivo. Pseudomonas aeruginosa is an opportunistic pathogen capable of causing both acute and chronic infections in susceptible hosts. Chronic P. aeruginosa infections are thought to be caused by bacterial biofilms. Biofilms are highly structured, multicellular, microbial communities encased in an **extracellular matrix** that enable long-term survival in the host. The aim of this research was to develop an animal model that would allow an in vivo study of P. aeruginosa biofilm infections in a Drosophila melanogaster host. At 24 h post oral infection of Drosophila, P. aeruginosa biofilms localized to and were visualized in dissected Drosophila crops. These biofilms had a characteristic aggregate structure and an **extracellular matrix** composed of DNA and exopolysaccharide. P. aeruginosa cells recovered from in vivo grown biofilms had increased antibiotic resistance relative to planktonically grown cells. In vivo, biofilm formation was dependent on expression of the pel exopolysaccharide genes, as a pelB::lux mutant failed to form biofilms. The pelB::lux mutant was significantly more virulent than PAO1, while a hyperbiofilm strain (PAZHI3) demonstrated significantly less virulence than PAO1, as indicated by survival of infected flies at day 14 postinfection. Biofilm formation, by strains PAO1 and PAZHI3, in the crop was associated with induction of diptericin, cecropin A1 and drosomycin antimicrobial peptide gene expression 24 h postinfection. In contrast, infection with the non-biofilm forming strain pelB::lux resulted in decreased AMP gene expression in the fly. In summary, these results provide novel insights into host-pathogen interactions during P. aeruginosa oral infection of Drosophila and highlight the use of Drosophila as an infection model that permits the study of P. aeruginosa biofilms in vivo.

---

## DptB

- NCBI Gene
- GeneRIF
- Pubmed

| Search Term | Rank | Fields |
| --- | --- | --- |
| [Mm]igration | 6 | PubMed(2) |
| [Ee]xtracellular [Mm]atrix | 8 | PubMed(1) |

  
hide/show details for DptB

### [Mm]igration

#### PubMed

1. The unconventional myosin CRINKLED and its mammalian orthologue MYO7A regulate caspases in their signalling roles. Caspases provide vital links in non-apoptotic regulatory networks controlling inflammation, compensatory proliferation, morphology and cell **migration**. How caspases are activated under non-apoptotic conditions and process a selective set of substrates without killing the cell remain enigmatic. Here we find that the Drosophila unconventional myosin CRINKLED (CK) selectively interacts with the initiator caspase DRONC and regulates some of its non-apoptotic functions. Loss of CK in the arista, border cells or proneural clusters of the wing imaginal discs affects DRONC-dependent patterning. Our data indicate that CK acts as substrate adaptor, recruiting SHAGGY46/GSK3-β to DRONC, thereby facilitating caspase-mediated cleavage and localized modulation of kinase activity. Similarly, the mammalian CK counterpart, MYO7A, binds to and impinges on CASPASE-8, revealing a new regulatory axis affecting receptor interacting protein kinase-1 (RIPK1)>CASPASE-8 signalling. Together, our results expose a conserved role for unconventional myosins in transducing caspase-dependent regulation of kinases, allowing them to take part in specific signalling events.
2. The RhoGEF Zizimin-related acts in the Drosophila cellular immune response via the Rho GTPases Rac2 and Cdc42. Zizimin-related (Zir), a Rho guanine nucleotide exchange factor (RhoGEF) homologous to the mammalian Dock-C/Zizimin-related family, was identified in a screen to find new genes involved in the Drosophila melanogaster cellular immune response against eggs from the parasitoid wasp Leptopilina boulardi. RhoGEFs activate Rho-family GTPases, which are known to be central regulators of cell **migration**, spreading and polarity. When a parasitoid wasp is recognized as foreign, multiple layers of circulating immunosurveillance cells (haemocytes) should attach to the egg. In Zir mutants this process is disrupted and lamellocytes, a haemocyte subtype, fail to properly encapsulate the wasp egg. Furthermore, macrophage-like plasmatocytes exhibit a strong reduction in their ability to phagocytise Escherichia coli and Staphylococcus aureus bacteria. During encapsulation and phagocytosis Zir genetically interacts with two Rho-family GTPases, Rac2 and Cdc42. Finally, Zir is dispensable for the humoral immune response against bacteria. We propose that Zir is necessary to activate the Rho-family GTPases Rac2 and Cdc42 during the Drosophila cellular immune response.

### [Ee]xtracellular [Mm]atrix

#### PubMed

1. Genome-wide transcriptional analysis of Drosophila larvae infected by entomopathogenic nematodes shows involvement of complement, recognition and **extracellular matrix** proteins. Heterorhabditis bacteriophora is an entomopathogenic nematode (EPN) which infects its host by accessing the hemolymph where it releases endosymbiotic bacteria of the species Photorhabdus luminescens. We performed a genome-wide transcriptional analysis of the Drosophila response to EPN infection at the time point at which the nematodes reached the hemolymph either via the cuticle or the gut and the bacteria had started to multiply. Many of the most strongly induced genes have been implicated in immune responses in other infection models. Mapping of the complete set of differentially regulated genes showed the hallmarks of a wound response, but also identified a large fraction of EPN-specific transcripts. Several genes identified by transcriptome profiling or their homologues play protective roles during nematode infections. Genes that positively contribute to controlling nematobacterial infections encode: a homolog of thioester-containing complement protein 3, a basement membrane component (glutactin), a recognition protein (GNBP-like 3) and possibly several small peptides. Of note is that several of these genes have not previously been implicated in immune responses.

---

## AttA

- NCBI Gene
- GeneRIF
- Pubmed

| Search Term | Rank | Fields |
| --- | --- | --- |
| [Mm]igration | 6 | PubMed(1) |
| [Ee]xtracellular [Mm]atrix | 8 | PubMed(1) |

  
hide/show details for AttA

### [Mm]igration

#### PubMed

1. The unconventional myosin CRINKLED and its mammalian orthologue MYO7A regulate caspases in their signalling roles. Caspases provide vital links in non-apoptotic regulatory networks controlling inflammation, compensatory proliferation, morphology and cell **migration**. How caspases are activated under non-apoptotic conditions and process a selective set of substrates without killing the cell remain enigmatic. Here we find that the Drosophila unconventional myosin CRINKLED (CK) selectively interacts with the initiator caspase DRONC and regulates some of its non-apoptotic functions. Loss of CK in the arista, border cells or proneural clusters of the wing imaginal discs affects DRONC-dependent patterning. Our data indicate that CK acts as substrate adaptor, recruiting SHAGGY46/GSK3-β to DRONC, thereby facilitating caspase-mediated cleavage and localized modulation of kinase activity. Similarly, the mammalian CK counterpart, MYO7A, binds to and impinges on CASPASE-8, revealing a new regulatory axis affecting receptor interacting protein kinase-1 (RIPK1)>CASPASE-8 signalling. Together, our results expose a conserved role for unconventional myosins in transducing caspase-dependent regulation of kinases, allowing them to take part in specific signalling events.

### [Ee]xtracellular [Mm]atrix

#### PubMed

1. Genome-wide transcriptional analysis of Drosophila larvae infected by entomopathogenic nematodes shows involvement of complement, recognition and **extracellular matrix** proteins. Heterorhabditis bacteriophora is an entomopathogenic nematode (EPN) which infects its host by accessing the hemolymph where it releases endosymbiotic bacteria of the species Photorhabdus luminescens. We performed a genome-wide transcriptional analysis of the Drosophila response to EPN infection at the time point at which the nematodes reached the hemolymph either via the cuticle or the gut and the bacteria had started to multiply. Many of the most strongly induced genes have been implicated in immune responses in other infection models. Mapping of the complete set of differentially regulated genes showed the hallmarks of a wound response, but also identified a large fraction of EPN-specific transcripts. Several genes identified by transcriptome profiling or their homologues play protective roles during nematode infections. Genes that positively contribute to controlling nematobacterial infections encode: a homolog of thioester-containing complement protein 3, a basement membrane component (glutactin), a recognition protein (GNBP-like 3) and possibly several small peptides. Of note is that several of these genes have not previously been implicated in immune responses.

---

## AttB

- NCBI Gene
- GeneRIF
- Pubmed

| Search Term | Rank | Fields |
| --- | --- | --- |
| [Mm]igration | 6 | PubMed(1) |

  
hide/show details for AttB

### [Mm]igration

#### PubMed

1. The RhoGEF Zizimin-related acts in the Drosophila cellular immune response via the Rho GTPases Rac2 and Cdc42. Zizimin-related (Zir), a Rho guanine nucleotide exchange factor (RhoGEF) homologous to the mammalian Dock-C/Zizimin-related family, was identified in a screen to find new genes involved in the Drosophila melanogaster cellular immune response against eggs from the parasitoid wasp Leptopilina boulardi. RhoGEFs activate Rho-family GTPases, which are known to be central regulators of cell **migration**, spreading and polarity. When a parasitoid wasp is recognized as foreign, multiple layers of circulating immunosurveillance cells (haemocytes) should attach to the egg. In Zir mutants this process is disrupted and lamellocytes, a haemocyte subtype, fail to properly encapsulate the wasp egg. Furthermore, macrophage-like plasmatocytes exhibit a strong reduction in their ability to phagocytise Escherichia coli and Staphylococcus aureus bacteria. During encapsulation and phagocytosis Zir genetically interacts with two Rho-family GTPases, Rac2 and Cdc42. Finally, Zir is dispensable for the humoral immune response against bacteria. We propose that Zir is necessary to activate the Rho-family GTPases Rac2 and Cdc42 during the Drosophila cellular immune response.

---

## Mtk

- NCBI Gene
- GeneRIF
- Pubmed

| Search Term | Rank | Fields |
| --- | --- | --- |
| [Mm]igration | 6 | PubMed(1) |
| [Ee]xtracellular [Mm]atrix | 8 | PubMed(1) |

  
hide/show details for Mtk

### [Mm]igration

#### PubMed

1. Functional genomic analysis of the periodic transcriptome in the developing Drosophila wing. The eukaryotic cell cycle, driven by both transcriptional and posttranslational mechanisms, is the central molecular oscillator underlying tissue growth throughout animals. Although genome-wide studies have investigated cell-cycle-associated transcription in unicellular systems, global patterns of periodic transcription in multicellular tissues remain largely unexplored. Here we define the cell-cycle-associated transcriptome of the developing Drosophila wing epithelium and compare it with that of cultured Drosophila S2 cells, revealing a core set of periodic genes and a surprising degree of context specificity in periodic transcription. We further employ RNAi-mediated phenotypic profiling to define functional requirements for more than 300 periodic genes, with a focus on those required for cell proliferation in vivo. Finally, we investigate uncharacterized genes required for interkinetic nuclear **migration**. Combined, these findings provide a global perspective on cell-cycle control in vivo, and they highlight a critical need to understand the context-specific regulation of cell proliferation.

### [Ee]xtracellular [Mm]atrix

#### PubMed

1. Genome-wide transcriptional analysis of Drosophila larvae infected by entomopathogenic nematodes shows involvement of complement, recognition and **extracellular matrix** proteins. Heterorhabditis bacteriophora is an entomopathogenic nematode (EPN) which infects its host by accessing the hemolymph where it releases endosymbiotic bacteria of the species Photorhabdus luminescens. We performed a genome-wide transcriptional analysis of the Drosophila response to EPN infection at the time point at which the nematodes reached the hemolymph either via the cuticle or the gut and the bacteria had started to multiply. Many of the most strongly induced genes have been implicated in immune responses in other infection models. Mapping of the complete set of differentially regulated genes showed the hallmarks of a wound response, but also identified a large fraction of EPN-specific transcripts. Several genes identified by transcriptome profiling or their homologues play protective roles during nematode infections. Genes that positively contribute to controlling nematobacterial infections encode: a homolog of thioester-containing complement protein 3, a basement membrane component (glutactin), a recognition protein (GNBP-like 3) and possibly several small peptides. Of note is that several of these genes have not previously been implicated in immune responses.

---

## GNBP-like3

- NCBI Gene
- GeneRIF
- Pubmed

| Search Term | Rank | Fields |
| --- | --- | --- |
| [Ee]xtracellular [Mm]atrix | 8 | PubMed(1) |

  
hide/show details for GNBP-like3

### [Ee]xtracellular [Mm]atrix

#### PubMed

1. Genome-wide transcriptional analysis of Drosophila larvae infected by entomopathogenic nematodes shows involvement of complement, recognition and **extracellular matrix** proteins. Heterorhabditis bacteriophora is an entomopathogenic nematode (EPN) which infects its host by accessing the hemolymph where it releases endosymbiotic bacteria of the species Photorhabdus luminescens. We performed a genome-wide transcriptional analysis of the Drosophila response to EPN infection at the time point at which the nematodes reached the hemolymph either via the cuticle or the gut and the bacteria had started to multiply. Many of the most strongly induced genes have been implicated in immune responses in other infection models. Mapping of the complete set of differentially regulated genes showed the hallmarks of a wound response, but also identified a large fraction of EPN-specific transcripts. Several genes identified by transcriptome profiling or their homologues play protective roles during nematode infections. Genes that positively contribute to controlling nematobacterial infections encode: a homolog of thioester-containing complement protein 3, a basement membrane component (glutactin), a recognition protein (GNBP-like 3) and possibly several small peptides. Of note is that several of these genes have not previously been implicated in immune responses.

---

## IM18

- NCBI Gene
- GeneRIF
- Pubmed

| Search Term | Rank | Fields |
| --- | --- | --- |
| [Ee]xtracellular [Mm]atrix | 8 | PubMed(1) |

  
hide/show details for IM18

### [Ee]xtracellular [Mm]atrix

#### PubMed

1. Genome-wide transcriptional analysis of Drosophila larvae infected by entomopathogenic nematodes shows involvement of complement, recognition and **extracellular matrix** proteins. Heterorhabditis bacteriophora is an entomopathogenic nematode (EPN) which infects its host by accessing the hemolymph where it releases endosymbiotic bacteria of the species Photorhabdus luminescens. We performed a genome-wide transcriptional analysis of the Drosophila response to EPN infection at the time point at which the nematodes reached the hemolymph either via the cuticle or the gut and the bacteria had started to multiply. Many of the most strongly induced genes have been implicated in immune responses in other infection models. Mapping of the complete set of differentially regulated genes showed the hallmarks of a wound response, but also identified a large fraction of EPN-specific transcripts. Several genes identified by transcriptome profiling or their homologues play protective roles during nematode infections. Genes that positively contribute to controlling nematobacterial infections encode: a homolog of thioester-containing complement protein 3, a basement membrane component (glutactin), a recognition protein (GNBP-like 3) and possibly several small peptides. Of note is that several of these genes have not previously been implicated in immune responses.

---

## PGRP-SA

- NCBI Gene
- GeneRIF
- Pubmed

| Search Term | Rank | Fields |
| --- | --- | --- |
| [Ee]xtracellular [Mm]atrix | 8 | PubMed(1) |

  
hide/show details for PGRP-SA

### [Ee]xtracellular [Mm]atrix

#### PubMed

1. Genome-wide transcriptional analysis of Drosophila larvae infected by entomopathogenic nematodes shows involvement of complement, recognition and **extracellular matrix** proteins. Heterorhabditis bacteriophora is an entomopathogenic nematode (EPN) which infects its host by accessing the hemolymph where it releases endosymbiotic bacteria of the species Photorhabdus luminescens. We performed a genome-wide transcriptional analysis of the Drosophila response to EPN infection at the time point at which the nematodes reached the hemolymph either via the cuticle or the gut and the bacteria had started to multiply. Many of the most strongly induced genes have been implicated in immune responses in other infection models. Mapping of the complete set of differentially regulated genes showed the hallmarks of a wound response, but also identified a large fraction of EPN-specific transcripts. Several genes identified by transcriptome profiling or their homologues play protective roles during nematode infections. Genes that positively contribute to controlling nematobacterial infections encode: a homolog of thioester-containing complement protein 3, a basement membrane component (glutactin), a recognition protein (GNBP-like 3) and possibly several small peptides. Of note is that several of these genes have not previously been implicated in immune responses.

---

## AttD

- NCBI Gene
- GeneRIF
- Pubmed

| Search Term | Rank | Fields |
| --- | --- | --- |
| [Mm]igration | 6 | PubMed(1) |

  
hide/show details for AttD

### [Mm]igration

#### PubMed

1. The unconventional myosin CRINKLED and its mammalian orthologue MYO7A regulate caspases in their signalling roles. Caspases provide vital links in non-apoptotic regulatory networks controlling inflammation, compensatory proliferation, morphology and cell **migration**. How caspases are activated under non-apoptotic conditions and process a selective set of substrates without killing the cell remain enigmatic. Here we find that the Drosophila unconventional myosin CRINKLED (CK) selectively interacts with the initiator caspase DRONC and regulates some of its non-apoptotic functions. Loss of CK in the arista, border cells or proneural clusters of the wing imaginal discs affects DRONC-dependent patterning. Our data indicate that CK acts as substrate adaptor, recruiting SHAGGY46/GSK3-β to DRONC, thereby facilitating caspase-mediated cleavage and localized modulation of kinase activity. Similarly, the mammalian CK counterpart, MYO7A, binds to and impinges on CASPASE-8, revealing a new regulatory axis affecting receptor interacting protein kinase-1 (RIPK1)>CASPASE-8 signalling. Together, our results expose a conserved role for unconventional myosins in transducing caspase-dependent regulation of kinases, allowing them to take part in specific signalling events.

---

## PGRP-SB1

- NCBI Gene
- GeneRIF
- Pubmed

| Search Term | Rank | Fields |
| --- | --- | --- |
| [Ee]xtracellular [Mm]atrix | 8 | PubMed(1) |

  
hide/show details for PGRP-SB1

### [Ee]xtracellular [Mm]atrix

#### PubMed

1. Genome-wide transcriptional analysis of Drosophila larvae infected by entomopathogenic nematodes shows involvement of complement, recognition and **extracellular matrix** proteins. Heterorhabditis bacteriophora is an entomopathogenic nematode (EPN) which infects its host by accessing the hemolymph where it releases endosymbiotic bacteria of the species Photorhabdus luminescens. We performed a genome-wide transcriptional analysis of the Drosophila response to EPN infection at the time point at which the nematodes reached the hemolymph either via the cuticle or the gut and the bacteria had started to multiply. Many of the most strongly induced genes have been implicated in immune responses in other infection models. Mapping of the complete set of differentially regulated genes showed the hallmarks of a wound response, but also identified a large fraction of EPN-specific transcripts. Several genes identified by transcriptome profiling or their homologues play protective roles during nematode infections. Genes that positively contribute to controlling nematobacterial infections encode: a homolog of thioester-containing complement protein 3, a basement membrane component (glutactin), a recognition protein (GNBP-like 3) and possibly several small peptides. Of note is that several of these genes have not previously been implicated in immune responses.

---

## Drs

- NCBI Gene
- GeneRIF
- Pubmed

| Search Term | Rank | Fields |
| --- | --- | --- |
| [Mm]igration | 6 | PubMed(3) |
| [Ee]xtracellular [Mm]atrix | 8 | PubMed(2) |
| [Ii]nvasive | 10 | PubMed(1) |

  
hide/show details for Drs

### [Mm]igration

#### PubMed

1. The RhoGEF Zizimin-related acts in the Drosophila cellular immune response via the Rho GTPases Rac2 and Cdc42. Zizimin-related (Zir), a Rho guanine nucleotide exchange factor (RhoGEF) homologous to the mammalian Dock-C/Zizimin-related family, was identified in a screen to find new genes involved in the Drosophila melanogaster cellular immune response against eggs from the parasitoid wasp Leptopilina boulardi. RhoGEFs activate Rho-family GTPases, which are known to be central regulators of cell **migration**, spreading and polarity. When a parasitoid wasp is recognized as foreign, multiple layers of circulating immunosurveillance cells (haemocytes) should attach to the egg. In Zir mutants this process is disrupted and lamellocytes, a haemocyte subtype, fail to properly encapsulate the wasp egg. Furthermore, macrophage-like plasmatocytes exhibit a strong reduction in their ability to phagocytise Escherichia coli and Staphylococcus aureus bacteria. During encapsulation and phagocytosis Zir genetically interacts with two Rho-family GTPases, Rac2 and Cdc42. Finally, Zir is dispensable for the humoral immune response against bacteria. We propose that Zir is necessary to activate the Rho-family GTPases Rac2 and Cdc42 during the Drosophila cellular immune response.
2. Functional genomic analysis of the periodic transcriptome in the developing Drosophila wing. The eukaryotic cell cycle, driven by both transcriptional and posttranslational mechanisms, is the central molecular oscillator underlying tissue growth throughout animals. Although genome-wide studies have investigated cell-cycle-associated transcription in unicellular systems, global patterns of periodic transcription in multicellular tissues remain largely unexplored. Here we define the cell-cycle-associated transcriptome of the developing Drosophila wing epithelium and compare it with that of cultured Drosophila S2 cells, revealing a core set of periodic genes and a surprising degree of context specificity in periodic transcription. We further employ RNAi-mediated phenotypic profiling to define functional requirements for more than 300 periodic genes, with a focus on those required for cell proliferation in vivo. Finally, we investigate uncharacterized genes required for interkinetic nuclear **migration**. Combined, these findings provide a global perspective on cell-cycle control in vivo, and they highlight a critical need to understand the context-specific regulation of cell proliferation.
3. Loss of the starvation-induced gene Rack1 leads to glycogen deficiency and impaired autophagic responses in Drosophila. Autophagy delivers cytoplasmic material for lysosomal degradation in eukaryotic cells. Starvation induces high levels of autophagy to promote survival in the lack of nutrients. We compared genome-wide transcriptional profiles of fed and starved control, autophagy-deficient Atg7 and Atg1 null mutant Drosophila larvae to search for novel regulators of autophagy. Genes involved in catabolic processes including autophagy were transcriptionally upregulated in all cases. We also detected repression of genes involved in DNA replication in autophagy mutants compared with control animals. The expression of Rack1 (receptor of activated protein kinase C 1) increased 4.1- to 5.5-fold during nutrient deprivation in all three genotypes. The scaffold protein Rack1 plays a role in a wide range of processes including translation, cell adhesion and **migration**, cell survival and cancer. Loss of Rack1 led to attenuated autophagic response to starvation, and glycogen stores were decreased 11.8-fold in Rack1 mutant cells. Endogenous Rack1 partially colocalized with GFP-Atg8a and early autophagic structures on the ultrastructural level, suggesting its involvement in autophagosome formation. Endogenous Rack1 also showed a high degree of colocalization with glycogen particles in the larval fat body, and with Shaggy, the Drosophila homolog of glycogen synthase kinase 3B (GSK-3B). Our results, for the first time, demonstrated the fundamental role of Rack1 in autophagy and glycogen synthesis.

### [Ee]xtracellular [Mm]atrix

#### PubMed

1. Genome-wide transcriptional analysis of Drosophila larvae infected by entomopathogenic nematodes shows involvement of complement, recognition and **extracellular matrix** proteins. Heterorhabditis bacteriophora is an entomopathogenic nematode (EPN) which infects its host by accessing the hemolymph where it releases endosymbiotic bacteria of the species Photorhabdus luminescens. We performed a genome-wide transcriptional analysis of the Drosophila response to EPN infection at the time point at which the nematodes reached the hemolymph either via the cuticle or the gut and the bacteria had started to multiply. Many of the most strongly induced genes have been implicated in immune responses in other infection models. Mapping of the complete set of differentially regulated genes showed the hallmarks of a wound response, but also identified a large fraction of EPN-specific transcripts. Several genes identified by transcriptome profiling or their homologues play protective roles during nematode infections. Genes that positively contribute to controlling nematobacterial infections encode: a homolog of thioester-containing complement protein 3, a basement membrane component (glutactin), a recognition protein (GNBP-like 3) and possibly several small peptides. Of note is that several of these genes have not previously been implicated in immune responses.
2. Drosophila melanogaster as an animal model for the study of Pseudomonas aeruginosa biofilm infections in vivo. Pseudomonas aeruginosa is an opportunistic pathogen capable of causing both acute and chronic infections in susceptible hosts. Chronic P. aeruginosa infections are thought to be caused by bacterial biofilms. Biofilms are highly structured, multicellular, microbial communities encased in an **extracellular matrix** that enable long-term survival in the host. The aim of this research was to develop an animal model that would allow an in vivo study of P. aeruginosa biofilm infections in a Drosophila melanogaster host. At 24 h post oral infection of Drosophila, P. aeruginosa biofilms localized to and were visualized in dissected Drosophila crops. These biofilms had a characteristic aggregate structure and an **extracellular matrix** composed of DNA and exopolysaccharide. P. aeruginosa cells recovered from in vivo grown biofilms had increased antibiotic resistance relative to planktonically grown cells. In vivo, biofilm formation was dependent on expression of the pel exopolysaccharide genes, as a pelB::lux mutant failed to form biofilms. The pelB::lux mutant was significantly more virulent than PAO1, while a hyperbiofilm strain (PAZHI3) demonstrated significantly less virulence than PAO1, as indicated by survival of infected flies at day 14 postinfection. Biofilm formation, by strains PAO1 and PAZHI3, in the crop was associated with induction of diptericin, cecropin A1 and drosomycin antimicrobial peptide gene expression 24 h postinfection. In contrast, infection with the non-biofilm forming strain pelB::lux resulted in decreased AMP gene expression in the fly. In summary, these results provide novel insights into host-pathogen interactions during P. aeruginosa oral infection of Drosophila and highlight the use of Drosophila as an infection model that permits the study of P. aeruginosa biofilms in vivo.

### [Ii]nvasive

#### PubMed

1. Pathogen and host factors are needed to provoke a systemic host response to gastrointestinal infection of Drosophila larvae by Candida albicans. Candida albicans systemic dissemination in immunocompromised patients is thought to develop from initial gastrointestinal (GI) colonisation. It is unclear what components of the innate immune system are necessary for preventing C. albicans dissemination from the GI tract, but studies in mice have indicated that both neutropenia and GI mucosal damage are crucial for allowing widespread **invasive** C. albicans disease. Mouse models, however, provide limited applicability to genome-wide screens for pathogen or host factors - factors that might influence systemic dissemination following GI colonisation. For this reason we developed a Drosophila model to study intestinal infection by Candida. We found that commensal flora aided host survival following GI infection. Candida provoked extensive JNK-mediated death of gut cells and induced antimicrobial peptide expression in the fat body. From the side of the host, nitric oxide and blood cells influenced systemic antimicrobial responses. The secretion of SAP4 and SAP6 (secreted aspartyl proteases) from Candida was also essential for activating systemic Toll-dependent immunity.

---

## NijA

- NCBI Gene
- GeneRIF
- Pubmed

| Search Term | Rank | Fields |
| --- | --- | --- |
| [Mm]igration | 6 | PubMed(1) |
| ECM | 7 | PubMed(1) |
| [Ee]xtracellular [Mm]atrix | 8 | PubMed(2) |

  
hide/show details for NijA

### [Mm]igration

#### PubMed

1. Genes conserved in bilaterians but jointly lost with Myc during nematode evolution are enriched in cell proliferation and cell **migration** functions. Animals use a stereotypical set of developmental genes to build body architectures of varying sizes and organizational complexity. Some genes are critical to developmental patterning, while other genes are important to physiological control of growth. However, growth regulator genes may not be as important in small-bodied "micro-metazoans" such as nematodes. Nematodes use a simplified developmental strategy of lineage-based cell fate specifications to produce an adult bilaterian body composed of a few hundreds of cells. Nematodes also lost the MYC proto-oncogenic regulator of cell proliferation. To identify additional regulators of cell proliferation that were lost with MYC, we computationally screened and determined 839 high-confidence genes that are conserved in bilaterians/lost in nematodes (CIBLIN genes). We find that 30 % of all CIBLIN genes encode transcriptional regulators of cell proliferation, epithelial-to-mesenchyme transitions, and other processes. Over 50 % of CIBLIN genes are unnamed genes in Drosophila, suggesting that there are many understudied genes. Interestingly, CIBLIN genes include many Myc synthetic lethal (MycSL) hits from recent screens. CIBLIN genes include key regulators of heparan sulfate proteoglycan (HSPG) sulfation patterns, and lysyl oxidases involved in cross-linking and modification of the extracellular matrix (ECM). These genes and others suggest the CIBLIN repertoire services critical functions in ECM remodeling and cell **migration** in large-bodied bilaterians. Correspondingly, CIBLIN genes are co-expressed with Myc in cancer transcriptomes, and include a preponderance of known determinants of cancer progression and tumor aggression. We propose that CIBLIN gene research can improve our understanding of regulatory control of cellular growth in metazoans.

### ECM

#### PubMed

1. Genes conserved in bilaterians but jointly lost with Myc during nematode evolution are enriched in cell proliferation and cell migration functions. Animals use a stereotypical set of developmental genes to build body architectures of varying sizes and organizational complexity. Some genes are critical to developmental patterning, while other genes are important to physiological control of growth. However, growth regulator genes may not be as important in small-bodied "micro-metazoans" such as nematodes. Nematodes use a simplified developmental strategy of lineage-based cell fate specifications to produce an adult bilaterian body composed of a few hundreds of cells. Nematodes also lost the MYC proto-oncogenic regulator of cell proliferation. To identify additional regulators of cell proliferation that were lost with MYC, we computationally screened and determined 839 high-confidence genes that are conserved in bilaterians/lost in nematodes (CIBLIN genes). We find that 30 % of all CIBLIN genes encode transcriptional regulators of cell proliferation, epithelial-to-mesenchyme transitions, and other processes. Over 50 % of CIBLIN genes are unnamed genes in Drosophila, suggesting that there are many understudied genes. Interestingly, CIBLIN genes include many Myc synthetic lethal (MycSL) hits from recent screens. CIBLIN genes include key regulators of heparan sulfate proteoglycan (HSPG) sulfation patterns, and lysyl oxidases involved in cross-linking and modification of the extracellular matrix (**ECM**). These genes and others suggest the CIBLIN repertoire services critical functions in **ECM** remodeling and cell migration in large-bodied bilaterians. Correspondingly, CIBLIN genes are co-expressed with Myc in cancer transcriptomes, and include a preponderance of known determinants of cancer progression and tumor aggression. We propose that CIBLIN gene research can improve our understanding of regulatory control of cellular growth in metazoans.

### [Ee]xtracellular [Mm]atrix

#### PubMed

1. An MMP liberates the Ninjurin A ectodomain to signal a loss of cell adhesion. Matrix metalloproteinases (MMPs) are important for developmental tissue remodeling and for the inflammatory response. Although the vertebrate MMP family is large and functionally redundant, the fruitfly Drosophila melanogaster has only two MMPs, both essential genes. Our previous work demonstrated that Mmp1 is required for growth of the tracheal system, and we suggested that the mutant phenotype resulted from aberrant persistence of cell adhesion to the **extracellular matrix**. Here we report the identification of NijA, a transmembrane protein whose vertebrate homologs regulate cell adhesion, as a two-hybrid binding partner for Mmp1. The binding of Mmp1 and NijA was confirmed by coimmunoprecipitation of endogenous proteins from flies, and the endogenous proteins were found to colocalize at the tracheal cell surface in larvae. When NijA is expressed in S2 cells, they lose adhesion to surfaces; this adhesion-loss phenotype is dependent on the expression and catalytic activity of Mmp1. Our data indicate that Mmp1 releases the N-terminal extracellular domain of NijA. This liberated ectodomain promotes the loss of cell adhesion in a cell-nonautonomous manner. We suggest that tracheal cell adhesion is regulated by a novel mechanism utilizing an MMP and a ninjurin family member.
2. Genes conserved in bilaterians but jointly lost with Myc during nematode evolution are enriched in cell proliferation and cell migration functions. Animals use a stereotypical set of developmental genes to build body architectures of varying sizes and organizational complexity. Some genes are critical to developmental patterning, while other genes are important to physiological control of growth. However, growth regulator genes may not be as important in small-bodied "micro-metazoans" such as nematodes. Nematodes use a simplified developmental strategy of lineage-based cell fate specifications to produce an adult bilaterian body composed of a few hundreds of cells. Nematodes also lost the MYC proto-oncogenic regulator of cell proliferation. To identify additional regulators of cell proliferation that were lost with MYC, we computationally screened and determined 839 high-confidence genes that are conserved in bilaterians/lost in nematodes (CIBLIN genes). We find that 30 % of all CIBLIN genes encode transcriptional regulators of cell proliferation, epithelial-to-mesenchyme transitions, and other processes. Over 50 % of CIBLIN genes are unnamed genes in Drosophila, suggesting that there are many understudied genes. Interestingly, CIBLIN genes include many Myc synthetic lethal (MycSL) hits from recent screens. CIBLIN genes include key regulators of heparan sulfate proteoglycan (HSPG) sulfation patterns, and lysyl oxidases involved in cross-linking and modification of the **extracellular matrix** (ECM). These genes and others suggest the CIBLIN repertoire services critical functions in ECM remodeling and cell migration in large-bodied bilaterians. Correspondingly, CIBLIN genes are co-expressed with Myc in cancer transcriptomes, and include a preponderance of known determinants of cancer progression and tumor aggression. We propose that CIBLIN gene research can improve our understanding of regulatory control of cellular growth in metazoans.

---

## Spn28Dc

- NCBI Gene
- GeneRIF
- Pubmed

| Search Term | Rank | Fields |
| --- | --- | --- |
| [Mm]igration | 6 | PubMed(1) |
| ECM | 7 | PubMed(1) |
| [Ee]xtracellular [Mm]atrix | 8 | PubMed(1) |

  
hide/show details for Spn28Dc

### [Mm]igration

#### PubMed

1. Genes conserved in bilaterians but jointly lost with Myc during nematode evolution are enriched in cell proliferation and cell **migration** functions. Animals use a stereotypical set of developmental genes to build body architectures of varying sizes and organizational complexity. Some genes are critical to developmental patterning, while other genes are important to physiological control of growth. However, growth regulator genes may not be as important in small-bodied "micro-metazoans" such as nematodes. Nematodes use a simplified developmental strategy of lineage-based cell fate specifications to produce an adult bilaterian body composed of a few hundreds of cells. Nematodes also lost the MYC proto-oncogenic regulator of cell proliferation. To identify additional regulators of cell proliferation that were lost with MYC, we computationally screened and determined 839 high-confidence genes that are conserved in bilaterians/lost in nematodes (CIBLIN genes). We find that 30 % of all CIBLIN genes encode transcriptional regulators of cell proliferation, epithelial-to-mesenchyme transitions, and other processes. Over 50 % of CIBLIN genes are unnamed genes in Drosophila, suggesting that there are many understudied genes. Interestingly, CIBLIN genes include many Myc synthetic lethal (MycSL) hits from recent screens. CIBLIN genes include key regulators of heparan sulfate proteoglycan (HSPG) sulfation patterns, and lysyl oxidases involved in cross-linking and modification of the extracellular matrix (ECM). These genes and others suggest the CIBLIN repertoire services critical functions in ECM remodeling and cell **migration** in large-bodied bilaterians. Correspondingly, CIBLIN genes are co-expressed with Myc in cancer transcriptomes, and include a preponderance of known determinants of cancer progression and tumor aggression. We propose that CIBLIN gene research can improve our understanding of regulatory control of cellular growth in metazoans.

### ECM

#### PubMed

1. Genes conserved in bilaterians but jointly lost with Myc during nematode evolution are enriched in cell proliferation and cell migration functions. Animals use a stereotypical set of developmental genes to build body architectures of varying sizes and organizational complexity. Some genes are critical to developmental patterning, while other genes are important to physiological control of growth. However, growth regulator genes may not be as important in small-bodied "micro-metazoans" such as nematodes. Nematodes use a simplified developmental strategy of lineage-based cell fate specifications to produce an adult bilaterian body composed of a few hundreds of cells. Nematodes also lost the MYC proto-oncogenic regulator of cell proliferation. To identify additional regulators of cell proliferation that were lost with MYC, we computationally screened and determined 839 high-confidence genes that are conserved in bilaterians/lost in nematodes (CIBLIN genes). We find that 30 % of all CIBLIN genes encode transcriptional regulators of cell proliferation, epithelial-to-mesenchyme transitions, and other processes. Over 50 % of CIBLIN genes are unnamed genes in Drosophila, suggesting that there are many understudied genes. Interestingly, CIBLIN genes include many Myc synthetic lethal (MycSL) hits from recent screens. CIBLIN genes include key regulators of heparan sulfate proteoglycan (HSPG) sulfation patterns, and lysyl oxidases involved in cross-linking and modification of the extracellular matrix (**ECM**). These genes and others suggest the CIBLIN repertoire services critical functions in **ECM** remodeling and cell migration in large-bodied bilaterians. Correspondingly, CIBLIN genes are co-expressed with Myc in cancer transcriptomes, and include a preponderance of known determinants of cancer progression and tumor aggression. We propose that CIBLIN gene research can improve our understanding of regulatory control of cellular growth in metazoans.

### [Ee]xtracellular [Mm]atrix

#### PubMed

1. Genes conserved in bilaterians but jointly lost with Myc during nematode evolution are enriched in cell proliferation and cell migration functions. Animals use a stereotypical set of developmental genes to build body architectures of varying sizes and organizational complexity. Some genes are critical to developmental patterning, while other genes are important to physiological control of growth. However, growth regulator genes may not be as important in small-bodied "micro-metazoans" such as nematodes. Nematodes use a simplified developmental strategy of lineage-based cell fate specifications to produce an adult bilaterian body composed of a few hundreds of cells. Nematodes also lost the MYC proto-oncogenic regulator of cell proliferation. To identify additional regulators of cell proliferation that were lost with MYC, we computationally screened and determined 839 high-confidence genes that are conserved in bilaterians/lost in nematodes (CIBLIN genes). We find that 30 % of all CIBLIN genes encode transcriptional regulators of cell proliferation, epithelial-to-mesenchyme transitions, and other processes. Over 50 % of CIBLIN genes are unnamed genes in Drosophila, suggesting that there are many understudied genes. Interestingly, CIBLIN genes include many Myc synthetic lethal (MycSL) hits from recent screens. CIBLIN genes include key regulators of heparan sulfate proteoglycan (HSPG) sulfation patterns, and lysyl oxidases involved in cross-linking and modification of the **extracellular matrix** (ECM). These genes and others suggest the CIBLIN repertoire services critical functions in ECM remodeling and cell migration in large-bodied bilaterians. Correspondingly, CIBLIN genes are co-expressed with Myc in cancer transcriptomes, and include a preponderance of known determinants of cancer progression and tumor aggression. We propose that CIBLIN gene research can improve our understanding of regulatory control of cellular growth in metazoans.

---

## Mmp1

- NCBI Gene
- GeneRIF
- Pubmed

| Search Term | Rank | Fields |
| --- | --- | --- |
| [Mm]igration | 6 | PubMed(12); GeneRIFs(1) |
| ECM | 7 | PubMed(5) |
| [Ee]xtracellular [Mm]atrix | 8 | Process(1); PubMed(19); Component(1); Conserved Domains(1) |
| [Ii]nvasive | 10 | PubMed(17) |
| [Mm]etastasize | 11 | PubMed(2) |
| [Mm]etastasis | 12 | PubMed(13) |
| [Cc]ell [Ii]nvasion | 13 | PubMed(9) |

  
hide/show details for Mmp1

### [Mm]igration

#### PubMed

1. Bendless modulates JNK-mediated cell death and **migration** in Drosophila. The TNF-JNK pathway is a highly conserved signaling pathway that regulates a wide spectrum of biological processes including cell death and **migration**. To further delineate this pathway, we carried out a genetic screen for dominant modifiers of the cell death phenotype triggered by ectopic expression of Eiger (Egr), the Drosophila TNF ortholog. Here we show that Bendless (Ben), an E2 ubiquitin-conjugating enzyme, modulates Egr-induced JNK activation and cell death through dTRAF2. Furthermore, Ben physically interacts with dTRAF2 and regulates Egr-induced dTRAF2 polyubiquitination. Finally, Ben is required for JNK-dependent tumor progression, cell **migration**, oxidative stress resistance and longevity. Our results indicate that Ben constitutes an essential component of the evolutionarily conserved TNF-JNK pathway that modulates cell death and invasion, tumor progression, stress response and lifespan in metazoans.
2. A drosophila model for EGFR-Ras and PI3K-dependent human glioma. Gliomas, the most common malignant tumors of the nervous system, frequently harbor mutations that activate the epidermal growth factor receptor (EGFR) and phosphatidylinositol-3 kinase (PI3K) signaling pathways. To investigate the genetic basis of this disease, we developed a glioma model in Drosophila. We found that constitutive coactivation of EGFR-Ras and PI3K pathways in Drosophila glia and glial precursors gives rise to neoplastic, invasive glial cells that create transplantable tumor-like growths, mimicking human glioma. Our model represents a robust organotypic and cell-type-specific Drosophila cancer model in which malignant cells are created by mutations in signature genes and pathways thought to be driving forces in a homologous human cancer. Genetic analyses demonstrated that EGFR and PI3K initiate malignant neoplastic transformation via a combinatorial genetic network composed primarily of other pathways commonly mutated or activated in human glioma, including the Tor, Myc, G1 Cyclins-Cdks, and Rb-E2F pathways. This network acts synergistically to coordinately stimulate cell cycle entry and progression, protein translation, and inappropriate cellular growth and **migration**. In particular, we found that the fly orthologs of CyclinE, Cdc25, and Myc are key rate-limiting genes required for glial neoplasia. Moreover, orthologs of Sin1, Rictor, and Cdk4 are genes required only for abnormal neoplastic glial proliferation but not for glial development. These and other genes within this network may represent important therapeutic targets in human glioma.
3. Dynamics of the basement membrane in invasive epithelial clusters in Drosophila. The basement membrane (BM) represents a barrier to cell **migration**, which has to be degraded to promote invasion. However, the role and behaviour of the BM during the development of pre-invasive cells is only poorly understood. Drosophila border cells (BCs) provide an attractive genetic model in which to study the cellular mechanisms underlying the **migration** of mixed cohorts of epithelial cells. BCs are made of two different epithelial cell types appearing sequentially during oogenesis: the polar cells and the outer BCs. Here, we show that the pre-invasive polar cells undergo an unusual and asymmetrical apical capping with major basement membrane proteins, including the two Drosophila Collagen IV alpha chains, Laminin A and Perlecan. Capping of polar cells proceeds through a novel, basal-to-apical transcytosis mechanism that involves the small GTPase Drab5. Apical capping is transient and is followed by rapid shedding prior to the initiation of BC **migration**, suggesting that the apical cap blocks **migration**. Consistently, non-migratory polar cells remain capped. We further show that JAK/STAT signalling and recruitment of outer BCs are required for correct shedding and **migration**. The dynamics of the BM represents a marker of migratory BC, revealing a novel developmentally regulated behaviour of BM coupled to epithelial cell invasiveness.
4. Active JNK-dependent secretion of Drosophila Tyrosyl-tRNA synthetase by loser cells recruits haemocytes during cell competition. Cell competition is a process by which the slow dividing cells (losers) are recognized and eliminated from growing tissues. Loser cells are extruded from the epithelium and engulfed by the haemocytes, the Drosophila macrophages. However, how macrophages identify the dying loser cells is unclear. Here we show that apoptotic loser cells secrete Tyrosyl-tRNA synthetase (TyrRS), which is best known as a core component of the translational machinery. Secreted TyrRS is cleaved by matrix metalloproteinases generating MiniTyr and EMAP fragments. EMAP acts as a guiding cue for macrophage **migration** in the Drosophila larvae, as it attracts the haemocytes to the apoptotic loser cells. JNK signalling and Kish, a component of the secretory pathway, are autonomously required for the active secretion of TyrRS by the loser cells. Altogether, this mechanism guarantees effective removal of unfit cells from the growing tissue.
5. A Jnk-Rho-Actin remodeling positive feedback network directs Src-driven invasion. Current models of tumor cell invasion propose that oncogenic signaling converges upon key orchestrators of cytoskeletal dynamics, including c-Jun N-terminal kinase (Jnk) and RhoGTPase family members; these signals dynamically direct Actin remodeling proteins (ARPs) to catalyze the cytoskeletal changes required for **migration**. Src is a key driver of tumor aggression, metastasis and patient mortality. To clarify how Src regulates Actin dynamics to promote invasive **migration**, we performed a genetic modifier screen in a Drosophila model of invasion. Nine genes linked to Actin dynamics were identified that mediate invasion in situ. We found that ARPs were required for many oncogenic effects of Src including Mmp1 expression and initiation of apoptosis. Surprisingly, they were also regulators of Jnk pathway activity: both Src and the small GTPase Rho1 activated Jnk in a manner dependent on ARPs during invasion. Our results suggest that ARPs are not simply downstream executors of signal transduction pathways. Rather, they participate in a positive feedback network involving canonical oncogenic signaling pathways that promote tumor invasion.
6. A secreted MMP is required for reepithelialization during wound healing. Matrix metalloproteinases (MMPs) are extracellular proteases highly expressed at wound sites. However, the precise function of MMPs during reepithelialization in vivo has been elusive in mammalian models because of the high level of redundancy among the 24 mammalian MMPs. For this reason we used Drosophila melanogaster, whose genome encodes only two MMPs-one secreted type (Mmp1) and one membrane-anchored type (Mmp2)-to study the function and regulation of the secreted class of MMPs in vivo. In the absence of redundancy, we found that the Drosophila secreted MMP, Mmp1, is required in the epidermis to facilitate reepithelialization by remodeling the basement membrane, promoting cell elongation and actin cytoskeletal reorganization, and activating extracellular signal-regulated kinase signaling. In addition, we report that the jun N-terminal kinase (JNK) pathway upregulates Mmp1 expression after wounding, but that Mmp1 is expressed independent of the JNK pathway in unwounded epidermis. When the JNK pathway is ectopically activated to overexpress Mmp1, the rate of healing is accelerated in an Mmp1-dependent manner. A primary function of Mmp1, under the control of the JNK pathway, is to promote basement membrane repair, which in turn may permit cell **migration** and the restoration of a continuous tissue.
7. Rho1-Wnd signaling regulates loss-of-cell polarity-induced cell invasion in Drosophila. Both cell polarity and c-Jun N-terminal kinase (JNK) activity are essential to the maintenance of tissue homeostasis, and disruption of either is commonly seen in cancer progression. Despite the established connection between loss-of-cell polarity and JNK activation, much less is known about the molecular mechanism by which aberrant cell polarity induces JNK-mediated cell **migration** and tumor invasion. Here we show results from a genetic screen using an in vivo invasion model via knocking down cell polarity gene in Drosophila wing discs, and identify Rho1-Wnd signaling as an important molecular link that mediates loss-of-cell polarity-triggered JNK activation and cell invasion. We show that Wallenda (Wnd), a protein kinase of the mitogen-activated protein kinase kinase kinase family, by forming a complex with the GTPase Rho1, is both necessary and sufficient for Rho1-induced JNK-dependent cell invasion, MMP1 activation and epithelial-mesenchymal transition. Furthermore, Wnd promotes cell proliferation and tissue growth through wingless production when apoptosis is inhibited by p35. Finally, Wnd shows oncogenic cooperation with Ras(V12) to trigger tumor growth in eye discs and causes invasion into the ventral nerve cord. Together, our data not only provides a novel mechanistic insight on how cell polarity loss contributes to cell invasion, but also highlights the value of the Drosophila model system to explore human cancer biology.
8. RhoL controls invasion and Rap1 localization during immune cell trans**migration** in Drosophila. Human immune cells have to penetrate an endothelial barrier during their beneficial pursuit of infection and their destructive infiltration of tissues in autoimmune diseases. This trans**migration** requires Rap1 GTPase to activate integrin affinity. We define a new model system for this process by demonstrating, with live imaging and genetics, that during embryonic development Drosophila melanogaster immune cells penetrate an epithelial, Drosophila E-cadherin (DE-cadherin)-based tissue barrier. A mutant in RhoL, a GTPase homologue that is specifically expressed in haemocytes, blocks this invasive step but not other aspects of guided **migration**. RhoL mediates integrin adhesion caused by Drosophila Rap1 overexpression and moves Rap1 away from a concentration in the cytoplasm to the leading edge during invasive **migration**. These findings indicate that a programmed migratory step during Drosophila development bears striking molecular similarities to vertebrate immune cell trans**migration** during inflammation, and identify RhoL as a new regulator of invasion, adhesion and Rap1 localization. Our work establishes the utility of Drosophila for identifying novel components of immune cell trans**migration** and for understanding the in vivo interplay of immune cells with the barriers they penetrate.
9. Control of Gliotactin localization and levels by tyrosine phosphorylation and endocytosis is necessary for survival of polarized epithelia. The tricellular junction (TCJ) forms at the convergence of bicellular junctions from three adjacent cells in polarized epithelia and is necessary for maintaining the transepithelial barrier. In the fruitfly Drosophila, the TCJ is generated at the meeting point of bicellular septate junctions. Gliotactin was the first identified component of the TCJ and is necessary for TCJ and septate junction development. Gliotactin is a member of the neuroligin family and associates with the PDZ protein discs large. Beyond this interaction, little is known about the mechanisms underlying Gliotactin localization and function at the TCJ. In this study, we show that Gliotactin is phosphorylated at conserved tyrosine residues, a process necessary for endocytosis and targeting to late endosomes and lysosomes for degradation. Regulation of Gliotactin levels through phosphorylation and endocytosis is necessary as overexpression results in displacement of Gliotactin away from the TCJ throughout the septate junction domain. Excessive Gliotactin in polarized epithelia leads to delamination, paired with subsequent **migration**, and apoptosis. The apoptosis and the resulting compensatory proliferation resulting from high levels of Gliotactin are mediated by the Drosophila JNK pathway. Therefore, Gliotactin levels within the cell membrane are regulated to ensure correct protein localization and cell survival.
10. A role for the epithelial microenvironment at tumor boundaries: evidence from Drosophila and human squamous cell carcinomas. Recent work has shown an increasing appreciation for the importance of the tumor environment, most commonly the overlying stroma. Less emphasis has been placed on the importance of local communication between transformed cells and their neighbors within the epithelium at tumor boundaries. We previously reported a Drosophila model that highlighted the importance of local interactions within the epithelial microenvironment: Src-transformed cells (Csk-deficient) were influenced by their immediate normal neighbors. The result was a consistent change in 'border cells' at the edge of transformed patches including delocalized p120-catenin and E-cadherin as well as invasive **migration** through the basal lamina. Here we show that the invasive properties of the boundary cells depend on up-regulation of Drosophila matrix metalloproteinase-1 as assessed by promoter activity, protein levels, in situ enzymatic activity, and tests of genetic modifier activity. Further, we provide evidence that these events at tumor borders may be evolutionarily conserved. We detected changes in 'boundary cells' within histological sections of human squamous cell carcinomas that were similar to those observed in Drosophila: both E-cadherin and p120-catenin exhibited normal junctional localization at the centers of the tumors but were reduced or delocalized at the boundary. Further, matrix metalloproteinase-2 was up regulated within these same boundary cells. These results support the view that local cell-cell interactions within the epithelial microenvironment impact tumor invasion and progression.
11. Centrosomal kinase Nek2 cooperates with oncogenic pathways to promote metastasis. Centrosomal kinase Nek2 is overexpressed in different cancers, yet how it contributes toward tumorigenesis remains poorly understood. dNek2 overexpression in a Drosophila melanogaster model led to upregulation of Drosophila Wnt ortholog wingless (Wg), and alteration of cell **migration** markers-Rho1, Rac1 and E-cadherin (Ecad)-resulting in changes in cell shape and tissue morphogenesis. dNek2 overexpression cooperated with receptor tyrosine kinase and mitogen-activated protein kinase signaling to upregulate activated Akt, Diap1, Mmp1 and Wg protein to promote local invasion, distant seeding and metastasis. In tumor cell injection assays, dNek2 cooperated with Ras and Src signaling to promote aggressive colonization of tumors into different adult fly tissues. Inhibition of the PI3K pathway suppressed the cooperation of dNek2 with other growth pathways. Consistent with our fly studies, overexpression of human Nek2 in A549 lung adenocarcinoma and HEK293T cells led to activation of the Akt pathway and increase in β-catenin protein levels. Our computational approach identified a class of Nek2-inhibitory compounds and a novel drug-like pharmacophore that reversed the Nek2 overexpression phenotypes in flies and human cells. Our finding posits a novel role for Nek2 in promoting metastasis in addition to its currently defined role in promoting chromosomal instability. It provides a rationale for the selective advantage of centrosome amplification in cancer.
12. Oncogenic Ras diverts a host TNF tumor suppressor activity into tumor promoter. The roles of inflammatory cytokines and the immune response in cancer remain paradoxical. In the case of tumor necrosis factor (TNF), there is undisputed evidence indicating both protumor and antitumor activities. Recent work in Drosophila indicated that a TNF-dependent mechanism eliminates cells deficient for the polarity tumor suppressors dlg or scrib. In this study, however, we show that in tumors deficient for scrib that also expressed the Ras oncoprotein, the TNF signal was diverted into a protumor signal that enhanced tumor growth through larval arrest and stimulated invasive **migration**. In this case, TNF promoted malignancy and was detrimental to host survival. TNF was expressed at high levels by tumor-associated hemocytes recruited from the circulation. The expression of TNF by hemocytes was both necessary and sufficient to trigger TNF signaling in tumor cells. Our evidence suggests that tumors can evolve into malignancy through oncogenic Ras activation and the hijacking of TNF signaling.

#### GeneRIFs

1. A primary function of Mmp1, under the control of the JNK pathway, is to promote basement membrane repair, which in turn may permit cell **migration** and the restoration of a continuous tissue.

### ECM

#### PubMed

1. **ECM**-Regulator timp Is Required for Stem Cell Niche Organization and Cyst Production in the Drosophila Ovary. The extracellular matrix (**ECM**) is a pivotal component adult tissues and of many tissue-specific stem cell niches. It provides structural support and regulates niche signaling during tissue maintenance and regeneration. In many tissues, **ECM** remodeling depends on the regulation of MMP (matrix metalloproteinase) activity by inhibitory TIMP (tissue inhibitors of metalloproteinases) proteins. Here, we report that the only Drosophila timp gene is required for maintaining the normal organization and function of the germline stem cell niche in adult females. timp mutant ovaries show reduced levels of both Drosophila Collagen IV α chains. In addition, tissue stiffness and the cellular organization of the ovarian niche are affected in timp mutants. Finally, loss of timp impairs the ability of the germline stem cell niche to generate new cysts. Our results demonstrating a crucial role for timp in tissue organization and gamete production thus provide a link between the regulation of **EC**M metabolism and tissue homeostasis.
2. A matrix metalloproteinase mediates airway remodeling in Drosophila. Organ size typically increases dramatically during juvenile growth. This growth presents a fundamental tension, as organs need resiliency to resist stresses while still maintaining plasticity to accommodate growth. The extracellular matrix (**ECM**) is central to providing resiliency, but how **ECM** is remodeled to accommodate growth is poorly understood. We investigated remodeling of Drosophila respiratory tubes (tracheae) that elongate continually during larval growth, despite being lined with a rigid cuticular **ECM**. Cuticle is initially deposited with a characteristic pattern of repeating ridges and valleys known as taenidia. We find that for tubes to elongate, the extracellular protease Mmp1 is required for expansion of **ECM** between the taenidial ridges during each intermolt period. Mmp1 protein localizes in periodically spaced puncta that are in register with the taenidial spacing. Mmp1 also degrades old cuticle at molts, promotes apical membrane expansion in larval tracheae, and promotes tube elongation in embryonic tracheae. Whereas work in other developmental systems has demonstrated that MMPs are required for axial elongation occurring in localized growth zones, this study demonstrates that MMPs can also mediate interstitial matrix remodeling during growth of an organ system.
3. Requirement of matrix metalloproteinase-1 for intestinal homeostasis in the adult Drosophila midgut. Stem cells are tightly regulated by both intrinsic and extrinsic signals as well as the extracellular matrix (**ECM**) for tissue homeostasis and regenerative capacity. Matrix metalloproteinases (MMPs), proteolytic enzymes, modulate the turnover of numerous substrates, including cytokine precursors, growth factors, and **ECM** molecules. However, the roles of MMPs in the regulation of adult stem cells are poorly understood. In the present study, we utilize the Drosophila midgut, which is an excellent model system for studying stem cell biology, to show that Mmp1 is involved in the regulation of intestinal stem cells (ISCs). The results showed that Mmp1 is expressed in the adult midgut and that its expression increases with age and with exposure to oxidative stress. Mmp1 knockdown or Timp-overexpressing flies and flies heterozygous for a viable, hypomorphic Mmp1 allele increased ISC proliferation in the gut, as shown by staining with an anti-phospho-histone H3 antibody and BrdU incorporation assays. Reduced Mmp1 levels induced intestinal hyperplasia, and the Mmp1depletion-induced ISC proliferation was rescued by the suppression of the EGFR signaling pathway, suggesting that Mmp1 regulates ISC proliferation through the EGFR signaling pathway. Furthermore, adult gut-specific knockdown and whole-animal heterozygotes of Mmp1 increased additively sensitivity to paraquat-induced oxidative stress and shortened lifespan. Our data suggest that Drosophila Mmp1 is involved in the regulation of ISC proliferation for maintenance of gut homeostasis.
4. Glia **ECM** interactions are required to shape the Drosophila nervous system. Organs are characterized by a specific shape that is often remodeled during development. The dynamics of organ shape is in particular evident during the formation of the Drosophila nervous system. During embryonic stages the central nervous system compacts, whereas selective growth occurs during larval stages. The nervous system is covered by a layer of surface glial cells that form the blood brain barrier and a thick extracellular matrix called neural lamella. The size of the neural lamella is dynamically adjusted to the growing nervous system and we show here that perineurial glial cells secrete proteases to remodel this matrix. Moreover, an imbalance in proteolytic activity results in an abnormal shape of the nervous system. To identify further components controlling nervous system shape we performed an RNAi based screen and identified the gene nolo, which encodes an ADAMTS-like protein. We generated loss of function alleles and demonstrate a requirement in glial cells. Mutant nolo larvae, however, do not show an abnormal nervous system shape. The only predicted off-target of the nolo(dsRNA) is Oatp30B, which encodes an organic anion transporting protein characterized by an extracellular protease inhibitor domain. Loss of function mutants were generated and double mutant analyses demonstrate a genetic interaction between nolo and Oatp30B which prevented the generation of maternal zygotic mutant larvae.
5. Drosophila MMP2 regulates the matrix molecule faulty attraction (Frac) to promote motor axon targeting in Drosophila. Matrix metalloproteinases (MMPs) are widely hypothesized to regulate signaling events through processing of extracellular matrix (**ECM**) molecules. We previously demonstrated that membrane-associated Mmp2 is expressed in exit glia and contributes to motor axon targeting. To identify possible substrates, we undertook a yeast interaction screen for Mmp2-binding proteins and identified the novel **ECM** protein faulty attraction (Frac). Frac encodes a multidomain extracellular protein rich in epidermal growth factor (EGF) and calcium-binding EGF domains, related to the vertebrate Fibrillin and Fibulin gene families. It is expressed in mesodermal domains flanking Mmp2-positive glia. The juxtaposition of Mmp2 and Frac proteins raises the possibility that Frac is a proteolytic target of Mmp2. Consistent with this hypothesis, levels of full-length Frac are increased in Mmp2 loss-of-function (LOF) and decreased in Mmp2 gain-of-function (GOF) embryos, indicating that Frac cleavage is Mmp2 dependent. To test whether frac is necessary for axon targeting, we characterized guidance in frac LOF mutants. Motor axons in frac LOF embryos are loosely associated and project ectopically, a phenotype essentially equivalent to that of Mmp2 LOF. The phenotypic similarity between enzyme and substrate mutants argues that Mmp2 activates Frac. In addition, Mmp2 overexpression pathfinding phenotypes depend on frac activity, indicating that Mmp2 is genetically upstream of frac. Last, overexpression experiments suggest that Frac is unlikely to have intrinsic signaling activity, raising the possibility that an Mmp2-generated Frac fragment acts as a guidance cue cofactor. Indeed, we present genetic evidence that Frac regulates a non-canonical LIM kinase 1-dependent bone morphogenetic protein signaling pathway in motoneurons necessary for axon pathfinding during embryogenesis.

### [Ee]xtracellular [Mm]atrix

#### Process

1. **extracellular matrix** organization

#### PubMed

1. An MMP liberates the Ninjurin A ectodomain to signal a loss of cell adhesion. Matrix metalloproteinases (MMPs) are important for developmental tissue remodeling and for the inflammatory response. Although the vertebrate MMP family is large and functionally redundant, the fruitfly Drosophila melanogaster has only two MMPs, both essential genes. Our previous work demonstrated that Mmp1 is required for growth of the tracheal system, and we suggested that the mutant phenotype resulted from aberrant persistence of cell adhesion to the **extracellular matrix**. Here we report the identification of NijA, a transmembrane protein whose vertebrate homologs regulate cell adhesion, as a two-hybrid binding partner for Mmp1. The binding of Mmp1 and NijA was confirmed by coimmunoprecipitation of endogenous proteins from flies, and the endogenous proteins were found to colocalize at the tracheal cell surface in larvae. When NijA is expressed in S2 cells, they lose adhesion to surfaces; this adhesion-loss phenotype is dependent on the expression and catalytic activity of Mmp1. Our data indicate that Mmp1 releases the N-terminal extracellular domain of NijA. This liberated ectodomain promotes the loss of cell adhesion in a cell-nonautonomous manner. We suggest that tracheal cell adhesion is regulated by a novel mechanism utilizing an MMP and a ninjurin family member.
2. ECM-Regulator timp Is Required for Stem Cell Niche Organization and Cyst Production in the Drosophila Ovary. The **extracellular matrix** (ECM) is a pivotal component adult tissues and of many tissue-specific stem cell niches. It provides structural support and regulates niche signaling during tissue maintenance and regeneration. In many tissues, ECM remodeling depends on the regulation of MMP (matrix metalloproteinase) activity by inhibitory TIMP (tissue inhibitors of metalloproteinases) proteins. Here, we report that the only Drosophila timp gene is required for maintaining the normal organization and function of the germline stem cell niche in adult females. timp mutant ovaries show reduced levels of both Drosophila Collagen IV α chains. In addition, tissue stiffness and the cellular organization of the ovarian niche are affected in timp mutants. Finally, loss of timp impairs the ability of the germline stem cell niche to generate new cysts. Our results demonstrating a crucial role for timp in tissue organization and gamete production thus provide a link between the regulation of ECM metabolism and tissue homeostasis.
3. Matrix metalloproteinases promote motor axon fasciculation in the Drosophila embryo. Matrix metalloproteinases (MMPs) are a large conserved family of extracellular proteases, a number of which are expressed during neuronal development and upregulated in nervous system diseases. Primarily on the basis of studies using pharmaceutical inhibitors, MMPs have been proposed to degrade the **extracellular matrix** to allow growth cone advance during development and hence play largely permissive roles in axon extension. Here we show that MMPs are not required for axon extension in the Drosophila embryo, but rather are specifically required for the execution of several stereotyped motor axon pathfinding decisions. The Drosophila genome contains only two MMP homologs, Mmp1 and Mmp2. We isolated Mmp1 in a misexpression screen to identify molecules required for motoneuron development. Misexpression of either MMP inhibits the regulated separation/defasciculation of motor axons at defined choice points. Conversely, motor nerves in Mmp1 and Mmp2 single mutants and Mmp1 Mmp2 double mutant embryos are loosely bundled/fasciculated, with ectopic axonal projections. Quantification of these phenotypes reveals that the genetic requirement for Mmp1 and Mmp2 is distinct in different nerve branches, although generally Mmp2 plays the predominant role in pathfinding. Using both an endogenous MMP inhibitor and MMP dominant-negative constructs, we demonstrate that MMP catalytic activity is required for motor axon fasciculation. In support of the model that MMPs promote fasciculation, we find that the defasciculation observed when MMP activity is compromised is suppressed by otherwise elevating interaxonal adhesion -- either by overexpressing Fas2 or by reducing Sema-1a dosage. These data demonstrate that MMP activity is essential for embryonic motor axon fasciculation.
4. Two classes of matrix metalloproteinases reciprocally regulate synaptogenesis. Synaptogenesis requires orchestrated intercellular communication between synaptic partners, with trans-synaptic signals necessarily traversing the extracellular synaptomatrix separating presynaptic and postsynaptic cells. **Extracellular matrix** metalloproteinases (Mmps) regulated by secreted tissue inhibitors of metalloproteinases (Timps), cleave secreted and membrane-associated targets to sculpt the extracellular environment and modulate intercellular signaling. Here, we test the roles of Mmp at the neuromuscular junction (NMJ) model synapse in the reductionist Drosophila system, which contains just two Mmps (secreted Mmp1 and GPI-anchored Mmp2) and one secreted Timp. We found that all three matrix metalloproteome components co-dependently localize in the synaptomatrix and show that both Mmp1 and Mmp2 independently restrict synapse morphogenesis and functional differentiation. Surprisingly, either dual knockdown or simultaneous inhibition of the two Mmp classes together restores normal synapse development, identifying a reciprocal suppression mechanism. The two Mmp classes co-regulate a Wnt trans-synaptic signaling pathway modulating structural and functional synaptogenesis, including the GPI-anchored heparan sulfate proteoglycan (HSPG) Wnt co-receptor Dally-like protein (Dlp), cognate receptor Frizzled-2 (Frz2) and Wingless (Wg) ligand. Loss of either Mmp1 or Mmp2 reciprocally misregulates Dlp at the synapse, with normal signaling restored by co-removal of both Mmp classes. Correcting Wnt co-receptor Dlp levels in both Mmp mutants prevents structural and functional synaptogenic defects. Taken together, these results identify an Mmp mechanism that fine-tunes HSPG co-receptor function to modulate Wnt signaling to coordinate synapse structural and functional development.
5. Basigin/EMMPRIN/CD147 mediates neuron-glia interactions in the optic lamina of Drosophila. Basigin, an IgG family glycoprotein found on the surface of human metastatic tumors, stimulates fibroblasts to secrete matrix metalloproteases (MMPs) that remodel the **extracellular matrix**, and is thus also known as **Extracellular Matrix** MetalloPRotease Inducer (EMMPRIN). Using Drosophila we previously identified novel roles for basigin. Specifically, photoreceptors of flies with basigin eyes show misplaced nuclei, rough ER and mitochondria, and swollen axon terminals, suggesting cytoskeletal disruptions. Here we demonstrate that basigin is required for normal neuron-glia interactions in the Drosophila visual system. Flies with basigin mutant photoreceptors have misplaced epithelial glial cells within the first optic neuropile, or lamina. In addition, epithelial glia insert finger-like projections--capitate projections (CPs)--sites of vesicle endocytosis and possibly neurotransmitter recycling. When basigin is missing from photoreceptors terminals, CP formation between glia and photoreceptor terminals is disrupted. Visual system function is also altered in flies with basigin mutant eyes. While photoreceptors depolarize normally to light, synaptic transmission is greatly diminished, consistent with a defect in neurotransmitter release. Basigin expression in photoreceptor neurons is required for normal structure and placement of glia cells.
6. A matrix metalloproteinase mediates airway remodeling in Drosophila. Organ size typically increases dramatically during juvenile growth. This growth presents a fundamental tension, as organs need resiliency to resist stresses while still maintaining plasticity to accommodate growth. The **extracellular matrix** (ECM) is central to providing resiliency, but how ECM is remodeled to accommodate growth is poorly understood. We investigated remodeling of Drosophila respiratory tubes (tracheae) that elongate continually during larval growth, despite being lined with a rigid cuticular ECM. Cuticle is initially deposited with a characteristic pattern of repeating ridges and valleys known as taenidia. We find that for tubes to elongate, the extracellular protease Mmp1 is required for expansion of ECM between the taenidial ridges during each intermolt period. Mmp1 protein localizes in periodically spaced puncta that are in register with the taenidial spacing. Mmp1 also degrades old cuticle at molts, promotes apical membrane expansion in larval tracheae, and promotes tube elongation in embryonic tracheae. Whereas work in other developmental systems has demonstrated that MMPs are required for axial elongation occurring in localized growth zones, this study demonstrates that MMPs can also mediate interstitial matrix remodeling during growth of an organ system.
7. Regulation of Drosophila matrix metalloprotease Mmp2 is essential for wing imaginal disc:trachea association and air sac tubulogenesis. The Drosophila Dorsal Air Sac Primordium (ASP) is a tracheal tube that grows toward Branchless FGF-expressing cells in the wing imaginal disc. We show that the ASP arises from a tracheal branch that invades the basal lamina of the disc to juxtapose directly with disc cells. We examined the role of matrix metalloproteases (Mmps), and found that reducing Mmp2 activity perturbed disc-trachea association, altered peritracheal distributions of collagen IV and Perlecan, misregulated ASP growth, and abrogated development of the dorsal air sacs. Whereas the function of the membrane-tethered Mmp2 in the ASP is non-cell autonomous we find that it may have distinct tissue-specific roles in the ASP and disc. These findings demonstrate a critical role for Mmp2 in tubulogenesis post-induction, and implicate Mmp2 in regulating dynamic and essential changes to the **extracellular matrix**.
8. Immune response to bacteria induces dissemination of Ras-activated Drosophila hindgut cells. Although pathogenic bacteria are suspected contributors to colorectal cancer progression, cancer-promoting bacteria and their mode of action remain largely unknown. Here we report that sustained infection with the human intestinal colonizer Pseudomonas aeruginosa synergizes with the Ras1V12 oncogene to induce basal invasion and dissemination of hindgut cells to distant sites. Cross-talk between infection and dissemination requires sustained activation by the bacteria of the Imd-dTab2-dTak1 innate immune pathway, which converges with Ras1V12 signalling on JNK pathway activation, culminating in **extracellular matrix** degradation. Hindgut, but not midgut, cells are amenable to this cooperative dissemination, which is progressive and genetically and pharmacologically inhibitable. Thus, Drosophila hindgut provides a valuable system for the study of intestinal malignancies.
9. Requirement of matrix metalloproteinase-1 for intestinal homeostasis in the adult Drosophila midgut. Stem cells are tightly regulated by both intrinsic and extrinsic signals as well as the **extracellular matrix** (ECM) for tissue homeostasis and regenerative capacity. Matrix metalloproteinases (MMPs), proteolytic enzymes, modulate the turnover of numerous substrates, including cytokine precursors, growth factors, and ECM molecules. However, the roles of MMPs in the regulation of adult stem cells are poorly understood. In the present study, we utilize the Drosophila midgut, which is an excellent model system for studying stem cell biology, to show that Mmp1 is involved in the regulation of intestinal stem cells (ISCs). The results showed that Mmp1 is expressed in the adult midgut and that its expression increases with age and with exposure to oxidative stress. Mmp1 knockdown or Timp-overexpressing flies and flies heterozygous for a viable, hypomorphic Mmp1 allele increased ISC proliferation in the gut, as shown by staining with an anti-phospho-histone H3 antibody and BrdU incorporation assays. Reduced Mmp1 levels induced intestinal hyperplasia, and the Mmp1depletion-induced ISC proliferation was rescued by the suppression of the EGFR signaling pathway, suggesting that Mmp1 regulates ISC proliferation through the EGFR signaling pathway. Furthermore, adult gut-specific knockdown and whole-animal heterozygotes of Mmp1 increased additively sensitivity to paraquat-induced oxidative stress and shortened lifespan. Our data suggest that Drosophila Mmp1 is involved in the regulation of ISC proliferation for maintenance of gut homeostasis.
10. Structural and enzymatic characterization of Drosophila Dm2-MMP, a membrane-bound matrix metalloproteinase with tissue-specific expression. We report the isolation and characterization of a cDNA encoding Dm2-MMP, the second matrix metalloproteinase (MMP) identified in the Drosophila melanogaster genome. The cloned cDNA codes for a polypeptide of 758 residues that displays a domain organization similar to that of other MMPs, including signal peptide, propeptide, catalytic, and hemopexin domains. However, the structure of Dm2-MMP is unique because of the presence of an insertion of 214 amino acids between the catalytic and hemopexin domains that is not present in any of the previously described MMPs. Dm2-MMP also contains a C-terminal extension predicted to form a cleavable glycosylphosphatidylinositol anchor site. Western blot and immunofluorescence analysis of S2 cells transfected with the isolated cDNA confirmed that Dm2-MMP is localized at the cell surface. Production of the catalytic domain of Dm2-MMP in Escherichia coli and analysis of its enzymatic activity revealed that this proteinase cleaves several synthetic peptides used for analysis of vertebrate MMPs. This proteolytic activity was abolished by MMP inhibitors such as BB-94, confirming that the isolated cDNA codes for an enzymatically active metalloproteinase. Reverse transcription-PCR analysis showed that Dm2-MMP is expressed at low levels in all of the developmental stages of Drosophila as well as in adult flies. However, detailed in situ hybridization at the larval stage revealed a strong tissue-specific expression in discrete regions of the brain and eye imaginal discs. According to these results, we propose that Dm2-MMP plays both general proteolytic functions during Drosophila development and in adult tissues and specific roles in eye development and neural tissues through the degradation and remodeling of the **extracellular matrix**.
11. Distinct functions for the catalytic and hemopexin domains of a Drosophila matrix metalloproteinase. Human matrix metalloproteinases (MMPs) are believed to contribute to tumor progression. Therapies based on inhibiting the catalytic domain of MMPs have been unsuccessful, but these studies raise the question of whether other MMP domains might be appropriate targets. The genetic dissection of domain function has been stymied in mouse because there are 24 related and partially redundant MMP genes in the mouse genome. Here, we present a genetic dissection of the functions of the hemopexin and catalytic domains of a canonical MMP in Drosophila melanogaster, an organism with only 2 MMPs that function nonredundantly. We compare the phenotypes of Mmp1 null alleles with alleles that have specific hemopexin domain lesions, and we also examine phenotypes of dominant-negative mutants. We find that, although the catalytic domain appears to be required for all MMP functions including **extracellular matrix** remodeling of the tracheal system, the hemopexin domain is required specifically for tissue invasion events later in metamorphosis but not for tracheal remodeling. Thus, we find that this MMP hemopexin domain has an apparent specialization for tissue invasion events, a finding with potential implications for inhibitor therapies.
12. Basement membrane remodeling is essential for Drosophila disc eversion and tumor invasion. Organ and tissue integrity is often maintained in animals by a specialized **extracellular matrix** structure called the basement membrane (BM). Accumulated evidence indicates that BM remodeling occurs during development and tumor invasion. Although the BM organizes and functions at the organ level, most past studies have explored its biochemical and in vitro properties. In this study, we monitor the BM in vivo during developmental tissue invasion for disc eversion and tumor invasion in Drosophila and modulate BM integrity with genetic alterations affecting either the whole organism or the targeted discs or tumors. We observe that the degradation of BM by the discs or the tumors is an early event during invasion processes and that preventing BM degradation completely blocks both tissue and tumor invasion, indicating that modulation of BM is essential for developmental and tumor invasion. Furthermore, elements of the invasion machinery, including JNK-induced matrix metalloproteinase (MMP) expression, are shared by both disc eversion and tumor invasion processes. Moreover, we show that although expression of MMP inhibitor, TIMP, is sufficient to halt developmental invasion, inhibition of proteases by both TIMP and RECK are required to block tumor invasion. These data suggest that tumor cells have a more robust invasion mechanism and could acquire metastatic behavior by co-opting developmental invasion programs. This type of co-option may be a general feature contributing to the progression of tumors. Finally, although past efforts using MMP inhibitors have not yielded much success, our results strongly argue that BM modulation could be a critical target for cancer therapy.
13. Tissue design: how Drosophila tumors remodel their neighborhood. Drosophila genetics has long been appreciated as a powerful approach for discovering the normal functions of genes that act as oncogenes and tumor suppressors in human cancer. Recent studies have also highlighted its advantages for deciphering how such genes function during tumorigenesis itself. Here we detail studies relating to how tumors, generated in developing organs and adult stem cell-based tissues, remodel the tissue landscape to their benefit. Like mammalian tumors, insect tumors can dissolve **extracellular matrix**, recruit blood cells, migrate and invade other tissues. While much is known about how mammalian fibroblasts, immune cells and vasculature promote late tumorigenesis, less is understood about the very earliest stages of tumor development in mammals. Because Drosophila has fewer mitotic cells and a simpler tissue architecture, it affords easy detection and analysis of early clonal tumor growth. Drosophila studies have revealed both cooperative and competitive interactions between tumor and normal cells during early tumor growth. During development, these interactions typically occur with other proliferative progenitor cells, but in adult stem cell-based tissues, the stem cell niche can fuel tumor growth.
14. Glia ECM interactions are required to shape the Drosophila nervous system. Organs are characterized by a specific shape that is often remodeled during development. The dynamics of organ shape is in particular evident during the formation of the Drosophila nervous system. During embryonic stages the central nervous system compacts, whereas selective growth occurs during larval stages. The nervous system is covered by a layer of surface glial cells that form the blood brain barrier and a thick **extracellular matrix** called neural lamella. The size of the neural lamella is dynamically adjusted to the growing nervous system and we show here that perineurial glial cells secrete proteases to remodel this matrix. Moreover, an imbalance in proteolytic activity results in an abnormal shape of the nervous system. To identify further components controlling nervous system shape we performed an RNAi based screen and identified the gene nolo, which encodes an ADAMTS-like protein. We generated loss of function alleles and demonstrate a requirement in glial cells. Mutant nolo larvae, however, do not show an abnormal nervous system shape. The only predicted off-target of the nolo(dsRNA) is Oatp30B, which encodes an organic anion transporting protein characterized by an extracellular protease inhibitor domain. Loss of function mutants were generated and double mutant analyses demonstrate a genetic interaction between nolo and Oatp30B which prevented the generation of maternal zygotic mutant larvae.
15. Dendrite reshaping of adult Drosophila sensory neurons requires matrix metalloproteinase-mediated modification of the basement membranes. In response to changes in the environment, dendrites from certain neurons change their shape, yet the mechanism remains largely unknown. Here we show that dendritic arbors of adult Drosophila sensory neurons are rapidly reshaped from a radial shape to a lattice-like shape within 24 hr after eclosion. This radial-to-lattice reshaping arises from rearrangement of the existing radial branches into the lattice-like pattern, rather than extensive dendrite pruning followed by regrowth of the lattice-shaped arbors over the period. We also find that the dendrite reshaping is completely blocked in mutants for the matrix metalloproteinase (Mmp) 2. Further genetic analysis indicates that Mmp2 promotes the dendrite reshaping through local degradation of the basement membrane upon which dendrites of the sensory neurons innervate. These findings suggest that regulated proteolytic alteration of the **extracellular matrix** microenvironment might be a fundamental mechanism to drive a large-scale change of dendritic structures during reorganization of neuronal circuits.
16. Dm1-MMP, a matrix metalloproteinase from Drosophila with a potential role in **extracellular matrix** remodeling during neural development. We have cloned and characterized a cDNA encoding Dm1-MMP, the first matrix metalloproteinase (MMP) identified in Drosophila melanogaster. The isolated cDNA encodes a protein of 541 residues that has a domain organization identical to that of most vertebrate MMPs including a signal sequence, a prodomain with the activation locus, a catalytic domain with a zinc-binding site, and a COOH-terminal hemopexin domain. Northern blot analysis of Dm1-MMP expression in embryonic and larval adult tissues revealed a strong expression level in the developing embryo at 10-22 h, declining thereafter and being undetectable in adults. Western blot analysis confirmed the presence of pro- and active forms of Dm1-MMP in vivo during larval development. In situ hybridization experiments demonstrated that Dm1-MMP is expressed in a segmented pattern in cell clusters at the midline during embryonic stage 12-13, when neurons of the central nervous system start to arise. Recombinant Dm1-MMP produced in Escherichia coli exhibits a potent proteolytic activity against synthetic peptides used for analysis of vertebrate MMPs. This activity is inhibited by tissue inhibitors of metalloproteinases and by synthetic MMP inhibitors such as BB-94. Furthermore, Dm1-MMP is able to degrade the **extracellular matrix** and basement membrane proteins fibronectin and type IV collagen. On the basis of these data, together with the predominant expression of Dm1-MMP in embryonic neural cells, we propose that this enzyme may be involved in the **extracellular matrix** remodeling taking place during the development of the central nervous system in Drosophila.
17. Dendrite-specific remodeling of Drosophila sensory neurons requires matrix metalloproteases, ubiquitin-proteasome, and ecdysone signaling. During neuronal maturation, dendrites develop from immature neurites into mature arbors. In response to changes in the environment, dendrites from certain mature neurons can undergo large-scale morphologic remodeling. Here, we show a group of Drosophila peripheral sensory neurons, the class IV dendritic arborization (C4da) neurons, that completely degrade and regrow their elaborate dendrites. Larval dendrites of C4da neurons are first severed from the soma and subsequently degraded during metamorphosis. This process is controlled by both intracellular and extracellular mechanisms: The ecdysone pathway and ubiquitin-proteasome system (UPS) are cell-intrinsic signals that initiate dendrite breakage, and **extracellular matrix** metalloproteases are required to degrade the severed dendrites. Surprisingly, C4da neurons retain their axonal projections during concurrent dendrite degradation, despite activated ecdysone and UPS pathways. These results demonstrate that, in response to environmental changes, certain neurons have cell-intrinsic abilities to completely lose their dendrites but keep their axons and subsequently regrow their dendritic arbors.
18. Drosophila MMP2 regulates the matrix molecule faulty attraction (Frac) to promote motor axon targeting in Drosophila. Matrix metalloproteinases (MMPs) are widely hypothesized to regulate signaling events through processing of **extracellular matrix** (ECM) molecules. We previously demonstrated that membrane-associated Mmp2 is expressed in exit glia and contributes to motor axon targeting. To identify possible substrates, we undertook a yeast interaction screen for Mmp2-binding proteins and identified the novel ECM protein faulty attraction (Frac). Frac encodes a multidomain extracellular protein rich in epidermal growth factor (EGF) and calcium-binding EGF domains, related to the vertebrate Fibrillin and Fibulin gene families. It is expressed in mesodermal domains flanking Mmp2-positive glia. The juxtaposition of Mmp2 and Frac proteins raises the possibility that Frac is a proteolytic target of Mmp2. Consistent with this hypothesis, levels of full-length Frac are increased in Mmp2 loss-of-function (LOF) and decreased in Mmp2 gain-of-function (GOF) embryos, indicating that Frac cleavage is Mmp2 dependent. To test whether frac is necessary for axon targeting, we characterized guidance in frac LOF mutants. Motor axons in frac LOF embryos are loosely associated and project ectopically, a phenotype essentially equivalent to that of Mmp2 LOF. The phenotypic similarity between enzyme and substrate mutants argues that Mmp2 activates Frac. In addition, Mmp2 overexpression pathfinding phenotypes depend on frac activity, indicating that Mmp2 is genetically upstream of frac. Last, overexpression experiments suggest that Frac is unlikely to have intrinsic signaling activity, raising the possibility that an Mmp2-generated Frac fragment acts as a guidance cue cofactor. Indeed, we present genetic evidence that Frac regulates a non-canonical LIM kinase 1-dependent bone morphogenetic protein signaling pathway in motoneurons necessary for axon pathfinding during embryogenesis.
19. The Gyc76C Receptor Guanylyl Cyclase and the Foraging cGMP-Dependent Kinase Regulate **Extracellular Matrix** Organization and BMP Signaling in the Developing Wing of Drosophila melanogaster. The developing crossveins of the wing of Drosophila melanogaster are specified by long-range BMP signaling and are especially sensitive to loss of extracellular modulators of BMP signaling such as the Chordin homolog Short gastrulation (Sog). However, the role of the **extracellular matrix** in BMP signaling and Sog activity in the crossveins has been poorly explored. Using a genetic mosaic screen for mutations that disrupt BMP signaling and posterior crossvein development, we identify Gyc76C, a member of the receptor guanylyl cyclase family that includes mammalian natriuretic peptide receptors. We show that Gyc76C and the soluble cGMP-dependent kinase Foraging, likely linked by cGMP, are necessary for normal refinement and maintenance of long-range BMP signaling in the posterior crossvein. This does not occur through cell-autonomous crosstalk between cGMP and BMP signal transduction, but likely through altered extracellular activity of Sog. We identify a novel pathway leading from Gyc76C to the organization of the wing **extracellular matrix** by matrix metalloproteinases, and show that both the **extracellular matrix** and BMP signaling effects are largely mediated by changes in the activity of matrix metalloproteinases. We discuss parallels and differences between this pathway and other examples of cGMP activity in both Drosophila melanogaster and mammalian cells and tissues.

#### Component

1. **extracellular matrix**

#### Conserved Domains

1. cd04278: ZnMc\_MMP; Zinc-dependent metalloprotease, matrix metalloproteinase (MMP) sub-family. MMPs are responsible for a great deal of pericellular proteolysis of **extracellular matrix** and cell surface molecules, playing crucial roles in morphogenesis, cell fate ...

### [Ii]nvasive

#### PubMed

1. Notch and Mef2 synergize to promote proliferation and metastasis through JNK signal activation in Drosophila. Genetic analyses in Drosophila revealed a synergy between Notch and the pleiotropic transcription factor Mef2 (myocyte enhancer factor 2), which profoundly influences proliferation and metastasis. We show that these hyperproliferative and **invasive** Drosophila phenotypes are attributed to upregulation of eiger, a member of the tumour necrosis factor superfamily of ligands, and the consequent activation of Jun N-terminal kinase signalling, which in turn triggers the expression of the **invasive** marker MMP1. Expression studies in human breast tumour samples demonstrate correlation between Notch and Mef2 paralogues and support the notion that Notch-MEF2 synergy may be significant for modulating human mammary oncogenesis.
2. A drosophila model for EGFR-Ras and PI3K-dependent human glioma. Gliomas, the most common malignant tumors of the nervous system, frequently harbor mutations that activate the epidermal growth factor receptor (EGFR) and phosphatidylinositol-3 kinase (PI3K) signaling pathways. To investigate the genetic basis of this disease, we developed a glioma model in Drosophila. We found that constitutive coactivation of EGFR-Ras and PI3K pathways in Drosophila glia and glial precursors gives rise to neoplastic, **invasive** glial cells that create transplantable tumor-like growths, mimicking human glioma. Our model represents a robust organotypic and cell-type-specific Drosophila cancer model in which malignant cells are created by mutations in signature genes and pathways thought to be driving forces in a homologous human cancer. Genetic analyses demonstrated that EGFR and PI3K initiate malignant neoplastic transformation via a combinatorial genetic network composed primarily of other pathways commonly mutated or activated in human glioma, including the Tor, Myc, G1 Cyclins-Cdks, and Rb-E2F pathways. This network acts synergistically to coordinately stimulate cell cycle entry and progression, protein translation, and inappropriate cellular growth and migration. In particular, we found that the fly orthologs of CyclinE, Cdc25, and Myc are key rate-limiting genes required for glial neoplasia. Moreover, orthologs of Sin1, Rictor, and Cdk4 are genes required only for abnormal neoplastic glial proliferation but not for glial development. These and other genes within this network may represent important therapeutic targets in human glioma.
3. Dynamics of the basement membrane in **invasive** epithelial clusters in Drosophila. The basement membrane (BM) represents a barrier to cell migration, which has to be degraded to promote invasion. However, the role and behaviour of the BM during the development of pre-**invasive** cells is only poorly understood. Drosophila border cells (BCs) provide an attractive genetic model in which to study the cellular mechanisms underlying the migration of mixed cohorts of epithelial cells. BCs are made of two different epithelial cell types appearing sequentially during oogenesis: the polar cells and the outer BCs. Here, we show that the pre-**invasive** polar cells undergo an unusual and asymmetrical apical capping with major basement membrane proteins, including the two Drosophila Collagen IV alpha chains, Laminin A and Perlecan. Capping of polar cells proceeds through a novel, basal-to-apical transcytosis mechanism that involves the small GTPase Drab5. Apical capping is transient and is followed by rapid shedding prior to the initiation of BC migration, suggesting that the apical cap blocks migration. Consistently, non-migratory polar cells remain capped. We further show that JAK/STAT signalling and recruitment of outer BCs are required for correct shedding and migration. The dynamics of the BM represents a marker of migratory BC, revealing a novel developmentally regulated behaviour of BM coupled to epithelial cell **invasive**ness.
4. The actin cross-linker Filamin/Cheerio mediates tumor malignancy downstream of JNK signaling. Cell shape dynamics, motility, and cell proliferation all depend on the actin cytoskeleton. Malignant cancer cells hijack the actin network to grow and migrate to secondary sites. Understanding the function of actin regulators is therefore of major interest. In the present study, we identify the actin cross-linking protein Filamin/Cheerio (Cher) as a mediator of malignancy in genetically defined Drosophila tumors. We show that in **invasive** tumors, resulting from cooperation of activated Ras with disrupted epithelial cell polarity, Cher is upregulated in a Jun N-terminal kinase (JNK)-dependent manner. Although dispensable in normal epithelium, Cher becomes required in the tumor cells for their growth and **invasive**ness. When deprived of Cher, these tumor clones lose their full potential to proliferate and breach tissue boundaries. Instead, the Cher-deficient clones remain confined within the limits of their source epithelium, permitting survival of the host animal. Through interaction with the myosin II heavy chain subunit, Cher is likely to strengthen the cortical actomyosin network and reinforce mechanical tension within the **invasive** tumors. Accordingly, Cher is required for aberrant expression of genes downstream of the Hippo/Yorkie signaling in the tumor tissue. Our study identifies Cher as a new target of JNK signaling that links cytoskeleton dynamics to tumor progression.
5. Elevated expression of the V-ATPase C subunit triggers JNK-dependent cell invasion and overgrowth in a Drosophila epithelium. The C subunit of the vacuolar H(+)-ATPase or V-ATPase regulates the activity and assembly of the proton pump at cellular membranes. It has been shown to be strongly upregulated in oral squamous cell carcinoma, a highly metastatic epithelial cancer. In addition, increased V-ATPase activity appears to correlate with **invasive**ness of cancer cells, but the underlying mechanism is largely unknown. Using the Drosophila wing imaginal epithelium as an in vivo model system, we demonstrate that overexpression of Vha44, the Drosophila orthologue of the C subunit, causes a tumor-like tissue transformation in cells of the wing epithelium. Overexpressing cells are excluded from the epithelium and acquire **invasive** properties while displaying high apoptotic rates. Blocking apoptosis in these cells unmasks a strong proliferation stimulus, leading to overgrowth. Furthermore, we show that excess Vha44 greatly increases acidification of endocytic compartments and interferes with endosomal trafficking. As a result, cargoes such as GFP-Lamp1 and Notch accumulate in highly acidified enlarged endolysosomal compartments. Consistent with previous reports on the endocytic activation of Eiger/JNK signaling, we find that V-ATPase stimulation by Vha44 causes JNK signaling activation whereas downmodulation of JNK signaling rescues the **invasive** phenotypes. In summary, our in vivo-findings demonstrate that increased levels of V-ATPase C subunit induce a Eiger/JNK-dependent cell transformation within an epithelial organ that recapitulates early carcinoma stages.
6. JNK- and Fos-regulated Mmp1 expression cooperates with Ras to induce **invasive** tumors in Drosophila. Loss of the epithelial polarity gene scribble in clones of Drosophila imaginal disc cells can cooperate with Ras signaling to induce malignant tumors. Such mutant tissue overproliferates, resists apoptosis, leaves its place of origin and invades other organs, ultimately causing lethality. We show that increased Jun N-terminal kinase (JNK) signaling resulting from the loss of scribble promotes the movement of transformed cells to secondary sites. This effect requires Fos-dependent transcriptional activation of a matrix metalloprotease gene mmp1 downstream of JNK. Expression of the Mmp inhibitor Timp or Mmp RNAi knockdown suppresses cell **invasive**ness. The pro**invasive** function of the JNK pathway is revealed in a tumor context when active Ras signaling prevents the apoptotic response to JNK activity as it occurs in nontransformed cells. Based on these results, we present a model that explains the oncogenic cooperation between JNK and Ras, and describes how aberrant regulation of cell survival, proliferation and mobilization cooperate to incite malignant tumor formation.
7. A Jnk-Rho-Actin remodeling positive feedback network directs Src-driven invasion. Current models of tumor cell invasion propose that oncogenic signaling converges upon key orchestrators of cytoskeletal dynamics, including c-Jun N-terminal kinase (Jnk) and RhoGTPase family members; these signals dynamically direct Actin remodeling proteins (ARPs) to catalyze the cytoskeletal changes required for migration. Src is a key driver of tumor aggression, metastasis and patient mortality. To clarify how Src regulates Actin dynamics to promote **invasive** migration, we performed a genetic modifier screen in a Drosophila model of invasion. Nine genes linked to Actin dynamics were identified that mediate invasion in situ. We found that ARPs were required for many oncogenic effects of Src including Mmp1 expression and initiation of apoptosis. Surprisingly, they were also regulators of Jnk pathway activity: both Src and the small GTPase Rho1 activated Jnk in a manner dependent on ARPs during invasion. Our results suggest that ARPs are not simply downstream executors of signal transduction pathways. Rather, they participate in a positive feedback network involving canonical oncogenic signaling pathways that promote tumor invasion.
8. RhoL controls invasion and Rap1 localization during immune cell transmigration in Drosophila. Human immune cells have to penetrate an endothelial barrier during their beneficial pursuit of infection and their destructive infiltration of tissues in autoimmune diseases. This transmigration requires Rap1 GTPase to activate integrin affinity. We define a new model system for this process by demonstrating, with live imaging and genetics, that during embryonic development Drosophila melanogaster immune cells penetrate an epithelial, Drosophila E-cadherin (DE-cadherin)-based tissue barrier. A mutant in RhoL, a GTPase homologue that is specifically expressed in haemocytes, blocks this **invasive** step but not other aspects of guided migration. RhoL mediates integrin adhesion caused by Drosophila Rap1 overexpression and moves Rap1 away from a concentration in the cytoplasm to the leading edge during **invasive** migration. These findings indicate that a programmed migratory step during Drosophila development bears striking molecular similarities to vertebrate immune cell transmigration during inflammation, and identify RhoL as a new regulator of invasion, adhesion and Rap1 localization. Our work establishes the utility of Drosophila for identifying novel components of immune cell transmigration and for understanding the in vivo interplay of immune cells with the barriers they penetrate.
9. APC loss-induced intestinal tumorigenesis in Drosophila: Roles of Ras in Wnt signaling activation and tumor progression. Adenomatous polyposis coli (APC) and K-ras are the two most frequently mutated genes found in human colorectal cancers. In human colorectal cancers, Wnt signaling activation after the loss of APC is hypothesized to be the key event for adenoma initiation, whereas additional mutations such as Ras activation are required for the progression from adenoma to carcinoma. However, accumulating data have led to conflicting views regarding the precise role of Ras in APC loss-induced tumorigenesis. Here, using Drosophila midgut as a model system, we show that in the absence of Ras, APC mutant epithelial cells cannot initiate hyperplasia, suggesting that Ras plays an essential role in tumor initiation. Conversely, activating Ras by expressing oncogenic Ras or Raf in APC-deficient cells led to a blockage of cell differentiation and to pre**invasive** tumor outgrowth, characteristics that are shared by advanced colorectal carcinoma in humans. Mechanistically, we find that Ras is not required for Wnt signaling activation after APC loss, although Ras hyperactivation is able to potentiate Wnt signaling by increasing the cytoplasmic and nuclear accumulation of Armadillo/β-catenin via mechanisms independent of JNK/Rac1 or PI3K-Akt signaling, partly owing to the downregulation of DE-cadherin. Together with the data from gene expression analyses, our results indicate that both parallel and cooperative mechanisms of Wnt and Ras signaling are responsible for the initiation and progression of intestinal tumorigenesis after APC loss.
10. A role for the epithelial microenvironment at tumor boundaries: evidence from Drosophila and human squamous cell carcinomas. Recent work has shown an increasing appreciation for the importance of the tumor environment, most commonly the overlying stroma. Less emphasis has been placed on the importance of local communication between transformed cells and their neighbors within the epithelium at tumor boundaries. We previously reported a Drosophila model that highlighted the importance of local interactions within the epithelial microenvironment: Src-transformed cells (Csk-deficient) were influenced by their immediate normal neighbors. The result was a consistent change in 'border cells' at the edge of transformed patches including delocalized p120-catenin and E-cadherin as well as **invasive** migration through the basal lamina. Here we show that the **invasive** properties of the boundary cells depend on up-regulation of Drosophila matrix metalloproteinase-1 as assessed by promoter activity, protein levels, in situ enzymatic activity, and tests of genetic modifier activity. Further, we provide evidence that these events at tumor borders may be evolutionarily conserved. We detected changes in 'boundary cells' within histological sections of human squamous cell carcinomas that were similar to those observed in Drosophila: both E-cadherin and p120-catenin exhibited normal junctional localization at the centers of the tumors but were reduced or delocalized at the boundary. Further, matrix metalloproteinase-2 was up regulated within these same boundary cells. These results support the view that local cell-cell interactions within the epithelial microenvironment impact tumor invasion and progression.
11. Aneuploidy-induced delaminating cells drive tumorigenesis in Drosophila epithelia. Genomic instability has been observed in essentially all sporadic carcinomas. Here we use Drosophila epithelial cells to address the role of chromosomal instability in cancer development as they have proved useful for elucidating the molecular mechanisms underlying tumorigenic growth. We first show that chromosomal instability leads to an apoptotic response. Interestingly, this response is p53 independent, as opposed to mammalian cells, and depends on the activation of the c-Jun N-terminal kinase (JNK) signaling cascade. When prevented from undergoing programmed cell death (PCD), chromosomal instability induces neoplasic overgrowth. These tumor-like tissues are able to grow extensively and metastasize when transplanted into the abdomen of adult hosts. Detailed analysis of the tumors allows us to identify a delaminating cell population as the critical one in driving tumorigenesis. Cells loose their apical-basal polarity, mislocalize DE-cadherin, and delaminate from the main epithelium. A JNK-dependent transcriptional program is activated specifically in delaminating cells and drives nonautonomous tissue overgrowth, basement membrane degradation, and **invasive**ness. These findings unravel a general and rapid tumorigenic potential of genomic instability, as opposed to its proposed role as a source of mutability to select specific tumor-prone aneuploid cells, and open unique avenues toward the understanding of the role of genomic instability in human cancer.
12. Oncogenic Ras diverts a host TNF tumor suppressor activity into tumor promoter. The roles of inflammatory cytokines and the immune response in cancer remain paradoxical. In the case of tumor necrosis factor (TNF), there is undisputed evidence indicating both protumor and antitumor activities. Recent work in Drosophila indicated that a TNF-dependent mechanism eliminates cells deficient for the polarity tumor suppressors dlg or scrib. In this study, however, we show that in tumors deficient for scrib that also expressed the Ras oncoprotein, the TNF signal was diverted into a protumor signal that enhanced tumor growth through larval arrest and stimulated **invasive** migration. In this case, TNF promoted malignancy and was detrimental to host survival. TNF was expressed at high levels by tumor-associated hemocytes recruited from the circulation. The expression of TNF by hemocytes was both necessary and sufficient to trigger TNF signaling in tumor cells. Our evidence suggests that tumors can evolve into malignancy through oncogenic Ras activation and the hijacking of TNF signaling.
13. Caspase signalling in the absence of apoptosis drives Jnk-dependent invasion. Tumours evolve several mechanisms to evade apoptosis, yet many resected carcinomas show significantly elevated caspase activity. Moreover, caspase activity is positively correlated with tumour aggression and adverse patient outcome. These observations indicate that caspases might have a functional role in promoting tumour invasion and metastasis. Using a Drosophila model of invasion, we show that precise effector caspase activity drives cell invasion without initiating apoptosis. Affected cells express the matrix metalloprotinase Mmp1 and invade by activating Jnk. Our results link Jnk and effector caspase signalling during the **invasive** process and suggest that tumours under apoptotic stresses from treatment, immune surveillance or intrinsic signals might be induced further along the metastatic cascade.
14. Sds22/PP1 links epithelial integrity and tumor suppression via regulation of myosin II and JNK signaling. Loss of epithelial integrity often correlates with the progression of malignant tumors. Sds22, a regulatory subunit of protein phosphatase 1 (PP1), has recently been linked to regulation of epithelial polarity in Drosophila. However, its role in tumorigenesis remains obscure. In this study, using Drosophila imaginal tissue as an in vivo model system, we show that sds22 is a new potential tumor suppressor gene in Drosophila. Without sds22, cells lose epithelial architecture, and become **invasive** and tumorigenic when combined with Ras overexpression; conversely, sds22 overexpression can largely suppress tumorigenic growth of Ras(V12)scrib(-/-) mutant cells. Mechanistically, we show that sds22 prevents cell invasion and metastasis by inhibiting myosin II and Jun N-terminal kinase (JNK) activity downstream of PP1. Loss of this inhibition causes cells to lose epithelial organization and promotes cell invasion. Finally, human Sds22 is focally deleted and downregulated in multiple carcinomas, and this downregulation correlates with tumor progression, suggesting that sds22 inactivation may contribute to tumorigenesis and metastatic potential in human cancers via a similar mechanism.
15. Drosophila endocytic neoplastic tumor suppressor genes regulate Sav/Wts/Hpo signaling and the c-Jun N-terminal kinase pathway. Genetic screens in the fruit fly Drosophila melanogaster have identified a class of neoplastic tumor suppressor genes (endocytic nTSGs), which encode proteins that localize to endosomes and facilitate the trafficking of membrane-bound receptors and adhesion molecules into the degradative lysosome. Loss of endocytic nTSGs transforms imaginal disc epithelia into highly proliferative, **invasive** tissues that fail to differentiate and display defects in cellular apicobasal polarity, adhesion and tissue architecture. As vertebrate homologs of some Drosophila nTSGs are linked to tumor formation, identifying molecular changes in signaling associated with nTSG loss could inform understanding of neoplastic transformation in vertebrates. Here we show that mutations in genes that act at multiple steps of the endolysosomal pathway lead to autonomous activation of the Sav/Wts/Hpo (SWH) transcriptional effector Yki (YAP/TAZ in vertebrates) and the Jun N-terminal kinase (JNK), which is known to promote Yki activity in cells with disrupted polarity. Yki and JNK activity are elevated by mutations at multiple steps in the endolysosomal pathway including mutations in the AP-2σ gene, which encodes a component of the AP-2 adaptor complex that recruits cargoes into clathrin-coated pits for subsequent internalization. Moreover, reduction of JNK activity can decrease elevated Yki-signaling caused by altered endocytosis. These studies reveal a broad requirement for components of the endocytic pathway in regulating SWH and JNK outputs, and place Drosophila endocytic nTSGs into a network that involving two major signaling pathways implicated in oncogenesis.
16. Sin3a acts through a multi-gene module to regulate invasion in Drosophila and human tumors. Chromatin remodeling proteins regulate multiple aspects of cell homeostasis, making them ideal candidates for misregulation in transformed cells. Here, we explore Sin3A, a member of the Sin3 family of proteins linked to tumorigenesis that are thought to regulate gene expression through their role as histone deacetylases (HDACs). We identified Drosophila Sin3a as an important mediator of oncogenic Ret receptor in a fly model of Multiple Endocrine Neoplasia Type 2. Reducing Drosophila Sin3a activity led to metastasis-like behavior and, in the presence of Diap1, secondary tumors distant from the site of origin. Genetic and Chip-Seq analyses identified previously undescribed Sin3a targets including genes involved in cell motility and actin dynamics, as well as signaling pathways including Src, Jnk and Rho. A key Sin3a oncogenic target, PP1B, regulates stability of β-Catenin/Armadillo: the outcome is to oppose T-cell factor (TCF) function and Wg/Wnt pathway signaling in both fly and mammalian cancer cells. Reducing Sin3A strongly increased the **invasiv**e behavior of A549 human lung adenocarcinoma cells. We show that Sin3A is downregulated in a variety of human tumors and that Src, JNK, RhoA and PP1B/β-Catenin are regulated in a manner analogous to our Drosophila models. Our data suggest that Sin3A influences a specific step of tumorigenesis by regulating a module of genes involved in cell invasion. Tumor progression may commonly rely on such 'modules of invasion' under the control of broad transcriptional regulators.
17. The Drosophila TNF receptor Grindelwald couples loss of cell polarity and neoplastic growth. Disruption of epithelial polarity is a key event in the acquisition of neoplastic growth. JNK signalling is known to play an important part in driving the malignant progression of many epithelial tumours, although the link between loss of polarity and JNK signalling remains elusive. In a Drosophila genome-wide genetic screen designed to identify molecules implicated in neoplastic growth, we identified grindelwald (grnd), a gene encoding a transmembrane protein with homology to members of the tumour necrosis factor receptor (TNFR) superfamily. Here we show that Grnd mediates the pro-apoptotic functions of Eiger (Egr), the unique Drosophila TNF, and that overexpression of an active form of Grnd lacking the extracellular domain is sufficient to activate JNK signalling in vivo. Grnd also promotes the **invasive**ness of Ras(V12)/scrib(-/-) tumours through Egr-dependent Matrix metalloprotease-1 (Mmp1) expression. Grnd localizes to the subapical membrane domain with the cell polarity determinant Crumbs (Crb) and couples Crb-induced loss of polarity with JNK activation and neoplastic growth through physical interaction with Veli (also known as Lin-7). Therefore, Grnd represents the first example of a TNFR that integrates signals from both Egr and apical polarity determinants to induce JNK-dependent cell death or tumour growth.

### [Mm]etastasize

#### PubMed

1. Metastatic ability of Drosophila tumors depends on MMP activity. We analyzed how cells from tumors caused by mutations in either lgl or brat use matrix metalloproteinases (MMPs) to facilitate metastasis in Drosophila. MMP1 accumulation is dramatically increased in lgl larval imaginal discs compared to both wild type and brat mutants. Removal of Mmp1 gene activity in lgl brain tumor cells reduced their frequency of ovarian micro-metastases after transplantation; whereas, removal of Mmp1 gene activity in brat tumor cells had no such effect. Host ovaries showed increased Mmp1 gene expression in response to transplantation of brat tumors but not of lgl tumors. Reduction of MMP activity in host ovaries by ectopic expression of TIMP significantly reduced both lgl and brat metastases in that organ. These results highlight the mechanisms that lgl and brat tumor cells use to **metastasize**. Our interpretation of these data is that secretion of MMP1 from lgl tumor cells facilitates their metastasis, while secretion of MMP1 from host ovaries facilitates brat tumor metastasis. This study is the first demonstration that Drosophila tumors utilize MMP activity to **metastasize**.
2. Aneuploidy-induced delaminating cells drive tumorigenesis in Drosophila epithelia. Genomic instability has been observed in essentially all sporadic carcinomas. Here we use Drosophila epithelial cells to address the role of chromosomal instability in cancer development as they have proved useful for elucidating the molecular mechanisms underlying tumorigenic growth. We first show that chromosomal instability leads to an apoptotic response. Interestingly, this response is p53 independent, as opposed to mammalian cells, and depends on the activation of the c-Jun N-terminal kinase (JNK) signaling cascade. When prevented from undergoing programmed cell death (PCD), chromosomal instability induces neoplasic overgrowth. These tumor-like tissues are able to grow extensively and **metastasize** when transplanted into the abdomen of adult hosts. Detailed analysis of the tumors allows us to identify a delaminating cell population as the critical one in driving tumorigenesis. Cells loose their apical-basal polarity, mislocalize DE-cadherin, and delaminate from the main epithelium. A JNK-dependent transcriptional program is activated specifically in delaminating cells and drives nonautonomous tissue overgrowth, basement membrane degradation, and invasiveness. These findings unravel a general and rapid tumorigenic potential of genomic instability, as opposed to its proposed role as a source of mutability to select specific tumor-prone aneuploid cells, and open unique avenues toward the understanding of the role of genomic instability in human cancer.

### [Mm]etastasis

#### PubMed

1. Notch and Mef2 synergize to promote proliferation and **metastasis** through JNK signal activation in Drosophila. Genetic analyses in Drosophila revealed a synergy between Notch and the pleiotropic transcription factor Mef2 (myocyte enhancer factor 2), which profoundly influences proliferation and **metastasis**. We show that these hyperproliferative and invasive Drosophila phenotypes are attributed to upregulation of eiger, a member of the tumour necrosis factor superfamily of ligands, and the consequent activation of Jun N-terminal kinase signalling, which in turn triggers the expression of the invasive marker MMP1. Expression studies in human breast tumour samples demonstrate correlation between Notch and Mef2 paralogues and support the notion that Notch-MEF2 synergy may be significant for modulating human mammary oncogenesis.
2. Drosophila Abelson kinase mediates cell invasion and proliferation through two distinct MAPK pathways. The Abelson (Abl) family of non-receptor tyrosine kinases has an important role in cell morphogenesis, motility, and proliferation. Although the function of Abl has been extensively studied in leukemia, its role in epithelial cell invasion remains obscure. Using the Drosophila wing epithelium as an in vivo model system, we show that overexpression (activation) of Drosophila Abl (dAbl) causes loss of epithelial apical/basal cell polarity and secretion of matrix metalloproteinases, resulting in a cellular invasion and apoptosis. Our in vivo data indicate that dAbl acts downstream of the Src kinases, which are known regulators of cell adhesion and invasion. Downstream of dAbl, Rac GTPases activate two distinct MAPK pathways: c-Jun N-terminal kinase signaling (required for cell invasion and apoptosis) and ERK signaling (inducing cell proliferation). Activated Abl also increases the activity of Src members through a positive feedback loop leading to signal amplification. Thus, targeting Src-Abl, using available dual inhibitors, could be of therapeutic importance in tumor cell **metastasis**.
3. Oncogenic programmes and Notch activity: an 'organized crime'? The inappropriate Notch signalling can influence virtually all aspect of cancer, including tumour-cell growth, survival, apoptosis, angiogenesis, invasion and **metastasis**, although it does not do this alone. Hence, elucidating the partners of Notch that are active in cancer is now the focus of much intense research activity. The genetic toolkits available, coupled to the small size and short life of the fruit fly Drosophila melanogaster, makes this an inexpensive and effective animal model, suited to large-scale cancer gene discovery studies. The fly eye is not only a non-vital organ but its stereotyped size and disposition also means it is easy to screen for mutations that cause tumours and metastases and provides ample opportunities to test cancer theories and to unravel unanticipated nexus between Notch and other cancer genes, or to discover unforeseen Notch's partners in cancer. These studies suggest that Notch's oncogenic capacity is brought about not simply by increasing signal strength but through partnerships, whereby oncogenes gain more by cooperating than acting individually, as in a ring 'organized crime'.
4. Metastatic ability of Drosophila tumors depends on MMP activity. We analyzed how cells from tumors caused by mutations in either lgl or brat use matrix metalloproteinases (MMPs) to facilitate **metastasis** in Drosophila. MMP1 accumulation is dramatically increased in lgl larval imaginal discs compared to both wild type and brat mutants. Removal of Mmp1 gene activity in lgl brain tumor cells reduced their frequency of ovarian micro-metastases after transplantation; whereas, removal of Mmp1 gene activity in brat tumor cells had no such effect. Host ovaries showed increased Mmp1 gene expression in response to transplantation of brat tumors but not of lgl tumors. Reduction of MMP activity in host ovaries by ectopic expression of TIMP significantly reduced both lgl and brat metastases in that organ. These results highlight the mechanisms that lgl and brat tumor cells use to metastasize. Our interpretation of these data is that secretion of MMP1 from lgl tumor cells facilitates their **metastasis**, while secretion of MMP1 from host ovaries facilitates brat tumor **metastasis**. This study is the first demonstration that Drosophila tumors utilize MMP activity to metastasize.
5. A Jnk-Rho-Actin remodeling positive feedback network directs Src-driven invasion. Current models of tumor cell invasion propose that oncogenic signaling converges upon key orchestrators of cytoskeletal dynamics, including c-Jun N-terminal kinase (Jnk) and RhoGTPase family members; these signals dynamically direct Actin remodeling proteins (ARPs) to catalyze the cytoskeletal changes required for migration. Src is a key driver of tumor aggression, **metastasis** and patient mortality. To clarify how Src regulates Actin dynamics to promote invasive migration, we performed a genetic modifier screen in a Drosophila model of invasion. Nine genes linked to Actin dynamics were identified that mediate invasion in situ. We found that ARPs were required for many oncogenic effects of Src including Mmp1 expression and initiation of apoptosis. Surprisingly, they were also regulators of Jnk pathway activity: both Src and the small GTPase Rho1 activated Jnk in a manner dependent on ARPs during invasion. Our results suggest that ARPs are not simply downstream executors of signal transduction pathways. Rather, they participate in a positive feedback network involving canonical oncogenic signaling pathways that promote tumor invasion.
6. Rabaptin-5 and Rabex-5 are neoplastic tumour suppressor genes that interact to modulate Rab5 dynamics in Drosophila melanogaster. Endocytosis plays an important role in the regulation of tumour growth and **metastasis**. In Drosophila, a number of endocytic neoplastic tumour suppressor genes have been identified that when mutated cause epithelial disruption and over-proliferation. Here we characterise the Drosophila homologue of the Rab5 effector Rabaptin-5, and show that it is a novel neoplastic tumour suppressor. Its ability to bind Rab5 and modulate early endosomal dynamics is conserved in Drosophila, as is its interaction with the Rab5 GEF Rabex5, for which we also demonstrate neoplastic tumour suppressor characteristics. Surprisingly, we do not observe disruption of apico-basal polarity in Rabaptin-5 and Rabex-5 mutant tissues; instead the tumour phenotype is associated with upregulation of Jun N-terminal Kinase (JNK) and Janus Kinase (JAK)/Signal Transducer and Activator of Transcription (STAT) signalling.
7. Centrosomal kinase Nek2 cooperates with oncogenic pathways to promote **metastasis**. Centrosomal kinase Nek2 is overexpressed in different cancers, yet how it contributes toward tumorigenesis remains poorly understood. dNek2 overexpression in a Drosophila melanogaster model led to upregulation of Drosophila Wnt ortholog wingless (Wg), and alteration of cell migration markers-Rho1, Rac1 and E-cadherin (Ecad)-resulting in changes in cell shape and tissue morphogenesis. dNek2 overexpression cooperated with receptor tyrosine kinase and mitogen-activated protein kinase signaling to upregulate activated Akt, Diap1, Mmp1 and Wg protein to promote local invasion, distant seeding and **metastasis**. In tumor cell injection assays, dNek2 cooperated with Ras and Src signaling to promote aggressive colonization of tumors into different adult fly tissues. Inhibition of the PI3K pathway suppressed the cooperation of dNek2 with other growth pathways. Consistent with our fly studies, overexpression of human Nek2 in A549 lung adenocarcinoma and HEK293T cells led to activation of the Akt pathway and increase in β-catenin protein levels. Our computational approach identified a class of Nek2-inhibitory compounds and a novel drug-like pharmacophore that reversed the Nek2 overexpression phenotypes in flies and human cells. Our finding posits a novel role for Nek2 in promoting **metastasi**s in addition to its currently defined role in promoting chromosomal instability. It provides a rationale for the selective advantage of centrosome amplification in cancer.
8. Caspase signalling in the absence of apoptosis drives Jnk-dependent invasion. Tumours evolve several mechanisms to evade apoptosis, yet many resected carcinomas show significantly elevated caspase activity. Moreover, caspase activity is positively correlated with tumour aggression and adverse patient outcome. These observations indicate that caspases might have a functional role in promoting tumour invasion and **metastasis**. Using a Drosophila model of invasion, we show that precise effector caspase activity drives cell invasion without initiating apoptosis. Affected cells express the matrix metalloprotinase Mmp1 and invade by activating Jnk. Our results link Jnk and effector caspase signalling during the invasive process and suggest that tumours under apoptotic stresses from treatment, immune surveillance or intrinsic signals might be induced further along the metastatic cascade.
9. Sds22/PP1 links epithelial integrity and tumor suppression via regulation of myosin II and JNK signaling. Loss of epithelial integrity often correlates with the progression of malignant tumors. Sds22, a regulatory subunit of protein phosphatase 1 (PP1), has recently been linked to regulation of epithelial polarity in Drosophila. However, its role in tumorigenesis remains obscure. In this study, using Drosophila imaginal tissue as an in vivo model system, we show that sds22 is a new potential tumor suppressor gene in Drosophila. Without sds22, cells lose epithelial architecture, and become invasive and tumorigenic when combined with Ras overexpression; conversely, sds22 overexpression can largely suppress tumorigenic growth of Ras(V12)scrib(-/-) mutant cells. Mechanistically, we show that sds22 prevents cell invasion and **metastasis** by inhibiting myosin II and Jun N-terminal kinase (JNK) activity downstream of PP1. Loss of this inhibition causes cells to lose epithelial organization and promotes cell invasion. Finally, human Sds22 is focally deleted and downregulated in multiple carcinomas, and this downregulation correlates with tumor progression, suggesting that sds22 inactivation may contribute to tumorigenesis and metastatic potential in human cancers via a similar mechanism.
10. Drosophila at the intersection of infection, inflammation, and cancer. Recent studies show that both cellular and humoral aspects of innate immunity play important roles during tumor progression. These interactions have traditionally been explored in vertebrate model systems. In recent years, Drosophila has emerged as a genetically tractable model system for studying key aspects of tumorigenesis including proliferation, invasion, and **metastasis**. The absence of adaptive immunity in Drosophila provides a unique opportunity to study the interactions between innate immune system and cancer in different genetic contexts. In this review, I discuss recent advances made by using Drosophila models of cancer to study the role of innate immune pathways Toll/Imd, JNK, and JAK-STAT, microbial infection and inflammation during tumor progression.
11. Modeling tumor invasion and **metastasis** in Drosophila. Conservation of major signaling pathways between humans and flies has made Drosophila a useful model organism for cancer research. Our understanding of the mechanisms regulating cell growth, differentiation and development has been considerably advanced by studies in Drosophila. Several recent high profile studies have examined the processes constraining the metastatic growth of tumor cells in fruit fly models. Cell invasion can be studied in the context of an in vivo setting in flies, enabling the genetic requirements of the microenvironment of tumor cells undergoing **metastasis** to be analyzed. This Perspective discusses the strengths and limitations of Drosophila models of cancer invasion and the unique tools that have enabled these studies. It also highlights several recent reports that together make a strong case for Drosophila as a system with the potential for both testing novel concepts in tumor progression and cell invasion, and for uncovering players in **metastasis**.
12. Sin3a acts through a multi-gene module to regulate invasion in Drosophila and human tumors. Chromatin remodeling proteins regulate multiple aspects of cell homeostasis, making them ideal candidates for misregulation in transformed cells. Here, we explore Sin3A, a member of the Sin3 family of proteins linked to tumorigenesis that are thought to regulate gene expression through their role as histone deacetylases (HDACs). We identified Drosophila Sin3a as an important mediator of oncogenic Ret receptor in a fly model of Multiple Endocrine Neoplasia Type 2. Reducing Drosophila Sin3a activity led to **metastasis**-like behavior and, in the presence of Diap1, secondary tumors distant from the site of origin. Genetic and Chip-Seq analyses identified previously undescribed Sin3a targets including genes involved in cell motility and actin dynamics, as well as signaling pathways including Src, Jnk and Rho. A key Sin3a oncogenic target, PP1B, regulates stability of β-Catenin/Armadillo: the outcome is to oppose T-cell factor (TCF) function and Wg/Wnt pathway signaling in both fly and mammalian cancer cells. Reducing Sin3A strongly increased the invasive behavior of A549 human lung adenocarcinoma cells. We show that Sin3A is downregulated in a variety of human tumors and that Src, JNK, RhoA and PP1B/β-Catenin are regulated in a manner analogous to our Drosophila models. Our data suggest that Sin3A influences a specific step of tumorigenesis by regulating a module of genes involved in cell invasion. Tumor progression may commonly rely on such 'modules of invasion' under the control of broad transcriptional regulators.
13. A novel link between FMR gene and the JNK pathway provides clues to possible role in malignant pleural mesothelioma. Malignant pleural mesothelioma (MPM) is an aggressive form of thoracic cancer with poor prognosis. While some studies have identified the molecular alterations associated with MPM, little is known about their role in MPM. For example, fragile X mental retardation (FMR) gene is up-regulated in MPM but its role in MPM is unknown. Here, utilizing Drosophila genetics, I investigate the possible role FMR may be playing in MPM. I provide evidence which suggests that FMR may contribute to tumorigenesis by up-regulating a matrix metalloprotease (MMP) and by degrading the basement membrane (BM), both important for tumor **metastasis**. I also demonstrate a novel link between FMR and the JNK pathway and suggest that the effects of FMR in MPM could in part be mediated by up-regulation of the JNK pathway.

### [Cc]ell [Ii]nvasion

#### PubMed

1. Drosophila Abelson kinase mediates **cell invasion** and proliferation through two distinct MAPK pathways. The Abelson (Abl) family of non-receptor tyrosine kinases has an important role in cell morphogenesis, motility, and proliferation. Although the function of Abl has been extensively studied in leukemia, its role in epithelial **cell invasion** remains obscure. Using the Drosophila wing epithelium as an in vivo model system, we show that overexpression (activation) of Drosophila Abl (dAbl) causes loss of epithelial apical/basal cell polarity and secretion of matrix metalloproteinases, resulting in a cellular invasion and apoptosis. Our in vivo data indicate that dAbl acts downstream of the Src kinases, which are known regulators of cell adhesion and invasion. Downstream of dAbl, Rac GTPases activate two distinct MAPK pathways: c-Jun N-terminal kinase signaling (required for **cell invasion** and apoptosis) and ERK signaling (inducing cell proliferation). Activated Abl also increases the activity of Src members through a positive feedback loop leading to signal amplification. Thus, targeting Src-Abl, using available dual inhibitors, could be of therapeutic importance in tumor cell metastasis.
2. Elevated expression of the V-ATPase C subunit triggers JNK-dependent **cell invasion** and overgrowth in a Drosophila epithelium. The C subunit of the vacuolar H(+)-ATPase or V-ATPase regulates the activity and assembly of the proton pump at cellular membranes. It has been shown to be strongly upregulated in oral squamous cell carcinoma, a highly metastatic epithelial cancer. In addition, increased V-ATPase activity appears to correlate with invasiveness of cancer cells, but the underlying mechanism is largely unknown. Using the Drosophila wing imaginal epithelium as an in vivo model system, we demonstrate that overexpression of Vha44, the Drosophila orthologue of the C subunit, causes a tumor-like tissue transformation in cells of the wing epithelium. Overexpressing cells are excluded from the epithelium and acquire invasive properties while displaying high apoptotic rates. Blocking apoptosis in these cells unmasks a strong proliferation stimulus, leading to overgrowth. Furthermore, we show that excess Vha44 greatly increases acidification of endocytic compartments and interferes with endosomal trafficking. As a result, cargoes such as GFP-Lamp1 and Notch accumulate in highly acidified enlarged endolysosomal compartments. Consistent with previous reports on the endocytic activation of Eiger/JNK signaling, we find that V-ATPase stimulation by Vha44 causes JNK signaling activation whereas downmodulation of JNK signaling rescues the invasive phenotypes. In summary, our in vivo-findings demonstrate that increased levels of V-ATPase C subunit induce a Eiger/JNK-dependent cell transformation within an epithelial organ that recapitulates early carcinoma stages.
3. A Jnk-Rho-Actin remodeling positive feedback network directs Src-driven invasion. Current models of tumor **cell invasion** propose that oncogenic signaling converges upon key orchestrators of cytoskeletal dynamics, including c-Jun N-terminal kinase (Jnk) and RhoGTPase family members; these signals dynamically direct Actin remodeling proteins (ARPs) to catalyze the cytoskeletal changes required for migration. Src is a key driver of tumor aggression, metastasis and patient mortality. To clarify how Src regulates Actin dynamics to promote invasive migration, we performed a genetic modifier screen in a Drosophila model of invasion. Nine genes linked to Actin dynamics were identified that mediate invasion in situ. We found that ARPs were required for many oncogenic effects of Src including Mmp1 expression and initiation of apoptosis. Surprisingly, they were also regulators of Jnk pathway activity: both Src and the small GTPase Rho1 activated Jnk in a manner dependent on ARPs during invasion. Our results suggest that ARPs are not simply downstream executors of signal transduction pathways. Rather, they participate in a positive feedback network involving canonical oncogenic signaling pathways that promote tumor invasion.
4. Rho1-Wnd signaling regulates loss-of-cell polarity-induced **cell invasion** in Drosophila. Both cell polarity and c-Jun N-terminal kinase (JNK) activity are essential to the maintenance of tissue homeostasis, and disruption of either is commonly seen in cancer progression. Despite the established connection between loss-of-cell polarity and JNK activation, much less is known about the molecular mechanism by which aberrant cell polarity induces JNK-mediated cell migration and tumor invasion. Here we show results from a genetic screen using an in vivo invasion model via knocking down cell polarity gene in Drosophila wing discs, and identify Rho1-Wnd signaling as an important molecular link that mediates loss-of-cell polarity-triggered JNK activation and **cell invasion**. We show that Wallenda (Wnd), a protein kinase of the mitogen-activated protein kinase kinase kinase family, by forming a complex with the GTPase Rho1, is both necessary and sufficient for Rho1-induced JNK-dependent **cell invasion**, MMP1 activation and epithelial-mesenchymal transition. Furthermore, Wnd promotes cell proliferation and tissue growth through wingless production when apoptosis is inhibited by p35. Finally, Wnd shows oncogenic cooperation with Ras(V12) to trigger tumor growth in eye discs and causes invasion into the ventral nerve cord. Together, our data not only provides a novel mechanistic insight on how cell polarity loss contributes to **cell invasion**, but also highlights the value of the Drosophila model system to explore human cancer biology.
5. dUev1a modulates TNF-JNK mediated tumor progression and cell death in Drosophila. Loss of cell polarity cooperates with oncogenic Ras to induce JNK-dependent tumor growth and invasion. To identify additional genes that modulate tumor progression, we have performed a genetic screen in Drosophila and found that loss of dUev1a, the ortholog of mammalian Uev1, suppressed lgl(-/-)/Ras(V12) induced JNK-mediated tumor growth and invasion. Furthermore, loss of dUev1a suppressed TNF ortholog Eiger-induced JNK-mediated **cell invasion** and cell death. Finally, dUev1a cooperated with Bendless to activate JNK signaling through dTRAF2. Together, our data indicate that dUev1a encodes an essential component of the evolutionary conserved TNF-JNK signaling pathway that modulates tumor progression and cell death in metazoan.
6. Caspase signalling in the absence of apoptosis drives Jnk-dependent invasion. Tumours evolve several mechanisms to evade apoptosis, yet many resected carcinomas show significantly elevated caspase activity. Moreover, caspase activity is positively correlated with tumour aggression and adverse patient outcome. These observations indicate that caspases might have a functional role in promoting tumour invasion and metastasis. Using a Drosophila model of invasion, we show that precise effector caspase activity drives **cell invasion** without initiating apoptosis. Affected cells express the matrix metalloprotinase Mmp1 and invade by activating Jnk. Our results link Jnk and effector caspase signalling during the invasive process and suggest that tumours under apoptotic stresses from treatment, immune surveillance or intrinsic signals might be induced further along the metastatic cascade.
7. Sds22/PP1 links epithelial integrity and tumor suppression via regulation of myosin II and JNK signaling. Loss of epithelial integrity often correlates with the progression of malignant tumors. Sds22, a regulatory subunit of protein phosphatase 1 (PP1), has recently been linked to regulation of epithelial polarity in Drosophila. However, its role in tumorigenesis remains obscure. In this study, using Drosophila imaginal tissue as an in vivo model system, we show that sds22 is a new potential tumor suppressor gene in Drosophila. Without sds22, cells lose epithelial architecture, and become invasive and tumorigenic when combined with Ras overexpression; conversely, sds22 overexpression can largely suppress tumorigenic growth of Ras(V12)scrib(-/-) mutant cells. Mechanistically, we show that sds22 prevents **cell invasion** and metastasis by inhibiting myosin II and Jun N-terminal kinase (JNK) activity downstream of PP1. Loss of this inhibition causes cells to lose epithelial organization and promotes **cell invasion**. Finally, human Sds22 is focally deleted and downregulated in multiple carcinomas, and this downregulation correlates with tumor progression, suggesting that sds22 inactivation may contribute to tumorigenesis and metastatic potential in human cancers via a similar mechanism.
8. Modeling tumor invasion and metastasis in Drosophila. Conservation of major signaling pathways between humans and flies has made Drosophila a useful model organism for cancer research. Our understanding of the mechanisms regulating cell growth, differentiation and development has been considerably advanced by studies in Drosophila. Several recent high profile studies have examined the processes constraining the metastatic growth of tumor cells in fruit fly models. **Cell invasion** can be studied in the context of an in vivo setting in flies, enabling the genetic requirements of the microenvironment of tumor cells undergoing metastasis to be analyzed. This Perspective discusses the strengths and limitations of Drosophila models of cancer invasion and the unique tools that have enabled these studies. It also highlights several recent reports that together make a strong case for Drosophila as a system with the potential for both testing novel concepts in tumor progression and **cell invasion**, and for uncovering players in metastasis.
9. Sin3a acts through a multi-gene module to regulate invasion in Drosophila and human tumors. Chromatin remodeling proteins regulate multiple aspects of cell homeostasis, making them ideal candidates for misregulation in transformed cells. Here, we explore Sin3A, a member of the Sin3 family of proteins linked to tumorigenesis that are thought to regulate gene expression through their role as histone deacetylases (HDACs). We identified Drosophila Sin3a as an important mediator of oncogenic Ret receptor in a fly model of Multiple Endocrine Neoplasia Type 2. Reducing Drosophila Sin3a activity led to metastasis-like behavior and, in the presence of Diap1, secondary tumors distant from the site of origin. Genetic and Chip-Seq analyses identified previously undescribed Sin3a targets including genes involved in cell motility and actin dynamics, as well as signaling pathways including Src, Jnk and Rho. A key Sin3a oncogenic target, PP1B, regulates stability of β-Catenin/Armadillo: the outcome is to oppose T-cell factor (TCF) function and Wg/Wnt pathway signaling in both fly and mammalian cancer cells. Reducing Sin3A strongly increased the invasive behavior of A549 human lung adenocarcinoma cells. We show that Sin3A is downregulated in a variety of human tumors and that Src, JNK, RhoA and PP1B/β-Catenin are regulated in a manner analogous to our Drosophila models. Our data suggest that Sin3A influences a specific step of tumorigenesis by regulating a module of genes involved i**n cell invasi**on. Tumor progression may commonly rely on such 'modules of invasion' under the control of broad transcriptional regulators.

---

## Ets21C

- NCBI Gene
- GeneRIF
- Pubmed

| Search Term | Rank | Fields |
| --- | --- | --- |
| [Ii]nvasive | 10 | PubMed(1) |

  
hide/show details for Ets21C

### [Ii]nvasive

#### PubMed

1. Interplay among Drosophila transcription factors Ets21c, Fos and Ftz-F1 drives JNK-mediated tumor malignancy. Cancer initiation and maintenance of the transformed cell state depend on altered cellular signaling and aberrant activities of transcription factors (TFs) that drive pathological gene expression in response to cooperating genetic lesions. Deciphering the roles of interacting TFs is therefore central to understanding carcinogenesis and for designing cancer therapies. Here, we use an unbiased genomic approach to define a TF network that triggers an abnormal gene expression program promoting malignancy of clonal tumors, generated in Drosophila imaginal disc epithelium by gain of oncogenic Ras (Ras(V12)) and loss of the tumor suppressor Scribble (scrib(1)). We show that malignant transformation of the ras(V12)scrib(1) tumors requires TFs of distinct families, namely the bZIP protein Fos, the ETS-domain factor Ets21c and the nuclear receptor Ftz-F1, all acting downstream of Jun-N-terminal kinase (JNK). Depleting any of the three TFs improves viability of tumor-bearing larvae, and this positive effect can be enhanced further by their combined removal. Although both Fos and Ftz-F1 synergistically contribute to ras(V12)scrib(1) tumor **invasive**ness, only Fos is required for JNK-induced differentiation defects and Matrix metalloprotease (MMP1) upregulation. In contrast, the Fos-dimerizing partner Jun is dispensable for JNK to exert its effects in ras(V12)scrib(1) tumors. Interestingly, Ets21c and Ftz-F1 are transcriptionally induced in these tumors in a JNK- and Fos-dependent manner, thereby demonstrating a hierarchy within the tripartite TF network, with Fos acting as the most upstream JNK effector. Of the three TFs, only Ets21c can efficiently substitute for loss of polarity and cooperate with Ras(V12) in inducing malignant clones that, like ras(V12)scrib(1) tumors, invade other tissues and overexpress MMP1 and the Drosophila insulin-like peptide 8 (Dilp8). While ras(V12)ets21c tumors require JNK for **invasive**ness, the JNK activity is dispensable for their growth. In conclusion, our study delineates both unique and overlapping functions of distinct TFs that cooperatively promote aberrant expression of target genes, leading to malignant tumor phenotypes.

---

## Rel

- NCBI Gene
- GeneRIF
- Pubmed

| Search Term | Rank | Fields |
| --- | --- | --- |
| [Mm]igration | 6 | PubMed(1) |
| ECM | 7 | PubMed(1) |
| [Ee]xtracellular [Mm]atrix | 8 | PubMed(2) |
| [Ii]nvasive | 10 | PubMed(2) |
| [Mm]etastasis | 12 | PubMed(1) |

  
hide/show details for Rel

### [Mm]igration

#### PubMed

1. Genes conserved in bilaterians but jointly lost with Myc during nematode evolution are enriched in cell proliferation and cell **migration** functions. Animals use a stereotypical set of developmental genes to build body architectures of varying sizes and organizational complexity. Some genes are critical to developmental patterning, while other genes are important to physiological control of growth. However, growth regulator genes may not be as important in small-bodied "micro-metazoans" such as nematodes. Nematodes use a simplified developmental strategy of lineage-based cell fate specifications to produce an adult bilaterian body composed of a few hundreds of cells. Nematodes also lost the MYC proto-oncogenic regulator of cell proliferation. To identify additional regulators of cell proliferation that were lost with MYC, we computationally screened and determined 839 high-confidence genes that are conserved in bilaterians/lost in nematodes (CIBLIN genes). We find that 30 % of all CIBLIN genes encode transcriptional regulators of cell proliferation, epithelial-to-mesenchyme transitions, and other processes. Over 50 % of CIBLIN genes are unnamed genes in Drosophila, suggesting that there are many understudied genes. Interestingly, CIBLIN genes include many Myc synthetic lethal (MycSL) hits from recent screens. CIBLIN genes include key regulators of heparan sulfate proteoglycan (HSPG) sulfation patterns, and lysyl oxidases involved in cross-linking and modification of the extracellular matrix (ECM). These genes and others suggest the CIBLIN repertoire services critical functions in ECM remodeling and cell **migration** in large-bodied bilaterians. Correspondingly, CIBLIN genes are co-expressed with Myc in cancer transcriptomes, and include a preponderance of known determinants of cancer progression and tumor aggression. We propose that CIBLIN gene research can improve our understanding of regulatory control of cellular growth in metazoans.

### ECM

#### PubMed

1. Genes conserved in bilaterians but jointly lost with Myc during nematode evolution are enriched in cell proliferation and cell migration functions. Animals use a stereotypical set of developmental genes to build body architectures of varying sizes and organizational complexity. Some genes are critical to developmental patterning, while other genes are important to physiological control of growth. However, growth regulator genes may not be as important in small-bodied "micro-metazoans" such as nematodes. Nematodes use a simplified developmental strategy of lineage-based cell fate specifications to produce an adult bilaterian body composed of a few hundreds of cells. Nematodes also lost the MYC proto-oncogenic regulator of cell proliferation. To identify additional regulators of cell proliferation that were lost with MYC, we computationally screened and determined 839 high-confidence genes that are conserved in bilaterians/lost in nematodes (CIBLIN genes). We find that 30 % of all CIBLIN genes encode transcriptional regulators of cell proliferation, epithelial-to-mesenchyme transitions, and other processes. Over 50 % of CIBLIN genes are unnamed genes in Drosophila, suggesting that there are many understudied genes. Interestingly, CIBLIN genes include many Myc synthetic lethal (MycSL) hits from recent screens. CIBLIN genes include key regulators of heparan sulfate proteoglycan (HSPG) sulfation patterns, and lysyl oxidases involved in cross-linking and modification of the extracellular matrix (**ECM**). These genes and others suggest the CIBLIN repertoire services critical functions in **ECM** remodeling and cell migration in large-bodied bilaterians. Correspondingly, CIBLIN genes are co-expressed with Myc in cancer transcriptomes, and include a preponderance of known determinants of cancer progression and tumor aggression. We propose that CIBLIN gene research can improve our understanding of regulatory control of cellular growth in metazoans.

### [Ee]xtracellular [Mm]atrix

#### PubMed

1. Immune response to bacteria induces dissemination of Ras-activated Drosophila hindgut cells. Although pathogenic bacteria are suspected contributors to colorectal cancer progression, cancer-promoting bacteria and their mode of action remain largely unknown. Here we report that sustained infection with the human intestinal colonizer Pseudomonas aeruginosa synergizes with the Ras1V12 oncogene to induce basal invasion and dissemination of hindgut cells to distant sites. Cross-talk between infection and dissemination requires sustained activation by the bacteria of the Imd-dTab2-dTak1 innate immune pathway, which converges with Ras1V12 signalling on JNK pathway activation, culminating in **extracellular matrix** degradation. Hindgut, but not midgut, cells are amenable to this cooperative dissemination, which is progressive and genetically and pharmacologically inhibitable. Thus, Drosophila hindgut provides a valuable system for the study of intestinal malignancies.
2. Genes conserved in bilaterians but jointly lost with Myc during nematode evolution are enriched in cell proliferation and cell migration functions. Animals use a stereotypical set of developmental genes to build body architectures of varying sizes and organizational complexity. Some genes are critical to developmental patterning, while other genes are important to physiological control of growth. However, growth regulator genes may not be as important in small-bodied "micro-metazoans" such as nematodes. Nematodes use a simplified developmental strategy of lineage-based cell fate specifications to produce an adult bilaterian body composed of a few hundreds of cells. Nematodes also lost the MYC proto-oncogenic regulator of cell proliferation. To identify additional regulators of cell proliferation that were lost with MYC, we computationally screened and determined 839 high-confidence genes that are conserved in bilaterians/lost in nematodes (CIBLIN genes). We find that 30 % of all CIBLIN genes encode transcriptional regulators of cell proliferation, epithelial-to-mesenchyme transitions, and other processes. Over 50 % of CIBLIN genes are unnamed genes in Drosophila, suggesting that there are many understudied genes. Interestingly, CIBLIN genes include many Myc synthetic lethal (MycSL) hits from recent screens. CIBLIN genes include key regulators of heparan sulfate proteoglycan (HSPG) sulfation patterns, and lysyl oxidases involved in cross-linking and modification of the **extracellular matrix** (ECM). These genes and others suggest the CIBLIN repertoire services critical functions in ECM remodeling and cell migration in large-bodied bilaterians. Correspondingly, CIBLIN genes are co-expressed with Myc in cancer transcriptomes, and include a preponderance of known determinants of cancer progression and tumor aggression. We propose that CIBLIN gene research can improve our understanding of regulatory control of cellular growth in metazoans.

### [Ii]nvasive

#### PubMed

1. **Invasive** and indigenous microbiota impact intestinal stem cell activity through multiple pathways in Drosophila. Gut homeostasis is controlled by both immune and developmental mechanisms, and its disruption can lead to inflammatory disorders or cancerous lesions of the intestine. While the impact of bacteria on the mucosal immune system is beginning to be precisely understood, little is known about the effects of bacteria on gut epithelium renewal. Here, we addressed how both infectious and indigenous bacteria modulate stem cell activity in Drosophila. We show that the increased epithelium renewal observed upon some bacterial infections is a consequence of the oxidative burst, a major defense of the Drosophila gut. Additionally, we provide evidence that the JAK-STAT (Janus kinase-signal transducers and activators of transcription) and JNK (c-Jun NH(2) terminal kinase) pathways are both required for bacteria-induced stem cell proliferation. Similarly, we demonstrate that indigenous gut microbiota activate the same, albeit reduced, program at basal levels. Altered control of gut microbiota in immune-deficient or aged flies correlates with increased epithelium renewal. Finally, we show that epithelium renewal is an essential component of Drosophila defense against oral bacterial infection. Altogether, these results indicate that gut homeostasis is achieved by a complex interregulation of the immune response, gut microbiota, and stem cell activity.
2. The multilayered innate immune defense of the gut. In the wild, the fruit fly Drosophila melanogaster thrives on rotten fruit. The digestive tract maintains a powerful gut immune barrier to regulate the ingested microbiota, including entomopathogenic bacteria. This gut immune barrier includes a chitinous peritrophic matrix that isolates the gut contents from the epithelial cells. In addition, the epithelial cells are tightly sealed by septate junctions and can mount an inducible immune response. This local response can be activated by **invasive** bacteria, or triggered by commensal bacteria in the gut lumen. As with chronic inflammation in mammals, constitutive activation of the gut innate immune response is detrimental to the health of flies. Accordingly, the Drosophila gut innate immune response is tightly regulated to maintain the endogenous microbiota, while preventing infections by pathogenic microorganisms.

### [Mm]etastasis

#### PubMed

1. Drosophila at the intersection of infection, inflammation, and cancer. Recent studies show that both cellular and humoral aspects of innate immunity play important roles during tumor progression. These interactions have traditionally been explored in vertebrate model systems. In recent years, Drosophila has emerged as a genetically tractable model system for studying key aspects of tumorigenesis including proliferation, invasion, and **metastasis**. The absence of adaptive immunity in Drosophila provides a unique opportunity to study the interactions between innate immune system and cancer in different genetic contexts. In this review, I discuss recent advances made by using Drosophila models of cancer to study the role of innate immune pathways Toll/Imd, JNK, and JAK-STAT, microbial infection and inflammation during tumor progression.

---

## pirk

- NCBI Gene
- GeneRIF
- Pubmed

| Search Term | Rank | Fields |
| --- | --- | --- |
| [Mm]igration | 6 | PubMed(1) |
| [Ii]nvasive | 10 | PubMed(1) |

  
hide/show details for pirk

### [Mm]igration

#### PubMed

1. The unconventional myosin CRINKLED and its mammalian orthologue MYO7A regulate caspases in their signalling roles. Caspases provide vital links in non-apoptotic regulatory networks controlling inflammation, compensatory proliferation, morphology and cell **migration**. How caspases are activated under non-apoptotic conditions and process a selective set of substrates without killing the cell remain enigmatic. Here we find that the Drosophila unconventional myosin CRINKLED (CK) selectively interacts with the initiator caspase DRONC and regulates some of its non-apoptotic functions. Loss of CK in the arista, border cells or proneural clusters of the wing imaginal discs affects DRONC-dependent patterning. Our data indicate that CK acts as substrate adaptor, recruiting SHAGGY46/GSK3-β to DRONC, thereby facilitating caspase-mediated cleavage and localized modulation of kinase activity. Similarly, the mammalian CK counterpart, MYO7A, binds to and impinges on CASPASE-8, revealing a new regulatory axis affecting receptor interacting protein kinase-1 (RIPK1)>CASPASE-8 signalling. Together, our results expose a conserved role for unconventional myosins in transducing caspase-dependent regulation of kinases, allowing them to take part in specific signalling events.

### [Ii]nvasive

#### PubMed

1. The multilayered innate immune defense of the gut. In the wild, the fruit fly Drosophila melanogaster thrives on rotten fruit. The digestive tract maintains a powerful gut immune barrier to regulate the ingested microbiota, including entomopathogenic bacteria. This gut immune barrier includes a chitinous peritrophic matrix that isolates the gut contents from the epithelial cells. In addition, the epithelial cells are tightly sealed by septate junctions and can mount an inducible immune response. This local response can be activated by **invasive** bacteria, or triggered by commensal bacteria in the gut lumen. As with chronic inflammation in mammals, constitutive activation of the gut innate immune response is detrimental to the health of flies. Accordingly, the Drosophila gut innate immune response is tightly regulated to maintain the endogenous microbiota, while preventing infections by pathogenic microorganisms.

---

## CG6675

- NCBI Gene
- GeneRIF
- Pubmed

| Search Term | Rank | Fields |
| --- | --- | --- |
| [Mm]igration | 6 | PubMed(1) |
| ECM | 7 | PubMed(1) |
| [Ee]xtracellular [Mm]atrix | 8 | PubMed(1) |

  
hide/show details for CG6675

### [Mm]igration

#### PubMed

1. Genes conserved in bilaterians but jointly lost with Myc during nematode evolution are enriched in cell proliferation and cell **migration** functions. Animals use a stereotypical set of developmental genes to build body architectures of varying sizes and organizational complexity. Some genes are critical to developmental patterning, while other genes are important to physiological control of growth. However, growth regulator genes may not be as important in small-bodied "micro-metazoans" such as nematodes. Nematodes use a simplified developmental strategy of lineage-based cell fate specifications to produce an adult bilaterian body composed of a few hundreds of cells. Nematodes also lost the MYC proto-oncogenic regulator of cell proliferation. To identify additional regulators of cell proliferation that were lost with MYC, we computationally screened and determined 839 high-confidence genes that are conserved in bilaterians/lost in nematodes (CIBLIN genes). We find that 30 % of all CIBLIN genes encode transcriptional regulators of cell proliferation, epithelial-to-mesenchyme transitions, and other processes. Over 50 % of CIBLIN genes are unnamed genes in Drosophila, suggesting that there are many understudied genes. Interestingly, CIBLIN genes include many Myc synthetic lethal (MycSL) hits from recent screens. CIBLIN genes include key regulators of heparan sulfate proteoglycan (HSPG) sulfation patterns, and lysyl oxidases involved in cross-linking and modification of the extracellular matrix (ECM). These genes and others suggest the CIBLIN repertoire services critical functions in ECM remodeling and cell **migration** in large-bodied bilaterians. Correspondingly, CIBLIN genes are co-expressed with Myc in cancer transcriptomes, and include a preponderance of known determinants of cancer progression and tumor aggression. We propose that CIBLIN gene research can improve our understanding of regulatory control of cellular growth in metazoans.

### ECM

#### PubMed

1. Genes conserved in bilaterians but jointly lost with Myc during nematode evolution are enriched in cell proliferation and cell migration functions. Animals use a stereotypical set of developmental genes to build body architectures of varying sizes and organizational complexity. Some genes are critical to developmental patterning, while other genes are important to physiological control of growth. However, growth regulator genes may not be as important in small-bodied "micro-metazoans" such as nematodes. Nematodes use a simplified developmental strategy of lineage-based cell fate specifications to produce an adult bilaterian body composed of a few hundreds of cells. Nematodes also lost the MYC proto-oncogenic regulator of cell proliferation. To identify additional regulators of cell proliferation that were lost with MYC, we computationally screened and determined 839 high-confidence genes that are conserved in bilaterians/lost in nematodes (CIBLIN genes). We find that 30 % of all CIBLIN genes encode transcriptional regulators of cell proliferation, epithelial-to-mesenchyme transitions, and other processes. Over 50 % of CIBLIN genes are unnamed genes in Drosophila, suggesting that there are many understudied genes. Interestingly, CIBLIN genes include many Myc synthetic lethal (MycSL) hits from recent screens. CIBLIN genes include key regulators of heparan sulfate proteoglycan (HSPG) sulfation patterns, and lysyl oxidases involved in cross-linking and modification of the extracellular matrix (**ECM**). These genes and others suggest the CIBLIN repertoire services critical functions in **ECM** remodeling and cell migration in large-bodied bilaterians. Correspondingly, CIBLIN genes are co-expressed with Myc in cancer transcriptomes, and include a preponderance of known determinants of cancer progression and tumor aggression. We propose that CIBLIN gene research can improve our understanding of regulatory control of cellular growth in metazoans.

### [Ee]xtracellular [Mm]atrix

#### PubMed

1. Genes conserved in bilaterians but jointly lost with Myc during nematode evolution are enriched in cell proliferation and cell migration functions. Animals use a stereotypical set of developmental genes to build body architectures of varying sizes and organizational complexity. Some genes are critical to developmental patterning, while other genes are important to physiological control of growth. However, growth regulator genes may not be as important in small-bodied "micro-metazoans" such as nematodes. Nematodes use a simplified developmental strategy of lineage-based cell fate specifications to produce an adult bilaterian body composed of a few hundreds of cells. Nematodes also lost the MYC proto-oncogenic regulator of cell proliferation. To identify additional regulators of cell proliferation that were lost with MYC, we computationally screened and determined 839 high-confidence genes that are conserved in bilaterians/lost in nematodes (CIBLIN genes). We find that 30 % of all CIBLIN genes encode transcriptional regulators of cell proliferation, epithelial-to-mesenchyme transitions, and other processes. Over 50 % of CIBLIN genes are unnamed genes in Drosophila, suggesting that there are many understudied genes. Interestingly, CIBLIN genes include many Myc synthetic lethal (MycSL) hits from recent screens. CIBLIN genes include key regulators of heparan sulfate proteoglycan (HSPG) sulfation patterns, and lysyl oxidases involved in cross-linking and modification of the **extracellular matrix** (ECM). These genes and others suggest the CIBLIN repertoire services critical functions in ECM remodeling and cell migration in large-bodied bilaterians. Correspondingly, CIBLIN genes are co-expressed with Myc in cancer transcriptomes, and include a preponderance of known determinants of cancer progression and tumor aggression. We propose that CIBLIN gene research can improve our understanding of regulatory control of cellular growth in metazoans.

---

## IM3

- NCBI Gene
- GeneRIF
- Pubmed

| Search Term | Rank | Fields |
| --- | --- | --- |
| [Ee]xtracellular [Mm]atrix | 8 | PubMed(1) |

  
hide/show details for IM3

### [Ee]xtracellular [Mm]atrix

#### PubMed

1. Genome-wide transcriptional analysis of Drosophila larvae infected by entomopathogenic nematodes shows involvement of complement, recognition and **extracellular matrix** proteins. Heterorhabditis bacteriophora is an entomopathogenic nematode (EPN) which infects its host by accessing the hemolymph where it releases endosymbiotic bacteria of the species Photorhabdus luminescens. We performed a genome-wide transcriptional analysis of the Drosophila response to EPN infection at the time point at which the nematodes reached the hemolymph either via the cuticle or the gut and the bacteria had started to multiply. Many of the most strongly induced genes have been implicated in immune responses in other infection models. Mapping of the complete set of differentially regulated genes showed the hallmarks of a wound response, but also identified a large fraction of EPN-specific transcripts. Several genes identified by transcriptome profiling or their homologues play protective roles during nematode infections. Genes that positively contribute to controlling nematobacterial infections encode: a homolog of thioester-containing complement protein 3, a basement membrane component (glutactin), a recognition protein (GNBP-like 3) and possibly several small peptides. Of note is that several of these genes have not previously been implicated in immune responses.

---

## Tig

- NCBI Gene
- GeneRIF
- Pubmed

| Search Term | Rank | Fields |
| --- | --- | --- |
| [Mm]igration | 6 | PubMed(10) |
| ECM | 7 | PubMed(14) |
| [Ee]xtracellular [Mm]atrix | 8 | PubMed(21); Component(3) |

  
hide/show details for Tig

### [Mm]igration

#### PubMed

1. Focal adhesions: what's new inside. The cytoplasmic side of focal adhesions is comprised of large molecular complexes that link transmembrane receptors, such as integrins, to the actin cytoskeleton and mediate signals modulating cell attachment, **migration**, proliferation, differentiation, and gene expression. These complexes are heterogeneous and dynamic structures that are apparent targets of regulatory signals that control the function of focal adhesions. Recent studies using genetic approaches in invertebrate and vertebrate systems have begun to reveal the structure and function of these complexes in vivo.
2. Slit coordinates cardiac morphogenesis in Drosophila. Slit is a secreted guidance cue that conveys repellent or attractive signals from target and guidepost cells. In Drosophila, responsive cells express one or more of three Robo receptors. The cardial cells of the developing heart express both Slit and Robo2. This is the first report of coincident expression of a Robo and its ligand. In slit mutants, cardial cell alignment, polarization and uniform **migration** are disrupted. The heart phenotype of robo2 mutants is similar, with fewer **migration** defects. In the guidance of neuronal growth cones in Drosophila, there is a phenotypic interaction between slit and robo heterozygotes, and also with genes required for Robo signaling. In contrast, in the heart, slit has little or no phenotypic interaction with Robo-related genes, including Robo2, Nck2, and Disabled. However, there is a strong phenotypic interaction with Integrin genes and their ligands, including Laminin and Collagen, and intracellular messengers, including Talin and ILK. This indicates that Slit participates in adhesion or adhesion signaling during heart development.
3. Analysis of integrin turnover in fly myotendinous junctions. Transient (short-term) cell adhesion underlies dynamic processes such as cell **migration**, whereas stable (long-term) cell adhesion maintains tissue architecture. Ongoing adhesion complex turnover is essential for transient cell adhesion, but it is not known whether turnover is also required for maintenance of long-term adhesion. We used fluorescence recovery after photobleaching to analyze the dynamics of an integrin adhesion complex (IAC) in a model of long-term cell-ECM adhesion, myotendinous junctions (MTJs), in fly embryos and larvae. We found that the IAC undergoes turnover in MTJs and that this process is mediated by clathrin-dependent endocytosis. Moreover, the small GTPase Rab5 can regulate the proportion of IAC components that undergo turnover. Also, altering Rab5 activity weakened MTJs, resulting in muscle defects. In addition, growth of MTJs was concomitant with a decrease in the proportion of IAC components undergoing turnover. We propose that IAC turnover is tightly regulated in long-term cell-ECM adhesions to allow normal tissue growth and maintenance.
4. Focal adhesion kinase is not required for integrin function or viability in Drosophila. The mammalian focal adhesion kinase (FAK) family of non-receptor protein-tyrosine kinases has been implicated in controlling a multitude of cellular responses to the engagement of cell-surface integrins and G-protein-coupled receptors. The high level of sequence conservation between the mammalian proteins and the Drosophila homologue of FAK, Fak56, suggested that it would have similar functions. However, we show here that Drosophila Fak56 is not essential for integrin functions in adhesion, **migration** or signaling in vivo. Furthermore, animals lacking Fak56 are viable and fertile, demonstrating that Fak56 is not essential for other developmental or physiological functions. Despite this, overexpressed Fak56 is a potent inhibitor of integrins binding to the extracellular matrix, suggesting that Fak56 may play a subtle role in the negative regulation of integrin adhesion.
5. Singling out Drosophila tendon cells: a dialogue between two distinct cell types. The precise match between somatic muscles and their epidermal attachment cells is achieved through a continuous dialogue between these two cell types. Whereas tendon cells direct myotube **migration** and final patterning, the muscles are essential for the maintenance of the fate of tendon cells. The Drosophila neuregulin-like ligand, Vein, and its receptor, the epidermal growth factor receptor (Egfr), are critical components in the inductive signaling process that takes place between muscles and tendon cells. Additional gene products that relay the Vein-Egfr effect in Drosophila are conserved in the vertebrate neuregulin-mediated cascade. This review describes genetic and molecular aspects of the muscle-tendon inductive processes in Drosophila, and compares them with the relevant mechanisms in the vertebrate embryo.
6. Slow as molasses is required for polarized membrane growth and germ cell **migration** in Drosophila. Drosophila germ cell **migration** is directed by attractive and repulsive guidance cues. We have identified a novel gene, slow as molasses (slam), which is required for germ cell **migration**. In slam zygotic mutants, germ cells fail to transit off the midgut into the mesoderm. We show that slam is required at this stage in parallel to HMG Coenzyme A reductase, a previously identified germ cell **migration** gene. Removal of both zygotic and maternal slam results in an earlier defect: a failure to form a cellular blastoderm. Consistent with this phenotype, we found that slam is one of the earliest genes to be transcribed in the embryo, and Slam protein localizes to the growing basal-lateral membrane during blastoderm formation, but Slam is not detected during later stages of embryogenesis. Because slam RNA and protein are expressed earlier than the time when we observe defects in germ cell **migration**, we propose that Slam is required for the localization of a signal to the basal side of blastoderm cells that is needed later in the posterior midgut to guide germ cells.
7. Drosophila importin-7 functions upstream of the Elmo signaling module to mediate the formation and stability of muscle attachments. Establishment and maintenance of stable muscle attachments is essential for coordinated body movement. Studies in Drosophila have pioneered a molecular understanding of the morphological events in the conserved process of muscle attachment formation, including myofiber **migration**, muscle-tendon signaling, and stable junctional adhesion between muscle cells and their corresponding target insertion sites. In both Drosophila and vertebrate models, integrin complexes play a key role in the biogenesis and stability of muscle attachments through the interactions of integrins with extracellular matrix (ECM) ligands. We show that Drosophila importin-7 (Dim7) is an upstream regulator of the conserved Elmo-Mbc→Rac signaling pathway in the formation of embryonic muscle attachment sites (MASs). Dim7 is encoded by the moleskin (msk) locus and was identified as an Elmo-interacting protein. Both Dim7 and Elmo localize to the ends of myofibers coincident with the timing of muscle-tendon attachment in late myogenesis. Phenotypic analysis of elmo mutants reveal muscle attachment defects similar to those previously described for integrin mutants. Furthermore, Elmo and Dim7 interact both biochemically and genetically in the developing musculature. The muscle detachment phenotype resulting from mutations in the msk locus can be rescued by components in the Elmo signaling pathway, including the Elmo-Mbc complex, an activated Elmo variant, or a constitutively active form of Rac. In larval muscles, the localization of Dim7 and activated Elmo to the sites of muscle attachment is attenuated upon RNAi knockdown of integrin heterodimer complex components. Our results show that integrins function as upstream signals to mediate Dim7-Elmo enrichment to the MASs.
8. Integrins are required for cardioblast polarisation in Drosophila. The formation of a tubular organ, such as the heart, requires the communication of positional and polarity signals between migratory cells. Key to this process is the establishment of a new luminal domain on the cell surface, generally from the apical domain of a migratory cell. This domain will also acquire basal properties, as it will produce a luminal extracellular matrix. Integrin receptors are the primary means of cell adhesion and adhesion signaling with the extracellular matrix. Here we characterise the requirement of Integrins in a genetic model of vasculogenesis, the formation of the heart in Drosophila.As with vertebrates, the Drosophila heart arises from lateral mesoderm that migrates medially to meet their contralateral partners, to then assemble a midline vessel. During **migration**, Integrins are among the first proteins restricted to the presumptive luminal domain of cardioblasts. Integrins are required for normal levels of leading edge membrane motility. Apical accumulation of Integrins is enhanced by Robo, and reciprocally, apicalisation of luminal factors like Slit and Robo requires Integrin function. Integrins may provide a template for the formation of a lumen by stabilising lumen factors like Robo. Subsequent to **migration**, Integrin is required for normal cardioblast alignment and lumen formation. This phenotype is most readily modified by other mutations that affect adhesion, such as Talin and extracellular matrix ligands.Our findings reveal an instructive role for Integrins in communicating polarising information to cells during **migration**, and during transition to an epithelial tube structure.
9. Vestigial is required during late-stage muscle differentiation in Drosophila melanogaster embryos. The somatic muscles of Drosophila develop in a complex pattern that is repeated in each embryonic hemi-segment. During early development, progenitor cells fuse to form a syncytial muscle, which further differentiates via expression of muscle-specific factors that induce specific responses to external signals to regulate late-stage processes such as **migration** and attachment. Initial communication between somatic muscles and the epidermal tendon cells is critical for both of these processes. However, later establishment of attachments between longitudinal muscles at the segmental borders is largely independent of the muscle-epidermal attachment signals, and relatively little is known about how this event is regulated. Using a combination of null mutations and a truncated version of Sd that binds Vg but not DNA, we show that Vestigial (Vg) is required in ventral longitudinal muscles to induce formation of stable intermuscular attachments. In several muscles, this activity may be independent of Sd. Furthermore, the cell-specific differentiation events induced by Vg in two cells fated to form attachments are coordinated by Drosophila epidermal growth factor signaling. Thus, Vg is a key factor to induce specific changes in ventral longitudinal muscles 1-4 identity and is required for these cells to be competent to form stable intermuscular attachments with each other.
10. Cell-cell adhesion via the ECM: integrin genetics in fly and worm. Integrins are essential for the development of the two genetically tractable invertebrate model organisms, the nematode worm Caenorhabditis elegans and the fruit fly Drosophila melanogaster. Just two integrins are present in C. elegans: one putative RGD binding integrin alphapat-2betapat-3, corresponding to Drosophila alphaPS2betaPS and vertebrate alpha5beta1, alphaVbeta1 and alpha8beta1, and one putative laminin binding integrin alphaina-1betapat-3, corresponding to Drosophila alphaPS1betaPS and vertebrate alpha3beta1, alpha6beta1 and alpha7beta1. In this review, the function of this minimal set of integrins during the development of these two invertebrates is compared. Despite the differences in bodyplan and developmental strategy, integrin adhesion to the extracellular matrix is required for similar processes: the formation of the link that translates muscle contraction into movement of the exoskeleton, cell **migration**, and morphogenetic interactions between epithelia. Other integrin functions, such as regulation of gene expression, have not yet been experimentally demonstrated in both organisms. Additional proteins have been characterised in each organism that are essential for integrin function, including extracellular matrix ligands and intracellular interacting proteins, but so far different proteins have been found in the two organisms. This in part represents the fact that the characterisation of the full set of interacting proteins is not complete in either system. However, in other cases different proteins appear to be used for similar functions in the two animals. The continued use of genetic approaches to identify proteins required for integrin function in these two model organisms should lead to the identification of the minimal set of conserved components that form integrin adhesive structures.

### ECM

#### PubMed

1. AlphaPS2 integrin-mediated muscle attachment in Drosophila requires the **ECM** protein Thrombospondin. During Drosophila embryogenesis, the attachment of somatic muscles to epidermal tendon cells requires heterodimeric PS-integrin proteins (alpha- and beta-subunits). The alpha-subunits are expressed complementarily, either tendon cell- or muscle-specific, whereas the beta-integrin subunit is expressed in both tissues. Mutations of beta-integrin cause a severe muscle detachment phenotype, whereas alpha-subunit mutations have weaker or only larval muscle detachment phenotypes. Furthermore, mutations of extracellular matrix (**ECM**) proteins known to act as integrin binding partners have comparatively weak effects only, suggesting the presence of additional integrin binding **ECM** proteins required for proper muscle attachment. Here, we report that mutations in the Drosophila gene thrombospondin (tsp) cause embryonic muscle detachment. tsp is specifically expressed in both developing and mature epidermal tendon cells. Its initial expression in segment border cells, the tendon precursors, is under the control of hedgehog-dependent signaling, whereas tsp expression in differentiated tendon cells depends on the transcription factor encoded by stripe. In the absence of tsp activity, no aspect of muscle pattern formation as well as the initial contact between muscle and tendon cells nor muscle-to-muscle attachments are affected. However, when muscle contractions occur during late embryogenesis, muscles detach from the tendon cells. The Tsp protein is localized to the tendon cell **ECM** where muscles attach. Genetic interaction studies indicate that Tsp specifically interacts with the alphaPS2 integrin and that this interaction is needed to withstand the forces of muscle contractions at the tendon cells.
2. Thrombospondin-mediated adhesion is essential for the formation of the myotendinous junction in Drosophila. Organogenesis of the somatic musculature in Drosophila is directed by the precise adhesion between migrating myotubes and their corresponding ectodermally derived tendon cells. Whereas the PS integrins mediate the adhesion between these two cell types, their extracellular matrix (**ECM**) ligands have been only partially characterized. We show that the **ECM** protein Thrombospondin (Tsp), produced by tendon cells, is essential for the formation of the integrin-mediated myotendinous junction. Tsp expression is induced by the tendon-specific transcription factor Stripe, and accumulates at the myotendinous junction following the association between the muscle and the tendon cell. In tsp mutant embryos, migrating somatic muscles fail to attach to tendon cells and often form hemiadherens junctions with their neighboring muscle cells, resulting in nonfunctional somatic musculature. Talin accumulation at the cytoplasmic faces of the muscles and tendons is greatly reduced, implicating Tsp as a potential integrin ligand. Consistently, purified Tsp C-terminal domain polypeptide mediates spreading of PS2 integrin-expressing S2 cells in a KGD- and PS2-integrin-dependent manner. We propose a model in which the myotendinous junction is formed by the specific association of Tsp with multiple muscle-specific PS2 integrin receptors and a subsequent consolidation of the junction by enhanced tendon-specific production of Tsp secreted into the junctional space.
3. Screening for Drosophila proteins with distinct expression patterns during development by use of monoclonal antibodies. Kc 167 is a cell line established from Drosophila embryonic hemocytes and has been shown to express many extracellular matrix (**ECM**) and other proteins important during development. We have screened monoclonal antibodies (mAbs) raised against heparin affinity purified proteins from conditioned medium of Kc 167 cells to identify novel proteins with important roles for development. One mAb recognized a protein expressed with temporary and tissue specific patterns during Drosophila embryogenesis and larval development. This approach is an alternative to screening of Expression Sequence Tag (EST) clones by in situ hybridization to initiate reverse genetics. In addition, a number of mAbs recognizing **ECM** proteins were also identified. These mAbs will be useful for biochemical and cell biological analyses of Drosophila **ECM** proteins.
4. In vivo functional analysis reveals specific roles for the integrin-binding sites of talin. Adhesion receptors play diverse roles during animal development and require precise spatiotemporal regulation, which is achieved through the activity of their binding partners. Integrins, adhesion receptors that mediate cell attachment to the extracellular matrix (**ECM**), connect to the intracellular environment through the cytoplasmic adapter protein talin. Talin has two essential functions: orchestrating the assembly of the intracellular adhesion complex (IAC), which associates with integrin, and regulating the affinity of integrins for the **ECM**. Talin can bind to integrins through two different integrin-binding sites (IBS-1 and IBS-2, respectively). Here, we have investigated the roles of each in the context of Drosophila development. We find that although IBS-1 and IBS-2 are partially redundant, they each have specialized roles during development: IBS-1 reinforces integrin attachment to the **ECM**, whereas IBS-2 reinforces the link between integrins and the IAC. Disruption of each IBS has different developmental consequences, illustrating how the functional diversity of integrin-mediated adhesion is achieved.
5. Analysis of integrin turnover in fly myotendinous junctions. Transient (short-term) cell adhesion underlies dynamic processes such as cell migration, whereas stable (long-term) cell adhesion maintains tissue architecture. Ongoing adhesion complex turnover is essential for transient cell adhesion, but it is not known whether turnover is also required for maintenance of long-term adhesion. We used fluorescence recovery after photobleaching to analyze the dynamics of an integrin adhesion complex (IAC) in a model of long-term cell-**ECM** adhesion, myotendinous junctions (MTJs), in fly embryos and larvae. We found that the IAC undergoes turnover in MTJs and that this process is mediated by clathrin-dependent endocytosis. Moreover, the small GTPase Rab5 can regulate the proportion of IAC components that undergo turnover. Also, altering Rab5 activity weakened MTJs, resulting in muscle defects. In addition, growth of MTJs was concomitant with a decrease in the proportion of IAC components undergoing turnover. We propose that IAC turnover is tightly regulated in long-term cell-**ECM** adhesions to allow normal tissue growth and maintenance.
6. The PS2 integrin ligand tiggrin is required for proper muscle function in Drosophila. Tiggrin is a novel extracellular matrix ligand for the Drosophila PS2 integrins. We have used flanking P elements to generate a precise deletion of tiggrin. Most flies lacking tiggrin die as larvae or pupae. A few adults do emerge and these appear to be relatively normal, displaying only misshapen abdomens and a low frequency of wing defects. Examination of larvae shows that muscle connections, function and morphology are defective in tiggrin mutants. Muscle contraction waves that extend the length of the larvae are much slower in tiggrin mutants. Direct examination of bodywall muscles shows defects in muscle attachment sites, where tiggrin is specifically localized, and muscles appear thinner. Transgenes expressing tiggrin are capable of rescuing tiggrin mutant phenotypes. Transgenes expressing a mutant tiggrin, whose Arg-Gly-Asp (RGD) integrin recognition sequence has been mutated to Leu-Gly-Ala (LGA) show much reduced, but significant, rescuing ability. Cell spreading assays detect no interactions of this mutant tiggrin with PS2 integrins. Therefore, while the RGD sequence is critical for PS2 interactions and full activity in the whole fly, the mutant tiggrin retains some function(s) that are probably mediated by interactions with other **ECM** molecules or cell surface receptors
7. Integrins modulate the Egfr signaling pathway to regulate tendon cell differentiation in the Drosophila embryo. Changes in the extracellular matrix (**ECM**) govern the differentiation of many cell types during embryogenesis. Integrins are cell matrix receptors that play a major role in cell-**ECM** adhesion and in transmitting signals from the **ECM** inside the cell to regulate gene expression. In this paper, it is shown that the PS integrins are required at the muscle attachment sites of the Drosophila embryo to regulate tendon cell differentiation. The analysis of the requirements of the individual alpha subunits, alphaPS1 and alphaPS2, demonstrates that both PS1 and PS2 integrins are involved in this process. In the absence of PS integrin function, the expression of tendon cell-specific genes such as stripe and beta1 tubulin is not maintained. In addition, embryos lacking the PS integrins also exhibit reduced levels of activated MAPK. This reduction is probably due to a downregulation of the Epidermal Growth Factor receptor (Egfr) pathway, since an activated form of the Egfr can rescue the phenotype of embryos mutant for the PS integrins. Furthermore, the levels of the Egfr ligand Vein at the muscle attachment sites are reduced in PS mutant embryos. Altogether, these results lead to a model in which integrin-mediated adhesion plays a role in regulating tendon cell differentiation by modulating the activity of the Egfr pathway at the level of its ligand Vein.
8. The talin head domain reinforces integrin-mediated adhesion by promoting adhesion complex stability and clustering. Talin serves an essential function during integrin-mediated adhesion in linking integrins to actin via the intracellular adhesion complex. In addition, the N-terminal head domain of talin regulates the affinity of integrins for their **ECM**-ligands, a process known as inside-out activation. We previously showed that in Drosophila, mutating the integrin binding site in the talin head domain resulted in weakened adhesion to the **ECM**. Intriguingly, subsequent studies showed that canonical inside-out activation of integrin might not take place in flies. Consistent with this, a mutation in talin that specifically blocks its ability to activate mammalian integrins does not significantly impinge on talin function during fly development. Here, we describe results suggesting that the talin head domain reinforces and stabilizes the integrin adhesion complex by promoting integrin clustering distinct from its ability to support inside-out activation. Specifically, we show that an allele of talin containing a mutation that disrupts intramolecular interactions within the talin head attenuates the assembly and reinforcement of the integrin adhesion complex. Importantly, we provide evidence that this mutation blocks integrin clustering in vivo. We propose that the talin head domain is essential for regulating integrin avidity in Drosophila and that this is crucial for integrin-mediated adhesion during animal development.
9. An interaction between integrin and the talin FERM domain mediates integrin activation but not linkage to the cytoskeleton. Transmembrane adhesion receptors, such as integrins, mediate cell adhesion by interacting with intracellular proteins that connect to the cytoskeleton. Talin, one such linker protein, is thought to have two roles: mediating inside-out activation of integrins, and connecting extracellular matrix (**ECM**)-bound integrins to the cytoskeleton. Talin's amino-terminal head, which consists of a FERM domain, binds an NPxY motif within the cytoplasmic tail of most integrin beta subunits. This is consistent with the role of FERM domains in recruiting other proteins to the plasma membrane. We tested the role of the talin-head-NPxY interaction in integrin function in Drosophila. We found that introduction of a mutation that perturbs this binding in vitro into the isolated talin head disrupts its recruitment by integrins in vivo. Surprisingly, when engineered into the full-length talin, this mutation did not disrupt talin recruitment by integrins nor its ability to connect integrins to the cytoskeleton. However, it reduced the ability of talin to strengthen integrin adhesion to the **ECM**, indicating that the function of the talin-head-NPxY interaction is solely to regulate integrin adhesion.
10. Drosophila importin-7 functions upstream of the Elmo signaling module to mediate the formation and stability of muscle attachments. Establishment and maintenance of stable muscle attachments is essential for coordinated body movement. Studies in Drosophila have pioneered a molecular understanding of the morphological events in the conserved process of muscle attachment formation, including myofiber migration, muscle-tendon signaling, and stable junctional adhesion between muscle cells and their corresponding target insertion sites. In both Drosophila and vertebrate models, integrin complexes play a key role in the biogenesis and stability of muscle attachments through the interactions of integrins with extracellular matrix (**ECM**) ligands. We show that Drosophila importin-7 (Dim7) is an upstream regulator of the conserved Elmo-Mbc→Rac signaling pathway in the formation of embryonic muscle attachment sites (MASs). Dim7 is encoded by the moleskin (msk) locus and was identified as an Elmo-interacting protein. Both Dim7 and Elmo localize to the ends of myofibers coincident with the timing of muscle-tendon attachment in late myogenesis. Phenotypic analysis of elmo mutants reveal muscle attachment defects similar to those previously described for integrin mutants. Furthermore, Elmo and Dim7 interact both biochemically and genetically in the developing musculature. The muscle detachment phenotype resulting from mutations in the msk locus can be rescued by components in the Elmo signaling pathway, including the Elmo-Mbc complex, an activated Elmo variant, or a constitutively active form of Rac. In larval muscles, the localization of Dim7 and activated Elmo to the sites of muscle attachment is attenuated upon RNAi knockdown of integrin heterodimer complex components. Our results show that integrins function as upstream signals to mediate Dim7-Elmo enrichment to the MASs.
11. A mucin-type O-glycosyltransferase modulates cell adhesion during Drosophila development. Cell-cell and cell-matrix adhesion are crucial during many stages of eukaryotic development. Here, we provide the first example that mucin-type O-linked glycosylation is involved in a developmentally regulated cell adhesion event in Drosophila melanogaster. Mutations in one member of the evolutionarily conserved family of enzymes that initiates O-linked glycosylation alter epithelial cell adhesion in the Drosophila wing blade. A transposon insertion mutation in pgant3 or RNA interference to pgant3 resulted in blistered wings, a phenotype characteristic of genes involved in integrin-mediated cell interactions. Expression of wild type pgant3 in the mutant background rescued the wing blistering phenotype, whereas expression of another family member (pgant35A) did not, revealing a unique requirement for pgant3. pgant3 mutants displayed reduced O-glycosylation along the basal surface of larval wing imaginal discs, which was restored with wild type pgant3 expression, suggesting that reduced glycosylation of basal proteins is responsible for disruption of adhesion in the adult wing blade. Glycosylation reactions demonstrated that PGANT3 glycosylates certain extracellular matrix (**ECM**) proteins. Immunoprecipitation experiments revealed that PGANT3 glycosylates tiggrin, an **ECM** protein known to bind integrin. We propose that this glycosyltransferase is uniquely responsible for glycosylating tiggrin in the wing disc, thus modulating proper cell adhesion through integrin-**ECM** interactions. This study provides the first evidence for the role of O-glycosylation in a developmentally regulated, integrin-mediated, cell adhesion event and reveals a novel player in wing blade formation during Drosophila development.
12. Cell-cell adhesion via the **ECM**: integrin genetics in fly and worm. Integrins are essential for the development of the two genetically tractable invertebrate model organisms, the nematode worm Caenorhabditis elegans and the fruit fly Drosophila melanogaster. Just two integrins are present in C. elegans: one putative RGD binding integrin alphapat-2betapat-3, corresponding to Drosophila alphaPS2betaPS and vertebrate alpha5beta1, alphaVbeta1 and alpha8beta1, and one putative laminin binding integrin alphaina-1betapat-3, corresponding to Drosophila alphaPS1betaPS and vertebrate alpha3beta1, alpha6beta1 and alpha7beta1. In this review, the function of this minimal set of integrins during the development of these two invertebrates is compared. Despite the differences in bodyplan and developmental strategy, integrin adhesion to the extracellular matrix is required for similar processes: the formation of the link that translates muscle contraction into movement of the exoskeleton, cell migration, and morphogenetic interactions between epithelia. Other integrin functions, such as regulation of gene expression, have not yet been experimentally demonstrated in both organisms. Additional proteins have been characterised in each organism that are essential for integrin function, including extracellular matrix ligands and intracellular interacting proteins, but so far different proteins have been found in the two organisms. This in part represents the fact that the characterisation of the full set of interacting proteins is not complete in either system. However, in other cases different proteins appear to be used for similar functions in the two animals. The continued use of genetic approaches to identify proteins required for integrin function in these two model organisms should lead to the identification of the minimal set of conserved components that form integrin adhesive structures.
13. Extracellular matrix and its receptors in Drosophila neural development. Extracellular matrix (**ECM**) and matrix receptors are intimately involved in most biological processes. The **ECM** plays fundamental developmental and physiological roles in health and disease, including processes underlying the development, maintenance, and regeneration of the nervous system. To understand the principles of **ECM**-mediated functions in the nervous system, genetic model organisms like Drosophila provide simple, malleable, and powerful experimental platforms. This article provides an overview of **ECM** proteins and receptors in Drosophila. It then focuses on their roles during three progressive phases of neural development: (1) neural progenitor proliferation, (2) axonal growth and pathfinding, and (3) synapse formation and function. Each section highlights known **ECM** and **ECM**-receptor components and recent studies done in mutant conditions to reveal their in vivo functions, all illustrating the enormous opportunities provided when merging work on the nervous system with systematic research into **ECM**-related gene functions.
14. Mutations in the Drosophila alphaPS2 integrin subunit uncover new features of adhesion site assembly. The Drosophila alphaPS2betaPS integrin is required for diverse development events, including muscle attachment. We characterized six unusual mutations in the alphaPS2 gene that cause a subset of the null phenotype. One mutation changes a residue in alphaPS2 that is equivalent to the residue in alphaV that contacts the arginine of RGD. This change severely reduced alphaPS2betaPS affinity for soluble ligand, abolished the ability of the integrin to recruit laminin to muscle attachment sites in the embryo and caused detachment of integrins and talin from the **ECM**. Three mutations that alter different parts of the alphaPS2 beta-propeller, plus a fourth that eliminated a late phase of alphaPS2 expression, all led to a strong decrease in alphaPS2betaPS at muscle ends, but, surprisingly, normal levels of talin were recruited. Thus, although talin recruitment requires alphaPS2betaPS, talin levels are not simply specified by the amount of integrin at the adhesive junction. These mutations caused detachment of talin and actin from integrins, suggesting that the integrin-talin link is weaker than the **ECM**-integrin link.

### [Ee]xtracellular [Mm]atrix

#### PubMed

1. The localized assembly of **extracellular matrix** integrin ligands requires cell-cell contact. The assembly of an organism requires the interaction between different layers of cells, in many cases via an **extracellular matrix**. In the developing Drosophila larva, muscles attach in an integrin-dependent manner to the epidermis, via a specialized **extracellular matrix** called tendon matrix. Tiggrin, a tendon matrix integrin ligand, is primarily synthesized by cells distant to the muscle attachment sites, yet it accumulates specifically at these sites. Previous work has shown that the PS integrins are not required for tiggrin localization, suggesting that there is redundancy among tiggrin receptors. We have examined this by testing whether the PS2 integrin can recruit tiggrin to ectopic locations within the Drosophila embryo. We found that neither the wild type nor modified forms of the PS2 integrin, which have higher affinity for tiggrin, can recruit tiggrin to new cellular contexts. Next, we genetically manipulated the fate of the muscles and the epidermal muscle attachment cells, which demonstrated that muscles have the primary role in recruiting tiggrin to the tendon matrix and that cell-cell contact is necessary for this recruitment. Thus we propose that the inherent polarity of the muscle cells leads to a molecular specialization of their ends, and interactions between the ends produces an integrin-independent tiggrin receptor. Thus, interaction between cells generates an extracellular environment capable of nucleating **extracellular matrix** assembly.
2. AlphaPS2 integrin-mediated muscle attachment in Drosophila requires the ECM protein Thrombospondin. During Drosophila embryogenesis, the attachment of somatic muscles to epidermal tendon cells requires heterodimeric PS-integrin proteins (alpha- and beta-subunits). The alpha-subunits are expressed complementarily, either tendon cell- or muscle-specific, whereas the beta-integrin subunit is expressed in both tissues. Mutations of beta-integrin cause a severe muscle detachment phenotype, whereas alpha-subunit mutations have weaker or only larval muscle detachment phenotypes. Furthermore, mutations of **extracellular matrix** (ECM) proteins known to act as integrin binding partners have comparatively weak effects only, suggesting the presence of additional integrin binding ECM proteins required for proper muscle attachment. Here, we report that mutations in the Drosophila gene thrombospondin (tsp) cause embryonic muscle detachment. tsp is specifically expressed in both developing and mature epidermal tendon cells. Its initial expression in segment border cells, the tendon precursors, is under the control of hedgehog-dependent signaling, whereas tsp expression in differentiated tendon cells depends on the transcription factor encoded by stripe. In the absence of tsp activity, no aspect of muscle pattern formation as well as the initial contact between muscle and tendon cells nor muscle-to-muscle attachments are affected. However, when muscle contractions occur during late embryogenesis, muscles detach from the tendon cells. The Tsp protein is localized to the tendon cell ECM where muscles attach. Genetic interaction studies indicate that Tsp specifically interacts with the alphaPS2 integrin and that this interaction is needed to withstand the forces of muscle contractions at the tendon cells.
3. Thrombospondin-mediated adhesion is essential for the formation of the myotendinous junction in Drosophila. Organogenesis of the somatic musculature in Drosophila is directed by the precise adhesion between migrating myotubes and their corresponding ectodermally derived tendon cells. Whereas the PS integrins mediate the adhesion between these two cell types, their **extracellular matrix** (ECM) ligands have been only partially characterized. We show that the ECM protein Thrombospondin (Tsp), produced by tendon cells, is essential for the formation of the integrin-mediated myotendinous junction. Tsp expression is induced by the tendon-specific transcription factor Stripe, and accumulates at the myotendinous junction following the association between the muscle and the tendon cell. In tsp mutant embryos, migrating somatic muscles fail to attach to tendon cells and often form hemiadherens junctions with their neighboring muscle cells, resulting in nonfunctional somatic musculature. Talin accumulation at the cytoplasmic faces of the muscles and tendons is greatly reduced, implicating Tsp as a potential integrin ligand. Consistently, purified Tsp C-terminal domain polypeptide mediates spreading of PS2 integrin-expressing S2 cells in a KGD- and PS2-integrin-dependent manner. We propose a model in which the myotendinous junction is formed by the specific association of Tsp with multiple muscle-specific PS2 integrin receptors and a subsequent consolidation of the junction by enhanced tendon-specific production of Tsp secreted into the junctional space.
4. An O-glycosyltransferase promotes cell adhesion during development by influencing secretion of an **extracellular matrix** integrin ligand. Protein secretion and localization are crucial during eukaryotic development, establishing local cell environments as well as mediating cell interactions, signaling, and adhesion. In this study, we demonstrate that the glycosyltransferase, pgant3, specifically modulates integrin-mediated cell adhesion by influencing the secretion and localization of the integrin ligand, Tiggrin. We demonstrate that Tiggrin is normally O-glycosylated and localized to the basal matrix where the dorsal and ventral cell layers adhere in wild type Drosophila wings. In pgant3 mutants, Tiggrin is no longer O-glycosylated and fails to be properly secreted to this basal cell layer interface, resulting in disruption of integrin-mediated cell adhesion in the wing. pgant3-mediated effects are dependent on enzymatic activity, as mutations that form a stable protein yet abrogate O-glycosyltransferase activity result in Tiggrin accumulation within the dorsal and ventral cells comprising the wing. Our results provide the first in vivo evidence for the role of O-glycosylation in the secretion of specific **extracellular matrix** proteins, thus altering the composition of the cellular "microenvironment" and thereby modulating developmentally regulated cell adhesion events. As alterations in cell adhesion are a hallmark of cancer progression, this work provides insight into the long-standing association between aberrant O-glycosylation and tumorigenesis.
5. Screening for Drosophila proteins with distinct expression patterns during development by use of monoclonal antibodies. Kc 167 is a cell line established from Drosophila embryonic hemocytes and has been shown to express many **extracellular matrix** (ECM) and other proteins important during development. We have screened monoclonal antibodies (mAbs) raised against heparin affinity purified proteins from conditioned medium of Kc 167 cells to identify novel proteins with important roles for development. One mAb recognized a protein expressed with temporary and tissue specific patterns during Drosophila embryogenesis and larval development. This approach is an alternative to screening of Expression Sequence Tag (EST) clones by in situ hybridization to initiate reverse genetics. In addition, a number of mAbs recognizing ECM proteins were also identified. These mAbs will be useful for biochemical and cell biological analyses of Drosophila ECM proteins.
6. Drosophila PS1 integrin is a laminin receptor and differs in ligand specificity from PS2. We have expressed Drosophila position-specific (PS) integrins on the surfaces of Schneider S2 cells and tested for adhesion and spreading on various matrix molecules. We report that PS1 integrin is a laminin receptor and that PS1 and PS2 integrins promote cell spreading on two different Drosophila **extracellular matrix** molecules, laminin and tiggrin, respectively. The differing ligand specificities of these two integrins, combined with data on the in vivo expression patterns of the integrins and their ligands, lead to a model for the structure of integrin-dependent attachments in the pupal wings and embryonic muscles of Drosophila.
7. In vivo functional analysis reveals specific roles for the integrin-binding sites of talin. Adhesion receptors play diverse roles during animal development and require precise spatiotemporal regulation, which is achieved through the activity of their binding partners. Integrins, adhesion receptors that mediate cell attachment to the **extracellular matrix** (ECM), connect to the intracellular environment through the cytoplasmic adapter protein talin. Talin has two essential functions: orchestrating the assembly of the intracellular adhesion complex (IAC), which associates with integrin, and regulating the affinity of integrins for the ECM. Talin can bind to integrins through two different integrin-binding sites (IBS-1 and IBS-2, respectively). Here, we have investigated the roles of each in the context of Drosophila development. We find that although IBS-1 and IBS-2 are partially redundant, they each have specialized roles during development: IBS-1 reinforces integrin attachment to the ECM, whereas IBS-2 reinforces the link between integrins and the IAC. Disruption of each IBS has different developmental consequences, illustrating how the functional diversity of integrin-mediated adhesion is achieved.
8. Distinct regulatory mechanisms control integrin adhesive processes during tissue morphogenesis. Cell adhesion must be precisely regulated to enable both dynamic morphogenetic processes and the subsequent transition to stable tissue maintenance. Integrins link the intracellular cytoskeleton and **extracellular matrix**, relaying bidirectional signals across the plasma membrane. In vitro studies have demonstrated that multiple mechanisms control integrin-mediated adhesion; however, their roles during development are poorly understood. We used mutations that activate or deactivate specific functions of vertebrate β-integrins in vitro to investigate how perturbing Drosophila βPS-integrin regulation in developing embryos regulation affects tissue morphogenesis and maintenance. We found that morphogenetic processes use various β-integrin regulatory mechanisms to differing degrees and that conformational changes associated with outside-in activation are essential for developmental integrin functions. Long-term adhesion is also sensitive to integrin dysregulation, suggesting integrins must be continuously regulated to support stable tissue maintenance. Altogether, in vivo phenotypic analyses allowed us to identify the importance of various β-integrin regulatory mechanisms during different morphogenetic processes.
9. The PS2 integrin ligand tiggrin is required for proper muscle function in Drosophila. Tiggrin is a novel **extracellular matrix** ligand for the Drosophila PS2 integrins. We have used flanking P elements to generate a precise deletion of tiggrin. Most flies lacking tiggrin die as larvae or pupae. A few adults do emerge and these appear to be relatively normal, displaying only misshapen abdomens and a low frequency of wing defects. Examination of larvae shows that muscle connections, function and morphology are defective in tiggrin mutants. Muscle contraction waves that extend the length of the larvae are much slower in tiggrin mutants. Direct examination of bodywall muscles shows defects in muscle attachment sites, where tiggrin is specifically localized, and muscles appear thinner. Transgenes expressing tiggrin are capable of rescuing tiggrin mutant phenotypes. Transgenes expressing a mutant tiggrin, whose Arg-Gly-Asp (RGD) integrin recognition sequence has been mutated to Leu-Gly-Ala (LGA) show much reduced, but significant, rescuing ability. Cell spreading assays detect no interactions of this mutant tiggrin with PS2 integrins. Therefore, while the RGD sequence is critical for PS2 interactions and full activity in the whole fly, the mutant tiggrin retains some function(s) that are probably mediated by interactions with other ECM molecules or cell surface receptors
10. DFak56 is a novel Drosophila melanogaster focal adhesion kinase. The mammalian focal adhesion kinase (FAK) family of nonreceptor protein-tyrosine kinases have been implicated in controlling a multitude of cellular responses to the engagement of cell surface integrins and G protein-coupled receptors. We describe here a Drosophila melanogaster FAK homologue, DFak56, which maps to band 56D on the right arm of the second chromosome. Full-length DFak56 cDNA encodes a phosphoprotein of 140 kDa, which shares strong sequence similarity not only with mammalian p125(FAK) but also with the more recently described mammalian Pyk2 (also known as CAKbeta, RAFTK, FAK2, and CADTK) FAK family member. DFak56 has intrinsic tyrosine kinase activity and is phosphorylated on tyrosine in vivo. As is the case for FAK, tyrosine phosphorylation of DFak56 is increased upon plating Drosophila embryo cells on **extracellular matrix** proteins. In situ hybridization and immunofluorescence staining analysis showed that DFak56 is ubiquitously expressed with particularly high levels within the developing central nervous system. We utilized the UAS-GAL4 expression system to express DFak56 and analyze its function in vivo. Overexpression of DFak56 in the wing imaginal disc results in wing blistering in adults, a phenotype also observed with both position-specific integrin loss of function and position-specific integrin overexpression. Our results imply a role for DFak56 in adhesion-dependent signaling pathways in vivo during D. melanogaster development.
11. Integrins modulate the Egfr signaling pathway to regulate tendon cell differentiation in the Drosophila embryo. Changes in the **extracellular matrix** (ECM) govern the differentiation of many cell types during embryogenesis. Integrins are cell matrix receptors that play a major role in cell-ECM adhesion and in transmitting signals from the ECM inside the cell to regulate gene expression. In this paper, it is shown that the PS integrins are required at the muscle attachment sites of the Drosophila embryo to regulate tendon cell differentiation. The analysis of the requirements of the individual alpha subunits, alphaPS1 and alphaPS2, demonstrates that both PS1 and PS2 integrins are involved in this process. In the absence of PS integrin function, the expression of tendon cell-specific genes such as stripe and beta1 tubulin is not maintained. In addition, embryos lacking the PS integrins also exhibit reduced levels of activated MAPK. This reduction is probably due to a downregulation of the Epidermal Growth Factor receptor (Egfr) pathway, since an activated form of the Egfr can rescue the phenotype of embryos mutant for the PS integrins. Furthermore, the levels of the Egfr ligand Vein at the muscle attachment sites are reduced in PS mutant embryos. Altogether, these results lead to a model in which integrin-mediated adhesion plays a role in regulating tendon cell differentiation by modulating the activity of the Egfr pathway at the level of its ligand Vein.
12. Focal adhesion kinase is not required for integrin function or viability in Drosophila. The mammalian focal adhesion kinase (FAK) family of non-receptor protein-tyrosine kinases has been implicated in controlling a multitude of cellular responses to the engagement of cell-surface integrins and G-protein-coupled receptors. The high level of sequence conservation between the mammalian proteins and the Drosophila homologue of FAK, Fak56, suggested that it would have similar functions. However, we show here that Drosophila Fak56 is not essential for integrin functions in adhesion, migration or signaling in vivo. Furthermore, animals lacking Fak56 are viable and fertile, demonstrating that Fak56 is not essential for other developmental or physiological functions. Despite this, overexpressed Fak56 is a potent inhibitor of integrins binding to the **extracellular matrix**, suggesting that Fak56 may play a subtle role in the negative regulation of integrin adhesion.
13. An interaction between integrin and the talin FERM domain mediates integrin activation but not linkage to the cytoskeleton. Transmembrane adhesion receptors, such as integrins, mediate cell adhesion by interacting with intracellular proteins that connect to the cytoskeleton. Talin, one such linker protein, is thought to have two roles: mediating inside-out activation of integrins, and connecting **extracellular matrix** (ECM)-bound integrins to the cytoskeleton. Talin's amino-terminal head, which consists of a FERM domain, binds an NPxY motif within the cytoplasmic tail of most integrin beta subunits. This is consistent with the role of FERM domains in recruiting other proteins to the plasma membrane. We tested the role of the talin-head-NPxY interaction in integrin function in Drosophila. We found that introduction of a mutation that perturbs this binding in vitro into the isolated talin head disrupts its recruitment by integrins in vivo. Surprisingly, when engineered into the full-length talin, this mutation did not disrupt talin recruitment by integrins nor its ability to connect integrins to the cytoskeleton. However, it reduced the ability of talin to strengthen integrin adhesion to the ECM, indicating that the function of the talin-head-NPxY interaction is solely to regulate integrin adhesion.
14. Tiggrin, a novel Drosophila **extracellular matrix** protein that functions as a ligand for Drosophila alpha PS2 beta PS integrins. Genetic and other studies of Drosophila integrins have implicated these **extracellular matrix** receptors in various morphogenetic events, but identification of their endogenous ligands has been elusive. We report the biochemical purification and cloning of tiggrin, a novel **extracellular matrix** protein from Drosophila. This 255 x 10(3) M(r) polypeptide contains the potential integrin recognition sequence Arg-Gly-Asp (RGD) and 16 repeats of a novel 73-77 amino acid motif. The tiggrin gene is at chromosome locus 26D1-2 and is expressed by embryonic hemocytes and fat body cells. Tiggrin protein is detected in matrices, especially at muscle attachment sites that also strongly express integrins. Tiggrin-coated surfaces support primary embryo cell culture and provide excellent substrates for alpha PS2 beta PS integrin-mediated cell spreading. Soluble RGD-peptides inhibit this cell spreading.
15. Drosophila importin-7 functions upstream of the Elmo signaling module to mediate the formation and stability of muscle attachments. Establishment and maintenance of stable muscle attachments is essential for coordinated body movement. Studies in Drosophila have pioneered a molecular understanding of the morphological events in the conserved process of muscle attachment formation, including myofiber migration, muscle-tendon signaling, and stable junctional adhesion between muscle cells and their corresponding target insertion sites. In both Drosophila and vertebrate models, integrin complexes play a key role in the biogenesis and stability of muscle attachments through the interactions of integrins with **extracellular matrix** (ECM) ligands. We show that Drosophila importin-7 (Dim7) is an upstream regulator of the conserved Elmo-Mbc→Rac signaling pathway in the formation of embryonic muscle attachment sites (MASs). Dim7 is encoded by the moleskin (msk) locus and was identified as an Elmo-interacting protein. Both Dim7 and Elmo localize to the ends of myofibers coincident with the timing of muscle-tendon attachment in late myogenesis. Phenotypic analysis of elmo mutants reveal muscle attachment defects similar to those previously described for integrin mutants. Furthermore, Elmo and Dim7 interact both biochemically and genetically in the developing musculature. The muscle detachment phenotype resulting from mutations in the msk locus can be rescued by components in the Elmo signaling pathway, including the Elmo-Mbc complex, an activated Elmo variant, or a constitutively active form of Rac. In larval muscles, the localization of Dim7 and activated Elmo to the sites of muscle attachment is attenuated upon RNAi knockdown of integrin heterodimer complex components. Our results show that integrins function as upstream signals to mediate Dim7-Elmo enrichment to the MASs.
16. A mucin-type O-glycosyltransferase modulates cell adhesion during Drosophila development. Cell-cell and cell-matrix adhesion are crucial during many stages of eukaryotic development. Here, we provide the first example that mucin-type O-linked glycosylation is involved in a developmentally regulated cell adhesion event in Drosophila melanogaster. Mutations in one member of the evolutionarily conserved family of enzymes that initiates O-linked glycosylation alter epithelial cell adhesion in the Drosophila wing blade. A transposon insertion mutation in pgant3 or RNA interference to pgant3 resulted in blistered wings, a phenotype characteristic of genes involved in integrin-mediated cell interactions. Expression of wild type pgant3 in the mutant background rescued the wing blistering phenotype, whereas expression of another family member (pgant35A) did not, revealing a unique requirement for pgant3. pgant3 mutants displayed reduced O-glycosylation along the basal surface of larval wing imaginal discs, which was restored with wild type pgant3 expression, suggesting that reduced glycosylation of basal proteins is responsible for disruption of adhesion in the adult wing blade. Glycosylation reactions demonstrated that PGANT3 glycosylates certain **extracellular matrix** (ECM) proteins. Immunoprecipitation experiments revealed that PGANT3 glycosylates tiggrin, an ECM protein known to bind integrin. We propose that this glycosyltransferase is uniquely responsible for glycosylating tiggrin in the wing disc, thus modulating proper cell adhesion through integrin-ECM interactions. This study provides the first evidence for the role of O-glycosylation in a developmentally regulated, integrin-mediated, cell adhesion event and reveals a novel player in wing blade formation during Drosophila development.
17. Integrins are required for cardioblast polarisation in Drosophila. The formation of a tubular organ, such as the heart, requires the communication of positional and polarity signals between migratory cells. Key to this process is the establishment of a new luminal domain on the cell surface, generally from the apical domain of a migratory cell. This domain will also acquire basal properties, as it will produce a luminal **extracellular matrix**. Integrin receptors are the primary means of cell adhesion and adhesion signaling with the **extracellular matrix**. Here we characterise the requirement of Integrins in a genetic model of vasculogenesis, the formation of the heart in Drosophila.As with vertebrates, the Drosophila heart arises from lateral mesoderm that migrates medially to meet their contralateral partners, to then assemble a midline vessel. During migration, Integrins are among the first proteins restricted to the presumptive luminal domain of cardioblasts. Integrins are required for normal levels of leading edge membrane motility. Apical accumulation of Integrins is enhanced by Robo, and reciprocally, apicalisation of luminal factors like Slit and Robo requires Integrin function. Integrins may provide a template for the formation of a lumen by stabilising lumen factors like Robo. Subsequent to migration, Integrin is required for normal cardioblast alignment and lumen formation. This phenotype is most readily modified by other mutations that affect adhesion, such as Talin and **extracellular matrix** ligands.Our findings reveal an instructive role for Integrins in communicating polarising information to cells during migration, and during transition to an epithelial tube structure.
18. Amino acid changes in Drosophila alphaPS2betaPS integrins that affect ligand affinity. We developed a ligand-mimetic antibody Fab fragment specific for Drosophila alphaPS2betaPS integrins to probe the ligand binding affinities of these invertebrate receptors. TWOW-1 was constructed by inserting a fragment of the **extracellular matrix** protein Tiggrin into the H-CDR3 of the alphavbeta3 ligand-mimetic antibody WOW-1. The specificity of alphaPS2betaPS binding to TWOW-1 was demonstrated by numerous tests used for other integrin-ligand interactions. Binding was decreased in the presence of EDTA or RGD peptides and by mutation of the TWOW-1 RGD sequence or the betaPS metal ion-dependent adhesion site (MIDAS) motif. TWOW-1 binding was increased by mutations in the alphaPS2 membrane-proximal cytoplasmic GFFNR sequence or by exposure to Mn2+. Although Mn2+ is sometimes assumed to promote maximal integrin activity, TWOW-1 binding in Mn2+ could be increased further by the alphaPS2 GFFNR --> GFANA mutation. A mutation in the betaPS I domain (betaPS-b58; V409D) greatly increased ligand binding affinity, explaining the increased cell spreading mediated by alphaPS2betaPS-b58. Further mutagenesis of this residue suggested that Val-409 normally stabilizes the closed head conformation. Mutations that potentially reduce interaction of the integrin beta subunit plexin-semaphorin-integrin (PSI) and stalk domains have been shown to have activating properties. We found that complete deletion of the betaPS PSI domain enhanced TWOW-1 binding. Moreover the PSI domain is dispensable for at least some other integrin functions because betaPS-DeltaPSI displayed an enhanced ability to mediate cell spreading. These studies establish a means to evaluate mechanisms and consequences of integrin affinity modulation in a tractable model genetic system.
19. Integrins modulate Sog activity in the Drosophila wing. Morphogenesis of the Drosophila wing depends on a series of cell-cell and cell-**extracellular matrix** interactions. During pupal wing development, two secreted proteins, encoded by the short gastrulation (sog) and decapentaplegic (dpp) genes, vie to position wing veins in the center of broad provein territories. Expression of the Bmp4 homolog dpp in vein cells is counteracted by expression of the Bmp antagonist sog in intervein cells, which results in the formation of straight veins of precise width. We screened for genetic interactions between sog and genes encoding a variety of extracellular components and uncovered interactions between sog and myospheroid (mys), multiple edematous wing (mew) and scab (scb), which encode betaPS, alphaPS1 and alphaPS3 integrin subunits, respectively. Clonal analysis reveals that integrin mutations affect the trajectory of veins inside the provein domain and/or their width and that misexpression of sog can alter the behavior of cells in such clones. In addition, we show that a low molecular weight form of Sog protein binds to alphaPS1betaPS. We find that Sog can diffuse from its intervein site of production into adjacent provein domains, but only on the dorsal surface of the wing, where Sog interacts functionally with integrins. Finally, we show that Sog diffusion into provein regions and the reticular pattern of extracellular Sog distribution in wild-type wings requires mys and mew function. We propose that integrins act by binding and possibly regulating the activity/availability of different forms of Sog during pupal development through an adhesion independent mechanism.
20. Cell-cell adhesion via the ECM: integrin genetics in fly and worm. Integrins are essential for the development of the two genetically tractable invertebrate model organisms, the nematode worm Caenorhabditis elegans and the fruit fly Drosophila melanogaster. Just two integrins are present in C. elegans: one putative RGD binding integrin alphapat-2betapat-3, corresponding to Drosophila alphaPS2betaPS and vertebrate alpha5beta1, alphaVbeta1 and alpha8beta1, and one putative laminin binding integrin alphaina-1betapat-3, corresponding to Drosophila alphaPS1betaPS and vertebrate alpha3beta1, alpha6beta1 and alpha7beta1. In this review, the function of this minimal set of integrins during the development of these two invertebrates is compared. Despite the differences in bodyplan and developmental strategy, integrin adhesion to the **extracellular matrix** is required for similar processes: the formation of the link that translates muscle contraction into movement of the exoskeleton, cell migration, and morphogenetic interactions between epithelia. Other integrin functions, such as regulation of gene expression, have not yet been experimentally demonstrated in both organisms. Additional proteins have been characterised in each organism that are essential for integrin function, including **extracellular matrix** ligands and intracellular interacting proteins, but so far different proteins have been found in the two organisms. This in part represents the fact that the characterisation of the full set of interacting proteins is not complete in either system. However, in other cases different proteins appear to be used for similar functions in the two animals. The continued use of genetic approaches to identify proteins required for integrin function in these two model organisms should lead to the identification of the minimal set of conserved components that form integrin adhesive structures.
21. **Extracellular matrix** and its receptors in Drosophila neural development. **Extracellular matrix** (ECM) and matrix receptors are intimately involved in most biological processes. The ECM plays fundamental developmental and physiological roles in health and disease, including processes underlying the development, maintenance, and regeneration of the nervous system. To understand the principles of ECM-mediated functions in the nervous system, genetic model organisms like Drosophila provide simple, malleable, and powerful experimental platforms. This article provides an overview of ECM proteins and receptors in Drosophila. It then focuses on their roles during three progressive phases of neural development: (1) neural progenitor proliferation, (2) axonal growth and pathfinding, and (3) synapse formation and function. Each section highlights known ECM and ECM-receptor components and recent studies done in mutant conditions to reveal their in vivo functions, all illustrating the enormous opportunities provided when merging work on the nervous system with systematic research into ECM-related gene functions.

#### Component

1. **extracellular matrix**
2. proteinaceous **extracellular matrix**
3. proteinaceous **extracellular matrix**

---

## IM2

- NCBI Gene
- GeneRIF
- Pubmed

| Search Term | Rank | Fields |
| --- | --- | --- |
| [Mm]igration | 6 | PubMed(1) |
| [Ee]xtracellular [Mm]atrix | 8 | PubMed(1) |

  
hide/show details for IM2

### [Mm]igration

#### PubMed

1. The RhoGEF Zizimin-related acts in the Drosophila cellular immune response via the Rho GTPases Rac2 and Cdc42. Zizimin-related (Zir), a Rho guanine nucleotide exchange factor (RhoGEF) homologous to the mammalian Dock-C/Zizimin-related family, was identified in a screen to find new genes involved in the Drosophila melanogaster cellular immune response against eggs from the parasitoid wasp Leptopilina boulardi. RhoGEFs activate Rho-family GTPases, which are known to be central regulators of cell **migration**, spreading and polarity. When a parasitoid wasp is recognized as foreign, multiple layers of circulating immunosurveillance cells (haemocytes) should attach to the egg. In Zir mutants this process is disrupted and lamellocytes, a haemocyte subtype, fail to properly encapsulate the wasp egg. Furthermore, macrophage-like plasmatocytes exhibit a strong reduction in their ability to phagocytise Escherichia coli and Staphylococcus aureus bacteria. During encapsulation and phagocytosis Zir genetically interacts with two Rho-family GTPases, Rac2 and Cdc42. Finally, Zir is dispensable for the humoral immune response against bacteria. We propose that Zir is necessary to activate the Rho-family GTPases Rac2 and Cdc42 during the Drosophila cellular immune response.

### [Ee]xtracellular [Mm]atrix

#### PubMed

1. Genome-wide transcriptional analysis of Drosophila larvae infected by entomopathogenic nematodes shows involvement of complement, recognition and **extracellular matrix** proteins. Heterorhabditis bacteriophora is an entomopathogenic nematode (EPN) which infects its host by accessing the hemolymph where it releases endosymbiotic bacteria of the species Photorhabdus luminescens. We performed a genome-wide transcriptional analysis of the Drosophila response to EPN infection at the time point at which the nematodes reached the hemolymph either via the cuticle or the gut and the bacteria had started to multiply. Many of the most strongly induced genes have been implicated in immune responses in other infection models. Mapping of the complete set of differentially regulated genes showed the hallmarks of a wound response, but also identified a large fraction of EPN-specific transcripts. Several genes identified by transcriptome profiling or their homologues play protective roles during nematode infections. Genes that positively contribute to controlling nematobacterial infections encode: a homolog of thioester-containing complement protein 3, a basement membrane component (glutactin), a recognition protein (GNBP-like 3) and possibly several small peptides. Of note is that several of these genes have not previously been implicated in immune responses.

---

## CG1299

- NCBI Gene
- GeneRIF
- Pubmed

| Search Term | Rank | Fields |
| --- | --- | --- |
| [Mm]igration | 6 | PubMed(1) |

  
hide/show details for CG1299

### [Mm]igration

#### PubMed

1. Gene expression profiling of Drosophila tracheal fusion cells. The Drosophila trachea is a premier genetic system to investigate the fundamental mechanisms of tubular organ formation. Tracheal fusion cells lead the branch fusion process to form an interconnected tubular network. Therefore, fusion cells in the Drosophila trachea will be an excellent model to study branch fusion in mammalian tubular organs, such as kidneys and blood vessels. The fusion process is a dynamic cellular process involving cell **migration**, adhesion, vesicle trafficking, cytoskeleton rearrangement, and membrane fusion. To understand how these cellular events are coordinated, we initiated the critical step to assemble a gene expression profile of fusion cells. For this study, we analyzed the expression of 234 potential tracheal-expressed genes in fusion cells during fusion cell development. 143 Tracheal genes were found to encode transcription factors, signal proteins, cytoskeleton and matrix proteins, transporters, and proteins with unknown function. These genes were divided into four subgroups based on their levels of expression in fusion cells compared to neighboring non-fusion cells revealed by in situ hybridization: (1) genes that have relative high abundance in fusion cells, (2) genes that are dynamically expressed in fusion cells, (3) genes that have relative low abundance in fusion cells, and (4) genes that are expressed at similar levels in fusion cells and non-fusion tracheal cells. This study identifies the expression profile of fusion cells and hypothetically suggests genes which are necessary for the fusion process and which play roles in distinct stages of fusion, as indicated by the location and timing of expression. These data will provide the basis for a comprehensive understanding of the molecular and cellular mechanisms of branch fusion.

---

## betaTub60D

- NCBI Gene
- GeneRIF
- Pubmed

| Search Term | Rank | Fields |
| --- | --- | --- |
| [Mm]igration | 6 | PubMed(9) |
| ECM | 7 | PubMed(1) |

  
hide/show details for betaTub60D

### [Mm]igration

#### PubMed

1. The proprioceptive and contractile systems in Drosophila are both patterned by the EGR family transcription factor Stripe. Coordinated locomotion of Drosophila larvae depends on accurate patterning and stable attachment to the cuticle of both muscles and proprioceptors (chordotonal organs). Unlike muscle spindles in mammals, the fly chordotonal organs are not embedded in the body-wall muscles. Yet, the contractile system (muscles and tendons) and the chordotonal organs constitute two parts of a single functional unit that controls locomotion, and thus must be patterned in full coordination. It is not known how such coordination is achieved. Here we show that the positioning and differentiation of the migrating chordotonal organs are instructed by Stripe, the same transcription factor that promotes tendon cell specification and differentiation and is required for normal patterning of the contractile system. Our data demonstrate that although chordotonal organs are patterned in a Stripe-dependent mechanism similarly to muscles, this mechanism is independent of Stripe activity in tendon cells. Thus, the two parts of the locomotive system use similar but independent patterning mechanisms that converge to form a functional unit. Stripe plays at least a dual role in chordotonal development. It is required within the ligament cells for terminal differentiation and proper **migration**, without which no induction of ligament attachment cells takes place. Stripe's activity is then necessary within the recruited cells for their differentiation as attachment cells. Similarly to the biphasic differentiation program of tendons, terminal differentiation of chordotonal attachment cells is associated with sequential activation of the two Stripe isoforms-Stripe B and Stripe A.
2. Comparative analysis of Hox downstream genes in Drosophila. Functional diversification of body parts is dependent on the formation of specialized structures along the various body axes. In animals, region-specific morphogenesis along the anteroposterior axis is controlled by a group of conserved transcription factors encoded by the Hox genes. Although it has long been assumed that Hox proteins carry out their function by regulating distinct sets of downstream genes, only a small number of such genes have been found, with very few having direct roles in controlling cellular behavior. We have quantitatively identified hundreds of Hox downstream genes in Drosophila by microarray analysis, and validated many of them by in situ hybridizations on loss- and gain-of-function mutants. One important finding is that Hox proteins, despite their similar DNA-binding properties in vitro, have highly specific effects on the transcriptome in vivo, because expression of many downstream genes respond primarily to a single Hox protein. In addition, a large fraction of downstream genes encodes realizator functions, which directly affect morphogenetic processes, such as orientation and rate of cell divisions, cell-cell adhesion and communication, cell shape and **migration**, or cell death. Focusing on these realizators, we provide a framework for the morphogenesis of the maxillary segment. As the genomic organization of Hox genes and the interaction of Hox proteins with specific co-factors are conserved in vertebrates and invertebrates, and similar classes of downstream genes are regulated by Hox proteins across the metazoan phylogeny, our findings represent a first step toward a mechanistic understanding of morphological diversification within a species as well as between species.
3. The role of LamininB2 (LanB2) during mesoderm differentiation in Drosophila. In Drosophila, four genes encode for laminin subunits and the formation of two laminin heterotrimers has been postulated. We report the identification of mutations in the Drosophila LamininB2 (LanB2) gene that encodes for the only laminin γ subunit and is found in both heterotrimers. We describe their effects on embryogenesis, in particular the differentiation of visceral tissues with respect to the ECM. Analysis of mesoderm endoderm interaction indicates disrupted basement membranes and defective endoderm **migratio**n, which finally interferes with visceral myotube stretching. Extracellular deposition of laminin is blocked due to the loss of the LanB2 subunit, resulting in an abnormal distribution of ECM components. Our data, concerning the different function of both trimers during organogenesis, suggest that these trimers might act in a cumulative way and probably at multiple steps during ECM assembly. We also observed genetic interactions with kon-tiki and thrombospondin, indicating a role for laminin during muscle attachment.
4. Cytoplasmic dynein is required for the nuclear attachment and **migration** of centrosomes during mitosis in Drosophila. Cytoplasmic dynein is a multisubunit minus-end-directed microtubule motor that serves multiple cellular functions. Genetic studies in Drosophila and mouse have demonstrated that dynein function is essential in metazoan organisms. However, whether the essential function of dynein reflects a mitotic requirement, and what specific mitotic tasks require dynein remains controversial. Drosophila is an excellent genetic system in which to analyze dynein function in mitosis, providing excellent cytology in embryonic and somatic cells. We have used previously characterized recessive lethal mutations in the dynein heavy chain gene, Dhc64C, to reveal the contributions of the dynein motor to mitotic centrosome behavior in the syncytial embryo. Embryos lacking wild-type cytoplasmic dynein heavy chain were analyzed by in vivo analysis of rhodamine-labeled microtubules, as well as by immunofluorescence in situ methods. Comparisons between wild-type and Dhc64C mutant embryos reveal that dynein function is required for the attachment and **migration** of centrosomes along the nuclear envelope during interphase/prophase, and to maintain the attachment of centrosomes to mitotic spindle poles. The disruption of these centrosome attachments in mutant embryos reveals a critical role for dynein function and centrosome positioning in the spatial organization of the syncytial cytoplasm of the developing embryo.
5. Fusion from myoblasts to myotubes is dependent on the rolling stone gene (rost) of Drosophila. The development and differentiation of the body wall musculature in Drosophila are accompanied by changes in gene expression and cellular architecture. We isolated a Drosophila gene, termed rolling stone (rost), which, when mutated, specifically blocks the fusion of mononucleated cells to myotubes in the body wall musculature. beta 3 tubulin, which is an early marker for the onset of mesoderm differentiation, is still expressed in these cells. Gastrulation and mesoderm formation, as well as the development of the epidermis and of the central and peripheral nervous systems, appear quite normal in homozygous rolling stone embryos. Embryonic development stops shortly before hatching in a P-element-induced mutant, as well as in 16 EMS-induced alleles. In mutant embryos, other mesodermal derivatives such as the visceral mesoderm and the dorsal vessel, develop fairly normally and defects are restricted to the body wall musculature. Myoblasts remain as single mononucleated cells, which express muscle myosin, showing that the developmental program of gene expression proceeds. These myoblasts occur at positions corresponding to the locations of dorsal, ventral and pleural muscles, showing that the gene rolling stone is involved in cell fusion, a process that is independent of cell **migration** in these mutants. This genetic analysis has set the stage for a molecular analysis to clarify where the rolling stone action is manifested in the fusion process and thus gives insight into the complex regulating network controlling the differentiation of the body wall musculature.
6. Determination and development of the larval muscle pattern in Drosophila melanogaster. This review describes briefly what is known about the early steps of mesoderm differentiation in the fruitfly Drosophila melanogaster. After a summary of general aspects including mesoderm differentiation, mesoderm cell **migration** and subdivision of the mesoderm, more detail is given about the specification of muscle progenitor cells, due to their role as the earliest obvious landmarks in muscle fiber development in Drosophila. Particular focus is given to recent results on the role of asymmetric cell division in muscle differentiation. Furthermore a short summary of myoblast fusion is provided.
7. The Hydra FGFR, Kringelchen, partially replaces the Drosophila Heartless FGFR. Fibroblast growth factor receptors (FGFR) are highly conserved receptor tyrosine kinases, and evolved early in metazoan evolution. In order to investigate their functional conservation, we asked whether the Kringelchen FGFR in the freshwater polyp Hydra vulgaris, is able to functionally replace FGFR in fly embryos. In Drosophila, two endogenous FGFR, Breathless (Btl) and Heartless (Htl), ensure formation of the tracheal system and mesodermal cell **migration** as well as formation of the heart. Using UAS-kringelchen-5xmyc transgenic flies and targeted expression, we show that Kringelchen is integrated correctly into the cell membrane of mesodermal and tracheal cells in Drosophila. Nevertheless, Kringelchen expression driven in tracheal cells failed to rescue the btl (LG19) mutant. The Hydra FGFR was able to substitute for Heartless in the htl (AB42) null mutant; however, this occurred only during early mesodermal cell **migration**. Our data provide evidence for functional conservation of this early-diverged FGFR across these distantly related phyla, but also selectivity for the Htl FGFR in the Drosophila system.
8. Fusion of circular and longitudinal muscles in Drosophila is independent of the endoderm but further visceral muscle differentiation requires a close contact between mesoderm and endoderm. In this study we describe the morphological and genetic analysis of the Drosophila mutant gürtelchen (gurt). gurt was identified by screening an EMS collection for novel mutations affecting visceral mesoderm development and was named after the distinct belt shaped visceral phenotype. Interestingly, determination of visceral cell identities and subsequent visceral myoblast fusion is not affected in mutant embryos indicating a later defect in visceral development. gurt is in fact a new huckebein (hkb) allele and as such exhibits nearly complete loss of endodermal derived structures. Targeted ablation of the endodermal primordia produces a phenotype that resembles the visceral defects observed in huckebein(gürtelchen) (hkb(gurt)) mutant embryos. It was shown previously that visceral mesoderm development requires complex interactions between visceral myoblasts and adjacent tissues. Signals from the neighbouring somatic myoblasts play an important role in cell type determination and are a prerequisite for visceral muscle fusion. Furthermore, the visceral mesoderm is known to influence endoderma**l migrati**on and midgut epithelium formation. Our analyses of the visceral phenotype of hkb(gurt) mutant embryos reveal that the adjacent endoderm plays a critical role in the later stages of visceral muscle development, and is required for visceral muscle elongation and outgrowth after proper myoblast fusion.
9. The transmembrane protein Kon-tiki couples to Dgrip to mediate myotube targeting in Drosophila. Directed cell **migration** and target recognition are critical for the development of both the nervous and muscular systems. Molecular mechanisms that control these processes in the nervous system have been intensively studied, whereas those that act during muscle development are still largely uncharacterized. Here we identify a transmembrane protein, Kon-tiki (Kon), that mediates myotube target recognition in the Drosophila embryo. Kon is expressed in a specific subset of myotubes and is required autonomously for these myotubes to recognize their tendon cell targets and to establish a stable connection. Kon is enriched at myotube tips during targeting and signals through the intracellular adaptor Dgrip in a conserved molecular pathway. Forced overexpression of Kon stimulates muscle motility. We propose that Kon promotes directed myotube **migration** and transduces a target-derived signal that initiates the formation of a stable connection.

### ECM

#### PubMed

1. The role of LamininB2 (LanB2) during mesoderm differentiation in Drosophila. In Drosophila, four genes encode for laminin subunits and the formation of two laminin heterotrimers has been postulated. We report the identification of mutations in the Drosophila LamininB2 (LanB2) gene that encodes for the only laminin γ subunit and is found in both heterotrimers. We describe their effects on embryogenesis, in particular the differentiation of visceral tissues with respect to the **EC**M. Analysis of mesoderm endoderm interaction indicates disrupted basement membranes and defective endoderm migration, which finally interferes with visceral myotube stretching. Extracellular deposition of laminin is blocked due to the loss of the LanB2 subunit, resulting in an abnormal distribution of **EC**M components. Our data, concerning the different function of both trimers during organogenesis, suggest that these trimers might act in a cumulative way and probably at multiple steps during **EC**M assembly. We also observed genetic interactions with kon-tiki and thrombospondin, indicating a role for laminin during muscle attachment.

---

## Gnmt

- NCBI Gene
- GeneRIF
- Pubmed

| Search Term | Rank | Fields |
| --- | --- | --- |
| [Mm]igration | 6 | PubMed(1) |
| ECM | 7 | PubMed(1) |
| [Ee]xtracellular [Mm]atrix | 8 | PubMed(1) |

  
hide/show details for Gnmt

### [Mm]igration

#### PubMed

1. Genes conserved in bilaterians but jointly lost with Myc during nematode evolution are enriched in cell proliferation and cell **migration** functions. Animals use a stereotypical set of developmental genes to build body architectures of varying sizes and organizational complexity. Some genes are critical to developmental patterning, while other genes are important to physiological control of growth. However, growth regulator genes may not be as important in small-bodied "micro-metazoans" such as nematodes. Nematodes use a simplified developmental strategy of lineage-based cell fate specifications to produce an adult bilaterian body composed of a few hundreds of cells. Nematodes also lost the MYC proto-oncogenic regulator of cell proliferation. To identify additional regulators of cell proliferation that were lost with MYC, we computationally screened and determined 839 high-confidence genes that are conserved in bilaterians/lost in nematodes (CIBLIN genes). We find that 30 % of all CIBLIN genes encode transcriptional regulators of cell proliferation, epithelial-to-mesenchyme transitions, and other processes. Over 50 % of CIBLIN genes are unnamed genes in Drosophila, suggesting that there are many understudied genes. Interestingly, CIBLIN genes include many Myc synthetic lethal (MycSL) hits from recent screens. CIBLIN genes include key regulators of heparan sulfate proteoglycan (HSPG) sulfation patterns, and lysyl oxidases involved in cross-linking and modification of the extracellular matrix (ECM). These genes and others suggest the CIBLIN repertoire services critical functions in ECM remodeling and cell **migration** in large-bodied bilaterians. Correspondingly, CIBLIN genes are co-expressed with Myc in cancer transcriptomes, and include a preponderance of known determinants of cancer progression and tumor aggression. We propose that CIBLIN gene research can improve our understanding of regulatory control of cellular growth in metazoans.

### ECM

#### PubMed

1. Genes conserved in bilaterians but jointly lost with Myc during nematode evolution are enriched in cell proliferation and cell migration functions. Animals use a stereotypical set of developmental genes to build body architectures of varying sizes and organizational complexity. Some genes are critical to developmental patterning, while other genes are important to physiological control of growth. However, growth regulator genes may not be as important in small-bodied "micro-metazoans" such as nematodes. Nematodes use a simplified developmental strategy of lineage-based cell fate specifications to produce an adult bilaterian body composed of a few hundreds of cells. Nematodes also lost the MYC proto-oncogenic regulator of cell proliferation. To identify additional regulators of cell proliferation that were lost with MYC, we computationally screened and determined 839 high-confidence genes that are conserved in bilaterians/lost in nematodes (CIBLIN genes). We find that 30 % of all CIBLIN genes encode transcriptional regulators of cell proliferation, epithelial-to-mesenchyme transitions, and other processes. Over 50 % of CIBLIN genes are unnamed genes in Drosophila, suggesting that there are many understudied genes. Interestingly, CIBLIN genes include many Myc synthetic lethal (MycSL) hits from recent screens. CIBLIN genes include key regulators of heparan sulfate proteoglycan (HSPG) sulfation patterns, and lysyl oxidases involved in cross-linking and modification of the extracellular matrix (**ECM**). These genes and others suggest the CIBLIN repertoire services critical functions in **ECM** remodeling and cell migration in large-bodied bilaterians. Correspondingly, CIBLIN genes are co-expressed with Myc in cancer transcriptomes, and include a preponderance of known determinants of cancer progression and tumor aggression. We propose that CIBLIN gene research can improve our understanding of regulatory control of cellular growth in metazoans.

### [Ee]xtracellular [Mm]atrix

#### PubMed

1. Genes conserved in bilaterians but jointly lost with Myc during nematode evolution are enriched in cell proliferation and cell migration functions. Animals use a stereotypical set of developmental genes to build body architectures of varying sizes and organizational complexity. Some genes are critical to developmental patterning, while other genes are important to physiological control of growth. However, growth regulator genes may not be as important in small-bodied "micro-metazoans" such as nematodes. Nematodes use a simplified developmental strategy of lineage-based cell fate specifications to produce an adult bilaterian body composed of a few hundreds of cells. Nematodes also lost the MYC proto-oncogenic regulator of cell proliferation. To identify additional regulators of cell proliferation that were lost with MYC, we computationally screened and determined 839 high-confidence genes that are conserved in bilaterians/lost in nematodes (CIBLIN genes). We find that 30 % of all CIBLIN genes encode transcriptional regulators of cell proliferation, epithelial-to-mesenchyme transitions, and other processes. Over 50 % of CIBLIN genes are unnamed genes in Drosophila, suggesting that there are many understudied genes. Interestingly, CIBLIN genes include many Myc synthetic lethal (MycSL) hits from recent screens. CIBLIN genes include key regulators of heparan sulfate proteoglycan (HSPG) sulfation patterns, and lysyl oxidases involved in cross-linking and modification of the **extracellular matrix** (ECM). These genes and others suggest the CIBLIN repertoire services critical functions in ECM remodeling and cell migration in large-bodied bilaterians. Correspondingly, CIBLIN genes are co-expressed with Myc in cancer transcriptomes, and include a preponderance of known determinants of cancer progression and tumor aggression. We propose that CIBLIN gene research can improve our understanding of regulatory control of cellular growth in metazoans.

---

## e

- NCBI Gene
- GeneRIF
- Pubmed

| Search Term | Rank | Fields |
| --- | --- | --- |
| [Mm]igration | 6 | PubMed(2) |
| [Ee]xtracellular [Mm]atrix | 8 | PubMed(1) |

  
hide/show details for e

### [Mm]igration

#### PubMed

1. Identification of genes controlling germ cell **migration** and embryonic gonad formation in Drosophila. Gonadogenesis in the Drosophila embryo is a complex process involving numerous cellular migratory steps and cell-cell interactions. The mechanisms guiding germ cells to move through, recognize and adhere to specific cell types are poorly understood. In order to identify genes that are required for these processes, we have conducted an extensive mutagenesis of the third chromosome and screened for mutations disrupting germ cell **migration** at any point in embryonic development. Phenotypic analysis of these mutants demonstrates that germ cell **migration** can be broken down into discrete developmental steps, with each step requiring a specific set of genes. Many of these genes are involved in the development of gonadal mesoderm, the tissue that associates with germ cells to form the embryonic gonad. Moreover, mutations that we isolated affecting embryonic patterning as well as germ cell **migration** suggest that the origin of gonadal mesoderm lies within the eve domain of the developing mesoderm.
2. Filamin is required for ring canal assembly and actin organization during Drosophila oogenesis. The remodeling of the actin cytoskeleton is essential for cell **migration**, cell division, and cell morphogenesis. Actin-binding proteins play a pivotal role in reorganizing the actin cytoskeleton in response to signals exchanged between cells. In consequence, actin-binding proteins are increasingly a focus of investigations into effectors of cell signaling and the coordination of cellular behaviors within developmental processes. One of the first actin-binding proteins identified was filamin, or actin-binding protein 280 (ABP280). Filamin is required for cell **migration** (Cunningham et al. 1992), and mutations in human alpha-filamin (FLN1; Fox et al. 1998) are responsible for impaired **migration** of cerebral neurons and give rise to periventricular heterotopia, a disorder that leads to epilepsy and vascular disorders, as well as embryonic lethality. We report the identification and characterization of a mutation in Drosophila filamin, the homologue of human alpha-filamin. During oogenesis, filamin is concentrated in the ring canal structures that fortify arrested cleavage furrows and establish cytoplasmic bridges between cells of the germline. The major structural features common to other filamins are conserved in Drosophila filamin. Mutations in Drosophila filamin disrupt actin filament organization and compromise membrane integrity during oocyte development, resulting in female sterility. The genetic and molecular characterization of Drosophila filamin provides the first genetic model system for the analysis of filamin function and regulation during development.

### [Ee]xtracellular [Mm]atrix

#### PubMed

1. The detached locus encodes Drosophila Dystrophin, which acts with other components of the Dystrophin Associated Protein Complex to influence intercellular signalling in developing wing veins. Dystrophin and Dystroglycan are the two central components of the multimeric Dystrophin Associated Protein Complex, or DAPC, that is thought to provide a mechanical link between the **extracellular matrix** and the actin cytoskeleton, disruption of which leads to muscular dystrophy in humans. We present the characterization of the Drosophila 'crossveinless' mutation detached (det), and show that the gene encodes the fly ortholog of Dystrophin. Our genetic analysis shows that, in flies, Dystrophin is a non-essential gene, and the sole overt morphological defect associated with null mutations in the locus is the variable loss of the posterior crossvein that has been described for alleles of det. Null mutations in Drosophila Dystroglycan (Dg) are similarly viable and exhibit this crossvein defect, indicating that both of the central DAPC components have been co-opted for this atypical function of the complex. In the developing wing, the Drosophila DAPC affects the intercellular signalling pathways involved in vein specification. In det and Dg mutant wings, the early BMP signalling that initiates crossvein specification is not maintained, particularly in the pro-vein territories adjacent to the longitudinal veins, and this results in the production of a crossvein fragment in the intervein between the two longitudinal veins. Genetic interaction studies suggest that the DAPC may exert this effect indirectly by down-regulating Notch signalling in pro-vein territories, leading to enhanced BMP signalling in the intervein by diffusion of BMP ligands from the longitudinal veins.

---

## insc

- NCBI Gene
- GeneRIF
- Pubmed

| Search Term | Rank | Fields |
| --- | --- | --- |
| [Mm]igration | 6 | PubMed(7) |
| [Ee]xtracellular [Mm]atrix | 8 | PubMed(2) |

  
hide/show details for insc

### [Mm]igration

#### PubMed

1. Fusion from myoblasts to myotubes is dependent on the rolling stone gene (rost) of Drosophila. The development and differentiation of the body wall musculature in Drosophila are accompanied by changes in gene expression and cellular architecture. We isolated a Drosophila gene, termed rolling stone (rost), which, when mutated, specifically blocks the fusion of mononucleated cells to myotubes in the body wall musculature. beta 3 tubulin, which is an early marker for the onset of mesoderm differentiation, is still expressed in these cells. Gastrulation and mesoderm formation, as well as the development of the epidermis and of the central and peripheral nervous systems, appear quite normal in homozygous rolling stone embryos. Embryonic development stops shortly before hatching in a P-element-induced mutant, as well as in 16 EMS-induced alleles. In mutant embryos, other mesodermal derivatives such as the visceral mesoderm and the dorsal vessel, develop fairly normally and defects are restricted to the body wall musculature. Myoblasts remain as single mononucleated cells, which express muscle myosin, showing that the developmental program of gene expression proceeds. These myoblasts occur at positions corresponding to the locations of dorsal, ventral and pleural muscles, showing that the gene rolling stone is involved in cell fusion, a process that is independent of cell **migration** in these mutants. This genetic analysis has set the stage for a molecular analysis to clarify where the rolling stone action is manifested in the fusion process and thus gives insight into the complex regulating network controlling the differentiation of the body wall musculature.
2. Determination and development of the larval muscle pattern in Drosophila melanogaster. This review describes briefly what is known about the early steps of mesoderm differentiation in the fruitfly Drosophila melanogaster. After a summary of general aspects including mesoderm differentiation, mesoderm cell **migration** and subdivision of the mesoderm, more detail is given about the specification of muscle progenitor cells, due to their role as the earliest obvious landmarks in muscle fiber development in Drosophila. Particular focus is given to recent results on the role of asymmetric cell division in muscle differentiation. Furthermore a short summary of myoblast fusion is provided.
3. Muscle building; mechanisms of myotube guidance and attachment site selection. The complex muscle patterns of higher organisms arise as migrating myoblasts are guided toward and connect with specific attachment sites. We review here the current understanding of myotube **migration**, focusing on its dynamic nature and the few molecular cues that have been identified to date. Much of this knowledge comes from studies in Drosophila, where powerful methods for in vivo imaging and genetic manipulation can be used to tackle this important but largely unsolved problem in developmental biology.
4. The Hem protein mediates neuronal **migration** by inhibiting WAVE degradation and functions opposite of Abelson tyrosine kinase. In the nervous system, neurons form in different regions, then they migrate and occupy specific positions. We have previously shown that RP2/sib, a well-studied neuronal pair in the Drosophila ventral nerve cord (VNC), has a complex **migration** route. Here, we show that the Hem protein, via the WAVE complex, regulates **migration** of GMC-1 and its progeny RP2 neuron. In Hem or WAVE mutants, RP2 neuron either abnormally migrates, crossing the midline from one hemisegment to the contralateral hemisegment, or does not migrate at al and fail to send out its axon projection. We report that Hem regulates neuronal **migration** through stabilizing WAVE. Since Hem and WAVE normally form a complex, our data argues that in the absence of Hem, WAVE, which is presumably no longer in a complex, becomes susceptible to degradation. We also find that Abelson tyrosine kinase affects RP2 **migration** in a similar manner as Hem and WAVE, and appears to operate via WAVE. However, while Abl negatively regulates the levels of WAVE, it regulates **migration** via regulating the activity of WAVE. Our results also show that during the degradation of WAVE, Hem function is opposite to that of and downstream of Abl.
5. Microtubule-induced Pins/Galphai cortical polarity in Drosophila neuroblasts. Cortical polarity regulates cell division, **migration**, and differentiation. Microtubules induce cortical polarity in yeast, but few examples are known in metazoans. We show that astral microtubules, kinesin Khc-73, and Discs large (Dlg) induce cortical polarization of Pins/Galphai in Drosophila neuroblasts; this cortical domain is functional for generating spindle asymmetry, daughter-cell-size asymmetry, and distinct sibling fates. Khc-73 localizes to astral microtubule plus ends, and Dlg/Khc-73 and Dlg/Pins coimmunoprecipitate, suggesting that microtubules induce Pins/Galphai cortical polarity through Dlg/Khc-73 interactions. The microtubule/Khc-73/Dlg pathway acts in parallel to the well-characterized Inscuteable/Par pathway, but each provides unique spatial and temporal information: The Inscuteable/Par pathway initiates at prophase to coordinate neuroblast cortical polarity with CNS tissue polarity, whereas the microtubule/Khc-73/Dlg pathway functions at metaphase to coordinate neuroblast cortical polarity with the mitotic spindle axis. These results identify a role for microtubules in polarizing the neuroblast cortex, a fundamental step for generating cell diversity through asymmetric cell division.
6. Conservation of the RNA Transport Machineries and Their Coupling to Translation Control across Eukaryotes. Restriction of proteins to discrete subcellular regions is a common mechanism to establish cellular asymmetries and depends on a coordinated program of mRNA localization and translation control. Many processes from the budding of a yeast to the establishment of metazoan embryonic axes and the **migration** of human neurons, depend on this type of cell polarization. How factors controlling transport and translation assemble to regulate at the same time the movement and translation of transported mRNAs, and whether these mechanisms are conserved across kingdoms is not yet entirely understood. In this review we will focus on some of the best characterized examples of mRNA transport machineries, the "yeast locasome" as an example of RNA transport and translation control in unicellular eukaryotes, and on the Drosophila Bic-D/Egl/Dyn RNA localization machinery as an example of RNA transport in higher eukaryotes. This focus is motivated by the relatively advanced knowledge about the proteins that connect the localizing mRNAs to the transport motors and the many well studied proteins involved in translational control of specific transcripts that are moved by these machineries. We will also discuss whether the core of these RNA transport machineries and factors regulating mRNA localization and translation are conserved across eukaryotes.
7. The Drosophila Hem/Kette/Nap1 protein regulates asymmetric division of neural precursor cells by regulating localization of Inscuteable and Numb. The Hem/Kette/Nap1 protein is involved in many biological processes. We have recently reported that Hem is required for the normal **migration** of neurons in the Drosophila embryo. In this paper, we report that Hem regulates the asymmetric division of neural precursor cells. We find that a well-studied Hem/Kette mutant allele produces at least two main, but possibly more, phenotypic classes of mutant embryos, and these phenotypes correlate with variable levels of maternal wild type Hem protein in the developing embryo. While the weaker class exhibits weak axon guidance defect and the mis-**migration** of neurons, the stronger class causes severe axon guidance defects, mis-**migration** of neurons and symmetric division of ganglion mother cells (GMC) of the RP2/sib lineage. We also show that the basis for the loss of asymmetric division is due to non-localization of Inscuteable and Numb in GMC-1. A non-asymmetric Numb segregates to both daughter cells of GMC-1, which then prevents Notch signaling from specifying a sib fate. This causes both cells to adopt an RP2 fate. Furthermore, loss of function for Abelson tyrosine kinase also causes loss of asymmetric localization of Inscuteable and Numb and symmetric division of GMC-1, the loss of function for WAVE has a very weakly penetrant loss of asymmetry defect. These results define another role for Hem/Kette/Nap1 in a neural precursor cell during neurogenesis.

### [Ee]xtracellular [Mm]atrix

#### PubMed

1. Adherens junctions: new insight into assembly, modulation and function. Adherens junctions play pivotal roles in cell and tissue organization and patterning by mediating cell adhesion and cell signaling. These junctions consist of large multiprotein complexes that join the actin cytoskeleton to the plasma membrane to form adhesive contacts between cells or between cells and **extracellular matrix**. The best-known adherens junction is the zonula adherens (ZA) that forms a belt surrounding the apical pole of epithelial cells. Recent studies in Drosophila have further illuminated the structure of adherens junctions. Scaffolding proteins encoded by the stardust gene are novel components of the Crumbs complex, which plays a critical role in ZA assembly.1-3 The small GTPase Rap1 controls the symmetric re-assembly of the ZA after cell division.4 Finally, the asymmetric distribution of adherens junction material regulates spindle orientation during asymmetric cell division in the sensory organ lineage.
2. Transcription of the beta 1 tubulin (beta Tub56D) gene in apodemes is strictly dependent on muscle insertion during embryogenesis in Drosophila melanogaster. The insertion of the somatic musculature into the epidermis during embryogenesis of Drosophila melanogaster represents an excellent system for the investigation of cell-cell communication processes. Evidence from earlier experiments suggested that the expression of the beta 1 tubulin gene from D. melanogaster in the epidermal attachments may be dependent upon myotube insertion. Analysis of the transcription of the beta 1 tubulin gene in mutants for pair-rule or segment polarity genes revealed strong coupling of the induction process to myotube insertion. Involvement of **extracellular matrix** proteins of the integrin family could be excluded since beta 1 mRNA is detectable in the apodemes in mutants for the common subunit PS beta until muscles detach late in embryogenesis. Furthermore, the lack of specific muscles in the myogenic mutants rolling stone (rost) and not enough muscles (nem) eliminates beta 1 transcription exclusively in the corresponding apodemes. The data presented clearly show that the transcription of the beta 1 tubulin gene in the muscle attachment sites is activated by myotube insertion by a yet unidentified pathway.

---

## Hayan

- NCBI Gene
- GeneRIF
- Pubmed

| Search Term | Rank | Fields |
| --- | --- | --- |
| [Mm]etastasis | 12 | PubMed(1) |

  
hide/show details for Hayan

### [Mm]etastasis

#### PubMed

1. Drosophila as a model to study the role of blood cells in inflammation, innate immunity and cancer. Drosophila has a primitive yet effective blood system with three types of haemocytes which function throughout different developmental stages and environmental stimuli. Haemocytes play essential roles in tissue modeling during embryogenesis and morphogenesis, and also in innate immunity. The open circulatory system of Drosophila makes haemocytes ideal signal mediators to cells and tissues in response to events such as infection and wounding. The application of recently developed and sophisticated genetic tools to the relatively simple genome of Drosophila has made the fly a popular system for modeling human tumorigensis and **metastasis**. Drosophila is now used for screening and investigation of genes implicated in human leukemia and also in modeling development of solid tumors. This second line of research offers promising opportunities to determine the seemingly conflicting roles of blood cells in tumor progression and invasion. This review provides an overview of the signaling pathways conserved in Drosophila during haematopoiesis, haemostasis, innate immunity, wound healing and inflammation. We also review the most recent progress in the use of Drosophila as a cancer research model with an emphasis on the roles haemocytes can play in various cancer models and in the links between inflammation and cancer.

---

## Thor

- NCBI Gene
- GeneRIF
- Pubmed

| Search Term | Rank | Fields |
| --- | --- | --- |
| [Ff]ilopodia | 1 | PubMed(1) |
| [Mm]igration | 6 | PubMed(3) |
| ECM | 7 | PubMed(1) |
| [Ee]xtracellular [Mm]atrix | 8 | PubMed(2) |
| [Ii]nvasive | 10 | PubMed(2) |
| [Mm]etastasis | 12 | PubMed(1) |

  
hide/show details for Thor

### [Ff]ilopodia

#### PubMed

1. Gene regulatory networks controlling hematopoietic progenitor niche cell production and differentiation in the Drosophila lymph gland. Hematopoiesis occurs in two phases in Drosophila, with the first completed during embryogenesis and the second accomplished during larval development. The lymph gland serves as the venue for the final hematopoietic program, with this larval tissue well-studied as to its cellular organization and genetic regulation. While the medullary zone contains stem-like hematopoietic progenitors, the posterior signaling center (PSC) functions as a niche microenvironment essential for controlling the decision between progenitor maintenance versus cellular differentiation. In this report, we utilize a PSC-specific GAL4 driver and UAS-gene RNAi strains, to selectively knockdown individual gene functions in PSC cells. We assessed the effect of abrogating the function of 820 genes as to their requirement for niche cell production and differentiation. 100 genes were shown to be essential for normal niche development, with various loci placed into sub-groups based on the functions of their encoded protein products and known genetic interactions. For members of three of these groups, we characterized loss- and gain-of-function phenotypes. Gene function knockdown of members of the BAP chromatin-remodeling complex resulted in niche cells that do not express the hedgehog (hh) gene and fail to differentiate **filopodia** believed important for Hh signaling from the niche to progenitors. Abrogating gene function of various members of the insulin-like growth factor and TOR signaling pathways resulted in anomalous PSC cell production, leading to a defective niche organization. Further analysis of the Pten, TSC1, and TSC2 tumor suppressor genes demonstrated their loss-of-function condition resulted in severely altered blood cell homeostasis, including the abundant production of lamellocytes, specialized hemocytes involved in innate immune responses. Together, this cell-specific RNAi knockdown survey and mutant phenotype analyses identified multiple genes and their regulatory networks required for the normal organization and function of the hematopoietic progenitor niche within the lymph gland.

### [Mm]igration

#### PubMed

1. A drosophila model for EGFR-Ras and PI3K-dependent human glioma. Gliomas, the most common malignant tumors of the nervous system, frequently harbor mutations that activate the epidermal growth factor receptor (EGFR) and phosphatidylinositol-3 kinase (PI3K) signaling pathways. To investigate the genetic basis of this disease, we developed a glioma model in Drosophila. We found that constitutive coactivation of EGFR-Ras and PI3K pathways in Drosophila glia and glial precursors gives rise to neoplastic, invasive glial cells that create transplantable tumor-like growths, mimicking human glioma. Our model represents a robust organotypic and cell-type-specific Drosophila cancer model in which malignant cells are created by mutations in signature genes and pathways thought to be driving forces in a homologous human cancer. Genetic analyses demonstrated that EGFR and PI3K initiate malignant neoplastic transformation via a combinatorial genetic network composed primarily of other pathways commonly mutated or activated in human glioma, including the Tor, Myc, G1 Cyclins-Cdks, and Rb-E2F pathways. This network acts synergistically to coordinately stimulate cell cycle entry and progression, protein translation, and inappropriate cellular growth and **migration**. In particular, we found that the fly orthologs of CyclinE, Cdc25, and Myc are key rate-limiting genes required for glial neoplasia. Moreover, orthologs of Sin1, Rictor, and Cdk4 are genes required only for abnormal neoplastic glial proliferation but not for glial development. These and other genes within this network may represent important therapeutic targets in human glioma.
2. Genes conserved in bilaterians but jointly lost with Myc during nematode evolution are enriched in cell proliferation and cell **migration** functions. Animals use a stereotypical set of developmental genes to build body architectures of varying sizes and organizational complexity. Some genes are critical to developmental patterning, while other genes are important to physiological control of growth. However, growth regulator genes may not be as important in small-bodied "micro-metazoans" such as nematodes. Nematodes use a simplified developmental strategy of lineage-based cell fate specifications to produce an adult bilaterian body composed of a few hundreds of cells. Nematodes also lost the MYC proto-oncogenic regulator of cell proliferation. To identify additional regulators of cell proliferation that were lost with MYC, we computationally screened and determined 839 high-confidence genes that are conserved in bilaterians/lost in nematodes (CIBLIN genes). We find that 30 % of all CIBLIN genes encode transcriptional regulators of cell proliferation, epithelial-to-mesenchyme transitions, and other processes. Over 50 % of CIBLIN genes are unnamed genes in Drosophila, suggesting that there are many understudied genes. Interestingly, CIBLIN genes include many Myc synthetic lethal (MycSL) hits from recent screens. CIBLIN genes include key regulators of heparan sulfate proteoglycan (HSPG) sulfation patterns, and lysyl oxidases involved in cross-linking and modification of the extracellular matrix (ECM). These genes and others suggest the CIBLIN repertoire services critical functions in ECM remodeling and cell **migration** in large-bodied bilaterians. Correspondingly, CIBLIN genes are co-expressed with Myc in cancer transcriptomes, and include a preponderance of known determinants of cancer progression and tumor aggression. We propose that CIBLIN gene research can improve our understanding of regulatory control of cellular growth in metazoans.
3. A conserved role for syndecan family members in the regulation of whole-body energy metabolism. Syndecans are a family of type-I transmembrane proteins that are involved in cell-matrix adhesion, **migration**, neuronal development, and inflammation. Previous quantitative genetic studies pinpointed Drosophila Syndecan (dSdc) as a positional candidate gene affecting variation in fat storage between two Drosophila melanogaster strains. Here, we first used quantitative complementation tests with dSdc mutants to confirm that natural variation in this gene affects variability in Drosophila fat storage. Next, we examined the effects of a viable dSdc mutant on Drosophila whole-body energy metabolism and associated traits. We observed that young flies homozygous for the dSdc mutation had reduced fat storage and slept longer than homozygous wild-type flies. They also displayed significantly reduced metabolic rate, lower expression of spargel (the Drosophila homologue of PGC-1), and reduced mitochondrial respiration. Compared to control flies, dSdc mutants had lower expression of brain insulin-like peptides, were less fecund, more sensitive to starvation, and had reduced life span. Finally, we tested for association between single nucleotide polymorphisms (SNPs) in the human SDC4 gene and variation in body composition, metabolism, glucose homeostasis, and sleep traits in a cohort of healthy early pubertal children. We found that SNP rs4599 was significantly associated with resting energy expenditure (P = 0.001 after Bonferroni correction) and nominally associated with fasting glucose levels (P = 0.01) and sleep duration (P = 0.044). On average, children homozygous for the minor allele had lower levels of glucose, higher resting energy expenditure, and slept shorter than children homozygous for the common allele. We also observed that SNP rs1981429 was nominally associated with lean tissue mass (P = 0.035) and intra-abdominal fat (P = 0.049), and SNP rs2267871 with insulin sensitivity (P = 0.037). Collectively, our results in Drosophila and humans argue that syndecan family members play a key role in the regulation of body metabolism.

### ECM

#### PubMed

1. Genes conserved in bilaterians but jointly lost with Myc during nematode evolution are enriched in cell proliferation and cell migration functions. Animals use a stereotypical set of developmental genes to build body architectures of varying sizes and organizational complexity. Some genes are critical to developmental patterning, while other genes are important to physiological control of growth. However, growth regulator genes may not be as important in small-bodied "micro-metazoans" such as nematodes. Nematodes use a simplified developmental strategy of lineage-based cell fate specifications to produce an adult bilaterian body composed of a few hundreds of cells. Nematodes also lost the MYC proto-oncogenic regulator of cell proliferation. To identify additional regulators of cell proliferation that were lost with MYC, we computationally screened and determined 839 high-confidence genes that are conserved in bilaterians/lost in nematodes (CIBLIN genes). We find that 30 % of all CIBLIN genes encode transcriptional regulators of cell proliferation, epithelial-to-mesenchyme transitions, and other processes. Over 50 % of CIBLIN genes are unnamed genes in Drosophila, suggesting that there are many understudied genes. Interestingly, CIBLIN genes include many Myc synthetic lethal (MycSL) hits from recent screens. CIBLIN genes include key regulators of heparan sulfate proteoglycan (HSPG) sulfation patterns, and lysyl oxidases involved in cross-linking and modification of the extracellular matrix (**ECM**). These genes and others suggest the CIBLIN repertoire services critical functions in **ECM** remodeling and cell migration in large-bodied bilaterians. Correspondingly, CIBLIN genes are co-expressed with Myc in cancer transcriptomes, and include a preponderance of known determinants of cancer progression and tumor aggression. We propose that CIBLIN gene research can improve our understanding of regulatory control of cellular growth in metazoans.

### [Ee]xtracellular [Mm]atrix

#### PubMed

1. Genes conserved in bilaterians but jointly lost with Myc during nematode evolution are enriched in cell proliferation and cell migration functions. Animals use a stereotypical set of developmental genes to build body architectures of varying sizes and organizational complexity. Some genes are critical to developmental patterning, while other genes are important to physiological control of growth. However, growth regulator genes may not be as important in small-bodied "micro-metazoans" such as nematodes. Nematodes use a simplified developmental strategy of lineage-based cell fate specifications to produce an adult bilaterian body composed of a few hundreds of cells. Nematodes also lost the MYC proto-oncogenic regulator of cell proliferation. To identify additional regulators of cell proliferation that were lost with MYC, we computationally screened and determined 839 high-confidence genes that are conserved in bilaterians/lost in nematodes (CIBLIN genes). We find that 30 % of all CIBLIN genes encode transcriptional regulators of cell proliferation, epithelial-to-mesenchyme transitions, and other processes. Over 50 % of CIBLIN genes are unnamed genes in Drosophila, suggesting that there are many understudied genes. Interestingly, CIBLIN genes include many Myc synthetic lethal (MycSL) hits from recent screens. CIBLIN genes include key regulators of heparan sulfate proteoglycan (HSPG) sulfation patterns, and lysyl oxidases involved in cross-linking and modification of the **extracellular matrix** (ECM). These genes and others suggest the CIBLIN repertoire services critical functions in ECM remodeling and cell migration in large-bodied bilaterians. Correspondingly, CIBLIN genes are co-expressed with Myc in cancer transcriptomes, and include a preponderance of known determinants of cancer progression and tumor aggression. We propose that CIBLIN gene research can improve our understanding of regulatory control of cellular growth in metazoans.
2. **Extracellular matrix**-modulated Heartless signaling in Drosophila blood progenitors regulates their differentiation via a Ras/ETS/FOG pathway and target of rapamycin function. Maintenance of hematopoietic progenitors ensures a continuous supply of blood cells during the lifespan of an organism. Thus, understanding the molecular basis for progenitor maintenance is a continued focus of investigation. A large pool of undifferentiated blood progenitors are maintained in the Drosophila hematopoietic organ, the larval lymph gland, by a complex network of signaling pathways that are mediated by niche-, progenitor-, or differentiated hemocyte-derived signals. In this study we examined the function of the Drosophila fibroblast growth factor receptor (FGFR), Heartless, a critical regulator of early lymph gland progenitor specification in the late embryo, during larval lymph gland hematopoiesis. Activation of Heartless signaling in hemocyte progenitors by its two ligands, Pyramus and Thisbe, is both required and sufficient to induce progenitor differentiation and formation of the plasmatocyte-rich lymph gland cortical zone. We identify two transcriptional regulators that function downstream of Heartless signaling in lymph gland progenitors, the ETS protein, Pointed, and the Friend-of-GATA (FOG) protein, U-shaped, which are required for this Heartless-induced differentiation response. Furthermore, cross-talk of Heartless and target of rapamycin signaling in hemocyte progenitors is required for lamellocyte differentiation downstream of Thisbe-mediated Heartless activation. Finally, we identify the Drosophila heparan sulfate proteoglycan, Trol, as a critical negative regulator of Heartless ligand signaling in the lymph gland, demonstrating that sequestration of differentiation signals by the **extracellular matrix** is a unique mechanism employed in blood progenitor maintenance that is of potential relevance to many other stem cell niches.

### [Ii]nvasive

#### PubMed

1. A drosophila model for EGFR-Ras and PI3K-dependent human glioma. Gliomas, the most common malignant tumors of the nervous system, frequently harbor mutations that activate the epidermal growth factor receptor (EGFR) and phosphatidylinositol-3 kinase (PI3K) signaling pathways. To investigate the genetic basis of this disease, we developed a glioma model in Drosophila. We found that constitutive coactivation of EGFR-Ras and PI3K pathways in Drosophila glia and glial precursors gives rise to neoplastic, **invasive** glial cells that create transplantable tumor-like growths, mimicking human glioma. Our model represents a robust organotypic and cell-type-specific Drosophila cancer model in which malignant cells are created by mutations in signature genes and pathways thought to be driving forces in a homologous human cancer. Genetic analyses demonstrated that EGFR and PI3K initiate malignant neoplastic transformation via a combinatorial genetic network composed primarily of other pathways commonly mutated or activated in human glioma, including the Tor, Myc, G1 Cyclins-Cdks, and Rb-E2F pathways. This network acts synergistically to coordinately stimulate cell cycle entry and progression, protein translation, and inappropriate cellular growth and migration. In particular, we found that the fly orthologs of CyclinE, Cdc25, and Myc are key rate-limiting genes required for glial neoplasia. Moreover, orthologs of Sin1, Rictor, and Cdk4 are genes required only for abnormal neoplastic glial proliferation but not for glial development. These and other genes within this network may represent important therapeutic targets in human glioma.
2. Reproductive arrest and stress resistance in winter-acclimated Drosophila suzukii. Overwintering insects must survive the multiple-stress environment of winter, which includes low temperatures, reduced food and water availability, and cold-active pathogens. Many insects overwinter in diapause, a developmental arrest associated with high stress tolerance. Drosophila suzukii (Diptera: Drosophilidae), spotted wing drosophila, is an **invasive** agricultural pest worldwide. Its ability to overwinter and therefore establish in temperate regions could have severe implications for fruit crop industries. We demonstrate here that laboratory populations of Canadian D. suzukii larvae reared under short-day, low temperature, conditions develop into dark 'winter morph' adults similar to those reported globally from field captures, and observed by us in southern Ontario, Canada. These winter-acclimated adults have delayed reproductive maturity, enhanced cold tolerance, and can remain active at low temperatures, although they do not have the increased desiccation tolerance or survival of fungal pathogen challenges that might be expected from a more heavily melanised cuticle. Winter-acclimated female D. suzukii have underdeveloped ovaries and altered transcript levels of several genes associated with reproduction and stress. While superficially indicative of reproductive diapause, the delayed reproductive maturity of winter-acclimated D. suzukii appears to be temperature-dependent, not regulated by photoperiod, and is thus unlikely to be 'true' diapause. The traits of this 'winter morph', however, likely facilitate overwintering in southern Canada, and have probably contributed to the global success of this fly as an **invasive** species.

### [Mm]etastasis

#### PubMed

1. Drosophila as a model to study the role of blood cells in inflammation, innate immunity and cancer. Drosophila has a primitive yet effective blood system with three types of haemocytes which function throughout different developmental stages and environmental stimuli. Haemocytes play essential roles in tissue modeling during embryogenesis and morphogenesis, and also in innate immunity. The open circulatory system of Drosophila makes haemocytes ideal signal mediators to cells and tissues in response to events such as infection and wounding. The application of recently developed and sophisticated genetic tools to the relatively simple genome of Drosophila has made the fly a popular system for modeling human tumorigensis and **metastasis**. Drosophila is now used for screening and investigation of genes implicated in human leukemia and also in modeling development of solid tumors. This second line of research offers promising opportunities to determine the seemingly conflicting roles of blood cells in tumor progression and invasion. This review provides an overview of the signaling pathways conserved in Drosophila during haematopoiesis, haemostasis, innate immunity, wound healing and inflammation. We also review the most recent progress in the use of Drosophila as a cancer research model with an emphasis on the roles haemocytes can play in various cancer models and in the links between inflammation and cancer.

---

## Def

- NCBI Gene
- GeneRIF
- Pubmed

| Search Term | Rank | Fields |
| --- | --- | --- |
| [Mm]igration | 6 | PubMed(1) |
| [Mm]etastasis | 12 | PubMed(1) |

  
hide/show details for Def

### [Mm]igration

#### PubMed

1. The unconventional myosin CRINKLED and its mammalian orthologue MYO7A regulate caspases in their signalling roles. Caspases provide vital links in non-apoptotic regulatory networks controlling inflammation, compensatory proliferation, morphology and cell **migration**. How caspases are activated under non-apoptotic conditions and process a selective set of substrates without killing the cell remain enigmatic. Here we find that the Drosophila unconventional myosin CRINKLED (CK) selectively interacts with the initiator caspase DRONC and regulates some of its non-apoptotic functions. Loss of CK in the arista, border cells or proneural clusters of the wing imaginal discs affects DRONC-dependent patterning. Our data indicate that CK acts as substrate adaptor, recruiting SHAGGY46/GSK3-β to DRONC, thereby facilitating caspase-mediated cleavage and localized modulation of kinase activity. Similarly, the mammalian CK counterpart, MYO7A, binds to and impinges on CASPASE-8, revealing a new regulatory axis affecting receptor interacting protein kinase-1 (RIPK1)>CASPASE-8 signalling. Together, our results expose a conserved role for unconventional myosins in transducing caspase-dependent regulation of kinases, allowing them to take part in specific signalling events.

### [Mm]etastasis

#### PubMed

1. Drosophila as a model to study the role of blood cells in inflammation, innate immunity and cancer. Drosophila has a primitive yet effective blood system with three types of haemocytes which function throughout different developmental stages and environmental stimuli. Haemocytes play essential roles in tissue modeling during embryogenesis and morphogenesis, and also in innate immunity. The open circulatory system of Drosophila makes haemocytes ideal signal mediators to cells and tissues in response to events such as infection and wounding. The application of recently developed and sophisticated genetic tools to the relatively simple genome of Drosophila has made the fly a popular system for modeling human tumorigensis and **metastasis**. Drosophila is now used for screening and investigation of genes implicated in human leukemia and also in modeling development of solid tumors. This second line of research offers promising opportunities to determine the seemingly conflicting roles of blood cells in tumor progression and invasion. This review provides an overview of the signaling pathways conserved in Drosophila during haematopoiesis, haemostasis, innate immunity, wound healing and inflammation. We also review the most recent progress in the use of Drosophila as a cancer research model with an emphasis on the roles haemocytes can play in various cancer models and in the links between inflammation and cancer.

---

## GstD2

- NCBI Gene
- GeneRIF
- Pubmed

| Search Term | Rank | Fields |
| --- | --- | --- |
| [Ii]nvasive | 10 | PubMed(1) |

  
hide/show details for GstD2

### [Ii]nvasive

#### PubMed

1. Neurodegeneration of Drosophila drop-dead mutants is associated with hypoxia in the brain. The Drosophila drop-dead (drd) mutant undergoes massive brain degeneration, resulting in sudden death. drd encodes a multi-pass membrane protein possessing nose resistant to fluoxetine (NRF) and putative acyltransferase domains. However, the etiology of brain degeneration that occurs in drd mutant flies is still poorly understood. Herein, we show that drd neurodegeneration may be because of an oxygen deficit in the brain. We found that DRD protein is selectively expressed in cells secreting cuticular and eggshell layers. These layers exhibit blue fluorescence upon UV excitation, which is reduced in drd flies. The drd tracheal air sacs lacking blue fluorescence collapse, which likely contributes to hypoxia. Consistently, genes induced in hypoxia are up-regulated in drd flies. Feeding of anti-reactive oxygen species agents partially rescue the drd from sudden death. We propose that drd flies can provide a non-**invasive** animal model for hypoxia-induced cell death.

---

## CG9733

- NCBI Gene
- GeneRIF
- Pubmed

| Search Term | Rank | Fields |
| --- | --- | --- |
| [Ff]ilopodia | 1 | PubMed(1) |

  
hide/show details for CG9733

### [Ff]ilopodia

#### PubMed

1. Gene regulatory networks controlling hematopoietic progenitor niche cell production and differentiation in the Drosophila lymph gland. Hematopoiesis occurs in two phases in Drosophila, with the first completed during embryogenesis and the second accomplished during larval development. The lymph gland serves as the venue for the final hematopoietic program, with this larval tissue well-studied as to its cellular organization and genetic regulation. While the medullary zone contains stem-like hematopoietic progenitors, the posterior signaling center (PSC) functions as a niche microenvironment essential for controlling the decision between progenitor maintenance versus cellular differentiation. In this report, we utilize a PSC-specific GAL4 driver and UAS-gene RNAi strains, to selectively knockdown individual gene functions in PSC cells. We assessed the effect of abrogating the function of 820 genes as to their requirement for niche cell production and differentiation. 100 genes were shown to be essential for normal niche development, with various loci placed into sub-groups based on the functions of their encoded protein products and known genetic interactions. For members of three of these groups, we characterized loss- and gain-of-function phenotypes. Gene function knockdown of members of the BAP chromatin-remodeling complex resulted in niche cells that do not express the hedgehog (hh) gene and fail to differentiate **filopodia** believed important for Hh signaling from the niche to progenitors. Abrogating gene function of various members of the insulin-like growth factor and TOR signaling pathways resulted in anomalous PSC cell production, leading to a defective niche organization. Further analysis of the Pten, TSC1, and TSC2 tumor suppressor genes demonstrated their loss-of-function condition resulted in severely altered blood cell homeostasis, including the abundant production of lamellocytes, specialized hemocytes involved in innate immune responses. Together, this cell-specific RNAi knockdown survey and mutant phenotype analyses identified multiple genes and their regulatory networks required for the normal organization and function of the hematopoietic progenitor niche within the lymph gland.

---

## dl

- NCBI Gene
- GeneRIF
- Pubmed

| Search Term | Rank | Fields |
| --- | --- | --- |
| [Ff]ilopodia | 1 | PubMed(4); GeneRIFs(2) |
| [Mm]igration | 6 | Process(2); PubMed(26) |
| [Ee]xtracellular [Mm]atrix | 8 | PubMed(7) |
| [Ii]nvasive | 10 | PubMed(1) |
| [Mm]etastasis | 12 | PubMed(7) |
| [Cc]ell [Ii]nvasion | 13 | PubMed(2) |

  
hide/show details for dl

### [Ff]ilopodia

#### PubMed

1. Delta-promoted **filopodia** mediate long-range lateral inhibition in Drosophila. Drosophila thoracic mechanosensory bristles originate from cells that are singled out from 'proneural' groups of competent epithelial cells. Neural competence is restricted to individual sensory organ precursors (SOPs) by Delta/Notch-mediated 'lateral inhibition', whereas other cells in the proneural field adopt an epidermal fate. The precursors of the large macrochaetes differentiate separately from individual proneural clusters that comprise about 20-30 cells or as heterochronic pairs from groups of more than 100 cells, whereas the precursors of the small regularly spaced microchaetes emerge from even larger proneural fields. This indicates that lateral inhibition might act over several cell diameters; it was difficult to reconcile with the fact that the inhibitory ligand Delta is membrane-bound until the observation that SOPs frequently extend thin processes offered an attractive hypothesis. Here we show that the extension of these planar **filopodia**--a common attribute of wing imaginal disc cells--is promoted by Delta and that their experimental suppression reduces Notch signalling in distant cells and increases bristle density in large proneural groups, showing that these membrane specializations mediate long-range lateral inhibition.
2. How Notch establishes longitudinal axon connections between successive segments of the Drosophila CNS. Development of the segmented central nerve cords of vertebrates and invertebrates requires connecting successive neuromeres. Here, we show both how a pathway is constructed to guide pioneer axons between segments of the Drosophila CNS, and how motility of the pioneers along that pathway is promoted. First, canonical Notch signaling in specialized glial cells causes nearby differentiating neurons to extrude a mesh of fine projections, and shapes that mesh into a continuous carpet that bridges from segment to segment, hugging the glial surface. This is the direct substratum that pioneer axons follow as they grow. Simultaneously, Notch uses an alternate, non-canonical signaling pathway in the pioneer growth cones themselves, promoting their motility by suppressing Abl signaling to stimulate **filopodia**l growth while presumably reducing substratum adhesion. This propels the axons as they establish the connection between successive segments.
3. Dynamic **filopodia** transmit intermittent Delta-Notch signaling to drive pattern refinement during lateral inhibition. The organization of bristles on the Drosophila notum has long served as a popular model of robust tissue patterning. During this process, membrane-tethered Delta activates intracellular Notch signaling in neighboring epithelial cells, which inhibits Delta expression. This induces lateral inhibition, yielding a pattern in which each Delta-expressing mechanosensory organ precursor cell in the epithelium is surrounded on all sides by cells with active Notch signaling. Here, we show that conventional models of Delta-Notch signaling cannot account for bristle spacing or the gradual refinement of this pattern. Instead, the pattern refinement we observe using live imaging is dependent upon dynamic, basal actin-based **filopodia** and can be quantitatively reproduced by simulations of lateral inhibition incorporating Delta-Notch signaling by transient **filopodia**l contacts between nonneighboring cells. Significantly, the intermittent signaling induced by these **filopodia**l dynamics generates a type of structured noise that is uniquely suited to the generation of well-ordered, tissue-wide epithelial patterns.
4. Notch and Numb are required for normal migration of peripheral glia in Drosophila. A prominent feature of glial cells is their ability to migrate along axons to finally wrap and insulate them. In the embryonic Drosophila PNS, most glial cells are born in the CNS and have to migrate to reach their final destinations. To understand how migration of the peripheral glia is regulated, we have conducted a genetic screen looking for mutants that disrupt the normal glial pattern. Here we present an analysis of two of these mutants: Notch and numb. Complete loss of Notch function leads to an increase in the number of glial cells. Embryos hemizygous for the weak Notch(B-8X) allele display an irregular migration phenotype and mutant glial cells show an increased formation of **filopodia**-like structures. A similar phenotype occurs in embryos carrying the Notch(ts1) allele when shifted to the restrictive temperature during the glial cell migration phase, suggesting that Notch must be activated during glial migration. This is corroborated by the fact that cell-specific reduction of Notch activity in glial cells by directed numb expression also results in similar migration phenotypes. Since the glial migration phenotypes of Notch and numb mutants resemble each other, our data support a model where the precise temporal and quantitative regulation of Numb and Notch activity is not only required during fate decisions but also later during glial differentiation and migration.

#### GeneRIFs

1. simulations of lateral inhibition incorporating Delta-Notch signaling by transient **filopodia**l contacts between nonneighboring cells
2. extension of planar **filopodia** is promoted by Delta; experimental suppression reduces Notch signalling in distant cells and increases bristle density in large proneural groups, showing these membrane specializations mediate long-range lateral inhibition

### [Mm]igration

#### Process

1. border follicle cell **migration**
2. glial cell **migration**

#### PubMed

1. Identification of genes controlling germ cell **migration** and embryonic gonad formation in Drosophila. Gonadogenesis in the Drosophila embryo is a complex process involving numerous cellular migratory steps and cell-cell interactions. The mechanisms guiding germ cells to move through, recognize and adhere to specific cell types are poorly understood. In order to identify genes that are required for these processes, we have conducted an extensive mutagenesis of the third chromosome and screened for mutations disrupting germ cell **migration** at any point in embryonic development. Phenotypic analysis of these mutants demonstrates that germ cell **migration** can be broken down into discrete developmental steps, with each step requiring a specific set of genes. Many of these genes are involved in the development of gonadal mesoderm, the tissue that associates with germ cells to form the embryonic gonad. Moreover, mutations that we isolated affecting embryonic patterning as well as germ cell **migration** suggest that the origin of gonadal mesoderm lies within the eve domain of the developing mesoderm.
2. Muscle building; mechanisms of myotube guidance and attachment site selection. The complex muscle patterns of higher organisms arise as migrating myoblasts are guided toward and connect with specific attachment sites. We review here the current understanding of myotube **migration**, focusing on its dynamic nature and the few molecular cues that have been identified to date. Much of this knowledge comes from studies in Drosophila, where powerful methods for in vivo imaging and genetic manipulation can be used to tackle this important but largely unsolved problem in developmental biology.
3. Cell fate choices in Drosophila tracheal morphogenesis. The Drosophila tracheal system is a branched tubular structure that supplies air to target tissues. The elaborate tracheal morphology is shaped by two linked inductive processes, one involving the choice of cell fates, and the other a guided cell **migration**. We will describe the molecular basis for these processes, and the allocation of cell fate decisions to four temporal hierarchies. First, tracheal placodes are specified within the embryonic ectoderm. Subsequently, branch fates are allocated within the tracheal placodes, prior to **migration**. Localized presentation of the FGF ligand, Branchless, to tracheal cells that express the FGF receptor, Breathless, guides **migration**. Once cell **migration** is initiated, distinct cell fates are determined within each migrating branch. Finally, inhibitory feedback mechanisms ensure the correct assignment of these fates. Tracheal cell fate choices are determined by signaling cascades triggered by signals emanating from the tracheal cells, as well as by ligands produced by adjacent tissues.
4. Opposing interactions between Drosophila cut and the C/EBP encoded by slow border cells direct apical constriction and epithelial invagination. Stage 10 of Drosophila oogenesis can be subdivided into stages 10A and 10B based on a change in the morphology of the centripetal follicle cells (FC) from a columnar to an apically constricted shape. This coordinated cell shape change drives epithelial cell sheet involution between the oocyte and nurse cell complex which patterns the operculum structure of the mature eggshell. We have shown previously that proper centripetal FC **migration** requires transient expression of the C/EBP encoded by slow border cells (slbo) at 10A, due in part to Notch activation followed by slbo autorepression (Levine et al., 2007). Here we show that decreased slbo expression in the centripetal FC coincides with increased expression of the transcription factor Cut, a Cut/Cux/CDP family member, at 10B. The 10A/10B temporal switch from Slbo to Cut expression is refined by both cross repression between Slbo and Cut, Slbo auto repression and Cut auto activation. High Cut levels are necessary and sufficient to direct polarized, supracellular accumulation of Actin, DE-cadherin and Armadillo associated with apical constriction of the centripetal FC. Separately, Slbo in the border cell rosette and Cut in the pole cells have antagonistic interactions to restrict Fas2 accumulation to the pole cells, which is important for proper border cell **migration**. The opposing effects of Cut and Slbo in these two tissues reflect the opposing interactions between their respective mammalian homologs CAAT Displacement Protein (CDP; now CUX1) and CAAT Enhancer Binding Protein (C/EBP) in tissue culture.
5. Integration of epithelial patterning and morphogenesis in Drosophila ovarian follicle cells. Drosophila oogenesis involves the coordinated development of germ cells and an overlying follicular epithelium. The follicle cells provide a genetically tractable system to investigate the cell biology of patterning and morphogenesis. Follicle cells initially form a cuboidal epithelium surrounding a syncytium of nurse cells and oocyte. Epithelial structure is maintained as these cells reorganize to create the three dimensional architecture of the eggshell. Both long-range and short-range cell-cell communications pattern the domains of follicle cells that will create specific eggshell structures. After terminal differentiation to deposit the eggshell proteins, the follicle cells die. This review summarizes recent progress in understanding the cell-cell communication that orchestrates follicle cell patterning and **migration**s. DE-cadherin-mediated adhesion is important at several steps in egg chamber formation and follicle cell **migration**. Notch signaling is critical during each successive round of patterning and **migration**. Integration of bone morphogenetic protein (BMP) and epidermal growth factor (EGF) signals patterns the elaborate structures of the dorsal-anterior eggshell.
6. The ETS domain transcriptional repressor Anterior open inhibits MAP kinase and Wingless signaling to couple tracheal cell fate with branch identity. Cells at the tips of budding branches in the Drosophila tracheal system generate two morphologically different types of seamless tubes. Terminal cells (TCs) form branched lumenized extensions that mediate gas exchange at target tissues, whereas fusion cells (FCs) form ring-like connections between adjacent tracheal metameres. Each tracheal branch contains a specific set of TCs, FCs, or both, but the mechanisms that select between the two tip cell types in a branch-specific fashion are not clear. Here, we show that the ETS domain transcriptional repressor anterior open (aop) is dispensable for directed tracheal cell **migration**, but plays a key role in tracheal tip cell fate specification. Whereas aop globally inhibits TC and FC specification, MAPK signaling overcomes this inhibition by triggering degradation of Aop in tip cells. Loss of aop function causes excessive FC and TC specification, indicating that without Aop-mediated inhibition, all tracheal cells are competent to adopt a specialized fate. We demonstrate that Aop plays a dual role by inhibiting both MAPK and Wingless signaling, which induce TC and FC fate, respectively. In addition, the branch-specific choice between the two seamless tube types depends on the tracheal branch identity gene spalt major, which is sufficient to inhibit TC specification. Thus, a single repressor, Aop, integrates two different signals to couple tip cell fate selection with branch identity. The switch from a branching towards an anastomosing tip cell type may have evolved with the acquisition of a main tube that connects separate tracheal primordia to generate a tubular network.
7. Transcriptional control in embryonic Drosophila midline guidance assessed through a whole genome approach. During the development of the Drosophila central nervous system the process of midline crossing is orchestrated by a number of guidance receptors and ligands. Many key axon guidance molecules have been identified in both invertebrates and vertebrates, but the transcriptional regulation of growth cone guidance remains largely unknown. It is established that translational regulation plays a role in midline crossing, and there are indications that transcriptional regulation is also involved. To investigate this issue, we conducted a genome-wide study of transcription in Drosophila embryos using wild type and a number of well-characterized Drosophila guidance mutants and transgenics. We also analyzed a previously published microarray time course of Drosophila embryonic development with an axon guidance focus.Using hopach, a novel clustering method which is well suited to microarray data analysis, we identified groups of genes with similar expression patterns across guidance mutants and transgenics. We then systematically characterized the resulting clusters with respect to their relevance to axon guidance using two complementary controlled vocabularies: the Gene Ontology (GO) and anatomical annotations of the Atlas of Pattern of Gene Expression (APoGE) in situ hybridization database. The analysis indicates that regulation of gene expression does play a role in the process of axon guidance in Drosophila. We also find a strong link between axon guidance and hemocyte **migration**, a result that agrees with mounting evidence that axon guidance molecules are co-opted in vertebrate vascularization. Cell cyclin activity in the context of axon guidance is also suggested from our array data. RNA and protein expression patterns of cell cyclins in axon guidance mutants and transgenics support this possible link.This study provides important insights into the regulation of axon guidance in vivo.
8. The Drosophila toucan (toc) gene is required in germline cells for the somatic cell patterning during oogenesis. We have characterized a new gene, called toucan, that is expressed and required in germline cells to promote proper differentiation of the somatic follicle cells. toucan mutant ovaries are defective in (i) the enclosure of newly formed germline cysts by the follicle cells, (ii) the formation of interfollicular stalks, (iii) the **migration** of the follicle cells over the oocyte and (iv) the formation of the eggshell. Overexpression of a toucan cDNA in the germline leads to the production of longer interfollicular stalks than wild-type ovaries, a phenotype that is the exact opposite of the toucan mutant phenotype. This observation shows that the formation of the interfollicular stalks depends not only on interactions among the somatic cells but also requires a germline signal. Moreover, dominant interactions have been observed between toucan and certain alleles of the daughterless, Notch and Delta genes, each of which is required in the somatic cells for the formation of egg chambers. toucan encodes for a large protein with a coiled-coil domain but has no other homology with known proteins. We propose that toucan participates in the production or localization of a germline-specific signal(s) that is required for the patterning of the follicular epithelium.
9. Cell lineage: compartments and Capricious. Until recently, little was known about the mechanisms that prevent cell **migration** across compartment boundaries in Drosophila. A new report suggests that the lineage restriction between the dorsal and ventral compartments of the developing wing relies in part on the transmembrane proteins, Capricious and Tartan.
10. Bunched sets a boundary for Notch signaling to pattern anterior eggshell structures during Drosophila oogenesis. Organized boundaries between different cell fates are critical in patterning and organogenesis. In some tissues, long-range signals position a boundary, and local Notch signaling maintains it. How Notch activity is restricted to boundary regions is not well understood. During Drosophila oogenesis, the long-range signals EGF and Dpp regulate expression of bunched (bun), which encodes a homolog of mammalian transcription factors TSC-22 and GILZ. Here, we show that bun establishes a boundary for Notch signaling in the follicle cell epithelium. Notch signaling is active in anterior follicle cells and is required for concurrent follicle cell reorganizations including centripetal **migration** and operculum formation. bun is required in posterior columnar follicle cells to repress the centripetal **migration** fate, including gene expression, cell shape changes and accumulation of cytoskeletal components. bun mutant clones adjacent to the centripetally migrating follicle cells showed ectopic Notch responses. bun is necessary, but not sufficient, to down-regulate Serrate protein levels throughout the follicular epithelium. These data indicate that Notch signaling is necessary, but not sufficient, for centripetal **migration** and that bun regulates the level of Notch stimulation to position the boundary between centripetally migrating and stationary columnar follicle cells.
11. Delta signaling from the germ line controls the proliferation and differentiation of the somatic follicle cells during Drosophila oogenesis. The body axes of Drosophila are established during oogenesis through reciprocal interactions between the germ line cells and the somatic follicle cells that surround them. The Notch pathway is required at two stages in this process: first, for the **migration** of the follicle cells around the germ line cyst and, later, for the polarization of the anterior-posterior (A-P) axis of the oocyte. Its function in these events, however, has remained controversial. Using clonal analysis, we show that Notch signaling controls cell proliferation and differentiation in the whole follicular epithelium. Notch mutant follicle cells remain in a precursor state and fail to switch from the mitotic cell cycle to the endocycle. Furthermore, removal of Delta from the germ line produces an identical phenotype, showing that Delta signals from the germ cells to control the timing of follicle cell differentiation. This explains the axis formation defects in Notch mutants, which arise because undifferentiated posterior follicle cells cannot signal to polarize the oocyte. Delta also signals from the germ line to Notch in the soma earlier in oogenesis to control the differentiation of the polar and stalk follicle cells. The germ line therefore regulates the development of the follicle cells through two complementary signaling pathways: Gurken signals twice to control spatial patterning, whereas Delta signals twice to exert temporal control.
12. A mutation in dVps28 reveals a link between a subunit of the endosomal sorting complex required for transport-I complex and the actin cytoskeleton in Drosophila. Proteins that constitute the endosomal sorting complex required for transport (ESCRT) are necessary for the sorting of proteins into multivesicular bodies (MVBs) and the budding of several enveloped viruses, including HIV-1. The first of these complexes, ESCRT-I, consists of three proteins: Vps28p, Vps37p, and Vps23p or Tsg101 in mammals. Here, we characterize a mutation in the Drosophila homolog of vps28. The dVps28 gene is essential: homozygous mutants die at the transition from the first to second instar. Removal of maternally contributed dVps28 causes early embryonic lethality. In such embryos lacking dVps28, several processes that require the actin cytoskeleton are perturbed, including axial **migration** of nuclei, formation of transient furrows during cortical divisions in syncytial embryos, and the subsequent cellularization. Defects in actin cytoskeleton organization also become apparent during sperm individualization in dVps28 mutant testis. Because dVps28 mutant cells contained MVBs, these defects are unlikely to be a secondary consequence of disrupted MVB formation and suggest an interaction between the actin cytoskeleton and endosomal membranes in Drosophila embryos earlier than previously appreciated.
13. Mutations affecting the pattern of the PNS in Drosophila reveal novel aspects of neuronal development. Through a systematic genetic screen, we have identified 55 mutations that affect the development of the PNS of Drosophila embryos. These mutations specify 13 novel and 5 previously characterized genes and define new phenotypes for 2 other known genes. Five classes of mutant phenotypes were identified in the screen: gain of neurons, loss of neurons, abnormal position of chordotonal neurons, aberrant neuronal trajectories, and abnormal morphology of neurons. Phenotypic analyses of mutations identified in this study revealed three novel aspects of PNS development. First, we have identified a novel gene that may be required to define glial versus neuronal cell identity. Second, our data indicate that neuronal **migration** plays an important role in pattern formation in the embryonic PNS. Third, we have identified mutations that cause a lack of sensory organs, but unlike mutations in proneural genes, do not affect the formation of sensory organ precursors. These genes may be required for key aspects of neuronal differentiation. Our studies suggest that approximately 70 essential genes are required for proper PNS development in Drosophila embryos.
14. The tao of stem cells in the germline. Germline stem cells (GSCs) are the self-renewing population of germ cells that serve as the source for gametogenesis. GSCs exist in diverse forms, from those that undergo strict self-renewing asymmetric divisions in Drosophila to those that maintain their population by balancing between mitosis and differentiation in Caenorhabditis elegans. Most vertebrate spermatogonial GSCs appear to adopt an intermediate strategy. In most animals, GSCs are established during preadult gonadogenesis following the proliferation and **migration** of embryonic primordial germ cells. GSCs produce numerous gametes throughout the sexually active period of adult life. The establishment and self-renewing division of GSCs are controlled by extracellular signals such as hormones from the hypothalamic-pituitary axis and local interactions between GSCs and their neighboring cells. These extracellular signals may then influence differential gene expression, cell cycle machinery, and cytoskeletal organization of GSCs for their formation and/or divisional asymmetry. In addition, the GSC mechanism is related to that for germline and sex determination. Current knowledge has provided a solid framework for further study of GSCs and stem cells in general.
15. Apical junctions and cell signalling in epithelia. Genetic analysis in Drosophila has led to the identification of several proteins that mediate cell-cell interactions controlling the fate and proliferation of epithelial cells. These proteins are localized or enriched in the adherens and septate junctions at the apical end of the lateral membranes between cells. The proteins localized or enriched at adherens junctions include Notch, which is important for the cell interactions controlling neuroblast and bristle patterning; Boss and sevenless, which are required for the cell interaction that establishes the R7 photoreceptor cell; and Armadillo, required for the wingless-dependent cell interactions that control segment polarity and imaginal disc patterning. Proteins localized at septate junctions include the product of the tumor suppressor gene dlg, which is required for septate junction formation, apical basal cell polarity, and the cell interactions that control proliferation. The results suggest that the cell signalling events important for cell fate determination and for cell proliferation control in epithelia occur at the apical junctions. The **migration** of the nucleus to the apical surface of the epithelium for mitosis may enable it to interact directly with the junction-associated signalling mechanisms.
16. A core transcriptional network for early mesoderm development in Drosophila melanogaster. Embryogenesis is controlled by large gene-regulatory networks, which generate spatially and temporally refined patterns of gene expression. Here, we report the characteristics of the regulatory network orchestrating early mesodermal development in the fruitfly Drosophila, where the transcription factor Twist is both necessary and sufficient to drive development. Through the integration of chromatin immunoprecipitation followed by microarray analysis (ChIP-on-chip) experiments during discrete time periods with computational approaches, we identified >2000 Twist-bound cis-regulatory modules (CRMs) and almost 500 direct target genes. Unexpectedly, Twist regulates an almost complete cassette of genes required for cell proliferation in addition to genes essential for morophogenesis and cell **migration**. Twist targets almost 25% of all annotated Drosophila transcription factors, which may represent the entire set of regulators necessary for the early development of this system. By combining in vivo binding data from Twist, Mef2, Tinman, and Dorsal we have constructed an initial transcriptional network of early mesoderm development. The network topology reveals extensive combinatorial binding, feed-forward regulation, and complex logical outputs as prevalent features. In addition to binary activation and repression, we suggest that Twist binds to almost all mesodermal CRMs to provide the competence to integrate inputs from more specialized transcription factors.
17. Drosophila awd, the homolog of human nm23, regulates FGF receptor levels and functions synergistically with shi/dynamin during tracheal development. Human nm23 has been implicated in suppression of metastasis in various cancers, but the underlying mechanism of such activity has not been fully understood. Using Drosophila tracheal system as a genetic model, we examined the function of the Drosophila homolog of nm23, the awd gene, in cell **migration**. We show that loss of Drosophila awd results in dysregulated tracheal cell motility. This phenotype can be suppressed by reducing the dosage of the chemotactic FGF receptor (FGFR) homolog, breathless (btl), indicating that btl and awd are functionally antagonists. In addition, mutants of shi/dynamin show similar tracheal phenotypes as in awd and exacerbate those in awd mutant, suggesting defects in vesicle-mediated turnover of FGFR in the awd mutant. Consistent with this, Btl-GFP chimera expressed from a cognate btl promoter-driven system accumulate at high levels on tracheal cell membrane of awd mutants as well as in awd RNA duplex-treated cultured cells. Thus, we propose that awd regulates tracheal cell motility by modulating the FGFR levels, through a dynamin-mediated pathway.
18. Notch and Numb are required for normal **migration** of peripheral glia in Drosophila. A prominent feature of glial cells is their ability to migrate along axons to finally wrap and insulate them. In the embryonic Drosophila PNS, most glial cells are born in the CNS and have to migrate to reach their final destinations. To understand how **migration** of the peripheral glia is regulated, we have conducted a genetic screen looking for mutants that disrupt the normal glial pattern. Here we present an analysis of two of these mutants: Notch and numb. Complete loss of Notch function leads to an increase in the number of glial cells. Embryos hemizygous for the weak Notch(B-8X) allele display an irregular **migration** phenotype and mutant glial cells show an increased formation of filopodia-like structures. A similar phenotype occurs in embryos carrying the Notch(ts1) allele when shifted to the restrictive temperature during the glial cell **migration** phase, suggesting that Notch must be activated during glial **migration**. This is corroborated by the fact that cell-specific reduction of Notch activity in glial cells by directed numb expression also results in similar **migration** phenotypes. Since the glial **migration** phenotypes of Notch and numb mutants resemble each other, our data support a model where the precise temporal and quantitative regulation of Numb and Notch activity is not only required during fate decisions but also later during glial differentiation and **migration**.
19. The Rap1 GTPase functions as a regulator of morphogenesis in vivo. The Ras-related Rap GTPases are highly conserved across diverse species but their normal biological function is not well understood. Initial studies in mammalian cells suggested a role for Rap as a Ras antagonist. More recent experiments indicate functions in calcium- and cAMP-mediated signaling and it has been proposed that protein kinase A-mediated phosphorylation activates Rap in vivo. We show that Ras1-mediated signaling pathways in Drosophila are not influenced by Rap1 levels, suggesting that Ras1 and Rap1 function via distinct pathways. Moreover, a mutation that abolishes the putative cAMP-dependent kinase phosphorylation site of Drosophila Rap1 can still rescue the Rap1 mutant phenotype. Our experiments show that Rap1 is not needed for cell proliferation and cell-fate specification but demonstrate a critical function for Rap1 in regulating normal morphogenesis in the eye disk, the ovary and the embryo. Rap1 mutations also disrupt cell **migration**s and cause abnormalities in cell shape. These findings indicate a role for Rap proteins as regulators of morphogenesis in vivo.
20. Genetic modifier screens reveal new components that interact with the Drosophila dystroglycan-dystrophin complex. The Dystroglycan-Dystrophin (Dg-Dys) complex has a capacity to transmit information from the extracellular matrix to the cytoskeleton inside the cell. It is proposed that this interaction is under tight regulation; however the signaling/regulatory components of Dg-Dys complex remain elusive. Understanding the regulation of the complex is critical since defects in this complex cause muscular dystrophy in humans. To reveal new regulators of the Dg-Dys complex, we used a model organism Drosophila melanogaster and performed genetic interaction screens to identify modifiers of Dg and Dys mutants in Drosophila wing veins. These mutant screens revealed that the Dg-Dys complex interacts with genes involved in muscle function and components of Notch, TGF-beta and EGFR signaling pathways. In addition, components of pathways that are required for cellular and/or axonal **migration** through cytoskeletal regulation, such as Semaphorin-Plexin, Frazzled-Netrin and Slit-Robo pathways show interactions with Dys and/or Dg. These data suggest that the Dg-Dys complex and the other pathways regulating extracellular information transfer to the cytoskeletal dynamics are more intercalated than previously thought.
21. Branching morphogenesis of the Drosophila tracheal system. Many organs including the mammalian lung and vascular system consist of branched tubular networks that transport essential gases or fluids, but the genetic programs that control the development of these complex three-dimensional structures are not well understood. The Drosophila melanogaster tracheal (respiratory) system is a network of interconnected epithelial tubes that transports oxygen and other gases in the body and provides a paradigm of branching morphogenesis. It develops by sequential sprouting of primary, secondary, and terminal branches from an epithelial sac of approximately 80 cells in each body segment of the embryo. Mapping of the cell movements and shape changes during the sprouting process has revealed that distinct mechanisms of epithelial **migration** and tube formation are used at each stage of branching. Genetic dissection of the process has identified a general program in which a fibroblast growth factor (FGF) and fibroblast growth factor receptor (FGFR) are used repeatedly to control branch budding and outgrowth. At each stage of branching, the mechanisms controlling FGF expression and the downstream signal transduction pathway change, altering the pattern and structure of the branches that form. During terminal branching, FGF expression is regulated by hypoxia, ensuring that tracheal structure matches cellular oxygen need. A branch diversification program operates in parallel to the general budding program: Regional signals locally modify the general program, conferring specific structural features and other properties on individual branches, such as their substrate outgrowth preferences, differences in tube size and shape, and the ability to fuse to other branches to interconnect the network.
22. Control of tracheal tubulogenesis by Wingless signaling. The tubular epithelium of the Drosophila tracheal system forms a network with a stereotyped pattern consisting of cells and branches with distinct identity. The tracheal primordium undergoes primary branching induced by the FGF homolog Branchless, differentiates cells with specialized functions such as fusion cells, which perform target recognition and adhesion during branch fusion, and extends branches toward specific targets. Specification of a unique identity for each primary branch is essential for directed **migration**, as a defect in either the EGFR or the Dpp pathway leads to a loss of branch identity and the misguidance of tracheal cell **migration**. Here, we investigate the role of Wingless signaling in the specification of cell and branch identity in the tracheal system. Wingless and its intracellular signal transducer, Armadillo, have multiple functions, including specifying the dorsal trunk through activation of Spalt expression and inducing differentiation of fusion cells in all fusion branches. Moreover, we show that Wingless signaling regulates Notch signaling by stimulating delta expression at the tip of primary branches. These activities of Wingless signaling together specify the shape of the dorsal trunk and other fusion branches.
23. A role for the mesoderm in endodermal **migration** and morphogenesis in Drosophila. The endodermal midgut arises from two primordia, the anterior midgut (AMG) primordium and the posterior midgut (PMG) primordium, which are separated by almost the entire length of the Drosophila embryo. To form the midgut, these two parts have to extend towards each other and to fuse laterally on both sides of the yolk. Shortly before and during that movement, AMG and PMG are arranged as mesenchymal cell masses, but later the midgut cells form an epithelium. We show that these two aspects of midgut development, **migration** of AMG and PMG and transition to an epithelium, depend on the mesoderm. The extension of the midgut primordia is achieved by cell **migration** along the visceral mesoderm which forms a continuous layer of cells within the germ band. In mutant embryos lacking the entire mesoderm or failing to differentiate the visceral mesoderm, AMG and PMG are formed but do not migrate properly. In addition, they fail to form an epithelium and instead either remain as compact cell masses anterior and posterior to the yolk (in twist and snail mutant embryos) or only occasionally wrap around the yolk before embryogenesis is completed (in tinman-deficient embryos). We conclude that the visceral mesoderm serves as a substratum for the migrating endodermal cells and that the contact between visceral mesoderm and endoderm is required for the latter to become an epithelium.
24. Spatially localized Kuzbanian required for specific activation of Notch during border cell **migration**. The transmembrane receptor Notch is used repeatedly during development for a variety of essential functions. During Drosophila oogenesis, Notch activity is required first to specify particular follicle cell fates, then to promote the differentiation of all follicle cell types, to promote border cell **migration**, and then to form dorsal appendages, raising the question as to how Notch activity is spatially and temporally regulated. Here we show the Notch activity pattern during oogenesis. Notch activation was found in many follicle cells at stage 6 but then at stage 9 was restricted to migrating border cells, despite uniform expression of Delta. Expression of Kuzbanian (KUZ), a metalloproteinase that can activate Notch as well as cleave other substrates, is enriched in border cells at stage 9; and dominant-negative KUZ caused a strong border cell **migration** defect, without affecting expression of markers of border cell fate or follicle cell differentiation. Constitutively active Notch rescued the **migration** defect due to dominant-negative KUZ, and conditional alleles of Delta and Notch also exhibited border cell **migration** defects. Expression of two different reporters of Notch activity was lost upon expression of dominant-negative KUZ. Taken together these results show that Notch activation and KUZ expression are restricted to border cells at stage 9 of oogenesis and are required for **migration**, but not differentiation, of these cells. This represents a previously unrecognized mechanism for achieving spatial restriction of Notch signaling.
25. Interplay of Notch and FGF signaling restricts cell fate and MAPK activation in the Drosophila trachea. The patterned branching in the Drosophila tracheal system is triggered by the FGF-like ligand Branchless that activates a receptor tyrosine kinase Breathless and the MAP kinase pathway. A single fusion cell at the tip of each fusion branch expresses the zinc-finger gene escargot, leads branch **migration** in a stereotypical pattern and contacts with another fusion cell to mediate fusion of the branches. A high level of MAP kinase activation is also limited to the tip of the branches. Restriction of such cell specialization events to the tip is essential for tracheal tubulogenesis. Here we show that Notch signaling plays crucial roles in the singling out process of the fusion cell. We found that Notch is activated in tracheal cells by Branchless signaling through stimulation of &Dgr; expression at the tip of tracheal branches and that activated Notch represses the fate of the fusion cell. In addition, Notch is required to restrict activation of MAP kinase to the tip of the branches, in part through the negative regulation of Branchless expression. Notch-mediated lateral inhibition in sending and receiving cells is thus essential to restrict the inductive influence of Branchless on the tracheal tubulogenesis.
26. TspanC8 tetraspanins regulate ADAM10/Kuzbanian trafficking and promote Notch activation in flies and mammals. The metalloprotease ADAM10/Kuzbanian catalyzes the ligand-dependent ectodomain shedding of Notch receptors and activates Notch. Here, we show that the human tetraspanins of the evolutionary conserved TspanC8 subfamily (Tspan5, Tspan10, Tspan14, Tspan15, Tspan17, and Tspan33) directly interact with ADAM10, regulate its exit from the endoplasmic reticulum, and that four of them regulate ADAM10 surface expression levels. In an independent RNAi screen in Drosophila, two TspanC8 genes were identified as Notch regulators. Functional analysis of the three Drosophila TspanC8 genes (Tsp3A, Tsp86D, and Tsp26D) indicated that these genes act redundantly to promote Notch signaling. During oogenesis, TspanC8 genes were up-regulated in border cells and regulated Kuzbanian distribution, Notch activity, and cell **migration**. Furthermore, the human TspanC8 tetraspanins Tspan5 and Tspan14 positively regulated ligand-induced ADAM10-dependent Notch1 signaling. We conclude that TspanC8 tetraspanins have a conserved function in the regulation of ADAM10 trafficking and activity, thereby positively regulating Notch receptor activation.

### [Ee]xtracellular [Mm]atrix

#### PubMed

1. The detached locus encodes Drosophila Dystrophin, which acts with other components of the Dystrophin Associated Protein Complex to influence intercellular signalling in developing wing veins. Dystrophin and Dystroglycan are the two central components of the multimeric Dystrophin Associated Protein Complex, or DAPC, that is thought to provide a mechanical link between the **extracellular matrix** and the actin cytoskeleton, disruption of which leads to muscular dystrophy in humans. We present the characterization of the Drosophila 'crossveinless' mutation detached (det), and show that the gene encodes the fly ortholog of Dystrophin. Our genetic analysis shows that, in flies, Dystrophin is a non-essential gene, and the sole overt morphological defect associated with null mutations in the locus is the variable loss of the posterior crossvein that has been described for alleles of det. Null mutations in Drosophila Dystroglycan (Dg) are similarly viable and exhibit this crossvein defect, indicating that both of the central DAPC components have been co-opted for this atypical function of the complex. In the developing wing, the Drosophila DAPC affects the intercellular signalling pathways involved in vein specification. In det and Dg mutant wings, the early BMP signalling that initiates crossvein specification is not maintained, particularly in the pro-vein territories adjacent to the longitudinal veins, and this results in the production of a crossvein fragment in the intervein between the two longitudinal veins. Genetic interaction studies suggest that the DAPC may exert this effect indirectly by down-regulating Notch signalling in pro-vein territories, leading to enhanced BMP signalling in the intervein by diffusion of BMP ligands from the longitudinal veins.
2. Dose-sensitive autosomal modifiers identify candidate genes for tissue autonomous and tissue nonautonomous regulation by the Drosophila nuclear zinc-finger protein, hindsight. The nuclear zinc-finger protein encoded by the hindsight (hnt) locus regulates several cellular processes in Drosophila epithelia, including the Jun N-terminal kinase (JNK) signaling pathway and actin polymerization. Defects in these molecular pathways may underlie the abnormal cellular interactions, loss of epithelial integrity, and apoptosis that occurs in hnt mutants, in turn causing failure of morphogenetic processes such as germ band retraction and dorsal closure in the embryo. To define the genetic pathways regulated by hnt, 124 deficiencies on the second and third chromosomes and 14 duplications on the second chromosome were assayed for dose-sensitive modification of a temperature-sensitive rough eye phenotype caused by the viable allele, hntpeb; 29 interacting regions were identified. Subsequently, 438 P-element-induced lethal mutations mapping to these regions and 12 candidate genes were tested for genetic interaction, leading to identification of 63 dominant modifier loci. A subset of the identified mutants also dominantly modify hnt308-induced embryonic lethality and thus represent general rather than tissue-specific interactors. General interactors include loci encoding transcription factors, actin-binding proteins, signal transduction proteins, and components of the **extracellular matrix**. Expression of several interactors was assessed in hnt mutant tissue. Five genes--apontic (apt), Delta (Dl), decapentaplegic (dpp), karst (kst), and puckered (puc)--are regulated tissue autonomously and, thus, may be direct transcriptional targets of HNT. Three of these genes--apt, Dl, and dpp--are also regulated nonautonomously in adjacent non-HNT-expressing tissues. The expression of several additional interactors--viking (vkg), Cg25, and laminin-alpha (LanA)-is affected only in a nonautonomous manner.
3. Finding a niche: studies from the Drosophila ovary. Specialized microenvironments called niches help maintain stem cells in an undifferentiated and self-renewing state. The existence of niches has long been predicted from mammalian studies, but identifying stem cells in their native environments in vivo has remained a challenge in most vertebrates. Many of the mechanistic insights into how niches regulate stem cell maintenance have been obtained using invertebrate models such as Drosophila. Here, we focus on the Drosophila ovarian germline stem cell niche and review recent studies that have begun to reveal how intricate crosstalk between various signaling pathways regulates stem cell maintenance, how the **extracellular matrix** modulates the signaling output of the niche and how epigenetic programming influences cell development and function both inside and outside the niche to ensure proper tissue homeostasis. These insights will probably inform the study of mammalian niches and how their malfunction contributes to human disease.
4. Regulation of Notch signaling by Drosophila heparan sulfate 3-O sulfotransferase. Heparan sulfate (HS) regulates the activity of various ligands and is involved in molecular recognition events on the cell surface and in the **extracellular matrix**. Specific binding of HS to different ligand proteins depends on the sulfation pattern of HS. For example, the interaction between antithrombin and a particular 3-O sulfated HS motif is thought to modulate blood coagulation. However, a recent study of mice defective for this modification suggested that 3-O sulfation plays other biological roles. Here, we show that Drosophila melanogaster HS 3-O sulfotransferase-b (Hs3st-B), which catalyzes HS 3-O sulfation, is a novel component of the Notch pathway. Reduction of Hs3st-B function by transgenic RNA interference compromised Notch signaling, producing neurogenic phenotypes. We also show that levels of Notch protein on the cell surface were markedly decreased by loss of Hs3st-B. These findings suggest that Hs3st-B is involved in Notch signaling by affecting stability or intracellular trafficking of Notch protein.
5. Presenilin-based genetic screens in Drosophila melanogaster identify novel notch pathway modifiers. Presenilin is the enzymatic component of gamma-secretase, a multisubunit intramembrane protease that processes several transmembrane receptors, such as the amyloid precursor protein (APP). Mutations in human Presenilins lead to altered APP cleavage and early-onset Alzheimer's disease. Presenilins also play an essential role in Notch receptor cleavage and signaling. The Notch pathway is a highly conserved signaling pathway that functions during the development of multicellular organisms, including vertebrates, Drosophila, and C. elegans. Recent studies have shown that Notch signaling is sensitive to perturbations in subcellular trafficking, although the specific mechanisms are largely unknown. To identify genes that regulate Notch pathway function, we have performed two genetic screens in Drosophila for modifiers of Presenilin-dependent Notch phenotypes. We describe here the cloning and identification of 19 modifiers, including nicastrin and several genes with previously undescribed involvement in Notch biology. The predicted functions of these newly identified genes are consistent with **extracellular matrix** and vesicular trafficking mechanisms in Presenilin and Notch pathway regulation and suggest a novel role for gamma-tubulin in the pathway.
6. Genetic modifier screens reveal new components that interact with the Drosophila dystroglycan-dystrophin complex. The Dystroglycan-Dystrophin (Dg-Dys) complex has a capacity to transmit information from the **extracellular matrix** to the cytoskeleton inside the cell. It is proposed that this interaction is under tight regulation; however the signaling/regulatory components of Dg-Dys complex remain elusive. Understanding the regulation of the complex is critical since defects in this complex cause muscular dystrophy in humans. To reveal new regulators of the Dg-Dys complex, we used a model organism Drosophila melanogaster and performed genetic interaction screens to identify modifiers of Dg and Dys mutants in Drosophila wing veins. These mutant screens revealed that the Dg-Dys complex interacts with genes involved in muscle function and components of Notch, TGF-beta and EGFR signaling pathways. In addition, components of pathways that are required for cellular and/or axonal migration through cytoskeletal regulation, such as Semaphorin-Plexin, Frazzled-Netrin and Slit-Robo pathways show interactions with Dys and/or Dg. These data suggest that the Dg-Dys complex and the other pathways regulating extracellular information transfer to the cytoskeletal dynamics are more intercalated than previously thought.
7. Extracellular O-linked β-N-acetylglucosamine: Its biology and relationship to human disease. The O-linked β-N-acetylglucosamine (O-GlcNAc)ylation of cytoplasmic and nuclear proteins regulates basic cellular functions and is involved in the etiology of neurodegeneration and diabetes. Intracellular O-GlcNAcylation is catalyzed by a single O-GlcNAc transferase, O-GlcNAc transferase (OGT). Recently, an atypical O-GlcNAc transferase, extracellular O-linked β-N-acetylglucosamine (EOGT), which is responsible for the modification of extracellular O-GlcNAc, was identified. Although both OGT and EOGT are regulated through the common hexosamine biosynthesis pathway, EOGT localizes to the lumen of the endoplasmic reticulum and transfers GlcNAc to epidermal growth factor-like domains in an OGT-independent manner. In Drosophila, loss of Eogt gives phenotypes similar to those caused by defects in the apic**al extracellular mat**rix. Dumpy, a membrane-anchored apic**al extracellular mat**rix protein, was identified as a major O-GlcNAcylated protein, and EOGT mediates Dumpy-dependent cell adhesion. In mammals, extracellular O-GlcNAc was detected on extracellular proteins including heparan sulfate proteoglycan 2, Nell1, laminin subunit alpha-5, Pamr1, and transmembrane proteins, including Notch receptors. Although the physiological function of O-GlcNAc in mammals has not yet been elucidated, exome sequencing identified homozygous EOGT mutations in patients with Adams-Oliver syndrome, a rare congenital disorder characterized by aplasia cutis congenita and terminal transverse limb defects. This review summarizes the current knowledge of extracellular O-GlcNAc and its implications in the pathological processes in Adams-Oliver syndrome.

### [Ii]nvasive

#### PubMed

1. Negative feedback regulation of Met-dependent **invasive** growth by Notch. The hepatocyte growth factor (HGF) receptor encoded by the Met oncogene controls a genetic program-known as "**invasive** growth"-responsible for several developmental processes and involved in cancer invasion and metastasis. This program functions through several regulatory gene products, as yet largely unknown, both upstream and downstream of Met. Here we show that activation of the Notch receptor results in transcriptional down-regulation of Met, suppression of HGF-dependent Ras signaling, and impairment of HGF-dependent cellular responses. In turn, Met activation leads to transcriptional induction of the Notch ligand Delta and the Notch effector HES-1, indicating that Met is able to self-tune its own protein levels and the ensuing biochemical and biological outputs through stimulation of the Notch pathway. By using branching morphogenesis of the tracheal system in Drosophila as a readout of **invasive** growth, we also show that exogenous expression of a constitutively active form of human Met induces enhanced sprouting of the tracheal tree, a phenotype that is further increased in embryos lacking Notch function. These results unravel an in-built mechanism of negative feedback regulation in which Met activation leads to transcriptional induction of Notch function, which in turn limits HGF activity through repression of the Met oncogene.

### [Mm]etastasis

#### PubMed

1. Oncogenic programmes and Notch activity: an 'organized crime'? The inappropriate Notch signalling can influence virtually all aspect of cancer, including tumour-cell growth, survival, apoptosis, angiogenesis, invasion and **metastasis**, although it does not do this alone. Hence, elucidating the partners of Notch that are active in cancer is now the focus of much intense research activity. The genetic toolkits available, coupled to the small size and short life of the fruit fly Drosophila melanogaster, makes this an inexpensive and effective animal model, suited to large-scale cancer gene discovery studies. The fly eye is not only a non-vital organ but its stereotyped size and disposition also means it is easy to screen for mutations that cause tumours and metastases and provides ample opportunities to test cancer theories and to unravel unanticipated nexus between Notch and other cancer genes, or to discover unforeseen Notch's partners in cancer. These studies suggest that Notch's oncogenic capacity is brought about not simply by increasing signal strength but through partnerships, whereby oncogenes gain more by cooperating than acting individually, as in a ring 'organized crime'.
2. Modeling tumor invasion and **metastasis** in Drosophila. Conservation of major signaling pathways between humans and flies has made Drosophila a useful model organism for cancer research. Our understanding of the mechanisms regulating cell growth, differentiation and development has been considerably advanced by studies in Drosophila. Several recent high profile studies have examined the processes constraining the metastatic growth of tumor cells in fruit fly models. Cell invasion can be studied in the context of an in vivo setting in flies, enabling the genetic requirements of the microenvironment of tumor cells undergoing **metastasis** to be analyzed. This Perspective discusses the strengths and limitations of Drosophila models of cancer invasion and the unique tools that have enabled these studies. It also highlights several recent reports that together make a strong case for Drosophila as a system with the potential for both testing novel concepts in tumor progression and cell invasion, and for uncovering players in **metastasis**.
3. Eyeing tumorigenesis: Notch signaling and epigenetic silencing of Rb in Drosophila. Notch signaling plays an essential role in the processes of embryogenesis and cellular differentiation, and it is believed that the oncogenic effects of dysregulated Notch signaling are an anomalous reflection of the normal functions of this cascade. Nonetheless, the cellular events associated with oncogenic Notch signaling have thus far remained elusive. In a recent report, Ferres-Marco et al. described how they used the Drosophila eye as a model system and found that elevated Notch signaling in combination with activation of components of the Polycomb complex of transcriptional repressors led to metastatic growth of tumors through epigenetic silencing of the Rbf gene. Rbf is the Drosophila homologue of the retinoblastoma tumor-suppressor gene (Rb), thus it represents a novel link between Notch signaling, tumor growth and **metastasis**.
4. Synergy between bacterial infection and genetic predisposition in intestinal dysplasia. Accumulating evidence suggests that hyperproliferating intestinal stem cells (SCs) and progenitors drive cancer initiation, maintenance, and **metastasis**. In addition, chronic inflammation and infection have been increasingly recognized for their roles in cancer. Nevertheless, the mechanisms by which bacterial infections can initiate SC-mediated tumorigenesis remain elusive. Using a Drosophila model of gut pathogenesis, we show that intestinal infection with Pseudomonas aeruginosa, a human opportunistic bacterial pathogen, activates the c-Jun N-terminal kinase (JNK) pathway, a hallmark of the host stress response. This, in turn, causes apoptosis of enterocytes, the largest class of differentiated intestinal cells, and promotes a dramatic proliferation of SCs and progenitors that serves as a homeostatic compensatory mechanism to replenish the apoptotic enterocytes. However, we find that this homeostatic mechanism can lead to massive over-proliferation of intestinal cells when infection occurs in animals with a latent oncogenic form of the Ras1 oncogene. The affected intestines develop excess layers of cells with altered apicobasal polarity reminiscent of dysplasia, suggesting that infection can directly synergize with the genetic background in predisposed individuals to initiate SC-mediated tumorigenesis. Our results provide a framework for the study of intestinal bacterial infections and their effects on undifferentiated and mature enteric epithelial cells in the initial stages of intestinal cancer. Assessment of progenitor cell responses to pathogenic intestinal bacteria could provide a measure of predisposition for apoptotic enterocyte-assisted intestinal dysplasias in humans.
5. Negative feedback regulation of Met-dependent invasive growth by Notch. The hepatocyte growth factor (HGF) receptor encoded by the Met oncogene controls a genetic program-known as "invasive growth"-responsible for several developmental processes and involved in cancer invasion and **metastasis**. This program functions through several regulatory gene products, as yet largely unknown, both upstream and downstream of Met. Here we show that activation of the Notch receptor results in transcriptional down-regulation of Met, suppression of HGF-dependent Ras signaling, and impairment of HGF-dependent cellular responses. In turn, Met activation leads to transcriptional induction of the Notch ligand Delta and the Notch effector HES-1, indicating that Met is able to self-tune its own protein levels and the ensuing biochemical and biological outputs through stimulation of the Notch pathway. By using branching morphogenesis of the tracheal system in Drosophila as a readout of invasive growth, we also show that exogenous expression of a constitutively active form of human Met induces enhanced sprouting of the tracheal tree, a phenotype that is further increased in embryos lacking Notch function. These results unravel an in-built mechanism of negative feedback regulation in which Met activation leads to transcriptional induction of Notch function, which in turn limits HGF activity through repression of the Met oncogene.
6. Targeting Notch signalling by the conserved miR-8/200 microRNA family in development and cancer cells. Notch signalling is crucial for the correct development and growth of numerous organs and tissues, and when subverted it can cause cancer. Loss of miR-8/200 microRNAs (miRNAs) is commonly observed in advanced tumours and correlates with their invasion and acquisition of stem-like properties. Here, we show that this miRNA family controls Notch signalling activation in Drosophila and human cells. In an overexpression screen, we identified the Drosophila miR-8 as a potent inhibitor of Notch-induced overgrowth and tumour **metastasis**. Gain and loss of mir-8 provoked developmental defects reminiscent of impaired Notch signalling and we demonstrated that miR-8 directly inhibits Notch ligand Serrate. Likewise, miR-200c and miR-141 directly inhibited JAGGED1, impeding proliferation of human metastatic prostate cancer cells. It has been suggested that JAGGED1 may also be important for metastases. Although in metastatic cancer cells, JAGGED1 modestly regulated ZEB1, the miR-200c's target in invasion, studies in Drosophila revealed that only concurrent overexpression of Notch and Zfh1/ZEB1 induced tumour metastases. Together, these data define a new way to attenuate or boost Notch signalling that may have clinical interest.
7. Drosophila awd, the homolog of human nm23, regulates FGF receptor levels and functions synergistically with shi/dynamin during tracheal development. Human nm23 has been implicated in suppression of **metastasis** in various cancers, but the underlying mechanism of such activity has not been fully understood. Using Drosophila tracheal system as a genetic model, we examined the function of the Drosophila homolog of nm23, the awd gene, in cell migration. We show that loss of Drosophila awd results in dysregulated tracheal cell motility. This phenotype can be suppressed by reducing the dosage of the chemotactic FGF receptor (FGFR) homolog, breathless (btl), indicating that btl and awd are functionally antagonists. In addition, mutants of shi/dynamin show similar tracheal phenotypes as in awd and exacerbate those in awd mutant, suggesting defects in vesicle-mediated turnover of FGFR in the awd mutant. Consistent with this, Btl-GFP chimera expressed from a cognate btl promoter-driven system accumulate at high levels on tracheal cell membrane of awd mutants as well as in awd RNA duplex-treated cultured cells. Thus, we propose that awd regulates tracheal cell motility by modulating the FGFR levels, through a dynamin-mediated pathway.

### [Cc]ell [Ii]nvasion

#### PubMed

1. Modeling tumor invasion and metastasis in Drosophila. Conservation of major signaling pathways between humans and flies has made Drosophila a useful model organism for cancer research. Our understanding of the mechanisms regulating cell growth, differentiation and development has been considerably advanced by studies in Drosophila. Several recent high profile studies have examined the processes constraining the metastatic growth of tumor cells in fruit fly models. **Cell invasion** can be studied in the context of an in vivo setting in flies, enabling the genetic requirements of the microenvironment of tumor cells undergoing metastasis to be analyzed. This Perspective discusses the strengths and limitations of Drosophila models of cancer invasion and the unique tools that have enabled these studies. It also highlights several recent reports that together make a strong case for Drosophila as a system with the potential for both testing novel concepts in tumor progression and **cell invasion**, and for uncovering players in metastasis.
2. Substrate specificity of rhomboid intramembrane proteases is governed by helix-breaking residues in the substrate transmembrane domain. Rhomboid intramembrane proteases initiate cell signaling during Drosophila development and Providencia bacterial growth by cleaving transmembrane ligand precursors. We have determined how specificity is achieved: Drosophila Rhomboid-1 is a site-specific protease that recognizes its substrate Spitz by a small region of the Spitz transmembrane domain (TMD). This substrate motif is necessary and sufficient for cleavage and is composed of residues known to disrupt helices. Rhomboids from diverse organisms including bacteria and vertebrates recognize the same substrate motif, suggesting that they use a universal targeting strategy. We used this information to search for other rhomboid substrates and identified a family of adhesion proteins from the human parasite Toxoplasma gondii, the TMDs of which were efficient substrates for rhomboid proteases. Intramembrane cleavage of these proteins is required for host **cell invasion**. These results provide an explanation of how rhomboid proteases achieve specificity, and allow some rhomboid substrates to be predicted from sequence information.

---

## dl

- NCBI Gene
- GeneRIF
- Pubmed

| Search Term | Rank | Fields |
| --- | --- | --- |
| [Mm]igration | 6 | Process(1); PubMed(9) |
| ECM | 7 | PubMed(1) |
| [Ee]xtracellular [Mm]atrix | 8 | PubMed(1) |
| [Ii]nvasive | 10 | PubMed(1) |
| [Mm]etastasis | 12 | PubMed(4) |

  
hide/show details for dl

### [Mm]igration

#### Process

1. germ cell **migration**

#### PubMed

1. Genes conserved in bilaterians but jointly lost with Myc during nematode evolution are enriched in cell proliferation and cell **migration** functions. Animals use a stereotypical set of developmental genes to build body architectures of varying sizes and organizational complexity. Some genes are critical to developmental patterning, while other genes are important to physiological control of growth. However, growth regulator genes may not be as important in small-bodied "micro-metazoans" such as nematodes. Nematodes use a simplified developmental strategy of lineage-based cell fate specifications to produce an adult bilaterian body composed of a few hundreds of cells. Nematodes also lost the MYC proto-oncogenic regulator of cell proliferation. To identify additional regulators of cell proliferation that were lost with MYC, we computationally screened and determined 839 high-confidence genes that are conserved in bilaterians/lost in nematodes (CIBLIN genes). We find that 30 % of all CIBLIN genes encode transcriptional regulators of cell proliferation, epithelial-to-mesenchyme transitions, and other processes. Over 50 % of CIBLIN genes are unnamed genes in Drosophila, suggesting that there are many understudied genes. Interestingly, CIBLIN genes include many Myc synthetic lethal (MycSL) hits from recent screens. CIBLIN genes include key regulators of heparan sulfate proteoglycan (HSPG) sulfation patterns, and lysyl oxidases involved in cross-linking and modification of the extracellular matrix (ECM). These genes and others suggest the CIBLIN repertoire services critical functions in ECM remodeling and cell **migration** in large-bodied bilaterians. Correspondingly, CIBLIN genes are co-expressed with Myc in cancer transcriptomes, and include a preponderance of known determinants of cancer progression and tumor aggression. We propose that CIBLIN gene research can improve our understanding of regulatory control of cellular growth in metazoans.
2. Determination and development of the larval muscle pattern in Drosophila melanogaster. This review describes briefly what is known about the early steps of mesoderm differentiation in the fruitfly Drosophila melanogaster. After a summary of general aspects including mesoderm differentiation, mesoderm cell **migration** and subdivision of the mesoderm, more detail is given about the specification of muscle progenitor cells, due to their role as the earliest obvious landmarks in muscle fiber development in Drosophila. Particular focus is given to recent results on the role of asymmetric cell division in muscle differentiation. Furthermore a short summary of myoblast fusion is provided.
3. Germline development in vertebrates and invertebrates. In all animals information is passed from parent to offspring via the germline, which segregates from the soma early in development and undergoes a complex developmental program to give rise to the adult gametes. Many aspects of germline development have been conserved throughout the animal kingdom. Here we review the unique properties of germ cells, the initial determination of germ cell fates, the maintenance of germ cell identity, the **migration** of germ cells to the somatic gonadal primordia and the proliferation of germ cells during development invertebrates and invertebrates. Similarities in germline development in such diverse organisms as Drosophila melanogaster, Caenorhabditis elegans, Xenopus laevis and Mus musculus will be highlighted.
4. In vivo RNAi screen identifies candidate signaling genes required for collective cell **migration** in Drosophila ovary. Collective **migration** of loosely or closely associated cell groups is prevalent in animal development, physiological events, and cancer metastasis. However, our understanding of the mechanisms of collective cell **migration** is incomplete. Drosophila border cells provide a powerful in vivo genetic model to study collective **migration** and identify essential genes for this process. Using border cell-specific RNAi-silencing in Drosophila, we knocked down 360 conserved signaling transduction genes in adult flies to identify essential pathways and genes for border cell **migration**. We uncovered a plethora of signaling genes, a large proportion of which had not been reported for border cells, including Rack1 (Receptor of activated C kinase) and brk (brinker), mad (mother against dpp), and sax (saxophone), which encode three components of TGF-β signaling. The RNAi knock down phenotype was validated by clonal analysis of Rack1 mutants. Our data suggest that inhibition of Src activity by Rack1 may be important for border cell **migratio**n and cluster cohesion maintenance. Lastly, results from our screen not only would shed light on signaling pathways involved in collective **migratio**n during embryogenesis and organogenesis in general, but also could help our understanding for the functions of conserved human genes involved in cancer metastasis.
5. Primordial germ cell **migration** and the assembly of the Drosophila embryonic gonad. During embryogenesis Drosophila pole cells, like germ cells in many other vertebrates and invertebrates, actively migrate before assembling into the gonad. Five separate steps can be distinguished: an initial passive displacement of the germ cells by gastrulation movements, an amoeboid migratory phase during which the pole cells pass through the endoderm, **migration** on endoderm toward mesoderm, separation into two bilateral groups associated with the gonadal mesoderm precursors, followed by condensation into the gonad itself. We have analyzed gonad assembly in embryonic pattern mutants to determine whether distinct cues are required in this process. We show that the initiation of **migration** does not require the presence of the mesoderm, the eventual target of the germ cells. Rather, **migration** is triggered as a consequence of the differentiation of the endoderm. Examination of embryos mutant for maternal genes of the terminal group suggests that a primary role of the endoderm in this process is to allow the germ cell access to the interior of the embryo. In addition, we show that normal gonad assembly requires a region of the embryo that includes the posterior compartment of the fifth and the sixth abdominal segments.
6. Identifying loci required for follicular patterning using directed mosaics. We have developed a 'directed mosaic' system in Drosophila by using the GAL4 system to control the expression of the yeast recombinase, FLP, in a spatial and temporal fashion. By directing FLP expression, we show that it is possible to efficiently and specifically target loss-of-function studies for vital loci to the developmental pathway of interest. A simple F1 adult phenotypic screen demonstrated that most adult tissues can be analyzed with this approach. Using GAL4 lines expressed during oogenesis, we have refined the system to examine the roles of vital loci in the development of the follicular epithelium. We have identified essential genes involved in egg chamber organization, cell **migration** and cell shape. Further, we have used this technique to gain insights into the role of the Drosophila EGF receptor pathway in establishing the egg axes. Finally, using different UAS-FLP, GAL4 and existing FRT lines, we have built stocks that permit the analysis of approximately 95% of the genome in follicular mosaics.
7. The polarisation of the anterior-posterior and dorsal-ventral axes during Drosophila oogenesis. Recent work on Drosophila oogenesis has begun to reveal how the first asymmetries in development arise and how these relate to the later events that localise the positional cues which define the embryonic axes. The Cadherin-dependent positioning of the oocyte creates an anterior-posterior polarity that is transmitted to the embryo through the localisation and localised translation of bicoid, oskar, and nanos mRNA. In contrast, dorsal-ventral polarity arises from the random **migration** of the nucleus to the anterior of the oocyte, where it determines where gurken mRNA is translated and localised. Gurken signalling then defines the embryonic dorsal-ventral axis by restricting pipe expression to the ventral follicle cells, where Pipe regulates the production of an unidentified cue that activates the Toll signalling pathway.
8. Fragmented forests, evolving flies: molecular variation in African populations of Drosophila teissieri. Microsatellite variation from eight loci was studied in five populations of Drosophila teissieri, a fruit-fly found only in the rain forests of sub-Saharan Africa. Five noncontiguous rain forest sites (from Tanzania, Gabon and Ivory Coast) were sampled to measure the effects of historical forest fragmentation on population structure in an obligatory forest-dwelling species. The Ivory Coast and Gabon populations showed a wider range of alleles, different modal alleles and had a higher genetic diversity than the three East African populations. As could be expected, genetic differentiation (FST) was significantly correlated with physical distance, but the westernmost population (Ivory Coast) showed values that were intermediate between the central (Gabon) and Eastern (Tanzania) populations. A **migration**-drift equilibrium in a stable continuum of populations did not appear adequate to describe the observed distribution. It seems probable that the species has undergone abrupt changes involving isolation, merging and **migration** of populations, as a consequence of repeated waves of forest fragmentation and coalescence.
9. Null alleles reveal novel requirements for Bic-D during Drosophila oogenesis and zygotic development. In the Drosophila ovary, the Bicaudal-D (Bic-D) gene is required for the differentiation of one of 16 interconnected cystocyte sister cells into an oocyte. A new class of Bic-Dnull alleles reveals a novel requirement for Bic-D for zygotic viability. In the germ line, the null mutations show that developmental processes that take place in germarial region 1, even those that create asymmetry, are independent of Bic-D function. Bic-D is then required to establish oocyte identity in one cystocyte and is essential, not only for the oocyte-specific accumulation of all oocyte markers that we have tested so far, but also for the posterior **migration** of the oocyte. In addition, normal polarity amongst the nurse cells requires Bic-D, indicating that the creation of different nurse cell identities may depend on oocyte determination. Our results show that different processes in early oogenesis require different amounts of Bic-D in a process-specific way and certain later processes can proceed at low levels of Bic-D. This suggests that the patterning of the female germ line and the development of an oocyte depend on differential responses to a single activity that is capable of initiating distinct oogenesis processes and can establish different cell fates.

### ECM

#### PubMed

1. Genes conserved in bilaterians but jointly lost with Myc during nematode evolution are enriched in cell proliferation and cell migration functions. Animals use a stereotypical set of developmental genes to build body architectures of varying sizes and organizational complexity. Some genes are critical to developmental patterning, while other genes are important to physiological control of growth. However, growth regulator genes may not be as important in small-bodied "micro-metazoans" such as nematodes. Nematodes use a simplified developmental strategy of lineage-based cell fate specifications to produce an adult bilaterian body composed of a few hundreds of cells. Nematodes also lost the MYC proto-oncogenic regulator of cell proliferation. To identify additional regulators of cell proliferation that were lost with MYC, we computationally screened and determined 839 high-confidence genes that are conserved in bilaterians/lost in nematodes (CIBLIN genes). We find that 30 % of all CIBLIN genes encode transcriptional regulators of cell proliferation, epithelial-to-mesenchyme transitions, and other processes. Over 50 % of CIBLIN genes are unnamed genes in Drosophila, suggesting that there are many understudied genes. Interestingly, CIBLIN genes include many Myc synthetic lethal (MycSL) hits from recent screens. CIBLIN genes include key regulators of heparan sulfate proteoglycan (HSPG) sulfation patterns, and lysyl oxidases involved in cross-linking and modification of the extracellular matrix (**ECM**). These genes and others suggest the CIBLIN repertoire services critical functions in **ECM** remodeling and cell migration in large-bodied bilaterians. Correspondingly, CIBLIN genes are co-expressed with Myc in cancer transcriptomes, and include a preponderance of known determinants of cancer progression and tumor aggression. We propose that CIBLIN gene research can improve our understanding of regulatory control of cellular growth in metazoans.

### [Ee]xtracellular [Mm]atrix

#### PubMed

1. Genes conserved in bilaterians but jointly lost with Myc during nematode evolution are enriched in cell proliferation and cell migration functions. Animals use a stereotypical set of developmental genes to build body architectures of varying sizes and organizational complexity. Some genes are critical to developmental patterning, while other genes are important to physiological control of growth. However, growth regulator genes may not be as important in small-bodied "micro-metazoans" such as nematodes. Nematodes use a simplified developmental strategy of lineage-based cell fate specifications to produce an adult bilaterian body composed of a few hundreds of cells. Nematodes also lost the MYC proto-oncogenic regulator of cell proliferation. To identify additional regulators of cell proliferation that were lost with MYC, we computationally screened and determined 839 high-confidence genes that are conserved in bilaterians/lost in nematodes (CIBLIN genes). We find that 30 % of all CIBLIN genes encode transcriptional regulators of cell proliferation, epithelial-to-mesenchyme transitions, and other processes. Over 50 % of CIBLIN genes are unnamed genes in Drosophila, suggesting that there are many understudied genes. Interestingly, CIBLIN genes include many Myc synthetic lethal (MycSL) hits from recent screens. CIBLIN genes include key regulators of heparan sulfate proteoglycan (HSPG) sulfation patterns, and lysyl oxidases involved in cross-linking and modification of the **extracellular matrix** (ECM). These genes and others suggest the CIBLIN repertoire services critical functions in ECM remodeling and cell migration in large-bodied bilaterians. Correspondingly, CIBLIN genes are co-expressed with Myc in cancer transcriptomes, and include a preponderance of known determinants of cancer progression and tumor aggression. We propose that CIBLIN gene research can improve our understanding of regulatory control of cellular growth in metazoans.

### [Ii]nvasive

#### PubMed

1. The Drosophila short gastrulation gene prevents Dpp from autoactivating and suppressing neurogenesis in the neuroectoderm. The short gastrulation (sog) gene is expressed in broad lateral stripes comprising the neuroectoderm of the Drosophila blastoderm embryo. sog encodes a predicted secreted protein that functions nonautonomously to antagonize the activity of the TGF-beta-like Decapentaplegic (Dpp) signaling pathway in the dorsal region of the embryo. Recently, it has been shown that sog and dpp are functionally equivalent to their respective Xenopus homologs chordin and BMP-4. In this report we provide the first direct evidence that sog plays a local role in the lateral region of the blastoderm embryo to oppose Dpp activity in the neuroectoderm. In the dorsal region, Dpp signaling both suppresses neurogenesis and maintains expression of genes that promote dorsal cell fates (dorsalization). We show that Dpp also can perform both of these functions in the neuroectoderm. In wild-type embryos, the ability of Dpp to induce expression of dorsal markers including itself (autoactivation) in the neuroectoderm is blocked by sog. We propose that Sog protects the neuroectoderm from an **invasive** positive feedback loop created by Dpp diffusion and autoactivation. We show that the two functions of Dpp signaling, neural suppression and dorsalization, are triggered by distinct thresholds of Dpp activity. Epistasis experiments reveal that all observed sog activity can be accounted for by Sog functioning as a dedicated Dpp antagonist. Finally, we provide evidence that Sog functions as a diffusible morphogen in the blastoderm embryo. These data strongly support the view that the primary phylogenetically conserved function of the Drosophila sog and dpp genes and the homologous Xenopus chordin and BMP-4 genes is to subdivide the primitive embryonic ectoderm into neural versus non-neural domains.

### [Mm]etastasis

#### PubMed

1. Drosophila at the intersection of infection, inflammation, and cancer. Recent studies show that both cellular and humoral aspects of innate immunity play important roles during tumor progression. These interactions have traditionally been explored in vertebrate model systems. In recent years, Drosophila has emerged as a genetically tractable model system for studying key aspects of tumorigenesis including proliferation, invasion, and **metastasis**. The absence of adaptive immunity in Drosophila provides a unique opportunity to study the interactions between innate immune system and cancer in different genetic contexts. In this review, I discuss recent advances made by using Drosophila models of cancer to study the role of innate immune pathways Toll/Imd, JNK, and JAK-STAT, microbial infection and inflammation during tumor progression.
2. Drosophila as a model to study the role of blood cells in inflammation, innate immunity and cancer. Drosophila has a primitive yet effective blood system with three types of haemocytes which function throughout different developmental stages and environmental stimuli. Haemocytes play essential roles in tissue modeling during embryogenesis and morphogenesis, and also in innate immunity. The open circulatory system of Drosophila makes haemocytes ideal signal mediators to cells and tissues in response to events such as infection and wounding. The application of recently developed and sophisticated genetic tools to the relatively simple genome of Drosophila has made the fly a popular system for modeling human tumorigensis and **metastasis**. Drosophila is now used for screening and investigation of genes implicated in human leukemia and also in modeling development of solid tumors. This second line of research offers promising opportunities to determine the seemingly conflicting roles of blood cells in tumor progression and invasion. This review provides an overview of the signaling pathways conserved in Drosophila during haematopoiesis, haemostasis, innate immunity, wound healing and inflammation. We also review the most recent progress in the use of Drosophila as a cancer research model with an emphasis on the roles haemocytes can play in various cancer models and in the links between inflammation and cancer.
3. In vivo RNAi screen identifies candidate signaling genes required for collective cell migration in Drosophila ovary. Collective migration of loosely or closely associated cell groups is prevalent in animal development, physiological events, and cancer **metastasis**. However, our understanding of the mechanisms of collective cell migration is incomplete. Drosophila border cells provide a powerful in vivo genetic model to study collective migration and identify essential genes for this process. Using border cell-specific RNAi-silencing in Drosophila, we knocked down 360 conserved signaling transduction genes in adult flies to identify essential pathways and genes for border cell migration. We uncovered a plethora of signaling genes, a large proportion of which had not been reported for border cells, including Rack1 (Receptor of activated C kinase) and brk (brinker), mad (mother against dpp), and sax (saxophone), which encode three components of TGF-β signaling. The RNAi knock down phenotype was validated by clonal analysis of Rack1 mutants. Our data suggest that inhibition of Src activity by Rack1 may be important for border cell migration and cluster cohesion maintenance. Lastly, results from our screen not only would shed light on signaling pathways involved in collective migration during embryogenesis and organogenesis in general, but also could help our understanding for the functions of conserved human genes involved in cancer **metastasi**s.
4. A conserved role for Snail as a potentiator of active transcription. The transcription factors of the Snail family are key regulators of epithelial-mesenchymal transitions, cell morphogenesis, and tumor **metastasis**. Since its discovery in Drosophila ∼25 years ago, Snail has been extensively studied for its role as a transcriptional repressor. Here we demonstrate that Drosophila Snail can positively modulate transcriptional activation. By combining information on in vivo occupancy with expression profiling of hand-selected, staged snail mutant embryos, we identified 106 genes that are potentially directly regulated by Snail during mesoderm development. In addition to the expected Snail-repressed genes, almost 50% of Snail targets showed an unanticipated activation. The majority of "Snail-activated" genes have enhancer elements cobound by Twist and are expressed in the mesoderm at the stages of Snail occupancy. Snail can potentiate Twist-mediated enhancer activation in vitro and is essential for enhancer activity in vivo. Using a machine learning approach, we show that differentially enriched motifs are sufficient to predict Snail's regulatory response. In silico mutagenesis revealed a likely causative motif, which we demonstrate is essential for enhancer activation. Taken together, these data indicate that Snail can potentiate enhancer activation by collaborating with different activators, providing a new mechanism by which Snail regulates development.

---

## CG5966

- NCBI Gene
- GeneRIF
- Pubmed

| Search Term | Rank | Fields |
| --- | --- | --- |
| [Mm]igration | 6 | PubMed(1) |
| ECM | 7 | PubMed(1) |
| [Ee]xtracellular [Mm]atrix | 8 | PubMed(1) |

  
hide/show details for CG5966

### [Mm]igration

#### PubMed

1. Genes conserved in bilaterians but jointly lost with Myc during nematode evolution are enriched in cell proliferation and cell **migration** functions. Animals use a stereotypical set of developmental genes to build body architectures of varying sizes and organizational complexity. Some genes are critical to developmental patterning, while other genes are important to physiological control of growth. However, growth regulator genes may not be as important in small-bodied "micro-metazoans" such as nematodes. Nematodes use a simplified developmental strategy of lineage-based cell fate specifications to produce an adult bilaterian body composed of a few hundreds of cells. Nematodes also lost the MYC proto-oncogenic regulator of cell proliferation. To identify additional regulators of cell proliferation that were lost with MYC, we computationally screened and determined 839 high-confidence genes that are conserved in bilaterians/lost in nematodes (CIBLIN genes). We find that 30 % of all CIBLIN genes encode transcriptional regulators of cell proliferation, epithelial-to-mesenchyme transitions, and other processes. Over 50 % of CIBLIN genes are unnamed genes in Drosophila, suggesting that there are many understudied genes. Interestingly, CIBLIN genes include many Myc synthetic lethal (MycSL) hits from recent screens. CIBLIN genes include key regulators of heparan sulfate proteoglycan (HSPG) sulfation patterns, and lysyl oxidases involved in cross-linking and modification of the extracellular matrix (ECM). These genes and others suggest the CIBLIN repertoire services critical functions in ECM remodeling and cell **migration** in large-bodied bilaterians. Correspondingly, CIBLIN genes are co-expressed with Myc in cancer transcriptomes, and include a preponderance of known determinants of cancer progression and tumor aggression. We propose that CIBLIN gene research can improve our understanding of regulatory control of cellular growth in metazoans.

### ECM

#### PubMed

1. Genes conserved in bilaterians but jointly lost with Myc during nematode evolution are enriched in cell proliferation and cell migration functions. Animals use a stereotypical set of developmental genes to build body architectures of varying sizes and organizational complexity. Some genes are critical to developmental patterning, while other genes are important to physiological control of growth. However, growth regulator genes may not be as important in small-bodied "micro-metazoans" such as nematodes. Nematodes use a simplified developmental strategy of lineage-based cell fate specifications to produce an adult bilaterian body composed of a few hundreds of cells. Nematodes also lost the MYC proto-oncogenic regulator of cell proliferation. To identify additional regulators of cell proliferation that were lost with MYC, we computationally screened and determined 839 high-confidence genes that are conserved in bilaterians/lost in nematodes (CIBLIN genes). We find that 30 % of all CIBLIN genes encode transcriptional regulators of cell proliferation, epithelial-to-mesenchyme transitions, and other processes. Over 50 % of CIBLIN genes are unnamed genes in Drosophila, suggesting that there are many understudied genes. Interestingly, CIBLIN genes include many Myc synthetic lethal (MycSL) hits from recent screens. CIBLIN genes include key regulators of heparan sulfate proteoglycan (HSPG) sulfation patterns, and lysyl oxidases involved in cross-linking and modification of the extracellular matrix (**ECM**). These genes and others suggest the CIBLIN repertoire services critical functions in **ECM** remodeling and cell migration in large-bodied bilaterians. Correspondingly, CIBLIN genes are co-expressed with Myc in cancer transcriptomes, and include a preponderance of known determinants of cancer progression and tumor aggression. We propose that CIBLIN gene research can improve our understanding of regulatory control of cellular growth in metazoans.

### [Ee]xtracellular [Mm]atrix

#### PubMed

1. Genes conserved in bilaterians but jointly lost with Myc during nematode evolution are enriched in cell proliferation and cell migration functions. Animals use a stereotypical set of developmental genes to build body architectures of varying sizes and organizational complexity. Some genes are critical to developmental patterning, while other genes are important to physiological control of growth. However, growth regulator genes may not be as important in small-bodied "micro-metazoans" such as nematodes. Nematodes use a simplified developmental strategy of lineage-based cell fate specifications to produce an adult bilaterian body composed of a few hundreds of cells. Nematodes also lost the MYC proto-oncogenic regulator of cell proliferation. To identify additional regulators of cell proliferation that were lost with MYC, we computationally screened and determined 839 high-confidence genes that are conserved in bilaterians/lost in nematodes (CIBLIN genes). We find that 30 % of all CIBLIN genes encode transcriptional regulators of cell proliferation, epithelial-to-mesenchyme transitions, and other processes. Over 50 % of CIBLIN genes are unnamed genes in Drosophila, suggesting that there are many understudied genes. Interestingly, CIBLIN genes include many Myc synthetic lethal (MycSL) hits from recent screens. CIBLIN genes include key regulators of heparan sulfate proteoglycan (HSPG) sulfation patterns, and lysyl oxidases involved in cross-linking and modification of the **extracellular matrix** (ECM). These genes and others suggest the CIBLIN repertoire services critical functions in ECM remodeling and cell migration in large-bodied bilaterians. Correspondingly, CIBLIN genes are co-expressed with Myc in cancer transcriptomes, and include a preponderance of known determinants of cancer progression and tumor aggression. We propose that CIBLIN gene research can improve our understanding of regulatory control of cellular growth in metazoans.

---

## RhoL

- NCBI Gene
- GeneRIF
- Pubmed

| Search Term | Rank | Fields |
| --- | --- | --- |
| [Ff]ilopodia | 1 | PubMed(1) |
| [Mm]igration | 6 | Process(2); PubMed(12) |
| [Ee]xtracellular [Mm]atrix | 8 | PubMed(2) |
| [Ii]nvasive | 10 | PubMed(2) |
| [Mm]etastasis | 12 | PubMed(3) |

  
hide/show details for RhoL

### [Ff]ilopodia

#### PubMed

1. Function of Rho GTPases in embryonic blood cell migration in Drosophila. Hemocyte development in the Drosophila embryo is a genetic model to study blood cell differentiation, cell migration and phagocytosis. Macrophages, which make up the majority of embryonic hemocytes, migrate extensively as individual cells on basement membrane-covered surfaces. The molecular mechanisms that contribute to this migration process are currently not well understood. We report the generation, by P element replacement, of two Gal4 lines that drive expression of UAS-controlled target genes during early (gcm-Gal4) or late (Coll-Gal4) stages of macrophage migration. gcm-Gal4 is used for live imaging analysis showing that macrophages extend large, dynamic lamellipodia as their main protrusions as well as **filopodia**. We use both Gal4 lines to express dominantnegative and constitutively active isoforms of the Rho GTPases Rac1, Cdc42, Rho1 and RhoL in macrophages, and complement these experiments by analyzing embryos mutant for Rho GTPases. Our findings suggest that Rac1 and Rac2 act redundantly in controlling migration and lamellipodia formation in Drosophila macrophages, and that the third Drosophila Rac gene, Mtl, makes no significant contribution to macrophage migration. Cdc42 appears not to be required within macrophages but in other tissues of the embryo to guide macrophages to the ventral trunk region. No evidence was found for a requirement of Rho1 or RhoL in macrophage migration. Finally, to estimate the number of genes whose zygotic expression is required for macrophage migration we analyzed 208 chromosomal deletions that cover most of the Drosophila genome. We find eight deletions that cause defects in macrophage migration suggesting the existence of approximately ten zygotic genes essential for macrophage migration.

### [Mm]igration

#### Process

1. border follicle cell **migration**
2. hemocyte **migration**

#### PubMed

1. RhoL controls invasion and Rap1 localization during immune cell trans**migration** in Drosophila. Human immune cells have to penetrate an endothelial barrier during their beneficial pursuit of infection and their destructive infiltration of tissues in autoimmune diseases. This trans**migration** requires Rap1 GTPase to activate integrin affinity. We define a new model system for this process by demonstrating, with live imaging and genetics, that during embryonic development Drosophila melanogaster immune cells penetrate an epithelial, Drosophila E-cadherin (DE-cadherin)-based tissue barrier. A mutant in RhoL, a GTPase homologue that is specifically expressed in haemocytes, blocks this invasive step but not other aspects of guided **migration**. RhoL mediates integrin adhesion caused by Drosophila Rap1 overexpression and moves Rap1 away from a concentration in the cytoplasm to the leading edge during invasive **migration**. These findings indicate that a programmed migratory step during Drosophila development bears striking molecular similarities to vertebrate immune cell trans**migration** during inflammation, and identify RhoL as a new regulator of invasion, adhesion and Rap1 localization. Our work establishes the utility of Drosophila for identifying novel components of immune cell trans**migration** and for understanding the in vivo interplay of immune cells with the barriers they penetrate.
2. Integration of epithelial patterning and morphogenesis in Drosophila ovarian follicle cells. Drosophila oogenesis involves the coordinated development of germ cells and an overlying follicular epithelium. The follicle cells provide a genetically tractable system to investigate the cell biology of patterning and morphogenesis. Follicle cells initially form a cuboidal epithelium surrounding a syncytium of nurse cells and oocyte. Epithelial structure is maintained as these cells reorganize to create the three dimensional architecture of the eggshell. Both long-range and short-range cell-cell communications pattern the domains of follicle cells that will create specific eggshell structures. After terminal differentiation to deposit the eggshell proteins, the follicle cells die. This review summarizes recent progress in understanding the cell-cell communication that orchestrates follicle cell patterning and **migration**s. DE-cadherin-mediated adhesion is important at several steps in egg chamber formation and follicle cell **migration**. Notch signaling is critical during each successive round of patterning and **migration**. Integration of bone morphogenetic protein (BMP) and epidermal growth factor (EGF) signals patterns the elaborate structures of the dorsal-anterior eggshell.
3. Dynamics of the basement membrane in invasive epithelial clusters in Drosophila. The basement membrane (BM) represents a barrier to cell **migration**, which has to be degraded to promote invasion. However, the role and behaviour of the BM during the development of pre-invasive cells is only poorly understood. Drosophila border cells (BCs) provide an attractive genetic model in which to study the cellular mechanisms underlying the **migration** of mixed cohorts of epithelial cells. BCs are made of two different epithelial cell types appearing sequentially during oogenesis: the polar cells and the outer BCs. Here, we show that the pre-invasive polar cells undergo an unusual and asymmetrical apical capping with major basement membrane proteins, including the two Drosophila Collagen IV alpha chains, Laminin A and Perlecan. Capping of polar cells proceeds through a novel, basal-to-apical transcytosis mechanism that involves the small GTPase Drab5. Apical capping is transient and is followed by rapid shedding prior to the initiation of BC **migration**, suggesting that the apical cap blocks **migration**. Consistently, non-migratory polar cells remain capped. We further show that JAK/STAT signalling and recruitment of outer BCs are required for correct shedding and **migration**. The dynamics of the BM represents a marker of migratory BC, revealing a novel developmentally regulated behaviour of BM coupled to epithelial cell invasiveness.
4. Molecular requirements for actin-based lamella formation in Drosophila S2 cells. Cell **migration** occurs through the protrusion of the actin-enriched lamella. Here, we investigated the effects of RNAi depletion of approximately 90 proteins implicated in actin function on lamella formation in Drosophila S2 cells. Similar to in vitro reconstitution studies of actin-based Listeria movement, we find that lamellae formation requires a relatively small set of proteins that participate in actin nucleation (Arp2/3 and SCAR), barbed end capping (capping protein), filament depolymerization (cofilin and Aip1), and actin monomer binding (profilin and cyclase-associated protein). Lamellae are initiated by parallel and partially redundant signaling pathways involving Rac GTPases and the adaptor protein Nck, which stimulate SCAR, an Arp2/3 activator. We also show that RNAi of three proteins (kette, Abi, and Sra-1) known to copurify with and inhibit SCAR in vitro leads to SCAR degradation, revealing a novel function of this protein complex in SCAR stability. Our results have identified an essential set of proteins involved in actin dynamics during lamella formation in Drosophila S2 cells.
5. Tre1, a G protein-coupled receptor, directs transepithelial **migration** of Drosophila germ cells. In most organisms, germ cells are formed distant from the somatic part of the gonad and thus have to migrate along and through a variety of tissues to reach the gonad. Transepithelial **migration** through the posterior midgut (PMG) is the first active step during Drosophila germ cell **migration**. Here we report the identification of a novel G protein-coupled receptor (GPCR), Tre1, that is essential for this **migration** step. Maternal tre1 RNA is localized to germ cells, and tre1 is required cell autonomously in germ cells. In tre1 mutant embryos, most germ cells do not exit the PMG. The few germ cells that do leave the midgut early migrate normally to the gonad, suggesting that this gene is specifically required for transepithelial **migration** and that mutant germ cells are still able to recognize other guidance cues. Additionally, inhibiting small Rho GTPases in germ cells affects transepithelial **migration**, suggesting that Tre1 signals through Rho1. We propose that Tre1 acts in a manner similar to chemokine receptors required during transepithelial **migration** of leukocytes, implying an evolutionarily conserved mechanism of transepithelial **migration**. Recently, the chemokine receptor CXCR4 was shown to direct **migration** in vertebrate germ cells. Thus, germ cells may more generally use GPCR signaling to navigate the embryo toward their target.
6. Genes that drive invasion and **migration** in Drosophila. Successful cell **migration** depends on the careful regulation of the timing of movement, the guidance of motile cells, and cytoskeletal and adhesive changes within the cells. This review focuses on genes that act cell-autonomously to promote these aspects of cell **migration** in Drosophila. We discuss recent advances in understanding the **migration** of the ovarian border cells, embryonic blood cells, primordial germ cells, somatic gonadal precursors, and tracheal cells. Comparison of genes that regulate these processes to those that promote tumorigenesis and metastasis in mammals demonstrates that studies in fruit flies are uncovering new genes highly relevant to cancer biology.
7. Regulation of the Rac GTPase pathway by the multifunctional Rho GEF Pebble is essential for mesoderm **migration** in the Drosophila gastrula. The Drosophila guanine nucleotide exchange factor Pebble (Pbl) is essential for cytokinesis and cell **migration** during gastrulation. In dividing cells, Pbl promotes Rho1 activation at the cell cortex, leading to formation of the contractile actin-myosin ring. The role of Pbl in fibroblast growth factor-triggered mesoderm spreading during gastrulation is less well understood and its targets and subcellular localization are unknown. To address these issues we performed a domain-function study in the embryo. We show that Pbl is localized to the nucleus and the cell cortex in migrating mesoderm cells and found that, in addition to the PH domain, the conserved C-terminal tail of the protein is crucial for cortical localization. Moreover, we show that the Rac pathway plays an essential role during mesoderm **migration**. Genetic and biochemical interactions indicate that during mesoderm **migration**, Pbl functions by activating a Rac-dependent pathway. Furthermore, gain-of-function and rescue experiments suggest an important regulatory role of the C-terminal tail of Pbl for the selective activation of Rho1-versus Rac-dependent pathways.
8. Drosophila blood cell chemotaxis. Drosophila melanogaster contains a population of blood cells called hemocytes that represent the functional equivalent of vertebrate macrophages. These cells undergo directed **migration**s to disperse during development and reach sites of tissue damage or altered self. These chemotactic behaviors are controlled by the expression of PDGF/Vegf-related ligands in developing embryos and local production of hydrogen peroxide at wounds. Recent work reveals that many molecules important in vertebrate cell motility, including integrins, formins, Ena/VASP proteins and the SCAR/WAVE complex, have a conserved function in these innate immune cells. The use of this model organism has elucidated how damage signals are activated by calcium signaling during inflammation and that the steroid hormone ecdysone activates immune competence at key developmental stages.
9. Cell type-specific roles for Cdc42, Rac, and RhoL in Drosophila oogenesis. The Rho subfamily of GTPases has been shown to regulate cellular morphology. We report the discovery of a new member of the Rho family, named RhoL, which is equally similar to Rac, Rho, and Cdc42. Expression of a dominant-negative RhoL transgene in the Drosophila ovary caused nurse cells to collapse and fuse together. Mutant forms of Cdc42 mimicked this effect. Expression of constitutively active RhoL led to nurse cell subcortical actin breakdown and disruption of nurse cell-follicle cell contacts, followed by germ cell apoptosis. In contrast, Rac activity was specifically required for **migration** of a subset of follicle cells called border cells. All three activities were necessary for normal transfer of nurse cell cytoplasm to the oocyte. These results suggest that Rho protein activities have cell type-specific effects on morphogenesis.
10. Wash functions downstream of Rho1 GTPase in a subset of Drosophila immune cell developmental **migration**s. Drosophila immune cells, the hemocytes, undergo four stereotypical developmental **migration**s to populate the embryo, where they provide immune reconnoitering, as well as a number of non-immune-related functions necessary for proper embryogenesis. Here, we describe a role for Rho1 in one of these developmental **migration**s in which posteriorly located hemocytes migrate toward the head. This **migration** requires the interaction of Rho1 with its downstream effector Wash, a Wiskott-Aldrich syndrome family protein. Both Wash knockdown and a Rho1 transgene harboring a mutation that prevents Wash binding exhibit the same developmental migratory defect as Rho1 knockdown. Wash activates the Arp2/3 complex, whose activity is needed for this **migration**, whereas members of the WASH regulatory complex (SWIP, Strumpellin, and CCDC53) are not. Our results suggest a WASH complex-independent signaling pathway to regulate the cytoskeleton during a subset of hemocyte developmental **migration**s.
11. Function of Rho GTPases in embryonic blood cell **migration** in Drosophila. Hemocyte development in the Drosophila embryo is a genetic model to study blood cell differentiation, cell **migration** and phagocytosis. Macrophages, which make up the majority of embryonic hemocytes, migrate extensively as individual cells on basement membrane-covered surfaces. The molecular mechanisms that contribute to this **migration** process are currently not well understood. We report the generation, by P element replacement, of two Gal4 lines that drive expression of UAS-controlled target genes during early (gcm-Gal4) or late (Coll-Gal4) stages of macrophage **migration**. gcm-Gal4 is used for live imaging analysis showing that macrophages extend large, dynamic lamellipodia as their main protrusions as well as filopodia. We use both Gal4 lines to express dominantnegative and constitutively active isoforms of the Rho GTPases Rac1, Cdc42, Rho1 and RhoL in macrophages, and complement these experiments by analyzing embryos mutant for Rho GTPases. Our findings suggest that Rac1 and Rac2 act redundantly in controlling **migration** and lamellipodia formation in Drosophila macrophages, and that the third Drosophila Rac gene, Mtl, makes no significant contribution to macrophage **migration**. Cdc42 appears not to be required within macrophages but in other tissues of the embryo to guide macrophages to the ventral trunk region. No evidence was found for a requirement of Rho1 or RhoL in macrophage **migration**. Finally, to estimate the number of genes whose zygotic expression is required for macrophage **migration** we analyzed 208 chromosomal deletions that cover most of the Drosophila genome. We find eight deletions that cause defects in macrophage **migration** suggesting the existence of approximately ten zygotic genes essential for macrophage **migration**.
12. Genetic analysis of the actin cytoskeleton in the Drosophila ovary. The Drosophila ovary provides a favorable model system in which to study cellular morphogenesis. The development of a mature egg involves a syncytium of 16 germline cells and over 1000 somatically derived follicle cells. Intercellular transport, stable intercellular bridges, cell **migration**s, cell shape changes, and specific subcellular localization of many embryonic patterning determinants contribute to egg development and require a dynamic cytoskeleton. We discuss many of the recent genetic and cell biological studies that have led to insights into how the actin cytoskeleton is assembled and regulated during the morphogenesis of the Drosophila egg.

### [Ee]xtracellular [Mm]atrix

#### PubMed

1. A directed miniscreen for genes involved in the Drosophila anti-parasitoid immune response. Drosophila larvae react against eggs from the endoparasitoid wasp Leptopilina boulardi by surrounding them in a multilayered cellular capsule. Once a wasp egg is recognized as foreign, circulating macrophage-like cells, known as plasmatocytes, adhere to the invader. After spreading around the wasp egg, plasmatocytes form cellular junctions between the cells, effectively separating the egg from the hemocoel. Next, a second sub-type of circulating immunosurveillance cell (hemocyte), known as lamellocytes, adhere to either the wasp egg or more likely the plasmatocytes surrounding the egg. From these events, it is obvious that adhesion and cell shape change are an essential part of Drosophila's cellular immune response against parasitoid wasp eggs. To date, very few genes have been described as being necessary for a proper anti-parasitization response in Drosophila. With this in mind, we performed a directed genetic miniscreen to discover new genes required for this response. Many of the genes with an encapsulation defect have mammalian homologues involved in cellular adhesion, wound healing, and thrombosis, including **extracellular matrix** proteins, cellular adhesion molecules, and small GTPases.
2. Cdc42 and formin activity control non-muscle myosin dynamics during Drosophila heart morphogenesis. During heart formation, a network of transcription factors and signaling pathways guide cardiac cell fate and differentiation, but the genetic mechanisms orchestrating heart assembly and lumen formation remain unclear. Here, we show that the small GTPase Cdc42 is essential for Drosophila melanogaster heart morphogenesis and lumen formation. Cdc42 genetically interacts with the cardiogenic transcription factor tinman; with dDAAM which belongs to the family of actin organizing formins; and with zipper, which encodes nonmuscle myosin II. Zipper is required for heart lumen formation, and its spatiotemporal activity at the prospective luminal surface is controlled by Cdc42. Heart-specific expression of activated Cdc42, or the regulatory formins dDAAM and Diaphanous caused mislocalization of Zipper and induced ectopic heart lumina, as characterized by luminal markers such as the **extracellular matrix** protein Slit. Placement of Slit at the lumen surface depends on Cdc42 and formin function. Thus, Cdc42 and formins play pivotal roles in heart lumen formation through the spatiotemporal regulation of the actomyosin network.

### [Ii]nvasive

#### PubMed

1. RhoL controls invasion and Rap1 localization during immune cell transmigration in Drosophila. Human immune cells have to penetrate an endothelial barrier during their beneficial pursuit of infection and their destructive infiltration of tissues in autoimmune diseases. This transmigration requires Rap1 GTPase to activate integrin affinity. We define a new model system for this process by demonstrating, with live imaging and genetics, that during embryonic development Drosophila melanogaster immune cells penetrate an epithelial, Drosophila E-cadherin (DE-cadherin)-based tissue barrier. A mutant in RhoL, a GTPase homologue that is specifically expressed in haemocytes, blocks this **invasive** step but not other aspects of guided migration. RhoL mediates integrin adhesion caused by Drosophila Rap1 overexpression and moves Rap1 away from a concentration in the cytoplasm to the leading edge during **invasive** migration. These findings indicate that a programmed migratory step during Drosophila development bears striking molecular similarities to vertebrate immune cell transmigration during inflammation, and identify RhoL as a new regulator of invasion, adhesion and Rap1 localization. Our work establishes the utility of Drosophila for identifying novel components of immune cell transmigration and for understanding the in vivo interplay of immune cells with the barriers they penetrate.
2. Dynamics of the basement membrane in **invasive** epithelial clusters in Drosophila. The basement membrane (BM) represents a barrier to cell migration, which has to be degraded to promote invasion. However, the role and behaviour of the BM during the development of pre-**invasive** cells is only poorly understood. Drosophila border cells (BCs) provide an attractive genetic model in which to study the cellular mechanisms underlying the migration of mixed cohorts of epithelial cells. BCs are made of two different epithelial cell types appearing sequentially during oogenesis: the polar cells and the outer BCs. Here, we show that the pre-**invasive** polar cells undergo an unusual and asymmetrical apical capping with major basement membrane proteins, including the two Drosophila Collagen IV alpha chains, Laminin A and Perlecan. Capping of polar cells proceeds through a novel, basal-to-apical transcytosis mechanism that involves the small GTPase Drab5. Apical capping is transient and is followed by rapid shedding prior to the initiation of BC migration, suggesting that the apical cap blocks migration. Consistently, non-migratory polar cells remain capped. We further show that JAK/STAT signalling and recruitment of outer BCs are required for correct shedding and migration. The dynamics of the BM represents a marker of migratory BC, revealing a novel developmentally regulated behaviour of BM coupled to epithelial cell **invasive**ness.

### [Mm]etastasis

#### PubMed

1. A conserved role for Snail as a potentiator of active transcription. The transcription factors of the Snail family are key regulators of epithelial-mesenchymal transitions, cell morphogenesis, and tumor **metastasis**. Since its discovery in Drosophila ∼25 years ago, Snail has been extensively studied for its role as a transcriptional repressor. Here we demonstrate that Drosophila Snail can positively modulate transcriptional activation. By combining information on in vivo occupancy with expression profiling of hand-selected, staged snail mutant embryos, we identified 106 genes that are potentially directly regulated by Snail during mesoderm development. In addition to the expected Snail-repressed genes, almost 50% of Snail targets showed an unanticipated activation. The majority of "Snail-activated" genes have enhancer elements cobound by Twist and are expressed in the mesoderm at the stages of Snail occupancy. Snail can potentiate Twist-mediated enhancer activation in vitro and is essential for enhancer activity in vivo. Using a machine learning approach, we show that differentially enriched motifs are sufficient to predict Snail's regulatory response. In silico mutagenesis revealed a likely causative motif, which we demonstrate is essential for enhancer activation. Taken together, these data indicate that Snail can potentiate enhancer activation by collaborating with different activators, providing a new mechanism by which Snail regulates development.
2. Drosophila as a model to study the role of blood cells in inflammation, innate immunity and cancer. Drosophila has a primitive yet effective blood system with three types of haemocytes which function throughout different developmental stages and environmental stimuli. Haemocytes play essential roles in tissue modeling during embryogenesis and morphogenesis, and also in innate immunity. The open circulatory system of Drosophila makes haemocytes ideal signal mediators to cells and tissues in response to events such as infection and wounding. The application of recently developed and sophisticated genetic tools to the relatively simple genome of Drosophila has made the fly a popular system for modeling human tumorigensis and **metastasis**. Drosophila is now used for screening and investigation of genes implicated in human leukemia and also in modeling development of solid tumors. This second line of research offers promising opportunities to determine the seemingly conflicting roles of blood cells in tumor progression and invasion. This review provides an overview of the signaling pathways conserved in Drosophila during haematopoiesis, haemostasis, innate immunity, wound healing and inflammation. We also review the most recent progress in the use of Drosophila as a cancer research model with an emphasis on the roles haemocytes can play in various cancer models and in the links between inflammation and cancer.
3. Genes that drive invasion and migration in Drosophila. Successful cell migration depends on the careful regulation of the timing of movement, the guidance of motile cells, and cytoskeletal and adhesive changes within the cells. This review focuses on genes that act cell-autonomously to promote these aspects of cell migration in Drosophila. We discuss recent advances in understanding the migration of the ovarian border cells, embryonic blood cells, primordial germ cells, somatic gonadal precursors, and tracheal cells. Comparison of genes that regulate these processes to those that promote tumorigenesis and **metastasis** in mammals demonstrates that studies in fruit flies are uncovering new genes highly relevant to cancer biology.

---

## Sp7

- NCBI Gene
- GeneRIF
- Pubmed

| Search Term | Rank | Fields |
| --- | --- | --- |
| [Ii]nvasive | 10 | PubMed(1) |
| [Mm]etastasis | 12 | PubMed(1) |

  
hide/show details for Sp7

### [Ii]nvasive

#### PubMed

1. Serine protease MP2 activates prophenoloxidase in the melanization immune response of Drosophila melanogaster. In arthropods, melanization plays a major role in the innate immune response to encapsulate and kill the **invasive** organisms. It is mediated by a serine protease cascade and is regulated by serpins. The identification of the molecular components of melanization and the regulation of those components are still unclear in Drosophila melanogaster, although some genetic research on the activation of melanization has been reported. Here we report that Drosophila serine protease MP2 directly cleaves both recombinant and native prophenoloxidase-1. Overexpression or repression of MP2 in flies resulted in increased and decreased rates of cleavage, respectively, of prophenoloxidase-1. Moreover, serine protease inhibitor Spn27A formed SDS-stable complexes with MP2, both in vitro and in vivo. The amidase activity of MP2 was inhibited efficiently by Spn27A. Spn27A also prevented MP2 from cleaving prophenoloxidase-1. Taken together, these results indicate that under our experimental conditions MP2 functions as a prophenoloxidase-activating protease, and that this function is inhibited by Spn27A. MP2 and Spn27A thus constitute a regulatory unit in the prophenoloxidase activation cascade in Drosophila. The combination of genetic, molecular genetic and biochemical approaches should allow further advances in our understanding of the prophenoloxidase-activating cascade in insects and indirectly shed further light on protease-cascades in humans and other vertebrates.

### [Mm]etastasis

#### PubMed

1. Drosophila as a model to study the role of blood cells in inflammation, innate immunity and cancer. Drosophila has a primitive yet effective blood system with three types of haemocytes which function throughout different developmental stages and environmental stimuli. Haemocytes play essential roles in tissue modeling during embryogenesis and morphogenesis, and also in innate immunity. The open circulatory system of Drosophila makes haemocytes ideal signal mediators to cells and tissues in response to events such as infection and wounding. The application of recently developed and sophisticated genetic tools to the relatively simple genome of Drosophila has made the fly a popular system for modeling human tumorigensis and **metastasis**. Drosophila is now used for screening and investigation of genes implicated in human leukemia and also in modeling development of solid tumors. This second line of research offers promising opportunities to determine the seemingly conflicting roles of blood cells in tumor progression and invasion. This review provides an overview of the signaling pathways conserved in Drosophila during haematopoiesis, haemostasis, innate immunity, wound healing and inflammation. We also review the most recent progress in the use of Drosophila as a cancer research model with an emphasis on the roles haemocytes can play in various cancer models and in the links between inflammation and cancer.

---

## Tep4

- NCBI Gene
- GeneRIF
- Pubmed

| Search Term | Rank | Fields |
| --- | --- | --- |
| [Ee]xtracellular [Mm]atrix | 8 | PubMed(1) |

  
hide/show details for Tep4

### [Ee]xtracellular [Mm]atrix

#### PubMed

1. Genome-wide transcriptional analysis of Drosophila larvae infected by entomopathogenic nematodes shows involvement of complement, recognition and **extracellular matrix** proteins. Heterorhabditis bacteriophora is an entomopathogenic nematode (EPN) which infects its host by accessing the hemolymph where it releases endosymbiotic bacteria of the species Photorhabdus luminescens. We performed a genome-wide transcriptional analysis of the Drosophila response to EPN infection at the time point at which the nematodes reached the hemolymph either via the cuticle or the gut and the bacteria had started to multiply. Many of the most strongly induced genes have been implicated in immune responses in other infection models. Mapping of the complete set of differentially regulated genes showed the hallmarks of a wound response, but also identified a large fraction of EPN-specific transcripts. Several genes identified by transcriptome profiling or their homologues play protective roles during nematode infections. Genes that positively contribute to controlling nematobacterial infections encode: a homolog of thioester-containing complement protein 3, a basement membrane component (glutactin), a recognition protein (GNBP-like 3) and possibly several small peptides. Of note is that several of these genes have not previously been implicated in immune responses.

---

## Irc

- NCBI Gene
- GeneRIF
- Pubmed

| Search Term | Rank | Fields |
| --- | --- | --- |
| [Ii]nvasive | 10 | PubMed(1) |

  
hide/show details for Irc

### [Ii]nvasive

#### PubMed

1. **Invasive** and indigenous microbiota impact intestinal stem cell activity through multiple pathways in Drosophila. Gut homeostasis is controlled by both immune and developmental mechanisms, and its disruption can lead to inflammatory disorders or cancerous lesions of the intestine. While the impact of bacteria on the mucosal immune system is beginning to be precisely understood, little is known about the effects of bacteria on gut epithelium renewal. Here, we addressed how both infectious and indigenous bacteria modulate stem cell activity in Drosophila. We show that the increased epithelium renewal observed upon some bacterial infections is a consequence of the oxidative burst, a major defense of the Drosophila gut. Additionally, we provide evidence that the JAK-STAT (Janus kinase-signal transducers and activators of transcription) and JNK (c-Jun NH(2) terminal kinase) pathways are both required for bacteria-induced stem cell proliferation. Similarly, we demonstrate that indigenous gut microbiota activate the same, albeit reduced, program at basal levels. Altered control of gut microbiota in immune-deficient or aged flies correlates with increased epithelium renewal. Finally, we show that epithelium renewal is an essential component of Drosophila defense against oral bacterial infection. Altogether, these results indicate that gut homeostasis is achieved by a complex interregulation of the immune response, gut microbiota, and stem cell activity.

---

## spz

- NCBI Gene
- GeneRIF
- Pubmed

| Search Term | Rank | Fields |
| --- | --- | --- |
| [Mm]igration | 6 | PubMed(2) |
| [Ee]xtracellular [Mm]atrix | 8 | PubMed(2) |
| [Ii]nvasive | 10 | PubMed(1) |
| [Mm]etastasis | 12 | PubMed(1) |

  
hide/show details for spz

### [Mm]igration

#### PubMed

1. The polarisation of the anterior-posterior and dorsal-ventral axes during Drosophila oogenesis. Recent work on Drosophila oogenesis has begun to reveal how the first asymmetries in development arise and how these relate to the later events that localise the positional cues which define the embryonic axes. The Cadherin-dependent positioning of the oocyte creates an anterior-posterior polarity that is transmitted to the embryo through the localisation and localised translation of bicoid, oskar, and nanos mRNA. In contrast, dorsal-ventral polarity arises from the random **migration** of the nucleus to the anterior of the oocyte, where it determines where gurken mRNA is translated and localised. Gurken signalling then defines the embryonic dorsal-ventral axis by restricting pipe expression to the ventral follicle cells, where Pipe regulates the production of an unidentified cue that activates the Toll signalling pathway.
2. Comparative analysis of Hox downstream genes in Drosophila. Functional diversification of body parts is dependent on the formation of specialized structures along the various body axes. In animals, region-specific morphogenesis along the anteroposterior axis is controlled by a group of conserved transcription factors encoded by the Hox genes. Although it has long been assumed that Hox proteins carry out their function by regulating distinct sets of downstream genes, only a small number of such genes have been found, with very few having direct roles in controlling cellular behavior. We have quantitatively identified hundreds of Hox downstream genes in Drosophila by microarray analysis, and validated many of them by in situ hybridizations on loss- and gain-of-function mutants. One important finding is that Hox proteins, despite their similar DNA-binding properties in vitro, have highly specific effects on the transcriptome in vivo, because expression of many downstream genes respond primarily to a single Hox protein. In addition, a large fraction of downstream genes encodes realizator functions, which directly affect morphogenetic processes, such as orientation and rate of cell divisions, cell-cell adhesion and communication, cell shape and **migration**, or cell death. Focusing on these realizators, we provide a framework for the morphogenesis of the maxillary segment. As the genomic organization of Hox genes and the interaction of Hox proteins with specific co-factors are conserved in vertebrates and invertebrates, and similar classes of downstream genes are regulated by Hox proteins across the metazoan phylogeny, our findings represent a first step toward a mechanistic understanding of morphological diversification within a species as well as between species.

### [Ee]xtracellular [Mm]atrix

#### PubMed

1. Functional binding of secreted molecules to heparan sulfate proteoglycans in Drosophila. Heparan sulfate proteoglycans (HSPGs) are associated with the cell surface and covalently linked to a small number of long unbranched chains of repeating disaccharides. Numerous biochemical studies of these **extracellular matrix** molecules have implicated them in a variety of biological phenomena, in particular cell-cell interactions. Recent genetic studies in Drosophila have begun to clarify the function of HSPGs in vivo and recent findings have implicated HSPGs in Wnt, Hedgehog, fibroblast growth factor and transforming growth factor-beta signaling pathways during development.
2. Developmental roles of heparan sulfate proteoglycans: a comparative review in Drosophila, mouse and human. In recent years, progress in the fields of development and proteoglycan biology have produced converging evidence of the role of proteoglycans in morphogenesis. Numerous studies have demonstrated that proteoglycans are involved in several distinct morphogenetic pathways upon which they act at different levels. In particular, proteoglycans can determine the generation of morphogen gradients and be required for their signal transduction. The surface of most cells and the **extracellular matrix** are decorated by heparan sulfates which are the most common glycosaminoglycans, normally present as heparan sulfate proteoglycans. Considerable structural heterogeneity is generated in proteoglycans by the biosynthetic modification of their heparan sulfate chains as well as by the diverse nature of their different core proteins. This heterogeneity provides an impressive potential for protein-protein and protein-carbohydrate interactions, and can partly explain the diversity of proteoglycan involvement in different morphogenetic pathways. In this review, we summarize the current knowledge about mutations affecting heparan sulfate proteoglycans that influence the function of growth factor pathways essential for tissue assembly, differentiation and development. The comparison of data obtained in Drosophila, rodents and humans reveals that mutations affecting the proteoglycan core proteins or one of the biosynthetic enzymes of their heparan sulfate chains have profound effects on growth and morphogenesis. Further research will complete the picture, but current evidence shows that at the very least, heparan sulfate proteoglycans need to be counted as legitimate elements of morphogenetic pathways that have been maintained throughout evolution as determinant mechanisms of pattern formation.

### [Ii]nvasive

#### PubMed

1. Pathogen and host factors are needed to provoke a systemic host response to gastrointestinal infection of Drosophila larvae by Candida albicans. Candida albicans systemic dissemination in immunocompromised patients is thought to develop from initial gastrointestinal (GI) colonisation. It is unclear what components of the innate immune system are necessary for preventing C. albicans dissemination from the GI tract, but studies in mice have indicated that both neutropenia and GI mucosal damage are crucial for allowing widespread **invasive** C. albicans disease. Mouse models, however, provide limited applicability to genome-wide screens for pathogen or host factors - factors that might influence systemic dissemination following GI colonisation. For this reason we developed a Drosophila model to study intestinal infection by Candida. We found that commensal flora aided host survival following GI infection. Candida provoked extensive JNK-mediated death of gut cells and induced antimicrobial peptide expression in the fat body. From the side of the host, nitric oxide and blood cells influenced systemic antimicrobial responses. The secretion of SAP4 and SAP6 (secreted aspartyl proteases) from Candida was also essential for activating systemic Toll-dependent immunity.

### [Mm]etastasis

#### PubMed

1. Drosophila at the intersection of infection, inflammation, and cancer. Recent studies show that both cellular and humoral aspects of innate immunity play important roles during tumor progression. These interactions have traditionally been explored in vertebrate model systems. In recent years, Drosophila has emerged as a genetically tractable model system for studying key aspects of tumorigenesis including proliferation, invasion, and **metastasis**. The absence of adaptive immunity in Drosophila provides a unique opportunity to study the interactions between innate immune system and cancer in different genetic contexts. In this review, I discuss recent advances made by using Drosophila models of cancer to study the role of innate immune pathways Toll/Imd, JNK, and JAK-STAT, microbial infection and inflammation during tumor progression.

---

## scaf

- NCBI Gene
- GeneRIF
- Pubmed

| Search Term | Rank | Fields |
| --- | --- | --- |
| [Mm]igration | 6 | PubMed(1) |

  
hide/show details for scaf

### [Mm]igration

#### PubMed

1. The Drosophila serine protease homologue Scarface regulates JNK signalling in a negative-feedback loop during epithelial morphogenesis. In Drosophila melanogaster, dorsal closure is a model of tissue morphogenesis leading to the dorsal **migration** and sealing of the embryonic ectoderm. The activation of the JNK signal transduction pathway, specifically in the leading edge cells, is essential to this process. In a genome-wide microarray screen, we identified new JNK target genes during dorsal closure. One of them is the gene scarface (scaf), which belongs to the large family of trypsin-like serine proteases. Some proteins of this family, like Scaf, bear an inactive catalytic site, representing a subgroup of serine protease homologues (SPH) whose functions are poorly understood. Here, we show that scaf is a general transcriptional target of the JNK pathway coding for a secreted SPH. scaf loss-of-function induces defects in JNK-controlled morphogenetic events such as embryonic dorsal closure and adult male terminalia rotation. Live imaging of the latter process reveals that, like for dorsal closure, JNK directs the dorsal fusion of two epithelial layers in the pupal genital disc. Genetic data show that scaf loss-of-function mimics JNK over-activity. Moreover, scaf ectopic expression aggravates the effect of the JNK negative regulator puc on male genitalia rotation. We finally demonstrate that scaf acts as an antagonist by negatively regulating JNK activity. Overall, our results identify the SPH-encoding gene scaf as a new transcriptional target of JNK signalling and reveal the first secreted regulator of the JNK pathway acting in a negative-feedback loop during epithelial morphogenesis.

---

## pain

- NCBI Gene
- GeneRIF
- Pubmed

| Search Term | Rank | Fields |
| --- | --- | --- |
| [Mm]igration | 6 | PubMed(4) |

  
hide/show details for pain

### [Mm]igration

#### PubMed

1. Subtypes of glial cells in the Drosophila embryonic ventral nerve cord as related to lineage and gene expression. In the Drosophila embryonic CNS several subtypes of glial cells develop, which arrange themselves at characteristic positions and presumably fulfil specific functions. The mechanisms leading to the specification and differentiation of glial subtypes are largely unknown. By DiI labelling in glia-specific Gal4 lines we have clarified the lineages of the lateral glia in the embryonic ventral nerve cord and linked each glial cell to a specific stem cell. For the lineage of the longitudinal glioblast we show that it consists of 9 cells, which acquire at least four different identities. A large collection of molecular markers (many of them representing transcription factors and potential Gcm target genes) reveals that individual glial cells express specific combinations of markers. However, cluster analysis uncovers similar combinatorial codes for cells within, and significant differences between the categories of surface-associated, cortex-associated, and longitudinal glia. Glial cells derived from the same stem cell may be homogeneous (though not identical; stem cells NB1-1, NB5-6, NB6-4, LGB) or heterogeneous (NB7-4, NB1-3) with regard to gene expression. In addition to providing a powerful tool to analyse the fate of individual glial cells in different genetic backgrounds, each of these marker genes represents a candidate factor involved in glial specification or differentiation. We demonstrate this by the analysis of a castor loss of function mutation, which affects the number and **migration** of specific glial cells.
2. Roundabout 2 regulates **migration** of sensory neurons by signaling in trans. Roundabout (Robo) receptors and their ligand Slit are important regulators of axon guidance and cell **migration**. The development of Drosophila embryonic sense organs provides a neuronal **migration** paradigm where the in vivo roles of Slit and Robo can be assayed using genetics.Here we show that Slit-Robo signaling controls **migration** of Drosophila larval sensory neurons that are part of the Chordotonal (Cho) stretch receptor organs. We used live imaging to show that abdominal Cho organs normally migrate ventrally during development, whereas thoracic Cho organs do not. Robo2 overexpression in cis (in the sensory neurons) or in trans (on neighboring visceral mesoderm) transforms abdominal organs to a thoracic morphology and position by blocking **migration**, while loss of Slit-Robo signaling produces a reverse transformation in which thoracic organs migrate ectopically. Rescue and tissue-specific knockout experiments indicate that trans signaling by Robo2 contributes to the normal positioning of the thoracic Cho organs. The differential positioning of Cho organs between the thorax and abdomen is known to be regulated by Hox genes, and we show that the essential Hox cofactor Homothorax, represses Robo2 expression in the abdominal visceral mesoderm.Our results suggest that segment-specific neuronal **migration** patterns are directed through a novel signaling complex (the "Slit sandwich") in which Robo2 on the thoracic visceral mesoderm binds to Slit and presents it to Robo receptors on Cho neurons. The differential positioning of Cho organs between thorax and abdomen may be determined by Hox gene-mediated repression of robo2.
3. Drosophila TRPA channel modulates sugar-stimulated neural excitation, avoidance and social response. Drosophila melanogaster postfeeding larvae show food-averse **migration** toward food-free habitats before metamorphosis. This developmental switching from food attraction to aversion is regulated by a neuropeptide Y (NPY)-related brain signaling peptide. We used the fly larva model to delineate the neurobiological basis of age-restricted response to environmental stimuli. Here we provide evidence for a fructose-responsive chemosensory pathway that modulates food-averse migratory and social behaviors. We found that fructose potently elicited larval food-averse behaviors, and painless (pain), a transient receptor potential channel that is responsive to noxious stimuli, was required for the fructose response. A subset of pain-expressing sensory neurons have been identified that show pain-dependent excitation by fructose. Although evolutionarily conserved avoidance mechanisms are widely appreciated for their roles in stress coping and survival, their biological importance in animal physiology and development remains unknown. Our findings demonstrate how an avoidance mechanism is recruited to facilitate animal development.
4. Response to mechanical stress is mediated by the TRPA channel painless in the Drosophila heart. Mechanotransduction modulates cellular functions as diverse as **migration**, proliferation, differentiation, and apoptosis. It is crucial for organ development and homeostasis and leads to pathologies when defective. However, despite considerable efforts made in the past, the molecular basis of mechanotransduction remains poorly understood. Here, we have investigated the genetic basis of mechanotransduction in Drosophila. We show that the fly heart senses and responds to mechanical forces by regulating cardiac activity. In particular, pauses in heart activity are observed under acute mechanical constraints in vivo. We further confirm by a variety of in situ tests that these cardiac arrests constitute the biological force-induced response. In order to identify molecular components of the mechanotransduction pathway, we carried out a genetic screen based on the dependence of cardiac activity upon mechanical constraints and identified Painless, a TRPA channel. We observe a clear absence of in vivo cardiac arrest following inactivation of painless and further demonstrate that painless is autonomously required in the heart to mediate the response to mechanical stress. Furthermore, direct activation of Painless is sufficient to produce pauses in heartbeat, mimicking the pressure-induced response. Painless thus constitutes part of a mechanosensitive pathway that adjusts cardiac muscle activity to mechanical constraints. This constitutes the first in vivo demonstration that a TRPA channel can mediate cardiac mechanotransduction. Furthermore, by establishing a high-throughput system to identify the molecular players involved in mechanotransduction in the cardiovascular system, our study paves the way for understanding the mechanisms underlying a mechanotransduction pathway.

---

## Socs36E

- NCBI Gene
- GeneRIF
- Pubmed

| Search Term | Rank | Fields |
| --- | --- | --- |
| [Mm]igration | 6 | Process(1); PubMed(6) |
| ECM | 7 | PubMed(2) |
| [Ee]xtracellular [Mm]atrix | 8 | PubMed(3) |
| [Ii]nvasive | 10 | PubMed(3) |
| [Mm]etastasis | 12 | PubMed(1) |

  
hide/show details for Socs36E

### [Mm]igration

#### Process

1. border follicle cell **migration**

#### PubMed

1. Requirement for JAK/STAT signaling throughout border cell **migration** in Drosophila. The evolutionarily conserved JAK/STAT signaling pathway is essential for the proliferation, survival and differentiation of many cells including cancer cells. Recent studies have implicated this transcriptional pathway in the process of cell **migration** in humans, mice, Drosophila and Dictyostelium. In the Drosophila ovary, JAK/STAT signaling is necessary and sufficient for the specification and **migration** of a group of cells called the border cells; however, it is not clear to what extent the requirement for cell fate is distinct from that for cell **migration**. We found that STAT protein is enriched in the migrating border cells throughout their **migration** and is an indicator of cells with highest JAK/STAT activity. In addition, stat(ts) mutants exhibited border cell **migration** defects after just 30 minutes at the non-permissive temperature, prior to any detectable change in the expression of cell fate markers. At later times, cell fate changes became evident, indicating that border cell fate is labile. JAK/STAT signaling was also required for organization of the border cell cluster. Finally, we show that both the accumulation of STAT protein and nuclear accumulation are positively regulated by JAK/STAT activity. The activity of the pathway is negatively regulated by overexpression of a SOCS protein and by blocking endocytosis. Together, our findings suggest that the requirement for STAT in border cells extends beyond the initial specification and delamination of cells from the epithelium.
2. Socs36E attenuates STAT signaling to optimize motile cell specification in the Drosophila ovary. The Janus kinase/Signal transducers and activators of transcription (JAK/STAT) pathway determines cell fates by regulating gene expression. One example is the specification of the motile cells called border cells during Drosophila oogenesis. It has been established that too much or too little STAT activity disrupts follicle cell identity and cell motility, which suggests the signaling must be precisely regulated. Here, we find that Suppressor of cytokine signaling at 36E (Socs36E) is a necessary negative regulator of JAK/STAT signaling during border cell specification. We find when STAT signaling is too low to induce **migration** in the presumptive border cell population, nearby follicle cells uncharacteristically become invasive to enable efficient **migration** of the cluster. We generated a genetic null allele that reveals Socs36E is required in the anterior follicle cells to limit invasive behavior to an optimal number of cells. We further show Socs36E genetically interacts with the required STAT feedback inhibitor apontic (apt) and APT's downstream target, mir-279, and provide evidence that suggests APT directly regulates Socs36E transcriptionally. Our work shows Socs36E plays a critical role in a genetic circuit that establishes a boundary between the motile border cell cluster and its non-invasive epithelial neighbors through STAT attenuation.
3. The Jak/STAT pathway in model organisms: emerging roles in cell movement. The JAK/STAT pathway was originally identified in mammals. Studies of this pathway in the mouse have revealed that JAK/STAT signaling plays a central role during hematopoeisis and other developmental processes. The role of JAK/STAT signaling in blood appears to be conserved throughout evolution, as it is also required during fly hematopoeisis. Studies in Dictyostelium, Drosophila, and zebrafish have shown that the JAK/STAT pathway is also required in an unusually broad set of developmental decisions, including cell proliferation, cell fate determination, cell **migration**, planar polarity, convergent extension, and immunity. There is increasing evidence that the versatility of this pathway relies on its cooperation with other signal transduction pathways. In this review, we discuss the components of the JAK/STAT pathway in model organisms and what is known about its requirement in cellular and developmental processes. In particular, we emphasize recent insights into the role that this pathway plays in the control of cell movement.
4. The Maf factor Traffic jam both enables and inhibits collective cell **migration** in Drosophila oogenesis. Border cell cluster (BCC) **migration** in the Drosophila ovary is an excellent system to study the gene regulatory network that enables collective cell **migration**. Here, we identify the large Maf transcription factor Traffic jam (Tj) as an important regulator of BCC **migration**. Tj has a multifaceted impact on the known core cascade that enables BCC motility, consisting of the Jak/Stat signaling pathway, the C/EBP factor Slow border cells (Slbo), and the downstream effector DE-cadherin (DEcad). The initiation of BCC **migration** coincides with a Slbo-dependent decrease in Tj expression. This reduction of Tj is required for normal BCC motility, as high Tj expression strongly impedes **migration**. At high concentration, Tj has a tripartite negative effect on the core pathway: a decrease in Slbo, an increase in the Jak/Stat inhibitor Socs36E, and a Slbo-independent reduction of DEcad. However, maintenance of a low expression level of Tj in the BCC during **migration** is equally important, as loss of tj function also results in a significant delay in **migration** concomitant with a reduction of Slbo and consequently of DEcad. Taken together, we conclude that the regulatory feedback loop between Tj and Slbo is necessary for achieving the correct activity levels of **migration**-regulating factors to ensure proper BCC motility.
5. JAK-STAT pathway in Drosophila morphogenesis: From organ selector to cell behavior regulator. One of the main contributions of Drosophila to the JAK-STAT field is the study of morphogenesis. JAK-STAT signaling controls the formation of many different structures through surprisingly different morphogenetic behaviors that include induction of cell rearrangements, invagination, folding of tissues, modulation of cell shape, and **migration**. This variability may be explained by the many transcription factors and signaling molecules STAT regulates at early stages of development. But is STAT just acting as an upstream inducer of morphogenesis or does it have a more direct role in controlling cell behaviors? Here we review what is known about how the canonical phosphorylation of STAT contributes to shaping the embryonic and imaginal structures.
6. Socs36E limits STAT signaling via Cullin2 and a SOCS-box independent mechanism in the Drosophila egg chamber. The Suppressor of Cytokine Signaling (SOCS) proteins are critical, highly conserved feedback inhibitors of signal transduction cascades. The family of SOCS proteins is divided into two groups: ancestral and vertebrate-specific SOCS proteins. Vertebrate-specific SOCS proteins have been heavily studied as a result of their strong mutant phenotypes. However, the ancestral clade remains less studied, a potential result of genetic redundancies in mammals. Use of the genetically tractable organism Drosophila melanogaster enables in vivo assessment of signaling components and mechanisms with less concern about the functional redundancy observed in mammals. In this study, we investigated how the SOCS family member Suppressor of Cytokine Signaling at 36E (Socs36E) attenuates Janus Kinase/Signal Transducer and Activator of Transcription (Jak/STAT) activation during specification of motile border cells in Drosophila oogenesis. We found that Socs36E genetically interacts with the Cullin2 (Cul2) scaffolding protein. Like Socs36E, Cul2 is required to limit the number of motile cells in egg chambers. We demonstrated that loss of Cul2 in the follicle cells significantly increased nuclear STAT protein levels, which resulted in additional cells acquiring invasive properties. Further, reduction of Cul2 suppressed border cell **migration** defects that occur in a Stat92E-sensitized genetic background. Our data incorporated Cul2 into a previously described Jak/STAT-directed genetic regulatory network that is required to generate a discrete boundary between cell fates. We also found that Socs36E is able to attenuate STAT activity in the egg chamber when it does not have a functional SOCS box. Collectively, this work contributes mechanistic insight to a Jak/STAT regulatory genetic circuit, and suggests that Socs36E regulates Jak/STAT signaling via a Cul2-dependent mechanism, as well as by a Cullin-independent manner, in vivo.

### ECM

#### PubMed

1. Hs3st-A and Hs3st-B regulate intestinal homeostasis in Drosophila adult midgut. Intrinsic and extrinsic signals as well as the extracellular matrix (**ECM**) tightly regulate stem cells for tissue homeostasis and regenerative capacity. Little is known about the regulation of tissue homeostasis by the **ECM**. Heparan sulfate proteoglycans (HSPGs), important components of the **ECM**, are involved in a variety of biological events. Two heparin sulfate 3-O sulfotransferase (Hs3st) genes, Hs3st-A and Hs3st-B, encode the modification enzymes in heparan sulfate (HS) biosynthesis. Here we demonstrate that Hs3st-A and Hs3st-B are required for adult midgut homeostasis. Depletion of Hs3st-A in enterocytes (ECs) results in increased intestinal stem cell (ISC) proliferation and tissue homeostasis loss. Moreover, increased ISC proliferation is also observed in Hs3st-B null mutant alone, or in combination with Hs3st-A RNAi. Hs3st-A depletion-induced ISC proliferation is effectively suppressed by simultaneous inhibition of the EGFR signaling pathway, suggesting that tissue homeostasis loss in Hs3st-A-deficient intestines is due to increased EGFR signaling. Furthermore, we find that Hs3st-A-depleted ECs are unhealthy and prone to death, while ectopic expression of the antiapoptotic p35 is able to greatly suppress tissue homeostasis loss in these intestines. Together, our data suggest that Drosophila Hs3st-A and Hs3st-B are involved in the regulation of ISC proliferation and midgut homeostasis maintenance.
2. Differential activities of the Drosophila JAK/STAT pathway ligands Upd, Upd2 and Upd3. JAK/STAT signalling in vertebrates is activated by multiple cytokines and growth factors. By contrast, the Drosophila genome encodes for only three related JAK/STAT ligands, Upd, Upd2 and Upd3. Identifying the differences between these three ligands will ultimately lead to a greater understanding of this disease-related signalling pathway and its roles in development. Here, we describe the analysis of the least well characterised of the Upd-like ligands, Upd3. We show that in tissue culture-based assays Upd3-GFP is secreted from cells and appears to interact with the extracellular matrix (**ECM**) in a similar manner to Upd, while still non-autonomously activating JAK/STAT signalling. Quantification of each of the Upd-like ligands in conditioned media has allowed us to determine the activity of equal amounts of each ligand on JAK/STAT ex vivo and reveals that Upd is the most potent ligand in this system. Finally, investigations into the effects of ectopic expression of Upd3 in vivo have confirmed its ability to activate pathway signalling at long-distance.

### [Ee]xtracellular [Mm]atrix

#### PubMed

1. Tissue design: how Drosophila tumors remodel their neighborhood. Drosophila genetics has long been appreciated as a powerful approach for discovering the normal functions of genes that act as oncogenes and tumor suppressors in human cancer. Recent studies have also highlighted its advantages for deciphering how such genes function during tumorigenesis itself. Here we detail studies relating to how tumors, generated in developing organs and adult stem cell-based tissues, remodel the tissue landscape to their benefit. Like mammalian tumors, insect tumors can dissolve **extracellular matrix**, recruit blood cells, migrate and invade other tissues. While much is known about how mammalian fibroblasts, immune cells and vasculature promote late tumorigenesis, less is understood about the very earliest stages of tumor development in mammals. Because Drosophila has fewer mitotic cells and a simpler tissue architecture, it affords easy detection and analysis of early clonal tumor growth. Drosophila studies have revealed both cooperative and competitive interactions between tumor and normal cells during early tumor growth. During development, these interactions typically occur with other proliferative progenitor cells, but in adult stem cell-based tissues, the stem cell niche can fuel tumor growth.
2. Hs3st-A and Hs3st-B regulate intestinal homeostasis in Drosophila adult midgut. Intrinsic and extrinsic signals as well as the **extracellular matrix** (ECM) tightly regulate stem cells for tissue homeostasis and regenerative capacity. Little is known about the regulation of tissue homeostasis by the ECM. Heparan sulfate proteoglycans (HSPGs), important components of the ECM, are involved in a variety of biological events. Two heparin sulfate 3-O sulfotransferase (Hs3st) genes, Hs3st-A and Hs3st-B, encode the modification enzymes in heparan sulfate (HS) biosynthesis. Here we demonstrate that Hs3st-A and Hs3st-B are required for adult midgut homeostasis. Depletion of Hs3st-A in enterocytes (ECs) results in increased intestinal stem cell (ISC) proliferation and tissue homeostasis loss. Moreover, increased ISC proliferation is also observed in Hs3st-B null mutant alone, or in combination with Hs3st-A RNAi. Hs3st-A depletion-induced ISC proliferation is effectively suppressed by simultaneous inhibition of the EGFR signaling pathway, suggesting that tissue homeostasis loss in Hs3st-A-deficient intestines is due to increased EGFR signaling. Furthermore, we find that Hs3st-A-depleted ECs are unhealthy and prone to death, while ectopic expression of the antiapoptotic p35 is able to greatly suppress tissue homeostasis loss in these intestines. Together, our data suggest that Drosophila Hs3st-A and Hs3st-B are involved in the regulation of ISC proliferation and midgut homeostasis maintenance.
3. Differential activities of the Drosophila JAK/STAT pathway ligands Upd, Upd2 and Upd3. JAK/STAT signalling in vertebrates is activated by multiple cytokines and growth factors. By contrast, the Drosophila genome encodes for only three related JAK/STAT ligands, Upd, Upd2 and Upd3. Identifying the differences between these three ligands will ultimately lead to a greater understanding of this disease-related signalling pathway and its roles in development. Here, we describe the analysis of the least well characterised of the Upd-like ligands, Upd3. We show that in tissue culture-based assays Upd3-GFP is secreted from cells and appears to interact with the **extracellular matrix** (ECM) in a similar manner to Upd, while still non-autonomously activating JAK/STAT signalling. Quantification of each of the Upd-like ligands in conditioned media has allowed us to determine the activity of equal amounts of each ligand on JAK/STAT ex vivo and reveals that Upd is the most potent ligand in this system. Finally, investigations into the effects of ectopic expression of Upd3 in vivo have confirmed its ability to activate pathway signalling at long-distance.

### [Ii]nvasive

#### PubMed

1. **Invasive** and indigenous microbiota impact intestinal stem cell activity through multiple pathways in Drosophila. Gut homeostasis is controlled by both immune and developmental mechanisms, and its disruption can lead to inflammatory disorders or cancerous lesions of the intestine. While the impact of bacteria on the mucosal immune system is beginning to be precisely understood, little is known about the effects of bacteria on gut epithelium renewal. Here, we addressed how both infectious and indigenous bacteria modulate stem cell activity in Drosophila. We show that the increased epithelium renewal observed upon some bacterial infections is a consequence of the oxidative burst, a major defense of the Drosophila gut. Additionally, we provide evidence that the JAK-STAT (Janus kinase-signal transducers and activators of transcription) and JNK (c-Jun NH(2) terminal kinase) pathways are both required for bacteria-induced stem cell proliferation. Similarly, we demonstrate that indigenous gut microbiota activate the same, albeit reduced, program at basal levels. Altered control of gut microbiota in immune-deficient or aged flies correlates with increased epithelium renewal. Finally, we show that epithelium renewal is an essential component of Drosophila defense against oral bacterial infection. Altogether, these results indicate that gut homeostasis is achieved by a complex interregulation of the immune response, gut microbiota, and stem cell activity.
2. Socs36E attenuates STAT signaling to optimize motile cell specification in the Drosophila ovary. The Janus kinase/Signal transducers and activators of transcription (JAK/STAT) pathway determines cell fates by regulating gene expression. One example is the specification of the motile cells called border cells during Drosophila oogenesis. It has been established that too much or too little STAT activity disrupts follicle cell identity and cell motility, which suggests the signaling must be precisely regulated. Here, we find that Suppressor of cytokine signaling at 36E (Socs36E) is a necessary negative regulator of JAK/STAT signaling during border cell specification. We find when STAT signaling is too low to induce migration in the presumptive border cell population, nearby follicle cells uncharacteristically become **invasive** to enable efficient migration of the cluster. We generated a genetic null allele that reveals Socs36E is required in the anterior follicle cells to limit **invasive** behavior to an optimal number of cells. We further show Socs36E genetically interacts with the required STAT feedback inhibitor apontic (apt) and APT's downstream target, mir-279, and provide evidence that suggests APT directly regulates Socs36E transcriptionally. Our work shows Socs36E plays a critical role in a genetic circuit that establishes a boundary between the motile border cell cluster and its non-**invasive** epithelial neighbors through STAT attenuation.
3. Socs36E limits STAT signaling via Cullin2 and a SOCS-box independent mechanism in the Drosophila egg chamber. The Suppressor of Cytokine Signaling (SOCS) proteins are critical, highly conserved feedback inhibitors of signal transduction cascades. The family of SOCS proteins is divided into two groups: ancestral and vertebrate-specific SOCS proteins. Vertebrate-specific SOCS proteins have been heavily studied as a result of their strong mutant phenotypes. However, the ancestral clade remains less studied, a potential result of genetic redundancies in mammals. Use of the genetically tractable organism Drosophila melanogaster enables in vivo assessment of signaling components and mechanisms with less concern about the functional redundancy observed in mammals. In this study, we investigated how the SOCS family member Suppressor of Cytokine Signaling at 36E (Socs36E) attenuates Janus Kinase/Signal Transducer and Activator of Transcription (Jak/STAT) activation during specification of motile border cells in Drosophila oogenesis. We found that Socs36E genetically interacts with the Cullin2 (Cul2) scaffolding protein. Like Socs36E, Cul2 is required to limit the number of motile cells in egg chambers. We demonstrated that loss of Cul2 in the follicle cells significantly increased nuclear STAT protein levels, which resulted in additional cells acquiring **invasive** properties. Further, reduction of Cul2 suppressed border cell migration defects that occur in a Stat92E-sensitized genetic background. Our data incorporated Cul2 into a previously described Jak/STAT-directed genetic regulatory network that is required to generate a discrete boundary between cell fates. We also found that Socs36E is able to attenuate STAT activity in the egg chamber when it does not have a functional SOCS box. Collectively, this work contributes mechanistic insight to a Jak/STAT regulatory genetic circuit, and suggests that Socs36E regulates Jak/STAT signaling via a Cul2-dependent mechanism, as well as by a Cullin-independent manner, in vivo.

### [Mm]etastasis

#### PubMed

1. Oncogenic cooperation between SOCS family proteins and EGFR identified using a Drosophila epithelial transformation model. MicroRNAs (miRNAs) are emerging as cooperating factors that promote the activity of oncogenes in tumor formation and disease progression. This poses the challenge of identifying the miRNA targets responsible for these interactions. In this study, we identify the growth regulatory miRNA bantam and its target, Socs36E, as cooperating factors in EGFR-driven tumorigenesis and **metastasis** in a Drosophila model of epithelial transformation. bantam promotes growth by limiting expression of Socs36E, which functions as a negative growth regulator. Socs36E has only a modest effect on growth on its own, but behaves as a tumor suppressor in combination with EGFR activation. The human ortholog of SOCS36E, SOCS5, behaves as a candidate tumor suppressor in cellular transformation in cooperation with EGFR/RAS pathway activation.

---

## CG30059

- NCBI Gene
- GeneRIF
- Pubmed

| Search Term | Rank | Fields |
| --- | --- | --- |
| [Mm]igration | 6 | PubMed(1) |
| ECM | 7 | PubMed(1) |
| [Ee]xtracellular [Mm]atrix | 8 | PubMed(1) |

  
hide/show details for CG30059

### [Mm]igration

#### PubMed

1. Genes conserved in bilaterians but jointly lost with Myc during nematode evolution are enriched in cell proliferation and cell **migration** functions. Animals use a stereotypical set of developmental genes to build body architectures of varying sizes and organizational complexity. Some genes are critical to developmental patterning, while other genes are important to physiological control of growth. However, growth regulator genes may not be as important in small-bodied "micro-metazoans" such as nematodes. Nematodes use a simplified developmental strategy of lineage-based cell fate specifications to produce an adult bilaterian body composed of a few hundreds of cells. Nematodes also lost the MYC proto-oncogenic regulator of cell proliferation. To identify additional regulators of cell proliferation that were lost with MYC, we computationally screened and determined 839 high-confidence genes that are conserved in bilaterians/lost in nematodes (CIBLIN genes). We find that 30 % of all CIBLIN genes encode transcriptional regulators of cell proliferation, epithelial-to-mesenchyme transitions, and other processes. Over 50 % of CIBLIN genes are unnamed genes in Drosophila, suggesting that there are many understudied genes. Interestingly, CIBLIN genes include many Myc synthetic lethal (MycSL) hits from recent screens. CIBLIN genes include key regulators of heparan sulfate proteoglycan (HSPG) sulfation patterns, and lysyl oxidases involved in cross-linking and modification of the extracellular matrix (ECM). These genes and others suggest the CIBLIN repertoire services critical functions in ECM remodeling and cell **migration** in large-bodied bilaterians. Correspondingly, CIBLIN genes are co-expressed with Myc in cancer transcriptomes, and include a preponderance of known determinants of cancer progression and tumor aggression. We propose that CIBLIN gene research can improve our understanding of regulatory control of cellular growth in metazoans.

### ECM

#### PubMed

1. Genes conserved in bilaterians but jointly lost with Myc during nematode evolution are enriched in cell proliferation and cell migration functions. Animals use a stereotypical set of developmental genes to build body architectures of varying sizes and organizational complexity. Some genes are critical to developmental patterning, while other genes are important to physiological control of growth. However, growth regulator genes may not be as important in small-bodied "micro-metazoans" such as nematodes. Nematodes use a simplified developmental strategy of lineage-based cell fate specifications to produce an adult bilaterian body composed of a few hundreds of cells. Nematodes also lost the MYC proto-oncogenic regulator of cell proliferation. To identify additional regulators of cell proliferation that were lost with MYC, we computationally screened and determined 839 high-confidence genes that are conserved in bilaterians/lost in nematodes (CIBLIN genes). We find that 30 % of all CIBLIN genes encode transcriptional regulators of cell proliferation, epithelial-to-mesenchyme transitions, and other processes. Over 50 % of CIBLIN genes are unnamed genes in Drosophila, suggesting that there are many understudied genes. Interestingly, CIBLIN genes include many Myc synthetic lethal (MycSL) hits from recent screens. CIBLIN genes include key regulators of heparan sulfate proteoglycan (HSPG) sulfation patterns, and lysyl oxidases involved in cross-linking and modification of the extracellular matrix (**ECM**). These genes and others suggest the CIBLIN repertoire services critical functions in **ECM** remodeling and cell migration in large-bodied bilaterians. Correspondingly, CIBLIN genes are co-expressed with Myc in cancer transcriptomes, and include a preponderance of known determinants of cancer progression and tumor aggression. We propose that CIBLIN gene research can improve our understanding of regulatory control of cellular growth in metazoans.

### [Ee]xtracellular [Mm]atrix

#### PubMed

1. Genes conserved in bilaterians but jointly lost with Myc during nematode evolution are enriched in cell proliferation and cell migration functions. Animals use a stereotypical set of developmental genes to build body architectures of varying sizes and organizational complexity. Some genes are critical to developmental patterning, while other genes are important to physiological control of growth. However, growth regulator genes may not be as important in small-bodied "micro-metazoans" such as nematodes. Nematodes use a simplified developmental strategy of lineage-based cell fate specifications to produce an adult bilaterian body composed of a few hundreds of cells. Nematodes also lost the MYC proto-oncogenic regulator of cell proliferation. To identify additional regulators of cell proliferation that were lost with MYC, we computationally screened and determined 839 high-confidence genes that are conserved in bilaterians/lost in nematodes (CIBLIN genes). We find that 30 % of all CIBLIN genes encode transcriptional regulators of cell proliferation, epithelial-to-mesenchyme transitions, and other processes. Over 50 % of CIBLIN genes are unnamed genes in Drosophila, suggesting that there are many understudied genes. Interestingly, CIBLIN genes include many Myc synthetic lethal (MycSL) hits from recent screens. CIBLIN genes include key regulators of heparan sulfate proteoglycan (HSPG) sulfation patterns, and lysyl oxidases involved in cross-linking and modification of the **extracellular matrix** (ECM). These genes and others suggest the CIBLIN repertoire services critical functions in ECM remodeling and cell migration in large-bodied bilaterians. Correspondingly, CIBLIN genes are co-expressed with Myc in cancer transcriptomes, and include a preponderance of known determinants of cancer progression and tumor aggression. We propose that CIBLIN gene research can improve our understanding of regulatory control of cellular growth in metazoans.

---

## Swim

- NCBI Gene
- GeneRIF
- Pubmed

| Search Term | Rank | Fields |
| --- | --- | --- |
| [Mm]igration | 6 | PubMed(1) |
| [Ee]xtracellular [Mm]atrix | 8 | PubMed(2) |

  
hide/show details for Swim

### [Mm]igration

#### PubMed

1. Minor proteins and enzymes of the Drosophila eggshell matrix. The Drosophila eggshell provides an in vivo model system for extracellular matrix assembly, in which programmed gene expression, cell **migration**s, extracellular protein trafficking, proteolytic processing, and cross-linking are all required to generate a multi-layered and regionally complex architecture. While abundant structural components of the eggshell are known and are being characterized, less is known about non-abundant structural, regulatory, and enzymatic components that are likely to play critical roles in eggshell assembly. We have used sensitive mass spectrometry-based analyses of fractionated eggshell matrices to validate six previously predicted eggshell proteins and to identify eleven novel components, and have characterized the expression patterns of many of their mRNAs. Among these are several putative structural or regulatory (non-enzymatic) proteins, most larger in mass than the major eggshell proteins and often showing preferential expression in follicle cells overlying specific structural features of the eggshell. Of particular note are the putative enzymes, some likely to be involved in matrix cross-linking (two yellow family members previously implicated in eggshell integrity, a heme peroxidase, and a small-molecule oxidoreductase) and others possibly involved in matrix proteolysis or adhesion (proteins related to cathepsins B and D). This work provides a framework for future molecular studies of eggshell assembly.

### [Ee]xtracellular [Mm]atrix

#### PubMed

1. Secreted Wingless-interacting molecule (Swim) promotes long-range signaling by maintaining Wingless solubility. Lipid-modified Wnt/Wingless (Wg) proteins can signal to their target cells in a short- or long-range manner. How these hydrophobic proteins travel through the extracellular environment remains an outstanding question. Here, we report on a Wg binding protein, Secreted Wg-interacting molecule (Swim), that facilitates Wg diffusion through the **extracellular matrix**. Swim, a putative member of the Lipocalin family of extracellular transport proteins, binds to Wg with nanomolar affinity in a lipid-dependent manner. In quantitative signaling assays, Swim is sufficient to maintain the solubility and activity of purified Wg. In Drosophila, swim RNAi phenotypes resemble wg loss-of-function phenotypes in long-range signaling. We propose that Swim is a cofactor that promotes long-range Wg signaling in vivo by maintaining the solubility of Wg.
2. Minor proteins and enzymes of the Drosophila eggshell matrix. The Drosophila eggshell provides an in vivo model system for **extracellular matrix** assembly, in which programmed gene expression, cell migrations, extracellular protein trafficking, proteolytic processing, and cross-linking are all required to generate a multi-layered and regionally complex architecture. While abundant structural components of the eggshell are known and are being characterized, less is known about non-abundant structural, regulatory, and enzymatic components that are likely to play critical roles in eggshell assembly. We have used sensitive mass spectrometry-based analyses of fractionated eggshell matrices to validate six previously predicted eggshell proteins and to identify eleven novel components, and have characterized the expression patterns of many of their mRNAs. Among these are several putative structural or regulatory (non-enzymatic) proteins, most larger in mass than the major eggshell proteins and often showing preferential expression in follicle cells overlying specific structural features of the eggshell. Of particular note are the putative enzymes, some likely to be involved in matrix cross-linking (two yellow family members previously implicated in eggshell integrity, a heme peroxidase, and a small-molecule oxidoreductase) and others possibly involved in matrix proteolysis or adhesion (proteins related to cathepsins B and D). This work provides a framework for future molecular studies of eggshell assembly.

---

## cher

- NCBI Gene
- GeneRIF
- Pubmed

| Search Term | Rank | Fields |
| --- | --- | --- |
| [Ff]ilopodia | 1 | PubMed(2) |
| [Mm]igration | 6 | PubMed(8) |
| ECM | 7 | PubMed(2) |
| [Ee]xtracellular [Mm]atrix | 8 | PubMed(2) |
| [Ii]nvasive | 10 | PubMed(2) |

  
hide/show details for cher

### [Ff]ilopodia

#### PubMed

1. Fascins, and their roles in cell structure and function. The fascins are a structurally unique and evolutionarily conserved group of actin cross-linking proteins. Fascins function in the organisation of two major forms of actin-based structures: dynamic, cortical cell protrusions and cytoplasmic microfilament bundles. The cortical structures, which include **filopodia**, spikes, lamellipodial ribs, oocyte microvilli and the dendrites of dendritic cells, have roles in cell-matrix adhesion, cell interactions and cell migration, whereas the cytoplasmic actin bundles appear to participate in cell architecture. We discuss the current understanding of the cellular mechanisms that regulate the binding of fascin to actin and how these processes contribute to the organisation or disassembly of cell protrusions. Although the in vivo roles of fascin have been studied principally in Drosophila, several human diseases are associated with inherited or acquired alterations in the expression of fascins. Strategies to modulate fascin-containing protrusions and thereby cell adhesive and migratory behaviour could have potential for therapeutic intervention in these conditions. The supplementary material referred to in this section can be found at http://www.interscience.wiley.com/jpages/0265-9247/suppmat/2002/v24.350.html
2. **Filopodia**-like actin cables position nuclei in association with perinuclear actin in Drosophila nurse cells. Controlling the position of the nucleus is vital for a number of cellular processes from yeast to humans. In Drosophila nurse cells, nuclear positioning is crucial during dumping, when nurse cells contract and expel their contents into the oocyte. We provide evidence that in nurse cells, continuous **filopodia**-like actin cables, growing from the plasma membrane and extending to the nucleus, achieve nuclear positioning. These actin cables move nuclei away from ring canals. When nurse cells contract, actin cables associate laterally with the nuclei, in some cases inducing nuclear turning so that actin cables become partially wound around the nuclei. Our data suggest that a perinuclear actin meshwork connects actin cables to nuclei via actin-crosslinking proteins such as the filamin Cheerio. We provide a revised model for how actin structures position nuclei in nurse cells, employing evolutionary conserved machinery.

### [Mm]igration

#### PubMed

1. Genetic analysis of the actin cytoskeleton in the Drosophila ovary. The Drosophila ovary provides a favorable model system in which to study cellular morphogenesis. The development of a mature egg involves a syncytium of 16 germline cells and over 1000 somatically derived follicle cells. Intercellular transport, stable intercellular bridges, cell **migration**s, cell shape changes, and specific subcellular localization of many embryonic patterning determinants contribute to egg development and require a dynamic cytoskeleton. We discuss many of the recent genetic and cell biological studies that have led to insights into how the actin cytoskeleton is assembled and regulated during the morphogenesis of the Drosophila egg.
2. Molecular requirements for actin-based lamella formation in Drosophila S2 cells. Cell **migration** occurs through the protrusion of the actin-enriched lamella. Here, we investigated the effects of RNAi depletion of approximately 90 proteins implicated in actin function on lamella formation in Drosophila S2 cells. Similar to in vitro reconstitution studies of actin-based Listeria movement, we find that lamellae formation requires a relatively small set of proteins that participate in actin nucleation (Arp2/3 and SCAR), barbed end capping (capping protein), filament depolymerization (cofilin and Aip1), and actin monomer binding (profilin and cyclase-associated protein). Lamellae are initiated by parallel and partially redundant signaling pathways involving Rac GTPases and the adaptor protein Nck, which stimulate SCAR, an Arp2/3 activator. We also show that RNAi of three proteins (kette, Abi, and Sra-1) known to copurify with and inhibit SCAR in vitro leads to SCAR degradation, revealing a novel function of this protein complex in SCAR stability. Our results have identified an essential set of proteins involved in actin dynamics during lamella formation in Drosophila S2 cells.
3. Filamin is required for ring canal assembly and actin organization during Drosophila oogenesis. The remodeling of the actin cytoskeleton is essential for cell **migration**, cell division, and cell morphogenesis. Actin-binding proteins play a pivotal role in reorganizing the actin cytoskeleton in response to signals exchanged between cells. In consequence, actin-binding proteins are increasingly a focus of investigations into effectors of cell signaling and the coordination of cellular behaviors within developmental processes. One of the first actin-binding proteins identified was filamin, or actin-binding protein 280 (ABP280). Filamin is required for cell **migration** (Cunningham et al. 1992), and mutations in human alpha-filamin (FLN1; Fox et al. 1998) are responsible for impaired **migration** of cerebral neurons and give rise to periventricular heterotopia, a disorder that leads to epilepsy and vascular disorders, as well as embryonic lethality. We report the identification and characterization of a mutation in Drosophila filamin, the homologue of human alpha-filamin. During oogenesis, filamin is concentrated in the ring canal structures that fortify arrested cleavage furrows and establish cytoplasmic bridges between cells of the germline. The major structural features common to other filamins are conserved in Drosophila filamin. Mutations in Drosophila filamin disrupt actin filament organization and compromise membrane integrity during oocyte development, resulting in female sterility. The genetic and molecular characterization of Drosophila filamin provides the first genetic model system for the analysis of filamin function and regulation during development.
4. Drosophila sosie functions with β(H)-Spectrin and actin organizers in cell **migratio**n, epithelial morphogenesis and cortical stability. Morphogenesis in multicellular organisms requires the careful coordination of cytoskeletal elements, dynamic regulation of cell adhesion and extensive cell **migratio**n. sosie (sie) is a novel gene required in various morphogenesis processes in Drosophila oogenesis. Lack of sie interferes with normal egg chamber packaging, maintenance of epithelial integrity and control of follicle cell **migratio**n, indicating that sie is involved in controlling epithelial integrity and cell **migratio**n. For these functions sie is required both in the germ line and in the soma. Consistent with this, Sosie localizes to plasma membranes in the germ line and in the somatic follicle cells and is predicted to present an EGF-like domain on the extracellular side. Two positively charged residues, C-terminal to the predicted transmembrane domain (on the cytoplasmic side), are required for normal plasma membrane localization of Sosie. Because sie also contributes to normal cortical localization of β(H)-Spectrin, it appears that cortical β(H)-Spectrin mediates some of the functions of sosie. sie also interacts with the genes coding for the actin organizers Filamin and Profilin and, in the absence of sie function, F-actin is less well organized and nurse cells frequently fuse.
5. Regulation of cell **migration** during tracheal development in Drosophila melanogaster. Most of the knowledge concerning the intracellular mechanisms involved in cell locomotion have been obtained from in vitro studies of cells in culture. Many of the concepts derived from these studies have been partially confirmed in in vivo systems but numerous questions regarding the developmental control of cell **migration** remain to be addressed. Tracheal morphogenesis in Drosophila melanogaster embryos represents an in vivo model system to study the genetic control of cell **migration**. We review what is known about tracheal development and regulation of tracheal cell **migration**. We try to link these in vivo studies and the movement of cells over two dimensional substrates and elaborate on important questions which remain to be addressed in the future.
6. Drosophila filamin is required for follicle cell motility during oogenesis. The Filamin family of actin binding proteins is required to maintain cell shape and promote cell locomotion. Using the Drosophila ovary, we provide a detailed description of Filamin-deficient cells during morphogenesis. Reduced expression of Filamin in follicle cells causes defects in the initial encapsulation of germline cysts and in the **migration** of border cells through the germline cyst. However, follicle cell morphogenesis is unaffected by point mutations that produce truncated Filamin proteins. In addition, mutant follicle cell movements can be partially rescued by a transgene encoding only the actin-binding domain and the first six filamin repeats. These data show that Filamin function in cell motility can be provided by a truncated Filamin protein that resembles Dictyostelium Actin Binding Protein-120.
7. Fascins, and their roles in cell structure and function. The fascins are a structurally unique and evolutionarily conserved group of actin cross-linking proteins. Fascins function in the organisation of two major forms of actin-based structures: dynamic, cortical cell protrusions and cytoplasmic microfilament bundles. The cortical structures, which include filopodia, spikes, lamellipodial ribs, oocyte microvilli and the dendrites of dendritic cells, have roles in cell-matrix adhesion, cell interactions and cell **migration**, whereas the cytoplasmic actin bundles appear to participate in cell architecture. We discuss the current understanding of the cellular mechanisms that regulate the binding of fascin to actin and how these processes contribute to the organisation or disassembly of cell protrusions. Although the in vivo roles of fascin have been studied principally in Drosophila, several human diseases are associated with inherited or acquired alterations in the expression of fascins. Strategies to modulate fascin-containing protrusions and thereby cell adhesive and migratory behaviour could have potential for therapeutic intervention in these conditions. The supplementary material referred to in this section can be found at http://www.interscience.wiley.com/jpages/0265-9247/suppmat/2002/v24.350.html
8. Morphogenesis in the absence of integrins: mutation of both Drosophila beta subunits prevents midgut **migration**. Two integrin beta subunits are encoded in the Drosophila genome. The betaPS subunit is widely expressed and heterodimers containing this subunit are required for many developmental processes. The second betasubunit, betanu, is a divergent integrin expressed primarily in the midgut endoderm. To elucidate its function, we generated null mutations in the gene encoding betanu. We find that betanu is not required for viability or fertility, and overall the mutant flies are normal in appearance. However, we could observe betanu function in the absence of betaPS. Consistent with its expression, removal of betanu only enhanced the phenotype of betaPS in the developing midgut. In embryos lacking the zygotic contribution of betaPS, loss of betanu resulted in enhanced separation between the midgut and the surrounding visceral mesoderm. In the absence of both maternal and zygotic betaPS, a delay in midgut **migration** was observed, but removing betanu as well blocked **migration** completely. These results demonstrate that the second beta subunit can partially compensate for loss of betaPS integrins, and that integrins are essential for **migration** of the primordial midgut cells. The two beta subunits mediate midgut **migration** by distinct mechanisms: one that requires talin and one that does not. Other examples of developmental cell **migration**, such as that of the primordial germ cells, occurred normally in the absence of integrins. Having generated the tools to eliminate integrin function completely, we confirm that Drosophila integrins do not control proliferation as they do in mammals, and have identified alphaPS3 as a heterodimeric partner for betanu.

### ECM

#### PubMed

1. Extracellular matrix and its receptors in Drosophila neural development. Extracellular matrix (**ECM**) and matrix receptors are intimately involved in most biological processes. The **ECM** plays fundamental developmental and physiological roles in health and disease, including processes underlying the development, maintenance, and regeneration of the nervous system. To understand the principles of **ECM**-mediated functions in the nervous system, genetic model organisms like Drosophila provide simple, malleable, and powerful experimental platforms. This article provides an overview of **ECM** proteins and receptors in Drosophila. It then focuses on their roles during three progressive phases of neural development: (1) neural progenitor proliferation, (2) axonal growth and pathfinding, and (3) synapse formation and function. Each section highlights known **ECM** and **ECM**-receptor components and recent studies done in mutant conditions to reveal their in vivo functions, all illustrating the enormous opportunities provided when merging work on the nervous system with systematic research into **ECM**-related gene functions.
2. Integrin-dependent anchoring of a stem-cell niche. Interactions between stem cells and their surrounding microenvironment, or niche, are critical for the establishment and maintenance of stem-cell properties. The adult Drosophila testis contains a morphologically discrete stem-cell niche, the 'hub'. The small cluster of non-dividing, somatic hub cells at the anterior tip of the fly testis is contacted by the germline stem cells (GSCs), which retain their stem-cell character through the direct association with the hub. Here we show that integrin-mediated adhesion is important for maintaining the correct position of embryonic hub cells during gonad morphogenesis. The misplaced hub in integrin-deficient embryos directs the orientation of cell divisions in the presumptive GSCs, a hallmark of the active germline stem-cell niche. A decrease in integrin-mediated adhesion in adult testes, which resulted in a loss of the hub and the stem-cell population, revealed the importance of hub-cell anchoring. Finally, we show that an extracellular matrix (**ECM**) is present around the gonad during late embryogenesis and that this **ECM** is defective in integrin-deficient gonads. On the basis of our data, we propose that integrins are required for the attachment of the hub cells to the **ECM**, which is essential for maintaining the stem-cell niche.

### [Ee]xtracellular [Mm]atrix

#### PubMed

1. **Extracellular matrix** and its receptors in Drosophila neural development. **Extracellular matrix** (ECM) and matrix receptors are intimately involved in most biological processes. The ECM plays fundamental developmental and physiological roles in health and disease, including processes underlying the development, maintenance, and regeneration of the nervous system. To understand the principles of ECM-mediated functions in the nervous system, genetic model organisms like Drosophila provide simple, malleable, and powerful experimental platforms. This article provides an overview of ECM proteins and receptors in Drosophila. It then focuses on their roles during three progressive phases of neural development: (1) neural progenitor proliferation, (2) axonal growth and pathfinding, and (3) synapse formation and function. Each section highlights known ECM and ECM-receptor components and recent studies done in mutant conditions to reveal their in vivo functions, all illustrating the enormous opportunities provided when merging work on the nervous system with systematic research into ECM-related gene functions.
2. Integrin-dependent anchoring of a stem-cell niche. Interactions between stem cells and their surrounding microenvironment, or niche, are critical for the establishment and maintenance of stem-cell properties. The adult Drosophila testis contains a morphologically discrete stem-cell niche, the 'hub'. The small cluster of non-dividing, somatic hub cells at the anterior tip of the fly testis is contacted by the germline stem cells (GSCs), which retain their stem-cell character through the direct association with the hub. Here we show that integrin-mediated adhesion is important for maintaining the correct position of embryonic hub cells during gonad morphogenesis. The misplaced hub in integrin-deficient embryos directs the orientation of cell divisions in the presumptive GSCs, a hallmark of the active germline stem-cell niche. A decrease in integrin-mediated adhesion in adult testes, which resulted in a loss of the hub and the stem-cell population, revealed the importance of hub-cell anchoring. Finally, we show that an **extracellular matrix** (ECM) is present around the gonad during late embryogenesis and that this ECM is defective in integrin-deficient gonads. On the basis of our data, we propose that integrins are required for the attachment of the hub cells to the ECM, which is essential for maintaining the stem-cell niche.

### [Ii]nvasive

#### PubMed

1. The actin cross-linker Filamin/Cheerio mediates tumor malignancy downstream of JNK signaling. Cell shape dynamics, motility, and cell proliferation all depend on the actin cytoskeleton. Malignant cancer cells hijack the actin network to grow and migrate to secondary sites. Understanding the function of actin regulators is therefore of major interest. In the present study, we identify the actin cross-linking protein Filamin/Cheerio (Cher) as a mediator of malignancy in genetically defined Drosophila tumors. We show that in **invasive** tumors, resulting from cooperation of activated Ras with disrupted epithelial cell polarity, Cher is upregulated in a Jun N-terminal kinase (JNK)-dependent manner. Although dispensable in normal epithelium, Cher becomes required in the tumor cells for their growth and **invasive**ness. When deprived of Cher, these tumor clones lose their full potential to proliferate and breach tissue boundaries. Instead, the Cher-deficient clones remain confined within the limits of their source epithelium, permitting survival of the host animal. Through interaction with the myosin II heavy chain subunit, Cher is likely to strengthen the cortical actomyosin network and reinforce mechanical tension within the **invasive** tumors. Accordingly, Cher is required for aberrant expression of genes downstream of the Hippo/Yorkie signaling in the tumor tissue. Our study identifies Cher as a new target of JNK signaling that links cytoskeleton dynamics to tumor progression.
2. The actin cross-linker Filamin/Cheerio mediates tumor malignancy downstream of JNK signaling. Cell shape dynamics, motility, and cell proliferation all depend on the actin cytoskeleton. Malignant cancer cells hijack the actin network to grow and migrate to secondary sites. Understanding the function of actin regulators is therefore of major interest. In the present study, we identify the actin cross-linking protein Filamin/Cheerio (Cher) as a mediator of malignancy in genetically defined Drosophila tumors. We show that in **invasive** tumors, resulting from cooperation of activated Ras with disrupted epithelial cell polarity, Cher is upregulated in a Jun N-terminal kinase (JNK)-dependent manner. Although dispensable in normal epithelium, Cher becomes required in the tumor cells for their growth and **invasive**ness. When deprived of Cher, these tumor clones lose their full potential to proliferate and breach tissue boundaries. Instead, the Cher-deficient clones remain confined within the limits of their source epithelium, permitting survival of the host animal. Through interaction with the myosin II heavy chain subunit, Cher is likely to strengthen the cortical actomyosin network and reinforce mechanical tension within the **invasive** tumors. Accordingly, Cher is required for aberrant expression of genes downstream of the Hippo/Yorkie signaling in the tumor tissue. Our study identifies Cher as a new target of JNK signaling that links cytoskeleton dynamics to tumor progression.

---

## CG4267

- NCBI Gene
- GeneRIF
- Pubmed

| Search Term | Rank | Fields |
| --- | --- | --- |
| [Mm]igration | 6 | PubMed(1) |
| ECM | 7 | PubMed(1) |
| [Ee]xtracellular [Mm]atrix | 8 | PubMed(1) |

  
hide/show details for CG4267

### [Mm]igration

#### PubMed

1. Genes conserved in bilaterians but jointly lost with Myc during nematode evolution are enriched in cell proliferation and cell **migration** functions. Animals use a stereotypical set of developmental genes to build body architectures of varying sizes and organizational complexity. Some genes are critical to developmental patterning, while other genes are important to physiological control of growth. However, growth regulator genes may not be as important in small-bodied "micro-metazoans" such as nematodes. Nematodes use a simplified developmental strategy of lineage-based cell fate specifications to produce an adult bilaterian body composed of a few hundreds of cells. Nematodes also lost the MYC proto-oncogenic regulator of cell proliferation. To identify additional regulators of cell proliferation that were lost with MYC, we computationally screened and determined 839 high-confidence genes that are conserved in bilaterians/lost in nematodes (CIBLIN genes). We find that 30 % of all CIBLIN genes encode transcriptional regulators of cell proliferation, epithelial-to-mesenchyme transitions, and other processes. Over 50 % of CIBLIN genes are unnamed genes in Drosophila, suggesting that there are many understudied genes. Interestingly, CIBLIN genes include many Myc synthetic lethal (MycSL) hits from recent screens. CIBLIN genes include key regulators of heparan sulfate proteoglycan (HSPG) sulfation patterns, and lysyl oxidases involved in cross-linking and modification of the extracellular matrix (ECM). These genes and others suggest the CIBLIN repertoire services critical functions in ECM remodeling and cell **migration** in large-bodied bilaterians. Correspondingly, CIBLIN genes are co-expressed with Myc in cancer transcriptomes, and include a preponderance of known determinants of cancer progression and tumor aggression. We propose that CIBLIN gene research can improve our understanding of regulatory control of cellular growth in metazoans.

### ECM

#### PubMed

1. Genes conserved in bilaterians but jointly lost with Myc during nematode evolution are enriched in cell proliferation and cell migration functions. Animals use a stereotypical set of developmental genes to build body architectures of varying sizes and organizational complexity. Some genes are critical to developmental patterning, while other genes are important to physiological control of growth. However, growth regulator genes may not be as important in small-bodied "micro-metazoans" such as nematodes. Nematodes use a simplified developmental strategy of lineage-based cell fate specifications to produce an adult bilaterian body composed of a few hundreds of cells. Nematodes also lost the MYC proto-oncogenic regulator of cell proliferation. To identify additional regulators of cell proliferation that were lost with MYC, we computationally screened and determined 839 high-confidence genes that are conserved in bilaterians/lost in nematodes (CIBLIN genes). We find that 30 % of all CIBLIN genes encode transcriptional regulators of cell proliferation, epithelial-to-mesenchyme transitions, and other processes. Over 50 % of CIBLIN genes are unnamed genes in Drosophila, suggesting that there are many understudied genes. Interestingly, CIBLIN genes include many Myc synthetic lethal (MycSL) hits from recent screens. CIBLIN genes include key regulators of heparan sulfate proteoglycan (HSPG) sulfation patterns, and lysyl oxidases involved in cross-linking and modification of the extracellular matrix (**ECM**). These genes and others suggest the CIBLIN repertoire services critical functions in **ECM** remodeling and cell migration in large-bodied bilaterians. Correspondingly, CIBLIN genes are co-expressed with Myc in cancer transcriptomes, and include a preponderance of known determinants of cancer progression and tumor aggression. We propose that CIBLIN gene research can improve our understanding of regulatory control of cellular growth in metazoans.

### [Ee]xtracellular [Mm]atrix

#### PubMed

1. Genes conserved in bilaterians but jointly lost with Myc during nematode evolution are enriched in cell proliferation and cell migration functions. Animals use a stereotypical set of developmental genes to build body architectures of varying sizes and organizational complexity. Some genes are critical to developmental patterning, while other genes are important to physiological control of growth. However, growth regulator genes may not be as important in small-bodied "micro-metazoans" such as nematodes. Nematodes use a simplified developmental strategy of lineage-based cell fate specifications to produce an adult bilaterian body composed of a few hundreds of cells. Nematodes also lost the MYC proto-oncogenic regulator of cell proliferation. To identify additional regulators of cell proliferation that were lost with MYC, we computationally screened and determined 839 high-confidence genes that are conserved in bilaterians/lost in nematodes (CIBLIN genes). We find that 30 % of all CIBLIN genes encode transcriptional regulators of cell proliferation, epithelial-to-mesenchyme transitions, and other processes. Over 50 % of CIBLIN genes are unnamed genes in Drosophila, suggesting that there are many understudied genes. Interestingly, CIBLIN genes include many Myc synthetic lethal (MycSL) hits from recent screens. CIBLIN genes include key regulators of heparan sulfate proteoglycan (HSPG) sulfation patterns, and lysyl oxidases involved in cross-linking and modification of the **extracellular matrix** (ECM). These genes and others suggest the CIBLIN repertoire services critical functions in ECM remodeling and cell migration in large-bodied bilaterians. Correspondingly, CIBLIN genes are co-expressed with Myc in cancer transcriptomes, and include a preponderance of known determinants of cancer progression and tumor aggression. We propose that CIBLIN gene research can improve our understanding of regulatory control of cellular growth in metazoans.

---

## CrebA

- NCBI Gene
- GeneRIF
- Pubmed

| Search Term | Rank | Fields |
| --- | --- | --- |
| [Mm]igration | 6 | PubMed(11) |
| ECM | 7 | PubMed(1) |
| [Ee]xtracellular [Mm]atrix | 8 | PubMed(2) |

  
hide/show details for CrebA

### [Mm]igration

#### PubMed

1. Gene expression profiling of Drosophila tracheal fusion cells. The Drosophila trachea is a premier genetic system to investigate the fundamental mechanisms of tubular organ formation. Tracheal fusion cells lead the branch fusion process to form an interconnected tubular network. Therefore, fusion cells in the Drosophila trachea will be an excellent model to study branch fusion in mammalian tubular organs, such as kidneys and blood vessels. The fusion process is a dynamic cellular process involving cell **migration**, adhesion, vesicle trafficking, cytoskeleton rearrangement, and membrane fusion. To understand how these cellular events are coordinated, we initiated the critical step to assemble a gene expression profile of fusion cells. For this study, we analyzed the expression of 234 potential tracheal-expressed genes in fusion cells during fusion cell development. 143 Tracheal genes were found to encode transcription factors, signal proteins, cytoskeleton and matrix proteins, transporters, and proteins with unknown function. These genes were divided into four subgroups based on their levels of expression in fusion cells compared to neighboring non-fusion cells revealed by in situ hybridization: (1) genes that have relative high abundance in fusion cells, (2) genes that are dynamically expressed in fusion cells, (3) genes that have relative low abundance in fusion cells, and (4) genes that are expressed at similar levels in fusion cells and non-fusion tracheal cells. This study identifies the expression profile of fusion cells and hypothetically suggests genes which are necessary for the fusion process and which play roles in distinct stages of fusion, as indicated by the location and timing of expression. These data will provide the basis for a comprehensive understanding of the molecular and cellular mechanisms of branch fusion.
2. Fork head prevents apoptosis and promotes cell shape change during formation of the Drosophila salivary glands. The secretory tubes of the Drosophila salivary glands are formed by the regulated, sequential internalization of the primordia. Secretory cell invagination occurs by a change in cell shape that includes basal nuclear **migration** and apical membrane constriction. In embryos mutant for fork head (fkh), which encodes a transcription factor homologous to mammalian hepatocyte nuclear factor 3beta (HNF-3beta), the secretory primordia are not internalized and secretory tubes do not form. Here, we show that secretory cells of fkh mutant embryos undergo extensive apoptotic cell death following the elevated expression of the apoptotic activator genes, reaper and head involution defective. We rescue the secretory cell death in the fkh mutants and show that the rescued cells still do not invaginate. The rescued fkh secretory cells undergo basal nuclear **migration** in the same spatial and temporal pattern as in wild-type secretory cells, but do not constrict their apical surface membranes. Our findings suggest at least two roles for fkh in formation of the embryonic salivary glands: an early role in promoting survival of the secretory cells, and a later role in secretory cell invagination, specifically in the constriction of the apical surface membrane.
3. A Rho GTPase signaling pathway is used reiteratively in epithelial folding and potentially selects the outcome of Rho activation. A single Rho GTPase family member is capable of initiating several different processes, including cell cycle regulation, cytokinesis, cell **migration**, and transcriptional regulation . It is not clear, however, how the Rho protein selects which of these processes to initiate. Guanine nucleotide exchange factors (GEFs), proteins that activate Rho GTPases, could be important in making this selection. We show here that in vivo, DRhoGEF2, a GEF that is ubiquitously expressed and specific for Rho1, is reiteratively required for epithelial folding and invagination, but not for other processes regulated by Rho. The limitation of DRhoGEF2 function supports the hypothesis that the GEF selects the outcome of Rho activation. DRhoGEF2 exerts its effects in gastrulation through the regulation of Myosin II to orchestrate coordinated apical cell constriction. Apical myosin localization is also regulated by Concertina (Cta), a Galpha(12/13) family member that is thought to activate DRhoGEF2 and is itself activated by a putative ligand, Folded gastrulation (Fog). Fog and Cta also play a role in the morphogenetic events requiring DRhoGEF2, suggesting the existence of a conserved signaling pathway in which Fog, Cta, and DRhoGEF2 locally activate Myosin for epithelial invagination and folding.
4. Posterior **migration** of the salivary gland requires an intact visceral mesoderm and integrin function. The final overall shape of an organ and its position within the developing embryo arise as a consequence of both its intrinsic properties and its interactions with surrounding tissues. Here, we focus on the role of directed cell **migration** in shaping and positioning the Drosophila salivary gland. We demonstrate that the salivary gland turns and migrates along the visceral mesoderm to become properly oriented with respect to the overall embryo. We show that salivary gland posterior **migration** requires the activities of genes that position the visceral mesoderm precursors, such as heartless, thickveins, and tinman, but does not require a differentiated visceral mesoderm. We also demonstrate a role for integrin function in salivary gland **migration**. Although the mutations affecting salivary gland motility and directional **migration** cause defects in the final positioning of the salivary gland, most do not affect the length or diameter of the salivary gland tube. These findings suggest that salivary tube dimensions may be an intrinsic property of salivary gland cells.
5. Rho GTPase controls invagination and cohesive **migration** of the Drosophila salivary gland through Crumbs and Rho-kinase. Coordinated cell movements shape simple epithelia into functional tissues and organs during embryogenesis. Regulators and effectors of the small GTPase Rho have been shown to be essential for epithelial morphogenesis in cell culture; however, the mechanism by which Rho GTPase and its downstream effectors control coordinated movement of epithelia in a developing tissue or organ is largely unknown. Here, we show that Rho1 GTPase activity is required for the invagination of Drosophila embryonic salivary gland epithelia and for directed **migration** of the internalized gland. We demonstrate that the absence of zygotic function of Rho1 results in the selective loss of the apical proteins, Crumbs (Crb), Drosophila atypical PKC and Stardust during gland invagination and that this is partially due to reduced crb RNA levels and apical localization. In parallel to regulation of crb RNA and protein, Rho1 activity also signals through Rho-kinase (Rok) to induce apical constriction and cell shape change during invagination. After invagination, Rho-Rok signaling is required again for the coordinated contraction and dorsal **migration** of the proximal half of the gland. We also show that Rho1 activity is required for proper development of the circular visceral mesoderm upon which the gland migrates. Our genetic and live-imaging analyses provide novel evidence that the proximal gland cells play an essential and active role in salivary gland **migration** that propels the entire gland to turn and migrate posteriorly.
6. Organ formation in Drosophila: specification and morphogenesis of the salivary gland. The Drosophila salivary gland has emerged as an outstanding model system for the process of organ formation. Many of the component steps, from initial regional specification through cell specialization and morphogenesis, are known and many of the genes required for these different processes have been identified. The salivary gland is a relatively simple organ; the entire gland comprises of only two major cell types, which derive from a single contiguous primordium. Salivary cells cease dividing once they are specified, and organ growth is achieved simply by an increase in size of individual cells, thus eliminating concerns about the potential unequal distribution of determinants during mitosis. Drosophila salivary glands form by the same cellular mechanisms as organs in higher organisms, including regulated cell shape changes, cell intercalation and directed cell **migration**. Thus, learning how these events are coordinated for tissue morphogenesis in an organism for which the genetic and molecular tools are unsurpassed should provide excellent paradigms for dissecting related processes in the more intricate organs of more complicated species.
7. Deficiency screen identifies a novel role for beta 2 tubulin in salivary gland and myoblast **migration** in the Drosophila embryo. The Drosophila embryonic salivary gland is an epithelial organ formed by the coordinated invagination and **migration** of primordial cells. To identify genes that regulate gland **migration** we performed a deficiency screen of the third chromosome. Here, we report on the analysis of the beta 2 tubulin isoform (beta2t) that maps at 85D15. We show that, in beta2t mutant embryos, salivary glands did not complete their posterior **migration** and that **migration** of fusion competent myoblasts and longitudinal visceral muscle founder cells between the gland and circular visceral mesoderm was delayed. We also demonstrate that gland **migration** defects correlate with reduced betaPS and alphaPS2 integrin expression in the surrounding mesoderm and that beta2t genetically interacts with genes encoding integrin alphaPS1 and alphaPS2 subunits. Our studies reveal for the first time that beta2t is expressed in embryogenesis and that beta2t plays an important role in salivary gland and myoblast **migration**, possibly through proper regulation of integrin adhesion proteins.
8. Dysfusion transcriptional control of Drosophila tracheal **migration**, adhesion, and fusion. The Drosophila dysfusion basic-helix-loop-helix-PAS transcription factor gene is expressed in specialized fusion cells that reside at the tips of migrating tracheal branches. dysfusion mutants were isolated, and genetic analysis of live embryos revealed that mutant tracheal branches migrate to close proximity but fail to recognize and adhere to each other. Misexpression of dysfusion throughout the trachea further indicated that dysfusion has the ability to both inhibit cell **migration** and promote ectopic tracheal fusion. Nineteen genes whose expression either increases or decreases in fusion cells during development were analyzed in dysfusion mutant embryos. dysfusion upregulates the levels of four genes, including the shotgun cell adhesion protein gene and the zona pellucida family transmembrane protein gene, CG13196. Misexpression experiments with CG13196 result in ectopic tracheal fusion events, suggesting that it also encodes a cell adhesion protein. Another target gene of dysfusion is members only, which inhibits protein nuclear export and influences tracheal fusion. dysfusion also indirectly downregulates protein levels of Trachealess, an important regulator of tracheal development. These results indicate that fusion cells undergo dynamic changes in gene expression as they switch from migratory to fusion modes and that dysfusion regulates a discrete, but important, set of these genes.
9. Rac function in epithelial tube morphogenesis. Epithelial cell **migration** and morphogenesis require dynamic remodeling of the actin cytoskeleton and cell-cell adhesion complexes. Numerous studies in cell culture and in model organisms have demonstrated the small GTPase Rac to be a critical regulator of these processes; however, little is known about Rac function in the morphogenic movements that drive epithelial tube formation. Here, we use the embryonic salivary glands of Drosophila to understand the role of Rac in epithelial tube morphogenesis. We show that inhibition of Rac function, either through loss of function mutations or dominant-negative mutations, disrupts salivary gland invagination and posterior **migration**. In contrast, constitutive activation of Rac induces motile behavior and subsequent cell death. We further show that Rac regulation of salivary gland morphogenesis occurs through modulation of cell-cell adhesion mediated by the E-cadherin/beta-catenin complex and that shibire, the Drosophila homolog of dynamin, functions downstream of Rac in regulating beta-catenin localization during gland morphogenesis. Our results demonstrate that regulation of cadherin-based adherens junctions by Rac is critical for salivary gland morphogenesis and that this regulation occurs through dynamin-mediated endocytosis.
10. ribbon encodes a novel BTB/POZ protein required for directed cell **migration** in Drosophila melanogaster. During development, directed cell **migration** is crucial for achieving proper shape and function of organs. One well-studied example is the embryonic development of the larval tracheal system of Drosophila, in which at least four signaling pathways coordinate cell **migration** to form an elaborate branched network essential for oxygen delivery throughout the larva. FGF signaling is required for guided **migration** of all tracheal branches, whereas the DPP, EGF receptor, and Wingless/WNT signaling pathways each mediate the formation of specific subsets of branches. Here, we characterize ribbon, which encodes a BTB/POZ-containing protein required for specific tracheal branch **migration**. In ribbon mutant tracheae, the dorsal trunk fails to form, and ventral branches are stunted; however, directed **migration**s of the dorsal and visceral branches are largely unaffected. The dorsal trunk also fails to form when FGF or Wingless/WNT signaling is lost, and we show that ribbon functions downstream of, or parallel to, these pathways to promote anterior-posterior **migration**. Directed cell **migration** of the salivary gland and dorsal epidermis are also affected in ribbon mutants, suggesting that conserved mechanisms may be employed to orient cell **migration**s in multiple tissues during development.
11. Drosophila laminins act as key regulators of basement membrane assembly and morphogenesis. Laminins are heterotrimeric molecules found in all basement membranes. In mammals, they have been involved in diverse developmental processes, from gastrulation to tissue maintenance. The Drosophila genome encodes two laminin alpha chains, one beta and one Gamma, which form two distinct laminin trimers. So far, only mutations affecting one or other trimer have been analysed. In order to study embryonic development in the complete absence of laminins, we mutated the gene encoding the sole laminin beta chain in Drosophila, LanB1, so that no trimers can be made. We show that LanB1 mutant embryos develop until the end of embryogenesis. Electron microscopy analysis of mutant embryos reveals that the basement membranes are absent and the remaining extracellular material appears disorganised and diffuse. Accordingly, abnormal accumulation of major basement membrane components, such as Collagen IV and Perlecan, is observed in mutant tissues. In addition, we show that elimination of LanB1 prevents the normal morphogenesis of most organs and tissues, including the gut, trachea, muscles and nervous system. In spite of the above structural roles for laminins, our results unravel novel functions in cell adhesion, **migration** and rearrangement. We propose that while an early function of laminins in gastrulation is not conserved in Drosophila and mammals, their function in basement membrane assembly and organogenesis seems to be maintained throughout evolution.

### ECM

#### PubMed

1. The Alg5 ortholog Wollknäuel is essential for correct epidermal differentiation during Drosophila late embryogenesis. The formation of an extracellular matrix **(EC**M) presupposes an ordered delivery of its components to ensure its stereotypic architecture. The Drosophila cuticle is an **EC**M produced by the epidermis at its apical site and is characterized by a layered organization. To understand the mechanisms of cuticle assembly during development, we have investigated early aspects of protein N-glycosylation, i.e. the attachment of a dolichol-linked oligosaccharide to distinct Asn sites of a protein known to be essential for sorting in the secretory pathway. Mutations in the Drosophila alg5 gene wollknäuel (wol) that codes for an enzyme initiating the glucosylation of the dolichol-linked oligosaccharide decrease, as expected, glucosylation and the amounts of N-glycosylated proteins such as the cuticle-organizing factor Knickkopf, without affecting their correct localization. At the same time, the polarity determinants Crumbs and atypical protein kinase C accumulate at the apical plasma membrane in wol deficient embryos. In part, these perturbations may also be caused by the unfolded protein response, which is commonly triggered by ER stress and downsizes transcription and translation in general. In any case, they are associated with the loss of cuticle layering and aberrant apical plasma membrane organization suggesting that glucosylation, either directly or indirectly through controlling protein degradation, is important for the efficient and balanced deployment of the biochemical functions of secreted and membrane-associated proteins during epidermal differentiation.

### [Ee]xtracellular [Mm]atrix

#### PubMed

1. An orchestrated program regulating secretory pathway genes and cargos by the transmembrane transcription factor CREB-H. CREB3 proteins comprise a set of ER-localized bZip transcription factors defined by the presence of a transmembrane domain. They are regulated by inter-compartmental transport, Golgi cleavage and nuclear transport where they promote appropriate transcriptional responses. Although CREB3 proteins play key roles in differentiation, inflammation and metabolism, a general framework relating their defining features to these diverse activities is lacking. We identify unique features of CREB3 organization including the ATB domain, which we show it is essential for transcriptional activity. This domain is absent in all other human bZip factors, but conserved in Drosophila CREBA, which controls secretory pathway genes (SPGs). Furthermore, each of the five human CREB3 factors was capable of activating SPGs in Drosophila, dependent upon the ATB domain. Expression of the CREB3 protein, CREB-H, in 293 cells, upregulated genes involved in secretory capacity, **extracellular matrix** formation and lipid metabolism and increased secretion of specific cargos. In liver cells, which normally express CREB-H, the active form specifically induced secretion of apolipoproteins, including ApoA-IV, ApoAI, consistent with data implicating CREB-H in metabolic homeostasis. Based on these data and other recent studies, we propose a general role for the CREB3 family in regulating secretory capacity, with particular relevance to specialized cargos.
2. The Alg5 ortholog Wollknäuel is essential for correct epidermal differentiation during Drosophila late embryogenesis. The formation of an **extracellular matri**x (ECM) presupposes an ordered delivery of its components to ensure its stereotypic architecture. The Drosophila cuticle is an ECM produced by the epidermis at its apical site and is characterized by a layered organization. To understand the mechanisms of cuticle assembly during development, we have investigated early aspects of protein N-glycosylation, i.e. the attachment of a dolichol-linked oligosaccharide to distinct Asn sites of a protein known to be essential for sorting in the secretory pathway. Mutations in the Drosophila alg5 gene wollknäuel (wol) that codes for an enzyme initiating the glucosylation of the dolichol-linked oligosaccharide decrease, as expected, glucosylation and the amounts of N-glycosylated proteins such as the cuticle-organizing factor Knickkopf, without affecting their correct localization. At the same time, the polarity determinants Crumbs and atypical protein kinase C accumulate at the apical plasma membrane in wol deficient embryos. In part, these perturbations may also be caused by the unfolded protein response, which is commonly triggered by ER stress and downsizes transcription and translation in general. In any case, they are associated with the loss of cuticle layering and aberrant apical plasma membrane organization suggesting that glucosylation, either directly or indirectly through controlling protein degradation, is important for the efficient and balanced deployment of the biochemical functions of secreted and membrane-associated proteins during epidermal differentiation.

---

## Pak3

- NCBI Gene
- GeneRIF
- Pubmed

| Search Term | Rank | Fields |
| --- | --- | --- |
| [Ff]ilopodia | 1 | PubMed(2) |
| [Pp]odosome(s)? | 5 | PubMed(1); GeneRIFs(1) |
| [Mm]igration | 6 | PubMed(5) |
| ECM | 7 | PubMed(1) |
| [Ee]xtracellular [Mm]atrix | 8 | PubMed(1) |
| [Mm]etastasis | 12 | PubMed(1) |

  
hide/show details for Pak3

### [Ff]ilopodia

#### PubMed

1. The leading edge during dorsal closure as a model for epithelial plasticity: Pak is required for recruitment of the Scribble complex and septate junction formation. Dorsal closure (DC) of the Drosophila embryo is a model for the study of wound healing and developmental epithelial fusions, and involves the sealing of a hole in the epidermis through the migration of the epidermal flanks over the tissue occupying the hole, the amnioserosa. During DC, the cells at the edge of the migrating epidermis extend Rac- and Cdc42-dependent actin-based lamellipodia and **filopodia** from their leading edge (LE), which exhibits a breakdown in apicobasal polarity as adhesions are severed with the neighbouring amnioserosa cells. Studies using mammalian cells have demonstrated that Scribble (Scrib), an important determinant of apicobasal polarity that functions in a protein complex, controls polarized cell migration through recruitment of Rac, Cdc42 and the serine/threonine kinase Pak, an effector for Rac and Cdc42, to the LE. We have used DC and the follicular epithelium to study the relationship between Pak and the Scrib complex at epithelial membranes undergoing changes in apicobasal polarity and adhesion during development. We propose that, during DC, the LE membrane undergoes an epithelial-to-mesenchymal-like transition to initiate epithelial sheet migration, followed by a mesenchymal-to-epithelial-like transition as the epithelial sheets meet up and restore cell-cell adhesion. This latter event requires integrin-localized Pak, which recruits the Scrib complex in septate junction formation. We conclude that there are bidirectional interactions between Pak and the Scrib complex modulating epithelial plasticity. Scrib can recruit Pak to the LE for polarized cell migration but, as migratory cells meet up, Pak can recruit the Scrib complex to restore apicobasal polarity and cell-cell adhesion.
2. Loss of Drosophila melanogaster p21-activated kinase 3 suppresses defects in synapse structure and function caused by spastin mutations. Microtubules are dynamic structures that must elongate, disassemble, and be cleaved into smaller pieces for proper neuronal development and function. The AAA ATPase Spastin severs microtubules along their lengths and is thought to regulate the balance between long, stable filaments and shorter fragments that seed extension or are transported. In both Drosophila and humans, loss of Spastin function results in reduction of synaptic connections and disabling motor defects. To gain insight into how spastin is regulated, we screened the Drosophila melanogaster genome for deletions that modify a spastin overexpression phenotype, eye size reduction. One suppressor region deleted p21-activated kinase 3 (pak3), which encodes a member of the Pak family of actin-regulatory enzymes, but whose in vivo function is unknown. We show that pak3 mutants have only mild synaptic defects at the larval neuromuscular junction, but exhibit a potent genetic interaction with spastin mutations. Aberrant bouton morphology, microtubule distribution, and synaptic transmission caused by spastin loss of function are all restored to wild type when pak3 is simultaneously reduced. Neuronal overexpression of pak3 induces actin-rich thin projections, suggesting that it functions in vivo to promote **filopodia** during presynaptic terminal arborization. pak3 therefore regulates synapse development in vivo, and when mutated, suppresses the synaptic defects that result from spastin loss.

### [Pp]odosome(s)?

#### PubMed

1. Group I PAKs function downstream of Rac to promote **podosome** invasion during myoblast fusion in vivo. The p21-activated kinases (PAKs) play essential roles in diverse cellular processes and are required for cell proliferation, apoptosis, polarity establishment, migration, and cell shape changes. Here, we have identified a novel function for the group I PAKs in cell-cell fusion. We show that the two Drosophila group I PAKs, DPak3 and DPak1, have partially redundant functions in myoblast fusion in vivo, with DPak3 playing a major role. DPak3 is enriched at the site of fusion colocalizing with the F-actin focus within a **podosome**-like structure (PLS), and promotes actin filament assembly during PLS invasion. Although the small GTPase Rac is involved in DPak3 activation and recruitment to the PLS, the kinase activity of DPak3 is required for effective PLS invasion. We propose a model whereby group I PAKs act downstream of Rac to organize the actin filaments within the PLS into a dense focus, which in turn promotes PLS invasion and fusion pore initiation during myoblast fusion.

#### GeneRIFs

1. The DPak3 is enriched at the site of fusion colocalizing with the F-actin focus within a **podosome**-like structure (PLS), and promotes actin filament assembly during PLS invasion.

### [Mm]igration

#### PubMed

1. dPak is required for integrity of the leading edge cytoskeleton during Drosophila dorsal closure but does not signal through the JNK cascade. The Pak kinases are effectors for the small GTPases Rac and Cdc42 and are divided into two subfamilies. Group I Paks possess an autoinhibitory domain that can suppress their kinase activity in trans. In Drosophila, two Group I kinases have been identified, dPak and Pak3. Rac and Cdc42 participate in dorsal closure of the embryo, a process in which a hole in the dorsal epidermis is sealed through **migration** of the epidermal flanks over a tissue called the amnioserosa. Dorsal closure is driven in part by an actomyosin contractile apparatus at the leading edge of the epidermis, and is regulated by a Jun amino terminal kinase (JNK) cascade. Impairment of dPak function using either loss-of-function mutations or expression of a transgene encoding the autoinhibitory domain of dPak led to disruption of the leading edge cytoskeleton and defects in dorsal closure but did not affect the JNK cascade. Group I Pak kinase activity in the amnioserosa is required for correct morphogenesis of the epidermis, and may be a component of the signaling known to occur between these two tissues. We conclude that dorsal closure requires Group I Pak function in both the amnioserosa and the epidermis.
2. The leading edge during dorsal closure as a model for epithelial plasticity: Pak is required for recruitment of the Scribble complex and septate junction formation. Dorsal closure (DC) of the Drosophila embryo is a model for the study of wound healing and developmental epithelial fusions, and involves the sealing of a hole in the epidermis through the **migration** of the epidermal flanks over the tissue occupying the hole, the amnioserosa. During DC, the cells at the edge of the migrating epidermis extend Rac- and Cdc42-dependent actin-based lamellipodia and filopodia from their leading edge (LE), which exhibits a breakdown in apicobasal polarity as adhesions are severed with the neighbouring amnioserosa cells. Studies using mammalian cells have demonstrated that Scribble (Scrib), an important determinant of apicobasal polarity that functions in a protein complex, controls polarized cell **migration** through recruitment of Rac, Cdc42 and the serine/threonine kinase Pak, an effector for Rac and Cdc42, to the LE. We have used DC and the follicular epithelium to study the relationship between Pak and the Scrib complex at epithelial membranes undergoing changes in apicobasal polarity and adhesion during development. We propose that, during DC, the LE membrane undergoes an epithelial-to-mesenchymal-like transition to initiate epithelial sheet **migration**, followed by a mesenchymal-to-epithelial-like transition as the epithelial sheets meet up and restore cell-cell adhesion. This latter event requires integrin-localized Pak, which recruits the Scrib complex in septate junction formation. We conclude that there are bidirectional interactions between Pak and the Scrib complex modulating epithelial plasticity. Scrib can recruit Pak to the LE for polarized cell **migration** but, as migratory cells meet up, Pak can recruit the Scrib complex to restore apicobasal polarity and cell-cell adhesion.
3. Pak3 regulates apical-basal polarity in migrating border cells during Drosophila oogenesis. Group cell **migration** is a highly coordinated process that is involved in a number of physiological events such as morphogenesis, wound healing and tumor metastasis. Unlike single cells, collectively moving cells are physically attached to each other and retain some degree of apical-basal polarity during the migratory phase. Although much is known about direction sensing, how polarity is regulated in multicellular movement remains unclear. Here we report the role of the protein kinase Pak3 in maintaining apical-basal polarity in migrating border cell clusters during Drosophila oogenesis. Pak3 is enriched in border cells and downregulation of its function impedes border cell movement. Time-lapse imaging suggests that Pak3 affects protrusive behavior of the border cell cluster, specifically regulating the stability and directionality of protrusions. Pak3 functions downstream of guidance receptor signaling to regulate the level and distribution of F-actin in migrating border cells. We also provide evidence that Pak3 genetically interacts with the lateral polarity marker Scribble and that it regulates JNK signaling in the moving border cells. Since Pak3 depletion results in mislocalization of several apical-basal polarity markers and overexpression of Jra rescues the polarity of the Pak3-depleted cluster, we propose that Pak3 functions through JNK signaling to modulate apical-basal polarity of the migrating border cell cluster. We also observe loss of apical-basal polarity in Rac1-depleted border cell clusters, suggesting that guidance receptor signaling functions through Rac GTPase and Pak3 to regulate the overall polarity of the cluster and mediate efficient collective movement of the border cells to the oocyte boundary.
4. Requirement for Pak3 in Rac1-induced organization of actin and myosin during Drosophila larval wound healing. Rho-family small GTPases regulate epithelial cell sheet **migration** by organizing actin and myosin during wound healing. Here, we report that Pak3, but not Pak1, is a downstream target protein for Rac1 in wound closure of the Drosophila larval epidermis. Pak3-deficient larvae failed to close a wound hole and this defect was not rescued by Pak1 expression, indicating differential functions of the two proteins. Pak3 localized to the wound margin, which selectively required Rac1. Pak3-deficient larvae showed severe defects in actin-myosin organization at the wound margin and in submarginal cells, which was reminiscent of the phenotypes of Rac1-deficient larvae. These results suggest that Pak3 specifically mediates Rac1 signaling in organizing actin and myosin during Drosophila epidermal wound healing.
5. Group I PAKs function downstream of Rac to promote podosome invasion during myoblast fusion in vivo. The p21-activated kinases (PAKs) play essential roles in diverse cellular processes and are required for cell proliferation, apoptosis, polarity establishment, **migration**, and cell shape changes. Here, we have identified a novel function for the group I PAKs in cell-cell fusion. We show that the two Drosophila group I PAKs, DPak3 and DPak1, have partially redundant functions in myoblast fusion in vivo, with DPak3 playing a major role. DPak3 is enriched at the site of fusion colocalizing with the F-actin focus within a podosome-like structure (PLS), and promotes actin filament assembly during PLS invasion. Although the small GTPase Rac is involved in DPak3 activation and recruitment to the PLS, the kinase activity of DPak3 is required for effective PLS invasion. We propose a model whereby group I PAKs act downstream of Rac to organize the actin filaments within the PLS into a dense focus, which in turn promotes PLS invasion and fusion pore initiation during myoblast fusion.

### ECM

#### PubMed

1. Induction of focal adhesions and motility in Drosophila S2 cells. Focal adhesions are dynamic structures that interact with the extracellular matrix on the cell exterior and actin filaments on the cell interior, enabling cells to adhere and crawl along surfaces. We describe a system for inducing the formation of focal adhesions in normally non-**ECM**-adherent, nonmotile Drosophila S2 cells. These focal adhesions contain the expected molecular markers such as talin, vinculin, and p130Cas, and they require talin for their formation. The S2 cells with induced focal adhesions also display a nonpolarized form of motility on vitronectin-coated substrates. Consistent with findings in mammalian cells, the degree of motility can be tuned by changing the stiffness of the substrate and was increased after the depletion of PAK3, a p21-activated kinase. A subset of nonmotile, nonpolarized cells also exhibited focal adhesions that rapidly assembled and disassembled around the cell perimeter. Such cooperative and dynamic fluctuations of focal adhesions were decreased by RNA interference (RNAi) depletion of myosin II and focal adhesion kinase, suggesting that this behavior requires force and focal adhesion maturation. These results demonstrate that S2 cells, a cell line that is well studied for cytoskeletal dynamics and readily amenable to protein manipulation by RNAi, can be used to study the assembly and dynamics of focal adhesions and mechanosensitive cell motility.

### [Ee]xtracellular [Mm]atrix

#### PubMed

1. Induction of focal adhesions and motility in Drosophila S2 cells. Focal adhesions are dynamic structures that interact with the **extracellular matrix** on the cell exterior and actin filaments on the cell interior, enabling cells to adhere and crawl along surfaces. We describe a system for inducing the formation of focal adhesions in normally non-ECM-adherent, nonmotile Drosophila S2 cells. These focal adhesions contain the expected molecular markers such as talin, vinculin, and p130Cas, and they require talin for their formation. The S2 cells with induced focal adhesions also display a nonpolarized form of motility on vitronectin-coated substrates. Consistent with findings in mammalian cells, the degree of motility can be tuned by changing the stiffness of the substrate and was increased after the depletion of PAK3, a p21-activated kinase. A subset of nonmotile, nonpolarized cells also exhibited focal adhesions that rapidly assembled and disassembled around the cell perimeter. Such cooperative and dynamic fluctuations of focal adhesions were decreased by RNA interference (RNAi) depletion of myosin II and focal adhesion kinase, suggesting that this behavior requires force and focal adhesion maturation. These results demonstrate that S2 cells, a cell line that is well studied for cytoskeletal dynamics and readily amenable to protein manipulation by RNAi, can be used to study the assembly and dynamics of focal adhesions and mechanosensitive cell motility.

### [Mm]etastasis

#### PubMed

1. Pak3 regulates apical-basal polarity in migrating border cells during Drosophila oogenesis. Group cell migration is a highly coordinated process that is involved in a number of physiological events such as morphogenesis, wound healing and tumor **metastasis**. Unlike single cells, collectively moving cells are physically attached to each other and retain some degree of apical-basal polarity during the migratory phase. Although much is known about direction sensing, how polarity is regulated in multicellular movement remains unclear. Here we report the role of the protein kinase Pak3 in maintaining apical-basal polarity in migrating border cell clusters during Drosophila oogenesis. Pak3 is enriched in border cells and downregulation of its function impedes border cell movement. Time-lapse imaging suggests that Pak3 affects protrusive behavior of the border cell cluster, specifically regulating the stability and directionality of protrusions. Pak3 functions downstream of guidance receptor signaling to regulate the level and distribution of F-actin in migrating border cells. We also provide evidence that Pak3 genetically interacts with the lateral polarity marker Scribble and that it regulates JNK signaling in the moving border cells. Since Pak3 depletion results in mislocalization of several apical-basal polarity markers and overexpression of Jra rescues the polarity of the Pak3-depleted cluster, we propose that Pak3 functions through JNK signaling to modulate apical-basal polarity of the migrating border cell cluster. We also observe loss of apical-basal polarity in Rac1-depleted border cell clusters, suggesting that guidance receptor signaling functions through Rac GTPase and Pak3 to regulate the overall polarity of the cluster and mediate efficient collective movement of the border cells to the oocyte boundary.

---

## Tep2

- NCBI Gene
- GeneRIF
- Pubmed

| Search Term | Rank | Fields |
| --- | --- | --- |
| [Ee]xtracellular [Mm]atrix | 8 | PubMed(1) |

  
hide/show details for Tep2

### [Ee]xtracellular [Mm]atrix

#### PubMed

1. Genome-wide transcriptional analysis of Drosophila larvae infected by entomopathogenic nematodes shows involvement of complement, recognition and **extracellular matrix** proteins. Heterorhabditis bacteriophora is an entomopathogenic nematode (EPN) which infects its host by accessing the hemolymph where it releases endosymbiotic bacteria of the species Photorhabdus luminescens. We performed a genome-wide transcriptional analysis of the Drosophila response to EPN infection at the time point at which the nematodes reached the hemolymph either via the cuticle or the gut and the bacteria had started to multiply. Many of the most strongly induced genes have been implicated in immune responses in other infection models. Mapping of the complete set of differentially regulated genes showed the hallmarks of a wound response, but also identified a large fraction of EPN-specific transcripts. Several genes identified by transcriptome profiling or their homologues play protective roles during nematode infections. Genes that positively contribute to controlling nematobacterial infections encode: a homolog of thioester-containing complement protein 3, a basement membrane component (glutactin), a recognition protein (GNBP-like 3) and possibly several small peptides. Of note is that several of these genes have not previously been implicated in immune responses.

---

## h

- NCBI Gene
- GeneRIF
- Pubmed

| Search Term | Rank | Fields |
| --- | --- | --- |
| [Mm]igration | 6 | PubMed(5) |
| ECM | 7 | PubMed(1) |
| [Ee]xtracellular [Mm]atrix | 8 | PubMed(1) |

  
hide/show details for h

### [Mm]igration

#### PubMed

1. Identification of genes controlling germ cell **migration** and embryonic gonad formation in Drosophila. Gonadogenesis in the Drosophila embryo is a complex process involving numerous cellular migratory steps and cell-cell interactions. The mechanisms guiding germ cells to move through, recognize and adhere to specific cell types are poorly understood. In order to identify genes that are required for these processes, we have conducted an extensive mutagenesis of the third chromosome and screened for mutations disrupting germ cell **migration** at any point in embryonic development. Phenotypic analysis of these mutants demonstrates that germ cell **migration** can be broken down into discrete developmental steps, with each step requiring a specific set of genes. Many of these genes are involved in the development of gonadal mesoderm, the tissue that associates with germ cells to form the embryonic gonad. Moreover, mutations that we isolated affecting embryonic patterning as well as germ cell **migration** suggest that the origin of gonadal mesoderm lies within the eve domain of the developing mesoderm.
2. Genes conserved in bilaterians but jointly lost with Myc during nematode evolution are enriched in cell proliferation and cell **migration** functions. Animals use a stereotypical set of developmental genes to build body architectures of varying sizes and organizational complexity. Some genes are critical to developmental patterning, while other genes are important to physiological control of growth. However, growth regulator genes may not be as important in small-bodied "micro-metazoans" such as nematodes. Nematodes use a simplified developmental strategy of lineage-based cell fate specifications to produce an adult bilaterian body composed of a few hundreds of cells. Nematodes also lost the MYC proto-oncogenic regulator of cell proliferation. To identify additional regulators of cell proliferation that were lost with MYC, we computationally screened and determined 839 high-confidence genes that are conserved in bilaterians/lost in nematodes (CIBLIN genes). We find that 30 % of all CIBLIN genes encode transcriptional regulators of cell proliferation, epithelial-to-mesenchyme transitions, and other processes. Over 50 % of CIBLIN genes are unnamed genes in Drosophila, suggesting that there are many understudied genes. Interestingly, CIBLIN genes include many Myc synthetic lethal (MycSL) hits from recent screens. CIBLIN genes include key regulators of heparan sulfate proteoglycan (HSPG) sulfation patterns, and lysyl oxidases involved in cross-linking and modification of the extracellular matrix (ECM). These genes and others suggest the CIBLIN repertoire services critical functions in ECM remodeling and cell **migration** in large-bodied bilaterians. Correspondingly, CIBLIN genes are co-expressed with Myc in cancer transcriptomes, and include a preponderance of known determinants of cancer progression and tumor aggression. We propose that CIBLIN gene research can improve our understanding of regulatory control of cellular growth in metazoans.
3. A molecular link between FGF and Dpp signaling in branch-specific **migration** of the Drosophila trachea. The tracheal system of Drosophila embryos achieves its archetypal branching pattern through a series of cell **migration** events requiring the FGF, Dpp, and Wg/WNT signaling pathways. To gain insight into tracheal cell **migration**, we performed an F4 EMS mutagenesis screen to generate and characterize new mutations resulting in tracheal defects. From 2591 mutagenized third chromosome lines, we identified 33 mutations with defects in tracheal development, corresponding to 12 distinct complementation groups. The new mutations included novel hypomorphic alleles of the FGF receptor gene, breathless, and the ETS-domain transcription factor gene, pointed. We show that reduced function of either breathless or pointed specifically affects **migration** of the dorsal and ventral tracheal branches, more specific functions than previously described for these genes. Our analysis reveals that breathless and pointed control dorsal branch **migration** through transcriptional regulation of the Dpp pathway effectors, Knirps and Knirps-related, which are necessary for **migration** of this branch. We further show that expression of knirps or knirps-related rescues dorsal but not ventral branch **migration** in the breathless hypomorph. These studies support a model in which both the Dpp- and the FGF-signaling pathways control expression of knirps and knirps-related, thereby regulating cell **migration** during dorsal branch formation.
4. Extramacrochaetae imposes order on the Drosophila eye by refining the activity of the Hedgehog signaling gradient. The compound eye of Drosophila melanogaster is configured by a differentiating wave, the morphogenetic furrow, that sweeps across the eye imaginal disc and transforms thousands of undifferentiated cells into a precisely ordered repetitive array of 800 ommatidia. The initiation of the furrow at the posterior margin of the epithelium and its subsequent movement across the eye field is controlled by the activity of the Hedgehog (Hh) signaling pathway. Differentiating photoreceptors that lie behind the furrow produce and secrete the Hh morphogen, which is captured by cells within the furrow itself. This leads to the stabilization of the full-length form of the zinc-finger transcription factor Cubitus interruptus (Ci(155)), the main effector of Hh signaling. Ci(155) functions as a transcriptional activator of a number of downstream targets, including decapentaplegic (dpp), a TGFβ homolog. In this report, we describe a mechanism that is in place within the fly retina to limit Hh pathway activity within and ahead of the furrow. We demonstrate that the helix-loop-helix (HLH) protein Extramacrochaetae (Emc) regulates Ci(155) levels. Loss of emc leads to an increase in Ci(155) levels, nuclear **migratio**n, apical cell constriction and an acceleration of the furrow. We find that these roles are distinct from the bHLH protein Hairy (H), which we show restricts atonal (ato) expression ahead of the furrow. Secondary furrow initiation along the dorsal and ventral margins is blocked by the activity of the Wingless (Wg) pathway. We also show that Emc regulates and cooperates with Wg signaling to inhibit lateral furrow initiation.
5. Tube morphogenesis: closure, but many openings remain. Epithelial and endothelial tubes form the basic structure of many organs and tissues in the fruit fly Drosophila melanogaster, the nematode Caenorhabditis elegans, zebrafish and mammals. Comparison of how tubes form during development defines several pathways that generate a single unbranched tube or dichotomously branching tubular networks. The formation of tubes can be induced directly by intrinsic signals within epithelial primordia or by inductive signaling between adjacent epithelia and the mesenchyme. Both processes are hierarchically controlled by master transcriptional regulators, growth factors and their receptors, directed cell **migration** and cellular reorganization, which is controlled by changes in the cytoskeleton and protein trafficking. This review provides a summary of these pathways based upon articles published in the Tube Morphogenesis Series in Trends in Cell Biology.

### ECM

#### PubMed

1. Genes conserved in bilaterians but jointly lost with Myc during nematode evolution are enriched in cell proliferation and cell migration functions. Animals use a stereotypical set of developmental genes to build body architectures of varying sizes and organizational complexity. Some genes are critical to developmental patterning, while other genes are important to physiological control of growth. However, growth regulator genes may not be as important in small-bodied "micro-metazoans" such as nematodes. Nematodes use a simplified developmental strategy of lineage-based cell fate specifications to produce an adult bilaterian body composed of a few hundreds of cells. Nematodes also lost the MYC proto-oncogenic regulator of cell proliferation. To identify additional regulators of cell proliferation that were lost with MYC, we computationally screened and determined 839 high-confidence genes that are conserved in bilaterians/lost in nematodes (CIBLIN genes). We find that 30 % of all CIBLIN genes encode transcriptional regulators of cell proliferation, epithelial-to-mesenchyme transitions, and other processes. Over 50 % of CIBLIN genes are unnamed genes in Drosophila, suggesting that there are many understudied genes. Interestingly, CIBLIN genes include many Myc synthetic lethal (MycSL) hits from recent screens. CIBLIN genes include key regulators of heparan sulfate proteoglycan (HSPG) sulfation patterns, and lysyl oxidases involved in cross-linking and modification of the extracellular matrix (**ECM**). These genes and others suggest the CIBLIN repertoire services critical functions in **ECM** remodeling and cell migration in large-bodied bilaterians. Correspondingly, CIBLIN genes are co-expressed with Myc in cancer transcriptomes, and include a preponderance of known determinants of cancer progression and tumor aggression. We propose that CIBLIN gene research can improve our understanding of regulatory control of cellular growth in metazoans.

### [Ee]xtracellular [Mm]atrix

#### PubMed

1. Genes conserved in bilaterians but jointly lost with Myc during nematode evolution are enriched in cell proliferation and cell migration functions. Animals use a stereotypical set of developmental genes to build body architectures of varying sizes and organizational complexity. Some genes are critical to developmental patterning, while other genes are important to physiological control of growth. However, growth regulator genes may not be as important in small-bodied "micro-metazoans" such as nematodes. Nematodes use a simplified developmental strategy of lineage-based cell fate specifications to produce an adult bilaterian body composed of a few hundreds of cells. Nematodes also lost the MYC proto-oncogenic regulator of cell proliferation. To identify additional regulators of cell proliferation that were lost with MYC, we computationally screened and determined 839 high-confidence genes that are conserved in bilaterians/lost in nematodes (CIBLIN genes). We find that 30 % of all CIBLIN genes encode transcriptional regulators of cell proliferation, epithelial-to-mesenchyme transitions, and other processes. Over 50 % of CIBLIN genes are unnamed genes in Drosophila, suggesting that there are many understudied genes. Interestingly, CIBLIN genes include many Myc synthetic lethal (MycSL) hits from recent screens. CIBLIN genes include key regulators of heparan sulfate proteoglycan (HSPG) sulfation patterns, and lysyl oxidases involved in cross-linking and modification of the **extracellular matrix** (ECM). These genes and others suggest the CIBLIN repertoire services critical functions in ECM remodeling and cell migration in large-bodied bilaterians. Correspondingly, CIBLIN genes are co-expressed with Myc in cancer transcriptomes, and include a preponderance of known determinants of cancer progression and tumor aggression. We propose that CIBLIN gene research can improve our understanding of regulatory control of cellular growth in metazoans.

---

## h

- NCBI Gene
- GeneRIF
- Pubmed

| Search Term | Rank | Fields |
| --- | --- | --- |
| [Mm]igration | 6 | PubMed(1) |
| [Ee]xtracellular [Mm]atrix | 8 | PubMed(2) |

  
hide/show details for h

### [Mm]igration

#### PubMed

1. Delta signaling from the germ line controls the proliferation and differentiation of the somatic follicle cells during Drosophila oogenesis. The body axes of Drosophila are established during oogenesis through reciprocal interactions between the germ line cells and the somatic follicle cells that surround them. The Notch pathway is required at two stages in this process: first, for the **migration** of the follicle cells around the germ line cyst and, later, for the polarization of the anterior-posterior (A-P) axis of the oocyte. Its function in these events, however, has remained controversial. Using clonal analysis, we show that Notch signaling controls cell proliferation and differentiation in the whole follicular epithelium. Notch mutant follicle cells remain in a precursor state and fail to switch from the mitotic cell cycle to the endocycle. Furthermore, removal of Delta from the germ line produces an identical phenotype, showing that Delta signals from the germ cells to control the timing of follicle cell differentiation. This explains the axis formation defects in Notch mutants, which arise because undifferentiated posterior follicle cells cannot signal to polarize the oocyte. Delta also signals from the germ line to Notch in the soma earlier in oogenesis to control the differentiation of the polar and stalk follicle cells. The germ line therefore regulates the development of the follicle cells through two complementary signaling pathways: Gurken signals twice to control spatial patterning, whereas Delta signals twice to exert temporal control.

### [Ee]xtracellular [Mm]atrix

#### PubMed

1. Presenilin-based genetic screens in Drosophila melanogaster identify novel notch pathway modifiers. Presenilin is the enzymatic component of gamma-secretase, a multisubunit intramembrane protease that processes several transmembrane receptors, such as the amyloid precursor protein (APP). Mutations in human Presenilins lead to altered APP cleavage and early-onset Alzheimer's disease. Presenilins also play an essential role in Notch receptor cleavage and signaling. The Notch pathway is a highly conserved signaling pathway that functions during the development of multicellular organisms, including vertebrates, Drosophila, and C. elegans. Recent studies have shown that Notch signaling is sensitive to perturbations in subcellular trafficking, although the specific mechanisms are largely unknown. To identify genes that regulate Notch pathway function, we have performed two genetic screens in Drosophila for modifiers of Presenilin-dependent Notch phenotypes. We describe here the cloning and identification of 19 modifiers, including nicastrin and several genes with previously undescribed involvement in Notch biology. The predicted functions of these newly identified genes are consistent with **extracellular matrix** and vesicular trafficking mechanisms in Presenilin and Notch pathway regulation and suggest a novel role for gamma-tubulin in the pathway.
2. Modifiers of notch transcriptional activity identified by genome-wide RNAi. The Notch signaling pathway regulates a diverse array of developmental processes, and aberrant Notch signaling can lead to diseases, including cancer. To obtain a more comprehensive understanding of the genetic network that integrates into Notch signaling, we performed a genome-wide RNAi screen in Drosophila cell culture to identify genes that modify Notch-dependent transcription.Employing complementary data analyses, we found 399 putative modifiers: 189 promoting and 210 antagonizing Notch activated transcription. These modifiers included several known Notch interactors, validating the robustness of the assay. Many novel modifiers were also identified, covering a range of cellular localizations from the **extracellular matrix** to the nucleus, as well as a large number of proteins with unknown function. Chromatin-modifying proteins represent a major class of genes identified, including histone deacetylase and demethylase complex components and other chromatin modifying, remodeling and replacement factors. A protein-protein interaction map of the Notch-dependent transcription modifiers revealed that a large number of the identified proteins interact physically with these core chromatin components.The genome-wide RNAi screen identified many genes that can modulate Notch transcriptional output. A protein interaction map of the identified genes highlighted a network of chromatin-modifying enzymes and remodelers that regulate Notch transcription. Our results open new avenues to explore the mechanisms of Notch signal regulation and the integration of this pathway into diverse cellular processes.

---

## Myo31DF

- NCBI Gene
- GeneRIF
- Pubmed

| Search Term | Rank | Fields |
| --- | --- | --- |
| [Mm]igration | 6 | PubMed(1) |
| ECM | 7 | PubMed(1) |
| [Ee]xtracellular [Mm]atrix | 8 | PubMed(1) |
| [Mm]etastasis | 12 | PubMed(1) |
| [Cc]ell [Ii]nvasion | 13 | PubMed(1) |

  
hide/show details for Myo31DF

### [Mm]igration

#### PubMed

1. Molecular requirements for actin-based lamella formation in Drosophila S2 cells. Cell **migration** occurs through the protrusion of the actin-enriched lamella. Here, we investigated the effects of RNAi depletion of approximately 90 proteins implicated in actin function on lamella formation in Drosophila S2 cells. Similar to in vitro reconstitution studies of actin-based Listeria movement, we find that lamellae formation requires a relatively small set of proteins that participate in actin nucleation (Arp2/3 and SCAR), barbed end capping (capping protein), filament depolymerization (cofilin and Aip1), and actin monomer binding (profilin and cyclase-associated protein). Lamellae are initiated by parallel and partially redundant signaling pathways involving Rac GTPases and the adaptor protein Nck, which stimulate SCAR, an Arp2/3 activator. We also show that RNAi of three proteins (kette, Abi, and Sra-1) known to copurify with and inhibit SCAR in vitro leads to SCAR degradation, revealing a novel function of this protein complex in SCAR stability. Our results have identified an essential set of proteins involved in actin dynamics during lamella formation in Drosophila S2 cells.

### ECM

#### PubMed

1. Requirement of matrix metalloproteinase-1 for intestinal homeostasis in the adult Drosophila midgut. Stem cells are tightly regulated by both intrinsic and extrinsic signals as well as the extracellular matrix (**ECM**) for tissue homeostasis and regenerative capacity. Matrix metalloproteinases (MMPs), proteolytic enzymes, modulate the turnover of numerous substrates, including cytokine precursors, growth factors, and **ECM** molecules. However, the roles of MMPs in the regulation of adult stem cells are poorly understood. In the present study, we utilize the Drosophila midgut, which is an excellent model system for studying stem cell biology, to show that Mmp1 is involved in the regulation of intestinal stem cells (ISCs). The results showed that Mmp1 is expressed in the adult midgut and that its expression increases with age and with exposure to oxidative stress. Mmp1 knockdown or Timp-overexpressing flies and flies heterozygous for a viable, hypomorphic Mmp1 allele increased ISC proliferation in the gut, as shown by staining with an anti-phospho-histone H3 antibody and BrdU incorporation assays. Reduced Mmp1 levels induced intestinal hyperplasia, and the Mmp1depletion-induced ISC proliferation was rescued by the suppression of the EGFR signaling pathway, suggesting that Mmp1 regulates ISC proliferation through the EGFR signaling pathway. Furthermore, adult gut-specific knockdown and whole-animal heterozygotes of Mmp1 increased additively sensitivity to paraquat-induced oxidative stress and shortened lifespan. Our data suggest that Drosophila Mmp1 is involved in the regulation of ISC proliferation for maintenance of gut homeostasis.

### [Ee]xtracellular [Mm]atrix

#### PubMed

1. Requirement of matrix metalloproteinase-1 for intestinal homeostasis in the adult Drosophila midgut. Stem cells are tightly regulated by both intrinsic and extrinsic signals as well as the **extracellular matrix** (ECM) for tissue homeostasis and regenerative capacity. Matrix metalloproteinases (MMPs), proteolytic enzymes, modulate the turnover of numerous substrates, including cytokine precursors, growth factors, and ECM molecules. However, the roles of MMPs in the regulation of adult stem cells are poorly understood. In the present study, we utilize the Drosophila midgut, which is an excellent model system for studying stem cell biology, to show that Mmp1 is involved in the regulation of intestinal stem cells (ISCs). The results showed that Mmp1 is expressed in the adult midgut and that its expression increases with age and with exposure to oxidative stress. Mmp1 knockdown or Timp-overexpressing flies and flies heterozygous for a viable, hypomorphic Mmp1 allele increased ISC proliferation in the gut, as shown by staining with an anti-phospho-histone H3 antibody and BrdU incorporation assays. Reduced Mmp1 levels induced intestinal hyperplasia, and the Mmp1depletion-induced ISC proliferation was rescued by the suppression of the EGFR signaling pathway, suggesting that Mmp1 regulates ISC proliferation through the EGFR signaling pathway. Furthermore, adult gut-specific knockdown and whole-animal heterozygotes of Mmp1 increased additively sensitivity to paraquat-induced oxidative stress and shortened lifespan. Our data suggest that Drosophila Mmp1 is involved in the regulation of ISC proliferation for maintenance of gut homeostasis.

### [Mm]etastasis

#### PubMed

1. Synergy between bacterial infection and genetic predisposition in intestinal dysplasia. Accumulating evidence suggests that hyperproliferating intestinal stem cells (SCs) and progenitors drive cancer initiation, maintenance, and **metastasis**. In addition, chronic inflammation and infection have been increasingly recognized for their roles in cancer. Nevertheless, the mechanisms by which bacterial infections can initiate SC-mediated tumorigenesis remain elusive. Using a Drosophila model of gut pathogenesis, we show that intestinal infection with Pseudomonas aeruginosa, a human opportunistic bacterial pathogen, activates the c-Jun N-terminal kinase (JNK) pathway, a hallmark of the host stress response. This, in turn, causes apoptosis of enterocytes, the largest class of differentiated intestinal cells, and promotes a dramatic proliferation of SCs and progenitors that serves as a homeostatic compensatory mechanism to replenish the apoptotic enterocytes. However, we find that this homeostatic mechanism can lead to massive over-proliferation of intestinal cells when infection occurs in animals with a latent oncogenic form of the Ras1 oncogene. The affected intestines develop excess layers of cells with altered apicobasal polarity reminiscent of dysplasia, suggesting that infection can directly synergize with the genetic background in predisposed individuals to initiate SC-mediated tumorigenesis. Our results provide a framework for the study of intestinal bacterial infections and their effects on undifferentiated and mature enteric epithelial cells in the initial stages of intestinal cancer. Assessment of progenitor cell responses to pathogenic intestinal bacteria could provide a measure of predisposition for apoptotic enterocyte-assisted intestinal dysplasias in humans.

### [Cc]ell [Ii]nvasion

#### PubMed

1. Rickettsia parkeri invasion of diverse host cells involves an Arp2/3 complex, WAVE complex and Rho-family GTPase-dependent pathway. Rickettsiae are obligate intracellular pathogens that are transmitted to humans by arthropod vectors and cause diseases such as spotted fever and typhus. Although rickettsiae require the host cell actin cytoskeleton for invasion, the cytoskeletal proteins that mediate this process have not been completely described. To identify the host factors important during **cell invasion** by Rickettsia parkeri, a member of the spotted fever group (SFG), we performed an RNAi screen targeting 105 proteins in Drosophila melanogaster S2R+ cells. The screen identified 21 core proteins important for invasion, including the GTPases Rac1 and Rac2, the WAVE nucleation-promoting factor complex and the Arp2/3 complex. In mammalian cells, including endothelial cells, the natural targets of R. parkeri, the Arp2/3 complex was also crucial for invasion, while requirements for WAVE2 as well as Rho GTPases depended on the particular cell type. We propose that R. parkeri invades S2R+ arthropod cells through a primary pathway leading to actin nucleation, whereas invasion of mammalian endothelial cells occurs via redundant pathways that converge on the host Arp2/3 complex. Our results reveal a key role for the WAVE and Arp2/3 complexes, as well as a higher degree of variation than previously appreciated in actin nucleation pathways activated during Rickettsia invasion.

---

## CG18278

- NCBI Gene
- GeneRIF
- Pubmed

| Search Term | Rank | Fields |
| --- | --- | --- |
| [Mm]igration | 6 | PubMed(1) |
| ECM | 7 | PubMed(1) |
| [Ee]xtracellular [Mm]atrix | 8 | PubMed(1) |

  
hide/show details for CG18278

### [Mm]igration

#### PubMed

1. Genes conserved in bilaterians but jointly lost with Myc during nematode evolution are enriched in cell proliferation and cell **migration** functions. Animals use a stereotypical set of developmental genes to build body architectures of varying sizes and organizational complexity. Some genes are critical to developmental patterning, while other genes are important to physiological control of growth. However, growth regulator genes may not be as important in small-bodied "micro-metazoans" such as nematodes. Nematodes use a simplified developmental strategy of lineage-based cell fate specifications to produce an adult bilaterian body composed of a few hundreds of cells. Nematodes also lost the MYC proto-oncogenic regulator of cell proliferation. To identify additional regulators of cell proliferation that were lost with MYC, we computationally screened and determined 839 high-confidence genes that are conserved in bilaterians/lost in nematodes (CIBLIN genes). We find that 30 % of all CIBLIN genes encode transcriptional regulators of cell proliferation, epithelial-to-mesenchyme transitions, and other processes. Over 50 % of CIBLIN genes are unnamed genes in Drosophila, suggesting that there are many understudied genes. Interestingly, CIBLIN genes include many Myc synthetic lethal (MycSL) hits from recent screens. CIBLIN genes include key regulators of heparan sulfate proteoglycan (HSPG) sulfation patterns, and lysyl oxidases involved in cross-linking and modification of the extracellular matrix (ECM). These genes and others suggest the CIBLIN repertoire services critical functions in ECM remodeling and cell **migration** in large-bodied bilaterians. Correspondingly, CIBLIN genes are co-expressed with Myc in cancer transcriptomes, and include a preponderance of known determinants of cancer progression and tumor aggression. We propose that CIBLIN gene research can improve our understanding of regulatory control of cellular growth in metazoans.

### ECM

#### PubMed

1. Genes conserved in bilaterians but jointly lost with Myc during nematode evolution are enriched in cell proliferation and cell migration functions. Animals use a stereotypical set of developmental genes to build body architectures of varying sizes and organizational complexity. Some genes are critical to developmental patterning, while other genes are important to physiological control of growth. However, growth regulator genes may not be as important in small-bodied "micro-metazoans" such as nematodes. Nematodes use a simplified developmental strategy of lineage-based cell fate specifications to produce an adult bilaterian body composed of a few hundreds of cells. Nematodes also lost the MYC proto-oncogenic regulator of cell proliferation. To identify additional regulators of cell proliferation that were lost with MYC, we computationally screened and determined 839 high-confidence genes that are conserved in bilaterians/lost in nematodes (CIBLIN genes). We find that 30 % of all CIBLIN genes encode transcriptional regulators of cell proliferation, epithelial-to-mesenchyme transitions, and other processes. Over 50 % of CIBLIN genes are unnamed genes in Drosophila, suggesting that there are many understudied genes. Interestingly, CIBLIN genes include many Myc synthetic lethal (MycSL) hits from recent screens. CIBLIN genes include key regulators of heparan sulfate proteoglycan (HSPG) sulfation patterns, and lysyl oxidases involved in cross-linking and modification of the extracellular matrix (**ECM**). These genes and others suggest the CIBLIN repertoire services critical functions in **ECM** remodeling and cell migration in large-bodied bilaterians. Correspondingly, CIBLIN genes are co-expressed with Myc in cancer transcriptomes, and include a preponderance of known determinants of cancer progression and tumor aggression. We propose that CIBLIN gene research can improve our understanding of regulatory control of cellular growth in metazoans.

### [Ee]xtracellular [Mm]atrix

#### PubMed

1. Genes conserved in bilaterians but jointly lost with Myc during nematode evolution are enriched in cell proliferation and cell migration functions. Animals use a stereotypical set of developmental genes to build body architectures of varying sizes and organizational complexity. Some genes are critical to developmental patterning, while other genes are important to physiological control of growth. However, growth regulator genes may not be as important in small-bodied "micro-metazoans" such as nematodes. Nematodes use a simplified developmental strategy of lineage-based cell fate specifications to produce an adult bilaterian body composed of a few hundreds of cells. Nematodes also lost the MYC proto-oncogenic regulator of cell proliferation. To identify additional regulators of cell proliferation that were lost with MYC, we computationally screened and determined 839 high-confidence genes that are conserved in bilaterians/lost in nematodes (CIBLIN genes). We find that 30 % of all CIBLIN genes encode transcriptional regulators of cell proliferation, epithelial-to-mesenchyme transitions, and other processes. Over 50 % of CIBLIN genes are unnamed genes in Drosophila, suggesting that there are many understudied genes. Interestingly, CIBLIN genes include many Myc synthetic lethal (MycSL) hits from recent screens. CIBLIN genes include key regulators of heparan sulfate proteoglycan (HSPG) sulfation patterns, and lysyl oxidases involved in cross-linking and modification of the **extracellular matrix** (ECM). These genes and others suggest the CIBLIN repertoire services critical functions in ECM remodeling and cell migration in large-bodied bilaterians. Correspondingly, CIBLIN genes are co-expressed with Myc in cancer transcriptomes, and include a preponderance of known determinants of cancer progression and tumor aggression. We propose that CIBLIN gene research can improve our understanding of regulatory control of cellular growth in metazoans.

---

## sog

- NCBI Gene
- GeneRIF
- Pubmed

| Search Term | Rank | Fields |
| --- | --- | --- |
| [Mm]igration | 6 | PubMed(3) |
| ECM | 7 | PubMed(2) |
| [Ee]xtracellular [Mm]atrix | 8 | PubMed(8) |
| [Ii]nvasive | 10 | PubMed(1) |
| [Mm]etastasis | 12 | PubMed(1) |

  
hide/show details for sog

### [Mm]igration

#### PubMed

1. Transcriptional repression via antilooping in the Drosophila embryo. Transcriptional repressors are thought to inhibit gene expression by interfering with the binding or function of RNA Polymerase II, perhaps by promoting local chromatin condensation. Here, we present evidence for a distinctive mechanism of repression, whereby sequence-specific repressors prevent the looping of distal enhancers to the promoter. Particular efforts focus on the Snail repressor, which plays a conserved role in promoting epithelial-mesenchyme transitions in both invertebrates and vertebrates, including mesoderm invagination in Drosophila, neural crest **migration** in vertebrates, and tumorigenesis in mammals. Chromosome conformation capture experiments were used to examine enhancer looping at Snail target genes in wild-type and mutant embryos. These studies suggest that the Snail repressor blocks the formation of fruitful enhancer-promoter interactions when bound to a distal enhancer. This higher-order mechanism of transcriptional repression has broad implications for the control of gene activity in metazoan development.
2. Interactions between the EGF receptor and DPP pathways establish distinct cell fates in the tracheal placodes. The formation of the tracheal network in Drosophila is driven by stereotyped **migration** of cells from the tracheal pits. No cell divisions take place during tracheal **migration** and the number of cells in each branch is fixed. This work examines the basis for the determination of tracheal branch fates, prior to the onset of **migration**. We show that the EGF receptor pathway is activated by localized processing of the ligand SPITZ in the tracheal placodes and is responsible for the capacity to form the dorsal trunk and visceral branch. The DPP pathway, on the contrary, is induced in the tracheal pit by local presentation of DPP from the adjacent dorsal and ventral ectodermal cells. This pathway patterns the dorsal and lateral branches. Elimination of both pathways blocks **migration** of all tracheal branches. Antagonistic interactions between the two pathways are demonstrated. The opposing activities of two pathways may refine the final determination of tracheal branch fates.
3. Embryonic development of the Drosophila corpus cardiacum, a neuroendocrine gland with similarity to the vertebrate pituitary, is controlled by sine oculis and glass. We have investigated the development of the Drosophila neuroendocrine gland, the corpus cardiacum (CC), and identified the role of regulatory genes and signaling pathways in CC morphogenesis. CC progenitors segregate from the blastoderm as part of the anterior lip of the ventral furrow. Among the early genetic determinants expressed and required in this domain are the genes giant (gt) and sine oculis (so). During the extended germ band stage, CC progenitor cells form a paired cluster of 6-8 cells sandwiched in between the inner surface of the protocerebrum and the foregut. While flanking the protocerebrum, CC progenitors are in direct contact with the neural precursors that give rise to the pars intercerebralis, the part of the brain whose neurons later innervate the CC. At this stage, the CC progenitors turn on the homeobox gene glass (gl), which is essential for the differentiation of the CC. During germ band retraction, CC progenitors increase in number and migrate posteriorly, passing underneath the brain commissure and attaching themselves to the primordia of the corpora allata (CA). During dorsal closure, the CC and CA move around the anterior aorta to become the ring gland. Signaling pathways that shape the determination and morphogenesis of the CC are decapentaplegic (dpp) and its antagonist short gastrulation (sog), as well as hedgehog (hh) and heartless (htl; a Drosophila FGFR homolog). Sog is expressed in the midventral domain from where CC progenitors originate, and these cells are completely absent in sog mutants. Dpp and hh are expressed in the anterior visceral head mesoderm and the foregut, respectively; both of these tissues flank the CC. Loss of hh and dpp results in defects in CC proliferation and **migration**. Htl appears in the somatic mesoderm of the head and trunk. Although mutations of htl do not cause direct effects on the early development of the CC, the later formation of the ring gland is highly abnormal due to the absence of the aorta in these mutants. Defects in the CC are also caused by mutations that severely reduce the protocerebrum, including tailless (tll), suggesting that additional signaling events exist between brain and CC progenitors. We discuss the parallels between neuroendocrine development in Drosophila and vertebrates.

### ECM

#### PubMed

1. Synthetic enzyme-substrate tethering obviates the Tolloid-**ECM** interaction during Drosophila BMP gradient formation. Members of the Tolloid family of metalloproteinases liberate BMPs from inhibitory complexes to regulate BMP gradient formation during embryonic dorsal-ventral axis patterning. Here, we determine mechanistically how Tolloid activity is regulated by its non-catalytic CUB domains in the Drosophila embryo. We show that Tolloid, via its N-terminal CUB domains, interacts with Collagen IV, which enhances Tolloid activity towards its substrate Sog, and facilitates Tsg-dependent stimulation of cleavage. In contrast, the two most C-terminal Tld CUB domains mediate Sog interaction to facilitate its processing as, based on our structural data, Tolloid curvature positions bound Sog in proximity to the protease domain. Having ascribed functions to the Tolloid non-catalytic domains, we recapitulate embryonic BMP gradient formation in their absence, by artificially tethering the Tld protease domain to Sog. Our studies highlight how the bipartite function of Tolloid CUB domains, in substrate and **ECM** interactions, fine-tune protease activity to a particular developmental context.
2. Peak BMP Responses in the Drosophila Embryo Are Dependent on the Activation of Integrin Signaling. Within a 3D tissue, cells need to integrate signals from growth factors, such as BMPs, and the extracellular matrix (**ECM**) to coordinate growth and differentiation. Here, we use the Drosophila embryo as a model to investigate how BMP responses are influenced by a cell's local **ECM** environment. We show that integrins, which are **ECM** receptors, are absolutely required for peak BMP signaling. This stimulatory effect of integrins requires their intracellular signaling function, which is activated by the **ECM** protein collagen IV. Mechanistically, integrins interact with the BMP receptor and stimulate phosphorylation of the downstream Mad transcription factor. The BMP-pathway-enhancing function of integrins is independent of focal adhesion kinase, but it requires conserved NPXY motifs in the β-integrin cytoplasmic tail. Furthermore, we show that an α-integrin subunit is a BMP target gene, identifying positive feedback between integrin signaling and BMP pathway activity that may contribute to robust cell fate decisions.

### [Ee]xtracellular [Mm]atrix

#### PubMed

1. The Gyc76C Receptor Guanylyl Cyclase and the Foraging cGMP-Dependent Kinase Regulate **Extracellular Matrix** Organization and BMP Signaling in the Developing Wing of Drosophila melanogaster. The developing crossveins of the wing of Drosophila melanogaster are specified by long-range BMP signaling and are especially sensitive to loss of extracellular modulators of BMP signaling such as the Chordin homolog Short gastrulation (Sog). However, the role of the **extracellular matrix** in BMP signaling and Sog activity in the crossveins has been poorly explored. Using a genetic mosaic screen for mutations that disrupt BMP signaling and posterior crossvein development, we identify Gyc76C, a member of the receptor guanylyl cyclase family that includes mammalian natriuretic peptide receptors. We show that Gyc76C and the soluble cGMP-dependent kinase Foraging, likely linked by cGMP, are necessary for normal refinement and maintenance of long-range BMP signaling in the posterior crossvein. This does not occur through cell-autonomous crosstalk between cGMP and BMP signal transduction, but likely through altered extracellular activity of Sog. We identify a novel pathway leading from Gyc76C to the organization of the wing **extracellular matrix** by matrix metalloproteinases, and show that both the **extracellular matrix** and BMP signaling effects are largely mediated by changes in the activity of matrix metalloproteinases. We discuss parallels and differences between this pathway and other examples of cGMP activity in both Drosophila melanogaster and mammalian cells and tissues.
2. Integrins modulate Sog activity in the Drosophila wing. Morphogenesis of the Drosophila wing depends on a series of cell-cell and cell-**extracellular matrix** interactions. During pupal wing development, two secreted proteins, encoded by the short gastrulation (sog) and decapentaplegic (dpp) genes, vie to position wing veins in the center of broad provein territories. Expression of the Bmp4 homolog dpp in vein cells is counteracted by expression of the Bmp antagonist sog in intervein cells, which results in the formation of straight veins of precise width. We screened for genetic interactions between sog and genes encoding a variety of extracellular components and uncovered interactions between sog and myospheroid (mys), multiple edematous wing (mew) and scab (scb), which encode betaPS, alphaPS1 and alphaPS3 integrin subunits, respectively. Clonal analysis reveals that integrin mutations affect the trajectory of veins inside the provein domain and/or their width and that misexpression of sog can alter the behavior of cells in such clones. In addition, we show that a low molecular weight form of Sog protein binds to alphaPS1betaPS. We find that Sog can diffuse from its intervein site of production into adjacent provein domains, but only on the dorsal surface of the wing, where Sog interacts functionally with integrins. Finally, we show that Sog diffusion into provein regions and the reticular pattern of extracellular Sog distribution in wild-type wings requires mys and mew function. We propose that integrins act by binding and possibly regulating the activity/availability of different forms of Sog during pupal development through an adhesion independent mechanism.
3. Integrins modulate Sog activity in the Drosophila wing. Morphogenesis of the Drosophila wing depends on a series of cell-cell and cell-**extracellular matrix** interactions. During pupal wing development, two secreted proteins, encoded by the short gastrulation (sog) and decapentaplegic (dpp) genes, vie to position wing veins in the center of broad provein territories. Expression of the Bmp4 homolog dpp in vein cells is counteracted by expression of the Bmp antagonist sog in intervein cells, which results in the formation of straight veins of precise width. We screened for genetic interactions between sog and genes encoding a variety of extracellular components and uncovered interactions between sog and myospheroid (mys), multiple edematous wing (mew) and scab (scb), which encode betaPS, alphaPS1 and alphaPS3 integrin subunits, respectively. Clonal analysis reveals that integrin mutations affect the trajectory of veins inside the provein domain and/or their width and that misexpression of sog can alter the behavior of cells in such clones. In addition, we show that a low molecular weight form of Sog protein binds to alphaPS1betaPS. We find that Sog can diffuse from its intervein site of production into adjacent provein domains, but only on the dorsal surface of the wing, where Sog interacts functionally with integrins. Finally, we show that Sog diffusion into provein regions and the reticular pattern of extracellular Sog distribution in wild-type wings requires mys and mew function. We propose that integrins act by binding and possibly regulating the activity/availability of different forms of Sog during pupal development through an adhesion independent mechanism.
4. The Gyc76C Receptor Guanylyl Cyclase and the Foraging cGMP-Dependent Kinase Regulate **Extracellular Matrix** Organization and BMP Signaling in the Developing Wing of Drosophila melanogaster. The developing crossveins of the wing of Drosophila melanogaster are specified by long-range BMP signaling and are especially sensitive to loss of extracellular modulators of BMP signaling such as the Chordin homolog Short gastrulation (Sog). However, the role of the **extracellular matrix** in BMP signaling and Sog activity in the crossveins has been poorly explored. Using a genetic mosaic screen for mutations that disrupt BMP signaling and posterior crossvein development, we identify Gyc76C, a member of the receptor guanylyl cyclase family that includes mammalian natriuretic peptide receptors. We show that Gyc76C and the soluble cGMP-dependent kinase Foraging, likely linked by cGMP, are necessary for normal refinement and maintenance of long-range BMP signaling in the posterior crossvein. This does not occur through cell-autonomous crosstalk between cGMP and BMP signal transduction, but likely through altered extracellular activity of Sog. We identify a novel pathway leading from Gyc76C to the organization of the wing **extracellular matrix** by matrix metalloproteinases, and show that both the **extracellular matrix** and BMP signaling effects are largely mediated by changes in the activity of matrix metalloproteinases. We discuss parallels and differences between this pathway and other examples of cGMP activity in both Drosophila melanogaster and mammalian cells and tissues.
5. Multistep molecular mechanism for bone morphogenetic protein extracellular transport in the Drosophila embryo. In the Drosophila embryo, formation of a bone morphogenetic protein (BMP) morphogen gradient requires transport of a heterodimer of the BMPs Decapentaplegic (Dpp) and Screw (Scw) in a protein shuttling complex. Although the core components of the shuttling complex--Short Gastrulation (Sog) and Twisted Gastrulation (Tsg)--have been identified, key aspects of this shuttling system remain mechanistically unresolved. Recently, we discovered that the **extracellular matrix** protein collagen IV is important for BMP gradient formation. Here, we formulate a molecular mechanism of BMP shuttling that is catalyzed by collagen IV. We show that Dpp is the only BMP ligand in Drosophila that binds collagen IV. A collagen IV binding-deficient Dpp mutant signals at longer range in vivo, indicating that collagen IV functions to immobilize free Dpp in the embryo. We also provide in vivo evidence that collagen IV functions as a scaffold to promote shuttling complex assembly in a multistep process. After binding of Dpp/Scw and Sog to collagen IV, protein interactions are remodeled, generating an intermediate complex in which Dpp/Scw-Sog is poised for release by Tsg through specific disruption of a collagen IV-Sog interaction. Because all components are evolutionarily conserved, we propose that regulation of BMP shuttling and immobilization through **extracellular matrix** interactions is widely used, both during development and in tissue homeostasis, to achieve a precise extracellular BMP distribution.
6. Peak BMP Responses in the Drosophila Embryo Are Dependent on the Activation of Integrin Signaling. Within a 3D tissue, cells need to integrate signals from growth factors, such as BMPs, and the **extracellular matrix** (ECM) to coordinate growth and differentiation. Here, we use the Drosophila embryo as a model to investigate how BMP responses are influenced by a cell's local ECM environment. We show that integrins, which are ECM receptors, are absolutely required for peak BMP signaling. This stimulatory effect of integrins requires their intracellular signaling function, which is activated by the ECM protein collagen IV. Mechanistically, integrins interact with the BMP receptor and stimulate phosphorylation of the downstream Mad transcription factor. The BMP-pathway-enhancing function of integrins is independent of focal adhesion kinase, but it requires conserved NPXY motifs in the β-integrin cytoplasmic tail. Furthermore, we show that an α-integrin subunit is a BMP target gene, identifying positive feedback between integrin signaling and BMP pathway activity that may contribute to robust cell fate decisions.
7. Cleavage of the Drosophila screw prodomain is critical for a dynamic BMP morphogen gradient in embryogenesis. Dorsoventral patterning of the Drosophila embryo is regulated by graded distribution of bone morphogenetic proteins (BMPs) composed of two ligands, decapentaplegic (Dpp) a BMP2/4 ortholog and screw (Scw) a BMP5/6/7/8 family member. scw(E1) encodes an unusual allele that was isolated as a dominant enhancer of partial loss-of-function mutations in dpp. However, the molecular mechanisms that underlie this genetic interaction remain to be addressed. Here we show that scw(E1) contains a mutation at the furin cleavage site within the prodomain that is crucial for ligand production. Furthermore, our data show that Scw(E1) preferentially forms heterodimers with Dpp rather than homotypic dimers, providing a possible explanation for the dominant negative phenotype of scw(E1) alleles. The unprocessed prodomain of Scw(E1) remains in a complex with the Dpp:Scw heterodimer, and thus could interfere with interaction of the ligand with the **extracellular matrix**, or the kinetics of processing/secretion of the ligand in vivo. These data reveal novel mechanisms by which post-translational regulation of Scw can modulate Dpp signaling activity.
8. Type IV collagens regulate BMP signalling in Drosophila. Dorsal-ventral patterning in vertebrate and invertebrate embryos is mediated by a conserved system of secreted proteins that establishes a bone morphogenetic protein (BMP) gradient. Although the Drosophila embryonic Decapentaplegic (Dpp) gradient has served as a model to understand how morphogen gradients are established, no role for the **extracellular matrix** has been previously described. Here we show that type IV collagen **extracellular matrix** proteins bind Dpp and regulate its signalling in both the Drosophila embryo and ovary. We provide evidence that the interaction between Dpp and type IV collagen augments Dpp signalling in the embryo by promoting gradient formation, yet it restricts the signalling range in the ovary through sequestration of the Dpp ligand. Together, these results identify a critical function of type IV collagens in modulating Dpp in the extracellular space during Drosophila development. On the basis of our findings that human type IV collagen binds BMP4, we predict that this role of type IV collagens will be conserved.

### [Ii]nvasive

#### PubMed

1. The Drosophila short gastrulation gene prevents Dpp from autoactivating and suppressing neurogenesis in the neuroectoderm. The short gastrulation (sog) gene is expressed in broad lateral stripes comprising the neuroectoderm of the Drosophila blastoderm embryo. sog encodes a predicted secreted protein that functions nonautonomously to antagonize the activity of the TGF-beta-like Decapentaplegic (Dpp) signaling pathway in the dorsal region of the embryo. Recently, it has been shown that sog and dpp are functionally equivalent to their respective Xenopus homologs chordin and BMP-4. In this report we provide the first direct evidence that sog plays a local role in the lateral region of the blastoderm embryo to oppose Dpp activity in the neuroectoderm. In the dorsal region, Dpp signaling both suppresses neurogenesis and maintains expression of genes that promote dorsal cell fates (dorsalization). We show that Dpp also can perform both of these functions in the neuroectoderm. In wild-type embryos, the ability of Dpp to induce expression of dorsal markers including itself (autoactivation) in the neuroectoderm is blocked by sog. We propose that Sog protects the neuroectoderm from an **invasive** positive feedback loop created by Dpp diffusion and autoactivation. We show that the two functions of Dpp signaling, neural suppression and dorsalization, are triggered by distinct thresholds of Dpp activity. Epistasis experiments reveal that all observed sog activity can be accounted for by Sog functioning as a dedicated Dpp antagonist. Finally, we provide evidence that Sog functions as a diffusible morphogen in the blastoderm embryo. These data strongly support the view that the primary phylogenetically conserved function of the Drosophila sog and dpp genes and the homologous Xenopus chordin and BMP-4 genes is to subdivide the primitive embryonic ectoderm into neural versus non-neural domains.

### [Mm]etastasis

#### PubMed

1. A conserved role for Snail as a potentiator of active transcription. The transcription factors of the Snail family are key regulators of epithelial-mesenchymal transitions, cell morphogenesis, and tumor **metastasis**. Since its discovery in Drosophila ∼25 years ago, Snail has been extensively studied for its role as a transcriptional repressor. Here we demonstrate that Drosophila Snail can positively modulate transcriptional activation. By combining information on in vivo occupancy with expression profiling of hand-selected, staged snail mutant embryos, we identified 106 genes that are potentially directly regulated by Snail during mesoderm development. In addition to the expected Snail-repressed genes, almost 50% of Snail targets showed an unanticipated activation. The majority of "Snail-activated" genes have enhancer elements cobound by Twist and are expressed in the mesoderm at the stages of Snail occupancy. Snail can potentiate Twist-mediated enhancer activation in vitro and is essential for enhancer activity in vivo. Using a machine learning approach, we show that differentially enriched motifs are sufficient to predict Snail's regulatory response. In silico mutagenesis revealed a likely causative motif, which we demonstrate is essential for enhancer activation. Taken together, these data indicate that Snail can potentiate enhancer activation by collaborating with different activators, providing a new mechanism by which Snail regulates development.

---

## Gadd45

- NCBI Gene
- GeneRIF
- Pubmed

| Search Term | Rank | Fields |
| --- | --- | --- |
| [Mm]etastasis | 12 | PubMed(1) |

  
hide/show details for Gadd45

### [Mm]etastasis

#### PubMed

1. Drosophila as a model to study the role of blood cells in inflammation, innate immunity and cancer. Drosophila has a primitive yet effective blood system with three types of haemocytes which function throughout different developmental stages and environmental stimuli. Haemocytes play essential roles in tissue modeling during embryogenesis and morphogenesis, and also in innate immunity. The open circulatory system of Drosophila makes haemocytes ideal signal mediators to cells and tissues in response to events such as infection and wounding. The application of recently developed and sophisticated genetic tools to the relatively simple genome of Drosophila has made the fly a popular system for modeling human tumorigensis and **metastasis**. Drosophila is now used for screening and investigation of genes implicated in human leukemia and also in modeling development of solid tumors. This second line of research offers promising opportunities to determine the seemingly conflicting roles of blood cells in tumor progression and invasion. This review provides an overview of the signaling pathways conserved in Drosophila during haematopoiesis, haemostasis, innate immunity, wound healing and inflammation. We also review the most recent progress in the use of Drosophila as a cancer research model with an emphasis on the roles haemocytes can play in various cancer models and in the links between inflammation and cancer.

---

## Galphaf

- NCBI Gene
- GeneRIF
- Pubmed

| Search Term | Rank | Fields |
| --- | --- | --- |
| [Ff]ilopodia | 1 | PubMed(1) |
| [Ff]ilopodium | 2 | Process(1) |
| [Ii]nvasive | 10 | PubMed(1) |
| [Mm]etastasis | 12 | PubMed(1) |

  
hide/show details for Galphaf

### [Ff]ilopodia

#### PubMed

1. Gα73B is a downstream effector of JAK/STAT signalling and a regulator of Rho1 in Drosophila haematopoiesis. JAK/STAT signalling regulates many essential developmental processes including cell proliferation and haematopoiesis, whereas its inappropriate activation is associated with the majority of myeloproliferative neoplasias and numerous cancers. Furthermore, high levels of JAK/STAT pathway signalling have also been associated with enhanced metastatic invasion by cancerous cells. Strikingly, gain-of-function mutations in the single Drosophila JAK homologue, Hopscotch, result in haemocyte neoplasia, inappropriate differentiation and the formation of melanised haemocyte-derived 'tumour' masses; phenotypes that are partly orthologous to human gain-of-function JAK2-associated pathologies. Here we show that Gα73B, a novel JAK/STAT pathway target gene, is necessary for JAK/STAT-mediated tumour formation in flies. In addition, although Gα73B does not affect haemocyte differentiation, it does regulate haemocyte morphology and motility under non-pathological conditions. We show that Gα73B is required for constitutive, but not injury-induced, activation of Rho1 and for the localisation of Rho1 i**nto filop**odia upon haemocyte activation. Consistent with these results, we also show that Rho1 interacts genetically with JAK/STAT signalling, and that wild-type levels of Rho1 are necessary for tumour formation. Our findings link JAK/STAT transcriptional outputs, Gα73B activity and Rho1-dependent cytoskeletal rearrangements and cell motility, therefore connecting a pathway associated with cancer with a marker indicative of invasiveness. As such, we suggest a mechanism by which JAK/STAT pathway signalling may promote metastasis.

### [Ff]ilopodium

#### Process

1. regulation of **filopodium** assembly

### [Ii]nvasive

#### PubMed

1. Gα73B is a downstream effector of JAK/STAT signalling and a regulator of Rho1 in Drosophila haematopoiesis. JAK/STAT signalling regulates many essential developmental processes including cell proliferation and haematopoiesis, whereas its inappropriate activation is associated with the majority of myeloproliferative neoplasias and numerous cancers. Furthermore, high levels of JAK/STAT pathway signalling have also been associated with enhanced metastatic invasion by cancerous cells. Strikingly, gain-of-function mutations in the single Drosophila JAK homologue, Hopscotch, result in haemocyte neoplasia, inappropriate differentiation and the formation of melanised haemocyte-derived 'tumour' masses; phenotypes that are partly orthologous to human gain-of-function JAK2-associated pathologies. Here we show that Gα73B, a novel JAK/STAT pathway target gene, is necessary for JAK/STAT-mediated tumour formation in flies. In addition, although Gα73B does not affect haemocyte differentiation, it does regulate haemocyte morphology and motility under non-pathological conditions. We show that Gα73B is required for constitutive, but not injury-induced, activation of Rho1 and for the localisation of Rho1 into filopodia upon haemocyte activation. Consistent with these results, we also show that Rho1 interacts genetically with JAK/STAT signalling, and that wild-type levels of Rho1 are necessary for tumour formation. Our findings link JAK/STAT transcriptional outputs, Gα73B activity and Rho1-dependent cytoskeletal rearrangements and cell motility, therefore connecting a pathway associated with cancer with a marker indicativ**e of inv**asiveness. As such, we suggest a mechanism by which JAK/STAT pathway signalling may promote metastasis.

### [Mm]etastasis

#### PubMed

1. Gα73B is a downstream effector of JAK/STAT signalling and a regulator of Rho1 in Drosophila haematopoiesis. JAK/STAT signalling regulates many essential developmental processes including cell proliferation and haematopoiesis, whereas its inappropriate activation is associated with the majority of myeloproliferative neoplasias and numerous cancers. Furthermore, high levels of JAK/STAT pathway signalling have also been associated with enhanced metastatic invasion by cancerous cells. Strikingly, gain-of-function mutations in the single Drosophila JAK homologue, Hopscotch, result in haemocyte neoplasia, inappropriate differentiation and the formation of melanised haemocyte-derived 'tumour' masses; phenotypes that are partly orthologous to human gain-of-function JAK2-associated pathologies. Here we show that Gα73B, a novel JAK/STAT pathway target gene, is necessary for JAK/STAT-mediated tumour formation in flies. In addition, although Gα73B does not affect haemocyte differentiation, it does regulate haemocyte morphology and motility under non-pathological conditions. We show that Gα73B is required for constitutive, but not injury-induced, activation of Rho1 and for the localisation of Rho1 into filopodia upon haemocyte activation. Consistent with these results, we also show that Rho1 interacts genetically with JAK/STAT signalling, and that wild-type levels of Rho1 are necessary for tumour formation. Our findings link JAK/STAT transcriptional outputs, Gα73B activity and Rho1-dependent cytoskeletal rearrangements and cell motility, therefore connecting a pathway associated with cancer with a marker indicative of invasiveness. As such, we suggest a mechanism by which JAK/STAT pathway signalling may pro**mote metas**tasis.

---

## Pu

- NCBI Gene
- GeneRIF
- Pubmed

| Search Term | Rank | Fields |
| --- | --- | --- |
| [Mm]igration | 6 | Process(1); PubMed(2) |

  
hide/show details for Pu

### [Mm]igration

#### Process

1. regulation of epithelial cell **migration**, open tracheal system

#### PubMed

1. Drosophila dopamine synthesis pathway genes regulate tracheal morphogenesis. While studying the developmental functions of the Drosophila dopamine synthesis pathway genes, we noted interesting and unexpected mutant phenotypes in the developing trachea, a tubule network that has been studied as a model for branching morphogenesis. Specifically, Punch (Pu) and pale (ple) mutants with reduced dopamine synthesis show ectopic/aberrant **migration**, while Catecholamines up (Catsup) mutants that over-express dopamine show a characteristic loss of **migration** phenotype. We also demonstrate expression of Punch, Ple, Catsup and dopamine in tracheal cells. The dopamine pathway mutant phenotypes can be reproduced by pharmacological treatments of dopamine and a pathway inhibitor 3-iodotyrosine (3-IT), implicating dopamine as a direct mediator of the regulatory function. Furthermore, we show that these mutants genetically interact with components of the endocytic pathway, namely shibire/dynamin and awd/nm23, that promote endocytosis of the chemotactic signaling receptor Btl/FGFR. Consistent with the genetic results, the surface and total cellular levels of a Btl-GFP fusion protein in the tracheal cells and in cultured S2 cells are reduced upon dopamine treatment, and increased in the presence of 3-IT. Moreover, the transducer of Btl signaling, MAP kinase, is hyper-activated throughout the tracheal tube in the Pu mutant. Finally we show that dopamine regulates endocytosis via controlling the dynamin protein level.
2. Function of the Drosophila POU domain transcription factor drifter as an upstream regulator of breathless receptor tyrosine kinase expression in developing trachea. Organogenesis of the Drosophila tracheal system involves extensive directed cell **migration**s leading to a stereotypic series of interconnected tubules. Although numerous gene products have been shown to be essential for tracheal morphogenesis, direct functional relationships between participants have not been previously established. Both the breathless gene, encoding a Drosophila fibroblast growth factor receptor tyrosine kinase homologue, and the POU-domain transcription factor gene, drifter, are expressed in all tracheal cells and are essential for directed cell **migration**s. We demonstrate here that ubiquitously expressed Breathless protein under control of a heterologous heat-shock promoter is able to rescue the severely disrupted tracheal phenotype associated with drifter loss-of-function mutations. In the absence of Drifter function, breathless expression is initiated normally but transcript levels fall drastically to undetectable levels as tracheal differentiation proceeds. In addition, breathless regulatory DNA contains seven high affinity Drifter binding sites similar to previously identified Drifter recognition elements. These results suggest that the Drifter protein, which maintains its own expression through a tracheal-specific autoregulatory enhancer, is not necessary for initiation of breathless expression but functions as a direct transcriptional regulator necessary for maintenance of breathless transcripts at high levels during tracheal cell **migration**. This example of a mechanism for maintenance of a committed cell fate offers a model for understanding how essential gene activities can be maintained throughout organogenesis.

---

## Spn27A

- NCBI Gene
- GeneRIF
- Pubmed

| Search Term | Rank | Fields |
| --- | --- | --- |
| [Ii]nvasive | 10 | PubMed(1) |

  
hide/show details for Spn27A

### [Ii]nvasive

#### PubMed

1. Serine protease MP2 activates prophenoloxidase in the melanization immune response of Drosophila melanogaster. In arthropods, melanization plays a major role in the innate immune response to encapsulate and kill the **invasive** organisms. It is mediated by a serine protease cascade and is regulated by serpins. The identification of the molecular components of melanization and the regulation of those components are still unclear in Drosophila melanogaster, although some genetic research on the activation of melanization has been reported. Here we report that Drosophila serine protease MP2 directly cleaves both recombinant and native prophenoloxidase-1. Overexpression or repression of MP2 in flies resulted in increased and decreased rates of cleavage, respectively, of prophenoloxidase-1. Moreover, serine protease inhibitor Spn27A formed SDS-stable complexes with MP2, both in vitro and in vivo. The amidase activity of MP2 was inhibited efficiently by Spn27A. Spn27A also prevented MP2 from cleaving prophenoloxidase-1. Taken together, these results indicate that under our experimental conditions MP2 functions as a prophenoloxidase-activating protease, and that this function is inhibited by Spn27A. MP2 and Spn27A thus constitute a regulatory unit in the prophenoloxidase activation cascade in Drosophila. The combination of genetic, molecular genetic and biochemical approaches should allow further advances in our understanding of the prophenoloxidase-activating cascade in insects and indirectly shed further light on protease-cascades in humans and other vertebrates.

---

## rho

- NCBI Gene
- GeneRIF
- Pubmed

| Search Term | Rank | Fields |
| --- | --- | --- |
| [Ff]ilopodia | 1 | PubMed(1) |
| [Mm]igration | 6 | PubMed(36) |
| ECM | 7 | PubMed(1) |
| [Ee]xtracellular [Mm]atrix | 8 | PubMed(5) |
| [Ii]nvasive | 10 | PubMed(3) |
| [Mm]etastasis | 12 | PubMed(3) |
| [Cc]ell [Ii]nvasion | 13 | PubMed(3) |

  
hide/show details for rho

### [Ff]ilopodia

#### PubMed

1. Cellular mechanisms governing synaptic development in Drosophila melanogaster. The neuromuscular connections of Drosophila are ideally suited for studying synaptic function and development. Hypotheses about cell recognition can be tested in a simple array of pre- and postsynaptic elements. Drosophila muscle fibers are multiply innervated by individually identifiable motoneurons. The neurons express several synaptic cotransmitters, including glutamate, proctolin, and octopamine, and are specialized by their synaptic morphology, neurotransmitters, and connectivity. During larval development the initial motoneuron endings grow extensively over the surface of the muscle fibers, and differentiate synaptic boutons of characteristic morphology. While considerable growth occurs postembryonically, the initial wiring of motoneurons to muscle fibers is accomplished during mid-to-late embryogenesis (stages 15-17). Efferent growth cones sample multiple muscle fibers with rapidly moving **filopodia**. Upon reaching their target muscle fibers, the growth cones rapidly differentiate into synaptic contacts whose morphology prefigures that of the larval junction. Mismatch experiments show that growth cones recognize specific muscle fibers, and can do so when the surrounding musculature is radically altered. However, when denied their normal targets, motoneurons can establish functional synapses on alternate muscle fibers. Blocking synaptic activity with either injected toxins or ion channel mutants does not derange synaptogenesis, but may influence the number of motor ending processes. The molecular mechanisms governing cellular recognition during synaptogenesis remain to be identified. However, several cell surface glycoproteins known to mediate cellular adhesion events in vitro are expressed by the developing synapses. Furthermore, enhancer detector lines have identified genes with expression restricted to small subsets of muscle fibers and/or motoneurons during the period of synaptogenesis. These observations suggest that in Drosophila a mechanism of target chemoaffinity may be involved in the genesis of stereotypic synaptic wiring.

### [Mm]igration

#### PubMed

1. Cell fate choices in Drosophila tracheal morphogenesis. The Drosophila tracheal system is a branched tubular structure that supplies air to target tissues. The elaborate tracheal morphology is shaped by two linked inductive processes, one involving the choice of cell fates, and the other a guided cell **migration**. We will describe the molecular basis for these processes, and the allocation of cell fate decisions to four temporal hierarchies. First, tracheal placodes are specified within the embryonic ectoderm. Subsequently, branch fates are allocated within the tracheal placodes, prior to **migration**. Localized presentation of the FGF ligand, Branchless, to tracheal cells that express the FGF receptor, Breathless, guides **migration**. Once cell **migration** is initiated, distinct cell fates are determined within each migrating branch. Finally, inhibitory feedback mechanisms ensure the correct assignment of these fates. Tracheal cell fate choices are determined by signaling cascades triggered by signals emanating from the tracheal cells, as well as by ligands produced by adjacent tissues.
2. Integration of epithelial patterning and morphogenesis in Drosophila ovarian follicle cells. Drosophila oogenesis involves the coordinated development of germ cells and an overlying follicular epithelium. The follicle cells provide a genetically tractable system to investigate the cell biology of patterning and morphogenesis. Follicle cells initially form a cuboidal epithelium surrounding a syncytium of nurse cells and oocyte. Epithelial structure is maintained as these cells reorganize to create the three dimensional architecture of the eggshell. Both long-range and short-range cell-cell communications pattern the domains of follicle cells that will create specific eggshell structures. After terminal differentiation to deposit the eggshell proteins, the follicle cells die. This review summarizes recent progress in understanding the cell-cell communication that orchestrates follicle cell patterning and **migration**s. DE-cadherin-mediated adhesion is important at several steps in egg chamber formation and follicle cell **migration**. Notch signaling is critical during each successive round of patterning and **migration**. Integration of bone morphogenetic protein (BMP) and epidermal growth factor (EGF) signals patterns the elaborate structures of the dorsal-anterior eggshell.
3. Drosophila blood cell chemotaxis. Drosophila melanogaster contains a population of blood cells called hemocytes that represent the functional equivalent of vertebrate macrophages. These cells undergo directed **migration**s to disperse during development and reach sites of tissue damage or altered self. These chemotactic behaviors are controlled by the expression of PDGF/Vegf-related ligands in developing embryos and local production of hydrogen peroxide at wounds. Recent work reveals that many molecules important in vertebrate cell motility, including integrins, formins, Ena/VASP proteins and the SCAR/WAVE complex, have a conserved function in these innate immune cells. The use of this model organism has elucidated how damage signals are activated by calcium signaling during inflammation and that the steroid hormone ecdysone activates immune competence at key developmental stages.
4. A molecular link between FGF and Dpp signaling in branch-specific **migration** of the Drosophila trachea. The tracheal system of Drosophila embryos achieves its archetypal branching pattern through a series of cell **migration** events requiring the FGF, Dpp, and Wg/WNT signaling pathways. To gain insight into tracheal cell **migration**, we performed an F4 EMS mutagenesis screen to generate and characterize new mutations resulting in tracheal defects. From 2591 mutagenized third chromosome lines, we identified 33 mutations with defects in tracheal development, corresponding to 12 distinct complementation groups. The new mutations included novel hypomorphic alleles of the FGF receptor gene, breathless, and the ETS-domain transcription factor gene, pointed. We show that reduced function of either breathless or pointed specifically affects **migration** of the dorsal and ventral tracheal branches, more specific functions than previously described for these genes. Our analysis reveals that breathless and pointed control dorsal branch **migration** through transcriptional regulation of the Dpp pathway effectors, Knirps and Knirps-related, which are necessary for **migration** of this branch. We further show that expression of knirps or knirps-related rescues dorsal but not ventral branch **migration** in the breathless hypomorph. These studies support a model in which both the Dpp- and the FGF-signaling pathways control expression of knirps and knirps-related, thereby regulating cell **migration** during dorsal branch formation.
5. ribbon encodes a novel BTB/POZ protein required for directed cell **migration** in Drosophila melanogaster. During development, directed cell **migration** is crucial for achieving proper shape and function of organs. One well-studied example is the embryonic development of the larval tracheal system of Drosophila, in which at least four signaling pathways coordinate cell **migration** to form an elaborate branched network essential for oxygen delivery throughout the larva. FGF signaling is required for guided **migration** of all tracheal branches, whereas the DPP, EGF receptor, and Wingless/WNT signaling pathways each mediate the formation of specific subsets of branches. Here, we characterize ribbon, which encodes a BTB/POZ-containing protein required for specific tracheal branch **migration**. In ribbon mutant tracheae, the dorsal trunk fails to form, and ventral branches are stunted; however, directed **migration**s of the dorsal and visceral branches are largely unaffected. The dorsal trunk also fails to form when FGF or Wingless/WNT signaling is lost, and we show that ribbon functions downstream of, or parallel to, these pathways to promote anterior-posterior **migration**. Directed cell **migration** of the salivary gland and dorsal epidermis are also affected in ribbon mutants, suggesting that conserved mechanisms may be employed to orient cell **migration**s in multiple tissues during development.
6. A core transcriptional network for early mesoderm development in Drosophila melanogaster. Embryogenesis is controlled by large gene-regulatory networks, which generate spatially and temporally refined patterns of gene expression. Here, we report the characteristics of the regulatory network orchestrating early mesodermal development in the fruitfly Drosophila, where the transcription factor Twist is both necessary and sufficient to drive development. Through the integration of chromatin immunoprecipitation followed by microarray analysis (ChIP-on-chip) experiments during discrete time periods with computational approaches, we identified >2000 Twist-bound cis-regulatory modules (CRMs) and almost 500 direct target genes. Unexpectedly, Twist regulates an almost complete cassette of genes required for cell proliferation in addition to genes essential for morophogenesis and cell **migration**. Twist targets almost 25% of all annotated Drosophila transcription factors, which may represent the entire set of regulators necessary for the early development of this system. By combining in vivo binding data from Twist, Mef2, Tinman, and Dorsal we have constructed an initial transcriptional network of early mesoderm development. The network topology reveals extensive combinatorial binding, feed-forward regulation, and complex logical outputs as prevalent features. In addition to binary activation and repression, we suggest that Twist binds to almost all mesodermal CRMs to provide the competence to integrate inputs from more specialized transcription factors.
7. Molecular requirements for actin-based lamella formation in Drosophila S2 cells. Cell **migration** occurs through the protrusion of the actin-enriched lamella. Here, we investigated the effects of RNAi depletion of approximately 90 proteins implicated in actin function on lamella formation in Drosophila S2 cells. Similar to in vitro reconstitution studies of actin-based Listeria movement, we find that lamellae formation requires a relatively small set of proteins that participate in actin nucleation (Arp2/3 and SCAR), barbed end capping (capping protein), filament depolymerization (cofilin and Aip1), and actin monomer binding (profilin and cyclase-associated protein). Lamellae are initiated by parallel and partially redundant signaling pathways involving Rac GTPases and the adaptor protein Nck, which stimulate SCAR, an Arp2/3 activator. We also show that RNAi of three proteins (kette, Abi, and Sra-1) known to copurify with and inhibit SCAR in vitro leads to SCAR degradation, revealing a novel function of this protein complex in SCAR stability. Our results have identified an essential set of proteins involved in actin dynamics during lamella formation in Drosophila S2 cells.
8. Primordial germ cell **migration** and the assembly of the Drosophila embryonic gonad. During embryogenesis Drosophila pole cells, like germ cells in many other vertebrates and invertebrates, actively migrate before assembling into the gonad. Five separate steps can be distinguished: an initial passive displacement of the germ cells by gastrulation movements, an amoeboid migratory phase during which the pole cells pass through the endoderm, **migration** on endoderm toward mesoderm, separation into two bilateral groups associated with the gonadal mesoderm precursors, followed by condensation into the gonad itself. We have analyzed gonad assembly in embryonic pattern mutants to determine whether distinct cues are required in this process. We show that the initiation of **migration** does not require the presence of the mesoderm, the eventual target of the germ cells. Rather, **migration** is triggered as a consequence of the differentiation of the endoderm. Examination of embryos mutant for maternal genes of the terminal group suggests that a primary role of the endoderm in this process is to allow the germ cell access to the interior of the embryo. In addition, we show that normal gonad assembly requires a region of the embryo that includes the posterior compartment of the fifth and the sixth abdominal segments.
9. Regulation of cell **migration** during tracheal development in Drosophila melanogaster. Most of the knowledge concerning the intracellular mechanisms involved in cell locomotion have been obtained from in vitro studies of cells in culture. Many of the concepts derived from these studies have been partially confirmed in in vivo systems but numerous questions regarding the developmental control of cell **migration** remain to be addressed. Tracheal morphogenesis in Drosophila melanogaster embryos represents an in vivo model system to study the genetic control of cell **migration**. We review what is known about tracheal development and regulation of tracheal cell **migration**. We try to link these in vivo studies and the movement of cells over two dimensional substrates and elaborate on important questions which remain to be addressed in the future.
10. Mutations affecting the pattern of the PNS in Drosophila reveal novel aspects of neuronal development. Through a systematic genetic screen, we have identified 55 mutations that affect the development of the PNS of Drosophila embryos. These mutations specify 13 novel and 5 previously characterized genes and define new phenotypes for 2 other known genes. Five classes of mutant phenotypes were identified in the screen: gain of neurons, loss of neurons, abnormal position of chordotonal neurons, aberrant neuronal trajectories, and abnormal morphology of neurons. Phenotypic analyses of mutations identified in this study revealed three novel aspects of PNS development. First, we have identified a novel gene that may be required to define glial versus neuronal cell identity. Second, our data indicate that neuronal **migration** plays an important role in pattern formation in the embryonic PNS. Third, we have identified mutations that cause a lack of sensory organs, but unlike mutations in proneural genes, do not affect the formation of sensory organ precursors. These genes may be required for key aspects of neuronal differentiation. Our studies suggest that approximately 70 essential genes are required for proper PNS development in Drosophila embryos.
11. The proprioceptive and contractile systems in Drosophila are both patterned by the EGR family transcription factor Stripe. Coordinated locomotion of Drosophila larvae depends on accurate patterning and stable attachment to the cuticle of both muscles and proprioceptors (chordotonal organs). Unlike muscle spindles in mammals, the fly chordotonal organs are not embedded in the body-wall muscles. Yet, the contractile system (muscles and tendons) and the chordotonal organs constitute two parts of a single functional unit that controls locomotion, and thus must be patterned in full coordination. It is not known how such coordination is achieved. Here we show that the positioning and differentiation of the migrating chordotonal organs are instructed by Stripe, the same transcription factor that promotes tendon cell specification and differentiation and is required for normal patterning of the contractile system. Our data demonstrate that although chordotonal organs are patterned in a Stripe-dependent mechanism similarly to muscles, this mechanism is independent of Stripe activity in tendon cells. Thus, the two parts of the locomotive system use similar but independent patterning mechanisms that converge to form a functional unit. Stripe plays at least a dual role in chordotonal development. It is required within the ligament cells for terminal differentiation and proper **migration**, without which no induction of ligament attachment cells takes place. Stripe's activity is then necessary within the recruited cells for their differentiation as attachment cells. Similarly to the biphasic differentiation program of tendons, terminal differentiation of chordotonal attachment cells is associated with sequential activation of the two Stripe isoforms-Stripe B and Stripe A.
12. The polarisation of the anterior-posterior and dorsal-ventral axes during Drosophila oogenesis. Recent work on Drosophila oogenesis has begun to reveal how the first asymmetries in development arise and how these relate to the later events that localise the positional cues which define the embryonic axes. The Cadherin-dependent positioning of the oocyte creates an anterior-posterior polarity that is transmitted to the embryo through the localisation and localised translation of bicoid, oskar, and nanos mRNA. In contrast, dorsal-ventral polarity arises from the random **migration** of the nucleus to the anterior of the oocyte, where it determines where gurken mRNA is translated and localised. Gurken signalling then defines the embryonic dorsal-ventral axis by restricting pipe expression to the ventral follicle cells, where Pipe regulates the production of an unidentified cue that activates the Toll signalling pathway.
13. Organ formation in Drosophila: specification and morphogenesis of the salivary gland. The Drosophila salivary gland has emerged as an outstanding model system for the process of organ formation. Many of the component steps, from initial regional specification through cell specialization and morphogenesis, are known and many of the genes required for these different processes have been identified. The salivary gland is a relatively simple organ; the entire gland comprises of only two major cell types, which derive from a single contiguous primordium. Salivary cells cease dividing once they are specified, and organ growth is achieved simply by an increase in size of individual cells, thus eliminating concerns about the potential unequal distribution of determinants during mitosis. Drosophila salivary glands form by the same cellular mechanisms as organs in higher organisms, including regulated cell shape changes, cell intercalation and directed cell **migration**. Thus, learning how these events are coordinated for tissue morphogenesis in an organism for which the genetic and molecular tools are unsurpassed should provide excellent paradigms for dissecting related processes in the more intricate organs of more complicated species.
14. Interactions between the EGF receptor and DPP pathways establish distinct cell fates in the tracheal placodes. The formation of the tracheal network in Drosophila is driven by stereotyped **migration** of cells from the tracheal pits. No cell divisions take place during tracheal **migration** and the number of cells in each branch is fixed. This work examines the basis for the determination of tracheal branch fates, prior to the onset of **migration**. We show that the EGF receptor pathway is activated by localized processing of the ligand SPITZ in the tracheal placodes and is responsible for the capacity to form the dorsal trunk and visceral branch. The DPP pathway, on the contrary, is induced in the tracheal pit by local presentation of DPP from the adjacent dorsal and ventral ectodermal cells. This pathway patterns the dorsal and lateral branches. Elimination of both pathways blocks **migration** of all tracheal branches. Antagonistic interactions between the two pathways are demonstrated. The opposing activities of two pathways may refine the final determination of tracheal branch fates.
15. Tube morphogenesis: closure, but many openings remain. Epithelial and endothelial tubes form the basic structure of many organs and tissues in the fruit fly Drosophila melanogaster, the nematode Caenorhabditis elegans, zebrafish and mammals. Comparison of how tubes form during development defines several pathways that generate a single unbranched tube or dichotomously branching tubular networks. The formation of tubes can be induced directly by intrinsic signals within epithelial primordia or by inductive signaling between adjacent epithelia and the mesenchyme. Both processes are hierarchically controlled by master transcriptional regulators, growth factors and their receptors, directed cell **migration** and cellular reorganization, which is controlled by changes in the cytoskeleton and protein trafficking. This review provides a summary of these pathways based upon articles published in the Tube Morphogenesis Series in Trends in Cell Biology.
16. Branching morphogenesis of the Drosophila tracheal system. Many organs including the mammalian lung and vascular system consist of branched tubular networks that transport essential gases or fluids, but the genetic programs that control the development of these complex three-dimensional structures are not well understood. The Drosophila melanogaster tracheal (respiratory) system is a network of interconnected epithelial tubes that transports oxygen and other gases in the body and provides a paradigm of branching morphogenesis. It develops by sequential sprouting of primary, secondary, and terminal branches from an epithelial sac of approximately 80 cells in each body segment of the embryo. Mapping of the cell movements and shape changes during the sprouting process has revealed that distinct mechanisms of epithelial **migration** and tube formation are used at each stage of branching. Genetic dissection of the process has identified a general program in which a fibroblast growth factor (FGF) and fibroblast growth factor receptor (FGFR) are used repeatedly to control branch budding and outgrowth. At each stage of branching, the mechanisms controlling FGF expression and the downstream signal transduction pathway change, altering the pattern and structure of the branches that form. During terminal branching, FGF expression is regulated by hypoxia, ensuring that tracheal structure matches cellular oxygen need. A branch diversification program operates in parallel to the general budding program: Regional signals locally modify the general program, conferring specific structural features and other properties on individual branches, such as their substrate outgrowth preferences, differences in tube size and shape, and the ability to fuse to other branches to interconnect the network.
17. The interdependence of the Rho GTPases and apicobasal cell polarity. Signaling via the Rho GTPases provides crucial regulation of numerous cell polarization events, including apicobasal (AB) polarity, polarized cell **migration**, polarized cell division and neuronal polarity. Here we review the relationships between the Rho family GTPases and epithelial AB polarization events, focusing on the 3 best-characterized members: Rho, Rac and Cdc42. We discuss a multitude of processes that are important for AB polarization, including lumen formation, apical membrane specification, cell-cell junction assembly and maintenance, as well as tissue polarity. Our discussions aim to highlight the immensely complex regulatory mechanisms that encompass Rho GTPase signaling during AB polarization. More specifically, in this review we discuss several emerging common themes, that include: 1) the need for Rho GTPase activities to be carefully balanced in both a spatial and temporal manner through a multitude of mechanisms; 2) the existence of signaling feedback loops and crosstalk to create robust cellular responses; and 3) the frequent multifunctionality that exists among AB polarity regulators. Regarding this latter theme, we provide further discussion of the potential plasticity of the cell polarity machinery and as a result the possible implications for human disease.
18. Tre1 GPCR initiates germ cell transepithelial **migration** by regulating Drosophila melanogaster E-cadherin. Despite significant progress in identifying the guidance pathways that control cell **migration**, how a cell starts to move within an intact organism, acquires motility, and loses contact with its neighbors is poorly understood. We show that activation of the G protein-coupled receptor (GPCR) trapped in endoderm 1 (Tre1) directs the redistribution of the G protein Gbeta as well as adherens junction proteins and Rho guanosine triphosphatase from the cell periphery to the lagging tail of germ cells at the onset of Drosophila melanogaster germ cell **migration**. Subsequently, Tre1 activity triggers germ cell dispersal and orients them toward the midgut for directed transepithelial **migration**. A transition toward invasive **migration** is also a prerequisite for metastasis formation, which often correlates with down-regulation of adhesion proteins. We show that uniform down-regulation of E-cadherin causes germ cell dispersal but is not sufficient for transepithelial **migration** in the absence of Tre1. Our findings therefore suggest a new mechanism for GPCR function that links cell polarity, modulation of cell adhesion, and invasion.
19. Drosophila tracheal morphogenesis: intricate cellular solutions to basic plumbing problems. The cellular architecture of tubular organs suggests striking similarities in the mechanisms of tubulogenesis between species. The formation of the Drosophila respiratory organ (trachea) highlights the basic principles of branch patterning and tube growth that generate a highly elaborate but stereotyped epithelial tubular network. Oriented cell **migration**, changes in cell shape, selective growth of the apical cell membrane and intracellular lumen formation are essential events in this process. These morphogenetic processes build four structurally distinct classes of tubes that facilitate optimal airflow and gas exchange with target tissues. The molecular players in these plots include attractant and repellent signals, differentiation factors that cause a high diversity of cell fates within the epithelium, and determinants of tube formation and dimensions.
20. Guidance of cell **migration** by EGF receptor signaling during Drosophila oogenesis. Directed cell **migration** is important for many aspects of normal animal development, but little is known about how cell **migration**s are guided or the mechanisms by which guidance cues are translated into directed cell movement. Here we present evidence that signaling mediated by the epidermal growth factor receptor (EGFR) guides dorsal **migration** of border cells during Drosophila oogenesis. The transforming growth factor-alpha (TGF-alpha)-like ligand Gurken appears to serve as the guidance cue. To mediate this guidance function, EGFR signals via a pathway that is independent of Raf-MAP kinase and receptor-specific.
21. The transcription factors KNIRPS and KNIRPS RELATED control cell **migration** and branch morphogenesis during Drosophila tracheal development. Cell **migration** during embryonic tracheal system development in Drosophila requires DPP and EGF signaling to generate the archetypal branching pattern. We show that two genes encoding the transcription factors KNIRPS and KNIRPS RELATED possess multiple and redundant functions during tracheal development. knirps/knirps related activity is necessary to mediate DPP signaling which is required for tracheal cell **migration** and formation of the dorsal and ventral branches. Ectopic knirps or knirps related expression in lateral tracheal cells respecifies their anteroposterior to a dorsoventral **migration** behavior, similar to that observed in the case of ectopic DPP expression. In dorsal tracheal cells knirps/knirps related activity represses the transcription factor SPALT; this repression is essential for secondary and terminal branch formation. However, in cells of the dorsal trunk, spalt expression is required for normal anteroposterior cell **migration** and morphogenesis. spalt expression is maintained by the EGF receptor pathway and, hence, some of the opposing activities of the EGF and DPP signaling pathways are mediated by spalt and knirps/knirps related. Furthermore, we provide evidence that the border between cells acquiring dorsal branch and dorsal trunk identity is established by the direct interaction of KNIRPS with a spalt cis-regulatory element.
22. TGF-α ligands can substitute for the neuregulin Vein in Drosophila development. ErbB receptors, including the epidermal growth factor receptor (Egfr), are activated by EGF ligands to govern cell proliferation, survival, **migratio**n and differentiation. The different EGF-induced cell responses in development are regulated by deployment of multiple ligands. These inputs, however, engage only a limited number of intracellular pathways and are thought to elicit specific responses by regulating the amplitude or duration of the intracellular signal. The single Drosophila Egfr has four ligands: three of the TGF-α-type and a single neuregulin-like called vein (vn). Here, we used mutant combinations and gene replacement to determine the constraints of ligand specificity in development. Mutant analysis revealed extensive ligand redundancy in embryogenesis and wing development. Surprisingly, we found that the essential role of vn in development could be largely replaced by expression of any TGF-α ligand, including spitz (spi), in the endogenous vn pattern. vn mutants die as white undifferentiated pupae, but the rescued individuals showed global differentiation of adult body parts. Spi is more potent than Vn, and the best morphological rescue occurred when Spi expression was reduced to achieve an intracellular signaling level comparable to that produced by Vn. Our results show that the developmental repertoire of a strong ligand like Spi is flexible and at the appropriate level can emulate the activity of a weak ligand like Vn. These findings align with a model whereby cells respond similarly to an equivalent quantitative level of an intracellular signal generated by two distinct ligands regardless of ligand identity.
23. The Drosophila spitz gene encodes a putative EGF-like growth factor involved in dorsal-ventral axis formation and neurogenesis. We describe the molecular characterization of the Drosophila gene spitz (spi), which encodes a putative 26-kD, EGF-like transmembrane protein that is structurally similar to TGF-alpha. Temporal and spatial expression patterns of spi transcripts indicate that spi is expressed throughout the embryo. Examination of mutant embryos reveals that spi is involved in a number of unrelated developmental choices, for example, dorsal-ventral axis formation, glial **migration**, sensory organ determination, and muscle development. We propose that spi may act as a ligand for cell-specific receptors, possibly rhomboid and/or the Drosophila EGF receptor homolog.
24. Following the 'tracks': Tramtrack69 regulates epithelial tube expansion in the Drosophila ovary through Paxillin, Dynamin, and the homeobox protein Mirror. Epithelial tubes are the infrastructure for organs and tissues, and tube morphogenesis requires precise orchestration of cell signaling, shape, **migration**, and adhesion. Follicle cells in the Drosophila ovary form a pair of epithelial tubes whose lumens act as molds for the eggshell respiratory filaments, or dorsal appendages (DAs). DA formation is a robust and accessible model for studying the patterning, formation, and expansion of epithelial tubes. Tramtrack69 (TTK69), a transcription factor that exhibits a variable embryonic DNA-binding preference, controls DA lumen volume and shape by promoting tube expansion; the tramtrack mutation twin peaks (ttk(twk)) reduces TTK69 levels late in oogenesis, inhibiting this expansion. Microarray analysis of wild-type and ttk(twk) ovaries, followed by in situ hybridization and RNAi of candidate genes, identified the Phospholipase B-like protein Lamina ancestor (LAMA), the scaffold protein Paxillin, the endocytotic regulator Shibire (Dynamin), and the homeodomain transcription factor Mirror, as TTK69 effectors of DA-tube expansion. These genes displayed enriched expression in DA-tube cells, except lama, which was expressed in all follicle cells. All four genes showed reduced expression in ttk(twk) mutants and exhibited RNAi phenotypes that were enhanced in a ttk(twk)/+ background, indicating ttk(twk) genetic interactions. Although previous studies show that Mirror patterns the follicular epithelium prior to DA tubulogenesis, we show that Mirror has an independent, novel role in tube expansion, involving positive regulation of Paxillin. Thus, characterization of ttk(twk)-differentially expressed genes expands the network of TTK69 effectors, identifies novel epithelial tube-expansion regulators, and significantly advances our understanding of this vital developmental process.
25. Cellular and molecular mechanisms of single and collective cell **migration**s in Drosophila: themes and variations. The process of cell **migration** is essential throughout life, driving embryonic morphogenesis and ensuring homeostasis in adults. Defects in cell **migration** are a major cause of human disease, with excessive **migration** causing autoimmune diseases and cancer metastasis, whereas reduced capacity for **migration** leads to birth defects and immunodeficiencies. Myriad studies in vitro have established a consensus view that cell **migration**s require cell polarization, Rho GTPase-mediated cytoskeletal rearrangements, and myosin-mediated contractility. However, in vivo studies later revealed a more complex picture, including the discovery that cells migrate not only as single units but also as clusters, strands, and sheets. In particular, the role of E-Cadherin in cell motility appears to be more complex than previously appreciated. Here, we discuss recent advances achieved by combining the plethora of genetic tools available to the Drosophila geneticist with live imaging and biophysical techniques. Finally, we discuss the emerging themes such studies have revealed and ponder the puzzles that remain to be solved.
26. The Drosophila gene pointed encodes two ETS-like proteins which are involved in the development of the midline glial cells. Glial-neuronal cell interactions at the ventral midline are necessary for the proper elaboration of commissures in the embryonic CNS of Drosophila. In particular, migrating midline glial cells are required for the separation of segmental commissures. During this process the glial cells recognize specific neuronal cells at the midline, they migrate posteriorly along their cell processes and thereby separate the segmental commissures. The gene pointed (pnt) is required for this glial-neuronal cell interaction, as loss of function mutations lead to a change in the **migration** behavior of the midline glial cells. As a consequence, anterior and posterior commissures do not become separated and appear fused. Molecular analysis of pointed has revealed two differently spliced types of transcripts, which are encoded in a region extending over 55 kb of genomic sequence. In the CNS both transcript classes are expressed in cells of the midline, including the midline glial cells. Sequence analysis of cDNA clones corresponding to both transcript types reveals two different pointed proteins which share an ETS domain common to a number of transcription factors related to the vertebrate ets oncogene. Furthermore, one pointed protein form contains an additional domain of homology of approx. 80 amino acids in length, which is shared by only a subset of the ETS protein family.
27. Combined activities of Gurken and decapentaplegic specify dorsal chorion structures of the Drosophila egg. During Drosophila oogenesis Gurken, associated with the oocyte nucleus, activates the Drosophila EGF receptor in the follicular epithelium. Gurken first specifies posterior follicle cells, which in turn signal back to the oocyte to induce the **migration** of the oocyte nucleus from a posterior to an anterior-dorsal position. Here, Gurken signals again to specify dorsal follicle cells, which give rise to dorsal chorion structures including the dorsal appendages. If Gurken signaling is delayed and starts after stage 6 of oogenesis the nucleus remains at the posterior pole of the oocyte. Eggs develop with a posterior ring of dorsal appendage material that is produced by main-body follicle cells expressing the gene Broad-Complex. They encircle terminal follicle cells expressing variable amounts of the TGFbeta homologue, decapentaplegic. By ectopically expressing decapentaplegic and clonal analysis with Mothers against dpp we show that Decapentaplegic signaling is required for Broad-Complex expression. Thus, the specification and positioning of dorsal appendages along the anterior-posterior axis depends on the intersection of both Gurken and Decapentaplegic signaling. This intersection also induces rhomboid expression and thereby initiates the positive feedback loop of EGF receptor activation, which positions the dorsal appendages along the dorsal-ventral egg axis.
28. Structural determinants of RGS-RhoGEF signaling critical to Entamoeba histolytica pathogenesis. G protein signaling pathways, as key components of physiologic responsiveness and timing, are frequent targets for pharmacologic intervention. Here, we identify an effector for heterotrimeric G protein α subunit (EhGα1) signaling from Entamoeba histolytica, the causative agent of amoebic colitis. EhGα1 interacts with this effector and guanosine triphosphatase-accelerating protein, EhRGS-RhoGEF, in a nucleotide state-selective fashion. Coexpression of EhRGS-RhoGEF with constitutively active EhGα1 and EhRacC leads to Rac-dependent spreading in Drosophila S2 cells. EhRGS-RhoGEF overexpression in E. histolytica trophozoites leads to re**duced mig**ration toward serum and lower cysteine protease activity, as well as reduced attachment to, and killing of, host cells. A 2.3 Å crystal structure of the full-length EhRGS-RhoGEF reveals a putative inhibitory helix engaging the Dbl homology domain Rho-binding surface and the pleckstrin homology domain. Mutational analysis of the EhGα1/EhRGS-RhoGEF interface confirms a canonical "regulator of G protein signaling" domain rather than a RhoGEF-RGS ("rgRGS") domain, suggesting a convergent evolution toward heterotrimeric and small G protein cross-talk.
29. Snail/slug family of repressors: slowly going into the fast lane of development and cancer. The existence of homologous genes in diverse species is intriguing. A detailed comparison of the structure and function of gene families may provide important insights into gene regulation and evolution. An unproven assumption is that homologous genes have a common ancestor. During evolution, the original function of the ancestral gene might be retained in the different species which evolved along separate courses. In addition, new functions could have developed as the sequence began to diverge. This may also explain partly the presence of multipurpose genes, which have multiple functions at different stages of development and in different tissues. The Drosophila gene snail is a multipurpose gene; it has been demonstrated that snail is critical for mesoderm formation, for CNS development, and for wing cell fate determination. The related vertebrate Snail and Slug genes have also been proposed to participate in mesoderm formation, neural crest cell **migration**, carcinogenesis, and apoptosis. In this review, we will discuss the Snail/Slug family of regulators in species ranging from insect to human. We will present the protein structures, expression patterns, and functions based on molecular genetic analyses. We will also include the studies that helped to elucidate the molecular mechanisms of repression and the relationship between the conserved and divergent functions of these genes. Moreover, the studies may enable us to trace the evolution of this gene family.
30. Signaling by the Drosophila epidermal growth factor receptor pathway during development. In 1997 we wrote a review entitled "A thousand and one roles for the Drosophila epidermal growth factor (EGF) receptor (DER/EGFR)." We are not there yet in terms of the number of developmental roles assigned to this receptor in Drosophila. Nevertheless, DER has certainly emerged as one of the key players in development, since it is used repeatedly to direct cell fate choices, cell division, cell survival, and **migration**. A battery of activating ligands and an inhibitory ligand achieves this versatility. For the ligands that are produced as membrane-bound precursors, trafficking and processing are the key regulatory steps, determining the eventual temporal and spatial pattern of receptor activation. In most cases DER is activated at a short range, in the cells adjacent to the ones producing the active ligand. This activation dictates a binary choice. In some instances DER is also activated over a longer range, and multiple cell fate choices may be induced, according to its level of activation. A battery of negative feedback loops assures the limited range of DER induction. The distinct responses to DER activation in the different tissues depend upon combinatorial interactions with other signaling pathways and tissue-specific factors, at the level of target-gene regulation.
31. EGF signalling regulates cell invagination as well as cell **migration** during formation of tracheal system in Drosophila. The Drosophila tracheal system is a network of epithelial tubes that arises from the tracheal placodes, lateral clusters of ectodermal cells in ten embryonic segments. The cells of each cluster invaginate and subsequent formation of the tracheal tree occurs by cell **migration** and fusion of tracheal branches, without cell division. The combined action of the Decapentaplegic (Dpp), Epidermal growth factor (EGF) and breathless/branchless pathways are thought to be responsible for the pattern of tracheal branches. We ask how these transduction pathways regulate cell **migration** and we analyse the consequences on cell behaviour of the Dpp and EGF pathways. We find that rhomboid (rho) mutant embryos display defects not only in tracheal cell **migration** but also in tracheal cell invagination unveiling a new role for EGF signalling in the formation of the tracheal system. These results indicate that the transduction pathways that control tracheal cell **migration** are active in different steps of tracheal formation, beginning at invagination. We discuss how the consecutive steps of tracheal morphogenesis might affect the final branching pattern.
32. Inhibition of ERK-MAP kinase signaling by RSK during Drosophila development. Although p90 ribosomal S6 kinase (RSK) is known as an important downstream effector of the ribosomal protein S6 kinase/extracellular signal-regulated kinase (Ras/ERK) pathway, its endogenous role, and precise molecular function remain unclear. Using gain-of-function and null mutants of RSK, its physiological role was successfully characterized in Drosophila. Surprisingly, RSK-null mutants were viable, but exhibited developmental abnormalities related to an enhanced ERK-dependent cellular differentiation such as ectopic photoreceptor- and vein-cell formation. Conversely, overexpression of RSK dramatically suppressed the ERK-dependent differentiation, which was further augmented by mutations in the Ras/ERK pathway. Consistent with these physiological phenotypes, RSK negatively regulated ERK-mediated developmental processes and gene expressions by blocking the nuclear localization of ERK in a kinase activity-independent manner. In addition, we further demonstrated that the RSK-dependent inhibition of ERK nuclear **migration** is mediated by the physical association between ERK and RSK. Collectively, our study reveals a novel regulatory mechanism of the Ras/ERK pathway by RSK, which negatively regulates ERK activity by acting as a cytoplasmic anchor in Drosophila.
33. ventral veinless, a POU domain transcription factor, regulates different transduction pathways required for tracheal branching in Drosophila. Cell **migration** is an important step in a variety of developmental processes in many multicellular organisms. A particularly appropriate model to address the study of cell **migration** is the tracheal system of Drosophila, whose formation occurs by **migration** and fusion from clusters of ectodermal cells specified in each side of ten embryonic segments. Morphogenesis of the tracheal tree requires the activity of many genes, among them breathless (btl) and ventral veinless (vvl) whose mutations abolish tracheal cell **migration**. Activation of the btl receptor by branchless (bnl), its putative ligand, exerts an instructive role in the process of guiding tracheal cell **migration**. vvl has been shown to be required for the maintenance of btl expression during tracheal tree formation. Here we show that, in addition, vvl is independently required for the specific expression in the tracheal cells of thick veins (tkv) and rhomboid (rho), two genes whose mutations disrupt only particular branches of the tracheal system. Indeed, we show that expression in the tracheal cells of an activated form of tkv, the putative decapentaplegic (dpp) receptor, is able to induce shifts in their **migration**, asserting the role of the dpp pathway in establishing the branching pattern of the tracheal tree. In addition, by ubiquitous expression of the btl and tkv genes in vvl mutant embryos we show that both genes contribute to vvl function. These results indicate that through activation of its target genes, vvl makes the tracheal cells competent to further signalling and suggest that the btl transduction pathway could collaborate with other transduction pathways also regulated by vvl to specify the tracheal branching pattern.
34. The midline of the Drosophila central nervous system: a model for the genetic analysis of cell fate, cell **migration**, and growth cone guidance. A row of mesectodermal cells separates the two lateral neurogenic regions in the Drosophila embryo and generates a discrete set of glia and neurons. Most CNS growth cones initially head straight toward the midline, suggesting that these midline cells play a key role in the formation of the axon commissures. We have used antibodies that stain the first axons, beta-galactosidase enhancer trap lines that selectively stain the different midline cells, and electron microscopic studies to elucidate the cells and interactions that mediate the normal formation of the two major commissures in each segment. This analysis has led to a model that proposes a series of sequential cell interactions controlling the development of the axon commissures. A genetic test of this model has utilized a number of mutations that, by either eliminating or altering the differentiation of various midline cells, perturb the development of the axon commissures in a predictable fashion.
35. Cad74A is regulated by BR and is required for robust dorsal appendage formation in Drosophila oogenesis. Drosophila egg development is an established model for studying epithelial patterning and morphogenesis, but the connection between signaling pathways and egg morphology is still incompletely understood. We have identified a non-classical cadherin, Cad74A, as a putative adhesion gene that bridges epithelial patterning and morphogenesis in the follicle cells. Starting in mid-oogenesis, Cad74A is expressed in the follicle cells that contact the oocyte, including the border cells and most of the columnar follicle cells. However, Cad74A is repressed in two dorsolateral patches of follicle cells, which participate in the formation of tubular respiratory appendages. We show genetically that Cad74A is downstream of the EGFR and BMP signaling pathways and is repressed by the Zn-finger transcription factor Broad. The correlation of Cad74A repression in the cells that bend out of the plane of the follicular epithelium is preserved across Drosophila species and mutant backgrounds exhibiting a range of eggshell phenotypes. Complete removal of Cad74A from the follicle cells causes defects in dorsal appendage formation. Ectopic expression of Cad74A in the roof cells results in shortened, flattened appendages due to the hindered **migration** of the roof cells. Based on these results, we propose that Cad74A is part of the adhesive machinery that enables robust dorsal appendage formation, and as such provides a link between the patterning of the follicle cells and eggshell morphogenesis.
36. Regulation of cofilin phosphorylation and asymmetry in collective cell **migration** during morphogenesis. During Drosophila oogenesis, two actin dynamics regulators, cofilin and Rac, are required for the collective **migration** of a coherent cluster of cells called border cells. Cell culture data have shown that Rac and cofilin are both essential for lamellipodium formation, but Rac signaling results in phosphorylation and hence inactivation of cofilin. So it remains unclear whether cofilin phosphorylation plays a promoting or inhibitory role during cell **migration**. We show here that cofilin is required for F-actin turnover and lamellipodial protrusion in the border cells. Interestingly, reducing the dosage of cofilin by half or expressing a phospho-mimetic mutant form, S3E, partially rescues the **migration** and protrusion defects of Rac-deficient border cells. Moreover, cofilin exhibits moderate accumulation in border cells at the migratory front of the cluster, whereas phospho-cofilin has a robust and uniform distribution pattern in all the outer border cells. Blocking or overactivating Rac signaling in border cells greatly reduces or increases cofilin phosphorylation, respectively, and each abolishes cell **migration**. Furthermore, Rac may signal through Pak and LIMK to result in uniform phosphorylation of cofilin in all the outer border cells, whereas the guidance receptor Pvr (PDGF/VEGF receptor) mediates the asymmetric localization of cofilin in the cluster but does not affect its phosphorylation. Our study provides one of the first models of how cofilin functions and is regulated in the collective **migration** of a group of cells in vivo.

### ECM

#### PubMed

1. Changing distributions of extracellular matrix components during early wing morphogenesis in Drosophila. A new monoclonal antibody, specific to an epitope in the carboxyl terminus of the Drosophila collagen IV molecule (basement membrane collagen) was identified. The distributions of collagen IV, laminin, and an additional extracellular molecule, the 2G2 antigen (2G2-Ag), were followed immunocytochemically during early wing development. In late third instar larvae, collagen IV and laminin surround the entire wing disc, whereas the 2G2-Ag is limited to the region of the future wing pouch. For the first few hours following eversion of the disc, all three **ECM** components line the basal surfaces of all epithelial cells in the wing pouch, both those destined to line the wing veins and those destined to become tightly apposed in the large intervein regions. Collagen IV and laminin persist on these cells during the two initial rounds of apposition of dorsal and ventral wing surfaces; later, they become restricted to the cells lining the veins. The 2G2-Ag disappears completely quite early in the pupal period. Collagen IV appears to be synthesized at least twice, once in the larva and a second time in the pupa; in between it is enzymatically cleaved and may be eliminated, probably by hemocytes. In an extreme allele of blistered the wing is ballooned to form a single internal space. Collagen IV and laminin line all basal wing cell surfaces early in pupal development as they do in the wild type. Later, however, they continue to line the entire cavity of the mutant wing rather than assuming a restricted distribution. In a completely veinless wing (rhomboidveinletvein), collagen IV and laminin are also present generally on basal surfaces at early times, but are completely absent between the tightly apposed wing layers later. The **ECM** distributions both in wild type wings and in mutants suggest that the matrix plays a role in the establishment of the wing venation pattern. One possibility, strengthened by recent findings regarding **ECM** receptors in Drosophila, is their involvement in dorsal-ventral wing layer adhesion. Our findings also lead us to suggest that certain sets of features which distinguish vein from intervein cells may be linked during cell differentiation and thus help to define these cell phenotypes. The features include cytoskeletal specializations and certain cell surface and **ECM** molecules.

### [Ee]xtracellular [Mm]atrix

#### PubMed

1. Integrins modulate Sog activity in the Drosophila wing. Morphogenesis of the Drosophila wing depends on a series of cell-cell and cell-**extracellular matrix** interactions. During pupal wing development, two secreted proteins, encoded by the short gastrulation (sog) and decapentaplegic (dpp) genes, vie to position wing veins in the center of broad provein territories. Expression of the Bmp4 homolog dpp in vein cells is counteracted by expression of the Bmp antagonist sog in intervein cells, which results in the formation of straight veins of precise width. We screened for genetic interactions between sog and genes encoding a variety of extracellular components and uncovered interactions between sog and myospheroid (mys), multiple edematous wing (mew) and scab (scb), which encode betaPS, alphaPS1 and alphaPS3 integrin subunits, respectively. Clonal analysis reveals that integrin mutations affect the trajectory of veins inside the provein domain and/or their width and that misexpression of sog can alter the behavior of cells in such clones. In addition, we show that a low molecular weight form of Sog protein binds to alphaPS1betaPS. We find that Sog can diffuse from its intervein site of production into adjacent provein domains, but only on the dorsal surface of the wing, where Sog interacts functionally with integrins. Finally, we show that Sog diffusion into provein regions and the reticular pattern of extracellular Sog distribution in wild-type wings requires mys and mew function. We propose that integrins act by binding and possibly regulating the activity/availability of different forms of Sog during pupal development through an adhesion independent mechanism.
2. Finding a niche: studies from the Drosophila ovary. Specialized microenvironments called niches help maintain stem cells in an undifferentiated and self-renewing state. The existence of niches has long been predicted from mammalian studies, but identifying stem cells in their native environments in vivo has remained a challenge in most vertebrates. Many of the mechanistic insights into how niches regulate stem cell maintenance have been obtained using invertebrate models such as Drosophila. Here, we focus on the Drosophila ovarian germline stem cell niche and review recent studies that have begun to reveal how intricate crosstalk between various signaling pathways regulates stem cell maintenance, how the **extracellular matrix** modulates the signaling output of the niche and how epigenetic programming influences cell development and function both inside and outside the niche to ensure proper tissue homeostasis. These insights will probably inform the study of mammalian niches and how their malfunction contributes to human disease.
3. Changing distributions of **extracellular matrix** components during early wing morphogenesis in Drosophila. A new monoclonal antibody, specific to an epitope in the carboxyl terminus of the Drosophila collagen IV molecule (basement membrane collagen) was identified. The distributions of collagen IV, laminin, and an additional extracellular molecule, the 2G2 antigen (2G2-Ag), were followed immunocytochemically during early wing development. In late third instar larvae, collagen IV and laminin surround the entire wing disc, whereas the 2G2-Ag is limited to the region of the future wing pouch. For the first few hours following eversion of the disc, all three ECM components line the basal surfaces of all epithelial cells in the wing pouch, both those destined to line the wing veins and those destined to become tightly apposed in the large intervein regions. Collagen IV and laminin persist on these cells during the two initial rounds of apposition of dorsal and ventral wing surfaces; later, they become restricted to the cells lining the veins. The 2G2-Ag disappears completely quite early in the pupal period. Collagen IV appears to be synthesized at least twice, once in the larva and a second time in the pupa; in between it is enzymatically cleaved and may be eliminated, probably by hemocytes. In an extreme allele of blistered the wing is ballooned to form a single internal space. Collagen IV and laminin line all basal wing cell surfaces early in pupal development as they do in the wild type. Later, however, they continue to line the entire cavity of the mutant wing rather than assuming a restricted distribution. In a completely veinless wing (rhomboidveinletvein), collagen IV and laminin are also present generally on basal surfaces at early times, but are completely absent between the tightly apposed wing layers later. The ECM distributions both in wild type wings and in mutants suggest that the matrix plays a role in the establishment of the wing venation pattern. One possibility, strengthened by recent findings regarding ECM receptors in Drosophila, is their involvement in dorsal-ventral wing layer adhesion. Our findings also lead us to suggest that certain sets of features which distinguish vein from intervein cells may be linked during cell differentiation and thus help to define these cell phenotypes. The features include cytoskeletal specializations and certain cell surface and ECM molecules.
4. Shavenbaby couples patterning to epidermal cell shape control. It is well established that developmental programs act during embryogenesis to determine animal morphogenesis. How these developmental cues produce specific cell shape during morphogenesis, however, has remained elusive. We addressed this question by studying the morphological differentiation of the Drosophila epidermis, governed by a well-known circuit of regulators leading to a stereotyped pattern of smooth cells and cells forming actin-rich extensions (trichomes). It was shown that the transcription factor Shavenbaby plays a pivotal role in the formation of trichomes and underlies all examined cases of the evolutionary diversification of their pattern. To gain insight into the mechanisms of morphological differentiation, we sought to identify shavenbaby's downstream targets. We show here that Shavenbaby controls epidermal cell shape, through the transcriptional activation of different classes of cellular effectors, directly contributing to the organization of actin filaments, regulation of the **extracellular matrix**, and modification of the cuticle. Individual inactivation of shavenbaby's targets produces distinct trichome defects and only their simultaneous inactivation prevent trichome formation. Our data show that shavenbaby governs an evolutionarily conserved developmental module consisting of a set of genes collectively responsible for trichome formation, shedding new light on molecular mechanisms acting during morphogenesis and the way they can influence evolution of animal forms.
5. Confrontation of scabrous expressing and non-expressing cells is essential for normal ommatidial spacing in the Drosophila eye. The establishment of neural precursor cells in Drosophila depends on cell-cell interactions and lateral inhibition. Scabrous (sca) is involved in this process by preventing an excess of cells from adopting a neural precursor fate. Specifically in eye development, Sca protein function has been implicated in the spacing pattern that is essential for the ordered appearance of the ommatidial array. During this process sca expression is restricted to neurogenic groups of cells and later to the neural precursors. We report that ectopic sca expression in the morphogenetic furrow results in a rough eye phenotype with oversized and fused ommatidia. These defects in adult eyes are due to the generation of too many ommatidial preclusters in the morphogenetic furrow. Strikingly, sca loss-of-function mutants have an almost identical phenotype. Our results suggest that Sca plays a positive role in establishing the spacing pattern within the furrow and that the quantitative difference in sca expression between neighboring groups of cells is a determining factor in this process. Ectopic expression of Sca also represses endogenous sca expression in the furrow, suggesting that Sca is involved in a feedback loop affecting its own transcription. Interestingly, sca shares homology to a group of **extracellular matrix** proteins that have been implicated in neuronal differentiation. We present a model for sca function based on its phenotypic and molecular features.

### [Ii]nvasive

#### PubMed

1. The Drosophila short gastrulation gene prevents Dpp from autoactivating and suppressing neurogenesis in the neuroectoderm. The short gastrulation (sog) gene is expressed in broad lateral stripes comprising the neuroectoderm of the Drosophila blastoderm embryo. sog encodes a predicted secreted protein that functions nonautonomously to antagonize the activity of the TGF-beta-like Decapentaplegic (Dpp) signaling pathway in the dorsal region of the embryo. Recently, it has been shown that sog and dpp are functionally equivalent to their respective Xenopus homologs chordin and BMP-4. In this report we provide the first direct evidence that sog plays a local role in the lateral region of the blastoderm embryo to oppose Dpp activity in the neuroectoderm. In the dorsal region, Dpp signaling both suppresses neurogenesis and maintains expression of genes that promote dorsal cell fates (dorsalization). We show that Dpp also can perform both of these functions in the neuroectoderm. In wild-type embryos, the ability of Dpp to induce expression of dorsal markers including itself (autoactivation) in the neuroectoderm is blocked by sog. We propose that Sog protects the neuroectoderm from an **invasive** positive feedback loop created by Dpp diffusion and autoactivation. We show that the two functions of Dpp signaling, neural suppression and dorsalization, are triggered by distinct thresholds of Dpp activity. Epistasis experiments reveal that all observed sog activity can be accounted for by Sog functioning as a dedicated Dpp antagonist. Finally, we provide evidence that Sog functions as a diffusible morphogen in the blastoderm embryo. These data strongly support the view that the primary phylogenetically conserved function of the Drosophila sog and dpp genes and the homologous Xenopus chordin and BMP-4 genes is to subdivide the primitive embryonic ectoderm into neural versus non-neural domains.
2. The actin cross-linker Filamin/Cheerio mediates tumor malignancy downstream of JNK signaling. Cell shape dynamics, motility, and cell proliferation all depend on the actin cytoskeleton. Malignant cancer cells hijack the actin network to grow and migrate to secondary sites. Understanding the function of actin regulators is therefore of major interest. In the present study, we identify the actin cross-linking protein Filamin/Cheerio (Cher) as a mediator of malignancy in genetically defined Drosophila tumors. We show that in **invasive** tumors, resulting from cooperation of activated Ras with disrupted epithelial cell polarity, Cher is upregulated in a Jun N-terminal kinase (JNK)-dependent manner. Although dispensable in normal epithelium, Cher becomes required in the tumor cells for their growth and **invasive**ness. When deprived of Cher, these tumor clones lose their full potential to proliferate and breach tissue boundaries. Instead, the Cher-deficient clones remain confined within the limits of their source epithelium, permitting survival of the host animal. Through interaction with the myosin II heavy chain subunit, Cher is likely to strengthen the cortical actomyosin network and reinforce mechanical tension within the **invasive** tumors. Accordingly, Cher is required for aberrant expression of genes downstream of the Hippo/Yorkie signaling in the tumor tissue. Our study identifies Cher as a new target of JNK signaling that links cytoskeleton dynamics to tumor progression.
3. Tre1 GPCR initiates germ cell transepithelial migration by regulating Drosophila melanogaster E-cadherin. Despite significant progress in identifying the guidance pathways that control cell migration, how a cell starts to move within an intact organism, acquires motility, and loses contact with its neighbors is poorly understood. We show that activation of the G protein-coupled receptor (GPCR) trapped in endoderm 1 (Tre1) directs the redistribution of the G protein Gbeta as well as adherens junction proteins and Rho guanosine triphosphatase from the cell periphery to the lagging tail of germ cells at the onset of Drosophila melanogaster germ cell migration. Subsequently, Tre1 activity triggers germ cell dispersal and orients them toward the midgut for directed transepithelial migration. A transition toward **invasive** migration is also a prerequisite for metastasis formation, which often correlates with down-regulation of adhesion proteins. We show that uniform down-regulation of E-cadherin causes germ cell dispersal but is not sufficient for transepithelial migration in the absence of Tre1. Our findings therefore suggest a new mechanism for GPCR function that links cell polarity, modulation of cell adhesion, and invasion.

### [Mm]etastasis

#### PubMed

1. A conserved role for Snail as a potentiator of active transcription. The transcription factors of the Snail family are key regulators of epithelial-mesenchymal transitions, cell morphogenesis, and tumor **metastasis**. Since its discovery in Drosophila ∼25 years ago, Snail has been extensively studied for its role as a transcriptional repressor. Here we demonstrate that Drosophila Snail can positively modulate transcriptional activation. By combining information on in vivo occupancy with expression profiling of hand-selected, staged snail mutant embryos, we identified 106 genes that are potentially directly regulated by Snail during mesoderm development. In addition to the expected Snail-repressed genes, almost 50% of Snail targets showed an unanticipated activation. The majority of "Snail-activated" genes have enhancer elements cobound by Twist and are expressed in the mesoderm at the stages of Snail occupancy. Snail can potentiate Twist-mediated enhancer activation in vitro and is essential for enhancer activity in vivo. Using a machine learning approach, we show that differentially enriched motifs are sufficient to predict Snail's regulatory response. In silico mutagenesis revealed a likely causative motif, which we demonstrate is essential for enhancer activation. Taken together, these data indicate that Snail can potentiate enhancer activation by collaborating with different activators, providing a new mechanism by which Snail regulates development.
2. Tre1 GPCR initiates germ cell transepithelial migration by regulating Drosophila melanogaster E-cadherin. Despite significant progress in identifying the guidance pathways that control cell migration, how a cell starts to move within an intact organism, acquires motility, and loses contact with its neighbors is poorly understood. We show that activation of the G protein-coupled receptor (GPCR) trapped in endoderm 1 (Tre1) directs the redistribution of the G protein Gbeta as well as adherens junction proteins and Rho guanosine triphosphatase from the cell periphery to the lagging tail of germ cells at the onset of Drosophila melanogaster germ cell migration. Subsequently, Tre1 activity triggers germ cell dispersal and orients them toward the midgut for directed transepithelial migration. A transition toward invasive migration is also a prerequisite for **metastasis** formation, which often correlates with down-regulation of adhesion proteins. We show that uniform down-regulation of E-cadherin causes germ cell dispersal but is not sufficient for transepithelial migration in the absence of Tre1. Our findings therefore suggest a new mechanism for GPCR function that links cell polarity, modulation of cell adhesion, and invasion.
3. Cellular and molecular mechanisms of single and collective cell migrations in Drosophila: themes and variations. The process of cell migration is essential throughout life, driving embryonic morphogenesis and ensuring homeostasis in adults. Defects in cell migration are a major cause of human disease, with excessive migration causing autoimmune diseases and cancer **metastasis**, whereas reduced capacity for migration leads to birth defects and immunodeficiencies. Myriad studies in vitro have established a consensus view that cell migrations require cell polarization, Rho GTPase-mediated cytoskeletal rearrangements, and myosin-mediated contractility. However, in vivo studies later revealed a more complex picture, including the discovery that cells migrate not only as single units but also as clusters, strands, and sheets. In particular, the role of E-Cadherin in cell motility appears to be more complex than previously appreciated. Here, we discuss recent advances achieved by combining the plethora of genetic tools available to the Drosophila geneticist with live imaging and biophysical techniques. Finally, we discuss the emerging themes such studies have revealed and ponder the puzzles that remain to be solved.

### [Cc]ell [Ii]nvasion

#### PubMed

1. Substrate specificity of rhomboid intramembrane proteases is governed by helix-breaking residues in the substrate transmembrane domain. Rhomboid intramembrane proteases initiate cell signaling during Drosophila development and Providencia bacterial growth by cleaving transmembrane ligand precursors. We have determined how specificity is achieved: Drosophila Rhomboid-1 is a site-specific protease that recognizes its substrate Spitz by a small region of the Spitz transmembrane domain (TMD). This substrate motif is necessary and sufficient for cleavage and is composed of residues known to disrupt helices. Rhomboids from diverse organisms including bacteria and vertebrates recognize the same substrate motif, suggesting that they use a universal targeting strategy. We used this information to search for other rhomboid substrates and identified a family of adhesion proteins from the human parasite Toxoplasma gondii, the TMDs of which were efficient substrates for rhomboid proteases. Intramembrane cleavage of these proteins is required for host **cell invasion**. These results provide an explanation of how rhomboid proteases achieve specificity, and allow some rhomboid substrates to be predicted from sequence information.
2. Substrate specificity of rhomboid intramembrane proteases is governed by helix-breaking residues in the substrate transmembrane domain. Rhomboid intramembrane proteases initiate cell signaling during Drosophila development and Providencia bacterial growth by cleaving transmembrane ligand precursors. We have determined how specificity is achieved: Drosophila Rhomboid-1 is a site-specific protease that recognizes its substrate Spitz by a small region of the Spitz transmembrane domain (TMD). This substrate motif is necessary and sufficient for cleavage and is composed of residues known to disrupt helices. Rhomboids from diverse organisms including bacteria and vertebrates recognize the same substrate motif, suggesting that they use a universal targeting strategy. We used this information to search for other rhomboid substrates and identified a family of adhesion proteins from the human parasite Toxoplasma gondii, the TMDs of which were efficient substrates for rhomboid proteases. Intramembrane cleavage of these proteins is required for host **cell invasion**. These results provide an explanation of how rhomboid proteases achieve specificity, and allow some rhomboid substrates to be predicted from sequence information.
3. A spatially localized rhomboid protease cleaves cell surface adhesins essential for invasion by Toxoplasma. Apicomplexan parasites cause serious human and animal diseases, the treatment of which requires identification of new therapeutic targets. Host-**cell invasion** culminates in the essential cleavage of parasite adhesins, and although the cleavage site for several adhesins maps within their transmembrane domains, the protease responsible for this processing has not been discovered. We have identified, cloned, and characterized the five nonmitochondrial rhomboid intramembrane proteases encoded in the recently completed genome of Toxoplasma gondii. Four T. gondii rhomboids (TgROMs) were active proteases with similar substrate specificity. TgROM1, TgROM4, and TgROM5 were expressed in the tachyzoite stage responsible for the disease, whereas TgROM2 and TgROM3 were expressed in the oocyst stage involved in transmission. Although both TgROM5 and TgROM4 localized to the cell surface in tachyzoites, TgROM5 was primarily at the posterior of the parasite, whereas adhesins were sequestered in internal micronemes. Upon microneme secretion, as occurs during invasion, the MIC2 adhesin was secreted to the apical end and translocated to the posterior, the site of cleavage, where it colocalized only with TgROM5. Moreover, only TgROM5 was able to cleave MIC adhesins in a cell-based assay, indicating that it likely provides the key protease activity necessary for invasion. T. gondii rhomboids have clear homologues in other apicomplexans including malaria; thus, our findings provide a model for studying invasion by this deadly pathogen and offer a target for therapeutic intervention.

---

## cact

- NCBI Gene
- GeneRIF
- Pubmed

| Search Term | Rank | Fields |
| --- | --- | --- |
| [Mm]igration | 6 | PubMed(3) |
| [Ee]xtracellular [Mm]atrix | 8 | PubMed(1) |

  
hide/show details for cact

### [Mm]igration

#### PubMed

1. A core transcriptional network for early mesoderm development in Drosophila melanogaster. Embryogenesis is controlled by large gene-regulatory networks, which generate spatially and temporally refined patterns of gene expression. Here, we report the characteristics of the regulatory network orchestrating early mesodermal development in the fruitfly Drosophila, where the transcription factor Twist is both necessary and sufficient to drive development. Through the integration of chromatin immunoprecipitation followed by microarray analysis (ChIP-on-chip) experiments during discrete time periods with computational approaches, we identified >2000 Twist-bound cis-regulatory modules (CRMs) and almost 500 direct target genes. Unexpectedly, Twist regulates an almost complete cassette of genes required for cell proliferation in addition to genes essential for morophogenesis and cell **migration**. Twist targets almost 25% of all annotated Drosophila transcription factors, which may represent the entire set of regulators necessary for the early development of this system. By combining in vivo binding data from Twist, Mef2, Tinman, and Dorsal we have constructed an initial transcriptional network of early mesoderm development. The network topology reveals extensive combinatorial binding, feed-forward regulation, and complex logical outputs as prevalent features. In addition to binary activation and repression, we suggest that Twist binds to almost all mesodermal CRMs to provide the competence to integrate inputs from more specialized transcription factors.
2. The polarisation of the anterior-posterior and dorsal-ventral axes during Drosophila oogenesis. Recent work on Drosophila oogenesis has begun to reveal how the first asymmetries in development arise and how these relate to the later events that localise the positional cues which define the embryonic axes. The Cadherin-dependent positioning of the oocyte creates an anterior-posterior polarity that is transmitted to the embryo through the localisation and localised translation of bicoid, oskar, and nanos mRNA. In contrast, dorsal-ventral polarity arises from the random **migration** of the nucleus to the anterior of the oocyte, where it determines where gurken mRNA is translated and localised. Gurken signalling then defines the embryonic dorsal-ventral axis by restricting pipe expression to the ventral follicle cells, where Pipe regulates the production of an unidentified cue that activates the Toll signalling pathway.
3. A functional RNAi screen for regulators of receptor tyrosine kinase and ERK signalling. Receptor tyrosine kinase (RTK) signalling through extracellular-signal-regulated kinases (ERKs) has pivotal roles during metazoan development, underlying processes as diverse as fate determination, differentiation, proliferation, survival, **migration** and growth. Abnormal RTK/ERK signalling has been extensively documented to contribute to developmental disorders and disease, most notably in oncogenic transformation by mutant RTKs or downstream pathway components such as Ras and Raf. Although the core RTK/ERK signalling cassette has been characterized by decades of research using mammalian cell culture and forward genetic screens in model organisms, signal propagation through this pathway is probably regulated by a larger network of moderate, context-specific proteins. The genes encoding these proteins may not have been discovered through traditional screens owing, in particular, to the requirement for visible phenotypes. To obtain a global view of RTK/ERK signalling, we performed an unbiased, RNA interference (RNAi), genome-wide, high-throughput screen in Drosophila cells using a novel, quantitative, cellular assay monitoring ERK activation. Here we show that ERK pathway output integrates a wide array of conserved cellular processes. Further analysis of selected components-in multiple cell types with different RTK ligands and oncogenic stimuli-validates and classifies 331 pathway regulators. The relevance of these genes is highlighted by our isolation of a Ste20-like kinase and a PPM-family phosphatase that seem to regulate RTK/ERK signalling in vivo and in mammalian cells. Novel regulators that modulate specific pathway outputs may be selective targets for drug discovery.

### [Ee]xtracellular [Mm]atrix

#### PubMed

1. Dose-sensitive autosomal modifiers identify candidate genes for tissue autonomous and tissue nonautonomous regulation by the Drosophila nuclear zinc-finger protein, hindsight. The nuclear zinc-finger protein encoded by the hindsight (hnt) locus regulates several cellular processes in Drosophila epithelia, including the Jun N-terminal kinase (JNK) signaling pathway and actin polymerization. Defects in these molecular pathways may underlie the abnormal cellular interactions, loss of epithelial integrity, and apoptosis that occurs in hnt mutants, in turn causing failure of morphogenetic processes such as germ band retraction and dorsal closure in the embryo. To define the genetic pathways regulated by hnt, 124 deficiencies on the second and third chromosomes and 14 duplications on the second chromosome were assayed for dose-sensitive modification of a temperature-sensitive rough eye phenotype caused by the viable allele, hntpeb; 29 interacting regions were identified. Subsequently, 438 P-element-induced lethal mutations mapping to these regions and 12 candidate genes were tested for genetic interaction, leading to identification of 63 dominant modifier loci. A subset of the identified mutants also dominantly modify hnt308-induced embryonic lethality and thus represent general rather than tissue-specific interactors. General interactors include loci encoding transcription factors, actin-binding proteins, signal transduction proteins, and components of the **extracellular matrix**. Expression of several interactors was assessed in hnt mutant tissue. Five genes--apontic (apt), Delta (Dl), decapentaplegic (dpp), karst (kst), and puckered (puc)--are regulated tissue autonomously and, thus, may be direct transcriptional targets of HNT. Three of these genes--apt, Dl, and dpp--are also regulated nonautonomously in adjacent non-HNT-expressing tissues. The expression of several additional interactors--viking (vkg), Cg25, and laminin-alpha (LanA)-is affected only in a nonautonomous manner.

---

## vir-1

- NCBI Gene
- GeneRIF
- Pubmed

| Search Term | Rank | Fields |
| --- | --- | --- |
| [Mm]igration | 6 | PubMed(1) |
| [Ee]xtracellular [Mm]atrix | 8 | PubMed(1) |

  
hide/show details for vir-1

### [Mm]igration

#### PubMed

1. Unwrapping glial biology: Gcm target genes regulating glial development, diversification, and function. Glia are the most abundant cell type in the mammalian brain. They regulate neuronal development and function, CNS immune surveillance, and stem cell biology, yet we know surprisingly little about glia in any organism. Here we identify over 40 new Drosophila glial genes. We use glial cells missing (gcm) mutants and misexpression to verify they are Gcm regulated in vivo. Many genes show unique spatiotemporal responsiveness to Gcm in the CNS, and thus glial subtype diversification requires spatially or temporally restricted Gcm cofactors. These genes provide insights into glial biology: we show unc-5 (a repulsive netrin receptor) orients glial **migration**s and the draper gene mediates glial engulfment of apoptotic neurons and larval locomotion. Many identified Drosophila glial genes have homologs expressed in mammalian glia, revealing conserved molecular features of glial cells. 80% of these Drosophila glial genes have mammalian homologs; these are now excellent candidates for regulating human glial development, function, or disease.

### [Ee]xtracellular [Mm]atrix

#### PubMed

1. An MMP liberates the Ninjurin A ectodomain to signal a loss of cell adhesion. Matrix metalloproteinases (MMPs) are important for developmental tissue remodeling and for the inflammatory response. Although the vertebrate MMP family is large and functionally redundant, the fruitfly Drosophila melanogaster has only two MMPs, both essential genes. Our previous work demonstrated that Mmp1 is required for growth of the tracheal system, and we suggested that the mutant phenotype resulted from aberrant persistence of cell adhesion to the **extracellular matrix**. Here we report the identification of NijA, a transmembrane protein whose vertebrate homologs regulate cell adhesion, as a two-hybrid binding partner for Mmp1. The binding of Mmp1 and NijA was confirmed by coimmunoprecipitation of endogenous proteins from flies, and the endogenous proteins were found to colocalize at the tracheal cell surface in larvae. When NijA is expressed in S2 cells, they lose adhesion to surfaces; this adhesion-loss phenotype is dependent on the expression and catalytic activity of Mmp1. Our data indicate that Mmp1 releases the N-terminal extracellular domain of NijA. This liberated ectodomain promotes the loss of cell adhesion in a cell-nonautonomous manner. We suggest that tracheal cell adhesion is regulated by a novel mechanism utilizing an MMP and a ninjurin family member.

---

## prage

- NCBI Gene
- GeneRIF
- Pubmed

| Search Term | Rank | Fields |
| --- | --- | --- |
| [Mm]igration | 6 | PubMed(1) |

  
hide/show details for prage

### [Mm]igration

#### PubMed

1. Gene expression profiling of Drosophila tracheal fusion cells. The Drosophila trachea is a premier genetic system to investigate the fundamental mechanisms of tubular organ formation. Tracheal fusion cells lead the branch fusion process to form an interconnected tubular network. Therefore, fusion cells in the Drosophila trachea will be an excellent model to study branch fusion in mammalian tubular organs, such as kidneys and blood vessels. The fusion process is a dynamic cellular process involving cell **migration**, adhesion, vesicle trafficking, cytoskeleton rearrangement, and membrane fusion. To understand how these cellular events are coordinated, we initiated the critical step to assemble a gene expression profile of fusion cells. For this study, we analyzed the expression of 234 potential tracheal-expressed genes in fusion cells during fusion cell development. 143 Tracheal genes were found to encode transcription factors, signal proteins, cytoskeleton and matrix proteins, transporters, and proteins with unknown function. These genes were divided into four subgroups based on their levels of expression in fusion cells compared to neighboring non-fusion cells revealed by in situ hybridization: (1) genes that have relative high abundance in fusion cells, (2) genes that are dynamically expressed in fusion cells, (3) genes that have relative low abundance in fusion cells, and (4) genes that are expressed at similar levels in fusion cells and non-fusion tracheal cells. This study identifies the expression profile of fusion cells and hypothetically suggests genes which are necessary for the fusion process and which play roles in distinct stages of fusion, as indicated by the location and timing of expression. These data will provide the basis for a comprehensive understanding of the molecular and cellular mechanisms of branch fusion.

---

## aay

- NCBI Gene
- GeneRIF
- Pubmed

| Search Term | Rank | Fields |
| --- | --- | --- |
| [Ii]nvasive | 10 | PubMed(1) |

  
hide/show details for aay

### [Ii]nvasive

#### PubMed

1. Neurodegeneration of Drosophila drop-dead mutants is associated with hypoxia in the brain. The Drosophila drop-dead (drd) mutant undergoes massive brain degeneration, resulting in sudden death. drd encodes a multi-pass membrane protein possessing nose resistant to fluoxetine (NRF) and putative acyltransferase domains. However, the etiology of brain degeneration that occurs in drd mutant flies is still poorly understood. Herein, we show that drd neurodegeneration may be because of an oxygen deficit in the brain. We found that DRD protein is selectively expressed in cells secreting cuticular and eggshell layers. These layers exhibit blue fluorescence upon UV excitation, which is reduced in drd flies. The drd tracheal air sacs lacking blue fluorescence collapse, which likely contributes to hypoxia. Consistently, genes induced in hypoxia are up-regulated in drd flies. Feeding of anti-reactive oxygen species agents partially rescue the drd from sudden death. We propose that drd flies can provide a non-**invasive** animal model for hypoxia-induced cell death.

---

## per

- NCBI Gene
- GeneRIF
- Pubmed

| Search Term | Rank | Fields |
| --- | --- | --- |
| [Mm]igration | 6 | PubMed(3) |
| [Ii]nvasive | 10 | PubMed(2) |

  
hide/show details for per

### [Mm]igration

#### PubMed

1. Heartless, a Drosophila FGF receptor homolog, is essential for cell **migration** and establishment of several mesodermal lineages. A Drosophila FGF receptor homolog (DFGF-R2/DFRI) termed Heartless (Htl) is expressed in the embryonic mesoderm. The phenotypes of null mutant embryos demonstrated that Htl is a central player that is required for the development of several mesodermal lineages. No abnormalities in the primary specification of the mesoderm were observed. The first defects were seen as irregular **migration** and spreading of the mesoderm over the ectoderm. Subsequently, cell fates were not induced in several lineages including the visceral mesoderm, heart, and the dorsal somatic muscles. The defects in the induction of cell fates are likely to result from failure of the mesoderm to spread over the ectoderm and receive patterning signals. The defective spreading could be circumvented in htl mutant embryos by providing an ectopic Dpp patterning signal, leading to the formation of heart and dorsal muscle cells. Htl appears to be required also subsequently during the **migration** and morphogenesis of the different lineages. Expression of a dominant-negative htl construct after the initial induction of cell fates gave rise to aberrant **migration** and organization of the visceral mesoderm, heart, and somatic muscles. Thus, a common role for Htl in cell **migration** and tissue organization may account for the pleiotropic defects of the htl mutation.
2. Distinguishing **migration** from isolation using genes with intragenic recombination: detecting introgression in the Drosophila simulans species complex. Determining the presence or absence of gene flow between populations is the target of some statistical methods in population genetics. Until recently, these methods either avoided the use of recombining genes, or treated recombination as a nuisance parameter. However, genes with recombination contribute additional information for the detection of gene flow (i.e. through linkage disequilibrium).We present three summary statistics based on the spatial arrangement of fixed differences, and shared and exclusive polymorphisms that are sensitive to the presence and direction of gene flow. Power and false positive rate for tests based on these statistics are studied by simulation.The application of these tests to populations from the Drosophila simulans species complex yielded results consistent with **migration** between D. simulans and its two endemic sister species D. mauritiana and D. sechellia, and between populations D. mauritiana on the islands of the Mauritius and Rodrigues.We demonstrate the sensitivity of the developed statistics to the presence and direction of gene flow, and characterize their power as a function of differentiation level and recombination rate. The properties of these statistics make them especially suitable for analyzing high-throughput sequencing data or for their integration within the approximate Bayesian computation framework.
3. Drosophila flies in "Evolution Canyon" as a model for incipient sympatric speciation. The genetic basis of population divergence leading to adaptive radiation and speciation is a major unresolved problem of evolutionary biology. Molecular elucidation of "speciation genes" advanced recently, yet it remains without clear identification of the gene complexes participating in reproductive isolation between natural populations, particularly, in sympatry. Genetic divergence was discovered between Drosophila melanogaster populations inhabiting ecologically contrasting, opposite slopes in "Evolution Canyon" (EC), Mt. Carmel, Israel. Interslope **migration** of flies is easy and verified. Nevertheless, significant interslope D. melanogaster population divergence was established at EC involving habitat choice, mate choice, thermal and drought tolerances, adaptive genes, and mobile elements. Parallel patterns of stress tolerance, habitat choice, and mate choice were demonstrated in Drosophila simulans at EC, although on a smaller scale. However, some tests for interslope genetic differentiation in Drosophila, derived from the opposite EC slopes, gave somewhat controversial results. Here we present new empirical data on interslope genetic divergence of Drosophila at EC, and summarize previous supporting and controversial results. We suggest that Drosophila populations at EC represent a rare example, demonstrating how selection overrides **migration**, and propose an ad hoc ecological model of incipient sympatric divergence.

### [Ii]nvasive

#### PubMed

1. Quantitative analysis of Drosophila period gene transcription in living animals. To determine the in vivo regulatory pattern of the clock gene period (per), the authors recently developed transgenic Drosophila carrying a luciferase cDNA fused to the promoter region of per. They have now carried out non**invasive**, high time-resolution experiments allowing high-throughput monitoring of circadian bioluminescence rhythms in individual living adults for several days. This immediately solved several problems (resulting directly from individual asynchrony within a population) that have accompanied previous biochemical experiments in which groups of animals were sacrificed at each time point. Furthermore, the authors have developed numerical analysis methods for automatically determining rhythmicity associated with bioluminescence records from single flies. This has revealed some features of per gene transcription that were previously unappreciated and provides a general strategy for the analysis of rhythmic time series in the study of molecular rhythms.
2. Novel features of drosophila period Transcription revealed by real-time luciferase reporting. The rapid turnover of luciferase and the sensitive, non-**invasive** nature of its assay make this reporter gene uniquely situated for temporal gene expression studies. To determine the in vivo regulatory pattern of the Drosophila clock gene period (per), we generated transgenic strains carrying a luciferase cDNA fused to the promoter region of the per gene. This has allowed us to monitor circadian rhythms of bioluminescence from pacemaker cells within the head for several days in individual living adults. These high time-resolution experiments permitted neuronal per transcription and opens the door to vastly simplified experiments in general chronobiology and studies of temporally regulated transcription in a wide range of experimental systems.

---

## pll

- NCBI Gene
- GeneRIF
- Pubmed

| Search Term | Rank | Fields |
| --- | --- | --- |
| [Mm]igration | 6 | PubMed(2) |
| [Mm]etastasis | 12 | PubMed(1) |

  
hide/show details for pll

### [Mm]igration

#### PubMed

1. The polarisation of the anterior-posterior and dorsal-ventral axes during Drosophila oogenesis. Recent work on Drosophila oogenesis has begun to reveal how the first asymmetries in development arise and how these relate to the later events that localise the positional cues which define the embryonic axes. The Cadherin-dependent positioning of the oocyte creates an anterior-posterior polarity that is transmitted to the embryo through the localisation and localised translation of bicoid, oskar, and nanos mRNA. In contrast, dorsal-ventral polarity arises from the random **migration** of the nucleus to the anterior of the oocyte, where it determines where gurken mRNA is translated and localised. Gurken signalling then defines the embryonic dorsal-ventral axis by restricting pipe expression to the ventral follicle cells, where Pipe regulates the production of an unidentified cue that activates the Toll signalling pathway.
2. In vivo RNAi screen identifies candidate signaling genes required for collective cell **migration** in Drosophila ovary. Collective **migration** of loosely or closely associated cell groups is prevalent in animal development, physiological events, and cancer metastasis. However, our understanding of the mechanisms of collective cell **migration** is incomplete. Drosophila border cells provide a powerful in vivo genetic model to study collective **migration** and identify essential genes for this process. Using border cell-specific RNAi-silencing in Drosophila, we knocked down 360 conserved signaling transduction genes in adult flies to identify essential pathways and genes for border cell **migration**. We uncovered a plethora of signaling genes, a large proportion of which had not been reported for border cells, including Rack1 (Receptor of activated C kinase) and brk (brinker), mad (mother against dpp), and sax (saxophone), which encode three components of TGF-β signaling. The RNAi knock down phenotype was validated by clonal analysis of Rack1 mutants. Our data suggest that inhibition of Src activity by Rack1 may be important for border cell **migratio**n and cluster cohesion maintenance. Lastly, results from our screen not only would shed light on signaling pathways involved in collective **migratio**n during embryogenesis and organogenesis in general, but also could help our understanding for the functions of conserved human genes involved in cancer metastasis.

### [Mm]etastasis

#### PubMed

1. In vivo RNAi screen identifies candidate signaling genes required for collective cell migration in Drosophila ovary. Collective migration of loosely or closely associated cell groups is prevalent in animal development, physiological events, and cancer **metastasis**. However, our understanding of the mechanisms of collective cell migration is incomplete. Drosophila border cells provide a powerful in vivo genetic model to study collective migration and identify essential genes for this process. Using border cell-specific RNAi-silencing in Drosophila, we knocked down 360 conserved signaling transduction genes in adult flies to identify essential pathways and genes for border cell migration. We uncovered a plethora of signaling genes, a large proportion of which had not been reported for border cells, including Rack1 (Receptor of activated C kinase) and brk (brinker), mad (mother against dpp), and sax (saxophone), which encode three components of TGF-β signaling. The RNAi knock down phenotype was validated by clonal analysis of Rack1 mutants. Our data suggest that inhibition of Src activity by Rack1 may be important for border cell migration and cluster cohesion maintenance. Lastly, results from our screen not only would shed light on signaling pathways involved in collective migration during embryogenesis and organogenesis in general, but also could help our understanding for the functions of conserved human genes involved in cancer **metastasi**s.

---

## Vinc

- NCBI Gene
- GeneRIF
- Pubmed

| Search Term | Rank | Fields |
| --- | --- | --- |
| [Mm]igration | 6 | PubMed(6) |
| ECM | 7 | PubMed(3) |
| [Ee]xtracellular [Mm]atrix | 8 | PubMed(3) |

  
hide/show details for Vinc

### [Mm]igration

#### PubMed

1. Focal adhesions: what's new inside. The cytoplasmic side of focal adhesions is comprised of large molecular complexes that link transmembrane receptors, such as integrins, to the actin cytoskeleton and mediate signals modulating cell attachment, **migration**, proliferation, differentiation, and gene expression. These complexes are heterogeneous and dynamic structures that are apparent targets of regulatory signals that control the function of focal adhesions. Recent studies using genetic approaches in invertebrate and vertebrate systems have begun to reveal the structure and function of these complexes in vivo.
2. Regulation of cell **migration** during tracheal development in Drosophila melanogaster. Most of the knowledge concerning the intracellular mechanisms involved in cell locomotion have been obtained from in vitro studies of cells in culture. Many of the concepts derived from these studies have been partially confirmed in in vivo systems but numerous questions regarding the developmental control of cell **migration** remain to be addressed. Tracheal morphogenesis in Drosophila melanogaster embryos represents an in vivo model system to study the genetic control of cell **migration**. We review what is known about tracheal development and regulation of tracheal cell **migration**. We try to link these in vivo studies and the movement of cells over two dimensional substrates and elaborate on important questions which remain to be addressed in the future.
3. Cell-cell adhesion via the ECM: integrin genetics in fly and worm. Integrins are essential for the development of the two genetically tractable invertebrate model organisms, the nematode worm Caenorhabditis elegans and the fruit fly Drosophila melanogaster. Just two integrins are present in C. elegans: one putative RGD binding integrin alphapat-2betapat-3, corresponding to Drosophila alphaPS2betaPS and vertebrate alpha5beta1, alphaVbeta1 and alpha8beta1, and one putative laminin binding integrin alphaina-1betapat-3, corresponding to Drosophila alphaPS1betaPS and vertebrate alpha3beta1, alpha6beta1 and alpha7beta1. In this review, the function of this minimal set of integrins during the development of these two invertebrates is compared. Despite the differences in bodyplan and developmental strategy, integrin adhesion to the extracellular matrix is required for similar processes: the formation of the link that translates muscle contraction into movement of the exoskeleton, cell **migration**, and morphogenetic interactions between epithelia. Other integrin functions, such as regulation of gene expression, have not yet been experimentally demonstrated in both organisms. Additional proteins have been characterised in each organism that are essential for integrin function, including extracellular matrix ligands and intracellular interacting proteins, but so far different proteins have been found in the two organisms. This in part represents the fact that the characterisation of the full set of interacting proteins is not complete in either system. However, in other cases different proteins appear to be used for similar functions in the two animals. The continued use of genetic approaches to identify proteins required for integrin function in these two model organisms should lead to the identification of the minimal set of conserved components that form integrin adhesive structures.
4. Molecular requirements for actin-based lamella formation in Drosophila S2 cells. Cell **migration** occurs through the protrusion of the actin-enriched lamella. Here, we investigated the effects of RNAi depletion of approximately 90 proteins implicated in actin function on lamella formation in Drosophila S2 cells. Similar to in vitro reconstitution studies of actin-based Listeria movement, we find that lamellae formation requires a relatively small set of proteins that participate in actin nucleation (Arp2/3 and SCAR), barbed end capping (capping protein), filament depolymerization (cofilin and Aip1), and actin monomer binding (profilin and cyclase-associated protein). Lamellae are initiated by parallel and partially redundant signaling pathways involving Rac GTPases and the adaptor protein Nck, which stimulate SCAR, an Arp2/3 activator. We also show that RNAi of three proteins (kette, Abi, and Sra-1) known to copurify with and inhibit SCAR in vitro leads to SCAR degradation, revealing a novel function of this protein complex in SCAR stability. Our results have identified an essential set of proteins involved in actin dynamics during lamella formation in Drosophila S2 cells.
5. The cloning, genomic organization and expression of the focal contact protein paxillin in Drosophila. Paxillin is a focal adhesion scaffolding protein, which has been proposed to play a role in focal adhesion dynamics. We have isolated a cDNA clone of the Drosophila homologue of paxillin. Comparison of the Drosophila paxillin sequence with those of vertebrate paxillins shows strong conservation of the LIM domains and LD repeats. Using the Drosophila genomic sequence we have identified two partial curated transcripts and deduced the structure of the paxillin gene. No homologues of other members of the paxillin family such as HIC-5 or leupaxin are to be found in the Drosophila genome. Surprisingly paxillin mRNA is expressed in a restricted pattern during embryogenesis. In particular it is strongly expressed in cells and tissues undergoing cell shape changes or cell **migration**. Many of the sites of expression are also known to be sites of integrin function or FAK expression. The data support a role for paxillin as an adapter and/or signaling protein during developmental processes involving integrin-mediated adhesion.
6. Drosophila integrin adhesion complexes are essential for hemocyte **migration** in vivo. Cell **migration** is an important biological process which has been intensively studied in the past decades. Numerous techniques, mainly involving two-dimensional cell culture systems, have contributed to dissecting the essential mechanisms underlying this process. However, the development of three-dimensional cell culture and in vivo systems has shown some differences with what was previously believed to be well-established cell **migration** mechanisms, suggesting that two-dimensional cell motility would be a poor predictor of in vivo behaviour. Drosophila is a widely recognized model organism to study developmental and homeostatic processes and has been widely used to investigate cell **migration**. Here, we focus on the **migration** of small groups of pupal hemocytes that accumulate during larval stages in dorsal patches. We show that integrins, and other known nascent adhesion-related proteins such as Rhea and Fermitin 1, are crucial for this process and that their depletion does not affect polarization in response to environmental cues. We also present evidence for the importance of adhesion maturation-related proteins in hemocyte **migration**, namely Zyxin. Zyxin depletion in hemocytes leads to a significant increase of cell speed without affecting their response to a chemotactic cue. This is the first report of a systematic analysis using Drosophila melanogaster hemocytes to study adhesion-related proteins and their function in cell **migration** in vivo. Our data point to mechanisms of cell **migration** similar to those described in three-dimensional in vitro systems and other in vivo model organisms.

### ECM

#### PubMed

1. Induction of focal adhesions and motility in Drosophila S2 cells. Focal adhesions are dynamic structures that interact with the extracellular matrix on the cell exterior and actin filaments on the cell interior, enabling cells to adhere and crawl along surfaces. We describe a system for inducing the formation of focal adhesions in normally non-**ECM**-adherent, nonmotile Drosophila S2 cells. These focal adhesions contain the expected molecular markers such as talin, vinculin, and p130Cas, and they require talin for their formation. The S2 cells with induced focal adhesions also display a nonpolarized form of motility on vitronectin-coated substrates. Consistent with findings in mammalian cells, the degree of motility can be tuned by changing the stiffness of the substrate and was increased after the depletion of PAK3, a p21-activated kinase. A subset of nonmotile, nonpolarized cells also exhibited focal adhesions that rapidly assembled and disassembled around the cell perimeter. Such cooperative and dynamic fluctuations of focal adhesions were decreased by RNA interference (RNAi) depletion of myosin II and focal adhesion kinase, suggesting that this behavior requires force and focal adhesion maturation. These results demonstrate that S2 cells, a cell line that is well studied for cytoskeletal dynamics and readily amenable to protein manipulation by RNAi, can be used to study the assembly and dynamics of focal adhesions and mechanosensitive cell motility.
2. Cell-cell adhesion via the **ECM**: integrin genetics in fly and worm. Integrins are essential for the development of the two genetically tractable invertebrate model organisms, the nematode worm Caenorhabditis elegans and the fruit fly Drosophila melanogaster. Just two integrins are present in C. elegans: one putative RGD binding integrin alphapat-2betapat-3, corresponding to Drosophila alphaPS2betaPS and vertebrate alpha5beta1, alphaVbeta1 and alpha8beta1, and one putative laminin binding integrin alphaina-1betapat-3, corresponding to Drosophila alphaPS1betaPS and vertebrate alpha3beta1, alpha6beta1 and alpha7beta1. In this review, the function of this minimal set of integrins during the development of these two invertebrates is compared. Despite the differences in bodyplan and developmental strategy, integrin adhesion to the extracellular matrix is required for similar processes: the formation of the link that translates muscle contraction into movement of the exoskeleton, cell migration, and morphogenetic interactions between epithelia. Other integrin functions, such as regulation of gene expression, have not yet been experimentally demonstrated in both organisms. Additional proteins have been characterised in each organism that are essential for integrin function, including extracellular matrix ligands and intracellular interacting proteins, but so far different proteins have been found in the two organisms. This in part represents the fact that the characterisation of the full set of interacting proteins is not complete in either system. However, in other cases different proteins appear to be used for similar functions in the two animals. The continued use of genetic approaches to identify proteins required for integrin function in these two model organisms should lead to the identification of the minimal set of conserved components that form integrin adhesive structures.
3. Extracellular matrix and its receptors in Drosophila neural development. Extracellular matrix (**ECM**) and matrix receptors are intimately involved in most biological processes. The **ECM** plays fundamental developmental and physiological roles in health and disease, including processes underlying the development, maintenance, and regeneration of the nervous system. To understand the principles of **ECM**-mediated functions in the nervous system, genetic model organisms like Drosophila provide simple, malleable, and powerful experimental platforms. This article provides an overview of **ECM** proteins and receptors in Drosophila. It then focuses on their roles during three progressive phases of neural development: (1) neural progenitor proliferation, (2) axonal growth and pathfinding, and (3) synapse formation and function. Each section highlights known **ECM** and **ECM**-receptor components and recent studies done in mutant conditions to reveal their in vivo functions, all illustrating the enormous opportunities provided when merging work on the nervous system with systematic research into **ECM**-related gene functions.

### [Ee]xtracellular [Mm]atrix

#### PubMed

1. Induction of focal adhesions and motility in Drosophila S2 cells. Focal adhesions are dynamic structures that interact with the **extracellular matrix** on the cell exterior and actin filaments on the cell interior, enabling cells to adhere and crawl along surfaces. We describe a system for inducing the formation of focal adhesions in normally non-ECM-adherent, nonmotile Drosophila S2 cells. These focal adhesions contain the expected molecular markers such as talin, vinculin, and p130Cas, and they require talin for their formation. The S2 cells with induced focal adhesions also display a nonpolarized form of motility on vitronectin-coated substrates. Consistent with findings in mammalian cells, the degree of motility can be tuned by changing the stiffness of the substrate and was increased after the depletion of PAK3, a p21-activated kinase. A subset of nonmotile, nonpolarized cells also exhibited focal adhesions that rapidly assembled and disassembled around the cell perimeter. Such cooperative and dynamic fluctuations of focal adhesions were decreased by RNA interference (RNAi) depletion of myosin II and focal adhesion kinase, suggesting that this behavior requires force and focal adhesion maturation. These results demonstrate that S2 cells, a cell line that is well studied for cytoskeletal dynamics and readily amenable to protein manipulation by RNAi, can be used to study the assembly and dynamics of focal adhesions and mechanosensitive cell motility.
2. Cell-cell adhesion via the ECM: integrin genetics in fly and worm. Integrins are essential for the development of the two genetically tractable invertebrate model organisms, the nematode worm Caenorhabditis elegans and the fruit fly Drosophila melanogaster. Just two integrins are present in C. elegans: one putative RGD binding integrin alphapat-2betapat-3, corresponding to Drosophila alphaPS2betaPS and vertebrate alpha5beta1, alphaVbeta1 and alpha8beta1, and one putative laminin binding integrin alphaina-1betapat-3, corresponding to Drosophila alphaPS1betaPS and vertebrate alpha3beta1, alpha6beta1 and alpha7beta1. In this review, the function of this minimal set of integrins during the development of these two invertebrates is compared. Despite the differences in bodyplan and developmental strategy, integrin adhesion to the **extracellular matrix** is required for similar processes: the formation of the link that translates muscle contraction into movement of the exoskeleton, cell migration, and morphogenetic interactions between epithelia. Other integrin functions, such as regulation of gene expression, have not yet been experimentally demonstrated in both organisms. Additional proteins have been characterised in each organism that are essential for integrin function, including **extracellular matrix** ligands and intracellular interacting proteins, but so far different proteins have been found in the two organisms. This in part represents the fact that the characterisation of the full set of interacting proteins is not complete in either system. However, in other cases different proteins appear to be used for similar functions in the two animals. The continued use of genetic approaches to identify proteins required for integrin function in these two model organisms should lead to the identification of the minimal set of conserved components that form integrin adhesive structures.
3. **Extracellular matrix** and its receptors in Drosophila neural development. **Extracellular matrix** (ECM) and matrix receptors are intimately involved in most biological processes. The ECM plays fundamental developmental and physiological roles in health and disease, including processes underlying the development, maintenance, and regeneration of the nervous system. To understand the principles of ECM-mediated functions in the nervous system, genetic model organisms like Drosophila provide simple, malleable, and powerful experimental platforms. This article provides an overview of ECM proteins and receptors in Drosophila. It then focuses on their roles during three progressive phases of neural development: (1) neural progenitor proliferation, (2) axonal growth and pathfinding, and (3) synapse formation and function. Each section highlights known ECM and ECM-receptor components and recent studies done in mutant conditions to reveal their in vivo functions, all illustrating the enormous opportunities provided when merging work on the nervous system with systematic research into ECM-related gene functions.

---

## scb

- NCBI Gene
- GeneRIF
- Pubmed

| Search Term | Rank | Fields |
| --- | --- | --- |
| [Mm]igration | 6 | Process(2); PubMed(22) |
| ECM | 7 | PubMed(11) |
| [Ee]xtracellular [Mm]atrix | 8 | PubMed(19) |

  
hide/show details for scb

### [Mm]igration

#### Process

1. cell **migration**
2. negative regulation of cell **migration**

#### PubMed

1. Integrins are required for cardioblast polarisation in Drosophila. The formation of a tubular organ, such as the heart, requires the communication of positional and polarity signals between migratory cells. Key to this process is the establishment of a new luminal domain on the cell surface, generally from the apical domain of a migratory cell. This domain will also acquire basal properties, as it will produce a luminal extracellular matrix. Integrin receptors are the primary means of cell adhesion and adhesion signaling with the extracellular matrix. Here we characterise the requirement of Integrins in a genetic model of vasculogenesis, the formation of the heart in Drosophila.As with vertebrates, the Drosophila heart arises from lateral mesoderm that migrates medially to meet their contralateral partners, to then assemble a midline vessel. During **migration**, Integrins are among the first proteins restricted to the presumptive luminal domain of cardioblasts. Integrins are required for normal levels of leading edge membrane motility. Apical accumulation of Integrins is enhanced by Robo, and reciprocally, apicalisation of luminal factors like Slit and Robo requires Integrin function. Integrins may provide a template for the formation of a lumen by stabilising lumen factors like Robo. Subsequent to **migration**, Integrin is required for normal cardioblast alignment and lumen formation. This phenotype is most readily modified by other mutations that affect adhesion, such as Talin and extracellular matrix ligands.Our findings reveal an instructive role for Integrins in communicating polarising information to cells during **migration**, and during transition to an epithelial tube structure.
2. Slit coordinates cardiac morphogenesis in Drosophila. Slit is a secreted guidance cue that conveys repellent or attractive signals from target and guidepost cells. In Drosophila, responsive cells express one or more of three Robo receptors. The cardial cells of the developing heart express both Slit and Robo2. This is the first report of coincident expression of a Robo and its ligand. In slit mutants, cardial cell alignment, polarization and uniform **migration** are disrupted. The heart phenotype of robo2 mutants is similar, with fewer **migration** defects. In the guidance of neuronal growth cones in Drosophila, there is a phenotypic interaction between slit and robo heterozygotes, and also with genes required for Robo signaling. In contrast, in the heart, slit has little or no phenotypic interaction with Robo-related genes, including Robo2, Nck2, and Disabled. However, there is a strong phenotypic interaction with Integrin genes and their ligands, including Laminin and Collagen, and intracellular messengers, including Talin and ILK. This indicates that Slit participates in adhesion or adhesion signaling during heart development.
3. Posterior **migration** of the salivary gland requires an intact visceral mesoderm and integrin function. The final overall shape of an organ and its position within the developing embryo arise as a consequence of both its intrinsic properties and its interactions with surrounding tissues. Here, we focus on the role of directed cell **migration** in shaping and positioning the Drosophila salivary gland. We demonstrate that the salivary gland turns and migrates along the visceral mesoderm to become properly oriented with respect to the overall embryo. We show that salivary gland posterior **migration** requires the activities of genes that position the visceral mesoderm precursors, such as heartless, thickveins, and tinman, but does not require a differentiated visceral mesoderm. We also demonstrate a role for integrin function in salivary gland **migration**. Although the mutations affecting salivary gland motility and directional **migration** cause defects in the final positioning of the salivary gland, most do not affect the length or diameter of the salivary gland tube. These findings suggest that salivary tube dimensions may be an intrinsic property of salivary gland cells.
4. Focal adhesions: what's new inside. The cytoplasmic side of focal adhesions is comprised of large molecular complexes that link transmembrane receptors, such as integrins, to the actin cytoskeleton and mediate signals modulating cell attachment, **migration**, proliferation, differentiation, and gene expression. These complexes are heterogeneous and dynamic structures that are apparent targets of regulatory signals that control the function of focal adhesions. Recent studies using genetic approaches in invertebrate and vertebrate systems have begun to reveal the structure and function of these complexes in vivo.
5. Morphogenesis in the absence of integrins: mutation of both Drosophila beta subunits prevents midgut **migration**. Two integrin beta subunits are encoded in the Drosophila genome. The betaPS subunit is widely expressed and heterodimers containing this subunit are required for many developmental processes. The second betasubunit, betanu, is a divergent integrin expressed primarily in the midgut endoderm. To elucidate its function, we generated null mutations in the gene encoding betanu. We find that betanu is not required for viability or fertility, and overall the mutant flies are normal in appearance. However, we could observe betanu function in the absence of betaPS. Consistent with its expression, removal of betanu only enhanced the phenotype of betaPS in the developing midgut. In embryos lacking the zygotic contribution of betaPS, loss of betanu resulted in enhanced separation between the midgut and the surrounding visceral mesoderm. In the absence of both maternal and zygotic betaPS, a delay in midgut **migration** was observed, but removing betanu as well blocked **migration** completely. These results demonstrate that the second beta subunit can partially compensate for loss of betaPS integrins, and that integrins are essential for **migration** of the primordial midgut cells. The two beta subunits mediate midgut **migration** by distinct mechanisms: one that requires talin and one that does not. Other examples of developmental cell **migration**, such as that of the primordial germ cells, occurred normally in the absence of integrins. Having generated the tools to eliminate integrin function completely, we confirm that Drosophila integrins do not control proliferation as they do in mammals, and have identified alphaPS3 as a heterodimeric partner for betanu.
6. Integrins are required for cardioblast polarisation in Drosophila. The formation of a tubular organ, such as the heart, requires the communication of positional and polarity signals between migratory cells. Key to this process is the establishment of a new luminal domain on the cell surface, generally from the apical domain of a migratory cell. This domain will also acquire basal properties, as it will produce a luminal extracellular matrix. Integrin receptors are the primary means of cell adhesion and adhesion signaling with the extracellular matrix. Here we characterise the requirement of Integrins in a genetic model of vasculogenesis, the formation of the heart in Drosophila.As with vertebrates, the Drosophila heart arises from lateral mesoderm that migrates medially to meet their contralateral partners, to then assemble a midline vessel. During **migration**, Integrins are among the first proteins restricted to the presumptive luminal domain of cardioblasts. Integrins are required for normal levels of leading edge membrane motility. Apical accumulation of Integrins is enhanced by Robo, and reciprocally, apicalisation of luminal factors like Slit and Robo requires Integrin function. Integrins may provide a template for the formation of a lumen by stabilising lumen factors like Robo. Subsequent to **migration**, Integrin is required for normal cardioblast alignment and lumen formation. This phenotype is most readily modified by other mutations that affect adhesion, such as Talin and extracellular matrix ligands.Our findings reveal an instructive role for Integrins in communicating polarising information to cells during **migration**, and during transition to an epithelial tube structure.
7. Cell-cell adhesion via the ECM: integrin genetics in fly and worm. Integrins are essential for the development of the two genetically tractable invertebrate model organisms, the nematode worm Caenorhabditis elegans and the fruit fly Drosophila melanogaster. Just two integrins are present in C. elegans: one putative RGD binding integrin alphapat-2betapat-3, corresponding to Drosophila alphaPS2betaPS and vertebrate alpha5beta1, alphaVbeta1 and alpha8beta1, and one putative laminin binding integrin alphaina-1betapat-3, corresponding to Drosophila alphaPS1betaPS and vertebrate alpha3beta1, alpha6beta1 and alpha7beta1. In this review, the function of this minimal set of integrins during the development of these two invertebrates is compared. Despite the differences in bodyplan and developmental strategy, integrin adhesion to the extracellular matrix is required for similar processes: the formation of the link that translates muscle contraction into movement of the exoskeleton, cell **migration**, and morphogenetic interactions between epithelia. Other integrin functions, such as regulation of gene expression, have not yet been experimentally demonstrated in both organisms. Additional proteins have been characterised in each organism that are essential for integrin function, including extracellular matrix ligands and intracellular interacting proteins, but so far different proteins have been found in the two organisms. This in part represents the fact that the characterisation of the full set of interacting proteins is not complete in either system. However, in other cases different proteins appear to be used for similar functions in the two animals. The continued use of genetic approaches to identify proteins required for integrin function in these two model organisms should lead to the identification of the minimal set of conserved components that form integrin adhesive structures.
8. Loss of focal adhesions in glia disrupts both glial and photoreceptor axon **migration** in the Drosophila visual system. Many aspects of glial development are regulated by extracellular signals, including those from the extracellular matrix (ECM). Signals from the ECM are received by cell surface receptors, including the integrin family. Previously, we have shown that Drosophila integrins form adhesion complexes with Integrin-linked kinase and talin in the peripheral nerve glia and have conserved roles in glial sheath formation. However, integrin function in other aspects of glial development is unclear. The Drosophila eye imaginal disc (ED) and optic stalk (OS) complex is an excellent model with which to study glial **migration**, differentiation and glia-neuron interactions. We studied the roles of the integrin complexes in these glial developmental processes during OS/eye development. The common beta subunit βPS and two alpha subunits, αPS2 and αPS3, are located in puncta at both glia-glia and glia-ECM interfaces. Depletion of βPS integrin and talin by RNAi impaired **the migra**tion and distribution of glia within the OS resulting in morphological defects. Reduction of integrin or talin in the glia also disrupted photoreceptor axon outgrowth leading to axon stalling in the OS and ED. The neuronal defects were correlated with a disruption of the carpet glia tube paired with invasion of glia into the core of the OS and the formation of a glial cap. Our results suggest that integrin-mediated extracellular signals are important for multiple aspects of glial development and non-autonomously affect axo**nal migra**tion during Drosophila eye development.
9. Abelson kinase regulates epithelial morphogenesis in Drosophila. Activation of the nonreceptor tyrosine kinase Abelson (Abl) contributes to the development of leukemia, but the complex roles of Abl in normal development are not fully understood. Drosophila Abl links neural axon guidance receptors to the cytoskeleton. Here we report a novel role for Drosophila Abl in epithelial cells, where it is critical for morphogenesis. Embryos completely lacking both maternal and zygotic Abl die with defects in several morphogenetic processes requiring cell shape changes and cell **migration**. We describe the cellular defects that underlie these problems, focusing on dorsal closure as an example. Further, we show that the Abl target Enabled (Ena), a modulator of actin dynamics, is involved with Abl in morphogenesis. We find that Ena localizes to adherens junctions of most epithelial cells, and that it genetically interacts with the adherens junction protein Armadillo (Arm) during morphogenesis. The defects of abl mutants are strongly enhanced by heterozygosity for shotgun, which encodes DE-cadherin. Finally, loss of Abl reduces Arm and alpha-catenin accumulation in adherens junctions, while having little or no effect on other components of the cytoskeleton or cell polarity machinery. We discuss possible models for Abl function during epithelial morphogenesis in light of these data.
10. Signaling pathways directing the movement and fusion of epithelial sheets: lessons from dorsal closure in Drosophila. Wound healing in embryos and various developmental events in metazoans require the spreading and fusion of epithelial sheets. The complex signaling pathways regulating these processes are being pieced together through genetic, cell biological, and biochemical approaches. At present, dorsal closure of the Drosophila embryo is the best-characterized example of epithelial sheet movement. Dorsal closure involves **migration** of the lateral epidermal flanks to close a hole in the dorsal epidermis occupied by an epithelium called the amnioserosa. Detailed genetic studies have revealed a network of interacting signaling molecules regulating this process. At the center of this network is a Jun N-terminal kinase cascade acting at the leading edge of the migrating epidermis that triggers signaling by the TGF-beta superfamily member Decapentaplegic and which interacts with the Wingless pathway. These signaling modules regulate the cytoskeletal reorganization and cell shape change necessary to drive dorsal closure. Activation of this network requires signals from the amnioserosa and input from a variety of proteins at cell-cell junctions. The Rho family of small GTPases is also instrumental, both in activation of signaling and regulation of the cytoskeleton. Many of the proteins regulating dorsal closure have been implicated in epithelial movement in other organisms, and dorsal closure has emerged as an ideal model system for the study of the **migration** and fusion of epithelial sheets.
11. Drosophila PS2 and PS3 integrins play distinct roles in retinal photoreceptors-glia interactions. Cellular **migration** and differentiation are important developmental processes that require dynamic cellular adhesion. Integrins are heterodimeric transmembrane receptors that play key roles in adhesion plasticity. Here, we explore the developing visual system of Drosophila to study the roles of integrin heterodimers in glia development. Our data show that αPS2 is essential for retinal glia **migratio**n from the brain into the eye disc and that glial cells have a role in the maintenance of the fenestrated membrane (Laminin-rich ECM layer) in the disc. Interestingly, the absence of glial cells in the eye disc did not affect the targeting of retinal axons to the optic stalk. In contrast, αPS3 is not required for retinal gli**a migrati**on, but together with Talin, it functions in glial cells to allow photoreceptor axons to target the optic stalk. Thus, we present evidence that αPS2 and αPS3 integrin have different and specific functions in the development of retinal glia.
12. [Dorsal closure in Drosophila. A genetic model for wound healing?]. Dorsal closure (DC) is a morphogenetic movement that establishes the dorsal ectoderm of the drosophila embryo. During this process, the two lateral epithelia stretch toward the dorsal midline, the suture line of the two leading edges. Cell **migration** during DC relies both on cell shape change controlled by the activity of the JNK pathway in the leading edge cells and modification of cell adhesiveness, probably dependent upon activation of the Dpp (TGF-beta) pathway. Coupling of the JNK and TGF-beta pathways is essential. The sequence of the cellular and molecular events of DC highlights interesting common features with wound healing in vertebrates. Like DC, wound healing relies on the **migration** of epithelia bordered by leading edges controlling the direction and speed of the movement. This review summarizes recent data concerning the control of epithelial morphogenesis during DC and the bases of wound healing. The molecular and cellular events that underlie these two analogous migratory processes are detailed, discussed and compared. We suggest that DC is a good genetic model for wound healing studying.
13. A misexpression screen to identify regulators of Drosophila larval hemocyte development. In Drosophila, defense against foreign pathogens is mediated by an effective innate immune system, the cellular arm of which is composed of circulating hemocytes that engulf bacteria and encapsulate larger foreign particles. Three hemocyte types occur: plasmatocytes, crystal cells, and lamellocytes. The most abundant larval hemocyte type is the plasmatocyte, which is responsible for phagocytosis and is present either in circulation or in adherent sessile domains under the larval cuticle. The mechanisms controlling differentiation of plasmatocytes and their **migration** toward these sessile compartments are unclear. To address these questions we have conducted a misexpression screen using the plasmatocyte-expressed GAL4 driver Peroxidasin-GAL4 (Pxn-GAL4) and existing enhancer-promoter (EP) and EP yellow (EY) transposon libraries to systematically misexpress approximately 20% of Drosophila genes in larval hemocytes. The Pxn-GAL4 strain also contains a UAS-GFP reporter enabling hemocyte phenotypes to be visualized in the semitransparent larvae. Among 3412 insertions screened we uncovered 101 candidate hemocyte regulators. Some of these are known to control hemocyte development, but the majority either have no characterized function or are proteins of known function not previously implicated in hemocyte development. We have further analyzed three candidate genes for changes in hemocyte morphology, cell-cell adhesion properties, phagocytosis activity, and melanotic tumor formation.
14. Hox transcription factors: modulators of cell-cell and cell-extracellular matrix adhesion. Hox genes encode homeodomain-containing transcription factors that determine cell and tissue identities in the embryo during development. Hox genes are also expressed in various adult tissues and cancer cells. In Drosophila, expression of cell adhesion molecules, cadherins and integrins, is regulated by Hox proteins operating in hierarchical molecular pathways and plays a crucial role in segment-specific organogenesis. A number of studies using mammalian cultured cells have revealed that cell adhesion molecules responsible for cell-cell and cell-extracellular matrix interactions are downstream targets of Hox proteins. However, whether Hox transcription factors regulate expression of cell adhesion molecules during vertebrate development is still not fully understood. In this review, the potential roles Hox proteins play in cell adhesion and **migration** during vertebrate body patterning are discussed.
15. Branch-specific **migration** cues in the Drosophila tracheal system. The Drosophila tracheal system forms by highly stereotyped **migration** of the tracheal cells, generating an elaborate network of interconnected tubes supplying oxygen to all tissues. A major guiding system in the **migration** process of all branches is the dynamic and localized expression of Branchless (Bnl), an FGF-like molecule. Bnl triggers the activation of the FGF receptor Breathless (Btl) locally in all tracheal cells. Is this the only guiding cue, or do additional local signals provide distinct inputs to each branch? Several recent papers identify such local signals, relying on contacts with specific cell types and with the matrix encountered by the migrating tracheal branches. In particular, the paper by Boube et al(1) demonstrates a role for PS integrins in promoting **migration** of a specific tracheal branch.
16. **Migration** of the Drosophila primordial midgut cells requires coordination of diverse PS integrin functions. Cell **migration** during embryogenesis involves two populations of cells: the migrating cells and the underlying cells that provide the substratum for **migration**. The formation of the Drosophila larval midgut involves the **migration** of the primordial midgut cells along a visceral mesoderm substratum. We show that integrin adhesion receptors are required in both populations of cells for normal rates of **migration**. In the absence of the PS integrins, the visceral mesoderm is disorganised, the primordial midgut cells do not display their normal motile appearance and their **migration** is delayed by 2 hours. Removing PS integrin function from the visceral mesoderm alone results in visceral mesoderm disorganization, but only causes a modest delay in **migration** and does not affect the appearance of the migrating cells. Removing PS integrin function from the migrating cells causes as severe a delay in **migration** as the complete loss of PS integrin function. The functions of PS1 and PS2 are specific in the two tissues, endoderm and mesoderm, since they cannot substitute for each other. In addition there is a partial redundancy in the function of the two PS integrins expressed in the endoderm, PS1 (alphaPS1betaPS) and PS3 (alphaPS3betaPS), since loss of just one alpha subunit in the midgut results in either a modest delay (alphaPS1) or no effect (alphaPS3). We have also examined the roles of small GTPases in promoting **migration** of the primordial midgut cells. We find that dominant negative (N17) versions of Rac and Cdc42 cause a very similar defect in **migration** as loss of integrins, while those of Rho and Ras have no effect. Thus integrins are involved in mediating **migration** by creating an optimal substratum for adhesion, adhering to that substratum and possibly by activating Rac and Cdc42.
17. Fak56 functions downstream of integrin alphaPS3betanu and suppresses MAPK activation in neuromuscular junction growth. Focal adhesion kinase (FAK) functions in cell **migration** and signaling through activation of the mitogen-activated protein kinase (MAPK) signaling cascade. Neuronal function of FAK has been suggested to control axonal branching; however, the underlying mechanism in this process is not clear.We have generated mutants for the Drosophila FAK gene, Fak56. Null Fak56 mutants display overgrowth of larval neuromuscular junctions (NMJs). Localization of phospho-FAK and rescue experiments suggest that Fak56 is required in presynapses to restrict NMJ growth. Genetic analyses imply that FAK mediates the signaling pathway of the integrin alphaPS3betanu heterodimer and functions redundantly with Src. At NMJs, Fak56 downregulates ERK activity, as shown by diphospho-ERK accumulation in Fak56 mutants, and suppression of Fak56 mutant NMJ phenotypes by reducing ERK activity.We conclude that Fak56 is required to restrict NMJ growth during NMJ development. Fak56 mediates an extracellular signal through the integrin receptor. Unlike its conventional role in activating MAPK/ERK, Fak56 suppresses ERK activation in this process. These results suggest that Fak56 mediates a specific neuronal signaling pathway distinct from that in other cellular processes.
18. Polarization of the epithelial layer and apical localization of integrins are required for engulfment of apoptotic cells in the Drosophila ovary. Inefficient clearance of dead cells or debris by epithelial cells can lead to or exacerbate debilitating conditions such as retinitis pigmentosa, macular degeneration, chronic obstructive pulmonary disease and asthma. Despite the importance of engulfment by epithelial cells, little is known about the molecular changes that are required within these cells. The misregulation of integrins has previously been associated with disease states, suggesting that a better understanding of the regulation of receptor trafficking could be key to treating diseases caused by defects in phagocytosis. Here, we demonstrate that the integrin heterodimer αPS3/βPS becomes apically enriched and is required for engulfment by the epithelial follicle cells of the Drosophila ovary. We found that integrin heterodimer localization and function is largely directed by the α-subunit. Moreover, proper cell polarity promotes asymmetric integrin enrichment, suggesting that αPS3/βPS trafficking occurs in a polarized fashion. We show that several genes previously known for their roles in trafficking and **cell migr**ation are also required for engulfment. Moreover, as in mammals, the same α-integrin subunit is required by professional and non-professional phagocytes and migrating cells in Drosophila. Our findings suggest that migrating and engulfing cells use common machinery, and demonstrate a crucial role for integrin function and polarized trafficking of integrin subunits during engulfment. This study also establishes the epithelial follicle cells of the Drosophila ovary as a powerful model for understanding the molecular changes required for engulfment by a polarized epithelium.
19. Ectopic expression screen identifies genes affecting Drosophila mesoderm development including the HSPG Trol. Gastrulation of the embryo involves coordinate cell movements likely supported by multiple signaling pathways, adhesion molecules, and extracellular matrix components. Fibroblast growth factors (FGFs) have a major role in Drosophila melanogaster mesoderm **migration**; however, few other inputs are known and the mechanism supporting cell movement is unclear. To provide insight, we performed an ectopic expression screen to identify secreted or membrane-associated molecules that act to support mesoderm **migration**. Twenty-four UAS insertions were identified that cause lethality when expressed in either the mesoderm (Twi-Gal4) or the ectoderm (69B-Gal4). The list was narrowed to a subset of 10 genes that were shown to exhibit loss-of-function mutant phenotypes specifically affecting mesoderm **migration**. These include the FGF ligand Pyramus, α-integrins, E-cadherin, Cueball, EGFR, JAK/STAT signaling components, as well as the heparan sulfate proteoglycan (HSPG) Terribly reduced optic lobes (Trol). Trol encodes the ortholog of mammalian HSPG Perlecan, a demonstrated FGF signaling cofactor. Here, we examine the role of Trol in Drosophila mesoderm **migratio**n and compare and contrast its role with that of Syndecan (Sdc), another HSPG previously implicated in this process. Embryos mutant for Trol or Sdc were obtained and analyzed. Our data support the view that both HSPGs function to support FGF-dependent processes in the early embryo as they share phenotypes with FGF mutants: Trol in terms of effects on mesoderm **migratio**n and caudal visceral mesoderm (CVM) **migratio**n and Sdc in terms of dorsal mesoderm specification. The differential roles uncovered for these two HSPGs suggest that HSPG cofactor choice may modify FGF-signaling outputs.
20. Integrin alpha chains exhibit distinct temporal and spatial localization patterns in epithelial cells of the Drosophila ovary. Integrins are heterodimeric transmembrane receptors that modulate cell adhesion, **migration**, and signaling. Multiple integrin chains contribute to development and morphogenesis of a given tissue. Here, we analyze the expression of Drosophila integrin alpha chains in the ovarian follicular epithelium, a model for tissue morphogenesis and cell **migration**. We find expression throughout development of the beta chain, betaPS. Alpha chains, however, exhibit both spatial and temporal expression differences. alphaPS1 and alphaPS2 integrins are detected during early and mid-oogenesis on apical, lateral, and basal membranes with the betaPS chain, whereas alphaPS3-family integrins (alphaPS3, alphaPS4, alphaPS5) are expressed in anterior cells late in oogenesis. Surprisingly, we find that alphaPS3-family integrins are dispensable for dorsal appendage morphogenesis but play a role in the final length of the egg, suggesting redundant functions of integrins in a simple tissue. We also demonstrate roles for alphaPS3betaPS integrin in border cell **migration** and in stretch cells.
21. PS integrins and laminins: key regulators of cell **migration** during Drosophila embryogenesis. During embryonic development, there are numerous cases where organ or tissue formation depends upon the **migration** of primordial cells. In the Drosophila embryo, the visceral mesoderm (vm) acts as a substrate for the **migration** of several cell populations of epithelial origin, including the endoderm, the trachea and the salivary glands. These migratory processes require both integrins and laminins. The current model is that αPS1βPS (PS1) and/or αPS3βPS (PS3) integrins are required in migrating cells, whereas αPS2βPS (PS2) integrin is required in the vm, where it performs an as yet unidentified function. Here, we show that PS1 integrins are also required fo**r the mig**ration over the vm of cells of mesodermal origin, the caudal visceral mesoderm (CVM). These results support a model in which PS1 might have evolved to acquire the migratory function of integrins, irrespective of the origin of the tissue. This integrin function is highly specific and its specificity resides mainly in the extracellular domain. In addition, we have identified the Laminin α1,2 trimer, as the key extracellular matrix (ECM) component regulati**ng CVM mi**gration. Furthermore, we show that, as it is the case in vertebrates, integrins, and specifically PS2, contributes to CVM movement by participating in the correct assembly of the ECM that serves as trac**ks for mi**gration.
22. Integrins in development: moving on, responding to, and sticking to the extracellular matrix. Integrins are cell surface receptors of the extracellular matrix present in all animals. Genetic analysis in worms, flies, and vertebrates has revealed integrin involvement in key developmental processes, and we focus here on examples of integrin functions that are comparable across these model organisms. Integrins contribute to cell movement by providing traction to migrating cells, through assembly of extracellular matrices that can serve as tracks for **migration**, and by transmitting guidance signals that direct cells or cell processes to their targets. Integrins also participate in signaling events that govern tissue differentiation and organogenesis. Finally, adhesion by integrin-mediated junctions allows tissues to withstand mechanical load and is essential for tissue integrity.

### ECM

#### PubMed

1. Extracellular matrix and its receptors in Drosophila neural development. Extracellular matrix (**ECM**) and matrix receptors are intimately involved in most biological processes. The **ECM** plays fundamental developmental and physiological roles in health and disease, including processes underlying the development, maintenance, and regeneration of the nervous system. To understand the principles of **ECM**-mediated functions in the nervous system, genetic model organisms like Drosophila provide simple, malleable, and powerful experimental platforms. This article provides an overview of **ECM** proteins and receptors in Drosophila. It then focuses on their roles during three progressive phases of neural development: (1) neural progenitor proliferation, (2) axonal growth and pathfinding, and (3) synapse formation and function. Each section highlights known **ECM** and **ECM**-receptor components and recent studies done in mutant conditions to reveal their in vivo functions, all illustrating the enormous opportunities provided when merging work on the nervous system with systematic research into **ECM**-related gene functions.
2. Integrin-dependent anchoring of a stem-cell niche. Interactions between stem cells and their surrounding microenvironment, or niche, are critical for the establishment and maintenance of stem-cell properties. The adult Drosophila testis contains a morphologically discrete stem-cell niche, the 'hub'. The small cluster of non-dividing, somatic hub cells at the anterior tip of the fly testis is contacted by the germline stem cells (GSCs), which retain their stem-cell character through the direct association with the hub. Here we show that integrin-mediated adhesion is important for maintaining the correct position of embryonic hub cells during gonad morphogenesis. The misplaced hub in integrin-deficient embryos directs the orientation of cell divisions in the presumptive GSCs, a hallmark of the active germline stem-cell niche. A decrease in integrin-mediated adhesion in adult testes, which resulted in a loss of the hub and the stem-cell population, revealed the importance of hub-cell anchoring. Finally, we show that an extracellular matrix (**ECM**) is present around the gonad during late embryogenesis and that this **ECM** is defective in integrin-deficient gonads. On the basis of our data, we propose that integrins are required for the attachment of the hub cells to the **ECM**, which is essential for maintaining the stem-cell niche.
3. Peak BMP Responses in the Drosophila Embryo Are Dependent on the Activation of Integrin Signaling. Within a 3D tissue, cells need to integrate signals from growth factors, such as BMPs, and the extracellular matrix (**ECM**) to coordinate growth and differentiation. Here, we use the Drosophila embryo as a model to investigate how BMP responses are influenced by a cell's local **ECM** environment. We show that integrins, which are **ECM** receptors, are absolutely required for peak BMP signaling. This stimulatory effect of integrins requires their intracellular signaling function, which is activated by the **ECM** protein collagen IV. Mechanistically, integrins interact with the BMP receptor and stimulate phosphorylation of the downstream Mad transcription factor. The BMP-pathway-enhancing function of integrins is independent of focal adhesion kinase, but it requires conserved NPXY motifs in the β-integrin cytoplasmic tail. Furthermore, we show that an α-integrin subunit is a BMP target gene, identifying positive feedback between integrin signaling and BMP pathway activity that may contribute to robust cell fate decisions.
4. A mucin-type O-glycosyltransferase modulates cell adhesion during Drosophila development. Cell-cell and cell-matrix adhesion are crucial during many stages of eukaryotic development. Here, we provide the first example that mucin-type O-linked glycosylation is involved in a developmentally regulated cell adhesion event in Drosophila melanogaster. Mutations in one member of the evolutionarily conserved family of enzymes that initiates O-linked glycosylation alter epithelial cell adhesion in the Drosophila wing blade. A transposon insertion mutation in pgant3 or RNA interference to pgant3 resulted in blistered wings, a phenotype characteristic of genes involved in integrin-mediated cell interactions. Expression of wild type pgant3 in the mutant background rescued the wing blistering phenotype, whereas expression of another family member (pgant35A) did not, revealing a unique requirement for pgant3. pgant3 mutants displayed reduced O-glycosylation along the basal surface of larval wing imaginal discs, which was restored with wild type pgant3 expression, suggesting that reduced glycosylation of basal proteins is responsible for disruption of adhesion in the adult wing blade. Glycosylation reactions demonstrated that PGANT3 glycosylates certain extracellular matrix (**ECM**) proteins. Immunoprecipitation experiments revealed that PGANT3 glycosylates tiggrin, an **ECM** protein known to bind integrin. We propose that this glycosyltransferase is uniquely responsible for glycosylating tiggrin in the wing disc, thus modulating proper cell adhesion through integrin-**ECM** interactions. This study provides the first evidence for the role of O-glycosylation in a developmentally regulated, integrin-mediated, cell adhesion event and reveals a novel player in wing blade formation during Drosophila development.
5. Cell-cell adhesion via the **ECM**: integrin genetics in fly and worm. Integrins are essential for the development of the two genetically tractable invertebrate model organisms, the nematode worm Caenorhabditis elegans and the fruit fly Drosophila melanogaster. Just two integrins are present in C. elegans: one putative RGD binding integrin alphapat-2betapat-3, corresponding to Drosophila alphaPS2betaPS and vertebrate alpha5beta1, alphaVbeta1 and alpha8beta1, and one putative laminin binding integrin alphaina-1betapat-3, corresponding to Drosophila alphaPS1betaPS and vertebrate alpha3beta1, alpha6beta1 and alpha7beta1. In this review, the function of this minimal set of integrins during the development of these two invertebrates is compared. Despite the differences in bodyplan and developmental strategy, integrin adhesion to the extracellular matrix is required for similar processes: the formation of the link that translates muscle contraction into movement of the exoskeleton, cell migration, and morphogenetic interactions between epithelia. Other integrin functions, such as regulation of gene expression, have not yet been experimentally demonstrated in both organisms. Additional proteins have been characterised in each organism that are essential for integrin function, including extracellular matrix ligands and intracellular interacting proteins, but so far different proteins have been found in the two organisms. This in part represents the fact that the characterisation of the full set of interacting proteins is not complete in either system. However, in other cases different proteins appear to be used for similar functions in the two animals. The continued use of genetic approaches to identify proteins required for integrin function in these two model organisms should lead to the identification of the minimal set of conserved components that form integrin adhesive structures.
6. Loss of focal adhesions in glia disrupts both glial and photoreceptor axon migration in the Drosophila visual system. Many aspects of glial development are regulated by extracellular signals, including those from the extracellular matrix (**ECM**). Signals from the **ECM** are received by cell surface receptors, including the integrin family. Previously, we have shown that Drosophila integrins form adhesion complexes with Integrin-linked kinase and talin in the peripheral nerve glia and have conserved roles in glial sheath formation. However, integrin function in other aspects of glial development is unclear. The Drosophila eye imaginal disc (ED) and optic stalk (OS) complex is an excellent model with which to study glial migration, differentiation and glia-neuron interactions. We studied the roles of the integrin complexes in these glial developmental processes during OS/eye development. The common beta subunit βPS and two alpha subunits, αPS2 and αPS3, are located in puncta at both glia-glia and gl**ia-**ECM interfaces. Depletion of βPS integrin and talin by RNAi impaired the migration and distribution of glia within the OS resulting in morphological defects. Reduction of integrin or talin in the glia also disrupted photoreceptor axon outgrowth leading to axon stalling in the OS and ED. The neuronal defects were correlated with a disruption of the carpet glia tube paired with invasion of glia into the core of the OS and the formation of a glial cap. Our results suggest that integrin-mediated extracellular signals are important for multiple aspects of glial development and non-autonomously affect axonal migration during Drosophila eye development.
7. Drosophila PS2 and PS3 integrins play distinct roles in retinal photoreceptors-glia interactions. Cellular migration and differentiation are important developmental processes that require dynamic cellular adhesion. Integrins are heterodimeric transmembrane receptors that play key roles in adhesion plasticity. Here, we explore the developing visual system of Drosophila to study the roles of integrin heterodimers in glia development. Our data show that αPS2 is essential for retinal glia migration from the brain into the eye disc and that glial cells have a role in the maintenance of the fenestrated membrane (Laminin-rich **EC**M layer) in the disc. Interestingly, the absence of glial cells in the eye disc did not affect the targeting of retinal axons to the optic stalk. In contrast, αPS3 is not required for retinal glia migration, but together with Talin, it functions in glial cells to allow photoreceptor axons to target the optic stalk. Thus, we present evidence that αPS2 and αPS3 integrin have different and specific functions in the development of retinal glia.
8. Integrins are necessary for the development and maintenance of the glial layers in the Drosophila peripheral nerve. Peripheral nerve development involves multiple classes of glia that cooperate to form overlapping glial layers paired with the deposition of a surrounding extracellular matrix (**ECM**). The formation of this tubular structure protects the ensheathed axons from physical and pathogenic damage and from changes in the ionic environment. Integrins, a major family of **ECM** receptors, play a number of roles in the development of myelinating Schwann cells, one class of glia ensheathing the peripheral nerves of vertebrates. However, the identity and the role of the integrin complexes utilized by the other classes of peripheral nerve glia have not been determined in any animal. Here, we show that, in the peripheral nerves of Drosophila melanogaster, two integrin complexes (αPS2βPS and αPS3βPS) are expressed in the different glial layers and form adhesion complexes with integrin-linked kinase and Talin. Knockdown of the common beta subunit (βPS) using inducible RNAi in all glial cells results in lethality and glial defects. Analysis of integrin complex function in specific glial layers showed that loss of βPS in the outermost layer (the perineurial glia) results in a failure to wrap the nerve, a phenotype similar to that of Matrix metalloproteinase 2-mediated degradation o**f t**he ECM. Knockdown of βPS integrin in the innermost wrapping glia causes a loss of glial processes around axons. Together, our data suggest that integrins are employed in different glial layers to mediate the development and maintenance of the protective glial sheath in Drosophila peripheral nerves.
9. Integrin signaling is required for maintenance and proliferation of intestinal stem cells in Drosophila. Tissue-specific stem cells are maintained by both local secreted signals and cell adhesion molecules that position the stem cells in the niche microenvironment. In the Drosophila midgut, multipotent intestinal stem cells (ISCs) are located basally along a thin layer of basement membrane that composed of extracellular matrix (**ECM**), which separates ISCs from the surrounding visceral musculature: the muscle cells constitute a regulatory niche for ISCs by producing multiple secreted signals that directly regulate ISC maintenance and proliferation. Here we show that integrin-mediated cell adhesion, which connects the **ECM** and intracellular cytoskeleton, is required for ISC anchorage to the basement membrane. Specifically, the α-integrin subunits including αPS1 encoded by mew and αPS3 encoded by scb, and the β-integrin subunit encoded by mys are richly expressed in ISCs and are required for the maintenance, rather than their survival or multiple lineage differentiation. Furthermore, ISC maintenance also requires the intercellular and intracellular integrin signaling components including Talin, Integrin-linked kinase (Ilk), and the ligand, Laminin A. Notably, integrin mutant ISCs are also less proliferative, and genetic interaction studies suggest that proper integrin signaling is a pre-requisite for ISC proliferation in response to various proliferative signals and for the initiation of intestinal hyperplasia after loss of adenomatous polyposis coli (Apc). Our studies suggest that integrin not only functions to anchor ISCs to the basement membrane, but also serves as an essential element for ISC proliferation during normal homeostasis and in response to oncogenic mutations.
10. The integrin adhesion complex changes its composition and function during morphogenesis of an epithelium. Cell adhesion to the extracellular matrix (**ECM**) is mediated by the integrin family of transmembrane receptors. Integrins link **ECM** ligands to the cytoskeleton, providing strong attachment to enable cell-shape change and tissue integrity. This connection is made possible by an intracellular complex of proteins, which links to actin filaments and controls signalling cascades that regulate cytoskeletal rearrangements. We have identified stress-fibre-associated focal adhesions that change their composition during tissue morphogenesis. Early expression of alphaPS1betaPS integrin decreases the levels of the actin-nucleating factors Enabled, Diaphanous and profilin, as well as downregulating the amount of F-actin incorporated into the stress fibres. As follicle cells mature in their developmental pathway and become squamous, the integrin in the focal adhesions changes from alphaPS1betaPS to alphaPS2betaPS. During the switch, stress fibres increase their length and change orientation, first changing by 90 degrees and then reorienting back. The normal rapid reorientation requires new expression of alphaPS2betaPS, which also permits recruitment of the adaptor protein tensin. Unexpectedly, it is the extracellular portion of the alphaPS2 subunit that provides the specificity for intracellular recruitment of tensin. Molecular variation of the integrin complex is thus a key component of developmentally programmed morphogenesis.
11. PS integrins and laminins: key regulators of cell migration during Drosophila embryogenesis. During embryonic development, there are numerous cases where organ or tissue formation depends upon the migration of primordial cells. In the Drosophila embryo, the visceral mesoderm (vm) acts as a substrate for the migration of several cell populations of epithelial origin, including the endoderm, the trachea and the salivary glands. These migratory processes require both integrins and laminins. The current model is that αPS1βPS (PS1) and/or αPS3βPS (PS3) integrins are required in migrating cells, whereas αPS2βPS (PS2) integrin is required in the vm, where it performs an as yet unidentified function. Here, we show that PS1 integrins are also required for the migration over the vm of cells of mesodermal origin, the caudal visceral mesoderm (CVM). These results support a model in which PS1 might have evolved to acquire the migratory function of integrins, irrespective of the origin of the tissue. This integrin function is highly specific and its specificity resides mainly in the extracellular domain. In addition, we have identified the Laminin α1,2 trimer, as the key extracellular m**atr**ix (ECM) component regulating CVM migration. Furthermore, we show that, as it is the case in vertebrates, integrins, and specifically PS2, contributes to CVM movement by participating in the correct assembly **of** the ECM that serves as tracks for migration.

### [Ee]xtracellular [Mm]atrix

#### PubMed

1. Integrins are required for cardioblast polarisation in Drosophila. The formation of a tubular organ, such as the heart, requires the communication of positional and polarity signals between migratory cells. Key to this process is the establishment of a new luminal domain on the cell surface, generally from the apical domain of a migratory cell. This domain will also acquire basal properties, as it will produce a luminal **extracellular matrix**. Integrin receptors are the primary means of cell adhesion and adhesion signaling with the **extracellular matrix**. Here we characterise the requirement of Integrins in a genetic model of vasculogenesis, the formation of the heart in Drosophila.As with vertebrates, the Drosophila heart arises from lateral mesoderm that migrates medially to meet their contralateral partners, to then assemble a midline vessel. During migration, Integrins are among the first proteins restricted to the presumptive luminal domain of cardioblasts. Integrins are required for normal levels of leading edge membrane motility. Apical accumulation of Integrins is enhanced by Robo, and reciprocally, apicalisation of luminal factors like Slit and Robo requires Integrin function. Integrins may provide a template for the formation of a lumen by stabilising lumen factors like Robo. Subsequent to migration, Integrin is required for normal cardioblast alignment and lumen formation. This phenotype is most readily modified by other mutations that affect adhesion, such as Talin and **extracellular matrix** ligands.Our findings reveal an instructive role for Integrins in communicating polarising information to cells during migration, and during transition to an epithelial tube structure.
2. **Extracellular matrix** and its receptors in Drosophila neural development. **Extracellular matrix** (ECM) and matrix receptors are intimately involved in most biological processes. The ECM plays fundamental developmental and physiological roles in health and disease, including processes underlying the development, maintenance, and regeneration of the nervous system. To understand the principles of ECM-mediated functions in the nervous system, genetic model organisms like Drosophila provide simple, malleable, and powerful experimental platforms. This article provides an overview of ECM proteins and receptors in Drosophila. It then focuses on their roles during three progressive phases of neural development: (1) neural progenitor proliferation, (2) axonal growth and pathfinding, and (3) synapse formation and function. Each section highlights known ECM and ECM-receptor components and recent studies done in mutant conditions to reveal their in vivo functions, all illustrating the enormous opportunities provided when merging work on the nervous system with systematic research into ECM-related gene functions.
3. Integrin-dependent anchoring of a stem-cell niche. Interactions between stem cells and their surrounding microenvironment, or niche, are critical for the establishment and maintenance of stem-cell properties. The adult Drosophila testis contains a morphologically discrete stem-cell niche, the 'hub'. The small cluster of non-dividing, somatic hub cells at the anterior tip of the fly testis is contacted by the germline stem cells (GSCs), which retain their stem-cell character through the direct association with the hub. Here we show that integrin-mediated adhesion is important for maintaining the correct position of embryonic hub cells during gonad morphogenesis. The misplaced hub in integrin-deficient embryos directs the orientation of cell divisions in the presumptive GSCs, a hallmark of the active germline stem-cell niche. A decrease in integrin-mediated adhesion in adult testes, which resulted in a loss of the hub and the stem-cell population, revealed the importance of hub-cell anchoring. Finally, we show that an **extracellular matrix** (ECM) is present around the gonad during late embryogenesis and that this ECM is defective in integrin-deficient gonads. On the basis of our data, we propose that integrins are required for the attachment of the hub cells to the ECM, which is essential for maintaining the stem-cell niche.
4. Integrins modulate Sog activity in the Drosophila wing. Morphogenesis of the Drosophila wing depends on a series of cell-cell and cell-**extracellular matrix** interactions. During pupal wing development, two secreted proteins, encoded by the short gastrulation (sog) and decapentaplegic (dpp) genes, vie to position wing veins in the center of broad provein territories. Expression of the Bmp4 homolog dpp in vein cells is counteracted by expression of the Bmp antagonist sog in intervein cells, which results in the formation of straight veins of precise width. We screened for genetic interactions between sog and genes encoding a variety of extracellular components and uncovered interactions between sog and myospheroid (mys), multiple edematous wing (mew) and scab (scb), which encode betaPS, alphaPS1 and alphaPS3 integrin subunits, respectively. Clonal analysis reveals that integrin mutations affect the trajectory of veins inside the provein domain and/or their width and that misexpression of sog can alter the behavior of cells in such clones. In addition, we show that a low molecular weight form of Sog protein binds to alphaPS1betaPS. We find that Sog can diffuse from its intervein site of production into adjacent provein domains, but only on the dorsal surface of the wing, where Sog interacts functionally with integrins. Finally, we show that Sog diffusion into provein regions and the reticular pattern of extracellular Sog distribution in wild-type wings requires mys and mew function. We propose that integrins act by binding and possibly regulating the activity/availability of different forms of Sog during pupal development through an adhesion independent mechanism.
5. Peak BMP Responses in the Drosophila Embryo Are Dependent on the Activation of Integrin Signaling. Within a 3D tissue, cells need to integrate signals from growth factors, such as BMPs, and the **extracellular matrix** (ECM) to coordinate growth and differentiation. Here, we use the Drosophila embryo as a model to investigate how BMP responses are influenced by a cell's local ECM environment. We show that integrins, which are ECM receptors, are absolutely required for peak BMP signaling. This stimulatory effect of integrins requires their intracellular signaling function, which is activated by the ECM protein collagen IV. Mechanistically, integrins interact with the BMP receptor and stimulate phosphorylation of the downstream Mad transcription factor. The BMP-pathway-enhancing function of integrins is independent of focal adhesion kinase, but it requires conserved NPXY motifs in the β-integrin cytoplasmic tail. Furthermore, we show that an α-integrin subunit is a BMP target gene, identifying positive feedback between integrin signaling and BMP pathway activity that may contribute to robust cell fate decisions.
6. A mucin-type O-glycosyltransferase modulates cell adhesion during Drosophila development. Cell-cell and cell-matrix adhesion are crucial during many stages of eukaryotic development. Here, we provide the first example that mucin-type O-linked glycosylation is involved in a developmentally regulated cell adhesion event in Drosophila melanogaster. Mutations in one member of the evolutionarily conserved family of enzymes that initiates O-linked glycosylation alter epithelial cell adhesion in the Drosophila wing blade. A transposon insertion mutation in pgant3 or RNA interference to pgant3 resulted in blistered wings, a phenotype characteristic of genes involved in integrin-mediated cell interactions. Expression of wild type pgant3 in the mutant background rescued the wing blistering phenotype, whereas expression of another family member (pgant35A) did not, revealing a unique requirement for pgant3. pgant3 mutants displayed reduced O-glycosylation along the basal surface of larval wing imaginal discs, which was restored with wild type pgant3 expression, suggesting that reduced glycosylation of basal proteins is responsible for disruption of adhesion in the adult wing blade. Glycosylation reactions demonstrated that PGANT3 glycosylates certain **extracellular matrix** (ECM) proteins. Immunoprecipitation experiments revealed that PGANT3 glycosylates tiggrin, an ECM protein known to bind integrin. We propose that this glycosyltransferase is uniquely responsible for glycosylating tiggrin in the wing disc, thus modulating proper cell adhesion through integrin-ECM interactions. This study provides the first evidence for the role of O-glycosylation in a developmentally regulated, integrin-mediated, cell adhesion event and reveals a novel player in wing blade formation during Drosophila development.
7. Integrins are required for cardioblast polarisation in Drosophila. The formation of a tubular organ, such as the heart, requires the communication of positional and polarity signals between migratory cells. Key to this process is the establishment of a new luminal domain on the cell surface, generally from the apical domain of a migratory cell. This domain will also acquire basal properties, as it will produce a luminal **extracellular matrix**. Integrin receptors are the primary means of cell adhesion and adhesion signaling with the **extracellular matrix**. Here we characterise the requirement of Integrins in a genetic model of vasculogenesis, the formation of the heart in Drosophila.As with vertebrates, the Drosophila heart arises from lateral mesoderm that migrates medially to meet their contralateral partners, to then assemble a midline vessel. During migration, Integrins are among the first proteins restricted to the presumptive luminal domain of cardioblasts. Integrins are required for normal levels of leading edge membrane motility. Apical accumulation of Integrins is enhanced by Robo, and reciprocally, apicalisation of luminal factors like Slit and Robo requires Integrin function. Integrins may provide a template for the formation of a lumen by stabilising lumen factors like Robo. Subsequent to migration, Integrin is required for normal cardioblast alignment and lumen formation. This phenotype is most readily modified by other mutations that affect adhesion, such as Talin and **extracellular matrix** ligands.Our findings reveal an instructive role for Integrins in communicating polarising information to cells during migration, and during transition to an epithelial tube structure.
8. Cell-cell adhesion via the ECM: integrin genetics in fly and worm. Integrins are essential for the development of the two genetically tractable invertebrate model organisms, the nematode worm Caenorhabditis elegans and the fruit fly Drosophila melanogaster. Just two integrins are present in C. elegans: one putative RGD binding integrin alphapat-2betapat-3, corresponding to Drosophila alphaPS2betaPS and vertebrate alpha5beta1, alphaVbeta1 and alpha8beta1, and one putative laminin binding integrin alphaina-1betapat-3, corresponding to Drosophila alphaPS1betaPS and vertebrate alpha3beta1, alpha6beta1 and alpha7beta1. In this review, the function of this minimal set of integrins during the development of these two invertebrates is compared. Despite the differences in bodyplan and developmental strategy, integrin adhesion to the **extracellular matrix** is required for similar processes: the formation of the link that translates muscle contraction into movement of the exoskeleton, cell migration, and morphogenetic interactions between epithelia. Other integrin functions, such as regulation of gene expression, have not yet been experimentally demonstrated in both organisms. Additional proteins have been characterised in each organism that are essential for integrin function, including **extracellular matrix** ligands and intracellular interacting proteins, but so far different proteins have been found in the two organisms. This in part represents the fact that the characterisation of the full set of interacting proteins is not complete in either system. However, in other cases different proteins appear to be used for similar functions in the two animals. The continued use of genetic approaches to identify proteins required for integrin function in these two model organisms should lead to the identification of the minimal set of conserved components that form integrin adhesive structures.
9. Loss of focal adhesions in glia disrupts both glial and photoreceptor axon migration in the Drosophila visual system. Many aspects of glial development are regulated by extracellular signals, including those from the **extracellular matrix** (ECM). Signals from the ECM are received by cell surface receptors, including the integrin family. Previously, we have shown that Drosophila integrins form adhesion complexes with Integrin-linked kinase and talin in the peripheral nerve glia and have conserved roles in glial sheath formation. However, integrin function in other aspects of glial development is unclear. The Drosophila eye imaginal disc (ED) and optic stalk (OS) complex is an excellent model with which to study glial migration, differentiation and glia-neuron interactions. We studied the roles of the integrin complexes in these glial developmental processes during OS/eye development. The common beta subunit βPS and two alpha subunits, αPS2 and αPS3, are located in puncta at both glia-glia and glia-ECM interfaces. Depletion of βPS integrin and talin by RNAi impaired the migration and distribution of glia within the OS resulting in morphological defects. Reduction of integrin or talin in the glia also disrupted photoreceptor axon outgrowth leading to axon stalling in the OS and ED. The neuronal defects were correlated with a disruption of the carpet glia tube paired with invasion of glia into the core of the OS and the formation of a glial cap. Our results suggest that integrin-mediated extracellular signals are important for multiple aspects of glial development and non-autonomously affect axonal migration during Drosophila eye development.
10. Differentiation, **extracellular matrix** synthesis, and integrin assembly by Drosophila embryo cells cultured on vitronectin and laminin substrates. Two contrasting substrates, Drosophila laminin and human vitronectin, caused determined primary Drosophila embryo cells to follow alternate intermediate differentiation steps without affecting the final outcome of differentiation. Integrin alpha PS2 beta PS3 was essential for the initial spreading of myocytes on vitronectin: focal contacts rich in beta PS3 integrins formed and were connected by actin- and myosin-containing stress fibers. While alpha PS2 beta PS3 was unnecessary for myotube formation on laminin, it was required for the subsequent change to a sarcomeric cytoarchitecture. The differentiating primary cultures synthesized integrins and assembled them into detergent-insoluble, cytoskeleton-associated complexes. Collagen IV, laminin, glutactin, papilin, and other **extracellular matrix** proteins were made primarily by hemocytes and were secreted into the medium. Further differentiation within the cultures was influenced by secreted components and by later addition of vitronectin or bovine serum. Comparison of the differentiation of various cell types on the two substrates showed that vitronectin provided a selective advantage for the differentiation of myocytes, with enrichment over epithelia, epidermal cells, and neurites.
11. The integrins. The integrins are a superfamily of cell adhesion receptors that bind to **extracellular matrix** ligands, cell-surface ligands, and soluble ligands. They are transmembrane alphabeta heterodimers and at least 18 alpha and eight beta subunits are known in humans, generating 24 heterodimers. Members of this family have been found in mammals, chicken and zebrafish, as well as lower eukaryotes, including sponges, the nematode Caenorhabditis elegans (two alpha and one beta subunits, generating two integrins) and the fruitfly Drosophila melanogaster (five alpha and one beta, generating five integrins). The alpha and beta subunits have distinct domain structures, with extracellular domains from each subunit contributing to the ligand-binding site of the heterodimer. The sequence arginine-glycine-aspartic acid (RGD) was identified as a general integrin-binding motif, but individual integrins are also specific for particular protein ligands. Immunologically important integrin ligands are the intercellular adhesion molecules (ICAMs), immunoglobulin superfamily members present on inflamed endothelium and antigen-presenting cells. On ligand binding, integrins transduce signals into the cell interior; they can also receive intracellular signals that regulate their ligand-binding affinity. Here we provide a brief overview that concentrates mostly on the organization, structure and function of mammalian integrins, which have been more extensively studied than integrins in other organisms.
12. Integrins are necessary for the development and maintenance of the glial layers in the Drosophila peripheral nerve. Peripheral nerve development involves multiple classes of glia that cooperate to form overlapping glial layers paired with the deposition of a surrounding **extracellular matrix** (ECM). The formation of this tubular structure protects the ensheathed axons from physical and pathogenic damage and from changes in the ionic environment. Integrins, a major family of ECM receptors, play a number of roles in the development of myelinating Schwann cells, one class of glia ensheathing the peripheral nerves of vertebrates. However, the identity and the role of the integrin complexes utilized by the other classes of peripheral nerve glia have not been determined in any animal. Here, we show that, in the peripheral nerves of Drosophila melanogaster, two integrin complexes (αPS2βPS and αPS3βPS) are expressed in the different glial layers and form adhesion complexes with integrin-linked kinase and Talin. Knockdown of the common beta subunit (βPS) using inducible RNAi in all glial cells results in lethality and glial defects. Analysis of integrin complex function in specific glial layers showed that loss of βPS in the outermost layer (the perineurial glia) results in a failure to wrap the nerve, a phenotype similar to that of Matrix metalloproteinase 2-mediated degradation of the ECM. Knockdown of βPS integrin in the innermost wrapping glia causes a loss of glial processes around axons. Together, our data suggest that integrins are employed in different glial layers to mediate the development and maintenance of the protective glial sheath in Drosophila peripheral nerves.
13. Hox transcription factors: modulators of cell-cell and cell-**extracellular matrix** adhesion. Hox genes encode homeodomain-containing transcription factors that determine cell and tissue identities in the embryo during development. Hox genes are also expressed in various adult tissues and cancer cells. In Drosophila, expression of cell adhesion molecules, cadherins and integrins, is regulated by Hox proteins operating in hierarchical molecular pathways and plays a crucial role in segment-specific organogenesis. A number of studies using mammalian cultured cells have revealed that cell adhesion molecules responsible for cell-cell and cell-**extracellular matrix** interactions are downstream targets of Hox proteins. However, whether Hox transcription factors regulate expression of cell adhesion molecules during vertebrate development is still not fully understood. In this review, the potential roles Hox proteins play in cell adhesion and migration during vertebrate body patterning are discussed.
14. Integrin signaling is required for maintenance and proliferation of intestinal stem cells in Drosophila. Tissue-specific stem cells are maintained by both local secreted signals and cell adhesion molecules that position the stem cells in the niche microenvironment. In the Drosophila midgut, multipotent intestinal stem cells (ISCs) are located basally along a thin layer of basement membrane that composed of **extracellular matrix** (ECM), which separates ISCs from the surrounding visceral musculature: the muscle cells constitute a regulatory niche for ISCs by producing multiple secreted signals that directly regulate ISC maintenance and proliferation. Here we show that integrin-mediated cell adhesion, which connects the ECM and intracellular cytoskeleton, is required for ISC anchorage to the basement membrane. Specifically, the α-integrin subunits including αPS1 encoded by mew and αPS3 encoded by scb, and the β-integrin subunit encoded by mys are richly expressed in ISCs and are required for the maintenance, rather than their survival or multiple lineage differentiation. Furthermore, ISC maintenance also requires the intercellular and intracellular integrin signaling components including Talin, Integrin-linked kinase (Ilk), and the ligand, Laminin A. Notably, integrin mutant ISCs are also less proliferative, and genetic interaction studies suggest that proper integrin signaling is a pre-requisite for ISC proliferation in response to various proliferative signals and for the initiation of intestinal hyperplasia after loss of adenomatous polyposis coli (Apc). Our studies suggest that integrin not only functions to anchor ISCs to the basement membrane, but also serves as an essential element for ISC proliferation during normal homeostasis and in response to oncogenic mutations.
15. The integrin adhesion complex changes its composition and function during morphogenesis of an epithelium. Cell adhesion to the **extracellular matrix** (ECM) is mediated by the integrin family of transmembrane receptors. Integrins link ECM ligands to the cytoskeleton, providing strong attachment to enable cell-shape change and tissue integrity. This connection is made possible by an intracellular complex of proteins, which links to actin filaments and controls signalling cascades that regulate cytoskeletal rearrangements. We have identified stress-fibre-associated focal adhesions that change their composition during tissue morphogenesis. Early expression of alphaPS1betaPS integrin decreases the levels of the actin-nucleating factors Enabled, Diaphanous and profilin, as well as downregulating the amount of F-actin incorporated into the stress fibres. As follicle cells mature in their developmental pathway and become squamous, the integrin in the focal adhesions changes from alphaPS1betaPS to alphaPS2betaPS. During the switch, stress fibres increase their length and change orientation, first changing by 90 degrees and then reorienting back. The normal rapid reorientation requires new expression of alphaPS2betaPS, which also permits recruitment of the adaptor protein tensin. Unexpectedly, it is the extracellular portion of the alphaPS2 subunit that provides the specificity for intracellular recruitment of tensin. Molecular variation of the integrin complex is thus a key component of developmentally programmed morphogenesis.
16. Ectopic expression screen identifies genes affecting Drosophila mesoderm development including the HSPG Trol. Gastrulation of the embryo involves coordinate cell movements likely supported by multiple signaling pathways, adhesion molecules, and **extracellular matrix** components. Fibroblast growth factors (FGFs) have a major role in Drosophila melanogaster mesoderm migration; however, few other inputs are known and the mechanism supporting cell movement is unclear. To provide insight, we performed an ectopic expression screen to identify secreted or membrane-associated molecules that act to support mesoderm migration. Twenty-four UAS insertions were identified that cause lethality when expressed in either the mesoderm (Twi-Gal4) or the ectoderm (69B-Gal4). The list was narrowed to a subset of 10 genes that were shown to exhibit loss-of-function mutant phenotypes specifically affecting mesoderm migration. These include the FGF ligand Pyramus, α-integrins, E-cadherin, Cueball, EGFR, JAK/STAT signaling components, as well as the heparan sulfate proteoglycan (HSPG) Terribly reduced optic lobes (Trol). Trol encodes the ortholog of mammalian HSPG Perlecan, a demonstrated FGF signaling cofactor. Here, we examine the role of Trol in Drosophila mesoderm migration and compare and contrast its role with that of Syndecan (Sdc), another HSPG previously implicated in this process. Embryos mutant for Trol or Sdc were obtained and analyzed. Our data support the view that both HSPGs function to support FGF-dependent processes in the early embryo as they share phenotypes with FGF mutants: Trol in terms of effects on mesoderm migration and caudal visceral mesoderm (CVM) migration and Sdc in terms of dorsal mesoderm specification. The differential roles uncovered for these two HSPGs suggest that HSPG cofactor choice may modify FGF-signaling outputs.
17. PS integrins and laminins: key regulators of cell migration during Drosophila embryogenesis. During embryonic development, there are numerous cases where organ or tissue formation depends upon the migration of primordial cells. In the Drosophila embryo, the visceral mesoderm (vm) acts as a substrate for the migration of several cell populations of epithelial origin, including the endoderm, the trachea and the salivary glands. These migratory processes require both integrins and laminins. The current model is that αPS1βPS (PS1) and/or αPS3βPS (PS3) integrins are required in migrating cells, whereas αPS2βPS (PS2) integrin is required in the vm, where it performs an as yet unidentified function. Here, we show that PS1 integrins are also required for the migration over the vm of cells of mesodermal origin, the caudal visceral mesoderm (CVM). These results support a model in which PS1 might have evolved to acquire the migratory function of integrins, irrespective of the origin of the tissue. This integrin function is highly specific and its specificity resides mainly in the extracellular domain. In addition, we have identified the Laminin α1,2 trimer, as t**he key extracellular** matrix (ECM) component regulating CVM migration. Furthermore, we show that, as it is the case in vertebrates, integrins, and specifically PS2, contributes to CVM movement by participating in the correct assembly of the ECM that serves as tracks for migration.
18. Integrins in development: moving on, responding to, and sticking to the **extracellular matrix**. Integrins are cell surface receptors of the **extracellular matrix** present in all animals. Genetic analysis in worms, flies, and vertebrates has revealed integrin involvement in key developmental processes, and we focus here on examples of integrin functions that are comparable across these model organisms. Integrins contribute to cell movement by providing traction to migrating cells, through assembly of extracellular matrices that can serve as tracks for migration, and by transmitting guidance signals that direct cells or cell processes to their targets. Integrins also participate in signaling events that govern tissue differentiation and organogenesis. Finally, adhesion by integrin-mediated junctions allows tissues to withstand mechanical load and is essential for tissue integrity.
19. Novel functions for integrins in epithelial morphogenesis. Dorsal closure during Drosophila embryogenesis provides a valuable model for epithelial morphogenesis and wound healing. Previous studies have focused on two cell populations, the dorsal epidermis and the extraembryonic amnioserosa. Here, we demonstrate that there is an additional player, the large yolk cell. We find that integrins are expressed in the amnioserosa and yolk cell membrane and that they are required for three processes: (1) assembly of an intervening **extracellular matrix**, (2) attachment between these two cell layers, and (3) contraction of the amnioserosa cells. We also provide evidence for integrin-**extracellular matrix** interactions occurring between the lateral surfaces of the amnioserosa cell and the leading edge epidermis that effectively mediate cell-cell adhesion. Thus, dorsal closure shares mechanistic similarities with vertebrate epithelial morphogenetic events, including epiboly, that also employ an underlying substrate.

---

## Fas3

- NCBI Gene
- GeneRIF
- Pubmed

| Search Term | Rank | Fields |
| --- | --- | --- |
| [Ff]ilopodia | 1 | PubMed(6) |
| [Ff]ilopodium | 2 | PubMed(1) |
| [Mm]igration | 6 | PubMed(83) |
| ECM | 7 | PubMed(8) |
| [Ee]xtracellular [Mm]atrix | 8 | PubMed(11) |
| [Ii]nvasive | 10 | PubMed(13) |
| [Mm]etastasize | 11 | PubMed(1) |
| [Mm]etastasis | 12 | PubMed(6) |
| [Cc]ell [Ii]nvasion | 13 | PubMed(2) |

  
hide/show details for Fas3

### [Ff]ilopodia

#### PubMed

1. Cellular mechanisms governing synaptic development in Drosophila melanogaster. The neuromuscular connections of Drosophila are ideally suited for studying synaptic function and development. Hypotheses about cell recognition can be tested in a simple array of pre- and postsynaptic elements. Drosophila muscle fibers are multiply innervated by individually identifiable motoneurons. The neurons express several synaptic cotransmitters, including glutamate, proctolin, and octopamine, and are specialized by their synaptic morphology, neurotransmitters, and connectivity. During larval development the initial motoneuron endings grow extensively over the surface of the muscle fibers, and differentiate synaptic boutons of characteristic morphology. While considerable growth occurs postembryonically, the initial wiring of motoneurons to muscle fibers is accomplished during mid-to-late embryogenesis (stages 15-17). Efferent growth cones sample multiple muscle fibers with rapidly moving **filopodia**. Upon reaching their target muscle fibers, the growth cones rapidly differentiate into synaptic contacts whose morphology prefigures that of the larval junction. Mismatch experiments show that growth cones recognize specific muscle fibers, and can do so when the surrounding musculature is radically altered. However, when denied their normal targets, motoneurons can establish functional synapses on alternate muscle fibers. Blocking synaptic activity with either injected toxins or ion channel mutants does not derange synaptogenesis, but may influence the number of motor ending processes. The molecular mechanisms governing cellular recognition during synaptogenesis remain to be identified. However, several cell surface glycoproteins known to mediate cellular adhesion events in vitro are expressed by the developing synapses. Furthermore, enhancer detector lines have identified genes with expression restricted to small subsets of muscle fibers and/or motoneurons during the period of synaptogenesis. These observations suggest that in Drosophila a mechanism of target chemoaffinity may be involved in the genesis of stereotypic synaptic wiring.
2. The leading edge during dorsal closure as a model for epithelial plasticity: Pak is required for recruitment of the Scribble complex and septate junction formation. Dorsal closure (DC) of the Drosophila embryo is a model for the study of wound healing and developmental epithelial fusions, and involves the sealing of a hole in the epidermis through the migration of the epidermal flanks over the tissue occupying the hole, the amnioserosa. During DC, the cells at the edge of the migrating epidermis extend Rac- and Cdc42-dependent actin-based lamellipodia and **filopodia** from their leading edge (LE), which exhibits a breakdown in apicobasal polarity as adhesions are severed with the neighbouring amnioserosa cells. Studies using mammalian cells have demonstrated that Scribble (Scrib), an important determinant of apicobasal polarity that functions in a protein complex, controls polarized cell migration through recruitment of Rac, Cdc42 and the serine/threonine kinase Pak, an effector for Rac and Cdc42, to the LE. We have used DC and the follicular epithelium to study the relationship between Pak and the Scrib complex at epithelial membranes undergoing changes in apicobasal polarity and adhesion during development. We propose that, during DC, the LE membrane undergoes an epithelial-to-mesenchymal-like transition to initiate epithelial sheet migration, followed by a mesenchymal-to-epithelial-like transition as the epithelial sheets meet up and restore cell-cell adhesion. This latter event requires integrin-localized Pak, which recruits the Scrib complex in septate junction formation. We conclude that there are bidirectional interactions between Pak and the Scrib complex modulating epithelial plasticity. Scrib can recruit Pak to the LE for polarized cell migration but, as migratory cells meet up, Pak can recruit the Scrib complex to restore apicobasal polarity and cell-cell adhesion.
3. Transcriptional regulation of Profilin during wound closure in Drosophila larvae. Injury is an inevitable part of life, making wound healing essential for survival. In postembryonic skin, wound closure requires that epidermal cells recognize the presence of a gap and change their behavior to migrate across it. In Drosophila larvae, wound closure requires two signaling pathways [the Jun N-terminal kinase (JNK) pathway and the Pvr receptor tyrosine kinase signaling pathway] and regulation of the actin cytoskeleton. In this and other systems, it remains unclear how the signaling pathways that initiate wound closure connect to the actin regulators that help execute wound-induced cell migrations. Here, we show that chickadee, which encodes the Drosophila Profilin, a protein important for actin filament recycling and cell migration during development, is required for the physiological process of larval epidermal wound closure. After injury, chickadee is transcriptionally upregulated in cells proximal to the wound. We found that JNK, but not Pvr, mediates the increase in chic transcription through the Jun and Fos transcription factors. Finally, we show that chic-deficient larvae fail to form a robust actin cable along the wound edge and also fail to form normal **filopodia**l and lamellipodial extensions into the wound gap. Our results thus connect a factor that regulates actin monomer recycling to the JNK signaling pathway during wound closure. They also reveal a physiological function for an important developmental regulator of actin and begin to tease out the logic of how the wound repair response is organized.
4. Growth cone behavior underlying the development of stereotypic synaptic connections in Drosophila embryos. Each muscle fiber in the segmented body wall of Drosophila larvae is innervated by anatomically stereotyped neuromuscular junctions. These synapses arise through the selective choices of motoneuronal growth cones at their peripheral targets. Using digital optical microscopy of staged intracellular dye fills, we have singly identified embryonic motoneurons and have examined individual growth cones when they contact and differentiate at the target cells. There is a precise connectivity between motoneuron and muscle fiber, which is the direct consequence of growth cone behavior. We have also found that Drosophila muscle fibers possess molecularly heterogeneous cell surfaces that may be involved in growth cone recognition of appropriate targets. Fasciclin III, a homophilic adhesion molecule, is coexpressed by several of the efferent growth cones and in a site-specific fashion by the target muscle fiber's membrane. The fasciclin III expression is transient, corresponding to the period in embryogenesis when the first neuromuscular contacts are made. Upon encountering the target cell surface, the growth cones can sprout stereotypically arrayed **filopodia**l processes, orient along the anterior-posterior axis, and turn in predictable directions. Subsequently, terminal branches are established in a nonrandom order. These phenomena were found to occur in two motoneurons that innervate adjacent muscle fiber targets, and may be general features of neuromuscular synaptogenesis in Drosophila.
5. Cadherin-mediated cell adhesion and cell motility in Drosophila trachea regulated by the transcription factor Escargot. Coordination of cell motility and adhesion is essential for concerted movement of tissues during animal morphogenesis. The Drosophila tracheal network is formed by branching, migration and fusion of tubular ectodermal epithelia. Tracheal tip cells, located at the end of each branch that is going to fuse, extend **filopodia** to search for targets and later change their cell shape to a seamless ring to allow passage of lumen. The cell adhesion molecule DE-cadherin accumulates at the site of contact to form a ring that marks the site of lumen entry and is essential for the fusion. DE-cadherin expression in tip cells of a subset of branches is dependent on escargot, a zinc finger gene expressed in all tip cells. Such escargot mutant tip cells failed to adhere to each other and continued to search for alternative targets by extending long **filopodia**. We present evidence indicating escargot positively regulates transcription of the DE-cadherin gene, shotgun. Overexpression of DE-cadherin rescued the defect in one of the fusion points in escargot mutants, demonstrating an essential role of DE-cadherin in target recognition and identifying escargot as a key regulator of cell adhesion and motility in tracheal morphogenesis.
6. JNK signaling pathway required for wound healing in regenerating Drosophila wing imaginal discs. We have examined wound healing during regeneration of Drosophila wing imaginal discs fragments by confocal microscopy and assessed the role of components of the JNK pathway in this process. After cutting, columnar and peripodial epithelia cells at the wound edge start to close the wound through formation and contraction of an actin cable. This is followed by a zipping process through **filopodia**l protrusions from both epithelia knitting the wound edges from proximal to distal areas of the disc. Activation of the JNK pathway is involved in such process. puckered (puc) expression is induced in several rows of cells at the edge of the wound, whereas absence of JNK pathway activity brought about by hemipterous, basket, and Dfos mutants impair wound healing. These defects are accompanied by lowered or loss of expression of puc. In support of a role of puc in wound healing, hep mutant phenotypes are rescued by reducing puc function, whereas overexpression of puc inhibits wound healing. Altogether, these results demonstrate a role for the JNK pathway in imaginal disc wound healing, similar to that reported for other healing processes such as embryonic dorsal closure, thoracic closure, and adult epithelial wound healing in Drosophila. Differences with such processes are also highlighted.

### [Ff]ilopodium

#### PubMed

1. M6 membrane protein plays an essential role in Drosophila oogenesis. We had previously shown that the transmembrane glycoprotein M6a, a member of the proteolipid protein (PLP) family, regulates neurite/**filopodium** outgrowth, hence, M6a might be involved in neuronal remodeling and differentiation. In this work we focused on M6, the only PLP family member present in Drosophila, and ortholog to M6a. Unexpectedly, we found that decreased expression of M6 leads to female sterility. M6 is expressed in the membrane of the follicular epithelium in ovarioles throughout oogenesis. Phenotypes triggered by M6 downregulation in hypomorphic mutants included egg collapse and egg permeability, thus suggesting M6 involvement in eggshell biosynthesis. In addition, RNAi-mediated M6 knockdown targeted specifically to follicle cells induced an arrest of egg chamber development, revealing that M6 is essential in oogenesis. Interestingly, M6-associated phenotypes evidenced abnormal changes of the follicle cell shape and disrupted follicular epithelium in mid- and late-stage egg chambers. Therefore, we propose that M6 plays a role in follicular epithelium maintenance involving membrane cell remodeling during oogenesis in Drosophila.

### [Mm]igration

#### PubMed

1. TspanC8 tetraspanins regulate ADAM10/Kuzbanian trafficking and promote Notch activation in flies and mammals. The metalloprotease ADAM10/Kuzbanian catalyzes the ligand-dependent ectodomain shedding of Notch receptors and activates Notch. Here, we show that the human tetraspanins of the evolutionary conserved TspanC8 subfamily (Tspan5, Tspan10, Tspan14, Tspan15, Tspan17, and Tspan33) directly interact with ADAM10, regulate its exit from the endoplasmic reticulum, and that four of them regulate ADAM10 surface expression levels. In an independent RNAi screen in Drosophila, two TspanC8 genes were identified as Notch regulators. Functional analysis of the three Drosophila TspanC8 genes (Tsp3A, Tsp86D, and Tsp26D) indicated that these genes act redundantly to promote Notch signaling. During oogenesis, TspanC8 genes were up-regulated in border cells and regulated Kuzbanian distribution, Notch activity, and cell **migration**. Furthermore, the human TspanC8 tetraspanins Tspan5 and Tspan14 positively regulated ligand-induced ADAM10-dependent Notch1 signaling. We conclude that TspanC8 tetraspanins have a conserved function in the regulation of ADAM10 trafficking and activity, thereby positively regulating Notch receptor activation.
2. Socs36E attenuates STAT signaling to optimize motile cell specification in the Drosophila ovary. The Janus kinase/Signal transducers and activators of transcription (JAK/STAT) pathway determines cell fates by regulating gene expression. One example is the specification of the motile cells called border cells during Drosophila oogenesis. It has been established that too much or too little STAT activity disrupts follicle cell identity and cell motility, which suggests the signaling must be precisely regulated. Here, we find that Suppressor of cytokine signaling at 36E (Socs36E) is a necessary negative regulator of JAK/STAT signaling during border cell specification. We find when STAT signaling is too low to induce **migration** in the presumptive border cell population, nearby follicle cells uncharacteristically become invasive to enable efficient **migration** of the cluster. We generated a genetic null allele that reveals Socs36E is required in the anterior follicle cells to limit invasive behavior to an optimal number of cells. We further show Socs36E genetically interacts with the required STAT feedback inhibitor apontic (apt) and APT's downstream target, mir-279, and provide evidence that suggests APT directly regulates Socs36E transcriptionally. Our work shows Socs36E plays a critical role in a genetic circuit that establishes a boundary between the motile border cell cluster and its non-invasive epithelial neighbors through STAT attenuation.
3. Integration of epithelial patterning and morphogenesis in Drosophila ovarian follicle cells. Drosophila oogenesis involves the coordinated development of germ cells and an overlying follicular epithelium. The follicle cells provide a genetically tractable system to investigate the cell biology of patterning and morphogenesis. Follicle cells initially form a cuboidal epithelium surrounding a syncytium of nurse cells and oocyte. Epithelial structure is maintained as these cells reorganize to create the three dimensional architecture of the eggshell. Both long-range and short-range cell-cell communications pattern the domains of follicle cells that will create specific eggshell structures. After terminal differentiation to deposit the eggshell proteins, the follicle cells die. This review summarizes recent progress in understanding the cell-cell communication that orchestrates follicle cell patterning and **migration**s. DE-cadherin-mediated adhesion is important at several steps in egg chamber formation and follicle cell **migration**. Notch signaling is critical during each successive round of patterning and **migration**. Integration of bone morphogenetic protein (BMP) and epidermal growth factor (EGF) signals patterns the elaborate structures of the dorsal-anterior eggshell.
4. Morphogenesis in the absence of integrins: mutation of both Drosophila beta subunits prevents midgut **migration**. Two integrin beta subunits are encoded in the Drosophila genome. The betaPS subunit is widely expressed and heterodimers containing this subunit are required for many developmental processes. The second betasubunit, betanu, is a divergent integrin expressed primarily in the midgut endoderm. To elucidate its function, we generated null mutations in the gene encoding betanu. We find that betanu is not required for viability or fertility, and overall the mutant flies are normal in appearance. However, we could observe betanu function in the absence of betaPS. Consistent with its expression, removal of betanu only enhanced the phenotype of betaPS in the developing midgut. In embryos lacking the zygotic contribution of betaPS, loss of betanu resulted in enhanced separation between the midgut and the surrounding visceral mesoderm. In the absence of both maternal and zygotic betaPS, a delay in midgut **migration** was observed, but removing betanu as well blocked **migration** completely. These results demonstrate that the second beta subunit can partially compensate for loss of betaPS integrins, and that integrins are essential for **migration** of the primordial midgut cells. The two beta subunits mediate midgut **migration** by distinct mechanisms: one that requires talin and one that does not. Other examples of developmental cell **migration**, such as that of the primordial germ cells, occurred normally in the absence of integrins. Having generated the tools to eliminate integrin function completely, we confirm that Drosophila integrins do not control proliferation as they do in mammals, and have identified alphaPS3 as a heterodimeric partner for betanu.
5. Posterior **migration** of the salivary gland requires an intact visceral mesoderm and integrin function. The final overall shape of an organ and its position within the developing embryo arise as a consequence of both its intrinsic properties and its interactions with surrounding tissues. Here, we focus on the role of directed cell **migration** in shaping and positioning the Drosophila salivary gland. We demonstrate that the salivary gland turns and migrates along the visceral mesoderm to become properly oriented with respect to the overall embryo. We show that salivary gland posterior **migration** requires the activities of genes that position the visceral mesoderm precursors, such as heartless, thickveins, and tinman, but does not require a differentiated visceral mesoderm. We also demonstrate a role for integrin function in salivary gland **migration**. Although the mutations affecting salivary gland motility and directional **migration** cause defects in the final positioning of the salivary gland, most do not affect the length or diameter of the salivary gland tube. These findings suggest that salivary tube dimensions may be an intrinsic property of salivary gland cells.
6. The role of LamininB2 (LanB2) during mesoderm differentiation in Drosophila. In Drosophila, four genes encode for laminin subunits and the formation of two laminin heterotrimers has been postulated. We report the identification of mutations in the Drosophila LamininB2 (LanB2) gene that encodes for the only laminin γ subunit and is found in both heterotrimers. We describe their effects on embryogenesis, in particular the differentiation of visceral tissues with respect to the ECM. Analysis of mesoderm endoderm interaction indicates disrupted basement membranes and defective endoderm **migratio**n, which finally interferes with visceral myotube stretching. Extracellular deposition of laminin is blocked due to the loss of the LanB2 subunit, resulting in an abnormal distribution of ECM components. Our data, concerning the different function of both trimers during organogenesis, suggest that these trimers might act in a cumulative way and probably at multiple steps during ECM assembly. We also observed genetic interactions with kon-tiki and thrombospondin, indicating a role for laminin during muscle attachment.
7. The Maf factor Traffic jam both enables and inhibits collective cell **migration** in Drosophila oogenesis. Border cell cluster (BCC) **migration** in the Drosophila ovary is an excellent system to study the gene regulatory network that enables collective cell **migration**. Here, we identify the large Maf transcription factor Traffic jam (Tj) as an important regulator of BCC **migration**. Tj has a multifaceted impact on the known core cascade that enables BCC motility, consisting of the Jak/Stat signaling pathway, the C/EBP factor Slow border cells (Slbo), and the downstream effector DE-cadherin (DEcad). The initiation of BCC **migration** coincides with a Slbo-dependent decrease in Tj expression. This reduction of Tj is required for normal BCC motility, as high Tj expression strongly impedes **migration**. At high concentration, Tj has a tripartite negative effect on the core pathway: a decrease in Slbo, an increase in the Jak/Stat inhibitor Socs36E, and a Slbo-independent reduction of DEcad. However, maintenance of a low expression level of Tj in the BCC during **migration** is equally important, as loss of tj function also results in a significant delay in **migration** concomitant with a reduction of Slbo and consequently of DEcad. Taken together, we conclude that the regulatory feedback loop between Tj and Slbo is necessary for achieving the correct activity levels of **migration**-regulating factors to ensure proper BCC motility.
8. Drosophila sosie functions with β(H)-Spectrin and actin organizers in cell **migratio**n, epithelial morphogenesis and cortical stability. Morphogenesis in multicellular organisms requires the careful coordination of cytoskeletal elements, dynamic regulation of cell adhesion and extensive cell **migratio**n. sosie (sie) is a novel gene required in various morphogenesis processes in Drosophila oogenesis. Lack of sie interferes with normal egg chamber packaging, maintenance of epithelial integrity and control of follicle cell **migratio**n, indicating that sie is involved in controlling epithelial integrity and cell **migratio**n. For these functions sie is required both in the germ line and in the soma. Consistent with this, Sosie localizes to plasma membranes in the germ line and in the somatic follicle cells and is predicted to present an EGF-like domain on the extracellular side. Two positively charged residues, C-terminal to the predicted transmembrane domain (on the cytoplasmic side), are required for normal plasma membrane localization of Sosie. Because sie also contributes to normal cortical localization of β(H)-Spectrin, it appears that cortical β(H)-Spectrin mediates some of the functions of sosie. sie also interacts with the genes coding for the actin organizers Filamin and Profilin and, in the absence of sie function, F-actin is less well organized and nurse cells frequently fuse.
9. Requirement for JAK/STAT signaling throughout border cell **migration** in Drosophila. The evolutionarily conserved JAK/STAT signaling pathway is essential for the proliferation, survival and differentiation of many cells including cancer cells. Recent studies have implicated this transcriptional pathway in the process of cell **migration** in humans, mice, Drosophila and Dictyostelium. In the Drosophila ovary, JAK/STAT signaling is necessary and sufficient for the specification and **migration** of a group of cells called the border cells; however, it is not clear to what extent the requirement for cell fate is distinct from that for cell **migration**. We found that STAT protein is enriched in the migrating border cells throughout their **migration** and is an indicator of cells with highest JAK/STAT activity. In addition, stat(ts) mutants exhibited border cell **migration** defects after just 30 minutes at the non-permissive temperature, prior to any detectable change in the expression of cell fate markers. At later times, cell fate changes became evident, indicating that border cell fate is labile. JAK/STAT signaling was also required for organization of the border cell cluster. Finally, we show that both the accumulation of STAT protein and nuclear accumulation are positively regulated by JAK/STAT activity. The activity of the pathway is negatively regulated by overexpression of a SOCS protein and by blocking endocytosis. Together, our findings suggest that the requirement for STAT in border cells extends beyond the initial specification and delamination of cells from the epithelium.
10. Apical junctions and cell signalling in epithelia. Genetic analysis in Drosophila has led to the identification of several proteins that mediate cell-cell interactions controlling the fate and proliferation of epithelial cells. These proteins are localized or enriched in the adherens and septate junctions at the apical end of the lateral membranes between cells. The proteins localized or enriched at adherens junctions include Notch, which is important for the cell interactions controlling neuroblast and bristle patterning; Boss and sevenless, which are required for the cell interaction that establishes the R7 photoreceptor cell; and Armadillo, required for the wingless-dependent cell interactions that control segment polarity and imaginal disc patterning. Proteins localized at septate junctions include the product of the tumor suppressor gene dlg, which is required for septate junction formation, apical basal cell polarity, and the cell interactions that control proliferation. The results suggest that the cell signalling events important for cell fate determination and for cell proliferation control in epithelia occur at the apical junctions. The **migration** of the nucleus to the apical surface of the epithelium for mitosis may enable it to interact directly with the junction-associated signalling mechanisms.
11. **Migration** of the Drosophila primordial midgut cells requires coordination of diverse PS integrin functions. Cell **migration** during embryogenesis involves two populations of cells: the migrating cells and the underlying cells that provide the substratum for **migration**. The formation of the Drosophila larval midgut involves the **migration** of the primordial midgut cells along a visceral mesoderm substratum. We show that integrin adhesion receptors are required in both populations of cells for normal rates of **migration**. In the absence of the PS integrins, the visceral mesoderm is disorganised, the primordial midgut cells do not display their normal motile appearance and their **migration** is delayed by 2 hours. Removing PS integrin function from the visceral mesoderm alone results in visceral mesoderm disorganization, but only causes a modest delay in **migration** and does not affect the appearance of the migrating cells. Removing PS integrin function from the migrating cells causes as severe a delay in **migration** as the complete loss of PS integrin function. The functions of PS1 and PS2 are specific in the two tissues, endoderm and mesoderm, since they cannot substitute for each other. In addition there is a partial redundancy in the function of the two PS integrins expressed in the endoderm, PS1 (alphaPS1betaPS) and PS3 (alphaPS3betaPS), since loss of just one alpha subunit in the midgut results in either a modest delay (alphaPS1) or no effect (alphaPS3). We have also examined the roles of small GTPases in promoting **migration** of the primordial midgut cells. We find that dominant negative (N17) versions of Rac and Cdc42 cause a very similar defect in **migration** as loss of integrins, while those of Rho and Ras have no effect. Thus integrins are involved in mediating **migration** by creating an optimal substratum for adhesion, adhering to that substratum and possibly by activating Rac and Cdc42.
12. JAK-STAT pathway in Drosophila morphogenesis: From organ selector to cell behavior regulator. One of the main contributions of Drosophila to the JAK-STAT field is the study of morphogenesis. JAK-STAT signaling controls the formation of many different structures through surprisingly different morphogenetic behaviors that include induction of cell rearrangements, invagination, folding of tissues, modulation of cell shape, and **migration**. This variability may be explained by the many transcription factors and signaling molecules STAT regulates at early stages of development. But is STAT just acting as an upstream inducer of morphogenesis or does it have a more direct role in controlling cell behaviors? Here we review what is known about how the canonical phosphorylation of STAT contributes to shaping the embryonic and imaginal structures.
13. Signaling pathways directing the movement and fusion of epithelial sheets: lessons from dorsal closure in Drosophila. Wound healing in embryos and various developmental events in metazoans require the spreading and fusion of epithelial sheets. The complex signaling pathways regulating these processes are being pieced together through genetic, cell biological, and biochemical approaches. At present, dorsal closure of the Drosophila embryo is the best-characterized example of epithelial sheet movement. Dorsal closure involves **migration** of the lateral epidermal flanks to close a hole in the dorsal epidermis occupied by an epithelium called the amnioserosa. Detailed genetic studies have revealed a network of interacting signaling molecules regulating this process. At the center of this network is a Jun N-terminal kinase cascade acting at the leading edge of the migrating epidermis that triggers signaling by the TGF-beta superfamily member Decapentaplegic and which interacts with the Wingless pathway. These signaling modules regulate the cytoskeletal reorganization and cell shape change necessary to drive dorsal closure. Activation of this network requires signals from the amnioserosa and input from a variety of proteins at cell-cell junctions. The Rho family of small GTPases is also instrumental, both in activation of signaling and regulation of the cytoskeleton. Many of the proteins regulating dorsal closure have been implicated in epithelial movement in other organisms, and dorsal closure has emerged as an ideal model system for the study of the **migration** and fusion of epithelial sheets.
14. Cellular and molecular mechanisms of single and collective cell **migration**s in Drosophila: themes and variations. The process of cell **migration** is essential throughout life, driving embryonic morphogenesis and ensuring homeostasis in adults. Defects in cell **migration** are a major cause of human disease, with excessive **migration** causing autoimmune diseases and cancer metastasis, whereas reduced capacity for **migration** leads to birth defects and immunodeficiencies. Myriad studies in vitro have established a consensus view that cell **migration**s require cell polarization, Rho GTPase-mediated cytoskeletal rearrangements, and myosin-mediated contractility. However, in vivo studies later revealed a more complex picture, including the discovery that cells migrate not only as single units but also as clusters, strands, and sheets. In particular, the role of E-Cadherin in cell motility appears to be more complex than previously appreciated. Here, we discuss recent advances achieved by combining the plethora of genetic tools available to the Drosophila geneticist with live imaging and biophysical techniques. Finally, we discuss the emerging themes such studies have revealed and ponder the puzzles that remain to be solved.
15. Focal adhesion kinase is not required for integrin function or viability in Drosophila. The mammalian focal adhesion kinase (FAK) family of non-receptor protein-tyrosine kinases has been implicated in controlling a multitude of cellular responses to the engagement of cell-surface integrins and G-protein-coupled receptors. The high level of sequence conservation between the mammalian proteins and the Drosophila homologue of FAK, Fak56, suggested that it would have similar functions. However, we show here that Drosophila Fak56 is not essential for integrin functions in adhesion, **migration** or signaling in vivo. Furthermore, animals lacking Fak56 are viable and fertile, demonstrating that Fak56 is not essential for other developmental or physiological functions. Despite this, overexpressed Fak56 is a potent inhibitor of integrins binding to the extracellular matrix, suggesting that Fak56 may play a subtle role in the negative regulation of integrin adhesion.
16. A role for the mesoderm in endodermal **migration** and morphogenesis in Drosophila. The endodermal midgut arises from two primordia, the anterior midgut (AMG) primordium and the posterior midgut (PMG) primordium, which are separated by almost the entire length of the Drosophila embryo. To form the midgut, these two parts have to extend towards each other and to fuse laterally on both sides of the yolk. Shortly before and during that movement, AMG and PMG are arranged as mesenchymal cell masses, but later the midgut cells form an epithelium. We show that these two aspects of midgut development, **migration** of AMG and PMG and transition to an epithelium, depend on the mesoderm. The extension of the midgut primordia is achieved by cell **migration** along the visceral mesoderm which forms a continuous layer of cells within the germ band. In mutant embryos lacking the entire mesoderm or failing to differentiate the visceral mesoderm, AMG and PMG are formed but do not migrate properly. In addition, they fail to form an epithelium and instead either remain as compact cell masses anterior and posterior to the yolk (in twist and snail mutant embryos) or only occasionally wrap around the yolk before embryogenesis is completed (in tinman-deficient embryos). We conclude that the visceral mesoderm serves as a substratum for the migrating endodermal cells and that the contact between visceral mesoderm and endoderm is required for the latter to become an epithelium.
17. Tre1, a G protein-coupled receptor, directs transepithelial **migration** of Drosophila germ cells. In most organisms, germ cells are formed distant from the somatic part of the gonad and thus have to migrate along and through a variety of tissues to reach the gonad. Transepithelial **migration** through the posterior midgut (PMG) is the first active step during Drosophila germ cell **migration**. Here we report the identification of a novel G protein-coupled receptor (GPCR), Tre1, that is essential for this **migration** step. Maternal tre1 RNA is localized to germ cells, and tre1 is required cell autonomously in germ cells. In tre1 mutant embryos, most germ cells do not exit the PMG. The few germ cells that do leave the midgut early migrate normally to the gonad, suggesting that this gene is specifically required for transepithelial **migration** and that mutant germ cells are still able to recognize other guidance cues. Additionally, inhibiting small Rho GTPases in germ cells affects transepithelial **migration**, suggesting that Tre1 signals through Rho1. We propose that Tre1 acts in a manner similar to chemokine receptors required during transepithelial **migration** of leukocytes, implying an evolutionarily conserved mechanism of transepithelial **migration**. Recently, the chemokine receptor CXCR4 was shown to direct **migration** in vertebrate germ cells. Thus, germ cells may more generally use GPCR signaling to navigate the embryo toward their target.
18. Heartless, a Drosophila FGF receptor homolog, is essential for cell **migration** and establishment of several mesodermal lineages. A Drosophila FGF receptor homolog (DFGF-R2/DFRI) termed Heartless (Htl) is expressed in the embryonic mesoderm. The phenotypes of null mutant embryos demonstrated that Htl is a central player that is required for the development of several mesodermal lineages. No abnormalities in the primary specification of the mesoderm were observed. The first defects were seen as irregular **migration** and spreading of the mesoderm over the ectoderm. Subsequently, cell fates were not induced in several lineages including the visceral mesoderm, heart, and the dorsal somatic muscles. The defects in the induction of cell fates are likely to result from failure of the mesoderm to spread over the ectoderm and receive patterning signals. The defective spreading could be circumvented in htl mutant embryos by providing an ectopic Dpp patterning signal, leading to the formation of heart and dorsal muscle cells. Htl appears to be required also subsequently during the **migration** and morphogenesis of the different lineages. Expression of a dominant-negative htl construct after the initial induction of cell fates gave rise to aberrant **migration** and organization of the visceral mesoderm, heart, and somatic muscles. Thus, a common role for Htl in cell **migration** and tissue organization may account for the pleiotropic defects of the htl mutation.
19. Fusion of circular and longitudinal muscles in Drosophila is independent of the endoderm but further visceral muscle differentiation requires a close contact between mesoderm and endoderm. In this study we describe the morphological and genetic analysis of the Drosophila mutant gürtelchen (gurt). gurt was identified by screening an EMS collection for novel mutations affecting visceral mesoderm development and was named after the distinct belt shaped visceral phenotype. Interestingly, determination of visceral cell identities and subsequent visceral myoblast fusion is not affected in mutant embryos indicating a later defect in visceral development. gurt is in fact a new huckebein (hkb) allele and as such exhibits nearly complete loss of endodermal derived structures. Targeted ablation of the endodermal primordia produces a phenotype that resembles the visceral defects observed in huckebein(gürtelchen) (hkb(gurt)) mutant embryos. It was shown previously that visceral mesoderm development requires complex interactions between visceral myoblasts and adjacent tissues. Signals from the neighbouring somatic myoblasts play an important role in cell type determination and are a prerequisite for visceral muscle fusion. Furthermore, the visceral mesoderm is known to influence endoderma**l migrati**on and midgut epithelium formation. Our analyses of the visceral phenotype of hkb(gurt) mutant embryos reveal that the adjacent endoderm plays a critical role in the later stages of visceral muscle development, and is required for visceral muscle elongation and outgrowth after proper myoblast fusion.
20. Drosophila filamin is required for follicle cell motility during oogenesis. The Filamin family of actin binding proteins is required to maintain cell shape and promote cell locomotion. Using the Drosophila ovary, we provide a detailed description of Filamin-deficient cells during morphogenesis. Reduced expression of Filamin in follicle cells causes defects in the initial encapsulation of germline cysts and in the **migration** of border cells through the germline cyst. However, follicle cell morphogenesis is unaffected by point mutations that produce truncated Filamin proteins. In addition, mutant follicle cell movements can be partially rescued by a transgene encoding only the actin-binding domain and the first six filamin repeats. These data show that Filamin function in cell motility can be provided by a truncated Filamin protein that resembles Dictyostelium Actin Binding Protein-120.
21. Delta signaling from the germ line controls the proliferation and differentiation of the somatic follicle cells during Drosophila oogenesis. The body axes of Drosophila are established during oogenesis through reciprocal interactions between the germ line cells and the somatic follicle cells that surround them. The Notch pathway is required at two stages in this process: first, for the **migration** of the follicle cells around the germ line cyst and, later, for the polarization of the anterior-posterior (A-P) axis of the oocyte. Its function in these events, however, has remained controversial. Using clonal analysis, we show that Notch signaling controls cell proliferation and differentiation in the whole follicular epithelium. Notch mutant follicle cells remain in a precursor state and fail to switch from the mitotic cell cycle to the endocycle. Furthermore, removal of Delta from the germ line produces an identical phenotype, showing that Delta signals from the germ cells to control the timing of follicle cell differentiation. This explains the axis formation defects in Notch mutants, which arise because undifferentiated posterior follicle cells cannot signal to polarize the oocyte. Delta also signals from the germ line to Notch in the soma earlier in oogenesis to control the differentiation of the polar and stalk follicle cells. The germ line therefore regulates the development of the follicle cells through two complementary signaling pathways: Gurken signals twice to control spatial patterning, whereas Delta signals twice to exert temporal control.
22. Comparative analysis of Hox downstream genes in Drosophila. Functional diversification of body parts is dependent on the formation of specialized structures along the various body axes. In animals, region-specific morphogenesis along the anteroposterior axis is controlled by a group of conserved transcription factors encoded by the Hox genes. Although it has long been assumed that Hox proteins carry out their function by regulating distinct sets of downstream genes, only a small number of such genes have been found, with very few having direct roles in controlling cellular behavior. We have quantitatively identified hundreds of Hox downstream genes in Drosophila by microarray analysis, and validated many of them by in situ hybridizations on loss- and gain-of-function mutants. One important finding is that Hox proteins, despite their similar DNA-binding properties in vitro, have highly specific effects on the transcriptome in vivo, because expression of many downstream genes respond primarily to a single Hox protein. In addition, a large fraction of downstream genes encodes realizator functions, which directly affect morphogenetic processes, such as orientation and rate of cell divisions, cell-cell adhesion and communication, cell shape and **migration**, or cell death. Focusing on these realizators, we provide a framework for the morphogenesis of the maxillary segment. As the genomic organization of Hox genes and the interaction of Hox proteins with specific co-factors are conserved in vertebrates and invertebrates, and similar classes of downstream genes are regulated by Hox proteins across the metazoan phylogeny, our findings represent a first step toward a mechanistic understanding of morphological diversification within a species as well as between species.
23. Deficiency screen identifies a novel role for beta 2 tubulin in salivary gland and myoblast **migration** in the Drosophila embryo. The Drosophila embryonic salivary gland is an epithelial organ formed by the coordinated invagination and **migration** of primordial cells. To identify genes that regulate gland **migration** we performed a deficiency screen of the third chromosome. Here, we report on the analysis of the beta 2 tubulin isoform (beta2t) that maps at 85D15. We show that, in beta2t mutant embryos, salivary glands did not complete their posterior **migration** and that **migration** of fusion competent myoblasts and longitudinal visceral muscle founder cells between the gland and circular visceral mesoderm was delayed. We also demonstrate that gland **migration** defects correlate with reduced betaPS and alphaPS2 integrin expression in the surrounding mesoderm and that beta2t genetically interacts with genes encoding integrin alphaPS1 and alphaPS2 subunits. Our studies reveal for the first time that beta2t is expressed in embryogenesis and that beta2t plays an important role in salivary gland and myoblast **migration**, possibly through proper regulation of integrin adhesion proteins.
24. Spatially localized Kuzbanian required for specific activation of Notch during border cell **migration**. The transmembrane receptor Notch is used repeatedly during development for a variety of essential functions. During Drosophila oogenesis, Notch activity is required first to specify particular follicle cell fates, then to promote the differentiation of all follicle cell types, to promote border cell **migration**, and then to form dorsal appendages, raising the question as to how Notch activity is spatially and temporally regulated. Here we show the Notch activity pattern during oogenesis. Notch activation was found in many follicle cells at stage 6 but then at stage 9 was restricted to migrating border cells, despite uniform expression of Delta. Expression of Kuzbanian (KUZ), a metalloproteinase that can activate Notch as well as cleave other substrates, is enriched in border cells at stage 9; and dominant-negative KUZ caused a strong border cell **migration** defect, without affecting expression of markers of border cell fate or follicle cell differentiation. Constitutively active Notch rescued the **migration** defect due to dominant-negative KUZ, and conditional alleles of Delta and Notch also exhibited border cell **migration** defects. Expression of two different reporters of Notch activity was lost upon expression of dominant-negative KUZ. Taken together these results show that Notch activation and KUZ expression are restricted to border cells at stage 9 of oogenesis and are required for **migration**, but not differentiation, of these cells. This represents a previously unrecognized mechanism for achieving spatial restriction of Notch signaling.
25. The leading edge during dorsal closure as a model for epithelial plasticity: Pak is required for recruitment of the Scribble complex and septate junction formation. Dorsal closure (DC) of the Drosophila embryo is a model for the study of wound healing and developmental epithelial fusions, and involves the sealing of a hole in the epidermis through the **migration** of the epidermal flanks over the tissue occupying the hole, the amnioserosa. During DC, the cells at the edge of the migrating epidermis extend Rac- and Cdc42-dependent actin-based lamellipodia and filopodia from their leading edge (LE), which exhibits a breakdown in apicobasal polarity as adhesions are severed with the neighbouring amnioserosa cells. Studies using mammalian cells have demonstrated that Scribble (Scrib), an important determinant of apicobasal polarity that functions in a protein complex, controls polarized cell **migration** through recruitment of Rac, Cdc42 and the serine/threonine kinase Pak, an effector for Rac and Cdc42, to the LE. We have used DC and the follicular epithelium to study the relationship between Pak and the Scrib complex at epithelial membranes undergoing changes in apicobasal polarity and adhesion during development. We propose that, during DC, the LE membrane undergoes an epithelial-to-mesenchymal-like transition to initiate epithelial sheet **migration**, followed by a mesenchymal-to-epithelial-like transition as the epithelial sheets meet up and restore cell-cell adhesion. This latter event requires integrin-localized Pak, which recruits the Scrib complex in septate junction formation. We conclude that there are bidirectional interactions between Pak and the Scrib complex modulating epithelial plasticity. Scrib can recruit Pak to the LE for polarized cell **migration** but, as migratory cells meet up, Pak can recruit the Scrib complex to restore apicobasal polarity and cell-cell adhesion.
26. Drosophila laminins act as key regulators of basement membrane assembly and morphogenesis. Laminins are heterotrimeric molecules found in all basement membranes. In mammals, they have been involved in diverse developmental processes, from gastrulation to tissue maintenance. The Drosophila genome encodes two laminin alpha chains, one beta and one Gamma, which form two distinct laminin trimers. So far, only mutations affecting one or other trimer have been analysed. In order to study embryonic development in the complete absence of laminins, we mutated the gene encoding the sole laminin beta chain in Drosophila, LanB1, so that no trimers can be made. We show that LanB1 mutant embryos develop until the end of embryogenesis. Electron microscopy analysis of mutant embryos reveals that the basement membranes are absent and the remaining extracellular material appears disorganised and diffuse. Accordingly, abnormal accumulation of major basement membrane components, such as Collagen IV and Perlecan, is observed in mutant tissues. In addition, we show that elimination of LanB1 prevents the normal morphogenesis of most organs and tissues, including the gut, trachea, muscles and nervous system. In spite of the above structural roles for laminins, our results unravel novel functions in cell adhesion, **migration** and rearrangement. We propose that while an early function of laminins in gastrulation is not conserved in Drosophila and mammals, their function in basement membrane assembly and organogenesis seems to be maintained throughout evolution.
27. The level of C/EBP protein is critical for cell **migration** during Drosophila oogenesis and is tightly controlled by regulated degradation. The C/EBP transcription factor, Slbo, is required for **migration** of border cells during Drosophila oogenesis. Unexpectedly, we find that neither increase nor decrease of Slbo activity is tolerated in border cells. Correct protein level is in part ensured by cell type-specific regulated turnover of Slbo protein. Through genetic screening, we identify two genes that are involved in this regulation. The Ubp64 ubiquitin hydrolase acts as a stabilizer of Slbo protein. A novel gene, tribbles, is a negative regulator of slbo in vivo. Tribbles acts by specifically targeting Slbo for rapid degradation via ubiquitination.
28. Formation of Neuronal Circuits by Interactions between Neuronal Populations Derived from Different Origins in the Drosophila Visual Center. A wide variety of neurons, including populations derived from different origins, are precisely arranged and correctly connected with their partner to establish a functional neural circuit during brain development. The molecular mechanisms that orchestrate the production and arrangement of these neurons have been obscure. Here, we demonstrate that cell-cell interactions play an important role in establishing the arrangement of neurons of different origins in the Drosophila visual center. Specific types of neurons born outside the medulla primordium migrate tangentially into the developing medulla cortex. During their tangential **migration**, these neurons express the repellent ligand Slit, and the two layers that the neurons intercalate between express the receptors Robo2 and Robo3. Genetic analysis suggests that Slit-Robo signaling may control the positioning of the layer cells or their processes to form a path for **migration**. Our results suggest that conserved axon guidance signaling is involved in the interactions between neurons of different origins during brain development.
29. The FGF8-related signals Pyramus and Thisbe promote pathfinding, substrate adhesion, and survival of migrating longitudinal gut muscle founder cells. Fibroblast growth factors (FGFs) frequently fulfill prominent roles in the regulation of cell **migration** in various contexts. In Drosophila, the FGF8-like ligands Pyramus (Pyr) and Thisbe (Ths), which signal through their receptor Heartless (Htl), are known to regulate early mesodermal cell **migration** after gastrulation as well as glial cell **migration** during eye development. Herein, we show that Pyr and Ths also exert key roles during the long-distance **migration** of a specific sub-population of mesodermal cells that migrate from the caudal visceral mesoderm within stereotypic bilateral paths along the trunk visceral mesoderm toward the anterior. These cells constitute the founder myoblasts of the longitudinal midgut muscles. In a forward genetic screen for regulators of this morphogenetic process we identified loss of function alleles for pyr. We show that pyr and ths are expressed along the paths of **migration** in the trunk visceral mesoderm and endoderm and act largely redundantly to help guide the founder myoblasts reliably onto and along their substrate of **migration**. Ectopically-provided Pyr and Ths signals can efficiently re-rout the migrating cells, both in the presence and absence of endogenous signals. Our data indicate that the guidance functions of these FGFs must act in concert with other important attractive or adhesive activities of the trunk visceral mesoderm. Apart from their guidance functions, the Pyr and Ths signals play an obligatory role for the survival of the migrating cells. Without these signals, essentially all of these cells enter cell death and detach from the **migration** substrate during early **migration**. We present experiments that allowed us to dissect the roles of these FGFs as guidance cues versus trophic activities during the **migration** of the longitudinal visceral muscle founders.
30. Jelly belly: a Drosophila LDL receptor repeat-containing signal required for mesoderm **migration** and differentiation. Inductive interactions subdivide the Drosophila mesoderm into visceral, somatic, and heart muscle precursors. The muscle precursors form organs by executing tissue-specific **migration**s and cell fusions. We identified a novel gene, jelly belly (jeb), which is required for visceral mesoderm development. jeb encodes a secreted protein that contains an LDL receptor repeat. In jeb mutants, visceral mesoderm precursors form, but they fail to migrate or differentiate normally; no visceral muscles develop. Jeb protein is produced in somatic muscle precursors and taken up by visceral muscle precursors. jeb reveals a signaling process in which somatic muscle precursors support the proper **migration** and differentiation of visceral muscle cells. Later in embryogenesis, jeb is transcribed in neurons and Jeb protein is found in axons.
31. PAR-1 kinase regulates epithelial detachment and directional protrusion of migrating border cells. Many cells that migrate during normal embryonic development or in metastatic cancer first detach from an epithelium. However, this step is often difficult to observe directly in vivo, and the mechanisms controlling the ability of cells to leave the epithelium are poorly understood. In addition, once cells detach, they must assume a migratory phenotype, involving changes in cytoskeletal and signaling dynamics. Drosophila border cells provide a model system in which a combination of forward genetics and live-cell imaging can allow researchers to investigate the cellular and molecular mechanisms of epithelial cell detachment and **migration** in vivo.We identified the Drosophila homolog of the serine/threonine kinase PAR-1 (MARK/Kin1) in a screen for mutations that disrupt border cell **migration**. Previous studies identified two proteins, Apontic and Notch, that indirectly affect border cell detachment by regulating transcription of downstream targets. In contrast, PAR-1 directly modulates apical-basal polarity between border cells and epithelial cells to promote detachment. Furthermore, PAR-1, but not the apical polarity complex protein PAR-3, promotes the directionality of transient cell protrusions, which border cells require for sensing the chemoattractant gradient.We conclude that PAR-1-dependent apical-basal polarity is required for proper detachment of migratory border cells from neighboring epithelial cells. Moreover, polarity controlled by PAR-1 influences the ability of migratory cells to sense direction, a critical feature of **migration**. Thus, this work reveals new insights into two distinct, but essential, steps of epithelial cell **migration**.
32. Microtubules and Lis-1/NudE/dynein regulate invasive cell-on-cell **migration** in Drosophila. The environment through which cells migrate in vivo differs considerably from the in vitro environment where cell **migration** is often studied. In vivo many cells migrate in crowded and complex 3-dimensional tissues and may use other cells as the substratum on which they move. This includes neurons, glia and their progenitors in the brain. Here we use a Drosophila model of invasive, collective **migration** in a cellular environment to investigate the roles of microtubules and microtubule regulators in this type of cell movement. Border cells are of epithelial origin and have no visible microtubule organizing center (MTOC). Interestingly, microtubule plus-end growth was biased away from the leading edge. General perturbation of the microtubule cytoskeleton and analysis by live imaging showed that microtubules in both the migrating cells and the substrate cells affect movement. Also, whole-tissue and cell autonomous deletion of the microtubule regulator Stathmin had distinct effects. A screen of 67 genes encoding microtubule interacting proteins uncovered cell autonomous requirements for Lis-1, NudE and Dynein in border cell **migration**. Net cluster **migration** was decreased, with initiation of **migration** and formation of dominant front cell protrusion being most dramatically affected. Organization of cells within the cluster and localization of cell-cell adhesion molecules were also abnormal. Given the established role of Lis-1 in migrating neurons, this could indicate a general role of Lis-1/NudE, Dynein and microtubules, in cell-on-cell **migration**. Spatial regulation of cell-cell adhesion may be a common theme, consistent with observing both cell autonomous and non-autonomous requirements in both systems.
33. The small GTPase Rac plays multiple roles in epithelial sheet fusion--dynamic studies of Drosophila dorsal closure. The coordinated **migration** and fusion of epithelial sheets is a crucial morphogenetic tool used on numerous occasions during the normal development of an embryo and re-activated as part of the wound healing response. Drosophila dorsal closure, whereby a hole in the embryonic epithelium is zipped closed late in embryogenesis, serves as an excellent, genetically tractable model for epithelial **migration**. Using live confocal imaging, we have dissected multiple roles for the small GTPase Rac in this process. We show that constitutive activation of Rac1 leads to excessive assembly of lamellipodia and precocious halting of epithelial sweeping, possibly through premature activation of contact-inhibition machinery. Conversely, blocking Rac activity, either by loss-of-function mutations or expression of dominant negative Rac1, disables the assembly of both actin cable and protrusions by epithelial cells. Movies of mutant embryos show that continued contraction of the amnioserosa is sufficient to draw the epithelial edges towards one another, allowing the zipper machinery to bypass non-functioning regions of leading edge. In addition to illustrating the key role of Rac in organization of leading edge actin, loss-of-function mutants also provide substantive proof that Rac acts upstream in the Jun N-terminal kinase (JNK) cascade to direct epithelial cell shape changes during dorsal closure.
34. The Drosophila cytokine receptor Domeless controls border cell **migration** and epithelial polarization during oogenesis. In mammals, the JAK/STAT (Janus Kinase/Signal Transducer and Activator of Transcription) signaling pathway is activated in response to cytokines and growth factors to control blood cell development, proliferation and cell determination. In Drosophila, a conserved JAK/STAT signaling pathway controls segmentation in embryos, as well as blood cell development and other processes in larvae and adults. During embryogenesis, transduction of the Unpaired [Upd; also known as Outstretched (Os)] ligand through the JAK/STAT pathway requires Domeless, a putative membrane protein with distant homology to vertebrate type I cytokine receptors. We have isolated domeless (dome) in a screen to identify genes essential in epithelial morphogenesis during oogenesis. The level of dome activity is critical for proper border cell **migration** and is controlled in part through a negative feedback loop. In addition to its essential role in border cells, we show that dome is required in the germarium for the polarization of follicle cells during encapsulation of germline cells. In this process, dome controls the expression of the apical determinant Crumbs. In contrast to the ligand Upd, whose expression is limited to a pair of polar cells at both ends of the egg chamber, dome is expressed in all germline and follicle cells. However, the Dome protein is specifically localized at apicolateral membranes and undergoes ligand-dependent internalization in the follicle cells. dome mutations interact genetically with JAK/STAT pathway genes in border cell **migration** and abolish the nuclear translocation of Stat92E in vivo. We also show that dome functions downstream of upd and that both the extracellular and intracellular domains of Dome are required for JAK/STAT signaling. Altogether, our data indicate that Dome is an essential receptor molecule for Upd and JAK/STAT signaling during oogenesis.
35. DE-Cadherin is required for intercellular motility during Drosophila oogenesis. Cadherins are involved in a variety of morphogenetic movements during animal development. However, it has been difficult to pinpoint the precise function of cadherins in morphogenetic processes due to the multifunctional nature of cadherin requirement. The data presented here indicate that homophilic adhesion promoted by Drosophila E-cadherin (DE-cadherin) mediates two cell **migration** events during Drosophila oogenesis. In Drosophila follicles, two groups of follicle cells, the border cells and the centripetal cells migrate on the surface of germline cells. We show that the border cells migrate as an epithelial patch in which two centrally located cells retain epithelial polarity and peripheral cells are partially depolarized. Both follicle cells and germline cells express DE-cadherin, and border cells and centripetal cells strongly upregulate the expression of DE-cadherin shortly before and during their **migration**. Removing DE-cadherin from either the follicle cells or the germline cells blocks **migration** of border cells and centripetal cells on the surface of germline cells. The function of DE-cadherin in border cells appears to be specific for **migration** as the formation of the border cell cluster and the adhesion between border cells are not disrupted in the absence of DE-cadherin. The speed of **migration** depends on the level of DE-cadherin expression, as border cells migrate more slowly when DE-cadherin activity is reduced. Finally, we show that the upregulation of DE-cadherin expression in border cells depends on the activity of the Drosophila C/EBP transcription factor that is essential for border cell **migration**.
36. Wunen, a Drosophila lipid phosphate phosphatase, is required for septate junction-mediated barrier function. Lipid phosphate phosphatases (LPPs) are integral membrane enzymes that regulate the levels of bioactive lipids such as sphingosine 1-phosphate and lysophosphatidic acid. The Drosophila LPPs Wunen (Wun) and Wunen-2 (Wun2) have a well-established role in regulating the survival and **migration** of germ cells. We now show that wun has an essential tissue-autonomous role in development of the trachea: the catalytic activity of Wun is required to maintain septate junction (SJ) paracellular barrier function, loss of which causes failure to accumulate crucial luminal components, suggesting a role for phospholipids in SJ function. We find that the integrity of the blood-brain barrier is also lost in wun mutants, indicating that loss of SJ function is not restricted to the tracheal system. Furthermore, by comparing the rescue ability of different LPP homologs we show that wun function in the trachea is distinct from its role in germ cell **migration**.
37. **Migration** of Drosophila intestinal stem cells across organ boundaries. All components of the Drosophila intestinal tract, including the endodermal midgut and ectodermal hindgut/Malpighian tubules, maintain populations of dividing stem cells. In the midgut and hindgut, these stem cells originate from within larger populations of intestinal progenitors that proliferate during the larval stage and form the adult intestine during metamorphosis. The origin of stem cells found in the excretory Malpighian tubules ('renal stem cells') has not been established. In this paper, we investigate the **migration** patterns of intestinal progenitors that take place during metamorphosis. Our data demonstrate that a subset of adult midgut progenitors (AMPs) move posteriorly to form the adult ureters and, consecutively, the renal stem cells. Inhibiting cell **migration** by AMP-directed expression of a dominant-negative form of Rac1 protein results in the absence of stem cells in the Malpighian tubules. As the majority of the hindgut progenitor cells migrate posteriorly and differentiate into hindgut enterocytes, a group of the progenitor cells, unexpectedly, invades anteriorly into the midgut territory. Consequently, these progenitor cells differentiate into midgut enterocytes. The midgut determinant GATAe is required for the differentiation of midgut enterocytes derived from hindgut progenitors. Wingless signaling acts to balance the proportion of hindgut progenitors that differentiate as midgut versus hindgut enterocytes. Our findings indicate that a stable boundary between midgut and hindgut/Malpighian tubules is not established during early embryonic development; instead, pluripotent progenitor populations cross in between these organs in both directions, and are able to adopt the fate of the organ in which they come to reside.
38. Auxilin is required for formation of Golgi-derived clathrin-coated vesicles during Drosophila spermatogenesis. Clathrin has previously been implicated in Drosophila male fertility and spermatid individualization. To understand further the role of membrane transport in this process, we analyzed the phenotypes of mutations in Drosophila auxilin (aux), a regulator of clathrin function, in spermatogenesis. Like partial loss-of-function Clathrin heavy chain (Chc) mutants, aux mutant males are sterile and produce no mature sperm. The reproductive defects of aux males were rescued by male germ cell-specific expression of aux, indicating that auxilin function is required autonomously in the germ cells. Furthermore, this rescue depends on both the clathrin-binding and J domains, suggesting that the ability of Aux to bind clathrin and the Hsc70 ATPase is essential for sperm formation. aux mutant spermatids show a deficit in formation of the plasma membrane during elongation, which probably disrupts the subsequent coordinated **migration** of investment cones during individualization. In wild-type germ cells, GFP-tagged clathrin localized to clusters of vesicular structures near the Golgi. These structures also contained the Golgi-associated clathrin adaptor AP-1, suggesting that they were Golgi-derived. By contrast, in aux mutant cells, clathrin localized to abnormal patches surrounding the Golgi and its colocalization with AP-1 was disrupted. Based on these results, we propose that Golgi-derived clathrin-positive vesicles are normally required for sustaining the plasma membrane increase necessary for spermatid differentiation. Our data suggest that Aux participates in forming these Golgi-derived clathrin-positive vesicles and that Aux, therefore, has a role in the secretory pathway.
39. Transcriptional regulation of Profilin during wound closure in Drosophila larvae. Injury is an inevitable part of life, making wound healing essential for survival. In postembryonic skin, wound closure requires that epidermal cells recognize the presence of a gap and change their behavior to migrate across it. In Drosophila larvae, wound closure requires two signaling pathways [the Jun N-terminal kinase (JNK) pathway and the Pvr receptor tyrosine kinase signaling pathway] and regulation of the actin cytoskeleton. In this and other systems, it remains unclear how the signaling pathways that initiate wound closure connect to the actin regulators that help execute wound-induced cell **migration**s. Here, we show that chickadee, which encodes the Drosophila Profilin, a protein important for actin filament recycling and cell **migration** during development, is required for the physiological process of larval epidermal wound closure. After injury, chickadee is transcriptionally upregulated in cells proximal to the wound. We found that JNK, but not Pvr, mediates the increase in chic transcription through the Jun and Fos transcription factors. Finally, we show that chic-deficient larvae fail to form a robust actin cable along the wound edge and also fail to form normal filopodial and lamellipodial extensions into the wound gap. Our results thus connect a factor that regulates actin monomer recycling to the JNK signaling pathway during wound closure. They also reveal a physiological function for an important developmental regulator of actin and begin to tease out the logic of how the wound repair response is organized.
40. On the role of PDZ domain-encoding genes in Drosophila border cell **migration**. Cells often move as collective groups during normal embryonic development and wound healing, although the mechanisms governing this type of **migration** are poorly understood. The Drosophila melanogaster border cells migrate as a cluster during late oogenesis and serve as a powerful in vivo genetic model for collective cell **migration**. To discover new genes that participate in border cell **migration**, 64 out of 66 genes that encode PDZ domain-containing proteins were systematically targeted by in vivo RNAi knockdown. The PDZ domain is one of the largest families of protein-protein interaction domains found in eukaryotes. Proteins that contain PDZ domains participate in a variety of biological processes, including signal transduction and establishment of epithelial apical-basal polarity. Targeting PDZ proteins effectively assesses a larger number of genes via the protein complexes and pathways through which these proteins function. par-6, a known regulator of border cell **migration**, was a positive hit and thus validated the approach. Knockdown of 14 PDZ domain genes disrupted **migration** with multiple RNAi lines. The candidate genes have diverse predicted cellular functions and are anticipated to provide new insights into the mechanisms that control border cell movement. As a test of this concept, two genes that disrupted **migration** were characterized in more detail: big bang and the Dlg5 homolog CG6509. We present evidence that Big bang regulates JAK/STAT signaling, whereas Dlg5/CG6509 maintains cluster cohesion. Moreover, these results demonstrate that targeting a selected class of genes by RNAi can uncover novel regulators of collective cell **migration**.
41. The Drosophila tricellular junction protein Gliotactin regulates its own mRNA levels through BMP-mediated induction of miR-184. Epithelial bicellular and tricellular junctions are essential for establishing and maintaining permeability barriers. Tricellular junctions are formed by the convergence of three bicellular junctions at the corners of neighbouring epithelia. Gliotactin, a member of the Neuroligin family, is located at theDrosophilatricellular junction, and is crucial for the formation of tricellular and septate junctions, as well as permeability barrier function. Gliotactin protein levels are tightly controlled by phosphorylation at tyrosine residues and endocytosis. Blocking endocytosis or overexpressing Gliotactin results in the spread of Gliotactin from the tricellular junction, resulting in apoptosis, delamination and **migration** of epithelial cells. We show that Gliotactin levels are also regulated at the mRNA level by micro (mi)RNA-mediated degradation and that miRNAs are targeted to a short region in the 3'UTR that includes a conserved miR-184 target site. miR-184 also targets a suite of septate junction proteins, including NrxIV, coracle and Mcr. miR-184 expression is triggered when Gliotactin is overexpressed, leading to activation of the BMP signalling pathway. Gliotactin specifically interferes with Dad, an inhibitory SMAD, leading to activation of the Tkv type-I receptor and activation of Mad to elevate the biogenesis and expression of miR-184.
42. Heartbroken is a specific downstream mediator of FGF receptor signalling in Drosophila. Drosophila possesses two FGF receptors which are encoded by the heartless and breathless genes. HEARTLESS is essential for early **migration** and patterning of the embryonic mesoderm, while BREATHLESS is required for proper branching of the tracheal system. We have identified a new gene, heartbroken, that participates in the signalling pathways of both FGF receptors. Mutations in heartbroken are associated with defects in the **migration** and later specification of mesodermal and tracheal cells. Genetic interaction and epistasis experiments indicate that heartbroken acts downstream of the two FGF receptors but either upstream of or parallel to RAS1. Furthermore, heartbroken is involved in both the HEARTLESS- and BREATHLESS-dependent activation of MAPK. In contrast, EGF receptor-dependent embryonic functions and MAPK activation are not perturbed in heartbroken mutant embryos. A strong heartbroken allele also suppresses the effects of hyperactivated FGF but not EGF receptors. Thus, heartbroken may contribute to the specificity of developmental responses elicited by FGF receptor signalling.
43. Dcas supports cell polarization and cell-cell adhesion complexes in development. Mammalian Cas proteins regulate cell **migration**, division and survival, and are often deregulated in cancer. However, the presence of four paralogous Cas family members in mammals (BCAR1/p130Cas, EFS/Sin1, NEDD9/HEF1/Cas-L, and CASS4/HEPL) has limited their analysis in development. We deleted the single Drosophila Cas gene, Dcas, to probe the developmental function of Dcas. Loss of Dcas had limited effect on embryonal development. However, we found that Dcas is an important modulator of the severity of the developmental phenotypes of mutations affecting integrins (If and mew) and their downstream effectors Fak56D or Src42A. Strikingly, embryonic lethal Fak56D-Dcas double mutant embryos had extensive cell polarity defects, including mislocalization and reduced expression of E-cadherin. Further genetic analysis established that loss of Dcas modified the embryonal lethal phenotypes of embryos with mutations in E-cadherin (Shg) or its signaling partners p120- and beta-catenin (Arm). These results support an important role for Cas proteins in cell-cell adhesion signaling in development.
44. From growth cone to synapse: the life history of the RP3 motor neuron. In Drosophila, the ability to analyze the development of individually identified neurons with a variety of imaging and biophysical techniques can be complemented by sophisticated genetics and molecular biology. This powerful combination is allowing the development and function of single neurons and their synaptic connections to be unraveled at an unparalleled level of resolution. In this article, we focus on a single, identified motoneuron--RP3--arguably the best understood neuron in the fruitfly. Many events in the life history of RP3 are well characterized, including cell **migration**, axon outgrowth and pathfinding within the central nervous system, pathfinding in the periphery to its appropriate muscle target domain, the specific recognition of its muscle targets, the events of synapse formation and maturation, and its mature function in the locomotion of the fly larva. Genetic analysis has revealed mutations in a number of different genes which affect specific aspects of RP3 development from axon outgrowth to synapse formation.
45. Drosophila female sterile mutation spoonbill interferes with multiple pathways in oogenesis. spoonbill is a Drosophila female-sterile mutation, which displays a range of eggshell and egg chamber patterning defects. Previous analysis has shown that the mutation interfered with the function of two major signaling pathways, GRK/EGFR and DPP. In this report, the nature of spoonbill was further investigated to examine whether it was associated with additional pathways in oogenesis. Clonal analysis, presented here, demonstrated that most of the aberrant phenotypes associated with spoonbill were dependent on a mutant germline. Nevertheless, SPOONBILL may function also in the soma to ensure proper polarization and **migration** of the border-cell-cluster. Further, genetic interaction studies implicated spoonbill in additional unrelated pathways such as the one(s) involved in actin polymerization/depolymerization. Based on the previous data and the results presented here, it is anticipated that spoonbill may encode a multifunctional protein that perhaps coordinately regulated the activity of multiple signaling pathways during oogenesis.
46. NETRIN and SLIT guide salivary gland **migration**. Directed **migration** is pivotal for the proper placement and function of nearly all organs. The majority of known guidance molecules involved in directed **migration** have been identified from studies of migrating axons during nervous system development. Here, we show that at least two of these axon guidance molecules, NETRIN and SLIT, act through their canonical receptors, to guide Drosophila embryonic salivary glands. NETRIN serves as a chemo-attractant while SLIT functions antagonistically to NETRIN as a chemo-repellent during salivary gland **migration**. CNS midline expression of both NETRIN and SLIT directs the glands to move unswervingly parallel to the CNS. NETRIN expression is also required in the visceral mesoderm, along which the glands move during their **migration**. We propose that analogous to axon guidance, a balance between chemo-attractants and chemo-repellents is required for the proper migratory path of the developing salivary glands.
47. Lasp anchors the Drosophila male stem cell niche and mediates spermatid individualization. Lasp family proteins contain an amino-terminal LIM domain, two actin-binding nebulin repeats and a carboxyl-terminal SH3 domain. Vertebrate Lasp-1 localizes to focal adhesions and the leading edge of migrating cells, and is required for cell **migration**. To assess the in vivo function of Lasp, we generated a null mutant in Drosophila Lasp. Lasp(1) is homozygous viable, but male sterile. In Lasp mutants the stem cell niche is no longer anchored to the apical tip of the testis, and actin cone **migration** is perturbed resulting in improper spermatid individualization. Hub cell mislocalization can by phenocopied by expressing Lasp or betaPS integrin RNAi transgenes in somatic cells, and Lasp genetically interacts with betaPS integrin, demonstrating that Lasp functions together with integrins in hub cells to anchor the stem cell niche. Finally, we show that the stem cell niche is maintained even if it is not properly localized.
48. The AF-6 homolog canoe acts as a Rap1 effector during dorsal closure of the Drosophila embryo. Rap1 belongs to the highly conserved Ras subfamily of small GTPases. In Drosophila, Rap1 plays a critical role in many different morphogenetic processes, but the molecular mechanisms executing its function are unknown. Here, we demonstrate that Canoe (Cno), the Drosophila homolog of mammalian junctional protein AF-6, acts as an effector of Rap1 in vivo. Cno binds to the activated form of Rap1 in a yeast two-hybrid assay, the two molecules colocalize to the adherens junction, and they display very similar phenotypes in embryonic dorsal closure (DC), a process that relies on the elongation and **migration** of epithelial cell sheets. Genetic interaction experiments show that Rap1 and Cno act in the same molecular pathway during DC and that the function of both molecules in DC depends on their ability to interact. We further show that Rap1 acts upstream of Cno, but that Rap1, unlike Cno, is not involved in the stimulation of JNK pathway activity, indicating that Cno has both a Rap1-dependent and a Rap1-independent function in the DC process.
49. Eyes absent, a key repressor of polar cell fate during Drosophila oogenesis. Throughout Drosophila oogenesis, specialized somatic follicle cells perform crucial functions in egg chamber formation and in signaling between somatic and germline cells. In the ovary, at least three types of somatic follicle cells, polar cells, stalk cells and main body epithelial follicle cells, can be distinguished when egg chambers bud from the germarium. Although specification of these three somatic cell types is important for normal oogenesis and subsequent embryogenesis, the molecular basis for establishment of their cell fates is not completely understood. Our studies reveal the gene eyes absent (eya) to be a key repressor of polar cell fate. EYA is a nuclear protein that is normally excluded from polar and stalk cells, and the absence of EYA is sufficient to cause epithelial follicle cells to develop as polar cells. Furthermore, ectopic expression of EYA is capable of suppressing normal polar cell fate and compromising the normal functions of polar cells, such as promotion of border cell **migration**. Finally, we show that ectopic Hedgehog signaling, which is known to cause ectopic polar cell formation, does so by repressing eya expression in epithelial follicle cells.
50. Cadherin-mediated cell adhesion and cell motility in Drosophila trachea regulated by the transcription factor Escargot. Coordination of cell motility and adhesion is essential for concerted movement of tissues during animal morphogenesis. The Drosophila tracheal network is formed by branching, **migration** and fusion of tubular ectodermal epithelia. Tracheal tip cells, located at the end of each branch that is going to fuse, extend filopodia to search for targets and later change their cell shape to a seamless ring to allow passage of lumen. The cell adhesion molecule DE-cadherin accumulates at the site of contact to form a ring that marks the site of lumen entry and is essential for the fusion. DE-cadherin expression in tip cells of a subset of branches is dependent on escargot, a zinc finger gene expressed in all tip cells. Such escargot mutant tip cells failed to adhere to each other and continued to search for alternative targets by extending long filopodia. We present evidence indicating escargot positively regulates transcription of the DE-cadherin gene, shotgun. Overexpression of DE-cadherin rescued the defect in one of the fusion points in escargot mutants, demonstrating an essential role of DE-cadherin in target recognition and identifying escargot as a key regulator of cell adhesion and motility in tracheal morphogenesis.
51. Left-right asymmetric morphogenesis of the anterior midgut depends on the activation of a non-muscle myosin II in Drosophila. Many animals exhibit stereotypical left-right (LR) asymmetry in their internal organs. The mechanisms of LR axis formation required for the subsequent LR asymmetric development are well understood, especially in some vertebrates. However, the molecular mechanisms underlying LR asymmetric morphogenesis, particularly how mechanical force is integrated into the LR asymmetric morphogenesis of organs, are poorly understood. Here, we identified zipper (zip), encoding a Drosophila non-muscle myosin II (myosin II) heavy chain, as a gene required for LR asymmetric development of the embryonic anterior midgut (AMG). Myosin II is known to directly generate mechanical force in various types of cells during morphogenesis and cell **migration**. We found that myosin II was involved in two events in the LR asymmetric development of the AMG. First, it introduced an LR bias to the directional position of circular visceral muscle (CVMU) cells, which externally cover the midgut epithelium. Second, it was required for the LR-biased rotation of the AMG. Our results suggest that myosin II in CVMU cells plays a crucial role in generating the force leading to LR asymmetric morphogenesis. Taken together with previous studies in vertebrates, the involvement of myosin II in LR asymmetric morphogenesis might be conserved evolutionarily.
52. Planar cell polarity signaling in collective cell movements during morphogenesis and disease. Collective and directed cell movements are crucial for diverse developmental processes in the animal kingdom, but they are also involved in wound repair and disease. During these processes groups of cells are oriented within the tissue plane, which is referred to as planar cell polarity (PCP). This requires a tight regulation that is in part conducted by the PCP pathway. Although this pathway was initially characterized in flies, subsequent studies in vertebrates revealed a set of conserved core factors but also effector molecules and signal modulators, which build the fundamental PCP machinery. The PCP pathway in Drosophila regulates several developmental processes involving collective cell movements such as border cell **migration** during oogenesis, ommatidial rotation during eye development, and embryonic dorsal closure. During vertebrate embryogenesis, PCP signaling also controls collective and directed cell movements including convergent extension during gastrulation, neural tube closure, neural crest cell **migration**, or heart morphogenesis. Similarly, PCP signaling is linked to processes such as wound repair, and cancer invasion and metastasis in adults. As a consequence, disruption of PCP signaling leads to pathological conditions. In this review, we will summarize recent findings about the role of PCP signaling in collective cell movements in flies and vertebrates. In addition, we will focus on how studies in Drosophila have been relevant to our understanding of the PCP molecular machinery and will describe several developmental defects and human disorders in which PCP signaling is compromised. Therefore, new discoveries about the contribution of this pathway to collective cell movements could provide new potential diagnostic and therapeutic targets for these disorders.
53. Organ positioning in Drosophila requires complex tissue-tissue interactions. Positioning an organ with respect to other tissues is a complex process necessary for proper anatomical development and organ function. The local environment surrounding an organ can serve both as a substrate for **migration** and as a source of guidance cues that direct **migration**. Little is known about the factors guiding Drosophila salivary gland movement or about the contacts the glands establish along their migratory path. Here, we provide a detailed description of the spatial and temporal interactions between the salivary glands and surrounding tissues during embryogenesis. The glands directly contact five other tissues: the visceral mesoderm, gastric caecae, somatic mesoderm, fat body, and central nervous system. Mutational analysis reveals that all of the tissues tested in this study are important for normal salivary gland positioning; proper differentiation of the visceral and somatic mesoderm is necessary for the glands to attain their final correct position. We also provide evidence that the segment-polarity gene, gooseberry (gsb), controls expression of signals from the developing fat body that direct posterior **migration** of the glands. These data further the understanding of how organ morphology and position are determined by three-dimensional constraints and guidance cues provided by neighboring tissues.
54. Cellular basis of the dynamic behavior of the imaginal thoracic discs during Drosophila metamorphosis. The eversion, **migration**, spreading, and fusion of the thoracic imaginal discs during metamorphosis of Drosophila are described using timed whole-mount preparations and several molecular markers. The leading edge of the migrating disc epithelia consists of two groups of cells, stalk cells (S cells) and specialized imaginal cells (I cells), that both express the gene puckered. With this and other markers, opening of the stalk, eversion of the discs, **migration** of the leading edges, and fusion of the imaginal epithelia can be visualized in detail. Fusion is initiated by S cells that migrate over the larval epithelium and constitute a bridge between two imaginal epithelia. S cells are subsequently lost and imaginal fusion is mediated by the I cells that remain at the site of fusion. The possible cellular basis of this process is discussed. Fusion along the dorsal midline of the notum from the mesothoracic wing discs occurs earlier than that of the prothoracic and metathoracic discs, which remain in a lateral position. For a relatively long period (30 h) the mesothoracic epithelium becomes attached to the head and abdomen, causing a temporary local discontinuity of the order of segments. Later the pro- and metathoracic discs intercalate between head and mesothorax and between abdomen and mesothorax, respectively, to reestablish the normal order.
55. Phylogenetic conservation of the regulatory and functional properties of the Vav oncoprotein family. Vav proteins are phosphorylation-dependent GDP/GTP exchange factors for Rho/Rac GTPases. Despite intense characterization of mammalian Vav proteins both biochemically and genetically, there is little information regarding the conservation of their biological properties in lower organisms. To approach this issue, we have performed a characterization of the regulatory, catalytic, and functional properties of the single Vav family member of Drosophila melanogaster. These analyses have shown that the intramolecular mechanisms controlling the enzyme activity of mammalian Vav proteins are already present in Drosophila, suggesting that such properties have been set up before the divergence between protostomes and deuterostomes during evolution. We also show that Drosophila and mammalian Vav proteins have similar catalytic specificities. As a consequence, Drosophila Vav can trigger oncogenic transformation, morphological change, and enhanced cell motility in mammalian cells. Gain-of-function studies using transgenic flies support the implication of this protein in cytoskeletal-dependent processes such as embryonic dorsal closure, myoblast fusion, tracheal development, and the **migration**/guidance of different cell types. These results highlight the important roles of Vav proteins in the signal transduction pathways regulating cytoskeletal dynamics. Moreover, they indicate that the foundations for the regulatory and enzymatic activities of this protein family have been set up very early during evolution.
56. faint sausage encodes a novel extracellular protein of the immunoglobulin superfamily required for cell **migration** and the establishment of normal axonal pathways in the Drosophila nervous system. We examined the structure of the nervous system in Drosophila embryos homozygous for a null mutation in the faint sausage (fas) gene. In the peripheral nervous system (PNS) of fas mutants, neurons fail to delaminate from the ectodermal epithelium; in the central nervous system (CNS), the positions of neuronal cell bodies and glial cells are abnormal and normal axonal pathways do not form. Sequence analysis of fas cDNAs revealed that the fas protein product has characteristics of an extracellular protein and that it is a novel member of the immunoglobulin (Ig) superfamily. In situ hybridization demonstrated that fas transcripts are expressed throughout the embryo but they are in relatively high concentrations in the lateral ectoderm, from which the peripheral nervous system delaminates and in the CNS. Antiserum directed against Fas protein was found to stain neurons but not glia in the CNS. We conclude that fas encodes a protein that, in the developing nervous system, is present on the surface of neurons and is essential for nerve cell **migration** and the establishment of axonal pathways.
57. Bazooka is a permissive factor for the invasive behavior of discs large tumor cells in Drosophila ovarian follicular epithelia. Drosophila Bazooka and atypical protein kinase C are essential for epithelial polarity and adhesion. We show here that wild-type bazooka function is required during cell invasion of epithelial follicle cells mutant for the tumor suppressor discs large. Clonal studies indicate that follicle cell Bazooka acts as a permissive factor during cell invasion, possibly by stabilizing adhesion between the invading somatic cells and their substratum, the germline cells. Genetic epistasis experiments demonstrate that bazooka acts downstream of discs large in tumor cell invasion. In contrast, during the **migration** of border cells, Bazooka function is dispensable for cell invasion and motility, but rather is required cell-autonomously in mediating cell adhesion within the migrating border cell cluster. Taken together, these studies reveal Bazooka functions distinctly in different types of invasive behaviors of epithelial follicle cells, potentially by regulating adhesion between follicle cells or between follicle cells and their germline substratum.
58. Activated armadillo/beta-catenin does not play a general role in cell **migration** and process extension in Drosophila. Human beta-catenin and its fly homolog Armadillo are best known for their roles in cadherin-based cell-cell adhesion and in transduction of Wingless/Wnt signals. It has been hypothesized that beta-catenin may also regulate cell **migration** and cell shape changes, possibly by regulating the microtubule cytoskeleton via interactions with APC. This hypothesis was based on experiments in which a hyperstable mutant form of beta-catenin was expressed in MDCK cells, where it altered their migratory properties and their ability to send out long cellular processes. We tested the generality of this hypothesis in vivo in Drosophila. We utilized three model systems in which cell **migration** and/or process extension are known to play key roles during development: the **migration** of the border cells during oogenesis, the extension of axons in the nervous system, and the **migration** and cell process extension of tracheal cells. In all cases, cells expressing activated Armadillo were able to migrate and extend cell processes essentially normally. The one alteration from normal involved an apparent cell fate change in certain tracheal cells. These results suggest that only certain cells are affected by activation of Armadillo/beta-catenin, and that Armadillo/beta-catenin does not play a general role in inhibiting cell **migration** or process extension.
59. Identification of mutations that cause cell **migration** defects in mosaic clones. Cell movement is an important feature of animal development, wound healing and tumor metastasis; however, the mechanisms underlying cell motility remain to be elucidated. To further our understanding, it would be useful to identify all of the proteins that are essential for a cell to migrate, yet such information is not currently available for any cell type. We have carried out a screen for mutations affecting border cell **migration** in Drosophila. Mutations that cause defects in mosaic clones were identified, so that genes that are also required for viability could be detected. From 6000 mutagenized lines, 20 mutations on chromosome 2R were isolated that cause defects in border cell position. One of the mutations was dominant while all of the recessive mutations appeared to be homozygous lethal. This lethality was used to place the mutations into 16 complementation groups. Many of the mutations failed to complement cytologically characterized deficiencies, allowing their rapid mapping. Mutations in three loci altered expression of a marker gene in the border cells, whereas the remaining mutations did not. One mutation, which caused production of supernumerary border cells, was found to disrupt the costal-2 locus, indicating a role for Hedgehog signaling in border cell development. This screen identified many new loci required for border cell **migration** and our results suggest that this is a useful approach for elucidating the mechanisms involved in cell motility.
60. Compartmentalization of visual centers in the Drosophila brain requires Slit and Robo proteins. Brain morphogenesis depends on the maintenance of boundaries between populations of non-intermingling cells. We used molecular markers to characterize a boundary within the optic lobe of the Drosophila brain and found that Slit and the Robo family of receptors, well-known regulators of axon guidance and neuronal **migration**, inhibit the mixing of adjacent cell populations in the developing optic lobe. Our data suggest that Slit is needed in the lamina to prevent inappropriate invasion of Robo-expressing neurons from the lobula cortex. We show that Slit protein surrounds lamina glia, while the distal cell neurons in the lobula cortex express all three Drosophila Robos. We examine the function of these proteins in the visual system by isolating a novel allele of slit that preferentially disrupts visual system expression of Slit and by creating transgenic RNA interference flies to inhibit the function of each Drosophila Robo in a tissue-specific fashion. We find that loss of Slit or simultaneous knockdown of Robo, Robo2 and Robo3 causes distal cell neurons to invade the lamina, resulting in cell mixing across the lamina/lobula cortex boundary. This boundary disruption appears to lead to alterations in patterns of axon navigation in the visual system. We propose that Slit and Robo-family proteins act to maintain the distinct cellular composition of the lamina and the lobula cortex.
61. Functions for Drosophila brachyenteron and forkhead in mesoderm specification and cell signalling. The visceral musculature of the larval midgut of Drosophila has a lattice-type structure and consists of an inner stratum of circular fibers and an outer stratum of longitudinal fibers. The longitudinal fibers originate from the posterior tip of the mesoderm anlage, which has been termed the caudal visceral mesoderm (CVM). In this study, we investigate the specification of the CVM and particularly the role of the Drosophila Brachyury-homologue brachyenteron. Supported by fork head, brachyenteron mediates the early specification of the CVM along with zinc-finger homeodomain protein-1. This is the first function described for brachyenteron or fork head in the mesoderm of Drosophila. The mode of cooperation resembles the interaction of the Xenopus homologues Xbra and Pintallavis. Another function of brachyenteron is to establish the surface properties of the CVM cells, which are essential for their orderly **migration** along the trunk-derived visceral mesoderm. During this movement, the CVM cells, under the control of brachyenteron, induce the formation of one muscle/pericardial precursor cell in each parasegment. We propose that the functions of brachyenteron in mesodermal development of Drosophila are comparable to the roles of the vertebrate Brachyury genes during gastrulation.
62. The epithelial-mesenchymal transition of the Drosophila mesoderm requires the Rho GTP exchange factor Pebble. Drosophila pebble (pbl) encodes a Rho-family GTP exchange factor (GEF) required for cytokinesis. The accumulation of high levels of PBL protein during interphase and the developmentally regulated expression of pbl in mesodermal tissues suggested that the primary cytokinetic mutant phenotype might be masking other roles. Using various muscle differentiation markers, we found that Even skipped (EVE) expression in the dorsal mesoderm is greatly reduced in pbl mutant embryos. EVE expression in the dorsalmost mesodermal cells is induced in response to DPP secreted by the dorsal epidermal cells. Further analysis revealed that this phenotype is likely to be a consequence of an earlier defect. pbl mutant mesodermal cells fail to undergo the normal epithelial-mesenchymal transition (EMT) and dorsal **migration** that follows ventral furrow formation. This phenotype is not a secondary consequence of failed cytokinesis, as it is rescued by a mutant form of pbl that does not rescue the cytokinetic defect. In wild-type embryos, newly invaginated cells at the lateral edges of the mesoderm extend numerous protrusions. In pbl mutant embryos, however, cells appear more tightly adhered to their neighbours and extend very few protrusions. Consistent with the dependence of the mesoderm EMT and cytokinesis on actin organisation, the GTP exchange function of the PBL RhoGEF is required for both processes. By contrast, the N-terminal BRCT domains of PBL are required only for the cytokinetic function of PBL. These studies reveal that a novel PBL-mediated intracellular signalling pathway operates in mesodermal cells during the transition from an epithelial to migratory mesenchymal morphology during gastrulation.
63. heartless encodes a fibroblast growth factor receptor (DFR1/DFGF-R2) involved in the directional **migration** of early mesodermal cells in the Drosophila embryo. After invagination of the mesodermal primordium in the gastrulating Drosophila embryo, the internalized cells migrate in a dorsolateral direction along the overlying ectoderm. This movement generates a stereotyped arrangement of mesodermal cells that is essential for their correct patterning by later position-specific inductive signals. We now report that proper mesodermal cell **migration** is dependent on the function of a fibroblast growth factor (FGF) receptor encoded by heartless (htl). In htl mutant embryos, the mesoderm forms normally but fails to undergo its usual dorsolateral **migration**. As a result, cardiac, visceral, and dorsal somatic muscle fates are not induced by Decapentaplegic (Dpp), a transforming growth factor beta family member that is derived from the dorsal ectoderm. Visceral mesoderm can nevertheless be induced by Dpp in the absence of htl function. Ras1 is an important downstream effector of Htl signaling because an activated form of Ras1 partially rescues the htl mutant phenotype. The evolutionary conservation of htl function is suggested by the strikingly similar mesodermal **migration** and patterning phenotypes associated with FGF receptor mutations in species as diverse as nematode and mouse. These studies establish that Htl signaling provides a vital connection between initial formation of the embryonic mesoderm in Drosophila and subsequent cell-fate specification within this germ layer.
64. A role for the chaperone Hsp70 in the regulation of border cell **migration** in the Drosophila ovary. Unravelling the molecular mechanisms that govern cell **migration** is of great importance towards understanding both normal embryogenesis and physiological and pathological processes occurring in the adult. **Migration** of border cells (BCs) during Drosophila oogenesis provides a simple and attractive model in which to address this problem. Here, we show that the molecular chaperone Hsp70 is required for BC **migration**. Thus, BCs lacking all Hsp70 genes present in the fly genome fail to reorganize their actin cytoskeleton, resulting in **migration** defects. Similar defects are found when the Hsp70 co-chaperone DnaJ-1, the Drosophila homolog of the human Hsp40, is overexpressed specifically in BCs. In addition, we provide biochemical and genetic evidence for an interaction between DnaJ-1 and PDGF/VEGF receptor (PVR), which is also required for actin-mediated BC **migration**. Furthermore, our results showing that PVR also interacts genetically with Hsp70 suggest that a mechanism by which the DnaJ-1/Hsp70 chaperone complex regulates BC **migration** is by modulating PVR function.
65. Regulation of cell adhesion and collective cell **migration** by hindsight and its human homolog RREB1. Cell movements represent a major driving force in embryonic development, tissue repair, and tumor metastasis [1]. The **migration** of single cells has been well studied, predominantly in cell culture [2, 3]; however, in vivo, a greater variety of modes of cell movement occur, including the movements of cells in clusters, strands, sheets, and tubes, also known as collective cell **migration**s [4, 5]. In spite of the relevance of these types of movements in both normal and pathological conditions, the molecular mechanisms that control them remain predominantly unknown. Epithelial follicle cells of the Drosophila ovary undergo several dynamic morphological changes, providing a genetically tractable model [6]. We found that anterior follicle cells, including border cells, mutant for the gene hindsight (hnt) accumulated excess cell-cell adhesion molecules and failed to undergo their normal collective movements. In addition, HNT affected border cell cluster cohesion and motility via effects on the JNK and STAT pathways, respectively. Interestingly, reduction of expression of the mammalian homolog of HNT, RREB1, by siRNA inhibited collective cell **migration** in a scratch-wound healing assay of MCF10A mammary epithelial cells, suppressed surface activity, retarded cell spreading after plating, and led to the formation of immobile, tightly adherent cell colonies. We propose that HNT and RREB1 are essential to reduce cell-cell adhesion when epithelial cells within an interconnected group undergo dynamic changes in cell shape.
66. Moving muscle. Jeb signaling in Drosophila. A recent study identifies a novel nonautonomous signaling pathway that regulates cell **migration** and differentiation in early Drosophila mesodermal tissues.
67. Myosin VI is required for E-cadherin-mediated border cell **migration**. Myosin VI (MyoVI) is a pointed-end-directed, actin-based motor protein, and mutations in the gene result in disorganization of hair cell stereocilia and cause deafness in mice. MyoVI also localizes to the leading edges of growth-factor-stimulated fibroblast cells and has been suggested to be involved in cell motility. There has been no direct test of this hypothesis, however. Drosophila melanogaster MyoVI is expressed in a small group of migratory follicle cells, known as border cells. Here we show that depletion of MyoVI specifically from border cells severely inhibited their **migration**. Similar to MyoVI, E-cadherin is required for border cell **migration**. We found that E-cadherin and Armadillo (Arm, Drosophila beta-catenin) protein levels were specifically reduced in cells lacking MyoVI, whereas other proteins were not. In addition, MyoVI protein levels were reduced in cells lacking DE-cadherin or Arm. MyoVI and Arm co-immunoprecipitated from ovarian protein extracts. These data suggest that MyoVI is required for border cell **migration** where it stabilizes E-cadherin and Arm. Mutations in MyoVIIA, another unconventional myosin protein, also lead to deafness, and MyoVIIA interacts with E-cadherin through a membrane protein called vezatin. Multiple biochemical mechanisms may exist, therefore, for cadherins to associate with diverse unconventional myosins that are required for normal stereocilium formation or maintenance.
68. Drosophila Yurt is a new protein-4.1-like protein required for epithelial morphogenesis. Proteins of the 4.1 family play a key role in the integrity of the cytoskeleton and in epithelial tissue movement, as shown by the disruption of the actin cytoskeleton in human erythrocytes caused by genetic loss of protein 4.1, and the failure of epithelial tissue **migration** during Drosophila embryogenesis caused by genetic loss of the 4.1 homolog Coracle. Here we report the genetic characterization of Yurt, a novel protein 4.1 family member in Drosophila that is associated with the plasma membrane of epithelial cells. Homozygous loss-of-function mutations in the yurt gene cause failure of germ-band retraction, dorsal closure, and head involution, associated with degeneration of the amnioserosa and followed by embryonic lethality. A mammalian homolog of Yurt is up-regulated in metastatic melanoma cells. These novel cytoskeletal proteins appear to play important roles in epithelial cell movements and in the morphogenetic tissue changes that depend on them.
69. The salvador-warts-hippo pathway is required for epithelial proliferation and axis specification in Drosophila. In Drosophila, the body axes are specified during oogenesis through interactions between the germline and the overlying somatic follicle cells [1-5]. A Gurken/TGF-alpha signal from the oocyte to the adjacent follicle cells assigns them a posterior identity [6, 7]. These posterior cells then signal back to the oocyte, thereby inducing the repolarization of the microtubule cytoskeleton, the **migration** of the oocyte nucleus, and the localization of the axis specifying mRNAs [8-10]. However, little is known about the signaling pathways within or from the follicle cells responsible for these patterning events. We show that the Salvador Warts Hippo (SWH) tumor-suppressor pathway is required in the follicle cells in order to induce their Gurken- and Notch-dependent differentiation and to limit their proliferation. The SWH pathway is also required in the follicle cells to induce axis specification in the oocyte, by inducing the **migration** of the oocyte nucleus, the reorganization of the cytoskeleton, and the localization of the mRNAs that specify the anterior-posterior and dorsal-ventral axes of the embryo. This work highlights a novel connection between cell proliferation, cell growth, and axis specification in egg chambers.
70. Paracrine signaling through the JAK/STAT pathway activates invasive behavior of ovarian epithelial cells in Drosophila. The JAK/STAT signaling pathway, renowned for its effects on cell proliferation and survival, is constitutively active in various human cancers, including ovarian. We have found that JAK and STAT are required to convert the border cells in the Drosophila ovary from stationary, epithelial cells to migratory, invasive cells. The ligand for this pathway, Unpaired (UPD), is expressed by two central cells within the migratory cell cluster. Mutations in upd or jak cause defects in **migration** and a reduction in the number of cells recruited to the cluster. Ectopic expression of either UPD or JAK is sufficient to induce extra epithelial cells to migrate. Thus, a localized signal activates the JAK/STAT pathway in neighboring epithelial cells, causing them to become invasive.
71. Jing: a downstream target of slbo required for developmental control of border cell **migration**. Epithelial to mesenchymal transitions and cell **migration** are important features of embryonic development and tumor metastasis. We are employing a systematic genetic approach to study the border cells in the Drosophila ovary, as a simple model for these cellular behaviors. Previously we found that expression of the basic-region/leucine zipper transcription factor, C/EBP, is required for the border cells to initiate their **migration**. Here we report the identification of a second nuclear factor, named JING (which means 'still'), that is required for initiation of border cell **migration**. The jing locus was identified in a screen for mutations that cause border cell **migration** defects in mosaic clones. The jing mutant phenotype resembles that of slbo mutations, which disrupt the Drosophila C/EBP gene, but is distinct from other classes of border cell **migration** mutants. Expression of a jing-lacZ reporter in border cells requires C/EBP. Moreover, expression of jing from a heat-inducible promoter rescues the border cell **migration** defects of hypomorphic slbo mutants. The JING protein is most closely related to a mouse protein, AEBP2, which was identified on the basis of its ability to bind a small regulatory sequence within the adipocyte AP2 gene to which mammalian C/EBP also binds. We propose that the need to coordinate cell differentiation with nutritional status may be the link between mammalian adipocytes and Drosophila border cells that led to the conservation of C/EBP and AEBP2.
72. Different Wnt signals act through the Frizzled and RYK receptors during Drosophila salivary gland **migration**. Guided cell **migration** is necessary for the proper function and development of many tissues, one of which is the Drosophila embryonic salivary gland. Here we show that two distinct Wnt signaling pathways regulate salivary gland **migration**. Early in **migration**, the salivary gland responds to a WNT4-Frizzled signal for proper positioning within the embryo. Disruption of this signal, through mutations in Wnt4, frizzled or frizzled 2, results in misguided salivary glands that curve ventrally. Furthermore, disruption of downstream components of the canonical Wnt pathway, such as dishevelled or Tcf, also results in ventrally curved salivary glands. Analysis of a second Wnt signal, which acts through the atypical Wnt receptor Derailed, indicates a requirement for Wnt5 signaling late in salivary gland **migration**. WNT5 is expressed in the central nervous system and acts as a repulsive signal, needed to keep the migrating salivary gland on course. The receptor for WNT5, Derailed, is expressed in the actively migrating tip of the salivary glands. In embryos mutant for derailed or Wnt5, salivary gland **migration** is disrupted; the tip of the gland migrates abnormally toward the central nervous system. Our results suggest that both the Wnt4-frizzled pathway and a separate Wnt5-derailed pathway are needed for proper salivary gland **migration**.
73. Specific tracheal **migration** is mediated by complementary expression of cell surface proteins. **Migration** of the Drosophila tracheal cells relies on cues provided by nearby cells; however, little is known about how these signals specify a migratory path. Here we investigate the role of cell surface proteins in the definition of such a pathway. We have found that the PS1 integrin is required in the tracheal cells of the visceral branch, whereas the PS2 integrin is required in the visceral mesoderm; both integrins are necessary for the spreading of the visceral branch over its substratum. This is the first identification of a cell surface molecule with expression restricted to a subset of tracheal cells that all migrate in a given direction. We have also found that expression of PS1 in the visceral branch is regulated by the genes that direct tracheal cell **migration**, showing that integrin expression is part of the cell-fate program that they specify. These results support a model in which signal transduction determines the tracheal migratory pathways by regulating the expression of cell surface proteins, which in turn interact with surface molecules on the surrounding cell population.
74. Requirement for Par-6 and Bazooka in Drosophila border cell **migration**. Polarized epithelial cells convert into migratory invasive cells during a number of developmental processes, as well as when tumors metastasize. Much has been learned recently concerning the molecules and mechanisms that are responsible for generating and maintaining epithelial cell polarity. However, less is known about what becomes of epithelial polarity proteins when various cell types become migratory and invasive. Here, we report the localization of several apical epithelial proteins, Par-6, Par-3/Bazooka and aPKC, during border cell **migration** in the Drosophila ovary. All of these proteins remained asymmetrically distributed throughout **migration**. Moreover, depletion of either Par-6 or Par-3/Bazooka by RNAi resulted in disorganization of the border cell cluster and impaired **migration**. The distributions of several transmembrane proteins required for **migration** were abnormal following Par-6 or Par-3/Bazooka downregulation, possibly accounting for the **migration** defects. Taken together, these results indicate that cells need not lose apical/basal polarity in order to invade neighboring tissues and in some cases even require such polarity for proper motility.
75. Inhibition of patterned cell shape change and cell invasion by Discs large during Drosophila oogenesis. Drosophila Discs large (Dlg) is a tumor suppressor gene whose loss in epithelial tissues causes disrupted cell polarity and increased cell proliferation. A human Dlg homolog, hDlg, has been implicated in tumorigenic processes via its association with the product of the Adenomatous Polyposis Coli (APC) gene. We show for the first time that Drosophila Dlg is required to block cell invasion. Loss of dlg activity during oogenesis causes follicle cells to change shape and invade in a pattern similar to border cells, a small population of cells that break from the post-mitotic follicular epithelium during wild-type oogenesis, yet dlg mutant cells have not adopted a border cell fate. Both functional and morphological evidence indicates that cooperation between germ cell and follicle cell Dlg, probably mediated by Dlg PDZ domains, is crucial for regulating cell mixing, suggesting a novel developmental mechanism and mode of action for the Dlg family of molecules. These findings suggest that Dlg does not simply inhibit individual cell behaviors during oogenesis, but rather acts in a developmental pathway essential for blocking cell proliferation and **migration** in a spatio-temporally defined manner. A model for Dlg action in blocking cell invasion is presented.
76. A Fasciclin 2 morphogenetic switch organizes epithelial cell cluster polarity and motility. Little is known about how intercellular communication is regulated in epithelial cell clusters to control delamination and **migration**. We investigate this problem using Drosophila border cells as a model. We find that just preceding cell cluster delamination, expression of transmembrane immunoglobulin superfamily member, Fasciclin 2, is lost in outer border cells, but not in inner polar cells of the cluster. Loss of Fasciclin 2 expression in outer border cells permits a switch in Fasciclin 2 polarity in the inner polar cells. This polarity switch, which is organized in collaboration with neoplastic tumor suppressors Discs large and Lethal-giant-larvae, directs cluster asymmetry essential for timing delamination from the epithelium. Fas2-mediated communication between polar and border cells maintains localization of Discs large and Lethal-giant-larvae in border cells to inhibit the rate of cluster **migration**. These findings are the first to show how a switch in cell adhesion molecule polarity regulates asymmetry and delamination of an epithelial cell cluster. The finding that Discs large and Lethal-giant-larvae inhibit the rate of normal cell cluster movement suggests that their loss in metastatic tumors may directly contribute to tumor motility. Furthermore, our results provide novel insight into the intimate link between epithelial polarity and acquisition of motile polarity that has important implications for development of invasive carcinomas.
77. Function of the ETS transcription factor Yan in border cell **migration**. Invasive cell **migration** in both normal development and metastatic cancer is regulated by various signaling pathways, transcription factors and cell-adhesion molecules. The coordination between these activities in the context of cell **migration** is poorly understood. During Drosophila oogenesis, a small group of cells called border cells exit the follicular epithelium to perform a stereotypic, invasive **migration**. We find that the ETS transcription factor Yan is required for border cell **migration** and that Yan expression is spatiotemporally regulated as border cells migrate from the anterior pole of the egg chamber towards the nurse cell-oocyte boundary. Yan expression is dependent on inputs from the JAK/STAT, Notch and Receptor Tyrosine Kinase pathways in border cells. Mechanistically, Yan functions to modulate the turnover of DE-Cadherin-dependent adhesive complexes to facilitate border cell **migration**. Our results suggest that Yan acts as a pivotal link between signal transduction, cell adhesion and invasive cell **migration** in Drosophila border cells.
78. Systematic analysis of the transcriptional switch inducing **migration** of border cells. Cell **migration** within a natural context is tightly controlled, often by specific transcription factors. However, the switch from stationary to migratory behavior is poorly understood. Border cells perform a spatially and temporally controlled invasive **migration** during Drosophila oogenesis. Slbo, a C/EBP family transcriptional activator, is required for them to become migratory. We purified wild-type and slbo mutant border cells as well as nonmigratory follicle cells and performed comparative whole-genome expression profiling, followed by functional tests of the contributions of identified targets to **migration**. About 300 genes were significantly upregulated in border cells, many dependent on Slbo. Among these, the microtubule regulator Stathmin was strongly upregulated and was required for normal **migration**. Actin cytoskeleton regulators were also induced, including, surprisingly, a large cluster of "muscle-specific" genes. We conclude that Slbo induces multiple cytoskeletal effectors, and that each contributes to the behavioral changes in border cells.
79. Tousled-like kinase regulates cytokine-mediated communication between cooperating cell types during collective border cell **migration**. Collective cell **migration** is emerging as a major contributor to normal development and disease. Collective movement of border cells in the Drosophila ovary requires cooperation between two distinct cell types: four to six migratory cells surrounding two immotile cells called polar cells. Polar cells secrete a cytokine, Unpaired (Upd), which activates JAK/STAT signaling in neighboring cells, stimulating their motility. Without Upd, **migration** fails, causing sterility. Ectopic Upd expression is sufficient to stimulate motility in otherwise immobile cells. Thus regulation of Upd is key. Here we report a limited RNAi screen for nuclear proteins required for border cell **migration**, which revealed that the gene encoding Tousled-like kinase (Tlk) is required in polar cells for Upd expression without affecting polar cell fate. In the absence of Tlk, fewer border cells are recruited and motility is impaired, similar to inhibition of JAK/STAT signaling. We further show that Tlk in polar cells is required for JAK/STAT activation in border cells. Genetic interactions further confirmed Tlk as a new regulator of Upd/JAK/STAT signaling. These findings shed light on the molecular mechanisms regulating the cooperation of motile and nonmotile cells during collective invasion, a phenomenon that may also drive metastatic cancer.
80. Drosophila Pxt: a cyclooxygenase-like facilitator of follicle maturation. Prostaglandins are local transient hormones that mediate a wide variety of biological events, including reproduction. The study of prostaglandin biology in a genetically tractable invertebrate model organism has been limited by the lack of clearly identified prostaglandin-mediated biological processes and prostaglandin metabolic genes, particularly analogs of cyclooxygenase (COX), the rate-limiting step in vertebrate prostaglandin synthesis. Here, we present pharmacological data that Drosophila ovarian follicle maturation requires COX-like activity and genetic evidence that this activity is supplied in vivo by the Drosophila peroxidase Pxt. pxt mutant females are sterile, and maturing follicles show defects in actin filament formation, nurse cell membrane stability and border cell **migration**. Maturation of pxt follicles in vitro is stimulated by prostaglandin treatment and fertility is restored in vivo to pxt mutants by expressing mammalian Cox1 protein. Our experiments suggest that prostaglandins promote Drosophila follicle maturation, in part by modulating the actin cytoskeleton, and establish Drosophila oogenesis as a model for understanding these critical biological regulators.
81. Asymmetric localisation of cytokine mRNA is essential for JAK/STAT activation during cell invasiveness. The transition from immotile epithelial cells to migrating cells occurs in all organisms during normal embryonic development, as well as during tumour metastasis. During Drosophila oogenesis, border cells (BCs) are recruited and delaminate from the follicular epithelium. This process is triggered by the polar cells (PCs), which secrete the cytokine Unpaired (Upd) and activate the JAK/STAT pathway in neighbouring cells, turning them into invasive BCs. Interestingly, either a decrease or an increase in BC number alters **migration**, indicating that mechanisms controlling the level of JAK/STAT signalling are crucial in this process. Here, we show that PCs have a highly stable and polarised network of microtubules along which upd transcripts are asymmetrically transported in a Dynein-dependent manner. We demonstrate that in the absence of upd mRNA localisation the ligand is no longer efficiently secreted, leading to a loss of signalling strength as well as recruitment and **migration** defects. These findings reveal a novel post-transcriptional regulatory mechanism of JAK/STAT signalling in the control of epithelial cell invasiveness.
82. Investigating the function of follicular subpopulations during Drosophila oogenesis through hormone-dependent enhancer-targeted cell ablation. Although it is known that the establishment of polarity during Drosophila oogenesis is initiated by signalling from the oocyte to the overlying follicle cells, much less is understood about the role of specific follicular subpopulations. One powerful approach for addressing this question, toxigenic cell ablation of specific subpopulations, has not previously been applicable to studying follicular subpopulations because many of the genes and Gal4 enhancer trap insertions that are expressed in the ovary are also expressed at earlier times in development. To overcome this problem, we have utilized a fusion protein between Gal4 and the human estrogen receptor to achieve hormone-dependent, tissue-specific gene expression of UAS-linked transgenes in flies. We used this system to study the role of the polar subpopulations of follicle cells during oogenesis by expressing within them a modified form of diphtheria toxin that causes cell death. Our results confirmed previous functions ascribed to these cells, and also demonstrated a previously undescribed role for the border cells in facilitating the **migration** of the anterior Fasciclin III-expressing polar pair cells to the edge of the oocyte.
83. Bicaudal-D is essential for egg chamber formation and cytoskeletal organization in drosophila oogenesis. Bicaudal-D (Bic-D) is required for the transport of determinant mRNAs and proteins to the presumptive oocyte, an essential step in the differentiation of the oocyte. Bic-D protein contains four well-defined heptad repeat domains characteristic of intermediate filament proteins. We characterized the ovarian phenotypes of females expressing mutant Bic-D proteins (Bic-D(H)) deleted for each of the heptad repeat domains. The altered **migration** of follicle cells we observe in mutant ovaries suggests that Bic-D functions in the germline and directs the inward **migration** of somatic follicle cells. In the germarium Bic-D is required for the organization of the egg chamber and the structural integrity of the oocyte and nurse cells. Examination of the polarized microtubule network in Bic-D(H) ovaries shows that Bic-D function is required for both the establishment of the polarized microtubule network and its maintenance throughout oogenesis. To explain the multiple functions suggested by the pleiotropic Bic-D phenotype, we propose that Bic-D protein could form itself a filamentous structure and represent an integral, essential part of the cytoskeleton.

### ECM

#### PubMed

1. The role of LamininB2 (LanB2) during mesoderm differentiation in Drosophila. In Drosophila, four genes encode for laminin subunits and the formation of two laminin heterotrimers has been postulated. We report the identification of mutations in the Drosophila LamininB2 (LanB2) gene that encodes for the only laminin γ subunit and is found in both heterotrimers. We describe their effects on embryogenesis, in particular the differentiation of visceral tissues with respect to the **EC**M. Analysis of mesoderm endoderm interaction indicates disrupted basement membranes and defective endoderm migration, which finally interferes with visceral myotube stretching. Extracellular deposition of laminin is blocked due to the loss of the LanB2 subunit, resulting in an abnormal distribution of **EC**M components. Our data, concerning the different function of both trimers during organogenesis, suggest that these trimers might act in a cumulative way and probably at multiple steps during **EC**M assembly. We also observed genetic interactions with kon-tiki and thrombospondin, indicating a role for laminin during muscle attachment.
2. The Alg5 ortholog Wollknäuel is essential for correct epidermal differentiation during Drosophila late embryogenesis. The formation of an extracellular matrix **(EC**M) presupposes an ordered delivery of its components to ensure its stereotypic architecture. The Drosophila cuticle is an **EC**M produced by the epidermis at its apical site and is characterized by a layered organization. To understand the mechanisms of cuticle assembly during development, we have investigated early aspects of protein N-glycosylation, i.e. the attachment of a dolichol-linked oligosaccharide to distinct Asn sites of a protein known to be essential for sorting in the secretory pathway. Mutations in the Drosophila alg5 gene wollknäuel (wol) that codes for an enzyme initiating the glucosylation of the dolichol-linked oligosaccharide decrease, as expected, glucosylation and the amounts of N-glycosylated proteins such as the cuticle-organizing factor Knickkopf, without affecting their correct localization. At the same time, the polarity determinants Crumbs and atypical protein kinase C accumulate at the apical plasma membrane in wol deficient embryos. In part, these perturbations may also be caused by the unfolded protein response, which is commonly triggered by ER stress and downsizes transcription and translation in general. In any case, they are associated with the loss of cuticle layering and aberrant apical plasma membrane organization suggesting that glucosylation, either directly or indirectly through controlling protein degradation, is important for the efficient and balanced deployment of the biochemical functions of secreted and membrane-associated proteins during epidermal differentiation.
3. Hormonal regulation of mummy is needed for apical extracellular matrix formation and epithelial morphogenesis in Drosophila. Many epithelia produce apical extracellular matrices (a**ECM**) that are crucial for organ morphogenesis or physiology. Apical **ECM** formation relies on coordinated synthesis and modification of constituting components, to enable their subcellular targeting and extracellular assembly into functional matrices. The exoskeleton of Drosophila, the cuticle, is a stratified a**ECM** containing ordered chitin polysaccharide lamellae and proteinaceous layers, and is suited for studies of molecular functions needed for a**ECM** assembly. Here, we show that Drosophila mummy (mmy) mutants display defects in epithelial organisation in conjunction with aberrant deposition of the cuticle and an apical matrix needed for tracheal tubulogenesis. We find that mmy encodes the UDP-N-acetylglucosamine pyrophosphorylase, which catalyses the production of UDP-N-acetylglucosamine, an obligate substrate for chitin synthases as well as for protein glycosylation and GPI-anchor formation. Consequently, in mmy mutants GlcNAc-groups including chitin are severely reduced and modification and subcellular localisation of proteins designated for extracellular space is defective. Moreover, mmy expression is selectively upregulated in epithelia at the time they actively deposit a**ECM**, and is altered by the moulting hormone 20-Hydroxyecdysone, suggesting that mmy is part of a developmental genetic programme to promote a**ECM** formation.
4. Trafficking through COPII stabilises cell polarity and drives secretion during Drosophila epidermal differentiation. The differentiation of an extracellular matrix (**ECM**) at the apical side of epithelial cells implies massive polarised secretion and membrane trafficking. An epithelial cell is hence engaged in coordinating secretion and cell polarity for a correct and efficient **ECM** formation.We are studying the molecular mechanisms that Drosophila tracheal and epidermal cells deploy to form their specific apical **ECM** during differentiation. In this work we demonstrate that the two genetically identified factors haunted and ghost are essential for polarity maintenance, membrane topology as well as for secretion of the tracheal luminal matrix and the cuticle. We show that they code for the Drosophila COPII vesicle-coating components Sec23 and Sec24, respectively, that organise vesicle transport from the ER to the Golgi apparatus.Taken together, epithelial differentiation during Drosophila embryogenesis is a concerted action of **ECM** formation, plasma membrane remodelling and maintenance of cell polarity that all three rely mainly, if not absolutely, on the canonical secretory pathway from the ER over the Golgi apparatus to the plasma membrane. Our results indicate that COPII vesicles constitute a central hub for these processes.
5. The receptor-like tyrosine phosphatase lar is required for epithelial planar polarity and for axis determination within drosophila ovarian follicles. The follicle cell monolayer that encircles each developing Drosophila oocyte contributes actively to egg development and patterning, and also represents a model stem cell-derived epithelium. We have identified mutations in the receptor-like transmembrane tyrosine phosphatase Lar that disorganize follicle formation, block egg chamber elongation and disrupt Oskar localization, which is an indicator of oocyte anterior-posterior polarity. Alterations in actin filament organization correlate with these defects. Actin filaments in the basal follicle cell domain normally become polarized during stage 6 around the anterior-posterior axis defined by the polar cells, but mutations in Lar frequently disrupt polar cell differentiation and actin polarization. Lar function is only needed in somatic cells, and (for Oskar localization) its action is autonomous to posterior follicle cells. Polarity signals may be laid down by these cells within the extracellular matrix (**ECM**), possibly in the distribution of the candidate Lar ligand Laminin A, and read out at the time Oskar is localized in a Lar-dependent manner. Lar is not required autonomously to polarize somatic cell actin during stages 6. We show that Lar acts somatically early in oogenesis, during follicle formation, and postulate that it functions in germarium intercyst cells that are required for polar cell specification and differentiation. Our studies suggest that positional information can be stored transiently in the **ECM**. A major function of Lar may be to transduce such signals.
6. Talin is essential for integrin function in Drosophila. We show that the Drosophila gene rhea, isolated because its wing blister phenotype is typical of mutants affecting integrin function, encodes talin. Embryos deficient in talin have very similar phenotypes to integrin (betaPS) null embryos, including failure in germ band retraction and muscle detachment. We demonstrate that talin is not required for the presence of integrins on the cell surface or their localization at muscle termini. However, talin is required for formation of focal adhesion-like clusters of integrins on the basal surface of imaginal disc epithelia and junctional plaques between muscle and tendon cells. These results indicate that talin is essential for integrin function and acts by stably linking clusters of **ECM**-linked integrins to the cytoskeleton.
7. The conserved ADAMTS-like protein lonely heart mediates matrix formation and cardiac tissue integrity. Here we report on the identification and functional characterization of the ADAMTS-like homolog lonely heart (loh) in Drosophila melanogaster. Loh displays all hallmarks of ADAMTSL proteins including several thrombospondin type 1 repeats (TSR1), and acts in concert with the collagen Pericardin (Prc). Loss of either loh or prc causes progressive cardiac damage peaking in the abolishment of heart function. We show that both proteins are integral components of the cardiac **ECM** mediating cellular adhesion between the cardiac tube and the pericardial cells. Loss of **ECM** integrity leads to an altered myo-fibrillar organization in cardiac cells massively influencing heart beat pattern. We show evidence that Loh acts as a secreted receptor for Prc and works as a crucial determinant to allow the formation of a cell and tissue specific **ECM**, while it does not influence the accumulation of other matrix proteins like Nidogen or Perlecan. Our findings demonstrate that the function of ADAMTS-like proteins is conserved throughout evolution and reveal a previously unknown interaction of these proteins with collagens.
8. Differential expression of Dystroglycan-spliceforms with and without the mucin-like domain during Drosophila embryogenesis. Dystroglycan (DG) is a widely expressed extracellular matrix (**ECM**) receptor required for muscle viability, synaptogenesis, basement-membrane formation and epithelial development. As an integral component of the Dystrophin-associated glycoprotein complex, DG plays a central role in linking the **ECM** and the cytoskeleton. Disruption of this linkage in skeletal muscle is the underlying cause in various types of muscular dystrophies (MD). One particular type of MD is caused by alterations of O-linked glycosylation in the mucin-like domain of DG, which is required for binding of the **ECM** molecules Laminin and Perlecan. In epithelial cells, reduced expression of DG is associated with increased invasiveness of cancer cells and loss of cell polarity. Drosophila Dg is, in contrast to vertebrate Dg, subjected to differential splicing of the mRNA. Interestingly, the shorter DG splice forms lack the mucin‑like domain. Here, we describe the embryonic expression patterns of full-length DG and a short variant of DG. We find that differential splicing of Dg is developmentally regulated and tissue-specific. In some tissues, e.g., hindgut, midgut constrictions, gonads, both DG variants can be detected. For the long form, we detected specific expression at the blastoderm stage, in the epidermis and in the tracheal pits. The short form showed exclusive expression in dorsal vessel cells, chordotonal organs and dorsal median cells. In the nervous system, the long form is predominantly expressed on axons, while the short form is present on glial cells. Our findings further support the idea that DG forms lacking the mucin-like domain serve a specific function in Drosophila.

### [Ee]xtracellular [Mm]atrix

#### PubMed

1. The Alg5 ortholog Wollknäuel is essential for correct epidermal differentiation during Drosophila late embryogenesis. The formation of an **extracellular matri**x (ECM) presupposes an ordered delivery of its components to ensure its stereotypic architecture. The Drosophila cuticle is an ECM produced by the epidermis at its apical site and is characterized by a layered organization. To understand the mechanisms of cuticle assembly during development, we have investigated early aspects of protein N-glycosylation, i.e. the attachment of a dolichol-linked oligosaccharide to distinct Asn sites of a protein known to be essential for sorting in the secretory pathway. Mutations in the Drosophila alg5 gene wollknäuel (wol) that codes for an enzyme initiating the glucosylation of the dolichol-linked oligosaccharide decrease, as expected, glucosylation and the amounts of N-glycosylated proteins such as the cuticle-organizing factor Knickkopf, without affecting their correct localization. At the same time, the polarity determinants Crumbs and atypical protein kinase C accumulate at the apical plasma membrane in wol deficient embryos. In part, these perturbations may also be caused by the unfolded protein response, which is commonly triggered by ER stress and downsizes transcription and translation in general. In any case, they are associated with the loss of cuticle layering and aberrant apical plasma membrane organization suggesting that glucosylation, either directly or indirectly through controlling protein degradation, is important for the efficient and balanced deployment of the biochemical functions of secreted and membrane-associated proteins during epidermal differentiation.
2. Focal adhesion kinase is not required for integrin function or viability in Drosophila. The mammalian focal adhesion kinase (FAK) family of non-receptor protein-tyrosine kinases has been implicated in controlling a multitude of cellular responses to the engagement of cell-surface integrins and G-protein-coupled receptors. The high level of sequence conservation between the mammalian proteins and the Drosophila homologue of FAK, Fak56, suggested that it would have similar functions. However, we show here that Drosophila Fak56 is not essential for integrin functions in adhesion, migration or signaling in vivo. Furthermore, animals lacking Fak56 are viable and fertile, demonstrating that Fak56 is not essential for other developmental or physiological functions. Despite this, overexpressed Fak56 is a potent inhibitor of integrins binding to the **extracellular matrix**, suggesting that Fak56 may play a subtle role in the negative regulation of integrin adhesion.
3. An O-glycosyltransferase promotes cell adhesion during development by influencing secretion of an **extracellular matrix** integrin ligand. Protein secretion and localization are crucial during eukaryotic development, establishing local cell environments as well as mediating cell interactions, signaling, and adhesion. In this study, we demonstrate that the glycosyltransferase, pgant3, specifically modulates integrin-mediated cell adhesion by influencing the secretion and localization of the integrin ligand, Tiggrin. We demonstrate that Tiggrin is normally O-glycosylated and localized to the basal matrix where the dorsal and ventral cell layers adhere in wild type Drosophila wings. In pgant3 mutants, Tiggrin is no longer O-glycosylated and fails to be properly secreted to this basal cell layer interface, resulting in disruption of integrin-mediated cell adhesion in the wing. pgant3-mediated effects are dependent on enzymatic activity, as mutations that form a stable protein yet abrogate O-glycosyltransferase activity result in Tiggrin accumulation within the dorsal and ventral cells comprising the wing. Our results provide the first in vivo evidence for the role of O-glycosylation in the secretion of specific **extracellular matrix** proteins, thus altering the composition of the cellular "microenvironment" and thereby modulating developmentally regulated cell adhesion events. As alterations in cell adhesion are a hallmark of cancer progression, this work provides insight into the long-standing association between aberrant O-glycosylation and tumorigenesis.
4. Hormonal regulation of mummy is needed for apical **extracellular matrix** formation and epithelial morphogenesis in Drosophila. Many epithelia produce apical extracellular matrices (aECM) that are crucial for organ morphogenesis or physiology. Apical ECM formation relies on coordinated synthesis and modification of constituting components, to enable their subcellular targeting and extracellular assembly into functional matrices. The exoskeleton of Drosophila, the cuticle, is a stratified aECM containing ordered chitin polysaccharide lamellae and proteinaceous layers, and is suited for studies of molecular functions needed for aECM assembly. Here, we show that Drosophila mummy (mmy) mutants display defects in epithelial organisation in conjunction with aberrant deposition of the cuticle and an apical matrix needed for tracheal tubulogenesis. We find that mmy encodes the UDP-N-acetylglucosamine pyrophosphorylase, which catalyses the production of UDP-N-acetylglucosamine, an obligate substrate for chitin synthases as well as for protein glycosylation and GPI-anchor formation. Consequently, in mmy mutants GlcNAc-groups including chitin are severely reduced and modification and subcellular localisation of proteins designated for extracellular space is defective. Moreover, mmy expression is selectively upregulated in epithelia at the time they actively deposit aECM, and is altered by the moulting hormone 20-Hydroxyecdysone, suggesting that mmy is part of a developmental genetic programme to promote aECM formation.
5. Trafficking through COPII stabilises cell polarity and drives secretion during Drosophila epidermal differentiation. The differentiation of an **extracellular matrix** (ECM) at the apical side of epithelial cells implies massive polarised secretion and membrane trafficking. An epithelial cell is hence engaged in coordinating secretion and cell polarity for a correct and efficient ECM formation.We are studying the molecular mechanisms that Drosophila tracheal and epidermal cells deploy to form their specific apical ECM during differentiation. In this work we demonstrate that the two genetically identified factors haunted and ghost are essential for polarity maintenance, membrane topology as well as for secretion of the tracheal luminal matrix and the cuticle. We show that they code for the Drosophila COPII vesicle-coating components Sec23 and Sec24, respectively, that organise vesicle transport from the ER to the Golgi apparatus.Taken together, epithelial differentiation during Drosophila embryogenesis is a concerted action of ECM formation, plasma membrane remodelling and maintenance of cell polarity that all three rely mainly, if not absolutely, on the canonical secretory pathway from the ER over the Golgi apparatus to the plasma membrane. Our results indicate that COPII vesicles constitute a central hub for these processes.
6. The receptor-like tyrosine phosphatase lar is required for epithelial planar polarity and for axis determination within drosophila ovarian follicles. The follicle cell monolayer that encircles each developing Drosophila oocyte contributes actively to egg development and patterning, and also represents a model stem cell-derived epithelium. We have identified mutations in the receptor-like transmembrane tyrosine phosphatase Lar that disorganize follicle formation, block egg chamber elongation and disrupt Oskar localization, which is an indicator of oocyte anterior-posterior polarity. Alterations in actin filament organization correlate with these defects. Actin filaments in the basal follicle cell domain normally become polarized during stage 6 around the anterior-posterior axis defined by the polar cells, but mutations in Lar frequently disrupt polar cell differentiation and actin polarization. Lar function is only needed in somatic cells, and (for Oskar localization) its action is autonomous to posterior follicle cells. Polarity signals may be laid down by these cells within the **extracellular matrix** (ECM), possibly in the distribution of the candidate Lar ligand Laminin A, and read out at the time Oskar is localized in a Lar-dependent manner. Lar is not required autonomously to polarize somatic cell actin during stages 6. We show that Lar acts somatically early in oogenesis, during follicle formation, and postulate that it functions in germarium intercyst cells that are required for polar cell specification and differentiation. Our studies suggest that positional information can be stored transiently in the ECM. A major function of Lar may be to transduce such signals.
7. Differential expression of Dystroglycan-spliceforms with and without the mucin-like domain during Drosophila embryogenesis. Dystroglycan (DG) is a widely expressed **extracellular matrix** (ECM) receptor required for muscle viability, synaptogenesis, basement-membrane formation and epithelial development. As an integral component of the Dystrophin-associated glycoprotein complex, DG plays a central role in linking the ECM and the cytoskeleton. Disruption of this linkage in skeletal muscle is the underlying cause in various types of muscular dystrophies (MD). One particular type of MD is caused by alterations of O-linked glycosylation in the mucin-like domain of DG, which is required for binding of the ECM molecules Laminin and Perlecan. In epithelial cells, reduced expression of DG is associated with increased invasiveness of cancer cells and loss of cell polarity. Drosophila Dg is, in contrast to vertebrate Dg, subjected to differential splicing of the mRNA. Interestingly, the shorter DG splice forms lack the mucin‑like domain. Here, we describe the embryonic expression patterns of full-length DG and a short variant of DG. We find that differential splicing of Dg is developmentally regulated and tissue-specific. In some tissues, e.g., hindgut, midgut constrictions, gonads, both DG variants can be detected. For the long form, we detected specific expression at the blastoderm stage, in the epidermis and in the tracheal pits. The short form showed exclusive expression in dorsal vessel cells, chordotonal organs and dorsal median cells. In the nervous system, the long form is predominantly expressed on axons, while the short form is present on glial cells. Our findings further support the idea that DG forms lacking the mucin-like domain serve a specific function in Drosophila.
8. GBF1 (Gartenzwerg)-dependent secretion is required for Drosophila tubulogenesis. Here we report on the generation and in vivo analysis of a series of loss-of-function mutants for the Drosophila ArfGEF, Gartenzwerg. The Drosophila gene gartenzwerg (garz) encodes the orthologue of mammalian GBF1. garz is expressed ubiquitously in embryos with substantially higher abundance in cells forming diverse tubular structures such as salivary glands, trachea, proventriculus or hindgut. In the absence of functional Garz protein, the integrity of the Golgi complex is impaired. As a result, both vesicle transport of cargo proteins and directed apical membrane delivery are severely disrupted. Dysfunction of the Arf1-COPI machinery caused by a loss of Garz leads to perturbations in establishing a polarized epithelial architecture of tubular organs. Furthermore, insufficient apical transport of proteins and other membrane components causes incomplete luminal diameter expansion and deficiencies in **extracellular matrix** assembly. The fact that homologues of Garz are present in every annotated metazoan genome indicates that secretion processes mediated by the GBF-type ArfGEFs play a universal role in animal development.
9. Crag regulates epithelial architecture and polarized deposition of basement membrane proteins in Drosophila. The polarized architecture of epithelia relies on an interplay between the cytoskeleton, the trafficking machinery, and cell-cell and cell-matrix adhesion. Specifically, contact with the basement membrane (BM), an **extracellular matrix** underlying the basal side of epithelia, is important for cell polarity. However, little is known about how BM proteins themselves achieve a polarized distribution. In a genetic screen in the Drosophila follicular epithelium, we identified mutations in Crag, which encodes a conserved protein with domains implicated in membrane trafficking. Follicle cells mutant for Crag lose epithelial integrity and frequently become invasive. The loss of Crag leads to the anomalous accumulation of BM components on both sides of epithelial cells without directly affecting the distribution of apical or basolateral membrane proteins. This defect is not generally observed in mutants affecting epithelial integrity. We propose that Crag plays a unique role in organizing epithelial architecture by regulating the polarized secretion of BM proteins.
10. DWnt4 regulates cell movement and focal adhesion kinase during Drosophila ovarian morphogenesis. Cell motility is regulated by extracellular cues and by intracellular factors that accumulate at sites of contact between cells and the **extracellular matrix**. One of these factors, focal adhesion kinase (FAK), regulates the cycle of focal adhesion formation and disassembly that is required for cell movement to occur. Recently, Wnt signaling has also been implicated in the control of cell movement in vertebrates, but the mechanism through which Wnt proteins influence motility is unclear. We demonstrate that Drosphila Wnt4 is required for cell movement and FAK regulation during ovarian morphogenesis. Dfrizzled2, Disheveled, and protein kinase C are also required. The DWnt4 cell motility pathway is distinct from both the canonical Wnt pathway and the planar polarity pathway. Our data suggest that DWnt4 facilitates motility through regulation of focal adhesions.
11. Tenectin, a novel **extracellular matrix** protein expressed during Drosophila melanogaster embryonic development. During Drosophila embryonic development, various morphogenetic processes require the remodeling of the **extracellular matrix**. In a previous study, we have identified and characterized a cDNA encoding a novel putative **extracellular matrix** protein named tenebrin, in the beetle Tenebrio molitor. Here, we examine the expression of the Drosophila ortholog, referred to as Tenectin (Tnc), during embryonic development. Tnc is expressed in the majority of tissues of neuroectodermic origin such as hindgut, foregut, tracheal system, anal plate, and CNS. In the CNS, the Tnc transcript is restricted to a few cells, whereas the protein is located in the dorsal part of the axonal tracts. In the hindgut and the trachea, Tnc protein is expressed on the apical pole of the cells. Tnc is an **extracellular matrix** protein secreted in a polarized way in different organs of Drosophila embryos.

### [Ii]nvasive

#### PubMed

1. Socs36E attenuates STAT signaling to optimize motile cell specification in the Drosophila ovary. The Janus kinase/Signal transducers and activators of transcription (JAK/STAT) pathway determines cell fates by regulating gene expression. One example is the specification of the motile cells called border cells during Drosophila oogenesis. It has been established that too much or too little STAT activity disrupts follicle cell identity and cell motility, which suggests the signaling must be precisely regulated. Here, we find that Suppressor of cytokine signaling at 36E (Socs36E) is a necessary negative regulator of JAK/STAT signaling during border cell specification. We find when STAT signaling is too low to induce migration in the presumptive border cell population, nearby follicle cells uncharacteristically become **invasive** to enable efficient migration of the cluster. We generated a genetic null allele that reveals Socs36E is required in the anterior follicle cells to limit **invasive** behavior to an optimal number of cells. We further show Socs36E genetically interacts with the required STAT feedback inhibitor apontic (apt) and APT's downstream target, mir-279, and provide evidence that suggests APT directly regulates Socs36E transcriptionally. Our work shows Socs36E plays a critical role in a genetic circuit that establishes a boundary between the motile border cell cluster and its non-**invasive** epithelial neighbors through STAT attenuation.
2. The actin cross-linker Filamin/Cheerio mediates tumor malignancy downstream of JNK signaling. Cell shape dynamics, motility, and cell proliferation all depend on the actin cytoskeleton. Malignant cancer cells hijack the actin network to grow and migrate to secondary sites. Understanding the function of actin regulators is therefore of major interest. In the present study, we identify the actin cross-linking protein Filamin/Cheerio (Cher) as a mediator of malignancy in genetically defined Drosophila tumors. We show that in **invasive** tumors, resulting from cooperation of activated Ras with disrupted epithelial cell polarity, Cher is upregulated in a Jun N-terminal kinase (JNK)-dependent manner. Although dispensable in normal epithelium, Cher becomes required in the tumor cells for their growth and **invasive**ness. When deprived of Cher, these tumor clones lose their full potential to proliferate and breach tissue boundaries. Instead, the Cher-deficient clones remain confined within the limits of their source epithelium, permitting survival of the host animal. Through interaction with the myosin II heavy chain subunit, Cher is likely to strengthen the cortical actomyosin network and reinforce mechanical tension within the **invasive** tumors. Accordingly, Cher is required for aberrant expression of genes downstream of the Hippo/Yorkie signaling in the tumor tissue. Our study identifies Cher as a new target of JNK signaling that links cytoskeleton dynamics to tumor progression.
3. Extrinsic and intrinsic mechanisms directing epithelial cell sheet replacement during Drosophila metamorphosis. The fusion of epithelial sheets is an essential morphogenetic event. Here, we study the development of the abdomen of Drosophila as a model of bounded epithelia expansion and uncover a complex multistep process for the generation of the adult epidermis from histoblasts, founder cells that replace the larval cells during metamorphosis. We find that histoblasts experience a biphasic cell cycle and emit apical projections that direct their **invasive** planar intercalation in between larval cells. Coordinately, the larval cells extrude from the epithelia by apical constriction of an actomyosin ring and as a consequence die by apoptosis and are removed by circulating haemocytes. We demonstrate that the proliferation of histoblasts and the death of larval cells are triggered by two independent extrinsic Ecdysone hormonal pulses. Finally, we show that histoblast spreading and the death of larval cells depend on a mutual exchange of signals and are non-autonomous processes.
4. Microtubules and Lis-1/NudE/dynein regulate **invasive** cell-on-cell migration in Drosophila. The environment through which cells migrate in vivo differs considerably from the in vitro environment where cell migration is often studied. In vivo many cells migrate in crowded and complex 3-dimensional tissues and may use other cells as the substratum on which they move. This includes neurons, glia and their progenitors in the brain. Here we use a Drosophila model of **invasive**, collective migration in a cellular environment to investigate the roles of microtubules and microtubule regulators in this type of cell movement. Border cells are of epithelial origin and have no visible microtubule organizing center (MTOC). Interestingly, microtubule plus-end growth was biased away from the leading edge. General perturbation of the microtubule cytoskeleton and analysis by live imaging showed that microtubules in both the migrating cells and the substrate cells affect movement. Also, whole-tissue and cell autonomous deletion of the microtubule regulator Stathmin had distinct effects. A screen of 67 genes encoding microtubule interacting proteins uncovered cell autonomous requirements for Lis-1, NudE and Dynein in border cell migration. Net cluster migration was decreased, with initiation of migration and formation of dominant front cell protrusion being most dramatically affected. Organization of cells within the cluster and localization of cell-cell adhesion molecules were also abnormal. Given the established role of Lis-1 in migrating neurons, this could indicate a general role of Lis-1/NudE, Dynein and microtubules, in cell-on-cell migration. Spatial regulation of cell-cell adhesion may be a common theme, consistent with observing both cell autonomous and non-autonomous requirements in both systems.
5. Differential expression of Dystroglycan-spliceforms with and without the mucin-like domain during Drosophila embryogenesis. Dystroglycan (DG) is a widely expressed extracellular matrix (ECM) receptor required for muscle viability, synaptogenesis, basement-membrane formation and epithelial development. As an integral component of the Dystrophin-associated glycoprotein complex, DG plays a central role in linking the ECM and the cytoskeleton. Disruption of this linkage in skeletal muscle is the underlying cause in various types of muscular dystrophies (MD). One particular type of MD is caused by alterations of O-linked glycosylation in the mucin-like domain of DG, which is required for binding of the ECM molecules Laminin and Perlecan. In epithelial cells, reduced expression of DG is associated with increased **invasive**ness of cancer cells and loss of cell polarity. Drosophila Dg is, in contrast to vertebrate Dg, subjected to differential splicing of the mRNA. Interestingly, the shorter DG splice forms lack the mucin‑like domain. Here, we describe the embryonic expression patterns of full-length DG and a short variant of DG. We find that differential splicing of Dg is developmentally regulated and tissue-specific. In some tissues, e.g., hindgut, midgut constrictions, gonads, both DG variants can be detected. For the long form, we detected specific expression at the blastoderm stage, in the epidermis and in the tracheal pits. The short form showed exclusive expression in dorsal vessel cells, chordotonal organs and dorsal median cells. In the nervous system, the long form is predominantly expressed on axons, while the short form is present on glial cells. Our findings further support the idea that DG forms lacking the mucin-like domain serve a specific function in Drosophila.
6. Bazooka is a permissive factor for the **invasive** behavior of discs large tumor cells in Drosophila ovarian follicular epithelia. Drosophila Bazooka and atypical protein kinase C are essential for epithelial polarity and adhesion. We show here that wild-type bazooka function is required during cell invasion of epithelial follicle cells mutant for the tumor suppressor discs large. Clonal studies indicate that follicle cell Bazooka acts as a permissive factor during cell invasion, possibly by stabilizing adhesion between the invading somatic cells and their substratum, the germline cells. Genetic epistasis experiments demonstrate that bazooka acts downstream of discs large in tumor cell invasion. In contrast, during the migration of border cells, Bazooka function is dispensable for cell invasion and motility, but rather is required cell-autonomously in mediating cell adhesion within the migrating border cell cluster. Taken together, these studies reveal Bazooka functions distinctly in different types of **invasive** behaviors of epithelial follicle cells, potentially by regulating adhesion between follicle cells or between follicle cells and their germline substratum.
7. Crag regulates epithelial architecture and polarized deposition of basement membrane proteins in Drosophila. The polarized architecture of epithelia relies on an interplay between the cytoskeleton, the trafficking machinery, and cell-cell and cell-matrix adhesion. Specifically, contact with the basement membrane (BM), an extracellular matrix underlying the basal side of epithelia, is important for cell polarity. However, little is known about how BM proteins themselves achieve a polarized distribution. In a genetic screen in the Drosophila follicular epithelium, we identified mutations in Crag, which encodes a conserved protein with domains implicated in membrane trafficking. Follicle cells mutant for Crag lose epithelial integrity and frequently become **invasive**. The loss of Crag leads to the anomalous accumulation of BM components on both sides of epithelial cells without directly affecting the distribution of apical or basolateral membrane proteins. This defect is not generally observed in mutants affecting epithelial integrity. We propose that Crag plays a unique role in organizing epithelial architecture by regulating the polarized secretion of BM proteins.
8. Paracrine signaling through the JAK/STAT pathway activates **invasive** behavior of ovarian epithelial cells in Drosophila. The JAK/STAT signaling pathway, renowned for its effects on cell proliferation and survival, is constitutively active in various human cancers, including ovarian. We have found that JAK and STAT are required to convert the border cells in the Drosophila ovary from stationary, epithelial cells to migratory, **invasive** cells. The ligand for this pathway, Unpaired (UPD), is expressed by two central cells within the migratory cell cluster. Mutations in upd or jak cause defects in migration and a reduction in the number of cells recruited to the cluster. Ectopic expression of either UPD or JAK is sufficient to induce extra epithelial cells to migrate. Thus, a localized signal activates the JAK/STAT pathway in neighboring epithelial cells, causing them to become **invasive**.
9. Requirement for Par-6 and Bazooka in Drosophila border cell migration. Polarized epithelial cells convert into migratory **invasive** cells during a number of developmental processes, as well as when tumors metastasize. Much has been learned recently concerning the molecules and mechanisms that are responsible for generating and maintaining epithelial cell polarity. However, less is known about what becomes of epithelial polarity proteins when various cell types become migratory and **invasive**. Here, we report the localization of several apical epithelial proteins, Par-6, Par-3/Bazooka and aPKC, during border cell migration in the Drosophila ovary. All of these proteins remained asymmetrically distributed throughout migration. Moreover, depletion of either Par-6 or Par-3/Bazooka by RNAi resulted in disorganization of the border cell cluster and impaired migration. The distributions of several transmembrane proteins required for migration were abnormal following Par-6 or Par-3/Bazooka downregulation, possibly accounting for the migration defects. Taken together, these results indicate that cells need not lose apical/basal polarity in order to invade neighboring tissues and in some cases even require such polarity for proper motility.
10. A Fasciclin 2 morphogenetic switch organizes epithelial cell cluster polarity and motility. Little is known about how intercellular communication is regulated in epithelial cell clusters to control delamination and migration. We investigate this problem using Drosophila border cells as a model. We find that just preceding cell cluster delamination, expression of transmembrane immunoglobulin superfamily member, Fasciclin 2, is lost in outer border cells, but not in inner polar cells of the cluster. Loss of Fasciclin 2 expression in outer border cells permits a switch in Fasciclin 2 polarity in the inner polar cells. This polarity switch, which is organized in collaboration with neoplastic tumor suppressors Discs large and Lethal-giant-larvae, directs cluster asymmetry essential for timing delamination from the epithelium. Fas2-mediated communication between polar and border cells maintains localization of Discs large and Lethal-giant-larvae in border cells to inhibit the rate of cluster migration. These findings are the first to show how a switch in cell adhesion molecule polarity regulates asymmetry and delamination of an epithelial cell cluster. The finding that Discs large and Lethal-giant-larvae inhibit the rate of normal cell cluster movement suggests that their loss in metastatic tumors may directly contribute to tumor motility. Furthermore, our results provide novel insight into the intimate link between epithelial polarity and acquisition of motile polarity that has important implications for development of **invasive** carcinomas.
11. Function of the ETS transcription factor Yan in border cell migration. **Invasive** cell migration in both normal development and metastatic cancer is regulated by various signaling pathways, transcription factors and cell-adhesion molecules. The coordination between these activities in the context of cell migration is poorly understood. During Drosophila oogenesis, a small group of cells called border cells exit the follicular epithelium to perform a stereotypic, **invasive** migration. We find that the ETS transcription factor Yan is required for border cell migration and that Yan expression is spatiotemporally regulated as border cells migrate from the anterior pole of the egg chamber towards the nurse cell-oocyte boundary. Yan expression is dependent on inputs from the JAK/STAT, Notch and Receptor Tyrosine Kinase pathways in border cells. Mechanistically, Yan functions to modulate the turnover of DE-Cadherin-dependent adhesive complexes to facilitate border cell migration. Our results suggest that Yan acts as a pivotal link between signal transduction, cell adhesion and **invasive** cell migration in Drosophila border cells.
12. Systematic analysis of the transcriptional switch inducing migration of border cells. Cell migration within a natural context is tightly controlled, often by specific transcription factors. However, the switch from stationary to migratory behavior is poorly understood. Border cells perform a spatially and temporally controlled **invasive** migration during Drosophila oogenesis. Slbo, a C/EBP family transcriptional activator, is required for them to become migratory. We purified wild-type and slbo mutant border cells as well as nonmigratory follicle cells and performed comparative whole-genome expression profiling, followed by functional tests of the contributions of identified targets to migration. About 300 genes were significantly upregulated in border cells, many dependent on Slbo. Among these, the microtubule regulator Stathmin was strongly upregulated and was required for normal migration. Actin cytoskeleton regulators were also induced, including, surprisingly, a large cluster of "muscle-specific" genes. We conclude that Slbo induces multiple cytoskeletal effectors, and that each contributes to the behavioral changes in border cells.
13. Asymmetric localisation of cytokine mRNA is essential for JAK/STAT activation during cell **invasive**ness. The transition from immotile epithelial cells to migrating cells occurs in all organisms during normal embryonic development, as well as during tumour metastasis. During Drosophila oogenesis, border cells (BCs) are recruited and delaminate from the follicular epithelium. This process is triggered by the polar cells (PCs), which secrete the cytokine Unpaired (Upd) and activate the JAK/STAT pathway in neighbouring cells, turning them into **invasive** BCs. Interestingly, either a decrease or an increase in BC number alters migration, indicating that mechanisms controlling the level of JAK/STAT signalling are crucial in this process. Here, we show that PCs have a highly stable and polarised network of microtubules along which upd transcripts are asymmetrically transported in a Dynein-dependent manner. We demonstrate that in the absence of upd mRNA localisation the ligand is no longer efficiently secreted, leading to a loss of signalling strength as well as recruitment and migration defects. These findings reveal a novel post-transcriptional regulatory mechanism of JAK/STAT signalling in the control of epithelial cell **invasive**ness.

### [Mm]etastasize

#### PubMed

1. Requirement for Par-6 and Bazooka in Drosophila border cell migration. Polarized epithelial cells convert into migratory invasive cells during a number of developmental processes, as well as when tumors **metastasize**. Much has been learned recently concerning the molecules and mechanisms that are responsible for generating and maintaining epithelial cell polarity. However, less is known about what becomes of epithelial polarity proteins when various cell types become migratory and invasive. Here, we report the localization of several apical epithelial proteins, Par-6, Par-3/Bazooka and aPKC, during border cell migration in the Drosophila ovary. All of these proteins remained asymmetrically distributed throughout migration. Moreover, depletion of either Par-6 or Par-3/Bazooka by RNAi resulted in disorganization of the border cell cluster and impaired migration. The distributions of several transmembrane proteins required for migration were abnormal following Par-6 or Par-3/Bazooka downregulation, possibly accounting for the migration defects. Taken together, these results indicate that cells need not lose apical/basal polarity in order to invade neighboring tissues and in some cases even require such polarity for proper motility.

### [Mm]etastasis

#### PubMed

1. Cellular and molecular mechanisms of single and collective cell migrations in Drosophila: themes and variations. The process of cell migration is essential throughout life, driving embryonic morphogenesis and ensuring homeostasis in adults. Defects in cell migration are a major cause of human disease, with excessive migration causing autoimmune diseases and cancer **metastasis**, whereas reduced capacity for migration leads to birth defects and immunodeficiencies. Myriad studies in vitro have established a consensus view that cell migrations require cell polarization, Rho GTPase-mediated cytoskeletal rearrangements, and myosin-mediated contractility. However, in vivo studies later revealed a more complex picture, including the discovery that cells migrate not only as single units but also as clusters, strands, and sheets. In particular, the role of E-Cadherin in cell motility appears to be more complex than previously appreciated. Here, we discuss recent advances achieved by combining the plethora of genetic tools available to the Drosophila geneticist with live imaging and biophysical techniques. Finally, we discuss the emerging themes such studies have revealed and ponder the puzzles that remain to be solved.
2. Planar cell polarity signaling in collective cell movements during morphogenesis and disease. Collective and directed cell movements are crucial for diverse developmental processes in the animal kingdom, but they are also involved in wound repair and disease. During these processes groups of cells are oriented within the tissue plane, which is referred to as planar cell polarity (PCP). This requires a tight regulation that is in part conducted by the PCP pathway. Although this pathway was initially characterized in flies, subsequent studies in vertebrates revealed a set of conserved core factors but also effector molecules and signal modulators, which build the fundamental PCP machinery. The PCP pathway in Drosophila regulates several developmental processes involving collective cell movements such as border cell migration during oogenesis, ommatidial rotation during eye development, and embryonic dorsal closure. During vertebrate embryogenesis, PCP signaling also controls collective and directed cell movements including convergent extension during gastrulation, neural tube closure, neural crest cell migration, or heart morphogenesis. Similarly, PCP signaling is linked to processes such as wound repair, and cancer invasion and **metastasis** in adults. As a consequence, disruption of PCP signaling leads to pathological conditions. In this review, we will summarize recent findings about the role of PCP signaling in collective cell movements in flies and vertebrates. In addition, we will focus on how studies in Drosophila have been relevant to our understanding of the PCP molecular machinery and will describe several developmental defects and human disorders in which PCP signaling is compromised. Therefore, new discoveries about the contribution of this pathway to collective cell movements could provide new potential diagnostic and therapeutic targets for these disorders.
3. Identification of mutations that cause cell migration defects in mosaic clones. Cell movement is an important feature of animal development, wound healing and tumor **metastasis**; however, the mechanisms underlying cell motility remain to be elucidated. To further our understanding, it would be useful to identify all of the proteins that are essential for a cell to migrate, yet such information is not currently available for any cell type. We have carried out a screen for mutations affecting border cell migration in Drosophila. Mutations that cause defects in mosaic clones were identified, so that genes that are also required for viability could be detected. From 6000 mutagenized lines, 20 mutations on chromosome 2R were isolated that cause defects in border cell position. One of the mutations was dominant while all of the recessive mutations appeared to be homozygous lethal. This lethality was used to place the mutations into 16 complementation groups. Many of the mutations failed to complement cytologically characterized deficiencies, allowing their rapid mapping. Mutations in three loci altered expression of a marker gene in the border cells, whereas the remaining mutations did not. One mutation, which caused production of supernumerary border cells, was found to disrupt the costal-2 locus, indicating a role for Hedgehog signaling in border cell development. This screen identified many new loci required for border cell migration and our results suggest that this is a useful approach for elucidating the mechanisms involved in cell motility.
4. Regulation of cell adhesion and collective cell migration by hindsight and its human homolog RREB1. Cell movements represent a major driving force in embryonic development, tissue repair, and tumor **metastasis** [1]. The migration of single cells has been well studied, predominantly in cell culture [2, 3]; however, in vivo, a greater variety of modes of cell movement occur, including the movements of cells in clusters, strands, sheets, and tubes, also known as collective cell migrations [4, 5]. In spite of the relevance of these types of movements in both normal and pathological conditions, the molecular mechanisms that control them remain predominantly unknown. Epithelial follicle cells of the Drosophila ovary undergo several dynamic morphological changes, providing a genetically tractable model [6]. We found that anterior follicle cells, including border cells, mutant for the gene hindsight (hnt) accumulated excess cell-cell adhesion molecules and failed to undergo their normal collective movements. In addition, HNT affected border cell cluster cohesion and motility via effects on the JNK and STAT pathways, respectively. Interestingly, reduction of expression of the mammalian homolog of HNT, RREB1, by siRNA inhibited collective cell migration in a scratch-wound healing assay of MCF10A mammary epithelial cells, suppressed surface activity, retarded cell spreading after plating, and led to the formation of immobile, tightly adherent cell colonies. We propose that HNT and RREB1 are essential to reduce cell-cell adhesion when epithelial cells within an interconnected group undergo dynamic changes in cell shape.
5. Jing: a downstream target of slbo required for developmental control of border cell migration. Epithelial to mesenchymal transitions and cell migration are important features of embryonic development and tumor **metastasis**. We are employing a systematic genetic approach to study the border cells in the Drosophila ovary, as a simple model for these cellular behaviors. Previously we found that expression of the basic-region/leucine zipper transcription factor, C/EBP, is required for the border cells to initiate their migration. Here we report the identification of a second nuclear factor, named JING (which means 'still'), that is required for initiation of border cell migration. The jing locus was identified in a screen for mutations that cause border cell migration defects in mosaic clones. The jing mutant phenotype resembles that of slbo mutations, which disrupt the Drosophila C/EBP gene, but is distinct from other classes of border cell migration mutants. Expression of a jing-lacZ reporter in border cells requires C/EBP. Moreover, expression of jing from a heat-inducible promoter rescues the border cell migration defects of hypomorphic slbo mutants. The JING protein is most closely related to a mouse protein, AEBP2, which was identified on the basis of its ability to bind a small regulatory sequence within the adipocyte AP2 gene to which mammalian C/EBP also binds. We propose that the need to coordinate cell differentiation with nutritional status may be the link between mammalian adipocytes and Drosophila border cells that led to the conservation of C/EBP and AEBP2.
6. Asymmetric localisation of cytokine mRNA is essential for JAK/STAT activation during cell invasiveness. The transition from immotile epithelial cells to migrating cells occurs in all organisms during normal embryonic development, as well as during tumour **metastasis**. During Drosophila oogenesis, border cells (BCs) are recruited and delaminate from the follicular epithelium. This process is triggered by the polar cells (PCs), which secrete the cytokine Unpaired (Upd) and activate the JAK/STAT pathway in neighbouring cells, turning them into invasive BCs. Interestingly, either a decrease or an increase in BC number alters migration, indicating that mechanisms controlling the level of JAK/STAT signalling are crucial in this process. Here, we show that PCs have a highly stable and polarised network of microtubules along which upd transcripts are asymmetrically transported in a Dynein-dependent manner. We demonstrate that in the absence of upd mRNA localisation the ligand is no longer efficiently secreted, leading to a loss of signalling strength as well as recruitment and migration defects. These findings reveal a novel post-transcriptional regulatory mechanism of JAK/STAT signalling in the control of epithelial cell invasiveness.

### [Cc]ell [Ii]nvasion

#### PubMed

1. Bazooka is a permissive factor for the invasive behavior of discs large tumor cells in Drosophila ovarian follicular epithelia. Drosophila Bazooka and atypical protein kinase C are essential for epithelial polarity and adhesion. We show here that wild-type bazooka function is required during **cell invasion** of epithelial follicle cells mutant for the tumor suppressor discs large. Clonal studies indicate that follicle cell Bazooka acts as a permissive factor during **cell invasion**, possibly by stabilizing adhesion between the invading somatic cells and their substratum, the germline cells. Genetic epistasis experiments demonstrate that bazooka acts downstream of discs large in tumor **cell invasion**. In contrast, during the migration of border cells, Bazooka function is dispensable for **cell invasion** and motility, but rather is required cell-autonomously in mediating cell adhesion within the migrating border cell cluster. Taken together, these studies reveal Bazooka functions distinctly in different types of invasive behaviors of epithelial follicle cells, potentially by regulating adhesion between follicle cells or between follicle cells and their germline substratum.
2. Inhibition of patterned cell shape change and **cell invasion** by Discs large during Drosophila oogenesis. Drosophila Discs large (Dlg) is a tumor suppressor gene whose loss in epithelial tissues causes disrupted cell polarity and increased cell proliferation. A human Dlg homolog, hDlg, has been implicated in tumorigenic processes via its association with the product of the Adenomatous Polyposis Coli (APC) gene. We show for the first time that Drosophila Dlg is required to block **cell invasion**. Loss of dlg activity during oogenesis causes follicle cells to change shape and invade in a pattern similar to border cells, a small population of cells that break from the post-mitotic follicular epithelium during wild-type oogenesis, yet dlg mutant cells have not adopted a border cell fate. Both functional and morphological evidence indicates that cooperation between germ cell and follicle cell Dlg, probably mediated by Dlg PDZ domains, is crucial for regulating cell mixing, suggesting a novel developmental mechanism and mode of action for the Dlg family of molecules. These findings suggest that Dlg does not simply inhibit individual cell behaviors during oogenesis, but rather acts in a developmental pathway essential for blocking cell proliferation and migration in a spatio-temporally defined manner. A model for Dlg action in blocking **cell invasion** is presented.

---

## babos

- NCBI Gene
- GeneRIF
- Pubmed

| Search Term | Rank | Fields |
| --- | --- | --- |
| [Mm]igration | 6 | PubMed(2) |

  
hide/show details for babos

### [Mm]igration

#### PubMed

1. A misexpression screen to identify regulators of Drosophila larval hemocyte development. In Drosophila, defense against foreign pathogens is mediated by an effective innate immune system, the cellular arm of which is composed of circulating hemocytes that engulf bacteria and encapsulate larger foreign particles. Three hemocyte types occur: plasmatocytes, crystal cells, and lamellocytes. The most abundant larval hemocyte type is the plasmatocyte, which is responsible for phagocytosis and is present either in circulation or in adherent sessile domains under the larval cuticle. The mechanisms controlling differentiation of plasmatocytes and their **migration** toward these sessile compartments are unclear. To address these questions we have conducted a misexpression screen using the plasmatocyte-expressed GAL4 driver Peroxidasin-GAL4 (Pxn-GAL4) and existing enhancer-promoter (EP) and EP yellow (EY) transposon libraries to systematically misexpress approximately 20% of Drosophila genes in larval hemocytes. The Pxn-GAL4 strain also contains a UAS-GFP reporter enabling hemocyte phenotypes to be visualized in the semitransparent larvae. Among 3412 insertions screened we uncovered 101 candidate hemocyte regulators. Some of these are known to control hemocyte development, but the majority either have no characterized function or are proteins of known function not previously implicated in hemocyte development. We have further analyzed three candidate genes for changes in hemocyte morphology, cell-cell adhesion properties, phagocytosis activity, and melanotic tumor formation.
2. Gene expression profiling of Drosophila tracheal fusion cells. The Drosophila trachea is a premier genetic system to investigate the fundamental mechanisms of tubular organ formation. Tracheal fusion cells lead the branch fusion process to form an interconnected tubular network. Therefore, fusion cells in the Drosophila trachea will be an excellent model to study branch fusion in mammalian tubular organs, such as kidneys and blood vessels. The fusion process is a dynamic cellular process involving cell **migration**, adhesion, vesicle trafficking, cytoskeleton rearrangement, and membrane fusion. To understand how these cellular events are coordinated, we initiated the critical step to assemble a gene expression profile of fusion cells. For this study, we analyzed the expression of 234 potential tracheal-expressed genes in fusion cells during fusion cell development. 143 Tracheal genes were found to encode transcription factors, signal proteins, cytoskeleton and matrix proteins, transporters, and proteins with unknown function. These genes were divided into four subgroups based on their levels of expression in fusion cells compared to neighboring non-fusion cells revealed by in situ hybridization: (1) genes that have relative high abundance in fusion cells, (2) genes that are dynamically expressed in fusion cells, (3) genes that have relative low abundance in fusion cells, and (4) genes that are expressed at similar levels in fusion cells and non-fusion tracheal cells. This study identifies the expression profile of fusion cells and hypothetically suggests genes which are necessary for the fusion process and which play roles in distinct stages of fusion, as indicated by the location and timing of expression. These data will provide the basis for a comprehensive understanding of the molecular and cellular mechanisms of branch fusion.

---

## RhoGAP18B

- NCBI Gene
- GeneRIF
- Pubmed

| Search Term | Rank | Fields |
| --- | --- | --- |
| [Mm]igration | 6 | PubMed(2) |

  
hide/show details for RhoGAP18B

### [Mm]igration

#### PubMed

1. Gene expression profiling of Drosophila tracheal fusion cells. The Drosophila trachea is a premier genetic system to investigate the fundamental mechanisms of tubular organ formation. Tracheal fusion cells lead the branch fusion process to form an interconnected tubular network. Therefore, fusion cells in the Drosophila trachea will be an excellent model to study branch fusion in mammalian tubular organs, such as kidneys and blood vessels. The fusion process is a dynamic cellular process involving cell **migration**, adhesion, vesicle trafficking, cytoskeleton rearrangement, and membrane fusion. To understand how these cellular events are coordinated, we initiated the critical step to assemble a gene expression profile of fusion cells. For this study, we analyzed the expression of 234 potential tracheal-expressed genes in fusion cells during fusion cell development. 143 Tracheal genes were found to encode transcription factors, signal proteins, cytoskeleton and matrix proteins, transporters, and proteins with unknown function. These genes were divided into four subgroups based on their levels of expression in fusion cells compared to neighboring non-fusion cells revealed by in situ hybridization: (1) genes that have relative high abundance in fusion cells, (2) genes that are dynamically expressed in fusion cells, (3) genes that have relative low abundance in fusion cells, and (4) genes that are expressed at similar levels in fusion cells and non-fusion tracheal cells. This study identifies the expression profile of fusion cells and hypothetically suggests genes which are necessary for the fusion process and which play roles in distinct stages of fusion, as indicated by the location and timing of expression. These data will provide the basis for a comprehensive understanding of the molecular and cellular mechanisms of branch fusion.
2. Transcriptional control in embryonic Drosophila midline guidance assessed through a whole genome approach. During the development of the Drosophila central nervous system the process of midline crossing is orchestrated by a number of guidance receptors and ligands. Many key axon guidance molecules have been identified in both invertebrates and vertebrates, but the transcriptional regulation of growth cone guidance remains largely unknown. It is established that translational regulation plays a role in midline crossing, and there are indications that transcriptional regulation is also involved. To investigate this issue, we conducted a genome-wide study of transcription in Drosophila embryos using wild type and a number of well-characterized Drosophila guidance mutants and transgenics. We also analyzed a previously published microarray time course of Drosophila embryonic development with an axon guidance focus.Using hopach, a novel clustering method which is well suited to microarray data analysis, we identified groups of genes with similar expression patterns across guidance mutants and transgenics. We then systematically characterized the resulting clusters with respect to their relevance to axon guidance using two complementary controlled vocabularies: the Gene Ontology (GO) and anatomical annotations of the Atlas of Pattern of Gene Expression (APoGE) in situ hybridization database. The analysis indicates that regulation of gene expression does play a role in the process of axon guidance in Drosophila. We also find a strong link between axon guidance and hemocyte **migration**, a result that agrees with mounting evidence that axon guidance molecules are co-opted in vertebrate vascularization. Cell cyclin activity in the context of axon guidance is also suggested from our array data. RNA and protein expression patterns of cell cyclins in axon guidance mutants and transgenics support this possible link.This study provides important insights into the regulation of axon guidance in vivo.

---

## bbg

- NCBI Gene
- GeneRIF
- Pubmed

| Search Term | Rank | Fields |
| --- | --- | --- |
| [Mm]igration | 6 | Process(1); PubMed(1) |
| [Ii]nvasive | 10 | PubMed(1) |

  
hide/show details for bbg

### [Mm]igration

#### Process

1. border follicle cell **migration**

#### PubMed

1. On the role of PDZ domain-encoding genes in Drosophila border cell **migration**. Cells often move as collective groups during normal embryonic development and wound healing, although the mechanisms governing this type of **migration** are poorly understood. The Drosophila melanogaster border cells migrate as a cluster during late oogenesis and serve as a powerful in vivo genetic model for collective cell **migration**. To discover new genes that participate in border cell **migration**, 64 out of 66 genes that encode PDZ domain-containing proteins were systematically targeted by in vivo RNAi knockdown. The PDZ domain is one of the largest families of protein-protein interaction domains found in eukaryotes. Proteins that contain PDZ domains participate in a variety of biological processes, including signal transduction and establishment of epithelial apical-basal polarity. Targeting PDZ proteins effectively assesses a larger number of genes via the protein complexes and pathways through which these proteins function. par-6, a known regulator of border cell **migration**, was a positive hit and thus validated the approach. Knockdown of 14 PDZ domain genes disrupted **migration** with multiple RNAi lines. The candidate genes have diverse predicted cellular functions and are anticipated to provide new insights into the mechanisms that control border cell movement. As a test of this concept, two genes that disrupted **migration** were characterized in more detail: big bang and the Dlg5 homolog CG6509. We present evidence that Big bang regulates JAK/STAT signaling, whereas Dlg5/CG6509 maintains cluster cohesion. Moreover, these results demonstrate that targeting a selected class of genes by RNAi can uncover novel regulators of collective cell **migration**.

### [Ii]nvasive

#### PubMed

1. The multilayered innate immune defense of the gut. In the wild, the fruit fly Drosophila melanogaster thrives on rotten fruit. The digestive tract maintains a powerful gut immune barrier to regulate the ingested microbiota, including entomopathogenic bacteria. This gut immune barrier includes a chitinous peritrophic matrix that isolates the gut contents from the epithelial cells. In addition, the epithelial cells are tightly sealed by septate junctions and can mount an inducible immune response. This local response can be activated by **invasive** bacteria, or triggered by commensal bacteria in the gut lumen. As with chronic inflammation in mammals, constitutive activation of the gut innate immune response is detrimental to the health of flies. Accordingly, the Drosophila gut innate immune response is tightly regulated to maintain the endogenous microbiota, while preventing infections by pathogenic microorganisms.

---

## drpr

- NCBI Gene
- GeneRIF
- Pubmed

| Search Term | Rank | Fields |
| --- | --- | --- |
| [Mm]igration | 6 | PubMed(4) |
| [Ee]xtracellular [Mm]atrix | 8 | PubMed(1) |
| [Mm]etastasis | 12 | PubMed(2) |

  
hide/show details for drpr

### [Mm]igration

#### PubMed

1. Polarization of the epithelial layer and apical localization of integrins are required for engulfment of apoptotic cells in the Drosophila ovary. Inefficient clearance of dead cells or debris by epithelial cells can lead to or exacerbate debilitating conditions such as retinitis pigmentosa, macular degeneration, chronic obstructive pulmonary disease and asthma. Despite the importance of engulfment by epithelial cells, little is known about the molecular changes that are required within these cells. The misregulation of integrins has previously been associated with disease states, suggesting that a better understanding of the regulation of receptor trafficking could be key to treating diseases caused by defects in phagocytosis. Here, we demonstrate that the integrin heterodimer αPS3/βPS becomes apically enriched and is required for engulfment by the epithelial follicle cells of the Drosophila ovary. We found that integrin heterodimer localization and function is largely directed by the α-subunit. Moreover, proper cell polarity promotes asymmetric integrin enrichment, suggesting that αPS3/βPS trafficking occurs in a polarized fashion. We show that several genes previously known for their roles in trafficking and **cell migr**ation are also required for engulfment. Moreover, as in mammals, the same α-integrin subunit is required by professional and non-professional phagocytes and migrating cells in Drosophila. Our findings suggest that migrating and engulfing cells use common machinery, and demonstrate a crucial role for integrin function and polarized trafficking of integrin subunits during engulfment. This study also establishes the epithelial follicle cells of the Drosophila ovary as a powerful model for understanding the molecular changes required for engulfment by a polarized epithelium.
2. Unwrapping glial biology: Gcm target genes regulating glial development, diversification, and function. Glia are the most abundant cell type in the mammalian brain. They regulate neuronal development and function, CNS immune surveillance, and stem cell biology, yet we know surprisingly little about glia in any organism. Here we identify over 40 new Drosophila glial genes. We use glial cells missing (gcm) mutants and misexpression to verify they are Gcm regulated in vivo. Many genes show unique spatiotemporal responsiveness to Gcm in the CNS, and thus glial subtype diversification requires spatially or temporally restricted Gcm cofactors. These genes provide insights into glial biology: we show unc-5 (a repulsive netrin receptor) orients glial **migration**s and the draper gene mediates glial engulfment of apoptotic neurons and larval locomotion. Many identified Drosophila glial genes have homologs expressed in mammalian glia, revealing conserved molecular features of glial cells. 80% of these Drosophila glial genes have mammalian homologs; these are now excellent candidates for regulating human glial development, function, or disease.
3. Comparative analysis of Hox downstream genes in Drosophila. Functional diversification of body parts is dependent on the formation of specialized structures along the various body axes. In animals, region-specific morphogenesis along the anteroposterior axis is controlled by a group of conserved transcription factors encoded by the Hox genes. Although it has long been assumed that Hox proteins carry out their function by regulating distinct sets of downstream genes, only a small number of such genes have been found, with very few having direct roles in controlling cellular behavior. We have quantitatively identified hundreds of Hox downstream genes in Drosophila by microarray analysis, and validated many of them by in situ hybridizations on loss- and gain-of-function mutants. One important finding is that Hox proteins, despite their similar DNA-binding properties in vitro, have highly specific effects on the transcriptome in vivo, because expression of many downstream genes respond primarily to a single Hox protein. In addition, a large fraction of downstream genes encodes realizator functions, which directly affect morphogenetic processes, such as orientation and rate of cell divisions, cell-cell adhesion and communication, cell shape and **migration**, or cell death. Focusing on these realizators, we provide a framework for the morphogenesis of the maxillary segment. As the genomic organization of Hox genes and the interaction of Hox proteins with specific co-factors are conserved in vertebrates and invertebrates, and similar classes of downstream genes are regulated by Hox proteins across the metazoan phylogeny, our findings represent a first step toward a mechanistic understanding of morphological diversification within a species as well as between species.
4. Draper/CED-1 mediates an ancient damage response to control inflammatory blood cell **migration** in vivo. Tissue damage leads to a robust and rapid inflammatory response whereby leukocytes are actively drawn toward the wound. Hydrogen peroxide (H2O2) has been shown to be an immediate damage signal essential for the recruitment of these inflammatory blood cells to wound sites in both Drosophila and vertebrates [1, 2]. Recent studies in zebrafish have shown that wound-induced H2O2 is detected by the redox-sensitive Src family kinase (SFK) Lyn within the responding blood cells [3]. Here, we show the same signaling occurs in Drosophila inflammatory cells in response to wound-induced H2O2 with mutants for the Lyn homolog Src42A displaying impaired inflammatory **migration** to wounds. We go on to show that activation of Src42A is necessary to trigger a signaling cascade within the inflammatory cells involving the ITAM domain-containing protein Draper-I (a member of the CED-1 family of apoptotic cell clearance receptors) and a downstream kinase, Shark, that is required for **migration** to wounds. The Src42A-Draper-Shark-mediated signaling axis is homologous to the well-established SFK-ITAM-Syk-signaling pathway used in vertebrate adaptive immune responses. Consequently, our results suggest that adaptive immunoreceptor-signaling pathways important in distinguishing self from non-self appear to have evolved from a more-ancient damage response. Furthermore, this changes the role of H2O2 from an inflammatory chemoattractant to an activator signal that primes immune cells to respond to damage cues via the activation of damage receptors such as Draper.

### [Ee]xtracellular [Mm]atrix

#### PubMed

1. Tissue design: how Drosophila tumors remodel their neighborhood. Drosophila genetics has long been appreciated as a powerful approach for discovering the normal functions of genes that act as oncogenes and tumor suppressors in human cancer. Recent studies have also highlighted its advantages for deciphering how such genes function during tumorigenesis itself. Here we detail studies relating to how tumors, generated in developing organs and adult stem cell-based tissues, remodel the tissue landscape to their benefit. Like mammalian tumors, insect tumors can dissolve **extracellular matrix**, recruit blood cells, migrate and invade other tissues. While much is known about how mammalian fibroblasts, immune cells and vasculature promote late tumorigenesis, less is understood about the very earliest stages of tumor development in mammals. Because Drosophila has fewer mitotic cells and a simpler tissue architecture, it affords easy detection and analysis of early clonal tumor growth. Drosophila studies have revealed both cooperative and competitive interactions between tumor and normal cells during early tumor growth. During development, these interactions typically occur with other proliferative progenitor cells, but in adult stem cell-based tissues, the stem cell niche can fuel tumor growth.

### [Mm]etastasis

#### PubMed

1. Drosophila as a model to study the role of blood cells in inflammation, innate immunity and cancer. Drosophila has a primitive yet effective blood system with three types of haemocytes which function throughout different developmental stages and environmental stimuli. Haemocytes play essential roles in tissue modeling during embryogenesis and morphogenesis, and also in innate immunity. The open circulatory system of Drosophila makes haemocytes ideal signal mediators to cells and tissues in response to events such as infection and wounding. The application of recently developed and sophisticated genetic tools to the relatively simple genome of Drosophila has made the fly a popular system for modeling human tumorigensis and **metastasis**. Drosophila is now used for screening and investigation of genes implicated in human leukemia and also in modeling development of solid tumors. This second line of research offers promising opportunities to determine the seemingly conflicting roles of blood cells in tumor progression and invasion. This review provides an overview of the signaling pathways conserved in Drosophila during haematopoiesis, haemostasis, innate immunity, wound healing and inflammation. We also review the most recent progress in the use of Drosophila as a cancer research model with an emphasis on the roles haemocytes can play in various cancer models and in the links between inflammation and cancer.
2. Cell Competition Drives the Formation of Metastatic Tumors in a Drosophila Model of Epithelial Tumor Formation. Cell competition is a homeostatic process in which proliferating cells compete for survival. Elimination of otherwise normal healthy cells through competition is important during development and has recently been shown to contribute to maintaining tissue health during organismal aging. The mechanisms that allow for ongoing cell competition during adult life could, in principle, contribute to tumorigenesis. However, direct evidence supporting this hypothesis has been lacking. Here, we provide evidence that cell competition drives tumor formation in a Drosophila model of epithelial cancer. Cells expressing EGFR together with the conserved microRNA miR-8 acquire the properties of supercompetitors. Neoplastic transformation and **metastasis** depend on the ability of these cells to induce apoptosis and engulf nearby cells. miR-8 expression causes genome instability by downregulating expression of the Septin family protein Peanut. Cytokinesis failure due to downregulation of Peanut is required for tumorigenesis. This study provides evidence that the cellular mechanisms that drive cell competition during normal tissue growth can be co-opted to drive tumor formation and **metastasis**. Analogous mechanisms for cytokinesis failure may lead to polyploid intermediates in tumorigenesis in mammalian cancer models.

---

## Myc

- NCBI Gene
- GeneRIF
- Pubmed

| Search Term | Rank | Fields |
| --- | --- | --- |
| [Ff]ilopodia | 1 | PubMed(1) |
| [Mm]igration | 6 | PubMed(6) |
| ECM | 7 | PubMed(2) |
| [Ee]xtracellular [Mm]atrix | 8 | PubMed(4) |
| [Ii]nvasive | 10 | PubMed(6) |
| [Mm]etastasis | 12 | PubMed(3) |
| [Cc]ell [Ii]nvasion | 13 | PubMed(1) |

  
hide/show details for Myc

### [Ff]ilopodia

#### PubMed

1. Muscle niche-driven Insulin-Notch-Myc cascade reactivates dormant Adult Muscle Precursors in Drosophila. How stem cells specified during development keep their non-differentiated quiescent state, and how they are reactivated, remain poorly understood. Here, we applied a Drosophila model to follow in vivo behavior of adult muscle precursors (AMPs), the transient fruit fly muscle stem cells. We report that emerging AMPs send out thin **filopodia** that make contact with neighboring muscles. AMPs keep their **filopodia**-based association with muscles throughout their dormant state but also when they start to proliferate, suggesting that muscles could play a role in AMP reactivation. Indeed, our genetic analyses indicate that muscles send inductive dIlp6 signals that switch the Insulin pathway ON in closely associated AMPs. This leads to the activation of Notch, which regulates AMP proliferation via dMyc. Altogether, we report that Drosophila AMPs display homing behavior to muscle niche and that the niche-driven Insulin-Notch-dMyc cascade plays a key role in setting the activated state of AMPs.

### [Mm]igration

#### PubMed

1. Active JNK-dependent secretion of Drosophila Tyrosyl-tRNA synthetase by loser cells recruits haemocytes during cell competition. Cell competition is a process by which the slow dividing cells (losers) are recognized and eliminated from growing tissues. Loser cells are extruded from the epithelium and engulfed by the haemocytes, the Drosophila macrophages. However, how macrophages identify the dying loser cells is unclear. Here we show that apoptotic loser cells secrete Tyrosyl-tRNA synthetase (TyrRS), which is best known as a core component of the translational machinery. Secreted TyrRS is cleaved by matrix metalloproteinases generating MiniTyr and EMAP fragments. EMAP acts as a guiding cue for macrophage **migration** in the Drosophila larvae, as it attracts the haemocytes to the apoptotic loser cells. JNK signalling and Kish, a component of the secretory pathway, are autonomously required for the active secretion of TyrRS by the loser cells. Altogether, this mechanism guarantees effective removal of unfit cells from the growing tissue.
2. A core transcriptional network for early mesoderm development in Drosophila melanogaster. Embryogenesis is controlled by large gene-regulatory networks, which generate spatially and temporally refined patterns of gene expression. Here, we report the characteristics of the regulatory network orchestrating early mesodermal development in the fruitfly Drosophila, where the transcription factor Twist is both necessary and sufficient to drive development. Through the integration of chromatin immunoprecipitation followed by microarray analysis (ChIP-on-chip) experiments during discrete time periods with computational approaches, we identified >2000 Twist-bound cis-regulatory modules (CRMs) and almost 500 direct target genes. Unexpectedly, Twist regulates an almost complete cassette of genes required for cell proliferation in addition to genes essential for morophogenesis and cell **migration**. Twist targets almost 25% of all annotated Drosophila transcription factors, which may represent the entire set of regulators necessary for the early development of this system. By combining in vivo binding data from Twist, Mef2, Tinman, and Dorsal we have constructed an initial transcriptional network of early mesoderm development. The network topology reveals extensive combinatorial binding, feed-forward regulation, and complex logical outputs as prevalent features. In addition to binary activation and repression, we suggest that Twist binds to almost all mesodermal CRMs to provide the competence to integrate inputs from more specialized transcription factors.
3. Genes conserved in bilaterians but jointly lost with Myc during nematode evolution are enriched in cell proliferation and cell **migration** functions. Animals use a stereotypical set of developmental genes to build body architectures of varying sizes and organizational complexity. Some genes are critical to developmental patterning, while other genes are important to physiological control of growth. However, growth regulator genes may not be as important in small-bodied "micro-metazoans" such as nematodes. Nematodes use a simplified developmental strategy of lineage-based cell fate specifications to produce an adult bilaterian body composed of a few hundreds of cells. Nematodes also lost the MYC proto-oncogenic regulator of cell proliferation. To identify additional regulators of cell proliferation that were lost with MYC, we computationally screened and determined 839 high-confidence genes that are conserved in bilaterians/lost in nematodes (CIBLIN genes). We find that 30 % of all CIBLIN genes encode transcriptional regulators of cell proliferation, epithelial-to-mesenchyme transitions, and other processes. Over 50 % of CIBLIN genes are unnamed genes in Drosophila, suggesting that there are many understudied genes. Interestingly, CIBLIN genes include many Myc synthetic lethal (MycSL) hits from recent screens. CIBLIN genes include key regulators of heparan sulfate proteoglycan (HSPG) sulfation patterns, and lysyl oxidases involved in cross-linking and modification of the extracellular matrix (ECM). These genes and others suggest the CIBLIN repertoire services critical functions in ECM remodeling and cell **migration** in large-bodied bilaterians. Correspondingly, CIBLIN genes are co-expressed with Myc in cancer transcriptomes, and include a preponderance of known determinants of cancer progression and tumor aggression. We propose that CIBLIN gene research can improve our understanding of regulatory control of cellular growth in metazoans.
4. A drosophila model for EGFR-Ras and PI3K-dependent human glioma. Gliomas, the most common malignant tumors of the nervous system, frequently harbor mutations that activate the epidermal growth factor receptor (EGFR) and phosphatidylinositol-3 kinase (PI3K) signaling pathways. To investigate the genetic basis of this disease, we developed a glioma model in Drosophila. We found that constitutive coactivation of EGFR-Ras and PI3K pathways in Drosophila glia and glial precursors gives rise to neoplastic, invasive glial cells that create transplantable tumor-like growths, mimicking human glioma. Our model represents a robust organotypic and cell-type-specific Drosophila cancer model in which malignant cells are created by mutations in signature genes and pathways thought to be driving forces in a homologous human cancer. Genetic analyses demonstrated that EGFR and PI3K initiate malignant neoplastic transformation via a combinatorial genetic network composed primarily of other pathways commonly mutated or activated in human glioma, including the Tor, Myc, G1 Cyclins-Cdks, and Rb-E2F pathways. This network acts synergistically to coordinately stimulate cell cycle entry and progression, protein translation, and inappropriate cellular growth and **migration**. In particular, we found that the fly orthologs of CyclinE, Cdc25, and Myc are key rate-limiting genes required for glial neoplasia. Moreover, orthologs of Sin1, Rictor, and Cdk4 are genes required only for abnormal neoplastic glial proliferation but not for glial development. These and other genes within this network may represent important therapeutic targets in human glioma.
5. Colorectal cancer progression: lessons from Drosophila? Human colorectal cancers arise as benign adenomas, tumours that retain their epithelial character, and then progress to malignant adenocarcinomas and carcinomas in which the epithelium becomes disrupted. Carcinomas often exhibit transcriptional downregulation of E-cadherin and other epithelial genes in an epithelial-to-mesenchymal transition (EMT), a mechanism first discovered in Drosophila to be mediated by the transcription factors Twist and Snail. In contrast, adenocarcinomas retain expression of E-cadherin and disruption of the epithelium occurs through formation of progressively smaller epithelial cysts with apical Crumbs/CRB3, Stardust/PALS1, and Bazooka/PAR3 localised to the inner lumen. Results from Drosophila show that morphologically similar cysts form upon induction of clonal heterogeneity in Wnt, Smad, or Ras signalling levels, which causes extrusion of epithelial cells at clonal boundaries. Thus, intratumour heterogeneity might also promote formation of adenocarcinomas in humans. Finally, epithelial cysts can collectively migrate, as in the case of Drosophila border cells, a potential model system for the invasive **migration** of adenocarcinoma cells.
6. A genetic screen in Drosophila for metastatic behavior. A genetic screen was designed in Drosophila to interrogate its genome for mutations sufficient to cause noninvasive tumors of the eye disc to invade neighboring or distant tissues. We found that cooperation between oncogenic RasV12 expression and inactivation of any one of a number of genes affecting cell polarity leads to metastatic behavior, including basement membrane degradation, loss of E-cadherin expression, **migration**, invasion, and secondary tumor formation. Inactivation of these cell polarity genes cannot drive metastatic behavior alone or in combination with other tumor-initiating alterations. These findings suggest that the oncogenic background of tissues makes a distinct contribution toward metastatic development.

### ECM

#### PubMed

1. Genes conserved in bilaterians but jointly lost with Myc during nematode evolution are enriched in cell proliferation and cell migration functions. Animals use a stereotypical set of developmental genes to build body architectures of varying sizes and organizational complexity. Some genes are critical to developmental patterning, while other genes are important to physiological control of growth. However, growth regulator genes may not be as important in small-bodied "micro-metazoans" such as nematodes. Nematodes use a simplified developmental strategy of lineage-based cell fate specifications to produce an adult bilaterian body composed of a few hundreds of cells. Nematodes also lost the MYC proto-oncogenic regulator of cell proliferation. To identify additional regulators of cell proliferation that were lost with MYC, we computationally screened and determined 839 high-confidence genes that are conserved in bilaterians/lost in nematodes (CIBLIN genes). We find that 30 % of all CIBLIN genes encode transcriptional regulators of cell proliferation, epithelial-to-mesenchyme transitions, and other processes. Over 50 % of CIBLIN genes are unnamed genes in Drosophila, suggesting that there are many understudied genes. Interestingly, CIBLIN genes include many Myc synthetic lethal (MycSL) hits from recent screens. CIBLIN genes include key regulators of heparan sulfate proteoglycan (HSPG) sulfation patterns, and lysyl oxidases involved in cross-linking and modification of the extracellular matrix (**ECM**). These genes and others suggest the CIBLIN repertoire services critical functions in **ECM** remodeling and cell migration in large-bodied bilaterians. Correspondingly, CIBLIN genes are co-expressed with Myc in cancer transcriptomes, and include a preponderance of known determinants of cancer progression and tumor aggression. We propose that CIBLIN gene research can improve our understanding of regulatory control of cellular growth in metazoans.
2. The microRNA bantam regulates a developmental transition in epithelial cells that restricts sensory dendrite growth. As animals grow, many early born structures grow by cell expansion rather than cell addition; thus growth of distinct structures must be coordinated to maintain proportionality. This phenomenon is particularly widespread in the nervous system, with dendrite arbors of many neurons expanding in concert with their substrate to sustain connectivity and maintain receptive field coverage as animals grow. After rapidly growing to establish body wall coverage, dendrites of Drosophila class IV dendrite arborization (C4da) neurons grow synchronously with their substrate, the body wall epithelium, providing a system to study how proportionality is maintained during animal growth. Here, we show that the microRNA bantam (ban) ensures coordinated growth of C4da dendrites and the epithelium through regulation of epithelial endoreplication, a modified cell cycle that entails genome amplification without cell division. In Drosophila larvae, epithelial endoreplication leads to progressive changes in dendrite-extracellular matrix (**ECM**) and dendrite-epithelium contacts, coupling dendrite/substrate expansion and restricting dendrite growth beyond established boundaries. Moreover, changes in epithelial expression of cell adhesion molecules, including the beta-integrin myospheroid (mys), accompany this developmental transition. Finally, endoreplication and the accompanying changes in epithelial mys expression are required to constrain late-stage dendrite growth and structural plasticity. Hence, modulating epithelium-**ECM** attachment probably influences substrate permissivity for dendrite growth and contributes to the dendrite-substrate coupling that ensures proportional expansion of the two cell types.

### [Ee]xtracellular [Mm]atrix

#### PubMed

1. Genes conserved in bilaterians but jointly lost with Myc during nematode evolution are enriched in cell proliferation and cell migration functions. Animals use a stereotypical set of developmental genes to build body architectures of varying sizes and organizational complexity. Some genes are critical to developmental patterning, while other genes are important to physiological control of growth. However, growth regulator genes may not be as important in small-bodied "micro-metazoans" such as nematodes. Nematodes use a simplified developmental strategy of lineage-based cell fate specifications to produce an adult bilaterian body composed of a few hundreds of cells. Nematodes also lost the MYC proto-oncogenic regulator of cell proliferation. To identify additional regulators of cell proliferation that were lost with MYC, we computationally screened and determined 839 high-confidence genes that are conserved in bilaterians/lost in nematodes (CIBLIN genes). We find that 30 % of all CIBLIN genes encode transcriptional regulators of cell proliferation, epithelial-to-mesenchyme transitions, and other processes. Over 50 % of CIBLIN genes are unnamed genes in Drosophila, suggesting that there are many understudied genes. Interestingly, CIBLIN genes include many Myc synthetic lethal (MycSL) hits from recent screens. CIBLIN genes include key regulators of heparan sulfate proteoglycan (HSPG) sulfation patterns, and lysyl oxidases involved in cross-linking and modification of the **extracellular matrix** (ECM). These genes and others suggest the CIBLIN repertoire services critical functions in ECM remodeling and cell migration in large-bodied bilaterians. Correspondingly, CIBLIN genes are co-expressed with Myc in cancer transcriptomes, and include a preponderance of known determinants of cancer progression and tumor aggression. We propose that CIBLIN gene research can improve our understanding of regulatory control of cellular growth in metazoans.
2. Tissue design: how Drosophila tumors remodel their neighborhood. Drosophila genetics has long been appreciated as a powerful approach for discovering the normal functions of genes that act as oncogenes and tumor suppressors in human cancer. Recent studies have also highlighted its advantages for deciphering how such genes function during tumorigenesis itself. Here we detail studies relating to how tumors, generated in developing organs and adult stem cell-based tissues, remodel the tissue landscape to their benefit. Like mammalian tumors, insect tumors can dissolve **extracellular matrix**, recruit blood cells, migrate and invade other tissues. While much is known about how mammalian fibroblasts, immune cells and vasculature promote late tumorigenesis, less is understood about the very earliest stages of tumor development in mammals. Because Drosophila has fewer mitotic cells and a simpler tissue architecture, it affords easy detection and analysis of early clonal tumor growth. Drosophila studies have revealed both cooperative and competitive interactions between tumor and normal cells during early tumor growth. During development, these interactions typically occur with other proliferative progenitor cells, but in adult stem cell-based tissues, the stem cell niche can fuel tumor growth.
3. Finding a niche: studies from the Drosophila ovary. Specialized microenvironments called niches help maintain stem cells in an undifferentiated and self-renewing state. The existence of niches has long been predicted from mammalian studies, but identifying stem cells in their native environments in vivo has remained a challenge in most vertebrates. Many of the mechanistic insights into how niches regulate stem cell maintenance have been obtained using invertebrate models such as Drosophila. Here, we focus on the Drosophila ovarian germline stem cell niche and review recent studies that have begun to reveal how intricate crosstalk between various signaling pathways regulates stem cell maintenance, how the **extracellular matrix** modulates the signaling output of the niche and how epigenetic programming influences cell development and function both inside and outside the niche to ensure proper tissue homeostasis. These insights will probably inform the study of mammalian niches and how their malfunction contributes to human disease.
4. The microRNA bantam regulates a developmental transition in epithelial cells that restricts sensory dendrite growth. As animals grow, many early born structures grow by cell expansion rather than cell addition; thus growth of distinct structures must be coordinated to maintain proportionality. This phenomenon is particularly widespread in the nervous system, with dendrite arbors of many neurons expanding in concert with their substrate to sustain connectivity and maintain receptive field coverage as animals grow. After rapidly growing to establish body wall coverage, dendrites of Drosophila class IV dendrite arborization (C4da) neurons grow synchronously with their substrate, the body wall epithelium, providing a system to study how proportionality is maintained during animal growth. Here, we show that the microRNA bantam (ban) ensures coordinated growth of C4da dendrites and the epithelium through regulation of epithelial endoreplication, a modified cell cycle that entails genome amplification without cell division. In Drosophila larvae, epithelial endoreplication leads to progressive changes in dendrite-**extracellular matrix** (ECM) and dendrite-epithelium contacts, coupling dendrite/substrate expansion and restricting dendrite growth beyond established boundaries. Moreover, changes in epithelial expression of cell adhesion molecules, including the beta-integrin myospheroid (mys), accompany this developmental transition. Finally, endoreplication and the accompanying changes in epithelial mys expression are required to constrain late-stage dendrite growth and structural plasticity. Hence, modulating epithelium-ECM attachment probably influences substrate permissivity for dendrite growth and contributes to the dendrite-substrate coupling that ensures proportional expansion of the two cell types.

### [Ii]nvasive

#### PubMed

1. A drosophila model for EGFR-Ras and PI3K-dependent human glioma. Gliomas, the most common malignant tumors of the nervous system, frequently harbor mutations that activate the epidermal growth factor receptor (EGFR) and phosphatidylinositol-3 kinase (PI3K) signaling pathways. To investigate the genetic basis of this disease, we developed a glioma model in Drosophila. We found that constitutive coactivation of EGFR-Ras and PI3K pathways in Drosophila glia and glial precursors gives rise to neoplastic, **invasive** glial cells that create transplantable tumor-like growths, mimicking human glioma. Our model represents a robust organotypic and cell-type-specific Drosophila cancer model in which malignant cells are created by mutations in signature genes and pathways thought to be driving forces in a homologous human cancer. Genetic analyses demonstrated that EGFR and PI3K initiate malignant neoplastic transformation via a combinatorial genetic network composed primarily of other pathways commonly mutated or activated in human glioma, including the Tor, Myc, G1 Cyclins-Cdks, and Rb-E2F pathways. This network acts synergistically to coordinately stimulate cell cycle entry and progression, protein translation, and inappropriate cellular growth and migration. In particular, we found that the fly orthologs of CyclinE, Cdc25, and Myc are key rate-limiting genes required for glial neoplasia. Moreover, orthologs of Sin1, Rictor, and Cdk4 are genes required only for abnormal neoplastic glial proliferation but not for glial development. These and other genes within this network may represent important therapeutic targets in human glioma.
2. Sin3a acts through a multi-gene module to regulate invasion in Drosophila and human tumors. Chromatin remodeling proteins regulate multiple aspects of cell homeostasis, making them ideal candidates for misregulation in transformed cells. Here, we explore Sin3A, a member of the Sin3 family of proteins linked to tumorigenesis that are thought to regulate gene expression through their role as histone deacetylases (HDACs). We identified Drosophila Sin3a as an important mediator of oncogenic Ret receptor in a fly model of Multiple Endocrine Neoplasia Type 2. Reducing Drosophila Sin3a activity led to metastasis-like behavior and, in the presence of Diap1, secondary tumors distant from the site of origin. Genetic and Chip-Seq analyses identified previously undescribed Sin3a targets including genes involved in cell motility and actin dynamics, as well as signaling pathways including Src, Jnk and Rho. A key Sin3a oncogenic target, PP1B, regulates stability of β-Catenin/Armadillo: the outcome is to oppose T-cell factor (TCF) function and Wg/Wnt pathway signaling in both fly and mammalian cancer cells. Reducing Sin3A strongly increased the **invasiv**e behavior of A549 human lung adenocarcinoma cells. We show that Sin3A is downregulated in a variety of human tumors and that Src, JNK, RhoA and PP1B/β-Catenin are regulated in a manner analogous to our Drosophila models. Our data suggest that Sin3A influences a specific step of tumorigenesis by regulating a module of genes involved in cell invasion. Tumor progression may commonly rely on such 'modules of invasion' under the control of broad transcriptional regulators.
3. Cell mixing induced by myc is required for competitive tissue invasion and destruction. Cell-cell intercalation is used in several developmental processes to shape the normal body plan. There is no clear evidence that intercalation is involved in pathologies. Here we use the proto-oncogene myc to study a process analogous to early phase of tumour expansion: myc-induced cell competition. Cell competition is a conserved mechanism driving the elimination of slow-proliferating cells (so-called 'losers') by faster-proliferating neighbours (so-called 'winners') through apoptosis and is important in preventing developmental malformations and maintain tissue fitness. Here we show, using long-term live imaging of myc-driven competition in the Drosophila pupal notum and in the wing imaginal disc, that the probability of elimination of loser cells correlates with the surface of contact shared with winners. As such, modifying loser-winner interface morphology can modulate the strength of competition. We further show that elimination of loser clones requires winner-loser cell mixing through cell-cell intercalation. Cell mixing is driven by differential growth and the high tension at winner-winner interfaces relative to winner-loser and loser-loser interfaces, which leads to a preferential stabilization of winner-loser contacts and reduction of clone compactness over time. Differences in tension are generated by a relative difference in F-actin levels between loser and winner junctions, induced by differential levels of the membrane lipid phosphatidylinositol (3,4,5)-trisphosphate. Our results establish the first link between cell-cell intercalation induced by a proto-oncogene and how it promotes **invasive**ness and destruction of healthy tissues.
4. The lethal giant larvae tumour suppressor mutation requires dMyc oncoprotein to promote clonal malignancy. Neoplastic overgrowth depends on the cooperation of several mutations ultimately leading to major rearrangements in cellular behaviour. Precancerous cells are often removed by cell death from normal tissues in the early steps of the tumourigenic process, but the molecules responsible for such a fundamental safeguard process remain in part elusive. With the aim to investigate the molecular crosstalk occurring between precancerous and normal cells in vivo, we took advantage of the clonal analysis methods that are available in Drosophila for studying the phenotypes due to lethal giant larvae (lgl) neoplastic mutation induced in different backgrounds and tissues.We observed that lgl mutant cells growing in wild-type imaginal wing discs show poor viability and are eliminated by Jun N-terminal Kinase (JNK)-dependent cell death. Furthermore, they express very low levels of dMyc oncoprotein compared with those found in the surrounding normal tissue. Evidence that this is a cause of lgl mutant cells elimination was obtained by increasing dMyc levels in lgl mutant clones: their overgrowth potential was indeed re-established, with mutant cells overwhelming the neighbouring tissue and forming tumourous masses displaying several cancer hallmarks. Moreover, when lgl mutant clones were induced in backgrounds of slow-dividing cells, they upregulated dMyc, lost apical-basal cell polarity and were able to overgrow. Those phenotypes were abolished by reducing dMyc levels in the mutant clones, thereby confirming its key role in lgl-induced tumourigenesis. Furthermore, we show that the eiger-dependent Intrinsic Tumour Suppressor pathway plays only a minor role in eliminating lgl mutant cells in the wing pouch; lgl-/- clonal death in this region is instead driven mainly by dMyc-induced Cell Competition.Our results provide the first evidence that dMyc oncoprotein is required in lgl tumour suppressor mutant tissue to promote **invasive** overgrowth in larval and adult epithelial tissues. Moreover, we show that dMyc abundance inside versus outside the mutant clones plays a key role in driving neoplastic overgrowth.
5. Colorectal cancer progression: lessons from Drosophila? Human colorectal cancers arise as benign adenomas, tumours that retain their epithelial character, and then progress to malignant adenocarcinomas and carcinomas in which the epithelium becomes disrupted. Carcinomas often exhibit transcriptional downregulation of E-cadherin and other epithelial genes in an epithelial-to-mesenchymal transition (EMT), a mechanism first discovered in Drosophila to be mediated by the transcription factors Twist and Snail. In contrast, adenocarcinomas retain expression of E-cadherin and disruption of the epithelium occurs through formation of progressively smaller epithelial cysts with apical Crumbs/CRB3, Stardust/PALS1, and Bazooka/PAR3 localised to the inner lumen. Results from Drosophila show that morphologically similar cysts form upon induction of clonal heterogeneity in Wnt, Smad, or Ras signalling levels, which causes extrusion of epithelial cells at clonal boundaries. Thus, intratumour heterogeneity might also promote formation of adenocarcinomas in humans. Finally, epithelial cysts can collectively migrate, as in the case of Drosophila border cells, a potential model system for the **invasive** migration of adenocarcinoma cells.
6. A genetic screen in Drosophila for metastatic behavior. A genetic screen was designed in Drosophila to interrogate its genome for mutations sufficient to cause non**invasive** tumors of the eye disc to invade neighboring or distant tissues. We found that cooperation between oncogenic RasV12 expression and inactivation of any one of a number of genes affecting cell polarity leads to metastatic behavior, including basement membrane degradation, loss of E-cadherin expression, migration, invasion, and secondary tumor formation. Inactivation of these cell polarity genes cannot drive metastatic behavior alone or in combination with other tumor-initiating alterations. These findings suggest that the oncogenic background of tissues makes a distinct contribution toward metastatic development.

### [Mm]etastasis

#### PubMed

1. Sin3a acts through a multi-gene module to regulate invasion in Drosophila and human tumors. Chromatin remodeling proteins regulate multiple aspects of cell homeostasis, making them ideal candidates for misregulation in transformed cells. Here, we explore Sin3A, a member of the Sin3 family of proteins linked to tumorigenesis that are thought to regulate gene expression through their role as histone deacetylases (HDACs). We identified Drosophila Sin3a as an important mediator of oncogenic Ret receptor in a fly model of Multiple Endocrine Neoplasia Type 2. Reducing Drosophila Sin3a activity led to **metastasis**-like behavior and, in the presence of Diap1, secondary tumors distant from the site of origin. Genetic and Chip-Seq analyses identified previously undescribed Sin3a targets including genes involved in cell motility and actin dynamics, as well as signaling pathways including Src, Jnk and Rho. A key Sin3a oncogenic target, PP1B, regulates stability of β-Catenin/Armadillo: the outcome is to oppose T-cell factor (TCF) function and Wg/Wnt pathway signaling in both fly and mammalian cancer cells. Reducing Sin3A strongly increased the invasive behavior of A549 human lung adenocarcinoma cells. We show that Sin3A is downregulated in a variety of human tumors and that Src, JNK, RhoA and PP1B/β-Catenin are regulated in a manner analogous to our Drosophila models. Our data suggest that Sin3A influences a specific step of tumorigenesis by regulating a module of genes involved in cell invasion. Tumor progression may commonly rely on such 'modules of invasion' under the control of broad transcriptional regulators.
2. Disruption of lysosome function promotes tumor growth and **metastasis** in Drosophila. Lysosome function is essential to many physiological processes. It has been suggested that deregulation of lysosome function could contribute to cancer. Through a genetic screen in Drosophila, we have discovered that mutations disrupting lysosomal degradation pathway components contribute to tumor development and progression. Loss-of-function mutations in the Class C vacuolar protein sorting (VPS) gene, deep orange (dor), dramatically promote tumor overgrowth and invasion of the Ras(V12) cells. Knocking down either of the two other components of the Class C VPS complex, carnation (car) and vps16A, also renders Ras(V12) cells capable for uncontrolled growth and metastatic behavior. Finally, chemical disruption of the lysosomal function by feeding animals with antimalarial drugs, chloroquine or monensin, leads to malignant tumor growth of the Ras(V12) cells. Taken together, our data provide evidence for a causative role of lysosome dysfunction in tumor growth and invasion and indicate that members of the Class C VPS complex behave as tumor suppressors.
3. Drosophila models for cancer research. Drosophila is a model system for cancer research. Investigation with fruit flies has facilitated a number of important recent discoveries in the field: the hippo signaling pathway, which coordinates cell proliferation and death to achieve normal tissue size; 'social' behaviors of cells, including cell competition and apoptosis-induced compensatory proliferation, that help ensure normal tissue size; and a growing understanding of how oncogenes and tumor suppressors cooperate to achieve tumor growth and **metastasis** in situ. In the future, Drosophila models can be extended beyond basic research in the search for human therapeutics.

### [Cc]ell [Ii]nvasion

#### PubMed

1. Sin3a acts through a multi-gene module to regulate invasion in Drosophila and human tumors. Chromatin remodeling proteins regulate multiple aspects of cell homeostasis, making them ideal candidates for misregulation in transformed cells. Here, we explore Sin3A, a member of the Sin3 family of proteins linked to tumorigenesis that are thought to regulate gene expression through their role as histone deacetylases (HDACs). We identified Drosophila Sin3a as an important mediator of oncogenic Ret receptor in a fly model of Multiple Endocrine Neoplasia Type 2. Reducing Drosophila Sin3a activity led to metastasis-like behavior and, in the presence of Diap1, secondary tumors distant from the site of origin. Genetic and Chip-Seq analyses identified previously undescribed Sin3a targets including genes involved in cell motility and actin dynamics, as well as signaling pathways including Src, Jnk and Rho. A key Sin3a oncogenic target, PP1B, regulates stability of β-Catenin/Armadillo: the outcome is to oppose T-cell factor (TCF) function and Wg/Wnt pathway signaling in both fly and mammalian cancer cells. Reducing Sin3A strongly increased the invasive behavior of A549 human lung adenocarcinoma cells. We show that Sin3A is downregulated in a variety of human tumors and that Src, JNK, RhoA and PP1B/β-Catenin are regulated in a manner analogous to our Drosophila models. Our data suggest that Sin3A influences a specific step of tumorigenesis by regulating a module of genes involved i**n cell invasi**on. Tumor progression may commonly rely on such 'modules of invasion' under the control of broad transcriptional regulators.

---

## CG30280

- NCBI Gene
- GeneRIF
- Pubmed

| Search Term | Rank | Fields |
| --- | --- | --- |
| [Mm]igration | 6 | PubMed(1) |
| ECM | 7 | PubMed(1) |
| [Ee]xtracellular [Mm]atrix | 8 | PubMed(1) |

  
hide/show details for CG30280

### [Mm]igration

#### PubMed

1. Genes conserved in bilaterians but jointly lost with Myc during nematode evolution are enriched in cell proliferation and cell **migration** functions. Animals use a stereotypical set of developmental genes to build body architectures of varying sizes and organizational complexity. Some genes are critical to developmental patterning, while other genes are important to physiological control of growth. However, growth regulator genes may not be as important in small-bodied "micro-metazoans" such as nematodes. Nematodes use a simplified developmental strategy of lineage-based cell fate specifications to produce an adult bilaterian body composed of a few hundreds of cells. Nematodes also lost the MYC proto-oncogenic regulator of cell proliferation. To identify additional regulators of cell proliferation that were lost with MYC, we computationally screened and determined 839 high-confidence genes that are conserved in bilaterians/lost in nematodes (CIBLIN genes). We find that 30 % of all CIBLIN genes encode transcriptional regulators of cell proliferation, epithelial-to-mesenchyme transitions, and other processes. Over 50 % of CIBLIN genes are unnamed genes in Drosophila, suggesting that there are many understudied genes. Interestingly, CIBLIN genes include many Myc synthetic lethal (MycSL) hits from recent screens. CIBLIN genes include key regulators of heparan sulfate proteoglycan (HSPG) sulfation patterns, and lysyl oxidases involved in cross-linking and modification of the extracellular matrix (ECM). These genes and others suggest the CIBLIN repertoire services critical functions in ECM remodeling and cell **migration** in large-bodied bilaterians. Correspondingly, CIBLIN genes are co-expressed with Myc in cancer transcriptomes, and include a preponderance of known determinants of cancer progression and tumor aggression. We propose that CIBLIN gene research can improve our understanding of regulatory control of cellular growth in metazoans.

### ECM

#### PubMed

1. Genes conserved in bilaterians but jointly lost with Myc during nematode evolution are enriched in cell proliferation and cell migration functions. Animals use a stereotypical set of developmental genes to build body architectures of varying sizes and organizational complexity. Some genes are critical to developmental patterning, while other genes are important to physiological control of growth. However, growth regulator genes may not be as important in small-bodied "micro-metazoans" such as nematodes. Nematodes use a simplified developmental strategy of lineage-based cell fate specifications to produce an adult bilaterian body composed of a few hundreds of cells. Nematodes also lost the MYC proto-oncogenic regulator of cell proliferation. To identify additional regulators of cell proliferation that were lost with MYC, we computationally screened and determined 839 high-confidence genes that are conserved in bilaterians/lost in nematodes (CIBLIN genes). We find that 30 % of all CIBLIN genes encode transcriptional regulators of cell proliferation, epithelial-to-mesenchyme transitions, and other processes. Over 50 % of CIBLIN genes are unnamed genes in Drosophila, suggesting that there are many understudied genes. Interestingly, CIBLIN genes include many Myc synthetic lethal (MycSL) hits from recent screens. CIBLIN genes include key regulators of heparan sulfate proteoglycan (HSPG) sulfation patterns, and lysyl oxidases involved in cross-linking and modification of the extracellular matrix (**ECM**). These genes and others suggest the CIBLIN repertoire services critical functions in **ECM** remodeling and cell migration in large-bodied bilaterians. Correspondingly, CIBLIN genes are co-expressed with Myc in cancer transcriptomes, and include a preponderance of known determinants of cancer progression and tumor aggression. We propose that CIBLIN gene research can improve our understanding of regulatory control of cellular growth in metazoans.

### [Ee]xtracellular [Mm]atrix

#### PubMed

1. Genes conserved in bilaterians but jointly lost with Myc during nematode evolution are enriched in cell proliferation and cell migration functions. Animals use a stereotypical set of developmental genes to build body architectures of varying sizes and organizational complexity. Some genes are critical to developmental patterning, while other genes are important to physiological control of growth. However, growth regulator genes may not be as important in small-bodied "micro-metazoans" such as nematodes. Nematodes use a simplified developmental strategy of lineage-based cell fate specifications to produce an adult bilaterian body composed of a few hundreds of cells. Nematodes also lost the MYC proto-oncogenic regulator of cell proliferation. To identify additional regulators of cell proliferation that were lost with MYC, we computationally screened and determined 839 high-confidence genes that are conserved in bilaterians/lost in nematodes (CIBLIN genes). We find that 30 % of all CIBLIN genes encode transcriptional regulators of cell proliferation, epithelial-to-mesenchyme transitions, and other processes. Over 50 % of CIBLIN genes are unnamed genes in Drosophila, suggesting that there are many understudied genes. Interestingly, CIBLIN genes include many Myc synthetic lethal (MycSL) hits from recent screens. CIBLIN genes include key regulators of heparan sulfate proteoglycan (HSPG) sulfation patterns, and lysyl oxidases involved in cross-linking and modification of the **extracellular matrix** (ECM). These genes and others suggest the CIBLIN repertoire services critical functions in ECM remodeling and cell migration in large-bodied bilaterians. Correspondingly, CIBLIN genes are co-expressed with Myc in cancer transcriptomes, and include a preponderance of known determinants of cancer progression and tumor aggression. We propose that CIBLIN gene research can improve our understanding of regulatory control of cellular growth in metazoans.

---

## puc

- NCBI Gene
- GeneRIF
- Pubmed

| Search Term | Rank | Fields |
| --- | --- | --- |
| [Ff]ilopodia | 1 | PubMed(3) |
| [Mm]igration | 6 | Process(2); PubMed(31) |
| ECM | 7 | PubMed(1) |
| [Ee]xtracellular [Mm]atrix | 8 | PubMed(4) |
| [Ii]nvasive | 10 | PubMed(14) |
| [Mm]etastasize | 11 | PubMed(1) |
| [Mm]etastasis | 12 | PubMed(13) |
| [Cc]ell [Ii]nvasion | 13 | PubMed(8) |

  
hide/show details for puc

### [Ff]ilopodia

#### PubMed

1. JNK signaling pathway required for wound healing in regenerating Drosophila wing imaginal discs. We have examined wound healing during regeneration of Drosophila wing imaginal discs fragments by confocal microscopy and assessed the role of components of the JNK pathway in this process. After cutting, columnar and peripodial epithelia cells at the wound edge start to close the wound through formation and contraction of an actin cable. This is followed by a zipping process through **filopodia**l protrusions from both epithelia knitting the wound edges from proximal to distal areas of the disc. Activation of the JNK pathway is involved in such process. puckered (puc) expression is induced in several rows of cells at the edge of the wound, whereas absence of JNK pathway activity brought about by hemipterous, basket, and Dfos mutants impair wound healing. These defects are accompanied by lowered or loss of expression of puc. In support of a role of puc in wound healing, hep mutant phenotypes are rescued by reducing puc function, whereas overexpression of puc inhibits wound healing. Altogether, these results demonstrate a role for the JNK pathway in imaginal disc wound healing, similar to that reported for other healing processes such as embryonic dorsal closure, thoracic closure, and adult epithelial wound healing in Drosophila. Differences with such processes are also highlighted.
2. JNK and decapentaplegic signaling control adhesiveness and cytoskeleton dynamics during thorax closure in Drosophila. One of the fundamental events in metamorphosis in insects is the replacement of larval tissues by imaginal tissues. Shortly after pupariation the imaginal discs evaginate to assume their positions at the surface of the prepupal animal. This is a very precise process that is only beginning to be understood. In Drosophila, during embryonic dorsal closure, the epithelial cells push the amnioserosa cells, which contract and eventually invaginate in the body cavity. In contrast, we find that during pupariation the imaginal cells crawl over the passive larval tissue following a very accurate temporal and spatial pattern. Spreading is driven by **filopodia** and actin bridges that, protruding from the leading edge, mediate the stretching of the imaginal epithelia. Although interfering with JNK (Jun N-terminal kinase) and dpp (decapentaplegic) produces similar phenotypic effects suppressing closure, their effects at the cellular level are different. The loss of JNK activity alters the adhesion properties of larval cells and leads to the detachment of the imaginal and larval tissues. The absence of dpp signaling affects the actin cytoskeleton, blocks the emission of **filopodia**, and promotes the collapse of the leading edge of the imaginal tissues. Interestingly, these effects are very similar to those observed after interfering with JNK and dpp signaling during embryonic dorsal closure.
3. Cell adhesion molecule Echinoid associates with unconventional myosin VI/Jaguar motor to regulate cell morphology during dorsal closure in Drosophila. Echinoid (Ed) is a homophilic immunoglobulin domain-containing cell adhesion molecule (CAM) that localizes to adherens junctions (AJs) and cooperates with Drosophila melanogaster epithelial (DE)-cadherin to mediate cell adhesion. Here we show that Ed takes part in many processes of dorsal closure, a morphogenetic movement driven by coordinated cell shape changes and migration of epidermal cells to cover the underlying amnioserosa. Ed is differentially expressed, appearing in epidermis but not in amnioserosa cells. Ed functions independently from the JNK signaling pathway and is required to regulate cell morphology, and for assembly of actomyosin cable, **filopodia**l protrusion and coordinated cell migration in dorsal-most epidermal cells. The effect of Ed on cell morphology requires the presence of the intracellular domain (Ed(intra)). Interestingly, Ed forms homodimers in vivo and Ed(intra) monomer directly associates with unconventional myosin VI/Jaguar (Jar) motor protein. We further show that ed genetically interacts with jar to control cell morphology. It has previously been shown that myosin VI is monomeric in vitro and that its dimeric form can associate with and travel processively along actin filaments. Thus, we propose that Ed mediates the dimerization of myosin VI/Jar in vivo which in turn regulates the reorganization and/or contraction of actin filaments to control changes in cell shape. Consistent with this, we found that ectopic ed expression in the amnioserosa induces myosin VI/Jar-dependent apical constriction of this tissue.

### [Mm]igration

#### Process

1. border follicle cell **migration**
2. positive regulation of border follicle cell **migration**

#### PubMed

1. Integration of epithelial patterning and morphogenesis in Drosophila ovarian follicle cells. Drosophila oogenesis involves the coordinated development of germ cells and an overlying follicular epithelium. The follicle cells provide a genetically tractable system to investigate the cell biology of patterning and morphogenesis. Follicle cells initially form a cuboidal epithelium surrounding a syncytium of nurse cells and oocyte. Epithelial structure is maintained as these cells reorganize to create the three dimensional architecture of the eggshell. Both long-range and short-range cell-cell communications pattern the domains of follicle cells that will create specific eggshell structures. After terminal differentiation to deposit the eggshell proteins, the follicle cells die. This review summarizes recent progress in understanding the cell-cell communication that orchestrates follicle cell patterning and **migration**s. DE-cadherin-mediated adhesion is important at several steps in egg chamber formation and follicle cell **migration**. Notch signaling is critical during each successive round of patterning and **migration**. Integration of bone morphogenetic protein (BMP) and epidermal growth factor (EGF) signals patterns the elaborate structures of the dorsal-anterior eggshell.
2. A role for the epithelial microenvironment at tumor boundaries: evidence from Drosophila and human squamous cell carcinomas. Recent work has shown an increasing appreciation for the importance of the tumor environment, most commonly the overlying stroma. Less emphasis has been placed on the importance of local communication between transformed cells and their neighbors within the epithelium at tumor boundaries. We previously reported a Drosophila model that highlighted the importance of local interactions within the epithelial microenvironment: Src-transformed cells (Csk-deficient) were influenced by their immediate normal neighbors. The result was a consistent change in 'border cells' at the edge of transformed patches including delocalized p120-catenin and E-cadherin as well as invasive **migration** through the basal lamina. Here we show that the invasive properties of the boundary cells depend on up-regulation of Drosophila matrix metalloproteinase-1 as assessed by promoter activity, protein levels, in situ enzymatic activity, and tests of genetic modifier activity. Further, we provide evidence that these events at tumor borders may be evolutionarily conserved. We detected changes in 'boundary cells' within histological sections of human squamous cell carcinomas that were similar to those observed in Drosophila: both E-cadherin and p120-catenin exhibited normal junctional localization at the centers of the tumors but were reduced or delocalized at the boundary. Further, matrix metalloproteinase-2 was up regulated within these same boundary cells. These results support the view that local cell-cell interactions within the epithelial microenvironment impact tumor invasion and progression.
3. Regulation of cell adhesion and collective cell **migration** by hindsight and its human homolog RREB1. Cell movements represent a major driving force in embryonic development, tissue repair, and tumor metastasis [1]. The **migration** of single cells has been well studied, predominantly in cell culture [2, 3]; however, in vivo, a greater variety of modes of cell movement occur, including the movements of cells in clusters, strands, sheets, and tubes, also known as collective cell **migration**s [4, 5]. In spite of the relevance of these types of movements in both normal and pathological conditions, the molecular mechanisms that control them remain predominantly unknown. Epithelial follicle cells of the Drosophila ovary undergo several dynamic morphological changes, providing a genetically tractable model [6]. We found that anterior follicle cells, including border cells, mutant for the gene hindsight (hnt) accumulated excess cell-cell adhesion molecules and failed to undergo their normal collective movements. In addition, HNT affected border cell cluster cohesion and motility via effects on the JNK and STAT pathways, respectively. Interestingly, reduction of expression of the mammalian homolog of HNT, RREB1, by siRNA inhibited collective cell **migration** in a scratch-wound healing assay of MCF10A mammary epithelial cells, suppressed surface activity, retarded cell spreading after plating, and led to the formation of immobile, tightly adherent cell colonies. We propose that HNT and RREB1 are essential to reduce cell-cell adhesion when epithelial cells within an interconnected group undergo dynamic changes in cell shape.
4. [Dorsal closure in Drosophila. A genetic model for wound healing?]. Dorsal closure (DC) is a morphogenetic movement that establishes the dorsal ectoderm of the drosophila embryo. During this process, the two lateral epithelia stretch toward the dorsal midline, the suture line of the two leading edges. Cell **migration** during DC relies both on cell shape change controlled by the activity of the JNK pathway in the leading edge cells and modification of cell adhesiveness, probably dependent upon activation of the Dpp (TGF-beta) pathway. Coupling of the JNK and TGF-beta pathways is essential. The sequence of the cellular and molecular events of DC highlights interesting common features with wound healing in vertebrates. Like DC, wound healing relies on the **migration** of epithelia bordered by leading edges controlling the direction and speed of the movement. This review summarizes recent data concerning the control of epithelial morphogenesis during DC and the bases of wound healing. The molecular and cellular events that underlie these two analogous migratory processes are detailed, discussed and compared. We suggest that DC is a good genetic model for wound healing studying.
5. Drosophila female sterile mutation spoonbill interferes with multiple pathways in oogenesis. spoonbill is a Drosophila female-sterile mutation, which displays a range of eggshell and egg chamber patterning defects. Previous analysis has shown that the mutation interfered with the function of two major signaling pathways, GRK/EGFR and DPP. In this report, the nature of spoonbill was further investigated to examine whether it was associated with additional pathways in oogenesis. Clonal analysis, presented here, demonstrated that most of the aberrant phenotypes associated with spoonbill were dependent on a mutant germline. Nevertheless, SPOONBILL may function also in the soma to ensure proper polarization and **migration** of the border-cell-cluster. Further, genetic interaction studies implicated spoonbill in additional unrelated pathways such as the one(s) involved in actin polymerization/depolymerization. Based on the previous data and the results presented here, it is anticipated that spoonbill may encode a multifunctional protein that perhaps coordinately regulated the activity of multiple signaling pathways during oogenesis.
6. Mutations affecting the pattern of the PNS in Drosophila reveal novel aspects of neuronal development. Through a systematic genetic screen, we have identified 55 mutations that affect the development of the PNS of Drosophila embryos. These mutations specify 13 novel and 5 previously characterized genes and define new phenotypes for 2 other known genes. Five classes of mutant phenotypes were identified in the screen: gain of neurons, loss of neurons, abnormal position of chordotonal neurons, aberrant neuronal trajectories, and abnormal morphology of neurons. Phenotypic analyses of mutations identified in this study revealed three novel aspects of PNS development. First, we have identified a novel gene that may be required to define glial versus neuronal cell identity. Second, our data indicate that neuronal **migration** plays an important role in pattern formation in the embryonic PNS. Third, we have identified mutations that cause a lack of sensory organs, but unlike mutations in proneural genes, do not affect the formation of sensory organ precursors. These genes may be required for key aspects of neuronal differentiation. Our studies suggest that approximately 70 essential genes are required for proper PNS development in Drosophila embryos.
7. Left-right asymmetric morphogenesis of the anterior midgut depends on the activation of a non-muscle myosin II in Drosophila. Many animals exhibit stereotypical left-right (LR) asymmetry in their internal organs. The mechanisms of LR axis formation required for the subsequent LR asymmetric development are well understood, especially in some vertebrates. However, the molecular mechanisms underlying LR asymmetric morphogenesis, particularly how mechanical force is integrated into the LR asymmetric morphogenesis of organs, are poorly understood. Here, we identified zipper (zip), encoding a Drosophila non-muscle myosin II (myosin II) heavy chain, as a gene required for LR asymmetric development of the embryonic anterior midgut (AMG). Myosin II is known to directly generate mechanical force in various types of cells during morphogenesis and cell **migration**. We found that myosin II was involved in two events in the LR asymmetric development of the AMG. First, it introduced an LR bias to the directional position of circular visceral muscle (CVMU) cells, which externally cover the midgut epithelium. Second, it was required for the LR-biased rotation of the AMG. Our results suggest that myosin II in CVMU cells plays a crucial role in generating the force leading to LR asymmetric morphogenesis. Taken together with previous studies in vertebrates, the involvement of myosin II in LR asymmetric morphogenesis might be conserved evolutionarily.
8. Bendless modulates JNK-mediated cell death and **migration** in Drosophila. The TNF-JNK pathway is a highly conserved signaling pathway that regulates a wide spectrum of biological processes including cell death and **migration**. To further delineate this pathway, we carried out a genetic screen for dominant modifiers of the cell death phenotype triggered by ectopic expression of Eiger (Egr), the Drosophila TNF ortholog. Here we show that Bendless (Ben), an E2 ubiquitin-conjugating enzyme, modulates Egr-induced JNK activation and cell death through dTRAF2. Furthermore, Ben physically interacts with dTRAF2 and regulates Egr-induced dTRAF2 polyubiquitination. Finally, Ben is required for JNK-dependent tumor progression, cell **migration**, oxidative stress resistance and longevity. Our results indicate that Ben constitutes an essential component of the evolutionarily conserved TNF-JNK pathway that modulates cell death and invasion, tumor progression, stress response and lifespan in metazoans.
9. Genes conserved in bilaterians but jointly lost with Myc during nematode evolution are enriched in cell proliferation and cell **migration** functions. Animals use a stereotypical set of developmental genes to build body architectures of varying sizes and organizational complexity. Some genes are critical to developmental patterning, while other genes are important to physiological control of growth. However, growth regulator genes may not be as important in small-bodied "micro-metazoans" such as nematodes. Nematodes use a simplified developmental strategy of lineage-based cell fate specifications to produce an adult bilaterian body composed of a few hundreds of cells. Nematodes also lost the MYC proto-oncogenic regulator of cell proliferation. To identify additional regulators of cell proliferation that were lost with MYC, we computationally screened and determined 839 high-confidence genes that are conserved in bilaterians/lost in nematodes (CIBLIN genes). We find that 30 % of all CIBLIN genes encode transcriptional regulators of cell proliferation, epithelial-to-mesenchyme transitions, and other processes. Over 50 % of CIBLIN genes are unnamed genes in Drosophila, suggesting that there are many understudied genes. Interestingly, CIBLIN genes include many Myc synthetic lethal (MycSL) hits from recent screens. CIBLIN genes include key regulators of heparan sulfate proteoglycan (HSPG) sulfation patterns, and lysyl oxidases involved in cross-linking and modification of the extracellular matrix (ECM). These genes and others suggest the CIBLIN repertoire services critical functions in ECM remodeling and cell **migration** in large-bodied bilaterians. Correspondingly, CIBLIN genes are co-expressed with Myc in cancer transcriptomes, and include a preponderance of known determinants of cancer progression and tumor aggression. We propose that CIBLIN gene research can improve our understanding of regulatory control of cellular growth in metazoans.
10. Rho1-Wnd signaling regulates loss-of-cell polarity-induced cell invasion in Drosophila. Both cell polarity and c-Jun N-terminal kinase (JNK) activity are essential to the maintenance of tissue homeostasis, and disruption of either is commonly seen in cancer progression. Despite the established connection between loss-of-cell polarity and JNK activation, much less is known about the molecular mechanism by which aberrant cell polarity induces JNK-mediated cell **migration** and tumor invasion. Here we show results from a genetic screen using an in vivo invasion model via knocking down cell polarity gene in Drosophila wing discs, and identify Rho1-Wnd signaling as an important molecular link that mediates loss-of-cell polarity-triggered JNK activation and cell invasion. We show that Wallenda (Wnd), a protein kinase of the mitogen-activated protein kinase kinase kinase family, by forming a complex with the GTPase Rho1, is both necessary and sufficient for Rho1-induced JNK-dependent cell invasion, MMP1 activation and epithelial-mesenchymal transition. Furthermore, Wnd promotes cell proliferation and tissue growth through wingless production when apoptosis is inhibited by p35. Finally, Wnd shows oncogenic cooperation with Ras(V12) to trigger tumor growth in eye discs and causes invasion into the ventral nerve cord. Together, our data not only provides a novel mechanistic insight on how cell polarity loss contributes to cell invasion, but also highlights the value of the Drosophila model system to explore human cancer biology.
11. Signaling pathways directing the movement and fusion of epithelial sheets: lessons from dorsal closure in Drosophila. Wound healing in embryos and various developmental events in metazoans require the spreading and fusion of epithelial sheets. The complex signaling pathways regulating these processes are being pieced together through genetic, cell biological, and biochemical approaches. At present, dorsal closure of the Drosophila embryo is the best-characterized example of epithelial sheet movement. Dorsal closure involves **migration** of the lateral epidermal flanks to close a hole in the dorsal epidermis occupied by an epithelium called the amnioserosa. Detailed genetic studies have revealed a network of interacting signaling molecules regulating this process. At the center of this network is a Jun N-terminal kinase cascade acting at the leading edge of the migrating epidermis that triggers signaling by the TGF-beta superfamily member Decapentaplegic and which interacts with the Wingless pathway. These signaling modules regulate the cytoskeletal reorganization and cell shape change necessary to drive dorsal closure. Activation of this network requires signals from the amnioserosa and input from a variety of proteins at cell-cell junctions. The Rho family of small GTPases is also instrumental, both in activation of signaling and regulation of the cytoskeleton. Many of the proteins regulating dorsal closure have been implicated in epithelial movement in other organisms, and dorsal closure has emerged as an ideal model system for the study of the **migration** and fusion of epithelial sheets.
12. Control of Gliotactin localization and levels by tyrosine phosphorylation and endocytosis is necessary for survival of polarized epithelia. The tricellular junction (TCJ) forms at the convergence of bicellular junctions from three adjacent cells in polarized epithelia and is necessary for maintaining the transepithelial barrier. In the fruitfly Drosophila, the TCJ is generated at the meeting point of bicellular septate junctions. Gliotactin was the first identified component of the TCJ and is necessary for TCJ and septate junction development. Gliotactin is a member of the neuroligin family and associates with the PDZ protein discs large. Beyond this interaction, little is known about the mechanisms underlying Gliotactin localization and function at the TCJ. In this study, we show that Gliotactin is phosphorylated at conserved tyrosine residues, a process necessary for endocytosis and targeting to late endosomes and lysosomes for degradation. Regulation of Gliotactin levels through phosphorylation and endocytosis is necessary as overexpression results in displacement of Gliotactin away from the TCJ throughout the septate junction domain. Excessive Gliotactin in polarized epithelia leads to delamination, paired with subsequent **migration**, and apoptosis. The apoptosis and the resulting compensatory proliferation resulting from high levels of Gliotactin are mediated by the Drosophila JNK pathway. Therefore, Gliotactin levels within the cell membrane are regulated to ensure correct protein localization and cell survival.
13. The Drosophila serine protease homologue Scarface regulates JNK signalling in a negative-feedback loop during epithelial morphogenesis. In Drosophila melanogaster, dorsal closure is a model of tissue morphogenesis leading to the dorsal **migration** and sealing of the embryonic ectoderm. The activation of the JNK signal transduction pathway, specifically in the leading edge cells, is essential to this process. In a genome-wide microarray screen, we identified new JNK target genes during dorsal closure. One of them is the gene scarface (scaf), which belongs to the large family of trypsin-like serine proteases. Some proteins of this family, like Scaf, bear an inactive catalytic site, representing a subgroup of serine protease homologues (SPH) whose functions are poorly understood. Here, we show that scaf is a general transcriptional target of the JNK pathway coding for a secreted SPH. scaf loss-of-function induces defects in JNK-controlled morphogenetic events such as embryonic dorsal closure and adult male terminalia rotation. Live imaging of the latter process reveals that, like for dorsal closure, JNK directs the dorsal fusion of two epithelial layers in the pupal genital disc. Genetic data show that scaf loss-of-function mimics JNK over-activity. Moreover, scaf ectopic expression aggravates the effect of the JNK negative regulator puc on male genitalia rotation. We finally demonstrate that scaf acts as an antagonist by negatively regulating JNK activity. Overall, our results identify the SPH-encoding gene scaf as a new transcriptional target of JNK signalling and reveal the first secreted regulator of the JNK pathway acting in a negative-feedback loop during epithelial morphogenesis.
14. A functional RNAi screen for regulators of receptor tyrosine kinase and ERK signalling. Receptor tyrosine kinase (RTK) signalling through extracellular-signal-regulated kinases (ERKs) has pivotal roles during metazoan development, underlying processes as diverse as fate determination, differentiation, proliferation, survival, **migration** and growth. Abnormal RTK/ERK signalling has been extensively documented to contribute to developmental disorders and disease, most notably in oncogenic transformation by mutant RTKs or downstream pathway components such as Ras and Raf. Although the core RTK/ERK signalling cassette has been characterized by decades of research using mammalian cell culture and forward genetic screens in model organisms, signal propagation through this pathway is probably regulated by a larger network of moderate, context-specific proteins. The genes encoding these proteins may not have been discovered through traditional screens owing, in particular, to the requirement for visible phenotypes. To obtain a global view of RTK/ERK signalling, we performed an unbiased, RNA interference (RNAi), genome-wide, high-throughput screen in Drosophila cells using a novel, quantitative, cellular assay monitoring ERK activation. Here we show that ERK pathway output integrates a wide array of conserved cellular processes. Further analysis of selected components-in multiple cell types with different RTK ligands and oncogenic stimuli-validates and classifies 331 pathway regulators. The relevance of these genes is highlighted by our isolation of a Ste20-like kinase and a PPM-family phosphatase that seem to regulate RTK/ERK signalling in vivo and in mammalian cells. Novel regulators that modulate specific pathway outputs may be selective targets for drug discovery.
15. The Drosophila dysfusion basic helix-loop-helix (bHLH)-PAS gene controls tracheal fusion and levels of the trachealess bHLH-PAS protein. The development of the mature insect trachea requires a complex series of cellular events, including tracheal cell specification, cell **migration**, tubule branching, and tubule fusion. Here we describe the identification of the Drosophila melanogaster dysfusion gene, which encodes a novel basic helix-loop-helix (bHLH)-PAS protein conserved between Caenorhabditis elegans, insects, and humans, and controls tracheal fusion events. The Dysfusion protein functions as a heterodimer with the Tango bHLH-PAS protein in vivo to form a putative DNA-binding complex. The dysfusion gene is expressed in a variety of embryonic cell types, including tracheal-fusion, leading-edge, foregut atrium cells, nervous system, hindgut, and anal pad cells. RNAi experiments indicate that dysfusion is required for dorsal branch, lateral trunk, and ganglionic branch fusion but not for fusion of the dorsal trunk. The escargot gene, which is also expressed in fusion cells and is required for tracheal fusion, precedes dysfusion expression. Analysis of escargot mutants indicates a complex pattern of dysfusion regulation, such that dysfusion expression is dependent on escargot in the dorsal and ganglionic branches but not the dorsal trunk. Early in tracheal development, the Trachealess bHLH-PAS protein is present at uniformly high levels in all tracheal cells, but since the levels of Dysfusion rise in wild-type fusion cells, the levels of Trachealess in fusion cells decline. The downregulation of Trachealess is dependent on dysfusion function. These results suggest the possibility that competitive interactions between basic helix-loop-helix-PAS proteins (Dysfusion, Trachealess, and possibly Similar) may be important for the proper development of the trachea.
16. POSH is involved in Eiger-Basket (TNF-JNK) signaling and embryogenesis in Drosophila. TNFα can trigger different signaling pathways, including the JNK pathway, to regulate various biological functions such as cell death, differentiation and proliferation. The scaffold protein POSH (Plenty of SH3 Domains) has been shown to be an important regulator of the JNK pathway, but whether it is involved in TNF-signaling has not been reported. Although POSH has been implicated to play a role in development in zebrafish, it has not been studied in null mutants and the underlying mechanism of its effects is still not clear. In this study, we provide evidence that the JNK pathway scaffold protein, POSH, is involved in TNF (Eiger) signaling in Drosophila. POSH is likely to act downstream of dTAB2 and upstream of dTAK1 in the TNF-JNK signaling pathway. In addition, we found that POSH is essential during Drosophila embryogenesis, including epidermal dorsal closure, similar to other JNK pathway components such as Silpper, Hemipterous, and Basket. We observed defects in F-actin accumulation and adherens junction formation during dorsal closure in different posh null mutants, suggesting that POSH is required for epidermal cell **migratio**n and cell-shape change during epidermal dorsal closure.
17. Src42A modulates tumor invasion and cell death via Ben/dUev1a-mediated JNK activation in Drosophila. Loss of the cell polarity gene could cooperate with oncogenic Ras to drive tumor growth and invasion, which critically depends on the c-Jun N-terminal Kinase (JNK) signaling pathway in Drosophila. By performing a genetic screen, we have identified Src42A, the ortholog of mammalian Src, as a key modulator of both Ras(V12)/lgl(-/-) triggered tumor invasion and loss of cell polarity gene-induced cell **migration**. Our genetic study further demonstrated that the Bendless (Ben)/dUev1a ubiquitin E2 complex is an essential regulator of Src42A-induced, JNK-mediated cell **migration**. Furthermore, we showed that ectopic Ben/dUev1a expression induced invasive cell **migration** along with increased MMP1 production in wing disc epithelia. Moreover, Ben/dUev1a could cooperate with Ras(V12) to promote tumor overgrowth and invasion. In addition, we found that the Ben/dUev1a complex is required for ectopic Src42A-triggered cell death and endogenous Src42A-dependent thorax closure. Our data not only provide a mechanistic insight into the role of Src in development and disease but also propose a potential oncogenic function for Ubc13 and Uev1a, the mammalian homologs of Ben and dUev1a.
18. Csk-deficient boundary cells are eliminated from normal Drosophila epithelia by exclusion, **migration**, and apoptosis. The construction and maintenance of normal epithelia relies on local signals that guide cells into their proper niches and remove unwanted cells. Failure to execute this process properly may result in aberrant development or diseases, including cancer and associated metastasis. Here, we show that local environment influences the behavior of dCsk-deficient cells. Broad loss of dCsk led to enlarged and mispatterned tissues due to overproliferation, a block in apoptosis, and decreased cadherin-mediated adhesion. Loss of dCsk in discrete patches led to a different outcome: epithelial exclusion, invasive **migration**, and apoptotic death. These latter phenotypes required sharp differences in dCsk activity between neighbors; dE-cadherin, P120-catenin, Rho1, JNK, and MMP2 mediated this signal. Together, our data demonstrate how the cellular microenvironment plays a central role in determining the outcome of altered dCsk activity, and reveal a role for P120-catenin in a mechanism that protects epithelial integrity by removing abnormal cells.
19. The integrin effector PINCH regulates JNK activity and epithelial **migration** in concert with Ras suppressor 1. Cell adhesion and **migration** are dynamic processes requiring the coordinated action of multiple signaling pathways, but the mechanisms underlying signal integration have remained elusive. Drosophila embryonic dorsal closure (DC) requires both integrin function and c-Jun amino-terminal kinase (JNK) signaling for opposed epithelial sheets to migrate, meet, and suture. Here, we show that PINCH, a protein required for integrin-dependent cell adhesion and actin-membrane anchorage, is present at the leading edge of these migrating epithelia and is required for DC. By analysis of native protein complexes, we identify RSU-1, a regulator of Ras signaling in mammalian cells, as a novel PINCH binding partner that contributes to PINCH stability. Mutation of the gene encoding RSU-1 results in wing blistering in Drosophila, demonstrating its role in integrin-dependent cell adhesion. Genetic interaction analyses reveal that both PINCH and RSU-1 antagonize JNK signaling during DC. Our results suggest that PINCH and RSU-1 contribute to the integration of JNK and integrin functions during Drosophila development.
20. Sec61alpha is required for dorsal closure during Drosophila embryogenesis through its regulation of Dpp signaling. During dorsal closure in Drosophila, signaling events in the dorsalmost row of epidermal cells (DME cells) direct the **migration** of lateral epidermal sheets towards the dorsal midline where they fuse to enclose the embryo. A Jun amino-terminal kinase (JNK) cascade in the DME cells induces the expression of Decapentaplegic (Dpp). Dpp signaling then regulates the cytoskeleton in the DME cells and amnioserosa to affect the cell shape changes necessary to complete dorsal closure. We identified a mutation in Sec61alpha that specifically perturbs dorsal closure. Sec61alpha encodes the main subunit of the translocon complex for co-translational import of proteins into the ER. JNK signaling is normal in Sec61alpha mutant embryos, but Dpp signaling is attenuated and the DME cells fail to maintain an actinomyosin cable as epithelial **migration** fails. Consistent with this model, dorsal closure is rescued in Sec61alpha mutant embryos by an activated form of the Dpp receptor Thick veins.
21. Downregulation of Jun kinase signaling in the amnioserosa is essential for dorsal closure of the Drosophila embryo. During Drosophila embryogenesis, Jun kinase (JNK) signaling has been shown to play a key role in regulating the morphogenetic process of dorsal closure, which also serves as a model for epithelial sheet fusion during wound repair. During dorsal closure the JNK signaling cascade in the dorsal-most (leading edge) cells of the epidermis activates the AP-1 transcription factor comprised of DJUN and DFOS that, in turn, upregulates the expression of the dpp gene. DPP is a secreted morphogen that signals lateral epidermal cells to elongate along the dorsoventral axis. The leading edge cells contact the peripheral cells of a monolayer extraembryonic epithelium, the amnioserosa, which lies on the dorsal side of the embryo. Focal complexes are present at the dorsal-most membrane of the leading edge cells, where they contact the amnioserosa.We show that the JNK signaling cascade is initially active in both the amnioserosa and the leading edge of the epidermis. JNK signaling is downregulated in the amnioserosa, but not in the leading edge, prior to dorsal closure. The subcellular localization of DFOS and DJUN is responsive to JNK signaling in the amnioserosa: JNK activation results in nuclear localization of DFOS and DJUN; the downregulation of JNK signaling results in the relocalization of DFOS and DJUN to the cytoplasm. The HINDSIGHT (HNT) Zn-finger protein and the PUCKERED (PUC) JNK phosphatase are essential for downregulation of the JNK cascade in the amnioserosa. Persistent JNK activity in the amnioserosa leads to defective focal complexes in the adjacent leading edge cells and to the failure of dorsal closure.Focal complexes are assembled at the boundary between high and low JNK activity. In the absence of focal complexes, miscommunication between the amnioserosa and the leading edge may lead to a premature "stop" signal that halts dorsalward **migration** of the leading edge. Spatial and temporal regulation of the JNK signaling cascade may be a general mechanism that controls tissue remodeling during morphogenesis and wound healing.
22. Light-mediated activation reveals a key role for Rac in collective guidance of cell movement in vivo. The small GTPase Rac induces actin polymerization, membrane ruffling and focal contact formation in cultured single cells but can either repress or stimulate motility in epithelial cells depending on the conditions. The role of Rac in collective epithelial cell movements in vivo, which are important for both morphogenesis and metastasis, is therefore difficult to predict. Recently, photoactivatable analogues of Rac (PA-Rac) have been developed, allowing rapid and reversible activation or inactivation of Rac using light. In cultured single cells, light-activated Rac leads to focal membrane ruffling, protrusion and **migration**. Here we show that focal activation of Rac is also sufficient to polarize an entire group of cells in vivo, specifically the border cells of the Drosophila ovary. Moreover, activation or inactivation of Rac in one cell of the cluster caused a dramatic response in the other cells, suggesting that the cells sense direction as a group according to relative levels of Rac activity. Communication between cells of the cluster required Jun amino-terminal kinase (JNK) but not guidance receptor signalling. These studies further show that photoactivatable proteins are effective tools in vivo.
23. PVR plays a critical role via JNK activation in thorax closure during Drosophila metamorphosis. PVR, the Drosophila homolog of the PDGF/VEGF receptor, has been implicated in border cell **migration** during oogenesis and hemocyte **migration** during embryogenesis. It was earlier shown that Mbc, a CDM family protein, and its effector, Rac, transduced the guidance signal from PVR during border cell **migration**. Here we demonstrate that PVR is also required for the morphogenetic process, thorax closure, during metamorphosis. The results of genetic and biochemical experiments indicate that PVR activates the JNK pathway. We present evidence showing Crk (an adaptor molecule), Mbc, ELMO (a homolog of Caenorhabditis elegans CED-12 and mammalian ELMO), and Rac to be mediators of JNK activation by PVR. In addition, we suppose that not only Rac but also Cdc42 is activated and involved in JNK activation downstream of PVR.
24. The control of cell motility and epithelial morphogenesis by Jun kinases. Originally identified as stress-activated protein kinases that control cell survival and proliferation through transcription factor c-Jun, the Jun N-terminal kinase (JNK) subgroup of MAP kinases (MAPKs) have recently emerged as crucial regulators of cell **migration** and the morphogenetic movement of epithelial sheets. In Drosophila, a well-orchestrated JNK signaling pathway controls formation of actin stress fibers and cell shape changes, which are required for the sealing of embryonic epidermis in a process known as dorsal closure. The JNK pathway is also involved in morphogenetic processes in mice including closure of the eyelid, neural tube and optic fissure. This article focuses on recent advances in understanding the role of JNK pathway in the regulation of cell **migration**, cytoskeleton rearrangement and the morphogenesis of epithelial sheets.
25. Coupling of Jun amino-terminal kinase and Decapentaplegic signaling pathways in Drosophila morphogenesis. Dorsal closure in Drosophila embryos involves the **migration** of two lateral epithelia toward the dorsal midline to establish the dorsal ectoderm. Previous work showed that this morphogenetic movement depends on the activities of a Jun amino (N)-terminal kinase kinase (JNKK) encoded by the hemipterous (hep) gene, and of a JNK encoded by basket. Hep is required for cell determination in the leading edge of migrating epithelia, by controlling specific expression of the puckered (puc) gene in these cells. During dorsal closure, decapentaplegic (dpp), a member of the transforming growth factor-beta (TGF-beta) superfamily, is expressed in the row of cells making up the leading edge of the epithelia. Here, we show that the small GTPases Dcdc42, Drac1, and the Hep JNKK control dpp expression in this migratory process. Appropriate dpp and puc expression in the leading edge also depends on the inhibitory function of the puc gene. Further, our data suggest that the leading edge is the source of a JNK autocrine signal, and exclude a role of Dpp as such a ligand. Dorsal closure couples JNK and dpp signaling pathways, a situation that may be conserved in vertebrate development.
26. The Sac1 lipid phosphatase regulates cell shape change and the JNK cascade during dorsal closure in Drosophila. The Sac1 lipid phosphatase dephosphorylates several phosphatidylinositol (PtdIns) phosphates and, in yeast, regulates a diverse range of cellular processes including organization of the actin cytoskeleton and secretion. We have identified mutations in the gene encoding Drosophila Sac1. sac1 mutants die as embryos with defects in dorsal closure (DC). DC involves the **migration** of the epidermis to close a hole in the dorsal surface of the embryo occupied by the amnioserosa. It requires cell shape change in both the epidermis and amnioserosa and activation of a Jun N-terminal kinase (JNK) MAPK cascade in the leading edge cells of the epidermis [2]. Loss of Sac1 leads to the improper activation of two key events in DC: cell shape change in the amnioserosa and JNK signaling. sac1 interacts genetically with other participants in these two events, and our data suggest that loss of Sac1 leads to upregulation of one or more signals controlling DC. This study is the first report of a role for Sac1 in the development of a multicellular organism.
27. Wallenda regulates JNK-mediated cell death in Drosophila. The c-Jun N-terminal kinase (JNK) pathway plays essential roles in regulating a variety of cellular processes including proliferation, **migration** and survival. Previous genetic studies in Drosophila have identified numerous cell death regulating genes, providing new insights into the mechanisms for related diseases. Despite the known role of the small GTPase Rac1 in regulating cell death, the downstream components and underlying mechanism remain largely elusive. Here, we show that Rac1 promotes JNK-dependent cell death through Wallenda (Wnd). In addition, we find that Wnd triggers JNK activation and cell death via its kinase domain. Moreover, we show that both MKK4 and Hep are critical for Wnd-induced cell death. Furthermore, Wnd is essential for ectopic Egr- or Rho1-induced JNK activation and cell death. Finally, Wnd is physiologically required for loss of scribble-induced JNK-dependent cell death. Thus, our data suggest that wnd encodes a novel essential cell death regulator in Drosophila.
28. Pericardin, a Drosophila type IV collagen-like protein is involved in the morphogenesis and maintenance of the heart epithelium during dorsal ectoderm closure. The steps that lead to the formation of a single primitive heart tube are highly conserved in vertebrate and invertebrate embryos. Concerted **migration** of the two lateral cardiogenic regions of the mesoderm and endoderm (or ectoderm in invertebrates) is required for their fusion at the midline of the embryo. Morphogenetic signals are involved in this process and the extracellular matrix has been proposed to serve as a link between the two layers of cells. Pericardin (Prc), a novel Drosophila extracellular matrix protein is a good candidate to participate in heart tube formation. The protein has the hallmarks of a type IV collagen alpha-chain and is mainly expressed in the pericardial cells at the onset of dorsal closure. As dorsal closure progresses, Pericardin expression becomes concentrated at the basal surface of the cardioblasts and around the pericardial cells, in close proximity to the dorsal ectoderm. Pericardin is absent from the lumen of the dorsal vessel. Genetic evidence suggests that Prc promotes the proper **migration** and alignment of heart cells. Df(3)vin6 embryos, as well as embryos in which prc has been silenced via RNAi, exhibit similar and significant defects in the formation of the heart epithelium. In these embryos, the heart epithelium appears disorganized during its **migration** to the dorsal midline. By the end of embryonic development, cardial and pericardial cells are misaligned such that small clusters of both cell types appear in the heart; these clusters of cells are associated with holes in the walls of the heart. A prc transgene can partially rescue each of these phenotypes, suggesting that prc regulates these events. Our results support, for the first time, the function of a collagen-like protein in the coordinated **migration** of dorsal ectoderm and heart cells.
29. A sensitized PiggyBac-based screen for regulators of border cell **migration** in Drosophila. **Migration** of border cells during Drosophila melanogaster oogenesis is a good model system for investigating the genetic requirements for cell **migration** in vivo. We present a sensitized loss-of-function screen used to identify new genes required in border cells for their **migration**. Chromosomes bearing FRTs on all four major autosomal arms were mutagenized by insertions of the transposable element PiggyBac, allowing multiple parallel clonal screens and easy identification of the mutated gene. For border cells, we analyzed homozygous mutant clones positively marked with lacZ and sensitized by expression of dominant-negative PVR, the guidance receptor. We identified new alleles of genes already known to be required for border cell **migration**, including aop/yan, DIAP1, and taiman as well as a conserved Slbo-regulated enhancer downstream of shg/DE-cadherin. Mutations in genes not previously described to be required in border cells were also uncovered: hrp48, vir, rme-8, kismet, and puckered. puckered was unique in that the **migration** defects were observed only when PVR signaling was reduced. We present evidence that an excess of JNK signaling is deleterious for **migration** in the absence of PVR activity at least in part through Fos transcriptional activity and possibly through antagonistic effects on DIAP1.
30. Cell adhesion molecule Echinoid associates with unconventional myosin VI/Jaguar motor to regulate cell morphology during dorsal closure in Drosophila. Echinoid (Ed) is a homophilic immunoglobulin domain-containing cell adhesion molecule (CAM) that localizes to adherens junctions (AJs) and cooperates with Drosophila melanogaster epithelial (DE)-cadherin to mediate cell adhesion. Here we show that Ed takes part in many processes of dorsal closure, a morphogenetic movement driven by coordinated cell shape changes and **migration** of epidermal cells to cover the underlying amnioserosa. Ed is differentially expressed, appearing in epidermis but not in amnioserosa cells. Ed functions independently from the JNK signaling pathway and is required to regulate cell morphology, and for assembly of actomyosin cable, filopodial protrusion and coordinated cell **migration** in dorsal-most epidermal cells. The effect of Ed on cell morphology requires the presence of the intracellular domain (Ed(intra)). Interestingly, Ed forms homodimers in vivo and Ed(intra) monomer directly associates with unconventional myosin VI/Jaguar (Jar) motor protein. We further show that ed genetically interacts with jar to control cell morphology. It has previously been shown that myosin VI is monomeric in vitro and that its dimeric form can associate with and travel processively along actin filaments. Thus, we propose that Ed mediates the dimerization of myosin VI/Jar in vivo which in turn regulates the reorganization and/or contraction of actin filaments to control changes in cell shape. Consistent with this, we found that ectopic ed expression in the amnioserosa induces myosin VI/Jar-dependent apical constriction of this tissue.
31. JNK signaling controls border cell cluster integrity and collective cell **migration**. Collective cell movement is a mechanism for invasion identified in many developmental events. Examples include the movement of lateral-line neurons in Zebrafish, cells in the inner blastocyst, and metastasis of epithelial tumors [1]. One key model to study collective **migration** is the movement of border cell clusters in Drosophila. Drosophila egg chambers contain 15 nurse cells and a single oocyte surrounded by somatic follicle cells. At their anterior end, polar cells recruit several neighboring follicle cells to form the border cell cluster [2]. By stage 9, and over 6 hr, border cells migrate as a cohort between nurse cells toward the oocyte. The specification and directionality of border cell movement are regulated by hormonal and signaling mechanisms [3]. However, how border cells are held together while they migrate is not known. Here, we show that a negative-feedback loop controlling JNK activity regulates border cell cluster integrity. JNK signaling modulates contacts between border cells and between border cells and substratum to sustain collective **migration** by regulating several effectors including the polarity factor Bazooka and the cytoskeletal adaptor D-Paxillin. We anticipate a role for the JNK pathway in controlling collective cell movements in other morphogenetic and clinical models.

### ECM

#### PubMed

1. Genes conserved in bilaterians but jointly lost with Myc during nematode evolution are enriched in cell proliferation and cell migration functions. Animals use a stereotypical set of developmental genes to build body architectures of varying sizes and organizational complexity. Some genes are critical to developmental patterning, while other genes are important to physiological control of growth. However, growth regulator genes may not be as important in small-bodied "micro-metazoans" such as nematodes. Nematodes use a simplified developmental strategy of lineage-based cell fate specifications to produce an adult bilaterian body composed of a few hundreds of cells. Nematodes also lost the MYC proto-oncogenic regulator of cell proliferation. To identify additional regulators of cell proliferation that were lost with MYC, we computationally screened and determined 839 high-confidence genes that are conserved in bilaterians/lost in nematodes (CIBLIN genes). We find that 30 % of all CIBLIN genes encode transcriptional regulators of cell proliferation, epithelial-to-mesenchyme transitions, and other processes. Over 50 % of CIBLIN genes are unnamed genes in Drosophila, suggesting that there are many understudied genes. Interestingly, CIBLIN genes include many Myc synthetic lethal (MycSL) hits from recent screens. CIBLIN genes include key regulators of heparan sulfate proteoglycan (HSPG) sulfation patterns, and lysyl oxidases involved in cross-linking and modification of the extracellular matrix (**ECM**). These genes and others suggest the CIBLIN repertoire services critical functions in **ECM** remodeling and cell migration in large-bodied bilaterians. Correspondingly, CIBLIN genes are co-expressed with Myc in cancer transcriptomes, and include a preponderance of known determinants of cancer progression and tumor aggression. We propose that CIBLIN gene research can improve our understanding of regulatory control of cellular growth in metazoans.

### [Ee]xtracellular [Mm]atrix

#### PubMed

1. Dose-sensitive autosomal modifiers identify candidate genes for tissue autonomous and tissue nonautonomous regulation by the Drosophila nuclear zinc-finger protein, hindsight. The nuclear zinc-finger protein encoded by the hindsight (hnt) locus regulates several cellular processes in Drosophila epithelia, including the Jun N-terminal kinase (JNK) signaling pathway and actin polymerization. Defects in these molecular pathways may underlie the abnormal cellular interactions, loss of epithelial integrity, and apoptosis that occurs in hnt mutants, in turn causing failure of morphogenetic processes such as germ band retraction and dorsal closure in the embryo. To define the genetic pathways regulated by hnt, 124 deficiencies on the second and third chromosomes and 14 duplications on the second chromosome were assayed for dose-sensitive modification of a temperature-sensitive rough eye phenotype caused by the viable allele, hntpeb; 29 interacting regions were identified. Subsequently, 438 P-element-induced lethal mutations mapping to these regions and 12 candidate genes were tested for genetic interaction, leading to identification of 63 dominant modifier loci. A subset of the identified mutants also dominantly modify hnt308-induced embryonic lethality and thus represent general rather than tissue-specific interactors. General interactors include loci encoding transcription factors, actin-binding proteins, signal transduction proteins, and components of the **extracellular matrix**. Expression of several interactors was assessed in hnt mutant tissue. Five genes--apontic (apt), Delta (Dl), decapentaplegic (dpp), karst (kst), and puckered (puc)--are regulated tissue autonomously and, thus, may be direct transcriptional targets of HNT. Three of these genes--apt, Dl, and dpp--are also regulated nonautonomously in adjacent non-HNT-expressing tissues. The expression of several additional interactors--viking (vkg), Cg25, and laminin-alpha (LanA)-is affected only in a nonautonomous manner.
2. Immune response to bacteria induces dissemination of Ras-activated Drosophila hindgut cells. Although pathogenic bacteria are suspected contributors to colorectal cancer progression, cancer-promoting bacteria and their mode of action remain largely unknown. Here we report that sustained infection with the human intestinal colonizer Pseudomonas aeruginosa synergizes with the Ras1V12 oncogene to induce basal invasion and dissemination of hindgut cells to distant sites. Cross-talk between infection and dissemination requires sustained activation by the bacteria of the Imd-dTab2-dTak1 innate immune pathway, which converges with Ras1V12 signalling on JNK pathway activation, culminating in **extracellular matrix** degradation. Hindgut, but not midgut, cells are amenable to this cooperative dissemination, which is progressive and genetically and pharmacologically inhibitable. Thus, Drosophila hindgut provides a valuable system for the study of intestinal malignancies.
3. Genes conserved in bilaterians but jointly lost with Myc during nematode evolution are enriched in cell proliferation and cell migration functions. Animals use a stereotypical set of developmental genes to build body architectures of varying sizes and organizational complexity. Some genes are critical to developmental patterning, while other genes are important to physiological control of growth. However, growth regulator genes may not be as important in small-bodied "micro-metazoans" such as nematodes. Nematodes use a simplified developmental strategy of lineage-based cell fate specifications to produce an adult bilaterian body composed of a few hundreds of cells. Nematodes also lost the MYC proto-oncogenic regulator of cell proliferation. To identify additional regulators of cell proliferation that were lost with MYC, we computationally screened and determined 839 high-confidence genes that are conserved in bilaterians/lost in nematodes (CIBLIN genes). We find that 30 % of all CIBLIN genes encode transcriptional regulators of cell proliferation, epithelial-to-mesenchyme transitions, and other processes. Over 50 % of CIBLIN genes are unnamed genes in Drosophila, suggesting that there are many understudied genes. Interestingly, CIBLIN genes include many Myc synthetic lethal (MycSL) hits from recent screens. CIBLIN genes include key regulators of heparan sulfate proteoglycan (HSPG) sulfation patterns, and lysyl oxidases involved in cross-linking and modification of the **extracellular matrix** (ECM). These genes and others suggest the CIBLIN repertoire services critical functions in ECM remodeling and cell migration in large-bodied bilaterians. Correspondingly, CIBLIN genes are co-expressed with Myc in cancer transcriptomes, and include a preponderance of known determinants of cancer progression and tumor aggression. We propose that CIBLIN gene research can improve our understanding of regulatory control of cellular growth in metazoans.
4. Pericardin, a Drosophila type IV collagen-like protein is involved in the morphogenesis and maintenance of the heart epithelium during dorsal ectoderm closure. The steps that lead to the formation of a single primitive heart tube are highly conserved in vertebrate and invertebrate embryos. Concerted migration of the two lateral cardiogenic regions of the mesoderm and endoderm (or ectoderm in invertebrates) is required for their fusion at the midline of the embryo. Morphogenetic signals are involved in this process and the **extracellular matrix** has been proposed to serve as a link between the two layers of cells. Pericardin (Prc), a novel Drosophila **extracellular matrix** protein is a good candidate to participate in heart tube formation. The protein has the hallmarks of a type IV collagen alpha-chain and is mainly expressed in the pericardial cells at the onset of dorsal closure. As dorsal closure progresses, Pericardin expression becomes concentrated at the basal surface of the cardioblasts and around the pericardial cells, in close proximity to the dorsal ectoderm. Pericardin is absent from the lumen of the dorsal vessel. Genetic evidence suggests that Prc promotes the proper migration and alignment of heart cells. Df(3)vin6 embryos, as well as embryos in which prc has been silenced via RNAi, exhibit similar and significant defects in the formation of the heart epithelium. In these embryos, the heart epithelium appears disorganized during its migration to the dorsal midline. By the end of embryonic development, cardial and pericardial cells are misaligned such that small clusters of both cell types appear in the heart; these clusters of cells are associated with holes in the walls of the heart. A prc transgene can partially rescue each of these phenotypes, suggesting that prc regulates these events. Our results support, for the first time, the function of a collagen-like protein in the coordinated migration of dorsal ectoderm and heart cells.

### [Ii]nvasive

#### PubMed

1. Elevated expression of the V-ATPase C subunit triggers JNK-dependent cell invasion and overgrowth in a Drosophila epithelium. The C subunit of the vacuolar H(+)-ATPase or V-ATPase regulates the activity and assembly of the proton pump at cellular membranes. It has been shown to be strongly upregulated in oral squamous cell carcinoma, a highly metastatic epithelial cancer. In addition, increased V-ATPase activity appears to correlate with **invasive**ness of cancer cells, but the underlying mechanism is largely unknown. Using the Drosophila wing imaginal epithelium as an in vivo model system, we demonstrate that overexpression of Vha44, the Drosophila orthologue of the C subunit, causes a tumor-like tissue transformation in cells of the wing epithelium. Overexpressing cells are excluded from the epithelium and acquire **invasive** properties while displaying high apoptotic rates. Blocking apoptosis in these cells unmasks a strong proliferation stimulus, leading to overgrowth. Furthermore, we show that excess Vha44 greatly increases acidification of endocytic compartments and interferes with endosomal trafficking. As a result, cargoes such as GFP-Lamp1 and Notch accumulate in highly acidified enlarged endolysosomal compartments. Consistent with previous reports on the endocytic activation of Eiger/JNK signaling, we find that V-ATPase stimulation by Vha44 causes JNK signaling activation whereas downmodulation of JNK signaling rescues the **invasive** phenotypes. In summary, our in vivo-findings demonstrate that increased levels of V-ATPase C subunit induce a Eiger/JNK-dependent cell transformation within an epithelial organ that recapitulates early carcinoma stages.
2. A role for the epithelial microenvironment at tumor boundaries: evidence from Drosophila and human squamous cell carcinomas. Recent work has shown an increasing appreciation for the importance of the tumor environment, most commonly the overlying stroma. Less emphasis has been placed on the importance of local communication between transformed cells and their neighbors within the epithelium at tumor boundaries. We previously reported a Drosophila model that highlighted the importance of local interactions within the epithelial microenvironment: Src-transformed cells (Csk-deficient) were influenced by their immediate normal neighbors. The result was a consistent change in 'border cells' at the edge of transformed patches including delocalized p120-catenin and E-cadherin as well as **invasive** migration through the basal lamina. Here we show that the **invasive** properties of the boundary cells depend on up-regulation of Drosophila matrix metalloproteinase-1 as assessed by promoter activity, protein levels, in situ enzymatic activity, and tests of genetic modifier activity. Further, we provide evidence that these events at tumor borders may be evolutionarily conserved. We detected changes in 'boundary cells' within histological sections of human squamous cell carcinomas that were similar to those observed in Drosophila: both E-cadherin and p120-catenin exhibited normal junctional localization at the centers of the tumors but were reduced or delocalized at the boundary. Further, matrix metalloproteinase-2 was up regulated within these same boundary cells. These results support the view that local cell-cell interactions within the epithelial microenvironment impact tumor invasion and progression.
3. Sds22/PP1 links epithelial integrity and tumor suppression via regulation of myosin II and JNK signaling. Loss of epithelial integrity often correlates with the progression of malignant tumors. Sds22, a regulatory subunit of protein phosphatase 1 (PP1), has recently been linked to regulation of epithelial polarity in Drosophila. However, its role in tumorigenesis remains obscure. In this study, using Drosophila imaginal tissue as an in vivo model system, we show that sds22 is a new potential tumor suppressor gene in Drosophila. Without sds22, cells lose epithelial architecture, and become **invasive** and tumorigenic when combined with Ras overexpression; conversely, sds22 overexpression can largely suppress tumorigenic growth of Ras(V12)scrib(-/-) mutant cells. Mechanistically, we show that sds22 prevents cell invasion and metastasis by inhibiting myosin II and Jun N-terminal kinase (JNK) activity downstream of PP1. Loss of this inhibition causes cells to lose epithelial organization and promotes cell invasion. Finally, human Sds22 is focally deleted and downregulated in multiple carcinomas, and this downregulation correlates with tumor progression, suggesting that sds22 inactivation may contribute to tumorigenesis and metastatic potential in human cancers via a similar mechanism.
4. **Invasive** and indigenous microbiota impact intestinal stem cell activity through multiple pathways in Drosophila. Gut homeostasis is controlled by both immune and developmental mechanisms, and its disruption can lead to inflammatory disorders or cancerous lesions of the intestine. While the impact of bacteria on the mucosal immune system is beginning to be precisely understood, little is known about the effects of bacteria on gut epithelium renewal. Here, we addressed how both infectious and indigenous bacteria modulate stem cell activity in Drosophila. We show that the increased epithelium renewal observed upon some bacterial infections is a consequence of the oxidative burst, a major defense of the Drosophila gut. Additionally, we provide evidence that the JAK-STAT (Janus kinase-signal transducers and activators of transcription) and JNK (c-Jun NH(2) terminal kinase) pathways are both required for bacteria-induced stem cell proliferation. Similarly, we demonstrate that indigenous gut microbiota activate the same, albeit reduced, program at basal levels. Altered control of gut microbiota in immune-deficient or aged flies correlates with increased epithelium renewal. Finally, we show that epithelium renewal is an essential component of Drosophila defense against oral bacterial infection. Altogether, these results indicate that gut homeostasis is achieved by a complex interregulation of the immune response, gut microbiota, and stem cell activity.
5. Aneuploidy-induced delaminating cells drive tumorigenesis in Drosophila epithelia. Genomic instability has been observed in essentially all sporadic carcinomas. Here we use Drosophila epithelial cells to address the role of chromosomal instability in cancer development as they have proved useful for elucidating the molecular mechanisms underlying tumorigenic growth. We first show that chromosomal instability leads to an apoptotic response. Interestingly, this response is p53 independent, as opposed to mammalian cells, and depends on the activation of the c-Jun N-terminal kinase (JNK) signaling cascade. When prevented from undergoing programmed cell death (PCD), chromosomal instability induces neoplasic overgrowth. These tumor-like tissues are able to grow extensively and metastasize when transplanted into the abdomen of adult hosts. Detailed analysis of the tumors allows us to identify a delaminating cell population as the critical one in driving tumorigenesis. Cells loose their apical-basal polarity, mislocalize DE-cadherin, and delaminate from the main epithelium. A JNK-dependent transcriptional program is activated specifically in delaminating cells and drives nonautonomous tissue overgrowth, basement membrane degradation, and **invasive**ness. These findings unravel a general and rapid tumorigenic potential of genomic instability, as opposed to its proposed role as a source of mutability to select specific tumor-prone aneuploid cells, and open unique avenues toward the understanding of the role of genomic instability in human cancer.
6. Sin3a acts through a multi-gene module to regulate invasion in Drosophila and human tumors. Chromatin remodeling proteins regulate multiple aspects of cell homeostasis, making them ideal candidates for misregulation in transformed cells. Here, we explore Sin3A, a member of the Sin3 family of proteins linked to tumorigenesis that are thought to regulate gene expression through their role as histone deacetylases (HDACs). We identified Drosophila Sin3a as an important mediator of oncogenic Ret receptor in a fly model of Multiple Endocrine Neoplasia Type 2. Reducing Drosophila Sin3a activity led to metastasis-like behavior and, in the presence of Diap1, secondary tumors distant from the site of origin. Genetic and Chip-Seq analyses identified previously undescribed Sin3a targets including genes involved in cell motility and actin dynamics, as well as signaling pathways including Src, Jnk and Rho. A key Sin3a oncogenic target, PP1B, regulates stability of β-Catenin/Armadillo: the outcome is to oppose T-cell factor (TCF) function and Wg/Wnt pathway signaling in both fly and mammalian cancer cells. Reducing Sin3A strongly increased the **invasiv**e behavior of A549 human lung adenocarcinoma cells. We show that Sin3A is downregulated in a variety of human tumors and that Src, JNK, RhoA and PP1B/β-Catenin are regulated in a manner analogous to our Drosophila models. Our data suggest that Sin3A influences a specific step of tumorigenesis by regulating a module of genes involved in cell invasion. Tumor progression may commonly rely on such 'modules of invasion' under the control of broad transcriptional regulators.
7. The actin cross-linker Filamin/Cheerio mediates tumor malignancy downstream of JNK signaling. Cell shape dynamics, motility, and cell proliferation all depend on the actin cytoskeleton. Malignant cancer cells hijack the actin network to grow and migrate to secondary sites. Understanding the function of actin regulators is therefore of major interest. In the present study, we identify the actin cross-linking protein Filamin/Cheerio (Cher) as a mediator of malignancy in genetically defined Drosophila tumors. We show that in **invasive** tumors, resulting from cooperation of activated Ras with disrupted epithelial cell polarity, Cher is upregulated in a Jun N-terminal kinase (JNK)-dependent manner. Although dispensable in normal epithelium, Cher becomes required in the tumor cells for their growth and **invasive**ness. When deprived of Cher, these tumor clones lose their full potential to proliferate and breach tissue boundaries. Instead, the Cher-deficient clones remain confined within the limits of their source epithelium, permitting survival of the host animal. Through interaction with the myosin II heavy chain subunit, Cher is likely to strengthen the cortical actomyosin network and reinforce mechanical tension within the **invasive** tumors. Accordingly, Cher is required for aberrant expression of genes downstream of the Hippo/Yorkie signaling in the tumor tissue. Our study identifies Cher as a new target of JNK signaling that links cytoskeleton dynamics to tumor progression.
8. JNK- and Fos-regulated Mmp1 expression cooperates with Ras to induce **invasive** tumors in Drosophila. Loss of the epithelial polarity gene scribble in clones of Drosophila imaginal disc cells can cooperate with Ras signaling to induce malignant tumors. Such mutant tissue overproliferates, resists apoptosis, leaves its place of origin and invades other organs, ultimately causing lethality. We show that increased Jun N-terminal kinase (JNK) signaling resulting from the loss of scribble promotes the movement of transformed cells to secondary sites. This effect requires Fos-dependent transcriptional activation of a matrix metalloprotease gene mmp1 downstream of JNK. Expression of the Mmp inhibitor Timp or Mmp RNAi knockdown suppresses cell **invasive**ness. The pro**invasive** function of the JNK pathway is revealed in a tumor context when active Ras signaling prevents the apoptotic response to JNK activity as it occurs in nontransformed cells. Based on these results, we present a model that explains the oncogenic cooperation between JNK and Ras, and describes how aberrant regulation of cell survival, proliferation and mobilization cooperate to incite malignant tumor formation.
9. Pathogen and host factors are needed to provoke a systemic host response to gastrointestinal infection of Drosophila larvae by Candida albicans. Candida albicans systemic dissemination in immunocompromised patients is thought to develop from initial gastrointestinal (GI) colonisation. It is unclear what components of the innate immune system are necessary for preventing C. albicans dissemination from the GI tract, but studies in mice have indicated that both neutropenia and GI mucosal damage are crucial for allowing widespread **invasive** C. albicans disease. Mouse models, however, provide limited applicability to genome-wide screens for pathogen or host factors - factors that might influence systemic dissemination following GI colonisation. For this reason we developed a Drosophila model to study intestinal infection by Candida. We found that commensal flora aided host survival following GI infection. Candida provoked extensive JNK-mediated death of gut cells and induced antimicrobial peptide expression in the fat body. From the side of the host, nitric oxide and blood cells influenced systemic antimicrobial responses. The secretion of SAP4 and SAP6 (secreted aspartyl proteases) from Candida was also essential for activating systemic Toll-dependent immunity.
10. Caspase signalling in the absence of apoptosis drives Jnk-dependent invasion. Tumours evolve several mechanisms to evade apoptosis, yet many resected carcinomas show significantly elevated caspase activity. Moreover, caspase activity is positively correlated with tumour aggression and adverse patient outcome. These observations indicate that caspases might have a functional role in promoting tumour invasion and metastasis. Using a Drosophila model of invasion, we show that precise effector caspase activity drives cell invasion without initiating apoptosis. Affected cells express the matrix metalloprotinase Mmp1 and invade by activating Jnk. Our results link Jnk and effector caspase signalling during the **invasive** process and suggest that tumours under apoptotic stresses from treatment, immune surveillance or intrinsic signals might be induced further along the metastatic cascade.
11. The Drosophila TNF receptor Grindelwald couples loss of cell polarity and neoplastic growth. Disruption of epithelial polarity is a key event in the acquisition of neoplastic growth. JNK signalling is known to play an important part in driving the malignant progression of many epithelial tumours, although the link between loss of polarity and JNK signalling remains elusive. In a Drosophila genome-wide genetic screen designed to identify molecules implicated in neoplastic growth, we identified grindelwald (grnd), a gene encoding a transmembrane protein with homology to members of the tumour necrosis factor receptor (TNFR) superfamily. Here we show that Grnd mediates the pro-apoptotic functions of Eiger (Egr), the unique Drosophila TNF, and that overexpression of an active form of Grnd lacking the extracellular domain is sufficient to activate JNK signalling in vivo. Grnd also promotes the **invasive**ness of Ras(V12)/scrib(-/-) tumours through Egr-dependent Matrix metalloprotease-1 (Mmp1) expression. Grnd localizes to the subapical membrane domain with the cell polarity determinant Crumbs (Crb) and couples Crb-induced loss of polarity with JNK activation and neoplastic growth through physical interaction with Veli (also known as Lin-7). Therefore, Grnd represents the first example of a TNFR that integrates signals from both Egr and apical polarity determinants to induce JNK-dependent cell death or tumour growth.
12. **Invasive** cell behavior during Drosophila imaginal disc eversion is mediated by the JNK signaling cascade. Drosophila imaginal discs are monolayered epithelial invaginations that grow during larval stages and evert at metamorphosis to assemble the adult exoskeleton. They consist of columnar cells, forming the imaginal epithelium, as well as squamous cells, which constitute the peripodial epithelium and stalk (PS). Here, we uncover a new morphogenetic/cellular mechanism for disc eversion. We show that imaginal discs evert by apposing their peripodial side to the larval epidermis and through the invasion of the larval epidermis by PS cells, which undergo a pseudo-epithelial-mesenchymal transition (PEMT). As a consequence, the PS/larval bilayer is perforated and the imaginal epithelia protrude, a process reminiscent of other developmental events, such as epithelial perforation in chordates. When eversion is completed, PS cells localize to the leading front, heading disc expansion. We found that the JNK pathway is necessary for PS/larval cells apposition, the PEMT, and the motile activity of leading front cells.
13. Src42A modulates tumor invasion and cell death via Ben/dUev1a-mediated JNK activation in Drosophila. Loss of the cell polarity gene could cooperate with oncogenic Ras to drive tumor growth and invasion, which critically depends on the c-Jun N-terminal Kinase (JNK) signaling pathway in Drosophila. By performing a genetic screen, we have identified Src42A, the ortholog of mammalian Src, as a key modulator of both Ras(V12)/lgl(-/-) triggered tumor invasion and loss of cell polarity gene-induced cell migration. Our genetic study further demonstrated that the Bendless (Ben)/dUev1a ubiquitin E2 complex is an essential regulator of Src42A-induced, JNK-mediated cell migration. Furthermore, we showed that ectopic Ben/dUev1a expression induced **invasive** cell migration along with increased MMP1 production in wing disc epithelia. Moreover, Ben/dUev1a could cooperate with Ras(V12) to promote tumor overgrowth and invasion. In addition, we found that the Ben/dUev1a complex is required for ectopic Src42A-triggered cell death and endogenous Src42A-dependent thorax closure. Our data not only provide a mechanistic insight into the role of Src in development and disease but also propose a potential oncogenic function for Ubc13 and Uev1a, the mammalian homologs of Ben and dUev1a.
14. Csk-deficient boundary cells are eliminated from normal Drosophila epithelia by exclusion, migration, and apoptosis. The construction and maintenance of normal epithelia relies on local signals that guide cells into their proper niches and remove unwanted cells. Failure to execute this process properly may result in aberrant development or diseases, including cancer and associated metastasis. Here, we show that local environment influences the behavior of dCsk-deficient cells. Broad loss of dCsk led to enlarged and mispatterned tissues due to overproliferation, a block in apoptosis, and decreased cadherin-mediated adhesion. Loss of dCsk in discrete patches led to a different outcome: epithelial exclusion, **invasive** migration, and apoptotic death. These latter phenotypes required sharp differences in dCsk activity between neighbors; dE-cadherin, P120-catenin, Rho1, JNK, and MMP2 mediated this signal. Together, our data demonstrate how the cellular microenvironment plays a central role in determining the outcome of altered dCsk activity, and reveal a role for P120-catenin in a mechanism that protects epithelial integrity by removing abnormal cells.

### [Mm]etastasize

#### PubMed

1. Aneuploidy-induced delaminating cells drive tumorigenesis in Drosophila epithelia. Genomic instability has been observed in essentially all sporadic carcinomas. Here we use Drosophila epithelial cells to address the role of chromosomal instability in cancer development as they have proved useful for elucidating the molecular mechanisms underlying tumorigenic growth. We first show that chromosomal instability leads to an apoptotic response. Interestingly, this response is p53 independent, as opposed to mammalian cells, and depends on the activation of the c-Jun N-terminal kinase (JNK) signaling cascade. When prevented from undergoing programmed cell death (PCD), chromosomal instability induces neoplasic overgrowth. These tumor-like tissues are able to grow extensively and **metastasize** when transplanted into the abdomen of adult hosts. Detailed analysis of the tumors allows us to identify a delaminating cell population as the critical one in driving tumorigenesis. Cells loose their apical-basal polarity, mislocalize DE-cadherin, and delaminate from the main epithelium. A JNK-dependent transcriptional program is activated specifically in delaminating cells and drives nonautonomous tissue overgrowth, basement membrane degradation, and invasiveness. These findings unravel a general and rapid tumorigenic potential of genomic instability, as opposed to its proposed role as a source of mutability to select specific tumor-prone aneuploid cells, and open unique avenues toward the understanding of the role of genomic instability in human cancer.

### [Mm]etastasis

#### PubMed

1. Drosophila Abelson kinase mediates cell invasion and proliferation through two distinct MAPK pathways. The Abelson (Abl) family of non-receptor tyrosine kinases has an important role in cell morphogenesis, motility, and proliferation. Although the function of Abl has been extensively studied in leukemia, its role in epithelial cell invasion remains obscure. Using the Drosophila wing epithelium as an in vivo model system, we show that overexpression (activation) of Drosophila Abl (dAbl) causes loss of epithelial apical/basal cell polarity and secretion of matrix metalloproteinases, resulting in a cellular invasion and apoptosis. Our in vivo data indicate that dAbl acts downstream of the Src kinases, which are known regulators of cell adhesion and invasion. Downstream of dAbl, Rac GTPases activate two distinct MAPK pathways: c-Jun N-terminal kinase signaling (required for cell invasion and apoptosis) and ERK signaling (inducing cell proliferation). Activated Abl also increases the activity of Src members through a positive feedback loop leading to signal amplification. Thus, targeting Src-Abl, using available dual inhibitors, could be of therapeutic importance in tumor cell **metastasis**.
2. Regulation of cell adhesion and collective cell migration by hindsight and its human homolog RREB1. Cell movements represent a major driving force in embryonic development, tissue repair, and tumor **metastasis** [1]. The migration of single cells has been well studied, predominantly in cell culture [2, 3]; however, in vivo, a greater variety of modes of cell movement occur, including the movements of cells in clusters, strands, sheets, and tubes, also known as collective cell migrations [4, 5]. In spite of the relevance of these types of movements in both normal and pathological conditions, the molecular mechanisms that control them remain predominantly unknown. Epithelial follicle cells of the Drosophila ovary undergo several dynamic morphological changes, providing a genetically tractable model [6]. We found that anterior follicle cells, including border cells, mutant for the gene hindsight (hnt) accumulated excess cell-cell adhesion molecules and failed to undergo their normal collective movements. In addition, HNT affected border cell cluster cohesion and motility via effects on the JNK and STAT pathways, respectively. Interestingly, reduction of expression of the mammalian homolog of HNT, RREB1, by siRNA inhibited collective cell migration in a scratch-wound healing assay of MCF10A mammary epithelial cells, suppressed surface activity, retarded cell spreading after plating, and led to the formation of immobile, tightly adherent cell colonies. We propose that HNT and RREB1 are essential to reduce cell-cell adhesion when epithelial cells within an interconnected group undergo dynamic changes in cell shape.
3. Sds22/PP1 links epithelial integrity and tumor suppression via regulation of myosin II and JNK signaling. Loss of epithelial integrity often correlates with the progression of malignant tumors. Sds22, a regulatory subunit of protein phosphatase 1 (PP1), has recently been linked to regulation of epithelial polarity in Drosophila. However, its role in tumorigenesis remains obscure. In this study, using Drosophila imaginal tissue as an in vivo model system, we show that sds22 is a new potential tumor suppressor gene in Drosophila. Without sds22, cells lose epithelial architecture, and become invasive and tumorigenic when combined with Ras overexpression; conversely, sds22 overexpression can largely suppress tumorigenic growth of Ras(V12)scrib(-/-) mutant cells. Mechanistically, we show that sds22 prevents cell invasion and **metastasis** by inhibiting myosin II and Jun N-terminal kinase (JNK) activity downstream of PP1. Loss of this inhibition causes cells to lose epithelial organization and promotes cell invasion. Finally, human Sds22 is focally deleted and downregulated in multiple carcinomas, and this downregulation correlates with tumor progression, suggesting that sds22 inactivation may contribute to tumorigenesis and metastatic potential in human cancers via a similar mechanism.
4. Sin3a acts through a multi-gene module to regulate invasion in Drosophila and human tumors. Chromatin remodeling proteins regulate multiple aspects of cell homeostasis, making them ideal candidates for misregulation in transformed cells. Here, we explore Sin3A, a member of the Sin3 family of proteins linked to tumorigenesis that are thought to regulate gene expression through their role as histone deacetylases (HDACs). We identified Drosophila Sin3a as an important mediator of oncogenic Ret receptor in a fly model of Multiple Endocrine Neoplasia Type 2. Reducing Drosophila Sin3a activity led to **metastasis**-like behavior and, in the presence of Diap1, secondary tumors distant from the site of origin. Genetic and Chip-Seq analyses identified previously undescribed Sin3a targets including genes involved in cell motility and actin dynamics, as well as signaling pathways including Src, Jnk and Rho. A key Sin3a oncogenic target, PP1B, regulates stability of β-Catenin/Armadillo: the outcome is to oppose T-cell factor (TCF) function and Wg/Wnt pathway signaling in both fly and mammalian cancer cells. Reducing Sin3A strongly increased the invasive behavior of A549 human lung adenocarcinoma cells. We show that Sin3A is downregulated in a variety of human tumors and that Src, JNK, RhoA and PP1B/β-Catenin are regulated in a manner analogous to our Drosophila models. Our data suggest that Sin3A influences a specific step of tumorigenesis by regulating a module of genes involved in cell invasion. Tumor progression may commonly rely on such 'modules of invasion' under the control of broad transcriptional regulators.
5. A novel link between FMR gene and the JNK pathway provides clues to possible role in malignant pleural mesothelioma. Malignant pleural mesothelioma (MPM) is an aggressive form of thoracic cancer with poor prognosis. While some studies have identified the molecular alterations associated with MPM, little is known about their role in MPM. For example, fragile X mental retardation (FMR) gene is up-regulated in MPM but its role in MPM is unknown. Here, utilizing Drosophila genetics, I investigate the possible role FMR may be playing in MPM. I provide evidence which suggests that FMR may contribute to tumorigenesis by up-regulating a matrix metalloprotease (MMP) and by degrading the basement membrane (BM), both important for tumor **metastasis**. I also demonstrate a novel link between FMR and the JNK pathway and suggest that the effects of FMR in MPM could in part be mediated by up-regulation of the JNK pathway.
6. Synergy between bacterial infection and genetic predisposition in intestinal dysplasia. Accumulating evidence suggests that hyperproliferating intestinal stem cells (SCs) and progenitors drive cancer initiation, maintenance, and **metastasis**. In addition, chronic inflammation and infection have been increasingly recognized for their roles in cancer. Nevertheless, the mechanisms by which bacterial infections can initiate SC-mediated tumorigenesis remain elusive. Using a Drosophila model of gut pathogenesis, we show that intestinal infection with Pseudomonas aeruginosa, a human opportunistic bacterial pathogen, activates the c-Jun N-terminal kinase (JNK) pathway, a hallmark of the host stress response. This, in turn, causes apoptosis of enterocytes, the largest class of differentiated intestinal cells, and promotes a dramatic proliferation of SCs and progenitors that serves as a homeostatic compensatory mechanism to replenish the apoptotic enterocytes. However, we find that this homeostatic mechanism can lead to massive over-proliferation of intestinal cells when infection occurs in animals with a latent oncogenic form of the Ras1 oncogene. The affected intestines develop excess layers of cells with altered apicobasal polarity reminiscent of dysplasia, suggesting that infection can directly synergize with the genetic background in predisposed individuals to initiate SC-mediated tumorigenesis. Our results provide a framework for the study of intestinal bacterial infections and their effects on undifferentiated and mature enteric epithelial cells in the initial stages of intestinal cancer. Assessment of progenitor cell responses to pathogenic intestinal bacteria could provide a measure of predisposition for apoptotic enterocyte-assisted intestinal dysplasias in humans.
7. Modeling tumor invasion and **metastasis** in Drosophila. Conservation of major signaling pathways between humans and flies has made Drosophila a useful model organism for cancer research. Our understanding of the mechanisms regulating cell growth, differentiation and development has been considerably advanced by studies in Drosophila. Several recent high profile studies have examined the processes constraining the metastatic growth of tumor cells in fruit fly models. Cell invasion can be studied in the context of an in vivo setting in flies, enabling the genetic requirements of the microenvironment of tumor cells undergoing **metastasis** to be analyzed. This Perspective discusses the strengths and limitations of Drosophila models of cancer invasion and the unique tools that have enabled these studies. It also highlights several recent reports that together make a strong case for Drosophila as a system with the potential for both testing novel concepts in tumor progression and cell invasion, and for uncovering players in **metastasis**.
8. Caspase signalling in the absence of apoptosis drives Jnk-dependent invasion. Tumours evolve several mechanisms to evade apoptosis, yet many resected carcinomas show significantly elevated caspase activity. Moreover, caspase activity is positively correlated with tumour aggression and adverse patient outcome. These observations indicate that caspases might have a functional role in promoting tumour invasion and **metastasis**. Using a Drosophila model of invasion, we show that precise effector caspase activity drives cell invasion without initiating apoptosis. Affected cells express the matrix metalloprotinase Mmp1 and invade by activating Jnk. Our results link Jnk and effector caspase signalling during the invasive process and suggest that tumours under apoptotic stresses from treatment, immune surveillance or intrinsic signals might be induced further along the metastatic cascade.
9. Loss of cell polarity drives tumor growth and invasion through JNK activation in Drosophila. Apparent defects in cell polarity are often seen in human cancer. However, the underlying mechanisms of how cell polarity disruption contributes to tumor progression are unknown. Here, using a Drosophila genetic model for Ras-induced tumor progression, we show a molecular link between loss of cell polarity and tumor malignancy. Mutation of different apicobasal polarity genes activates c-Jun N-terminal kinase (JNK) signaling and downregulates the E-cadherin/beta-catenin adhesion complex, both of which are necessary and sufficient to cause oncogenic Ras(V12)-induced benign tumors in the developing eye to exhibit metastatic behavior. Furthermore, activated JNK and Ras signaling cooperate in promoting tumor growth cell autonomously, as JNK signaling switches its proapoptotic role to a progrowth effect in the presence of oncogenic Ras. Our finding that such context-dependent alterations promote both tumor growth and metastatic behavior suggests that **metastasis**-promoting mutations may be selected for based primarily on their growth-promoting capabilities. Similar oncogenic cooperation mediated through these evolutionarily conserved signaling pathways could contribute to human cancer progression.
10. Csk-deficient boundary cells are eliminated from normal Drosophila epithelia by exclusion, migration, and apoptosis. The construction and maintenance of normal epithelia relies on local signals that guide cells into their proper niches and remove unwanted cells. Failure to execute this process properly may result in aberrant development or diseases, including cancer and associated **metastasis**. Here, we show that local environment influences the behavior of dCsk-deficient cells. Broad loss of dCsk led to enlarged and mispatterned tissues due to overproliferation, a block in apoptosis, and decreased cadherin-mediated adhesion. Loss of dCsk in discrete patches led to a different outcome: epithelial exclusion, invasive migration, and apoptotic death. These latter phenotypes required sharp differences in dCsk activity between neighbors; dE-cadherin, P120-catenin, Rho1, JNK, and MMP2 mediated this signal. Together, our data demonstrate how the cellular microenvironment plays a central role in determining the outcome of altered dCsk activity, and reveal a role for P120-catenin in a mechanism that protects epithelial integrity by removing abnormal cells.
11. Light-mediated activation reveals a key role for Rac in collective guidance of cell movement in vivo. The small GTPase Rac induces actin polymerization, membrane ruffling and focal contact formation in cultured single cells but can either repress or stimulate motility in epithelial cells depending on the conditions. The role of Rac in collective epithelial cell movements in vivo, which are important for both morphogenesis and **metastasis**, is therefore difficult to predict. Recently, photoactivatable analogues of Rac (PA-Rac) have been developed, allowing rapid and reversible activation or inactivation of Rac using light. In cultured single cells, light-activated Rac leads to focal membrane ruffling, protrusion and migration. Here we show that focal activation of Rac is also sufficient to polarize an entire group of cells in vivo, specifically the border cells of the Drosophila ovary. Moreover, activation or inactivation of Rac in one cell of the cluster caused a dramatic response in the other cells, suggesting that the cells sense direction as a group according to relative levels of Rac activity. Communication between cells of the cluster required Jun amino-terminal kinase (JNK) but not guidance receptor signalling. These studies further show that photoactivatable proteins are effective tools in vivo.
12. Transgenic expression of the Helicobacter pylori virulence factor CagA promotes apoptosis or tumorigenesis through JNK activation in Drosophila. Gastric cancer development is strongly correlated with infection by Helicobacter pylori possessing the effector protein CagA. Using a transgenic Drosophila melanogaster model, we show that CagA expression in the simple model epithelium of the larval wing imaginal disc causes dramatic tissue perturbations and apoptosis when CagA-expressing and non-expressing cells are juxtaposed. This cell death phenotype occurs through activation of JNK signaling and is enhanced by loss of the neoplastic tumor suppressors in CagA-expressing cells or loss of the TNF homolog Eiger in wild type neighboring cells. We further explored the effects of CagA-mediated JNK pathway activation on an epithelium in the context of oncogenic Ras activation, using a Drosophila model of **metastasis**. In this model, CagA expression in epithelial cells enhances the growth and invasion of tumors in a JNK-dependent manner. These data suggest a potential role for CagA-mediated JNK pathway activation in promoting gastric cancer progression.
13. JNK signaling controls border cell cluster integrity and collective cell migration. Collective cell movement is a mechanism for invasion identified in many developmental events. Examples include the movement of lateral-line neurons in Zebrafish, cells in the inner blastocyst, and **metastasis** of epithelial tumors [1]. One key model to study collective migration is the movement of border cell clusters in Drosophila. Drosophila egg chambers contain 15 nurse cells and a single oocyte surrounded by somatic follicle cells. At their anterior end, polar cells recruit several neighboring follicle cells to form the border cell cluster [2]. By stage 9, and over 6 hr, border cells migrate as a cohort between nurse cells toward the oocyte. The specification and directionality of border cell movement are regulated by hormonal and signaling mechanisms [3]. However, how border cells are held together while they migrate is not known. Here, we show that a negative-feedback loop controlling JNK activity regulates border cell cluster integrity. JNK signaling modulates contacts between border cells and between border cells and substratum to sustain collective migration by regulating several effectors including the polarity factor Bazooka and the cytoskeletal adaptor D-Paxillin. We anticipate a role for the JNK pathway in controlling collective cell movements in other morphogenetic and clinical models.

### [Cc]ell [Ii]nvasion

#### PubMed

1. Drosophila Abelson kinase mediates **cell invasion** and proliferation through two distinct MAPK pathways. The Abelson (Abl) family of non-receptor tyrosine kinases has an important role in cell morphogenesis, motility, and proliferation. Although the function of Abl has been extensively studied in leukemia, its role in epithelial **cell invasion** remains obscure. Using the Drosophila wing epithelium as an in vivo model system, we show that overexpression (activation) of Drosophila Abl (dAbl) causes loss of epithelial apical/basal cell polarity and secretion of matrix metalloproteinases, resulting in a cellular invasion and apoptosis. Our in vivo data indicate that dAbl acts downstream of the Src kinases, which are known regulators of cell adhesion and invasion. Downstream of dAbl, Rac GTPases activate two distinct MAPK pathways: c-Jun N-terminal kinase signaling (required for **cell invasion** and apoptosis) and ERK signaling (inducing cell proliferation). Activated Abl also increases the activity of Src members through a positive feedback loop leading to signal amplification. Thus, targeting Src-Abl, using available dual inhibitors, could be of therapeutic importance in tumor cell metastasis.
2. Elevated expression of the V-ATPase C subunit triggers JNK-dependent **cell invasion** and overgrowth in a Drosophila epithelium. The C subunit of the vacuolar H(+)-ATPase or V-ATPase regulates the activity and assembly of the proton pump at cellular membranes. It has been shown to be strongly upregulated in oral squamous cell carcinoma, a highly metastatic epithelial cancer. In addition, increased V-ATPase activity appears to correlate with invasiveness of cancer cells, but the underlying mechanism is largely unknown. Using the Drosophila wing imaginal epithelium as an in vivo model system, we demonstrate that overexpression of Vha44, the Drosophila orthologue of the C subunit, causes a tumor-like tissue transformation in cells of the wing epithelium. Overexpressing cells are excluded from the epithelium and acquire invasive properties while displaying high apoptotic rates. Blocking apoptosis in these cells unmasks a strong proliferation stimulus, leading to overgrowth. Furthermore, we show that excess Vha44 greatly increases acidification of endocytic compartments and interferes with endosomal trafficking. As a result, cargoes such as GFP-Lamp1 and Notch accumulate in highly acidified enlarged endolysosomal compartments. Consistent with previous reports on the endocytic activation of Eiger/JNK signaling, we find that V-ATPase stimulation by Vha44 causes JNK signaling activation whereas downmodulation of JNK signaling rescues the invasive phenotypes. In summary, our in vivo-findings demonstrate that increased levels of V-ATPase C subunit induce a Eiger/JNK-dependent cell transformation within an epithelial organ that recapitulates early carcinoma stages.
3. Sds22/PP1 links epithelial integrity and tumor suppression via regulation of myosin II and JNK signaling. Loss of epithelial integrity often correlates with the progression of malignant tumors. Sds22, a regulatory subunit of protein phosphatase 1 (PP1), has recently been linked to regulation of epithelial polarity in Drosophila. However, its role in tumorigenesis remains obscure. In this study, using Drosophila imaginal tissue as an in vivo model system, we show that sds22 is a new potential tumor suppressor gene in Drosophila. Without sds22, cells lose epithelial architecture, and become invasive and tumorigenic when combined with Ras overexpression; conversely, sds22 overexpression can largely suppress tumorigenic growth of Ras(V12)scrib(-/-) mutant cells. Mechanistically, we show that sds22 prevents **cell invasion** and metastasis by inhibiting myosin II and Jun N-terminal kinase (JNK) activity downstream of PP1. Loss of this inhibition causes cells to lose epithelial organization and promotes **cell invasion**. Finally, human Sds22 is focally deleted and downregulated in multiple carcinomas, and this downregulation correlates with tumor progression, suggesting that sds22 inactivation may contribute to tumorigenesis and metastatic potential in human cancers via a similar mechanism.
4. dUev1a modulates TNF-JNK mediated tumor progression and cell death in Drosophila. Loss of cell polarity cooperates with oncogenic Ras to induce JNK-dependent tumor growth and invasion. To identify additional genes that modulate tumor progression, we have performed a genetic screen in Drosophila and found that loss of dUev1a, the ortholog of mammalian Uev1, suppressed lgl(-/-)/Ras(V12) induced JNK-mediated tumor growth and invasion. Furthermore, loss of dUev1a suppressed TNF ortholog Eiger-induced JNK-mediated **cell invasion** and cell death. Finally, dUev1a cooperated with Bendless to activate JNK signaling through dTRAF2. Together, our data indicate that dUev1a encodes an essential component of the evolutionary conserved TNF-JNK signaling pathway that modulates tumor progression and cell death in metazoan.
5. Sin3a acts through a multi-gene module to regulate invasion in Drosophila and human tumors. Chromatin remodeling proteins regulate multiple aspects of cell homeostasis, making them ideal candidates for misregulation in transformed cells. Here, we explore Sin3A, a member of the Sin3 family of proteins linked to tumorigenesis that are thought to regulate gene expression through their role as histone deacetylases (HDACs). We identified Drosophila Sin3a as an important mediator of oncogenic Ret receptor in a fly model of Multiple Endocrine Neoplasia Type 2. Reducing Drosophila Sin3a activity led to metastasis-like behavior and, in the presence of Diap1, secondary tumors distant from the site of origin. Genetic and Chip-Seq analyses identified previously undescribed Sin3a targets including genes involved in cell motility and actin dynamics, as well as signaling pathways including Src, Jnk and Rho. A key Sin3a oncogenic target, PP1B, regulates stability of β-Catenin/Armadillo: the outcome is to oppose T-cell factor (TCF) function and Wg/Wnt pathway signaling in both fly and mammalian cancer cells. Reducing Sin3A strongly increased the invasive behavior of A549 human lung adenocarcinoma cells. We show that Sin3A is downregulated in a variety of human tumors and that Src, JNK, RhoA and PP1B/β-Catenin are regulated in a manner analogous to our Drosophila models. Our data suggest that Sin3A influences a specific step of tumorigenesis by regulating a module of genes involved i**n cell invasi**on. Tumor progression may commonly rely on such 'modules of invasion' under the control of broad transcriptional regulators.
6. Rho1-Wnd signaling regulates loss-of-cell polarity-induced **cell invasion** in Drosophila. Both cell polarity and c-Jun N-terminal kinase (JNK) activity are essential to the maintenance of tissue homeostasis, and disruption of either is commonly seen in cancer progression. Despite the established connection between loss-of-cell polarity and JNK activation, much less is known about the molecular mechanism by which aberrant cell polarity induces JNK-mediated cell migration and tumor invasion. Here we show results from a genetic screen using an in vivo invasion model via knocking down cell polarity gene in Drosophila wing discs, and identify Rho1-Wnd signaling as an important molecular link that mediates loss-of-cell polarity-triggered JNK activation and **cell invasion**. We show that Wallenda (Wnd), a protein kinase of the mitogen-activated protein kinase kinase kinase family, by forming a complex with the GTPase Rho1, is both necessary and sufficient for Rho1-induced JNK-dependent **cell invasion**, MMP1 activation and epithelial-mesenchymal transition. Furthermore, Wnd promotes cell proliferation and tissue growth through wingless production when apoptosis is inhibited by p35. Finally, Wnd shows oncogenic cooperation with Ras(V12) to trigger tumor growth in eye discs and causes invasion into the ventral nerve cord. Together, our data not only provides a novel mechanistic insight on how cell polarity loss contributes to **cell invasion**, but also highlights the value of the Drosophila model system to explore human cancer biology.
7. Modeling tumor invasion and metastasis in Drosophila. Conservation of major signaling pathways between humans and flies has made Drosophila a useful model organism for cancer research. Our understanding of the mechanisms regulating cell growth, differentiation and development has been considerably advanced by studies in Drosophila. Several recent high profile studies have examined the processes constraining the metastatic growth of tumor cells in fruit fly models. **Cell invasion** can be studied in the context of an in vivo setting in flies, enabling the genetic requirements of the microenvironment of tumor cells undergoing metastasis to be analyzed. This Perspective discusses the strengths and limitations of Drosophila models of cancer invasion and the unique tools that have enabled these studies. It also highlights several recent reports that together make a strong case for Drosophila as a system with the potential for both testing novel concepts in tumor progression and **cell invasion**, and for uncovering players in metastasis.
8. Caspase signalling in the absence of apoptosis drives Jnk-dependent invasion. Tumours evolve several mechanisms to evade apoptosis, yet many resected carcinomas show significantly elevated caspase activity. Moreover, caspase activity is positively correlated with tumour aggression and adverse patient outcome. These observations indicate that caspases might have a functional role in promoting tumour invasion and metastasis. Using a Drosophila model of invasion, we show that precise effector caspase activity drives **cell invasion** without initiating apoptosis. Affected cells express the matrix metalloprotinase Mmp1 and invade by activating Jnk. Our results link Jnk and effector caspase signalling during the invasive process and suggest that tumours under apoptotic stresses from treatment, immune surveillance or intrinsic signals might be induced further along the metastatic cascade.

---

## Tsp42Ea

- NCBI Gene
- GeneRIF
- Pubmed

| Search Term | Rank | Fields |
| --- | --- | --- |
| [Mm]etastasis | 12 | PubMed(1) |

  
hide/show details for Tsp42Ea

### [Mm]etastasis

#### PubMed

1. Genomewide analysis of the Drosophila tetraspanins reveals a subset with similar function in the formation of the embryonic synapse. Tetraspanins encode a large conserved family of proteins that span the membrane four times and are expressed in a variety of eukaryotic tissues. They are part of membrane complexes that are involved in such diverse processes as intracellular signaling, cellular motility, **metastasis**, and tumor suppression. The single fly tetraspanin characterized to date, late bloomer (lbm), is expressed on the axons, terminal arbors, and growth cones of motoneurons. In embryos lacking Lbm protein, motoneurons reach their muscle targets, but initially fail to form synaptic terminals. During larval stages, however, functional contacts are formed. The newly available genomic sequence of Drosophila melanogaster indicates the existence of 34 additional members of the tetraspanin family in the fly. To address the possibility that other tetraspanins with functions that might compensate for a lack of lbm exist, we determined the expression domains of the Drosophila tetraspanin gene family members by RNA in situ analysis. We found two other tetraspanins also expressed in motoneurons and subsequently generated a small chromosomal deletion that removes all three motoneuron-specific tetraspanins. The deletion results in a significant enhancement in the lbm phenotype, indicating that the two additional motoneuron-expressed tetraspanins can, at least in part, compensate for the absence of lbm during the formation of the embryonic synapse.

---

## CG30281

- NCBI Gene
- GeneRIF
- Pubmed

| Search Term | Rank | Fields |
| --- | --- | --- |
| [Mm]igration | 6 | PubMed(1) |
| ECM | 7 | PubMed(1) |
| [Ee]xtracellular [Mm]atrix | 8 | PubMed(1) |

  
hide/show details for CG30281

### [Mm]igration

#### PubMed

1. Genes conserved in bilaterians but jointly lost with Myc during nematode evolution are enriched in cell proliferation and cell **migration** functions. Animals use a stereotypical set of developmental genes to build body architectures of varying sizes and organizational complexity. Some genes are critical to developmental patterning, while other genes are important to physiological control of growth. However, growth regulator genes may not be as important in small-bodied "micro-metazoans" such as nematodes. Nematodes use a simplified developmental strategy of lineage-based cell fate specifications to produce an adult bilaterian body composed of a few hundreds of cells. Nematodes also lost the MYC proto-oncogenic regulator of cell proliferation. To identify additional regulators of cell proliferation that were lost with MYC, we computationally screened and determined 839 high-confidence genes that are conserved in bilaterians/lost in nematodes (CIBLIN genes). We find that 30 % of all CIBLIN genes encode transcriptional regulators of cell proliferation, epithelial-to-mesenchyme transitions, and other processes. Over 50 % of CIBLIN genes are unnamed genes in Drosophila, suggesting that there are many understudied genes. Interestingly, CIBLIN genes include many Myc synthetic lethal (MycSL) hits from recent screens. CIBLIN genes include key regulators of heparan sulfate proteoglycan (HSPG) sulfation patterns, and lysyl oxidases involved in cross-linking and modification of the extracellular matrix (ECM). These genes and others suggest the CIBLIN repertoire services critical functions in ECM remodeling and cell **migration** in large-bodied bilaterians. Correspondingly, CIBLIN genes are co-expressed with Myc in cancer transcriptomes, and include a preponderance of known determinants of cancer progression and tumor aggression. We propose that CIBLIN gene research can improve our understanding of regulatory control of cellular growth in metazoans.

### ECM

#### PubMed

1. Genes conserved in bilaterians but jointly lost with Myc during nematode evolution are enriched in cell proliferation and cell migration functions. Animals use a stereotypical set of developmental genes to build body architectures of varying sizes and organizational complexity. Some genes are critical to developmental patterning, while other genes are important to physiological control of growth. However, growth regulator genes may not be as important in small-bodied "micro-metazoans" such as nematodes. Nematodes use a simplified developmental strategy of lineage-based cell fate specifications to produce an adult bilaterian body composed of a few hundreds of cells. Nematodes also lost the MYC proto-oncogenic regulator of cell proliferation. To identify additional regulators of cell proliferation that were lost with MYC, we computationally screened and determined 839 high-confidence genes that are conserved in bilaterians/lost in nematodes (CIBLIN genes). We find that 30 % of all CIBLIN genes encode transcriptional regulators of cell proliferation, epithelial-to-mesenchyme transitions, and other processes. Over 50 % of CIBLIN genes are unnamed genes in Drosophila, suggesting that there are many understudied genes. Interestingly, CIBLIN genes include many Myc synthetic lethal (MycSL) hits from recent screens. CIBLIN genes include key regulators of heparan sulfate proteoglycan (HSPG) sulfation patterns, and lysyl oxidases involved in cross-linking and modification of the extracellular matrix (**ECM**). These genes and others suggest the CIBLIN repertoire services critical functions in **ECM** remodeling and cell migration in large-bodied bilaterians. Correspondingly, CIBLIN genes are co-expressed with Myc in cancer transcriptomes, and include a preponderance of known determinants of cancer progression and tumor aggression. We propose that CIBLIN gene research can improve our understanding of regulatory control of cellular growth in metazoans.

### [Ee]xtracellular [Mm]atrix

#### PubMed

1. Genes conserved in bilaterians but jointly lost with Myc during nematode evolution are enriched in cell proliferation and cell migration functions. Animals use a stereotypical set of developmental genes to build body architectures of varying sizes and organizational complexity. Some genes are critical to developmental patterning, while other genes are important to physiological control of growth. However, growth regulator genes may not be as important in small-bodied "micro-metazoans" such as nematodes. Nematodes use a simplified developmental strategy of lineage-based cell fate specifications to produce an adult bilaterian body composed of a few hundreds of cells. Nematodes also lost the MYC proto-oncogenic regulator of cell proliferation. To identify additional regulators of cell proliferation that were lost with MYC, we computationally screened and determined 839 high-confidence genes that are conserved in bilaterians/lost in nematodes (CIBLIN genes). We find that 30 % of all CIBLIN genes encode transcriptional regulators of cell proliferation, epithelial-to-mesenchyme transitions, and other processes. Over 50 % of CIBLIN genes are unnamed genes in Drosophila, suggesting that there are many understudied genes. Interestingly, CIBLIN genes include many Myc synthetic lethal (MycSL) hits from recent screens. CIBLIN genes include key regulators of heparan sulfate proteoglycan (HSPG) sulfation patterns, and lysyl oxidases involved in cross-linking and modification of the **extracellular matrix** (ECM). These genes and others suggest the CIBLIN repertoire services critical functions in ECM remodeling and cell migration in large-bodied bilaterians. Correspondingly, CIBLIN genes are co-expressed with Myc in cancer transcriptomes, and include a preponderance of known determinants of cancer progression and tumor aggression. We propose that CIBLIN gene research can improve our understanding of regulatory control of cellular growth in metazoans.

---

## Rac2

- NCBI Gene
- GeneRIF
- Pubmed

| Search Term | Rank | Fields |
| --- | --- | --- |
| [Ff]ilopodia | 1 | PubMed(9) |
| [Ff]ilopodium | 2 | PubMed(1) |
| [Pp]odosome(s)? | 5 | PubMed(3) |
| [Mm]igration | 6 | Process(2); PubMed(35) |
| ECM | 7 | PubMed(1) |
| [Ee]xtracellular [Mm]atrix | 8 | PubMed(5) |
| [Ii]nvasive | 10 | PubMed(2) |
| [Mm]etastasis | 12 | PubMed(4) |
| [Cc]ell [Ii]nvasion | 13 | PubMed(2) |

  
hide/show details for Rac2

### [Ff]ilopodia

#### PubMed

1. Function of Rho GTPases in embryonic blood cell migration in Drosophila. Hemocyte development in the Drosophila embryo is a genetic model to study blood cell differentiation, cell migration and phagocytosis. Macrophages, which make up the majority of embryonic hemocytes, migrate extensively as individual cells on basement membrane-covered surfaces. The molecular mechanisms that contribute to this migration process are currently not well understood. We report the generation, by P element replacement, of two Gal4 lines that drive expression of UAS-controlled target genes during early (gcm-Gal4) or late (Coll-Gal4) stages of macrophage migration. gcm-Gal4 is used for live imaging analysis showing that macrophages extend large, dynamic lamellipodia as their main protrusions as well as **filopodia**. We use both Gal4 lines to express dominantnegative and constitutively active isoforms of the Rho GTPases Rac1, Cdc42, Rho1 and RhoL in macrophages, and complement these experiments by analyzing embryos mutant for Rho GTPases. Our findings suggest that Rac1 and Rac2 act redundantly in controlling migration and lamellipodia formation in Drosophila macrophages, and that the third Drosophila Rac gene, Mtl, makes no significant contribution to macrophage migration. Cdc42 appears not to be required within macrophages but in other tissues of the embryo to guide macrophages to the ventral trunk region. No evidence was found for a requirement of Rho1 or RhoL in macrophage migration. Finally, to estimate the number of genes whose zygotic expression is required for macrophage migration we analyzed 208 chromosomal deletions that cover most of the Drosophila genome. We find eight deletions that cause defects in macrophage migration suggesting the existence of approximately ten zygotic genes essential for macrophage migration.
2. The Formin Diaphanous Regulates Myoblast Fusion through Actin Polymerization and Arp2/3 Regulation. The formation of multinucleated muscle cells through cell-cell fusion is a conserved process from fruit flies to humans. Numerous studies have shown the importance of Arp2/3, its regulators, and branched actin for the formation of an actin structure, the F-actin focus, at the fusion site. This F-actin focus forms the core of an invasive podosome-like structure that is required for myoblast fusion. In this study, we find that the formin Diaphanous (Dia), which nucleates and facilitates the elongation of actin filaments, is essential for Drosophila myoblast fusion. Following cell recognition and adhesion, Dia is enriched at the myoblast fusion site, concomitant with, and having the same dynamics as, the F-actin focus. Through analysis of Dia loss-of-function conditions using mutant alleles but particularly a dominant negative Dia transgene, we demonstrate that reduction in Dia activity in myoblasts leads to a fusion block. Significantly, no actin focus is detected, and neither branched actin regulators, SCAR or WASp, accumulate at the fusion site when Dia levels are reduced. Expression of constitutively active Dia also causes a fusion block that is associated with an increase in highly dynamic **filopodia**, altered actin turnover rates and F-actin distribution, and mislocalization of SCAR and WASp at the fusion site. Together our data indicate that Dia plays two roles during invasive podosome formation at the fusion site: it dictates the level of linear F-actin polymerization, and it is required for appropriate branched actin polymerization via localization of SCAR and WASp. These studies provide new insight to the mechanisms of cell-cell fusion, the relationship between different regulators of actin polymerization, and invasive podosome formation that occurs in normal development and in disease.
3. Wound healing recapitulates morphogenesis in Drosophila embryos. The capacity to repair a wound is a fundamental survival mechanism that is activated at any site of damage throughout embryonic and adult life. To study the cell biology and genetics of this process, we have developed a wounding model in Drosophila melanogaster embryos that allows live imaging of rearrangements and changes in cell shape, and of the cytoskeletal machinery that draws closed an in vivo wound. Using embryos expressing green fluorescent protein (GFP) fusion proteins, we show that two cytoskeletal-dependent elements -- an actin cable and dynamic **filopodia**l/lamellipodial protrusions -- are expressed by epithelial cells at the wound edge and are pivotal for repair. Modulating the activities of the small GTPases Rho and Cdc42 demonstrates that these actin-dependent elements have differing cellular functions, but that either alone can drive wound closure. The actin cable operates as a 'purse-string' to draw the hole closed, whereas **filopodia** are essential for the final 'knitting' together of epithelial cells at the end of repair. Our data suggest a more complex model for epithelial repair than previously envisaged and highlight remarkable similarities with the well-characterized morphogenetic movement of dorsal closure in Drosophila.
4. RhoA and Rac1 GTPases mediate the dynamic rearrangement of actin in peripheral glia. Peripheral glial cells in both vertebrates and insects are born centrally and travel large distances to ensheathe axons in the periphery. There is very little known about how this migration is carried out. In other cells, it is known that rearrangement of the Actin cytoskeleton is an integral part of cell motility, yet the distribution of Actin in peripheral glial cell migration in vivo has not been previously characterized. To gain an understanding of how glia migrate, we specifically labeled the peripheral glia of Drosophila melanogaster using an Actin-GFP marker and analyzed their development in the embryonic PNS. It was found that Actin cytoskeleton is dynamically rearranged during glial cell migration. The peripheral glia were observed to migrate as a continuous chain of cells, with the leading glial cells appearing to participate to the greatest extent in exploring the extracellular surroundings with **filopodia**-like Actin containing projections. We hypothesized that the small GTPases Rho, Rac and Cdc42 are involved in Actin cytoskeletal rearrangements that underlie peripheral glial migration and nerve ensheathement. To test this, transgenic forms of the GTPases were ectopically expressed specifically in the peripheral glia during their migration and wrapping phases. The effects on glial Actin-GFP distribution and the overall effects on glial cell migration and morphological development were assessed. We found that RhoA and Rac1 have distinct roles in peripheral glial cell migration and nerve ensheathement; however, Cdc42 does not have a significant role in peripheral glial development. RhoA and Rac1 gain-of-function and loss-of-function mutants had both disruption of glial cell development and secondary effects on sensory axon fasciculation. Together, Actin cytoskeletal dynamics is an integral part of peripheral glial migration and nerve ensheathement, and is mediated by RhoA and Rac1.
5. The Formin DAAM Functions as Molecular Effector of the Planar Cell Polarity Pathway during Axonal Development in Drosophila. Recent studies established that the planar cell polarity (PCP) pathway is critical for various aspects of nervous system development and function, including axonal guidance. Although it seems clear that PCP signaling regulates actin dynamics, the mechanisms through which this occurs remain elusive. Here, we establish a functional link between the PCP system and one specific actin regulator, the formin DAAM, which has previously been shown to be required for embryonic axonal morphogenesis and **filopodia** formation in the growth cone. We show that dDAAM also plays a pivotal role during axonal growth and guidance in the adult Drosophila mushroom body, a brain center for learning and memory. By using a combination of genetic and biochemical assays, we demonstrate that Wnt5 and the PCP signaling proteins Frizzled, Strabismus, and Dishevelled act in concert with the small GTPase Rac1 to activate the actin assembly functions of dDAAM essential for correct targeting of mushroom body axons. Collectively, these data suggest that dDAAM is used as a major molecular effector of the PCP guidance pathway. By uncovering a signaling system from the Wnt5 guidance cue to an actin assembly factor, we propose that the Wnt5/PCP navigation system is linked by dDAAM to the regulation of the growth cone actin cytoskeleton, and thereby growth cone behavior, in a direct way.
6. Abi, Sra1, and Kette control the stability and localization of SCAR/WAVE to regulate the formation of actin-based protrusions. In animal cells, GTPase signaling pathways are thought to generate cellular protrusions by modulating the activity of downstream actin-regulatory proteins. Although the molecular events linking activation of a GTPase to the formation of an actin-based process with a characteristic morphology are incompletely understood, Rac-GTP is thought to promote the activation of SCAR/WAVE, whereas Cdc42 is thought to initiate the formation of **filopodia** through WASP. SCAR and WASP then activate the Arp2/3 complex to nucleate the formation of new actin filaments, which through polymerization exert a protrusive force on the membrane.Using RNAi to screen for genes regulating cell form in an adherent Drosophila cell line, we identified a set of genes, including Abi/E3B1, that are absolutely required for the formation of dynamic protrusions. These genes delineate a pathway from Cdc42 and Rac to SCAR and the Arp2/3 complex. Efforts to place Abi in this signaling hierarchy revealed that Abi and two components of a recently identified SCAR complex, Sra1 (p140/PIR121/CYFIP) and Kette (Nap1/Hem), protect SCAR from proteasome-mediated degradation and are critical for SCAR localization and for the generation of Arp2/3-dependent protrusions.In Drosophila cells, SCAR is regulated by Abi, Kette, and Sra1, components of a conserved regulatory SCAR complex. By controlling the stability, localization, and function of SCAR, these proteins may help to ensure that Arp2/3 activation and the generation of actin-based protrusions remain strictly dependant on local GTPase signaling.
7. Formin proteins of the DAAM subfamily play a role during axon growth. The regulation of growth cone actin dynamics is a critical aspect of axonal growth control. Among the proteins that are directly involved in the regulation of actin dynamics, actin nucleation factors play a pivotal role by promoting the formation of novel actin filaments. However, the essential nucleation factors in developing neurons have so far not been clearly identified. Here, we show expression data, and use true loss-of-function analysis and targeted expression of activated constructs to demonstrate that the Drosophila formin DAAM plays a critical role in axonal morphogenesis. In agreement with this finding, we show that dDAAM is required for **filopodia** formation at axonal growth cones. Our genetic interaction, immunoprecipitation and protein localization studies argue that dDAAM acts in concert with Rac GTPases, Profilin and Enabled during axonal growth regulation. We also show that mouse Daam1 rescues the CNS defects observed in dDAAM mutant flies to a high degree, and vice versa, that Drosophila DAAM induces the formation of neurite-like protrusions when expressed in mouse P19 cells, strongly suggesting that the function of DAAM in developing neurons has been conserved during evolution.
8. The actin-binding protein Canoe/AF-6 forms a complex with Robo and is required for Slit-Robo signaling during axon pathfinding at the CNS midline. Axon guidance is a key process during nervous system development and regeneration. One of the best established paradigms to study the mechanisms underlying this process is the axon decision of whether or not to cross the midline in the Drosophila CNS. An essential regulator of that decision is the well conserved Slit-Robo signaling pathway. Slit guidance cues act through Robo receptors to repel axons from the midline. Despite good progress in our knowledge about these proteins, the intracellular mechanisms associated with Robo function remain poorly defined. In this work, we found that the scaffolding protein Canoe (Cno), the Drosophila orthologue of AF-6/Afadin, is essential for Slit-Robo signaling. Cno is expressed along longitudinal axonal pioneer tracts, and longitudinal Robo/Fasciclin2-positive axons aberrantly cross the midline in cno mutant embryos. cno mutant primary neurons show a significant reduction of Robo localized in growth cone **filopodia** and Cno forms a complex with Robo in vivo. Moreover, the commissureless (comm) phenotype (i.e., lack of commissures due to constitutive surface presentation of Robo in all neurons) is suppressed in comm, cno double-mutant embryos. Specific genetic interactions between cno, slit, robo, and genes encoding other components of the Robo pathway, such as Neurexin-IV, Syndecan, and Rac GTPases, further confirm that Cno functionally interacts with the Slit-Robo pathway. Our data argue that Cno is a novel regulator of the Slit-Robo signaling pathway, crucial for regulating the subcellular localization of Robo and for transducing its signaling to the actin cytoskeleton during axon guidance at the midline.
9. Reciprocal regulation of Rac1 and Rho1 in Drosophila circulating immune surveillance cells. In many cell types it is evident that the small GTPases Rac and Rho regulate each other's activities. What is unclear is exactly how this regulation occurs. To further elucidate this interaction we examined the activities of Rac1 and Rho1 in Drosophila cellular immune surveillance cells. In larvae the cellular immune response involves circulating cells (hemocytes) that can be recruited from a hematopoietic organ located behind the brain, as well as a sessile population found just underneath the larval cuticle. We demonstrate for the first time that Rho-kinase activation requires both Rho1 and the Drosophila c-Jun N-terminal kinase (Basket). We also show that Rac1, via Basket, regulates Rho1 activity, possibly by inhibiting RhoGAPp190. In the reciprocal pathway, co-expression of dominant negative Rho-kinase and constitutive active Rho1 induces a Rac1-like phenotype. This induction requires the formin Diaphanous. Co-expression of dominant negative Rho-kinase and constitutive active Rho1 also induces **filopodia** formation, with Diaphanous enriched at the tips. The Rac1-like phenotypes, and **filopodia** formation, could be blocked by co-expression of dominant negative Rac1. Finally, though dominant negative Rac1 is able to block **filopodia** formation in the overexpression experiments, only Rac2 is necessary for **filopodia** formed by hemocytes after parasitization.

### [Ff]ilopodium

#### PubMed

1. Bifocal is a downstream target of the Ste20-like serine/threonine kinase misshapen in regulating photoreceptor growth cone targeting in Drosophila. Misshapen (Msn) has been proposed to shut down Drosophila photoreceptor (R cell) growth cone motility in response to targeting signals linked by the SH2/SH3 adaptor protein Dock. Here, we show that Bifocal (Bif), a putative cytoskeletal regulator, is a component of the Msn pathway for regulating R cell growth cone targeting. bif displays strong genetic interaction with msn. Phenotypic analysis indicates a specific role for Bif to terminate R1-R6 growth cones. Biochemical studies show that Msn associates directly with Bif and phosphorylates Bif in vitro. Cell culture studies demonstrate that Msn interacts with Bif to regulate F-actin structure and **filopodium** formation. We propose that Bif functions downstream of Msn to reorganize actin cytoskeleton in decelerating R cell growth cone motility at the target region.

### [Pp]odosome(s)?

#### PubMed

1. Group I PAKs function downstream of Rac to promote **podosome** invasion during myoblast fusion in vivo. The p21-activated kinases (PAKs) play essential roles in diverse cellular processes and are required for cell proliferation, apoptosis, polarity establishment, migration, and cell shape changes. Here, we have identified a novel function for the group I PAKs in cell-cell fusion. We show that the two Drosophila group I PAKs, DPak3 and DPak1, have partially redundant functions in myoblast fusion in vivo, with DPak3 playing a major role. DPak3 is enriched at the site of fusion colocalizing with the F-actin focus within a **podosome**-like structure (PLS), and promotes actin filament assembly during PLS invasion. Although the small GTPase Rac is involved in DPak3 activation and recruitment to the PLS, the kinase activity of DPak3 is required for effective PLS invasion. We propose a model whereby group I PAKs act downstream of Rac to organize the actin filaments within the PLS into a dense focus, which in turn promotes PLS invasion and fusion pore initiation during myoblast fusion.
2. The Formin Diaphanous Regulates Myoblast Fusion through Actin Polymerization and Arp2/3 Regulation. The formation of multinucleated muscle cells through cell-cell fusion is a conserved process from fruit flies to humans. Numerous studies have shown the importance of Arp2/3, its regulators, and branched actin for the formation of an actin structure, the F-actin focus, at the fusion site. This F-actin focus forms the core of an invasive **podosome**-like structure that is required for myoblast fusion. In this study, we find that the formin Diaphanous (Dia), which nucleates and facilitates the elongation of actin filaments, is essential for Drosophila myoblast fusion. Following cell recognition and adhesion, Dia is enriched at the myoblast fusion site, concomitant with, and having the same dynamics as, the F-actin focus. Through analysis of Dia loss-of-function conditions using mutant alleles but particularly a dominant negative Dia transgene, we demonstrate that reduction in Dia activity in myoblasts leads to a fusion block. Significantly, no actin focus is detected, and neither branched actin regulators, SCAR or WASp, accumulate at the fusion site when Dia levels are reduced. Expression of constitutively active Dia also causes a fusion block that is associated with an increase in highly dynamic filopodia, altered actin turnover rates and F-actin distribution, and mislocalization of SCAR and WASp at the fusion site. Together our data indicate that Dia plays two roles during invasive **podosome** formation at the fusion site: it dictates the level of linear F-actin polymerization, and it is required for appropriate branched actin polymerization via localization of SCAR and WASp. These studies provide new insight to the mechanisms of cell-cell fusion, the relationship between different regulators of actin polymerization, and invasive **podosome** formation that occurs in normal development and in disease.
3. Asymmetric Mbc, active Rac1 and F-actin foci in the fusion-competent myoblasts during myoblast fusion in Drosophila. Myoblast fusion is an intricate process that is initiated by cell recognition and adhesion, and culminates in cell membrane breakdown and formation of multinucleate syncytia. In the Drosophila embryo, this process occurs asymmetrically between founder cells that pattern the musculature and fusion-competent myoblasts (FCMs) that account for the bulk of the myoblasts. The present studies clarify and amplify current models of myoblast fusion in several important ways. We demonstrate that the non-conventional guanine nucleotide exchange factor (GEF) Mbc plays a fundamental role in the FCMs, where it functions to activate Rac1, but is not required in the founder cells for fusion. Mbc, active Rac1 and F-actin foci are highly enriched in the FCMs, where they localize to the Sns:Kirre junction. Furthermore, Mbc is crucial for the integrity of the F-actin foci and the FCM cytoskeleton, presumably via its activation of Rac1 in these cells. Finally, the local asymmetric distribution of these proteins at adhesion sites is reminiscent of invasive **podosomes** and, consistent with this model, they are enriched at sites of membrane deformation, where the FCM protrudes into the founder cell/myotube. These data are consistent with models promoting actin polymerization as the driving force for myoblast fusion.

### [Mm]igration

#### Process

1. hemocyte **migration**
2. hemocyte **migration**

#### PubMed

1. The RhoGEF Zizimin-related acts in the Drosophila cellular immune response via the Rho GTPases Rac2 and Cdc42. Zizimin-related (Zir), a Rho guanine nucleotide exchange factor (RhoGEF) homologous to the mammalian Dock-C/Zizimin-related family, was identified in a screen to find new genes involved in the Drosophila melanogaster cellular immune response against eggs from the parasitoid wasp Leptopilina boulardi. RhoGEFs activate Rho-family GTPases, which are known to be central regulators of cell **migration**, spreading and polarity. When a parasitoid wasp is recognized as foreign, multiple layers of circulating immunosurveillance cells (haemocytes) should attach to the egg. In Zir mutants this process is disrupted and lamellocytes, a haemocyte subtype, fail to properly encapsulate the wasp egg. Furthermore, macrophage-like plasmatocytes exhibit a strong reduction in their ability to phagocytise Escherichia coli and Staphylococcus aureus bacteria. During encapsulation and phagocytosis Zir genetically interacts with two Rho-family GTPases, Rac2 and Cdc42. Finally, Zir is dispensable for the humoral immune response against bacteria. We propose that Zir is necessary to activate the Rho-family GTPases Rac2 and Cdc42 during the Drosophila cellular immune response.
2. Genes that drive invasion and **migration** in Drosophila. Successful cell **migration** depends on the careful regulation of the timing of movement, the guidance of motile cells, and cytoskeletal and adhesive changes within the cells. This review focuses on genes that act cell-autonomously to promote these aspects of cell **migration** in Drosophila. We discuss recent advances in understanding the **migration** of the ovarian border cells, embryonic blood cells, primordial germ cells, somatic gonadal precursors, and tracheal cells. Comparison of genes that regulate these processes to those that promote tumorigenesis and metastasis in mammals demonstrates that studies in fruit flies are uncovering new genes highly relevant to cancer biology.
3. In vivo RNAi screen identifies candidate signaling genes required for collective cell **migration** in Drosophila ovary. Collective **migration** of loosely or closely associated cell groups is prevalent in animal development, physiological events, and cancer metastasis. However, our understanding of the mechanisms of collective cell **migration** is incomplete. Drosophila border cells provide a powerful in vivo genetic model to study collective **migration** and identify essential genes for this process. Using border cell-specific RNAi-silencing in Drosophila, we knocked down 360 conserved signaling transduction genes in adult flies to identify essential pathways and genes for border cell **migration**. We uncovered a plethora of signaling genes, a large proportion of which had not been reported for border cells, including Rack1 (Receptor of activated C kinase) and brk (brinker), mad (mother against dpp), and sax (saxophone), which encode three components of TGF-β signaling. The RNAi knock down phenotype was validated by clonal analysis of Rack1 mutants. Our data suggest that inhibition of Src activity by Rack1 may be important for border cell **migratio**n and cluster cohesion maintenance. Lastly, results from our screen not only would shed light on signaling pathways involved in collective **migratio**n during embryogenesis and organogenesis in general, but also could help our understanding for the functions of conserved human genes involved in cancer metastasis.
4. Following the 'tracks': Tramtrack69 regulates epithelial tube expansion in the Drosophila ovary through Paxillin, Dynamin, and the homeobox protein Mirror. Epithelial tubes are the infrastructure for organs and tissues, and tube morphogenesis requires precise orchestration of cell signaling, shape, **migration**, and adhesion. Follicle cells in the Drosophila ovary form a pair of epithelial tubes whose lumens act as molds for the eggshell respiratory filaments, or dorsal appendages (DAs). DA formation is a robust and accessible model for studying the patterning, formation, and expansion of epithelial tubes. Tramtrack69 (TTK69), a transcription factor that exhibits a variable embryonic DNA-binding preference, controls DA lumen volume and shape by promoting tube expansion; the tramtrack mutation twin peaks (ttk(twk)) reduces TTK69 levels late in oogenesis, inhibiting this expansion. Microarray analysis of wild-type and ttk(twk) ovaries, followed by in situ hybridization and RNAi of candidate genes, identified the Phospholipase B-like protein Lamina ancestor (LAMA), the scaffold protein Paxillin, the endocytotic regulator Shibire (Dynamin), and the homeodomain transcription factor Mirror, as TTK69 effectors of DA-tube expansion. These genes displayed enriched expression in DA-tube cells, except lama, which was expressed in all follicle cells. All four genes showed reduced expression in ttk(twk) mutants and exhibited RNAi phenotypes that were enhanced in a ttk(twk)/+ background, indicating ttk(twk) genetic interactions. Although previous studies show that Mirror patterns the follicular epithelium prior to DA tubulogenesis, we show that Mirror has an independent, novel role in tube expansion, involving positive regulation of Paxillin. Thus, characterization of ttk(twk)-differentially expressed genes expands the network of TTK69 effectors, identifies novel epithelial tube-expansion regulators, and significantly advances our understanding of this vital developmental process.
5. Unwrapping glial biology: Gcm target genes regulating glial development, diversification, and function. Glia are the most abundant cell type in the mammalian brain. They regulate neuronal development and function, CNS immune surveillance, and stem cell biology, yet we know surprisingly little about glia in any organism. Here we identify over 40 new Drosophila glial genes. We use glial cells missing (gcm) mutants and misexpression to verify they are Gcm regulated in vivo. Many genes show unique spatiotemporal responsiveness to Gcm in the CNS, and thus glial subtype diversification requires spatially or temporally restricted Gcm cofactors. These genes provide insights into glial biology: we show unc-5 (a repulsive netrin receptor) orients glial **migration**s and the draper gene mediates glial engulfment of apoptotic neurons and larval locomotion. Many identified Drosophila glial genes have homologs expressed in mammalian glia, revealing conserved molecular features of glial cells. 80% of these Drosophila glial genes have mammalian homologs; these are now excellent candidates for regulating human glial development, function, or disease.
6. The RhoGEF Zizimin-related acts in the Drosophila cellular immune response via the Rho GTPases Rac2 and Cdc42. Zizimin-related (Zir), a Rho guanine nucleotide exchange factor (RhoGEF) homologous to the mammalian Dock-C/Zizimin-related family, was identified in a screen to find new genes involved in the Drosophila melanogaster cellular immune response against eggs from the parasitoid wasp Leptopilina boulardi. RhoGEFs activate Rho-family GTPases, which are known to be central regulators of cell **migration**, spreading and polarity. When a parasitoid wasp is recognized as foreign, multiple layers of circulating immunosurveillance cells (haemocytes) should attach to the egg. In Zir mutants this process is disrupted and lamellocytes, a haemocyte subtype, fail to properly encapsulate the wasp egg. Furthermore, macrophage-like plasmatocytes exhibit a strong reduction in their ability to phagocytise Escherichia coli and Staphylococcus aureus bacteria. During encapsulation and phagocytosis Zir genetically interacts with two Rho-family GTPases, Rac2 and Cdc42. Finally, Zir is dispensable for the humoral immune response against bacteria. We propose that Zir is necessary to activate the Rho-family GTPases Rac2 and Cdc42 during the Drosophila cellular immune response.
7. Regulation of the Rac GTPase pathway by the multifunctional Rho GEF Pebble is essential for mesoderm **migration** in the Drosophila gastrula. The Drosophila guanine nucleotide exchange factor Pebble (Pbl) is essential for cytokinesis and cell **migration** during gastrulation. In dividing cells, Pbl promotes Rho1 activation at the cell cortex, leading to formation of the contractile actin-myosin ring. The role of Pbl in fibroblast growth factor-triggered mesoderm spreading during gastrulation is less well understood and its targets and subcellular localization are unknown. To address these issues we performed a domain-function study in the embryo. We show that Pbl is localized to the nucleus and the cell cortex in migrating mesoderm cells and found that, in addition to the PH domain, the conserved C-terminal tail of the protein is crucial for cortical localization. Moreover, we show that the Rac pathway plays an essential role during mesoderm **migration**. Genetic and biochemical interactions indicate that during mesoderm **migration**, Pbl functions by activating a Rac-dependent pathway. Furthermore, gain-of-function and rescue experiments suggest an important regulatory role of the C-terminal tail of Pbl for the selective activation of Rho1-versus Rac-dependent pathways.
8. Requirement for Pak3 in Rac1-induced organization of actin and myosin during Drosophila larval wound healing. Rho-family small GTPases regulate epithelial cell sheet **migration** by organizing actin and myosin during wound healing. Here, we report that Pak3, but not Pak1, is a downstream target protein for Rac1 in wound closure of the Drosophila larval epidermis. Pak3-deficient larvae failed to close a wound hole and this defect was not rescued by Pak1 expression, indicating differential functions of the two proteins. Pak3 localized to the wound margin, which selectively required Rac1. Pak3-deficient larvae showed severe defects in actin-myosin organization at the wound margin and in submarginal cells, which was reminiscent of the phenotypes of Rac1-deficient larvae. These results suggest that Pak3 specifically mediates Rac1 signaling in organizing actin and myosin during Drosophila epidermal wound healing.
9. PVR plays a critical role via JNK activation in thorax closure during Drosophila metamorphosis. PVR, the Drosophila homolog of the PDGF/VEGF receptor, has been implicated in border cell **migration** during oogenesis and hemocyte **migration** during embryogenesis. It was earlier shown that Mbc, a CDM family protein, and its effector, Rac, transduced the guidance signal from PVR during border cell **migration**. Here we demonstrate that PVR is also required for the morphogenetic process, thorax closure, during metamorphosis. The results of genetic and biochemical experiments indicate that PVR activates the JNK pathway. We present evidence showing Crk (an adaptor molecule), Mbc, ELMO (a homolog of Caenorhabditis elegans CED-12 and mammalian ELMO), and Rac to be mediators of JNK activation by PVR. In addition, we suppose that not only Rac but also Cdc42 is activated and involved in JNK activation downstream of PVR.
10. Signaling pathways directing the movement and fusion of epithelial sheets: lessons from dorsal closure in Drosophila. Wound healing in embryos and various developmental events in metazoans require the spreading and fusion of epithelial sheets. The complex signaling pathways regulating these processes are being pieced together through genetic, cell biological, and biochemical approaches. At present, dorsal closure of the Drosophila embryo is the best-characterized example of epithelial sheet movement. Dorsal closure involves **migration** of the lateral epidermal flanks to close a hole in the dorsal epidermis occupied by an epithelium called the amnioserosa. Detailed genetic studies have revealed a network of interacting signaling molecules regulating this process. At the center of this network is a Jun N-terminal kinase cascade acting at the leading edge of the migrating epidermis that triggers signaling by the TGF-beta superfamily member Decapentaplegic and which interacts with the Wingless pathway. These signaling modules regulate the cytoskeletal reorganization and cell shape change necessary to drive dorsal closure. Activation of this network requires signals from the amnioserosa and input from a variety of proteins at cell-cell junctions. The Rho family of small GTPases is also instrumental, both in activation of signaling and regulation of the cytoskeleton. Many of the proteins regulating dorsal closure have been implicated in epithelial movement in other organisms, and dorsal closure has emerged as an ideal model system for the study of the **migration** and fusion of epithelial sheets.
11. Regulation of cell **migration** during tracheal development in Drosophila melanogaster. Most of the knowledge concerning the intracellular mechanisms involved in cell locomotion have been obtained from in vitro studies of cells in culture. Many of the concepts derived from these studies have been partially confirmed in in vivo systems but numerous questions regarding the developmental control of cell **migration** remain to be addressed. Tracheal morphogenesis in Drosophila melanogaster embryos represents an in vivo model system to study the genetic control of cell **migration**. We review what is known about tracheal development and regulation of tracheal cell **migration**. We try to link these in vivo studies and the movement of cells over two dimensional substrates and elaborate on important questions which remain to be addressed in the future.
12. The small GTPase Rac plays multiple roles in epithelial sheet fusion--dynamic studies of Drosophila dorsal closure. The coordinated **migration** and fusion of epithelial sheets is a crucial morphogenetic tool used on numerous occasions during the normal development of an embryo and re-activated as part of the wound healing response. Drosophila dorsal closure, whereby a hole in the embryonic epithelium is zipped closed late in embryogenesis, serves as an excellent, genetically tractable model for epithelial **migration**. Using live confocal imaging, we have dissected multiple roles for the small GTPase Rac in this process. We show that constitutive activation of Rac1 leads to excessive assembly of lamellipodia and precocious halting of epithelial sweeping, possibly through premature activation of contact-inhibition machinery. Conversely, blocking Rac activity, either by loss-of-function mutations or expression of dominant negative Rac1, disables the assembly of both actin cable and protrusions by epithelial cells. Movies of mutant embryos show that continued contraction of the amnioserosa is sufficient to draw the epithelial edges towards one another, allowing the zipper machinery to bypass non-functioning regions of leading edge. In addition to illustrating the key role of Rac in organization of leading edge actin, loss-of-function mutants also provide substantive proof that Rac acts upstream in the Jun N-terminal kinase (JNK) cascade to direct epithelial cell shape changes during dorsal closure.
13. Transcriptional control in embryonic Drosophila midline guidance assessed through a whole genome approach. During the development of the Drosophila central nervous system the process of midline crossing is orchestrated by a number of guidance receptors and ligands. Many key axon guidance molecules have been identified in both invertebrates and vertebrates, but the transcriptional regulation of growth cone guidance remains largely unknown. It is established that translational regulation plays a role in midline crossing, and there are indications that transcriptional regulation is also involved. To investigate this issue, we conducted a genome-wide study of transcription in Drosophila embryos using wild type and a number of well-characterized Drosophila guidance mutants and transgenics. We also analyzed a previously published microarray time course of Drosophila embryonic development with an axon guidance focus.Using hopach, a novel clustering method which is well suited to microarray data analysis, we identified groups of genes with similar expression patterns across guidance mutants and transgenics. We then systematically characterized the resulting clusters with respect to their relevance to axon guidance using two complementary controlled vocabularies: the Gene Ontology (GO) and anatomical annotations of the Atlas of Pattern of Gene Expression (APoGE) in situ hybridization database. The analysis indicates that regulation of gene expression does play a role in the process of axon guidance in Drosophila. We also find a strong link between axon guidance and hemocyte **migration**, a result that agrees with mounting evidence that axon guidance molecules are co-opted in vertebrate vascularization. Cell cyclin activity in the context of axon guidance is also suggested from our array data. RNA and protein expression patterns of cell cyclins in axon guidance mutants and transgenics support this possible link.This study provides important insights into the regulation of axon guidance in vivo.
14. Function of Rho GTPases in embryonic blood cell **migration** in Drosophila. Hemocyte development in the Drosophila embryo is a genetic model to study blood cell differentiation, cell **migration** and phagocytosis. Macrophages, which make up the majority of embryonic hemocytes, migrate extensively as individual cells on basement membrane-covered surfaces. The molecular mechanisms that contribute to this **migration** process are currently not well understood. We report the generation, by P element replacement, of two Gal4 lines that drive expression of UAS-controlled target genes during early (gcm-Gal4) or late (Coll-Gal4) stages of macrophage **migration**. gcm-Gal4 is used for live imaging analysis showing that macrophages extend large, dynamic lamellipodia as their main protrusions as well as filopodia. We use both Gal4 lines to express dominantnegative and constitutively active isoforms of the Rho GTPases Rac1, Cdc42, Rho1 and RhoL in macrophages, and complement these experiments by analyzing embryos mutant for Rho GTPases. Our findings suggest that Rac1 and Rac2 act redundantly in controlling **migration** and lamellipodia formation in Drosophila macrophages, and that the third Drosophila Rac gene, Mtl, makes no significant contribution to macrophage **migration**. Cdc42 appears not to be required within macrophages but in other tissues of the embryo to guide macrophages to the ventral trunk region. No evidence was found for a requirement of Rho1 or RhoL in macrophage **migration**. Finally, to estimate the number of genes whose zygotic expression is required for macrophage **migration** we analyzed 208 chromosomal deletions that cover most of the Drosophila genome. We find eight deletions that cause defects in macrophage **migration** suggesting the existence of approximately ten zygotic genes essential for macrophage **migration**.
15. Group I PAKs function downstream of Rac to promote podosome invasion during myoblast fusion in vivo. The p21-activated kinases (PAKs) play essential roles in diverse cellular processes and are required for cell proliferation, apoptosis, polarity establishment, **migration**, and cell shape changes. Here, we have identified a novel function for the group I PAKs in cell-cell fusion. We show that the two Drosophila group I PAKs, DPak3 and DPak1, have partially redundant functions in myoblast fusion in vivo, with DPak3 playing a major role. DPak3 is enriched at the site of fusion colocalizing with the F-actin focus within a podosome-like structure (PLS), and promotes actin filament assembly during PLS invasion. Although the small GTPase Rac is involved in DPak3 activation and recruitment to the PLS, the kinase activity of DPak3 is required for effective PLS invasion. We propose a model whereby group I PAKs act downstream of Rac to organize the actin filaments within the PLS into a dense focus, which in turn promotes PLS invasion and fusion pore initiation during myoblast fusion.
16. Rac function in epithelial tube morphogenesis. Epithelial cell **migration** and morphogenesis require dynamic remodeling of the actin cytoskeleton and cell-cell adhesion complexes. Numerous studies in cell culture and in model organisms have demonstrated the small GTPase Rac to be a critical regulator of these processes; however, little is known about Rac function in the morphogenic movements that drive epithelial tube formation. Here, we use the embryonic salivary glands of Drosophila to understand the role of Rac in epithelial tube morphogenesis. We show that inhibition of Rac function, either through loss of function mutations or dominant-negative mutations, disrupts salivary gland invagination and posterior **migration**. In contrast, constitutive activation of Rac induces motile behavior and subsequent cell death. We further show that Rac regulation of salivary gland morphogenesis occurs through modulation of cell-cell adhesion mediated by the E-cadherin/beta-catenin complex and that shibire, the Drosophila homolog of dynamin, functions downstream of Rac in regulating beta-catenin localization during gland morphogenesis. Our results demonstrate that regulation of cadherin-based adherens junctions by Rac is critical for salivary gland morphogenesis and that this regulation occurs through dynamin-mediated endocytosis.
17. Drosophila importin-7 functions upstream of the Elmo signaling module to mediate the formation and stability of muscle attachments. Establishment and maintenance of stable muscle attachments is essential for coordinated body movement. Studies in Drosophila have pioneered a molecular understanding of the morphological events in the conserved process of muscle attachment formation, including myofiber **migration**, muscle-tendon signaling, and stable junctional adhesion between muscle cells and their corresponding target insertion sites. In both Drosophila and vertebrate models, integrin complexes play a key role in the biogenesis and stability of muscle attachments through the interactions of integrins with extracellular matrix (ECM) ligands. We show that Drosophila importin-7 (Dim7) is an upstream regulator of the conserved Elmo-Mbc→Rac signaling pathway in the formation of embryonic muscle attachment sites (MASs). Dim7 is encoded by the moleskin (msk) locus and was identified as an Elmo-interacting protein. Both Dim7 and Elmo localize to the ends of myofibers coincident with the timing of muscle-tendon attachment in late myogenesis. Phenotypic analysis of elmo mutants reveal muscle attachment defects similar to those previously described for integrin mutants. Furthermore, Elmo and Dim7 interact both biochemically and genetically in the developing musculature. The muscle detachment phenotype resulting from mutations in the msk locus can be rescued by components in the Elmo signaling pathway, including the Elmo-Mbc complex, an activated Elmo variant, or a constitutively active form of Rac. In larval muscles, the localization of Dim7 and activated Elmo to the sites of muscle attachment is attenuated upon RNAi knockdown of integrin heterodimer complex components. Our results show that integrins function as upstream signals to mediate Dim7-Elmo enrichment to the MASs.
18. A role for Drosophila IAP1-mediated caspase inhibition in Rac-dependent cell **migration**. Border cell **migration** in the Drosophila ovary is a relatively simple and genetically tractable model for studying the conversion of epithelial cells to migratory cells. Like many cell **migration**s, border cell **migration** is inhibited by a dominant-negative form of the GTPase Rac. To identify new genes that function in Rac-dependent cell motility, we screened for genes that when overexpressed suppressed the **migration** defect caused by dominant-negative Rac. Overexpression of the Drosophila inhibitor of apoptosis 1 (DIAP1), which is encoded by the thread (th) gene, suppressed the **migration** defect. Moreover, loss-of-function mutations in th caused **migration** defects but, surprisingly, did not cause apoptosis. Mutations affecting the Dark protein, an activator of the upstream caspase Dronc, also rescued RacN17 **migration** defects. These results indicate an apoptosis-independent role for DIAP1-mediated Dronc inhibition in Rac-mediated cell motility.
19. The Ste20-like kinase misshapen functions together with Bicaudal-D and dynein in driving nuclear **migration** in the developing drosophila eye. Nuclear translocation, driven by the motility apparatus consisting of the cytoplasmic dynein motor and microtubules, is essential for cell **migration** during embryonic development. Bicaudal-D (Bic-D), an evolutionarily conserved dynein-interacting protein, is required for developmental control of nuclear **migration** in Drosophila. Nothing is known about the signaling events that coordinate the function of Bic-D and dynein during development. Here, we show that Misshapen (Msn), the fly homolog of the vertebrate Nck-interacting kinase is a component of a novel signaling pathway that regulates photoreceptor (R-cell) nuclear **migration** in the developing Drosophila compound eye. Msn, like Bic-D, is required for the apical **migration** of differentiating R-cell precursor nuclei. msn displays strong genetic interaction with Bic-D. Biochemical studies demonstrate that Msn increases the phosphorylation of Bic-D, which appears to be necessary for the apical accumulation of both Bic-D and dynein in developing R-cell precursor cells. We propose that Msn functions together with Bic-D to regulate the apical localization of dynein in generating directed nuclear **migration** within differentiating R-cell precursor cells.
20. The social lives of migrating cells in Drosophila. Studies of cell **migration** in Drosophila are yielding insights into the complex interactions migrating cells have with each other and with the cells in their environment. Intriguing links between factors that promote cell **migration** and those that control cell survival have been reported recently. For example, migrating germ cells compete with the surrounding somatic tissue for the substrate of the lipid phosphate phosphatases encoded by the genes Wunen and Wunen2. Germ cells take up the dephosphorylated lipid and require it for their survival. In addition, the secreted growth factors called PVFs, previously thought to guide the **migration**s of hemocytes in the embryo, were found to function instead predominantly as survival factors. And in border cells, DIAP1 and Dronc, two proteins known mainly for their ability to regulate cell death, were found to control cell **migration**.
21. Vilse, a conserved Rac/Cdc42 GAP mediating Robo repulsion in tracheal cells and axons. Slit proteins steer the **migration** of many cell types through their binding to Robo receptors, but how Robo controls cell motility is not clear. We describe the functional analysis of vilse, a Drosophila gene required for Robo repulsion in epithelial cells and axons. Vilse defines a conserved family of RhoGAPs (Rho GTPase-activating proteins), with representatives in flies and vertebrates. The phenotypes of vilse mutants resemble the tracheal and axonal phenotypes of Slit and Robo mutants at the CNS midline. Dosage-sensitive genetic interactions between vilse, slit, and robo mutants suggest that vilse is a component of robo signaling. Moreover, overexpression of Vilse in the trachea of robo mutants ameliorates the phenotypes of robo, indicating that Vilse acts downstream of Robo to mediate midline repulsion. Vilse and its human homolog bind directly to the intracellular domains of the corresponding Robo receptors and promote the hydrolysis of RacGTP and, less efficiently, of Cdc42GTP. These results together with genetic interaction experiments with robo, vilse, and rac mutants suggest a mechanism whereby Robo repulsion is mediated by the localized inactivation of Rac through Vilse.
22. RhoA and Rac1 GTPases mediate the dynamic rearrangement of actin in peripheral glia. Peripheral glial cells in both vertebrates and insects are born centrally and travel large distances to ensheathe axons in the periphery. There is very little known about how this **migration** is carried out. In other cells, it is known that rearrangement of the Actin cytoskeleton is an integral part of cell motility, yet the distribution of Actin in peripheral glial cell **migration** in vivo has not been previously characterized. To gain an understanding of how glia migrate, we specifically labeled the peripheral glia of Drosophila melanogaster using an Actin-GFP marker and analyzed their development in the embryonic PNS. It was found that Actin cytoskeleton is dynamically rearranged during glial cell **migration**. The peripheral glia were observed to migrate as a continuous chain of cells, with the leading glial cells appearing to participate to the greatest extent in exploring the extracellular surroundings with filopodia-like Actin containing projections. We hypothesized that the small GTPases Rho, Rac and Cdc42 are involved in Actin cytoskeletal rearrangements that underlie peripheral glial **migration** and nerve ensheathement. To test this, transgenic forms of the GTPases were ectopically expressed specifically in the peripheral glia during their **migration** and wrapping phases. The effects on glial Actin-GFP distribution and the overall effects on glial cell **migration** and morphological development were assessed. We found that RhoA and Rac1 have distinct roles in peripheral glial cell **migration** and nerve ensheathement; however, Cdc42 does not have a significant role in peripheral glial development. RhoA and Rac1 gain-of-function and loss-of-function mutants had both disruption of glial cell development and secondary effects on sensory axon fasciculation. Together, Actin cytoskeletal dynamics is an integral part of peripheral glial **migration** and nerve ensheathement, and is mediated by RhoA and Rac1.
23. Rho-LIM kinase signaling regulates ecdysone-induced gene expression and morphogenesis during Drosophila metamorphosis. The steroid hormone 20-hydroxyecdysone (ecdysone) is the key regulator of postembryonic developmental transitions in insects and controls metamorphosis by triggering the morphogenesis of adult tissues from larvae. The Rho GTPase, which mediates cell shape change and **migration**, is also an essential regulator of tissue morphogenesis during development. Rho activity can modulate gene expression, in part, by activating LIM kinase (LIMK) and consequently affecting actin-induced SRF transcriptional activity. We have established a link between Rho-LIMK-SRF signaling and the ecdysone-induced transcriptional response during Drosophila development. Specifically, we determined that the Rho GTPase, via LIMK, regulates the expression of several ecdysone-responsive genes, including those encoding the ecdysone receptor itself, a downstream transcription factor (Br-C), and Stubble, a transmembrane protease required for proper leg formation. Stubble and Br-C mutants exhibit strong genetic interactions with several Rho pathway components in the formation of adult structures, but not with Rac or Cdc42. In cultured SL2 cells, inhibition of Rho, F-actin assembly, or SRF blocks the transcriptional response to ecdysone. Together, these findings indicate a link between Rho-LIMK signaling and steroid hormone-induced gene expression in the context of metamorphosis and thereby establish a novel role for the Rho GTPase in development.
24. The Trio family of guanine-nucleotide-exchange factors: regulators of axon guidance. Axon guidance requires the integration of diverse guidance signals presented by numerous extracellular cues and cell-cell interactions. The molecular mechanisms that interpret these signals involve networks of intracellular signaling proteins that coordinate a variety of responses to the environment, including remodeling and assembly of the actin cytoskeleton. Although it has been clear for some time that Rho family GTPases play a central role in the orchestration of cytoskeletal assembly, our understanding of the components that regulate these important molecules is far more primitive. Recent functional studies of the Trio family of guanine-nucleotide-exchange factors reveal that Trio proteins play a vital role in neuronal cell **migration** and axon guidance. Although the molecular analysis of Trio proteins is still in its infancy, accumulated evidence suggests that Trio proteins function as integrators of multiple upstream inputs and as activators of multiple downstream pathways. Future studies of these mechanisms promise to yield insights not only into neural development but also into the ongoing function and remodeling of the adult nervous system.
25. The CDM protein DOCK2 in lymphocyte **migration**. T and B lymphocytes migrate hundreds of micrometers each day to survey the body's lymphoid tissues for antigens. No other mammalian cell type undergoes such extensive and continual movement, raising the question of whether lymphocytes have specializations to support their migratory behavior. This possibility has recently gained support from studies of mice deficient in DOCK2, a member of the Caenorhabditis elegans Ced-5, mammalian DOCK180 and Drosophila melanogaster myoblast city (CDM) family of scaffolding proteins. **Migration** of lymphocytes, but not other cell types, is severely disrupted in DOCK2-deficient mice. Despite the conserved role of CDM molecules in regulating Rac activation and actin assembly, relatively little is known about how these molecules function. Here, we review the role of DOCK2 in lymphocyte homing to lymphoid tissues and discuss recent findings for other CDM family molecules that provide a basis for understanding how DOCK2 might function in lymphocytes.
26. The SCAR and WASp nucleation-promoting factors act sequentially to mediate Drosophila myoblast fusion. The actin nucleation-promoting factors SCAR/WAVE and WASp, together with associated elements, mediate the formation of muscle fibres through myoblast fusion during Drosophila embryogenesis. Our phenotypic analysis, following the disruption of these two pathways, suggests that they function in a sequential manner. Suppressor of cyclic AMP receptor (SCAR) activity is required before the formation of pores in the membranes of fusing cells, whereas Wiskott-Aldrich syndrome protein (WASp) promotes the expansion of nascent pores and completion of the fusion process. Genetic epistasis experiments are consistent with this step-wise temporal progression. Our observations further imply a separate, Rac-dependent role for the SCAR complex in promoting myoblast **migration**. In keeping with the sequential utilization of the two systems, we observe abnormal accumulations of filamentous actin at the fusion sites when both pathways are disrupted, resembling those present when only SCAR-complex function is impaired. This observation further suggests that actin-filament accumulation at the fusion sites might not depend on Arp2/3 activity altogether.
27. The role of the Rho GTPases in neuronal development. Our brain serves as a center for cognitive function and neurons within the brain relay and store information about our surroundings and experiences. Modulation of this complex neuronal circuitry allows us to process that information and respond appropriately. Proper development of neurons is therefore vital to the mental health of an individual, and perturbations in their signaling or morphology are likely to result in cognitive impairment. The development of a neuron requires a series of steps that begins with **migration** from its birth place and initiation of process outgrowth, and ultimately leads to differentiation and the formation of connections that allow it to communicate with appropriate targets. Over the past several years, it has become clear that the Rho family of GTPases and related molecules play an important role in various aspects of neuronal development, including neurite outgrowth and differentiation, axon pathfinding, and dendritic spine formation and maintenance. Given the importance of these molecules in these processes, it is therefore not surprising that mutations in genes encoding a number of regulators and effectors of the Rho GTPases have been associated with human neurological diseases. This review will focus on the role of the Rho GTPases and their associated signaling molecules throughout neuronal development and discuss how perturbations in Rho GTPase signaling may lead to cognitive disorders.
28. Genetic interaction screens identify a role for hedgehog signaling in Drosophila border cell **migration**. Cell motility is essential for embryonic development and physiological processes such as the immune response, but also contributes to pathological conditions such as tumor progression and inflammation. However, our understanding of the mechanisms underlying migratory processes is incomplete. Drosophila border cells provide a powerful genetic model to identify the roles of genes that contribute to cell **migration**.Members of the Hedgehog signaling pathway were uncovered in two independent screens for interactions with the small GTPase Rac and the polarity protein Par-1 in border cell **migration**. Consistent with a role in **migration**, multiple Hh signaling components were enriched in the migratory border cells. Interference with Hh signaling by several different methods resulted in incomplete cell **migration**. Moreover, the polarized distribution of E-Cadherin and a marker of tyrosine kinase activity were altered when Hh signaling was disrupted. Conservation of Hh-Rac and Hh-Par-1 signaling was illustrated in the wing, in which Hh-dependent phenotypes were enhanced by loss of Rac or par-1.We identified a pathway by which Hh signaling connects to Rac and Par-1 in cell **migration**. These results further highlight the importance of modifier screens in the identification of new genes that function in developmental pathways.
29. Live imaging of wound inflammation in Drosophila embryos reveals key roles for small GTPases during in vivo cell **migration**. Aa robust inflammatory response to tissue damage and infection is conserved across almost all animal phyla. Neutrophils and macrophages, or their equivalents, are drawn to the wound site where they engulf cell and matrix debris and release signals that direct components of the repair process. This orchestrated cell **migration** is clinically important, and yet, to date, leukocyte chemotaxis has largely been studied in vitro. Here, we describe a genetically tractable in vivo wound model of inflammation in the Drosophila melanogaster embryo that is amenable to cinemicroscopy. For the first time, we are able to examine the roles of Rho-family small GTPases during inflammation in vivo and show that Rac-mediated lamellae are essential for hemocyte motility and Rho signaling is necessary for cells to retract from sites of matrix- and cell-cell contacts. Cdc42 is necessary for maintaining cellular polarity and yet, despite in vitro evidence, is dispensable for sensing and crawling toward wound cues.
30. Psidin, a conserved protein that regulates protrusion dynamics and cell **migration**. Dynamic assembly and disassembly of actin filaments is a major driving force for cell movements. Border cells in the Drosophila ovary provide a simple and genetically tractable model to study the mechanisms regulating cell **migration**. To identify new genes that regulate cell movement in vivo, we screened lethal mutations on chromosome 3R for defects in border cell **migration** and identified two alleles of the gene psidin (psid). In vitro, purified Psid protein bound F-actin and inhibited the interaction of tropomyosin with F-actin. In vivo, psid mutations exhibited genetic interactions with the genes encoding tropomyosin and cofilin. Border cells overexpressing Psid together with GFP-actin exhibited altered protrusion/retraction dynamics. Psid knockdown in cultured S2 cells reduced, and Psid overexpression enhanced, lamellipodial dynamics. Knockdown of the human homolog of Psid reduced the speed and directionality of **migration** in wounded MCF10A breast epithelial monolayers, whereas overexpression of the protein increased **migration** speed and altered protrusion dynamics in EGF-stimulated cells. These results indicate that Psid is an actin regulatory protein that plays a conserved role in protrusion dynamics and cell **migration**.
31. Real-time analysis of Drosophila post-embryonic haemocyte behaviour. The larval stage of the model organism Drosophila is frequently used to study host-pathogen interactions. During embryogenesis the cellular arm of the immune response, consisting of macrophage-like cells known as plasmatocytes, is extremely motile and functions to phagocytise pathogens and apoptotic bodies, as well as produce extracellular matrix. The cellular branch of the larval (post-embryonic) innate immune system consists of three cell types--plasmatocytes, crystal cells and lamellocytes--which are involved in the phagocytosis, encapsulation and melanisation of invading pathogens. Post-embryonic haemocyte motility is poorly understood thus further characterisation is required, for the purpose of standardisation.In order to examine post-embryonic haemocyte cytoskeletal dynamics or **migration**, the most commonly used system is in vitro cell lines. The current study employs an ex vivo system (an adaptation of in vitro cell incubation using primary cells), in which primary larval or pre-pupal haemocytes are isolated for short term analysis, in order to discover various aspects of their behaviour during events requiring cytoskeleton dynamics.The ex vivo method allows for real-time analysis and manipulation of primary post-embryonic haemocytes. This technique was used to characterise, and potentially standardised, larval and pre-pupal haemocyte cytoskeleton dynamics, assayed on different extracellular matrices. Using this method it was determined that, while larval haemocytes are unable to migrate, haemocytes recovered from pre-pupae are capable of **migration**.
32. Rac1 GTPase acts downstream of αPS1βPS integrin to control collectiv**e migrati**on and lumen size in the Drosophila salivary gland. During collectiv**e migrati**on of the Drosophila embryonic salivary gland, the distal gland cells mediate integrin-based contacts with surrounding tissues while proximal gland cells change shape and rearrange. Here, we show that αPS1βPS integrin controls salivary gl**and migra**tion through Rac1 GTPase which downregulates E-cadherin in proximal and distal gland cells, and promotes extension of actin-rich basal membrane protrusions in the distal cells. In embryos mutant for multiple edematous wings (mew), which encodes the αPS1 subunit of the αPS1βPS integrin heterodimer, or rac1 and rac2 GTPases, salivary gland cells failed to migrate, to downregulate E-cadherin and to extend basal membrane protrusions. Selective inhibition of Rac1 in just the proximal or distal gland cells demonstrate that proximal gland cells play an active role in the coll**ective mi**gration of the whole gland and that con**tinued mi**gration of the distal cells depends on the proximal cells. Loss of rac1rac2 also affected gland lumen length and width whereas, loss of mew affected lumen length only. Activation of rac1 in mew mutant embryos significantly rescued the **gland mi**gration, lumen length and basal membrane protrusion defects and partially rescued the E-cadherin defects. Independent of mew, Rac regulates cell shape change and rearrangement in the proximal gland, which is importa**nt for mi**gration and lumen width. Our studies shed novel insight into a Rac1-mediated link between integrin and cadherin adhesion proteins in vivo, control of lumen length and width and how activities of proximal and distal gland cells are coordinated to result in the coll**ective mi**gration of the entire salivary gland.
33. In Drosophila melanogaster, the rolling pebbles isoform 6 (Rols6) is essential for proper Malpighian tubule morphology. During myoblast fusion, cell-cell recognition along with cell **migration** and adhesion are essential biological processes. The factors involved in these processes include members of the immunoglobulin superfamily like Sticks and stones (Sns), Dumbfounded (Duf) and Hibris (Hbs), SH3 domain-containing adaptor molecules like Myoblast city (Mbc) and multidomain proteins like Rolling pebbles (Rols). For rolling pebbles, two differentially expressed transcripts have been defined (rols7 and rols6). However, to date, only a muscle fusion phenotype has been described and assigned to the lack of the mesoderm-specific expressed rols7 transcript. Here, we show that a loss of the second rolling pebbles transcript, rols6, which is expressed from the early bud to later embryonic stages during Malpighian tubule (MpT) development, leads to an abnormal MpT morphology that is not due to defects in cell determination or proliferation but to aberrant morphogenesis. In addition, when Myoblast city or Rac are knocked out, a similar phenotype is observed. Myoblast city and Rac are essentially involved in the development of the somatic muscles and were proposed to be interaction partners of Rols7. Because of the predicted structural similarities of the Rols7 and Rols6 proteins, we argue that genetic interaction of rols6, mbc and rac might lead to proper MpT morphology. We also propose that these interactions result in stable cell connections due to rearrangement of the cytoskeleton.
34. Unique and Overlapping Functions of Formins Frl and DAAM During Ommatidial Rotation and Neuronal Development in Drosophila. The noncanonical Frizzled/planar cell polarity (PCP) pathway regulates establishment of polarity within the plane of an epithelium to generate diversity of cell fates, asymmetric, but highly aligned structures, or to orchestrate the directional **migration** of cells during convergent extension during vertebrate gastrulation. In Drosophila, PCP signaling is essential to orient actin wing hairs and to align ommatidia in the eye, in part by coordinating the movement of groups of photoreceptor cells during ommatidial rotation. Importantly, the coordination of PCP signaling with changes in the cytoskeleton is essential for proper epithelial polarity. Formins polymerize linear actin filaments and are key regulators of the actin cytoskeleton. Here, we show that the diaphanous-related formin, Frl, the single fly member of the FMNL (formin related in leukocytes/formin-like) formin subfamily affects ommatidial rotation in the Drosophila eye and is controlled by the Rho family GTPase Cdc42. Interestingly, we also found that frl mutants exhibit an axon growth phenotype in the mushroom body, a center for olfactory learning in the Drosophila brain, which is also affected in a subset of PCP genes. Significantly, Frl cooperates with Cdc42 and another formin, DAAM, during mushroom body formation. This study thus suggests that different formins can cooperate or act independently in distinct tissues, likely integrating various signaling inputs with the regulation of the cytoskeleton. It furthermore highlights the importance and complexity of formin-dependent cytoskeletal regulation in multiple organs and developmental contexts.
35. Drosophila ELMO/CED-12 interacts with Myoblast city to direct myoblast fusion and ommatidial organization. Members of the CDM (CED-5, Dock180, Myoblast city) superfamily of guanine nucleotide exchange factors function in diverse processes that include cell **migration** and myoblast fusion. Previous studies have shown that the SH3, DHR1 and DHR2 domains of Myoblast city (MBC) are essential for it to direct myoblast fusion in the Drosophila embryo, while the conserved DCrk-binding proline rich region is expendable. Herein, we describe the isolation of Drosophila ELMO/CED-12, an approximately 82 kDa protein with a pleckstrin homology (PH) and proline-rich domain, by interaction with the MBC SH3 domain. Mass spectrometry confirms the presence of an MBC/ELMO complex within the embryonic musculature at the time of myoblast fusion and embryos maternally and/or zygotically mutant for elmo exhibit defects in myoblast fusion. Overexpression of MBC and ELMO in the embryonic mesoderm causes defects in myoblast fusion reminiscent of those seen with constitutively-activated Rac1, supporting the previous finding that both the absence of and an excess of Rac activity are deleterious to myoblast fusion. Overexpression of MBC and ELMO/CED-12 in the eye causes perturbations in ommatidial organization that are suppressed by mutations in Rac1 and Rac2, demonstrating genetically that MBC and ELMO/CED-12 cooperate to activate these small GTPases in Drosophila.

### ECM

#### PubMed

1. Drosophila importin-7 functions upstream of the Elmo signaling module to mediate the formation and stability of muscle attachments. Establishment and maintenance of stable muscle attachments is essential for coordinated body movement. Studies in Drosophila have pioneered a molecular understanding of the morphological events in the conserved process of muscle attachment formation, including myofiber migration, muscle-tendon signaling, and stable junctional adhesion between muscle cells and their corresponding target insertion sites. In both Drosophila and vertebrate models, integrin complexes play a key role in the biogenesis and stability of muscle attachments through the interactions of integrins with extracellular matrix (**ECM**) ligands. We show that Drosophila importin-7 (Dim7) is an upstream regulator of the conserved Elmo-Mbc→Rac signaling pathway in the formation of embryonic muscle attachment sites (MASs). Dim7 is encoded by the moleskin (msk) locus and was identified as an Elmo-interacting protein. Both Dim7 and Elmo localize to the ends of myofibers coincident with the timing of muscle-tendon attachment in late myogenesis. Phenotypic analysis of elmo mutants reveal muscle attachment defects similar to those previously described for integrin mutants. Furthermore, Elmo and Dim7 interact both biochemically and genetically in the developing musculature. The muscle detachment phenotype resulting from mutations in the msk locus can be rescued by components in the Elmo signaling pathway, including the Elmo-Mbc complex, an activated Elmo variant, or a constitutively active form of Rac. In larval muscles, the localization of Dim7 and activated Elmo to the sites of muscle attachment is attenuated upon RNAi knockdown of integrin heterodimer complex components. Our results show that integrins function as upstream signals to mediate Dim7-Elmo enrichment to the MASs.

### [Ee]xtracellular [Mm]atrix

#### PubMed

1. A directed miniscreen for genes involved in the Drosophila anti-parasitoid immune response. Drosophila larvae react against eggs from the endoparasitoid wasp Leptopilina boulardi by surrounding them in a multilayered cellular capsule. Once a wasp egg is recognized as foreign, circulating macrophage-like cells, known as plasmatocytes, adhere to the invader. After spreading around the wasp egg, plasmatocytes form cellular junctions between the cells, effectively separating the egg from the hemocoel. Next, a second sub-type of circulating immunosurveillance cell (hemocyte), known as lamellocytes, adhere to either the wasp egg or more likely the plasmatocytes surrounding the egg. From these events, it is obvious that adhesion and cell shape change are an essential part of Drosophila's cellular immune response against parasitoid wasp eggs. To date, very few genes have been described as being necessary for a proper anti-parasitization response in Drosophila. With this in mind, we performed a directed genetic miniscreen to discover new genes required for this response. Many of the genes with an encapsulation defect have mammalian homologues involved in cellular adhesion, wound healing, and thrombosis, including **extracellular matrix** proteins, cellular adhesion molecules, and small GTPases.
2. Drosophila importin-7 functions upstream of the Elmo signaling module to mediate the formation and stability of muscle attachments. Establishment and maintenance of stable muscle attachments is essential for coordinated body movement. Studies in Drosophila have pioneered a molecular understanding of the morphological events in the conserved process of muscle attachment formation, including myofiber migration, muscle-tendon signaling, and stable junctional adhesion between muscle cells and their corresponding target insertion sites. In both Drosophila and vertebrate models, integrin complexes play a key role in the biogenesis and stability of muscle attachments through the interactions of integrins with **extracellular matrix** (ECM) ligands. We show that Drosophila importin-7 (Dim7) is an upstream regulator of the conserved Elmo-Mbc→Rac signaling pathway in the formation of embryonic muscle attachment sites (MASs). Dim7 is encoded by the moleskin (msk) locus and was identified as an Elmo-interacting protein. Both Dim7 and Elmo localize to the ends of myofibers coincident with the timing of muscle-tendon attachment in late myogenesis. Phenotypic analysis of elmo mutants reveal muscle attachment defects similar to those previously described for integrin mutants. Furthermore, Elmo and Dim7 interact both biochemically and genetically in the developing musculature. The muscle detachment phenotype resulting from mutations in the msk locus can be rescued by components in the Elmo signaling pathway, including the Elmo-Mbc complex, an activated Elmo variant, or a constitutively active form of Rac. In larval muscles, the localization of Dim7 and activated Elmo to the sites of muscle attachment is attenuated upon RNAi knockdown of integrin heterodimer complex components. Our results show that integrins function as upstream signals to mediate Dim7-Elmo enrichment to the MASs.
3. The serine/threonine kinase dPak is required for polarized assembly of F-actin bundles and apical-basal polarity in the Drosophila follicular epithelium. During epithelial development cells become polarized along their apical-basal axis and some epithelia also exhibit polarity in the plane of the tissue. Mutations in the gene encoding a Drosophila Pak family serine/threonine kinase, dPak, disrupt the follicular epithelium that covers developing egg chambers during oogenesis. The follicular epithelium normally exhibits planar polarized organization of basal F-actin bundles such that they lie perpendicular to the anterior-posterior axis of the egg chamber, and requires contact with the basement membrane for apical-basal polarization. During oogenesis, dPak becomes localized to the basal end of follicle cells and is required for polarized organization of the basal actin cytoskeleton and for epithelial integrity and apical-basal polarity. The receptor protein tyrosine phosphatase Dlar and integrins, all receptors for **extracellular matrix** proteins, are required for polarization of the basal F-actin bundles, and for correct dPak localization in follicle cells. dpak mutant follicle cells show increased beta(Heavy)-spectrin levels, and we speculate that dPak regulation of beta(Heavy)-spectrin, a known participant in the maintenance of membrane domains, is required for correct apical-basal polarization of the membrane. We propose that dPak mediates communication between the basement membrane and intracellular proteins required for polarization of the basal F-actin and for apical-basal polarity.
4. The Ret receptor regulates sensory neuron dendrite growth and integrin mediated adhesion. Neurons develop highly stereotyped receptive fields by coordinated growth of their dendrites. Although cell surface cues play a major role in this process, few dendrite specific signals have been identified to date. We conducted an in vivo RNAi screen in Drosophila class IV dendritic arborization (C4da) neurons and identified the conserved Ret receptor, known to play a role in axon guidance, as an important regulator of dendrite development. The loss of Ret results in severe dendrite defects due to loss of **extracellular matrix** adhesion, thus impairing growth within a 2D plane. We provide evidence that Ret interacts with integrins to regulate dendrite adhesion via rac1. In addition, Ret is required for dendrite stability and normal F-actin distribution suggesting it has an essential role in dendrite maintenance. We propose novel functions for Ret as a regulator in dendrite patterning and adhesion distinct from its role in axon guidance.
5. Real-time analysis of Drosophila post-embryonic haemocyte behaviour. The larval stage of the model organism Drosophila is frequently used to study host-pathogen interactions. During embryogenesis the cellular arm of the immune response, consisting of macrophage-like cells known as plasmatocytes, is extremely motile and functions to phagocytise pathogens and apoptotic bodies, as well as produce **extracellular matrix**. The cellular branch of the larval (post-embryonic) innate immune system consists of three cell types--plasmatocytes, crystal cells and lamellocytes--which are involved in the phagocytosis, encapsulation and melanisation of invading pathogens. Post-embryonic haemocyte motility is poorly understood thus further characterisation is required, for the purpose of standardisation.In order to examine post-embryonic haemocyte cytoskeletal dynamics or migration, the most commonly used system is in vitro cell lines. The current study employs an ex vivo system (an adaptation of in vitro cell incubation using primary cells), in which primary larval or pre-pupal haemocytes are isolated for short term analysis, in order to discover various aspects of their behaviour during events requiring cytoskeleton dynamics.The ex vivo method allows for real-time analysis and manipulation of primary post-embryonic haemocytes. This technique was used to characterise, and potentially standardised, larval and pre-pupal haemocyte cytoskeleton dynamics, assayed on different extracellular matrices. Using this method it was determined that, while larval haemocytes are unable to migrate, haemocytes recovered from pre-pupae are capable of migration.

### [Ii]nvasive

#### PubMed

1. The Formin Diaphanous Regulates Myoblast Fusion through Actin Polymerization and Arp2/3 Regulation. The formation of multinucleated muscle cells through cell-cell fusion is a conserved process from fruit flies to humans. Numerous studies have shown the importance of Arp2/3, its regulators, and branched actin for the formation of an actin structure, the F-actin focus, at the fusion site. This F-actin focus forms the core of an **invasive** podosome-like structure that is required for myoblast fusion. In this study, we find that the formin Diaphanous (Dia), which nucleates and facilitates the elongation of actin filaments, is essential for Drosophila myoblast fusion. Following cell recognition and adhesion, Dia is enriched at the myoblast fusion site, concomitant with, and having the same dynamics as, the F-actin focus. Through analysis of Dia loss-of-function conditions using mutant alleles but particularly a dominant negative Dia transgene, we demonstrate that reduction in Dia activity in myoblasts leads to a fusion block. Significantly, no actin focus is detected, and neither branched actin regulators, SCAR or WASp, accumulate at the fusion site when Dia levels are reduced. Expression of constitutively active Dia also causes a fusion block that is associated with an increase in highly dynamic filopodia, altered actin turnover rates and F-actin distribution, and mislocalization of SCAR and WASp at the fusion site. Together our data indicate that Dia plays two roles during **invasive** podosome formation at the fusion site: it dictates the level of linear F-actin polymerization, and it is required for appropriate branched actin polymerization via localization of SCAR and WASp. These studies provide new insight to the mechanisms of cell-cell fusion, the relationship between different regulators of actin polymerization, and **invasive** podosome formation that occurs in normal development and in disease.
2. Asymmetric Mbc, active Rac1 and F-actin foci in the fusion-competent myoblasts during myoblast fusion in Drosophila. Myoblast fusion is an intricate process that is initiated by cell recognition and adhesion, and culminates in cell membrane breakdown and formation of multinucleate syncytia. In the Drosophila embryo, this process occurs asymmetrically between founder cells that pattern the musculature and fusion-competent myoblasts (FCMs) that account for the bulk of the myoblasts. The present studies clarify and amplify current models of myoblast fusion in several important ways. We demonstrate that the non-conventional guanine nucleotide exchange factor (GEF) Mbc plays a fundamental role in the FCMs, where it functions to activate Rac1, but is not required in the founder cells for fusion. Mbc, active Rac1 and F-actin foci are highly enriched in the FCMs, where they localize to the Sns:Kirre junction. Furthermore, Mbc is crucial for the integrity of the F-actin foci and the FCM cytoskeleton, presumably via its activation of Rac1 in these cells. Finally, the local asymmetric distribution of these proteins at adhesion sites is reminiscent of **invasive** podosomes and, consistent with this model, they are enriched at sites of membrane deformation, where the FCM protrudes into the founder cell/myotube. These data are consistent with models promoting actin polymerization as the driving force for myoblast fusion.

### [Mm]etastasis

#### PubMed

1. Drosophila Abelson kinase mediates cell invasion and proliferation through two distinct MAPK pathways. The Abelson (Abl) family of non-receptor tyrosine kinases has an important role in cell morphogenesis, motility, and proliferation. Although the function of Abl has been extensively studied in leukemia, its role in epithelial cell invasion remains obscure. Using the Drosophila wing epithelium as an in vivo model system, we show that overexpression (activation) of Drosophila Abl (dAbl) causes loss of epithelial apical/basal cell polarity and secretion of matrix metalloproteinases, resulting in a cellular invasion and apoptosis. Our in vivo data indicate that dAbl acts downstream of the Src kinases, which are known regulators of cell adhesion and invasion. Downstream of dAbl, Rac GTPases activate two distinct MAPK pathways: c-Jun N-terminal kinase signaling (required for cell invasion and apoptosis) and ERK signaling (inducing cell proliferation). Activated Abl also increases the activity of Src members through a positive feedback loop leading to signal amplification. Thus, targeting Src-Abl, using available dual inhibitors, could be of therapeutic importance in tumor cell **metastasis**.
2. Genes that drive invasion and migration in Drosophila. Successful cell migration depends on the careful regulation of the timing of movement, the guidance of motile cells, and cytoskeletal and adhesive changes within the cells. This review focuses on genes that act cell-autonomously to promote these aspects of cell migration in Drosophila. We discuss recent advances in understanding the migration of the ovarian border cells, embryonic blood cells, primordial germ cells, somatic gonadal precursors, and tracheal cells. Comparison of genes that regulate these processes to those that promote tumorigenesis and **metastasis** in mammals demonstrates that studies in fruit flies are uncovering new genes highly relevant to cancer biology.
3. In vivo RNAi screen identifies candidate signaling genes required for collective cell migration in Drosophila ovary. Collective migration of loosely or closely associated cell groups is prevalent in animal development, physiological events, and cancer **metastasis**. However, our understanding of the mechanisms of collective cell migration is incomplete. Drosophila border cells provide a powerful in vivo genetic model to study collective migration and identify essential genes for this process. Using border cell-specific RNAi-silencing in Drosophila, we knocked down 360 conserved signaling transduction genes in adult flies to identify essential pathways and genes for border cell migration. We uncovered a plethora of signaling genes, a large proportion of which had not been reported for border cells, including Rack1 (Receptor of activated C kinase) and brk (brinker), mad (mother against dpp), and sax (saxophone), which encode three components of TGF-β signaling. The RNAi knock down phenotype was validated by clonal analysis of Rack1 mutants. Our data suggest that inhibition of Src activity by Rack1 may be important for border cell migration and cluster cohesion maintenance. Lastly, results from our screen not only would shed light on signaling pathways involved in collective migration during embryogenesis and organogenesis in general, but also could help our understanding for the functions of conserved human genes involved in cancer **metastasi**s.
4. Drosophila as a model to study the role of blood cells in inflammation, innate immunity and cancer. Drosophila has a primitive yet effective blood system with three types of haemocytes which function throughout different developmental stages and environmental stimuli. Haemocytes play essential roles in tissue modeling during embryogenesis and morphogenesis, and also in innate immunity. The open circulatory system of Drosophila makes haemocytes ideal signal mediators to cells and tissues in response to events such as infection and wounding. The application of recently developed and sophisticated genetic tools to the relatively simple genome of Drosophila has made the fly a popular system for modeling human tumorigensis and **metastasis**. Drosophila is now used for screening and investigation of genes implicated in human leukemia and also in modeling development of solid tumors. This second line of research offers promising opportunities to determine the seemingly conflicting roles of blood cells in tumor progression and invasion. This review provides an overview of the signaling pathways conserved in Drosophila during haematopoiesis, haemostasis, innate immunity, wound healing and inflammation. We also review the most recent progress in the use of Drosophila as a cancer research model with an emphasis on the roles haemocytes can play in various cancer models and in the links between inflammation and cancer.

### [Cc]ell [Ii]nvasion

#### PubMed

1. Drosophila Abelson kinase mediates **cell invasion** and proliferation through two distinct MAPK pathways. The Abelson (Abl) family of non-receptor tyrosine kinases has an important role in cell morphogenesis, motility, and proliferation. Although the function of Abl has been extensively studied in leukemia, its role in epithelial **cell invasion** remains obscure. Using the Drosophila wing epithelium as an in vivo model system, we show that overexpression (activation) of Drosophila Abl (dAbl) causes loss of epithelial apical/basal cell polarity and secretion of matrix metalloproteinases, resulting in a cellular invasion and apoptosis. Our in vivo data indicate that dAbl acts downstream of the Src kinases, which are known regulators of cell adhesion and invasion. Downstream of dAbl, Rac GTPases activate two distinct MAPK pathways: c-Jun N-terminal kinase signaling (required for **cell invasion** and apoptosis) and ERK signaling (inducing cell proliferation). Activated Abl also increases the activity of Src members through a positive feedback loop leading to signal amplification. Thus, targeting Src-Abl, using available dual inhibitors, could be of therapeutic importance in tumor cell metastasis.
2. Rickettsia parkeri invasion of diverse host cells involves an Arp2/3 complex, WAVE complex and Rho-family GTPase-dependent pathway. Rickettsiae are obligate intracellular pathogens that are transmitted to humans by arthropod vectors and cause diseases such as spotted fever and typhus. Although rickettsiae require the host cell actin cytoskeleton for invasion, the cytoskeletal proteins that mediate this process have not been completely described. To identify the host factors important during **cell invasion** by Rickettsia parkeri, a member of the spotted fever group (SFG), we performed an RNAi screen targeting 105 proteins in Drosophila melanogaster S2R+ cells. The screen identified 21 core proteins important for invasion, including the GTPases Rac1 and Rac2, the WAVE nucleation-promoting factor complex and the Arp2/3 complex. In mammalian cells, including endothelial cells, the natural targets of R. parkeri, the Arp2/3 complex was also crucial for invasion, while requirements for WAVE2 as well as Rho GTPases depended on the particular cell type. We propose that R. parkeri invades S2R+ arthropod cells through a primary pathway leading to actin nucleation, whereas invasion of mammalian endothelial cells occurs via redundant pathways that converge on the host Arp2/3 complex. Our results reveal a key role for the WAVE and Arp2/3 complexes, as well as a higher degree of variation than previously appreciated in actin nucleation pathways activated during Rickettsia invasion.

---

## nec

- NCBI Gene
- GeneRIF
- Pubmed

| Search Term | Rank | Fields |
| --- | --- | --- |
| [Mm]igration | 6 | PubMed(1) |
| ECM | 7 | PubMed(1) |
| [Ee]xtracellular [Mm]atrix | 8 | PubMed(1) |

  
hide/show details for nec

### [Mm]igration

#### PubMed

1. Genes conserved in bilaterians but jointly lost with Myc during nematode evolution are enriched in cell proliferation and cell **migration** functions. Animals use a stereotypical set of developmental genes to build body architectures of varying sizes and organizational complexity. Some genes are critical to developmental patterning, while other genes are important to physiological control of growth. However, growth regulator genes may not be as important in small-bodied "micro-metazoans" such as nematodes. Nematodes use a simplified developmental strategy of lineage-based cell fate specifications to produce an adult bilaterian body composed of a few hundreds of cells. Nematodes also lost the MYC proto-oncogenic regulator of cell proliferation. To identify additional regulators of cell proliferation that were lost with MYC, we computationally screened and determined 839 high-confidence genes that are conserved in bilaterians/lost in nematodes (CIBLIN genes). We find that 30 % of all CIBLIN genes encode transcriptional regulators of cell proliferation, epithelial-to-mesenchyme transitions, and other processes. Over 50 % of CIBLIN genes are unnamed genes in Drosophila, suggesting that there are many understudied genes. Interestingly, CIBLIN genes include many Myc synthetic lethal (MycSL) hits from recent screens. CIBLIN genes include key regulators of heparan sulfate proteoglycan (HSPG) sulfation patterns, and lysyl oxidases involved in cross-linking and modification of the extracellular matrix (ECM). These genes and others suggest the CIBLIN repertoire services critical functions in ECM remodeling and cell **migration** in large-bodied bilaterians. Correspondingly, CIBLIN genes are co-expressed with Myc in cancer transcriptomes, and include a preponderance of known determinants of cancer progression and tumor aggression. We propose that CIBLIN gene research can improve our understanding of regulatory control of cellular growth in metazoans.

### ECM

#### PubMed

1. Genes conserved in bilaterians but jointly lost with Myc during nematode evolution are enriched in cell proliferation and cell migration functions. Animals use a stereotypical set of developmental genes to build body architectures of varying sizes and organizational complexity. Some genes are critical to developmental patterning, while other genes are important to physiological control of growth. However, growth regulator genes may not be as important in small-bodied "micro-metazoans" such as nematodes. Nematodes use a simplified developmental strategy of lineage-based cell fate specifications to produce an adult bilaterian body composed of a few hundreds of cells. Nematodes also lost the MYC proto-oncogenic regulator of cell proliferation. To identify additional regulators of cell proliferation that were lost with MYC, we computationally screened and determined 839 high-confidence genes that are conserved in bilaterians/lost in nematodes (CIBLIN genes). We find that 30 % of all CIBLIN genes encode transcriptional regulators of cell proliferation, epithelial-to-mesenchyme transitions, and other processes. Over 50 % of CIBLIN genes are unnamed genes in Drosophila, suggesting that there are many understudied genes. Interestingly, CIBLIN genes include many Myc synthetic lethal (MycSL) hits from recent screens. CIBLIN genes include key regulators of heparan sulfate proteoglycan (HSPG) sulfation patterns, and lysyl oxidases involved in cross-linking and modification of the extracellular matrix (**ECM**). These genes and others suggest the CIBLIN repertoire services critical functions in **ECM** remodeling and cell migration in large-bodied bilaterians. Correspondingly, CIBLIN genes are co-expressed with Myc in cancer transcriptomes, and include a preponderance of known determinants of cancer progression and tumor aggression. We propose that CIBLIN gene research can improve our understanding of regulatory control of cellular growth in metazoans.

### [Ee]xtracellular [Mm]atrix

#### PubMed

1. Genes conserved in bilaterians but jointly lost with Myc during nematode evolution are enriched in cell proliferation and cell migration functions. Animals use a stereotypical set of developmental genes to build body architectures of varying sizes and organizational complexity. Some genes are critical to developmental patterning, while other genes are important to physiological control of growth. However, growth regulator genes may not be as important in small-bodied "micro-metazoans" such as nematodes. Nematodes use a simplified developmental strategy of lineage-based cell fate specifications to produce an adult bilaterian body composed of a few hundreds of cells. Nematodes also lost the MYC proto-oncogenic regulator of cell proliferation. To identify additional regulators of cell proliferation that were lost with MYC, we computationally screened and determined 839 high-confidence genes that are conserved in bilaterians/lost in nematodes (CIBLIN genes). We find that 30 % of all CIBLIN genes encode transcriptional regulators of cell proliferation, epithelial-to-mesenchyme transitions, and other processes. Over 50 % of CIBLIN genes are unnamed genes in Drosophila, suggesting that there are many understudied genes. Interestingly, CIBLIN genes include many Myc synthetic lethal (MycSL) hits from recent screens. CIBLIN genes include key regulators of heparan sulfate proteoglycan (HSPG) sulfation patterns, and lysyl oxidases involved in cross-linking and modification of the **extracellular matrix** (ECM). These genes and others suggest the CIBLIN repertoire services critical functions in ECM remodeling and cell migration in large-bodied bilaterians. Correspondingly, CIBLIN genes are co-expressed with Myc in cancer transcriptomes, and include a preponderance of known determinants of cancer progression and tumor aggression. We propose that CIBLIN gene research can improve our understanding of regulatory control of cellular growth in metazoans.

---

## Spn55B

- NCBI Gene
- GeneRIF
- Pubmed

| Search Term | Rank | Fields |
| --- | --- | --- |
| [Mm]igration | 6 | PubMed(1) |
| ECM | 7 | PubMed(1) |
| [Ee]xtracellular [Mm]atrix | 8 | PubMed(1) |
| [Ii]nvasive | 10 | PubMed(1) |

  
hide/show details for Spn55B

### [Mm]igration

#### PubMed

1. Genes conserved in bilaterians but jointly lost with Myc during nematode evolution are enriched in cell proliferation and cell **migration** functions. Animals use a stereotypical set of developmental genes to build body architectures of varying sizes and organizational complexity. Some genes are critical to developmental patterning, while other genes are important to physiological control of growth. However, growth regulator genes may not be as important in small-bodied "micro-metazoans" such as nematodes. Nematodes use a simplified developmental strategy of lineage-based cell fate specifications to produce an adult bilaterian body composed of a few hundreds of cells. Nematodes also lost the MYC proto-oncogenic regulator of cell proliferation. To identify additional regulators of cell proliferation that were lost with MYC, we computationally screened and determined 839 high-confidence genes that are conserved in bilaterians/lost in nematodes (CIBLIN genes). We find that 30 % of all CIBLIN genes encode transcriptional regulators of cell proliferation, epithelial-to-mesenchyme transitions, and other processes. Over 50 % of CIBLIN genes are unnamed genes in Drosophila, suggesting that there are many understudied genes. Interestingly, CIBLIN genes include many Myc synthetic lethal (MycSL) hits from recent screens. CIBLIN genes include key regulators of heparan sulfate proteoglycan (HSPG) sulfation patterns, and lysyl oxidases involved in cross-linking and modification of the extracellular matrix (ECM). These genes and others suggest the CIBLIN repertoire services critical functions in ECM remodeling and cell **migration** in large-bodied bilaterians. Correspondingly, CIBLIN genes are co-expressed with Myc in cancer transcriptomes, and include a preponderance of known determinants of cancer progression and tumor aggression. We propose that CIBLIN gene research can improve our understanding of regulatory control of cellular growth in metazoans.

### ECM

#### PubMed

1. Genes conserved in bilaterians but jointly lost with Myc during nematode evolution are enriched in cell proliferation and cell migration functions. Animals use a stereotypical set of developmental genes to build body architectures of varying sizes and organizational complexity. Some genes are critical to developmental patterning, while other genes are important to physiological control of growth. However, growth regulator genes may not be as important in small-bodied "micro-metazoans" such as nematodes. Nematodes use a simplified developmental strategy of lineage-based cell fate specifications to produce an adult bilaterian body composed of a few hundreds of cells. Nematodes also lost the MYC proto-oncogenic regulator of cell proliferation. To identify additional regulators of cell proliferation that were lost with MYC, we computationally screened and determined 839 high-confidence genes that are conserved in bilaterians/lost in nematodes (CIBLIN genes). We find that 30 % of all CIBLIN genes encode transcriptional regulators of cell proliferation, epithelial-to-mesenchyme transitions, and other processes. Over 50 % of CIBLIN genes are unnamed genes in Drosophila, suggesting that there are many understudied genes. Interestingly, CIBLIN genes include many Myc synthetic lethal (MycSL) hits from recent screens. CIBLIN genes include key regulators of heparan sulfate proteoglycan (HSPG) sulfation patterns, and lysyl oxidases involved in cross-linking and modification of the extracellular matrix (**ECM**). These genes and others suggest the CIBLIN repertoire services critical functions in **ECM** remodeling and cell migration in large-bodied bilaterians. Correspondingly, CIBLIN genes are co-expressed with Myc in cancer transcriptomes, and include a preponderance of known determinants of cancer progression and tumor aggression. We propose that CIBLIN gene research can improve our understanding of regulatory control of cellular growth in metazoans.

### [Ee]xtracellular [Mm]atrix

#### PubMed

1. Genes conserved in bilaterians but jointly lost with Myc during nematode evolution are enriched in cell proliferation and cell migration functions. Animals use a stereotypical set of developmental genes to build body architectures of varying sizes and organizational complexity. Some genes are critical to developmental patterning, while other genes are important to physiological control of growth. However, growth regulator genes may not be as important in small-bodied "micro-metazoans" such as nematodes. Nematodes use a simplified developmental strategy of lineage-based cell fate specifications to produce an adult bilaterian body composed of a few hundreds of cells. Nematodes also lost the MYC proto-oncogenic regulator of cell proliferation. To identify additional regulators of cell proliferation that were lost with MYC, we computationally screened and determined 839 high-confidence genes that are conserved in bilaterians/lost in nematodes (CIBLIN genes). We find that 30 % of all CIBLIN genes encode transcriptional regulators of cell proliferation, epithelial-to-mesenchyme transitions, and other processes. Over 50 % of CIBLIN genes are unnamed genes in Drosophila, suggesting that there are many understudied genes. Interestingly, CIBLIN genes include many Myc synthetic lethal (MycSL) hits from recent screens. CIBLIN genes include key regulators of heparan sulfate proteoglycan (HSPG) sulfation patterns, and lysyl oxidases involved in cross-linking and modification of the **extracellular matrix** (ECM). These genes and others suggest the CIBLIN repertoire services critical functions in ECM remodeling and cell migration in large-bodied bilaterians. Correspondingly, CIBLIN genes are co-expressed with Myc in cancer transcriptomes, and include a preponderance of known determinants of cancer progression and tumor aggression. We propose that CIBLIN gene research can improve our understanding of regulatory control of cellular growth in metazoans.

### [Ii]nvasive

#### PubMed

1. The Spn4 gene of Drosophila encodes a potent furin-directed secretory pathway serpin. Proprotein convertases (PCs) are an important class of host-cell serine endoproteases implicated in many physiological and pathological processes. Owing to their expanding roles in the proteolytic events required for generating infectious microbial pathogens and for tumor growth and **invasive**ness, there is increasing interest in identifying endogenous PC inhibitors. Here we report the identification of Spn4A, a previously uncharacterized secretory pathway serine protease inhibitor (serpin) from Drosophila melanogaster that contains a consensus furin cleavage site, -Arg(P4)-Arg-Lys-Arg(P1) downsream-, in its reactive site loop (RSL). Our biochemical and kinetics analysis revealed that recombinant Spn4A inhibits human furin (K(i), 13 pM; k(ass), 3.2 x 10(7) M(-1) x s(-1)) and Drosophila PC2 (K(i), 3.5 nM; k(ass), 9.2 x 10(4) M(-1) x s(-1)) by a slow-binding mechanism characteristic of serpin molecules and forms a kinetically trapped SDS-stable complex with each enzyme. For both PCs, the stoichiometry of inhibition by Spn4A is nearly 1, which is characteristic of known physiological serpin-protease interactions. Mass analysis of furin-Spn4A reaction products identified the actual reactive site center of Spn4A to be -Arg(P4)-Arg-Lys-Arg(P1)-downstream-. Moreover, we demonstrate that Spn4A's highly effective PC inhibition properties are critically dependent on the unusual length of its RSL, which is composed of 18 aa instead of the typical 17-residue RSL found in most other inhibitory serpins. The identification of Spn4A, the most potent and effective natural serpin of PCs identified to date, suggests that Spn4A could be a prototype of endogenous serpins involved in the precise regulation of PC-dependent proteolytic cleavage events in the secretory pathway of eukaryotic cells.

---

## Jra

- NCBI Gene
- GeneRIF
- Pubmed

| Search Term | Rank | Fields |
| --- | --- | --- |
| [Ff]ilopodia | 1 | PubMed(2) |
| [Mm]igration | 6 | PubMed(18) |
| ECM | 7 | PubMed(1) |
| [Ee]xtracellular [Mm]atrix | 8 | PubMed(3) |
| [Ii]nvasive | 10 | PubMed(4) |
| [Mm]etastasis | 12 | PubMed(3) |
| [Cc]ell [Ii]nvasion | 13 | PubMed(1) |

  
hide/show details for Jra

### [Ff]ilopodia

#### PubMed

1. Transcriptional regulation of Profilin during wound closure in Drosophila larvae. Injury is an inevitable part of life, making wound healing essential for survival. In postembryonic skin, wound closure requires that epidermal cells recognize the presence of a gap and change their behavior to migrate across it. In Drosophila larvae, wound closure requires two signaling pathways [the Jun N-terminal kinase (JNK) pathway and the Pvr receptor tyrosine kinase signaling pathway] and regulation of the actin cytoskeleton. In this and other systems, it remains unclear how the signaling pathways that initiate wound closure connect to the actin regulators that help execute wound-induced cell migrations. Here, we show that chickadee, which encodes the Drosophila Profilin, a protein important for actin filament recycling and cell migration during development, is required for the physiological process of larval epidermal wound closure. After injury, chickadee is transcriptionally upregulated in cells proximal to the wound. We found that JNK, but not Pvr, mediates the increase in chic transcription through the Jun and Fos transcription factors. Finally, we show that chic-deficient larvae fail to form a robust actin cable along the wound edge and also fail to form normal **filopodia**l and lamellipodial extensions into the wound gap. Our results thus connect a factor that regulates actin monomer recycling to the JNK signaling pathway during wound closure. They also reveal a physiological function for an important developmental regulator of actin and begin to tease out the logic of how the wound repair response is organized.
2. Gene regulatory networks controlling hematopoietic progenitor niche cell production and differentiation in the Drosophila lymph gland. Hematopoiesis occurs in two phases in Drosophila, with the first completed during embryogenesis and the second accomplished during larval development. The lymph gland serves as the venue for the final hematopoietic program, with this larval tissue well-studied as to its cellular organization and genetic regulation. While the medullary zone contains stem-like hematopoietic progenitors, the posterior signaling center (PSC) functions as a niche microenvironment essential for controlling the decision between progenitor maintenance versus cellular differentiation. In this report, we utilize a PSC-specific GAL4 driver and UAS-gene RNAi strains, to selectively knockdown individual gene functions in PSC cells. We assessed the effect of abrogating the function of 820 genes as to their requirement for niche cell production and differentiation. 100 genes were shown to be essential for normal niche development, with various loci placed into sub-groups based on the functions of their encoded protein products and known genetic interactions. For members of three of these groups, we characterized loss- and gain-of-function phenotypes. Gene function knockdown of members of the BAP chromatin-remodeling complex resulted in niche cells that do not express the hedgehog (hh) gene and fail to differentiate **filopodia** believed important for Hh signaling from the niche to progenitors. Abrogating gene function of various members of the insulin-like growth factor and TOR signaling pathways resulted in anomalous PSC cell production, leading to a defective niche organization. Further analysis of the Pten, TSC1, and TSC2 tumor suppressor genes demonstrated their loss-of-function condition resulted in severely altered blood cell homeostasis, including the abundant production of lamellocytes, specialized hemocytes involved in innate immune responses. Together, this cell-specific RNAi knockdown survey and mutant phenotype analyses identified multiple genes and their regulatory networks required for the normal organization and function of the hematopoietic progenitor niche within the lymph gland.

### [Mm]igration

#### PubMed

1. The level of C/EBP protein is critical for cell **migration** during Drosophila oogenesis and is tightly controlled by regulated degradation. The C/EBP transcription factor, Slbo, is required for **migration** of border cells during Drosophila oogenesis. Unexpectedly, we find that neither increase nor decrease of Slbo activity is tolerated in border cells. Correct protein level is in part ensured by cell type-specific regulated turnover of Slbo protein. Through genetic screening, we identify two genes that are involved in this regulation. The Ubp64 ubiquitin hydrolase acts as a stabilizer of Slbo protein. A novel gene, tribbles, is a negative regulator of slbo in vivo. Tribbles acts by specifically targeting Slbo for rapid degradation via ubiquitination.
2. Transcriptional regulation of Profilin during wound closure in Drosophila larvae. Injury is an inevitable part of life, making wound healing essential for survival. In postembryonic skin, wound closure requires that epidermal cells recognize the presence of a gap and change their behavior to migrate across it. In Drosophila larvae, wound closure requires two signaling pathways [the Jun N-terminal kinase (JNK) pathway and the Pvr receptor tyrosine kinase signaling pathway] and regulation of the actin cytoskeleton. In this and other systems, it remains unclear how the signaling pathways that initiate wound closure connect to the actin regulators that help execute wound-induced cell **migration**s. Here, we show that chickadee, which encodes the Drosophila Profilin, a protein important for actin filament recycling and cell **migration** during development, is required for the physiological process of larval epidermal wound closure. After injury, chickadee is transcriptionally upregulated in cells proximal to the wound. We found that JNK, but not Pvr, mediates the increase in chic transcription through the Jun and Fos transcription factors. Finally, we show that chic-deficient larvae fail to form a robust actin cable along the wound edge and also fail to form normal filopodial and lamellipodial extensions into the wound gap. Our results thus connect a factor that regulates actin monomer recycling to the JNK signaling pathway during wound closure. They also reveal a physiological function for an important developmental regulator of actin and begin to tease out the logic of how the wound repair response is organized.
3. [Dorsal closure in Drosophila. A genetic model for wound healing?]. Dorsal closure (DC) is a morphogenetic movement that establishes the dorsal ectoderm of the drosophila embryo. During this process, the two lateral epithelia stretch toward the dorsal midline, the suture line of the two leading edges. Cell **migration** during DC relies both on cell shape change controlled by the activity of the JNK pathway in the leading edge cells and modification of cell adhesiveness, probably dependent upon activation of the Dpp (TGF-beta) pathway. Coupling of the JNK and TGF-beta pathways is essential. The sequence of the cellular and molecular events of DC highlights interesting common features with wound healing in vertebrates. Like DC, wound healing relies on the **migration** of epithelia bordered by leading edges controlling the direction and speed of the movement. This review summarizes recent data concerning the control of epithelial morphogenesis during DC and the bases of wound healing. The molecular and cellular events that underlie these two analogous migratory processes are detailed, discussed and compared. We suggest that DC is a good genetic model for wound healing studying.
4. Drosophila female sterile mutation spoonbill interferes with multiple pathways in oogenesis. spoonbill is a Drosophila female-sterile mutation, which displays a range of eggshell and egg chamber patterning defects. Previous analysis has shown that the mutation interfered with the function of two major signaling pathways, GRK/EGFR and DPP. In this report, the nature of spoonbill was further investigated to examine whether it was associated with additional pathways in oogenesis. Clonal analysis, presented here, demonstrated that most of the aberrant phenotypes associated with spoonbill were dependent on a mutant germline. Nevertheless, SPOONBILL may function also in the soma to ensure proper polarization and **migration** of the border-cell-cluster. Further, genetic interaction studies implicated spoonbill in additional unrelated pathways such as the one(s) involved in actin polymerization/depolymerization. Based on the previous data and the results presented here, it is anticipated that spoonbill may encode a multifunctional protein that perhaps coordinately regulated the activity of multiple signaling pathways during oogenesis.
5. Downregulation of Jun kinase signaling in the amnioserosa is essential for dorsal closure of the Drosophila embryo. During Drosophila embryogenesis, Jun kinase (JNK) signaling has been shown to play a key role in regulating the morphogenetic process of dorsal closure, which also serves as a model for epithelial sheet fusion during wound repair. During dorsal closure the JNK signaling cascade in the dorsal-most (leading edge) cells of the epidermis activates the AP-1 transcription factor comprised of DJUN and DFOS that, in turn, upregulates the expression of the dpp gene. DPP is a secreted morphogen that signals lateral epidermal cells to elongate along the dorsoventral axis. The leading edge cells contact the peripheral cells of a monolayer extraembryonic epithelium, the amnioserosa, which lies on the dorsal side of the embryo. Focal complexes are present at the dorsal-most membrane of the leading edge cells, where they contact the amnioserosa.We show that the JNK signaling cascade is initially active in both the amnioserosa and the leading edge of the epidermis. JNK signaling is downregulated in the amnioserosa, but not in the leading edge, prior to dorsal closure. The subcellular localization of DFOS and DJUN is responsive to JNK signaling in the amnioserosa: JNK activation results in nuclear localization of DFOS and DJUN; the downregulation of JNK signaling results in the relocalization of DFOS and DJUN to the cytoplasm. The HINDSIGHT (HNT) Zn-finger protein and the PUCKERED (PUC) JNK phosphatase are essential for downregulation of the JNK cascade in the amnioserosa. Persistent JNK activity in the amnioserosa leads to defective focal complexes in the adjacent leading edge cells and to the failure of dorsal closure.Focal complexes are assembled at the boundary between high and low JNK activity. In the absence of focal complexes, miscommunication between the amnioserosa and the leading edge may lead to a premature "stop" signal that halts dorsalward **migration** of the leading edge. Spatial and temporal regulation of the JNK signaling cascade may be a general mechanism that controls tissue remodeling during morphogenesis and wound healing.
6. A functional RNAi screen for regulators of receptor tyrosine kinase and ERK signalling. Receptor tyrosine kinase (RTK) signalling through extracellular-signal-regulated kinases (ERKs) has pivotal roles during metazoan development, underlying processes as diverse as fate determination, differentiation, proliferation, survival, **migration** and growth. Abnormal RTK/ERK signalling has been extensively documented to contribute to developmental disorders and disease, most notably in oncogenic transformation by mutant RTKs or downstream pathway components such as Ras and Raf. Although the core RTK/ERK signalling cassette has been characterized by decades of research using mammalian cell culture and forward genetic screens in model organisms, signal propagation through this pathway is probably regulated by a larger network of moderate, context-specific proteins. The genes encoding these proteins may not have been discovered through traditional screens owing, in particular, to the requirement for visible phenotypes. To obtain a global view of RTK/ERK signalling, we performed an unbiased, RNA interference (RNAi), genome-wide, high-throughput screen in Drosophila cells using a novel, quantitative, cellular assay monitoring ERK activation. Here we show that ERK pathway output integrates a wide array of conserved cellular processes. Further analysis of selected components-in multiple cell types with different RTK ligands and oncogenic stimuli-validates and classifies 331 pathway regulators. The relevance of these genes is highlighted by our isolation of a Ste20-like kinase and a PPM-family phosphatase that seem to regulate RTK/ERK signalling in vivo and in mammalian cells. Novel regulators that modulate specific pathway outputs may be selective targets for drug discovery.
7. Signaling pathways directing the movement and fusion of epithelial sheets: lessons from dorsal closure in Drosophila. Wound healing in embryos and various developmental events in metazoans require the spreading and fusion of epithelial sheets. The complex signaling pathways regulating these processes are being pieced together through genetic, cell biological, and biochemical approaches. At present, dorsal closure of the Drosophila embryo is the best-characterized example of epithelial sheet movement. Dorsal closure involves **migration** of the lateral epidermal flanks to close a hole in the dorsal epidermis occupied by an epithelium called the amnioserosa. Detailed genetic studies have revealed a network of interacting signaling molecules regulating this process. At the center of this network is a Jun N-terminal kinase cascade acting at the leading edge of the migrating epidermis that triggers signaling by the TGF-beta superfamily member Decapentaplegic and which interacts with the Wingless pathway. These signaling modules regulate the cytoskeletal reorganization and cell shape change necessary to drive dorsal closure. Activation of this network requires signals from the amnioserosa and input from a variety of proteins at cell-cell junctions. The Rho family of small GTPases is also instrumental, both in activation of signaling and regulation of the cytoskeleton. Many of the proteins regulating dorsal closure have been implicated in epithelial movement in other organisms, and dorsal closure has emerged as an ideal model system for the study of the **migration** and fusion of epithelial sheets.
8. The control of cell motility and epithelial morphogenesis by Jun kinases. Originally identified as stress-activated protein kinases that control cell survival and proliferation through transcription factor c-Jun, the Jun N-terminal kinase (JNK) subgroup of MAP kinases (MAPKs) have recently emerged as crucial regulators of cell **migration** and the morphogenetic movement of epithelial sheets. In Drosophila, a well-orchestrated JNK signaling pathway controls formation of actin stress fibers and cell shape changes, which are required for the sealing of embryonic epidermis in a process known as dorsal closure. The JNK pathway is also involved in morphogenetic processes in mice including closure of the eyelid, neural tube and optic fissure. This article focuses on recent advances in understanding the role of JNK pathway in the regulation of cell **migration**, cytoskeleton rearrangement and the morphogenesis of epithelial sheets.
9. A sensitized PiggyBac-based screen for regulators of border cell **migration** in Drosophila. **Migration** of border cells during Drosophila melanogaster oogenesis is a good model system for investigating the genetic requirements for cell **migration** in vivo. We present a sensitized loss-of-function screen used to identify new genes required in border cells for their **migration**. Chromosomes bearing FRTs on all four major autosomal arms were mutagenized by insertions of the transposable element PiggyBac, allowing multiple parallel clonal screens and easy identification of the mutated gene. For border cells, we analyzed homozygous mutant clones positively marked with lacZ and sensitized by expression of dominant-negative PVR, the guidance receptor. We identified new alleles of genes already known to be required for border cell **migration**, including aop/yan, DIAP1, and taiman as well as a conserved Slbo-regulated enhancer downstream of shg/DE-cadherin. Mutations in genes not previously described to be required in border cells were also uncovered: hrp48, vir, rme-8, kismet, and puckered. puckered was unique in that the **migration** defects were observed only when PVR signaling was reduced. We present evidence that an excess of JNK signaling is deleterious for **migration** in the absence of PVR activity at least in part through Fos transcriptional activity and possibly through antagonistic effects on DIAP1.
10. JNK signaling controls border cell cluster integrity and collective cell **migration**. Collective cell movement is a mechanism for invasion identified in many developmental events. Examples include the movement of lateral-line neurons in Zebrafish, cells in the inner blastocyst, and metastasis of epithelial tumors [1]. One key model to study collective **migration** is the movement of border cell clusters in Drosophila. Drosophila egg chambers contain 15 nurse cells and a single oocyte surrounded by somatic follicle cells. At their anterior end, polar cells recruit several neighboring follicle cells to form the border cell cluster [2]. By stage 9, and over 6 hr, border cells migrate as a cohort between nurse cells toward the oocyte. The specification and directionality of border cell movement are regulated by hormonal and signaling mechanisms [3]. However, how border cells are held together while they migrate is not known. Here, we show that a negative-feedback loop controlling JNK activity regulates border cell cluster integrity. JNK signaling modulates contacts between border cells and between border cells and substratum to sustain collective **migration** by regulating several effectors including the polarity factor Bazooka and the cytoskeletal adaptor D-Paxillin. We anticipate a role for the JNK pathway in controlling collective cell movements in other morphogenetic and clinical models.
11. Regulation of cell adhesion and collective cell **migration** by hindsight and its human homolog RREB1. Cell movements represent a major driving force in embryonic development, tissue repair, and tumor metastasis [1]. The **migration** of single cells has been well studied, predominantly in cell culture [2, 3]; however, in vivo, a greater variety of modes of cell movement occur, including the movements of cells in clusters, strands, sheets, and tubes, also known as collective cell **migration**s [4, 5]. In spite of the relevance of these types of movements in both normal and pathological conditions, the molecular mechanisms that control them remain predominantly unknown. Epithelial follicle cells of the Drosophila ovary undergo several dynamic morphological changes, providing a genetically tractable model [6]. We found that anterior follicle cells, including border cells, mutant for the gene hindsight (hnt) accumulated excess cell-cell adhesion molecules and failed to undergo their normal collective movements. In addition, HNT affected border cell cluster cohesion and motility via effects on the JNK and STAT pathways, respectively. Interestingly, reduction of expression of the mammalian homolog of HNT, RREB1, by siRNA inhibited collective cell **migration** in a scratch-wound healing assay of MCF10A mammary epithelial cells, suppressed surface activity, retarded cell spreading after plating, and led to the formation of immobile, tightly adherent cell colonies. We propose that HNT and RREB1 are essential to reduce cell-cell adhesion when epithelial cells within an interconnected group undergo dynamic changes in cell shape.
12. Dcdc42 acts in TGF-beta signaling during Drosophila morphogenesis: distinct roles for the Drac1/JNK and Dcdc42/TGF-beta cascades in cytoskeletal regulation. During Drosophila embryogenesis the two halves of the lateral epidermis migrate dorsally over a surface of flattened cells, the amnioserosa, and meet at the dorsal midline in order to form the continuous sheet of the larval epidermis. During this process of epithelial **migration**, known as dorsal closure, signaling from a Jun-amino-terminal-kinase cascade causes the production of the secreted transforming-growth-factor-beta-like ligand, Decapentaplegic. Binding of Decapentaplegic to the putative transforming-growth-factor-beta-like receptors Thickveins and Punt activates a transforming-growth-factor-beta-like pathway that is also required for dorsal closure. Mutations in genes involved in either the Jun-amino-terminal-kinase cascade or the transforming-growth-factor-beta-like signaling pathway can disrupt dorsal closure. Our findings show that although these pathways are linked they are not equivalent in function. Signaling by the Jun-amino-terminal-kinase cascade may be initiated by the small Ras-like GTPase Drac1 and acts to assemble the cytoskeleton and specify the identity of the first row of cells of the epidermis prior to the onset of dorsal closure. Signaling in the transforming-growth-factor-beta-like pathway is mediated by Dcdc42, and acts during the closure process to control the mechanics of the **migration** process, most likely via its putative effector kinase DPAK.
13. A targeted UAS-RNAi screen in Drosophila larvae identifies wound closure genes regulating distinct cellular processes. Robust mechanisms for tissue repair are critical for survival of multicellular organisms. Efficient cutaneous wound repair requires the **migration** of cells at the wound edge and farther back within the epidermal sheet, but the genes that control and coordinate these **migration**s remain obscure. This is in part because a systematic screening approach for in vivo identification and classification of postembryonic wound closure genes has yet to be developed. Here, we performed a proof-of-principle reporter-based in vivo RNAi screen in the Drosophila melanogaster larval epidermis to identify genes required for normal wound closure. Among the candidate genes tested were kinases and transcriptional mediators of the Jun N-terminal kinase (JNK) signaling pathway shown to be required for epithelial sheet **migration** during development. Also targeted were genes involved in actin cytoskeletal remodeling. Importantly, RNAi knockdown of both canonical and noncanonical members of the JNK pathway caused open wounds, as did several genes involved in actin cytoskeletal remodeling. Our analysis of JNK pathway components reveals redundancy among the upstream activating kinases and distinct roles for the downstream transcription factors DJun and DFos. Quantitative and qualitative morphological classification of the open wound phenotypes and evaluation of JNK activation suggest that multiple cellular processes are required in the migrating epidermal cells, including functions specific to cells at the wound edge and others specific to cells farther back within the epidermal sheet. Together, our results identify a new set of conserved wound closure genes, determine putative functional roles for these genes within the migrating epidermal sheet, and provide a template for a broader in vivo RNAi screen to discover the full complement of genes required for wound closure during larval epidermal wound healing.
14. The Drosophila female sterile mutation twin peaks is a novel allele of tramtrack and reveals a requirement for Ttk69 in epithelial morphogenesis. The Drosophila gene tramtrack (ttk) encodes two transcriptional repressors, Ttk69 and Ttk88, which are required for normal embryogenesis and imaginal disc development. Here, we characterize a novel female sterile allele of tramtrack called twin peaks (ttk(twk)) that, unlike othertramtrack alleles, has no effect on viability and produces no obvious morphological defects, except during oogenesis. Females homozygous for twin peaks produce small eggs with thin eggshells and short dorsal respiratory appendages. Complementation analyses, immunolocalization, and rescue data demonstrate that these defects are due to loss of Ttk69, which is expressed in the follicle cells and is required for normal chorion production and dorsal follicle-cell **migration**. Analyses of phenotypes produced by mutations in other loci that regulate eggshell synthesis suggest that the chorion production and follicle-cell **migration** defects are independent. We present evidence that twin peaks disrupts a promoter or promoters required for late-stage follicle-cell expression of Ttk69. We hypothesize that loss of Ttk69 in all follicle cells disrupts chorion gene expression and lack of function in dorsal anterior follicle cells inhibits morphogenetic changes required for elongating the dorsal appendages.
15. The Ste20 kinase misshapen is essential for the invasive behaviour of ovarian epithelial cells in Drosophila. Stationary-to-migratory transitions of epithelial cells have a key role in development and tumour progression. Border cell **migration** is a powerful system in which to investigate this transition in living organisms. Here, we identify the Ste20-like kinase misshapen (msn) as a novel regulator of border-cell **migration** in Drosophila. Expression of msn in border cells is independent of the transcription factor slow border cells and of inputs from all pathways that are known to control border-cell **migration**. The msn gene functions to modulate the levels and/or distribution of Drosophila E-cadherin to promote the invasive migratory behaviour of border cells.
16. Embryonic morphogenesis signaling pathway mediated by JNK targets the transcription factor JUN and the TGF-beta homologue decapentaplegic. The dorsal surface of the Drosophila embryo is formed by the **migration** of the lateral epithelial cells to cover the amnioserosa. The Drosophila cJun-N-terminal kinase (DJNK) is essential for this process. Mutations in DJNK or the DJNK activator hemipterous (HEP) lead to incomplete dorsal closure, resulting in a hole in the dorsal cuticle. The molecules downstream of DJNK in this signaling pathway have not been established. Here we demonstrate that the basket1 (bsk1) mutation of DJNK causes decreased interaction with DJUN. Expression of decapentaplegic (DPP), a TGF-beta homologue, in the leading edge of the dorsal epithelium, is identified as a genetic target of the JNK pathway. A constitutive allele of JUN is able to rescue the dorsal closure defect of bsk1 and restores DPP expression. Furthermore, ectopic DPP rescues the defects in dorsal closure caused by bsk1. These data indicate that the interaction of DJNK with DJUN contributes to the dorsal closure signaling pathway and targets DPP expression.
17. The Dpp/TGFβ-dependent corepressor Schnurri protects epithelial cells from JNK-induced apoptosis in drosophila embryos. Jun N-terminal kinase (JNK) often mediates apoptosis in response to cellular stress. However, during normal development, JNK signaling controls a variety of live cell behaviors, such as during dorsal closure in Drosophila embryos. During this process, the latent proapoptotic activity of JNK becomes apparent following Dpp signaling suppression, which leads to JNK-dependent transcriptional activation of the proapoptotic gene reaper. Dpp signaling also protects cells from JNK-dependent apoptosis caused by epithelial disruption. We find that repression of reaper transcription by Dpp is mediated by Schnurri. Moreover, reporter gene analysis shows that a transcriptional regulatory module comprising AP-1 and Schnurri binding sites located upstream of reaper integrate the activities of JNK and Dpp. This arrangement allows JNK to control a migratory behavior without triggering apoptosis. Dpp plays a dual role during dorsal closure. It cooperates with JNK in stimulating cell **migratio**n and also prevents JNK from inducing apoptosis.
18. bullwinkle and shark regulate dorsal-appendage morphogenesis in Drosophila oogenesis. bullwinkle (bwk) regulates embryonic anteroposterior patterning and, through a novel germline-to-soma signal, morphogenesis of the eggshell dorsal appendages. We screened for dominant modifiers of the bullwinkle mooseantler eggshell phenotype and identified shark, which encodes an SH2-domain, ankyrin-repeat tyrosine kinase. At the onset of dorsal-appendage formation, shark is expressed in a punctate pattern in the squamous stretch cells overlying the nurse cells. Confocal microscopy with cell-type-specific markers demonstrates that the stretch cells act as a substrate for the migrating dorsal-appendage-forming cells and extend cellular projections towards them. Mosaic analyses reveal that shark is required in follicle cells for cell **migration** and chorion deposition. Proper shark RNA expression in the stretch cells requires bwk activity, while restoration of shark expression in the stretch cells suppresses the bwk dorsal-appendage phenotype. These results suggest that shark plays an important downstream role in the bwk-signaling pathway. Candidate testing implicates Src42A in a similar role, suggesting conservation with a vertebrate signaling pathway involving non-receptor tyrosine kinases.

### ECM

#### PubMed

1. Extracellular matrix and its receptors in Drosophila neural development. Extracellular matrix (**ECM**) and matrix receptors are intimately involved in most biological processes. The **ECM** plays fundamental developmental and physiological roles in health and disease, including processes underlying the development, maintenance, and regeneration of the nervous system. To understand the principles of **ECM**-mediated functions in the nervous system, genetic model organisms like Drosophila provide simple, malleable, and powerful experimental platforms. This article provides an overview of **ECM** proteins and receptors in Drosophila. It then focuses on their roles during three progressive phases of neural development: (1) neural progenitor proliferation, (2) axonal growth and pathfinding, and (3) synapse formation and function. Each section highlights known **ECM** and **ECM**-receptor components and recent studies done in mutant conditions to reveal their in vivo functions, all illustrating the enormous opportunities provided when merging work on the nervous system with systematic research into **ECM**-related gene functions.

### [Ee]xtracellular [Mm]atrix

#### PubMed

1. Dose-sensitive autosomal modifiers identify candidate genes for tissue autonomous and tissue nonautonomous regulation by the Drosophila nuclear zinc-finger protein, hindsight. The nuclear zinc-finger protein encoded by the hindsight (hnt) locus regulates several cellular processes in Drosophila epithelia, including the Jun N-terminal kinase (JNK) signaling pathway and actin polymerization. Defects in these molecular pathways may underlie the abnormal cellular interactions, loss of epithelial integrity, and apoptosis that occurs in hnt mutants, in turn causing failure of morphogenetic processes such as germ band retraction and dorsal closure in the embryo. To define the genetic pathways regulated by hnt, 124 deficiencies on the second and third chromosomes and 14 duplications on the second chromosome were assayed for dose-sensitive modification of a temperature-sensitive rough eye phenotype caused by the viable allele, hntpeb; 29 interacting regions were identified. Subsequently, 438 P-element-induced lethal mutations mapping to these regions and 12 candidate genes were tested for genetic interaction, leading to identification of 63 dominant modifier loci. A subset of the identified mutants also dominantly modify hnt308-induced embryonic lethality and thus represent general rather than tissue-specific interactors. General interactors include loci encoding transcription factors, actin-binding proteins, signal transduction proteins, and components of the **extracellular matrix**. Expression of several interactors was assessed in hnt mutant tissue. Five genes--apontic (apt), Delta (Dl), decapentaplegic (dpp), karst (kst), and puckered (puc)--are regulated tissue autonomously and, thus, may be direct transcriptional targets of HNT. Three of these genes--apt, Dl, and dpp--are also regulated nonautonomously in adjacent non-HNT-expressing tissues. The expression of several additional interactors--viking (vkg), Cg25, and laminin-alpha (LanA)-is affected only in a nonautonomous manner.
2. Immune response to bacteria induces dissemination of Ras-activated Drosophila hindgut cells. Although pathogenic bacteria are suspected contributors to colorectal cancer progression, cancer-promoting bacteria and their mode of action remain largely unknown. Here we report that sustained infection with the human intestinal colonizer Pseudomonas aeruginosa synergizes with the Ras1V12 oncogene to induce basal invasion and dissemination of hindgut cells to distant sites. Cross-talk between infection and dissemination requires sustained activation by the bacteria of the Imd-dTab2-dTak1 innate immune pathway, which converges with Ras1V12 signalling on JNK pathway activation, culminating in **extracellular matrix** degradation. Hindgut, but not midgut, cells are amenable to this cooperative dissemination, which is progressive and genetically and pharmacologically inhibitable. Thus, Drosophila hindgut provides a valuable system for the study of intestinal malignancies.
3. **Extracellular matrix** and its receptors in Drosophila neural development. **Extracellular matrix** (ECM) and matrix receptors are intimately involved in most biological processes. The ECM plays fundamental developmental and physiological roles in health and disease, including processes underlying the development, maintenance, and regeneration of the nervous system. To understand the principles of ECM-mediated functions in the nervous system, genetic model organisms like Drosophila provide simple, malleable, and powerful experimental platforms. This article provides an overview of ECM proteins and receptors in Drosophila. It then focuses on their roles during three progressive phases of neural development: (1) neural progenitor proliferation, (2) axonal growth and pathfinding, and (3) synapse formation and function. Each section highlights known ECM and ECM-receptor components and recent studies done in mutant conditions to reveal their in vivo functions, all illustrating the enormous opportunities provided when merging work on the nervous system with systematic research into ECM-related gene functions.

### [Ii]nvasive

#### PubMed

1. **Invasive** and indigenous microbiota impact intestinal stem cell activity through multiple pathways in Drosophila. Gut homeostasis is controlled by both immune and developmental mechanisms, and its disruption can lead to inflammatory disorders or cancerous lesions of the intestine. While the impact of bacteria on the mucosal immune system is beginning to be precisely understood, little is known about the effects of bacteria on gut epithelium renewal. Here, we addressed how both infectious and indigenous bacteria modulate stem cell activity in Drosophila. We show that the increased epithelium renewal observed upon some bacterial infections is a consequence of the oxidative burst, a major defense of the Drosophila gut. Additionally, we provide evidence that the JAK-STAT (Janus kinase-signal transducers and activators of transcription) and JNK (c-Jun NH(2) terminal kinase) pathways are both required for bacteria-induced stem cell proliferation. Similarly, we demonstrate that indigenous gut microbiota activate the same, albeit reduced, program at basal levels. Altered control of gut microbiota in immune-deficient or aged flies correlates with increased epithelium renewal. Finally, we show that epithelium renewal is an essential component of Drosophila defense against oral bacterial infection. Altogether, these results indicate that gut homeostasis is achieved by a complex interregulation of the immune response, gut microbiota, and stem cell activity.
2. Interplay among Drosophila transcription factors Ets21c, Fos and Ftz-F1 drives JNK-mediated tumor malignancy. Cancer initiation and maintenance of the transformed cell state depend on altered cellular signaling and aberrant activities of transcription factors (TFs) that drive pathological gene expression in response to cooperating genetic lesions. Deciphering the roles of interacting TFs is therefore central to understanding carcinogenesis and for designing cancer therapies. Here, we use an unbiased genomic approach to define a TF network that triggers an abnormal gene expression program promoting malignancy of clonal tumors, generated in Drosophila imaginal disc epithelium by gain of oncogenic Ras (Ras(V12)) and loss of the tumor suppressor Scribble (scrib(1)). We show that malignant transformation of the ras(V12)scrib(1) tumors requires TFs of distinct families, namely the bZIP protein Fos, the ETS-domain factor Ets21c and the nuclear receptor Ftz-F1, all acting downstream of Jun-N-terminal kinase (JNK). Depleting any of the three TFs improves viability of tumor-bearing larvae, and this positive effect can be enhanced further by their combined removal. Although both Fos and Ftz-F1 synergistically contribute to ras(V12)scrib(1) tumor **invasive**ness, only Fos is required for JNK-induced differentiation defects and Matrix metalloprotease (MMP1) upregulation. In contrast, the Fos-dimerizing partner Jun is dispensable for JNK to exert its effects in ras(V12)scrib(1) tumors. Interestingly, Ets21c and Ftz-F1 are transcriptionally induced in these tumors in a JNK- and Fos-dependent manner, thereby demonstrating a hierarchy within the tripartite TF network, with Fos acting as the most upstream JNK effector. Of the three TFs, only Ets21c can efficiently substitute for loss of polarity and cooperate with Ras(V12) in inducing malignant clones that, like ras(V12)scrib(1) tumors, invade other tissues and overexpress MMP1 and the Drosophila insulin-like peptide 8 (Dilp8). While ras(V12)ets21c tumors require JNK for **invasive**ness, the JNK activity is dispensable for their growth. In conclusion, our study delineates both unique and overlapping functions of distinct TFs that cooperatively promote aberrant expression of target genes, leading to malignant tumor phenotypes.
3. Sin3a acts through a multi-gene module to regulate invasion in Drosophila and human tumors. Chromatin remodeling proteins regulate multiple aspects of cell homeostasis, making them ideal candidates for misregulation in transformed cells. Here, we explore Sin3A, a member of the Sin3 family of proteins linked to tumorigenesis that are thought to regulate gene expression through their role as histone deacetylases (HDACs). We identified Drosophila Sin3a as an important mediator of oncogenic Ret receptor in a fly model of Multiple Endocrine Neoplasia Type 2. Reducing Drosophila Sin3a activity led to metastasis-like behavior and, in the presence of Diap1, secondary tumors distant from the site of origin. Genetic and Chip-Seq analyses identified previously undescribed Sin3a targets including genes involved in cell motility and actin dynamics, as well as signaling pathways including Src, Jnk and Rho. A key Sin3a oncogenic target, PP1B, regulates stability of β-Catenin/Armadillo: the outcome is to oppose T-cell factor (TCF) function and Wg/Wnt pathway signaling in both fly and mammalian cancer cells. Reducing Sin3A strongly increased the **invasiv**e behavior of A549 human lung adenocarcinoma cells. We show that Sin3A is downregulated in a variety of human tumors and that Src, JNK, RhoA and PP1B/β-Catenin are regulated in a manner analogous to our Drosophila models. Our data suggest that Sin3A influences a specific step of tumorigenesis by regulating a module of genes involved in cell invasion. Tumor progression may commonly rely on such 'modules of invasion' under the control of broad transcriptional regulators.
4. The Ste20 kinase misshapen is essential for the **invasive** behaviour of ovarian epithelial cells in Drosophila. Stationary-to-migratory transitions of epithelial cells have a key role in development and tumour progression. Border cell migration is a powerful system in which to investigate this transition in living organisms. Here, we identify the Ste20-like kinase misshapen (msn) as a novel regulator of border-cell migration in Drosophila. Expression of msn in border cells is independent of the transcription factor slow border cells and of inputs from all pathways that are known to control border-cell migration. The msn gene functions to modulate the levels and/or distribution of Drosophila E-cadherin to promote the **invasive** migratory behaviour of border cells.

### [Mm]etastasis

#### PubMed

1. Sin3a acts through a multi-gene module to regulate invasion in Drosophila and human tumors. Chromatin remodeling proteins regulate multiple aspects of cell homeostasis, making them ideal candidates for misregulation in transformed cells. Here, we explore Sin3A, a member of the Sin3 family of proteins linked to tumorigenesis that are thought to regulate gene expression through their role as histone deacetylases (HDACs). We identified Drosophila Sin3a as an important mediator of oncogenic Ret receptor in a fly model of Multiple Endocrine Neoplasia Type 2. Reducing Drosophila Sin3a activity led to **metastasis**-like behavior and, in the presence of Diap1, secondary tumors distant from the site of origin. Genetic and Chip-Seq analyses identified previously undescribed Sin3a targets including genes involved in cell motility and actin dynamics, as well as signaling pathways including Src, Jnk and Rho. A key Sin3a oncogenic target, PP1B, regulates stability of β-Catenin/Armadillo: the outcome is to oppose T-cell factor (TCF) function and Wg/Wnt pathway signaling in both fly and mammalian cancer cells. Reducing Sin3A strongly increased the invasive behavior of A549 human lung adenocarcinoma cells. We show that Sin3A is downregulated in a variety of human tumors and that Src, JNK, RhoA and PP1B/β-Catenin are regulated in a manner analogous to our Drosophila models. Our data suggest that Sin3A influences a specific step of tumorigenesis by regulating a module of genes involved in cell invasion. Tumor progression may commonly rely on such 'modules of invasion' under the control of broad transcriptional regulators.
2. JNK signaling controls border cell cluster integrity and collective cell migration. Collective cell movement is a mechanism for invasion identified in many developmental events. Examples include the movement of lateral-line neurons in Zebrafish, cells in the inner blastocyst, and **metastasis** of epithelial tumors [1]. One key model to study collective migration is the movement of border cell clusters in Drosophila. Drosophila egg chambers contain 15 nurse cells and a single oocyte surrounded by somatic follicle cells. At their anterior end, polar cells recruit several neighboring follicle cells to form the border cell cluster [2]. By stage 9, and over 6 hr, border cells migrate as a cohort between nurse cells toward the oocyte. The specification and directionality of border cell movement are regulated by hormonal and signaling mechanisms [3]. However, how border cells are held together while they migrate is not known. Here, we show that a negative-feedback loop controlling JNK activity regulates border cell cluster integrity. JNK signaling modulates contacts between border cells and between border cells and substratum to sustain collective migration by regulating several effectors including the polarity factor Bazooka and the cytoskeletal adaptor D-Paxillin. We anticipate a role for the JNK pathway in controlling collective cell movements in other morphogenetic and clinical models.
3. Regulation of cell adhesion and collective cell migration by hindsight and its human homolog RREB1. Cell movements represent a major driving force in embryonic development, tissue repair, and tumor **metastasis** [1]. The migration of single cells has been well studied, predominantly in cell culture [2, 3]; however, in vivo, a greater variety of modes of cell movement occur, including the movements of cells in clusters, strands, sheets, and tubes, also known as collective cell migrations [4, 5]. In spite of the relevance of these types of movements in both normal and pathological conditions, the molecular mechanisms that control them remain predominantly unknown. Epithelial follicle cells of the Drosophila ovary undergo several dynamic morphological changes, providing a genetically tractable model [6]. We found that anterior follicle cells, including border cells, mutant for the gene hindsight (hnt) accumulated excess cell-cell adhesion molecules and failed to undergo their normal collective movements. In addition, HNT affected border cell cluster cohesion and motility via effects on the JNK and STAT pathways, respectively. Interestingly, reduction of expression of the mammalian homolog of HNT, RREB1, by siRNA inhibited collective cell migration in a scratch-wound healing assay of MCF10A mammary epithelial cells, suppressed surface activity, retarded cell spreading after plating, and led to the formation of immobile, tightly adherent cell colonies. We propose that HNT and RREB1 are essential to reduce cell-cell adhesion when epithelial cells within an interconnected group undergo dynamic changes in cell shape.

### [Cc]ell [Ii]nvasion

#### PubMed

1. Sin3a acts through a multi-gene module to regulate invasion in Drosophila and human tumors. Chromatin remodeling proteins regulate multiple aspects of cell homeostasis, making them ideal candidates for misregulation in transformed cells. Here, we explore Sin3A, a member of the Sin3 family of proteins linked to tumorigenesis that are thought to regulate gene expression through their role as histone deacetylases (HDACs). We identified Drosophila Sin3a as an important mediator of oncogenic Ret receptor in a fly model of Multiple Endocrine Neoplasia Type 2. Reducing Drosophila Sin3a activity led to metastasis-like behavior and, in the presence of Diap1, secondary tumors distant from the site of origin. Genetic and Chip-Seq analyses identified previously undescribed Sin3a targets including genes involved in cell motility and actin dynamics, as well as signaling pathways including Src, Jnk and Rho. A key Sin3a oncogenic target, PP1B, regulates stability of β-Catenin/Armadillo: the outcome is to oppose T-cell factor (TCF) function and Wg/Wnt pathway signaling in both fly and mammalian cancer cells. Reducing Sin3A strongly increased the invasive behavior of A549 human lung adenocarcinoma cells. We show that Sin3A is downregulated in a variety of human tumors and that Src, JNK, RhoA and PP1B/β-Catenin are regulated in a manner analogous to our Drosophila models. Our data suggest that Sin3A influences a specific step of tumorigenesis by regulating a module of genes involved i**n cell invasi**on. Tumor progression may commonly rely on such 'modules of invasion' under the control of broad transcriptional regulators.

---

## kay

- NCBI Gene
- GeneRIF
- Pubmed

| Search Term | Rank | Fields |
| --- | --- | --- |
| [Mm]igration | 6 | PubMed(1) |

  
hide/show details for kay

### [Mm]igration

#### PubMed

1. Top-DER- and Dpp-dependent requirements for the Drosophila fos/kayak gene in follicular epithelium morphogenesis. The Drosophila fos (Dfos)/kayak gene has been previously identified as a key regulator of epithelial cell morphogenesis during dorsal closure of the embryo and fusion of the adult thorax. We show here that it is also required for two morphogenetic movements of the follicular epithelium during oogenesis. Firstly, it is necessary for the proper posteriorward **migration** of main body follicle cells during stage 9. Secondly, it controls, from stage 11 onwards, the morphogenetic reorganization of the follicle cells that are committed to secrete the respiratory appendages. We demonstrate that DER pathway activation and a critical level of Dpp/TGFbeta signalling are required to pattern a high level of transcription of Dfos at the anterior and dorsal edges of the two groups of cells that will give rise to the respiratory appendages. In addition, we provide evidence that, within the dorsal-anterior territory, the level of paracrine Dpp/TGFbeta signalling controls the commitment of follicle cells towards either an operculum or an appendage secretion fate. Finally, we show that Dfos is required in follicle cells for the dumping of the nurse cell cytoplasm into the oocyte and the subsequent apoptosis of nurse cells. This suggests that in somatic follicle cells, Dfos controls the expression of one or several factors that are necessary for these processes in underlying germinal nurse cells.

---

## kay

- NCBI Gene
- GeneRIF
- Pubmed

| Search Term | Rank | Fields |
| --- | --- | --- |
| [Ff]ilopodia | 1 | PubMed(2) |
| [Mm]igration | 6 | Process(2); PubMed(19); GeneRIFs(1) |
| ECM | 7 | PubMed(1) |
| [Ee]xtracellular [Mm]atrix | 8 | PubMed(2) |
| [Ii]nvasive | 10 | PubMed(4) |
| [Mm]etastasis | 12 | PubMed(1) |

  
hide/show details for kay

### [Ff]ilopodia

#### PubMed

1. Transcriptional regulation of Profilin during wound closure in Drosophila larvae. Injury is an inevitable part of life, making wound healing essential for survival. In postembryonic skin, wound closure requires that epidermal cells recognize the presence of a gap and change their behavior to migrate across it. In Drosophila larvae, wound closure requires two signaling pathways [the Jun N-terminal kinase (JNK) pathway and the Pvr receptor tyrosine kinase signaling pathway] and regulation of the actin cytoskeleton. In this and other systems, it remains unclear how the signaling pathways that initiate wound closure connect to the actin regulators that help execute wound-induced cell migrations. Here, we show that chickadee, which encodes the Drosophila Profilin, a protein important for actin filament recycling and cell migration during development, is required for the physiological process of larval epidermal wound closure. After injury, chickadee is transcriptionally upregulated in cells proximal to the wound. We found that JNK, but not Pvr, mediates the increase in chic transcription through the Jun and Fos transcription factors. Finally, we show that chic-deficient larvae fail to form a robust actin cable along the wound edge and also fail to form normal **filopodia**l and lamellipodial extensions into the wound gap. Our results thus connect a factor that regulates actin monomer recycling to the JNK signaling pathway during wound closure. They also reveal a physiological function for an important developmental regulator of actin and begin to tease out the logic of how the wound repair response is organized.
2. JNK signaling pathway required for wound healing in regenerating Drosophila wing imaginal discs. We have examined wound healing during regeneration of Drosophila wing imaginal discs fragments by confocal microscopy and assessed the role of components of the JNK pathway in this process. After cutting, columnar and peripodial epithelia cells at the wound edge start to close the wound through formation and contraction of an actin cable. This is followed by a zipping process through **filopodia**l protrusions from both epithelia knitting the wound edges from proximal to distal areas of the disc. Activation of the JNK pathway is involved in such process. puckered (puc) expression is induced in several rows of cells at the edge of the wound, whereas absence of JNK pathway activity brought about by hemipterous, basket, and Dfos mutants impair wound healing. These defects are accompanied by lowered or loss of expression of puc. In support of a role of puc in wound healing, hep mutant phenotypes are rescued by reducing puc function, whereas overexpression of puc inhibits wound healing. Altogether, these results demonstrate a role for the JNK pathway in imaginal disc wound healing, similar to that reported for other healing processes such as embryonic dorsal closure, thoracic closure, and adult epithelial wound healing in Drosophila. Differences with such processes are also highlighted.

### [Mm]igration

#### Process

1. border follicle cell **migration**
2. ovarian follicle cell **migration**

#### PubMed

1. A sensitized PiggyBac-based screen for regulators of border cell **migration** in Drosophila. **Migration** of border cells during Drosophila melanogaster oogenesis is a good model system for investigating the genetic requirements for cell **migration** in vivo. We present a sensitized loss-of-function screen used to identify new genes required in border cells for their **migration**. Chromosomes bearing FRTs on all four major autosomal arms were mutagenized by insertions of the transposable element PiggyBac, allowing multiple parallel clonal screens and easy identification of the mutated gene. For border cells, we analyzed homozygous mutant clones positively marked with lacZ and sensitized by expression of dominant-negative PVR, the guidance receptor. We identified new alleles of genes already known to be required for border cell **migration**, including aop/yan, DIAP1, and taiman as well as a conserved Slbo-regulated enhancer downstream of shg/DE-cadherin. Mutations in genes not previously described to be required in border cells were also uncovered: hrp48, vir, rme-8, kismet, and puckered. puckered was unique in that the **migration** defects were observed only when PVR signaling was reduced. We present evidence that an excess of JNK signaling is deleterious for **migration** in the absence of PVR activity at least in part through Fos transcriptional activity and possibly through antagonistic effects on DIAP1.
2. [Dorsal closure in Drosophila. A genetic model for wound healing?]. Dorsal closure (DC) is a morphogenetic movement that establishes the dorsal ectoderm of the drosophila embryo. During this process, the two lateral epithelia stretch toward the dorsal midline, the suture line of the two leading edges. Cell **migration** during DC relies both on cell shape change controlled by the activity of the JNK pathway in the leading edge cells and modification of cell adhesiveness, probably dependent upon activation of the Dpp (TGF-beta) pathway. Coupling of the JNK and TGF-beta pathways is essential. The sequence of the cellular and molecular events of DC highlights interesting common features with wound healing in vertebrates. Like DC, wound healing relies on the **migration** of epithelia bordered by leading edges controlling the direction and speed of the movement. This review summarizes recent data concerning the control of epithelial morphogenesis during DC and the bases of wound healing. The molecular and cellular events that underlie these two analogous migratory processes are detailed, discussed and compared. We suggest that DC is a good genetic model for wound healing studying.
3. Dcdc42 acts in TGF-beta signaling during Drosophila morphogenesis: distinct roles for the Drac1/JNK and Dcdc42/TGF-beta cascades in cytoskeletal regulation. During Drosophila embryogenesis the two halves of the lateral epidermis migrate dorsally over a surface of flattened cells, the amnioserosa, and meet at the dorsal midline in order to form the continuous sheet of the larval epidermis. During this process of epithelial **migration**, known as dorsal closure, signaling from a Jun-amino-terminal-kinase cascade causes the production of the secreted transforming-growth-factor-beta-like ligand, Decapentaplegic. Binding of Decapentaplegic to the putative transforming-growth-factor-beta-like receptors Thickveins and Punt activates a transforming-growth-factor-beta-like pathway that is also required for dorsal closure. Mutations in genes involved in either the Jun-amino-terminal-kinase cascade or the transforming-growth-factor-beta-like signaling pathway can disrupt dorsal closure. Our findings show that although these pathways are linked they are not equivalent in function. Signaling by the Jun-amino-terminal-kinase cascade may be initiated by the small Ras-like GTPase Drac1 and acts to assemble the cytoskeleton and specify the identity of the first row of cells of the epidermis prior to the onset of dorsal closure. Signaling in the transforming-growth-factor-beta-like pathway is mediated by Dcdc42, and acts during the closure process to control the mechanics of the **migration** process, most likely via its putative effector kinase DPAK.
4. Top-DER- and Dpp-dependent requirements for the Drosophila fos/kayak gene in follicular epithelium morphogenesis. The Drosophila fos (Dfos)/kayak gene has been previously identified as a key regulator of epithelial cell morphogenesis during dorsal closure of the embryo and fusion of the adult thorax. We show here that it is also required for two morphogenetic movements of the follicular epithelium during oogenesis. Firstly, it is necessary for the proper posteriorward **migration** of main body follicle cells during stage 9. Secondly, it controls, from stage 11 onwards, the morphogenetic reorganization of the follicle cells that are committed to secrete the respiratory appendages. We demonstrate that DER pathway activation and a critical level of Dpp/TGFbeta signalling are required to pattern a high level of transcription of Dfos at the anterior and dorsal edges of the two groups of cells that will give rise to the respiratory appendages. In addition, we provide evidence that, within the dorsal-anterior territory, the level of paracrine Dpp/TGFbeta signalling controls the commitment of follicle cells towards either an operculum or an appendage secretion fate. Finally, we show that Dfos is required in follicle cells for the dumping of the nurse cell cytoplasm into the oocyte and the subsequent apoptosis of nurse cells. This suggests that in somatic follicle cells, Dfos controls the expression of one or several factors that are necessary for these processes in underlying germinal nurse cells.
5. Transcriptional regulation of Profilin during wound closure in Drosophila larvae. Injury is an inevitable part of life, making wound healing essential for survival. In postembryonic skin, wound closure requires that epidermal cells recognize the presence of a gap and change their behavior to migrate across it. In Drosophila larvae, wound closure requires two signaling pathways [the Jun N-terminal kinase (JNK) pathway and the Pvr receptor tyrosine kinase signaling pathway] and regulation of the actin cytoskeleton. In this and other systems, it remains unclear how the signaling pathways that initiate wound closure connect to the actin regulators that help execute wound-induced cell **migration**s. Here, we show that chickadee, which encodes the Drosophila Profilin, a protein important for actin filament recycling and cell **migration** during development, is required for the physiological process of larval epidermal wound closure. After injury, chickadee is transcriptionally upregulated in cells proximal to the wound. We found that JNK, but not Pvr, mediates the increase in chic transcription through the Jun and Fos transcription factors. Finally, we show that chic-deficient larvae fail to form a robust actin cable along the wound edge and also fail to form normal filopodial and lamellipodial extensions into the wound gap. Our results thus connect a factor that regulates actin monomer recycling to the JNK signaling pathway during wound closure. They also reveal a physiological function for an important developmental regulator of actin and begin to tease out the logic of how the wound repair response is organized.
6. A misexpression screen to identify regulators of Drosophila larval hemocyte development. In Drosophila, defense against foreign pathogens is mediated by an effective innate immune system, the cellular arm of which is composed of circulating hemocytes that engulf bacteria and encapsulate larger foreign particles. Three hemocyte types occur: plasmatocytes, crystal cells, and lamellocytes. The most abundant larval hemocyte type is the plasmatocyte, which is responsible for phagocytosis and is present either in circulation or in adherent sessile domains under the larval cuticle. The mechanisms controlling differentiation of plasmatocytes and their **migration** toward these sessile compartments are unclear. To address these questions we have conducted a misexpression screen using the plasmatocyte-expressed GAL4 driver Peroxidasin-GAL4 (Pxn-GAL4) and existing enhancer-promoter (EP) and EP yellow (EY) transposon libraries to systematically misexpress approximately 20% of Drosophila genes in larval hemocytes. The Pxn-GAL4 strain also contains a UAS-GFP reporter enabling hemocyte phenotypes to be visualized in the semitransparent larvae. Among 3412 insertions screened we uncovered 101 candidate hemocyte regulators. Some of these are known to control hemocyte development, but the majority either have no characterized function or are proteins of known function not previously implicated in hemocyte development. We have further analyzed three candidate genes for changes in hemocyte morphology, cell-cell adhesion properties, phagocytosis activity, and melanotic tumor formation.
7. Genes conserved in bilaterians but jointly lost with Myc during nematode evolution are enriched in cell proliferation and cell **migration** functions. Animals use a stereotypical set of developmental genes to build body architectures of varying sizes and organizational complexity. Some genes are critical to developmental patterning, while other genes are important to physiological control of growth. However, growth regulator genes may not be as important in small-bodied "micro-metazoans" such as nematodes. Nematodes use a simplified developmental strategy of lineage-based cell fate specifications to produce an adult bilaterian body composed of a few hundreds of cells. Nematodes also lost the MYC proto-oncogenic regulator of cell proliferation. To identify additional regulators of cell proliferation that were lost with MYC, we computationally screened and determined 839 high-confidence genes that are conserved in bilaterians/lost in nematodes (CIBLIN genes). We find that 30 % of all CIBLIN genes encode transcriptional regulators of cell proliferation, epithelial-to-mesenchyme transitions, and other processes. Over 50 % of CIBLIN genes are unnamed genes in Drosophila, suggesting that there are many understudied genes. Interestingly, CIBLIN genes include many Myc synthetic lethal (MycSL) hits from recent screens. CIBLIN genes include key regulators of heparan sulfate proteoglycan (HSPG) sulfation patterns, and lysyl oxidases involved in cross-linking and modification of the extracellular matrix (ECM). These genes and others suggest the CIBLIN repertoire services critical functions in ECM remodeling and cell **migration** in large-bodied bilaterians. Correspondingly, CIBLIN genes are co-expressed with Myc in cancer transcriptomes, and include a preponderance of known determinants of cancer progression and tumor aggression. We propose that CIBLIN gene research can improve our understanding of regulatory control of cellular growth in metazoans.
8. Downregulation of Jun kinase signaling in the amnioserosa is essential for dorsal closure of the Drosophila embryo. During Drosophila embryogenesis, Jun kinase (JNK) signaling has been shown to play a key role in regulating the morphogenetic process of dorsal closure, which also serves as a model for epithelial sheet fusion during wound repair. During dorsal closure the JNK signaling cascade in the dorsal-most (leading edge) cells of the epidermis activates the AP-1 transcription factor comprised of DJUN and DFOS that, in turn, upregulates the expression of the dpp gene. DPP is a secreted morphogen that signals lateral epidermal cells to elongate along the dorsoventral axis. The leading edge cells contact the peripheral cells of a monolayer extraembryonic epithelium, the amnioserosa, which lies on the dorsal side of the embryo. Focal complexes are present at the dorsal-most membrane of the leading edge cells, where they contact the amnioserosa.We show that the JNK signaling cascade is initially active in both the amnioserosa and the leading edge of the epidermis. JNK signaling is downregulated in the amnioserosa, but not in the leading edge, prior to dorsal closure. The subcellular localization of DFOS and DJUN is responsive to JNK signaling in the amnioserosa: JNK activation results in nuclear localization of DFOS and DJUN; the downregulation of JNK signaling results in the relocalization of DFOS and DJUN to the cytoplasm. The HINDSIGHT (HNT) Zn-finger protein and the PUCKERED (PUC) JNK phosphatase are essential for downregulation of the JNK cascade in the amnioserosa. Persistent JNK activity in the amnioserosa leads to defective focal complexes in the adjacent leading edge cells and to the failure of dorsal closure.Focal complexes are assembled at the boundary between high and low JNK activity. In the absence of focal complexes, miscommunication between the amnioserosa and the leading edge may lead to a premature "stop" signal that halts dorsalward **migration** of the leading edge. Spatial and temporal regulation of the JNK signaling cascade may be a general mechanism that controls tissue remodeling during morphogenesis and wound healing.
9. Primordial germ cell **migration** and the assembly of the Drosophila embryonic gonad. During embryogenesis Drosophila pole cells, like germ cells in many other vertebrates and invertebrates, actively migrate before assembling into the gonad. Five separate steps can be distinguished: an initial passive displacement of the germ cells by gastrulation movements, an amoeboid migratory phase during which the pole cells pass through the endoderm, **migration** on endoderm toward mesoderm, separation into two bilateral groups associated with the gonadal mesoderm precursors, followed by condensation into the gonad itself. We have analyzed gonad assembly in embryonic pattern mutants to determine whether distinct cues are required in this process. We show that the initiation of **migration** does not require the presence of the mesoderm, the eventual target of the germ cells. Rather, **migration** is triggered as a consequence of the differentiation of the endoderm. Examination of embryos mutant for maternal genes of the terminal group suggests that a primary role of the endoderm in this process is to allow the germ cell access to the interior of the embryo. In addition, we show that normal gonad assembly requires a region of the embryo that includes the posterior compartment of the fifth and the sixth abdominal segments.
10. A targeted UAS-RNAi screen in Drosophila larvae identifies wound closure genes regulating distinct cellular processes. Robust mechanisms for tissue repair are critical for survival of multicellular organisms. Efficient cutaneous wound repair requires the **migration** of cells at the wound edge and farther back within the epidermal sheet, but the genes that control and coordinate these **migration**s remain obscure. This is in part because a systematic screening approach for in vivo identification and classification of postembryonic wound closure genes has yet to be developed. Here, we performed a proof-of-principle reporter-based in vivo RNAi screen in the Drosophila melanogaster larval epidermis to identify genes required for normal wound closure. Among the candidate genes tested were kinases and transcriptional mediators of the Jun N-terminal kinase (JNK) signaling pathway shown to be required for epithelial sheet **migration** during development. Also targeted were genes involved in actin cytoskeletal remodeling. Importantly, RNAi knockdown of both canonical and noncanonical members of the JNK pathway caused open wounds, as did several genes involved in actin cytoskeletal remodeling. Our analysis of JNK pathway components reveals redundancy among the upstream activating kinases and distinct roles for the downstream transcription factors DJun and DFos. Quantitative and qualitative morphological classification of the open wound phenotypes and evaluation of JNK activation suggest that multiple cellular processes are required in the migrating epidermal cells, including functions specific to cells at the wound edge and others specific to cells farther back within the epidermal sheet. Together, our results identify a new set of conserved wound closure genes, determine putative functional roles for these genes within the migrating epidermal sheet, and provide a template for a broader in vivo RNAi screen to discover the full complement of genes required for wound closure during larval epidermal wound healing.
11. Signaling pathways directing the movement and fusion of epithelial sheets: lessons from dorsal closure in Drosophila. Wound healing in embryos and various developmental events in metazoans require the spreading and fusion of epithelial sheets. The complex signaling pathways regulating these processes are being pieced together through genetic, cell biological, and biochemical approaches. At present, dorsal closure of the Drosophila embryo is the best-characterized example of epithelial sheet movement. Dorsal closure involves **migration** of the lateral epidermal flanks to close a hole in the dorsal epidermis occupied by an epithelium called the amnioserosa. Detailed genetic studies have revealed a network of interacting signaling molecules regulating this process. At the center of this network is a Jun N-terminal kinase cascade acting at the leading edge of the migrating epidermis that triggers signaling by the TGF-beta superfamily member Decapentaplegic and which interacts with the Wingless pathway. These signaling modules regulate the cytoskeletal reorganization and cell shape change necessary to drive dorsal closure. Activation of this network requires signals from the amnioserosa and input from a variety of proteins at cell-cell junctions. The Rho family of small GTPases is also instrumental, both in activation of signaling and regulation of the cytoskeleton. Many of the proteins regulating dorsal closure have been implicated in epithelial movement in other organisms, and dorsal closure has emerged as an ideal model system for the study of the **migration** and fusion of epithelial sheets.
12. The control of cell motility and epithelial morphogenesis by Jun kinases. Originally identified as stress-activated protein kinases that control cell survival and proliferation through transcription factor c-Jun, the Jun N-terminal kinase (JNK) subgroup of MAP kinases (MAPKs) have recently emerged as crucial regulators of cell **migration** and the morphogenetic movement of epithelial sheets. In Drosophila, a well-orchestrated JNK signaling pathway controls formation of actin stress fibers and cell shape changes, which are required for the sealing of embryonic epidermis in a process known as dorsal closure. The JNK pathway is also involved in morphogenetic processes in mice including closure of the eyelid, neural tube and optic fissure. This article focuses on recent advances in understanding the role of JNK pathway in the regulation of cell **migration**, cytoskeleton rearrangement and the morphogenesis of epithelial sheets.
13. The small GTPase Rac plays multiple roles in epithelial sheet fusion--dynamic studies of Drosophila dorsal closure. The coordinated **migration** and fusion of epithelial sheets is a crucial morphogenetic tool used on numerous occasions during the normal development of an embryo and re-activated as part of the wound healing response. Drosophila dorsal closure, whereby a hole in the embryonic epithelium is zipped closed late in embryogenesis, serves as an excellent, genetically tractable model for epithelial **migration**. Using live confocal imaging, we have dissected multiple roles for the small GTPase Rac in this process. We show that constitutive activation of Rac1 leads to excessive assembly of lamellipodia and precocious halting of epithelial sweeping, possibly through premature activation of contact-inhibition machinery. Conversely, blocking Rac activity, either by loss-of-function mutations or expression of dominant negative Rac1, disables the assembly of both actin cable and protrusions by epithelial cells. Movies of mutant embryos show that continued contraction of the amnioserosa is sufficient to draw the epithelial edges towards one another, allowing the zipper machinery to bypass non-functioning regions of leading edge. In addition to illustrating the key role of Rac in organization of leading edge actin, loss-of-function mutants also provide substantive proof that Rac acts upstream in the Jun N-terminal kinase (JNK) cascade to direct epithelial cell shape changes during dorsal closure.
14. Drosophila female sterile mutation spoonbill interferes with multiple pathways in oogenesis. spoonbill is a Drosophila female-sterile mutation, which displays a range of eggshell and egg chamber patterning defects. Previous analysis has shown that the mutation interfered with the function of two major signaling pathways, GRK/EGFR and DPP. In this report, the nature of spoonbill was further investigated to examine whether it was associated with additional pathways in oogenesis. Clonal analysis, presented here, demonstrated that most of the aberrant phenotypes associated with spoonbill were dependent on a mutant germline. Nevertheless, SPOONBILL may function also in the soma to ensure proper polarization and **migration** of the border-cell-cluster. Further, genetic interaction studies implicated spoonbill in additional unrelated pathways such as the one(s) involved in actin polymerization/depolymerization. Based on the previous data and the results presented here, it is anticipated that spoonbill may encode a multifunctional protein that perhaps coordinately regulated the activity of multiple signaling pathways during oogenesis.
15. A functional RNAi screen for regulators of receptor tyrosine kinase and ERK signalling. Receptor tyrosine kinase (RTK) signalling through extracellular-signal-regulated kinases (ERKs) has pivotal roles during metazoan development, underlying processes as diverse as fate determination, differentiation, proliferation, survival, **migration** and growth. Abnormal RTK/ERK signalling has been extensively documented to contribute to developmental disorders and disease, most notably in oncogenic transformation by mutant RTKs or downstream pathway components such as Ras and Raf. Although the core RTK/ERK signalling cassette has been characterized by decades of research using mammalian cell culture and forward genetic screens in model organisms, signal propagation through this pathway is probably regulated by a larger network of moderate, context-specific proteins. The genes encoding these proteins may not have been discovered through traditional screens owing, in particular, to the requirement for visible phenotypes. To obtain a global view of RTK/ERK signalling, we performed an unbiased, RNA interference (RNAi), genome-wide, high-throughput screen in Drosophila cells using a novel, quantitative, cellular assay monitoring ERK activation. Here we show that ERK pathway output integrates a wide array of conserved cellular processes. Further analysis of selected components-in multiple cell types with different RTK ligands and oncogenic stimuli-validates and classifies 331 pathway regulators. The relevance of these genes is highlighted by our isolation of a Ste20-like kinase and a PPM-family phosphatase that seem to regulate RTK/ERK signalling in vivo and in mammalian cells. Novel regulators that modulate specific pathway outputs may be selective targets for drug discovery.
16. The Dpp/TGFβ-dependent corepressor Schnurri protects epithelial cells from JNK-induced apoptosis in drosophila embryos. Jun N-terminal kinase (JNK) often mediates apoptosis in response to cellular stress. However, during normal development, JNK signaling controls a variety of live cell behaviors, such as during dorsal closure in Drosophila embryos. During this process, the latent proapoptotic activity of JNK becomes apparent following Dpp signaling suppression, which leads to JNK-dependent transcriptional activation of the proapoptotic gene reaper. Dpp signaling also protects cells from JNK-dependent apoptosis caused by epithelial disruption. We find that repression of reaper transcription by Dpp is mediated by Schnurri. Moreover, reporter gene analysis shows that a transcriptional regulatory module comprising AP-1 and Schnurri binding sites located upstream of reaper integrate the activities of JNK and Dpp. This arrangement allows JNK to control a migratory behavior without triggering apoptosis. Dpp plays a dual role during dorsal closure. It cooperates with JNK in stimulating cell **migratio**n and also prevents JNK from inducing apoptosis.
17. A sensitized PiggyBac-based screen for regulators of border cell **migration** in Drosophila. **Migration** of border cells during Drosophila melanogaster oogenesis is a good model system for investigating the genetic requirements for cell **migration** in vivo. We present a sensitized loss-of-function screen used to identify new genes required in border cells for their **migration**. Chromosomes bearing FRTs on all four major autosomal arms were mutagenized by insertions of the transposable element PiggyBac, allowing multiple parallel clonal screens and easy identification of the mutated gene. For border cells, we analyzed homozygous mutant clones positively marked with lacZ and sensitized by expression of dominant-negative PVR, the guidance receptor. We identified new alleles of genes already known to be required for border cell **migration**, including aop/yan, DIAP1, and taiman as well as a conserved Slbo-regulated enhancer downstream of shg/DE-cadherin. Mutations in genes not previously described to be required in border cells were also uncovered: hrp48, vir, rme-8, kismet, and puckered. puckered was unique in that the **migration** defects were observed only when PVR signaling was reduced. We present evidence that an excess of JNK signaling is deleterious for **migration** in the absence of PVR activity at least in part through Fos transcriptional activity and possibly through antagonistic effects on DIAP1.
18. bullwinkle and shark regulate dorsal-appendage morphogenesis in Drosophila oogenesis. bullwinkle (bwk) regulates embryonic anteroposterior patterning and, through a novel germline-to-soma signal, morphogenesis of the eggshell dorsal appendages. We screened for dominant modifiers of the bullwinkle mooseantler eggshell phenotype and identified shark, which encodes an SH2-domain, ankyrin-repeat tyrosine kinase. At the onset of dorsal-appendage formation, shark is expressed in a punctate pattern in the squamous stretch cells overlying the nurse cells. Confocal microscopy with cell-type-specific markers demonstrates that the stretch cells act as a substrate for the migrating dorsal-appendage-forming cells and extend cellular projections towards them. Mosaic analyses reveal that shark is required in follicle cells for cell **migration** and chorion deposition. Proper shark RNA expression in the stretch cells requires bwk activity, while restoration of shark expression in the stretch cells suppresses the bwk dorsal-appendage phenotype. These results suggest that shark plays an important downstream role in the bwk-signaling pathway. Candidate testing implicates Src42A in a similar role, suggesting conservation with a vertebrate signaling pathway involving non-receptor tyrosine kinases.
19. JNK signaling controls border cell cluster integrity and collective cell **migration**. Collective cell movement is a mechanism for invasion identified in many developmental events. Examples include the movement of lateral-line neurons in Zebrafish, cells in the inner blastocyst, and metastasis of epithelial tumors [1]. One key model to study collective **migration** is the movement of border cell clusters in Drosophila. Drosophila egg chambers contain 15 nurse cells and a single oocyte surrounded by somatic follicle cells. At their anterior end, polar cells recruit several neighboring follicle cells to form the border cell cluster [2]. By stage 9, and over 6 hr, border cells migrate as a cohort between nurse cells toward the oocyte. The specification and directionality of border cell movement are regulated by hormonal and signaling mechanisms [3]. However, how border cells are held together while they migrate is not known. Here, we show that a negative-feedback loop controlling JNK activity regulates border cell cluster integrity. JNK signaling modulates contacts between border cells and between border cells and substratum to sustain collective **migration** by regulating several effectors including the polarity factor Bazooka and the cytoskeletal adaptor D-Paxillin. We anticipate a role for the JNK pathway in controlling collective cell movements in other morphogenetic and clinical models.

#### GeneRIFs

1. excess of JNK signaling is deleterious for **migration** in the absence of PVR activity at least in part through Fos transcriptional activity and possibly through antagonistic effects on DIAP1.

### ECM

#### PubMed

1. Genes conserved in bilaterians but jointly lost with Myc during nematode evolution are enriched in cell proliferation and cell migration functions. Animals use a stereotypical set of developmental genes to build body architectures of varying sizes and organizational complexity. Some genes are critical to developmental patterning, while other genes are important to physiological control of growth. However, growth regulator genes may not be as important in small-bodied "micro-metazoans" such as nematodes. Nematodes use a simplified developmental strategy of lineage-based cell fate specifications to produce an adult bilaterian body composed of a few hundreds of cells. Nematodes also lost the MYC proto-oncogenic regulator of cell proliferation. To identify additional regulators of cell proliferation that were lost with MYC, we computationally screened and determined 839 high-confidence genes that are conserved in bilaterians/lost in nematodes (CIBLIN genes). We find that 30 % of all CIBLIN genes encode transcriptional regulators of cell proliferation, epithelial-to-mesenchyme transitions, and other processes. Over 50 % of CIBLIN genes are unnamed genes in Drosophila, suggesting that there are many understudied genes. Interestingly, CIBLIN genes include many Myc synthetic lethal (MycSL) hits from recent screens. CIBLIN genes include key regulators of heparan sulfate proteoglycan (HSPG) sulfation patterns, and lysyl oxidases involved in cross-linking and modification of the extracellular matrix (**ECM**). These genes and others suggest the CIBLIN repertoire services critical functions in **ECM** remodeling and cell migration in large-bodied bilaterians. Correspondingly, CIBLIN genes are co-expressed with Myc in cancer transcriptomes, and include a preponderance of known determinants of cancer progression and tumor aggression. We propose that CIBLIN gene research can improve our understanding of regulatory control of cellular growth in metazoans.

### [Ee]xtracellular [Mm]atrix

#### PubMed

1. Modifiers of notch transcriptional activity identified by genome-wide RNAi. The Notch signaling pathway regulates a diverse array of developmental processes, and aberrant Notch signaling can lead to diseases, including cancer. To obtain a more comprehensive understanding of the genetic network that integrates into Notch signaling, we performed a genome-wide RNAi screen in Drosophila cell culture to identify genes that modify Notch-dependent transcription.Employing complementary data analyses, we found 399 putative modifiers: 189 promoting and 210 antagonizing Notch activated transcription. These modifiers included several known Notch interactors, validating the robustness of the assay. Many novel modifiers were also identified, covering a range of cellular localizations from the **extracellular matrix** to the nucleus, as well as a large number of proteins with unknown function. Chromatin-modifying proteins represent a major class of genes identified, including histone deacetylase and demethylase complex components and other chromatin modifying, remodeling and replacement factors. A protein-protein interaction map of the Notch-dependent transcription modifiers revealed that a large number of the identified proteins interact physically with these core chromatin components.The genome-wide RNAi screen identified many genes that can modulate Notch transcriptional output. A protein interaction map of the identified genes highlighted a network of chromatin-modifying enzymes and remodelers that regulate Notch transcription. Our results open new avenues to explore the mechanisms of Notch signal regulation and the integration of this pathway into diverse cellular processes.
2. Genes conserved in bilaterians but jointly lost with Myc during nematode evolution are enriched in cell proliferation and cell migration functions. Animals use a stereotypical set of developmental genes to build body architectures of varying sizes and organizational complexity. Some genes are critical to developmental patterning, while other genes are important to physiological control of growth. However, growth regulator genes may not be as important in small-bodied "micro-metazoans" such as nematodes. Nematodes use a simplified developmental strategy of lineage-based cell fate specifications to produce an adult bilaterian body composed of a few hundreds of cells. Nematodes also lost the MYC proto-oncogenic regulator of cell proliferation. To identify additional regulators of cell proliferation that were lost with MYC, we computationally screened and determined 839 high-confidence genes that are conserved in bilaterians/lost in nematodes (CIBLIN genes). We find that 30 % of all CIBLIN genes encode transcriptional regulators of cell proliferation, epithelial-to-mesenchyme transitions, and other processes. Over 50 % of CIBLIN genes are unnamed genes in Drosophila, suggesting that there are many understudied genes. Interestingly, CIBLIN genes include many Myc synthetic lethal (MycSL) hits from recent screens. CIBLIN genes include key regulators of heparan sulfate proteoglycan (HSPG) sulfation patterns, and lysyl oxidases involved in cross-linking and modification of the **extracellular matrix** (ECM). These genes and others suggest the CIBLIN repertoire services critical functions in ECM remodeling and cell migration in large-bodied bilaterians. Correspondingly, CIBLIN genes are co-expressed with Myc in cancer transcriptomes, and include a preponderance of known determinants of cancer progression and tumor aggression. We propose that CIBLIN gene research can improve our understanding of regulatory control of cellular growth in metazoans.

### [Ii]nvasive

#### PubMed

1. Interplay among Drosophila transcription factors Ets21c, Fos and Ftz-F1 drives JNK-mediated tumor malignancy. Cancer initiation and maintenance of the transformed cell state depend on altered cellular signaling and aberrant activities of transcription factors (TFs) that drive pathological gene expression in response to cooperating genetic lesions. Deciphering the roles of interacting TFs is therefore central to understanding carcinogenesis and for designing cancer therapies. Here, we use an unbiased genomic approach to define a TF network that triggers an abnormal gene expression program promoting malignancy of clonal tumors, generated in Drosophila imaginal disc epithelium by gain of oncogenic Ras (Ras(V12)) and loss of the tumor suppressor Scribble (scrib(1)). We show that malignant transformation of the ras(V12)scrib(1) tumors requires TFs of distinct families, namely the bZIP protein Fos, the ETS-domain factor Ets21c and the nuclear receptor Ftz-F1, all acting downstream of Jun-N-terminal kinase (JNK). Depleting any of the three TFs improves viability of tumor-bearing larvae, and this positive effect can be enhanced further by their combined removal. Although both Fos and Ftz-F1 synergistically contribute to ras(V12)scrib(1) tumor **invasive**ness, only Fos is required for JNK-induced differentiation defects and Matrix metalloprotease (MMP1) upregulation. In contrast, the Fos-dimerizing partner Jun is dispensable for JNK to exert its effects in ras(V12)scrib(1) tumors. Interestingly, Ets21c and Ftz-F1 are transcriptionally induced in these tumors in a JNK- and Fos-dependent manner, thereby demonstrating a hierarchy within the tripartite TF network, with Fos acting as the most upstream JNK effector. Of the three TFs, only Ets21c can efficiently substitute for loss of polarity and cooperate with Ras(V12) in inducing malignant clones that, like ras(V12)scrib(1) tumors, invade other tissues and overexpress MMP1 and the Drosophila insulin-like peptide 8 (Dilp8). While ras(V12)ets21c tumors require JNK for **invasive**ness, the JNK activity is dispensable for their growth. In conclusion, our study delineates both unique and overlapping functions of distinct TFs that cooperatively promote aberrant expression of target genes, leading to malignant tumor phenotypes.
2. The actin cross-linker Filamin/Cheerio mediates tumor malignancy downstream of JNK signaling. Cell shape dynamics, motility, and cell proliferation all depend on the actin cytoskeleton. Malignant cancer cells hijack the actin network to grow and migrate to secondary sites. Understanding the function of actin regulators is therefore of major interest. In the present study, we identify the actin cross-linking protein Filamin/Cheerio (Cher) as a mediator of malignancy in genetically defined Drosophila tumors. We show that in **invasive** tumors, resulting from cooperation of activated Ras with disrupted epithelial cell polarity, Cher is upregulated in a Jun N-terminal kinase (JNK)-dependent manner. Although dispensable in normal epithelium, Cher becomes required in the tumor cells for their growth and **invasive**ness. When deprived of Cher, these tumor clones lose their full potential to proliferate and breach tissue boundaries. Instead, the Cher-deficient clones remain confined within the limits of their source epithelium, permitting survival of the host animal. Through interaction with the myosin II heavy chain subunit, Cher is likely to strengthen the cortical actomyosin network and reinforce mechanical tension within the **invasive** tumors. Accordingly, Cher is required for aberrant expression of genes downstream of the Hippo/Yorkie signaling in the tumor tissue. Our study identifies Cher as a new target of JNK signaling that links cytoskeleton dynamics to tumor progression.
3. Interplay among Drosophila transcription factors Ets21c, Fos and Ftz-F1 drives JNK-mediated tumor malignancy. Cancer initiation and maintenance of the transformed cell state depend on altered cellular signaling and aberrant activities of transcription factors (TFs) that drive pathological gene expression in response to cooperating genetic lesions. Deciphering the roles of interacting TFs is therefore central to understanding carcinogenesis and for designing cancer therapies. Here, we use an unbiased genomic approach to define a TF network that triggers an abnormal gene expression program promoting malignancy of clonal tumors, generated in Drosophila imaginal disc epithelium by gain of oncogenic Ras (Ras(V12)) and loss of the tumor suppressor Scribble (scrib(1)). We show that malignant transformation of the ras(V12)scrib(1) tumors requires TFs of distinct families, namely the bZIP protein Fos, the ETS-domain factor Ets21c and the nuclear receptor Ftz-F1, all acting downstream of Jun-N-terminal kinase (JNK). Depleting any of the three TFs improves viability of tumor-bearing larvae, and this positive effect can be enhanced further by their combined removal. Although both Fos and Ftz-F1 synergistically contribute to ras(V12)scrib(1) tumor **invasive**ness, only Fos is required for JNK-induced differentiation defects and Matrix metalloprotease (MMP1) upregulation. In contrast, the Fos-dimerizing partner Jun is dispensable for JNK to exert its effects in ras(V12)scrib(1) tumors. Interestingly, Ets21c and Ftz-F1 are transcriptionally induced in these tumors in a JNK- and Fos-dependent manner, thereby demonstrating a hierarchy within the tripartite TF network, with Fos acting as the most upstream JNK effector. Of the three TFs, only Ets21c can efficiently substitute for loss of polarity and cooperate with Ras(V12) in inducing malignant clones that, like ras(V12)scrib(1) tumors, invade other tissues and overexpress MMP1 and the Drosophila insulin-like peptide 8 (Dilp8). While ras(V12)ets21c tumors require JNK for **invasive**ness, the JNK activity is dispensable for their growth. In conclusion, our study delineates both unique and overlapping functions of distinct TFs that cooperatively promote aberrant expression of target genes, leading to malignant tumor phenotypes.
4. JNK- and Fos-regulated Mmp1 expression cooperates with Ras to induce **invasive** tumors in Drosophila. Loss of the epithelial polarity gene scribble in clones of Drosophila imaginal disc cells can cooperate with Ras signaling to induce malignant tumors. Such mutant tissue overproliferates, resists apoptosis, leaves its place of origin and invades other organs, ultimately causing lethality. We show that increased Jun N-terminal kinase (JNK) signaling resulting from the loss of scribble promotes the movement of transformed cells to secondary sites. This effect requires Fos-dependent transcriptional activation of a matrix metalloprotease gene mmp1 downstream of JNK. Expression of the Mmp inhibitor Timp or Mmp RNAi knockdown suppresses cell **invasive**ness. The pro**invasive** function of the JNK pathway is revealed in a tumor context when active Ras signaling prevents the apoptotic response to JNK activity as it occurs in nontransformed cells. Based on these results, we present a model that explains the oncogenic cooperation between JNK and Ras, and describes how aberrant regulation of cell survival, proliferation and mobilization cooperate to incite malignant tumor formation.

### [Mm]etastasis

#### PubMed

1. JNK signaling controls border cell cluster integrity and collective cell migration. Collective cell movement is a mechanism for invasion identified in many developmental events. Examples include the movement of lateral-line neurons in Zebrafish, cells in the inner blastocyst, and **metastasis** of epithelial tumors [1]. One key model to study collective migration is the movement of border cell clusters in Drosophila. Drosophila egg chambers contain 15 nurse cells and a single oocyte surrounded by somatic follicle cells. At their anterior end, polar cells recruit several neighboring follicle cells to form the border cell cluster [2]. By stage 9, and over 6 hr, border cells migrate as a cohort between nurse cells toward the oocyte. The specification and directionality of border cell movement are regulated by hormonal and signaling mechanisms [3]. However, how border cells are held together while they migrate is not known. Here, we show that a negative-feedback loop controlling JNK activity regulates border cell cluster integrity. JNK signaling modulates contacts between border cells and between border cells and substratum to sustain collective migration by regulating several effectors including the polarity factor Bazooka and the cytoskeletal adaptor D-Paxillin. We anticipate a role for the JNK pathway in controlling collective cell movements in other morphogenetic and clinical models.

---

## Swip-1

- NCBI Gene
- GeneRIF
- Pubmed

| Search Term | Rank | Fields |
| --- | --- | --- |
| [Mm]igration | 6 | PubMed(1) |

  
hide/show details for Swip-1

### [Mm]igration

#### PubMed

1. Regulation of Drosophila mesoderm **migration** by phosphoinositides and the PH domain of the Rho GTP exchange factor Pebble. The Drosophila RhoGEF Pebble (Pbl) is required for cytokinesis and **migration** of mesodermal cells. In a screen for genes that could suppress **migration** defects in pbl mutants we identified the phosphatidylinositol phosphate (PtdInsP) regulator pi5k59B. Genetic interaction tests with other PtdInsP regulators suggested that PtdIns(4,5)P2 levels are important for mesoderm **migration** when Pbl is depleted. Consistent with this, the leading front of migrating mesodermal cells was enriched for PtdIns(4,5)P2. Given that Pbl contains a Pleckstrin Homology (PH) domain, a known PtdInsP-binding motif, we examined PtdInsP-binding of Pbl and the importance of the PH domain for Pbl function. In vitro lipid blot assays showed that Pbl binds promiscuously to PtdInsPs, with binding strength associated with the degree of phosphorylation. Pbl was also able to bind lipid vesicles containing PtdIns(4,5)P2 but binding was strongly reduced upon deletion of the PH domain. Similarly, in vivo, loss of the PH domain prevented localisation of Pbl to the cell cortex and severely affected several aspects of early mesoderm development, including flattening of the invaginated tube onto the ectoderm, extension of protrusions, and dorsal **migration** to form a monolayer. Pbl lacking the PH domain could still localise to the cytokinetic furrow, however, and cytokinesis failure was reduced in pbl(ΔPH) mutants. Taken together, our results support a model in which interaction of the PH-domain of Pbl with PtdIns(4,5)P2 helps localise it to the plasma membrane which is important for mesoderm **migratio**n.

---

## Hsp23

- NCBI Gene
- GeneRIF
- Pubmed

| Search Term | Rank | Fields |
| --- | --- | --- |
| [Mm]igration | 6 | PubMed(2) |
| [Ii]nvasive | 10 | PubMed(1) |

  
hide/show details for Hsp23

### [Mm]igration

#### PubMed

1. Comparative analysis of Hox downstream genes in Drosophila. Functional diversification of body parts is dependent on the formation of specialized structures along the various body axes. In animals, region-specific morphogenesis along the anteroposterior axis is controlled by a group of conserved transcription factors encoded by the Hox genes. Although it has long been assumed that Hox proteins carry out their function by regulating distinct sets of downstream genes, only a small number of such genes have been found, with very few having direct roles in controlling cellular behavior. We have quantitatively identified hundreds of Hox downstream genes in Drosophila by microarray analysis, and validated many of them by in situ hybridizations on loss- and gain-of-function mutants. One important finding is that Hox proteins, despite their similar DNA-binding properties in vitro, have highly specific effects on the transcriptome in vivo, because expression of many downstream genes respond primarily to a single Hox protein. In addition, a large fraction of downstream genes encodes realizator functions, which directly affect morphogenetic processes, such as orientation and rate of cell divisions, cell-cell adhesion and communication, cell shape and **migration**, or cell death. Focusing on these realizators, we provide a framework for the morphogenesis of the maxillary segment. As the genomic organization of Hox genes and the interaction of Hox proteins with specific co-factors are conserved in vertebrates and invertebrates, and similar classes of downstream genes are regulated by Hox proteins across the metazoan phylogeny, our findings represent a first step toward a mechanistic understanding of morphological diversification within a species as well as between species.
2. Drosophila flies in "Evolution Canyon" as a model for incipient sympatric speciation. The genetic basis of population divergence leading to adaptive radiation and speciation is a major unresolved problem of evolutionary biology. Molecular elucidation of "speciation genes" advanced recently, yet it remains without clear identification of the gene complexes participating in reproductive isolation between natural populations, particularly, in sympatry. Genetic divergence was discovered between Drosophila melanogaster populations inhabiting ecologically contrasting, opposite slopes in "Evolution Canyon" (EC), Mt. Carmel, Israel. Interslope **migration** of flies is easy and verified. Nevertheless, significant interslope D. melanogaster population divergence was established at EC involving habitat choice, mate choice, thermal and drought tolerances, adaptive genes, and mobile elements. Parallel patterns of stress tolerance, habitat choice, and mate choice were demonstrated in Drosophila simulans at EC, although on a smaller scale. However, some tests for interslope genetic differentiation in Drosophila, derived from the opposite EC slopes, gave somewhat controversial results. Here we present new empirical data on interslope genetic divergence of Drosophila at EC, and summarize previous supporting and controversial results. We suggest that Drosophila populations at EC represent a rare example, demonstrating how selection overrides **migration**, and propose an ad hoc ecological model of incipient sympatric divergence.

### [Ii]nvasive

#### PubMed

1. Reproductive arrest and stress resistance in winter-acclimated Drosophila suzukii. Overwintering insects must survive the multiple-stress environment of winter, which includes low temperatures, reduced food and water availability, and cold-active pathogens. Many insects overwinter in diapause, a developmental arrest associated with high stress tolerance. Drosophila suzukii (Diptera: Drosophilidae), spotted wing drosophila, is an **invasive** agricultural pest worldwide. Its ability to overwinter and therefore establish in temperate regions could have severe implications for fruit crop industries. We demonstrate here that laboratory populations of Canadian D. suzukii larvae reared under short-day, low temperature, conditions develop into dark 'winter morph' adults similar to those reported globally from field captures, and observed by us in southern Ontario, Canada. These winter-acclimated adults have delayed reproductive maturity, enhanced cold tolerance, and can remain active at low temperatures, although they do not have the increased desiccation tolerance or survival of fungal pathogen challenges that might be expected from a more heavily melanised cuticle. Winter-acclimated female D. suzukii have underdeveloped ovaries and altered transcript levels of several genes associated with reproduction and stress. While superficially indicative of reproductive diapause, the delayed reproductive maturity of winter-acclimated D. suzukii appears to be temperature-dependent, not regulated by photoperiod, and is thus unlikely to be 'true' diapause. The traits of this 'winter morph', however, likely facilitate overwintering in southern Canada, and have probably contributed to the global success of this fly as an **invasive** species.

---

## TRAM

- NCBI Gene
- GeneRIF
- Pubmed

| Search Term | Rank | Fields |
| --- | --- | --- |
| [Mm]igration | 6 | PubMed(1) |
| [Ii]nvasive | 10 | PubMed(1) |

  
hide/show details for TRAM

### [Mm]igration

#### PubMed

1. A genetic screen in Drosophila for regulators of human prostate cancer progression. To uncover the mechanism by which human prostate cancer progresses, we performed a genetic screen for regulators of human prostate cancer progression using the Drosophila accessory gland, a functional homolog of the mammalian prostate. Cell growth and **migration** of secondary cells in the adult male accessory gland were found to be regulated by paired, N-cadherin, and E-cadherin, which are Drosophila homologues of regulators of human prostate cancer progression. Using this screening system, we also identified three genes that promoted growth and **migration** of secondary cells in the accessory gland. The human homologues of these candidate genes - MRGBP, CNPY2, and MEP1A - were found to be expressed in human prostate cancer model cells and to promote replication and invasiveness in these cells. These findings suggest that the development of the Drosophila accessory gland and human prostate cancer cell growth and invasion are partly regulated through a common mechanism. The screening system using the Drosophila accessory gland can be a useful tool for uncovering the mechanisms of human prostate cancer progression.

### [Ii]nvasive

#### PubMed

1. A genetic screen in Drosophila for regulators of human prostate cancer progression. To uncover the mechanism by which human prostate cancer progresses, we performed a genetic screen for regulators of human prostate cancer progression using the Drosophila accessory gland, a functional homolog of the mammalian prostate. Cell growth and migration of secondary cells in the adult male accessory gland were found to be regulated by paired, N-cadherin, and E-cadherin, which are Drosophila homologues of regulators of human prostate cancer progression. Using this screening system, we also identified three genes that promoted growth and migration of secondary cells in the accessory gland. The human homologues of these candidate genes - MRGBP, CNPY2, and MEP1A - were found to be expressed in human prostate cancer model cells and to promote replication and **invasive**ness in these cells. These findings suggest that the development of the Drosophila accessory gland and human prostate cancer cell growth and invasion are partly regulated through a common mechanism. The screening system using the Drosophila accessory gland can be a useful tool for uncovering the mechanisms of human prostate cancer progression.

---

## CG5002

- NCBI Gene
- GeneRIF
- Pubmed

| Search Term | Rank | Fields |
| --- | --- | --- |
| [Mm]igration | 6 | PubMed(1) |
| ECM | 7 | PubMed(1) |
| [Ee]xtracellular [Mm]atrix | 8 | PubMed(1) |

  
hide/show details for CG5002

### [Mm]igration

#### PubMed

1. Genes conserved in bilaterians but jointly lost with Myc during nematode evolution are enriched in cell proliferation and cell **migration** functions. Animals use a stereotypical set of developmental genes to build body architectures of varying sizes and organizational complexity. Some genes are critical to developmental patterning, while other genes are important to physiological control of growth. However, growth regulator genes may not be as important in small-bodied "micro-metazoans" such as nematodes. Nematodes use a simplified developmental strategy of lineage-based cell fate specifications to produce an adult bilaterian body composed of a few hundreds of cells. Nematodes also lost the MYC proto-oncogenic regulator of cell proliferation. To identify additional regulators of cell proliferation that were lost with MYC, we computationally screened and determined 839 high-confidence genes that are conserved in bilaterians/lost in nematodes (CIBLIN genes). We find that 30 % of all CIBLIN genes encode transcriptional regulators of cell proliferation, epithelial-to-mesenchyme transitions, and other processes. Over 50 % of CIBLIN genes are unnamed genes in Drosophila, suggesting that there are many understudied genes. Interestingly, CIBLIN genes include many Myc synthetic lethal (MycSL) hits from recent screens. CIBLIN genes include key regulators of heparan sulfate proteoglycan (HSPG) sulfation patterns, and lysyl oxidases involved in cross-linking and modification of the extracellular matrix (ECM). These genes and others suggest the CIBLIN repertoire services critical functions in ECM remodeling and cell **migration** in large-bodied bilaterians. Correspondingly, CIBLIN genes are co-expressed with Myc in cancer transcriptomes, and include a preponderance of known determinants of cancer progression and tumor aggression. We propose that CIBLIN gene research can improve our understanding of regulatory control of cellular growth in metazoans.

### ECM

#### PubMed

1. Genes conserved in bilaterians but jointly lost with Myc during nematode evolution are enriched in cell proliferation and cell migration functions. Animals use a stereotypical set of developmental genes to build body architectures of varying sizes and organizational complexity. Some genes are critical to developmental patterning, while other genes are important to physiological control of growth. However, growth regulator genes may not be as important in small-bodied "micro-metazoans" such as nematodes. Nematodes use a simplified developmental strategy of lineage-based cell fate specifications to produce an adult bilaterian body composed of a few hundreds of cells. Nematodes also lost the MYC proto-oncogenic regulator of cell proliferation. To identify additional regulators of cell proliferation that were lost with MYC, we computationally screened and determined 839 high-confidence genes that are conserved in bilaterians/lost in nematodes (CIBLIN genes). We find that 30 % of all CIBLIN genes encode transcriptional regulators of cell proliferation, epithelial-to-mesenchyme transitions, and other processes. Over 50 % of CIBLIN genes are unnamed genes in Drosophila, suggesting that there are many understudied genes. Interestingly, CIBLIN genes include many Myc synthetic lethal (MycSL) hits from recent screens. CIBLIN genes include key regulators of heparan sulfate proteoglycan (HSPG) sulfation patterns, and lysyl oxidases involved in cross-linking and modification of the extracellular matrix (**ECM**). These genes and others suggest the CIBLIN repertoire services critical functions in **ECM** remodeling and cell migration in large-bodied bilaterians. Correspondingly, CIBLIN genes are co-expressed with Myc in cancer transcriptomes, and include a preponderance of known determinants of cancer progression and tumor aggression. We propose that CIBLIN gene research can improve our understanding of regulatory control of cellular growth in metazoans.

### [Ee]xtracellular [Mm]atrix

#### PubMed

1. Genes conserved in bilaterians but jointly lost with Myc during nematode evolution are enriched in cell proliferation and cell migration functions. Animals use a stereotypical set of developmental genes to build body architectures of varying sizes and organizational complexity. Some genes are critical to developmental patterning, while other genes are important to physiological control of growth. However, growth regulator genes may not be as important in small-bodied "micro-metazoans" such as nematodes. Nematodes use a simplified developmental strategy of lineage-based cell fate specifications to produce an adult bilaterian body composed of a few hundreds of cells. Nematodes also lost the MYC proto-oncogenic regulator of cell proliferation. To identify additional regulators of cell proliferation that were lost with MYC, we computationally screened and determined 839 high-confidence genes that are conserved in bilaterians/lost in nematodes (CIBLIN genes). We find that 30 % of all CIBLIN genes encode transcriptional regulators of cell proliferation, epithelial-to-mesenchyme transitions, and other processes. Over 50 % of CIBLIN genes are unnamed genes in Drosophila, suggesting that there are many understudied genes. Interestingly, CIBLIN genes include many Myc synthetic lethal (MycSL) hits from recent screens. CIBLIN genes include key regulators of heparan sulfate proteoglycan (HSPG) sulfation patterns, and lysyl oxidases involved in cross-linking and modification of the **extracellular matrix** (ECM). These genes and others suggest the CIBLIN repertoire services critical functions in ECM remodeling and cell migration in large-bodied bilaterians. Correspondingly, CIBLIN genes are co-expressed with Myc in cancer transcriptomes, and include a preponderance of known determinants of cancer progression and tumor aggression. We propose that CIBLIN gene research can improve our understanding of regulatory control of cellular growth in metazoans.

---

## Ptp4E

- NCBI Gene
- GeneRIF
- Pubmed

| Search Term | Rank | Fields |
| --- | --- | --- |
| [Ee]xtracellular [Mm]atrix | 8 | PubMed(1) |

  
hide/show details for Ptp4E

### [Ee]xtracellular [Mm]atrix

#### PubMed

1. Protein tyrosine phosphatases and neural development. During neural development, cells interact dynamically with each other and with the **extracellular matrix**, using cell signaling to control differentiation, axonogenesis, and survival. Enzymes that regulate protein tyrosine phosphorylation often lie at the core of such cell signaling. Protein tyrosine phosphatases (PTPases) are recognized as being of central importance here, and a growing family of PTPases are now known to be expressed in embryonic neurons and glia. Both receptor-like and cytoplasmic enzymes have been identified. The receptor family includes immunoglobulin superfamily members that influence cell-cell adhesion, proteoglycans that control neurite growth, and enzymes in Drosophila that regulate axon guidance and target cell recognition. Cytoplasmic PTPases are implicated in nerve cell commitment and potentially in the regulation of cell survival. This review outlines what we currently know about PTPases in the nervous system and presents concepts concerning their possible modes of action.

---

## Tre1

- NCBI Gene
- GeneRIF
- Pubmed

| Search Term | Rank | Fields |
| --- | --- | --- |
| [Ff]ilopodia | 1 | PubMed(1) |
| [Mm]igration | 6 | Process(3); PubMed(19); GeneRIFs(4) |
| ECM | 7 | PubMed(1) |
| [Ee]xtracellular [Mm]atrix | 8 | PubMed(1) |
| [Ii]nvasive | 10 | PubMed(4) |
| [Mm]etastasis | 12 | PubMed(5) |

  
hide/show details for Tre1

### [Ff]ilopodia

#### PubMed

1. Drosophila gene tao-1 encodes proteins with and without a Ste20 kinase domain that affect cytoskeletal architecture and cell migration differently. Tao-1, the single representative of the Sterile 20 kinase subfamily in Drosophila, is best known for destabilizing microtubules at the actin-rich cortex, regulating the cytoskeletal architecture of cells. More recently, Tao-1 was shown to act in the Salvador-Warts-Hippo pathway by phosphorylating Hippo, regulating cell growth as well as cell polarity. Here, we show that tao-1 encodes two proteins, one with the Sterile 20 kinase domain (Tao-L) and one without it (Tao-S), and that they act in an antagonistic manner. Tao-L expression causes lamellipodia-like cell protrusions, whereas Tao-S expression results in **filopodia**-like structures that make cells stick to the surface they attach to. Ectopic Tao-1 expression in the anterior region of Drosophila embryos results in pole cell formation as normally observed at the posterior end. Tao-S expression causes primordial germ cells (PGCs) to adhere to the inner wall of the gut primordia and prevents proper transepithelial migration to the gonads. Conversely, RNAi knockdowns of Tao-1 cause disordered migration of PGCs out of the gut epithelium, their dispersal within the embryo and cell death. The results reveal a novel function of Tao-1 in cell migration, which is based on antagonistic activities of two proteins encoded by a single gene.

### [Mm]igration

#### Process

1. germ cell **migration**
2. germ cell **migration**
3. pole cell **migration**

#### PubMed

1. Tre1 GPCR initiates germ cell transepithelial **migration** by regulating Drosophila melanogaster E-cadherin. Despite significant progress in identifying the guidance pathways that control cell **migration**, how a cell starts to move within an intact organism, acquires motility, and loses contact with its neighbors is poorly understood. We show that activation of the G protein-coupled receptor (GPCR) trapped in endoderm 1 (Tre1) directs the redistribution of the G protein Gbeta as well as adherens junction proteins and Rho guanosine triphosphatase from the cell periphery to the lagging tail of germ cells at the onset of Drosophila melanogaster germ cell **migration**. Subsequently, Tre1 activity triggers germ cell dispersal and orients them toward the midgut for directed transepithelial **migration**. A transition toward invasive **migration** is also a prerequisite for metastasis formation, which often correlates with down-regulation of adhesion proteins. We show that uniform down-regulation of E-cadherin causes germ cell dispersal but is not sufficient for transepithelial **migration** in the absence of Tre1. Our findings therefore suggest a new mechanism for GPCR function that links cell polarity, modulation of cell adhesion, and invasion.
2. Tre1, a G protein-coupled receptor, directs transepithelial **migration** of Drosophila germ cells. In most organisms, germ cells are formed distant from the somatic part of the gonad and thus have to migrate along and through a variety of tissues to reach the gonad. Transepithelial **migration** through the posterior midgut (PMG) is the first active step during Drosophila germ cell **migration**. Here we report the identification of a novel G protein-coupled receptor (GPCR), Tre1, that is essential for this **migration** step. Maternal tre1 RNA is localized to germ cells, and tre1 is required cell autonomously in germ cells. In tre1 mutant embryos, most germ cells do not exit the PMG. The few germ cells that do leave the midgut early migrate normally to the gonad, suggesting that this gene is specifically required for transepithelial **migration** and that mutant germ cells are still able to recognize other guidance cues. Additionally, inhibiting small Rho GTPases in germ cells affects transepithelial **migration**, suggesting that Tre1 signals through Rho1. We propose that Tre1 acts in a manner similar to chemokine receptors required during transepithelial **migration** of leukocytes, implying an evolutionarily conserved mechanism of transepithelial **migration**. Recently, the chemokine receptor CXCR4 was shown to direct **migration** in vertebrate germ cells. Thus, germ cells may more generally use GPCR signaling to navigate the embryo toward their target.
3. Genes conserved in bilaterians but jointly lost with Myc during nematode evolution are enriched in cell proliferation and cell **migration** functions. Animals use a stereotypical set of developmental genes to build body architectures of varying sizes and organizational complexity. Some genes are critical to developmental patterning, while other genes are important to physiological control of growth. However, growth regulator genes may not be as important in small-bodied "micro-metazoans" such as nematodes. Nematodes use a simplified developmental strategy of lineage-based cell fate specifications to produce an adult bilaterian body composed of a few hundreds of cells. Nematodes also lost the MYC proto-oncogenic regulator of cell proliferation. To identify additional regulators of cell proliferation that were lost with MYC, we computationally screened and determined 839 high-confidence genes that are conserved in bilaterians/lost in nematodes (CIBLIN genes). We find that 30 % of all CIBLIN genes encode transcriptional regulators of cell proliferation, epithelial-to-mesenchyme transitions, and other processes. Over 50 % of CIBLIN genes are unnamed genes in Drosophila, suggesting that there are many understudied genes. Interestingly, CIBLIN genes include many Myc synthetic lethal (MycSL) hits from recent screens. CIBLIN genes include key regulators of heparan sulfate proteoglycan (HSPG) sulfation patterns, and lysyl oxidases involved in cross-linking and modification of the extracellular matrix (ECM). These genes and others suggest the CIBLIN repertoire services critical functions in ECM remodeling and cell **migration** in large-bodied bilaterians. Correspondingly, CIBLIN genes are co-expressed with Myc in cancer transcriptomes, and include a preponderance of known determinants of cancer progression and tumor aggression. We propose that CIBLIN gene research can improve our understanding of regulatory control of cellular growth in metazoans.
4. Tre1, a G protein-coupled receptor, directs transepithelial **migration** of Drosophila germ cells. In most organisms, germ cells are formed distant from the somatic part of the gonad and thus have to migrate along and through a variety of tissues to reach the gonad. Transepithelial **migration** through the posterior midgut (PMG) is the first active step during Drosophila germ cell **migration**. Here we report the identification of a novel G protein-coupled receptor (GPCR), Tre1, that is essential for this **migration** step. Maternal tre1 RNA is localized to germ cells, and tre1 is required cell autonomously in germ cells. In tre1 mutant embryos, most germ cells do not exit the PMG. The few germ cells that do leave the midgut early migrate normally to the gonad, suggesting that this gene is specifically required for transepithelial **migration** and that mutant germ cells are still able to recognize other guidance cues. Additionally, inhibiting small Rho GTPases in germ cells affects transepithelial **migration**, suggesting that Tre1 signals through Rho1. We propose that Tre1 acts in a manner similar to chemokine receptors required during transepithelial **migration** of leukocytes, implying an evolutionarily conserved mechanism of transepithelial **migration**. Recently, the chemokine receptor CXCR4 was shown to direct **migration** in vertebrate germ cells. Thus, germ cells may more generally use GPCR signaling to navigate the embryo toward their target.
5. Genes that drive invasion and **migration** in Drosophila. Successful cell **migration** depends on the careful regulation of the timing of movement, the guidance of motile cells, and cytoskeletal and adhesive changes within the cells. This review focuses on genes that act cell-autonomously to promote these aspects of cell **migration** in Drosophila. We discuss recent advances in understanding the **migration** of the ovarian border cells, embryonic blood cells, primordial germ cells, somatic gonadal precursors, and tracheal cells. Comparison of genes that regulate these processes to those that promote tumorigenesis and metastasis in mammals demonstrates that studies in fruit flies are uncovering new genes highly relevant to cancer biology.
6. The social lives of migrating cells in Drosophila. Studies of cell **migration** in Drosophila are yielding insights into the complex interactions migrating cells have with each other and with the cells in their environment. Intriguing links between factors that promote cell **migration** and those that control cell survival have been reported recently. For example, migrating germ cells compete with the surrounding somatic tissue for the substrate of the lipid phosphate phosphatases encoded by the genes Wunen and Wunen2. Germ cells take up the dephosphorylated lipid and require it for their survival. In addition, the secreted growth factors called PVFs, previously thought to guide the **migration**s of hemocytes in the embryo, were found to function instead predominantly as survival factors. And in border cells, DIAP1 and Dronc, two proteins known mainly for their ability to regulate cell death, were found to control cell **migration**.
7. Tre1 GPCR initiates germ cell transepithelial **migration** by regulating Drosophila melanogaster E-cadherin. Despite significant progress in identifying the guidance pathways that control cell **migration**, how a cell starts to move within an intact organism, acquires motility, and loses contact with its neighbors is poorly understood. We show that activation of the G protein-coupled receptor (GPCR) trapped in endoderm 1 (Tre1) directs the redistribution of the G protein Gbeta as well as adherens junction proteins and Rho guanosine triphosphatase from the cell periphery to the lagging tail of germ cells at the onset of Drosophila melanogaster germ cell **migration**. Subsequently, Tre1 activity triggers germ cell dispersal and orients them toward the midgut for directed transepithelial **migration**. A transition toward invasive **migration** is also a prerequisite for metastasis formation, which often correlates with down-regulation of adhesion proteins. We show that uniform down-regulation of E-cadherin causes germ cell dispersal but is not sufficient for transepithelial **migration** in the absence of Tre1. Our findings therefore suggest a new mechanism for GPCR function that links cell polarity, modulation of cell adhesion, and invasion.
8. Cell **migration** and programmed cell death of Drosophila germ cells. Cell **migration** and programmed cell death are essential components of animal development and homeostasis, and the germ cells of Drosophila provide a simple genetic system to study the molecular mechanisms that govern these important cellular processes. Detailed descriptions of germ cell **migration** in Drosophila were accomplished long ago, but most genetic and molecular analyses of the process have occurred within the past 10 years. A few of the genes required for germ cell **migration** have been identified, and a very interesting picture is emerging. However, a process as complex as cell **migration** must involve the functions of many more molecules. In addition, cell **migration** and cell death mechanisms are often linked, as it is important to eliminate cells that are misplaced and could present a danger to the organism. In Drosophila, genes involved in germ cell **migration** can also affect programmed cell death. Currently, very little is known about how germ cells ectopic to the gonads are eliminated. To date, only four genes have been reported with roles in germ cell death, and three of these have additional functions in germ cell pathfinding. The nature of the cell death program has not been elucidated. Here, I provide a brief review of Drosophila germ cell **migration** and programmed cell death at both the descriptive and molecular levels. Many questions remain to be answered, but advances made in recent years are providing useful insights into these critical biological phenomena.
9. Neurl4 contributes to germ cell formation and integrity in Drosophila. Primordial germ cells (PGCs) form at the posterior pole of the Drosophila embryo, and then migrate to their final destination in the gonad where they will produce eggs or sperm. Studies of the different stages in this process, including assembly of germ plasm in the oocyte during oogenesis, specification of a subset of syncytial embryonic nuclei as PGCs, and **migration**, have been informed by genetic analyses. Mutants have defined steps in the process, and the identities of the affected genes have suggested biochemical mechanisms. Here we describe a novel PGC phenotype. When Neurl4 activity is reduced, newly formed PGCs frequently adopt irregular shapes and appear to bud off vesicles. PGC number is also reduced, an effect exacerbated by a separate role for Neurl4 in germ plasm formation during oogenesis. Like its mammalian homolog, Drosophila Neurl4 protein is concentrated in centrosomes and downregulates centrosomal protein CP110. Reducing CP110 activity suppresses the abnormal PGC morphology of Neurl4 mutants. These results extend prior analyses of Neurl4 in cultured cells, revealing a heightened requirement for Neurl4 in germ-line cells in Drosophila.
10. An evolutionarily conserved arginine is essential for Tre1 G protein-coupled receptor function during germ cell **migration** in Drosophila melanogaster. G protein-coupled receptors (GPCRs) play central roles in mediating cellular responses to environmental signals leading to changes in cell physiology and behaviors, including cell **migration**. Numerous clinical pathologies including metastasis, an invasive form of cell **migration**, have been linked to abnormal GPCR signaling. While the structures of some GPCRs have been defined, the in vivo roles of conserved amino acid residues and their relationships to receptor function are not fully understood. Trapped in endoderm 1 (Tre1) is an orphan receptor of the rhodopsin class that is necessary for primordial germ cell **migration** in Drosophila melanogaster embryos. In this study, we employ molecular genetic approaches to identify residues in Tre1 that are critical to its functions in germ cell **migration**.First, we show that the previously reported scattershot mutation is an allele of tre1. The scattershot allele results in an in-frame deletion of 8 amino acids at the junction of the third transmembrane domain and the second intracellular loop of Tre1 that dramatically impairs the function of this GPCR in germ cell **migration**. To further refine the molecular basis for this phenotype, we assayed the effects of single amino acid substitutions in transgenic animals and determined that the arginine within the evolutionarily conserved E/N/DRY motif is critical for receptor function in mediating germ cell **migration** within an intact developing embryo.These structure-function studies of GPCR signaling in native contexts will inform future studies into the basic biology of this large and clinically important family of receptors.
11. Germ cell specification and **migration** in Drosophila and beyond. The passage of an individual's genome to future generations is essential for the maintenance of species and is mediated by highly specialized cells, the germ cells. Genetic studies in a number of model organisms have provided insight into the molecular mechanisms that control specification, **migration** and survival of early germ cells. Focusing on Drosophila, we will discuss the mechanisms by which germ cells initially form and remain transcriptionally silent while somatic cells are transcriptionally active. We will further discuss three separate attractive and repellent guidance pathways, mediated by a G-protein coupled receptor, two lipid phosphate phosphohydrolases, and isoprenylation. We will compare and contrast these findings with those obtained in other organisms, in particular zebrafish and mice. While aspects of germ cell specification are strikingly different between these species, germ cell specific gene functions have been conserved. In particular, mechanisms that sense directional cues during germ cell **migration** seem to be shared between invertebrates and vertebrates.
12. Identification of X-linked genes required for **migration** and programmed cell death of Drosophila melanogaster germ cells. Drosophila germ cells form at the posterior pole of the embryo and migrate to the somatic gonad. Approximately 50% of the germ cells that form reach their target. The errant cells within the embryo undergo developmentally regulated cell death. Prior studies have identified some autosomal genes that regulate germ cell **migration**, but the genes that control germ cell death are not known. To identify X-linked genes required for germ cell **migration** and/or death, we performed a screen for mutations that disrupt these processes. Here we report the identification of scattershot and outsiders, two genes that regulate the programmed death of germ cells. The scattershot gene is defined by a mutation that disrupts both germ cell **migration** and the death of germ cells ectopic to the gonad. Maternal and zygotic expression of scattershot is required, but the **migration** and cell death functions can be genetically uncoupled. Zygotic expression of wild-type scattershot rescues germ cell pathfinding, but does not restore the programmed death of errant cells. The outsiders gene is required zygotically. In outsiders mutant embryos, the appropriate number of germ cells is incorporated into the gonad, but germ cells ectopic to the gonad persist.
13. Guidance of primordial germ cell **migration**. Primordial germ cells (PGCs), the progenitors of the gametes, migrate from the position where they are specified towards the region where the gonad develops. To reach their target, the PGCs obtain directional cues from cells positioned along their **migration** path. One such cue, the chemokine SDF-1, has recently been found to be critical for proper PGC **migration** in zebrafish and in mice. In Drosophila, too, a molecule that is structurally related to chemokine receptors and is important for PGC **migration** has been identified. The ability to visualize chemokine-guided **migration** at a high resolution in vivo in these model organisms provides a unique opportunity to study this process, which is relevant for many events in normal development and disease.
14. Drosophila gene tao-1 encodes proteins with and without a Ste20 kinase domain that affect cytoskeletal architecture and cell **migration** differently. Tao-1, the single representative of the Sterile 20 kinase subfamily in Drosophila, is best known for destabilizing microtubules at the actin-rich cortex, regulating the cytoskeletal architecture of cells. More recently, Tao-1 was shown to act in the Salvador-Warts-Hippo pathway by phosphorylating Hippo, regulating cell growth as well as cell polarity. Here, we show that tao-1 encodes two proteins, one with the Sterile 20 kinase domain (Tao-L) and one without it (Tao-S), and that they act in an antagonistic manner. Tao-L expression causes lamellipodia-like cell protrusions, whereas Tao-S expression results in filopodia-like structures that make cells stick to the surface they attach to. Ectopic Tao-1 expression in the anterior region of Drosophila embryos results in pole cell formation as normally observed at the posterior end. Tao-S expression causes primordial germ cells (PGCs) to adhere to the inner wall of the gut primordia and prevents proper transepithelial **migration** to the gonads. Conversely, RNAi knockdowns of Tao-1 cause disordered **migration** of PGCs out of the gut epithelium, their dispersal within the embryo and cell death. The results reveal a novel function of Tao-1 in cell **migration**, which is based on antagonistic activities of two proteins encoded by a single gene.
15. A Novel Gene Controlling the Timing of Courtship Initiation in Drosophila melanogaster. Over the past 35 years, developmental geneticists have made impressive progress toward an understanding of how genes specify morphology and function, particularly as they relate to the specification of each physical component of an organism. In the last 20 years, male courtship behavior in Drosophila melanogaster has emerged as a robust model system for the study of genetic specification of behavior. Courtship behavior is both complex and innate, and a single gene, fruitless (fru), is both necessary and sufficient for all aspects of the courtship ritual. Typically, loss of male-specific Fruitless protein function results in male flies that perform the courtship ritual incorrectly, slowly, or not at all. Here we describe a novel requirement for fru: we have identified a group of cells in which male Fru proteins are required to reduce the speed of courtship initiation. In addition, we have identified a gene, Trapped in endoderm 1 (Tre1), which is required in these cells for normal courtship and mating behavior. Tre1 encodes a G-protein-coupled receptor required for establishment of cell polarity and cell **migration** and has previously not been shown to be involved in courtship behavior. We describe the results of feminization of the Tre1-expressing neurons, as well as the effects on courtship behavior of mutation of Tre1. In addition, we show that Tre1 is expressed in a sexually dimorphic pattern in the central and peripheral nervous systems and investigate the role of the Tre1 cells in mate identification.
16. Follow the fatty brick road: lipid signaling in cell **migration**. Lysophospholipids play important roles in the **migration** of lymphocytes, smooth muscle cells and germ cells in vertebrates and invertebrates. In vertebrates, the migratory responses are mediated by specific G-protein-coupled receptors. These are expressed in both migrating lymphocyte and smooth muscle cells, and in their surrounding cells. In Drosophila germ cell **migration**, lipid phosphatases also act in both the surrounding and the migrating cells. In all three scenarios, the contributions of these genes in the stationary and migrating cells are being teased apart by genetic studies and direct observation, with exciting results.
17. Drosophila primordial germ cell **migration** requires epithelial remodeling of the endoderm. Trans-epithelial **migration** describes the ability of migrating cells to cross epithelial tissues and occurs during development, infection, inflammation, immune surveillance, wound healing and cancer metastasis. Here we investigate Drosophila primordial germ cells (PGCs), which migrate through the endodermal epithelium. Through live imaging and genetic experimentation we demonstrate that PGCs take advantage of endodermal tissue remodeling to gain access to the gonadal mesoderm and are unable to migrate through intact epithelial tissues. These results are in contrast to the behavior of leukocytes, which actively loosen epithelial junctions to migrate, and raise the possibility that in other contexts in which migrating cells appear to breach tissue barriers, they are actually exploiting existing tissue permeability. Therefore, the use of active invasive programs is not the sole mechanism to infiltrate tissues.
18. Molecular dynamics simulations on the Tre1 G protein-coupled receptor: exploring the role of the arginine of the NRY motif in Tre1 structure. The arginine of the D/E/NRY motif in Rhodopsin family G protein-coupled receptors (GPCRs) is conserved in 96% of these proteins. In some GPCRs, this arginine in transmembrane 3 can form a salt bridge with an aspartic acid or glutamic acid in transmembrane 6. The Drosophila melanogaster GPCR Trapped in endoderm-1 (Tre1) is required for normal primordial germ cell **migration**. In a mutant form of the protein, Tre1sctt, eight amino acids RYILIACH are missing, resulting in a severe disruption of primordial germ cell development. The impact of the loss of these amino acids on Tre1 structure is unknown. Since the missing amino acids in Tre1sctt include the arginine that is part of the D/E/NRY motif in Tre1, molecular dynamics simulations were performed to explore the hypothesis that these amino acids are involved in salt bridge formation and help maintain Tre1 structure.Structural predictions of wild type Tre1 (Tre1+) and Tre1sctt were subjected to over 250 ns of molecular dynamics simulations. The ability of the model systems to form a salt bridge between the arginine of the D/E/NRY motif and an aspartic acid residue in transmembrane 6 was analyzed. The results indicate that a stable salt bridge can form in the Tre1+ systems and a weak salt bridge or no salt bridge, using an alternative arginine, is likely in the Tre1sctt systems.The weak salt bridge or lack of a salt bridge in the Tre1sctt systems could be one possible explanation for the disrupted function of Tre1sctt in primordial germ cell **migratio**n. These results provide a framework for studying the importance of the arginine of the D/E/NRY motif in the structure and function of other GPCRs that are involved in cell **migratio**n, such as CXCR4 in the mouse, zebrafish, and chicken.
19. Functional analysis of the Drosophila embryonic germ cell transcriptome by RNA interference. In Drosophila melanogaster, primordial germ cells are specified at the posterior pole of the very early embryo. This process is regulated by the posterior localized germ plasm that contains a large number of RNAs of maternal origin. Transcription in the primordial germ cells is actively down-regulated until germ cell fate is established. Bulk expression of the zygotic genes commences concomitantly with the degradation of the maternal transcripts. Thus, during embryogenesis, maternally provided and zygotically transcribed mRNAs determine germ cell development collectively. In an effort to identify novel genes involved in the regulation of germ cell behavior, we carried out a large-scale RNAi screen targeting both maternal and zygotic components of the embryonic germ line transcriptome. We identified 48 genes necessary for distinct stages in germ cell development. We found pebble and fascetto to be essential for germ cell **migration** and germ cell division, respectively. Our data uncover a previously unanticipated role of mei-P26 in maintenance of embryonic germ cell fate. We also performed systematic co-RNAi experiments, through which we found a low rate of functional redundancy among homologous gene pairs. As our data indicate a high degree of evolutionary conservation in genetic regulation of germ cell development, they are likely to provide valuable insights into the biology of the germ line in general.

#### GeneRIFs

1. The scattershot allele results in an in-frame deletion of 8 amino acids at the junction of the third transmembrane domain and the second intracellular loop of Tre1 that dramatically impairs the function of this GPCR in germ cell **migration**.
2. plays an important role in regulation of germ cell **migration**, polarity , cell adhesion, and cadherin binding. (review)
3. Down-regulation of E-cadherin causes germ cell dispersal but is not sufficient for transepithelial **migration** in the absence of Tre1, suggesting a new mechanism for Tre1 GPCR function that links cell polarity, modulation of cell adhesion, and invasion.
4. Tre1 is a G protein-coupled receptor that directs transepithelial **migration** of Drosophila germ cells

### ECM

#### PubMed

1. Genes conserved in bilaterians but jointly lost with Myc during nematode evolution are enriched in cell proliferation and cell migration functions. Animals use a stereotypical set of developmental genes to build body architectures of varying sizes and organizational complexity. Some genes are critical to developmental patterning, while other genes are important to physiological control of growth. However, growth regulator genes may not be as important in small-bodied "micro-metazoans" such as nematodes. Nematodes use a simplified developmental strategy of lineage-based cell fate specifications to produce an adult bilaterian body composed of a few hundreds of cells. Nematodes also lost the MYC proto-oncogenic regulator of cell proliferation. To identify additional regulators of cell proliferation that were lost with MYC, we computationally screened and determined 839 high-confidence genes that are conserved in bilaterians/lost in nematodes (CIBLIN genes). We find that 30 % of all CIBLIN genes encode transcriptional regulators of cell proliferation, epithelial-to-mesenchyme transitions, and other processes. Over 50 % of CIBLIN genes are unnamed genes in Drosophila, suggesting that there are many understudied genes. Interestingly, CIBLIN genes include many Myc synthetic lethal (MycSL) hits from recent screens. CIBLIN genes include key regulators of heparan sulfate proteoglycan (HSPG) sulfation patterns, and lysyl oxidases involved in cross-linking and modification of the extracellular matrix (**ECM**). These genes and others suggest the CIBLIN repertoire services critical functions in **ECM** remodeling and cell migration in large-bodied bilaterians. Correspondingly, CIBLIN genes are co-expressed with Myc in cancer transcriptomes, and include a preponderance of known determinants of cancer progression and tumor aggression. We propose that CIBLIN gene research can improve our understanding of regulatory control of cellular growth in metazoans.

### [Ee]xtracellular [Mm]atrix

#### PubMed

1. Genes conserved in bilaterians but jointly lost with Myc during nematode evolution are enriched in cell proliferation and cell migration functions. Animals use a stereotypical set of developmental genes to build body architectures of varying sizes and organizational complexity. Some genes are critical to developmental patterning, while other genes are important to physiological control of growth. However, growth regulator genes may not be as important in small-bodied "micro-metazoans" such as nematodes. Nematodes use a simplified developmental strategy of lineage-based cell fate specifications to produce an adult bilaterian body composed of a few hundreds of cells. Nematodes also lost the MYC proto-oncogenic regulator of cell proliferation. To identify additional regulators of cell proliferation that were lost with MYC, we computationally screened and determined 839 high-confidence genes that are conserved in bilaterians/lost in nematodes (CIBLIN genes). We find that 30 % of all CIBLIN genes encode transcriptional regulators of cell proliferation, epithelial-to-mesenchyme transitions, and other processes. Over 50 % of CIBLIN genes are unnamed genes in Drosophila, suggesting that there are many understudied genes. Interestingly, CIBLIN genes include many Myc synthetic lethal (MycSL) hits from recent screens. CIBLIN genes include key regulators of heparan sulfate proteoglycan (HSPG) sulfation patterns, and lysyl oxidases involved in cross-linking and modification of the **extracellular matrix** (ECM). These genes and others suggest the CIBLIN repertoire services critical functions in ECM remodeling and cell migration in large-bodied bilaterians. Correspondingly, CIBLIN genes are co-expressed with Myc in cancer transcriptomes, and include a preponderance of known determinants of cancer progression and tumor aggression. We propose that CIBLIN gene research can improve our understanding of regulatory control of cellular growth in metazoans.

### [Ii]nvasive

#### PubMed

1. Tre1 GPCR initiates germ cell transepithelial migration by regulating Drosophila melanogaster E-cadherin. Despite significant progress in identifying the guidance pathways that control cell migration, how a cell starts to move within an intact organism, acquires motility, and loses contact with its neighbors is poorly understood. We show that activation of the G protein-coupled receptor (GPCR) trapped in endoderm 1 (Tre1) directs the redistribution of the G protein Gbeta as well as adherens junction proteins and Rho guanosine triphosphatase from the cell periphery to the lagging tail of germ cells at the onset of Drosophila melanogaster germ cell migration. Subsequently, Tre1 activity triggers germ cell dispersal and orients them toward the midgut for directed transepithelial migration. A transition toward **invasive** migration is also a prerequisite for metastasis formation, which often correlates with down-regulation of adhesion proteins. We show that uniform down-regulation of E-cadherin causes germ cell dispersal but is not sufficient for transepithelial migration in the absence of Tre1. Our findings therefore suggest a new mechanism for GPCR function that links cell polarity, modulation of cell adhesion, and invasion.
2. Tre1 GPCR initiates germ cell transepithelial migration by regulating Drosophila melanogaster E-cadherin. Despite significant progress in identifying the guidance pathways that control cell migration, how a cell starts to move within an intact organism, acquires motility, and loses contact with its neighbors is poorly understood. We show that activation of the G protein-coupled receptor (GPCR) trapped in endoderm 1 (Tre1) directs the redistribution of the G protein Gbeta as well as adherens junction proteins and Rho guanosine triphosphatase from the cell periphery to the lagging tail of germ cells at the onset of Drosophila melanogaster germ cell migration. Subsequently, Tre1 activity triggers germ cell dispersal and orients them toward the midgut for directed transepithelial migration. A transition toward **invasive** migration is also a prerequisite for metastasis formation, which often correlates with down-regulation of adhesion proteins. We show that uniform down-regulation of E-cadherin causes germ cell dispersal but is not sufficient for transepithelial migration in the absence of Tre1. Our findings therefore suggest a new mechanism for GPCR function that links cell polarity, modulation of cell adhesion, and invasion.
3. An evolutionarily conserved arginine is essential for Tre1 G protein-coupled receptor function during germ cell migration in Drosophila melanogaster. G protein-coupled receptors (GPCRs) play central roles in mediating cellular responses to environmental signals leading to changes in cell physiology and behaviors, including cell migration. Numerous clinical pathologies including metastasis, an **invasive** form of cell migration, have been linked to abnormal GPCR signaling. While the structures of some GPCRs have been defined, the in vivo roles of conserved amino acid residues and their relationships to receptor function are not fully understood. Trapped in endoderm 1 (Tre1) is an orphan receptor of the rhodopsin class that is necessary for primordial germ cell migration in Drosophila melanogaster embryos. In this study, we employ molecular genetic approaches to identify residues in Tre1 that are critical to its functions in germ cell migration.First, we show that the previously reported scattershot mutation is an allele of tre1. The scattershot allele results in an in-frame deletion of 8 amino acids at the junction of the third transmembrane domain and the second intracellular loop of Tre1 that dramatically impairs the function of this GPCR in germ cell migration. To further refine the molecular basis for this phenotype, we assayed the effects of single amino acid substitutions in transgenic animals and determined that the arginine within the evolutionarily conserved E/N/DRY motif is critical for receptor function in mediating germ cell migration within an intact developing embryo.These structure-function studies of GPCR signaling in native contexts will inform future studies into the basic biology of this large and clinically important family of receptors.
4. Drosophila primordial germ cell migration requires epithelial remodeling of the endoderm. Trans-epithelial migration describes the ability of migrating cells to cross epithelial tissues and occurs during development, infection, inflammation, immune surveillance, wound healing and cancer metastasis. Here we investigate Drosophila primordial germ cells (PGCs), which migrate through the endodermal epithelium. Through live imaging and genetic experimentation we demonstrate that PGCs take advantage of endodermal tissue remodeling to gain access to the gonadal mesoderm and are unable to migrate through intact epithelial tissues. These results are in contrast to the behavior of leukocytes, which actively loosen epithelial junctions to migrate, and raise the possibility that in other contexts in which migrating cells appear to breach tissue barriers, they are actually exploiting existing tissue permeability. Therefore, the use of active **invasive** programs is not the sole mechanism to infiltrate tissues.

### [Mm]etastasis

#### PubMed

1. Tre1 GPCR initiates germ cell transepithelial migration by regulating Drosophila melanogaster E-cadherin. Despite significant progress in identifying the guidance pathways that control cell migration, how a cell starts to move within an intact organism, acquires motility, and loses contact with its neighbors is poorly understood. We show that activation of the G protein-coupled receptor (GPCR) trapped in endoderm 1 (Tre1) directs the redistribution of the G protein Gbeta as well as adherens junction proteins and Rho guanosine triphosphatase from the cell periphery to the lagging tail of germ cells at the onset of Drosophila melanogaster germ cell migration. Subsequently, Tre1 activity triggers germ cell dispersal and orients them toward the midgut for directed transepithelial migration. A transition toward invasive migration is also a prerequisite for **metastasis** formation, which often correlates with down-regulation of adhesion proteins. We show that uniform down-regulation of E-cadherin causes germ cell dispersal but is not sufficient for transepithelial migration in the absence of Tre1. Our findings therefore suggest a new mechanism for GPCR function that links cell polarity, modulation of cell adhesion, and invasion.
2. Genes that drive invasion and migration in Drosophila. Successful cell migration depends on the careful regulation of the timing of movement, the guidance of motile cells, and cytoskeletal and adhesive changes within the cells. This review focuses on genes that act cell-autonomously to promote these aspects of cell migration in Drosophila. We discuss recent advances in understanding the migration of the ovarian border cells, embryonic blood cells, primordial germ cells, somatic gonadal precursors, and tracheal cells. Comparison of genes that regulate these processes to those that promote tumorigenesis and **metastasis** in mammals demonstrates that studies in fruit flies are uncovering new genes highly relevant to cancer biology.
3. Tre1 GPCR initiates germ cell transepithelial migration by regulating Drosophila melanogaster E-cadherin. Despite significant progress in identifying the guidance pathways that control cell migration, how a cell starts to move within an intact organism, acquires motility, and loses contact with its neighbors is poorly understood. We show that activation of the G protein-coupled receptor (GPCR) trapped in endoderm 1 (Tre1) directs the redistribution of the G protein Gbeta as well as adherens junction proteins and Rho guanosine triphosphatase from the cell periphery to the lagging tail of germ cells at the onset of Drosophila melanogaster germ cell migration. Subsequently, Tre1 activity triggers germ cell dispersal and orients them toward the midgut for directed transepithelial migration. A transition toward invasive migration is also a prerequisite for **metastasis** formation, which often correlates with down-regulation of adhesion proteins. We show that uniform down-regulation of E-cadherin causes germ cell dispersal but is not sufficient for transepithelial migration in the absence of Tre1. Our findings therefore suggest a new mechanism for GPCR function that links cell polarity, modulation of cell adhesion, and invasion.
4. An evolutionarily conserved arginine is essential for Tre1 G protein-coupled receptor function during germ cell migration in Drosophila melanogaster. G protein-coupled receptors (GPCRs) play central roles in mediating cellular responses to environmental signals leading to changes in cell physiology and behaviors, including cell migration. Numerous clinical pathologies including **metastasis**, an invasive form of cell migration, have been linked to abnormal GPCR signaling. While the structures of some GPCRs have been defined, the in vivo roles of conserved amino acid residues and their relationships to receptor function are not fully understood. Trapped in endoderm 1 (Tre1) is an orphan receptor of the rhodopsin class that is necessary for primordial germ cell migration in Drosophila melanogaster embryos. In this study, we employ molecular genetic approaches to identify residues in Tre1 that are critical to its functions in germ cell migration.First, we show that the previously reported scattershot mutation is an allele of tre1. The scattershot allele results in an in-frame deletion of 8 amino acids at the junction of the third transmembrane domain and the second intracellular loop of Tre1 that dramatically impairs the function of this GPCR in germ cell migration. To further refine the molecular basis for this phenotype, we assayed the effects of single amino acid substitutions in transgenic animals and determined that the arginine within the evolutionarily conserved E/N/DRY motif is critical for receptor function in mediating germ cell migration within an intact developing embryo.These structure-function studies of GPCR signaling in native contexts will inform future studies into the basic biology of this large and clinically important family of receptors.
5. Drosophila primordial germ cell migration requires epithelial remodeling of the endoderm. Trans-epithelial migration describes the ability of migrating cells to cross epithelial tissues and occurs during development, infection, inflammation, immune surveillance, wound healing and cancer **metastasis**. Here we investigate Drosophila primordial germ cells (PGCs), which migrate through the endodermal epithelium. Through live imaging and genetic experimentation we demonstrate that PGCs take advantage of endodermal tissue remodeling to gain access to the gonadal mesoderm and are unable to migrate through intact epithelial tissues. These results are in contrast to the behavior of leukocytes, which actively loosen epithelial junctions to migrate, and raise the possibility that in other contexts in which migrating cells appear to breach tissue barriers, they are actually exploiting existing tissue permeability. Therefore, the use of active invasive programs is not the sole mechanism to infiltrate tissues.

---

## sktl

- NCBI Gene
- GeneRIF
- Pubmed

| Search Term | Rank | Fields |
| --- | --- | --- |
| [Mm]igration | 6 | PubMed(2) |
| [Ee]xtracellular [Mm]atrix | 8 | PubMed(1) |

  
hide/show details for sktl

### [Mm]igration

#### PubMed

1. Regulation of Drosophila mesoderm **migration** by phosphoinositides and the PH domain of the Rho GTP exchange factor Pebble. The Drosophila RhoGEF Pebble (Pbl) is required for cytokinesis and **migration** of mesodermal cells. In a screen for genes that could suppress **migration** defects in pbl mutants we identified the phosphatidylinositol phosphate (PtdInsP) regulator pi5k59B. Genetic interaction tests with other PtdInsP regulators suggested that PtdIns(4,5)P2 levels are important for mesoderm **migration** when Pbl is depleted. Consistent with this, the leading front of migrating mesodermal cells was enriched for PtdIns(4,5)P2. Given that Pbl contains a Pleckstrin Homology (PH) domain, a known PtdInsP-binding motif, we examined PtdInsP-binding of Pbl and the importance of the PH domain for Pbl function. In vitro lipid blot assays showed that Pbl binds promiscuously to PtdInsPs, with binding strength associated with the degree of phosphorylation. Pbl was also able to bind lipid vesicles containing PtdIns(4,5)P2 but binding was strongly reduced upon deletion of the PH domain. Similarly, in vivo, loss of the PH domain prevented localisation of Pbl to the cell cortex and severely affected several aspects of early mesoderm development, including flattening of the invaginated tube onto the ectoderm, extension of protrusions, and dorsal **migration** to form a monolayer. Pbl lacking the PH domain could still localise to the cytokinetic furrow, however, and cytokinesis failure was reduced in pbl(ΔPH) mutants. Taken together, our results support a model in which interaction of the PH-domain of Pbl with PtdIns(4,5)P2 helps localise it to the plasma membrane which is important for mesoderm **migratio**n.
2. Regulation of cell **migration** during tracheal development in Drosophila melanogaster. Most of the knowledge concerning the intracellular mechanisms involved in cell locomotion have been obtained from in vitro studies of cells in culture. Many of the concepts derived from these studies have been partially confirmed in in vivo systems but numerous questions regarding the developmental control of cell **migration** remain to be addressed. Tracheal morphogenesis in Drosophila melanogaster embryos represents an in vivo model system to study the genetic control of cell **migration**. We review what is known about tracheal development and regulation of tracheal cell **migration**. We try to link these in vivo studies and the movement of cells over two dimensional substrates and elaborate on important questions which remain to be addressed in the future.

### [Ee]xtracellular [Mm]atrix

#### PubMed

1. Polarized deposition of basement membrane proteins depends on Phosphatidylinositol synthase and the levels of Phosphatidylinositol 4,5-bisphosphate. The basement membrane (BM), a specialized sheet of the **extracellular matrix** contacting the basal side of epithelial tissues, plays an important role in the control of the polarized structure of epithelial cells. However, little is known about how BM proteins themselves achieve a polarized distribution. Here, we identify phosphatidylinositol 4,5-bisphosphate (PIP2) as a critical regulator of the polarized secretion of BM proteins. A decrease of PIP2 levels, in particular through mutations in Phosphatidylinositol synthase (Pis) and other members of the phosphoinositide pathway, leads to the aberrant accumulation of BM components at the apical side of the cell without primarily affecting the distribution of apical and basolateral polarity proteins. In addition, PIP2 controls the apical and lateral localization of Crag (Calmodulin-binding protein related to a Rab3 GDP/GTP exchange protein), a factor specifically required to prevent aberrant apical secretion of BM. We propose that PIP2, through the control of Crag's subcellular localization, restricts the secretion of BM proteins to the basal side.

---

## slp2

- NCBI Gene
- GeneRIF
- Pubmed

| Search Term | Rank | Fields |
| --- | --- | --- |
| [Mm]igration | 6 | PubMed(3) |
| [Ee]xtracellular [Mm]atrix | 8 | PubMed(1) |

  
hide/show details for slp2

### [Mm]igration

#### PubMed

1. A core transcriptional network for early mesoderm development in Drosophila melanogaster. Embryogenesis is controlled by large gene-regulatory networks, which generate spatially and temporally refined patterns of gene expression. Here, we report the characteristics of the regulatory network orchestrating early mesodermal development in the fruitfly Drosophila, where the transcription factor Twist is both necessary and sufficient to drive development. Through the integration of chromatin immunoprecipitation followed by microarray analysis (ChIP-on-chip) experiments during discrete time periods with computational approaches, we identified >2000 Twist-bound cis-regulatory modules (CRMs) and almost 500 direct target genes. Unexpectedly, Twist regulates an almost complete cassette of genes required for cell proliferation in addition to genes essential for morophogenesis and cell **migration**. Twist targets almost 25% of all annotated Drosophila transcription factors, which may represent the entire set of regulators necessary for the early development of this system. By combining in vivo binding data from Twist, Mef2, Tinman, and Dorsal we have constructed an initial transcriptional network of early mesoderm development. The network topology reveals extensive combinatorial binding, feed-forward regulation, and complex logical outputs as prevalent features. In addition to binary activation and repression, we suggest that Twist binds to almost all mesodermal CRMs to provide the competence to integrate inputs from more specialized transcription factors.
2. Transcriptional control in embryonic Drosophila midline guidance assessed through a whole genome approach. During the development of the Drosophila central nervous system the process of midline crossing is orchestrated by a number of guidance receptors and ligands. Many key axon guidance molecules have been identified in both invertebrates and vertebrates, but the transcriptional regulation of growth cone guidance remains largely unknown. It is established that translational regulation plays a role in midline crossing, and there are indications that transcriptional regulation is also involved. To investigate this issue, we conducted a genome-wide study of transcription in Drosophila embryos using wild type and a number of well-characterized Drosophila guidance mutants and transgenics. We also analyzed a previously published microarray time course of Drosophila embryonic development with an axon guidance focus.Using hopach, a novel clustering method which is well suited to microarray data analysis, we identified groups of genes with similar expression patterns across guidance mutants and transgenics. We then systematically characterized the resulting clusters with respect to their relevance to axon guidance using two complementary controlled vocabularies: the Gene Ontology (GO) and anatomical annotations of the Atlas of Pattern of Gene Expression (APoGE) in situ hybridization database. The analysis indicates that regulation of gene expression does play a role in the process of axon guidance in Drosophila. We also find a strong link between axon guidance and hemocyte **migration**, a result that agrees with mounting evidence that axon guidance molecules are co-opted in vertebrate vascularization. Cell cyclin activity in the context of axon guidance is also suggested from our array data. RNA and protein expression patterns of cell cyclins in axon guidance mutants and transgenics support this possible link.This study provides important insights into the regulation of axon guidance in vivo.
3. Determination and development of the larval muscle pattern in Drosophila melanogaster. This review describes briefly what is known about the early steps of mesoderm differentiation in the fruitfly Drosophila melanogaster. After a summary of general aspects including mesoderm differentiation, mesoderm cell **migration** and subdivision of the mesoderm, more detail is given about the specification of muscle progenitor cells, due to their role as the earliest obvious landmarks in muscle fiber development in Drosophila. Particular focus is given to recent results on the role of asymmetric cell division in muscle differentiation. Furthermore a short summary of myoblast fusion is provided.

### [Ee]xtracellular [Mm]atrix

#### PubMed

1. Tenm, a Drosophila gene related to tenascin, is a new pair-rule gene. We describe the molecular characterization of the Drosophila gene tenm, a large transcription unit spanning > 110 kb of DNA. tenm encodes a large extracellular protein of 2515 amino acids related to the **extracellular matrix** molecule tenascin. The Tenm protein is found in seven stripes during the blastoderm stage, and each stripe overlaps with the even-skipped stripes. tenm mutants show a phenotype resembling that of odd-paired (opa), a member of the pair-rule class of segmentation genes. Thus, Tenm is the first example of a pair-rule gene product acting from outside the cell. While the Tenm protein is under the control of fushi tarazu and even-skipped, but not of opa, at least two pair-rule genes, paired (prd) and sloppy paired (slp), and all segment-polarity genes analysed to date are under the control of tenm. Our data suggest that Tenm initiates a signal transduction cascade which acts, via or in concert with opa, on downstream targets such as prd, slp, gooseberry, engrailed and wingless, leading to an opa-like phenotype.

---

## Pkg21D

- NCBI Gene
- GeneRIF
- Pubmed

| Search Term | Rank | Fields |
| --- | --- | --- |
| [Mm]igration | 6 | PubMed(1) |
| [Ee]xtracellular [Mm]atrix | 8 | PubMed(2) |

  
hide/show details for Pkg21D

### [Mm]igration

#### PubMed

1. Receptor guanylyl cyclase Gyc76C is required for invagination, collective **migration** and lumen shape in the Drosophila embryonic salivary gland. The Drosophila embryonic salivary gland is formed by the invagination and collective **migration** of cells. Here, we report on a novel developmental role for receptor-type guanylyl cyclase at 76C, Gyc76C, in morphogenesis of the salivary gland. We demonstrate that Gyc76C and downstream cGMP-dependent protein kinase 1 (DG1) function in the gland and surrounding mesoderm to control invagination, collective **migration** and lumen shape. Loss of gyc76C resulted in glands that failed to invaginate, complete posterior **migration** and had branched lumens. Salivary gland **migration** defects of gyc76C mutant embryos were rescued by expression of wild-type gyc76C specifically in the gland or surrounding mesoderm, whereas invagination defects were rescued primarily by expression in the gland. In migrating salivary glands of gyc76C mutant embryos, integrin subunits localized normally to gland-mesoderm contact sites but talin localization in the surrounding circular visceral mesoderm and fat body was altered. The extracellular matrix protein, laminin, also failed to accumulate around the migrating salivary gland of gyc76C mutant embryos, and gyc76C and laminin genetically interacted in gland **migration**. Our studies suggest that gyc76C controls salivary gland invagination, collective **migration** and lumen shape, in part by regulating the localization of talin and the laminin matrix.

### [Ee]xtracellular [Mm]atrix

#### PubMed

1. The Gyc76C Receptor Guanylyl Cyclase and the Foraging cGMP-Dependent Kinase Regulate **Extracellular Matrix** Organization and BMP Signaling in the Developing Wing of Drosophila melanogaster. The developing crossveins of the wing of Drosophila melanogaster are specified by long-range BMP signaling and are especially sensitive to loss of extracellular modulators of BMP signaling such as the Chordin homolog Short gastrulation (Sog). However, the role of the **extracellular matrix** in BMP signaling and Sog activity in the crossveins has been poorly explored. Using a genetic mosaic screen for mutations that disrupt BMP signaling and posterior crossvein development, we identify Gyc76C, a member of the receptor guanylyl cyclase family that includes mammalian natriuretic peptide receptors. We show that Gyc76C and the soluble cGMP-dependent kinase Foraging, likely linked by cGMP, are necessary for normal refinement and maintenance of long-range BMP signaling in the posterior crossvein. This does not occur through cell-autonomous crosstalk between cGMP and BMP signal transduction, but likely through altered extracellular activity of Sog. We identify a novel pathway leading from Gyc76C to the organization of the wing **extracellular matrix** by matrix metalloproteinases, and show that both the **extracellular matrix** and BMP signaling effects are largely mediated by changes in the activity of matrix metalloproteinases. We discuss parallels and differences between this pathway and other examples of cGMP activity in both Drosophila melanogaster and mammalian cells and tissues.
2. Receptor guanylyl cyclase Gyc76C is required for invagination, collective migration and lumen shape in the Drosophila embryonic salivary gland. The Drosophila embryonic salivary gland is formed by the invagination and collective migration of cells. Here, we report on a novel developmental role for receptor-type guanylyl cyclase at 76C, Gyc76C, in morphogenesis of the salivary gland. We demonstrate that Gyc76C and downstream cGMP-dependent protein kinase 1 (DG1) function in the gland and surrounding mesoderm to control invagination, collective migration and lumen shape. Loss of gyc76C resulted in glands that failed to invaginate, complete posterior migration and had branched lumens. Salivary gland migration defects of gyc76C mutant embryos were rescued by expression of wild-type gyc76C specifically in the gland or surrounding mesoderm, whereas invagination defects were rescued primarily by expression in the gland. In migrating salivary glands of gyc76C mutant embryos, integrin subunits localized normally to gland-mesoderm contact sites but talin localization in the surrounding circular visceral mesoderm and fat body was altered. The **extracellular matrix** protein, laminin, also failed to accumulate around the migrating salivary gland of gyc76C mutant embryos, and gyc76C and laminin genetically interacted in gland migration. Our studies suggest that gyc76C controls salivary gland invagination, collective migration and lumen shape, in part by regulating the localization of talin and the laminin matrix.

---

## Dif

- NCBI Gene
- GeneRIF
- Pubmed

| Search Term | Rank | Fields |
| --- | --- | --- |
| [Mm]igration | 6 | PubMed(1) |
| ECM | 7 | PubMed(1) |
| [Ee]xtracellular [Mm]atrix | 8 | PubMed(1) |
| [Ii]nvasive | 10 | PubMed(1) |
| [Mm]etastasis | 12 | PubMed(2) |

  
hide/show details for Dif

### [Mm]igration

#### PubMed

1. Genes conserved in bilaterians but jointly lost with Myc during nematode evolution are enriched in cell proliferation and cell **migration** functions. Animals use a stereotypical set of developmental genes to build body architectures of varying sizes and organizational complexity. Some genes are critical to developmental patterning, while other genes are important to physiological control of growth. However, growth regulator genes may not be as important in small-bodied "micro-metazoans" such as nematodes. Nematodes use a simplified developmental strategy of lineage-based cell fate specifications to produce an adult bilaterian body composed of a few hundreds of cells. Nematodes also lost the MYC proto-oncogenic regulator of cell proliferation. To identify additional regulators of cell proliferation that were lost with MYC, we computationally screened and determined 839 high-confidence genes that are conserved in bilaterians/lost in nematodes (CIBLIN genes). We find that 30 % of all CIBLIN genes encode transcriptional regulators of cell proliferation, epithelial-to-mesenchyme transitions, and other processes. Over 50 % of CIBLIN genes are unnamed genes in Drosophila, suggesting that there are many understudied genes. Interestingly, CIBLIN genes include many Myc synthetic lethal (MycSL) hits from recent screens. CIBLIN genes include key regulators of heparan sulfate proteoglycan (HSPG) sulfation patterns, and lysyl oxidases involved in cross-linking and modification of the extracellular matrix (ECM). These genes and others suggest the CIBLIN repertoire services critical functions in ECM remodeling and cell **migration** in large-bodied bilaterians. Correspondingly, CIBLIN genes are co-expressed with Myc in cancer transcriptomes, and include a preponderance of known determinants of cancer progression and tumor aggression. We propose that CIBLIN gene research can improve our understanding of regulatory control of cellular growth in metazoans.

### ECM

#### PubMed

1. Genes conserved in bilaterians but jointly lost with Myc during nematode evolution are enriched in cell proliferation and cell migration functions. Animals use a stereotypical set of developmental genes to build body architectures of varying sizes and organizational complexity. Some genes are critical to developmental patterning, while other genes are important to physiological control of growth. However, growth regulator genes may not be as important in small-bodied "micro-metazoans" such as nematodes. Nematodes use a simplified developmental strategy of lineage-based cell fate specifications to produce an adult bilaterian body composed of a few hundreds of cells. Nematodes also lost the MYC proto-oncogenic regulator of cell proliferation. To identify additional regulators of cell proliferation that were lost with MYC, we computationally screened and determined 839 high-confidence genes that are conserved in bilaterians/lost in nematodes (CIBLIN genes). We find that 30 % of all CIBLIN genes encode transcriptional regulators of cell proliferation, epithelial-to-mesenchyme transitions, and other processes. Over 50 % of CIBLIN genes are unnamed genes in Drosophila, suggesting that there are many understudied genes. Interestingly, CIBLIN genes include many Myc synthetic lethal (MycSL) hits from recent screens. CIBLIN genes include key regulators of heparan sulfate proteoglycan (HSPG) sulfation patterns, and lysyl oxidases involved in cross-linking and modification of the extracellular matrix (**ECM**). These genes and others suggest the CIBLIN repertoire services critical functions in **ECM** remodeling and cell migration in large-bodied bilaterians. Correspondingly, CIBLIN genes are co-expressed with Myc in cancer transcriptomes, and include a preponderance of known determinants of cancer progression and tumor aggression. We propose that CIBLIN gene research can improve our understanding of regulatory control of cellular growth in metazoans.

### [Ee]xtracellular [Mm]atrix

#### PubMed

1. Genes conserved in bilaterians but jointly lost with Myc during nematode evolution are enriched in cell proliferation and cell migration functions. Animals use a stereotypical set of developmental genes to build body architectures of varying sizes and organizational complexity. Some genes are critical to developmental patterning, while other genes are important to physiological control of growth. However, growth regulator genes may not be as important in small-bodied "micro-metazoans" such as nematodes. Nematodes use a simplified developmental strategy of lineage-based cell fate specifications to produce an adult bilaterian body composed of a few hundreds of cells. Nematodes also lost the MYC proto-oncogenic regulator of cell proliferation. To identify additional regulators of cell proliferation that were lost with MYC, we computationally screened and determined 839 high-confidence genes that are conserved in bilaterians/lost in nematodes (CIBLIN genes). We find that 30 % of all CIBLIN genes encode transcriptional regulators of cell proliferation, epithelial-to-mesenchyme transitions, and other processes. Over 50 % of CIBLIN genes are unnamed genes in Drosophila, suggesting that there are many understudied genes. Interestingly, CIBLIN genes include many Myc synthetic lethal (MycSL) hits from recent screens. CIBLIN genes include key regulators of heparan sulfate proteoglycan (HSPG) sulfation patterns, and lysyl oxidases involved in cross-linking and modification of the **extracellular matrix** (ECM). These genes and others suggest the CIBLIN repertoire services critical functions in ECM remodeling and cell migration in large-bodied bilaterians. Correspondingly, CIBLIN genes are co-expressed with Myc in cancer transcriptomes, and include a preponderance of known determinants of cancer progression and tumor aggression. We propose that CIBLIN gene research can improve our understanding of regulatory control of cellular growth in metazoans.

### [Ii]nvasive

#### PubMed

1. Pathogen and host factors are needed to provoke a systemic host response to gastrointestinal infection of Drosophila larvae by Candida albicans. Candida albicans systemic dissemination in immunocompromised patients is thought to develop from initial gastrointestinal (GI) colonisation. It is unclear what components of the innate immune system are necessary for preventing C. albicans dissemination from the GI tract, but studies in mice have indicated that both neutropenia and GI mucosal damage are crucial for allowing widespread **invasive** C. albicans disease. Mouse models, however, provide limited applicability to genome-wide screens for pathogen or host factors - factors that might influence systemic dissemination following GI colonisation. For this reason we developed a Drosophila model to study intestinal infection by Candida. We found that commensal flora aided host survival following GI infection. Candida provoked extensive JNK-mediated death of gut cells and induced antimicrobial peptide expression in the fat body. From the side of the host, nitric oxide and blood cells influenced systemic antimicrobial responses. The secretion of SAP4 and SAP6 (secreted aspartyl proteases) from Candida was also essential for activating systemic Toll-dependent immunity.

### [Mm]etastasis

#### PubMed

1. Drosophila as a model to study the role of blood cells in inflammation, innate immunity and cancer. Drosophila has a primitive yet effective blood system with three types of haemocytes which function throughout different developmental stages and environmental stimuli. Haemocytes play essential roles in tissue modeling during embryogenesis and morphogenesis, and also in innate immunity. The open circulatory system of Drosophila makes haemocytes ideal signal mediators to cells and tissues in response to events such as infection and wounding. The application of recently developed and sophisticated genetic tools to the relatively simple genome of Drosophila has made the fly a popular system for modeling human tumorigensis and **metastasis**. Drosophila is now used for screening and investigation of genes implicated in human leukemia and also in modeling development of solid tumors. This second line of research offers promising opportunities to determine the seemingly conflicting roles of blood cells in tumor progression and invasion. This review provides an overview of the signaling pathways conserved in Drosophila during haematopoiesis, haemostasis, innate immunity, wound healing and inflammation. We also review the most recent progress in the use of Drosophila as a cancer research model with an emphasis on the roles haemocytes can play in various cancer models and in the links between inflammation and cancer.
2. Drosophila at the intersection of infection, inflammation, and cancer. Recent studies show that both cellular and humoral aspects of innate immunity play important roles during tumor progression. These interactions have traditionally been explored in vertebrate model systems. In recent years, Drosophila has emerged as a genetically tractable model system for studying key aspects of tumorigenesis including proliferation, invasion, and **metastasis**. The absence of adaptive immunity in Drosophila provides a unique opportunity to study the interactions between innate immune system and cancer in different genetic contexts. In this review, I discuss recent advances made by using Drosophila models of cancer to study the role of innate immune pathways Toll/Imd, JNK, and JAK-STAT, microbial infection and inflammation during tumor progression.

---

## Lac

- NCBI Gene
- GeneRIF
- Pubmed

| Search Term | Rank | Fields |
| --- | --- | --- |
| [Mm]igration | 6 | PubMed(2) |
| ECM | 7 | PubMed(1) |
| [Ee]xtracellular [Mm]atrix | 8 | PubMed(3) |

  
hide/show details for Lac

### [Mm]igration

#### PubMed

1. Unwrapping glial biology: Gcm target genes regulating glial development, diversification, and function. Glia are the most abundant cell type in the mammalian brain. They regulate neuronal development and function, CNS immune surveillance, and stem cell biology, yet we know surprisingly little about glia in any organism. Here we identify over 40 new Drosophila glial genes. We use glial cells missing (gcm) mutants and misexpression to verify they are Gcm regulated in vivo. Many genes show unique spatiotemporal responsiveness to Gcm in the CNS, and thus glial subtype diversification requires spatially or temporally restricted Gcm cofactors. These genes provide insights into glial biology: we show unc-5 (a repulsive netrin receptor) orients glial **migration**s and the draper gene mediates glial engulfment of apoptotic neurons and larval locomotion. Many identified Drosophila glial genes have homologs expressed in mammalian glia, revealing conserved molecular features of glial cells. 80% of these Drosophila glial genes have mammalian homologs; these are now excellent candidates for regulating human glial development, function, or disease.
2. Gene expression profiling of Drosophila tracheal fusion cells. The Drosophila trachea is a premier genetic system to investigate the fundamental mechanisms of tubular organ formation. Tracheal fusion cells lead the branch fusion process to form an interconnected tubular network. Therefore, fusion cells in the Drosophila trachea will be an excellent model to study branch fusion in mammalian tubular organs, such as kidneys and blood vessels. The fusion process is a dynamic cellular process involving cell **migration**, adhesion, vesicle trafficking, cytoskeleton rearrangement, and membrane fusion. To understand how these cellular events are coordinated, we initiated the critical step to assemble a gene expression profile of fusion cells. For this study, we analyzed the expression of 234 potential tracheal-expressed genes in fusion cells during fusion cell development. 143 Tracheal genes were found to encode transcription factors, signal proteins, cytoskeleton and matrix proteins, transporters, and proteins with unknown function. These genes were divided into four subgroups based on their levels of expression in fusion cells compared to neighboring non-fusion cells revealed by in situ hybridization: (1) genes that have relative high abundance in fusion cells, (2) genes that are dynamically expressed in fusion cells, (3) genes that have relative low abundance in fusion cells, and (4) genes that are expressed at similar levels in fusion cells and non-fusion tracheal cells. This study identifies the expression profile of fusion cells and hypothetically suggests genes which are necessary for the fusion process and which play roles in distinct stages of fusion, as indicated by the location and timing of expression. These data will provide the basis for a comprehensive understanding of the molecular and cellular mechanisms of branch fusion.

### ECM

#### PubMed

1. Epithelial polarity proteins regulate Drosophila tracheal tube size in parallel to the luminal matrix pathway. Regulation of epithelial tube size is critical for organ function. However, the mechanisms of tube size control remain poorly understood. In the Drosophila trachea, tube dimensions are regulated by a luminal extracellular matrix (**ECM**). **ECM** organization requires apical (luminal) secretion of the protein Vermiform (Verm), which depends on the basolateral septate junction (SJ). Here, we show that apical and basolateral epithelial polarity proteins interact to control tracheal tube size independently of the Verm pathway. Mutations in yurt (yrt) and scribble (scrib), which encode SJ-associated polarity proteins, cause an expansion of tracheal tubes but do not disrupt Verm secretion. Reducing activity of the apical polarity protein Crumbs (Crb) suppresses the length defects in yrt but not scrib mutants, suggesting that Yrt acts by negatively regulating Crb. Conversely, Crb overexpression increases tracheal tube dimensions. Reducing crb dosage also rescues tracheal size defects caused by mutations in coracle (cora), which encodes an SJ-associated polarity protein. In addition, crb mutations suppress cora length defects without restoring Verm secretion. Together, these data indicate that Yrt, Cora, Crb, and Scrib operate independently of the Verm pathway. Our data support a model in which Cora and Yrt act through Crb to regulate epithelial tube size.

### [Ee]xtracellular [Mm]atrix

#### PubMed

1. A junctional problem of apical proportions: epithelial tube-size control by septate junctions in the Drosophila tracheal system. The size of epithelial tubes is critical for the function of organs such as the lung, kidney and vascular system. However, the molecular mechanisms regulating tube size are largely unknown. Recent work in the Drosophila tracheal system reveals that septate junctions play a previously unsuspected role in tube-size control. Surprisingly, this tube-size function is distinct from the established diffusion barrier function of septate junctions, and involves regulation of cell shape rather than cell number. Possible tube-size functions of septate junctions include patterning of the apical **extracellular matrix** and regulation of conserved cell polarity genes such as Scribble and Discs Large.
2. Organization and function of the blood-brain barrier in Drosophila. The function of a complex nervous system depends on an intricate interplay between neuronal and glial cell types. One of the many functions of glial cells is to provide an efficient insulation of the nervous system and thereby allowing a fine tuned homeostasis of ions and other small molecules. Here, we present a detailed cellular analysis of the glial cell complement constituting the blood-brain barrier in Drosophila. Using electron microscopic analysis and single cell-labeling experiments, we characterize different glial cell layers at the surface of the nervous system, the perineurial glial layer, the subperineurial glial layer, the wrapping glial cell layer, and a thick layer of **extracellular matrix**, the neural lamella. To test the functional roles of these sheaths we performed a series of dye penetration experiments in the nervous systems of wild-type and mutant embryos. Comparing the kinetics of uptake of different sized fluorescently labeled dyes in different mutants allowed to conclude that most of the barrier function is mediated by the septate junctions formed by the subperineurial cells, whereas the perineurial glial cell layer and the neural lamella contribute to barrier selectivity against much larger particles (i.e., the size of proteins). We further compare the requirements of different septate junction components for the integrity of the blood-brain barrier and provide evidence that two of the six Claudin-like proteins found in Drosophila are needed for normal blood-brain barrier function.
3. Epithelial polarity proteins regulate Drosophila tracheal tube size in parallel to the luminal matrix pathway. Regulation of epithelial tube size is critical for organ function. However, the mechanisms of tube size control remain poorly understood. In the Drosophila trachea, tube dimensions are regulated by a luminal **extracellular matrix** (ECM). ECM organization requires apical (luminal) secretion of the protein Vermiform (Verm), which depends on the basolateral septate junction (SJ). Here, we show that apical and basolateral epithelial polarity proteins interact to control tracheal tube size independently of the Verm pathway. Mutations in yurt (yrt) and scribble (scrib), which encode SJ-associated polarity proteins, cause an expansion of tracheal tubes but do not disrupt Verm secretion. Reducing activity of the apical polarity protein Crumbs (Crb) suppresses the length defects in yrt but not scrib mutants, suggesting that Yrt acts by negatively regulating Crb. Conversely, Crb overexpression increases tracheal tube dimensions. Reducing crb dosage also rescues tracheal size defects caused by mutations in coracle (cora), which encodes an SJ-associated polarity protein. In addition, crb mutations suppress cora length defects without restoring Verm secretion. Together, these data indicate that Yrt, Cora, Crb, and Scrib operate independently of the Verm pathway. Our data support a model in which Cora and Yrt act through Crb to regulate epithelial tube size.

---

## b

- NCBI Gene
- GeneRIF
- Pubmed

| Search Term | Rank | Fields |
| --- | --- | --- |
| [Mm]igration | 6 | PubMed(1) |

  
hide/show details for b

### [Mm]igration

#### PubMed

1. Slow as molasses is required for polarized membrane growth and germ cell **migration** in Drosophila. Drosophila germ cell **migration** is directed by attractive and repulsive guidance cues. We have identified a novel gene, slow as molasses (slam), which is required for germ cell **migration**. In slam zygotic mutants, germ cells fail to transit off the midgut into the mesoderm. We show that slam is required at this stage in parallel to HMG Coenzyme A reductase, a previously identified germ cell **migration** gene. Removal of both zygotic and maternal slam results in an earlier defect: a failure to form a cellular blastoderm. Consistent with this phenotype, we found that slam is one of the earliest genes to be transcribed in the embryo, and Slam protein localizes to the growing basal-lateral membrane during blastoderm formation, but Slam is not detected during later stages of embryogenesis. Because slam RNA and protein are expressed earlier than the time when we observe defects in germ cell **migration**, we propose that Slam is required for the localization of a signal to the basal side of blastoderm cells that is needed later in the posterior midgut to guide germ cells.

---

## CG3168

- NCBI Gene
- GeneRIF
- Pubmed

| Search Term | Rank | Fields |
| --- | --- | --- |
| [Mm]igration | 6 | PubMed(2) |
| ECM | 7 | PubMed(1) |
| [Ee]xtracellular [Mm]atrix | 8 | PubMed(1) |

  
hide/show details for CG3168

### [Mm]igration

#### PubMed

1. Functional genomic analysis of the periodic transcriptome in the developing Drosophila wing. The eukaryotic cell cycle, driven by both transcriptional and posttranslational mechanisms, is the central molecular oscillator underlying tissue growth throughout animals. Although genome-wide studies have investigated cell-cycle-associated transcription in unicellular systems, global patterns of periodic transcription in multicellular tissues remain largely unexplored. Here we define the cell-cycle-associated transcriptome of the developing Drosophila wing epithelium and compare it with that of cultured Drosophila S2 cells, revealing a core set of periodic genes and a surprising degree of context specificity in periodic transcription. We further employ RNAi-mediated phenotypic profiling to define functional requirements for more than 300 periodic genes, with a focus on those required for cell proliferation in vivo. Finally, we investigate uncharacterized genes required for interkinetic nuclear **migration**. Combined, these findings provide a global perspective on cell-cycle control in vivo, and they highlight a critical need to understand the context-specific regulation of cell proliferation.
2. Genes conserved in bilaterians but jointly lost with Myc during nematode evolution are enriched in cell proliferation and cell **migration** functions. Animals use a stereotypical set of developmental genes to build body architectures of varying sizes and organizational complexity. Some genes are critical to developmental patterning, while other genes are important to physiological control of growth. However, growth regulator genes may not be as important in small-bodied "micro-metazoans" such as nematodes. Nematodes use a simplified developmental strategy of lineage-based cell fate specifications to produce an adult bilaterian body composed of a few hundreds of cells. Nematodes also lost the MYC proto-oncogenic regulator of cell proliferation. To identify additional regulators of cell proliferation that were lost with MYC, we computationally screened and determined 839 high-confidence genes that are conserved in bilaterians/lost in nematodes (CIBLIN genes). We find that 30 % of all CIBLIN genes encode transcriptional regulators of cell proliferation, epithelial-to-mesenchyme transitions, and other processes. Over 50 % of CIBLIN genes are unnamed genes in Drosophila, suggesting that there are many understudied genes. Interestingly, CIBLIN genes include many Myc synthetic lethal (MycSL) hits from recent screens. CIBLIN genes include key regulators of heparan sulfate proteoglycan (HSPG) sulfation patterns, and lysyl oxidases involved in cross-linking and modification of the extracellular matrix (ECM). These genes and others suggest the CIBLIN repertoire services critical functions in ECM remodeling and cell **migration** in large-bodied bilaterians. Correspondingly, CIBLIN genes are co-expressed with Myc in cancer transcriptomes, and include a preponderance of known determinants of cancer progression and tumor aggression. We propose that CIBLIN gene research can improve our understanding of regulatory control of cellular growth in metazoans.

### ECM

#### PubMed

1. Genes conserved in bilaterians but jointly lost with Myc during nematode evolution are enriched in cell proliferation and cell migration functions. Animals use a stereotypical set of developmental genes to build body architectures of varying sizes and organizational complexity. Some genes are critical to developmental patterning, while other genes are important to physiological control of growth. However, growth regulator genes may not be as important in small-bodied "micro-metazoans" such as nematodes. Nematodes use a simplified developmental strategy of lineage-based cell fate specifications to produce an adult bilaterian body composed of a few hundreds of cells. Nematodes also lost the MYC proto-oncogenic regulator of cell proliferation. To identify additional regulators of cell proliferation that were lost with MYC, we computationally screened and determined 839 high-confidence genes that are conserved in bilaterians/lost in nematodes (CIBLIN genes). We find that 30 % of all CIBLIN genes encode transcriptional regulators of cell proliferation, epithelial-to-mesenchyme transitions, and other processes. Over 50 % of CIBLIN genes are unnamed genes in Drosophila, suggesting that there are many understudied genes. Interestingly, CIBLIN genes include many Myc synthetic lethal (MycSL) hits from recent screens. CIBLIN genes include key regulators of heparan sulfate proteoglycan (HSPG) sulfation patterns, and lysyl oxidases involved in cross-linking and modification of the extracellular matrix (**ECM**). These genes and others suggest the CIBLIN repertoire services critical functions in **ECM** remodeling and cell migration in large-bodied bilaterians. Correspondingly, CIBLIN genes are co-expressed with Myc in cancer transcriptomes, and include a preponderance of known determinants of cancer progression and tumor aggression. We propose that CIBLIN gene research can improve our understanding of regulatory control of cellular growth in metazoans.

### [Ee]xtracellular [Mm]atrix

#### PubMed

1. Genes conserved in bilaterians but jointly lost with Myc during nematode evolution are enriched in cell proliferation and cell migration functions. Animals use a stereotypical set of developmental genes to build body architectures of varying sizes and organizational complexity. Some genes are critical to developmental patterning, while other genes are important to physiological control of growth. However, growth regulator genes may not be as important in small-bodied "micro-metazoans" such as nematodes. Nematodes use a simplified developmental strategy of lineage-based cell fate specifications to produce an adult bilaterian body composed of a few hundreds of cells. Nematodes also lost the MYC proto-oncogenic regulator of cell proliferation. To identify additional regulators of cell proliferation that were lost with MYC, we computationally screened and determined 839 high-confidence genes that are conserved in bilaterians/lost in nematodes (CIBLIN genes). We find that 30 % of all CIBLIN genes encode transcriptional regulators of cell proliferation, epithelial-to-mesenchyme transitions, and other processes. Over 50 % of CIBLIN genes are unnamed genes in Drosophila, suggesting that there are many understudied genes. Interestingly, CIBLIN genes include many Myc synthetic lethal (MycSL) hits from recent screens. CIBLIN genes include key regulators of heparan sulfate proteoglycan (HSPG) sulfation patterns, and lysyl oxidases involved in cross-linking and modification of the **extracellular matrix** (ECM). These genes and others suggest the CIBLIN repertoire services critical functions in ECM remodeling and cell migration in large-bodied bilaterians. Correspondingly, CIBLIN genes are co-expressed with Myc in cancer transcriptomes, and include a preponderance of known determinants of cancer progression and tumor aggression. We propose that CIBLIN gene research can improve our understanding of regulatory control of cellular growth in metazoans.

---

## CG5004

- NCBI Gene
- GeneRIF
- Pubmed

| Search Term | Rank | Fields |
| --- | --- | --- |
| [Mm]igration | 6 | PubMed(1) |
| ECM | 7 | PubMed(1) |
| [Ee]xtracellular [Mm]atrix | 8 | PubMed(1) |

  
hide/show details for CG5004

### [Mm]igration

#### PubMed

1. Genes conserved in bilaterians but jointly lost with Myc during nematode evolution are enriched in cell proliferation and cell **migration** functions. Animals use a stereotypical set of developmental genes to build body architectures of varying sizes and organizational complexity. Some genes are critical to developmental patterning, while other genes are important to physiological control of growth. However, growth regulator genes may not be as important in small-bodied "micro-metazoans" such as nematodes. Nematodes use a simplified developmental strategy of lineage-based cell fate specifications to produce an adult bilaterian body composed of a few hundreds of cells. Nematodes also lost the MYC proto-oncogenic regulator of cell proliferation. To identify additional regulators of cell proliferation that were lost with MYC, we computationally screened and determined 839 high-confidence genes that are conserved in bilaterians/lost in nematodes (CIBLIN genes). We find that 30 % of all CIBLIN genes encode transcriptional regulators of cell proliferation, epithelial-to-mesenchyme transitions, and other processes. Over 50 % of CIBLIN genes are unnamed genes in Drosophila, suggesting that there are many understudied genes. Interestingly, CIBLIN genes include many Myc synthetic lethal (MycSL) hits from recent screens. CIBLIN genes include key regulators of heparan sulfate proteoglycan (HSPG) sulfation patterns, and lysyl oxidases involved in cross-linking and modification of the extracellular matrix (ECM). These genes and others suggest the CIBLIN repertoire services critical functions in ECM remodeling and cell **migration** in large-bodied bilaterians. Correspondingly, CIBLIN genes are co-expressed with Myc in cancer transcriptomes, and include a preponderance of known determinants of cancer progression and tumor aggression. We propose that CIBLIN gene research can improve our understanding of regulatory control of cellular growth in metazoans.

### ECM

#### PubMed

1. Genes conserved in bilaterians but jointly lost with Myc during nematode evolution are enriched in cell proliferation and cell migration functions. Animals use a stereotypical set of developmental genes to build body architectures of varying sizes and organizational complexity. Some genes are critical to developmental patterning, while other genes are important to physiological control of growth. However, growth regulator genes may not be as important in small-bodied "micro-metazoans" such as nematodes. Nematodes use a simplified developmental strategy of lineage-based cell fate specifications to produce an adult bilaterian body composed of a few hundreds of cells. Nematodes also lost the MYC proto-oncogenic regulator of cell proliferation. To identify additional regulators of cell proliferation that were lost with MYC, we computationally screened and determined 839 high-confidence genes that are conserved in bilaterians/lost in nematodes (CIBLIN genes). We find that 30 % of all CIBLIN genes encode transcriptional regulators of cell proliferation, epithelial-to-mesenchyme transitions, and other processes. Over 50 % of CIBLIN genes are unnamed genes in Drosophila, suggesting that there are many understudied genes. Interestingly, CIBLIN genes include many Myc synthetic lethal (MycSL) hits from recent screens. CIBLIN genes include key regulators of heparan sulfate proteoglycan (HSPG) sulfation patterns, and lysyl oxidases involved in cross-linking and modification of the extracellular matrix (**ECM**). These genes and others suggest the CIBLIN repertoire services critical functions in **ECM** remodeling and cell migration in large-bodied bilaterians. Correspondingly, CIBLIN genes are co-expressed with Myc in cancer transcriptomes, and include a preponderance of known determinants of cancer progression and tumor aggression. We propose that CIBLIN gene research can improve our understanding of regulatory control of cellular growth in metazoans.

### [Ee]xtracellular [Mm]atrix

#### PubMed

1. Genes conserved in bilaterians but jointly lost with Myc during nematode evolution are enriched in cell proliferation and cell migration functions. Animals use a stereotypical set of developmental genes to build body architectures of varying sizes and organizational complexity. Some genes are critical to developmental patterning, while other genes are important to physiological control of growth. However, growth regulator genes may not be as important in small-bodied "micro-metazoans" such as nematodes. Nematodes use a simplified developmental strategy of lineage-based cell fate specifications to produce an adult bilaterian body composed of a few hundreds of cells. Nematodes also lost the MYC proto-oncogenic regulator of cell proliferation. To identify additional regulators of cell proliferation that were lost with MYC, we computationally screened and determined 839 high-confidence genes that are conserved in bilaterians/lost in nematodes (CIBLIN genes). We find that 30 % of all CIBLIN genes encode transcriptional regulators of cell proliferation, epithelial-to-mesenchyme transitions, and other processes. Over 50 % of CIBLIN genes are unnamed genes in Drosophila, suggesting that there are many understudied genes. Interestingly, CIBLIN genes include many Myc synthetic lethal (MycSL) hits from recent screens. CIBLIN genes include key regulators of heparan sulfate proteoglycan (HSPG) sulfation patterns, and lysyl oxidases involved in cross-linking and modification of the **extracellular matrix** (ECM). These genes and others suggest the CIBLIN repertoire services critical functions in ECM remodeling and cell migration in large-bodied bilaterians. Correspondingly, CIBLIN genes are co-expressed with Myc in cancer transcriptomes, and include a preponderance of known determinants of cancer progression and tumor aggression. We propose that CIBLIN gene research can improve our understanding of regulatory control of cellular growth in metazoans.

---

## Pez

- NCBI Gene
- GeneRIF
- Pubmed

| Search Term | Rank | Fields |
| --- | --- | --- |
| [Mm]igration | 6 | PubMed(3) |

  
hide/show details for Pez

### [Mm]igration

#### PubMed

1. Slow as molasses is required for polarized membrane growth and germ cell **migration** in Drosophila. Drosophila germ cell **migration** is directed by attractive and repulsive guidance cues. We have identified a novel gene, slow as molasses (slam), which is required for germ cell **migration**. In slam zygotic mutants, germ cells fail to transit off the midgut into the mesoderm. We show that slam is required at this stage in parallel to HMG Coenzyme A reductase, a previously identified germ cell **migration** gene. Removal of both zygotic and maternal slam results in an earlier defect: a failure to form a cellular blastoderm. Consistent with this phenotype, we found that slam is one of the earliest genes to be transcribed in the embryo, and Slam protein localizes to the growing basal-lateral membrane during blastoderm formation, but Slam is not detected during later stages of embryogenesis. Because slam RNA and protein are expressed earlier than the time when we observe defects in germ cell **migration**, we propose that Slam is required for the localization of a signal to the basal side of blastoderm cells that is needed later in the posterior midgut to guide germ cells.
2. KIBRA: In the brain and beyond. In mammals, the KIBRA locus has been associated with memory performance and cognition by genome-wide single nucleotide polymorphism screening. Genetic studies in Drosophila and human cells have identified KIBRA as a novel regulator of the Hippo signaling pathway, which plays a critical role in human tumorigenesis. Recent studies also indicated that KIBRA is involved in other physiological processes including cell polarity, membrane/vesicular trafficking, mitosis and cell **migration**. At the biochemical level, KIBRA protein is highly phosphorylated by various kinases in epithelial cells. Here, we discuss the updates concerning the function and regulation of KIBRA in the brain and beyond.
3. The Bro1-domain-containing protein Myopic/HDPTP coordinates with Rab4 to regulate cell adhesion and **migration**. Protein tyrosine phosphatases (PTPs) are a group of tightly regulated enzymes that coordinate with protein tyrosine kinases to control protein phosphorylation during various cellular processes. Using genetic analysis in Drosophila non-transmembrane PTPs, we identified one role that Myopic (Mop), the Drosophila homolog of the human His domain phosphotyrosine phosphatase (HDPTP), plays in cell adhesion. Depletion of Mop results in aberrant integrin distribution and border cell dissociation during Drosophila oogenesis. Interestingly, Mop phosphatase activity is not required for its role in maintaining border cell cluster integrity. We further identified Rab4 GTPase as a Mop interactor in a yeast two-hybrid screen. Expression of the Rab4 dominant-negative mutant leads to border cell dissociation and suppression of Mop-induced wing-blade adhesion defects, suggesting a critical role of Rab4 in Mop-mediated signaling. In mammals, it has been shown that Rab4-dependent recycling of integrins is necessary for cell adhesion and **migration**. We found that human HDPTP regulates the spatial distribution of Rab4 and integrin trafficking. Depletion of HDPTP resulted in actin reorganization and increased cell motility. Together, our findings suggest an evolutionarily conserved function of HDPTP-Rab4 in the regulation of endocytic trafficking, cell adhesion and **migration**.

---

## Tsp42Ed

- NCBI Gene
- GeneRIF
- Pubmed

| Search Term | Rank | Fields |
| --- | --- | --- |
| [Mm]etastasis | 12 | PubMed(1) |

  
hide/show details for Tsp42Ed

### [Mm]etastasis

#### PubMed

1. Genomewide analysis of the Drosophila tetraspanins reveals a subset with similar function in the formation of the embryonic synapse. Tetraspanins encode a large conserved family of proteins that span the membrane four times and are expressed in a variety of eukaryotic tissues. They are part of membrane complexes that are involved in such diverse processes as intracellular signaling, cellular motility, **metastasis**, and tumor suppression. The single fly tetraspanin characterized to date, late bloomer (lbm), is expressed on the axons, terminal arbors, and growth cones of motoneurons. In embryos lacking Lbm protein, motoneurons reach their muscle targets, but initially fail to form synaptic terminals. During larval stages, however, functional contacts are formed. The newly available genomic sequence of Drosophila melanogaster indicates the existence of 34 additional members of the tetraspanin family in the fly. To address the possibility that other tetraspanins with functions that might compensate for a lack of lbm exist, we determined the expression domains of the Drosophila tetraspanin gene family members by RNA in situ analysis. We found two other tetraspanins also expressed in motoneurons and subsequently generated a small chromosomal deletion that removes all three motoneuron-specific tetraspanins. The deletion results in a significant enhancement in the lbm phenotype, indicating that the two additional motoneuron-expressed tetraspanins can, at least in part, compensate for the absence of lbm during the formation of the embryonic synapse.

---

## Mes2

- NCBI Gene
- GeneRIF
- Pubmed

| Search Term | Rank | Fields |
| --- | --- | --- |
| [Mm]igration | 6 | PubMed(1) |

  
hide/show details for Mes2

### [Mm]igration

#### PubMed

1. Unwrapping glial biology: Gcm target genes regulating glial development, diversification, and function. Glia are the most abundant cell type in the mammalian brain. They regulate neuronal development and function, CNS immune surveillance, and stem cell biology, yet we know surprisingly little about glia in any organism. Here we identify over 40 new Drosophila glial genes. We use glial cells missing (gcm) mutants and misexpression to verify they are Gcm regulated in vivo. Many genes show unique spatiotemporal responsiveness to Gcm in the CNS, and thus glial subtype diversification requires spatially or temporally restricted Gcm cofactors. These genes provide insights into glial biology: we show unc-5 (a repulsive netrin receptor) orients glial **migration**s and the draper gene mediates glial engulfment of apoptotic neurons and larval locomotion. Many identified Drosophila glial genes have homologs expressed in mammalian glia, revealing conserved molecular features of glial cells. 80% of these Drosophila glial genes have mammalian homologs; these are now excellent candidates for regulating human glial development, function, or disease.

---

## AnxB11

- NCBI Gene
- GeneRIF
- Pubmed

| Search Term | Rank | Fields |
| --- | --- | --- |
| [Ff]ilopodia | 1 | PubMed(1) |
| [Mm]igration | 6 | PubMed(2) |
| [Ii]nvasive | 10 | PubMed(1) |

  
hide/show details for AnxB11

### [Ff]ilopodia

#### PubMed

1. Cytonemes are required for the establishment of a normal Hedgehog morphogen gradient in Drosophila epithelia. Hedgehog (Hh) signalling is important in development, stem cell biology and disease. In a variety of tissues, Hh acts as a morphogen to regulate growth and cell fate specification. Several hypotheses have been proposed to explain morphogen movement, one of which is transport along **filopodia**-like protrusions called cytonemes. Here, we analyse the mechanism underlying Hh movement in the wing disc and the abdominal epidermis of Drosophila melanogaster. We show that, in both epithelia, cells generate cytonemes in regions of Hh signalling. These protrusions are actin-based and span several cell diameters. Various Hh signalling components localize to cytonemes, as well as to punctate structures that move along cytonemes and are probably exovesicles. Using in vivo imaging, we show that cytonemes are dynamic structures and that Hh gradient establishment correlates with cytoneme formation in space and time. Indeed, mutant conditions that affect cytoneme formation reduce both cytoneme length and Hh gradient length. Our results suggest that cytoneme-mediated Hh transport is the mechanistic basis for Hh gradient formation.

### [Mm]igration

#### PubMed

1. A genetic screen in Drosophila for regulators of human prostate cancer progression. To uncover the mechanism by which human prostate cancer progresses, we performed a genetic screen for regulators of human prostate cancer progression using the Drosophila accessory gland, a functional homolog of the mammalian prostate. Cell growth and **migration** of secondary cells in the adult male accessory gland were found to be regulated by paired, N-cadherin, and E-cadherin, which are Drosophila homologues of regulators of human prostate cancer progression. Using this screening system, we also identified three genes that promoted growth and **migration** of secondary cells in the accessory gland. The human homologues of these candidate genes - MRGBP, CNPY2, and MEP1A - were found to be expressed in human prostate cancer model cells and to promote replication and invasiveness in these cells. These findings suggest that the development of the Drosophila accessory gland and human prostate cancer cell growth and invasion are partly regulated through a common mechanism. The screening system using the Drosophila accessory gland can be a useful tool for uncovering the mechanisms of human prostate cancer progression.
2. Exosomes as Hedgehog carriers in cytoneme-mediated transport and secretion. The Hedgehog signalling pathway is crucial for development, adult stem cell maintenance, cell **migration** and axon guidance in a wide range of organisms. During development, the Hh morphogen directs tissue patterning according to a concentration gradient. Lipid modifications on Hh are needed to achieve graded distribution, leading to debate about how Hh is transported to target cells despite being membrane-tethered. Cytonemes in the region of Hh signalling have been shown to be essential for gradient formation, but the carrier of the morphogen is yet to be defined. Here we show that Hh and its co-receptor Ihog are in exovesicles transported via cytonemes. These exovesicles present protein markers and other features of exosomes. Moreover, the cell machinery for exosome formation is necessary for normal Hh secretion and graded signalling. We propose Hh transport via exosomes along cytonemes as a significant mechanism for the restricted distribution of a lipid-modified morphogen.

### [Ii]nvasive

#### PubMed

1. A genetic screen in Drosophila for regulators of human prostate cancer progression. To uncover the mechanism by which human prostate cancer progresses, we performed a genetic screen for regulators of human prostate cancer progression using the Drosophila accessory gland, a functional homolog of the mammalian prostate. Cell growth and migration of secondary cells in the adult male accessory gland were found to be regulated by paired, N-cadherin, and E-cadherin, which are Drosophila homologues of regulators of human prostate cancer progression. Using this screening system, we also identified three genes that promoted growth and migration of secondary cells in the accessory gland. The human homologues of these candidate genes - MRGBP, CNPY2, and MEP1A - were found to be expressed in human prostate cancer model cells and to promote replication and **invasive**ness in these cells. These findings suggest that the development of the Drosophila accessory gland and human prostate cancer cell growth and invasion are partly regulated through a common mechanism. The screening system using the Drosophila accessory gland can be a useful tool for uncovering the mechanisms of human prostate cancer progression.

---

## zfh1

- NCBI Gene
- GeneRIF
- Pubmed

| Search Term | Rank | Fields |
| --- | --- | --- |
| [Mm]igration | 6 | Process(3); PubMed(20) |
| ECM | 7 | PubMed(1) |
| [Ee]xtracellular [Mm]atrix | 8 | PubMed(5) |
| [Ii]nvasive | 10 | PubMed(2) |
| [Mm]etastasis | 12 | PubMed(3) |

  
hide/show details for zfh1

### [Mm]igration

#### Process

1. germ cell **migration**
2. germ cell **migration**
3. pole cell **migration**

#### PubMed

1. Subtypes of glial cells in the Drosophila embryonic ventral nerve cord as related to lineage and gene expression. In the Drosophila embryonic CNS several subtypes of glial cells develop, which arrange themselves at characteristic positions and presumably fulfil specific functions. The mechanisms leading to the specification and differentiation of glial subtypes are largely unknown. By DiI labelling in glia-specific Gal4 lines we have clarified the lineages of the lateral glia in the embryonic ventral nerve cord and linked each glial cell to a specific stem cell. For the lineage of the longitudinal glioblast we show that it consists of 9 cells, which acquire at least four different identities. A large collection of molecular markers (many of them representing transcription factors and potential Gcm target genes) reveals that individual glial cells express specific combinations of markers. However, cluster analysis uncovers similar combinatorial codes for cells within, and significant differences between the categories of surface-associated, cortex-associated, and longitudinal glia. Glial cells derived from the same stem cell may be homogeneous (though not identical; stem cells NB1-1, NB5-6, NB6-4, LGB) or heterogeneous (NB7-4, NB1-3) with regard to gene expression. In addition to providing a powerful tool to analyse the fate of individual glial cells in different genetic backgrounds, each of these marker genes represents a candidate factor involved in glial specification or differentiation. We demonstrate this by the analysis of a castor loss of function mutation, which affects the number and **migration** of specific glial cells.
2. The Hem protein mediates neuronal **migration** by inhibiting WAVE degradation and functions opposite of Abelson tyrosine kinase. In the nervous system, neurons form in different regions, then they migrate and occupy specific positions. We have previously shown that RP2/sib, a well-studied neuronal pair in the Drosophila ventral nerve cord (VNC), has a complex **migration** route. Here, we show that the Hem protein, via the WAVE complex, regulates **migration** of GMC-1 and its progeny RP2 neuron. In Hem or WAVE mutants, RP2 neuron either abnormally migrates, crossing the midline from one hemisegment to the contralateral hemisegment, or does not migrate at al and fail to send out its axon projection. We report that Hem regulates neuronal **migration** through stabilizing WAVE. Since Hem and WAVE normally form a complex, our data argues that in the absence of Hem, WAVE, which is presumably no longer in a complex, becomes susceptible to degradation. We also find that Abelson tyrosine kinase affects RP2 **migration** in a similar manner as Hem and WAVE, and appears to operate via WAVE. However, while Abl negatively regulates the levels of WAVE, it regulates **migration** via regulating the activity of WAVE. Our results also show that during the degradation of WAVE, Hem function is opposite to that of and downstream of Abl.
3. Germline development in vertebrates and invertebrates. In all animals information is passed from parent to offspring via the germline, which segregates from the soma early in development and undergoes a complex developmental program to give rise to the adult gametes. Many aspects of germline development have been conserved throughout the animal kingdom. Here we review the unique properties of germ cells, the initial determination of germ cell fates, the maintenance of germ cell identity, the **migration** of germ cells to the somatic gonadal primordia and the proliferation of germ cells during development invertebrates and invertebrates. Similarities in germline development in such diverse organisms as Drosophila melanogaster, Caenorhabditis elegans, Xenopus laevis and Mus musculus will be highlighted.
4. Slow as molasses is required for polarized membrane growth and germ cell **migration** in Drosophila. Drosophila germ cell **migration** is directed by attractive and repulsive guidance cues. We have identified a novel gene, slow as molasses (slam), which is required for germ cell **migration**. In slam zygotic mutants, germ cells fail to transit off the midgut into the mesoderm. We show that slam is required at this stage in parallel to HMG Coenzyme A reductase, a previously identified germ cell **migration** gene. Removal of both zygotic and maternal slam results in an earlier defect: a failure to form a cellular blastoderm. Consistent with this phenotype, we found that slam is one of the earliest genes to be transcribed in the embryo, and Slam protein localizes to the growing basal-lateral membrane during blastoderm formation, but Slam is not detected during later stages of embryogenesis. Because slam RNA and protein are expressed earlier than the time when we observe defects in germ cell **migration**, we propose that Slam is required for the localization of a signal to the basal side of blastoderm cells that is needed later in the posterior midgut to guide germ cells.
5. Identification of genes controlling germ cell **migration** and embryonic gonad formation in Drosophila. Gonadogenesis in the Drosophila embryo is a complex process involving numerous cellular migratory steps and cell-cell interactions. The mechanisms guiding germ cells to move through, recognize and adhere to specific cell types are poorly understood. In order to identify genes that are required for these processes, we have conducted an extensive mutagenesis of the third chromosome and screened for mutations disrupting germ cell **migration** at any point in embryonic development. Phenotypic analysis of these mutants demonstrates that germ cell **migration** can be broken down into discrete developmental steps, with each step requiring a specific set of genes. Many of these genes are involved in the development of gonadal mesoderm, the tissue that associates with germ cells to form the embryonic gonad. Moreover, mutations that we isolated affecting embryonic patterning as well as germ cell **migration** suggest that the origin of gonadal mesoderm lies within the eve domain of the developing mesoderm.
6. A functional RNAi screen for regulators of receptor tyrosine kinase and ERK signalling. Receptor tyrosine kinase (RTK) signalling through extracellular-signal-regulated kinases (ERKs) has pivotal roles during metazoan development, underlying processes as diverse as fate determination, differentiation, proliferation, survival, **migration** and growth. Abnormal RTK/ERK signalling has been extensively documented to contribute to developmental disorders and disease, most notably in oncogenic transformation by mutant RTKs or downstream pathway components such as Ras and Raf. Although the core RTK/ERK signalling cassette has been characterized by decades of research using mammalian cell culture and forward genetic screens in model organisms, signal propagation through this pathway is probably regulated by a larger network of moderate, context-specific proteins. The genes encoding these proteins may not have been discovered through traditional screens owing, in particular, to the requirement for visible phenotypes. To obtain a global view of RTK/ERK signalling, we performed an unbiased, RNA interference (RNAi), genome-wide, high-throughput screen in Drosophila cells using a novel, quantitative, cellular assay monitoring ERK activation. Here we show that ERK pathway output integrates a wide array of conserved cellular processes. Further analysis of selected components-in multiple cell types with different RTK ligands and oncogenic stimuli-validates and classifies 331 pathway regulators. The relevance of these genes is highlighted by our isolation of a Ste20-like kinase and a PPM-family phosphatase that seem to regulate RTK/ERK signalling in vivo and in mammalian cells. Novel regulators that modulate specific pathway outputs may be selective targets for drug discovery.
7. Germ cell specification and **migration** in Drosophila and beyond. The passage of an individual's genome to future generations is essential for the maintenance of species and is mediated by highly specialized cells, the germ cells. Genetic studies in a number of model organisms have provided insight into the molecular mechanisms that control specification, **migration** and survival of early germ cells. Focusing on Drosophila, we will discuss the mechanisms by which germ cells initially form and remain transcriptionally silent while somatic cells are transcriptionally active. We will further discuss three separate attractive and repellent guidance pathways, mediated by a G-protein coupled receptor, two lipid phosphate phosphohydrolases, and isoprenylation. We will compare and contrast these findings with those obtained in other organisms, in particular zebrafish and mice. While aspects of germ cell specification are strikingly different between these species, germ cell specific gene functions have been conserved. In particular, mechanisms that sense directional cues during germ cell **migration** seem to be shared between invertebrates and vertebrates.
8. Functions for Drosophila brachyenteron and forkhead in mesoderm specification and cell signalling. The visceral musculature of the larval midgut of Drosophila has a lattice-type structure and consists of an inner stratum of circular fibers and an outer stratum of longitudinal fibers. The longitudinal fibers originate from the posterior tip of the mesoderm anlage, which has been termed the caudal visceral mesoderm (CVM). In this study, we investigate the specification of the CVM and particularly the role of the Drosophila Brachyury-homologue brachyenteron. Supported by fork head, brachyenteron mediates the early specification of the CVM along with zinc-finger homeodomain protein-1. This is the first function described for brachyenteron or fork head in the mesoderm of Drosophila. The mode of cooperation resembles the interaction of the Xenopus homologues Xbra and Pintallavis. Another function of brachyenteron is to establish the surface properties of the CVM cells, which are essential for their orderly **migration** along the trunk-derived visceral mesoderm. During this movement, the CVM cells, under the control of brachyenteron, induce the formation of one muscle/pericardial precursor cell in each parasegment. We propose that the functions of brachyenteron in mesodermal development of Drosophila are comparable to the roles of the vertebrate Brachyury genes during gastrulation.
9. Integrins are required for cardioblast polarisation in Drosophila. The formation of a tubular organ, such as the heart, requires the communication of positional and polarity signals between migratory cells. Key to this process is the establishment of a new luminal domain on the cell surface, generally from the apical domain of a migratory cell. This domain will also acquire basal properties, as it will produce a luminal extracellular matrix. Integrin receptors are the primary means of cell adhesion and adhesion signaling with the extracellular matrix. Here we characterise the requirement of Integrins in a genetic model of vasculogenesis, the formation of the heart in Drosophila.As with vertebrates, the Drosophila heart arises from lateral mesoderm that migrates medially to meet their contralateral partners, to then assemble a midline vessel. During **migration**, Integrins are among the first proteins restricted to the presumptive luminal domain of cardioblasts. Integrins are required for normal levels of leading edge membrane motility. Apical accumulation of Integrins is enhanced by Robo, and reciprocally, apicalisation of luminal factors like Slit and Robo requires Integrin function. Integrins may provide a template for the formation of a lumen by stabilising lumen factors like Robo. Subsequent to **migration**, Integrin is required for normal cardioblast alignment and lumen formation. This phenotype is most readily modified by other mutations that affect adhesion, such as Talin and extracellular matrix ligands.Our findings reveal an instructive role for Integrins in communicating polarising information to cells during **migration**, and during transition to an epithelial tube structure.
10. Determination and development of the larval muscle pattern in Drosophila melanogaster. This review describes briefly what is known about the early steps of mesoderm differentiation in the fruitfly Drosophila melanogaster. After a summary of general aspects including mesoderm differentiation, mesoderm cell **migration** and subdivision of the mesoderm, more detail is given about the specification of muscle progenitor cells, due to their role as the earliest obvious landmarks in muscle fiber development in Drosophila. Particular focus is given to recent results on the role of asymmetric cell division in muscle differentiation. Furthermore a short summary of myoblast fusion is provided.
11. The Drosophila Hem/Kette/Nap1 protein regulates asymmetric division of neural precursor cells by regulating localization of Inscuteable and Numb. The Hem/Kette/Nap1 protein is involved in many biological processes. We have recently reported that Hem is required for the normal **migration** of neurons in the Drosophila embryo. In this paper, we report that Hem regulates the asymmetric division of neural precursor cells. We find that a well-studied Hem/Kette mutant allele produces at least two main, but possibly more, phenotypic classes of mutant embryos, and these phenotypes correlate with variable levels of maternal wild type Hem protein in the developing embryo. While the weaker class exhibits weak axon guidance defect and the mis-**migration** of neurons, the stronger class causes severe axon guidance defects, mis-**migration** of neurons and symmetric division of ganglion mother cells (GMC) of the RP2/sib lineage. We also show that the basis for the loss of asymmetric division is due to non-localization of Inscuteable and Numb in GMC-1. A non-asymmetric Numb segregates to both daughter cells of GMC-1, which then prevents Notch signaling from specifying a sib fate. This causes both cells to adopt an RP2 fate. Furthermore, loss of function for Abelson tyrosine kinase also causes loss of asymmetric localization of Inscuteable and Numb and symmetric division of GMC-1, the loss of function for WAVE has a very weakly penetrant loss of asymmetry defect. These results define another role for Hem/Kette/Nap1 in a neural precursor cell during neurogenesis.
12. Germ plasm assembly and germ cell **migration** in Drosophila. Many aspects of germ cell behavior, **migration**, and gonad formation are shared between vertebrate and invertebrate species. For example, a specialized germ plasm has been observed in many species including Caenorhabditis elegans and Xenopus. Furthermore, the fact that Vasa marks germ cells in many species suggests that even certain molecular aspects of germ cells may be common between different organisms. In most organisms, germ cells initially form at a location away from their target mesodermal tissues and have to migrate to reach the mesoderm. Further genetic studies will reveal the extent to which molecular aspects of germ cell **migration** and gonad formation are conserved.
13. Cellular interactions during heart morphogenesis in the Drosophila embryo. The formation of the dorsal vessel or heart in a Drosophila melanogaster embryo can be divided into three main steps: i) the determination step allows individualization of heart precursor cells from the dorsal mesoderm. They are arranged in clusters of seven to nine cells, located in each of the eleven segments of the trunk. Preliminary observations suggest that the gene Notch could participate in the choice of fate that the cardioblasts and the pericardial cells will adopt within the cardiogenic region. In the same line, a new gene, whose expression, as revealed by a P-lacZ insertion, is initiated at gastrulation in the developing mesoderm and becomes restricted within the mesoderm to the myogenic lineages, could participate in the determination of the cardioblasts identity; ii) once the cardioblasts have separated from the dorsal mesoderm, they reorganize to form an epithelial monolayer. The gene coding for the alpha-subunit of the transduction protein Go, which is expressed in the cardioblasts shortly before this step, could be involved in this process. Indeed, mutants in the Go alpha gene are affected in the formation of the cardiac endothelium; and iii) the last step consists of the **migration** of the cardiac epithelium towards the dorsal midline of the embryo to form the dorsal vessel by apposition of the two layers of cardioblasts. We show that an extracellular matrix component is specifically expressed at the surface of the dorsal vessel and could participate in the interaction between the dorsalmost ectodermal cells and the heart during this **migration** step.
14. zfh-1 is required for germ cell **migration** and gonadal mesoderm development in Drosophila. In Drosophila as well as many vertebrate systems, germ cells form extraembryonically and migrate into the embryo before navigating toward gonadal mesodermal cells. How the gonadal mesoderm attracts migratory germ cells is not understood in any system. We have taken a genetic approach to identify genes required for germ cell **migration** in Drosophila. Here we describe the role of zfh-1 in germ cell **migration** to the gonadal mesoderm. In zfh-1 mutant embryos, the initial association of germ cells and gonadal mesoderm is blocked. Loss of zfh-1 activity disrupts the development of two distinct mesodermal populations: the caudal visceral mesoderm and the gonadal mesoderm. We demonstrate that the caudal visceral mesoderm facilitates the **migration** of germ cells from the endoderm to the mesoderm. Zfh-1 is also expressed in the gonadal mesoderm throughout the development of this tissue. Ectopic expression of Zfh-1 is sufficient to induce additional gonadal mesodermal cells and to alter the temporal course of gene expression within these cells. Finally, through analysis of a tinman zfh-1 double mutant, we show that zfh-1 acts in conjunction with tinman, another homeodomain protein, in the specification of lateral mesodermal derivatives, including the gonadal mesoderm.
15. Moving towards the next generation. In most organisms, primordial germ cells are set aside from the cells of the body early in development. To form an embryonic gonad, germ cells often have to migrate along complex routes through and along diverse tissues until they reach the somatic part of the gonad. Recent advances have been made in the genetic analysis of these early stages of germ line development. Here we review findings from Drosophila, zebrafish, and mouse; each organism provides unique insight into the mechanisms that determine germ cell fate and the cues that may guide their **migration**.
16. The alternative migratory pathways of the Drosophila tracheal cells are associated with distinct subsets of mesodermal cells. The Drosophila tracheal system is a model for the study of the mechanisms that guide cell **migration**. The general conclusion from many studies is that **migration** of tracheal cells relies on directional cues provided by nearby cells. However, very little is known about which paths are followed by the migrating tracheal cells and what kind of interactions they establish to move in the appropriate direction. Here we analyze how tracheal cells migrate relative to their surroundings and which tissues participate in tracheal cell **migration**. We find that cells in different branches exploit different strategies for their **migration**; while some migrate through preexisting grooves, others make their way through homogeneous cell populations. We also find that alternative migratory pathways of tracheal cells are associated with distinct subsets of mesodermal cells and propose a model for the allocation of groups of tracheal cells to different branches. These results show how adjacent tissues influence morphogenesis of the tracheal system and offer a model for understanding how organ formation is determined by its genetic program and by the surrounding topological constraints.
17. Wingless activity in the precursor cells specifies neuronal migratory behavior in the Drosophila nerve cord. Neurons and their precursor cells are formed in different regions within the developing CNS, but they migrate and occupy very specific sites in the mature CNS. The ultimate position of neurons is crucial for establishing proper synaptic connectivity in the brain. In Drosophila, despite its extensive use as a model system to study neurogenesis, we know almost nothing about neuronal **migration** or its regulation. In this paper, I show that one of the most studied neuronal pairs in the Drosophila nerve cord, RP2/sib, has a complicated migratory route. Based on my studies on Wingless (Wg) signaling, I report that the neuronal migratory pattern is determined at the precursor cell stage level. The results show that Wg activity in the precursor neuroectodermal and neuroblast levels specify neuronal migratory pattern two divisions later, thus, well ahead of the actual migratory event. Moreover, at least two downstream genes, Cut and Zfh1, are involved in this process but their role is at the downstream neuronal level. The functional importance of normal neuronal **migration** and the requirement of Wg signaling for the process are indicated by the finding that mislocated RP2 neurons in embryos mutant for Wg-signaling fail to properly send out their axon projection.
18. HLH54F is required for the specification and **migration** of longitudinal gut muscle founders from the caudal mesoderm of Drosophila. HLH54F, the Drosophila ortholog of the vertebrate basic helix-loop-helix domain-encoding genes capsulin and musculin, is expressed in the founder cells and developing muscle fibers of the longitudinal midgut muscles. These cells descend from the posterior-most portion of the mesoderm, termed the caudal visceral mesoderm (CVM), and migrate onto the trunk visceral mesoderm prior to undergoing myoblast fusion and muscle fiber formation. We show that HLH54F expression in the CVM is regulated by a combination of terminal patterning genes and snail. We generated HLH54F mutations and show that this gene is crucial for the specification, **migration** and survival of the CVM cells and the longitudinal midgut muscle founders. HLH54F mutant embryos, larvae, and adults lack all longitudinal midgut muscles, which causes defects in gut morphology and integrity. The function of HLH54F as a direct activator of gene expression is exemplified by our analysis of a CVM-specific enhancer from the Dorsocross locus, which requires combined inputs from HLH54F and Biniou in a feed-forward fashion. We conclude that HLH54F is the earliest specific regulator of CVM development and that it plays a pivotal role in all major aspects of development and differentiation of this largely twist-independent population of mesodermal cells.
19. Lateral positioning at the dorsal midline: Slit and Roundabout receptors guide Drosophila heart cell **migration**. Heart morphogenesis requires the coordinated regulation of cell movements and cell-cell interactions between distinct populations of cardiac precursor cells. Little is known about the mechanisms that organize cardiac cells into this complex structure. In this study, we analyzed the role of Slit, an extracellular matrix protein and its transmembrane receptors Roundabout (Robo) and Roundabout2 (Robo2) during morphogenesis of the Drosophila heart tube, a process analogous to early heart formation in vertebrates. During heart assembly, two types of progenitor cells align into rows and coordinately migrate to the dorsal midline of the embryo, where they merge to assemble a linear heart tube. Here we show that cardiac-specific expression of Slit is required to maintain adhesion between cells within each row during dorsal **migration**. Moreover, differential Robo expression determines the relative distance each row is positioned from the dorsal midline. The innermost CBs express only Robo, whereas the flanking pericardial cells express both receptors. Removal of robo2 causes pericardial cells to shift toward the midline, whereas ectopic robo2 in CBs drives them laterally, resulting in an unfused heart tube. We propose a model in which Slit has a dual role during assembly of the linear heart tube, functioning to regulate both cell positioning and adhesive interactions between migrating cardiac precursor cells.
20. 14-3-3ε Is required for germ cell **migratio**n in Drosophila. Although 14-3-3 proteins participate in multiple biological processes, isoform-specific specialized functions, as well as functional redundancy are emerging with tissue and developmental stage-specificity. Accordingly, the two 14-3-3ε proteins in Drosophila exhibit functional specificity and redundancy. Homozygotes for loss of function alleles of D14-3-3ε contain significantly fewer germ line cells (pole cells) in their gonads, a phenotype not shared by mutants in the other 14-3-3 gene leo. We show that although D14-3-3ε is enriched within pole cells it is required in mesodermal somatic gonad precursor cells which guide pole cells in th**eir migra**tion through the mesoderm and coalesce with them to form the embryonic gonad. Loss of D14-3-3ε results in defective pole **cell migr**ation, reduced pole cell number. We present evidence that D14-3-3ε loss results in reduction or loss of the transcription factor Zfh-1, one of the main regulatory molecules of the pole **cell mig**ration, from the somatic gonad precursor cells.

### ECM

#### PubMed

1. Integrin-dependent anchoring of a stem-cell niche. Interactions between stem cells and their surrounding microenvironment, or niche, are critical for the establishment and maintenance of stem-cell properties. The adult Drosophila testis contains a morphologically discrete stem-cell niche, the 'hub'. The small cluster of non-dividing, somatic hub cells at the anterior tip of the fly testis is contacted by the germline stem cells (GSCs), which retain their stem-cell character through the direct association with the hub. Here we show that integrin-mediated adhesion is important for maintaining the correct position of embryonic hub cells during gonad morphogenesis. The misplaced hub in integrin-deficient embryos directs the orientation of cell divisions in the presumptive GSCs, a hallmark of the active germline stem-cell niche. A decrease in integrin-mediated adhesion in adult testes, which resulted in a loss of the hub and the stem-cell population, revealed the importance of hub-cell anchoring. Finally, we show that an extracellular matrix (**ECM**) is present around the gonad during late embryogenesis and that this **ECM** is defective in integrin-deficient gonads. On the basis of our data, we propose that integrins are required for the attachment of the hub cells to the **ECM**, which is essential for maintaining the stem-cell niche.

### [Ee]xtracellular [Mm]atrix

#### PubMed

1. Cdc42 and formin activity control non-muscle myosin dynamics during Drosophila heart morphogenesis. During heart formation, a network of transcription factors and signaling pathways guide cardiac cell fate and differentiation, but the genetic mechanisms orchestrating heart assembly and lumen formation remain unclear. Here, we show that the small GTPase Cdc42 is essential for Drosophila melanogaster heart morphogenesis and lumen formation. Cdc42 genetically interacts with the cardiogenic transcription factor tinman; with dDAAM which belongs to the family of actin organizing formins; and with zipper, which encodes nonmuscle myosin II. Zipper is required for heart lumen formation, and its spatiotemporal activity at the prospective luminal surface is controlled by Cdc42. Heart-specific expression of activated Cdc42, or the regulatory formins dDAAM and Diaphanous caused mislocalization of Zipper and induced ectopic heart lumina, as characterized by luminal markers such as the **extracellular matrix** protein Slit. Placement of Slit at the lumen surface depends on Cdc42 and formin function. Thus, Cdc42 and formins play pivotal roles in heart lumen formation through the spatiotemporal regulation of the actomyosin network.
2. Integrin-dependent anchoring of a stem-cell niche. Interactions between stem cells and their surrounding microenvironment, or niche, are critical for the establishment and maintenance of stem-cell properties. The adult Drosophila testis contains a morphologically discrete stem-cell niche, the 'hub'. The small cluster of non-dividing, somatic hub cells at the anterior tip of the fly testis is contacted by the germline stem cells (GSCs), which retain their stem-cell character through the direct association with the hub. Here we show that integrin-mediated adhesion is important for maintaining the correct position of embryonic hub cells during gonad morphogenesis. The misplaced hub in integrin-deficient embryos directs the orientation of cell divisions in the presumptive GSCs, a hallmark of the active germline stem-cell niche. A decrease in integrin-mediated adhesion in adult testes, which resulted in a loss of the hub and the stem-cell population, revealed the importance of hub-cell anchoring. Finally, we show that an **extracellular matrix** (ECM) is present around the gonad during late embryogenesis and that this ECM is defective in integrin-deficient gonads. On the basis of our data, we propose that integrins are required for the attachment of the hub cells to the ECM, which is essential for maintaining the stem-cell niche.
3. Integrins are required for cardioblast polarisation in Drosophila. The formation of a tubular organ, such as the heart, requires the communication of positional and polarity signals between migratory cells. Key to this process is the establishment of a new luminal domain on the cell surface, generally from the apical domain of a migratory cell. This domain will also acquire basal properties, as it will produce a luminal **extracellular matrix**. Integrin receptors are the primary means of cell adhesion and adhesion signaling with the **extracellular matrix**. Here we characterise the requirement of Integrins in a genetic model of vasculogenesis, the formation of the heart in Drosophila.As with vertebrates, the Drosophila heart arises from lateral mesoderm that migrates medially to meet their contralateral partners, to then assemble a midline vessel. During migration, Integrins are among the first proteins restricted to the presumptive luminal domain of cardioblasts. Integrins are required for normal levels of leading edge membrane motility. Apical accumulation of Integrins is enhanced by Robo, and reciprocally, apicalisation of luminal factors like Slit and Robo requires Integrin function. Integrins may provide a template for the formation of a lumen by stabilising lumen factors like Robo. Subsequent to migration, Integrin is required for normal cardioblast alignment and lumen formation. This phenotype is most readily modified by other mutations that affect adhesion, such as Talin and **extracellular matrix** ligands.Our findings reveal an instructive role for Integrins in communicating polarising information to cells during migration, and during transition to an epithelial tube structure.
4. Cellular interactions during heart morphogenesis in the Drosophila embryo. The formation of the dorsal vessel or heart in a Drosophila melanogaster embryo can be divided into three main steps: i) the determination step allows individualization of heart precursor cells from the dorsal mesoderm. They are arranged in clusters of seven to nine cells, located in each of the eleven segments of the trunk. Preliminary observations suggest that the gene Notch could participate in the choice of fate that the cardioblasts and the pericardial cells will adopt within the cardiogenic region. In the same line, a new gene, whose expression, as revealed by a P-lacZ insertion, is initiated at gastrulation in the developing mesoderm and becomes restricted within the mesoderm to the myogenic lineages, could participate in the determination of the cardioblasts identity; ii) once the cardioblasts have separated from the dorsal mesoderm, they reorganize to form an epithelial monolayer. The gene coding for the alpha-subunit of the transduction protein Go, which is expressed in the cardioblasts shortly before this step, could be involved in this process. Indeed, mutants in the Go alpha gene are affected in the formation of the cardiac endothelium; and iii) the last step consists of the migration of the cardiac epithelium towards the dorsal midline of the embryo to form the dorsal vessel by apposition of the two layers of cardioblasts. We show that an **extracellular matrix** component is specifically expressed at the surface of the dorsal vessel and could participate in the interaction between the dorsalmost ectodermal cells and the heart during this migration step.
5. Lateral positioning at the dorsal midline: Slit and Roundabout receptors guide Drosophila heart cell migration. Heart morphogenesis requires the coordinated regulation of cell movements and cell-cell interactions between distinct populations of cardiac precursor cells. Little is known about the mechanisms that organize cardiac cells into this complex structure. In this study, we analyzed the role of Slit, an **extracellular matrix** protein and its transmembrane receptors Roundabout (Robo) and Roundabout2 (Robo2) during morphogenesis of the Drosophila heart tube, a process analogous to early heart formation in vertebrates. During heart assembly, two types of progenitor cells align into rows and coordinately migrate to the dorsal midline of the embryo, where they merge to assemble a linear heart tube. Here we show that cardiac-specific expression of Slit is required to maintain adhesion between cells within each row during dorsal migration. Moreover, differential Robo expression determines the relative distance each row is positioned from the dorsal midline. The innermost CBs express only Robo, whereas the flanking pericardial cells express both receptors. Removal of robo2 causes pericardial cells to shift toward the midline, whereas ectopic robo2 in CBs drives them laterally, resulting in an unfused heart tube. We propose a model in which Slit has a dual role during assembly of the linear heart tube, functioning to regulate both cell positioning and adhesive interactions between migrating cardiac precursor cells.

### [Ii]nvasive

#### PubMed

1. Genome-wide polycomb target gene prediction in Drosophila melanogaster. As key epigenetic regulators, polycomb group (PcG) proteins are responsible for the control of cell proliferation and differentiation as well as stem cell pluripotency and self-renewal. Aberrant epigenetic modification by PcG is strongly correlated with the severity and **invasive**ness of many types of cancers. Unfortunately, the molecular mechanism of PcG-mediated epigenetic regulation remained elusive, partly due to the extremely limited pool of experimentally confirmed PcG target genes. In order to facilitate experimental identification of PcG target genes, here we propose a novel computational method, EpiPredictor, that achieved significantly higher matching ratios with several recent chromatin immunoprecipitation studies than jPREdictor, an existing computational method. We further validated a subset of genes that were uniquely predicted by EpiPredictor by cross-referencing existing literature and by experimental means. Our data suggest that multiple transcription factor networking at the cis-regulatory elements is critical for PcG recruitment, while high GC content and high conservation level are also important features of PcG target genes. EpiPredictor should substantially expedite experimental discovery of PcG target genes by providing an effective initial screening tool. From a computational standpoint, our strategy of modelling transcription factor interaction with a non-linear kernel is original, effective and transferable to many other applications.
2. Robust intestinal homeostasis relies on cellular plasticity in enteroblasts mediated by miR-8-Escargot switch. The intestinal epithelium is remarkably robust despite perturbations and demand uncertainty. Here, we investigate the basis of such robustness using novel tracing methods that allow simultaneously capturing the dynamics of stem and committed progenitor cells (called enteroblasts) and intestinal cell turnover with spatiotemporal resolution. We found that intestinal stem cells (ISCs) divide "ahead" of demand during Drosophila midgut homeostasis. Their newborn enteroblasts, on the other hand, take on a highly polarized shape, acquire **invasive** properties and motility. They extend long membrane protrusions that make cell-cell contact with mature cells, while exercising a capacity to delay their final differentiation until a local demand materializes. This cellular plasticity is mechanistically linked to the epithelial-mesenchymal transition (EMT) programme mediated by escargot, a snail family gene. Activation of the conserved microRNA miR-8/miR-200 in "pausing" enteroblasts in response to a local cell loss promotes timely terminal differentiation via a reverse MET by antagonizing escargot. Our findings unveil that robust intestinal renewal relies on hitherto unrecognized plasticity in enteroblasts and reveal their active role in sensing and/or responding to local demand.

### [Mm]etastasis

#### PubMed

1. A conserved role for Snail as a potentiator of active transcription. The transcription factors of the Snail family are key regulators of epithelial-mesenchymal transitions, cell morphogenesis, and tumor **metastasis**. Since its discovery in Drosophila ∼25 years ago, Snail has been extensively studied for its role as a transcriptional repressor. Here we demonstrate that Drosophila Snail can positively modulate transcriptional activation. By combining information on in vivo occupancy with expression profiling of hand-selected, staged snail mutant embryos, we identified 106 genes that are potentially directly regulated by Snail during mesoderm development. In addition to the expected Snail-repressed genes, almost 50% of Snail targets showed an unanticipated activation. The majority of "Snail-activated" genes have enhancer elements cobound by Twist and are expressed in the mesoderm at the stages of Snail occupancy. Snail can potentiate Twist-mediated enhancer activation in vitro and is essential for enhancer activity in vivo. Using a machine learning approach, we show that differentially enriched motifs are sufficient to predict Snail's regulatory response. In silico mutagenesis revealed a likely causative motif, which we demonstrate is essential for enhancer activation. Taken together, these data indicate that Snail can potentiate enhancer activation by collaborating with different activators, providing a new mechanism by which Snail regulates development.
2. Oncogenic programmes and Notch activity: an 'organized crime'? The inappropriate Notch signalling can influence virtually all aspect of cancer, including tumour-cell growth, survival, apoptosis, angiogenesis, invasion and **metastasis**, although it does not do this alone. Hence, elucidating the partners of Notch that are active in cancer is now the focus of much intense research activity. The genetic toolkits available, coupled to the small size and short life of the fruit fly Drosophila melanogaster, makes this an inexpensive and effective animal model, suited to large-scale cancer gene discovery studies. The fly eye is not only a non-vital organ but its stereotyped size and disposition also means it is easy to screen for mutations that cause tumours and metastases and provides ample opportunities to test cancer theories and to unravel unanticipated nexus between Notch and other cancer genes, or to discover unforeseen Notch's partners in cancer. These studies suggest that Notch's oncogenic capacity is brought about not simply by increasing signal strength but through partnerships, whereby oncogenes gain more by cooperating than acting individually, as in a ring 'organized crime'.
3. Targeting Notch signalling by the conserved miR-8/200 microRNA family in development and cancer cells. Notch signalling is crucial for the correct development and growth of numerous organs and tissues, and when subverted it can cause cancer. Loss of miR-8/200 microRNAs (miRNAs) is commonly observed in advanced tumours and correlates with their invasion and acquisition of stem-like properties. Here, we show that this miRNA family controls Notch signalling activation in Drosophila and human cells. In an overexpression screen, we identified the Drosophila miR-8 as a potent inhibitor of Notch-induced overgrowth and tumour **metastasis**. Gain and loss of mir-8 provoked developmental defects reminiscent of impaired Notch signalling and we demonstrated that miR-8 directly inhibits Notch ligand Serrate. Likewise, miR-200c and miR-141 directly inhibited JAGGED1, impeding proliferation of human metastatic prostate cancer cells. It has been suggested that JAGGED1 may also be important for metastases. Although in metastatic cancer cells, JAGGED1 modestly regulated ZEB1, the miR-200c's target in invasion, studies in Drosophila revealed that only concurrent overexpression of Notch and Zfh1/ZEB1 induced tumour metastases. Together, these data define a new way to attenuate or boost Notch signalling that may have clinical interest.

---

## Exn

- NCBI Gene
- GeneRIF
- Pubmed

| Search Term | Rank | Fields |
| --- | --- | --- |
| [Ee]xtracellular [Mm]atrix | 8 | PubMed(1) |

  
hide/show details for Exn

### [Ee]xtracellular [Mm]atrix

#### PubMed

1. A directed miniscreen for genes involved in the Drosophila anti-parasitoid immune response. Drosophila larvae react against eggs from the endoparasitoid wasp Leptopilina boulardi by surrounding them in a multilayered cellular capsule. Once a wasp egg is recognized as foreign, circulating macrophage-like cells, known as plasmatocytes, adhere to the invader. After spreading around the wasp egg, plasmatocytes form cellular junctions between the cells, effectively separating the egg from the hemocoel. Next, a second sub-type of circulating immunosurveillance cell (hemocyte), known as lamellocytes, adhere to either the wasp egg or more likely the plasmatocytes surrounding the egg. From these events, it is obvious that adhesion and cell shape change are an essential part of Drosophila's cellular immune response against parasitoid wasp eggs. To date, very few genes have been described as being necessary for a proper anti-parasitization response in Drosophila. With this in mind, we performed a directed genetic miniscreen to discover new genes required for this response. Many of the genes with an encapsulation defect have mammalian homologues involved in cellular adhesion, wound healing, and thrombosis, including **extracellular matrix** proteins, cellular adhesion molecules, and small GTPases.

---

## CalpB

- NCBI Gene
- GeneRIF
- Pubmed

| Search Term | Rank | Fields |
| --- | --- | --- |
| [Mm]igration | 6 | Process(1); PubMed(2); GeneRIFs(1) |

  
hide/show details for CalpB

### [Mm]igration

#### Process

1. border follicle cell **migration**

#### PubMed

1. Mutations in CAPN1 Cause Autosomal-Recessive Hereditary Spastic Paraplegia. Hereditary spastic paraplegia (HSP) is a genetically and clinically heterogeneous disease characterized by spasticity and weakness of the lower limbs with or without additional neurological symptoms. Although more than 70 genes and genetic loci have been implicated in HSP, many families remain genetically undiagnosed, suggesting that other genetic causes of HSP are still to be identified. HSP can be inherited in an autosomal-dominant, autosomal-recessive, or X-linked manner. In the current study, we performed whole-exome sequencing to analyze a total of nine affected individuals in three families with autosomal-recessive HSP. Rare homozygous and compound-heterozygous nonsense, missense, frameshift, and splice-site mutations in CAPN1 were identified in all affected individuals, and sequencing in additional family members confirmed the segregation of these mutations with the disease (spastic paraplegia 76 [SPG76]). CAPN1 encodes calpain 1, a protease that is widely present in the CNS. Calpain 1 is involved in synaptic plasticity, synaptic restructuring, and axon maturation and maintenance. Three models of calpain 1 deficiency were further studied. In Caenorhabditis elegans, loss of calpain 1 function resulted in neuronal and axonal dysfunction and degeneration. Similarly, loss-of-function of the Drosophila melanogaster ortholog calpain B caused locomotor defects and axonal anomalies. Knockdown of calpain 1a, a CAPN1 ortholog in Danio rerio, resulted in abnormal branchiomotor neuron **migration** and disorganized acetylated-tubulin axonal networks in the brain. The identification of mutations in CAPN1 in HSP expands our understanding of the disease causes and potential mechanisms.
2. CalpB modulates border cell **migration** in Drosophila egg chambers. Calpains are calcium regulated intracellular cysteine proteases implicated in a variety of physiological functions and pathological conditions. The Drosophila melanogaster genome contains only two genes, CalpA and CalpB coding for canonical, active calpain enzymes. The movement of the border cells in Drosophila egg chambers is a well characterized model of the eukaryotic cell **migration**. Using this genetically pliable model we can investigate the physiological role of calpains in cell motility.We demonstrate at the whole organism level that CalpB is implicated in cell **migration**, while the structurally related CalpA paralog can not fulfill the same function. The downregulation of the CalpB gene by mutations or RNA interference results in a delayed **migration** of the border cells in Drosophila egg chambers. This phenotype is significantly enhanced when the focal adhesion complex genes encoding for α-PS2 integrin ( if), β-PS integrin (mys) and talin (rhea) are silenced. The reduction of CalpB activity diminishes the release of integrins from the rear end of the border cells. The delaye**d migrati**on and the reduced integrin release phenotypes can be suppressed by expressing wild-type talin-head in the border cells but not talin-head(R367A), a mutant form which is not able to bind β-PS integrin. CalpB can cleave talin in vitro, and the two proteins coimmunoprecipitate from Drosophila extracts.The physiological function of CalpB in border cell motility has been demonstrated in vivo. The genetic interaction between the CalpB and the if, mys, as well as rhea genes, the involvement of active talin head-domains in the process, and the fact that CalpB and talin interact with each other collectively suggest that the limited proteolytic cleavage of talin is one of the possible mechanisms through which CalpB regulates ce**ll migrat**ion.

#### GeneRIFs

1. Study demonstrates that CalpB but not the structurally related CalpA has an important modulator function during in vivo cell **migration**.

---

## mys

- NCBI Gene
- GeneRIF
- Pubmed

| Search Term | Rank | Fields |
| --- | --- | --- |
| [Ff]ilopodia | 1 | PubMed(5) |
| [Mm]igration | 6 | Process(6); PubMed(69); GeneRIFs(2) |
| ECM | 7 | PubMed(43) |
| [Ee]xtracellular [Mm]atrix | 8 | Function(1); PubMed(85); GeneRIFs(1) |
| [Ii]nvasive | 10 | PubMed(4) |
| [Mm]etastasize | 11 | PubMed(1) |
| [Mm]etastasis | 12 | PubMed(6) |
| [Cc]ell [Ii]nvasion | 13 | PubMed(1) |

  
hide/show details for mys

### [Ff]ilopodia

#### PubMed

1. The leading edge during dorsal closure as a model for epithelial plasticity: Pak is required for recruitment of the Scribble complex and septate junction formation. Dorsal closure (DC) of the Drosophila embryo is a model for the study of wound healing and developmental epithelial fusions, and involves the sealing of a hole in the epidermis through the migration of the epidermal flanks over the tissue occupying the hole, the amnioserosa. During DC, the cells at the edge of the migrating epidermis extend Rac- and Cdc42-dependent actin-based lamellipodia and **filopodia** from their leading edge (LE), which exhibits a breakdown in apicobasal polarity as adhesions are severed with the neighbouring amnioserosa cells. Studies using mammalian cells have demonstrated that Scribble (Scrib), an important determinant of apicobasal polarity that functions in a protein complex, controls polarized cell migration through recruitment of Rac, Cdc42 and the serine/threonine kinase Pak, an effector for Rac and Cdc42, to the LE. We have used DC and the follicular epithelium to study the relationship between Pak and the Scrib complex at epithelial membranes undergoing changes in apicobasal polarity and adhesion during development. We propose that, during DC, the LE membrane undergoes an epithelial-to-mesenchymal-like transition to initiate epithelial sheet migration, followed by a mesenchymal-to-epithelial-like transition as the epithelial sheets meet up and restore cell-cell adhesion. This latter event requires integrin-localized Pak, which recruits the Scrib complex in septate junction formation. We conclude that there are bidirectional interactions between Pak and the Scrib complex modulating epithelial plasticity. Scrib can recruit Pak to the LE for polarized cell migration but, as migratory cells meet up, Pak can recruit the Scrib complex to restore apicobasal polarity and cell-cell adhesion.
2. Muscle niche-driven Insulin-Notch-Myc cascade reactivates dormant Adult Muscle Precursors in Drosophila. How stem cells specified during development keep their non-differentiated quiescent state, and how they are reactivated, remain poorly understood. Here, we applied a Drosophila model to follow in vivo behavior of adult muscle precursors (AMPs), the transient fruit fly muscle stem cells. We report that emerging AMPs send out thin **filopodia** that make contact with neighboring muscles. AMPs keep their **filopodia**-based association with muscles throughout their dormant state but also when they start to proliferate, suggesting that muscles could play a role in AMP reactivation. Indeed, our genetic analyses indicate that muscles send inductive dIlp6 signals that switch the Insulin pathway ON in closely associated AMPs. This leads to the activation of Notch, which regulates AMP proliferation via dMyc. Altogether, we report that Drosophila AMPs display homing behavior to muscle niche and that the niche-driven Insulin-Notch-dMyc cascade plays a key role in setting the activated state of AMPs.
3. Guidance of subcellular tubulogenesis by actin under the control of a synaptotagmin-like protein and Moesin. Apical membranes in many polarized epithelial cells show specialized morphological adaptations that fulfil distinct physiological functions. The air-transporting tubules of Drosophila tracheal terminal cells represent an extreme case of membrane specialization. Here we show that Bitesize (Btsz), a synaptotagmin-like protein family member, is needed for luminal membrane morphogenesis. Unlike in multicellular tubes and other epithelia, where it influences apical integrity by affecting adherens junctions, Btsz here acts at a distance from junctions. Localized at the luminal membrane through its tandem C2 domain, it recruits activated Moesin. Both proteins are needed for the integrity of the actin cytoskeleton at the luminal membrane, but not for other pools of F-actin in the cell, nor do actin-dependent processes at the outer membrane, such as **filopodia**l activity or membrane growth depend on Btsz. Btsz and Moesin guide luminal membrane morphogenesis through organizing actin and allowing the incorporation of membrane containing the apical determinant Crumbs.
4. Tissue-specific laminin expression facilitates integrin-dependent association of the embryonic wing disc with the trachea in Drosophila. The interaction of heterologous tissues involves cell adhesion mediated by the extracellular matrix and its receptor integrins. The Drosophila wing disc is an ectodermal invagination that contacts specific tracheal branches at the basolateral cell surface. We show that an alpha subunit of laminin, encoded by wing blister (wb), is essential for the establishment of the interaction between the wing and trachea. During embryogenesis, wing disc cells present Wb at their basolateral surface and extend posteriorly, expanding their association to more posteriorly located tracheal branches. These migratory processes are impaired in the absence of the trachea, Wb, or integrins. Time-lapse and transmission electron microscopy analyses suggest that Wb facilitates integrin-dependent contact over a large surface and controls the cellular behavior of the wing cells, including their exploratory **filopodia**l activity. Our data identify Wb laminin as an extracellular matrix ligand that is essential for integrin-dependent cellular migration in Drosophila.
5. Polarity proteins and Rho GTPases cooperate to spatially organise epithelial actin-based protrusions. Different actin-filament-based structures co-exist in many cells. Here, we characterise dynamic actin-based protrusions that form at distinct positions within columnar epithelial cells, focusing on basal **filopodia** and sheet-like intermediate-level protrusions that extend between surrounding epithelial cells. Using a genetic analysis, we found that the form and distribution of these actin-filament-based structures depends on the activities of apical polarity determinants, not on basal integrin signalling. Bazooka/Par3 acts upstream of the RacGEF Sif/TIAM1 to limit **filopodia** to the basal domain, whereas Cdc42, aPKC and Par6 are required for normal protrusion morphology and dynamics. Downstream of these polarity regulators, Sif/TIAM1, Rac, SCAR and Arp2/3 complexes catalyse actin nucleation to generate lamellipodia and **filopodia**, whose form depends on the level of Rac activation. Taken together, these data reveal a role for Baz/Par3 in the establishment of an intercellular gradient of Rac inhibition, from apical to basal, and an intimate association between different apically concentrated Par proteins and Rho-family GTPases in the regulation of the distribution and structure of the polarised epithelial actin cytoskeleton.

### [Mm]igration

#### Process

1. border follicle cell **migration**
2. cell **migration**
3. epithelial cell **migration**, open tracheal system
4. hemocyte **migration**
5. negative regulation of cell **migration**
6. substrate-dependent cell **migration**, cell extension

#### PubMed

1. Rac1 GTPase acts downstream of αPS1βPS integrin to control collectiv**e migrati**on and lumen size in the Drosophila salivary gland. During collectiv**e migrati**on of the Drosophila embryonic salivary gland, the distal gland cells mediate integrin-based contacts with surrounding tissues while proximal gland cells change shape and rearrange. Here, we show that αPS1βPS integrin controls salivary gl**and migra**tion through Rac1 GTPase which downregulates E-cadherin in proximal and distal gland cells, and promotes extension of actin-rich basal membrane protrusions in the distal cells. In embryos mutant for multiple edematous wings (mew), which encodes the αPS1 subunit of the αPS1βPS integrin heterodimer, or rac1 and rac2 GTPases, salivary gland cells failed to migrate, to downregulate E-cadherin and to extend basal membrane protrusions. Selective inhibition of Rac1 in just the proximal or distal gland cells demonstrate that proximal gland cells play an active role in the coll**ective mi**gration of the whole gland and that con**tinued mi**gration of the distal cells depends on the proximal cells. Loss of rac1rac2 also affected gland lumen length and width whereas, loss of mew affected lumen length only. Activation of rac1 in mew mutant embryos significantly rescued the **gland mi**gration, lumen length and basal membrane protrusion defects and partially rescued the E-cadherin defects. Independent of mew, Rac regulates cell shape change and rearrangement in the proximal gland, which is importa**nt for mi**gration and lumen width. Our studies shed novel insight into a Rac1-mediated link between integrin and cadherin adhesion proteins in vivo, control of lumen length and width and how activities of proximal and distal gland cells are coordinated to result in the coll**ective mi**gration of the entire salivary gland.
2. Integrins are required for cardioblast polarisation in Drosophila. The formation of a tubular organ, such as the heart, requires the communication of positional and polarity signals between migratory cells. Key to this process is the establishment of a new luminal domain on the cell surface, generally from the apical domain of a migratory cell. This domain will also acquire basal properties, as it will produce a luminal extracellular matrix. Integrin receptors are the primary means of cell adhesion and adhesion signaling with the extracellular matrix. Here we characterise the requirement of Integrins in a genetic model of vasculogenesis, the formation of the heart in Drosophila.As with vertebrates, the Drosophila heart arises from lateral mesoderm that migrates medially to meet their contralateral partners, to then assemble a midline vessel. During **migration**, Integrins are among the first proteins restricted to the presumptive luminal domain of cardioblasts. Integrins are required for normal levels of leading edge membrane motility. Apical accumulation of Integrins is enhanced by Robo, and reciprocally, apicalisation of luminal factors like Slit and Robo requires Integrin function. Integrins may provide a template for the formation of a lumen by stabilising lumen factors like Robo. Subsequent to **migration**, Integrin is required for normal cardioblast alignment and lumen formation. This phenotype is most readily modified by other mutations that affect adhesion, such as Talin and extracellular matrix ligands.Our findings reveal an instructive role for Integrins in communicating polarising information to cells during **migration**, and during transition to an epithelial tube structure.
3. The Bro1-domain-containing protein Myopic/HDPTP coordinates with Rab4 to regulate cell adhesion and **migration**. Protein tyrosine phosphatases (PTPs) are a group of tightly regulated enzymes that coordinate with protein tyrosine kinases to control protein phosphorylation during various cellular processes. Using genetic analysis in Drosophila non-transmembrane PTPs, we identified one role that Myopic (Mop), the Drosophila homolog of the human His domain phosphotyrosine phosphatase (HDPTP), plays in cell adhesion. Depletion of Mop results in aberrant integrin distribution and border cell dissociation during Drosophila oogenesis. Interestingly, Mop phosphatase activity is not required for its role in maintaining border cell cluster integrity. We further identified Rab4 GTPase as a Mop interactor in a yeast two-hybrid screen. Expression of the Rab4 dominant-negative mutant leads to border cell dissociation and suppression of Mop-induced wing-blade adhesion defects, suggesting a critical role of Rab4 in Mop-mediated signaling. In mammals, it has been shown that Rab4-dependent recycling of integrins is necessary for cell adhesion and **migration**. We found that human HDPTP regulates the spatial distribution of Rab4 and integrin trafficking. Depletion of HDPTP resulted in actin reorganization and increased cell motility. Together, our findings suggest an evolutionarily conserved function of HDPTP-Rab4 in the regulation of endocytic trafficking, cell adhesion and **migration**.
4. Posterior **migration** of the salivary gland requires an intact visceral mesoderm and integrin function. The final overall shape of an organ and its position within the developing embryo arise as a consequence of both its intrinsic properties and its interactions with surrounding tissues. Here, we focus on the role of directed cell **migration** in shaping and positioning the Drosophila salivary gland. We demonstrate that the salivary gland turns and migrates along the visceral mesoderm to become properly oriented with respect to the overall embryo. We show that salivary gland posterior **migration** requires the activities of genes that position the visceral mesoderm precursors, such as heartless, thickveins, and tinman, but does not require a differentiated visceral mesoderm. We also demonstrate a role for integrin function in salivary gland **migration**. Although the mutations affecting salivary gland motility and directional **migration** cause defects in the final positioning of the salivary gland, most do not affect the length or diameter of the salivary gland tube. These findings suggest that salivary tube dimensions may be an intrinsic property of salivary gland cells.
5. The integrin effector PINCH regulates JNK activity and epithelial **migration** in concert with Ras suppressor 1. Cell adhesion and **migration** are dynamic processes requiring the coordinated action of multiple signaling pathways, but the mechanisms underlying signal integration have remained elusive. Drosophila embryonic dorsal closure (DC) requires both integrin function and c-Jun amino-terminal kinase (JNK) signaling for opposed epithelial sheets to migrate, meet, and suture. Here, we show that PINCH, a protein required for integrin-dependent cell adhesion and actin-membrane anchorage, is present at the leading edge of these migrating epithelia and is required for DC. By analysis of native protein complexes, we identify RSU-1, a regulator of Ras signaling in mammalian cells, as a novel PINCH binding partner that contributes to PINCH stability. Mutation of the gene encoding RSU-1 results in wing blistering in Drosophila, demonstrating its role in integrin-dependent cell adhesion. Genetic interaction analyses reveal that both PINCH and RSU-1 antagonize JNK signaling during DC. Our results suggest that PINCH and RSU-1 contribute to the integration of JNK and integrin functions during Drosophila development.
6. Fak56 functions downstream of integrin alphaPS3betanu and suppresses MAPK activation in neuromuscular junction growth. Focal adhesion kinase (FAK) functions in cell **migration** and signaling through activation of the mitogen-activated protein kinase (MAPK) signaling cascade. Neuronal function of FAK has been suggested to control axonal branching; however, the underlying mechanism in this process is not clear.We have generated mutants for the Drosophila FAK gene, Fak56. Null Fak56 mutants display overgrowth of larval neuromuscular junctions (NMJs). Localization of phospho-FAK and rescue experiments suggest that Fak56 is required in presynapses to restrict NMJ growth. Genetic analyses imply that FAK mediates the signaling pathway of the integrin alphaPS3betanu heterodimer and functions redundantly with Src. At NMJs, Fak56 downregulates ERK activity, as shown by diphospho-ERK accumulation in Fak56 mutants, and suppression of Fak56 mutant NMJ phenotypes by reducing ERK activity.We conclude that Fak56 is required to restrict NMJ growth during NMJ development. Fak56 mediates an extracellular signal through the integrin receptor. Unlike its conventional role in activating MAPK/ERK, Fak56 suppresses ERK activation in this process. These results suggest that Fak56 mediates a specific neuronal signaling pathway distinct from that in other cellular processes.
7. A misexpression screen to identify regulators of Drosophila larval hemocyte development. In Drosophila, defense against foreign pathogens is mediated by an effective innate immune system, the cellular arm of which is composed of circulating hemocytes that engulf bacteria and encapsulate larger foreign particles. Three hemocyte types occur: plasmatocytes, crystal cells, and lamellocytes. The most abundant larval hemocyte type is the plasmatocyte, which is responsible for phagocytosis and is present either in circulation or in adherent sessile domains under the larval cuticle. The mechanisms controlling differentiation of plasmatocytes and their **migration** toward these sessile compartments are unclear. To address these questions we have conducted a misexpression screen using the plasmatocyte-expressed GAL4 driver Peroxidasin-GAL4 (Pxn-GAL4) and existing enhancer-promoter (EP) and EP yellow (EY) transposon libraries to systematically misexpress approximately 20% of Drosophila genes in larval hemocytes. The Pxn-GAL4 strain also contains a UAS-GFP reporter enabling hemocyte phenotypes to be visualized in the semitransparent larvae. Among 3412 insertions screened we uncovered 101 candidate hemocyte regulators. Some of these are known to control hemocyte development, but the majority either have no characterized function or are proteins of known function not previously implicated in hemocyte development. We have further analyzed three candidate genes for changes in hemocyte morphology, cell-cell adhesion properties, phagocytosis activity, and melanotic tumor formation.
8. CalpB modulates border cell **migration** in Drosophila egg chambers. Calpains are calcium regulated intracellular cysteine proteases implicated in a variety of physiological functions and pathological conditions. The Drosophila melanogaster genome contains only two genes, CalpA and CalpB coding for canonical, active calpain enzymes. The movement of the border cells in Drosophila egg chambers is a well characterized model of the eukaryotic cell **migration**. Using this genetically pliable model we can investigate the physiological role of calpains in cell motility.We demonstrate at the whole organism level that CalpB is implicated in cell **migration**, while the structurally related CalpA paralog can not fulfill the same function. The downregulation of the CalpB gene by mutations or RNA interference results in a delayed **migration** of the border cells in Drosophila egg chambers. This phenotype is significantly enhanced when the focal adhesion complex genes encoding for α-PS2 integrin ( if), β-PS integrin (mys) and talin (rhea) are silenced. The reduction of CalpB activity diminishes the release of integrins from the rear end of the border cells. The delaye**d migrati**on and the reduced integrin release phenotypes can be suppressed by expressing wild-type talin-head in the border cells but not talin-head(R367A), a mutant form which is not able to bind β-PS integrin. CalpB can cleave talin in vitro, and the two proteins coimmunoprecipitate from Drosophila extracts.The physiological function of CalpB in border cell motility has been demonstrated in vivo. The genetic interaction between the CalpB and the if, mys, as well as rhea genes, the involvement of active talin head-domains in the process, and the fact that CalpB and talin interact with each other collectively suggest that the limited proteolytic cleavage of talin is one of the possible mechanisms through which CalpB regulates ce**ll migrat**ion.
9. The cloning, genomic organization and expression of the focal contact protein paxillin in Drosophila. Paxillin is a focal adhesion scaffolding protein, which has been proposed to play a role in focal adhesion dynamics. We have isolated a cDNA clone of the Drosophila homologue of paxillin. Comparison of the Drosophila paxillin sequence with those of vertebrate paxillins shows strong conservation of the LIM domains and LD repeats. Using the Drosophila genomic sequence we have identified two partial curated transcripts and deduced the structure of the paxillin gene. No homologues of other members of the paxillin family such as HIC-5 or leupaxin are to be found in the Drosophila genome. Surprisingly paxillin mRNA is expressed in a restricted pattern during embryogenesis. In particular it is strongly expressed in cells and tissues undergoing cell shape changes or cell **migration**. Many of the sites of expression are also known to be sites of integrin function or FAK expression. The data support a role for paxillin as an adapter and/or signaling protein during developmental processes involving integrin-mediated adhesion.
10. Morphogenesis in the absence of integrins: mutation of both Drosophila beta subunits prevents midgut **migration**. Two integrin beta subunits are encoded in the Drosophila genome. The betaPS subunit is widely expressed and heterodimers containing this subunit are required for many developmental processes. The second betasubunit, betanu, is a divergent integrin expressed primarily in the midgut endoderm. To elucidate its function, we generated null mutations in the gene encoding betanu. We find that betanu is not required for viability or fertility, and overall the mutant flies are normal in appearance. However, we could observe betanu function in the absence of betaPS. Consistent with its expression, removal of betanu only enhanced the phenotype of betaPS in the developing midgut. In embryos lacking the zygotic contribution of betaPS, loss of betanu resulted in enhanced separation between the midgut and the surrounding visceral mesoderm. In the absence of both maternal and zygotic betaPS, a delay in midgut **migration** was observed, but removing betanu as well blocked **migration** completely. These results demonstrate that the second beta subunit can partially compensate for loss of betaPS integrins, and that integrins are essential for **migration** of the primordial midgut cells. The two beta subunits mediate midgut **migration** by distinct mechanisms: one that requires talin and one that does not. Other examples of developmental cell **migration**, such as that of the primordial germ cells, occurred normally in the absence of integrins. Having generated the tools to eliminate integrin function completely, we confirm that Drosophila integrins do not control proliferation as they do in mammals, and have identified alphaPS3 as a heterodimeric partner for betanu.
11. Drosophila blood cell chemotaxis. Drosophila melanogaster contains a population of blood cells called hemocytes that represent the functional equivalent of vertebrate macrophages. These cells undergo directed **migration**s to disperse during development and reach sites of tissue damage or altered self. These chemotactic behaviors are controlled by the expression of PDGF/Vegf-related ligands in developing embryos and local production of hydrogen peroxide at wounds. Recent work reveals that many molecules important in vertebrate cell motility, including integrins, formins, Ena/VASP proteins and the SCAR/WAVE complex, have a conserved function in these innate immune cells. The use of this model organism has elucidated how damage signals are activated by calcium signaling during inflammation and that the steroid hormone ecdysone activates immune competence at key developmental stages.
12. Slit coordinates cardiac morphogenesis in Drosophila. Slit is a secreted guidance cue that conveys repellent or attractive signals from target and guidepost cells. In Drosophila, responsive cells express one or more of three Robo receptors. The cardial cells of the developing heart express both Slit and Robo2. This is the first report of coincident expression of a Robo and its ligand. In slit mutants, cardial cell alignment, polarization and uniform **migration** are disrupted. The heart phenotype of robo2 mutants is similar, with fewer **migration** defects. In the guidance of neuronal growth cones in Drosophila, there is a phenotypic interaction between slit and robo heterozygotes, and also with genes required for Robo signaling. In contrast, in the heart, slit has little or no phenotypic interaction with Robo-related genes, including Robo2, Nck2, and Disabled. However, there is a strong phenotypic interaction with Integrin genes and their ligands, including Laminin and Collagen, and intracellular messengers, including Talin and ILK. This indicates that Slit participates in adhesion or adhesion signaling during heart development.
13. Drosophila importin-7 functions upstream of the Elmo signaling module to mediate the formation and stability of muscle attachments. Establishment and maintenance of stable muscle attachments is essential for coordinated body movement. Studies in Drosophila have pioneered a molecular understanding of the morphological events in the conserved process of muscle attachment formation, including myofiber **migration**, muscle-tendon signaling, and stable junctional adhesion between muscle cells and their corresponding target insertion sites. In both Drosophila and vertebrate models, integrin complexes play a key role in the biogenesis and stability of muscle attachments through the interactions of integrins with extracellular matrix (ECM) ligands. We show that Drosophila importin-7 (Dim7) is an upstream regulator of the conserved Elmo-Mbc→Rac signaling pathway in the formation of embryonic muscle attachment sites (MASs). Dim7 is encoded by the moleskin (msk) locus and was identified as an Elmo-interacting protein. Both Dim7 and Elmo localize to the ends of myofibers coincident with the timing of muscle-tendon attachment in late myogenesis. Phenotypic analysis of elmo mutants reveal muscle attachment defects similar to those previously described for integrin mutants. Furthermore, Elmo and Dim7 interact both biochemically and genetically in the developing musculature. The muscle detachment phenotype resulting from mutations in the msk locus can be rescued by components in the Elmo signaling pathway, including the Elmo-Mbc complex, an activated Elmo variant, or a constitutively active form of Rac. In larval muscles, the localization of Dim7 and activated Elmo to the sites of muscle attachment is attenuated upon RNAi knockdown of integrin heterodimer complex components. Our results show that integrins function as upstream signals to mediate Dim7-Elmo enrichment to the MASs.
14. Branch-specific **migration** cues in the Drosophila tracheal system. The Drosophila tracheal system forms by highly stereotyped **migration** of the tracheal cells, generating an elaborate network of interconnected tubes supplying oxygen to all tissues. A major guiding system in the **migration** process of all branches is the dynamic and localized expression of Branchless (Bnl), an FGF-like molecule. Bnl triggers the activation of the FGF receptor Breathless (Btl) locally in all tracheal cells. Is this the only guiding cue, or do additional local signals provide distinct inputs to each branch? Several recent papers identify such local signals, relying on contacts with specific cell types and with the matrix encountered by the migrating tracheal branches. In particular, the paper by Boube et al(1) demonstrates a role for PS integrins in promoting **migration** of a specific tracheal branch.
15. The proprioceptive and contractile systems in Drosophila are both patterned by the EGR family transcription factor Stripe. Coordinated locomotion of Drosophila larvae depends on accurate patterning and stable attachment to the cuticle of both muscles and proprioceptors (chordotonal organs). Unlike muscle spindles in mammals, the fly chordotonal organs are not embedded in the body-wall muscles. Yet, the contractile system (muscles and tendons) and the chordotonal organs constitute two parts of a single functional unit that controls locomotion, and thus must be patterned in full coordination. It is not known how such coordination is achieved. Here we show that the positioning and differentiation of the migrating chordotonal organs are instructed by Stripe, the same transcription factor that promotes tendon cell specification and differentiation and is required for normal patterning of the contractile system. Our data demonstrate that although chordotonal organs are patterned in a Stripe-dependent mechanism similarly to muscles, this mechanism is independent of Stripe activity in tendon cells. Thus, the two parts of the locomotive system use similar but independent patterning mechanisms that converge to form a functional unit. Stripe plays at least a dual role in chordotonal development. It is required within the ligament cells for terminal differentiation and proper **migration**, without which no induction of ligament attachment cells takes place. Stripe's activity is then necessary within the recruited cells for their differentiation as attachment cells. Similarly to the biphasic differentiation program of tendons, terminal differentiation of chordotonal attachment cells is associated with sequential activation of the two Stripe isoforms-Stripe B and Stripe A.
16. [Dorsal closure in Drosophila. A genetic model for wound healing?]. Dorsal closure (DC) is a morphogenetic movement that establishes the dorsal ectoderm of the drosophila embryo. During this process, the two lateral epithelia stretch toward the dorsal midline, the suture line of the two leading edges. Cell **migration** during DC relies both on cell shape change controlled by the activity of the JNK pathway in the leading edge cells and modification of cell adhesiveness, probably dependent upon activation of the Dpp (TGF-beta) pathway. Coupling of the JNK and TGF-beta pathways is essential. The sequence of the cellular and molecular events of DC highlights interesting common features with wound healing in vertebrates. Like DC, wound healing relies on the **migration** of epithelia bordered by leading edges controlling the direction and speed of the movement. This review summarizes recent data concerning the control of epithelial morphogenesis during DC and the bases of wound healing. The molecular and cellular events that underlie these two analogous migratory processes are detailed, discussed and compared. We suggest that DC is a good genetic model for wound healing studying.
17. Singling out Drosophila tendon cells: a dialogue between two distinct cell types. The precise match between somatic muscles and their epidermal attachment cells is achieved through a continuous dialogue between these two cell types. Whereas tendon cells direct myotube **migration** and final patterning, the muscles are essential for the maintenance of the fate of tendon cells. The Drosophila neuregulin-like ligand, Vein, and its receptor, the epidermal growth factor receptor (Egfr), are critical components in the inductive signaling process that takes place between muscles and tendon cells. Additional gene products that relay the Vein-Egfr effect in Drosophila are conserved in the vertebrate neuregulin-mediated cascade. This review describes genetic and molecular aspects of the muscle-tendon inductive processes in Drosophila, and compares them with the relevant mechanisms in the vertebrate embryo.
18. Polarization of the epithelial layer and apical localization of integrins are required for engulfment of apoptotic cells in the Drosophila ovary. Inefficient clearance of dead cells or debris by epithelial cells can lead to or exacerbate debilitating conditions such as retinitis pigmentosa, macular degeneration, chronic obstructive pulmonary disease and asthma. Despite the importance of engulfment by epithelial cells, little is known about the molecular changes that are required within these cells. The misregulation of integrins has previously been associated with disease states, suggesting that a better understanding of the regulation of receptor trafficking could be key to treating diseases caused by defects in phagocytosis. Here, we demonstrate that the integrin heterodimer αPS3/βPS becomes apically enriched and is required for engulfment by the epithelial follicle cells of the Drosophila ovary. We found that integrin heterodimer localization and function is largely directed by the α-subunit. Moreover, proper cell polarity promotes asymmetric integrin enrichment, suggesting that αPS3/βPS trafficking occurs in a polarized fashion. We show that several genes previously known for their roles in trafficking and **cell migr**ation are also required for engulfment. Moreover, as in mammals, the same α-integrin subunit is required by professional and non-professional phagocytes and migrating cells in Drosophila. Our findings suggest that migrating and engulfing cells use common machinery, and demonstrate a crucial role for integrin function and polarized trafficking of integrin subunits during engulfment. This study also establishes the epithelial follicle cells of the Drosophila ovary as a powerful model for understanding the molecular changes required for engulfment by a polarized epithelium.
19. Dcas supports cell polarization and cell-cell adhesion complexes in development. Mammalian Cas proteins regulate cell **migration**, division and survival, and are often deregulated in cancer. However, the presence of four paralogous Cas family members in mammals (BCAR1/p130Cas, EFS/Sin1, NEDD9/HEF1/Cas-L, and CASS4/HEPL) has limited their analysis in development. We deleted the single Drosophila Cas gene, Dcas, to probe the developmental function of Dcas. Loss of Dcas had limited effect on embryonal development. However, we found that Dcas is an important modulator of the severity of the developmental phenotypes of mutations affecting integrins (If and mew) and their downstream effectors Fak56D or Src42A. Strikingly, embryonic lethal Fak56D-Dcas double mutant embryos had extensive cell polarity defects, including mislocalization and reduced expression of E-cadherin. Further genetic analysis established that loss of Dcas modified the embryonal lethal phenotypes of embryos with mutations in E-cadherin (Shg) or its signaling partners p120- and beta-catenin (Arm). These results support an important role for Cas proteins in cell-cell adhesion signaling in development.
20. Lasp anchors the Drosophila male stem cell niche and mediates spermatid individualization. Lasp family proteins contain an amino-terminal LIM domain, two actin-binding nebulin repeats and a carboxyl-terminal SH3 domain. Vertebrate Lasp-1 localizes to focal adhesions and the leading edge of migrating cells, and is required for cell **migration**. To assess the in vivo function of Lasp, we generated a null mutant in Drosophila Lasp. Lasp(1) is homozygous viable, but male sterile. In Lasp mutants the stem cell niche is no longer anchored to the apical tip of the testis, and actin cone **migration** is perturbed resulting in improper spermatid individualization. Hub cell mislocalization can by phenocopied by expressing Lasp or betaPS integrin RNAi transgenes in somatic cells, and Lasp genetically interacts with betaPS integrin, demonstrating that Lasp functions together with integrins in hub cells to anchor the stem cell niche. Finally, we show that the stem cell niche is maintained even if it is not properly localized.
21. Identifying loci required for follicular patterning using directed mosaics. We have developed a 'directed mosaic' system in Drosophila by using the GAL4 system to control the expression of the yeast recombinase, FLP, in a spatial and temporal fashion. By directing FLP expression, we show that it is possible to efficiently and specifically target loss-of-function studies for vital loci to the developmental pathway of interest. A simple F1 adult phenotypic screen demonstrated that most adult tissues can be analyzed with this approach. Using GAL4 lines expressed during oogenesis, we have refined the system to examine the roles of vital loci in the development of the follicular epithelium. We have identified essential genes involved in egg chamber organization, cell **migration** and cell shape. Further, we have used this technique to gain insights into the role of the Drosophila EGF receptor pathway in establishing the egg axes. Finally, using different UAS-FLP, GAL4 and existing FRT lines, we have built stocks that permit the analysis of approximately 95% of the genome in follicular mosaics.
22. Receptor guanylyl cyclase Gyc76C is required for invagination, collective **migration** and lumen shape in the Drosophila embryonic salivary gland. The Drosophila embryonic salivary gland is formed by the invagination and collective **migration** of cells. Here, we report on a novel developmental role for receptor-type guanylyl cyclase at 76C, Gyc76C, in morphogenesis of the salivary gland. We demonstrate that Gyc76C and downstream cGMP-dependent protein kinase 1 (DG1) function in the gland and surrounding mesoderm to control invagination, collective **migration** and lumen shape. Loss of gyc76C resulted in glands that failed to invaginate, complete posterior **migration** and had branched lumens. Salivary gland **migration** defects of gyc76C mutant embryos were rescued by expression of wild-type gyc76C specifically in the gland or surrounding mesoderm, whereas invagination defects were rescued primarily by expression in the gland. In migrating salivary glands of gyc76C mutant embryos, integrin subunits localized normally to gland-mesoderm contact sites but talin localization in the surrounding circular visceral mesoderm and fat body was altered. The extracellular matrix protein, laminin, also failed to accumulate around the migrating salivary gland of gyc76C mutant embryos, and gyc76C and laminin genetically interacted in gland **migration**. Our studies suggest that gyc76C controls salivary gland invagination, collective **migration** and lumen shape, in part by regulating the localization of talin and the laminin matrix.
23. Integrins are required for cardioblast polarisation in Drosophila. The formation of a tubular organ, such as the heart, requires the communication of positional and polarity signals between migratory cells. Key to this process is the establishment of a new luminal domain on the cell surface, generally from the apical domain of a migratory cell. This domain will also acquire basal properties, as it will produce a luminal extracellular matrix. Integrin receptors are the primary means of cell adhesion and adhesion signaling with the extracellular matrix. Here we characterise the requirement of Integrins in a genetic model of vasculogenesis, the formation of the heart in Drosophila.As with vertebrates, the Drosophila heart arises from lateral mesoderm that migrates medially to meet their contralateral partners, to then assemble a midline vessel. During **migration**, Integrins are among the first proteins restricted to the presumptive luminal domain of cardioblasts. Integrins are required for normal levels of leading edge membrane motility. Apical accumulation of Integrins is enhanced by Robo, and reciprocally, apicalisation of luminal factors like Slit and Robo requires Integrin function. Integrins may provide a template for the formation of a lumen by stabilising lumen factors like Robo. Subsequent to **migration**, Integrin is required for normal cardioblast alignment and lumen formation. This phenotype is most readily modified by other mutations that affect adhesion, such as Talin and extracellular matrix ligands.Our findings reveal an instructive role for Integrins in communicating polarising information to cells during **migration**, and during transition to an epithelial tube structure.
24. Integrins in development: moving on, responding to, and sticking to the extracellular matrix. Integrins are cell surface receptors of the extracellular matrix present in all animals. Genetic analysis in worms, flies, and vertebrates has revealed integrin involvement in key developmental processes, and we focus here on examples of integrin functions that are comparable across these model organisms. Integrins contribute to cell movement by providing traction to migrating cells, through assembly of extracellular matrices that can serve as tracks for **migration**, and by transmitting guidance signals that direct cells or cell processes to their targets. Integrins also participate in signaling events that govern tissue differentiation and organogenesis. Finally, adhesion by integrin-mediated junctions allows tissues to withstand mechanical load and is essential for tissue integrity.
25. **Migration** of the Drosophila primordial midgut cells requires coordination of diverse PS integrin functions. Cell **migration** during embryogenesis involves two populations of cells: the migrating cells and the underlying cells that provide the substratum for **migration**. The formation of the Drosophila larval midgut involves the **migration** of the primordial midgut cells along a visceral mesoderm substratum. We show that integrin adhesion receptors are required in both populations of cells for normal rates of **migration**. In the absence of the PS integrins, the visceral mesoderm is disorganised, the primordial midgut cells do not display their normal motile appearance and their **migration** is delayed by 2 hours. Removing PS integrin function from the visceral mesoderm alone results in visceral mesoderm disorganization, but only causes a modest delay in **migration** and does not affect the appearance of the migrating cells. Removing PS integrin function from the migrating cells causes as severe a delay in **migration** as the complete loss of PS integrin function. The functions of PS1 and PS2 are specific in the two tissues, endoderm and mesoderm, since they cannot substitute for each other. In addition there is a partial redundancy in the function of the two PS integrins expressed in the endoderm, PS1 (alphaPS1betaPS) and PS3 (alphaPS3betaPS), since loss of just one alpha subunit in the midgut results in either a modest delay (alphaPS1) or no effect (alphaPS3). We have also examined the roles of small GTPases in promoting **migration** of the primordial midgut cells. We find that dominant negative (N17) versions of Rac and Cdc42 cause a very similar defect in **migration** as loss of integrins, while those of Rho and Ras have no effect. Thus integrins are involved in mediating **migration** by creating an optimal substratum for adhesion, adhering to that substratum and possibly by activating Rac and Cdc42.
26. Loss of focal adhesions in glia disrupts both glial and photoreceptor axon **migration** in the Drosophila visual system. Many aspects of glial development are regulated by extracellular signals, including those from the extracellular matrix (ECM). Signals from the ECM are received by cell surface receptors, including the integrin family. Previously, we have shown that Drosophila integrins form adhesion complexes with Integrin-linked kinase and talin in the peripheral nerve glia and have conserved roles in glial sheath formation. However, integrin function in other aspects of glial development is unclear. The Drosophila eye imaginal disc (ED) and optic stalk (OS) complex is an excellent model with which to study glial **migration**, differentiation and glia-neuron interactions. We studied the roles of the integrin complexes in these glial developmental processes during OS/eye development. The common beta subunit βPS and two alpha subunits, αPS2 and αPS3, are located in puncta at both glia-glia and glia-ECM interfaces. Depletion of βPS integrin and talin by RNAi impaired **the migra**tion and distribution of glia within the OS resulting in morphological defects. Reduction of integrin or talin in the glia also disrupted photoreceptor axon outgrowth leading to axon stalling in the OS and ED. The neuronal defects were correlated with a disruption of the carpet glia tube paired with invasion of glia into the core of the OS and the formation of a glial cap. Our results suggest that integrin-mediated extracellular signals are important for multiple aspects of glial development and non-autonomously affect axo**nal migra**tion during Drosophila eye development.
27. Analysis of integrin turnover in fly myotendinous junctions. Transient (short-term) cell adhesion underlies dynamic processes such as cell **migration**, whereas stable (long-term) cell adhesion maintains tissue architecture. Ongoing adhesion complex turnover is essential for transient cell adhesion, but it is not known whether turnover is also required for maintenance of long-term adhesion. We used fluorescence recovery after photobleaching to analyze the dynamics of an integrin adhesion complex (IAC) in a model of long-term cell-ECM adhesion, myotendinous junctions (MTJs), in fly embryos and larvae. We found that the IAC undergoes turnover in MTJs and that this process is mediated by clathrin-dependent endocytosis. Moreover, the small GTPase Rab5 can regulate the proportion of IAC components that undergo turnover. Also, altering Rab5 activity weakened MTJs, resulting in muscle defects. In addition, growth of MTJs was concomitant with a decrease in the proportion of IAC components undergoing turnover. We propose that IAC turnover is tightly regulated in long-term cell-ECM adhesions to allow normal tissue growth and maintenance.
28. A secreted MMP is required for reepithelialization during wound healing. Matrix metalloproteinases (MMPs) are extracellular proteases highly expressed at wound sites. However, the precise function of MMPs during reepithelialization in vivo has been elusive in mammalian models because of the high level of redundancy among the 24 mammalian MMPs. For this reason we used Drosophila melanogaster, whose genome encodes only two MMPs-one secreted type (Mmp1) and one membrane-anchored type (Mmp2)-to study the function and regulation of the secreted class of MMPs in vivo. In the absence of redundancy, we found that the Drosophila secreted MMP, Mmp1, is required in the epidermis to facilitate reepithelialization by remodeling the basement membrane, promoting cell elongation and actin cytoskeletal reorganization, and activating extracellular signal-regulated kinase signaling. In addition, we report that the jun N-terminal kinase (JNK) pathway upregulates Mmp1 expression after wounding, but that Mmp1 is expressed independent of the JNK pathway in unwounded epidermis. When the JNK pathway is ectopically activated to overexpress Mmp1, the rate of healing is accelerated in an Mmp1-dependent manner. A primary function of Mmp1, under the control of the JNK pathway, is to promote basement membrane repair, which in turn may permit cell **migration** and the restoration of a continuous tissue.
29. Signaling pathways directing the movement and fusion of epithelial sheets: lessons from dorsal closure in Drosophila. Wound healing in embryos and various developmental events in metazoans require the spreading and fusion of epithelial sheets. The complex signaling pathways regulating these processes are being pieced together through genetic, cell biological, and biochemical approaches. At present, dorsal closure of the Drosophila embryo is the best-characterized example of epithelial sheet movement. Dorsal closure involves **migration** of the lateral epidermal flanks to close a hole in the dorsal epidermis occupied by an epithelium called the amnioserosa. Detailed genetic studies have revealed a network of interacting signaling molecules regulating this process. At the center of this network is a Jun N-terminal kinase cascade acting at the leading edge of the migrating epidermis that triggers signaling by the TGF-beta superfamily member Decapentaplegic and which interacts with the Wingless pathway. These signaling modules regulate the cytoskeletal reorganization and cell shape change necessary to drive dorsal closure. Activation of this network requires signals from the amnioserosa and input from a variety of proteins at cell-cell junctions. The Rho family of small GTPases is also instrumental, both in activation of signaling and regulation of the cytoskeleton. Many of the proteins regulating dorsal closure have been implicated in epithelial movement in other organisms, and dorsal closure has emerged as an ideal model system for the study of the **migration** and fusion of epithelial sheets.
30. Focal adhesion kinase is not required for integrin function or viability in Drosophila. The mammalian focal adhesion kinase (FAK) family of non-receptor protein-tyrosine kinases has been implicated in controlling a multitude of cellular responses to the engagement of cell-surface integrins and G-protein-coupled receptors. The high level of sequence conservation between the mammalian proteins and the Drosophila homologue of FAK, Fak56, suggested that it would have similar functions. However, we show here that Drosophila Fak56 is not essential for integrin functions in adhesion, **migration** or signaling in vivo. Furthermore, animals lacking Fak56 are viable and fertile, demonstrating that Fak56 is not essential for other developmental or physiological functions. Despite this, overexpressed Fak56 is a potent inhibitor of integrins binding to the extracellular matrix, suggesting that Fak56 may play a subtle role in the negative regulation of integrin adhesion.
31. A role for the mesoderm in endodermal **migration** and morphogenesis in Drosophila. The endodermal midgut arises from two primordia, the anterior midgut (AMG) primordium and the posterior midgut (PMG) primordium, which are separated by almost the entire length of the Drosophila embryo. To form the midgut, these two parts have to extend towards each other and to fuse laterally on both sides of the yolk. Shortly before and during that movement, AMG and PMG are arranged as mesenchymal cell masses, but later the midgut cells form an epithelium. We show that these two aspects of midgut development, **migration** of AMG and PMG and transition to an epithelium, depend on the mesoderm. The extension of the midgut primordia is achieved by cell **migration** along the visceral mesoderm which forms a continuous layer of cells within the germ band. In mutant embryos lacking the entire mesoderm or failing to differentiate the visceral mesoderm, AMG and PMG are formed but do not migrate properly. In addition, they fail to form an epithelium and instead either remain as compact cell masses anterior and posterior to the yolk (in twist and snail mutant embryos) or only occasionally wrap around the yolk before embryogenesis is completed (in tinman-deficient embryos). We conclude that the visceral mesoderm serves as a substratum for the migrating endodermal cells and that the contact between visceral mesoderm and endoderm is required for the latter to become an epithelium.
32. Rac1 GTPase acts downstream of αPS1βPS integrin to control collectiv**e migrati**on and lumen size in the Drosophila salivary gland. During collectiv**e migrati**on of the Drosophila embryonic salivary gland, the distal gland cells mediate integrin-based contacts with surrounding tissues while proximal gland cells change shape and rearrange. Here, we show that αPS1βPS integrin controls salivary gl**and migra**tion through Rac1 GTPase which downregulates E-cadherin in proximal and distal gland cells, and promotes extension of actin-rich basal membrane protrusions in the distal cells. In embryos mutant for multiple edematous wings (mew), which encodes the αPS1 subunit of the αPS1βPS integrin heterodimer, or rac1 and rac2 GTPases, salivary gland cells failed to migrate, to downregulate E-cadherin and to extend basal membrane protrusions. Selective inhibition of Rac1 in just the proximal or distal gland cells demonstrate that proximal gland cells play an active role in the coll**ective mi**gration of the whole gland and that con**tinued mi**gration of the distal cells depends on the proximal cells. Loss of rac1rac2 also affected gland lumen length and width whereas, loss of mew affected lumen length only. Activation of rac1 in mew mutant embryos significantly rescued the **gland mi**gration, lumen length and basal membrane protrusion defects and partially rescued the E-cadherin defects. Independent of mew, Rac regulates cell shape change and rearrangement in the proximal gland, which is importa**nt for mi**gration and lumen width. Our studies shed novel insight into a Rac1-mediated link between integrin and cadherin adhesion proteins in vivo, control of lumen length and width and how activities of proximal and distal gland cells are coordinated to result in the coll**ective mi**gration of the entire salivary gland.
33. Drosophila PS2 and PS3 integrins play distinct roles in retinal photoreceptors-glia interactions. Cellular **migration** and differentiation are important developmental processes that require dynamic cellular adhesion. Integrins are heterodimeric transmembrane receptors that play key roles in adhesion plasticity. Here, we explore the developing visual system of Drosophila to study the roles of integrin heterodimers in glia development. Our data show that αPS2 is essential for retinal glia **migratio**n from the brain into the eye disc and that glial cells have a role in the maintenance of the fenestrated membrane (Laminin-rich ECM layer) in the disc. Interestingly, the absence of glial cells in the eye disc did not affect the targeting of retinal axons to the optic stalk. In contrast, αPS3 is not required for retinal gli**a migrati**on, but together with Talin, it functions in glial cells to allow photoreceptor axons to target the optic stalk. Thus, we present evidence that αPS2 and αPS3 integrin have different and specific functions in the development of retinal glia.
34. Drosophila integrin adhesion complexes are essential for hemocyte **migration** in vivo. Cell **migration** is an important biological process which has been intensively studied in the past decades. Numerous techniques, mainly involving two-dimensional cell culture systems, have contributed to dissecting the essential mechanisms underlying this process. However, the development of three-dimensional cell culture and in vivo systems has shown some differences with what was previously believed to be well-established cell **migration** mechanisms, suggesting that two-dimensional cell motility would be a poor predictor of in vivo behaviour. Drosophila is a widely recognized model organism to study developmental and homeostatic processes and has been widely used to investigate cell **migration**. Here, we focus on the **migration** of small groups of pupal hemocytes that accumulate during larval stages in dorsal patches. We show that integrins, and other known nascent adhesion-related proteins such as Rhea and Fermitin 1, are crucial for this process and that their depletion does not affect polarization in response to environmental cues. We also present evidence for the importance of adhesion maturation-related proteins in hemocyte **migration**, namely Zyxin. Zyxin depletion in hemocytes leads to a significant increase of cell speed without affecting their response to a chemotactic cue. This is the first report of a systematic analysis using Drosophila melanogaster hemocytes to study adhesion-related proteins and their function in cell **migration** in vivo. Our data point to mechanisms of cell **migration** similar to those described in three-dimensional in vitro systems and other in vivo model organisms.
35. JNK signaling controls border cell cluster integrity and collective cell **migration**. Collective cell movement is a mechanism for invasion identified in many developmental events. Examples include the movement of lateral-line neurons in Zebrafish, cells in the inner blastocyst, and metastasis of epithelial tumors [1]. One key model to study collective **migration** is the movement of border cell clusters in Drosophila. Drosophila egg chambers contain 15 nurse cells and a single oocyte surrounded by somatic follicle cells. At their anterior end, polar cells recruit several neighboring follicle cells to form the border cell cluster [2]. By stage 9, and over 6 hr, border cells migrate as a cohort between nurse cells toward the oocyte. The specification and directionality of border cell movement are regulated by hormonal and signaling mechanisms [3]. However, how border cells are held together while they migrate is not known. Here, we show that a negative-feedback loop controlling JNK activity regulates border cell cluster integrity. JNK signaling modulates contacts between border cells and between border cells and substratum to sustain collective **migration** by regulating several effectors including the polarity factor Bazooka and the cytoskeletal adaptor D-Paxillin. We anticipate a role for the JNK pathway in controlling collective cell movements in other morphogenetic and clinical models.
36. Hox transcription factors: modulators of cell-cell and cell-extracellular matrix adhesion. Hox genes encode homeodomain-containing transcription factors that determine cell and tissue identities in the embryo during development. Hox genes are also expressed in various adult tissues and cancer cells. In Drosophila, expression of cell adhesion molecules, cadherins and integrins, is regulated by Hox proteins operating in hierarchical molecular pathways and plays a crucial role in segment-specific organogenesis. A number of studies using mammalian cultured cells have revealed that cell adhesion molecules responsible for cell-cell and cell-extracellular matrix interactions are downstream targets of Hox proteins. However, whether Hox transcription factors regulate expression of cell adhesion molecules during vertebrate development is still not fully understood. In this review, the potential roles Hox proteins play in cell adhesion and **migration** during vertebrate body patterning are discussed.
37. Branching morphogenesis of the Drosophila tracheal system. Many organs including the mammalian lung and vascular system consist of branched tubular networks that transport essential gases or fluids, but the genetic programs that control the development of these complex three-dimensional structures are not well understood. The Drosophila melanogaster tracheal (respiratory) system is a network of interconnected epithelial tubes that transports oxygen and other gases in the body and provides a paradigm of branching morphogenesis. It develops by sequential sprouting of primary, secondary, and terminal branches from an epithelial sac of approximately 80 cells in each body segment of the embryo. Mapping of the cell movements and shape changes during the sprouting process has revealed that distinct mechanisms of epithelial **migration** and tube formation are used at each stage of branching. Genetic dissection of the process has identified a general program in which a fibroblast growth factor (FGF) and fibroblast growth factor receptor (FGFR) are used repeatedly to control branch budding and outgrowth. At each stage of branching, the mechanisms controlling FGF expression and the downstream signal transduction pathway change, altering the pattern and structure of the branches that form. During terminal branching, FGF expression is regulated by hypoxia, ensuring that tracheal structure matches cellular oxygen need. A branch diversification program operates in parallel to the general budding program: Regional signals locally modify the general program, conferring specific structural features and other properties on individual branches, such as their substrate outgrowth preferences, differences in tube size and shape, and the ability to fuse to other branches to interconnect the network.
38. Focal adhesions: what's new inside. The cytoplasmic side of focal adhesions is comprised of large molecular complexes that link transmembrane receptors, such as integrins, to the actin cytoskeleton and mediate signals modulating cell attachment, **migration**, proliferation, differentiation, and gene expression. These complexes are heterogeneous and dynamic structures that are apparent targets of regulatory signals that control the function of focal adhesions. Recent studies using genetic approaches in invertebrate and vertebrate systems have begun to reveal the structure and function of these complexes in vivo.
39. PS integrins and laminins: key regulators of cell **migration** during Drosophila embryogenesis. During embryonic development, there are numerous cases where organ or tissue formation depends upon the **migration** of primordial cells. In the Drosophila embryo, the visceral mesoderm (vm) acts as a substrate for the **migration** of several cell populations of epithelial origin, including the endoderm, the trachea and the salivary glands. These migratory processes require both integrins and laminins. The current model is that αPS1βPS (PS1) and/or αPS3βPS (PS3) integrins are required in migrating cells, whereas αPS2βPS (PS2) integrin is required in the vm, where it performs an as yet unidentified function. Here, we show that PS1 integrins are also required fo**r the mig**ration over the vm of cells of mesodermal origin, the caudal visceral mesoderm (CVM). These results support a model in which PS1 might have evolved to acquire the migratory function of integrins, irrespective of the origin of the tissue. This integrin function is highly specific and its specificity resides mainly in the extracellular domain. In addition, we have identified the Laminin α1,2 trimer, as the key extracellular matrix (ECM) component regulati**ng CVM mi**gration. Furthermore, we show that, as it is the case in vertebrates, integrins, and specifically PS2, contributes to CVM movement by participating in the correct assembly of the ECM that serves as trac**ks for mi**gration.
40. Specific tracheal **migration** is mediated by complementary expression of cell surface proteins. **Migration** of the Drosophila tracheal cells relies on cues provided by nearby cells; however, little is known about how these signals specify a migratory path. Here we investigate the role of cell surface proteins in the definition of such a pathway. We have found that the PS1 integrin is required in the tracheal cells of the visceral branch, whereas the PS2 integrin is required in the visceral mesoderm; both integrins are necessary for the spreading of the visceral branch over its substratum. This is the first identification of a cell surface molecule with expression restricted to a subset of tracheal cells that all migrate in a given direction. We have also found that expression of PS1 in the visceral branch is regulated by the genes that direct tracheal cell **migration**, showing that integrin expression is part of the cell-fate program that they specify. These results support a model in which signal transduction determines the tracheal migratory pathways by regulating the expression of cell surface proteins, which in turn interact with surface molecules on the surrounding cell population.
41. Requirement for Par-6 and Bazooka in Drosophila border cell **migration**. Polarized epithelial cells convert into migratory invasive cells during a number of developmental processes, as well as when tumors metastasize. Much has been learned recently concerning the molecules and mechanisms that are responsible for generating and maintaining epithelial cell polarity. However, less is known about what becomes of epithelial polarity proteins when various cell types become migratory and invasive. Here, we report the localization of several apical epithelial proteins, Par-6, Par-3/Bazooka and aPKC, during border cell **migration** in the Drosophila ovary. All of these proteins remained asymmetrically distributed throughout **migration**. Moreover, depletion of either Par-6 or Par-3/Bazooka by RNAi resulted in disorganization of the border cell cluster and impaired **migration**. The distributions of several transmembrane proteins required for **migration** were abnormal following Par-6 or Par-3/Bazooka downregulation, possibly accounting for the **migration** defects. Taken together, these results indicate that cells need not lose apical/basal polarity in order to invade neighboring tissues and in some cases even require such polarity for proper motility.
42. Cellular and molecular mechanisms of single and collective cell **migration**s in Drosophila: themes and variations. The process of cell **migration** is essential throughout life, driving embryonic morphogenesis and ensuring homeostasis in adults. Defects in cell **migration** are a major cause of human disease, with excessive **migration** causing autoimmune diseases and cancer metastasis, whereas reduced capacity for **migration** leads to birth defects and immunodeficiencies. Myriad studies in vitro have established a consensus view that cell **migration**s require cell polarization, Rho GTPase-mediated cytoskeletal rearrangements, and myosin-mediated contractility. However, in vivo studies later revealed a more complex picture, including the discovery that cells migrate not only as single units but also as clusters, strands, and sheets. In particular, the role of E-Cadherin in cell motility appears to be more complex than previously appreciated. Here, we discuss recent advances achieved by combining the plethora of genetic tools available to the Drosophila geneticist with live imaging and biophysical techniques. Finally, we discuss the emerging themes such studies have revealed and ponder the puzzles that remain to be solved.
43. Deficiency screen identifies a novel role for beta 2 tubulin in salivary gland and myoblast **migration** in the Drosophila embryo. The Drosophila embryonic salivary gland is an epithelial organ formed by the coordinated invagination and **migration** of primordial cells. To identify genes that regulate gland **migration** we performed a deficiency screen of the third chromosome. Here, we report on the analysis of the beta 2 tubulin isoform (beta2t) that maps at 85D15. We show that, in beta2t mutant embryos, salivary glands did not complete their posterior **migration** and that **migration** of fusion competent myoblasts and longitudinal visceral muscle founder cells between the gland and circular visceral mesoderm was delayed. We also demonstrate that gland **migration** defects correlate with reduced betaPS and alphaPS2 integrin expression in the surrounding mesoderm and that beta2t genetically interacts with genes encoding integrin alphaPS1 and alphaPS2 subunits. Our studies reveal for the first time that beta2t is expressed in embryogenesis and that beta2t plays an important role in salivary gland and myoblast **migration**, possibly through proper regulation of integrin adhesion proteins.
44. Vestigial is required during late-stage muscle differentiation in Drosophila melanogaster embryos. The somatic muscles of Drosophila develop in a complex pattern that is repeated in each embryonic hemi-segment. During early development, progenitor cells fuse to form a syncytial muscle, which further differentiates via expression of muscle-specific factors that induce specific responses to external signals to regulate late-stage processes such as **migration** and attachment. Initial communication between somatic muscles and the epidermal tendon cells is critical for both of these processes. However, later establishment of attachments between longitudinal muscles at the segmental borders is largely independent of the muscle-epidermal attachment signals, and relatively little is known about how this event is regulated. Using a combination of null mutations and a truncated version of Sd that binds Vg but not DNA, we show that Vestigial (Vg) is required in ventral longitudinal muscles to induce formation of stable intermuscular attachments. In several muscles, this activity may be independent of Sd. Furthermore, the cell-specific differentiation events induced by Vg in two cells fated to form attachments are coordinated by Drosophila epidermal growth factor signaling. Thus, Vg is a key factor to induce specific changes in ventral longitudinal muscles 1-4 identity and is required for these cells to be competent to form stable intermuscular attachments with each other.
45. Integrin alpha chains exhibit distinct temporal and spatial localization patterns in epithelial cells of the Drosophila ovary. Integrins are heterodimeric transmembrane receptors that modulate cell adhesion, **migration**, and signaling. Multiple integrin chains contribute to development and morphogenesis of a given tissue. Here, we analyze the expression of Drosophila integrin alpha chains in the ovarian follicular epithelium, a model for tissue morphogenesis and cell **migration**. We find expression throughout development of the beta chain, betaPS. Alpha chains, however, exhibit both spatial and temporal expression differences. alphaPS1 and alphaPS2 integrins are detected during early and mid-oogenesis on apical, lateral, and basal membranes with the betaPS chain, whereas alphaPS3-family integrins (alphaPS3, alphaPS4, alphaPS5) are expressed in anterior cells late in oogenesis. Surprisingly, we find that alphaPS3-family integrins are dispensable for dorsal appendage morphogenesis but play a role in the final length of the egg, suggesting redundant functions of integrins in a simple tissue. We also demonstrate roles for alphaPS3betaPS integrin in border cell **migration** and in stretch cells.
46. The leading edge during dorsal closure as a model for epithelial plasticity: Pak is required for recruitment of the Scribble complex and septate junction formation. Dorsal closure (DC) of the Drosophila embryo is a model for the study of wound healing and developmental epithelial fusions, and involves the sealing of a hole in the epidermis through the **migration** of the epidermal flanks over the tissue occupying the hole, the amnioserosa. During DC, the cells at the edge of the migrating epidermis extend Rac- and Cdc42-dependent actin-based lamellipodia and filopodia from their leading edge (LE), which exhibits a breakdown in apicobasal polarity as adhesions are severed with the neighbouring amnioserosa cells. Studies using mammalian cells have demonstrated that Scribble (Scrib), an important determinant of apicobasal polarity that functions in a protein complex, controls polarized cell **migration** through recruitment of Rac, Cdc42 and the serine/threonine kinase Pak, an effector for Rac and Cdc42, to the LE. We have used DC and the follicular epithelium to study the relationship between Pak and the Scrib complex at epithelial membranes undergoing changes in apicobasal polarity and adhesion during development. We propose that, during DC, the LE membrane undergoes an epithelial-to-mesenchymal-like transition to initiate epithelial sheet **migration**, followed by a mesenchymal-to-epithelial-like transition as the epithelial sheets meet up and restore cell-cell adhesion. This latter event requires integrin-localized Pak, which recruits the Scrib complex in septate junction formation. We conclude that there are bidirectional interactions between Pak and the Scrib complex modulating epithelial plasticity. Scrib can recruit Pak to the LE for polarized cell **migration** but, as migratory cells meet up, Pak can recruit the Scrib complex to restore apicobasal polarity and cell-cell adhesion.
47. Cell-cell adhesion via the ECM: integrin genetics in fly and worm. Integrins are essential for the development of the two genetically tractable invertebrate model organisms, the nematode worm Caenorhabditis elegans and the fruit fly Drosophila melanogaster. Just two integrins are present in C. elegans: one putative RGD binding integrin alphapat-2betapat-3, corresponding to Drosophila alphaPS2betaPS and vertebrate alpha5beta1, alphaVbeta1 and alpha8beta1, and one putative laminin binding integrin alphaina-1betapat-3, corresponding to Drosophila alphaPS1betaPS and vertebrate alpha3beta1, alpha6beta1 and alpha7beta1. In this review, the function of this minimal set of integrins during the development of these two invertebrates is compared. Despite the differences in bodyplan and developmental strategy, integrin adhesion to the extracellular matrix is required for similar processes: the formation of the link that translates muscle contraction into movement of the exoskeleton, cell **migration**, and morphogenetic interactions between epithelia. Other integrin functions, such as regulation of gene expression, have not yet been experimentally demonstrated in both organisms. Additional proteins have been characterised in each organism that are essential for integrin function, including extracellular matrix ligands and intracellular interacting proteins, but so far different proteins have been found in the two organisms. This in part represents the fact that the characterisation of the full set of interacting proteins is not complete in either system. However, in other cases different proteins appear to be used for similar functions in the two animals. The continued use of genetic approaches to identify proteins required for integrin function in these two model organisms should lead to the identification of the minimal set of conserved components that form integrin adhesive structures.
48. Drosophila laminins act as key regulators of basement membrane assembly and morphogenesis. Laminins are heterotrimeric molecules found in all basement membranes. In mammals, they have been involved in diverse developmental processes, from gastrulation to tissue maintenance. The Drosophila genome encodes two laminin alpha chains, one beta and one Gamma, which form two distinct laminin trimers. So far, only mutations affecting one or other trimer have been analysed. In order to study embryonic development in the complete absence of laminins, we mutated the gene encoding the sole laminin beta chain in Drosophila, LanB1, so that no trimers can be made. We show that LanB1 mutant embryos develop until the end of embryogenesis. Electron microscopy analysis of mutant embryos reveals that the basement membranes are absent and the remaining extracellular material appears disorganised and diffuse. Accordingly, abnormal accumulation of major basement membrane components, such as Collagen IV and Perlecan, is observed in mutant tissues. In addition, we show that elimination of LanB1 prevents the normal morphogenesis of most organs and tissues, including the gut, trachea, muscles and nervous system. In spite of the above structural roles for laminins, our results unravel novel functions in cell adhesion, **migration** and rearrangement. We propose that while an early function of laminins in gastrulation is not conserved in Drosophila and mammals, their function in basement membrane assembly and organogenesis seems to be maintained throughout evolution.
49. Regulation of Integrin β 1 recycling to lipid rafts by Rab1a to promote cell **migratio**n. Rab1a is a member of the Rab family of small GTPases with a well characterized function in the regulation of vesicle trafficking from the endoplasmic reticulum to the Golgi apparatus and within Golgi compartments. The integrin family heterodimeric transmembrane proteins serve as major receptors for extracellular matrix proteins, which play essential roles in cell adhesion and **migratio**n. Although effects on intracellular trafficking of integrins or other key cargos by Rab1a could influence cell **migratio**n, the regulatory mechanisms linking Rab1a to cell **migratio**n are not well understood. Here, we report identification of Rab1a as a novel regulator of cell **migratio**n using an unbiased RNAi screen targeting GTPases. Inhibition of Rab1a reduced integrin-mediated cell adhesion and spreading on fibronectins, reduced integrin β1 localization to lipid rafts, and decreased recycling of integrin β1 to the plasma membrane. Analysis of Rab1a effector molecules showed that p115 mediated Rab1a regulation of integrin recycling and lipid raft localization in ce**ll migrat**ion. Taken together, these results suggest a novel function for Rab1a in the regulation of ce**ll migrat**ion through controlling integrin β1 recycling and localization to lipid rafts via a specific downstream effector pathway.
50. The PDZ-GEF dizzy regulates cell shape of migrating macrophages via Rap1 and integrins in the Drosophila embryo. In Drosophila embryos, macrophages originate from the cephalic mesoderm and perform a complex **migration** throughout the entire embryo. The molecular mechanisms regulating this cell **migration** remain largely unknown. We identified the Drosophila PDZ G-nucleotide exchange factor (PDZ-GEF) Dizzy as a component essential for normal macrophage **migration**. In mutants lacking Dizzy, macrophages have smaller cellular protrusions, and their **migration** is slowed down significantly. This phenotype appears to be cell-autonomous, as it is also observed in embryos with a dsRNA-induced reduction of dizzy function in macrophages. In a complementary fashion, macrophages overexpressing Dizzy are vastly extended and form very long protrusions. These cell shape changes depend on the function of the small GTPase Rap1: in rap1 mutants, Dizzy is unable to induce the large protrusions. Furthermore, forced expression of a dominant-active form of Rap1, but not of the wild-type form, induces similar cell shape changes as Dizzy does overexpression. These findings suggest that Dizzy acts through Rap1. We propose that integrin-dependent adhesion is a Rap1-mediated target of Dizzy activity: in integrin mutants, neither Dizzy nor Rap1 can induce cell shape changes in macrophages. These data provide the first link between a PDZ-GEF, the corresponding small GTPase and integrin-dependent cell adhesion during cell **migration** in embryonic development.
51. Basolateral junctions are sufficient to suppress epithelial invasion during Drosophila oogenesis. Epithelial junctions play crucial roles during metazoan evolution and development by facilitating tissue formation, maintenance, and function. Little is known about the role of distinct types of junctions in controlling epithelial transformations leading to invasion of neighboring tissues. Discovering the key junction complexes that control these processes and how they function may also provide mechanistic insight into carcinoma cell invasion. Here, using the Drosophila ovary as a model, we show that four proteins of the basolateral junction (BLJ), Fasciclin-2, Neuroglian, Discs-large, and Lethal-giant-larvae, but not proteins of other epithelial junctions, directly suppress epithelial tumorigenesis and invasion. Remarkably, the expression pattern of Fasciclin-2 predicts which cells will invade. We compared the apicobasal polarity of BLJ tumor cells to border cells (BCs), an epithelium-derived cluster that normally migrates during mid-oogenesis. Both tumor cells and BCs differentiate a lateralized membrane pattern that is necessary but not sufficient for invasion. Independent of lateralization, derepression of motility pathways is also necessary, as indicated by a strong linear correlation between faster BC **migration** and an increased incidence of tumor invasion. However, without membrane lateralization, derepression of motility pathways is also not sufficient for invasion. Our results demonstrate that spatiotemporal patterns of basolateral junction activity directly suppress epithelial invasion by organizing the cooperative activity of distinct polarity and motility pathways.
52. Egfr signaling regulates ommatidial rotation and cell motility in the Drosophila eye via MAPK/Pnt signaling and the Ras effector Canoe/AF6. Epidermal Growth Factor-receptor (Egfr) signaling is evolutionarily conserved and controls a variety of different cellular processes. In Drosophila these include proliferation, patterning, cell-fate determination, **migration** and survival. Here we provide evidence for a new role of Egfr signaling in controlling ommatidial rotation during planar cell polarity (PCP) establishment in the Drosophila eye. Although the signaling pathways involved in PCP establishment and photoreceptor cell-type specification are beginning to be unraveled, very little is known about the associated 90 degrees rotation process. One of the few rotation-specific mutations known is roulette (rlt) in which ommatidia rotate to a random degree, often more than 90 degrees. Here we show that rlt is a rotation-specific allele of the inhibitory Egfr ligand Argos and that modulation of Egfr activity shows defects in ommatidial rotation. Our data indicate that, beside the Raf/MAPK cascade, the Ras effector Canoe/AF6 acts downstream of Egfr/Ras and provides a link from Egfr to cytoskeletal elements in this developmentally regulated cell motility process. We provide further evidence for an involvement of cadherins and non-muscle myosin II as downstream components controlling rotation. In particular, the involvement of the cadherin Flamingo, a PCP gene, downstream of Egfr signaling provides the first link between PCP establishment and the Egfr pathway.
53. Functions for PS integrins in tissue adhesion, **migration**, and shape changes during early embryonic development in Drosophila. The position-specific (PS) integrins of Drosophila are a family of cell surface heterodimers that function as receptors for extracellular matrix molecules. In this study we used time-lapse videomicroscopy to examine living embryos lacking both maternal and zygotic expression of the genes that encode the alpha PS1, alpha PS2, and beta PS integrin subunits. We demonstrate roles for these molecules as early as gastrulation. Abnormalities in mutant embryos include: separation and twisting of the embryonic germband, abnormal shape and **migration** of midgut primordia, irregular visceral mesoderm, detachment of amnioserosa cells, rupture of the cuticle along the dorsal midline, lack of midgut constriction, and detachment of somatic muscles. These observations suggest multiple roles for PS integrins in the adhesion of cells and in the formation, organization, and **migration** of embryonic tissues. We also show that although alpha PS1 beta PS and alpha PS2 beta PS are often expressed in adjacent embryonic tissues, this distribution does not necessarily reflect equivalent requirements. Furthermore, the complete loss of both alpha subunits does not produce all of the phenotypes observed in embryos lacking beta PS. This suggests that alpha PS1 beta PS and alpha PS2 beta PS are not required in all embryonic processes utilizing PS integrins.
54. Integrin-ECM interactions regulate the changes in cell shape driving the morphogenesis of the Drosophila wing epithelium. During development, morphogenesis involves **migration** and changes in the shape of epithelial sheets, both of which require coordination of cell adhesion. Thus, while modulation of integrin-mediated adhesion to the ECM regulates epithelial motility, cell-cell adhesion via cadherins controls the remodelling of epithelial sheets. We have used the Drosophila wing epithelium to demonstrate that cell-ECM interactions mediated by integrins also regulate the changes in cell shape that underly epithelial morphogenesis. We show that integrins control the transitions from columnar to cuboidal cell shape underlying wing formation, and we demonstrate that eliminating the ECM has the same effect on cell shape as inhibiting integrin function. Furthermore, lack of integrin activity also induces detachment of the basal lamina and failure to assemble the basal matrix. Hence, we propose that integrins control epithelial cell shape by mediating adherence of these cells to the ECM. Finally, we show that the ECM has an instructive rather than a structural role, because inhibition of Raf reverses the cell shape changes caused by perturbing integrins.
55. Focal adhesion kinase controls morphogenesis of the Drosophila optic stalk. Photoreceptor cell axons (R axons) innervate optic ganglia in the Drosophila brain through the tubular optic stalk. This structure consists of surface glia (SG) and forms independently of R axon projection. In a screen for genes involved in optic stalk formation, we identified Fak56D encoding a Drosophila homolog of mammalian focal adhesion kinase (FAK). FAK is a main component of the focal adhesion signaling that regulates various cellular events, including cell **migration** and morphology. We show that Fak56D mutation causes severe disruption of the optic stalk structure. These phenotypes were completely rescued by Fak56D transgene expression in the SG cells but not in photoreceptor cells. Moreover, Fak56D genetically interacts with myospheroid, which encodes an integrin beta subunit. In addition, we found that CdGAPr is also required for optic stalk formation and genetically interacts with Fak56D. CdGAPr encodes a GTPase-activating domain that is homologous to that of mammalian CdGAP, which functions in focal adhesion signaling. Hence the optic stalk is a simple monolayered structure that can serve as an ideal system for studying glial cell morphogenesis and the developmental role(s) of focal adhesion signaling.
56. The function of PS integrins in Drosophila wing morphogenesis. Integrins are found on many cell types during the development of most organisms. In Drosophila their functions can be analysed genetically. An analysis of lethal mutations in a PS integrin gene showed that the integrins were required for muscle attachment and for certain cell sheet **migration**s during embryogenesis. In this paper we use viable mutations in integrin component genes to look at integrin function in the later stages of development of one adult structure, the wing. We show that two known viable mutations, one which has its primary effect on the fly's escape response, the other on wing morphogenesis, are mutations in the beta and PS2alpha subunits, respectively, of the PS integrins. The mutation non-jumper (mys(mj42)) in the beta subunit leads to wasting of the thoracic jump muscles. Flies in which the dosage of this allele is reduced (and no wildtype copy is present) show defects also in wing morphogenesis. The two surfaces of the wing fail to connect properly, resulting in 'blistering' of the wing and the formation of extra crossveins. The mutation in the gene for the PS2alpha integrin subunit, inflated, also leads to a failure in wing surface apposition and consequent wing blistering. When the two mutations are combined, the mutant phenotype is greatly enhanced. Thus, one of the roles of the PS integrins in late Drosophila development is to ensure the correct apposition and patterning of the wing epithelia.
57. LRT, a tendon-specific leucine-rich repeat protein, promotes muscle-tendon targeting through its interaction with Robo. Correct muscle **migration** towards tendon cells, and the adhesion of these two cell types, form the basis for contractile tissue assembly in the Drosophila embryo. While molecules promoting the attraction of muscles towards tendon cells have been described, signals involved in the arrest of muscle **migration** following the arrival of myotubes at their corresponding tendon cells have yet to be elucidated. Here, we describe a novel tendon-specific transmembrane protein, which we named LRT due to the presence of a leucine-rich repeat domain (LRR) in its extracellular region. Our analysis suggests that LRT acts non-autonomously to better target the muscle and/or arrest its **migration** upon arrival at its corresponding tendon cell. Muscles in embryos lacking LRT exhibited continuous formation of membrane extensions despite arrival at their corresponding tendon cells, and a partial failure of muscles to target their correct tendon cells. In addition, overexpression of LRT in tendon cells often stalled muscles located close to the tendon cells. LRT formed a protein complex with Robo, and we detected a functional genetic interaction between Robo and LRT at the level of muscle **migration** behavior. Taken together, our data suggest a novel mechanism by which muscles are targeted towards tendon cells as a result of LRT-Robo interactions. This mechanism may apply to the Robo-dependent **migration** of a wide variety of cell types.
58. Mesoderm **migration** in Drosophila is a multi-step process requiring FGF signaling and integrin activity. **Migration** is a complex, dynamic process that has largely been studied using qualitative or static approaches. As technology has improved, we can now take quantitative approaches towards understanding cell **migration** using in vivo imaging and tracking analyses. In this manner, we have established a four-step model of mesoderm **migration** during Drosophila gastrulation: (I) mesodermal tube formation, (II) collapse of the mesoderm, (III) dorsal **migration** and spreading and (IV) monolayer formation. Our data provide evidence that these steps are temporally distinct and that each might require different chemical inputs. To support this, we analyzed the role of fibroblast growth factor (FGF) signaling, in particular the function of two Drosophila FGF ligands, Pyramus and Thisbe, during mesoderm **migration**. We determined that FGF signaling through both ligands controls movements in the radial direction. Thisbe is required for the initial collapse of the mesoderm onto the ectoderm, whereas both Pyramus and Thisbe are required for monolayer formation. In addition, we uncovered that the GTPase Rap1 regulates radial movement of cells and localization of the beta-integrin subunit, Myospheroid, which is also required for monolayer formation. Our analyses suggest that distinct signals influence particular movements, as we found that FGF signaling is involved in controlling collapse and monolayer formation but not dorsal movement, whereas integrins are required to support monolayer formation only and not earlier movements. Our work demonstrates that complex cell **migration** is not necessarily a fluid process, but suggests instead that different types of movements are directed by distinct inputs in a stepwise manner.
59. Dynamin-mediated endocytosis is required for tube closure, cell intercalation, and biased apical expansion during epithelial tubulogenesis in the Drosophila ovary. Most metazoans are able to grow beyond a few hundred cells and to support differentiated tissues because they elaborate multicellular, epithelial tubes that are indispensable for nutrient and gas exchange. To identify and characterize the cellular behaviors and molecular mechanisms required for the morphogenesis of epithelial tubes (i.e., tubulogenesis), we have turned to the D. melanogaster ovary. Here, epithelia surrounding the developing egg chambers first pattern, then form and extend a set of simple, paired, epithelial tubes, the dorsal appendage (DA) tubes, and they create these structures in the absence of cell division or cell death. This genetically tractable system lets us assess the relative contributions that coordinated changes in cell shape, adhesion, orientation, and **migration** make to basic epithelial tubulogenesis. We find that Dynamin, a conserved regulator of endocytosis and the cytoskeleton, serves a key role in DA tubulogenesis. We demonstrate that Dynamin is required for distinct aspects of DA tubulogenesis: DA-tube closure, DA-tube-cell intercalation, and biased apical-luminal cell expansion. We provide evidence that Dynamin promotes these processes by facilitating endocytosis of cell-cell and cell-matrix adhesion complexes, and we find that precise levels and sub-cellular distribution of E-Cadherin and specific Integrin subunits impact DA tubulogenesis. Thus, our studies identify novel morphogenetic roles (i.e., tube closure and biased apical expansion), and expand upon established roles (i.e., cell intercalation and adhesion remodeling), for Dynamin in tubulogenesis.
60. Tissue-specific laminin expression facilitates integrin-dependent association of the embryonic wing disc with the trachea in Drosophila. The interaction of heterologous tissues involves cell adhesion mediated by the extracellular matrix and its receptor integrins. The Drosophila wing disc is an ectodermal invagination that contacts specific tracheal branches at the basolateral cell surface. We show that an alpha subunit of laminin, encoded by wing blister (wb), is essential for the establishment of the interaction between the wing and trachea. During embryogenesis, wing disc cells present Wb at their basolateral surface and extend posteriorly, expanding their association to more posteriorly located tracheal branches. These migratory processes are impaired in the absence of the trachea, Wb, or integrins. Time-lapse and transmission electron microscopy analyses suggest that Wb facilitates integrin-dependent contact over a large surface and controls the cellular behavior of the wing cells, including their exploratory filopodial activity. Our data identify Wb laminin as an extracellular matrix ligand that is essential for integrin-dependent cellular **migration** in Drosophila.
61. Round and round gets you somewhere: collective cell **migration** and planar polarity in elongating Drosophila egg chambers. Planar polarity is a developmental mechanism wherein individual cell behaviors are coordinated across a two-dimensional plane. A great deal of attention has been paid to the roles that the Frizzled/Strabismus and Fat/Dachsous signaling pathways play in this process; however, it is becoming increasingly clear that planar polarity can also be generated through alternate mechanisms. This review focuses on an unconventional form of planar polarity found within the follicular epithelium of the Drosophila egg chamber that helps to create the elongated shape of the egg. We highlight recent studies showing that the planar polarity in this system arises through collective **migration** of the follicle cells and the resulting rotational motion of the egg chamber.
62. Diaphanous regulates myosin and adherens junctions to control cell contractility and protrusive behavior during morphogenesis. Formins are key regulators of actin nucleation and elongation. Diaphanous-related formins, the best-known subclass, are activated by Rho and play essential roles in cytokinesis. In cultured cells, Diaphanous-related formins also regulate cell adhesion, polarity and microtubules, suggesting that they may be key regulators of cell shape change and **migration** during development. However, their essential roles in cytokinesis hamper our ability to test this hypothesis. We used loss- and gain-of-function approaches to examine the role of Diaphanous in Drosophila morphogenesis. We found that Diaphanous has a dynamic expression pattern consistent with a role in regulating cell shape change. We used constitutively active Diaphanous to examine its roles in morphogenesis and its mechanisms of action. This revealed an unexpected role in regulating myosin levels and activity at adherens junctions during cell shape change, suggesting that Diaphanous helps coordinate adhesion and contractility of the underlying actomyosin ring. We tested this hypothesis by reducing Diaphanous function, revealing striking roles in stabilizing adherens junctions and inhibiting cell protrusiveness. These effects also are mediated through coordinated effects on myosin activity and adhesion, suggesting a common mechanism for Diaphanous action during morphogenesis.
63. Fibroblast growth factor signalling controls successive cell behaviours during mesoderm layer formation in Drosophila. Fibroblast growth factor (FGF)-dependent epithelial-mesenchymal transitions and cell **migration** contribute to the establishment of germ layers in vertebrates and other animals, but a comprehensive demonstration of the cellular activities that FGF controls to mediate these events has not been provided for any system. The establishment of the Drosophila mesoderm layer from an epithelial primordium involves a transition to a mesenchymal state and the dispersal of cells away from the site of internalisation in a FGF-dependent fashion. We show here that FGF plays multiple roles at successive stages of mesoderm morphogenesis in Drosophila. It is first required for the mesoderm primordium to lose its epithelial polarity. An intimate, FGF-dependent contact is established and maintained between the germ layers through mesoderm cell protrusions. These protrusions extend deep into the underlying ectoderm epithelium and are associated with high levels of E-cadherin at the germ layer interface. Finally, FGF directs distinct hitherto unrecognised and partially redundant protrusive behaviours during later mesoderm spreading. Cells first move radially towards the ectoderm, and then switch to a dorsally directed movement across its surface. We show that both movements are important for layer formation and present evidence suggesting that they are controlled by genetically distinct mechanisms.
64. Spatial restriction of receptor tyrosine kinase activity through a polarized endocytic cycle controls border cell **migration**. Border cell **migration** is a stereotyped **migration** occurring during the development of the Drosophila egg chamber. During this process, a cluster composed of six to eight follicle cells migrates between nurse cells toward the oocyte. Receptor tyrosine kinases (RTKs) are enriched at the leading edge of the follicle cells and establish the directionality of their **migration**. Endocytosis has been shown to play a role in the maintenance of this polarization; however, the mechanisms involved are largely unknown. In this study, we show that border cell **migration** requires the function of the small GTPases Rab5 and Rab11 that regulate trafficking through the early and the recycling endosome, respectively. Expression of a dominant negative form of rab11 induces a loss of the polarization of RTK activity, which correlates with a severe **migration** phenotype. In addition, we demonstrate that the exocyst component Sec15 is distributed in structures that are polarized during the **migration** process in a Rab11-dependent manner and that the down-regulation of different subunits of the exocyst also affects **migration**. Together, our data demonstrate a fundamental role for a plasma membrane-endosome trafficking cycle in the maintenance of active RTK at the leading edge of border cells during their **migration**.
65. Netrins and Frazzled/DCC promote the **migration** and mesenchymal to epithelial transition of Drosophila midgut cells. Mesenchymal-epithelial transitions (METs) are important in both development and the growth of secondary tumours. Although the molecular basis for epithelial polarity is well studied, less is known about the cues that induce MET. Here we show that Netrins, well known as chemotropic guidance factors, provide a basal polarising cue during the Drosophila midgut MET. Both netrinA and netrinB are expressed in the visceral mesoderm, the substrate upon which midgut cells migrate, while their receptor frazzled (fra) is expressed in midgut cells. Netrins are required to polarise Fra to the basal surface, and Netrins and Fra undergo mutually-dependent endocytosis, with Fra subsequently trafficking to late endosomes. Mutations to fra and netrins affect both **migration** and MET but to different degrees. Loss of fra strongly delays **migration**, midgut cells fail to extend protrusions, and apico-basal polarisation of proteins and epithelium formation is inhibited. In netrin mutants, the **migration** phenotype is weaker and cells still extend protrusions. However, apico-basal polarisation of proteins, including Fra, and FActin is greatly disrupted and a monolayer fails to form. Delocalised accumulations of FActin are prevalent in netrin mutants but not fra mutants suggesting delocalised Fra may disrupt the MET. βPS localisation is also affected in netrin mutants in that a basal gradient is reduced while localisation to the midgut/VM interface is increased. Since a similar effect is seen when endocytosis is inhibited, Netrin and Fra may regulate Integrin turnover. The results suggest Netrin-dependent basal polarisation of Fra is critical for the formation of an epithelium.
66. Dpp signaling directs cell motility and invasiveness during epithelial morphogenesis. Tissue remodeling in development and disease involves the coordinated invasion of neighboring territories and/or the replacement of entire cell populations. Cell guidance, cell matching, transitions from passive to migratory epithelia, cell growth and death, and extracellular matrix remodeling all impinge on epithelial spreading. Significantly, the extracellular signals that direct these activities and the specific cellular elements and mechanisms regulated by these signals remain in most cases to be identified. To address these issues, we performed an analysis of histoblasts (Drosophila abdominal epithelial founder cells) on their transition from a dormant state to active **migration** replacing obsolete larval epidermal cells (LECs). We found that during expansion, Decapentaplegic (Dpp) secreted from surrounding LECs leads to graded pathway activation in cells at the periphery of histoblast nests. Across nests, Dpp activity confers differential cellular behavior and motility by modulating cell-cell contacts, the organization and activity of the cytoskeleton, and histoblast attachment to the substrate. Furthermore, Dpp also prevents the premature death of LECs, allowing the coordination of histoblast expansion to LEC delamination. Dpp signaling activity directing histoblast spreading and invasiveness mimics transforming growth factor-beta and bone morphogenetic proteins' role in enhancing the motility and invasiveness of cancer cells, resulting in the promotion of metastasis.
67. A dual role for the βPS integrin myospheroid in mediating Drosophila embryonic macrophage **migratio**n. Throughout embryonic development, macrophages not only act as the first line of defence against infection but also help to sculpt organs and tissues of the embryo by removing dead cells and secreting extracellular matrix components. Key to their function is the ability of embryonic macrophages to migrate and disperse throughout the embryo. Despite these important developmental functions, little is known about the molecular mechanisms underlying embryonic macrophage **migratio**n in vivo. Integrins are key regulators of many of the adult macrophage responses, but their role in embryonic macrophages remains poorly characterized. Here, we have used Drosophila macrophages (haemocytes) as a model system to address the role of integrins during embryonic macrophage dispersal in vivo. We show that the main βPS integrin, myospheroid, affects haemocyt**e migrati**on in two ways; by shaping the three-dimensional environment in which haemocytes migrate and by regulating th**e migrati**on of haemocytes themselves. Live imaging revealed a requirement for myospheroid within haemocytes to coordinate the microtubule and actin dynamics, and to enable haemocyte developmental dispersal, contact repulsion and inflammator**y migrati**on towards wounds.
68. A new member of the spectrin superfamily may participate in the formation of embryonic muscle attachments in Drosophila. Myotube **migration** and the formation of muscle attachments are crucial events for the proper development of muscle patterning in the Drosophila embryo. This paper describes the identification of a new embryonic muscle-specific protein, MSP-300, in Drosophila. This protein is initially expressed by muscle precursors at muscle-ectoderm and muscle-muscle attachment sites. As development continues, MSP-300 becomes associated with muscle myofibrillar network. Studies of the subcellular localization of this muscle-specific protein in primary embryonic cultured myotubes show that MSP-300 decorates actin filaments, and that it is specifically enriched in sites where actin microfilaments are linked to the plasma membrane. Migrating myotubes exhibit high levels of this protein at their leading edge while, in myotubes that have already developed sarcomeric architecture, the protein is localized mainly at the Z-discs. Sequence of a partial 3.9 kb cDNA clone and molecular analysis of the predicted protein sequence of this protein indicates that it encodes a high relative molecular mass protein (approximately 300 x 10(3), which exhibits at least five spectrin-like repeats. Several properties are shared by MSP-300 and members of the spectrin superfamily: it is associated with actin microfilaments, its sequence exhibits spectrin-like repeats and it is localized at sites where actin is linked to the plasma membrane. This protein could have a developmental role in the formation of muscle-ectoderm attachments and may be involved in myotube **migration** on the ectoderm.
69. Misshapen decreases integrin levels to promote epithelial motility and planar polarity in Drosophila. Complex organ shapes arise from the coordinate actions of individual cells. The Drosophila egg chamber is an organ-like structure that lengthens along its anterior-posterior axis as it grows. This morphogenesis depends on an unusual form of planar polarity in the organ's outer epithelial layer, the follicle cells. Interestingly, this epithelium also undergoes a directed **migration** that causes the egg chamber to rotate around its anterior-posterior axis. However, the functional relationship between planar polarity and **migration** in this tissue is unknown. We have previously reported that mutations in the Misshapen kinase disrupt follicle cell planar polarity. Here we show that Misshapen's primary role in this system is to promote individual cell motility. Misshapen decreases integrin levels at the basal surface, which may facilitate detachment of each cell's trailing edge. These data provide mechanistic insight into Misshapen's conserved role in cell **migration** and suggest that follicle cell planar polarity may be an emergent property of individual cell migratory behaviors within the epithelium.

#### GeneRIFs

1. alphaPS1betaPS integrin controls salivary gland **migration** through Rac1 GTPase which downregulates E-cadherin in proximal and distal gland cells.
2. Results suggest a novel function for Rab1a in the regulation of cell **migration** through controlling integrin beta1 recycling and localization to lipid rafts via a specific downstream effector pathway.

### ECM

#### PubMed

1. Peak BMP Responses in the Drosophila Embryo Are Dependent on the Activation of Integrin Signaling. Within a 3D tissue, cells need to integrate signals from growth factors, such as BMPs, and the extracellular matrix (**ECM**) to coordinate growth and differentiation. Here, we use the Drosophila embryo as a model to investigate how BMP responses are influenced by a cell's local **ECM** environment. We show that integrins, which are **ECM** receptors, are absolutely required for peak BMP signaling. This stimulatory effect of integrins requires their intracellular signaling function, which is activated by the **ECM** protein collagen IV. Mechanistically, integrins interact with the BMP receptor and stimulate phosphorylation of the downstream Mad transcription factor. The BMP-pathway-enhancing function of integrins is independent of focal adhesion kinase, but it requires conserved NPXY motifs in the β-integrin cytoplasmic tail. Furthermore, we show that an α-integrin subunit is a BMP target gene, identifying positive feedback between integrin signaling and BMP pathway activity that may contribute to robust cell fate decisions.
2. Drosophila importin-7 functions upstream of the Elmo signaling module to mediate the formation and stability of muscle attachments. Establishment and maintenance of stable muscle attachments is essential for coordinated body movement. Studies in Drosophila have pioneered a molecular understanding of the morphological events in the conserved process of muscle attachment formation, including myofiber migration, muscle-tendon signaling, and stable junctional adhesion between muscle cells and their corresponding target insertion sites. In both Drosophila and vertebrate models, integrin complexes play a key role in the biogenesis and stability of muscle attachments through the interactions of integrins with extracellular matrix (**ECM**) ligands. We show that Drosophila importin-7 (Dim7) is an upstream regulator of the conserved Elmo-Mbc→Rac signaling pathway in the formation of embryonic muscle attachment sites (MASs). Dim7 is encoded by the moleskin (msk) locus and was identified as an Elmo-interacting protein. Both Dim7 and Elmo localize to the ends of myofibers coincident with the timing of muscle-tendon attachment in late myogenesis. Phenotypic analysis of elmo mutants reveal muscle attachment defects similar to those previously described for integrin mutants. Furthermore, Elmo and Dim7 interact both biochemically and genetically in the developing musculature. The muscle detachment phenotype resulting from mutations in the msk locus can be rescued by components in the Elmo signaling pathway, including the Elmo-Mbc complex, an activated Elmo variant, or a constitutively active form of Rac. In larval muscles, the localization of Dim7 and activated Elmo to the sites of muscle attachment is attenuated upon RNAi knockdown of integrin heterodimer complex components. Our results show that integrins function as upstream signals to mediate Dim7-Elmo enrichment to the MASs.
3. Integrins are necessary for the development and maintenance of the glial layers in the Drosophila peripheral nerve. Peripheral nerve development involves multiple classes of glia that cooperate to form overlapping glial layers paired with the deposition of a surrounding extracellular matrix (**ECM**). The formation of this tubular structure protects the ensheathed axons from physical and pathogenic damage and from changes in the ionic environment. Integrins, a major family of **ECM** receptors, play a number of roles in the development of myelinating Schwann cells, one class of glia ensheathing the peripheral nerves of vertebrates. However, the identity and the role of the integrin complexes utilized by the other classes of peripheral nerve glia have not been determined in any animal. Here, we show that, in the peripheral nerves of Drosophila melanogaster, two integrin complexes (αPS2βPS and αPS3βPS) are expressed in the different glial layers and form adhesion complexes with integrin-linked kinase and Talin. Knockdown of the common beta subunit (βPS) using inducible RNAi in all glial cells results in lethality and glial defects. Analysis of integrin complex function in specific glial layers showed that loss of βPS in the outermost layer (the perineurial glia) results in a failure to wrap the nerve, a phenotype similar to that of Matrix metalloproteinase 2-mediated degradation o**f t**he ECM. Knockdown of βPS integrin in the innermost wrapping glia causes a loss of glial processes around axons. Together, our data suggest that integrins are employed in different glial layers to mediate the development and maintenance of the protective glial sheath in Drosophila peripheral nerves.
4. The integrin adhesion complex changes its composition and function during morphogenesis of an epithelium. Cell adhesion to the extracellular matrix (**ECM**) is mediated by the integrin family of transmembrane receptors. Integrins link **ECM** ligands to the cytoskeleton, providing strong attachment to enable cell-shape change and tissue integrity. This connection is made possible by an intracellular complex of proteins, which links to actin filaments and controls signalling cascades that regulate cytoskeletal rearrangements. We have identified stress-fibre-associated focal adhesions that change their composition during tissue morphogenesis. Early expression of alphaPS1betaPS integrin decreases the levels of the actin-nucleating factors Enabled, Diaphanous and profilin, as well as downregulating the amount of F-actin incorporated into the stress fibres. As follicle cells mature in their developmental pathway and become squamous, the integrin in the focal adhesions changes from alphaPS1betaPS to alphaPS2betaPS. During the switch, stress fibres increase their length and change orientation, first changing by 90 degrees and then reorienting back. The normal rapid reorientation requires new expression of alphaPS2betaPS, which also permits recruitment of the adaptor protein tensin. Unexpectedly, it is the extracellular portion of the alphaPS2 subunit that provides the specificity for intracellular recruitment of tensin. Molecular variation of the integrin complex is thus a key component of developmentally programmed morphogenesis.
5. AlphaPS2 integrin-mediated muscle attachment in Drosophila requires the **ECM** protein Thrombospondin. During Drosophila embryogenesis, the attachment of somatic muscles to epidermal tendon cells requires heterodimeric PS-integrin proteins (alpha- and beta-subunits). The alpha-subunits are expressed complementarily, either tendon cell- or muscle-specific, whereas the beta-integrin subunit is expressed in both tissues. Mutations of beta-integrin cause a severe muscle detachment phenotype, whereas alpha-subunit mutations have weaker or only larval muscle detachment phenotypes. Furthermore, mutations of extracellular matrix (**ECM**) proteins known to act as integrin binding partners have comparatively weak effects only, suggesting the presence of additional integrin binding **ECM** proteins required for proper muscle attachment. Here, we report that mutations in the Drosophila gene thrombospondin (tsp) cause embryonic muscle detachment. tsp is specifically expressed in both developing and mature epidermal tendon cells. Its initial expression in segment border cells, the tendon precursors, is under the control of hedgehog-dependent signaling, whereas tsp expression in differentiated tendon cells depends on the transcription factor encoded by stripe. In the absence of tsp activity, no aspect of muscle pattern formation as well as the initial contact between muscle and tendon cells nor muscle-to-muscle attachments are affected. However, when muscle contractions occur during late embryogenesis, muscles detach from the tendon cells. The Tsp protein is localized to the tendon cell **ECM** where muscles attach. Genetic interaction studies indicate that Tsp specifically interacts with the alphaPS2 integrin and that this interaction is needed to withstand the forces of muscle contractions at the tendon cells.
6. Thrombospondin-mediated adhesion is essential for the formation of the myotendinous junction in Drosophila. Organogenesis of the somatic musculature in Drosophila is directed by the precise adhesion between migrating myotubes and their corresponding ectodermally derived tendon cells. Whereas the PS integrins mediate the adhesion between these two cell types, their extracellular matrix (**ECM**) ligands have been only partially characterized. We show that the **ECM** protein Thrombospondin (Tsp), produced by tendon cells, is essential for the formation of the integrin-mediated myotendinous junction. Tsp expression is induced by the tendon-specific transcription factor Stripe, and accumulates at the myotendinous junction following the association between the muscle and the tendon cell. In tsp mutant embryos, migrating somatic muscles fail to attach to tendon cells and often form hemiadherens junctions with their neighboring muscle cells, resulting in nonfunctional somatic musculature. Talin accumulation at the cytoplasmic faces of the muscles and tendons is greatly reduced, implicating Tsp as a potential integrin ligand. Consistently, purified Tsp C-terminal domain polypeptide mediates spreading of PS2 integrin-expressing S2 cells in a KGD- and PS2-integrin-dependent manner. We propose a model in which the myotendinous junction is formed by the specific association of Tsp with multiple muscle-specific PS2 integrin receptors and a subsequent consolidation of the junction by enhanced tendon-specific production of Tsp secreted into the junctional space.
7. Talin is essential for integrin function in Drosophila. We show that the Drosophila gene rhea, isolated because its wing blister phenotype is typical of mutants affecting integrin function, encodes talin. Embryos deficient in talin have very similar phenotypes to integrin (betaPS) null embryos, including failure in germ band retraction and muscle detachment. We demonstrate that talin is not required for the presence of integrins on the cell surface or their localization at muscle termini. However, talin is required for formation of focal adhesion-like clusters of integrins on the basal surface of imaginal disc epithelia and junctional plaques between muscle and tendon cells. These results indicate that talin is essential for integrin function and acts by stably linking clusters of **ECM**-linked integrins to the cytoskeleton.
8. The microRNA bantam regulates a developmental transition in epithelial cells that restricts sensory dendrite growth. As animals grow, many early born structures grow by cell expansion rather than cell addition; thus growth of distinct structures must be coordinated to maintain proportionality. This phenomenon is particularly widespread in the nervous system, with dendrite arbors of many neurons expanding in concert with their substrate to sustain connectivity and maintain receptive field coverage as animals grow. After rapidly growing to establish body wall coverage, dendrites of Drosophila class IV dendrite arborization (C4da) neurons grow synchronously with their substrate, the body wall epithelium, providing a system to study how proportionality is maintained during animal growth. Here, we show that the microRNA bantam (ban) ensures coordinated growth of C4da dendrites and the epithelium through regulation of epithelial endoreplication, a modified cell cycle that entails genome amplification without cell division. In Drosophila larvae, epithelial endoreplication leads to progressive changes in dendrite-extracellular matrix (**ECM**) and dendrite-epithelium contacts, coupling dendrite/substrate expansion and restricting dendrite growth beyond established boundaries. Moreover, changes in epithelial expression of cell adhesion molecules, including the beta-integrin myospheroid (mys), accompany this developmental transition. Finally, endoreplication and the accompanying changes in epithelial mys expression are required to constrain late-stage dendrite growth and structural plasticity. Hence, modulating epithelium-**ECM** attachment probably influences substrate permissivity for dendrite growth and contributes to the dendrite-substrate coupling that ensures proportional expansion of the two cell types.
9. The conserved ADAMTS-like protein lonely heart mediates matrix formation and cardiac tissue integrity. Here we report on the identification and functional characterization of the ADAMTS-like homolog lonely heart (loh) in Drosophila melanogaster. Loh displays all hallmarks of ADAMTSL proteins including several thrombospondin type 1 repeats (TSR1), and acts in concert with the collagen Pericardin (Prc). Loss of either loh or prc causes progressive cardiac damage peaking in the abolishment of heart function. We show that both proteins are integral components of the cardiac **ECM** mediating cellular adhesion between the cardiac tube and the pericardial cells. Loss of **ECM** integrity leads to an altered myo-fibrillar organization in cardiac cells massively influencing heart beat pattern. We show evidence that Loh acts as a secreted receptor for Prc and works as a crucial determinant to allow the formation of a cell and tissue specific **ECM**, while it does not influence the accumulation of other matrix proteins like Nidogen or Perlecan. Our findings demonstrate that the function of ADAMTS-like proteins is conserved throughout evolution and reveal a previously unknown interaction of these proteins with collagens.
10. The PS2 integrin ligand tiggrin is required for proper muscle function in Drosophila. Tiggrin is a novel extracellular matrix ligand for the Drosophila PS2 integrins. We have used flanking P elements to generate a precise deletion of tiggrin. Most flies lacking tiggrin die as larvae or pupae. A few adults do emerge and these appear to be relatively normal, displaying only misshapen abdomens and a low frequency of wing defects. Examination of larvae shows that muscle connections, function and morphology are defective in tiggrin mutants. Muscle contraction waves that extend the length of the larvae are much slower in tiggrin mutants. Direct examination of bodywall muscles shows defects in muscle attachment sites, where tiggrin is specifically localized, and muscles appear thinner. Transgenes expressing tiggrin are capable of rescuing tiggrin mutant phenotypes. Transgenes expressing a mutant tiggrin, whose Arg-Gly-Asp (RGD) integrin recognition sequence has been mutated to Leu-Gly-Ala (LGA) show much reduced, but significant, rescuing ability. Cell spreading assays detect no interactions of this mutant tiggrin with PS2 integrins. Therefore, while the RGD sequence is critical for PS2 interactions and full activity in the whole fly, the mutant tiggrin retains some function(s) that are probably mediated by interactions with other **ECM** molecules or cell surface receptors
11. Mutations in the Drosophila alphaPS2 integrin subunit uncover new features of adhesion site assembly. The Drosophila alphaPS2betaPS integrin is required for diverse development events, including muscle attachment. We characterized six unusual mutations in the alphaPS2 gene that cause a subset of the null phenotype. One mutation changes a residue in alphaPS2 that is equivalent to the residue in alphaV that contacts the arginine of RGD. This change severely reduced alphaPS2betaPS affinity for soluble ligand, abolished the ability of the integrin to recruit laminin to muscle attachment sites in the embryo and caused detachment of integrins and talin from the **ECM**. Three mutations that alter different parts of the alphaPS2 beta-propeller, plus a fourth that eliminated a late phase of alphaPS2 expression, all led to a strong decrease in alphaPS2betaPS at muscle ends, but, surprisingly, normal levels of talin were recruited. Thus, although talin recruitment requires alphaPS2betaPS, talin levels are not simply specified by the amount of integrin at the adhesive junction. These mutations caused detachment of talin and actin from integrins, suggesting that the integrin-talin link is weaker than the **ECM**-integrin link.
12. In vivo functional analysis reveals specific roles for the integrin-binding sites of talin. Adhesion receptors play diverse roles during animal development and require precise spatiotemporal regulation, which is achieved through the activity of their binding partners. Integrins, adhesion receptors that mediate cell attachment to the extracellular matrix (**ECM**), connect to the intracellular environment through the cytoplasmic adapter protein talin. Talin has two essential functions: orchestrating the assembly of the intracellular adhesion complex (IAC), which associates with integrin, and regulating the affinity of integrins for the **ECM**. Talin can bind to integrins through two different integrin-binding sites (IBS-1 and IBS-2, respectively). Here, we have investigated the roles of each in the context of Drosophila development. We find that although IBS-1 and IBS-2 are partially redundant, they each have specialized roles during development: IBS-1 reinforces integrin attachment to the **ECM**, whereas IBS-2 reinforces the link between integrins and the IAC. Disruption of each IBS has different developmental consequences, illustrating how the functional diversity of integrin-mediated adhesion is achieved.
13. Integrin signaling is required for maintenance and proliferation of intestinal stem cells in Drosophila. Tissue-specific stem cells are maintained by both local secreted signals and cell adhesion molecules that position the stem cells in the niche microenvironment. In the Drosophila midgut, multipotent intestinal stem cells (ISCs) are located basally along a thin layer of basement membrane that composed of extracellular matrix (**ECM**), which separates ISCs from the surrounding visceral musculature: the muscle cells constitute a regulatory niche for ISCs by producing multiple secreted signals that directly regulate ISC maintenance and proliferation. Here we show that integrin-mediated cell adhesion, which connects the **ECM** and intracellular cytoskeleton, is required for ISC anchorage to the basement membrane. Specifically, the α-integrin subunits including αPS1 encoded by mew and αPS3 encoded by scb, and the β-integrin subunit encoded by mys are richly expressed in ISCs and are required for the maintenance, rather than their survival or multiple lineage differentiation. Furthermore, ISC maintenance also requires the intercellular and intracellular integrin signaling components including Talin, Integrin-linked kinase (Ilk), and the ligand, Laminin A. Notably, integrin mutant ISCs are also less proliferative, and genetic interaction studies suggest that proper integrin signaling is a pre-requisite for ISC proliferation in response to various proliferative signals and for the initiation of intestinal hyperplasia after loss of adenomatous polyposis coli (Apc). Our studies suggest that integrin not only functions to anchor ISCs to the basement membrane, but also serves as an essential element for ISC proliferation during normal homeostasis and in response to oncogenic mutations.
14. Glia **ECM** interactions are required to shape the Drosophila nervous system. Organs are characterized by a specific shape that is often remodeled during development. The dynamics of organ shape is in particular evident during the formation of the Drosophila nervous system. During embryonic stages the central nervous system compacts, whereas selective growth occurs during larval stages. The nervous system is covered by a layer of surface glial cells that form the blood brain barrier and a thick extracellular matrix called neural lamella. The size of the neural lamella is dynamically adjusted to the growing nervous system and we show here that perineurial glial cells secrete proteases to remodel this matrix. Moreover, an imbalance in proteolytic activity results in an abnormal shape of the nervous system. To identify further components controlling nervous system shape we performed an RNAi based screen and identified the gene nolo, which encodes an ADAMTS-like protein. We generated loss of function alleles and demonstrate a requirement in glial cells. Mutant nolo larvae, however, do not show an abnormal nervous system shape. The only predicted off-target of the nolo(dsRNA) is Oatp30B, which encodes an organic anion transporting protein characterized by an extracellular protease inhibitor domain. Loss of function mutants were generated and double mutant analyses demonstrate a genetic interaction between nolo and Oatp30B which prevented the generation of maternal zygotic mutant larvae.
15. Integrin-dependent anchoring of a stem-cell niche. Interactions between stem cells and their surrounding microenvironment, or niche, are critical for the establishment and maintenance of stem-cell properties. The adult Drosophila testis contains a morphologically discrete stem-cell niche, the 'hub'. The small cluster of non-dividing, somatic hub cells at the anterior tip of the fly testis is contacted by the germline stem cells (GSCs), which retain their stem-cell character through the direct association with the hub. Here we show that integrin-mediated adhesion is important for maintaining the correct position of embryonic hub cells during gonad morphogenesis. The misplaced hub in integrin-deficient embryos directs the orientation of cell divisions in the presumptive GSCs, a hallmark of the active germline stem-cell niche. A decrease in integrin-mediated adhesion in adult testes, which resulted in a loss of the hub and the stem-cell population, revealed the importance of hub-cell anchoring. Finally, we show that an extracellular matrix (**ECM**) is present around the gonad during late embryogenesis and that this **ECM** is defective in integrin-deficient gonads. On the basis of our data, we propose that integrins are required for the attachment of the hub cells to the **ECM**, which is essential for maintaining the stem-cell niche.
16. Loss of focal adhesions in glia disrupts both glial and photoreceptor axon migration in the Drosophila visual system. Many aspects of glial development are regulated by extracellular signals, including those from the extracellular matrix (**ECM**). Signals from the **ECM** are received by cell surface receptors, including the integrin family. Previously, we have shown that Drosophila integrins form adhesion complexes with Integrin-linked kinase and talin in the peripheral nerve glia and have conserved roles in glial sheath formation. However, integrin function in other aspects of glial development is unclear. The Drosophila eye imaginal disc (ED) and optic stalk (OS) complex is an excellent model with which to study glial migration, differentiation and glia-neuron interactions. We studied the roles of the integrin complexes in these glial developmental processes during OS/eye development. The common beta subunit βPS and two alpha subunits, αPS2 and αPS3, are located in puncta at both glia-glia and gl**ia-**ECM interfaces. Depletion of βPS integrin and talin by RNAi impaired the migration and distribution of glia within the OS resulting in morphological defects. Reduction of integrin or talin in the glia also disrupted photoreceptor axon outgrowth leading to axon stalling in the OS and ED. The neuronal defects were correlated with a disruption of the carpet glia tube paired with invasion of glia into the core of the OS and the formation of a glial cap. Our results suggest that integrin-mediated extracellular signals are important for multiple aspects of glial development and non-autonomously affect axonal migration during Drosophila eye development.
17. Analysis of integrin turnover in fly myotendinous junctions. Transient (short-term) cell adhesion underlies dynamic processes such as cell migration, whereas stable (long-term) cell adhesion maintains tissue architecture. Ongoing adhesion complex turnover is essential for transient cell adhesion, but it is not known whether turnover is also required for maintenance of long-term adhesion. We used fluorescence recovery after photobleaching to analyze the dynamics of an integrin adhesion complex (IAC) in a model of long-term cell-**ECM** adhesion, myotendinous junctions (MTJs), in fly embryos and larvae. We found that the IAC undergoes turnover in MTJs and that this process is mediated by clathrin-dependent endocytosis. Moreover, the small GTPase Rab5 can regulate the proportion of IAC components that undergo turnover. Also, altering Rab5 activity weakened MTJs, resulting in muscle defects. In addition, growth of MTJs was concomitant with a decrease in the proportion of IAC components undergoing turnover. We propose that IAC turnover is tightly regulated in long-term cell-**ECM** adhesions to allow normal tissue growth and maintenance.
18. An interaction between integrin and the talin FERM domain mediates integrin activation but not linkage to the cytoskeleton. Transmembrane adhesion receptors, such as integrins, mediate cell adhesion by interacting with intracellular proteins that connect to the cytoskeleton. Talin, one such linker protein, is thought to have two roles: mediating inside-out activation of integrins, and connecting extracellular matrix (**ECM**)-bound integrins to the cytoskeleton. Talin's amino-terminal head, which consists of a FERM domain, binds an NPxY motif within the cytoplasmic tail of most integrin beta subunits. This is consistent with the role of FERM domains in recruiting other proteins to the plasma membrane. We tested the role of the talin-head-NPxY interaction in integrin function in Drosophila. We found that introduction of a mutation that perturbs this binding in vitro into the isolated talin head disrupts its recruitment by integrins in vivo. Surprisingly, when engineered into the full-length talin, this mutation did not disrupt talin recruitment by integrins nor its ability to connect integrins to the cytoskeleton. However, it reduced the ability of talin to strengthen integrin adhesion to the **ECM**, indicating that the function of the talin-head-NPxY interaction is solely to regulate integrin adhesion.
19. Drosophila PS2 and PS3 integrins play distinct roles in retinal photoreceptors-glia interactions. Cellular migration and differentiation are important developmental processes that require dynamic cellular adhesion. Integrins are heterodimeric transmembrane receptors that play key roles in adhesion plasticity. Here, we explore the developing visual system of Drosophila to study the roles of integrin heterodimers in glia development. Our data show that αPS2 is essential for retinal glia migration from the brain into the eye disc and that glial cells have a role in the maintenance of the fenestrated membrane (Laminin-rich **EC**M layer) in the disc. Interestingly, the absence of glial cells in the eye disc did not affect the targeting of retinal axons to the optic stalk. In contrast, αPS3 is not required for retinal glia migration, but together with Talin, it functions in glial cells to allow photoreceptor axons to target the optic stalk. Thus, we present evidence that αPS2 and αPS3 integrin have different and specific functions in the development of retinal glia.
20. PS integrins and laminins: key regulators of cell migration during Drosophila embryogenesis. During embryonic development, there are numerous cases where organ or tissue formation depends upon the migration of primordial cells. In the Drosophila embryo, the visceral mesoderm (vm) acts as a substrate for the migration of several cell populations of epithelial origin, including the endoderm, the trachea and the salivary glands. These migratory processes require both integrins and laminins. The current model is that αPS1βPS (PS1) and/or αPS3βPS (PS3) integrins are required in migrating cells, whereas αPS2βPS (PS2) integrin is required in the vm, where it performs an as yet unidentified function. Here, we show that PS1 integrins are also required for the migration over the vm of cells of mesodermal origin, the caudal visceral mesoderm (CVM). These results support a model in which PS1 might have evolved to acquire the migratory function of integrins, irrespective of the origin of the tissue. This integrin function is highly specific and its specificity resides mainly in the extracellular domain. In addition, we have identified the Laminin α1,2 trimer, as the key extracellular m**atr**ix (ECM) component regulating CVM migration. Furthermore, we show that, as it is the case in vertebrates, integrins, and specifically PS2, contributes to CVM movement by participating in the correct assembly **of** the ECM that serves as tracks for migration.
21. Cell-cell adhesion via the **ECM**: integrin genetics in fly and worm. Integrins are essential for the development of the two genetically tractable invertebrate model organisms, the nematode worm Caenorhabditis elegans and the fruit fly Drosophila melanogaster. Just two integrins are present in C. elegans: one putative RGD binding integrin alphapat-2betapat-3, corresponding to Drosophila alphaPS2betaPS and vertebrate alpha5beta1, alphaVbeta1 and alpha8beta1, and one putative laminin binding integrin alphaina-1betapat-3, corresponding to Drosophila alphaPS1betaPS and vertebrate alpha3beta1, alpha6beta1 and alpha7beta1. In this review, the function of this minimal set of integrins during the development of these two invertebrates is compared. Despite the differences in bodyplan and developmental strategy, integrin adhesion to the extracellular matrix is required for similar processes: the formation of the link that translates muscle contraction into movement of the exoskeleton, cell migration, and morphogenetic interactions between epithelia. Other integrin functions, such as regulation of gene expression, have not yet been experimentally demonstrated in both organisms. Additional proteins have been characterised in each organism that are essential for integrin function, including extracellular matrix ligands and intracellular interacting proteins, but so far different proteins have been found in the two organisms. This in part represents the fact that the characterisation of the full set of interacting proteins is not complete in either system. However, in other cases different proteins appear to be used for similar functions in the two animals. The continued use of genetic approaches to identify proteins required for integrin function in these two model organisms should lead to the identification of the minimal set of conserved components that form integrin adhesive structures.
22. Integrins modulate the Egfr signaling pathway to regulate tendon cell differentiation in the Drosophila embryo. Changes in the extracellular matrix (**ECM**) govern the differentiation of many cell types during embryogenesis. Integrins are cell matrix receptors that play a major role in cell-**ECM** adhesion and in transmitting signals from the **ECM** inside the cell to regulate gene expression. In this paper, it is shown that the PS integrins are required at the muscle attachment sites of the Drosophila embryo to regulate tendon cell differentiation. The analysis of the requirements of the individual alpha subunits, alphaPS1 and alphaPS2, demonstrates that both PS1 and PS2 integrins are involved in this process. In the absence of PS integrin function, the expression of tendon cell-specific genes such as stripe and beta1 tubulin is not maintained. In addition, embryos lacking the PS integrins also exhibit reduced levels of activated MAPK. This reduction is probably due to a downregulation of the Epidermal Growth Factor receptor (Egfr) pathway, since an activated form of the Egfr can rescue the phenotype of embryos mutant for the PS integrins. Furthermore, the levels of the Egfr ligand Vein at the muscle attachment sites are reduced in PS mutant embryos. Altogether, these results lead to a model in which integrin-mediated adhesion plays a role in regulating tendon cell differentiation by modulating the activity of the Egfr pathway at the level of its ligand Vein.
23. Extracellular matrix and its receptors in Drosophila neural development. Extracellular matrix (**ECM**) and matrix receptors are intimately involved in most biological processes. The **ECM** plays fundamental developmental and physiological roles in health and disease, including processes underlying the development, maintenance, and regeneration of the nervous system. To understand the principles of **ECM**-mediated functions in the nervous system, genetic model organisms like Drosophila provide simple, malleable, and powerful experimental platforms. This article provides an overview of **ECM** proteins and receptors in Drosophila. It then focuses on their roles during three progressive phases of neural development: (1) neural progenitor proliferation, (2) axonal growth and pathfinding, and (3) synapse formation and function. Each section highlights known **ECM** and **ECM**-receptor components and recent studies done in mutant conditions to reveal their in vivo functions, all illustrating the enormous opportunities provided when merging work on the nervous system with systematic research into **ECM**-related gene functions.
24. Drosophila miR-9a targets the **ECM** receptor Dystroglycan to canalize myotendinous junction formation. Establishment of intercellular interactions between various cell types of different origin is vital for organism development and tissue maintenance. Therefore, precise timing, expression pattern, and amounts of extracellular matrix (**ECM**) proteins must be tightly regulated. Particularly, the **ECM** is important for the development and function of myotendinous junctions (MTJs). We find that precise levels of the **ECM** receptor Dystroglycan (Dg) are required for MTJ formation in Drosophila and that Dg levels in this process are controlled by miR-9a. In the embryo, Dg is enriched at the termini of the growing muscles facing the tendon matrix and absent from miR-9a-expressing tendons. This gradient of Dg expression is crucial for proper muscle-tendon attachments and is adjusted by miR-9a. In addition to Dg, miR-9a regulates the expression of several other critical muscle genes, and we therefore propose that during embryogenesis, miR-9a specifically controls the expression of mesodermal genes to canalize MTJ morphogenesis.
25. Extracellular matrix and its receptors during development. Extracellular matrix (**ECM**) components are essential for morphogenesis of virtually all tissues. The **ECM** interacts with the cell surface by binding to specific receptors. The first family of receptors for the **ECM** that was identified was the integrin family. Integrins are composed of an alpha and a beta-chain, both of which are single pass transmembrane proteins. In muscle cells the dystroglycan complex forms another important receptor system for **ECM**. It is a complex composed of many proteins. Recent studies have shown that dystroglycan is expressed by embryonic epithelial cells as well. The nature of constituents of the dystroglycan complex is well known for muscle, whereas the detailed composition of the dystroglycan complex in embryonic epithelium is not yet well known. We here review the evidence that binding of **ECM** to integrins and the dystroglycan complex could be essential for muscle and epithelial cell development and function. It is likely that integrins and the dystroglycan complex have distinct roles during development. It will be an interesting task to study the signal transduction pathways elicited by the interactions between **ECM** and the two receptor systems during muscle and epithelial morphogenesis.
26. Integrins regulate repulsion-mediated dendritic patterning of drosophila sensory neurons by restricting dendrites in a 2D space. Dendrites of the same neuron usually avoid each other. Some neurons also repel similar neurons through dendrite-dendrite interaction to tile the receptive field. Nonoverlapping coverage based on such contact-dependent repulsion requires dendrites to compete for limited space. Here we show that Drosophila class IV dendritic arborization (da) neurons, which tile the larval body wall, grow their dendrites mainly in a 2D space on the extracellular matrix (**ECM**) secreted by the epidermis. Removing neuronal integrins or blocking epidermal laminin production causes dendrites to grow into the epidermis, suggesting that integrin-laminin interaction attaches dendrites to the **ECM**. We further show that some of the previously identified tiling mutants fail to confine dendrites in a 2D plane. Expansion of these mutant dendrites in three dimensions results in overlap of dendritic fields. Moreover, overexpression of integrins in these mutant neurons effectively reduces dendritic crossing and restores tiling, revealing an additional mechanism for tiling.
27. Presynaptic secretion of mind-the-gap organizes the synaptic extracellular matrix-integrin interface and postsynaptic environments. Mind-the-Gap (MTG) is required during synaptogenesis of the Drosophila glutamatergic neuromuscular junction (NMJ) to organize the postsynaptic domain. Here, we generate MTG::GFP transgenic animals to demonstrate MTG is synaptically targeted, secreted, and localized to punctate domains in the synaptic extracellular matrix (**ECM**). Drosophila NMJs form specialized **ECM** carbohydrate domains, with carbohydrate moieties and integrin **ECM** receptors occupying overlapping territories. Presynaptically secreted MTG recruits and reorganizes secreted carbohydrates, and acts to recruit synaptic integrins and **ECM** glycans. Transgenic MTG::GFP expression rescues hatching, movement, and synaptogenic defects in embryonic-lethal mtg null mutants. Targeted neuronal MTG expression rescues mutant synaptogenesis defects, and increases rescue of adult viability, supporting an essential neuronal function. These results indicate that presynaptically secreted MTG regulates the **ECM**-integrin interface, and drives an inductive mechanism for the functional differentiation of the postsynaptic domain of glutamatergic synapses. We suggest that MTG pioneers a novel protein family involved in **ECM**-dependent synaptic differentiation.
28. Integrin-mediated adhesion maintains sarcomeric integrity. Integrin-mediated adhesion to the **ECM** is essential for normal development of animal tissues. During muscle development, integrins provide the structural stability required to construct such a highly tensile, force generating tissue. Mutations that disrupt integrin-mediated adhesion in skeletal muscles give rise to a myopathy in humans and mice. To determine if this is due to defects in formation or defects in maintenance of muscle tissue, we used an inducible, targeted RNAi based approach to disrupt integrin-mediated adhesion in fully formed adult fly muscles. A decrease in integrin-mediated adhesion in adult muscles led to a progressive loss of muscle function due to a failure to maintain normal sarcomeric cytoarchitecture. This defect was due to a gradual, age dependent disorganization of the sarcomeric actin, Z-line, and M-line. Electron microscopic analysis showed that reduction in integrin-mediated adhesion resulted in detachment of actin filaments from the Z-lines, separation of the Z-lines from the membrane, and eventually to disintegration of the Z-lines. Our results show that integrin-mediated adhesion is essential for maintaining sarcomeric integrity and illustrate that the seemingly stable adhesive contacts underlying sarcomeric architecture are inherently dynamic.
29. Integrin-**ECM** interactions regulate the changes in cell shape driving the morphogenesis of the Drosophila wing epithelium. During development, morphogenesis involves migration and changes in the shape of epithelial sheets, both of which require coordination of cell adhesion. Thus, while modulation of integrin-mediated adhesion to the **ECM** regulates epithelial motility, cell-cell adhesion via cadherins controls the remodelling of epithelial sheets. We have used the Drosophila wing epithelium to demonstrate that cell-**ECM** interactions mediated by integrins also regulate the changes in cell shape that underly epithelial morphogenesis. We show that integrins control the transitions from columnar to cuboidal cell shape underlying wing formation, and we demonstrate that eliminating the **ECM** has the same effect on cell shape as inhibiting integrin function. Furthermore, lack of integrin activity also induces detachment of the basal lamina and failure to assemble the basal matrix. Hence, we propose that integrins control epithelial cell shape by mediating adherence of these cells to the **ECM**. Finally, we show that the **ECM** has an instructive rather than a structural role, because inhibition of Raf reverses the cell shape changes caused by perturbing integrins.
30. Perlecan and Dystroglycan act at the basal side of the Drosophila follicular epithelium to maintain epithelial organization. Dystroglycan (Dg) is a widely expressed extracellular matrix (**ECM**) receptor required for muscle viability, synaptogenesis, basementmembrane formation and epithelial development. As an integral component of the Dystrophin-associated glycoprotein complex, Dg plays a central role in linking the **ECM** and the cytoskeleton. Disruption of this linkage in skeletal muscle leads to various types of muscular dystrophies. In epithelial cells, reduced expression of Dg is associated with increased invasiveness of cancer cells. We have previously shown that Dg is required for epithelial cell polarity in Drosophila, but the mechanisms of this polarizing activity and upstream/downstream components are largely unknown. Using the Drosophila follicle-cell epithelium (FCE) as a model system, we show that the **ECM** molecule Perlecan (Pcan) is required for maintenance of epithelial-cell polarity. Follicle cells that lack Pcan develop polarity defects similar to those of Dg mutant cells. Furthermore, Dg depends on Pcan but not on Laminin A for its localization in the basal-cell membrane, and the two proteins bind in vitro. Interestingly, the Dg form that interacts with Pcan in the FCE lacks the mucin-like domain, which is thought to be essential for Dg ligand binding activity. Finally, we describe two examples of how Dg promotes the differentiation of the basal membrane domain: (1) by recruiting/anchoring the cytoplasmic protein Dystrophin; and (2) by excluding the transmembrane protein Neurexin. We suggest that the interaction of Pcan and Dg at the basal side of the epithelium promotes basal membrane differentiation and is required for maintenance of cell polarity in the FCE.
31. M-spondin, a novel **ECM** protein highly homologous to vertebrate F-spondin, is localized at the muscle attachment sites in the Drosophila embryo. The muscle attachment site (MAS) in Drosophila provides a unique and excellent model system to study the mechanism of cell-matrix adhesion in developing organisms. Here, we report on the isolation and characterization of a novel extracellular matrix (**ECM**) molecule localized at the MAS, encoded by the M-spondin (mspo) gene. M-spondin protein contains a thrombospondin type I repeat (TSR) previously found in a variety of **ECM** molecules. Furthermore, it shares two conserved domains with F-spondin, a vertebrate **ECM** molecule with TSRs. The presence of TSR(s) and the two homologous domains thus defines a novel gene family of **ECM** molecules. The mspo mRNA was expressed by a large subset of muscles in the embryonic body wall. Secreted M-spondin protein diffused and eventually became immobilized at the MAS in late embryos. When expressed in S2 cells, the protein was secreted and became concentrated in the matrix on the surface of the culture dish. Genetic analysis revealed that both deletion mutants and misexpression mutants suffered no obvious developmental defects. We propose that M-spondin, although its function is redundant, is a component of the **ECM** and mediates mechanical linkage between the muscles and apodemes.
32. Global tissue revolutions in a morphogenetic movement controlling elongation. Polarized cell behaviors drive axis elongation in animal embryos, but the mechanisms underlying elongation of many tissues remain unknown. Eggs of Drosophila undergo elongation from a sphere to an ellipsoid during oogenesis. We used live imaging of follicles (developing eggs) to elucidate the cellular basis of egg elongation. We find that elongating follicles undergo repeated rounds of circumferential rotation around their long axes. Follicle epithelia mutant for integrin or collagen IV fail to rotate and elongate, which results in round eggs. We present evidence that polarized rotation is required to build a polarized, fibrillar extracellular matrix (**ECM**) that constrains tissue shape. Thus, global tissue rotation is a morphogenetic behavior that uses planar polarity information in the **ECM** to control tissue elongation.
33. Integrins contribute to the establishment and maintenance of cell polarity in the follicular epithelium of the Drosophila ovary. The generation of epithelial cell polarity is a key process during development. Although the induction and orientation of cell polarity by cell-cell and cell-extracellular matrix (**ECM**) interactions is well established, the molecular mechanisms by which signals from the **ECM** control cell polarity in developing epithelial tissues remain poorly understood. Here, we have used the follicular epithelium of the Drosophila ovary to investigate the role that integrins, the main cell-**ECM** receptors, play in the establishment of apicobasal polarity. Mature follicle cells have an apical side facing the germ line and a basal side in contact with a basement membrane. Our results show that integrins - presumably via interactions with the basement membrane - play a reinforcing role in follicle cell polarization, as they are required to establish and/or maintain follicle cell membrane asymmetry only when contact with the germ line is prevented. We suggest that the primary cue for polarization of the follicular epithelium is contact with the germline cells. In addition, while interfering with apical and lateral polarization cues leads to apoptosis, we show here that inhibition of contact with the basement membrane mediated by integrins does not affect cell survival. Finally, we provide evidence to suggest that integrins are required to orientate epithelial polarity in vivo.
34. Mechanical force regulates integrin turnover in Drosophila in vivo. Regulated assembly and disassembly, or turnover, of integrin-mediated cell-extracellular matrix (**ECM**) adhesions is essential for dynamic cell movements and long-term tissue maintenance. For example, in Drosophila, misregulation of integrin turnover disrupts muscle-tendon attachment at myotendinous junctions (MTJs). We demonstrate that mechanical force, which modulates integrin activity, also regulates integrin and intracellular adhesion complex (IAC) turnover in vivo. Using conditional mutants to alter the tensile force on MTJs, we found that the proportion of IAC components undergoing turnover inversely correlated with the force applied on MTJs. This effect was disrupted by point mutations in β-integrin that interfere with **EC**M-induced conformational changes and activation of β-integrin or integrin-mediated cytoplasmic signalling. These mutants also disrupted integrin dynamics at MTJs during larval development. Together, these data suggest that specific β-integrin-mediated signals regulate adhesion turnover in response to tension during tissue formation. We propose that integr**in-**ECM adhesive stability is continuously controlled by force in vivo through integrin-dependent auto-regulatory feedback mechanisms so that tissues can quickly adapt to and withstand mechanical stresses.
35. Epidermis-Derived Semaphorin Promotes Dendrite Self-Avoidance by Regulating Dendrite-Substrate Adhesion in Drosophila Sensory Neurons. Precise patterning of dendritic arbors is critical for the wiring and function of neural circuits. Dendrite-extracellular matrix (**ECM**) adhesion ensures that the dendrites of Drosophila dendritic arborization (da) sensory neurons are properly restricted in a 2D space, and thereby facilitates contact-mediated dendritic self-avoidance and tiling. However, the mechanisms regulating dendrite-**ECM** adhesion in vivo are poorly understood. Here, we show that mutations in the semaphorin ligand sema-2b lead to a dramatic increase in self-crossing of dendrites due to defects in dendrite-**ECM** adhesion, resulting in a failure to confine dendrites to a 2D plane. Furthermore, we find that Sema-2b is secreted from the epidermis and signals through the Plexin B receptor in neighboring neurons. Importantly, we find that Sema-2b/PlexB genetically and physically interacts with TORC2 complex, Tricornered (Trc) kinase, and integrins. These results reveal a novel role for semaphorins in dendrite patterning and illustrate how epidermal-derived cues regulate neural circuit assembly.
36. Tenectin is a novel alphaPS2betaPS integrin ligand required for wing morphogenesis and male genital looping in Drosophila. Morphogenesis of the adult structures of holometabolous insects is regulated by ecdysteroids and juvenile hormones and involves cell-cell interactions mediated in part by the cell surface integrin receptors and their extracellular matrix (**ECM**) ligands. These adhesion molecules and their regulation by hormones are not well characterized. We describe the gene structure of a newly described **ECM** molecule, tenectin, and demonstrate that it is a hormonally regulated **ECM** protein required for proper morphogenesis of the adult wing and male genitalia. Tenectin's function as a new ligand of the PS2 integrins is demonstrated by both genetic interactions in the fly and by cell spreading and cell adhesion assays in cultured cells. Its interaction with the PS2 integrins is dependent on RGD and RGD-like motifs. Tenectin's function in looping morphogenesis in the development of the male genitalia led to experiments that demonstrate a role for PS integrins in the execution of left-right asymmetry.
37. In vivo regulation of integrin turnover by outside-in activation. The development of three-dimensional tissue architecture requires precise control over the attachment of cells to the extracellular matrix (**ECM**). Integrins, the main **ECM**-binding receptors in animals, are regulated in multiple ways to modulate cell-**ECM** adhesion. One example is the conformational activation of integrins by extracellular signals ('outside-in activation') or by intracellular signals ('inside-out activation'), whereas another is the modulation of integrin turnover. We demonstrate that outside-in activation regulates integrin turnover to stabilize tissue architecture in vivo Treating Drosophila embryos with Mg(2+) and Mn(2+), known to induce outside-in activation, resulted in decreased integrin turnover. Mathematical modeling combined with mutational analysis provides mechanistic insight into the stabilization of integrins at the membrane. We show that as tissues mature, outside-in activation is crucial for regulating the stabilization of integrin-mediated adhesions. This data identifies a new in vivo role for outside-in activation and sheds light on the key transition between tissue morphogenesis and maintenance.
38. Zasp regulates integrin activation. Integrins are heterodimeric adhesion receptors that link the extracellular matrix (**ECM**) to the cytoskeleton. Binding of the scaffold protein, talin, to the cytoplasmic tail of β-integrin causes a conformational change of the extracellular domains of the integrin heterodimer, thus allowing high-affinity binding of **EC**M ligands. This essential process is called integrin activation. Here we report that the Z-band alternatively spliced PDZ-motif-containing protein (Zasp) cooperates with talin to activate α5β1 integrins in mammalian tissue culture and αPS2βPS integrins in Drosophila. Zasp is a PDZ-LIM-domain-containing protein mutated in human cardiomyopathies previously thought to function primarily in assembly and maintenance of the muscle contractile machinery. Notably, Zasp is the first protein shown to co-activate α5β1 integrins with talin and appears to do so in a manner distinct from known αIIbβ3 integrin co-activators.
39. IPP Complex Reinforces Adhesion by Relaying Tension-Dependent Signals to Inhibit Integrin Turnover. Cytoskeleton-mediated forces regulate the assembly and function of integrin adhesions; however, the underlying mechanisms remain unclear. The tripartite IPP complex, comprising ILK, Parvin, and PINCH, mediates the integrin-actin link at Drosophila embryo muscle attachment sites (MASs). Here, we demonstrate a developmentally earlier function for the IPP complex: to reinforce integrin-extracellular matrix (**ECM**) adhesion in response to tension. In IPP-complex mutants, the integrin-**ECM** linkage at MASs breaks in response to intense muscle contractility. Mechanistically, the IPP complex is required to relay force-elicited signals that decelerate integrin turnover at the plasma membrane so that the integrin immobile fraction is adequate to withstand tension. Epistasis analysis shows that alleviation of muscle contractility, downregulation of endocytosis, and enhanced integrin binding to the **ECM** are sufficient to restore integrin-**ECM** adhesion and maintain integrin-adhesome organization in IPP-complex mutants. Our findings reveal a role for the IPP complex as an essential mechanosensitive regulatory switch of integrin turnover in vivo.
40. Papillote and Piopio: Drosophila ZP-domain proteins required for cell adhesion to the apical extracellular matrix and microtubule organization. Adhesion between epithelial cells and extracellular substrates is normally mediated through basal adhesion complexes. However, some cells also possess comparable junctions on their apical surface. Here, we describe two new Drosophila proteins, Piopio and Papillote, that are required for the link between the apical epithelial surface and the overlying apical extracellular matrix (a**ECM**). The two proteins share a zona pellucida (ZP) domain with mammalian a**ECM** components, including the tectorins found in the vertebrate inner ear. Tagged versions of both proteins localized to the apical epithelial surface. Mutations in piopio, papillote and dumpy (another gene encoding a ZP-domain protein) cause defects in the innermost layer of the a**ECM** and its detachment from the epidermis. Loss of Piopio, but not Papillote or Dumpy, causes the absence of specialized microtubule bundles from pupal wings, suggesting that Piopio plays a role in microtubule organization. Thus, ZP domain-containing proteins may have shared functions within the a**ECM**, while also exhibiting specific interactions with the cytoskeleton.
41. The receptor tyrosine phosphatase Dlar and integrins organize actin filaments in the Drosophila follicular epithelium. Regulation of actin structures is instrumental in maintaining proper cytoarchitecture in many tissues. In the follicular epithelium of Drosophila ovaries, a system of actin filaments is coordinated across the basal surface of cells encircling the oocyte. These filaments have been postulated to regulate oocyte elongation; however, the molecular components that control this cytoskeletal array are not yet understood.We find that the receptor tyrosine phosphatase (RPTP) Dlar and integrins are involved in organizing basal actin filaments in follicle cells. Mutations in Dlar and the common beta-integrin subunit mys cause a failure in oocyte elongation, which is correlated with a loss of proper actin filament organization. Immunolocalization shows that early in oogenesis Dlar is polarized to membranes where filaments terminate but becomes generally distributed late in development, at which time beta-integrin and Enabled specifically associate with actin filament terminals. Rescue experiments point to the early period of polar Dlar localization as critical for its function. Furthermore, clonal analysis shows that loss of Dlar or mys influences actin filament polarity in wild-type cells that surround mutant tissues, suggesting that communication between neighboring cells regulates cytoskeletal organization. Finally, we find that two integrin alpha subunits encoded by mew and if are required for proper oocyte elongation, implying that multiple components of the **ECM** are instructive in coordinating actin fiber polarity.Dlar cooperates with integrins to coordinate actin filaments at the basal surface of the follicular epithelium. To our knowledge, this is the first direct demonstration of an RPTP's influence on the actin cytoskeleton.
42. Drosophila perlecan regulates intestinal stem cell activity via cell-matrix attachment. Stem cells require specialized local microenvironments, termed niches, for normal retention, proliferation, and multipotency. Niches are composed of cells together with their associated extracellular matrix (**ECM**). Currently, the roles of **ECM** in regulating niche functions are poorly understood. Here, we demonstrate that Perlecan (Pcan), a highly conserved **ECM** component, controls intestinal stem cell (ISC) activities and ISC-**ECM** attachment in Drosophila adult posterior midgut. Loss of Pcan from ISCs, but not other surrounding cells, causes ISCs to detach from underlying **EC**M, lose their identity, and fail to proliferate. These defects are not a result of a loss of epidermal growth factor receptor (EGFR) or Janus kinase/signal transducer and activator of transcription (JAK/STAT) signaling activity but partially depend on integrin signaling activity. We propose that Pcan secreted by ISCs confers niche properties to the adjacen**t E**CM that is required for ISC maintenance of stem cell identity, activity, and anchorage to the niche.
43. Moleskin is essential for the formation of the myotendinous junction in Drosophila. It is the precise connectivity between skeletal muscles and their corresponding tendon cells to form a functional myotendinous junction (MTJ) that allows for the force generation required for muscle contraction and organismal movement. The Drosophila MTJ is composed of secreted extracellular matrix (**ECM**) proteins deposited between integrin-mediated hemi-adherens junctions on the surface of muscle and tendon cells. In this paper, we have identified a novel, cytoplasmic role for the canonical nuclear import protein Moleskin (Msk) in Drosophila embryonic somatic muscle attachment. Msk protein is enriched at muscle attachment sites in late embryogenesis and msk mutant embryos exhibit a failure in muscle-tendon cell attachment. Although the muscle-tendon attachment sites are reduced in size, components of the integrin complexes and **ECM** proteins are properly localized in msk mutant embryos. However, msk mutants fail to localize phosphorylated focal adhesion kinase (pFAK) to the sites of muscle-tendon cell junctions. In addition, the tendon cell specific proteins Stripe (Sr) and activated mitogen-activated protein kinase (MAPK) are reduced in msk mutant embryos. Our rescue experiments demonstrate that Msk is required in the muscle cell, but not in the tendon cells. Moreover, muscle attachment defects due to loss of Msk are rescued by an activated form of MAPK or the secreted epidermal growth factor receptor (Egfr) ligand Vein. Taken together, these findings provide strong evidence that Msk signals non-autonomously through the Vein-Egfr signaling pathway for late tendon cell late differentiation and/or maintenance.

### [Ee]xtracellular [Mm]atrix

#### Function

1. **extracellular matrix** protein binding

#### PubMed

1. Integrins are required for cardioblast polarisation in Drosophila. The formation of a tubular organ, such as the heart, requires the communication of positional and polarity signals between migratory cells. Key to this process is the establishment of a new luminal domain on the cell surface, generally from the apical domain of a migratory cell. This domain will also acquire basal properties, as it will produce a luminal **extracellular matrix**. Integrin receptors are the primary means of cell adhesion and adhesion signaling with the **extracellular matrix**. Here we characterise the requirement of Integrins in a genetic model of vasculogenesis, the formation of the heart in Drosophila.As with vertebrates, the Drosophila heart arises from lateral mesoderm that migrates medially to meet their contralateral partners, to then assemble a midline vessel. During migration, Integrins are among the first proteins restricted to the presumptive luminal domain of cardioblasts. Integrins are required for normal levels of leading edge membrane motility. Apical accumulation of Integrins is enhanced by Robo, and reciprocally, apicalisation of luminal factors like Slit and Robo requires Integrin function. Integrins may provide a template for the formation of a lumen by stabilising lumen factors like Robo. Subsequent to migration, Integrin is required for normal cardioblast alignment and lumen formation. This phenotype is most readily modified by other mutations that affect adhesion, such as Talin and **extracellular matrix** ligands.Our findings reveal an instructive role for Integrins in communicating polarising information to cells during migration, and during transition to an epithelial tube structure.
2. Transcription of the beta 1 tubulin (beta Tub56D) gene in apodemes is strictly dependent on muscle insertion during embryogenesis in Drosophila melanogaster. The insertion of the somatic musculature into the epidermis during embryogenesis of Drosophila melanogaster represents an excellent system for the investigation of cell-cell communication processes. Evidence from earlier experiments suggested that the expression of the beta 1 tubulin gene from D. melanogaster in the epidermal attachments may be dependent upon myotube insertion. Analysis of the transcription of the beta 1 tubulin gene in mutants for pair-rule or segment polarity genes revealed strong coupling of the induction process to myotube insertion. Involvement of **extracellular matrix** proteins of the integrin family could be excluded since beta 1 mRNA is detectable in the apodemes in mutants for the common subunit PS beta until muscles detach late in embryogenesis. Furthermore, the lack of specific muscles in the myogenic mutants rolling stone (rost) and not enough muscles (nem) eliminates beta 1 transcription exclusively in the corresponding apodemes. The data presented clearly show that the transcription of the beta 1 tubulin gene in the muscle attachment sites is activated by myotube insertion by a yet unidentified pathway.
3. Peak BMP Responses in the Drosophila Embryo Are Dependent on the Activation of Integrin Signaling. Within a 3D tissue, cells need to integrate signals from growth factors, such as BMPs, and the **extracellular matrix** (ECM) to coordinate growth and differentiation. Here, we use the Drosophila embryo as a model to investigate how BMP responses are influenced by a cell's local ECM environment. We show that integrins, which are ECM receptors, are absolutely required for peak BMP signaling. This stimulatory effect of integrins requires their intracellular signaling function, which is activated by the ECM protein collagen IV. Mechanistically, integrins interact with the BMP receptor and stimulate phosphorylation of the downstream Mad transcription factor. The BMP-pathway-enhancing function of integrins is independent of focal adhesion kinase, but it requires conserved NPXY motifs in the β-integrin cytoplasmic tail. Furthermore, we show that an α-integrin subunit is a BMP target gene, identifying positive feedback between integrin signaling and BMP pathway activity that may contribute to robust cell fate decisions.
4. Drosophila importin-7 functions upstream of the Elmo signaling module to mediate the formation and stability of muscle attachments. Establishment and maintenance of stable muscle attachments is essential for coordinated body movement. Studies in Drosophila have pioneered a molecular understanding of the morphological events in the conserved process of muscle attachment formation, including myofiber migration, muscle-tendon signaling, and stable junctional adhesion between muscle cells and their corresponding target insertion sites. In both Drosophila and vertebrate models, integrin complexes play a key role in the biogenesis and stability of muscle attachments through the interactions of integrins with **extracellular matrix** (ECM) ligands. We show that Drosophila importin-7 (Dim7) is an upstream regulator of the conserved Elmo-Mbc→Rac signaling pathway in the formation of embryonic muscle attachment sites (MASs). Dim7 is encoded by the moleskin (msk) locus and was identified as an Elmo-interacting protein. Both Dim7 and Elmo localize to the ends of myofibers coincident with the timing of muscle-tendon attachment in late myogenesis. Phenotypic analysis of elmo mutants reveal muscle attachment defects similar to those previously described for integrin mutants. Furthermore, Elmo and Dim7 interact both biochemically and genetically in the developing musculature. The muscle detachment phenotype resulting from mutations in the msk locus can be rescued by components in the Elmo signaling pathway, including the Elmo-Mbc complex, an activated Elmo variant, or a constitutively active form of Rac. In larval muscles, the localization of Dim7 and activated Elmo to the sites of muscle attachment is attenuated upon RNAi knockdown of integrin heterodimer complex components. Our results show that integrins function as upstream signals to mediate Dim7-Elmo enrichment to the MASs.
5. An O-glycosyltransferase promotes cell adhesion during development by influencing secretion of an **extracellular matrix** integrin ligand. Protein secretion and localization are crucial during eukaryotic development, establishing local cell environments as well as mediating cell interactions, signaling, and adhesion. In this study, we demonstrate that the glycosyltransferase, pgant3, specifically modulates integrin-mediated cell adhesion by influencing the secretion and localization of the integrin ligand, Tiggrin. We demonstrate that Tiggrin is normally O-glycosylated and localized to the basal matrix where the dorsal and ventral cell layers adhere in wild type Drosophila wings. In pgant3 mutants, Tiggrin is no longer O-glycosylated and fails to be properly secreted to this basal cell layer interface, resulting in disruption of integrin-mediated cell adhesion in the wing. pgant3-mediated effects are dependent on enzymatic activity, as mutations that form a stable protein yet abrogate O-glycosyltransferase activity result in Tiggrin accumulation within the dorsal and ventral cells comprising the wing. Our results provide the first in vivo evidence for the role of O-glycosylation in the secretion of specific **extracellular matrix** proteins, thus altering the composition of the cellular "microenvironment" and thereby modulating developmentally regulated cell adhesion events. As alterations in cell adhesion are a hallmark of cancer progression, this work provides insight into the long-standing association between aberrant O-glycosylation and tumorigenesis.
6. Drosophila PS1 integrin is a laminin receptor and differs in ligand specificity from PS2. We have expressed Drosophila position-specific (PS) integrins on the surfaces of Schneider S2 cells and tested for adhesion and spreading on various matrix molecules. We report that PS1 integrin is a laminin receptor and that PS1 and PS2 integrins promote cell spreading on two different Drosophila **extracellular matrix** molecules, laminin and tiggrin, respectively. The differing ligand specificities of these two integrins, combined with data on the in vivo expression patterns of the integrins and their ligands, lead to a model for the structure of integrin-dependent attachments in the pupal wings and embryonic muscles of Drosophila.
7. Integrins are necessary for the development and maintenance of the glial layers in the Drosophila peripheral nerve. Peripheral nerve development involves multiple classes of glia that cooperate to form overlapping glial layers paired with the deposition of a surrounding **extracellular matrix** (ECM). The formation of this tubular structure protects the ensheathed axons from physical and pathogenic damage and from changes in the ionic environment. Integrins, a major family of ECM receptors, play a number of roles in the development of myelinating Schwann cells, one class of glia ensheathing the peripheral nerves of vertebrates. However, the identity and the role of the integrin complexes utilized by the other classes of peripheral nerve glia have not been determined in any animal. Here, we show that, in the peripheral nerves of Drosophila melanogaster, two integrin complexes (αPS2βPS and αPS3βPS) are expressed in the different glial layers and form adhesion complexes with integrin-linked kinase and Talin. Knockdown of the common beta subunit (βPS) using inducible RNAi in all glial cells results in lethality and glial defects. Analysis of integrin complex function in specific glial layers showed that loss of βPS in the outermost layer (the perineurial glia) results in a failure to wrap the nerve, a phenotype similar to that of Matrix metalloproteinase 2-mediated degradation of the ECM. Knockdown of βPS integrin in the innermost wrapping glia causes a loss of glial processes around axons. Together, our data suggest that integrins are employed in different glial layers to mediate the development and maintenance of the protective glial sheath in Drosophila peripheral nerves.
8. The integrin adhesion complex changes its composition and function during morphogenesis of an epithelium. Cell adhesion to the **extracellular matrix** (ECM) is mediated by the integrin family of transmembrane receptors. Integrins link ECM ligands to the cytoskeleton, providing strong attachment to enable cell-shape change and tissue integrity. This connection is made possible by an intracellular complex of proteins, which links to actin filaments and controls signalling cascades that regulate cytoskeletal rearrangements. We have identified stress-fibre-associated focal adhesions that change their composition during tissue morphogenesis. Early expression of alphaPS1betaPS integrin decreases the levels of the actin-nucleating factors Enabled, Diaphanous and profilin, as well as downregulating the amount of F-actin incorporated into the stress fibres. As follicle cells mature in their developmental pathway and become squamous, the integrin in the focal adhesions changes from alphaPS1betaPS to alphaPS2betaPS. During the switch, stress fibres increase their length and change orientation, first changing by 90 degrees and then reorienting back. The normal rapid reorientation requires new expression of alphaPS2betaPS, which also permits recruitment of the adaptor protein tensin. Unexpectedly, it is the extracellular portion of the alphaPS2 subunit that provides the specificity for intracellular recruitment of tensin. Molecular variation of the integrin complex is thus a key component of developmentally programmed morphogenesis.
9. AlphaPS2 integrin-mediated muscle attachment in Drosophila requires the ECM protein Thrombospondin. During Drosophila embryogenesis, the attachment of somatic muscles to epidermal tendon cells requires heterodimeric PS-integrin proteins (alpha- and beta-subunits). The alpha-subunits are expressed complementarily, either tendon cell- or muscle-specific, whereas the beta-integrin subunit is expressed in both tissues. Mutations of beta-integrin cause a severe muscle detachment phenotype, whereas alpha-subunit mutations have weaker or only larval muscle detachment phenotypes. Furthermore, mutations of **extracellular matrix** (ECM) proteins known to act as integrin binding partners have comparatively weak effects only, suggesting the presence of additional integrin binding ECM proteins required for proper muscle attachment. Here, we report that mutations in the Drosophila gene thrombospondin (tsp) cause embryonic muscle detachment. tsp is specifically expressed in both developing and mature epidermal tendon cells. Its initial expression in segment border cells, the tendon precursors, is under the control of hedgehog-dependent signaling, whereas tsp expression in differentiated tendon cells depends on the transcription factor encoded by stripe. In the absence of tsp activity, no aspect of muscle pattern formation as well as the initial contact between muscle and tendon cells nor muscle-to-muscle attachments are affected. However, when muscle contractions occur during late embryogenesis, muscles detach from the tendon cells. The Tsp protein is localized to the tendon cell ECM where muscles attach. Genetic interaction studies indicate that Tsp specifically interacts with the alphaPS2 integrin and that this interaction is needed to withstand the forces of muscle contractions at the tendon cells.
10. Thrombospondin-mediated adhesion is essential for the formation of the myotendinous junction in Drosophila. Organogenesis of the somatic musculature in Drosophila is directed by the precise adhesion between migrating myotubes and their corresponding ectodermally derived tendon cells. Whereas the PS integrins mediate the adhesion between these two cell types, their **extracellular matrix** (ECM) ligands have been only partially characterized. We show that the ECM protein Thrombospondin (Tsp), produced by tendon cells, is essential for the formation of the integrin-mediated myotendinous junction. Tsp expression is induced by the tendon-specific transcription factor Stripe, and accumulates at the myotendinous junction following the association between the muscle and the tendon cell. In tsp mutant embryos, migrating somatic muscles fail to attach to tendon cells and often form hemiadherens junctions with their neighboring muscle cells, resulting in nonfunctional somatic musculature. Talin accumulation at the cytoplasmic faces of the muscles and tendons is greatly reduced, implicating Tsp as a potential integrin ligand. Consistently, purified Tsp C-terminal domain polypeptide mediates spreading of PS2 integrin-expressing S2 cells in a KGD- and PS2-integrin-dependent manner. We propose a model in which the myotendinous junction is formed by the specific association of Tsp with multiple muscle-specific PS2 integrin receptors and a subsequent consolidation of the junction by enhanced tendon-specific production of Tsp secreted into the junctional space.
11. The integrins. The integrins are a superfamily of cell adhesion receptors that bind to **extracellular matrix** ligands, cell-surface ligands, and soluble ligands. They are transmembrane alphabeta heterodimers and at least 18 alpha and eight beta subunits are known in humans, generating 24 heterodimers. Members of this family have been found in mammals, chicken and zebrafish, as well as lower eukaryotes, including sponges, the nematode Caenorhabditis elegans (two alpha and one beta subunits, generating two integrins) and the fruitfly Drosophila melanogaster (five alpha and one beta, generating five integrins). The alpha and beta subunits have distinct domain structures, with extracellular domains from each subunit contributing to the ligand-binding site of the heterodimer. The sequence arginine-glycine-aspartic acid (RGD) was identified as a general integrin-binding motif, but individual integrins are also specific for particular protein ligands. Immunologically important integrin ligands are the intercellular adhesion molecules (ICAMs), immunoglobulin superfamily members present on inflamed endothelium and antigen-presenting cells. On ligand binding, integrins transduce signals into the cell interior; they can also receive intracellular signals that regulate their ligand-binding affinity. Here we provide a brief overview that concentrates mostly on the organization, structure and function of mammalian integrins, which have been more extensively studied than integrins in other organisms.
12. The serine/threonine kinase dPak is required for polarized assembly of F-actin bundles and apical-basal polarity in the Drosophila follicular epithelium. During epithelial development cells become polarized along their apical-basal axis and some epithelia also exhibit polarity in the plane of the tissue. Mutations in the gene encoding a Drosophila Pak family serine/threonine kinase, dPak, disrupt the follicular epithelium that covers developing egg chambers during oogenesis. The follicular epithelium normally exhibits planar polarized organization of basal F-actin bundles such that they lie perpendicular to the anterior-posterior axis of the egg chamber, and requires contact with the basement membrane for apical-basal polarization. During oogenesis, dPak becomes localized to the basal end of follicle cells and is required for polarized organization of the basal actin cytoskeleton and for epithelial integrity and apical-basal polarity. The receptor protein tyrosine phosphatase Dlar and integrins, all receptors for **extracellular matrix** proteins, are required for polarization of the basal F-actin bundles, and for correct dPak localization in follicle cells. dpak mutant follicle cells show increased beta(Heavy)-spectrin levels, and we speculate that dPak regulation of beta(Heavy)-spectrin, a known participant in the maintenance of membrane domains, is required for correct apical-basal polarization of the membrane. We propose that dPak mediates communication between the basement membrane and intracellular proteins required for polarization of the basal F-actin and for apical-basal polarity.
13. The Gyc76C Receptor Guanylyl Cyclase and the Foraging cGMP-Dependent Kinase Regulate **Extracellular Matrix** Organization and BMP Signaling in the Developing Wing of Drosophila melanogaster. The developing crossveins of the wing of Drosophila melanogaster are specified by long-range BMP signaling and are especially sensitive to loss of extracellular modulators of BMP signaling such as the Chordin homolog Short gastrulation (Sog). However, the role of the **extracellular matrix** in BMP signaling and Sog activity in the crossveins has been poorly explored. Using a genetic mosaic screen for mutations that disrupt BMP signaling and posterior crossvein development, we identify Gyc76C, a member of the receptor guanylyl cyclase family that includes mammalian natriuretic peptide receptors. We show that Gyc76C and the soluble cGMP-dependent kinase Foraging, likely linked by cGMP, are necessary for normal refinement and maintenance of long-range BMP signaling in the posterior crossvein. This does not occur through cell-autonomous crosstalk between cGMP and BMP signal transduction, but likely through altered extracellular activity of Sog. We identify a novel pathway leading from Gyc76C to the organization of the wing **extracellular matrix** by matrix metalloproteinases, and show that both the **extracellular matrix** and BMP signaling effects are largely mediated by changes in the activity of matrix metalloproteinases. We discuss parallels and differences between this pathway and other examples of cGMP activity in both Drosophila melanogaster and mammalian cells and tissues.
14. The microRNA bantam regulates a developmental transition in epithelial cells that restricts sensory dendrite growth. As animals grow, many early born structures grow by cell expansion rather than cell addition; thus growth of distinct structures must be coordinated to maintain proportionality. This phenomenon is particularly widespread in the nervous system, with dendrite arbors of many neurons expanding in concert with their substrate to sustain connectivity and maintain receptive field coverage as animals grow. After rapidly growing to establish body wall coverage, dendrites of Drosophila class IV dendrite arborization (C4da) neurons grow synchronously with their substrate, the body wall epithelium, providing a system to study how proportionality is maintained during animal growth. Here, we show that the microRNA bantam (ban) ensures coordinated growth of C4da dendrites and the epithelium through regulation of epithelial endoreplication, a modified cell cycle that entails genome amplification without cell division. In Drosophila larvae, epithelial endoreplication leads to progressive changes in dendrite-**extracellular matrix** (ECM) and dendrite-epithelium contacts, coupling dendrite/substrate expansion and restricting dendrite growth beyond established boundaries. Moreover, changes in epithelial expression of cell adhesion molecules, including the beta-integrin myospheroid (mys), accompany this developmental transition. Finally, endoreplication and the accompanying changes in epithelial mys expression are required to constrain late-stage dendrite growth and structural plasticity. Hence, modulating epithelium-ECM attachment probably influences substrate permissivity for dendrite growth and contributes to the dendrite-substrate coupling that ensures proportional expansion of the two cell types.
15. The PS2 integrin ligand tiggrin is required for proper muscle function in Drosophila. Tiggrin is a novel **extracellular matrix** ligand for the Drosophila PS2 integrins. We have used flanking P elements to generate a precise deletion of tiggrin. Most flies lacking tiggrin die as larvae or pupae. A few adults do emerge and these appear to be relatively normal, displaying only misshapen abdomens and a low frequency of wing defects. Examination of larvae shows that muscle connections, function and morphology are defective in tiggrin mutants. Muscle contraction waves that extend the length of the larvae are much slower in tiggrin mutants. Direct examination of bodywall muscles shows defects in muscle attachment sites, where tiggrin is specifically localized, and muscles appear thinner. Transgenes expressing tiggrin are capable of rescuing tiggrin mutant phenotypes. Transgenes expressing a mutant tiggrin, whose Arg-Gly-Asp (RGD) integrin recognition sequence has been mutated to Leu-Gly-Ala (LGA) show much reduced, but significant, rescuing ability. Cell spreading assays detect no interactions of this mutant tiggrin with PS2 integrins. Therefore, while the RGD sequence is critical for PS2 interactions and full activity in the whole fly, the mutant tiggrin retains some function(s) that are probably mediated by interactions with other ECM molecules or cell surface receptors
16. Integrins modulate Sog activity in the Drosophila wing. Morphogenesis of the Drosophila wing depends on a series of cell-cell and cell-**extracellular matrix** interactions. During pupal wing development, two secreted proteins, encoded by the short gastrulation (sog) and decapentaplegic (dpp) genes, vie to position wing veins in the center of broad provein territories. Expression of the Bmp4 homolog dpp in vein cells is counteracted by expression of the Bmp antagonist sog in intervein cells, which results in the formation of straight veins of precise width. We screened for genetic interactions between sog and genes encoding a variety of extracellular components and uncovered interactions between sog and myospheroid (mys), multiple edematous wing (mew) and scab (scb), which encode betaPS, alphaPS1 and alphaPS3 integrin subunits, respectively. Clonal analysis reveals that integrin mutations affect the trajectory of veins inside the provein domain and/or their width and that misexpression of sog can alter the behavior of cells in such clones. In addition, we show that a low molecular weight form of Sog protein binds to alphaPS1betaPS. We find that Sog can diffuse from its intervein site of production into adjacent provein domains, but only on the dorsal surface of the wing, where Sog interacts functionally with integrins. Finally, we show that Sog diffusion into provein regions and the reticular pattern of extracellular Sog distribution in wild-type wings requires mys and mew function. We propose that integrins act by binding and possibly regulating the activity/availability of different forms of Sog during pupal development through an adhesion independent mechanism.
17. In vivo functional analysis reveals specific roles for the integrin-binding sites of talin. Adhesion receptors play diverse roles during animal development and require precise spatiotemporal regulation, which is achieved through the activity of their binding partners. Integrins, adhesion receptors that mediate cell attachment to the **extracellular matrix** (ECM), connect to the intracellular environment through the cytoplasmic adapter protein talin. Talin has two essential functions: orchestrating the assembly of the intracellular adhesion complex (IAC), which associates with integrin, and regulating the affinity of integrins for the ECM. Talin can bind to integrins through two different integrin-binding sites (IBS-1 and IBS-2, respectively). Here, we have investigated the roles of each in the context of Drosophila development. We find that although IBS-1 and IBS-2 are partially redundant, they each have specialized roles during development: IBS-1 reinforces integrin attachment to the ECM, whereas IBS-2 reinforces the link between integrins and the IAC. Disruption of each IBS has different developmental consequences, illustrating how the functional diversity of integrin-mediated adhesion is achieved.
18. Integrin signaling is required for maintenance and proliferation of intestinal stem cells in Drosophila. Tissue-specific stem cells are maintained by both local secreted signals and cell adhesion molecules that position the stem cells in the niche microenvironment. In the Drosophila midgut, multipotent intestinal stem cells (ISCs) are located basally along a thin layer of basement membrane that composed of **extracellular matrix** (ECM), which separates ISCs from the surrounding visceral musculature: the muscle cells constitute a regulatory niche for ISCs by producing multiple secreted signals that directly regulate ISC maintenance and proliferation. Here we show that integrin-mediated cell adhesion, which connects the ECM and intracellular cytoskeleton, is required for ISC anchorage to the basement membrane. Specifically, the α-integrin subunits including αPS1 encoded by mew and αPS3 encoded by scb, and the β-integrin subunit encoded by mys are richly expressed in ISCs and are required for the maintenance, rather than their survival or multiple lineage differentiation. Furthermore, ISC maintenance also requires the intercellular and intracellular integrin signaling components including Talin, Integrin-linked kinase (Ilk), and the ligand, Laminin A. Notably, integrin mutant ISCs are also less proliferative, and genetic interaction studies suggest that proper integrin signaling is a pre-requisite for ISC proliferation in response to various proliferative signals and for the initiation of intestinal hyperplasia after loss of adenomatous polyposis coli (Apc). Our studies suggest that integrin not only functions to anchor ISCs to the basement membrane, but also serves as an essential element for ISC proliferation during normal homeostasis and in response to oncogenic mutations.
19. Glia ECM interactions are required to shape the Drosophila nervous system. Organs are characterized by a specific shape that is often remodeled during development. The dynamics of organ shape is in particular evident during the formation of the Drosophila nervous system. During embryonic stages the central nervous system compacts, whereas selective growth occurs during larval stages. The nervous system is covered by a layer of surface glial cells that form the blood brain barrier and a thick **extracellular matrix** called neural lamella. The size of the neural lamella is dynamically adjusted to the growing nervous system and we show here that perineurial glial cells secrete proteases to remodel this matrix. Moreover, an imbalance in proteolytic activity results in an abnormal shape of the nervous system. To identify further components controlling nervous system shape we performed an RNAi based screen and identified the gene nolo, which encodes an ADAMTS-like protein. We generated loss of function alleles and demonstrate a requirement in glial cells. Mutant nolo larvae, however, do not show an abnormal nervous system shape. The only predicted off-target of the nolo(dsRNA) is Oatp30B, which encodes an organic anion transporting protein characterized by an extracellular protease inhibitor domain. Loss of function mutants were generated and double mutant analyses demonstrate a genetic interaction between nolo and Oatp30B which prevented the generation of maternal zygotic mutant larvae.
20. The Ret receptor regulates sensory neuron dendrite growth and integrin mediated adhesion. Neurons develop highly stereotyped receptive fields by coordinated growth of their dendrites. Although cell surface cues play a major role in this process, few dendrite specific signals have been identified to date. We conducted an in vivo RNAi screen in Drosophila class IV dendritic arborization (C4da) neurons and identified the conserved Ret receptor, known to play a role in axon guidance, as an important regulator of dendrite development. The loss of Ret results in severe dendrite defects due to loss of **extracellular matrix** adhesion, thus impairing growth within a 2D plane. We provide evidence that Ret interacts with integrins to regulate dendrite adhesion via rac1. In addition, Ret is required for dendrite stability and normal F-actin distribution suggesting it has an essential role in dendrite maintenance. We propose novel functions for Ret as a regulator in dendrite patterning and adhesion distinct from its role in axon guidance.
21. Receptor guanylyl cyclase Gyc76C is required for invagination, collective migration and lumen shape in the Drosophila embryonic salivary gland. The Drosophila embryonic salivary gland is formed by the invagination and collective migration of cells. Here, we report on a novel developmental role for receptor-type guanylyl cyclase at 76C, Gyc76C, in morphogenesis of the salivary gland. We demonstrate that Gyc76C and downstream cGMP-dependent protein kinase 1 (DG1) function in the gland and surrounding mesoderm to control invagination, collective migration and lumen shape. Loss of gyc76C resulted in glands that failed to invaginate, complete posterior migration and had branched lumens. Salivary gland migration defects of gyc76C mutant embryos were rescued by expression of wild-type gyc76C specifically in the gland or surrounding mesoderm, whereas invagination defects were rescued primarily by expression in the gland. In migrating salivary glands of gyc76C mutant embryos, integrin subunits localized normally to gland-mesoderm contact sites but talin localization in the surrounding circular visceral mesoderm and fat body was altered. The **extracellular matrix** protein, laminin, also failed to accumulate around the migrating salivary gland of gyc76C mutant embryos, and gyc76C and laminin genetically interacted in gland migration. Our studies suggest that gyc76C controls salivary gland invagination, collective migration and lumen shape, in part by regulating the localization of talin and the laminin matrix.
22. Integrins are required for cardioblast polarisation in Drosophila. The formation of a tubular organ, such as the heart, requires the communication of positional and polarity signals between migratory cells. Key to this process is the establishment of a new luminal domain on the cell surface, generally from the apical domain of a migratory cell. This domain will also acquire basal properties, as it will produce a luminal **extracellular matrix**. Integrin receptors are the primary means of cell adhesion and adhesion signaling with the **extracellular matrix**. Here we characterise the requirement of Integrins in a genetic model of vasculogenesis, the formation of the heart in Drosophila.As with vertebrates, the Drosophila heart arises from lateral mesoderm that migrates medially to meet their contralateral partners, to then assemble a midline vessel. During migration, Integrins are among the first proteins restricted to the presumptive luminal domain of cardioblasts. Integrins are required for normal levels of leading edge membrane motility. Apical accumulation of Integrins is enhanced by Robo, and reciprocally, apicalisation of luminal factors like Slit and Robo requires Integrin function. Integrins may provide a template for the formation of a lumen by stabilising lumen factors like Robo. Subsequent to migration, Integrin is required for normal cardioblast alignment and lumen formation. This phenotype is most readily modified by other mutations that affect adhesion, such as Talin and **extracellular matrix** ligands.Our findings reveal an instructive role for Integrins in communicating polarising information to cells during migration, and during transition to an epithelial tube structure.
23. Integrin-dependent anchoring of a stem-cell niche. Interactions between stem cells and their surrounding microenvironment, or niche, are critical for the establishment and maintenance of stem-cell properties. The adult Drosophila testis contains a morphologically discrete stem-cell niche, the 'hub'. The small cluster of non-dividing, somatic hub cells at the anterior tip of the fly testis is contacted by the germline stem cells (GSCs), which retain their stem-cell character through the direct association with the hub. Here we show that integrin-mediated adhesion is important for maintaining the correct position of embryonic hub cells during gonad morphogenesis. The misplaced hub in integrin-deficient embryos directs the orientation of cell divisions in the presumptive GSCs, a hallmark of the active germline stem-cell niche. A decrease in integrin-mediated adhesion in adult testes, which resulted in a loss of the hub and the stem-cell population, revealed the importance of hub-cell anchoring. Finally, we show that an **extracellular matrix** (ECM) is present around the gonad during late embryogenesis and that this ECM is defective in integrin-deficient gonads. On the basis of our data, we propose that integrins are required for the attachment of the hub cells to the ECM, which is essential for maintaining the stem-cell niche.
24. Tiggrin, a novel Drosophila **extracellular matrix** protein that functions as a ligand for Drosophila alpha PS2 beta PS integrins. Genetic and other studies of Drosophila integrins have implicated these **extracellular matrix** receptors in various morphogenetic events, but identification of their endogenous ligands has been elusive. We report the biochemical purification and cloning of tiggrin, a novel **extracellular matrix** protein from Drosophila. This 255 x 10(3) M(r) polypeptide contains the potential integrin recognition sequence Arg-Gly-Asp (RGD) and 16 repeats of a novel 73-77 amino acid motif. The tiggrin gene is at chromosome locus 26D1-2 and is expressed by embryonic hemocytes and fat body cells. Tiggrin protein is detected in matrices, especially at muscle attachment sites that also strongly express integrins. Tiggrin-coated surfaces support primary embryo cell culture and provide excellent substrates for alpha PS2 beta PS integrin-mediated cell spreading. Soluble RGD-peptides inhibit this cell spreading.
25. Amino acid changes in Drosophila alphaPS2betaPS integrins that affect ligand affinity. We developed a ligand-mimetic antibody Fab fragment specific for Drosophila alphaPS2betaPS integrins to probe the ligand binding affinities of these invertebrate receptors. TWOW-1 was constructed by inserting a fragment of the **extracellular matrix** protein Tiggrin into the H-CDR3 of the alphavbeta3 ligand-mimetic antibody WOW-1. The specificity of alphaPS2betaPS binding to TWOW-1 was demonstrated by numerous tests used for other integrin-ligand interactions. Binding was decreased in the presence of EDTA or RGD peptides and by mutation of the TWOW-1 RGD sequence or the betaPS metal ion-dependent adhesion site (MIDAS) motif. TWOW-1 binding was increased by mutations in the alphaPS2 membrane-proximal cytoplasmic GFFNR sequence or by exposure to Mn2+. Although Mn2+ is sometimes assumed to promote maximal integrin activity, TWOW-1 binding in Mn2+ could be increased further by the alphaPS2 GFFNR --> GFANA mutation. A mutation in the betaPS I domain (betaPS-b58; V409D) greatly increased ligand binding affinity, explaining the increased cell spreading mediated by alphaPS2betaPS-b58. Further mutagenesis of this residue suggested that Val-409 normally stabilizes the closed head conformation. Mutations that potentially reduce interaction of the integrin beta subunit plexin-semaphorin-integrin (PSI) and stalk domains have been shown to have activating properties. We found that complete deletion of the betaPS PSI domain enhanced TWOW-1 binding. Moreover the PSI domain is dispensable for at least some other integrin functions because betaPS-DeltaPSI displayed an enhanced ability to mediate cell spreading. These studies establish a means to evaluate mechanisms and consequences of integrin affinity modulation in a tractable model genetic system.
26. Integrins in development: moving on, responding to, and sticking to the **extracellular matrix**. Integrins are cell surface receptors of the **extracellular matrix** present in all animals. Genetic analysis in worms, flies, and vertebrates has revealed integrin involvement in key developmental processes, and we focus here on examples of integrin functions that are comparable across these model organisms. Integrins contribute to cell movement by providing traction to migrating cells, through assembly of extracellular matrices that can serve as tracks for migration, and by transmitting guidance signals that direct cells or cell processes to their targets. Integrins also participate in signaling events that govern tissue differentiation and organogenesis. Finally, adhesion by integrin-mediated junctions allows tissues to withstand mechanical load and is essential for tissue integrity.
27. Loss of focal adhesions in glia disrupts both glial and photoreceptor axon migration in the Drosophila visual system. Many aspects of glial development are regulated by extracellular signals, including those from the **extracellular matrix** (ECM). Signals from the ECM are received by cell surface receptors, including the integrin family. Previously, we have shown that Drosophila integrins form adhesion complexes with Integrin-linked kinase and talin in the peripheral nerve glia and have conserved roles in glial sheath formation. However, integrin function in other aspects of glial development is unclear. The Drosophila eye imaginal disc (ED) and optic stalk (OS) complex is an excellent model with which to study glial migration, differentiation and glia-neuron interactions. We studied the roles of the integrin complexes in these glial developmental processes during OS/eye development. The common beta subunit βPS and two alpha subunits, αPS2 and αPS3, are located in puncta at both glia-glia and glia-ECM interfaces. Depletion of βPS integrin and talin by RNAi impaired the migration and distribution of glia within the OS resulting in morphological defects. Reduction of integrin or talin in the glia also disrupted photoreceptor axon outgrowth leading to axon stalling in the OS and ED. The neuronal defects were correlated with a disruption of the carpet glia tube paired with invasion of glia into the core of the OS and the formation of a glial cap. Our results suggest that integrin-mediated extracellular signals are important for multiple aspects of glial development and non-autonomously affect axonal migration during Drosophila eye development.
28. Differentiation, **extracellular matrix** synthesis, and integrin assembly by Drosophila embryo cells cultured on vitronectin and laminin substrates. Two contrasting substrates, Drosophila laminin and human vitronectin, caused determined primary Drosophila embryo cells to follow alternate intermediate differentiation steps without affecting the final outcome of differentiation. Integrin alpha PS2 beta PS3 was essential for the initial spreading of myocytes on vitronectin: focal contacts rich in beta PS3 integrins formed and were connected by actin- and myosin-containing stress fibers. While alpha PS2 beta PS3 was unnecessary for myotube formation on laminin, it was required for the subsequent change to a sarcomeric cytoarchitecture. The differentiating primary cultures synthesized integrins and assembled them into detergent-insoluble, cytoskeleton-associated complexes. Collagen IV, laminin, glutactin, papilin, and other **extracellular matrix** proteins were made primarily by hemocytes and were secreted into the medium. Further differentiation within the cultures was influenced by secreted components and by later addition of vitronectin or bovine serum. Comparison of the differentiation of various cell types on the two substrates showed that vitronectin provided a selective advantage for the differentiation of myocytes, with enrichment over epithelia, epidermal cells, and neurites.
29. Focal adhesion kinase is not required for integrin function or viability in Drosophila. The mammalian focal adhesion kinase (FAK) family of non-receptor protein-tyrosine kinases has been implicated in controlling a multitude of cellular responses to the engagement of cell-surface integrins and G-protein-coupled receptors. The high level of sequence conservation between the mammalian proteins and the Drosophila homologue of FAK, Fak56, suggested that it would have similar functions. However, we show here that Drosophila Fak56 is not essential for integrin functions in adhesion, migration or signaling in vivo. Furthermore, animals lacking Fak56 are viable and fertile, demonstrating that Fak56 is not essential for other developmental or physiological functions. Despite this, overexpressed Fak56 is a potent inhibitor of integrins binding to the **extracellular matrix**, suggesting that Fak56 may play a subtle role in the negative regulation of integrin adhesion.
30. An interaction between integrin and the talin FERM domain mediates integrin activation but not linkage to the cytoskeleton. Transmembrane adhesion receptors, such as integrins, mediate cell adhesion by interacting with intracellular proteins that connect to the cytoskeleton. Talin, one such linker protein, is thought to have two roles: mediating inside-out activation of integrins, and connecting **extracellular matrix** (ECM)-bound integrins to the cytoskeleton. Talin's amino-terminal head, which consists of a FERM domain, binds an NPxY motif within the cytoplasmic tail of most integrin beta subunits. This is consistent with the role of FERM domains in recruiting other proteins to the plasma membrane. We tested the role of the talin-head-NPxY interaction in integrin function in Drosophila. We found that introduction of a mutation that perturbs this binding in vitro into the isolated talin head disrupts its recruitment by integrins in vivo. Surprisingly, when engineered into the full-length talin, this mutation did not disrupt talin recruitment by integrins nor its ability to connect integrins to the cytoskeleton. However, it reduced the ability of talin to strengthen integrin adhesion to the ECM, indicating that the function of the talin-head-NPxY interaction is solely to regulate integrin adhesion.
31. The localized assembly of **extracellular matrix** integrin ligands requires cell-cell contact. The assembly of an organism requires the interaction between different layers of cells, in many cases via an **extracellular matrix**. In the developing Drosophila larva, muscles attach in an integrin-dependent manner to the epidermis, via a specialized **extracellular matrix** called tendon matrix. Tiggrin, a tendon matrix integrin ligand, is primarily synthesized by cells distant to the muscle attachment sites, yet it accumulates specifically at these sites. Previous work has shown that the PS integrins are not required for tiggrin localization, suggesting that there is redundancy among tiggrin receptors. We have examined this by testing whether the PS2 integrin can recruit tiggrin to ectopic locations within the Drosophila embryo. We found that neither the wild type nor modified forms of the PS2 integrin, which have higher affinity for tiggrin, can recruit tiggrin to new cellular contexts. Next, we genetically manipulated the fate of the muscles and the epidermal muscle attachment cells, which demonstrated that muscles have the primary role in recruiting tiggrin to the tendon matrix and that cell-cell contact is necessary for this recruitment. Thus we propose that the inherent polarity of the muscle cells leads to a molecular specialization of their ends, and interactions between the ends produces an integrin-independent tiggrin receptor. Thus, interaction between cells generates an extracellular environment capable of nucleating **extracellular matrix** assembly.
32. Crag regulates epithelial architecture and polarized deposition of basement membrane proteins in Drosophila. The polarized architecture of epithelia relies on an interplay between the cytoskeleton, the trafficking machinery, and cell-cell and cell-matrix adhesion. Specifically, contact with the basement membrane (BM), an **extracellular matrix** underlying the basal side of epithelia, is important for cell polarity. However, little is known about how BM proteins themselves achieve a polarized distribution. In a genetic screen in the Drosophila follicular epithelium, we identified mutations in Crag, which encodes a conserved protein with domains implicated in membrane trafficking. Follicle cells mutant for Crag lose epithelial integrity and frequently become invasive. The loss of Crag leads to the anomalous accumulation of BM components on both sides of epithelial cells without directly affecting the distribution of apical or basolateral membrane proteins. This defect is not generally observed in mutants affecting epithelial integrity. We propose that Crag plays a unique role in organizing epithelial architecture by regulating the polarized secretion of BM proteins.
33. Distinct regulatory mechanisms control integrin adhesive processes during tissue morphogenesis. Cell adhesion must be precisely regulated to enable both dynamic morphogenetic processes and the subsequent transition to stable tissue maintenance. Integrins link the intracellular cytoskeleton and **extracellular matrix**, relaying bidirectional signals across the plasma membrane. In vitro studies have demonstrated that multiple mechanisms control integrin-mediated adhesion; however, their roles during development are poorly understood. We used mutations that activate or deactivate specific functions of vertebrate β-integrins in vitro to investigate how perturbing Drosophila βPS-integrin regulation in developing embryos regulation affects tissue morphogenesis and maintenance. We found that morphogenetic processes use various β-integrin regulatory mechanisms to differing degrees and that conformational changes associated with outside-in activation are essential for developmental integrin functions. Long-term adhesion is also sensitive to integrin dysregulation, suggesting integrins must be continuously regulated to support stable tissue maintenance. Altogether, in vivo phenotypic analyses allowed us to identify the importance of various β-integrin regulatory mechanisms during different morphogenetic processes.
34. Hox transcription factors: modulators of cell-cell and cell-**extracellular matrix** adhesion. Hox genes encode homeodomain-containing transcription factors that determine cell and tissue identities in the embryo during development. Hox genes are also expressed in various adult tissues and cancer cells. In Drosophila, expression of cell adhesion molecules, cadherins and integrins, is regulated by Hox proteins operating in hierarchical molecular pathways and plays a crucial role in segment-specific organogenesis. A number of studies using mammalian cultured cells have revealed that cell adhesion molecules responsible for cell-cell and cell-**extracellular matrix** interactions are downstream targets of Hox proteins. However, whether Hox transcription factors regulate expression of cell adhesion molecules during vertebrate development is still not fully understood. In this review, the potential roles Hox proteins play in cell adhesion and migration during vertebrate body patterning are discussed.
35. PS integrins and laminins: key regulators of cell migration during Drosophila embryogenesis. During embryonic development, there are numerous cases where organ or tissue formation depends upon the migration of primordial cells. In the Drosophila embryo, the visceral mesoderm (vm) acts as a substrate for the migration of several cell populations of epithelial origin, including the endoderm, the trachea and the salivary glands. These migratory processes require both integrins and laminins. The current model is that αPS1βPS (PS1) and/or αPS3βPS (PS3) integrins are required in migrating cells, whereas αPS2βPS (PS2) integrin is required in the vm, where it performs an as yet unidentified function. Here, we show that PS1 integrins are also required for the migration over the vm of cells of mesodermal origin, the caudal visceral mesoderm (CVM). These results support a model in which PS1 might have evolved to acquire the migratory function of integrins, irrespective of the origin of the tissue. This integrin function is highly specific and its specificity resides mainly in the extracellular domain. In addition, we have identified the Laminin α1,2 trimer, as t**he key extracellular** matrix (ECM) component regulating CVM migration. Furthermore, we show that, as it is the case in vertebrates, integrins, and specifically PS2, contributes to CVM movement by participating in the correct assembly of the ECM that serves as tracks for migration.
36. Novel functions for integrins in epithelial morphogenesis. Dorsal closure during Drosophila embryogenesis provides a valuable model for epithelial morphogenesis and wound healing. Previous studies have focused on two cell populations, the dorsal epidermis and the extraembryonic amnioserosa. Here, we demonstrate that there is an additional player, the large yolk cell. We find that integrins are expressed in the amnioserosa and yolk cell membrane and that they are required for three processes: (1) assembly of an intervening **extracellular matrix**, (2) attachment between these two cell layers, and (3) contraction of the amnioserosa cells. We also provide evidence for integrin-**extracellular matrix** interactions occurring between the lateral surfaces of the amnioserosa cell and the leading edge epidermis that effectively mediate cell-cell adhesion. Thus, dorsal closure shares mechanistic similarities with vertebrate epithelial morphogenetic events, including epiboly, that also employ an underlying substrate.
37. Cell-cell adhesion via the ECM: integrin genetics in fly and worm. Integrins are essential for the development of the two genetically tractable invertebrate model organisms, the nematode worm Caenorhabditis elegans and the fruit fly Drosophila melanogaster. Just two integrins are present in C. elegans: one putative RGD binding integrin alphapat-2betapat-3, corresponding to Drosophila alphaPS2betaPS and vertebrate alpha5beta1, alphaVbeta1 and alpha8beta1, and one putative laminin binding integrin alphaina-1betapat-3, corresponding to Drosophila alphaPS1betaPS and vertebrate alpha3beta1, alpha6beta1 and alpha7beta1. In this review, the function of this minimal set of integrins during the development of these two invertebrates is compared. Despite the differences in bodyplan and developmental strategy, integrin adhesion to the **extracellular matrix** is required for similar processes: the formation of the link that translates muscle contraction into movement of the exoskeleton, cell migration, and morphogenetic interactions between epithelia. Other integrin functions, such as regulation of gene expression, have not yet been experimentally demonstrated in both organisms. Additional proteins have been characterised in each organism that are essential for integrin function, including **extracellular matrix** ligands and intracellular interacting proteins, but so far different proteins have been found in the two organisms. This in part represents the fact that the characterisation of the full set of interacting proteins is not complete in either system. However, in other cases different proteins appear to be used for similar functions in the two animals. The continued use of genetic approaches to identify proteins required for integrin function in these two model organisms should lead to the identification of the minimal set of conserved components that form integrin adhesive structures.
38. Integrins modulate the Egfr signaling pathway to regulate tendon cell differentiation in the Drosophila embryo. Changes in the **extracellular matrix** (ECM) govern the differentiation of many cell types during embryogenesis. Integrins are cell matrix receptors that play a major role in cell-ECM adhesion and in transmitting signals from the ECM inside the cell to regulate gene expression. In this paper, it is shown that the PS integrins are required at the muscle attachment sites of the Drosophila embryo to regulate tendon cell differentiation. The analysis of the requirements of the individual alpha subunits, alphaPS1 and alphaPS2, demonstrates that both PS1 and PS2 integrins are involved in this process. In the absence of PS integrin function, the expression of tendon cell-specific genes such as stripe and beta1 tubulin is not maintained. In addition, embryos lacking the PS integrins also exhibit reduced levels of activated MAPK. This reduction is probably due to a downregulation of the Epidermal Growth Factor receptor (Egfr) pathway, since an activated form of the Egfr can rescue the phenotype of embryos mutant for the PS integrins. Furthermore, the levels of the Egfr ligand Vein at the muscle attachment sites are reduced in PS mutant embryos. Altogether, these results lead to a model in which integrin-mediated adhesion plays a role in regulating tendon cell differentiation by modulating the activity of the Egfr pathway at the level of its ligand Vein.
39. **Extracellular matrix** and its receptors in Drosophila neural development. **Extracellular matrix** (ECM) and matrix receptors are intimately involved in most biological processes. The ECM plays fundamental developmental and physiological roles in health and disease, including processes underlying the development, maintenance, and regeneration of the nervous system. To understand the principles of ECM-mediated functions in the nervous system, genetic model organisms like Drosophila provide simple, malleable, and powerful experimental platforms. This article provides an overview of ECM proteins and receptors in Drosophila. It then focuses on their roles during three progressive phases of neural development: (1) neural progenitor proliferation, (2) axonal growth and pathfinding, and (3) synapse formation and function. Each section highlights known ECM and ECM-receptor components and recent studies done in mutant conditions to reveal their in vivo functions, all illustrating the enormous opportunities provided when merging work on the nervous system with systematic research into ECM-related gene functions.
40. Regulation of Integrin β 1 recycling to lipid rafts by Rab1a to promote cell migration. Rab1a is a member of the Rab family of small GTPases with a well characterized function in the regulation of vesicle trafficking from the endoplasmic reticulum to the Golgi apparatus and within Golgi compartments. The integrin family heterodimeric transmembrane proteins serve as major receptors for **extracellular matri**x proteins, which play essential roles in cell adhesion and migration. Although effects on intracellular trafficking of integrins or other key cargos by Rab1a could influence cell migration, the regulatory mechanisms linking Rab1a to cell migration are not well understood. Here, we report identification of Rab1a as a novel regulator of cell migration using an unbiased RNAi screen targeting GTPases. Inhibition of Rab1a reduced integrin-mediated cell adhesion and spreading on fibronectins, reduced integrin β1 localization to lipid rafts, and decreased recycling of integrin β1 to the plasma membrane. Analysis of Rab1a effector molecules showed that p115 mediated Rab1a regulation of integrin recycling and lipid raft localization in cell migration. Taken together, these results suggest a novel function for Rab1a in the regulation of cell migration through controlling integrin β1 recycling and localization to lipid rafts via a specific downstream effector pathway.
41. Identification of integrin beta subunit mutations that alter affinity for **extracellular matrix** ligand. We examined over 50 mutations in the Drosophila βPS integrin subunit that alter integrin function in situ for their ability to bind a soluble monovalent ligand, TWOW-1. Surprisingly, very few of the mutations, which were selected for conditional lethality in the fly, reduce the ligand binding ability of the integrin. The most prevalent class of mutations activates the integrin heterodimer. These findings emphasize the importance of integrin affinity regulation and point out how molecular interactions throughout the integrin molecule are important in keeping the integrin in a low affinity state. Mutations strongly support the controversial deadbolt hypothesis, where the CD loop in the β tail domain acts to restrain the I domain in the inactive, bent conformation. Site-directed mutations in the cytoplasmic domains of βPS and αPS2C reveal different effects on ligand binding from those observed for αIIbβ3 integrins and identify for the first time a cytoplasmic cysteine residue, conserved in three human integrins, as being important in affinity regulation. In the fly, we find that genetic interactions of the βPS mutations with reduction in talin function are consistent with the integrin affinity differences measured in cells. Additionally, these genetic interactions report on increased and decreased integrin functions that do not result in affinity changes in the PS2C integrin measured in cultured cells.
42. dGRASP-mediated noncanonical integrin secretion is required for Drosophila epithelial remodeling. Integral plasma membrane proteins are typically transported in the secretory pathway from the endoplasmic reticulum and the Golgi complex. Here we show that at specific stages of Drosophila development corresponding to morphological changes in epithelia, apposed basolateral membranes separate slightly, allowing new plasma membrane contacts with basal **extracellular matrix**. At these sites, newly synthesized integrin alpha subunits are deposited via a mechanism that appears to bypass the Golgi. We show that the Drosophila Golgi resident protein dGRASP localizes to these membrane domains and that, in the absence of dGRASP, the integrin subunit is retained intracellularly in both follicular and wing epithelia that are found disrupted. We propose that this dGRASP-mediated noncanonical secretion route allows for developmental regulation of integrin function upon epithelial remodeling. We speculate that this mechanism might be used during development as a means of targeting a specific subset of transmembrane proteins to the plasma membrane.
43. O-linked-N-acetylglucosamine on extracellular protein domains mediates epithelial cell-matrix interactions. The O-linked-N-acetylglucosamine (O-GlcNAc) modification of cytoplasmic and nuclear proteins regulates basic cellular functions and is involved in the aetiology of diabetes and neurodegeneration. This intracellular O-GlcNAcylation is catalyzed by a single O-GlcNAc transferase, OGT. Here we report a novel OGT, EOGT, responsible for extracellular O-GlcNAcylation. Although both OGT and EOGT are regulated by hexosamine flux, EOGT localizes to the lumen of the endoplasmic reticulum and transfers GlcNAc to epidermal growth factor-like domains in an OGT-independent manner. Loss of Eogt gives phenotypes similar to those caused by defects in the apical **extracellular matrix**. Dumpy (Dp), a membrane-anchored extracellular protein, is O-GlcNAcylated, and EOGT is required for Dp-dependent epithelial cell-matrix interactions. Thus, O-GlcNAcylation of secreted and membrane glycoproteins is a novel mediator of cell-cell or cell-matrix interactions at the cell surface.
44. Drosophila miR-9a targets the ECM receptor Dystroglycan to canalize myotendinous junction formation. Establishment of intercellular interactions between various cell types of different origin is vital for organism development and tissue maintenance. Therefore, precise timing, expression pattern, and amounts of **extracellular matrix** (ECM) proteins must be tightly regulated. Particularly, the ECM is important for the development and function of myotendinous junctions (MTJs). We find that precise levels of the ECM receptor Dystroglycan (Dg) are required for MTJ formation in Drosophila and that Dg levels in this process are controlled by miR-9a. In the embryo, Dg is enriched at the termini of the growing muscles facing the tendon matrix and absent from miR-9a-expressing tendons. This gradient of Dg expression is crucial for proper muscle-tendon attachments and is adjusted by miR-9a. In addition to Dg, miR-9a regulates the expression of several other critical muscle genes, and we therefore propose that during embryogenesis, miR-9a specifically controls the expression of mesodermal genes to canalize MTJ morphogenesis.
45. Laminin is required for heart, somatic muscles, and gut development in the Drosophila embryo. Laminin, an **extracellular matrix** glycoprotein, has been implicated in a wide array of biological activities. Its specific roles during development, however, remain to be elucidated. In this report we describe the specific contribution of laminin to histogenesis and organogenesis during Drosophila embryogenesis. In particular, the function of laminin during mesoderm development and morphogenesis was dissected by analyzing embryos deficient in laminin A chain alone or in both laminin A and B2 chains. We show that laminin function is not required for the early phases of mesoderm patterning and morphogenesis in embryos at the extended germ band phase (stage 11). However, laminin function is required for the proper morphogenesis of the heart, somatic mesoderm, and the gut during later stages of embryonic development. In laminin A-deficient embryos, the heart is "broken" as a result of dissociation of the pericardial cells. The somatic myotubes are defective and do not extend properly, and the ventral oblique muscles never reach their proper attachment sites. Finally, during midgut formation, the endoderm fails to undergo the initial columnar polarization in the absence of functional laminin. These studies indicate that the primary function of laminin is to provide structural support for the various cell types, thereby enabling their proper organization into the various organs.
46. Integrin signaling regulates spindle orientation in Drosophila to preserve the follicular-epithelium monolayer. Epithelia act as important physiological barriers and as structural components of tissues and organs. In the Drosophila ovary, follicle cells envelop the germline cysts to form a monolayer epithelium. During division, the orientation of the mitotic spindle in follicle cells is such that both daughter cells remain within the same plane, and the simple structure of the follicular epithelium is thus preserved. Here we show that integrins, heterodimeric transmembrane receptors that connect the **extracellular matrix** to the cell's cytoskeleton [1, 2], are required for maintaining the ovarian monolayer epithelium in Drosophila. Mosaic egg chambers containing integrin mutant follicle cells develop stratified epithelia at both poles. This stratification is due neither to abnormal cell proliferation nor to defects in the apical-basal polarity of the mutant cells. Instead, integrin function is required for the correct orientation of the mitotic apparatus both in mutant cells and in their immediately adjacent wild-type neighbors. We further demonstrate that integrin-mediated signaling, rather than adhesion, is sufficient for maintaining the integrity of the follicular epithelium. The above data show that integrins are necessary for preserving the simple organization of a specialized epithelium and link integrin-mediated signaling to the correct orientation of the mitotic spindle in this epithelial cell type.
47. **Extracellular matrix** and its receptors during development. **Extracellular matrix** (ECM) components are essential for morphogenesis of virtually all tissues. The ECM interacts with the cell surface by binding to specific receptors. The first family of receptors for the ECM that was identified was the integrin family. Integrins are composed of an alpha and a beta-chain, both of which are single pass transmembrane proteins. In muscle cells the dystroglycan complex forms another important receptor system for ECM. It is a complex composed of many proteins. Recent studies have shown that dystroglycan is expressed by embryonic epithelial cells as well. The nature of constituents of the dystroglycan complex is well known for muscle, whereas the detailed composition of the dystroglycan complex in embryonic epithelium is not yet well known. We here review the evidence that binding of ECM to integrins and the dystroglycan complex could be essential for muscle and epithelial cell development and function. It is likely that integrins and the dystroglycan complex have distinct roles during development. It will be an interesting task to study the signal transduction pathways elicited by the interactions between ECM and the two receptor systems during muscle and epithelial morphogenesis.
48. Integrins regulate repulsion-mediated dendritic patterning of drosophila sensory neurons by restricting dendrites in a 2D space. Dendrites of the same neuron usually avoid each other. Some neurons also repel similar neurons through dendrite-dendrite interaction to tile the receptive field. Nonoverlapping coverage based on such contact-dependent repulsion requires dendrites to compete for limited space. Here we show that Drosophila class IV dendritic arborization (da) neurons, which tile the larval body wall, grow their dendrites mainly in a 2D space on the **extracellular matrix** (ECM) secreted by the epidermis. Removing neuronal integrins or blocking epidermal laminin production causes dendrites to grow into the epidermis, suggesting that integrin-laminin interaction attaches dendrites to the ECM. We further show that some of the previously identified tiling mutants fail to confine dendrites in a 2D plane. Expansion of these mutant dendrites in three dimensions results in overlap of dendritic fields. Moreover, overexpression of integrins in these mutant neurons effectively reduces dendritic crossing and restores tiling, revealing an additional mechanism for tiling.
49. Presynaptic secretion of mind-the-gap organizes the synaptic **extracellular matrix**-integrin interface and postsynaptic environments. Mind-the-Gap (MTG) is required during synaptogenesis of the Drosophila glutamatergic neuromuscular junction (NMJ) to organize the postsynaptic domain. Here, we generate MTG::GFP transgenic animals to demonstrate MTG is synaptically targeted, secreted, and localized to punctate domains in the synaptic **extracellular matrix** (ECM). Drosophila NMJs form specialized ECM carbohydrate domains, with carbohydrate moieties and integrin ECM receptors occupying overlapping territories. Presynaptically secreted MTG recruits and reorganizes secreted carbohydrates, and acts to recruit synaptic integrins and ECM glycans. Transgenic MTG::GFP expression rescues hatching, movement, and synaptogenic defects in embryonic-lethal mtg null mutants. Targeted neuronal MTG expression rescues mutant synaptogenesis defects, and increases rescue of adult viability, supporting an essential neuronal function. These results indicate that presynaptically secreted MTG regulates the ECM-integrin interface, and drives an inductive mechanism for the functional differentiation of the postsynaptic domain of glutamatergic synapses. We suggest that MTG pioneers a novel protein family involved in ECM-dependent synaptic differentiation.
50. Drosophila integrin-linked kinase is required at sites of integrin adhesion to link the cytoskeleton to the plasma membrane. Integrin-linked kinase (ILK) was identified by its interaction with the cytoplasmic tail of human beta1 integrin and previous data suggest that ILK is a component of diverse signaling pathways, including integrin, Wnt, and protein kinase B. Here we show that the absence of ILK function in Drosophila causes defects similar to loss of integrin adhesion, but not similar to loss of these signaling pathways. ILK mutations cause embryonic lethality and defects in muscle attachment, and clones of cells lacking ILK in the adult wing fail to adhere, forming wing blisters. Consistent with this, an ILK-green fluorescent protein fusion protein colocalizes with the position-specific integrins at sites of integrin function: muscle attachment sites and the basal junctions of the wing epithelium. Surprisingly, mutations in the kinase domain shown to inactivate the kinase activity of human ILK do not show any phenotype in Drosophila, suggesting a kinase-independent function for ILK. The muscle detachment in ILK mutants is associated with detachment of the actin filaments from the muscle ends, unlike integrin mutants, in which the primary defect is detachment of the plasma membrane from the **extracellular matrix**. Our data suggest that ILK is a component of the structure linking the cytoskeleton and the plasma membrane at sites of integrin-mediated adhesion.
51. The conserved transmembrane proteoglycan Perdido/Kon-tiki is essential for myofibrillogenesis and sarcomeric structure in Drosophila. Muscle differentiation requires the assembly of high-order structures called myofibrils, composed of sarcomeres. Even though the molecular organization of sarcomeres is well known, the mechanisms underlying myofibrillogenesis are poorly understood. It has been proposed that integrin-dependent adhesion nucleates myofibrils at the periphery of the muscle cell to sustain sarcomere assembly. Here, we report a role for the gene perdido (perd, also known as kon-tiki, a transmembrane chondroitin proteoglycan) in myofibrillogenesis. Expression of perd RNAi in muscles, prior to adult myogenesis, can induce misorientation and detachment of Drosophila adult abdominal muscles. In comparison to controls, perd-depleted muscles contain fewer myofibrils, which are localized at the cell periphery. These myofibrils are detached from each other and display a defective sarcomeric structure. Our results demonstrate that the **extracellular matrix** receptor Perd has a specific role in the assembly of myofibrils and in sarcomeric organization. We suggest that Perd acts downstream or in parallel to integrins to enable the connection of nascent myofibrils to the Z-bands. Our work identifies the Drosophila adult abdominal muscles as a model to investigate in vivo the mechanisms behind myofibrillogenesis.
52. Functions for PS integrins in tissue adhesion, migration, and shape changes during early embryonic development in Drosophila. The position-specific (PS) integrins of Drosophila are a family of cell surface heterodimers that function as receptors for **extracellular matrix** molecules. In this study we used time-lapse videomicroscopy to examine living embryos lacking both maternal and zygotic expression of the genes that encode the alpha PS1, alpha PS2, and beta PS integrin subunits. We demonstrate roles for these molecules as early as gastrulation. Abnormalities in mutant embryos include: separation and twisting of the embryonic germband, abnormal shape and migration of midgut primordia, irregular visceral mesoderm, detachment of amnioserosa cells, rupture of the cuticle along the dorsal midline, lack of midgut constriction, and detachment of somatic muscles. These observations suggest multiple roles for PS integrins in the adhesion of cells and in the formation, organization, and migration of embryonic tissues. We also show that although alpha PS1 beta PS and alpha PS2 beta PS are often expressed in adjacent embryonic tissues, this distribution does not necessarily reflect equivalent requirements. Furthermore, the complete loss of both alpha subunits does not produce all of the phenotypes observed in embryos lacking beta PS. This suggests that alpha PS1 beta PS and alpha PS2 beta PS are not required in all embryonic processes utilizing PS integrins.
53. Slowdown promotes muscle integrity by modulating integrin-mediated adhesion at the myotendinous junction. The correct assembly of the myotendinous junction (MTJ) is crucial for proper muscle function. In Drosophila, this junction comprises hemi-adherens junctions that are formed upon arrival of muscles at their corresponding tendon cells. The MTJ mainly comprises muscle-specific alphaPS2betaPS integrin receptors and their tendon-derived **extracellular matrix** ligand Thrombospondin (Tsp). We report the identification and functional analysis of a novel tendon-derived secreted protein named Slowdown (Slow). Homozygous slow mutant larvae exhibit muscle or tendon rupture, sluggish larval movement, partial lethality, and the surviving adult flies are unable to fly. These defects result from improper assembly of the embryonic MTJ. In slow mutants, Tsp prematurely accumulates at muscle ends, the morphology of the muscle leading edge changes and the MTJ architecture is aberrant. Slow was found to form a protein complex with Tsp. This complex is biologically active and capable of altering the morphology and directionality of muscle ends. Our analysis implicates Slow as an essential component of the MTJ, crucial for ensuring muscle and tendon integrity during larval locomotion.
54. Integrins and the development of three-dimensional structure in the Drosophila compound eye. A stereotyped, three-dimensional network of cell-cell contacts mediated by adherens junctions and cell-**extracellular matrix** contacts mediated by focal adhesions defines the architecture of the Drosophila ommatidium. Developmental reconstruction shows that this network is built in an incremental and generally conservative sequence; contacts established early in eye development typically persist into adulthood. Reconstructions show that photoreceptor apical surfaces are involuted into the retinal epithelium and are subsequently elaborated to form the photosensitive rhabdomeres. Rhabdomeres become aligned to the ommatidial optical axis via their anchorage to the retinal floor at the cone cell plate, a specialized nexus of cell-cell and cell-**extracellular matrix** contacts. Parallel reconstructions of retinal development in integrin mutants show that several eye phenotypes trace their origin to the structural failure of the cone cell plate.
55. Analysis of adhesion molecules and basement membrane contributions to synaptic adhesion at the Drosophila embryonic NMJ. Synapse formation and maintenance crucially underlie brain function in health and disease. Both processes are believed to depend on cell adhesion molecules (CAMs). Many different classes of CAMs localise to synapses, including cadherins, protocadherins, neuroligins, neurexins, integrins, and immunoglobulin adhesion proteins, and further contributions come from the **extracellular matrix** and its receptors. Most of these factors have been scrutinised by loss-of-function analyses in animal models. However, which adhesion factors establish the essential physical links across synaptic clefts and allow the assembly of synaptic machineries at the contact site in vivo is still unclear. To investigate these key questions, we have used the neuromuscular junction (NMJ) of Drosophila embryos as a genetically amenable model synapse. Our ultrastructural analyses of NMJs lacking different classes of CAMs revealed that loss of all neurexins, all classical cadherins or all glutamate receptors, as well as combinations between these or with a Laminin deficiency, failed to reveal structural phenotypes. These results are compatible with a view that these CAMs might have no structural role at this model synapse. However, we consider it far more likely that they operate in a redundant or well buffered context. We propose a model based on a multi-adaptor principle to explain this phenomenon. Furthermore, we report a new CAM-independent adhesion mechanism that involves the basement membranes (BM) covering neuromuscular terminals. Thus, motorneuronal terminals show strong partial detachment of the junction when BM-to-cell surface attachment is impaired by removing Laminin A, or when BMs lose their structural integrity upon loss of type IV collagens. We conclude that BMs are essential to tie embryonic motorneuronal terminals to the muscle surface, lending CAM-independent structural support to their adhesion. Therefore, future developmental studies of these synaptic junctions in Drosophila need to consider the important contribution made by BM-dependent mechanisms, in addition to CAM-dependent adhesion.
56. The distribution of PS integrins, laminin A and F-actin during key stages in Drosophila wing development. We first summarize wing development during metamorphosis of Drosophila and identify four critical steps in the conversion of a folded single layered wing disc to a flat bilayered wing. Each step occurs twice, once during the 12 hour prepupal period and again during the 84 hour pupal period. (1) Apposition in which basal surfaces of dorsal and ventral epithelia come close together. (2) Adhesion in which basal junctions form between the apposed basal surfaces. (3) Expansion in which wing area increases as a result of cells flattening. (4) Separation in which dorsal and ventral epithelia are separated by a bulky **extracellular matrix** but remain connected by slender cytoplasmic processes containing the microtubules and microfilaments of the transalar cytoskeleton. Disc ultrastructure is correlated with the distribution of the beta chain of integrin, laminin A, and filamentous actin for each key stage of pupal development. Integrin and laminin exhibit a mutually exclusive distribution from the adhesion stage onwards. Integrin is present on the basal surface of intervein cells but not on vein cells whereas laminin A is absent from the basal surfaces of intervein cells but is present on vein cells. We conclude that laminin is not a ligand for integrin in this context. During apposition and adhesion stages integrin is broadly distributed over the basal and lateral surfaces of intervein cells but subsequently becomes localized to small basal foci. These foci correspond to basal contact zones between transalar processes. The distribution of filamentous actin is dynamic, changing from an apical distribution during hair morphogenesis to a basal distribution as the transalar cytoskeleton develops. Basal adherens-type junctions are first evident during the adhesion stage and become closely associated with the transalar cytoskeleton during the separation stage. Thus, basal junction formation occurs in two discrete steps; intercellular connections are established first and junction/cytoskeletal connections are formed about 20 hours later. These observations provide a basis for future investigations of integrin mediated adhesion in vivo.
57. Perlecan and Dystroglycan act at the basal side of the Drosophila follicular epithelium to maintain epithelial organization. Dystroglycan (Dg) is a widely expressed **extracellular matrix** (ECM) receptor required for muscle viability, synaptogenesis, basementmembrane formation and epithelial development. As an integral component of the Dystrophin-associated glycoprotein complex, Dg plays a central role in linking the ECM and the cytoskeleton. Disruption of this linkage in skeletal muscle leads to various types of muscular dystrophies. In epithelial cells, reduced expression of Dg is associated with increased invasiveness of cancer cells. We have previously shown that Dg is required for epithelial cell polarity in Drosophila, but the mechanisms of this polarizing activity and upstream/downstream components are largely unknown. Using the Drosophila follicle-cell epithelium (FCE) as a model system, we show that the ECM molecule Perlecan (Pcan) is required for maintenance of epithelial-cell polarity. Follicle cells that lack Pcan develop polarity defects similar to those of Dg mutant cells. Furthermore, Dg depends on Pcan but not on Laminin A for its localization in the basal-cell membrane, and the two proteins bind in vitro. Interestingly, the Dg form that interacts with Pcan in the FCE lacks the mucin-like domain, which is thought to be essential for Dg ligand binding activity. Finally, we describe two examples of how Dg promotes the differentiation of the basal membrane domain: (1) by recruiting/anchoring the cytoplasmic protein Dystrophin; and (2) by excluding the transmembrane protein Neurexin. We suggest that the interaction of Pcan and Dg at the basal side of the epithelium promotes basal membrane differentiation and is required for maintenance of cell polarity in the FCE.
58. blistery encodes Drosophila tensin protein and interacts with integrin and the JNK signaling pathway during wing development. Tensin is an actin-binding protein that is localized in focal adhesions. At focal adhesion sites, tensin participates in the protein complex that establishes transmembrane linkage between the **extracellular matrix** and cytoskeletal actin filaments. Even though there have been many studies on tensin as an adaptor protein, the role of tensin during development has not yet been clearly elucidated. Thus, this study was designed to dissect the developmental role of tensin by isolating Drosophila tensin mutants and characterizing its role in wing development. The Drosophila tensin loss-of-function mutations resulted in the formation of blisters in the wings, which was due to a defective wing unfolding process. Interestingly, by(1)-the mutant allele of the gene blistery (by)-also showed a blistered wing phenotype, but failed to complement the wing blister phenotype of the Drosophila tensin mutants, and contains Y62N/T163R point mutations in Drosophila tensin coding sequences. These results demonstrate that by encodes Drosophila tensin protein and that the Drosophila tensin mutants are alleles of by. Using a genetic approach, we have demonstrated that tensin interacts with integrin and also with the components of the JNK signaling pathway during wing development; overexpression of by in wing imaginal discs significantly increased JNK activity and induced apoptotic cell death. Collectively, our data suggest that tensin relays signals from the **extracellular matrix** to the cytoskeleton through interaction with integrin, and through the modulation of the JNK signal transduction pathway during Drosophila wing development.
59. Tissue-specific laminin expression facilitates integrin-dependent association of the embryonic wing disc with the trachea in Drosophila. The interaction of heterologous tissues involves cell adhesion mediated by the **extracellular matrix** and its receptor integrins. The Drosophila wing disc is an ectodermal invagination that contacts specific tracheal branches at the basolateral cell surface. We show that an alpha subunit of laminin, encoded by wing blister (wb), is essential for the establishment of the interaction between the wing and trachea. During embryogenesis, wing disc cells present Wb at their basolateral surface and extend posteriorly, expanding their association to more posteriorly located tracheal branches. These migratory processes are impaired in the absence of the trachea, Wb, or integrins. Time-lapse and transmission electron microscopy analyses suggest that Wb facilitates integrin-dependent contact over a large surface and controls the cellular behavior of the wing cells, including their exploratory filopodial activity. Our data identify Wb laminin as an **extracellular matrix** ligand that is essential for integrin-dependent cellular migration in Drosophila.
60. M-spondin, a novel ECM protein highly homologous to vertebrate F-spondin, is localized at the muscle attachment sites in the Drosophila embryo. The muscle attachment site (MAS) in Drosophila provides a unique and excellent model system to study the mechanism of cell-matrix adhesion in developing organisms. Here, we report on the isolation and characterization of a novel **extracellular matrix** (ECM) molecule localized at the MAS, encoded by the M-spondin (mspo) gene. M-spondin protein contains a thrombospondin type I repeat (TSR) previously found in a variety of ECM molecules. Furthermore, it shares two conserved domains with F-spondin, a vertebrate ECM molecule with TSRs. The presence of TSR(s) and the two homologous domains thus defines a novel gene family of ECM molecules. The mspo mRNA was expressed by a large subset of muscles in the embryonic body wall. Secreted M-spondin protein diffused and eventually became immobilized at the MAS in late embryos. When expressed in S2 cells, the protein was secreted and became concentrated in the matrix on the surface of the culture dish. Genetic analysis revealed that both deletion mutants and misexpression mutants suffered no obvious developmental defects. We propose that M-spondin, although its function is redundant, is a component of the ECM and mediates mechanical linkage between the muscles and apodemes.
61. The **extracellular matrix** proteoglycan perlecan facilitates transmembrane semaphorin-mediated repulsive guidance. The Drosophila transmembrane semaphorin-1a (Sema-1a) is a repulsive guidance cue that uses the Plexin A (PlexA) receptor during neural development. Sema-1a is required in axons to facilitate motor axon defasciculation at guidance choice points. We found that mutations in the trol gene strongly suppress Sema-1a-mediated repulsive axon guidance. trol encodes the phylogenetically conserved secreted heparan sulfate proteoglycan (HSPG) perlecan, a component of the **extracellular matrix**. Motor axon guidance defects in perlecan mutants resemble those observed in Sema-1a- and PlexA-null mutant embryos, and perlecan mutants genetically interact with PlexA and Sema-1a. Perlecan protein is found in both the CNS and the periphery, with higher expression levels in close proximity to motor axon trajectories and pathway choice points. Restoring perlecan to mutant motor neurons rescues perlecan axon guidance defects. Perlecan augments the reduction in phospho-focal adhesion kinase (phospho-FAK) levels that result from treating insect cells in vitro with Sema-1a, and genetic interactions among integrin, Sema-1a, and FAK in vivo support an antagonistic relationship between Sema-1a and integrin signaling. Therefore, perlecan is required for Sema-1a-PlexA-mediated repulsive guidance, revealing roles for **extracellular matrix** proteoglycans in modulating transmembrane guidance cue signaling during neural development.
62. Requirement for integrins during Drosophila wing development. The position-specific (PS) integrins of Drosophila are highly homologous to vertebrate integrins, most of which are cell-surface receptors for **extracellular matrix** components. Integrins are heterodimers, each consisting of noncovalently associated alpha- and beta-subunits. As for the subfamilies of vertebrate integrins, the same beta-subunit is found in both Drosophila PS integrins, combined with a specific alpha-subunit to generate either a complete functional PS1 or PS2 integrin. Both alpha- and beta-subunits are large transmembrane proteins (relative molecular masses greater than 100,000). Either one or both of these two PS integrins are expressed in most fly tissues during development. A particularly intriguing pattern of expression is found in the mature wing imaginal disc, where the PS1 integrin is expressed primarily on the presumptive dorsal wing epithelium, and the PS2 integrin is found almost exclusively on the ventral epithelium. Immediately after pupariation, the central wing pouch evaginates, folding along its centre to appose the epithelia that will secret the dorsal and ventral surfaces of the adult wing blade. Here we report the results of a genetic analysis indicating that both of the PS integrins are required to maintain the close apposition of the dorsal and ventral wing epithelia during morphogenesis. Also, we conclude that the integrins are not necessary for the maintenance of the cell lineage restriction between the two presumptive wing surfaces in the developing imaginal disc.
63. Global tissue revolutions in a morphogenetic movement controlling elongation. Polarized cell behaviors drive axis elongation in animal embryos, but the mechanisms underlying elongation of many tissues remain unknown. Eggs of Drosophila undergo elongation from a sphere to an ellipsoid during oogenesis. We used live imaging of follicles (developing eggs) to elucidate the cellular basis of egg elongation. We find that elongating follicles undergo repeated rounds of circumferential rotation around their long axes. Follicle epithelia mutant for integrin or collagen IV fail to rotate and elongate, which results in round eggs. We present evidence that polarized rotation is required to build a polarized, fibrillar **extracellular matrix** (ECM) that constrains tissue shape. Thus, global tissue rotation is a morphogenetic behavior that uses planar polarity information in the ECM to control tissue elongation.
64. The function of PS integrins during Drosophila embryogenesis. The Drosophila position-specific (PS) antigens are homologous to the vertebrate fibronectin receptor family, or integrins. A Drosophila gene required for embryonic morphogenesis, l(1)myospheroid, codes for a product homologous to the beta subunit of the vertebrate integrins. l(1)myospheroid mutants die during embryogenesis. We show here that they lack the beta subunit of the PS antigens. In the absence of the beta subunit in mutant embryos, the PS alpha subunits are not expressed on the cell surface. We conclude that the l(1)myospheroid phenotype represents the lack-of-function phenotype for these Drosophila integrins. In wild-type embryos, PS antigens are found at the interface between mesoderm and ectoderm, and later mainly at the attachment sites of muscles to the epidermis and gut. Together these results indicate that during embryogenesis, Drosophila integrins are used to attach mesoderm to ectoderm, and are required for the proper assembly of the **extracellular matrix** and for muscle attachment.
65. Integrins contribute to the establishment and maintenance of cell polarity in the follicular epithelium of the Drosophila ovary. The generation of epithelial cell polarity is a key process during development. Although the induction and orientation of cell polarity by cell-cell and cell-**extracellular matrix** (ECM) interactions is well established, the molecular mechanisms by which signals from the ECM control cell polarity in developing epithelial tissues remain poorly understood. Here, we have used the follicular epithelium of the Drosophila ovary to investigate the role that integrins, the main cell-ECM receptors, play in the establishment of apicobasal polarity. Mature follicle cells have an apical side facing the germ line and a basal side in contact with a basement membrane. Our results show that integrins - presumably via interactions with the basement membrane - play a reinforcing role in follicle cell polarization, as they are required to establish and/or maintain follicle cell membrane asymmetry only when contact with the germ line is prevented. We suggest that the primary cue for polarization of the follicular epithelium is contact with the germline cells. In addition, while interfering with apical and lateral polarization cues leads to apoptosis, we show here that inhibition of contact with the basement membrane mediated by integrins does not affect cell survival. Finally, we provide evidence to suggest that integrins are required to orientate epithelial polarity in vivo.
66. Mechanical force regulates integrin turnover in Drosophila in vivo. Regulated assembly and disassembly, or turnover, of integrin-mediated cell-**extracellular matrix** (ECM) adhesions is essential for dynamic cell movements and long-term tissue maintenance. For example, in Drosophila, misregulation of integrin turnover disrupts muscle-tendon attachment at myotendinous junctions (MTJs). We demonstrate that mechanical force, which modulates integrin activity, also regulates integrin and intracellular adhesion complex (IAC) turnover in vivo. Using conditional mutants to alter the tensile force on MTJs, we found that the proportion of IAC components undergoing turnover inversely correlated with the force applied on MTJs. This effect was disrupted by point mutations in β-integrin that interfere with ECM-induced conformational changes and activation of β-integrin or integrin-mediated cytoplasmic signalling. These mutants also disrupted integrin dynamics at MTJs during larval development. Together, these data suggest that specific β-integrin-mediated signals regulate adhesion turnover in response to tension during tissue formation. We propose that integrin-ECM adhesive stability is continuously controlled by force in vivo through integrin-dependent auto-regulatory feedback mechanisms so that tissues can quickly adapt to and withstand mechanical stresses.
67. Epidermis-Derived Semaphorin Promotes Dendrite Self-Avoidance by Regulating Dendrite-Substrate Adhesion in Drosophila Sensory Neurons. Precise patterning of dendritic arbors is critical for the wiring and function of neural circuits. Dendrite-**extracellular matrix** (ECM) adhesion ensures that the dendrites of Drosophila dendritic arborization (da) sensory neurons are properly restricted in a 2D space, and thereby facilitates contact-mediated dendritic self-avoidance and tiling. However, the mechanisms regulating dendrite-ECM adhesion in vivo are poorly understood. Here, we show that mutations in the semaphorin ligand sema-2b lead to a dramatic increase in self-crossing of dendrites due to defects in dendrite-ECM adhesion, resulting in a failure to confine dendrites to a 2D plane. Furthermore, we find that Sema-2b is secreted from the epidermis and signals through the Plexin B receptor in neighboring neurons. Importantly, we find that Sema-2b/PlexB genetically and physically interacts with TORC2 complex, Tricornered (Trc) kinase, and integrins. These results reveal a novel role for semaphorins in dendrite patterning and illustrate how epidermal-derived cues regulate neural circuit assembly.
68. Tenectin is a novel alphaPS2betaPS integrin ligand required for wing morphogenesis and male genital looping in Drosophila. Morphogenesis of the adult structures of holometabolous insects is regulated by ecdysteroids and juvenile hormones and involves cell-cell interactions mediated in part by the cell surface integrin receptors and their **extracellular matrix** (ECM) ligands. These adhesion molecules and their regulation by hormones are not well characterized. We describe the gene structure of a newly described ECM molecule, tenectin, and demonstrate that it is a hormonally regulated ECM protein required for proper morphogenesis of the adult wing and male genitalia. Tenectin's function as a new ligand of the PS2 integrins is demonstrated by both genetic interactions in the fly and by cell spreading and cell adhesion assays in cultured cells. Its interaction with the PS2 integrins is dependent on RGD and RGD-like motifs. Tenectin's function in looping morphogenesis in the development of the male genitalia led to experiments that demonstrate a role for PS integrins in the execution of left-right asymmetry.
69. Integrins and morphogenesis. The Drosophila position specific (PS) integrins consist of two cell surface heterodimers, PS1 (alpha PS1 beta PS) and PS2 (alpha OS2 beta PS), which are expressed on complementary sides of attachments between cell layers and are essential for these attachments. Current evidence suggests that the PS integrins bind to components of the **extracellular matrix**, similar to the majority of vertebrate integrins, but specific Drosophila ligands have not yet been identified. In the embryo PS1 is found on the surface of the epidermis and endoderm, while PS2 is restricted to the mesoderm. The integrins are concentrated at the sites where the somatic muscles attach to the epidermis and at the interface between the visceral mesoderm and the endoderm. In myospheroid mutant embryos, which lack the beta PS subunit, the adhesion between the mesoderm and the other cell layers fails. The PS integrins are also required for the adhesion of the dorsal to the ventral surface of the wing during metamorphosis. PS1 is expressed on the basal surface of the dorsal cells and PS2 is expressed on the ventral cells. Loss of PS integrin function in the wing results in balloon shaped wings because of the failure of the two surfaces of the wing blade to adhere to each other. These and other aspects of the phenotypes of mutations in the genes encoding the PS integrins indicate that integrins play an important role in the adhesion of different cell layers to each other and thus an essential role in the morphogenesis of the organism. The use of **extracellular matrix** receptors in this role may aid in keeping the different cell layers distinct.
70. Cloning and characterization of Dfak56, a homolog of focal adhesion kinase, in Drosophila melanogaster. The focal adhesion kinase (FAK) protein-tyrosine kinase plays important roles in cell adhesion in vertebrates. Using polymerase chain reaction-based cloning strategy, we cloned a Drosophila gene that is homologous to the vertebrate FAK family of protein-tyrosine kinases. We designated this gene Dfak56 and characterized its gene product. The overall protein structure and deduced amino acid sequence of Dfak56 show significant similarity to those of FAK and PYK2. Dfak56 has in vitro autophosphorylation activity at tyrosine residues. Expression of the Dfak56 mRNA and the protein was observed in the central nervous system and the muscle-epidermis attachment site in the embryo, where Drosophila position-specific integrins are localized. The results suggest that like FAK in vertebrates, Dfak56 functions downstream of integrins. Dfak56 was tyrosine-phosphorylated upon integrin-dependent attachment of the cell to the **extracellular matrix**. We conclude that the Dfak56 tyrosine kinase is involved in integrin-mediated cell adhesion signaling and thus is a functional homolog of vertebrate FAK.
71. In vivo regulation of integrin turnover by outside-in activation. The development of three-dimensional tissue architecture requires precise control over the attachment of cells to the **extracellular matrix** (ECM). Integrins, the main ECM-binding receptors in animals, are regulated in multiple ways to modulate cell-ECM adhesion. One example is the conformational activation of integrins by extracellular signals ('outside-in activation') or by intracellular signals ('inside-out activation'), whereas another is the modulation of integrin turnover. We demonstrate that outside-in activation regulates integrin turnover to stabilize tissue architecture in vivo Treating Drosophila embryos with Mg(2+) and Mn(2+), known to induce outside-in activation, resulted in decreased integrin turnover. Mathematical modeling combined with mutational analysis provides mechanistic insight into the stabilization of integrins at the membrane. We show that as tissues mature, outside-in activation is crucial for regulating the stabilization of integrin-mediated adhesions. This data identifies a new in vivo role for outside-in activation and sheds light on the key transition between tissue morphogenesis and maintenance.
72. Zasp regulates integrin activation. Integrins are heterodimeric adhesion receptors that link the **extracellular matrix** (ECM) to the cytoskeleton. Binding of the scaffold protein, talin, to the cytoplasmic tail of β-integrin causes a conformational change of the extracellular domains of the integrin heterodimer, thus allowing high-affinity binding of ECM ligands. This essential process is called integrin activation. Here we report that the Z-band alternatively spliced PDZ-motif-containing protein (Zasp) cooperates with talin to activate α5β1 integrins in mammalian tissue culture and αPS2βPS integrins in Drosophila. Zasp is a PDZ-LIM-domain-containing protein mutated in human cardiomyopathies previously thought to function primarily in assembly and maintenance of the muscle contractile machinery. Notably, Zasp is the first protein shown to co-activate α5β1 integrins with talin and appears to do so in a manner distinct from known αIIbβ3 integrin co-activators.
73. Dpp signaling directs cell motility and invasiveness during epithelial morphogenesis. Tissue remodeling in development and disease involves the coordinated invasion of neighboring territories and/or the replacement of entire cell populations. Cell guidance, cell matching, transitions from passive to migratory epithelia, cell growth and death, and **extracellular matrix** remodeling all impinge on epithelial spreading. Significantly, the extracellular signals that direct these activities and the specific cellular elements and mechanisms regulated by these signals remain in most cases to be identified. To address these issues, we performed an analysis of histoblasts (Drosophila abdominal epithelial founder cells) on their transition from a dormant state to active migration replacing obsolete larval epidermal cells (LECs). We found that during expansion, Decapentaplegic (Dpp) secreted from surrounding LECs leads to graded pathway activation in cells at the periphery of histoblast nests. Across nests, Dpp activity confers differential cellular behavior and motility by modulating cell-cell contacts, the organization and activity of the cytoskeleton, and histoblast attachment to the substrate. Furthermore, Dpp also prevents the premature death of LECs, allowing the coordination of histoblast expansion to LEC delamination. Dpp signaling activity directing histoblast spreading and invasiveness mimics transforming growth factor-beta and bone morphogenetic proteins' role in enhancing the motility and invasiveness of cancer cells, resulting in the promotion of metastasis.
74. Null mutations in the alpha PS2 and beta PS integrin subunit genes have distinct phenotypes. The two Drosophila position-specific (PS) integrins are expressed on complementary sides of sites where different cell layers adhere to each other, such as the attachments of the embryonic muscles to the epidermis. While there is suggestive evidence that the PS integrin-mediated adhesion is via the **extracellular matrix**, it is also possible that it occurs through the direct interaction of the two integrins, alpha PS1 beta PS and alpha PS2 beta PS. To help distinguish between these possibilities a comparison between the phenotypes caused by the absence of the beta PS subunit and the absence of one of the PS alpha subunits, alpha PS2, has been made. Two pieces of evidence are provided that prove that the alpha PS2 subunit is encoded by the locus inflated (if). Firstly, three new if alleles have been isolated, each of which is associated with a molecular lesion in the alpha PS2 gene, and each of which results in the complete loss of if activity. Secondly, a 39 kb fragment of genomic DNA that encompasses the alpha PS2 gene completely rescues if mutations when introduced into the germline by P-element-mediated transformation. A comparison of the null inflated phenotype with that of the locus that encodes the beta PS subunit, myospheroid (mys), reveals that while the beta PS subunit is required for the adhesion of the epidermis along the dorsal midline, the alpha PS2 subunit is not. In if mutant embryos, the muscles remain attached to the other cell layers significantly longer than in a mys mutant embryo. This shows that the alpha PS2 beta PS integrin only contributes part of the adhesive activity at the sites of PS integrin adhesion, and rules out a model where PS integrin function occurs solely by the direct interaction of the two PS integrins.
75. Basigin (EMMPRIN/CD147) interacts with integrin to affect cellular architecture. Basigin, an IgG family glycoprotein found on the surface of human metastatic tumors, stimulates fibroblasts to secrete matrix metalloproteases that remodel the **extracellular matrix**. Using Drosophila melanogaster we identify intracellular, matrix metalloprotease-independent, roles for basigin. Specifically, we found that basigin, interacting with integrin, is required for normal cell architecture in some cell types. Basigin promotes cytoskeletal rearrangements and the formation of lamellipodia in cultured insect cells. Loss of basigin from photoreceptors leads to misplaced nuclei, rough ER and mitochondria, as well as to swollen axon terminals. These changes in intracellular structure suggest cytoskeletal disruptions. These defects can be rescued by either fly or mouse basigin. Basigin and integrin colocalize to cultured cells and to the visual system. Basigin-mediated changes in the architecture of cultured cells require integrin binding activity. Basigin and integrin interact genetically to affect cell structure in the animal, possibly by forming complexes at cell contacts that help organize internal cell structure.
76. Distinct developmental roles for direct and indirect talin-mediated linkage to actin. Transmembrane adhesion receptors, such as integrins, mediate cell adhesion by interacting with intracellular proteins that connect to the cytoskeleton. Talin, one such linker protein, is essential to connect **extracellular matrix**-bound integrins to the cytoskeleton. Talin can connect to the cytoskeleton either directly, through its actin-binding motifs, or indirectly, by recruiting other actin-binding proteins. Talin's carboxy-terminal end contains a well-characterized actin-binding domain (ABD). We tested the role of the C-terminal ABD of talin in integrin function in Drosophila. We found that introduction of mutations that reduced actin binding in vitro into the isolated C-terminal Talin-ABD impaired actin binding in vivo. Moreover, when engineered into full-length talin, these mutations disrupted a subset of integrin-mediated adhesion-dependent developmental events. Specifically, morphogenetic processes that involve dynamic, short-term integrin-mediated adhesion were particularly sensitive to impaired function of the C-terminal Talin-ABD. We propose that during development talin connects integrins to the cytoskeleton in distinct ways in different types of integrin-mediated adhesion: directly in transient adhesions and indirectly in stable long-lasting adhesions. Our results provide insight into how a similar array of molecular components can contribute to diverse adhesive processes throughout development.
77. The lethal myospheroid gene of Drosophila encodes a membrane protein homologous to vertebrate integrin beta subunits. A mutant of Drosophila melanogaster carrying the lethal(1) myospheroid mutation [l(1)mys] has a defective musculature and a phenotype that suggests a defect of basement membranes. The genomic region that is interrupted by an insertion in a mutant carrying l(1)mys was used to isolate cDNA clones, and their sequences are presented here. The cDNA sequence predicts a cysteine-rich integral membrane protein that displays 45% sequence identity to chicken integrin and the human fibronectin receptor beta subunit and much greater similarity over localized segments. These similarities extend to other vertebrate integrin beta subunits, and we conclude that the myospheroid protein is an integrin beta subunit of Drosophila. This implies evolutionary conservation of a group of transmembrane proteins that are receptors for **extracellular matrix** and, coupled with the myospheroid phenotype, indicates an important role for the interaction of cells with **extracellular matrix** during development.
78. Absence of PS integrins or laminin A affects extracellular adhesion, but not intracellular assembly, of hemiadherens and neuromuscular junctions in Drosophila embryos. We have examined the role of integrins in the formation of the cell junctions that connect muscles to epidermis (muscle attachments) and muscles to neurons (neuromuscular junctions). To this end we have analyzed muscle attachments and neuromuscular junctions ultrastructurally in single or double mutant Drosophila embryos lacking PS1 integrin (alphaPS1betaPS), PS2 integrin (alphaPS2betaPS), and/or their potential extracellular ligand laminin A. At the muscle attachments PS integrins are essential for the adhesion of hemiadherens junctions (HAJs) to **extracellular matrix**, but not for their intracellular link to the cytoskeleton. The PS2 integrin is only expressed in the muscles, but it is essential for the adhesion of muscle and epidermal HAJs to electron dense **extracellular matrix**. It is also required for adhesion of muscle HAJs to a less electron dense form of **extracellular matrix**, the basement membrane. The PS1 integrin is expressed in epidermal cells and can mediate adhesion of the epidermal HAJs to the basement membrane. The ligands involved in adhesion mediated by both PS integrins seem distinct because adhesion mediated by PS1 appears to require the **extracellular matrix** component laminin A, while adhesion mediated by PS2 integrin does not. At neuromuscular junctions the formation of functional synapses occurs normally in embryos lacking PS integrins and/or laminin A, but the extent of contact between neuronal and muscle surfaces is altered significantly. We suggest that neuromuscular contact in part requires basement membrane adhesion to the general muscle surface, and this form of adhesion is completely abolished in the absence of laminin A.
79. IPP Complex Reinforces Adhesion by Relaying Tension-Dependent Signals to Inhibit Integrin Turnover. Cytoskeleton-mediated forces regulate the assembly and function of integrin adhesions; however, the underlying mechanisms remain unclear. The tripartite IPP complex, comprising ILK, Parvin, and PINCH, mediates the integrin-actin link at Drosophila embryo muscle attachment sites (MASs). Here, we demonstrate a developmentally earlier function for the IPP complex: to reinforce integrin-**extracellular matrix** (ECM) adhesion in response to tension. In IPP-complex mutants, the integrin-ECM linkage at MASs breaks in response to intense muscle contractility. Mechanistically, the IPP complex is required to relay force-elicited signals that decelerate integrin turnover at the plasma membrane so that the integrin immobile fraction is adequate to withstand tension. Epistasis analysis shows that alleviation of muscle contractility, downregulation of endocytosis, and enhanced integrin binding to the ECM are sufficient to restore integrin-ECM adhesion and maintain integrin-adhesome organization in IPP-complex mutants. Our findings reveal a role for the IPP complex as an essential mechanosensitive regulatory switch of integrin turnover in vivo.
80. A dual role for the βPS integrin myospheroid in mediating Drosophila embryonic macrophage migration. Throughout embryonic development, macrophages not only act as the first line of defence against infection but also help to sculpt organs and tissues of the embryo by removing dead cells and secreting **extracellular matri**x components. Key to their function is the ability of embryonic macrophages to migrate and disperse throughout the embryo. Despite these important developmental functions, little is known about the molecular mechanisms underlying embryonic macrophage migration in vivo. Integrins are key regulators of many of the adult macrophage responses, but their role in embryonic macrophages remains poorly characterized. Here, we have used Drosophila macrophages (haemocytes) as a model system to address the role of integrins during embryonic macrophage dispersal in vivo. We show that the main βPS integrin, myospheroid, affects haemocyte migration in two ways; by shaping the three-dimensional environment in which haemocytes migrate and by regulating the migration of haemocytes themselves. Live imaging revealed a requirement for myospheroid within haemocytes to coordinate the microtubule and actin dynamics, and to enable haemocyte developmental dispersal, contact repulsion and inflammatory migration towards wounds.
81. Papillote and Piopio: Drosophila ZP-domain proteins required for cell adhesion to the apical **extracellular matrix** and microtubule organization. Adhesion between epithelial cells and extracellular substrates is normally mediated through basal adhesion complexes. However, some cells also possess comparable junctions on their apical surface. Here, we describe two new Drosophila proteins, Piopio and Papillote, that are required for the link between the apical epithelial surface and the overlying apical **extracellular matrix** (aECM). The two proteins share a zona pellucida (ZP) domain with mammalian aECM components, including the tectorins found in the vertebrate inner ear. Tagged versions of both proteins localized to the apical epithelial surface. Mutations in piopio, papillote and dumpy (another gene encoding a ZP-domain protein) cause defects in the innermost layer of the aECM and its detachment from the epidermis. Loss of Piopio, but not Papillote or Dumpy, causes the absence of specialized microtubule bundles from pupal wings, suggesting that Piopio plays a role in microtubule organization. Thus, ZP domain-containing proteins may have shared functions within the aECM, while also exhibiting specific interactions with the cytoskeleton.
82. miRNA-based buffering of the cobblestone-lissencephaly-associated **extracellular matrix** receptor dystroglycan via its alternative 3'-UTR. Many proteins are expressed dynamically during different stages of cellular life and the accuracy of protein amounts is critical for cell endurance. Therefore, cells should have a perceptive system that notifies about fluctuations in the amounts of certain components and an executive system that efficiently restores their precise levels. At least one mechanism that evolution has employed for this task is regulation of 3'-UTR length for microRNA targeting. Here we show that in Drosophila the microRNA complex miR-310s acts as an executive mechanism to buffer levels of the muscular dystrophy-associated **extracellular matrix** receptor dystroglycan via its alternative 3'-UTR. miR-310s gene expression fluctuates depending on dystroglycan amounts and nitric oxide signalling, which perceives dystroglycan levels and regulates microRNA gene expression. Aberrant levels of dystroglycan or deficiencies in miR-310s and nitric oxide signalling result in cobblestone brain appearance, resembling human lissencephaly type II phenotype.
83. Drosophila perlecan regulates intestinal stem cell activity via cell-matrix attachment. Stem cells require specialized local microenvironments, termed niches, for normal retention, proliferation, and multipotency. Niches are composed of cells together with their associated **extracellular matrix** (ECM). Currently, the roles of ECM in regulating niche functions are poorly understood. Here, we demonstrate that Perlecan (Pcan), a highly conserved ECM component, controls intestinal stem cell (ISC) activities and ISC-ECM attachment in Drosophila adult posterior midgut. Loss of Pcan from ISCs, but not other surrounding cells, causes ISCs to detach from underlying ECM, lose their identity, and fail to proliferate. These defects are not a result of a loss of epidermal growth factor receptor (EGFR) or Janus kinase/signal transducer and activator of transcription (JAK/STAT) signaling activity but partially depend on integrin signaling activity. We propose that Pcan secreted by ISCs confers niche properties to the adjacent ECM that is required for ISC maintenance of stem cell identity, activity, and anchorage to the niche.
84. Moleskin is essential for the formation of the myotendinous junction in Drosophila. It is the precise connectivity between skeletal muscles and their corresponding tendon cells to form a functional myotendinous junction (MTJ) that allows for the force generation required for muscle contraction and organismal movement. The Drosophila MTJ is composed of secreted **extracellular matrix** (ECM) proteins deposited between integrin-mediated hemi-adherens junctions on the surface of muscle and tendon cells. In this paper, we have identified a novel, cytoplasmic role for the canonical nuclear import protein Moleskin (Msk) in Drosophila embryonic somatic muscle attachment. Msk protein is enriched at muscle attachment sites in late embryogenesis and msk mutant embryos exhibit a failure in muscle-tendon cell attachment. Although the muscle-tendon attachment sites are reduced in size, components of the integrin complexes and ECM proteins are properly localized in msk mutant embryos. However, msk mutants fail to localize phosphorylated focal adhesion kinase (pFAK) to the sites of muscle-tendon cell junctions. In addition, the tendon cell specific proteins Stripe (Sr) and activated mitogen-activated protein kinase (MAPK) are reduced in msk mutant embryos. Our rescue experiments demonstrate that Msk is required in the muscle cell, but not in the tendon cells. Moreover, muscle attachment defects due to loss of Msk are rescued by an activated form of MAPK or the secreted epidermal growth factor receptor (Egfr) ligand Vein. Taken together, these findings provide strong evidence that Msk signals non-autonomously through the Vein-Egfr signaling pathway for late tendon cell late differentiation and/or maintenance.
85. The Rho-family GTPase Rac1 regulates integrin localization in Drosophila immunosurveillance cells. When the parasitoid wasp Leptopilina boulardi lays an egg in a Drosophila larva, phagocytic cells called plasmatocytes and specialized cells known as lamellocytes encapsulate the egg. The Drosophila β-integrin Myospheroid (Mys) is necessary for lamellocytes to adhere to the cellular capsule surrounding L. boulardi eggs. Integrins are heterodimeric adhesion receptors consisting of α and β subunits, and similar to other plasma membrane receptors undergo ligand-dependent endocytosis. In mammalian cells it is known that integrin binding to t**he extracellular mat**rix induces the activation of Rac GTPases, and we have previously shown that Rac1 and Rac2 are necessary for a proper encapsulation response in Drosophila larvae. We wanted to test the possibility that Myospheroid and Rac GTPases interact during the Drosophila anti-parasitoid immune response.In the current study we demonstrate that Rac1 is required for the proper localization of Myospheroid to the cell periphery of haemocytes after parasitization. Interestingly, the mislocalization of Myospheroid in Rac1 mutants is rescued by hyperthermia, involving the heat shock protein Hsp83. From these results we conclude that Rac1 and Hsp83 are required for the proper localization of Mys after parasitization.We show for the first time that the small GTPase Rac1 is required for Mysopheroid localization. Interestingly, the necessity of Rac1 in Mys localization was negated by hyperthermia. This presents a problem, in Drosophila we quite often raise larvae at 29°C when using the GAL4/UAS misexpression system. If hyperthermia rescues receptor endosomal recycling defects, raising larvae in hyperthermic conditions may mask potentially interesting phenotypes.

#### GeneRIFs

1. analysis of integrin beta subunit mutations that alter affinity for **extracellular matrix** ligand

### [Ii]nvasive

#### PubMed

1. Crag regulates epithelial architecture and polarized deposition of basement membrane proteins in Drosophila. The polarized architecture of epithelia relies on an interplay between the cytoskeleton, the trafficking machinery, and cell-cell and cell-matrix adhesion. Specifically, contact with the basement membrane (BM), an extracellular matrix underlying the basal side of epithelia, is important for cell polarity. However, little is known about how BM proteins themselves achieve a polarized distribution. In a genetic screen in the Drosophila follicular epithelium, we identified mutations in Crag, which encodes a conserved protein with domains implicated in membrane trafficking. Follicle cells mutant for Crag lose epithelial integrity and frequently become **invasive**. The loss of Crag leads to the anomalous accumulation of BM components on both sides of epithelial cells without directly affecting the distribution of apical or basolateral membrane proteins. This defect is not generally observed in mutants affecting epithelial integrity. We propose that Crag plays a unique role in organizing epithelial architecture by regulating the polarized secretion of BM proteins.
2. Requirement for Par-6 and Bazooka in Drosophila border cell migration. Polarized epithelial cells convert into migratory **invasive** cells during a number of developmental processes, as well as when tumors metastasize. Much has been learned recently concerning the molecules and mechanisms that are responsible for generating and maintaining epithelial cell polarity. However, less is known about what becomes of epithelial polarity proteins when various cell types become migratory and **invasive**. Here, we report the localization of several apical epithelial proteins, Par-6, Par-3/Bazooka and aPKC, during border cell migration in the Drosophila ovary. All of these proteins remained asymmetrically distributed throughout migration. Moreover, depletion of either Par-6 or Par-3/Bazooka by RNAi resulted in disorganization of the border cell cluster and impaired migration. The distributions of several transmembrane proteins required for migration were abnormal following Par-6 or Par-3/Bazooka downregulation, possibly accounting for the migration defects. Taken together, these results indicate that cells need not lose apical/basal polarity in order to invade neighboring tissues and in some cases even require such polarity for proper motility.
3. Perlecan and Dystroglycan act at the basal side of the Drosophila follicular epithelium to maintain epithelial organization. Dystroglycan (Dg) is a widely expressed extracellular matrix (ECM) receptor required for muscle viability, synaptogenesis, basementmembrane formation and epithelial development. As an integral component of the Dystrophin-associated glycoprotein complex, Dg plays a central role in linking the ECM and the cytoskeleton. Disruption of this linkage in skeletal muscle leads to various types of muscular dystrophies. In epithelial cells, reduced expression of Dg is associated with increased **invasive**ness of cancer cells. We have previously shown that Dg is required for epithelial cell polarity in Drosophila, but the mechanisms of this polarizing activity and upstream/downstream components are largely unknown. Using the Drosophila follicle-cell epithelium (FCE) as a model system, we show that the ECM molecule Perlecan (Pcan) is required for maintenance of epithelial-cell polarity. Follicle cells that lack Pcan develop polarity defects similar to those of Dg mutant cells. Furthermore, Dg depends on Pcan but not on Laminin A for its localization in the basal-cell membrane, and the two proteins bind in vitro. Interestingly, the Dg form that interacts with Pcan in the FCE lacks the mucin-like domain, which is thought to be essential for Dg ligand binding activity. Finally, we describe two examples of how Dg promotes the differentiation of the basal membrane domain: (1) by recruiting/anchoring the cytoplasmic protein Dystrophin; and (2) by excluding the transmembrane protein Neurexin. We suggest that the interaction of Pcan and Dg at the basal side of the epithelium promotes basal membrane differentiation and is required for maintenance of cell polarity in the FCE.
4. Dpp signaling directs cell motility and **invasive**ness during epithelial morphogenesis. Tissue remodeling in development and disease involves the coordinated invasion of neighboring territories and/or the replacement of entire cell populations. Cell guidance, cell matching, transitions from passive to migratory epithelia, cell growth and death, and extracellular matrix remodeling all impinge on epithelial spreading. Significantly, the extracellular signals that direct these activities and the specific cellular elements and mechanisms regulated by these signals remain in most cases to be identified. To address these issues, we performed an analysis of histoblasts (Drosophila abdominal epithelial founder cells) on their transition from a dormant state to active migration replacing obsolete larval epidermal cells (LECs). We found that during expansion, Decapentaplegic (Dpp) secreted from surrounding LECs leads to graded pathway activation in cells at the periphery of histoblast nests. Across nests, Dpp activity confers differential cellular behavior and motility by modulating cell-cell contacts, the organization and activity of the cytoskeleton, and histoblast attachment to the substrate. Furthermore, Dpp also prevents the premature death of LECs, allowing the coordination of histoblast expansion to LEC delamination. Dpp signaling activity directing histoblast spreading and **invasive**ness mimics transforming growth factor-beta and bone morphogenetic proteins' role in enhancing the motility and **invasive**ness of cancer cells, resulting in the promotion of metastasis.

### [Mm]etastasize

#### PubMed

1. Requirement for Par-6 and Bazooka in Drosophila border cell migration. Polarized epithelial cells convert into migratory invasive cells during a number of developmental processes, as well as when tumors **metastasize**. Much has been learned recently concerning the molecules and mechanisms that are responsible for generating and maintaining epithelial cell polarity. However, less is known about what becomes of epithelial polarity proteins when various cell types become migratory and invasive. Here, we report the localization of several apical epithelial proteins, Par-6, Par-3/Bazooka and aPKC, during border cell migration in the Drosophila ovary. All of these proteins remained asymmetrically distributed throughout migration. Moreover, depletion of either Par-6 or Par-3/Bazooka by RNAi resulted in disorganization of the border cell cluster and impaired migration. The distributions of several transmembrane proteins required for migration were abnormal following Par-6 or Par-3/Bazooka downregulation, possibly accounting for the migration defects. Taken together, these results indicate that cells need not lose apical/basal polarity in order to invade neighboring tissues and in some cases even require such polarity for proper motility.

### [Mm]etastasis

#### PubMed

1. Drosophila as a model to study the role of blood cells in inflammation, innate immunity and cancer. Drosophila has a primitive yet effective blood system with three types of haemocytes which function throughout different developmental stages and environmental stimuli. Haemocytes play essential roles in tissue modeling during embryogenesis and morphogenesis, and also in innate immunity. The open circulatory system of Drosophila makes haemocytes ideal signal mediators to cells and tissues in response to events such as infection and wounding. The application of recently developed and sophisticated genetic tools to the relatively simple genome of Drosophila has made the fly a popular system for modeling human tumorigensis and **metastasis**. Drosophila is now used for screening and investigation of genes implicated in human leukemia and also in modeling development of solid tumors. This second line of research offers promising opportunities to determine the seemingly conflicting roles of blood cells in tumor progression and invasion. This review provides an overview of the signaling pathways conserved in Drosophila during haematopoiesis, haemostasis, innate immunity, wound healing and inflammation. We also review the most recent progress in the use of Drosophila as a cancer research model with an emphasis on the roles haemocytes can play in various cancer models and in the links between inflammation and cancer.
2. JNK signaling controls border cell cluster integrity and collective cell migration. Collective cell movement is a mechanism for invasion identified in many developmental events. Examples include the movement of lateral-line neurons in Zebrafish, cells in the inner blastocyst, and **metastasis** of epithelial tumors [1]. One key model to study collective migration is the movement of border cell clusters in Drosophila. Drosophila egg chambers contain 15 nurse cells and a single oocyte surrounded by somatic follicle cells. At their anterior end, polar cells recruit several neighboring follicle cells to form the border cell cluster [2]. By stage 9, and over 6 hr, border cells migrate as a cohort between nurse cells toward the oocyte. The specification and directionality of border cell movement are regulated by hormonal and signaling mechanisms [3]. However, how border cells are held together while they migrate is not known. Here, we show that a negative-feedback loop controlling JNK activity regulates border cell cluster integrity. JNK signaling modulates contacts between border cells and between border cells and substratum to sustain collective migration by regulating several effectors including the polarity factor Bazooka and the cytoskeletal adaptor D-Paxillin. We anticipate a role for the JNK pathway in controlling collective cell movements in other morphogenetic and clinical models.
3. Cellular and molecular mechanisms of single and collective cell migrations in Drosophila: themes and variations. The process of cell migration is essential throughout life, driving embryonic morphogenesis and ensuring homeostasis in adults. Defects in cell migration are a major cause of human disease, with excessive migration causing autoimmune diseases and cancer **metastasis**, whereas reduced capacity for migration leads to birth defects and immunodeficiencies. Myriad studies in vitro have established a consensus view that cell migrations require cell polarization, Rho GTPase-mediated cytoskeletal rearrangements, and myosin-mediated contractility. However, in vivo studies later revealed a more complex picture, including the discovery that cells migrate not only as single units but also as clusters, strands, and sheets. In particular, the role of E-Cadherin in cell motility appears to be more complex than previously appreciated. Here, we discuss recent advances achieved by combining the plethora of genetic tools available to the Drosophila geneticist with live imaging and biophysical techniques. Finally, we discuss the emerging themes such studies have revealed and ponder the puzzles that remain to be solved.
4. The Drosophila **metastasis** suppressor gene Nm23 homolog, awd, regulates epithelial integrity during oogenesis. The expression levels of the **metastasis** suppressor gene Nm23 have been shown to correlate positively or inversely with prognosis in different cancer cohorts. This indicates that Nm23 may be needed at different expression levels and may function differently in various tissues. Here we report a novel epithelial function of the Drosophila melanogaster homolog of human Nm23, abnormal wing discs (awd). We show a dynamic expression pattern of the Awd protein during morphogenesis of the Drosophila follicle cells during oogenesis. Loss-of-function awd mutant cells result in the accumulation and spreading of adherens junction components, such as Drosophila E-cadherin, beta-catenin/Armadillo, and alpha-spectrin, and the disruption of epithelial integrity, including breaking up of the epithelial sheet and piling up of follicle cells. In contrast, overexpression of awd diminishes adherens junction components and induces a mesenchymal-cell-like cell shape change. The gain-of-function phenotype is consistent with a potential oncogenic function of this **metastasis** suppressor gene. Interestingly, we demonstrate that the epithelial function of awd is mediated by Rab5 and show that the Rab5 expression level is downregulated in awd mutant cells. Therefore, awd modulates the level and localization of adherens junction components via endocytosis. This is the first demonstration of an in vivo function of Nm23 family genes in regulating epithelial morphogenesis.
5. Mutations in the polycomb group gene polyhomeotic lead to epithelial instability in both the ovary and wing imaginal disc in Drosophila. Most human cancers originate from epithelial tissues and cell polarity and adhesion defects can lead to **metastasis**. The Polycomb-Group of chromatin factors were first characterized in Drosophila as repressors of homeotic genes during development, while studies in mammals indicate a conserved role in body plan organization, as well as an implication in other processes such as stem cell maintenance, cell proliferation, and tumorigenesis. We have analyzed the function of the Drosophila Polycomb-Group gene polyhomeotic in epithelial cells of two different organs, the ovary and the wing imaginal disc.Clonal analysis of loss and gain of function of polyhomeotic resulted in segregation between mutant and wild-type cells in both the follicular and wing imaginal disc epithelia, without excessive cell proliferation. Both basal and apical expulsion of mutant cells was observed, the former characterized by specific reorganization of cell adhesion and polarity proteins, the latter by complete cytoplasmic diffusion of these proteins. Among several candidate target genes tested, only the homeotic gene Abdominal-B was a target of PH in both ovarian and wing disc cells. Although overexpression of Abdominal-B was sufficient to cause cell segregation in the wing disc, epistatic analysis indicated that the presence of Abdominal-B is not necessary for expulsion of polyhomeotic mutant epithelial cells suggesting that additional polyhomeotic targets are implicated in this phenomenon.Our results indicate that polyhomeotic mutations have a direct effect on epithelial integrity that can be uncoupled from overproliferation. We show that cells in an epithelium expressing different levels of polyhomeotic sort out indicating differential adhesive properties between the cell populations. Interestingly, we found distinct modalities between apical and basal expulsion of ph mutant cells and further studies of this phenomenon should allow parallels to be made with the modified adhesive and polarity properties of different types of epithelial tumors.
6. Dpp signaling directs cell motility and invasiveness during epithelial morphogenesis. Tissue remodeling in development and disease involves the coordinated invasion of neighboring territories and/or the replacement of entire cell populations. Cell guidance, cell matching, transitions from passive to migratory epithelia, cell growth and death, and extracellular matrix remodeling all impinge on epithelial spreading. Significantly, the extracellular signals that direct these activities and the specific cellular elements and mechanisms regulated by these signals remain in most cases to be identified. To address these issues, we performed an analysis of histoblasts (Drosophila abdominal epithelial founder cells) on their transition from a dormant state to active migration replacing obsolete larval epidermal cells (LECs). We found that during expansion, Decapentaplegic (Dpp) secreted from surrounding LECs leads to graded pathway activation in cells at the periphery of histoblast nests. Across nests, Dpp activity confers differential cellular behavior and motility by modulating cell-cell contacts, the organization and activity of the cytoskeleton, and histoblast attachment to the substrate. Furthermore, Dpp also prevents the premature death of LECs, allowing the coordination of histoblast expansion to LEC delamination. Dpp signaling activity directing histoblast spreading and invasiveness mimics transforming growth factor-beta and bone morphogenetic proteins' role in enhancing the motility and invasiveness of cancer cells, resulting in the promotion of **metastasis**.

### [Cc]ell [Ii]nvasion

#### PubMed

1. Basolateral junctions are sufficient to suppress epithelial invasion during Drosophila oogenesis. Epithelial junctions play crucial roles during metazoan evolution and development by facilitating tissue formation, maintenance, and function. Little is known about the role of distinct types of junctions in controlling epithelial transformations leading to invasion of neighboring tissues. Discovering the key junction complexes that control these processes and how they function may also provide mechanistic insight into carcinoma **cell invasion**. Here, using the Drosophila ovary as a model, we show that four proteins of the basolateral junction (BLJ), Fasciclin-2, Neuroglian, Discs-large, and Lethal-giant-larvae, but not proteins of other epithelial junctions, directly suppress epithelial tumorigenesis and invasion. Remarkably, the expression pattern of Fasciclin-2 predicts which cells will invade. We compared the apicobasal polarity of BLJ tumor cells to border cells (BCs), an epithelium-derived cluster that normally migrates during mid-oogenesis. Both tumor cells and BCs differentiate a lateralized membrane pattern that is necessary but not sufficient for invasion. Independent of lateralization, derepression of motility pathways is also necessary, as indicated by a strong linear correlation between faster BC migration and an increased incidence of tumor invasion. However, without membrane lateralization, derepression of motility pathways is also not sufficient for invasion. Our results demonstrate that spatiotemporal patterns of basolateral junction activity directly suppress epithelial invasion by organizing the cooperative activity of distinct polarity and motility pathways.

---

## slow

- NCBI Gene
- GeneRIF
- Pubmed

| Search Term | Rank | Fields |
| --- | --- | --- |
| [Mm]igration | 6 | PubMed(2) |
| ECM | 7 | PubMed(1) |
| [Ee]xtracellular [Mm]atrix | 8 | PubMed(3) |

  
hide/show details for slow

### [Mm]igration

#### PubMed

1. Genes conserved in bilaterians but jointly lost with Myc during nematode evolution are enriched in cell proliferation and cell **migration** functions. Animals use a stereotypical set of developmental genes to build body architectures of varying sizes and organizational complexity. Some genes are critical to developmental patterning, while other genes are important to physiological control of growth. However, growth regulator genes may not be as important in small-bodied "micro-metazoans" such as nematodes. Nematodes use a simplified developmental strategy of lineage-based cell fate specifications to produce an adult bilaterian body composed of a few hundreds of cells. Nematodes also lost the MYC proto-oncogenic regulator of cell proliferation. To identify additional regulators of cell proliferation that were lost with MYC, we computationally screened and determined 839 high-confidence genes that are conserved in bilaterians/lost in nematodes (CIBLIN genes). We find that 30 % of all CIBLIN genes encode transcriptional regulators of cell proliferation, epithelial-to-mesenchyme transitions, and other processes. Over 50 % of CIBLIN genes are unnamed genes in Drosophila, suggesting that there are many understudied genes. Interestingly, CIBLIN genes include many Myc synthetic lethal (MycSL) hits from recent screens. CIBLIN genes include key regulators of heparan sulfate proteoglycan (HSPG) sulfation patterns, and lysyl oxidases involved in cross-linking and modification of the extracellular matrix (ECM). These genes and others suggest the CIBLIN repertoire services critical functions in ECM remodeling and cell **migration** in large-bodied bilaterians. Correspondingly, CIBLIN genes are co-expressed with Myc in cancer transcriptomes, and include a preponderance of known determinants of cancer progression and tumor aggression. We propose that CIBLIN gene research can improve our understanding of regulatory control of cellular growth in metazoans.
2. Comparative analysis of Hox downstream genes in Drosophila. Functional diversification of body parts is dependent on the formation of specialized structures along the various body axes. In animals, region-specific morphogenesis along the anteroposterior axis is controlled by a group of conserved transcription factors encoded by the Hox genes. Although it has long been assumed that Hox proteins carry out their function by regulating distinct sets of downstream genes, only a small number of such genes have been found, with very few having direct roles in controlling cellular behavior. We have quantitatively identified hundreds of Hox downstream genes in Drosophila by microarray analysis, and validated many of them by in situ hybridizations on loss- and gain-of-function mutants. One important finding is that Hox proteins, despite their similar DNA-binding properties in vitro, have highly specific effects on the transcriptome in vivo, because expression of many downstream genes respond primarily to a single Hox protein. In addition, a large fraction of downstream genes encodes realizator functions, which directly affect morphogenetic processes, such as orientation and rate of cell divisions, cell-cell adhesion and communication, cell shape and **migration**, or cell death. Focusing on these realizators, we provide a framework for the morphogenesis of the maxillary segment. As the genomic organization of Hox genes and the interaction of Hox proteins with specific co-factors are conserved in vertebrates and invertebrates, and similar classes of downstream genes are regulated by Hox proteins across the metazoan phylogeny, our findings represent a first step toward a mechanistic understanding of morphological diversification within a species as well as between species.

### ECM

#### PubMed

1. Genes conserved in bilaterians but jointly lost with Myc during nematode evolution are enriched in cell proliferation and cell migration functions. Animals use a stereotypical set of developmental genes to build body architectures of varying sizes and organizational complexity. Some genes are critical to developmental patterning, while other genes are important to physiological control of growth. However, growth regulator genes may not be as important in small-bodied "micro-metazoans" such as nematodes. Nematodes use a simplified developmental strategy of lineage-based cell fate specifications to produce an adult bilaterian body composed of a few hundreds of cells. Nematodes also lost the MYC proto-oncogenic regulator of cell proliferation. To identify additional regulators of cell proliferation that were lost with MYC, we computationally screened and determined 839 high-confidence genes that are conserved in bilaterians/lost in nematodes (CIBLIN genes). We find that 30 % of all CIBLIN genes encode transcriptional regulators of cell proliferation, epithelial-to-mesenchyme transitions, and other processes. Over 50 % of CIBLIN genes are unnamed genes in Drosophila, suggesting that there are many understudied genes. Interestingly, CIBLIN genes include many Myc synthetic lethal (MycSL) hits from recent screens. CIBLIN genes include key regulators of heparan sulfate proteoglycan (HSPG) sulfation patterns, and lysyl oxidases involved in cross-linking and modification of the extracellular matrix (**ECM**). These genes and others suggest the CIBLIN repertoire services critical functions in **ECM** remodeling and cell migration in large-bodied bilaterians. Correspondingly, CIBLIN genes are co-expressed with Myc in cancer transcriptomes, and include a preponderance of known determinants of cancer progression and tumor aggression. We propose that CIBLIN gene research can improve our understanding of regulatory control of cellular growth in metazoans.

### [Ee]xtracellular [Mm]atrix

#### PubMed

1. Slowdown promotes muscle integrity by modulating integrin-mediated adhesion at the myotendinous junction. The correct assembly of the myotendinous junction (MTJ) is crucial for proper muscle function. In Drosophila, this junction comprises hemi-adherens junctions that are formed upon arrival of muscles at their corresponding tendon cells. The MTJ mainly comprises muscle-specific alphaPS2betaPS integrin receptors and their tendon-derived **extracellular matrix** ligand Thrombospondin (Tsp). We report the identification and functional analysis of a novel tendon-derived secreted protein named Slowdown (Slow). Homozygous slow mutant larvae exhibit muscle or tendon rupture, sluggish larval movement, partial lethality, and the surviving adult flies are unable to fly. These defects result from improper assembly of the embryonic MTJ. In slow mutants, Tsp prematurely accumulates at muscle ends, the morphology of the muscle leading edge changes and the MTJ architecture is aberrant. Slow was found to form a protein complex with Tsp. This complex is biologically active and capable of altering the morphology and directionality of muscle ends. Our analysis implicates Slow as an essential component of the MTJ, crucial for ensuring muscle and tendon integrity during larval locomotion.
2. Genes conserved in bilaterians but jointly lost with Myc during nematode evolution are enriched in cell proliferation and cell migration functions. Animals use a stereotypical set of developmental genes to build body architectures of varying sizes and organizational complexity. Some genes are critical to developmental patterning, while other genes are important to physiological control of growth. However, growth regulator genes may not be as important in small-bodied "micro-metazoans" such as nematodes. Nematodes use a simplified developmental strategy of lineage-based cell fate specifications to produce an adult bilaterian body composed of a few hundreds of cells. Nematodes also lost the MYC proto-oncogenic regulator of cell proliferation. To identify additional regulators of cell proliferation that were lost with MYC, we computationally screened and determined 839 high-confidence genes that are conserved in bilaterians/lost in nematodes (CIBLIN genes). We find that 30 % of all CIBLIN genes encode transcriptional regulators of cell proliferation, epithelial-to-mesenchyme transitions, and other processes. Over 50 % of CIBLIN genes are unnamed genes in Drosophila, suggesting that there are many understudied genes. Interestingly, CIBLIN genes include many Myc synthetic lethal (MycSL) hits from recent screens. CIBLIN genes include key regulators of heparan sulfate proteoglycan (HSPG) sulfation patterns, and lysyl oxidases involved in cross-linking and modification of the **extracellular matrix** (ECM). These genes and others suggest the CIBLIN repertoire services critical functions in ECM remodeling and cell migration in large-bodied bilaterians. Correspondingly, CIBLIN genes are co-expressed with Myc in cancer transcriptomes, and include a preponderance of known determinants of cancer progression and tumor aggression. We propose that CIBLIN gene research can improve our understanding of regulatory control of cellular growth in metazoans.
3. Slowdown promotes muscle integrity by modulating integrin-mediated adhesion at the myotendinous junction. The correct assembly of the myotendinous junction (MTJ) is crucial for proper muscle function. In Drosophila, this junction comprises hemi-adherens junctions that are formed upon arrival of muscles at their corresponding tendon cells. The MTJ mainly comprises muscle-specific alphaPS2betaPS integrin receptors and their tendon-derived **extracellular matrix** ligand Thrombospondin (Tsp). We report the identification and functional analysis of a novel tendon-derived secreted protein named Slowdown (Slow). Homozygous slow mutant larvae exhibit muscle or tendon rupture, sluggish larval movement, partial lethality, and the surviving adult flies are unable to fly. These defects result from improper assembly of the embryonic MTJ. In slow mutants, Tsp prematurely accumulates at muscle ends, the morphology of the muscle leading edge changes and the MTJ architecture is aberrant. Slow was found to form a protein complex with Tsp. This complex is biologically active and capable of altering the morphology and directionality of muscle ends. Our analysis implicates Slow as an essential component of the MTJ, crucial for ensuring muscle and tendon integrity during larval locomotion.

---

## Prx2540-2

- NCBI Gene
- GeneRIF
- Pubmed

| Search Term | Rank | Fields |
| --- | --- | --- |
| [Mm]igration | 6 | PubMed(1) |

  
hide/show details for Prx2540-2

### [Mm]igration

#### PubMed

1. Methylmercury activates enhancer-of-split and bearded complex genes independent of the notch receptor. Methylmercury (MeHg) is a persistent environmental toxin that has targeted effects on fetal neural development. Although a number of cytotoxic mechanisms of MeHg have been characterized in cultured cells, its mode of action in the developing nervous system in vivo is less clear. Studies of MeHg-affected rodent and human brains show disrupted cortical and cerebellar architecture suggestive of mechanisms that augment cell signaling pathways potentially affecting cell **migration** and proliferation. We previously identified the Notch receptor pathway, a highly conserved signaling mechanism fundamental for neural development, as a target for MeHg-induced signaling in Drosophila neural cell lines. Here we have expanded our use of the Drosophila model to resolve a broader spectrum of transcriptional changes resulting from MeHg exposure in vivo and in vitro. Several Notch target genes within the Enhancer-of-split (E(spl)C) and Bearded (BrdC) complexes are upregulated with MeHg exposure in the embryo and in cultured neural cells. However, the profile of MeHg-induced E(spl)C and BrdC gene expression differs significantly from that seen with activation of the Notch receptor. Targeted knockdown of Notch and of the downstream coactivator Suppressor of Hairless (Su(H)), shows no effect on MeHg-induced transcription, indicating a novel Notch-independent mechanism of action for MeHg. MeHg transcriptional activation is partially mimicked by iodoacetamide but not by N-ethylmaleimide, two thiol-specific electrophiles, revealing a degree of specificity of cellular thiol targets in MeHg-induced transcriptional events.

---

## S

- NCBI Gene
- GeneRIF
- Pubmed

| Search Term | Rank | Fields |
| --- | --- | --- |
| [Mm]igration | 6 | PubMed(9) |
| [Ee]xtracellular [Mm]atrix | 8 | PubMed(1) |
| [Cc]ell [Ii]nvasion | 13 | PubMed(1) |

  
hide/show details for S

### [Mm]igration

#### PubMed

1. Signaling by the Drosophila epidermal growth factor receptor pathway during development. In 1997 we wrote a review entitled "A thousand and one roles for the Drosophila epidermal growth factor (EGF) receptor (DER/EGFR)." We are not there yet in terms of the number of developmental roles assigned to this receptor in Drosophila. Nevertheless, DER has certainly emerged as one of the key players in development, since it is used repeatedly to direct cell fate choices, cell division, cell survival, and **migration**. A battery of activating ligands and an inhibitory ligand achieves this versatility. For the ligands that are produced as membrane-bound precursors, trafficking and processing are the key regulatory steps, determining the eventual temporal and spatial pattern of receptor activation. In most cases DER is activated at a short range, in the cells adjacent to the ones producing the active ligand. This activation dictates a binary choice. In some instances DER is also activated over a longer range, and multiple cell fate choices may be induced, according to its level of activation. A battery of negative feedback loops assures the limited range of DER induction. The distinct responses to DER activation in the different tissues depend upon combinatorial interactions with other signaling pathways and tissue-specific factors, at the level of target-gene regulation.
2. Egfr signaling regulates ommatidial rotation and cell motility in the Drosophila eye via MAPK/Pnt signaling and the Ras effector Canoe/AF6. Epidermal Growth Factor-receptor (Egfr) signaling is evolutionarily conserved and controls a variety of different cellular processes. In Drosophila these include proliferation, patterning, cell-fate determination, **migration** and survival. Here we provide evidence for a new role of Egfr signaling in controlling ommatidial rotation during planar cell polarity (PCP) establishment in the Drosophila eye. Although the signaling pathways involved in PCP establishment and photoreceptor cell-type specification are beginning to be unraveled, very little is known about the associated 90 degrees rotation process. One of the few rotation-specific mutations known is roulette (rlt) in which ommatidia rotate to a random degree, often more than 90 degrees. Here we show that rlt is a rotation-specific allele of the inhibitory Egfr ligand Argos and that modulation of Egfr activity shows defects in ommatidial rotation. Our data indicate that, beside the Raf/MAPK cascade, the Ras effector Canoe/AF6 acts downstream of Egfr/Ras and provides a link from Egfr to cytoskeletal elements in this developmentally regulated cell motility process. We provide further evidence for an involvement of cadherins and non-muscle myosin II as downstream components controlling rotation. In particular, the involvement of the cadherin Flamingo, a PCP gene, downstream of Egfr signaling provides the first link between PCP establishment and the Egfr pathway.
3. The midline of the Drosophila central nervous system: a model for the genetic analysis of cell fate, cell **migration**, and growth cone guidance. A row of mesectodermal cells separates the two lateral neurogenic regions in the Drosophila embryo and generates a discrete set of glia and neurons. Most CNS growth cones initially head straight toward the midline, suggesting that these midline cells play a key role in the formation of the axon commissures. We have used antibodies that stain the first axons, beta-galactosidase enhancer trap lines that selectively stain the different midline cells, and electron microscopic studies to elucidate the cells and interactions that mediate the normal formation of the two major commissures in each segment. This analysis has led to a model that proposes a series of sequential cell interactions controlling the development of the axon commissures. A genetic test of this model has utilized a number of mutations that, by either eliminating or altering the differentiation of various midline cells, perturb the development of the axon commissures in a predictable fashion.
4. Primordial germ cell **migration** and the assembly of the Drosophila embryonic gonad. During embryogenesis Drosophila pole cells, like germ cells in many other vertebrates and invertebrates, actively migrate before assembling into the gonad. Five separate steps can be distinguished: an initial passive displacement of the germ cells by gastrulation movements, an amoeboid migratory phase during which the pole cells pass through the endoderm, **migration** on endoderm toward mesoderm, separation into two bilateral groups associated with the gonadal mesoderm precursors, followed by condensation into the gonad itself. We have analyzed gonad assembly in embryonic pattern mutants to determine whether distinct cues are required in this process. We show that the initiation of **migration** does not require the presence of the mesoderm, the eventual target of the germ cells. Rather, **migration** is triggered as a consequence of the differentiation of the endoderm. Examination of embryos mutant for maternal genes of the terminal group suggests that a primary role of the endoderm in this process is to allow the germ cell access to the interior of the embryo. In addition, we show that normal gonad assembly requires a region of the embryo that includes the posterior compartment of the fifth and the sixth abdominal segments.
5. The Drosophila gene pointed encodes two ETS-like proteins which are involved in the development of the midline glial cells. Glial-neuronal cell interactions at the ventral midline are necessary for the proper elaboration of commissures in the embryonic CNS of Drosophila. In particular, migrating midline glial cells are required for the separation of segmental commissures. During this process the glial cells recognize specific neuronal cells at the midline, they migrate posteriorly along their cell processes and thereby separate the segmental commissures. The gene pointed (pnt) is required for this glial-neuronal cell interaction, as loss of function mutations lead to a change in the **migration** behavior of the midline glial cells. As a consequence, anterior and posterior commissures do not become separated and appear fused. Molecular analysis of pointed has revealed two differently spliced types of transcripts, which are encoded in a region extending over 55 kb of genomic sequence. In the CNS both transcript classes are expressed in cells of the midline, including the midline glial cells. Sequence analysis of cDNA clones corresponding to both transcript types reveals two different pointed proteins which share an ETS domain common to a number of transcription factors related to the vertebrate ets oncogene. Furthermore, one pointed protein form contains an additional domain of homology of approx. 80 amino acids in length, which is shared by only a subset of the ETS protein family.
6. Cell fate choices in Drosophila tracheal morphogenesis. The Drosophila tracheal system is a branched tubular structure that supplies air to target tissues. The elaborate tracheal morphology is shaped by two linked inductive processes, one involving the choice of cell fates, and the other a guided cell **migration**. We will describe the molecular basis for these processes, and the allocation of cell fate decisions to four temporal hierarchies. First, tracheal placodes are specified within the embryonic ectoderm. Subsequently, branch fates are allocated within the tracheal placodes, prior to **migration**. Localized presentation of the FGF ligand, Branchless, to tracheal cells that express the FGF receptor, Breathless, guides **migration**. Once cell **migration** is initiated, distinct cell fates are determined within each migrating branch. Finally, inhibitory feedback mechanisms ensure the correct assignment of these fates. Tracheal cell fate choices are determined by signaling cascades triggered by signals emanating from the tracheal cells, as well as by ligands produced by adjacent tissues.
7. Interactions between the EGF receptor and DPP pathways establish distinct cell fates in the tracheal placodes. The formation of the tracheal network in Drosophila is driven by stereotyped **migration** of cells from the tracheal pits. No cell divisions take place during tracheal **migration** and the number of cells in each branch is fixed. This work examines the basis for the determination of tracheal branch fates, prior to the onset of **migration**. We show that the EGF receptor pathway is activated by localized processing of the ligand SPITZ in the tracheal placodes and is responsible for the capacity to form the dorsal trunk and visceral branch. The DPP pathway, on the contrary, is induced in the tracheal pit by local presentation of DPP from the adjacent dorsal and ventral ectodermal cells. This pathway patterns the dorsal and lateral branches. Elimination of both pathways blocks **migration** of all tracheal branches. Antagonistic interactions between the two pathways are demonstrated. The opposing activities of two pathways may refine the final determination of tracheal branch fates.
8. A new allele uncovers the role of echinus in the control of ommatidial rotation in the Drosophila eye. The precise orientation of the ommatidia in the Drosophila eye is achieved through a specialized process of cell **migration** taking place in the third-instar eye imaginal disc when ommatidial clusters rotate by 90 degrees. This process is strictly coordinated with the establishment of planar cell polarity (PCP), but it relies on a specific set of genes that control its mechanism independently from PCP signaling. Recently, the epidermal growth factor receptor (EGFR) pathway has been implicated in determining ommatidial rotation. We have isolated a new allele of echinus, a gene known to control the patterning and number of interommatidial cells. We show that echinus displays defects in the rotation of ommatidia that are not evident until mid-pupal stages, and we propose that echinus action is that of opposing EGFR by an unknown mechanism and that this can explain both its influence in ommatidial rotation and lattice programmed cell death (PCD).
9. Spalt modifies EGFR-mediated induction of chordotonal precursors in the embryonic PNS of Drosophila promoting the development of oenocytes. Genes of the spalt family encode nuclear zinc finger proteins. In Drosophila melanogaster, they are necessary for the establishment of head/trunk identity, correct tracheal **migration** and patterning of the wing imaginal disc. Spalt proteins display a predominant pattern of expression in the nervous system, not only in Drosophila but also in species of fish, mouse, frog and human, suggesting an evolutionarily conserved role for these proteins in nervous system development. Here we show that Spalt works as a cell fate switch between two EGFR-induced cell types, the oenocytes and the precursors of the pentascolopodial organ in the embryonic peripheral nervous system. We show that removal of spalt increases the number of scolopodia, as a result of extra secondary recruitment of precursor cells at the expense of the oenocytes. In addition, the absence of spalt causes defects in the normal **migration** of the pentascolopodial organ. The dual function of spalt in the development of this organ, recruitment of precursors and **migration**, is reminiscent of its role in tracheal formation and of the role of a spalt homologue, sem-4, in the Caenorhabditis elegans nervous system.

### [Ee]xtracellular [Mm]atrix

#### PubMed

1. Presenilin-based genetic screens in Drosophila melanogaster identify novel notch pathway modifiers. Presenilin is the enzymatic component of gamma-secretase, a multisubunit intramembrane protease that processes several transmembrane receptors, such as the amyloid precursor protein (APP). Mutations in human Presenilins lead to altered APP cleavage and early-onset Alzheimer's disease. Presenilins also play an essential role in Notch receptor cleavage and signaling. The Notch pathway is a highly conserved signaling pathway that functions during the development of multicellular organisms, including vertebrates, Drosophila, and C. elegans. Recent studies have shown that Notch signaling is sensitive to perturbations in subcellular trafficking, although the specific mechanisms are largely unknown. To identify genes that regulate Notch pathway function, we have performed two genetic screens in Drosophila for modifiers of Presenilin-dependent Notch phenotypes. We describe here the cloning and identification of 19 modifiers, including nicastrin and several genes with previously undescribed involvement in Notch biology. The predicted functions of these newly identified genes are consistent with **extracellular matrix** and vesicular trafficking mechanisms in Presenilin and Notch pathway regulation and suggest a novel role for gamma-tubulin in the pathway.

### [Cc]ell [Ii]nvasion

#### PubMed

1. Substrate specificity of rhomboid intramembrane proteases is governed by helix-breaking residues in the substrate transmembrane domain. Rhomboid intramembrane proteases initiate cell signaling during Drosophila development and Providencia bacterial growth by cleaving transmembrane ligand precursors. We have determined how specificity is achieved: Drosophila Rhomboid-1 is a site-specific protease that recognizes its substrate Spitz by a small region of the Spitz transmembrane domain (TMD). This substrate motif is necessary and sufficient for cleavage and is composed of residues known to disrupt helices. Rhomboids from diverse organisms including bacteria and vertebrates recognize the same substrate motif, suggesting that they use a universal targeting strategy. We used this information to search for other rhomboid substrates and identified a family of adhesion proteins from the human parasite Toxoplasma gondii, the TMDs of which were efficient substrates for rhomboid proteases. Intramembrane cleavage of these proteins is required for host **cell invasion**. These results provide an explanation of how rhomboid proteases achieve specificity, and allow some rhomboid substrates to be predicted from sequence information.

---

## Mlc-c

- NCBI Gene
- GeneRIF
- Pubmed

| Search Term | Rank | Fields |
| --- | --- | --- |
| [Mm]igration | 6 | PubMed(1) |

  
hide/show details for Mlc-c

### [Mm]igration

#### PubMed

1. Calcium-activated Myosin V closes the Drosophila pupil. Approximately 40 years ago, an elegant automatic-gain control was revealed in compound eye photoreceptors: In bright light, an assembly of small pigment granules migrates to the cytoplasmic face of the photosensitive membrane organelle, the rhabdomere, where they attenuate waveguide propagation along the rhabdomere. This **migration** results in a "longitudinal pupil" that reduces rhodopsin exposure by a factor of 0.8 log units. Light-induced elevation of cytosolic free Ca(2+) triggers the **migration** of pigment granules, and pigment granules fail to migrate in a mutant deficient in photoactivated TRP calcium channels. However, the mechanism that moves photoreceptor pigment granules remains elusive. Are the granules actively pulled toward the rhabdomere upon light, or are they instead actively pulled into the cytoplasm in the absence of light? Here we show that Ca(2+)-activated Myosin V (MyoV) pulls pigment granules to the rhabdomere. Thus, one of MyoV's several functions is also as a sensory-adaptation motor. In vitro, Ca(2+) both activates and inhibits MyoV motility; in vivo, its role is undetermined. This first demonstration of an in vivo role for Ca(2+) in MyoV activity shows that in Drosophila photoreceptors, Ca(2+) stimulates MyoV motility.

---

## CG6701

- NCBI Gene
- GeneRIF
- Pubmed

| Search Term | Rank | Fields |
| --- | --- | --- |
| [Mm]igration | 6 | PubMed(1) |

  
hide/show details for CG6701

### [Mm]igration

#### PubMed

1. A functional RNAi screen for regulators of receptor tyrosine kinase and ERK signalling. Receptor tyrosine kinase (RTK) signalling through extracellular-signal-regulated kinases (ERKs) has pivotal roles during metazoan development, underlying processes as diverse as fate determination, differentiation, proliferation, survival, **migration** and growth. Abnormal RTK/ERK signalling has been extensively documented to contribute to developmental disorders and disease, most notably in oncogenic transformation by mutant RTKs or downstream pathway components such as Ras and Raf. Although the core RTK/ERK signalling cassette has been characterized by decades of research using mammalian cell culture and forward genetic screens in model organisms, signal propagation through this pathway is probably regulated by a larger network of moderate, context-specific proteins. The genes encoding these proteins may not have been discovered through traditional screens owing, in particular, to the requirement for visible phenotypes. To obtain a global view of RTK/ERK signalling, we performed an unbiased, RNA interference (RNAi), genome-wide, high-throughput screen in Drosophila cells using a novel, quantitative, cellular assay monitoring ERK activation. Here we show that ERK pathway output integrates a wide array of conserved cellular processes. Further analysis of selected components-in multiple cell types with different RTK ligands and oncogenic stimuli-validates and classifies 331 pathway regulators. The relevance of these genes is highlighted by our isolation of a Ste20-like kinase and a PPM-family phosphatase that seem to regulate RTK/ERK signalling in vivo and in mammalian cells. Novel regulators that modulate specific pathway outputs may be selective targets for drug discovery.

---

## AdamTS-A

- NCBI Gene
- GeneRIF
- Pubmed

| Search Term | Rank | Fields |
| --- | --- | --- |
| [Mm]igration | 6 | Process(3); PubMed(1) |
| ECM | 7 | PubMed(1) |
| [Ee]xtracellular [Mm]atrix | 8 | Process(1); PubMed(2); Component(1); GeneRIFs(1) |

  
hide/show details for AdamTS-A

### [Mm]igration

#### Process

1. epithelial cell **migration**, open tracheal system
2. mesodermal cell **migration**
3. pole cell **migration**

#### PubMed

1. The secreted AdamTS-A metalloprotease is required for collective cell **migration**. Members of the ADAMTS family of secreted metalloproteases play crucial roles in modulating the extracellular matrix (ECM) in development and disease. Here, we show that ADAMTS-A, the Drosophila ortholog of human ADAMTS 9 and ADAMTS 20, and of C. elegans GON-1, is required for cell **migration** during embryogenesis. AdamTS-A is expressed in multiple migratory cell types, including hemocytes, caudal visceral mesoderm (CVM), the visceral branch of the trachea (VBs) and the secretory portion of the salivary gland (SG). Loss of AdamTS-A causes defects in germ cell, CVM and VB **migration** and, depending on the tissue, AdamTS-A functions both autonomously and non-autonomously. In the highly polarized collective of the SG epithelium, loss of AdamTS-A causes apical surface irregularities and cell elongation defects. We provide evidence that ADAMTS-A is secreted into the SG lumen where it functions to release cells from the apical ECM, consistent with the defects observed in AdamTS-A mutant SGs. We show that loss of the apically localized protocadherin Cad99C rescues the SG defects, suggesting that Cad99C serves as a link between the SG apical membrane and the secreted apical ECM component(s) cleaved by ADAMTS-A. Our analysis of AdamTS-A function in the SG suggests a novel role for ADAMTS proteins in detaching cells from the apical ECM, facilitating tube elongation during collective cell **migration**.

### ECM

#### PubMed

1. The secreted AdamTS-A metalloprotease is required for collective cell migration. Members of the ADAMTS family of secreted metalloproteases play crucial roles in modulating the extracellular matrix (**ECM**) in development and disease. Here, we show that ADAMTS-A, the Drosophila ortholog of human ADAMTS 9 and ADAMTS 20, and of C. elegans GON-1, is required for cell migration during embryogenesis. AdamTS-A is expressed in multiple migratory cell types, including hemocytes, caudal visceral mesoderm (CVM), the visceral branch of the trachea (VBs) and the secretory portion of the salivary gland (SG). Loss of AdamTS-A causes defects in germ cell, CVM and VB migration and, depending on the tissue, AdamTS-A functions both autonomously and non-autonomously. In the highly polarized collective of the SG epithelium, loss of AdamTS-A causes apical surface irregularities and cell elongation defects. We provide evidence that ADAMTS-A is secreted into the SG lumen where it functions to release cells from the apical **ECM**, consistent with the defects observed in AdamTS-A mutant SGs. We show that loss of the apically localized protocadherin Cad99C rescues the SG defects, suggesting that Cad99C serves as a link between the SG apical membrane and the secreted apical **ECM** component(s) cleaved by ADAMTS-A. Our analysis of AdamTS-A function in the SG suggests a novel role for ADAMTS proteins in detaching cells from the apical **ECM**, facilitating tube elongation during collective cell migration.

### [Ee]xtracellular [Mm]atrix

#### Process

1. **extracellular matrix** organization

#### PubMed

1. Functional evolution of ADAMTS genes: evidence from analyses of phylogeny and gene organization. The ADAMTS (A Disintegrin-like and Metalloprotease with Thrombospondin motifs) proteins are a family of metalloproteases with sequence similarity to the ADAM proteases, that contain the thrombospondin type 1 sequence repeat motifs (TSRs) common to **extracellular matrix** proteins. ADAMTS proteins have recently gained attention with the discovery of their role in a variety of diseases, including tissue and blood disorders, cancer, osteoarthritis, Alzheimer's and the genetic syndromes Weill-Marchesani syndrome (ADAMTS10), thrombotic thrombocytopenic purpura (ADAMTS13), and Ehlers-Danlos syndrome type VIIC (ADAMTS2) in humans and belted white-spotting mutation in mice (ADAMTS20).Phylogenetic analysis and comparison of the exon/intron organization of vertebrate (Homo, Mus, Fugu), chordate (Ciona) and invertebrate (Drosophila and Caenorhabditis) ADAMTS homologs has elucidated the evolutionary relationships of this important gene family, which comprises 19 members in humans.The evolutionary history of ADAMTS genes in vertebrate genomes has been marked by rampant gene duplication, including a retrotransposition that gave rise to a distinct ADAMTS subfamily (ADAMTS1, -4, -5, -8, -15) that may have distinct aggrecanase and angiogenesis functions.
2. The secreted AdamTS-A metalloprotease is required for collective cell migration. Members of the ADAMTS family of secreted metalloproteases play crucial roles in modulating the **extracellular matrix** (ECM) in development and disease. Here, we show that ADAMTS-A, the Drosophila ortholog of human ADAMTS 9 and ADAMTS 20, and of C. elegans GON-1, is required for cell migration during embryogenesis. AdamTS-A is expressed in multiple migratory cell types, including hemocytes, caudal visceral mesoderm (CVM), the visceral branch of the trachea (VBs) and the secretory portion of the salivary gland (SG). Loss of AdamTS-A causes defects in germ cell, CVM and VB migration and, depending on the tissue, AdamTS-A functions both autonomously and non-autonomously. In the highly polarized collective of the SG epithelium, loss of AdamTS-A causes apical surface irregularities and cell elongation defects. We provide evidence that ADAMTS-A is secreted into the SG lumen where it functions to release cells from the apical ECM, consistent with the defects observed in AdamTS-A mutant SGs. We show that loss of the apically localized protocadherin Cad99C rescues the SG defects, suggesting that Cad99C serves as a link between the SG apical membrane and the secreted apical ECM component(s) cleaved by ADAMTS-A. Our analysis of AdamTS-A function in the SG suggests a novel role for ADAMTS proteins in detaching cells from the apical ECM, facilitating tube elongation during collective cell migration.

#### Component

1. proteinaceous **extracellular matrix**

#### GeneRIFs

1. ADAMTS-A detaches salivary gland cells from the apical **extracellular matrix**.

---

## Idgf1

- NCBI Gene
- GeneRIF
- Pubmed

| Search Term | Rank | Fields |
| --- | --- | --- |
| ECM | 7 | PubMed(1) |
| [Ee]xtracellular [Mm]atrix | 8 | PubMed(1) |

  
hide/show details for Idgf1

### ECM

#### PubMed

1. Chitinases and Imaginal disc growth factors organize the extracellular matrix formation at barrier tissues in insects. The cuticle forms an apical extracellular-matrix (**ECM**) that covers exposed organs, such as epidermis, trachea and gut, for organizing morphogenesis and protection of insects. Recently, we reported that cuticle proteins and chitin are involved in **ECM** formation. However, molecular mechanisms that control assembly, maturation and replacement of the **ECM** and its components are not well known. Here we investigated the poorly described glyco-18-domain hydrolase family in Drosophila and identified the Chitinases (Chts) and imaginal-disc-growth-factors (Idgfs) that are essential for larval and adult molting. We demonstrate that Cht and idgf depletion results in deformed cuticles, larval and adult molting defects, and insufficient protection against wounding and bacterial infection, which altogether leads to early lethality. We show that Cht2/Cht5/Cht7/Cht9/Cht12 and idgf1/idgf3/idgf4/idgf5/idgf6 are needed for organizing proteins and chitin-matrix at the apical cell surface. Our data indicate that normal **ECM** formation requires Chts, which potentially hydrolyze chitin-polymers. We further suggest that the non-enzymatic idgfs act as structural proteins to maintain the **ECM** scaffold against chitinolytic degradation. Conservation of Chts and Idgfs proposes analogous roles in **ECM** dynamics across the insect taxa, indicating that Chts/Idgfs are new targets for species specific pest control.

### [Ee]xtracellular [Mm]atrix

#### PubMed

1. Chitinases and Imaginal disc growth factors organize the **extracellular matrix** formation at barrier tissues in insects. The cuticle forms an apical extracellular-matrix (ECM) that covers exposed organs, such as epidermis, trachea and gut, for organizing morphogenesis and protection of insects. Recently, we reported that cuticle proteins and chitin are involved in ECM formation. However, molecular mechanisms that control assembly, maturation and replacement of the ECM and its components are not well known. Here we investigated the poorly described glyco-18-domain hydrolase family in Drosophila and identified the Chitinases (Chts) and imaginal-disc-growth-factors (Idgfs) that are essential for larval and adult molting. We demonstrate that Cht and idgf depletion results in deformed cuticles, larval and adult molting defects, and insufficient protection against wounding and bacterial infection, which altogether leads to early lethality. We show that Cht2/Cht5/Cht7/Cht9/Cht12 and idgf1/idgf3/idgf4/idgf5/idgf6 are needed for organizing proteins and chitin-matrix at the apical cell surface. Our data indicate that normal ECM formation requires Chts, which potentially hydrolyze chitin-polymers. We further suggest that the non-enzymatic idgfs act as structural proteins to maintain the ECM scaffold against chitinolytic degradation. Conservation of Chts and Idgfs proposes analogous roles in ECM dynamics across the insect taxa, indicating that Chts/Idgfs are new targets for species specific pest control.

---

## Exn

- NCBI Gene
- GeneRIF
- Pubmed

| Search Term | Rank | Fields |
| --- | --- | --- |
| [Ee]xtracellular [Mm]atrix | 8 | PubMed(1) |

  
hide/show details for Exn

### [Ee]xtracellular [Mm]atrix

#### PubMed

1. A directed miniscreen for genes involved in the Drosophila anti-parasitoid immune response. Drosophila larvae react against eggs from the endoparasitoid wasp Leptopilina boulardi by surrounding them in a multilayered cellular capsule. Once a wasp egg is recognized as foreign, circulating macrophage-like cells, known as plasmatocytes, adhere to the invader. After spreading around the wasp egg, plasmatocytes form cellular junctions between the cells, effectively separating the egg from the hemocoel. Next, a second sub-type of circulating immunosurveillance cell (hemocyte), known as lamellocytes, adhere to either the wasp egg or more likely the plasmatocytes surrounding the egg. From these events, it is obvious that adhesion and cell shape change are an essential part of Drosophila's cellular immune response against parasitoid wasp eggs. To date, very few genes have been described as being necessary for a proper anti-parasitization response in Drosophila. With this in mind, we performed a directed genetic miniscreen to discover new genes required for this response. Many of the genes with an encapsulation defect have mammalian homologues involved in cellular adhesion, wound healing, and thrombosis, including **extracellular matrix** proteins, cellular adhesion molecules, and small GTPases.

---

## Ilp6

- NCBI Gene
- GeneRIF
- Pubmed

| Search Term | Rank | Fields |
| --- | --- | --- |
| [Ff]ilopodia | 1 | PubMed(1) |

  
hide/show details for Ilp6

### [Ff]ilopodia

#### PubMed

1. Muscle niche-driven Insulin-Notch-Myc cascade reactivates dormant Adult Muscle Precursors in Drosophila. How stem cells specified during development keep their non-differentiated quiescent state, and how they are reactivated, remain poorly understood. Here, we applied a Drosophila model to follow in vivo behavior of adult muscle precursors (AMPs), the transient fruit fly muscle stem cells. We report that emerging AMPs send out thin **filopodia** that make contact with neighboring muscles. AMPs keep their **filopodia**-based association with muscles throughout their dormant state but also when they start to proliferate, suggesting that muscles could play a role in AMP reactivation. Indeed, our genetic analyses indicate that muscles send inductive dIlp6 signals that switch the Insulin pathway ON in closely associated AMPs. This leads to the activation of Notch, which regulates AMP proliferation via dMyc. Altogether, we report that Drosophila AMPs display homing behavior to muscle niche and that the niche-driven Insulin-Notch-dMyc cascade plays a key role in setting the activated state of AMPs.

---

## Pvr

- NCBI Gene
- GeneRIF
- Pubmed

| Search Term | Rank | Fields |
| --- | --- | --- |
| [Ff]ilopodia | 1 | PubMed(3) |
| [Mm]igration | 6 | Process(5); PubMed(61); GeneRIFs(3) |
| [Ee]xtracellular [Mm]atrix | 8 | PubMed(2) |
| [Ii]nvasive | 10 | PubMed(7) |
| [Mm]etastasize | 11 | PubMed(1) |
| [Mm]etastasis | 12 | PubMed(13) |
| [Cc]ell [Ii]nvasion | 13 | PubMed(1) |

  
hide/show details for Pvr

### [Ff]ilopodia

#### PubMed

1. Transcriptional regulation of Profilin during wound closure in Drosophila larvae. Injury is an inevitable part of life, making wound healing essential for survival. In postembryonic skin, wound closure requires that epidermal cells recognize the presence of a gap and change their behavior to migrate across it. In Drosophila larvae, wound closure requires two signaling pathways [the Jun N-terminal kinase (JNK) pathway and the Pvr receptor tyrosine kinase signaling pathway] and regulation of the actin cytoskeleton. In this and other systems, it remains unclear how the signaling pathways that initiate wound closure connect to the actin regulators that help execute wound-induced cell migrations. Here, we show that chickadee, which encodes the Drosophila Profilin, a protein important for actin filament recycling and cell migration during development, is required for the physiological process of larval epidermal wound closure. After injury, chickadee is transcriptionally upregulated in cells proximal to the wound. We found that JNK, but not Pvr, mediates the increase in chic transcription through the Jun and Fos transcription factors. Finally, we show that chic-deficient larvae fail to form a robust actin cable along the wound edge and also fail to form normal **filopodia**l and lamellipodial extensions into the wound gap. Our results thus connect a factor that regulates actin monomer recycling to the JNK signaling pathway during wound closure. They also reveal a physiological function for an important developmental regulator of actin and begin to tease out the logic of how the wound repair response is organized.
2. Function of Rho GTPases in embryonic blood cell migration in Drosophila. Hemocyte development in the Drosophila embryo is a genetic model to study blood cell differentiation, cell migration and phagocytosis. Macrophages, which make up the majority of embryonic hemocytes, migrate extensively as individual cells on basement membrane-covered surfaces. The molecular mechanisms that contribute to this migration process are currently not well understood. We report the generation, by P element replacement, of two Gal4 lines that drive expression of UAS-controlled target genes during early (gcm-Gal4) or late (Coll-Gal4) stages of macrophage migration. gcm-Gal4 is used for live imaging analysis showing that macrophages extend large, dynamic lamellipodia as their main protrusions as well as **filopodia**. We use both Gal4 lines to express dominantnegative and constitutively active isoforms of the Rho GTPases Rac1, Cdc42, Rho1 and RhoL in macrophages, and complement these experiments by analyzing embryos mutant for Rho GTPases. Our findings suggest that Rac1 and Rac2 act redundantly in controlling migration and lamellipodia formation in Drosophila macrophages, and that the third Drosophila Rac gene, Mtl, makes no significant contribution to macrophage migration. Cdc42 appears not to be required within macrophages but in other tissues of the embryo to guide macrophages to the ventral trunk region. No evidence was found for a requirement of Rho1 or RhoL in macrophage migration. Finally, to estimate the number of genes whose zygotic expression is required for macrophage migration we analyzed 208 chromosomal deletions that cover most of the Drosophila genome. We find eight deletions that cause defects in macrophage migration suggesting the existence of approximately ten zygotic genes essential for macrophage migration.
3. Cortactin modulates cell migration and ring canal morphogenesis during Drosophila oogenesis. Cortactin is a Src substrate that interacts with F-actin and can stimulate actin polymerization by direct interaction with the Arp2/3 complex. We have isolated complete loss-of-function mutants of the single Drosophila cortactin gene. Mutants are viable and fertile, showing that cortactin is not an essential gene. However, cortactin mutants show distinct defects during oogenesis. During oogenesis, Cortactin protein is enriched at the F-actin rich ring canals in the germ line, and in migrating border cells. In cortactin mutants, the ring canals are smaller than normal. A similar phenotype has been observed in Src64 mutants and in mutants for genes encoding Arp2/3 complex components, supporting that these protein products act together to control specific processes in vivo. Cortactin mutants also show impaired border cell migration. This invasive cell migration is guided by Drosophila EGFR and PDGF/VEGF receptor (PVR). We find that accumulation of Cortactin protein is positively regulated by PVR. Also, overexpression of Cortactin can by itself induce F-actin accumulation and ectopic **filopodia** formation in epithelial cells. We present evidence that Cortactin is one of the factors acting downstream of PVR and Src to stimulate F-actin accumulation. Cortactin is a minor contributor in this regulation, consistent with the cortactin gene not being essential for development.

### [Mm]igration

#### Process

1. border follicle cell **migration**
2. border follicle cell **migration**
3. border follicle cell **migration**
4. hemocyte **migration**
5. hemocyte **migration**

#### PubMed

1. Spatial restriction of receptor tyrosine kinase activity through a polarized endocytic cycle controls border cell **migration**. Border cell **migration** is a stereotyped **migration** occurring during the development of the Drosophila egg chamber. During this process, a cluster composed of six to eight follicle cells migrates between nurse cells toward the oocyte. Receptor tyrosine kinases (RTKs) are enriched at the leading edge of the follicle cells and establish the directionality of their **migration**. Endocytosis has been shown to play a role in the maintenance of this polarization; however, the mechanisms involved are largely unknown. In this study, we show that border cell **migration** requires the function of the small GTPases Rab5 and Rab11 that regulate trafficking through the early and the recycling endosome, respectively. Expression of a dominant negative form of rab11 induces a loss of the polarization of RTK activity, which correlates with a severe **migration** phenotype. In addition, we demonstrate that the exocyst component Sec15 is distributed in structures that are polarized during the **migration** process in a Rab11-dependent manner and that the down-regulation of different subunits of the exocyst also affects **migration**. Together, our data demonstrate a fundamental role for a plasma membrane-endosome trafficking cycle in the maintenance of active RTK at the leading edge of border cells during their **migration**.
2. Regulation of cofilin phosphorylation and asymmetry in collective cell **migration** during morphogenesis. During Drosophila oogenesis, two actin dynamics regulators, cofilin and Rac, are required for the collective **migration** of a coherent cluster of cells called border cells. Cell culture data have shown that Rac and cofilin are both essential for lamellipodium formation, but Rac signaling results in phosphorylation and hence inactivation of cofilin. So it remains unclear whether cofilin phosphorylation plays a promoting or inhibitory role during cell **migration**. We show here that cofilin is required for F-actin turnover and lamellipodial protrusion in the border cells. Interestingly, reducing the dosage of cofilin by half or expressing a phospho-mimetic mutant form, S3E, partially rescues the **migration** and protrusion defects of Rac-deficient border cells. Moreover, cofilin exhibits moderate accumulation in border cells at the migratory front of the cluster, whereas phospho-cofilin has a robust and uniform distribution pattern in all the outer border cells. Blocking or overactivating Rac signaling in border cells greatly reduces or increases cofilin phosphorylation, respectively, and each abolishes cell **migration**. Furthermore, Rac may signal through Pak and LIMK to result in uniform phosphorylation of cofilin in all the outer border cells, whereas the guidance receptor Pvr (PDGF/VEGF receptor) mediates the asymmetric localization of cofilin in the cluster but does not affect its phosphorylation. Our study provides one of the first models of how cofilin functions and is regulated in the collective **migration** of a group of cells in vivo.
3. A sensitized PiggyBac-based screen for regulators of border cell **migration** in Drosophila. **Migration** of border cells during Drosophila melanogaster oogenesis is a good model system for investigating the genetic requirements for cell **migration** in vivo. We present a sensitized loss-of-function screen used to identify new genes required in border cells for their **migration**. Chromosomes bearing FRTs on all four major autosomal arms were mutagenized by insertions of the transposable element PiggyBac, allowing multiple parallel clonal screens and easy identification of the mutated gene. For border cells, we analyzed homozygous mutant clones positively marked with lacZ and sensitized by expression of dominant-negative PVR, the guidance receptor. We identified new alleles of genes already known to be required for border cell **migration**, including aop/yan, DIAP1, and taiman as well as a conserved Slbo-regulated enhancer downstream of shg/DE-cadherin. Mutations in genes not previously described to be required in border cells were also uncovered: hrp48, vir, rme-8, kismet, and puckered. puckered was unique in that the **migration** defects were observed only when PVR signaling was reduced. We present evidence that an excess of JNK signaling is deleterious for **migration** in the absence of PVR activity at least in part through Fos transcriptional activity and possibly through antagonistic effects on DIAP1.
4. PVR plays a critical role via JNK activation in thorax closure during Drosophila metamorphosis. PVR, the Drosophila homolog of the PDGF/VEGF receptor, has been implicated in border cell **migration** during oogenesis and hemocyte **migration** during embryogenesis. It was earlier shown that Mbc, a CDM family protein, and its effector, Rac, transduced the guidance signal from PVR during border cell **migration**. Here we demonstrate that PVR is also required for the morphogenetic process, thorax closure, during metamorphosis. The results of genetic and biochemical experiments indicate that PVR activates the JNK pathway. We present evidence showing Crk (an adaptor molecule), Mbc, ELMO (a homolog of Caenorhabditis elegans CED-12 and mammalian ELMO), and Rac to be mediators of JNK activation by PVR. In addition, we suppose that not only Rac but also Cdc42 is activated and involved in JNK activation downstream of PVR.
5. Signaling by the Drosophila epidermal growth factor receptor pathway during development. In 1997 we wrote a review entitled "A thousand and one roles for the Drosophila epidermal growth factor (EGF) receptor (DER/EGFR)." We are not there yet in terms of the number of developmental roles assigned to this receptor in Drosophila. Nevertheless, DER has certainly emerged as one of the key players in development, since it is used repeatedly to direct cell fate choices, cell division, cell survival, and **migration**. A battery of activating ligands and an inhibitory ligand achieves this versatility. For the ligands that are produced as membrane-bound precursors, trafficking and processing are the key regulatory steps, determining the eventual temporal and spatial pattern of receptor activation. In most cases DER is activated at a short range, in the cells adjacent to the ones producing the active ligand. This activation dictates a binary choice. In some instances DER is also activated over a longer range, and multiple cell fate choices may be induced, according to its level of activation. A battery of negative feedback loops assures the limited range of DER induction. The distinct responses to DER activation in the different tissues depend upon combinatorial interactions with other signaling pathways and tissue-specific factors, at the level of target-gene regulation.
6. Genes that drive invasion and **migration** in Drosophila. Successful cell **migration** depends on the careful regulation of the timing of movement, the guidance of motile cells, and cytoskeletal and adhesive changes within the cells. This review focuses on genes that act cell-autonomously to promote these aspects of cell **migration** in Drosophila. We discuss recent advances in understanding the **migration** of the ovarian border cells, embryonic blood cells, primordial germ cells, somatic gonadal precursors, and tracheal cells. Comparison of genes that regulate these processes to those that promote tumorigenesis and metastasis in mammals demonstrates that studies in fruit flies are uncovering new genes highly relevant to cancer biology.
7. The social lives of migrating cells in Drosophila. Studies of cell **migration** in Drosophila are yielding insights into the complex interactions migrating cells have with each other and with the cells in their environment. Intriguing links between factors that promote cell **migration** and those that control cell survival have been reported recently. For example, migrating germ cells compete with the surrounding somatic tissue for the substrate of the lipid phosphate phosphatases encoded by the genes Wunen and Wunen2. Germ cells take up the dephosphorylated lipid and require it for their survival. In addition, the secreted growth factors called PVFs, previously thought to guide the **migration**s of hemocytes in the embryo, were found to function instead predominantly as survival factors. And in border cells, DIAP1 and Dronc, two proteins known mainly for their ability to regulate cell death, were found to control cell **migration**.
8. Transcriptional regulation of Profilin during wound closure in Drosophila larvae. Injury is an inevitable part of life, making wound healing essential for survival. In postembryonic skin, wound closure requires that epidermal cells recognize the presence of a gap and change their behavior to migrate across it. In Drosophila larvae, wound closure requires two signaling pathways [the Jun N-terminal kinase (JNK) pathway and the Pvr receptor tyrosine kinase signaling pathway] and regulation of the actin cytoskeleton. In this and other systems, it remains unclear how the signaling pathways that initiate wound closure connect to the actin regulators that help execute wound-induced cell **migration**s. Here, we show that chickadee, which encodes the Drosophila Profilin, a protein important for actin filament recycling and cell **migration** during development, is required for the physiological process of larval epidermal wound closure. After injury, chickadee is transcriptionally upregulated in cells proximal to the wound. We found that JNK, but not Pvr, mediates the increase in chic transcription through the Jun and Fos transcription factors. Finally, we show that chic-deficient larvae fail to form a robust actin cable along the wound edge and also fail to form normal filopodial and lamellipodial extensions into the wound gap. Our results thus connect a factor that regulates actin monomer recycling to the JNK signaling pathway during wound closure. They also reveal a physiological function for an important developmental regulator of actin and begin to tease out the logic of how the wound repair response is organized.
9. Light-mediated activation reveals a key role for Rac in collective guidance of cell movement in vivo. The small GTPase Rac induces actin polymerization, membrane ruffling and focal contact formation in cultured single cells but can either repress or stimulate motility in epithelial cells depending on the conditions. The role of Rac in collective epithelial cell movements in vivo, which are important for both morphogenesis and metastasis, is therefore difficult to predict. Recently, photoactivatable analogues of Rac (PA-Rac) have been developed, allowing rapid and reversible activation or inactivation of Rac using light. In cultured single cells, light-activated Rac leads to focal membrane ruffling, protrusion and **migration**. Here we show that focal activation of Rac is also sufficient to polarize an entire group of cells in vivo, specifically the border cells of the Drosophila ovary. Moreover, activation or inactivation of Rac in one cell of the cluster caused a dramatic response in the other cells, suggesting that the cells sense direction as a group according to relative levels of Rac activity. Communication between cells of the cluster required Jun amino-terminal kinase (JNK) but not guidance receptor signalling. These studies further show that photoactivatable proteins are effective tools in vivo.
10. A functional RNAi screen for regulators of receptor tyrosine kinase and ERK signalling. Receptor tyrosine kinase (RTK) signalling through extracellular-signal-regulated kinases (ERKs) has pivotal roles during metazoan development, underlying processes as diverse as fate determination, differentiation, proliferation, survival, **migration** and growth. Abnormal RTK/ERK signalling has been extensively documented to contribute to developmental disorders and disease, most notably in oncogenic transformation by mutant RTKs or downstream pathway components such as Ras and Raf. Although the core RTK/ERK signalling cassette has been characterized by decades of research using mammalian cell culture and forward genetic screens in model organisms, signal propagation through this pathway is probably regulated by a larger network of moderate, context-specific proteins. The genes encoding these proteins may not have been discovered through traditional screens owing, in particular, to the requirement for visible phenotypes. To obtain a global view of RTK/ERK signalling, we performed an unbiased, RNA interference (RNAi), genome-wide, high-throughput screen in Drosophila cells using a novel, quantitative, cellular assay monitoring ERK activation. Here we show that ERK pathway output integrates a wide array of conserved cellular processes. Further analysis of selected components-in multiple cell types with different RTK ligands and oncogenic stimuli-validates and classifies 331 pathway regulators. The relevance of these genes is highlighted by our isolation of a Ste20-like kinase and a PPM-family phosphatase that seem to regulate RTK/ERK signalling in vivo and in mammalian cells. Novel regulators that modulate specific pathway outputs may be selective targets for drug discovery.
11. Function of Rho GTPases in embryonic blood cell **migration** in Drosophila. Hemocyte development in the Drosophila embryo is a genetic model to study blood cell differentiation, cell **migration** and phagocytosis. Macrophages, which make up the majority of embryonic hemocytes, migrate extensively as individual cells on basement membrane-covered surfaces. The molecular mechanisms that contribute to this **migration** process are currently not well understood. We report the generation, by P element replacement, of two Gal4 lines that drive expression of UAS-controlled target genes during early (gcm-Gal4) or late (Coll-Gal4) stages of macrophage **migration**. gcm-Gal4 is used for live imaging analysis showing that macrophages extend large, dynamic lamellipodia as their main protrusions as well as filopodia. We use both Gal4 lines to express dominantnegative and constitutively active isoforms of the Rho GTPases Rac1, Cdc42, Rho1 and RhoL in macrophages, and complement these experiments by analyzing embryos mutant for Rho GTPases. Our findings suggest that Rac1 and Rac2 act redundantly in controlling **migration** and lamellipodia formation in Drosophila macrophages, and that the third Drosophila Rac gene, Mtl, makes no significant contribution to macrophage **migration**. Cdc42 appears not to be required within macrophages but in other tissues of the embryo to guide macrophages to the ventral trunk region. No evidence was found for a requirement of Rho1 or RhoL in macrophage **migration**. Finally, to estimate the number of genes whose zygotic expression is required for macrophage **migration** we analyzed 208 chromosomal deletions that cover most of the Drosophila genome. We find eight deletions that cause defects in macrophage **migration** suggesting the existence of approximately ten zygotic genes essential for macrophage **migration**.
12. PVR plays a critical role via JNK activation in thorax closure during Drosophila metamorphosis. PVR, the Drosophila homolog of the PDGF/VEGF receptor, has been implicated in border cell **migration** during oogenesis and hemocyte **migration** during embryogenesis. It was earlier shown that Mbc, a CDM family protein, and its effector, Rac, transduced the guidance signal from PVR during border cell **migration**. Here we demonstrate that PVR is also required for the morphogenetic process, thorax closure, during metamorphosis. The results of genetic and biochemical experiments indicate that PVR activates the JNK pathway. We present evidence showing Crk (an adaptor molecule), Mbc, ELMO (a homolog of Caenorhabditis elegans CED-12 and mammalian ELMO), and Rac to be mediators of JNK activation by PVR. In addition, we suppose that not only Rac but also Cdc42 is activated and involved in JNK activation downstream of PVR.
13. RhoL controls invasion and Rap1 localization during immune cell trans**migration** in Drosophila. Human immune cells have to penetrate an endothelial barrier during their beneficial pursuit of infection and their destructive infiltration of tissues in autoimmune diseases. This trans**migration** requires Rap1 GTPase to activate integrin affinity. We define a new model system for this process by demonstrating, with live imaging and genetics, that during embryonic development Drosophila melanogaster immune cells penetrate an epithelial, Drosophila E-cadherin (DE-cadherin)-based tissue barrier. A mutant in RhoL, a GTPase homologue that is specifically expressed in haemocytes, blocks this invasive step but not other aspects of guided **migration**. RhoL mediates integrin adhesion caused by Drosophila Rap1 overexpression and moves Rap1 away from a concentration in the cytoplasm to the leading edge during invasive **migration**. These findings indicate that a programmed migratory step during Drosophila development bears striking molecular similarities to vertebrate immune cell trans**migration** during inflammation, and identify RhoL as a new regulator of invasion, adhesion and Rap1 localization. Our work establishes the utility of Drosophila for identifying novel components of immune cell trans**migration** and for understanding the in vivo interplay of immune cells with the barriers they penetrate.
14. Function of the ETS transcription factor Yan in border cell **migration**. Invasive cell **migration** in both normal development and metastatic cancer is regulated by various signaling pathways, transcription factors and cell-adhesion molecules. The coordination between these activities in the context of cell **migration** is poorly understood. During Drosophila oogenesis, a small group of cells called border cells exit the follicular epithelium to perform a stereotypic, invasive **migration**. We find that the ETS transcription factor Yan is required for border cell **migration** and that Yan expression is spatiotemporally regulated as border cells migrate from the anterior pole of the egg chamber towards the nurse cell-oocyte boundary. Yan expression is dependent on inputs from the JAK/STAT, Notch and Receptor Tyrosine Kinase pathways in border cells. Mechanistically, Yan functions to modulate the turnover of DE-Cadherin-dependent adhesive complexes to facilitate border cell **migration**. Our results suggest that Yan acts as a pivotal link between signal transduction, cell adhesion and invasive cell **migration** in Drosophila border cells.
15. Cellular and molecular mechanisms of single and collective cell **migration**s in Drosophila: themes and variations. The process of cell **migration** is essential throughout life, driving embryonic morphogenesis and ensuring homeostasis in adults. Defects in cell **migration** are a major cause of human disease, with excessive **migration** causing autoimmune diseases and cancer metastasis, whereas reduced capacity for **migration** leads to birth defects and immunodeficiencies. Myriad studies in vitro have established a consensus view that cell **migration**s require cell polarization, Rho GTPase-mediated cytoskeletal rearrangements, and myosin-mediated contractility. However, in vivo studies later revealed a more complex picture, including the discovery that cells migrate not only as single units but also as clusters, strands, and sheets. In particular, the role of E-Cadherin in cell motility appears to be more complex than previously appreciated. Here, we discuss recent advances achieved by combining the plethora of genetic tools available to the Drosophila geneticist with live imaging and biophysical techniques. Finally, we discuss the emerging themes such studies have revealed and ponder the puzzles that remain to be solved.
16. In vivo RNAi screen identifies candidate signaling genes required for collective cell **migration** in Drosophila ovary. Collective **migration** of loosely or closely associated cell groups is prevalent in animal development, physiological events, and cancer metastasis. However, our understanding of the mechanisms of collective cell **migration** is incomplete. Drosophila border cells provide a powerful in vivo genetic model to study collective **migration** and identify essential genes for this process. Using border cell-specific RNAi-silencing in Drosophila, we knocked down 360 conserved signaling transduction genes in adult flies to identify essential pathways and genes for border cell **migration**. We uncovered a plethora of signaling genes, a large proportion of which had not been reported for border cells, including Rack1 (Receptor of activated C kinase) and brk (brinker), mad (mother against dpp), and sax (saxophone), which encode three components of TGF-β signaling. The RNAi knock down phenotype was validated by clonal analysis of Rack1 mutants. Our data suggest that inhibition of Src activity by Rack1 may be important for border cell **migratio**n and cluster cohesion maintenance. Lastly, results from our screen not only would shed light on signaling pathways involved in collective **migratio**n during embryogenesis and organogenesis in general, but also could help our understanding for the functions of conserved human genes involved in cancer metastasis.
17. Drosophila blood cell chemotaxis. Drosophila melanogaster contains a population of blood cells called hemocytes that represent the functional equivalent of vertebrate macrophages. These cells undergo directed **migration**s to disperse during development and reach sites of tissue damage or altered self. These chemotactic behaviors are controlled by the expression of PDGF/Vegf-related ligands in developing embryos and local production of hydrogen peroxide at wounds. Recent work reveals that many molecules important in vertebrate cell motility, including integrins, formins, Ena/VASP proteins and the SCAR/WAVE complex, have a conserved function in these innate immune cells. The use of this model organism has elucidated how damage signals are activated by calcium signaling during inflammation and that the steroid hormone ecdysone activates immune competence at key developmental stages.
18. A role for Drosophila IAP1-mediated caspase inhibition in Rac-dependent cell **migration**. Border cell **migration** in the Drosophila ovary is a relatively simple and genetically tractable model for studying the conversion of epithelial cells to migratory cells. Like many cell **migration**s, border cell **migration** is inhibited by a dominant-negative form of the GTPase Rac. To identify new genes that function in Rac-dependent cell motility, we screened for genes that when overexpressed suppressed the **migration** defect caused by dominant-negative Rac. Overexpression of the Drosophila inhibitor of apoptosis 1 (DIAP1), which is encoded by the thread (th) gene, suppressed the **migration** defect. Moreover, loss-of-function mutations in th caused **migration** defects but, surprisingly, did not cause apoptosis. Mutations affecting the Dark protein, an activator of the upstream caspase Dronc, also rescued RacN17 **migration** defects. These results indicate an apoptosis-independent role for DIAP1-mediated Dronc inhibition in Rac-mediated cell motility.
19. A role for the chaperone Hsp70 in the regulation of border cell **migration** in the Drosophila ovary. Unravelling the molecular mechanisms that govern cell **migration** is of great importance towards understanding both normal embryogenesis and physiological and pathological processes occurring in the adult. **Migration** of border cells (BCs) during Drosophila oogenesis provides a simple and attractive model in which to address this problem. Here, we show that the molecular chaperone Hsp70 is required for BC **migration**. Thus, BCs lacking all Hsp70 genes present in the fly genome fail to reorganize their actin cytoskeleton, resulting in **migration** defects. Similar defects are found when the Hsp70 co-chaperone DnaJ-1, the Drosophila homolog of the human Hsp40, is overexpressed specifically in BCs. In addition, we provide biochemical and genetic evidence for an interaction between DnaJ-1 and PDGF/VEGF receptor (PVR), which is also required for actin-mediated BC **migration**. Furthermore, our results showing that PVR also interacts genetically with Hsp70 suggest that a mechanism by which the DnaJ-1/Hsp70 chaperone complex regulates BC **migration** is by modulating PVR function.
20. Regulation of cofilin phosphorylation and asymmetry in collective cell **migration** during morphogenesis. During Drosophila oogenesis, two actin dynamics regulators, cofilin and Rac, are required for the collective **migration** of a coherent cluster of cells called border cells. Cell culture data have shown that Rac and cofilin are both essential for lamellipodium formation, but Rac signaling results in phosphorylation and hence inactivation of cofilin. So it remains unclear whether cofilin phosphorylation plays a promoting or inhibitory role during cell **migration**. We show here that cofilin is required for F-actin turnover and lamellipodial protrusion in the border cells. Interestingly, reducing the dosage of cofilin by half or expressing a phospho-mimetic mutant form, S3E, partially rescues the **migration** and protrusion defects of Rac-deficient border cells. Moreover, cofilin exhibits moderate accumulation in border cells at the migratory front of the cluster, whereas phospho-cofilin has a robust and uniform distribution pattern in all the outer border cells. Blocking or overactivating Rac signaling in border cells greatly reduces or increases cofilin phosphorylation, respectively, and each abolishes cell **migration**. Furthermore, Rac may signal through Pak and LIMK to result in uniform phosphorylation of cofilin in all the outer border cells, whereas the guidance receptor Pvr (PDGF/VEGF receptor) mediates the asymmetric localization of cofilin in the cluster but does not affect its phosphorylation. Our study provides one of the first models of how cofilin functions and is regulated in the collective **migration** of a group of cells in vivo.
21. A sensitized PiggyBac-based screen for regulators of border cell **migration** in Drosophila. **Migration** of border cells during Drosophila melanogaster oogenesis is a good model system for investigating the genetic requirements for cell **migration** in vivo. We present a sensitized loss-of-function screen used to identify new genes required in border cells for their **migration**. Chromosomes bearing FRTs on all four major autosomal arms were mutagenized by insertions of the transposable element PiggyBac, allowing multiple parallel clonal screens and easy identification of the mutated gene. For border cells, we analyzed homozygous mutant clones positively marked with lacZ and sensitized by expression of dominant-negative PVR, the guidance receptor. We identified new alleles of genes already known to be required for border cell **migration**, including aop/yan, DIAP1, and taiman as well as a conserved Slbo-regulated enhancer downstream of shg/DE-cadherin. Mutations in genes not previously described to be required in border cells were also uncovered: hrp48, vir, rme-8, kismet, and puckered. puckered was unique in that the **migration** defects were observed only when PVR signaling was reduced. We present evidence that an excess of JNK signaling is deleterious for **migration** in the absence of PVR activity at least in part through Fos transcriptional activity and possibly through antagonistic effects on DIAP1.
22. Pak3 regulates apical-basal polarity in migrating border cells during Drosophila oogenesis. Group cell **migration** is a highly coordinated process that is involved in a number of physiological events such as morphogenesis, wound healing and tumor metastasis. Unlike single cells, collectively moving cells are physically attached to each other and retain some degree of apical-basal polarity during the migratory phase. Although much is known about direction sensing, how polarity is regulated in multicellular movement remains unclear. Here we report the role of the protein kinase Pak3 in maintaining apical-basal polarity in migrating border cell clusters during Drosophila oogenesis. Pak3 is enriched in border cells and downregulation of its function impedes border cell movement. Time-lapse imaging suggests that Pak3 affects protrusive behavior of the border cell cluster, specifically regulating the stability and directionality of protrusions. Pak3 functions downstream of guidance receptor signaling to regulate the level and distribution of F-actin in migrating border cells. We also provide evidence that Pak3 genetically interacts with the lateral polarity marker Scribble and that it regulates JNK signaling in the moving border cells. Since Pak3 depletion results in mislocalization of several apical-basal polarity markers and overexpression of Jra rescues the polarity of the Pak3-depleted cluster, we propose that Pak3 functions through JNK signaling to modulate apical-basal polarity of the migrating border cell cluster. We also observe loss of apical-basal polarity in Rac1-depleted border cell clusters, suggesting that guidance receptor signaling functions through Rac GTPase and Pak3 to regulate the overall polarity of the cluster and mediate efficient collective movement of the border cells to the oocyte boundary.
23. Regulation of cell adhesion and collective cell **migration** by hindsight and its human homolog RREB1. Cell movements represent a major driving force in embryonic development, tissue repair, and tumor metastasis [1]. The **migration** of single cells has been well studied, predominantly in cell culture [2, 3]; however, in vivo, a greater variety of modes of cell movement occur, including the movements of cells in clusters, strands, sheets, and tubes, also known as collective cell **migration**s [4, 5]. In spite of the relevance of these types of movements in both normal and pathological conditions, the molecular mechanisms that control them remain predominantly unknown. Epithelial follicle cells of the Drosophila ovary undergo several dynamic morphological changes, providing a genetically tractable model [6]. We found that anterior follicle cells, including border cells, mutant for the gene hindsight (hnt) accumulated excess cell-cell adhesion molecules and failed to undergo their normal collective movements. In addition, HNT affected border cell cluster cohesion and motility via effects on the JNK and STAT pathways, respectively. Interestingly, reduction of expression of the mammalian homolog of HNT, RREB1, by siRNA inhibited collective cell **migration** in a scratch-wound healing assay of MCF10A mammary epithelial cells, suppressed surface activity, retarded cell spreading after plating, and led to the formation of immobile, tightly adherent cell colonies. We propose that HNT and RREB1 are essential to reduce cell-cell adhesion when epithelial cells within an interconnected group undergo dynamic changes in cell shape.
24. Regulators of endocytosis maintain localized receptor tyrosine kinase signaling in guided **migration**. Guidance receptors detect extracellular cues and instruct migrating cells how to orient in space. Border cells perform a directional invasive **migration** during Drosophila oogenesis and use two receptor tyrosine kinases (RTKs), EGFR and PVR (PDGF/VEGF Receptor), to read guidance cues. We find that spatial localization of RTK signaling within these migrating cells is actively controlled. Border cells lacking Cbl, an RTK-associated E3 ubiquitin ligase, have delocalized guidance signaling, resulting in severe **migration** defects. Absence of Sprint, a receptor-recruited, Ras-activated Rab5 guanine exchange factor, gives related defects. In contrast, increasing the level of RTK signaling by receptor overexpression or removing Hrs and thereby decreasing RTK degradation does not perturb **migration**. Cbl and Sprint both regulate early steps of RTK endocytosis. Thus, a physiological role of RTK endocytosis is to ensure localized intracellular response to guidance cues by stimulating spatial restriction of signaling.
25. The PDGF/VEGF receptor controls blood cell survival in Drosophila. The Drosophila PDGF/VEGF receptor (PVR) has known functions in the guidance of cell **migration**. We now demonstrate that during embryonic hematopoiesis, PVR has a role in the control of antiapoptotic cell survival. In Pvr mutants, a large fraction of the embryonic hemocyte population undergoes apoptosis, and the remaining blood cells cannibalistically phagocytose their dying peers. Consequently, total hemocyte numbers drop dramatically during embryogenesis, and large aggregates of engorged macrophages carrying multiple apoptotic corpses form. Hemocyte-specific expression of the pan-caspase inhibitor p35 in Pvr mutants eliminates hemocyte aggregates and restores blood cell counts and morphology. Additional rescue experiments suggest involvement of the Ras pathway in PVR-mediated blood cell survival. In cell culture, we demonstrate that PVR directly controls survival of a hemocyte cell line. This function of PVR shows striking conservation with mammalian hematopoiesis and establishes Drosophila as a model to study hematopoietic cell survival in development and disease.
26. Invasive cell **migration** is initiated by guided growth of long cellular extensions. The **migration** of border cells during Drosophila melanogaster oogenesis is a simple and powerful system for studying invasive cell **migration** in vivo. Border cells are somatic cells that delaminate from the follicular epithelium of an egg chamber and invade the germ line cluster. They migrate between the nurse cells to reach the oocyte, using DE-cadherin for adhesion to the substratum. Border cells take approximately 6 h to migrate a distance of 100 microm. The **migration** is guided by EGFR (epidermal growth factor receptor) and PVR (platelet-derived growth factor (PDGF)/vascular endothelial growth factor (VEGF) receptor). Here, we show that a single long cellular extension (LCE), several cell diameters in length, is formed at the initiation of **migration**. The LCE may function as a 'pathfinder' in response to guidance cues. LCE growth requires directional guidance signals and specific adhesion to the substratum. Interference with actin-myosin interactions allows continued LCE growth while preventing translocation of the cell bodies. We discuss similarities between LCEs and axons and the use of LCE-like structures as a general mechanism for initiating invasive **migration** in vivo.
27. Involvement of CD9 and PDGFR in **migration** is evolutionarily conserved from Drosophila glia to human glioma. Platelet-derived growth factor receptor (PDGFR) signaling plays an important role in the biology of malignant gliomas. To investigate mechanisms modulating PDGFR signaling in gliomagenesis, we employed a Drosophila glioma model and genetic screen to identify genes interacting with Pvr, the fly homolog of PDGFRs. Glial expression of constitutively activated Pvr (λPvr) led to glial over **migratio**n and lethality at late larval stage. Among 3316 dsRNA strains crossed against the tester strain, 128 genes shifted lethality to pupal stage, including tetraspanin 2A (tsp2A). In a second step knockdown of all Drosophila tetraspanins was investigated. Of all tetraspanin dsRNA strains only knockdown of tsp2A partially rescued the Pvr-induced phenotype. Human CD9 (TSPAN29/MRP-1), a close homolog of tsp2A, was found to be expressed in glioma cell lines A172 and U343MG as well as in the majority of glioblastoma samples (16/22, 73 %). Furthermore, in situ proximity ligation assay revealed close association of CD9 with PDGFR α and β. In U343MG cells, knockdown of CD9 blocked PDGF-BB stimula**ted migra**tion. In conclusion, modulation of PDGFR signaling by CD9 is evolutionarily conserved from Drosophila glia to human glioma and plays a role in g**lia migra**tion.
28. The Drosophila VEGF receptor homolog is expressed in hemocytes. Several signalling pathways have been defined by studies of genes originally characterised in Drosophila. However, some mammalian signalling systems have so far escaped discovery in the fly. Here, we describe the identification and characterisation of fly homologs for the mammalian vascular endothelial growth factor/platelet derived growth factor (VEGF/PDGF) and the VEGF receptor. The Drosophila factor (DmVEGF-1) gene has two splice variants and is expressed during all stages, the signal distribution during embryogenesis being ubiquitous. The receptor (DmVEGFR) gene has several splice variants; the variations affecting only the extracellular domain. The most prominent form is expressed in cells of the embryonic haematopoietic cell lineage, starting in the mesodermal area of the head around stage 10 of embryogenesis. Expression persists in hemocytes as embryonic development proceeds and the cells migrate posteriorly. In a fly strain carrying a deletion uncovering the DmVEGFR gene, hemocytes are still present, but their **migration** is hampered and the hemocytes remain mainly in the anterior end close to their origin. These data suggest that the VEGF/PDGF signalling system may regulate the **migration** of the Drosophila embryonic haemocyte precursor cells.
29. Two signals are better than one: border cell **migration** in Drosophila. Recent studies of border cell **migration** during Drosophila oogenesis demonstrate that the EGFR and PDGFR signaling pathways act in a partially redundant manner to guide this process. Evidence presented shows that PDGFR signaling directs border cell **migration** via Rac and the Rac activator Mbc/CED-5/Dock180.
30. Signaling systems, guided cell **migration**, and organogenesis: insights from genetic studies in Drosophila. During development, cells change their position extensively. Although the basic cellular mechanisms involved in cell locomotion have been studied mostly in cultured cells, genetic and molecular approaches using model organisms are starting to shed light on the complex events influencing cell **migration** during development. Recent technical advances in following and analyzing migrating cells inside the living embryo offer the possibility of understanding how different signaling systems regulate the fundamental cellular processes underlying guided cell **migration** in vivo. In Drosophila melanogaster, studies of migrating cells have concentrated mainly on hemocytes, germ cells, border cells, and tracheal cells. Interestingly, most of these cells were recently shown to make different cellular extensions and to use receptor tyrosine kinases to sense the chemoattractive signal. This review describes our current understanding of how different signaling networks control guided **migration** in these four systems and discusses the impact of novel imaging techniques on the study of guided cell **migration** during development.
31. Direct detection of guidance receptor activity during border cell **migration**. Guidance receptor signaling is crucial for steering migrating cells. Despite this, we generally lack direct measurements of such signaling. Border cells in Drosophila migrate as a tightly associated group, but dynamically, with front and rear cells exchanging places. They use the receptor tyrosine kinase (RTK) PDGF/VEGF receptor (PVR) as a guidance receptor, perceiving the attractant Pvf1. Here we determine the spatial distribution of PVR signaling by generating an antibody that specifically detects activated PVR in situ. PVR activity is very low in migrating border cells, due to strong activity of cellular phosphatases. Measurements of signal at the cell cortex show variability but a strong bias for both total active PVR and specific activity of PVR to be elevated at the front versus side of the leading cell, often with several-fold difference in signal levels. This polarized active PVR signal requires the E3 ubiquitin ligase Cbl and the recycling regulator Rab11, indicating a dependency on receptor trafficking. The endogenous ligand gradient contributes to shaping of signaling by increasing the specific activity of PVR toward the source in front cells. Surprisingly, signaling is also elevated at the back versus the side of rear cells. This distally polarized distribution of active PVR is ligand independent. Thus the actual guidance signal transmitted in border cells appears to integrate perceived ligand distribution with cell polarity or cell orientation with respect to the cluster. A general implication is that both group configuration and extrinsic cues can directly modulate guidance receptor signaling during collective cell **migration**.
32. Guidance of cell **migration** by the Drosophila PDGF/VEGF receptor. Directed cell **migration**s are important for development, but the signaling pathways and mechanisms responsible for guiding cell **migration** in vivo are poorly understood. **Migration** of border cells during Drosophila oogenesis is a simple and attractive model system in which to address these questions. We demonstrate that PVR, a receptor tyrosine kinase related to mammalian PDGF and VEGF receptors, acts in border cells to guide them to the oocyte. The oocyte is the source of a ligand for PVR, PDGF/VEGF factor 1 (PVF1). Intriguingly, the guidance function of PVR is largely redundant with that of EGFR. We present evidence implicating Rac and the Rac activator Mbc/DOCK180/CED-5 as mediators of the guidance signal.
33. Condensation of the central nervous system in embryonic Drosophila is inhibited by blocking hemocyte **migration** or neural activity. Condensation is a process whereby a tissue undergoes a coordinated decrease in size and increase in cellular density during development. Although it occurs in many developmental contexts, the mechanisms underlying this process are largely unknown. Here, we investigate condensation in the embryonic Drosophila ventral nerve cord (VNC). Two major events coincide with condensation during embryogenesis: the deposition of extracellular matrix by hemocytes, and the onset of central nervous system activity. We find that preventing hemocyte **migration** by removing the function of the Drosophila VEGF receptor homologue, Pvr, or by disrupting Rac1 function in these cells, inhibits condensation. In the absence of hemocytes migrating adjacent to the developing VNC, the extracellular matrix components Collagen IV, Viking and Peroxidasin are not deposited around this tissue. Blocking neural activity by targeted expression of tetanus toxin light chain or an inwardly rectifying potassium channel also inhibits condensation. We find that disrupting Rac1 function in either glia or neurons, including those located in the nerve cord, causes a similar phenotype. Our data suggest that condensation of the VNC during Drosophila embryogenesis depends on both hemocyte-deposited extracellular matrix and neural activity, and allow us to propose a mechanism whereby these processes work together to shape the developing central nervous system.
34. Distinct roles for two receptor tyrosine kinases in epithelial branching morphogenesis in Drosophila. Branching morphogenesis is a widespread mechanism used to increase the surface area of epithelial organs. Many signaling systems steer development of branched organs, but it is still unclear which cellular processes are regulated by the different pathways. We have used the development of the air sacs of the dorsal thorax of Drosophila to study cellular events and their regulation via cell-cell signaling. We find that two receptor tyrosine kinases play important but distinct roles in air sac outgrowth. Fgf signaling directs cell **migration** at the tip of the structure, while Egf signaling is instrumental for cell division and cell survival in the growing epithelial structure. Interestingly, we find that Fgf signaling requires Ras, the Mapk pathway, and Pointed to direct **migration**, suggesting that both cytoskeletal and nuclear events are downstream of receptor activation. Ras and the Mapk pathway are also needed for Egf-regulated cell division/survival, but Pointed is dispensable.
35. Lamellipodin and the Scar/WAVE complex cooperate to promote cell **migration** in vivo. Cell **migration** is essential for development, but its deregulation causes metastasis. The Scar/WAVE complex is absolutely required for lamellipodia and is a key effector in cell **migration**, but its regulation in vivo is enigmatic. Lamellipodin (Lpd) controls lamellipodium formation through an unknown mechanism. Here, we report that Lpd directly binds active Rac, which regulates a direct interaction between Lpd and the Scar/WAVE complex via Abi. Consequently, Lpd controls lamellipodium size, cell **migration** speed, and persistence via Scar/WAVE in vitro. Moreover, Lpd knockout mice display defective pigmentation because fewer migrating neural crest-derived melanoblasts reach their target during development. Consistently, Lpd regulates mesenchymal neural crest cell **migration** cell autonomously in Xenopus laevis via the Scar/WAVE complex. Further, Lpd's Drosophila melanogaster orthologue Pico binds Scar, and both regulate collective epithelial border cell **migration**. Pico also controls directed cell protrusions of border cell clusters in a Scar-dependent manner. Taken together, Lpd is an essential, evolutionary conserved regulator of the Scar/WAVE complex during cell **migration** in vivo.
36. PVF1, a PDGF/VEGF homolog, is sufficient to guide border cells and interacts genetically with Taiman. The border cells of the Drosophila ovary undergo a well-defined and developmentally regulated cell **migration**. Two signals have previously been shown to control where and when the cells migrate. The steroid hormone ecdysone, acting through its receptor and a coactivator known as Taiman, contributes to regulating the timing of border cell **migration**. PVF1, a growth factor related to platelet-derived growth factor and vascular-endothelial growth factor, contributes to guiding the border cells to the oocyte. To probe the mechanisms controlling border cell **migration** further, we performed a screen for genes that exhibit dominant genetic interactions with taiman. We identified 14 genomic regions that interact with taiman. Within one region, we identified Pvf1 as the gene responsible for the interaction. Signaling by PVF1 has been proposed to guide the border cells to their proper target, but ectopic PVF1 has not been tested for its ability to redirect the border cells. We tested the ability of PVF1, as well as other factors such as Gurken, to guide the border cells to new targets. Our results demonstrate that ectopic expression of PVF1 is sufficient to redirect border cells in some egg chambers but that the other factors tested are not. These data suggest that the guidance of border cell **migration** is robust and that there are likely to be additional factors that contribute to long-range guidance of these cells. In addition, we find that taiman and Pvf1 regulate the dynamic localization of E-cadherin in the border cells, possibly accounting for the interaction between these two pathways.
37. Border-cell **migration** requires integration of spatial and temporal signals by the BTB protein Abrupt. During development, elaborate patterns of cell differentiation and movement must occur in the correct locations and at the proper times. Developmental timing has been studied less than spatial pattern formation, and the mechanisms integrating the two are poorly understood. Border-cell **migration** in the Drosophila ovary occurs specifically at stage 9. Timing of the **migration** is regulated by the steroid hormone ecdysone, whereas spatial patterning of the migratory population requires localized activity of the JAK-STAT pathway. Ecdysone signalling is patterned spatially as well as temporally, although the mechanisms are not well understood. In stage 9 egg chambers, ecdysone signalling is highest in anterior follicle cells including the border cells. We identify the gene abrupt as a repressor of ecdysone signalling and border-cell **migration**. Abrupt protein is normally lost from border-cell nuclei during stage 9, in response to JAK-STAT activity. This contributes to the spatial pattern of the ecdysone response. Abrupt attenuates ecdysone signalling by means of a direct interaction with the basic helix-loop-helix (bHLH) domain of the P160 ecdysone receptor coactivator Taiman (Tai). Taken together, these findings provide a molecular mechanism by which spatial and temporal cues are integrated.
38. Hemocyte-secreted type IV collagen enhances BMP signaling to guide renal tubule morphogenesis in Drosophila. Details of the mechanisms that determine the shape and positioning of organs in the body cavity remain largely obscure. We show that stereotypic positioning of outgrowing Drosophila renal tubules depends on signaling in a subset of tubule cells and results from enhanced sensitivity to guidance signals by targeted matrix deposition. VEGF/PDGF ligands from the tubules attract hemocytes, which secrete components of the basement membrane to ensheath them. Collagen IV sensitizes tubule cells to localized BMP guidance cues. Signaling results in pathway activation in a subset of tubule cells that lead outgrowth through the body cavity. Failure of hemocyte **migration**, loss of collagen IV, or abrogation of BMP signaling results in tubule misrouting and defective organ shape and positioning. Such regulated interplay between cell-cell and cell-matrix interactions is likely to have wide relevance in organogenesis and congenital disease.
39. Increased centrosome amplification in aged stem cells of the Drosophila midgut. Age-related changes in long-lived tissue-resident stem cells may be tightly linked to aging and age-related diseases such as cancer. Centrosomes play key roles in cell proliferation, differentiation and **migration**. Supernumerary centrosomes are known to be an early event in tumorigenesis and senescence. However, the age-related changes of centrosome duplication in tissue-resident stem cells in vivo remain unknown. Here, using anti-γ-tubulin and anti-PH3, we analyzed mitotic intestinal stem cells with supernumerary centrosomes in the adult Drosophila midgut, which may be a versatile model system for stem cell biology. The results showed increased centrosome amplification in intestinal stem cells of aged and oxidatively stressed Drosophila midguts. Increased centrosome amplification was detected by overexpression of PVR, EGFR, and AKT in intestinal stem cells/enteroblasts, known to mimic age-related changes including hyperproliferation of intestinal stem cells and hyperplasia in the midgut. Our data show the first direct evidence for the age-related increase of centrosome amplification in intestinal stem cells and suggest that the Drosophila midgut is an excellent model for studying molecular mechanisms underlying centrosome amplification in aging adult stem cells in vivo.
40. Homothorax plays autonomous and nonautonomous roles in proximodistal axis formation and **migration** of the Drosophila renal tubules. The Drosophila Malpighian tubules (MpTs) serve as a functional equivalent of the mammalian renal tubules. The MpTs are composed of two pairs of epithelial tubes that bud from the midgut-hindgut boundary during embryogenesis. The MpT primordia grow, elongate and migrate through the body cavity to assume their final position and shape. The stereotypic pattern of MpT **migration** is regulated by multiple intrinsic and extrinsic signals, many of which are still obscure. In this work, we implicate the TALE-class homeoprotein Homothorax (Hth) in MpT patterning. We show that in the absence of Hth the tubules fail to rearrange and migrate. Hth plays both autonomous and nonautonomous roles in this developmental process. Within the tubules Hth is required for convergent extension and for defining distal versus proximal cell identities. The difference between distal and proximal cell identities seems to be required for proper formation of the leading loop. Outside the tubules, wide-range mesodermal expression of Hth is required for directing anterior **migration**. The nonautonomous effects of Hth on MpT **migration** can be partially attributed to its effects on homeotic determination along the anterior posterior axis of the embryo and to its effects on stellate cell (SC) incorporation into the MpT.
41. Multiple EGFR ligands participate in guiding migrating border cells. Cell **migration** is an important feature of embryonic development as well as tumor metastasis. Border cells in the Drosophila ovary have emerged as a useful in vivo model for uncovering the molecular mechanisms that control many aspects of cell **migration** including guidance. It was previously shown that two receptor tyrosine kinases, epidermal growth factor receptor (EGFR) and PDGF- and VEGF-related receptor (PVR), together contribute to border cell **migration**. Whereas the ligand for PVR, PVF1, is known to guide border cells, it is unclear which of the four activating EGFR ligands function in this process. We developed an assay to detect the ability of secreted factors to reroute migrating border cells in vivo and tested the activity of EGFR ligands compared to PVF1. Two ligands, Keren and Spitz, guided border cells whereas the other ligands, Gurken and Vein, did not. In addition, only Keren and Spitz were expressed at the appropriate stage in the oocyte, the target of border cell **migration**. Therefore, a complex combination of EGFR and PVR ligands together guide border cells to the oocyte.
42. GTP exchange factor Vav regulates guided cell **migration** by coupling guidance receptor signalling to local Rac activation. Guided cell **migration** is a key mechanism for cell positioning in morphogenesis. The current model suggests that the spatially controlled activation of receptor tyrosine kinases (RTKs) by guidance cues limits Rac activity at the leading edge, which is crucial for establishing and maintaining polarized cell protrusions at the front. However, little is known about the mechanisms by which RTKs control the local activation of Rac. Here, using a multidisciplinary approach, we identify the GTP exchange factor (GEF) Vav as a key regulator of Rac activity downstream of RTKs in a developmentally regulated cell **migration** event, that of the Drosophila border cells (BCs). We show that elimination of the vav gene impairs BC **migration**. Live imaging analysis reveals that vav is required for the stabilization and maintenance of protrusions at the front of the BC cluster. In addition, activation of the PDGF/VEGF-related receptor (PVR) by its ligand the PDGF/PVF1 factor brings about activation of Vav protein by direct interaction with the intracellular domain of PVR. Finally, FRET analyses demonstrate that Vav is required in BCs for the asymmetric distribution of Rac activity at the front. Our results unravel an important role for the Vav proteins as signal transducers that couple signalling downstream of RTKs with local Rac activation during morphogenetic movements.
43. Two distinct modes of guidance signalling during collective **migration** of border cells. Although directed **migration** is a feature of both individual cells and cell groups, guided **migration** has been studied most extensively for single cells in simple environments. Collective guidance of cell groups remains poorly understood, despite its relevance for development and metastasis. Neural crest cells and neuronal precursors migrate as loosely organized streams of individual cells, whereas cells of the fish lateral line, Drosophila tracheal tubes and border-cell clusters migrate as more coherent groups. Here we use Drosophila border cells to examine how collective guidance is performed. We report that border cells migrate in two phases using distinct mechanisms. Genetic analysis combined with live imaging shows that polarized cell behaviour is critical for the initial phase of **migration**, whereas dynamic collective behaviour dominates later. PDGF- and VEGF-related receptor and epidermal growth factor receptor act in both phases, but use different effector pathways in each. The myoblast city (Mbc, also known as DOCK180) and engulfment and cell motility (ELMO, also known as Ced-12) pathway is required for the early phase, in which guidance depends on subcellular localization of signalling within a leading cell. During the later phase, mitogen-activated protein kinase and phospholipase Cgamma are used redundantly, and we find that the cluster makes use of the difference in signal levels between cells to guide **migration**. Thus, information processing at the multicellular level is used to guide collective behaviour of a cell group.
44. Effective guidance of collective **migration** based on differences in cell states. Directed cell **migration** is important for normal animal development and physiology. The process can also be subverted by tumor cells to invade other tissues and to metastasize. Some cells, such as leukocytes, migrate individually; other cells migrate together in groups or sheets, called collective cell **migration**. Guidance of individually migrating cells depends critically on subcellularly localized perception and transduction of signals. For collective cell **migration**, guidance could result from cells within a group achieving different signaling levels, with directionality then encoded in the collective rather than in individual cells. Here we subject this collective guidance hypothesis to direct tests, using **migration** of border cells during Drosophila oogenesis as our model system. These cells normally use two receptor tyrosine kinases (RTKs), PDGF/VEGF-related receptor (PVR) and EGFR, to read guidance cues secreted by the oocyte. Elevated but delocalized RTK signaling in one cell of the cluster was achieved by overexpression of PVR in the absence of ligand or by overexpression of fusion receptors unable to detect Drosophila ligands; alternatively, Rac was photoactivated centrally within a single cell. In each case, one cell within the group was in a high signal state, whereas others were in low signal states. The high signal cell directed cluster movement effectively. We conclude that differences in cell signaling states are sufficient to direct collective **migration** and are likely a substantial contributor to normal guidance. Cell signaling states could manifest as differences in gene expression or metabolite levels and thus differ substantially from factors normally considered when analyzing eukaryotic cell guidance.
45. Cortactin modulates cell **migration** and ring canal morphogenesis during Drosophila oogenesis. Cortactin is a Src substrate that interacts with F-actin and can stimulate actin polymerization by direct interaction with the Arp2/3 complex. We have isolated complete loss-of-function mutants of the single Drosophila cortactin gene. Mutants are viable and fertile, showing that cortactin is not an essential gene. However, cortactin mutants show distinct defects during oogenesis. During oogenesis, Cortactin protein is enriched at the F-actin rich ring canals in the germ line, and in migrating border cells. In cortactin mutants, the ring canals are smaller than normal. A similar phenotype has been observed in Src64 mutants and in mutants for genes encoding Arp2/3 complex components, supporting that these protein products act together to control specific processes in vivo. Cortactin mutants also show impaired border cell **migration**. This invasive cell **migration** is guided by Drosophila EGFR and PDGF/VEGF receptor (PVR). We find that accumulation of Cortactin protein is positively regulated by PVR. Also, overexpression of Cortactin can by itself induce F-actin accumulation and ectopic filopodia formation in epithelial cells. We present evidence that Cortactin is one of the factors acting downstream of PVR and Src to stimulate F-actin accumulation. Cortactin is a minor contributor in this regulation, consistent with the cortactin gene not being essential for development.
46. Awd, the homolog of metastasis suppressor gene Nm23, regulates Drosophila epithelial cell invasion. Border cell **migration** during Drosophila melanogaster oogenesis is a highly pliable model for studying epithelial to mesenchymal transition and directional cell **migration**. The process involves delamination of a group of 6 to 10 follicle cells from the epithelium followed by guided **migration** and invasion through the nurse cell complex toward the oocyte. The guidance cue is mainly provided by the homolog of platelet-derived growth factor/vascular endothelial growth factor family of growth factor, or Pvf, emanating from the oocyte, although Drosophila epidermal growth factor receptor signaling also plays an auxiliary role. Earlier studies implicated a stringent control of the strength of Pvf-mediated signaling since both down-regulation of Pvf and overexpression of active Pvf receptor (Pvr) resulted in stalled border cell **migration**. Here we show that the metastasis suppressor gene homolog Nm23/awd is a negative regulator of border cell **migration**. Its down-regulation allows for optimal spatial signaling from two crucial pathways, Pvr and JAK/STAT. Its overexpression in the border cells results in stalled **migration** and can revert the phenotype of overexpressing constitutive Pvr or dominant-negative dynamin. This is a rare example demonstrating the relevance of a metastasis suppressor gene function utilized in a developmental process involving cell invasion.
47. Analysis of cell **migration** using whole-genome expression profiling of migratory cells in the Drosophila ovary. Cell **migration** contributes to normal development and homeostasis as well as to pathological processes such as inflammation and tumor metastasis. Previous genetic screens have revealed signaling pathways that govern follicle cell **migration**s in the Drosophila ovary, but few downstream targets of the critical transcriptional regulators have been identified. To characterize the gene expression profile of two migratory cell populations and identify Slbo targets, we purified border cells and centripetal cells expressing the mouse CD8 antigen and carried out whole-genome microarray analysis. Genes predicted to control actin dynamics and the endocytic and secretory pathways were overrepresented in the migratory cell transcriptome. Mutations in five genes, including ttk, failed to complement previously isolated mutations that cause cell **migration** defects in mosaic clones. Functional analysis revealed a role for the Notch-activating protease Kuzbanian in border cell **migration** and identified Tie as a guidance receptor for the border cells.
48. A blood-borne PDGF/VEGF-like ligand initiates wound-induced epidermal cell **migration** in Drosophila larvae. Epidermal cell **migration** is critical for restoration of tissue structure and function after damage. However, the mechanisms by which differentiated cells neighboring the wound sense the wound and assume a motile phenotype remain unclear. Here, we show that Pvr, a receptor tyrosine kinase (RTK) related to platelet-derived growth factor (PDGF) and vascular endothelial growth factor (VEGF) receptors, and one of its ligands, Pvf1, are required for epidermal wound closure. Morphological comparison of wound-edge cells lacking Pvr or the Jun N-terminal kinase (JNK) signaling pathway previously implicated in larval wound closure suggests that Pvr signaling leads wound-margin epidermal cells to extend actin-based cell processes into the wound gap while JNK mediates transient dedifferentiation of cells at the wound margin. Genetic epistasis experiments reinforce the conclusion that the JNK and Pvr signaling pathways act in parallel. Tissue-specific knockdown and rescue experiments suggest that epidermally derived Pvf1 may be sequestered in the blood and that tissue damage exposes blood-borne Pvf1 to Pvr receptors on wound-edge epidermal cells and initiates the extension of cell processes into the wound gap. These results uncover a novel mechanism of sensing tissue damage and suggest that PDGF/VEGF ligands and receptors may play a conserved autocrine role in epidermal wound closure.
49. Roles of PDGF in animal development. Recent advances in genetic manipulation have greatly expanded our understanding of cellular responses to platelet-derived growth factors (PDGFs) during animal development. In addition to driving mesenchymal proliferation, PDGFs have been shown to direct the **migration**, differentiation and function of a variety of specialized mesenchymal and migratory cell types, both during development and in the adult animal. Furthermore, the availability of genomic sequence data has facilitated the identification of novel PDGF and PDGF receptor (PDGFR) family members in C. elegans, Drosophila, Xenopus, zebrafish and mouse. Early data from these different systems suggest that some functions of PDGFs have been evolutionarily conserved.
50. Macrophage-mediated corpse engulfment is required for normal Drosophila CNS morphogenesis. Cell death plays an essential role in development, and the removal of cell corpses presents an important challenge for the developing organism. Macrophages are largely responsible for the clearance of cell corpses in Drosophila melanogaster and mammalian systems. We have examined the developmental requirement for macrophages in Drosophila and find that macrophage function is essential for central nervous system (CNS) morphogenesis. We generate and analyze mutations in the Pvr locus, which encodes a receptor tyrosine kinase of the PDGF/VEGF family that is required for hemocyte **migration**. We find that loss of Pvr function causes the mispositioning of glia within the CNS and the disruption of the CNS axon scaffold. We further find that inhibition of hemocyte development or of Croquemort, a receptor required for macrophage-mediated corpse engulfment, causes similar CNS defects. These data indicate that macrophage-mediated clearance of cell corpses is required for proper morphogenesis of the Drosophila CNS.
51. Initiating and guiding **migration**: lessons from border cells. Cell **migration** occurs in many different contexts. Amoebae and other isolated cells migrate in culture. In animals, 'professional' migratory cells of the immune system constantly survey the body for intruders, whereas other cell types perform specific developmentally regulated **migration**s. One simple model for the latter type of event is **migration** of border cells during Drosophila oogenesis. Recent findings have shed light on how border cell fate is induced and on how the **migration** is guided. This article discusses the implications of these studies and compares (invasive) **migration** through a tissue with what is known about cells crawling on a flat substratum.
52. Guidance receptor promotes the asymmetric distribution of exocyst and recycling endosome during collective cell **migration**. During collective **migration**, guidance receptors signal downstream to result in a polarized distribution of molecules, including cytoskeletal regulators and guidance receptors themselves, in response to an extracellular gradient of chemotactic factors. However, the underlying mechanism of asymmetry generation in the context of the **migration** of a group of cells is not well understood. Using border cells in the Drosophila ovary as a model system for collective **migration**, we found that the receptor tyrosine kinase (RTK) PDGF/VEGF receptor (PVR) is required for a polarized distribution of recycling endosome and exocyst in the leading cells of the border cell cluster. Interestingly, PVR signaled through the small GTPase Rac to positively affect the levels of Rab11-labeled recycling endosomes, probably in an F-actin-dependent manner. Conversely, the exocyst complex component Sec3 was required for the asymmetric localization of RTK activity and F-actin, similar to that previously reported for the function of Rab11. Together, these results suggested a positive-feedback loop in border cells, in which RTKs such as PVR act to induce a higher level of vesicle recycling and tethering activity in the leading cells, which in turn enables RTK activity to be distributed in a more polarized fashion at the front. We also provided evidence that E-cadherin, the major adhesion molecule for border cell **migration**, is a specific cargo in the Rab11-labeled recycling endosomes and that Sec3 is required for the delivery of the E-cadherin-containing vesicles to the membrane.
53. CZH proteins: a new family of Rho-GEFs. The Rho family of small GTPases are important regulators of multiple cellular activities and, most notably, reorganization of the actin cytoskeleton. Dbl-homology (DH)-domain-containing proteins are the classical guanine nucleotide exchange factors (GEFs) responsible for activation of Rho GTPases. However, members of a newly discovered family can also act as Rho-GEFs. These CZH proteins include: CDM (Ced-5, Dock180 and Myoblast city) proteins, which activate Rac; and zizimin proteins, which activate Cdc42. The family contains 11 mammalian proteins and has members in many other eukaryotes. The GEF activity is carried out by a novel, DH-unrelated domain named the DOCKER, CZH2 or DHR2 domain. CZH proteins have been implicated in cell **migration**, phagocytosis of apoptotic cells, T-cell activation and neurite outgrowth, and probably arose relatively early in eukaryotic evolution.
54. Distinct mechanisms regulate hemocyte chemotaxis during development and wound healing in Drosophila melanogaster. Drosophila melanogaster hemocytes are highly motile macrophage-like cells that undergo a stereotypic pattern of **migration** to populate the whole embryo by late embryogenesis. We demonstrate that the migratory patterns of hemocytes at the embryonic ventral midline are orchestrated by chemotactic signals from the PDGF/VEGF ligands Pvf2 and -3 and that these directed **migration**s occur independently of phosphoinositide 3-kinase (PI3K) signaling. In contrast, using both laser ablation and a novel wounding assay that allows localized treatment with inhibitory drugs, we show that PI3K is essential for hemocyte chemotaxis toward wounds and that Pvf signals and PDGF/VEGF receptor expression are not required for this rapid chemotactic response. Our results demonstrate that at least two separate mechanisms operate in D. melanogaster embryos to direct hemocyte **migration** and show that although PI3K is crucial for hemocytes to sense a chemotactic gradient from a wound, it is not required to sense the growth factor signals that coordinate their developmental **migration**s along the ventral midline during embryogenesis.
55. Cellular and molecular mechanisms of border cell **migration** analyzed using time-lapse live-cell imaging. Border cells in the Drosophila ovary originate within an epithelium, detach from it, invade neighboring nurse cells, and migrate as a coherent cluster. This **migration** has served as a useful genetic model for understanding epithelial cell motility. The prevailing model of growth factor-mediated chemotaxis in general, and of border cells in particular, posits that receptor activation promotes cellular protrusion at the leading edge. Here we report the time-lapse video imaging of border cell **migration**, allowing us to test this model. Reducing the activities of the guidance receptors EGFR and PVR did not result in the expected inhibition of protrusion, but instead resulted in protrusion in all directions. In contrast, reduction in Notch activity resulted in failure of the cells to detach from the epithelium without affecting direction sensing. These observations provide new insight into the cellular dynamics and molecular mechanisms of cell **migration** in vivo.
56. Developmental control of blood cell **migration** by the Drosophila VEGF pathway. We show that a vascular endothelial growth factor (VEGF) pathway controls embryonic **migration**s of blood cells (hemocytes) in Drosophila. The VEGF receptor homolog is expressed in hemocytes, and three VEGF homologs are expressed along hemocyte **migration** routes. A receptor mutation arrests progression of blood cell movement. Mutations in Vegf17E or Vegf27Cb have no effect, but simultaneous inactivation of all three Vegf genes phenocopied the receptor mutant, and ectopic expression of Vegf27Cb redirected **migration**. Genetic experiments indicate that the VEGF pathway functions independently of pathways governing hemocyte homing on apoptotic cells. The results suggest that the Drosophila VEGF pathway guides developmental **migration**s of blood cells, and we speculate that the ancestral function of VEGF pathways was to guide blood cell movement.
57. Gliatrophic and gliatropic roles of PVF/PVR signaling during axon guidance. Evidence of molecular and functional homology between vertebrate and Drosophila glia is limited, restricting the power of Drosophila as a model system to unravel the molecular basis of glial function. Like in vertebrates, in the Drosophila central nervous system glial cells are produced in excess and surplus glia are eliminated by apoptosis adjusting final glial number to axons. The underlying molecular mechanisms are largely unknown, as the only gliatrophic pathway known to date in flies is the EGFR and its ligands. The PDGFR signaling pathway plays a major role in regulating oligodendrocyte **migration** and number in vertebrates. Here, we show that the Drosophila PDGFR/VEGFR homologue PVR is required in midline glia during axon guidance for glial survival and **migration**, ultimately enabling axonal enwrapment. The midline glia migrate aided by the VUM and the MP1 midline neurons--sources of PVF ligands--and concomitantly interactions with neurons maintain midline glia survival. Upon loss of function for PVF/PVR signaling midline glia apoptosis increases, and gain of function induces supernumerary midline glia. Midline glial cells are displaced towards ectopic sources of PVF ligands. PVR signaling promotes midline glia survival through AKT and ERK pathways. This work shows that the PVR/PDGFR pathway plays conserved gliatrophic and gliatropic roles in subsets of glial cells in flies and vertebrates.
58. Two ligands signal through the Drosophila PDGF/VEGF receptor to ensure proper salivary gland positioning. The Drosophila embryonic salivary gland is a migrating tissue that undergoes a stereotypic pattern of **migration** into the embryo. We demonstrate that the migratory path of the salivary gland requires the PDGF/VEGF pathway. The PDGF/VEGF receptor, Pvr, is strongly expressed in the salivary glands, and Pvr mutations cause abnormal ventral curving of the glands, suggesting that Pvr is involved in gland **migration**. Although the Pvr ligands, Pvf1 and Pvf2, have distinct expression patterns in the Drosophila embryo, mutations for either one of the ligands result in salivary gland **migration** defects similar to those seen in embryos that lack Pvr. Rescue experiments indicate that the PDGF/VEGF pathway functions autonomously in the salivary gland. The results of this study demonstrate that the Drosophila PDGF/VEGF pathway is essential for proper positioning of the salivary glands.
59. Mechanical feedback through E-cadherin promotes direction sensing during collective cell **migration**. E-cadherin is a major homophilic cell-cell adhesion molecule that inhibits motility of individual cells on matrix. However, its contribution to **migration** of cells through cell-rich tissues is less clear. We developed an in vivo sensor of mechanical tension across E-cadherin molecules, which we combined with cell-type-specific RNAi, photoactivatable Rac, and morphodynamic profiling, to interrogate how E-cadherin contributes to collective **migration** of cells between other cells. Using the Drosophila ovary as a model, we found that adhesion between border cells and their substrate, the nurse cells, functions in a positive feedback loop with Rac and actin assembly to stabilize forward-directed protrusion and directionally persistent movement. Adhesion between individual border cells communicates direction from the lead cell to the followers. Adhesion between motile cells and polar cells holds the cluster together and polarizes each individual cell. Thus, E-cadherin is an integral component of the guidance mechanisms that orchestrate collective chemotaxis in vivo.
60. Modeling glioma growth and invasion in Drosophila melanogaster. Glioblastoma is the most common and most malignant intrinsic human brain tumor, characterized by extensive invasion and proliferation of glial (astrocytic) tumor cells, frequent activation of tyrosine kinase receptor signaling pathways, relative resistance to chemotherapy and radiotherapy, and poor prognosis. Using the Gal4-UAS system, we have produced glioma models in Drosophila by overexpressing homologs of human tyrosine kinase receptors under control of the glia-specific promoter reversed polarity (repo). Glial overexpression of activated epidermal growth factor receptor (EGFR) resulted in enhanced proliferation and **migration** of larval glial cells with increased numbers in the eye imaginal disc, diffuse tumor-like enlargement of the optic stalk, and marked ectopic invasion of glial cells along the optic nerve. Glial overexpression of the downstream kinase PI3K showed similar pathology. Overexpression of activated pvr (platelet-derived growth factor receptor/vascular endothelial growth factor receptor homolog) led to **migration** of glial cells along the optic nerve, whereas expression of activated htl (fibroblast growth factor receptor 1 homolog) and INR (insulin receptor) showed markedly elevated numbers of glial cells in the optic stalk. The EGFR/phosphatidylinositol 3-phosphate kinase (PI3K) phenotype was partly reverted by the administration of the EGFR tyrosine kinase inhibitor gefitinib and completely rescued by the PI3K inhibitor wortmannin and the Akt inhibitor triciribine. We suggest that Drosophila models will be useful for deciphering signaling cascades underlying abnormal behavior of glioma cells for genetic screens to reveal interacting genes involved in gliomagenesis and for experimental therapy approaches.
61. Cell behaviors regulated by guidance cues in collective **migration** of border cells. Border cells perform a collective, invasive, and directed **migration** during Drosophila melanogaster oogenesis. Two receptor tyrosine kinases (RTKs), the platelet-derived growth factor/vascular endothelial growth factor-related receptor (PVR) and the epidermal growth factor receptor (EGFR), are important for reading guidance cues, but how these cues steer **migration** is not well understood. During collective **migration**, front, back, and side extensions dynamically project from individual cells within the group. We find that guidance input from both RTKs affects the presence and size of these extensions, primarily by favoring the persistence of front extensions. Guidance cues also control the productivity of extensions, specifically rendering back extensions nonproductive. Early and late phases of border cell **migration** differ in efficiency of forward cluster movement, although motility of individual cells appears constant. This is caused by differences in behavioral effects of the RTKs: PVR dominantly induces large persistent front extensions and efficient streamlined group movement, whereas EGFR does not. Thus, guidance receptors steer movement of this cell group by differentially affecting multiple **migration**-related features.

#### GeneRIFs

1. Data show that expression of a dominant negative form of rab11 induces a loss of the polarization of RTK activity, which correlates with a severe **migration** phenotype.
2. excess of JNK signaling is deleterious for **migration** in the absence of PVR activity at least in part through Fos transcriptional activity and possibly through antagonistic effects on DIAP1.
3. The VEGF receptor homolog is expressed in hemocytes, and three VEGF homologs are expressed along hemocyte **migration** routes.

### [Ee]xtracellular [Mm]atrix

#### PubMed

1. Tissue design: how Drosophila tumors remodel their neighborhood. Drosophila genetics has long been appreciated as a powerful approach for discovering the normal functions of genes that act as oncogenes and tumor suppressors in human cancer. Recent studies have also highlighted its advantages for deciphering how such genes function during tumorigenesis itself. Here we detail studies relating to how tumors, generated in developing organs and adult stem cell-based tissues, remodel the tissue landscape to their benefit. Like mammalian tumors, insect tumors can dissolve **extracellular matrix**, recruit blood cells, migrate and invade other tissues. While much is known about how mammalian fibroblasts, immune cells and vasculature promote late tumorigenesis, less is understood about the very earliest stages of tumor development in mammals. Because Drosophila has fewer mitotic cells and a simpler tissue architecture, it affords easy detection and analysis of early clonal tumor growth. Drosophila studies have revealed both cooperative and competitive interactions between tumor and normal cells during early tumor growth. During development, these interactions typically occur with other proliferative progenitor cells, but in adult stem cell-based tissues, the stem cell niche can fuel tumor growth.
2. Condensation of the central nervous system in embryonic Drosophila is inhibited by blocking hemocyte migration or neural activity. Condensation is a process whereby a tissue undergoes a coordinated decrease in size and increase in cellular density during development. Although it occurs in many developmental contexts, the mechanisms underlying this process are largely unknown. Here, we investigate condensation in the embryonic Drosophila ventral nerve cord (VNC). Two major events coincide with condensation during embryogenesis: the deposition of **extracellular matrix** by hemocytes, and the onset of central nervous system activity. We find that preventing hemocyte migration by removing the function of the Drosophila VEGF receptor homologue, Pvr, or by disrupting Rac1 function in these cells, inhibits condensation. In the absence of hemocytes migrating adjacent to the developing VNC, the **extracellular matrix** components Collagen IV, Viking and Peroxidasin are not deposited around this tissue. Blocking neural activity by targeted expression of tetanus toxin light chain or an inwardly rectifying potassium channel also inhibits condensation. We find that disrupting Rac1 function in either glia or neurons, including those located in the nerve cord, causes a similar phenotype. Our data suggest that condensation of the VNC during Drosophila embryogenesis depends on both hemocyte-deposited **extracellular matrix** and neural activity, and allow us to propose a mechanism whereby these processes work together to shape the developing central nervous system.

### [Ii]nvasive

#### PubMed

1. RhoL controls invasion and Rap1 localization during immune cell transmigration in Drosophila. Human immune cells have to penetrate an endothelial barrier during their beneficial pursuit of infection and their destructive infiltration of tissues in autoimmune diseases. This transmigration requires Rap1 GTPase to activate integrin affinity. We define a new model system for this process by demonstrating, with live imaging and genetics, that during embryonic development Drosophila melanogaster immune cells penetrate an epithelial, Drosophila E-cadherin (DE-cadherin)-based tissue barrier. A mutant in RhoL, a GTPase homologue that is specifically expressed in haemocytes, blocks this **invasive** step but not other aspects of guided migration. RhoL mediates integrin adhesion caused by Drosophila Rap1 overexpression and moves Rap1 away from a concentration in the cytoplasm to the leading edge during **invasive** migration. These findings indicate that a programmed migratory step during Drosophila development bears striking molecular similarities to vertebrate immune cell transmigration during inflammation, and identify RhoL as a new regulator of invasion, adhesion and Rap1 localization. Our work establishes the utility of Drosophila for identifying novel components of immune cell transmigration and for understanding the in vivo interplay of immune cells with the barriers they penetrate.
2. Function of the ETS transcription factor Yan in border cell migration. **Invasive** cell migration in both normal development and metastatic cancer is regulated by various signaling pathways, transcription factors and cell-adhesion molecules. The coordination between these activities in the context of cell migration is poorly understood. During Drosophila oogenesis, a small group of cells called border cells exit the follicular epithelium to perform a stereotypic, **invasive** migration. We find that the ETS transcription factor Yan is required for border cell migration and that Yan expression is spatiotemporally regulated as border cells migrate from the anterior pole of the egg chamber towards the nurse cell-oocyte boundary. Yan expression is dependent on inputs from the JAK/STAT, Notch and Receptor Tyrosine Kinase pathways in border cells. Mechanistically, Yan functions to modulate the turnover of DE-Cadherin-dependent adhesive complexes to facilitate border cell migration. Our results suggest that Yan acts as a pivotal link between signal transduction, cell adhesion and **invasive** cell migration in Drosophila border cells.
3. Regulators of endocytosis maintain localized receptor tyrosine kinase signaling in guided migration. Guidance receptors detect extracellular cues and instruct migrating cells how to orient in space. Border cells perform a directional **invasive** migration during Drosophila oogenesis and use two receptor tyrosine kinases (RTKs), EGFR and PVR (PDGF/VEGF Receptor), to read guidance cues. We find that spatial localization of RTK signaling within these migrating cells is actively controlled. Border cells lacking Cbl, an RTK-associated E3 ubiquitin ligase, have delocalized guidance signaling, resulting in severe migration defects. Absence of Sprint, a receptor-recruited, Ras-activated Rab5 guanine exchange factor, gives related defects. In contrast, increasing the level of RTK signaling by receptor overexpression or removing Hrs and thereby decreasing RTK degradation does not perturb migration. Cbl and Sprint both regulate early steps of RTK endocytosis. Thus, a physiological role of RTK endocytosis is to ensure localized intracellular response to guidance cues by stimulating spatial restriction of signaling.
4. **Invasive** cell migration is initiated by guided growth of long cellular extensions. The migration of border cells during Drosophila melanogaster oogenesis is a simple and powerful system for studying **invasive** cell migration in vivo. Border cells are somatic cells that delaminate from the follicular epithelium of an egg chamber and invade the germ line cluster. They migrate between the nurse cells to reach the oocyte, using DE-cadherin for adhesion to the substratum. Border cells take approximately 6 h to migrate a distance of 100 microm. The migration is guided by EGFR (epidermal growth factor receptor) and PVR (platelet-derived growth factor (PDGF)/vascular endothelial growth factor (VEGF) receptor). Here, we show that a single long cellular extension (LCE), several cell diameters in length, is formed at the initiation of migration. The LCE may function as a 'pathfinder' in response to guidance cues. LCE growth requires directional guidance signals and specific adhesion to the substratum. Interference with actin-myosin interactions allows continued LCE growth while preventing translocation of the cell bodies. We discuss similarities between LCEs and axons and the use of LCE-like structures as a general mechanism for initiating **invasive** migration in vivo.
5. Cortactin modulates cell migration and ring canal morphogenesis during Drosophila oogenesis. Cortactin is a Src substrate that interacts with F-actin and can stimulate actin polymerization by direct interaction with the Arp2/3 complex. We have isolated complete loss-of-function mutants of the single Drosophila cortactin gene. Mutants are viable and fertile, showing that cortactin is not an essential gene. However, cortactin mutants show distinct defects during oogenesis. During oogenesis, Cortactin protein is enriched at the F-actin rich ring canals in the germ line, and in migrating border cells. In cortactin mutants, the ring canals are smaller than normal. A similar phenotype has been observed in Src64 mutants and in mutants for genes encoding Arp2/3 complex components, supporting that these protein products act together to control specific processes in vivo. Cortactin mutants also show impaired border cell migration. This **invasive** cell migration is guided by Drosophila EGFR and PDGF/VEGF receptor (PVR). We find that accumulation of Cortactin protein is positively regulated by PVR. Also, overexpression of Cortactin can by itself induce F-actin accumulation and ectopic filopodia formation in epithelial cells. We present evidence that Cortactin is one of the factors acting downstream of PVR and Src to stimulate F-actin accumulation. Cortactin is a minor contributor in this regulation, consistent with the cortactin gene not being essential for development.
6. Initiating and guiding migration: lessons from border cells. Cell migration occurs in many different contexts. Amoebae and other isolated cells migrate in culture. In animals, 'professional' migratory cells of the immune system constantly survey the body for intruders, whereas other cell types perform specific developmentally regulated migrations. One simple model for the latter type of event is migration of border cells during Drosophila oogenesis. Recent findings have shed light on how border cell fate is induced and on how the migration is guided. This article discusses the implications of these studies and compares (**invasive**) migration through a tissue with what is known about cells crawling on a flat substratum.
7. Cell behaviors regulated by guidance cues in collective migration of border cells. Border cells perform a collective, **invasive**, and directed migration during Drosophila melanogaster oogenesis. Two receptor tyrosine kinases (RTKs), the platelet-derived growth factor/vascular endothelial growth factor-related receptor (PVR) and the epidermal growth factor receptor (EGFR), are important for reading guidance cues, but how these cues steer migration is not well understood. During collective migration, front, back, and side extensions dynamically project from individual cells within the group. We find that guidance input from both RTKs affects the presence and size of these extensions, primarily by favoring the persistence of front extensions. Guidance cues also control the productivity of extensions, specifically rendering back extensions nonproductive. Early and late phases of border cell migration differ in efficiency of forward cluster movement, although motility of individual cells appears constant. This is caused by differences in behavioral effects of the RTKs: PVR dominantly induces large persistent front extensions and efficient streamlined group movement, whereas EGFR does not. Thus, guidance receptors steer movement of this cell group by differentially affecting multiple migration-related features.

### [Mm]etastasize

#### PubMed

1. Effective guidance of collective migration based on differences in cell states. Directed cell migration is important for normal animal development and physiology. The process can also be subverted by tumor cells to invade other tissues and to **metastasize**. Some cells, such as leukocytes, migrate individually; other cells migrate together in groups or sheets, called collective cell migration. Guidance of individually migrating cells depends critically on subcellularly localized perception and transduction of signals. For collective cell migration, guidance could result from cells within a group achieving different signaling levels, with directionality then encoded in the collective rather than in individual cells. Here we subject this collective guidance hypothesis to direct tests, using migration of border cells during Drosophila oogenesis as our model system. These cells normally use two receptor tyrosine kinases (RTKs), PDGF/VEGF-related receptor (PVR) and EGFR, to read guidance cues secreted by the oocyte. Elevated but delocalized RTK signaling in one cell of the cluster was achieved by overexpression of PVR in the absence of ligand or by overexpression of fusion receptors unable to detect Drosophila ligands; alternatively, Rac was photoactivated centrally within a single cell. In each case, one cell within the group was in a high signal state, whereas others were in low signal states. The high signal cell directed cluster movement effectively. We conclude that differences in cell signaling states are sufficient to direct collective migration and are likely a substantial contributor to normal guidance. Cell signaling states could manifest as differences in gene expression or metabolite levels and thus differ substantially from factors normally considered when analyzing eukaryotic cell guidance.

### [Mm]etastasis

#### PubMed

1. Loss of cell polarity drives tumor growth and invasion through JNK activation in Drosophila. Apparent defects in cell polarity are often seen in human cancer. However, the underlying mechanisms of how cell polarity disruption contributes to tumor progression are unknown. Here, using a Drosophila genetic model for Ras-induced tumor progression, we show a molecular link between loss of cell polarity and tumor malignancy. Mutation of different apicobasal polarity genes activates c-Jun N-terminal kinase (JNK) signaling and downregulates the E-cadherin/beta-catenin adhesion complex, both of which are necessary and sufficient to cause oncogenic Ras(V12)-induced benign tumors in the developing eye to exhibit metastatic behavior. Furthermore, activated JNK and Ras signaling cooperate in promoting tumor growth cell autonomously, as JNK signaling switches its proapoptotic role to a progrowth effect in the presence of oncogenic Ras. Our finding that such context-dependent alterations promote both tumor growth and metastatic behavior suggests that **metastasis**-promoting mutations may be selected for based primarily on their growth-promoting capabilities. Similar oncogenic cooperation mediated through these evolutionarily conserved signaling pathways could contribute to human cancer progression.
2. Genes that drive invasion and migration in Drosophila. Successful cell migration depends on the careful regulation of the timing of movement, the guidance of motile cells, and cytoskeletal and adhesive changes within the cells. This review focuses on genes that act cell-autonomously to promote these aspects of cell migration in Drosophila. We discuss recent advances in understanding the migration of the ovarian border cells, embryonic blood cells, primordial germ cells, somatic gonadal precursors, and tracheal cells. Comparison of genes that regulate these processes to those that promote tumorigenesis and **metastasis** in mammals demonstrates that studies in fruit flies are uncovering new genes highly relevant to cancer biology.
3. Light-mediated activation reveals a key role for Rac in collective guidance of cell movement in vivo. The small GTPase Rac induces actin polymerization, membrane ruffling and focal contact formation in cultured single cells but can either repress or stimulate motility in epithelial cells depending on the conditions. The role of Rac in collective epithelial cell movements in vivo, which are important for both morphogenesis and **metastasis**, is therefore difficult to predict. Recently, photoactivatable analogues of Rac (PA-Rac) have been developed, allowing rapid and reversible activation or inactivation of Rac using light. In cultured single cells, light-activated Rac leads to focal membrane ruffling, protrusion and migration. Here we show that focal activation of Rac is also sufficient to polarize an entire group of cells in vivo, specifically the border cells of the Drosophila ovary. Moreover, activation or inactivation of Rac in one cell of the cluster caused a dramatic response in the other cells, suggesting that the cells sense direction as a group according to relative levels of Rac activity. Communication between cells of the cluster required Jun amino-terminal kinase (JNK) but not guidance receptor signalling. These studies further show that photoactivatable proteins are effective tools in vivo.
4. Cellular and molecular mechanisms of single and collective cell migrations in Drosophila: themes and variations. The process of cell migration is essential throughout life, driving embryonic morphogenesis and ensuring homeostasis in adults. Defects in cell migration are a major cause of human disease, with excessive migration causing autoimmune diseases and cancer **metastasis**, whereas reduced capacity for migration leads to birth defects and immunodeficiencies. Myriad studies in vitro have established a consensus view that cell migrations require cell polarization, Rho GTPase-mediated cytoskeletal rearrangements, and myosin-mediated contractility. However, in vivo studies later revealed a more complex picture, including the discovery that cells migrate not only as single units but also as clusters, strands, and sheets. In particular, the role of E-Cadherin in cell motility appears to be more complex than previously appreciated. Here, we discuss recent advances achieved by combining the plethora of genetic tools available to the Drosophila geneticist with live imaging and biophysical techniques. Finally, we discuss the emerging themes such studies have revealed and ponder the puzzles that remain to be solved.
5. In vivo RNAi screen identifies candidate signaling genes required for collective cell migration in Drosophila ovary. Collective migration of loosely or closely associated cell groups is prevalent in animal development, physiological events, and cancer **metastasis**. However, our understanding of the mechanisms of collective cell migration is incomplete. Drosophila border cells provide a powerful in vivo genetic model to study collective migration and identify essential genes for this process. Using border cell-specific RNAi-silencing in Drosophila, we knocked down 360 conserved signaling transduction genes in adult flies to identify essential pathways and genes for border cell migration. We uncovered a plethora of signaling genes, a large proportion of which had not been reported for border cells, including Rack1 (Receptor of activated C kinase) and brk (brinker), mad (mother against dpp), and sax (saxophone), which encode three components of TGF-β signaling. The RNAi knock down phenotype was validated by clonal analysis of Rack1 mutants. Our data suggest that inhibition of Src activity by Rack1 may be important for border cell migration and cluster cohesion maintenance. Lastly, results from our screen not only would shed light on signaling pathways involved in collective migration during embryogenesis and organogenesis in general, but also could help our understanding for the functions of conserved human genes involved in cancer **metastasi**s.
6. Drosophila as a model to study the role of blood cells in inflammation, innate immunity and cancer. Drosophila has a primitive yet effective blood system with three types of haemocytes which function throughout different developmental stages and environmental stimuli. Haemocytes play essential roles in tissue modeling during embryogenesis and morphogenesis, and also in innate immunity. The open circulatory system of Drosophila makes haemocytes ideal signal mediators to cells and tissues in response to events such as infection and wounding. The application of recently developed and sophisticated genetic tools to the relatively simple genome of Drosophila has made the fly a popular system for modeling human tumorigensis and **metastasis**. Drosophila is now used for screening and investigation of genes implicated in human leukemia and also in modeling development of solid tumors. This second line of research offers promising opportunities to determine the seemingly conflicting roles of blood cells in tumor progression and invasion. This review provides an overview of the signaling pathways conserved in Drosophila during haematopoiesis, haemostasis, innate immunity, wound healing and inflammation. We also review the most recent progress in the use of Drosophila as a cancer research model with an emphasis on the roles haemocytes can play in various cancer models and in the links between inflammation and cancer.
7. Pak3 regulates apical-basal polarity in migrating border cells during Drosophila oogenesis. Group cell migration is a highly coordinated process that is involved in a number of physiological events such as morphogenesis, wound healing and tumor **metastasis**. Unlike single cells, collectively moving cells are physically attached to each other and retain some degree of apical-basal polarity during the migratory phase. Although much is known about direction sensing, how polarity is regulated in multicellular movement remains unclear. Here we report the role of the protein kinase Pak3 in maintaining apical-basal polarity in migrating border cell clusters during Drosophila oogenesis. Pak3 is enriched in border cells and downregulation of its function impedes border cell movement. Time-lapse imaging suggests that Pak3 affects protrusive behavior of the border cell cluster, specifically regulating the stability and directionality of protrusions. Pak3 functions downstream of guidance receptor signaling to regulate the level and distribution of F-actin in migrating border cells. We also provide evidence that Pak3 genetically interacts with the lateral polarity marker Scribble and that it regulates JNK signaling in the moving border cells. Since Pak3 depletion results in mislocalization of several apical-basal polarity markers and overexpression of Jra rescues the polarity of the Pak3-depleted cluster, we propose that Pak3 functions through JNK signaling to modulate apical-basal polarity of the migrating border cell cluster. We also observe loss of apical-basal polarity in Rac1-depleted border cell clusters, suggesting that guidance receptor signaling functions through Rac GTPase and Pak3 to regulate the overall polarity of the cluster and mediate efficient collective movement of the border cells to the oocyte boundary.
8. Regulation of cell adhesion and collective cell migration by hindsight and its human homolog RREB1. Cell movements represent a major driving force in embryonic development, tissue repair, and tumor **metastasis** [1]. The migration of single cells has been well studied, predominantly in cell culture [2, 3]; however, in vivo, a greater variety of modes of cell movement occur, including the movements of cells in clusters, strands, sheets, and tubes, also known as collective cell migrations [4, 5]. In spite of the relevance of these types of movements in both normal and pathological conditions, the molecular mechanisms that control them remain predominantly unknown. Epithelial follicle cells of the Drosophila ovary undergo several dynamic morphological changes, providing a genetically tractable model [6]. We found that anterior follicle cells, including border cells, mutant for the gene hindsight (hnt) accumulated excess cell-cell adhesion molecules and failed to undergo their normal collective movements. In addition, HNT affected border cell cluster cohesion and motility via effects on the JNK and STAT pathways, respectively. Interestingly, reduction of expression of the mammalian homolog of HNT, RREB1, by siRNA inhibited collective cell migration in a scratch-wound healing assay of MCF10A mammary epithelial cells, suppressed surface activity, retarded cell spreading after plating, and led to the formation of immobile, tightly adherent cell colonies. We propose that HNT and RREB1 are essential to reduce cell-cell adhesion when epithelial cells within an interconnected group undergo dynamic changes in cell shape.
9. Lamellipodin and the Scar/WAVE complex cooperate to promote cell migration in vivo. Cell migration is essential for development, but its deregulation causes **metastasis**. The Scar/WAVE complex is absolutely required for lamellipodia and is a key effector in cell migration, but its regulation in vivo is enigmatic. Lamellipodin (Lpd) controls lamellipodium formation through an unknown mechanism. Here, we report that Lpd directly binds active Rac, which regulates a direct interaction between Lpd and the Scar/WAVE complex via Abi. Consequently, Lpd controls lamellipodium size, cell migration speed, and persistence via Scar/WAVE in vitro. Moreover, Lpd knockout mice display defective pigmentation because fewer migrating neural crest-derived melanoblasts reach their target during development. Consistently, Lpd regulates mesenchymal neural crest cell migration cell autonomously in Xenopus laevis via the Scar/WAVE complex. Further, Lpd's Drosophila melanogaster orthologue Pico binds Scar, and both regulate collective epithelial border cell migration. Pico also controls directed cell protrusions of border cell clusters in a Scar-dependent manner. Taken together, Lpd is an essential, evolutionary conserved regulator of the Scar/WAVE complex during cell migration in vivo.
10. Multiple EGFR ligands participate in guiding migrating border cells. Cell migration is an important feature of embryonic development as well as tumor **metastasis**. Border cells in the Drosophila ovary have emerged as a useful in vivo model for uncovering the molecular mechanisms that control many aspects of cell migration including guidance. It was previously shown that two receptor tyrosine kinases, epidermal growth factor receptor (EGFR) and PDGF- and VEGF-related receptor (PVR), together contribute to border cell migration. Whereas the ligand for PVR, PVF1, is known to guide border cells, it is unclear which of the four activating EGFR ligands function in this process. We developed an assay to detect the ability of secreted factors to reroute migrating border cells in vivo and tested the activity of EGFR ligands compared to PVF1. Two ligands, Keren and Spitz, guided border cells whereas the other ligands, Gurken and Vein, did not. In addition, only Keren and Spitz were expressed at the appropriate stage in the oocyte, the target of border cell migration. Therefore, a complex combination of EGFR and PVR ligands together guide border cells to the oocyte.
11. Two distinct modes of guidance signalling during collective migration of border cells. Although directed migration is a feature of both individual cells and cell groups, guided migration has been studied most extensively for single cells in simple environments. Collective guidance of cell groups remains poorly understood, despite its relevance for development and **metastasis**. Neural crest cells and neuronal precursors migrate as loosely organized streams of individual cells, whereas cells of the fish lateral line, Drosophila tracheal tubes and border-cell clusters migrate as more coherent groups. Here we use Drosophila border cells to examine how collective guidance is performed. We report that border cells migrate in two phases using distinct mechanisms. Genetic analysis combined with live imaging shows that polarized cell behaviour is critical for the initial phase of migration, whereas dynamic collective behaviour dominates later. PDGF- and VEGF-related receptor and epidermal growth factor receptor act in both phases, but use different effector pathways in each. The myoblast city (Mbc, also known as DOCK180) and engulfment and cell motility (ELMO, also known as Ced-12) pathway is required for the early phase, in which guidance depends on subcellular localization of signalling within a leading cell. During the later phase, mitogen-activated protein kinase and phospholipase Cgamma are used redundantly, and we find that the cluster makes use of the difference in signal levels between cells to guide migration. Thus, information processing at the multicellular level is used to guide collective behaviour of a cell group.
12. Awd, the homolog of **metastasis** suppressor gene Nm23, regulates Drosophila epithelial cell invasion. Border cell migration during Drosophila melanogaster oogenesis is a highly pliable model for studying epithelial to mesenchymal transition and directional cell migration. The process involves delamination of a group of 6 to 10 follicle cells from the epithelium followed by guided migration and invasion through the nurse cell complex toward the oocyte. The guidance cue is mainly provided by the homolog of platelet-derived growth factor/vascular endothelial growth factor family of growth factor, or Pvf, emanating from the oocyte, although Drosophila epidermal growth factor receptor signaling also plays an auxiliary role. Earlier studies implicated a stringent control of the strength of Pvf-mediated signaling since both down-regulation of Pvf and overexpression of active Pvf receptor (Pvr) resulted in stalled border cell migration. Here we show that the **metastasis** suppressor gene homolog Nm23/awd is a negative regulator of border cell migration. Its down-regulation allows for optimal spatial signaling from two crucial pathways, Pvr and JAK/STAT. Its overexpression in the border cells results in stalled migration and can revert the phenotype of overexpressing constitutive Pvr or dominant-negative dynamin. This is a rare example demonstrating the relevance of a **metastasis** suppressor gene function utilized in a developmental process involving cell invasion.
13. Analysis of cell migration using whole-genome expression profiling of migratory cells in the Drosophila ovary. Cell migration contributes to normal development and homeostasis as well as to pathological processes such as inflammation and tumor **metastasis**. Previous genetic screens have revealed signaling pathways that govern follicle cell migrations in the Drosophila ovary, but few downstream targets of the critical transcriptional regulators have been identified. To characterize the gene expression profile of two migratory cell populations and identify Slbo targets, we purified border cells and centripetal cells expressing the mouse CD8 antigen and carried out whole-genome microarray analysis. Genes predicted to control actin dynamics and the endocytic and secretory pathways were overrepresented in the migratory cell transcriptome. Mutations in five genes, including ttk, failed to complement previously isolated mutations that cause cell migration defects in mosaic clones. Functional analysis revealed a role for the Notch-activating protease Kuzbanian in border cell migration and identified Tie as a guidance receptor for the border cells.

### [Cc]ell [Ii]nvasion

#### PubMed

1. Awd, the homolog of metastasis suppressor gene Nm23, regulates Drosophila epithelial **cell invasion**. Border cell migration during Drosophila melanogaster oogenesis is a highly pliable model for studying epithelial to mesenchymal transition and directional cell migration. The process involves delamination of a group of 6 to 10 follicle cells from the epithelium followed by guided migration and invasion through the nurse cell complex toward the oocyte. The guidance cue is mainly provided by the homolog of platelet-derived growth factor/vascular endothelial growth factor family of growth factor, or Pvf, emanating from the oocyte, although Drosophila epidermal growth factor receptor signaling also plays an auxiliary role. Earlier studies implicated a stringent control of the strength of Pvf-mediated signaling since both down-regulation of Pvf and overexpression of active Pvf receptor (Pvr) resulted in stalled border cell migration. Here we show that the metastasis suppressor gene homolog Nm23/awd is a negative regulator of border cell migration. Its down-regulation allows for optimal spatial signaling from two crucial pathways, Pvr and JAK/STAT. Its overexpression in the border cells results in stalled migration and can revert the phenotype of overexpressing constitutive Pvr or dominant-negative dynamin. This is a rare example demonstrating the relevance of a metastasis suppressor gene function utilized in a developmental process involving **cell invasion**.

---

## Oseg4

- NCBI Gene
- GeneRIF
- Pubmed

| Search Term | Rank | Fields |
| --- | --- | --- |
| [Mm]igration | 6 | PubMed(1) |

  
hide/show details for Oseg4

### [Mm]igration

#### PubMed

1. Unwrapping glial biology: Gcm target genes regulating glial development, diversification, and function. Glia are the most abundant cell type in the mammalian brain. They regulate neuronal development and function, CNS immune surveillance, and stem cell biology, yet we know surprisingly little about glia in any organism. Here we identify over 40 new Drosophila glial genes. We use glial cells missing (gcm) mutants and misexpression to verify they are Gcm regulated in vivo. Many genes show unique spatiotemporal responsiveness to Gcm in the CNS, and thus glial subtype diversification requires spatially or temporally restricted Gcm cofactors. These genes provide insights into glial biology: we show unc-5 (a repulsive netrin receptor) orients glial **migration**s and the draper gene mediates glial engulfment of apoptotic neurons and larval locomotion. Many identified Drosophila glial genes have homologs expressed in mammalian glia, revealing conserved molecular features of glial cells. 80% of these Drosophila glial genes have mammalian homologs; these are now excellent candidates for regulating human glial development, function, or disease.

---

## CG8547

- NCBI Gene
- GeneRIF
- Pubmed

| Search Term | Rank | Fields |
| --- | --- | --- |
| [Ee]xtracellular [Mm]atrix | 8 | PubMed(1) |

  
hide/show details for CG8547

### [Ee]xtracellular [Mm]atrix

#### PubMed

1. Drosophila convoluted/dALS is an essential gene required for tracheal tube morphogenesis and apical matrix organization. Insulin-like growth factors (IGFs) control cell and organism growth through evolutionarily conserved signaling pathways. The mammalian acid-labile subunit (ALS) is a secreted protein that complexes with IGFs to modulate their activity. Recent work has shown that a Drosophila homolog of ALS, dALS, can also complex with and modulate the activity of a Drosophila IGF. Here we report the first mutations in the gene encoding dALS. Unexpectedly, we find that these mutations are allelic to a previously described mutation in convoluted (conv), a gene required for epithelial morphogenesis. In conv mutants, the tubes of the Drosophila tracheal system become abnormally elongated without altering tracheal cell number. conv null mutations cause larval lethality, but do not disrupt several processes required for tracheal tube size control, including septate junction formation, deposition of a lumenal/apical **extracellular matrix**, and lumenal secretion of Vermiform and Serpentine, two putative matrix-modifying proteins. Clearance of lumenal matrix and subcellular localization of clathrin also appear normal in conv mutants. However, we show that Conv/dALS is required for the dynamic organization of the transient lumenal matrix and normal structure of the cuticle that lines the tracheal lumen. These and other data suggest that the Conv/dALS-dependent tube size control mechanism is distinct from other known processes involved in tracheal tube size regulation. Moreover, we present evidence indicating that Conv/dALS has a novel, IGF-signaling independent function in tracheal morphogenesis.

---

## Syb

- NCBI Gene
- GeneRIF
- Pubmed

| Search Term | Rank | Fields |
| --- | --- | --- |
| [Ff]ilopodia | 1 | PubMed(1) |
| [Mm]igration | 6 | PubMed(2) |

  
hide/show details for Syb

### [Ff]ilopodia

#### PubMed

1. Gene regulatory networks controlling hematopoietic progenitor niche cell production and differentiation in the Drosophila lymph gland. Hematopoiesis occurs in two phases in Drosophila, with the first completed during embryogenesis and the second accomplished during larval development. The lymph gland serves as the venue for the final hematopoietic program, with this larval tissue well-studied as to its cellular organization and genetic regulation. While the medullary zone contains stem-like hematopoietic progenitors, the posterior signaling center (PSC) functions as a niche microenvironment essential for controlling the decision between progenitor maintenance versus cellular differentiation. In this report, we utilize a PSC-specific GAL4 driver and UAS-gene RNAi strains, to selectively knockdown individual gene functions in PSC cells. We assessed the effect of abrogating the function of 820 genes as to their requirement for niche cell production and differentiation. 100 genes were shown to be essential for normal niche development, with various loci placed into sub-groups based on the functions of their encoded protein products and known genetic interactions. For members of three of these groups, we characterized loss- and gain-of-function phenotypes. Gene function knockdown of members of the BAP chromatin-remodeling complex resulted in niche cells that do not express the hedgehog (hh) gene and fail to differentiate **filopodia** believed important for Hh signaling from the niche to progenitors. Abrogating gene function of various members of the insulin-like growth factor and TOR signaling pathways resulted in anomalous PSC cell production, leading to a defective niche organization. Further analysis of the Pten, TSC1, and TSC2 tumor suppressor genes demonstrated their loss-of-function condition resulted in severely altered blood cell homeostasis, including the abundant production of lamellocytes, specialized hemocytes involved in innate immune responses. Together, this cell-specific RNAi knockdown survey and mutant phenotype analyses identified multiple genes and their regulatory networks required for the normal organization and function of the hematopoietic progenitor niche within the lymph gland.

### [Mm]igration

#### PubMed

1. Branching morphogenesis of the Drosophila tracheal system. Many organs including the mammalian lung and vascular system consist of branched tubular networks that transport essential gases or fluids, but the genetic programs that control the development of these complex three-dimensional structures are not well understood. The Drosophila melanogaster tracheal (respiratory) system is a network of interconnected epithelial tubes that transports oxygen and other gases in the body and provides a paradigm of branching morphogenesis. It develops by sequential sprouting of primary, secondary, and terminal branches from an epithelial sac of approximately 80 cells in each body segment of the embryo. Mapping of the cell movements and shape changes during the sprouting process has revealed that distinct mechanisms of epithelial **migration** and tube formation are used at each stage of branching. Genetic dissection of the process has identified a general program in which a fibroblast growth factor (FGF) and fibroblast growth factor receptor (FGFR) are used repeatedly to control branch budding and outgrowth. At each stage of branching, the mechanisms controlling FGF expression and the downstream signal transduction pathway change, altering the pattern and structure of the branches that form. During terminal branching, FGF expression is regulated by hypoxia, ensuring that tracheal structure matches cellular oxygen need. A branch diversification program operates in parallel to the general budding program: Regional signals locally modify the general program, conferring specific structural features and other properties on individual branches, such as their substrate outgrowth preferences, differences in tube size and shape, and the ability to fuse to other branches to interconnect the network.
2. The histone acetyltransferase Elp3 plays in active role in the control of synaptic bouton expansion and sleep in Drosophila. The histone acetyltransferase Elp3 (Elongator Protein 3) is the catalytic subunit of the highly conserved Elongator complex. Elp3 is essential for the complex functions of Elongator in both the nucleus and cytoplasm of neurons, including the epigenetic control of neuronal motility genes and the acetylation of α-tubulin that affects axonal branching and cortical neuron **migratio**n. Accordingly, misregulation of Elp3 has been implicated in human disorders that specifically affect neuronal function, including familial dysautonomia, a disease characterized by degeneration of the sensory and autonomic nervous system, and the motor neuron degenerative disorder amyotrophic lateral sclerosis. These studies underscore the importance of Elp3 in neurodevelopment and disease, and the need to further characterize the multiple nuclear and cytoplasmic based roles of ELP3 required for neurogenesis in animal models, in vivo. In this report, we investigate the behavioral and morphological consequences that result from targeted reduction of ELP3 specifically in the developing Drosophila nervous system. We demonstrate that loss of Elp3 during neurodevelopment leads to a hyperactive phenotype and sleep loss in the adult flies, a significant expansion in synaptic bouton number and axonal length and branching in the larval neuromuscular junction as well as the misregulation of certain genes known to be involved in these processes. Our results uncover a novel role for Elp3 in the regulation of synaptic bouton expansion during neurogenesis that may be linked with a requirement for sleep.

---

## E(spl)m3-HLH

- NCBI Gene
- GeneRIF
- Pubmed

| Search Term | Rank | Fields |
| --- | --- | --- |
| [Mm]igration | 6 | PubMed(1) |

  
hide/show details for E(spl)m3-HLH

### [Mm]igration

#### PubMed

1. Methylmercury activates enhancer-of-split and bearded complex genes independent of the notch receptor. Methylmercury (MeHg) is a persistent environmental toxin that has targeted effects on fetal neural development. Although a number of cytotoxic mechanisms of MeHg have been characterized in cultured cells, its mode of action in the developing nervous system in vivo is less clear. Studies of MeHg-affected rodent and human brains show disrupted cortical and cerebellar architecture suggestive of mechanisms that augment cell signaling pathways potentially affecting cell **migration** and proliferation. We previously identified the Notch receptor pathway, a highly conserved signaling mechanism fundamental for neural development, as a target for MeHg-induced signaling in Drosophila neural cell lines. Here we have expanded our use of the Drosophila model to resolve a broader spectrum of transcriptional changes resulting from MeHg exposure in vivo and in vitro. Several Notch target genes within the Enhancer-of-split (E(spl)C) and Bearded (BrdC) complexes are upregulated with MeHg exposure in the embryo and in cultured neural cells. However, the profile of MeHg-induced E(spl)C and BrdC gene expression differs significantly from that seen with activation of the Notch receptor. Targeted knockdown of Notch and of the downstream coactivator Suppressor of Hairless (Su(H)), shows no effect on MeHg-induced transcription, indicating a novel Notch-independent mechanism of action for MeHg. MeHg transcriptional activation is partially mimicked by iodoacetamide but not by N-ethylmaleimide, two thiol-specific electrophiles, revealing a degree of specificity of cellular thiol targets in MeHg-induced transcriptional events.

---

## GMF

- NCBI Gene
- GeneRIF
- Pubmed

| Search Term | Rank | Fields |
| --- | --- | --- |
| [Mm]igration | 6 | Process(1); PubMed(1) |

  
hide/show details for GMF

### [Mm]igration

#### Process

1. border follicle cell **migration**

#### PubMed

1. GMF promotes leading-edge dynamics and collective cell **migration** in vivo. Lamellipodia are dynamic actin-rich cellular extensions that drive advancement of the leading edge during cell **migration**. Lamellipodia undergo periodic extension and retraction cycles, but the molecular mechanisms underlying these dynamics and their role in cell **migration** have remained obscure. We show that glia-maturation factor (GMF), which is an Arp2/3 complex inhibitor and actin filament debranching factor, regulates lamellipodial protrusion dynamics in living cells. In cultured S2R(+) cells, GMF silencing resulted in an increase in the width of lamellipodial actin filament arrays. Importantly, live-cell imaging of mutant Drosophila egg chambers revealed that the dynamics of actin-rich protrusions in migrating border cells is diminished in the absence of GMF. Consequently, velocity of border cell clusters undergoing guided **migration** was reduced in GMF mutant flies. Furthermore, genetic studies demonstrated that GMF cooperates with the Drosophila homolog of Aip1 (flare) in promoting disassembly of Arp2/3-nucleated actin filament networks and driving border cell **migration**. These data suggest that GMF functions in vivo to promote the disassembly of Arp2/3-nucleated actin filament arrays, making an important contribution to cell **migration** within a 3D tissue environment.

---

## aos

- NCBI Gene
- GeneRIF
- Pubmed

| Search Term | Rank | Fields |
| --- | --- | --- |
| [Mm]igration | 6 | PubMed(14) |
| [Ee]xtracellular [Mm]atrix | 8 | PubMed(2) |
| [Ii]nvasive | 10 | PubMed(1) |
| [Mm]etastasis | 12 | PubMed(1) |

  
hide/show details for aos

### [Mm]igration

#### PubMed

1. Genetic modifier screens reveal new components that interact with the Drosophila dystroglycan-dystrophin complex. The Dystroglycan-Dystrophin (Dg-Dys) complex has a capacity to transmit information from the extracellular matrix to the cytoskeleton inside the cell. It is proposed that this interaction is under tight regulation; however the signaling/regulatory components of Dg-Dys complex remain elusive. Understanding the regulation of the complex is critical since defects in this complex cause muscular dystrophy in humans. To reveal new regulators of the Dg-Dys complex, we used a model organism Drosophila melanogaster and performed genetic interaction screens to identify modifiers of Dg and Dys mutants in Drosophila wing veins. These mutant screens revealed that the Dg-Dys complex interacts with genes involved in muscle function and components of Notch, TGF-beta and EGFR signaling pathways. In addition, components of pathways that are required for cellular and/or axonal **migration** through cytoskeletal regulation, such as Semaphorin-Plexin, Frazzled-Netrin and Slit-Robo pathways show interactions with Dys and/or Dg. These data suggest that the Dg-Dys complex and the other pathways regulating extracellular information transfer to the cytoskeletal dynamics are more intercalated than previously thought.
2. Dynamics of the basement membrane in invasive epithelial clusters in Drosophila. The basement membrane (BM) represents a barrier to cell **migration**, which has to be degraded to promote invasion. However, the role and behaviour of the BM during the development of pre-invasive cells is only poorly understood. Drosophila border cells (BCs) provide an attractive genetic model in which to study the cellular mechanisms underlying the **migration** of mixed cohorts of epithelial cells. BCs are made of two different epithelial cell types appearing sequentially during oogenesis: the polar cells and the outer BCs. Here, we show that the pre-invasive polar cells undergo an unusual and asymmetrical apical capping with major basement membrane proteins, including the two Drosophila Collagen IV alpha chains, Laminin A and Perlecan. Capping of polar cells proceeds through a novel, basal-to-apical transcytosis mechanism that involves the small GTPase Drab5. Apical capping is transient and is followed by rapid shedding prior to the initiation of BC **migration**, suggesting that the apical cap blocks **migration**. Consistently, non-migratory polar cells remain capped. We further show that JAK/STAT signalling and recruitment of outer BCs are required for correct shedding and **migration**. The dynamics of the BM represents a marker of migratory BC, revealing a novel developmentally regulated behaviour of BM coupled to epithelial cell invasiveness.
3. Signaling by the Drosophila epidermal growth factor receptor pathway during development. In 1997 we wrote a review entitled "A thousand and one roles for the Drosophila epidermal growth factor (EGF) receptor (DER/EGFR)." We are not there yet in terms of the number of developmental roles assigned to this receptor in Drosophila. Nevertheless, DER has certainly emerged as one of the key players in development, since it is used repeatedly to direct cell fate choices, cell division, cell survival, and **migration**. A battery of activating ligands and an inhibitory ligand achieves this versatility. For the ligands that are produced as membrane-bound precursors, trafficking and processing are the key regulatory steps, determining the eventual temporal and spatial pattern of receptor activation. In most cases DER is activated at a short range, in the cells adjacent to the ones producing the active ligand. This activation dictates a binary choice. In some instances DER is also activated over a longer range, and multiple cell fate choices may be induced, according to its level of activation. A battery of negative feedback loops assures the limited range of DER induction. The distinct responses to DER activation in the different tissues depend upon combinatorial interactions with other signaling pathways and tissue-specific factors, at the level of target-gene regulation.
4. Egfr signaling regulates ommatidial rotation and cell motility in the Drosophila eye via MAPK/Pnt signaling and the Ras effector Canoe/AF6. Epidermal Growth Factor-receptor (Egfr) signaling is evolutionarily conserved and controls a variety of different cellular processes. In Drosophila these include proliferation, patterning, cell-fate determination, **migration** and survival. Here we provide evidence for a new role of Egfr signaling in controlling ommatidial rotation during planar cell polarity (PCP) establishment in the Drosophila eye. Although the signaling pathways involved in PCP establishment and photoreceptor cell-type specification are beginning to be unraveled, very little is known about the associated 90 degrees rotation process. One of the few rotation-specific mutations known is roulette (rlt) in which ommatidia rotate to a random degree, often more than 90 degrees. Here we show that rlt is a rotation-specific allele of the inhibitory Egfr ligand Argos and that modulation of Egfr activity shows defects in ommatidial rotation. Our data indicate that, beside the Raf/MAPK cascade, the Ras effector Canoe/AF6 acts downstream of Egfr/Ras and provides a link from Egfr to cytoskeletal elements in this developmentally regulated cell motility process. We provide further evidence for an involvement of cadherins and non-muscle myosin II as downstream components controlling rotation. In particular, the involvement of the cadherin Flamingo, a PCP gene, downstream of Egfr signaling provides the first link between PCP establishment and the Egfr pathway.
5. A new allele uncovers the role of echinus in the control of ommatidial rotation in the Drosophila eye. The precise orientation of the ommatidia in the Drosophila eye is achieved through a specialized process of cell **migration** taking place in the third-instar eye imaginal disc when ommatidial clusters rotate by 90 degrees. This process is strictly coordinated with the establishment of planar cell polarity (PCP), but it relies on a specific set of genes that control its mechanism independently from PCP signaling. Recently, the epidermal growth factor receptor (EGFR) pathway has been implicated in determining ommatidial rotation. We have isolated a new allele of echinus, a gene known to control the patterning and number of interommatidial cells. We show that echinus displays defects in the rotation of ommatidia that are not evident until mid-pupal stages, and we propose that echinus action is that of opposing EGFR by an unknown mechanism and that this can explain both its influence in ommatidial rotation and lattice programmed cell death (PCD).
6. The polarisation of the anterior-posterior and dorsal-ventral axes during Drosophila oogenesis. Recent work on Drosophila oogenesis has begun to reveal how the first asymmetries in development arise and how these relate to the later events that localise the positional cues which define the embryonic axes. The Cadherin-dependent positioning of the oocyte creates an anterior-posterior polarity that is transmitted to the embryo through the localisation and localised translation of bicoid, oskar, and nanos mRNA. In contrast, dorsal-ventral polarity arises from the random **migration** of the nucleus to the anterior of the oocyte, where it determines where gurken mRNA is translated and localised. Gurken signalling then defines the embryonic dorsal-ventral axis by restricting pipe expression to the ventral follicle cells, where Pipe regulates the production of an unidentified cue that activates the Toll signalling pathway.
7. Transcriptional control in embryonic Drosophila midline guidance assessed through a whole genome approach. During the development of the Drosophila central nervous system the process of midline crossing is orchestrated by a number of guidance receptors and ligands. Many key axon guidance molecules have been identified in both invertebrates and vertebrates, but the transcriptional regulation of growth cone guidance remains largely unknown. It is established that translational regulation plays a role in midline crossing, and there are indications that transcriptional regulation is also involved. To investigate this issue, we conducted a genome-wide study of transcription in Drosophila embryos using wild type and a number of well-characterized Drosophila guidance mutants and transgenics. We also analyzed a previously published microarray time course of Drosophila embryonic development with an axon guidance focus.Using hopach, a novel clustering method which is well suited to microarray data analysis, we identified groups of genes with similar expression patterns across guidance mutants and transgenics. We then systematically characterized the resulting clusters with respect to their relevance to axon guidance using two complementary controlled vocabularies: the Gene Ontology (GO) and anatomical annotations of the Atlas of Pattern of Gene Expression (APoGE) in situ hybridization database. The analysis indicates that regulation of gene expression does play a role in the process of axon guidance in Drosophila. We also find a strong link between axon guidance and hemocyte **migration**, a result that agrees with mounting evidence that axon guidance molecules are co-opted in vertebrate vascularization. Cell cyclin activity in the context of axon guidance is also suggested from our array data. RNA and protein expression patterns of cell cyclins in axon guidance mutants and transgenics support this possible link.This study provides important insights into the regulation of axon guidance in vivo.
8. Integration of epithelial patterning and morphogenesis in Drosophila ovarian follicle cells. Drosophila oogenesis involves the coordinated development of germ cells and an overlying follicular epithelium. The follicle cells provide a genetically tractable system to investigate the cell biology of patterning and morphogenesis. Follicle cells initially form a cuboidal epithelium surrounding a syncytium of nurse cells and oocyte. Epithelial structure is maintained as these cells reorganize to create the three dimensional architecture of the eggshell. Both long-range and short-range cell-cell communications pattern the domains of follicle cells that will create specific eggshell structures. After terminal differentiation to deposit the eggshell proteins, the follicle cells die. This review summarizes recent progress in understanding the cell-cell communication that orchestrates follicle cell patterning and **migration**s. DE-cadherin-mediated adhesion is important at several steps in egg chamber formation and follicle cell **migration**. Notch signaling is critical during each successive round of patterning and **migration**. Integration of bone morphogenetic protein (BMP) and epidermal growth factor (EGF) signals patterns the elaborate structures of the dorsal-anterior eggshell.
9. Ectopic expression screen identifies genes affecting Drosophila mesoderm development including the HSPG Trol. Gastrulation of the embryo involves coordinate cell movements likely supported by multiple signaling pathways, adhesion molecules, and extracellular matrix components. Fibroblast growth factors (FGFs) have a major role in Drosophila melanogaster mesoderm **migration**; however, few other inputs are known and the mechanism supporting cell movement is unclear. To provide insight, we performed an ectopic expression screen to identify secreted or membrane-associated molecules that act to support mesoderm **migration**. Twenty-four UAS insertions were identified that cause lethality when expressed in either the mesoderm (Twi-Gal4) or the ectoderm (69B-Gal4). The list was narrowed to a subset of 10 genes that were shown to exhibit loss-of-function mutant phenotypes specifically affecting mesoderm **migration**. These include the FGF ligand Pyramus, α-integrins, E-cadherin, Cueball, EGFR, JAK/STAT signaling components, as well as the heparan sulfate proteoglycan (HSPG) Terribly reduced optic lobes (Trol). Trol encodes the ortholog of mammalian HSPG Perlecan, a demonstrated FGF signaling cofactor. Here, we examine the role of Trol in Drosophila mesoderm **migratio**n and compare and contrast its role with that of Syndecan (Sdc), another HSPG previously implicated in this process. Embryos mutant for Trol or Sdc were obtained and analyzed. Our data support the view that both HSPGs function to support FGF-dependent processes in the early embryo as they share phenotypes with FGF mutants: Trol in terms of effects on mesoderm **migratio**n and caudal visceral mesoderm (CVM) **migratio**n and Sdc in terms of dorsal mesoderm specification. The differential roles uncovered for these two HSPGs suggest that HSPG cofactor choice may modify FGF-signaling outputs.
10. Unique and Overlapping Functions of Formins Frl and DAAM During Ommatidial Rotation and Neuronal Development in Drosophila. The noncanonical Frizzled/planar cell polarity (PCP) pathway regulates establishment of polarity within the plane of an epithelium to generate diversity of cell fates, asymmetric, but highly aligned structures, or to orchestrate the directional **migration** of cells during convergent extension during vertebrate gastrulation. In Drosophila, PCP signaling is essential to orient actin wing hairs and to align ommatidia in the eye, in part by coordinating the movement of groups of photoreceptor cells during ommatidial rotation. Importantly, the coordination of PCP signaling with changes in the cytoskeleton is essential for proper epithelial polarity. Formins polymerize linear actin filaments and are key regulators of the actin cytoskeleton. Here, we show that the diaphanous-related formin, Frl, the single fly member of the FMNL (formin related in leukocytes/formin-like) formin subfamily affects ommatidial rotation in the Drosophila eye and is controlled by the Rho family GTPase Cdc42. Interestingly, we also found that frl mutants exhibit an axon growth phenotype in the mushroom body, a center for olfactory learning in the Drosophila brain, which is also affected in a subset of PCP genes. Significantly, Frl cooperates with Cdc42 and another formin, DAAM, during mushroom body formation. This study thus suggests that different formins can cooperate or act independently in distinct tissues, likely integrating various signaling inputs with the regulation of the cytoskeleton. It furthermore highlights the importance and complexity of formin-dependent cytoskeletal regulation in multiple organs and developmental contexts.
11. FGF coordinates air sac development by activation of the EGF ligand Vein through the transcription factor PntP2. How several signaling pathways are coordinated to generate complex organs through regulation of tissue growth and patterning is a fundamental question in developmental biology. The larval trachea of Drosophila is composed of differentiated functional cells and groups of imaginal tracheoblasts that build the adult trachea during metamorphosis. Air sac primordium cells (ASP) are tracheal imaginal cells that form the dorsal air sacs that supply oxygen to the flight muscles of the Drosophila adult. The ASP emerges from the tracheal branch that connects to the wing disc by the activation of both Bnl-FGF/Btl and EGFR signaling pathways. Together, these pathways promote cell **migration** and proliferation. In this study we demonstrate that Vein (vn) is the EGF ligand responsible for the activation of the EGFR pathway in the ASP. We also find that the Bnl-FGF/Btl pathway regulates the expression of vn through the transcription factor PointedP2 (PntP2). Furthermore, we show that the FGF target gene escargot (esg) attenuates EGFR signaling at the tip cells of the developing ASP, reducing their mitotic rate to allow proper **migration**. Altogether, our results reveal a link between Bnl-FGF/Btl and EGFR signaling and provide novel insight into how the crosstalk of these pathways regulates **migration** and growth.
12. Nemo regulates cell dynamics and represses the expression of miple, a midkine/pleiotrophin cytokine, during ommatidial rotation. Ommatidial rotation is one of the most important events for correct patterning of the Drosophila eye. Although several signaling pathways are involved in this process, few genes have been shown to specifically affect it. One of them is nemo (nmo), which encodes a MAP-like protein kinase that regulates the rate of rotation throughout the entire process, and serves as a link between core planar cell polarity (PCP) factors and the E-cadherin-β-catenin complex. To determine more precisely the role of nmo in ommatidial rotation, live-imaging analyses in nmo mutant and wild-type early pupal eye discs were performed. We demonstrate that ommatidial rotation is not a continuous process, and that rotating and non-rotating interommatidial cells are very dynamic. Our in vivo analyses also show that nmo regulates the speed of rotation and is required in cone cells for correct ommatidial rotation, and that these cells as well as interommatidial cells are less dynamic in nmo mutants. Furthermore, microarray analyses of nmo and wild-type larval eye discs led us to identify new genes and signaling pathways related to nmo function during this process. One of them, miple, encodes the Drosophila ortholog of the midkine/pleiotrophin secreted cytokines that are involved in cell **migratio**n processes. miple is highly up-regulated in nmo mutant discs. Indeed, phenotypic analyses reveal that miple overexpression leads to ommatidial rotation defects. Genetic interaction assays suggest that miple is signaling through Ptp99A, the Drosophila ortholog of the vertebrate midkine/pleiotrophin PTPζ receptor. Accordingly, we propose that one of the roles of Nmo during ommatial rotation is to repress miple expression, which may in turn affect the dynamics in E-cadherin-β-catenin complexes.
13. Time-lapse imaging reveals stereotypical patterns of Drosophila midline glial **migration**. The Drosophila CNS midline glia (MG) are multifunctional cells that ensheath and provide trophic support to commissural axons, and direct embryonic development by employing a variety of signaling molecules. These glia consist of two functionally distinct populations: the anterior MG (AMG) and posterior MG (PMG). Only the AMG ensheath axon commissures, whereas the function of the non-ensheathing PMG is unknown. The Drosophila MG have proven to be an excellent system for studying glial proliferation, cell fate, apoptosis, and axon-glial interactions. However, insight into how AMG migrate and acquire their specific positions within the axon-glial scaffold has been lacking. In this paper, we use time-lapse imaging, single-cell analysis, and embryo staining to comprehensively describe the proliferation, **migration**, and apoptosis of the Drosophila MG. We identified 3 groups of MG that differed in the trajectories of their initial inward **migration**: AMG that migrate inward and to the anterior before undergoing apoptosis, AMG that migrate inward and to the posterior to ensheath commissural axons, and PMG that migrate inward and to the anterior to contact the commissural axons before undergoing apoptosis. In a second phase of their **migration**, the surviving AMG stereotypically migrated posteriorly to specific positions surrounding the commissures, and their final position was correlated with their location prior to **migration**. Most noteworthy are AMG that migrated between the commissures from a ventral to a dorsal position. Single-cell analysis indicated that individual AMG possessed wide-ranging and elaborate membrane extensions that partially ensheathed both commissures. These results provide a strong foundation for future genetic experiments to identify mutants affecting MG development, particularly in guidance cues that may direct **migration**. Drosophila MG are homologous in structure and function to the glial-like cells that populate the vertebrate CNS floorplate, and study of Drosophila MG will provide useful insights into floorplate development and function.
14. The Hrs/Stam complex acts as a positive and negative regulator of RTK signaling during Drosophila development. Endocytosis is a key regulatory step of diverse signalling pathways, including receptor tyrosine kinase (RTK) signalling. Hrs and Stam constitute the ESCRT-0 complex that controls the initial selection of ubiquitinated proteins, which will subsequently be degraded in lysosomes. It has been well established ex vivo and during Drosophila embryogenesis that Hrs promotes EGFR down regulation. We have recently isolated the first mutations of stam in flies and shown that Stam is required for air sac morphogenesis, a larval respiratory structure whose formation critically depends on finely tuned levels of FGFR activity. This suggest that Stam, putatively within the ESCRT-0 complex, modulates FGF signalling, a possibility that has not been examined in Drosophila yet.Here, we assessed the role of the Hrs/Stam complex in the regulation of signalling activity during Drosophila development. We show that stam and hrs are required for efficient FGFR signalling in the tracheal system, both during cell **migration** in the air sac primordium and during the formation of fine cytoplasmic extensions in terminal cells. We find that stam and hrs mutant cells display altered FGFR/Btl localisation, likely contributing to impaired signalling levels. Electron microscopy analyses indicate that endosome maturation is impaired at distinct steps by hrs and stam mutations. These somewhat unexpected results prompted us to further explore the function of stam and hrs in EGFR signalling. We show that while stam and hrs together downregulate EGFR signalling in the embryo, they are required for full activation of EGFR signalling during wing development.Our study shows that the ESCRT-0 complex differentially regulates RTK signalling, either positively or negatively depending on tissues and developmental stages, further highlighting the importance of endocytosis in modulating signalling pathways during development.

### [Ee]xtracellular [Mm]atrix

#### PubMed

1. Genetic modifier screens reveal new components that interact with the Drosophila dystroglycan-dystrophin complex. The Dystroglycan-Dystrophin (Dg-Dys) complex has a capacity to transmit information from the **extracellular matrix** to the cytoskeleton inside the cell. It is proposed that this interaction is under tight regulation; however the signaling/regulatory components of Dg-Dys complex remain elusive. Understanding the regulation of the complex is critical since defects in this complex cause muscular dystrophy in humans. To reveal new regulators of the Dg-Dys complex, we used a model organism Drosophila melanogaster and performed genetic interaction screens to identify modifiers of Dg and Dys mutants in Drosophila wing veins. These mutant screens revealed that the Dg-Dys complex interacts with genes involved in muscle function and components of Notch, TGF-beta and EGFR signaling pathways. In addition, components of pathways that are required for cellular and/or axonal migration through cytoskeletal regulation, such as Semaphorin-Plexin, Frazzled-Netrin and Slit-Robo pathways show interactions with Dys and/or Dg. These data suggest that the Dg-Dys complex and the other pathways regulating extracellular information transfer to the cytoskeletal dynamics are more intercalated than previously thought.
2. Ectopic expression screen identifies genes affecting Drosophila mesoderm development including the HSPG Trol. Gastrulation of the embryo involves coordinate cell movements likely supported by multiple signaling pathways, adhesion molecules, and **extracellular matrix** components. Fibroblast growth factors (FGFs) have a major role in Drosophila melanogaster mesoderm migration; however, few other inputs are known and the mechanism supporting cell movement is unclear. To provide insight, we performed an ectopic expression screen to identify secreted or membrane-associated molecules that act to support mesoderm migration. Twenty-four UAS insertions were identified that cause lethality when expressed in either the mesoderm (Twi-Gal4) or the ectoderm (69B-Gal4). The list was narrowed to a subset of 10 genes that were shown to exhibit loss-of-function mutant phenotypes specifically affecting mesoderm migration. These include the FGF ligand Pyramus, α-integrins, E-cadherin, Cueball, EGFR, JAK/STAT signaling components, as well as the heparan sulfate proteoglycan (HSPG) Terribly reduced optic lobes (Trol). Trol encodes the ortholog of mammalian HSPG Perlecan, a demonstrated FGF signaling cofactor. Here, we examine the role of Trol in Drosophila mesoderm migration and compare and contrast its role with that of Syndecan (Sdc), another HSPG previously implicated in this process. Embryos mutant for Trol or Sdc were obtained and analyzed. Our data support the view that both HSPGs function to support FGF-dependent processes in the early embryo as they share phenotypes with FGF mutants: Trol in terms of effects on mesoderm migration and caudal visceral mesoderm (CVM) migration and Sdc in terms of dorsal mesoderm specification. The differential roles uncovered for these two HSPGs suggest that HSPG cofactor choice may modify FGF-signaling outputs.

### [Ii]nvasive

#### PubMed

1. Dynamics of the basement membrane in **invasive** epithelial clusters in Drosophila. The basement membrane (BM) represents a barrier to cell migration, which has to be degraded to promote invasion. However, the role and behaviour of the BM during the development of pre-**invasive** cells is only poorly understood. Drosophila border cells (BCs) provide an attractive genetic model in which to study the cellular mechanisms underlying the migration of mixed cohorts of epithelial cells. BCs are made of two different epithelial cell types appearing sequentially during oogenesis: the polar cells and the outer BCs. Here, we show that the pre-**invasive** polar cells undergo an unusual and asymmetrical apical capping with major basement membrane proteins, including the two Drosophila Collagen IV alpha chains, Laminin A and Perlecan. Capping of polar cells proceeds through a novel, basal-to-apical transcytosis mechanism that involves the small GTPase Drab5. Apical capping is transient and is followed by rapid shedding prior to the initiation of BC migration, suggesting that the apical cap blocks migration. Consistently, non-migratory polar cells remain capped. We further show that JAK/STAT signalling and recruitment of outer BCs are required for correct shedding and migration. The dynamics of the BM represents a marker of migratory BC, revealing a novel developmentally regulated behaviour of BM coupled to epithelial cell **invasive**ness.

### [Mm]etastasis

#### PubMed

1. Egfr/Ras pathway mediates interactions between peripodial and disc proper cells in Drosophila wing discs. All imaginal discs in Drosophila are made up of a layer of columnar epithelium or the disc proper and a layer of squamous epithelium called the peripodial membrane. Although the developmental and molecular events in columnar epithelium or the disc proper are well understood, the peripodial membrane has gained attention only recently. Using the technique of lineage tracing, we show that peripodial and disc proper cells arise from a common set of precursors cells in the embryo, and that these cells diverge in the early larval stages. However, peripodial and disc proper cells maintain a spatial relationship even after the separation of their lineages. The peripodial membrane plays a significant role during the regional subdivision of the wing disc into presumptive wing, notum and hinge. The Egfr/Ras pathway mediates this function of the peripodial membrane. These results on signaling between squamous and columnar epithelia are particularly significant in the context of in vitro studies using human cell lines that suggest a role for the Egfr/Ras pathway in **metastasis** and tumour progression.

---

## REPTOR

- NCBI Gene
- GeneRIF
- Pubmed

| Search Term | Rank | Fields |
| --- | --- | --- |
| [Mm]igration | 6 | PubMed(2) |
| ECM | 7 | PubMed(1) |
| [Ee]xtracellular [Mm]atrix | 8 | PubMed(1) |

  
hide/show details for REPTOR

### [Mm]igration

#### PubMed

1. Transcriptional control in embryonic Drosophila midline guidance assessed through a whole genome approach. During the development of the Drosophila central nervous system the process of midline crossing is orchestrated by a number of guidance receptors and ligands. Many key axon guidance molecules have been identified in both invertebrates and vertebrates, but the transcriptional regulation of growth cone guidance remains largely unknown. It is established that translational regulation plays a role in midline crossing, and there are indications that transcriptional regulation is also involved. To investigate this issue, we conducted a genome-wide study of transcription in Drosophila embryos using wild type and a number of well-characterized Drosophila guidance mutants and transgenics. We also analyzed a previously published microarray time course of Drosophila embryonic development with an axon guidance focus.Using hopach, a novel clustering method which is well suited to microarray data analysis, we identified groups of genes with similar expression patterns across guidance mutants and transgenics. We then systematically characterized the resulting clusters with respect to their relevance to axon guidance using two complementary controlled vocabularies: the Gene Ontology (GO) and anatomical annotations of the Atlas of Pattern of Gene Expression (APoGE) in situ hybridization database. The analysis indicates that regulation of gene expression does play a role in the process of axon guidance in Drosophila. We also find a strong link between axon guidance and hemocyte **migration**, a result that agrees with mounting evidence that axon guidance molecules are co-opted in vertebrate vascularization. Cell cyclin activity in the context of axon guidance is also suggested from our array data. RNA and protein expression patterns of cell cyclins in axon guidance mutants and transgenics support this possible link.This study provides important insights into the regulation of axon guidance in vivo.
2. Genes conserved in bilaterians but jointly lost with Myc during nematode evolution are enriched in cell proliferation and cell **migration** functions. Animals use a stereotypical set of developmental genes to build body architectures of varying sizes and organizational complexity. Some genes are critical to developmental patterning, while other genes are important to physiological control of growth. However, growth regulator genes may not be as important in small-bodied "micro-metazoans" such as nematodes. Nematodes use a simplified developmental strategy of lineage-based cell fate specifications to produce an adult bilaterian body composed of a few hundreds of cells. Nematodes also lost the MYC proto-oncogenic regulator of cell proliferation. To identify additional regulators of cell proliferation that were lost with MYC, we computationally screened and determined 839 high-confidence genes that are conserved in bilaterians/lost in nematodes (CIBLIN genes). We find that 30 % of all CIBLIN genes encode transcriptional regulators of cell proliferation, epithelial-to-mesenchyme transitions, and other processes. Over 50 % of CIBLIN genes are unnamed genes in Drosophila, suggesting that there are many understudied genes. Interestingly, CIBLIN genes include many Myc synthetic lethal (MycSL) hits from recent screens. CIBLIN genes include key regulators of heparan sulfate proteoglycan (HSPG) sulfation patterns, and lysyl oxidases involved in cross-linking and modification of the extracellular matrix (ECM). These genes and others suggest the CIBLIN repertoire services critical functions in ECM remodeling and cell **migration** in large-bodied bilaterians. Correspondingly, CIBLIN genes are co-expressed with Myc in cancer transcriptomes, and include a preponderance of known determinants of cancer progression and tumor aggression. We propose that CIBLIN gene research can improve our understanding of regulatory control of cellular growth in metazoans.

### ECM

#### PubMed

1. Genes conserved in bilaterians but jointly lost with Myc during nematode evolution are enriched in cell proliferation and cell migration functions. Animals use a stereotypical set of developmental genes to build body architectures of varying sizes and organizational complexity. Some genes are critical to developmental patterning, while other genes are important to physiological control of growth. However, growth regulator genes may not be as important in small-bodied "micro-metazoans" such as nematodes. Nematodes use a simplified developmental strategy of lineage-based cell fate specifications to produce an adult bilaterian body composed of a few hundreds of cells. Nematodes also lost the MYC proto-oncogenic regulator of cell proliferation. To identify additional regulators of cell proliferation that were lost with MYC, we computationally screened and determined 839 high-confidence genes that are conserved in bilaterians/lost in nematodes (CIBLIN genes). We find that 30 % of all CIBLIN genes encode transcriptional regulators of cell proliferation, epithelial-to-mesenchyme transitions, and other processes. Over 50 % of CIBLIN genes are unnamed genes in Drosophila, suggesting that there are many understudied genes. Interestingly, CIBLIN genes include many Myc synthetic lethal (MycSL) hits from recent screens. CIBLIN genes include key regulators of heparan sulfate proteoglycan (HSPG) sulfation patterns, and lysyl oxidases involved in cross-linking and modification of the extracellular matrix (**ECM**). These genes and others suggest the CIBLIN repertoire services critical functions in **ECM** remodeling and cell migration in large-bodied bilaterians. Correspondingly, CIBLIN genes are co-expressed with Myc in cancer transcriptomes, and include a preponderance of known determinants of cancer progression and tumor aggression. We propose that CIBLIN gene research can improve our understanding of regulatory control of cellular growth in metazoans.

### [Ee]xtracellular [Mm]atrix

#### PubMed

1. Genes conserved in bilaterians but jointly lost with Myc during nematode evolution are enriched in cell proliferation and cell migration functions. Animals use a stereotypical set of developmental genes to build body architectures of varying sizes and organizational complexity. Some genes are critical to developmental patterning, while other genes are important to physiological control of growth. However, growth regulator genes may not be as important in small-bodied "micro-metazoans" such as nematodes. Nematodes use a simplified developmental strategy of lineage-based cell fate specifications to produce an adult bilaterian body composed of a few hundreds of cells. Nematodes also lost the MYC proto-oncogenic regulator of cell proliferation. To identify additional regulators of cell proliferation that were lost with MYC, we computationally screened and determined 839 high-confidence genes that are conserved in bilaterians/lost in nematodes (CIBLIN genes). We find that 30 % of all CIBLIN genes encode transcriptional regulators of cell proliferation, epithelial-to-mesenchyme transitions, and other processes. Over 50 % of CIBLIN genes are unnamed genes in Drosophila, suggesting that there are many understudied genes. Interestingly, CIBLIN genes include many Myc synthetic lethal (MycSL) hits from recent screens. CIBLIN genes include key regulators of heparan sulfate proteoglycan (HSPG) sulfation patterns, and lysyl oxidases involved in cross-linking and modification of the **extracellular matrix** (ECM). These genes and others suggest the CIBLIN repertoire services critical functions in ECM remodeling and cell migration in large-bodied bilaterians. Correspondingly, CIBLIN genes are co-expressed with Myc in cancer transcriptomes, and include a preponderance of known determinants of cancer progression and tumor aggression. We propose that CIBLIN gene research can improve our understanding of regulatory control of cellular growth in metazoans.

---

## Act42A

- NCBI Gene
- GeneRIF
- Pubmed

| Search Term | Rank | Fields |
| --- | --- | --- |
| [Ff]ilopodia | 1 | PubMed(2) |
| [Mm]igration | 6 | PubMed(9) |
| [Ii]nvasive | 10 | PubMed(2) |
| [Mm]etastasis | 12 | PubMed(1) |

  
hide/show details for Act42A

### [Ff]ilopodia

#### PubMed

1. JNK signaling pathway required for wound healing in regenerating Drosophila wing imaginal discs. We have examined wound healing during regeneration of Drosophila wing imaginal discs fragments by confocal microscopy and assessed the role of components of the JNK pathway in this process. After cutting, columnar and peripodial epithelia cells at the wound edge start to close the wound through formation and contraction of an actin cable. This is followed by a zipping process through **filopodia**l protrusions from both epithelia knitting the wound edges from proximal to distal areas of the disc. Activation of the JNK pathway is involved in such process. puckered (puc) expression is induced in several rows of cells at the edge of the wound, whereas absence of JNK pathway activity brought about by hemipterous, basket, and Dfos mutants impair wound healing. These defects are accompanied by lowered or loss of expression of puc. In support of a role of puc in wound healing, hep mutant phenotypes are rescued by reducing puc function, whereas overexpression of puc inhibits wound healing. Altogether, these results demonstrate a role for the JNK pathway in imaginal disc wound healing, similar to that reported for other healing processes such as embryonic dorsal closure, thoracic closure, and adult epithelial wound healing in Drosophila. Differences with such processes are also highlighted.
2. Quantitative analysis of cytoskeletal reorganization during epithelial tissue sealing by large-volume electron tomography. The closure of epidermal openings is an essential biological process that causes major developmental problems such as spina bifida in humans if it goes awry. At present, the mechanism of closure remains elusive. Therefore, we reconstructed a model closure event, dorsal closure in fly embryos, by large-volume correlative electron tomography. We present a comprehensive, quantitative analysis of the cytoskeletal reorganization, enabling separated epidermal cells to seal the epithelium. After establishing contact through actin-driven exploratory **filopodia**, cells use a single lamella to generate 'roof tile'-like overlaps. These shorten to produce the force, 'zipping' the tissue closed. The shortening overlaps lack detectable actin filament ensembles but are crowded with microtubules. Cortical accumulation of shrinking microtubule ends suggests a force generation mechanism in which cortical motors pull on microtubule ends as for mitotic spindle positioning. In addition, microtubules orient **filopodia** and lamellae before zipping. Our 4D electron microscopy picture describes an entire developmental process and provides fundamental insight into epidermal closure.

### [Mm]igration

#### PubMed

1. Focal adhesions: what's new inside. The cytoplasmic side of focal adhesions is comprised of large molecular complexes that link transmembrane receptors, such as integrins, to the actin cytoskeleton and mediate signals modulating cell attachment, **migration**, proliferation, differentiation, and gene expression. These complexes are heterogeneous and dynamic structures that are apparent targets of regulatory signals that control the function of focal adhesions. Recent studies using genetic approaches in invertebrate and vertebrate systems have begun to reveal the structure and function of these complexes in vivo.
2. The control of cell motility and epithelial morphogenesis by Jun kinases. Originally identified as stress-activated protein kinases that control cell survival and proliferation through transcription factor c-Jun, the Jun N-terminal kinase (JNK) subgroup of MAP kinases (MAPKs) have recently emerged as crucial regulators of cell **migration** and the morphogenetic movement of epithelial sheets. In Drosophila, a well-orchestrated JNK signaling pathway controls formation of actin stress fibers and cell shape changes, which are required for the sealing of embryonic epidermis in a process known as dorsal closure. The JNK pathway is also involved in morphogenetic processes in mice including closure of the eyelid, neural tube and optic fissure. This article focuses on recent advances in understanding the role of JNK pathway in the regulation of cell **migration**, cytoskeleton rearrangement and the morphogenesis of epithelial sheets.
3. Function of the ETS transcription factor Yan in border cell **migration**. Invasive cell **migration** in both normal development and metastatic cancer is regulated by various signaling pathways, transcription factors and cell-adhesion molecules. The coordination between these activities in the context of cell **migration** is poorly understood. During Drosophila oogenesis, a small group of cells called border cells exit the follicular epithelium to perform a stereotypic, invasive **migration**. We find that the ETS transcription factor Yan is required for border cell **migration** and that Yan expression is spatiotemporally regulated as border cells migrate from the anterior pole of the egg chamber towards the nurse cell-oocyte boundary. Yan expression is dependent on inputs from the JAK/STAT, Notch and Receptor Tyrosine Kinase pathways in border cells. Mechanistically, Yan functions to modulate the turnover of DE-Cadherin-dependent adhesive complexes to facilitate border cell **migration**. Our results suggest that Yan acts as a pivotal link between signal transduction, cell adhesion and invasive cell **migration** in Drosophila border cells.
4. Systematic analysis of the transcriptional switch inducing **migration** of border cells. Cell **migration** within a natural context is tightly controlled, often by specific transcription factors. However, the switch from stationary to migratory behavior is poorly understood. Border cells perform a spatially and temporally controlled invasive **migration** during Drosophila oogenesis. Slbo, a C/EBP family transcriptional activator, is required for them to become migratory. We purified wild-type and slbo mutant border cells as well as nonmigratory follicle cells and performed comparative whole-genome expression profiling, followed by functional tests of the contributions of identified targets to **migration**. About 300 genes were significantly upregulated in border cells, many dependent on Slbo. Among these, the microtubule regulator Stathmin was strongly upregulated and was required for normal **migration**. Actin cytoskeleton regulators were also induced, including, surprisingly, a large cluster of "muscle-specific" genes. We conclude that Slbo induces multiple cytoskeletal effectors, and that each contributes to the behavioral changes in border cells.
5. Regulation of Drosophila mesoderm **migration** by phosphoinositides and the PH domain of the Rho GTP exchange factor Pebble. The Drosophila RhoGEF Pebble (Pbl) is required for cytokinesis and **migration** of mesodermal cells. In a screen for genes that could suppress **migration** defects in pbl mutants we identified the phosphatidylinositol phosphate (PtdInsP) regulator pi5k59B. Genetic interaction tests with other PtdInsP regulators suggested that PtdIns(4,5)P2 levels are important for mesoderm **migration** when Pbl is depleted. Consistent with this, the leading front of migrating mesodermal cells was enriched for PtdIns(4,5)P2. Given that Pbl contains a Pleckstrin Homology (PH) domain, a known PtdInsP-binding motif, we examined PtdInsP-binding of Pbl and the importance of the PH domain for Pbl function. In vitro lipid blot assays showed that Pbl binds promiscuously to PtdInsPs, with binding strength associated with the degree of phosphorylation. Pbl was also able to bind lipid vesicles containing PtdIns(4,5)P2 but binding was strongly reduced upon deletion of the PH domain. Similarly, in vivo, loss of the PH domain prevented localisation of Pbl to the cell cortex and severely affected several aspects of early mesoderm development, including flattening of the invaginated tube onto the ectoderm, extension of protrusions, and dorsal **migration** to form a monolayer. Pbl lacking the PH domain could still localise to the cytokinetic furrow, however, and cytokinesis failure was reduced in pbl(ΔPH) mutants. Taken together, our results support a model in which interaction of the PH-domain of Pbl with PtdIns(4,5)P2 helps localise it to the plasma membrane which is important for mesoderm **migratio**n.
6. A requirement for the Abnormal Spindle protein to organise microtubules of the central spindle for cytokinesis in Drosophila. Drosophila abnormal spindle (asp) mutants exhibit a mitotic metaphase checkpoint arrest with abnormal spindle poles, which reflects a requirement for Asp for the integrity of microtubule organising centres (MTOCs). In male meiosis, the absence of a strong spindle integrity checkpoint enables asp mutant cells to proceed through anaphase and telophase. However, the central spindle region is not correctly organised and cells frequently fail to complete cytokinesis. This contrasts with meiosis in wild-type males where at late anaphase a dense array of microtubules forms in the central spindle region that has Asp localised at its border. We speculate that Asp is associated with the minus ends of microtubules that have been released from the spindle poles to form the central spindle. A parallel situation arises in female meiosis where Asp not only associates with the minus ends of microtubules at the acentriolar poles but also with the central spindle pole body that forms between the two tandem spindles of meiosis II. Upon fertilisation, Asp is also recruited to the MTOC that nucleates the sperm aster. Asp is required for growth of the microtubules of the sperm aster, which in asp mutants remains diminutive and so prevents **migration** of the pronuclei.
7. IKK epsilon regulates F actin assembly and interacts with Drosophila IAP1 in cellular morphogenesis. Differentiated cells assume complex shapes through polarized cell **migration** and growth. These processes require the restricted organization of the actin cytoskeleton at limited subcellular regions. IKK epsilon is a member of the IkappaB kinase family, and its developmental role has not been clear. Drosophila IKK epsilon was localized to the ruffling membrane of cultured cells and was required for F actin turnover at the cell margin. In IKK epsilon mutants, tracheal terminal cells, bristles, and arista laterals, which require accurate F actin assembly for their polarized elongation, all exhibited aberrantly branched morphology. These phenotypes were sensitive to a change in the dosage of Drosophila inhibitor of apoptosis protein 1 (DIAP1) and the caspase DRONC without apparent change in cell viability. In contrast to this, hyperactivation of IKK epsilon destabilized F actin-based structures. Expression of a dominant-negative form of IKK epsilon increased the amount of DIAP1. The results suggest that at the physiological level, IKK epsilon acts as a negative regulator of F actin assembly and maintains the fidelity of polarized elongation during cell morphogenesis. This IKK epsilon function involves the negative regulation of the nonapoptotic activity of DIAP1.
8. Drosophila klaroid encodes a SUN domain protein required for Klarsicht localization to the nuclear envelope and nuclear **migration** in the eye. KASH (Klarsicht/Anc-1/Syne homology) domain proteins are cytoskeleton-associated proteins localized uniquely to the outer nuclear membrane. Klarsicht is a KASH protein required for nuclear **migration** in differentiating cells of the Drosophila eye. The C-terminal KASH domain of Klarsicht resides in the perinuclear space, and the cytoplasmic moiety connects to the microtubule organizing center. In C. elegans and vertebrate cells, SUN (Sad1/UNC-84) domain proteins reside in the inner nuclear membrane and tether KASH proteins to the outer nuclear membrane. Is there a Drosophila SUN protein that performs a similar function, and if so, is it like Klarsicht, obviously essential for nuclear positioning only in the eye? Here, we identify Drosophila Klaroid, a SUN protein that tethers Klarsicht. klaroid loss-of-function mutants are indistinguishable phenotypically from klarsicht mutants. Remarkably, neither gene is essential for Drosophila viability or fertility, and even in klaroid klorsicht double mutants, the only obvious external morphological defect is rough eyes. In addition, we find that klaroid and klarsicht are required for nuclear **migration** in differentiating neurons and in non-neural cells. Finally, while perinuclear Klaroid is ubiquitous in the eye, Klarsicht expression is limited to differentiating cells and may be part of the trigger for apical nuclear **migration**.
9. Interkinetic nuclear **migration** is a broadly conserved feature of cell division in pseudostratified epithelia. Animal development requires tight integration between the processes of proliferative growth and epithelial morphogenesis, both of which play out at the level of individual cells. In this respect, not only must polarized epithelial cells assume complex morphologies, these distinct forms must be radically and repeatedly transformed to permit mitosis. A dramatic illustration of this integration between epithelial morphogenesis and cell proliferation is interkinetic nuclear **migration** (IKNM), wherein the nuclei of pseudostratified epithelial cells translocate to the apical epithelial surface to execute cell division. IKNM is widely considered a hallmark of pseudostratified vertebrate neuroepithelia, and prior investigations have proposed both actomyosin- and microtubule-dependent mechanisms for apical localization of the mitotic nucleus. Here, using comparative functional analysis in arthropod and cnidarian systems (Drosophila melanogaster and Nematostella vectensis), we show that actomyosin-dependent IKNM is likely to be a general feature of mitosis in pseudostratified epithelia throughout Eumetazoa. Furthermore, our studies suggest a mechanistic link between IKNM and the fundamental process of mitotic cell rounding.

### [Ii]nvasive

#### PubMed

1. Function of the ETS transcription factor Yan in border cell migration. **Invasive** cell migration in both normal development and metastatic cancer is regulated by various signaling pathways, transcription factors and cell-adhesion molecules. The coordination between these activities in the context of cell migration is poorly understood. During Drosophila oogenesis, a small group of cells called border cells exit the follicular epithelium to perform a stereotypic, **invasive** migration. We find that the ETS transcription factor Yan is required for border cell migration and that Yan expression is spatiotemporally regulated as border cells migrate from the anterior pole of the egg chamber towards the nurse cell-oocyte boundary. Yan expression is dependent on inputs from the JAK/STAT, Notch and Receptor Tyrosine Kinase pathways in border cells. Mechanistically, Yan functions to modulate the turnover of DE-Cadherin-dependent adhesive complexes to facilitate border cell migration. Our results suggest that Yan acts as a pivotal link between signal transduction, cell adhesion and **invasive** cell migration in Drosophila border cells.
2. Systematic analysis of the transcriptional switch inducing migration of border cells. Cell migration within a natural context is tightly controlled, often by specific transcription factors. However, the switch from stationary to migratory behavior is poorly understood. Border cells perform a spatially and temporally controlled **invasive** migration during Drosophila oogenesis. Slbo, a C/EBP family transcriptional activator, is required for them to become migratory. We purified wild-type and slbo mutant border cells as well as nonmigratory follicle cells and performed comparative whole-genome expression profiling, followed by functional tests of the contributions of identified targets to migration. About 300 genes were significantly upregulated in border cells, many dependent on Slbo. Among these, the microtubule regulator Stathmin was strongly upregulated and was required for normal migration. Actin cytoskeleton regulators were also induced, including, surprisingly, a large cluster of "muscle-specific" genes. We conclude that Slbo induces multiple cytoskeletal effectors, and that each contributes to the behavioral changes in border cells.

### [Mm]etastasis

#### PubMed

1. The Drosophila TIPE family member Sigmar interacts with the Ste20-like kinase Misshapen and modulates JNK signaling, cytoskeletal remodeling and autophagy. TNFAIP8 and other mammalian TIPE family proteins have attracted increased interest due to their associations with disease-related processes including oncogenic transformation, **metastasis**, and inflammation. The molecular and cellular functions of TIPE family proteins are still not well understood. Here we report the molecular and genetic characterization of the Drosophila TNFAIP8 homolog, CG4091/sigmar. Previous gene expression studies revealed dynamic expression of sigmar in larval salivary glands prior to histolysis. Here we demonstrate that in sigmar loss-of-function mutants, the salivary glands are morphologically abnormal with defects in the tubulin network and decreased autophagic flux. Sigmar localizes subcellularly to microtubule-containing projections in Drosophila S2 cells, and co-immunoprecipitates with the Ste20-like kinase Misshapen, a regulator of the JNK pathway. Further, the Drosophila TNF ligand Eiger can induce sigmar expression, and sigmar loss-of-function leads to altered localization of pDJNK in salivary glands. Together, these findings link Sigmar to the JNK pathway, cytoskeletal remodeling and autophagy activity during salivary gland development, and provide new insights into TIPE family member function.

---

## wmd

- NCBI Gene
- GeneRIF
- Pubmed

| Search Term | Rank | Fields |
| --- | --- | --- |
| ECM | 7 | PubMed(1) |
| [Ee]xtracellular [Mm]atrix | 8 | PubMed(1) |

  
hide/show details for wmd

### ECM

#### PubMed

1. Conditional switches for extracellular matrix patterning in Drosophila melanogaster. An F(1) mutagenesis strategy was developed to identify conditional mutations affecting extracellular matrix (**ECM**) patterning. Tubulogenesis requires coordinated movement of epithelial cells and deposition of a multilayered **ECM**. In the Drosophila ovary, an epithelium of follicle cells creates the eggshells, including the paired tubular dorsal appendages (DAs) that act as breathing tubes for the embryo. A P-element mutagenesis strategy allowed for conditional overexpression of hundreds of genes in follicle cells. Conditional phenotypes were scored at the level of individual mutant (F(1)) female flies. **ECM** pattern regulators were readily identified including MAPK signaling gene ets domain lacking (fused DAs), Wnt pathway genes frizzled 3 and osa (long DAs), Hh pathway gene debra (branched DAs), and transcription factor genes sima/HIF-1alpha, ush, lilli, Tfb1, broad, and foxo. In moving cells the [Ca(2+)]/calcineurin pathway can regulate adhesion to **ECM** while adherens junctions link cells together. Accordingly, thin eggshell and DA phenotypes were identified for the calcineurin regulator calreticulin and the adherens junction component arc. Finally a tubulogenesis defect phenotype was identified for the gene pterodactyl, homologous to the mammalian serine/threonine receptor-associated protein (STRAP) that integrates the TGF-beta and PI3K/AKT signaling pathways. Because phenotypes can be scored in each mutant fly before and after gene induction, this F(1) conditional mutagenesis strategy should allow for increased scale in screens for mutations affecting repeated (reiterated) events in adult animals, including gametogenesis, movement, behavior, and learning.

### [Ee]xtracellular [Mm]atrix

#### PubMed

1. Conditional switches for **extracellular matrix** patterning in Drosophila melanogaster. An F(1) mutagenesis strategy was developed to identify conditional mutations affecting **extracellular matrix** (ECM) patterning. Tubulogenesis requires coordinated movement of epithelial cells and deposition of a multilayered ECM. In the Drosophila ovary, an epithelium of follicle cells creates the eggshells, including the paired tubular dorsal appendages (DAs) that act as breathing tubes for the embryo. A P-element mutagenesis strategy allowed for conditional overexpression of hundreds of genes in follicle cells. Conditional phenotypes were scored at the level of individual mutant (F(1)) female flies. ECM pattern regulators were readily identified including MAPK signaling gene ets domain lacking (fused DAs), Wnt pathway genes frizzled 3 and osa (long DAs), Hh pathway gene debra (branched DAs), and transcription factor genes sima/HIF-1alpha, ush, lilli, Tfb1, broad, and foxo. In moving cells the [Ca(2+)]/calcineurin pathway can regulate adhesion to ECM while adherens junctions link cells together. Accordingly, thin eggshell and DA phenotypes were identified for the calcineurin regulator calreticulin and the adherens junction component arc. Finally a tubulogenesis defect phenotype was identified for the gene pterodactyl, homologous to the mammalian serine/threonine receptor-associated protein (STRAP) that integrates the TGF-beta and PI3K/AKT signaling pathways. Because phenotypes can be scored in each mutant fly before and after gene induction, this F(1) conditional mutagenesis strategy should allow for increased scale in screens for mutations affecting repeated (reiterated) events in adult animals, including gametogenesis, movement, behavior, and learning.

---

## Reg-5

- NCBI Gene
- GeneRIF
- Pubmed

| Search Term | Rank | Fields |
| --- | --- | --- |
| [Mm]igration | 6 | PubMed(1) |

  
hide/show details for Reg-5

### [Mm]igration

#### PubMed

1. Gene expression profiling of Drosophila tracheal fusion cells. The Drosophila trachea is a premier genetic system to investigate the fundamental mechanisms of tubular organ formation. Tracheal fusion cells lead the branch fusion process to form an interconnected tubular network. Therefore, fusion cells in the Drosophila trachea will be an excellent model to study branch fusion in mammalian tubular organs, such as kidneys and blood vessels. The fusion process is a dynamic cellular process involving cell **migration**, adhesion, vesicle trafficking, cytoskeleton rearrangement, and membrane fusion. To understand how these cellular events are coordinated, we initiated the critical step to assemble a gene expression profile of fusion cells. For this study, we analyzed the expression of 234 potential tracheal-expressed genes in fusion cells during fusion cell development. 143 Tracheal genes were found to encode transcription factors, signal proteins, cytoskeleton and matrix proteins, transporters, and proteins with unknown function. These genes were divided into four subgroups based on their levels of expression in fusion cells compared to neighboring non-fusion cells revealed by in situ hybridization: (1) genes that have relative high abundance in fusion cells, (2) genes that are dynamically expressed in fusion cells, (3) genes that have relative low abundance in fusion cells, and (4) genes that are expressed at similar levels in fusion cells and non-fusion tracheal cells. This study identifies the expression profile of fusion cells and hypothetically suggests genes which are necessary for the fusion process and which play roles in distinct stages of fusion, as indicated by the location and timing of expression. These data will provide the basis for a comprehensive understanding of the molecular and cellular mechanisms of branch fusion.

---

## Diap1

- NCBI Gene
- GeneRIF
- Pubmed

| Search Term | Rank | Fields |
| --- | --- | --- |
| [Ff]ilopodia | 1 | PubMed(1) |
| [Mm]igration | 6 | Process(3); PubMed(20); GeneRIFs(2) |
| ECM | 7 | PubMed(1) |
| [Ee]xtracellular [Mm]atrix | 8 | PubMed(4) |
| [Ii]nvasive | 10 | PubMed(7) |
| [Mm]etastasis | 12 | PubMed(8) |
| [Cc]ell [Ii]nvasion | 13 | PubMed(4) |

  
hide/show details for Diap1

### [Ff]ilopodia

#### PubMed

1. Distinct presynaptic and postsynaptic dismantling processes of Drosophila neuromuscular junctions during metamorphosis. Synapse remodeling is a widespread and fundamental process that underlies the formation of neuronal circuitry during development and in adaptation to physiological and/or environmental changes. However, the mechanisms of synapse remodeling are poorly understood. Synapses at the neuromuscular junction (NMJ) in Drosophila larvae undergo dramatic and extensive remodeling during metamorphosis to generate adult-specific synapses. To explore the molecular and cellular processes of synapse elimination, we performed confocal microscopy, live imaging, and electron microscopy (EM) of NMJ synapses during the early stages of metamorphosis in Drosophila in which the expressions of selected genes were genetically altered. We report that the localization of the postsynaptic scaffold protein Disc large (Dlg) becomes diffuse first and then undetectable, as larval muscles undergo histolysis, whereas presynaptic vesicles aggregate and are retrogradely transported along axons in synchrony with the formation of **filopodia**-like structures along NMJ elaborations and retraction of the presynaptic plasma membrane. EM revealed that the postsynaptic subsynaptic reticulum vacuolizes in the early stages of synapse dismantling concomitant with diffuse localization of Dlg. Ecdysone is the major hormone that drives metamorphosis. Blockade of the ecdysone signaling specifically in presynaptic neurons by expression of a dominant-negative form of ecdysone receptors delayed presynaptic but not postsynaptic dismantling. However, inhibition of ecdysone signaling, as well as ubiquitination pathway or apoptosis specifically in postsynaptic muscles, arrested both presynaptic and postsynaptic dismantling. These results demonstrate that presynaptic and postsynaptic dismantling takes place through different mechanisms and that the postsynaptic side plays an instructive role in synapse dismantling.

### [Mm]igration

#### Process

1. border follicle cell **migration**
2. border follicle cell **migration**
3. germ cell **migration**

#### PubMed

1. A sensitized PiggyBac-based screen for regulators of border cell **migration** in Drosophila. **Migration** of border cells during Drosophila melanogaster oogenesis is a good model system for investigating the genetic requirements for cell **migration** in vivo. We present a sensitized loss-of-function screen used to identify new genes required in border cells for their **migration**. Chromosomes bearing FRTs on all four major autosomal arms were mutagenized by insertions of the transposable element PiggyBac, allowing multiple parallel clonal screens and easy identification of the mutated gene. For border cells, we analyzed homozygous mutant clones positively marked with lacZ and sensitized by expression of dominant-negative PVR, the guidance receptor. We identified new alleles of genes already known to be required for border cell **migration**, including aop/yan, DIAP1, and taiman as well as a conserved Slbo-regulated enhancer downstream of shg/DE-cadherin. Mutations in genes not previously described to be required in border cells were also uncovered: hrp48, vir, rme-8, kismet, and puckered. puckered was unique in that the **migration** defects were observed only when PVR signaling was reduced. We present evidence that an excess of JNK signaling is deleterious for **migration** in the absence of PVR activity at least in part through Fos transcriptional activity and possibly through antagonistic effects on DIAP1.
2. A role for Drosophila IAP1-mediated caspase inhibition in Rac-dependent cell **migration**. Border cell **migration** in the Drosophila ovary is a relatively simple and genetically tractable model for studying the conversion of epithelial cells to migratory cells. Like many cell **migration**s, border cell **migration** is inhibited by a dominant-negative form of the GTPase Rac. To identify new genes that function in Rac-dependent cell motility, we screened for genes that when overexpressed suppressed the **migration** defect caused by dominant-negative Rac. Overexpression of the Drosophila inhibitor of apoptosis 1 (DIAP1), which is encoded by the thread (th) gene, suppressed the **migration** defect. Moreover, loss-of-function mutations in th caused **migration** defects but, surprisingly, did not cause apoptosis. Mutations affecting the Dark protein, an activator of the upstream caspase Dronc, also rescued RacN17 **migration** defects. These results indicate an apoptosis-independent role for DIAP1-mediated Dronc inhibition in Rac-mediated cell motility.
3. IKK epsilon regulates F actin assembly and interacts with Drosophila IAP1 in cellular morphogenesis. Differentiated cells assume complex shapes through polarized cell **migration** and growth. These processes require the restricted organization of the actin cytoskeleton at limited subcellular regions. IKK epsilon is a member of the IkappaB kinase family, and its developmental role has not been clear. Drosophila IKK epsilon was localized to the ruffling membrane of cultured cells and was required for F actin turnover at the cell margin. In IKK epsilon mutants, tracheal terminal cells, bristles, and arista laterals, which require accurate F actin assembly for their polarized elongation, all exhibited aberrantly branched morphology. These phenotypes were sensitive to a change in the dosage of Drosophila inhibitor of apoptosis protein 1 (DIAP1) and the caspase DRONC without apparent change in cell viability. In contrast to this, hyperactivation of IKK epsilon destabilized F actin-based structures. Expression of a dominant-negative form of IKK epsilon increased the amount of DIAP1. The results suggest that at the physiological level, IKK epsilon acts as a negative regulator of F actin assembly and maintains the fidelity of polarized elongation during cell morphogenesis. This IKK epsilon function involves the negative regulation of the nonapoptotic activity of DIAP1.
4. Cell **migration** and programmed cell death of Drosophila germ cells. Cell **migration** and programmed cell death are essential components of animal development and homeostasis, and the germ cells of Drosophila provide a simple genetic system to study the molecular mechanisms that govern these important cellular processes. Detailed descriptions of germ cell **migration** in Drosophila were accomplished long ago, but most genetic and molecular analyses of the process have occurred within the past 10 years. A few of the genes required for germ cell **migration** have been identified, and a very interesting picture is emerging. However, a process as complex as cell **migration** must involve the functions of many more molecules. In addition, cell **migration** and cell death mechanisms are often linked, as it is important to eliminate cells that are misplaced and could present a danger to the organism. In Drosophila, genes involved in germ cell **migration** can also affect programmed cell death. Currently, very little is known about how germ cells ectopic to the gonads are eliminated. To date, only four genes have been reported with roles in germ cell death, and three of these have additional functions in germ cell pathfinding. The nature of the cell death program has not been elucidated. Here, I provide a brief review of Drosophila germ cell **migration** and programmed cell death at both the descriptive and molecular levels. Many questions remain to be answered, but advances made in recent years are providing useful insights into these critical biological phenomena.
5. A role for Drosophila IAP1-mediated caspase inhibition in Rac-dependent cell **migration**. Border cell **migration** in the Drosophila ovary is a relatively simple and genetically tractable model for studying the conversion of epithelial cells to migratory cells. Like many cell **migration**s, border cell **migration** is inhibited by a dominant-negative form of the GTPase Rac. To identify new genes that function in Rac-dependent cell motility, we screened for genes that when overexpressed suppressed the **migration** defect caused by dominant-negative Rac. Overexpression of the Drosophila inhibitor of apoptosis 1 (DIAP1), which is encoded by the thread (th) gene, suppressed the **migration** defect. Moreover, loss-of-function mutations in th caused **migration** defects but, surprisingly, did not cause apoptosis. Mutations affecting the Dark protein, an activator of the upstream caspase Dronc, also rescued RacN17 **migration** defects. These results indicate an apoptosis-independent role for DIAP1-mediated Dronc inhibition in Rac-mediated cell motility.
6. Identification of genes controlling germ cell **migration** and embryonic gonad formation in Drosophila. Gonadogenesis in the Drosophila embryo is a complex process involving numerous cellular migratory steps and cell-cell interactions. The mechanisms guiding germ cells to move through, recognize and adhere to specific cell types are poorly understood. In order to identify genes that are required for these processes, we have conducted an extensive mutagenesis of the third chromosome and screened for mutations disrupting germ cell **migration** at any point in embryonic development. Phenotypic analysis of these mutants demonstrates that germ cell **migration** can be broken down into discrete developmental steps, with each step requiring a specific set of genes. Many of these genes are involved in the development of gonadal mesoderm, the tissue that associates with germ cells to form the embryonic gonad. Moreover, mutations that we isolated affecting embryonic patterning as well as germ cell **migration** suggest that the origin of gonadal mesoderm lies within the eve domain of the developing mesoderm.
7. The unconventional myosin CRINKLED and its mammalian orthologue MYO7A regulate caspases in their signalling roles. Caspases provide vital links in non-apoptotic regulatory networks controlling inflammation, compensatory proliferation, morphology and cell **migration**. How caspases are activated under non-apoptotic conditions and process a selective set of substrates without killing the cell remain enigmatic. Here we find that the Drosophila unconventional myosin CRINKLED (CK) selectively interacts with the initiator caspase DRONC and regulates some of its non-apoptotic functions. Loss of CK in the arista, border cells or proneural clusters of the wing imaginal discs affects DRONC-dependent patterning. Our data indicate that CK acts as substrate adaptor, recruiting SHAGGY46/GSK3-β to DRONC, thereby facilitating caspase-mediated cleavage and localized modulation of kinase activity. Similarly, the mammalian CK counterpart, MYO7A, binds to and impinges on CASPASE-8, revealing a new regulatory axis affecting receptor interacting protein kinase-1 (RIPK1)>CASPASE-8 signalling. Together, our results expose a conserved role for unconventional myosins in transducing caspase-dependent regulation of kinases, allowing them to take part in specific signalling events.
8. A sensitized PiggyBac-based screen for regulators of border cell **migration** in Drosophila. **Migration** of border cells during Drosophila melanogaster oogenesis is a good model system for investigating the genetic requirements for cell **migration** in vivo. We present a sensitized loss-of-function screen used to identify new genes required in border cells for their **migration**. Chromosomes bearing FRTs on all four major autosomal arms were mutagenized by insertions of the transposable element PiggyBac, allowing multiple parallel clonal screens and easy identification of the mutated gene. For border cells, we analyzed homozygous mutant clones positively marked with lacZ and sensitized by expression of dominant-negative PVR, the guidance receptor. We identified new alleles of genes already known to be required for border cell **migration**, including aop/yan, DIAP1, and taiman as well as a conserved Slbo-regulated enhancer downstream of shg/DE-cadherin. Mutations in genes not previously described to be required in border cells were also uncovered: hrp48, vir, rme-8, kismet, and puckered. puckered was unique in that the **migration** defects were observed only when PVR signaling was reduced. We present evidence that an excess of JNK signaling is deleterious for **migration** in the absence of PVR activity at least in part through Fos transcriptional activity and possibly through antagonistic effects on DIAP1.
9. The social lives of migrating cells in Drosophila. Studies of cell **migration** in Drosophila are yielding insights into the complex interactions migrating cells have with each other and with the cells in their environment. Intriguing links between factors that promote cell **migration** and those that control cell survival have been reported recently. For example, migrating germ cells compete with the surrounding somatic tissue for the substrate of the lipid phosphate phosphatases encoded by the genes Wunen and Wunen2. Germ cells take up the dephosphorylated lipid and require it for their survival. In addition, the secreted growth factors called PVFs, previously thought to guide the **migration**s of hemocytes in the embryo, were found to function instead predominantly as survival factors. And in border cells, DIAP1 and Dronc, two proteins known mainly for their ability to regulate cell death, were found to control cell **migration**.
10. IKK epsilon regulates F actin assembly and interacts with Drosophila IAP1 in cellular morphogenesis. Differentiated cells assume complex shapes through polarized cell **migration** and growth. These processes require the restricted organization of the actin cytoskeleton at limited subcellular regions. IKK epsilon is a member of the IkappaB kinase family, and its developmental role has not been clear. Drosophila IKK epsilon was localized to the ruffling membrane of cultured cells and was required for F actin turnover at the cell margin. In IKK epsilon mutants, tracheal terminal cells, bristles, and arista laterals, which require accurate F actin assembly for their polarized elongation, all exhibited aberrantly branched morphology. These phenotypes were sensitive to a change in the dosage of Drosophila inhibitor of apoptosis protein 1 (DIAP1) and the caspase DRONC without apparent change in cell viability. In contrast to this, hyperactivation of IKK epsilon destabilized F actin-based structures. Expression of a dominant-negative form of IKK epsilon increased the amount of DIAP1. The results suggest that at the physiological level, IKK epsilon acts as a negative regulator of F actin assembly and maintains the fidelity of polarized elongation during cell morphogenesis. This IKK epsilon function involves the negative regulation of the nonapoptotic activity of DIAP1.
11. Genetic interaction screens identify a role for hedgehog signaling in Drosophila border cell **migration**. Cell motility is essential for embryonic development and physiological processes such as the immune response, but also contributes to pathological conditions such as tumor progression and inflammation. However, our understanding of the mechanisms underlying migratory processes is incomplete. Drosophila border cells provide a powerful genetic model to identify the roles of genes that contribute to cell **migration**.Members of the Hedgehog signaling pathway were uncovered in two independent screens for interactions with the small GTPase Rac and the polarity protein Par-1 in border cell **migration**. Consistent with a role in **migration**, multiple Hh signaling components were enriched in the migratory border cells. Interference with Hh signaling by several different methods resulted in incomplete cell **migration**. Moreover, the polarized distribution of E-Cadherin and a marker of tyrosine kinase activity were altered when Hh signaling was disrupted. Conservation of Hh-Rac and Hh-Par-1 signaling was illustrated in the wing, in which Hh-dependent phenotypes were enhanced by loss of Rac or par-1.We identified a pathway by which Hh signaling connects to Rac and Par-1 in cell **migration**. These results further highlight the importance of modifier screens in the identification of new genes that function in developmental pathways.
12. Genes conserved in bilaterians but jointly lost with Myc during nematode evolution are enriched in cell proliferation and cell **migration** functions. Animals use a stereotypical set of developmental genes to build body architectures of varying sizes and organizational complexity. Some genes are critical to developmental patterning, while other genes are important to physiological control of growth. However, growth regulator genes may not be as important in small-bodied "micro-metazoans" such as nematodes. Nematodes use a simplified developmental strategy of lineage-based cell fate specifications to produce an adult bilaterian body composed of a few hundreds of cells. Nematodes also lost the MYC proto-oncogenic regulator of cell proliferation. To identify additional regulators of cell proliferation that were lost with MYC, we computationally screened and determined 839 high-confidence genes that are conserved in bilaterians/lost in nematodes (CIBLIN genes). We find that 30 % of all CIBLIN genes encode transcriptional regulators of cell proliferation, epithelial-to-mesenchyme transitions, and other processes. Over 50 % of CIBLIN genes are unnamed genes in Drosophila, suggesting that there are many understudied genes. Interestingly, CIBLIN genes include many Myc synthetic lethal (MycSL) hits from recent screens. CIBLIN genes include key regulators of heparan sulfate proteoglycan (HSPG) sulfation patterns, and lysyl oxidases involved in cross-linking and modification of the extracellular matrix (ECM). These genes and others suggest the CIBLIN repertoire services critical functions in ECM remodeling and cell **migration** in large-bodied bilaterians. Correspondingly, CIBLIN genes are co-expressed with Myc in cancer transcriptomes, and include a preponderance of known determinants of cancer progression and tumor aggression. We propose that CIBLIN gene research can improve our understanding of regulatory control of cellular growth in metazoans.
13. Csk-deficient boundary cells are eliminated from normal Drosophila epithelia by exclusion, **migration**, and apoptosis. The construction and maintenance of normal epithelia relies on local signals that guide cells into their proper niches and remove unwanted cells. Failure to execute this process properly may result in aberrant development or diseases, including cancer and associated metastasis. Here, we show that local environment influences the behavior of dCsk-deficient cells. Broad loss of dCsk led to enlarged and mispatterned tissues due to overproliferation, a block in apoptosis, and decreased cadherin-mediated adhesion. Loss of dCsk in discrete patches led to a different outcome: epithelial exclusion, invasive **migration**, and apoptotic death. These latter phenotypes required sharp differences in dCsk activity between neighbors; dE-cadherin, P120-catenin, Rho1, JNK, and MMP2 mediated this signal. Together, our data demonstrate how the cellular microenvironment plays a central role in determining the outcome of altered dCsk activity, and reveal a role for P120-catenin in a mechanism that protects epithelial integrity by removing abnormal cells.
14. Centrosomal kinase Nek2 cooperates with oncogenic pathways to promote metastasis. Centrosomal kinase Nek2 is overexpressed in different cancers, yet how it contributes toward tumorigenesis remains poorly understood. dNek2 overexpression in a Drosophila melanogaster model led to upregulation of Drosophila Wnt ortholog wingless (Wg), and alteration of cell **migration** markers-Rho1, Rac1 and E-cadherin (Ecad)-resulting in changes in cell shape and tissue morphogenesis. dNek2 overexpression cooperated with receptor tyrosine kinase and mitogen-activated protein kinase signaling to upregulate activated Akt, Diap1, Mmp1 and Wg protein to promote local invasion, distant seeding and metastasis. In tumor cell injection assays, dNek2 cooperated with Ras and Src signaling to promote aggressive colonization of tumors into different adult fly tissues. Inhibition of the PI3K pathway suppressed the cooperation of dNek2 with other growth pathways. Consistent with our fly studies, overexpression of human Nek2 in A549 lung adenocarcinoma and HEK293T cells led to activation of the Akt pathway and increase in β-catenin protein levels. Our computational approach identified a class of Nek2-inhibitory compounds and a novel drug-like pharmacophore that reversed the Nek2 overexpression phenotypes in flies and human cells. Our finding posits a novel role for Nek2 in promoting metastasis in addition to its currently defined role in promoting chromosomal instability. It provides a rationale for the selective advantage of centrosome amplification in cancer.
15. More than cell death: caspases and caspase inhibitors on the move. It is becoming clear that "apoptotic" caspases can effect cellular processes other than cell death. A recent paper in Cell points to a novel role of the Drosophila caspase inhibitor DIAP1 as a determinant of cell **migration**.
16. Transcriptional output of the Salvador/warts/hippo pathway is controlled in distinct fashions in Drosophila melanogaster and mammalian cell lines. The Salvador/Warts/Hippo (SWH) pathway is an important modulator of organ size, and deregulation of pathway activity can lead to cancer. Several SWH pathway components are mutated or expressed at altered levels in different human tumors including NF2, LATS1, LATS2, SAV1, and YAP. The SWH pathway regulates tissue growth by restricting the activity of the transcriptional coactivator protein known as Yorkie (Yki) in Drosophila melanogaster and Yes-associated protein (YAP) in mammals. Yki/YAP drives tissue growth in partnership with the Scalloped (Sd)/TEAD1-4 transcription factors. Yki/YAP also possesses two WW domains, which contact several proteins that have been suggested to either promote or inhibit the ability of Yki to induce transcription. To investigate the regulatory role of the Yki/YAP WW domains, we analyzed the functional consequence of mutating these domains. WW domain mutant YAP promoted transformation and **migration** of breast epithelial cells with increased potency, suggesting that WW domains mediate the inhibitory regulation of YAP in these cells. By contrast, the WW domains were required for YAP to promote NIH-3T3 cell transformation and for the ability of Yki to drive tissue growth in D. melanogaster and optimally activate Sd. This shows that Yki/YAP WW domains have distinct regulatory roles in different cell types and implies the existence of proteins that promote tissue growth in collaboration with Yki and Sd.
17. Basolateral junctions utilize warts signaling to control epithelial-mesenchymal transition and proliferation crucial for **migration** and invasion of Drosophila ovarian epithelial cells. Fasciclin2 (Fas2) and Discslarge (Dlg) localize to the basolateral junction (BLJ) of Drosophila follicle epithelial cells and inhibit their proliferation and invasion. To identify a BLJ signaling pathway we completed a genomewide screen for mutants that enhance dlg tumorigenesis. We identified two genes that encode known BLJ scaffolding proteins, lethal giant larvae (lgl) and scribble (scrib), and several not previously associated with BLJ function, including warts (wts) and roughened eye (roe), which encode a serine-threonine kinase and a transcription factor, respectively. Like scrib, wts and roe also enhance Fas2 and lgl tumorigenesis. Further, scrib, wts, and roe block border cell **migration**, and cause noninvasive tumors that resemble dlg partial loss of function, suggesting that the BLJ utilizes Wts signaling to repress EMT and proliferation, but not motility. Apicolateral junction proteins Fat (Ft), Expanded (Ex), and Merlin (Mer) either are not involved in these processes, or have highly spatio-temporally restricted roles, diminishing their significance as upstream inputs to Wts in follicle cells. This is further indicated in that Wts targets, CyclinE and DIAP1, are elevated in Fas2, dlg, lgl, wts, and roe cells, but not Fat, ex, or mer cells. Thus, the BLJ appears to regulate epithelial polarity and dynamics not only as a localized scaffold, but also by communicating signals to the nucleus. Wts may be regulated by distinct junction inputs depending on developmental context.
18. Regulation of the Drosophila apoptosome through feedback inhibition. Apoptosis is induced by caspases, which are members of the cysteine protease family. Caspases are synthesized as inactive zymogens and initiator caspases first gain activity by associating with an oligomeric complex of their adaptor proteins, such as the apoptosome. Activated initiator caspases subsequently cleave and activate effector caspases. Although such a proteolytic cascade would predict that a small number of active caspases could irreversibly amplify caspase activity and trigger apoptosis, many cells can maintain moderate levels of caspase activity to perform non-apoptotic roles in cellular differentiation, shape change and **migration**. Here we show that the Drosophila melanogaster apoptosome engages in a feedback inhibitory loop, which moderates its activation level in vivo. Specifically, the adaptor protein Apaf-1 lowers the level of its associated initiator caspase Dronc, without triggering apoptosis. Conversely, Dronc lowers Apaf-1 protein levels. This mutual suppression depends on the catalytic site of Dronc and a caspase cleavage site within Apaf-1. Moreover, the Drosophila inhibitor of apoptosis protein 1 (Diap1) is required for this process. We speculate that this feedback inhibition allows cells to regulate the degree of caspase activation for apoptotic and non-apoptotic purposes.
19. Lamellipodia-based **migration**s of larval epithelial cells are required for normal closure of the adult epidermis of Drosophila. Cell **migration**s are an important feature of animal development. They are, furthermore, essential to wound healing and tumour progression. Despite recent progress, it is still mysterious how cell **migration** is spatially and temporally regulated during morphogenesis and how cell **migration** is coordinated with other cellular behaviours to shape tissues and organs. The formation of the abdominal epithelium of Drosophila during metamorphosis provides an attractive system to study morphogenesis. Here, the diploid adult histoblasts replace the polyploid larval epithelial cells (LECs). Using in vivo 4D microscopy, I show that, besides apical constriction and apoptosis, the LECs undergo extensive coordinated **migration**s. The **migration**s follow a transition from a stationary (epithelial) to a migratory mode. The migratory behaviour is stimulated by autocrine Dpp signalling. Directed apical lamellipodia-like protrusions propel the cells. Initially, planar cell polarity determines the orientation of LEC **migration**. While LECs are migrating they also constrict apically, and changes in activity of the small GTPase Rho1 can favour one behaviour over the other. This study shows that the LECs play a more active role in morphogenesis than previously thought, with their **migration**s contributing to abdominal closure. It furthermore provides insights into how the migratory behaviour of cells is regulated during morphogenesis.
20. Homeostatic interactions at the front of **migration** control the integrity and the efficiency of a migratory glial chain. In metazoans, cell **migration** often occurs in a collective manner: the cells move while physically and functionally connected to their neighbors. The coordinated and timely movement of the cells eventually ensures the proper organization of tissues, and deregulation in such a process contributes to the development of severe diseases. Thus, understanding the cellular mechanisms underlying coordinated cell movement is of great interest in basic and medical science. The developing Drosophila wing provides an excellent model to follow the chain **migration** of glial cells in vivo. Cells at the tip of the glial collective have been shown to control the timely movement of the chain. In the present study, we show that while pioneers trigger chain **migration**, they cannot move as single cells. We also show that isolating cell clusters at the chain tip restores the formation of smaller migratory communities. Interestingly, the migratory efficiency of these de novo formed communities depends on the number of cells and progressively improves as the size of the cluster increases. Thus, homeostatic events at the migratory front control community integrity, efficiency, and coordination, emphasizing the importance of interactions and cell counting in fine-tuning collective processes.

#### GeneRIFs

1. excess of JNK signaling is deleterious for **migration** in the absence of PVR activity at least in part through Fos transcriptional activity and possibly through antagonistic effects on DIAP1.
2. Overexpression of IAP1 suppressed cell **migration** and did not cause apoptosis.

### ECM

#### PubMed

1. Genes conserved in bilaterians but jointly lost with Myc during nematode evolution are enriched in cell proliferation and cell migration functions. Animals use a stereotypical set of developmental genes to build body architectures of varying sizes and organizational complexity. Some genes are critical to developmental patterning, while other genes are important to physiological control of growth. However, growth regulator genes may not be as important in small-bodied "micro-metazoans" such as nematodes. Nematodes use a simplified developmental strategy of lineage-based cell fate specifications to produce an adult bilaterian body composed of a few hundreds of cells. Nematodes also lost the MYC proto-oncogenic regulator of cell proliferation. To identify additional regulators of cell proliferation that were lost with MYC, we computationally screened and determined 839 high-confidence genes that are conserved in bilaterians/lost in nematodes (CIBLIN genes). We find that 30 % of all CIBLIN genes encode transcriptional regulators of cell proliferation, epithelial-to-mesenchyme transitions, and other processes. Over 50 % of CIBLIN genes are unnamed genes in Drosophila, suggesting that there are many understudied genes. Interestingly, CIBLIN genes include many Myc synthetic lethal (MycSL) hits from recent screens. CIBLIN genes include key regulators of heparan sulfate proteoglycan (HSPG) sulfation patterns, and lysyl oxidases involved in cross-linking and modification of the extracellular matrix (**ECM**). These genes and others suggest the CIBLIN repertoire services critical functions in **ECM** remodeling and cell migration in large-bodied bilaterians. Correspondingly, CIBLIN genes are co-expressed with Myc in cancer transcriptomes, and include a preponderance of known determinants of cancer progression and tumor aggression. We propose that CIBLIN gene research can improve our understanding of regulatory control of cellular growth in metazoans.

### [Ee]xtracellular [Mm]atrix

#### PubMed

1. Genes conserved in bilaterians but jointly lost with Myc during nematode evolution are enriched in cell proliferation and cell migration functions. Animals use a stereotypical set of developmental genes to build body architectures of varying sizes and organizational complexity. Some genes are critical to developmental patterning, while other genes are important to physiological control of growth. However, growth regulator genes may not be as important in small-bodied "micro-metazoans" such as nematodes. Nematodes use a simplified developmental strategy of lineage-based cell fate specifications to produce an adult bilaterian body composed of a few hundreds of cells. Nematodes also lost the MYC proto-oncogenic regulator of cell proliferation. To identify additional regulators of cell proliferation that were lost with MYC, we computationally screened and determined 839 high-confidence genes that are conserved in bilaterians/lost in nematodes (CIBLIN genes). We find that 30 % of all CIBLIN genes encode transcriptional regulators of cell proliferation, epithelial-to-mesenchyme transitions, and other processes. Over 50 % of CIBLIN genes are unnamed genes in Drosophila, suggesting that there are many understudied genes. Interestingly, CIBLIN genes include many Myc synthetic lethal (MycSL) hits from recent screens. CIBLIN genes include key regulators of heparan sulfate proteoglycan (HSPG) sulfation patterns, and lysyl oxidases involved in cross-linking and modification of the **extracellular matrix** (ECM). These genes and others suggest the CIBLIN repertoire services critical functions in ECM remodeling and cell migration in large-bodied bilaterians. Correspondingly, CIBLIN genes are co-expressed with Myc in cancer transcriptomes, and include a preponderance of known determinants of cancer progression and tumor aggression. We propose that CIBLIN gene research can improve our understanding of regulatory control of cellular growth in metazoans.
2. Joint development in the Drosophila leg: cell movements and cell populations. Flexible joints separate the rigid sections of the insect leg, allowing them to move. In Drosophila, the initial patterning of these joints is apparent in the larval imaginal discs from which the adult legs will develop. Here, we describe the later patterning and morphogenesis of the joints, which occurs after pupariation (AP). In the tibial/tarsal joint, the apodeme insertion site provides a fixed marker for the boundary between proximal and distal joint territories (the P/D boundary). Cells on either side of this boundary behave differently during morphogenesis. Morphogenesis begins with the apical constriction of distal joint cells, about 24 h AP. Distal cells then become columnar, causing distal tissue nearest the P/D boundary to fold into the leg. In the last stage of joint morphogenesis, the proximal joint cells closest to the P/D boundary align and elongate to form a "palisade" (a row of columnar cells) over the distal joint cells. The proximal and distal joint territories are characterised by the differential organisation of cytoskeletal and **extracellular matrix** proteins, and by the differential expression of enhancer trap lines and other gene markers. These markers also define a number of more localised territories within the pupal joint.
3. Non-canonical roles for Yorkie and Drosophila Inhibitor of Apoptosis 1 in epithelial tube size control. Precise control of epithelial tube size is critical for organ function, yet the molecular mechanisms remain poorly understood. Here, we examine the roles of cell growth and a highly conserved organ growth regulatory pathway in controlling the dimensions of the Drosophila tracheal (airway) system, a well-characterized system for investigating epithelial tube morphogenesis. We find that tracheal tube-size is regulated in unexpected ways by the transcription factor Yorkie (Yki, homolog of mammalian YAP and TAZ) and the Salvador/Warts/Hippo (SWH) kinase pathway. Yki activity typically promotes cell division, inhibits apoptosis, and can promote cell growth. However, reducing Yki activity in developing embryos increases rather than decreases the length of the major tracheal tubes, the dorsal trunks (DTs). Similarly, reduction of Hippo pathway activity, which antagonizes Yki, shortens tracheal DTs. yki mutations do not alter DT cell volume or cell number, indicating that Yki and the Hippo pathway regulate cell shape and apical surface area, but not volume. Yki does not appear to act through known tracheal pathways of apical **extracellular matrix**, septate junctions (SJs), basolateral or tubular polarity. Instead, the Hippo pathway and Yki appear to act downstream or in parallel to SJs because a double mutant combination of an upstream Hippo pathway activator, kibra, and the SJ component sinu have the short tracheal phenotype of a kibra mutant. We demonstrate that the critical target of Yki in tube size control is Drosophila Inhibitor of Apoptosis 1 (DIAP1), which in turn antagonizes the Drosophila effector caspase, Ice. Strikingly, there is no change in tracheal cell number in DIAP1 or Ice mutants, thus epithelial tube size regulation defines new non-apoptotic roles for Yki, DIAP1 and Ice.
4. Genetic and chemical modifiers of a CUG toxicity model in Drosophila. Non-coding CUG repeat expansions interfere with the activity of human Muscleblind-like (MBNL) proteins contributing to myotonic dystrophy 1 (DM1). To understand this toxic RNA gain-of-function mechanism we developed a Drosophila model expressing 60 pure and 480 interrupted CUG repeats in the context of a non-translatable RNA. These flies reproduced aspects of the DM1 pathology, most notably nuclear accumulation of CUG transcripts, muscle degeneration, splicing misregulation, and diminished Muscleblind function in vivo. Reduced Muscleblind activity was evident from the sensitivity of CUG-induced phenotypes to a decrease in muscleblind genetic dosage and rescue by MBNL1 expression, and further supported by the co-localization of Muscleblind and CUG repeat RNA in ribonuclear foci. Targeted expression of CUG repeats to the developing eye and brain mushroom bodies was toxic leading to rough eyes and semilethality, respectively. These phenotypes were utilized to identify genetic and chemical modifiers of the CUG-induced toxicity. 15 genetic modifiers of the rough eye phenotype were isolated. These genes identify putative cellular processes unknown to be altered by CUG repeat RNA, and they include mRNA export factor Aly, apoptosis inhibitor Thread, chromatin remodelling factor Nurf-38, and **extracellular matrix** structural component Viking. Ten chemical compounds suppressed the semilethal phenotype. These compounds significantly improved viability of CUG expressing flies and included non-steroidal anti-inflammatory agents (ketoprofen), muscarinic, cholinergic and histamine receptor inhibitors (orphenadrine), and drugs that can affect sodium and calcium metabolism such as clenbuterol and spironolactone. These findings provide new insights into the DM1 phenotype, and suggest novel candidates for DM1 treatments.

### [Ii]nvasive

#### PubMed

1. The lethal giant larvae tumour suppressor mutation requires dMyc oncoprotein to promote clonal malignancy. Neoplastic overgrowth depends on the cooperation of several mutations ultimately leading to major rearrangements in cellular behaviour. Precancerous cells are often removed by cell death from normal tissues in the early steps of the tumourigenic process, but the molecules responsible for such a fundamental safeguard process remain in part elusive. With the aim to investigate the molecular crosstalk occurring between precancerous and normal cells in vivo, we took advantage of the clonal analysis methods that are available in Drosophila for studying the phenotypes due to lethal giant larvae (lgl) neoplastic mutation induced in different backgrounds and tissues.We observed that lgl mutant cells growing in wild-type imaginal wing discs show poor viability and are eliminated by Jun N-terminal Kinase (JNK)-dependent cell death. Furthermore, they express very low levels of dMyc oncoprotein compared with those found in the surrounding normal tissue. Evidence that this is a cause of lgl mutant cells elimination was obtained by increasing dMyc levels in lgl mutant clones: their overgrowth potential was indeed re-established, with mutant cells overwhelming the neighbouring tissue and forming tumourous masses displaying several cancer hallmarks. Moreover, when lgl mutant clones were induced in backgrounds of slow-dividing cells, they upregulated dMyc, lost apical-basal cell polarity and were able to overgrow. Those phenotypes were abolished by reducing dMyc levels in the mutant clones, thereby confirming its key role in lgl-induced tumourigenesis. Furthermore, we show that the eiger-dependent Intrinsic Tumour Suppressor pathway plays only a minor role in eliminating lgl mutant cells in the wing pouch; lgl-/- clonal death in this region is instead driven mainly by dMyc-induced Cell Competition.Our results provide the first evidence that dMyc oncoprotein is required in lgl tumour suppressor mutant tissue to promote **invasive** overgrowth in larval and adult epithelial tissues. Moreover, we show that dMyc abundance inside versus outside the mutant clones plays a key role in driving neoplastic overgrowth.
2. Sin3a acts through a multi-gene module to regulate invasion in Drosophila and human tumors. Chromatin remodeling proteins regulate multiple aspects of cell homeostasis, making them ideal candidates for misregulation in transformed cells. Here, we explore Sin3A, a member of the Sin3 family of proteins linked to tumorigenesis that are thought to regulate gene expression through their role as histone deacetylases (HDACs). We identified Drosophila Sin3a as an important mediator of oncogenic Ret receptor in a fly model of Multiple Endocrine Neoplasia Type 2. Reducing Drosophila Sin3a activity led to metastasis-like behavior and, in the presence of Diap1, secondary tumors distant from the site of origin. Genetic and Chip-Seq analyses identified previously undescribed Sin3a targets including genes involved in cell motility and actin dynamics, as well as signaling pathways including Src, Jnk and Rho. A key Sin3a oncogenic target, PP1B, regulates stability of β-Catenin/Armadillo: the outcome is to oppose T-cell factor (TCF) function and Wg/Wnt pathway signaling in both fly and mammalian cancer cells. Reducing Sin3A strongly increased the **invasiv**e behavior of A549 human lung adenocarcinoma cells. We show that Sin3A is downregulated in a variety of human tumors and that Src, JNK, RhoA and PP1B/β-Catenin are regulated in a manner analogous to our Drosophila models. Our data suggest that Sin3A influences a specific step of tumorigenesis by regulating a module of genes involved in cell invasion. Tumor progression may commonly rely on such 'modules of invasion' under the control of broad transcriptional regulators.
3. Notch and Mef2 synergize to promote proliferation and metastasis through JNK signal activation in Drosophila. Genetic analyses in Drosophila revealed a synergy between Notch and the pleiotropic transcription factor Mef2 (myocyte enhancer factor 2), which profoundly influences proliferation and metastasis. We show that these hyperproliferative and **invasive** Drosophila phenotypes are attributed to upregulation of eiger, a member of the tumour necrosis factor superfamily of ligands, and the consequent activation of Jun N-terminal kinase signalling, which in turn triggers the expression of the **invasive** marker MMP1. Expression studies in human breast tumour samples demonstrate correlation between Notch and Mef2 paralogues and support the notion that Notch-MEF2 synergy may be significant for modulating human mammary oncogenesis.
4. Csk-deficient boundary cells are eliminated from normal Drosophila epithelia by exclusion, migration, and apoptosis. The construction and maintenance of normal epithelia relies on local signals that guide cells into their proper niches and remove unwanted cells. Failure to execute this process properly may result in aberrant development or diseases, including cancer and associated metastasis. Here, we show that local environment influences the behavior of dCsk-deficient cells. Broad loss of dCsk led to enlarged and mispatterned tissues due to overproliferation, a block in apoptosis, and decreased cadherin-mediated adhesion. Loss of dCsk in discrete patches led to a different outcome: epithelial exclusion, **invasive** migration, and apoptotic death. These latter phenotypes required sharp differences in dCsk activity between neighbors; dE-cadherin, P120-catenin, Rho1, JNK, and MMP2 mediated this signal. Together, our data demonstrate how the cellular microenvironment plays a central role in determining the outcome of altered dCsk activity, and reveal a role for P120-catenin in a mechanism that protects epithelial integrity by removing abnormal cells.
5. Cell mixing induced by myc is required for competitive tissue invasion and destruction. Cell-cell intercalation is used in several developmental processes to shape the normal body plan. There is no clear evidence that intercalation is involved in pathologies. Here we use the proto-oncogene myc to study a process analogous to early phase of tumour expansion: myc-induced cell competition. Cell competition is a conserved mechanism driving the elimination of slow-proliferating cells (so-called 'losers') by faster-proliferating neighbours (so-called 'winners') through apoptosis and is important in preventing developmental malformations and maintain tissue fitness. Here we show, using long-term live imaging of myc-driven competition in the Drosophila pupal notum and in the wing imaginal disc, that the probability of elimination of loser cells correlates with the surface of contact shared with winners. As such, modifying loser-winner interface morphology can modulate the strength of competition. We further show that elimination of loser clones requires winner-loser cell mixing through cell-cell intercalation. Cell mixing is driven by differential growth and the high tension at winner-winner interfaces relative to winner-loser and loser-loser interfaces, which leads to a preferential stabilization of winner-loser contacts and reduction of clone compactness over time. Differences in tension are generated by a relative difference in F-actin levels between loser and winner junctions, induced by differential levels of the membrane lipid phosphatidylinositol (3,4,5)-trisphosphate. Our results establish the first link between cell-cell intercalation induced by a proto-oncogene and how it promotes **invasive**ness and destruction of healthy tissues.
6. Caspase signalling in the absence of apoptosis drives Jnk-dependent invasion. Tumours evolve several mechanisms to evade apoptosis, yet many resected carcinomas show significantly elevated caspase activity. Moreover, caspase activity is positively correlated with tumour aggression and adverse patient outcome. These observations indicate that caspases might have a functional role in promoting tumour invasion and metastasis. Using a Drosophila model of invasion, we show that precise effector caspase activity drives cell invasion without initiating apoptosis. Affected cells express the matrix metalloprotinase Mmp1 and invade by activating Jnk. Our results link Jnk and effector caspase signalling during the **invasive** process and suggest that tumours under apoptotic stresses from treatment, immune surveillance or intrinsic signals might be induced further along the metastatic cascade.
7. Basolateral junctions utilize warts signaling to control epithelial-mesenchymal transition and proliferation crucial for migration and invasion of Drosophila ovarian epithelial cells. Fasciclin2 (Fas2) and Discslarge (Dlg) localize to the basolateral junction (BLJ) of Drosophila follicle epithelial cells and inhibit their proliferation and invasion. To identify a BLJ signaling pathway we completed a genomewide screen for mutants that enhance dlg tumorigenesis. We identified two genes that encode known BLJ scaffolding proteins, lethal giant larvae (lgl) and scribble (scrib), and several not previously associated with BLJ function, including warts (wts) and roughened eye (roe), which encode a serine-threonine kinase and a transcription factor, respectively. Like scrib, wts and roe also enhance Fas2 and lgl tumorigenesis. Further, scrib, wts, and roe block border cell migration, and cause non**invasive** tumors that resemble dlg partial loss of function, suggesting that the BLJ utilizes Wts signaling to repress EMT and proliferation, but not motility. Apicolateral junction proteins Fat (Ft), Expanded (Ex), and Merlin (Mer) either are not involved in these processes, or have highly spatio-temporally restricted roles, diminishing their significance as upstream inputs to Wts in follicle cells. This is further indicated in that Wts targets, CyclinE and DIAP1, are elevated in Fas2, dlg, lgl, wts, and roe cells, but not Fat, ex, or mer cells. Thus, the BLJ appears to regulate epithelial polarity and dynamics not only as a localized scaffold, but also by communicating signals to the nucleus. Wts may be regulated by distinct junction inputs depending on developmental context.

### [Mm]etastasis

#### PubMed

1. Sin3a acts through a multi-gene module to regulate invasion in Drosophila and human tumors. Chromatin remodeling proteins regulate multiple aspects of cell homeostasis, making them ideal candidates for misregulation in transformed cells. Here, we explore Sin3A, a member of the Sin3 family of proteins linked to tumorigenesis that are thought to regulate gene expression through their role as histone deacetylases (HDACs). We identified Drosophila Sin3a as an important mediator of oncogenic Ret receptor in a fly model of Multiple Endocrine Neoplasia Type 2. Reducing Drosophila Sin3a activity led to **metastasis**-like behavior and, in the presence of Diap1, secondary tumors distant from the site of origin. Genetic and Chip-Seq analyses identified previously undescribed Sin3a targets including genes involved in cell motility and actin dynamics, as well as signaling pathways including Src, Jnk and Rho. A key Sin3a oncogenic target, PP1B, regulates stability of β-Catenin/Armadillo: the outcome is to oppose T-cell factor (TCF) function and Wg/Wnt pathway signaling in both fly and mammalian cancer cells. Reducing Sin3A strongly increased the invasive behavior of A549 human lung adenocarcinoma cells. We show that Sin3A is downregulated in a variety of human tumors and that Src, JNK, RhoA and PP1B/β-Catenin are regulated in a manner analogous to our Drosophila models. Our data suggest that Sin3A influences a specific step of tumorigenesis by regulating a module of genes involved in cell invasion. Tumor progression may commonly rely on such 'modules of invasion' under the control of broad transcriptional regulators.
2. Notch and Mef2 synergize to promote proliferation and **metastasis** through JNK signal activation in Drosophila. Genetic analyses in Drosophila revealed a synergy between Notch and the pleiotropic transcription factor Mef2 (myocyte enhancer factor 2), which profoundly influences proliferation and **metastasis**. We show that these hyperproliferative and invasive Drosophila phenotypes are attributed to upregulation of eiger, a member of the tumour necrosis factor superfamily of ligands, and the consequent activation of Jun N-terminal kinase signalling, which in turn triggers the expression of the invasive marker MMP1. Expression studies in human breast tumour samples demonstrate correlation between Notch and Mef2 paralogues and support the notion that Notch-MEF2 synergy may be significant for modulating human mammary oncogenesis.
3. Cell Competition Drives the Formation of Metastatic Tumors in a Drosophila Model of Epithelial Tumor Formation. Cell competition is a homeostatic process in which proliferating cells compete for survival. Elimination of otherwise normal healthy cells through competition is important during development and has recently been shown to contribute to maintaining tissue health during organismal aging. The mechanisms that allow for ongoing cell competition during adult life could, in principle, contribute to tumorigenesis. However, direct evidence supporting this hypothesis has been lacking. Here, we provide evidence that cell competition drives tumor formation in a Drosophila model of epithelial cancer. Cells expressing EGFR together with the conserved microRNA miR-8 acquire the properties of supercompetitors. Neoplastic transformation and **metastasis** depend on the ability of these cells to induce apoptosis and engulf nearby cells. miR-8 expression causes genome instability by downregulating expression of the Septin family protein Peanut. Cytokinesis failure due to downregulation of Peanut is required for tumorigenesis. This study provides evidence that the cellular mechanisms that drive cell competition during normal tissue growth can be co-opted to drive tumor formation and **metastasis**. Analogous mechanisms for cytokinesis failure may lead to polyploid intermediates in tumorigenesis in mammalian cancer models.
4. Drosophila models for cancer research. Drosophila is a model system for cancer research. Investigation with fruit flies has facilitated a number of important recent discoveries in the field: the hippo signaling pathway, which coordinates cell proliferation and death to achieve normal tissue size; 'social' behaviors of cells, including cell competition and apoptosis-induced compensatory proliferation, that help ensure normal tissue size; and a growing understanding of how oncogenes and tumor suppressors cooperate to achieve tumor growth and **metastasis** in situ. In the future, Drosophila models can be extended beyond basic research in the search for human therapeutics.
5. Csk-deficient boundary cells are eliminated from normal Drosophila epithelia by exclusion, migration, and apoptosis. The construction and maintenance of normal epithelia relies on local signals that guide cells into their proper niches and remove unwanted cells. Failure to execute this process properly may result in aberrant development or diseases, including cancer and associated **metastasis**. Here, we show that local environment influences the behavior of dCsk-deficient cells. Broad loss of dCsk led to enlarged and mispatterned tissues due to overproliferation, a block in apoptosis, and decreased cadherin-mediated adhesion. Loss of dCsk in discrete patches led to a different outcome: epithelial exclusion, invasive migration, and apoptotic death. These latter phenotypes required sharp differences in dCsk activity between neighbors; dE-cadherin, P120-catenin, Rho1, JNK, and MMP2 mediated this signal. Together, our data demonstrate how the cellular microenvironment plays a central role in determining the outcome of altered dCsk activity, and reveal a role for P120-catenin in a mechanism that protects epithelial integrity by removing abnormal cells.
6. Centrosomal kinase Nek2 cooperates with oncogenic pathways to promote **metastasis**. Centrosomal kinase Nek2 is overexpressed in different cancers, yet how it contributes toward tumorigenesis remains poorly understood. dNek2 overexpression in a Drosophila melanogaster model led to upregulation of Drosophila Wnt ortholog wingless (Wg), and alteration of cell migration markers-Rho1, Rac1 and E-cadherin (Ecad)-resulting in changes in cell shape and tissue morphogenesis. dNek2 overexpression cooperated with receptor tyrosine kinase and mitogen-activated protein kinase signaling to upregulate activated Akt, Diap1, Mmp1 and Wg protein to promote local invasion, distant seeding and **metastasis**. In tumor cell injection assays, dNek2 cooperated with Ras and Src signaling to promote aggressive colonization of tumors into different adult fly tissues. Inhibition of the PI3K pathway suppressed the cooperation of dNek2 with other growth pathways. Consistent with our fly studies, overexpression of human Nek2 in A549 lung adenocarcinoma and HEK293T cells led to activation of the Akt pathway and increase in β-catenin protein levels. Our computational approach identified a class of Nek2-inhibitory compounds and a novel drug-like pharmacophore that reversed the Nek2 overexpression phenotypes in flies and human cells. Our finding posits a novel role for Nek2 in promoting **metastasi**s in addition to its currently defined role in promoting chromosomal instability. It provides a rationale for the selective advantage of centrosome amplification in cancer.
7. Caspase signalling in the absence of apoptosis drives Jnk-dependent invasion. Tumours evolve several mechanisms to evade apoptosis, yet many resected carcinomas show significantly elevated caspase activity. Moreover, caspase activity is positively correlated with tumour aggression and adverse patient outcome. These observations indicate that caspases might have a functional role in promoting tumour invasion and **metastasis**. Using a Drosophila model of invasion, we show that precise effector caspase activity drives cell invasion without initiating apoptosis. Affected cells express the matrix metalloprotinase Mmp1 and invade by activating Jnk. Our results link Jnk and effector caspase signalling during the invasive process and suggest that tumours under apoptotic stresses from treatment, immune surveillance or intrinsic signals might be induced further along the metastatic cascade.
8. Modeling tumor invasion and **metastasis** in Drosophila. Conservation of major signaling pathways between humans and flies has made Drosophila a useful model organism for cancer research. Our understanding of the mechanisms regulating cell growth, differentiation and development has been considerably advanced by studies in Drosophila. Several recent high profile studies have examined the processes constraining the metastatic growth of tumor cells in fruit fly models. Cell invasion can be studied in the context of an in vivo setting in flies, enabling the genetic requirements of the microenvironment of tumor cells undergoing **metastasis** to be analyzed. This Perspective discusses the strengths and limitations of Drosophila models of cancer invasion and the unique tools that have enabled these studies. It also highlights several recent reports that together make a strong case for Drosophila as a system with the potential for both testing novel concepts in tumor progression and cell invasion, and for uncovering players in **metastasis**.

### [Cc]ell [Ii]nvasion

#### PubMed

1. Sin3a acts through a multi-gene module to regulate invasion in Drosophila and human tumors. Chromatin remodeling proteins regulate multiple aspects of cell homeostasis, making them ideal candidates for misregulation in transformed cells. Here, we explore Sin3A, a member of the Sin3 family of proteins linked to tumorigenesis that are thought to regulate gene expression through their role as histone deacetylases (HDACs). We identified Drosophila Sin3a as an important mediator of oncogenic Ret receptor in a fly model of Multiple Endocrine Neoplasia Type 2. Reducing Drosophila Sin3a activity led to metastasis-like behavior and, in the presence of Diap1, secondary tumors distant from the site of origin. Genetic and Chip-Seq analyses identified previously undescribed Sin3a targets including genes involved in cell motility and actin dynamics, as well as signaling pathways including Src, Jnk and Rho. A key Sin3a oncogenic target, PP1B, regulates stability of β-Catenin/Armadillo: the outcome is to oppose T-cell factor (TCF) function and Wg/Wnt pathway signaling in both fly and mammalian cancer cells. Reducing Sin3A strongly increased the invasive behavior of A549 human lung adenocarcinoma cells. We show that Sin3A is downregulated in a variety of human tumors and that Src, JNK, RhoA and PP1B/β-Catenin are regulated in a manner analogous to our Drosophila models. Our data suggest that Sin3A influences a specific step of tumorigenesis by regulating a module of genes involved i**n cell invasi**on. Tumor progression may commonly rely on such 'modules of invasion' under the control of broad transcriptional regulators.
2. Caspase signalling in the absence of apoptosis drives Jnk-dependent invasion. Tumours evolve several mechanisms to evade apoptosis, yet many resected carcinomas show significantly elevated caspase activity. Moreover, caspase activity is positively correlated with tumour aggression and adverse patient outcome. These observations indicate that caspases might have a functional role in promoting tumour invasion and metastasis. Using a Drosophila model of invasion, we show that precise effector caspase activity drives **cell invasion** without initiating apoptosis. Affected cells express the matrix metalloprotinase Mmp1 and invade by activating Jnk. Our results link Jnk and effector caspase signalling during the invasive process and suggest that tumours under apoptotic stresses from treatment, immune surveillance or intrinsic signals might be induced further along the metastatic cascade.
3. Modeling tumor invasion and metastasis in Drosophila. Conservation of major signaling pathways between humans and flies has made Drosophila a useful model organism for cancer research. Our understanding of the mechanisms regulating cell growth, differentiation and development has been considerably advanced by studies in Drosophila. Several recent high profile studies have examined the processes constraining the metastatic growth of tumor cells in fruit fly models. **Cell invasion** can be studied in the context of an in vivo setting in flies, enabling the genetic requirements of the microenvironment of tumor cells undergoing metastasis to be analyzed. This Perspective discusses the strengths and limitations of Drosophila models of cancer invasion and the unique tools that have enabled these studies. It also highlights several recent reports that together make a strong case for Drosophila as a system with the potential for both testing novel concepts in tumor progression and **cell invasion**, and for uncovering players in metastasis.
4. Live-cell delamination counterbalances epithelial growth to limit tissue overcrowding. The development and maintenance of an epithelium requires finely balanced rates of growth and cell death. However, the mechanical and biochemical mechanisms that ensure proper feedback control of tissue growth, which when deregulated contribute to tumorigenesis, are poorly understood. Here we use the fly notum as a model system to identify a novel process of crowding-induced cell delamination that balances growth to ensure the development of well-ordered cell packing. In crowded regions of the tissue, a proportion of cells undergo a serial loss of cell-cell junctions and a progressive loss of apical area, before being squeezed out by their neighbours. This path of delamination is recapitulated by a simple computational model of epithelial mechanics, in which stochastic cell loss relieves overcrowding as the system tends towards equilibrium. We show that this process of delamination is mechanistically distinct from apoptosis-mediated cell extrusion and precedes the first signs of cell death. Overall, this analysis reveals a simple mechanism that buffers epithelia against variations in growth. Because live-cell delamination constitutes a mechanistic link between epithelial hyperplasia and **cell invasion**, this is likely to have important implications for our understanding of the early stages of cancer development.

---

## capu

- NCBI Gene
- GeneRIF
- Pubmed

| Search Term | Rank | Fields |
| --- | --- | --- |
| [Ff]ilopodia | 1 | PubMed(1) |
| [Mm]igration | 6 | PubMed(9) |
| [Ii]nvasive | 10 | PubMed(2) |
| [Mm]etastasis | 12 | PubMed(1) |
| [Cc]ell [Ii]nvasion | 13 | PubMed(1) |

  
hide/show details for capu

### [Ff]ilopodia

#### PubMed

1. Formin3 is required for assembly of the F-actin structure that mediates tracheal fusion in Drosophila. During tracheal development in Drosophila, some branches join to form a continuous luminal network. Specialized cells at the branch tip, called fusion cells, extend **filopodia** to make contact and become doughnut shaped to allow passage of the lumen. These morphogenetic processes accompany the highly regulated cytoskeletal reorganization of fusion cells. We identified the Drosophila formin3 (form3) gene that encodes a novel formin and plays a role in tracheal fusion. Formins are a family of proteins characterized by highly conserved formin homology (FH) domains. The formin family functions in various actin-based processes, including cytokinesis and cell polarity. During embryogenesis, form3 mRNA is expressed mainly in the tracheal system. In form3 mutant embryos, the tracheal fusion does not occur at some points. This phenotype is rescued by the forced expression of form3 in the trachea. We used live imaging of GFP-moesin during tracheal fusion to show that an F-actin structure that spans the adjoining fusion cells and mediates the luminal connection does not form at abnormal anastomosis sites in form3 mutants. These results suggested that Form3 plays a role in the F-actin assembly, which is essential for cellular rearrangement during tracheal fusion.

### [Mm]igration

#### PubMed

1. The Ste20-like kinase misshapen functions together with Bicaudal-D and dynein in driving nuclear **migration** in the developing drosophila eye. Nuclear translocation, driven by the motility apparatus consisting of the cytoplasmic dynein motor and microtubules, is essential for cell **migration** during embryonic development. Bicaudal-D (Bic-D), an evolutionarily conserved dynein-interacting protein, is required for developmental control of nuclear **migration** in Drosophila. Nothing is known about the signaling events that coordinate the function of Bic-D and dynein during development. Here, we show that Misshapen (Msn), the fly homolog of the vertebrate Nck-interacting kinase is a component of a novel signaling pathway that regulates photoreceptor (R-cell) nuclear **migration** in the developing Drosophila compound eye. Msn, like Bic-D, is required for the apical **migration** of differentiating R-cell precursor nuclei. msn displays strong genetic interaction with Bic-D. Biochemical studies demonstrate that Msn increases the phosphorylation of Bic-D, which appears to be necessary for the apical accumulation of both Bic-D and dynein in developing R-cell precursor cells. We propose that Msn functions together with Bic-D to regulate the apical localization of dynein in generating directed nuclear **migration** within differentiating R-cell precursor cells.
2. Drosophila female sterile mutation spoonbill interferes with multiple pathways in oogenesis. spoonbill is a Drosophila female-sterile mutation, which displays a range of eggshell and egg chamber patterning defects. Previous analysis has shown that the mutation interfered with the function of two major signaling pathways, GRK/EGFR and DPP. In this report, the nature of spoonbill was further investigated to examine whether it was associated with additional pathways in oogenesis. Clonal analysis, presented here, demonstrated that most of the aberrant phenotypes associated with spoonbill were dependent on a mutant germline. Nevertheless, SPOONBILL may function also in the soma to ensure proper polarization and **migration** of the border-cell-cluster. Further, genetic interaction studies implicated spoonbill in additional unrelated pathways such as the one(s) involved in actin polymerization/depolymerization. Based on the previous data and the results presented here, it is anticipated that spoonbill may encode a multifunctional protein that perhaps coordinately regulated the activity of multiple signaling pathways during oogenesis.
3. Genetic analysis of the actin cytoskeleton in the Drosophila ovary. The Drosophila ovary provides a favorable model system in which to study cellular morphogenesis. The development of a mature egg involves a syncytium of 16 germline cells and over 1000 somatically derived follicle cells. Intercellular transport, stable intercellular bridges, cell **migration**s, cell shape changes, and specific subcellular localization of many embryonic patterning determinants contribute to egg development and require a dynamic cytoskeleton. We discuss many of the recent genetic and cell biological studies that have led to insights into how the actin cytoskeleton is assembled and regulated during the morphogenesis of the Drosophila egg.
4. Molecular requirements for actin-based lamella formation in Drosophila S2 cells. Cell **migration** occurs through the protrusion of the actin-enriched lamella. Here, we investigated the effects of RNAi depletion of approximately 90 proteins implicated in actin function on lamella formation in Drosophila S2 cells. Similar to in vitro reconstitution studies of actin-based Listeria movement, we find that lamellae formation requires a relatively small set of proteins that participate in actin nucleation (Arp2/3 and SCAR), barbed end capping (capping protein), filament depolymerization (cofilin and Aip1), and actin monomer binding (profilin and cyclase-associated protein). Lamellae are initiated by parallel and partially redundant signaling pathways involving Rac GTPases and the adaptor protein Nck, which stimulate SCAR, an Arp2/3 activator. We also show that RNAi of three proteins (kette, Abi, and Sra-1) known to copurify with and inhibit SCAR in vitro leads to SCAR degradation, revealing a novel function of this protein complex in SCAR stability. Our results have identified an essential set of proteins involved in actin dynamics during lamella formation in Drosophila S2 cells.
5. A Jnk-Rho-Actin remodeling positive feedback network directs Src-driven invasion. Current models of tumor cell invasion propose that oncogenic signaling converges upon key orchestrators of cytoskeletal dynamics, including c-Jun N-terminal kinase (Jnk) and RhoGTPase family members; these signals dynamically direct Actin remodeling proteins (ARPs) to catalyze the cytoskeletal changes required for **migration**. Src is a key driver of tumor aggression, metastasis and patient mortality. To clarify how Src regulates Actin dynamics to promote invasive **migration**, we performed a genetic modifier screen in a Drosophila model of invasion. Nine genes linked to Actin dynamics were identified that mediate invasion in situ. We found that ARPs were required for many oncogenic effects of Src including Mmp1 expression and initiation of apoptosis. Surprisingly, they were also regulators of Jnk pathway activity: both Src and the small GTPase Rho1 activated Jnk in a manner dependent on ARPs during invasion. Our results suggest that ARPs are not simply downstream executors of signal transduction pathways. Rather, they participate in a positive feedback network involving canonical oncogenic signaling pathways that promote tumor invasion.
6. Basolateral junctions utilize warts signaling to control epithelial-mesenchymal transition and proliferation crucial for **migration** and invasion of Drosophila ovarian epithelial cells. Fasciclin2 (Fas2) and Discslarge (Dlg) localize to the basolateral junction (BLJ) of Drosophila follicle epithelial cells and inhibit their proliferation and invasion. To identify a BLJ signaling pathway we completed a genomewide screen for mutants that enhance dlg tumorigenesis. We identified two genes that encode known BLJ scaffolding proteins, lethal giant larvae (lgl) and scribble (scrib), and several not previously associated with BLJ function, including warts (wts) and roughened eye (roe), which encode a serine-threonine kinase and a transcription factor, respectively. Like scrib, wts and roe also enhance Fas2 and lgl tumorigenesis. Further, scrib, wts, and roe block border cell **migration**, and cause noninvasive tumors that resemble dlg partial loss of function, suggesting that the BLJ utilizes Wts signaling to repress EMT and proliferation, but not motility. Apicolateral junction proteins Fat (Ft), Expanded (Ex), and Merlin (Mer) either are not involved in these processes, or have highly spatio-temporally restricted roles, diminishing their significance as upstream inputs to Wts in follicle cells. This is further indicated in that Wts targets, CyclinE and DIAP1, are elevated in Fas2, dlg, lgl, wts, and roe cells, but not Fat, ex, or mer cells. Thus, the BLJ appears to regulate epithelial polarity and dynamics not only as a localized scaffold, but also by communicating signals to the nucleus. Wts may be regulated by distinct junction inputs depending on developmental context.
7. Germ cell development in Drosophila. More than 100 years have passed since Weismann first recognized the role of germ cells in the continuity of a species. Today, it remains unclear how a germ cell is initially set aside from somatic cells and how it chooses its unique developmental path. In this review, we address various aspects of germ cell development in Drosophila, such as germ cell determination, germ cell **migration**, gonad formation, sex determination, and gametogenesis. Many aspects of germ cell development, including the morphology of germ cells, their migratory behavior, as well as the processes of gonad formation and gametogenesis, show striking similarities among organisms. Considering the conservation of factors that regulate somatic development, it is likely that some aspects of germ cell development are shared not only on a morphological but also on the molecular level between Drosophila and other organisms.
8. A genetic screen for suppressors and enhancers of the Drosophila cdk1-cyclin B identifies maternal factors that regulate microtubule and microfilament stability. Coordination between cell-cycle progression and cytoskeletal dynamics is important for faithful transmission of genetic information. In early Drosophila embryos, increasing maternal cyclin B leads to higher Cdk1-CycB activity, shorter microtubules, and slower nuclear movement during cycles 5-7 and delays in nuclear **migration** to the cortex at cycle 10. Later during cycle 14 interphase of six cycB embryos, we observed patches of mitotic nuclei, chromosome bridges, abnormal nuclear distribution, and small and large nuclei. These phenotypes indicate disrupted coordination between the cell-cycle machinery and cytoskeletal function. Using these sensitized phenotypes, we performed a dosage-sensitive genetic screen to identify maternal proteins involved in this process. We identified 10 suppressors classified into three groups: (1) gene products regulating Cdk1 activities, cdk1 and cyclin A; (2) gene products interacting with both microtubules and microfilaments, Actin-related protein 87C; and (3) gene products interacting with microfilaments, chickadee, diaphanous, Cdc42, quail, spaghetti-squash, zipper, and scrambled. Interestingly, most of the suppressors that rescue the astral microtubule phenotype also reduce Cdk1-CycB activities and are microfilament-related genes. This suggests that the major mechanism of suppression relies on the interactions among Cdk1-CycB, microtubule, and microfilament networks. Our results indicate that the balance among these different components is vital for normal early cell cycles and for embryonic development. Our observations also indicate that microtubules and cortical microfilaments antagonize each other during the preblastoderm stage.
9. mago nashi mediates the posterior follicle cell-to-oocyte signal to organize axis formation in Drosophila. Establishment of the anteroposterior and dorsoventral axes in the Drosophila egg chamber requires reciprocal signaling between the germ line and soma. Upon activation of the Drosophila EGF receptor in the posterior follicle cells, these cells signal back to the oocyte, resulting in a reorganization of the oocyte cytoplasm and anterodorsal **migration** of the oocyte nucleus. We demonstrate that the gene mago nashi (mago) encodes an evolutionarily conserved protein that must be localized within the posterior pole plasm for germ-plasm assembly and Caenorhabditis elegans mago is a functional homologue of Drosophila mago. In the absence of mago+ function during oogenesis, the anteroposterior and dorsoventral coordinates of the oocyte are not specified and the germ plasm fails to assemble.

### [Ii]nvasive

#### PubMed

1. A Jnk-Rho-Actin remodeling positive feedback network directs Src-driven invasion. Current models of tumor cell invasion propose that oncogenic signaling converges upon key orchestrators of cytoskeletal dynamics, including c-Jun N-terminal kinase (Jnk) and RhoGTPase family members; these signals dynamically direct Actin remodeling proteins (ARPs) to catalyze the cytoskeletal changes required for migration. Src is a key driver of tumor aggression, metastasis and patient mortality. To clarify how Src regulates Actin dynamics to promote **invasive** migration, we performed a genetic modifier screen in a Drosophila model of invasion. Nine genes linked to Actin dynamics were identified that mediate invasion in situ. We found that ARPs were required for many oncogenic effects of Src including Mmp1 expression and initiation of apoptosis. Surprisingly, they were also regulators of Jnk pathway activity: both Src and the small GTPase Rho1 activated Jnk in a manner dependent on ARPs during invasion. Our results suggest that ARPs are not simply downstream executors of signal transduction pathways. Rather, they participate in a positive feedback network involving canonical oncogenic signaling pathways that promote tumor invasion.
2. Basolateral junctions utilize warts signaling to control epithelial-mesenchymal transition and proliferation crucial for migration and invasion of Drosophila ovarian epithelial cells. Fasciclin2 (Fas2) and Discslarge (Dlg) localize to the basolateral junction (BLJ) of Drosophila follicle epithelial cells and inhibit their proliferation and invasion. To identify a BLJ signaling pathway we completed a genomewide screen for mutants that enhance dlg tumorigenesis. We identified two genes that encode known BLJ scaffolding proteins, lethal giant larvae (lgl) and scribble (scrib), and several not previously associated with BLJ function, including warts (wts) and roughened eye (roe), which encode a serine-threonine kinase and a transcription factor, respectively. Like scrib, wts and roe also enhance Fas2 and lgl tumorigenesis. Further, scrib, wts, and roe block border cell migration, and cause non**invasive** tumors that resemble dlg partial loss of function, suggesting that the BLJ utilizes Wts signaling to repress EMT and proliferation, but not motility. Apicolateral junction proteins Fat (Ft), Expanded (Ex), and Merlin (Mer) either are not involved in these processes, or have highly spatio-temporally restricted roles, diminishing their significance as upstream inputs to Wts in follicle cells. This is further indicated in that Wts targets, CyclinE and DIAP1, are elevated in Fas2, dlg, lgl, wts, and roe cells, but not Fat, ex, or mer cells. Thus, the BLJ appears to regulate epithelial polarity and dynamics not only as a localized scaffold, but also by communicating signals to the nucleus. Wts may be regulated by distinct junction inputs depending on developmental context.

### [Mm]etastasis

#### PubMed

1. A Jnk-Rho-Actin remodeling positive feedback network directs Src-driven invasion. Current models of tumor cell invasion propose that oncogenic signaling converges upon key orchestrators of cytoskeletal dynamics, including c-Jun N-terminal kinase (Jnk) and RhoGTPase family members; these signals dynamically direct Actin remodeling proteins (ARPs) to catalyze the cytoskeletal changes required for migration. Src is a key driver of tumor aggression, **metastasis** and patient mortality. To clarify how Src regulates Actin dynamics to promote invasive migration, we performed a genetic modifier screen in a Drosophila model of invasion. Nine genes linked to Actin dynamics were identified that mediate invasion in situ. We found that ARPs were required for many oncogenic effects of Src including Mmp1 expression and initiation of apoptosis. Surprisingly, they were also regulators of Jnk pathway activity: both Src and the small GTPase Rho1 activated Jnk in a manner dependent on ARPs during invasion. Our results suggest that ARPs are not simply downstream executors of signal transduction pathways. Rather, they participate in a positive feedback network involving canonical oncogenic signaling pathways that promote tumor invasion.

### [Cc]ell [Ii]nvasion

#### PubMed

1. A Jnk-Rho-Actin remodeling positive feedback network directs Src-driven invasion. Current models of tumor **cell invasion** propose that oncogenic signaling converges upon key orchestrators of cytoskeletal dynamics, including c-Jun N-terminal kinase (Jnk) and RhoGTPase family members; these signals dynamically direct Actin remodeling proteins (ARPs) to catalyze the cytoskeletal changes required for migration. Src is a key driver of tumor aggression, metastasis and patient mortality. To clarify how Src regulates Actin dynamics to promote invasive migration, we performed a genetic modifier screen in a Drosophila model of invasion. Nine genes linked to Actin dynamics were identified that mediate invasion in situ. We found that ARPs were required for many oncogenic effects of Src including Mmp1 expression and initiation of apoptosis. Surprisingly, they were also regulators of Jnk pathway activity: both Src and the small GTPase Rho1 activated Jnk in a manner dependent on ARPs during invasion. Our results suggest that ARPs are not simply downstream executors of signal transduction pathways. Rather, they participate in a positive feedback network involving canonical oncogenic signaling pathways that promote tumor invasion.

---

## Frl

- NCBI Gene
- GeneRIF
- Pubmed

| Search Term | Rank | Fields |
| --- | --- | --- |
| [Ff]ilopodia | 1 | PubMed(1) |
| [Mm]igration | 6 | PubMed(4) |

  
hide/show details for Frl

### [Ff]ilopodia

#### PubMed

1. Formin3 is required for assembly of the F-actin structure that mediates tracheal fusion in Drosophila. During tracheal development in Drosophila, some branches join to form a continuous luminal network. Specialized cells at the branch tip, called fusion cells, extend **filopodia** to make contact and become doughnut shaped to allow passage of the lumen. These morphogenetic processes accompany the highly regulated cytoskeletal reorganization of fusion cells. We identified the Drosophila formin3 (form3) gene that encodes a novel formin and plays a role in tracheal fusion. Formins are a family of proteins characterized by highly conserved formin homology (FH) domains. The formin family functions in various actin-based processes, including cytokinesis and cell polarity. During embryogenesis, form3 mRNA is expressed mainly in the tracheal system. In form3 mutant embryos, the tracheal fusion does not occur at some points. This phenotype is rescued by the forced expression of form3 in the trachea. We used live imaging of GFP-moesin during tracheal fusion to show that an F-actin structure that spans the adjoining fusion cells and mediates the luminal connection does not form at abnormal anastomosis sites in form3 mutants. These results suggested that Form3 plays a role in the F-actin assembly, which is essential for cellular rearrangement during tracheal fusion.

### [Mm]igration

#### PubMed

1. Unique and Overlapping Functions of Formins Frl and DAAM During Ommatidial Rotation and Neuronal Development in Drosophila. The noncanonical Frizzled/planar cell polarity (PCP) pathway regulates establishment of polarity within the plane of an epithelium to generate diversity of cell fates, asymmetric, but highly aligned structures, or to orchestrate the directional **migration** of cells during convergent extension during vertebrate gastrulation. In Drosophila, PCP signaling is essential to orient actin wing hairs and to align ommatidia in the eye, in part by coordinating the movement of groups of photoreceptor cells during ommatidial rotation. Importantly, the coordination of PCP signaling with changes in the cytoskeleton is essential for proper epithelial polarity. Formins polymerize linear actin filaments and are key regulators of the actin cytoskeleton. Here, we show that the diaphanous-related formin, Frl, the single fly member of the FMNL (formin related in leukocytes/formin-like) formin subfamily affects ommatidial rotation in the Drosophila eye and is controlled by the Rho family GTPase Cdc42. Interestingly, we also found that frl mutants exhibit an axon growth phenotype in the mushroom body, a center for olfactory learning in the Drosophila brain, which is also affected in a subset of PCP genes. Significantly, Frl cooperates with Cdc42 and another formin, DAAM, during mushroom body formation. This study thus suggests that different formins can cooperate or act independently in distinct tissues, likely integrating various signaling inputs with the regulation of the cytoskeleton. It furthermore highlights the importance and complexity of formin-dependent cytoskeletal regulation in multiple organs and developmental contexts.
2. Neurl4 contributes to germ cell formation and integrity in Drosophila. Primordial germ cells (PGCs) form at the posterior pole of the Drosophila embryo, and then migrate to their final destination in the gonad where they will produce eggs or sperm. Studies of the different stages in this process, including assembly of germ plasm in the oocyte during oogenesis, specification of a subset of syncytial embryonic nuclei as PGCs, and **migration**, have been informed by genetic analyses. Mutants have defined steps in the process, and the identities of the affected genes have suggested biochemical mechanisms. Here we describe a novel PGC phenotype. When Neurl4 activity is reduced, newly formed PGCs frequently adopt irregular shapes and appear to bud off vesicles. PGC number is also reduced, an effect exacerbated by a separate role for Neurl4 in germ plasm formation during oogenesis. Like its mammalian homolog, Drosophila Neurl4 protein is concentrated in centrosomes and downregulates centrosomal protein CP110. Reducing CP110 activity suppresses the abnormal PGC morphology of Neurl4 mutants. These results extend prior analyses of Neurl4 in cultured cells, revealing a heightened requirement for Neurl4 in germ-line cells in Drosophila.
3. Unique and Overlapping Functions of Formins Frl and DAAM During Ommatidial Rotation and Neuronal Development in Drosophila. The noncanonical Frizzled/planar cell polarity (PCP) pathway regulates establishment of polarity within the plane of an epithelium to generate diversity of cell fates, asymmetric, but highly aligned structures, or to orchestrate the directional **migration** of cells during convergent extension during vertebrate gastrulation. In Drosophila, PCP signaling is essential to orient actin wing hairs and to align ommatidia in the eye, in part by coordinating the movement of groups of photoreceptor cells during ommatidial rotation. Importantly, the coordination of PCP signaling with changes in the cytoskeleton is essential for proper epithelial polarity. Formins polymerize linear actin filaments and are key regulators of the actin cytoskeleton. Here, we show that the diaphanous-related formin, Frl, the single fly member of the FMNL (formin related in leukocytes/formin-like) formin subfamily affects ommatidial rotation in the Drosophila eye and is controlled by the Rho family GTPase Cdc42. Interestingly, we also found that frl mutants exhibit an axon growth phenotype in the mushroom body, a center for olfactory learning in the Drosophila brain, which is also affected in a subset of PCP genes. Significantly, Frl cooperates with Cdc42 and another formin, DAAM, during mushroom body formation. This study thus suggests that different formins can cooperate or act independently in distinct tissues, likely integrating various signaling inputs with the regulation of the cytoskeleton. It furthermore highlights the importance and complexity of formin-dependent cytoskeletal regulation in multiple organs and developmental contexts.
4. Identification and characterization of a set of conserved and new regulators of cytoskeletal organization, cell morphology and **migration**. Cell **migration** is essential during development and in human disease progression including cancer. Most cell **migration** studies concentrate on known or predicted components of **migration** pathways.Here we use data from a genome-wide RNAi morphology screen in Drosophila melanogaster cells together with bioinformatics to identify 26 new regulators of morphology and cytoskeletal organization in human cells. These include genes previously implicated in a wide range of functions, from mental retardation, Down syndrome and Huntington's disease to RNA and DNA-binding genes. We classify these genes into seven groups according to phenotype and identify those that affect cell **migration**. We further characterize a subset of seven genes, FAM40A, FAM40B, ARC, FMNL3, FNBP3/FBP11, LIMD1 and ZRANB1, each of which has a different effect on cell shape, actin filament distribution and cell **migration**. Interestingly, in several instances closely related isoforms with a single Drosophila homologue have distinct phenotypes. For example, FAM40B depletion induces cell elongation and tail retraction defects, whereas FAM40A depletion reduces cell spreading.Our results identify multiple regulators of cell **migration** and cytoskeletal signalling that are highly conserved between Drosophila and humans, and show that closely related paralogues can have very different functions in these processes.

---

## CG30108

- NCBI Gene
- GeneRIF
- Pubmed

| Search Term | Rank | Fields |
| --- | --- | --- |
| [Mm]igration | 6 | PubMed(1) |
| ECM | 7 | PubMed(1) |
| [Ee]xtracellular [Mm]atrix | 8 | PubMed(1) |

  
hide/show details for CG30108

### [Mm]igration

#### PubMed

1. Genes conserved in bilaterians but jointly lost with Myc during nematode evolution are enriched in cell proliferation and cell **migration** functions. Animals use a stereotypical set of developmental genes to build body architectures of varying sizes and organizational complexity. Some genes are critical to developmental patterning, while other genes are important to physiological control of growth. However, growth regulator genes may not be as important in small-bodied "micro-metazoans" such as nematodes. Nematodes use a simplified developmental strategy of lineage-based cell fate specifications to produce an adult bilaterian body composed of a few hundreds of cells. Nematodes also lost the MYC proto-oncogenic regulator of cell proliferation. To identify additional regulators of cell proliferation that were lost with MYC, we computationally screened and determined 839 high-confidence genes that are conserved in bilaterians/lost in nematodes (CIBLIN genes). We find that 30 % of all CIBLIN genes encode transcriptional regulators of cell proliferation, epithelial-to-mesenchyme transitions, and other processes. Over 50 % of CIBLIN genes are unnamed genes in Drosophila, suggesting that there are many understudied genes. Interestingly, CIBLIN genes include many Myc synthetic lethal (MycSL) hits from recent screens. CIBLIN genes include key regulators of heparan sulfate proteoglycan (HSPG) sulfation patterns, and lysyl oxidases involved in cross-linking and modification of the extracellular matrix (ECM). These genes and others suggest the CIBLIN repertoire services critical functions in ECM remodeling and cell **migration** in large-bodied bilaterians. Correspondingly, CIBLIN genes are co-expressed with Myc in cancer transcriptomes, and include a preponderance of known determinants of cancer progression and tumor aggression. We propose that CIBLIN gene research can improve our understanding of regulatory control of cellular growth in metazoans.

### ECM

#### PubMed

1. Genes conserved in bilaterians but jointly lost with Myc during nematode evolution are enriched in cell proliferation and cell migration functions. Animals use a stereotypical set of developmental genes to build body architectures of varying sizes and organizational complexity. Some genes are critical to developmental patterning, while other genes are important to physiological control of growth. However, growth regulator genes may not be as important in small-bodied "micro-metazoans" such as nematodes. Nematodes use a simplified developmental strategy of lineage-based cell fate specifications to produce an adult bilaterian body composed of a few hundreds of cells. Nematodes also lost the MYC proto-oncogenic regulator of cell proliferation. To identify additional regulators of cell proliferation that were lost with MYC, we computationally screened and determined 839 high-confidence genes that are conserved in bilaterians/lost in nematodes (CIBLIN genes). We find that 30 % of all CIBLIN genes encode transcriptional regulators of cell proliferation, epithelial-to-mesenchyme transitions, and other processes. Over 50 % of CIBLIN genes are unnamed genes in Drosophila, suggesting that there are many understudied genes. Interestingly, CIBLIN genes include many Myc synthetic lethal (MycSL) hits from recent screens. CIBLIN genes include key regulators of heparan sulfate proteoglycan (HSPG) sulfation patterns, and lysyl oxidases involved in cross-linking and modification of the extracellular matrix (**ECM**). These genes and others suggest the CIBLIN repertoire services critical functions in **ECM** remodeling and cell migration in large-bodied bilaterians. Correspondingly, CIBLIN genes are co-expressed with Myc in cancer transcriptomes, and include a preponderance of known determinants of cancer progression and tumor aggression. We propose that CIBLIN gene research can improve our understanding of regulatory control of cellular growth in metazoans.

### [Ee]xtracellular [Mm]atrix

#### PubMed

1. Genes conserved in bilaterians but jointly lost with Myc during nematode evolution are enriched in cell proliferation and cell migration functions. Animals use a stereotypical set of developmental genes to build body architectures of varying sizes and organizational complexity. Some genes are critical to developmental patterning, while other genes are important to physiological control of growth. However, growth regulator genes may not be as important in small-bodied "micro-metazoans" such as nematodes. Nematodes use a simplified developmental strategy of lineage-based cell fate specifications to produce an adult bilaterian body composed of a few hundreds of cells. Nematodes also lost the MYC proto-oncogenic regulator of cell proliferation. To identify additional regulators of cell proliferation that were lost with MYC, we computationally screened and determined 839 high-confidence genes that are conserved in bilaterians/lost in nematodes (CIBLIN genes). We find that 30 % of all CIBLIN genes encode transcriptional regulators of cell proliferation, epithelial-to-mesenchyme transitions, and other processes. Over 50 % of CIBLIN genes are unnamed genes in Drosophila, suggesting that there are many understudied genes. Interestingly, CIBLIN genes include many Myc synthetic lethal (MycSL) hits from recent screens. CIBLIN genes include key regulators of heparan sulfate proteoglycan (HSPG) sulfation patterns, and lysyl oxidases involved in cross-linking and modification of the **extracellular matrix** (ECM). These genes and others suggest the CIBLIN repertoire services critical functions in ECM remodeling and cell migration in large-bodied bilaterians. Correspondingly, CIBLIN genes are co-expressed with Myc in cancer transcriptomes, and include a preponderance of known determinants of cancer progression and tumor aggression. We propose that CIBLIN gene research can improve our understanding of regulatory control of cellular growth in metazoans.

---

## cwo

- NCBI Gene
- GeneRIF
- Pubmed

| Search Term | Rank | Fields |
| --- | --- | --- |
| [Ff]ilopodia | 1 | PubMed(1) |

  
hide/show details for cwo

### [Ff]ilopodia

#### PubMed

1. Gene regulatory networks controlling hematopoietic progenitor niche cell production and differentiation in the Drosophila lymph gland. Hematopoiesis occurs in two phases in Drosophila, with the first completed during embryogenesis and the second accomplished during larval development. The lymph gland serves as the venue for the final hematopoietic program, with this larval tissue well-studied as to its cellular organization and genetic regulation. While the medullary zone contains stem-like hematopoietic progenitors, the posterior signaling center (PSC) functions as a niche microenvironment essential for controlling the decision between progenitor maintenance versus cellular differentiation. In this report, we utilize a PSC-specific GAL4 driver and UAS-gene RNAi strains, to selectively knockdown individual gene functions in PSC cells. We assessed the effect of abrogating the function of 820 genes as to their requirement for niche cell production and differentiation. 100 genes were shown to be essential for normal niche development, with various loci placed into sub-groups based on the functions of their encoded protein products and known genetic interactions. For members of three of these groups, we characterized loss- and gain-of-function phenotypes. Gene function knockdown of members of the BAP chromatin-remodeling complex resulted in niche cells that do not express the hedgehog (hh) gene and fail to differentiate **filopodia** believed important for Hh signaling from the niche to progenitors. Abrogating gene function of various members of the insulin-like growth factor and TOR signaling pathways resulted in anomalous PSC cell production, leading to a defective niche organization. Further analysis of the Pten, TSC1, and TSC2 tumor suppressor genes demonstrated their loss-of-function condition resulted in severely altered blood cell homeostasis, including the abundant production of lamellocytes, specialized hemocytes involved in innate immune responses. Together, this cell-specific RNAi knockdown survey and mutant phenotype analyses identified multiple genes and their regulatory networks required for the normal organization and function of the hematopoietic progenitor niche within the lymph gland.

---

## oys

- NCBI Gene
- GeneRIF
- Pubmed

| Search Term | Rank | Fields |
| --- | --- | --- |
| [Mm]igration | 6 | Process(1) |

  
hide/show details for oys

### [Mm]igration

#### Process

1. germ cell **migration**

---

## pot

- NCBI Gene
- GeneRIF
- Pubmed

| Search Term | Rank | Fields |
| --- | --- | --- |
| ECM | 7 | PubMed(1) |
| [Ee]xtracellular [Mm]atrix | 8 | PubMed(1); GeneRIFs(1) |

  
hide/show details for pot

### ECM

#### PubMed

1. Papillote and Piopio: Drosophila ZP-domain proteins required for cell adhesion to the apical extracellular matrix and microtubule organization. Adhesion between epithelial cells and extracellular substrates is normally mediated through basal adhesion complexes. However, some cells also possess comparable junctions on their apical surface. Here, we describe two new Drosophila proteins, Piopio and Papillote, that are required for the link between the apical epithelial surface and the overlying apical extracellular matrix (a**ECM**). The two proteins share a zona pellucida (ZP) domain with mammalian a**ECM** components, including the tectorins found in the vertebrate inner ear. Tagged versions of both proteins localized to the apical epithelial surface. Mutations in piopio, papillote and dumpy (another gene encoding a ZP-domain protein) cause defects in the innermost layer of the a**ECM** and its detachment from the epidermis. Loss of Piopio, but not Papillote or Dumpy, causes the absence of specialized microtubule bundles from pupal wings, suggesting that Piopio plays a role in microtubule organization. Thus, ZP domain-containing proteins may have shared functions within the a**ECM**, while also exhibiting specific interactions with the cytoskeleton.

### [Ee]xtracellular [Mm]atrix

#### PubMed

1. Papillote and Piopio: Drosophila ZP-domain proteins required for cell adhesion to the apical **extracellular matrix** and microtubule organization. Adhesion between epithelial cells and extracellular substrates is normally mediated through basal adhesion complexes. However, some cells also possess comparable junctions on their apical surface. Here, we describe two new Drosophila proteins, Piopio and Papillote, that are required for the link between the apical epithelial surface and the overlying apical **extracellular matrix** (aECM). The two proteins share a zona pellucida (ZP) domain with mammalian aECM components, including the tectorins found in the vertebrate inner ear. Tagged versions of both proteins localized to the apical epithelial surface. Mutations in piopio, papillote and dumpy (another gene encoding a ZP-domain protein) cause defects in the innermost layer of the aECM and its detachment from the epidermis. Loss of Piopio, but not Papillote or Dumpy, causes the absence of specialized microtubule bundles from pupal wings, suggesting that Piopio plays a role in microtubule organization. Thus, ZP domain-containing proteins may have shared functions within the aECM, while also exhibiting specific interactions with the cytoskeleton.

#### GeneRIFs

1. Papillote protein is required for the link between the apical epithelial surface and the overlying apical **extracellular matrix**.

---

## pot

- NCBI Gene
- GeneRIF
- Pubmed

| Search Term | Rank | Fields |
| --- | --- | --- |
| [Mm]igration | 6 | PubMed(1) |
| ECM | 7 | PubMed(2) |
| [Ee]xtracellular [Mm]atrix | 8 | PubMed(2) |

  
hide/show details for pot

### [Mm]igration

#### PubMed

1. Wispy, the Drosophila homolog of GLD-2, is required during oogenesis and egg activation. Egg activation is the process that modifies mature, arrested oocytes so that embryo development can proceed. One key aspect of egg activation is the cytoplasmic polyadenylation of certain maternal mRNAs to permit or enhance their translation. wispy (wisp) maternal-effect mutations in Drosophila block development during the egg-to-embryo transition. We show here that the wisp gene encodes a member of the GLD-2 family of cytoplasmic poly(A) polymerases (PAPs). The WISP protein is required for poly(A) tail elongation of bicoid, Toll, and torso mRNAs upon egg activation. In Drosophila, WISP and Smaug (SMG) have previously been reported to be required to trigger the destabilization of maternal mRNAs during egg activation. SMG is the major regulator of this activity. We report here that SMG is still translated in activated eggs from wisp mutant mothers, indicating that WISP does not regulate mRNA stability by controlling the translation of smg mRNA. We have also analyzed in detail the very early developmental arrest associated with wisp mutations. Pronuclear **migration** does not occur in activated eggs laid by wisp mutant females. Finally, we find that WISP function is also needed during oogenesis to regulate the poly(A) tail length of dmos during oocyte maturation and to maintain a high level of active (phospho-) mitogen-activated protein kinases (MAPKs).

### ECM

#### PubMed

1. Papillote and Piopio: Drosophila ZP-domain proteins required for cell adhesion to the apical extracellular matrix and microtubule organization. Adhesion between epithelial cells and extracellular substrates is normally mediated through basal adhesion complexes. However, some cells also possess comparable junctions on their apical surface. Here, we describe two new Drosophila proteins, Piopio and Papillote, that are required for the link between the apical epithelial surface and the overlying apical extracellular matrix (a**ECM**). The two proteins share a zona pellucida (ZP) domain with mammalian a**ECM** components, including the tectorins found in the vertebrate inner ear. Tagged versions of both proteins localized to the apical epithelial surface. Mutations in piopio, papillote and dumpy (another gene encoding a ZP-domain protein) cause defects in the innermost layer of the a**ECM** and its detachment from the epidermis. Loss of Piopio, but not Papillote or Dumpy, causes the absence of specialized microtubule bundles from pupal wings, suggesting that Piopio plays a role in microtubule organization. Thus, ZP domain-containing proteins may have shared functions within the a**ECM**, while also exhibiting specific interactions with the cytoskeleton.
2. The transcription factor Grainy head and the steroid hormone ecdysone cooperate during differentiation of the skin of Drosophila melanogaster. The arthropod epidermis is an epithelium that deposits the apical cuticle, which is a stratified extracellular matrix (**ECM**) protecting the animal against pathogens, preventing dehydration and also serving as an exoskeleton. Differentiation of the cuticle conceivably implies coordinated production, secretion and localization of its components. The underlying molecular mechanisms are poorly explored. In this work, we show that the transcription factor Grainy head and the steroid hormone ecdysone drive the production of two partially overlapping sets of cuticle factors. Nevertheless, Grainy head is needed to modulate the expression of ecdysone signalling factors; the significance of this cross-talk is yet unclear. In addition, we found that ecdysone signalling negatively regulates its own impact. In conclusion, our findings suggest that at least two independently triggered pathways have evolved in parallel to cooperatively ensure the stereotypic implementation of the cuticle. As Grainy head is also essential for epithelial differentiation in vertebrates, we speculate that it acts to decode the ancient skin programme common to all animals. Full differentiation of the skin necessitates a second, complementing taxon-specific programme that requires its own decoder, which is represented by ecdysone in arthropods, whereas the vertebrate specific one remains to be identified.

### [Ee]xtracellular [Mm]atrix

#### PubMed

1. Papillote and Piopio: Drosophila ZP-domain proteins required for cell adhesion to the apical **extracellular matrix** and microtubule organization. Adhesion between epithelial cells and extracellular substrates is normally mediated through basal adhesion complexes. However, some cells also possess comparable junctions on their apical surface. Here, we describe two new Drosophila proteins, Piopio and Papillote, that are required for the link between the apical epithelial surface and the overlying apical **extracellular matrix** (aECM). The two proteins share a zona pellucida (ZP) domain with mammalian aECM components, including the tectorins found in the vertebrate inner ear. Tagged versions of both proteins localized to the apical epithelial surface. Mutations in piopio, papillote and dumpy (another gene encoding a ZP-domain protein) cause defects in the innermost layer of the aECM and its detachment from the epidermis. Loss of Piopio, but not Papillote or Dumpy, causes the absence of specialized microtubule bundles from pupal wings, suggesting that Piopio plays a role in microtubule organization. Thus, ZP domain-containing proteins may have shared functions within the aECM, while also exhibiting specific interactions with the cytoskeleton.
2. The transcription factor Grainy head and the steroid hormone ecdysone cooperate during differentiation of the skin of Drosophila melanogaster. The arthropod epidermis is an epithelium that deposits the apical cuticle, which is a stratified **extracellular matrix** (ECM) protecting the animal against pathogens, preventing dehydration and also serving as an exoskeleton. Differentiation of the cuticle conceivably implies coordinated production, secretion and localization of its components. The underlying molecular mechanisms are poorly explored. In this work, we show that the transcription factor Grainy head and the steroid hormone ecdysone drive the production of two partially overlapping sets of cuticle factors. Nevertheless, Grainy head is needed to modulate the expression of ecdysone signalling factors; the significance of this cross-talk is yet unclear. In addition, we found that ecdysone signalling negatively regulates its own impact. In conclusion, our findings suggest that at least two independently triggered pathways have evolved in parallel to cooperatively ensure the stereotypic implementation of the cuticle. As Grainy head is also essential for epithelial differentiation in vertebrates, we speculate that it acts to decode the ancient skin programme common to all animals. Full differentiation of the skin necessitates a second, complementing taxon-specific programme that requires its own decoder, which is represented by ecdysone in arthropods, whereas the vertebrate specific one remains to be identified.

---

## edl

- NCBI Gene
- GeneRIF
- Pubmed

| Search Term | Rank | Fields |
| --- | --- | --- |
| [Mm]igration | 6 | PubMed(2) |
| ECM | 7 | PubMed(1) |
| [Ee]xtracellular [Mm]atrix | 8 | PubMed(1) |

  
hide/show details for edl

### [Mm]igration

#### PubMed

1. FGF coordinates air sac development by activation of the EGF ligand Vein through the transcription factor PntP2. How several signaling pathways are coordinated to generate complex organs through regulation of tissue growth and patterning is a fundamental question in developmental biology. The larval trachea of Drosophila is composed of differentiated functional cells and groups of imaginal tracheoblasts that build the adult trachea during metamorphosis. Air sac primordium cells (ASP) are tracheal imaginal cells that form the dorsal air sacs that supply oxygen to the flight muscles of the Drosophila adult. The ASP emerges from the tracheal branch that connects to the wing disc by the activation of both Bnl-FGF/Btl and EGFR signaling pathways. Together, these pathways promote cell **migration** and proliferation. In this study we demonstrate that Vein (vn) is the EGF ligand responsible for the activation of the EGFR pathway in the ASP. We also find that the Bnl-FGF/Btl pathway regulates the expression of vn through the transcription factor PointedP2 (PntP2). Furthermore, we show that the FGF target gene escargot (esg) attenuates EGFR signaling at the tip cells of the developing ASP, reducing their mitotic rate to allow proper **migration**. Altogether, our results reveal a link between Bnl-FGF/Btl and EGFR signaling and provide novel insight into how the crosstalk of these pathways regulates **migration** and growth.
2. Cad74A is regulated by BR and is required for robust dorsal appendage formation in Drosophila oogenesis. Drosophila egg development is an established model for studying epithelial patterning and morphogenesis, but the connection between signaling pathways and egg morphology is still incompletely understood. We have identified a non-classical cadherin, Cad74A, as a putative adhesion gene that bridges epithelial patterning and morphogenesis in the follicle cells. Starting in mid-oogenesis, Cad74A is expressed in the follicle cells that contact the oocyte, including the border cells and most of the columnar follicle cells. However, Cad74A is repressed in two dorsolateral patches of follicle cells, which participate in the formation of tubular respiratory appendages. We show genetically that Cad74A is downstream of the EGFR and BMP signaling pathways and is repressed by the Zn-finger transcription factor Broad. The correlation of Cad74A repression in the cells that bend out of the plane of the follicular epithelium is preserved across Drosophila species and mutant backgrounds exhibiting a range of eggshell phenotypes. Complete removal of Cad74A from the follicle cells causes defects in dorsal appendage formation. Ectopic expression of Cad74A in the roof cells results in shortened, flattened appendages due to the hindered **migration** of the roof cells. Based on these results, we propose that Cad74A is part of the adhesive machinery that enables robust dorsal appendage formation, and as such provides a link between the patterning of the follicle cells and eggshell morphogenesis.

### ECM

#### PubMed

1. Conditional switches for extracellular matrix patterning in Drosophila melanogaster. An F(1) mutagenesis strategy was developed to identify conditional mutations affecting extracellular matrix (**ECM**) patterning. Tubulogenesis requires coordinated movement of epithelial cells and deposition of a multilayered **ECM**. In the Drosophila ovary, an epithelium of follicle cells creates the eggshells, including the paired tubular dorsal appendages (DAs) that act as breathing tubes for the embryo. A P-element mutagenesis strategy allowed for conditional overexpression of hundreds of genes in follicle cells. Conditional phenotypes were scored at the level of individual mutant (F(1)) female flies. **ECM** pattern regulators were readily identified including MAPK signaling gene ets domain lacking (fused DAs), Wnt pathway genes frizzled 3 and osa (long DAs), Hh pathway gene debra (branched DAs), and transcription factor genes sima/HIF-1alpha, ush, lilli, Tfb1, broad, and foxo. In moving cells the [Ca(2+)]/calcineurin pathway can regulate adhesion to **ECM** while adherens junctions link cells together. Accordingly, thin eggshell and DA phenotypes were identified for the calcineurin regulator calreticulin and the adherens junction component arc. Finally a tubulogenesis defect phenotype was identified for the gene pterodactyl, homologous to the mammalian serine/threonine receptor-associated protein (STRAP) that integrates the TGF-beta and PI3K/AKT signaling pathways. Because phenotypes can be scored in each mutant fly before and after gene induction, this F(1) conditional mutagenesis strategy should allow for increased scale in screens for mutations affecting repeated (reiterated) events in adult animals, including gametogenesis, movement, behavior, and learning.

### [Ee]xtracellular [Mm]atrix

#### PubMed

1. Conditional switches for **extracellular matrix** patterning in Drosophila melanogaster. An F(1) mutagenesis strategy was developed to identify conditional mutations affecting **extracellular matrix** (ECM) patterning. Tubulogenesis requires coordinated movement of epithelial cells and deposition of a multilayered ECM. In the Drosophila ovary, an epithelium of follicle cells creates the eggshells, including the paired tubular dorsal appendages (DAs) that act as breathing tubes for the embryo. A P-element mutagenesis strategy allowed for conditional overexpression of hundreds of genes in follicle cells. Conditional phenotypes were scored at the level of individual mutant (F(1)) female flies. ECM pattern regulators were readily identified including MAPK signaling gene ets domain lacking (fused DAs), Wnt pathway genes frizzled 3 and osa (long DAs), Hh pathway gene debra (branched DAs), and transcription factor genes sima/HIF-1alpha, ush, lilli, Tfb1, broad, and foxo. In moving cells the [Ca(2+)]/calcineurin pathway can regulate adhesion to ECM while adherens junctions link cells together. Accordingly, thin eggshell and DA phenotypes were identified for the calcineurin regulator calreticulin and the adherens junction component arc. Finally a tubulogenesis defect phenotype was identified for the gene pterodactyl, homologous to the mammalian serine/threonine receptor-associated protein (STRAP) that integrates the TGF-beta and PI3K/AKT signaling pathways. Because phenotypes can be scored in each mutant fly before and after gene induction, this F(1) conditional mutagenesis strategy should allow for increased scale in screens for mutations affecting repeated (reiterated) events in adult animals, including gametogenesis, movement, behavior, and learning.

---

## sqh

- NCBI Gene
- GeneRIF
- Pubmed

| Search Term | Rank | Fields |
| --- | --- | --- |
| [Ff]ilopodia | 1 | PubMed(6) |
| [Mm]igration | 6 | Process(1); PubMed(32) |
| ECM | 7 | PubMed(3) |
| [Ee]xtracellular [Mm]atrix | 8 | PubMed(7) |
| [Ii]nvasive | 10 | PubMed(9) |
| [Mm]etastasis | 12 | PubMed(5) |
| [Cc]ell [Ii]nvasion | 13 | PubMed(1) |

  
hide/show details for sqh

### [Ff]ilopodia

#### PubMed

1. Quantitative analysis of cytoskeletal reorganization during epithelial tissue sealing by large-volume electron tomography. The closure of epidermal openings is an essential biological process that causes major developmental problems such as spina bifida in humans if it goes awry. At present, the mechanism of closure remains elusive. Therefore, we reconstructed a model closure event, dorsal closure in fly embryos, by large-volume correlative electron tomography. We present a comprehensive, quantitative analysis of the cytoskeletal reorganization, enabling separated epidermal cells to seal the epithelium. After establishing contact through actin-driven exploratory **filopodia**, cells use a single lamella to generate 'roof tile'-like overlaps. These shorten to produce the force, 'zipping' the tissue closed. The shortening overlaps lack detectable actin filament ensembles but are crowded with microtubules. Cortical accumulation of shrinking microtubule ends suggests a force generation mechanism in which cortical motors pull on microtubule ends as for mitotic spindle positioning. In addition, microtubules orient **filopodia** and lamellae before zipping. Our 4D electron microscopy picture describes an entire developmental process and provides fundamental insight into epidermal closure.
2. Wound healing recapitulates morphogenesis in Drosophila embryos. The capacity to repair a wound is a fundamental survival mechanism that is activated at any site of damage throughout embryonic and adult life. To study the cell biology and genetics of this process, we have developed a wounding model in Drosophila melanogaster embryos that allows live imaging of rearrangements and changes in cell shape, and of the cytoskeletal machinery that draws closed an in vivo wound. Using embryos expressing green fluorescent protein (GFP) fusion proteins, we show that two cytoskeletal-dependent elements -- an actin cable and dynamic **filopodia**l/lamellipodial protrusions -- are expressed by epithelial cells at the wound edge and are pivotal for repair. Modulating the activities of the small GTPases Rho and Cdc42 demonstrates that these actin-dependent elements have differing cellular functions, but that either alone can drive wound closure. The actin cable operates as a 'purse-string' to draw the hole closed, whereas **filopodia** are essential for the final 'knitting' together of epithelial cells at the end of repair. Our data suggest a more complex model for epithelial repair than previously envisaged and highlight remarkable similarities with the well-characterized morphogenetic movement of dorsal closure in Drosophila.
3. Distinct tissue distributions and subcellular localizations of differently phosphorylated forms of the myosin regulatory light chain in Drosophila. Nonmuscle myosin II (myosin hereafter) has well-established roles in generating contractile force on actin filaments during morphogenetic processes in all metazoans. Myosin activation is regulated by phosphorylation of the myosin regulatory light chain (MRLC, encoded by spaghettisquash or sqh in Drosophila) first on Ser21 and subsequently on Thr20. These phosphorylation events are positively controlled by a variety of kinases including myosin light chain kinase, Rho kinase, citron kinase, and AMP kinase and are negatively regulated by myosin phosphatase. The activation of myosin is thus highly regulated and likely developmentally controlled. In order to monitor the activity of myosin during development, we have generated antibodies against the monophosphorylated (Sqh1P) and diphosphorylated (Sqh2P) forms of Sqh. We first show that the antibodies are highly specific. We then used these antibodies to monitor myosin activation in wild type Drosophila tissues. Interestingly, Sqh1P and Sqh2P show distinct patterns of expression in embryos. Sqh1P is expressed nearly ubiquitously and outlines cells consistent with a junctional localization, whereas Sqh2P is strongly expressed on the apical surfaces and in **filopodia** of tissues undergoing extensive cell shape change or cell movements including the invaginating fore- and hindgut, the invaginating tracheal system, the dorsal pouch and the dorsal most row of epidermal (DME) cells during dorsal closure. In imaginal discs, Sqh1P predominantly localizes in the adherens junction, whereas Sqh2P locates to the apical domain. These antibodies thus have the potential to be very useful in monitoring myosin activation for functional studies of morphogenesis in Drosophila.
4. Recapitulation of morphogenetic cell shape changes enables wound re-epithelialisation. Wound repair is a fundamental, conserved mechanism for maintaining tissue homeostasis and shares many parallels with embryonic morphogenesis. Small wounds in simple epithelia rapidly assemble a contractile actomyosin cable at their leading edge, as well as dynamic **filopodia** that finally knit the wound edges together. Most studies of wound re-epithelialisation have focused on the actin machineries that assemble in the leading edge of front row cells and that resemble the contractile mechanisms that drive morphogenetic episodes, including Drosophila dorsal closure, but, clearly, multiple cell rows back must also contribute for efficient repair of the wound. Here, we examine the role of cells back from the wound edge and show that they also stretch towards the wound and cells anterior-posterior to the wound edge rearrange their junctions with neighbours to drive cell intercalation events. This process in anterior-posterior cells is active and dependent on pulses of actomyosin that lead to ratcheted shrinkage of junctions; the actomyosin pulses are targeted to breaks in the cell polarity protein Par3 at cell vertices. Inhibiting actomyosin dynamics back from the leading edge prevents junction shrinkage and inhibits the wound edge from advancing. These events recapitulate cell rearrangements that occur during germband extension, in which intercalation events drive the elongation of tissues.
5. Drosophila growth cones: a genetically tractable platform for the analysis of axonal growth dynamics. The formation of neuronal networks, during development and regeneration, requires outgrowth of axons along reproducible paths toward their appropriate postsynaptic target cells. Axonal extension occurs at growth cones (GCs) at the tips of axons. GC advance and navigation requires the activity of their cytoskeletal networks, comprising filamentous actin (F-actin) in lamellipodia and **filopodia** as well as dynamic microtubules (MTs) emanating from bundles of the axonal core. The molecular mechanisms governing these two cytoskeletal networks, their cross-talk, and their response to extracellular signaling cues are only partially understood, hindering our conceptual understanding of how regulated changes in GC behavior are controlled. Here, we introduce Drosophila GCs as a suitable model to address these mechanisms. Morphological and cytoskeletal readouts of Drosophila GCs are similar to those of other models, including mammals, as demonstrated here for MT and F-actin dynamics, axonal growth rates, **filopodia**l structure and motility, organizational principles of MT networks, and subcellular marker localization. Therefore, we expect fundamental insights gained in Drosophila to be translatable into vertebrate biology. The advantage of the Drosophila model over others is its enormous amenability to combinatorial genetics as a powerful strategy to address the complexity of regulatory networks governing axonal growth. Thus, using pharmacological and genetic manipulations, we demonstrate a role of the actin cytoskeleton in a specific form of MT organization (loop formation), known to regulate GC pausing behavior. We demonstrate these events to be mediated by the actin-MT linking factor Short stop, thus identifying an essential molecular player in this context.
6. F-actin-based extensions of the head cyst cell adhere to the maturing spermatids to maintain them in a tight bundle and prevent their premature release in Drosophila testis. In Drosophila, all the 64 clonally derived spermatocytes differentiate in syncytium inside two somatic-origin cyst cells. They elongate to form slender spermatids, which are individualized and then released into the seminal vesicle. During individualization, differentiating spermatids are organized in a tight bundle inside the cyst, which is expected to play an important role in sperm selection. However, actual significance of this process and its underlying mechanism are unclear.We show that dynamic F-actin-based processes extend from the head cyst cell at the start of individualization, filling the interstitial space at the rostral ends of the maturing spermatid bundle. In addition to actin, these structures contained lamin, beta-catenin, dynamin, myosin VI and several other **filopodia**l components. Further, pharmacological and genetic analyses showed that cytoskeletal stability and dynamin function are essential for their maintenance. Disruption of these F-actin based processes was associated with spermatid bundle disassembly and premature sperm release inside the testis.Altogether, our data suggests that the head cyst cell adheres to the maturing spermatid heads through F-actin-based extensions, thus maintaining them in a tight bundle. This is likely to regulate mature sperm release into the seminal vesicle. Overall, this process bears resemblance to mammalian spermiation.

### [Mm]igration

#### Process

1. border follicle cell **migration**

#### PubMed

1. Distinguishing **migration** from isolation using genes with intragenic recombination: detecting introgression in the Drosophila simulans species complex. Determining the presence or absence of gene flow between populations is the target of some statistical methods in population genetics. Until recently, these methods either avoided the use of recombining genes, or treated recombination as a nuisance parameter. However, genes with recombination contribute additional information for the detection of gene flow (i.e. through linkage disequilibrium).We present three summary statistics based on the spatial arrangement of fixed differences, and shared and exclusive polymorphisms that are sensitive to the presence and direction of gene flow. Power and false positive rate for tests based on these statistics are studied by simulation.The application of these tests to populations from the Drosophila simulans species complex yielded results consistent with **migration** between D. simulans and its two endemic sister species D. mauritiana and D. sechellia, and between populations D. mauritiana on the islands of the Mauritius and Rodrigues.We demonstrate the sensitivity of the developed statistics to the presence and direction of gene flow, and characterize their power as a function of differentiation level and recombination rate. The properties of these statistics make them especially suitable for analyzing high-throughput sequencing data or for their integration within the approximate Bayesian computation framework.
2. A genetic screen for suppressors and enhancers of the Drosophila cdk1-cyclin B identifies maternal factors that regulate microtubule and microfilament stability. Coordination between cell-cycle progression and cytoskeletal dynamics is important for faithful transmission of genetic information. In early Drosophila embryos, increasing maternal cyclin B leads to higher Cdk1-CycB activity, shorter microtubules, and slower nuclear movement during cycles 5-7 and delays in nuclear **migration** to the cortex at cycle 10. Later during cycle 14 interphase of six cycB embryos, we observed patches of mitotic nuclei, chromosome bridges, abnormal nuclear distribution, and small and large nuclei. These phenotypes indicate disrupted coordination between the cell-cycle machinery and cytoskeletal function. Using these sensitized phenotypes, we performed a dosage-sensitive genetic screen to identify maternal proteins involved in this process. We identified 10 suppressors classified into three groups: (1) gene products regulating Cdk1 activities, cdk1 and cyclin A; (2) gene products interacting with both microtubules and microfilaments, Actin-related protein 87C; and (3) gene products interacting with microfilaments, chickadee, diaphanous, Cdc42, quail, spaghetti-squash, zipper, and scrambled. Interestingly, most of the suppressors that rescue the astral microtubule phenotype also reduce Cdk1-CycB activities and are microfilament-related genes. This suggests that the major mechanism of suppression relies on the interactions among Cdk1-CycB, microtubule, and microfilament networks. Our results indicate that the balance among these different components is vital for normal early cell cycles and for embryonic development. Our observations also indicate that microtubules and cortical microfilaments antagonize each other during the preblastoderm stage.
3. A Rho GTPase signaling pathway is used reiteratively in epithelial folding and potentially selects the outcome of Rho activation. A single Rho GTPase family member is capable of initiating several different processes, including cell cycle regulation, cytokinesis, cell **migration**, and transcriptional regulation . It is not clear, however, how the Rho protein selects which of these processes to initiate. Guanine nucleotide exchange factors (GEFs), proteins that activate Rho GTPases, could be important in making this selection. We show here that in vivo, DRhoGEF2, a GEF that is ubiquitously expressed and specific for Rho1, is reiteratively required for epithelial folding and invagination, but not for other processes regulated by Rho. The limitation of DRhoGEF2 function supports the hypothesis that the GEF selects the outcome of Rho activation. DRhoGEF2 exerts its effects in gastrulation through the regulation of Myosin II to orchestrate coordinated apical cell constriction. Apical myosin localization is also regulated by Concertina (Cta), a Galpha(12/13) family member that is thought to activate DRhoGEF2 and is itself activated by a putative ligand, Folded gastrulation (Fog). Fog and Cta also play a role in the morphogenetic events requiring DRhoGEF2, suggesting the existence of a conserved signaling pathway in which Fog, Cta, and DRhoGEF2 locally activate Myosin for epithelial invagination and folding.
4. Functional genomic analysis of the periodic transcriptome in the developing Drosophila wing. The eukaryotic cell cycle, driven by both transcriptional and posttranslational mechanisms, is the central molecular oscillator underlying tissue growth throughout animals. Although genome-wide studies have investigated cell-cycle-associated transcription in unicellular systems, global patterns of periodic transcription in multicellular tissues remain largely unexplored. Here we define the cell-cycle-associated transcriptome of the developing Drosophila wing epithelium and compare it with that of cultured Drosophila S2 cells, revealing a core set of periodic genes and a surprising degree of context specificity in periodic transcription. We further employ RNAi-mediated phenotypic profiling to define functional requirements for more than 300 periodic genes, with a focus on those required for cell proliferation in vivo. Finally, we investigate uncharacterized genes required for interkinetic nuclear **migration**. Combined, these findings provide a global perspective on cell-cycle control in vivo, and they highlight a critical need to understand the context-specific regulation of cell proliferation.
5. Rho GTPase controls invagination and cohesive **migration** of the Drosophila salivary gland through Crumbs and Rho-kinase. Coordinated cell movements shape simple epithelia into functional tissues and organs during embryogenesis. Regulators and effectors of the small GTPase Rho have been shown to be essential for epithelial morphogenesis in cell culture; however, the mechanism by which Rho GTPase and its downstream effectors control coordinated movement of epithelia in a developing tissue or organ is largely unknown. Here, we show that Rho1 GTPase activity is required for the invagination of Drosophila embryonic salivary gland epithelia and for directed **migration** of the internalized gland. We demonstrate that the absence of zygotic function of Rho1 results in the selective loss of the apical proteins, Crumbs (Crb), Drosophila atypical PKC and Stardust during gland invagination and that this is partially due to reduced crb RNA levels and apical localization. In parallel to regulation of crb RNA and protein, Rho1 activity also signals through Rho-kinase (Rok) to induce apical constriction and cell shape change during invagination. After invagination, Rho-Rok signaling is required again for the coordinated contraction and dorsal **migration** of the proximal half of the gland. We also show that Rho1 activity is required for proper development of the circular visceral mesoderm upon which the gland migrates. Our genetic and live-imaging analyses provide novel evidence that the proximal gland cells play an essential and active role in salivary gland **migration** that propels the entire gland to turn and migrate posteriorly.
6. Sec61alpha is required for dorsal closure during Drosophila embryogenesis through its regulation of Dpp signaling. During dorsal closure in Drosophila, signaling events in the dorsalmost row of epidermal cells (DME cells) direct the **migration** of lateral epidermal sheets towards the dorsal midline where they fuse to enclose the embryo. A Jun amino-terminal kinase (JNK) cascade in the DME cells induces the expression of Decapentaplegic (Dpp). Dpp signaling then regulates the cytoskeleton in the DME cells and amnioserosa to affect the cell shape changes necessary to complete dorsal closure. We identified a mutation in Sec61alpha that specifically perturbs dorsal closure. Sec61alpha encodes the main subunit of the translocon complex for co-translational import of proteins into the ER. JNK signaling is normal in Sec61alpha mutant embryos, but Dpp signaling is attenuated and the DME cells fail to maintain an actinomyosin cable as epithelial **migration** fails. Consistent with this model, dorsal closure is rescued in Sec61alpha mutant embryos by an activated form of the Dpp receptor Thick veins.
7. Integration of epithelial patterning and morphogenesis in Drosophila ovarian follicle cells. Drosophila oogenesis involves the coordinated development of germ cells and an overlying follicular epithelium. The follicle cells provide a genetically tractable system to investigate the cell biology of patterning and morphogenesis. Follicle cells initially form a cuboidal epithelium surrounding a syncytium of nurse cells and oocyte. Epithelial structure is maintained as these cells reorganize to create the three dimensional architecture of the eggshell. Both long-range and short-range cell-cell communications pattern the domains of follicle cells that will create specific eggshell structures. After terminal differentiation to deposit the eggshell proteins, the follicle cells die. This review summarizes recent progress in understanding the cell-cell communication that orchestrates follicle cell patterning and **migration**s. DE-cadherin-mediated adhesion is important at several steps in egg chamber formation and follicle cell **migration**. Notch signaling is critical during each successive round of patterning and **migration**. Integration of bone morphogenetic protein (BMP) and epidermal growth factor (EGF) signals patterns the elaborate structures of the dorsal-anterior eggshell.
8. Diaphanous regulates myosin and adherens junctions to control cell contractility and protrusive behavior during morphogenesis. Formins are key regulators of actin nucleation and elongation. Diaphanous-related formins, the best-known subclass, are activated by Rho and play essential roles in cytokinesis. In cultured cells, Diaphanous-related formins also regulate cell adhesion, polarity and microtubules, suggesting that they may be key regulators of cell shape change and **migration** during development. However, their essential roles in cytokinesis hamper our ability to test this hypothesis. We used loss- and gain-of-function approaches to examine the role of Diaphanous in Drosophila morphogenesis. We found that Diaphanous has a dynamic expression pattern consistent with a role in regulating cell shape change. We used constitutively active Diaphanous to examine its roles in morphogenesis and its mechanisms of action. This revealed an unexpected role in regulating myosin levels and activity at adherens junctions during cell shape change, suggesting that Diaphanous helps coordinate adhesion and contractility of the underlying actomyosin ring. We tested this hypothesis by reducing Diaphanous function, revealing striking roles in stabilizing adherens junctions and inhibiting cell protrusiveness. These effects also are mediated through coordinated effects on myosin activity and adhesion, suggesting a common mechanism for Diaphanous action during morphogenesis.
9. The role of the Rho GTPases in neuronal development. Our brain serves as a center for cognitive function and neurons within the brain relay and store information about our surroundings and experiences. Modulation of this complex neuronal circuitry allows us to process that information and respond appropriately. Proper development of neurons is therefore vital to the mental health of an individual, and perturbations in their signaling or morphology are likely to result in cognitive impairment. The development of a neuron requires a series of steps that begins with **migration** from its birth place and initiation of process outgrowth, and ultimately leads to differentiation and the formation of connections that allow it to communicate with appropriate targets. Over the past several years, it has become clear that the Rho family of GTPases and related molecules play an important role in various aspects of neuronal development, including neurite outgrowth and differentiation, axon pathfinding, and dendritic spine formation and maintenance. Given the importance of these molecules in these processes, it is therefore not surprising that mutations in genes encoding a number of regulators and effectors of the Rho GTPases have been associated with human neurological diseases. This review will focus on the role of the Rho GTPases and their associated signaling molecules throughout neuronal development and discuss how perturbations in Rho GTPase signaling may lead to cognitive disorders.
10. Cellular and molecular mechanisms of single and collective cell **migration**s in Drosophila: themes and variations. The process of cell **migration** is essential throughout life, driving embryonic morphogenesis and ensuring homeostasis in adults. Defects in cell **migration** are a major cause of human disease, with excessive **migration** causing autoimmune diseases and cancer metastasis, whereas reduced capacity for **migration** leads to birth defects and immunodeficiencies. Myriad studies in vitro have established a consensus view that cell **migration**s require cell polarization, Rho GTPase-mediated cytoskeletal rearrangements, and myosin-mediated contractility. However, in vivo studies later revealed a more complex picture, including the discovery that cells migrate not only as single units but also as clusters, strands, and sheets. In particular, the role of E-Cadherin in cell motility appears to be more complex than previously appreciated. Here, we discuss recent advances achieved by combining the plethora of genetic tools available to the Drosophila geneticist with live imaging and biophysical techniques. Finally, we discuss the emerging themes such studies have revealed and ponder the puzzles that remain to be solved.
11. Systematic analysis of the transcriptional switch inducing **migration** of border cells. Cell **migration** within a natural context is tightly controlled, often by specific transcription factors. However, the switch from stationary to migratory behavior is poorly understood. Border cells perform a spatially and temporally controlled invasive **migration** during Drosophila oogenesis. Slbo, a C/EBP family transcriptional activator, is required for them to become migratory. We purified wild-type and slbo mutant border cells as well as nonmigratory follicle cells and performed comparative whole-genome expression profiling, followed by functional tests of the contributions of identified targets to **migration**. About 300 genes were significantly upregulated in border cells, many dependent on Slbo. Among these, the microtubule regulator Stathmin was strongly upregulated and was required for normal **migration**. Actin cytoskeleton regulators were also induced, including, surprisingly, a large cluster of "muscle-specific" genes. We conclude that Slbo induces multiple cytoskeletal effectors, and that each contributes to the behavioral changes in border cells.
12. Invasive cell **migration** is initiated by guided growth of long cellular extensions. The **migration** of border cells during Drosophila melanogaster oogenesis is a simple and powerful system for studying invasive cell **migration** in vivo. Border cells are somatic cells that delaminate from the follicular epithelium of an egg chamber and invade the germ line cluster. They migrate between the nurse cells to reach the oocyte, using DE-cadherin for adhesion to the substratum. Border cells take approximately 6 h to migrate a distance of 100 microm. The **migration** is guided by EGFR (epidermal growth factor receptor) and PVR (platelet-derived growth factor (PDGF)/vascular endothelial growth factor (VEGF) receptor). Here, we show that a single long cellular extension (LCE), several cell diameters in length, is formed at the initiation of **migration**. The LCE may function as a 'pathfinder' in response to guidance cues. LCE growth requires directional guidance signals and specific adhesion to the substratum. Interference with actin-myosin interactions allows continued LCE growth while preventing translocation of the cell bodies. We discuss similarities between LCEs and axons and the use of LCE-like structures as a general mechanism for initiating invasive **migration** in vivo.
13. Left-right asymmetric morphogenesis of the anterior midgut depends on the activation of a non-muscle myosin II in Drosophila. Many animals exhibit stereotypical left-right (LR) asymmetry in their internal organs. The mechanisms of LR axis formation required for the subsequent LR asymmetric development are well understood, especially in some vertebrates. However, the molecular mechanisms underlying LR asymmetric morphogenesis, particularly how mechanical force is integrated into the LR asymmetric morphogenesis of organs, are poorly understood. Here, we identified zipper (zip), encoding a Drosophila non-muscle myosin II (myosin II) heavy chain, as a gene required for LR asymmetric development of the embryonic anterior midgut (AMG). Myosin II is known to directly generate mechanical force in various types of cells during morphogenesis and cell **migration**. We found that myosin II was involved in two events in the LR asymmetric development of the AMG. First, it introduced an LR bias to the directional position of circular visceral muscle (CVMU) cells, which externally cover the midgut epithelium. Second, it was required for the LR-biased rotation of the AMG. Our results suggest that myosin II in CVMU cells plays a crucial role in generating the force leading to LR asymmetric morphogenesis. Taken together with previous studies in vertebrates, the involvement of myosin II in LR asymmetric morphogenesis might be conserved evolutionarily.
14. Bunched sets a boundary for Notch signaling to pattern anterior eggshell structures during Drosophila oogenesis. Organized boundaries between different cell fates are critical in patterning and organogenesis. In some tissues, long-range signals position a boundary, and local Notch signaling maintains it. How Notch activity is restricted to boundary regions is not well understood. During Drosophila oogenesis, the long-range signals EGF and Dpp regulate expression of bunched (bun), which encodes a homolog of mammalian transcription factors TSC-22 and GILZ. Here, we show that bun establishes a boundary for Notch signaling in the follicle cell epithelium. Notch signaling is active in anterior follicle cells and is required for concurrent follicle cell reorganizations including centripetal **migration** and operculum formation. bun is required in posterior columnar follicle cells to repress the centripetal **migration** fate, including gene expression, cell shape changes and accumulation of cytoskeletal components. bun mutant clones adjacent to the centripetally migrating follicle cells showed ectopic Notch responses. bun is necessary, but not sufficient, to down-regulate Serrate protein levels throughout the follicular epithelium. These data indicate that Notch signaling is necessary, but not sufficient, for centripetal **migration** and that bun regulates the level of Notch stimulation to position the boundary between centripetally migrating and stationary columnar follicle cells.
15. The microcephaly protein Asp regulates neuroepithelium morphogenesis by controlling the spatial distribution of myosin II. Mutations in ASPM are the most frequent cause of microcephaly, a disorder characterized by reduced brain size at birth. ASPM is recognized as a major regulator of brain size, yet its role during neural development remains poorly understood. Moreover, the role of ASPM proteins in invertebrate brain morphogenesis has never been investigated. Here, we characterized the function of the Drosophila ASPM orthologue, Asp, and found that asp mutants present severe defects in brain size and neuroepithelium morphogenesis. We show that size reduction depends on the mitotic function of Asp, whereas regulation of tissue shape depends on an uncharacterized function. Asp interacts with myosin II regulating its polarized distribution along the apico-basal axis. In the absence of Asp, mislocalization of myosin II results in interkinetic nucl**ear migra**tion and tissue architecture defects. We propose that Asp regulates neuroepithelium morphogenesis through myosin-II-mediated structural and mechanical processes to maintain force balance and tissue cohesiveness.
16. Tip-cell **migration** controls stalk-cell intercalation during Drosophila tracheal tube elongation. Branching morphogenesis remodels epithelial tissues into tubular networks. This process is crucial to many organs, from the insect trachea to the vertebrate vasculature. Although Drosophila tracheal development has been well characterized morphologically and genetically, very little is known about the forces involved during morphogenesis. The repertoire of cell behaviors underlying tracheal primary branch remodeling is limited to cell **migration**, cell-shape changes, and stalk-cell intercalation (SCI), a process in which cells insert in between cells previously in contact with each other.Here, we identify the major forces that contribute to tracheal primary branch remodeling by using genetic and microsurgery experiments. As the tip cells migrate, they elongate the branches and create a tensile stress. This tensile stress triggers SCI, which, in turn, allows the branches to further elongate.The mechanism that we describe contrasts with "convergent extension by cell intercalation" acting during Drosophila germ band extension (GBE), where cell intercalation is the cause of epithelium elongation. Surprisingly, in tracheal branches, one or two leading cells produce enough mechanical power to intercalate many lagging cells. This may apply to other tubular networks.
17. Oscillation and polarity of E-cadherin asymmetries control actomyosin flow patterns during morphogenesis. Actomyosin flows are involved in a variety of cellular processes, including cytokinesis, cell **migration**, polarization, and morphogenesis. In epithelia, flow polarization orients cell deformations. It is unclear, however, how flows are polarized and how global patterns of junction remodeling emerge from flow polarization locally. We address this question during intercalation-driving extension of the Drosophila germband. Intercalation is associated with polarized junction remodeling, whereby actomyosin pulses flow anisotropically toward dorsal-ventral junctions and shrink them. Here, we show that planar polarization of flows emerges from polarized fluctuations in the levels of E-cadherin clusters that produce transient and oscillating asymmetries of coupling. These fluctuations are triggered by polarized E-cadherin endocytosis and are amplified by flow itself. This work suggests that fluctuations and mechanical instability are not the consequences of limited control over the systems key parameters, but rather that they define the axis of symmetry breaking.
18. The genetics of cell **migration** in Drosophila melanogaster and Caenorhabditis elegans development. Cell **migration**s are found throughout the animal kingdom and are among the most dramatic and complex of cellular behaviors. Historically, the mechanics of cell **migration** have been studied primarily in vitro, where cells can be readily viewed and manipulated. However, genetic approaches in relatively simple model organisms are yielding additional insights into the molecular mechanisms underlying cell movements and their regulation during development. This review will focus on these simple model systems where we understand some of the signaling and receptor molecules that stimulate and guide cell movements. The chemotactic guidance factor encoded by the Caenorhabditis elegans unc-6 locus, whose mammalian homolog is Netrin, is perhaps the best known of the cell **migration** guidance factors. In addition, receptor tyrosine kinases (RTKs), and FGF receptors in particular, have emerged as key mediators of cell **migration** in vivo, confirming the importance of molecules that were initially identified and studied in cell culture. Somewhat surprisingly, screens for mutations that affect primordial germ cell **migration** in Drosophila have revealed that enzymes involved in lipid metabolism play a role in guiding cell **migration** in vivo, possibly by producing and/or degrading lipid chemoattractants or chemorepellents. Cell adhesion molecules, such as integrins, have been extensively characterized with respect to their contribution to cell **migration** in vitro and genetic evidence now supports a role for these receptors in certain instances in vivo as well. The role for non-muscle myosin in cell motility was controversial, but has now been demonstrated genetically, at least in some cell types. Currently the best characterized link between membrane receptor signaling and regulation of the actin cytoskeleton is that provided by the Rho family of small GTPases. Members of this family are clearly essential for the **migration**s of some cells; however, key questions remain concerning how chemoattractant and chemorepellent signals are integrated within the cell and transduced to the cytoskeleton to produce directed cell **migration**. New types of genetic screens promise to fill in some of these gaps in the near future.
19. Command and control: regulatory pathways controlling invasive behavior of the border cells. The invasiveness of cancer cells resembles the normal behavior of cells that migrate into surrounding tissues during development. For example, the border cells in the Drosophila ovary undergo a partial epithelial to mesenchymal transition and invade the neighboring cluster of germline cells, migrating to the oocyte border. Once there, they provide patterning information to the oocyte and produce an eggshell specialization known as the micropyle. Border cell **migration** has been subjected to extensive genetic analyses using a variety of screening approaches. Recent findings demonstrate that conversion of the border cells from a stationary group of epithelial cells to invasive cells requires integration of the activities of at least two transcriptional regulatory pathways. One such pathway requires the slbo gene, which encodes Drosophila C/EBP, a basic region/leucine zipper transcriptional activator that is required for elevated expression of a number of downstream targets, including DE-cadherin and focal adhesion kinase (FAK). An independent pathway requires the activity of the ecdysone receptor and a recently identified co-activator for the ecdysone receptor known as Taiman (abbreviated TAI, pronounced ti-maan', meaning too slow). Ecdysone is produced in the Drosophila ovary in response to adequate nutrition and is required for progression of oogenesis through stage 9, when border cell **migration** occurs. Border cells mutant for tai accumulate abnormally high levels of adhesion complexes at their surfaces, which may account for their inability to migrate. Thus border cell **migration** requires a differentiation program mediated by the C/EBP pathway, which is required for elevated expression of a number of proteins required for motility. In addition, **migration** requires a hormonal signal that relays information regarding nutritional status and appears to be required for regulation of the proper localization of some of the C/EBP targets. These findings suggest that steroid hormones can regulate cell motility relatively directly, independent of the effects on proliferation. This may contribute to the metastatic effects of steroid hormones on certain cancers and the inhibition of metastasis by steroid hormone antagonists such as tamoxifen.
20. The role of apoptosis in shaping the tracheal system in the Drosophila embryo. The tubular network of the tracheal system in the Drosophila embryo is created from a set of epithelial placodes by cell **migration**, rearrangements, fusions and shape changes. A designated number of cells is initially allocated to each branch of the system. We show here that the final cell number in the dorsal branches is not only determined by early patterning events and subsequent cell rearrangements but also by elimination of cells from the developing branch. Extruded cells die and are engulfed by macrophages. Our results suggest that the pattern of cell extrusion and death is not hard-wired, but is determined by environmental cues.
21. Drosophila nonmuscle myosin II is required for rapid cytoplasmic transport during oogenesis and for axial nuclear **migration** in early embryos. The X-linked Drosophila gene spaghetti squash (sqh) encodes the regulatory light chain of nonmuscle myosin II. To assess the requirement for myosin II in oogenesis and early embryogenesis, we induced homozygous germline clones of the hypomorphic mutation sqh1 in otherwise heterozygous mothers. Developing oocytes in such sqh1 germline clones often failed to attain full size due to a defect in 'dumping', the rapid phase of cytoplasmic transport from nurse cells. In contrast to other dumpless mutants described to date, sqh1 egg chambers showed no evidence of ring canal obstruction, and no obvious alteration in the actin network. However the distribution of myosin II was abnormal. We conclude that the molecular motor responsible for cytoplasmic dumping is supplied largely, if not exclusively, by nurse cell myosin II and we suggest that regulation of myosin activity is one means by which cytoplasmic transport may be controlled during oocyte development. The eggs resulting from sqh1 clones, though smaller than normal, began development but exhibited an early defect in axial **migration** of cleavage nuclei towards the posterior pole of the embryo, in a similar manner to that seen in early cleavage eggs in which the actin cytoskeleton is disrupted. Thus both nurse cell dumping and axial **migration** require a maternally supplied myosin II.
22. Dynamic myosin activation promotes collective morphology and **migration** by locally balancing oppositional forces from surrounding tissue. Migrating cells need to overcome physical constraints from the local microenvironment to navigate their way through tissues. Cells that move collectively have the additional challenge of negotiating complex environments in vivo while maintaining cohesion of the group as a whole. The mechanisms by which collectives maintain a migratory morphology while resisting physical constraints from the surrounding tissue are poorly understood. Drosophila border cells represent a genetic model of collective **migration** within a cell-dense tissue. Border cells move as a cohesive group of 6-10 cells, traversing a network of large germ line-derived nurse cells within the ovary. Here we show that the border cell cluster is compact and round throughout their entire **migration**, a shape that is maintained despite the mechanical pressure imposed by the surrounding nurse cells. Nonmuscle myosin II (Myo-II) activity at the cluster periphery becomes elevated in response to increased constriction by nurse cells. Furthermore, the distinctive border cell collective morphology requires highly dynamic and localized enrichment of Myo-II. Thus, activated Myo-II promotes cortical tension at the outer edge of the migrating border cell cluster to resist compressive forces from nurse cells. We propose that dynamic actomyosin tension at the periphery of collectives facilitates their movement through restrictive tissues.
23. Drosophila PATJ supports adherens junction stability by modulating Myosin light chain activity. The assembly and consolidation of the adherens junctions (AJs) are key events in the establishment of an intact epithelium. However, AJs are further modified to obtain flexibility for cell **migration** and morphogenetic movements. Intact AJs in turn are a prerequisite for the establishment and maintenance of apical-basal polarity in epithelial cells. In this study, we report that the conserved PDZ (PSD95, Discs large, ZO-1) domain-containing protein PATJ (Pals1-associated tight junction protein) was not per se crucial for the maintenance of apical-basal polarity in Drosophila melanogaster epithelial cells but rather regulated Myosin localization and phosphorylation. PATJ directly bound to the Myosin-binding subunit of Myosin phosphatase and decreased Myosin dephosphorylation, resulting in activated Myosin. Thereby, PATJ supports the stability of the Zonula Adherens. Notably, weakening of AJ in a PATJ mutant epithelium led first to a loss of Myosin from the AJ, subsequently to a disassembly of the AJ, and finally, to a loss of apical-basal polarity and disruption of the tissue.
24. Cell rearrangements, cell divisions and cell death in a migrating epithelial sheet in the abdomen of Drosophila. During morphogenesis, cell movements, cell divisions and cell death work together to form complex patterns and to shape organs. These events are the outcome of decisions made by many individual cells, but how these decisions are controlled and coordinated is elusive. The adult abdominal epidermis of Drosophila is formed during metamorphosis by divisions and extensive cell **migration**s of the diploid histoblasts, which replace the polyploid larval cells. Using in vivo 4D microscopy, we have studied the behaviour of the histoblasts and analysed in detail how they reach their final position and to what extent they rearrange during their spreading. Tracking individual cells, we show that the cells migrate in two phases that differ in speed, direction and amount of cellular rearrangement. Cells of the anterior (A) and posterior (P) compartments differ in their behaviour. Cells near the A/P border are more likely to change their neighbours during **migration**. The mitoses do not show any preferential orientation. After mitosis, the sisters become preferentially aligned with the direction of movement. Thus, in the abdomen, it is the extensive cell **migration**s that appear to contribute most to morphogenesis. This contrasts with other developing epithelia, such as the wing imaginal disc and the embryonic germband in Drosophila, where oriented mitoses and local cell rearrangements appear to direct morphogenesis. Furthermore, our results suggest that an active force created by the histoblasts contributes to the formation of the adult epidermis. Finally, we show that histoblasts occasionally undergo apoptosis.
25. Essential roles of myosin phosphatase in the maintenance of epithelial cell integrity of Drosophila imaginal disc cells. Reorganization of the actin cytoskeleton and contraction of actomyosin play pivotal roles in controlling cell shape changes and motility in epithelial morphogenesis. Dephosphorylation of the myosin regulatory light chain (MRLC) by myosin phosphatase is one of the key events involved. Allelic combinations producing intermediate strength mutants of the Drosophila myosin-binding subunit (DMBS) of myosin phosphatase showed imaginal discs with multilayered disrupted morphologies, and extremely mislocated cells, suggesting that DMBS is required to maintain proper epithelial organization. Clonal analyses revealed that DMBS null mutant cells appear to retract basally and localization of apical junction markers such as DE-cadherin is indetectable in most cells, whereas phosphorylated MRLC and F-actin become heavily concentrated apically, indicating misconfiguration of the apical cytoskeleton. In agreement with these findings, DMBS was found to concentrate at the apical domain suggesting its function is localized. Phenotypes similar to DMBS mutants including increased **migration** of cells were obtained by overexpressing the constitutive active form of MRLC or Rho-associated kinase signifying that the phenotypes are indeed caused through activation of Myosin II. The requirement of DMBS for the integrity of static epithelial cells in imaginal discs suggests that the regulation of Myosin II by DMBS has a role more general than its previously demonstrated functions in morphogenetic events.
26. Par-1 controls myosin-II activity through myosin phosphatase to regulate border cell **migration**. Localized actomyosin contraction couples with actin polymerization and cell-matrix adhesion to regulate cell protrusions and retract trailing edges of migrating cells. Although many cells migrate in collective groups during tissue morphogenesis, mechanisms that coordinate actomyosin dynamics in collective cell **migration** are poorly understood. **Migration** of Drosophila border cells, a genetically tractable model for collective cell **migration**, requires nonmuscle myosin-II (Myo-II). How Myo-II specifically controls border cell **migration** and how Myo-II is itself regulated is largely unknown.We show that Myo-II regulates two essential features of border cell **migration**: (1) initial detachment of the border cell cluster from the follicular epithelium and (2) the dynamics of cellular protrusions. We further demonstrate that the cell polarity protein Par-1 (MARK), a serine-threonine kinase, regulates the localization and activation of Myo-II in border cells. Par-1 binds to myosin phosphatase and phosphorylates it at a known inactivating site. Par-1 thus promotes phosphorylated myosin regulatory light chain, thereby increasing Myo-II activity. Furthermore, Par-1 localizes to and increases active Myo-II at the cluster rear to promote detachment; in the absence of Par-1, spatially distinct active Myo-II is lost.We identify a critical new role for Par-1 kinase: spatiotemporal regulation of Myo-II activity within the border cell cluster through localized inhibition of myosin phosphatase. Polarity proteins such as Par-1, which intrinsically localize, can thus directly modulate the actomyosin dynamics required for border cell detachment and **migration**. Such a link between polarity proteins and cytoskeletal dynamics may also occur in other collective cell **migration**s.
27. Drosophila nonmuscle myosin II has multiple essential roles in imaginal disc and egg chamber morphogenesis. Morphogenesis is characterized by orchestrated changes in the shape and position of individual cells. Many of these movements are thought to be powered by motor proteins. However, in metazoans, it is often difficult to match specific motors with the movements they drive. The nonmuscle myosin II heavy chain (MHC encoded by zipper is required for cell sheet movements in Drosophila embryos. To determine if myosin II is required for other processes, we examined the phenotypes of strong and weak larval lethal mutations in spaghetti squash (sqh), which encodes the nonmuscle myosin II regulatory light chain (RLC). sqh mutants can be rescued to adulthood by daily induction of a sqh cDNA transgene driven by the hsp70 promoter. By transiently ceasing induction of the cDNA, we depleted RLC at specific times during development. When RLC is transiently depleted in larvae, the resulting adult phenotypes demonstrate that RLC is required in a stage-specific fashion for proper development of eye and leg imaginal discs. When RLC is depleted in adult females, oogenesis is reversibly disrupted. Without RLC induction, developing egg chambers display a succession of phenotypes that demonstrate roles for myosin II in morphogenesis of the interfollicular stalks, three morphologically and mechanistically distinct types of follicle cell **migration**, and completion of nurse cell cytoplasm transport (dumping). Finally, we show that in sqh mutant tissues, MHC is abnormally localized in punctate structures that do not contain appreciable amounts of filamentous actin or the myosin tail-binding protein p127. This suggests that sqh mutant phenotypes are chiefly caused by sequestration of myosin into inactive aggregates. These results show that myosin II is responsible for a surprisingly diverse array of cell shape changes throughout development.
28. Nonmuscle myosin II localization is regulated by JNK during Drosophila larval wound healing. We investigated cell shape changes during wound closure in the Drosophila larval epidermis. During reepithelialization, epidermal cells permanently change shape from pentagonal or hexagonal to irregular forms. This process requires zipper, a gene encoding the Drosophila nonmuscle myosin II heavy chain. Following wounding, myosin II is localized at the wound margin and at the rear end of individual cells located within several rows from the wound hole. The c-Jun N-terminal kinase (JNK) pathway is essential for this myosin II localization. These results suggest that not only the wound leading edge but also the cells lying distal to the leading edge cells actively participate in epithelial cell sheet **migration** during wound hole closure.
29. Epithelial rotation promotes the global alignment of contractile actin bundles during Drosophila egg chamber elongation. Tissues use numerous mechanisms to change shape during development. The Drosophila egg chamber is an organ-like structure that elongates to form an elliptical egg. During elongation the follicular epithelial cells undergo a collective **migration** that causes the egg chamber to rotate within its surrounding basement membrane. Rotation coincides with the formation of a 'molecular corset', in which actin bundles in the epithelium and fibrils in the basement membrane are all aligned perpendicular to the elongation axis. Here we show that rotation plays a critical role in building the actin-based component of the corset. Rotation begins shortly after egg chamber formation and requires lamellipodial protrusions at each follicle cell's leading edge. During early stages, rotation is necessary for tissue-level actin bundle alignment, but it becomes dispensable after the basement membrane is polarized. This work highlights how collective cell **migration** can be used to build a polarized tissue organization for organ morphogenesis.
30. Excessive Myosin activity in mbs mutants causes photoreceptor movement out of the Drosophila eye disc epithelium. Neuronal cells must extend a motile growth cone while maintaining the cell body in its original position. In migrating cells, myosin contraction provides the driving force that pulls the rear of the cell toward the leading edge. We have characterized the function of myosin light chain phosphatase, which down-regulates myosin activity, in Drosophila photoreceptor neurons. Mutations in the gene encoding the myosin binding subunit of this enzyme cause photoreceptors to drop out of the eye disc epithelium and move toward and through the optic stalk. We show that this phenotype is due to excessive phosphorylation of the myosin regulatory light chain Spaghetti squash rather than another potential substrate, Moesin, and that it requires the nonmuscle myosin II heavy chain Zipper. Myosin binding subunit mutant cells continue to express apical epithelial markers and do not undergo ectopic apical constriction. In addition, mutant cells in the wing disc remain within the epithelium and differentiate abnormal wing hairs. We suggest that excessive myosin activity in photoreceptor neurons may pull the cell bodies toward the growth cones in a process resembling normal cell **migration**.
31. The Hippo pathway polarizes the actin cytoskeleton during collective **migration** of Drosophila border cells. Collective **migration** of Drosophila border cells depends on a dynamic actin cytoskeleton that is highly polarized such that it concentrates around the outer rim of the migrating cluster of cells. How the actin cytoskeleton becomes polarized in these cells to enable collective movement remains unknown. Here we show that the Hippo signaling pathway links determinants of cell polarity to polarization of the actin cytoskeleton in border cells. Upstream Hippo pathway components localize to contacts between border cells inside the cluster and signal through the Hippo and Warts kinases to polarize actin and promote border cell **migration**. Phosphorylation of the transcriptional coactivator Yorkie (Yki)/YAP by Warts does not mediate the function of this pathway in promoting border cell **migration**, but rather provides negative feedback to limit the speed of **migration**. Instead, Warts phosphorylates and inhibits the actin regulator Ena to activate F-actin Capping protein activity on inner membranes and thereby restricts F-actin polymerization mainly to the outer rim of the migrating cluster.
32. Second-site noncomplementation identifies genomic regions required for Drosophila nonmuscle myosin function during morphogenesis. Drosophila is an ideal metazoan model system for analyzing the role of nonmuscle myosin-II (henceforth, myosin) during development. In Drosophila, myosin function is required for cytokinesis and morphogenesis driven by cell **migration** and/or cell shape changes during oogenesis, embryogenesis, larval development and pupal metamorphosis. The mechanisms that regulate myosin function and the supramolecular structures into which myosin incorporates have not been systematically characterized. The genetic screens described here identify genomic regions that uncover loci that facilitate myosin function. The nonmuscle myosin heavy chain is encoded by a single locus, zipper. Contiguous chromosomal deficiencies that represent approximately 70% of the euchromatic genome were screened for genetic interactions with two recessive lethal alleles of zipper in a second-site noncomplementation assay for the malformed phenotype. Malformation in the adult leg reflects aberrations in cell shape changes driven by myosin-based contraction during leg morphogenesis. Of the 158 deficiencies tested, 47 behaved as second-site noncomplementors of zipper. Two of the deficiencies are strong interactors, 17 are intermediate and 28 are weak. Finer genetic mapping reveals that mutations in cytoplasmic tropomyosin and viking (collagen IV) behave as second-site noncomplementors of zipper during leg morphogenesis and that zipper function requires a previously uncharacterized locus, E3.10/J3.8, for leg morphogenesis and viability.

### ECM

#### PubMed

1. Direct laser manipulation reveals the mechanics of cell contacts in vivo. Cell-generated forces produce a variety of tissue movements and tissue shape changes. The cytoskeletal elements that underlie these dynamics act at cell-cell and cell-**ECM** contacts to apply local forces on adhesive structures. In epithelia, force imbalance at cell contacts induces cell shape changes, such as apical constriction or polarized junction remodeling, driving tissue morphogenesis. The dynamics of these processes are well-characterized; however, the mechanical basis of cell shape changes is largely unknown because of a lack of mechanical measurements in vivo. We have developed an approach combining optical tweezers with light-sheet microscopy to probe the mechanical properties of epithelial cell junctions in the early Drosophila embryo. We show that optical trapping can efficiently deform cell-cell interfaces and measure tension at cell junctions, which is on the order of 100 pN. We show that tension at cell junctions equilibrates over a few seconds, a short timescale compared with the contractile events that drive morphogenetic movements. We also show that tension increases along cell interfaces during early tissue morphogenesis and becomes anisotropic as cells intercalate during germ-band extension. By performing pull-and-release experiments, we identify time-dependent properties of junctional mechanics consistent with a simple viscoelastic model. Integrating this constitutive law into a tissue-scale model, we predict quantitatively how local deformations propagate throughout the tissue.
2. Dynamic shape changes of **ECM**-producing cells drive morphogenesis of ball-and-socket joints in the fly leg. Animal body shape is framed by the skeleton, which is composed of extracellular matrix (**ECM**). Although how the body plan manifests in skeletal morphology has been studied intensively, cellular mechanisms that directly control skeletal **ECM** morphology remain elusive. In particular, how dynamic behaviors of **ECM**-secreting cells, such as shape changes and movements, contribute to **ECM** morphogenesis is unclear. Strict control of **ECM** morphology is crucial in the joints, where opposing sides of the skeleton must have precisely reciprocal shapes to fit each other. Here we found that, in the development of ball-and-socket joints in the Drosophila leg, the two sides of **ECM** form sequentially. We show that distinct cell populations produce the 'ball' and the 'socket', and that these cells undergo extensive shape changes while depositing **ECM**. We propose that shape changes of **ECM**-producing cells enable the sequential **ECM** formation to allow the morphological coupling of adjacent components. Our results highlight the importance of dynamic cell behaviors in precise shaping of skeletal **ECM** architecture.
3. Tissue elongation requires oscillating contractions of a basal actomyosin network. Understanding how molecular dynamics leads to cellular behaviours that ultimately sculpt organs and tissues is a major challenge not only in basic developmental biology but also in tissue engineering and regenerative medicine. Here we use live imaging to show that the basal surfaces of Drosophila follicle cells undergo a series of directional, oscillating contractions driven by periodic myosin accumulation on a polarized actin network. Inhibition of the actomyosin contractions or their coupling to extracellular matrix (**ECM**) blocked elongation of the whole tissue, whereas enhancement of the contractions exaggerated it. Myosin accumulated in a periodic manner before each contraction and was regulated by the small GTPase Rho, its downstream kinase, ROCK, and cytosolic calcium. Disrupting the link between the actin cytoskeleton and the **ECM** decreased the amplitude and period of the contractions, whereas enhancing cell-**ECM** adhesion increased them. In contrast, disrupting cell-cell adhesions resulted in loss of the actin network. Our findings reveal a mechanism controlling organ shape and an experimental model for the study of the effects of oscillatory actomyosin activity within a coherent cell sheet.

### [Ee]xtracellular [Mm]atrix

#### PubMed

1. Fluorescent labeling of Drosophila heart structures. The Drosophila melanogaster dorsal vessel, or heart, is a tubular structure comprised of a single layer of contractile cardiomyocytes, pericardial cells that align along each side of the heart wall, supportive alary muscles and, in adults, a layer of ventral longitudinal muscle cells. The contractile fibers house conserved constituents of the muscle cytoarchitecture including densely packed bundles of myofibrils and cytoskeletal/submembranous protein complexes, which interact with homologous components of the **extracellular matrix**. Here we describe a protocol for the fixation and the fluorescent labeling of particular myocardial elements from the hearts of dissected larvae and semi-intact adult Drosophila. Specifically, we demonstrate the labeling of sarcomeric F-actin and of alpha-actinin in larval hearts. Additionally, we perform labeling of F-actin and alpha-actinin in myosin-GFP expressing adult flies and of alpha-actinin and pericardin, a type IV **extracellular matrix** collagen, in wild type adult hearts. Particular attention is given to a mounting strategy for semi-intact adult hearts that minimizes handling and optimizes the opportunity for maintaining the integrity of the cardiac tubes and the associated tissues. These preparations are suitable for imaging via fluorescent and confocal microscopy. Overall, this procedure allows for careful and detailed analysis of the structural characteristics of the heart from a powerful genetically tractable model system.
2. Dystroglycan and perlecan provide a basal cue required for epithelial polarity during energetic stress. Dystroglycan localizes to the basal domain of epithelial cells and has been reported to play a role in apical-basal polarity. Here, we show that Dystroglycan null mutant follicle cells have normal apical-basal polarity, but lose the planar polarity of their basal actin stress fibers, a phenotype it shares with Dystrophin mutants. However, unlike Dystrophin mutants, mutants in Dystroglycan or in its **extracellular matrix** ligand Perlecan lose polarity under energetic stress. The maintenance of epithelial polarity under energetic stress requires the activation of Myosin II by the cellular energy sensor AMPK. Starved Dystroglycan or Perlecan null cells activate AMPK normally, but do not activate Myosin II. Thus, Perlecan signaling through Dystroglycan may determine where Myosin II can be activated by AMPK, thereby providing the basal polarity cue for the low-energy epithelial polarity pathway. Since Dystroglycan is often downregulated in tumors, loss of this pathway may play a role in cancer progression.
3. The actomyosin machinery is required for Drosophila retinal lumen formation. Multicellular tubes consist of polarized cells wrapped around a central lumen and are essential structures underlying many developmental and physiological functions. In Drosophila compound eyes, each ommatidium forms a luminal matrix, the inter-rhabdomeral space, to shape and separate the key phototransduction organelles, the rhabdomeres, for proper visual perception. In an enhancer screen to define mechanisms of retina lumen formation, we identified Actin5C as a key molecule. Our results demonstrate that the disruption of lumen formation upon the reduction of Actin5C is not linked to any discernible defect in microvillus formation, the rhabdomere terminal web (RTW), or the overall morphogenesis and basal extension of the rhabdomere. Second, the failure of proper lumen formation is not the result of previously identified processes of retinal lumen formation: Prominin localization, expansion of the apical membrane, or secretion of the luminal matrix. Rather, the phenotype observed with Actin5C is phenocopied upon the decrease of the individual components of non-muscle myosin II (MyoII) and its upstream activators. In photoreceptor cells MyoII localizes to the base of the rhabdomeres, overlapping with the actin filaments of the RTW. Consistent with the well-established roll of actomyosin-mediated cellular contraction, reduction of MyoII results in reduced distance between apical membranes as measured by a decrease in lumen diameter. Together, our results indicate the actomyosin machinery coordinates with the localization of apical membrane components and the secretion of an **extracellular matrix** to overcome apical membrane adhesion to initiate and expand the retinal lumen.
4. Dynamic shape changes of ECM-producing cells drive morphogenesis of ball-and-socket joints in the fly leg. Animal body shape is framed by the skeleton, which is composed of **extracellular matrix** (ECM). Although how the body plan manifests in skeletal morphology has been studied intensively, cellular mechanisms that directly control skeletal ECM morphology remain elusive. In particular, how dynamic behaviors of ECM-secreting cells, such as shape changes and movements, contribute to ECM morphogenesis is unclear. Strict control of ECM morphology is crucial in the joints, where opposing sides of the skeleton must have precisely reciprocal shapes to fit each other. Here we found that, in the development of ball-and-socket joints in the Drosophila leg, the two sides of ECM form sequentially. We show that distinct cell populations produce the 'ball' and the 'socket', and that these cells undergo extensive shape changes while depositing ECM. We propose that shape changes of ECM-producing cells enable the sequential ECM formation to allow the morphological coupling of adjacent components. Our results highlight the importance of dynamic cell behaviors in precise shaping of skeletal ECM architecture.
5. miR-8 modulates cytoskeletal regulators to influence cell survival and epithelial organization in Drosophila wings. The miR-200 microRNA family plays important tumor suppressive roles. The sole Drosophila miR-200 ortholog, miR-8 plays conserved roles in Wingless, Notch and Insulin signaling - pathways linked to tumorigenesis, yet homozygous null animals are viable and often appear morphologically normal. We observed that wing tissues mosaic for miR-8 levels by genetic loss or gain of function exhibited patterns of cell death consistent with a role for miR-8 in modulating cell survival in vivo. Here we show that miR-8 levels impact several actin cytoskeletal regulators that can affect cell survival and epithelial organization. We show that loss of miR-8 can confer resistance to apoptosis independent of an epithelial to mesenchymal transition while the persistence of cells expressing high levels of miR-8 in the wing epithelium leads to increased JNK signaling, aberrant expression of **extracellular matrix** remodeling proteins and disruption of proper wing epithelial organization. Altogether our results suggest that very low as well as very high levels of miR-8 can contribute to hallmarks associated with cancer, suggesting approaches to increase miR-200 microRNAs in cancer treatment should be moderate.
6. The making of a fly leg: a model for epithelial morphogenesis. Epithelial development dictates the shape of an organism. The metamorphic development of a Drosophila leg precursor into an adult leg is a well-defined example of epithelial morphogenesis that can be analyzed from the perspectives of genetics and molecular and cell biology. The steroid hormone 20-hydroxyecdysone induces and regulates the entire process. Mutants affecting Drosophila leg morphogenesis characteristically have short thick legs (the malformed phenotype) resulting from a failure to execute normal cell shape changes at a specific stage of development. Mutations that cause the malformed phenotype have already led to the identification and cloning of genes encoding transcription factors, a transmembrane serine protease presumably required for modification of the apical **extracellular matrix**, and components of the contractile cytoskeleton and adherens junctions. All of these products are required for the execution of normal changes in leg cell shape.
7. Tissue elongation requires oscillating contractions of a basal actomyosin network. Understanding how molecular dynamics leads to cellular behaviours that ultimately sculpt organs and tissues is a major challenge not only in basic developmental biology but also in tissue engineering and regenerative medicine. Here we use live imaging to show that the basal surfaces of Drosophila follicle cells undergo a series of directional, oscillating contractions driven by periodic myosin accumulation on a polarized actin network. Inhibition of the actomyosin contractions or their coupling to **extracellular matrix** (ECM) blocked elongation of the whole tissue, whereas enhancement of the contractions exaggerated it. Myosin accumulated in a periodic manner before each contraction and was regulated by the small GTPase Rho, its downstream kinase, ROCK, and cytosolic calcium. Disrupting the link between the actin cytoskeleton and the ECM decreased the amplitude and period of the contractions, whereas enhancing cell-ECM adhesion increased them. In contrast, disrupting cell-cell adhesions resulted in loss of the actin network. Our findings reveal a mechanism controlling organ shape and an experimental model for the study of the effects of oscillatory actomyosin activity within a coherent cell sheet.

### [Ii]nvasive

#### PubMed

1. Cell mixing induced by myc is required for competitive tissue invasion and destruction. Cell-cell intercalation is used in several developmental processes to shape the normal body plan. There is no clear evidence that intercalation is involved in pathologies. Here we use the proto-oncogene myc to study a process analogous to early phase of tumour expansion: myc-induced cell competition. Cell competition is a conserved mechanism driving the elimination of slow-proliferating cells (so-called 'losers') by faster-proliferating neighbours (so-called 'winners') through apoptosis and is important in preventing developmental malformations and maintain tissue fitness. Here we show, using long-term live imaging of myc-driven competition in the Drosophila pupal notum and in the wing imaginal disc, that the probability of elimination of loser cells correlates with the surface of contact shared with winners. As such, modifying loser-winner interface morphology can modulate the strength of competition. We further show that elimination of loser clones requires winner-loser cell mixing through cell-cell intercalation. Cell mixing is driven by differential growth and the high tension at winner-winner interfaces relative to winner-loser and loser-loser interfaces, which leads to a preferential stabilization of winner-loser contacts and reduction of clone compactness over time. Differences in tension are generated by a relative difference in F-actin levels between loser and winner junctions, induced by differential levels of the membrane lipid phosphatidylinositol (3,4,5)-trisphosphate. Our results establish the first link between cell-cell intercalation induced by a proto-oncogene and how it promotes **invasive**ness and destruction of healthy tissues.
2. Extrinsic and intrinsic mechanisms directing epithelial cell sheet replacement during Drosophila metamorphosis. The fusion of epithelial sheets is an essential morphogenetic event. Here, we study the development of the abdomen of Drosophila as a model of bounded epithelia expansion and uncover a complex multistep process for the generation of the adult epidermis from histoblasts, founder cells that replace the larval cells during metamorphosis. We find that histoblasts experience a biphasic cell cycle and emit apical projections that direct their **invasive** planar intercalation in between larval cells. Coordinately, the larval cells extrude from the epithelia by apical constriction of an actomyosin ring and as a consequence die by apoptosis and are removed by circulating haemocytes. We demonstrate that the proliferation of histoblasts and the death of larval cells are triggered by two independent extrinsic Ecdysone hormonal pulses. Finally, we show that histoblast spreading and the death of larval cells depend on a mutual exchange of signals and are non-autonomous processes.
3. The actin cross-linker Filamin/Cheerio mediates tumor malignancy downstream of JNK signaling. Cell shape dynamics, motility, and cell proliferation all depend on the actin cytoskeleton. Malignant cancer cells hijack the actin network to grow and migrate to secondary sites. Understanding the function of actin regulators is therefore of major interest. In the present study, we identify the actin cross-linking protein Filamin/Cheerio (Cher) as a mediator of malignancy in genetically defined Drosophila tumors. We show that in **invasive** tumors, resulting from cooperation of activated Ras with disrupted epithelial cell polarity, Cher is upregulated in a Jun N-terminal kinase (JNK)-dependent manner. Although dispensable in normal epithelium, Cher becomes required in the tumor cells for their growth and **invasive**ness. When deprived of Cher, these tumor clones lose their full potential to proliferate and breach tissue boundaries. Instead, the Cher-deficient clones remain confined within the limits of their source epithelium, permitting survival of the host animal. Through interaction with the myosin II heavy chain subunit, Cher is likely to strengthen the cortical actomyosin network and reinforce mechanical tension within the **invasive** tumors. Accordingly, Cher is required for aberrant expression of genes downstream of the Hippo/Yorkie signaling in the tumor tissue. Our study identifies Cher as a new target of JNK signaling that links cytoskeleton dynamics to tumor progression.
4. Systematic analysis of the transcriptional switch inducing migration of border cells. Cell migration within a natural context is tightly controlled, often by specific transcription factors. However, the switch from stationary to migratory behavior is poorly understood. Border cells perform a spatially and temporally controlled **invasive** migration during Drosophila oogenesis. Slbo, a C/EBP family transcriptional activator, is required for them to become migratory. We purified wild-type and slbo mutant border cells as well as nonmigratory follicle cells and performed comparative whole-genome expression profiling, followed by functional tests of the contributions of identified targets to migration. About 300 genes were significantly upregulated in border cells, many dependent on Slbo. Among these, the microtubule regulator Stathmin was strongly upregulated and was required for normal migration. Actin cytoskeleton regulators were also induced, including, surprisingly, a large cluster of "muscle-specific" genes. We conclude that Slbo induces multiple cytoskeletal effectors, and that each contributes to the behavioral changes in border cells.
5. **Invasive** cell migration is initiated by guided growth of long cellular extensions. The migration of border cells during Drosophila melanogaster oogenesis is a simple and powerful system for studying **invasive** cell migration in vivo. Border cells are somatic cells that delaminate from the follicular epithelium of an egg chamber and invade the germ line cluster. They migrate between the nurse cells to reach the oocyte, using DE-cadherin for adhesion to the substratum. Border cells take approximately 6 h to migrate a distance of 100 microm. The migration is guided by EGFR (epidermal growth factor receptor) and PVR (platelet-derived growth factor (PDGF)/vascular endothelial growth factor (VEGF) receptor). Here, we show that a single long cellular extension (LCE), several cell diameters in length, is formed at the initiation of migration. The LCE may function as a 'pathfinder' in response to guidance cues. LCE growth requires directional guidance signals and specific adhesion to the substratum. Interference with actin-myosin interactions allows continued LCE growth while preventing translocation of the cell bodies. We discuss similarities between LCEs and axons and the use of LCE-like structures as a general mechanism for initiating **invasive** migration in vivo.
6. Sds22/PP1 links epithelial integrity and tumor suppression via regulation of myosin II and JNK signaling. Loss of epithelial integrity often correlates with the progression of malignant tumors. Sds22, a regulatory subunit of protein phosphatase 1 (PP1), has recently been linked to regulation of epithelial polarity in Drosophila. However, its role in tumorigenesis remains obscure. In this study, using Drosophila imaginal tissue as an in vivo model system, we show that sds22 is a new potential tumor suppressor gene in Drosophila. Without sds22, cells lose epithelial architecture, and become **invasive** and tumorigenic when combined with Ras overexpression; conversely, sds22 overexpression can largely suppress tumorigenic growth of Ras(V12)scrib(-/-) mutant cells. Mechanistically, we show that sds22 prevents cell invasion and metastasis by inhibiting myosin II and Jun N-terminal kinase (JNK) activity downstream of PP1. Loss of this inhibition causes cells to lose epithelial organization and promotes cell invasion. Finally, human Sds22 is focally deleted and downregulated in multiple carcinomas, and this downregulation correlates with tumor progression, suggesting that sds22 inactivation may contribute to tumorigenesis and metastatic potential in human cancers via a similar mechanism.
7. Bayesian inference of force dynamics during morphogenesis. During morphogenesis, cells push and pull each other to trigger precise deformations of a tissue to shape the body. Therefore, to understand the development of animal forms, it is essential to analyze how mechanical forces coordinate behaviors of individual cells that underlie tissue deformations. However, the lack of a direct and non-**invasive** force-measurement method has hampered our ability to identify the underlying physical principles required to regulate morphogenesis. In this study, by employing Bayesian statistics, we develop a novel inverse problem framework to estimate the pressure of each cell and the tension of each contact surface from the observed geometry of the cells. We confirmed that the true and estimated values of forces fit well in artificially generated data sets. Moreover, estimates of forces in Drosophila epithelial tissues are consistent with other readouts of forces obtained by indirect or **invasive** methods such as laser-induced destruction of cortical actin cables. Using the method, we clarify the developmental changes in the patterns of tensile force in the Drosophila dorsal thorax. In summary, the batch and non**invasive** nature of the described force-estimation method will enable us to analyze the mechanical control of morphogenesis at an unprecedented quantitative level.
8. Command and control: regulatory pathways controlling **invasive** behavior of the border cells. The **invasive**ness of cancer cells resembles the normal behavior of cells that migrate into surrounding tissues during development. For example, the border cells in the Drosophila ovary undergo a partial epithelial to mesenchymal transition and invade the neighboring cluster of germline cells, migrating to the oocyte border. Once there, they provide patterning information to the oocyte and produce an eggshell specialization known as the micropyle. Border cell migration has been subjected to extensive genetic analyses using a variety of screening approaches. Recent findings demonstrate that conversion of the border cells from a stationary group of epithelial cells to **invasive** cells requires integration of the activities of at least two transcriptional regulatory pathways. One such pathway requires the slbo gene, which encodes Drosophila C/EBP, a basic region/leucine zipper transcriptional activator that is required for elevated expression of a number of downstream targets, including DE-cadherin and focal adhesion kinase (FAK). An independent pathway requires the activity of the ecdysone receptor and a recently identified co-activator for the ecdysone receptor known as Taiman (abbreviated TAI, pronounced ti-maan', meaning too slow). Ecdysone is produced in the Drosophila ovary in response to adequate nutrition and is required for progression of oogenesis through stage 9, when border cell migration occurs. Border cells mutant for tai accumulate abnormally high levels of adhesion complexes at their surfaces, which may account for their inability to migrate. Thus border cell migration requires a differentiation program mediated by the C/EBP pathway, which is required for elevated expression of a number of proteins required for motility. In addition, migration requires a hormonal signal that relays information regarding nutritional status and appears to be required for regulation of the proper localization of some of the C/EBP targets. These findings suggest that steroid hormones can regulate cell motility relatively directly, independent of the effects on proliferation. This may contribute to the metastatic effects of steroid hormones on certain cancers and the inhibition of metastasis by steroid hormone antagonists such as tamoxifen.
9. Mechanical tension drives cell membrane fusion. Membrane fusion is an energy-consuming process that requires tight juxtaposition of two lipid bilayers. Little is known about how cells overcome energy barriers to bring their membranes together for fusion. Previously, we have shown that cell-cell fusion is an asymmetric process in which an "attacking" cell drills finger-like protrusions into the "receiving" cell to promote cell fusion. Here, we show that the receiving cell mounts a Myosin II (MyoII)-mediated mechanosensory response to its **invasive** fusion partner. MyoII acts as a mechanosensor, which directs its force-induced recruitment to the fusion site, and the mechanosensory response of MyoII is amplified by chemical signaling initiated by cell adhesion molecules. The accumulated MyoII, in turn, increases cortical tension and promotes fusion pore formation. We propose that the protrusive and resisting forces from fusion partners put the fusogenic synapse under high mechanical tension, which helps to overcome energy barriers for membrane apposition and drives cell membrane fusion.

### [Mm]etastasis

#### PubMed

1. Cellular and molecular mechanisms of single and collective cell migrations in Drosophila: themes and variations. The process of cell migration is essential throughout life, driving embryonic morphogenesis and ensuring homeostasis in adults. Defects in cell migration are a major cause of human disease, with excessive migration causing autoimmune diseases and cancer **metastasis**, whereas reduced capacity for migration leads to birth defects and immunodeficiencies. Myriad studies in vitro have established a consensus view that cell migrations require cell polarization, Rho GTPase-mediated cytoskeletal rearrangements, and myosin-mediated contractility. However, in vivo studies later revealed a more complex picture, including the discovery that cells migrate not only as single units but also as clusters, strands, and sheets. In particular, the role of E-Cadherin in cell motility appears to be more complex than previously appreciated. Here, we discuss recent advances achieved by combining the plethora of genetic tools available to the Drosophila geneticist with live imaging and biophysical techniques. Finally, we discuss the emerging themes such studies have revealed and ponder the puzzles that remain to be solved.
2. Sds22/PP1 links epithelial integrity and tumor suppression via regulation of myosin II and JNK signaling. Loss of epithelial integrity often correlates with the progression of malignant tumors. Sds22, a regulatory subunit of protein phosphatase 1 (PP1), has recently been linked to regulation of epithelial polarity in Drosophila. However, its role in tumorigenesis remains obscure. In this study, using Drosophila imaginal tissue as an in vivo model system, we show that sds22 is a new potential tumor suppressor gene in Drosophila. Without sds22, cells lose epithelial architecture, and become invasive and tumorigenic when combined with Ras overexpression; conversely, sds22 overexpression can largely suppress tumorigenic growth of Ras(V12)scrib(-/-) mutant cells. Mechanistically, we show that sds22 prevents cell invasion and **metastasis** by inhibiting myosin II and Jun N-terminal kinase (JNK) activity downstream of PP1. Loss of this inhibition causes cells to lose epithelial organization and promotes cell invasion. Finally, human Sds22 is focally deleted and downregulated in multiple carcinomas, and this downregulation correlates with tumor progression, suggesting that sds22 inactivation may contribute to tumorigenesis and metastatic potential in human cancers via a similar mechanism.
3. Distinct functions for Rho1 in maintaining adherens junctions and apical tension in remodeling epithelia. Maintenance and remodeling of adherens junctions (AJs) and cell shape in epithelia are necessary for the development of functional epithelia and are commonly altered during cancer progression/**metastasis**. Although formation of nascent AJs has received much attention, whether shared mechanisms are responsible for the maintenance and remodeling of AJs in dynamic epithelia, particularly in vivo, is not clear. Using clonal analysis in the postmitotic Drosophila melanogaster pupal eye epithelium, we demonstrate that Rho1 is required to maintain AJ integrity independent of its role in sustaining apical cell tension. Rho1 depletion in a remodeling postmitotic epithelium disrupts AJs but only when depleted in adjacent cells. Surprisingly, neither of the Rho effectors, Rok or Dia, is necessary downstream of Rho1 to maintain AJs; instead, Rho1 maintains AJs by inhibiting Drosophila epithelial cadherin endocytosis in a Cdc42/Par6-dependent manner. In contrast, depletion of Rho1 in single cells decreases apical tension, and Rok and myosin are necessary, while Dia function also contributes, downstream of Rho1 to sustain apical cell tension.
4. Command and control: regulatory pathways controlling invasive behavior of the border cells. The invasiveness of cancer cells resembles the normal behavior of cells that migrate into surrounding tissues during development. For example, the border cells in the Drosophila ovary undergo a partial epithelial to mesenchymal transition and invade the neighboring cluster of germline cells, migrating to the oocyte border. Once there, they provide patterning information to the oocyte and produce an eggshell specialization known as the micropyle. Border cell migration has been subjected to extensive genetic analyses using a variety of screening approaches. Recent findings demonstrate that conversion of the border cells from a stationary group of epithelial cells to invasive cells requires integration of the activities of at least two transcriptional regulatory pathways. One such pathway requires the slbo gene, which encodes Drosophila C/EBP, a basic region/leucine zipper transcriptional activator that is required for elevated expression of a number of downstream targets, including DE-cadherin and focal adhesion kinase (FAK). An independent pathway requires the activity of the ecdysone receptor and a recently identified co-activator for the ecdysone receptor known as Taiman (abbreviated TAI, pronounced ti-maan', meaning too slow). Ecdysone is produced in the Drosophila ovary in response to adequate nutrition and is required for progression of oogenesis through stage 9, when border cell migration occurs. Border cells mutant for tai accumulate abnormally high levels of adhesion complexes at their surfaces, which may account for their inability to migrate. Thus border cell migration requires a differentiation program mediated by the C/EBP pathway, which is required for elevated expression of a number of proteins required for motility. In addition, migration requires a hormonal signal that relays information regarding nutritional status and appears to be required for regulation of the proper localization of some of the C/EBP targets. These findings suggest that steroid hormones can regulate cell motility relatively directly, independent of the effects on proliferation. This may contribute to the metastatic effects of steroid hormones on certain cancers and the inhibition of **metastasis** by steroid hormone antagonists such as tamoxifen.
5. Small is beautiful: what flies tell us about ERM protein function in development. Actin dynamics is recognized as being a determinant in many developmental processes and pathologies, such as cell polarity, morphogenesis and tumour **metastasis**. However, how actin interacts with the plasma membrane is poorly understood. Although numerous studies in cell culture point to the crucial role of Ezrin, Radixin and Moesin (ERM) proteins in the actin-membrane link, genetic approaches in mice have not yet revealed their activity during development. Drosophila has recently become an alternative and promising system for the genetic study of ERM protein function. This article focuses on advances made in flies, providing evidence for the evolutionary conservation of functional properties of ERM proteins, in addition to shedding new light on their importance for development.

### [Cc]ell [Ii]nvasion

#### PubMed

1. Sds22/PP1 links epithelial integrity and tumor suppression via regulation of myosin II and JNK signaling. Loss of epithelial integrity often correlates with the progression of malignant tumors. Sds22, a regulatory subunit of protein phosphatase 1 (PP1), has recently been linked to regulation of epithelial polarity in Drosophila. However, its role in tumorigenesis remains obscure. In this study, using Drosophila imaginal tissue as an in vivo model system, we show that sds22 is a new potential tumor suppressor gene in Drosophila. Without sds22, cells lose epithelial architecture, and become invasive and tumorigenic when combined with Ras overexpression; conversely, sds22 overexpression can largely suppress tumorigenic growth of Ras(V12)scrib(-/-) mutant cells. Mechanistically, we show that sds22 prevents **cell invasion** and metastasis by inhibiting myosin II and Jun N-terminal kinase (JNK) activity downstream of PP1. Loss of this inhibition causes cells to lose epithelial organization and promotes **cell invasion**. Finally, human Sds22 is focally deleted and downregulated in multiple carcinomas, and this downregulation correlates with tumor progression, suggesting that sds22 inactivation may contribute to tumorigenesis and metastatic potential in human cancers via a similar mechanism.

---

## InR

- NCBI Gene
- GeneRIF
- Pubmed

| Search Term | Rank | Fields |
| --- | --- | --- |
| [Ff]ilopodia | 1 | PubMed(2) |
| [Mm]igration | 6 | PubMed(7) |
| [Ee]xtracellular [Mm]atrix | 8 | PubMed(2) |
| [Ii]nvasive | 10 | PubMed(1) |
| [Mm]etastasis | 12 | PubMed(1) |

  
hide/show details for InR

### [Ff]ilopodia

#### PubMed

1. Gene regulatory networks controlling hematopoietic progenitor niche cell production and differentiation in the Drosophila lymph gland. Hematopoiesis occurs in two phases in Drosophila, with the first completed during embryogenesis and the second accomplished during larval development. The lymph gland serves as the venue for the final hematopoietic program, with this larval tissue well-studied as to its cellular organization and genetic regulation. While the medullary zone contains stem-like hematopoietic progenitors, the posterior signaling center (PSC) functions as a niche microenvironment essential for controlling the decision between progenitor maintenance versus cellular differentiation. In this report, we utilize a PSC-specific GAL4 driver and UAS-gene RNAi strains, to selectively knockdown individual gene functions in PSC cells. We assessed the effect of abrogating the function of 820 genes as to their requirement for niche cell production and differentiation. 100 genes were shown to be essential for normal niche development, with various loci placed into sub-groups based on the functions of their encoded protein products and known genetic interactions. For members of three of these groups, we characterized loss- and gain-of-function phenotypes. Gene function knockdown of members of the BAP chromatin-remodeling complex resulted in niche cells that do not express the hedgehog (hh) gene and fail to differentiate **filopodia** believed important for Hh signaling from the niche to progenitors. Abrogating gene function of various members of the insulin-like growth factor and TOR signaling pathways resulted in anomalous PSC cell production, leading to a defective niche organization. Further analysis of the Pten, TSC1, and TSC2 tumor suppressor genes demonstrated their loss-of-function condition resulted in severely altered blood cell homeostasis, including the abundant production of lamellocytes, specialized hemocytes involved in innate immune responses. Together, this cell-specific RNAi knockdown survey and mutant phenotype analyses identified multiple genes and their regulatory networks required for the normal organization and function of the hematopoietic progenitor niche within the lymph gland.
2. Muscle niche-driven Insulin-Notch-Myc cascade reactivates dormant Adult Muscle Precursors in Drosophila. How stem cells specified during development keep their non-differentiated quiescent state, and how they are reactivated, remain poorly understood. Here, we applied a Drosophila model to follow in vivo behavior of adult muscle precursors (AMPs), the transient fruit fly muscle stem cells. We report that emerging AMPs send out thin **filopodia** that make contact with neighboring muscles. AMPs keep their **filopodia**-based association with muscles throughout their dormant state but also when they start to proliferate, suggesting that muscles could play a role in AMP reactivation. Indeed, our genetic analyses indicate that muscles send inductive dIlp6 signals that switch the Insulin pathway ON in closely associated AMPs. This leads to the activation of Notch, which regulates AMP proliferation via dMyc. Altogether, we report that Drosophila AMPs display homing behavior to muscle niche and that the niche-driven Insulin-Notch-dMyc cascade plays a key role in setting the activated state of AMPs.

### [Mm]igration

#### PubMed

1. A misexpression screen to identify regulators of Drosophila larval hemocyte development. In Drosophila, defense against foreign pathogens is mediated by an effective innate immune system, the cellular arm of which is composed of circulating hemocytes that engulf bacteria and encapsulate larger foreign particles. Three hemocyte types occur: plasmatocytes, crystal cells, and lamellocytes. The most abundant larval hemocyte type is the plasmatocyte, which is responsible for phagocytosis and is present either in circulation or in adherent sessile domains under the larval cuticle. The mechanisms controlling differentiation of plasmatocytes and their **migration** toward these sessile compartments are unclear. To address these questions we have conducted a misexpression screen using the plasmatocyte-expressed GAL4 driver Peroxidasin-GAL4 (Pxn-GAL4) and existing enhancer-promoter (EP) and EP yellow (EY) transposon libraries to systematically misexpress approximately 20% of Drosophila genes in larval hemocytes. The Pxn-GAL4 strain also contains a UAS-GFP reporter enabling hemocyte phenotypes to be visualized in the semitransparent larvae. Among 3412 insertions screened we uncovered 101 candidate hemocyte regulators. Some of these are known to control hemocyte development, but the majority either have no characterized function or are proteins of known function not previously implicated in hemocyte development. We have further analyzed three candidate genes for changes in hemocyte morphology, cell-cell adhesion properties, phagocytosis activity, and melanotic tumor formation.
2. Signaling pathways directing the movement and fusion of epithelial sheets: lessons from dorsal closure in Drosophila. Wound healing in embryos and various developmental events in metazoans require the spreading and fusion of epithelial sheets. The complex signaling pathways regulating these processes are being pieced together through genetic, cell biological, and biochemical approaches. At present, dorsal closure of the Drosophila embryo is the best-characterized example of epithelial sheet movement. Dorsal closure involves **migration** of the lateral epidermal flanks to close a hole in the dorsal epidermis occupied by an epithelium called the amnioserosa. Detailed genetic studies have revealed a network of interacting signaling molecules regulating this process. At the center of this network is a Jun N-terminal kinase cascade acting at the leading edge of the migrating epidermis that triggers signaling by the TGF-beta superfamily member Decapentaplegic and which interacts with the Wingless pathway. These signaling modules regulate the cytoskeletal reorganization and cell shape change necessary to drive dorsal closure. Activation of this network requires signals from the amnioserosa and input from a variety of proteins at cell-cell junctions. The Rho family of small GTPases is also instrumental, both in activation of signaling and regulation of the cytoskeleton. Many of the proteins regulating dorsal closure have been implicated in epithelial movement in other organisms, and dorsal closure has emerged as an ideal model system for the study of the **migration** and fusion of epithelial sheets.
3. Dynamics of the basement membrane in invasive epithelial clusters in Drosophila. The basement membrane (BM) represents a barrier to cell **migration**, which has to be degraded to promote invasion. However, the role and behaviour of the BM during the development of pre-invasive cells is only poorly understood. Drosophila border cells (BCs) provide an attractive genetic model in which to study the cellular mechanisms underlying the **migration** of mixed cohorts of epithelial cells. BCs are made of two different epithelial cell types appearing sequentially during oogenesis: the polar cells and the outer BCs. Here, we show that the pre-invasive polar cells undergo an unusual and asymmetrical apical capping with major basement membrane proteins, including the two Drosophila Collagen IV alpha chains, Laminin A and Perlecan. Capping of polar cells proceeds through a novel, basal-to-apical transcytosis mechanism that involves the small GTPase Drab5. Apical capping is transient and is followed by rapid shedding prior to the initiation of BC **migration**, suggesting that the apical cap blocks **migration**. Consistently, non-migratory polar cells remain capped. We further show that JAK/STAT signalling and recruitment of outer BCs are required for correct shedding and **migration**. The dynamics of the BM represents a marker of migratory BC, revealing a novel developmentally regulated behaviour of BM coupled to epithelial cell invasiveness.
4. Modeling glioma growth and invasion in Drosophila melanogaster. Glioblastoma is the most common and most malignant intrinsic human brain tumor, characterized by extensive invasion and proliferation of glial (astrocytic) tumor cells, frequent activation of tyrosine kinase receptor signaling pathways, relative resistance to chemotherapy and radiotherapy, and poor prognosis. Using the Gal4-UAS system, we have produced glioma models in Drosophila by overexpressing homologs of human tyrosine kinase receptors under control of the glia-specific promoter reversed polarity (repo). Glial overexpression of activated epidermal growth factor receptor (EGFR) resulted in enhanced proliferation and **migration** of larval glial cells with increased numbers in the eye imaginal disc, diffuse tumor-like enlargement of the optic stalk, and marked ectopic invasion of glial cells along the optic nerve. Glial overexpression of the downstream kinase PI3K showed similar pathology. Overexpression of activated pvr (platelet-derived growth factor receptor/vascular endothelial growth factor receptor homolog) led to **migration** of glial cells along the optic nerve, whereas expression of activated htl (fibroblast growth factor receptor 1 homolog) and INR (insulin receptor) showed markedly elevated numbers of glial cells in the optic stalk. The EGFR/phosphatidylinositol 3-phosphate kinase (PI3K) phenotype was partly reverted by the administration of the EGFR tyrosine kinase inhibitor gefitinib and completely rescued by the PI3K inhibitor wortmannin and the Akt inhibitor triciribine. We suggest that Drosophila models will be useful for deciphering signaling cascades underlying abnormal behavior of glioma cells for genetic screens to reveal interacting genes involved in gliomagenesis and for experimental therapy approaches.
5. A functional RNAi screen for regulators of receptor tyrosine kinase and ERK signalling. Receptor tyrosine kinase (RTK) signalling through extracellular-signal-regulated kinases (ERKs) has pivotal roles during metazoan development, underlying processes as diverse as fate determination, differentiation, proliferation, survival, **migration** and growth. Abnormal RTK/ERK signalling has been extensively documented to contribute to developmental disorders and disease, most notably in oncogenic transformation by mutant RTKs or downstream pathway components such as Ras and Raf. Although the core RTK/ERK signalling cassette has been characterized by decades of research using mammalian cell culture and forward genetic screens in model organisms, signal propagation through this pathway is probably regulated by a larger network of moderate, context-specific proteins. The genes encoding these proteins may not have been discovered through traditional screens owing, in particular, to the requirement for visible phenotypes. To obtain a global view of RTK/ERK signalling, we performed an unbiased, RNA interference (RNAi), genome-wide, high-throughput screen in Drosophila cells using a novel, quantitative, cellular assay monitoring ERK activation. Here we show that ERK pathway output integrates a wide array of conserved cellular processes. Further analysis of selected components-in multiple cell types with different RTK ligands and oncogenic stimuli-validates and classifies 331 pathway regulators. The relevance of these genes is highlighted by our isolation of a Ste20-like kinase and a PPM-family phosphatase that seem to regulate RTK/ERK signalling in vivo and in mammalian cells. Novel regulators that modulate specific pathway outputs may be selective targets for drug discovery.
6. PTEN affects cell size, cell proliferation and apoptosis during Drosophila eye development. Mutations in the tumor suppressor gene PTEN (MMAC1/TEP1) are associated with a large number of human cancers and several autosomal-dominant disorders. Mice mutant for PTEN die at early embryonic stages and the mutant embryonic fibroblasts display decreased sensitivity to cell death. Overexpression of PTEN in different mammalian tissue culture cells affects various processes including cell proliferation, cell death and cell **migration**. We have characterized the Drosophila PTEN gene and present evidence that both inactivation and overexpression of PTEN affect cell size, while overexpression of PTEN also inhibits cell cycle progression at early mitosis and promotes cell death during eye development in a context-dependent manner. Furthermore, we have shown that PTEN acts in the insulin signaling pathway and all signals from the insulin receptor can be antagonized by either Drosophila or human PTEN, suggesting a potential means for alleviating symptoms associated with altered insulin signaling.
7. Dissecting muscle and neuronal disorders in a Drosophila model of muscular dystrophy. Perturbation in the Dystroglycan (Dg)-Dystrophin (Dys) complex results in muscular dystrophies and brain abnormalities in human. Here we report that Drosophila is an excellent genetically tractable model to study muscular dystrophies and neuronal abnormalities caused by defects in this complex. Using a fluorescence polarization assay, we show a high conservation in Dg-Dys interaction between human and Drosophila. Genetic and RNAi-induced perturbations of Dg and Dys in Drosophila cause cell polarity and muscular dystrophy phenotypes: decreased mobility, age-dependent muscle degeneration and defective photoreceptor path-finding. Dg and Dys are required in targeting glial cells and neurons for correct neuronal **migration**. Importantly, we now report that Dg interacts with insulin receptor and Nck/Dock SH2/SH3-adaptor molecule in photoreceptor path-finding. This is the first demonstration of a genetic interaction between Dg and InR.

### [Ee]xtracellular [Mm]atrix

#### PubMed

1. Tissue design: how Drosophila tumors remodel their neighborhood. Drosophila genetics has long been appreciated as a powerful approach for discovering the normal functions of genes that act as oncogenes and tumor suppressors in human cancer. Recent studies have also highlighted its advantages for deciphering how such genes function during tumorigenesis itself. Here we detail studies relating to how tumors, generated in developing organs and adult stem cell-based tissues, remodel the tissue landscape to their benefit. Like mammalian tumors, insect tumors can dissolve **extracellular matrix**, recruit blood cells, migrate and invade other tissues. While much is known about how mammalian fibroblasts, immune cells and vasculature promote late tumorigenesis, less is understood about the very earliest stages of tumor development in mammals. Because Drosophila has fewer mitotic cells and a simpler tissue architecture, it affords easy detection and analysis of early clonal tumor growth. Drosophila studies have revealed both cooperative and competitive interactions between tumor and normal cells during early tumor growth. During development, these interactions typically occur with other proliferative progenitor cells, but in adult stem cell-based tissues, the stem cell niche can fuel tumor growth.
2. Finding a niche: studies from the Drosophila ovary. Specialized microenvironments called niches help maintain stem cells in an undifferentiated and self-renewing state. The existence of niches has long been predicted from mammalian studies, but identifying stem cells in their native environments in vivo has remained a challenge in most vertebrates. Many of the mechanistic insights into how niches regulate stem cell maintenance have been obtained using invertebrate models such as Drosophila. Here, we focus on the Drosophila ovarian germline stem cell niche and review recent studies that have begun to reveal how intricate crosstalk between various signaling pathways regulates stem cell maintenance, how the **extracellular matrix** modulates the signaling output of the niche and how epigenetic programming influences cell development and function both inside and outside the niche to ensure proper tissue homeostasis. These insights will probably inform the study of mammalian niches and how their malfunction contributes to human disease.

### [Ii]nvasive

#### PubMed

1. Dynamics of the basement membrane in **invasive** epithelial clusters in Drosophila. The basement membrane (BM) represents a barrier to cell migration, which has to be degraded to promote invasion. However, the role and behaviour of the BM during the development of pre-**invasive** cells is only poorly understood. Drosophila border cells (BCs) provide an attractive genetic model in which to study the cellular mechanisms underlying the migration of mixed cohorts of epithelial cells. BCs are made of two different epithelial cell types appearing sequentially during oogenesis: the polar cells and the outer BCs. Here, we show that the pre-**invasive** polar cells undergo an unusual and asymmetrical apical capping with major basement membrane proteins, including the two Drosophila Collagen IV alpha chains, Laminin A and Perlecan. Capping of polar cells proceeds through a novel, basal-to-apical transcytosis mechanism that involves the small GTPase Drab5. Apical capping is transient and is followed by rapid shedding prior to the initiation of BC migration, suggesting that the apical cap blocks migration. Consistently, non-migratory polar cells remain capped. We further show that JAK/STAT signalling and recruitment of outer BCs are required for correct shedding and migration. The dynamics of the BM represents a marker of migratory BC, revealing a novel developmentally regulated behaviour of BM coupled to epithelial cell **invasive**ness.

### [Mm]etastasis

#### PubMed

1. Disruption of lysosome function promotes tumor growth and **metastasis** in Drosophila. Lysosome function is essential to many physiological processes. It has been suggested that deregulation of lysosome function could contribute to cancer. Through a genetic screen in Drosophila, we have discovered that mutations disrupting lysosomal degradation pathway components contribute to tumor development and progression. Loss-of-function mutations in the Class C vacuolar protein sorting (VPS) gene, deep orange (dor), dramatically promote tumor overgrowth and invasion of the Ras(V12) cells. Knocking down either of the two other components of the Class C VPS complex, carnation (car) and vps16A, also renders Ras(V12) cells capable for uncontrolled growth and metastatic behavior. Finally, chemical disruption of the lysosomal function by feeding animals with antimalarial drugs, chloroquine or monensin, leads to malignant tumor growth of the Ras(V12) cells. Taken together, our data provide evidence for a causative role of lysosome dysfunction in tumor growth and invasion and indicate that members of the Class C VPS complex behave as tumor suppressors.

---

## CG33234

- NCBI Gene
- GeneRIF
- Pubmed

| Search Term | Rank | Fields |
| --- | --- | --- |
| [Mm]igration | 6 | PubMed(1) |
| ECM | 7 | PubMed(1) |
| [Ee]xtracellular [Mm]atrix | 8 | PubMed(1) |

  
hide/show details for CG33234

### [Mm]igration

#### PubMed

1. Genes conserved in bilaterians but jointly lost with Myc during nematode evolution are enriched in cell proliferation and cell **migration** functions. Animals use a stereotypical set of developmental genes to build body architectures of varying sizes and organizational complexity. Some genes are critical to developmental patterning, while other genes are important to physiological control of growth. However, growth regulator genes may not be as important in small-bodied "micro-metazoans" such as nematodes. Nematodes use a simplified developmental strategy of lineage-based cell fate specifications to produce an adult bilaterian body composed of a few hundreds of cells. Nematodes also lost the MYC proto-oncogenic regulator of cell proliferation. To identify additional regulators of cell proliferation that were lost with MYC, we computationally screened and determined 839 high-confidence genes that are conserved in bilaterians/lost in nematodes (CIBLIN genes). We find that 30 % of all CIBLIN genes encode transcriptional regulators of cell proliferation, epithelial-to-mesenchyme transitions, and other processes. Over 50 % of CIBLIN genes are unnamed genes in Drosophila, suggesting that there are many understudied genes. Interestingly, CIBLIN genes include many Myc synthetic lethal (MycSL) hits from recent screens. CIBLIN genes include key regulators of heparan sulfate proteoglycan (HSPG) sulfation patterns, and lysyl oxidases involved in cross-linking and modification of the extracellular matrix (ECM). These genes and others suggest the CIBLIN repertoire services critical functions in ECM remodeling and cell **migration** in large-bodied bilaterians. Correspondingly, CIBLIN genes are co-expressed with Myc in cancer transcriptomes, and include a preponderance of known determinants of cancer progression and tumor aggression. We propose that CIBLIN gene research can improve our understanding of regulatory control of cellular growth in metazoans.

### ECM

#### PubMed

1. Genes conserved in bilaterians but jointly lost with Myc during nematode evolution are enriched in cell proliferation and cell migration functions. Animals use a stereotypical set of developmental genes to build body architectures of varying sizes and organizational complexity. Some genes are critical to developmental patterning, while other genes are important to physiological control of growth. However, growth regulator genes may not be as important in small-bodied "micro-metazoans" such as nematodes. Nematodes use a simplified developmental strategy of lineage-based cell fate specifications to produce an adult bilaterian body composed of a few hundreds of cells. Nematodes also lost the MYC proto-oncogenic regulator of cell proliferation. To identify additional regulators of cell proliferation that were lost with MYC, we computationally screened and determined 839 high-confidence genes that are conserved in bilaterians/lost in nematodes (CIBLIN genes). We find that 30 % of all CIBLIN genes encode transcriptional regulators of cell proliferation, epithelial-to-mesenchyme transitions, and other processes. Over 50 % of CIBLIN genes are unnamed genes in Drosophila, suggesting that there are many understudied genes. Interestingly, CIBLIN genes include many Myc synthetic lethal (MycSL) hits from recent screens. CIBLIN genes include key regulators of heparan sulfate proteoglycan (HSPG) sulfation patterns, and lysyl oxidases involved in cross-linking and modification of the extracellular matrix (**ECM**). These genes and others suggest the CIBLIN repertoire services critical functions in **ECM** remodeling and cell migration in large-bodied bilaterians. Correspondingly, CIBLIN genes are co-expressed with Myc in cancer transcriptomes, and include a preponderance of known determinants of cancer progression and tumor aggression. We propose that CIBLIN gene research can improve our understanding of regulatory control of cellular growth in metazoans.

### [Ee]xtracellular [Mm]atrix

#### PubMed

1. Genes conserved in bilaterians but jointly lost with Myc during nematode evolution are enriched in cell proliferation and cell migration functions. Animals use a stereotypical set of developmental genes to build body architectures of varying sizes and organizational complexity. Some genes are critical to developmental patterning, while other genes are important to physiological control of growth. However, growth regulator genes may not be as important in small-bodied "micro-metazoans" such as nematodes. Nematodes use a simplified developmental strategy of lineage-based cell fate specifications to produce an adult bilaterian body composed of a few hundreds of cells. Nematodes also lost the MYC proto-oncogenic regulator of cell proliferation. To identify additional regulators of cell proliferation that were lost with MYC, we computationally screened and determined 839 high-confidence genes that are conserved in bilaterians/lost in nematodes (CIBLIN genes). We find that 30 % of all CIBLIN genes encode transcriptional regulators of cell proliferation, epithelial-to-mesenchyme transitions, and other processes. Over 50 % of CIBLIN genes are unnamed genes in Drosophila, suggesting that there are many understudied genes. Interestingly, CIBLIN genes include many Myc synthetic lethal (MycSL) hits from recent screens. CIBLIN genes include key regulators of heparan sulfate proteoglycan (HSPG) sulfation patterns, and lysyl oxidases involved in cross-linking and modification of the **extracellular matrix** (ECM). These genes and others suggest the CIBLIN repertoire services critical functions in ECM remodeling and cell migration in large-bodied bilaterians. Correspondingly, CIBLIN genes are co-expressed with Myc in cancer transcriptomes, and include a preponderance of known determinants of cancer progression and tumor aggression. We propose that CIBLIN gene research can improve our understanding of regulatory control of cellular growth in metazoans.

---

## Dat

- NCBI Gene
- GeneRIF
- Pubmed

| Search Term | Rank | Fields |
| --- | --- | --- |
| [Mm]igration | 6 | PubMed(1) |

  
hide/show details for Dat

### [Mm]igration

#### PubMed

1. Unwrapping glial biology: Gcm target genes regulating glial development, diversification, and function. Glia are the most abundant cell type in the mammalian brain. They regulate neuronal development and function, CNS immune surveillance, and stem cell biology, yet we know surprisingly little about glia in any organism. Here we identify over 40 new Drosophila glial genes. We use glial cells missing (gcm) mutants and misexpression to verify they are Gcm regulated in vivo. Many genes show unique spatiotemporal responsiveness to Gcm in the CNS, and thus glial subtype diversification requires spatially or temporally restricted Gcm cofactors. These genes provide insights into glial biology: we show unc-5 (a repulsive netrin receptor) orients glial **migration**s and the draper gene mediates glial engulfment of apoptotic neurons and larval locomotion. Many identified Drosophila glial genes have homologs expressed in mammalian glia, revealing conserved molecular features of glial cells. 80% of these Drosophila glial genes have mammalian homologs; these are now excellent candidates for regulating human glial development, function, or disease.

---

## a

- NCBI Gene
- GeneRIF
- Pubmed

| Search Term | Rank | Fields |
| --- | --- | --- |
| ECM | 7 | PubMed(1) |
| [Ee]xtracellular [Mm]atrix | 8 | PubMed(1) |

  
hide/show details for a

### ECM

#### PubMed

1. Conditional switches for extracellular matrix patterning in Drosophila melanogaster. An F(1) mutagenesis strategy was developed to identify conditional mutations affecting extracellular matrix (**ECM**) patterning. Tubulogenesis requires coordinated movement of epithelial cells and deposition of a multilayered **ECM**. In the Drosophila ovary, an epithelium of follicle cells creates the eggshells, including the paired tubular dorsal appendages (DAs) that act as breathing tubes for the embryo. A P-element mutagenesis strategy allowed for conditional overexpression of hundreds of genes in follicle cells. Conditional phenotypes were scored at the level of individual mutant (F(1)) female flies. **ECM** pattern regulators were readily identified including MAPK signaling gene ets domain lacking (fused DAs), Wnt pathway genes frizzled 3 and osa (long DAs), Hh pathway gene debra (branched DAs), and transcription factor genes sima/HIF-1alpha, ush, lilli, Tfb1, broad, and foxo. In moving cells the [Ca(2+)]/calcineurin pathway can regulate adhesion to **ECM** while adherens junctions link cells together. Accordingly, thin eggshell and DA phenotypes were identified for the calcineurin regulator calreticulin and the adherens junction component arc. Finally a tubulogenesis defect phenotype was identified for the gene pterodactyl, homologous to the mammalian serine/threonine receptor-associated protein (STRAP) that integrates the TGF-beta and PI3K/AKT signaling pathways. Because phenotypes can be scored in each mutant fly before and after gene induction, this F(1) conditional mutagenesis strategy should allow for increased scale in screens for mutations affecting repeated (reiterated) events in adult animals, including gametogenesis, movement, behavior, and learning.

### [Ee]xtracellular [Mm]atrix

#### PubMed

1. Conditional switches for **extracellular matrix** patterning in Drosophila melanogaster. An F(1) mutagenesis strategy was developed to identify conditional mutations affecting **extracellular matrix** (ECM) patterning. Tubulogenesis requires coordinated movement of epithelial cells and deposition of a multilayered ECM. In the Drosophila ovary, an epithelium of follicle cells creates the eggshells, including the paired tubular dorsal appendages (DAs) that act as breathing tubes for the embryo. A P-element mutagenesis strategy allowed for conditional overexpression of hundreds of genes in follicle cells. Conditional phenotypes were scored at the level of individual mutant (F(1)) female flies. ECM pattern regulators were readily identified including MAPK signaling gene ets domain lacking (fused DAs), Wnt pathway genes frizzled 3 and osa (long DAs), Hh pathway gene debra (branched DAs), and transcription factor genes sima/HIF-1alpha, ush, lilli, Tfb1, broad, and foxo. In moving cells the [Ca(2+)]/calcineurin pathway can regulate adhesion to ECM while adherens junctions link cells together. Accordingly, thin eggshell and DA phenotypes were identified for the calcineurin regulator calreticulin and the adherens junction component arc. Finally a tubulogenesis defect phenotype was identified for the gene pterodactyl, homologous to the mammalian serine/threonine receptor-associated protein (STRAP) that integrates the TGF-beta and PI3K/AKT signaling pathways. Because phenotypes can be scored in each mutant fly before and after gene induction, this F(1) conditional mutagenesis strategy should allow for increased scale in screens for mutations affecting repeated (reiterated) events in adult animals, including gametogenesis, movement, behavior, and learning.

---

## Spn77Ba

- NCBI Gene
- GeneRIF
- Pubmed

| Search Term | Rank | Fields |
| --- | --- | --- |
| [Ii]nvasive | 10 | PubMed(1) |

  
hide/show details for Spn77Ba

### [Ii]nvasive

#### PubMed

1. Serine protease MP2 activates prophenoloxidase in the melanization immune response of Drosophila melanogaster. In arthropods, melanization plays a major role in the innate immune response to encapsulate and kill the **invasive** organisms. It is mediated by a serine protease cascade and is regulated by serpins. The identification of the molecular components of melanization and the regulation of those components are still unclear in Drosophila melanogaster, although some genetic research on the activation of melanization has been reported. Here we report that Drosophila serine protease MP2 directly cleaves both recombinant and native prophenoloxidase-1. Overexpression or repression of MP2 in flies resulted in increased and decreased rates of cleavage, respectively, of prophenoloxidase-1. Moreover, serine protease inhibitor Spn27A formed SDS-stable complexes with MP2, both in vitro and in vivo. The amidase activity of MP2 was inhibited efficiently by Spn27A. Spn27A also prevented MP2 from cleaving prophenoloxidase-1. Taken together, these results indicate that under our experimental conditions MP2 functions as a prophenoloxidase-activating protease, and that this function is inhibited by Spn27A. MP2 and Spn27A thus constitute a regulatory unit in the prophenoloxidase activation cascade in Drosophila. The combination of genetic, molecular genetic and biochemical approaches should allow further advances in our understanding of the prophenoloxidase-activating cascade in insects and indirectly shed further light on protease-cascades in humans and other vertebrates.

---

## Traf-like

- NCBI Gene
- GeneRIF
- Pubmed

| Search Term | Rank | Fields |
| --- | --- | --- |
| [Mm]igration | 6 | PubMed(1) |
| ECM | 7 | PubMed(1) |
| [Ee]xtracellular [Mm]atrix | 8 | PubMed(1) |

  
hide/show details for Traf-like

### [Mm]igration

#### PubMed

1. Genes conserved in bilaterians but jointly lost with Myc during nematode evolution are enriched in cell proliferation and cell **migration** functions. Animals use a stereotypical set of developmental genes to build body architectures of varying sizes and organizational complexity. Some genes are critical to developmental patterning, while other genes are important to physiological control of growth. However, growth regulator genes may not be as important in small-bodied "micro-metazoans" such as nematodes. Nematodes use a simplified developmental strategy of lineage-based cell fate specifications to produce an adult bilaterian body composed of a few hundreds of cells. Nematodes also lost the MYC proto-oncogenic regulator of cell proliferation. To identify additional regulators of cell proliferation that were lost with MYC, we computationally screened and determined 839 high-confidence genes that are conserved in bilaterians/lost in nematodes (CIBLIN genes). We find that 30 % of all CIBLIN genes encode transcriptional regulators of cell proliferation, epithelial-to-mesenchyme transitions, and other processes. Over 50 % of CIBLIN genes are unnamed genes in Drosophila, suggesting that there are many understudied genes. Interestingly, CIBLIN genes include many Myc synthetic lethal (MycSL) hits from recent screens. CIBLIN genes include key regulators of heparan sulfate proteoglycan (HSPG) sulfation patterns, and lysyl oxidases involved in cross-linking and modification of the extracellular matrix (ECM). These genes and others suggest the CIBLIN repertoire services critical functions in ECM remodeling and cell **migration** in large-bodied bilaterians. Correspondingly, CIBLIN genes are co-expressed with Myc in cancer transcriptomes, and include a preponderance of known determinants of cancer progression and tumor aggression. We propose that CIBLIN gene research can improve our understanding of regulatory control of cellular growth in metazoans.

### ECM

#### PubMed

1. Genes conserved in bilaterians but jointly lost with Myc during nematode evolution are enriched in cell proliferation and cell migration functions. Animals use a stereotypical set of developmental genes to build body architectures of varying sizes and organizational complexity. Some genes are critical to developmental patterning, while other genes are important to physiological control of growth. However, growth regulator genes may not be as important in small-bodied "micro-metazoans" such as nematodes. Nematodes use a simplified developmental strategy of lineage-based cell fate specifications to produce an adult bilaterian body composed of a few hundreds of cells. Nematodes also lost the MYC proto-oncogenic regulator of cell proliferation. To identify additional regulators of cell proliferation that were lost with MYC, we computationally screened and determined 839 high-confidence genes that are conserved in bilaterians/lost in nematodes (CIBLIN genes). We find that 30 % of all CIBLIN genes encode transcriptional regulators of cell proliferation, epithelial-to-mesenchyme transitions, and other processes. Over 50 % of CIBLIN genes are unnamed genes in Drosophila, suggesting that there are many understudied genes. Interestingly, CIBLIN genes include many Myc synthetic lethal (MycSL) hits from recent screens. CIBLIN genes include key regulators of heparan sulfate proteoglycan (HSPG) sulfation patterns, and lysyl oxidases involved in cross-linking and modification of the extracellular matrix (**ECM**). These genes and others suggest the CIBLIN repertoire services critical functions in **ECM** remodeling and cell migration in large-bodied bilaterians. Correspondingly, CIBLIN genes are co-expressed with Myc in cancer transcriptomes, and include a preponderance of known determinants of cancer progression and tumor aggression. We propose that CIBLIN gene research can improve our understanding of regulatory control of cellular growth in metazoans.

### [Ee]xtracellular [Mm]atrix

#### PubMed

1. Genes conserved in bilaterians but jointly lost with Myc during nematode evolution are enriched in cell proliferation and cell migration functions. Animals use a stereotypical set of developmental genes to build body architectures of varying sizes and organizational complexity. Some genes are critical to developmental patterning, while other genes are important to physiological control of growth. However, growth regulator genes may not be as important in small-bodied "micro-metazoans" such as nematodes. Nematodes use a simplified developmental strategy of lineage-based cell fate specifications to produce an adult bilaterian body composed of a few hundreds of cells. Nematodes also lost the MYC proto-oncogenic regulator of cell proliferation. To identify additional regulators of cell proliferation that were lost with MYC, we computationally screened and determined 839 high-confidence genes that are conserved in bilaterians/lost in nematodes (CIBLIN genes). We find that 30 % of all CIBLIN genes encode transcriptional regulators of cell proliferation, epithelial-to-mesenchyme transitions, and other processes. Over 50 % of CIBLIN genes are unnamed genes in Drosophila, suggesting that there are many understudied genes. Interestingly, CIBLIN genes include many Myc synthetic lethal (MycSL) hits from recent screens. CIBLIN genes include key regulators of heparan sulfate proteoglycan (HSPG) sulfation patterns, and lysyl oxidases involved in cross-linking and modification of the **extracellular matrix** (ECM). These genes and others suggest the CIBLIN repertoire services critical functions in ECM remodeling and cell migration in large-bodied bilaterians. Correspondingly, CIBLIN genes are co-expressed with Myc in cancer transcriptomes, and include a preponderance of known determinants of cancer progression and tumor aggression. We propose that CIBLIN gene research can improve our understanding of regulatory control of cellular growth in metazoans.

---

## Vrp1

- NCBI Gene
- GeneRIF
- Pubmed

| Search Term | Rank | Fields |
| --- | --- | --- |
| [Ff]ilopodia | 1 | PubMed(2) |
| [Ff]ilopodium | 2 | Component(1) |
| [Pp]odosome(s)? | 5 | PubMed(3) |
| [Mm]igration | 6 | PubMed(4) |
| [Ii]nvasive | 10 | PubMed(3) |
| [Cc]ell [Ii]nvasion | 13 | PubMed(1) |

  
hide/show details for Vrp1

### [Ff]ilopodia

#### PubMed

1. Adhesion and Fusion of Muscle Cells Are Promoted by **Filopodia**. Indirect flight muscles (IFMs) in Drosophila are generated during pupariation by fusion of hundreds of myoblasts with larval muscle templates (myotubes). Live observation of these muscles during the fusion process revealed multiple long actin-based protrusions that emanate from the myotube surface and require Enabled and IRSp53 for their generation and maintenance. Fusion is blocked when formation of these **filopodia** is compromised. While **filopodia** are not required for the signaling process underlying critical myoblast cell-fate changes prior to fusion, myotube-myoblast adhesion appears to be **filopodia** dependent. Without **filopodia**, close apposition between the cell membranes is not achieved, the cell-adhesion molecule Duf is not recruited to the myotube surface, and adhesion-dependent actin foci do not form. We therefore propose that the **filopodia** are necessary to prime the heterotypic adhesion process between the two cell types, possibly by recruiting the cell-adhesion molecule Sns to discrete patches on the myoblast cell surface.
2. Live imaging provides new insights on dynamic F-actin **filopodia** and differential endocytosis during myoblast fusion in Drosophila. The process of myogenesis includes the recognition, adhesion, and fusion of committed myoblasts into multinucleate syncytia. In the larval body wall muscles of Drosophila, this elaborate process is initiated by Founder Cells and Fusion-Competent Myoblasts (FCMs), and cell adhesion molecules Kin-of-IrreC (Kirre) and Sticks-and-stones (Sns) on their respective surfaces. The FCMs appear to provide the driving force for fusion, via the assembly of protrusions associated with branched F-actin and the WASp, SCAR and Arp2/3 pathways. In the present study, we utilize the dorsal pharyngeal musculature that forms in the Drosophila embryo as a model to explore myoblast fusion and visualize the fusion process in live embryos. These muscles rely on the same cell types and genes as the body wall muscles, but are amenable to live imaging since they do not undergo extensive morphogenetic movement during formation. Time-lapse imaging with F-actin and membrane markers revealed dynamic FCM-associated actin-enriched protrusions that rapidly extend and retract into the myotube from different sites within the actin focus. Ultrastructural analysis of this actin-enriched area showed that they have two morphologically distinct structures: wider invasions and/or narrow **filopodia** that contain long linear filaments. Consistent with this, formin Diaphanous (Dia) and branched actin nucleator, Arp3, are found decorating the **filopodia** or enriched at the actin focus, respectively, indicating that linear actin is present along with branched actin at sites of fusion in the FCM. Gain-of-function Dia and loss-of-function Arp3 both lead to fusion defects, a decrease of F-actin foci and prominent **filopodia** from the FCMs. We also observed differential endocytosis of cell surface components at sites of fusion, with actin reorganizing factors, WASp and SCAR, and Kirre remaining on the myotube surface and Sns preferentially taken up with other membrane proteins into early endosomes and lysosomes in the myotube.

### [Ff]ilopodium

#### Component

1. **filopodium** tip

### [Pp]odosome(s)?

#### PubMed

1. Group I PAKs function downstream of Rac to promote **podosome** invasion during myoblast fusion in vivo. The p21-activated kinases (PAKs) play essential roles in diverse cellular processes and are required for cell proliferation, apoptosis, polarity establishment, migration, and cell shape changes. Here, we have identified a novel function for the group I PAKs in cell-cell fusion. We show that the two Drosophila group I PAKs, DPak3 and DPak1, have partially redundant functions in myoblast fusion in vivo, with DPak3 playing a major role. DPak3 is enriched at the site of fusion colocalizing with the F-actin focus within a **podosome**-like structure (PLS), and promotes actin filament assembly during PLS invasion. Although the small GTPase Rac is involved in DPak3 activation and recruitment to the PLS, the kinase activity of DPak3 is required for effective PLS invasion. We propose a model whereby group I PAKs act downstream of Rac to organize the actin filaments within the PLS into a dense focus, which in turn promotes PLS invasion and fusion pore initiation during myoblast fusion.
2. An invasive **podosome**-like structure promotes fusion pore formation during myoblast fusion. Recent studies in Drosophila have implicated actin cytoskeletal remodeling in myoblast fusion, but the cellular mechanisms underlying this process remain poorly understood. Here we show that actin polymerization occurs in an asymmetric and cell type-specific manner between a muscle founder cell and a fusion-competent myoblast (FCM). In the FCM, a dense F-actin-enriched focus forms at the site of fusion, whereas a thin sheath of F-actin is induced along the apposing founder cell membrane. The FCM-specific actin focus invades the apposing founder cell with multiple finger-like protrusions, leading to the formation of a single-channel macro fusion pore between the two muscle cells. Two actin nucleation-promoting factors of the Arp2/3 complex, WASP and Scar, are required for the formation of the F-actin foci, whereas WASP but not Scar promotes efficient foci invasion. Our studies uncover a novel invasive **podosome**-like structure (PLS) in a developing tissue and reveal a previously unrecognized function of PLSs in facilitating cell membrane juxtaposition and fusion.
3. Competition between Blown fuse and WASP for WIP binding regulates the dynamics of WASP-dependent actin polymerization in vivo. Dynamic rearrangements of the actin cytoskeleton play a key role in numerous cellular processes. In Drosophila, fusion between a muscle founder cell and a fusion competent myoblast (FCM) is mediated by an invasive, F-actin-enriched **podosome**-like structure (PLS). Here, we show that the dynamics of the PLS is controlled by Blown fuse (Blow), a cytoplasmic protein required for myoblast fusion but whose molecular function has been elusive. We demonstrate that Blow is an FCM-specific protein that colocalizes with WASP, WIP/Solitary, and the actin focus within the PLS. Biochemically, Blow modulates the stability of the WASP-WIP complex by competing with WASP for WIP binding, leading to a rapid exchange of WASP, WIP and G-actin within the PLS, which, in turn, actively invades the adjacent founder cell to promote fusion pore formation. These studies identify a regulatory protein that modulates the actin cytoskeletal dynamics by controlling the stability of the WASP-WIP complex.

### [Mm]igration

#### PubMed

1. The SCAR and WASp nucleation-promoting factors act sequentially to mediate Drosophila myoblast fusion. The actin nucleation-promoting factors SCAR/WAVE and WASp, together with associated elements, mediate the formation of muscle fibres through myoblast fusion during Drosophila embryogenesis. Our phenotypic analysis, following the disruption of these two pathways, suggests that they function in a sequential manner. Suppressor of cyclic AMP receptor (SCAR) activity is required before the formation of pores in the membranes of fusing cells, whereas Wiskott-Aldrich syndrome protein (WASp) promotes the expansion of nascent pores and completion of the fusion process. Genetic epistasis experiments are consistent with this step-wise temporal progression. Our observations further imply a separate, Rac-dependent role for the SCAR complex in promoting myoblast **migration**. In keeping with the sequential utilization of the two systems, we observe abnormal accumulations of filamentous actin at the fusion sites when both pathways are disrupted, resembling those present when only SCAR-complex function is impaired. This observation further suggests that actin-filament accumulation at the fusion sites might not depend on Arp2/3 activity altogether.
2. Fusion of circular and longitudinal muscles in Drosophila is independent of the endoderm but further visceral muscle differentiation requires a close contact between mesoderm and endoderm. In this study we describe the morphological and genetic analysis of the Drosophila mutant gürtelchen (gurt). gurt was identified by screening an EMS collection for novel mutations affecting visceral mesoderm development and was named after the distinct belt shaped visceral phenotype. Interestingly, determination of visceral cell identities and subsequent visceral myoblast fusion is not affected in mutant embryos indicating a later defect in visceral development. gurt is in fact a new huckebein (hkb) allele and as such exhibits nearly complete loss of endodermal derived structures. Targeted ablation of the endodermal primordia produces a phenotype that resembles the visceral defects observed in huckebein(gürtelchen) (hkb(gurt)) mutant embryos. It was shown previously that visceral mesoderm development requires complex interactions between visceral myoblasts and adjacent tissues. Signals from the neighbouring somatic myoblasts play an important role in cell type determination and are a prerequisite for visceral muscle fusion. Furthermore, the visceral mesoderm is known to influence endoderma**l migrati**on and midgut epithelium formation. Our analyses of the visceral phenotype of hkb(gurt) mutant embryos reveal that the adjacent endoderm plays a critical role in the later stages of visceral muscle development, and is required for visceral muscle elongation and outgrowth after proper myoblast fusion.
3. Group I PAKs function downstream of Rac to promote podosome invasion during myoblast fusion in vivo. The p21-activated kinases (PAKs) play essential roles in diverse cellular processes and are required for cell proliferation, apoptosis, polarity establishment, **migration**, and cell shape changes. Here, we have identified a novel function for the group I PAKs in cell-cell fusion. We show that the two Drosophila group I PAKs, DPak3 and DPak1, have partially redundant functions in myoblast fusion in vivo, with DPak3 playing a major role. DPak3 is enriched at the site of fusion colocalizing with the F-actin focus within a podosome-like structure (PLS), and promotes actin filament assembly during PLS invasion. Although the small GTPase Rac is involved in DPak3 activation and recruitment to the PLS, the kinase activity of DPak3 is required for effective PLS invasion. We propose a model whereby group I PAKs act downstream of Rac to organize the actin filaments within the PLS into a dense focus, which in turn promotes PLS invasion and fusion pore initiation during myoblast fusion.
4. Distinct genetic programs guide Drosophila circular and longitudinal visceral myoblast fusion. The visceral musculature of Drosophila larvae comprises circular visceral muscles tightly interwoven with longitudinal visceral muscles. During myogenesis, the circular muscles arise by one-to-one fusion of a circular visceral founder cell (FC) with a visceral fusion-competent myoblast (FCM) from the trunk visceral mesoderm, and longitudinal muscles arise from FCs of the caudal visceral mesoderm. Longitudinal FCs migrate anteriorly under guidance of fibroblast growth factors during embryogenesis; it is proposed that they fuse with FCMs from the trunk visceral mesoderm to give rise to syncytia containing up to six nuclei.Using fluorescence in situ hybridization and immunochemical analyses, we investigated whether these fusion events during **migration** use the same molecular repertoire and cellular components as fusion-restricted myogenic adhesive structure (FuRMAS), the adhesive signaling center that mediates myoblast fusion in the somatic mesoderm. Longitudinal muscles were formed by the fusion of one FC with Sns-positive FCMs, and defects in FCM specification led to defects in longitudinal muscle formation. At the fusion sites, Duf/Kirre and the adaptor protein Rols7 accumulated in longitudinal FCs, and Blow and F-actin accumulated in FCMs. The accumulation of these four proteins at the fusion sites argues for FuRMAS-like adhesion and signaling centers. Longitudinal fusion was disturbed in rols and blow single, and scar wip double mutants. Mutants of wasp or its interaction partner wip had no defects in longitudinal fusion.Our results indicated that all embryonic fusion events depend on the same cell-adhesion molecules, but that the need for Rols7 and regulators of F-actin distinctly differs. Rols7 was required for longitudinal visceral and somatic myoblast fusion but not for circular visceral fusion. Importantly, longitudinal fusion depended on Kette and SCAR/Wave but was independent of WASp-dependent Arp2/3 activation. Thus, the complexity of the players involved in muscle formation increases from binucleated circular muscles to longitudinal visceral muscles to somatic muscles.

### [Ii]nvasive

#### PubMed

1. Mechanical tension drives cell membrane fusion. Membrane fusion is an energy-consuming process that requires tight juxtaposition of two lipid bilayers. Little is known about how cells overcome energy barriers to bring their membranes together for fusion. Previously, we have shown that cell-cell fusion is an asymmetric process in which an "attacking" cell drills finger-like protrusions into the "receiving" cell to promote cell fusion. Here, we show that the receiving cell mounts a Myosin II (MyoII)-mediated mechanosensory response to its **invasive** fusion partner. MyoII acts as a mechanosensor, which directs its force-induced recruitment to the fusion site, and the mechanosensory response of MyoII is amplified by chemical signaling initiated by cell adhesion molecules. The accumulated MyoII, in turn, increases cortical tension and promotes fusion pore formation. We propose that the protrusive and resisting forces from fusion partners put the fusogenic synapse under high mechanical tension, which helps to overcome energy barriers for membrane apposition and drives cell membrane fusion.
2. An **invasive** podosome-like structure promotes fusion pore formation during myoblast fusion. Recent studies in Drosophila have implicated actin cytoskeletal remodeling in myoblast fusion, but the cellular mechanisms underlying this process remain poorly understood. Here we show that actin polymerization occurs in an asymmetric and cell type-specific manner between a muscle founder cell and a fusion-competent myoblast (FCM). In the FCM, a dense F-actin-enriched focus forms at the site of fusion, whereas a thin sheath of F-actin is induced along the apposing founder cell membrane. The FCM-specific actin focus invades the apposing founder cell with multiple finger-like protrusions, leading to the formation of a single-channel macro fusion pore between the two muscle cells. Two actin nucleation-promoting factors of the Arp2/3 complex, WASP and Scar, are required for the formation of the F-actin foci, whereas WASP but not Scar promotes efficient foci invasion. Our studies uncover a novel **invasive** podosome-like structure (PLS) in a developing tissue and reveal a previously unrecognized function of PLSs in facilitating cell membrane juxtaposition and fusion.
3. Competition between Blown fuse and WASP for WIP binding regulates the dynamics of WASP-dependent actin polymerization in vivo. Dynamic rearrangements of the actin cytoskeleton play a key role in numerous cellular processes. In Drosophila, fusion between a muscle founder cell and a fusion competent myoblast (FCM) is mediated by an **invasive**, F-actin-enriched podosome-like structure (PLS). Here, we show that the dynamics of the PLS is controlled by Blown fuse (Blow), a cytoplasmic protein required for myoblast fusion but whose molecular function has been elusive. We demonstrate that Blow is an FCM-specific protein that colocalizes with WASP, WIP/Solitary, and the actin focus within the PLS. Biochemically, Blow modulates the stability of the WASP-WIP complex by competing with WASP for WIP binding, leading to a rapid exchange of WASP, WIP and G-actin within the PLS, which, in turn, actively invades the adjacent founder cell to promote fusion pore formation. These studies identify a regulatory protein that modulates the actin cytoskeletal dynamics by controlling the stability of the WASP-WIP complex.

### [Cc]ell [Ii]nvasion

#### PubMed

1. Rickettsia parkeri invasion of diverse host cells involves an Arp2/3 complex, WAVE complex and Rho-family GTPase-dependent pathway. Rickettsiae are obligate intracellular pathogens that are transmitted to humans by arthropod vectors and cause diseases such as spotted fever and typhus. Although rickettsiae require the host cell actin cytoskeleton for invasion, the cytoskeletal proteins that mediate this process have not been completely described. To identify the host factors important during **cell invasion** by Rickettsia parkeri, a member of the spotted fever group (SFG), we performed an RNAi screen targeting 105 proteins in Drosophila melanogaster S2R+ cells. The screen identified 21 core proteins important for invasion, including the GTPases Rac1 and Rac2, the WAVE nucleation-promoting factor complex and the Arp2/3 complex. In mammalian cells, including endothelial cells, the natural targets of R. parkeri, the Arp2/3 complex was also crucial for invasion, while requirements for WAVE2 as well as Rho GTPases depended on the particular cell type. We propose that R. parkeri invades S2R+ arthropod cells through a primary pathway leading to actin nucleation, whereas invasion of mammalian endothelial cells occurs via redundant pathways that converge on the host Arp2/3 complex. Our results reveal a key role for the WAVE and Arp2/3 complexes, as well as a higher degree of variation than previously appreciated in actin nucleation pathways activated during Rickettsia invasion.

---

## ALiX

- NCBI Gene
- GeneRIF
- Pubmed

| Search Term | Rank | Fields |
| --- | --- | --- |
| [Ff]ilopodia | 1 | PubMed(1) |
| [Mm]igration | 6 | PubMed(2) |

  
hide/show details for ALiX

### [Ff]ilopodia

#### PubMed

1. Cytonemes are required for the establishment of a normal Hedgehog morphogen gradient in Drosophila epithelia. Hedgehog (Hh) signalling is important in development, stem cell biology and disease. In a variety of tissues, Hh acts as a morphogen to regulate growth and cell fate specification. Several hypotheses have been proposed to explain morphogen movement, one of which is transport along **filopodia**-like protrusions called cytonemes. Here, we analyse the mechanism underlying Hh movement in the wing disc and the abdominal epidermis of Drosophila melanogaster. We show that, in both epithelia, cells generate cytonemes in regions of Hh signalling. These protrusions are actin-based and span several cell diameters. Various Hh signalling components localize to cytonemes, as well as to punctate structures that move along cytonemes and are probably exovesicles. Using in vivo imaging, we show that cytonemes are dynamic structures and that Hh gradient establishment correlates with cytoneme formation in space and time. Indeed, mutant conditions that affect cytoneme formation reduce both cytoneme length and Hh gradient length. Our results suggest that cytoneme-mediated Hh transport is the mechanistic basis for Hh gradient formation.

### [Mm]igration

#### PubMed

1. Molecular requirements for actin-based lamella formation in Drosophila S2 cells. Cell **migration** occurs through the protrusion of the actin-enriched lamella. Here, we investigated the effects of RNAi depletion of approximately 90 proteins implicated in actin function on lamella formation in Drosophila S2 cells. Similar to in vitro reconstitution studies of actin-based Listeria movement, we find that lamellae formation requires a relatively small set of proteins that participate in actin nucleation (Arp2/3 and SCAR), barbed end capping (capping protein), filament depolymerization (cofilin and Aip1), and actin monomer binding (profilin and cyclase-associated protein). Lamellae are initiated by parallel and partially redundant signaling pathways involving Rac GTPases and the adaptor protein Nck, which stimulate SCAR, an Arp2/3 activator. We also show that RNAi of three proteins (kette, Abi, and Sra-1) known to copurify with and inhibit SCAR in vitro leads to SCAR degradation, revealing a novel function of this protein complex in SCAR stability. Our results have identified an essential set of proteins involved in actin dynamics during lamella formation in Drosophila S2 cells.
2. Exosomes as Hedgehog carriers in cytoneme-mediated transport and secretion. The Hedgehog signalling pathway is crucial for development, adult stem cell maintenance, cell **migration** and axon guidance in a wide range of organisms. During development, the Hh morphogen directs tissue patterning according to a concentration gradient. Lipid modifications on Hh are needed to achieve graded distribution, leading to debate about how Hh is transported to target cells despite being membrane-tethered. Cytonemes in the region of Hh signalling have been shown to be essential for gradient formation, but the carrier of the morphogen is yet to be defined. Here we show that Hh and its co-receptor Ihog are in exovesicles transported via cytonemes. These exovesicles present protein markers and other features of exosomes. Moreover, the cell machinery for exosome formation is necessary for normal Hh secretion and graded signalling. We propose Hh transport via exosomes along cytonemes as a significant mechanism for the restricted distribution of a lipid-modified morphogen.

---

## CG17111

- NCBI Gene
- GeneRIF
- Pubmed

| Search Term | Rank | Fields |
| --- | --- | --- |
| [Ee]xtracellular [Mm]atrix | 8 | PubMed(2) |

  
hide/show details for CG17111

### [Ee]xtracellular [Mm]atrix

#### PubMed

1. Zona pellucida domain proteins remodel the apical compartment for localized cell shape changes. The zona pellucida domain (ZPD) defines a conserved family of membrane-anchored matrix proteins that are, as yet, poorly characterized with respect to their functions during development. Using genetic approaches in flies, we show here that a set of eight ZPD proteins is required for the localized reorganization of embryonic epidermal cells during morphogenesis. Despite varying degrees of sequence conservation, these ZPD proteins exert specific and nonredundant functions in the remodeling of epidermal cell shape. Each one accumulates in a restricted subregion of the apical compartment, where it organizes local interactions between the membrane and the **extracellular matrix**. In addition, ZPD proteins are required to sculpture the actin-rich cell extensions and maintain appropriate organization of the apical compartment. These results on ZPD proteins therefore reveal a functional subcompartmentalization of the apical membrane and its role in the polarized control of epithelial cell shape during development.
2. nompA encodes a PNS-specific, ZP domain protein required to connect mechanosensory dendrites to sensory structures. Mutations in the no-mechanoreceptor-potential A (nompA) gene, which eliminate transduction in Drosophila mechanosensory organs, disrupt contacts between neuronal sensory endings and cuticular structures. nompA encodes a transmembrane protein with a large, modular extracellular segment that includes a zona pellucida (ZP) domain and several plasminogen N-terminal (PAN) modules. It is specifically expressed in type I sense organs of the peripheral nervous system by the support cells that ensheath the neuronal sensory process. A green fluorescent protein (GFP)-NompA fusion protein is localized to the dendritic cap, an **extracellular matrix** that covers the ciliary outer segment of the sensory process and that shows organizational defects in nompA mutants. The structure and location of NompA suggest that it forms part of a mechanical linkage required to transmit mechanical stimuli to the transduction apparatus.

---

## Act5C

- NCBI Gene
- GeneRIF
- Pubmed

| Search Term | Rank | Fields |
| --- | --- | --- |
| [Ff]ilopodia | 1 | PubMed(18) |
| [Ff]ilopodium | 2 | PubMed(1) |
| [Pp]odosome(s)? | 5 | PubMed(3) |
| [Mm]igration | 6 | PubMed(40) |
| ECM | 7 | PubMed(1) |
| [Ee]xtracellular [Mm]atrix | 8 | PubMed(4) |
| [Ii]nvasive | 10 | PubMed(10) |
| [Mm]etastasis | 12 | PubMed(2) |

  
hide/show details for Act5C

### [Ff]ilopodia

#### PubMed

1. Quantitative analysis of cytoskeletal reorganization during epithelial tissue sealing by large-volume electron tomography. The closure of epidermal openings is an essential biological process that causes major developmental problems such as spina bifida in humans if it goes awry. At present, the mechanism of closure remains elusive. Therefore, we reconstructed a model closure event, dorsal closure in fly embryos, by large-volume correlative electron tomography. We present a comprehensive, quantitative analysis of the cytoskeletal reorganization, enabling separated epidermal cells to seal the epithelium. After establishing contact through actin-driven exploratory **filopodia**, cells use a single lamella to generate 'roof tile'-like overlaps. These shorten to produce the force, 'zipping' the tissue closed. The shortening overlaps lack detectable actin filament ensembles but are crowded with microtubules. Cortical accumulation of shrinking microtubule ends suggests a force generation mechanism in which cortical motors pull on microtubule ends as for mitotic spindle positioning. In addition, microtubules orient **filopodia** and lamellae before zipping. Our 4D electron microscopy picture describes an entire developmental process and provides fundamental insight into epidermal closure.
2. Loss of Drosophila melanogaster p21-activated kinase 3 suppresses defects in synapse structure and function caused by spastin mutations. Microtubules are dynamic structures that must elongate, disassemble, and be cleaved into smaller pieces for proper neuronal development and function. The AAA ATPase Spastin severs microtubules along their lengths and is thought to regulate the balance between long, stable filaments and shorter fragments that seed extension or are transported. In both Drosophila and humans, loss of Spastin function results in reduction of synaptic connections and disabling motor defects. To gain insight into how spastin is regulated, we screened the Drosophila melanogaster genome for deletions that modify a spastin overexpression phenotype, eye size reduction. One suppressor region deleted p21-activated kinase 3 (pak3), which encodes a member of the Pak family of actin-regulatory enzymes, but whose in vivo function is unknown. We show that pak3 mutants have only mild synaptic defects at the larval neuromuscular junction, but exhibit a potent genetic interaction with spastin mutations. Aberrant bouton morphology, microtubule distribution, and synaptic transmission caused by spastin loss of function are all restored to wild type when pak3 is simultaneously reduced. Neuronal overexpression of pak3 induces actin-rich thin projections, suggesting that it functions in vivo to promote **filopodia** during presynaptic terminal arborization. pak3 therefore regulates synapse development in vivo, and when mutated, suppresses the synaptic defects that result from spastin loss.
3. Wound healing recapitulates morphogenesis in Drosophila embryos. The capacity to repair a wound is a fundamental survival mechanism that is activated at any site of damage throughout embryonic and adult life. To study the cell biology and genetics of this process, we have developed a wounding model in Drosophila melanogaster embryos that allows live imaging of rearrangements and changes in cell shape, and of the cytoskeletal machinery that draws closed an in vivo wound. Using embryos expressing green fluorescent protein (GFP) fusion proteins, we show that two cytoskeletal-dependent elements -- an actin cable and dynamic **filopodia**l/lamellipodial protrusions -- are expressed by epithelial cells at the wound edge and are pivotal for repair. Modulating the activities of the small GTPases Rho and Cdc42 demonstrates that these actin-dependent elements have differing cellular functions, but that either alone can drive wound closure. The actin cable operates as a 'purse-string' to draw the hole closed, whereas **filopodia** are essential for the final 'knitting' together of epithelial cells at the end of repair. Our data suggest a more complex model for epithelial repair than previously envisaged and highlight remarkable similarities with the well-characterized morphogenetic movement of dorsal closure in Drosophila.
4. JNK signaling pathway required for wound healing in regenerating Drosophila wing imaginal discs. We have examined wound healing during regeneration of Drosophila wing imaginal discs fragments by confocal microscopy and assessed the role of components of the JNK pathway in this process. After cutting, columnar and peripodial epithelia cells at the wound edge start to close the wound through formation and contraction of an actin cable. This is followed by a zipping process through **filopodia**l protrusions from both epithelia knitting the wound edges from proximal to distal areas of the disc. Activation of the JNK pathway is involved in such process. puckered (puc) expression is induced in several rows of cells at the edge of the wound, whereas absence of JNK pathway activity brought about by hemipterous, basket, and Dfos mutants impair wound healing. These defects are accompanied by lowered or loss of expression of puc. In support of a role of puc in wound healing, hep mutant phenotypes are rescued by reducing puc function, whereas overexpression of puc inhibits wound healing. Altogether, these results demonstrate a role for the JNK pathway in imaginal disc wound healing, similar to that reported for other healing processes such as embryonic dorsal closure, thoracic closure, and adult epithelial wound healing in Drosophila. Differences with such processes are also highlighted.
5. RhoA and Rac1 GTPases mediate the dynamic rearrangement of actin in peripheral glia. Peripheral glial cells in both vertebrates and insects are born centrally and travel large distances to ensheathe axons in the periphery. There is very little known about how this migration is carried out. In other cells, it is known that rearrangement of the Actin cytoskeleton is an integral part of cell motility, yet the distribution of Actin in peripheral glial cell migration in vivo has not been previously characterized. To gain an understanding of how glia migrate, we specifically labeled the peripheral glia of Drosophila melanogaster using an Actin-GFP marker and analyzed their development in the embryonic PNS. It was found that Actin cytoskeleton is dynamically rearranged during glial cell migration. The peripheral glia were observed to migrate as a continuous chain of cells, with the leading glial cells appearing to participate to the greatest extent in exploring the extracellular surroundings with **filopodia**-like Actin containing projections. We hypothesized that the small GTPases Rho, Rac and Cdc42 are involved in Actin cytoskeletal rearrangements that underlie peripheral glial migration and nerve ensheathement. To test this, transgenic forms of the GTPases were ectopically expressed specifically in the peripheral glia during their migration and wrapping phases. The effects on glial Actin-GFP distribution and the overall effects on glial cell migration and morphological development were assessed. We found that RhoA and Rac1 have distinct roles in peripheral glial cell migration and nerve ensheathement; however, Cdc42 does not have a significant role in peripheral glial development. RhoA and Rac1 gain-of-function and loss-of-function mutants had both disruption of glial cell development and secondary effects on sensory axon fasciculation. Together, Actin cytoskeletal dynamics is an integral part of peripheral glial migration and nerve ensheathement, and is mediated by RhoA and Rac1.
6. How Notch establishes longitudinal axon connections between successive segments of the Drosophila CNS. Development of the segmented central nerve cords of vertebrates and invertebrates requires connecting successive neuromeres. Here, we show both how a pathway is constructed to guide pioneer axons between segments of the Drosophila CNS, and how motility of the pioneers along that pathway is promoted. First, canonical Notch signaling in specialized glial cells causes nearby differentiating neurons to extrude a mesh of fine projections, and shapes that mesh into a continuous carpet that bridges from segment to segment, hugging the glial surface. This is the direct substratum that pioneer axons follow as they grow. Simultaneously, Notch uses an alternate, non-canonical signaling pathway in the pioneer growth cones themselves, promoting their motility by suppressing Abl signaling to stimulate **filopodia**l growth while presumably reducing substratum adhesion. This propels the axons as they establish the connection between successive segments.
7. Notch and Numb are required for normal migration of peripheral glia in Drosophila. A prominent feature of glial cells is their ability to migrate along axons to finally wrap and insulate them. In the embryonic Drosophila PNS, most glial cells are born in the CNS and have to migrate to reach their final destinations. To understand how migration of the peripheral glia is regulated, we have conducted a genetic screen looking for mutants that disrupt the normal glial pattern. Here we present an analysis of two of these mutants: Notch and numb. Complete loss of Notch function leads to an increase in the number of glial cells. Embryos hemizygous for the weak Notch(B-8X) allele display an irregular migration phenotype and mutant glial cells show an increased formation of **filopodia**-like structures. A similar phenotype occurs in embryos carrying the Notch(ts1) allele when shifted to the restrictive temperature during the glial cell migration phase, suggesting that Notch must be activated during glial migration. This is corroborated by the fact that cell-specific reduction of Notch activity in glial cells by directed numb expression also results in similar migration phenotypes. Since the glial migration phenotypes of Notch and numb mutants resemble each other, our data support a model where the precise temporal and quantitative regulation of Numb and Notch activity is not only required during fate decisions but also later during glial differentiation and migration.
8. F-actin-based extensions of the head cyst cell adhere to the maturing spermatids to maintain them in a tight bundle and prevent their premature release in Drosophila testis. In Drosophila, all the 64 clonally derived spermatocytes differentiate in syncytium inside two somatic-origin cyst cells. They elongate to form slender spermatids, which are individualized and then released into the seminal vesicle. During individualization, differentiating spermatids are organized in a tight bundle inside the cyst, which is expected to play an important role in sperm selection. However, actual significance of this process and its underlying mechanism are unclear.We show that dynamic F-actin-based processes extend from the head cyst cell at the start of individualization, filling the interstitial space at the rostral ends of the maturing spermatid bundle. In addition to actin, these structures contained lamin, beta-catenin, dynamin, myosin VI and several other **filopodia**l components. Further, pharmacological and genetic analyses showed that cytoskeletal stability and dynamin function are essential for their maintenance. Disruption of these F-actin based processes was associated with spermatid bundle disassembly and premature sperm release inside the testis.Altogether, our data suggests that the head cyst cell adheres to the maturing spermatid heads through F-actin-based extensions, thus maintaining them in a tight bundle. This is likely to regulate mature sperm release into the seminal vesicle. Overall, this process bears resemblance to mammalian spermiation.
9. **Filopodia**-like actin cables position nuclei in association with perinuclear actin in Drosophila nurse cells. Controlling the position of the nucleus is vital for a number of cellular processes from yeast to humans. In Drosophila nurse cells, nuclear positioning is crucial during dumping, when nurse cells contract and expel their contents into the oocyte. We provide evidence that in nurse cells, continuous **filopodia**-like actin cables, growing from the plasma membrane and extending to the nucleus, achieve nuclear positioning. These actin cables move nuclei away from ring canals. When nurse cells contract, actin cables associate laterally with the nuclei, in some cases inducing nuclear turning so that actin cables become partially wound around the nuclei. Our data suggest that a perinuclear actin meshwork connects actin cables to nuclei via actin-crosslinking proteins such as the filamin Cheerio. We provide a revised model for how actin structures position nuclei in nurse cells, employing evolutionary conserved machinery.
10. Calcium/calmodulin-dependent protein kinase II alters structural plasticity and cytoskeletal dynamics in Drosophila. Drosophila dendritic arborization (da) neurons contain subclasses of neurons with distinct dendritic morphologies. We investigated calcium/calmodulin-dependent protein kinase II (CaMKII) regulation of dendritic structure and dynamics in vivo using optically transparent Drosophila larvae. CaMKII increases the dynamic nature and formation of dendritic **filopodia** throughout larval development but only affects neurons that normally contain dendritic **filopodia**. In parallel, we examined the effects of Rac1 activity on dendritic structure to explore signaling specificity. In contrast to CaMKII activity, Rac1 does not alter **filopodia** stability but instead causes de novo **filopodia** formation on all da neurons. Although both mediators increase cytoskeletal turnover, measured by fluorescence recovery after photobleaching experiments, only CaMKII increases the dynamic nature of dendritic **filopodia**. CaMKII signaling thus appears to use mechanisms and machinery distinct from Rac1 signaling. This study illustrates a molecular means of uncoupling cytoskeletal regulation from morphological regulation. Our results suggest that Drosophila dendritic **filopodia** may share some cytoskeletal regulatory mechanisms with mammalian dendritic **filopodia**. Furthermore, general dendrite cytoskeletal compartmentalization is conserved in multipolar neurons.
11. A novel forward genetic screen for identifying mutations affecting larval neuronal dendrite development in Drosophila melanogaster. Vertebrate and invertebrate dendrites are information-processing compartments that can be found on both central and peripheral neurons. Elucidating the molecular underpinnings of information processing in the nervous system ultimately requires an understanding of the genetic pathways that regulate dendrite formation and maintenance. Despite the importance of dendrite development, few forward genetic approaches have been used to analyze the latest stages of dendrite development, including the formation of F-actin-rich dendritic **filopodia** or dendritic spines. We developed a forward genetic screen utilizing transgenic Drosophila second instar larvae expressing an actin, green fluorescent protein (GFP) fusion protein (actin::GFP) in subsets of sensory neurons. Utilizing this fluorescent transgenic reporter, we conducted a forward genetic screen of >4000 mutagenized chromosomes bearing lethal mutations that affected multiple aspects of larval dendrite development. We isolated 13 mutations on the X and second chromosomes composing 11 complementation groups affecting dendrite outgrowth/branching, dendritic **filopodia** formation, or actin::GFP localization within dendrites in vivo. In a fortuitous observation, we observed that the structure of dendritic arborization (da) neuron dendritic **filopodia** changes in response to a changing environment.
12. Sisyphus, the Drosophila myosin XV homolog, traffics within **filopodia** transporting key sensory and adhesion cargos. Unconventional myosin proteins of the MyTH-FERM superclass are involved in intra**filopodia**l trafficking, are thought to be mediators of membrane-cytoskeleton interactions, and are linked to several forms of deafness in mammals. Here we show that the Drosophila myosin XV homolog, Sisyphus, is expressed at high levels in leading edge cells and their cellular protrusions during the morphogenetic process of dorsal closure. Sisyphus is required for the correct alignment of cells on opposing sides of the fusing epithelial sheets, as well as for adhesion of the cells during the final zippering/fusion phase. We have identified several putative Sisyphus cargos, including DE-cadherin (also known as Shotgun) and the microtubule-linked proteins Katanin-60, EB1, Milton and aPKC. These cargos bind to the Sisyphus FERM domain, and their binding is in some cases mutually exclusive. Our data suggest a mechanism for Sisyphus in which it maintains a balance between actin and microtubule cytoskeleton components, thereby contributing to cytoskeletal cross-talk necessary for regulating **filopodia**l dynamics during dorsal closure.
13. FGF is an essential mitogen and chemoattractant for the air sacs of the drosophila tracheal system. The Drosophila adult has a complex tracheal system that forms during the pupal period. We have studied the derivation of part of this system, the air sacs of the dorsal thorax. During the third larval instar, air sac precursor cells bud from a tracheal branch in response to FGF, and then they proliferate and migrate to the adepithelial layer of the wing imaginal disc. In addition, FGF induces these air sac precursors to extend cytoneme-like **filopodia** to FGF-expressing cells. These findings provide evidence that FGF is a mitogen in Drosophila, correlate growth factor signaling with **filopodia**l contact between signaling and responding cells, and suggest that FGF can act on differentiated tracheal cells to induce a novel behavior and role.
14. Time-lapse and cell ablation reveal the role of cell interactions in fly glia migration and proliferation. Migration and proliferation have been mostly explored in culture systems or fixed preparations. We present a simple genetic model, the chains of glia moving along fly wing nerves, to follow such dynamic processes by time-lapse in the whole animal. We show that glia undergo extensive cytoskeleton and mitotic apparatus rearrangements during division and migration. Single cell labelling identifies different glia: pioneers with high **filopodia**l, exploratory, activity and, less active followers. In combination with time-lapse, altering this cellular environment by genetic means or cell ablation has allowed to us define the role of specific cell-cell interactions. First, neurone-glia interactions are not necessary for glia motility but do affect the direction of migration. Second, repulsive interactions between glia control the extent of movement. Finally, autonomous cues control proliferation.
15. In vivo imaging reveals different cellular functions for FGF and Dpp signaling in tracheal branching morphogenesis. In the developing tracheal system of Drosophila melanogaster, six major branches arise by guided cell migration from a sac-like structure. The chemoattractant Branchless/FGF (Bnl) appears to guide cell migration and is essential for the formation of all tracheal branches, while Decapentaplegic (Dpp) signaling is strictly required for the formation of a subset of branches, the dorsal and ventral branches. Using in vivo confocal video microscopy, we find that the two signaling systems affect different cellular functions required for branching morphogenesis. Bnl/FGF signaling affects the formation of dynamic **filopodia**, possibly controlling cytoskeletal activity and motility as such, and Dpp controls cellular functions allowing branch morphogenesis and outgrowth.
16. Exploring the roles of diaphanous and enabled activity in shaping the balance between **filopodia** and lamellipodia. During migration cell protrusions power cell extension and sample the environment. Different cells produce different protrusions, from keratocytes dominated by lamellipodia, to growth cones combining **filopodia** and lamellipodia, to dendritic spines. One key challenge is to determine how the toolkit of actin regulators are coordinated to generate these diverse protrusive arrays. Here we use Drosophila leading-edge (LE) cells to explore how Diaphanous (Dia)-related formins and Ena/VASP proteins cooperate in this process. We first dissect the Dia regulatory region, revealing novel roles for the GTPase-binding and FH3 domains in cortical localization, **filopodia**l initiation, and lengthening. Second, we provide evidence that activating Dia mobilizes Ena from storage places near the LE to act at the LE. Further, Dia and Ena coIP and can recruit one another to new locations, suggesting cooperation is key to their mechanisms of action. Third, we directly explore the functional relationship between Dia and Ena, varying their levels and activity separately in the same cell type. Surprisingly, although each is sufficient to induce **filopodia**, together they induce lamellipodia. Our data suggest they work together in a complex and nonadditive way, with the ratio between active Dia and Ena being one factor that modulates the balance between **filopodia** and lamellipodia.
17. Dynamic analysis of actin cable function during Drosophila dorsal closure. Throughout development, a series of epithelial movements and fusions occur that collectively shape the embryo. They are dependent on coordinated reorganizations and contractions of the actin cytoskeleton within defined populations of epithelial cells. One paradigm morphogenetic movement, dorsal closure in the Drosophila embryo, involves closure of a dorsal epithelial hole by sweeping of epithelium from the two sides of the embryo over the exposed extraembryonic amnioserosa to form a seam where the two epithelial edges fuse together. The front row cells exhibit a thick actin cable at their leading edge. Here, we test the function of this cable by live analysis of GFP-actin-expressing embryos in which the cable is disrupted by modulating Rho1 signaling or by loss of non-muscle myosin (Zipper) function. We show that the cable serves a dual role during dorsal closure. It is contractile and thus can operate as a "purse string," but it also restricts forward movement of the leading edge and excess activity of **filopodia**/lamellipodia. Stripes of epithelium in which cable assembly is disrupted gain a migrational advantage over their wild-type neighbors, suggesting that the cable acts to restrain front row cells, thus maintaining a taut, free edge for efficient zippering together of the epithelial sheets.
18. Dynamic actin-based epithelial adhesion and cell matching during Drosophila dorsal closure. The adhesion of two epithelial sheets is a fundamental process that occurs throughout embryogenesis and during wound repair. Sealing of the dorsal epidermis along the midline of the Drosophila embryo provides a genetically tractable model to analyse the closure of such holes. Several studies indicate that the actin cytoskeleton plays a critical role in dorsal closure. Although many components of the signalling cascade directing this process have been identified, the precise cell-biological events upon which these signals act remain poorly described.By confocal imaging of living fly embryos expressing green fluorescent protein (GFP)-tagged actin, we found that dorsal closure relies on the activity of dynamic **filopodia** and lamellipodia that extend from front-row cells to actively zipper the epithelial sheets together. As these epithelial fronts approach one another, we observed long, thin **filopodia**, apparently 'sampling' cells on the opposing face. When the assembly of these actin-based protrusions was blocked (by interfering with the activities of Cdc42 and Jun N-terminal kinase signalling), the adhesion and fusion of opposing epithelial cells was prevented and their ability to 'sense' correct partners was also blocked, leading to segment misalignment along the midline seam.Dynamic, actin-based protrusions (**filopodia** and lamellae) are critical, both in the mechanics of epithelial adhesion during dorsal closure and in the correct 'matching' of opposing cells along the fusion seam.

### [Ff]ilopodium

#### PubMed

1. Fascin controls neuronal class-specific dendrite arbor morphology. The branched morphology of dendrites represents a functional hallmark of distinct neuronal types. Nonetheless, how diverse neuronal class-specific dendrite branches are generated is not understood. We investigated specific classes of sensory neurons of Drosophila larvae to address the fundamental mechanisms underlying the formation of distinct branch types. We addressed the function of fascin, a conserved actin-bundling protein involved in **filopodium** formation, in class III and class IV sensory neurons. We found that the terminal branchlets of different classes of neurons have distinctive dynamics and are formed on the basis of molecularly separable mechanisms; in particular, class III neurons require fascin for terminal branching whereas class IV neurons do not. In class III neurons, fascin controls the formation and dynamics of terminal branchlets. Previous studies have shown that transcription factor combinations define dendrite patterns; we find that fascin represents a downstream component of such programs, as it is a major effector of the transcription factor Cut in defining class III-specific dendrite morphology. Furthermore, fascin defines the morphological distinction between class III and class IV neurons. In fact, loss of fascin function leads to a partial conversion of class III neurons to class IV characteristics, while the reverse effect is obtained by fascin overexpression in class IV neurons. We propose that dedicated molecular mechanisms underlie the formation and dynamics of distinct dendrite branch types to elicit the accurate establishment of neuronal circuits.

### [Pp]odosome(s)?

#### PubMed

1. An invasive **podosome**-like structure promotes fusion pore formation during myoblast fusion. Recent studies in Drosophila have implicated actin cytoskeletal remodeling in myoblast fusion, but the cellular mechanisms underlying this process remain poorly understood. Here we show that actin polymerization occurs in an asymmetric and cell type-specific manner between a muscle founder cell and a fusion-competent myoblast (FCM). In the FCM, a dense F-actin-enriched focus forms at the site of fusion, whereas a thin sheath of F-actin is induced along the apposing founder cell membrane. The FCM-specific actin focus invades the apposing founder cell with multiple finger-like protrusions, leading to the formation of a single-channel macro fusion pore between the two muscle cells. Two actin nucleation-promoting factors of the Arp2/3 complex, WASP and Scar, are required for the formation of the F-actin foci, whereas WASP but not Scar promotes efficient foci invasion. Our studies uncover a novel invasive **podosome**-like structure (PLS) in a developing tissue and reveal a previously unrecognized function of PLSs in facilitating cell membrane juxtaposition and fusion.
2. Competition between Blown fuse and WASP for WIP binding regulates the dynamics of WASP-dependent actin polymerization in vivo. Dynamic rearrangements of the actin cytoskeleton play a key role in numerous cellular processes. In Drosophila, fusion between a muscle founder cell and a fusion competent myoblast (FCM) is mediated by an invasive, F-actin-enriched **podosome**-like structure (PLS). Here, we show that the dynamics of the PLS is controlled by Blown fuse (Blow), a cytoplasmic protein required for myoblast fusion but whose molecular function has been elusive. We demonstrate that Blow is an FCM-specific protein that colocalizes with WASP, WIP/Solitary, and the actin focus within the PLS. Biochemically, Blow modulates the stability of the WASP-WIP complex by competing with WASP for WIP binding, leading to a rapid exchange of WASP, WIP and G-actin within the PLS, which, in turn, actively invades the adjacent founder cell to promote fusion pore formation. These studies identify a regulatory protein that modulates the actin cytoskeletal dynamics by controlling the stability of the WASP-WIP complex.
3. Group I PAKs function downstream of Rac to promote **podosome** invasion during myoblast fusion in vivo. The p21-activated kinases (PAKs) play essential roles in diverse cellular processes and are required for cell proliferation, apoptosis, polarity establishment, migration, and cell shape changes. Here, we have identified a novel function for the group I PAKs in cell-cell fusion. We show that the two Drosophila group I PAKs, DPak3 and DPak1, have partially redundant functions in myoblast fusion in vivo, with DPak3 playing a major role. DPak3 is enriched at the site of fusion colocalizing with the F-actin focus within a **podosome**-like structure (PLS), and promotes actin filament assembly during PLS invasion. Although the small GTPase Rac is involved in DPak3 activation and recruitment to the PLS, the kinase activity of DPak3 is required for effective PLS invasion. We propose a model whereby group I PAKs act downstream of Rac to organize the actin filaments within the PLS into a dense focus, which in turn promotes PLS invasion and fusion pore initiation during myoblast fusion.

### [Mm]igration

#### PubMed

1. The control of cell motility and epithelial morphogenesis by Jun kinases. Originally identified as stress-activated protein kinases that control cell survival and proliferation through transcription factor c-Jun, the Jun N-terminal kinase (JNK) subgroup of MAP kinases (MAPKs) have recently emerged as crucial regulators of cell **migration** and the morphogenetic movement of epithelial sheets. In Drosophila, a well-orchestrated JNK signaling pathway controls formation of actin stress fibers and cell shape changes, which are required for the sealing of embryonic epidermis in a process known as dorsal closure. The JNK pathway is also involved in morphogenetic processes in mice including closure of the eyelid, neural tube and optic fissure. This article focuses on recent advances in understanding the role of JNK pathway in the regulation of cell **migration**, cytoskeleton rearrangement and the morphogenesis of epithelial sheets.
2. The small GTPase Rac plays multiple roles in epithelial sheet fusion--dynamic studies of Drosophila dorsal closure. The coordinated **migration** and fusion of epithelial sheets is a crucial morphogenetic tool used on numerous occasions during the normal development of an embryo and re-activated as part of the wound healing response. Drosophila dorsal closure, whereby a hole in the embryonic epithelium is zipped closed late in embryogenesis, serves as an excellent, genetically tractable model for epithelial **migration**. Using live confocal imaging, we have dissected multiple roles for the small GTPase Rac in this process. We show that constitutive activation of Rac1 leads to excessive assembly of lamellipodia and precocious halting of epithelial sweeping, possibly through premature activation of contact-inhibition machinery. Conversely, blocking Rac activity, either by loss-of-function mutations or expression of dominant negative Rac1, disables the assembly of both actin cable and protrusions by epithelial cells. Movies of mutant embryos show that continued contraction of the amnioserosa is sufficient to draw the epithelial edges towards one another, allowing the zipper machinery to bypass non-functioning regions of leading edge. In addition to illustrating the key role of Rac in organization of leading edge actin, loss-of-function mutants also provide substantive proof that Rac acts upstream in the Jun N-terminal kinase (JNK) cascade to direct epithelial cell shape changes during dorsal closure.
3. Interkinetic nuclear **migration** is a broadly conserved feature of cell division in pseudostratified epithelia. Animal development requires tight integration between the processes of proliferative growth and epithelial morphogenesis, both of which play out at the level of individual cells. In this respect, not only must polarized epithelial cells assume complex morphologies, these distinct forms must be radically and repeatedly transformed to permit mitosis. A dramatic illustration of this integration between epithelial morphogenesis and cell proliferation is interkinetic nuclear **migration** (IKNM), wherein the nuclei of pseudostratified epithelial cells translocate to the apical epithelial surface to execute cell division. IKNM is widely considered a hallmark of pseudostratified vertebrate neuroepithelia, and prior investigations have proposed both actomyosin- and microtubule-dependent mechanisms for apical localization of the mitotic nucleus. Here, using comparative functional analysis in arthropod and cnidarian systems (Drosophila melanogaster and Nematostella vectensis), we show that actomyosin-dependent IKNM is likely to be a general feature of mitosis in pseudostratified epithelia throughout Eumetazoa. Furthermore, our studies suggest a mechanistic link between IKNM and the fundamental process of mitotic cell rounding.
4. Initiating and guiding **migration**: lessons from border cells. Cell **migration** occurs in many different contexts. Amoebae and other isolated cells migrate in culture. In animals, 'professional' migratory cells of the immune system constantly survey the body for intruders, whereas other cell types perform specific developmentally regulated **migration**s. One simple model for the latter type of event is **migration** of border cells during Drosophila oogenesis. Recent findings have shed light on how border cell fate is induced and on how the **migration** is guided. This article discusses the implications of these studies and compares (invasive) **migration** through a tissue with what is known about cells crawling on a flat substratum.
5. Psidin, a conserved protein that regulates protrusion dynamics and cell **migration**. Dynamic assembly and disassembly of actin filaments is a major driving force for cell movements. Border cells in the Drosophila ovary provide a simple and genetically tractable model to study the mechanisms regulating cell **migration**. To identify new genes that regulate cell movement in vivo, we screened lethal mutations on chromosome 3R for defects in border cell **migration** and identified two alleles of the gene psidin (psid). In vitro, purified Psid protein bound F-actin and inhibited the interaction of tropomyosin with F-actin. In vivo, psid mutations exhibited genetic interactions with the genes encoding tropomyosin and cofilin. Border cells overexpressing Psid together with GFP-actin exhibited altered protrusion/retraction dynamics. Psid knockdown in cultured S2 cells reduced, and Psid overexpression enhanced, lamellipodial dynamics. Knockdown of the human homolog of Psid reduced the speed and directionality of **migration** in wounded MCF10A breast epithelial monolayers, whereas overexpression of the protein increased **migration** speed and altered protrusion dynamics in EGF-stimulated cells. These results indicate that Psid is an actin regulatory protein that plays a conserved role in protrusion dynamics and cell **migration**.
6. Dysfusion transcriptional control of Drosophila tracheal **migration**, adhesion, and fusion. The Drosophila dysfusion basic-helix-loop-helix-PAS transcription factor gene is expressed in specialized fusion cells that reside at the tips of migrating tracheal branches. dysfusion mutants were isolated, and genetic analysis of live embryos revealed that mutant tracheal branches migrate to close proximity but fail to recognize and adhere to each other. Misexpression of dysfusion throughout the trachea further indicated that dysfusion has the ability to both inhibit cell **migration** and promote ectopic tracheal fusion. Nineteen genes whose expression either increases or decreases in fusion cells during development were analyzed in dysfusion mutant embryos. dysfusion upregulates the levels of four genes, including the shotgun cell adhesion protein gene and the zona pellucida family transmembrane protein gene, CG13196. Misexpression experiments with CG13196 result in ectopic tracheal fusion events, suggesting that it also encodes a cell adhesion protein. Another target gene of dysfusion is members only, which inhibits protein nuclear export and influences tracheal fusion. dysfusion also indirectly downregulates protein levels of Trachealess, an important regulator of tracheal development. These results indicate that fusion cells undergo dynamic changes in gene expression as they switch from migratory to fusion modes and that dysfusion regulates a discrete, but important, set of these genes.
7. A role for Drosophila IAP1-mediated caspase inhibition in Rac-dependent cell **migration**. Border cell **migration** in the Drosophila ovary is a relatively simple and genetically tractable model for studying the conversion of epithelial cells to migratory cells. Like many cell **migration**s, border cell **migration** is inhibited by a dominant-negative form of the GTPase Rac. To identify new genes that function in Rac-dependent cell motility, we screened for genes that when overexpressed suppressed the **migration** defect caused by dominant-negative Rac. Overexpression of the Drosophila inhibitor of apoptosis 1 (DIAP1), which is encoded by the thread (th) gene, suppressed the **migration** defect. Moreover, loss-of-function mutations in th caused **migration** defects but, surprisingly, did not cause apoptosis. Mutations affecting the Dark protein, an activator of the upstream caspase Dronc, also rescued RacN17 **migration** defects. These results indicate an apoptosis-independent role for DIAP1-mediated Dronc inhibition in Rac-mediated cell motility.
8. Time-lapse imaging reveals stereotypical patterns of Drosophila midline glial **migration**. The Drosophila CNS midline glia (MG) are multifunctional cells that ensheath and provide trophic support to commissural axons, and direct embryonic development by employing a variety of signaling molecules. These glia consist of two functionally distinct populations: the anterior MG (AMG) and posterior MG (PMG). Only the AMG ensheath axon commissures, whereas the function of the non-ensheathing PMG is unknown. The Drosophila MG have proven to be an excellent system for studying glial proliferation, cell fate, apoptosis, and axon-glial interactions. However, insight into how AMG migrate and acquire their specific positions within the axon-glial scaffold has been lacking. In this paper, we use time-lapse imaging, single-cell analysis, and embryo staining to comprehensively describe the proliferation, **migration**, and apoptosis of the Drosophila MG. We identified 3 groups of MG that differed in the trajectories of their initial inward **migration**: AMG that migrate inward and to the anterior before undergoing apoptosis, AMG that migrate inward and to the posterior to ensheath commissural axons, and PMG that migrate inward and to the anterior to contact the commissural axons before undergoing apoptosis. In a second phase of their **migration**, the surviving AMG stereotypically migrated posteriorly to specific positions surrounding the commissures, and their final position was correlated with their location prior to **migration**. Most noteworthy are AMG that migrated between the commissures from a ventral to a dorsal position. Single-cell analysis indicated that individual AMG possessed wide-ranging and elaborate membrane extensions that partially ensheathed both commissures. These results provide a strong foundation for future genetic experiments to identify mutants affecting MG development, particularly in guidance cues that may direct **migration**. Drosophila MG are homologous in structure and function to the glial-like cells that populate the vertebrate CNS floorplate, and study of Drosophila MG will provide useful insights into floorplate development and function.
9. Diaphanous regulates myosin and adherens junctions to control cell contractility and protrusive behavior during morphogenesis. Formins are key regulators of actin nucleation and elongation. Diaphanous-related formins, the best-known subclass, are activated by Rho and play essential roles in cytokinesis. In cultured cells, Diaphanous-related formins also regulate cell adhesion, polarity and microtubules, suggesting that they may be key regulators of cell shape change and **migration** during development. However, their essential roles in cytokinesis hamper our ability to test this hypothesis. We used loss- and gain-of-function approaches to examine the role of Diaphanous in Drosophila morphogenesis. We found that Diaphanous has a dynamic expression pattern consistent with a role in regulating cell shape change. We used constitutively active Diaphanous to examine its roles in morphogenesis and its mechanisms of action. This revealed an unexpected role in regulating myosin levels and activity at adherens junctions during cell shape change, suggesting that Diaphanous helps coordinate adhesion and contractility of the underlying actomyosin ring. We tested this hypothesis by reducing Diaphanous function, revealing striking roles in stabilizing adherens junctions and inhibiting cell protrusiveness. These effects also are mediated through coordinated effects on myosin activity and adhesion, suggesting a common mechanism for Diaphanous action during morphogenesis.
10. Wash functions downstream of Rho1 GTPase in a subset of Drosophila immune cell developmental **migration**s. Drosophila immune cells, the hemocytes, undergo four stereotypical developmental **migration**s to populate the embryo, where they provide immune reconnoitering, as well as a number of non-immune-related functions necessary for proper embryogenesis. Here, we describe a role for Rho1 in one of these developmental **migration**s in which posteriorly located hemocytes migrate toward the head. This **migration** requires the interaction of Rho1 with its downstream effector Wash, a Wiskott-Aldrich syndrome family protein. Both Wash knockdown and a Rho1 transgene harboring a mutation that prevents Wash binding exhibit the same developmental migratory defect as Rho1 knockdown. Wash activates the Arp2/3 complex, whose activity is needed for this **migration**, whereas members of the WASH regulatory complex (SWIP, Strumpellin, and CCDC53) are not. Our results suggest a WASH complex-independent signaling pathway to regulate the cytoskeleton during a subset of hemocyte developmental **migration**s.
11. Distinct mechanisms regulate hemocyte chemotaxis during development and wound healing in Drosophila melanogaster. Drosophila melanogaster hemocytes are highly motile macrophage-like cells that undergo a stereotypic pattern of **migration** to populate the whole embryo by late embryogenesis. We demonstrate that the migratory patterns of hemocytes at the embryonic ventral midline are orchestrated by chemotactic signals from the PDGF/VEGF ligands Pvf2 and -3 and that these directed **migration**s occur independently of phosphoinositide 3-kinase (PI3K) signaling. In contrast, using both laser ablation and a novel wounding assay that allows localized treatment with inhibitory drugs, we show that PI3K is essential for hemocyte chemotaxis toward wounds and that Pvf signals and PDGF/VEGF receptor expression are not required for this rapid chemotactic response. Our results demonstrate that at least two separate mechanisms operate in D. melanogaster embryos to direct hemocyte **migration** and show that although PI3K is crucial for hemocytes to sense a chemotactic gradient from a wound, it is not required to sense the growth factor signals that coordinate their developmental **migration**s along the ventral midline during embryogenesis.
12. Tip-cell **migration** controls stalk-cell intercalation during Drosophila tracheal tube elongation. Branching morphogenesis remodels epithelial tissues into tubular networks. This process is crucial to many organs, from the insect trachea to the vertebrate vasculature. Although Drosophila tracheal development has been well characterized morphologically and genetically, very little is known about the forces involved during morphogenesis. The repertoire of cell behaviors underlying tracheal primary branch remodeling is limited to cell **migration**, cell-shape changes, and stalk-cell intercalation (SCI), a process in which cells insert in between cells previously in contact with each other.Here, we identify the major forces that contribute to tracheal primary branch remodeling by using genetic and microsurgery experiments. As the tip cells migrate, they elongate the branches and create a tensile stress. This tensile stress triggers SCI, which, in turn, allows the branches to further elongate.The mechanism that we describe contrasts with "convergent extension by cell intercalation" acting during Drosophila germ band extension (GBE), where cell intercalation is the cause of epithelium elongation. Surprisingly, in tracheal branches, one or two leading cells produce enough mechanical power to intercalate many lagging cells. This may apply to other tubular networks.
13. The PDZ-GEF dizzy regulates cell shape of migrating macrophages via Rap1 and integrins in the Drosophila embryo. In Drosophila embryos, macrophages originate from the cephalic mesoderm and perform a complex **migration** throughout the entire embryo. The molecular mechanisms regulating this cell **migration** remain largely unknown. We identified the Drosophila PDZ G-nucleotide exchange factor (PDZ-GEF) Dizzy as a component essential for normal macrophage **migration**. In mutants lacking Dizzy, macrophages have smaller cellular protrusions, and their **migration** is slowed down significantly. This phenotype appears to be cell-autonomous, as it is also observed in embryos with a dsRNA-induced reduction of dizzy function in macrophages. In a complementary fashion, macrophages overexpressing Dizzy are vastly extended and form very long protrusions. These cell shape changes depend on the function of the small GTPase Rap1: in rap1 mutants, Dizzy is unable to induce the large protrusions. Furthermore, forced expression of a dominant-active form of Rap1, but not of the wild-type form, induces similar cell shape changes as Dizzy does overexpression. These findings suggest that Dizzy acts through Rap1. We propose that integrin-dependent adhesion is a Rap1-mediated target of Dizzy activity: in integrin mutants, neither Dizzy nor Rap1 can induce cell shape changes in macrophages. These data provide the first link between a PDZ-GEF, the corresponding small GTPase and integrin-dependent cell adhesion during cell **migration** in embryonic development.
14. IKK epsilon regulates F actin assembly and interacts with Drosophila IAP1 in cellular morphogenesis. Differentiated cells assume complex shapes through polarized cell **migration** and growth. These processes require the restricted organization of the actin cytoskeleton at limited subcellular regions. IKK epsilon is a member of the IkappaB kinase family, and its developmental role has not been clear. Drosophila IKK epsilon was localized to the ruffling membrane of cultured cells and was required for F actin turnover at the cell margin. In IKK epsilon mutants, tracheal terminal cells, bristles, and arista laterals, which require accurate F actin assembly for their polarized elongation, all exhibited aberrantly branched morphology. These phenotypes were sensitive to a change in the dosage of Drosophila inhibitor of apoptosis protein 1 (DIAP1) and the caspase DRONC without apparent change in cell viability. In contrast to this, hyperactivation of IKK epsilon destabilized F actin-based structures. Expression of a dominant-negative form of IKK epsilon increased the amount of DIAP1. The results suggest that at the physiological level, IKK epsilon acts as a negative regulator of F actin assembly and maintains the fidelity of polarized elongation during cell morphogenesis. This IKK epsilon function involves the negative regulation of the nonapoptotic activity of DIAP1.
15. Fibroblast growth factor signalling controls successive cell behaviours during mesoderm layer formation in Drosophila. Fibroblast growth factor (FGF)-dependent epithelial-mesenchymal transitions and cell **migration** contribute to the establishment of germ layers in vertebrates and other animals, but a comprehensive demonstration of the cellular activities that FGF controls to mediate these events has not been provided for any system. The establishment of the Drosophila mesoderm layer from an epithelial primordium involves a transition to a mesenchymal state and the dispersal of cells away from the site of internalisation in a FGF-dependent fashion. We show here that FGF plays multiple roles at successive stages of mesoderm morphogenesis in Drosophila. It is first required for the mesoderm primordium to lose its epithelial polarity. An intimate, FGF-dependent contact is established and maintained between the germ layers through mesoderm cell protrusions. These protrusions extend deep into the underlying ectoderm epithelium and are associated with high levels of E-cadherin at the germ layer interface. Finally, FGF directs distinct hitherto unrecognised and partially redundant protrusive behaviours during later mesoderm spreading. Cells first move radially towards the ectoderm, and then switch to a dorsally directed movement across its surface. We show that both movements are important for layer formation and present evidence suggesting that they are controlled by genetically distinct mechanisms.
16. A blood-borne PDGF/VEGF-like ligand initiates wound-induced epidermal cell **migration** in Drosophila larvae. Epidermal cell **migration** is critical for restoration of tissue structure and function after damage. However, the mechanisms by which differentiated cells neighboring the wound sense the wound and assume a motile phenotype remain unclear. Here, we show that Pvr, a receptor tyrosine kinase (RTK) related to platelet-derived growth factor (PDGF) and vascular endothelial growth factor (VEGF) receptors, and one of its ligands, Pvf1, are required for epidermal wound closure. Morphological comparison of wound-edge cells lacking Pvr or the Jun N-terminal kinase (JNK) signaling pathway previously implicated in larval wound closure suggests that Pvr signaling leads wound-margin epidermal cells to extend actin-based cell processes into the wound gap while JNK mediates transient dedifferentiation of cells at the wound margin. Genetic epistasis experiments reinforce the conclusion that the JNK and Pvr signaling pathways act in parallel. Tissue-specific knockdown and rescue experiments suggest that epidermally derived Pvf1 may be sequestered in the blood and that tissue damage exposes blood-borne Pvf1 to Pvr receptors on wound-edge epidermal cells and initiates the extension of cell processes into the wound gap. These results uncover a novel mechanism of sensing tissue damage and suggest that PDGF/VEGF ligands and receptors may play a conserved autocrine role in epidermal wound closure.
17. Genetic interaction screens identify a role for hedgehog signaling in Drosophila border cell **migration**. Cell motility is essential for embryonic development and physiological processes such as the immune response, but also contributes to pathological conditions such as tumor progression and inflammation. However, our understanding of the mechanisms underlying migratory processes is incomplete. Drosophila border cells provide a powerful genetic model to identify the roles of genes that contribute to cell **migration**.Members of the Hedgehog signaling pathway were uncovered in two independent screens for interactions with the small GTPase Rac and the polarity protein Par-1 in border cell **migration**. Consistent with a role in **migration**, multiple Hh signaling components were enriched in the migratory border cells. Interference with Hh signaling by several different methods resulted in incomplete cell **migration**. Moreover, the polarized distribution of E-Cadherin and a marker of tyrosine kinase activity were altered when Hh signaling was disrupted. Conservation of Hh-Rac and Hh-Par-1 signaling was illustrated in the wing, in which Hh-dependent phenotypes were enhanced by loss of Rac or par-1.We identified a pathway by which Hh signaling connects to Rac and Par-1 in cell **migration**. These results further highlight the importance of modifier screens in the identification of new genes that function in developmental pathways.
18. RhoA and Rac1 GTPases mediate the dynamic rearrangement of actin in peripheral glia. Peripheral glial cells in both vertebrates and insects are born centrally and travel large distances to ensheathe axons in the periphery. There is very little known about how this **migration** is carried out. In other cells, it is known that rearrangement of the Actin cytoskeleton is an integral part of cell motility, yet the distribution of Actin in peripheral glial cell **migration** in vivo has not been previously characterized. To gain an understanding of how glia migrate, we specifically labeled the peripheral glia of Drosophila melanogaster using an Actin-GFP marker and analyzed their development in the embryonic PNS. It was found that Actin cytoskeleton is dynamically rearranged during glial cell **migration**. The peripheral glia were observed to migrate as a continuous chain of cells, with the leading glial cells appearing to participate to the greatest extent in exploring the extracellular surroundings with filopodia-like Actin containing projections. We hypothesized that the small GTPases Rho, Rac and Cdc42 are involved in Actin cytoskeletal rearrangements that underlie peripheral glial **migration** and nerve ensheathement. To test this, transgenic forms of the GTPases were ectopically expressed specifically in the peripheral glia during their **migration** and wrapping phases. The effects on glial Actin-GFP distribution and the overall effects on glial cell **migration** and morphological development were assessed. We found that RhoA and Rac1 have distinct roles in peripheral glial cell **migration** and nerve ensheathement; however, Cdc42 does not have a significant role in peripheral glial development. RhoA and Rac1 gain-of-function and loss-of-function mutants had both disruption of glial cell development and secondary effects on sensory axon fasciculation. Together, Actin cytoskeletal dynamics is an integral part of peripheral glial **migration** and nerve ensheathement, and is mediated by RhoA and Rac1.
19. The epithelial-mesenchymal transition of the Drosophila mesoderm requires the Rho GTP exchange factor Pebble. Drosophila pebble (pbl) encodes a Rho-family GTP exchange factor (GEF) required for cytokinesis. The accumulation of high levels of PBL protein during interphase and the developmentally regulated expression of pbl in mesodermal tissues suggested that the primary cytokinetic mutant phenotype might be masking other roles. Using various muscle differentiation markers, we found that Even skipped (EVE) expression in the dorsal mesoderm is greatly reduced in pbl mutant embryos. EVE expression in the dorsalmost mesodermal cells is induced in response to DPP secreted by the dorsal epidermal cells. Further analysis revealed that this phenotype is likely to be a consequence of an earlier defect. pbl mutant mesodermal cells fail to undergo the normal epithelial-mesenchymal transition (EMT) and dorsal **migration** that follows ventral furrow formation. This phenotype is not a secondary consequence of failed cytokinesis, as it is rescued by a mutant form of pbl that does not rescue the cytokinetic defect. In wild-type embryos, newly invaginated cells at the lateral edges of the mesoderm extend numerous protrusions. In pbl mutant embryos, however, cells appear more tightly adhered to their neighbours and extend very few protrusions. Consistent with the dependence of the mesoderm EMT and cytokinesis on actin organisation, the GTP exchange function of the PBL RhoGEF is required for both processes. By contrast, the N-terminal BRCT domains of PBL are required only for the cytokinetic function of PBL. These studies reveal that a novel PBL-mediated intracellular signalling pathway operates in mesodermal cells during the transition from an epithelial to migratory mesenchymal morphology during gastrulation.
20. Invasive cell **migration** is initiated by guided growth of long cellular extensions. The **migration** of border cells during Drosophila melanogaster oogenesis is a simple and powerful system for studying invasive cell **migration** in vivo. Border cells are somatic cells that delaminate from the follicular epithelium of an egg chamber and invade the germ line cluster. They migrate between the nurse cells to reach the oocyte, using DE-cadherin for adhesion to the substratum. Border cells take approximately 6 h to migrate a distance of 100 microm. The **migration** is guided by EGFR (epidermal growth factor receptor) and PVR (platelet-derived growth factor (PDGF)/vascular endothelial growth factor (VEGF) receptor). Here, we show that a single long cellular extension (LCE), several cell diameters in length, is formed at the initiation of **migration**. The LCE may function as a 'pathfinder' in response to guidance cues. LCE growth requires directional guidance signals and specific adhesion to the substratum. Interference with actin-myosin interactions allows continued LCE growth while preventing translocation of the cell bodies. We discuss similarities between LCEs and axons and the use of LCE-like structures as a general mechanism for initiating invasive **migration** in vivo.
21. Notch and Numb are required for normal **migration** of peripheral glia in Drosophila. A prominent feature of glial cells is their ability to migrate along axons to finally wrap and insulate them. In the embryonic Drosophila PNS, most glial cells are born in the CNS and have to migrate to reach their final destinations. To understand how **migration** of the peripheral glia is regulated, we have conducted a genetic screen looking for mutants that disrupt the normal glial pattern. Here we present an analysis of two of these mutants: Notch and numb. Complete loss of Notch function leads to an increase in the number of glial cells. Embryos hemizygous for the weak Notch(B-8X) allele display an irregular **migration** phenotype and mutant glial cells show an increased formation of filopodia-like structures. A similar phenotype occurs in embryos carrying the Notch(ts1) allele when shifted to the restrictive temperature during the glial cell **migration** phase, suggesting that Notch must be activated during glial **migration**. This is corroborated by the fact that cell-specific reduction of Notch activity in glial cells by directed numb expression also results in similar **migration** phenotypes. Since the glial **migration** phenotypes of Notch and numb mutants resemble each other, our data support a model where the precise temporal and quantitative regulation of Numb and Notch activity is not only required during fate decisions but also later during glial differentiation and **migration**.
22. A requirement for the Abnormal Spindle protein to organise microtubules of the central spindle for cytokinesis in Drosophila. Drosophila abnormal spindle (asp) mutants exhibit a mitotic metaphase checkpoint arrest with abnormal spindle poles, which reflects a requirement for Asp for the integrity of microtubule organising centres (MTOCs). In male meiosis, the absence of a strong spindle integrity checkpoint enables asp mutant cells to proceed through anaphase and telophase. However, the central spindle region is not correctly organised and cells frequently fail to complete cytokinesis. This contrasts with meiosis in wild-type males where at late anaphase a dense array of microtubules forms in the central spindle region that has Asp localised at its border. We speculate that Asp is associated with the minus ends of microtubules that have been released from the spindle poles to form the central spindle. A parallel situation arises in female meiosis where Asp not only associates with the minus ends of microtubules at the acentriolar poles but also with the central spindle pole body that forms between the two tandem spindles of meiosis II. Upon fertilisation, Asp is also recruited to the MTOC that nucleates the sperm aster. Asp is required for growth of the microtubules of the sperm aster, which in asp mutants remains diminutive and so prevents **migration** of the pronuclei.
23. Focal adhesions: what's new inside. The cytoplasmic side of focal adhesions is comprised of large molecular complexes that link transmembrane receptors, such as integrins, to the actin cytoskeleton and mediate signals modulating cell attachment, **migration**, proliferation, differentiation, and gene expression. These complexes are heterogeneous and dynamic structures that are apparent targets of regulatory signals that control the function of focal adhesions. Recent studies using genetic approaches in invertebrate and vertebrate systems have begun to reveal the structure and function of these complexes in vivo.
24. Function of the ETS transcription factor Yan in border cell **migration**. Invasive cell **migration** in both normal development and metastatic cancer is regulated by various signaling pathways, transcription factors and cell-adhesion molecules. The coordination between these activities in the context of cell **migration** is poorly understood. During Drosophila oogenesis, a small group of cells called border cells exit the follicular epithelium to perform a stereotypic, invasive **migration**. We find that the ETS transcription factor Yan is required for border cell **migration** and that Yan expression is spatiotemporally regulated as border cells migrate from the anterior pole of the egg chamber towards the nurse cell-oocyte boundary. Yan expression is dependent on inputs from the JAK/STAT, Notch and Receptor Tyrosine Kinase pathways in border cells. Mechanistically, Yan functions to modulate the turnover of DE-Cadherin-dependent adhesive complexes to facilitate border cell **migration**. Our results suggest that Yan acts as a pivotal link between signal transduction, cell adhesion and invasive cell **migration** in Drosophila border cells.
25. Distinct roles for two receptor tyrosine kinases in epithelial branching morphogenesis in Drosophila. Branching morphogenesis is a widespread mechanism used to increase the surface area of epithelial organs. Many signaling systems steer development of branched organs, but it is still unclear which cellular processes are regulated by the different pathways. We have used the development of the air sacs of the dorsal thorax of Drosophila to study cellular events and their regulation via cell-cell signaling. We find that two receptor tyrosine kinases play important but distinct roles in air sac outgrowth. Fgf signaling directs cell **migration** at the tip of the structure, while Egf signaling is instrumental for cell division and cell survival in the growing epithelial structure. Interestingly, we find that Fgf signaling requires Ras, the Mapk pathway, and Pointed to direct **migration**, suggesting that both cytoskeletal and nuclear events are downstream of receptor activation. Ras and the Mapk pathway are also needed for Egf-regulated cell division/survival, but Pointed is dispensable.
26. Drosophila klaroid encodes a SUN domain protein required for Klarsicht localization to the nuclear envelope and nuclear **migration** in the eye. KASH (Klarsicht/Anc-1/Syne homology) domain proteins are cytoskeleton-associated proteins localized uniquely to the outer nuclear membrane. Klarsicht is a KASH protein required for nuclear **migration** in differentiating cells of the Drosophila eye. The C-terminal KASH domain of Klarsicht resides in the perinuclear space, and the cytoplasmic moiety connects to the microtubule organizing center. In C. elegans and vertebrate cells, SUN (Sad1/UNC-84) domain proteins reside in the inner nuclear membrane and tether KASH proteins to the outer nuclear membrane. Is there a Drosophila SUN protein that performs a similar function, and if so, is it like Klarsicht, obviously essential for nuclear positioning only in the eye? Here, we identify Drosophila Klaroid, a SUN protein that tethers Klarsicht. klaroid loss-of-function mutants are indistinguishable phenotypically from klarsicht mutants. Remarkably, neither gene is essential for Drosophila viability or fertility, and even in klaroid klorsicht double mutants, the only obvious external morphological defect is rough eyes. In addition, we find that klaroid and klarsicht are required for nuclear **migration** in differentiating neurons and in non-neural cells. Finally, while perinuclear Klaroid is ubiquitous in the eye, Klarsicht expression is limited to differentiating cells and may be part of the trigger for apical nuclear **migration**.
27. Dpp signaling directs cell motility and invasiveness during epithelial morphogenesis. Tissue remodeling in development and disease involves the coordinated invasion of neighboring territories and/or the replacement of entire cell populations. Cell guidance, cell matching, transitions from passive to migratory epithelia, cell growth and death, and extracellular matrix remodeling all impinge on epithelial spreading. Significantly, the extracellular signals that direct these activities and the specific cellular elements and mechanisms regulated by these signals remain in most cases to be identified. To address these issues, we performed an analysis of histoblasts (Drosophila abdominal epithelial founder cells) on their transition from a dormant state to active **migration** replacing obsolete larval epidermal cells (LECs). We found that during expansion, Decapentaplegic (Dpp) secreted from surrounding LECs leads to graded pathway activation in cells at the periphery of histoblast nests. Across nests, Dpp activity confers differential cellular behavior and motility by modulating cell-cell contacts, the organization and activity of the cytoskeleton, and histoblast attachment to the substrate. Furthermore, Dpp also prevents the premature death of LECs, allowing the coordination of histoblast expansion to LEC delamination. Dpp signaling activity directing histoblast spreading and invasiveness mimics transforming growth factor-beta and bone morphogenetic proteins' role in enhancing the motility and invasiveness of cancer cells, resulting in the promotion of metastasis.
28. Group I PAKs function downstream of Rac to promote podosome invasion during myoblast fusion in vivo. The p21-activated kinases (PAKs) play essential roles in diverse cellular processes and are required for cell proliferation, apoptosis, polarity establishment, **migration**, and cell shape changes. Here, we have identified a novel function for the group I PAKs in cell-cell fusion. We show that the two Drosophila group I PAKs, DPak3 and DPak1, have partially redundant functions in myoblast fusion in vivo, with DPak3 playing a major role. DPak3 is enriched at the site of fusion colocalizing with the F-actin focus within a podosome-like structure (PLS), and promotes actin filament assembly during PLS invasion. Although the small GTPase Rac is involved in DPak3 activation and recruitment to the PLS, the kinase activity of DPak3 is required for effective PLS invasion. We propose a model whereby group I PAKs act downstream of Rac to organize the actin filaments within the PLS into a dense focus, which in turn promotes PLS invasion and fusion pore initiation during myoblast fusion.
29. Enabled and Capping protein play important roles in shaping cell behavior during Drosophila oogenesis. During development, cells craft an impressive array of actin-based structures, mediating events as diverse as cytokinesis, apical constriction, and cell **migration**. One challenge is to determine how cells regulate actin assembly and disassembly to carry out these cell behaviors. During Drosophila oogenesis diverse cell behaviors are seen in the soma and germline. We used oogenesis to explore developmental roles of two important actin regulators: Enabled/VASP proteins and Capping protein. We found that Enabled plays an important role in cortical integrity of nurse cells, formation of robust bundled actin filaments in late nurse cells that facilitate nurse cell dumping, and **migration** of somatic border cells. During nurse cell dumping, Enabled localizes to barbed ends of the nurse cell actin filaments, suggesting its mechanism of action. We further pursued this mechanism using mutant Enabled proteins, each affecting one of its protein domains. These data suggest critical roles for the EVH2 domain and its tetramerization subdomain, while the EVH1 domain appears less critical. Enabled appears to be negatively regulated during oogenesis by Abelson kinase. We also explored the function of Capping protein. This revealed important roles in oocyte determination, nurse cell cortical integrity and nurse cell dumping, and support the idea that Capping protein and Enabled act antagonistically during dumping. Together these data reveal places that these actin regulators shape oogenesis.
30. The core and conserved role of MAL is homeostatic regulation of actin levels. The transcription cofactor MAL is regulated by free actin levels and thus by actin dynamics. MAL, together with its DNA-binding partner, SRF, is required for invasive cell **migration** and in experimental metastasis. Although MAL/SRF has many targets, we provide genetic evidence in both Drosophila and human cellular models that actin is the key target that must be regulated by MAL/SRF for invasive cell **migration**. By regulating MAL/SRF activity, actin protein feeds back on production of actin mRNA to ensure sufficient supply of actin. This constitutes a dedicated homeostatic feedback system that provides a foundation for cellular actin dynamics.
31. Time-lapse and cell ablation reveal the role of cell interactions in fly glia **migration** and proliferation. **Migration** and proliferation have been mostly explored in culture systems or fixed preparations. We present a simple genetic model, the chains of glia moving along fly wing nerves, to follow such dynamic processes by time-lapse in the whole animal. We show that glia undergo extensive cytoskeleton and mitotic apparatus rearrangements during division and **migration**. Single cell labelling identifies different glia: pioneers with high filopodial, exploratory, activity and, less active followers. In combination with time-lapse, altering this cellular environment by genetic means or cell ablation has allowed to us define the role of specific cell-cell interactions. First, neurone-glia interactions are not necessary for glia motility but do affect the direction of **migration**. Second, repulsive interactions between glia control the extent of movement. Finally, autonomous cues control proliferation.
32. In vivo imaging reveals different cellular functions for FGF and Dpp signaling in tracheal branching morphogenesis. In the developing tracheal system of Drosophila melanogaster, six major branches arise by guided cell **migration** from a sac-like structure. The chemoattractant Branchless/FGF (Bnl) appears to guide cell **migration** and is essential for the formation of all tracheal branches, while Decapentaplegic (Dpp) signaling is strictly required for the formation of a subset of branches, the dorsal and ventral branches. Using in vivo confocal video microscopy, we find that the two signaling systems affect different cellular functions required for branching morphogenesis. Bnl/FGF signaling affects the formation of dynamic filopodia, possibly controlling cytoskeletal activity and motility as such, and Dpp controls cellular functions allowing branch morphogenesis and outgrowth.
33. The Drosophila Dead end Arf-like3 GTPase controls vesicle trafficking during tracheal fusion cell morphogenesis. The Drosophila larval tracheal system consists of a highly branched tubular organ that becomes interconnected by **migration**-fusion events during embryonic development. Fusion cells at the tip of each branch guide **migration**, adhere, and then undergo extensive remodeling as the tracheal lumen extends between the two branches. The Drosophila dead end gene is expressed in fusion cells, and encodes an Arf-like3 GTPase. Analyses of dead end RNAi and mutant embryos reveal that the lumen fails to connect between the two branches. Expression of a constitutively active form of Dead end in S2 cells reveals that it influences the state of actin polymerization, and is present on particles that traffic along actin/microtubule-containing processes. Imaging experiments in vivo reveal that Dead end-containing vesicles are associated with recycling endosomes and the exocyst, and control exocyst localization in fusion cells. These results indicate that the Dead end GTPase plays an important role in trafficking membrane components involved in tracheal fusion cell morphogenesis and lumenal development.
34. Exploring the roles of diaphanous and enabled activity in shaping the balance between filopodia and lamellipodia. During **migration** cell protrusions power cell extension and sample the environment. Different cells produce different protrusions, from keratocytes dominated by lamellipodia, to growth cones combining filopodia and lamellipodia, to dendritic spines. One key challenge is to determine how the toolkit of actin regulators are coordinated to generate these diverse protrusive arrays. Here we use Drosophila leading-edge (LE) cells to explore how Diaphanous (Dia)-related formins and Ena/VASP proteins cooperate in this process. We first dissect the Dia regulatory region, revealing novel roles for the GTPase-binding and FH3 domains in cortical localization, filopodial initiation, and lengthening. Second, we provide evidence that activating Dia mobilizes Ena from storage places near the LE to act at the LE. Further, Dia and Ena coIP and can recruit one another to new locations, suggesting cooperation is key to their mechanisms of action. Third, we directly explore the functional relationship between Dia and Ena, varying their levels and activity separately in the same cell type. Surprisingly, although each is sufficient to induce filopodia, together they induce lamellipodia. Our data suggest they work together in a complex and nonadditive way, with the ratio between active Dia and Ena being one factor that modulates the balance between filopodia and lamellipodia.
35. Aster **migration** determines the length scale of nuclear separation in the Drosophila syncytial embryo. In the early embryo of many species, comparatively small spindles are positioned near the cell center for subsequent cytokinesis. In most insects, however, rapid nuclear divisions occur in the absence of cytokinesis, and nuclei distribute rapidly throughout the large syncytial embryo. Even distribution and anchoring of nuclei at the embryo cortex are crucial for cellularization of the blastoderm embryo. The principles underlying nuclear dispersal in a syncytium are unclear. We established a cell-free system from individual Drosophila melanogaster embryos that supports successive nuclear division cycles with native characteristics. This allowed us to investigate nuclear separation in predefined volumes. Encapsulating nuclei in microchambers revealed that the early cytoplasm is programmed to separate nuclei a distinct distance. Laser microsurgery revealed an important role of microtubule aster **migration** through cytoplasmic space, which depended on F-actin and cooperated with anaphase spindle elongation. These activities define a characteristic separation length scale that appears to be a conserved property of developing insect embryos.
36. Dynamic analysis of actin cable function during Drosophila dorsal closure. Throughout development, a series of epithelial movements and fusions occur that collectively shape the embryo. They are dependent on coordinated reorganizations and contractions of the actin cytoskeleton within defined populations of epithelial cells. One paradigm morphogenetic movement, dorsal closure in the Drosophila embryo, involves closure of a dorsal epithelial hole by sweeping of epithelium from the two sides of the embryo over the exposed extraembryonic amnioserosa to form a seam where the two epithelial edges fuse together. The front row cells exhibit a thick actin cable at their leading edge. Here, we test the function of this cable by live analysis of GFP-actin-expressing embryos in which the cable is disrupted by modulating Rho1 signaling or by loss of non-muscle myosin (Zipper) function. We show that the cable serves a dual role during dorsal closure. It is contractile and thus can operate as a "purse string," but it also restricts forward movement of the leading edge and excess activity of filopodia/lamellipodia. Stripes of epithelium in which cable assembly is disrupted gain a **migration**al advantage over their wild-type neighbors, suggesting that the cable acts to restrain front row cells, thus maintaining a taut, free edge for efficient zippering together of the epithelial sheets.
37. Reciprocal interactions between neurons and glia are required for Drosophila peripheral nervous system development. A major developmental role of peripheral glia is to mediate sensory axon guidance; however, it is not known whether sensory neurons influence peripheral glial development. To determine whether glia and neurons reciprocally interact during embryonic development, we ablated each cell type by overexpressing the apoptosis gene, grim, and observed the effects on peripheral nervous system (PNS) development. When neurons are ablated, glial defects occur as a secondary effect, and vice versa. Therefore glia and neurons are codependent during embryogenesis. To further explore glial-neuronal interactions, we genetically disrupted glial **migration** or differentiation and observed the secondary effects on sensory neuron development. Glial **migration** and ensheathment of PNS axons was blocked by overexpression of activated Rho GTPase, a regulator of actin dynamics. Here, sensory axons extended to the CNS without exhibiting gross pathfinding errors. In contrast, disrupting differentiation by expression of dominant-negative Ras GTPase in glia resulted in major sensory axon pathfinding errors, similar to those seen in glial ablations. Glial overexpression of transgenic components of the epidermal growth factor receptor (EGFR) signaling pathway yielded similar sensory neuron defects and also downregulated the expression of the glial marker Neuroglian. Mutant analysis also suggested that the EGFR ligands Spitz and Vein play roles in peripheral glial development. The observations support a model in which glia express genes necessary for sensory neuron development, and these genes are potentially under the control of the EGFR/Ras signaling pathway.
38. Actin dynamics in lamellipodia of migrating border cells in the Drosophila ovary revealed by a GFP-actin fusion protein. Directional **migration** of border cells in the Drosophila egg chambers is a developmentally regulated event that requires dynamic cellular functions. In this study, the electron microscopic observation of migrating border cells revealed loose actin bundles in forepart lamellipodia and numerous microvilli extending from nurse cells and providing multiple adhesive contacts with border cells. To analyze the dynamics of actin in migrating border cells in vivo, we constructed a green fluorescent protein-actin fusion protein and induced its expression in Drosophila using the GAL4/UAS system. The green fluorescent protein-actin was incorporated into the actin bundles and it enabled visualization of the rapid cytoskeletal changes in border cell lamellipodia. During the growth of the lamellipodia, the actin bundles that increased in number and size radiated from the bundle-organizing center. Quantification of the fluorescence intensity showed that an accumulation of bundle-associated and spotted green fluorescent protein-actin signals took place during their centripetal movement. Our results favored a treadmilling model for actin behavior in border cell lamellipodia.
39. Genetic control of cell intercalation during tracheal morphogenesis in Drosophila. Branching morphogenesis transforms an epithelial sheet into a tubular network with distinct features regarding the length and diameter of individual tubes. Branching is controlled by several signaling pathways, but the molecular consequences of these pathways in the responding cells are poorly understood.We have undertaken a detailed characterization of cell rearrangements during tracheal branching morphogenesis in Drosophila embryos with a GFP fusion protein labeling the adherens junctions (AJs) and high-resolution live imaging. To analyze the branching process at the cellular level, we further developed an imaging approach that allows us to follow single cells during the branching process. We find that controlled cell intercalation, which requires extensive AJ remodeling, is key to the formation of tracheal branches of different cellular complexities. In particular, most branches consist of tubes with individual cells wrapped around the lumen. These branches form through cell intercalation, which requires the transformation of most of the initial intercellular AJs into autocellular AJs. We propose a step-wise model explaining how this AJ remodeling occurs and use this model to better understand defects in various mutants. We find that Dpp and Wnt signaling control cell intercalation by regulating the expression of Spalt, a zinc finger transcription factor; Spalt inhibits intercalation, leading to the formation of large, multicellular tubes.Tracheal morphogenesis is regulated by an interplay of different signaling systems that control cell **migration** and cell intercalation, respectively. Only the combined action of these signaling systems allows efficient branch elongation and the formation of morphologically distinct branches.
40. MICAL, the flavoenzyme participating in cytoskeleton dynamics. MICAL (from the Molecule Interacting with CasL) indicates a family of recently discovered cytosolic, multidomain proteins, which uniquely couple an N-terminal FAD-containing monooxygenase-like domain to typical calponine homology, LIM and coiled-coil protein-interaction modules. Genetic and cell biology approaches have demonstrated an essential role of the catalytic activity of the monooxygenase-like domain in transducing the signal initiated by semaphorins interaction with their plexin receptors, which results in local actin cytoskeleton disassembly as part of fundamental processes that include differentiation, **migration** and cell-cell contacts in neuronal and non-neuronal cell types. This review focuses on the structure-function relations of the MICAL monooxygenase-like domain as they are emerging from the available in vitro studies on mouse, human and Drosophila MICAL forms that demonstrated a NADPH-dependent actin depolymerizing activity of MICAL. With Drosophila MICAL forms, actin depolymerization was demonstrated to be associated to conversion of Met44 to methionine sulfone through a postulated hydroxylating reaction. Arguments supporting the concept that MICAL effect on F-actin may be reversible will be discussed.

### ECM

#### PubMed

1. Tissue elongation requires oscillating contractions of a basal actomyosin network. Understanding how molecular dynamics leads to cellular behaviours that ultimately sculpt organs and tissues is a major challenge not only in basic developmental biology but also in tissue engineering and regenerative medicine. Here we use live imaging to show that the basal surfaces of Drosophila follicle cells undergo a series of directional, oscillating contractions driven by periodic myosin accumulation on a polarized actin network. Inhibition of the actomyosin contractions or their coupling to extracellular matrix (**ECM**) blocked elongation of the whole tissue, whereas enhancement of the contractions exaggerated it. Myosin accumulated in a periodic manner before each contraction and was regulated by the small GTPase Rho, its downstream kinase, ROCK, and cytosolic calcium. Disrupting the link between the actin cytoskeleton and the **ECM** decreased the amplitude and period of the contractions, whereas enhancing cell-**ECM** adhesion increased them. In contrast, disrupting cell-cell adhesions resulted in loss of the actin network. Our findings reveal a mechanism controlling organ shape and an experimental model for the study of the effects of oscillatory actomyosin activity within a coherent cell sheet.

### [Ee]xtracellular [Mm]atrix

#### PubMed

1. The actomyosin machinery is required for Drosophila retinal lumen formation. Multicellular tubes consist of polarized cells wrapped around a central lumen and are essential structures underlying many developmental and physiological functions. In Drosophila compound eyes, each ommatidium forms a luminal matrix, the inter-rhabdomeral space, to shape and separate the key phototransduction organelles, the rhabdomeres, for proper visual perception. In an enhancer screen to define mechanisms of retina lumen formation, we identified Actin5C as a key molecule. Our results demonstrate that the disruption of lumen formation upon the reduction of Actin5C is not linked to any discernible defect in microvillus formation, the rhabdomere terminal web (RTW), or the overall morphogenesis and basal extension of the rhabdomere. Second, the failure of proper lumen formation is not the result of previously identified processes of retinal lumen formation: Prominin localization, expansion of the apical membrane, or secretion of the luminal matrix. Rather, the phenotype observed with Actin5C is phenocopied upon the decrease of the individual components of non-muscle myosin II (MyoII) and its upstream activators. In photoreceptor cells MyoII localizes to the base of the rhabdomeres, overlapping with the actin filaments of the RTW. Consistent with the well-established roll of actomyosin-mediated cellular contraction, reduction of MyoII results in reduced distance between apical membranes as measured by a decrease in lumen diameter. Together, our results indicate the actomyosin machinery coordinates with the localization of apical membrane components and the secretion of an **extracellular matrix** to overcome apical membrane adhesion to initiate and expand the retinal lumen.
2. Tissue elongation requires oscillating contractions of a basal actomyosin network. Understanding how molecular dynamics leads to cellular behaviours that ultimately sculpt organs and tissues is a major challenge not only in basic developmental biology but also in tissue engineering and regenerative medicine. Here we use live imaging to show that the basal surfaces of Drosophila follicle cells undergo a series of directional, oscillating contractions driven by periodic myosin accumulation on a polarized actin network. Inhibition of the actomyosin contractions or their coupling to **extracellular matrix** (ECM) blocked elongation of the whole tissue, whereas enhancement of the contractions exaggerated it. Myosin accumulated in a periodic manner before each contraction and was regulated by the small GTPase Rho, its downstream kinase, ROCK, and cytosolic calcium. Disrupting the link between the actin cytoskeleton and the ECM decreased the amplitude and period of the contractions, whereas enhancing cell-ECM adhesion increased them. In contrast, disrupting cell-cell adhesions resulted in loss of the actin network. Our findings reveal a mechanism controlling organ shape and an experimental model for the study of the effects of oscillatory actomyosin activity within a coherent cell sheet.
3. Dpp signaling directs cell motility and invasiveness during epithelial morphogenesis. Tissue remodeling in development and disease involves the coordinated invasion of neighboring territories and/or the replacement of entire cell populations. Cell guidance, cell matching, transitions from passive to migratory epithelia, cell growth and death, and **extracellular matrix** remodeling all impinge on epithelial spreading. Significantly, the extracellular signals that direct these activities and the specific cellular elements and mechanisms regulated by these signals remain in most cases to be identified. To address these issues, we performed an analysis of histoblasts (Drosophila abdominal epithelial founder cells) on their transition from a dormant state to active migration replacing obsolete larval epidermal cells (LECs). We found that during expansion, Decapentaplegic (Dpp) secreted from surrounding LECs leads to graded pathway activation in cells at the periphery of histoblast nests. Across nests, Dpp activity confers differential cellular behavior and motility by modulating cell-cell contacts, the organization and activity of the cytoskeleton, and histoblast attachment to the substrate. Furthermore, Dpp also prevents the premature death of LECs, allowing the coordination of histoblast expansion to LEC delamination. Dpp signaling activity directing histoblast spreading and invasiveness mimics transforming growth factor-beta and bone morphogenetic proteins' role in enhancing the motility and invasiveness of cancer cells, resulting in the promotion of metastasis.
4. Epithelial tube morphogenesis during Drosophila tracheal development requires Piopio, a luminal ZP protein. The formation of branched epithelial networks is fundamental to the development of many organs, such as the lung, the kidney or the vasculature. Little is known about the mechanisms that control cell rearrangements during tubulogenesis and regulate the size of individual tubes. Recent studies indicate that whereas the basal surface of tube cells interacts with the surrounding tissues and helps to shape the ramification pattern of tubular organs, the apical surface has an important role in the regulation of tube diameter and tube growth. Here we report that two proteins, Piopio (Pio) and Dumpy (Dp), containing a zona pellucida (ZP) domain are essential for the generation of the interconnected tracheal network in Drosophila melanogaster. Pio is secreted apically, accumulates in the tracheal lumen and possibly interacts with Dp through the ZP domains. In the absence of Pio and Dp, multicellular tubes do not rearrange through cell elongation and cell intercalation to form narrow tubes with autocellular junctions; instead they are transformed into multicellular cysts, which leads to a severe disruption of the branched pattern. We propose that an **extracellular matrix** containing Pio and Dp provides a structural network in the luminal space, around which cell rearrangements can take place in an ordered fashion without losing interconnections. Our results suggest that a similar structural role might be attributed to other ZP-domain proteins in the formation of different branched organs.

### [Ii]nvasive

#### PubMed

1. Extrinsic and intrinsic mechanisms directing epithelial cell sheet replacement during Drosophila metamorphosis. The fusion of epithelial sheets is an essential morphogenetic event. Here, we study the development of the abdomen of Drosophila as a model of bounded epithelia expansion and uncover a complex multistep process for the generation of the adult epidermis from histoblasts, founder cells that replace the larval cells during metamorphosis. We find that histoblasts experience a biphasic cell cycle and emit apical projections that direct their **invasive** planar intercalation in between larval cells. Coordinately, the larval cells extrude from the epithelia by apical constriction of an actomyosin ring and as a consequence die by apoptosis and are removed by circulating haemocytes. We demonstrate that the proliferation of histoblasts and the death of larval cells are triggered by two independent extrinsic Ecdysone hormonal pulses. Finally, we show that histoblast spreading and the death of larval cells depend on a mutual exchange of signals and are non-autonomous processes.
2. Initiating and guiding migration: lessons from border cells. Cell migration occurs in many different contexts. Amoebae and other isolated cells migrate in culture. In animals, 'professional' migratory cells of the immune system constantly survey the body for intruders, whereas other cell types perform specific developmentally regulated migrations. One simple model for the latter type of event is migration of border cells during Drosophila oogenesis. Recent findings have shed light on how border cell fate is induced and on how the migration is guided. This article discusses the implications of these studies and compares (**invasive**) migration through a tissue with what is known about cells crawling on a flat substratum.
3. Cell mixing induced by myc is required for competitive tissue invasion and destruction. Cell-cell intercalation is used in several developmental processes to shape the normal body plan. There is no clear evidence that intercalation is involved in pathologies. Here we use the proto-oncogene myc to study a process analogous to early phase of tumour expansion: myc-induced cell competition. Cell competition is a conserved mechanism driving the elimination of slow-proliferating cells (so-called 'losers') by faster-proliferating neighbours (so-called 'winners') through apoptosis and is important in preventing developmental malformations and maintain tissue fitness. Here we show, using long-term live imaging of myc-driven competition in the Drosophila pupal notum and in the wing imaginal disc, that the probability of elimination of loser cells correlates with the surface of contact shared with winners. As such, modifying loser-winner interface morphology can modulate the strength of competition. We further show that elimination of loser clones requires winner-loser cell mixing through cell-cell intercalation. Cell mixing is driven by differential growth and the high tension at winner-winner interfaces relative to winner-loser and loser-loser interfaces, which leads to a preferential stabilization of winner-loser contacts and reduction of clone compactness over time. Differences in tension are generated by a relative difference in F-actin levels between loser and winner junctions, induced by differential levels of the membrane lipid phosphatidylinositol (3,4,5)-trisphosphate. Our results establish the first link between cell-cell intercalation induced by a proto-oncogene and how it promotes **invasive**ness and destruction of healthy tissues.
4. **Invasive** cell migration is initiated by guided growth of long cellular extensions. The migration of border cells during Drosophila melanogaster oogenesis is a simple and powerful system for studying **invasive** cell migration in vivo. Border cells are somatic cells that delaminate from the follicular epithelium of an egg chamber and invade the germ line cluster. They migrate between the nurse cells to reach the oocyte, using DE-cadherin for adhesion to the substratum. Border cells take approximately 6 h to migrate a distance of 100 microm. The migration is guided by EGFR (epidermal growth factor receptor) and PVR (platelet-derived growth factor (PDGF)/vascular endothelial growth factor (VEGF) receptor). Here, we show that a single long cellular extension (LCE), several cell diameters in length, is formed at the initiation of migration. The LCE may function as a 'pathfinder' in response to guidance cues. LCE growth requires directional guidance signals and specific adhesion to the substratum. Interference with actin-myosin interactions allows continued LCE growth while preventing translocation of the cell bodies. We discuss similarities between LCEs and axons and the use of LCE-like structures as a general mechanism for initiating **invasive** migration in vivo.
5. An **invasive** podosome-like structure promotes fusion pore formation during myoblast fusion. Recent studies in Drosophila have implicated actin cytoskeletal remodeling in myoblast fusion, but the cellular mechanisms underlying this process remain poorly understood. Here we show that actin polymerization occurs in an asymmetric and cell type-specific manner between a muscle founder cell and a fusion-competent myoblast (FCM). In the FCM, a dense F-actin-enriched focus forms at the site of fusion, whereas a thin sheath of F-actin is induced along the apposing founder cell membrane. The FCM-specific actin focus invades the apposing founder cell with multiple finger-like protrusions, leading to the formation of a single-channel macro fusion pore between the two muscle cells. Two actin nucleation-promoting factors of the Arp2/3 complex, WASP and Scar, are required for the formation of the F-actin foci, whereas WASP but not Scar promotes efficient foci invasion. Our studies uncover a novel **invasive** podosome-like structure (PLS) in a developing tissue and reveal a previously unrecognized function of PLSs in facilitating cell membrane juxtaposition and fusion.
6. Competition between Blown fuse and WASP for WIP binding regulates the dynamics of WASP-dependent actin polymerization in vivo. Dynamic rearrangements of the actin cytoskeleton play a key role in numerous cellular processes. In Drosophila, fusion between a muscle founder cell and a fusion competent myoblast (FCM) is mediated by an **invasive**, F-actin-enriched podosome-like structure (PLS). Here, we show that the dynamics of the PLS is controlled by Blown fuse (Blow), a cytoplasmic protein required for myoblast fusion but whose molecular function has been elusive. We demonstrate that Blow is an FCM-specific protein that colocalizes with WASP, WIP/Solitary, and the actin focus within the PLS. Biochemically, Blow modulates the stability of the WASP-WIP complex by competing with WASP for WIP binding, leading to a rapid exchange of WASP, WIP and G-actin within the PLS, which, in turn, actively invades the adjacent founder cell to promote fusion pore formation. These studies identify a regulatory protein that modulates the actin cytoskeletal dynamics by controlling the stability of the WASP-WIP complex.
7. Function of the ETS transcription factor Yan in border cell migration. **Invasive** cell migration in both normal development and metastatic cancer is regulated by various signaling pathways, transcription factors and cell-adhesion molecules. The coordination between these activities in the context of cell migration is poorly understood. During Drosophila oogenesis, a small group of cells called border cells exit the follicular epithelium to perform a stereotypic, **invasive** migration. We find that the ETS transcription factor Yan is required for border cell migration and that Yan expression is spatiotemporally regulated as border cells migrate from the anterior pole of the egg chamber towards the nurse cell-oocyte boundary. Yan expression is dependent on inputs from the JAK/STAT, Notch and Receptor Tyrosine Kinase pathways in border cells. Mechanistically, Yan functions to modulate the turnover of DE-Cadherin-dependent adhesive complexes to facilitate border cell migration. Our results suggest that Yan acts as a pivotal link between signal transduction, cell adhesion and **invasive** cell migration in Drosophila border cells.
8. Dpp signaling directs cell motility and **invasive**ness during epithelial morphogenesis. Tissue remodeling in development and disease involves the coordinated invasion of neighboring territories and/or the replacement of entire cell populations. Cell guidance, cell matching, transitions from passive to migratory epithelia, cell growth and death, and extracellular matrix remodeling all impinge on epithelial spreading. Significantly, the extracellular signals that direct these activities and the specific cellular elements and mechanisms regulated by these signals remain in most cases to be identified. To address these issues, we performed an analysis of histoblasts (Drosophila abdominal epithelial founder cells) on their transition from a dormant state to active migration replacing obsolete larval epidermal cells (LECs). We found that during expansion, Decapentaplegic (Dpp) secreted from surrounding LECs leads to graded pathway activation in cells at the periphery of histoblast nests. Across nests, Dpp activity confers differential cellular behavior and motility by modulating cell-cell contacts, the organization and activity of the cytoskeleton, and histoblast attachment to the substrate. Furthermore, Dpp also prevents the premature death of LECs, allowing the coordination of histoblast expansion to LEC delamination. Dpp signaling activity directing histoblast spreading and **invasive**ness mimics transforming growth factor-beta and bone morphogenetic proteins' role in enhancing the motility and **invasive**ness of cancer cells, resulting in the promotion of metastasis.
9. The core and conserved role of MAL is homeostatic regulation of actin levels. The transcription cofactor MAL is regulated by free actin levels and thus by actin dynamics. MAL, together with its DNA-binding partner, SRF, is required for **invasive** cell migration and in experimental metastasis. Although MAL/SRF has many targets, we provide genetic evidence in both Drosophila and human cellular models that actin is the key target that must be regulated by MAL/SRF for **invasive** cell migration. By regulating MAL/SRF activity, actin protein feeds back on production of actin mRNA to ensure sufficient supply of actin. This constitutes a dedicated homeostatic feedback system that provides a foundation for cellular actin dynamics.
10. A Cathepsin-L is required for **invasive** behavior during Air Sac Primordium development in Drosophila melanogaster. The Drosophila Air Sac Primordium (ASP) has emerged as an important structure where cellular, genetic and molecular events responsible for **invasive** behavior and branching morphogenesis can be studied. In this report we present data which demonstrate that a Cathepsin-L encoded by the gene CP1 in Drosophila is necessary for **invasive** behavior during ASP development. We find that CP1 is expressed in ASP and knockdown of CP1 results in suppression of migratory and **invasive** behavior observed during ASP development. We further show that CP1 possibly regulates **invasive** behavior by promoting degradation of Basement Membrane. Our data provide clues to the possible role of Cathepsin L in human lung development and tumor invasion, especially, given the similarities between human lung and Drosophila ASP development.

### [Mm]etastasis

#### PubMed

1. Dpp signaling directs cell motility and invasiveness during epithelial morphogenesis. Tissue remodeling in development and disease involves the coordinated invasion of neighboring territories and/or the replacement of entire cell populations. Cell guidance, cell matching, transitions from passive to migratory epithelia, cell growth and death, and extracellular matrix remodeling all impinge on epithelial spreading. Significantly, the extracellular signals that direct these activities and the specific cellular elements and mechanisms regulated by these signals remain in most cases to be identified. To address these issues, we performed an analysis of histoblasts (Drosophila abdominal epithelial founder cells) on their transition from a dormant state to active migration replacing obsolete larval epidermal cells (LECs). We found that during expansion, Decapentaplegic (Dpp) secreted from surrounding LECs leads to graded pathway activation in cells at the periphery of histoblast nests. Across nests, Dpp activity confers differential cellular behavior and motility by modulating cell-cell contacts, the organization and activity of the cytoskeleton, and histoblast attachment to the substrate. Furthermore, Dpp also prevents the premature death of LECs, allowing the coordination of histoblast expansion to LEC delamination. Dpp signaling activity directing histoblast spreading and invasiveness mimics transforming growth factor-beta and bone morphogenetic proteins' role in enhancing the motility and invasiveness of cancer cells, resulting in the promotion of **metastasis**.
2. The core and conserved role of MAL is homeostatic regulation of actin levels. The transcription cofactor MAL is regulated by free actin levels and thus by actin dynamics. MAL, together with its DNA-binding partner, SRF, is required for invasive cell migration and in experimental **metastasis**. Although MAL/SRF has many targets, we provide genetic evidence in both Drosophila and human cellular models that actin is the key target that must be regulated by MAL/SRF for invasive cell migration. By regulating MAL/SRF activity, actin protein feeds back on production of actin mRNA to ensure sufficient supply of actin. This constitutes a dedicated homeostatic feedback system that provides a foundation for cellular actin dynamics.

---

## ush

- NCBI Gene
- GeneRIF
- Pubmed

| Search Term | Rank | Fields |
| --- | --- | --- |
| [Ff]ilopodia | 1 | PubMed(1) |
| [Mm]igration | 6 | PubMed(6) |
| ECM | 7 | PubMed(3) |
| [Ee]xtracellular [Mm]atrix | 8 | PubMed(4) |
| [Ii]nvasive | 10 | PubMed(1) |
| [Mm]etastasis | 12 | PubMed(2) |

  
hide/show details for ush

### [Ff]ilopodia

#### PubMed

1. Gα73B is a downstream effector of JAK/STAT signalling and a regulator of Rho1 in Drosophila haematopoiesis. JAK/STAT signalling regulates many essential developmental processes including cell proliferation and haematopoiesis, whereas its inappropriate activation is associated with the majority of myeloproliferative neoplasias and numerous cancers. Furthermore, high levels of JAK/STAT pathway signalling have also been associated with enhanced metastatic invasion by cancerous cells. Strikingly, gain-of-function mutations in the single Drosophila JAK homologue, Hopscotch, result in haemocyte neoplasia, inappropriate differentiation and the formation of melanised haemocyte-derived 'tumour' masses; phenotypes that are partly orthologous to human gain-of-function JAK2-associated pathologies. Here we show that Gα73B, a novel JAK/STAT pathway target gene, is necessary for JAK/STAT-mediated tumour formation in flies. In addition, although Gα73B does not affect haemocyte differentiation, it does regulate haemocyte morphology and motility under non-pathological conditions. We show that Gα73B is required for constitutive, but not injury-induced, activation of Rho1 and for the localisation of Rho1 i**nto filop**odia upon haemocyte activation. Consistent with these results, we also show that Rho1 interacts genetically with JAK/STAT signalling, and that wild-type levels of Rho1 are necessary for tumour formation. Our findings link JAK/STAT transcriptional outputs, Gα73B activity and Rho1-dependent cytoskeletal rearrangements and cell motility, therefore connecting a pathway associated with cancer with a marker indicative of invasiveness. As such, we suggest a mechanism by which JAK/STAT pathway signalling may promote metastasis.

### [Mm]igration

#### PubMed

1. Cellular basis of the dynamic behavior of the imaginal thoracic discs during Drosophila metamorphosis. The eversion, **migration**, spreading, and fusion of the thoracic imaginal discs during metamorphosis of Drosophila are described using timed whole-mount preparations and several molecular markers. The leading edge of the migrating disc epithelia consists of two groups of cells, stalk cells (S cells) and specialized imaginal cells (I cells), that both express the gene puckered. With this and other markers, opening of the stalk, eversion of the discs, **migration** of the leading edges, and fusion of the imaginal epithelia can be visualized in detail. Fusion is initiated by S cells that migrate over the larval epithelium and constitute a bridge between two imaginal epithelia. S cells are subsequently lost and imaginal fusion is mediated by the I cells that remain at the site of fusion. The possible cellular basis of this process is discussed. Fusion along the dorsal midline of the notum from the mesothoracic wing discs occurs earlier than that of the prothoracic and metathoracic discs, which remain in a lateral position. For a relatively long period (30 h) the mesothoracic epithelium becomes attached to the head and abdomen, causing a temporary local discontinuity of the order of segments. Later the pro- and metathoracic discs intercalate between head and mesothorax and between abdomen and mesothorax, respectively, to reestablish the normal order.
2. Primordial germ cell **migration** and the assembly of the Drosophila embryonic gonad. During embryogenesis Drosophila pole cells, like germ cells in many other vertebrates and invertebrates, actively migrate before assembling into the gonad. Five separate steps can be distinguished: an initial passive displacement of the germ cells by gastrulation movements, an amoeboid migratory phase during which the pole cells pass through the endoderm, **migration** on endoderm toward mesoderm, separation into two bilateral groups associated with the gonadal mesoderm precursors, followed by condensation into the gonad itself. We have analyzed gonad assembly in embryonic pattern mutants to determine whether distinct cues are required in this process. We show that the initiation of **migration** does not require the presence of the mesoderm, the eventual target of the germ cells. Rather, **migration** is triggered as a consequence of the differentiation of the endoderm. Examination of embryos mutant for maternal genes of the terminal group suggests that a primary role of the endoderm in this process is to allow the germ cell access to the interior of the embryo. In addition, we show that normal gonad assembly requires a region of the embryo that includes the posterior compartment of the fifth and the sixth abdominal segments.
3. Signaling pathways directing the movement and fusion of epithelial sheets: lessons from dorsal closure in Drosophila. Wound healing in embryos and various developmental events in metazoans require the spreading and fusion of epithelial sheets. The complex signaling pathways regulating these processes are being pieced together through genetic, cell biological, and biochemical approaches. At present, dorsal closure of the Drosophila embryo is the best-characterized example of epithelial sheet movement. Dorsal closure involves **migration** of the lateral epidermal flanks to close a hole in the dorsal epidermis occupied by an epithelium called the amnioserosa. Detailed genetic studies have revealed a network of interacting signaling molecules regulating this process. At the center of this network is a Jun N-terminal kinase cascade acting at the leading edge of the migrating epidermis that triggers signaling by the TGF-beta superfamily member Decapentaplegic and which interacts with the Wingless pathway. These signaling modules regulate the cytoskeletal reorganization and cell shape change necessary to drive dorsal closure. Activation of this network requires signals from the amnioserosa and input from a variety of proteins at cell-cell junctions. The Rho family of small GTPases is also instrumental, both in activation of signaling and regulation of the cytoskeleton. Many of the proteins regulating dorsal closure have been implicated in epithelial movement in other organisms, and dorsal closure has emerged as an ideal model system for the study of the **migration** and fusion of epithelial sheets.
4. A role for the mesoderm in endodermal **migration** and morphogenesis in Drosophila. The endodermal midgut arises from two primordia, the anterior midgut (AMG) primordium and the posterior midgut (PMG) primordium, which are separated by almost the entire length of the Drosophila embryo. To form the midgut, these two parts have to extend towards each other and to fuse laterally on both sides of the yolk. Shortly before and during that movement, AMG and PMG are arranged as mesenchymal cell masses, but later the midgut cells form an epithelium. We show that these two aspects of midgut development, **migration** of AMG and PMG and transition to an epithelium, depend on the mesoderm. The extension of the midgut primordia is achieved by cell **migration** along the visceral mesoderm which forms a continuous layer of cells within the germ band. In mutant embryos lacking the entire mesoderm or failing to differentiate the visceral mesoderm, AMG and PMG are formed but do not migrate properly. In addition, they fail to form an epithelium and instead either remain as compact cell masses anterior and posterior to the yolk (in twist and snail mutant embryos) or only occasionally wrap around the yolk before embryogenesis is completed (in tinman-deficient embryos). We conclude that the visceral mesoderm serves as a substratum for the migrating endodermal cells and that the contact between visceral mesoderm and endoderm is required for the latter to become an epithelium.
5. Conserved cardiogenic functions of the multitype zinc-finger proteins: U-shaped and FOG-2. Multitype zinc-finger proteins murine Friend of GATA-2 (FOG-2) and Drosophila U-shaped (Ush) are required for heart development. Both FOG proteins participate in signal transduction pathways that are essential for cardiogenesis. FOG-2 regulates signaling from the myocardium, which is required for the production of the coronary vasculature. Ush functions in a common pathway with the Heartless (Htl) fibroblast growth factor (FGF) receptor to control mesodermal cell **migration**, which is required for cardiogenic cell fate commitment. In vitro studies have demonstrated that both FOG proteins repress GATA factor transcriptional activation of cardiac promoters. These similarities provide further evidence for the conservation of gene functions during cardiogenesis in Drosophila and higher eukaryotes.
6. The multitype zinc-finger protein U-shaped functions in heart cell specification in the Drosophila embryo. Multitype zinc-finger proteins of the Friend of GATA/U-shaped (Ush) class function as transcriptional regulators of gene expression through their modulation of GATA factor activity. To better understand intrinsic properties of these proteins, we investigated the expression and function of the ush gene during Drosophila embryogenesis. ush is dynamically expressed in the embryo, including several cell types present within the mesoderm. The gene is active in the cardiogenic mesoderm, and a loss of function results in an overproduction of both cardial and pericardial cells, indicating a requirement for the gene in the formation of these distinct cardiac cell types. Conversely, ectopic expression of ush results in a decrease in the number of cardioblasts in the heart and the inhibition of a cardial cell enhancer normally regulated by the synergistic activity of the Pannier and Tinman cardiogenic factors. These findings suggest that, similar to its known function in thoracic bristle patterning, Ush functions in the control of heart cell specification through its modulation of Pannier transcriptional activity. ush is also required for mesodermal cell **migration** early in embryogenesis, where it shows a genetic interaction with the Heartless fibroblast growth factor receptor gene. Taken together, these results demonstrate a critical role for the Ush transcriptional regulator in several diverse processes of mesoderm differentiation and heart formation.

### ECM

#### PubMed

1. Conditional switches for extracellular matrix patterning in Drosophila melanogaster. An F(1) mutagenesis strategy was developed to identify conditional mutations affecting extracellular matrix (**ECM**) patterning. Tubulogenesis requires coordinated movement of epithelial cells and deposition of a multilayered **ECM**. In the Drosophila ovary, an epithelium of follicle cells creates the eggshells, including the paired tubular dorsal appendages (DAs) that act as breathing tubes for the embryo. A P-element mutagenesis strategy allowed for conditional overexpression of hundreds of genes in follicle cells. Conditional phenotypes were scored at the level of individual mutant (F(1)) female flies. **ECM** pattern regulators were readily identified including MAPK signaling gene ets domain lacking (fused DAs), Wnt pathway genes frizzled 3 and osa (long DAs), Hh pathway gene debra (branched DAs), and transcription factor genes sima/HIF-1alpha, ush, lilli, Tfb1, broad, and foxo. In moving cells the [Ca(2+)]/calcineurin pathway can regulate adhesion to **ECM** while adherens junctions link cells together. Accordingly, thin eggshell and DA phenotypes were identified for the calcineurin regulator calreticulin and the adherens junction component arc. Finally a tubulogenesis defect phenotype was identified for the gene pterodactyl, homologous to the mammalian serine/threonine receptor-associated protein (STRAP) that integrates the TGF-beta and PI3K/AKT signaling pathways. Because phenotypes can be scored in each mutant fly before and after gene induction, this F(1) conditional mutagenesis strategy should allow for increased scale in screens for mutations affecting repeated (reiterated) events in adult animals, including gametogenesis, movement, behavior, and learning.
2. Peak BMP Responses in the Drosophila Embryo Are Dependent on the Activation of Integrin Signaling. Within a 3D tissue, cells need to integrate signals from growth factors, such as BMPs, and the extracellular matrix (**ECM**) to coordinate growth and differentiation. Here, we use the Drosophila embryo as a model to investigate how BMP responses are influenced by a cell's local **ECM** environment. We show that integrins, which are **ECM** receptors, are absolutely required for peak BMP signaling. This stimulatory effect of integrins requires their intracellular signaling function, which is activated by the **ECM** protein collagen IV. Mechanistically, integrins interact with the BMP receptor and stimulate phosphorylation of the downstream Mad transcription factor. The BMP-pathway-enhancing function of integrins is independent of focal adhesion kinase, but it requires conserved NPXY motifs in the β-integrin cytoplasmic tail. Furthermore, we show that an α-integrin subunit is a BMP target gene, identifying positive feedback between integrin signaling and BMP pathway activity that may contribute to robust cell fate decisions.
3. Synthetic enzyme-substrate tethering obviates the Tolloid-**ECM** interaction during Drosophila BMP gradient formation. Members of the Tolloid family of metalloproteinases liberate BMPs from inhibitory complexes to regulate BMP gradient formation during embryonic dorsal-ventral axis patterning. Here, we determine mechanistically how Tolloid activity is regulated by its non-catalytic CUB domains in the Drosophila embryo. We show that Tolloid, via its N-terminal CUB domains, interacts with Collagen IV, which enhances Tolloid activity towards its substrate Sog, and facilitates Tsg-dependent stimulation of cleavage. In contrast, the two most C-terminal Tld CUB domains mediate Sog interaction to facilitate its processing as, based on our structural data, Tolloid curvature positions bound Sog in proximity to the protease domain. Having ascribed functions to the Tolloid non-catalytic domains, we recapitulate embryonic BMP gradient formation in their absence, by artificially tethering the Tld protease domain to Sog. Our studies highlight how the bipartite function of Tolloid CUB domains, in substrate and **ECM** interactions, fine-tune protease activity to a particular developmental context.

### [Ee]xtracellular [Mm]atrix

#### PubMed

1. Conditional switches for **extracellular matrix** patterning in Drosophila melanogaster. An F(1) mutagenesis strategy was developed to identify conditional mutations affecting **extracellular matrix** (ECM) patterning. Tubulogenesis requires coordinated movement of epithelial cells and deposition of a multilayered ECM. In the Drosophila ovary, an epithelium of follicle cells creates the eggshells, including the paired tubular dorsal appendages (DAs) that act as breathing tubes for the embryo. A P-element mutagenesis strategy allowed for conditional overexpression of hundreds of genes in follicle cells. Conditional phenotypes were scored at the level of individual mutant (F(1)) female flies. ECM pattern regulators were readily identified including MAPK signaling gene ets domain lacking (fused DAs), Wnt pathway genes frizzled 3 and osa (long DAs), Hh pathway gene debra (branched DAs), and transcription factor genes sima/HIF-1alpha, ush, lilli, Tfb1, broad, and foxo. In moving cells the [Ca(2+)]/calcineurin pathway can regulate adhesion to ECM while adherens junctions link cells together. Accordingly, thin eggshell and DA phenotypes were identified for the calcineurin regulator calreticulin and the adherens junction component arc. Finally a tubulogenesis defect phenotype was identified for the gene pterodactyl, homologous to the mammalian serine/threonine receptor-associated protein (STRAP) that integrates the TGF-beta and PI3K/AKT signaling pathways. Because phenotypes can be scored in each mutant fly before and after gene induction, this F(1) conditional mutagenesis strategy should allow for increased scale in screens for mutations affecting repeated (reiterated) events in adult animals, including gametogenesis, movement, behavior, and learning.
2. **Extracellular matrix**-modulated Heartless signaling in Drosophila blood progenitors regulates their differentiation via a Ras/ETS/FOG pathway and target of rapamycin function. Maintenance of hematopoietic progenitors ensures a continuous supply of blood cells during the lifespan of an organism. Thus, understanding the molecular basis for progenitor maintenance is a continued focus of investigation. A large pool of undifferentiated blood progenitors are maintained in the Drosophila hematopoietic organ, the larval lymph gland, by a complex network of signaling pathways that are mediated by niche-, progenitor-, or differentiated hemocyte-derived signals. In this study we examined the function of the Drosophila fibroblast growth factor receptor (FGFR), Heartless, a critical regulator of early lymph gland progenitor specification in the late embryo, during larval lymph gland hematopoiesis. Activation of Heartless signaling in hemocyte progenitors by its two ligands, Pyramus and Thisbe, is both required and sufficient to induce progenitor differentiation and formation of the plasmatocyte-rich lymph gland cortical zone. We identify two transcriptional regulators that function downstream of Heartless signaling in lymph gland progenitors, the ETS protein, Pointed, and the Friend-of-GATA (FOG) protein, U-shaped, which are required for this Heartless-induced differentiation response. Furthermore, cross-talk of Heartless and target of rapamycin signaling in hemocyte progenitors is required for lamellocyte differentiation downstream of Thisbe-mediated Heartless activation. Finally, we identify the Drosophila heparan sulfate proteoglycan, Trol, as a critical negative regulator of Heartless ligand signaling in the lymph gland, demonstrating that sequestration of differentiation signals by the **extracellular matrix** is a unique mechanism employed in blood progenitor maintenance that is of potential relevance to many other stem cell niches.
3. Type IV collagens regulate BMP signalling in Drosophila. Dorsal-ventral patterning in vertebrate and invertebrate embryos is mediated by a conserved system of secreted proteins that establishes a bone morphogenetic protein (BMP) gradient. Although the Drosophila embryonic Decapentaplegic (Dpp) gradient has served as a model to understand how morphogen gradients are established, no role for the **extracellular matrix** has been previously described. Here we show that type IV collagen **extracellular matrix** proteins bind Dpp and regulate its signalling in both the Drosophila embryo and ovary. We provide evidence that the interaction between Dpp and type IV collagen augments Dpp signalling in the embryo by promoting gradient formation, yet it restricts the signalling range in the ovary through sequestration of the Dpp ligand. Together, these results identify a critical function of type IV collagens in modulating Dpp in the extracellular space during Drosophila development. On the basis of our findings that human type IV collagen binds BMP4, we predict that this role of type IV collagens will be conserved.
4. Peak BMP Responses in the Drosophila Embryo Are Dependent on the Activation of Integrin Signaling. Within a 3D tissue, cells need to integrate signals from growth factors, such as BMPs, and the **extracellular matrix** (ECM) to coordinate growth and differentiation. Here, we use the Drosophila embryo as a model to investigate how BMP responses are influenced by a cell's local ECM environment. We show that integrins, which are ECM receptors, are absolutely required for peak BMP signaling. This stimulatory effect of integrins requires their intracellular signaling function, which is activated by the ECM protein collagen IV. Mechanistically, integrins interact with the BMP receptor and stimulate phosphorylation of the downstream Mad transcription factor. The BMP-pathway-enhancing function of integrins is independent of focal adhesion kinase, but it requires conserved NPXY motifs in the β-integrin cytoplasmic tail. Furthermore, we show that an α-integrin subunit is a BMP target gene, identifying positive feedback between integrin signaling and BMP pathway activity that may contribute to robust cell fate decisions.

### [Ii]nvasive

#### PubMed

1. Gα73B is a downstream effector of JAK/STAT signalling and a regulator of Rho1 in Drosophila haematopoiesis. JAK/STAT signalling regulates many essential developmental processes including cell proliferation and haematopoiesis, whereas its inappropriate activation is associated with the majority of myeloproliferative neoplasias and numerous cancers. Furthermore, high levels of JAK/STAT pathway signalling have also been associated with enhanced metastatic invasion by cancerous cells. Strikingly, gain-of-function mutations in the single Drosophila JAK homologue, Hopscotch, result in haemocyte neoplasia, inappropriate differentiation and the formation of melanised haemocyte-derived 'tumour' masses; phenotypes that are partly orthologous to human gain-of-function JAK2-associated pathologies. Here we show that Gα73B, a novel JAK/STAT pathway target gene, is necessary for JAK/STAT-mediated tumour formation in flies. In addition, although Gα73B does not affect haemocyte differentiation, it does regulate haemocyte morphology and motility under non-pathological conditions. We show that Gα73B is required for constitutive, but not injury-induced, activation of Rho1 and for the localisation of Rho1 into filopodia upon haemocyte activation. Consistent with these results, we also show that Rho1 interacts genetically with JAK/STAT signalling, and that wild-type levels of Rho1 are necessary for tumour formation. Our findings link JAK/STAT transcriptional outputs, Gα73B activity and Rho1-dependent cytoskeletal rearrangements and cell motility, therefore connecting a pathway associated with cancer with a marker indicativ**e of inv**asiveness. As such, we suggest a mechanism by which JAK/STAT pathway signalling may promote metastasis.

### [Mm]etastasis

#### PubMed

1. Gα73B is a downstream effector of JAK/STAT signalling and a regulator of Rho1 in Drosophila haematopoiesis. JAK/STAT signalling regulates many essential developmental processes including cell proliferation and haematopoiesis, whereas its inappropriate activation is associated with the majority of myeloproliferative neoplasias and numerous cancers. Furthermore, high levels of JAK/STAT pathway signalling have also been associated with enhanced metastatic invasion by cancerous cells. Strikingly, gain-of-function mutations in the single Drosophila JAK homologue, Hopscotch, result in haemocyte neoplasia, inappropriate differentiation and the formation of melanised haemocyte-derived 'tumour' masses; phenotypes that are partly orthologous to human gain-of-function JAK2-associated pathologies. Here we show that Gα73B, a novel JAK/STAT pathway target gene, is necessary for JAK/STAT-mediated tumour formation in flies. In addition, although Gα73B does not affect haemocyte differentiation, it does regulate haemocyte morphology and motility under non-pathological conditions. We show that Gα73B is required for constitutive, but not injury-induced, activation of Rho1 and for the localisation of Rho1 into filopodia upon haemocyte activation. Consistent with these results, we also show that Rho1 interacts genetically with JAK/STAT signalling, and that wild-type levels of Rho1 are necessary for tumour formation. Our findings link JAK/STAT transcriptional outputs, Gα73B activity and Rho1-dependent cytoskeletal rearrangements and cell motility, therefore connecting a pathway associated with cancer with a marker indicative of invasiveness. As such, we suggest a mechanism by which JAK/STAT pathway signalling may pro**mote metas**tasis.
2. Drosophila as a model to study the role of blood cells in inflammation, innate immunity and cancer. Drosophila has a primitive yet effective blood system with three types of haemocytes which function throughout different developmental stages and environmental stimuli. Haemocytes play essential roles in tissue modeling during embryogenesis and morphogenesis, and also in innate immunity. The open circulatory system of Drosophila makes haemocytes ideal signal mediators to cells and tissues in response to events such as infection and wounding. The application of recently developed and sophisticated genetic tools to the relatively simple genome of Drosophila has made the fly a popular system for modeling human tumorigensis and **metastasis**. Drosophila is now used for screening and investigation of genes implicated in human leukemia and also in modeling development of solid tumors. This second line of research offers promising opportunities to determine the seemingly conflicting roles of blood cells in tumor progression and invasion. This review provides an overview of the signaling pathways conserved in Drosophila during haematopoiesis, haemostasis, innate immunity, wound healing and inflammation. We also review the most recent progress in the use of Drosophila as a cancer research model with an emphasis on the roles haemocytes can play in various cancer models and in the links between inflammation and cancer.

---

## rau

- NCBI Gene
- GeneRIF
- Pubmed

| Search Term | Rank | Fields |
| --- | --- | --- |
| [Mm]igration | 6 | PubMed(2) |

  
hide/show details for rau

### [Mm]igration

#### PubMed

1. Unwrapping glial biology: Gcm target genes regulating glial development, diversification, and function. Glia are the most abundant cell type in the mammalian brain. They regulate neuronal development and function, CNS immune surveillance, and stem cell biology, yet we know surprisingly little about glia in any organism. Here we identify over 40 new Drosophila glial genes. We use glial cells missing (gcm) mutants and misexpression to verify they are Gcm regulated in vivo. Many genes show unique spatiotemporal responsiveness to Gcm in the CNS, and thus glial subtype diversification requires spatially or temporally restricted Gcm cofactors. These genes provide insights into glial biology: we show unc-5 (a repulsive netrin receptor) orients glial **migration**s and the draper gene mediates glial engulfment of apoptotic neurons and larval locomotion. Many identified Drosophila glial genes have homologs expressed in mammalian glia, revealing conserved molecular features of glial cells. 80% of these Drosophila glial genes have mammalian homologs; these are now excellent candidates for regulating human glial development, function, or disease.
2. Subtypes of glial cells in the Drosophila embryonic ventral nerve cord as related to lineage and gene expression. In the Drosophila embryonic CNS several subtypes of glial cells develop, which arrange themselves at characteristic positions and presumably fulfil specific functions. The mechanisms leading to the specification and differentiation of glial subtypes are largely unknown. By DiI labelling in glia-specific Gal4 lines we have clarified the lineages of the lateral glia in the embryonic ventral nerve cord and linked each glial cell to a specific stem cell. For the lineage of the longitudinal glioblast we show that it consists of 9 cells, which acquire at least four different identities. A large collection of molecular markers (many of them representing transcription factors and potential Gcm target genes) reveals that individual glial cells express specific combinations of markers. However, cluster analysis uncovers similar combinatorial codes for cells within, and significant differences between the categories of surface-associated, cortex-associated, and longitudinal glia. Glial cells derived from the same stem cell may be homogeneous (though not identical; stem cells NB1-1, NB5-6, NB6-4, LGB) or heterogeneous (NB7-4, NB1-3) with regard to gene expression. In addition to providing a powerful tool to analyse the fate of individual glial cells in different genetic backgrounds, each of these marker genes represents a candidate factor involved in glial specification or differentiation. We demonstrate this by the analysis of a castor loss of function mutation, which affects the number and **migration** of specific glial cells.

---

## stc

- NCBI Gene
- GeneRIF
- Pubmed

| Search Term | Rank | Fields |
| --- | --- | --- |
| [Mm]igration | 6 | PubMed(1) |

  
hide/show details for stc

### [Mm]igration

#### PubMed

1. The Drosophila zygotic lethal gene shuttle craft is required maternally for proper embryonic development. The Drosophila gene shuttle craft (stc) is expressed zygotically in the embryonic central nervous system (CNS) where it is required to maintain the proper morphology of motoneuronal axon nerve routes following their **migration** from the ventral cord. Here, we report that a prominent maternal source of STC protein is also present throughout both oogenesis and embryogenesis. To determine whether this maternal component is required in the ovary and/or embryo, we used the Drosophila autosomal dominant female sterile technique to generate germ-line clones that lacked the stc maternal function. Our results demonstrate that a maternally derived source of STC protein is required during embryogenesis but not oogenesis. In contrast to the zygotic phenotype, the primary defect in embryos derived from stc germ-line clones affects segmentation by causing disruptions and deletions in distinct thoracic (T1-T3) and abdominal (A4-A8) segments. These localized defects are responsible for additional phenotypes observed later in development which include gaps in the ventral nerve cord and deletions of denticle belts in the cuticle. An additional phenotype occurring in all other neuromeric segments consists of the misguided **migration** of motoneuronal axons as they project out of the ventral nerve cord. Thus, the stc zygotic function is required later in development and cannot correct the segmentation and subsequent CNS abnormalities associated with loss of its earlier acting maternally derived activity.

---

## Timp

- NCBI Gene
- GeneRIF
- Pubmed

| Search Term | Rank | Fields |
| --- | --- | --- |
| [Mm]igration | 6 | PubMed(5) |
| ECM | 7 | PubMed(5); GeneRIFs(1) |
| [Ee]xtracellular [Mm]atrix | 8 | PubMed(20); Component(1) |
| [Ii]nvasive | 10 | PubMed(6) |
| [Mm]etastasize | 11 | PubMed(1) |
| [Mm]etastasis | 12 | PubMed(4) |
| [Cc]ell [Ii]nvasion | 13 | PubMed(3) |

  
hide/show details for Timp

### [Mm]igration

#### PubMed

1. RhoL controls invasion and Rap1 localization during immune cell trans**migration** in Drosophila. Human immune cells have to penetrate an endothelial barrier during their beneficial pursuit of infection and their destructive infiltration of tissues in autoimmune diseases. This trans**migration** requires Rap1 GTPase to activate integrin affinity. We define a new model system for this process by demonstrating, with live imaging and genetics, that during embryonic development Drosophila melanogaster immune cells penetrate an epithelial, Drosophila E-cadherin (DE-cadherin)-based tissue barrier. A mutant in RhoL, a GTPase homologue that is specifically expressed in haemocytes, blocks this invasive step but not other aspects of guided **migration**. RhoL mediates integrin adhesion caused by Drosophila Rap1 overexpression and moves Rap1 away from a concentration in the cytoplasm to the leading edge during invasive **migration**. These findings indicate that a programmed migratory step during Drosophila development bears striking molecular similarities to vertebrate immune cell trans**migration** during inflammation, and identify RhoL as a new regulator of invasion, adhesion and Rap1 localization. Our work establishes the utility of Drosophila for identifying novel components of immune cell trans**migration** and for understanding the in vivo interplay of immune cells with the barriers they penetrate.
2. Dynamics of the basement membrane in invasive epithelial clusters in Drosophila. The basement membrane (BM) represents a barrier to cell **migration**, which has to be degraded to promote invasion. However, the role and behaviour of the BM during the development of pre-invasive cells is only poorly understood. Drosophila border cells (BCs) provide an attractive genetic model in which to study the cellular mechanisms underlying the **migration** of mixed cohorts of epithelial cells. BCs are made of two different epithelial cell types appearing sequentially during oogenesis: the polar cells and the outer BCs. Here, we show that the pre-invasive polar cells undergo an unusual and asymmetrical apical capping with major basement membrane proteins, including the two Drosophila Collagen IV alpha chains, Laminin A and Perlecan. Capping of polar cells proceeds through a novel, basal-to-apical transcytosis mechanism that involves the small GTPase Drab5. Apical capping is transient and is followed by rapid shedding prior to the initiation of BC **migration**, suggesting that the apical cap blocks **migration**. Consistently, non-migratory polar cells remain capped. We further show that JAK/STAT signalling and recruitment of outer BCs are required for correct shedding and **migration**. The dynamics of the BM represents a marker of migratory BC, revealing a novel developmentally regulated behaviour of BM coupled to epithelial cell invasiveness.
3. A secreted MMP is required for reepithelialization during wound healing. Matrix metalloproteinases (MMPs) are extracellular proteases highly expressed at wound sites. However, the precise function of MMPs during reepithelialization in vivo has been elusive in mammalian models because of the high level of redundancy among the 24 mammalian MMPs. For this reason we used Drosophila melanogaster, whose genome encodes only two MMPs-one secreted type (Mmp1) and one membrane-anchored type (Mmp2)-to study the function and regulation of the secreted class of MMPs in vivo. In the absence of redundancy, we found that the Drosophila secreted MMP, Mmp1, is required in the epidermis to facilitate reepithelialization by remodeling the basement membrane, promoting cell elongation and actin cytoskeletal reorganization, and activating extracellular signal-regulated kinase signaling. In addition, we report that the jun N-terminal kinase (JNK) pathway upregulates Mmp1 expression after wounding, but that Mmp1 is expressed independent of the JNK pathway in unwounded epidermis. When the JNK pathway is ectopically activated to overexpress Mmp1, the rate of healing is accelerated in an Mmp1-dependent manner. A primary function of Mmp1, under the control of the JNK pathway, is to promote basement membrane repair, which in turn may permit cell **migration** and the restoration of a continuous tissue.
4. The orthologous Tbx transcription factors Omb and TBX2 induce epithelial cell **migration** and extrusion in vivo without involvement of matrix metalloproteinases. The transcription factors TBX2 and TBX3 are overexpressed in various human cancers. Here, we investigated the effect of overexpressing the orthologous Tbx genes Drosophila optomotor-blind (omb) and human TBX2 in the epithelium of the Drosophila wing imaginal disc and observed two types of cell motility. Omb/TBX2 overexpressing cells could move within the plane of the epithelium. Invasive cells migrated long-distance as single cells retaining or regaining normal cell shape and apico-basal polarity in spite of attenuated apical DE-cadherin concentration. Inappropriate levels of DE-cadherin were sufficient to drive cell **migration** in the wing disc epithelium. Omb/TBX2 overexpression and reduced DE-cadherin-dependent adhesion caused the formation of actin-rich lateral cell protrusions. Omb/TBX2 overexpressing cells could also delaminate basally, penetrating the basal lamina, however, without degradation of extracellular matrix. Expression of Timp, an inhibitor of matrix metalloproteases, blocked neither intraepithelial motility nor basal extrusion. Our results reveal an MMP-independent mechanism of cell invasion and suggest a conserved role of Tbx2-related proteins in cell invasion and metastasis-related processes.
5. Tissue remodeling during maturation of the Drosophila wing. The final step in morphogenesis of the adult fly is wing maturation, a process not well understood at the cellular level due to the impermeable and refractive nature of cuticle synthesized some 30 h prior to eclosion from the pupal case. Advances in GFP technology now make it possible to visualize cells using fluorescence after cuticle synthesis is complete. We find that, between eclosion and wing expansion, the epithelia within the folded wing begin to delaminate from the cuticle and that delamination is complete when the wing has fully expanded. After expansion, epithelial cells lose contact with each other, adherens junctions are disrupted, and nuclei become pycnotic. The cells then change shape, elongate, and migrate from the wing into the thorax. During wing maturation, the Timp gene product, tissue inhibitor of metalloproteinases, and probably other components of an extracellular matrix are expressed that bond the dorsal and ventral cuticular surfaces of the wing following **migration** of the cells. These steps are dissected using the batone and Timp genes and ectopic expression of alphaPS integrin, inhibitors of Armadillo/beta-catenin nuclear activity and baculovirus caspase inhibitor p35. We conclude that an epithelial-mesenchymal transition is responsible for epithelial delamination and dissolution.

### ECM

#### PubMed

1. **ECM**-Regulator timp Is Required for Stem Cell Niche Organization and Cyst Production in the Drosophila Ovary. The extracellular matrix (**ECM**) is a pivotal component adult tissues and of many tissue-specific stem cell niches. It provides structural support and regulates niche signaling during tissue maintenance and regeneration. In many tissues, **ECM** remodeling depends on the regulation of MMP (matrix metalloproteinase) activity by inhibitory TIMP (tissue inhibitors of metalloproteinases) proteins. Here, we report that the only Drosophila timp gene is required for maintaining the normal organization and function of the germline stem cell niche in adult females. timp mutant ovaries show reduced levels of both Drosophila Collagen IV α chains. In addition, tissue stiffness and the cellular organization of the ovarian niche are affected in timp mutants. Finally, loss of timp impairs the ability of the germline stem cell niche to generate new cysts. Our results demonstrating a crucial role for timp in tissue organization and gamete production thus provide a link between the regulation of **EC**M metabolism and tissue homeostasis.
2. **ECM**-Regulator timp Is Required for Stem Cell Niche Organization and Cyst Production in the Drosophila Ovary. The extracellular matrix (**ECM**) is a pivotal component adult tissues and of many tissue-specific stem cell niches. It provides structural support and regulates niche signaling during tissue maintenance and regeneration. In many tissues, **ECM** remodeling depends on the regulation of MMP (matrix metalloproteinase) activity by inhibitory TIMP (tissue inhibitors of metalloproteinases) proteins. Here, we report that the only Drosophila timp gene is required for maintaining the normal organization and function of the germline stem cell niche in adult females. timp mutant ovaries show reduced levels of both Drosophila Collagen IV α chains. In addition, tissue stiffness and the cellular organization of the ovarian niche are affected in timp mutants. Finally, loss of timp impairs the ability of the germline stem cell niche to generate new cysts. Our results demonstrating a crucial role for timp in tissue organization and gamete production thus provide a link between the regulation of **EC**M metabolism and tissue homeostasis.
3. Glia **ECM** interactions are required to shape the Drosophila nervous system. Organs are characterized by a specific shape that is often remodeled during development. The dynamics of organ shape is in particular evident during the formation of the Drosophila nervous system. During embryonic stages the central nervous system compacts, whereas selective growth occurs during larval stages. The nervous system is covered by a layer of surface glial cells that form the blood brain barrier and a thick extracellular matrix called neural lamella. The size of the neural lamella is dynamically adjusted to the growing nervous system and we show here that perineurial glial cells secrete proteases to remodel this matrix. Moreover, an imbalance in proteolytic activity results in an abnormal shape of the nervous system. To identify further components controlling nervous system shape we performed an RNAi based screen and identified the gene nolo, which encodes an ADAMTS-like protein. We generated loss of function alleles and demonstrate a requirement in glial cells. Mutant nolo larvae, however, do not show an abnormal nervous system shape. The only predicted off-target of the nolo(dsRNA) is Oatp30B, which encodes an organic anion transporting protein characterized by an extracellular protease inhibitor domain. Loss of function mutants were generated and double mutant analyses demonstrate a genetic interaction between nolo and Oatp30B which prevented the generation of maternal zygotic mutant larvae.
4. Requirement of matrix metalloproteinase-1 for intestinal homeostasis in the adult Drosophila midgut. Stem cells are tightly regulated by both intrinsic and extrinsic signals as well as the extracellular matrix (**ECM**) for tissue homeostasis and regenerative capacity. Matrix metalloproteinases (MMPs), proteolytic enzymes, modulate the turnover of numerous substrates, including cytokine precursors, growth factors, and **ECM** molecules. However, the roles of MMPs in the regulation of adult stem cells are poorly understood. In the present study, we utilize the Drosophila midgut, which is an excellent model system for studying stem cell biology, to show that Mmp1 is involved in the regulation of intestinal stem cells (ISCs). The results showed that Mmp1 is expressed in the adult midgut and that its expression increases with age and with exposure to oxidative stress. Mmp1 knockdown or Timp-overexpressing flies and flies heterozygous for a viable, hypomorphic Mmp1 allele increased ISC proliferation in the gut, as shown by staining with an anti-phospho-histone H3 antibody and BrdU incorporation assays. Reduced Mmp1 levels induced intestinal hyperplasia, and the Mmp1depletion-induced ISC proliferation was rescued by the suppression of the EGFR signaling pathway, suggesting that Mmp1 regulates ISC proliferation through the EGFR signaling pathway. Furthermore, adult gut-specific knockdown and whole-animal heterozygotes of Mmp1 increased additively sensitivity to paraquat-induced oxidative stress and shortened lifespan. Our data suggest that Drosophila Mmp1 is involved in the regulation of ISC proliferation for maintenance of gut homeostasis.
5. Inflated wings, tissue autolysis and early death in tissue inhibitor of metalloproteinases mutants of Drosophila. In vertebrates, tissue inhibitors of metalloproteinases (TIMPs) play key roles in extracellular matrix (**ECM**) homeostasis and growth control. Deletion of the recently cloned Timp gene of Drosophila results in a subviable phenotype. Adult flies display inflated wings similar to integrin mutants, suffer from a bloated gut and progressive dissolution of internal tissues, and die prematurely. Our results demonstrate that the Timp gene product controls selective aspects of **ECM** function in Drosophila, and suggest that it is involved in cell adhesion/cell signaling pathways. Hence, Drosophila Timp mutants may prove useful as a model system for a wide variety of pathological conditions related to **ECM** dysregulation.

#### GeneRIFs

1. Our results demonstrating a crucial role for timp in tissue organization and gamete production thus provide a link between the regulation of **ECM** metabolism and tissue homeostasis.

### [Ee]xtracellular [Mm]atrix

#### PubMed

1. ECM-Regulator timp Is Required for Stem Cell Niche Organization and Cyst Production in the Drosophila Ovary. The **extracellular matrix** (ECM) is a pivotal component adult tissues and of many tissue-specific stem cell niches. It provides structural support and regulates niche signaling during tissue maintenance and regeneration. In many tissues, ECM remodeling depends on the regulation of MMP (matrix metalloproteinase) activity by inhibitory TIMP (tissue inhibitors of metalloproteinases) proteins. Here, we report that the only Drosophila timp gene is required for maintaining the normal organization and function of the germline stem cell niche in adult females. timp mutant ovaries show reduced levels of both Drosophila Collagen IV α chains. In addition, tissue stiffness and the cellular organization of the ovarian niche are affected in timp mutants. Finally, loss of timp impairs the ability of the germline stem cell niche to generate new cysts. Our results demonstrating a crucial role for timp in tissue organization and gamete production thus provide a link between the regulation of ECM metabolism and tissue homeostasis.
2. An MMP liberates the Ninjurin A ectodomain to signal a loss of cell adhesion. Matrix metalloproteinases (MMPs) are important for developmental tissue remodeling and for the inflammatory response. Although the vertebrate MMP family is large and functionally redundant, the fruitfly Drosophila melanogaster has only two MMPs, both essential genes. Our previous work demonstrated that Mmp1 is required for growth of the tracheal system, and we suggested that the mutant phenotype resulted from aberrant persistence of cell adhesion to the **extracellular matrix**. Here we report the identification of NijA, a transmembrane protein whose vertebrate homologs regulate cell adhesion, as a two-hybrid binding partner for Mmp1. The binding of Mmp1 and NijA was confirmed by coimmunoprecipitation of endogenous proteins from flies, and the endogenous proteins were found to colocalize at the tracheal cell surface in larvae. When NijA is expressed in S2 cells, they lose adhesion to surfaces; this adhesion-loss phenotype is dependent on the expression and catalytic activity of Mmp1. Our data indicate that Mmp1 releases the N-terminal extracellular domain of NijA. This liberated ectodomain promotes the loss of cell adhesion in a cell-nonautonomous manner. We suggest that tracheal cell adhesion is regulated by a novel mechanism utilizing an MMP and a ninjurin family member.
3. Regulation of Drosophila matrix metalloprotease Mmp2 is essential for wing imaginal disc:trachea association and air sac tubulogenesis. The Drosophila Dorsal Air Sac Primordium (ASP) is a tracheal tube that grows toward Branchless FGF-expressing cells in the wing imaginal disc. We show that the ASP arises from a tracheal branch that invades the basal lamina of the disc to juxtapose directly with disc cells. We examined the role of matrix metalloproteases (Mmps), and found that reducing Mmp2 activity perturbed disc-trachea association, altered peritracheal distributions of collagen IV and Perlecan, misregulated ASP growth, and abrogated development of the dorsal air sacs. Whereas the function of the membrane-tethered Mmp2 in the ASP is non-cell autonomous we find that it may have distinct tissue-specific roles in the ASP and disc. These findings demonstrate a critical role for Mmp2 in tubulogenesis post-induction, and implicate Mmp2 in regulating dynamic and essential changes to the **extracellular matrix**.
4. Tissue design: how Drosophila tumors remodel their neighborhood. Drosophila genetics has long been appreciated as a powerful approach for discovering the normal functions of genes that act as oncogenes and tumor suppressors in human cancer. Recent studies have also highlighted its advantages for deciphering how such genes function during tumorigenesis itself. Here we detail studies relating to how tumors, generated in developing organs and adult stem cell-based tissues, remodel the tissue landscape to their benefit. Like mammalian tumors, insect tumors can dissolve **extracellular matrix**, recruit blood cells, migrate and invade other tissues. While much is known about how mammalian fibroblasts, immune cells and vasculature promote late tumorigenesis, less is understood about the very earliest stages of tumor development in mammals. Because Drosophila has fewer mitotic cells and a simpler tissue architecture, it affords easy detection and analysis of early clonal tumor growth. Drosophila studies have revealed both cooperative and competitive interactions between tumor and normal cells during early tumor growth. During development, these interactions typically occur with other proliferative progenitor cells, but in adult stem cell-based tissues, the stem cell niche can fuel tumor growth.
5. ECM-Regulator timp Is Required for Stem Cell Niche Organization and Cyst Production in the Drosophila Ovary. The **extracellular matrix** (ECM) is a pivotal component adult tissues and of many tissue-specific stem cell niches. It provides structural support and regulates niche signaling during tissue maintenance and regeneration. In many tissues, ECM remodeling depends on the regulation of MMP (matrix metalloproteinase) activity by inhibitory TIMP (tissue inhibitors of metalloproteinases) proteins. Here, we report that the only Drosophila timp gene is required for maintaining the normal organization and function of the germline stem cell niche in adult females. timp mutant ovaries show reduced levels of both Drosophila Collagen IV α chains. In addition, tissue stiffness and the cellular organization of the ovarian niche are affected in timp mutants. Finally, loss of timp impairs the ability of the germline stem cell niche to generate new cysts. Our results demonstrating a crucial role for timp in tissue organization and gamete production thus provide a link between the regulation of ECM metabolism and tissue homeostasis.
6. Dendrite-specific remodeling of Drosophila sensory neurons requires matrix metalloproteases, ubiquitin-proteasome, and ecdysone signaling. During neuronal maturation, dendrites develop from immature neurites into mature arbors. In response to changes in the environment, dendrites from certain mature neurons can undergo large-scale morphologic remodeling. Here, we show a group of Drosophila peripheral sensory neurons, the class IV dendritic arborization (C4da) neurons, that completely degrade and regrow their elaborate dendrites. Larval dendrites of C4da neurons are first severed from the soma and subsequently degraded during metamorphosis. This process is controlled by both intracellular and extracellular mechanisms: The ecdysone pathway and ubiquitin-proteasome system (UPS) are cell-intrinsic signals that initiate dendrite breakage, and **extracellular matrix** metalloproteases are required to degrade the severed dendrites. Surprisingly, C4da neurons retain their axonal projections during concurrent dendrite degradation, despite activated ecdysone and UPS pathways. These results demonstrate that, in response to environmental changes, certain neurons have cell-intrinsic abilities to completely lose their dendrites but keep their axons and subsequently regrow their dendritic arbors.
7. Matrix metalloproteinases promote motor axon fasciculation in the Drosophila embryo. Matrix metalloproteinases (MMPs) are a large conserved family of extracellular proteases, a number of which are expressed during neuronal development and upregulated in nervous system diseases. Primarily on the basis of studies using pharmaceutical inhibitors, MMPs have been proposed to degrade the **extracellular matrix** to allow growth cone advance during development and hence play largely permissive roles in axon extension. Here we show that MMPs are not required for axon extension in the Drosophila embryo, but rather are specifically required for the execution of several stereotyped motor axon pathfinding decisions. The Drosophila genome contains only two MMP homologs, Mmp1 and Mmp2. We isolated Mmp1 in a misexpression screen to identify molecules required for motoneuron development. Misexpression of either MMP inhibits the regulated separation/defasciculation of motor axons at defined choice points. Conversely, motor nerves in Mmp1 and Mmp2 single mutants and Mmp1 Mmp2 double mutant embryos are loosely bundled/fasciculated, with ectopic axonal projections. Quantification of these phenotypes reveals that the genetic requirement for Mmp1 and Mmp2 is distinct in different nerve branches, although generally Mmp2 plays the predominant role in pathfinding. Using both an endogenous MMP inhibitor and MMP dominant-negative constructs, we demonstrate that MMP catalytic activity is required for motor axon fasciculation. In support of the model that MMPs promote fasciculation, we find that the defasciculation observed when MMP activity is compromised is suppressed by otherwise elevating interaxonal adhesion -- either by overexpressing Fas2 or by reducing Sema-1a dosage. These data demonstrate that MMP activity is essential for embryonic motor axon fasciculation.
8. Glia ECM interactions are required to shape the Drosophila nervous system. Organs are characterized by a specific shape that is often remodeled during development. The dynamics of organ shape is in particular evident during the formation of the Drosophila nervous system. During embryonic stages the central nervous system compacts, whereas selective growth occurs during larval stages. The nervous system is covered by a layer of surface glial cells that form the blood brain barrier and a thick **extracellular matrix** called neural lamella. The size of the neural lamella is dynamically adjusted to the growing nervous system and we show here that perineurial glial cells secrete proteases to remodel this matrix. Moreover, an imbalance in proteolytic activity results in an abnormal shape of the nervous system. To identify further components controlling nervous system shape we performed an RNAi based screen and identified the gene nolo, which encodes an ADAMTS-like protein. We generated loss of function alleles and demonstrate a requirement in glial cells. Mutant nolo larvae, however, do not show an abnormal nervous system shape. The only predicted off-target of the nolo(dsRNA) is Oatp30B, which encodes an organic anion transporting protein characterized by an extracellular protease inhibitor domain. Loss of function mutants were generated and double mutant analyses demonstrate a genetic interaction between nolo and Oatp30B which prevented the generation of maternal zygotic mutant larvae.
9. Requirement of matrix metalloproteinase-1 for intestinal homeostasis in the adult Drosophila midgut. Stem cells are tightly regulated by both intrinsic and extrinsic signals as well as the **extracellular matrix** (ECM) for tissue homeostasis and regenerative capacity. Matrix metalloproteinases (MMPs), proteolytic enzymes, modulate the turnover of numerous substrates, including cytokine precursors, growth factors, and ECM molecules. However, the roles of MMPs in the regulation of adult stem cells are poorly understood. In the present study, we utilize the Drosophila midgut, which is an excellent model system for studying stem cell biology, to show that Mmp1 is involved in the regulation of intestinal stem cells (ISCs). The results showed that Mmp1 is expressed in the adult midgut and that its expression increases with age and with exposure to oxidative stress. Mmp1 knockdown or Timp-overexpressing flies and flies heterozygous for a viable, hypomorphic Mmp1 allele increased ISC proliferation in the gut, as shown by staining with an anti-phospho-histone H3 antibody and BrdU incorporation assays. Reduced Mmp1 levels induced intestinal hyperplasia, and the Mmp1depletion-induced ISC proliferation was rescued by the suppression of the EGFR signaling pathway, suggesting that Mmp1 regulates ISC proliferation through the EGFR signaling pathway. Furthermore, adult gut-specific knockdown and whole-animal heterozygotes of Mmp1 increased additively sensitivity to paraquat-induced oxidative stress and shortened lifespan. Our data suggest that Drosophila Mmp1 is involved in the regulation of ISC proliferation for maintenance of gut homeostasis.
10. The Gyc76C Receptor Guanylyl Cyclase and the Foraging cGMP-Dependent Kinase Regulate **Extracellular Matrix** Organization and BMP Signaling in the Developing Wing of Drosophila melanogaster. The developing crossveins of the wing of Drosophila melanogaster are specified by long-range BMP signaling and are especially sensitive to loss of extracellular modulators of BMP signaling such as the Chordin homolog Short gastrulation (Sog). However, the role of the **extracellular matrix** in BMP signaling and Sog activity in the crossveins has been poorly explored. Using a genetic mosaic screen for mutations that disrupt BMP signaling and posterior crossvein development, we identify Gyc76C, a member of the receptor guanylyl cyclase family that includes mammalian natriuretic peptide receptors. We show that Gyc76C and the soluble cGMP-dependent kinase Foraging, likely linked by cGMP, are necessary for normal refinement and maintenance of long-range BMP signaling in the posterior crossvein. This does not occur through cell-autonomous crosstalk between cGMP and BMP signal transduction, but likely through altered extracellular activity of Sog. We identify a novel pathway leading from Gyc76C to the organization of the wing **extracellular matrix** by matrix metalloproteinases, and show that both the **extracellular matrix** and BMP signaling effects are largely mediated by changes in the activity of matrix metalloproteinases. We discuss parallels and differences between this pathway and other examples of cGMP activity in both Drosophila melanogaster and mammalian cells and tissues.
11. Two classes of matrix metalloproteinases reciprocally regulate synaptogenesis. Synaptogenesis requires orchestrated intercellular communication between synaptic partners, with trans-synaptic signals necessarily traversing the extracellular synaptomatrix separating presynaptic and postsynaptic cells. **Extracellular matrix** metalloproteinases (Mmps) regulated by secreted tissue inhibitors of metalloproteinases (Timps), cleave secreted and membrane-associated targets to sculpt the extracellular environment and modulate intercellular signaling. Here, we test the roles of Mmp at the neuromuscular junction (NMJ) model synapse in the reductionist Drosophila system, which contains just two Mmps (secreted Mmp1 and GPI-anchored Mmp2) and one secreted Timp. We found that all three matrix metalloproteome components co-dependently localize in the synaptomatrix and show that both Mmp1 and Mmp2 independently restrict synapse morphogenesis and functional differentiation. Surprisingly, either dual knockdown or simultaneous inhibition of the two Mmp classes together restores normal synapse development, identifying a reciprocal suppression mechanism. The two Mmp classes co-regulate a Wnt trans-synaptic signaling pathway modulating structural and functional synaptogenesis, including the GPI-anchored heparan sulfate proteoglycan (HSPG) Wnt co-receptor Dally-like protein (Dlp), cognate receptor Frizzled-2 (Frz2) and Wingless (Wg) ligand. Loss of either Mmp1 or Mmp2 reciprocally misregulates Dlp at the synapse, with normal signaling restored by co-removal of both Mmp classes. Correcting Wnt co-receptor Dlp levels in both Mmp mutants prevents structural and functional synaptogenic defects. Taken together, these results identify an Mmp mechanism that fine-tunes HSPG co-receptor function to modulate Wnt signaling to coordinate synapse structural and functional development.
12. Immune response to bacteria induces dissemination of Ras-activated Drosophila hindgut cells. Although pathogenic bacteria are suspected contributors to colorectal cancer progression, cancer-promoting bacteria and their mode of action remain largely unknown. Here we report that sustained infection with the human intestinal colonizer Pseudomonas aeruginosa synergizes with the Ras1V12 oncogene to induce basal invasion and dissemination of hindgut cells to distant sites. Cross-talk between infection and dissemination requires sustained activation by the bacteria of the Imd-dTab2-dTak1 innate immune pathway, which converges with Ras1V12 signalling on JNK pathway activation, culminating in **extracellular matrix** degradation. Hindgut, but not midgut, cells are amenable to this cooperative dissemination, which is progressive and genetically and pharmacologically inhibitable. Thus, Drosophila hindgut provides a valuable system for the study of intestinal malignancies.
13. Distinct functions for the catalytic and hemopexin domains of a Drosophila matrix metalloproteinase. Human matrix metalloproteinases (MMPs) are believed to contribute to tumor progression. Therapies based on inhibiting the catalytic domain of MMPs have been unsuccessful, but these studies raise the question of whether other MMP domains might be appropriate targets. The genetic dissection of domain function has been stymied in mouse because there are 24 related and partially redundant MMP genes in the mouse genome. Here, we present a genetic dissection of the functions of the hemopexin and catalytic domains of a canonical MMP in Drosophila melanogaster, an organism with only 2 MMPs that function nonredundantly. We compare the phenotypes of Mmp1 null alleles with alleles that have specific hemopexin domain lesions, and we also examine phenotypes of dominant-negative mutants. We find that, although the catalytic domain appears to be required for all MMP functions including **extracellular matrix** remodeling of the tracheal system, the hemopexin domain is required specifically for tissue invasion events later in metamorphosis but not for tracheal remodeling. Thus, we find that this MMP hemopexin domain has an apparent specialization for tissue invasion events, a finding with potential implications for inhibitor therapies.
14. Basement membrane remodeling is essential for Drosophila disc eversion and tumor invasion. Organ and tissue integrity is often maintained in animals by a specialized **extracellular matrix** structure called the basement membrane (BM). Accumulated evidence indicates that BM remodeling occurs during development and tumor invasion. Although the BM organizes and functions at the organ level, most past studies have explored its biochemical and in vitro properties. In this study, we monitor the BM in vivo during developmental tissue invasion for disc eversion and tumor invasion in Drosophila and modulate BM integrity with genetic alterations affecting either the whole organism or the targeted discs or tumors. We observe that the degradation of BM by the discs or the tumors is an early event during invasion processes and that preventing BM degradation completely blocks both tissue and tumor invasion, indicating that modulation of BM is essential for developmental and tumor invasion. Furthermore, elements of the invasion machinery, including JNK-induced matrix metalloproteinase (MMP) expression, are shared by both disc eversion and tumor invasion processes. Moreover, we show that although expression of MMP inhibitor, TIMP, is sufficient to halt developmental invasion, inhibition of proteases by both TIMP and RECK are required to block tumor invasion. These data suggest that tumor cells have a more robust invasion mechanism and could acquire metastatic behavior by co-opting developmental invasion programs. This type of co-option may be a general feature contributing to the progression of tumors. Finally, although past efforts using MMP inhibitors have not yielded much success, our results strongly argue that BM modulation could be a critical target for cancer therapy.
15. The orthologous Tbx transcription factors Omb and TBX2 induce epithelial cell migration and extrusion in vivo without involvement of matrix metalloproteinases. The transcription factors TBX2 and TBX3 are overexpressed in various human cancers. Here, we investigated the effect of overexpressing the orthologous Tbx genes Drosophila optomotor-blind (omb) and human TBX2 in the epithelium of the Drosophila wing imaginal disc and observed two types of cell motility. Omb/TBX2 overexpressing cells could move within the plane of the epithelium. Invasive cells migrated long-distance as single cells retaining or regaining normal cell shape and apico-basal polarity in spite of attenuated apical DE-cadherin concentration. Inappropriate levels of DE-cadherin were sufficient to drive cell migration in the wing disc epithelium. Omb/TBX2 overexpression and reduced DE-cadherin-dependent adhesion caused the formation of actin-rich lateral cell protrusions. Omb/TBX2 overexpressing cells could also delaminate basally, penetrating the basal lamina, however, without degradation of **extracellular matrix**. Expression of Timp, an inhibitor of matrix metalloproteases, blocked neither intraepithelial motility nor basal extrusion. Our results reveal an MMP-independent mechanism of cell invasion and suggest a conserved role of Tbx2-related proteins in cell invasion and metastasis-related processes.
16. Tissue remodeling during maturation of the Drosophila wing. The final step in morphogenesis of the adult fly is wing maturation, a process not well understood at the cellular level due to the impermeable and refractive nature of cuticle synthesized some 30 h prior to eclosion from the pupal case. Advances in GFP technology now make it possible to visualize cells using fluorescence after cuticle synthesis is complete. We find that, between eclosion and wing expansion, the epithelia within the folded wing begin to delaminate from the cuticle and that delamination is complete when the wing has fully expanded. After expansion, epithelial cells lose contact with each other, adherens junctions are disrupted, and nuclei become pycnotic. The cells then change shape, elongate, and migrate from the wing into the thorax. During wing maturation, the Timp gene product, tissue inhibitor of metalloproteinases, and probably other components of an **extracellular matrix** are expressed that bond the dorsal and ventral cuticular surfaces of the wing following migration of the cells. These steps are dissected using the batone and Timp genes and ectopic expression of alphaPS integrin, inhibitors of Armadillo/beta-catenin nuclear activity and baculovirus caspase inhibitor p35. We conclude that an epithelial-mesenchymal transition is responsible for epithelial delamination and dissolution.
17. Invertebrate tissue inhibitor of metalloproteinase: structure and nested gene organization within the synapsin locus is conserved from Drosophila to human. Vertebrate tissue inhibitors of metalloproteinases (TIMPs) regulate **extracellular matrix** metalloproteinases and are thus involved in a wide variety of developmental and physiological processes. By identifying cDNAs of a transcript detected within an intron of the Drosophila synapsin gene we have cloned the Drosophila TIMP gene (Timp), which represents the first invertebrate member of the TIMP gene family. Sequence analysis revealed an open reading frame of 210 amino acids with 35% identity to human TIMPs and a conserved exon-intron structure. Analysis of sequence data from the Sanger Centre demonstrated that the human TIMP3 gene is encoded within intron V of the human synapsin-III gene, indicating that the nested organization of TIMP and synapsin genes may be a general feature conserved in evolution. We therefore speculate that the human TIMP4 gene will be located in intron V of the human synapsin-II gene on chromosome 3p25, and we present preliminary evidence that a human synapsin-IV gene is located near the TIMP2 gene on chromosome 17q23-q25.
18. Tissue inhibitors of metalloproteinases. Orchestration of the growth and remodeling of tissues and responses of cells to their extracellular environment is mediated by metalloproteinases of the Metzincin clan. This group of proteins comprises several families of endopeptidases in which a zinc atom is liganded at the catalytic site to three histidine residues and an invariant methionine residue. Tissue inhibitors of metalloproteinases (TIMPs) are endogenous protein regulators of the matrix metalloproteinase (MMPs) family, and also of families such as the disintegrin metalloproteinases (ADAM and ADAMTS). TIMPs therefore have a pivotal role in determining the influence of the **extracellular matrix**, of cell adhesion molecules, and of many cytokines, chemokines and growth factors on cell phenotype. The TIMP family is an ancient one, with a single representative in lower eukaryotes and four members in mammals. Although much is known about their mechanism of action in proteinase regulation in mammalian cells, less is known about their functions in lower organisms. Recently, non-inhibitory functions of TIMPs have been identified in mammalian cells, including signaling roles downstream of specific receptors. There are clearly still questions to be answered with regard to their overall roles in biology.
19. Inflated wings, tissue autolysis and early death in tissue inhibitor of metalloproteinases mutants of Drosophila. In vertebrates, tissue inhibitors of metalloproteinases (TIMPs) play key roles in **extracellular matrix** (ECM) homeostasis and growth control. Deletion of the recently cloned Timp gene of Drosophila results in a subviable phenotype. Adult flies display inflated wings similar to integrin mutants, suffer from a bloated gut and progressive dissolution of internal tissues, and die prematurely. Our results demonstrate that the Timp gene product controls selective aspects of ECM function in Drosophila, and suggest that it is involved in cell adhesion/cell signaling pathways. Hence, Drosophila Timp mutants may prove useful as a model system for a wide variety of pathological conditions related to ECM dysregulation.
20. The Dorsocross T-box transcription factors promote tissue morphogenesis in the Drosophila wing imaginal disc. The Drosophila wing imaginal disc is subdivided into notum, hinge and blade territories during the third larval instar by formation of several deep apical folds. The molecular mechanisms of these subdivisions and the subsequent initiation of morphogenic processes during metamorphosis are poorly understood. Here, we demonstrate that the Dorsocross (Doc) T-box genes promote the progression of epithelial folds that not only separate the hinge and blade regions of the wing disc but also contribute to metamorphic development by changing cell shapes and bending the wing disc. We found that Doc expression was restricted by two inhibitors, Vestigial and Homothorax, leading to two narrow Doc stripes where the folds separating hinge and blade are forming. Doc mutant clones prevented the lateral extension and deepening of these folds at the larval stage and delayed wing disc bending in the early pupal stage. Ectopic Doc expression was sufficient to generate deep apical folds by causing a basolateral redistribution of the apical microtubule web and a shortening of cells. Cells of both the endogenous blade/hinge folds and of folds elicited by ectopic Doc expression expressed Matrix metalloproteinase 2 (Mmp2). In these folds, integrins and **extracellular matrix** proteins were depleted. Overexpression of Doc along the blade/hinge folds caused precocious wing disc bending, which could be suppressed by co-expressing MMP2RNAi.

#### Component

1. proteinaceous **extracellular matrix**

### [Ii]nvasive

#### PubMed

1. RhoL controls invasion and Rap1 localization during immune cell transmigration in Drosophila. Human immune cells have to penetrate an endothelial barrier during their beneficial pursuit of infection and their destructive infiltration of tissues in autoimmune diseases. This transmigration requires Rap1 GTPase to activate integrin affinity. We define a new model system for this process by demonstrating, with live imaging and genetics, that during embryonic development Drosophila melanogaster immune cells penetrate an epithelial, Drosophila E-cadherin (DE-cadherin)-based tissue barrier. A mutant in RhoL, a GTPase homologue that is specifically expressed in haemocytes, blocks this **invasive** step but not other aspects of guided migration. RhoL mediates integrin adhesion caused by Drosophila Rap1 overexpression and moves Rap1 away from a concentration in the cytoplasm to the leading edge during **invasive** migration. These findings indicate that a programmed migratory step during Drosophila development bears striking molecular similarities to vertebrate immune cell transmigration during inflammation, and identify RhoL as a new regulator of invasion, adhesion and Rap1 localization. Our work establishes the utility of Drosophila for identifying novel components of immune cell transmigration and for understanding the in vivo interplay of immune cells with the barriers they penetrate.
2. APC loss-induced intestinal tumorigenesis in Drosophila: Roles of Ras in Wnt signaling activation and tumor progression. Adenomatous polyposis coli (APC) and K-ras are the two most frequently mutated genes found in human colorectal cancers. In human colorectal cancers, Wnt signaling activation after the loss of APC is hypothesized to be the key event for adenoma initiation, whereas additional mutations such as Ras activation are required for the progression from adenoma to carcinoma. However, accumulating data have led to conflicting views regarding the precise role of Ras in APC loss-induced tumorigenesis. Here, using Drosophila midgut as a model system, we show that in the absence of Ras, APC mutant epithelial cells cannot initiate hyperplasia, suggesting that Ras plays an essential role in tumor initiation. Conversely, activating Ras by expressing oncogenic Ras or Raf in APC-deficient cells led to a blockage of cell differentiation and to pre**invasive** tumor outgrowth, characteristics that are shared by advanced colorectal carcinoma in humans. Mechanistically, we find that Ras is not required for Wnt signaling activation after APC loss, although Ras hyperactivation is able to potentiate Wnt signaling by increasing the cytoplasmic and nuclear accumulation of Armadillo/β-catenin via mechanisms independent of JNK/Rac1 or PI3K-Akt signaling, partly owing to the downregulation of DE-cadherin. Together with the data from gene expression analyses, our results indicate that both parallel and cooperative mechanisms of Wnt and Ras signaling are responsible for the initiation and progression of intestinal tumorigenesis after APC loss.
3. Dynamics of the basement membrane in **invasive** epithelial clusters in Drosophila. The basement membrane (BM) represents a barrier to cell migration, which has to be degraded to promote invasion. However, the role and behaviour of the BM during the development of pre-**invasive** cells is only poorly understood. Drosophila border cells (BCs) provide an attractive genetic model in which to study the cellular mechanisms underlying the migration of mixed cohorts of epithelial cells. BCs are made of two different epithelial cell types appearing sequentially during oogenesis: the polar cells and the outer BCs. Here, we show that the pre-**invasive** polar cells undergo an unusual and asymmetrical apical capping with major basement membrane proteins, including the two Drosophila Collagen IV alpha chains, Laminin A and Perlecan. Capping of polar cells proceeds through a novel, basal-to-apical transcytosis mechanism that involves the small GTPase Drab5. Apical capping is transient and is followed by rapid shedding prior to the initiation of BC migration, suggesting that the apical cap blocks migration. Consistently, non-migratory polar cells remain capped. We further show that JAK/STAT signalling and recruitment of outer BCs are required for correct shedding and migration. The dynamics of the BM represents a marker of migratory BC, revealing a novel developmentally regulated behaviour of BM coupled to epithelial cell **invasive**ness.
4. JNK- and Fos-regulated Mmp1 expression cooperates with Ras to induce **invasive** tumors in Drosophila. Loss of the epithelial polarity gene scribble in clones of Drosophila imaginal disc cells can cooperate with Ras signaling to induce malignant tumors. Such mutant tissue overproliferates, resists apoptosis, leaves its place of origin and invades other organs, ultimately causing lethality. We show that increased Jun N-terminal kinase (JNK) signaling resulting from the loss of scribble promotes the movement of transformed cells to secondary sites. This effect requires Fos-dependent transcriptional activation of a matrix metalloprotease gene mmp1 downstream of JNK. Expression of the Mmp inhibitor Timp or Mmp RNAi knockdown suppresses cell **invasive**ness. The pro**invasive** function of the JNK pathway is revealed in a tumor context when active Ras signaling prevents the apoptotic response to JNK activity as it occurs in nontransformed cells. Based on these results, we present a model that explains the oncogenic cooperation between JNK and Ras, and describes how aberrant regulation of cell survival, proliferation and mobilization cooperate to incite malignant tumor formation.
5. Sds22/PP1 links epithelial integrity and tumor suppression via regulation of myosin II and JNK signaling. Loss of epithelial integrity often correlates with the progression of malignant tumors. Sds22, a regulatory subunit of protein phosphatase 1 (PP1), has recently been linked to regulation of epithelial polarity in Drosophila. However, its role in tumorigenesis remains obscure. In this study, using Drosophila imaginal tissue as an in vivo model system, we show that sds22 is a new potential tumor suppressor gene in Drosophila. Without sds22, cells lose epithelial architecture, and become **invasive** and tumorigenic when combined with Ras overexpression; conversely, sds22 overexpression can largely suppress tumorigenic growth of Ras(V12)scrib(-/-) mutant cells. Mechanistically, we show that sds22 prevents cell invasion and metastasis by inhibiting myosin II and Jun N-terminal kinase (JNK) activity downstream of PP1. Loss of this inhibition causes cells to lose epithelial organization and promotes cell invasion. Finally, human Sds22 is focally deleted and downregulated in multiple carcinomas, and this downregulation correlates with tumor progression, suggesting that sds22 inactivation may contribute to tumorigenesis and metastatic potential in human cancers via a similar mechanism.
6. The orthologous Tbx transcription factors Omb and TBX2 induce epithelial cell migration and extrusion in vivo without involvement of matrix metalloproteinases. The transcription factors TBX2 and TBX3 are overexpressed in various human cancers. Here, we investigated the effect of overexpressing the orthologous Tbx genes Drosophila optomotor-blind (omb) and human TBX2 in the epithelium of the Drosophila wing imaginal disc and observed two types of cell motility. Omb/TBX2 overexpressing cells could move within the plane of the epithelium. **Invasive** cells migrated long-distance as single cells retaining or regaining normal cell shape and apico-basal polarity in spite of attenuated apical DE-cadherin concentration. Inappropriate levels of DE-cadherin were sufficient to drive cell migration in the wing disc epithelium. Omb/TBX2 overexpression and reduced DE-cadherin-dependent adhesion caused the formation of actin-rich lateral cell protrusions. Omb/TBX2 overexpressing cells could also delaminate basally, penetrating the basal lamina, however, without degradation of extracellular matrix. Expression of Timp, an inhibitor of matrix metalloproteases, blocked neither intraepithelial motility nor basal extrusion. Our results reveal an MMP-independent mechanism of cell invasion and suggest a conserved role of Tbx2-related proteins in cell invasion and metastasis-related processes.

### [Mm]etastasize

#### PubMed

1. Metastatic ability of Drosophila tumors depends on MMP activity. We analyzed how cells from tumors caused by mutations in either lgl or brat use matrix metalloproteinases (MMPs) to facilitate metastasis in Drosophila. MMP1 accumulation is dramatically increased in lgl larval imaginal discs compared to both wild type and brat mutants. Removal of Mmp1 gene activity in lgl brain tumor cells reduced their frequency of ovarian micro-metastases after transplantation; whereas, removal of Mmp1 gene activity in brat tumor cells had no such effect. Host ovaries showed increased Mmp1 gene expression in response to transplantation of brat tumors but not of lgl tumors. Reduction of MMP activity in host ovaries by ectopic expression of TIMP significantly reduced both lgl and brat metastases in that organ. These results highlight the mechanisms that lgl and brat tumor cells use to **metastasize**. Our interpretation of these data is that secretion of MMP1 from lgl tumor cells facilitates their metastasis, while secretion of MMP1 from host ovaries facilitates brat tumor metastasis. This study is the first demonstration that Drosophila tumors utilize MMP activity to **metastasize**.

### [Mm]etastasis

#### PubMed

1. Metastatic ability of Drosophila tumors depends on MMP activity. We analyzed how cells from tumors caused by mutations in either lgl or brat use matrix metalloproteinases (MMPs) to facilitate **metastasis** in Drosophila. MMP1 accumulation is dramatically increased in lgl larval imaginal discs compared to both wild type and brat mutants. Removal of Mmp1 gene activity in lgl brain tumor cells reduced their frequency of ovarian micro-metastases after transplantation; whereas, removal of Mmp1 gene activity in brat tumor cells had no such effect. Host ovaries showed increased Mmp1 gene expression in response to transplantation of brat tumors but not of lgl tumors. Reduction of MMP activity in host ovaries by ectopic expression of TIMP significantly reduced both lgl and brat metastases in that organ. These results highlight the mechanisms that lgl and brat tumor cells use to metastasize. Our interpretation of these data is that secretion of MMP1 from lgl tumor cells facilitates their **metastasis**, while secretion of MMP1 from host ovaries facilitates brat tumor **metastasis**. This study is the first demonstration that Drosophila tumors utilize MMP activity to metastasize.
2. Modeling tumor invasion and **metastasis** in Drosophila. Conservation of major signaling pathways between humans and flies has made Drosophila a useful model organism for cancer research. Our understanding of the mechanisms regulating cell growth, differentiation and development has been considerably advanced by studies in Drosophila. Several recent high profile studies have examined the processes constraining the metastatic growth of tumor cells in fruit fly models. Cell invasion can be studied in the context of an in vivo setting in flies, enabling the genetic requirements of the microenvironment of tumor cells undergoing **metastasis** to be analyzed. This Perspective discusses the strengths and limitations of Drosophila models of cancer invasion and the unique tools that have enabled these studies. It also highlights several recent reports that together make a strong case for Drosophila as a system with the potential for both testing novel concepts in tumor progression and cell invasion, and for uncovering players in **metastasis**.
3. Sds22/PP1 links epithelial integrity and tumor suppression via regulation of myosin II and JNK signaling. Loss of epithelial integrity often correlates with the progression of malignant tumors. Sds22, a regulatory subunit of protein phosphatase 1 (PP1), has recently been linked to regulation of epithelial polarity in Drosophila. However, its role in tumorigenesis remains obscure. In this study, using Drosophila imaginal tissue as an in vivo model system, we show that sds22 is a new potential tumor suppressor gene in Drosophila. Without sds22, cells lose epithelial architecture, and become invasive and tumorigenic when combined with Ras overexpression; conversely, sds22 overexpression can largely suppress tumorigenic growth of Ras(V12)scrib(-/-) mutant cells. Mechanistically, we show that sds22 prevents cell invasion and **metastasis** by inhibiting myosin II and Jun N-terminal kinase (JNK) activity downstream of PP1. Loss of this inhibition causes cells to lose epithelial organization and promotes cell invasion. Finally, human Sds22 is focally deleted and downregulated in multiple carcinomas, and this downregulation correlates with tumor progression, suggesting that sds22 inactivation may contribute to tumorigenesis and metastatic potential in human cancers via a similar mechanism.
4. The orthologous Tbx transcription factors Omb and TBX2 induce epithelial cell migration and extrusion in vivo without involvement of matrix metalloproteinases. The transcription factors TBX2 and TBX3 are overexpressed in various human cancers. Here, we investigated the effect of overexpressing the orthologous Tbx genes Drosophila optomotor-blind (omb) and human TBX2 in the epithelium of the Drosophila wing imaginal disc and observed two types of cell motility. Omb/TBX2 overexpressing cells could move within the plane of the epithelium. Invasive cells migrated long-distance as single cells retaining or regaining normal cell shape and apico-basal polarity in spite of attenuated apical DE-cadherin concentration. Inappropriate levels of DE-cadherin were sufficient to drive cell migration in the wing disc epithelium. Omb/TBX2 overexpression and reduced DE-cadherin-dependent adhesion caused the formation of actin-rich lateral cell protrusions. Omb/TBX2 overexpressing cells could also delaminate basally, penetrating the basal lamina, however, without degradation of extracellular matrix. Expression of Timp, an inhibitor of matrix metalloproteases, blocked neither intraepithelial motility nor basal extrusion. Our results reveal an MMP-independent mechanism of cell invasion and suggest a conserved role of Tbx2-related proteins in cell invasion and **metastasis**-related processes.

### [Cc]ell [Ii]nvasion

#### PubMed

1. Modeling tumor invasion and metastasis in Drosophila. Conservation of major signaling pathways between humans and flies has made Drosophila a useful model organism for cancer research. Our understanding of the mechanisms regulating cell growth, differentiation and development has been considerably advanced by studies in Drosophila. Several recent high profile studies have examined the processes constraining the metastatic growth of tumor cells in fruit fly models. **Cell invasion** can be studied in the context of an in vivo setting in flies, enabling the genetic requirements of the microenvironment of tumor cells undergoing metastasis to be analyzed. This Perspective discusses the strengths and limitations of Drosophila models of cancer invasion and the unique tools that have enabled these studies. It also highlights several recent reports that together make a strong case for Drosophila as a system with the potential for both testing novel concepts in tumor progression and **cell invasion**, and for uncovering players in metastasis.
2. Sds22/PP1 links epithelial integrity and tumor suppression via regulation of myosin II and JNK signaling. Loss of epithelial integrity often correlates with the progression of malignant tumors. Sds22, a regulatory subunit of protein phosphatase 1 (PP1), has recently been linked to regulation of epithelial polarity in Drosophila. However, its role in tumorigenesis remains obscure. In this study, using Drosophila imaginal tissue as an in vivo model system, we show that sds22 is a new potential tumor suppressor gene in Drosophila. Without sds22, cells lose epithelial architecture, and become invasive and tumorigenic when combined with Ras overexpression; conversely, sds22 overexpression can largely suppress tumorigenic growth of Ras(V12)scrib(-/-) mutant cells. Mechanistically, we show that sds22 prevents **cell invasion** and metastasis by inhibiting myosin II and Jun N-terminal kinase (JNK) activity downstream of PP1. Loss of this inhibition causes cells to lose epithelial organization and promotes **cell invasion**. Finally, human Sds22 is focally deleted and downregulated in multiple carcinomas, and this downregulation correlates with tumor progression, suggesting that sds22 inactivation may contribute to tumorigenesis and metastatic potential in human cancers via a similar mechanism.
3. The orthologous Tbx transcription factors Omb and TBX2 induce epithelial cell migration and extrusion in vivo without involvement of matrix metalloproteinases. The transcription factors TBX2 and TBX3 are overexpressed in various human cancers. Here, we investigated the effect of overexpressing the orthologous Tbx genes Drosophila optomotor-blind (omb) and human TBX2 in the epithelium of the Drosophila wing imaginal disc and observed two types of cell motility. Omb/TBX2 overexpressing cells could move within the plane of the epithelium. Invasive cells migrated long-distance as single cells retaining or regaining normal cell shape and apico-basal polarity in spite of attenuated apical DE-cadherin concentration. Inappropriate levels of DE-cadherin were sufficient to drive cell migration in the wing disc epithelium. Omb/TBX2 overexpression and reduced DE-cadherin-dependent adhesion caused the formation of actin-rich lateral cell protrusions. Omb/TBX2 overexpressing cells could also delaminate basally, penetrating the basal lamina, however, without degradation of extracellular matrix. Expression of Timp, an inhibitor of matrix metalloproteases, blocked neither intraepithelial motility nor basal extrusion. Our results reveal an MMP-independent mechanism of **cell invasion** and suggest a conserved role of Tbx2-related proteins in **cell invasion** and metastasis-related processes.

---

## CG6928

- NCBI Gene
- GeneRIF
- Pubmed

| Search Term | Rank | Fields |
| --- | --- | --- |
| [Mm]igration | 6 | PubMed(1) |
| ECM | 7 | PubMed(1) |
| [Ee]xtracellular [Mm]atrix | 8 | PubMed(1) |

  
hide/show details for CG6928

### [Mm]igration

#### PubMed

1. Genes conserved in bilaterians but jointly lost with Myc during nematode evolution are enriched in cell proliferation and cell **migration** functions. Animals use a stereotypical set of developmental genes to build body architectures of varying sizes and organizational complexity. Some genes are critical to developmental patterning, while other genes are important to physiological control of growth. However, growth regulator genes may not be as important in small-bodied "micro-metazoans" such as nematodes. Nematodes use a simplified developmental strategy of lineage-based cell fate specifications to produce an adult bilaterian body composed of a few hundreds of cells. Nematodes also lost the MYC proto-oncogenic regulator of cell proliferation. To identify additional regulators of cell proliferation that were lost with MYC, we computationally screened and determined 839 high-confidence genes that are conserved in bilaterians/lost in nematodes (CIBLIN genes). We find that 30 % of all CIBLIN genes encode transcriptional regulators of cell proliferation, epithelial-to-mesenchyme transitions, and other processes. Over 50 % of CIBLIN genes are unnamed genes in Drosophila, suggesting that there are many understudied genes. Interestingly, CIBLIN genes include many Myc synthetic lethal (MycSL) hits from recent screens. CIBLIN genes include key regulators of heparan sulfate proteoglycan (HSPG) sulfation patterns, and lysyl oxidases involved in cross-linking and modification of the extracellular matrix (ECM). These genes and others suggest the CIBLIN repertoire services critical functions in ECM remodeling and cell **migration** in large-bodied bilaterians. Correspondingly, CIBLIN genes are co-expressed with Myc in cancer transcriptomes, and include a preponderance of known determinants of cancer progression and tumor aggression. We propose that CIBLIN gene research can improve our understanding of regulatory control of cellular growth in metazoans.

### ECM

#### PubMed

1. Genes conserved in bilaterians but jointly lost with Myc during nematode evolution are enriched in cell proliferation and cell migration functions. Animals use a stereotypical set of developmental genes to build body architectures of varying sizes and organizational complexity. Some genes are critical to developmental patterning, while other genes are important to physiological control of growth. However, growth regulator genes may not be as important in small-bodied "micro-metazoans" such as nematodes. Nematodes use a simplified developmental strategy of lineage-based cell fate specifications to produce an adult bilaterian body composed of a few hundreds of cells. Nematodes also lost the MYC proto-oncogenic regulator of cell proliferation. To identify additional regulators of cell proliferation that were lost with MYC, we computationally screened and determined 839 high-confidence genes that are conserved in bilaterians/lost in nematodes (CIBLIN genes). We find that 30 % of all CIBLIN genes encode transcriptional regulators of cell proliferation, epithelial-to-mesenchyme transitions, and other processes. Over 50 % of CIBLIN genes are unnamed genes in Drosophila, suggesting that there are many understudied genes. Interestingly, CIBLIN genes include many Myc synthetic lethal (MycSL) hits from recent screens. CIBLIN genes include key regulators of heparan sulfate proteoglycan (HSPG) sulfation patterns, and lysyl oxidases involved in cross-linking and modification of the extracellular matrix (**ECM**). These genes and others suggest the CIBLIN repertoire services critical functions in **ECM** remodeling and cell migration in large-bodied bilaterians. Correspondingly, CIBLIN genes are co-expressed with Myc in cancer transcriptomes, and include a preponderance of known determinants of cancer progression and tumor aggression. We propose that CIBLIN gene research can improve our understanding of regulatory control of cellular growth in metazoans.

### [Ee]xtracellular [Mm]atrix

#### PubMed

1. Genes conserved in bilaterians but jointly lost with Myc during nematode evolution are enriched in cell proliferation and cell migration functions. Animals use a stereotypical set of developmental genes to build body architectures of varying sizes and organizational complexity. Some genes are critical to developmental patterning, while other genes are important to physiological control of growth. However, growth regulator genes may not be as important in small-bodied "micro-metazoans" such as nematodes. Nematodes use a simplified developmental strategy of lineage-based cell fate specifications to produce an adult bilaterian body composed of a few hundreds of cells. Nematodes also lost the MYC proto-oncogenic regulator of cell proliferation. To identify additional regulators of cell proliferation that were lost with MYC, we computationally screened and determined 839 high-confidence genes that are conserved in bilaterians/lost in nematodes (CIBLIN genes). We find that 30 % of all CIBLIN genes encode transcriptional regulators of cell proliferation, epithelial-to-mesenchyme transitions, and other processes. Over 50 % of CIBLIN genes are unnamed genes in Drosophila, suggesting that there are many understudied genes. Interestingly, CIBLIN genes include many Myc synthetic lethal (MycSL) hits from recent screens. CIBLIN genes include key regulators of heparan sulfate proteoglycan (HSPG) sulfation patterns, and lysyl oxidases involved in cross-linking and modification of the **extracellular matrix** (ECM). These genes and others suggest the CIBLIN repertoire services critical functions in ECM remodeling and cell migration in large-bodied bilaterians. Correspondingly, CIBLIN genes are co-expressed with Myc in cancer transcriptomes, and include a preponderance of known determinants of cancer progression and tumor aggression. We propose that CIBLIN gene research can improve our understanding of regulatory control of cellular growth in metazoans.

---

## Fim

- NCBI Gene
- GeneRIF
- Pubmed

| Search Term | Rank | Fields |
| --- | --- | --- |
| [Ff]ilopodia | 1 | PubMed(1) |
| [Mm]igration | 6 | PubMed(1) |
| [Ii]nvasive | 10 | PubMed(1) |
| [Cc]ell [Ii]nvasion | 13 | PubMed(1) |

  
hide/show details for Fim

### [Ff]ilopodia

#### PubMed

1. **Filopodia**-like actin cables position nuclei in association with perinuclear actin in Drosophila nurse cells. Controlling the position of the nucleus is vital for a number of cellular processes from yeast to humans. In Drosophila nurse cells, nuclear positioning is crucial during dumping, when nurse cells contract and expel their contents into the oocyte. We provide evidence that in nurse cells, continuous **filopodia**-like actin cables, growing from the plasma membrane and extending to the nucleus, achieve nuclear positioning. These actin cables move nuclei away from ring canals. When nurse cells contract, actin cables associate laterally with the nuclei, in some cases inducing nuclear turning so that actin cables become partially wound around the nuclei. Our data suggest that a perinuclear actin meshwork connects actin cables to nuclei via actin-crosslinking proteins such as the filamin Cheerio. We provide a revised model for how actin structures position nuclei in nurse cells, employing evolutionary conserved machinery.

### [Mm]igration

#### PubMed

1. Molecular requirements for actin-based lamella formation in Drosophila S2 cells. Cell **migration** occurs through the protrusion of the actin-enriched lamella. Here, we investigated the effects of RNAi depletion of approximately 90 proteins implicated in actin function on lamella formation in Drosophila S2 cells. Similar to in vitro reconstitution studies of actin-based Listeria movement, we find that lamellae formation requires a relatively small set of proteins that participate in actin nucleation (Arp2/3 and SCAR), barbed end capping (capping protein), filament depolymerization (cofilin and Aip1), and actin monomer binding (profilin and cyclase-associated protein). Lamellae are initiated by parallel and partially redundant signaling pathways involving Rac GTPases and the adaptor protein Nck, which stimulate SCAR, an Arp2/3 activator. We also show that RNAi of three proteins (kette, Abi, and Sra-1) known to copurify with and inhibit SCAR in vitro leads to SCAR degradation, revealing a novel function of this protein complex in SCAR stability. Our results have identified an essential set of proteins involved in actin dynamics during lamella formation in Drosophila S2 cells.

### [Ii]nvasive

#### PubMed

1. Mechanical tension drives cell membrane fusion. Membrane fusion is an energy-consuming process that requires tight juxtaposition of two lipid bilayers. Little is known about how cells overcome energy barriers to bring their membranes together for fusion. Previously, we have shown that cell-cell fusion is an asymmetric process in which an "attacking" cell drills finger-like protrusions into the "receiving" cell to promote cell fusion. Here, we show that the receiving cell mounts a Myosin II (MyoII)-mediated mechanosensory response to its **invasive** fusion partner. MyoII acts as a mechanosensor, which directs its force-induced recruitment to the fusion site, and the mechanosensory response of MyoII is amplified by chemical signaling initiated by cell adhesion molecules. The accumulated MyoII, in turn, increases cortical tension and promotes fusion pore formation. We propose that the protrusive and resisting forces from fusion partners put the fusogenic synapse under high mechanical tension, which helps to overcome energy barriers for membrane apposition and drives cell membrane fusion.

### [Cc]ell [Ii]nvasion

#### PubMed

1. Rickettsia parkeri invasion of diverse host cells involves an Arp2/3 complex, WAVE complex and Rho-family GTPase-dependent pathway. Rickettsiae are obligate intracellular pathogens that are transmitted to humans by arthropod vectors and cause diseases such as spotted fever and typhus. Although rickettsiae require the host cell actin cytoskeleton for invasion, the cytoskeletal proteins that mediate this process have not been completely described. To identify the host factors important during **cell invasion** by Rickettsia parkeri, a member of the spotted fever group (SFG), we performed an RNAi screen targeting 105 proteins in Drosophila melanogaster S2R+ cells. The screen identified 21 core proteins important for invasion, including the GTPases Rac1 and Rac2, the WAVE nucleation-promoting factor complex and the Arp2/3 complex. In mammalian cells, including endothelial cells, the natural targets of R. parkeri, the Arp2/3 complex was also crucial for invasion, while requirements for WAVE2 as well as Rho GTPases depended on the particular cell type. We propose that R. parkeri invades S2R+ arthropod cells through a primary pathway leading to actin nucleation, whereas invasion of mammalian endothelial cells occurs via redundant pathways that converge on the host Arp2/3 complex. Our results reveal a key role for the WAVE and Arp2/3 complexes, as well as a higher degree of variation than previously appreciated in actin nucleation pathways activated during Rickettsia invasion.

---

## RtGEF

- NCBI Gene
- GeneRIF
- Pubmed

| Search Term | Rank | Fields |
| --- | --- | --- |
| [Ff]ilopodia | 1 | PubMed(1) |
| [Mm]igration | 6 | PubMed(1) |
| [Ee]xtracellular [Mm]atrix | 8 | PubMed(1) |
| [Ii]nvasive | 10 | PubMed(1) |
| [Mm]etastasis | 12 | PubMed(1) |
| [Cc]ell [Ii]nvasion | 13 | PubMed(1) |

  
hide/show details for RtGEF

### [Ff]ilopodia

#### PubMed

1. The leading edge during dorsal closure as a model for epithelial plasticity: Pak is required for recruitment of the Scribble complex and septate junction formation. Dorsal closure (DC) of the Drosophila embryo is a model for the study of wound healing and developmental epithelial fusions, and involves the sealing of a hole in the epidermis through the migration of the epidermal flanks over the tissue occupying the hole, the amnioserosa. During DC, the cells at the edge of the migrating epidermis extend Rac- and Cdc42-dependent actin-based lamellipodia and **filopodia** from their leading edge (LE), which exhibits a breakdown in apicobasal polarity as adhesions are severed with the neighbouring amnioserosa cells. Studies using mammalian cells have demonstrated that Scribble (Scrib), an important determinant of apicobasal polarity that functions in a protein complex, controls polarized cell migration through recruitment of Rac, Cdc42 and the serine/threonine kinase Pak, an effector for Rac and Cdc42, to the LE. We have used DC and the follicular epithelium to study the relationship between Pak and the Scrib complex at epithelial membranes undergoing changes in apicobasal polarity and adhesion during development. We propose that, during DC, the LE membrane undergoes an epithelial-to-mesenchymal-like transition to initiate epithelial sheet migration, followed by a mesenchymal-to-epithelial-like transition as the epithelial sheets meet up and restore cell-cell adhesion. This latter event requires integrin-localized Pak, which recruits the Scrib complex in septate junction formation. We conclude that there are bidirectional interactions between Pak and the Scrib complex modulating epithelial plasticity. Scrib can recruit Pak to the LE for polarized cell migration but, as migratory cells meet up, Pak can recruit the Scrib complex to restore apicobasal polarity and cell-cell adhesion.

### [Mm]igration

#### PubMed

1. The leading edge during dorsal closure as a model for epithelial plasticity: Pak is required for recruitment of the Scribble complex and septate junction formation. Dorsal closure (DC) of the Drosophila embryo is a model for the study of wound healing and developmental epithelial fusions, and involves the sealing of a hole in the epidermis through the **migration** of the epidermal flanks over the tissue occupying the hole, the amnioserosa. During DC, the cells at the edge of the migrating epidermis extend Rac- and Cdc42-dependent actin-based lamellipodia and filopodia from their leading edge (LE), which exhibits a breakdown in apicobasal polarity as adhesions are severed with the neighbouring amnioserosa cells. Studies using mammalian cells have demonstrated that Scribble (Scrib), an important determinant of apicobasal polarity that functions in a protein complex, controls polarized cell **migration** through recruitment of Rac, Cdc42 and the serine/threonine kinase Pak, an effector for Rac and Cdc42, to the LE. We have used DC and the follicular epithelium to study the relationship between Pak and the Scrib complex at epithelial membranes undergoing changes in apicobasal polarity and adhesion during development. We propose that, during DC, the LE membrane undergoes an epithelial-to-mesenchymal-like transition to initiate epithelial sheet **migration**, followed by a mesenchymal-to-epithelial-like transition as the epithelial sheets meet up and restore cell-cell adhesion. This latter event requires integrin-localized Pak, which recruits the Scrib complex in septate junction formation. We conclude that there are bidirectional interactions between Pak and the Scrib complex modulating epithelial plasticity. Scrib can recruit Pak to the LE for polarized cell **migration** but, as migratory cells meet up, Pak can recruit the Scrib complex to restore apicobasal polarity and cell-cell adhesion.

### [Ee]xtracellular [Mm]atrix

#### PubMed

1. Presynaptic establishment of the synaptic cleft **extracellular matrix** is required for post-synaptic differentiation. Formation and regulation of excitatory glutamatergic synapses is essential for shaping neural circuits throughout development. In a Drosophila genetic screen for synaptogenesis mutants, we identified mind the gap (mtg), which encodes a secreted, extracellular N-glycosaminoglycan-binding protein. MTG is expressed neuronally and detected in the synaptic cleft, and is required to form the specialized transsynaptic matrix that links the presynaptic active zone with the post-synaptic glutamate receptor (GluR) domain. Null mtg embryonic mutant synapses exhibit greatly reduced GluR function, and a corresponding loss of localized GluR domains. All known post-synaptic signaling/scaffold proteins functioning upstream of GluR localization are also grossly reduced or mislocalized in mtg mutants, including the dPix-dPak-Dock cascade and the Dlg/PSD-95 scaffold. Ubiquitous or neuronally targeted mtg RNA interference (RNAi) similarly reduce post-synaptic assembly, whereas post-synaptically targeted RNAi has no effect, indicating that presynaptic MTG induces and maintains the post-synaptic pathways driving GluR domain formation. These findings suggest that MTG is secreted from the presynaptic terminal to shape the extracellular synaptic cleft domain, and that the cleft domain functions to mediate transsynaptic signals required for post-synaptic development.

### [Ii]nvasive

#### PubMed

1. Sin3a acts through a multi-gene module to regulate invasion in Drosophila and human tumors. Chromatin remodeling proteins regulate multiple aspects of cell homeostasis, making them ideal candidates for misregulation in transformed cells. Here, we explore Sin3A, a member of the Sin3 family of proteins linked to tumorigenesis that are thought to regulate gene expression through their role as histone deacetylases (HDACs). We identified Drosophila Sin3a as an important mediator of oncogenic Ret receptor in a fly model of Multiple Endocrine Neoplasia Type 2. Reducing Drosophila Sin3a activity led to metastasis-like behavior and, in the presence of Diap1, secondary tumors distant from the site of origin. Genetic and Chip-Seq analyses identified previously undescribed Sin3a targets including genes involved in cell motility and actin dynamics, as well as signaling pathways including Src, Jnk and Rho. A key Sin3a oncogenic target, PP1B, regulates stability of β-Catenin/Armadillo: the outcome is to oppose T-cell factor (TCF) function and Wg/Wnt pathway signaling in both fly and mammalian cancer cells. Reducing Sin3A strongly increased the **invasiv**e behavior of A549 human lung adenocarcinoma cells. We show that Sin3A is downregulated in a variety of human tumors and that Src, JNK, RhoA and PP1B/β-Catenin are regulated in a manner analogous to our Drosophila models. Our data suggest that Sin3A influences a specific step of tumorigenesis by regulating a module of genes involved in cell invasion. Tumor progression may commonly rely on such 'modules of invasion' under the control of broad transcriptional regulators.

### [Mm]etastasis

#### PubMed

1. Sin3a acts through a multi-gene module to regulate invasion in Drosophila and human tumors. Chromatin remodeling proteins regulate multiple aspects of cell homeostasis, making them ideal candidates for misregulation in transformed cells. Here, we explore Sin3A, a member of the Sin3 family of proteins linked to tumorigenesis that are thought to regulate gene expression through their role as histone deacetylases (HDACs). We identified Drosophila Sin3a as an important mediator of oncogenic Ret receptor in a fly model of Multiple Endocrine Neoplasia Type 2. Reducing Drosophila Sin3a activity led to **metastasis**-like behavior and, in the presence of Diap1, secondary tumors distant from the site of origin. Genetic and Chip-Seq analyses identified previously undescribed Sin3a targets including genes involved in cell motility and actin dynamics, as well as signaling pathways including Src, Jnk and Rho. A key Sin3a oncogenic target, PP1B, regulates stability of β-Catenin/Armadillo: the outcome is to oppose T-cell factor (TCF) function and Wg/Wnt pathway signaling in both fly and mammalian cancer cells. Reducing Sin3A strongly increased the invasive behavior of A549 human lung adenocarcinoma cells. We show that Sin3A is downregulated in a variety of human tumors and that Src, JNK, RhoA and PP1B/β-Catenin are regulated in a manner analogous to our Drosophila models. Our data suggest that Sin3A influences a specific step of tumorigenesis by regulating a module of genes involved in cell invasion. Tumor progression may commonly rely on such 'modules of invasion' under the control of broad transcriptional regulators.

### [Cc]ell [Ii]nvasion

#### PubMed

1. Sin3a acts through a multi-gene module to regulate invasion in Drosophila and human tumors. Chromatin remodeling proteins regulate multiple aspects of cell homeostasis, making them ideal candidates for misregulation in transformed cells. Here, we explore Sin3A, a member of the Sin3 family of proteins linked to tumorigenesis that are thought to regulate gene expression through their role as histone deacetylases (HDACs). We identified Drosophila Sin3a as an important mediator of oncogenic Ret receptor in a fly model of Multiple Endocrine Neoplasia Type 2. Reducing Drosophila Sin3a activity led to metastasis-like behavior and, in the presence of Diap1, secondary tumors distant from the site of origin. Genetic and Chip-Seq analyses identified previously undescribed Sin3a targets including genes involved in cell motility and actin dynamics, as well as signaling pathways including Src, Jnk and Rho. A key Sin3a oncogenic target, PP1B, regulates stability of β-Catenin/Armadillo: the outcome is to oppose T-cell factor (TCF) function and Wg/Wnt pathway signaling in both fly and mammalian cancer cells. Reducing Sin3A strongly increased the invasive behavior of A549 human lung adenocarcinoma cells. We show that Sin3A is downregulated in a variety of human tumors and that Src, JNK, RhoA and PP1B/β-Catenin are regulated in a manner analogous to our Drosophila models. Our data suggest that Sin3A influences a specific step of tumorigenesis by regulating a module of genes involved i**n cell invasi**on. Tumor progression may commonly rely on such 'modules of invasion' under the control of broad transcriptional regulators.

---

## flr

- NCBI Gene
- GeneRIF
- Pubmed

| Search Term | Rank | Fields |
| --- | --- | --- |
| [Mm]igration | 6 | Process(1); PubMed(1) |

  
hide/show details for flr

### [Mm]igration

#### Process

1. border follicle cell **migration**

#### PubMed

1. GMF promotes leading-edge dynamics and collective cell **migration** in vivo. Lamellipodia are dynamic actin-rich cellular extensions that drive advancement of the leading edge during cell **migration**. Lamellipodia undergo periodic extension and retraction cycles, but the molecular mechanisms underlying these dynamics and their role in cell **migration** have remained obscure. We show that glia-maturation factor (GMF), which is an Arp2/3 complex inhibitor and actin filament debranching factor, regulates lamellipodial protrusion dynamics in living cells. In cultured S2R(+) cells, GMF silencing resulted in an increase in the width of lamellipodial actin filament arrays. Importantly, live-cell imaging of mutant Drosophila egg chambers revealed that the dynamics of actin-rich protrusions in migrating border cells is diminished in the absence of GMF. Consequently, velocity of border cell clusters undergoing guided **migration** was reduced in GMF mutant flies. Furthermore, genetic studies demonstrated that GMF cooperates with the Drosophila homolog of Aip1 (flare) in promoting disassembly of Arp2/3-nucleated actin filament networks and driving border cell **migration**. These data suggest that GMF functions in vivo to promote the disassembly of Arp2/3-nucleated actin filament arrays, making an important contribution to cell **migration** within a 3D tissue environment.

---

## ics

- NCBI Gene
- GeneRIF
- Pubmed

| Search Term | Rank | Fields |
| --- | --- | --- |
| [Mm]igration | 6 | PubMed(2) |

  
hide/show details for ics

### [Mm]igration

#### PubMed

1. The integrin effector PINCH regulates JNK activity and epithelial **migration** in concert with Ras suppressor 1. Cell adhesion and **migration** are dynamic processes requiring the coordinated action of multiple signaling pathways, but the mechanisms underlying signal integration have remained elusive. Drosophila embryonic dorsal closure (DC) requires both integrin function and c-Jun amino-terminal kinase (JNK) signaling for opposed epithelial sheets to migrate, meet, and suture. Here, we show that PINCH, a protein required for integrin-dependent cell adhesion and actin-membrane anchorage, is present at the leading edge of these migrating epithelia and is required for DC. By analysis of native protein complexes, we identify RSU-1, a regulator of Ras signaling in mammalian cells, as a novel PINCH binding partner that contributes to PINCH stability. Mutation of the gene encoding RSU-1 results in wing blistering in Drosophila, demonstrating its role in integrin-dependent cell adhesion. Genetic interaction analyses reveal that both PINCH and RSU-1 antagonize JNK signaling during DC. Our results suggest that PINCH and RSU-1 contribute to the integration of JNK and integrin functions during Drosophila development.
2. The integrin effector PINCH regulates JNK activity and epithelial **migration** in concert with Ras suppressor 1. Cell adhesion and **migration** are dynamic processes requiring the coordinated action of multiple signaling pathways, but the mechanisms underlying signal integration have remained elusive. Drosophila embryonic dorsal closure (DC) requires both integrin function and c-Jun amino-terminal kinase (JNK) signaling for opposed epithelial sheets to migrate, meet, and suture. Here, we show that PINCH, a protein required for integrin-dependent cell adhesion and actin-membrane anchorage, is present at the leading edge of these migrating epithelia and is required for DC. By analysis of native protein complexes, we identify RSU-1, a regulator of Ras signaling in mammalian cells, as a novel PINCH binding partner that contributes to PINCH stability. Mutation of the gene encoding RSU-1 results in wing blistering in Drosophila, demonstrating its role in integrin-dependent cell adhesion. Genetic interaction analyses reveal that both PINCH and RSU-1 antagonize JNK signaling during DC. Our results suggest that PINCH and RSU-1 contribute to the integration of JNK and integrin functions during Drosophila development.

---

## Asap

- NCBI Gene
- GeneRIF
- Pubmed

| Search Term | Rank | Fields |
| --- | --- | --- |
| [Mm]igration | 6 | PubMed(1) |

  
hide/show details for Asap

### [Mm]igration

#### PubMed

1. Regulation of Drosophila mesoderm **migration** by phosphoinositides and the PH domain of the Rho GTP exchange factor Pebble. The Drosophila RhoGEF Pebble (Pbl) is required for cytokinesis and **migration** of mesodermal cells. In a screen for genes that could suppress **migration** defects in pbl mutants we identified the phosphatidylinositol phosphate (PtdInsP) regulator pi5k59B. Genetic interaction tests with other PtdInsP regulators suggested that PtdIns(4,5)P2 levels are important for mesoderm **migration** when Pbl is depleted. Consistent with this, the leading front of migrating mesodermal cells was enriched for PtdIns(4,5)P2. Given that Pbl contains a Pleckstrin Homology (PH) domain, a known PtdInsP-binding motif, we examined PtdInsP-binding of Pbl and the importance of the PH domain for Pbl function. In vitro lipid blot assays showed that Pbl binds promiscuously to PtdInsPs, with binding strength associated with the degree of phosphorylation. Pbl was also able to bind lipid vesicles containing PtdIns(4,5)P2 but binding was strongly reduced upon deletion of the PH domain. Similarly, in vivo, loss of the PH domain prevented localisation of Pbl to the cell cortex and severely affected several aspects of early mesoderm development, including flattening of the invaginated tube onto the ectoderm, extension of protrusions, and dorsal **migration** to form a monolayer. Pbl lacking the PH domain could still localise to the cytokinetic furrow, however, and cytokinesis failure was reduced in pbl(ΔPH) mutants. Taken together, our results support a model in which interaction of the PH-domain of Pbl with PtdIns(4,5)P2 helps localise it to the plasma membrane which is important for mesoderm **migratio**n.

---

## moody

- NCBI Gene
- GeneRIF
- Pubmed

| Search Term | Rank | Fields |
| --- | --- | --- |
| [Mm]igration | 6 | PubMed(5) |
| ECM | 7 | PubMed(1) |
| [Ee]xtracellular [Mm]atrix | 8 | PubMed(2) |

  
hide/show details for moody

### [Mm]igration

#### PubMed

1. Unwrapping glial biology: Gcm target genes regulating glial development, diversification, and function. Glia are the most abundant cell type in the mammalian brain. They regulate neuronal development and function, CNS immune surveillance, and stem cell biology, yet we know surprisingly little about glia in any organism. Here we identify over 40 new Drosophila glial genes. We use glial cells missing (gcm) mutants and misexpression to verify they are Gcm regulated in vivo. Many genes show unique spatiotemporal responsiveness to Gcm in the CNS, and thus glial subtype diversification requires spatially or temporally restricted Gcm cofactors. These genes provide insights into glial biology: we show unc-5 (a repulsive netrin receptor) orients glial **migration**s and the draper gene mediates glial engulfment of apoptotic neurons and larval locomotion. Many identified Drosophila glial genes have homologs expressed in mammalian glia, revealing conserved molecular features of glial cells. 80% of these Drosophila glial genes have mammalian homologs; these are now excellent candidates for regulating human glial development, function, or disease.
2. Genes conserved in bilaterians but jointly lost with Myc during nematode evolution are enriched in cell proliferation and cell **migration** functions. Animals use a stereotypical set of developmental genes to build body architectures of varying sizes and organizational complexity. Some genes are critical to developmental patterning, while other genes are important to physiological control of growth. However, growth regulator genes may not be as important in small-bodied "micro-metazoans" such as nematodes. Nematodes use a simplified developmental strategy of lineage-based cell fate specifications to produce an adult bilaterian body composed of a few hundreds of cells. Nematodes also lost the MYC proto-oncogenic regulator of cell proliferation. To identify additional regulators of cell proliferation that were lost with MYC, we computationally screened and determined 839 high-confidence genes that are conserved in bilaterians/lost in nematodes (CIBLIN genes). We find that 30 % of all CIBLIN genes encode transcriptional regulators of cell proliferation, epithelial-to-mesenchyme transitions, and other processes. Over 50 % of CIBLIN genes are unnamed genes in Drosophila, suggesting that there are many understudied genes. Interestingly, CIBLIN genes include many Myc synthetic lethal (MycSL) hits from recent screens. CIBLIN genes include key regulators of heparan sulfate proteoglycan (HSPG) sulfation patterns, and lysyl oxidases involved in cross-linking and modification of the extracellular matrix (ECM). These genes and others suggest the CIBLIN repertoire services critical functions in ECM remodeling and cell **migration** in large-bodied bilaterians. Correspondingly, CIBLIN genes are co-expressed with Myc in cancer transcriptomes, and include a preponderance of known determinants of cancer progression and tumor aggression. We propose that CIBLIN gene research can improve our understanding of regulatory control of cellular growth in metazoans.
3. Tre1, a G protein-coupled receptor, directs transepithelial **migration** of Drosophila germ cells. In most organisms, germ cells are formed distant from the somatic part of the gonad and thus have to migrate along and through a variety of tissues to reach the gonad. Transepithelial **migration** through the posterior midgut (PMG) is the first active step during Drosophila germ cell **migration**. Here we report the identification of a novel G protein-coupled receptor (GPCR), Tre1, that is essential for this **migration** step. Maternal tre1 RNA is localized to germ cells, and tre1 is required cell autonomously in germ cells. In tre1 mutant embryos, most germ cells do not exit the PMG. The few germ cells that do leave the midgut early migrate normally to the gonad, suggesting that this gene is specifically required for transepithelial **migration** and that mutant germ cells are still able to recognize other guidance cues. Additionally, inhibiting small Rho GTPases in germ cells affects transepithelial **migration**, suggesting that Tre1 signals through Rho1. We propose that Tre1 acts in a manner similar to chemokine receptors required during transepithelial **migration** of leukocytes, implying an evolutionarily conserved mechanism of transepithelial **migration**. Recently, the chemokine receptor CXCR4 was shown to direct **migration** in vertebrate germ cells. Thus, germ cells may more generally use GPCR signaling to navigate the embryo toward their target.
4. Subtypes of glial cells in the Drosophila embryonic ventral nerve cord as related to lineage and gene expression. In the Drosophila embryonic CNS several subtypes of glial cells develop, which arrange themselves at characteristic positions and presumably fulfil specific functions. The mechanisms leading to the specification and differentiation of glial subtypes are largely unknown. By DiI labelling in glia-specific Gal4 lines we have clarified the lineages of the lateral glia in the embryonic ventral nerve cord and linked each glial cell to a specific stem cell. For the lineage of the longitudinal glioblast we show that it consists of 9 cells, which acquire at least four different identities. A large collection of molecular markers (many of them representing transcription factors and potential Gcm target genes) reveals that individual glial cells express specific combinations of markers. However, cluster analysis uncovers similar combinatorial codes for cells within, and significant differences between the categories of surface-associated, cortex-associated, and longitudinal glia. Glial cells derived from the same stem cell may be homogeneous (though not identical; stem cells NB1-1, NB5-6, NB6-4, LGB) or heterogeneous (NB7-4, NB1-3) with regard to gene expression. In addition to providing a powerful tool to analyse the fate of individual glial cells in different genetic backgrounds, each of these marker genes represents a candidate factor involved in glial specification or differentiation. We demonstrate this by the analysis of a castor loss of function mutation, which affects the number and **migration** of specific glial cells.
5. Hedgehog does not guide migrating Drosophila germ cells. In many species, the germ cells, precursors of sperm and egg, migrate during embryogenesis. The signals that regulate this **migration** are thus essential for fertility. In flies, lipid signals have been shown to affect germ cell guidance. In particular, the synthesis of geranylgeranyl pyrophosphate through the 3-hydroxy-3-methyl-glutaryl coenzyme A reductase (Hmgcr) pathway is critical for attracting germ cells to their target tissue. In a genetic analysis of signaling pathways known to affect cell **migration** of other migratory cells, we failed to find a role for the Hedgehog (Hh) pathway in germ cell **migration**. However, previous reports had implicated Hh as a germ cell attractant in flies and suggested that Hh signaling is enhanced through the action of the Hmgcr pathway. We therefore repeated several critical experiments and carried out further experiments to test specifically whether Hh is a germ cell attractant in flies. In contrast to previously reported findings and consistent with findings in zebrafish our data do not support the notion that Hh has a direct role in the guidance of migrating germ cells in flies.

### ECM

#### PubMed

1. Genes conserved in bilaterians but jointly lost with Myc during nematode evolution are enriched in cell proliferation and cell migration functions. Animals use a stereotypical set of developmental genes to build body architectures of varying sizes and organizational complexity. Some genes are critical to developmental patterning, while other genes are important to physiological control of growth. However, growth regulator genes may not be as important in small-bodied "micro-metazoans" such as nematodes. Nematodes use a simplified developmental strategy of lineage-based cell fate specifications to produce an adult bilaterian body composed of a few hundreds of cells. Nematodes also lost the MYC proto-oncogenic regulator of cell proliferation. To identify additional regulators of cell proliferation that were lost with MYC, we computationally screened and determined 839 high-confidence genes that are conserved in bilaterians/lost in nematodes (CIBLIN genes). We find that 30 % of all CIBLIN genes encode transcriptional regulators of cell proliferation, epithelial-to-mesenchyme transitions, and other processes. Over 50 % of CIBLIN genes are unnamed genes in Drosophila, suggesting that there are many understudied genes. Interestingly, CIBLIN genes include many Myc synthetic lethal (MycSL) hits from recent screens. CIBLIN genes include key regulators of heparan sulfate proteoglycan (HSPG) sulfation patterns, and lysyl oxidases involved in cross-linking and modification of the extracellular matrix (**ECM**). These genes and others suggest the CIBLIN repertoire services critical functions in **ECM** remodeling and cell migration in large-bodied bilaterians. Correspondingly, CIBLIN genes are co-expressed with Myc in cancer transcriptomes, and include a preponderance of known determinants of cancer progression and tumor aggression. We propose that CIBLIN gene research can improve our understanding of regulatory control of cellular growth in metazoans.

### [Ee]xtracellular [Mm]atrix

#### PubMed

1. Organization and function of the blood-brain barrier in Drosophila. The function of a complex nervous system depends on an intricate interplay between neuronal and glial cell types. One of the many functions of glial cells is to provide an efficient insulation of the nervous system and thereby allowing a fine tuned homeostasis of ions and other small molecules. Here, we present a detailed cellular analysis of the glial cell complement constituting the blood-brain barrier in Drosophila. Using electron microscopic analysis and single cell-labeling experiments, we characterize different glial cell layers at the surface of the nervous system, the perineurial glial layer, the subperineurial glial layer, the wrapping glial cell layer, and a thick layer of **extracellular matrix**, the neural lamella. To test the functional roles of these sheaths we performed a series of dye penetration experiments in the nervous systems of wild-type and mutant embryos. Comparing the kinetics of uptake of different sized fluorescently labeled dyes in different mutants allowed to conclude that most of the barrier function is mediated by the septate junctions formed by the subperineurial cells, whereas the perineurial glial cell layer and the neural lamella contribute to barrier selectivity against much larger particles (i.e., the size of proteins). We further compare the requirements of different septate junction components for the integrity of the blood-brain barrier and provide evidence that two of the six Claudin-like proteins found in Drosophila are needed for normal blood-brain barrier function.
2. Genes conserved in bilaterians but jointly lost with Myc during nematode evolution are enriched in cell proliferation and cell migration functions. Animals use a stereotypical set of developmental genes to build body architectures of varying sizes and organizational complexity. Some genes are critical to developmental patterning, while other genes are important to physiological control of growth. However, growth regulator genes may not be as important in small-bodied "micro-metazoans" such as nematodes. Nematodes use a simplified developmental strategy of lineage-based cell fate specifications to produce an adult bilaterian body composed of a few hundreds of cells. Nematodes also lost the MYC proto-oncogenic regulator of cell proliferation. To identify additional regulators of cell proliferation that were lost with MYC, we computationally screened and determined 839 high-confidence genes that are conserved in bilaterians/lost in nematodes (CIBLIN genes). We find that 30 % of all CIBLIN genes encode transcriptional regulators of cell proliferation, epithelial-to-mesenchyme transitions, and other processes. Over 50 % of CIBLIN genes are unnamed genes in Drosophila, suggesting that there are many understudied genes. Interestingly, CIBLIN genes include many Myc synthetic lethal (MycSL) hits from recent screens. CIBLIN genes include key regulators of heparan sulfate proteoglycan (HSPG) sulfation patterns, and lysyl oxidases involved in cross-linking and modification of the **extracellular matrix** (ECM). These genes and others suggest the CIBLIN repertoire services critical functions in ECM remodeling and cell migration in large-bodied bilaterians. Correspondingly, CIBLIN genes are co-expressed with Myc in cancer transcriptomes, and include a preponderance of known determinants of cancer progression and tumor aggression. We propose that CIBLIN gene research can improve our understanding of regulatory control of cellular growth in metazoans.

---

## dnr1

- NCBI Gene
- GeneRIF
- Pubmed

| Search Term | Rank | Fields |
| --- | --- | --- |
| [Mm]igration | 6 | PubMed(1) |
| ECM | 7 | PubMed(1) |
| [Ee]xtracellular [Mm]atrix | 8 | PubMed(1) |

  
hide/show details for dnr1

### [Mm]igration

#### PubMed

1. Genes conserved in bilaterians but jointly lost with Myc during nematode evolution are enriched in cell proliferation and cell **migration** functions. Animals use a stereotypical set of developmental genes to build body architectures of varying sizes and organizational complexity. Some genes are critical to developmental patterning, while other genes are important to physiological control of growth. However, growth regulator genes may not be as important in small-bodied "micro-metazoans" such as nematodes. Nematodes use a simplified developmental strategy of lineage-based cell fate specifications to produce an adult bilaterian body composed of a few hundreds of cells. Nematodes also lost the MYC proto-oncogenic regulator of cell proliferation. To identify additional regulators of cell proliferation that were lost with MYC, we computationally screened and determined 839 high-confidence genes that are conserved in bilaterians/lost in nematodes (CIBLIN genes). We find that 30 % of all CIBLIN genes encode transcriptional regulators of cell proliferation, epithelial-to-mesenchyme transitions, and other processes. Over 50 % of CIBLIN genes are unnamed genes in Drosophila, suggesting that there are many understudied genes. Interestingly, CIBLIN genes include many Myc synthetic lethal (MycSL) hits from recent screens. CIBLIN genes include key regulators of heparan sulfate proteoglycan (HSPG) sulfation patterns, and lysyl oxidases involved in cross-linking and modification of the extracellular matrix (ECM). These genes and others suggest the CIBLIN repertoire services critical functions in ECM remodeling and cell **migration** in large-bodied bilaterians. Correspondingly, CIBLIN genes are co-expressed with Myc in cancer transcriptomes, and include a preponderance of known determinants of cancer progression and tumor aggression. We propose that CIBLIN gene research can improve our understanding of regulatory control of cellular growth in metazoans.

### ECM

#### PubMed

1. Genes conserved in bilaterians but jointly lost with Myc during nematode evolution are enriched in cell proliferation and cell migration functions. Animals use a stereotypical set of developmental genes to build body architectures of varying sizes and organizational complexity. Some genes are critical to developmental patterning, while other genes are important to physiological control of growth. However, growth regulator genes may not be as important in small-bodied "micro-metazoans" such as nematodes. Nematodes use a simplified developmental strategy of lineage-based cell fate specifications to produce an adult bilaterian body composed of a few hundreds of cells. Nematodes also lost the MYC proto-oncogenic regulator of cell proliferation. To identify additional regulators of cell proliferation that were lost with MYC, we computationally screened and determined 839 high-confidence genes that are conserved in bilaterians/lost in nematodes (CIBLIN genes). We find that 30 % of all CIBLIN genes encode transcriptional regulators of cell proliferation, epithelial-to-mesenchyme transitions, and other processes. Over 50 % of CIBLIN genes are unnamed genes in Drosophila, suggesting that there are many understudied genes. Interestingly, CIBLIN genes include many Myc synthetic lethal (MycSL) hits from recent screens. CIBLIN genes include key regulators of heparan sulfate proteoglycan (HSPG) sulfation patterns, and lysyl oxidases involved in cross-linking and modification of the extracellular matrix (**ECM**). These genes and others suggest the CIBLIN repertoire services critical functions in **ECM** remodeling and cell migration in large-bodied bilaterians. Correspondingly, CIBLIN genes are co-expressed with Myc in cancer transcriptomes, and include a preponderance of known determinants of cancer progression and tumor aggression. We propose that CIBLIN gene research can improve our understanding of regulatory control of cellular growth in metazoans.

### [Ee]xtracellular [Mm]atrix

#### PubMed

1. Genes conserved in bilaterians but jointly lost with Myc during nematode evolution are enriched in cell proliferation and cell migration functions. Animals use a stereotypical set of developmental genes to build body architectures of varying sizes and organizational complexity. Some genes are critical to developmental patterning, while other genes are important to physiological control of growth. However, growth regulator genes may not be as important in small-bodied "micro-metazoans" such as nematodes. Nematodes use a simplified developmental strategy of lineage-based cell fate specifications to produce an adult bilaterian body composed of a few hundreds of cells. Nematodes also lost the MYC proto-oncogenic regulator of cell proliferation. To identify additional regulators of cell proliferation that were lost with MYC, we computationally screened and determined 839 high-confidence genes that are conserved in bilaterians/lost in nematodes (CIBLIN genes). We find that 30 % of all CIBLIN genes encode transcriptional regulators of cell proliferation, epithelial-to-mesenchyme transitions, and other processes. Over 50 % of CIBLIN genes are unnamed genes in Drosophila, suggesting that there are many understudied genes. Interestingly, CIBLIN genes include many Myc synthetic lethal (MycSL) hits from recent screens. CIBLIN genes include key regulators of heparan sulfate proteoglycan (HSPG) sulfation patterns, and lysyl oxidases involved in cross-linking and modification of the **extracellular matrix** (ECM). These genes and others suggest the CIBLIN repertoire services critical functions in ECM remodeling and cell migration in large-bodied bilaterians. Correspondingly, CIBLIN genes are co-expressed with Myc in cancer transcriptomes, and include a preponderance of known determinants of cancer progression and tumor aggression. We propose that CIBLIN gene research can improve our understanding of regulatory control of cellular growth in metazoans.

---

## dos

- NCBI Gene
- GeneRIF
- Pubmed

| Search Term | Rank | Fields |
| --- | --- | --- |
| [Mm]igration | 6 | PubMed(2) |
| [Ee]xtracellular [Mm]atrix | 8 | PubMed(1) |

  
hide/show details for dos

### [Mm]igration

#### PubMed

1. Guidance of cell **migration** by the Drosophila PDGF/VEGF receptor. Directed cell **migration**s are important for development, but the signaling pathways and mechanisms responsible for guiding cell **migration** in vivo are poorly understood. **Migration** of border cells during Drosophila oogenesis is a simple and attractive model system in which to address these questions. We demonstrate that PVR, a receptor tyrosine kinase related to mammalian PDGF and VEGF receptors, acts in border cells to guide them to the oocyte. The oocyte is the source of a ligand for PVR, PDGF/VEGF factor 1 (PVF1). Intriguingly, the guidance function of PVR is largely redundant with that of EGFR. We present evidence implicating Rac and the Rac activator Mbc/DOCK180/CED-5 as mediators of the guidance signal.
2. Shp-2 tyrosine phosphatase: signaling one cell or many. Shp-2, a widely expressed cytoplasmic tyrosine phosphatase with two src-homology 2 (SH2) domains, has received much attention in the signal transduction field recently. Significant progress has been made in understanding the structure and function of this phosphatase, together with its Drosophila homologue, Corkscrew, as well as the close relative Shp-1 tyrosine phosphatase. The crystal structure of Shp-2 revealed an autoinhibitory mechanism of the catalytic activity by the N-terminal SH2 domain. Shp-2 apparently participates in signaling events downstream of receptors for growth factors, cytokines, hormones, antigens, and extracellular matrixes in the control of cell growth, differentiation, **migration**, and death. Shp-2 is an important molecule that integrates signals among various cytoplasmic pathways and may also couple intracellular and intercellular information flow.

### [Ee]xtracellular [Mm]atrix

#### PubMed

1. Shp-2 tyrosine phosphatase: signaling one cell or many. Shp-2, a widely expressed cytoplasmic tyrosine phosphatase with two src-homology 2 (SH2) domains, has received much attention in the signal transduction field recently. Significant progress has been made in understanding the structure and function of this phosphatase, together with its Drosophila homologue, Corkscrew, as well as the close relative Shp-1 tyrosine phosphatase. The crystal structure of Shp-2 revealed an autoinhibitory mechanism of the catalytic activity by the N-terminal SH2 domain. Shp-2 apparently participates in signaling events downstream of receptors for growth factors, cytokines, hormones, antigens, and **extracellular matrix**es in the control of cell growth, differentiation, migration, and death. Shp-2 is an important molecule that integrates signals among various cytoplasmic pathways and may also couple intracellular and intercellular information flow.

---

## pbl

- NCBI Gene
- GeneRIF
- Pubmed

| Search Term | Rank | Fields |
| --- | --- | --- |
| [Mm]igration | 6 | Process(2); PubMed(12); GeneRIFs(2) |
| [Ee]xtracellular [Mm]atrix | 8 | PubMed(1) |
| [Ii]nvasive | 10 | PubMed(1) |
| [Mm]etastasis | 12 | PubMed(1) |

  
hide/show details for pbl

### [Mm]igration

#### Process

1. mesoderm **migration** involved in gastrulation
2. mesodermal cell **migration**

#### PubMed

1. Regulation of Drosophila mesoderm **migration** by phosphoinositides and the PH domain of the Rho GTP exchange factor Pebble. The Drosophila RhoGEF Pebble (Pbl) is required for cytokinesis and **migration** of mesodermal cells. In a screen for genes that could suppress **migration** defects in pbl mutants we identified the phosphatidylinositol phosphate (PtdInsP) regulator pi5k59B. Genetic interaction tests with other PtdInsP regulators suggested that PtdIns(4,5)P2 levels are important for mesoderm **migration** when Pbl is depleted. Consistent with this, the leading front of migrating mesodermal cells was enriched for PtdIns(4,5)P2. Given that Pbl contains a Pleckstrin Homology (PH) domain, a known PtdInsP-binding motif, we examined PtdInsP-binding of Pbl and the importance of the PH domain for Pbl function. In vitro lipid blot assays showed that Pbl binds promiscuously to PtdInsPs, with binding strength associated with the degree of phosphorylation. Pbl was also able to bind lipid vesicles containing PtdIns(4,5)P2 but binding was strongly reduced upon deletion of the PH domain. Similarly, in vivo, loss of the PH domain prevented localisation of Pbl to the cell cortex and severely affected several aspects of early mesoderm development, including flattening of the invaginated tube onto the ectoderm, extension of protrusions, and dorsal **migration** to form a monolayer. Pbl lacking the PH domain could still localise to the cytokinetic furrow, however, and cytokinesis failure was reduced in pbl(ΔPH) mutants. Taken together, our results support a model in which interaction of the PH-domain of Pbl with PtdIns(4,5)P2 helps localise it to the plasma membrane which is important for mesoderm **migratio**n.
2. Regulation of the Rac GTPase pathway by the multifunctional Rho GEF Pebble is essential for mesoderm **migration** in the Drosophila gastrula. The Drosophila guanine nucleotide exchange factor Pebble (Pbl) is essential for cytokinesis and cell **migration** during gastrulation. In dividing cells, Pbl promotes Rho1 activation at the cell cortex, leading to formation of the contractile actin-myosin ring. The role of Pbl in fibroblast growth factor-triggered mesoderm spreading during gastrulation is less well understood and its targets and subcellular localization are unknown. To address these issues we performed a domain-function study in the embryo. We show that Pbl is localized to the nucleus and the cell cortex in migrating mesoderm cells and found that, in addition to the PH domain, the conserved C-terminal tail of the protein is crucial for cortical localization. Moreover, we show that the Rac pathway plays an essential role during mesoderm **migration**. Genetic and biochemical interactions indicate that during mesoderm **migration**, Pbl functions by activating a Rac-dependent pathway. Furthermore, gain-of-function and rescue experiments suggest an important regulatory role of the C-terminal tail of Pbl for the selective activation of Rho1-versus Rac-dependent pathways.
3. The epithelial-mesenchymal transition of the Drosophila mesoderm requires the Rho GTP exchange factor Pebble. Drosophila pebble (pbl) encodes a Rho-family GTP exchange factor (GEF) required for cytokinesis. The accumulation of high levels of PBL protein during interphase and the developmentally regulated expression of pbl in mesodermal tissues suggested that the primary cytokinetic mutant phenotype might be masking other roles. Using various muscle differentiation markers, we found that Even skipped (EVE) expression in the dorsal mesoderm is greatly reduced in pbl mutant embryos. EVE expression in the dorsalmost mesodermal cells is induced in response to DPP secreted by the dorsal epidermal cells. Further analysis revealed that this phenotype is likely to be a consequence of an earlier defect. pbl mutant mesodermal cells fail to undergo the normal epithelial-mesenchymal transition (EMT) and dorsal **migration** that follows ventral furrow formation. This phenotype is not a secondary consequence of failed cytokinesis, as it is rescued by a mutant form of pbl that does not rescue the cytokinetic defect. In wild-type embryos, newly invaginated cells at the lateral edges of the mesoderm extend numerous protrusions. In pbl mutant embryos, however, cells appear more tightly adhered to their neighbours and extend very few protrusions. Consistent with the dependence of the mesoderm EMT and cytokinesis on actin organisation, the GTP exchange function of the PBL RhoGEF is required for both processes. By contrast, the N-terminal BRCT domains of PBL are required only for the cytokinetic function of PBL. These studies reveal that a novel PBL-mediated intracellular signalling pathway operates in mesodermal cells during the transition from an epithelial to migratory mesenchymal morphology during gastrulation.
4. A core transcriptional network for early mesoderm development in Drosophila melanogaster. Embryogenesis is controlled by large gene-regulatory networks, which generate spatially and temporally refined patterns of gene expression. Here, we report the characteristics of the regulatory network orchestrating early mesodermal development in the fruitfly Drosophila, where the transcription factor Twist is both necessary and sufficient to drive development. Through the integration of chromatin immunoprecipitation followed by microarray analysis (ChIP-on-chip) experiments during discrete time periods with computational approaches, we identified >2000 Twist-bound cis-regulatory modules (CRMs) and almost 500 direct target genes. Unexpectedly, Twist regulates an almost complete cassette of genes required for cell proliferation in addition to genes essential for morophogenesis and cell **migration**. Twist targets almost 25% of all annotated Drosophila transcription factors, which may represent the entire set of regulators necessary for the early development of this system. By combining in vivo binding data from Twist, Mef2, Tinman, and Dorsal we have constructed an initial transcriptional network of early mesoderm development. The network topology reveals extensive combinatorial binding, feed-forward regulation, and complex logical outputs as prevalent features. In addition to binary activation and repression, we suggest that Twist binds to almost all mesodermal CRMs to provide the competence to integrate inputs from more specialized transcription factors.
5. Regulation of the Rac GTPase pathway by the multifunctional Rho GEF Pebble is essential for mesoderm **migration** in the Drosophila gastrula. The Drosophila guanine nucleotide exchange factor Pebble (Pbl) is essential for cytokinesis and cell **migration** during gastrulation. In dividing cells, Pbl promotes Rho1 activation at the cell cortex, leading to formation of the contractile actin-myosin ring. The role of Pbl in fibroblast growth factor-triggered mesoderm spreading during gastrulation is less well understood and its targets and subcellular localization are unknown. To address these issues we performed a domain-function study in the embryo. We show that Pbl is localized to the nucleus and the cell cortex in migrating mesoderm cells and found that, in addition to the PH domain, the conserved C-terminal tail of the protein is crucial for cortical localization. Moreover, we show that the Rac pathway plays an essential role during mesoderm **migration**. Genetic and biochemical interactions indicate that during mesoderm **migration**, Pbl functions by activating a Rac-dependent pathway. Furthermore, gain-of-function and rescue experiments suggest an important regulatory role of the C-terminal tail of Pbl for the selective activation of Rho1-versus Rac-dependent pathways.
6. Cellular and molecular mechanisms of single and collective cell **migration**s in Drosophila: themes and variations. The process of cell **migration** is essential throughout life, driving embryonic morphogenesis and ensuring homeostasis in adults. Defects in cell **migration** are a major cause of human disease, with excessive **migration** causing autoimmune diseases and cancer metastasis, whereas reduced capacity for **migration** leads to birth defects and immunodeficiencies. Myriad studies in vitro have established a consensus view that cell **migration**s require cell polarization, Rho GTPase-mediated cytoskeletal rearrangements, and myosin-mediated contractility. However, in vivo studies later revealed a more complex picture, including the discovery that cells migrate not only as single units but also as clusters, strands, and sheets. In particular, the role of E-Cadherin in cell motility appears to be more complex than previously appreciated. Here, we discuss recent advances achieved by combining the plethora of genetic tools available to the Drosophila geneticist with live imaging and biophysical techniques. Finally, we discuss the emerging themes such studies have revealed and ponder the puzzles that remain to be solved.
7. The epithelial-mesenchymal transition of the Drosophila mesoderm requires the Rho GTP exchange factor Pebble. Drosophila pebble (pbl) encodes a Rho-family GTP exchange factor (GEF) required for cytokinesis. The accumulation of high levels of PBL protein during interphase and the developmentally regulated expression of pbl in mesodermal tissues suggested that the primary cytokinetic mutant phenotype might be masking other roles. Using various muscle differentiation markers, we found that Even skipped (EVE) expression in the dorsal mesoderm is greatly reduced in pbl mutant embryos. EVE expression in the dorsalmost mesodermal cells is induced in response to DPP secreted by the dorsal epidermal cells. Further analysis revealed that this phenotype is likely to be a consequence of an earlier defect. pbl mutant mesodermal cells fail to undergo the normal epithelial-mesenchymal transition (EMT) and dorsal **migration** that follows ventral furrow formation. This phenotype is not a secondary consequence of failed cytokinesis, as it is rescued by a mutant form of pbl that does not rescue the cytokinetic defect. In wild-type embryos, newly invaginated cells at the lateral edges of the mesoderm extend numerous protrusions. In pbl mutant embryos, however, cells appear more tightly adhered to their neighbours and extend very few protrusions. Consistent with the dependence of the mesoderm EMT and cytokinesis on actin organisation, the GTP exchange function of the PBL RhoGEF is required for both processes. By contrast, the N-terminal BRCT domains of PBL are required only for the cytokinetic function of PBL. These studies reveal that a novel PBL-mediated intracellular signalling pathway operates in mesodermal cells during the transition from an epithelial to migratory mesenchymal morphology during gastrulation.
8. Regulation of Drosophila mesoderm **migration** by phosphoinositides and the PH domain of the Rho GTP exchange factor Pebble. The Drosophila RhoGEF Pebble (Pbl) is required for cytokinesis and **migration** of mesodermal cells. In a screen for genes that could suppress **migration** defects in pbl mutants we identified the phosphatidylinositol phosphate (PtdInsP) regulator pi5k59B. Genetic interaction tests with other PtdInsP regulators suggested that PtdIns(4,5)P2 levels are important for mesoderm **migration** when Pbl is depleted. Consistent with this, the leading front of migrating mesodermal cells was enriched for PtdIns(4,5)P2. Given that Pbl contains a Pleckstrin Homology (PH) domain, a known PtdInsP-binding motif, we examined PtdInsP-binding of Pbl and the importance of the PH domain for Pbl function. In vitro lipid blot assays showed that Pbl binds promiscuously to PtdInsPs, with binding strength associated with the degree of phosphorylation. Pbl was also able to bind lipid vesicles containing PtdIns(4,5)P2 but binding was strongly reduced upon deletion of the PH domain. Similarly, in vivo, loss of the PH domain prevented localisation of Pbl to the cell cortex and severely affected several aspects of early mesoderm development, including flattening of the invaginated tube onto the ectoderm, extension of protrusions, and dorsal **migration** to form a monolayer. Pbl lacking the PH domain could still localise to the cytokinetic furrow, however, and cytokinesis failure was reduced in pbl(ΔPH) mutants. Taken together, our results support a model in which interaction of the PH-domain of Pbl with PtdIns(4,5)P2 helps localise it to the plasma membrane which is important for mesoderm **migratio**n.
9. Functional analysis of the Drosophila embryonic germ cell transcriptome by RNA interference. In Drosophila melanogaster, primordial germ cells are specified at the posterior pole of the very early embryo. This process is regulated by the posterior localized germ plasm that contains a large number of RNAs of maternal origin. Transcription in the primordial germ cells is actively down-regulated until germ cell fate is established. Bulk expression of the zygotic genes commences concomitantly with the degradation of the maternal transcripts. Thus, during embryogenesis, maternally provided and zygotically transcribed mRNAs determine germ cell development collectively. In an effort to identify novel genes involved in the regulation of germ cell behavior, we carried out a large-scale RNAi screen targeting both maternal and zygotic components of the embryonic germ line transcriptome. We identified 48 genes necessary for distinct stages in germ cell development. We found pebble and fascetto to be essential for germ cell **migration** and germ cell division, respectively. Our data uncover a previously unanticipated role of mei-P26 in maintenance of embryonic germ cell fate. We also performed systematic co-RNAi experiments, through which we found a low rate of functional redundancy among homologous gene pairs. As our data indicate a high degree of evolutionary conservation in genetic regulation of germ cell development, they are likely to provide valuable insights into the biology of the germ line in general.
10. Signalling through the RhoGEF Pebble in Drosophila. Small GTPase pathways of the Ras superfamily are implicated in a wide range of signalling processes in animal cells. Small GTPases control pathways by acting as molecular switches. They are converted from an inactive GDP-bound form to an active GTP-bound form by GTP exchange factors (GEFs). The spatial and temporal regulation of GEFs is a major component of the regulation of small GTPases. Here we review the role of the Drosophila RhoGEF, Pebble (the Drosophila ortholog of mammalian ECT2). We discuss its roles in cytokinesis and cell **migration**, highlighting the diversity with which Rho family signalling pathways operate in biological systems.
11. Snail-dependent repression of the RhoGEF pebble is required for gastrulation consistency in Drosophila melanogaster. The Rho GTP exchange factor, Pebble (Pbl), long recognised as an essential activator of Rho during cytokinesis, also regulates mesoderm **migration** at gastrulation. Like other cell cycle components, pbl expression patterns broadly correlate with proliferative tissue. Surprisingly, in spite of its role in the early mesoderm, pbl is downregulated in the presumptive mesoderm before ventral furrow formation. Here, we show that this mesoderm-specific repression of pbl is dependent on the transcriptional repressor Snail (Sna). pbl repression was lost in sna mutants but was unaffected when Sna was ectopically expressed, showing that Sna is necessary, but not sufficient, for pbl repression. Using DamID, the first intron of pbl was identified as a Sna-binding region. Nine sites with the Sna-binding consensus motif CAGGT[GA] were identified in this intron. Mutating these to TAGGC[GA] abolished the ventral repression of pbl. Surprisingly, Sna-dependent repression of pbl was not essential for viability or fertility. Loss of repression did, however, increase the frequency of low-penetrance gastrulation defects. Consistent with this, expression of a pbl-GFP transgene in the presumptive mesoderm generated similar gastrulation defects. Finally, we show that a cluster of Snail-binding sites in the middle of the first intron of pbl orthologues is a conserved feature in the other 11 sequenced Drosophila species. We conclude that pbl levels are precisely regulated to ensure that there is enough protein available for its role in early mesoderm development but not so much as to inhibit the orderly progression of gastrulation.
12. The RhoGEF Pebble is required for cell shape changes during cell **migration** triggered by the Drosophila FGF receptor Heartless. The FGF receptor Heartless (HTL) is required for mesodermal cell **migration** in the Drosophila gastrula. We show that mesoderm cells undergo different phases of specific cell shape changes during mesoderm **migration**. During the migratory phase, the cells adhere to the basal surface of the ectoderm and exhibit extensive protrusive activity. HTL is required for the protrusive activity of the mesoderm cells. Moreover, the early phenotype of htl mutants suggests that HTL is required for the adhesion of mesoderm cells to the ectoderm. In a genetic screen we identified pebble (pbl) as a novel gene required for mesoderm **migration**. pbl encodes a guanyl nucleotide exchange factor (GEF) for RHO1 and is known as an essential regulator of cytokinesis. We show that the function of PBL in cell **migration** is independent of the function of PBL in cytokinesis. Although RHO1 acts as a substrate for PBL in cytokinesis, compromising RHO1 function in the mesoderm does not block cell **migration**. These data suggest that the function of PBL in cell **migration** might be mediated through a pathway distinct from RHO1. This idea is supported by allele-specific differences in the expressivity of the cytokinesis and cell **migration** phenotypes of different pbl mutants. We show that PBL is autonomously required in the mesoderm for cell **migration**. Like HTL, PBL is required for early cell shape changes during mesoderm **migration**. Expression of a constitutively active form of HTL is unable to rescue the early cellular defects in pbl mutants, suggesting that PBL is required for the ability of HTL to trigger these cell shape changes. These results provide evidence for a novel function of the Rho-GEF PBL in HTL-dependent mesodermal cell **migration**.

#### GeneRIFs

1. interaction of the PH-domain of Pbl with PtdIns(4,5)P2 helps localise it to the plasma membrane which is important for mesoderm **migration**.
2. PBL is autonomously required in the mesoderm for cell **migration**.PBL is required for the ability of HTL to trigger cell shape changes

### [Ee]xtracellular [Mm]atrix

#### PubMed

1. A directed miniscreen for genes involved in the Drosophila anti-parasitoid immune response. Drosophila larvae react against eggs from the endoparasitoid wasp Leptopilina boulardi by surrounding them in a multilayered cellular capsule. Once a wasp egg is recognized as foreign, circulating macrophage-like cells, known as plasmatocytes, adhere to the invader. After spreading around the wasp egg, plasmatocytes form cellular junctions between the cells, effectively separating the egg from the hemocoel. Next, a second sub-type of circulating immunosurveillance cell (hemocyte), known as lamellocytes, adhere to either the wasp egg or more likely the plasmatocytes surrounding the egg. From these events, it is obvious that adhesion and cell shape change are an essential part of Drosophila's cellular immune response against parasitoid wasp eggs. To date, very few genes have been described as being necessary for a proper anti-parasitization response in Drosophila. With this in mind, we performed a directed genetic miniscreen to discover new genes required for this response. Many of the genes with an encapsulation defect have mammalian homologues involved in cellular adhesion, wound healing, and thrombosis, including **extracellular matrix** proteins, cellular adhesion molecules, and small GTPases.

### [Ii]nvasive

#### PubMed

1. Identification of novel Ras-cooperating oncogenes in Drosophila melanogaster: a RhoGEF/Rho-family/JNK pathway is a central driver of tumorigenesis. We have shown previously that mutations in the apico-basal cell polarity regulators cooperate with oncogenic Ras (Ras(ACT)) to promote tumorigenesis in Drosophila melanogaster and mammalian cells. To identify novel genes that cooperate with Ras(ACT) in tumorigenesis, we carried out a genome-wide screen for genes that when overexpressed throughout the developing Drosophila eye enhance Ras(ACT)-driven hyperplasia. Ras(ACT)-cooperating genes identified were Rac1 Rho1, RhoGEF2, pbl, rib, and east, which encode cell morphology regulators. In a clonal setting, which reveals genes conferring a competitive advantage over wild-type cells, only Rac1, an activated allele of Rho1 (Rho1(ACT)), RhoGEF2, and pbl cooperated with Ras(ACT), resulting in reduced differentiation and large **invasive** tumors. Expression of RhoGEF2 or Rac1 with Ras(ACT) upregulated Jun kinase (JNK) activity, and JNK upregulation was essential for cooperation. However, in the whole-tissue system, upregulation of JNK alone was not sufficient for cooperation with Ras(ACT), while in the clonal setting, JNK upregulation was sufficient for Ras(ACT)-mediated tumorigenesis. JNK upregulation was also sufficient to confer **invasive** growth of Ras(V12)-expressing mammalian MCF10A breast epithelial cells. Consistent with this, HER2(+) human breast cancers (where human epidermal growth factor 2 is overexpressed and Ras signaling upregulated) show a significant correlation with a signature representing JNK pathway activation. Moreover, our genetic analysis in Drosophila revealed that Rho1 and Rac are important for the cooperation of RhoGEF2 or Pbl overexpression and of mutants in polarity regulators, Dlg and aPKC, with Ras(ACT) in the whole-tissue context. Collectively our analysis reveals the importance of the RhoGEF/Rho-family/JNK pathway in cooperative tumorigenesis with Ras(ACT).

### [Mm]etastasis

#### PubMed

1. Cellular and molecular mechanisms of single and collective cell migrations in Drosophila: themes and variations. The process of cell migration is essential throughout life, driving embryonic morphogenesis and ensuring homeostasis in adults. Defects in cell migration are a major cause of human disease, with excessive migration causing autoimmune diseases and cancer **metastasis**, whereas reduced capacity for migration leads to birth defects and immunodeficiencies. Myriad studies in vitro have established a consensus view that cell migrations require cell polarization, Rho GTPase-mediated cytoskeletal rearrangements, and myosin-mediated contractility. However, in vivo studies later revealed a more complex picture, including the discovery that cells migrate not only as single units but also as clusters, strands, and sheets. In particular, the role of E-Cadherin in cell motility appears to be more complex than previously appreciated. Here, we discuss recent advances achieved by combining the plethora of genetic tools available to the Drosophila geneticist with live imaging and biophysical techniques. Finally, we discuss the emerging themes such studies have revealed and ponder the puzzles that remain to be solved.

---

## p38a

- NCBI Gene
- GeneRIF
- Pubmed

| Search Term | Rank | Fields |
| --- | --- | --- |
| [Mm]igration | 6 | PubMed(1) |
| [Ii]nvasive | 10 | PubMed(1) |

  
hide/show details for p38a

### [Mm]igration

#### PubMed

1. Invadolysin, a conserved lipid-droplet-associated metalloproteinase, is required for mitochondrial function in Drosophila. Mitochondria are the main producers of ATP, the principal energy source of the cell, and reactive oxygen species (ROS), important signaling molecules. Mitochondrial morphogenesis and function depend on a hierarchical network of mechanisms in which proteases appear to be center stage. The invadolysin gene encodes an essential conserved metalloproteinase of the M8 family that is necessary for mitosis and cell **migration** during Drosophila development. We previously demonstrated that invadolysin is found associated with lipid droplets in cells. Here, we present data demonstrating that invadolysin interacts physically with three mitochondrial ATP synthase subunits. Our studies have focused on the genetic phenotypes of invadolysin and bellwether, the Drosophila homolog of ATP synthase α, mutants. The invadolysin mutation presents defects in mitochondrial physiology similar to those observed in bellwether mutants. The invadolysin and bellwether mutants have parallel phenotypes that affect lipid storage and mitochondrial electron transport chain activity, which result in a reduction in ATP production and an accumulation of ROS. As a consequence, invadolysin mutant larvae show lower energetic status and higher oxidative stress. Our data demonstrate an essential role for invadolysin in mitochondrial function that is crucial for normal development and survival.

### [Ii]nvasive

#### PubMed

1. The multilayered innate immune defense of the gut. In the wild, the fruit fly Drosophila melanogaster thrives on rotten fruit. The digestive tract maintains a powerful gut immune barrier to regulate the ingested microbiota, including entomopathogenic bacteria. This gut immune barrier includes a chitinous peritrophic matrix that isolates the gut contents from the epithelial cells. In addition, the epithelial cells are tightly sealed by septate junctions and can mount an inducible immune response. This local response can be activated by **invasive** bacteria, or triggered by commensal bacteria in the gut lumen. As with chronic inflammation in mammals, constitutive activation of the gut innate immune response is detrimental to the health of flies. Accordingly, the Drosophila gut innate immune response is tightly regulated to maintain the endogenous microbiota, while preventing infections by pathogenic microorganisms.

---

## Tollo

- NCBI Gene
- GeneRIF
- Pubmed

| Search Term | Rank | Fields |
| --- | --- | --- |
| [Mm]igration | 6 | PubMed(1) |
| [Ee]xtracellular [Mm]atrix | 8 | PubMed(1) |

  
hide/show details for Tollo

### [Mm]igration

#### PubMed

1. Ectopic expression screen identifies genes affecting Drosophila mesoderm development including the HSPG Trol. Gastrulation of the embryo involves coordinate cell movements likely supported by multiple signaling pathways, adhesion molecules, and extracellular matrix components. Fibroblast growth factors (FGFs) have a major role in Drosophila melanogaster mesoderm **migration**; however, few other inputs are known and the mechanism supporting cell movement is unclear. To provide insight, we performed an ectopic expression screen to identify secreted or membrane-associated molecules that act to support mesoderm **migration**. Twenty-four UAS insertions were identified that cause lethality when expressed in either the mesoderm (Twi-Gal4) or the ectoderm (69B-Gal4). The list was narrowed to a subset of 10 genes that were shown to exhibit loss-of-function mutant phenotypes specifically affecting mesoderm **migration**. These include the FGF ligand Pyramus, α-integrins, E-cadherin, Cueball, EGFR, JAK/STAT signaling components, as well as the heparan sulfate proteoglycan (HSPG) Terribly reduced optic lobes (Trol). Trol encodes the ortholog of mammalian HSPG Perlecan, a demonstrated FGF signaling cofactor. Here, we examine the role of Trol in Drosophila mesoderm **migratio**n and compare and contrast its role with that of Syndecan (Sdc), another HSPG previously implicated in this process. Embryos mutant for Trol or Sdc were obtained and analyzed. Our data support the view that both HSPGs function to support FGF-dependent processes in the early embryo as they share phenotypes with FGF mutants: Trol in terms of effects on mesoderm **migratio**n and caudal visceral mesoderm (CVM) **migratio**n and Sdc in terms of dorsal mesoderm specification. The differential roles uncovered for these two HSPGs suggest that HSPG cofactor choice may modify FGF-signaling outputs.

### [Ee]xtracellular [Mm]atrix

#### PubMed

1. Ectopic expression screen identifies genes affecting Drosophila mesoderm development including the HSPG Trol. Gastrulation of the embryo involves coordinate cell movements likely supported by multiple signaling pathways, adhesion molecules, and **extracellular matrix** components. Fibroblast growth factors (FGFs) have a major role in Drosophila melanogaster mesoderm migration; however, few other inputs are known and the mechanism supporting cell movement is unclear. To provide insight, we performed an ectopic expression screen to identify secreted or membrane-associated molecules that act to support mesoderm migration. Twenty-four UAS insertions were identified that cause lethality when expressed in either the mesoderm (Twi-Gal4) or the ectoderm (69B-Gal4). The list was narrowed to a subset of 10 genes that were shown to exhibit loss-of-function mutant phenotypes specifically affecting mesoderm migration. These include the FGF ligand Pyramus, α-integrins, E-cadherin, Cueball, EGFR, JAK/STAT signaling components, as well as the heparan sulfate proteoglycan (HSPG) Terribly reduced optic lobes (Trol). Trol encodes the ortholog of mammalian HSPG Perlecan, a demonstrated FGF signaling cofactor. Here, we examine the role of Trol in Drosophila mesoderm migration and compare and contrast its role with that of Syndecan (Sdc), another HSPG previously implicated in this process. Embryos mutant for Trol or Sdc were obtained and analyzed. Our data support the view that both HSPGs function to support FGF-dependent processes in the early embryo as they share phenotypes with FGF mutants: Trol in terms of effects on mesoderm migration and caudal visceral mesoderm (CVM) migration and Sdc in terms of dorsal mesoderm specification. The differential roles uncovered for these two HSPGs suggest that HSPG cofactor choice may modify FGF-signaling outputs.

---

## Fit1

- NCBI Gene
- GeneRIF
- Pubmed

| Search Term | Rank | Fields |
| --- | --- | --- |
| [Mm]igration | 6 | PubMed(2) |

  
hide/show details for Fit1

### [Mm]igration

#### PubMed

1. Drosophila integrin adhesion complexes are essential for hemocyte **migration** in vivo. Cell **migration** is an important biological process which has been intensively studied in the past decades. Numerous techniques, mainly involving two-dimensional cell culture systems, have contributed to dissecting the essential mechanisms underlying this process. However, the development of three-dimensional cell culture and in vivo systems has shown some differences with what was previously believed to be well-established cell **migration** mechanisms, suggesting that two-dimensional cell motility would be a poor predictor of in vivo behaviour. Drosophila is a widely recognized model organism to study developmental and homeostatic processes and has been widely used to investigate cell **migration**. Here, we focus on the **migration** of small groups of pupal hemocytes that accumulate during larval stages in dorsal patches. We show that integrins, and other known nascent adhesion-related proteins such as Rhea and Fermitin 1, are crucial for this process and that their depletion does not affect polarization in response to environmental cues. We also present evidence for the importance of adhesion maturation-related proteins in hemocyte **migration**, namely Zyxin. Zyxin depletion in hemocytes leads to a significant increase of cell speed without affecting their response to a chemotactic cue. This is the first report of a systematic analysis using Drosophila melanogaster hemocytes to study adhesion-related proteins and their function in cell **migration** in vivo. Our data point to mechanisms of cell **migration** similar to those described in three-dimensional in vitro systems and other in vivo model organisms.
2. Drosophila blood cell chemotaxis. Drosophila melanogaster contains a population of blood cells called hemocytes that represent the functional equivalent of vertebrate macrophages. These cells undergo directed **migration**s to disperse during development and reach sites of tissue damage or altered self. These chemotactic behaviors are controlled by the expression of PDGF/Vegf-related ligands in developing embryos and local production of hydrogen peroxide at wounds. Recent work reveals that many molecules important in vertebrate cell motility, including integrins, formins, Ena/VASP proteins and the SCAR/WAVE complex, have a conserved function in these innate immune cells. The use of this model organism has elucidated how damage signals are activated by calcium signaling during inflammation and that the steroid hormone ecdysone activates immune competence at key developmental stages.

---

## Dredd

- NCBI Gene
- GeneRIF
- Pubmed

| Search Term | Rank | Fields |
| --- | --- | --- |
| [Mm]igration | 6 | PubMed(1) |
| ECM | 7 | PubMed(1) |
| [Ee]xtracellular [Mm]atrix | 8 | PubMed(2) |

  
hide/show details for Dredd

### [Mm]igration

#### PubMed

1. Genes conserved in bilaterians but jointly lost with Myc during nematode evolution are enriched in cell proliferation and cell **migration** functions. Animals use a stereotypical set of developmental genes to build body architectures of varying sizes and organizational complexity. Some genes are critical to developmental patterning, while other genes are important to physiological control of growth. However, growth regulator genes may not be as important in small-bodied "micro-metazoans" such as nematodes. Nematodes use a simplified developmental strategy of lineage-based cell fate specifications to produce an adult bilaterian body composed of a few hundreds of cells. Nematodes also lost the MYC proto-oncogenic regulator of cell proliferation. To identify additional regulators of cell proliferation that were lost with MYC, we computationally screened and determined 839 high-confidence genes that are conserved in bilaterians/lost in nematodes (CIBLIN genes). We find that 30 % of all CIBLIN genes encode transcriptional regulators of cell proliferation, epithelial-to-mesenchyme transitions, and other processes. Over 50 % of CIBLIN genes are unnamed genes in Drosophila, suggesting that there are many understudied genes. Interestingly, CIBLIN genes include many Myc synthetic lethal (MycSL) hits from recent screens. CIBLIN genes include key regulators of heparan sulfate proteoglycan (HSPG) sulfation patterns, and lysyl oxidases involved in cross-linking and modification of the extracellular matrix (ECM). These genes and others suggest the CIBLIN repertoire services critical functions in ECM remodeling and cell **migration** in large-bodied bilaterians. Correspondingly, CIBLIN genes are co-expressed with Myc in cancer transcriptomes, and include a preponderance of known determinants of cancer progression and tumor aggression. We propose that CIBLIN gene research can improve our understanding of regulatory control of cellular growth in metazoans.

### ECM

#### PubMed

1. Genes conserved in bilaterians but jointly lost with Myc during nematode evolution are enriched in cell proliferation and cell migration functions. Animals use a stereotypical set of developmental genes to build body architectures of varying sizes and organizational complexity. Some genes are critical to developmental patterning, while other genes are important to physiological control of growth. However, growth regulator genes may not be as important in small-bodied "micro-metazoans" such as nematodes. Nematodes use a simplified developmental strategy of lineage-based cell fate specifications to produce an adult bilaterian body composed of a few hundreds of cells. Nematodes also lost the MYC proto-oncogenic regulator of cell proliferation. To identify additional regulators of cell proliferation that were lost with MYC, we computationally screened and determined 839 high-confidence genes that are conserved in bilaterians/lost in nematodes (CIBLIN genes). We find that 30 % of all CIBLIN genes encode transcriptional regulators of cell proliferation, epithelial-to-mesenchyme transitions, and other processes. Over 50 % of CIBLIN genes are unnamed genes in Drosophila, suggesting that there are many understudied genes. Interestingly, CIBLIN genes include many Myc synthetic lethal (MycSL) hits from recent screens. CIBLIN genes include key regulators of heparan sulfate proteoglycan (HSPG) sulfation patterns, and lysyl oxidases involved in cross-linking and modification of the extracellular matrix (**ECM**). These genes and others suggest the CIBLIN repertoire services critical functions in **ECM** remodeling and cell migration in large-bodied bilaterians. Correspondingly, CIBLIN genes are co-expressed with Myc in cancer transcriptomes, and include a preponderance of known determinants of cancer progression and tumor aggression. We propose that CIBLIN gene research can improve our understanding of regulatory control of cellular growth in metazoans.

### [Ee]xtracellular [Mm]atrix

#### PubMed

1. Genes conserved in bilaterians but jointly lost with Myc during nematode evolution are enriched in cell proliferation and cell migration functions. Animals use a stereotypical set of developmental genes to build body architectures of varying sizes and organizational complexity. Some genes are critical to developmental patterning, while other genes are important to physiological control of growth. However, growth regulator genes may not be as important in small-bodied "micro-metazoans" such as nematodes. Nematodes use a simplified developmental strategy of lineage-based cell fate specifications to produce an adult bilaterian body composed of a few hundreds of cells. Nematodes also lost the MYC proto-oncogenic regulator of cell proliferation. To identify additional regulators of cell proliferation that were lost with MYC, we computationally screened and determined 839 high-confidence genes that are conserved in bilaterians/lost in nematodes (CIBLIN genes). We find that 30 % of all CIBLIN genes encode transcriptional regulators of cell proliferation, epithelial-to-mesenchyme transitions, and other processes. Over 50 % of CIBLIN genes are unnamed genes in Drosophila, suggesting that there are many understudied genes. Interestingly, CIBLIN genes include many Myc synthetic lethal (MycSL) hits from recent screens. CIBLIN genes include key regulators of heparan sulfate proteoglycan (HSPG) sulfation patterns, and lysyl oxidases involved in cross-linking and modification of the **extracellular matrix** (ECM). These genes and others suggest the CIBLIN repertoire services critical functions in ECM remodeling and cell migration in large-bodied bilaterians. Correspondingly, CIBLIN genes are co-expressed with Myc in cancer transcriptomes, and include a preponderance of known determinants of cancer progression and tumor aggression. We propose that CIBLIN gene research can improve our understanding of regulatory control of cellular growth in metazoans.
2. Immune response to bacteria induces dissemination of Ras-activated Drosophila hindgut cells. Although pathogenic bacteria are suspected contributors to colorectal cancer progression, cancer-promoting bacteria and their mode of action remain largely unknown. Here we report that sustained infection with the human intestinal colonizer Pseudomonas aeruginosa synergizes with the Ras1V12 oncogene to induce basal invasion and dissemination of hindgut cells to distant sites. Cross-talk between infection and dissemination requires sustained activation by the bacteria of the Imd-dTab2-dTak1 innate immune pathway, which converges with Ras1V12 signalling on JNK pathway activation, culminating in **extracellular matrix** degradation. Hindgut, but not midgut, cells are amenable to this cooperative dissemination, which is progressive and genetically and pharmacologically inhibitable. Thus, Drosophila hindgut provides a valuable system for the study of intestinal malignancies.

---

## CG12012

- NCBI Gene
- GeneRIF
- Pubmed

| Search Term | Rank | Fields |
| --- | --- | --- |
| [Mm]igration | 6 | PubMed(1) |
| ECM | 7 | PubMed(1) |
| [Ee]xtracellular [Mm]atrix | 8 | PubMed(1) |

  
hide/show details for CG12012

### [Mm]igration

#### PubMed

1. Genes conserved in bilaterians but jointly lost with Myc during nematode evolution are enriched in cell proliferation and cell **migration** functions. Animals use a stereotypical set of developmental genes to build body architectures of varying sizes and organizational complexity. Some genes are critical to developmental patterning, while other genes are important to physiological control of growth. However, growth regulator genes may not be as important in small-bodied "micro-metazoans" such as nematodes. Nematodes use a simplified developmental strategy of lineage-based cell fate specifications to produce an adult bilaterian body composed of a few hundreds of cells. Nematodes also lost the MYC proto-oncogenic regulator of cell proliferation. To identify additional regulators of cell proliferation that were lost with MYC, we computationally screened and determined 839 high-confidence genes that are conserved in bilaterians/lost in nematodes (CIBLIN genes). We find that 30 % of all CIBLIN genes encode transcriptional regulators of cell proliferation, epithelial-to-mesenchyme transitions, and other processes. Over 50 % of CIBLIN genes are unnamed genes in Drosophila, suggesting that there are many understudied genes. Interestingly, CIBLIN genes include many Myc synthetic lethal (MycSL) hits from recent screens. CIBLIN genes include key regulators of heparan sulfate proteoglycan (HSPG) sulfation patterns, and lysyl oxidases involved in cross-linking and modification of the extracellular matrix (ECM). These genes and others suggest the CIBLIN repertoire services critical functions in ECM remodeling and cell **migration** in large-bodied bilaterians. Correspondingly, CIBLIN genes are co-expressed with Myc in cancer transcriptomes, and include a preponderance of known determinants of cancer progression and tumor aggression. We propose that CIBLIN gene research can improve our understanding of regulatory control of cellular growth in metazoans.

### ECM

#### PubMed

1. Genes conserved in bilaterians but jointly lost with Myc during nematode evolution are enriched in cell proliferation and cell migration functions. Animals use a stereotypical set of developmental genes to build body architectures of varying sizes and organizational complexity. Some genes are critical to developmental patterning, while other genes are important to physiological control of growth. However, growth regulator genes may not be as important in small-bodied "micro-metazoans" such as nematodes. Nematodes use a simplified developmental strategy of lineage-based cell fate specifications to produce an adult bilaterian body composed of a few hundreds of cells. Nematodes also lost the MYC proto-oncogenic regulator of cell proliferation. To identify additional regulators of cell proliferation that were lost with MYC, we computationally screened and determined 839 high-confidence genes that are conserved in bilaterians/lost in nematodes (CIBLIN genes). We find that 30 % of all CIBLIN genes encode transcriptional regulators of cell proliferation, epithelial-to-mesenchyme transitions, and other processes. Over 50 % of CIBLIN genes are unnamed genes in Drosophila, suggesting that there are many understudied genes. Interestingly, CIBLIN genes include many Myc synthetic lethal (MycSL) hits from recent screens. CIBLIN genes include key regulators of heparan sulfate proteoglycan (HSPG) sulfation patterns, and lysyl oxidases involved in cross-linking and modification of the extracellular matrix (**ECM**). These genes and others suggest the CIBLIN repertoire services critical functions in **ECM** remodeling and cell migration in large-bodied bilaterians. Correspondingly, CIBLIN genes are co-expressed with Myc in cancer transcriptomes, and include a preponderance of known determinants of cancer progression and tumor aggression. We propose that CIBLIN gene research can improve our understanding of regulatory control of cellular growth in metazoans.

### [Ee]xtracellular [Mm]atrix

#### PubMed

1. Genes conserved in bilaterians but jointly lost with Myc during nematode evolution are enriched in cell proliferation and cell migration functions. Animals use a stereotypical set of developmental genes to build body architectures of varying sizes and organizational complexity. Some genes are critical to developmental patterning, while other genes are important to physiological control of growth. However, growth regulator genes may not be as important in small-bodied "micro-metazoans" such as nematodes. Nematodes use a simplified developmental strategy of lineage-based cell fate specifications to produce an adult bilaterian body composed of a few hundreds of cells. Nematodes also lost the MYC proto-oncogenic regulator of cell proliferation. To identify additional regulators of cell proliferation that were lost with MYC, we computationally screened and determined 839 high-confidence genes that are conserved in bilaterians/lost in nematodes (CIBLIN genes). We find that 30 % of all CIBLIN genes encode transcriptional regulators of cell proliferation, epithelial-to-mesenchyme transitions, and other processes. Over 50 % of CIBLIN genes are unnamed genes in Drosophila, suggesting that there are many understudied genes. Interestingly, CIBLIN genes include many Myc synthetic lethal (MycSL) hits from recent screens. CIBLIN genes include key regulators of heparan sulfate proteoglycan (HSPG) sulfation patterns, and lysyl oxidases involved in cross-linking and modification of the **extracellular matrix** (ECM). These genes and others suggest the CIBLIN repertoire services critical functions in ECM remodeling and cell migration in large-bodied bilaterians. Correspondingly, CIBLIN genes are co-expressed with Myc in cancer transcriptomes, and include a preponderance of known determinants of cancer progression and tumor aggression. We propose that CIBLIN gene research can improve our understanding of regulatory control of cellular growth in metazoans.

---

## Rok

- NCBI Gene
- GeneRIF
- Pubmed

| Search Term | Rank | Fields |
| --- | --- | --- |
| [Ff]ilopodia | 1 | PubMed(1) |
| [Mm]igration | 6 | PubMed(21); GeneRIFs(1) |
| ECM | 7 | PubMed(1) |
| [Ee]xtracellular [Mm]atrix | 8 | PubMed(4) |
| [Ii]nvasive | 10 | PubMed(4) |
| [Mm]etastasis | 12 | PubMed(4) |
| [Cc]ell [Ii]nvasion | 13 | PubMed(1) |

  
hide/show details for Rok

### [Ff]ilopodia

#### PubMed

1. Reciprocal regulation of Rac1 and Rho1 in Drosophila circulating immune surveillance cells. In many cell types it is evident that the small GTPases Rac and Rho regulate each other's activities. What is unclear is exactly how this regulation occurs. To further elucidate this interaction we examined the activities of Rac1 and Rho1 in Drosophila cellular immune surveillance cells. In larvae the cellular immune response involves circulating cells (hemocytes) that can be recruited from a hematopoietic organ located behind the brain, as well as a sessile population found just underneath the larval cuticle. We demonstrate for the first time that Rho-kinase activation requires both Rho1 and the Drosophila c-Jun N-terminal kinase (Basket). We also show that Rac1, via Basket, regulates Rho1 activity, possibly by inhibiting RhoGAPp190. In the reciprocal pathway, co-expression of dominant negative Rho-kinase and constitutive active Rho1 induces a Rac1-like phenotype. This induction requires the formin Diaphanous. Co-expression of dominant negative Rho-kinase and constitutive active Rho1 also induces **filopodia** formation, with Diaphanous enriched at the tips. The Rac1-like phenotypes, and **filopodia** formation, could be blocked by co-expression of dominant negative Rac1. Finally, though dominant negative Rac1 is able to block **filopodia** formation in the overexpression experiments, only Rac2 is necessary for **filopodia** formed by hemocytes after parasitization.

### [Mm]igration

#### PubMed

1. Rho GTPase controls invagination and cohesive **migration** of the Drosophila salivary gland through Crumbs and Rho-kinase. Coordinated cell movements shape simple epithelia into functional tissues and organs during embryogenesis. Regulators and effectors of the small GTPase Rho have been shown to be essential for epithelial morphogenesis in cell culture; however, the mechanism by which Rho GTPase and its downstream effectors control coordinated movement of epithelia in a developing tissue or organ is largely unknown. Here, we show that Rho1 GTPase activity is required for the invagination of Drosophila embryonic salivary gland epithelia and for directed **migration** of the internalized gland. We demonstrate that the absence of zygotic function of Rho1 results in the selective loss of the apical proteins, Crumbs (Crb), Drosophila atypical PKC and Stardust during gland invagination and that this is partially due to reduced crb RNA levels and apical localization. In parallel to regulation of crb RNA and protein, Rho1 activity also signals through Rho-kinase (Rok) to induce apical constriction and cell shape change during invagination. After invagination, Rho-Rok signaling is required again for the coordinated contraction and dorsal **migration** of the proximal half of the gland. We also show that Rho1 activity is required for proper development of the circular visceral mesoderm upon which the gland migrates. Our genetic and live-imaging analyses provide novel evidence that the proximal gland cells play an essential and active role in salivary gland **migration** that propels the entire gland to turn and migrate posteriorly.
2. Planar cell polarity signaling in collective cell movements during morphogenesis and disease. Collective and directed cell movements are crucial for diverse developmental processes in the animal kingdom, but they are also involved in wound repair and disease. During these processes groups of cells are oriented within the tissue plane, which is referred to as planar cell polarity (PCP). This requires a tight regulation that is in part conducted by the PCP pathway. Although this pathway was initially characterized in flies, subsequent studies in vertebrates revealed a set of conserved core factors but also effector molecules and signal modulators, which build the fundamental PCP machinery. The PCP pathway in Drosophila regulates several developmental processes involving collective cell movements such as border cell **migration** during oogenesis, ommatidial rotation during eye development, and embryonic dorsal closure. During vertebrate embryogenesis, PCP signaling also controls collective and directed cell movements including convergent extension during gastrulation, neural tube closure, neural crest cell **migration**, or heart morphogenesis. Similarly, PCP signaling is linked to processes such as wound repair, and cancer invasion and metastasis in adults. As a consequence, disruption of PCP signaling leads to pathological conditions. In this review, we will summarize recent findings about the role of PCP signaling in collective cell movements in flies and vertebrates. In addition, we will focus on how studies in Drosophila have been relevant to our understanding of the PCP molecular machinery and will describe several developmental defects and human disorders in which PCP signaling is compromised. Therefore, new discoveries about the contribution of this pathway to collective cell movements could provide new potential diagnostic and therapeutic targets for these disorders.
3. Rho GTPase controls invagination and cohesive **migration** of the Drosophila salivary gland through Crumbs and Rho-kinase. Coordinated cell movements shape simple epithelia into functional tissues and organs during embryogenesis. Regulators and effectors of the small GTPase Rho have been shown to be essential for epithelial morphogenesis in cell culture; however, the mechanism by which Rho GTPase and its downstream effectors control coordinated movement of epithelia in a developing tissue or organ is largely unknown. Here, we show that Rho1 GTPase activity is required for the invagination of Drosophila embryonic salivary gland epithelia and for directed **migration** of the internalized gland. We demonstrate that the absence of zygotic function of Rho1 results in the selective loss of the apical proteins, Crumbs (Crb), Drosophila atypical PKC and Stardust during gland invagination and that this is partially due to reduced crb RNA levels and apical localization. In parallel to regulation of crb RNA and protein, Rho1 activity also signals through Rho-kinase (Rok) to induce apical constriction and cell shape change during invagination. After invagination, Rho-Rok signaling is required again for the coordinated contraction and dorsal **migration** of the proximal half of the gland. We also show that Rho1 activity is required for proper development of the circular visceral mesoderm upon which the gland migrates. Our genetic and live-imaging analyses provide novel evidence that the proximal gland cells play an essential and active role in salivary gland **migration** that propels the entire gland to turn and migrate posteriorly.
4. Molecular requirements for actin-based lamella formation in Drosophila S2 cells. Cell **migration** occurs through the protrusion of the actin-enriched lamella. Here, we investigated the effects of RNAi depletion of approximately 90 proteins implicated in actin function on lamella formation in Drosophila S2 cells. Similar to in vitro reconstitution studies of actin-based Listeria movement, we find that lamellae formation requires a relatively small set of proteins that participate in actin nucleation (Arp2/3 and SCAR), barbed end capping (capping protein), filament depolymerization (cofilin and Aip1), and actin monomer binding (profilin and cyclase-associated protein). Lamellae are initiated by parallel and partially redundant signaling pathways involving Rac GTPases and the adaptor protein Nck, which stimulate SCAR, an Arp2/3 activator. We also show that RNAi of three proteins (kette, Abi, and Sra-1) known to copurify with and inhibit SCAR in vitro leads to SCAR degradation, revealing a novel function of this protein complex in SCAR stability. Our results have identified an essential set of proteins involved in actin dynamics during lamella formation in Drosophila S2 cells.
5. Drosophila blood cell chemotaxis. Drosophila melanogaster contains a population of blood cells called hemocytes that represent the functional equivalent of vertebrate macrophages. These cells undergo directed **migration**s to disperse during development and reach sites of tissue damage or altered self. These chemotactic behaviors are controlled by the expression of PDGF/Vegf-related ligands in developing embryos and local production of hydrogen peroxide at wounds. Recent work reveals that many molecules important in vertebrate cell motility, including integrins, formins, Ena/VASP proteins and the SCAR/WAVE complex, have a conserved function in these innate immune cells. The use of this model organism has elucidated how damage signals are activated by calcium signaling during inflammation and that the steroid hormone ecdysone activates immune competence at key developmental stages.
6. Dynamic myosin activation promotes collective morphology and **migration** by locally balancing oppositional forces from surrounding tissue. Migrating cells need to overcome physical constraints from the local microenvironment to navigate their way through tissues. Cells that move collectively have the additional challenge of negotiating complex environments in vivo while maintaining cohesion of the group as a whole. The mechanisms by which collectives maintain a migratory morphology while resisting physical constraints from the surrounding tissue are poorly understood. Drosophila border cells represent a genetic model of collective **migration** within a cell-dense tissue. Border cells move as a cohesive group of 6-10 cells, traversing a network of large germ line-derived nurse cells within the ovary. Here we show that the border cell cluster is compact and round throughout their entire **migration**, a shape that is maintained despite the mechanical pressure imposed by the surrounding nurse cells. Nonmuscle myosin II (Myo-II) activity at the cluster periphery becomes elevated in response to increased constriction by nurse cells. Furthermore, the distinctive border cell collective morphology requires highly dynamic and localized enrichment of Myo-II. Thus, activated Myo-II promotes cortical tension at the outer edge of the migrating border cell cluster to resist compressive forces from nurse cells. We propose that dynamic actomyosin tension at the periphery of collectives facilitates their movement through restrictive tissues.
7. Rho-LIM kinase signaling regulates ecdysone-induced gene expression and morphogenesis during Drosophila metamorphosis. The steroid hormone 20-hydroxyecdysone (ecdysone) is the key regulator of postembryonic developmental transitions in insects and controls metamorphosis by triggering the morphogenesis of adult tissues from larvae. The Rho GTPase, which mediates cell shape change and **migration**, is also an essential regulator of tissue morphogenesis during development. Rho activity can modulate gene expression, in part, by activating LIM kinase (LIMK) and consequently affecting actin-induced SRF transcriptional activity. We have established a link between Rho-LIMK-SRF signaling and the ecdysone-induced transcriptional response during Drosophila development. Specifically, we determined that the Rho GTPase, via LIMK, regulates the expression of several ecdysone-responsive genes, including those encoding the ecdysone receptor itself, a downstream transcription factor (Br-C), and Stubble, a transmembrane protease required for proper leg formation. Stubble and Br-C mutants exhibit strong genetic interactions with several Rho pathway components in the formation of adult structures, but not with Rac or Cdc42. In cultured SL2 cells, inhibition of Rho, F-actin assembly, or SRF blocks the transcriptional response to ecdysone. Together, these findings indicate a link between Rho-LIMK signaling and steroid hormone-induced gene expression in the context of metamorphosis and thereby establish a novel role for the Rho GTPase in development.
8. Signaling pathways directing the movement and fusion of epithelial sheets: lessons from dorsal closure in Drosophila. Wound healing in embryos and various developmental events in metazoans require the spreading and fusion of epithelial sheets. The complex signaling pathways regulating these processes are being pieced together through genetic, cell biological, and biochemical approaches. At present, dorsal closure of the Drosophila embryo is the best-characterized example of epithelial sheet movement. Dorsal closure involves **migration** of the lateral epidermal flanks to close a hole in the dorsal epidermis occupied by an epithelium called the amnioserosa. Detailed genetic studies have revealed a network of interacting signaling molecules regulating this process. At the center of this network is a Jun N-terminal kinase cascade acting at the leading edge of the migrating epidermis that triggers signaling by the TGF-beta superfamily member Decapentaplegic and which interacts with the Wingless pathway. These signaling modules regulate the cytoskeletal reorganization and cell shape change necessary to drive dorsal closure. Activation of this network requires signals from the amnioserosa and input from a variety of proteins at cell-cell junctions. The Rho family of small GTPases is also instrumental, both in activation of signaling and regulation of the cytoskeleton. Many of the proteins regulating dorsal closure have been implicated in epithelial movement in other organisms, and dorsal closure has emerged as an ideal model system for the study of the **migration** and fusion of epithelial sheets.
9. Regulation of cell **migration** during tracheal development in Drosophila melanogaster. Most of the knowledge concerning the intracellular mechanisms involved in cell locomotion have been obtained from in vitro studies of cells in culture. Many of the concepts derived from these studies have been partially confirmed in in vivo systems but numerous questions regarding the developmental control of cell **migration** remain to be addressed. Tracheal morphogenesis in Drosophila melanogaster embryos represents an in vivo model system to study the genetic control of cell **migration**. We review what is known about tracheal development and regulation of tracheal cell **migration**. We try to link these in vivo studies and the movement of cells over two dimensional substrates and elaborate on important questions which remain to be addressed in the future.
10. The role of the Rho GTPases in neuronal development. Our brain serves as a center for cognitive function and neurons within the brain relay and store information about our surroundings and experiences. Modulation of this complex neuronal circuitry allows us to process that information and respond appropriately. Proper development of neurons is therefore vital to the mental health of an individual, and perturbations in their signaling or morphology are likely to result in cognitive impairment. The development of a neuron requires a series of steps that begins with **migration** from its birth place and initiation of process outgrowth, and ultimately leads to differentiation and the formation of connections that allow it to communicate with appropriate targets. Over the past several years, it has become clear that the Rho family of GTPases and related molecules play an important role in various aspects of neuronal development, including neurite outgrowth and differentiation, axon pathfinding, and dendritic spine formation and maintenance. Given the importance of these molecules in these processes, it is therefore not surprising that mutations in genes encoding a number of regulators and effectors of the Rho GTPases have been associated with human neurological diseases. This review will focus on the role of the Rho GTPases and their associated signaling molecules throughout neuronal development and discuss how perturbations in Rho GTPase signaling may lead to cognitive disorders.
11. Essential roles of myosin phosphatase in the maintenance of epithelial cell integrity of Drosophila imaginal disc cells. Reorganization of the actin cytoskeleton and contraction of actomyosin play pivotal roles in controlling cell shape changes and motility in epithelial morphogenesis. Dephosphorylation of the myosin regulatory light chain (MRLC) by myosin phosphatase is one of the key events involved. Allelic combinations producing intermediate strength mutants of the Drosophila myosin-binding subunit (DMBS) of myosin phosphatase showed imaginal discs with multilayered disrupted morphologies, and extremely mislocated cells, suggesting that DMBS is required to maintain proper epithelial organization. Clonal analyses revealed that DMBS null mutant cells appear to retract basally and localization of apical junction markers such as DE-cadherin is indetectable in most cells, whereas phosphorylated MRLC and F-actin become heavily concentrated apically, indicating misconfiguration of the apical cytoskeleton. In agreement with these findings, DMBS was found to concentrate at the apical domain suggesting its function is localized. Phenotypes similar to DMBS mutants including increased **migration** of cells were obtained by overexpressing the constitutive active form of MRLC or Rho-associated kinase signifying that the phenotypes are indeed caused through activation of Myosin II. The requirement of DMBS for the integrity of static epithelial cells in imaginal discs suggests that the regulation of Myosin II by DMBS has a role more general than its previously demonstrated functions in morphogenetic events.
12. Par-1 controls myosin-II activity through myosin phosphatase to regulate border cell **migration**. Localized actomyosin contraction couples with actin polymerization and cell-matrix adhesion to regulate cell protrusions and retract trailing edges of migrating cells. Although many cells migrate in collective groups during tissue morphogenesis, mechanisms that coordinate actomyosin dynamics in collective cell **migration** are poorly understood. **Migration** of Drosophila border cells, a genetically tractable model for collective cell **migration**, requires nonmuscle myosin-II (Myo-II). How Myo-II specifically controls border cell **migration** and how Myo-II is itself regulated is largely unknown.We show that Myo-II regulates two essential features of border cell **migration**: (1) initial detachment of the border cell cluster from the follicular epithelium and (2) the dynamics of cellular protrusions. We further demonstrate that the cell polarity protein Par-1 (MARK), a serine-threonine kinase, regulates the localization and activation of Myo-II in border cells. Par-1 binds to myosin phosphatase and phosphorylates it at a known inactivating site. Par-1 thus promotes phosphorylated myosin regulatory light chain, thereby increasing Myo-II activity. Furthermore, Par-1 localizes to and increases active Myo-II at the cluster rear to promote detachment; in the absence of Par-1, spatially distinct active Myo-II is lost.We identify a critical new role for Par-1 kinase: spatiotemporal regulation of Myo-II activity within the border cell cluster through localized inhibition of myosin phosphatase. Polarity proteins such as Par-1, which intrinsically localize, can thus directly modulate the actomyosin dynamics required for border cell detachment and **migration**. Such a link between polarity proteins and cytoskeletal dynamics may also occur in other collective cell **migration**s.
13. Interkinetic nuclear **migration** is a broadly conserved feature of cell division in pseudostratified epithelia. Animal development requires tight integration between the processes of proliferative growth and epithelial morphogenesis, both of which play out at the level of individual cells. In this respect, not only must polarized epithelial cells assume complex morphologies, these distinct forms must be radically and repeatedly transformed to permit mitosis. A dramatic illustration of this integration between epithelial morphogenesis and cell proliferation is interkinetic nuclear **migration** (IKNM), wherein the nuclei of pseudostratified epithelial cells translocate to the apical epithelial surface to execute cell division. IKNM is widely considered a hallmark of pseudostratified vertebrate neuroepithelia, and prior investigations have proposed both actomyosin- and microtubule-dependent mechanisms for apical localization of the mitotic nucleus. Here, using comparative functional analysis in arthropod and cnidarian systems (Drosophila melanogaster and Nematostella vectensis), we show that actomyosin-dependent IKNM is likely to be a general feature of mitosis in pseudostratified epithelia throughout Eumetazoa. Furthermore, our studies suggest a mechanistic link between IKNM and the fundamental process of mitotic cell rounding.
14. Excessive Myosin activity in mbs mutants causes photoreceptor movement out of the Drosophila eye disc epithelium. Neuronal cells must extend a motile growth cone while maintaining the cell body in its original position. In migrating cells, myosin contraction provides the driving force that pulls the rear of the cell toward the leading edge. We have characterized the function of myosin light chain phosphatase, which down-regulates myosin activity, in Drosophila photoreceptor neurons. Mutations in the gene encoding the myosin binding subunit of this enzyme cause photoreceptors to drop out of the eye disc epithelium and move toward and through the optic stalk. We show that this phenotype is due to excessive phosphorylation of the myosin regulatory light chain Spaghetti squash rather than another potential substrate, Moesin, and that it requires the nonmuscle myosin II heavy chain Zipper. Myosin binding subunit mutant cells continue to express apical epithelial markers and do not undergo ectopic apical constriction. In addition, mutant cells in the wing disc remain within the epithelium and differentiate abnormal wing hairs. We suggest that excessive myosin activity in photoreceptor neurons may pull the cell bodies toward the growth cones in a process resembling normal cell **migration**.
15. Regulation of cofilin phosphorylation and asymmetry in collective cell **migration** during morphogenesis. During Drosophila oogenesis, two actin dynamics regulators, cofilin and Rac, are required for the collective **migration** of a coherent cluster of cells called border cells. Cell culture data have shown that Rac and cofilin are both essential for lamellipodium formation, but Rac signaling results in phosphorylation and hence inactivation of cofilin. So it remains unclear whether cofilin phosphorylation plays a promoting or inhibitory role during cell **migration**. We show here that cofilin is required for F-actin turnover and lamellipodial protrusion in the border cells. Interestingly, reducing the dosage of cofilin by half or expressing a phospho-mimetic mutant form, S3E, partially rescues the **migration** and protrusion defects of Rac-deficient border cells. Moreover, cofilin exhibits moderate accumulation in border cells at the migratory front of the cluster, whereas phospho-cofilin has a robust and uniform distribution pattern in all the outer border cells. Blocking or overactivating Rac signaling in border cells greatly reduces or increases cofilin phosphorylation, respectively, and each abolishes cell **migration**. Furthermore, Rac may signal through Pak and LIMK to result in uniform phosphorylation of cofilin in all the outer border cells, whereas the guidance receptor Pvr (PDGF/VEGF receptor) mediates the asymmetric localization of cofilin in the cluster but does not affect its phosphorylation. Our study provides one of the first models of how cofilin functions and is regulated in the collective **migration** of a group of cells in vivo.
16. Integrins in development: moving on, responding to, and sticking to the extracellular matrix. Integrins are cell surface receptors of the extracellular matrix present in all animals. Genetic analysis in worms, flies, and vertebrates has revealed integrin involvement in key developmental processes, and we focus here on examples of integrin functions that are comparable across these model organisms. Integrins contribute to cell movement by providing traction to migrating cells, through assembly of extracellular matrices that can serve as tracks for **migration**, and by transmitting guidance signals that direct cells or cell processes to their targets. Integrins also participate in signaling events that govern tissue differentiation and organogenesis. Finally, adhesion by integrin-mediated junctions allows tissues to withstand mechanical load and is essential for tissue integrity.
17. Left-right asymmetric morphogenesis of the anterior midgut depends on the activation of a non-muscle myosin II in Drosophila. Many animals exhibit stereotypical left-right (LR) asymmetry in their internal organs. The mechanisms of LR axis formation required for the subsequent LR asymmetric development are well understood, especially in some vertebrates. However, the molecular mechanisms underlying LR asymmetric morphogenesis, particularly how mechanical force is integrated into the LR asymmetric morphogenesis of organs, are poorly understood. Here, we identified zipper (zip), encoding a Drosophila non-muscle myosin II (myosin II) heavy chain, as a gene required for LR asymmetric development of the embryonic anterior midgut (AMG). Myosin II is known to directly generate mechanical force in various types of cells during morphogenesis and cell **migration**. We found that myosin II was involved in two events in the LR asymmetric development of the AMG. First, it introduced an LR bias to the directional position of circular visceral muscle (CVMU) cells, which externally cover the midgut epithelium. Second, it was required for the LR-biased rotation of the AMG. Our results suggest that myosin II in CVMU cells plays a crucial role in generating the force leading to LR asymmetric morphogenesis. Taken together with previous studies in vertebrates, the involvement of myosin II in LR asymmetric morphogenesis might be conserved evolutionarily.
18. The Drosophila FHOD1-like formin Knittrig acts through Rok to promote stress fiber formation and directed macrophage **migration** during the cellular immune response. A tight spatiotemporal control of actin polymerization is important for many cellular processes that shape cells into a multicellular organism. The formation of unbranched F-actin is induced by several members of the formin family. Drosophila encodes six formin genes, representing six of the seven known mammalian subclasses. Knittrig, the Drosophila homolog of mammalian FHOD1, is specifically expressed in the developing central nervous system midline glia, the trachea, the wing and in macrophages. knittrig mutants exhibit mild tracheal defects but survive until late pupal stages and mainly die as pharate adult flies. knittrig mutant macrophages are smaller and show reduced cell spreading and cell **migration** in in vivo wounding experiments. Rescue experiments further demonstrate a cell-autonomous function of Knittrig in regulating actin dynamics and cell **migration**. Knittrig localizes at the rear of migrating macrophages in vivo, suggesting a cellular requirement of Knittrig in the retraction of the trailing edge. Supporting this notion, we found that Knittrig is a target of the Rho-dependent kinase Rok. Co-expression with Rok or expression of an activated form of Knittrig induces actin stress fibers in macrophages and in epithelial tissues. Thus, we propose a model in which Rok-induced phosphorylation of residues within the basic region mediates the activation of Knittrig in controlling macrophage **migration**.
19. Assessing the critical period for Rho kinase activity during Drosophila ventral furrow formation. Drosophila ventral furrow formation (VFF), which is the first morphogenetic event during embryo development, serves as a model for epithelial sheet folding. VFF can be subdivided into five cell shape changes: apical membrane flattening, apicobasal nuclear **migration**, apicobasal cell shortening, random apical constriction, and concerted apical constriction. These processes are generally believed to be driven by Rho kinase (Rok) activation of myosin II to stimulate the constriction of the apical actomyosin network. To test the role of Rok and its downstream target myosin II in VFF, timed injections of the Rok inhibitor, Y-27632, were performed.Embryos injected with Y-27632 before the concerted apical constriction phase of VFF were able to execute apicobasal nuclear **migration** and random apical constriction, but were unable to enter the concerted apical constriction phase. Embryos injected with Y-27632 during concerted apical constriction reverted to the transition point between random apical constriction and concerted apical constriction. Finally, embryos injected with Y-27632 upon the initiation of furrow ingression were able to complete VFF.Together these results suggest a critical period for Rok activity and presumably myosin II activation during the initiation of the concerted apical constriction phase of VFF.
20. Drosophila Rho-kinase (DRok) is required for tissue morphogenesis in diverse compartments of the egg chamber during oogenesis. The Rho-kinases are widely utilized downstream targets of the activated Rho GTPase that have been directly implicated in many aspects of Rho-dependent effects on F-actin assembly, acto-myosin contractility, and microtubule stability, and consequently play an essential role in regulating cell shape, **migration**, polarity, and division. We have determined that the single closely related Drosophila Rho-kinase ortholog, DRok, is required for several aspects of oogenesis, including maintaining the integrity of the oocyte cortex, actin-mediated tethering of nurse cell nuclei, "dumping" of nurse cell contents into the oocyte, establishment of oocyte polarity, and the trafficking of oocyte yolk granules. These defects are associated with abnormalities in DRok-dependent actin dynamics and appear to be mediated by multiple downstream effectors of activated DRok that have previously been implicated in oogenesis. DRok regulates at least one of these targets, the membrane cytoskeletal cross-linker DMoesin, via a direct phosphorylation that is required to promote localization of DMoesin to the oocyte cortex. The collective oogenesis defects associated with DRok deficiency reveal its essential role in multiple aspects of proper oocyte formation and suggest that DRok defines a novel class of oogenesis determinants that function as key regulators of several distinct actin-dependent processes required for proper tissue morphogenesis.
21. Drosophila pupal macrophages--a versatile tool for combined ex vivo and in vivo imaging of actin dynamics at high resolution. Molecular understanding of actin dynamics requires a genetically traceable model system that allows live cell imaging together with high-resolution microscopy techniques. Here, we used Drosophila pupal macrophages that combine many advantages of cultured cells with a genetic in vivo model system. Using structured illumination microscopy together with advanced spinning disk confocal microscopy we show that these cells provide a powerful system for single gene analysis. It allows forward genetic screens to characterize the regulatory network controlling cell shape and directed cell **migration** in a physiological context. We knocked down components regulating lamellipodia formation, including WAVE, single subunits of Arp2/3 complex and CPA, one of the two capping protein subunits and demonstrate the advantages of this model system by imaging mutant macrophages ex vivo as well as in vivo upon laser-induced wounding.

#### GeneRIFs

1. model in which Rok-induced phosphorylation of residues within the basic region mediates the activation of FHOD1 homolog Knittrig in controlling macrophage **migration**

### ECM

#### PubMed

1. Tissue elongation requires oscillating contractions of a basal actomyosin network. Understanding how molecular dynamics leads to cellular behaviours that ultimately sculpt organs and tissues is a major challenge not only in basic developmental biology but also in tissue engineering and regenerative medicine. Here we use live imaging to show that the basal surfaces of Drosophila follicle cells undergo a series of directional, oscillating contractions driven by periodic myosin accumulation on a polarized actin network. Inhibition of the actomyosin contractions or their coupling to extracellular matrix (**ECM**) blocked elongation of the whole tissue, whereas enhancement of the contractions exaggerated it. Myosin accumulated in a periodic manner before each contraction and was regulated by the small GTPase Rho, its downstream kinase, ROCK, and cytosolic calcium. Disrupting the link between the actin cytoskeleton and the **ECM** decreased the amplitude and period of the contractions, whereas enhancing cell-**ECM** adhesion increased them. In contrast, disrupting cell-cell adhesions resulted in loss of the actin network. Our findings reveal a mechanism controlling organ shape and an experimental model for the study of the effects of oscillatory actomyosin activity within a coherent cell sheet.

### [Ee]xtracellular [Mm]atrix

#### PubMed

1. The actomyosin machinery is required for Drosophila retinal lumen formation. Multicellular tubes consist of polarized cells wrapped around a central lumen and are essential structures underlying many developmental and physiological functions. In Drosophila compound eyes, each ommatidium forms a luminal matrix, the inter-rhabdomeral space, to shape and separate the key phototransduction organelles, the rhabdomeres, for proper visual perception. In an enhancer screen to define mechanisms of retina lumen formation, we identified Actin5C as a key molecule. Our results demonstrate that the disruption of lumen formation upon the reduction of Actin5C is not linked to any discernible defect in microvillus formation, the rhabdomere terminal web (RTW), or the overall morphogenesis and basal extension of the rhabdomere. Second, the failure of proper lumen formation is not the result of previously identified processes of retinal lumen formation: Prominin localization, expansion of the apical membrane, or secretion of the luminal matrix. Rather, the phenotype observed with Actin5C is phenocopied upon the decrease of the individual components of non-muscle myosin II (MyoII) and its upstream activators. In photoreceptor cells MyoII localizes to the base of the rhabdomeres, overlapping with the actin filaments of the RTW. Consistent with the well-established roll of actomyosin-mediated cellular contraction, reduction of MyoII results in reduced distance between apical membranes as measured by a decrease in lumen diameter. Together, our results indicate the actomyosin machinery coordinates with the localization of apical membrane components and the secretion of an **extracellular matrix** to overcome apical membrane adhesion to initiate and expand the retinal lumen.
2. A directed miniscreen for genes involved in the Drosophila anti-parasitoid immune response. Drosophila larvae react against eggs from the endoparasitoid wasp Leptopilina boulardi by surrounding them in a multilayered cellular capsule. Once a wasp egg is recognized as foreign, circulating macrophage-like cells, known as plasmatocytes, adhere to the invader. After spreading around the wasp egg, plasmatocytes form cellular junctions between the cells, effectively separating the egg from the hemocoel. Next, a second sub-type of circulating immunosurveillance cell (hemocyte), known as lamellocytes, adhere to either the wasp egg or more likely the plasmatocytes surrounding the egg. From these events, it is obvious that adhesion and cell shape change are an essential part of Drosophila's cellular immune response against parasitoid wasp eggs. To date, very few genes have been described as being necessary for a proper anti-parasitization response in Drosophila. With this in mind, we performed a directed genetic miniscreen to discover new genes required for this response. Many of the genes with an encapsulation defect have mammalian homologues involved in cellular adhesion, wound healing, and thrombosis, including **extracellular matrix** proteins, cellular adhesion molecules, and small GTPases.
3. Tissue elongation requires oscillating contractions of a basal actomyosin network. Understanding how molecular dynamics leads to cellular behaviours that ultimately sculpt organs and tissues is a major challenge not only in basic developmental biology but also in tissue engineering and regenerative medicine. Here we use live imaging to show that the basal surfaces of Drosophila follicle cells undergo a series of directional, oscillating contractions driven by periodic myosin accumulation on a polarized actin network. Inhibition of the actomyosin contractions or their coupling to **extracellular matrix** (ECM) blocked elongation of the whole tissue, whereas enhancement of the contractions exaggerated it. Myosin accumulated in a periodic manner before each contraction and was regulated by the small GTPase Rho, its downstream kinase, ROCK, and cytosolic calcium. Disrupting the link between the actin cytoskeleton and the ECM decreased the amplitude and period of the contractions, whereas enhancing cell-ECM adhesion increased them. In contrast, disrupting cell-cell adhesions resulted in loss of the actin network. Our findings reveal a mechanism controlling organ shape and an experimental model for the study of the effects of oscillatory actomyosin activity within a coherent cell sheet.
4. Integrins in development: moving on, responding to, and sticking to the **extracellular matrix**. Integrins are cell surface receptors of the **extracellular matrix** present in all animals. Genetic analysis in worms, flies, and vertebrates has revealed integrin involvement in key developmental processes, and we focus here on examples of integrin functions that are comparable across these model organisms. Integrins contribute to cell movement by providing traction to migrating cells, through assembly of extracellular matrices that can serve as tracks for migration, and by transmitting guidance signals that direct cells or cell processes to their targets. Integrins also participate in signaling events that govern tissue differentiation and organogenesis. Finally, adhesion by integrin-mediated junctions allows tissues to withstand mechanical load and is essential for tissue integrity.

### [Ii]nvasive

#### PubMed

1. Extrinsic and intrinsic mechanisms directing epithelial cell sheet replacement during Drosophila metamorphosis. The fusion of epithelial sheets is an essential morphogenetic event. Here, we study the development of the abdomen of Drosophila as a model of bounded epithelia expansion and uncover a complex multistep process for the generation of the adult epidermis from histoblasts, founder cells that replace the larval cells during metamorphosis. We find that histoblasts experience a biphasic cell cycle and emit apical projections that direct their **invasive** planar intercalation in between larval cells. Coordinately, the larval cells extrude from the epithelia by apical constriction of an actomyosin ring and as a consequence die by apoptosis and are removed by circulating haemocytes. We demonstrate that the proliferation of histoblasts and the death of larval cells are triggered by two independent extrinsic Ecdysone hormonal pulses. Finally, we show that histoblast spreading and the death of larval cells depend on a mutual exchange of signals and are non-autonomous processes.
2. Mechanical tension drives cell membrane fusion. Membrane fusion is an energy-consuming process that requires tight juxtaposition of two lipid bilayers. Little is known about how cells overcome energy barriers to bring their membranes together for fusion. Previously, we have shown that cell-cell fusion is an asymmetric process in which an "attacking" cell drills finger-like protrusions into the "receiving" cell to promote cell fusion. Here, we show that the receiving cell mounts a Myosin II (MyoII)-mediated mechanosensory response to its **invasive** fusion partner. MyoII acts as a mechanosensor, which directs its force-induced recruitment to the fusion site, and the mechanosensory response of MyoII is amplified by chemical signaling initiated by cell adhesion molecules. The accumulated MyoII, in turn, increases cortical tension and promotes fusion pore formation. We propose that the protrusive and resisting forces from fusion partners put the fusogenic synapse under high mechanical tension, which helps to overcome energy barriers for membrane apposition and drives cell membrane fusion.
3. Sin3a acts through a multi-gene module to regulate invasion in Drosophila and human tumors. Chromatin remodeling proteins regulate multiple aspects of cell homeostasis, making them ideal candidates for misregulation in transformed cells. Here, we explore Sin3A, a member of the Sin3 family of proteins linked to tumorigenesis that are thought to regulate gene expression through their role as histone deacetylases (HDACs). We identified Drosophila Sin3a as an important mediator of oncogenic Ret receptor in a fly model of Multiple Endocrine Neoplasia Type 2. Reducing Drosophila Sin3a activity led to metastasis-like behavior and, in the presence of Diap1, secondary tumors distant from the site of origin. Genetic and Chip-Seq analyses identified previously undescribed Sin3a targets including genes involved in cell motility and actin dynamics, as well as signaling pathways including Src, Jnk and Rho. A key Sin3a oncogenic target, PP1B, regulates stability of β-Catenin/Armadillo: the outcome is to oppose T-cell factor (TCF) function and Wg/Wnt pathway signaling in both fly and mammalian cancer cells. Reducing Sin3A strongly increased the **invasiv**e behavior of A549 human lung adenocarcinoma cells. We show that Sin3A is downregulated in a variety of human tumors and that Src, JNK, RhoA and PP1B/β-Catenin are regulated in a manner analogous to our Drosophila models. Our data suggest that Sin3A influences a specific step of tumorigenesis by regulating a module of genes involved in cell invasion. Tumor progression may commonly rely on such 'modules of invasion' under the control of broad transcriptional regulators.
4. Moesin functions antagonistically to the Rho pathway to maintain epithelial integrity. Two prominent characteristics of epithelial cells, apical-basal polarity and a highly ordered cytoskeleton, depend on the existence of precisely localized protein complexes associated with the apical plasma membrane, and on a separate machinery that regulates the spatial order of actin assembly. ERM (ezrin, radixin, moesin) proteins have been proposed to link transmembrane proteins to the actin cytoskeleton in the apical domain, suggesting a structural role in epithelial cells, and they have been implicated in signalling pathways. Here, we show that the sole Drosophila ERM protein Moesin functions to promote cortical actin assembly and apical-basal polarity. As a result, cells lacking Moesin lose epithelial characteristics and adopt **invasive** migratory behaviour. Our data demonstrate that Moesin facilitates epithelial morphology not by providing an essential structural function, but rather by antagonizing activity of the small GTPase Rho. Thus, Moesin functions in maintaining epithelial integrity by regulating cell-signalling events that affect actin organization and polarity. Furthermore, our results show that there is negative feedback between ERM activation and activity of the Rho pathway.

### [Mm]etastasis

#### PubMed

1. Planar cell polarity signaling in collective cell movements during morphogenesis and disease. Collective and directed cell movements are crucial for diverse developmental processes in the animal kingdom, but they are also involved in wound repair and disease. During these processes groups of cells are oriented within the tissue plane, which is referred to as planar cell polarity (PCP). This requires a tight regulation that is in part conducted by the PCP pathway. Although this pathway was initially characterized in flies, subsequent studies in vertebrates revealed a set of conserved core factors but also effector molecules and signal modulators, which build the fundamental PCP machinery. The PCP pathway in Drosophila regulates several developmental processes involving collective cell movements such as border cell migration during oogenesis, ommatidial rotation during eye development, and embryonic dorsal closure. During vertebrate embryogenesis, PCP signaling also controls collective and directed cell movements including convergent extension during gastrulation, neural tube closure, neural crest cell migration, or heart morphogenesis. Similarly, PCP signaling is linked to processes such as wound repair, and cancer invasion and **metastasis** in adults. As a consequence, disruption of PCP signaling leads to pathological conditions. In this review, we will summarize recent findings about the role of PCP signaling in collective cell movements in flies and vertebrates. In addition, we will focus on how studies in Drosophila have been relevant to our understanding of the PCP molecular machinery and will describe several developmental defects and human disorders in which PCP signaling is compromised. Therefore, new discoveries about the contribution of this pathway to collective cell movements could provide new potential diagnostic and therapeutic targets for these disorders.
2. Small is beautiful: what flies tell us about ERM protein function in development. Actin dynamics is recognized as being a determinant in many developmental processes and pathologies, such as cell polarity, morphogenesis and tumour **metastasis**. However, how actin interacts with the plasma membrane is poorly understood. Although numerous studies in cell culture point to the crucial role of Ezrin, Radixin and Moesin (ERM) proteins in the actin-membrane link, genetic approaches in mice have not yet revealed their activity during development. Drosophila has recently become an alternative and promising system for the genetic study of ERM protein function. This article focuses on advances made in flies, providing evidence for the evolutionary conservation of functional properties of ERM proteins, in addition to shedding new light on their importance for development.
3. Distinct functions for Rho1 in maintaining adherens junctions and apical tension in remodeling epithelia. Maintenance and remodeling of adherens junctions (AJs) and cell shape in epithelia are necessary for the development of functional epithelia and are commonly altered during cancer progression/**metastasis**. Although formation of nascent AJs has received much attention, whether shared mechanisms are responsible for the maintenance and remodeling of AJs in dynamic epithelia, particularly in vivo, is not clear. Using clonal analysis in the postmitotic Drosophila melanogaster pupal eye epithelium, we demonstrate that Rho1 is required to maintain AJ integrity independent of its role in sustaining apical cell tension. Rho1 depletion in a remodeling postmitotic epithelium disrupts AJs but only when depleted in adjacent cells. Surprisingly, neither of the Rho effectors, Rok or Dia, is necessary downstream of Rho1 to maintain AJs; instead, Rho1 maintains AJs by inhibiting Drosophila epithelial cadherin endocytosis in a Cdc42/Par6-dependent manner. In contrast, depletion of Rho1 in single cells decreases apical tension, and Rok and myosin are necessary, while Dia function also contributes, downstream of Rho1 to sustain apical cell tension.
4. Sin3a acts through a multi-gene module to regulate invasion in Drosophila and human tumors. Chromatin remodeling proteins regulate multiple aspects of cell homeostasis, making them ideal candidates for misregulation in transformed cells. Here, we explore Sin3A, a member of the Sin3 family of proteins linked to tumorigenesis that are thought to regulate gene expression through their role as histone deacetylases (HDACs). We identified Drosophila Sin3a as an important mediator of oncogenic Ret receptor in a fly model of Multiple Endocrine Neoplasia Type 2. Reducing Drosophila Sin3a activity led to **metastasis**-like behavior and, in the presence of Diap1, secondary tumors distant from the site of origin. Genetic and Chip-Seq analyses identified previously undescribed Sin3a targets including genes involved in cell motility and actin dynamics, as well as signaling pathways including Src, Jnk and Rho. A key Sin3a oncogenic target, PP1B, regulates stability of β-Catenin/Armadillo: the outcome is to oppose T-cell factor (TCF) function and Wg/Wnt pathway signaling in both fly and mammalian cancer cells. Reducing Sin3A strongly increased the invasive behavior of A549 human lung adenocarcinoma cells. We show that Sin3A is downregulated in a variety of human tumors and that Src, JNK, RhoA and PP1B/β-Catenin are regulated in a manner analogous to our Drosophila models. Our data suggest that Sin3A influences a specific step of tumorigenesis by regulating a module of genes involved in cell invasion. Tumor progression may commonly rely on such 'modules of invasion' under the control of broad transcriptional regulators.

### [Cc]ell [Ii]nvasion

#### PubMed

1. Sin3a acts through a multi-gene module to regulate invasion in Drosophila and human tumors. Chromatin remodeling proteins regulate multiple aspects of cell homeostasis, making them ideal candidates for misregulation in transformed cells. Here, we explore Sin3A, a member of the Sin3 family of proteins linked to tumorigenesis that are thought to regulate gene expression through their role as histone deacetylases (HDACs). We identified Drosophila Sin3a as an important mediator of oncogenic Ret receptor in a fly model of Multiple Endocrine Neoplasia Type 2. Reducing Drosophila Sin3a activity led to metastasis-like behavior and, in the presence of Diap1, secondary tumors distant from the site of origin. Genetic and Chip-Seq analyses identified previously undescribed Sin3a targets including genes involved in cell motility and actin dynamics, as well as signaling pathways including Src, Jnk and Rho. A key Sin3a oncogenic target, PP1B, regulates stability of β-Catenin/Armadillo: the outcome is to oppose T-cell factor (TCF) function and Wg/Wnt pathway signaling in both fly and mammalian cancer cells. Reducing Sin3A strongly increased the invasive behavior of A549 human lung adenocarcinoma cells. We show that Sin3A is downregulated in a variety of human tumors and that Src, JNK, RhoA and PP1B/β-Catenin are regulated in a manner analogous to our Drosophila models. Our data suggest that Sin3A influences a specific step of tumorigenesis by regulating a module of genes involved i**n cell invasi**on. Tumor progression may commonly rely on such 'modules of invasion' under the control of broad transcriptional regulators.

---

## DOR

- NCBI Gene
- GeneRIF
- Pubmed

| Search Term | Rank | Fields |
| --- | --- | --- |
| [Mm]igration | 6 | PubMed(2) |
| ECM | 7 | PubMed(1) |
| [Ee]xtracellular [Mm]atrix | 8 | PubMed(1) |

  
hide/show details for DOR

### [Mm]igration

#### PubMed

1. Transcriptional control in embryonic Drosophila midline guidance assessed through a whole genome approach. During the development of the Drosophila central nervous system the process of midline crossing is orchestrated by a number of guidance receptors and ligands. Many key axon guidance molecules have been identified in both invertebrates and vertebrates, but the transcriptional regulation of growth cone guidance remains largely unknown. It is established that translational regulation plays a role in midline crossing, and there are indications that transcriptional regulation is also involved. To investigate this issue, we conducted a genome-wide study of transcription in Drosophila embryos using wild type and a number of well-characterized Drosophila guidance mutants and transgenics. We also analyzed a previously published microarray time course of Drosophila embryonic development with an axon guidance focus.Using hopach, a novel clustering method which is well suited to microarray data analysis, we identified groups of genes with similar expression patterns across guidance mutants and transgenics. We then systematically characterized the resulting clusters with respect to their relevance to axon guidance using two complementary controlled vocabularies: the Gene Ontology (GO) and anatomical annotations of the Atlas of Pattern of Gene Expression (APoGE) in situ hybridization database. The analysis indicates that regulation of gene expression does play a role in the process of axon guidance in Drosophila. We also find a strong link between axon guidance and hemocyte **migration**, a result that agrees with mounting evidence that axon guidance molecules are co-opted in vertebrate vascularization. Cell cyclin activity in the context of axon guidance is also suggested from our array data. RNA and protein expression patterns of cell cyclins in axon guidance mutants and transgenics support this possible link.This study provides important insights into the regulation of axon guidance in vivo.
2. Genes conserved in bilaterians but jointly lost with Myc during nematode evolution are enriched in cell proliferation and cell **migration** functions. Animals use a stereotypical set of developmental genes to build body architectures of varying sizes and organizational complexity. Some genes are critical to developmental patterning, while other genes are important to physiological control of growth. However, growth regulator genes may not be as important in small-bodied "micro-metazoans" such as nematodes. Nematodes use a simplified developmental strategy of lineage-based cell fate specifications to produce an adult bilaterian body composed of a few hundreds of cells. Nematodes also lost the MYC proto-oncogenic regulator of cell proliferation. To identify additional regulators of cell proliferation that were lost with MYC, we computationally screened and determined 839 high-confidence genes that are conserved in bilaterians/lost in nematodes (CIBLIN genes). We find that 30 % of all CIBLIN genes encode transcriptional regulators of cell proliferation, epithelial-to-mesenchyme transitions, and other processes. Over 50 % of CIBLIN genes are unnamed genes in Drosophila, suggesting that there are many understudied genes. Interestingly, CIBLIN genes include many Myc synthetic lethal (MycSL) hits from recent screens. CIBLIN genes include key regulators of heparan sulfate proteoglycan (HSPG) sulfation patterns, and lysyl oxidases involved in cross-linking and modification of the extracellular matrix (ECM). These genes and others suggest the CIBLIN repertoire services critical functions in ECM remodeling and cell **migration** in large-bodied bilaterians. Correspondingly, CIBLIN genes are co-expressed with Myc in cancer transcriptomes, and include a preponderance of known determinants of cancer progression and tumor aggression. We propose that CIBLIN gene research can improve our understanding of regulatory control of cellular growth in metazoans.

### ECM

#### PubMed

1. Genes conserved in bilaterians but jointly lost with Myc during nematode evolution are enriched in cell proliferation and cell migration functions. Animals use a stereotypical set of developmental genes to build body architectures of varying sizes and organizational complexity. Some genes are critical to developmental patterning, while other genes are important to physiological control of growth. However, growth regulator genes may not be as important in small-bodied "micro-metazoans" such as nematodes. Nematodes use a simplified developmental strategy of lineage-based cell fate specifications to produce an adult bilaterian body composed of a few hundreds of cells. Nematodes also lost the MYC proto-oncogenic regulator of cell proliferation. To identify additional regulators of cell proliferation that were lost with MYC, we computationally screened and determined 839 high-confidence genes that are conserved in bilaterians/lost in nematodes (CIBLIN genes). We find that 30 % of all CIBLIN genes encode transcriptional regulators of cell proliferation, epithelial-to-mesenchyme transitions, and other processes. Over 50 % of CIBLIN genes are unnamed genes in Drosophila, suggesting that there are many understudied genes. Interestingly, CIBLIN genes include many Myc synthetic lethal (MycSL) hits from recent screens. CIBLIN genes include key regulators of heparan sulfate proteoglycan (HSPG) sulfation patterns, and lysyl oxidases involved in cross-linking and modification of the extracellular matrix (**ECM**). These genes and others suggest the CIBLIN repertoire services critical functions in **ECM** remodeling and cell migration in large-bodied bilaterians. Correspondingly, CIBLIN genes are co-expressed with Myc in cancer transcriptomes, and include a preponderance of known determinants of cancer progression and tumor aggression. We propose that CIBLIN gene research can improve our understanding of regulatory control of cellular growth in metazoans.

### [Ee]xtracellular [Mm]atrix

#### PubMed

1. Genes conserved in bilaterians but jointly lost with Myc during nematode evolution are enriched in cell proliferation and cell migration functions. Animals use a stereotypical set of developmental genes to build body architectures of varying sizes and organizational complexity. Some genes are critical to developmental patterning, while other genes are important to physiological control of growth. However, growth regulator genes may not be as important in small-bodied "micro-metazoans" such as nematodes. Nematodes use a simplified developmental strategy of lineage-based cell fate specifications to produce an adult bilaterian body composed of a few hundreds of cells. Nematodes also lost the MYC proto-oncogenic regulator of cell proliferation. To identify additional regulators of cell proliferation that were lost with MYC, we computationally screened and determined 839 high-confidence genes that are conserved in bilaterians/lost in nematodes (CIBLIN genes). We find that 30 % of all CIBLIN genes encode transcriptional regulators of cell proliferation, epithelial-to-mesenchyme transitions, and other processes. Over 50 % of CIBLIN genes are unnamed genes in Drosophila, suggesting that there are many understudied genes. Interestingly, CIBLIN genes include many Myc synthetic lethal (MycSL) hits from recent screens. CIBLIN genes include key regulators of heparan sulfate proteoglycan (HSPG) sulfation patterns, and lysyl oxidases involved in cross-linking and modification of the **extracellular matrix** (ECM). These genes and others suggest the CIBLIN repertoire services critical functions in ECM remodeling and cell migration in large-bodied bilaterians. Correspondingly, CIBLIN genes are co-expressed with Myc in cancer transcriptomes, and include a preponderance of known determinants of cancer progression and tumor aggression. We propose that CIBLIN gene research can improve our understanding of regulatory control of cellular growth in metazoans.

---

## DOR

- NCBI Gene
- GeneRIF
- Pubmed

| Search Term | Rank | Fields |
| --- | --- | --- |
| [Mm]igration | 6 | Process(1); PubMed(3) |
| [Ee]xtracellular [Mm]atrix | 8 | PubMed(1) |
| [Ii]nvasive | 10 | PubMed(1) |
| [Mm]etastasis | 12 | PubMed(3) |
| [Cc]ell [Ii]nvasion | 13 | PubMed(1) |

  
hide/show details for DOR

### [Mm]igration

#### Process

1. border follicle cell **migration**

#### PubMed

1. Dynamics of the basement membrane in invasive epithelial clusters in Drosophila. The basement membrane (BM) represents a barrier to cell **migration**, which has to be degraded to promote invasion. However, the role and behaviour of the BM during the development of pre-invasive cells is only poorly understood. Drosophila border cells (BCs) provide an attractive genetic model in which to study the cellular mechanisms underlying the **migration** of mixed cohorts of epithelial cells. BCs are made of two different epithelial cell types appearing sequentially during oogenesis: the polar cells and the outer BCs. Here, we show that the pre-invasive polar cells undergo an unusual and asymmetrical apical capping with major basement membrane proteins, including the two Drosophila Collagen IV alpha chains, Laminin A and Perlecan. Capping of polar cells proceeds through a novel, basal-to-apical transcytosis mechanism that involves the small GTPase Drab5. Apical capping is transient and is followed by rapid shedding prior to the initiation of BC **migration**, suggesting that the apical cap blocks **migration**. Consistently, non-migratory polar cells remain capped. We further show that JAK/STAT signalling and recruitment of outer BCs are required for correct shedding and **migration**. The dynamics of the BM represents a marker of migratory BC, revealing a novel developmentally regulated behaviour of BM coupled to epithelial cell invasiveness.
2. A mutation in dVps28 reveals a link between a subunit of the endosomal sorting complex required for transport-I complex and the actin cytoskeleton in Drosophila. Proteins that constitute the endosomal sorting complex required for transport (ESCRT) are necessary for the sorting of proteins into multivesicular bodies (MVBs) and the budding of several enveloped viruses, including HIV-1. The first of these complexes, ESCRT-I, consists of three proteins: Vps28p, Vps37p, and Vps23p or Tsg101 in mammals. Here, we characterize a mutation in the Drosophila homolog of vps28. The dVps28 gene is essential: homozygous mutants die at the transition from the first to second instar. Removal of maternally contributed dVps28 causes early embryonic lethality. In such embryos lacking dVps28, several processes that require the actin cytoskeleton are perturbed, including axial **migration** of nuclei, formation of transient furrows during cortical divisions in syncytial embryos, and the subsequent cellularization. Defects in actin cytoskeleton organization also become apparent during sperm individualization in dVps28 mutant testis. Because dVps28 mutant cells contained MVBs, these defects are unlikely to be a secondary consequence of disrupted MVB formation and suggest an interaction between the actin cytoskeleton and endosomal membranes in Drosophila embryos earlier than previously appreciated.
3. Hedgehog is a positive regulator of FGF signalling during embryonic tracheal cell **migration**. Cell **migration** is a widespread and complex process that is crucial for morphogenesis and for the underlying invasion and metastasis of human cancers. During **migration**, cells are steered toward target sites by guidance molecules that induce cell direction and movement through complex intracellular mechanisms. The spatio-temporal regulation of the expression of these guidance molecules is of extreme importance for both normal morphogenesis and human disease. One way to achieve this precise regulation is by combinatorial inputs of different transcription factors. Here we used Drosophila melanogaster mutants with **migration** defects in the ganglionic branches of the tracheal system to further clarify guidance regulation during cell **migration**. By studying the cellular consequences of overactivated Hh signalling, using ptc mutants, we found that Hh positively regulates Bnl/FGF levels during embryonic stages. Our results show that Hh modulates cell **migration** non-autonomously in the tissues surrounding the action of its activity. We further demonstrate that the Hh signalling pathway regulates bnl expression via Stripe (Sr), a zinc-finger transcription factor with homology to the Early Growth Response (EGR) family of vertebrate transcription factors. We propose that Hh modulates embryonic cell **migration** by participating in the spatio-temporal regulation of bnl expression in a permissive mode. By doing so, we provide a molecular link between the activation of Hh signalling and increased chemotactic responses during cell **migration**.

### [Ee]xtracellular [Mm]atrix

#### PubMed

1. Hedgehog lipid modifications are required for Hedgehog stabilization in the **extracellular matrix**. The Hedgehog (Hh) family of morphogenetic proteins has important instructional roles in metazoan development. Despite Hh being modified by Ct-cholesterol and Nt-palmitate adducts, Hh migrates far from its site of synthesis and programs cellular outcomes, depending on its local concentrations. We show that in the receiving cells of the Drosophila wing imaginal disc, lipid-unmodified Hh spreads across many more cell diameters than the wild type and this spreading leads to the activation of low but not high threshold responses. Unlipidated Hh forms become internalized through the apical plasma membrane, while wild-type Hh enters through the basolateral cell surface - in all cases via a dynamin-dependent mechanism. Full activation of the Hh pathway and the spread of Hh throughout the **extracellular matrix** depend on the ability of lipid-modified Hh to interact with heparan sulfate proteoglycans (HSPG). However, neither Hh-lipid modifications nor HSPG function are required to activate the targets that respond to low levels of Hh. All these data show that the interaction of lipid-modified Hh with HSPG is important both for precise Hh spreading through the epithelium surface and for correct Hh reception.

### [Ii]nvasive

#### PubMed

1. Dynamics of the basement membrane in **invasive** epithelial clusters in Drosophila. The basement membrane (BM) represents a barrier to cell migration, which has to be degraded to promote invasion. However, the role and behaviour of the BM during the development of pre-**invasive** cells is only poorly understood. Drosophila border cells (BCs) provide an attractive genetic model in which to study the cellular mechanisms underlying the migration of mixed cohorts of epithelial cells. BCs are made of two different epithelial cell types appearing sequentially during oogenesis: the polar cells and the outer BCs. Here, we show that the pre-**invasive** polar cells undergo an unusual and asymmetrical apical capping with major basement membrane proteins, including the two Drosophila Collagen IV alpha chains, Laminin A and Perlecan. Capping of polar cells proceeds through a novel, basal-to-apical transcytosis mechanism that involves the small GTPase Drab5. Apical capping is transient and is followed by rapid shedding prior to the initiation of BC migration, suggesting that the apical cap blocks migration. Consistently, non-migratory polar cells remain capped. We further show that JAK/STAT signalling and recruitment of outer BCs are required for correct shedding and migration. The dynamics of the BM represents a marker of migratory BC, revealing a novel developmentally regulated behaviour of BM coupled to epithelial cell **invasive**ness.

### [Mm]etastasis

#### PubMed

1. Modeling tumor invasion and **metastasis** in Drosophila. Conservation of major signaling pathways between humans and flies has made Drosophila a useful model organism for cancer research. Our understanding of the mechanisms regulating cell growth, differentiation and development has been considerably advanced by studies in Drosophila. Several recent high profile studies have examined the processes constraining the metastatic growth of tumor cells in fruit fly models. Cell invasion can be studied in the context of an in vivo setting in flies, enabling the genetic requirements of the microenvironment of tumor cells undergoing **metastasis** to be analyzed. This Perspective discusses the strengths and limitations of Drosophila models of cancer invasion and the unique tools that have enabled these studies. It also highlights several recent reports that together make a strong case for Drosophila as a system with the potential for both testing novel concepts in tumor progression and cell invasion, and for uncovering players in **metastasis**.
2. Disruption of lysosome function promotes tumor growth and **metastasis** in Drosophila. Lysosome function is essential to many physiological processes. It has been suggested that deregulation of lysosome function could contribute to cancer. Through a genetic screen in Drosophila, we have discovered that mutations disrupting lysosomal degradation pathway components contribute to tumor development and progression. Loss-of-function mutations in the Class C vacuolar protein sorting (VPS) gene, deep orange (dor), dramatically promote tumor overgrowth and invasion of the Ras(V12) cells. Knocking down either of the two other components of the Class C VPS complex, carnation (car) and vps16A, also renders Ras(V12) cells capable for uncontrolled growth and metastatic behavior. Finally, chemical disruption of the lysosomal function by feeding animals with antimalarial drugs, chloroquine or monensin, leads to malignant tumor growth of the Ras(V12) cells. Taken together, our data provide evidence for a causative role of lysosome dysfunction in tumor growth and invasion and indicate that members of the Class C VPS complex behave as tumor suppressors.
3. Hedgehog is a positive regulator of FGF signalling during embryonic tracheal cell migration. Cell migration is a widespread and complex process that is crucial for morphogenesis and for the underlying invasion and **metastasis** of human cancers. During migration, cells are steered toward target sites by guidance molecules that induce cell direction and movement through complex intracellular mechanisms. The spatio-temporal regulation of the expression of these guidance molecules is of extreme importance for both normal morphogenesis and human disease. One way to achieve this precise regulation is by combinatorial inputs of different transcription factors. Here we used Drosophila melanogaster mutants with migration defects in the ganglionic branches of the tracheal system to further clarify guidance regulation during cell migration. By studying the cellular consequences of overactivated Hh signalling, using ptc mutants, we found that Hh positively regulates Bnl/FGF levels during embryonic stages. Our results show that Hh modulates cell migration non-autonomously in the tissues surrounding the action of its activity. We further demonstrate that the Hh signalling pathway regulates bnl expression via Stripe (Sr), a zinc-finger transcription factor with homology to the Early Growth Response (EGR) family of vertebrate transcription factors. We propose that Hh modulates embryonic cell migration by participating in the spatio-temporal regulation of bnl expression in a permissive mode. By doing so, we provide a molecular link between the activation of Hh signalling and increased chemotactic responses during cell migration.

### [Cc]ell [Ii]nvasion

#### PubMed

1. Modeling tumor invasion and metastasis in Drosophila. Conservation of major signaling pathways between humans and flies has made Drosophila a useful model organism for cancer research. Our understanding of the mechanisms regulating cell growth, differentiation and development has been considerably advanced by studies in Drosophila. Several recent high profile studies have examined the processes constraining the metastatic growth of tumor cells in fruit fly models. **Cell invasion** can be studied in the context of an in vivo setting in flies, enabling the genetic requirements of the microenvironment of tumor cells undergoing metastasis to be analyzed. This Perspective discusses the strengths and limitations of Drosophila models of cancer invasion and the unique tools that have enabled these studies. It also highlights several recent reports that together make a strong case for Drosophila as a system with the potential for both testing novel concepts in tumor progression and **cell invasion**, and for uncovering players in metastasis.

---

## Ack-like

- NCBI Gene
- GeneRIF
- Pubmed

| Search Term | Rank | Fields |
| --- | --- | --- |
| [Mm]igration | 6 | Process(1) |
| [Mm]etastasis | 12 | PubMed(1) |

  
hide/show details for Ack-like

### [Mm]igration

#### Process

1. cell **migration**

### [Mm]etastasis

#### PubMed

1. Activated Cdc42 kinase regulates Dock localization in male germ cells during Drosophila spermatogenesis. Deregulation of the non-receptor tyrosine kinase ACK1 (Activated Cdc42-associated kinase) correlates with poor prognosis in cancers and has been implicated in promoting **metastasis**. To further understand its in vivo function, we have characterized the developmental defects of a null mutation in Drosophila Ack, which bears a high degree of sequence similarity to mammalian ACK1 but lacks a CRIB domain. We show that Ack, while not essential for viability, is critical for sperm formation. This function depends on Ack tyrosine kinase activity and is required cell autonomously in differentiating male germ cells at or after the spermatocyte stage. Ack associates predominantly with endocytic clathrin sites in spermatocytes, but disruption of Ack function has no apparent effect on clathrin localization and receptor-mediated internalization of Boss (Bride of sevenless) protein in eye discs. Instead, Ack is required for the subcellular distribution of Dock (dreadlocks), the Drosophila homolog of the SH2- and SH3-containing adaptor protein Nck. Moreover, Dock forms a complex with Ack, and the localization of Dock in male germ cells depends on its SH2 domain. Together, our results suggest that Ack-dependent tyrosine phosphorylation recruits Dock to promote sperm differentiation.

---

## CG2765

- NCBI Gene
- GeneRIF
- Pubmed

| Search Term | Rank | Fields |
| --- | --- | --- |
| [Ff]ilopodia | 1 | PubMed(1) |

  
hide/show details for CG2765

### [Ff]ilopodia

#### PubMed

1. Sisyphus, the Drosophila myosin XV homolog, traffics within **filopodia** transporting key sensory and adhesion cargos. Unconventional myosin proteins of the MyTH-FERM superclass are involved in intra**filopodia**l trafficking, are thought to be mediators of membrane-cytoskeleton interactions, and are linked to several forms of deafness in mammals. Here we show that the Drosophila myosin XV homolog, Sisyphus, is expressed at high levels in leading edge cells and their cellular protrusions during the morphogenetic process of dorsal closure. Sisyphus is required for the correct alignment of cells on opposing sides of the fusing epithelial sheets, as well as for adhesion of the cells during the final zippering/fusion phase. We have identified several putative Sisyphus cargos, including DE-cadherin (also known as Shotgun) and the microtubule-linked proteins Katanin-60, EB1, Milton and aPKC. These cargos bind to the Sisyphus FERM domain, and their binding is in some cases mutually exclusive. Our data suggest a mechanism for Sisyphus in which it maintains a balance between actin and microtubule cytoskeleton components, thereby contributing to cytoskeletal cross-talk necessary for regulating **filopodia**l dynamics during dorsal closure.

---

## trbl

- NCBI Gene
- GeneRIF
- Pubmed

| Search Term | Rank | Fields |
| --- | --- | --- |
| [Mm]igration | 6 | PubMed(6) |
| [Ii]nvasive | 10 | PubMed(1) |
| [Mm]etastasis | 12 | PubMed(1) |

  
hide/show details for trbl

### [Mm]igration

#### PubMed

1. The level of C/EBP protein is critical for cell **migration** during Drosophila oogenesis and is tightly controlled by regulated degradation. The C/EBP transcription factor, Slbo, is required for **migration** of border cells during Drosophila oogenesis. Unexpectedly, we find that neither increase nor decrease of Slbo activity is tolerated in border cells. Correct protein level is in part ensured by cell type-specific regulated turnover of Slbo protein. Through genetic screening, we identify two genes that are involved in this regulation. The Ubp64 ubiquitin hydrolase acts as a stabilizer of Slbo protein. A novel gene, tribbles, is a negative regulator of slbo in vivo. Tribbles acts by specifically targeting Slbo for rapid degradation via ubiquitination.
2. Command and control: regulatory pathways controlling invasive behavior of the border cells. The invasiveness of cancer cells resembles the normal behavior of cells that migrate into surrounding tissues during development. For example, the border cells in the Drosophila ovary undergo a partial epithelial to mesenchymal transition and invade the neighboring cluster of germline cells, migrating to the oocyte border. Once there, they provide patterning information to the oocyte and produce an eggshell specialization known as the micropyle. Border cell **migration** has been subjected to extensive genetic analyses using a variety of screening approaches. Recent findings demonstrate that conversion of the border cells from a stationary group of epithelial cells to invasive cells requires integration of the activities of at least two transcriptional regulatory pathways. One such pathway requires the slbo gene, which encodes Drosophila C/EBP, a basic region/leucine zipper transcriptional activator that is required for elevated expression of a number of downstream targets, including DE-cadherin and focal adhesion kinase (FAK). An independent pathway requires the activity of the ecdysone receptor and a recently identified co-activator for the ecdysone receptor known as Taiman (abbreviated TAI, pronounced ti-maan', meaning too slow). Ecdysone is produced in the Drosophila ovary in response to adequate nutrition and is required for progression of oogenesis through stage 9, when border cell **migration** occurs. Border cells mutant for tai accumulate abnormally high levels of adhesion complexes at their surfaces, which may account for their inability to migrate. Thus border cell **migration** requires a differentiation program mediated by the C/EBP pathway, which is required for elevated expression of a number of proteins required for motility. In addition, **migration** requires a hormonal signal that relays information regarding nutritional status and appears to be required for regulation of the proper localization of some of the C/EBP targets. These findings suggest that steroid hormones can regulate cell motility relatively directly, independent of the effects on proliferation. This may contribute to the metastatic effects of steroid hormones on certain cancers and the inhibition of metastasis by steroid hormone antagonists such as tamoxifen.
3. A core transcriptional network for early mesoderm development in Drosophila melanogaster. Embryogenesis is controlled by large gene-regulatory networks, which generate spatially and temporally refined patterns of gene expression. Here, we report the characteristics of the regulatory network orchestrating early mesodermal development in the fruitfly Drosophila, where the transcription factor Twist is both necessary and sufficient to drive development. Through the integration of chromatin immunoprecipitation followed by microarray analysis (ChIP-on-chip) experiments during discrete time periods with computational approaches, we identified >2000 Twist-bound cis-regulatory modules (CRMs) and almost 500 direct target genes. Unexpectedly, Twist regulates an almost complete cassette of genes required for cell proliferation in addition to genes essential for morophogenesis and cell **migration**. Twist targets almost 25% of all annotated Drosophila transcription factors, which may represent the entire set of regulators necessary for the early development of this system. By combining in vivo binding data from Twist, Mef2, Tinman, and Dorsal we have constructed an initial transcriptional network of early mesoderm development. The network topology reveals extensive combinatorial binding, feed-forward regulation, and complex logical outputs as prevalent features. In addition to binary activation and repression, we suggest that Twist binds to almost all mesodermal CRMs to provide the competence to integrate inputs from more specialized transcription factors.
4. The kinase domain of Drosophila Tribbles is required for turnover of fly C/EBP during cell **migration**. Drosophila Tribbles (Trbl) encodes the founding member of the Trib family of kinase-like proteins that regulate cell **migration**, proliferation, growth and homeostasis. Trbl was identified in a misexpression screen in the ovary as an antagonist of border cell **migration** and acts in part by directing turnover of the C/EBP protein encoded by the gene slow border cells (slbo). The ability of mammalian Trib isoforms to promote C/EBP turnover during tissue differentiation indicates that this function is highly conserved. To better understand the role of Trbl in cell **migration**, we tested specific Trbl antisera, a trbl null allele and Trbl transgenes bearing site-directed mutations. Trbl is expressed at high levels in the nuclei of follicle cell epithelia and is downregulated in delaminating epithelia as expression of Slbo (C/EBP) is upregulated. This complementary pattern of expression during subsequent cell **migration** is achieved by negative feedback whereby slbo represses Trbl expression and trbl is necessary and sufficient to promote Slbo protein turnover. A series of point mutations that scan the conserved kinase domain of Trbl reveal that the conserved DLK catalytic loop is required for Trbl-Slbo binding and turnover, as well as for interactions between Trbl subunits, suggesting a mechanism of Trbl function.
5. Tribbles, a cell-cycle brake that coordinates proliferation and morphogenesis during Drosophila gastrulation. The final shape and size of an organism is determined by both morphogenetic processes and cell proliferation and it is essential that these processes be properly coordinated. In particular, cell division is incompatible with certain types of morphogenetic cell behaviour, such as **migration**, adhesion and changes in cell shape. Mechanisms must therefore exist to ensure that one does not interfere with the other.We address here the coordination of proliferation and morphogenesis during the development of the mesoderm in Drosophila. We show that it is essential that mitosis be blocked in the mesoderm during early gastrulation, and identify the putative serine/threonine kinase Tribbles as controlling this block. In its absence, the mitotic block is lifted, resulting in severe defects during early gastrulation. Tribbles, a homologue of a group of vertebrate proteins of unknown function, acts in concert with another, as yet unidentified, factor to counteract the activity of the protein phosphatase Cdc25/String.In a finely tuned balance with Cdc25/String, Tribbles controls the timing of mitosis in the prospective mesoderm, allowing cell-shape changes to be completed. This mechanism for coordinating cell division and cell-shape changes may have helped Drosophila to evolve its mode of rapid early development.
6. Drosophila tribbles antagonizes insulin signaling-mediated growth and metabolism via interactions with Akt kinase. Drosophila Tribbles (Trbl) is the founding member of the Trib family of kinase-like docking proteins that modulate cell signaling during proliferation, **migration** and growth. In a wing misexpression screen for Trbl interacting proteins, we identified the Ser/Thr protein kinase Akt1. Given the central role of Akt1 in insulin signaling, we tested the function of Trbl in larval fat body, a tissue where rapid increases in size are exquisitely sensitive to insulin/insulin-like growth factor levels. Consistent with a role in antagonizing insulin-mediated growth, trbl RNAi knockdown in the fat body increased cell size, advanced the timing of pupation and increased levels of circulating triglyceride. Complementarily, overexpression of Trbl reduced fat body cell size, decreased overall larval size, delayed maturation and lowered levels of triglycerides, while circulating glucose levels increased. The conserved Trbl kinase domain is required for function in vivo and for interaction with Akt in a yeast two-hybrid assay. Consistent with direct regulation of Akt, overexpression of Trbl in the fat body decreased levels of activated Akt (pSer505-Akt) while misexpression of trbl RNAi increased phospho-Akt levels, and neither treatment affected total Akt levels. Trbl misexpression effectively suppressed Akt-mediated wing and muscle cell size increases and reduced phosphorylation of the Akt target FoxO (pSer256-FoxO). Taken together, these data show that Drosophila Trbl has a conserved role to bind Akt and block Akt-mediated insulin signaling, and implicate Trib proteins as novel sites of signaling pathway integration that link nutrient availability with cell growth and proliferation.

### [Ii]nvasive

#### PubMed

1. Command and control: regulatory pathways controlling **invasive** behavior of the border cells. The **invasive**ness of cancer cells resembles the normal behavior of cells that migrate into surrounding tissues during development. For example, the border cells in the Drosophila ovary undergo a partial epithelial to mesenchymal transition and invade the neighboring cluster of germline cells, migrating to the oocyte border. Once there, they provide patterning information to the oocyte and produce an eggshell specialization known as the micropyle. Border cell migration has been subjected to extensive genetic analyses using a variety of screening approaches. Recent findings demonstrate that conversion of the border cells from a stationary group of epithelial cells to **invasive** cells requires integration of the activities of at least two transcriptional regulatory pathways. One such pathway requires the slbo gene, which encodes Drosophila C/EBP, a basic region/leucine zipper transcriptional activator that is required for elevated expression of a number of downstream targets, including DE-cadherin and focal adhesion kinase (FAK). An independent pathway requires the activity of the ecdysone receptor and a recently identified co-activator for the ecdysone receptor known as Taiman (abbreviated TAI, pronounced ti-maan', meaning too slow). Ecdysone is produced in the Drosophila ovary in response to adequate nutrition and is required for progression of oogenesis through stage 9, when border cell migration occurs. Border cells mutant for tai accumulate abnormally high levels of adhesion complexes at their surfaces, which may account for their inability to migrate. Thus border cell migration requires a differentiation program mediated by the C/EBP pathway, which is required for elevated expression of a number of proteins required for motility. In addition, migration requires a hormonal signal that relays information regarding nutritional status and appears to be required for regulation of the proper localization of some of the C/EBP targets. These findings suggest that steroid hormones can regulate cell motility relatively directly, independent of the effects on proliferation. This may contribute to the metastatic effects of steroid hormones on certain cancers and the inhibition of metastasis by steroid hormone antagonists such as tamoxifen.

### [Mm]etastasis

#### PubMed

1. Command and control: regulatory pathways controlling invasive behavior of the border cells. The invasiveness of cancer cells resembles the normal behavior of cells that migrate into surrounding tissues during development. For example, the border cells in the Drosophila ovary undergo a partial epithelial to mesenchymal transition and invade the neighboring cluster of germline cells, migrating to the oocyte border. Once there, they provide patterning information to the oocyte and produce an eggshell specialization known as the micropyle. Border cell migration has been subjected to extensive genetic analyses using a variety of screening approaches. Recent findings demonstrate that conversion of the border cells from a stationary group of epithelial cells to invasive cells requires integration of the activities of at least two transcriptional regulatory pathways. One such pathway requires the slbo gene, which encodes Drosophila C/EBP, a basic region/leucine zipper transcriptional activator that is required for elevated expression of a number of downstream targets, including DE-cadherin and focal adhesion kinase (FAK). An independent pathway requires the activity of the ecdysone receptor and a recently identified co-activator for the ecdysone receptor known as Taiman (abbreviated TAI, pronounced ti-maan', meaning too slow). Ecdysone is produced in the Drosophila ovary in response to adequate nutrition and is required for progression of oogenesis through stage 9, when border cell migration occurs. Border cells mutant for tai accumulate abnormally high levels of adhesion complexes at their surfaces, which may account for their inability to migrate. Thus border cell migration requires a differentiation program mediated by the C/EBP pathway, which is required for elevated expression of a number of proteins required for motility. In addition, migration requires a hormonal signal that relays information regarding nutritional status and appears to be required for regulation of the proper localization of some of the C/EBP targets. These findings suggest that steroid hormones can regulate cell motility relatively directly, independent of the effects on proliferation. This may contribute to the metastatic effects of steroid hormones on certain cancers and the inhibition of **metastasis** by steroid hormone antagonists such as tamoxifen.

---

## Hsc70-3

- NCBI Gene
- GeneRIF
- Pubmed

| Search Term | Rank | Fields |
| --- | --- | --- |
| [Mm]igration | 6 | PubMed(1) |

  
hide/show details for Hsc70-3

### [Mm]igration

#### PubMed

1. The histone acetyltransferase Elp3 plays in active role in the control of synaptic bouton expansion and sleep in Drosophila. The histone acetyltransferase Elp3 (Elongator Protein 3) is the catalytic subunit of the highly conserved Elongator complex. Elp3 is essential for the complex functions of Elongator in both the nucleus and cytoplasm of neurons, including the epigenetic control of neuronal motility genes and the acetylation of α-tubulin that affects axonal branching and cortical neuron **migratio**n. Accordingly, misregulation of Elp3 has been implicated in human disorders that specifically affect neuronal function, including familial dysautonomia, a disease characterized by degeneration of the sensory and autonomic nervous system, and the motor neuron degenerative disorder amyotrophic lateral sclerosis. These studies underscore the importance of Elp3 in neurodevelopment and disease, and the need to further characterize the multiple nuclear and cytoplasmic based roles of ELP3 required for neurogenesis in animal models, in vivo. In this report, we investigate the behavioral and morphological consequences that result from targeted reduction of ELP3 specifically in the developing Drosophila nervous system. We demonstrate that loss of Elp3 during neurodevelopment leads to a hyperactive phenotype and sleep loss in the adult flies, a significant expansion in synaptic bouton number and axonal length and branching in the larval neuromuscular junction as well as the misregulation of certain genes known to be involved in these processes. Our results uncover a novel role for Elp3 in the regulation of synaptic bouton expansion during neurogenesis that may be linked with a requirement for sleep.

---

## Drice

- NCBI Gene
- GeneRIF
- Pubmed

| Search Term | Rank | Fields |
| --- | --- | --- |
| [Mm]igration | 6 | PubMed(5) |
| ECM | 7 | PubMed(1) |
| [Ee]xtracellular [Mm]atrix | 8 | PubMed(2) |
| [Ii]nvasive | 10 | PubMed(1) |
| [Mm]etastasis | 12 | PubMed(2) |
| [Cc]ell [Ii]nvasion | 13 | PubMed(1) |

  
hide/show details for Drice

### [Mm]igration

#### PubMed

1. The social lives of migrating cells in Drosophila. Studies of cell **migration** in Drosophila are yielding insights into the complex interactions migrating cells have with each other and with the cells in their environment. Intriguing links between factors that promote cell **migration** and those that control cell survival have been reported recently. For example, migrating germ cells compete with the surrounding somatic tissue for the substrate of the lipid phosphate phosphatases encoded by the genes Wunen and Wunen2. Germ cells take up the dephosphorylated lipid and require it for their survival. In addition, the secreted growth factors called PVFs, previously thought to guide the **migration**s of hemocytes in the embryo, were found to function instead predominantly as survival factors. And in border cells, DIAP1 and Dronc, two proteins known mainly for their ability to regulate cell death, were found to control cell **migration**.
2. The unconventional myosin CRINKLED and its mammalian orthologue MYO7A regulate caspases in their signalling roles. Caspases provide vital links in non-apoptotic regulatory networks controlling inflammation, compensatory proliferation, morphology and cell **migration**. How caspases are activated under non-apoptotic conditions and process a selective set of substrates without killing the cell remain enigmatic. Here we find that the Drosophila unconventional myosin CRINKLED (CK) selectively interacts with the initiator caspase DRONC and regulates some of its non-apoptotic functions. Loss of CK in the arista, border cells or proneural clusters of the wing imaginal discs affects DRONC-dependent patterning. Our data indicate that CK acts as substrate adaptor, recruiting SHAGGY46/GSK3-β to DRONC, thereby facilitating caspase-mediated cleavage and localized modulation of kinase activity. Similarly, the mammalian CK counterpart, MYO7A, binds to and impinges on CASPASE-8, revealing a new regulatory axis affecting receptor interacting protein kinase-1 (RIPK1)>CASPASE-8 signalling. Together, our results expose a conserved role for unconventional myosins in transducing caspase-dependent regulation of kinases, allowing them to take part in specific signalling events.
3. Genes conserved in bilaterians but jointly lost with Myc during nematode evolution are enriched in cell proliferation and cell **migration** functions. Animals use a stereotypical set of developmental genes to build body architectures of varying sizes and organizational complexity. Some genes are critical to developmental patterning, while other genes are important to physiological control of growth. However, growth regulator genes may not be as important in small-bodied "micro-metazoans" such as nematodes. Nematodes use a simplified developmental strategy of lineage-based cell fate specifications to produce an adult bilaterian body composed of a few hundreds of cells. Nematodes also lost the MYC proto-oncogenic regulator of cell proliferation. To identify additional regulators of cell proliferation that were lost with MYC, we computationally screened and determined 839 high-confidence genes that are conserved in bilaterians/lost in nematodes (CIBLIN genes). We find that 30 % of all CIBLIN genes encode transcriptional regulators of cell proliferation, epithelial-to-mesenchyme transitions, and other processes. Over 50 % of CIBLIN genes are unnamed genes in Drosophila, suggesting that there are many understudied genes. Interestingly, CIBLIN genes include many Myc synthetic lethal (MycSL) hits from recent screens. CIBLIN genes include key regulators of heparan sulfate proteoglycan (HSPG) sulfation patterns, and lysyl oxidases involved in cross-linking and modification of the extracellular matrix (ECM). These genes and others suggest the CIBLIN repertoire services critical functions in ECM remodeling and cell **migration** in large-bodied bilaterians. Correspondingly, CIBLIN genes are co-expressed with Myc in cancer transcriptomes, and include a preponderance of known determinants of cancer progression and tumor aggression. We propose that CIBLIN gene research can improve our understanding of regulatory control of cellular growth in metazoans.
4. Distinct roles for two receptor tyrosine kinases in epithelial branching morphogenesis in Drosophila. Branching morphogenesis is a widespread mechanism used to increase the surface area of epithelial organs. Many signaling systems steer development of branched organs, but it is still unclear which cellular processes are regulated by the different pathways. We have used the development of the air sacs of the dorsal thorax of Drosophila to study cellular events and their regulation via cell-cell signaling. We find that two receptor tyrosine kinases play important but distinct roles in air sac outgrowth. Fgf signaling directs cell **migration** at the tip of the structure, while Egf signaling is instrumental for cell division and cell survival in the growing epithelial structure. Interestingly, we find that Fgf signaling requires Ras, the Mapk pathway, and Pointed to direct **migration**, suggesting that both cytoskeletal and nuclear events are downstream of receptor activation. Ras and the Mapk pathway are also needed for Egf-regulated cell division/survival, but Pointed is dispensable.
5. Germ cell-autonomous Wunen2 is required for germline development in Drosophila embryos. In many animals, primordial germ cells (PGCs) migrate through the embryo towards the future gonad, a process guided by attractive and repulsive cues provided from surrounding somatic cells. In Drosophila, the two related lipid phosphate phosphatases (LPPs), Wunen (Wun) and Wun2, are thought to degrade extracellular substrates and to act redundantly in somatic cells to provide a repulsive environment to steer the **migration** of PGCs, or pole cells. Wun and Wun2 also affect the viability of pole cells, because overexpression of either one in somatic cells causes pole cell death. However, the means by which they regulate pole cell **migration** and survival remains elusive. We report that Wun2 has a maternal function required for the survival of pole cells during their **migration** to the gonad. Maternal wun2 RNA was found to be concentrated in pole cells and pole cell-specific expression of wun2 rescued the pole cell death phenotype of the maternal wun2 mutant, suggesting that wun2 activity in pole cells is required for their survival. Furthermore, we obtained genetic evidence that pole cell survival requires a proper balance of LPP activity in pole cells and somatic cells. We propose that Wun2 in pole cells competes with somatic Wun and Wun2 for a common lipid phosphate substrate, which is required by pole cells to produce their survival signal. In somatic cells, Wun and Wun2 may provide a repulsive environment for pole cell **migration** by depleting this extracellular substrate.

### ECM

#### PubMed

1. Genes conserved in bilaterians but jointly lost with Myc during nematode evolution are enriched in cell proliferation and cell migration functions. Animals use a stereotypical set of developmental genes to build body architectures of varying sizes and organizational complexity. Some genes are critical to developmental patterning, while other genes are important to physiological control of growth. However, growth regulator genes may not be as important in small-bodied "micro-metazoans" such as nematodes. Nematodes use a simplified developmental strategy of lineage-based cell fate specifications to produce an adult bilaterian body composed of a few hundreds of cells. Nematodes also lost the MYC proto-oncogenic regulator of cell proliferation. To identify additional regulators of cell proliferation that were lost with MYC, we computationally screened and determined 839 high-confidence genes that are conserved in bilaterians/lost in nematodes (CIBLIN genes). We find that 30 % of all CIBLIN genes encode transcriptional regulators of cell proliferation, epithelial-to-mesenchyme transitions, and other processes. Over 50 % of CIBLIN genes are unnamed genes in Drosophila, suggesting that there are many understudied genes. Interestingly, CIBLIN genes include many Myc synthetic lethal (MycSL) hits from recent screens. CIBLIN genes include key regulators of heparan sulfate proteoglycan (HSPG) sulfation patterns, and lysyl oxidases involved in cross-linking and modification of the extracellular matrix (**ECM**). These genes and others suggest the CIBLIN repertoire services critical functions in **ECM** remodeling and cell migration in large-bodied bilaterians. Correspondingly, CIBLIN genes are co-expressed with Myc in cancer transcriptomes, and include a preponderance of known determinants of cancer progression and tumor aggression. We propose that CIBLIN gene research can improve our understanding of regulatory control of cellular growth in metazoans.

### [Ee]xtracellular [Mm]atrix

#### PubMed

1. Genes conserved in bilaterians but jointly lost with Myc during nematode evolution are enriched in cell proliferation and cell migration functions. Animals use a stereotypical set of developmental genes to build body architectures of varying sizes and organizational complexity. Some genes are critical to developmental patterning, while other genes are important to physiological control of growth. However, growth regulator genes may not be as important in small-bodied "micro-metazoans" such as nematodes. Nematodes use a simplified developmental strategy of lineage-based cell fate specifications to produce an adult bilaterian body composed of a few hundreds of cells. Nematodes also lost the MYC proto-oncogenic regulator of cell proliferation. To identify additional regulators of cell proliferation that were lost with MYC, we computationally screened and determined 839 high-confidence genes that are conserved in bilaterians/lost in nematodes (CIBLIN genes). We find that 30 % of all CIBLIN genes encode transcriptional regulators of cell proliferation, epithelial-to-mesenchyme transitions, and other processes. Over 50 % of CIBLIN genes are unnamed genes in Drosophila, suggesting that there are many understudied genes. Interestingly, CIBLIN genes include many Myc synthetic lethal (MycSL) hits from recent screens. CIBLIN genes include key regulators of heparan sulfate proteoglycan (HSPG) sulfation patterns, and lysyl oxidases involved in cross-linking and modification of the **extracellular matrix** (ECM). These genes and others suggest the CIBLIN repertoire services critical functions in ECM remodeling and cell migration in large-bodied bilaterians. Correspondingly, CIBLIN genes are co-expressed with Myc in cancer transcriptomes, and include a preponderance of known determinants of cancer progression and tumor aggression. We propose that CIBLIN gene research can improve our understanding of regulatory control of cellular growth in metazoans.
2. Non-canonical roles for Yorkie and Drosophila Inhibitor of Apoptosis 1 in epithelial tube size control. Precise control of epithelial tube size is critical for organ function, yet the molecular mechanisms remain poorly understood. Here, we examine the roles of cell growth and a highly conserved organ growth regulatory pathway in controlling the dimensions of the Drosophila tracheal (airway) system, a well-characterized system for investigating epithelial tube morphogenesis. We find that tracheal tube-size is regulated in unexpected ways by the transcription factor Yorkie (Yki, homolog of mammalian YAP and TAZ) and the Salvador/Warts/Hippo (SWH) kinase pathway. Yki activity typically promotes cell division, inhibits apoptosis, and can promote cell growth. However, reducing Yki activity in developing embryos increases rather than decreases the length of the major tracheal tubes, the dorsal trunks (DTs). Similarly, reduction of Hippo pathway activity, which antagonizes Yki, shortens tracheal DTs. yki mutations do not alter DT cell volume or cell number, indicating that Yki and the Hippo pathway regulate cell shape and apical surface area, but not volume. Yki does not appear to act through known tracheal pathways of apical **extracellular matrix**, septate junctions (SJs), basolateral or tubular polarity. Instead, the Hippo pathway and Yki appear to act downstream or in parallel to SJs because a double mutant combination of an upstream Hippo pathway activator, kibra, and the SJ component sinu have the short tracheal phenotype of a kibra mutant. We demonstrate that the critical target of Yki in tube size control is Drosophila Inhibitor of Apoptosis 1 (DIAP1), which in turn antagonizes the Drosophila effector caspase, Ice. Strikingly, there is no change in tracheal cell number in DIAP1 or Ice mutants, thus epithelial tube size regulation defines new non-apoptotic roles for Yki, DIAP1 and Ice.

### [Ii]nvasive

#### PubMed

1. Caspase signalling in the absence of apoptosis drives Jnk-dependent invasion. Tumours evolve several mechanisms to evade apoptosis, yet many resected carcinomas show significantly elevated caspase activity. Moreover, caspase activity is positively correlated with tumour aggression and adverse patient outcome. These observations indicate that caspases might have a functional role in promoting tumour invasion and metastasis. Using a Drosophila model of invasion, we show that precise effector caspase activity drives cell invasion without initiating apoptosis. Affected cells express the matrix metalloprotinase Mmp1 and invade by activating Jnk. Our results link Jnk and effector caspase signalling during the **invasive** process and suggest that tumours under apoptotic stresses from treatment, immune surveillance or intrinsic signals might be induced further along the metastatic cascade.

### [Mm]etastasis

#### PubMed

1. Drosophila models for cancer research. Drosophila is a model system for cancer research. Investigation with fruit flies has facilitated a number of important recent discoveries in the field: the hippo signaling pathway, which coordinates cell proliferation and death to achieve normal tissue size; 'social' behaviors of cells, including cell competition and apoptosis-induced compensatory proliferation, that help ensure normal tissue size; and a growing understanding of how oncogenes and tumor suppressors cooperate to achieve tumor growth and **metastasis** in situ. In the future, Drosophila models can be extended beyond basic research in the search for human therapeutics.
2. Caspase signalling in the absence of apoptosis drives Jnk-dependent invasion. Tumours evolve several mechanisms to evade apoptosis, yet many resected carcinomas show significantly elevated caspase activity. Moreover, caspase activity is positively correlated with tumour aggression and adverse patient outcome. These observations indicate that caspases might have a functional role in promoting tumour invasion and **metastasis**. Using a Drosophila model of invasion, we show that precise effector caspase activity drives cell invasion without initiating apoptosis. Affected cells express the matrix metalloprotinase Mmp1 and invade by activating Jnk. Our results link Jnk and effector caspase signalling during the invasive process and suggest that tumours under apoptotic stresses from treatment, immune surveillance or intrinsic signals might be induced further along the metastatic cascade.

### [Cc]ell [Ii]nvasion

#### PubMed

1. Caspase signalling in the absence of apoptosis drives Jnk-dependent invasion. Tumours evolve several mechanisms to evade apoptosis, yet many resected carcinomas show significantly elevated caspase activity. Moreover, caspase activity is positively correlated with tumour aggression and adverse patient outcome. These observations indicate that caspases might have a functional role in promoting tumour invasion and metastasis. Using a Drosophila model of invasion, we show that precise effector caspase activity drives **cell invasion** without initiating apoptosis. Affected cells express the matrix metalloprotinase Mmp1 and invade by activating Jnk. Our results link Jnk and effector caspase signalling during the invasive process and suggest that tumours under apoptotic stresses from treatment, immune surveillance or intrinsic signals might be induced further along the metastatic cascade.

---

## siz

- NCBI Gene
- GeneRIF
- Pubmed

| Search Term | Rank | Fields |
| --- | --- | --- |
| [Ff]ilopodia | 1 | PubMed(1) |
| [Pp]odosome(s)? | 5 | PubMed(1) |
| [Mm]igration | 6 | PubMed(1) |
| [Ee]xtracellular [Mm]atrix | 8 | PubMed(1) |
| [Ii]nvasive | 10 | PubMed(1) |

  
hide/show details for siz

### [Ff]ilopodia

#### PubMed

1. The Formin Diaphanous Regulates Myoblast Fusion through Actin Polymerization and Arp2/3 Regulation. The formation of multinucleated muscle cells through cell-cell fusion is a conserved process from fruit flies to humans. Numerous studies have shown the importance of Arp2/3, its regulators, and branched actin for the formation of an actin structure, the F-actin focus, at the fusion site. This F-actin focus forms the core of an invasive podosome-like structure that is required for myoblast fusion. In this study, we find that the formin Diaphanous (Dia), which nucleates and facilitates the elongation of actin filaments, is essential for Drosophila myoblast fusion. Following cell recognition and adhesion, Dia is enriched at the myoblast fusion site, concomitant with, and having the same dynamics as, the F-actin focus. Through analysis of Dia loss-of-function conditions using mutant alleles but particularly a dominant negative Dia transgene, we demonstrate that reduction in Dia activity in myoblasts leads to a fusion block. Significantly, no actin focus is detected, and neither branched actin regulators, SCAR or WASp, accumulate at the fusion site when Dia levels are reduced. Expression of constitutively active Dia also causes a fusion block that is associated with an increase in highly dynamic **filopodia**, altered actin turnover rates and F-actin distribution, and mislocalization of SCAR and WASp at the fusion site. Together our data indicate that Dia plays two roles during invasive podosome formation at the fusion site: it dictates the level of linear F-actin polymerization, and it is required for appropriate branched actin polymerization via localization of SCAR and WASp. These studies provide new insight to the mechanisms of cell-cell fusion, the relationship between different regulators of actin polymerization, and invasive podosome formation that occurs in normal development and in disease.

### [Pp]odosome(s)?

#### PubMed

1. The Formin Diaphanous Regulates Myoblast Fusion through Actin Polymerization and Arp2/3 Regulation. The formation of multinucleated muscle cells through cell-cell fusion is a conserved process from fruit flies to humans. Numerous studies have shown the importance of Arp2/3, its regulators, and branched actin for the formation of an actin structure, the F-actin focus, at the fusion site. This F-actin focus forms the core of an invasive **podosome**-like structure that is required for myoblast fusion. In this study, we find that the formin Diaphanous (Dia), which nucleates and facilitates the elongation of actin filaments, is essential for Drosophila myoblast fusion. Following cell recognition and adhesion, Dia is enriched at the myoblast fusion site, concomitant with, and having the same dynamics as, the F-actin focus. Through analysis of Dia loss-of-function conditions using mutant alleles but particularly a dominant negative Dia transgene, we demonstrate that reduction in Dia activity in myoblasts leads to a fusion block. Significantly, no actin focus is detected, and neither branched actin regulators, SCAR or WASp, accumulate at the fusion site when Dia levels are reduced. Expression of constitutively active Dia also causes a fusion block that is associated with an increase in highly dynamic filopodia, altered actin turnover rates and F-actin distribution, and mislocalization of SCAR and WASp at the fusion site. Together our data indicate that Dia plays two roles during invasive **podosome** formation at the fusion site: it dictates the level of linear F-actin polymerization, and it is required for appropriate branched actin polymerization via localization of SCAR and WASp. These studies provide new insight to the mechanisms of cell-cell fusion, the relationship between different regulators of actin polymerization, and invasive **podosome** formation that occurs in normal development and in disease.

### [Mm]igration

#### PubMed

1. Neurons and glia: team players in axon guidance. In the developing nervous system, growth cones follow specific trajectories to reach their target area and ultimately connect with their correct postsynaptic partners. This review focuses on studies in both Drosophila and vertebrates to highlight that mutual interactions between neurons and glia are essential in forming specific neuronal connections. Glia signal to neurons to direct pathfinding and targeting of axons, as well as to stabilize and refine axonal branches within the target area. Equally, neurons provide crucial information to glia, supporting their **migration** and correct positioning.

### [Ee]xtracellular [Mm]atrix

#### PubMed

1. GBF1 (Gartenzwerg)-dependent secretion is required for Drosophila tubulogenesis. Here we report on the generation and in vivo analysis of a series of loss-of-function mutants for the Drosophila ArfGEF, Gartenzwerg. The Drosophila gene gartenzwerg (garz) encodes the orthologue of mammalian GBF1. garz is expressed ubiquitously in embryos with substantially higher abundance in cells forming diverse tubular structures such as salivary glands, trachea, proventriculus or hindgut. In the absence of functional Garz protein, the integrity of the Golgi complex is impaired. As a result, both vesicle transport of cargo proteins and directed apical membrane delivery are severely disrupted. Dysfunction of the Arf1-COPI machinery caused by a loss of Garz leads to perturbations in establishing a polarized epithelial architecture of tubular organs. Furthermore, insufficient apical transport of proteins and other membrane components causes incomplete luminal diameter expansion and deficiencies in **extracellular matrix** assembly. The fact that homologues of Garz are present in every annotated metazoan genome indicates that secretion processes mediated by the GBF-type ArfGEFs play a universal role in animal development.

### [Ii]nvasive

#### PubMed

1. The Formin Diaphanous Regulates Myoblast Fusion through Actin Polymerization and Arp2/3 Regulation. The formation of multinucleated muscle cells through cell-cell fusion is a conserved process from fruit flies to humans. Numerous studies have shown the importance of Arp2/3, its regulators, and branched actin for the formation of an actin structure, the F-actin focus, at the fusion site. This F-actin focus forms the core of an **invasive** podosome-like structure that is required for myoblast fusion. In this study, we find that the formin Diaphanous (Dia), which nucleates and facilitates the elongation of actin filaments, is essential for Drosophila myoblast fusion. Following cell recognition and adhesion, Dia is enriched at the myoblast fusion site, concomitant with, and having the same dynamics as, the F-actin focus. Through analysis of Dia loss-of-function conditions using mutant alleles but particularly a dominant negative Dia transgene, we demonstrate that reduction in Dia activity in myoblasts leads to a fusion block. Significantly, no actin focus is detected, and neither branched actin regulators, SCAR or WASp, accumulate at the fusion site when Dia levels are reduced. Expression of constitutively active Dia also causes a fusion block that is associated with an increase in highly dynamic filopodia, altered actin turnover rates and F-actin distribution, and mislocalization of SCAR and WASp at the fusion site. Together our data indicate that Dia plays two roles during **invasive** podosome formation at the fusion site: it dictates the level of linear F-actin polymerization, and it is required for appropriate branched actin polymerization via localization of SCAR and WASp. These studies provide new insight to the mechanisms of cell-cell fusion, the relationship between different regulators of actin polymerization, and **invasive** podosome formation that occurs in normal development and in disease.

---

## aop

- NCBI Gene
- GeneRIF
- Pubmed

| Search Term | Rank | Fields |
| --- | --- | --- |
| [Ff]ilopodia | 1 | PubMed(1) |
| [Mm]igration | 6 | Process(1); PubMed(16); GeneRIFs(1) |
| ECM | 7 | PubMed(1) |
| [Ee]xtracellular [Mm]atrix | 8 | PubMed(2) |
| [Ii]nvasive | 10 | PubMed(4) |
| [Mm]etastasis | 12 | PubMed(1) |

  
hide/show details for aop

### [Ff]ilopodia

#### PubMed

1. Fascin links Btl/FGFR signalling to the actin cytoskeleton during Drosophila tracheal morphogenesis. A key challenge in normal development and in disease is to elucidate the mechanisms of cell migration. Here we approach this question using the tracheal system of Drosophila as a model. Tracheal cell migration requires the Breathless/FGFR pathway; however, how the pathway induces migration remains poorly understood. We find that the Breathless pathway upregulates singed at the tip of tracheal branches, and that this regulation is functionally relevant. singed encodes Drosophila Fascin, which belongs to a conserved family of actin-bundling proteins involved in cancer progression and metastasis upon misregulation. We show that singed is required for **filopodia** stiffness and proper morphology of tracheal tip cells, defects that correlate with an abnormal actin organisation. We propose that singed-regulated **filopodia** and cell fronts are required for timely and guided branch migration and for terminal branching and branch fusion. We find that singed requirements rely on its actin-bundling activity controlled by phosphorylation, and that active Singed can promote tip cell features. Furthermore, we find that singed acts in concert with forked, another actin cross-linker. The absence of both cross-linkers further stresses the relevance of tip cell morphology and **filopodia** for tracheal development. In summary, our results on the one hand reveal a previously undescribed role for forked in the organisation of transient actin structures such as **filopodia**, and on the other hand identify singed as a new target of Breathless signal, establishing a link between guidance cues, the actin cytoskeleton and tracheal morphogenesis.

### [Mm]igration

#### Process

1. border follicle cell **migration**

#### PubMed

1. The ETS domain transcriptional repressor Anterior open inhibits MAP kinase and Wingless signaling to couple tracheal cell fate with branch identity. Cells at the tips of budding branches in the Drosophila tracheal system generate two morphologically different types of seamless tubes. Terminal cells (TCs) form branched lumenized extensions that mediate gas exchange at target tissues, whereas fusion cells (FCs) form ring-like connections between adjacent tracheal metameres. Each tracheal branch contains a specific set of TCs, FCs, or both, but the mechanisms that select between the two tip cell types in a branch-specific fashion are not clear. Here, we show that the ETS domain transcriptional repressor anterior open (aop) is dispensable for directed tracheal cell **migration**, but plays a key role in tracheal tip cell fate specification. Whereas aop globally inhibits TC and FC specification, MAPK signaling overcomes this inhibition by triggering degradation of Aop in tip cells. Loss of aop function causes excessive FC and TC specification, indicating that without Aop-mediated inhibition, all tracheal cells are competent to adopt a specialized fate. We demonstrate that Aop plays a dual role by inhibiting both MAPK and Wingless signaling, which induce TC and FC fate, respectively. In addition, the branch-specific choice between the two seamless tube types depends on the tracheal branch identity gene spalt major, which is sufficient to inhibit TC specification. Thus, a single repressor, Aop, integrates two different signals to couple tip cell fate selection with branch identity. The switch from a branching towards an anastomosing tip cell type may have evolved with the acquisition of a main tube that connects separate tracheal primordia to generate a tubular network.
2. Function of the ETS transcription factor Yan in border cell **migration**. Invasive cell **migration** in both normal development and metastatic cancer is regulated by various signaling pathways, transcription factors and cell-adhesion molecules. The coordination between these activities in the context of cell **migration** is poorly understood. During Drosophila oogenesis, a small group of cells called border cells exit the follicular epithelium to perform a stereotypic, invasive **migration**. We find that the ETS transcription factor Yan is required for border cell **migration** and that Yan expression is spatiotemporally regulated as border cells migrate from the anterior pole of the egg chamber towards the nurse cell-oocyte boundary. Yan expression is dependent on inputs from the JAK/STAT, Notch and Receptor Tyrosine Kinase pathways in border cells. Mechanistically, Yan functions to modulate the turnover of DE-Cadherin-dependent adhesive complexes to facilitate border cell **migration**. Our results suggest that Yan acts as a pivotal link between signal transduction, cell adhesion and invasive cell **migration** in Drosophila border cells.
3. Signaling by the Drosophila epidermal growth factor receptor pathway during development. In 1997 we wrote a review entitled "A thousand and one roles for the Drosophila epidermal growth factor (EGF) receptor (DER/EGFR)." We are not there yet in terms of the number of developmental roles assigned to this receptor in Drosophila. Nevertheless, DER has certainly emerged as one of the key players in development, since it is used repeatedly to direct cell fate choices, cell division, cell survival, and **migration**. A battery of activating ligands and an inhibitory ligand achieves this versatility. For the ligands that are produced as membrane-bound precursors, trafficking and processing are the key regulatory steps, determining the eventual temporal and spatial pattern of receptor activation. In most cases DER is activated at a short range, in the cells adjacent to the ones producing the active ligand. This activation dictates a binary choice. In some instances DER is also activated over a longer range, and multiple cell fate choices may be induced, according to its level of activation. A battery of negative feedback loops assures the limited range of DER induction. The distinct responses to DER activation in the different tissues depend upon combinatorial interactions with other signaling pathways and tissue-specific factors, at the level of target-gene regulation.
4. [Dorsal closure in Drosophila. A genetic model for wound healing?]. Dorsal closure (DC) is a morphogenetic movement that establishes the dorsal ectoderm of the drosophila embryo. During this process, the two lateral epithelia stretch toward the dorsal midline, the suture line of the two leading edges. Cell **migration** during DC relies both on cell shape change controlled by the activity of the JNK pathway in the leading edge cells and modification of cell adhesiveness, probably dependent upon activation of the Dpp (TGF-beta) pathway. Coupling of the JNK and TGF-beta pathways is essential. The sequence of the cellular and molecular events of DC highlights interesting common features with wound healing in vertebrates. Like DC, wound healing relies on the **migration** of epithelia bordered by leading edges controlling the direction and speed of the movement. This review summarizes recent data concerning the control of epithelial morphogenesis during DC and the bases of wound healing. The molecular and cellular events that underlie these two analogous migratory processes are detailed, discussed and compared. We suggest that DC is a good genetic model for wound healing studying.
5. The social lives of migrating cells in Drosophila. Studies of cell **migration** in Drosophila are yielding insights into the complex interactions migrating cells have with each other and with the cells in their environment. Intriguing links between factors that promote cell **migration** and those that control cell survival have been reported recently. For example, migrating germ cells compete with the surrounding somatic tissue for the substrate of the lipid phosphate phosphatases encoded by the genes Wunen and Wunen2. Germ cells take up the dephosphorylated lipid and require it for their survival. In addition, the secreted growth factors called PVFs, previously thought to guide the **migration**s of hemocytes in the embryo, were found to function instead predominantly as survival factors. And in border cells, DIAP1 and Dronc, two proteins known mainly for their ability to regulate cell death, were found to control cell **migration**.
6. Spalt modifies EGFR-mediated induction of chordotonal precursors in the embryonic PNS of Drosophila promoting the development of oenocytes. Genes of the spalt family encode nuclear zinc finger proteins. In Drosophila melanogaster, they are necessary for the establishment of head/trunk identity, correct tracheal **migration** and patterning of the wing imaginal disc. Spalt proteins display a predominant pattern of expression in the nervous system, not only in Drosophila but also in species of fish, mouse, frog and human, suggesting an evolutionarily conserved role for these proteins in nervous system development. Here we show that Spalt works as a cell fate switch between two EGFR-induced cell types, the oenocytes and the precursors of the pentascolopodial organ in the embryonic peripheral nervous system. We show that removal of spalt increases the number of scolopodia, as a result of extra secondary recruitment of precursor cells at the expense of the oenocytes. In addition, the absence of spalt causes defects in the normal **migration** of the pentascolopodial organ. The dual function of spalt in the development of this organ, recruitment of precursors and **migration**, is reminiscent of its role in tracheal formation and of the role of a spalt homologue, sem-4, in the Caenorhabditis elegans nervous system.
7. Branching morphogenesis of the Drosophila tracheal system. Many organs including the mammalian lung and vascular system consist of branched tubular networks that transport essential gases or fluids, but the genetic programs that control the development of these complex three-dimensional structures are not well understood. The Drosophila melanogaster tracheal (respiratory) system is a network of interconnected epithelial tubes that transports oxygen and other gases in the body and provides a paradigm of branching morphogenesis. It develops by sequential sprouting of primary, secondary, and terminal branches from an epithelial sac of approximately 80 cells in each body segment of the embryo. Mapping of the cell movements and shape changes during the sprouting process has revealed that distinct mechanisms of epithelial **migration** and tube formation are used at each stage of branching. Genetic dissection of the process has identified a general program in which a fibroblast growth factor (FGF) and fibroblast growth factor receptor (FGFR) are used repeatedly to control branch budding and outgrowth. At each stage of branching, the mechanisms controlling FGF expression and the downstream signal transduction pathway change, altering the pattern and structure of the branches that form. During terminal branching, FGF expression is regulated by hypoxia, ensuring that tracheal structure matches cellular oxygen need. A branch diversification program operates in parallel to the general budding program: Regional signals locally modify the general program, conferring specific structural features and other properties on individual branches, such as their substrate outgrowth preferences, differences in tube size and shape, and the ability to fuse to other branches to interconnect the network.
8. The ETS domain transcriptional repressor Anterior open inhibits MAP kinase and Wingless signaling to couple tracheal cell fate with branch identity. Cells at the tips of budding branches in the Drosophila tracheal system generate two morphologically different types of seamless tubes. Terminal cells (TCs) form branched lumenized extensions that mediate gas exchange at target tissues, whereas fusion cells (FCs) form ring-like connections between adjacent tracheal metameres. Each tracheal branch contains a specific set of TCs, FCs, or both, but the mechanisms that select between the two tip cell types in a branch-specific fashion are not clear. Here, we show that the ETS domain transcriptional repressor anterior open (aop) is dispensable for directed tracheal cell **migration**, but plays a key role in tracheal tip cell fate specification. Whereas aop globally inhibits TC and FC specification, MAPK signaling overcomes this inhibition by triggering degradation of Aop in tip cells. Loss of aop function causes excessive FC and TC specification, indicating that without Aop-mediated inhibition, all tracheal cells are competent to adopt a specialized fate. We demonstrate that Aop plays a dual role by inhibiting both MAPK and Wingless signaling, which induce TC and FC fate, respectively. In addition, the branch-specific choice between the two seamless tube types depends on the tracheal branch identity gene spalt major, which is sufficient to inhibit TC specification. Thus, a single repressor, Aop, integrates two different signals to couple tip cell fate selection with branch identity. The switch from a branching towards an anastomosing tip cell type may have evolved with the acquisition of a main tube that connects separate tracheal primordia to generate a tubular network.
9. Signaling pathways directing the movement and fusion of epithelial sheets: lessons from dorsal closure in Drosophila. Wound healing in embryos and various developmental events in metazoans require the spreading and fusion of epithelial sheets. The complex signaling pathways regulating these processes are being pieced together through genetic, cell biological, and biochemical approaches. At present, dorsal closure of the Drosophila embryo is the best-characterized example of epithelial sheet movement. Dorsal closure involves **migration** of the lateral epidermal flanks to close a hole in the dorsal epidermis occupied by an epithelium called the amnioserosa. Detailed genetic studies have revealed a network of interacting signaling molecules regulating this process. At the center of this network is a Jun N-terminal kinase cascade acting at the leading edge of the migrating epidermis that triggers signaling by the TGF-beta superfamily member Decapentaplegic and which interacts with the Wingless pathway. These signaling modules regulate the cytoskeletal reorganization and cell shape change necessary to drive dorsal closure. Activation of this network requires signals from the amnioserosa and input from a variety of proteins at cell-cell junctions. The Rho family of small GTPases is also instrumental, both in activation of signaling and regulation of the cytoskeleton. Many of the proteins regulating dorsal closure have been implicated in epithelial movement in other organisms, and dorsal closure has emerged as an ideal model system for the study of the **migration** and fusion of epithelial sheets.
10. Function of the ETS transcription factor Yan in border cell **migration**. Invasive cell **migration** in both normal development and metastatic cancer is regulated by various signaling pathways, transcription factors and cell-adhesion molecules. The coordination between these activities in the context of cell **migration** is poorly understood. During Drosophila oogenesis, a small group of cells called border cells exit the follicular epithelium to perform a stereotypic, invasive **migration**. We find that the ETS transcription factor Yan is required for border cell **migration** and that Yan expression is spatiotemporally regulated as border cells migrate from the anterior pole of the egg chamber towards the nurse cell-oocyte boundary. Yan expression is dependent on inputs from the JAK/STAT, Notch and Receptor Tyrosine Kinase pathways in border cells. Mechanistically, Yan functions to modulate the turnover of DE-Cadherin-dependent adhesive complexes to facilitate border cell **migration**. Our results suggest that Yan acts as a pivotal link between signal transduction, cell adhesion and invasive cell **migration** in Drosophila border cells.
11. Systematic analysis of the transcriptional switch inducing **migration** of border cells. Cell **migration** within a natural context is tightly controlled, often by specific transcription factors. However, the switch from stationary to migratory behavior is poorly understood. Border cells perform a spatially and temporally controlled invasive **migration** during Drosophila oogenesis. Slbo, a C/EBP family transcriptional activator, is required for them to become migratory. We purified wild-type and slbo mutant border cells as well as nonmigratory follicle cells and performed comparative whole-genome expression profiling, followed by functional tests of the contributions of identified targets to **migration**. About 300 genes were significantly upregulated in border cells, many dependent on Slbo. Among these, the microtubule regulator Stathmin was strongly upregulated and was required for normal **migration**. Actin cytoskeleton regulators were also induced, including, surprisingly, a large cluster of "muscle-specific" genes. We conclude that Slbo induces multiple cytoskeletal effectors, and that each contributes to the behavioral changes in border cells.
12. Genes conserved in bilaterians but jointly lost with Myc during nematode evolution are enriched in cell proliferation and cell **migration** functions. Animals use a stereotypical set of developmental genes to build body architectures of varying sizes and organizational complexity. Some genes are critical to developmental patterning, while other genes are important to physiological control of growth. However, growth regulator genes may not be as important in small-bodied "micro-metazoans" such as nematodes. Nematodes use a simplified developmental strategy of lineage-based cell fate specifications to produce an adult bilaterian body composed of a few hundreds of cells. Nematodes also lost the MYC proto-oncogenic regulator of cell proliferation. To identify additional regulators of cell proliferation that were lost with MYC, we computationally screened and determined 839 high-confidence genes that are conserved in bilaterians/lost in nematodes (CIBLIN genes). We find that 30 % of all CIBLIN genes encode transcriptional regulators of cell proliferation, epithelial-to-mesenchyme transitions, and other processes. Over 50 % of CIBLIN genes are unnamed genes in Drosophila, suggesting that there are many understudied genes. Interestingly, CIBLIN genes include many Myc synthetic lethal (MycSL) hits from recent screens. CIBLIN genes include key regulators of heparan sulfate proteoglycan (HSPG) sulfation patterns, and lysyl oxidases involved in cross-linking and modification of the extracellular matrix (ECM). These genes and others suggest the CIBLIN repertoire services critical functions in ECM remodeling and cell **migration** in large-bodied bilaterians. Correspondingly, CIBLIN genes are co-expressed with Myc in cancer transcriptomes, and include a preponderance of known determinants of cancer progression and tumor aggression. We propose that CIBLIN gene research can improve our understanding of regulatory control of cellular growth in metazoans.
13. Reciprocal interactions between neurons and glia are required for Drosophila peripheral nervous system development. A major developmental role of peripheral glia is to mediate sensory axon guidance; however, it is not known whether sensory neurons influence peripheral glial development. To determine whether glia and neurons reciprocally interact during embryonic development, we ablated each cell type by overexpressing the apoptosis gene, grim, and observed the effects on peripheral nervous system (PNS) development. When neurons are ablated, glial defects occur as a secondary effect, and vice versa. Therefore glia and neurons are codependent during embryogenesis. To further explore glial-neuronal interactions, we genetically disrupted glial **migration** or differentiation and observed the secondary effects on sensory neuron development. Glial **migration** and ensheathment of PNS axons was blocked by overexpression of activated Rho GTPase, a regulator of actin dynamics. Here, sensory axons extended to the CNS without exhibiting gross pathfinding errors. In contrast, disrupting differentiation by expression of dominant-negative Ras GTPase in glia resulted in major sensory axon pathfinding errors, similar to those seen in glial ablations. Glial overexpression of transgenic components of the epidermal growth factor receptor (EGFR) signaling pathway yielded similar sensory neuron defects and also downregulated the expression of the glial marker Neuroglian. Mutant analysis also suggested that the EGFR ligands Spitz and Vein play roles in peripheral glial development. The observations support a model in which glia express genes necessary for sensory neuron development, and these genes are potentially under the control of the EGFR/Ras signaling pathway.
14. A sensitized PiggyBac-based screen for regulators of border cell **migration** in Drosophila. **Migration** of border cells during Drosophila melanogaster oogenesis is a good model system for investigating the genetic requirements for cell **migration** in vivo. We present a sensitized loss-of-function screen used to identify new genes required in border cells for their **migration**. Chromosomes bearing FRTs on all four major autosomal arms were mutagenized by insertions of the transposable element PiggyBac, allowing multiple parallel clonal screens and easy identification of the mutated gene. For border cells, we analyzed homozygous mutant clones positively marked with lacZ and sensitized by expression of dominant-negative PVR, the guidance receptor. We identified new alleles of genes already known to be required for border cell **migration**, including aop/yan, DIAP1, and taiman as well as a conserved Slbo-regulated enhancer downstream of shg/DE-cadherin. Mutations in genes not previously described to be required in border cells were also uncovered: hrp48, vir, rme-8, kismet, and puckered. puckered was unique in that the **migration** defects were observed only when PVR signaling was reduced. We present evidence that an excess of JNK signaling is deleterious for **migration** in the absence of PVR activity at least in part through Fos transcriptional activity and possibly through antagonistic effects on DIAP1.
15. Fascin links Btl/FGFR signalling to the actin cytoskeleton during Drosophila tracheal morphogenesis. A key challenge in normal development and in disease is to elucidate the mechanisms of cell **migration**. Here we approach this question using the tracheal system of Drosophila as a model. Tracheal cell **migration** requires the Breathless/FGFR pathway; however, how the pathway induces **migration** remains poorly understood. We find that the Breathless pathway upregulates singed at the tip of tracheal branches, and that this regulation is functionally relevant. singed encodes Drosophila Fascin, which belongs to a conserved family of actin-bundling proteins involved in cancer progression and metastasis upon misregulation. We show that singed is required for filopodia stiffness and proper morphology of tracheal tip cells, defects that correlate with an abnormal actin organisation. We propose that singed-regulated filopodia and cell fronts are required for timely and guided branch **migration** and for terminal branching and branch fusion. We find that singed requirements rely on its actin-bundling activity controlled by phosphorylation, and that active Singed can promote tip cell features. Furthermore, we find that singed acts in concert with forked, another actin cross-linker. The absence of both cross-linkers further stresses the relevance of tip cell morphology and filopodia for tracheal development. In summary, our results on the one hand reveal a previously undescribed role for forked in the organisation of transient actin structures such as filopodia, and on the other hand identify singed as a new target of Breathless signal, establishing a link between guidance cues, the actin cytoskeleton and tracheal morphogenesis.
16. Systematic gain-of-function genetics in Drosophila. A modular misexpression system was used to carry out systematic gain-of-function genetic screens in Drosophila. The system is based on inducible expression of genes tagged by insertion of a P-element vector carrying a GAL4-regulated promoter oriented to transcribe flanking genomic sequences. To identify genes involved in eye and wing development, the 2300 independent lines were screened for dominant phenotypes. Among many novel genes, the screen identified known genes, including hedgehog and decapentaplegic, implicated in these processes. A genetic interaction screen for suppressors of a cell **migration** defect in a hypomorphic slow border cells mutant identified known genes with likely roles in tyrosine kinase signaling and control of actin cytoskeleton, among many novel genes. These studies demonstrate the ability of the modular misexpression system to identify developmentally important genes and suggest that it will be generally useful for genetic interaction screens.

#### GeneRIFs

1. Yan functions to modulate the turnover of DE-Cadherin-dependent adhesive complexes to facilitate border cell **migration**

### ECM

#### PubMed

1. Genes conserved in bilaterians but jointly lost with Myc during nematode evolution are enriched in cell proliferation and cell migration functions. Animals use a stereotypical set of developmental genes to build body architectures of varying sizes and organizational complexity. Some genes are critical to developmental patterning, while other genes are important to physiological control of growth. However, growth regulator genes may not be as important in small-bodied "micro-metazoans" such as nematodes. Nematodes use a simplified developmental strategy of lineage-based cell fate specifications to produce an adult bilaterian body composed of a few hundreds of cells. Nematodes also lost the MYC proto-oncogenic regulator of cell proliferation. To identify additional regulators of cell proliferation that were lost with MYC, we computationally screened and determined 839 high-confidence genes that are conserved in bilaterians/lost in nematodes (CIBLIN genes). We find that 30 % of all CIBLIN genes encode transcriptional regulators of cell proliferation, epithelial-to-mesenchyme transitions, and other processes. Over 50 % of CIBLIN genes are unnamed genes in Drosophila, suggesting that there are many understudied genes. Interestingly, CIBLIN genes include many Myc synthetic lethal (MycSL) hits from recent screens. CIBLIN genes include key regulators of heparan sulfate proteoglycan (HSPG) sulfation patterns, and lysyl oxidases involved in cross-linking and modification of the extracellular matrix (**ECM**). These genes and others suggest the CIBLIN repertoire services critical functions in **ECM** remodeling and cell migration in large-bodied bilaterians. Correspondingly, CIBLIN genes are co-expressed with Myc in cancer transcriptomes, and include a preponderance of known determinants of cancer progression and tumor aggression. We propose that CIBLIN gene research can improve our understanding of regulatory control of cellular growth in metazoans.

### [Ee]xtracellular [Mm]atrix

#### PubMed

1. Dose-sensitive autosomal modifiers identify candidate genes for tissue autonomous and tissue nonautonomous regulation by the Drosophila nuclear zinc-finger protein, hindsight. The nuclear zinc-finger protein encoded by the hindsight (hnt) locus regulates several cellular processes in Drosophila epithelia, including the Jun N-terminal kinase (JNK) signaling pathway and actin polymerization. Defects in these molecular pathways may underlie the abnormal cellular interactions, loss of epithelial integrity, and apoptosis that occurs in hnt mutants, in turn causing failure of morphogenetic processes such as germ band retraction and dorsal closure in the embryo. To define the genetic pathways regulated by hnt, 124 deficiencies on the second and third chromosomes and 14 duplications on the second chromosome were assayed for dose-sensitive modification of a temperature-sensitive rough eye phenotype caused by the viable allele, hntpeb; 29 interacting regions were identified. Subsequently, 438 P-element-induced lethal mutations mapping to these regions and 12 candidate genes were tested for genetic interaction, leading to identification of 63 dominant modifier loci. A subset of the identified mutants also dominantly modify hnt308-induced embryonic lethality and thus represent general rather than tissue-specific interactors. General interactors include loci encoding transcription factors, actin-binding proteins, signal transduction proteins, and components of the **extracellular matrix**. Expression of several interactors was assessed in hnt mutant tissue. Five genes--apontic (apt), Delta (Dl), decapentaplegic (dpp), karst (kst), and puckered (puc)--are regulated tissue autonomously and, thus, may be direct transcriptional targets of HNT. Three of these genes--apt, Dl, and dpp--are also regulated nonautonomously in adjacent non-HNT-expressing tissues. The expression of several additional interactors--viking (vkg), Cg25, and laminin-alpha (LanA)-is affected only in a nonautonomous manner.
2. Genes conserved in bilaterians but jointly lost with Myc during nematode evolution are enriched in cell proliferation and cell migration functions. Animals use a stereotypical set of developmental genes to build body architectures of varying sizes and organizational complexity. Some genes are critical to developmental patterning, while other genes are important to physiological control of growth. However, growth regulator genes may not be as important in small-bodied "micro-metazoans" such as nematodes. Nematodes use a simplified developmental strategy of lineage-based cell fate specifications to produce an adult bilaterian body composed of a few hundreds of cells. Nematodes also lost the MYC proto-oncogenic regulator of cell proliferation. To identify additional regulators of cell proliferation that were lost with MYC, we computationally screened and determined 839 high-confidence genes that are conserved in bilaterians/lost in nematodes (CIBLIN genes). We find that 30 % of all CIBLIN genes encode transcriptional regulators of cell proliferation, epithelial-to-mesenchyme transitions, and other processes. Over 50 % of CIBLIN genes are unnamed genes in Drosophila, suggesting that there are many understudied genes. Interestingly, CIBLIN genes include many Myc synthetic lethal (MycSL) hits from recent screens. CIBLIN genes include key regulators of heparan sulfate proteoglycan (HSPG) sulfation patterns, and lysyl oxidases involved in cross-linking and modification of the **extracellular matrix** (ECM). These genes and others suggest the CIBLIN repertoire services critical functions in ECM remodeling and cell migration in large-bodied bilaterians. Correspondingly, CIBLIN genes are co-expressed with Myc in cancer transcriptomes, and include a preponderance of known determinants of cancer progression and tumor aggression. We propose that CIBLIN gene research can improve our understanding of regulatory control of cellular growth in metazoans.

### [Ii]nvasive

#### PubMed

1. Function of the ETS transcription factor Yan in border cell migration. **Invasive** cell migration in both normal development and metastatic cancer is regulated by various signaling pathways, transcription factors and cell-adhesion molecules. The coordination between these activities in the context of cell migration is poorly understood. During Drosophila oogenesis, a small group of cells called border cells exit the follicular epithelium to perform a stereotypic, **invasive** migration. We find that the ETS transcription factor Yan is required for border cell migration and that Yan expression is spatiotemporally regulated as border cells migrate from the anterior pole of the egg chamber towards the nurse cell-oocyte boundary. Yan expression is dependent on inputs from the JAK/STAT, Notch and Receptor Tyrosine Kinase pathways in border cells. Mechanistically, Yan functions to modulate the turnover of DE-Cadherin-dependent adhesive complexes to facilitate border cell migration. Our results suggest that Yan acts as a pivotal link between signal transduction, cell adhesion and **invasive** cell migration in Drosophila border cells.
2. Function of the ETS transcription factor Yan in border cell migration. **Invasive** cell migration in both normal development and metastatic cancer is regulated by various signaling pathways, transcription factors and cell-adhesion molecules. The coordination between these activities in the context of cell migration is poorly understood. During Drosophila oogenesis, a small group of cells called border cells exit the follicular epithelium to perform a stereotypic, **invasive** migration. We find that the ETS transcription factor Yan is required for border cell migration and that Yan expression is spatiotemporally regulated as border cells migrate from the anterior pole of the egg chamber towards the nurse cell-oocyte boundary. Yan expression is dependent on inputs from the JAK/STAT, Notch and Receptor Tyrosine Kinase pathways in border cells. Mechanistically, Yan functions to modulate the turnover of DE-Cadherin-dependent adhesive complexes to facilitate border cell migration. Our results suggest that Yan acts as a pivotal link between signal transduction, cell adhesion and **invasive** cell migration in Drosophila border cells.
3. Systematic analysis of the transcriptional switch inducing migration of border cells. Cell migration within a natural context is tightly controlled, often by specific transcription factors. However, the switch from stationary to migratory behavior is poorly understood. Border cells perform a spatially and temporally controlled **invasive** migration during Drosophila oogenesis. Slbo, a C/EBP family transcriptional activator, is required for them to become migratory. We purified wild-type and slbo mutant border cells as well as nonmigratory follicle cells and performed comparative whole-genome expression profiling, followed by functional tests of the contributions of identified targets to migration. About 300 genes were significantly upregulated in border cells, many dependent on Slbo. Among these, the microtubule regulator Stathmin was strongly upregulated and was required for normal migration. Actin cytoskeleton regulators were also induced, including, surprisingly, a large cluster of "muscle-specific" genes. We conclude that Slbo induces multiple cytoskeletal effectors, and that each contributes to the behavioral changes in border cells.
4. The expression of the Drosophila awd gene during normal development and in neoplastic brain tumors caused by lgl mutations. The abnormal wing discs (awd) gene of Drosophila is homologous to the nm23 gene of mammals, a gene whose expression is altered in metastatic tumors. Both awd and nm23 encode nucleoside diphosphate kinases (NDP kinases). We have examined the accumulation of AWD/NDP kinase during normal development by assaying enzyme activity in extracts. There is a nearly constant level of activity throughout larval and pupal development. We have examined the tissue-specific transcription of the awd gene by RNA in situ hybridization and by reporter gene expression. In imaginal discs and brains there is no detectable awd gene expression until the beginning of the third larval instar, despite the constant level of enzyme activity measured in extracts of larvae and pupae. The most intense awd gene expression in imaginal discs and brains occurs after the end of larval development. We have also examined awd gene expression in neoplastic brain tumors caused by mutations in the lethal giant larvae (lgl) gene. In lgl mutant brains, as in normal brains, awd gene expression begins during the third larval instar. No tumors form in brains from lgl-; awd- double mutant larva, so awd gene expression is required for tumor formation and/or proliferation. There is more accumulation of AWD/NDP kinase in lgl- mutant brains than there is in normal brains. Using an awd reporter gene, we show that this is a consequence of an increased proportion of awd gene-expressing cells in mutant brains. Using the same awd reporter gene as a marker of donor cells, we have confirmed the **invasive**ness of lgl-induced neuroblastomas.

### [Mm]etastasis

#### PubMed

1. Fascin links Btl/FGFR signalling to the actin cytoskeleton during Drosophila tracheal morphogenesis. A key challenge in normal development and in disease is to elucidate the mechanisms of cell migration. Here we approach this question using the tracheal system of Drosophila as a model. Tracheal cell migration requires the Breathless/FGFR pathway; however, how the pathway induces migration remains poorly understood. We find that the Breathless pathway upregulates singed at the tip of tracheal branches, and that this regulation is functionally relevant. singed encodes Drosophila Fascin, which belongs to a conserved family of actin-bundling proteins involved in cancer progression and **metastasis** upon misregulation. We show that singed is required for filopodia stiffness and proper morphology of tracheal tip cells, defects that correlate with an abnormal actin organisation. We propose that singed-regulated filopodia and cell fronts are required for timely and guided branch migration and for terminal branching and branch fusion. We find that singed requirements rely on its actin-bundling activity controlled by phosphorylation, and that active Singed can promote tip cell features. Furthermore, we find that singed acts in concert with forked, another actin cross-linker. The absence of both cross-linkers further stresses the relevance of tip cell morphology and filopodia for tracheal development. In summary, our results on the one hand reveal a previously undescribed role for forked in the organisation of transient actin structures such as filopodia, and on the other hand identify singed as a new target of Breathless signal, establishing a link between guidance cues, the actin cytoskeleton and tracheal morphogenesis.

---

## CG10420

- NCBI Gene
- GeneRIF
- Pubmed

| Search Term | Rank | Fields |
| --- | --- | --- |
| [Mm]igration | 6 | PubMed(1) |
| ECM | 7 | PubMed(1) |
| [Ee]xtracellular [Mm]atrix | 8 | PubMed(1) |

  
hide/show details for CG10420

### [Mm]igration

#### PubMed

1. Genes conserved in bilaterians but jointly lost with Myc during nematode evolution are enriched in cell proliferation and cell **migration** functions. Animals use a stereotypical set of developmental genes to build body architectures of varying sizes and organizational complexity. Some genes are critical to developmental patterning, while other genes are important to physiological control of growth. However, growth regulator genes may not be as important in small-bodied "micro-metazoans" such as nematodes. Nematodes use a simplified developmental strategy of lineage-based cell fate specifications to produce an adult bilaterian body composed of a few hundreds of cells. Nematodes also lost the MYC proto-oncogenic regulator of cell proliferation. To identify additional regulators of cell proliferation that were lost with MYC, we computationally screened and determined 839 high-confidence genes that are conserved in bilaterians/lost in nematodes (CIBLIN genes). We find that 30 % of all CIBLIN genes encode transcriptional regulators of cell proliferation, epithelial-to-mesenchyme transitions, and other processes. Over 50 % of CIBLIN genes are unnamed genes in Drosophila, suggesting that there are many understudied genes. Interestingly, CIBLIN genes include many Myc synthetic lethal (MycSL) hits from recent screens. CIBLIN genes include key regulators of heparan sulfate proteoglycan (HSPG) sulfation patterns, and lysyl oxidases involved in cross-linking and modification of the extracellular matrix (ECM). These genes and others suggest the CIBLIN repertoire services critical functions in ECM remodeling and cell **migration** in large-bodied bilaterians. Correspondingly, CIBLIN genes are co-expressed with Myc in cancer transcriptomes, and include a preponderance of known determinants of cancer progression and tumor aggression. We propose that CIBLIN gene research can improve our understanding of regulatory control of cellular growth in metazoans.

### ECM

#### PubMed

1. Genes conserved in bilaterians but jointly lost with Myc during nematode evolution are enriched in cell proliferation and cell migration functions. Animals use a stereotypical set of developmental genes to build body architectures of varying sizes and organizational complexity. Some genes are critical to developmental patterning, while other genes are important to physiological control of growth. However, growth regulator genes may not be as important in small-bodied "micro-metazoans" such as nematodes. Nematodes use a simplified developmental strategy of lineage-based cell fate specifications to produce an adult bilaterian body composed of a few hundreds of cells. Nematodes also lost the MYC proto-oncogenic regulator of cell proliferation. To identify additional regulators of cell proliferation that were lost with MYC, we computationally screened and determined 839 high-confidence genes that are conserved in bilaterians/lost in nematodes (CIBLIN genes). We find that 30 % of all CIBLIN genes encode transcriptional regulators of cell proliferation, epithelial-to-mesenchyme transitions, and other processes. Over 50 % of CIBLIN genes are unnamed genes in Drosophila, suggesting that there are many understudied genes. Interestingly, CIBLIN genes include many Myc synthetic lethal (MycSL) hits from recent screens. CIBLIN genes include key regulators of heparan sulfate proteoglycan (HSPG) sulfation patterns, and lysyl oxidases involved in cross-linking and modification of the extracellular matrix (**ECM**). These genes and others suggest the CIBLIN repertoire services critical functions in **ECM** remodeling and cell migration in large-bodied bilaterians. Correspondingly, CIBLIN genes are co-expressed with Myc in cancer transcriptomes, and include a preponderance of known determinants of cancer progression and tumor aggression. We propose that CIBLIN gene research can improve our understanding of regulatory control of cellular growth in metazoans.

### [Ee]xtracellular [Mm]atrix

#### PubMed

1. Genes conserved in bilaterians but jointly lost with Myc during nematode evolution are enriched in cell proliferation and cell migration functions. Animals use a stereotypical set of developmental genes to build body architectures of varying sizes and organizational complexity. Some genes are critical to developmental patterning, while other genes are important to physiological control of growth. However, growth regulator genes may not be as important in small-bodied "micro-metazoans" such as nematodes. Nematodes use a simplified developmental strategy of lineage-based cell fate specifications to produce an adult bilaterian body composed of a few hundreds of cells. Nematodes also lost the MYC proto-oncogenic regulator of cell proliferation. To identify additional regulators of cell proliferation that were lost with MYC, we computationally screened and determined 839 high-confidence genes that are conserved in bilaterians/lost in nematodes (CIBLIN genes). We find that 30 % of all CIBLIN genes encode transcriptional regulators of cell proliferation, epithelial-to-mesenchyme transitions, and other processes. Over 50 % of CIBLIN genes are unnamed genes in Drosophila, suggesting that there are many understudied genes. Interestingly, CIBLIN genes include many Myc synthetic lethal (MycSL) hits from recent screens. CIBLIN genes include key regulators of heparan sulfate proteoglycan (HSPG) sulfation patterns, and lysyl oxidases involved in cross-linking and modification of the **extracellular matrix** (ECM). These genes and others suggest the CIBLIN repertoire services critical functions in ECM remodeling and cell migration in large-bodied bilaterians. Correspondingly, CIBLIN genes are co-expressed with Myc in cancer transcriptomes, and include a preponderance of known determinants of cancer progression and tumor aggression. We propose that CIBLIN gene research can improve our understanding of regulatory control of cellular growth in metazoans.

---

## Keap1

- NCBI Gene
- GeneRIF
- Pubmed

| Search Term | Rank | Fields |
| --- | --- | --- |
| [Mm]igration | 6 | PubMed(1) |
| ECM | 7 | PubMed(1) |
| [Ee]xtracellular [Mm]atrix | 8 | PubMed(1) |

  
hide/show details for Keap1

### [Mm]igration

#### PubMed

1. Genes conserved in bilaterians but jointly lost with Myc during nematode evolution are enriched in cell proliferation and cell **migration** functions. Animals use a stereotypical set of developmental genes to build body architectures of varying sizes and organizational complexity. Some genes are critical to developmental patterning, while other genes are important to physiological control of growth. However, growth regulator genes may not be as important in small-bodied "micro-metazoans" such as nematodes. Nematodes use a simplified developmental strategy of lineage-based cell fate specifications to produce an adult bilaterian body composed of a few hundreds of cells. Nematodes also lost the MYC proto-oncogenic regulator of cell proliferation. To identify additional regulators of cell proliferation that were lost with MYC, we computationally screened and determined 839 high-confidence genes that are conserved in bilaterians/lost in nematodes (CIBLIN genes). We find that 30 % of all CIBLIN genes encode transcriptional regulators of cell proliferation, epithelial-to-mesenchyme transitions, and other processes. Over 50 % of CIBLIN genes are unnamed genes in Drosophila, suggesting that there are many understudied genes. Interestingly, CIBLIN genes include many Myc synthetic lethal (MycSL) hits from recent screens. CIBLIN genes include key regulators of heparan sulfate proteoglycan (HSPG) sulfation patterns, and lysyl oxidases involved in cross-linking and modification of the extracellular matrix (ECM). These genes and others suggest the CIBLIN repertoire services critical functions in ECM remodeling and cell **migration** in large-bodied bilaterians. Correspondingly, CIBLIN genes are co-expressed with Myc in cancer transcriptomes, and include a preponderance of known determinants of cancer progression and tumor aggression. We propose that CIBLIN gene research can improve our understanding of regulatory control of cellular growth in metazoans.

### ECM

#### PubMed

1. Genes conserved in bilaterians but jointly lost with Myc during nematode evolution are enriched in cell proliferation and cell migration functions. Animals use a stereotypical set of developmental genes to build body architectures of varying sizes and organizational complexity. Some genes are critical to developmental patterning, while other genes are important to physiological control of growth. However, growth regulator genes may not be as important in small-bodied "micro-metazoans" such as nematodes. Nematodes use a simplified developmental strategy of lineage-based cell fate specifications to produce an adult bilaterian body composed of a few hundreds of cells. Nematodes also lost the MYC proto-oncogenic regulator of cell proliferation. To identify additional regulators of cell proliferation that were lost with MYC, we computationally screened and determined 839 high-confidence genes that are conserved in bilaterians/lost in nematodes (CIBLIN genes). We find that 30 % of all CIBLIN genes encode transcriptional regulators of cell proliferation, epithelial-to-mesenchyme transitions, and other processes. Over 50 % of CIBLIN genes are unnamed genes in Drosophila, suggesting that there are many understudied genes. Interestingly, CIBLIN genes include many Myc synthetic lethal (MycSL) hits from recent screens. CIBLIN genes include key regulators of heparan sulfate proteoglycan (HSPG) sulfation patterns, and lysyl oxidases involved in cross-linking and modification of the extracellular matrix (**ECM**). These genes and others suggest the CIBLIN repertoire services critical functions in **ECM** remodeling and cell migration in large-bodied bilaterians. Correspondingly, CIBLIN genes are co-expressed with Myc in cancer transcriptomes, and include a preponderance of known determinants of cancer progression and tumor aggression. We propose that CIBLIN gene research can improve our understanding of regulatory control of cellular growth in metazoans.

### [Ee]xtracellular [Mm]atrix

#### PubMed

1. Genes conserved in bilaterians but jointly lost with Myc during nematode evolution are enriched in cell proliferation and cell migration functions. Animals use a stereotypical set of developmental genes to build body architectures of varying sizes and organizational complexity. Some genes are critical to developmental patterning, while other genes are important to physiological control of growth. However, growth regulator genes may not be as important in small-bodied "micro-metazoans" such as nematodes. Nematodes use a simplified developmental strategy of lineage-based cell fate specifications to produce an adult bilaterian body composed of a few hundreds of cells. Nematodes also lost the MYC proto-oncogenic regulator of cell proliferation. To identify additional regulators of cell proliferation that were lost with MYC, we computationally screened and determined 839 high-confidence genes that are conserved in bilaterians/lost in nematodes (CIBLIN genes). We find that 30 % of all CIBLIN genes encode transcriptional regulators of cell proliferation, epithelial-to-mesenchyme transitions, and other processes. Over 50 % of CIBLIN genes are unnamed genes in Drosophila, suggesting that there are many understudied genes. Interestingly, CIBLIN genes include many Myc synthetic lethal (MycSL) hits from recent screens. CIBLIN genes include key regulators of heparan sulfate proteoglycan (HSPG) sulfation patterns, and lysyl oxidases involved in cross-linking and modification of the **extracellular matrix** (ECM). These genes and others suggest the CIBLIN repertoire services critical functions in ECM remodeling and cell migration in large-bodied bilaterians. Correspondingly, CIBLIN genes are co-expressed with Myc in cancer transcriptomes, and include a preponderance of known determinants of cancer progression and tumor aggression. We propose that CIBLIN gene research can improve our understanding of regulatory control of cellular growth in metazoans.

---

## dome

- NCBI Gene
- GeneRIF
- Pubmed

| Search Term | Rank | Fields |
| --- | --- | --- |
| [Ff]ilopodia | 1 | PubMed(2) |
| [Mm]igration | 6 | Process(2); PubMed(18) |
| ECM | 7 | PubMed(1) |
| [Ee]xtracellular [Mm]atrix | 8 | PubMed(4) |
| [Ii]nvasive | 10 | PubMed(6) |
| [Mm]etastasize | 11 | PubMed(1) |
| [Mm]etastasis | 12 | PubMed(6) |
| [Cc]ell [Ii]nvasion | 13 | PubMed(3) |

  
hide/show details for dome

### [Ff]ilopodia

#### PubMed

1. Gene regulatory networks controlling hematopoietic progenitor niche cell production and differentiation in the Drosophila lymph gland. Hematopoiesis occurs in two phases in Drosophila, with the first completed during embryogenesis and the second accomplished during larval development. The lymph gland serves as the venue for the final hematopoietic program, with this larval tissue well-studied as to its cellular organization and genetic regulation. While the medullary zone contains stem-like hematopoietic progenitors, the posterior signaling center (PSC) functions as a niche microenvironment essential for controlling the decision between progenitor maintenance versus cellular differentiation. In this report, we utilize a PSC-specific GAL4 driver and UAS-gene RNAi strains, to selectively knockdown individual gene functions in PSC cells. We assessed the effect of abrogating the function of 820 genes as to their requirement for niche cell production and differentiation. 100 genes were shown to be essential for normal niche development, with various loci placed into sub-groups based on the functions of their encoded protein products and known genetic interactions. For members of three of these groups, we characterized loss- and gain-of-function phenotypes. Gene function knockdown of members of the BAP chromatin-remodeling complex resulted in niche cells that do not express the hedgehog (hh) gene and fail to differentiate **filopodia** believed important for Hh signaling from the niche to progenitors. Abrogating gene function of various members of the insulin-like growth factor and TOR signaling pathways resulted in anomalous PSC cell production, leading to a defective niche organization. Further analysis of the Pten, TSC1, and TSC2 tumor suppressor genes demonstrated their loss-of-function condition resulted in severely altered blood cell homeostasis, including the abundant production of lamellocytes, specialized hemocytes involved in innate immune responses. Together, this cell-specific RNAi knockdown survey and mutant phenotype analyses identified multiple genes and their regulatory networks required for the normal organization and function of the hematopoietic progenitor niche within the lymph gland.
2. JAK/STAT signalling in Drosophila controls cell motility during germ cell migration. The gonad is formed from two populations of cells originating at different locations: the primordial germ cells (PGCs), giving rise to either sperm or oocytes, and the somatic gonadal mesoderm precursors (SGPs), which support development of the gametes. Following the PGCs' migration during gastrulation, these two populations meet, forming the immature gonad. We present evidence that during embryonic development, the PGCs require the canonical JAK/STAT signalling cascade to migrate efficiently towards the SGPs. Loss of function for any element of the JAK/STAT pathway causes frequent germ cell mislocalisation. We have found that wild-type germ cells produce **filopodia** while they migrate through the mesoderm towards the gonad. Our observations suggest that PGCs use **filopodia** to migrate and to keep contact with each other. Interestingly, activation of the JAK/STAT pathway is required for these **filopodia** to form, and ectopic JAK/STAT activation enhances their formation.

### [Mm]igration

#### Process

1. border follicle cell **migration**
2. regulation of cell **migration**

#### PubMed

1. Awd, the homolog of metastasis suppressor gene Nm23, regulates Drosophila epithelial cell invasion. Border cell **migration** during Drosophila melanogaster oogenesis is a highly pliable model for studying epithelial to mesenchymal transition and directional cell **migration**. The process involves delamination of a group of 6 to 10 follicle cells from the epithelium followed by guided **migration** and invasion through the nurse cell complex toward the oocyte. The guidance cue is mainly provided by the homolog of platelet-derived growth factor/vascular endothelial growth factor family of growth factor, or Pvf, emanating from the oocyte, although Drosophila epidermal growth factor receptor signaling also plays an auxiliary role. Earlier studies implicated a stringent control of the strength of Pvf-mediated signaling since both down-regulation of Pvf and overexpression of active Pvf receptor (Pvr) resulted in stalled border cell **migration**. Here we show that the metastasis suppressor gene homolog Nm23/awd is a negative regulator of border cell **migration**. Its down-regulation allows for optimal spatial signaling from two crucial pathways, Pvr and JAK/STAT. Its overexpression in the border cells results in stalled **migration** and can revert the phenotype of overexpressing constitutive Pvr or dominant-negative dynamin. This is a rare example demonstrating the relevance of a metastasis suppressor gene function utilized in a developmental process involving cell invasion.
2. The Drosophila cytokine receptor Domeless controls border cell **migration** and epithelial polarization during oogenesis. In mammals, the JAK/STAT (Janus Kinase/Signal Transducer and Activator of Transcription) signaling pathway is activated in response to cytokines and growth factors to control blood cell development, proliferation and cell determination. In Drosophila, a conserved JAK/STAT signaling pathway controls segmentation in embryos, as well as blood cell development and other processes in larvae and adults. During embryogenesis, transduction of the Unpaired [Upd; also known as Outstretched (Os)] ligand through the JAK/STAT pathway requires Domeless, a putative membrane protein with distant homology to vertebrate type I cytokine receptors. We have isolated domeless (dome) in a screen to identify genes essential in epithelial morphogenesis during oogenesis. The level of dome activity is critical for proper border cell **migration** and is controlled in part through a negative feedback loop. In addition to its essential role in border cells, we show that dome is required in the germarium for the polarization of follicle cells during encapsulation of germline cells. In this process, dome controls the expression of the apical determinant Crumbs. In contrast to the ligand Upd, whose expression is limited to a pair of polar cells at both ends of the egg chamber, dome is expressed in all germline and follicle cells. However, the Dome protein is specifically localized at apicolateral membranes and undergoes ligand-dependent internalization in the follicle cells. dome mutations interact genetically with JAK/STAT pathway genes in border cell **migration** and abolish the nuclear translocation of Stat92E in vivo. We also show that dome functions downstream of upd and that both the extracellular and intracellular domains of Dome are required for JAK/STAT signaling. Altogether, our data indicate that Dome is an essential receptor molecule for Upd and JAK/STAT signaling during oogenesis.
3. Dynamics of the basement membrane in invasive epithelial clusters in Drosophila. The basement membrane (BM) represents a barrier to cell **migration**, which has to be degraded to promote invasion. However, the role and behaviour of the BM during the development of pre-invasive cells is only poorly understood. Drosophila border cells (BCs) provide an attractive genetic model in which to study the cellular mechanisms underlying the **migration** of mixed cohorts of epithelial cells. BCs are made of two different epithelial cell types appearing sequentially during oogenesis: the polar cells and the outer BCs. Here, we show that the pre-invasive polar cells undergo an unusual and asymmetrical apical capping with major basement membrane proteins, including the two Drosophila Collagen IV alpha chains, Laminin A and Perlecan. Capping of polar cells proceeds through a novel, basal-to-apical transcytosis mechanism that involves the small GTPase Drab5. Apical capping is transient and is followed by rapid shedding prior to the initiation of BC **migration**, suggesting that the apical cap blocks **migration**. Consistently, non-migratory polar cells remain capped. We further show that JAK/STAT signalling and recruitment of outer BCs are required for correct shedding and **migration**. The dynamics of the BM represents a marker of migratory BC, revealing a novel developmentally regulated behaviour of BM coupled to epithelial cell invasiveness.
4. Genes that drive invasion and **migration** in Drosophila. Successful cell **migration** depends on the careful regulation of the timing of movement, the guidance of motile cells, and cytoskeletal and adhesive changes within the cells. This review focuses on genes that act cell-autonomously to promote these aspects of cell **migration** in Drosophila. We discuss recent advances in understanding the **migration** of the ovarian border cells, embryonic blood cells, primordial germ cells, somatic gonadal precursors, and tracheal cells. Comparison of genes that regulate these processes to those that promote tumorigenesis and metastasis in mammals demonstrates that studies in fruit flies are uncovering new genes highly relevant to cancer biology.
5. The social lives of migrating cells in Drosophila. Studies of cell **migration** in Drosophila are yielding insights into the complex interactions migrating cells have with each other and with the cells in their environment. Intriguing links between factors that promote cell **migration** and those that control cell survival have been reported recently. For example, migrating germ cells compete with the surrounding somatic tissue for the substrate of the lipid phosphate phosphatases encoded by the genes Wunen and Wunen2. Germ cells take up the dephosphorylated lipid and require it for their survival. In addition, the secreted growth factors called PVFs, previously thought to guide the **migration**s of hemocytes in the embryo, were found to function instead predominantly as survival factors. And in border cells, DIAP1 and Dronc, two proteins known mainly for their ability to regulate cell death, were found to control cell **migration**.
6. Branching morphogenesis of the Drosophila tracheal system. Many organs including the mammalian lung and vascular system consist of branched tubular networks that transport essential gases or fluids, but the genetic programs that control the development of these complex three-dimensional structures are not well understood. The Drosophila melanogaster tracheal (respiratory) system is a network of interconnected epithelial tubes that transports oxygen and other gases in the body and provides a paradigm of branching morphogenesis. It develops by sequential sprouting of primary, secondary, and terminal branches from an epithelial sac of approximately 80 cells in each body segment of the embryo. Mapping of the cell movements and shape changes during the sprouting process has revealed that distinct mechanisms of epithelial **migration** and tube formation are used at each stage of branching. Genetic dissection of the process has identified a general program in which a fibroblast growth factor (FGF) and fibroblast growth factor receptor (FGFR) are used repeatedly to control branch budding and outgrowth. At each stage of branching, the mechanisms controlling FGF expression and the downstream signal transduction pathway change, altering the pattern and structure of the branches that form. During terminal branching, FGF expression is regulated by hypoxia, ensuring that tracheal structure matches cellular oxygen need. A branch diversification program operates in parallel to the general budding program: Regional signals locally modify the general program, conferring specific structural features and other properties on individual branches, such as their substrate outgrowth preferences, differences in tube size and shape, and the ability to fuse to other branches to interconnect the network.
7. Paracrine signaling through the JAK/STAT pathway activates invasive behavior of ovarian epithelial cells in Drosophila. The JAK/STAT signaling pathway, renowned for its effects on cell proliferation and survival, is constitutively active in various human cancers, including ovarian. We have found that JAK and STAT are required to convert the border cells in the Drosophila ovary from stationary, epithelial cells to migratory, invasive cells. The ligand for this pathway, Unpaired (UPD), is expressed by two central cells within the migratory cell cluster. Mutations in upd or jak cause defects in **migration** and a reduction in the number of cells recruited to the cluster. Ectopic expression of either UPD or JAK is sufficient to induce extra epithelial cells to migrate. Thus, a localized signal activates the JAK/STAT pathway in neighboring epithelial cells, causing them to become invasive.
8. JAK-STAT pathway in Drosophila morphogenesis: From organ selector to cell behavior regulator. One of the main contributions of Drosophila to the JAK-STAT field is the study of morphogenesis. JAK-STAT signaling controls the formation of many different structures through surprisingly different morphogenetic behaviors that include induction of cell rearrangements, invagination, folding of tissues, modulation of cell shape, and **migration**. This variability may be explained by the many transcription factors and signaling molecules STAT regulates at early stages of development. But is STAT just acting as an upstream inducer of morphogenesis or does it have a more direct role in controlling cell behaviors? Here we review what is known about how the canonical phosphorylation of STAT contributes to shaping the embryonic and imaginal structures.
9. Requirement for Par-6 and Bazooka in Drosophila border cell **migration**. Polarized epithelial cells convert into migratory invasive cells during a number of developmental processes, as well as when tumors metastasize. Much has been learned recently concerning the molecules and mechanisms that are responsible for generating and maintaining epithelial cell polarity. However, less is known about what becomes of epithelial polarity proteins when various cell types become migratory and invasive. Here, we report the localization of several apical epithelial proteins, Par-6, Par-3/Bazooka and aPKC, during border cell **migration** in the Drosophila ovary. All of these proteins remained asymmetrically distributed throughout **migration**. Moreover, depletion of either Par-6 or Par-3/Bazooka by RNAi resulted in disorganization of the border cell cluster and impaired **migration**. The distributions of several transmembrane proteins required for **migration** were abnormal following Par-6 or Par-3/Bazooka downregulation, possibly accounting for the **migration** defects. Taken together, these results indicate that cells need not lose apical/basal polarity in order to invade neighboring tissues and in some cases even require such polarity for proper motility.
10. Initiating and guiding **migration**: lessons from border cells. Cell **migration** occurs in many different contexts. Amoebae and other isolated cells migrate in culture. In animals, 'professional' migratory cells of the immune system constantly survey the body for intruders, whereas other cell types perform specific developmentally regulated **migration**s. One simple model for the latter type of event is **migration** of border cells during Drosophila oogenesis. Recent findings have shed light on how border cell fate is induced and on how the **migration** is guided. This article discusses the implications of these studies and compares (invasive) **migration** through a tissue with what is known about cells crawling on a flat substratum.
11. The Drosophila cytokine receptor Domeless controls border cell **migration** and epithelial polarization during oogenesis. In mammals, the JAK/STAT (Janus Kinase/Signal Transducer and Activator of Transcription) signaling pathway is activated in response to cytokines and growth factors to control blood cell development, proliferation and cell determination. In Drosophila, a conserved JAK/STAT signaling pathway controls segmentation in embryos, as well as blood cell development and other processes in larvae and adults. During embryogenesis, transduction of the Unpaired [Upd; also known as Outstretched (Os)] ligand through the JAK/STAT pathway requires Domeless, a putative membrane protein with distant homology to vertebrate type I cytokine receptors. We have isolated domeless (dome) in a screen to identify genes essential in epithelial morphogenesis during oogenesis. The level of dome activity is critical for proper border cell **migration** and is controlled in part through a negative feedback loop. In addition to its essential role in border cells, we show that dome is required in the germarium for the polarization of follicle cells during encapsulation of germline cells. In this process, dome controls the expression of the apical determinant Crumbs. In contrast to the ligand Upd, whose expression is limited to a pair of polar cells at both ends of the egg chamber, dome is expressed in all germline and follicle cells. However, the Dome protein is specifically localized at apicolateral membranes and undergoes ligand-dependent internalization in the follicle cells. dome mutations interact genetically with JAK/STAT pathway genes in border cell **migration** and abolish the nuclear translocation of Stat92E in vivo. We also show that dome functions downstream of upd and that both the extracellular and intracellular domains of Dome are required for JAK/STAT signaling. Altogether, our data indicate that Dome is an essential receptor molecule for Upd and JAK/STAT signaling during oogenesis.
12. Requirement for JAK/STAT signaling throughout border cell **migration** in Drosophila. The evolutionarily conserved JAK/STAT signaling pathway is essential for the proliferation, survival and differentiation of many cells including cancer cells. Recent studies have implicated this transcriptional pathway in the process of cell **migration** in humans, mice, Drosophila and Dictyostelium. In the Drosophila ovary, JAK/STAT signaling is necessary and sufficient for the specification and **migration** of a group of cells called the border cells; however, it is not clear to what extent the requirement for cell fate is distinct from that for cell **migration**. We found that STAT protein is enriched in the migrating border cells throughout their **migration** and is an indicator of cells with highest JAK/STAT activity. In addition, stat(ts) mutants exhibited border cell **migration** defects after just 30 minutes at the non-permissive temperature, prior to any detectable change in the expression of cell fate markers. At later times, cell fate changes became evident, indicating that border cell fate is labile. JAK/STAT signaling was also required for organization of the border cell cluster. Finally, we show that both the accumulation of STAT protein and nuclear accumulation are positively regulated by JAK/STAT activity. The activity of the pathway is negatively regulated by overexpression of a SOCS protein and by blocking endocytosis. Together, our findings suggest that the requirement for STAT in border cells extends beyond the initial specification and delamination of cells from the epithelium.
13. The Jak/STAT pathway in model organisms: emerging roles in cell movement. The JAK/STAT pathway was originally identified in mammals. Studies of this pathway in the mouse have revealed that JAK/STAT signaling plays a central role during hematopoeisis and other developmental processes. The role of JAK/STAT signaling in blood appears to be conserved throughout evolution, as it is also required during fly hematopoeisis. Studies in Dictyostelium, Drosophila, and zebrafish have shown that the JAK/STAT pathway is also required in an unusually broad set of developmental decisions, including cell proliferation, cell fate determination, cell **migration**, planar polarity, convergent extension, and immunity. There is increasing evidence that the versatility of this pathway relies on its cooperation with other signal transduction pathways. In this review, we discuss the components of the JAK/STAT pathway in model organisms and what is known about its requirement in cellular and developmental processes. In particular, we emphasize recent insights into the role that this pathway plays in the control of cell movement.
14. Awd, the homolog of metastasis suppressor gene Nm23, regulates Drosophila epithelial cell invasion. Border cell **migration** during Drosophila melanogaster oogenesis is a highly pliable model for studying epithelial to mesenchymal transition and directional cell **migration**. The process involves delamination of a group of 6 to 10 follicle cells from the epithelium followed by guided **migration** and invasion through the nurse cell complex toward the oocyte. The guidance cue is mainly provided by the homolog of platelet-derived growth factor/vascular endothelial growth factor family of growth factor, or Pvf, emanating from the oocyte, although Drosophila epidermal growth factor receptor signaling also plays an auxiliary role. Earlier studies implicated a stringent control of the strength of Pvf-mediated signaling since both down-regulation of Pvf and overexpression of active Pvf receptor (Pvr) resulted in stalled border cell **migration**. Here we show that the metastasis suppressor gene homolog Nm23/awd is a negative regulator of border cell **migration**. Its down-regulation allows for optimal spatial signaling from two crucial pathways, Pvr and JAK/STAT. Its overexpression in the border cells results in stalled **migration** and can revert the phenotype of overexpressing constitutive Pvr or dominant-negative dynamin. This is a rare example demonstrating the relevance of a metastasis suppressor gene function utilized in a developmental process involving cell invasion.
15. Ectopic expression screen identifies genes affecting Drosophila mesoderm development including the HSPG Trol. Gastrulation of the embryo involves coordinate cell movements likely supported by multiple signaling pathways, adhesion molecules, and extracellular matrix components. Fibroblast growth factors (FGFs) have a major role in Drosophila melanogaster mesoderm **migration**; however, few other inputs are known and the mechanism supporting cell movement is unclear. To provide insight, we performed an ectopic expression screen to identify secreted or membrane-associated molecules that act to support mesoderm **migration**. Twenty-four UAS insertions were identified that cause lethality when expressed in either the mesoderm (Twi-Gal4) or the ectoderm (69B-Gal4). The list was narrowed to a subset of 10 genes that were shown to exhibit loss-of-function mutant phenotypes specifically affecting mesoderm **migration**. These include the FGF ligand Pyramus, α-integrins, E-cadherin, Cueball, EGFR, JAK/STAT signaling components, as well as the heparan sulfate proteoglycan (HSPG) Terribly reduced optic lobes (Trol). Trol encodes the ortholog of mammalian HSPG Perlecan, a demonstrated FGF signaling cofactor. Here, we examine the role of Trol in Drosophila mesoderm **migratio**n and compare and contrast its role with that of Syndecan (Sdc), another HSPG previously implicated in this process. Embryos mutant for Trol or Sdc were obtained and analyzed. Our data support the view that both HSPGs function to support FGF-dependent processes in the early embryo as they share phenotypes with FGF mutants: Trol in terms of effects on mesoderm **migratio**n and caudal visceral mesoderm (CVM) **migratio**n and Sdc in terms of dorsal mesoderm specification. The differential roles uncovered for these two HSPGs suggest that HSPG cofactor choice may modify FGF-signaling outputs.
16. JAK/STAT signalling in Drosophila controls cell motility during germ cell **migration**. The gonad is formed from two populations of cells originating at different locations: the primordial germ cells (PGCs), giving rise to either sperm or oocytes, and the somatic gonadal mesoderm precursors (SGPs), which support development of the gametes. Following the PGCs' **migration** during gastrulation, these two populations meet, forming the immature gonad. We present evidence that during embryonic development, the PGCs require the canonical JAK/STAT signalling cascade to migrate efficiently towards the SGPs. Loss of function for any element of the JAK/STAT pathway causes frequent germ cell mislocalisation. We have found that wild-type germ cells produce filopodia while they migrate through the mesoderm towards the gonad. Our observations suggest that PGCs use filopodia to migrate and to keep contact with each other. Interestingly, activation of the JAK/STAT pathway is required for these filopodia to form, and ectopic JAK/STAT activation enhances their formation.
17. Spinster controls Dpp signaling during glial **migration** in the Drosophila eye. The development of multicellular organisms requires the well balanced and coordinated **migration** of many cell types. This is of particular importance within the developing nervous system, where glial cells often move long distances to reach their targets. The majority of glial cells in the peripheral nervous system of the Drosophila embryo is derived from the CNS and migrates along motor axons toward their targets. In the developing Drosophila eye, CNS-derived glial cells move outward toward the nascent photoreceptor cells, but the molecular mechanisms coupling the **migration** of glial cells with the growth of the eye imaginal disc are mostly unknown. Here, we used an enhancer trap approach to identify the gene spinster, which encodes a multipass transmembrane protein involved in endosome-lysosome trafficking, as being expressed in many glial cells. spinster mutants are characterized by glial over**migration**. Genetic experiments demonstrate that Spinster modulates the activity of several signaling cascades. Within the migrating perineurial glial cells, Spinster is required to downregulate Dpp (Decapentaplegic) signaling activity, which ceases migratory abilities. In addition, Spinster affects the growth of the carpet cell, which indirectly modulates glial **migration**.
18. Requirements of Lgl in cell differentiation and motility during Drosophila ovarian follicular epithelium morphogenesis. The epithelial follicle cell layer over the egg chamber in Drosophila ovary undergoes patterning and morphogenesis at oogenesis. These developmental processes are essential for constructing the eggshell and establishing the body axes of the egg and resultant embryo, thereby being crucial for the egg development. We have previously shown that lethal(2)giant larvae (lgl), a Drosophila neoplastic tumor suppressor gene (nTSG) is required for the posterior follicle cell (PFC) fate induction during antero-posterior pattern formation of the follicular epithelium. In this report, we further characterize lgl in this epithelium patterning and the morphogenetic changes of specified border cells. Genetic interactions of lgl with discs large (dlg) and scribble (scrib), another two nTSGs in specifying the PFC fate reveal a cooperative role of this group of genes. Meanwhile, we find that loss of lgl function causes failure of follicle cells at the anterior to differentiate properly. The clonal analysis further indicates that lgl is necessary not only for the border cell differentiation, but also for control of the collective border cell **migration** via presumably modulating the apico-basal polarity and cell adhesion. Overall, we identify Lgl as an essential factor in regulating differentiation and morphogenetic movement of the ovarian epithelial follicle cells.

### ECM

#### PubMed

1. Differential activities of the Drosophila JAK/STAT pathway ligands Upd, Upd2 and Upd3. JAK/STAT signalling in vertebrates is activated by multiple cytokines and growth factors. By contrast, the Drosophila genome encodes for only three related JAK/STAT ligands, Upd, Upd2 and Upd3. Identifying the differences between these three ligands will ultimately lead to a greater understanding of this disease-related signalling pathway and its roles in development. Here, we describe the analysis of the least well characterised of the Upd-like ligands, Upd3. We show that in tissue culture-based assays Upd3-GFP is secreted from cells and appears to interact with the extracellular matrix (**ECM**) in a similar manner to Upd, while still non-autonomously activating JAK/STAT signalling. Quantification of each of the Upd-like ligands in conditioned media has allowed us to determine the activity of equal amounts of each ligand on JAK/STAT ex vivo and reveals that Upd is the most potent ligand in this system. Finally, investigations into the effects of ectopic expression of Upd3 in vivo have confirmed its ability to activate pathway signalling at long-distance.

### [Ee]xtracellular [Mm]atrix

#### PubMed

1. Tissue design: how Drosophila tumors remodel their neighborhood. Drosophila genetics has long been appreciated as a powerful approach for discovering the normal functions of genes that act as oncogenes and tumor suppressors in human cancer. Recent studies have also highlighted its advantages for deciphering how such genes function during tumorigenesis itself. Here we detail studies relating to how tumors, generated in developing organs and adult stem cell-based tissues, remodel the tissue landscape to their benefit. Like mammalian tumors, insect tumors can dissolve **extracellular matrix**, recruit blood cells, migrate and invade other tissues. While much is known about how mammalian fibroblasts, immune cells and vasculature promote late tumorigenesis, less is understood about the very earliest stages of tumor development in mammals. Because Drosophila has fewer mitotic cells and a simpler tissue architecture, it affords easy detection and analysis of early clonal tumor growth. Drosophila studies have revealed both cooperative and competitive interactions between tumor and normal cells during early tumor growth. During development, these interactions typically occur with other proliferative progenitor cells, but in adult stem cell-based tissues, the stem cell niche can fuel tumor growth.
2. Differential activities of the Drosophila JAK/STAT pathway ligands Upd, Upd2 and Upd3. JAK/STAT signalling in vertebrates is activated by multiple cytokines and growth factors. By contrast, the Drosophila genome encodes for only three related JAK/STAT ligands, Upd, Upd2 and Upd3. Identifying the differences between these three ligands will ultimately lead to a greater understanding of this disease-related signalling pathway and its roles in development. Here, we describe the analysis of the least well characterised of the Upd-like ligands, Upd3. We show that in tissue culture-based assays Upd3-GFP is secreted from cells and appears to interact with the **extracellular matrix** (ECM) in a similar manner to Upd, while still non-autonomously activating JAK/STAT signalling. Quantification of each of the Upd-like ligands in conditioned media has allowed us to determine the activity of equal amounts of each ligand on JAK/STAT ex vivo and reveals that Upd is the most potent ligand in this system. Finally, investigations into the effects of ectopic expression of Upd3 in vivo have confirmed its ability to activate pathway signalling at long-distance.
3. Ectopic expression screen identifies genes affecting Drosophila mesoderm development including the HSPG Trol. Gastrulation of the embryo involves coordinate cell movements likely supported by multiple signaling pathways, adhesion molecules, and **extracellular matrix** components. Fibroblast growth factors (FGFs) have a major role in Drosophila melanogaster mesoderm migration; however, few other inputs are known and the mechanism supporting cell movement is unclear. To provide insight, we performed an ectopic expression screen to identify secreted or membrane-associated molecules that act to support mesoderm migration. Twenty-four UAS insertions were identified that cause lethality when expressed in either the mesoderm (Twi-Gal4) or the ectoderm (69B-Gal4). The list was narrowed to a subset of 10 genes that were shown to exhibit loss-of-function mutant phenotypes specifically affecting mesoderm migration. These include the FGF ligand Pyramus, α-integrins, E-cadherin, Cueball, EGFR, JAK/STAT signaling components, as well as the heparan sulfate proteoglycan (HSPG) Terribly reduced optic lobes (Trol). Trol encodes the ortholog of mammalian HSPG Perlecan, a demonstrated FGF signaling cofactor. Here, we examine the role of Trol in Drosophila mesoderm migration and compare and contrast its role with that of Syndecan (Sdc), another HSPG previously implicated in this process. Embryos mutant for Trol or Sdc were obtained and analyzed. Our data support the view that both HSPGs function to support FGF-dependent processes in the early embryo as they share phenotypes with FGF mutants: Trol in terms of effects on mesoderm migration and caudal visceral mesoderm (CVM) migration and Sdc in terms of dorsal mesoderm specification. The differential roles uncovered for these two HSPGs suggest that HSPG cofactor choice may modify FGF-signaling outputs.
4. Characterisation of Upd2, a Drosophila JAK/STAT pathway ligand. The characterisation of ligands that activate the JAK/STAT pathway has the potential to throw light onto a comparatively poorly understood aspect of this important signal transduction cascade. Here, we describe our analysis of the only invertebrate JAK/STAT pathway ligands identified to date, the Drosophila unpaired-like family. We show that upd2 is expressed in a pattern essentially identical to that of upd and demonstrate that the proteins encoded by this region activate JAK/STAT pathway signalling. Mutational analysis demonstrates a mutual semi-redundancy that can be visualised in multiple tissues known to require JAK/STAT signalling. In order to better characterise the in vivo function of these ligands, we developed a reporter based on a natural JAK/STAT pathway responsive enhancer and show that ectopic upd2 expression can effectively activate the JAK/STAT pathway. While both Upd and Upd2 are secreted JAK/STAT pathway agonists, tissue culture assays show that the signal-sequences of Upd and Upd2 confer distinct properties, with Upd associated primarily with the **extracellular matrix** and Upd2 secreted into the media. The differing biophysical characteristics identified for Upd-like molecules have implications for their function in vivo and adds another aspect to our understanding of cytokine signalling in Drosophila.

### [Ii]nvasive

#### PubMed

1. Dynamics of the basement membrane in **invasive** epithelial clusters in Drosophila. The basement membrane (BM) represents a barrier to cell migration, which has to be degraded to promote invasion. However, the role and behaviour of the BM during the development of pre-**invasive** cells is only poorly understood. Drosophila border cells (BCs) provide an attractive genetic model in which to study the cellular mechanisms underlying the migration of mixed cohorts of epithelial cells. BCs are made of two different epithelial cell types appearing sequentially during oogenesis: the polar cells and the outer BCs. Here, we show that the pre-**invasive** polar cells undergo an unusual and asymmetrical apical capping with major basement membrane proteins, including the two Drosophila Collagen IV alpha chains, Laminin A and Perlecan. Capping of polar cells proceeds through a novel, basal-to-apical transcytosis mechanism that involves the small GTPase Drab5. Apical capping is transient and is followed by rapid shedding prior to the initiation of BC migration, suggesting that the apical cap blocks migration. Consistently, non-migratory polar cells remain capped. We further show that JAK/STAT signalling and recruitment of outer BCs are required for correct shedding and migration. The dynamics of the BM represents a marker of migratory BC, revealing a novel developmentally regulated behaviour of BM coupled to epithelial cell **invasive**ness.
2. **Invasive** and indigenous microbiota impact intestinal stem cell activity through multiple pathways in Drosophila. Gut homeostasis is controlled by both immune and developmental mechanisms, and its disruption can lead to inflammatory disorders or cancerous lesions of the intestine. While the impact of bacteria on the mucosal immune system is beginning to be precisely understood, little is known about the effects of bacteria on gut epithelium renewal. Here, we addressed how both infectious and indigenous bacteria modulate stem cell activity in Drosophila. We show that the increased epithelium renewal observed upon some bacterial infections is a consequence of the oxidative burst, a major defense of the Drosophila gut. Additionally, we provide evidence that the JAK-STAT (Janus kinase-signal transducers and activators of transcription) and JNK (c-Jun NH(2) terminal kinase) pathways are both required for bacteria-induced stem cell proliferation. Similarly, we demonstrate that indigenous gut microbiota activate the same, albeit reduced, program at basal levels. Altered control of gut microbiota in immune-deficient or aged flies correlates with increased epithelium renewal. Finally, we show that epithelium renewal is an essential component of Drosophila defense against oral bacterial infection. Altogether, these results indicate that gut homeostasis is achieved by a complex interregulation of the immune response, gut microbiota, and stem cell activity.
3. Paracrine signaling through the JAK/STAT pathway activates **invasive** behavior of ovarian epithelial cells in Drosophila. The JAK/STAT signaling pathway, renowned for its effects on cell proliferation and survival, is constitutively active in various human cancers, including ovarian. We have found that JAK and STAT are required to convert the border cells in the Drosophila ovary from stationary, epithelial cells to migratory, **invasive** cells. The ligand for this pathway, Unpaired (UPD), is expressed by two central cells within the migratory cell cluster. Mutations in upd or jak cause defects in migration and a reduction in the number of cells recruited to the cluster. Ectopic expression of either UPD or JAK is sufficient to induce extra epithelial cells to migrate. Thus, a localized signal activates the JAK/STAT pathway in neighboring epithelial cells, causing them to become **invasive**.
4. Requirement for Par-6 and Bazooka in Drosophila border cell migration. Polarized epithelial cells convert into migratory **invasive** cells during a number of developmental processes, as well as when tumors metastasize. Much has been learned recently concerning the molecules and mechanisms that are responsible for generating and maintaining epithelial cell polarity. However, less is known about what becomes of epithelial polarity proteins when various cell types become migratory and **invasive**. Here, we report the localization of several apical epithelial proteins, Par-6, Par-3/Bazooka and aPKC, during border cell migration in the Drosophila ovary. All of these proteins remained asymmetrically distributed throughout migration. Moreover, depletion of either Par-6 or Par-3/Bazooka by RNAi resulted in disorganization of the border cell cluster and impaired migration. The distributions of several transmembrane proteins required for migration were abnormal following Par-6 or Par-3/Bazooka downregulation, possibly accounting for the migration defects. Taken together, these results indicate that cells need not lose apical/basal polarity in order to invade neighboring tissues and in some cases even require such polarity for proper motility.
5. Initiating and guiding migration: lessons from border cells. Cell migration occurs in many different contexts. Amoebae and other isolated cells migrate in culture. In animals, 'professional' migratory cells of the immune system constantly survey the body for intruders, whereas other cell types perform specific developmentally regulated migrations. One simple model for the latter type of event is migration of border cells during Drosophila oogenesis. Recent findings have shed light on how border cell fate is induced and on how the migration is guided. This article discusses the implications of these studies and compares (**invasive**) migration through a tissue with what is known about cells crawling on a flat substratum.
6. Sin3a acts through a multi-gene module to regulate invasion in Drosophila and human tumors. Chromatin remodeling proteins regulate multiple aspects of cell homeostasis, making them ideal candidates for misregulation in transformed cells. Here, we explore Sin3A, a member of the Sin3 family of proteins linked to tumorigenesis that are thought to regulate gene expression through their role as histone deacetylases (HDACs). We identified Drosophila Sin3a as an important mediator of oncogenic Ret receptor in a fly model of Multiple Endocrine Neoplasia Type 2. Reducing Drosophila Sin3a activity led to metastasis-like behavior and, in the presence of Diap1, secondary tumors distant from the site of origin. Genetic and Chip-Seq analyses identified previously undescribed Sin3a targets including genes involved in cell motility and actin dynamics, as well as signaling pathways including Src, Jnk and Rho. A key Sin3a oncogenic target, PP1B, regulates stability of β-Catenin/Armadillo: the outcome is to oppose T-cell factor (TCF) function and Wg/Wnt pathway signaling in both fly and mammalian cancer cells. Reducing Sin3A strongly increased the **invasiv**e behavior of A549 human lung adenocarcinoma cells. We show that Sin3A is downregulated in a variety of human tumors and that Src, JNK, RhoA and PP1B/β-Catenin are regulated in a manner analogous to our Drosophila models. Our data suggest that Sin3A influences a specific step of tumorigenesis by regulating a module of genes involved in cell invasion. Tumor progression may commonly rely on such 'modules of invasion' under the control of broad transcriptional regulators.

### [Mm]etastasize

#### PubMed

1. Requirement for Par-6 and Bazooka in Drosophila border cell migration. Polarized epithelial cells convert into migratory invasive cells during a number of developmental processes, as well as when tumors **metastasize**. Much has been learned recently concerning the molecules and mechanisms that are responsible for generating and maintaining epithelial cell polarity. However, less is known about what becomes of epithelial polarity proteins when various cell types become migratory and invasive. Here, we report the localization of several apical epithelial proteins, Par-6, Par-3/Bazooka and aPKC, during border cell migration in the Drosophila ovary. All of these proteins remained asymmetrically distributed throughout migration. Moreover, depletion of either Par-6 or Par-3/Bazooka by RNAi resulted in disorganization of the border cell cluster and impaired migration. The distributions of several transmembrane proteins required for migration were abnormal following Par-6 or Par-3/Bazooka downregulation, possibly accounting for the migration defects. Taken together, these results indicate that cells need not lose apical/basal polarity in order to invade neighboring tissues and in some cases even require such polarity for proper motility.

### [Mm]etastasis

#### PubMed

1. Awd, the homolog of **metastasis** suppressor gene Nm23, regulates Drosophila epithelial cell invasion. Border cell migration during Drosophila melanogaster oogenesis is a highly pliable model for studying epithelial to mesenchymal transition and directional cell migration. The process involves delamination of a group of 6 to 10 follicle cells from the epithelium followed by guided migration and invasion through the nurse cell complex toward the oocyte. The guidance cue is mainly provided by the homolog of platelet-derived growth factor/vascular endothelial growth factor family of growth factor, or Pvf, emanating from the oocyte, although Drosophila epidermal growth factor receptor signaling also plays an auxiliary role. Earlier studies implicated a stringent control of the strength of Pvf-mediated signaling since both down-regulation of Pvf and overexpression of active Pvf receptor (Pvr) resulted in stalled border cell migration. Here we show that the **metastasis** suppressor gene homolog Nm23/awd is a negative regulator of border cell migration. Its down-regulation allows for optimal spatial signaling from two crucial pathways, Pvr and JAK/STAT. Its overexpression in the border cells results in stalled migration and can revert the phenotype of overexpressing constitutive Pvr or dominant-negative dynamin. This is a rare example demonstrating the relevance of a **metastasis** suppressor gene function utilized in a developmental process involving cell invasion.
2. Genes that drive invasion and migration in Drosophila. Successful cell migration depends on the careful regulation of the timing of movement, the guidance of motile cells, and cytoskeletal and adhesive changes within the cells. This review focuses on genes that act cell-autonomously to promote these aspects of cell migration in Drosophila. We discuss recent advances in understanding the migration of the ovarian border cells, embryonic blood cells, primordial germ cells, somatic gonadal precursors, and tracheal cells. Comparison of genes that regulate these processes to those that promote tumorigenesis and **metastasis** in mammals demonstrates that studies in fruit flies are uncovering new genes highly relevant to cancer biology.
3. Oncogenic cooperation between SOCS family proteins and EGFR identified using a Drosophila epithelial transformation model. MicroRNAs (miRNAs) are emerging as cooperating factors that promote the activity of oncogenes in tumor formation and disease progression. This poses the challenge of identifying the miRNA targets responsible for these interactions. In this study, we identify the growth regulatory miRNA bantam and its target, Socs36E, as cooperating factors in EGFR-driven tumorigenesis and **metastasis** in a Drosophila model of epithelial transformation. bantam promotes growth by limiting expression of Socs36E, which functions as a negative growth regulator. Socs36E has only a modest effect on growth on its own, but behaves as a tumor suppressor in combination with EGFR activation. The human ortholog of SOCS36E, SOCS5, behaves as a candidate tumor suppressor in cellular transformation in cooperation with EGFR/RAS pathway activation.
4. Sin3a acts through a multi-gene module to regulate invasion in Drosophila and human tumors. Chromatin remodeling proteins regulate multiple aspects of cell homeostasis, making them ideal candidates for misregulation in transformed cells. Here, we explore Sin3A, a member of the Sin3 family of proteins linked to tumorigenesis that are thought to regulate gene expression through their role as histone deacetylases (HDACs). We identified Drosophila Sin3a as an important mediator of oncogenic Ret receptor in a fly model of Multiple Endocrine Neoplasia Type 2. Reducing Drosophila Sin3a activity led to **metastasis**-like behavior and, in the presence of Diap1, secondary tumors distant from the site of origin. Genetic and Chip-Seq analyses identified previously undescribed Sin3a targets including genes involved in cell motility and actin dynamics, as well as signaling pathways including Src, Jnk and Rho. A key Sin3a oncogenic target, PP1B, regulates stability of β-Catenin/Armadillo: the outcome is to oppose T-cell factor (TCF) function and Wg/Wnt pathway signaling in both fly and mammalian cancer cells. Reducing Sin3A strongly increased the invasive behavior of A549 human lung adenocarcinoma cells. We show that Sin3A is downregulated in a variety of human tumors and that Src, JNK, RhoA and PP1B/β-Catenin are regulated in a manner analogous to our Drosophila models. Our data suggest that Sin3A influences a specific step of tumorigenesis by regulating a module of genes involved in cell invasion. Tumor progression may commonly rely on such 'modules of invasion' under the control of broad transcriptional regulators.
5. Drosophila as a model to study the role of blood cells in inflammation, innate immunity and cancer. Drosophila has a primitive yet effective blood system with three types of haemocytes which function throughout different developmental stages and environmental stimuli. Haemocytes play essential roles in tissue modeling during embryogenesis and morphogenesis, and also in innate immunity. The open circulatory system of Drosophila makes haemocytes ideal signal mediators to cells and tissues in response to events such as infection and wounding. The application of recently developed and sophisticated genetic tools to the relatively simple genome of Drosophila has made the fly a popular system for modeling human tumorigensis and **metastasis**. Drosophila is now used for screening and investigation of genes implicated in human leukemia and also in modeling development of solid tumors. This second line of research offers promising opportunities to determine the seemingly conflicting roles of blood cells in tumor progression and invasion. This review provides an overview of the signaling pathways conserved in Drosophila during haematopoiesis, haemostasis, innate immunity, wound healing and inflammation. We also review the most recent progress in the use of Drosophila as a cancer research model with an emphasis on the roles haemocytes can play in various cancer models and in the links between inflammation and cancer.
6. Awd, the homolog of **metastasis** suppressor gene Nm23, regulates Drosophila epithelial cell invasion. Border cell migration during Drosophila melanogaster oogenesis is a highly pliable model for studying epithelial to mesenchymal transition and directional cell migration. The process involves delamination of a group of 6 to 10 follicle cells from the epithelium followed by guided migration and invasion through the nurse cell complex toward the oocyte. The guidance cue is mainly provided by the homolog of platelet-derived growth factor/vascular endothelial growth factor family of growth factor, or Pvf, emanating from the oocyte, although Drosophila epidermal growth factor receptor signaling also plays an auxiliary role. Earlier studies implicated a stringent control of the strength of Pvf-mediated signaling since both down-regulation of Pvf and overexpression of active Pvf receptor (Pvr) resulted in stalled border cell migration. Here we show that the **metastasis** suppressor gene homolog Nm23/awd is a negative regulator of border cell migration. Its down-regulation allows for optimal spatial signaling from two crucial pathways, Pvr and JAK/STAT. Its overexpression in the border cells results in stalled migration and can revert the phenotype of overexpressing constitutive Pvr or dominant-negative dynamin. This is a rare example demonstrating the relevance of a **metastasis** suppressor gene function utilized in a developmental process involving cell invasion.

### [Cc]ell [Ii]nvasion

#### PubMed

1. Awd, the homolog of metastasis suppressor gene Nm23, regulates Drosophila epithelial **cell invasion**. Border cell migration during Drosophila melanogaster oogenesis is a highly pliable model for studying epithelial to mesenchymal transition and directional cell migration. The process involves delamination of a group of 6 to 10 follicle cells from the epithelium followed by guided migration and invasion through the nurse cell complex toward the oocyte. The guidance cue is mainly provided by the homolog of platelet-derived growth factor/vascular endothelial growth factor family of growth factor, or Pvf, emanating from the oocyte, although Drosophila epidermal growth factor receptor signaling also plays an auxiliary role. Earlier studies implicated a stringent control of the strength of Pvf-mediated signaling since both down-regulation of Pvf and overexpression of active Pvf receptor (Pvr) resulted in stalled border cell migration. Here we show that the metastasis suppressor gene homolog Nm23/awd is a negative regulator of border cell migration. Its down-regulation allows for optimal spatial signaling from two crucial pathways, Pvr and JAK/STAT. Its overexpression in the border cells results in stalled migration and can revert the phenotype of overexpressing constitutive Pvr or dominant-negative dynamin. This is a rare example demonstrating the relevance of a metastasis suppressor gene function utilized in a developmental process involving **cell invasion**.
2. Sin3a acts through a multi-gene module to regulate invasion in Drosophila and human tumors. Chromatin remodeling proteins regulate multiple aspects of cell homeostasis, making them ideal candidates for misregulation in transformed cells. Here, we explore Sin3A, a member of the Sin3 family of proteins linked to tumorigenesis that are thought to regulate gene expression through their role as histone deacetylases (HDACs). We identified Drosophila Sin3a as an important mediator of oncogenic Ret receptor in a fly model of Multiple Endocrine Neoplasia Type 2. Reducing Drosophila Sin3a activity led to metastasis-like behavior and, in the presence of Diap1, secondary tumors distant from the site of origin. Genetic and Chip-Seq analyses identified previously undescribed Sin3a targets including genes involved in cell motility and actin dynamics, as well as signaling pathways including Src, Jnk and Rho. A key Sin3a oncogenic target, PP1B, regulates stability of β-Catenin/Armadillo: the outcome is to oppose T-cell factor (TCF) function and Wg/Wnt pathway signaling in both fly and mammalian cancer cells. Reducing Sin3A strongly increased the invasive behavior of A549 human lung adenocarcinoma cells. We show that Sin3A is downregulated in a variety of human tumors and that Src, JNK, RhoA and PP1B/β-Catenin are regulated in a manner analogous to our Drosophila models. Our data suggest that Sin3A influences a specific step of tumorigenesis by regulating a module of genes involved i**n cell invasi**on. Tumor progression may commonly rely on such 'modules of invasion' under the control of broad transcriptional regulators.
3. Awd, the homolog of metastasis suppressor gene Nm23, regulates Drosophila epithelial **cell invasion**. Border cell migration during Drosophila melanogaster oogenesis is a highly pliable model for studying epithelial to mesenchymal transition and directional cell migration. The process involves delamination of a group of 6 to 10 follicle cells from the epithelium followed by guided migration and invasion through the nurse cell complex toward the oocyte. The guidance cue is mainly provided by the homolog of platelet-derived growth factor/vascular endothelial growth factor family of growth factor, or Pvf, emanating from the oocyte, although Drosophila epidermal growth factor receptor signaling also plays an auxiliary role. Earlier studies implicated a stringent control of the strength of Pvf-mediated signaling since both down-regulation of Pvf and overexpression of active Pvf receptor (Pvr) resulted in stalled border cell migration. Here we show that the metastasis suppressor gene homolog Nm23/awd is a negative regulator of border cell migration. Its down-regulation allows for optimal spatial signaling from two crucial pathways, Pvr and JAK/STAT. Its overexpression in the border cells results in stalled migration and can revert the phenotype of overexpressing constitutive Pvr or dominant-negative dynamin. This is a rare example demonstrating the relevance of a metastasis suppressor gene function utilized in a developmental process involving **cell invasion**.

---

## CG32982

- NCBI Gene
- GeneRIF
- Pubmed

| Search Term | Rank | Fields |
| --- | --- | --- |
| [Mm]igration | 6 | PubMed(1) |

  
hide/show details for CG32982

### [Mm]igration

#### PubMed

1. A core transcriptional network for early mesoderm development in Drosophila melanogaster. Embryogenesis is controlled by large gene-regulatory networks, which generate spatially and temporally refined patterns of gene expression. Here, we report the characteristics of the regulatory network orchestrating early mesodermal development in the fruitfly Drosophila, where the transcription factor Twist is both necessary and sufficient to drive development. Through the integration of chromatin immunoprecipitation followed by microarray analysis (ChIP-on-chip) experiments during discrete time periods with computational approaches, we identified >2000 Twist-bound cis-regulatory modules (CRMs) and almost 500 direct target genes. Unexpectedly, Twist regulates an almost complete cassette of genes required for cell proliferation in addition to genes essential for morophogenesis and cell **migration**. Twist targets almost 25% of all annotated Drosophila transcription factors, which may represent the entire set of regulators necessary for the early development of this system. By combining in vivo binding data from Twist, Mef2, Tinman, and Dorsal we have constructed an initial transcriptional network of early mesoderm development. The network topology reveals extensive combinatorial binding, feed-forward regulation, and complex logical outputs as prevalent features. In addition to binary activation and repression, we suggest that Twist binds to almost all mesodermal CRMs to provide the competence to integrate inputs from more specialized transcription factors.

---

## betaggt-II

- NCBI Gene
- GeneRIF
- Pubmed

| Search Term | Rank | Fields |
| --- | --- | --- |
| [Mm]igration | 6 | PubMed(1) |

  
hide/show details for betaggt-II

### [Mm]igration

#### PubMed

1. Isoprenoids control germ cell **migration** downstream of HMGCoA reductase. 3-hydroxy-3-methylglutaryl coenzyme A reductase (HMGCoAr) provides attractive cues to Drosophila germ cells, guiding them toward the embryonic gonad. However, it remains unclear how HMGCoAr mediates this attraction. In a genomic analysis of the HMGCoAr pathway, we found that the fly genome lacks several enzymes required for cholesterol biosynthesis, ruling out cholesterol and cholesterol-derived proteins as mediators of PGC **migration**. Genetic analysis of the pathway revealed that two enzymes, farnesyl-diphosphate synthase and geranylgeranyl-diphosphate synthase, required for the production of isoprenoids, act downstream of HMGCoAr in germ cell **migration**. Consistent with a role in geranylgeranylation, embryos deficient in geranylgeranyl transferase type I show germ cell **migration** defects. Our data, together with similar findings in zebrafish, implicate an isoprenylated protein in germ cell attraction. The specificity and evolutionary conservation of the HMGCoAr pathway for germ cells suggest that an attractant common to invertebrates and vertebrates guides germ cells in early embryos.

---

## Su(Tpl)

- NCBI Gene
- GeneRIF
- Pubmed

| Search Term | Rank | Fields |
| --- | --- | --- |
| [Mm]igration | 6 | PubMed(1) |
| ECM | 7 | PubMed(1) |
| [Ee]xtracellular [Mm]atrix | 8 | PubMed(1) |

  
hide/show details for Su(Tpl)

### [Mm]igration

#### PubMed

1. Genes conserved in bilaterians but jointly lost with Myc during nematode evolution are enriched in cell proliferation and cell **migration** functions. Animals use a stereotypical set of developmental genes to build body architectures of varying sizes and organizational complexity. Some genes are critical to developmental patterning, while other genes are important to physiological control of growth. However, growth regulator genes may not be as important in small-bodied "micro-metazoans" such as nematodes. Nematodes use a simplified developmental strategy of lineage-based cell fate specifications to produce an adult bilaterian body composed of a few hundreds of cells. Nematodes also lost the MYC proto-oncogenic regulator of cell proliferation. To identify additional regulators of cell proliferation that were lost with MYC, we computationally screened and determined 839 high-confidence genes that are conserved in bilaterians/lost in nematodes (CIBLIN genes). We find that 30 % of all CIBLIN genes encode transcriptional regulators of cell proliferation, epithelial-to-mesenchyme transitions, and other processes. Over 50 % of CIBLIN genes are unnamed genes in Drosophila, suggesting that there are many understudied genes. Interestingly, CIBLIN genes include many Myc synthetic lethal (MycSL) hits from recent screens. CIBLIN genes include key regulators of heparan sulfate proteoglycan (HSPG) sulfation patterns, and lysyl oxidases involved in cross-linking and modification of the extracellular matrix (ECM). These genes and others suggest the CIBLIN repertoire services critical functions in ECM remodeling and cell **migration** in large-bodied bilaterians. Correspondingly, CIBLIN genes are co-expressed with Myc in cancer transcriptomes, and include a preponderance of known determinants of cancer progression and tumor aggression. We propose that CIBLIN gene research can improve our understanding of regulatory control of cellular growth in metazoans.

### ECM

#### PubMed

1. Genes conserved in bilaterians but jointly lost with Myc during nematode evolution are enriched in cell proliferation and cell migration functions. Animals use a stereotypical set of developmental genes to build body architectures of varying sizes and organizational complexity. Some genes are critical to developmental patterning, while other genes are important to physiological control of growth. However, growth regulator genes may not be as important in small-bodied "micro-metazoans" such as nematodes. Nematodes use a simplified developmental strategy of lineage-based cell fate specifications to produce an adult bilaterian body composed of a few hundreds of cells. Nematodes also lost the MYC proto-oncogenic regulator of cell proliferation. To identify additional regulators of cell proliferation that were lost with MYC, we computationally screened and determined 839 high-confidence genes that are conserved in bilaterians/lost in nematodes (CIBLIN genes). We find that 30 % of all CIBLIN genes encode transcriptional regulators of cell proliferation, epithelial-to-mesenchyme transitions, and other processes. Over 50 % of CIBLIN genes are unnamed genes in Drosophila, suggesting that there are many understudied genes. Interestingly, CIBLIN genes include many Myc synthetic lethal (MycSL) hits from recent screens. CIBLIN genes include key regulators of heparan sulfate proteoglycan (HSPG) sulfation patterns, and lysyl oxidases involved in cross-linking and modification of the extracellular matrix (**ECM**). These genes and others suggest the CIBLIN repertoire services critical functions in **ECM** remodeling and cell migration in large-bodied bilaterians. Correspondingly, CIBLIN genes are co-expressed with Myc in cancer transcriptomes, and include a preponderance of known determinants of cancer progression and tumor aggression. We propose that CIBLIN gene research can improve our understanding of regulatory control of cellular growth in metazoans.

### [Ee]xtracellular [Mm]atrix

#### PubMed

1. Genes conserved in bilaterians but jointly lost with Myc during nematode evolution are enriched in cell proliferation and cell migration functions. Animals use a stereotypical set of developmental genes to build body architectures of varying sizes and organizational complexity. Some genes are critical to developmental patterning, while other genes are important to physiological control of growth. However, growth regulator genes may not be as important in small-bodied "micro-metazoans" such as nematodes. Nematodes use a simplified developmental strategy of lineage-based cell fate specifications to produce an adult bilaterian body composed of a few hundreds of cells. Nematodes also lost the MYC proto-oncogenic regulator of cell proliferation. To identify additional regulators of cell proliferation that were lost with MYC, we computationally screened and determined 839 high-confidence genes that are conserved in bilaterians/lost in nematodes (CIBLIN genes). We find that 30 % of all CIBLIN genes encode transcriptional regulators of cell proliferation, epithelial-to-mesenchyme transitions, and other processes. Over 50 % of CIBLIN genes are unnamed genes in Drosophila, suggesting that there are many understudied genes. Interestingly, CIBLIN genes include many Myc synthetic lethal (MycSL) hits from recent screens. CIBLIN genes include key regulators of heparan sulfate proteoglycan (HSPG) sulfation patterns, and lysyl oxidases involved in cross-linking and modification of the **extracellular matrix** (ECM). These genes and others suggest the CIBLIN repertoire services critical functions in ECM remodeling and cell migration in large-bodied bilaterians. Correspondingly, CIBLIN genes are co-expressed with Myc in cancer transcriptomes, and include a preponderance of known determinants of cancer progression and tumor aggression. We propose that CIBLIN gene research can improve our understanding of regulatory control of cellular growth in metazoans.

---

## SdhAL

- NCBI Gene
- GeneRIF
- Pubmed

| Search Term | Rank | Fields |
| --- | --- | --- |
| [Mm]igration | 6 | PubMed(1) |

  
hide/show details for SdhAL

### [Mm]igration

#### PubMed

1. A core transcriptional network for early mesoderm development in Drosophila melanogaster. Embryogenesis is controlled by large gene-regulatory networks, which generate spatially and temporally refined patterns of gene expression. Here, we report the characteristics of the regulatory network orchestrating early mesodermal development in the fruitfly Drosophila, where the transcription factor Twist is both necessary and sufficient to drive development. Through the integration of chromatin immunoprecipitation followed by microarray analysis (ChIP-on-chip) experiments during discrete time periods with computational approaches, we identified >2000 Twist-bound cis-regulatory modules (CRMs) and almost 500 direct target genes. Unexpectedly, Twist regulates an almost complete cassette of genes required for cell proliferation in addition to genes essential for morophogenesis and cell **migration**. Twist targets almost 25% of all annotated Drosophila transcription factors, which may represent the entire set of regulators necessary for the early development of this system. By combining in vivo binding data from Twist, Mef2, Tinman, and Dorsal we have constructed an initial transcriptional network of early mesoderm development. The network topology reveals extensive combinatorial binding, feed-forward regulation, and complex logical outputs as prevalent features. In addition to binary activation and repression, we suggest that Twist binds to almost all mesodermal CRMs to provide the competence to integrate inputs from more specialized transcription factors.

---

## Pabp2

- NCBI Gene
- GeneRIF
- Pubmed

| Search Term | Rank | Fields |
| --- | --- | --- |
| [Mm]igration | 6 | PubMed(1) |

  
hide/show details for Pabp2

### [Mm]igration

#### PubMed

1. Gain-of-function screen for genes that affect Drosophila muscle pattern formation. This article reports the production of an EP-element insertion library with more than 3,700 unique target sites within the Drosophila melanogaster genome and its use to systematically identify genes that affect embryonic muscle pattern formation. We designed a UAS/GAL4 system to drive GAL4-responsive expression of the EP-targeted genes in developing apodeme cells to which migrating myotubes finally attach and in an intrasegmental pattern of cells that serve myotubes as a **migration** substrate on their way towards the apodemes. The results suggest that misexpression of more than 1.5% of the Drosophila genes can interfere with proper myotube guidance and/or muscle attachment. In addition to factors already known to participate in these processes, we identified a number of enzymes that participate in the synthesis or modification of protein carbohydrate side chains and in Ubiquitin modifications and/or the Ubiquitin-dependent degradation of proteins, suggesting that these processes are relevant for muscle pattern formation.

---

## Cyp310a1

- NCBI Gene
- GeneRIF
- Pubmed

| Search Term | Rank | Fields |
| --- | --- | --- |
| [Mm]igration | 6 | PubMed(1) |
| ECM | 7 | PubMed(1) |
| [Ee]xtracellular [Mm]atrix | 8 | PubMed(1) |

  
hide/show details for Cyp310a1

### [Mm]igration

#### PubMed

1. Genes conserved in bilaterians but jointly lost with Myc during nematode evolution are enriched in cell proliferation and cell **migration** functions. Animals use a stereotypical set of developmental genes to build body architectures of varying sizes and organizational complexity. Some genes are critical to developmental patterning, while other genes are important to physiological control of growth. However, growth regulator genes may not be as important in small-bodied "micro-metazoans" such as nematodes. Nematodes use a simplified developmental strategy of lineage-based cell fate specifications to produce an adult bilaterian body composed of a few hundreds of cells. Nematodes also lost the MYC proto-oncogenic regulator of cell proliferation. To identify additional regulators of cell proliferation that were lost with MYC, we computationally screened and determined 839 high-confidence genes that are conserved in bilaterians/lost in nematodes (CIBLIN genes). We find that 30 % of all CIBLIN genes encode transcriptional regulators of cell proliferation, epithelial-to-mesenchyme transitions, and other processes. Over 50 % of CIBLIN genes are unnamed genes in Drosophila, suggesting that there are many understudied genes. Interestingly, CIBLIN genes include many Myc synthetic lethal (MycSL) hits from recent screens. CIBLIN genes include key regulators of heparan sulfate proteoglycan (HSPG) sulfation patterns, and lysyl oxidases involved in cross-linking and modification of the extracellular matrix (ECM). These genes and others suggest the CIBLIN repertoire services critical functions in ECM remodeling and cell **migration** in large-bodied bilaterians. Correspondingly, CIBLIN genes are co-expressed with Myc in cancer transcriptomes, and include a preponderance of known determinants of cancer progression and tumor aggression. We propose that CIBLIN gene research can improve our understanding of regulatory control of cellular growth in metazoans.

### ECM

#### PubMed

1. Genes conserved in bilaterians but jointly lost with Myc during nematode evolution are enriched in cell proliferation and cell migration functions. Animals use a stereotypical set of developmental genes to build body architectures of varying sizes and organizational complexity. Some genes are critical to developmental patterning, while other genes are important to physiological control of growth. However, growth regulator genes may not be as important in small-bodied "micro-metazoans" such as nematodes. Nematodes use a simplified developmental strategy of lineage-based cell fate specifications to produce an adult bilaterian body composed of a few hundreds of cells. Nematodes also lost the MYC proto-oncogenic regulator of cell proliferation. To identify additional regulators of cell proliferation that were lost with MYC, we computationally screened and determined 839 high-confidence genes that are conserved in bilaterians/lost in nematodes (CIBLIN genes). We find that 30 % of all CIBLIN genes encode transcriptional regulators of cell proliferation, epithelial-to-mesenchyme transitions, and other processes. Over 50 % of CIBLIN genes are unnamed genes in Drosophila, suggesting that there are many understudied genes. Interestingly, CIBLIN genes include many Myc synthetic lethal (MycSL) hits from recent screens. CIBLIN genes include key regulators of heparan sulfate proteoglycan (HSPG) sulfation patterns, and lysyl oxidases involved in cross-linking and modification of the extracellular matrix (**ECM**). These genes and others suggest the CIBLIN repertoire services critical functions in **ECM** remodeling and cell migration in large-bodied bilaterians. Correspondingly, CIBLIN genes are co-expressed with Myc in cancer transcriptomes, and include a preponderance of known determinants of cancer progression and tumor aggression. We propose that CIBLIN gene research can improve our understanding of regulatory control of cellular growth in metazoans.

### [Ee]xtracellular [Mm]atrix

#### PubMed

1. Genes conserved in bilaterians but jointly lost with Myc during nematode evolution are enriched in cell proliferation and cell migration functions. Animals use a stereotypical set of developmental genes to build body architectures of varying sizes and organizational complexity. Some genes are critical to developmental patterning, while other genes are important to physiological control of growth. However, growth regulator genes may not be as important in small-bodied "micro-metazoans" such as nematodes. Nematodes use a simplified developmental strategy of lineage-based cell fate specifications to produce an adult bilaterian body composed of a few hundreds of cells. Nematodes also lost the MYC proto-oncogenic regulator of cell proliferation. To identify additional regulators of cell proliferation that were lost with MYC, we computationally screened and determined 839 high-confidence genes that are conserved in bilaterians/lost in nematodes (CIBLIN genes). We find that 30 % of all CIBLIN genes encode transcriptional regulators of cell proliferation, epithelial-to-mesenchyme transitions, and other processes. Over 50 % of CIBLIN genes are unnamed genes in Drosophila, suggesting that there are many understudied genes. Interestingly, CIBLIN genes include many Myc synthetic lethal (MycSL) hits from recent screens. CIBLIN genes include key regulators of heparan sulfate proteoglycan (HSPG) sulfation patterns, and lysyl oxidases involved in cross-linking and modification of the **extracellular matrix** (ECM). These genes and others suggest the CIBLIN repertoire services critical functions in ECM remodeling and cell migration in large-bodied bilaterians. Correspondingly, CIBLIN genes are co-expressed with Myc in cancer transcriptomes, and include a preponderance of known determinants of cancer progression and tumor aggression. We propose that CIBLIN gene research can improve our understanding of regulatory control of cellular growth in metazoans.

---

## cpa

- NCBI Gene
- GeneRIF
- Pubmed

| Search Term | Rank | Fields |
| --- | --- | --- |
| [Ff]ilopodia | 1 | PubMed(2) |
| [Ff]ilopodium | 2 | Process(2) |
| [Mm]igration | 6 | PubMed(6) |
| [Ii]nvasive | 10 | PubMed(1) |
| [Mm]etastasis | 12 | PubMed(1) |
| [Cc]ell [Ii]nvasion | 13 | PubMed(1) |

  
hide/show details for cpa

### [Ff]ilopodia

#### PubMed

1. Cytonemes are required for the establishment of a normal Hedgehog morphogen gradient in Drosophila epithelia. Hedgehog (Hh) signalling is important in development, stem cell biology and disease. In a variety of tissues, Hh acts as a morphogen to regulate growth and cell fate specification. Several hypotheses have been proposed to explain morphogen movement, one of which is transport along **filopodia**-like protrusions called cytonemes. Here, we analyse the mechanism underlying Hh movement in the wing disc and the abdominal epidermis of Drosophila melanogaster. We show that, in both epithelia, cells generate cytonemes in regions of Hh signalling. These protrusions are actin-based and span several cell diameters. Various Hh signalling components localize to cytonemes, as well as to punctate structures that move along cytonemes and are probably exovesicles. Using in vivo imaging, we show that cytonemes are dynamic structures and that Hh gradient establishment correlates with cytoneme formation in space and time. Indeed, mutant conditions that affect cytoneme formation reduce both cytoneme length and Hh gradient length. Our results suggest that cytoneme-mediated Hh transport is the mechanistic basis for Hh gradient formation.
2. Dissecting regulatory networks of **filopodia** formation in a Drosophila growth cone model. F-actin networks are important structural determinants of cell shape and morphogenesis. They are regulated through a number of actin-binding proteins. The function of many of these proteins is well understood, but very little is known about how they cooperate and integrate their activities in cellular contexts. Here, we have focussed on the cellular roles of actin regulators in controlling **filopodia**l dynamics. **Filopodia** are needle-shaped, actin-driven cell protrusions with characteristic features that are well conserved amongst vertebrates and invertebrates. However, existing models of **filopodia** formation are still incomplete and controversial, pieced together from a wide range of different organisms and cell types. Therefore, we used embryonic Drosophila primary neurons as one consistent cellular model to study **filopodia** regulation. Our data for loss-of-function of capping proteins, enabled, different Arp2/3 complex components, the formin DAAM and profilin reveal characteristic changes in **filopodia** number and length, providing a promising starting point to study their functional relationships in the cellular context. Furthermore, the results are consistent with effects reported for the respective vertebrate homologues, demonstrating the conserved nature of our Drosophila model system. Using combinatorial genetics, we demonstrate that different classes of nucleators cooperate in **filopodia** formation. In the absence of Arp2/3 or DAAM **filopodia** numbers are reduced, in their combined absence **filopodia** are eliminated, and in genetic assays they display strong functional interactions with regard to **filopodia** formation. The two nucleators also genetically interact with enabled, but not with profilin. In contrast, enabled shows strong genetic interaction with profilin, although loss of profilin alone does not affect **filopodia** numbers. Our genetic data support a model in which Arp2/3 and DAAM cooperate in a common mechanism of **filopodia** formation that essentially depends on enabled, and is regulated through profilin activity at different steps.

### [Ff]ilopodium

#### Process

1. negative regulation of **filopodium** assembly
2. regulation of **filopodium** assembly

### [Mm]igration

#### PubMed

1. Enabled and Capping protein play important roles in shaping cell behavior during Drosophila oogenesis. During development, cells craft an impressive array of actin-based structures, mediating events as diverse as cytokinesis, apical constriction, and cell **migration**. One challenge is to determine how cells regulate actin assembly and disassembly to carry out these cell behaviors. During Drosophila oogenesis diverse cell behaviors are seen in the soma and germline. We used oogenesis to explore developmental roles of two important actin regulators: Enabled/VASP proteins and Capping protein. We found that Enabled plays an important role in cortical integrity of nurse cells, formation of robust bundled actin filaments in late nurse cells that facilitate nurse cell dumping, and **migration** of somatic border cells. During nurse cell dumping, Enabled localizes to barbed ends of the nurse cell actin filaments, suggesting its mechanism of action. We further pursued this mechanism using mutant Enabled proteins, each affecting one of its protein domains. These data suggest critical roles for the EVH2 domain and its tetramerization subdomain, while the EVH1 domain appears less critical. Enabled appears to be negatively regulated during oogenesis by Abelson kinase. We also explored the function of Capping protein. This revealed important roles in oocyte determination, nurse cell cortical integrity and nurse cell dumping, and support the idea that Capping protein and Enabled act antagonistically during dumping. Together these data reveal places that these actin regulators shape oogenesis.
2. Enabled and Capping protein play important roles in shaping cell behavior during Drosophila oogenesis. During development, cells craft an impressive array of actin-based structures, mediating events as diverse as cytokinesis, apical constriction, and cell **migration**. One challenge is to determine how cells regulate actin assembly and disassembly to carry out these cell behaviors. During Drosophila oogenesis diverse cell behaviors are seen in the soma and germline. We used oogenesis to explore developmental roles of two important actin regulators: Enabled/VASP proteins and Capping protein. We found that Enabled plays an important role in cortical integrity of nurse cells, formation of robust bundled actin filaments in late nurse cells that facilitate nurse cell dumping, and **migration** of somatic border cells. During nurse cell dumping, Enabled localizes to barbed ends of the nurse cell actin filaments, suggesting its mechanism of action. We further pursued this mechanism using mutant Enabled proteins, each affecting one of its protein domains. These data suggest critical roles for the EVH2 domain and its tetramerization subdomain, while the EVH1 domain appears less critical. Enabled appears to be negatively regulated during oogenesis by Abelson kinase. We also explored the function of Capping protein. This revealed important roles in oocyte determination, nurse cell cortical integrity and nurse cell dumping, and support the idea that Capping protein and Enabled act antagonistically during dumping. Together these data reveal places that these actin regulators shape oogenesis.
3. The Hippo pathway polarizes the actin cytoskeleton during collective **migration** of Drosophila border cells. Collective **migration** of Drosophila border cells depends on a dynamic actin cytoskeleton that is highly polarized such that it concentrates around the outer rim of the migrating cluster of cells. How the actin cytoskeleton becomes polarized in these cells to enable collective movement remains unknown. Here we show that the Hippo signaling pathway links determinants of cell polarity to polarization of the actin cytoskeleton in border cells. Upstream Hippo pathway components localize to contacts between border cells inside the cluster and signal through the Hippo and Warts kinases to polarize actin and promote border cell **migration**. Phosphorylation of the transcriptional coactivator Yorkie (Yki)/YAP by Warts does not mediate the function of this pathway in promoting border cell **migration**, but rather provides negative feedback to limit the speed of **migration**. Instead, Warts phosphorylates and inhibits the actin regulator Ena to activate F-actin Capping protein activity on inner membranes and thereby restricts F-actin polymerization mainly to the outer rim of the migrating cluster.
4. A Jnk-Rho-Actin remodeling positive feedback network directs Src-driven invasion. Current models of tumor cell invasion propose that oncogenic signaling converges upon key orchestrators of cytoskeletal dynamics, including c-Jun N-terminal kinase (Jnk) and RhoGTPase family members; these signals dynamically direct Actin remodeling proteins (ARPs) to catalyze the cytoskeletal changes required for **migration**. Src is a key driver of tumor aggression, metastasis and patient mortality. To clarify how Src regulates Actin dynamics to promote invasive **migration**, we performed a genetic modifier screen in a Drosophila model of invasion. Nine genes linked to Actin dynamics were identified that mediate invasion in situ. We found that ARPs were required for many oncogenic effects of Src including Mmp1 expression and initiation of apoptosis. Surprisingly, they were also regulators of Jnk pathway activity: both Src and the small GTPase Rho1 activated Jnk in a manner dependent on ARPs during invasion. Our results suggest that ARPs are not simply downstream executors of signal transduction pathways. Rather, they participate in a positive feedback network involving canonical oncogenic signaling pathways that promote tumor invasion.
5. Drosophila pupal macrophages--a versatile tool for combined ex vivo and in vivo imaging of actin dynamics at high resolution. Molecular understanding of actin dynamics requires a genetically traceable model system that allows live cell imaging together with high-resolution microscopy techniques. Here, we used Drosophila pupal macrophages that combine many advantages of cultured cells with a genetic in vivo model system. Using structured illumination microscopy together with advanced spinning disk confocal microscopy we show that these cells provide a powerful system for single gene analysis. It allows forward genetic screens to characterize the regulatory network controlling cell shape and directed cell **migration** in a physiological context. We knocked down components regulating lamellipodia formation, including WAVE, single subunits of Arp2/3 complex and CPA, one of the two capping protein subunits and demonstrate the advantages of this model system by imaging mutant macrophages ex vivo as well as in vivo upon laser-induced wounding.
6. Regulation of Drosophila mesoderm **migration** by phosphoinositides and the PH domain of the Rho GTP exchange factor Pebble. The Drosophila RhoGEF Pebble (Pbl) is required for cytokinesis and **migration** of mesodermal cells. In a screen for genes that could suppress **migration** defects in pbl mutants we identified the phosphatidylinositol phosphate (PtdInsP) regulator pi5k59B. Genetic interaction tests with other PtdInsP regulators suggested that PtdIns(4,5)P2 levels are important for mesoderm **migration** when Pbl is depleted. Consistent with this, the leading front of migrating mesodermal cells was enriched for PtdIns(4,5)P2. Given that Pbl contains a Pleckstrin Homology (PH) domain, a known PtdInsP-binding motif, we examined PtdInsP-binding of Pbl and the importance of the PH domain for Pbl function. In vitro lipid blot assays showed that Pbl binds promiscuously to PtdInsPs, with binding strength associated with the degree of phosphorylation. Pbl was also able to bind lipid vesicles containing PtdIns(4,5)P2 but binding was strongly reduced upon deletion of the PH domain. Similarly, in vivo, loss of the PH domain prevented localisation of Pbl to the cell cortex and severely affected several aspects of early mesoderm development, including flattening of the invaginated tube onto the ectoderm, extension of protrusions, and dorsal **migration** to form a monolayer. Pbl lacking the PH domain could still localise to the cytokinetic furrow, however, and cytokinesis failure was reduced in pbl(ΔPH) mutants. Taken together, our results support a model in which interaction of the PH-domain of Pbl with PtdIns(4,5)P2 helps localise it to the plasma membrane which is important for mesoderm **migratio**n.

### [Ii]nvasive

#### PubMed

1. A Jnk-Rho-Actin remodeling positive feedback network directs Src-driven invasion. Current models of tumor cell invasion propose that oncogenic signaling converges upon key orchestrators of cytoskeletal dynamics, including c-Jun N-terminal kinase (Jnk) and RhoGTPase family members; these signals dynamically direct Actin remodeling proteins (ARPs) to catalyze the cytoskeletal changes required for migration. Src is a key driver of tumor aggression, metastasis and patient mortality. To clarify how Src regulates Actin dynamics to promote **invasive** migration, we performed a genetic modifier screen in a Drosophila model of invasion. Nine genes linked to Actin dynamics were identified that mediate invasion in situ. We found that ARPs were required for many oncogenic effects of Src including Mmp1 expression and initiation of apoptosis. Surprisingly, they were also regulators of Jnk pathway activity: both Src and the small GTPase Rho1 activated Jnk in a manner dependent on ARPs during invasion. Our results suggest that ARPs are not simply downstream executors of signal transduction pathways. Rather, they participate in a positive feedback network involving canonical oncogenic signaling pathways that promote tumor invasion.

### [Mm]etastasis

#### PubMed

1. A Jnk-Rho-Actin remodeling positive feedback network directs Src-driven invasion. Current models of tumor cell invasion propose that oncogenic signaling converges upon key orchestrators of cytoskeletal dynamics, including c-Jun N-terminal kinase (Jnk) and RhoGTPase family members; these signals dynamically direct Actin remodeling proteins (ARPs) to catalyze the cytoskeletal changes required for migration. Src is a key driver of tumor aggression, **metastasis** and patient mortality. To clarify how Src regulates Actin dynamics to promote invasive migration, we performed a genetic modifier screen in a Drosophila model of invasion. Nine genes linked to Actin dynamics were identified that mediate invasion in situ. We found that ARPs were required for many oncogenic effects of Src including Mmp1 expression and initiation of apoptosis. Surprisingly, they were also regulators of Jnk pathway activity: both Src and the small GTPase Rho1 activated Jnk in a manner dependent on ARPs during invasion. Our results suggest that ARPs are not simply downstream executors of signal transduction pathways. Rather, they participate in a positive feedback network involving canonical oncogenic signaling pathways that promote tumor invasion.

### [Cc]ell [Ii]nvasion

#### PubMed

1. A Jnk-Rho-Actin remodeling positive feedback network directs Src-driven invasion. Current models of tumor **cell invasion** propose that oncogenic signaling converges upon key orchestrators of cytoskeletal dynamics, including c-Jun N-terminal kinase (Jnk) and RhoGTPase family members; these signals dynamically direct Actin remodeling proteins (ARPs) to catalyze the cytoskeletal changes required for migration. Src is a key driver of tumor aggression, metastasis and patient mortality. To clarify how Src regulates Actin dynamics to promote invasive migration, we performed a genetic modifier screen in a Drosophila model of invasion. Nine genes linked to Actin dynamics were identified that mediate invasion in situ. We found that ARPs were required for many oncogenic effects of Src including Mmp1 expression and initiation of apoptosis. Surprisingly, they were also regulators of Jnk pathway activity: both Src and the small GTPase Rho1 activated Jnk in a manner dependent on ARPs during invasion. Our results suggest that ARPs are not simply downstream executors of signal transduction pathways. Rather, they participate in a positive feedback network involving canonical oncogenic signaling pathways that promote tumor invasion.

---

## cib

- NCBI Gene
- GeneRIF
- Pubmed

| Search Term | Rank | Fields |
| --- | --- | --- |
| [Mm]igration | 6 | PubMed(3) |
| [Mm]etastasis | 12 | PubMed(1) |

  
hide/show details for cib

### [Mm]igration

#### PubMed

1. Molecular requirements for actin-based lamella formation in Drosophila S2 cells. Cell **migration** occurs through the protrusion of the actin-enriched lamella. Here, we investigated the effects of RNAi depletion of approximately 90 proteins implicated in actin function on lamella formation in Drosophila S2 cells. Similar to in vitro reconstitution studies of actin-based Listeria movement, we find that lamellae formation requires a relatively small set of proteins that participate in actin nucleation (Arp2/3 and SCAR), barbed end capping (capping protein), filament depolymerization (cofilin and Aip1), and actin monomer binding (profilin and cyclase-associated protein). Lamellae are initiated by parallel and partially redundant signaling pathways involving Rac GTPases and the adaptor protein Nck, which stimulate SCAR, an Arp2/3 activator. We also show that RNAi of three proteins (kette, Abi, and Sra-1) known to copurify with and inhibit SCAR in vitro leads to SCAR degradation, revealing a novel function of this protein complex in SCAR stability. Our results have identified an essential set of proteins involved in actin dynamics during lamella formation in Drosophila S2 cells.
2. Gene expression profiling of Drosophila tracheal fusion cells. The Drosophila trachea is a premier genetic system to investigate the fundamental mechanisms of tubular organ formation. Tracheal fusion cells lead the branch fusion process to form an interconnected tubular network. Therefore, fusion cells in the Drosophila trachea will be an excellent model to study branch fusion in mammalian tubular organs, such as kidneys and blood vessels. The fusion process is a dynamic cellular process involving cell **migration**, adhesion, vesicle trafficking, cytoskeleton rearrangement, and membrane fusion. To understand how these cellular events are coordinated, we initiated the critical step to assemble a gene expression profile of fusion cells. For this study, we analyzed the expression of 234 potential tracheal-expressed genes in fusion cells during fusion cell development. 143 Tracheal genes were found to encode transcription factors, signal proteins, cytoskeleton and matrix proteins, transporters, and proteins with unknown function. These genes were divided into four subgroups based on their levels of expression in fusion cells compared to neighboring non-fusion cells revealed by in situ hybridization: (1) genes that have relative high abundance in fusion cells, (2) genes that are dynamically expressed in fusion cells, (3) genes that have relative low abundance in fusion cells, and (4) genes that are expressed at similar levels in fusion cells and non-fusion tracheal cells. This study identifies the expression profile of fusion cells and hypothetically suggests genes which are necessary for the fusion process and which play roles in distinct stages of fusion, as indicated by the location and timing of expression. These data will provide the basis for a comprehensive understanding of the molecular and cellular mechanisms of branch fusion.
3. Analysis of cell **migration** using whole-genome expression profiling of migratory cells in the Drosophila ovary. Cell **migration** contributes to normal development and homeostasis as well as to pathological processes such as inflammation and tumor metastasis. Previous genetic screens have revealed signaling pathways that govern follicle cell **migration**s in the Drosophila ovary, but few downstream targets of the critical transcriptional regulators have been identified. To characterize the gene expression profile of two migratory cell populations and identify Slbo targets, we purified border cells and centripetal cells expressing the mouse CD8 antigen and carried out whole-genome microarray analysis. Genes predicted to control actin dynamics and the endocytic and secretory pathways were overrepresented in the migratory cell transcriptome. Mutations in five genes, including ttk, failed to complement previously isolated mutations that cause cell **migration** defects in mosaic clones. Functional analysis revealed a role for the Notch-activating protease Kuzbanian in border cell **migration** and identified Tie as a guidance receptor for the border cells.

### [Mm]etastasis

#### PubMed

1. Analysis of cell migration using whole-genome expression profiling of migratory cells in the Drosophila ovary. Cell migration contributes to normal development and homeostasis as well as to pathological processes such as inflammation and tumor **metastasis**. Previous genetic screens have revealed signaling pathways that govern follicle cell migrations in the Drosophila ovary, but few downstream targets of the critical transcriptional regulators have been identified. To characterize the gene expression profile of two migratory cell populations and identify Slbo targets, we purified border cells and centripetal cells expressing the mouse CD8 antigen and carried out whole-genome microarray analysis. Genes predicted to control actin dynamics and the endocytic and secretory pathways were overrepresented in the migratory cell transcriptome. Mutations in five genes, including ttk, failed to complement previously isolated mutations that cause cell migration defects in mosaic clones. Functional analysis revealed a role for the Notch-activating protease Kuzbanian in border cell migration and identified Tie as a guidance receptor for the border cells.

---

## Hmgcr

- NCBI Gene
- GeneRIF
- Pubmed

| Search Term | Rank | Fields |
| --- | --- | --- |
| [Mm]igration | 6 | Process(5); PubMed(27) |
| [Mm]etastasis | 12 | PubMed(2) |

  
hide/show details for Hmgcr

### [Mm]igration

#### Process

1. germ cell **migration**
2. germ cell **migration**
3. pole cell **migration**
4. pole cell **migration**
5. pole cell **migration**

#### PubMed

1. Isoprenoids control germ cell **migration** downstream of HMGCoA reductase. 3-hydroxy-3-methylglutaryl coenzyme A reductase (HMGCoAr) provides attractive cues to Drosophila germ cells, guiding them toward the embryonic gonad. However, it remains unclear how HMGCoAr mediates this attraction. In a genomic analysis of the HMGCoAr pathway, we found that the fly genome lacks several enzymes required for cholesterol biosynthesis, ruling out cholesterol and cholesterol-derived proteins as mediators of PGC **migration**. Genetic analysis of the pathway revealed that two enzymes, farnesyl-diphosphate synthase and geranylgeranyl-diphosphate synthase, required for the production of isoprenoids, act downstream of HMGCoAr in germ cell **migration**. Consistent with a role in geranylgeranylation, embryos deficient in geranylgeranyl transferase type I show germ cell **migration** defects. Our data, together with similar findings in zebrafish, implicate an isoprenylated protein in germ cell attraction. The specificity and evolutionary conservation of the HMGCoAr pathway for germ cells suggest that an attractant common to invertebrates and vertebrates guides germ cells in early embryos.
2. Signaling systems, guided cell **migration**, and organogenesis: insights from genetic studies in Drosophila. During development, cells change their position extensively. Although the basic cellular mechanisms involved in cell locomotion have been studied mostly in cultured cells, genetic and molecular approaches using model organisms are starting to shed light on the complex events influencing cell **migration** during development. Recent technical advances in following and analyzing migrating cells inside the living embryo offer the possibility of understanding how different signaling systems regulate the fundamental cellular processes underlying guided cell **migration** in vivo. In Drosophila melanogaster, studies of migrating cells have concentrated mainly on hemocytes, germ cells, border cells, and tracheal cells. Interestingly, most of these cells were recently shown to make different cellular extensions and to use receptor tyrosine kinases to sense the chemoattractive signal. This review describes our current understanding of how different signaling networks control guided **migration** in these four systems and discusses the impact of novel imaging techniques on the study of guided cell **migration** during development.
3. Cell **migration** and programmed cell death of Drosophila germ cells. Cell **migration** and programmed cell death are essential components of animal development and homeostasis, and the germ cells of Drosophila provide a simple genetic system to study the molecular mechanisms that govern these important cellular processes. Detailed descriptions of germ cell **migration** in Drosophila were accomplished long ago, but most genetic and molecular analyses of the process have occurred within the past 10 years. A few of the genes required for germ cell **migration** have been identified, and a very interesting picture is emerging. However, a process as complex as cell **migration** must involve the functions of many more molecules. In addition, cell **migration** and cell death mechanisms are often linked, as it is important to eliminate cells that are misplaced and could present a danger to the organism. In Drosophila, genes involved in germ cell **migration** can also affect programmed cell death. Currently, very little is known about how germ cells ectopic to the gonads are eliminated. To date, only four genes have been reported with roles in germ cell death, and three of these have additional functions in germ cell pathfinding. The nature of the cell death program has not been elucidated. Here, I provide a brief review of Drosophila germ cell **migration** and programmed cell death at both the descriptive and molecular levels. Many questions remain to be answered, but advances made in recent years are providing useful insights into these critical biological phenomena.
4. Identification of genes controlling germ cell **migration** and embryonic gonad formation in Drosophila. Gonadogenesis in the Drosophila embryo is a complex process involving numerous cellular migratory steps and cell-cell interactions. The mechanisms guiding germ cells to move through, recognize and adhere to specific cell types are poorly understood. In order to identify genes that are required for these processes, we have conducted an extensive mutagenesis of the third chromosome and screened for mutations disrupting germ cell **migration** at any point in embryonic development. Phenotypic analysis of these mutants demonstrates that germ cell **migration** can be broken down into discrete developmental steps, with each step requiring a specific set of genes. Many of these genes are involved in the development of gonadal mesoderm, the tissue that associates with germ cells to form the embryonic gonad. Moreover, mutations that we isolated affecting embryonic patterning as well as germ cell **migration** suggest that the origin of gonadal mesoderm lies within the eve domain of the developing mesoderm.
5. Germ plasm assembly and germ cell **migration** in Drosophila. Many aspects of germ cell behavior, **migration**, and gonad formation are shared between vertebrate and invertebrate species. For example, a specialized germ plasm has been observed in many species including Caenorhabditis elegans and Xenopus. Furthermore, the fact that Vasa marks germ cells in many species suggests that even certain molecular aspects of germ cells may be common between different organisms. In most organisms, germ cells initially form at a location away from their target mesodermal tissues and have to migrate to reach the mesoderm. Further genetic studies will reveal the extent to which molecular aspects of germ cell **migration** and gonad formation are conserved.
6. Isoprenoids control germ cell **migration** downstream of HMGCoA reductase. 3-hydroxy-3-methylglutaryl coenzyme A reductase (HMGCoAr) provides attractive cues to Drosophila germ cells, guiding them toward the embryonic gonad. However, it remains unclear how HMGCoAr mediates this attraction. In a genomic analysis of the HMGCoAr pathway, we found that the fly genome lacks several enzymes required for cholesterol biosynthesis, ruling out cholesterol and cholesterol-derived proteins as mediators of PGC **migration**. Genetic analysis of the pathway revealed that two enzymes, farnesyl-diphosphate synthase and geranylgeranyl-diphosphate synthase, required for the production of isoprenoids, act downstream of HMGCoAr in germ cell **migration**. Consistent with a role in geranylgeranylation, embryos deficient in geranylgeranyl transferase type I show germ cell **migration** defects. Our data, together with similar findings in zebrafish, implicate an isoprenylated protein in germ cell attraction. The specificity and evolutionary conservation of the HMGCoAr pathway for germ cells suggest that an attractant common to invertebrates and vertebrates guides germ cells in early embryos.
7. Neurl4 contributes to germ cell formation and integrity in Drosophila. Primordial germ cells (PGCs) form at the posterior pole of the Drosophila embryo, and then migrate to their final destination in the gonad where they will produce eggs or sperm. Studies of the different stages in this process, including assembly of germ plasm in the oocyte during oogenesis, specification of a subset of syncytial embryonic nuclei as PGCs, and **migration**, have been informed by genetic analyses. Mutants have defined steps in the process, and the identities of the affected genes have suggested biochemical mechanisms. Here we describe a novel PGC phenotype. When Neurl4 activity is reduced, newly formed PGCs frequently adopt irregular shapes and appear to bud off vesicles. PGC number is also reduced, an effect exacerbated by a separate role for Neurl4 in germ plasm formation during oogenesis. Like its mammalian homolog, Drosophila Neurl4 protein is concentrated in centrosomes and downregulates centrosomal protein CP110. Reducing CP110 activity suppresses the abnormal PGC morphology of Neurl4 mutants. These results extend prior analyses of Neurl4 in cultured cells, revealing a heightened requirement for Neurl4 in germ-line cells in Drosophila.
8. Moving towards the next generation. In most organisms, primordial germ cells are set aside from the cells of the body early in development. To form an embryonic gonad, germ cells often have to migrate along complex routes through and along diverse tissues until they reach the somatic part of the gonad. Recent advances have been made in the genetic analysis of these early stages of germ line development. Here we review findings from Drosophila, zebrafish, and mouse; each organism provides unique insight into the mechanisms that determine germ cell fate and the cues that may guide their **migration**.
9. Germline development in vertebrates and invertebrates. In all animals information is passed from parent to offspring via the germline, which segregates from the soma early in development and undergoes a complex developmental program to give rise to the adult gametes. Many aspects of germline development have been conserved throughout the animal kingdom. Here we review the unique properties of germ cells, the initial determination of germ cell fates, the maintenance of germ cell identity, the **migration** of germ cells to the somatic gonadal primordia and the proliferation of germ cells during development invertebrates and invertebrates. Similarities in germline development in such diverse organisms as Drosophila melanogaster, Caenorhabditis elegans, Xenopus laevis and Mus musculus will be highlighted.
10. Germ cell specification and **migration** in Drosophila and beyond. The passage of an individual's genome to future generations is essential for the maintenance of species and is mediated by highly specialized cells, the germ cells. Genetic studies in a number of model organisms have provided insight into the molecular mechanisms that control specification, **migration** and survival of early germ cells. Focusing on Drosophila, we will discuss the mechanisms by which germ cells initially form and remain transcriptionally silent while somatic cells are transcriptionally active. We will further discuss three separate attractive and repellent guidance pathways, mediated by a G-protein coupled receptor, two lipid phosphate phosphohydrolases, and isoprenylation. We will compare and contrast these findings with those obtained in other organisms, in particular zebrafish and mice. While aspects of germ cell specification are strikingly different between these species, germ cell specific gene functions have been conserved. In particular, mechanisms that sense directional cues during germ cell **migration** seem to be shared between invertebrates and vertebrates.
11. Guidance of primordial germ cell **migration**. Primordial germ cells (PGCs), the progenitors of the gametes, migrate from the position where they are specified towards the region where the gonad develops. To reach their target, the PGCs obtain directional cues from cells positioned along their **migration** path. One such cue, the chemokine SDF-1, has recently been found to be critical for proper PGC **migration** in zebrafish and in mice. In Drosophila, too, a molecule that is structurally related to chemokine receptors and is important for PGC **migration** has been identified. The ability to visualize chemokine-guided **migration** at a high resolution in vivo in these model organisms provides a unique opportunity to study this process, which is relevant for many events in normal development and disease.
12. Slow as molasses is required for polarized membrane growth and germ cell **migration** in Drosophila. Drosophila germ cell **migration** is directed by attractive and repulsive guidance cues. We have identified a novel gene, slow as molasses (slam), which is required for germ cell **migration**. In slam zygotic mutants, germ cells fail to transit off the midgut into the mesoderm. We show that slam is required at this stage in parallel to HMG Coenzyme A reductase, a previously identified germ cell **migration** gene. Removal of both zygotic and maternal slam results in an earlier defect: a failure to form a cellular blastoderm. Consistent with this phenotype, we found that slam is one of the earliest genes to be transcribed in the embryo, and Slam protein localizes to the growing basal-lateral membrane during blastoderm formation, but Slam is not detected during later stages of embryogenesis. Because slam RNA and protein are expressed earlier than the time when we observe defects in germ cell **migration**, we propose that Slam is required for the localization of a signal to the basal side of blastoderm cells that is needed later in the posterior midgut to guide germ cells.
13. The social lives of migrating cells in Drosophila. Studies of cell **migration** in Drosophila are yielding insights into the complex interactions migrating cells have with each other and with the cells in their environment. Intriguing links between factors that promote cell **migration** and those that control cell survival have been reported recently. For example, migrating germ cells compete with the surrounding somatic tissue for the substrate of the lipid phosphate phosphatases encoded by the genes Wunen and Wunen2. Germ cells take up the dephosphorylated lipid and require it for their survival. In addition, the secreted growth factors called PVFs, previously thought to guide the **migration**s of hemocytes in the embryo, were found to function instead predominantly as survival factors. And in border cells, DIAP1 and Dronc, two proteins known mainly for their ability to regulate cell death, were found to control cell **migration**.
14. The genetics of cell **migration** in Drosophila melanogaster and Caenorhabditis elegans development. Cell **migration**s are found throughout the animal kingdom and are among the most dramatic and complex of cellular behaviors. Historically, the mechanics of cell **migration** have been studied primarily in vitro, where cells can be readily viewed and manipulated. However, genetic approaches in relatively simple model organisms are yielding additional insights into the molecular mechanisms underlying cell movements and their regulation during development. This review will focus on these simple model systems where we understand some of the signaling and receptor molecules that stimulate and guide cell movements. The chemotactic guidance factor encoded by the Caenorhabditis elegans unc-6 locus, whose mammalian homolog is Netrin, is perhaps the best known of the cell **migration** guidance factors. In addition, receptor tyrosine kinases (RTKs), and FGF receptors in particular, have emerged as key mediators of cell **migration** in vivo, confirming the importance of molecules that were initially identified and studied in cell culture. Somewhat surprisingly, screens for mutations that affect primordial germ cell **migration** in Drosophila have revealed that enzymes involved in lipid metabolism play a role in guiding cell **migration** in vivo, possibly by producing and/or degrading lipid chemoattractants or chemorepellents. Cell adhesion molecules, such as integrins, have been extensively characterized with respect to their contribution to cell **migration** in vitro and genetic evidence now supports a role for these receptors in certain instances in vivo as well. The role for non-muscle myosin in cell motility was controversial, but has now been demonstrated genetically, at least in some cell types. Currently the best characterized link between membrane receptor signaling and regulation of the actin cytoskeleton is that provided by the Rho family of small GTPases. Members of this family are clearly essential for the **migration**s of some cells; however, key questions remain concerning how chemoattractant and chemorepellent signals are integrated within the cell and transduced to the cytoskeleton to produce directed cell **migration**. New types of genetic screens promise to fill in some of these gaps in the near future.
15. Hedgehog does not guide migrating Drosophila germ cells. In many species, the germ cells, precursors of sperm and egg, migrate during embryogenesis. The signals that regulate this **migration** are thus essential for fertility. In flies, lipid signals have been shown to affect germ cell guidance. In particular, the synthesis of geranylgeranyl pyrophosphate through the 3-hydroxy-3-methyl-glutaryl coenzyme A reductase (Hmgcr) pathway is critical for attracting germ cells to their target tissue. In a genetic analysis of signaling pathways known to affect cell **migration** of other migratory cells, we failed to find a role for the Hedgehog (Hh) pathway in germ cell **migration**. However, previous reports had implicated Hh as a germ cell attractant in flies and suggested that Hh signaling is enhanced through the action of the Hmgcr pathway. We therefore repeated several critical experiments and carried out further experiments to test specifically whether Hh is a germ cell attractant in flies. In contrast to previously reported findings and consistent with findings in zebrafish our data do not support the notion that Hh has a direct role in the guidance of migrating germ cells in flies.
16. Cellular and molecular mechanisms of single and collective cell **migration**s in Drosophila: themes and variations. The process of cell **migration** is essential throughout life, driving embryonic morphogenesis and ensuring homeostasis in adults. Defects in cell **migration** are a major cause of human disease, with excessive **migration** causing autoimmune diseases and cancer metastasis, whereas reduced capacity for **migration** leads to birth defects and immunodeficiencies. Myriad studies in vitro have established a consensus view that cell **migration**s require cell polarization, Rho GTPase-mediated cytoskeletal rearrangements, and myosin-mediated contractility. However, in vivo studies later revealed a more complex picture, including the discovery that cells migrate not only as single units but also as clusters, strands, and sheets. In particular, the role of E-Cadherin in cell motility appears to be more complex than previously appreciated. Here, we discuss recent advances achieved by combining the plethora of genetic tools available to the Drosophila geneticist with live imaging and biophysical techniques. Finally, we discuss the emerging themes such studies have revealed and ponder the puzzles that remain to be solved.
17. HMGCoA reductase potentiates hedgehog signaling in Drosophila melanogaster. Drosophila HMGCoA reductase (hmgcr) catalyzes the biosynthesis of a mevalonate precursor for isoprenoids and has been implicated in the production of a signal by the somatic gonadal precursor cells (SGPs) that attracts migrating germ cells. Here, we show that hmgcr functions in the hedgehog (hh) signaling pathway. When hmgcr activity is reduced, high levels of Hh accumulate in hh-expressing cells in each parasegment, while the adjacent "Hh-receiving" cells cannot sustain wg expression and fail to relocalize the Smoothened (Smo) receptor. Conversely, ectopic Hmgcr upregulates Hh signaling when it is produced in hh-expressing cells, but has no effect when produced in the receiving cells. These findings suggest that Hmgcr might orchestrate germ cell **migration** by promoting the release and/or transport of Hh from the SGPs. Consistent with this model, there are substantial germ cell **migration** defects in trans combinations between hmgcr and mutations in different components of the hh pathway.
18. toutvelu, a regulator of heparan sulfate proteoglycan biosynthesis, controls guidance cues for germ-cell **migration**. The primitive embryonic gonad in Drosophila melanogaster is composed of germ cells and somatic gonadal precursor cells (SGPs). The assembly of a functional gonad involves a complex series of germ-cell **migration** events, which are thought to be guided by attractive and repulsive cues. Here, we demonstrate a novel role for toutvelu (ttv), a regulator of heparan sulfate proteoglycan biosynthesis during this process. Germline clonal analysis suggests that maternal deposition of ttv is required for proper germ-cell **migration**. Conversely, ectopic expression of ttv in early embryos results in severe germ-cell **migration** defects and inappropriate spreading of Hh protein. Moreover, overexpression of ttv in only the receiving cells, rather than in the sending cells, leads to phenotypic consequences. Finally, supporting the claim that the signaling molecule Hedgehog (Hh) may function as a chemoattractant to guide germ cells, errant germ cells are found localized near pockets containing high concentrations of Hh protein.
19. Cell **migration** in Drosophila. Recent genetic studies in Drosophila have identified signals that direct cell movement, mechanisms that transduce such signals within migrating cells and some of the molecular machinery underlying cell motility. Activation of the fibroblast growth factor receptor signaling pathway is required for **migration** of the cells of the developing respiratory system and mesoderm. A signal dependent on 3-hydroxy-3-methylglutanyl Coenzyme A reductase attracts migrating primordial germ cells to the somatic gonad, whereas the phosphohydrolase, phosphatidic acid phosphatase type 2, repels germ cells. In the female germline, the migratory path of border cells is directed by the homophilic adhesion molecule E cadherin.
20. HMG-CoA reductase guides migrating primordial germ cells. The enzyme 3-hydroxy-3-methylglutaryl coenzyme A (HMG-CoA) reductase is best known for catalysing a rate-limiting step in cholesterol biosynthesis, but it also participates in the production of a wide variety of other compounds. Some clinical benefits attributed to inhibitors of HMG-CoA reductase are now thought to be independent of any serum cholesterol-lowering effect. Here we describe a new cholesterol-independent role for HMG-CoA reductase, in regulating a developmental process: primordial germ cell **migration**. We show that in Drosophila this enzyme is highly expressed in the somatic gonad and that it is necessary for primordial germ cells to migrate to this tissue. Misexpression of HMG-CoA reductase is sufficient to attract primordial germ cells to tissues other than the gonadal mesoderm. We conclude that the regulated expression of HMG-CoA reductase has a critical developmental function in providing spatial information to guide migrating primordial germ cells.
21. Control of lateral **migration** and germ cell elimination by the Drosophila melanogaster lipid phosphate phosphatases Wunen and Wunen 2. In most organisms, primordial germ cells (PGCs) arise far from the region where somatic gonadal precursors (SGPs) are specified. Although PGCs in general originate as a single cluster of cells, the somatic parts of the gonad form on each site of the embryo. Thus, to reach the gonad, PGCs not only migrate from their site of origin but also split into two groups. Taking advantage of high-resolution real-time imaging, we show that in Drosophila melanogaster PGCs are polarized and migrate directionally toward the SGPs, avoiding the midline. Unexpectedly, neither PGC attractants synthesized in the SGPs nor known midline repellents for axon guidance were required to sort PGCs bilaterally. Repellent activity provided by wunen (wun) and wunen-2 (wun-2) expressed in the central nervous system, however, is essential in this **migration** process and controls PGC survival. Our results suggest that expression of wun/wun-2 repellents along the migratory paths provides faithful control over the sorting of PGCs into two gonads and eliminates PGCs left in the middle of the embryo.
22. Germ cell **migration**: as slow as molasses. In Drosophila embryos, germ cells and somatic cells are formed separately. A recent analysis of the slow as molasses (slam) gene provides a potential link between somatic cell formation and germ cell **migration**.
23. An ABC transporter controls export of a Drosophila germ cell attractant. Directed cell **migration**, which is critical for embryonic development, leukocyte trafficking, and cell metastasis, depends on chemoattraction. 3-hydroxy-3-methylglutaryl coenzyme A reductase regulates the production of an attractant for Drosophila germ cells that may itself be geranylated. Chemoattractants are commonly secreted through a classical, signal peptide-dependent pathway, but a geranyl-modified attractant would require an alternative pathway. In budding yeast, pheromones produced by a-cells are farnesylated and secreted in a signal peptide-independent manner, requiring the adenosine triphosphate-binding cassette (ABC) transporter Ste6p. Here we show that Drosophila germ cell **migration** uses a similar pathway, demonstrating that invertebrate germ cells, like yeast cells, are attracted to lipid-modified peptides. Components of this unconventional export pathway are highly conserved, suggesting that this pathway may control the production of similarly modified chemoattractants in organisms ranging from yeast to humans.
24. Ggamma1, a downstream target for the hmgcr-isoprenoid biosynthetic pathway, is required for releasing the Hedgehog ligand and directing germ cell **migration**. The isoprenoid biosynthetic pathway leading from the production of mevalonate by HMGCoA reductase (Hmgcr) to the geranylation of the G protein subunit, Ggamma1, plays an important role in cardiac development in the fly. Hmgcr has also been implicated in the release of the signaling molecule Hedgehog (Hh) from hh expressing cells and in the production of an attractant that directs primordial germ cells to migrate to the somatic gonadal precursor cells (SGPs). The studies reported here indicate that this same hmgcr-->Ggamma1 pathway provides a novel post-translational mechanism for modulating the range and activity of the Hh signal produced by hh expressing cells. We show that, like hmgcr, ggamma1 and quemao (which encodes the enzyme, geranylgeranyl diphosphate synthetase, that produces the substrate for geranylation of Ggamma1) are components of the hh signaling pathway and are required for the efficient release of the Hh ligand from hh expressing cells. We also show that the hmgcr-->Ggamma1 pathway is linked to production of the germ cell attractant by the SGPs through its ability to enhance the potency of the Hh signal. We show that germ cell **migration** is disrupted by the loss or gain of ggamma1 activity, by trans-heterozygous combinations between ggamma1 and either hmgcr or hh mutations, and by ectopic expression of dominant negative Ggamma1 proteins that cannot be geranylated.
25. The hedgehog pathway gene shifted functions together with the hmgcr-dependent isoprenoid biosynthetic pathway to orchestrate germ cell **migration**. The Drosophila embryonic gonad is assembled from two distinct cell types, the Primordial Germ Cells (PGCs) and the Somatic Gonadal Precursor cells (SGPs). The PGCs form at the posterior of blastoderm stage embryos and are subsequently carried inside the embryo during gastrulation. To reach the SGPs, the PGCs must traverse the midgut wall and then migrate through the mesoderm. A combination of local repulsive cues and attractive signals emanating from the SGPs guide **migration**. We have investigated the role of the hedgehog (hh) pathway gene shifted (shf) in directing PGC **migration**. shf encodes a secreted protein that facilitates the long distance transmission of Hh through the proteoglycan matrix after it is released from basolateral membranes of Hh expressing cells in the wing imaginal disc. shf is expressed in the gonadal mesoderm, and loss- and gain-of-function experiments demonstrate that it is required for PGC **migration**. Previous studies have established that the hmgcr-dependent isoprenoid biosynthetic pathway plays a pivotal role in generating the PGC attractant both by the SGPs and by other tissues when hmgcr is ectopically expressed. We show that production of this PGC attractant depends upon shf as well as a second hh pathway gene gγ1. Further linking the PGC attractant to Hh, we present evidence indicating that ectopic expression of hmgcr in the nervous system promotes the release/transmission of the Hh ligand from these cells into and through the underlying mesodermal cell layer, where Hh can contact migrating PGCs. Finally, potentiation of Hh by hmgcr appears to depend upon cholesterol modification.
26. Live imaging of Drosophila gonad formation reveals roles for Six4 in regulating germline and somatic cell **migration**. Movement of cells, either as amoeboid individuals or in organised groups, is a key feature of organ formation. Both modes of **migration** occur during Drosophila embryonic gonad development, which therefore provides a paradigm for understanding the contribution of these processes to organ morphogenesis. Gonads of Drosophila are formed from three distinct cell types: primordial germ cells (PGCs), somatic gonadal precursors (SGPs), and in males, male-specific somatic gonadal precursors (msSGPs). These originate in distinct locations and migrate to associate in two intermingled clusters which then compact to form the spherical primitive gonads. PGC movements are well studied, but much less is known of the migratory events and other interactions undergone by their somatic partners. These appear to move in organised groups like, for example, lateral line cells in zebra fish or Drosophila ovarian border cells.We have used time-lapse fluorescence imaging to characterise gonadal cell behaviour in wild type and mutant embryos. We show that the homeodomain transcription factor Six4 is required for the **migration** of the PGCs and the msSGPs towards the SGPs. We have identified a likely cause of this in the case of PGCs as we have found that Six4 is required for expression of Hmgcr which codes for HMGCoA reductase and is necessary for attraction of PGCs by SGPs. Six4 affects msSGP **migration** by a different pathway as these move normally in Hmgcr mutant embryos. Additionally, embryos lacking fully functional Six4 show a novel phenotype in which the SGPs, which originate in distinct clusters, fail to coalesce to form unified gonads.Our work establishes the Drosophila gonad as a model system for the analysis of coordinated cell **migration**s and morphogenesis using live imaging and demonstrates that Six4 is a key regulator of somatic cell function during gonadogenesis. Our data suggest that the initial association of SGP clusters is under distinct control from the movements that drive gonad compaction.
27. Germ cells. Recently, cell signaling has been shown to be required for the formation of germ cells in the mouse embryo, direct observation of germ cell **migration** in living mouse embryos has been achieved, novel genes that control germ-cell **migration** have been identified in Drosophila, and the roles of many components of germ plasm in several species have become clearer.

### [Mm]etastasis

#### PubMed

1. Cellular and molecular mechanisms of single and collective cell migrations in Drosophila: themes and variations. The process of cell migration is essential throughout life, driving embryonic morphogenesis and ensuring homeostasis in adults. Defects in cell migration are a major cause of human disease, with excessive migration causing autoimmune diseases and cancer **metastasis**, whereas reduced capacity for migration leads to birth defects and immunodeficiencies. Myriad studies in vitro have established a consensus view that cell migrations require cell polarization, Rho GTPase-mediated cytoskeletal rearrangements, and myosin-mediated contractility. However, in vivo studies later revealed a more complex picture, including the discovery that cells migrate not only as single units but also as clusters, strands, and sheets. In particular, the role of E-Cadherin in cell motility appears to be more complex than previously appreciated. Here, we discuss recent advances achieved by combining the plethora of genetic tools available to the Drosophila geneticist with live imaging and biophysical techniques. Finally, we discuss the emerging themes such studies have revealed and ponder the puzzles that remain to be solved.
2. An ABC transporter controls export of a Drosophila germ cell attractant. Directed cell migration, which is critical for embryonic development, leukocyte trafficking, and cell **metastasis**, depends on chemoattraction. 3-hydroxy-3-methylglutaryl coenzyme A reductase regulates the production of an attractant for Drosophila germ cells that may itself be geranylated. Chemoattractants are commonly secreted through a classical, signal peptide-dependent pathway, but a geranyl-modified attractant would require an alternative pathway. In budding yeast, pheromones produced by a-cells are farnesylated and secreted in a signal peptide-independent manner, requiring the adenosine triphosphate-binding cassette (ABC) transporter Ste6p. Here we show that Drosophila germ cell migration uses a similar pathway, demonstrating that invertebrate germ cells, like yeast cells, are attracted to lipid-modified peptides. Components of this unconventional export pathway are highly conserved, suggesting that this pathway may control the production of similarly modified chemoattractants in organisms ranging from yeast to humans.

---

## CG45050

- NCBI Gene
- GeneRIF
- Pubmed

| Search Term | Rank | Fields |
| --- | --- | --- |
| ECM | 7 | PubMed(1) |
| [Ee]xtracellular [Mm]atrix | 8 | PubMed(1) |

  
hide/show details for CG45050

### ECM

#### PubMed

1. Conditional switches for extracellular matrix patterning in Drosophila melanogaster. An F(1) mutagenesis strategy was developed to identify conditional mutations affecting extracellular matrix (**ECM**) patterning. Tubulogenesis requires coordinated movement of epithelial cells and deposition of a multilayered **ECM**. In the Drosophila ovary, an epithelium of follicle cells creates the eggshells, including the paired tubular dorsal appendages (DAs) that act as breathing tubes for the embryo. A P-element mutagenesis strategy allowed for conditional overexpression of hundreds of genes in follicle cells. Conditional phenotypes were scored at the level of individual mutant (F(1)) female flies. **ECM** pattern regulators were readily identified including MAPK signaling gene ets domain lacking (fused DAs), Wnt pathway genes frizzled 3 and osa (long DAs), Hh pathway gene debra (branched DAs), and transcription factor genes sima/HIF-1alpha, ush, lilli, Tfb1, broad, and foxo. In moving cells the [Ca(2+)]/calcineurin pathway can regulate adhesion to **ECM** while adherens junctions link cells together. Accordingly, thin eggshell and DA phenotypes were identified for the calcineurin regulator calreticulin and the adherens junction component arc. Finally a tubulogenesis defect phenotype was identified for the gene pterodactyl, homologous to the mammalian serine/threonine receptor-associated protein (STRAP) that integrates the TGF-beta and PI3K/AKT signaling pathways. Because phenotypes can be scored in each mutant fly before and after gene induction, this F(1) conditional mutagenesis strategy should allow for increased scale in screens for mutations affecting repeated (reiterated) events in adult animals, including gametogenesis, movement, behavior, and learning.

### [Ee]xtracellular [Mm]atrix

#### PubMed

1. Conditional switches for **extracellular matrix** patterning in Drosophila melanogaster. An F(1) mutagenesis strategy was developed to identify conditional mutations affecting **extracellular matrix** (ECM) patterning. Tubulogenesis requires coordinated movement of epithelial cells and deposition of a multilayered ECM. In the Drosophila ovary, an epithelium of follicle cells creates the eggshells, including the paired tubular dorsal appendages (DAs) that act as breathing tubes for the embryo. A P-element mutagenesis strategy allowed for conditional overexpression of hundreds of genes in follicle cells. Conditional phenotypes were scored at the level of individual mutant (F(1)) female flies. ECM pattern regulators were readily identified including MAPK signaling gene ets domain lacking (fused DAs), Wnt pathway genes frizzled 3 and osa (long DAs), Hh pathway gene debra (branched DAs), and transcription factor genes sima/HIF-1alpha, ush, lilli, Tfb1, broad, and foxo. In moving cells the [Ca(2+)]/calcineurin pathway can regulate adhesion to ECM while adherens junctions link cells together. Accordingly, thin eggshell and DA phenotypes were identified for the calcineurin regulator calreticulin and the adherens junction component arc. Finally a tubulogenesis defect phenotype was identified for the gene pterodactyl, homologous to the mammalian serine/threonine receptor-associated protein (STRAP) that integrates the TGF-beta and PI3K/AKT signaling pathways. Because phenotypes can be scored in each mutant fly before and after gene induction, this F(1) conditional mutagenesis strategy should allow for increased scale in screens for mutations affecting repeated (reiterated) events in adult animals, including gametogenesis, movement, behavior, and learning.

---

## capt

- NCBI Gene
- GeneRIF
- Pubmed

| Search Term | Rank | Fields |
| --- | --- | --- |
| [Ff]ilopodia | 1 | PubMed(1) |
| [Mm]igration | 6 | PubMed(4) |
| [Ii]nvasive | 10 | PubMed(1) |
| [Mm]etastasis | 12 | PubMed(1) |
| [Cc]ell [Ii]nvasion | 13 | PubMed(1) |

  
hide/show details for capt

### [Ff]ilopodia

#### PubMed

1. A novel forward genetic screen for identifying mutations affecting larval neuronal dendrite development in Drosophila melanogaster. Vertebrate and invertebrate dendrites are information-processing compartments that can be found on both central and peripheral neurons. Elucidating the molecular underpinnings of information processing in the nervous system ultimately requires an understanding of the genetic pathways that regulate dendrite formation and maintenance. Despite the importance of dendrite development, few forward genetic approaches have been used to analyze the latest stages of dendrite development, including the formation of F-actin-rich dendritic **filopodia** or dendritic spines. We developed a forward genetic screen utilizing transgenic Drosophila second instar larvae expressing an actin, green fluorescent protein (GFP) fusion protein (actin::GFP) in subsets of sensory neurons. Utilizing this fluorescent transgenic reporter, we conducted a forward genetic screen of >4000 mutagenized chromosomes bearing lethal mutations that affected multiple aspects of larval dendrite development. We isolated 13 mutations on the X and second chromosomes composing 11 complementation groups affecting dendrite outgrowth/branching, dendritic **filopodia** formation, or actin::GFP localization within dendrites in vivo. In a fortuitous observation, we observed that the structure of dendritic arborization (da) neuron dendritic **filopodia** changes in response to a changing environment.

### [Mm]igration

#### PubMed

1. A Jnk-Rho-Actin remodeling positive feedback network directs Src-driven invasion. Current models of tumor cell invasion propose that oncogenic signaling converges upon key orchestrators of cytoskeletal dynamics, including c-Jun N-terminal kinase (Jnk) and RhoGTPase family members; these signals dynamically direct Actin remodeling proteins (ARPs) to catalyze the cytoskeletal changes required for **migration**. Src is a key driver of tumor aggression, metastasis and patient mortality. To clarify how Src regulates Actin dynamics to promote invasive **migration**, we performed a genetic modifier screen in a Drosophila model of invasion. Nine genes linked to Actin dynamics were identified that mediate invasion in situ. We found that ARPs were required for many oncogenic effects of Src including Mmp1 expression and initiation of apoptosis. Surprisingly, they were also regulators of Jnk pathway activity: both Src and the small GTPase Rho1 activated Jnk in a manner dependent on ARPs during invasion. Our results suggest that ARPs are not simply downstream executors of signal transduction pathways. Rather, they participate in a positive feedback network involving canonical oncogenic signaling pathways that promote tumor invasion.
2. Molecular requirements for actin-based lamella formation in Drosophila S2 cells. Cell **migration** occurs through the protrusion of the actin-enriched lamella. Here, we investigated the effects of RNAi depletion of approximately 90 proteins implicated in actin function on lamella formation in Drosophila S2 cells. Similar to in vitro reconstitution studies of actin-based Listeria movement, we find that lamellae formation requires a relatively small set of proteins that participate in actin nucleation (Arp2/3 and SCAR), barbed end capping (capping protein), filament depolymerization (cofilin and Aip1), and actin monomer binding (profilin and cyclase-associated protein). Lamellae are initiated by parallel and partially redundant signaling pathways involving Rac GTPases and the adaptor protein Nck, which stimulate SCAR, an Arp2/3 activator. We also show that RNAi of three proteins (kette, Abi, and Sra-1) known to copurify with and inhibit SCAR in vitro leads to SCAR degradation, revealing a novel function of this protein complex in SCAR stability. Our results have identified an essential set of proteins involved in actin dynamics during lamella formation in Drosophila S2 cells.
3. Egfr signaling regulates ommatidial rotation and cell motility in the Drosophila eye via MAPK/Pnt signaling and the Ras effector Canoe/AF6. Epidermal Growth Factor-receptor (Egfr) signaling is evolutionarily conserved and controls a variety of different cellular processes. In Drosophila these include proliferation, patterning, cell-fate determination, **migration** and survival. Here we provide evidence for a new role of Egfr signaling in controlling ommatidial rotation during planar cell polarity (PCP) establishment in the Drosophila eye. Although the signaling pathways involved in PCP establishment and photoreceptor cell-type specification are beginning to be unraveled, very little is known about the associated 90 degrees rotation process. One of the few rotation-specific mutations known is roulette (rlt) in which ommatidia rotate to a random degree, often more than 90 degrees. Here we show that rlt is a rotation-specific allele of the inhibitory Egfr ligand Argos and that modulation of Egfr activity shows defects in ommatidial rotation. Our data indicate that, beside the Raf/MAPK cascade, the Ras effector Canoe/AF6 acts downstream of Egfr/Ras and provides a link from Egfr to cytoskeletal elements in this developmentally regulated cell motility process. We provide further evidence for an involvement of cadherins and non-muscle myosin II as downstream components controlling rotation. In particular, the involvement of the cadherin Flamingo, a PCP gene, downstream of Egfr signaling provides the first link between PCP establishment and the Egfr pathway.
4. A clonal genetic screen for mutants causing defects in larval tracheal morphogenesis in Drosophila. The initial establishment of the tracheal network in the Drosophila embryo is beginning to be understood in great detail, both in its genetic control cascades and in its cell biological events. By contrast, the vast expansion of the system during larval growth, with its extensive ramification of preexisting tracheal branches, has been analyzed less well. The mutant phenotypes of many genes involved in this process are probably not easy to reveal, as these genes may be required for other functions at earlier developmental stages. We therefore conducted a screen for defects in individual clonal homozygous mutant cells in the tracheal network of heterozygous larvae using the mosaic analysis with a repressible cell marker (MARCM) system to generate marked, recombinant mitotic clones. We describe the identification of a set of mutants with distinct phenotypic effects. In particular we found a range of defects in terminal cells, including failure in lumen formation and reduced or extensive branching. Other mutations affect cell growth, cell shape, and cell **migration**.

### [Ii]nvasive

#### PubMed

1. A Jnk-Rho-Actin remodeling positive feedback network directs Src-driven invasion. Current models of tumor cell invasion propose that oncogenic signaling converges upon key orchestrators of cytoskeletal dynamics, including c-Jun N-terminal kinase (Jnk) and RhoGTPase family members; these signals dynamically direct Actin remodeling proteins (ARPs) to catalyze the cytoskeletal changes required for migration. Src is a key driver of tumor aggression, metastasis and patient mortality. To clarify how Src regulates Actin dynamics to promote **invasive** migration, we performed a genetic modifier screen in a Drosophila model of invasion. Nine genes linked to Actin dynamics were identified that mediate invasion in situ. We found that ARPs were required for many oncogenic effects of Src including Mmp1 expression and initiation of apoptosis. Surprisingly, they were also regulators of Jnk pathway activity: both Src and the small GTPase Rho1 activated Jnk in a manner dependent on ARPs during invasion. Our results suggest that ARPs are not simply downstream executors of signal transduction pathways. Rather, they participate in a positive feedback network involving canonical oncogenic signaling pathways that promote tumor invasion.

### [Mm]etastasis

#### PubMed

1. A Jnk-Rho-Actin remodeling positive feedback network directs Src-driven invasion. Current models of tumor cell invasion propose that oncogenic signaling converges upon key orchestrators of cytoskeletal dynamics, including c-Jun N-terminal kinase (Jnk) and RhoGTPase family members; these signals dynamically direct Actin remodeling proteins (ARPs) to catalyze the cytoskeletal changes required for migration. Src is a key driver of tumor aggression, **metastasis** and patient mortality. To clarify how Src regulates Actin dynamics to promote invasive migration, we performed a genetic modifier screen in a Drosophila model of invasion. Nine genes linked to Actin dynamics were identified that mediate invasion in situ. We found that ARPs were required for many oncogenic effects of Src including Mmp1 expression and initiation of apoptosis. Surprisingly, they were also regulators of Jnk pathway activity: both Src and the small GTPase Rho1 activated Jnk in a manner dependent on ARPs during invasion. Our results suggest that ARPs are not simply downstream executors of signal transduction pathways. Rather, they participate in a positive feedback network involving canonical oncogenic signaling pathways that promote tumor invasion.

### [Cc]ell [Ii]nvasion

#### PubMed

1. A Jnk-Rho-Actin remodeling positive feedback network directs Src-driven invasion. Current models of tumor **cell invasion** propose that oncogenic signaling converges upon key orchestrators of cytoskeletal dynamics, including c-Jun N-terminal kinase (Jnk) and RhoGTPase family members; these signals dynamically direct Actin remodeling proteins (ARPs) to catalyze the cytoskeletal changes required for migration. Src is a key driver of tumor aggression, metastasis and patient mortality. To clarify how Src regulates Actin dynamics to promote invasive migration, we performed a genetic modifier screen in a Drosophila model of invasion. Nine genes linked to Actin dynamics were identified that mediate invasion in situ. We found that ARPs were required for many oncogenic effects of Src including Mmp1 expression and initiation of apoptosis. Surprisingly, they were also regulators of Jnk pathway activity: both Src and the small GTPase Rho1 activated Jnk in a manner dependent on ARPs during invasion. Our results suggest that ARPs are not simply downstream executors of signal transduction pathways. Rather, they participate in a positive feedback network involving canonical oncogenic signaling pathways that promote tumor invasion.

---

## stv

- NCBI Gene
- GeneRIF
- Pubmed

| Search Term | Rank | Fields |
| --- | --- | --- |
| [Mm]igration | 6 | PubMed(1) |
| ECM | 7 | PubMed(1) |
| [Ee]xtracellular [Mm]atrix | 8 | PubMed(1) |

  
hide/show details for stv

### [Mm]igration

#### PubMed

1. Genes conserved in bilaterians but jointly lost with Myc during nematode evolution are enriched in cell proliferation and cell **migration** functions. Animals use a stereotypical set of developmental genes to build body architectures of varying sizes and organizational complexity. Some genes are critical to developmental patterning, while other genes are important to physiological control of growth. However, growth regulator genes may not be as important in small-bodied "micro-metazoans" such as nematodes. Nematodes use a simplified developmental strategy of lineage-based cell fate specifications to produce an adult bilaterian body composed of a few hundreds of cells. Nematodes also lost the MYC proto-oncogenic regulator of cell proliferation. To identify additional regulators of cell proliferation that were lost with MYC, we computationally screened and determined 839 high-confidence genes that are conserved in bilaterians/lost in nematodes (CIBLIN genes). We find that 30 % of all CIBLIN genes encode transcriptional regulators of cell proliferation, epithelial-to-mesenchyme transitions, and other processes. Over 50 % of CIBLIN genes are unnamed genes in Drosophila, suggesting that there are many understudied genes. Interestingly, CIBLIN genes include many Myc synthetic lethal (MycSL) hits from recent screens. CIBLIN genes include key regulators of heparan sulfate proteoglycan (HSPG) sulfation patterns, and lysyl oxidases involved in cross-linking and modification of the extracellular matrix (ECM). These genes and others suggest the CIBLIN repertoire services critical functions in ECM remodeling and cell **migration** in large-bodied bilaterians. Correspondingly, CIBLIN genes are co-expressed with Myc in cancer transcriptomes, and include a preponderance of known determinants of cancer progression and tumor aggression. We propose that CIBLIN gene research can improve our understanding of regulatory control of cellular growth in metazoans.

### ECM

#### PubMed

1. Genes conserved in bilaterians but jointly lost with Myc during nematode evolution are enriched in cell proliferation and cell migration functions. Animals use a stereotypical set of developmental genes to build body architectures of varying sizes and organizational complexity. Some genes are critical to developmental patterning, while other genes are important to physiological control of growth. However, growth regulator genes may not be as important in small-bodied "micro-metazoans" such as nematodes. Nematodes use a simplified developmental strategy of lineage-based cell fate specifications to produce an adult bilaterian body composed of a few hundreds of cells. Nematodes also lost the MYC proto-oncogenic regulator of cell proliferation. To identify additional regulators of cell proliferation that were lost with MYC, we computationally screened and determined 839 high-confidence genes that are conserved in bilaterians/lost in nematodes (CIBLIN genes). We find that 30 % of all CIBLIN genes encode transcriptional regulators of cell proliferation, epithelial-to-mesenchyme transitions, and other processes. Over 50 % of CIBLIN genes are unnamed genes in Drosophila, suggesting that there are many understudied genes. Interestingly, CIBLIN genes include many Myc synthetic lethal (MycSL) hits from recent screens. CIBLIN genes include key regulators of heparan sulfate proteoglycan (HSPG) sulfation patterns, and lysyl oxidases involved in cross-linking and modification of the extracellular matrix (**ECM**). These genes and others suggest the CIBLIN repertoire services critical functions in **ECM** remodeling and cell migration in large-bodied bilaterians. Correspondingly, CIBLIN genes are co-expressed with Myc in cancer transcriptomes, and include a preponderance of known determinants of cancer progression and tumor aggression. We propose that CIBLIN gene research can improve our understanding of regulatory control of cellular growth in metazoans.

### [Ee]xtracellular [Mm]atrix

#### PubMed

1. Genes conserved in bilaterians but jointly lost with Myc during nematode evolution are enriched in cell proliferation and cell migration functions. Animals use a stereotypical set of developmental genes to build body architectures of varying sizes and organizational complexity. Some genes are critical to developmental patterning, while other genes are important to physiological control of growth. However, growth regulator genes may not be as important in small-bodied "micro-metazoans" such as nematodes. Nematodes use a simplified developmental strategy of lineage-based cell fate specifications to produce an adult bilaterian body composed of a few hundreds of cells. Nematodes also lost the MYC proto-oncogenic regulator of cell proliferation. To identify additional regulators of cell proliferation that were lost with MYC, we computationally screened and determined 839 high-confidence genes that are conserved in bilaterians/lost in nematodes (CIBLIN genes). We find that 30 % of all CIBLIN genes encode transcriptional regulators of cell proliferation, epithelial-to-mesenchyme transitions, and other processes. Over 50 % of CIBLIN genes are unnamed genes in Drosophila, suggesting that there are many understudied genes. Interestingly, CIBLIN genes include many Myc synthetic lethal (MycSL) hits from recent screens. CIBLIN genes include key regulators of heparan sulfate proteoglycan (HSPG) sulfation patterns, and lysyl oxidases involved in cross-linking and modification of the **extracellular matrix** (ECM). These genes and others suggest the CIBLIN repertoire services critical functions in ECM remodeling and cell migration in large-bodied bilaterians. Correspondingly, CIBLIN genes are co-expressed with Myc in cancer transcriptomes, and include a preponderance of known determinants of cancer progression and tumor aggression. We propose that CIBLIN gene research can improve our understanding of regulatory control of cellular growth in metazoans.

---

## Ect3

- NCBI Gene
- GeneRIF
- Pubmed

| Search Term | Rank | Fields |
| --- | --- | --- |
| [Mm]igration | 6 | PubMed(1) |

  
hide/show details for Ect3

### [Mm]igration

#### PubMed

1. Functional genomic analysis of the periodic transcriptome in the developing Drosophila wing. The eukaryotic cell cycle, driven by both transcriptional and posttranslational mechanisms, is the central molecular oscillator underlying tissue growth throughout animals. Although genome-wide studies have investigated cell-cycle-associated transcription in unicellular systems, global patterns of periodic transcription in multicellular tissues remain largely unexplored. Here we define the cell-cycle-associated transcriptome of the developing Drosophila wing epithelium and compare it with that of cultured Drosophila S2 cells, revealing a core set of periodic genes and a surprising degree of context specificity in periodic transcription. We further employ RNAi-mediated phenotypic profiling to define functional requirements for more than 300 periodic genes, with a focus on those required for cell proliferation in vivo. Finally, we investigate uncharacterized genes required for interkinetic nuclear **migration**. Combined, these findings provide a global perspective on cell-cycle control in vivo, and they highlight a critical need to understand the context-specific regulation of cell proliferation.

---

## pins

- NCBI Gene
- GeneRIF
- Pubmed

| Search Term | Rank | Fields |
| --- | --- | --- |
| [Mm]igration | 6 | PubMed(1) |
| [Ee]xtracellular [Mm]atrix | 8 | PubMed(1) |
| [Mm]etastasis | 12 | PubMed(1) |
| [Cc]ell [Ii]nvasion | 13 | PubMed(1) |

  
hide/show details for pins

### [Mm]igration

#### PubMed

1. Microtubule-induced Pins/Galphai cortical polarity in Drosophila neuroblasts. Cortical polarity regulates cell division, **migration**, and differentiation. Microtubules induce cortical polarity in yeast, but few examples are known in metazoans. We show that astral microtubules, kinesin Khc-73, and Discs large (Dlg) induce cortical polarization of Pins/Galphai in Drosophila neuroblasts; this cortical domain is functional for generating spindle asymmetry, daughter-cell-size asymmetry, and distinct sibling fates. Khc-73 localizes to astral microtubule plus ends, and Dlg/Khc-73 and Dlg/Pins coimmunoprecipitate, suggesting that microtubules induce Pins/Galphai cortical polarity through Dlg/Khc-73 interactions. The microtubule/Khc-73/Dlg pathway acts in parallel to the well-characterized Inscuteable/Par pathway, but each provides unique spatial and temporal information: The Inscuteable/Par pathway initiates at prophase to coordinate neuroblast cortical polarity with CNS tissue polarity, whereas the microtubule/Khc-73/Dlg pathway functions at metaphase to coordinate neuroblast cortical polarity with the mitotic spindle axis. These results identify a role for microtubules in polarizing the neuroblast cortex, a fundamental step for generating cell diversity through asymmetric cell division.

### [Ee]xtracellular [Mm]atrix

#### PubMed

1. Adherens junctions: new insight into assembly, modulation and function. Adherens junctions play pivotal roles in cell and tissue organization and patterning by mediating cell adhesion and cell signaling. These junctions consist of large multiprotein complexes that join the actin cytoskeleton to the plasma membrane to form adhesive contacts between cells or between cells and **extracellular matrix**. The best-known adherens junction is the zonula adherens (ZA) that forms a belt surrounding the apical pole of epithelial cells. Recent studies in Drosophila have further illuminated the structure of adherens junctions. Scaffolding proteins encoded by the stardust gene are novel components of the Crumbs complex, which plays a critical role in ZA assembly.1-3 The small GTPase Rap1 controls the symmetric re-assembly of the ZA after cell division.4 Finally, the asymmetric distribution of adherens junction material regulates spindle orientation during asymmetric cell division in the sensory organ lineage.

### [Mm]etastasis

#### PubMed

1. Modeling tumor invasion and **metastasis** in Drosophila. Conservation of major signaling pathways between humans and flies has made Drosophila a useful model organism for cancer research. Our understanding of the mechanisms regulating cell growth, differentiation and development has been considerably advanced by studies in Drosophila. Several recent high profile studies have examined the processes constraining the metastatic growth of tumor cells in fruit fly models. Cell invasion can be studied in the context of an in vivo setting in flies, enabling the genetic requirements of the microenvironment of tumor cells undergoing **metastasis** to be analyzed. This Perspective discusses the strengths and limitations of Drosophila models of cancer invasion and the unique tools that have enabled these studies. It also highlights several recent reports that together make a strong case for Drosophila as a system with the potential for both testing novel concepts in tumor progression and cell invasion, and for uncovering players in **metastasis**.

### [Cc]ell [Ii]nvasion

#### PubMed

1. Modeling tumor invasion and metastasis in Drosophila. Conservation of major signaling pathways between humans and flies has made Drosophila a useful model organism for cancer research. Our understanding of the mechanisms regulating cell growth, differentiation and development has been considerably advanced by studies in Drosophila. Several recent high profile studies have examined the processes constraining the metastatic growth of tumor cells in fruit fly models. **Cell invasion** can be studied in the context of an in vivo setting in flies, enabling the genetic requirements of the microenvironment of tumor cells undergoing metastasis to be analyzed. This Perspective discusses the strengths and limitations of Drosophila models of cancer invasion and the unique tools that have enabled these studies. It also highlights several recent reports that together make a strong case for Drosophila as a system with the potential for both testing novel concepts in tumor progression and **cell invasion**, and for uncovering players in metastasis.

---

## Spn42Da

- NCBI Gene
- GeneRIF
- Pubmed

| Search Term | Rank | Fields |
| --- | --- | --- |
| [Mm]igration | 6 | PubMed(1) |
| ECM | 7 | PubMed(1) |
| [Ee]xtracellular [Mm]atrix | 8 | PubMed(1) |
| [Ii]nvasive | 10 | PubMed(3) |
| [Mm]etastasis | 12 | PubMed(1) |

  
hide/show details for Spn42Da

### [Mm]igration

#### PubMed

1. Genes conserved in bilaterians but jointly lost with Myc during nematode evolution are enriched in cell proliferation and cell **migration** functions. Animals use a stereotypical set of developmental genes to build body architectures of varying sizes and organizational complexity. Some genes are critical to developmental patterning, while other genes are important to physiological control of growth. However, growth regulator genes may not be as important in small-bodied "micro-metazoans" such as nematodes. Nematodes use a simplified developmental strategy of lineage-based cell fate specifications to produce an adult bilaterian body composed of a few hundreds of cells. Nematodes also lost the MYC proto-oncogenic regulator of cell proliferation. To identify additional regulators of cell proliferation that were lost with MYC, we computationally screened and determined 839 high-confidence genes that are conserved in bilaterians/lost in nematodes (CIBLIN genes). We find that 30 % of all CIBLIN genes encode transcriptional regulators of cell proliferation, epithelial-to-mesenchyme transitions, and other processes. Over 50 % of CIBLIN genes are unnamed genes in Drosophila, suggesting that there are many understudied genes. Interestingly, CIBLIN genes include many Myc synthetic lethal (MycSL) hits from recent screens. CIBLIN genes include key regulators of heparan sulfate proteoglycan (HSPG) sulfation patterns, and lysyl oxidases involved in cross-linking and modification of the extracellular matrix (ECM). These genes and others suggest the CIBLIN repertoire services critical functions in ECM remodeling and cell **migration** in large-bodied bilaterians. Correspondingly, CIBLIN genes are co-expressed with Myc in cancer transcriptomes, and include a preponderance of known determinants of cancer progression and tumor aggression. We propose that CIBLIN gene research can improve our understanding of regulatory control of cellular growth in metazoans.

### ECM

#### PubMed

1. Genes conserved in bilaterians but jointly lost with Myc during nematode evolution are enriched in cell proliferation and cell migration functions. Animals use a stereotypical set of developmental genes to build body architectures of varying sizes and organizational complexity. Some genes are critical to developmental patterning, while other genes are important to physiological control of growth. However, growth regulator genes may not be as important in small-bodied "micro-metazoans" such as nematodes. Nematodes use a simplified developmental strategy of lineage-based cell fate specifications to produce an adult bilaterian body composed of a few hundreds of cells. Nematodes also lost the MYC proto-oncogenic regulator of cell proliferation. To identify additional regulators of cell proliferation that were lost with MYC, we computationally screened and determined 839 high-confidence genes that are conserved in bilaterians/lost in nematodes (CIBLIN genes). We find that 30 % of all CIBLIN genes encode transcriptional regulators of cell proliferation, epithelial-to-mesenchyme transitions, and other processes. Over 50 % of CIBLIN genes are unnamed genes in Drosophila, suggesting that there are many understudied genes. Interestingly, CIBLIN genes include many Myc synthetic lethal (MycSL) hits from recent screens. CIBLIN genes include key regulators of heparan sulfate proteoglycan (HSPG) sulfation patterns, and lysyl oxidases involved in cross-linking and modification of the extracellular matrix (**ECM**). These genes and others suggest the CIBLIN repertoire services critical functions in **ECM** remodeling and cell migration in large-bodied bilaterians. Correspondingly, CIBLIN genes are co-expressed with Myc in cancer transcriptomes, and include a preponderance of known determinants of cancer progression and tumor aggression. We propose that CIBLIN gene research can improve our understanding of regulatory control of cellular growth in metazoans.

### [Ee]xtracellular [Mm]atrix

#### PubMed

1. Genes conserved in bilaterians but jointly lost with Myc during nematode evolution are enriched in cell proliferation and cell migration functions. Animals use a stereotypical set of developmental genes to build body architectures of varying sizes and organizational complexity. Some genes are critical to developmental patterning, while other genes are important to physiological control of growth. However, growth regulator genes may not be as important in small-bodied "micro-metazoans" such as nematodes. Nematodes use a simplified developmental strategy of lineage-based cell fate specifications to produce an adult bilaterian body composed of a few hundreds of cells. Nematodes also lost the MYC proto-oncogenic regulator of cell proliferation. To identify additional regulators of cell proliferation that were lost with MYC, we computationally screened and determined 839 high-confidence genes that are conserved in bilaterians/lost in nematodes (CIBLIN genes). We find that 30 % of all CIBLIN genes encode transcriptional regulators of cell proliferation, epithelial-to-mesenchyme transitions, and other processes. Over 50 % of CIBLIN genes are unnamed genes in Drosophila, suggesting that there are many understudied genes. Interestingly, CIBLIN genes include many Myc synthetic lethal (MycSL) hits from recent screens. CIBLIN genes include key regulators of heparan sulfate proteoglycan (HSPG) sulfation patterns, and lysyl oxidases involved in cross-linking and modification of the **extracellular matrix** (ECM). These genes and others suggest the CIBLIN repertoire services critical functions in ECM remodeling and cell migration in large-bodied bilaterians. Correspondingly, CIBLIN genes are co-expressed with Myc in cancer transcriptomes, and include a preponderance of known determinants of cancer progression and tumor aggression. We propose that CIBLIN gene research can improve our understanding of regulatory control of cellular growth in metazoans.

### [Ii]nvasive

#### PubMed

1. The Spn4 gene of Drosophila encodes a potent furin-directed secretory pathway serpin. Proprotein convertases (PCs) are an important class of host-cell serine endoproteases implicated in many physiological and pathological processes. Owing to their expanding roles in the proteolytic events required for generating infectious microbial pathogens and for tumor growth and **invasive**ness, there is increasing interest in identifying endogenous PC inhibitors. Here we report the identification of Spn4A, a previously uncharacterized secretory pathway serine protease inhibitor (serpin) from Drosophila melanogaster that contains a consensus furin cleavage site, -Arg(P4)-Arg-Lys-Arg(P1) downsream-, in its reactive site loop (RSL). Our biochemical and kinetics analysis revealed that recombinant Spn4A inhibits human furin (K(i), 13 pM; k(ass), 3.2 x 10(7) M(-1) x s(-1)) and Drosophila PC2 (K(i), 3.5 nM; k(ass), 9.2 x 10(4) M(-1) x s(-1)) by a slow-binding mechanism characteristic of serpin molecules and forms a kinetically trapped SDS-stable complex with each enzyme. For both PCs, the stoichiometry of inhibition by Spn4A is nearly 1, which is characteristic of known physiological serpin-protease interactions. Mass analysis of furin-Spn4A reaction products identified the actual reactive site center of Spn4A to be -Arg(P4)-Arg-Lys-Arg(P1)-downstream-. Moreover, we demonstrate that Spn4A's highly effective PC inhibition properties are critically dependent on the unusual length of its RSL, which is composed of 18 aa instead of the typical 17-residue RSL found in most other inhibitory serpins. The identification of Spn4A, the most potent and effective natural serpin of PCs identified to date, suggests that Spn4A could be a prototype of endogenous serpins involved in the precise regulation of PC-dependent proteolytic cleavage events in the secretory pathway of eukaryotic cells.
2. The Spn4 gene of Drosophila encodes a potent furin-directed secretory pathway serpin. Proprotein convertases (PCs) are an important class of host-cell serine endoproteases implicated in many physiological and pathological processes. Owing to their expanding roles in the proteolytic events required for generating infectious microbial pathogens and for tumor growth and **invasive**ness, there is increasing interest in identifying endogenous PC inhibitors. Here we report the identification of Spn4A, a previously uncharacterized secretory pathway serine protease inhibitor (serpin) from Drosophila melanogaster that contains a consensus furin cleavage site, -Arg(P4)-Arg-Lys-Arg(P1) downsream-, in its reactive site loop (RSL). Our biochemical and kinetics analysis revealed that recombinant Spn4A inhibits human furin (K(i), 13 pM; k(ass), 3.2 x 10(7) M(-1) x s(-1)) and Drosophila PC2 (K(i), 3.5 nM; k(ass), 9.2 x 10(4) M(-1) x s(-1)) by a slow-binding mechanism characteristic of serpin molecules and forms a kinetically trapped SDS-stable complex with each enzyme. For both PCs, the stoichiometry of inhibition by Spn4A is nearly 1, which is characteristic of known physiological serpin-protease interactions. Mass analysis of furin-Spn4A reaction products identified the actual reactive site center of Spn4A to be -Arg(P4)-Arg-Lys-Arg(P1)-downstream-. Moreover, we demonstrate that Spn4A's highly effective PC inhibition properties are critically dependent on the unusual length of its RSL, which is composed of 18 aa instead of the typical 17-residue RSL found in most other inhibitory serpins. The identification of Spn4A, the most potent and effective natural serpin of PCs identified to date, suggests that Spn4A could be a prototype of endogenous serpins involved in the precise regulation of PC-dependent proteolytic cleavage events in the secretory pathway of eukaryotic cells.
3. Repression of liver colorectal metastasis by the serpin Spn4A a naturally occurring inhibitor of the constitutive secretory proprotein convertases. Liver is the most common site of metastasis from colorectal cancers, and liver of patients with liver colorectal metastasis have abnormal levels of the proprotein convertases (PCs). These proteases are involved in the activation and/or expression of various colon cancer-related mediators, making them promising targets in colorectal liver metastasis therapy. Here, we revealed that the serpin Spn4 from Drosophila melanogaster inhibits the activity of all the PCs found in the constitutive secretory pathway and represses the metastatic potential of the colon cancer cells HT-29 and CT-26. In these cells, Spn4A inhibited the processing of the PCs substrates IGF-1R and PDGF-A that associated their reduced anchorage-independent growth, **invasive**ness and survival in response to apoptotic agents. In vivo, Spn4A-expressing tumor cells showed repressed subcutaneous tumor development and liver metastases formation in response to their intrasplenic inoculation. In these cells Spn4A induced the expression of molecules with anti-metastatic functions and inhibited expression of pro-tumorigenic molecules. Taken together, our findings identify Spn4A as the only endogenous inhibitor of all the constitutive secretory pathway PCs, which is able to repress the metastatic potential of colon cancer cells. These results suggest the potential use of Spn4A and/or derivates as a useful adduct colorectal liver metastasis prevention.

### [Mm]etastasis

#### PubMed

1. Repression of liver colorectal **metastasis** by the serpin Spn4A a naturally occurring inhibitor of the constitutive secretory proprotein convertases. Liver is the most common site of **metastasis** from colorectal cancers, and liver of patients with liver colorectal **metastasis** have abnormal levels of the proprotein convertases (PCs). These proteases are involved in the activation and/or expression of various colon cancer-related mediators, making them promising targets in colorectal liver **metastasis** therapy. Here, we revealed that the serpin Spn4 from Drosophila melanogaster inhibits the activity of all the PCs found in the constitutive secretory pathway and represses the metastatic potential of the colon cancer cells HT-29 and CT-26. In these cells, Spn4A inhibited the processing of the PCs substrates IGF-1R and PDGF-A that associated their reduced anchorage-independent growth, invasiveness and survival in response to apoptotic agents. In vivo, Spn4A-expressing tumor cells showed repressed subcutaneous tumor development and liver metastases formation in response to their intrasplenic inoculation. In these cells Spn4A induced the expression of molecules with anti-metastatic functions and inhibited expression of pro-tumorigenic molecules. Taken together, our findings identify Spn4A as the only endogenous inhibitor of all the constitutive secretory pathway PCs, which is able to repress the metastatic potential of colon cancer cells. These results suggest the potential use of Spn4A and/or derivates as a useful adduct colorectal liver **metastasis** prevention.

---

## Pi3K92E

- NCBI Gene
- GeneRIF
- Pubmed

| Search Term | Rank | Fields |
| --- | --- | --- |
| [Ff]ilopodia | 1 | PubMed(1) |
| [Mm]igration | 6 | PubMed(17) |
| [Ii]nvasive | 10 | PubMed(7) |
| [Mm]etastasis | 12 | PubMed(1) |

  
hide/show details for Pi3K92E

### [Ff]ilopodia

#### PubMed

1. Gene regulatory networks controlling hematopoietic progenitor niche cell production and differentiation in the Drosophila lymph gland. Hematopoiesis occurs in two phases in Drosophila, with the first completed during embryogenesis and the second accomplished during larval development. The lymph gland serves as the venue for the final hematopoietic program, with this larval tissue well-studied as to its cellular organization and genetic regulation. While the medullary zone contains stem-like hematopoietic progenitors, the posterior signaling center (PSC) functions as a niche microenvironment essential for controlling the decision between progenitor maintenance versus cellular differentiation. In this report, we utilize a PSC-specific GAL4 driver and UAS-gene RNAi strains, to selectively knockdown individual gene functions in PSC cells. We assessed the effect of abrogating the function of 820 genes as to their requirement for niche cell production and differentiation. 100 genes were shown to be essential for normal niche development, with various loci placed into sub-groups based on the functions of their encoded protein products and known genetic interactions. For members of three of these groups, we characterized loss- and gain-of-function phenotypes. Gene function knockdown of members of the BAP chromatin-remodeling complex resulted in niche cells that do not express the hedgehog (hh) gene and fail to differentiate **filopodia** believed important for Hh signaling from the niche to progenitors. Abrogating gene function of various members of the insulin-like growth factor and TOR signaling pathways resulted in anomalous PSC cell production, leading to a defective niche organization. Further analysis of the Pten, TSC1, and TSC2 tumor suppressor genes demonstrated their loss-of-function condition resulted in severely altered blood cell homeostasis, including the abundant production of lamellocytes, specialized hemocytes involved in innate immune responses. Together, this cell-specific RNAi knockdown survey and mutant phenotype analyses identified multiple genes and their regulatory networks required for the normal organization and function of the hematopoietic progenitor niche within the lymph gland.

### [Mm]igration

#### PubMed

1. The PDGF/VEGF receptor controls blood cell survival in Drosophila. The Drosophila PDGF/VEGF receptor (PVR) has known functions in the guidance of cell **migration**. We now demonstrate that during embryonic hematopoiesis, PVR has a role in the control of antiapoptotic cell survival. In Pvr mutants, a large fraction of the embryonic hemocyte population undergoes apoptosis, and the remaining blood cells cannibalistically phagocytose their dying peers. Consequently, total hemocyte numbers drop dramatically during embryogenesis, and large aggregates of engorged macrophages carrying multiple apoptotic corpses form. Hemocyte-specific expression of the pan-caspase inhibitor p35 in Pvr mutants eliminates hemocyte aggregates and restores blood cell counts and morphology. Additional rescue experiments suggest involvement of the Ras pathway in PVR-mediated blood cell survival. In cell culture, we demonstrate that PVR directly controls survival of a hemocyte cell line. This function of PVR shows striking conservation with mammalian hematopoiesis and establishes Drosophila as a model to study hematopoietic cell survival in development and disease.
2. A drosophila model for EGFR-Ras and PI3K-dependent human glioma. Gliomas, the most common malignant tumors of the nervous system, frequently harbor mutations that activate the epidermal growth factor receptor (EGFR) and phosphatidylinositol-3 kinase (PI3K) signaling pathways. To investigate the genetic basis of this disease, we developed a glioma model in Drosophila. We found that constitutive coactivation of EGFR-Ras and PI3K pathways in Drosophila glia and glial precursors gives rise to neoplastic, invasive glial cells that create transplantable tumor-like growths, mimicking human glioma. Our model represents a robust organotypic and cell-type-specific Drosophila cancer model in which malignant cells are created by mutations in signature genes and pathways thought to be driving forces in a homologous human cancer. Genetic analyses demonstrated that EGFR and PI3K initiate malignant neoplastic transformation via a combinatorial genetic network composed primarily of other pathways commonly mutated or activated in human glioma, including the Tor, Myc, G1 Cyclins-Cdks, and Rb-E2F pathways. This network acts synergistically to coordinately stimulate cell cycle entry and progression, protein translation, and inappropriate cellular growth and **migration**. In particular, we found that the fly orthologs of CyclinE, Cdc25, and Myc are key rate-limiting genes required for glial neoplasia. Moreover, orthologs of Sin1, Rictor, and Cdk4 are genes required only for abnormal neoplastic glial proliferation but not for glial development. These and other genes within this network may represent important therapeutic targets in human glioma.
3. Egfr signaling regulates ommatidial rotation and cell motility in the Drosophila eye via MAPK/Pnt signaling and the Ras effector Canoe/AF6. Epidermal Growth Factor-receptor (Egfr) signaling is evolutionarily conserved and controls a variety of different cellular processes. In Drosophila these include proliferation, patterning, cell-fate determination, **migration** and survival. Here we provide evidence for a new role of Egfr signaling in controlling ommatidial rotation during planar cell polarity (PCP) establishment in the Drosophila eye. Although the signaling pathways involved in PCP establishment and photoreceptor cell-type specification are beginning to be unraveled, very little is known about the associated 90 degrees rotation process. One of the few rotation-specific mutations known is roulette (rlt) in which ommatidia rotate to a random degree, often more than 90 degrees. Here we show that rlt is a rotation-specific allele of the inhibitory Egfr ligand Argos and that modulation of Egfr activity shows defects in ommatidial rotation. Our data indicate that, beside the Raf/MAPK cascade, the Ras effector Canoe/AF6 acts downstream of Egfr/Ras and provides a link from Egfr to cytoskeletal elements in this developmentally regulated cell motility process. We provide further evidence for an involvement of cadherins and non-muscle myosin II as downstream components controlling rotation. In particular, the involvement of the cadherin Flamingo, a PCP gene, downstream of Egfr signaling provides the first link between PCP establishment and the Egfr pathway.
4. Colorectal cancer progression: lessons from Drosophila? Human colorectal cancers arise as benign adenomas, tumours that retain their epithelial character, and then progress to malignant adenocarcinomas and carcinomas in which the epithelium becomes disrupted. Carcinomas often exhibit transcriptional downregulation of E-cadherin and other epithelial genes in an epithelial-to-mesenchymal transition (EMT), a mechanism first discovered in Drosophila to be mediated by the transcription factors Twist and Snail. In contrast, adenocarcinomas retain expression of E-cadherin and disruption of the epithelium occurs through formation of progressively smaller epithelial cysts with apical Crumbs/CRB3, Stardust/PALS1, and Bazooka/PAR3 localised to the inner lumen. Results from Drosophila show that morphologically similar cysts form upon induction of clonal heterogeneity in Wnt, Smad, or Ras signalling levels, which causes extrusion of epithelial cells at clonal boundaries. Thus, intratumour heterogeneity might also promote formation of adenocarcinomas in humans. Finally, epithelial cysts can collectively migrate, as in the case of Drosophila border cells, a potential model system for the invasive **migration** of adenocarcinoma cells.
5. Guidance of cell **migration** by EGF receptor signaling during Drosophila oogenesis. Directed cell **migration** is important for many aspects of normal animal development, but little is known about how cell **migration**s are guided or the mechanisms by which guidance cues are translated into directed cell movement. Here we present evidence that signaling mediated by the epidermal growth factor receptor (EGFR) guides dorsal **migration** of border cells during Drosophila oogenesis. The transforming growth factor-alpha (TGF-alpha)-like ligand Gurken appears to serve as the guidance cue. To mediate this guidance function, EGFR signals via a pathway that is independent of Raf-MAP kinase and receptor-specific.
6. PTEN affects cell size, cell proliferation and apoptosis during Drosophila eye development. Mutations in the tumor suppressor gene PTEN (MMAC1/TEP1) are associated with a large number of human cancers and several autosomal-dominant disorders. Mice mutant for PTEN die at early embryonic stages and the mutant embryonic fibroblasts display decreased sensitivity to cell death. Overexpression of PTEN in different mammalian tissue culture cells affects various processes including cell proliferation, cell death and cell **migration**. We have characterized the Drosophila PTEN gene and present evidence that both inactivation and overexpression of PTEN affect cell size, while overexpression of PTEN also inhibits cell cycle progression at early mitosis and promotes cell death during eye development in a context-dependent manner. Furthermore, we have shown that PTEN acts in the insulin signaling pathway and all signals from the insulin receptor can be antagonized by either Drosophila or human PTEN, suggesting a potential means for alleviating symptoms associated with altered insulin signaling.
7. Modeling glioma growth and invasion in Drosophila melanogaster. Glioblastoma is the most common and most malignant intrinsic human brain tumor, characterized by extensive invasion and proliferation of glial (astrocytic) tumor cells, frequent activation of tyrosine kinase receptor signaling pathways, relative resistance to chemotherapy and radiotherapy, and poor prognosis. Using the Gal4-UAS system, we have produced glioma models in Drosophila by overexpressing homologs of human tyrosine kinase receptors under control of the glia-specific promoter reversed polarity (repo). Glial overexpression of activated epidermal growth factor receptor (EGFR) resulted in enhanced proliferation and **migration** of larval glial cells with increased numbers in the eye imaginal disc, diffuse tumor-like enlargement of the optic stalk, and marked ectopic invasion of glial cells along the optic nerve. Glial overexpression of the downstream kinase PI3K showed similar pathology. Overexpression of activated pvr (platelet-derived growth factor receptor/vascular endothelial growth factor receptor homolog) led to **migration** of glial cells along the optic nerve, whereas expression of activated htl (fibroblast growth factor receptor 1 homolog) and INR (insulin receptor) showed markedly elevated numbers of glial cells in the optic stalk. The EGFR/phosphatidylinositol 3-phosphate kinase (PI3K) phenotype was partly reverted by the administration of the EGFR tyrosine kinase inhibitor gefitinib and completely rescued by the PI3K inhibitor wortmannin and the Akt inhibitor triciribine. We suggest that Drosophila models will be useful for deciphering signaling cascades underlying abnormal behavior of glioma cells for genetic screens to reveal interacting genes involved in gliomagenesis and for experimental therapy approaches.
8. Distinct mechanisms regulate hemocyte chemotaxis during development and wound healing in Drosophila melanogaster. Drosophila melanogaster hemocytes are highly motile macrophage-like cells that undergo a stereotypic pattern of **migration** to populate the whole embryo by late embryogenesis. We demonstrate that the migratory patterns of hemocytes at the embryonic ventral midline are orchestrated by chemotactic signals from the PDGF/VEGF ligands Pvf2 and -3 and that these directed **migration**s occur independently of phosphoinositide 3-kinase (PI3K) signaling. In contrast, using both laser ablation and a novel wounding assay that allows localized treatment with inhibitory drugs, we show that PI3K is essential for hemocyte chemotaxis toward wounds and that Pvf signals and PDGF/VEGF receptor expression are not required for this rapid chemotactic response. Our results demonstrate that at least two separate mechanisms operate in D. melanogaster embryos to direct hemocyte **migration** and show that although PI3K is crucial for hemocytes to sense a chemotactic gradient from a wound, it is not required to sense the growth factor signals that coordinate their developmental **migration**s along the ventral midline during embryogenesis.
9. Two signals are better than one: border cell **migration** in Drosophila. Recent studies of border cell **migration** during Drosophila oogenesis demonstrate that the EGFR and PDGFR signaling pathways act in a partially redundant manner to guide this process. Evidence presented shows that PDGFR signaling directs border cell **migration** via Rac and the Rac activator Mbc/CED-5/Dock180.
10. Drosophila tribbles antagonizes insulin signaling-mediated growth and metabolism via interactions with Akt kinase. Drosophila Tribbles (Trbl) is the founding member of the Trib family of kinase-like docking proteins that modulate cell signaling during proliferation, **migration** and growth. In a wing misexpression screen for Trbl interacting proteins, we identified the Ser/Thr protein kinase Akt1. Given the central role of Akt1 in insulin signaling, we tested the function of Trbl in larval fat body, a tissue where rapid increases in size are exquisitely sensitive to insulin/insulin-like growth factor levels. Consistent with a role in antagonizing insulin-mediated growth, trbl RNAi knockdown in the fat body increased cell size, advanced the timing of pupation and increased levels of circulating triglyceride. Complementarily, overexpression of Trbl reduced fat body cell size, decreased overall larval size, delayed maturation and lowered levels of triglycerides, while circulating glucose levels increased. The conserved Trbl kinase domain is required for function in vivo and for interaction with Akt in a yeast two-hybrid assay. Consistent with direct regulation of Akt, overexpression of Trbl in the fat body decreased levels of activated Akt (pSer505-Akt) while misexpression of trbl RNAi increased phospho-Akt levels, and neither treatment affected total Akt levels. Trbl misexpression effectively suppressed Akt-mediated wing and muscle cell size increases and reduced phosphorylation of the Akt target FoxO (pSer256-FoxO). Taken together, these data show that Drosophila Trbl has a conserved role to bind Akt and block Akt-mediated insulin signaling, and implicate Trib proteins as novel sites of signaling pathway integration that link nutrient availability with cell growth and proliferation.
11. Initiating and guiding **migration**: lessons from border cells. Cell **migration** occurs in many different contexts. Amoebae and other isolated cells migrate in culture. In animals, 'professional' migratory cells of the immune system constantly survey the body for intruders, whereas other cell types perform specific developmentally regulated **migration**s. One simple model for the latter type of event is **migration** of border cells during Drosophila oogenesis. Recent findings have shed light on how border cell fate is induced and on how the **migration** is guided. This article discusses the implications of these studies and compares (invasive) **migration** through a tissue with what is known about cells crawling on a flat substratum.
12. Gliatrophic and gliatropic roles of PVF/PVR signaling during axon guidance. Evidence of molecular and functional homology between vertebrate and Drosophila glia is limited, restricting the power of Drosophila as a model system to unravel the molecular basis of glial function. Like in vertebrates, in the Drosophila central nervous system glial cells are produced in excess and surplus glia are eliminated by apoptosis adjusting final glial number to axons. The underlying molecular mechanisms are largely unknown, as the only gliatrophic pathway known to date in flies is the EGFR and its ligands. The PDGFR signaling pathway plays a major role in regulating oligodendrocyte **migration** and number in vertebrates. Here, we show that the Drosophila PDGFR/VEGFR homologue PVR is required in midline glia during axon guidance for glial survival and **migration**, ultimately enabling axonal enwrapment. The midline glia migrate aided by the VUM and the MP1 midline neurons--sources of PVF ligands--and concomitantly interactions with neurons maintain midline glia survival. Upon loss of function for PVF/PVR signaling midline glia apoptosis increases, and gain of function induces supernumerary midline glia. Midline glial cells are displaced towards ectopic sources of PVF ligands. PVR signaling promotes midline glia survival through AKT and ERK pathways. This work shows that the PVR/PDGFR pathway plays conserved gliatrophic and gliatropic roles in subsets of glial cells in flies and vertebrates.
13. Invasive cell **migration** is initiated by guided growth of long cellular extensions. The **migration** of border cells during Drosophila melanogaster oogenesis is a simple and powerful system for studying invasive cell **migration** in vivo. Border cells are somatic cells that delaminate from the follicular epithelium of an egg chamber and invade the germ line cluster. They migrate between the nurse cells to reach the oocyte, using DE-cadherin for adhesion to the substratum. Border cells take approximately 6 h to migrate a distance of 100 microm. The **migration** is guided by EGFR (epidermal growth factor receptor) and PVR (platelet-derived growth factor (PDGF)/vascular endothelial growth factor (VEGF) receptor). Here, we show that a single long cellular extension (LCE), several cell diameters in length, is formed at the initiation of **migration**. The LCE may function as a 'pathfinder' in response to guidance cues. LCE growth requires directional guidance signals and specific adhesion to the substratum. Interference with actin-myosin interactions allows continued LCE growth while preventing translocation of the cell bodies. We discuss similarities between LCEs and axons and the use of LCE-like structures as a general mechanism for initiating invasive **migration** in vivo.
14. Regulation of Drosophila mesoderm **migration** by phosphoinositides and the PH domain of the Rho GTP exchange factor Pebble. The Drosophila RhoGEF Pebble (Pbl) is required for cytokinesis and **migration** of mesodermal cells. In a screen for genes that could suppress **migration** defects in pbl mutants we identified the phosphatidylinositol phosphate (PtdInsP) regulator pi5k59B. Genetic interaction tests with other PtdInsP regulators suggested that PtdIns(4,5)P2 levels are important for mesoderm **migration** when Pbl is depleted. Consistent with this, the leading front of migrating mesodermal cells was enriched for PtdIns(4,5)P2. Given that Pbl contains a Pleckstrin Homology (PH) domain, a known PtdInsP-binding motif, we examined PtdInsP-binding of Pbl and the importance of the PH domain for Pbl function. In vitro lipid blot assays showed that Pbl binds promiscuously to PtdInsPs, with binding strength associated with the degree of phosphorylation. Pbl was also able to bind lipid vesicles containing PtdIns(4,5)P2 but binding was strongly reduced upon deletion of the PH domain. Similarly, in vivo, loss of the PH domain prevented localisation of Pbl to the cell cortex and severely affected several aspects of early mesoderm development, including flattening of the invaginated tube onto the ectoderm, extension of protrusions, and dorsal **migration** to form a monolayer. Pbl lacking the PH domain could still localise to the cytokinetic furrow, however, and cytokinesis failure was reduced in pbl(ΔPH) mutants. Taken together, our results support a model in which interaction of the PH-domain of Pbl with PtdIns(4,5)P2 helps localise it to the plasma membrane which is important for mesoderm **migratio**n.
15. Fibroblast growth factor receptor-dependent morphogenesis of the Drosophila mesoderm. The Drosophila fibroblast growth factor (FGF) receptors Heartless and Breathless are required for the morphogenesis of the mesoderm and the tracheal system. In this article we discuss a number of questions relating to the morphogenesis of these tissues and speculate on poorly understood aspects of the underlying mechanisms. As yet a ligand has not been identified for Heartless, but in the case of Breathless the ligand may in some situations act as a chemotactic signal. We consider it unlikely that release of a distant chemotactic signal plays a role in the morphogenesis of the mesoderm. Instead we propose that the change in the mesoderm from an invaginated epithelium to a single layer of cells spread out on the ectoderm could be a result of the mesodermal cells trying to maximize their contact with the ectoderm. Exactly how the activation of the FGF receptors affects cell behaviour and leads to cell movement is not understood. The signal could simply be permissive, causing cells to become motile, or it could act as a directional signal for cells that are already motile, or perhaps provide both functions. Furthermore, it is unclear how signal transduction is coupled to morphological change. It seems unlikely that activation of transcription targets is essential for cell **migration** and it is possible that FGF signalling may have a direct effect on the cytoskeleton independent of the activation of the mitogen-activated protein kinase cascade. Analysis of the function of dof, which encodes a cytoplasmic protein required for FGF signal transduction may provide an insight into these issues.
16. Phototransduction: shedding light on translocation. Light induces the **migration** of arrestin to the photosensitive membrane in both vertebrate and invertebrate photoreceptors. New work has identified a phosphoinositide lipid binding domain in Drosophila arrestin and implicates PIP(3) in control of arrestin translocation.
17. Protein tyrosine phosphatase PTPN3 inhibits lung cancer cell proliferation and **migration** by promoting EGFR endocytic degradation. Epidermal growth factor receptor (EGFR) regulates multiple signaling cascades essential for cell proliferation, growth and differentiation. Using a genetic approach, we found that Drosophila FERM and PDZ domain-containing protein tyrosine phosphatase, dPtpmeg, negatively regulates border cell **migration** and inhibits the EGFR/Ras/mitogen-activated protein kinase signaling pathway during wing morphogenesis. We further identified EGFR pathway substrate 15 (Eps15) as a target of dPtpmeg and its human homolog PTPN3. Eps15 is a scaffolding adaptor protein known to be involved in EGFR endocytosis and trafficking. Interestingly, PTPN3-mediated tyrosine dephosphorylation of Eps15 promotes EGFR for lipid raft-mediated endocytosis and lysosomal degradation. PTPN3 and the Eps15 tyrosine phosphorylation-deficient mutant suppress non-small-cell lung cancer cell growth and **migration** in vitro and reduce lung tumor xenograft growth in vivo. Moreover, depletion of PTPN3 impairs the degradation of EGFR and enhances proliferation and tumorigenicity of lung cancer cells. Taken together, these results indicate that PTPN3 may act as a tumor suppressor in lung cancer through its modulation of EGFR signaling.

### [Ii]nvasive

#### PubMed

1. A drosophila model for EGFR-Ras and PI3K-dependent human glioma. Gliomas, the most common malignant tumors of the nervous system, frequently harbor mutations that activate the epidermal growth factor receptor (EGFR) and phosphatidylinositol-3 kinase (PI3K) signaling pathways. To investigate the genetic basis of this disease, we developed a glioma model in Drosophila. We found that constitutive coactivation of EGFR-Ras and PI3K pathways in Drosophila glia and glial precursors gives rise to neoplastic, **invasive** glial cells that create transplantable tumor-like growths, mimicking human glioma. Our model represents a robust organotypic and cell-type-specific Drosophila cancer model in which malignant cells are created by mutations in signature genes and pathways thought to be driving forces in a homologous human cancer. Genetic analyses demonstrated that EGFR and PI3K initiate malignant neoplastic transformation via a combinatorial genetic network composed primarily of other pathways commonly mutated or activated in human glioma, including the Tor, Myc, G1 Cyclins-Cdks, and Rb-E2F pathways. This network acts synergistically to coordinately stimulate cell cycle entry and progression, protein translation, and inappropriate cellular growth and migration. In particular, we found that the fly orthologs of CyclinE, Cdc25, and Myc are key rate-limiting genes required for glial neoplasia. Moreover, orthologs of Sin1, Rictor, and Cdk4 are genes required only for abnormal neoplastic glial proliferation but not for glial development. These and other genes within this network may represent important therapeutic targets in human glioma.
2. Cell mixing induced by myc is required for competitive tissue invasion and destruction. Cell-cell intercalation is used in several developmental processes to shape the normal body plan. There is no clear evidence that intercalation is involved in pathologies. Here we use the proto-oncogene myc to study a process analogous to early phase of tumour expansion: myc-induced cell competition. Cell competition is a conserved mechanism driving the elimination of slow-proliferating cells (so-called 'losers') by faster-proliferating neighbours (so-called 'winners') through apoptosis and is important in preventing developmental malformations and maintain tissue fitness. Here we show, using long-term live imaging of myc-driven competition in the Drosophila pupal notum and in the wing imaginal disc, that the probability of elimination of loser cells correlates with the surface of contact shared with winners. As such, modifying loser-winner interface morphology can modulate the strength of competition. We further show that elimination of loser clones requires winner-loser cell mixing through cell-cell intercalation. Cell mixing is driven by differential growth and the high tension at winner-winner interfaces relative to winner-loser and loser-loser interfaces, which leads to a preferential stabilization of winner-loser contacts and reduction of clone compactness over time. Differences in tension are generated by a relative difference in F-actin levels between loser and winner junctions, induced by differential levels of the membrane lipid phosphatidylinositol (3,4,5)-trisphosphate. Our results establish the first link between cell-cell intercalation induced by a proto-oncogene and how it promotes **invasive**ness and destruction of healthy tissues.
3. Colorectal cancer progression: lessons from Drosophila? Human colorectal cancers arise as benign adenomas, tumours that retain their epithelial character, and then progress to malignant adenocarcinomas and carcinomas in which the epithelium becomes disrupted. Carcinomas often exhibit transcriptional downregulation of E-cadherin and other epithelial genes in an epithelial-to-mesenchymal transition (EMT), a mechanism first discovered in Drosophila to be mediated by the transcription factors Twist and Snail. In contrast, adenocarcinomas retain expression of E-cadherin and disruption of the epithelium occurs through formation of progressively smaller epithelial cysts with apical Crumbs/CRB3, Stardust/PALS1, and Bazooka/PAR3 localised to the inner lumen. Results from Drosophila show that morphologically similar cysts form upon induction of clonal heterogeneity in Wnt, Smad, or Ras signalling levels, which causes extrusion of epithelial cells at clonal boundaries. Thus, intratumour heterogeneity might also promote formation of adenocarcinomas in humans. Finally, epithelial cysts can collectively migrate, as in the case of Drosophila border cells, a potential model system for the **invasive** migration of adenocarcinoma cells.
4. APC loss-induced intestinal tumorigenesis in Drosophila: Roles of Ras in Wnt signaling activation and tumor progression. Adenomatous polyposis coli (APC) and K-ras are the two most frequently mutated genes found in human colorectal cancers. In human colorectal cancers, Wnt signaling activation after the loss of APC is hypothesized to be the key event for adenoma initiation, whereas additional mutations such as Ras activation are required for the progression from adenoma to carcinoma. However, accumulating data have led to conflicting views regarding the precise role of Ras in APC loss-induced tumorigenesis. Here, using Drosophila midgut as a model system, we show that in the absence of Ras, APC mutant epithelial cells cannot initiate hyperplasia, suggesting that Ras plays an essential role in tumor initiation. Conversely, activating Ras by expressing oncogenic Ras or Raf in APC-deficient cells led to a blockage of cell differentiation and to pre**invasive** tumor outgrowth, characteristics that are shared by advanced colorectal carcinoma in humans. Mechanistically, we find that Ras is not required for Wnt signaling activation after APC loss, although Ras hyperactivation is able to potentiate Wnt signaling by increasing the cytoplasmic and nuclear accumulation of Armadillo/β-catenin via mechanisms independent of JNK/Rac1 or PI3K-Akt signaling, partly owing to the downregulation of DE-cadherin. Together with the data from gene expression analyses, our results indicate that both parallel and cooperative mechanisms of Wnt and Ras signaling are responsible for the initiation and progression of intestinal tumorigenesis after APC loss.
5. The lethal giant larvae tumour suppressor mutation requires dMyc oncoprotein to promote clonal malignancy. Neoplastic overgrowth depends on the cooperation of several mutations ultimately leading to major rearrangements in cellular behaviour. Precancerous cells are often removed by cell death from normal tissues in the early steps of the tumourigenic process, but the molecules responsible for such a fundamental safeguard process remain in part elusive. With the aim to investigate the molecular crosstalk occurring between precancerous and normal cells in vivo, we took advantage of the clonal analysis methods that are available in Drosophila for studying the phenotypes due to lethal giant larvae (lgl) neoplastic mutation induced in different backgrounds and tissues.We observed that lgl mutant cells growing in wild-type imaginal wing discs show poor viability and are eliminated by Jun N-terminal Kinase (JNK)-dependent cell death. Furthermore, they express very low levels of dMyc oncoprotein compared with those found in the surrounding normal tissue. Evidence that this is a cause of lgl mutant cells elimination was obtained by increasing dMyc levels in lgl mutant clones: their overgrowth potential was indeed re-established, with mutant cells overwhelming the neighbouring tissue and forming tumourous masses displaying several cancer hallmarks. Moreover, when lgl mutant clones were induced in backgrounds of slow-dividing cells, they upregulated dMyc, lost apical-basal cell polarity and were able to overgrow. Those phenotypes were abolished by reducing dMyc levels in the mutant clones, thereby confirming its key role in lgl-induced tumourigenesis. Furthermore, we show that the eiger-dependent Intrinsic Tumour Suppressor pathway plays only a minor role in eliminating lgl mutant cells in the wing pouch; lgl-/- clonal death in this region is instead driven mainly by dMyc-induced Cell Competition.Our results provide the first evidence that dMyc oncoprotein is required in lgl tumour suppressor mutant tissue to promote **invasive** overgrowth in larval and adult epithelial tissues. Moreover, we show that dMyc abundance inside versus outside the mutant clones plays a key role in driving neoplastic overgrowth.
6. Initiating and guiding migration: lessons from border cells. Cell migration occurs in many different contexts. Amoebae and other isolated cells migrate in culture. In animals, 'professional' migratory cells of the immune system constantly survey the body for intruders, whereas other cell types perform specific developmentally regulated migrations. One simple model for the latter type of event is migration of border cells during Drosophila oogenesis. Recent findings have shed light on how border cell fate is induced and on how the migration is guided. This article discusses the implications of these studies and compares (**invasive**) migration through a tissue with what is known about cells crawling on a flat substratum.
7. **Invasive** cell migration is initiated by guided growth of long cellular extensions. The migration of border cells during Drosophila melanogaster oogenesis is a simple and powerful system for studying **invasive** cell migration in vivo. Border cells are somatic cells that delaminate from the follicular epithelium of an egg chamber and invade the germ line cluster. They migrate between the nurse cells to reach the oocyte, using DE-cadherin for adhesion to the substratum. Border cells take approximately 6 h to migrate a distance of 100 microm. The migration is guided by EGFR (epidermal growth factor receptor) and PVR (platelet-derived growth factor (PDGF)/vascular endothelial growth factor (VEGF) receptor). Here, we show that a single long cellular extension (LCE), several cell diameters in length, is formed at the initiation of migration. The LCE may function as a 'pathfinder' in response to guidance cues. LCE growth requires directional guidance signals and specific adhesion to the substratum. Interference with actin-myosin interactions allows continued LCE growth while preventing translocation of the cell bodies. We discuss similarities between LCEs and axons and the use of LCE-like structures as a general mechanism for initiating **invasive** migration in vivo.

### [Mm]etastasis

#### PubMed

1. Crumbs controls epithelial integrity by inhibiting Rac1 and PI3K. Drosophila Crumbs (Crb) and its mammalian ortholog CRB3 control epithelial polarity through poorly understood molecular mechanisms. Elucidating these mechanisms is crucial, because the physiology of epithelia largely depends on the polarized architecture of individual epithelial cells. In addition, loss of CRB3 favors tumor cell growth, **metastasis** and epithelial to mesenchymal transition (EMT). Using Drosophila embryos, we report that Rac1 sustains PI3K signaling, which is required for Rac1 activation. Crb represses this positive-feedback loop. Notably, this property confers to Crb its ability to promote epithelial integrity in vivo, because attenuation of either Rac1 or PI3K activity rescues the crb mutant phenotype. Moreover, inhibition of Rac1 or PI3K results in Crb-dependent apical membrane growth, whereas Rac1 activation restricts membrane localization of Crb and interferes with apical domain formation. This illustrates that Crb and the Rac1-PI3K module are antagonists, and that the fine balance between the activities of these proteins is crucial to maintain epithelial organization and an appropriate apical to basolateral ratio. Together, our results elucidate a mechanism that mediates Crb function and further define the role of PI3K and Rac1 in epithelial morphogenesis, allowing for a better understanding of how distinct membrane domains are regulated in polarized epithelial cells.

---

## CG45186

- NCBI Gene
- GeneRIF
- Pubmed

| Search Term | Rank | Fields |
| --- | --- | --- |
| [Mm]igration | 6 | PubMed(1) |

  
hide/show details for CG45186

### [Mm]igration

#### PubMed

1. Molecular requirements for actin-based lamella formation in Drosophila S2 cells. Cell **migration** occurs through the protrusion of the actin-enriched lamella. Here, we investigated the effects of RNAi depletion of approximately 90 proteins implicated in actin function on lamella formation in Drosophila S2 cells. Similar to in vitro reconstitution studies of actin-based Listeria movement, we find that lamellae formation requires a relatively small set of proteins that participate in actin nucleation (Arp2/3 and SCAR), barbed end capping (capping protein), filament depolymerization (cofilin and Aip1), and actin monomer binding (profilin and cyclase-associated protein). Lamellae are initiated by parallel and partially redundant signaling pathways involving Rac GTPases and the adaptor protein Nck, which stimulate SCAR, an Arp2/3 activator. We also show that RNAi of three proteins (kette, Abi, and Sra-1) known to copurify with and inhibit SCAR in vitro leads to SCAR degradation, revealing a novel function of this protein complex in SCAR stability. Our results have identified an essential set of proteins involved in actin dynamics during lamella formation in Drosophila S2 cells.

---

## coro

- NCBI Gene
- GeneRIF
- Pubmed

| Search Term | Rank | Fields |
| --- | --- | --- |
| [Mm]igration | 6 | PubMed(2) |
| [Ee]xtracellular [Mm]atrix | 8 | PubMed(1) |
| [Mm]etastasis | 12 | PubMed(1) |

  
hide/show details for coro

### [Mm]igration

#### PubMed

1. Molecular requirements for actin-based lamella formation in Drosophila S2 cells. Cell **migration** occurs through the protrusion of the actin-enriched lamella. Here, we investigated the effects of RNAi depletion of approximately 90 proteins implicated in actin function on lamella formation in Drosophila S2 cells. Similar to in vitro reconstitution studies of actin-based Listeria movement, we find that lamellae formation requires a relatively small set of proteins that participate in actin nucleation (Arp2/3 and SCAR), barbed end capping (capping protein), filament depolymerization (cofilin and Aip1), and actin monomer binding (profilin and cyclase-associated protein). Lamellae are initiated by parallel and partially redundant signaling pathways involving Rac GTPases and the adaptor protein Nck, which stimulate SCAR, an Arp2/3 activator. We also show that RNAi of three proteins (kette, Abi, and Sra-1) known to copurify with and inhibit SCAR in vitro leads to SCAR degradation, revealing a novel function of this protein complex in SCAR stability. Our results have identified an essential set of proteins involved in actin dynamics during lamella formation in Drosophila S2 cells.
2. Regulation of Drosophila mesoderm **migration** by phosphoinositides and the PH domain of the Rho GTP exchange factor Pebble. The Drosophila RhoGEF Pebble (Pbl) is required for cytokinesis and **migration** of mesodermal cells. In a screen for genes that could suppress **migration** defects in pbl mutants we identified the phosphatidylinositol phosphate (PtdInsP) regulator pi5k59B. Genetic interaction tests with other PtdInsP regulators suggested that PtdIns(4,5)P2 levels are important for mesoderm **migration** when Pbl is depleted. Consistent with this, the leading front of migrating mesodermal cells was enriched for PtdIns(4,5)P2. Given that Pbl contains a Pleckstrin Homology (PH) domain, a known PtdInsP-binding motif, we examined PtdInsP-binding of Pbl and the importance of the PH domain for Pbl function. In vitro lipid blot assays showed that Pbl binds promiscuously to PtdInsPs, with binding strength associated with the degree of phosphorylation. Pbl was also able to bind lipid vesicles containing PtdIns(4,5)P2 but binding was strongly reduced upon deletion of the PH domain. Similarly, in vivo, loss of the PH domain prevented localisation of Pbl to the cell cortex and severely affected several aspects of early mesoderm development, including flattening of the invaginated tube onto the ectoderm, extension of protrusions, and dorsal **migration** to form a monolayer. Pbl lacking the PH domain could still localise to the cytokinetic furrow, however, and cytokinesis failure was reduced in pbl(ΔPH) mutants. Taken together, our results support a model in which interaction of the PH-domain of Pbl with PtdIns(4,5)P2 helps localise it to the plasma membrane which is important for mesoderm **migratio**n.

### [Ee]xtracellular [Mm]atrix

#### PubMed

1. Genetic and chemical modifiers of a CUG toxicity model in Drosophila. Non-coding CUG repeat expansions interfere with the activity of human Muscleblind-like (MBNL) proteins contributing to myotonic dystrophy 1 (DM1). To understand this toxic RNA gain-of-function mechanism we developed a Drosophila model expressing 60 pure and 480 interrupted CUG repeats in the context of a non-translatable RNA. These flies reproduced aspects of the DM1 pathology, most notably nuclear accumulation of CUG transcripts, muscle degeneration, splicing misregulation, and diminished Muscleblind function in vivo. Reduced Muscleblind activity was evident from the sensitivity of CUG-induced phenotypes to a decrease in muscleblind genetic dosage and rescue by MBNL1 expression, and further supported by the co-localization of Muscleblind and CUG repeat RNA in ribonuclear foci. Targeted expression of CUG repeats to the developing eye and brain mushroom bodies was toxic leading to rough eyes and semilethality, respectively. These phenotypes were utilized to identify genetic and chemical modifiers of the CUG-induced toxicity. 15 genetic modifiers of the rough eye phenotype were isolated. These genes identify putative cellular processes unknown to be altered by CUG repeat RNA, and they include mRNA export factor Aly, apoptosis inhibitor Thread, chromatin remodelling factor Nurf-38, and **extracellular matrix** structural component Viking. Ten chemical compounds suppressed the semilethal phenotype. These compounds significantly improved viability of CUG expressing flies and included non-steroidal anti-inflammatory agents (ketoprofen), muscarinic, cholinergic and histamine receptor inhibitors (orphenadrine), and drugs that can affect sodium and calcium metabolism such as clenbuterol and spironolactone. These findings provide new insights into the DM1 phenotype, and suggest novel candidates for DM1 treatments.

### [Mm]etastasis

#### PubMed

1. Egfr/Ras pathway mediates interactions between peripodial and disc proper cells in Drosophila wing discs. All imaginal discs in Drosophila are made up of a layer of columnar epithelium or the disc proper and a layer of squamous epithelium called the peripodial membrane. Although the developmental and molecular events in columnar epithelium or the disc proper are well understood, the peripodial membrane has gained attention only recently. Using the technique of lineage tracing, we show that peripodial and disc proper cells arise from a common set of precursors cells in the embryo, and that these cells diverge in the early larval stages. However, peripodial and disc proper cells maintain a spatial relationship even after the separation of their lineages. The peripodial membrane plays a significant role during the regional subdivision of the wing disc into presumptive wing, notum and hinge. The Egfr/Ras pathway mediates this function of the peripodial membrane. These results on signaling between squamous and columnar epithelia are particularly significant in the context of in vitro studies using human cell lines that suggest a role for the Egfr/Ras pathway in **metastasis** and tumour progression.

---

## Ddc

- NCBI Gene
- GeneRIF
- Pubmed

| Search Term | Rank | Fields |
| --- | --- | --- |
| [Mm]igration | 6 | PubMed(1) |
| ECM | 7 | PubMed(1) |
| [Ee]xtracellular [Mm]atrix | 8 | PubMed(2) |

  
hide/show details for Ddc

### [Mm]igration

#### PubMed

1. The Drosophila spitz gene encodes a putative EGF-like growth factor involved in dorsal-ventral axis formation and neurogenesis. We describe the molecular characterization of the Drosophila gene spitz (spi), which encodes a putative 26-kD, EGF-like transmembrane protein that is structurally similar to TGF-alpha. Temporal and spatial expression patterns of spi transcripts indicate that spi is expressed throughout the embryo. Examination of mutant embryos reveals that spi is involved in a number of unrelated developmental choices, for example, dorsal-ventral axis formation, glial **migration**, sensory organ determination, and muscle development. We propose that spi may act as a ligand for cell-specific receptors, possibly rhomboid and/or the Drosophila EGF receptor homolog.

### ECM

#### PubMed

1. The transcription factor Grainy head and the steroid hormone ecdysone cooperate during differentiation of the skin of Drosophila melanogaster. The arthropod epidermis is an epithelium that deposits the apical cuticle, which is a stratified extracellular matrix (**ECM**) protecting the animal against pathogens, preventing dehydration and also serving as an exoskeleton. Differentiation of the cuticle conceivably implies coordinated production, secretion and localization of its components. The underlying molecular mechanisms are poorly explored. In this work, we show that the transcription factor Grainy head and the steroid hormone ecdysone drive the production of two partially overlapping sets of cuticle factors. Nevertheless, Grainy head is needed to modulate the expression of ecdysone signalling factors; the significance of this cross-talk is yet unclear. In addition, we found that ecdysone signalling negatively regulates its own impact. In conclusion, our findings suggest that at least two independently triggered pathways have evolved in parallel to cooperatively ensure the stereotypic implementation of the cuticle. As Grainy head is also essential for epithelial differentiation in vertebrates, we speculate that it acts to decode the ancient skin programme common to all animals. Full differentiation of the skin necessitates a second, complementing taxon-specific programme that requires its own decoder, which is represented by ecdysone in arthropods, whereas the vertebrate specific one remains to be identified.

### [Ee]xtracellular [Mm]atrix

#### PubMed

1. Shavenbaby couples patterning to epidermal cell shape control. It is well established that developmental programs act during embryogenesis to determine animal morphogenesis. How these developmental cues produce specific cell shape during morphogenesis, however, has remained elusive. We addressed this question by studying the morphological differentiation of the Drosophila epidermis, governed by a well-known circuit of regulators leading to a stereotyped pattern of smooth cells and cells forming actin-rich extensions (trichomes). It was shown that the transcription factor Shavenbaby plays a pivotal role in the formation of trichomes and underlies all examined cases of the evolutionary diversification of their pattern. To gain insight into the mechanisms of morphological differentiation, we sought to identify shavenbaby's downstream targets. We show here that Shavenbaby controls epidermal cell shape, through the transcriptional activation of different classes of cellular effectors, directly contributing to the organization of actin filaments, regulation of the **extracellular matrix**, and modification of the cuticle. Individual inactivation of shavenbaby's targets produces distinct trichome defects and only their simultaneous inactivation prevent trichome formation. Our data show that shavenbaby governs an evolutionarily conserved developmental module consisting of a set of genes collectively responsible for trichome formation, shedding new light on molecular mechanisms acting during morphogenesis and the way they can influence evolution of animal forms.
2. The transcription factor Grainy head and the steroid hormone ecdysone cooperate during differentiation of the skin of Drosophila melanogaster. The arthropod epidermis is an epithelium that deposits the apical cuticle, which is a stratified **extracellular matrix** (ECM) protecting the animal against pathogens, preventing dehydration and also serving as an exoskeleton. Differentiation of the cuticle conceivably implies coordinated production, secretion and localization of its components. The underlying molecular mechanisms are poorly explored. In this work, we show that the transcription factor Grainy head and the steroid hormone ecdysone drive the production of two partially overlapping sets of cuticle factors. Nevertheless, Grainy head is needed to modulate the expression of ecdysone signalling factors; the significance of this cross-talk is yet unclear. In addition, we found that ecdysone signalling negatively regulates its own impact. In conclusion, our findings suggest that at least two independently triggered pathways have evolved in parallel to cooperatively ensure the stereotypic implementation of the cuticle. As Grainy head is also essential for epithelial differentiation in vertebrates, we speculate that it acts to decode the ancient skin programme common to all animals. Full differentiation of the skin necessitates a second, complementing taxon-specific programme that requires its own decoder, which is represented by ecdysone in arthropods, whereas the vertebrate specific one remains to be identified.

---

## vari

- NCBI Gene
- GeneRIF
- Pubmed

| Search Term | Rank | Fields |
| --- | --- | --- |
| [Mm]igration | 6 | PubMed(2) |
| ECM | 7 | PubMed(1) |
| [Ee]xtracellular [Mm]atrix | 8 | PubMed(2) |
| [Ii]nvasive | 10 | PubMed(1) |

  
hide/show details for vari

### [Mm]igration

#### PubMed

1. On the role of PDZ domain-encoding genes in Drosophila border cell **migration**. Cells often move as collective groups during normal embryonic development and wound healing, although the mechanisms governing this type of **migration** are poorly understood. The Drosophila melanogaster border cells migrate as a cluster during late oogenesis and serve as a powerful in vivo genetic model for collective cell **migration**. To discover new genes that participate in border cell **migration**, 64 out of 66 genes that encode PDZ domain-containing proteins were systematically targeted by in vivo RNAi knockdown. The PDZ domain is one of the largest families of protein-protein interaction domains found in eukaryotes. Proteins that contain PDZ domains participate in a variety of biological processes, including signal transduction and establishment of epithelial apical-basal polarity. Targeting PDZ proteins effectively assesses a larger number of genes via the protein complexes and pathways through which these proteins function. par-6, a known regulator of border cell **migration**, was a positive hit and thus validated the approach. Knockdown of 14 PDZ domain genes disrupted **migration** with multiple RNAi lines. The candidate genes have diverse predicted cellular functions and are anticipated to provide new insights into the mechanisms that control border cell movement. As a test of this concept, two genes that disrupted **migration** were characterized in more detail: big bang and the Dlg5 homolog CG6509. We present evidence that Big bang regulates JAK/STAT signaling, whereas Dlg5/CG6509 maintains cluster cohesion. Moreover, these results demonstrate that targeting a selected class of genes by RNAi can uncover novel regulators of collective cell **migration**.
2. A clonal genetic screen for mutants causing defects in larval tracheal morphogenesis in Drosophila. The initial establishment of the tracheal network in the Drosophila embryo is beginning to be understood in great detail, both in its genetic control cascades and in its cell biological events. By contrast, the vast expansion of the system during larval growth, with its extensive ramification of preexisting tracheal branches, has been analyzed less well. The mutant phenotypes of many genes involved in this process are probably not easy to reveal, as these genes may be required for other functions at earlier developmental stages. We therefore conducted a screen for defects in individual clonal homozygous mutant cells in the tracheal network of heterozygous larvae using the mosaic analysis with a repressible cell marker (MARCM) system to generate marked, recombinant mitotic clones. We describe the identification of a set of mutants with distinct phenotypic effects. In particular we found a range of defects in terminal cells, including failure in lumen formation and reduced or extensive branching. Other mutations affect cell growth, cell shape, and cell **migration**.

### ECM

#### PubMed

1. Epithelial polarity proteins regulate Drosophila tracheal tube size in parallel to the luminal matrix pathway. Regulation of epithelial tube size is critical for organ function. However, the mechanisms of tube size control remain poorly understood. In the Drosophila trachea, tube dimensions are regulated by a luminal extracellular matrix (**ECM**). **ECM** organization requires apical (luminal) secretion of the protein Vermiform (Verm), which depends on the basolateral septate junction (SJ). Here, we show that apical and basolateral epithelial polarity proteins interact to control tracheal tube size independently of the Verm pathway. Mutations in yurt (yrt) and scribble (scrib), which encode SJ-associated polarity proteins, cause an expansion of tracheal tubes but do not disrupt Verm secretion. Reducing activity of the apical polarity protein Crumbs (Crb) suppresses the length defects in yrt but not scrib mutants, suggesting that Yrt acts by negatively regulating Crb. Conversely, Crb overexpression increases tracheal tube dimensions. Reducing crb dosage also rescues tracheal size defects caused by mutations in coracle (cora), which encodes an SJ-associated polarity protein. In addition, crb mutations suppress cora length defects without restoring Verm secretion. Together, these data indicate that Yrt, Cora, Crb, and Scrib operate independently of the Verm pathway. Our data support a model in which Cora and Yrt act through Crb to regulate epithelial tube size.

### [Ee]xtracellular [Mm]atrix

#### PubMed

1. Epithelial polarity proteins regulate Drosophila tracheal tube size in parallel to the luminal matrix pathway. Regulation of epithelial tube size is critical for organ function. However, the mechanisms of tube size control remain poorly understood. In the Drosophila trachea, tube dimensions are regulated by a luminal **extracellular matrix** (ECM). ECM organization requires apical (luminal) secretion of the protein Vermiform (Verm), which depends on the basolateral septate junction (SJ). Here, we show that apical and basolateral epithelial polarity proteins interact to control tracheal tube size independently of the Verm pathway. Mutations in yurt (yrt) and scribble (scrib), which encode SJ-associated polarity proteins, cause an expansion of tracheal tubes but do not disrupt Verm secretion. Reducing activity of the apical polarity protein Crumbs (Crb) suppresses the length defects in yrt but not scrib mutants, suggesting that Yrt acts by negatively regulating Crb. Conversely, Crb overexpression increases tracheal tube dimensions. Reducing crb dosage also rescues tracheal size defects caused by mutations in coracle (cora), which encodes an SJ-associated polarity protein. In addition, crb mutations suppress cora length defects without restoring Verm secretion. Together, these data indicate that Yrt, Cora, Crb, and Scrib operate independently of the Verm pathway. Our data support a model in which Cora and Yrt act through Crb to regulate epithelial tube size.
2. Drosophila convoluted/dALS is an essential gene required for tracheal tube morphogenesis and apical matrix organization. Insulin-like growth factors (IGFs) control cell and organism growth through evolutionarily conserved signaling pathways. The mammalian acid-labile subunit (ALS) is a secreted protein that complexes with IGFs to modulate their activity. Recent work has shown that a Drosophila homolog of ALS, dALS, can also complex with and modulate the activity of a Drosophila IGF. Here we report the first mutations in the gene encoding dALS. Unexpectedly, we find that these mutations are allelic to a previously described mutation in convoluted (conv), a gene required for epithelial morphogenesis. In conv mutants, the tubes of the Drosophila tracheal system become abnormally elongated without altering tracheal cell number. conv null mutations cause larval lethality, but do not disrupt several processes required for tracheal tube size control, including septate junction formation, deposition of a lumenal/apical **extracellular matrix**, and lumenal secretion of Vermiform and Serpentine, two putative matrix-modifying proteins. Clearance of lumenal matrix and subcellular localization of clathrin also appear normal in conv mutants. However, we show that Conv/dALS is required for the dynamic organization of the transient lumenal matrix and normal structure of the cuticle that lines the tracheal lumen. These and other data suggest that the Conv/dALS-dependent tube size control mechanism is distinct from other known processes involved in tracheal tube size regulation. Moreover, we present evidence indicating that Conv/dALS has a novel, IGF-signaling independent function in tracheal morphogenesis.

### [Ii]nvasive

#### PubMed

1. The expression of the Drosophila awd gene during normal development and in neoplastic brain tumors caused by lgl mutations. The abnormal wing discs (awd) gene of Drosophila is homologous to the nm23 gene of mammals, a gene whose expression is altered in metastatic tumors. Both awd and nm23 encode nucleoside diphosphate kinases (NDP kinases). We have examined the accumulation of AWD/NDP kinase during normal development by assaying enzyme activity in extracts. There is a nearly constant level of activity throughout larval and pupal development. We have examined the tissue-specific transcription of the awd gene by RNA in situ hybridization and by reporter gene expression. In imaginal discs and brains there is no detectable awd gene expression until the beginning of the third larval instar, despite the constant level of enzyme activity measured in extracts of larvae and pupae. The most intense awd gene expression in imaginal discs and brains occurs after the end of larval development. We have also examined awd gene expression in neoplastic brain tumors caused by mutations in the lethal giant larvae (lgl) gene. In lgl mutant brains, as in normal brains, awd gene expression begins during the third larval instar. No tumors form in brains from lgl-; awd- double mutant larva, so awd gene expression is required for tumor formation and/or proliferation. There is more accumulation of AWD/NDP kinase in lgl- mutant brains than there is in normal brains. Using an awd reporter gene, we show that this is a consequence of an increased proportion of awd gene-expressing cells in mutant brains. Using the same awd reporter gene as a marker of donor cells, we have confirmed the **invasive**ness of lgl-induced neuroblastomas.

---

## nclb

- NCBI Gene
- GeneRIF
- Pubmed

| Search Term | Rank | Fields |
| --- | --- | --- |
| [Mm]igration | 6 | PubMed(1) |

  
hide/show details for nclb

### [Mm]igration

#### PubMed

1. Functional analysis of the Drosophila embryonic germ cell transcriptome by RNA interference. In Drosophila melanogaster, primordial germ cells are specified at the posterior pole of the very early embryo. This process is regulated by the posterior localized germ plasm that contains a large number of RNAs of maternal origin. Transcription in the primordial germ cells is actively down-regulated until germ cell fate is established. Bulk expression of the zygotic genes commences concomitantly with the degradation of the maternal transcripts. Thus, during embryogenesis, maternally provided and zygotically transcribed mRNAs determine germ cell development collectively. In an effort to identify novel genes involved in the regulation of germ cell behavior, we carried out a large-scale RNAi screen targeting both maternal and zygotic components of the embryonic germ line transcriptome. We identified 48 genes necessary for distinct stages in germ cell development. We found pebble and fascetto to be essential for germ cell **migration** and germ cell division, respectively. Our data uncover a previously unanticipated role of mei-P26 in maintenance of embryonic germ cell fate. We also performed systematic co-RNAi experiments, through which we found a low rate of functional redundancy among homologous gene pairs. As our data indicate a high degree of evolutionary conservation in genetic regulation of germ cell development, they are likely to provide valuable insights into the biology of the germ line in general.

---

## rhea

- NCBI Gene
- GeneRIF
- Pubmed

| Search Term | Rank | Fields |
| --- | --- | --- |
| [Mm]igration | 6 | Process(1); PubMed(22) |
| ECM | 7 | PubMed(27) |
| [Ee]xtracellular [Mm]atrix | 8 | PubMed(33); GeneRIFs(2) |
| [Mm]etastasis | 12 | PubMed(3) |

  
hide/show details for rhea

### [Mm]igration

#### Process

1. border follicle cell **migration**

#### PubMed

1. Slit coordinates cardiac morphogenesis in Drosophila. Slit is a secreted guidance cue that conveys repellent or attractive signals from target and guidepost cells. In Drosophila, responsive cells express one or more of three Robo receptors. The cardial cells of the developing heart express both Slit and Robo2. This is the first report of coincident expression of a Robo and its ligand. In slit mutants, cardial cell alignment, polarization and uniform **migration** are disrupted. The heart phenotype of robo2 mutants is similar, with fewer **migration** defects. In the guidance of neuronal growth cones in Drosophila, there is a phenotypic interaction between slit and robo heterozygotes, and also with genes required for Robo signaling. In contrast, in the heart, slit has little or no phenotypic interaction with Robo-related genes, including Robo2, Nck2, and Disabled. However, there is a strong phenotypic interaction with Integrin genes and their ligands, including Laminin and Collagen, and intracellular messengers, including Talin and ILK. This indicates that Slit participates in adhesion or adhesion signaling during heart development.
2. Drosophila integrin adhesion complexes are essential for hemocyte **migration** in vivo. Cell **migration** is an important biological process which has been intensively studied in the past decades. Numerous techniques, mainly involving two-dimensional cell culture systems, have contributed to dissecting the essential mechanisms underlying this process. However, the development of three-dimensional cell culture and in vivo systems has shown some differences with what was previously believed to be well-established cell **migration** mechanisms, suggesting that two-dimensional cell motility would be a poor predictor of in vivo behaviour. Drosophila is a widely recognized model organism to study developmental and homeostatic processes and has been widely used to investigate cell **migration**. Here, we focus on the **migration** of small groups of pupal hemocytes that accumulate during larval stages in dorsal patches. We show that integrins, and other known nascent adhesion-related proteins such as Rhea and Fermitin 1, are crucial for this process and that their depletion does not affect polarization in response to environmental cues. We also present evidence for the importance of adhesion maturation-related proteins in hemocyte **migration**, namely Zyxin. Zyxin depletion in hemocytes leads to a significant increase of cell speed without affecting their response to a chemotactic cue. This is the first report of a systematic analysis using Drosophila melanogaster hemocytes to study adhesion-related proteins and their function in cell **migration** in vivo. Our data point to mechanisms of cell **migration** similar to those described in three-dimensional in vitro systems and other in vivo model organisms.
3. A dual role for the βPS integrin myospheroid in mediating Drosophila embryonic macrophage **migratio**n. Throughout embryonic development, macrophages not only act as the first line of defence against infection but also help to sculpt organs and tissues of the embryo by removing dead cells and secreting extracellular matrix components. Key to their function is the ability of embryonic macrophages to migrate and disperse throughout the embryo. Despite these important developmental functions, little is known about the molecular mechanisms underlying embryonic macrophage **migratio**n in vivo. Integrins are key regulators of many of the adult macrophage responses, but their role in embryonic macrophages remains poorly characterized. Here, we have used Drosophila macrophages (haemocytes) as a model system to address the role of integrins during embryonic macrophage dispersal in vivo. We show that the main βPS integrin, myospheroid, affects haemocyt**e migrati**on in two ways; by shaping the three-dimensional environment in which haemocytes migrate and by regulating th**e migrati**on of haemocytes themselves. Live imaging revealed a requirement for myospheroid within haemocytes to coordinate the microtubule and actin dynamics, and to enable haemocyte developmental dispersal, contact repulsion and inflammator**y migrati**on towards wounds.
4. Polarization of the epithelial layer and apical localization of integrins are required for engulfment of apoptotic cells in the Drosophila ovary. Inefficient clearance of dead cells or debris by epithelial cells can lead to or exacerbate debilitating conditions such as retinitis pigmentosa, macular degeneration, chronic obstructive pulmonary disease and asthma. Despite the importance of engulfment by epithelial cells, little is known about the molecular changes that are required within these cells. The misregulation of integrins has previously been associated with disease states, suggesting that a better understanding of the regulation of receptor trafficking could be key to treating diseases caused by defects in phagocytosis. Here, we demonstrate that the integrin heterodimer αPS3/βPS becomes apically enriched and is required for engulfment by the epithelial follicle cells of the Drosophila ovary. We found that integrin heterodimer localization and function is largely directed by the α-subunit. Moreover, proper cell polarity promotes asymmetric integrin enrichment, suggesting that αPS3/βPS trafficking occurs in a polarized fashion. We show that several genes previously known for their roles in trafficking and **cell migr**ation are also required for engulfment. Moreover, as in mammals, the same α-integrin subunit is required by professional and non-professional phagocytes and migrating cells in Drosophila. Our findings suggest that migrating and engulfing cells use common machinery, and demonstrate a crucial role for integrin function and polarized trafficking of integrin subunits during engulfment. This study also establishes the epithelial follicle cells of the Drosophila ovary as a powerful model for understanding the molecular changes required for engulfment by a polarized epithelium.
5. Loss of focal adhesions in glia disrupts both glial and photoreceptor axon **migration** in the Drosophila visual system. Many aspects of glial development are regulated by extracellular signals, including those from the extracellular matrix (ECM). Signals from the ECM are received by cell surface receptors, including the integrin family. Previously, we have shown that Drosophila integrins form adhesion complexes with Integrin-linked kinase and talin in the peripheral nerve glia and have conserved roles in glial sheath formation. However, integrin function in other aspects of glial development is unclear. The Drosophila eye imaginal disc (ED) and optic stalk (OS) complex is an excellent model with which to study glial **migration**, differentiation and glia-neuron interactions. We studied the roles of the integrin complexes in these glial developmental processes during OS/eye development. The common beta subunit βPS and two alpha subunits, αPS2 and αPS3, are located in puncta at both glia-glia and glia-ECM interfaces. Depletion of βPS integrin and talin by RNAi impaired **the migra**tion and distribution of glia within the OS resulting in morphological defects. Reduction of integrin or talin in the glia also disrupted photoreceptor axon outgrowth leading to axon stalling in the OS and ED. The neuronal defects were correlated with a disruption of the carpet glia tube paired with invasion of glia into the core of the OS and the formation of a glial cap. Our results suggest that integrin-mediated extracellular signals are important for multiple aspects of glial development and non-autonomously affect axo**nal migra**tion during Drosophila eye development.
6. Molecular requirements for actin-based lamella formation in Drosophila S2 cells. Cell **migration** occurs through the protrusion of the actin-enriched lamella. Here, we investigated the effects of RNAi depletion of approximately 90 proteins implicated in actin function on lamella formation in Drosophila S2 cells. Similar to in vitro reconstitution studies of actin-based Listeria movement, we find that lamellae formation requires a relatively small set of proteins that participate in actin nucleation (Arp2/3 and SCAR), barbed end capping (capping protein), filament depolymerization (cofilin and Aip1), and actin monomer binding (profilin and cyclase-associated protein). Lamellae are initiated by parallel and partially redundant signaling pathways involving Rac GTPases and the adaptor protein Nck, which stimulate SCAR, an Arp2/3 activator. We also show that RNAi of three proteins (kette, Abi, and Sra-1) known to copurify with and inhibit SCAR in vitro leads to SCAR degradation, revealing a novel function of this protein complex in SCAR stability. Our results have identified an essential set of proteins involved in actin dynamics during lamella formation in Drosophila S2 cells.
7. Receptor guanylyl cyclase Gyc76C is required for invagination, collective **migration** and lumen shape in the Drosophila embryonic salivary gland. The Drosophila embryonic salivary gland is formed by the invagination and collective **migration** of cells. Here, we report on a novel developmental role for receptor-type guanylyl cyclase at 76C, Gyc76C, in morphogenesis of the salivary gland. We demonstrate that Gyc76C and downstream cGMP-dependent protein kinase 1 (DG1) function in the gland and surrounding mesoderm to control invagination, collective **migration** and lumen shape. Loss of gyc76C resulted in glands that failed to invaginate, complete posterior **migration** and had branched lumens. Salivary gland **migration** defects of gyc76C mutant embryos were rescued by expression of wild-type gyc76C specifically in the gland or surrounding mesoderm, whereas invagination defects were rescued primarily by expression in the gland. In migrating salivary glands of gyc76C mutant embryos, integrin subunits localized normally to gland-mesoderm contact sites but talin localization in the surrounding circular visceral mesoderm and fat body was altered. The extracellular matrix protein, laminin, also failed to accumulate around the migrating salivary gland of gyc76C mutant embryos, and gyc76C and laminin genetically interacted in gland **migration**. Our studies suggest that gyc76C controls salivary gland invagination, collective **migration** and lumen shape, in part by regulating the localization of talin and the laminin matrix.
8. CalpB modulates border cell **migration** in Drosophila egg chambers. Calpains are calcium regulated intracellular cysteine proteases implicated in a variety of physiological functions and pathological conditions. The Drosophila melanogaster genome contains only two genes, CalpA and CalpB coding for canonical, active calpain enzymes. The movement of the border cells in Drosophila egg chambers is a well characterized model of the eukaryotic cell **migration**. Using this genetically pliable model we can investigate the physiological role of calpains in cell motility.We demonstrate at the whole organism level that CalpB is implicated in cell **migration**, while the structurally related CalpA paralog can not fulfill the same function. The downregulation of the CalpB gene by mutations or RNA interference results in a delayed **migration** of the border cells in Drosophila egg chambers. This phenotype is significantly enhanced when the focal adhesion complex genes encoding for α-PS2 integrin ( if), β-PS integrin (mys) and talin (rhea) are silenced. The reduction of CalpB activity diminishes the release of integrins from the rear end of the border cells. The delaye**d migrati**on and the reduced integrin release phenotypes can be suppressed by expressing wild-type talin-head in the border cells but not talin-head(R367A), a mutant form which is not able to bind β-PS integrin. CalpB can cleave talin in vitro, and the two proteins coimmunoprecipitate from Drosophila extracts.The physiological function of CalpB in border cell motility has been demonstrated in vivo. The genetic interaction between the CalpB and the if, mys, as well as rhea genes, the involvement of active talin head-domains in the process, and the fact that CalpB and talin interact with each other collectively suggest that the limited proteolytic cleavage of talin is one of the possible mechanisms through which CalpB regulates ce**ll migrat**ion.
9. The cloning, genomic organization and expression of the focal contact protein paxillin in Drosophila. Paxillin is a focal adhesion scaffolding protein, which has been proposed to play a role in focal adhesion dynamics. We have isolated a cDNA clone of the Drosophila homologue of paxillin. Comparison of the Drosophila paxillin sequence with those of vertebrate paxillins shows strong conservation of the LIM domains and LD repeats. Using the Drosophila genomic sequence we have identified two partial curated transcripts and deduced the structure of the paxillin gene. No homologues of other members of the paxillin family such as HIC-5 or leupaxin are to be found in the Drosophila genome. Surprisingly paxillin mRNA is expressed in a restricted pattern during embryogenesis. In particular it is strongly expressed in cells and tissues undergoing cell shape changes or cell **migration**. Many of the sites of expression are also known to be sites of integrin function or FAK expression. The data support a role for paxillin as an adapter and/or signaling protein during developmental processes involving integrin-mediated adhesion.
10. Analysis of integrin turnover in fly myotendinous junctions. Transient (short-term) cell adhesion underlies dynamic processes such as cell **migration**, whereas stable (long-term) cell adhesion maintains tissue architecture. Ongoing adhesion complex turnover is essential for transient cell adhesion, but it is not known whether turnover is also required for maintenance of long-term adhesion. We used fluorescence recovery after photobleaching to analyze the dynamics of an integrin adhesion complex (IAC) in a model of long-term cell-ECM adhesion, myotendinous junctions (MTJs), in fly embryos and larvae. We found that the IAC undergoes turnover in MTJs and that this process is mediated by clathrin-dependent endocytosis. Moreover, the small GTPase Rab5 can regulate the proportion of IAC components that undergo turnover. Also, altering Rab5 activity weakened MTJs, resulting in muscle defects. In addition, growth of MTJs was concomitant with a decrease in the proportion of IAC components undergoing turnover. We propose that IAC turnover is tightly regulated in long-term cell-ECM adhesions to allow normal tissue growth and maintenance.
11. Morphogenesis in the absence of integrins: mutation of both Drosophila beta subunits prevents midgut **migration**. Two integrin beta subunits are encoded in the Drosophila genome. The betaPS subunit is widely expressed and heterodimers containing this subunit are required for many developmental processes. The second betasubunit, betanu, is a divergent integrin expressed primarily in the midgut endoderm. To elucidate its function, we generated null mutations in the gene encoding betanu. We find that betanu is not required for viability or fertility, and overall the mutant flies are normal in appearance. However, we could observe betanu function in the absence of betaPS. Consistent with its expression, removal of betanu only enhanced the phenotype of betaPS in the developing midgut. In embryos lacking the zygotic contribution of betaPS, loss of betanu resulted in enhanced separation between the midgut and the surrounding visceral mesoderm. In the absence of both maternal and zygotic betaPS, a delay in midgut **migration** was observed, but removing betanu as well blocked **migration** completely. These results demonstrate that the second beta subunit can partially compensate for loss of betaPS integrins, and that integrins are essential for **migration** of the primordial midgut cells. The two beta subunits mediate midgut **migration** by distinct mechanisms: one that requires talin and one that does not. Other examples of developmental cell **migration**, such as that of the primordial germ cells, occurred normally in the absence of integrins. Having generated the tools to eliminate integrin function completely, we confirm that Drosophila integrins do not control proliferation as they do in mammals, and have identified alphaPS3 as a heterodimeric partner for betanu.
12. Drosophila blood cell chemotaxis. Drosophila melanogaster contains a population of blood cells called hemocytes that represent the functional equivalent of vertebrate macrophages. These cells undergo directed **migration**s to disperse during development and reach sites of tissue damage or altered self. These chemotactic behaviors are controlled by the expression of PDGF/Vegf-related ligands in developing embryos and local production of hydrogen peroxide at wounds. Recent work reveals that many molecules important in vertebrate cell motility, including integrins, formins, Ena/VASP proteins and the SCAR/WAVE complex, have a conserved function in these innate immune cells. The use of this model organism has elucidated how damage signals are activated by calcium signaling during inflammation and that the steroid hormone ecdysone activates immune competence at key developmental stages.
13. Regulation of cell **migration** during tracheal development in Drosophila melanogaster. Most of the knowledge concerning the intracellular mechanisms involved in cell locomotion have been obtained from in vitro studies of cells in culture. Many of the concepts derived from these studies have been partially confirmed in in vivo systems but numerous questions regarding the developmental control of cell **migration** remain to be addressed. Tracheal morphogenesis in Drosophila melanogaster embryos represents an in vivo model system to study the genetic control of cell **migration**. We review what is known about tracheal development and regulation of tracheal cell **migration**. We try to link these in vivo studies and the movement of cells over two dimensional substrates and elaborate on important questions which remain to be addressed in the future.
14. Focal adhesion kinase is not required for integrin function or viability in Drosophila. The mammalian focal adhesion kinase (FAK) family of non-receptor protein-tyrosine kinases has been implicated in controlling a multitude of cellular responses to the engagement of cell-surface integrins and G-protein-coupled receptors. The high level of sequence conservation between the mammalian proteins and the Drosophila homologue of FAK, Fak56, suggested that it would have similar functions. However, we show here that Drosophila Fak56 is not essential for integrin functions in adhesion, **migration** or signaling in vivo. Furthermore, animals lacking Fak56 are viable and fertile, demonstrating that Fak56 is not essential for other developmental or physiological functions. Despite this, overexpressed Fak56 is a potent inhibitor of integrins binding to the extracellular matrix, suggesting that Fak56 may play a subtle role in the negative regulation of integrin adhesion.
15. Vestigial is required during late-stage muscle differentiation in Drosophila melanogaster embryos. The somatic muscles of Drosophila develop in a complex pattern that is repeated in each embryonic hemi-segment. During early development, progenitor cells fuse to form a syncytial muscle, which further differentiates via expression of muscle-specific factors that induce specific responses to external signals to regulate late-stage processes such as **migration** and attachment. Initial communication between somatic muscles and the epidermal tendon cells is critical for both of these processes. However, later establishment of attachments between longitudinal muscles at the segmental borders is largely independent of the muscle-epidermal attachment signals, and relatively little is known about how this event is regulated. Using a combination of null mutations and a truncated version of Sd that binds Vg but not DNA, we show that Vestigial (Vg) is required in ventral longitudinal muscles to induce formation of stable intermuscular attachments. In several muscles, this activity may be independent of Sd. Furthermore, the cell-specific differentiation events induced by Vg in two cells fated to form attachments are coordinated by Drosophila epidermal growth factor signaling. Thus, Vg is a key factor to induce specific changes in ventral longitudinal muscles 1-4 identity and is required for these cells to be competent to form stable intermuscular attachments with each other.
16. Focal adhesions: what's new inside. The cytoplasmic side of focal adhesions is comprised of large molecular complexes that link transmembrane receptors, such as integrins, to the actin cytoskeleton and mediate signals modulating cell attachment, **migration**, proliferation, differentiation, and gene expression. These complexes are heterogeneous and dynamic structures that are apparent targets of regulatory signals that control the function of focal adhesions. Recent studies using genetic approaches in invertebrate and vertebrate systems have begun to reveal the structure and function of these complexes in vivo.
17. Integrins are required for cardioblast polarisation in Drosophila. The formation of a tubular organ, such as the heart, requires the communication of positional and polarity signals between migratory cells. Key to this process is the establishment of a new luminal domain on the cell surface, generally from the apical domain of a migratory cell. This domain will also acquire basal properties, as it will produce a luminal extracellular matrix. Integrin receptors are the primary means of cell adhesion and adhesion signaling with the extracellular matrix. Here we characterise the requirement of Integrins in a genetic model of vasculogenesis, the formation of the heart in Drosophila.As with vertebrates, the Drosophila heart arises from lateral mesoderm that migrates medially to meet their contralateral partners, to then assemble a midline vessel. During **migration**, Integrins are among the first proteins restricted to the presumptive luminal domain of cardioblasts. Integrins are required for normal levels of leading edge membrane motility. Apical accumulation of Integrins is enhanced by Robo, and reciprocally, apicalisation of luminal factors like Slit and Robo requires Integrin function. Integrins may provide a template for the formation of a lumen by stabilising lumen factors like Robo. Subsequent to **migration**, Integrin is required for normal cardioblast alignment and lumen formation. This phenotype is most readily modified by other mutations that affect adhesion, such as Talin and extracellular matrix ligands.Our findings reveal an instructive role for Integrins in communicating polarising information to cells during **migration**, and during transition to an epithelial tube structure.
18. Integrin-ECM interactions regulate the changes in cell shape driving the morphogenesis of the Drosophila wing epithelium. During development, morphogenesis involves **migration** and changes in the shape of epithelial sheets, both of which require coordination of cell adhesion. Thus, while modulation of integrin-mediated adhesion to the ECM regulates epithelial motility, cell-cell adhesion via cadherins controls the remodelling of epithelial sheets. We have used the Drosophila wing epithelium to demonstrate that cell-ECM interactions mediated by integrins also regulate the changes in cell shape that underly epithelial morphogenesis. We show that integrins control the transitions from columnar to cuboidal cell shape underlying wing formation, and we demonstrate that eliminating the ECM has the same effect on cell shape as inhibiting integrin function. Furthermore, lack of integrin activity also induces detachment of the basal lamina and failure to assemble the basal matrix. Hence, we propose that integrins control epithelial cell shape by mediating adherence of these cells to the ECM. Finally, we show that the ECM has an instructive rather than a structural role, because inhibition of Raf reverses the cell shape changes caused by perturbing integrins.
19. Misshapen decreases integrin levels to promote epithelial motility and planar polarity in Drosophila. Complex organ shapes arise from the coordinate actions of individual cells. The Drosophila egg chamber is an organ-like structure that lengthens along its anterior-posterior axis as it grows. This morphogenesis depends on an unusual form of planar polarity in the organ's outer epithelial layer, the follicle cells. Interestingly, this epithelium also undergoes a directed **migration** that causes the egg chamber to rotate around its anterior-posterior axis. However, the functional relationship between planar polarity and **migration** in this tissue is unknown. We have previously reported that mutations in the Misshapen kinase disrupt follicle cell planar polarity. Here we show that Misshapen's primary role in this system is to promote individual cell motility. Misshapen decreases integrin levels at the basal surface, which may facilitate detachment of each cell's trailing edge. These data provide mechanistic insight into Misshapen's conserved role in cell **migration** and suggest that follicle cell planar polarity may be an emergent property of individual cell migratory behaviors within the epithelium.
20. Cell-cell adhesion via the ECM: integrin genetics in fly and worm. Integrins are essential for the development of the two genetically tractable invertebrate model organisms, the nematode worm Caenorhabditis elegans and the fruit fly Drosophila melanogaster. Just two integrins are present in C. elegans: one putative RGD binding integrin alphapat-2betapat-3, corresponding to Drosophila alphaPS2betaPS and vertebrate alpha5beta1, alphaVbeta1 and alpha8beta1, and one putative laminin binding integrin alphaina-1betapat-3, corresponding to Drosophila alphaPS1betaPS and vertebrate alpha3beta1, alpha6beta1 and alpha7beta1. In this review, the function of this minimal set of integrins during the development of these two invertebrates is compared. Despite the differences in bodyplan and developmental strategy, integrin adhesion to the extracellular matrix is required for similar processes: the formation of the link that translates muscle contraction into movement of the exoskeleton, cell **migration**, and morphogenetic interactions between epithelia. Other integrin functions, such as regulation of gene expression, have not yet been experimentally demonstrated in both organisms. Additional proteins have been characterised in each organism that are essential for integrin function, including extracellular matrix ligands and intracellular interacting proteins, but so far different proteins have been found in the two organisms. This in part represents the fact that the characterisation of the full set of interacting proteins is not complete in either system. However, in other cases different proteins appear to be used for similar functions in the two animals. The continued use of genetic approaches to identify proteins required for integrin function in these two model organisms should lead to the identification of the minimal set of conserved components that form integrin adhesive structures.
21. JNK signaling controls border cell cluster integrity and collective cell **migration**. Collective cell movement is a mechanism for invasion identified in many developmental events. Examples include the movement of lateral-line neurons in Zebrafish, cells in the inner blastocyst, and metastasis of epithelial tumors [1]. One key model to study collective **migration** is the movement of border cell clusters in Drosophila. Drosophila egg chambers contain 15 nurse cells and a single oocyte surrounded by somatic follicle cells. At their anterior end, polar cells recruit several neighboring follicle cells to form the border cell cluster [2]. By stage 9, and over 6 hr, border cells migrate as a cohort between nurse cells toward the oocyte. The specification and directionality of border cell movement are regulated by hormonal and signaling mechanisms [3]. However, how border cells are held together while they migrate is not known. Here, we show that a negative-feedback loop controlling JNK activity regulates border cell cluster integrity. JNK signaling modulates contacts between border cells and between border cells and substratum to sustain collective **migration** by regulating several effectors including the polarity factor Bazooka and the cytoskeletal adaptor D-Paxillin. We anticipate a role for the JNK pathway in controlling collective cell movements in other morphogenetic and clinical models.
22. Integrins in development: moving on, responding to, and sticking to the extracellular matrix. Integrins are cell surface receptors of the extracellular matrix present in all animals. Genetic analysis in worms, flies, and vertebrates has revealed integrin involvement in key developmental processes, and we focus here on examples of integrin functions that are comparable across these model organisms. Integrins contribute to cell movement by providing traction to migrating cells, through assembly of extracellular matrices that can serve as tracks for **migration**, and by transmitting guidance signals that direct cells or cell processes to their targets. Integrins also participate in signaling events that govern tissue differentiation and organogenesis. Finally, adhesion by integrin-mediated junctions allows tissues to withstand mechanical load and is essential for tissue integrity.

### ECM

#### PubMed

1. The talin head domain reinforces integrin-mediated adhesion by promoting adhesion complex stability and clustering. Talin serves an essential function during integrin-mediated adhesion in linking integrins to actin via the intracellular adhesion complex. In addition, the N-terminal head domain of talin regulates the affinity of integrins for their **ECM**-ligands, a process known as inside-out activation. We previously showed that in Drosophila, mutating the integrin binding site in the talin head domain resulted in weakened adhesion to the **ECM**. Intriguingly, subsequent studies showed that canonical inside-out activation of integrin might not take place in flies. Consistent with this, a mutation in talin that specifically blocks its ability to activate mammalian integrins does not significantly impinge on talin function during fly development. Here, we describe results suggesting that the talin head domain reinforces and stabilizes the integrin adhesion complex by promoting integrin clustering distinct from its ability to support inside-out activation. Specifically, we show that an allele of talin containing a mutation that disrupts intramolecular interactions within the talin head attenuates the assembly and reinforcement of the integrin adhesion complex. Importantly, we provide evidence that this mutation blocks integrin clustering in vivo. We propose that the talin head domain is essential for regulating integrin avidity in Drosophila and that this is crucial for integrin-mediated adhesion during animal development.
2. In vivo functional analysis reveals specific roles for the integrin-binding sites of talin. Adhesion receptors play diverse roles during animal development and require precise spatiotemporal regulation, which is achieved through the activity of their binding partners. Integrins, adhesion receptors that mediate cell attachment to the extracellular matrix (**ECM**), connect to the intracellular environment through the cytoplasmic adapter protein talin. Talin has two essential functions: orchestrating the assembly of the intracellular adhesion complex (IAC), which associates with integrin, and regulating the affinity of integrins for the **ECM**. Talin can bind to integrins through two different integrin-binding sites (IBS-1 and IBS-2, respectively). Here, we have investigated the roles of each in the context of Drosophila development. We find that although IBS-1 and IBS-2 are partially redundant, they each have specialized roles during development: IBS-1 reinforces integrin attachment to the **ECM**, whereas IBS-2 reinforces the link between integrins and the IAC. Disruption of each IBS has different developmental consequences, illustrating how the functional diversity of integrin-mediated adhesion is achieved.
3. Talin is essential for integrin function in Drosophila. We show that the Drosophila gene rhea, isolated because its wing blister phenotype is typical of mutants affecting integrin function, encodes talin. Embryos deficient in talin have very similar phenotypes to integrin (betaPS) null embryos, including failure in germ band retraction and muscle detachment. We demonstrate that talin is not required for the presence of integrins on the cell surface or their localization at muscle termini. However, talin is required for formation of focal adhesion-like clusters of integrins on the basal surface of imaginal disc epithelia and junctional plaques between muscle and tendon cells. These results indicate that talin is essential for integrin function and acts by stably linking clusters of **ECM**-linked integrins to the cytoskeleton.
4. In vivo regulation of integrin turnover by outside-in activation. The development of three-dimensional tissue architecture requires precise control over the attachment of cells to the extracellular matrix (**ECM**). Integrins, the main **ECM**-binding receptors in animals, are regulated in multiple ways to modulate cell-**ECM** adhesion. One example is the conformational activation of integrins by extracellular signals ('outside-in activation') or by intracellular signals ('inside-out activation'), whereas another is the modulation of integrin turnover. We demonstrate that outside-in activation regulates integrin turnover to stabilize tissue architecture in vivo Treating Drosophila embryos with Mg(2+) and Mn(2+), known to induce outside-in activation, resulted in decreased integrin turnover. Mathematical modeling combined with mutational analysis provides mechanistic insight into the stabilization of integrins at the membrane. We show that as tissues mature, outside-in activation is crucial for regulating the stabilization of integrin-mediated adhesions. This data identifies a new in vivo role for outside-in activation and sheds light on the key transition between tissue morphogenesis and maintenance.
5. Integrin-mediated adhesion maintains sarcomeric integrity. Integrin-mediated adhesion to the **ECM** is essential for normal development of animal tissues. During muscle development, integrins provide the structural stability required to construct such a highly tensile, force generating tissue. Mutations that disrupt integrin-mediated adhesion in skeletal muscles give rise to a myopathy in humans and mice. To determine if this is due to defects in formation or defects in maintenance of muscle tissue, we used an inducible, targeted RNAi based approach to disrupt integrin-mediated adhesion in fully formed adult fly muscles. A decrease in integrin-mediated adhesion in adult muscles led to a progressive loss of muscle function due to a failure to maintain normal sarcomeric cytoarchitecture. This defect was due to a gradual, age dependent disorganization of the sarcomeric actin, Z-line, and M-line. Electron microscopic analysis showed that reduction in integrin-mediated adhesion resulted in detachment of actin filaments from the Z-lines, separation of the Z-lines from the membrane, and eventually to disintegration of the Z-lines. Our results show that integrin-mediated adhesion is essential for maintaining sarcomeric integrity and illustrate that the seemingly stable adhesive contacts underlying sarcomeric architecture are inherently dynamic.
6. Loss of focal adhesions in glia disrupts both glial and photoreceptor axon migration in the Drosophila visual system. Many aspects of glial development are regulated by extracellular signals, including those from the extracellular matrix (**ECM**). Signals from the **ECM** are received by cell surface receptors, including the integrin family. Previously, we have shown that Drosophila integrins form adhesion complexes with Integrin-linked kinase and talin in the peripheral nerve glia and have conserved roles in glial sheath formation. However, integrin function in other aspects of glial development is unclear. The Drosophila eye imaginal disc (ED) and optic stalk (OS) complex is an excellent model with which to study glial migration, differentiation and glia-neuron interactions. We studied the roles of the integrin complexes in these glial developmental processes during OS/eye development. The common beta subunit βPS and two alpha subunits, αPS2 and αPS3, are located in puncta at both glia-glia and gl**ia-**ECM interfaces. Depletion of βPS integrin and talin by RNAi impaired the migration and distribution of glia within the OS resulting in morphological defects. Reduction of integrin or talin in the glia also disrupted photoreceptor axon outgrowth leading to axon stalling in the OS and ED. The neuronal defects were correlated with a disruption of the carpet glia tube paired with invasion of glia into the core of the OS and the formation of a glial cap. Our results suggest that integrin-mediated extracellular signals are important for multiple aspects of glial development and non-autonomously affect axonal migration during Drosophila eye development.
7. Integrin-dependent anchoring of a stem-cell niche. Interactions between stem cells and their surrounding microenvironment, or niche, are critical for the establishment and maintenance of stem-cell properties. The adult Drosophila testis contains a morphologically discrete stem-cell niche, the 'hub'. The small cluster of non-dividing, somatic hub cells at the anterior tip of the fly testis is contacted by the germline stem cells (GSCs), which retain their stem-cell character through the direct association with the hub. Here we show that integrin-mediated adhesion is important for maintaining the correct position of embryonic hub cells during gonad morphogenesis. The misplaced hub in integrin-deficient embryos directs the orientation of cell divisions in the presumptive GSCs, a hallmark of the active germline stem-cell niche. A decrease in integrin-mediated adhesion in adult testes, which resulted in a loss of the hub and the stem-cell population, revealed the importance of hub-cell anchoring. Finally, we show that an extracellular matrix (**ECM**) is present around the gonad during late embryogenesis and that this **ECM** is defective in integrin-deficient gonads. On the basis of our data, we propose that integrins are required for the attachment of the hub cells to the **ECM**, which is essential for maintaining the stem-cell niche.
8. Analysis of integrin turnover in fly myotendinous junctions. Transient (short-term) cell adhesion underlies dynamic processes such as cell migration, whereas stable (long-term) cell adhesion maintains tissue architecture. Ongoing adhesion complex turnover is essential for transient cell adhesion, but it is not known whether turnover is also required for maintenance of long-term adhesion. We used fluorescence recovery after photobleaching to analyze the dynamics of an integrin adhesion complex (IAC) in a model of long-term cell-**ECM** adhesion, myotendinous junctions (MTJs), in fly embryos and larvae. We found that the IAC undergoes turnover in MTJs and that this process is mediated by clathrin-dependent endocytosis. Moreover, the small GTPase Rab5 can regulate the proportion of IAC components that undergo turnover. Also, altering Rab5 activity weakened MTJs, resulting in muscle defects. In addition, growth of MTJs was concomitant with a decrease in the proportion of IAC components undergoing turnover. We propose that IAC turnover is tightly regulated in long-term cell-**ECM** adhesions to allow normal tissue growth and maintenance.
9. Mutations in the Drosophila alphaPS2 integrin subunit uncover new features of adhesion site assembly. The Drosophila alphaPS2betaPS integrin is required for diverse development events, including muscle attachment. We characterized six unusual mutations in the alphaPS2 gene that cause a subset of the null phenotype. One mutation changes a residue in alphaPS2 that is equivalent to the residue in alphaV that contacts the arginine of RGD. This change severely reduced alphaPS2betaPS affinity for soluble ligand, abolished the ability of the integrin to recruit laminin to muscle attachment sites in the embryo and caused detachment of integrins and talin from the **ECM**. Three mutations that alter different parts of the alphaPS2 beta-propeller, plus a fourth that eliminated a late phase of alphaPS2 expression, all led to a strong decrease in alphaPS2betaPS at muscle ends, but, surprisingly, normal levels of talin were recruited. Thus, although talin recruitment requires alphaPS2betaPS, talin levels are not simply specified by the amount of integrin at the adhesive junction. These mutations caused detachment of talin and actin from integrins, suggesting that the integrin-talin link is weaker than the **ECM**-integrin link.
10. In vivo functional analysis reveals specific roles for the integrin-binding sites of talin. Adhesion receptors play diverse roles during animal development and require precise spatiotemporal regulation, which is achieved through the activity of their binding partners. Integrins, adhesion receptors that mediate cell attachment to the extracellular matrix (**ECM**), connect to the intracellular environment through the cytoplasmic adapter protein talin. Talin has two essential functions: orchestrating the assembly of the intracellular adhesion complex (IAC), which associates with integrin, and regulating the affinity of integrins for the **ECM**. Talin can bind to integrins through two different integrin-binding sites (IBS-1 and IBS-2, respectively). Here, we have investigated the roles of each in the context of Drosophila development. We find that although IBS-1 and IBS-2 are partially redundant, they each have specialized roles during development: IBS-1 reinforces integrin attachment to the **ECM**, whereas IBS-2 reinforces the link between integrins and the IAC. Disruption of each IBS has different developmental consequences, illustrating how the functional diversity of integrin-mediated adhesion is achieved.
11. Integrins are necessary for the development and maintenance of the glial layers in the Drosophila peripheral nerve. Peripheral nerve development involves multiple classes of glia that cooperate to form overlapping glial layers paired with the deposition of a surrounding extracellular matrix (**ECM**). The formation of this tubular structure protects the ensheathed axons from physical and pathogenic damage and from changes in the ionic environment. Integrins, a major family of **ECM** receptors, play a number of roles in the development of myelinating Schwann cells, one class of glia ensheathing the peripheral nerves of vertebrates. However, the identity and the role of the integrin complexes utilized by the other classes of peripheral nerve glia have not been determined in any animal. Here, we show that, in the peripheral nerves of Drosophila melanogaster, two integrin complexes (αPS2βPS and αPS3βPS) are expressed in the different glial layers and form adhesion complexes with integrin-linked kinase and Talin. Knockdown of the common beta subunit (βPS) using inducible RNAi in all glial cells results in lethality and glial defects. Analysis of integrin complex function in specific glial layers showed that loss of βPS in the outermost layer (the perineurial glia) results in a failure to wrap the nerve, a phenotype similar to that of Matrix metalloproteinase 2-mediated degradation o**f t**he ECM. Knockdown of βPS integrin in the innermost wrapping glia causes a loss of glial processes around axons. Together, our data suggest that integrins are employed in different glial layers to mediate the development and maintenance of the protective glial sheath in Drosophila peripheral nerves.
12. Integrins contribute to the establishment and maintenance of cell polarity in the follicular epithelium of the Drosophila ovary. The generation of epithelial cell polarity is a key process during development. Although the induction and orientation of cell polarity by cell-cell and cell-extracellular matrix (**ECM**) interactions is well established, the molecular mechanisms by which signals from the **ECM** control cell polarity in developing epithelial tissues remain poorly understood. Here, we have used the follicular epithelium of the Drosophila ovary to investigate the role that integrins, the main cell-**ECM** receptors, play in the establishment of apicobasal polarity. Mature follicle cells have an apical side facing the germ line and a basal side in contact with a basement membrane. Our results show that integrins - presumably via interactions with the basement membrane - play a reinforcing role in follicle cell polarization, as they are required to establish and/or maintain follicle cell membrane asymmetry only when contact with the germ line is prevented. We suggest that the primary cue for polarization of the follicular epithelium is contact with the germline cells. In addition, while interfering with apical and lateral polarization cues leads to apoptosis, we show here that inhibition of contact with the basement membrane mediated by integrins does not affect cell survival. Finally, we provide evidence to suggest that integrins are required to orientate epithelial polarity in vivo.
13. An interaction between integrin and the talin FERM domain mediates integrin activation but not linkage to the cytoskeleton. Transmembrane adhesion receptors, such as integrins, mediate cell adhesion by interacting with intracellular proteins that connect to the cytoskeleton. Talin, one such linker protein, is thought to have two roles: mediating inside-out activation of integrins, and connecting extracellular matrix (**ECM**)-bound integrins to the cytoskeleton. Talin's amino-terminal head, which consists of a FERM domain, binds an NPxY motif within the cytoplasmic tail of most integrin beta subunits. This is consistent with the role of FERM domains in recruiting other proteins to the plasma membrane. We tested the role of the talin-head-NPxY interaction in integrin function in Drosophila. We found that introduction of a mutation that perturbs this binding in vitro into the isolated talin head disrupts its recruitment by integrins in vivo. Surprisingly, when engineered into the full-length talin, this mutation did not disrupt talin recruitment by integrins nor its ability to connect integrins to the cytoskeleton. However, it reduced the ability of talin to strengthen integrin adhesion to the **ECM**, indicating that the function of the talin-head-NPxY interaction is solely to regulate integrin adhesion.
14. Mechanical force regulates integrin turnover in Drosophila in vivo. Regulated assembly and disassembly, or turnover, of integrin-mediated cell-extracellular matrix (**ECM**) adhesions is essential for dynamic cell movements and long-term tissue maintenance. For example, in Drosophila, misregulation of integrin turnover disrupts muscle-tendon attachment at myotendinous junctions (MTJs). We demonstrate that mechanical force, which modulates integrin activity, also regulates integrin and intracellular adhesion complex (IAC) turnover in vivo. Using conditional mutants to alter the tensile force on MTJs, we found that the proportion of IAC components undergoing turnover inversely correlated with the force applied on MTJs. This effect was disrupted by point mutations in β-integrin that interfere with **EC**M-induced conformational changes and activation of β-integrin or integrin-mediated cytoplasmic signalling. These mutants also disrupted integrin dynamics at MTJs during larval development. Together, these data suggest that specific β-integrin-mediated signals regulate adhesion turnover in response to tension during tissue formation. We propose that integr**in-**ECM adhesive stability is continuously controlled by force in vivo through integrin-dependent auto-regulatory feedback mechanisms so that tissues can quickly adapt to and withstand mechanical stresses.
15. Integrin signaling is required for maintenance and proliferation of intestinal stem cells in Drosophila. Tissue-specific stem cells are maintained by both local secreted signals and cell adhesion molecules that position the stem cells in the niche microenvironment. In the Drosophila midgut, multipotent intestinal stem cells (ISCs) are located basally along a thin layer of basement membrane that composed of extracellular matrix (**ECM**), which separates ISCs from the surrounding visceral musculature: the muscle cells constitute a regulatory niche for ISCs by producing multiple secreted signals that directly regulate ISC maintenance and proliferation. Here we show that integrin-mediated cell adhesion, which connects the **ECM** and intracellular cytoskeleton, is required for ISC anchorage to the basement membrane. Specifically, the α-integrin subunits including αPS1 encoded by mew and αPS3 encoded by scb, and the β-integrin subunit encoded by mys are richly expressed in ISCs and are required for the maintenance, rather than their survival or multiple lineage differentiation. Furthermore, ISC maintenance also requires the intercellular and intracellular integrin signaling components including Talin, Integrin-linked kinase (Ilk), and the ligand, Laminin A. Notably, integrin mutant ISCs are also less proliferative, and genetic interaction studies suggest that proper integrin signaling is a pre-requisite for ISC proliferation in response to various proliferative signals and for the initiation of intestinal hyperplasia after loss of adenomatous polyposis coli (Apc). Our studies suggest that integrin not only functions to anchor ISCs to the basement membrane, but also serves as an essential element for ISC proliferation during normal homeostasis and in response to oncogenic mutations.
16. Moleskin is essential for the formation of the myotendinous junction in Drosophila. It is the precise connectivity between skeletal muscles and their corresponding tendon cells to form a functional myotendinous junction (MTJ) that allows for the force generation required for muscle contraction and organismal movement. The Drosophila MTJ is composed of secreted extracellular matrix (**ECM**) proteins deposited between integrin-mediated hemi-adherens junctions on the surface of muscle and tendon cells. In this paper, we have identified a novel, cytoplasmic role for the canonical nuclear import protein Moleskin (Msk) in Drosophila embryonic somatic muscle attachment. Msk protein is enriched at muscle attachment sites in late embryogenesis and msk mutant embryos exhibit a failure in muscle-tendon cell attachment. Although the muscle-tendon attachment sites are reduced in size, components of the integrin complexes and **ECM** proteins are properly localized in msk mutant embryos. However, msk mutants fail to localize phosphorylated focal adhesion kinase (pFAK) to the sites of muscle-tendon cell junctions. In addition, the tendon cell specific proteins Stripe (Sr) and activated mitogen-activated protein kinase (MAPK) are reduced in msk mutant embryos. Our rescue experiments demonstrate that Msk is required in the muscle cell, but not in the tendon cells. Moreover, muscle attachment defects due to loss of Msk are rescued by an activated form of MAPK or the secreted epidermal growth factor receptor (Egfr) ligand Vein. Taken together, these findings provide strong evidence that Msk signals non-autonomously through the Vein-Egfr signaling pathway for late tendon cell late differentiation and/or maintenance.
17. The integrin adhesion complex changes its composition and function during morphogenesis of an epithelium. Cell adhesion to the extracellular matrix (**ECM**) is mediated by the integrin family of transmembrane receptors. Integrins link **ECM** ligands to the cytoskeleton, providing strong attachment to enable cell-shape change and tissue integrity. This connection is made possible by an intracellular complex of proteins, which links to actin filaments and controls signalling cascades that regulate cytoskeletal rearrangements. We have identified stress-fibre-associated focal adhesions that change their composition during tissue morphogenesis. Early expression of alphaPS1betaPS integrin decreases the levels of the actin-nucleating factors Enabled, Diaphanous and profilin, as well as downregulating the amount of F-actin incorporated into the stress fibres. As follicle cells mature in their developmental pathway and become squamous, the integrin in the focal adhesions changes from alphaPS1betaPS to alphaPS2betaPS. During the switch, stress fibres increase their length and change orientation, first changing by 90 degrees and then reorienting back. The normal rapid reorientation requires new expression of alphaPS2betaPS, which also permits recruitment of the adaptor protein tensin. Unexpectedly, it is the extracellular portion of the alphaPS2 subunit that provides the specificity for intracellular recruitment of tensin. Molecular variation of the integrin complex is thus a key component of developmentally programmed morphogenesis.
18. The talin head domain reinforces integrin-mediated adhesion by promoting adhesion complex stability and clustering. Talin serves an essential function during integrin-mediated adhesion in linking integrins to actin via the intracellular adhesion complex. In addition, the N-terminal head domain of talin regulates the affinity of integrins for their **ECM**-ligands, a process known as inside-out activation. We previously showed that in Drosophila, mutating the integrin binding site in the talin head domain resulted in weakened adhesion to the **ECM**. Intriguingly, subsequent studies showed that canonical inside-out activation of integrin might not take place in flies. Consistent with this, a mutation in talin that specifically blocks its ability to activate mammalian integrins does not significantly impinge on talin function during fly development. Here, we describe results suggesting that the talin head domain reinforces and stabilizes the integrin adhesion complex by promoting integrin clustering distinct from its ability to support inside-out activation. Specifically, we show that an allele of talin containing a mutation that disrupts intramolecular interactions within the talin head attenuates the assembly and reinforcement of the integrin adhesion complex. Importantly, we provide evidence that this mutation blocks integrin clustering in vivo. We propose that the talin head domain is essential for regulating integrin avidity in Drosophila and that this is crucial for integrin-mediated adhesion during animal development.
19. Thrombospondin-mediated adhesion is essential for the formation of the myotendinous junction in Drosophila. Organogenesis of the somatic musculature in Drosophila is directed by the precise adhesion between migrating myotubes and their corresponding ectodermally derived tendon cells. Whereas the PS integrins mediate the adhesion between these two cell types, their extracellular matrix (**ECM**) ligands have been only partially characterized. We show that the **ECM** protein Thrombospondin (Tsp), produced by tendon cells, is essential for the formation of the integrin-mediated myotendinous junction. Tsp expression is induced by the tendon-specific transcription factor Stripe, and accumulates at the myotendinous junction following the association between the muscle and the tendon cell. In tsp mutant embryos, migrating somatic muscles fail to attach to tendon cells and often form hemiadherens junctions with their neighboring muscle cells, resulting in nonfunctional somatic musculature. Talin accumulation at the cytoplasmic faces of the muscles and tendons is greatly reduced, implicating Tsp as a potential integrin ligand. Consistently, purified Tsp C-terminal domain polypeptide mediates spreading of PS2 integrin-expressing S2 cells in a KGD- and PS2-integrin-dependent manner. We propose a model in which the myotendinous junction is formed by the specific association of Tsp with multiple muscle-specific PS2 integrin receptors and a subsequent consolidation of the junction by enhanced tendon-specific production of Tsp secreted into the junctional space.
20. Integrin-**ECM** interactions regulate the changes in cell shape driving the morphogenesis of the Drosophila wing epithelium. During development, morphogenesis involves migration and changes in the shape of epithelial sheets, both of which require coordination of cell adhesion. Thus, while modulation of integrin-mediated adhesion to the **ECM** regulates epithelial motility, cell-cell adhesion via cadherins controls the remodelling of epithelial sheets. We have used the Drosophila wing epithelium to demonstrate that cell-**ECM** interactions mediated by integrins also regulate the changes in cell shape that underly epithelial morphogenesis. We show that integrins control the transitions from columnar to cuboidal cell shape underlying wing formation, and we demonstrate that eliminating the **ECM** has the same effect on cell shape as inhibiting integrin function. Furthermore, lack of integrin activity also induces detachment of the basal lamina and failure to assemble the basal matrix. Hence, we propose that integrins control epithelial cell shape by mediating adherence of these cells to the **ECM**. Finally, we show that the **ECM** has an instructive rather than a structural role, because inhibition of Raf reverses the cell shape changes caused by perturbing integrins.
21. Induction of focal adhesions and motility in Drosophila S2 cells. Focal adhesions are dynamic structures that interact with the extracellular matrix on the cell exterior and actin filaments on the cell interior, enabling cells to adhere and crawl along surfaces. We describe a system for inducing the formation of focal adhesions in normally non-**ECM**-adherent, nonmotile Drosophila S2 cells. These focal adhesions contain the expected molecular markers such as talin, vinculin, and p130Cas, and they require talin for their formation. The S2 cells with induced focal adhesions also display a nonpolarized form of motility on vitronectin-coated substrates. Consistent with findings in mammalian cells, the degree of motility can be tuned by changing the stiffness of the substrate and was increased after the depletion of PAK3, a p21-activated kinase. A subset of nonmotile, nonpolarized cells also exhibited focal adhesions that rapidly assembled and disassembled around the cell perimeter. Such cooperative and dynamic fluctuations of focal adhesions were decreased by RNA interference (RNAi) depletion of myosin II and focal adhesion kinase, suggesting that this behavior requires force and focal adhesion maturation. These results demonstrate that S2 cells, a cell line that is well studied for cytoskeletal dynamics and readily amenable to protein manipulation by RNAi, can be used to study the assembly and dynamics of focal adhesions and mechanosensitive cell motility.
22. IPP Complex Reinforces Adhesion by Relaying Tension-Dependent Signals to Inhibit Integrin Turnover. Cytoskeleton-mediated forces regulate the assembly and function of integrin adhesions; however, the underlying mechanisms remain unclear. The tripartite IPP complex, comprising ILK, Parvin, and PINCH, mediates the integrin-actin link at Drosophila embryo muscle attachment sites (MASs). Here, we demonstrate a developmentally earlier function for the IPP complex: to reinforce integrin-extracellular matrix (**ECM**) adhesion in response to tension. In IPP-complex mutants, the integrin-**ECM** linkage at MASs breaks in response to intense muscle contractility. Mechanistically, the IPP complex is required to relay force-elicited signals that decelerate integrin turnover at the plasma membrane so that the integrin immobile fraction is adequate to withstand tension. Epistasis analysis shows that alleviation of muscle contractility, downregulation of endocytosis, and enhanced integrin binding to the **ECM** are sufficient to restore integrin-**ECM** adhesion and maintain integrin-adhesome organization in IPP-complex mutants. Our findings reveal a role for the IPP complex as an essential mechanosensitive regulatory switch of integrin turnover in vivo.
23. Tissue elongation requires oscillating contractions of a basal actomyosin network. Understanding how molecular dynamics leads to cellular behaviours that ultimately sculpt organs and tissues is a major challenge not only in basic developmental biology but also in tissue engineering and regenerative medicine. Here we use live imaging to show that the basal surfaces of Drosophila follicle cells undergo a series of directional, oscillating contractions driven by periodic myosin accumulation on a polarized actin network. Inhibition of the actomyosin contractions or their coupling to extracellular matrix (**ECM**) blocked elongation of the whole tissue, whereas enhancement of the contractions exaggerated it. Myosin accumulated in a periodic manner before each contraction and was regulated by the small GTPase Rho, its downstream kinase, ROCK, and cytosolic calcium. Disrupting the link between the actin cytoskeleton and the **ECM** decreased the amplitude and period of the contractions, whereas enhancing cell-**ECM** adhesion increased them. In contrast, disrupting cell-cell adhesions resulted in loss of the actin network. Our findings reveal a mechanism controlling organ shape and an experimental model for the study of the effects of oscillatory actomyosin activity within a coherent cell sheet.
24. Cell-cell adhesion via the **ECM**: integrin genetics in fly and worm. Integrins are essential for the development of the two genetically tractable invertebrate model organisms, the nematode worm Caenorhabditis elegans and the fruit fly Drosophila melanogaster. Just two integrins are present in C. elegans: one putative RGD binding integrin alphapat-2betapat-3, corresponding to Drosophila alphaPS2betaPS and vertebrate alpha5beta1, alphaVbeta1 and alpha8beta1, and one putative laminin binding integrin alphaina-1betapat-3, corresponding to Drosophila alphaPS1betaPS and vertebrate alpha3beta1, alpha6beta1 and alpha7beta1. In this review, the function of this minimal set of integrins during the development of these two invertebrates is compared. Despite the differences in bodyplan and developmental strategy, integrin adhesion to the extracellular matrix is required for similar processes: the formation of the link that translates muscle contraction into movement of the exoskeleton, cell migration, and morphogenetic interactions between epithelia. Other integrin functions, such as regulation of gene expression, have not yet been experimentally demonstrated in both organisms. Additional proteins have been characterised in each organism that are essential for integrin function, including extracellular matrix ligands and intracellular interacting proteins, but so far different proteins have been found in the two organisms. This in part represents the fact that the characterisation of the full set of interacting proteins is not complete in either system. However, in other cases different proteins appear to be used for similar functions in the two animals. The continued use of genetic approaches to identify proteins required for integrin function in these two model organisms should lead to the identification of the minimal set of conserved components that form integrin adhesive structures.
25. Extracellular matrix and its receptors in Drosophila neural development. Extracellular matrix (**ECM**) and matrix receptors are intimately involved in most biological processes. The **ECM** plays fundamental developmental and physiological roles in health and disease, including processes underlying the development, maintenance, and regeneration of the nervous system. To understand the principles of **ECM**-mediated functions in the nervous system, genetic model organisms like Drosophila provide simple, malleable, and powerful experimental platforms. This article provides an overview of **ECM** proteins and receptors in Drosophila. It then focuses on their roles during three progressive phases of neural development: (1) neural progenitor proliferation, (2) axonal growth and pathfinding, and (3) synapse formation and function. Each section highlights known **ECM** and **ECM**-receptor components and recent studies done in mutant conditions to reveal their in vivo functions, all illustrating the enormous opportunities provided when merging work on the nervous system with systematic research into **ECM**-related gene functions.
26. Talin is essential for integrin function in Drosophila. We show that the Drosophila gene rhea, isolated because its wing blister phenotype is typical of mutants affecting integrin function, encodes talin. Embryos deficient in talin have very similar phenotypes to integrin (betaPS) null embryos, including failure in germ band retraction and muscle detachment. We demonstrate that talin is not required for the presence of integrins on the cell surface or their localization at muscle termini. However, talin is required for formation of focal adhesion-like clusters of integrins on the basal surface of imaginal disc epithelia and junctional plaques between muscle and tendon cells. These results indicate that talin is essential for integrin function and acts by stably linking clusters of **ECM**-linked integrins to the cytoskeleton.
27. Talin autoinhibition is required for morphogenesis. The establishment of a multicellular body plan requires coordinating changes in cell adhesion and the cytoskeleton to ensure proper cell shape and position within a tissue. Cell adhesion to the extracellular matrix (**ECM**) via integrins plays diverse, essential roles during animal embryogenesis and therefore must be precisely regulated. Talin, a FERM-domain containing protein, forms a direct link between integrin adhesion receptors and the actin cytoskeleton and is an important regulator of integrin function. Similar to other FERM proteins, talin makes an intramolecular interaction that could autoinhibit its activity. However, the functional consequence of such an interaction has not been previously explored in vivo. Here, we demonstrate that targeted disruption of talin autoinhibition gives rise to morphogenetic defects during fly development and specifically that dorsal closure (DC), a process that resembles wound healing, is delayed. Impairment of autoinhibition leads to reduced talin turnover at and increased talin and integrin recruitment to sites of integrin-**ECM** attachment. Finally, we present evidence that talin autoinhibition is regulated by Rap1-dependent signaling. Based on our data, we propose that talin autoinhibition provides a switch for modulating adhesion turnover and adhesion stability that is essential for morphogenesis.

### [Ee]xtracellular [Mm]atrix

#### PubMed

1. In vivo functional analysis reveals specific roles for the integrin-binding sites of talin. Adhesion receptors play diverse roles during animal development and require precise spatiotemporal regulation, which is achieved through the activity of their binding partners. Integrins, adhesion receptors that mediate cell attachment to the **extracellular matrix** (ECM), connect to the intracellular environment through the cytoplasmic adapter protein talin. Talin has two essential functions: orchestrating the assembly of the intracellular adhesion complex (IAC), which associates with integrin, and regulating the affinity of integrins for the ECM. Talin can bind to integrins through two different integrin-binding sites (IBS-1 and IBS-2, respectively). Here, we have investigated the roles of each in the context of Drosophila development. We find that although IBS-1 and IBS-2 are partially redundant, they each have specialized roles during development: IBS-1 reinforces integrin attachment to the ECM, whereas IBS-2 reinforces the link between integrins and the IAC. Disruption of each IBS has different developmental consequences, illustrating how the functional diversity of integrin-mediated adhesion is achieved.
2. Distinct developmental roles for direct and indirect talin-mediated linkage to actin. Transmembrane adhesion receptors, such as integrins, mediate cell adhesion by interacting with intracellular proteins that connect to the cytoskeleton. Talin, one such linker protein, is essential to connect **extracellular matrix**-bound integrins to the cytoskeleton. Talin can connect to the cytoskeleton either directly, through its actin-binding motifs, or indirectly, by recruiting other actin-binding proteins. Talin's carboxy-terminal end contains a well-characterized actin-binding domain (ABD). We tested the role of the C-terminal ABD of talin in integrin function in Drosophila. We found that introduction of mutations that reduced actin binding in vitro into the isolated C-terminal Talin-ABD impaired actin binding in vivo. Moreover, when engineered into full-length talin, these mutations disrupted a subset of integrin-mediated adhesion-dependent developmental events. Specifically, morphogenetic processes that involve dynamic, short-term integrin-mediated adhesion were particularly sensitive to impaired function of the C-terminal Talin-ABD. We propose that during development talin connects integrins to the cytoskeleton in distinct ways in different types of integrin-mediated adhesion: directly in transient adhesions and indirectly in stable long-lasting adhesions. Our results provide insight into how a similar array of molecular components can contribute to diverse adhesive processes throughout development.
3. Distinct regulatory mechanisms control integrin adhesive processes during tissue morphogenesis. Cell adhesion must be precisely regulated to enable both dynamic morphogenetic processes and the subsequent transition to stable tissue maintenance. Integrins link the intracellular cytoskeleton and **extracellular matrix**, relaying bidirectional signals across the plasma membrane. In vitro studies have demonstrated that multiple mechanisms control integrin-mediated adhesion; however, their roles during development are poorly understood. We used mutations that activate or deactivate specific functions of vertebrate β-integrins in vitro to investigate how perturbing Drosophila βPS-integrin regulation in developing embryos regulation affects tissue morphogenesis and maintenance. We found that morphogenetic processes use various β-integrin regulatory mechanisms to differing degrees and that conformational changes associated with outside-in activation are essential for developmental integrin functions. Long-term adhesion is also sensitive to integrin dysregulation, suggesting integrins must be continuously regulated to support stable tissue maintenance. Altogether, in vivo phenotypic analyses allowed us to identify the importance of various β-integrin regulatory mechanisms during different morphogenetic processes.
4. Integrin signaling regulates spindle orientation in Drosophila to preserve the follicular-epithelium monolayer. Epithelia act as important physiological barriers and as structural components of tissues and organs. In the Drosophila ovary, follicle cells envelop the germline cysts to form a monolayer epithelium. During division, the orientation of the mitotic spindle in follicle cells is such that both daughter cells remain within the same plane, and the simple structure of the follicular epithelium is thus preserved. Here we show that integrins, heterodimeric transmembrane receptors that connect the **extracellular matrix** to the cell's cytoskeleton [1, 2], are required for maintaining the ovarian monolayer epithelium in Drosophila. Mosaic egg chambers containing integrin mutant follicle cells develop stratified epithelia at both poles. This stratification is due neither to abnormal cell proliferation nor to defects in the apical-basal polarity of the mutant cells. Instead, integrin function is required for the correct orientation of the mitotic apparatus both in mutant cells and in their immediately adjacent wild-type neighbors. We further demonstrate that integrin-mediated signaling, rather than adhesion, is sufficient for maintaining the integrity of the follicular epithelium. The above data show that integrins are necessary for preserving the simple organization of a specialized epithelium and link integrin-mediated signaling to the correct orientation of the mitotic spindle in this epithelial cell type.
5. In vivo regulation of integrin turnover by outside-in activation. The development of three-dimensional tissue architecture requires precise control over the attachment of cells to the **extracellular matrix** (ECM). Integrins, the main ECM-binding receptors in animals, are regulated in multiple ways to modulate cell-ECM adhesion. One example is the conformational activation of integrins by extracellular signals ('outside-in activation') or by intracellular signals ('inside-out activation'), whereas another is the modulation of integrin turnover. We demonstrate that outside-in activation regulates integrin turnover to stabilize tissue architecture in vivo Treating Drosophila embryos with Mg(2+) and Mn(2+), known to induce outside-in activation, resulted in decreased integrin turnover. Mathematical modeling combined with mutational analysis provides mechanistic insight into the stabilization of integrins at the membrane. We show that as tissues mature, outside-in activation is crucial for regulating the stabilization of integrin-mediated adhesions. This data identifies a new in vivo role for outside-in activation and sheds light on the key transition between tissue morphogenesis and maintenance.
6. The conserved transmembrane proteoglycan Perdido/Kon-tiki is essential for myofibrillogenesis and sarcomeric structure in Drosophila. Muscle differentiation requires the assembly of high-order structures called myofibrils, composed of sarcomeres. Even though the molecular organization of sarcomeres is well known, the mechanisms underlying myofibrillogenesis are poorly understood. It has been proposed that integrin-dependent adhesion nucleates myofibrils at the periphery of the muscle cell to sustain sarcomere assembly. Here, we report a role for the gene perdido (perd, also known as kon-tiki, a transmembrane chondroitin proteoglycan) in myofibrillogenesis. Expression of perd RNAi in muscles, prior to adult myogenesis, can induce misorientation and detachment of Drosophila adult abdominal muscles. In comparison to controls, perd-depleted muscles contain fewer myofibrils, which are localized at the cell periphery. These myofibrils are detached from each other and display a defective sarcomeric structure. Our results demonstrate that the **extracellular matrix** receptor Perd has a specific role in the assembly of myofibrils and in sarcomeric organization. We suggest that Perd acts downstream or in parallel to integrins to enable the connection of nascent myofibrils to the Z-bands. Our work identifies the Drosophila adult abdominal muscles as a model to investigate in vivo the mechanisms behind myofibrillogenesis.
7. A dual role for the βPS integrin myospheroid in mediating Drosophila embryonic macrophage migration. Throughout embryonic development, macrophages not only act as the first line of defence against infection but also help to sculpt organs and tissues of the embryo by removing dead cells and secreting **extracellular matri**x components. Key to their function is the ability of embryonic macrophages to migrate and disperse throughout the embryo. Despite these important developmental functions, little is known about the molecular mechanisms underlying embryonic macrophage migration in vivo. Integrins are key regulators of many of the adult macrophage responses, but their role in embryonic macrophages remains poorly characterized. Here, we have used Drosophila macrophages (haemocytes) as a model system to address the role of integrins during embryonic macrophage dispersal in vivo. We show that the main βPS integrin, myospheroid, affects haemocyte migration in two ways; by shaping the three-dimensional environment in which haemocytes migrate and by regulating the migration of haemocytes themselves. Live imaging revealed a requirement for myospheroid within haemocytes to coordinate the microtubule and actin dynamics, and to enable haemocyte developmental dispersal, contact repulsion and inflammatory migration towards wounds.
8. Loss of focal adhesions in glia disrupts both glial and photoreceptor axon migration in the Drosophila visual system. Many aspects of glial development are regulated by extracellular signals, including those from the **extracellular matrix** (ECM). Signals from the ECM are received by cell surface receptors, including the integrin family. Previously, we have shown that Drosophila integrins form adhesion complexes with Integrin-linked kinase and talin in the peripheral nerve glia and have conserved roles in glial sheath formation. However, integrin function in other aspects of glial development is unclear. The Drosophila eye imaginal disc (ED) and optic stalk (OS) complex is an excellent model with which to study glial migration, differentiation and glia-neuron interactions. We studied the roles of the integrin complexes in these glial developmental processes during OS/eye development. The common beta subunit βPS and two alpha subunits, αPS2 and αPS3, are located in puncta at both glia-glia and glia-ECM interfaces. Depletion of βPS integrin and talin by RNAi impaired the migration and distribution of glia within the OS resulting in morphological defects. Reduction of integrin or talin in the glia also disrupted photoreceptor axon outgrowth leading to axon stalling in the OS and ED. The neuronal defects were correlated with a disruption of the carpet glia tube paired with invasion of glia into the core of the OS and the formation of a glial cap. Our results suggest that integrin-mediated extracellular signals are important for multiple aspects of glial development and non-autonomously affect axonal migration during Drosophila eye development.
9. Receptor guanylyl cyclase Gyc76C is required for invagination, collective migration and lumen shape in the Drosophila embryonic salivary gland. The Drosophila embryonic salivary gland is formed by the invagination and collective migration of cells. Here, we report on a novel developmental role for receptor-type guanylyl cyclase at 76C, Gyc76C, in morphogenesis of the salivary gland. We demonstrate that Gyc76C and downstream cGMP-dependent protein kinase 1 (DG1) function in the gland and surrounding mesoderm to control invagination, collective migration and lumen shape. Loss of gyc76C resulted in glands that failed to invaginate, complete posterior migration and had branched lumens. Salivary gland migration defects of gyc76C mutant embryos were rescued by expression of wild-type gyc76C specifically in the gland or surrounding mesoderm, whereas invagination defects were rescued primarily by expression in the gland. In migrating salivary glands of gyc76C mutant embryos, integrin subunits localized normally to gland-mesoderm contact sites but talin localization in the surrounding circular visceral mesoderm and fat body was altered. The **extracellular matrix** protein, laminin, also failed to accumulate around the migrating salivary gland of gyc76C mutant embryos, and gyc76C and laminin genetically interacted in gland migration. Our studies suggest that gyc76C controls salivary gland invagination, collective migration and lumen shape, in part by regulating the localization of talin and the laminin matrix.
10. Integrin-dependent anchoring of a stem-cell niche. Interactions between stem cells and their surrounding microenvironment, or niche, are critical for the establishment and maintenance of stem-cell properties. The adult Drosophila testis contains a morphologically discrete stem-cell niche, the 'hub'. The small cluster of non-dividing, somatic hub cells at the anterior tip of the fly testis is contacted by the germline stem cells (GSCs), which retain their stem-cell character through the direct association with the hub. Here we show that integrin-mediated adhesion is important for maintaining the correct position of embryonic hub cells during gonad morphogenesis. The misplaced hub in integrin-deficient embryos directs the orientation of cell divisions in the presumptive GSCs, a hallmark of the active germline stem-cell niche. A decrease in integrin-mediated adhesion in adult testes, which resulted in a loss of the hub and the stem-cell population, revealed the importance of hub-cell anchoring. Finally, we show that an **extracellular matrix** (ECM) is present around the gonad during late embryogenesis and that this ECM is defective in integrin-deficient gonads. On the basis of our data, we propose that integrins are required for the attachment of the hub cells to the ECM, which is essential for maintaining the stem-cell niche.
11. In vivo functional analysis reveals specific roles for the integrin-binding sites of talin. Adhesion receptors play diverse roles during animal development and require precise spatiotemporal regulation, which is achieved through the activity of their binding partners. Integrins, adhesion receptors that mediate cell attachment to the **extracellular matrix** (ECM), connect to the intracellular environment through the cytoplasmic adapter protein talin. Talin has two essential functions: orchestrating the assembly of the intracellular adhesion complex (IAC), which associates with integrin, and regulating the affinity of integrins for the ECM. Talin can bind to integrins through two different integrin-binding sites (IBS-1 and IBS-2, respectively). Here, we have investigated the roles of each in the context of Drosophila development. We find that although IBS-1 and IBS-2 are partially redundant, they each have specialized roles during development: IBS-1 reinforces integrin attachment to the ECM, whereas IBS-2 reinforces the link between integrins and the IAC. Disruption of each IBS has different developmental consequences, illustrating how the functional diversity of integrin-mediated adhesion is achieved.
12. Integrins are necessary for the development and maintenance of the glial layers in the Drosophila peripheral nerve. Peripheral nerve development involves multiple classes of glia that cooperate to form overlapping glial layers paired with the deposition of a surrounding **extracellular matrix** (ECM). The formation of this tubular structure protects the ensheathed axons from physical and pathogenic damage and from changes in the ionic environment. Integrins, a major family of ECM receptors, play a number of roles in the development of myelinating Schwann cells, one class of glia ensheathing the peripheral nerves of vertebrates. However, the identity and the role of the integrin complexes utilized by the other classes of peripheral nerve glia have not been determined in any animal. Here, we show that, in the peripheral nerves of Drosophila melanogaster, two integrin complexes (αPS2βPS and αPS3βPS) are expressed in the different glial layers and form adhesion complexes with integrin-linked kinase and Talin. Knockdown of the common beta subunit (βPS) using inducible RNAi in all glial cells results in lethality and glial defects. Analysis of integrin complex function in specific glial layers showed that loss of βPS in the outermost layer (the perineurial glia) results in a failure to wrap the nerve, a phenotype similar to that of Matrix metalloproteinase 2-mediated degradation of the ECM. Knockdown of βPS integrin in the innermost wrapping glia causes a loss of glial processes around axons. Together, our data suggest that integrins are employed in different glial layers to mediate the development and maintenance of the protective glial sheath in Drosophila peripheral nerves.
13. Integrins contribute to the establishment and maintenance of cell polarity in the follicular epithelium of the Drosophila ovary. The generation of epithelial cell polarity is a key process during development. Although the induction and orientation of cell polarity by cell-cell and cell-**extracellular matrix** (ECM) interactions is well established, the molecular mechanisms by which signals from the ECM control cell polarity in developing epithelial tissues remain poorly understood. Here, we have used the follicular epithelium of the Drosophila ovary to investigate the role that integrins, the main cell-ECM receptors, play in the establishment of apicobasal polarity. Mature follicle cells have an apical side facing the germ line and a basal side in contact with a basement membrane. Our results show that integrins - presumably via interactions with the basement membrane - play a reinforcing role in follicle cell polarization, as they are required to establish and/or maintain follicle cell membrane asymmetry only when contact with the germ line is prevented. We suggest that the primary cue for polarization of the follicular epithelium is contact with the germline cells. In addition, while interfering with apical and lateral polarization cues leads to apoptosis, we show here that inhibition of contact with the basement membrane mediated by integrins does not affect cell survival. Finally, we provide evidence to suggest that integrins are required to orientate epithelial polarity in vivo.
14. Focal adhesion kinase is not required for integrin function or viability in Drosophila. The mammalian focal adhesion kinase (FAK) family of non-receptor protein-tyrosine kinases has been implicated in controlling a multitude of cellular responses to the engagement of cell-surface integrins and G-protein-coupled receptors. The high level of sequence conservation between the mammalian proteins and the Drosophila homologue of FAK, Fak56, suggested that it would have similar functions. However, we show here that Drosophila Fak56 is not essential for integrin functions in adhesion, migration or signaling in vivo. Furthermore, animals lacking Fak56 are viable and fertile, demonstrating that Fak56 is not essential for other developmental or physiological functions. Despite this, overexpressed Fak56 is a potent inhibitor of integrins binding to the **extracellular matrix**, suggesting that Fak56 may play a subtle role in the negative regulation of integrin adhesion.
15. An interaction between integrin and the talin FERM domain mediates integrin activation but not linkage to the cytoskeleton. Transmembrane adhesion receptors, such as integrins, mediate cell adhesion by interacting with intracellular proteins that connect to the cytoskeleton. Talin, one such linker protein, is thought to have two roles: mediating inside-out activation of integrins, and connecting **extracellular matrix** (ECM)-bound integrins to the cytoskeleton. Talin's amino-terminal head, which consists of a FERM domain, binds an NPxY motif within the cytoplasmic tail of most integrin beta subunits. This is consistent with the role of FERM domains in recruiting other proteins to the plasma membrane. We tested the role of the talin-head-NPxY interaction in integrin function in Drosophila. We found that introduction of a mutation that perturbs this binding in vitro into the isolated talin head disrupts its recruitment by integrins in vivo. Surprisingly, when engineered into the full-length talin, this mutation did not disrupt talin recruitment by integrins nor its ability to connect integrins to the cytoskeleton. However, it reduced the ability of talin to strengthen integrin adhesion to the ECM, indicating that the function of the talin-head-NPxY interaction is solely to regulate integrin adhesion.
16. Mechanical force regulates integrin turnover in Drosophila in vivo. Regulated assembly and disassembly, or turnover, of integrin-mediated cell-**extracellular matrix** (ECM) adhesions is essential for dynamic cell movements and long-term tissue maintenance. For example, in Drosophila, misregulation of integrin turnover disrupts muscle-tendon attachment at myotendinous junctions (MTJs). We demonstrate that mechanical force, which modulates integrin activity, also regulates integrin and intracellular adhesion complex (IAC) turnover in vivo. Using conditional mutants to alter the tensile force on MTJs, we found that the proportion of IAC components undergoing turnover inversely correlated with the force applied on MTJs. This effect was disrupted by point mutations in β-integrin that interfere with ECM-induced conformational changes and activation of β-integrin or integrin-mediated cytoplasmic signalling. These mutants also disrupted integrin dynamics at MTJs during larval development. Together, these data suggest that specific β-integrin-mediated signals regulate adhesion turnover in response to tension during tissue formation. We propose that integrin-ECM adhesive stability is continuously controlled by force in vivo through integrin-dependent auto-regulatory feedback mechanisms so that tissues can quickly adapt to and withstand mechanical stresses.
17. Integrin signaling is required for maintenance and proliferation of intestinal stem cells in Drosophila. Tissue-specific stem cells are maintained by both local secreted signals and cell adhesion molecules that position the stem cells in the niche microenvironment. In the Drosophila midgut, multipotent intestinal stem cells (ISCs) are located basally along a thin layer of basement membrane that composed of **extracellular matrix** (ECM), which separates ISCs from the surrounding visceral musculature: the muscle cells constitute a regulatory niche for ISCs by producing multiple secreted signals that directly regulate ISC maintenance and proliferation. Here we show that integrin-mediated cell adhesion, which connects the ECM and intracellular cytoskeleton, is required for ISC anchorage to the basement membrane. Specifically, the α-integrin subunits including αPS1 encoded by mew and αPS3 encoded by scb, and the β-integrin subunit encoded by mys are richly expressed in ISCs and are required for the maintenance, rather than their survival or multiple lineage differentiation. Furthermore, ISC maintenance also requires the intercellular and intracellular integrin signaling components including Talin, Integrin-linked kinase (Ilk), and the ligand, Laminin A. Notably, integrin mutant ISCs are also less proliferative, and genetic interaction studies suggest that proper integrin signaling is a pre-requisite for ISC proliferation in response to various proliferative signals and for the initiation of intestinal hyperplasia after loss of adenomatous polyposis coli (Apc). Our studies suggest that integrin not only functions to anchor ISCs to the basement membrane, but also serves as an essential element for ISC proliferation during normal homeostasis and in response to oncogenic mutations.
18. Moleskin is essential for the formation of the myotendinous junction in Drosophila. It is the precise connectivity between skeletal muscles and their corresponding tendon cells to form a functional myotendinous junction (MTJ) that allows for the force generation required for muscle contraction and organismal movement. The Drosophila MTJ is composed of secreted **extracellular matrix** (ECM) proteins deposited between integrin-mediated hemi-adherens junctions on the surface of muscle and tendon cells. In this paper, we have identified a novel, cytoplasmic role for the canonical nuclear import protein Moleskin (Msk) in Drosophila embryonic somatic muscle attachment. Msk protein is enriched at muscle attachment sites in late embryogenesis and msk mutant embryos exhibit a failure in muscle-tendon cell attachment. Although the muscle-tendon attachment sites are reduced in size, components of the integrin complexes and ECM proteins are properly localized in msk mutant embryos. However, msk mutants fail to localize phosphorylated focal adhesion kinase (pFAK) to the sites of muscle-tendon cell junctions. In addition, the tendon cell specific proteins Stripe (Sr) and activated mitogen-activated protein kinase (MAPK) are reduced in msk mutant embryos. Our rescue experiments demonstrate that Msk is required in the muscle cell, but not in the tendon cells. Moreover, muscle attachment defects due to loss of Msk are rescued by an activated form of MAPK or the secreted epidermal growth factor receptor (Egfr) ligand Vein. Taken together, these findings provide strong evidence that Msk signals non-autonomously through the Vein-Egfr signaling pathway for late tendon cell late differentiation and/or maintenance.
19. The integrin adhesion complex changes its composition and function during morphogenesis of an epithelium. Cell adhesion to the **extracellular matrix** (ECM) is mediated by the integrin family of transmembrane receptors. Integrins link ECM ligands to the cytoskeleton, providing strong attachment to enable cell-shape change and tissue integrity. This connection is made possible by an intracellular complex of proteins, which links to actin filaments and controls signalling cascades that regulate cytoskeletal rearrangements. We have identified stress-fibre-associated focal adhesions that change their composition during tissue morphogenesis. Early expression of alphaPS1betaPS integrin decreases the levels of the actin-nucleating factors Enabled, Diaphanous and profilin, as well as downregulating the amount of F-actin incorporated into the stress fibres. As follicle cells mature in their developmental pathway and become squamous, the integrin in the focal adhesions changes from alphaPS1betaPS to alphaPS2betaPS. During the switch, stress fibres increase their length and change orientation, first changing by 90 degrees and then reorienting back. The normal rapid reorientation requires new expression of alphaPS2betaPS, which also permits recruitment of the adaptor protein tensin. Unexpectedly, it is the extracellular portion of the alphaPS2 subunit that provides the specificity for intracellular recruitment of tensin. Molecular variation of the integrin complex is thus a key component of developmentally programmed morphogenesis.
20. The localized assembly of **extracellular matrix** integrin ligands requires cell-cell contact. The assembly of an organism requires the interaction between different layers of cells, in many cases via an **extracellular matrix**. In the developing Drosophila larva, muscles attach in an integrin-dependent manner to the epidermis, via a specialized **extracellular matrix** called tendon matrix. Tiggrin, a tendon matrix integrin ligand, is primarily synthesized by cells distant to the muscle attachment sites, yet it accumulates specifically at these sites. Previous work has shown that the PS integrins are not required for tiggrin localization, suggesting that there is redundancy among tiggrin receptors. We have examined this by testing whether the PS2 integrin can recruit tiggrin to ectopic locations within the Drosophila embryo. We found that neither the wild type nor modified forms of the PS2 integrin, which have higher affinity for tiggrin, can recruit tiggrin to new cellular contexts. Next, we genetically manipulated the fate of the muscles and the epidermal muscle attachment cells, which demonstrated that muscles have the primary role in recruiting tiggrin to the tendon matrix and that cell-cell contact is necessary for this recruitment. Thus we propose that the inherent polarity of the muscle cells leads to a molecular specialization of their ends, and interactions between the ends produces an integrin-independent tiggrin receptor. Thus, interaction between cells generates an extracellular environment capable of nucleating **extracellular matrix** assembly.
21. Distinct developmental roles for direct and indirect talin-mediated linkage to actin. Transmembrane adhesion receptors, such as integrins, mediate cell adhesion by interacting with intracellular proteins that connect to the cytoskeleton. Talin, one such linker protein, is essential to connect **extracellular matrix**-bound integrins to the cytoskeleton. Talin can connect to the cytoskeleton either directly, through its actin-binding motifs, or indirectly, by recruiting other actin-binding proteins. Talin's carboxy-terminal end contains a well-characterized actin-binding domain (ABD). We tested the role of the C-terminal ABD of talin in integrin function in Drosophila. We found that introduction of mutations that reduced actin binding in vitro into the isolated C-terminal Talin-ABD impaired actin binding in vivo. Moreover, when engineered into full-length talin, these mutations disrupted a subset of integrin-mediated adhesion-dependent developmental events. Specifically, morphogenetic processes that involve dynamic, short-term integrin-mediated adhesion were particularly sensitive to impaired function of the C-terminal Talin-ABD. We propose that during development talin connects integrins to the cytoskeleton in distinct ways in different types of integrin-mediated adhesion: directly in transient adhesions and indirectly in stable long-lasting adhesions. Our results provide insight into how a similar array of molecular components can contribute to diverse adhesive processes throughout development.
22. Thrombospondin-mediated adhesion is essential for the formation of the myotendinous junction in Drosophila. Organogenesis of the somatic musculature in Drosophila is directed by the precise adhesion between migrating myotubes and their corresponding ectodermally derived tendon cells. Whereas the PS integrins mediate the adhesion between these two cell types, their **extracellular matrix** (ECM) ligands have been only partially characterized. We show that the ECM protein Thrombospondin (Tsp), produced by tendon cells, is essential for the formation of the integrin-mediated myotendinous junction. Tsp expression is induced by the tendon-specific transcription factor Stripe, and accumulates at the myotendinous junction following the association between the muscle and the tendon cell. In tsp mutant embryos, migrating somatic muscles fail to attach to tendon cells and often form hemiadherens junctions with their neighboring muscle cells, resulting in nonfunctional somatic musculature. Talin accumulation at the cytoplasmic faces of the muscles and tendons is greatly reduced, implicating Tsp as a potential integrin ligand. Consistently, purified Tsp C-terminal domain polypeptide mediates spreading of PS2 integrin-expressing S2 cells in a KGD- and PS2-integrin-dependent manner. We propose a model in which the myotendinous junction is formed by the specific association of Tsp with multiple muscle-specific PS2 integrin receptors and a subsequent consolidation of the junction by enhanced tendon-specific production of Tsp secreted into the junctional space.
23. Integrins are required for cardioblast polarisation in Drosophila. The formation of a tubular organ, such as the heart, requires the communication of positional and polarity signals between migratory cells. Key to this process is the establishment of a new luminal domain on the cell surface, generally from the apical domain of a migratory cell. This domain will also acquire basal properties, as it will produce a luminal **extracellular matrix**. Integrin receptors are the primary means of cell adhesion and adhesion signaling with the **extracellular matrix**. Here we characterise the requirement of Integrins in a genetic model of vasculogenesis, the formation of the heart in Drosophila.As with vertebrates, the Drosophila heart arises from lateral mesoderm that migrates medially to meet their contralateral partners, to then assemble a midline vessel. During migration, Integrins are among the first proteins restricted to the presumptive luminal domain of cardioblasts. Integrins are required for normal levels of leading edge membrane motility. Apical accumulation of Integrins is enhanced by Robo, and reciprocally, apicalisation of luminal factors like Slit and Robo requires Integrin function. Integrins may provide a template for the formation of a lumen by stabilising lumen factors like Robo. Subsequent to migration, Integrin is required for normal cardioblast alignment and lumen formation. This phenotype is most readily modified by other mutations that affect adhesion, such as Talin and **extracellular matrix** ligands.Our findings reveal an instructive role for Integrins in communicating polarising information to cells during migration, and during transition to an epithelial tube structure.
24. Induction of focal adhesions and motility in Drosophila S2 cells. Focal adhesions are dynamic structures that interact with the **extracellular matrix** on the cell exterior and actin filaments on the cell interior, enabling cells to adhere and crawl along surfaces. We describe a system for inducing the formation of focal adhesions in normally non-ECM-adherent, nonmotile Drosophila S2 cells. These focal adhesions contain the expected molecular markers such as talin, vinculin, and p130Cas, and they require talin for their formation. The S2 cells with induced focal adhesions also display a nonpolarized form of motility on vitronectin-coated substrates. Consistent with findings in mammalian cells, the degree of motility can be tuned by changing the stiffness of the substrate and was increased after the depletion of PAK3, a p21-activated kinase. A subset of nonmotile, nonpolarized cells also exhibited focal adhesions that rapidly assembled and disassembled around the cell perimeter. Such cooperative and dynamic fluctuations of focal adhesions were decreased by RNA interference (RNAi) depletion of myosin II and focal adhesion kinase, suggesting that this behavior requires force and focal adhesion maturation. These results demonstrate that S2 cells, a cell line that is well studied for cytoskeletal dynamics and readily amenable to protein manipulation by RNAi, can be used to study the assembly and dynamics of focal adhesions and mechanosensitive cell motility.
25. IPP Complex Reinforces Adhesion by Relaying Tension-Dependent Signals to Inhibit Integrin Turnover. Cytoskeleton-mediated forces regulate the assembly and function of integrin adhesions; however, the underlying mechanisms remain unclear. The tripartite IPP complex, comprising ILK, Parvin, and PINCH, mediates the integrin-actin link at Drosophila embryo muscle attachment sites (MASs). Here, we demonstrate a developmentally earlier function for the IPP complex: to reinforce integrin-**extracellular matrix** (ECM) adhesion in response to tension. In IPP-complex mutants, the integrin-ECM linkage at MASs breaks in response to intense muscle contractility. Mechanistically, the IPP complex is required to relay force-elicited signals that decelerate integrin turnover at the plasma membrane so that the integrin immobile fraction is adequate to withstand tension. Epistasis analysis shows that alleviation of muscle contractility, downregulation of endocytosis, and enhanced integrin binding to the ECM are sufficient to restore integrin-ECM adhesion and maintain integrin-adhesome organization in IPP-complex mutants. Our findings reveal a role for the IPP complex as an essential mechanosensitive regulatory switch of integrin turnover in vivo.
26. Slowdown promotes muscle integrity by modulating integrin-mediated adhesion at the myotendinous junction. The correct assembly of the myotendinous junction (MTJ) is crucial for proper muscle function. In Drosophila, this junction comprises hemi-adherens junctions that are formed upon arrival of muscles at their corresponding tendon cells. The MTJ mainly comprises muscle-specific alphaPS2betaPS integrin receptors and their tendon-derived **extracellular matrix** ligand Thrombospondin (Tsp). We report the identification and functional analysis of a novel tendon-derived secreted protein named Slowdown (Slow). Homozygous slow mutant larvae exhibit muscle or tendon rupture, sluggish larval movement, partial lethality, and the surviving adult flies are unable to fly. These defects result from improper assembly of the embryonic MTJ. In slow mutants, Tsp prematurely accumulates at muscle ends, the morphology of the muscle leading edge changes and the MTJ architecture is aberrant. Slow was found to form a protein complex with Tsp. This complex is biologically active and capable of altering the morphology and directionality of muscle ends. Our analysis implicates Slow as an essential component of the MTJ, crucial for ensuring muscle and tendon integrity during larval locomotion.
27. Tissue elongation requires oscillating contractions of a basal actomyosin network. Understanding how molecular dynamics leads to cellular behaviours that ultimately sculpt organs and tissues is a major challenge not only in basic developmental biology but also in tissue engineering and regenerative medicine. Here we use live imaging to show that the basal surfaces of Drosophila follicle cells undergo a series of directional, oscillating contractions driven by periodic myosin accumulation on a polarized actin network. Inhibition of the actomyosin contractions or their coupling to **extracellular matrix** (ECM) blocked elongation of the whole tissue, whereas enhancement of the contractions exaggerated it. Myosin accumulated in a periodic manner before each contraction and was regulated by the small GTPase Rho, its downstream kinase, ROCK, and cytosolic calcium. Disrupting the link between the actin cytoskeleton and the ECM decreased the amplitude and period of the contractions, whereas enhancing cell-ECM adhesion increased them. In contrast, disrupting cell-cell adhesions resulted in loss of the actin network. Our findings reveal a mechanism controlling organ shape and an experimental model for the study of the effects of oscillatory actomyosin activity within a coherent cell sheet.
28. Cell-cell adhesion via the ECM: integrin genetics in fly and worm. Integrins are essential for the development of the two genetically tractable invertebrate model organisms, the nematode worm Caenorhabditis elegans and the fruit fly Drosophila melanogaster. Just two integrins are present in C. elegans: one putative RGD binding integrin alphapat-2betapat-3, corresponding to Drosophila alphaPS2betaPS and vertebrate alpha5beta1, alphaVbeta1 and alpha8beta1, and one putative laminin binding integrin alphaina-1betapat-3, corresponding to Drosophila alphaPS1betaPS and vertebrate alpha3beta1, alpha6beta1 and alpha7beta1. In this review, the function of this minimal set of integrins during the development of these two invertebrates is compared. Despite the differences in bodyplan and developmental strategy, integrin adhesion to the **extracellular matrix** is required for similar processes: the formation of the link that translates muscle contraction into movement of the exoskeleton, cell migration, and morphogenetic interactions between epithelia. Other integrin functions, such as regulation of gene expression, have not yet been experimentally demonstrated in both organisms. Additional proteins have been characterised in each organism that are essential for integrin function, including **extracellular matrix** ligands and intracellular interacting proteins, but so far different proteins have been found in the two organisms. This in part represents the fact that the characterisation of the full set of interacting proteins is not complete in either system. However, in other cases different proteins appear to be used for similar functions in the two animals. The continued use of genetic approaches to identify proteins required for integrin function in these two model organisms should lead to the identification of the minimal set of conserved components that form integrin adhesive structures.
29. **Extracellular matrix** and its receptors in Drosophila neural development. **Extracellular matrix** (ECM) and matrix receptors are intimately involved in most biological processes. The ECM plays fundamental developmental and physiological roles in health and disease, including processes underlying the development, maintenance, and regeneration of the nervous system. To understand the principles of ECM-mediated functions in the nervous system, genetic model organisms like Drosophila provide simple, malleable, and powerful experimental platforms. This article provides an overview of ECM proteins and receptors in Drosophila. It then focuses on their roles during three progressive phases of neural development: (1) neural progenitor proliferation, (2) axonal growth and pathfinding, and (3) synapse formation and function. Each section highlights known ECM and ECM-receptor components and recent studies done in mutant conditions to reveal their in vivo functions, all illustrating the enormous opportunities provided when merging work on the nervous system with systematic research into ECM-related gene functions.
30. Integrins in development: moving on, responding to, and sticking to the **extracellular matrix**. Integrins are cell surface receptors of the **extracellular matrix** present in all animals. Genetic analysis in worms, flies, and vertebrates has revealed integrin involvement in key developmental processes, and we focus here on examples of integrin functions that are comparable across these model organisms. Integrins contribute to cell movement by providing traction to migrating cells, through assembly of extracellular matrices that can serve as tracks for migration, and by transmitting guidance signals that direct cells or cell processes to their targets. Integrins also participate in signaling events that govern tissue differentiation and organogenesis. Finally, adhesion by integrin-mediated junctions allows tissues to withstand mechanical load and is essential for tissue integrity.
31. dumpy interacts with a large number of genes in the developing wing of Drosophila melanogaster. The complex Drosophila dumpy gene encodes a gigantic protein located in the apical **extracellular matrix** of epithelial cells. It has been shown to interact with several proteins notably during embryonic tracheal development. Here we examine Dumpy's interactions in vivo with mutations in 20 genes previously recovered in a screen for recessive lethals that generate blisters when somatic clones are produced by mitotic crossing over during wing development. Primarily using double mutants, we looked for both dominant effects of the wing blister mutants and the effects of blister mutant clones on dumpy expression. Sixteen of the mutants either suppressed or enhanced dumpy mutant phenotypes indicating the large Dumpy protein is a very important component of the epithelial **extracellular matrix** in the wing. Dumpy also interacts strongly with held out wings, which is involved in RNA localization and possibly alternative splicing.
32. Talin autoinhibition is required for morphogenesis. The establishment of a multicellular body plan requires coordinating changes in cell adhesion and the cytoskeleton to ensure proper cell shape and position within a tissue. Cell adhesion to the **extracellular matrix** (ECM) via integrins plays diverse, essential roles during animal embryogenesis and therefore must be precisely regulated. Talin, a FERM-domain containing protein, forms a direct link between integrin adhesion receptors and the actin cytoskeleton and is an important regulator of integrin function. Similar to other FERM proteins, talin makes an intramolecular interaction that could autoinhibit its activity. However, the functional consequence of such an interaction has not been previously explored in vivo. Here, we demonstrate that targeted disruption of talin autoinhibition gives rise to morphogenetic defects during fly development and specifically that dorsal closure (DC), a process that resembles wound healing, is delayed. Impairment of autoinhibition leads to reduced talin turnover at and increased talin and integrin recruitment to sites of integrin-ECM attachment. Finally, we present evidence that talin autoinhibition is regulated by Rap1-dependent signaling. Based on our data, we propose that talin autoinhibition provides a switch for modulating adhesion turnover and adhesion stability that is essential for morphogenesis.
33. Crystal structure of the talin integrin binding domain 2. Integrins are transmembrane receptors that mediate cell adhesion to the **extracellular matrix** and play essential roles in tissue development and maintenance. The cytoplasmic segment of integrin associates with talin, a large intracellular protein that links integrin to the actin cytoskeleton. Binding of talin via an integrin binding segment (IBS1) results in large conformational changes in the extracellular portion of integrin, which modulates the affinity of integrins for their **extracellular matrix** ligands. However, integrin binding also requires a second segment of talin (IBS2). Despite detailed descriptions of the integrin-IBS1 binding, the molecular determinants that drive the integrin-IBS2 association are poorly understood. Here, we describe the crystal structure of the talin IBS2 domain, which forms a five-helix bundle. The large structural homology with a vinculin binding domain hints at an ancient gene duplication and suggests that helix 4 may bind to vinculin if the bundle is unfolded. Mapping previous mutations on the surface highlights a likely binding interface for integrin.

#### GeneRIFs

1. talin integrin binding sites reinforce integrin attachment to the **extracellular matrix** and reinforce the link between integrins and the intracellular adhesion complex
2. talin is essential for integrin function and acts by stably linking clusters of **extracellular matrix**-linked integrins to the cytoskeleton.

### [Mm]etastasis

#### PubMed

1. Mutations in the polycomb group gene polyhomeotic lead to epithelial instability in both the ovary and wing imaginal disc in Drosophila. Most human cancers originate from epithelial tissues and cell polarity and adhesion defects can lead to **metastasis**. The Polycomb-Group of chromatin factors were first characterized in Drosophila as repressors of homeotic genes during development, while studies in mammals indicate a conserved role in body plan organization, as well as an implication in other processes such as stem cell maintenance, cell proliferation, and tumorigenesis. We have analyzed the function of the Drosophila Polycomb-Group gene polyhomeotic in epithelial cells of two different organs, the ovary and the wing imaginal disc.Clonal analysis of loss and gain of function of polyhomeotic resulted in segregation between mutant and wild-type cells in both the follicular and wing imaginal disc epithelia, without excessive cell proliferation. Both basal and apical expulsion of mutant cells was observed, the former characterized by specific reorganization of cell adhesion and polarity proteins, the latter by complete cytoplasmic diffusion of these proteins. Among several candidate target genes tested, only the homeotic gene Abdominal-B was a target of PH in both ovarian and wing disc cells. Although overexpression of Abdominal-B was sufficient to cause cell segregation in the wing disc, epistatic analysis indicated that the presence of Abdominal-B is not necessary for expulsion of polyhomeotic mutant epithelial cells suggesting that additional polyhomeotic targets are implicated in this phenomenon.Our results indicate that polyhomeotic mutations have a direct effect on epithelial integrity that can be uncoupled from overproliferation. We show that cells in an epithelium expressing different levels of polyhomeotic sort out indicating differential adhesive properties between the cell populations. Interestingly, we found distinct modalities between apical and basal expulsion of ph mutant cells and further studies of this phenomenon should allow parallels to be made with the modified adhesive and polarity properties of different types of epithelial tumors.
2. Drosophila as a model to study the role of blood cells in inflammation, innate immunity and cancer. Drosophila has a primitive yet effective blood system with three types of haemocytes which function throughout different developmental stages and environmental stimuli. Haemocytes play essential roles in tissue modeling during embryogenesis and morphogenesis, and also in innate immunity. The open circulatory system of Drosophila makes haemocytes ideal signal mediators to cells and tissues in response to events such as infection and wounding. The application of recently developed and sophisticated genetic tools to the relatively simple genome of Drosophila has made the fly a popular system for modeling human tumorigensis and **metastasis**. Drosophila is now used for screening and investigation of genes implicated in human leukemia and also in modeling development of solid tumors. This second line of research offers promising opportunities to determine the seemingly conflicting roles of blood cells in tumor progression and invasion. This review provides an overview of the signaling pathways conserved in Drosophila during haematopoiesis, haemostasis, innate immunity, wound healing and inflammation. We also review the most recent progress in the use of Drosophila as a cancer research model with an emphasis on the roles haemocytes can play in various cancer models and in the links between inflammation and cancer.
3. JNK signaling controls border cell cluster integrity and collective cell migration. Collective cell movement is a mechanism for invasion identified in many developmental events. Examples include the movement of lateral-line neurons in Zebrafish, cells in the inner blastocyst, and **metastasis** of epithelial tumors [1]. One key model to study collective migration is the movement of border cell clusters in Drosophila. Drosophila egg chambers contain 15 nurse cells and a single oocyte surrounded by somatic follicle cells. At their anterior end, polar cells recruit several neighboring follicle cells to form the border cell cluster [2]. By stage 9, and over 6 hr, border cells migrate as a cohort between nurse cells toward the oocyte. The specification and directionality of border cell movement are regulated by hormonal and signaling mechanisms [3]. However, how border cells are held together while they migrate is not known. Here, we show that a negative-feedback loop controlling JNK activity regulates border cell cluster integrity. JNK signaling modulates contacts between border cells and between border cells and substratum to sustain collective migration by regulating several effectors including the polarity factor Bazooka and the cytoskeletal adaptor D-Paxillin. We anticipate a role for the JNK pathway in controlling collective cell movements in other morphogenetic and clinical models.

---

## Arpc1

- NCBI Gene
- GeneRIF
- Pubmed

| Search Term | Rank | Fields |
| --- | --- | --- |
| [Ff]ilopodia | 1 | PubMed(2) |
| [Ff]ilopodium | 2 | Process(1) |
| [Mm]igration | 6 | PubMed(3) |
| [Ee]xtracellular [Mm]atrix | 8 | PubMed(1) |
| [Ii]nvasive | 10 | PubMed(1) |
| [Mm]etastasis | 12 | PubMed(1) |
| [Cc]ell [Ii]nvasion | 13 | PubMed(2) |

  
hide/show details for Arpc1

### [Ff]ilopodia

#### PubMed

1. Dissecting regulatory networks of **filopodia** formation in a Drosophila growth cone model. F-actin networks are important structural determinants of cell shape and morphogenesis. They are regulated through a number of actin-binding proteins. The function of many of these proteins is well understood, but very little is known about how they cooperate and integrate their activities in cellular contexts. Here, we have focussed on the cellular roles of actin regulators in controlling **filopodia**l dynamics. **Filopodia** are needle-shaped, actin-driven cell protrusions with characteristic features that are well conserved amongst vertebrates and invertebrates. However, existing models of **filopodia** formation are still incomplete and controversial, pieced together from a wide range of different organisms and cell types. Therefore, we used embryonic Drosophila primary neurons as one consistent cellular model to study **filopodia** regulation. Our data for loss-of-function of capping proteins, enabled, different Arp2/3 complex components, the formin DAAM and profilin reveal characteristic changes in **filopodia** number and length, providing a promising starting point to study their functional relationships in the cellular context. Furthermore, the results are consistent with effects reported for the respective vertebrate homologues, demonstrating the conserved nature of our Drosophila model system. Using combinatorial genetics, we demonstrate that different classes of nucleators cooperate in **filopodia** formation. In the absence of Arp2/3 or DAAM **filopodia** numbers are reduced, in their combined absence **filopodia** are eliminated, and in genetic assays they display strong functional interactions with regard to **filopodia** formation. The two nucleators also genetically interact with enabled, but not with profilin. In contrast, enabled shows strong genetic interaction with profilin, although loss of profilin alone does not affect **filopodia** numbers. Our genetic data support a model in which Arp2/3 and DAAM cooperate in a common mechanism of **filopodia** formation that essentially depends on enabled, and is regulated through profilin activity at different steps.
2. Drosophila growth cones: a genetically tractable platform for the analysis of axonal growth dynamics. The formation of neuronal networks, during development and regeneration, requires outgrowth of axons along reproducible paths toward their appropriate postsynaptic target cells. Axonal extension occurs at growth cones (GCs) at the tips of axons. GC advance and navigation requires the activity of their cytoskeletal networks, comprising filamentous actin (F-actin) in lamellipodia and **filopodia** as well as dynamic microtubules (MTs) emanating from bundles of the axonal core. The molecular mechanisms governing these two cytoskeletal networks, their cross-talk, and their response to extracellular signaling cues are only partially understood, hindering our conceptual understanding of how regulated changes in GC behavior are controlled. Here, we introduce Drosophila GCs as a suitable model to address these mechanisms. Morphological and cytoskeletal readouts of Drosophila GCs are similar to those of other models, including mammals, as demonstrated here for MT and F-actin dynamics, axonal growth rates, **filopodia**l structure and motility, organizational principles of MT networks, and subcellular marker localization. Therefore, we expect fundamental insights gained in Drosophila to be translatable into vertebrate biology. The advantage of the Drosophila model over others is its enormous amenability to combinatorial genetics as a powerful strategy to address the complexity of regulatory networks governing axonal growth. Thus, using pharmacological and genetic manipulations, we demonstrate a role of the actin cytoskeleton in a specific form of MT organization (loop formation), known to regulate GC pausing behavior. We demonstrate these events to be mediated by the actin-MT linking factor Short stop, thus identifying an essential molecular player in this context.

### [Ff]ilopodium

#### Process

1. positive regulation of **filopodium** assembly

### [Mm]igration

#### PubMed

1. A Jnk-Rho-Actin remodeling positive feedback network directs Src-driven invasion. Current models of tumor cell invasion propose that oncogenic signaling converges upon key orchestrators of cytoskeletal dynamics, including c-Jun N-terminal kinase (Jnk) and RhoGTPase family members; these signals dynamically direct Actin remodeling proteins (ARPs) to catalyze the cytoskeletal changes required for **migration**. Src is a key driver of tumor aggression, metastasis and patient mortality. To clarify how Src regulates Actin dynamics to promote invasive **migration**, we performed a genetic modifier screen in a Drosophila model of invasion. Nine genes linked to Actin dynamics were identified that mediate invasion in situ. We found that ARPs were required for many oncogenic effects of Src including Mmp1 expression and initiation of apoptosis. Surprisingly, they were also regulators of Jnk pathway activity: both Src and the small GTPase Rho1 activated Jnk in a manner dependent on ARPs during invasion. Our results suggest that ARPs are not simply downstream executors of signal transduction pathways. Rather, they participate in a positive feedback network involving canonical oncogenic signaling pathways that promote tumor invasion.
2. Wash functions downstream of Rho1 GTPase in a subset of Drosophila immune cell developmental **migration**s. Drosophila immune cells, the hemocytes, undergo four stereotypical developmental **migration**s to populate the embryo, where they provide immune reconnoitering, as well as a number of non-immune-related functions necessary for proper embryogenesis. Here, we describe a role for Rho1 in one of these developmental **migration**s in which posteriorly located hemocytes migrate toward the head. This **migration** requires the interaction of Rho1 with its downstream effector Wash, a Wiskott-Aldrich syndrome family protein. Both Wash knockdown and a Rho1 transgene harboring a mutation that prevents Wash binding exhibit the same developmental migratory defect as Rho1 knockdown. Wash activates the Arp2/3 complex, whose activity is needed for this **migration**, whereas members of the WASH regulatory complex (SWIP, Strumpellin, and CCDC53) are not. Our results suggest a WASH complex-independent signaling pathway to regulate the cytoskeleton during a subset of hemocyte developmental **migration**s.
3. Cortical actin dynamics facilitate early-stage centrosome separation. Proper centrosome separation is a prerequisite for positioning the bipolar spindle. Although studies demonstrate that microtubules (MTs) and their associated motors drive centrosome separation [1], the role of actin in centrosome separation remains less clear. Studies in tissue culture cells indicate that actin- and myosin-based cortical flow is primarily responsible for driving late centrosome separation [2], whereas other studies suggest that actin plays a more passive role by serving as an attachment site for astral MTs to pull centrosomes apart [3-6]. Here we demonstrate that prior to nuclear envelope breakdown (NEB) in Drosophila embryos, proper centrosome separation does not require myosin II but requires dynamic actin rearrangements at the growing edge of the interphase cap. Both Arp2/3- and Formin-mediated actin remodeling are required for separating the centrosome pairs before NEB. The Apc2-Armadillo complex appears to link cap expansion to centrosome separation. In contrast, the mechanisms driving centrosome separation after NEB are independent of the actin cytoskeleton and compensate for earlier separation defects. Our studies show that the dynamics of actin polymerization drive centrosome separation, and this has important implications for centrosome positioning during processes such as cell **migration** [7, 8], cell polarity maintenance [9, 10], and asymmetric cell division [11, 12].

### [Ee]xtracellular [Mm]atrix

#### PubMed

1. Shavenbaby couples patterning to epidermal cell shape control. It is well established that developmental programs act during embryogenesis to determine animal morphogenesis. How these developmental cues produce specific cell shape during morphogenesis, however, has remained elusive. We addressed this question by studying the morphological differentiation of the Drosophila epidermis, governed by a well-known circuit of regulators leading to a stereotyped pattern of smooth cells and cells forming actin-rich extensions (trichomes). It was shown that the transcription factor Shavenbaby plays a pivotal role in the formation of trichomes and underlies all examined cases of the evolutionary diversification of their pattern. To gain insight into the mechanisms of morphological differentiation, we sought to identify shavenbaby's downstream targets. We show here that Shavenbaby controls epidermal cell shape, through the transcriptional activation of different classes of cellular effectors, directly contributing to the organization of actin filaments, regulation of the **extracellular matrix**, and modification of the cuticle. Individual inactivation of shavenbaby's targets produces distinct trichome defects and only their simultaneous inactivation prevent trichome formation. Our data show that shavenbaby governs an evolutionarily conserved developmental module consisting of a set of genes collectively responsible for trichome formation, shedding new light on molecular mechanisms acting during morphogenesis and the way they can influence evolution of animal forms.

### [Ii]nvasive

#### PubMed

1. A Jnk-Rho-Actin remodeling positive feedback network directs Src-driven invasion. Current models of tumor cell invasion propose that oncogenic signaling converges upon key orchestrators of cytoskeletal dynamics, including c-Jun N-terminal kinase (Jnk) and RhoGTPase family members; these signals dynamically direct Actin remodeling proteins (ARPs) to catalyze the cytoskeletal changes required for migration. Src is a key driver of tumor aggression, metastasis and patient mortality. To clarify how Src regulates Actin dynamics to promote **invasive** migration, we performed a genetic modifier screen in a Drosophila model of invasion. Nine genes linked to Actin dynamics were identified that mediate invasion in situ. We found that ARPs were required for many oncogenic effects of Src including Mmp1 expression and initiation of apoptosis. Surprisingly, they were also regulators of Jnk pathway activity: both Src and the small GTPase Rho1 activated Jnk in a manner dependent on ARPs during invasion. Our results suggest that ARPs are not simply downstream executors of signal transduction pathways. Rather, they participate in a positive feedback network involving canonical oncogenic signaling pathways that promote tumor invasion.

### [Mm]etastasis

#### PubMed

1. A Jnk-Rho-Actin remodeling positive feedback network directs Src-driven invasion. Current models of tumor cell invasion propose that oncogenic signaling converges upon key orchestrators of cytoskeletal dynamics, including c-Jun N-terminal kinase (Jnk) and RhoGTPase family members; these signals dynamically direct Actin remodeling proteins (ARPs) to catalyze the cytoskeletal changes required for migration. Src is a key driver of tumor aggression, **metastasis** and patient mortality. To clarify how Src regulates Actin dynamics to promote invasive migration, we performed a genetic modifier screen in a Drosophila model of invasion. Nine genes linked to Actin dynamics were identified that mediate invasion in situ. We found that ARPs were required for many oncogenic effects of Src including Mmp1 expression and initiation of apoptosis. Surprisingly, they were also regulators of Jnk pathway activity: both Src and the small GTPase Rho1 activated Jnk in a manner dependent on ARPs during invasion. Our results suggest that ARPs are not simply downstream executors of signal transduction pathways. Rather, they participate in a positive feedback network involving canonical oncogenic signaling pathways that promote tumor invasion.

### [Cc]ell [Ii]nvasion

#### PubMed

1. A Jnk-Rho-Actin remodeling positive feedback network directs Src-driven invasion. Current models of tumor **cell invasion** propose that oncogenic signaling converges upon key orchestrators of cytoskeletal dynamics, including c-Jun N-terminal kinase (Jnk) and RhoGTPase family members; these signals dynamically direct Actin remodeling proteins (ARPs) to catalyze the cytoskeletal changes required for migration. Src is a key driver of tumor aggression, metastasis and patient mortality. To clarify how Src regulates Actin dynamics to promote invasive migration, we performed a genetic modifier screen in a Drosophila model of invasion. Nine genes linked to Actin dynamics were identified that mediate invasion in situ. We found that ARPs were required for many oncogenic effects of Src including Mmp1 expression and initiation of apoptosis. Surprisingly, they were also regulators of Jnk pathway activity: both Src and the small GTPase Rho1 activated Jnk in a manner dependent on ARPs during invasion. Our results suggest that ARPs are not simply downstream executors of signal transduction pathways. Rather, they participate in a positive feedback network involving canonical oncogenic signaling pathways that promote tumor invasion.
2. Rickettsia parkeri invasion of diverse host cells involves an Arp2/3 complex, WAVE complex and Rho-family GTPase-dependent pathway. Rickettsiae are obligate intracellular pathogens that are transmitted to humans by arthropod vectors and cause diseases such as spotted fever and typhus. Although rickettsiae require the host cell actin cytoskeleton for invasion, the cytoskeletal proteins that mediate this process have not been completely described. To identify the host factors important during **cell invasion** by Rickettsia parkeri, a member of the spotted fever group (SFG), we performed an RNAi screen targeting 105 proteins in Drosophila melanogaster S2R+ cells. The screen identified 21 core proteins important for invasion, including the GTPases Rac1 and Rac2, the WAVE nucleation-promoting factor complex and the Arp2/3 complex. In mammalian cells, including endothelial cells, the natural targets of R. parkeri, the Arp2/3 complex was also crucial for invasion, while requirements for WAVE2 as well as Rho GTPases depended on the particular cell type. We propose that R. parkeri invades S2R+ arthropod cells through a primary pathway leading to actin nucleation, whereas invasion of mammalian endothelial cells occurs via redundant pathways that converge on the host Arp2/3 complex. Our results reveal a key role for the WAVE and Arp2/3 complexes, as well as a higher degree of variation than previously appreciated in actin nucleation pathways activated during Rickettsia invasion.

---

## CG13185

- NCBI Gene
- GeneRIF
- Pubmed

| Search Term | Rank | Fields |
| --- | --- | --- |
| [Mm]igration | 6 | PubMed(2) |

  
hide/show details for CG13185

### [Mm]igration

#### PubMed

1. FGF ligands in Drosophila have distinct activities required to support cell **migration** and differentiation. Fibroblast growth factor (FGF) signaling controls a vast array of biological processes including cell differentiation and **migration**, wound healing and malignancy. In vertebrates, FGF signaling is complex, with over 100 predicted FGF ligand-receptor combinations. Drosophila melanogaster presents a simpler model system in which to study FGF signaling, with only three ligands and two FGF receptors (FGFRs) identified. Here we analyze the specificity of FGFR [Heartless (Htl) and Breathless (Btl)] activation by each of the FGF ligands [Pyramus (Pyr), Thisbe (Ths) and Branchless (Bnl)] in Drosophila. We confirm that both Pyr and Ths can activate Htl, and that only Bnl can activate Btl. To examine the role of each ligand in supporting activation of the Htl FGFR, we utilize genetic approaches that focus on the earliest stages of embryonic development. When pyr and ths are equivalently expressed using the Gal4 system, these ligands support qualitatively different FGFR signaling responses. Both Pyr and Ths function in a non-autonomous fashion to support mesoderm spreading during gastrulation, but Pyr exhibits a longer functional range. pyr and ths single mutants exhibit defects in mesoderm spreading during gastrulation, yet only pyr mutants exhibit severe defects in dorsal mesoderm specification. We demonstrate that the Drosophila FGFs have different activities and that cell **migration** and differentiation have different ligand requirements. Furthermore, these FGF ligands are not regulated solely by differential expression, but the sequences of these linked genes have evolved to serve different functions. We contend that inherent properties of FGF ligands make them suitable to support specific FGF-dependent processes, and that FGF ligands are not always interchangeable.
2. pyramus and thisbe: FGF genes that pattern the mesoderm of Drosophila embryos. The Heartless (Htl) FGF receptor is required for the differentiation of a variety of mesodermal tissues in the Drosophila embryo, yet its ligand is not known. Here we identify two new FGF genes, thisbe (ths) and pyramus (pyr), which probably encode the elusive ligands for this receptor. The two genes exhibit dynamic patterns of expression in epithelial tissues adjacent to Htl-expressing mesoderm derivatives, including the neurogenic ectoderm, stomadeum, and hindgut. Embryos that lack ths+ and pyr+ exhibit defects related to those seen in htl mutants, including delayed mesodermal **migration** during gastrulation and a loss of cardiac tissues and hindgut musculature. The misexpression of Ths in wild-type and mutant embryos suggests that FGF signaling is required for both cell **migration** and the transcriptional induction of cardiac gene expression. The characterization of htl and ths regulatory DNAs indicates that high levels of the maternal Dorsal gradient directly activate htl expression, whereas low levels activate ths. It is therefore possible to describe FGF signaling and other aspects of gastrulation as a direct manifestation of discrete threshold readouts of the Dorsal gradient.

---

## nuf

- NCBI Gene
- GeneRIF
- Pubmed

| Search Term | Rank | Fields |
| --- | --- | --- |
| [Mm]igration | 6 | PubMed(4) |

  
hide/show details for nuf

### [Mm]igration

#### PubMed

1. A core transcriptional network for early mesoderm development in Drosophila melanogaster. Embryogenesis is controlled by large gene-regulatory networks, which generate spatially and temporally refined patterns of gene expression. Here, we report the characteristics of the regulatory network orchestrating early mesodermal development in the fruitfly Drosophila, where the transcription factor Twist is both necessary and sufficient to drive development. Through the integration of chromatin immunoprecipitation followed by microarray analysis (ChIP-on-chip) experiments during discrete time periods with computational approaches, we identified >2000 Twist-bound cis-regulatory modules (CRMs) and almost 500 direct target genes. Unexpectedly, Twist regulates an almost complete cassette of genes required for cell proliferation in addition to genes essential for morophogenesis and cell **migration**. Twist targets almost 25% of all annotated Drosophila transcription factors, which may represent the entire set of regulators necessary for the early development of this system. By combining in vivo binding data from Twist, Mef2, Tinman, and Dorsal we have constructed an initial transcriptional network of early mesoderm development. The network topology reveals extensive combinatorial binding, feed-forward regulation, and complex logical outputs as prevalent features. In addition to binary activation and repression, we suggest that Twist binds to almost all mesodermal CRMs to provide the competence to integrate inputs from more specialized transcription factors.
2. A mutation in dVps28 reveals a link between a subunit of the endosomal sorting complex required for transport-I complex and the actin cytoskeleton in Drosophila. Proteins that constitute the endosomal sorting complex required for transport (ESCRT) are necessary for the sorting of proteins into multivesicular bodies (MVBs) and the budding of several enveloped viruses, including HIV-1. The first of these complexes, ESCRT-I, consists of three proteins: Vps28p, Vps37p, and Vps23p or Tsg101 in mammals. Here, we characterize a mutation in the Drosophila homolog of vps28. The dVps28 gene is essential: homozygous mutants die at the transition from the first to second instar. Removal of maternally contributed dVps28 causes early embryonic lethality. In such embryos lacking dVps28, several processes that require the actin cytoskeleton are perturbed, including axial **migration** of nuclei, formation of transient furrows during cortical divisions in syncytial embryos, and the subsequent cellularization. Defects in actin cytoskeleton organization also become apparent during sperm individualization in dVps28 mutant testis. Because dVps28 mutant cells contained MVBs, these defects are unlikely to be a secondary consequence of disrupted MVB formation and suggest an interaction between the actin cytoskeleton and endosomal membranes in Drosophila embryos earlier than previously appreciated.
3. Mutations affecting the cytoskeletal organization of syncytial Drosophila embryos. Cytoplasmic organization, nuclear **migration**, and nuclear division in the early syncytial Drosophila embryo are all modulated by the cytoskeleton. In an attempt to identify genes involved in cytoskeletal functions, we have examined a collection of maternal-effect lethal mutations induced by single P-element transposition for those that cause defects in nuclear movement, organization, or morphology during the syncytial embryonic divisions. We describe three mutations, grapes, scrambled, and nuclear-fallout, which define three previously uncharacterized genes. Females homozygous for these mutations produce embryos that exhibit extensive mitotic division errors only after the nuclei migrate to the surface. Analysis of the microfilament and microtubule organization in embryos derived from these newly identified mutations reveal disruptions in the cortical cytoskeleton. Each of the three mutations disrupts the actin-based pseudocleavage furrows and the cellularization furrows in a distinct fashion. In addition to identifying new genes involved in cytoskeletal organization, these mutations provide insights into cytoskeletal function during early Drosophila embryogenesis.
4. The Seckel syndrome and centrosomal protein Ninein localizes asymmetrically to stem cell centrosomes but is not required for normal development, behavior, or DNA damage response in Drosophila. Ninein (Nin) is a centrosomal protein whose gene is mutated in Seckel syndrome (SCKL, MIM 210600), an inherited recessive disease that results in primordial dwarfism, cognitive deficiencies, and increased sensitivity to genotoxic stress. Nin regulates neural stem cell self-renewal, interkinetic nuclear **migration**, and microtubule assembly in mammals. Nin is evolutionarily conserved, yet its role in cell division and development has not been investigated in a model organism. Here we characterize the single Nin orthologue in Drosophila Drosophila Nin localizes to the periphery of the centrosome but not at centriolar structures as in mammals. However, Nin shares the property of its mammalian orthologue of promoting microtubule assembly. In neural and germline stem cells, Nin localizes asymmetrically to the younger (daughter) centrosome, yet it is not required for the asymmetric division of stem cells. In wing epithelia and muscle, Nin localizes to noncentrosomal microtubule-organizing centers. Surprisingly, loss of nin expression from a nin mutant does not significantly affect embryonic and brain development, fertility, or locomotor performance of mutant flies or their survival upon exposure to DNA-damaging agents. Although it is not essential, our data suggest that Nin plays a supportive role in centrosomal and extracentrosomal microtubule organization and asymmetric stem cell division.

---

## shrb

- NCBI Gene
- GeneRIF
- Pubmed

| Search Term | Rank | Fields |
| --- | --- | --- |
| [Ff]ilopodia | 1 | PubMed(2) |
| ECM | 7 | PubMed(1) |
| [Ee]xtracellular [Mm]atrix | 8 | PubMed(2) |

  
hide/show details for shrb

### [Ff]ilopodia

#### PubMed

1. Muscle niche-driven Insulin-Notch-Myc cascade reactivates dormant Adult Muscle Precursors in Drosophila. How stem cells specified during development keep their non-differentiated quiescent state, and how they are reactivated, remain poorly understood. Here, we applied a Drosophila model to follow in vivo behavior of adult muscle precursors (AMPs), the transient fruit fly muscle stem cells. We report that emerging AMPs send out thin **filopodia** that make contact with neighboring muscles. AMPs keep their **filopodia**-based association with muscles throughout their dormant state but also when they start to proliferate, suggesting that muscles could play a role in AMP reactivation. Indeed, our genetic analyses indicate that muscles send inductive dIlp6 signals that switch the Insulin pathway ON in closely associated AMPs. This leads to the activation of Notch, which regulates AMP proliferation via dMyc. Altogether, we report that Drosophila AMPs display homing behavior to muscle niche and that the niche-driven Insulin-Notch-dMyc cascade plays a key role in setting the activated state of AMPs.
2. Gene regulatory networks controlling hematopoietic progenitor niche cell production and differentiation in the Drosophila lymph gland. Hematopoiesis occurs in two phases in Drosophila, with the first completed during embryogenesis and the second accomplished during larval development. The lymph gland serves as the venue for the final hematopoietic program, with this larval tissue well-studied as to its cellular organization and genetic regulation. While the medullary zone contains stem-like hematopoietic progenitors, the posterior signaling center (PSC) functions as a niche microenvironment essential for controlling the decision between progenitor maintenance versus cellular differentiation. In this report, we utilize a PSC-specific GAL4 driver and UAS-gene RNAi strains, to selectively knockdown individual gene functions in PSC cells. We assessed the effect of abrogating the function of 820 genes as to their requirement for niche cell production and differentiation. 100 genes were shown to be essential for normal niche development, with various loci placed into sub-groups based on the functions of their encoded protein products and known genetic interactions. For members of three of these groups, we characterized loss- and gain-of-function phenotypes. Gene function knockdown of members of the BAP chromatin-remodeling complex resulted in niche cells that do not express the hedgehog (hh) gene and fail to differentiate **filopodia** believed important for Hh signaling from the niche to progenitors. Abrogating gene function of various members of the insulin-like growth factor and TOR signaling pathways resulted in anomalous PSC cell production, leading to a defective niche organization. Further analysis of the Pten, TSC1, and TSC2 tumor suppressor genes demonstrated their loss-of-function condition resulted in severely altered blood cell homeostasis, including the abundant production of lamellocytes, specialized hemocytes involved in innate immune responses. Together, this cell-specific RNAi knockdown survey and mutant phenotype analyses identified multiple genes and their regulatory networks required for the normal organization and function of the hematopoietic progenitor niche within the lymph gland.

### ECM

#### PubMed

1. Balance between apical membrane growth and luminal matrix resistance determines epithelial tubule shape. The morphological stability of biological tubes is crucial for the efficient circulation of fluids and gases. Failure of this stability causes irregularly shaped tubes found in multiple pathological conditions. Here, we report that Drosophila mutants of the ESCRT III component Shrub/Vps32 exhibit a strikingly elongated sinusoidal tube phenotype. This is caused by excessive apical membrane synthesis accompanied by the ectopic accumulation and overactivation of Crumbs in swollen endosomes. Furthermore, we demonstrate that the apical extracellular matrix (a**ECM**) of the tracheal tube is a viscoelastic material coupled with the apical membrane. We present a simple mechanical model in which a**ECM** elasticity, apical membrane growth, and their interaction are three vital parameters determining the stability of biological tubes. Our findings demonstrate a mechanical role for the extracellular matrix and suggest that the interaction of the apical membrane and an elastic a**ECM** determines the final morphology of biological tubes independent of cell shape.

### [Ee]xtracellular [Mm]atrix

#### PubMed

1. Balance between apical membrane growth and luminal matrix resistance determines epithelial tubule shape. The morphological stability of biological tubes is crucial for the efficient circulation of fluids and gases. Failure of this stability causes irregularly shaped tubes found in multiple pathological conditions. Here, we report that Drosophila mutants of the ESCRT III component Shrub/Vps32 exhibit a strikingly elongated sinusoidal tube phenotype. This is caused by excessive apical membrane synthesis accompanied by the ectopic accumulation and overactivation of Crumbs in swollen endosomes. Furthermore, we demonstrate that the apical **extracellular matrix** (aECM) of the tracheal tube is a viscoelastic material coupled with the apical membrane. We present a simple mechanical model in which aECM elasticity, apical membrane growth, and their interaction are three vital parameters determining the stability of biological tubes. Our findings demonstrate a mechanical role for the **extracellular matrix** and suggest that the interaction of the apical membrane and an elastic aECM determines the final morphology of biological tubes independent of cell shape.
2. A fat body-derived apical **extracellular matrix** enzyme is transported to the tracheal lumen and is required for tube morphogenesis in Drosophila. The apical **extracellular matrix** plays a central role in epithelial tube morphogenesis. In the Drosophila tracheal system, Serpentine (Serp), a secreted chitin deacetylase expressed by the tracheal cells plays a key role in regulating tube length. Here, we show that the fly fat body, which is functionally equivalent to the mammalian liver, also contributes to tracheal morphogenesis. Serp was expressed by the fat body, and the secreted Serp was taken up by the tracheal cells and translocated to the lumen to functionally support normal tracheal development. This process was defective in rab9 and shrub/vps32 mutants and in wild-type embryos treated with a secretory pathway inhibitor, leading to an abundant accumulation of Serp in the fat body. We demonstrated that fat body-derived Serp reached the tracheal lumen after establishment of epithelial barrier function and was retained in the lumen in a chitin synthase-dependent manner. Our results thus reveal that the fat body, a mesodermal organ, actively contributes to tracheal development.

---

## Hrs

- NCBI Gene
- GeneRIF
- Pubmed

| Search Term | Rank | Fields |
| --- | --- | --- |
| [Ff]ilopodia | 1 | PubMed(1) |
| [Mm]igration | 6 | Process(2); PubMed(6) |
| [Ii]nvasive | 10 | PubMed(1) |
| [Mm]etastasis | 12 | PubMed(1) |

  
hide/show details for Hrs

### [Ff]ilopodia

#### PubMed

1. Cytonemes are required for the establishment of a normal Hedgehog morphogen gradient in Drosophila epithelia. Hedgehog (Hh) signalling is important in development, stem cell biology and disease. In a variety of tissues, Hh acts as a morphogen to regulate growth and cell fate specification. Several hypotheses have been proposed to explain morphogen movement, one of which is transport along **filopodia**-like protrusions called cytonemes. Here, we analyse the mechanism underlying Hh movement in the wing disc and the abdominal epidermis of Drosophila melanogaster. We show that, in both epithelia, cells generate cytonemes in regions of Hh signalling. These protrusions are actin-based and span several cell diameters. Various Hh signalling components localize to cytonemes, as well as to punctate structures that move along cytonemes and are probably exovesicles. Using in vivo imaging, we show that cytonemes are dynamic structures and that Hh gradient establishment correlates with cytoneme formation in space and time. Indeed, mutant conditions that affect cytoneme formation reduce both cytoneme length and Hh gradient length. Our results suggest that cytoneme-mediated Hh transport is the mechanistic basis for Hh gradient formation.

### [Mm]igration

#### Process

1. border follicle cell **migration**
2. regulation of epithelial cell **migration**, open tracheal system

#### PubMed

1. The Hrs/Stam complex acts as a positive and negative regulator of RTK signaling during Drosophila development. Endocytosis is a key regulatory step of diverse signalling pathways, including receptor tyrosine kinase (RTK) signalling. Hrs and Stam constitute the ESCRT-0 complex that controls the initial selection of ubiquitinated proteins, which will subsequently be degraded in lysosomes. It has been well established ex vivo and during Drosophila embryogenesis that Hrs promotes EGFR down regulation. We have recently isolated the first mutations of stam in flies and shown that Stam is required for air sac morphogenesis, a larval respiratory structure whose formation critically depends on finely tuned levels of FGFR activity. This suggest that Stam, putatively within the ESCRT-0 complex, modulates FGF signalling, a possibility that has not been examined in Drosophila yet.Here, we assessed the role of the Hrs/Stam complex in the regulation of signalling activity during Drosophila development. We show that stam and hrs are required for efficient FGFR signalling in the tracheal system, both during cell **migration** in the air sac primordium and during the formation of fine cytoplasmic extensions in terminal cells. We find that stam and hrs mutant cells display altered FGFR/Btl localisation, likely contributing to impaired signalling levels. Electron microscopy analyses indicate that endosome maturation is impaired at distinct steps by hrs and stam mutations. These somewhat unexpected results prompted us to further explore the function of stam and hrs in EGFR signalling. We show that while stam and hrs together downregulate EGFR signalling in the embryo, they are required for full activation of EGFR signalling during wing development.Our study shows that the ESCRT-0 complex differentially regulates RTK signalling, either positively or negatively depending on tissues and developmental stages, further highlighting the importance of endocytosis in modulating signalling pathways during development.
2. Regulators of endocytosis maintain localized receptor tyrosine kinase signaling in guided **migration**. Guidance receptors detect extracellular cues and instruct migrating cells how to orient in space. Border cells perform a directional invasive **migration** during Drosophila oogenesis and use two receptor tyrosine kinases (RTKs), EGFR and PVR (PDGF/VEGF Receptor), to read guidance cues. We find that spatial localization of RTK signaling within these migrating cells is actively controlled. Border cells lacking Cbl, an RTK-associated E3 ubiquitin ligase, have delocalized guidance signaling, resulting in severe **migration** defects. Absence of Sprint, a receptor-recruited, Ras-activated Rab5 guanine exchange factor, gives related defects. In contrast, increasing the level of RTK signaling by receptor overexpression or removing Hrs and thereby decreasing RTK degradation does not perturb **migration**. Cbl and Sprint both regulate early steps of RTK endocytosis. Thus, a physiological role of RTK endocytosis is to ensure localized intracellular response to guidance cues by stimulating spatial restriction of signaling.
3. Spinster controls Dpp signaling during glial **migration** in the Drosophila eye. The development of multicellular organisms requires the well balanced and coordinated **migration** of many cell types. This is of particular importance within the developing nervous system, where glial cells often move long distances to reach their targets. The majority of glial cells in the peripheral nervous system of the Drosophila embryo is derived from the CNS and migrates along motor axons toward their targets. In the developing Drosophila eye, CNS-derived glial cells move outward toward the nascent photoreceptor cells, but the molecular mechanisms coupling the **migration** of glial cells with the growth of the eye imaginal disc are mostly unknown. Here, we used an enhancer trap approach to identify the gene spinster, which encodes a multipass transmembrane protein involved in endosome-lysosome trafficking, as being expressed in many glial cells. spinster mutants are characterized by glial over**migration**. Genetic experiments demonstrate that Spinster modulates the activity of several signaling cascades. Within the migrating perineurial glial cells, Spinster is required to downregulate Dpp (Decapentaplegic) signaling activity, which ceases migratory abilities. In addition, Spinster affects the growth of the carpet cell, which indirectly modulates glial **migration**.
4. Two distinct modes of guidance signalling during collective **migration** of border cells. Although directed **migration** is a feature of both individual cells and cell groups, guided **migration** has been studied most extensively for single cells in simple environments. Collective guidance of cell groups remains poorly understood, despite its relevance for development and metastasis. Neural crest cells and neuronal precursors migrate as loosely organized streams of individual cells, whereas cells of the fish lateral line, Drosophila tracheal tubes and border-cell clusters migrate as more coherent groups. Here we use Drosophila border cells to examine how collective guidance is performed. We report that border cells migrate in two phases using distinct mechanisms. Genetic analysis combined with live imaging shows that polarized cell behaviour is critical for the initial phase of **migration**, whereas dynamic collective behaviour dominates later. PDGF- and VEGF-related receptor and epidermal growth factor receptor act in both phases, but use different effector pathways in each. The myoblast city (Mbc, also known as DOCK180) and engulfment and cell motility (ELMO, also known as Ced-12) pathway is required for the early phase, in which guidance depends on subcellular localization of signalling within a leading cell. During the later phase, mitogen-activated protein kinase and phospholipase Cgamma are used redundantly, and we find that the cluster makes use of the difference in signal levels between cells to guide **migration**. Thus, information processing at the multicellular level is used to guide collective behaviour of a cell group.
5. Exosomes as Hedgehog carriers in cytoneme-mediated transport and secretion. The Hedgehog signalling pathway is crucial for development, adult stem cell maintenance, cell **migration** and axon guidance in a wide range of organisms. During development, the Hh morphogen directs tissue patterning according to a concentration gradient. Lipid modifications on Hh are needed to achieve graded distribution, leading to debate about how Hh is transported to target cells despite being membrane-tethered. Cytonemes in the region of Hh signalling have been shown to be essential for gradient formation, but the carrier of the morphogen is yet to be defined. Here we show that Hh and its co-receptor Ihog are in exovesicles transported via cytonemes. These exovesicles present protein markers and other features of exosomes. Moreover, the cell machinery for exosome formation is necessary for normal Hh secretion and graded signalling. We propose Hh transport via exosomes along cytonemes as a significant mechanism for the restricted distribution of a lipid-modified morphogen.
6. Control of Gliotactin localization and levels by tyrosine phosphorylation and endocytosis is necessary for survival of polarized epithelia. The tricellular junction (TCJ) forms at the convergence of bicellular junctions from three adjacent cells in polarized epithelia and is necessary for maintaining the transepithelial barrier. In the fruitfly Drosophila, the TCJ is generated at the meeting point of bicellular septate junctions. Gliotactin was the first identified component of the TCJ and is necessary for TCJ and septate junction development. Gliotactin is a member of the neuroligin family and associates with the PDZ protein discs large. Beyond this interaction, little is known about the mechanisms underlying Gliotactin localization and function at the TCJ. In this study, we show that Gliotactin is phosphorylated at conserved tyrosine residues, a process necessary for endocytosis and targeting to late endosomes and lysosomes for degradation. Regulation of Gliotactin levels through phosphorylation and endocytosis is necessary as overexpression results in displacement of Gliotactin away from the TCJ throughout the septate junction domain. Excessive Gliotactin in polarized epithelia leads to delamination, paired with subsequent **migration**, and apoptosis. The apoptosis and the resulting compensatory proliferation resulting from high levels of Gliotactin are mediated by the Drosophila JNK pathway. Therefore, Gliotactin levels within the cell membrane are regulated to ensure correct protein localization and cell survival.

### [Ii]nvasive

#### PubMed

1. Regulators of endocytosis maintain localized receptor tyrosine kinase signaling in guided migration. Guidance receptors detect extracellular cues and instruct migrating cells how to orient in space. Border cells perform a directional **invasive** migration during Drosophila oogenesis and use two receptor tyrosine kinases (RTKs), EGFR and PVR (PDGF/VEGF Receptor), to read guidance cues. We find that spatial localization of RTK signaling within these migrating cells is actively controlled. Border cells lacking Cbl, an RTK-associated E3 ubiquitin ligase, have delocalized guidance signaling, resulting in severe migration defects. Absence of Sprint, a receptor-recruited, Ras-activated Rab5 guanine exchange factor, gives related defects. In contrast, increasing the level of RTK signaling by receptor overexpression or removing Hrs and thereby decreasing RTK degradation does not perturb migration. Cbl and Sprint both regulate early steps of RTK endocytosis. Thus, a physiological role of RTK endocytosis is to ensure localized intracellular response to guidance cues by stimulating spatial restriction of signaling.

### [Mm]etastasis

#### PubMed

1. Two distinct modes of guidance signalling during collective migration of border cells. Although directed migration is a feature of both individual cells and cell groups, guided migration has been studied most extensively for single cells in simple environments. Collective guidance of cell groups remains poorly understood, despite its relevance for development and **metastasis**. Neural crest cells and neuronal precursors migrate as loosely organized streams of individual cells, whereas cells of the fish lateral line, Drosophila tracheal tubes and border-cell clusters migrate as more coherent groups. Here we use Drosophila border cells to examine how collective guidance is performed. We report that border cells migrate in two phases using distinct mechanisms. Genetic analysis combined with live imaging shows that polarized cell behaviour is critical for the initial phase of migration, whereas dynamic collective behaviour dominates later. PDGF- and VEGF-related receptor and epidermal growth factor receptor act in both phases, but use different effector pathways in each. The myoblast city (Mbc, also known as DOCK180) and engulfment and cell motility (ELMO, also known as Ced-12) pathway is required for the early phase, in which guidance depends on subcellular localization of signalling within a leading cell. During the later phase, mitogen-activated protein kinase and phospholipase Cgamma are used redundantly, and we find that the cluster makes use of the difference in signal levels between cells to guide migration. Thus, information processing at the multicellular level is used to guide collective behaviour of a cell group.

---

## hdc

- NCBI Gene
- GeneRIF
- Pubmed

| Search Term | Rank | Fields |
| --- | --- | --- |
| [Mm]igration | 6 | PubMed(3) |

  
hide/show details for hdc

### [Mm]igration

#### PubMed

1. Cell fate choices in Drosophila tracheal morphogenesis. The Drosophila tracheal system is a branched tubular structure that supplies air to target tissues. The elaborate tracheal morphology is shaped by two linked inductive processes, one involving the choice of cell fates, and the other a guided cell **migration**. We will describe the molecular basis for these processes, and the allocation of cell fate decisions to four temporal hierarchies. First, tracheal placodes are specified within the embryonic ectoderm. Subsequently, branch fates are allocated within the tracheal placodes, prior to **migration**. Localized presentation of the FGF ligand, Branchless, to tracheal cells that express the FGF receptor, Breathless, guides **migration**. Once cell **migration** is initiated, distinct cell fates are determined within each migrating branch. Finally, inhibitory feedback mechanisms ensure the correct assignment of these fates. Tracheal cell fate choices are determined by signaling cascades triggered by signals emanating from the tracheal cells, as well as by ligands produced by adjacent tissues.
2. Branching morphogenesis of the Drosophila tracheal system. Many organs including the mammalian lung and vascular system consist of branched tubular networks that transport essential gases or fluids, but the genetic programs that control the development of these complex three-dimensional structures are not well understood. The Drosophila melanogaster tracheal (respiratory) system is a network of interconnected epithelial tubes that transports oxygen and other gases in the body and provides a paradigm of branching morphogenesis. It develops by sequential sprouting of primary, secondary, and terminal branches from an epithelial sac of approximately 80 cells in each body segment of the embryo. Mapping of the cell movements and shape changes during the sprouting process has revealed that distinct mechanisms of epithelial **migration** and tube formation are used at each stage of branching. Genetic dissection of the process has identified a general program in which a fibroblast growth factor (FGF) and fibroblast growth factor receptor (FGFR) are used repeatedly to control branch budding and outgrowth. At each stage of branching, the mechanisms controlling FGF expression and the downstream signal transduction pathway change, altering the pattern and structure of the branches that form. During terminal branching, FGF expression is regulated by hypoxia, ensuring that tracheal structure matches cellular oxygen need. A branch diversification program operates in parallel to the general budding program: Regional signals locally modify the general program, conferring specific structural features and other properties on individual branches, such as their substrate outgrowth preferences, differences in tube size and shape, and the ability to fuse to other branches to interconnect the network.
3. Dysfusion transcriptional control of Drosophila tracheal **migration**, adhesion, and fusion. The Drosophila dysfusion basic-helix-loop-helix-PAS transcription factor gene is expressed in specialized fusion cells that reside at the tips of migrating tracheal branches. dysfusion mutants were isolated, and genetic analysis of live embryos revealed that mutant tracheal branches migrate to close proximity but fail to recognize and adhere to each other. Misexpression of dysfusion throughout the trachea further indicated that dysfusion has the ability to both inhibit cell **migration** and promote ectopic tracheal fusion. Nineteen genes whose expression either increases or decreases in fusion cells during development were analyzed in dysfusion mutant embryos. dysfusion upregulates the levels of four genes, including the shotgun cell adhesion protein gene and the zona pellucida family transmembrane protein gene, CG13196. Misexpression experiments with CG13196 result in ectopic tracheal fusion events, suggesting that it also encodes a cell adhesion protein. Another target gene of dysfusion is members only, which inhibits protein nuclear export and influences tracheal fusion. dysfusion also indirectly downregulates protein levels of Trachealess, an important regulator of tracheal development. These results indicate that fusion cells undergo dynamic changes in gene expression as they switch from migratory to fusion modes and that dysfusion regulates a discrete, but important, set of these genes.

---

## comm

- NCBI Gene
- GeneRIF
- Pubmed

| Search Term | Rank | Fields |
| --- | --- | --- |
| [Ff]ilopodia | 1 | PubMed(1) |
| [Mm]igration | 6 | PubMed(9) |
| [Ee]xtracellular [Mm]atrix | 8 | PubMed(1) |

  
hide/show details for comm

### [Ff]ilopodia

#### PubMed

1. The actin-binding protein Canoe/AF-6 forms a complex with Robo and is required for Slit-Robo signaling during axon pathfinding at the CNS midline. Axon guidance is a key process during nervous system development and regeneration. One of the best established paradigms to study the mechanisms underlying this process is the axon decision of whether or not to cross the midline in the Drosophila CNS. An essential regulator of that decision is the well conserved Slit-Robo signaling pathway. Slit guidance cues act through Robo receptors to repel axons from the midline. Despite good progress in our knowledge about these proteins, the intracellular mechanisms associated with Robo function remain poorly defined. In this work, we found that the scaffolding protein Canoe (Cno), the Drosophila orthologue of AF-6/Afadin, is essential for Slit-Robo signaling. Cno is expressed along longitudinal axonal pioneer tracts, and longitudinal Robo/Fasciclin2-positive axons aberrantly cross the midline in cno mutant embryos. cno mutant primary neurons show a significant reduction of Robo localized in growth cone **filopodia** and Cno forms a complex with Robo in vivo. Moreover, the commissureless (comm) phenotype (i.e., lack of commissures due to constitutive surface presentation of Robo in all neurons) is suppressed in comm, cno double-mutant embryos. Specific genetic interactions between cno, slit, robo, and genes encoding other components of the Robo pathway, such as Neurexin-IV, Syndecan, and Rac GTPases, further confirm that Cno functionally interacts with the Slit-Robo pathway. Our data argue that Cno is a novel regulator of the Slit-Robo signaling pathway, crucial for regulating the subcellular localization of Robo and for transducing its signaling to the actin cytoskeleton during axon guidance at the midline.

### [Mm]igration

#### PubMed

1. Roundabout 2 regulates **migration** of sensory neurons by signaling in trans. Roundabout (Robo) receptors and their ligand Slit are important regulators of axon guidance and cell **migration**. The development of Drosophila embryonic sense organs provides a neuronal **migration** paradigm where the in vivo roles of Slit and Robo can be assayed using genetics.Here we show that Slit-Robo signaling controls **migration** of Drosophila larval sensory neurons that are part of the Chordotonal (Cho) stretch receptor organs. We used live imaging to show that abdominal Cho organs normally migrate ventrally during development, whereas thoracic Cho organs do not. Robo2 overexpression in cis (in the sensory neurons) or in trans (on neighboring visceral mesoderm) transforms abdominal organs to a thoracic morphology and position by blocking **migration**, while loss of Slit-Robo signaling produces a reverse transformation in which thoracic organs migrate ectopically. Rescue and tissue-specific knockout experiments indicate that trans signaling by Robo2 contributes to the normal positioning of the thoracic Cho organs. The differential positioning of Cho organs between the thorax and abdomen is known to be regulated by Hox genes, and we show that the essential Hox cofactor Homothorax, represses Robo2 expression in the abdominal visceral mesoderm.Our results suggest that segment-specific neuronal **migration** patterns are directed through a novel signaling complex (the "Slit sandwich") in which Robo2 on the thoracic visceral mesoderm binds to Slit and presents it to Robo receptors on Cho neurons. The differential positioning of Cho organs between thorax and abdomen may be determined by Hox gene-mediated repression of robo2.
2. Neurons and glia: team players in axon guidance. In the developing nervous system, growth cones follow specific trajectories to reach their target area and ultimately connect with their correct postsynaptic partners. This review focuses on studies in both Drosophila and vertebrates to highlight that mutual interactions between neurons and glia are essential in forming specific neuronal connections. Glia signal to neurons to direct pathfinding and targeting of axons, as well as to stabilize and refine axonal branches within the target area. Equally, neurons provide crucial information to glia, supporting their **migration** and correct positioning.
3. Transcriptional control in embryonic Drosophila midline guidance assessed through a whole genome approach. During the development of the Drosophila central nervous system the process of midline crossing is orchestrated by a number of guidance receptors and ligands. Many key axon guidance molecules have been identified in both invertebrates and vertebrates, but the transcriptional regulation of growth cone guidance remains largely unknown. It is established that translational regulation plays a role in midline crossing, and there are indications that transcriptional regulation is also involved. To investigate this issue, we conducted a genome-wide study of transcription in Drosophila embryos using wild type and a number of well-characterized Drosophila guidance mutants and transgenics. We also analyzed a previously published microarray time course of Drosophila embryonic development with an axon guidance focus.Using hopach, a novel clustering method which is well suited to microarray data analysis, we identified groups of genes with similar expression patterns across guidance mutants and transgenics. We then systematically characterized the resulting clusters with respect to their relevance to axon guidance using two complementary controlled vocabularies: the Gene Ontology (GO) and anatomical annotations of the Atlas of Pattern of Gene Expression (APoGE) in situ hybridization database. The analysis indicates that regulation of gene expression does play a role in the process of axon guidance in Drosophila. We also find a strong link between axon guidance and hemocyte **migration**, a result that agrees with mounting evidence that axon guidance molecules are co-opted in vertebrate vascularization. Cell cyclin activity in the context of axon guidance is also suggested from our array data. RNA and protein expression patterns of cell cyclins in axon guidance mutants and transgenics support this possible link.This study provides important insights into the regulation of axon guidance in vivo.
4. From growth cone to synapse: the life history of the RP3 motor neuron. In Drosophila, the ability to analyze the development of individually identified neurons with a variety of imaging and biophysical techniques can be complemented by sophisticated genetics and molecular biology. This powerful combination is allowing the development and function of single neurons and their synaptic connections to be unraveled at an unparalleled level of resolution. In this article, we focus on a single, identified motoneuron--RP3--arguably the best understood neuron in the fruitfly. Many events in the life history of RP3 are well characterized, including cell **migration**, axon outgrowth and pathfinding within the central nervous system, pathfinding in the periphery to its appropriate muscle target domain, the specific recognition of its muscle targets, the events of synapse formation and maturation, and its mature function in the locomotion of the fly larva. Genetic analysis has revealed mutations in a number of different genes which affect specific aspects of RP3 development from axon outgrowth to synapse formation.
5. NETRIN and SLIT guide salivary gland **migration**. Directed **migration** is pivotal for the proper placement and function of nearly all organs. The majority of known guidance molecules involved in directed **migration** have been identified from studies of migrating axons during nervous system development. Here, we show that at least two of these axon guidance molecules, NETRIN and SLIT, act through their canonical receptors, to guide Drosophila embryonic salivary glands. NETRIN serves as a chemo-attractant while SLIT functions antagonistically to NETRIN as a chemo-repellent during salivary gland **migration**. CNS midline expression of both NETRIN and SLIT directs the glands to move unswervingly parallel to the CNS. NETRIN expression is also required in the visceral mesoderm, along which the glands move during their **migration**. We propose that analogous to axon guidance, a balance between chemo-attractants and chemo-repellents is required for the proper migratory path of the developing salivary glands.
6. Drosophila syndecan regulates tracheal cell **migration** by stabilizing Robo levels. Here we identify a new role for Syndecan (Sdc), the only transmembrane heparan sulphate proteoglycan in Drosophila, in tracheal development. Sdc is required cell autonomously for efficient directed **migration** and fusion of dorsal branch cells, but not for dorsal branch formation per se. The cytoplasmic domain of Sdc is dispensable, indicating that Sdc does not transduce a signal by itself. Although the branch-specific phenotype of sdc mutants resembles those seen in the absence of Slit/Robo2 signalling, genetic interaction experiments indicate that Sdc also helps to suppress Slit/Robo2 signalling. We conclude that Sdc cell autonomously regulates Slit/Robo2 signalling in tracheal cells to guarantee ordered directional **migration** and branch fusion.
7. Slit is the midline repellent for the robo receptor in Drosophila. Previous studies suggested that Roundabout (Robo) is a repulsive guidance receptor on growth cones that binds to an unknown midline ligand. Here we present genetic evidence that Slit is the midline Robo ligand; a companion paper presents biochemical evidence that Slit binds Robo. Slit is a large extracellular matrix protein expressed by midline glia. In slit mutants, growth cones enter the midline but never leave it; they abnormally continue to express high levels of Robo while at the midline. slit and robo display dosage-sensitive genetic interactions, indicating that they function in the same pathway. slit is also required for **migration** of muscle precursors away from the midline. Slit appears to function as a short-range repellent controlling axon crossing of the midline and as a long-range chemorepellent controlling mesoderm **migration** away from the midline.
8. Interdependence of macrophage **migration** and ventral nerve cord development in Drosophila embryos. During embryonic development, Drosophila macrophages (haemocytes) undergo a series of stereotypical **migration**s to disperse throughout the embryo. One major migratory route is along the ventral nerve cord (VNC), where haemocytes are required for the correct development of this tissue. We show, for the first time, that a reciprocal relationship exists between haemocytes and the VNC and that defects in nerve cord development prevent haemocyte **migration** along this structure. Using live imaging, we demonstrate that the axonal guidance cue Slit and its receptor Robo are both required for haemocyte **migration**, but signalling is not autonomously required in haemocytes. We show that the failure of haemocyte **migration** along the VNC in slit mutants is not due to a lack of chemotactic signals within this structure, but rather to a failure in its detachment from the overlying epithelium, creating a physical barrier to haemocyte **migration**. This block of haemocyte **migration** in turn disrupts the formation of the dorsoventral channels within the VNC, further highlighting the importance of haemocyte **migration** for correct neural development. This study illustrates the important role played by the three-dimensional environment in directing cell **migration** in vivo and reveals an intriguing interplay between the developing nervous system and the blood cells within the fly, demonstrating that their development is both closely coupled and interdependent.
9. Slit proteins: key regulators of axon guidance, axonal branching, and cell **migration**. In the past year, Slit proteins have been identified as important regulators of axon guidance and cell **migration** in Drosophila and vertebrates. Remarkably, they were simultaneously identified as negative regulators, repelling various axonal and cell **migration**s in both invertebrates and vertebrates, and as positive regulators, stimulating branching and extension of at least one class of axons in vertebrates.

### [Ee]xtracellular [Mm]atrix

#### PubMed

1. Slit is the midline repellent for the robo receptor in Drosophila. Previous studies suggested that Roundabout (Robo) is a repulsive guidance receptor on growth cones that binds to an unknown midline ligand. Here we present genetic evidence that Slit is the midline Robo ligand; a companion paper presents biochemical evidence that Slit binds Robo. Slit is a large **extracellular matrix** protein expressed by midline glia. In slit mutants, growth cones enter the midline but never leave it; they abnormally continue to express high levels of Robo while at the midline. slit and robo display dosage-sensitive genetic interactions, indicating that they function in the same pathway. slit is also required for migration of muscle precursors away from the midline. Slit appears to function as a short-range repellent controlling axon crossing of the midline and as a long-range chemorepellent controlling mesoderm migration away from the midline.

---

## CG32668

- NCBI Gene
- GeneRIF
- Pubmed

| Search Term | Rank | Fields |
| --- | --- | --- |
| [Mm]igration | 6 | PubMed(1) |
| ECM | 7 | PubMed(1) |
| [Ee]xtracellular [Mm]atrix | 8 | PubMed(1) |

  
hide/show details for CG32668

### [Mm]igration

#### PubMed

1. Genes conserved in bilaterians but jointly lost with Myc during nematode evolution are enriched in cell proliferation and cell **migration** functions. Animals use a stereotypical set of developmental genes to build body architectures of varying sizes and organizational complexity. Some genes are critical to developmental patterning, while other genes are important to physiological control of growth. However, growth regulator genes may not be as important in small-bodied "micro-metazoans" such as nematodes. Nematodes use a simplified developmental strategy of lineage-based cell fate specifications to produce an adult bilaterian body composed of a few hundreds of cells. Nematodes also lost the MYC proto-oncogenic regulator of cell proliferation. To identify additional regulators of cell proliferation that were lost with MYC, we computationally screened and determined 839 high-confidence genes that are conserved in bilaterians/lost in nematodes (CIBLIN genes). We find that 30 % of all CIBLIN genes encode transcriptional regulators of cell proliferation, epithelial-to-mesenchyme transitions, and other processes. Over 50 % of CIBLIN genes are unnamed genes in Drosophila, suggesting that there are many understudied genes. Interestingly, CIBLIN genes include many Myc synthetic lethal (MycSL) hits from recent screens. CIBLIN genes include key regulators of heparan sulfate proteoglycan (HSPG) sulfation patterns, and lysyl oxidases involved in cross-linking and modification of the extracellular matrix (ECM). These genes and others suggest the CIBLIN repertoire services critical functions in ECM remodeling and cell **migration** in large-bodied bilaterians. Correspondingly, CIBLIN genes are co-expressed with Myc in cancer transcriptomes, and include a preponderance of known determinants of cancer progression and tumor aggression. We propose that CIBLIN gene research can improve our understanding of regulatory control of cellular growth in metazoans.

### ECM

#### PubMed

1. Genes conserved in bilaterians but jointly lost with Myc during nematode evolution are enriched in cell proliferation and cell migration functions. Animals use a stereotypical set of developmental genes to build body architectures of varying sizes and organizational complexity. Some genes are critical to developmental patterning, while other genes are important to physiological control of growth. However, growth regulator genes may not be as important in small-bodied "micro-metazoans" such as nematodes. Nematodes use a simplified developmental strategy of lineage-based cell fate specifications to produce an adult bilaterian body composed of a few hundreds of cells. Nematodes also lost the MYC proto-oncogenic regulator of cell proliferation. To identify additional regulators of cell proliferation that were lost with MYC, we computationally screened and determined 839 high-confidence genes that are conserved in bilaterians/lost in nematodes (CIBLIN genes). We find that 30 % of all CIBLIN genes encode transcriptional regulators of cell proliferation, epithelial-to-mesenchyme transitions, and other processes. Over 50 % of CIBLIN genes are unnamed genes in Drosophila, suggesting that there are many understudied genes. Interestingly, CIBLIN genes include many Myc synthetic lethal (MycSL) hits from recent screens. CIBLIN genes include key regulators of heparan sulfate proteoglycan (HSPG) sulfation patterns, and lysyl oxidases involved in cross-linking and modification of the extracellular matrix (**ECM**). These genes and others suggest the CIBLIN repertoire services critical functions in **ECM** remodeling and cell migration in large-bodied bilaterians. Correspondingly, CIBLIN genes are co-expressed with Myc in cancer transcriptomes, and include a preponderance of known determinants of cancer progression and tumor aggression. We propose that CIBLIN gene research can improve our understanding of regulatory control of cellular growth in metazoans.

### [Ee]xtracellular [Mm]atrix

#### PubMed

1. Genes conserved in bilaterians but jointly lost with Myc during nematode evolution are enriched in cell proliferation and cell migration functions. Animals use a stereotypical set of developmental genes to build body architectures of varying sizes and organizational complexity. Some genes are critical to developmental patterning, while other genes are important to physiological control of growth. However, growth regulator genes may not be as important in small-bodied "micro-metazoans" such as nematodes. Nematodes use a simplified developmental strategy of lineage-based cell fate specifications to produce an adult bilaterian body composed of a few hundreds of cells. Nematodes also lost the MYC proto-oncogenic regulator of cell proliferation. To identify additional regulators of cell proliferation that were lost with MYC, we computationally screened and determined 839 high-confidence genes that are conserved in bilaterians/lost in nematodes (CIBLIN genes). We find that 30 % of all CIBLIN genes encode transcriptional regulators of cell proliferation, epithelial-to-mesenchyme transitions, and other processes. Over 50 % of CIBLIN genes are unnamed genes in Drosophila, suggesting that there are many understudied genes. Interestingly, CIBLIN genes include many Myc synthetic lethal (MycSL) hits from recent screens. CIBLIN genes include key regulators of heparan sulfate proteoglycan (HSPG) sulfation patterns, and lysyl oxidases involved in cross-linking and modification of the **extracellular matrix** (ECM). These genes and others suggest the CIBLIN repertoire services critical functions in ECM remodeling and cell migration in large-bodied bilaterians. Correspondingly, CIBLIN genes are co-expressed with Myc in cancer transcriptomes, and include a preponderance of known determinants of cancer progression and tumor aggression. We propose that CIBLIN gene research can improve our understanding of regulatory control of cellular growth in metazoans.

---

## Axs

- NCBI Gene
- GeneRIF
- Pubmed

| Search Term | Rank | Fields |
| --- | --- | --- |
| [Mm]igration | 6 | PubMed(1) |
| ECM | 7 | PubMed(1) |
| [Ee]xtracellular [Mm]atrix | 8 | PubMed(1) |

  
hide/show details for Axs

### [Mm]igration

#### PubMed

1. Genes conserved in bilaterians but jointly lost with Myc during nematode evolution are enriched in cell proliferation and cell **migration** functions. Animals use a stereotypical set of developmental genes to build body architectures of varying sizes and organizational complexity. Some genes are critical to developmental patterning, while other genes are important to physiological control of growth. However, growth regulator genes may not be as important in small-bodied "micro-metazoans" such as nematodes. Nematodes use a simplified developmental strategy of lineage-based cell fate specifications to produce an adult bilaterian body composed of a few hundreds of cells. Nematodes also lost the MYC proto-oncogenic regulator of cell proliferation. To identify additional regulators of cell proliferation that were lost with MYC, we computationally screened and determined 839 high-confidence genes that are conserved in bilaterians/lost in nematodes (CIBLIN genes). We find that 30 % of all CIBLIN genes encode transcriptional regulators of cell proliferation, epithelial-to-mesenchyme transitions, and other processes. Over 50 % of CIBLIN genes are unnamed genes in Drosophila, suggesting that there are many understudied genes. Interestingly, CIBLIN genes include many Myc synthetic lethal (MycSL) hits from recent screens. CIBLIN genes include key regulators of heparan sulfate proteoglycan (HSPG) sulfation patterns, and lysyl oxidases involved in cross-linking and modification of the extracellular matrix (ECM). These genes and others suggest the CIBLIN repertoire services critical functions in ECM remodeling and cell **migration** in large-bodied bilaterians. Correspondingly, CIBLIN genes are co-expressed with Myc in cancer transcriptomes, and include a preponderance of known determinants of cancer progression and tumor aggression. We propose that CIBLIN gene research can improve our understanding of regulatory control of cellular growth in metazoans.

### ECM

#### PubMed

1. Genes conserved in bilaterians but jointly lost with Myc during nematode evolution are enriched in cell proliferation and cell migration functions. Animals use a stereotypical set of developmental genes to build body architectures of varying sizes and organizational complexity. Some genes are critical to developmental patterning, while other genes are important to physiological control of growth. However, growth regulator genes may not be as important in small-bodied "micro-metazoans" such as nematodes. Nematodes use a simplified developmental strategy of lineage-based cell fate specifications to produce an adult bilaterian body composed of a few hundreds of cells. Nematodes also lost the MYC proto-oncogenic regulator of cell proliferation. To identify additional regulators of cell proliferation that were lost with MYC, we computationally screened and determined 839 high-confidence genes that are conserved in bilaterians/lost in nematodes (CIBLIN genes). We find that 30 % of all CIBLIN genes encode transcriptional regulators of cell proliferation, epithelial-to-mesenchyme transitions, and other processes. Over 50 % of CIBLIN genes are unnamed genes in Drosophila, suggesting that there are many understudied genes. Interestingly, CIBLIN genes include many Myc synthetic lethal (MycSL) hits from recent screens. CIBLIN genes include key regulators of heparan sulfate proteoglycan (HSPG) sulfation patterns, and lysyl oxidases involved in cross-linking and modification of the extracellular matrix (**ECM**). These genes and others suggest the CIBLIN repertoire services critical functions in **ECM** remodeling and cell migration in large-bodied bilaterians. Correspondingly, CIBLIN genes are co-expressed with Myc in cancer transcriptomes, and include a preponderance of known determinants of cancer progression and tumor aggression. We propose that CIBLIN gene research can improve our understanding of regulatory control of cellular growth in metazoans.

### [Ee]xtracellular [Mm]atrix

#### PubMed

1. Genes conserved in bilaterians but jointly lost with Myc during nematode evolution are enriched in cell proliferation and cell migration functions. Animals use a stereotypical set of developmental genes to build body architectures of varying sizes and organizational complexity. Some genes are critical to developmental patterning, while other genes are important to physiological control of growth. However, growth regulator genes may not be as important in small-bodied "micro-metazoans" such as nematodes. Nematodes use a simplified developmental strategy of lineage-based cell fate specifications to produce an adult bilaterian body composed of a few hundreds of cells. Nematodes also lost the MYC proto-oncogenic regulator of cell proliferation. To identify additional regulators of cell proliferation that were lost with MYC, we computationally screened and determined 839 high-confidence genes that are conserved in bilaterians/lost in nematodes (CIBLIN genes). We find that 30 % of all CIBLIN genes encode transcriptional regulators of cell proliferation, epithelial-to-mesenchyme transitions, and other processes. Over 50 % of CIBLIN genes are unnamed genes in Drosophila, suggesting that there are many understudied genes. Interestingly, CIBLIN genes include many Myc synthetic lethal (MycSL) hits from recent screens. CIBLIN genes include key regulators of heparan sulfate proteoglycan (HSPG) sulfation patterns, and lysyl oxidases involved in cross-linking and modification of the **extracellular matrix** (ECM). These genes and others suggest the CIBLIN repertoire services critical functions in ECM remodeling and cell migration in large-bodied bilaterians. Correspondingly, CIBLIN genes are co-expressed with Myc in cancer transcriptomes, and include a preponderance of known determinants of cancer progression and tumor aggression. We propose that CIBLIN gene research can improve our understanding of regulatory control of cellular growth in metazoans.

---

## kraken

- NCBI Gene
- GeneRIF
- Pubmed

| Search Term | Rank | Fields |
| --- | --- | --- |
| [Mm]igration | 6 | PubMed(1) |
| ECM | 7 | PubMed(1) |
| [Ee]xtracellular [Mm]atrix | 8 | PubMed(1) |

  
hide/show details for kraken

### [Mm]igration

#### PubMed

1. Genes conserved in bilaterians but jointly lost with Myc during nematode evolution are enriched in cell proliferation and cell **migration** functions. Animals use a stereotypical set of developmental genes to build body architectures of varying sizes and organizational complexity. Some genes are critical to developmental patterning, while other genes are important to physiological control of growth. However, growth regulator genes may not be as important in small-bodied "micro-metazoans" such as nematodes. Nematodes use a simplified developmental strategy of lineage-based cell fate specifications to produce an adult bilaterian body composed of a few hundreds of cells. Nematodes also lost the MYC proto-oncogenic regulator of cell proliferation. To identify additional regulators of cell proliferation that were lost with MYC, we computationally screened and determined 839 high-confidence genes that are conserved in bilaterians/lost in nematodes (CIBLIN genes). We find that 30 % of all CIBLIN genes encode transcriptional regulators of cell proliferation, epithelial-to-mesenchyme transitions, and other processes. Over 50 % of CIBLIN genes are unnamed genes in Drosophila, suggesting that there are many understudied genes. Interestingly, CIBLIN genes include many Myc synthetic lethal (MycSL) hits from recent screens. CIBLIN genes include key regulators of heparan sulfate proteoglycan (HSPG) sulfation patterns, and lysyl oxidases involved in cross-linking and modification of the extracellular matrix (ECM). These genes and others suggest the CIBLIN repertoire services critical functions in ECM remodeling and cell **migration** in large-bodied bilaterians. Correspondingly, CIBLIN genes are co-expressed with Myc in cancer transcriptomes, and include a preponderance of known determinants of cancer progression and tumor aggression. We propose that CIBLIN gene research can improve our understanding of regulatory control of cellular growth in metazoans.

### ECM

#### PubMed

1. Genes conserved in bilaterians but jointly lost with Myc during nematode evolution are enriched in cell proliferation and cell migration functions. Animals use a stereotypical set of developmental genes to build body architectures of varying sizes and organizational complexity. Some genes are critical to developmental patterning, while other genes are important to physiological control of growth. However, growth regulator genes may not be as important in small-bodied "micro-metazoans" such as nematodes. Nematodes use a simplified developmental strategy of lineage-based cell fate specifications to produce an adult bilaterian body composed of a few hundreds of cells. Nematodes also lost the MYC proto-oncogenic regulator of cell proliferation. To identify additional regulators of cell proliferation that were lost with MYC, we computationally screened and determined 839 high-confidence genes that are conserved in bilaterians/lost in nematodes (CIBLIN genes). We find that 30 % of all CIBLIN genes encode transcriptional regulators of cell proliferation, epithelial-to-mesenchyme transitions, and other processes. Over 50 % of CIBLIN genes are unnamed genes in Drosophila, suggesting that there are many understudied genes. Interestingly, CIBLIN genes include many Myc synthetic lethal (MycSL) hits from recent screens. CIBLIN genes include key regulators of heparan sulfate proteoglycan (HSPG) sulfation patterns, and lysyl oxidases involved in cross-linking and modification of the extracellular matrix (**ECM**). These genes and others suggest the CIBLIN repertoire services critical functions in **ECM** remodeling and cell migration in large-bodied bilaterians. Correspondingly, CIBLIN genes are co-expressed with Myc in cancer transcriptomes, and include a preponderance of known determinants of cancer progression and tumor aggression. We propose that CIBLIN gene research can improve our understanding of regulatory control of cellular growth in metazoans.

### [Ee]xtracellular [Mm]atrix

#### PubMed

1. Genes conserved in bilaterians but jointly lost with Myc during nematode evolution are enriched in cell proliferation and cell migration functions. Animals use a stereotypical set of developmental genes to build body architectures of varying sizes and organizational complexity. Some genes are critical to developmental patterning, while other genes are important to physiological control of growth. However, growth regulator genes may not be as important in small-bodied "micro-metazoans" such as nematodes. Nematodes use a simplified developmental strategy of lineage-based cell fate specifications to produce an adult bilaterian body composed of a few hundreds of cells. Nematodes also lost the MYC proto-oncogenic regulator of cell proliferation. To identify additional regulators of cell proliferation that were lost with MYC, we computationally screened and determined 839 high-confidence genes that are conserved in bilaterians/lost in nematodes (CIBLIN genes). We find that 30 % of all CIBLIN genes encode transcriptional regulators of cell proliferation, epithelial-to-mesenchyme transitions, and other processes. Over 50 % of CIBLIN genes are unnamed genes in Drosophila, suggesting that there are many understudied genes. Interestingly, CIBLIN genes include many Myc synthetic lethal (MycSL) hits from recent screens. CIBLIN genes include key regulators of heparan sulfate proteoglycan (HSPG) sulfation patterns, and lysyl oxidases involved in cross-linking and modification of the **extracellular matrix** (ECM). These genes and others suggest the CIBLIN repertoire services critical functions in ECM remodeling and cell migration in large-bodied bilaterians. Correspondingly, CIBLIN genes are co-expressed with Myc in cancer transcriptomes, and include a preponderance of known determinants of cancer progression and tumor aggression. We propose that CIBLIN gene research can improve our understanding of regulatory control of cellular growth in metazoans.

---

## step

- NCBI Gene
- GeneRIF
- Pubmed

| Search Term | Rank | Fields |
| --- | --- | --- |
| [Mm]igration | 6 | PubMed(1) |

  
hide/show details for step

### [Mm]igration

#### PubMed

1. Regulation of Drosophila mesoderm **migration** by phosphoinositides and the PH domain of the Rho GTP exchange factor Pebble. The Drosophila RhoGEF Pebble (Pbl) is required for cytokinesis and **migration** of mesodermal cells. In a screen for genes that could suppress **migration** defects in pbl mutants we identified the phosphatidylinositol phosphate (PtdInsP) regulator pi5k59B. Genetic interaction tests with other PtdInsP regulators suggested that PtdIns(4,5)P2 levels are important for mesoderm **migration** when Pbl is depleted. Consistent with this, the leading front of migrating mesodermal cells was enriched for PtdIns(4,5)P2. Given that Pbl contains a Pleckstrin Homology (PH) domain, a known PtdInsP-binding motif, we examined PtdInsP-binding of Pbl and the importance of the PH domain for Pbl function. In vitro lipid blot assays showed that Pbl binds promiscuously to PtdInsPs, with binding strength associated with the degree of phosphorylation. Pbl was also able to bind lipid vesicles containing PtdIns(4,5)P2 but binding was strongly reduced upon deletion of the PH domain. Similarly, in vivo, loss of the PH domain prevented localisation of Pbl to the cell cortex and severely affected several aspects of early mesoderm development, including flattening of the invaginated tube onto the ectoderm, extension of protrusions, and dorsal **migration** to form a monolayer. Pbl lacking the PH domain could still localise to the cytokinetic furrow, however, and cytokinesis failure was reduced in pbl(ΔPH) mutants. Taken together, our results support a model in which interaction of the PH-domain of Pbl with PtdIns(4,5)P2 helps localise it to the plasma membrane which is important for mesoderm **migratio**n.

---

## luna

- NCBI Gene
- GeneRIF
- Pubmed

| Search Term | Rank | Fields |
| --- | --- | --- |
| [Mm]igration | 6 | PubMed(1) |
| ECM | 7 | PubMed(1) |
| [Ee]xtracellular [Mm]atrix | 8 | PubMed(1) |

  
hide/show details for luna

### [Mm]igration

#### PubMed

1. Genes conserved in bilaterians but jointly lost with Myc during nematode evolution are enriched in cell proliferation and cell **migration** functions. Animals use a stereotypical set of developmental genes to build body architectures of varying sizes and organizational complexity. Some genes are critical to developmental patterning, while other genes are important to physiological control of growth. However, growth regulator genes may not be as important in small-bodied "micro-metazoans" such as nematodes. Nematodes use a simplified developmental strategy of lineage-based cell fate specifications to produce an adult bilaterian body composed of a few hundreds of cells. Nematodes also lost the MYC proto-oncogenic regulator of cell proliferation. To identify additional regulators of cell proliferation that were lost with MYC, we computationally screened and determined 839 high-confidence genes that are conserved in bilaterians/lost in nematodes (CIBLIN genes). We find that 30 % of all CIBLIN genes encode transcriptional regulators of cell proliferation, epithelial-to-mesenchyme transitions, and other processes. Over 50 % of CIBLIN genes are unnamed genes in Drosophila, suggesting that there are many understudied genes. Interestingly, CIBLIN genes include many Myc synthetic lethal (MycSL) hits from recent screens. CIBLIN genes include key regulators of heparan sulfate proteoglycan (HSPG) sulfation patterns, and lysyl oxidases involved in cross-linking and modification of the extracellular matrix (ECM). These genes and others suggest the CIBLIN repertoire services critical functions in ECM remodeling and cell **migration** in large-bodied bilaterians. Correspondingly, CIBLIN genes are co-expressed with Myc in cancer transcriptomes, and include a preponderance of known determinants of cancer progression and tumor aggression. We propose that CIBLIN gene research can improve our understanding of regulatory control of cellular growth in metazoans.

### ECM

#### PubMed

1. Genes conserved in bilaterians but jointly lost with Myc during nematode evolution are enriched in cell proliferation and cell migration functions. Animals use a stereotypical set of developmental genes to build body architectures of varying sizes and organizational complexity. Some genes are critical to developmental patterning, while other genes are important to physiological control of growth. However, growth regulator genes may not be as important in small-bodied "micro-metazoans" such as nematodes. Nematodes use a simplified developmental strategy of lineage-based cell fate specifications to produce an adult bilaterian body composed of a few hundreds of cells. Nematodes also lost the MYC proto-oncogenic regulator of cell proliferation. To identify additional regulators of cell proliferation that were lost with MYC, we computationally screened and determined 839 high-confidence genes that are conserved in bilaterians/lost in nematodes (CIBLIN genes). We find that 30 % of all CIBLIN genes encode transcriptional regulators of cell proliferation, epithelial-to-mesenchyme transitions, and other processes. Over 50 % of CIBLIN genes are unnamed genes in Drosophila, suggesting that there are many understudied genes. Interestingly, CIBLIN genes include many Myc synthetic lethal (MycSL) hits from recent screens. CIBLIN genes include key regulators of heparan sulfate proteoglycan (HSPG) sulfation patterns, and lysyl oxidases involved in cross-linking and modification of the extracellular matrix (**ECM**). These genes and others suggest the CIBLIN repertoire services critical functions in **ECM** remodeling and cell migration in large-bodied bilaterians. Correspondingly, CIBLIN genes are co-expressed with Myc in cancer transcriptomes, and include a preponderance of known determinants of cancer progression and tumor aggression. We propose that CIBLIN gene research can improve our understanding of regulatory control of cellular growth in metazoans.

### [Ee]xtracellular [Mm]atrix

#### PubMed

1. Genes conserved in bilaterians but jointly lost with Myc during nematode evolution are enriched in cell proliferation and cell migration functions. Animals use a stereotypical set of developmental genes to build body architectures of varying sizes and organizational complexity. Some genes are critical to developmental patterning, while other genes are important to physiological control of growth. However, growth regulator genes may not be as important in small-bodied "micro-metazoans" such as nematodes. Nematodes use a simplified developmental strategy of lineage-based cell fate specifications to produce an adult bilaterian body composed of a few hundreds of cells. Nematodes also lost the MYC proto-oncogenic regulator of cell proliferation. To identify additional regulators of cell proliferation that were lost with MYC, we computationally screened and determined 839 high-confidence genes that are conserved in bilaterians/lost in nematodes (CIBLIN genes). We find that 30 % of all CIBLIN genes encode transcriptional regulators of cell proliferation, epithelial-to-mesenchyme transitions, and other processes. Over 50 % of CIBLIN genes are unnamed genes in Drosophila, suggesting that there are many understudied genes. Interestingly, CIBLIN genes include many Myc synthetic lethal (MycSL) hits from recent screens. CIBLIN genes include key regulators of heparan sulfate proteoglycan (HSPG) sulfation patterns, and lysyl oxidases involved in cross-linking and modification of the **extracellular matrix** (ECM). These genes and others suggest the CIBLIN repertoire services critical functions in ECM remodeling and cell migration in large-bodied bilaterians. Correspondingly, CIBLIN genes are co-expressed with Myc in cancer transcriptomes, and include a preponderance of known determinants of cancer progression and tumor aggression. We propose that CIBLIN gene research can improve our understanding of regulatory control of cellular growth in metazoans.

---

## Rab6

- NCBI Gene
- GeneRIF
- Pubmed

| Search Term | Rank | Fields |
| --- | --- | --- |
| [Mm]igration | 6 | PubMed(2) |
| [Mm]etastasis | 12 | PubMed(1) |

  
hide/show details for Rab6

### [Mm]igration

#### PubMed

1. Analysis of cell **migration** using whole-genome expression profiling of migratory cells in the Drosophila ovary. Cell **migration** contributes to normal development and homeostasis as well as to pathological processes such as inflammation and tumor metastasis. Previous genetic screens have revealed signaling pathways that govern follicle cell **migration**s in the Drosophila ovary, but few downstream targets of the critical transcriptional regulators have been identified. To characterize the gene expression profile of two migratory cell populations and identify Slbo targets, we purified border cells and centripetal cells expressing the mouse CD8 antigen and carried out whole-genome microarray analysis. Genes predicted to control actin dynamics and the endocytic and secretory pathways were overrepresented in the migratory cell transcriptome. Mutations in five genes, including ttk, failed to complement previously isolated mutations that cause cell **migration** defects in mosaic clones. Functional analysis revealed a role for the Notch-activating protease Kuzbanian in border cell **migration** and identified Tie as a guidance receptor for the border cells.
2. Rab-mediated vesicular transport is required for neuronal positioning in the developing Drosophila visual system. The establishment of tissue architecture in the nervous system requires the proper **migration** and positioning of newly born neurons during embryonic development. Defects in nuclear translocation, a key process in neuronal positioning, are associated with brain diseases such as lissencephaly in humans. Accumulated evidence suggests that the molecular mechanisms controlling neuronal movement are conserved throughout evolution. While the initial events of neuronal **migration** have been extensively studied, less is known about the molecular details underlying the establishment of neuronal architecture after initial **migration**.In a search for novel players in the control of photoreceptor (R cell) positioning in the developing fly visual system, we found that misexpression of the RabGAP RN-Tre disrupted the apical localization of R-cell nuclei. RN-Tre interacts with Rab5 and Rab11 in the fly eye. Genetic analysis shows that Rab5, Shi and Rab11 are required for maintaining apical localization of R-cell nuclei.We propose that Rab5, Shi and Rab11 function together in a vesicular transport pathway for regulating R-cell positioning in the developing eye.

### [Mm]etastasis

#### PubMed

1. Analysis of cell migration using whole-genome expression profiling of migratory cells in the Drosophila ovary. Cell migration contributes to normal development and homeostasis as well as to pathological processes such as inflammation and tumor **metastasis**. Previous genetic screens have revealed signaling pathways that govern follicle cell migrations in the Drosophila ovary, but few downstream targets of the critical transcriptional regulators have been identified. To characterize the gene expression profile of two migratory cell populations and identify Slbo targets, we purified border cells and centripetal cells expressing the mouse CD8 antigen and carried out whole-genome microarray analysis. Genes predicted to control actin dynamics and the endocytic and secretory pathways were overrepresented in the migratory cell transcriptome. Mutations in five genes, including ttk, failed to complement previously isolated mutations that cause cell migration defects in mosaic clones. Functional analysis revealed a role for the Notch-activating protease Kuzbanian in border cell migration and identified Tie as a guidance receptor for the border cells.

---

## CG3077

- NCBI Gene
- GeneRIF
- Pubmed

| Search Term | Rank | Fields |
| --- | --- | --- |
| [Mm]igration | 6 | PubMed(1) |
| ECM | 7 | PubMed(1) |
| [Ee]xtracellular [Mm]atrix | 8 | PubMed(1) |

  
hide/show details for CG3077

### [Mm]igration

#### PubMed

1. Genes conserved in bilaterians but jointly lost with Myc during nematode evolution are enriched in cell proliferation and cell **migration** functions. Animals use a stereotypical set of developmental genes to build body architectures of varying sizes and organizational complexity. Some genes are critical to developmental patterning, while other genes are important to physiological control of growth. However, growth regulator genes may not be as important in small-bodied "micro-metazoans" such as nematodes. Nematodes use a simplified developmental strategy of lineage-based cell fate specifications to produce an adult bilaterian body composed of a few hundreds of cells. Nematodes also lost the MYC proto-oncogenic regulator of cell proliferation. To identify additional regulators of cell proliferation that were lost with MYC, we computationally screened and determined 839 high-confidence genes that are conserved in bilaterians/lost in nematodes (CIBLIN genes). We find that 30 % of all CIBLIN genes encode transcriptional regulators of cell proliferation, epithelial-to-mesenchyme transitions, and other processes. Over 50 % of CIBLIN genes are unnamed genes in Drosophila, suggesting that there are many understudied genes. Interestingly, CIBLIN genes include many Myc synthetic lethal (MycSL) hits from recent screens. CIBLIN genes include key regulators of heparan sulfate proteoglycan (HSPG) sulfation patterns, and lysyl oxidases involved in cross-linking and modification of the extracellular matrix (ECM). These genes and others suggest the CIBLIN repertoire services critical functions in ECM remodeling and cell **migration** in large-bodied bilaterians. Correspondingly, CIBLIN genes are co-expressed with Myc in cancer transcriptomes, and include a preponderance of known determinants of cancer progression and tumor aggression. We propose that CIBLIN gene research can improve our understanding of regulatory control of cellular growth in metazoans.

### ECM

#### PubMed

1. Genes conserved in bilaterians but jointly lost with Myc during nematode evolution are enriched in cell proliferation and cell migration functions. Animals use a stereotypical set of developmental genes to build body architectures of varying sizes and organizational complexity. Some genes are critical to developmental patterning, while other genes are important to physiological control of growth. However, growth regulator genes may not be as important in small-bodied "micro-metazoans" such as nematodes. Nematodes use a simplified developmental strategy of lineage-based cell fate specifications to produce an adult bilaterian body composed of a few hundreds of cells. Nematodes also lost the MYC proto-oncogenic regulator of cell proliferation. To identify additional regulators of cell proliferation that were lost with MYC, we computationally screened and determined 839 high-confidence genes that are conserved in bilaterians/lost in nematodes (CIBLIN genes). We find that 30 % of all CIBLIN genes encode transcriptional regulators of cell proliferation, epithelial-to-mesenchyme transitions, and other processes. Over 50 % of CIBLIN genes are unnamed genes in Drosophila, suggesting that there are many understudied genes. Interestingly, CIBLIN genes include many Myc synthetic lethal (MycSL) hits from recent screens. CIBLIN genes include key regulators of heparan sulfate proteoglycan (HSPG) sulfation patterns, and lysyl oxidases involved in cross-linking and modification of the extracellular matrix (**ECM**). These genes and others suggest the CIBLIN repertoire services critical functions in **ECM** remodeling and cell migration in large-bodied bilaterians. Correspondingly, CIBLIN genes are co-expressed with Myc in cancer transcriptomes, and include a preponderance of known determinants of cancer progression and tumor aggression. We propose that CIBLIN gene research can improve our understanding of regulatory control of cellular growth in metazoans.

### [Ee]xtracellular [Mm]atrix

#### PubMed

1. Genes conserved in bilaterians but jointly lost with Myc during nematode evolution are enriched in cell proliferation and cell migration functions. Animals use a stereotypical set of developmental genes to build body architectures of varying sizes and organizational complexity. Some genes are critical to developmental patterning, while other genes are important to physiological control of growth. However, growth regulator genes may not be as important in small-bodied "micro-metazoans" such as nematodes. Nematodes use a simplified developmental strategy of lineage-based cell fate specifications to produce an adult bilaterian body composed of a few hundreds of cells. Nematodes also lost the MYC proto-oncogenic regulator of cell proliferation. To identify additional regulators of cell proliferation that were lost with MYC, we computationally screened and determined 839 high-confidence genes that are conserved in bilaterians/lost in nematodes (CIBLIN genes). We find that 30 % of all CIBLIN genes encode transcriptional regulators of cell proliferation, epithelial-to-mesenchyme transitions, and other processes. Over 50 % of CIBLIN genes are unnamed genes in Drosophila, suggesting that there are many understudied genes. Interestingly, CIBLIN genes include many Myc synthetic lethal (MycSL) hits from recent screens. CIBLIN genes include key regulators of heparan sulfate proteoglycan (HSPG) sulfation patterns, and lysyl oxidases involved in cross-linking and modification of the **extracellular matrix** (ECM). These genes and others suggest the CIBLIN repertoire services critical functions in ECM remodeling and cell migration in large-bodied bilaterians. Correspondingly, CIBLIN genes are co-expressed with Myc in cancer transcriptomes, and include a preponderance of known determinants of cancer progression and tumor aggression. We propose that CIBLIN gene research can improve our understanding of regulatory control of cellular growth in metazoans.

---

## ps

- NCBI Gene
- GeneRIF
- Pubmed

| Search Term | Rank | Fields |
| --- | --- | --- |
| [Mm]igration | 6 | PubMed(1) |
| [Ee]xtracellular [Mm]atrix | 8 | PubMed(1) |

  
hide/show details for ps

### [Mm]igration

#### PubMed

1. Fork head prevents apoptosis and promotes cell shape change during formation of the Drosophila salivary glands. The secretory tubes of the Drosophila salivary glands are formed by the regulated, sequential internalization of the primordia. Secretory cell invagination occurs by a change in cell shape that includes basal nuclear **migration** and apical membrane constriction. In embryos mutant for fork head (fkh), which encodes a transcription factor homologous to mammalian hepatocyte nuclear factor 3beta (HNF-3beta), the secretory primordia are not internalized and secretory tubes do not form. Here, we show that secretory cells of fkh mutant embryos undergo extensive apoptotic cell death following the elevated expression of the apoptotic activator genes, reaper and head involution defective. We rescue the secretory cell death in the fkh mutants and show that the rescued cells still do not invaginate. The rescued fkh secretory cells undergo basal nuclear **migration** in the same spatial and temporal pattern as in wild-type secretory cells, but do not constrict their apical surface membranes. Our findings suggest at least two roles for fkh in formation of the embryonic salivary glands: an early role in promoting survival of the secretory cells, and a later role in secretory cell invagination, specifically in the constriction of the apical surface membrane.

### [Ee]xtracellular [Mm]atrix

#### PubMed

1. Dose-sensitive autosomal modifiers identify candidate genes for tissue autonomous and tissue nonautonomous regulation by the Drosophila nuclear zinc-finger protein, hindsight. The nuclear zinc-finger protein encoded by the hindsight (hnt) locus regulates several cellular processes in Drosophila epithelia, including the Jun N-terminal kinase (JNK) signaling pathway and actin polymerization. Defects in these molecular pathways may underlie the abnormal cellular interactions, loss of epithelial integrity, and apoptosis that occurs in hnt mutants, in turn causing failure of morphogenetic processes such as germ band retraction and dorsal closure in the embryo. To define the genetic pathways regulated by hnt, 124 deficiencies on the second and third chromosomes and 14 duplications on the second chromosome were assayed for dose-sensitive modification of a temperature-sensitive rough eye phenotype caused by the viable allele, hntpeb; 29 interacting regions were identified. Subsequently, 438 P-element-induced lethal mutations mapping to these regions and 12 candidate genes were tested for genetic interaction, leading to identification of 63 dominant modifier loci. A subset of the identified mutants also dominantly modify hnt308-induced embryonic lethality and thus represent general rather than tissue-specific interactors. General interactors include loci encoding transcription factors, actin-binding proteins, signal transduction proteins, and components of the **extracellular matrix**. Expression of several interactors was assessed in hnt mutant tissue. Five genes--apontic (apt), Delta (Dl), decapentaplegic (dpp), karst (kst), and puckered (puc)--are regulated tissue autonomously and, thus, may be direct transcriptional targets of HNT. Three of these genes--apt, Dl, and dpp--are also regulated nonautonomously in adjacent non-HNT-expressing tissues. The expression of several additional interactors--viking (vkg), Cg25, and laminin-alpha (LanA)-is affected only in a nonautonomous manner.

---

## CG3097

- NCBI Gene
- GeneRIF
- Pubmed

| Search Term | Rank | Fields |
| --- | --- | --- |
| [Mm]igration | 6 | PubMed(1) |

  
hide/show details for CG3097

### [Mm]igration

#### PubMed

1. Comparative analysis of Hox downstream genes in Drosophila. Functional diversification of body parts is dependent on the formation of specialized structures along the various body axes. In animals, region-specific morphogenesis along the anteroposterior axis is controlled by a group of conserved transcription factors encoded by the Hox genes. Although it has long been assumed that Hox proteins carry out their function by regulating distinct sets of downstream genes, only a small number of such genes have been found, with very few having direct roles in controlling cellular behavior. We have quantitatively identified hundreds of Hox downstream genes in Drosophila by microarray analysis, and validated many of them by in situ hybridizations on loss- and gain-of-function mutants. One important finding is that Hox proteins, despite their similar DNA-binding properties in vitro, have highly specific effects on the transcriptome in vivo, because expression of many downstream genes respond primarily to a single Hox protein. In addition, a large fraction of downstream genes encodes realizator functions, which directly affect morphogenetic processes, such as orientation and rate of cell divisions, cell-cell adhesion and communication, cell shape and **migration**, or cell death. Focusing on these realizators, we provide a framework for the morphogenesis of the maxillary segment. As the genomic organization of Hox genes and the interaction of Hox proteins with specific co-factors are conserved in vertebrates and invertebrates, and similar classes of downstream genes are regulated by Hox proteins across the metazoan phylogeny, our findings represent a first step toward a mechanistic understanding of morphological diversification within a species as well as between species.

---

## Shc

- NCBI Gene
- GeneRIF
- Pubmed

| Search Term | Rank | Fields |
| --- | --- | --- |
| [Mm]igration | 6 | PubMed(4) |
| [Ee]xtracellular [Mm]atrix | 8 | PubMed(1) |
| [Mm]etastasis | 12 | PubMed(1) |

  
hide/show details for Shc

### [Mm]igration

#### PubMed

1. Two distinct modes of guidance signalling during collective **migration** of border cells. Although directed **migration** is a feature of both individual cells and cell groups, guided **migration** has been studied most extensively for single cells in simple environments. Collective guidance of cell groups remains poorly understood, despite its relevance for development and metastasis. Neural crest cells and neuronal precursors migrate as loosely organized streams of individual cells, whereas cells of the fish lateral line, Drosophila tracheal tubes and border-cell clusters migrate as more coherent groups. Here we use Drosophila border cells to examine how collective guidance is performed. We report that border cells migrate in two phases using distinct mechanisms. Genetic analysis combined with live imaging shows that polarized cell behaviour is critical for the initial phase of **migration**, whereas dynamic collective behaviour dominates later. PDGF- and VEGF-related receptor and epidermal growth factor receptor act in both phases, but use different effector pathways in each. The myoblast city (Mbc, also known as DOCK180) and engulfment and cell motility (ELMO, also known as Ced-12) pathway is required for the early phase, in which guidance depends on subcellular localization of signalling within a leading cell. During the later phase, mitogen-activated protein kinase and phospholipase Cgamma are used redundantly, and we find that the cluster makes use of the difference in signal levels between cells to guide **migration**. Thus, information processing at the multicellular level is used to guide collective behaviour of a cell group.
2. Guidance of cell **migration** by the Drosophila PDGF/VEGF receptor. Directed cell **migration**s are important for development, but the signaling pathways and mechanisms responsible for guiding cell **migration** in vivo are poorly understood. **Migration** of border cells during Drosophila oogenesis is a simple and attractive model system in which to address these questions. We demonstrate that PVR, a receptor tyrosine kinase related to mammalian PDGF and VEGF receptors, acts in border cells to guide them to the oocyte. The oocyte is the source of a ligand for PVR, PDGF/VEGF factor 1 (PVF1). Intriguingly, the guidance function of PVR is largely redundant with that of EGFR. We present evidence implicating Rac and the Rac activator Mbc/DOCK180/CED-5 as mediators of the guidance signal.
3. Distinct roles for two receptor tyrosine kinases in epithelial branching morphogenesis in Drosophila. Branching morphogenesis is a widespread mechanism used to increase the surface area of epithelial organs. Many signaling systems steer development of branched organs, but it is still unclear which cellular processes are regulated by the different pathways. We have used the development of the air sacs of the dorsal thorax of Drosophila to study cellular events and their regulation via cell-cell signaling. We find that two receptor tyrosine kinases play important but distinct roles in air sac outgrowth. Fgf signaling directs cell **migration** at the tip of the structure, while Egf signaling is instrumental for cell division and cell survival in the growing epithelial structure. Interestingly, we find that Fgf signaling requires Ras, the Mapk pathway, and Pointed to direct **migration**, suggesting that both cytoskeletal and nuclear events are downstream of receptor activation. Ras and the Mapk pathway are also needed for Egf-regulated cell division/survival, but Pointed is dispensable.
4. Developmental control of blood cell **migration** by the Drosophila VEGF pathway. We show that a vascular endothelial growth factor (VEGF) pathway controls embryonic **migration**s of blood cells (hemocytes) in Drosophila. The VEGF receptor homolog is expressed in hemocytes, and three VEGF homologs are expressed along hemocyte **migration** routes. A receptor mutation arrests progression of blood cell movement. Mutations in Vegf17E or Vegf27Cb have no effect, but simultaneous inactivation of all three Vegf genes phenocopied the receptor mutant, and ectopic expression of Vegf27Cb redirected **migration**. Genetic experiments indicate that the VEGF pathway functions independently of pathways governing hemocyte homing on apoptotic cells. The results suggest that the Drosophila VEGF pathway guides developmental **migration**s of blood cells, and we speculate that the ancestral function of VEGF pathways was to guide blood cell movement.

### [Ee]xtracellular [Mm]atrix

#### PubMed

1. A directed miniscreen for genes involved in the Drosophila anti-parasitoid immune response. Drosophila larvae react against eggs from the endoparasitoid wasp Leptopilina boulardi by surrounding them in a multilayered cellular capsule. Once a wasp egg is recognized as foreign, circulating macrophage-like cells, known as plasmatocytes, adhere to the invader. After spreading around the wasp egg, plasmatocytes form cellular junctions between the cells, effectively separating the egg from the hemocoel. Next, a second sub-type of circulating immunosurveillance cell (hemocyte), known as lamellocytes, adhere to either the wasp egg or more likely the plasmatocytes surrounding the egg. From these events, it is obvious that adhesion and cell shape change are an essential part of Drosophila's cellular immune response against parasitoid wasp eggs. To date, very few genes have been described as being necessary for a proper anti-parasitization response in Drosophila. With this in mind, we performed a directed genetic miniscreen to discover new genes required for this response. Many of the genes with an encapsulation defect have mammalian homologues involved in cellular adhesion, wound healing, and thrombosis, including **extracellular matrix** proteins, cellular adhesion molecules, and small GTPases.

### [Mm]etastasis

#### PubMed

1. Two distinct modes of guidance signalling during collective migration of border cells. Although directed migration is a feature of both individual cells and cell groups, guided migration has been studied most extensively for single cells in simple environments. Collective guidance of cell groups remains poorly understood, despite its relevance for development and **metastasis**. Neural crest cells and neuronal precursors migrate as loosely organized streams of individual cells, whereas cells of the fish lateral line, Drosophila tracheal tubes and border-cell clusters migrate as more coherent groups. Here we use Drosophila border cells to examine how collective guidance is performed. We report that border cells migrate in two phases using distinct mechanisms. Genetic analysis combined with live imaging shows that polarized cell behaviour is critical for the initial phase of migration, whereas dynamic collective behaviour dominates later. PDGF- and VEGF-related receptor and epidermal growth factor receptor act in both phases, but use different effector pathways in each. The myoblast city (Mbc, also known as DOCK180) and engulfment and cell motility (ELMO, also known as Ced-12) pathway is required for the early phase, in which guidance depends on subcellular localization of signalling within a leading cell. During the later phase, mitogen-activated protein kinase and phospholipase Cgamma are used redundantly, and we find that the cluster makes use of the difference in signal levels between cells to guide migration. Thus, information processing at the multicellular level is used to guide collective behaviour of a cell group.

---

## Moe

- NCBI Gene
- GeneRIF
- Pubmed

| Search Term | Rank | Fields |
| --- | --- | --- |
| [Ff]ilopodia | 1 | PubMed(19) |
| [Mm]igration | 6 | PubMed(45) |
| ECM | 7 | PubMed(3) |
| [Ee]xtracellular [Mm]atrix | 8 | PubMed(7) |
| [Ii]nvasive | 10 | PubMed(9) |
| [Mm]etastasize | 11 | PubMed(1) |
| [Mm]etastasis | 12 | PubMed(10) |

  
hide/show details for Moe

### [Ff]ilopodia

#### PubMed

1. Guidance of subcellular tubulogenesis by actin under the control of a synaptotagmin-like protein and Moesin. Apical membranes in many polarized epithelial cells show specialized morphological adaptations that fulfil distinct physiological functions. The air-transporting tubules of Drosophila tracheal terminal cells represent an extreme case of membrane specialization. Here we show that Bitesize (Btsz), a synaptotagmin-like protein family member, is needed for luminal membrane morphogenesis. Unlike in multicellular tubes and other epithelia, where it influences apical integrity by affecting adherens junctions, Btsz here acts at a distance from junctions. Localized at the luminal membrane through its tandem C2 domain, it recruits activated Moesin. Both proteins are needed for the integrity of the actin cytoskeleton at the luminal membrane, but not for other pools of F-actin in the cell, nor do actin-dependent processes at the outer membrane, such as **filopodia**l activity or membrane growth depend on Btsz. Btsz and Moesin guide luminal membrane morphogenesis through organizing actin and allowing the incorporation of membrane containing the apical determinant Crumbs.
2. Calcium/calmodulin-dependent protein kinase II alters structural plasticity and cytoskeletal dynamics in Drosophila. Drosophila dendritic arborization (da) neurons contain subclasses of neurons with distinct dendritic morphologies. We investigated calcium/calmodulin-dependent protein kinase II (CaMKII) regulation of dendritic structure and dynamics in vivo using optically transparent Drosophila larvae. CaMKII increases the dynamic nature and formation of dendritic **filopodia** throughout larval development but only affects neurons that normally contain dendritic **filopodia**. In parallel, we examined the effects of Rac1 activity on dendritic structure to explore signaling specificity. In contrast to CaMKII activity, Rac1 does not alter **filopodia** stability but instead causes de novo **filopodia** formation on all da neurons. Although both mediators increase cytoskeletal turnover, measured by fluorescence recovery after photobleaching experiments, only CaMKII increases the dynamic nature of dendritic **filopodia**. CaMKII signaling thus appears to use mechanisms and machinery distinct from Rac1 signaling. This study illustrates a molecular means of uncoupling cytoskeletal regulation from morphological regulation. Our results suggest that Drosophila dendritic **filopodia** may share some cytoskeletal regulatory mechanisms with mammalian dendritic **filopodia**. Furthermore, general dendrite cytoskeletal compartmentalization is conserved in multipolar neurons.
3. Guidance of subcellular tubulogenesis by actin under the control of a synaptotagmin-like protein and Moesin. Apical membranes in many polarized epithelial cells show specialized morphological adaptations that fulfil distinct physiological functions. The air-transporting tubules of Drosophila tracheal terminal cells represent an extreme case of membrane specialization. Here we show that Bitesize (Btsz), a synaptotagmin-like protein family member, is needed for luminal membrane morphogenesis. Unlike in multicellular tubes and other epithelia, where it influences apical integrity by affecting adherens junctions, Btsz here acts at a distance from junctions. Localized at the luminal membrane through its tandem C2 domain, it recruits activated Moesin. Both proteins are needed for the integrity of the actin cytoskeleton at the luminal membrane, but not for other pools of F-actin in the cell, nor do actin-dependent processes at the outer membrane, such as **filopodia**l activity or membrane growth depend on Btsz. Btsz and Moesin guide luminal membrane morphogenesis through organizing actin and allowing the incorporation of membrane containing the apical determinant Crumbs.
4. A novel forward genetic screen for identifying mutations affecting larval neuronal dendrite development in Drosophila melanogaster. Vertebrate and invertebrate dendrites are information-processing compartments that can be found on both central and peripheral neurons. Elucidating the molecular underpinnings of information processing in the nervous system ultimately requires an understanding of the genetic pathways that regulate dendrite formation and maintenance. Despite the importance of dendrite development, few forward genetic approaches have been used to analyze the latest stages of dendrite development, including the formation of F-actin-rich dendritic **filopodia** or dendritic spines. We developed a forward genetic screen utilizing transgenic Drosophila second instar larvae expressing an actin, green fluorescent protein (GFP) fusion protein (actin::GFP) in subsets of sensory neurons. Utilizing this fluorescent transgenic reporter, we conducted a forward genetic screen of >4000 mutagenized chromosomes bearing lethal mutations that affected multiple aspects of larval dendrite development. We isolated 13 mutations on the X and second chromosomes composing 11 complementation groups affecting dendrite outgrowth/branching, dendritic **filopodia** formation, or actin::GFP localization within dendrites in vivo. In a fortuitous observation, we observed that the structure of dendritic arborization (da) neuron dendritic **filopodia** changes in response to a changing environment.
5. Tissue-specific laminin expression facilitates integrin-dependent association of the embryonic wing disc with the trachea in Drosophila. The interaction of heterologous tissues involves cell adhesion mediated by the extracellular matrix and its receptor integrins. The Drosophila wing disc is an ectodermal invagination that contacts specific tracheal branches at the basolateral cell surface. We show that an alpha subunit of laminin, encoded by wing blister (wb), is essential for the establishment of the interaction between the wing and trachea. During embryogenesis, wing disc cells present Wb at their basolateral surface and extend posteriorly, expanding their association to more posteriorly located tracheal branches. These migratory processes are impaired in the absence of the trachea, Wb, or integrins. Time-lapse and transmission electron microscopy analyses suggest that Wb facilitates integrin-dependent contact over a large surface and controls the cellular behavior of the wing cells, including their exploratory **filopodia**l activity. Our data identify Wb laminin as an extracellular matrix ligand that is essential for integrin-dependent cellular migration in Drosophila.
6. Formin3 is required for assembly of the F-actin structure that mediates tracheal fusion in Drosophila. During tracheal development in Drosophila, some branches join to form a continuous luminal network. Specialized cells at the branch tip, called fusion cells, extend **filopodia** to make contact and become doughnut shaped to allow passage of the lumen. These morphogenetic processes accompany the highly regulated cytoskeletal reorganization of fusion cells. We identified the Drosophila formin3 (form3) gene that encodes a novel formin and plays a role in tracheal fusion. Formins are a family of proteins characterized by highly conserved formin homology (FH) domains. The formin family functions in various actin-based processes, including cytokinesis and cell polarity. During embryogenesis, form3 mRNA is expressed mainly in the tracheal system. In form3 mutant embryos, the tracheal fusion does not occur at some points. This phenotype is rescued by the forced expression of form3 in the trachea. We used live imaging of GFP-moesin during tracheal fusion to show that an F-actin structure that spans the adjoining fusion cells and mediates the luminal connection does not form at abnormal anastomosis sites in form3 mutants. These results suggested that Form3 plays a role in the F-actin assembly, which is essential for cellular rearrangement during tracheal fusion.
7. Cytonemes are required for the establishment of a normal Hedgehog morphogen gradient in Drosophila epithelia. Hedgehog (Hh) signalling is important in development, stem cell biology and disease. In a variety of tissues, Hh acts as a morphogen to regulate growth and cell fate specification. Several hypotheses have been proposed to explain morphogen movement, one of which is transport along **filopodia**-like protrusions called cytonemes. Here, we analyse the mechanism underlying Hh movement in the wing disc and the abdominal epidermis of Drosophila melanogaster. We show that, in both epithelia, cells generate cytonemes in regions of Hh signalling. These protrusions are actin-based and span several cell diameters. Various Hh signalling components localize to cytonemes, as well as to punctate structures that move along cytonemes and are probably exovesicles. Using in vivo imaging, we show that cytonemes are dynamic structures and that Hh gradient establishment correlates with cytoneme formation in space and time. Indeed, mutant conditions that affect cytoneme formation reduce both cytoneme length and Hh gradient length. Our results suggest that cytoneme-mediated Hh transport is the mechanistic basis for Hh gradient formation.
8. Cortactin modulates cell migration and ring canal morphogenesis during Drosophila oogenesis. Cortactin is a Src substrate that interacts with F-actin and can stimulate actin polymerization by direct interaction with the Arp2/3 complex. We have isolated complete loss-of-function mutants of the single Drosophila cortactin gene. Mutants are viable and fertile, showing that cortactin is not an essential gene. However, cortactin mutants show distinct defects during oogenesis. During oogenesis, Cortactin protein is enriched at the F-actin rich ring canals in the germ line, and in migrating border cells. In cortactin mutants, the ring canals are smaller than normal. A similar phenotype has been observed in Src64 mutants and in mutants for genes encoding Arp2/3 complex components, supporting that these protein products act together to control specific processes in vivo. Cortactin mutants also show impaired border cell migration. This invasive cell migration is guided by Drosophila EGFR and PDGF/VEGF receptor (PVR). We find that accumulation of Cortactin protein is positively regulated by PVR. Also, overexpression of Cortactin can by itself induce F-actin accumulation and ectopic **filopodia** formation in epithelial cells. We present evidence that Cortactin is one of the factors acting downstream of PVR and Src to stimulate F-actin accumulation. Cortactin is a minor contributor in this regulation, consistent with the cortactin gene not being essential for development.
9. Gα73B is a downstream effector of JAK/STAT signalling and a regulator of Rho1 in Drosophila haematopoiesis. JAK/STAT signalling regulates many essential developmental processes including cell proliferation and haematopoiesis, whereas its inappropriate activation is associated with the majority of myeloproliferative neoplasias and numerous cancers. Furthermore, high levels of JAK/STAT pathway signalling have also been associated with enhanced metastatic invasion by cancerous cells. Strikingly, gain-of-function mutations in the single Drosophila JAK homologue, Hopscotch, result in haemocyte neoplasia, inappropriate differentiation and the formation of melanised haemocyte-derived 'tumour' masses; phenotypes that are partly orthologous to human gain-of-function JAK2-associated pathologies. Here we show that Gα73B, a novel JAK/STAT pathway target gene, is necessary for JAK/STAT-mediated tumour formation in flies. In addition, although Gα73B does not affect haemocyte differentiation, it does regulate haemocyte morphology and motility under non-pathological conditions. We show that Gα73B is required for constitutive, but not injury-induced, activation of Rho1 and for the localisation of Rho1 i**nto filop**odia upon haemocyte activation. Consistent with these results, we also show that Rho1 interacts genetically with JAK/STAT signalling, and that wild-type levels of Rho1 are necessary for tumour formation. Our findings link JAK/STAT transcriptional outputs, Gα73B activity and Rho1-dependent cytoskeletal rearrangements and cell motility, therefore connecting a pathway associated with cancer with a marker indicative of invasiveness. As such, we suggest a mechanism by which JAK/STAT pathway signalling may promote metastasis.
10. Polarity proteins and Rho GTPases cooperate to spatially organise epithelial actin-based protrusions. Different actin-filament-based structures co-exist in many cells. Here, we characterise dynamic actin-based protrusions that form at distinct positions within columnar epithelial cells, focusing on basal **filopodia** and sheet-like intermediate-level protrusions that extend between surrounding epithelial cells. Using a genetic analysis, we found that the form and distribution of these actin-filament-based structures depends on the activities of apical polarity determinants, not on basal integrin signalling. Bazooka/Par3 acts upstream of the RacGEF Sif/TIAM1 to limit **filopodia** to the basal domain, whereas Cdc42, aPKC and Par6 are required for normal protrusion morphology and dynamics. Downstream of these polarity regulators, Sif/TIAM1, Rac, SCAR and Arp2/3 complexes catalyse actin nucleation to generate lamellipodia and **filopodia**, whose form depends on the level of Rac activation. Taken together, these data reveal a role for Baz/Par3 in the establishment of an intercellular gradient of Rac inhibition, from apical to basal, and an intimate association between different apically concentrated Par proteins and Rho-family GTPases in the regulation of the distribution and structure of the polarised epithelial actin cytoskeleton.
11. Dynamic **filopodia** transmit intermittent Delta-Notch signaling to drive pattern refinement during lateral inhibition. The organization of bristles on the Drosophila notum has long served as a popular model of robust tissue patterning. During this process, membrane-tethered Delta activates intracellular Notch signaling in neighboring epithelial cells, which inhibits Delta expression. This induces lateral inhibition, yielding a pattern in which each Delta-expressing mechanosensory organ precursor cell in the epithelium is surrounded on all sides by cells with active Notch signaling. Here, we show that conventional models of Delta-Notch signaling cannot account for bristle spacing or the gradual refinement of this pattern. Instead, the pattern refinement we observe using live imaging is dependent upon dynamic, basal actin-based **filopodia** and can be quantitatively reproduced by simulations of lateral inhibition incorporating Delta-Notch signaling by transient **filopodia**l contacts between nonneighboring cells. Significantly, the intermittent signaling induced by these **filopodia**l dynamics generates a type of structured noise that is uniquely suited to the generation of well-ordered, tissue-wide epithelial patterns.
12. Loss of Drosophila melanogaster p21-activated kinase 3 suppresses defects in synapse structure and function caused by spastin mutations. Microtubules are dynamic structures that must elongate, disassemble, and be cleaved into smaller pieces for proper neuronal development and function. The AAA ATPase Spastin severs microtubules along their lengths and is thought to regulate the balance between long, stable filaments and shorter fragments that seed extension or are transported. In both Drosophila and humans, loss of Spastin function results in reduction of synaptic connections and disabling motor defects. To gain insight into how spastin is regulated, we screened the Drosophila melanogaster genome for deletions that modify a spastin overexpression phenotype, eye size reduction. One suppressor region deleted p21-activated kinase 3 (pak3), which encodes a member of the Pak family of actin-regulatory enzymes, but whose in vivo function is unknown. We show that pak3 mutants have only mild synaptic defects at the larval neuromuscular junction, but exhibit a potent genetic interaction with spastin mutations. Aberrant bouton morphology, microtubule distribution, and synaptic transmission caused by spastin loss of function are all restored to wild type when pak3 is simultaneously reduced. Neuronal overexpression of pak3 induces actin-rich thin projections, suggesting that it functions in vivo to promote **filopodia** during presynaptic terminal arborization. pak3 therefore regulates synapse development in vivo, and when mutated, suppresses the synaptic defects that result from spastin loss.
13. Dendritic **filopodia**, Ripped Pocket, NOMPC, and NMDARs contribute to the sense of touch in Drosophila larvae. Among the Aristotelian senses, the subcellular and molecular mechanisms involved in the sense of touch are the most poorly understood.We demonstrate that specialized sensory neurons, the class II and class III multidendritic (md) neurons, are gentle touch sensors of Drosophila larvae. Genetic silencing of these cells significantly impairs gentle touch responses, optogenetic activation of these cells triggers behavioral touch-like responses, and optical recordings from these neurons show that they respond to force. The class III neurons possess highly dynamic dendritic protrusions rich in F-actin. Genetic manipulations that alter actin dynamics indicate that the actin-rich protrusions (termed sensory **filopodia**) on the class III neurons are required for behavioral sensitivity to gentle touch. Through a genome-wide RNAi screen of ion channels, we identified Ripped Pocket (rpk), No Mechanoreceptor Potential C (nompC), and NMDA Receptors 1 and 2 (Nmdars) as playing critical roles in both behavioral responses to touch and in the formation of the actin-rich sensory **filopodia**. Consistent with this requirement, reporters for rpk and nompC show expression in the class III neurons. A genetic null allele of rpk confirms its critical role in touch responses.Output from class II and class III md neurons of the Drosophila larvae is necessary and sufficient for eliciting behavioral touch responses. These cells show physiological responses to force. Ion channels in several force-sensing gene families are required for behavioral sensitivity to touch and for the formation of the actin-rich sensory **filopodia**.
14. Dynamic analysis of **filopodia**l interactions during the zippering phase of Drosophila dorsal closure. Dorsal closure is a paradigm epithelial fusion episode that occurs late in Drosophila embryogenesis and leads to sealing of a midline hole by bonding of two opposing epithelial sheets. The leading edge epithelial cells express **filopodia** and fusion is dependent on interdigitation of these **filopodia** to prime formation of adhesions. Since the opposing epithelia are molecularly patterned there must exist some mechanism for accurately aligning the two sheets across this fusion seam. To address this, we generated a fly in which RFP-Moesin and GFP-Moesin are expressed in mutually exclusive stripes within each segment using the engrailed and patched promoters. We observe mutually exclusive interactions between the **filopodia** of engrailed and patched cells. Interactions between **filopodia** from matching cells leads to formation of tethers between them, and these tethers can pull misaligned epithelial sheets into alignment. **Filopodia**l matching also occurs during repair of laser wounds in the ventral epithelium, and so this behaviour is not restricted to leading edge cells during dorsal closure. Finally, we characterise the behaviour of a patched-expressing cell that we observe within the engrailed region of segments A1-A5, and provide evidence that this cell contributes to cell matching.
15. Ena drives invasive macrophage migration in Drosophila embryos. It is seldom the primary tumour that proves fatal in cancer, with metastasis the fundamental pathological process for disease progression. Upregulation of Mena, a member of the evolutionarily conserved Ena/VASP family of actin cytoskeletal regulators, promotes metastasis and invasive motility of breast cancer cells in vivo. To complement in vitro studies of Ena/VASP function in fibroblasts, we manipulated levels of Ena, the Drosophila homologue of Mena, in migrating embryonic macrophages (haemocytes). Consistent with data from fibroblasts in vitro, Ena localises to regions of actin dynamics within migrating haemocytes, stimulates lamellipodial dynamics and positively regulates the number and length of **filopodia**. However, whereas Ena overexpression in fibroblasts reduces migration speeds, overexpressing Ena in haemocytes leads to a dramatic increase in migration speeds, more closely resembling the increased motility of breast cancer cells that overexpress Mena. We provide evidence that this key difference is due to spatial constraints imposed on cells within the three-dimensional environment of the embryo; this might explain how Mena can be used to promote aggressive migratory behaviour during cancer progression.
16. GFP-moesin illuminates actin cytoskeleton dynamics in living tissue and demonstrates cell shape changes during morphogenesis in Drosophila. Moesin, ezrin, and radixin (MER) are components of the cortical actin cytoskeleton and membrane processes such as **filopodia** and microvilli. Their C-terminal tails contain an extended region that is predicted to be helical, an actin binding domain, and a region(s) that participates in self-association. We engineered an in vivo fluorescent actin binding protein (GFP-moe) by joining sequences that encode the jellyfish green fluorescent protein (GFP) to sequences that encode the C-terminal end of the sole Drosophila MER homolog, moesin [Moesin-like gene product, referred to previously as the D17 MER-like protein; Edwards et al., 1994, Proc. Natl. Acad. Sci. USA 91, 4589], and Dmoesin [McCartney and Fehon, 1996, J. Cell Biol. 133, 843]. Transgenic flies expressing this fusion protein under control of the hsp70 promoter were generated and used for analysis of cell shape changes during morphogenesis of various developmental stages and tissues. Following heat shock, high levels of stable fusion protein are produced by all somatic tissues. GFP-moe localizes to the cortical actin cytoskeleton, providing a strong in vivo marker for cell shape and pattern during epithelial morphogenesis. The protein also becomes highly enriched in pseudopods, microvilli, axons, denticles, the border cell process, and other membrane projections, potentially by binding to endogenous moesin as well as actin. We show that GFP-moe can be used to examine the development and behavior of these dynamic structures in live specimens. We observe a bright green fluorescent, presumably actin-rich, polar cell proboscis that inserts itself into the forming micropyle and appears to maintain an opening for sperm passage around which the chorion is formed. We also confirm the existence of an actin-rich purse string at the leading edge of the lateral epidermis and provide a dynamic analysis of its behavior as it migrates during dorsal closure. Observations of embryos, larvae, and pupae show that GFP-moe is also useful for labeling the developing nervous system and will be a good general marker of dynamic cell behavior during morphogenesis in live tissues and demonstrate that fusion of a subcellular localization signal to GFP greatly increases its utility as a cell marker.
17. Fascin promotes **filopodia** formation independent of its role in actin bundling. Fascin is an evolutionarily conserved actin-binding protein that plays a key role in forming **filopodia**. It is widely thought that this function involves fascin directly bundling actin filaments, which is controlled by an N-terminal regulatory serine residue. In this paper, by studying cellular processes in Drosophila melanogaster that require fascin activity, we identify a regulatory residue within the C-terminal region of the protein (S289). Unexpectedly, although mutation (S289A) of this residue disrupted the actin-bundling capacity of fascin, fascin S289A fully rescued **filopodia** formation in fascin mutant flies. Live imaging of migrating macrophages in vivo revealed that this mutation restricted the localization of fascin to the distal ends of **filopodia**. The corresponding mutation of human fascin (S274) similarly affected its interaction with actin and altered **filopodia** dynamics within carcinoma cells. These data reveal an evolutionarily conserved role for this regulatory region and unveil a function for fascin, uncoupled from actin bundling, at the distal end of **filopodia**.
18. The importance of structured noise in the generation of self-organizing tissue patterns through contact-mediated cell-cell signalling. Lateral inhibition provides the basis for a self-organizing patterning system in which distinct cell states emerge from an otherwise uniform field of cells. The development of the microchaete bristle pattern on the notum of the fruitfly, Drosophila melanogaster, has long served as a popular model of this process. We recently showed that this bristle pattern depends upon a population of dynamic, basal actin-based **filopodia**, which span multiple cell diameters. These protrusions establish transient signalling contacts between non-neighbouring cells, generating a type of structured noise that helps to yield a well-ordered and spaced pattern of bristles. Here, we develop a general model of protrusion-based patterning to analyse the role of noise in this process. Using a simple asynchronous cellular automata rule-based model we show that this type of structured noise drives the gradual refinement of lateral inhibition-mediated patterning, as the system moves towards a stable configuration in which cells expressing the inhibitory signal are near-optimally packed. By analysing the effects of introducing thresholds required for signal detection in this model of lateral inhibition, our study shows how **filopodia**-mediated cell-cell communication can generate complex patterns of spots and stripes, which, in the presence of signalling noise, align themselves across a patterning field. Thus, intermittent protrusion-based signalling has the potential to yield robust self-organizing tissue-wide patterns without the need to invoke diffusion-mediated signalling.
19. Knot/Collier and cut control different aspects of dendrite cytoskeleton and synergize to define final arbor shape. In a complex nervous system, neuronal functional diversity is reflected in the wide variety of dendritic arbor shapes. Different neuronal classes are defined by class-specific transcription factor combinatorial codes. We show that the combination of the transcription factors Knot and Cut is particular to Drosophila class IV dendritic arborization (da) neurons. Knot and Cut control different aspects of the dendrite cytoskeleton, promoting microtubule- and actin-based dendritic arbors, respectively. Knot delineates class IV arbor morphology by simultaneously synergizing with Cut to promote complexity and repressing Cut-mediated promotion of dendritic **filopodia**/spikes. Knot increases dendritic arbor outgrowth through promoting the expression of Spastin, a microtubule-severing protein disrupted in autosomal dominant hereditary spastic paraplegia (AD-HSP). Knot and Cut may modulate cellular mechanisms that are conserved between Drosophila and vertebrates. Hence, this study gives significant general insight into how multiple transcription factors combine to control class-specific dendritic arbor morphology through controlling different aspects of the cytoskeleton.

### [Mm]igration

#### PubMed

1. Drosophila Rho-kinase (DRok) is required for tissue morphogenesis in diverse compartments of the egg chamber during oogenesis. The Rho-kinases are widely utilized downstream targets of the activated Rho GTPase that have been directly implicated in many aspects of Rho-dependent effects on F-actin assembly, acto-myosin contractility, and microtubule stability, and consequently play an essential role in regulating cell shape, **migration**, polarity, and division. We have determined that the single closely related Drosophila Rho-kinase ortholog, DRok, is required for several aspects of oogenesis, including maintaining the integrity of the oocyte cortex, actin-mediated tethering of nurse cell nuclei, "dumping" of nurse cell contents into the oocyte, establishment of oocyte polarity, and the trafficking of oocyte yolk granules. These defects are associated with abnormalities in DRok-dependent actin dynamics and appear to be mediated by multiple downstream effectors of activated DRok that have previously been implicated in oogenesis. DRok regulates at least one of these targets, the membrane cytoskeletal cross-linker DMoesin, via a direct phosphorylation that is required to promote localization of DMoesin to the oocyte cortex. The collective oogenesis defects associated with DRok deficiency reveal its essential role in multiple aspects of proper oocyte formation and suggest that DRok defines a novel class of oogenesis determinants that function as key regulators of several distinct actin-dependent processes required for proper tissue morphogenesis.
2. Control of lateral **migration** and germ cell elimination by the Drosophila melanogaster lipid phosphate phosphatases Wunen and Wunen 2. In most organisms, primordial germ cells (PGCs) arise far from the region where somatic gonadal precursors (SGPs) are specified. Although PGCs in general originate as a single cluster of cells, the somatic parts of the gonad form on each site of the embryo. Thus, to reach the gonad, PGCs not only migrate from their site of origin but also split into two groups. Taking advantage of high-resolution real-time imaging, we show that in Drosophila melanogaster PGCs are polarized and migrate directionally toward the SGPs, avoiding the midline. Unexpectedly, neither PGC attractants synthesized in the SGPs nor known midline repellents for axon guidance were required to sort PGCs bilaterally. Repellent activity provided by wunen (wun) and wunen-2 (wun-2) expressed in the central nervous system, however, is essential in this **migration** process and controls PGC survival. Our results suggest that expression of wun/wun-2 repellents along the migratory paths provides faithful control over the sorting of PGCs into two gonads and eliminates PGCs left in the middle of the embryo.
3. Tissue-specific laminin expression facilitates integrin-dependent association of the embryonic wing disc with the trachea in Drosophila. The interaction of heterologous tissues involves cell adhesion mediated by the extracellular matrix and its receptor integrins. The Drosophila wing disc is an ectodermal invagination that contacts specific tracheal branches at the basolateral cell surface. We show that an alpha subunit of laminin, encoded by wing blister (wb), is essential for the establishment of the interaction between the wing and trachea. During embryogenesis, wing disc cells present Wb at their basolateral surface and extend posteriorly, expanding their association to more posteriorly located tracheal branches. These migratory processes are impaired in the absence of the trachea, Wb, or integrins. Time-lapse and transmission electron microscopy analyses suggest that Wb facilitates integrin-dependent contact over a large surface and controls the cellular behavior of the wing cells, including their exploratory filopodial activity. Our data identify Wb laminin as an extracellular matrix ligand that is essential for integrin-dependent cellular **migration** in Drosophila.
4. IKK epsilon regulates F actin assembly and interacts with Drosophila IAP1 in cellular morphogenesis. Differentiated cells assume complex shapes through polarized cell **migration** and growth. These processes require the restricted organization of the actin cytoskeleton at limited subcellular regions. IKK epsilon is a member of the IkappaB kinase family, and its developmental role has not been clear. Drosophila IKK epsilon was localized to the ruffling membrane of cultured cells and was required for F actin turnover at the cell margin. In IKK epsilon mutants, tracheal terminal cells, bristles, and arista laterals, which require accurate F actin assembly for their polarized elongation, all exhibited aberrantly branched morphology. These phenotypes were sensitive to a change in the dosage of Drosophila inhibitor of apoptosis protein 1 (DIAP1) and the caspase DRONC without apparent change in cell viability. In contrast to this, hyperactivation of IKK epsilon destabilized F actin-based structures. Expression of a dominant-negative form of IKK epsilon increased the amount of DIAP1. The results suggest that at the physiological level, IKK epsilon acts as a negative regulator of F actin assembly and maintains the fidelity of polarized elongation during cell morphogenesis. This IKK epsilon function involves the negative regulation of the nonapoptotic activity of DIAP1.
5. RhoL controls invasion and Rap1 localization during immune cell trans**migration** in Drosophila. Human immune cells have to penetrate an endothelial barrier during their beneficial pursuit of infection and their destructive infiltration of tissues in autoimmune diseases. This trans**migration** requires Rap1 GTPase to activate integrin affinity. We define a new model system for this process by demonstrating, with live imaging and genetics, that during embryonic development Drosophila melanogaster immune cells penetrate an epithelial, Drosophila E-cadherin (DE-cadherin)-based tissue barrier. A mutant in RhoL, a GTPase homologue that is specifically expressed in haemocytes, blocks this invasive step but not other aspects of guided **migration**. RhoL mediates integrin adhesion caused by Drosophila Rap1 overexpression and moves Rap1 away from a concentration in the cytoplasm to the leading edge during invasive **migration**. These findings indicate that a programmed migratory step during Drosophila development bears striking molecular similarities to vertebrate immune cell trans**migration** during inflammation, and identify RhoL as a new regulator of invasion, adhesion and Rap1 localization. Our work establishes the utility of Drosophila for identifying novel components of immune cell trans**migration** and for understanding the in vivo interplay of immune cells with the barriers they penetrate.
6. Abelson kinase regulates epithelial morphogenesis in Drosophila. Activation of the nonreceptor tyrosine kinase Abelson (Abl) contributes to the development of leukemia, but the complex roles of Abl in normal development are not fully understood. Drosophila Abl links neural axon guidance receptors to the cytoskeleton. Here we report a novel role for Drosophila Abl in epithelial cells, where it is critical for morphogenesis. Embryos completely lacking both maternal and zygotic Abl die with defects in several morphogenetic processes requiring cell shape changes and cell **migration**. We describe the cellular defects that underlie these problems, focusing on dorsal closure as an example. Further, we show that the Abl target Enabled (Ena), a modulator of actin dynamics, is involved with Abl in morphogenesis. We find that Ena localizes to adherens junctions of most epithelial cells, and that it genetically interacts with the adherens junction protein Armadillo (Arm) during morphogenesis. The defects of abl mutants are strongly enhanced by heterozygosity for shotgun, which encodes DE-cadherin. Finally, loss of Abl reduces Arm and alpha-catenin accumulation in adherens junctions, while having little or no effect on other components of the cytoskeleton or cell polarity machinery. We discuss possible models for Abl function during epithelial morphogenesis in light of these data.
7. Following the 'tracks': Tramtrack69 regulates epithelial tube expansion in the Drosophila ovary through Paxillin, Dynamin, and the homeobox protein Mirror. Epithelial tubes are the infrastructure for organs and tissues, and tube morphogenesis requires precise orchestration of cell signaling, shape, **migration**, and adhesion. Follicle cells in the Drosophila ovary form a pair of epithelial tubes whose lumens act as molds for the eggshell respiratory filaments, or dorsal appendages (DAs). DA formation is a robust and accessible model for studying the patterning, formation, and expansion of epithelial tubes. Tramtrack69 (TTK69), a transcription factor that exhibits a variable embryonic DNA-binding preference, controls DA lumen volume and shape by promoting tube expansion; the tramtrack mutation twin peaks (ttk(twk)) reduces TTK69 levels late in oogenesis, inhibiting this expansion. Microarray analysis of wild-type and ttk(twk) ovaries, followed by in situ hybridization and RNAi of candidate genes, identified the Phospholipase B-like protein Lamina ancestor (LAMA), the scaffold protein Paxillin, the endocytotic regulator Shibire (Dynamin), and the homeodomain transcription factor Mirror, as TTK69 effectors of DA-tube expansion. These genes displayed enriched expression in DA-tube cells, except lama, which was expressed in all follicle cells. All four genes showed reduced expression in ttk(twk) mutants and exhibited RNAi phenotypes that were enhanced in a ttk(twk)/+ background, indicating ttk(twk) genetic interactions. Although previous studies show that Mirror patterns the follicular epithelium prior to DA tubulogenesis, we show that Mirror has an independent, novel role in tube expansion, involving positive regulation of Paxillin. Thus, characterization of ttk(twk)-differentially expressed genes expands the network of TTK69 effectors, identifies novel epithelial tube-expansion regulators, and significantly advances our understanding of this vital developmental process.
8. Molecular requirements for actin-based lamella formation in Drosophila S2 cells. Cell **migration** occurs through the protrusion of the actin-enriched lamella. Here, we investigated the effects of RNAi depletion of approximately 90 proteins implicated in actin function on lamella formation in Drosophila S2 cells. Similar to in vitro reconstitution studies of actin-based Listeria movement, we find that lamellae formation requires a relatively small set of proteins that participate in actin nucleation (Arp2/3 and SCAR), barbed end capping (capping protein), filament depolymerization (cofilin and Aip1), and actin monomer binding (profilin and cyclase-associated protein). Lamellae are initiated by parallel and partially redundant signaling pathways involving Rac GTPases and the adaptor protein Nck, which stimulate SCAR, an Arp2/3 activator. We also show that RNAi of three proteins (kette, Abi, and Sra-1) known to copurify with and inhibit SCAR in vitro leads to SCAR degradation, revealing a novel function of this protein complex in SCAR stability. Our results have identified an essential set of proteins involved in actin dynamics during lamella formation in Drosophila S2 cells.
9. The Ste20 kinase misshapen is essential for the invasive behaviour of ovarian epithelial cells in Drosophila. Stationary-to-migratory transitions of epithelial cells have a key role in development and tumour progression. Border cell **migration** is a powerful system in which to investigate this transition in living organisms. Here, we identify the Ste20-like kinase misshapen (msn) as a novel regulator of border-cell **migration** in Drosophila. Expression of msn in border cells is independent of the transcription factor slow border cells and of inputs from all pathways that are known to control border-cell **migration**. The msn gene functions to modulate the levels and/or distribution of Drosophila E-cadherin to promote the invasive migratory behaviour of border cells.
10. Diaphanous regulates myosin and adherens junctions to control cell contractility and protrusive behavior during morphogenesis. Formins are key regulators of actin nucleation and elongation. Diaphanous-related formins, the best-known subclass, are activated by Rho and play essential roles in cytokinesis. In cultured cells, Diaphanous-related formins also regulate cell adhesion, polarity and microtubules, suggesting that they may be key regulators of cell shape change and **migration** during development. However, their essential roles in cytokinesis hamper our ability to test this hypothesis. We used loss- and gain-of-function approaches to examine the role of Diaphanous in Drosophila morphogenesis. We found that Diaphanous has a dynamic expression pattern consistent with a role in regulating cell shape change. We used constitutively active Diaphanous to examine its roles in morphogenesis and its mechanisms of action. This revealed an unexpected role in regulating myosin levels and activity at adherens junctions during cell shape change, suggesting that Diaphanous helps coordinate adhesion and contractility of the underlying actomyosin ring. We tested this hypothesis by reducing Diaphanous function, revealing striking roles in stabilizing adherens junctions and inhibiting cell protrusiveness. These effects also are mediated through coordinated effects on myosin activity and adhesion, suggesting a common mechanism for Diaphanous action during morphogenesis.
11. Drosophila Yurt is a new protein-4.1-like protein required for epithelial morphogenesis. Proteins of the 4.1 family play a key role in the integrity of the cytoskeleton and in epithelial tissue movement, as shown by the disruption of the actin cytoskeleton in human erythrocytes caused by genetic loss of protein 4.1, and the failure of epithelial tissue **migration** during Drosophila embryogenesis caused by genetic loss of the 4.1 homolog Coracle. Here we report the genetic characterization of Yurt, a novel protein 4.1 family member in Drosophila that is associated with the plasma membrane of epithelial cells. Homozygous loss-of-function mutations in the yurt gene cause failure of germ-band retraction, dorsal closure, and head involution, associated with degeneration of the amnioserosa and followed by embryonic lethality. A mammalian homolog of Yurt is up-regulated in metastatic melanoma cells. These novel cytoskeletal proteins appear to play important roles in epithelial cell movements and in the morphogenetic tissue changes that depend on them.
12. The role of LamininB2 (LanB2) during mesoderm differentiation in Drosophila. In Drosophila, four genes encode for laminin subunits and the formation of two laminin heterotrimers has been postulated. We report the identification of mutations in the Drosophila LamininB2 (LanB2) gene that encodes for the only laminin γ subunit and is found in both heterotrimers. We describe their effects on embryogenesis, in particular the differentiation of visceral tissues with respect to the ECM. Analysis of mesoderm endoderm interaction indicates disrupted basement membranes and defective endoderm **migratio**n, which finally interferes with visceral myotube stretching. Extracellular deposition of laminin is blocked due to the loss of the LanB2 subunit, resulting in an abnormal distribution of ECM components. Our data, concerning the different function of both trimers during organogenesis, suggest that these trimers might act in a cumulative way and probably at multiple steps during ECM assembly. We also observed genetic interactions with kon-tiki and thrombospondin, indicating a role for laminin during muscle attachment.
13. Cellular and molecular mechanisms of single and collective cell **migration**s in Drosophila: themes and variations. The process of cell **migration** is essential throughout life, driving embryonic morphogenesis and ensuring homeostasis in adults. Defects in cell **migration** are a major cause of human disease, with excessive **migration** causing autoimmune diseases and cancer metastasis, whereas reduced capacity for **migration** leads to birth defects and immunodeficiencies. Myriad studies in vitro have established a consensus view that cell **migration**s require cell polarization, Rho GTPase-mediated cytoskeletal rearrangements, and myosin-mediated contractility. However, in vivo studies later revealed a more complex picture, including the discovery that cells migrate not only as single units but also as clusters, strands, and sheets. In particular, the role of E-Cadherin in cell motility appears to be more complex than previously appreciated. Here, we discuss recent advances achieved by combining the plethora of genetic tools available to the Drosophila geneticist with live imaging and biophysical techniques. Finally, we discuss the emerging themes such studies have revealed and ponder the puzzles that remain to be solved.
14. Distinct roles for two receptor tyrosine kinases in epithelial branching morphogenesis in Drosophila. Branching morphogenesis is a widespread mechanism used to increase the surface area of epithelial organs. Many signaling systems steer development of branched organs, but it is still unclear which cellular processes are regulated by the different pathways. We have used the development of the air sacs of the dorsal thorax of Drosophila to study cellular events and their regulation via cell-cell signaling. We find that two receptor tyrosine kinases play important but distinct roles in air sac outgrowth. Fgf signaling directs cell **migration** at the tip of the structure, while Egf signaling is instrumental for cell division and cell survival in the growing epithelial structure. Interestingly, we find that Fgf signaling requires Ras, the Mapk pathway, and Pointed to direct **migration**, suggesting that both cytoskeletal and nuclear events are downstream of receptor activation. Ras and the Mapk pathway are also needed for Egf-regulated cell division/survival, but Pointed is dispensable.
15. Hedgehog is a positive regulator of FGF signalling during embryonic tracheal cell **migration**. Cell **migration** is a widespread and complex process that is crucial for morphogenesis and for the underlying invasion and metastasis of human cancers. During **migration**, cells are steered toward target sites by guidance molecules that induce cell direction and movement through complex intracellular mechanisms. The spatio-temporal regulation of the expression of these guidance molecules is of extreme importance for both normal morphogenesis and human disease. One way to achieve this precise regulation is by combinatorial inputs of different transcription factors. Here we used Drosophila melanogaster mutants with **migration** defects in the ganglionic branches of the tracheal system to further clarify guidance regulation during cell **migration**. By studying the cellular consequences of overactivated Hh signalling, using ptc mutants, we found that Hh positively regulates Bnl/FGF levels during embryonic stages. Our results show that Hh modulates cell **migration** non-autonomously in the tissues surrounding the action of its activity. We further demonstrate that the Hh signalling pathway regulates bnl expression via Stripe (Sr), a zinc-finger transcription factor with homology to the Early Growth Response (EGR) family of vertebrate transcription factors. We propose that Hh modulates embryonic cell **migration** by participating in the spatio-temporal regulation of bnl expression in a permissive mode. By doing so, we provide a molecular link between the activation of Hh signalling and increased chemotactic responses during cell **migration**.
16. Cortactin modulates cell **migration** and ring canal morphogenesis during Drosophila oogenesis. Cortactin is a Src substrate that interacts with F-actin and can stimulate actin polymerization by direct interaction with the Arp2/3 complex. We have isolated complete loss-of-function mutants of the single Drosophila cortactin gene. Mutants are viable and fertile, showing that cortactin is not an essential gene. However, cortactin mutants show distinct defects during oogenesis. During oogenesis, Cortactin protein is enriched at the F-actin rich ring canals in the germ line, and in migrating border cells. In cortactin mutants, the ring canals are smaller than normal. A similar phenotype has been observed in Src64 mutants and in mutants for genes encoding Arp2/3 complex components, supporting that these protein products act together to control specific processes in vivo. Cortactin mutants also show impaired border cell **migration**. This invasive cell **migration** is guided by Drosophila EGFR and PDGF/VEGF receptor (PVR). We find that accumulation of Cortactin protein is positively regulated by PVR. Also, overexpression of Cortactin can by itself induce F-actin accumulation and ectopic filopodia formation in epithelial cells. We present evidence that Cortactin is one of the factors acting downstream of PVR and Src to stimulate F-actin accumulation. Cortactin is a minor contributor in this regulation, consistent with the cortactin gene not being essential for development.
17. An ABC transporter controls export of a Drosophila germ cell attractant. Directed cell **migration**, which is critical for embryonic development, leukocyte trafficking, and cell metastasis, depends on chemoattraction. 3-hydroxy-3-methylglutaryl coenzyme A reductase regulates the production of an attractant for Drosophila germ cells that may itself be geranylated. Chemoattractants are commonly secreted through a classical, signal peptide-dependent pathway, but a geranyl-modified attractant would require an alternative pathway. In budding yeast, pheromones produced by a-cells are farnesylated and secreted in a signal peptide-independent manner, requiring the adenosine triphosphate-binding cassette (ABC) transporter Ste6p. Here we show that Drosophila germ cell **migration** uses a similar pathway, demonstrating that invertebrate germ cells, like yeast cells, are attracted to lipid-modified peptides. Components of this unconventional export pathway are highly conserved, suggesting that this pathway may control the production of similarly modified chemoattractants in organisms ranging from yeast to humans.
18. Essential roles of myosin phosphatase in the maintenance of epithelial cell integrity of Drosophila imaginal disc cells. Reorganization of the actin cytoskeleton and contraction of actomyosin play pivotal roles in controlling cell shape changes and motility in epithelial morphogenesis. Dephosphorylation of the myosin regulatory light chain (MRLC) by myosin phosphatase is one of the key events involved. Allelic combinations producing intermediate strength mutants of the Drosophila myosin-binding subunit (DMBS) of myosin phosphatase showed imaginal discs with multilayered disrupted morphologies, and extremely mislocated cells, suggesting that DMBS is required to maintain proper epithelial organization. Clonal analyses revealed that DMBS null mutant cells appear to retract basally and localization of apical junction markers such as DE-cadherin is indetectable in most cells, whereas phosphorylated MRLC and F-actin become heavily concentrated apically, indicating misconfiguration of the apical cytoskeleton. In agreement with these findings, DMBS was found to concentrate at the apical domain suggesting its function is localized. Phenotypes similar to DMBS mutants including increased **migration** of cells were obtained by overexpressing the constitutive active form of MRLC or Rho-associated kinase signifying that the phenotypes are indeed caused through activation of Myosin II. The requirement of DMBS for the integrity of static epithelial cells in imaginal discs suggests that the regulation of Myosin II by DMBS has a role more general than its previously demonstrated functions in morphogenetic events.
19. Live imaging of wound inflammation in Drosophila embryos reveals key roles for small GTPases during in vivo cell **migration**. Aa robust inflammatory response to tissue damage and infection is conserved across almost all animal phyla. Neutrophils and macrophages, or their equivalents, are drawn to the wound site where they engulf cell and matrix debris and release signals that direct components of the repair process. This orchestrated cell **migration** is clinically important, and yet, to date, leukocyte chemotaxis has largely been studied in vitro. Here, we describe a genetically tractable in vivo wound model of inflammation in the Drosophila melanogaster embryo that is amenable to cinemicroscopy. For the first time, we are able to examine the roles of Rho-family small GTPases during inflammation in vivo and show that Rac-mediated lamellae are essential for hemocyte motility and Rho signaling is necessary for cells to retract from sites of matrix- and cell-cell contacts. Cdc42 is necessary for maintaining cellular polarity and yet, despite in vitro evidence, is dispensable for sensing and crawling toward wound cues.
20. Integrins are required for cardioblast polarisation in Drosophila. The formation of a tubular organ, such as the heart, requires the communication of positional and polarity signals between migratory cells. Key to this process is the establishment of a new luminal domain on the cell surface, generally from the apical domain of a migratory cell. This domain will also acquire basal properties, as it will produce a luminal extracellular matrix. Integrin receptors are the primary means of cell adhesion and adhesion signaling with the extracellular matrix. Here we characterise the requirement of Integrins in a genetic model of vasculogenesis, the formation of the heart in Drosophila.As with vertebrates, the Drosophila heart arises from lateral mesoderm that migrates medially to meet their contralateral partners, to then assemble a midline vessel. During **migration**, Integrins are among the first proteins restricted to the presumptive luminal domain of cardioblasts. Integrins are required for normal levels of leading edge membrane motility. Apical accumulation of Integrins is enhanced by Robo, and reciprocally, apicalisation of luminal factors like Slit and Robo requires Integrin function. Integrins may provide a template for the formation of a lumen by stabilising lumen factors like Robo. Subsequent to **migration**, Integrin is required for normal cardioblast alignment and lumen formation. This phenotype is most readily modified by other mutations that affect adhesion, such as Talin and extracellular matrix ligands.Our findings reveal an instructive role for Integrins in communicating polarising information to cells during **migration**, and during transition to an epithelial tube structure.
21. Excessive Myosin activity in mbs mutants causes photoreceptor movement out of the Drosophila eye disc epithelium. Neuronal cells must extend a motile growth cone while maintaining the cell body in its original position. In migrating cells, myosin contraction provides the driving force that pulls the rear of the cell toward the leading edge. We have characterized the function of myosin light chain phosphatase, which down-regulates myosin activity, in Drosophila photoreceptor neurons. Mutations in the gene encoding the myosin binding subunit of this enzyme cause photoreceptors to drop out of the eye disc epithelium and move toward and through the optic stalk. We show that this phenotype is due to excessive phosphorylation of the myosin regulatory light chain Spaghetti squash rather than another potential substrate, Moesin, and that it requires the nonmuscle myosin II heavy chain Zipper. Myosin binding subunit mutant cells continue to express apical epithelial markers and do not undergo ectopic apical constriction. In addition, mutant cells in the wing disc remain within the epithelium and differentiate abnormal wing hairs. We suggest that excessive myosin activity in photoreceptor neurons may pull the cell bodies toward the growth cones in a process resembling normal cell **migration**.
22. Tip-cell **migration** controls stalk-cell intercalation during Drosophila tracheal tube elongation. Branching morphogenesis remodels epithelial tissues into tubular networks. This process is crucial to many organs, from the insect trachea to the vertebrate vasculature. Although Drosophila tracheal development has been well characterized morphologically and genetically, very little is known about the forces involved during morphogenesis. The repertoire of cell behaviors underlying tracheal primary branch remodeling is limited to cell **migration**, cell-shape changes, and stalk-cell intercalation (SCI), a process in which cells insert in between cells previously in contact with each other.Here, we identify the major forces that contribute to tracheal primary branch remodeling by using genetic and microsurgery experiments. As the tip cells migrate, they elongate the branches and create a tensile stress. This tensile stress triggers SCI, which, in turn, allows the branches to further elongate.The mechanism that we describe contrasts with "convergent extension by cell intercalation" acting during Drosophila germ band extension (GBE), where cell intercalation is the cause of epithelium elongation. Surprisingly, in tracheal branches, one or two leading cells produce enough mechanical power to intercalate many lagging cells. This may apply to other tubular networks.
23. Cellular and molecular mechanisms of border cell **migration** analyzed using time-lapse live-cell imaging. Border cells in the Drosophila ovary originate within an epithelium, detach from it, invade neighboring nurse cells, and migrate as a coherent cluster. This **migration** has served as a useful genetic model for understanding epithelial cell motility. The prevailing model of growth factor-mediated chemotaxis in general, and of border cells in particular, posits that receptor activation promotes cellular protrusion at the leading edge. Here we report the time-lapse video imaging of border cell **migration**, allowing us to test this model. Reducing the activities of the guidance receptors EGFR and PVR did not result in the expected inhibition of protrusion, but instead resulted in protrusion in all directions. In contrast, reduction in Notch activity resulted in failure of the cells to detach from the epithelium without affecting direction sensing. These observations provide new insight into the cellular dynamics and molecular mechanisms of cell **migration** in vivo.
24. Time-lapse imaging reveals stereotypical patterns of Drosophila midline glial **migration**. The Drosophila CNS midline glia (MG) are multifunctional cells that ensheath and provide trophic support to commissural axons, and direct embryonic development by employing a variety of signaling molecules. These glia consist of two functionally distinct populations: the anterior MG (AMG) and posterior MG (PMG). Only the AMG ensheath axon commissures, whereas the function of the non-ensheathing PMG is unknown. The Drosophila MG have proven to be an excellent system for studying glial proliferation, cell fate, apoptosis, and axon-glial interactions. However, insight into how AMG migrate and acquire their specific positions within the axon-glial scaffold has been lacking. In this paper, we use time-lapse imaging, single-cell analysis, and embryo staining to comprehensively describe the proliferation, **migration**, and apoptosis of the Drosophila MG. We identified 3 groups of MG that differed in the trajectories of their initial inward **migration**: AMG that migrate inward and to the anterior before undergoing apoptosis, AMG that migrate inward and to the posterior to ensheath commissural axons, and PMG that migrate inward and to the anterior to contact the commissural axons before undergoing apoptosis. In a second phase of their **migration**, the surviving AMG stereotypically migrated posteriorly to specific positions surrounding the commissures, and their final position was correlated with their location prior to **migration**. Most noteworthy are AMG that migrated between the commissures from a ventral to a dorsal position. Single-cell analysis indicated that individual AMG possessed wide-ranging and elaborate membrane extensions that partially ensheathed both commissures. These results provide a strong foundation for future genetic experiments to identify mutants affecting MG development, particularly in guidance cues that may direct **migration**. Drosophila MG are homologous in structure and function to the glial-like cells that populate the vertebrate CNS floorplate, and study of Drosophila MG will provide useful insights into floorplate development and function.
25. Genetic control of cell intercalation during tracheal morphogenesis in Drosophila. Branching morphogenesis transforms an epithelial sheet into a tubular network with distinct features regarding the length and diameter of individual tubes. Branching is controlled by several signaling pathways, but the molecular consequences of these pathways in the responding cells are poorly understood.We have undertaken a detailed characterization of cell rearrangements during tracheal branching morphogenesis in Drosophila embryos with a GFP fusion protein labeling the adherens junctions (AJs) and high-resolution live imaging. To analyze the branching process at the cellular level, we further developed an imaging approach that allows us to follow single cells during the branching process. We find that controlled cell intercalation, which requires extensive AJ remodeling, is key to the formation of tracheal branches of different cellular complexities. In particular, most branches consist of tubes with individual cells wrapped around the lumen. These branches form through cell intercalation, which requires the transformation of most of the initial intercellular AJs into autocellular AJs. We propose a step-wise model explaining how this AJ remodeling occurs and use this model to better understand defects in various mutants. We find that Dpp and Wnt signaling control cell intercalation by regulating the expression of Spalt, a zinc finger transcription factor; Spalt inhibits intercalation, leading to the formation of large, multicellular tubes.Tracheal morphogenesis is regulated by an interplay of different signaling systems that control cell **migration** and cell intercalation, respectively. Only the combined action of these signaling systems allows efficient branch elongation and the formation of morphologically distinct branches.
26. Fusion of circular and longitudinal muscles in Drosophila is independent of the endoderm but further visceral muscle differentiation requires a close contact between mesoderm and endoderm. In this study we describe the morphological and genetic analysis of the Drosophila mutant gürtelchen (gurt). gurt was identified by screening an EMS collection for novel mutations affecting visceral mesoderm development and was named after the distinct belt shaped visceral phenotype. Interestingly, determination of visceral cell identities and subsequent visceral myoblast fusion is not affected in mutant embryos indicating a later defect in visceral development. gurt is in fact a new huckebein (hkb) allele and as such exhibits nearly complete loss of endodermal derived structures. Targeted ablation of the endodermal primordia produces a phenotype that resembles the visceral defects observed in huckebein(gürtelchen) (hkb(gurt)) mutant embryos. It was shown previously that visceral mesoderm development requires complex interactions between visceral myoblasts and adjacent tissues. Signals from the neighbouring somatic myoblasts play an important role in cell type determination and are a prerequisite for visceral muscle fusion. Furthermore, the visceral mesoderm is known to influence endoderma**l migrati**on and midgut epithelium formation. Our analyses of the visceral phenotype of hkb(gurt) mutant embryos reveal that the adjacent endoderm plays a critical role in the later stages of visceral muscle development, and is required for visceral muscle elongation and outgrowth after proper myoblast fusion.
27. The transmembrane protein Kon-tiki couples to Dgrip to mediate myotube targeting in Drosophila. Directed cell **migration** and target recognition are critical for the development of both the nervous and muscular systems. Molecular mechanisms that control these processes in the nervous system have been intensively studied, whereas those that act during muscle development are still largely uncharacterized. Here we identify a transmembrane protein, Kon-tiki (Kon), that mediates myotube target recognition in the Drosophila embryo. Kon is expressed in a specific subset of myotubes and is required autonomously for these myotubes to recognize their tendon cell targets and to establish a stable connection. Kon is enriched at myotube tips during targeting and signals through the intracellular adaptor Dgrip in a conserved molecular pathway. Forced overexpression of Kon stimulates muscle motility. We propose that Kon promotes directed myotube **migration** and transduces a target-derived signal that initiates the formation of a stable connection.
28. Left-right asymmetric morphogenesis of the anterior midgut depends on the activation of a non-muscle myosin II in Drosophila. Many animals exhibit stereotypical left-right (LR) asymmetry in their internal organs. The mechanisms of LR axis formation required for the subsequent LR asymmetric development are well understood, especially in some vertebrates. However, the molecular mechanisms underlying LR asymmetric morphogenesis, particularly how mechanical force is integrated into the LR asymmetric morphogenesis of organs, are poorly understood. Here, we identified zipper (zip), encoding a Drosophila non-muscle myosin II (myosin II) heavy chain, as a gene required for LR asymmetric development of the embryonic anterior midgut (AMG). Myosin II is known to directly generate mechanical force in various types of cells during morphogenesis and cell **migration**. We found that myosin II was involved in two events in the LR asymmetric development of the AMG. First, it introduced an LR bias to the directional position of circular visceral muscle (CVMU) cells, which externally cover the midgut epithelium. Second, it was required for the LR-biased rotation of the AMG. Our results suggest that myosin II in CVMU cells plays a crucial role in generating the force leading to LR asymmetric morphogenesis. Taken together with previous studies in vertebrates, the involvement of myosin II in LR asymmetric morphogenesis might be conserved evolutionarily.
29. Sequoia establishes tip-cell number in Drosophila trachea by regulating FGF levels. Competition and determination of leading and trailing cells during collective cell **migration** is a widespread phenomenon in development, wound healing and tumour invasion. Here, we analyse this issue during in vivo ganglionic branch cell **migration** in the Drosophila tracheal system. We identify Sequoia (Seq) as a negative transcriptional regulator of Branchless (Bnl), a Drosophila FGF homologue, and observe that modulation of Bnl levels determines how many cells will lead this migrating cluster, regardless of Notch lateral inhibition. Our results show that becoming a tip cell does not prevent others in the branch taking the same position, suggesting that leader choice does not depend only on sensing relative amounts of FGF receptor activity.
30. Emerging role for ERM proteins in cell adhesion and **migration**. The highly related ERM (Ezrin, Radixin, Moesin) proteins provide a regulated linkage between the membrane and the underlying actin cytoskeleton. They also provide a platform for the transmission of signals in responses to extracellular cues. Studies in different model organisms and in cultured cells have highlighted the importance of ERM proteins in the generation and maintenance of specific domains of the plasma membrane. A central question is how do ERM proteins coordinate actin filament organization and membrane protein transport/stability with signal transduction pathways to build up complex structures? Through their interaction with numerous partners including membrane proteins, actin cytoskeleton and signaling molecules, ERM proteins have the ability to organize multiprotein complexes in specific cellular compartments. Likewise, ERM proteins participate in diverse functions including cell morphogenesis, endocytosis/exocytosis, adhesion and **migration**. This review focuses on aspects still poorly understood related to the function of ERM proteins in epithelial cell adhesion and **migration**.
31. Manipulation of gene expression by infrared laser heat shock and its application to the study of tracheal development in Drosophila. Induction of gene expression in a specific cell and a defined time window is desirable to investigate gene function at the cellular level during morphogenesis. To achieve this, we attempted to introduce the infrared laser-evoked gene operator system (IR-LEGO, Kamei et al., 2009) in the Drosophila embryo. In this technique, infrared laser light illumination induces genes to be expressed under the control of heat shock promoters at the single cell level.We applied IR-LEGO to a transgenic fly stock, HS-eGFP, in which the enhanced green fluorescent protein (eGFP) gene is placed under the control of heat shock protein 70 promoter, and showed that eGFP expression can be induced in single cells within 1-2 hr after IR illumination. Furthermore, induction of HS-Branchless transgene encoding the Drosophila fibroblast growth factor (FGF) effectively altered the **migration** and branching patterns of the tracheal system.Our results indicated that IR-LEGO is a promising choice for the timely control of gene expression in a small group of cells in the Drosophila embryo. By using IR-LEGO, we further demonstrated that the tracheal terminal branching program is sensitive to localized expression of exogenous FGF.
32. Ena drives invasive macrophage **migration** in Drosophila embryos. It is seldom the primary tumour that proves fatal in cancer, with metastasis the fundamental pathological process for disease progression. Upregulation of Mena, a member of the evolutionarily conserved Ena/VASP family of actin cytoskeletal regulators, promotes metastasis and invasive motility of breast cancer cells in vivo. To complement in vitro studies of Ena/VASP function in fibroblasts, we manipulated levels of Ena, the Drosophila homologue of Mena, in migrating embryonic macrophages (haemocytes). Consistent with data from fibroblasts in vitro, Ena localises to regions of actin dynamics within migrating haemocytes, stimulates lamellipodial dynamics and positively regulates the number and length of filopodia. However, whereas Ena overexpression in fibroblasts reduces **migration** speeds, overexpressing Ena in haemocytes leads to a dramatic increase in **migration** speeds, more closely resembling the increased motility of breast cancer cells that overexpress Mena. We provide evidence that this key difference is due to spatial constraints imposed on cells within the three-dimensional environment of the embryo; this might explain how Mena can be used to promote aggressive migratory behaviour during cancer progression.
33. Light-regulated interaction of Dmoesin with TRP and TRPL channels is required for maintenance of photoreceptors. Recent studies in Drosophila melanogaster retina indicate that absorption of light causes the translocation of signaling molecules and actin from the photoreceptor's signaling membrane to the cytosol, but the underlying mechanisms are not fully understood. As ezrin-radixin-moesin (ERM) proteins are known to regulate actin-membrane interactions in a signal-dependent manner, we analyzed the role of Dmoesin, the unique D. melanogaster ERM, in response to light. We report that the illumination of dark-raised flies triggers the dissociation of Dmoesin from the light-sensitive transient receptor potential (TRP) and TRP-like channels, followed by the **migration** of Dmoesin from the membrane to the cytoplasm. Furthermore, we show that light-activated **migration** of Dmoesin results from the dephosphorylation of a conserved threonine in Dmoesin. The expression of a Dmoesin mutant form that impairs this phosphorylation inhibits Dmoesin movement and leads to light-induced retinal degeneration. Thus, our data strongly suggest that the light- and phosphorylation-dependent dynamic association of Dmoesin to membrane channels is involved in maintenance of the photoreceptor cells.
34. Fascin is required for blood cell **migration** during Drosophila embryogenesis. Fascin is well characterized in vitro as an actin-bundling protein and its increased expression is correlated with the invasiveness of various cancers. However, the actual roles and regulation of Fascin in vivo remain elusive. Here we show that Fascin is required for the invasive-like **migration** of blood cells in Drosophila embryos. Fascin expression is highly regulated during embryonic development and, within the blood lineage, is specific to the motile subpopulation of cells, which comprises macrophage-like plasmatocytes. We show that Fascin is required for plasmatocyte **migration**, both as these cells undergo developmental dispersal and during an inflammatory response to epithelial wounding. Live analyses further demonstrate that Fascin localizes to, and is essential for the assembly of, dynamic actin-rich microspikes within plasmatocyte lamellae that polarize towards the direction of **migration**. We show that a regulatory serine of Fascin identified from in vitro studies is not required for in vivo cell motility, but is crucial for the formation of actin bundles within epithelial bristles. Together, these results offer a first glimpse into the mechanisms regulating Fascin function during normal development, which might be relevant for understanding the impact of Fascin in cancers.
35. Hedgehog and Decapentaplegic instruct polarized growth of cell extensions in the Drosophila trachea. The **migration** of cellular extensions is guided by signals from tissues with which they contact. Many axon guidance molecules regulate growth cone **migration** by directly regulating actin cytoskeletal dynamics. Secreted morphogens control global patterns of cell fate decisions during organogenesis through transcriptional regulation, and constitute another class of guidance molecules. We have investigated the guidance roles of the morphogens Hedgehog and Decapentaplegic during directed outgrowth of cytoplasmic extensions in the Drosophila trachea. A subset of tracheal terminal cells adheres to the internal surface of the epidermis and elongates cytoplasmic processes called terminal branches. Hedgehog promotes terminal branch spreading and its extension over the posterior compartment of the epidermis. Decapentaplegic, which is expressed at the onset of terminal branching, restricts dorsal extension of the terminal branch and ensures its monopolar growth. Orthogonal expression of Hedgehog and Decapentaplegic in the epidermis instructs monopolar extension of the terminal branch along the posterior compartment, thereby matching the pattern of airway growth with that of the epidermis.
36. Oocyte and embryonic cytoskeletal defects caused by mutations in the Drosophila swallow gene. The maternal effect gene swallow ( swa) of Drosophila is required for bicoid and htsN4 mRNA localization during oogenesis. Swallow is also required for additional, poorly understood, functions in early embryogenesis. We have examined the cytoskeleton in swa mutant oocytes and embryos by immunocytochemistry and confocal microscopy. Mid- and late-stage swaoocytes have defective cytoplasmic actin networks. Stage-10 oocytes have solid actin clumps and hollow actin spheres in the subcortical layer, and late-stage oocytes have uniformly distributed hollow actin spheres in the subcortical layer and in deeper cytoplasm. Swa preblastoderm embryos have uneven and irregularly distributed actin at the cortex, and defective subcortical actin networks that contain hollow and solid spheres. In swa syncytial blastoderm embryos, the abnormal actin cytoskeleton is associated with defects in nuclear distribution, **migration** and cleavage. Actin cytoskeletal defects correlate with spindle defects, suggesting that the abnormal organization of the actin cytoskeleton allows interaction of mitotic spindles, which induces defective nuclear divisions and loss of nuclei from the surface of the embryo.
37. Clasp-mediated microtubule bundling regulates persistent motility and contact repulsion in Drosophila macrophages in vivo. Drosophila melanogaster macrophages are highly migratory cells that lend themselves beautifully to high resolution in vivo imaging experiments. By expressing fluorescent probes to reveal actin and microtubules, we can observe the dynamic interplay of these two cytoskeletal networks as macrophages migrate and interact with one another within a living organism. We show that before an episode of persistent motility, whether responding to developmental guidance or wound cues, macrophages assemble a polarized array of microtubules that bundle into a compass-like arm that appears to anticipate the direction of **migration**. Whenever cells collide with one another, their microtubule arms transiently align just before cell-cell repulsion, and we show that forcing depolymerization of microtubules by expression of Spastin leads to their defective polarity and failure to contact inhibit from one another. The same is true in orbit/clasp mutants, indicating a pivotal role for this microtubule-binding protein in the assembly and/or functioning of the microtubule arm during polarized **migration** and contact repulsion.
38. Essential requirement for RING finger E3 ubiquitin ligase Hakai in early embryonic development of Drosophila. Hakai is a RING finger type E3 ubiquitin ligase that is highly conserved in metazoans. Mammalian Hakai was shown to bind and ubiquitinate the intracellular domain of E-cadherin, and this activity is implicated in down-regulation of E-cadherin during v-Src-induced cellular transformation. To evaluate this model in vivo, we studied the function of the Drosophila homologue of Hakai. In cultured S2 cells, Drosophila Hakai and E-cadherin (Shotgun) formed a complex in a way distinct from the interaction described for mammalian counterparts. Hakai null mutants died during larval stages but this lethality could be offset by a HA-tagged Hakai construct. While zygotic Hakai function was dispensable for cell proliferation and differentiation in the wing disc epithelium, maternal Hakai mutants showed a variety of defects in epithelial integrity, including stochastic loss of E-cadherin expression and reduction of aPKC; defects in cell specification and cell **migration** were also observed. No increase of E-cadherin, however, was observed. Regulation of multiple target proteins under control of Hakai is, therefore, essential for early embryonic morphogenesis in Drosophila.
39. The Drosophila HEM-2/NAP1 homolog KETTE controls axonal pathfinding and cytoskeletal organization. In Drosophila, the correct formation of the segmental commissures depends on neuron-glial interactions at the midline. The VUM midline neurons extend axons along which glial cells migrate in between anterior and posterior commissures. Here, we show that the gene kette is required for the normal projection of the VUM axons and subsequently disrupts glial **migration**. Axonal projection defects are also found for many other moto- and interneurons. In addition, kette affects the cell morphology of mesodermal and epidermal derivatives, which show an abnormal actin cytoskeleton. The KETTE protein is homologous to the transmembrane protein HEM-2/NAP1 evolutionary conserved from worms to vertebrates. In vitro analysis has shown a specific interaction of the vertebrate HEM-2/NAP1 with the SH2-SH3 adapter protein NCK and the small GTPase RAC1, which both have been implicated in regulating cytoskeleton organization and axonal growth. Hypomorphic kette mutations lead to axonal defects similar to mutations in the Drosophila NCK homolog dreadlocks. Furthermore, we show that kette and dock mutants genetically interact. NCK is thought to interact with the small G proteins RAC1 and CDC42, which play a role in axonal growth. In line with these observations, a kette phenocopy can be obtained following directed expression of mutant DCDC42 or DRAC1 in the CNS midline. In addition, the kette mutant phenotype can be partially rescued by expression of an activated DRAC1 transgene. Our data suggest an important role of the HEM-2 protein in cytoskeletal organization during axonal pathfinding.
40. Lipid phosphate phosphatase activity regulates dispersal and bilateral sorting of embryonic germ cells in Drosophila. In Drosophila, germ cell survival and directionality of **migration** are controlled by two lipid phosphate phosphatases (LPP), wunen (wun) and wunen-2 (wun2). wun wun2 double mutant analysis reveals that the two genes, hereafter collectively called wunens, act redundantly in primordial germ cells. We find that wunens mediate germ cell-germ cell repulsion and that this repulsion is necessary for germ cell dispersal and proper transepithelial **migration** at the onset of **migration** and for the equal sorting of the germ cells between the two embryonic gonads during their **migration**. We propose that this dispersal function optimizes adult fecundity by assuring maximal germ cell occupancy of both gonads. Furthermore, we find that the requirement for wunens in germ cell survival can be eliminated by blocking germ cell **migration**. We suggest that this essential function of Wunen is needed to maintain cell integrity in actively migrating germ cells.
41. Netrin-dependent downregulation of Frazzled/DCC is required for the dissociation of the peripodial epithelium in Drosophila. Netrins are secreted chemoattractants with roles in axon guidance, cell **migration** and epithelial plasticity. Netrin-1 also promotes the survival of metastasized cells by inhibiting the pro-apoptotic effects of its receptor Deleted in Colorectal Carcinoma (DCC). Here we report that Netrins can also regulate epithelial dissociation during Drosophila wing eversion. During eversion, peripodial epithelial cells lose apico-basal polarity and adherens junctions, and become migratory and invasive--a process similar to an epithelial-mesenchymal transition. Loss of netrinA inhibits the breakdown of cell-cell junctions, leading to eversion failure. In contrast, the Netrin receptor Frazzled blocks eversion when overexpressed, whereas frazzled RNAi accelerates eversion in vitro. In peripodial cells Frazzled is endocytosed, and undergoes NetA-dependent degradation, which is required for eversion. Finally, we provide evidence that Frazzled acts through the ERM-family protein Moesin to inhibit eversion. This mechanism may also help explain the role of Netrin and DCC in cancer metastasis.
42. I-BAR protein antagonism of endocytosis mediates directional sensing during guided cell **migration**. Although directed cellular **migration** facilitates the coordinated movement of cells during development and repair, the mechanisms regulating such **migration** remain poorly understood. Missing-in-metastasis (MIM) is a defining member of the inverse Bin/Amphiphysin/Rvs domain (I-BAR) subfamily of lipid binding, cytoskeletal regulators whose levels are altered in a number of cancers. Here, we provide the first genetic evidence that an I-BAR protein regulates directed cell **migration** in vivo. Drosophila MIM (dmim) is involved in Drosophila border cell **migration**, with loss of dmim function resulting in a lack of directional movement by the border cell cluster. In vivo endocytosis assays combined with genetic analyses demonstrate that the dmim product regulates directed cell movement by inhibiting endocytosis and antagonizing the activities of the CD2-associated protein/cortactin complex in these cells. These studies demonstrate that DMIM antagonizes pro-endocytic components to facilitate polarity and localized guidance cue sensing during directional cell **migration**.
43. Rab11 regulates cell-cell communication during collective cell movements. Collective cell movements contribute to development and metastasis. The small GTPase Rac is a key regulator of actin dynamics and cell **migration** but the mechanisms that restrict Rac activation and localization in a group of collectively migrating cells are unknown. Here, we demonstrate that the small GTPases Rab5 and Rab11 regulate Rac activity and polarization during collective cell **migration**. We use photoactivatable forms of Rac to demonstrate that Rab11 acts on the entire group to ensure that Rac activity is properly restricted to the leading cell through regulation of cell-cell communication. In addition, we show that Rab11 binds to the actin cytoskeleton regulator Moesin and regulates its activation in vivo during **migration**. Accordingly, reducing the level of Moesin activity also affects cell-cell communication, whereas expressing active Moesin rescues loss of Rab11 function. Our model suggests that Rab11 controls the sensing of the relative levels of Rac activity in a group of cells, leading to the organization of individual cells in a coherent multicellular motile structure.
44. Peroxiredoxin stabilization of DE-cadherin promotes primordial germ cell adhesion. Regulated adhesion between cells and their environment is critical for normal cell **migration**. We have identified mutations in a gene encoding the Drosophila hydrogen peroxide (H₂O₂)-degrading enzyme Jafrac1, which lead to germ cell adhesion defects. During gastrulation, primordial germ cells (PGCs) associate tightly with the invaginating midgut primordium as it enters the embryo; however, in embryos from jafrac1 mutant mothers this association is disrupted, leaving some PGCs trailing on the outside of the embryo. We observed similar phenotypes in embryos from DE-cadherin/shotgun (shg) mutant mothers and were able to rescue the jafrac1 phenotype by increasing DE-cadherin levels. This and our biochemical evidence strongly suggest that Jafrac1-mediated reduction of H₂O₂ is required to maintain DE-cadherin protein levels in the early embryo. Our results present in vivo evidence of a peroxiredoxin regulating DE-cadherin-mediated adhesion.
45. A genetic mosaic analysis with a repressible cell marker screen to identify genes involved in tracheal cell **migration** during Drosophila air sac morphogenesis. Branching morphogenesis of the Drosophila tracheal system relies on the fibroblast growth factor receptor (FGFR) signaling pathway. The Drosophila FGF ligand Branchless (Bnl) and the FGFR Breathless (Btl/FGFR) are required for cell **migration** during the establishment of the interconnected network of tracheal tubes. However, due to an important maternal contribution of members of the FGFR pathway in the oocyte, a thorough genetic dissection of the role of components of the FGFR signaling cascade in tracheal cell **migration** is impossible in the embryo. To bypass this shortcoming, we studied tracheal cell **migration** in the dorsal air sac primordium, a structure that forms during late larval development. Using a mosaic analysis with a repressible cell marker (MARCM) clone approach in mosaic animals, combined with an ethyl methanesulfonate (EMS)-mutagenesis screen of the left arm of the second chromosome, we identified novel genes implicated in cell **migration**. We screened 1123 mutagenized lines and identified 47 lines displaying tracheal cell **migration** defects in the air sac primordium. Using complementation analyses based on lethality, mutations in 20 of these lines were genetically mapped to specific genomic areas. Three of the mutants were mapped to either the Mhc or the stam complementation groups. Further experiments confirmed that these genes are required for cell **migration** in the tracheal air sac primordium.

### ECM

#### PubMed

1. The role of LamininB2 (LanB2) during mesoderm differentiation in Drosophila. In Drosophila, four genes encode for laminin subunits and the formation of two laminin heterotrimers has been postulated. We report the identification of mutations in the Drosophila LamininB2 (LanB2) gene that encodes for the only laminin γ subunit and is found in both heterotrimers. We describe their effects on embryogenesis, in particular the differentiation of visceral tissues with respect to the **EC**M. Analysis of mesoderm endoderm interaction indicates disrupted basement membranes and defective endoderm migration, which finally interferes with visceral myotube stretching. Extracellular deposition of laminin is blocked due to the loss of the LanB2 subunit, resulting in an abnormal distribution of **EC**M components. Our data, concerning the different function of both trimers during organogenesis, suggest that these trimers might act in a cumulative way and probably at multiple steps during **EC**M assembly. We also observed genetic interactions with kon-tiki and thrombospondin, indicating a role for laminin during muscle attachment.
2. Dynamic shape changes of **ECM**-producing cells drive morphogenesis of ball-and-socket joints in the fly leg. Animal body shape is framed by the skeleton, which is composed of extracellular matrix (**ECM**). Although how the body plan manifests in skeletal morphology has been studied intensively, cellular mechanisms that directly control skeletal **ECM** morphology remain elusive. In particular, how dynamic behaviors of **ECM**-secreting cells, such as shape changes and movements, contribute to **ECM** morphogenesis is unclear. Strict control of **ECM** morphology is crucial in the joints, where opposing sides of the skeleton must have precisely reciprocal shapes to fit each other. Here we found that, in the development of ball-and-socket joints in the Drosophila leg, the two sides of **ECM** form sequentially. We show that distinct cell populations produce the 'ball' and the 'socket', and that these cells undergo extensive shape changes while depositing **ECM**. We propose that shape changes of **ECM**-producing cells enable the sequential **ECM** formation to allow the morphological coupling of adjacent components. Our results highlight the importance of dynamic cell behaviors in precise shaping of skeletal **ECM** architecture.
3. Tissue elongation requires oscillating contractions of a basal actomyosin network. Understanding how molecular dynamics leads to cellular behaviours that ultimately sculpt organs and tissues is a major challenge not only in basic developmental biology but also in tissue engineering and regenerative medicine. Here we use live imaging to show that the basal surfaces of Drosophila follicle cells undergo a series of directional, oscillating contractions driven by periodic myosin accumulation on a polarized actin network. Inhibition of the actomyosin contractions or their coupling to extracellular matrix (**ECM**) blocked elongation of the whole tissue, whereas enhancement of the contractions exaggerated it. Myosin accumulated in a periodic manner before each contraction and was regulated by the small GTPase Rho, its downstream kinase, ROCK, and cytosolic calcium. Disrupting the link between the actin cytoskeleton and the **ECM** decreased the amplitude and period of the contractions, whereas enhancing cell-**ECM** adhesion increased them. In contrast, disrupting cell-cell adhesions resulted in loss of the actin network. Our findings reveal a mechanism controlling organ shape and an experimental model for the study of the effects of oscillatory actomyosin activity within a coherent cell sheet.

### [Ee]xtracellular [Mm]atrix

#### PubMed

1. Tissue-specific laminin expression facilitates integrin-dependent association of the embryonic wing disc with the trachea in Drosophila. The interaction of heterologous tissues involves cell adhesion mediated by the **extracellular matrix** and its receptor integrins. The Drosophila wing disc is an ectodermal invagination that contacts specific tracheal branches at the basolateral cell surface. We show that an alpha subunit of laminin, encoded by wing blister (wb), is essential for the establishment of the interaction between the wing and trachea. During embryogenesis, wing disc cells present Wb at their basolateral surface and extend posteriorly, expanding their association to more posteriorly located tracheal branches. These migratory processes are impaired in the absence of the trachea, Wb, or integrins. Time-lapse and transmission electron microscopy analyses suggest that Wb facilitates integrin-dependent contact over a large surface and controls the cellular behavior of the wing cells, including their exploratory filopodial activity. Our data identify Wb laminin as an **extracellular matrix** ligand that is essential for integrin-dependent cellular migration in Drosophila.
2. Slowdown promotes muscle integrity by modulating integrin-mediated adhesion at the myotendinous junction. The correct assembly of the myotendinous junction (MTJ) is crucial for proper muscle function. In Drosophila, this junction comprises hemi-adherens junctions that are formed upon arrival of muscles at their corresponding tendon cells. The MTJ mainly comprises muscle-specific alphaPS2betaPS integrin receptors and their tendon-derived **extracellular matrix** ligand Thrombospondin (Tsp). We report the identification and functional analysis of a novel tendon-derived secreted protein named Slowdown (Slow). Homozygous slow mutant larvae exhibit muscle or tendon rupture, sluggish larval movement, partial lethality, and the surviving adult flies are unable to fly. These defects result from improper assembly of the embryonic MTJ. In slow mutants, Tsp prematurely accumulates at muscle ends, the morphology of the muscle leading edge changes and the MTJ architecture is aberrant. Slow was found to form a protein complex with Tsp. This complex is biologically active and capable of altering the morphology and directionality of muscle ends. Our analysis implicates Slow as an essential component of the MTJ, crucial for ensuring muscle and tendon integrity during larval locomotion.
3. Integrins are required for cardioblast polarisation in Drosophila. The formation of a tubular organ, such as the heart, requires the communication of positional and polarity signals between migratory cells. Key to this process is the establishment of a new luminal domain on the cell surface, generally from the apical domain of a migratory cell. This domain will also acquire basal properties, as it will produce a luminal **extracellular matrix**. Integrin receptors are the primary means of cell adhesion and adhesion signaling with the **extracellular matrix**. Here we characterise the requirement of Integrins in a genetic model of vasculogenesis, the formation of the heart in Drosophila.As with vertebrates, the Drosophila heart arises from lateral mesoderm that migrates medially to meet their contralateral partners, to then assemble a midline vessel. During migration, Integrins are among the first proteins restricted to the presumptive luminal domain of cardioblasts. Integrins are required for normal levels of leading edge membrane motility. Apical accumulation of Integrins is enhanced by Robo, and reciprocally, apicalisation of luminal factors like Slit and Robo requires Integrin function. Integrins may provide a template for the formation of a lumen by stabilising lumen factors like Robo. Subsequent to migration, Integrin is required for normal cardioblast alignment and lumen formation. This phenotype is most readily modified by other mutations that affect adhesion, such as Talin and **extracellular matrix** ligands.Our findings reveal an instructive role for Integrins in communicating polarising information to cells during migration, and during transition to an epithelial tube structure.
4. Dynamic shape changes of ECM-producing cells drive morphogenesis of ball-and-socket joints in the fly leg. Animal body shape is framed by the skeleton, which is composed of **extracellular matrix** (ECM). Although how the body plan manifests in skeletal morphology has been studied intensively, cellular mechanisms that directly control skeletal ECM morphology remain elusive. In particular, how dynamic behaviors of ECM-secreting cells, such as shape changes and movements, contribute to ECM morphogenesis is unclear. Strict control of ECM morphology is crucial in the joints, where opposing sides of the skeleton must have precisely reciprocal shapes to fit each other. Here we found that, in the development of ball-and-socket joints in the Drosophila leg, the two sides of ECM form sequentially. We show that distinct cell populations produce the 'ball' and the 'socket', and that these cells undergo extensive shape changes while depositing ECM. We propose that shape changes of ECM-producing cells enable the sequential ECM formation to allow the morphological coupling of adjacent components. Our results highlight the importance of dynamic cell behaviors in precise shaping of skeletal ECM architecture.
5. Tissue elongation requires oscillating contractions of a basal actomyosin network. Understanding how molecular dynamics leads to cellular behaviours that ultimately sculpt organs and tissues is a major challenge not only in basic developmental biology but also in tissue engineering and regenerative medicine. Here we use live imaging to show that the basal surfaces of Drosophila follicle cells undergo a series of directional, oscillating contractions driven by periodic myosin accumulation on a polarized actin network. Inhibition of the actomyosin contractions or their coupling to **extracellular matrix** (ECM) blocked elongation of the whole tissue, whereas enhancement of the contractions exaggerated it. Myosin accumulated in a periodic manner before each contraction and was regulated by the small GTPase Rho, its downstream kinase, ROCK, and cytosolic calcium. Disrupting the link between the actin cytoskeleton and the ECM decreased the amplitude and period of the contractions, whereas enhancing cell-ECM adhesion increased them. In contrast, disrupting cell-cell adhesions resulted in loss of the actin network. Our findings reveal a mechanism controlling organ shape and an experimental model for the study of the effects of oscillatory actomyosin activity within a coherent cell sheet.
6. Conversion of plasma membrane topology during epithelial tube connection requires Arf-like 3 small GTPase in Drosophila. The development of tubular organs often involves the hollowing of cells into a torus (doughnut shape), as observed in blood vessel formation in vertebrates and tracheal development in insects. During the fusion of Drosophila tracheal branches, fusion cells located at the tip of migrating branches contact each other and form intracellular luminal cavities on opposite sides of the cells that open to connect the tubule lumens. This process involves the intracellular fusion of plasma membranes associated with microtubule tracks. Here, we studied the function of an evolutionarily conserved small GTPase, Arf-like 3, in branch fusion. Arf-like 3 is N-terminally acetylated, and associates with both intracellular vesicles and microtubules. In Arf-like 3 mutants, the cell adhesion of fusion cells, specification of apical membrane domains, and secretion of luminal **extracellular matrix** proceeded normally, but the luminal cavities did not open due to the failure of intracellular fusion of the plasma membranes. We present evidence that the Arf-like 3 mutation impairs the localized assembly of the exocyst complex, suggesting that the targeting of exocytosis machinery to specific apical domains is the key step in converting the plasma membrane topology in fusion cells.
7. Apoptotic force and tissue dynamics during Drosophila embryogenesis. Understanding cell morphogenesis during metazoan development requires knowledge of how cells and the **extracellular matrix** produce and respond to forces. We investigated how apoptosis, which remodels tissue by eliminating supernumerary cells, also contributes forces to a tissue (the amnioserosa) that promotes cell-sheet fusion (dorsal closure) in the Drosophila embryo. We showed that expression in the amnioserosa of proteins that suppress or enhance apoptosis slows or speeds dorsal closure, respectively. These changes correlate with the forces produced by the amnioserosa and the rate of seam formation between the cell sheets (zipping), key processes that contribute to closure. This apoptotic force is used by the embryo to drive cell-sheet movements during development, a role not classically attributed to apoptosis.

### [Ii]nvasive

#### PubMed

1. Moesin functions antagonistically to the Rho pathway to maintain epithelial integrity. Two prominent characteristics of epithelial cells, apical-basal polarity and a highly ordered cytoskeleton, depend on the existence of precisely localized protein complexes associated with the apical plasma membrane, and on a separate machinery that regulates the spatial order of actin assembly. ERM (ezrin, radixin, moesin) proteins have been proposed to link transmembrane proteins to the actin cytoskeleton in the apical domain, suggesting a structural role in epithelial cells, and they have been implicated in signalling pathways. Here, we show that the sole Drosophila ERM protein Moesin functions to promote cortical actin assembly and apical-basal polarity. As a result, cells lacking Moesin lose epithelial characteristics and adopt **invasive** migratory behaviour. Our data demonstrate that Moesin facilitates epithelial morphology not by providing an essential structural function, but rather by antagonizing activity of the small GTPase Rho. Thus, Moesin functions in maintaining epithelial integrity by regulating cell-signalling events that affect actin organization and polarity. Furthermore, our results show that there is negative feedback between ERM activation and activity of the Rho pathway.
2. RhoL controls invasion and Rap1 localization during immune cell transmigration in Drosophila. Human immune cells have to penetrate an endothelial barrier during their beneficial pursuit of infection and their destructive infiltration of tissues in autoimmune diseases. This transmigration requires Rap1 GTPase to activate integrin affinity. We define a new model system for this process by demonstrating, with live imaging and genetics, that during embryonic development Drosophila melanogaster immune cells penetrate an epithelial, Drosophila E-cadherin (DE-cadherin)-based tissue barrier. A mutant in RhoL, a GTPase homologue that is specifically expressed in haemocytes, blocks this **invasive** step but not other aspects of guided migration. RhoL mediates integrin adhesion caused by Drosophila Rap1 overexpression and moves Rap1 away from a concentration in the cytoplasm to the leading edge during **invasive** migration. These findings indicate that a programmed migratory step during Drosophila development bears striking molecular similarities to vertebrate immune cell transmigration during inflammation, and identify RhoL as a new regulator of invasion, adhesion and Rap1 localization. Our work establishes the utility of Drosophila for identifying novel components of immune cell transmigration and for understanding the in vivo interplay of immune cells with the barriers they penetrate.
3. The Ste20 kinase misshapen is essential for the **invasive** behaviour of ovarian epithelial cells in Drosophila. Stationary-to-migratory transitions of epithelial cells have a key role in development and tumour progression. Border cell migration is a powerful system in which to investigate this transition in living organisms. Here, we identify the Ste20-like kinase misshapen (msn) as a novel regulator of border-cell migration in Drosophila. Expression of msn in border cells is independent of the transcription factor slow border cells and of inputs from all pathways that are known to control border-cell migration. The msn gene functions to modulate the levels and/or distribution of Drosophila E-cadherin to promote the **invasive** migratory behaviour of border cells.
4. Cortactin modulates cell migration and ring canal morphogenesis during Drosophila oogenesis. Cortactin is a Src substrate that interacts with F-actin and can stimulate actin polymerization by direct interaction with the Arp2/3 complex. We have isolated complete loss-of-function mutants of the single Drosophila cortactin gene. Mutants are viable and fertile, showing that cortactin is not an essential gene. However, cortactin mutants show distinct defects during oogenesis. During oogenesis, Cortactin protein is enriched at the F-actin rich ring canals in the germ line, and in migrating border cells. In cortactin mutants, the ring canals are smaller than normal. A similar phenotype has been observed in Src64 mutants and in mutants for genes encoding Arp2/3 complex components, supporting that these protein products act together to control specific processes in vivo. Cortactin mutants also show impaired border cell migration. This **invasive** cell migration is guided by Drosophila EGFR and PDGF/VEGF receptor (PVR). We find that accumulation of Cortactin protein is positively regulated by PVR. Also, overexpression of Cortactin can by itself induce F-actin accumulation and ectopic filopodia formation in epithelial cells. We present evidence that Cortactin is one of the factors acting downstream of PVR and Src to stimulate F-actin accumulation. Cortactin is a minor contributor in this regulation, consistent with the cortactin gene not being essential for development.
5. Moesin functions antagonistically to the Rho pathway to maintain epithelial integrity. Two prominent characteristics of epithelial cells, apical-basal polarity and a highly ordered cytoskeleton, depend on the existence of precisely localized protein complexes associated with the apical plasma membrane, and on a separate machinery that regulates the spatial order of actin assembly. ERM (ezrin, radixin, moesin) proteins have been proposed to link transmembrane proteins to the actin cytoskeleton in the apical domain, suggesting a structural role in epithelial cells, and they have been implicated in signalling pathways. Here, we show that the sole Drosophila ERM protein Moesin functions to promote cortical actin assembly and apical-basal polarity. As a result, cells lacking Moesin lose epithelial characteristics and adopt **invasive** migratory behaviour. Our data demonstrate that Moesin facilitates epithelial morphology not by providing an essential structural function, but rather by antagonizing activity of the small GTPase Rho. Thus, Moesin functions in maintaining epithelial integrity by regulating cell-signalling events that affect actin organization and polarity. Furthermore, our results show that there is negative feedback between ERM activation and activity of the Rho pathway.
6. Gα73B is a downstream effector of JAK/STAT signalling and a regulator of Rho1 in Drosophila haematopoiesis. JAK/STAT signalling regulates many essential developmental processes including cell proliferation and haematopoiesis, whereas its inappropriate activation is associated with the majority of myeloproliferative neoplasias and numerous cancers. Furthermore, high levels of JAK/STAT pathway signalling have also been associated with enhanced metastatic invasion by cancerous cells. Strikingly, gain-of-function mutations in the single Drosophila JAK homologue, Hopscotch, result in haemocyte neoplasia, inappropriate differentiation and the formation of melanised haemocyte-derived 'tumour' masses; phenotypes that are partly orthologous to human gain-of-function JAK2-associated pathologies. Here we show that Gα73B, a novel JAK/STAT pathway target gene, is necessary for JAK/STAT-mediated tumour formation in flies. In addition, although Gα73B does not affect haemocyte differentiation, it does regulate haemocyte morphology and motility under non-pathological conditions. We show that Gα73B is required for constitutive, but not injury-induced, activation of Rho1 and for the localisation of Rho1 into filopodia upon haemocyte activation. Consistent with these results, we also show that Rho1 interacts genetically with JAK/STAT signalling, and that wild-type levels of Rho1 are necessary for tumour formation. Our findings link JAK/STAT transcriptional outputs, Gα73B activity and Rho1-dependent cytoskeletal rearrangements and cell motility, therefore connecting a pathway associated with cancer with a marker indicativ**e of inv**asiveness. As such, we suggest a mechanism by which JAK/STAT pathway signalling may promote metastasis.
7. Ena drives **invasive** macrophage migration in Drosophila embryos. It is seldom the primary tumour that proves fatal in cancer, with metastasis the fundamental pathological process for disease progression. Upregulation of Mena, a member of the evolutionarily conserved Ena/VASP family of actin cytoskeletal regulators, promotes metastasis and **invasive** motility of breast cancer cells in vivo. To complement in vitro studies of Ena/VASP function in fibroblasts, we manipulated levels of Ena, the Drosophila homologue of Mena, in migrating embryonic macrophages (haemocytes). Consistent with data from fibroblasts in vitro, Ena localises to regions of actin dynamics within migrating haemocytes, stimulates lamellipodial dynamics and positively regulates the number and length of filopodia. However, whereas Ena overexpression in fibroblasts reduces migration speeds, overexpressing Ena in haemocytes leads to a dramatic increase in migration speeds, more closely resembling the increased motility of breast cancer cells that overexpress Mena. We provide evidence that this key difference is due to spatial constraints imposed on cells within the three-dimensional environment of the embryo; this might explain how Mena can be used to promote aggressive migratory behaviour during cancer progression.
8. Fascin is required for blood cell migration during Drosophila embryogenesis. Fascin is well characterized in vitro as an actin-bundling protein and its increased expression is correlated with the **invasive**ness of various cancers. However, the actual roles and regulation of Fascin in vivo remain elusive. Here we show that Fascin is required for the **invasive**-like migration of blood cells in Drosophila embryos. Fascin expression is highly regulated during embryonic development and, within the blood lineage, is specific to the motile subpopulation of cells, which comprises macrophage-like plasmatocytes. We show that Fascin is required for plasmatocyte migration, both as these cells undergo developmental dispersal and during an inflammatory response to epithelial wounding. Live analyses further demonstrate that Fascin localizes to, and is essential for the assembly of, dynamic actin-rich microspikes within plasmatocyte lamellae that polarize towards the direction of migration. We show that a regulatory serine of Fascin identified from in vitro studies is not required for in vivo cell motility, but is crucial for the formation of actin bundles within epithelial bristles. Together, these results offer a first glimpse into the mechanisms regulating Fascin function during normal development, which might be relevant for understanding the impact of Fascin in cancers.
9. Netrin-dependent downregulation of Frazzled/DCC is required for the dissociation of the peripodial epithelium in Drosophila. Netrins are secreted chemoattractants with roles in axon guidance, cell migration and epithelial plasticity. Netrin-1 also promotes the survival of metastasized cells by inhibiting the pro-apoptotic effects of its receptor Deleted in Colorectal Carcinoma (DCC). Here we report that Netrins can also regulate epithelial dissociation during Drosophila wing eversion. During eversion, peripodial epithelial cells lose apico-basal polarity and adherens junctions, and become migratory and **invasive**--a process similar to an epithelial-mesenchymal transition. Loss of netrinA inhibits the breakdown of cell-cell junctions, leading to eversion failure. In contrast, the Netrin receptor Frazzled blocks eversion when overexpressed, whereas frazzled RNAi accelerates eversion in vitro. In peripodial cells Frazzled is endocytosed, and undergoes NetA-dependent degradation, which is required for eversion. Finally, we provide evidence that Frazzled acts through the ERM-family protein Moesin to inhibit eversion. This mechanism may also help explain the role of Netrin and DCC in cancer metastasis.

### [Mm]etastasize

#### PubMed

1. Netrin-dependent downregulation of Frazzled/DCC is required for the dissociation of the peripodial epithelium in Drosophila. Netrins are secreted chemoattractants with roles in axon guidance, cell migration and epithelial plasticity. Netrin-1 also promotes the survival of **metastasize**d cells by inhibiting the pro-apoptotic effects of its receptor Deleted in Colorectal Carcinoma (DCC). Here we report that Netrins can also regulate epithelial dissociation during Drosophila wing eversion. During eversion, peripodial epithelial cells lose apico-basal polarity and adherens junctions, and become migratory and invasive--a process similar to an epithelial-mesenchymal transition. Loss of netrinA inhibits the breakdown of cell-cell junctions, leading to eversion failure. In contrast, the Netrin receptor Frazzled blocks eversion when overexpressed, whereas frazzled RNAi accelerates eversion in vitro. In peripodial cells Frazzled is endocytosed, and undergoes NetA-dependent degradation, which is required for eversion. Finally, we provide evidence that Frazzled acts through the ERM-family protein Moesin to inhibit eversion. This mechanism may also help explain the role of Netrin and DCC in cancer metastasis.

### [Mm]etastasis

#### PubMed

1. Cellular and molecular mechanisms of single and collective cell migrations in Drosophila: themes and variations. The process of cell migration is essential throughout life, driving embryonic morphogenesis and ensuring homeostasis in adults. Defects in cell migration are a major cause of human disease, with excessive migration causing autoimmune diseases and cancer **metastasis**, whereas reduced capacity for migration leads to birth defects and immunodeficiencies. Myriad studies in vitro have established a consensus view that cell migrations require cell polarization, Rho GTPase-mediated cytoskeletal rearrangements, and myosin-mediated contractility. However, in vivo studies later revealed a more complex picture, including the discovery that cells migrate not only as single units but also as clusters, strands, and sheets. In particular, the role of E-Cadherin in cell motility appears to be more complex than previously appreciated. Here, we discuss recent advances achieved by combining the plethora of genetic tools available to the Drosophila geneticist with live imaging and biophysical techniques. Finally, we discuss the emerging themes such studies have revealed and ponder the puzzles that remain to be solved.
2. Hedgehog is a positive regulator of FGF signalling during embryonic tracheal cell migration. Cell migration is a widespread and complex process that is crucial for morphogenesis and for the underlying invasion and **metastasis** of human cancers. During migration, cells are steered toward target sites by guidance molecules that induce cell direction and movement through complex intracellular mechanisms. The spatio-temporal regulation of the expression of these guidance molecules is of extreme importance for both normal morphogenesis and human disease. One way to achieve this precise regulation is by combinatorial inputs of different transcription factors. Here we used Drosophila melanogaster mutants with migration defects in the ganglionic branches of the tracheal system to further clarify guidance regulation during cell migration. By studying the cellular consequences of overactivated Hh signalling, using ptc mutants, we found that Hh positively regulates Bnl/FGF levels during embryonic stages. Our results show that Hh modulates cell migration non-autonomously in the tissues surrounding the action of its activity. We further demonstrate that the Hh signalling pathway regulates bnl expression via Stripe (Sr), a zinc-finger transcription factor with homology to the Early Growth Response (EGR) family of vertebrate transcription factors. We propose that Hh modulates embryonic cell migration by participating in the spatio-temporal regulation of bnl expression in a permissive mode. By doing so, we provide a molecular link between the activation of Hh signalling and increased chemotactic responses during cell migration.
3. An ABC transporter controls export of a Drosophila germ cell attractant. Directed cell migration, which is critical for embryonic development, leukocyte trafficking, and cell **metastasis**, depends on chemoattraction. 3-hydroxy-3-methylglutaryl coenzyme A reductase regulates the production of an attractant for Drosophila germ cells that may itself be geranylated. Chemoattractants are commonly secreted through a classical, signal peptide-dependent pathway, but a geranyl-modified attractant would require an alternative pathway. In budding yeast, pheromones produced by a-cells are farnesylated and secreted in a signal peptide-independent manner, requiring the adenosine triphosphate-binding cassette (ABC) transporter Ste6p. Here we show that Drosophila germ cell migration uses a similar pathway, demonstrating that invertebrate germ cells, like yeast cells, are attracted to lipid-modified peptides. Components of this unconventional export pathway are highly conserved, suggesting that this pathway may control the production of similarly modified chemoattractants in organisms ranging from yeast to humans.
4. Small is beautiful: what flies tell us about ERM protein function in development. Actin dynamics is recognized as being a determinant in many developmental processes and pathologies, such as cell polarity, morphogenesis and tumour **metastasis**. However, how actin interacts with the plasma membrane is poorly understood. Although numerous studies in cell culture point to the crucial role of Ezrin, Radixin and Moesin (ERM) proteins in the actin-membrane link, genetic approaches in mice have not yet revealed their activity during development. Drosophila has recently become an alternative and promising system for the genetic study of ERM protein function. This article focuses on advances made in flies, providing evidence for the evolutionary conservation of functional properties of ERM proteins, in addition to shedding new light on their importance for development.
5. Gα73B is a downstream effector of JAK/STAT signalling and a regulator of Rho1 in Drosophila haematopoiesis. JAK/STAT signalling regulates many essential developmental processes including cell proliferation and haematopoiesis, whereas its inappropriate activation is associated with the majority of myeloproliferative neoplasias and numerous cancers. Furthermore, high levels of JAK/STAT pathway signalling have also been associated with enhanced metastatic invasion by cancerous cells. Strikingly, gain-of-function mutations in the single Drosophila JAK homologue, Hopscotch, result in haemocyte neoplasia, inappropriate differentiation and the formation of melanised haemocyte-derived 'tumour' masses; phenotypes that are partly orthologous to human gain-of-function JAK2-associated pathologies. Here we show that Gα73B, a novel JAK/STAT pathway target gene, is necessary for JAK/STAT-mediated tumour formation in flies. In addition, although Gα73B does not affect haemocyte differentiation, it does regulate haemocyte morphology and motility under non-pathological conditions. We show that Gα73B is required for constitutive, but not injury-induced, activation of Rho1 and for the localisation of Rho1 into filopodia upon haemocyte activation. Consistent with these results, we also show that Rho1 interacts genetically with JAK/STAT signalling, and that wild-type levels of Rho1 are necessary for tumour formation. Our findings link JAK/STAT transcriptional outputs, Gα73B activity and Rho1-dependent cytoskeletal rearrangements and cell motility, therefore connecting a pathway associated with cancer with a marker indicative of invasiveness. As such, we suggest a mechanism by which JAK/STAT pathway signalling may pro**mote metas**tasis.
6. Ena drives invasive macrophage migration in Drosophila embryos. It is seldom the primary tumour that proves fatal in cancer, with **metastasis** the fundamental pathological process for disease progression. Upregulation of Mena, a member of the evolutionarily conserved Ena/VASP family of actin cytoskeletal regulators, promotes **metastasis** and invasive motility of breast cancer cells in vivo. To complement in vitro studies of Ena/VASP function in fibroblasts, we manipulated levels of Ena, the Drosophila homologue of Mena, in migrating embryonic macrophages (haemocytes). Consistent with data from fibroblasts in vitro, Ena localises to regions of actin dynamics within migrating haemocytes, stimulates lamellipodial dynamics and positively regulates the number and length of filopodia. However, whereas Ena overexpression in fibroblasts reduces migration speeds, overexpressing Ena in haemocytes leads to a dramatic increase in migration speeds, more closely resembling the increased motility of breast cancer cells that overexpress Mena. We provide evidence that this key difference is due to spatial constraints imposed on cells within the three-dimensional environment of the embryo; this might explain how Mena can be used to promote aggressive migratory behaviour during cancer progression.
7. Netrin-dependent downregulation of Frazzled/DCC is required for the dissociation of the peripodial epithelium in Drosophila. Netrins are secreted chemoattractants with roles in axon guidance, cell migration and epithelial plasticity. Netrin-1 also promotes the survival of metastasized cells by inhibiting the pro-apoptotic effects of its receptor Deleted in Colorectal Carcinoma (DCC). Here we report that Netrins can also regulate epithelial dissociation during Drosophila wing eversion. During eversion, peripodial epithelial cells lose apico-basal polarity and adherens junctions, and become migratory and invasive--a process similar to an epithelial-mesenchymal transition. Loss of netrinA inhibits the breakdown of cell-cell junctions, leading to eversion failure. In contrast, the Netrin receptor Frazzled blocks eversion when overexpressed, whereas frazzled RNAi accelerates eversion in vitro. In peripodial cells Frazzled is endocytosed, and undergoes NetA-dependent degradation, which is required for eversion. Finally, we provide evidence that Frazzled acts through the ERM-family protein Moesin to inhibit eversion. This mechanism may also help explain the role of Netrin and DCC in cancer **metastasis**.
8. I-BAR protein antagonism of endocytosis mediates directional sensing during guided cell migration. Although directed cellular migration facilitates the coordinated movement of cells during development and repair, the mechanisms regulating such migration remain poorly understood. Missing-in-**metastasis** (MIM) is a defining member of the inverse Bin/Amphiphysin/Rvs domain (I-BAR) subfamily of lipid binding, cytoskeletal regulators whose levels are altered in a number of cancers. Here, we provide the first genetic evidence that an I-BAR protein regulates directed cell migration in vivo. Drosophila MIM (dmim) is involved in Drosophila border cell migration, with loss of dmim function resulting in a lack of directional movement by the border cell cluster. In vivo endocytosis assays combined with genetic analyses demonstrate that the dmim product regulates directed cell movement by inhibiting endocytosis and antagonizing the activities of the CD2-associated protein/cortactin complex in these cells. These studies demonstrate that DMIM antagonizes pro-endocytic components to facilitate polarity and localized guidance cue sensing during directional cell migration.
9. Rab11 regulates cell-cell communication during collective cell movements. Collective cell movements contribute to development and **metastasis**. The small GTPase Rac is a key regulator of actin dynamics and cell migration but the mechanisms that restrict Rac activation and localization in a group of collectively migrating cells are unknown. Here, we demonstrate that the small GTPases Rab5 and Rab11 regulate Rac activity and polarization during collective cell migration. We use photoactivatable forms of Rac to demonstrate that Rab11 acts on the entire group to ensure that Rac activity is properly restricted to the leading cell through regulation of cell-cell communication. In addition, we show that Rab11 binds to the actin cytoskeleton regulator Moesin and regulates its activation in vivo during migration. Accordingly, reducing the level of Moesin activity also affects cell-cell communication, whereas expressing active Moesin rescues loss of Rab11 function. Our model suggests that Rab11 controls the sensing of the relative levels of Rac activity in a group of cells, leading to the organization of individual cells in a coherent multicellular motile structure.
10. The actin-binding ERM protein Moesin directly regulates spindle assembly and function during mitosis. Ezrin-Radixin-Moesin proteins are highly conserved, actin-binding cytoskeletal proteins that play an essential role in microvilli formation, T-cell activation, and tumor **metastasis** by linking actin filaments to the plasma membrane. Recent studies demonstrated that the only Ezrin-Radixin-Moesin protein of Drosophila melanogaster, Moesin, is involved in mitotic spindle function through stabilizing cell shape and microtubules at the cell cortex. We previously observed that Moesin localizes to the mitotic spindle; hence, we tested for the biological significance of this surprising localization and investigated whether it plays a direct role in spindle function. To separate the cortical and spindle functions of Moesin during mitosis we combined cell biological and genetic methods. We used early Drosophila embryos, in which mitosis occurs in the absence of a cell cortex, and found in vivo evidence for the direct requirement of Moesin in mitotic spindle assembly and function. We also found that the accumulation of Moesin precedes the construction of the microtubule spindle, and the fusiform structure formed by Moesin persists even after the microtubules have disassembled.

---

## CG4554

- NCBI Gene
- GeneRIF
- Pubmed

| Search Term | Rank | Fields |
| --- | --- | --- |
| [Mm]etastasis | 12 | Conserved Domains(1) |

  
hide/show details for CG4554

### [Mm]etastasis

#### Conserved Domains

1. pfam07539: DRIM; Down-regulated in **metastasis**

---

## LRR

- NCBI Gene
- GeneRIF
- Pubmed

| Search Term | Rank | Fields |
| --- | --- | --- |
| [Mm]igration | 6 | PubMed(1) |

  
hide/show details for LRR

### [Mm]igration

#### PubMed

1. Molecular requirements for actin-based lamella formation in Drosophila S2 cells. Cell **migration** occurs through the protrusion of the actin-enriched lamella. Here, we investigated the effects of RNAi depletion of approximately 90 proteins implicated in actin function on lamella formation in Drosophila S2 cells. Similar to in vitro reconstitution studies of actin-based Listeria movement, we find that lamellae formation requires a relatively small set of proteins that participate in actin nucleation (Arp2/3 and SCAR), barbed end capping (capping protein), filament depolymerization (cofilin and Aip1), and actin monomer binding (profilin and cyclase-associated protein). Lamellae are initiated by parallel and partially redundant signaling pathways involving Rac GTPases and the adaptor protein Nck, which stimulate SCAR, an Arp2/3 activator. We also show that RNAi of three proteins (kette, Abi, and Sra-1) known to copurify with and inhibit SCAR in vitro leads to SCAR degradation, revealing a novel function of this protein complex in SCAR stability. Our results have identified an essential set of proteins involved in actin dynamics during lamella formation in Drosophila S2 cells.

---

## CG42709

- NCBI Gene
- GeneRIF
- Pubmed

| Search Term | Rank | Fields |
| --- | --- | --- |
| [Ee]xtracellular [Mm]atrix | 8 | Component(1) |

  
hide/show details for CG42709

### [Ee]xtracellular [Mm]atrix

#### Component

1. proteinaceous **extracellular matrix**

---

## Slob

- NCBI Gene
- GeneRIF
- Pubmed

| Search Term | Rank | Fields |
| --- | --- | --- |
| [Mm]igration | 6 | PubMed(1) |
| ECM | 7 | PubMed(1) |
| [Ee]xtracellular [Mm]atrix | 8 | PubMed(1) |

  
hide/show details for Slob

### [Mm]igration

#### PubMed

1. Genes conserved in bilaterians but jointly lost with Myc during nematode evolution are enriched in cell proliferation and cell **migration** functions. Animals use a stereotypical set of developmental genes to build body architectures of varying sizes and organizational complexity. Some genes are critical to developmental patterning, while other genes are important to physiological control of growth. However, growth regulator genes may not be as important in small-bodied "micro-metazoans" such as nematodes. Nematodes use a simplified developmental strategy of lineage-based cell fate specifications to produce an adult bilaterian body composed of a few hundreds of cells. Nematodes also lost the MYC proto-oncogenic regulator of cell proliferation. To identify additional regulators of cell proliferation that were lost with MYC, we computationally screened and determined 839 high-confidence genes that are conserved in bilaterians/lost in nematodes (CIBLIN genes). We find that 30 % of all CIBLIN genes encode transcriptional regulators of cell proliferation, epithelial-to-mesenchyme transitions, and other processes. Over 50 % of CIBLIN genes are unnamed genes in Drosophila, suggesting that there are many understudied genes. Interestingly, CIBLIN genes include many Myc synthetic lethal (MycSL) hits from recent screens. CIBLIN genes include key regulators of heparan sulfate proteoglycan (HSPG) sulfation patterns, and lysyl oxidases involved in cross-linking and modification of the extracellular matrix (ECM). These genes and others suggest the CIBLIN repertoire services critical functions in ECM remodeling and cell **migration** in large-bodied bilaterians. Correspondingly, CIBLIN genes are co-expressed with Myc in cancer transcriptomes, and include a preponderance of known determinants of cancer progression and tumor aggression. We propose that CIBLIN gene research can improve our understanding of regulatory control of cellular growth in metazoans.

### ECM

#### PubMed

1. Genes conserved in bilaterians but jointly lost with Myc during nematode evolution are enriched in cell proliferation and cell migration functions. Animals use a stereotypical set of developmental genes to build body architectures of varying sizes and organizational complexity. Some genes are critical to developmental patterning, while other genes are important to physiological control of growth. However, growth regulator genes may not be as important in small-bodied "micro-metazoans" such as nematodes. Nematodes use a simplified developmental strategy of lineage-based cell fate specifications to produce an adult bilaterian body composed of a few hundreds of cells. Nematodes also lost the MYC proto-oncogenic regulator of cell proliferation. To identify additional regulators of cell proliferation that were lost with MYC, we computationally screened and determined 839 high-confidence genes that are conserved in bilaterians/lost in nematodes (CIBLIN genes). We find that 30 % of all CIBLIN genes encode transcriptional regulators of cell proliferation, epithelial-to-mesenchyme transitions, and other processes. Over 50 % of CIBLIN genes are unnamed genes in Drosophila, suggesting that there are many understudied genes. Interestingly, CIBLIN genes include many Myc synthetic lethal (MycSL) hits from recent screens. CIBLIN genes include key regulators of heparan sulfate proteoglycan (HSPG) sulfation patterns, and lysyl oxidases involved in cross-linking and modification of the extracellular matrix (**ECM**). These genes and others suggest the CIBLIN repertoire services critical functions in **ECM** remodeling and cell migration in large-bodied bilaterians. Correspondingly, CIBLIN genes are co-expressed with Myc in cancer transcriptomes, and include a preponderance of known determinants of cancer progression and tumor aggression. We propose that CIBLIN gene research can improve our understanding of regulatory control of cellular growth in metazoans.

### [Ee]xtracellular [Mm]atrix

#### PubMed

1. Genes conserved in bilaterians but jointly lost with Myc during nematode evolution are enriched in cell proliferation and cell migration functions. Animals use a stereotypical set of developmental genes to build body architectures of varying sizes and organizational complexity. Some genes are critical to developmental patterning, while other genes are important to physiological control of growth. However, growth regulator genes may not be as important in small-bodied "micro-metazoans" such as nematodes. Nematodes use a simplified developmental strategy of lineage-based cell fate specifications to produce an adult bilaterian body composed of a few hundreds of cells. Nematodes also lost the MYC proto-oncogenic regulator of cell proliferation. To identify additional regulators of cell proliferation that were lost with MYC, we computationally screened and determined 839 high-confidence genes that are conserved in bilaterians/lost in nematodes (CIBLIN genes). We find that 30 % of all CIBLIN genes encode transcriptional regulators of cell proliferation, epithelial-to-mesenchyme transitions, and other processes. Over 50 % of CIBLIN genes are unnamed genes in Drosophila, suggesting that there are many understudied genes. Interestingly, CIBLIN genes include many Myc synthetic lethal (MycSL) hits from recent screens. CIBLIN genes include key regulators of heparan sulfate proteoglycan (HSPG) sulfation patterns, and lysyl oxidases involved in cross-linking and modification of the **extracellular matrix** (ECM). These genes and others suggest the CIBLIN repertoire services critical functions in ECM remodeling and cell migration in large-bodied bilaterians. Correspondingly, CIBLIN genes are co-expressed with Myc in cancer transcriptomes, and include a preponderance of known determinants of cancer progression and tumor aggression. We propose that CIBLIN gene research can improve our understanding of regulatory control of cellular growth in metazoans.

---

## Nrt

- NCBI Gene
- GeneRIF
- Pubmed

| Search Term | Rank | Fields |
| --- | --- | --- |
| [Ff]ilopodium | 2 | PubMed(1) |
| [Mm]igration | 6 | PubMed(14) |
| ECM | 7 | PubMed(2) |
| [Ee]xtracellular [Mm]atrix | 8 | PubMed(2) |

  
hide/show details for Nrt

### [Ff]ilopodium

#### PubMed

1. Bifocal is a downstream target of the Ste20-like serine/threonine kinase misshapen in regulating photoreceptor growth cone targeting in Drosophila. Misshapen (Msn) has been proposed to shut down Drosophila photoreceptor (R cell) growth cone motility in response to targeting signals linked by the SH2/SH3 adaptor protein Dock. Here, we show that Bifocal (Bif), a putative cytoskeletal regulator, is a component of the Msn pathway for regulating R cell growth cone targeting. bif displays strong genetic interaction with msn. Phenotypic analysis indicates a specific role for Bif to terminate R1-R6 growth cones. Biochemical studies show that Msn associates directly with Bif and phosphorylates Bif in vitro. Cell culture studies demonstrate that Msn interacts with Bif to regulate F-actin structure and **filopodium** formation. We propose that Bif functions downstream of Msn to reorganize actin cytoskeleton in decelerating R cell growth cone motility at the target region.

### [Mm]igration

#### PubMed

1. The Rap1 GTPase functions as a regulator of morphogenesis in vivo. The Ras-related Rap GTPases are highly conserved across diverse species but their normal biological function is not well understood. Initial studies in mammalian cells suggested a role for Rap as a Ras antagonist. More recent experiments indicate functions in calcium- and cAMP-mediated signaling and it has been proposed that protein kinase A-mediated phosphorylation activates Rap in vivo. We show that Ras1-mediated signaling pathways in Drosophila are not influenced by Rap1 levels, suggesting that Ras1 and Rap1 function via distinct pathways. Moreover, a mutation that abolishes the putative cAMP-dependent kinase phosphorylation site of Drosophila Rap1 can still rescue the Rap1 mutant phenotype. Our experiments show that Rap1 is not needed for cell proliferation and cell-fate specification but demonstrate a critical function for Rap1 in regulating normal morphogenesis in the eye disk, the ovary and the embryo. Rap1 mutations also disrupt cell **migration**s and cause abnormalities in cell shape. These findings indicate a role for Rap proteins as regulators of morphogenesis in vivo.
2. Germ cell specification and **migration** in Drosophila and beyond. The passage of an individual's genome to future generations is essential for the maintenance of species and is mediated by highly specialized cells, the germ cells. Genetic studies in a number of model organisms have provided insight into the molecular mechanisms that control specification, **migration** and survival of early germ cells. Focusing on Drosophila, we will discuss the mechanisms by which germ cells initially form and remain transcriptionally silent while somatic cells are transcriptionally active. We will further discuss three separate attractive and repellent guidance pathways, mediated by a G-protein coupled receptor, two lipid phosphate phosphohydrolases, and isoprenylation. We will compare and contrast these findings with those obtained in other organisms, in particular zebrafish and mice. While aspects of germ cell specification are strikingly different between these species, germ cell specific gene functions have been conserved. In particular, mechanisms that sense directional cues during germ cell **migration** seem to be shared between invertebrates and vertebrates.
3. Slow as molasses is required for polarized membrane growth and germ cell **migration** in Drosophila. Drosophila germ cell **migration** is directed by attractive and repulsive guidance cues. We have identified a novel gene, slow as molasses (slam), which is required for germ cell **migration**. In slam zygotic mutants, germ cells fail to transit off the midgut into the mesoderm. We show that slam is required at this stage in parallel to HMG Coenzyme A reductase, a previously identified germ cell **migration** gene. Removal of both zygotic and maternal slam results in an earlier defect: a failure to form a cellular blastoderm. Consistent with this phenotype, we found that slam is one of the earliest genes to be transcribed in the embryo, and Slam protein localizes to the growing basal-lateral membrane during blastoderm formation, but Slam is not detected during later stages of embryogenesis. Because slam RNA and protein are expressed earlier than the time when we observe defects in germ cell **migration**, we propose that Slam is required for the localization of a signal to the basal side of blastoderm cells that is needed later in the posterior midgut to guide germ cells.
4. Rho GTPase controls invagination and cohesive **migration** of the Drosophila salivary gland through Crumbs and Rho-kinase. Coordinated cell movements shape simple epithelia into functional tissues and organs during embryogenesis. Regulators and effectors of the small GTPase Rho have been shown to be essential for epithelial morphogenesis in cell culture; however, the mechanism by which Rho GTPase and its downstream effectors control coordinated movement of epithelia in a developing tissue or organ is largely unknown. Here, we show that Rho1 GTPase activity is required for the invagination of Drosophila embryonic salivary gland epithelia and for directed **migration** of the internalized gland. We demonstrate that the absence of zygotic function of Rho1 results in the selective loss of the apical proteins, Crumbs (Crb), Drosophila atypical PKC and Stardust during gland invagination and that this is partially due to reduced crb RNA levels and apical localization. In parallel to regulation of crb RNA and protein, Rho1 activity also signals through Rho-kinase (Rok) to induce apical constriction and cell shape change during invagination. After invagination, Rho-Rok signaling is required again for the coordinated contraction and dorsal **migration** of the proximal half of the gland. We also show that Rho1 activity is required for proper development of the circular visceral mesoderm upon which the gland migrates. Our genetic and live-imaging analyses provide novel evidence that the proximal gland cells play an essential and active role in salivary gland **migration** that propels the entire gland to turn and migrate posteriorly.
5. Abelson kinase regulates epithelial morphogenesis in Drosophila. Activation of the nonreceptor tyrosine kinase Abelson (Abl) contributes to the development of leukemia, but the complex roles of Abl in normal development are not fully understood. Drosophila Abl links neural axon guidance receptors to the cytoskeleton. Here we report a novel role for Drosophila Abl in epithelial cells, where it is critical for morphogenesis. Embryos completely lacking both maternal and zygotic Abl die with defects in several morphogenetic processes requiring cell shape changes and cell **migration**. We describe the cellular defects that underlie these problems, focusing on dorsal closure as an example. Further, we show that the Abl target Enabled (Ena), a modulator of actin dynamics, is involved with Abl in morphogenesis. We find that Ena localizes to adherens junctions of most epithelial cells, and that it genetically interacts with the adherens junction protein Armadillo (Arm) during morphogenesis. The defects of abl mutants are strongly enhanced by heterozygosity for shotgun, which encodes DE-cadherin. Finally, loss of Abl reduces Arm and alpha-catenin accumulation in adherens junctions, while having little or no effect on other components of the cytoskeleton or cell polarity machinery. We discuss possible models for Abl function during epithelial morphogenesis in light of these data.
6. Slit coordinates cardiac morphogenesis in Drosophila. Slit is a secreted guidance cue that conveys repellent or attractive signals from target and guidepost cells. In Drosophila, responsive cells express one or more of three Robo receptors. The cardial cells of the developing heart express both Slit and Robo2. This is the first report of coincident expression of a Robo and its ligand. In slit mutants, cardial cell alignment, polarization and uniform **migration** are disrupted. The heart phenotype of robo2 mutants is similar, with fewer **migration** defects. In the guidance of neuronal growth cones in Drosophila, there is a phenotypic interaction between slit and robo heterozygotes, and also with genes required for Robo signaling. In contrast, in the heart, slit has little or no phenotypic interaction with Robo-related genes, including Robo2, Nck2, and Disabled. However, there is a strong phenotypic interaction with Integrin genes and their ligands, including Laminin and Collagen, and intracellular messengers, including Talin and ILK. This indicates that Slit participates in adhesion or adhesion signaling during heart development.
7. Fibroblast growth factor signalling controls successive cell behaviours during mesoderm layer formation in Drosophila. Fibroblast growth factor (FGF)-dependent epithelial-mesenchymal transitions and cell **migration** contribute to the establishment of germ layers in vertebrates and other animals, but a comprehensive demonstration of the cellular activities that FGF controls to mediate these events has not been provided for any system. The establishment of the Drosophila mesoderm layer from an epithelial primordium involves a transition to a mesenchymal state and the dispersal of cells away from the site of internalisation in a FGF-dependent fashion. We show here that FGF plays multiple roles at successive stages of mesoderm morphogenesis in Drosophila. It is first required for the mesoderm primordium to lose its epithelial polarity. An intimate, FGF-dependent contact is established and maintained between the germ layers through mesoderm cell protrusions. These protrusions extend deep into the underlying ectoderm epithelium and are associated with high levels of E-cadherin at the germ layer interface. Finally, FGF directs distinct hitherto unrecognised and partially redundant protrusive behaviours during later mesoderm spreading. Cells first move radially towards the ectoderm, and then switch to a dorsally directed movement across its surface. We show that both movements are important for layer formation and present evidence suggesting that they are controlled by genetically distinct mechanisms.
8. The RhoGEF Pebble is required for cell shape changes during cell **migration** triggered by the Drosophila FGF receptor Heartless. The FGF receptor Heartless (HTL) is required for mesodermal cell **migration** in the Drosophila gastrula. We show that mesoderm cells undergo different phases of specific cell shape changes during mesoderm **migration**. During the migratory phase, the cells adhere to the basal surface of the ectoderm and exhibit extensive protrusive activity. HTL is required for the protrusive activity of the mesoderm cells. Moreover, the early phenotype of htl mutants suggests that HTL is required for the adhesion of mesoderm cells to the ectoderm. In a genetic screen we identified pebble (pbl) as a novel gene required for mesoderm **migration**. pbl encodes a guanyl nucleotide exchange factor (GEF) for RHO1 and is known as an essential regulator of cytokinesis. We show that the function of PBL in cell **migration** is independent of the function of PBL in cytokinesis. Although RHO1 acts as a substrate for PBL in cytokinesis, compromising RHO1 function in the mesoderm does not block cell **migration**. These data suggest that the function of PBL in cell **migration** might be mediated through a pathway distinct from RHO1. This idea is supported by allele-specific differences in the expressivity of the cytokinesis and cell **migration** phenotypes of different pbl mutants. We show that PBL is autonomously required in the mesoderm for cell **migration**. Like HTL, PBL is required for early cell shape changes during mesoderm **migration**. Expression of a constitutively active form of HTL is unable to rescue the early cellular defects in pbl mutants, suggesting that PBL is required for the ability of HTL to trigger these cell shape changes. These results provide evidence for a novel function of the Rho-GEF PBL in HTL-dependent mesodermal cell **migration**.
9. Neurexin IV and Wrapper interactions mediate Drosophila midline glial **migration** and axonal ensheathment. Glia play crucial roles in ensheathing axons, a process that requires an intricate series of glia-neuron interactions. The membrane-anchored protein Wrapper is present in Drosophila midline glia and is required for ensheathment of commissural axons. By contrast, Neurexin IV is present on the membranes of neurons and commissural axons, and is highly concentrated at their interfaces with midline glia. Analysis of Neurexin IV and wrapper mutant embryos revealed identical defects in glial **migration**, ensheathment and glial subdivision of the commissures. Mutant and misexpression experiments indicated that Neurexin IV membrane localization is dependent on interactions with Wrapper. Cell culture aggregation assays and biochemical experiments demonstrated the ability of Neurexin IV to promote cell adhesion by binding to Wrapper. These results show that neuronal-expressed Neurexin IV and midline glial-expressed Wrapper act as heterophilic adhesion molecules that mediate multiple cellular events involved in glia-neuron interactions.
10. Haemocyte-derived SPARC is required for collagen-IV-dependent stability of basal laminae in Drosophila embryos. SPARC is an evolutionarily conserved collagen-binding extracellular matrix (ECM) glycoprotein whose morphogenetic contribution(s) to embryonic development remain elusive despite decades of research. We have therefore used Drosophila genetics to gain insight into the role of SPARC during embryogenesis. In Drosophila embryos, high levels of SPARC and other basal lamina components (such as network-forming collagen IV, laminin and perlecan) are synthesized and secreted by haemocytes, and assembled into basal laminae. A SPARC mutant was generated by P-element mutagenesis that is embryonic lethal because of multiple developmental defects. Whereas no differences in collagen IV immunostaining were observed in haemocytes between wild-type and SPARC-mutant embryos, collagen IV was not visible in basal laminae of SPARC-mutant embryos. In addition, the laminin network of SPARC-mutant embryos appeared fragmented and discontinuous by late embryogenesis. Transgenic expression of SPARC protein by haemocytes in SPARC-mutant embryos restored collagen IV and laminin continuity in basal laminae. However, transgenic expression of SPARC by neural cells failed to rescue collagen IV in basal laminae, indicating that the presence of collagen IV deposition requires SPARC expression by haemocytes. Our previous finding that haemocyte-derived SPARC protein levels are reduced in collagen-IV-mutant embryos and the observation that collagen-IV-mutant embryos showed a striking phenotypic similarity to SPARC-mutant embryos suggests a mutual dependence between these major basal laminae components during embryogenesis. Patterning defects and impaired condensation of the ventral nerve cord also resulted from the loss SPARC expression prior to haemocyte **migration**. Hence, SPARC is required for basal lamina maturation and condensation of the ventral nerve cord during Drosophila embryogenesis.
11. Photoactivatable GFP resolves Drosophila mesoderm **migration** behaviour. Mesoderm **migration** is a pivotal event in the early embryonic development of animals. One of the best-studied examples occurs during Drosophila gastrulation. Here, mesodermal cells invaginate, undergo an epithelial-to-mesenchymal transition (EMT), and spread out dorsally over the inner surface of the ectoderm. Although several genes required for spreading have been identified, our inability to visualise mesodermal cells in living embryos has left us to speculate about the cell rearrangements involved. Several mechanisms, such as chemotaxis towards a dorsally expressed attractant, differential affinity between mesodermal cells and the ectoderm, and convergent extension, have been proposed. Here we resolve the behaviour of Drosophila mesodermal cells in live embryos using photoactivatable-GFP fused to alpha-Tubulin (PAGFP-Tub). By photoactivating presumptive mesodermal cells before gastrulation, we could observe their **migration** over non-fluorescent ectodermal cells. We show that the outermost (outer) cells, which are in contact with the ectoderm, migrate dorsolaterally as a group but can be overtaken by more internal (inner) cells. Using laser-photoactivation of individual cells, we then show that inner cells adjacent to the centre of the furrow migrate dorsolaterally away from the midline to reach dorsal positions, while cells at the centre of the furrow disperse randomly across the mesoderm, before intercalating with outer cells. These movements are dependent on the FGF receptor Heartless. The results indicate that chemotactic movement and differential affinity are the primary drivers of mesodermal cell spreading. These characterisations pave the way for a more detailed analysis of gene function during early mesoderm development.
12. Molecular and functional analysis of Drosophila single-minded larval central brain expression. Developmental regulatory proteins are commonly utilized in multiple cell types throughout development. The Drosophila single-minded (sim) gene acts as master regulator of embryonic CNS midline cell development and transcription. However, it is also expressed in the brain during larval development. In this paper, we demonstrate that sim is expressed in three clusters of anterior central brain neurons: DAMv1/2, BAmas1/2, and TRdm and in three clusters of posterior central brain neurons: a subset of DPM neurons, and two previously unidentified clusters, which we term PLSC and PSC. In addition, sim is expressed in the lamina and medulla of the optic lobes. MARCM studies confirm that sim is expressed at high levels in neurons but is low or absent in neuroblasts (NBs) and ganglion mother cell (GMC) precursors. In the anterior brain, sim(+) neurons are detected in 1st and 2nd instar larvae but rapidly increase in number during the 3rd instar stage. To understand the regulation of sim brain transcription, 12 fragments encompassing 5'-flanking, intronic, and 3'-flanking regions were tested for the presence of enhancers that drive brain expression of a reporter gene. Three of these fragments drove expression in sim(+) brain cells, including all sim(+) neuronal clusters in the central brain and optic lobes. One fragment upstream of sim is autoregulatory and is expressed in all sim(+) brain cells. One intronic fragment drives expression in only the PSC and laminar neurons. Another downstream intronic fragment drives expression in all sim(+) brain neurons, except the PSC and lamina. Thus, together these two enhancers drive expression in all sim(+) brain neurons. Sequence analysis of existing sim mutant alleles identified three likely null alleles to utilize in MARCM experiments to examine sim brain function. Mutant clones of DAMv1/2 neurons revealed a consistent axonal fasciculation defect. Thus, unlike the embryonic roles of sim that control CNS midline neuron and glial formation and differentiation, postembryonic sim, instead, controls aspects of axon guidance in the brain. This resembles the roles of vertebrate sim that have an early role in neuronal **migration** and a later role in axonogenesis.
13. FGF8-like1 and FGF8-like2 encode putative ligands of the FGF receptor Htl and are required for mesoderm **migration** in the Drosophila gastrula. Mesoderm **migration** in the Drosophila gastrula depends on the fibroblast growth factor (FGF) receptor Heartless (Htl). During gastrulation Htl is required for adhesive interactions of the mesoderm with the ectoderm and for the generation of protrusive activity of the mesoderm cells during **migration**. After gastrulation Htl is essential for the differentiation of dorsal mesodermal derivatives. It is not known how Htl is activated, because its ligand has not yet been identified.We performed a genome-wide genetic screen for early zygotic genes and identified seven genomic regions that are required for normal **migration** of the mesoderm cells during gastrulation. One of these genomic intervals produces upon its deletion a phenocopy of the htl cell **migration** phenotype. Here we present the genetic and molecular mapping of this genomic region. We identified two genes, FGF8-like1 and FGF8-like2, that encode novel FGF homologs and were only partially annotated in the Drosophila genome. We show that FGF8-like1 and FGF8-like2 are expressed in the neuroectoderm during gastrulation and present evidence that both act in concert to direct cell shape changes during mesodermal cell **migration** and are required for the activation of the Htl signaling cascade during gastrulation.We conclude that FGF8-like1 and FGF8-like2 encode two novel Drosophila FGF homologs, which are required for mesodermal cell **migration** during gastrulation. Our results suggest that FGF8-like1 and FGF8-like2 represent ligands of the Htl FGF receptor.
14. Proteolytic processing and cell biological functions of the amyloid precursor protein. Recent research has identified some key players involved in the proteolytic processing of amyloid precursor protein (APP) to amyloid beta-peptide, the principal component of the amyloid plaques in Alzheimer patients. Interesting parallels exists with the proteolysis of other proteins involved in cell differentiation, cholesterol homeostasis and stress responses. Since the cytoplasmic domain of APP is anchored to a complex protein network that might function in axonal elongation, dendritic arborisation and neuronal cell **migration**, the proteolysis of APP might be critically involved in intracellular signalling events.

### ECM

#### PubMed

1. Haemocyte-derived SPARC is required for collagen-IV-dependent stability of basal laminae in Drosophila embryos. SPARC is an evolutionarily conserved collagen-binding extracellular matrix (**ECM**) glycoprotein whose morphogenetic contribution(s) to embryonic development remain elusive despite decades of research. We have therefore used Drosophila genetics to gain insight into the role of SPARC during embryogenesis. In Drosophila embryos, high levels of SPARC and other basal lamina components (such as network-forming collagen IV, laminin and perlecan) are synthesized and secreted by haemocytes, and assembled into basal laminae. A SPARC mutant was generated by P-element mutagenesis that is embryonic lethal because of multiple developmental defects. Whereas no differences in collagen IV immunostaining were observed in haemocytes between wild-type and SPARC-mutant embryos, collagen IV was not visible in basal laminae of SPARC-mutant embryos. In addition, the laminin network of SPARC-mutant embryos appeared fragmented and discontinuous by late embryogenesis. Transgenic expression of SPARC protein by haemocytes in SPARC-mutant embryos restored collagen IV and laminin continuity in basal laminae. However, transgenic expression of SPARC by neural cells failed to rescue collagen IV in basal laminae, indicating that the presence of collagen IV deposition requires SPARC expression by haemocytes. Our previous finding that haemocyte-derived SPARC protein levels are reduced in collagen-IV-mutant embryos and the observation that collagen-IV-mutant embryos showed a striking phenotypic similarity to SPARC-mutant embryos suggests a mutual dependence between these major basal laminae components during embryogenesis. Patterning defects and impaired condensation of the ventral nerve cord also resulted from the loss SPARC expression prior to haemocyte migration. Hence, SPARC is required for basal lamina maturation and condensation of the ventral nerve cord during Drosophila embryogenesis.
2. Dystroglycan is required for polarizing the epithelial cells and the oocyte in Drosophila. The transmembrane protein Dystroglycan is a central element of the dystrophin-associated glycoprotein complex, which is involved in the pathogenesis of many forms of muscular dystrophy. Dystroglycan is a receptor for multiple extracellular matrix (**ECM**) molecules such as Laminin, agrin and perlecan, and plays a role in linking the **ECM** to the actin cytoskeleton; however, how these interactions are regulated and their basic cellular functions are poorly understood. Using mosaic analysis and RNAi in the model organism Drosophila melanogaster, we show that Dystroglycan is required cell-autonomously for cellular polarity in two different cell types, the epithelial cells (apicobasal polarity) and the oocyte (anteroposterior polarity). Loss of Dystroglycan function in follicle and disc epithelia results in expansion of apical markers to the basal side of cells and overexpression results in a reduced apical localization of these same markers. In Dystroglycan germline clones early oocyte polarity markers fail to be localized to the posterior, and oocyte cortical F-actin organization is abnormal. Dystroglycan is also required non-cell-autonomously to organize the planar polarity of basal actin in follicle cells, possibly by organizing the Laminin **ECM**. These data suggest that the primary function of Dystroglycan in oogenesis is to organize cellular polarity; and this study sets the stage for analyzing the Dystroglycan complex by using the power of Drosophila molecular genetics.

### [Ee]xtracellular [Mm]atrix

#### PubMed

1. Haemocyte-derived SPARC is required for collagen-IV-dependent stability of basal laminae in Drosophila embryos. SPARC is an evolutionarily conserved collagen-binding **extracellular matrix** (ECM) glycoprotein whose morphogenetic contribution(s) to embryonic development remain elusive despite decades of research. We have therefore used Drosophila genetics to gain insight into the role of SPARC during embryogenesis. In Drosophila embryos, high levels of SPARC and other basal lamina components (such as network-forming collagen IV, laminin and perlecan) are synthesized and secreted by haemocytes, and assembled into basal laminae. A SPARC mutant was generated by P-element mutagenesis that is embryonic lethal because of multiple developmental defects. Whereas no differences in collagen IV immunostaining were observed in haemocytes between wild-type and SPARC-mutant embryos, collagen IV was not visible in basal laminae of SPARC-mutant embryos. In addition, the laminin network of SPARC-mutant embryos appeared fragmented and discontinuous by late embryogenesis. Transgenic expression of SPARC protein by haemocytes in SPARC-mutant embryos restored collagen IV and laminin continuity in basal laminae. However, transgenic expression of SPARC by neural cells failed to rescue collagen IV in basal laminae, indicating that the presence of collagen IV deposition requires SPARC expression by haemocytes. Our previous finding that haemocyte-derived SPARC protein levels are reduced in collagen-IV-mutant embryos and the observation that collagen-IV-mutant embryos showed a striking phenotypic similarity to SPARC-mutant embryos suggests a mutual dependence between these major basal laminae components during embryogenesis. Patterning defects and impaired condensation of the ventral nerve cord also resulted from the loss SPARC expression prior to haemocyte migration. Hence, SPARC is required for basal lamina maturation and condensation of the ventral nerve cord during Drosophila embryogenesis.
2. Dystroglycan is required for polarizing the epithelial cells and the oocyte in Drosophila. The transmembrane protein Dystroglycan is a central element of the dystrophin-associated glycoprotein complex, which is involved in the pathogenesis of many forms of muscular dystrophy. Dystroglycan is a receptor for multiple **extracellular matrix** (ECM) molecules such as Laminin, agrin and perlecan, and plays a role in linking the ECM to the actin cytoskeleton; however, how these interactions are regulated and their basic cellular functions are poorly understood. Using mosaic analysis and RNAi in the model organism Drosophila melanogaster, we show that Dystroglycan is required cell-autonomously for cellular polarity in two different cell types, the epithelial cells (apicobasal polarity) and the oocyte (anteroposterior polarity). Loss of Dystroglycan function in follicle and disc epithelia results in expansion of apical markers to the basal side of cells and overexpression results in a reduced apical localization of these same markers. In Dystroglycan germline clones early oocyte polarity markers fail to be localized to the posterior, and oocyte cortical F-actin organization is abnormal. Dystroglycan is also required non-cell-autonomously to organize the planar polarity of basal actin in follicle cells, possibly by organizing the Laminin ECM. These data suggest that the primary function of Dystroglycan in oogenesis is to organize cellular polarity; and this study sets the stage for analyzing the Dystroglycan complex by using the power of Drosophila molecular genetics.

---

## B4

- NCBI Gene
- GeneRIF
- Pubmed

| Search Term | Rank | Fields |
| --- | --- | --- |
| [Mm]igration | 6 | PubMed(1) |

  
hide/show details for B4

### [Mm]igration

#### PubMed

1. A functional RNAi screen for regulators of receptor tyrosine kinase and ERK signalling. Receptor tyrosine kinase (RTK) signalling through extracellular-signal-regulated kinases (ERKs) has pivotal roles during metazoan development, underlying processes as diverse as fate determination, differentiation, proliferation, survival, **migration** and growth. Abnormal RTK/ERK signalling has been extensively documented to contribute to developmental disorders and disease, most notably in oncogenic transformation by mutant RTKs or downstream pathway components such as Ras and Raf. Although the core RTK/ERK signalling cassette has been characterized by decades of research using mammalian cell culture and forward genetic screens in model organisms, signal propagation through this pathway is probably regulated by a larger network of moderate, context-specific proteins. The genes encoding these proteins may not have been discovered through traditional screens owing, in particular, to the requirement for visible phenotypes. To obtain a global view of RTK/ERK signalling, we performed an unbiased, RNA interference (RNAi), genome-wide, high-throughput screen in Drosophila cells using a novel, quantitative, cellular assay monitoring ERK activation. Here we show that ERK pathway output integrates a wide array of conserved cellular processes. Further analysis of selected components-in multiple cell types with different RTK ligands and oncogenic stimuli-validates and classifies 331 pathway regulators. The relevance of these genes is highlighted by our isolation of a Ste20-like kinase and a PPM-family phosphatase that seem to regulate RTK/ERK signalling in vivo and in mammalian cells. Novel regulators that modulate specific pathway outputs may be selective targets for drug discovery.

---

## Sk1

- NCBI Gene
- GeneRIF
- Pubmed

| Search Term | Rank | Fields |
| --- | --- | --- |
| [Mm]igration | 6 | PubMed(1) |

  
hide/show details for Sk1

### [Mm]igration

#### PubMed

1. Characterization of the Drosophila sphingosine kinases and requirement for Sk2 in normal reproductive function. Sphingosine kinase is a highly conserved enzyme that catalyzes the synthesis of sphingosine 1-phosphate and reduces cellular levels of sphingosine and ceramide. Although ceramide is pro-apoptotic and sphingosine is generally growth-inhibitory, sphingosine 1-phosphate signaling promotes cell proliferation, survival, and **migration**. Sphingosine kinase is thus in a strategic position to regulate important cell fate decisions which may contribute to normal animal development. To facilitate studies examining the potential role of sphingosine kinase and long chain base metabolism in Drosophila development, we characterized two putative Drosophila sphingosine kinase genes, Sk1 and Sk2. Both genes functionally and biochemically complement a yeast sphingosine kinase mutant, express predominantly cytosolic activities, and are capable of phosphorylating a range of endogenous and non-endogenous sphingoid base substrates. The two genes demonstrate overlapping but distinct temporal and spatial expression patterns in the Drosophila embryo, and timing of expression is consistent with observed changes in long chain base levels throughout development. A null Sk2 transposon insertion mutant demonstrated elevated long chain base levels, impaired flight performance, and diminished ovulation. This is the first reported mutation of a sphingosine kinase in an animal model; the associated phenotypes indicate that Sk1 and Sk2 are not redundant in biological function and that sphingosine kinase is essential for diverse physiological functions in this organism.

---

## l(2)gd1

- NCBI Gene
- GeneRIF
- Pubmed

| Search Term | Rank | Fields |
| --- | --- | --- |
| [Mm]etastasis | 12 | PubMed(1) |

  
hide/show details for l(2)gd1

### [Mm]etastasis

#### PubMed

1. Oncogenic programmes and Notch activity: an 'organized crime'? The inappropriate Notch signalling can influence virtually all aspect of cancer, including tumour-cell growth, survival, apoptosis, angiogenesis, invasion and **metastasis**, although it does not do this alone. Hence, elucidating the partners of Notch that are active in cancer is now the focus of much intense research activity. The genetic toolkits available, coupled to the small size and short life of the fruit fly Drosophila melanogaster, makes this an inexpensive and effective animal model, suited to large-scale cancer gene discovery studies. The fly eye is not only a non-vital organ but its stereotyped size and disposition also means it is easy to screen for mutations that cause tumours and metastases and provides ample opportunities to test cancer theories and to unravel unanticipated nexus between Notch and other cancer genes, or to discover unforeseen Notch's partners in cancer. These studies suggest that Notch's oncogenic capacity is brought about not simply by increasing signal strength but through partnerships, whereby oncogenes gain more by cooperating than acting individually, as in a ring 'organized crime'.

---

## raw

- NCBI Gene
- GeneRIF
- Pubmed

| Search Term | Rank | Fields |
| --- | --- | --- |
| [Mm]igration | 6 | PubMed(2) |

  
hide/show details for raw

### [Mm]igration

#### PubMed

1. Signaling pathways directing the movement and fusion of epithelial sheets: lessons from dorsal closure in Drosophila. Wound healing in embryos and various developmental events in metazoans require the spreading and fusion of epithelial sheets. The complex signaling pathways regulating these processes are being pieced together through genetic, cell biological, and biochemical approaches. At present, dorsal closure of the Drosophila embryo is the best-characterized example of epithelial sheet movement. Dorsal closure involves **migration** of the lateral epidermal flanks to close a hole in the dorsal epidermis occupied by an epithelium called the amnioserosa. Detailed genetic studies have revealed a network of interacting signaling molecules regulating this process. At the center of this network is a Jun N-terminal kinase cascade acting at the leading edge of the migrating epidermis that triggers signaling by the TGF-beta superfamily member Decapentaplegic and which interacts with the Wingless pathway. These signaling modules regulate the cytoskeletal reorganization and cell shape change necessary to drive dorsal closure. Activation of this network requires signals from the amnioserosa and input from a variety of proteins at cell-cell junctions. The Rho family of small GTPases is also instrumental, both in activation of signaling and regulation of the cytoskeleton. Many of the proteins regulating dorsal closure have been implicated in epithelial movement in other organisms, and dorsal closure has emerged as an ideal model system for the study of the **migration** and fusion of epithelial sheets.
2. A functional RNAi screen for regulators of receptor tyrosine kinase and ERK signalling. Receptor tyrosine kinase (RTK) signalling through extracellular-signal-regulated kinases (ERKs) has pivotal roles during metazoan development, underlying processes as diverse as fate determination, differentiation, proliferation, survival, **migration** and growth. Abnormal RTK/ERK signalling has been extensively documented to contribute to developmental disorders and disease, most notably in oncogenic transformation by mutant RTKs or downstream pathway components such as Ras and Raf. Although the core RTK/ERK signalling cassette has been characterized by decades of research using mammalian cell culture and forward genetic screens in model organisms, signal propagation through this pathway is probably regulated by a larger network of moderate, context-specific proteins. The genes encoding these proteins may not have been discovered through traditional screens owing, in particular, to the requirement for visible phenotypes. To obtain a global view of RTK/ERK signalling, we performed an unbiased, RNA interference (RNAi), genome-wide, high-throughput screen in Drosophila cells using a novel, quantitative, cellular assay monitoring ERK activation. Here we show that ERK pathway output integrates a wide array of conserved cellular processes. Further analysis of selected components-in multiple cell types with different RTK ligands and oncogenic stimuli-validates and classifies 331 pathway regulators. The relevance of these genes is highlighted by our isolation of a Ste20-like kinase and a PPM-family phosphatase that seem to regulate RTK/ERK signalling in vivo and in mammalian cells. Novel regulators that modulate specific pathway outputs may be selective targets for drug discovery.

---

## tkv

- NCBI Gene
- GeneRIF
- Pubmed

| Search Term | Rank | Fields |
| --- | --- | --- |
| [Ff]ilopodia | 1 | PubMed(3) |
| [Mm]igration | 6 | Process(2); PubMed(38); GeneRIFs(1) |
| ECM | 7 | PubMed(2) |
| [Ee]xtracellular [Mm]atrix | 8 | PubMed(11) |
| [Ii]nvasive | 10 | PubMed(2) |
| [Mm]etastasis | 12 | PubMed(2) |

  
hide/show details for tkv

### [Ff]ilopodia

#### PubMed

1. JNK and decapentaplegic signaling control adhesiveness and cytoskeleton dynamics during thorax closure in Drosophila. One of the fundamental events in metamorphosis in insects is the replacement of larval tissues by imaginal tissues. Shortly after pupariation the imaginal discs evaginate to assume their positions at the surface of the prepupal animal. This is a very precise process that is only beginning to be understood. In Drosophila, during embryonic dorsal closure, the epithelial cells push the amnioserosa cells, which contract and eventually invaginate in the body cavity. In contrast, we find that during pupariation the imaginal cells crawl over the passive larval tissue following a very accurate temporal and spatial pattern. Spreading is driven by **filopodia** and actin bridges that, protruding from the leading edge, mediate the stretching of the imaginal epithelia. Although interfering with JNK (Jun N-terminal kinase) and dpp (decapentaplegic) produces similar phenotypic effects suppressing closure, their effects at the cellular level are different. The loss of JNK activity alters the adhesion properties of larval cells and leads to the detachment of the imaginal and larval tissues. The absence of dpp signaling affects the actin cytoskeleton, blocks the emission of **filopodia**, and promotes the collapse of the leading edge of the imaginal tissues. Interestingly, these effects are very similar to those observed after interfering with JNK and dpp signaling during embryonic dorsal closure.
2. Dependence of Drosophila wing imaginal disc cytonemes on Decapentaplegic. The anterior/posterior (A/P) and dorsal/ventral (D/V) compartment borders that subdivide the wing imaginal discs of Drosophila third instar larvae are each associated with a developmental organizer. Decapentaplegic (Dpp), a member of the transforming growth factor-beta (TGF-beta) superfamily, embodies the activity of the A/P organizer. It is produced at the A/P organizer and distributes in a gradient of decreasing concentration to regulate target genes, functioning non-autonomously to regulate growth and patterning of both the anterior and posterior compartments. Wingless (Wg) is produced at the D/V organizer and embodies its activity. The mechanisms that distribute Dpp and Wg are not known, but proposed mechanisms include extracellular diffusion, successive transfers between neighbouring cells, vesicle-mediated movement, and direct transfer via cytonemes. Cytonemes are actin-based **filopodia**l extensions that have been found to orient towards the A/P organizer from outlying cells. Here we show that in the wing disc, cytonemes orient towards both the A/P and D/V organizers, and that their presence and orientation correlates with Dpp signalling. We also show that the Dpp receptor, Thickveins (Tkv), is present in punctae that move along cytonemes. These observations are consistent with a role for cytonemes in signal transduction.
3. Specificity of Drosophila cytonemes for distinct signaling pathways. Cytonemes are types of **filopodia** in the Drosophila wing imaginal disc that are proposed to serve as conduits in which morphogen signaling proteins move between producing and target cells. We investigated the specificity of cytonemes that are made by target cells. Cells in wing discs made cytonemes that responded specifically to Decapentaplegic (Dpp) and cells in eye discs made cytonemes that responded specifically to Spitz (the Drosophila epidermal growth factor protein). Tracheal cells had at least two types: one made in response to Branchless (a Drosophila fibroblast growth factor protein, Bnl), to which they segregate the Bnl receptor, and another to which they segregate the Dpp receptor. We conclude that cells can make several types of cytonemes, each of which responds specifically to a signaling pathway by means of the selective presence of a particular signaling protein receptor that has been localized to that cytoneme.

### [Mm]igration

#### Process

1. cell **migration**
2. germ cell **migration**

#### PubMed

1. Sec61alpha is required for dorsal closure during Drosophila embryogenesis through its regulation of Dpp signaling. During dorsal closure in Drosophila, signaling events in the dorsalmost row of epidermal cells (DME cells) direct the **migration** of lateral epidermal sheets towards the dorsal midline where they fuse to enclose the embryo. A Jun amino-terminal kinase (JNK) cascade in the DME cells induces the expression of Decapentaplegic (Dpp). Dpp signaling then regulates the cytoskeleton in the DME cells and amnioserosa to affect the cell shape changes necessary to complete dorsal closure. We identified a mutation in Sec61alpha that specifically perturbs dorsal closure. Sec61alpha encodes the main subunit of the translocon complex for co-translational import of proteins into the ER. JNK signaling is normal in Sec61alpha mutant embryos, but Dpp signaling is attenuated and the DME cells fail to maintain an actinomyosin cable as epithelial **migration** fails. Consistent with this model, dorsal closure is rescued in Sec61alpha mutant embryos by an activated form of the Dpp receptor Thick veins.
2. Posterior **migration** of the salivary gland requires an intact visceral mesoderm and integrin function. The final overall shape of an organ and its position within the developing embryo arise as a consequence of both its intrinsic properties and its interactions with surrounding tissues. Here, we focus on the role of directed cell **migration** in shaping and positioning the Drosophila salivary gland. We demonstrate that the salivary gland turns and migrates along the visceral mesoderm to become properly oriented with respect to the overall embryo. We show that salivary gland posterior **migration** requires the activities of genes that position the visceral mesoderm precursors, such as heartless, thickveins, and tinman, but does not require a differentiated visceral mesoderm. We also demonstrate a role for integrin function in salivary gland **migration**. Although the mutations affecting salivary gland motility and directional **migration** cause defects in the final positioning of the salivary gland, most do not affect the length or diameter of the salivary gland tube. These findings suggest that salivary tube dimensions may be an intrinsic property of salivary gland cells.
3. Dcdc42 acts in TGF-beta signaling during Drosophila morphogenesis: distinct roles for the Drac1/JNK and Dcdc42/TGF-beta cascades in cytoskeletal regulation. During Drosophila embryogenesis the two halves of the lateral epidermis migrate dorsally over a surface of flattened cells, the amnioserosa, and meet at the dorsal midline in order to form the continuous sheet of the larval epidermis. During this process of epithelial **migration**, known as dorsal closure, signaling from a Jun-amino-terminal-kinase cascade causes the production of the secreted transforming-growth-factor-beta-like ligand, Decapentaplegic. Binding of Decapentaplegic to the putative transforming-growth-factor-beta-like receptors Thickveins and Punt activates a transforming-growth-factor-beta-like pathway that is also required for dorsal closure. Mutations in genes involved in either the Jun-amino-terminal-kinase cascade or the transforming-growth-factor-beta-like signaling pathway can disrupt dorsal closure. Our findings show that although these pathways are linked they are not equivalent in function. Signaling by the Jun-amino-terminal-kinase cascade may be initiated by the small Ras-like GTPase Drac1 and acts to assemble the cytoskeleton and specify the identity of the first row of cells of the epidermis prior to the onset of dorsal closure. Signaling in the transforming-growth-factor-beta-like pathway is mediated by Dcdc42, and acts during the closure process to control the mechanics of the **migration** process, most likely via its putative effector kinase DPAK.
4. More than cell death: caspases and caspase inhibitors on the move. It is becoming clear that "apoptotic" caspases can effect cellular processes other than cell death. A recent paper in Cell points to a novel role of the Drosophila caspase inhibitor DIAP1 as a determinant of cell **migration**.
5. Posterior **migration** of the salivary gland requires an intact visceral mesoderm and integrin function. The final overall shape of an organ and its position within the developing embryo arise as a consequence of both its intrinsic properties and its interactions with surrounding tissues. Here, we focus on the role of directed cell **migration** in shaping and positioning the Drosophila salivary gland. We demonstrate that the salivary gland turns and migrates along the visceral mesoderm to become properly oriented with respect to the overall embryo. We show that salivary gland posterior **migration** requires the activities of genes that position the visceral mesoderm precursors, such as heartless, thickveins, and tinman, but does not require a differentiated visceral mesoderm. We also demonstrate a role for integrin function in salivary gland **migration**. Although the mutations affecting salivary gland motility and directional **migration** cause defects in the final positioning of the salivary gland, most do not affect the length or diameter of the salivary gland tube. These findings suggest that salivary tube dimensions may be an intrinsic property of salivary gland cells.
6. Integration of epithelial patterning and morphogenesis in Drosophila ovarian follicle cells. Drosophila oogenesis involves the coordinated development of germ cells and an overlying follicular epithelium. The follicle cells provide a genetically tractable system to investigate the cell biology of patterning and morphogenesis. Follicle cells initially form a cuboidal epithelium surrounding a syncytium of nurse cells and oocyte. Epithelial structure is maintained as these cells reorganize to create the three dimensional architecture of the eggshell. Both long-range and short-range cell-cell communications pattern the domains of follicle cells that will create specific eggshell structures. After terminal differentiation to deposit the eggshell proteins, the follicle cells die. This review summarizes recent progress in understanding the cell-cell communication that orchestrates follicle cell patterning and **migration**s. DE-cadherin-mediated adhesion is important at several steps in egg chamber formation and follicle cell **migration**. Notch signaling is critical during each successive round of patterning and **migration**. Integration of bone morphogenetic protein (BMP) and epidermal growth factor (EGF) signals patterns the elaborate structures of the dorsal-anterior eggshell.
7. A molecular link between FGF and Dpp signaling in branch-specific **migration** of the Drosophila trachea. The tracheal system of Drosophila embryos achieves its archetypal branching pattern through a series of cell **migration** events requiring the FGF, Dpp, and Wg/WNT signaling pathways. To gain insight into tracheal cell **migration**, we performed an F4 EMS mutagenesis screen to generate and characterize new mutations resulting in tracheal defects. From 2591 mutagenized third chromosome lines, we identified 33 mutations with defects in tracheal development, corresponding to 12 distinct complementation groups. The new mutations included novel hypomorphic alleles of the FGF receptor gene, breathless, and the ETS-domain transcription factor gene, pointed. We show that reduced function of either breathless or pointed specifically affects **migration** of the dorsal and ventral tracheal branches, more specific functions than previously described for these genes. Our analysis reveals that breathless and pointed control dorsal branch **migration** through transcriptional regulation of the Dpp pathway effectors, Knirps and Knirps-related, which are necessary for **migration** of this branch. We further show that expression of knirps or knirps-related rescues dorsal but not ventral branch **migration** in the breathless hypomorph. These studies support a model in which both the Dpp- and the FGF-signaling pathways control expression of knirps and knirps-related, thereby regulating cell **migration** during dorsal branch formation.
8. Coupling of Jun amino-terminal kinase and Decapentaplegic signaling pathways in Drosophila morphogenesis. Dorsal closure in Drosophila embryos involves the **migration** of two lateral epithelia toward the dorsal midline to establish the dorsal ectoderm. Previous work showed that this morphogenetic movement depends on the activities of a Jun amino (N)-terminal kinase kinase (JNKK) encoded by the hemipterous (hep) gene, and of a JNK encoded by basket. Hep is required for cell determination in the leading edge of migrating epithelia, by controlling specific expression of the puckered (puc) gene in these cells. During dorsal closure, decapentaplegic (dpp), a member of the transforming growth factor-beta (TGF-beta) superfamily, is expressed in the row of cells making up the leading edge of the epithelia. Here, we show that the small GTPases Dcdc42, Drac1, and the Hep JNKK control dpp expression in this migratory process. Appropriate dpp and puc expression in the leading edge also depends on the inhibitory function of the puc gene. Further, our data suggest that the leading edge is the source of a JNK autocrine signal, and exclude a role of Dpp as such a ligand. Dorsal closure couples JNK and dpp signaling pathways, a situation that may be conserved in vertebrate development.
9. Cell fate choices in Drosophila tracheal morphogenesis. The Drosophila tracheal system is a branched tubular structure that supplies air to target tissues. The elaborate tracheal morphology is shaped by two linked inductive processes, one involving the choice of cell fates, and the other a guided cell **migration**. We will describe the molecular basis for these processes, and the allocation of cell fate decisions to four temporal hierarchies. First, tracheal placodes are specified within the embryonic ectoderm. Subsequently, branch fates are allocated within the tracheal placodes, prior to **migration**. Localized presentation of the FGF ligand, Branchless, to tracheal cells that express the FGF receptor, Breathless, guides **migration**. Once cell **migration** is initiated, distinct cell fates are determined within each migrating branch. Finally, inhibitory feedback mechanisms ensure the correct assignment of these fates. Tracheal cell fate choices are determined by signaling cascades triggered by signals emanating from the tracheal cells, as well as by ligands produced by adjacent tissues.
10. [Dorsal closure in Drosophila. A genetic model for wound healing?]. Dorsal closure (DC) is a morphogenetic movement that establishes the dorsal ectoderm of the drosophila embryo. During this process, the two lateral epithelia stretch toward the dorsal midline, the suture line of the two leading edges. Cell **migration** during DC relies both on cell shape change controlled by the activity of the JNK pathway in the leading edge cells and modification of cell adhesiveness, probably dependent upon activation of the Dpp (TGF-beta) pathway. Coupling of the JNK and TGF-beta pathways is essential. The sequence of the cellular and molecular events of DC highlights interesting common features with wound healing in vertebrates. Like DC, wound healing relies on the **migration** of epithelia bordered by leading edges controlling the direction and speed of the movement. This review summarizes recent data concerning the control of epithelial morphogenesis during DC and the bases of wound healing. The molecular and cellular events that underlie these two analogous migratory processes are detailed, discussed and compared. We suggest that DC is a good genetic model for wound healing studying.
11. Primordial germ cell **migration** and the assembly of the Drosophila embryonic gonad. During embryogenesis Drosophila pole cells, like germ cells in many other vertebrates and invertebrates, actively migrate before assembling into the gonad. Five separate steps can be distinguished: an initial passive displacement of the germ cells by gastrulation movements, an amoeboid migratory phase during which the pole cells pass through the endoderm, **migration** on endoderm toward mesoderm, separation into two bilateral groups associated with the gonadal mesoderm precursors, followed by condensation into the gonad itself. We have analyzed gonad assembly in embryonic pattern mutants to determine whether distinct cues are required in this process. We show that the initiation of **migration** does not require the presence of the mesoderm, the eventual target of the germ cells. Rather, **migration** is triggered as a consequence of the differentiation of the endoderm. Examination of embryos mutant for maternal genes of the terminal group suggests that a primary role of the endoderm in this process is to allow the germ cell access to the interior of the embryo. In addition, we show that normal gonad assembly requires a region of the embryo that includes the posterior compartment of the fifth and the sixth abdominal segments.
12. Regulation of cell **migration** during tracheal development in Drosophila melanogaster. Most of the knowledge concerning the intracellular mechanisms involved in cell locomotion have been obtained from in vitro studies of cells in culture. Many of the concepts derived from these studies have been partially confirmed in in vivo systems but numerous questions regarding the developmental control of cell **migration** remain to be addressed. Tracheal morphogenesis in Drosophila melanogaster embryos represents an in vivo model system to study the genetic control of cell **migration**. We review what is known about tracheal development and regulation of tracheal cell **migration**. We try to link these in vivo studies and the movement of cells over two dimensional substrates and elaborate on important questions which remain to be addressed in the future.
13. The Drosophila tricellular junction protein Gliotactin regulates its own mRNA levels through BMP-mediated induction of miR-184. Epithelial bicellular and tricellular junctions are essential for establishing and maintaining permeability barriers. Tricellular junctions are formed by the convergence of three bicellular junctions at the corners of neighbouring epithelia. Gliotactin, a member of the Neuroligin family, is located at theDrosophilatricellular junction, and is crucial for the formation of tricellular and septate junctions, as well as permeability barrier function. Gliotactin protein levels are tightly controlled by phosphorylation at tyrosine residues and endocytosis. Blocking endocytosis or overexpressing Gliotactin results in the spread of Gliotactin from the tricellular junction, resulting in apoptosis, delamination and **migration** of epithelial cells. We show that Gliotactin levels are also regulated at the mRNA level by micro (mi)RNA-mediated degradation and that miRNAs are targeted to a short region in the 3'UTR that includes a conserved miR-184 target site. miR-184 also targets a suite of septate junction proteins, including NrxIV, coracle and Mcr. miR-184 expression is triggered when Gliotactin is overexpressed, leading to activation of the BMP signalling pathway. Gliotactin specifically interferes with Dad, an inhibitory SMAD, leading to activation of the Tkv type-I receptor and activation of Mad to elevate the biogenesis and expression of miR-184.
14. Lamellipodia-based **migration**s of larval epithelial cells are required for normal closure of the adult epidermis of Drosophila. Cell **migration**s are an important feature of animal development. They are, furthermore, essential to wound healing and tumour progression. Despite recent progress, it is still mysterious how cell **migration** is spatially and temporally regulated during morphogenesis and how cell **migration** is coordinated with other cellular behaviours to shape tissues and organs. The formation of the abdominal epithelium of Drosophila during metamorphosis provides an attractive system to study morphogenesis. Here, the diploid adult histoblasts replace the polyploid larval epithelial cells (LECs). Using in vivo 4D microscopy, I show that, besides apical constriction and apoptosis, the LECs undergo extensive coordinated **migration**s. The **migration**s follow a transition from a stationary (epithelial) to a migratory mode. The migratory behaviour is stimulated by autocrine Dpp signalling. Directed apical lamellipodia-like protrusions propel the cells. Initially, planar cell polarity determines the orientation of LEC **migration**. While LECs are migrating they also constrict apically, and changes in activity of the small GTPase Rho1 can favour one behaviour over the other. This study shows that the LECs play a more active role in morphogenesis than previously thought, with their **migration**s contributing to abdominal closure. It furthermore provides insights into how the migratory behaviour of cells is regulated during morphogenesis.
15. The Dpp/TGFβ-dependent corepressor Schnurri protects epithelial cells from JNK-induced apoptosis in drosophila embryos. Jun N-terminal kinase (JNK) often mediates apoptosis in response to cellular stress. However, during normal development, JNK signaling controls a variety of live cell behaviors, such as during dorsal closure in Drosophila embryos. During this process, the latent proapoptotic activity of JNK becomes apparent following Dpp signaling suppression, which leads to JNK-dependent transcriptional activation of the proapoptotic gene reaper. Dpp signaling also protects cells from JNK-dependent apoptosis caused by epithelial disruption. We find that repression of reaper transcription by Dpp is mediated by Schnurri. Moreover, reporter gene analysis shows that a transcriptional regulatory module comprising AP-1 and Schnurri binding sites located upstream of reaper integrate the activities of JNK and Dpp. This arrangement allows JNK to control a migratory behavior without triggering apoptosis. Dpp plays a dual role during dorsal closure. It cooperates with JNK in stimulating cell **migratio**n and also prevents JNK from inducing apoptosis.
16. ribbon encodes a novel BTB/POZ protein required for directed cell **migration** in Drosophila melanogaster. During development, directed cell **migration** is crucial for achieving proper shape and function of organs. One well-studied example is the embryonic development of the larval tracheal system of Drosophila, in which at least four signaling pathways coordinate cell **migration** to form an elaborate branched network essential for oxygen delivery throughout the larva. FGF signaling is required for guided **migration** of all tracheal branches, whereas the DPP, EGF receptor, and Wingless/WNT signaling pathways each mediate the formation of specific subsets of branches. Here, we characterize ribbon, which encodes a BTB/POZ-containing protein required for specific tracheal branch **migration**. In ribbon mutant tracheae, the dorsal trunk fails to form, and ventral branches are stunted; however, directed **migration**s of the dorsal and visceral branches are largely unaffected. The dorsal trunk also fails to form when FGF or Wingless/WNT signaling is lost, and we show that ribbon functions downstream of, or parallel to, these pathways to promote anterior-posterior **migration**. Directed cell **migration** of the salivary gland and dorsal epidermis are also affected in ribbon mutants, suggesting that conserved mechanisms may be employed to orient cell **migration**s in multiple tissues during development.
17. bullwinkle and shark regulate dorsal-appendage morphogenesis in Drosophila oogenesis. bullwinkle (bwk) regulates embryonic anteroposterior patterning and, through a novel germline-to-soma signal, morphogenesis of the eggshell dorsal appendages. We screened for dominant modifiers of the bullwinkle mooseantler eggshell phenotype and identified shark, which encodes an SH2-domain, ankyrin-repeat tyrosine kinase. At the onset of dorsal-appendage formation, shark is expressed in a punctate pattern in the squamous stretch cells overlying the nurse cells. Confocal microscopy with cell-type-specific markers demonstrates that the stretch cells act as a substrate for the migrating dorsal-appendage-forming cells and extend cellular projections towards them. Mosaic analyses reveal that shark is required in follicle cells for cell **migration** and chorion deposition. Proper shark RNA expression in the stretch cells requires bwk activity, while restoration of shark expression in the stretch cells suppresses the bwk dorsal-appendage phenotype. These results suggest that shark plays an important downstream role in the bwk-signaling pathway. Candidate testing implicates Src42A in a similar role, suggesting conservation with a vertebrate signaling pathway involving non-receptor tyrosine kinases.
18. The transcription factors KNIRPS and KNIRPS RELATED control cell **migration** and branch morphogenesis during Drosophila tracheal development. Cell **migration** during embryonic tracheal system development in Drosophila requires DPP and EGF signaling to generate the archetypal branching pattern. We show that two genes encoding the transcription factors KNIRPS and KNIRPS RELATED possess multiple and redundant functions during tracheal development. knirps/knirps related activity is necessary to mediate DPP signaling which is required for tracheal cell **migration** and formation of the dorsal and ventral branches. Ectopic knirps or knirps related expression in lateral tracheal cells respecifies their anteroposterior to a dorsoventral **migration** behavior, similar to that observed in the case of ectopic DPP expression. In dorsal tracheal cells knirps/knirps related activity represses the transcription factor SPALT; this repression is essential for secondary and terminal branch formation. However, in cells of the dorsal trunk, spalt expression is required for normal anteroposterior cell **migration** and morphogenesis. spalt expression is maintained by the EGF receptor pathway and, hence, some of the opposing activities of the EGF and DPP signaling pathways are mediated by spalt and knirps/knirps related. Furthermore, we provide evidence that the border between cells acquiring dorsal branch and dorsal trunk identity is established by the direct interaction of KNIRPS with a spalt cis-regulatory element.
19. Spinster controls Dpp signaling during glial **migration** in the Drosophila eye. The development of multicellular organisms requires the well balanced and coordinated **migration** of many cell types. This is of particular importance within the developing nervous system, where glial cells often move long distances to reach their targets. The majority of glial cells in the peripheral nervous system of the Drosophila embryo is derived from the CNS and migrates along motor axons toward their targets. In the developing Drosophila eye, CNS-derived glial cells move outward toward the nascent photoreceptor cells, but the molecular mechanisms coupling the **migration** of glial cells with the growth of the eye imaginal disc are mostly unknown. Here, we used an enhancer trap approach to identify the gene spinster, which encodes a multipass transmembrane protein involved in endosome-lysosome trafficking, as being expressed in many glial cells. spinster mutants are characterized by glial over**migration**. Genetic experiments demonstrate that Spinster modulates the activity of several signaling cascades. Within the migrating perineurial glial cells, Spinster is required to downregulate Dpp (Decapentaplegic) signaling activity, which ceases migratory abilities. In addition, Spinster affects the growth of the carpet cell, which indirectly modulates glial **migration**.
20. Signaling pathways directing the movement and fusion of epithelial sheets: lessons from dorsal closure in Drosophila. Wound healing in embryos and various developmental events in metazoans require the spreading and fusion of epithelial sheets. The complex signaling pathways regulating these processes are being pieced together through genetic, cell biological, and biochemical approaches. At present, dorsal closure of the Drosophila embryo is the best-characterized example of epithelial sheet movement. Dorsal closure involves **migration** of the lateral epidermal flanks to close a hole in the dorsal epidermis occupied by an epithelium called the amnioserosa. Detailed genetic studies have revealed a network of interacting signaling molecules regulating this process. At the center of this network is a Jun N-terminal kinase cascade acting at the leading edge of the migrating epidermis that triggers signaling by the TGF-beta superfamily member Decapentaplegic and which interacts with the Wingless pathway. These signaling modules regulate the cytoskeletal reorganization and cell shape change necessary to drive dorsal closure. Activation of this network requires signals from the amnioserosa and input from a variety of proteins at cell-cell junctions. The Rho family of small GTPases is also instrumental, both in activation of signaling and regulation of the cytoskeleton. Many of the proteins regulating dorsal closure have been implicated in epithelial movement in other organisms, and dorsal closure has emerged as an ideal model system for the study of the **migration** and fusion of epithelial sheets.
21. Organ formation in Drosophila: specification and morphogenesis of the salivary gland. The Drosophila salivary gland has emerged as an outstanding model system for the process of organ formation. Many of the component steps, from initial regional specification through cell specialization and morphogenesis, are known and many of the genes required for these different processes have been identified. The salivary gland is a relatively simple organ; the entire gland comprises of only two major cell types, which derive from a single contiguous primordium. Salivary cells cease dividing once they are specified, and organ growth is achieved simply by an increase in size of individual cells, thus eliminating concerns about the potential unequal distribution of determinants during mitosis. Drosophila salivary glands form by the same cellular mechanisms as organs in higher organisms, including regulated cell shape changes, cell intercalation and directed cell **migration**. Thus, learning how these events are coordinated for tissue morphogenesis in an organism for which the genetic and molecular tools are unsurpassed should provide excellent paradigms for dissecting related processes in the more intricate organs of more complicated species.
22. Interactions between the EGF receptor and DPP pathways establish distinct cell fates in the tracheal placodes. The formation of the tracheal network in Drosophila is driven by stereotyped **migration** of cells from the tracheal pits. No cell divisions take place during tracheal **migration** and the number of cells in each branch is fixed. This work examines the basis for the determination of tracheal branch fates, prior to the onset of **migration**. We show that the EGF receptor pathway is activated by localized processing of the ligand SPITZ in the tracheal placodes and is responsible for the capacity to form the dorsal trunk and visceral branch. The DPP pathway, on the contrary, is induced in the tracheal pit by local presentation of DPP from the adjacent dorsal and ventral ectodermal cells. This pathway patterns the dorsal and lateral branches. Elimination of both pathways blocks **migration** of all tracheal branches. Antagonistic interactions between the two pathways are demonstrated. The opposing activities of two pathways may refine the final determination of tracheal branch fates.
23. Genetic modifier screens reveal new components that interact with the Drosophila dystroglycan-dystrophin complex. The Dystroglycan-Dystrophin (Dg-Dys) complex has a capacity to transmit information from the extracellular matrix to the cytoskeleton inside the cell. It is proposed that this interaction is under tight regulation; however the signaling/regulatory components of Dg-Dys complex remain elusive. Understanding the regulation of the complex is critical since defects in this complex cause muscular dystrophy in humans. To reveal new regulators of the Dg-Dys complex, we used a model organism Drosophila melanogaster and performed genetic interaction screens to identify modifiers of Dg and Dys mutants in Drosophila wing veins. These mutant screens revealed that the Dg-Dys complex interacts with genes involved in muscle function and components of Notch, TGF-beta and EGFR signaling pathways. In addition, components of pathways that are required for cellular and/or axonal **migration** through cytoskeletal regulation, such as Semaphorin-Plexin, Frazzled-Netrin and Slit-Robo pathways show interactions with Dys and/or Dg. These data suggest that the Dg-Dys complex and the other pathways regulating extracellular information transfer to the cytoskeletal dynamics are more intercalated than previously thought.
24. EGF signalling regulates cell invagination as well as cell **migration** during formation of tracheal system in Drosophila. The Drosophila tracheal system is a network of epithelial tubes that arises from the tracheal placodes, lateral clusters of ectodermal cells in ten embryonic segments. The cells of each cluster invaginate and subsequent formation of the tracheal tree occurs by cell **migration** and fusion of tracheal branches, without cell division. The combined action of the Decapentaplegic (Dpp), Epidermal growth factor (EGF) and breathless/branchless pathways are thought to be responsible for the pattern of tracheal branches. We ask how these transduction pathways regulate cell **migration** and we analyse the consequences on cell behaviour of the Dpp and EGF pathways. We find that rhomboid (rho) mutant embryos display defects not only in tracheal cell **migration** but also in tracheal cell invagination unveiling a new role for EGF signalling in the formation of the tracheal system. These results indicate that the transduction pathways that control tracheal cell **migration** are active in different steps of tracheal formation, beginning at invagination. We discuss how the consecutive steps of tracheal morphogenesis might affect the final branching pattern.
25. Germ cell specification and **migration** in Drosophila and beyond. The passage of an individual's genome to future generations is essential for the maintenance of species and is mediated by highly specialized cells, the germ cells. Genetic studies in a number of model organisms have provided insight into the molecular mechanisms that control specification, **migration** and survival of early germ cells. Focusing on Drosophila, we will discuss the mechanisms by which germ cells initially form and remain transcriptionally silent while somatic cells are transcriptionally active. We will further discuss three separate attractive and repellent guidance pathways, mediated by a G-protein coupled receptor, two lipid phosphate phosphohydrolases, and isoprenylation. We will compare and contrast these findings with those obtained in other organisms, in particular zebrafish and mice. While aspects of germ cell specification are strikingly different between these species, germ cell specific gene functions have been conserved. In particular, mechanisms that sense directional cues during germ cell **migration** seem to be shared between invertebrates and vertebrates.
26. The genetics of cell **migration** in Drosophila melanogaster and Caenorhabditis elegans development. Cell **migration**s are found throughout the animal kingdom and are among the most dramatic and complex of cellular behaviors. Historically, the mechanics of cell **migration** have been studied primarily in vitro, where cells can be readily viewed and manipulated. However, genetic approaches in relatively simple model organisms are yielding additional insights into the molecular mechanisms underlying cell movements and their regulation during development. This review will focus on these simple model systems where we understand some of the signaling and receptor molecules that stimulate and guide cell movements. The chemotactic guidance factor encoded by the Caenorhabditis elegans unc-6 locus, whose mammalian homolog is Netrin, is perhaps the best known of the cell **migration** guidance factors. In addition, receptor tyrosine kinases (RTKs), and FGF receptors in particular, have emerged as key mediators of cell **migration** in vivo, confirming the importance of molecules that were initially identified and studied in cell culture. Somewhat surprisingly, screens for mutations that affect primordial germ cell **migration** in Drosophila have revealed that enzymes involved in lipid metabolism play a role in guiding cell **migration** in vivo, possibly by producing and/or degrading lipid chemoattractants or chemorepellents. Cell adhesion molecules, such as integrins, have been extensively characterized with respect to their contribution to cell **migration** in vitro and genetic evidence now supports a role for these receptors in certain instances in vivo as well. The role for non-muscle myosin in cell motility was controversial, but has now been demonstrated genetically, at least in some cell types. Currently the best characterized link between membrane receptor signaling and regulation of the actin cytoskeleton is that provided by the Rho family of small GTPases. Members of this family are clearly essential for the **migration**s of some cells; however, key questions remain concerning how chemoattractant and chemorepellent signals are integrated within the cell and transduced to the cytoskeleton to produce directed cell **migration**. New types of genetic screens promise to fill in some of these gaps in the near future.
27. Sec61alpha is required for dorsal closure during Drosophila embryogenesis through its regulation of Dpp signaling. During dorsal closure in Drosophila, signaling events in the dorsalmost row of epidermal cells (DME cells) direct the **migration** of lateral epidermal sheets towards the dorsal midline where they fuse to enclose the embryo. A Jun amino-terminal kinase (JNK) cascade in the DME cells induces the expression of Decapentaplegic (Dpp). Dpp signaling then regulates the cytoskeleton in the DME cells and amnioserosa to affect the cell shape changes necessary to complete dorsal closure. We identified a mutation in Sec61alpha that specifically perturbs dorsal closure. Sec61alpha encodes the main subunit of the translocon complex for co-translational import of proteins into the ER. JNK signaling is normal in Sec61alpha mutant embryos, but Dpp signaling is attenuated and the DME cells fail to maintain an actinomyosin cable as epithelial **migration** fails. Consistent with this model, dorsal closure is rescued in Sec61alpha mutant embryos by an activated form of the Dpp receptor Thick veins.
28. ventral veinless, a POU domain transcription factor, regulates different transduction pathways required for tracheal branching in Drosophila. Cell **migration** is an important step in a variety of developmental processes in many multicellular organisms. A particularly appropriate model to address the study of cell **migration** is the tracheal system of Drosophila, whose formation occurs by **migration** and fusion from clusters of ectodermal cells specified in each side of ten embryonic segments. Morphogenesis of the tracheal tree requires the activity of many genes, among them breathless (btl) and ventral veinless (vvl) whose mutations abolish tracheal cell **migration**. Activation of the btl receptor by branchless (bnl), its putative ligand, exerts an instructive role in the process of guiding tracheal cell **migration**. vvl has been shown to be required for the maintenance of btl expression during tracheal tree formation. Here we show that, in addition, vvl is independently required for the specific expression in the tracheal cells of thick veins (tkv) and rhomboid (rho), two genes whose mutations disrupt only particular branches of the tracheal system. Indeed, we show that expression in the tracheal cells of an activated form of tkv, the putative decapentaplegic (dpp) receptor, is able to induce shifts in their **migration**, asserting the role of the dpp pathway in establishing the branching pattern of the tracheal tree. In addition, by ubiquitous expression of the btl and tkv genes in vvl mutant embryos we show that both genes contribute to vvl function. These results indicate that through activation of its target genes, vvl makes the tracheal cells competent to further signalling and suggest that the btl transduction pathway could collaborate with other transduction pathways also regulated by vvl to specify the tracheal branching pattern.
29. Branching morphogenesis of the Drosophila tracheal system. Many organs including the mammalian lung and vascular system consist of branched tubular networks that transport essential gases or fluids, but the genetic programs that control the development of these complex three-dimensional structures are not well understood. The Drosophila melanogaster tracheal (respiratory) system is a network of interconnected epithelial tubes that transports oxygen and other gases in the body and provides a paradigm of branching morphogenesis. It develops by sequential sprouting of primary, secondary, and terminal branches from an epithelial sac of approximately 80 cells in each body segment of the embryo. Mapping of the cell movements and shape changes during the sprouting process has revealed that distinct mechanisms of epithelial **migration** and tube formation are used at each stage of branching. Genetic dissection of the process has identified a general program in which a fibroblast growth factor (FGF) and fibroblast growth factor receptor (FGFR) are used repeatedly to control branch budding and outgrowth. At each stage of branching, the mechanisms controlling FGF expression and the downstream signal transduction pathway change, altering the pattern and structure of the branches that form. During terminal branching, FGF expression is regulated by hypoxia, ensuring that tracheal structure matches cellular oxygen need. A branch diversification program operates in parallel to the general budding program: Regional signals locally modify the general program, conferring specific structural features and other properties on individual branches, such as their substrate outgrowth preferences, differences in tube size and shape, and the ability to fuse to other branches to interconnect the network.
30. The alternative migratory pathways of the Drosophila tracheal cells are associated with distinct subsets of mesodermal cells. The Drosophila tracheal system is a model for the study of the mechanisms that guide cell **migration**. The general conclusion from many studies is that **migration** of tracheal cells relies on directional cues provided by nearby cells. However, very little is known about which paths are followed by the migrating tracheal cells and what kind of interactions they establish to move in the appropriate direction. Here we analyze how tracheal cells migrate relative to their surroundings and which tissues participate in tracheal cell **migration**. We find that cells in different branches exploit different strategies for their **migration**; while some migrate through preexisting grooves, others make their way through homogeneous cell populations. We also find that alternative migratory pathways of tracheal cells are associated with distinct subsets of mesodermal cells and propose a model for the allocation of groups of tracheal cells to different branches. These results show how adjacent tissues influence morphogenesis of the tracheal system and offer a model for understanding how organ formation is determined by its genetic program and by the surrounding topological constraints.
31. Cad74A is regulated by BR and is required for robust dorsal appendage formation in Drosophila oogenesis. Drosophila egg development is an established model for studying epithelial patterning and morphogenesis, but the connection between signaling pathways and egg morphology is still incompletely understood. We have identified a non-classical cadherin, Cad74A, as a putative adhesion gene that bridges epithelial patterning and morphogenesis in the follicle cells. Starting in mid-oogenesis, Cad74A is expressed in the follicle cells that contact the oocyte, including the border cells and most of the columnar follicle cells. However, Cad74A is repressed in two dorsolateral patches of follicle cells, which participate in the formation of tubular respiratory appendages. We show genetically that Cad74A is downstream of the EGFR and BMP signaling pathways and is repressed by the Zn-finger transcription factor Broad. The correlation of Cad74A repression in the cells that bend out of the plane of the follicular epithelium is preserved across Drosophila species and mutant backgrounds exhibiting a range of eggshell phenotypes. Complete removal of Cad74A from the follicle cells causes defects in dorsal appendage formation. Ectopic expression of Cad74A in the roof cells results in shortened, flattened appendages due to the hindered **migration** of the roof cells. Based on these results, we propose that Cad74A is part of the adhesive machinery that enables robust dorsal appendage formation, and as such provides a link between the patterning of the follicle cells and eggshell morphogenesis.
32. Dpp signaling directs cell motility and invasiveness during epithelial morphogenesis. Tissue remodeling in development and disease involves the coordinated invasion of neighboring territories and/or the replacement of entire cell populations. Cell guidance, cell matching, transitions from passive to migratory epithelia, cell growth and death, and extracellular matrix remodeling all impinge on epithelial spreading. Significantly, the extracellular signals that direct these activities and the specific cellular elements and mechanisms regulated by these signals remain in most cases to be identified. To address these issues, we performed an analysis of histoblasts (Drosophila abdominal epithelial founder cells) on their transition from a dormant state to active **migration** replacing obsolete larval epidermal cells (LECs). We found that during expansion, Decapentaplegic (Dpp) secreted from surrounding LECs leads to graded pathway activation in cells at the periphery of histoblast nests. Across nests, Dpp activity confers differential cellular behavior and motility by modulating cell-cell contacts, the organization and activity of the cytoskeleton, and histoblast attachment to the substrate. Furthermore, Dpp also prevents the premature death of LECs, allowing the coordination of histoblast expansion to LEC delamination. Dpp signaling activity directing histoblast spreading and invasiveness mimics transforming growth factor-beta and bone morphogenetic proteins' role in enhancing the motility and invasiveness of cancer cells, resulting in the promotion of metastasis.
33. Genetic control of cell intercalation during tracheal morphogenesis in Drosophila. Branching morphogenesis transforms an epithelial sheet into a tubular network with distinct features regarding the length and diameter of individual tubes. Branching is controlled by several signaling pathways, but the molecular consequences of these pathways in the responding cells are poorly understood.We have undertaken a detailed characterization of cell rearrangements during tracheal branching morphogenesis in Drosophila embryos with a GFP fusion protein labeling the adherens junctions (AJs) and high-resolution live imaging. To analyze the branching process at the cellular level, we further developed an imaging approach that allows us to follow single cells during the branching process. We find that controlled cell intercalation, which requires extensive AJ remodeling, is key to the formation of tracheal branches of different cellular complexities. In particular, most branches consist of tubes with individual cells wrapped around the lumen. These branches form through cell intercalation, which requires the transformation of most of the initial intercellular AJs into autocellular AJs. We propose a step-wise model explaining how this AJ remodeling occurs and use this model to better understand defects in various mutants. We find that Dpp and Wnt signaling control cell intercalation by regulating the expression of Spalt, a zinc finger transcription factor; Spalt inhibits intercalation, leading to the formation of large, multicellular tubes.Tracheal morphogenesis is regulated by an interplay of different signaling systems that control cell **migration** and cell intercalation, respectively. Only the combined action of these signaling systems allows efficient branch elongation and the formation of morphologically distinct branches.
34. The tracheae defective gene encodes a bZIP protein that controls tracheal cell movement during Drosophila embryogenesis. The tracheae defective (tdf) gene is required for the formation of the tracheal system during Drosophila embryogenesis. It encodes a putative bZIP transcription factor (TDF). Antibodies directed against TDF detect a nuclear protein in all tracheal cells before invagination and throughout tracheal system morphogenesis. Examination of tdf mutants revealed that tdf activity is not necessary for determining tracheal cell identity but for subsequent morphogenetic cell movements. tdf activity is under the control of trachealess, the key regulator gene for tracheal development. In contrast, tdf activity is not dependent on and does not interfere with the fibroblast growth factor- (FGF) and Decapentaplegic- (DPP) mediated signalling that direct guided tracheal cell **migration**. Our results suggest that lack of tdf activity affects tracheal cell **migration** in general rather than specific aspects of cell **migration**. tdf activity involves a maternal and zygotic component and its requirement is not limited to tracheal system formation. The complex spatiotemporal patterns of TDF expression in the embryo correspond to defects, suggesting that cell **migration** is impaired. We propose that the bZIP protein TDF functions as a co-regulator of target genes that provide cells with the ability to migrate.
35. DPP controls tracheal cell **migration** along the dorsoventral body axis of the Drosophila embryo. We report that DPP signaling is required for directed tracheal cell **migration** during Drosophila embryogenesis. The failure of tracheal cells to receive the DPP signal from adjacent dorsal and ventral cells results in the absence of dorsal and ventral **migration**s. Ectopic DPP signaling can reprogram cells in the center of the placode to adopt a dorsoventral **migration** behavior. The effects observed in response to ectopic DPP signaling are also observed upon the tracheal-specific expression of a constitutive active DPP type I receptor (TKV(Q253D)), indicating that the DPP signal is received and transmitted in tracheal cells to control their **migration** behavior. DPP signaling determines localized gene expression patterns in the developing tracheal placode, and is also required for the dorsal expression of the recently identified BRANCHLESS (BNL) guidance molecule, the ligand of the BREATHLESS (BTL) receptor. Thus, DPP plays a dual role during tracheal cell **migration**. It is required to control the dorsal expression of the BNL ligand; in addition, the DPP signal recruits groups of dorsal and ventral tracheal cells and programs them to migrate in dorsal and ventral directions.
36. The protein kinase LKB1 negatively regulates bone morphogenetic protein receptor signaling. The protein kinase LKB1 regulates cell metabolism and growth and is implicated in intestinal and lung cancer. Bone morphogenetic protein (BMP) signaling regulates cell differentiation during development and tissue homeostasis. We demonstrate that LKB1 physically interacts with BMP type I receptors and requires Smad7 to promote downregulation of the receptor. Accordingly, LKB1 suppresses BMP-induced osteoblast differentiation and affects BMP signaling in Drosophila wing longitudinal vein morphogenesis. LKB1 protein expression and Smad1 phosphorylation analysis in a cohort of non-small cell lung cancer patients demonstrated a negative correlation predominantly in a subset enriched in adenocarcinomas. Lung cancer patient data analysis indicated strong correlation between LKB1 loss-of-function mutations and high BMP2 expression, and these two events further correlated with expression of a gene subset functionally linked to apoptosis and **migration**. This new mechanism of BMP receptor regulation by LKB1 has ramifications in physiological organogenesis and disease.
37. Dpp and Hedgehog mediate neuron-glia interactions in Drosophila eye development by promoting the proliferation and motility of subretinal glia. Neuron-glia interactions are crucial for the establishment of normal connectivity in the nervous system during development, but the molecular signals involved in these interactions are largely unknown. Here we show that differentiating photoreceptors in the developing Drosophila eye influence the proliferative and migratory behavior of the subretinal glia through the diffusible factors Decapentaplegic (Dpp) and Hedgehog (Hh). We demonstrate that proliferation and **migration** of the glia are separable processes, and that Dpp promotes both the proliferation and motility of the glia, whereas Hh appears to promote only their motility; neither specifies the direction of **migration**. We present evidence that Dpp and Hh act on the glia in parallel and through the regulation of transcription. Finally, we show that ectopic **migration** of subretinal glia can result in the ectopic projection of photoreceptor axons. Our study suggests a novel function for Hh in regulating migratory behavior and provides further evidence for a complex mutual dependence between glial and neuronal cells during development.
38. Elbow and Noc define a family of zinc finger proteins controlling morphogenesis of specific tracheal branches. The elbow (elB) gene encodes a conserved nuclear protein with a single zinc finger. Expression of ElB is restricted to a specific subset of tracheal cells, namely the dorsal branch and the lateral trunks. Stalled or aberrant **migration** of these branches is observed in elB mutant embryos. Conversely, ElB misexpression in the trachea gave rise to absence of the visceral branch and an increase in the number of cells forming the dorsal branch. These results imply that the restricted expression of ElB contributes to the specification of distinct branch fates, as reflected in their stereotypic pattern of **migration**. As elB loss-of-function tracheal phenotypes are reminiscent of defects in Dpp signaling, the relationship between ElB and the Dpp pathway was examined. By using pMad antibodies that detect the activation pattern of the Dpp pathway, we show that Dpp signaling in the trachea is not impaired in elB mutants. In addition, expression of the Dpp target gene kni was unaltered. The opposite is true as well, because expression of elB is independent of Dpp signaling. ElB thus defines a parallel input, which determines the identity of the lateral trunk and dorsal branch cells. No ocelli (Noc) is the Drosophila protein most similar to ElB. Mutations in noc give rise to a similar tracheal phenotype. Noc is capable of associating with ElB, suggesting that they can function as a heterodimer. ElB also associates with the Groucho protein, indicating that the complex has the capacity to repress transcription of target genes. Indeed, in elB or noc mutants, expanded expression of tracheal branch-specific genes was observed.

#### GeneRIFs

1. We show that salivary gland posterior **migration** requires the activities of genes that position the visceral mesoderm precursors, such as heartless, thickveins, and tinman, but does not require a differentiated visceral mesoderm.

### ECM

#### PubMed

1. Peak BMP Responses in the Drosophila Embryo Are Dependent on the Activation of Integrin Signaling. Within a 3D tissue, cells need to integrate signals from growth factors, such as BMPs, and the extracellular matrix (**ECM**) to coordinate growth and differentiation. Here, we use the Drosophila embryo as a model to investigate how BMP responses are influenced by a cell's local **ECM** environment. We show that integrins, which are **ECM** receptors, are absolutely required for peak BMP signaling. This stimulatory effect of integrins requires their intracellular signaling function, which is activated by the **ECM** protein collagen IV. Mechanistically, integrins interact with the BMP receptor and stimulate phosphorylation of the downstream Mad transcription factor. The BMP-pathway-enhancing function of integrins is independent of focal adhesion kinase, but it requires conserved NPXY motifs in the β-integrin cytoplasmic tail. Furthermore, we show that an α-integrin subunit is a BMP target gene, identifying positive feedback between integrin signaling and BMP pathway activity that may contribute to robust cell fate decisions.
2. Extracellular matrix and its receptors in Drosophila neural development. Extracellular matrix (**ECM**) and matrix receptors are intimately involved in most biological processes. The **ECM** plays fundamental developmental and physiological roles in health and disease, including processes underlying the development, maintenance, and regeneration of the nervous system. To understand the principles of **ECM**-mediated functions in the nervous system, genetic model organisms like Drosophila provide simple, malleable, and powerful experimental platforms. This article provides an overview of **ECM** proteins and receptors in Drosophila. It then focuses on their roles during three progressive phases of neural development: (1) neural progenitor proliferation, (2) axonal growth and pathfinding, and (3) synapse formation and function. Each section highlights known **ECM** and **ECM**-receptor components and recent studies done in mutant conditions to reveal their in vivo functions, all illustrating the enormous opportunities provided when merging work on the nervous system with systematic research into **ECM**-related gene functions.

### [Ee]xtracellular [Mm]atrix

#### PubMed

1. Dose-sensitive autosomal modifiers identify candidate genes for tissue autonomous and tissue nonautonomous regulation by the Drosophila nuclear zinc-finger protein, hindsight. The nuclear zinc-finger protein encoded by the hindsight (hnt) locus regulates several cellular processes in Drosophila epithelia, including the Jun N-terminal kinase (JNK) signaling pathway and actin polymerization. Defects in these molecular pathways may underlie the abnormal cellular interactions, loss of epithelial integrity, and apoptosis that occurs in hnt mutants, in turn causing failure of morphogenetic processes such as germ band retraction and dorsal closure in the embryo. To define the genetic pathways regulated by hnt, 124 deficiencies on the second and third chromosomes and 14 duplications on the second chromosome were assayed for dose-sensitive modification of a temperature-sensitive rough eye phenotype caused by the viable allele, hntpeb; 29 interacting regions were identified. Subsequently, 438 P-element-induced lethal mutations mapping to these regions and 12 candidate genes were tested for genetic interaction, leading to identification of 63 dominant modifier loci. A subset of the identified mutants also dominantly modify hnt308-induced embryonic lethality and thus represent general rather than tissue-specific interactors. General interactors include loci encoding transcription factors, actin-binding proteins, signal transduction proteins, and components of the **extracellular matrix**. Expression of several interactors was assessed in hnt mutant tissue. Five genes--apontic (apt), Delta (Dl), decapentaplegic (dpp), karst (kst), and puckered (puc)--are regulated tissue autonomously and, thus, may be direct transcriptional targets of HNT. Three of these genes--apt, Dl, and dpp--are also regulated nonautonomously in adjacent non-HNT-expressing tissues. The expression of several additional interactors--viking (vkg), Cg25, and laminin-alpha (LanA)-is affected only in a nonautonomous manner.
2. Peak BMP Responses in the Drosophila Embryo Are Dependent on the Activation of Integrin Signaling. Within a 3D tissue, cells need to integrate signals from growth factors, such as BMPs, and the **extracellular matrix** (ECM) to coordinate growth and differentiation. Here, we use the Drosophila embryo as a model to investigate how BMP responses are influenced by a cell's local ECM environment. We show that integrins, which are ECM receptors, are absolutely required for peak BMP signaling. This stimulatory effect of integrins requires their intracellular signaling function, which is activated by the ECM protein collagen IV. Mechanistically, integrins interact with the BMP receptor and stimulate phosphorylation of the downstream Mad transcription factor. The BMP-pathway-enhancing function of integrins is independent of focal adhesion kinase, but it requires conserved NPXY motifs in the β-integrin cytoplasmic tail. Furthermore, we show that an α-integrin subunit is a BMP target gene, identifying positive feedback between integrin signaling and BMP pathway activity that may contribute to robust cell fate decisions.
3. The Gyc76C Receptor Guanylyl Cyclase and the Foraging cGMP-Dependent Kinase Regulate **Extracellular Matrix** Organization and BMP Signaling in the Developing Wing of Drosophila melanogaster. The developing crossveins of the wing of Drosophila melanogaster are specified by long-range BMP signaling and are especially sensitive to loss of extracellular modulators of BMP signaling such as the Chordin homolog Short gastrulation (Sog). However, the role of the **extracellular matrix** in BMP signaling and Sog activity in the crossveins has been poorly explored. Using a genetic mosaic screen for mutations that disrupt BMP signaling and posterior crossvein development, we identify Gyc76C, a member of the receptor guanylyl cyclase family that includes mammalian natriuretic peptide receptors. We show that Gyc76C and the soluble cGMP-dependent kinase Foraging, likely linked by cGMP, are necessary for normal refinement and maintenance of long-range BMP signaling in the posterior crossvein. This does not occur through cell-autonomous crosstalk between cGMP and BMP signal transduction, but likely through altered extracellular activity of Sog. We identify a novel pathway leading from Gyc76C to the organization of the wing **extracellular matrix** by matrix metalloproteinases, and show that both the **extracellular matrix** and BMP signaling effects are largely mediated by changes in the activity of matrix metalloproteinases. We discuss parallels and differences between this pathway and other examples of cGMP activity in both Drosophila melanogaster and mammalian cells and tissues.
4. Integrins modulate Sog activity in the Drosophila wing. Morphogenesis of the Drosophila wing depends on a series of cell-cell and cell-**extracellular matrix** interactions. During pupal wing development, two secreted proteins, encoded by the short gastrulation (sog) and decapentaplegic (dpp) genes, vie to position wing veins in the center of broad provein territories. Expression of the Bmp4 homolog dpp in vein cells is counteracted by expression of the Bmp antagonist sog in intervein cells, which results in the formation of straight veins of precise width. We screened for genetic interactions between sog and genes encoding a variety of extracellular components and uncovered interactions between sog and myospheroid (mys), multiple edematous wing (mew) and scab (scb), which encode betaPS, alphaPS1 and alphaPS3 integrin subunits, respectively. Clonal analysis reveals that integrin mutations affect the trajectory of veins inside the provein domain and/or their width and that misexpression of sog can alter the behavior of cells in such clones. In addition, we show that a low molecular weight form of Sog protein binds to alphaPS1betaPS. We find that Sog can diffuse from its intervein site of production into adjacent provein domains, but only on the dorsal surface of the wing, where Sog interacts functionally with integrins. Finally, we show that Sog diffusion into provein regions and the reticular pattern of extracellular Sog distribution in wild-type wings requires mys and mew function. We propose that integrins act by binding and possibly regulating the activity/availability of different forms of Sog during pupal development through an adhesion independent mechanism.
5. The detached locus encodes Drosophila Dystrophin, which acts with other components of the Dystrophin Associated Protein Complex to influence intercellular signalling in developing wing veins. Dystrophin and Dystroglycan are the two central components of the multimeric Dystrophin Associated Protein Complex, or DAPC, that is thought to provide a mechanical link between the **extracellular matrix** and the actin cytoskeleton, disruption of which leads to muscular dystrophy in humans. We present the characterization of the Drosophila 'crossveinless' mutation detached (det), and show that the gene encodes the fly ortholog of Dystrophin. Our genetic analysis shows that, in flies, Dystrophin is a non-essential gene, and the sole overt morphological defect associated with null mutations in the locus is the variable loss of the posterior crossvein that has been described for alleles of det. Null mutations in Drosophila Dystroglycan (Dg) are similarly viable and exhibit this crossvein defect, indicating that both of the central DAPC components have been co-opted for this atypical function of the complex. In the developing wing, the Drosophila DAPC affects the intercellular signalling pathways involved in vein specification. In det and Dg mutant wings, the early BMP signalling that initiates crossvein specification is not maintained, particularly in the pro-vein territories adjacent to the longitudinal veins, and this results in the production of a crossvein fragment in the intervein between the two longitudinal veins. Genetic interaction studies suggest that the DAPC may exert this effect indirectly by down-regulating Notch signalling in pro-vein territories, leading to enhanced BMP signalling in the intervein by diffusion of BMP ligands from the longitudinal veins.
6. Genetic modifier screens reveal new components that interact with the Drosophila dystroglycan-dystrophin complex. The Dystroglycan-Dystrophin (Dg-Dys) complex has a capacity to transmit information from the **extracellular matrix** to the cytoskeleton inside the cell. It is proposed that this interaction is under tight regulation; however the signaling/regulatory components of Dg-Dys complex remain elusive. Understanding the regulation of the complex is critical since defects in this complex cause muscular dystrophy in humans. To reveal new regulators of the Dg-Dys complex, we used a model organism Drosophila melanogaster and performed genetic interaction screens to identify modifiers of Dg and Dys mutants in Drosophila wing veins. These mutant screens revealed that the Dg-Dys complex interacts with genes involved in muscle function and components of Notch, TGF-beta and EGFR signaling pathways. In addition, components of pathways that are required for cellular and/or axonal migration through cytoskeletal regulation, such as Semaphorin-Plexin, Frazzled-Netrin and Slit-Robo pathways show interactions with Dys and/or Dg. These data suggest that the Dg-Dys complex and the other pathways regulating extracellular information transfer to the cytoskeletal dynamics are more intercalated than previously thought.
7. Developmental roles of heparan sulfate proteoglycans: a comparative review in Drosophila, mouse and human. In recent years, progress in the fields of development and proteoglycan biology have produced converging evidence of the role of proteoglycans in morphogenesis. Numerous studies have demonstrated that proteoglycans are involved in several distinct morphogenetic pathways upon which they act at different levels. In particular, proteoglycans can determine the generation of morphogen gradients and be required for their signal transduction. The surface of most cells and the **extracellular matrix** are decorated by heparan sulfates which are the most common glycosaminoglycans, normally present as heparan sulfate proteoglycans. Considerable structural heterogeneity is generated in proteoglycans by the biosynthetic modification of their heparan sulfate chains as well as by the diverse nature of their different core proteins. This heterogeneity provides an impressive potential for protein-protein and protein-carbohydrate interactions, and can partly explain the diversity of proteoglycan involvement in different morphogenetic pathways. In this review, we summarize the current knowledge about mutations affecting heparan sulfate proteoglycans that influence the function of growth factor pathways essential for tissue assembly, differentiation and development. The comparison of data obtained in Drosophila, rodents and humans reveals that mutations affecting the proteoglycan core proteins or one of the biosynthetic enzymes of their heparan sulfate chains have profound effects on growth and morphogenesis. Further research will complete the picture, but current evidence shows that at the very least, heparan sulfate proteoglycans need to be counted as legitimate elements of morphogenetic pathways that have been maintained throughout evolution as determinant mechanisms of pattern formation.
8. Dpp signaling directs cell motility and invasiveness during epithelial morphogenesis. Tissue remodeling in development and disease involves the coordinated invasion of neighboring territories and/or the replacement of entire cell populations. Cell guidance, cell matching, transitions from passive to migratory epithelia, cell growth and death, and **extracellular matrix** remodeling all impinge on epithelial spreading. Significantly, the extracellular signals that direct these activities and the specific cellular elements and mechanisms regulated by these signals remain in most cases to be identified. To address these issues, we performed an analysis of histoblasts (Drosophila abdominal epithelial founder cells) on their transition from a dormant state to active migration replacing obsolete larval epidermal cells (LECs). We found that during expansion, Decapentaplegic (Dpp) secreted from surrounding LECs leads to graded pathway activation in cells at the periphery of histoblast nests. Across nests, Dpp activity confers differential cellular behavior and motility by modulating cell-cell contacts, the organization and activity of the cytoskeleton, and histoblast attachment to the substrate. Furthermore, Dpp also prevents the premature death of LECs, allowing the coordination of histoblast expansion to LEC delamination. Dpp signaling activity directing histoblast spreading and invasiveness mimics transforming growth factor-beta and bone morphogenetic proteins' role in enhancing the motility and invasiveness of cancer cells, resulting in the promotion of metastasis.
9. Finding a niche: studies from the Drosophila ovary. Specialized microenvironments called niches help maintain stem cells in an undifferentiated and self-renewing state. The existence of niches has long been predicted from mammalian studies, but identifying stem cells in their native environments in vivo has remained a challenge in most vertebrates. Many of the mechanistic insights into how niches regulate stem cell maintenance have been obtained using invertebrate models such as Drosophila. Here, we focus on the Drosophila ovarian germline stem cell niche and review recent studies that have begun to reveal how intricate crosstalk between various signaling pathways regulates stem cell maintenance, how the **extracellular matrix** modulates the signaling output of the niche and how epigenetic programming influences cell development and function both inside and outside the niche to ensure proper tissue homeostasis. These insights will probably inform the study of mammalian niches and how their malfunction contributes to human disease.
10. **Extracellular matrix** and its receptors in Drosophila neural development. **Extracellular matrix** (ECM) and matrix receptors are intimately involved in most biological processes. The ECM plays fundamental developmental and physiological roles in health and disease, including processes underlying the development, maintenance, and regeneration of the nervous system. To understand the principles of ECM-mediated functions in the nervous system, genetic model organisms like Drosophila provide simple, malleable, and powerful experimental platforms. This article provides an overview of ECM proteins and receptors in Drosophila. It then focuses on their roles during three progressive phases of neural development: (1) neural progenitor proliferation, (2) axonal growth and pathfinding, and (3) synapse formation and function. Each section highlights known ECM and ECM-receptor components and recent studies done in mutant conditions to reveal their in vivo functions, all illustrating the enormous opportunities provided when merging work on the nervous system with systematic research into ECM-related gene functions.
11. Dally regulates Dpp morphogen gradient formation by stabilizing Dpp on the cell surface. Decapentaplegic (Dpp), a Drosophila homologue of bone morphogenetic proteins, acts as a morphogen to regulate patterning along the anterior-posterior axis of the developing wing. Previous studies showed that Dally, a heparan sulfate proteoglycan, regulates both the distribution of Dpp morphogen and cellular responses to Dpp. However, the molecular mechanism by which Dally affects the Dpp morphogen gradient remains to be elucidated. Here, we characterized activity, stability, and gradient formation of a truncated form of Dpp (Dpp(Delta N)), which lacks a short domain at the N-terminus essential for its interaction with Dally. Dpp(Delta N) shows the same signaling activity and protein stability as wild-type Dpp in vitro but has a shorter half-life in vivo, suggesting that Dally stabilizes Dpp in the **extracellular matrix**. Furthermore, genetic interaction experiments revealed that Dally antagonizes the effect of Thickveins (Tkv; a Dpp type I receptor) on Dpp signaling. Given that Tkv can downregulate Dpp signaling by receptor-mediated endocytosis of Dpp, the ability of dally to antagonize tkv suggests that Dally inhibits this process. Based on these observations, we propose a model in which Dally regulates Dpp distribution and signaling by disrupting receptor-mediated internalization and degradation of the Dpp-receptor complex.

### [Ii]nvasive

#### PubMed

1. The Drosophila short gastrulation gene prevents Dpp from autoactivating and suppressing neurogenesis in the neuroectoderm. The short gastrulation (sog) gene is expressed in broad lateral stripes comprising the neuroectoderm of the Drosophila blastoderm embryo. sog encodes a predicted secreted protein that functions nonautonomously to antagonize the activity of the TGF-beta-like Decapentaplegic (Dpp) signaling pathway in the dorsal region of the embryo. Recently, it has been shown that sog and dpp are functionally equivalent to their respective Xenopus homologs chordin and BMP-4. In this report we provide the first direct evidence that sog plays a local role in the lateral region of the blastoderm embryo to oppose Dpp activity in the neuroectoderm. In the dorsal region, Dpp signaling both suppresses neurogenesis and maintains expression of genes that promote dorsal cell fates (dorsalization). We show that Dpp also can perform both of these functions in the neuroectoderm. In wild-type embryos, the ability of Dpp to induce expression of dorsal markers including itself (autoactivation) in the neuroectoderm is blocked by sog. We propose that Sog protects the neuroectoderm from an **invasive** positive feedback loop created by Dpp diffusion and autoactivation. We show that the two functions of Dpp signaling, neural suppression and dorsalization, are triggered by distinct thresholds of Dpp activity. Epistasis experiments reveal that all observed sog activity can be accounted for by Sog functioning as a dedicated Dpp antagonist. Finally, we provide evidence that Sog functions as a diffusible morphogen in the blastoderm embryo. These data strongly support the view that the primary phylogenetically conserved function of the Drosophila sog and dpp genes and the homologous Xenopus chordin and BMP-4 genes is to subdivide the primitive embryonic ectoderm into neural versus non-neural domains.
2. Dpp signaling directs cell motility and **invasive**ness during epithelial morphogenesis. Tissue remodeling in development and disease involves the coordinated invasion of neighboring territories and/or the replacement of entire cell populations. Cell guidance, cell matching, transitions from passive to migratory epithelia, cell growth and death, and extracellular matrix remodeling all impinge on epithelial spreading. Significantly, the extracellular signals that direct these activities and the specific cellular elements and mechanisms regulated by these signals remain in most cases to be identified. To address these issues, we performed an analysis of histoblasts (Drosophila abdominal epithelial founder cells) on their transition from a dormant state to active migration replacing obsolete larval epidermal cells (LECs). We found that during expansion, Decapentaplegic (Dpp) secreted from surrounding LECs leads to graded pathway activation in cells at the periphery of histoblast nests. Across nests, Dpp activity confers differential cellular behavior and motility by modulating cell-cell contacts, the organization and activity of the cytoskeleton, and histoblast attachment to the substrate. Furthermore, Dpp also prevents the premature death of LECs, allowing the coordination of histoblast expansion to LEC delamination. Dpp signaling activity directing histoblast spreading and **invasive**ness mimics transforming growth factor-beta and bone morphogenetic proteins' role in enhancing the motility and **invasive**ness of cancer cells, resulting in the promotion of metastasis.

### [Mm]etastasis

#### PubMed

1. Dpp signaling directs cell motility and invasiveness during epithelial morphogenesis. Tissue remodeling in development and disease involves the coordinated invasion of neighboring territories and/or the replacement of entire cell populations. Cell guidance, cell matching, transitions from passive to migratory epithelia, cell growth and death, and extracellular matrix remodeling all impinge on epithelial spreading. Significantly, the extracellular signals that direct these activities and the specific cellular elements and mechanisms regulated by these signals remain in most cases to be identified. To address these issues, we performed an analysis of histoblasts (Drosophila abdominal epithelial founder cells) on their transition from a dormant state to active migration replacing obsolete larval epidermal cells (LECs). We found that during expansion, Decapentaplegic (Dpp) secreted from surrounding LECs leads to graded pathway activation in cells at the periphery of histoblast nests. Across nests, Dpp activity confers differential cellular behavior and motility by modulating cell-cell contacts, the organization and activity of the cytoskeleton, and histoblast attachment to the substrate. Furthermore, Dpp also prevents the premature death of LECs, allowing the coordination of histoblast expansion to LEC delamination. Dpp signaling activity directing histoblast spreading and invasiveness mimics transforming growth factor-beta and bone morphogenetic proteins' role in enhancing the motility and invasiveness of cancer cells, resulting in the promotion of **metastasis**.
2. Tumor suppressor properties of the ESCRT-II complex component Vps25 in Drosophila. We have found that the Drosophila gene vps25 possesses several properties of a tumor suppressor. First, vps25 mutant cells activate Notch and Dpp receptor signaling, inducing ectopic organizers in developing eyes and limbs and consequent overproliferation of both mutant and nearby wild-type cells. Second, as the mutant cells proliferate, they lose their epithelial organization and undergo apoptosis. Strikingly, when apoptosis of mutant cells is blocked, tumor-like overgrowths are formed that are capable of **metastasis**. vps25 encodes a component of the ESCRT-II complex, which sorts membrane proteins into multivesicular bodies during endocytic trafficking to the lysosome. Activation of Notch and Dpp receptor signaling in mutant cells results from an endocytic blockage that causes accumulation of these receptors and other signaling components in endosomes. These results highlight the importance of endocytic trafficking in regulating signaling and epithelial organization and suggest a possible role for ESCRT components in human cancer.

---

## Map205

- NCBI Gene
- GeneRIF
- Pubmed

| Search Term | Rank | Fields |
| --- | --- | --- |
| [Mm]igration | 6 | PubMed(1) |
| [Mm]etastasis | 12 | PubMed(1) |

  
hide/show details for Map205

### [Mm]igration

#### PubMed

1. MHP1, an essential gene in Saccharomyces cerevisiae required for microtubule function. The gene for a microtubule-associated protein (MAP), termed MHP1 (MAP-Homologous Protein 1), was isolated from Saccharomyces cerevisiae by expression cloning using antibodies specific for the Drosophila 205K MAP. MHP1 encodes an essential protein of 1,398 amino acids that contains near its COOH-terminal end a sequence homologous to the microtubule-binding domain of MAP2, MAP4, and tau. While total disruptions are lethal, NH2-terminal deletion mutations of MHP1 are viable, and the expression of the COOH-terminal two-thirds of the protein is sufficient for vegetative growth. Nonviable deletion-disruption mutations of MHP1 can be partially complemented by the expression of the Drosophila 205K MAP. Mhp1p binds to microtubules in vitro, and it is the COOH-terminal region containing the tau-homologous motif that mediates microtubule binding. Antibodies directed against a COOH-terminal peptide of Mhp1p decorate cytoplasmic microtubules and mitotic spindles as revealed by immunofluorescence microscopy. The overexpression of an NH2-terminal deletion mutation of MHP1 results in an accumulation of large-budded cells with short spindles and disturbed nuclear **migration**. In asynchronously growing cells that overexpress MHP1 from a multicopy plasmid, the length and number of cytoplasmic microtubules is increased and the proportion of mitotic cells is decreased, while haploid cells in which the expression of MHP1 has been silenced exhibit few microtubules. These results suggest that MHP1 is essential for the formation and/or stabilization of microtubules.

### [Mm]etastasis

#### PubMed

1. The Drosophila TIPE family member Sigmar interacts with the Ste20-like kinase Misshapen and modulates JNK signaling, cytoskeletal remodeling and autophagy. TNFAIP8 and other mammalian TIPE family proteins have attracted increased interest due to their associations with disease-related processes including oncogenic transformation, **metastasis**, and inflammation. The molecular and cellular functions of TIPE family proteins are still not well understood. Here we report the molecular and genetic characterization of the Drosophila TNFAIP8 homolog, CG4091/sigmar. Previous gene expression studies revealed dynamic expression of sigmar in larval salivary glands prior to histolysis. Here we demonstrate that in sigmar loss-of-function mutants, the salivary glands are morphologically abnormal with defects in the tubulin network and decreased autophagic flux. Sigmar localizes subcellularly to microtubule-containing projections in Drosophila S2 cells, and co-immunoprecipitates with the Ste20-like kinase Misshapen, a regulator of the JNK pathway. Further, the Drosophila TNF ligand Eiger can induce sigmar expression, and sigmar loss-of-function leads to altered localization of pDJNK in salivary glands. Together, these findings link Sigmar to the JNK pathway, cytoskeletal remodeling and autophagy activity during salivary gland development, and provide new insights into TIPE family member function.

---

## stck

- NCBI Gene
- GeneRIF
- Pubmed

| Search Term | Rank | Fields |
| --- | --- | --- |
| [Mm]igration | 6 | PubMed(7) |
| ECM | 7 | PubMed(7) |
| [Ee]xtracellular [Mm]atrix | 8 | PubMed(9) |

  
hide/show details for stck

### [Mm]igration

#### PubMed

1. The integrin effector PINCH regulates JNK activity and epithelial **migration** in concert with Ras suppressor 1. Cell adhesion and **migration** are dynamic processes requiring the coordinated action of multiple signaling pathways, but the mechanisms underlying signal integration have remained elusive. Drosophila embryonic dorsal closure (DC) requires both integrin function and c-Jun amino-terminal kinase (JNK) signaling for opposed epithelial sheets to migrate, meet, and suture. Here, we show that PINCH, a protein required for integrin-dependent cell adhesion and actin-membrane anchorage, is present at the leading edge of these migrating epithelia and is required for DC. By analysis of native protein complexes, we identify RSU-1, a regulator of Ras signaling in mammalian cells, as a novel PINCH binding partner that contributes to PINCH stability. Mutation of the gene encoding RSU-1 results in wing blistering in Drosophila, demonstrating its role in integrin-dependent cell adhesion. Genetic interaction analyses reveal that both PINCH and RSU-1 antagonize JNK signaling during DC. Our results suggest that PINCH and RSU-1 contribute to the integration of JNK and integrin functions during Drosophila development.
2. Polarization of the epithelial layer and apical localization of integrins are required for engulfment of apoptotic cells in the Drosophila ovary. Inefficient clearance of dead cells or debris by epithelial cells can lead to or exacerbate debilitating conditions such as retinitis pigmentosa, macular degeneration, chronic obstructive pulmonary disease and asthma. Despite the importance of engulfment by epithelial cells, little is known about the molecular changes that are required within these cells. The misregulation of integrins has previously been associated with disease states, suggesting that a better understanding of the regulation of receptor trafficking could be key to treating diseases caused by defects in phagocytosis. Here, we demonstrate that the integrin heterodimer αPS3/βPS becomes apically enriched and is required for engulfment by the epithelial follicle cells of the Drosophila ovary. We found that integrin heterodimer localization and function is largely directed by the α-subunit. Moreover, proper cell polarity promotes asymmetric integrin enrichment, suggesting that αPS3/βPS trafficking occurs in a polarized fashion. We show that several genes previously known for their roles in trafficking and **cell migr**ation are also required for engulfment. Moreover, as in mammals, the same α-integrin subunit is required by professional and non-professional phagocytes and migrating cells in Drosophila. Our findings suggest that migrating and engulfing cells use common machinery, and demonstrate a crucial role for integrin function and polarized trafficking of integrin subunits during engulfment. This study also establishes the epithelial follicle cells of the Drosophila ovary as a powerful model for understanding the molecular changes required for engulfment by a polarized epithelium.
3. Focal adhesions: what's new inside. The cytoplasmic side of focal adhesions is comprised of large molecular complexes that link transmembrane receptors, such as integrins, to the actin cytoskeleton and mediate signals modulating cell attachment, **migration**, proliferation, differentiation, and gene expression. These complexes are heterogeneous and dynamic structures that are apparent targets of regulatory signals that control the function of focal adhesions. Recent studies using genetic approaches in invertebrate and vertebrate systems have begun to reveal the structure and function of these complexes in vivo.
4. Vestigial is required during late-stage muscle differentiation in Drosophila melanogaster embryos. The somatic muscles of Drosophila develop in a complex pattern that is repeated in each embryonic hemi-segment. During early development, progenitor cells fuse to form a syncytial muscle, which further differentiates via expression of muscle-specific factors that induce specific responses to external signals to regulate late-stage processes such as **migration** and attachment. Initial communication between somatic muscles and the epidermal tendon cells is critical for both of these processes. However, later establishment of attachments between longitudinal muscles at the segmental borders is largely independent of the muscle-epidermal attachment signals, and relatively little is known about how this event is regulated. Using a combination of null mutations and a truncated version of Sd that binds Vg but not DNA, we show that Vestigial (Vg) is required in ventral longitudinal muscles to induce formation of stable intermuscular attachments. In several muscles, this activity may be independent of Sd. Furthermore, the cell-specific differentiation events induced by Vg in two cells fated to form attachments are coordinated by Drosophila epidermal growth factor signaling. Thus, Vg is a key factor to induce specific changes in ventral longitudinal muscles 1-4 identity and is required for these cells to be competent to form stable intermuscular attachments with each other.
5. The integrin effector PINCH regulates JNK activity and epithelial **migration** in concert with Ras suppressor 1. Cell adhesion and **migration** are dynamic processes requiring the coordinated action of multiple signaling pathways, but the mechanisms underlying signal integration have remained elusive. Drosophila embryonic dorsal closure (DC) requires both integrin function and c-Jun amino-terminal kinase (JNK) signaling for opposed epithelial sheets to migrate, meet, and suture. Here, we show that PINCH, a protein required for integrin-dependent cell adhesion and actin-membrane anchorage, is present at the leading edge of these migrating epithelia and is required for DC. By analysis of native protein complexes, we identify RSU-1, a regulator of Ras signaling in mammalian cells, as a novel PINCH binding partner that contributes to PINCH stability. Mutation of the gene encoding RSU-1 results in wing blistering in Drosophila, demonstrating its role in integrin-dependent cell adhesion. Genetic interaction analyses reveal that both PINCH and RSU-1 antagonize JNK signaling during DC. Our results suggest that PINCH and RSU-1 contribute to the integration of JNK and integrin functions during Drosophila development.
6. Cell-cell adhesion via the ECM: integrin genetics in fly and worm. Integrins are essential for the development of the two genetically tractable invertebrate model organisms, the nematode worm Caenorhabditis elegans and the fruit fly Drosophila melanogaster. Just two integrins are present in C. elegans: one putative RGD binding integrin alphapat-2betapat-3, corresponding to Drosophila alphaPS2betaPS and vertebrate alpha5beta1, alphaVbeta1 and alpha8beta1, and one putative laminin binding integrin alphaina-1betapat-3, corresponding to Drosophila alphaPS1betaPS and vertebrate alpha3beta1, alpha6beta1 and alpha7beta1. In this review, the function of this minimal set of integrins during the development of these two invertebrates is compared. Despite the differences in bodyplan and developmental strategy, integrin adhesion to the extracellular matrix is required for similar processes: the formation of the link that translates muscle contraction into movement of the exoskeleton, cell **migration**, and morphogenetic interactions between epithelia. Other integrin functions, such as regulation of gene expression, have not yet been experimentally demonstrated in both organisms. Additional proteins have been characterised in each organism that are essential for integrin function, including extracellular matrix ligands and intracellular interacting proteins, but so far different proteins have been found in the two organisms. This in part represents the fact that the characterisation of the full set of interacting proteins is not complete in either system. However, in other cases different proteins appear to be used for similar functions in the two animals. The continued use of genetic approaches to identify proteins required for integrin function in these two model organisms should lead to the identification of the minimal set of conserved components that form integrin adhesive structures.
7. A genome-wide screen identifies conserved protein hubs required for cadherin-mediated cell-cell adhesion. Cadherins and associated catenins provide an important structural interface between neighboring cells, the actin cytoskeleton, and intracellular signaling pathways in a variety of cell types throughout the Metazoa. However, the full inventory of the proteins and pathways required for cadherin-mediated adhesion has not been established. To this end, we completed a genome-wide (~14,000 genes) ribonucleic acid interference (RNAi) screen that targeted Ca(2+)-dependent adhesion in DE-cadherin-expressing Drosophila melanogaster S2 cells in suspension culture. This novel screen eliminated Ca(2+)-independent cell-cell adhesion, integrin-based adhesion, cell spreading, and cell **migration**. We identified 17 interconnected regulatory hubs, based on protein functions and protein-protein interactions that regulate the levels of the core cadherin-catenin complex and coordinate cadherin-mediated cell-cell adhesion. Representative proteins from these hubs were analyzed further in Drosophila oogenesis, using targeted germline RNAi, and adhesion was analyzed in Madin-Darby canine kidney mammalian epithelial cell-cell adhesion. These experiments reveal roles for a diversity of cellular pathways that are required for cadherin function in Metazoa, including cytoskeleton organization, cell-substrate interactions, and nuclear and cytoplasmic signaling.

### ECM

#### PubMed

1. Integrin-dependent anchoring of a stem-cell niche. Interactions between stem cells and their surrounding microenvironment, or niche, are critical for the establishment and maintenance of stem-cell properties. The adult Drosophila testis contains a morphologically discrete stem-cell niche, the 'hub'. The small cluster of non-dividing, somatic hub cells at the anterior tip of the fly testis is contacted by the germline stem cells (GSCs), which retain their stem-cell character through the direct association with the hub. Here we show that integrin-mediated adhesion is important for maintaining the correct position of embryonic hub cells during gonad morphogenesis. The misplaced hub in integrin-deficient embryos directs the orientation of cell divisions in the presumptive GSCs, a hallmark of the active germline stem-cell niche. A decrease in integrin-mediated adhesion in adult testes, which resulted in a loss of the hub and the stem-cell population, revealed the importance of hub-cell anchoring. Finally, we show that an extracellular matrix (**ECM**) is present around the gonad during late embryogenesis and that this **ECM** is defective in integrin-deficient gonads. On the basis of our data, we propose that integrins are required for the attachment of the hub cells to the **ECM**, which is essential for maintaining the stem-cell niche.
2. IPP Complex Reinforces Adhesion by Relaying Tension-Dependent Signals to Inhibit Integrin Turnover. Cytoskeleton-mediated forces regulate the assembly and function of integrin adhesions; however, the underlying mechanisms remain unclear. The tripartite IPP complex, comprising ILK, Parvin, and PINCH, mediates the integrin-actin link at Drosophila embryo muscle attachment sites (MASs). Here, we demonstrate a developmentally earlier function for the IPP complex: to reinforce integrin-extracellular matrix (**ECM**) adhesion in response to tension. In IPP-complex mutants, the integrin-**ECM** linkage at MASs breaks in response to intense muscle contractility. Mechanistically, the IPP complex is required to relay force-elicited signals that decelerate integrin turnover at the plasma membrane so that the integrin immobile fraction is adequate to withstand tension. Epistasis analysis shows that alleviation of muscle contractility, downregulation of endocytosis, and enhanced integrin binding to the **ECM** are sufficient to restore integrin-**ECM** adhesion and maintain integrin-adhesome organization in IPP-complex mutants. Our findings reveal a role for the IPP complex as an essential mechanosensitive regulatory switch of integrin turnover in vivo.
3. An interaction between integrin and the talin FERM domain mediates integrin activation but not linkage to the cytoskeleton. Transmembrane adhesion receptors, such as integrins, mediate cell adhesion by interacting with intracellular proteins that connect to the cytoskeleton. Talin, one such linker protein, is thought to have two roles: mediating inside-out activation of integrins, and connecting extracellular matrix (**ECM**)-bound integrins to the cytoskeleton. Talin's amino-terminal head, which consists of a FERM domain, binds an NPxY motif within the cytoplasmic tail of most integrin beta subunits. This is consistent with the role of FERM domains in recruiting other proteins to the plasma membrane. We tested the role of the talin-head-NPxY interaction in integrin function in Drosophila. We found that introduction of a mutation that perturbs this binding in vitro into the isolated talin head disrupts its recruitment by integrins in vivo. Surprisingly, when engineered into the full-length talin, this mutation did not disrupt talin recruitment by integrins nor its ability to connect integrins to the cytoskeleton. However, it reduced the ability of talin to strengthen integrin adhesion to the **ECM**, indicating that the function of the talin-head-NPxY interaction is solely to regulate integrin adhesion.
4. The talin head domain reinforces integrin-mediated adhesion by promoting adhesion complex stability and clustering. Talin serves an essential function during integrin-mediated adhesion in linking integrins to actin via the intracellular adhesion complex. In addition, the N-terminal head domain of talin regulates the affinity of integrins for their **ECM**-ligands, a process known as inside-out activation. We previously showed that in Drosophila, mutating the integrin binding site in the talin head domain resulted in weakened adhesion to the **ECM**. Intriguingly, subsequent studies showed that canonical inside-out activation of integrin might not take place in flies. Consistent with this, a mutation in talin that specifically blocks its ability to activate mammalian integrins does not significantly impinge on talin function during fly development. Here, we describe results suggesting that the talin head domain reinforces and stabilizes the integrin adhesion complex by promoting integrin clustering distinct from its ability to support inside-out activation. Specifically, we show that an allele of talin containing a mutation that disrupts intramolecular interactions within the talin head attenuates the assembly and reinforcement of the integrin adhesion complex. Importantly, we provide evidence that this mutation blocks integrin clustering in vivo. We propose that the talin head domain is essential for regulating integrin avidity in Drosophila and that this is crucial for integrin-mediated adhesion during animal development.
5. The integrin adhesion complex changes its composition and function during morphogenesis of an epithelium. Cell adhesion to the extracellular matrix (**ECM**) is mediated by the integrin family of transmembrane receptors. Integrins link **ECM** ligands to the cytoskeleton, providing strong attachment to enable cell-shape change and tissue integrity. This connection is made possible by an intracellular complex of proteins, which links to actin filaments and controls signalling cascades that regulate cytoskeletal rearrangements. We have identified stress-fibre-associated focal adhesions that change their composition during tissue morphogenesis. Early expression of alphaPS1betaPS integrin decreases the levels of the actin-nucleating factors Enabled, Diaphanous and profilin, as well as downregulating the amount of F-actin incorporated into the stress fibres. As follicle cells mature in their developmental pathway and become squamous, the integrin in the focal adhesions changes from alphaPS1betaPS to alphaPS2betaPS. During the switch, stress fibres increase their length and change orientation, first changing by 90 degrees and then reorienting back. The normal rapid reorientation requires new expression of alphaPS2betaPS, which also permits recruitment of the adaptor protein tensin. Unexpectedly, it is the extracellular portion of the alphaPS2 subunit that provides the specificity for intracellular recruitment of tensin. Molecular variation of the integrin complex is thus a key component of developmentally programmed morphogenesis.
6. Cell-cell adhesion via the **ECM**: integrin genetics in fly and worm. Integrins are essential for the development of the two genetically tractable invertebrate model organisms, the nematode worm Caenorhabditis elegans and the fruit fly Drosophila melanogaster. Just two integrins are present in C. elegans: one putative RGD binding integrin alphapat-2betapat-3, corresponding to Drosophila alphaPS2betaPS and vertebrate alpha5beta1, alphaVbeta1 and alpha8beta1, and one putative laminin binding integrin alphaina-1betapat-3, corresponding to Drosophila alphaPS1betaPS and vertebrate alpha3beta1, alpha6beta1 and alpha7beta1. In this review, the function of this minimal set of integrins during the development of these two invertebrates is compared. Despite the differences in bodyplan and developmental strategy, integrin adhesion to the extracellular matrix is required for similar processes: the formation of the link that translates muscle contraction into movement of the exoskeleton, cell migration, and morphogenetic interactions between epithelia. Other integrin functions, such as regulation of gene expression, have not yet been experimentally demonstrated in both organisms. Additional proteins have been characterised in each organism that are essential for integrin function, including extracellular matrix ligands and intracellular interacting proteins, but so far different proteins have been found in the two organisms. This in part represents the fact that the characterisation of the full set of interacting proteins is not complete in either system. However, in other cases different proteins appear to be used for similar functions in the two animals. The continued use of genetic approaches to identify proteins required for integrin function in these two model organisms should lead to the identification of the minimal set of conserved components that form integrin adhesive structures.
7. Extracellular matrix and its receptors in Drosophila neural development. Extracellular matrix (**ECM**) and matrix receptors are intimately involved in most biological processes. The **ECM** plays fundamental developmental and physiological roles in health and disease, including processes underlying the development, maintenance, and regeneration of the nervous system. To understand the principles of **ECM**-mediated functions in the nervous system, genetic model organisms like Drosophila provide simple, malleable, and powerful experimental platforms. This article provides an overview of **ECM** proteins and receptors in Drosophila. It then focuses on their roles during three progressive phases of neural development: (1) neural progenitor proliferation, (2) axonal growth and pathfinding, and (3) synapse formation and function. Each section highlights known **ECM** and **ECM**-receptor components and recent studies done in mutant conditions to reveal their in vivo functions, all illustrating the enormous opportunities provided when merging work on the nervous system with systematic research into **ECM**-related gene functions.

### [Ee]xtracellular [Mm]atrix

#### PubMed

1. dGRASP-mediated noncanonical integrin secretion is required for Drosophila epithelial remodeling. Integral plasma membrane proteins are typically transported in the secretory pathway from the endoplasmic reticulum and the Golgi complex. Here we show that at specific stages of Drosophila development corresponding to morphological changes in epithelia, apposed basolateral membranes separate slightly, allowing new plasma membrane contacts with basal **extracellular matrix**. At these sites, newly synthesized integrin alpha subunits are deposited via a mechanism that appears to bypass the Golgi. We show that the Drosophila Golgi resident protein dGRASP localizes to these membrane domains and that, in the absence of dGRASP, the integrin subunit is retained intracellularly in both follicular and wing epithelia that are found disrupted. We propose that this dGRASP-mediated noncanonical secretion route allows for developmental regulation of integrin function upon epithelial remodeling. We speculate that this mechanism might be used during development as a means of targeting a specific subset of transmembrane proteins to the plasma membrane.
2. Integrin-dependent anchoring of a stem-cell niche. Interactions between stem cells and their surrounding microenvironment, or niche, are critical for the establishment and maintenance of stem-cell properties. The adult Drosophila testis contains a morphologically discrete stem-cell niche, the 'hub'. The small cluster of non-dividing, somatic hub cells at the anterior tip of the fly testis is contacted by the germline stem cells (GSCs), which retain their stem-cell character through the direct association with the hub. Here we show that integrin-mediated adhesion is important for maintaining the correct position of embryonic hub cells during gonad morphogenesis. The misplaced hub in integrin-deficient embryos directs the orientation of cell divisions in the presumptive GSCs, a hallmark of the active germline stem-cell niche. A decrease in integrin-mediated adhesion in adult testes, which resulted in a loss of the hub and the stem-cell population, revealed the importance of hub-cell anchoring. Finally, we show that an **extracellular matrix** (ECM) is present around the gonad during late embryogenesis and that this ECM is defective in integrin-deficient gonads. On the basis of our data, we propose that integrins are required for the attachment of the hub cells to the ECM, which is essential for maintaining the stem-cell niche.
3. Distinct regulatory mechanisms control integrin adhesive processes during tissue morphogenesis. Cell adhesion must be precisely regulated to enable both dynamic morphogenetic processes and the subsequent transition to stable tissue maintenance. Integrins link the intracellular cytoskeleton and **extracellular matrix**, relaying bidirectional signals across the plasma membrane. In vitro studies have demonstrated that multiple mechanisms control integrin-mediated adhesion; however, their roles during development are poorly understood. We used mutations that activate or deactivate specific functions of vertebrate β-integrins in vitro to investigate how perturbing Drosophila βPS-integrin regulation in developing embryos regulation affects tissue morphogenesis and maintenance. We found that morphogenetic processes use various β-integrin regulatory mechanisms to differing degrees and that conformational changes associated with outside-in activation are essential for developmental integrin functions. Long-term adhesion is also sensitive to integrin dysregulation, suggesting integrins must be continuously regulated to support stable tissue maintenance. Altogether, in vivo phenotypic analyses allowed us to identify the importance of various β-integrin regulatory mechanisms during different morphogenetic processes.
4. IPP Complex Reinforces Adhesion by Relaying Tension-Dependent Signals to Inhibit Integrin Turnover. Cytoskeleton-mediated forces regulate the assembly and function of integrin adhesions; however, the underlying mechanisms remain unclear. The tripartite IPP complex, comprising ILK, Parvin, and PINCH, mediates the integrin-actin link at Drosophila embryo muscle attachment sites (MASs). Here, we demonstrate a developmentally earlier function for the IPP complex: to reinforce integrin-**extracellular matrix** (ECM) adhesion in response to tension. In IPP-complex mutants, the integrin-ECM linkage at MASs breaks in response to intense muscle contractility. Mechanistically, the IPP complex is required to relay force-elicited signals that decelerate integrin turnover at the plasma membrane so that the integrin immobile fraction is adequate to withstand tension. Epistasis analysis shows that alleviation of muscle contractility, downregulation of endocytosis, and enhanced integrin binding to the ECM are sufficient to restore integrin-ECM adhesion and maintain integrin-adhesome organization in IPP-complex mutants. Our findings reveal a role for the IPP complex as an essential mechanosensitive regulatory switch of integrin turnover in vivo.
5. An interaction between integrin and the talin FERM domain mediates integrin activation but not linkage to the cytoskeleton. Transmembrane adhesion receptors, such as integrins, mediate cell adhesion by interacting with intracellular proteins that connect to the cytoskeleton. Talin, one such linker protein, is thought to have two roles: mediating inside-out activation of integrins, and connecting **extracellular matrix** (ECM)-bound integrins to the cytoskeleton. Talin's amino-terminal head, which consists of a FERM domain, binds an NPxY motif within the cytoplasmic tail of most integrin beta subunits. This is consistent with the role of FERM domains in recruiting other proteins to the plasma membrane. We tested the role of the talin-head-NPxY interaction in integrin function in Drosophila. We found that introduction of a mutation that perturbs this binding in vitro into the isolated talin head disrupts its recruitment by integrins in vivo. Surprisingly, when engineered into the full-length talin, this mutation did not disrupt talin recruitment by integrins nor its ability to connect integrins to the cytoskeleton. However, it reduced the ability of talin to strengthen integrin adhesion to the ECM, indicating that the function of the talin-head-NPxY interaction is solely to regulate integrin adhesion.
6. dumpy interacts with a large number of genes in the developing wing of Drosophila melanogaster. The complex Drosophila dumpy gene encodes a gigantic protein located in the apical **extracellular matrix** of epithelial cells. It has been shown to interact with several proteins notably during embryonic tracheal development. Here we examine Dumpy's interactions in vivo with mutations in 20 genes previously recovered in a screen for recessive lethals that generate blisters when somatic clones are produced by mitotic crossing over during wing development. Primarily using double mutants, we looked for both dominant effects of the wing blister mutants and the effects of blister mutant clones on dumpy expression. Sixteen of the mutants either suppressed or enhanced dumpy mutant phenotypes indicating the large Dumpy protein is a very important component of the epithelial **extracellular matrix** in the wing. Dumpy also interacts strongly with held out wings, which is involved in RNA localization and possibly alternative splicing.
7. The integrin adhesion complex changes its composition and function during morphogenesis of an epithelium. Cell adhesion to the **extracellular matrix** (ECM) is mediated by the integrin family of transmembrane receptors. Integrins link ECM ligands to the cytoskeleton, providing strong attachment to enable cell-shape change and tissue integrity. This connection is made possible by an intracellular complex of proteins, which links to actin filaments and controls signalling cascades that regulate cytoskeletal rearrangements. We have identified stress-fibre-associated focal adhesions that change their composition during tissue morphogenesis. Early expression of alphaPS1betaPS integrin decreases the levels of the actin-nucleating factors Enabled, Diaphanous and profilin, as well as downregulating the amount of F-actin incorporated into the stress fibres. As follicle cells mature in their developmental pathway and become squamous, the integrin in the focal adhesions changes from alphaPS1betaPS to alphaPS2betaPS. During the switch, stress fibres increase their length and change orientation, first changing by 90 degrees and then reorienting back. The normal rapid reorientation requires new expression of alphaPS2betaPS, which also permits recruitment of the adaptor protein tensin. Unexpectedly, it is the extracellular portion of the alphaPS2 subunit that provides the specificity for intracellular recruitment of tensin. Molecular variation of the integrin complex is thus a key component of developmentally programmed morphogenesis.
8. Cell-cell adhesion via the ECM: integrin genetics in fly and worm. Integrins are essential for the development of the two genetically tractable invertebrate model organisms, the nematode worm Caenorhabditis elegans and the fruit fly Drosophila melanogaster. Just two integrins are present in C. elegans: one putative RGD binding integrin alphapat-2betapat-3, corresponding to Drosophila alphaPS2betaPS and vertebrate alpha5beta1, alphaVbeta1 and alpha8beta1, and one putative laminin binding integrin alphaina-1betapat-3, corresponding to Drosophila alphaPS1betaPS and vertebrate alpha3beta1, alpha6beta1 and alpha7beta1. In this review, the function of this minimal set of integrins during the development of these two invertebrates is compared. Despite the differences in bodyplan and developmental strategy, integrin adhesion to the **extracellular matrix** is required for similar processes: the formation of the link that translates muscle contraction into movement of the exoskeleton, cell migration, and morphogenetic interactions between epithelia. Other integrin functions, such as regulation of gene expression, have not yet been experimentally demonstrated in both organisms. Additional proteins have been characterised in each organism that are essential for integrin function, including **extracellular matrix** ligands and intracellular interacting proteins, but so far different proteins have been found in the two organisms. This in part represents the fact that the characterisation of the full set of interacting proteins is not complete in either system. However, in other cases different proteins appear to be used for similar functions in the two animals. The continued use of genetic approaches to identify proteins required for integrin function in these two model organisms should lead to the identification of the minimal set of conserved components that form integrin adhesive structures.
9. **Extracellular matrix** and its receptors in Drosophila neural development. **Extracellular matrix** (ECM) and matrix receptors are intimately involved in most biological processes. The ECM plays fundamental developmental and physiological roles in health and disease, including processes underlying the development, maintenance, and regeneration of the nervous system. To understand the principles of ECM-mediated functions in the nervous system, genetic model organisms like Drosophila provide simple, malleable, and powerful experimental platforms. This article provides an overview of ECM proteins and receptors in Drosophila. It then focuses on their roles during three progressive phases of neural development: (1) neural progenitor proliferation, (2) axonal growth and pathfinding, and (3) synapse formation and function. Each section highlights known ECM and ECM-receptor components and recent studies done in mutant conditions to reveal their in vivo functions, all illustrating the enormous opportunities provided when merging work on the nervous system with systematic research into ECM-related gene functions.

---

## Kap-alpha1

- NCBI Gene
- GeneRIF
- Pubmed

| Search Term | Rank | Fields |
| --- | --- | --- |
| [Mm]igration | 6 | PubMed(1) |
| ECM | 7 | PubMed(1) |
| [Ee]xtracellular [Mm]atrix | 8 | PubMed(1) |

  
hide/show details for Kap-alpha1

### [Mm]igration

#### PubMed

1. Genes conserved in bilaterians but jointly lost with Myc during nematode evolution are enriched in cell proliferation and cell **migration** functions. Animals use a stereotypical set of developmental genes to build body architectures of varying sizes and organizational complexity. Some genes are critical to developmental patterning, while other genes are important to physiological control of growth. However, growth regulator genes may not be as important in small-bodied "micro-metazoans" such as nematodes. Nematodes use a simplified developmental strategy of lineage-based cell fate specifications to produce an adult bilaterian body composed of a few hundreds of cells. Nematodes also lost the MYC proto-oncogenic regulator of cell proliferation. To identify additional regulators of cell proliferation that were lost with MYC, we computationally screened and determined 839 high-confidence genes that are conserved in bilaterians/lost in nematodes (CIBLIN genes). We find that 30 % of all CIBLIN genes encode transcriptional regulators of cell proliferation, epithelial-to-mesenchyme transitions, and other processes. Over 50 % of CIBLIN genes are unnamed genes in Drosophila, suggesting that there are many understudied genes. Interestingly, CIBLIN genes include many Myc synthetic lethal (MycSL) hits from recent screens. CIBLIN genes include key regulators of heparan sulfate proteoglycan (HSPG) sulfation patterns, and lysyl oxidases involved in cross-linking and modification of the extracellular matrix (ECM). These genes and others suggest the CIBLIN repertoire services critical functions in ECM remodeling and cell **migration** in large-bodied bilaterians. Correspondingly, CIBLIN genes are co-expressed with Myc in cancer transcriptomes, and include a preponderance of known determinants of cancer progression and tumor aggression. We propose that CIBLIN gene research can improve our understanding of regulatory control of cellular growth in metazoans.

### ECM

#### PubMed

1. Genes conserved in bilaterians but jointly lost with Myc during nematode evolution are enriched in cell proliferation and cell migration functions. Animals use a stereotypical set of developmental genes to build body architectures of varying sizes and organizational complexity. Some genes are critical to developmental patterning, while other genes are important to physiological control of growth. However, growth regulator genes may not be as important in small-bodied "micro-metazoans" such as nematodes. Nematodes use a simplified developmental strategy of lineage-based cell fate specifications to produce an adult bilaterian body composed of a few hundreds of cells. Nematodes also lost the MYC proto-oncogenic regulator of cell proliferation. To identify additional regulators of cell proliferation that were lost with MYC, we computationally screened and determined 839 high-confidence genes that are conserved in bilaterians/lost in nematodes (CIBLIN genes). We find that 30 % of all CIBLIN genes encode transcriptional regulators of cell proliferation, epithelial-to-mesenchyme transitions, and other processes. Over 50 % of CIBLIN genes are unnamed genes in Drosophila, suggesting that there are many understudied genes. Interestingly, CIBLIN genes include many Myc synthetic lethal (MycSL) hits from recent screens. CIBLIN genes include key regulators of heparan sulfate proteoglycan (HSPG) sulfation patterns, and lysyl oxidases involved in cross-linking and modification of the extracellular matrix (**ECM**). These genes and others suggest the CIBLIN repertoire services critical functions in **ECM** remodeling and cell migration in large-bodied bilaterians. Correspondingly, CIBLIN genes are co-expressed with Myc in cancer transcriptomes, and include a preponderance of known determinants of cancer progression and tumor aggression. We propose that CIBLIN gene research can improve our understanding of regulatory control of cellular growth in metazoans.

### [Ee]xtracellular [Mm]atrix

#### PubMed

1. Genes conserved in bilaterians but jointly lost with Myc during nematode evolution are enriched in cell proliferation and cell migration functions. Animals use a stereotypical set of developmental genes to build body architectures of varying sizes and organizational complexity. Some genes are critical to developmental patterning, while other genes are important to physiological control of growth. However, growth regulator genes may not be as important in small-bodied "micro-metazoans" such as nematodes. Nematodes use a simplified developmental strategy of lineage-based cell fate specifications to produce an adult bilaterian body composed of a few hundreds of cells. Nematodes also lost the MYC proto-oncogenic regulator of cell proliferation. To identify additional regulators of cell proliferation that were lost with MYC, we computationally screened and determined 839 high-confidence genes that are conserved in bilaterians/lost in nematodes (CIBLIN genes). We find that 30 % of all CIBLIN genes encode transcriptional regulators of cell proliferation, epithelial-to-mesenchyme transitions, and other processes. Over 50 % of CIBLIN genes are unnamed genes in Drosophila, suggesting that there are many understudied genes. Interestingly, CIBLIN genes include many Myc synthetic lethal (MycSL) hits from recent screens. CIBLIN genes include key regulators of heparan sulfate proteoglycan (HSPG) sulfation patterns, and lysyl oxidases involved in cross-linking and modification of the **extracellular matrix** (ECM). These genes and others suggest the CIBLIN repertoire services critical functions in ECM remodeling and cell migration in large-bodied bilaterians. Correspondingly, CIBLIN genes are co-expressed with Myc in cancer transcriptomes, and include a preponderance of known determinants of cancer progression and tumor aggression. We propose that CIBLIN gene research can improve our understanding of regulatory control of cellular growth in metazoans.

---

## SK

- NCBI Gene
- GeneRIF
- Pubmed

| Search Term | Rank | Fields |
| --- | --- | --- |
| [Mm]igration | 6 | PubMed(1) |

  
hide/show details for SK

### [Mm]igration

#### PubMed

1. Response to mechanical stress is mediated by the TRPA channel painless in the Drosophila heart. Mechanotransduction modulates cellular functions as diverse as **migration**, proliferation, differentiation, and apoptosis. It is crucial for organ development and homeostasis and leads to pathologies when defective. However, despite considerable efforts made in the past, the molecular basis of mechanotransduction remains poorly understood. Here, we have investigated the genetic basis of mechanotransduction in Drosophila. We show that the fly heart senses and responds to mechanical forces by regulating cardiac activity. In particular, pauses in heart activity are observed under acute mechanical constraints in vivo. We further confirm by a variety of in situ tests that these cardiac arrests constitute the biological force-induced response. In order to identify molecular components of the mechanotransduction pathway, we carried out a genetic screen based on the dependence of cardiac activity upon mechanical constraints and identified Painless, a TRPA channel. We observe a clear absence of in vivo cardiac arrest following inactivation of painless and further demonstrate that painless is autonomously required in the heart to mediate the response to mechanical stress. Furthermore, direct activation of Painless is sufficient to produce pauses in heartbeat, mimicking the pressure-induced response. Painless thus constitutes part of a mechanosensitive pathway that adjusts cardiac muscle activity to mechanical constraints. This constitutes the first in vivo demonstration that a TRPA channel can mediate cardiac mechanotransduction. Furthermore, by establishing a high-throughput system to identify the molecular players involved in mechanotransduction in the cardiovascular system, our study paves the way for understanding the mechanisms underlying a mechanotransduction pathway.

---

## tnc

- NCBI Gene
- GeneRIF
- Pubmed

| Search Term | Rank | Fields |
| --- | --- | --- |
| ECM | 7 | PubMed(3) |
| [Ee]xtracellular [Mm]atrix | 8 | Function(1); PubMed(5); Component(2); GeneRIFs(1) |

  
hide/show details for tnc

### ECM

#### PubMed

1. Tenectin is a novel alphaPS2betaPS integrin ligand required for wing morphogenesis and male genital looping in Drosophila. Morphogenesis of the adult structures of holometabolous insects is regulated by ecdysteroids and juvenile hormones and involves cell-cell interactions mediated in part by the cell surface integrin receptors and their extracellular matrix (**ECM**) ligands. These adhesion molecules and their regulation by hormones are not well characterized. We describe the gene structure of a newly described **ECM** molecule, tenectin, and demonstrate that it is a hormonally regulated **ECM** protein required for proper morphogenesis of the adult wing and male genitalia. Tenectin's function as a new ligand of the PS2 integrins is demonstrated by both genetic interactions in the fly and by cell spreading and cell adhesion assays in cultured cells. Its interaction with the PS2 integrins is dependent on RGD and RGD-like motifs. Tenectin's function in looping morphogenesis in the development of the male genitalia led to experiments that demonstrate a role for PS integrins in the execution of left-right asymmetry.
2. Extracellular matrix and its receptors in Drosophila neural development. Extracellular matrix (**ECM**) and matrix receptors are intimately involved in most biological processes. The **ECM** plays fundamental developmental and physiological roles in health and disease, including processes underlying the development, maintenance, and regeneration of the nervous system. To understand the principles of **ECM**-mediated functions in the nervous system, genetic model organisms like Drosophila provide simple, malleable, and powerful experimental platforms. This article provides an overview of **ECM** proteins and receptors in Drosophila. It then focuses on their roles during three progressive phases of neural development: (1) neural progenitor proliferation, (2) axonal growth and pathfinding, and (3) synapse formation and function. Each section highlights known **ECM** and **ECM**-receptor components and recent studies done in mutant conditions to reveal their in vivo functions, all illustrating the enormous opportunities provided when merging work on the nervous system with systematic research into **ECM**-related gene functions.
3. Tenectin is a novel alphaPS2betaPS integrin ligand required for wing morphogenesis and male genital looping in Drosophila. Morphogenesis of the adult structures of holometabolous insects is regulated by ecdysteroids and juvenile hormones and involves cell-cell interactions mediated in part by the cell surface integrin receptors and their extracellular matrix (**ECM**) ligands. These adhesion molecules and their regulation by hormones are not well characterized. We describe the gene structure of a newly described **ECM** molecule, tenectin, and demonstrate that it is a hormonally regulated **ECM** protein required for proper morphogenesis of the adult wing and male genitalia. Tenectin's function as a new ligand of the PS2 integrins is demonstrated by both genetic interactions in the fly and by cell spreading and cell adhesion assays in cultured cells. Its interaction with the PS2 integrins is dependent on RGD and RGD-like motifs. Tenectin's function in looping morphogenesis in the development of the male genitalia led to experiments that demonstrate a role for PS integrins in the execution of left-right asymmetry.

### [Ee]xtracellular [Mm]atrix

#### Function

1. **extracellular matrix** structural constituent

#### PubMed

1. Tenectin is a novel alphaPS2betaPS integrin ligand required for wing morphogenesis and male genital looping in Drosophila. Morphogenesis of the adult structures of holometabolous insects is regulated by ecdysteroids and juvenile hormones and involves cell-cell interactions mediated in part by the cell surface integrin receptors and their **extracellular matrix** (ECM) ligands. These adhesion molecules and their regulation by hormones are not well characterized. We describe the gene structure of a newly described ECM molecule, tenectin, and demonstrate that it is a hormonally regulated ECM protein required for proper morphogenesis of the adult wing and male genitalia. Tenectin's function as a new ligand of the PS2 integrins is demonstrated by both genetic interactions in the fly and by cell spreading and cell adhesion assays in cultured cells. Its interaction with the PS2 integrins is dependent on RGD and RGD-like motifs. Tenectin's function in looping morphogenesis in the development of the male genitalia led to experiments that demonstrate a role for PS integrins in the execution of left-right asymmetry.
2. Tenectin, a novel **extracellular matrix** protein expressed during Drosophila melanogaster embryonic development. During Drosophila embryonic development, various morphogenetic processes require the remodeling of the **extracellular matrix**. In a previous study, we have identified and characterized a cDNA encoding a novel putative **extracellular matrix** protein named tenebrin, in the beetle Tenebrio molitor. Here, we examine the expression of the Drosophila ortholog, referred to as Tenectin (Tnc), during embryonic development. Tnc is expressed in the majority of tissues of neuroectodermic origin such as hindgut, foregut, tracheal system, anal plate, and CNS. In the CNS, the Tnc transcript is restricted to a few cells, whereas the protein is located in the dorsal part of the axonal tracts. In the hindgut and the trachea, Tnc protein is expressed on the apical pole of the cells. Tnc is an **extracellular matrix** protein secreted in a polarized way in different organs of Drosophila embryos.
3. **Extracellular matrix** and its receptors in Drosophila neural development. **Extracellular matrix** (ECM) and matrix receptors are intimately involved in most biological processes. The ECM plays fundamental developmental and physiological roles in health and disease, including processes underlying the development, maintenance, and regeneration of the nervous system. To understand the principles of ECM-mediated functions in the nervous system, genetic model organisms like Drosophila provide simple, malleable, and powerful experimental platforms. This article provides an overview of ECM proteins and receptors in Drosophila. It then focuses on their roles during three progressive phases of neural development: (1) neural progenitor proliferation, (2) axonal growth and pathfinding, and (3) synapse formation and function. Each section highlights known ECM and ECM-receptor components and recent studies done in mutant conditions to reveal their in vivo functions, all illustrating the enormous opportunities provided when merging work on the nervous system with systematic research into ECM-related gene functions.
4. Tenectin, a novel **extracellular matrix** protein expressed during Drosophila melanogaster embryonic development. During Drosophila embryonic development, various morphogenetic processes require the remodeling of the **extracellular matrix**. In a previous study, we have identified and characterized a cDNA encoding a novel putative **extracellular matrix** protein named tenebrin, in the beetle Tenebrio molitor. Here, we examine the expression of the Drosophila ortholog, referred to as Tenectin (Tnc), during embryonic development. Tnc is expressed in the majority of tissues of neuroectodermic origin such as hindgut, foregut, tracheal system, anal plate, and CNS. In the CNS, the Tnc transcript is restricted to a few cells, whereas the protein is located in the dorsal part of the axonal tracts. In the hindgut and the trachea, Tnc protein is expressed on the apical pole of the cells. Tnc is an **extracellular matrix** protein secreted in a polarized way in different organs of Drosophila embryos.
5. Tenectin is a novel alphaPS2betaPS integrin ligand required for wing morphogenesis and male genital looping in Drosophila. Morphogenesis of the adult structures of holometabolous insects is regulated by ecdysteroids and juvenile hormones and involves cell-cell interactions mediated in part by the cell surface integrin receptors and their **extracellular matrix** (ECM) ligands. These adhesion molecules and their regulation by hormones are not well characterized. We describe the gene structure of a newly described ECM molecule, tenectin, and demonstrate that it is a hormonally regulated ECM protein required for proper morphogenesis of the adult wing and male genitalia. Tenectin's function as a new ligand of the PS2 integrins is demonstrated by both genetic interactions in the fly and by cell spreading and cell adhesion assays in cultured cells. Its interaction with the PS2 integrins is dependent on RGD and RGD-like motifs. Tenectin's function in looping morphogenesis in the development of the male genitalia led to experiments that demonstrate a role for PS integrins in the execution of left-right asymmetry.

#### Component

1. **extracellular matrix**
2. **extracellular matrix**

#### GeneRIFs

1. Tnc is an **extracellular matrix** protein secreted in a polarized way in different organs of Drosophila embryos.

---

## cv-c

- NCBI Gene
- GeneRIF
- Pubmed

| Search Term | Rank | Fields |
| --- | --- | --- |
| [Mm]igration | 6 | Process(1); PubMed(2) |
| [Ee]xtracellular [Mm]atrix | 8 | PubMed(1) |

  
hide/show details for cv-c

### [Mm]igration

#### Process

1. epithelial cell **migration**, open tracheal system

#### PubMed

1. Transcriptional control in embryonic Drosophila midline guidance assessed through a whole genome approach. During the development of the Drosophila central nervous system the process of midline crossing is orchestrated by a number of guidance receptors and ligands. Many key axon guidance molecules have been identified in both invertebrates and vertebrates, but the transcriptional regulation of growth cone guidance remains largely unknown. It is established that translational regulation plays a role in midline crossing, and there are indications that transcriptional regulation is also involved. To investigate this issue, we conducted a genome-wide study of transcription in Drosophila embryos using wild type and a number of well-characterized Drosophila guidance mutants and transgenics. We also analyzed a previously published microarray time course of Drosophila embryonic development with an axon guidance focus.Using hopach, a novel clustering method which is well suited to microarray data analysis, we identified groups of genes with similar expression patterns across guidance mutants and transgenics. We then systematically characterized the resulting clusters with respect to their relevance to axon guidance using two complementary controlled vocabularies: the Gene Ontology (GO) and anatomical annotations of the Atlas of Pattern of Gene Expression (APoGE) in situ hybridization database. The analysis indicates that regulation of gene expression does play a role in the process of axon guidance in Drosophila. We also find a strong link between axon guidance and hemocyte **migration**, a result that agrees with mounting evidence that axon guidance molecules are co-opted in vertebrate vascularization. Cell cyclin activity in the context of axon guidance is also suggested from our array data. RNA and protein expression patterns of cell cyclins in axon guidance mutants and transgenics support this possible link.This study provides important insights into the regulation of axon guidance in vivo.
2. stumps, a Drosophila gene required for fibroblast growth factor (FGF)-directed **migration**s of tracheal and mesodermal cells. Fibroblast growth factors (FGFs) bind to FGF receptors, transmembrane tyrosine kinases that activate mitogenic, motogenic, and differentiative responses in different tissues. While there has been substantial progress in elucidating the Ras-MAP kinase pathway that mediates the differentiative responses, the signal transduction pathways that lead to directed cell **migration**s are not well defined. Here we describe a Drosophila gene called stumps that is required for FGF-dependent **migration**s of tracheal and mesodermal cells. These **migration**s are controlled by different FGF ligands and receptors, and they occur by different cellular mechanisms: the tracheal **migration**s occur as part of an epithelium whereas the mesodermal **migration**s are fibroblast-like. In the stumps mutant, tracheal cells fail to move out from the epithelial sacs, and only rudimentary tracheal branches form. Mesodermal cells fail in their dorsal **migration**s after gastrulation. The stumps mutation does not block all FGF signaling effects in these tissues: both random cell **migration**s and Ras-MAP kinase-mediated induction of FGF-specific effector genes occurred upon ectopic expression of the ligand or upon expression of a constitutively activated Ras protein in the migrating cells. The results suggest that stumps function promotes FGF-directed cell **migration**s, either by potentiating the FGF signaling process or by coupling the signal to the cellular machinery required for directed cell movement.

### [Ee]xtracellular [Mm]atrix

#### PubMed

1. The Gyc76C Receptor Guanylyl Cyclase and the Foraging cGMP-Dependent Kinase Regulate **Extracellular Matrix** Organization and BMP Signaling in the Developing Wing of Drosophila melanogaster. The developing crossveins of the wing of Drosophila melanogaster are specified by long-range BMP signaling and are especially sensitive to loss of extracellular modulators of BMP signaling such as the Chordin homolog Short gastrulation (Sog). However, the role of the **extracellular matrix** in BMP signaling and Sog activity in the crossveins has been poorly explored. Using a genetic mosaic screen for mutations that disrupt BMP signaling and posterior crossvein development, we identify Gyc76C, a member of the receptor guanylyl cyclase family that includes mammalian natriuretic peptide receptors. We show that Gyc76C and the soluble cGMP-dependent kinase Foraging, likely linked by cGMP, are necessary for normal refinement and maintenance of long-range BMP signaling in the posterior crossvein. This does not occur through cell-autonomous crosstalk between cGMP and BMP signal transduction, but likely through altered extracellular activity of Sog. We identify a novel pathway leading from Gyc76C to the organization of the wing **extracellular matrix** by matrix metalloproteinases, and show that both the **extracellular matrix** and BMP signaling effects are largely mediated by changes in the activity of matrix metalloproteinases. We discuss parallels and differences between this pathway and other examples of cGMP activity in both Drosophila melanogaster and mammalian cells and tissues.

---

## Stam

- NCBI Gene
- GeneRIF
- Pubmed

| Search Term | Rank | Fields |
| --- | --- | --- |
| [Mm]igration | 6 | Process(2); PubMed(3) |

  
hide/show details for Stam

### [Mm]igration

#### Process

1. epithelial cell **migration**, open tracheal system
2. regulation of epithelial cell **migration**, open tracheal system

#### PubMed

1. The Jak/STAT pathway in model organisms: emerging roles in cell movement. The JAK/STAT pathway was originally identified in mammals. Studies of this pathway in the mouse have revealed that JAK/STAT signaling plays a central role during hematopoeisis and other developmental processes. The role of JAK/STAT signaling in blood appears to be conserved throughout evolution, as it is also required during fly hematopoeisis. Studies in Dictyostelium, Drosophila, and zebrafish have shown that the JAK/STAT pathway is also required in an unusually broad set of developmental decisions, including cell proliferation, cell fate determination, cell **migration**, planar polarity, convergent extension, and immunity. There is increasing evidence that the versatility of this pathway relies on its cooperation with other signal transduction pathways. In this review, we discuss the components of the JAK/STAT pathway in model organisms and what is known about its requirement in cellular and developmental processes. In particular, we emphasize recent insights into the role that this pathway plays in the control of cell movement.
2. The Hrs/Stam complex acts as a positive and negative regulator of RTK signaling during Drosophila development. Endocytosis is a key regulatory step of diverse signalling pathways, including receptor tyrosine kinase (RTK) signalling. Hrs and Stam constitute the ESCRT-0 complex that controls the initial selection of ubiquitinated proteins, which will subsequently be degraded in lysosomes. It has been well established ex vivo and during Drosophila embryogenesis that Hrs promotes EGFR down regulation. We have recently isolated the first mutations of stam in flies and shown that Stam is required for air sac morphogenesis, a larval respiratory structure whose formation critically depends on finely tuned levels of FGFR activity. This suggest that Stam, putatively within the ESCRT-0 complex, modulates FGF signalling, a possibility that has not been examined in Drosophila yet.Here, we assessed the role of the Hrs/Stam complex in the regulation of signalling activity during Drosophila development. We show that stam and hrs are required for efficient FGFR signalling in the tracheal system, both during cell **migration** in the air sac primordium and during the formation of fine cytoplasmic extensions in terminal cells. We find that stam and hrs mutant cells display altered FGFR/Btl localisation, likely contributing to impaired signalling levels. Electron microscopy analyses indicate that endosome maturation is impaired at distinct steps by hrs and stam mutations. These somewhat unexpected results prompted us to further explore the function of stam and hrs in EGFR signalling. We show that while stam and hrs together downregulate EGFR signalling in the embryo, they are required for full activation of EGFR signalling during wing development.Our study shows that the ESCRT-0 complex differentially regulates RTK signalling, either positively or negatively depending on tissues and developmental stages, further highlighting the importance of endocytosis in modulating signalling pathways during development.
3. A genetic mosaic analysis with a repressible cell marker screen to identify genes involved in tracheal cell **migration** during Drosophila air sac morphogenesis. Branching morphogenesis of the Drosophila tracheal system relies on the fibroblast growth factor receptor (FGFR) signaling pathway. The Drosophila FGF ligand Branchless (Bnl) and the FGFR Breathless (Btl/FGFR) are required for cell **migration** during the establishment of the interconnected network of tracheal tubes. However, due to an important maternal contribution of members of the FGFR pathway in the oocyte, a thorough genetic dissection of the role of components of the FGFR signaling cascade in tracheal cell **migration** is impossible in the embryo. To bypass this shortcoming, we studied tracheal cell **migration** in the dorsal air sac primordium, a structure that forms during late larval development. Using a mosaic analysis with a repressible cell marker (MARCM) clone approach in mosaic animals, combined with an ethyl methanesulfonate (EMS)-mutagenesis screen of the left arm of the second chromosome, we identified novel genes implicated in cell **migration**. We screened 1123 mutagenized lines and identified 47 lines displaying tracheal cell **migration** defects in the air sac primordium. Using complementation analyses based on lethality, mutations in 20 of these lines were genetically mapped to specific genomic areas. Three of the mutants were mapped to either the Mhc or the stam complementation groups. Further experiments confirmed that these genes are required for cell **migration** in the tracheal air sac primordium.

---

## garz

- NCBI Gene
- GeneRIF
- Pubmed

| Search Term | Rank | Fields |
| --- | --- | --- |
| [Ee]xtracellular [Mm]atrix | 8 | PubMed(2) |

  
hide/show details for garz

### [Ee]xtracellular [Mm]atrix

#### PubMed

1. GBF1 (Gartenzwerg)-dependent secretion is required for Drosophila tubulogenesis. Here we report on the generation and in vivo analysis of a series of loss-of-function mutants for the Drosophila ArfGEF, Gartenzwerg. The Drosophila gene gartenzwerg (garz) encodes the orthologue of mammalian GBF1. garz is expressed ubiquitously in embryos with substantially higher abundance in cells forming diverse tubular structures such as salivary glands, trachea, proventriculus or hindgut. In the absence of functional Garz protein, the integrity of the Golgi complex is impaired. As a result, both vesicle transport of cargo proteins and directed apical membrane delivery are severely disrupted. Dysfunction of the Arf1-COPI machinery caused by a loss of Garz leads to perturbations in establishing a polarized epithelial architecture of tubular organs. Furthermore, insufficient apical transport of proteins and other membrane components causes incomplete luminal diameter expansion and deficiencies in **extracellular matrix** assembly. The fact that homologues of Garz are present in every annotated metazoan genome indicates that secretion processes mediated by the GBF-type ArfGEFs play a universal role in animal development.
2. GBF1 (Gartenzwerg)-dependent secretion is required for Drosophila tubulogenesis. Here we report on the generation and in vivo analysis of a series of loss-of-function mutants for the Drosophila ArfGEF, Gartenzwerg. The Drosophila gene gartenzwerg (garz) encodes the orthologue of mammalian GBF1. garz is expressed ubiquitously in embryos with substantially higher abundance in cells forming diverse tubular structures such as salivary glands, trachea, proventriculus or hindgut. In the absence of functional Garz protein, the integrity of the Golgi complex is impaired. As a result, both vesicle transport of cargo proteins and directed apical membrane delivery are severely disrupted. Dysfunction of the Arf1-COPI machinery caused by a loss of Garz leads to perturbations in establishing a polarized epithelial architecture of tubular organs. Furthermore, insufficient apical transport of proteins and other membrane components causes incomplete luminal diameter expansion and deficiencies in **extracellular matrix** assembly. The fact that homologues of Garz are present in every annotated metazoan genome indicates that secretion processes mediated by the GBF-type ArfGEFs play a universal role in animal development.

---

## Idgf3

- NCBI Gene
- GeneRIF
- Pubmed

| Search Term | Rank | Fields |
| --- | --- | --- |
| [Mm]igration | 6 | PubMed(1) |
| ECM | 7 | PubMed(1) |
| [Ee]xtracellular [Mm]atrix | 8 | PubMed(2) |

  
hide/show details for Idgf3

### [Mm]igration

#### PubMed

1. serpent, a GATA-like transcription factor gene, induces fat-cell development in Drosophila melanogaster. The GATA-like transcription factor gene serpent is necessary for embryonic fat-cell differentiation in Drosophila (Sam, S., Leise, W. and Hoshizaki, D. K. (1996) Mech. Dev. 60, 197-205) and has been proposed to function in a cell-fate choice between fat cell and somatic gonadal precursors (Moore, L. A., Broihier, H. T., Van Doren, M. and Lehmann, R. (1998) Development 125, 837-44; Riechmann, V., Irion, U., Wilson, R., Grosskortenhaus, R. and Leptin, M. (1997) Development 124, 2915-22). Here, we report that deregulated expression of serpent in the mesoderm induces the formation of ectopic fat cells and prevents the **migration** and coalescence of the somatic gonadal precursors. The ectopic fat cells do not arise from hyperproliferation of the primary fat-cell clusters but they do associate with the endogenous fat cells to form a fat body that is expanded in both the dorsal/ventral and anterior/posterior axes. Misexpression of serpent also affects the differentiation of muscle cells. Few body-wall muscle precursors are specified and there is a loss of most body-wall muscle fibers. The precursors of the visceral mesoderm are also absent and concomitantly the visceral muscle is absent. We suggest that the ectopic fat cells might originate from cells that have the potential, but do not normally, differentiate into fat cells or from cells that have acquired a fat-cell fate. In light of our results, we discuss the role of serpent in fat-cell specification and in cell fate choices.

### ECM

#### PubMed

1. Chitinases and Imaginal disc growth factors organize the extracellular matrix formation at barrier tissues in insects. The cuticle forms an apical extracellular-matrix (**ECM**) that covers exposed organs, such as epidermis, trachea and gut, for organizing morphogenesis and protection of insects. Recently, we reported that cuticle proteins and chitin are involved in **ECM** formation. However, molecular mechanisms that control assembly, maturation and replacement of the **ECM** and its components are not well known. Here we investigated the poorly described glyco-18-domain hydrolase family in Drosophila and identified the Chitinases (Chts) and imaginal-disc-growth-factors (Idgfs) that are essential for larval and adult molting. We demonstrate that Cht and idgf depletion results in deformed cuticles, larval and adult molting defects, and insufficient protection against wounding and bacterial infection, which altogether leads to early lethality. We show that Cht2/Cht5/Cht7/Cht9/Cht12 and idgf1/idgf3/idgf4/idgf5/idgf6 are needed for organizing proteins and chitin-matrix at the apical cell surface. Our data indicate that normal **ECM** formation requires Chts, which potentially hydrolyze chitin-polymers. We further suggest that the non-enzymatic idgfs act as structural proteins to maintain the **ECM** scaffold against chitinolytic degradation. Conservation of Chts and Idgfs proposes analogous roles in **ECM** dynamics across the insect taxa, indicating that Chts/Idgfs are new targets for species specific pest control.

### [Ee]xtracellular [Mm]atrix

#### PubMed

1. Chitinases and Imaginal disc growth factors organize the **extracellular matrix** formation at barrier tissues in insects. The cuticle forms an apical extracellular-matrix (ECM) that covers exposed organs, such as epidermis, trachea and gut, for organizing morphogenesis and protection of insects. Recently, we reported that cuticle proteins and chitin are involved in ECM formation. However, molecular mechanisms that control assembly, maturation and replacement of the ECM and its components are not well known. Here we investigated the poorly described glyco-18-domain hydrolase family in Drosophila and identified the Chitinases (Chts) and imaginal-disc-growth-factors (Idgfs) that are essential for larval and adult molting. We demonstrate that Cht and idgf depletion results in deformed cuticles, larval and adult molting defects, and insufficient protection against wounding and bacterial infection, which altogether leads to early lethality. We show that Cht2/Cht5/Cht7/Cht9/Cht12 and idgf1/idgf3/idgf4/idgf5/idgf6 are needed for organizing proteins and chitin-matrix at the apical cell surface. Our data indicate that normal ECM formation requires Chts, which potentially hydrolyze chitin-polymers. We further suggest that the non-enzymatic idgfs act as structural proteins to maintain the ECM scaffold against chitinolytic degradation. Conservation of Chts and Idgfs proposes analogous roles in ECM dynamics across the insect taxa, indicating that Chts/Idgfs are new targets for species specific pest control.
2. Genome-wide transcriptional analysis of Drosophila larvae infected by entomopathogenic nematodes shows involvement of complement, recognition and **extracellular matrix** proteins. Heterorhabditis bacteriophora is an entomopathogenic nematode (EPN) which infects its host by accessing the hemolymph where it releases endosymbiotic bacteria of the species Photorhabdus luminescens. We performed a genome-wide transcriptional analysis of the Drosophila response to EPN infection at the time point at which the nematodes reached the hemolymph either via the cuticle or the gut and the bacteria had started to multiply. Many of the most strongly induced genes have been implicated in immune responses in other infection models. Mapping of the complete set of differentially regulated genes showed the hallmarks of a wound response, but also identified a large fraction of EPN-specific transcripts. Several genes identified by transcriptome profiling or their homologues play protective roles during nematode infections. Genes that positively contribute to controlling nematobacterial infections encode: a homolog of thioester-containing complement protein 3, a basement membrane component (glutactin), a recognition protein (GNBP-like 3) and possibly several small peptides. Of note is that several of these genes have not previously been implicated in immune responses.

---

## spg

- NCBI Gene
- GeneRIF
- Pubmed

| Search Term | Rank | Fields |
| --- | --- | --- |
| [Pp]odosome(s)? | 5 | PubMed(1) |
| [Mm]igration | 6 | PubMed(3) |
| [Ii]nvasive | 10 | PubMed(1) |

  
hide/show details for spg

### [Pp]odosome(s)?

#### PubMed

1. Asymmetric Mbc, active Rac1 and F-actin foci in the fusion-competent myoblasts during myoblast fusion in Drosophila. Myoblast fusion is an intricate process that is initiated by cell recognition and adhesion, and culminates in cell membrane breakdown and formation of multinucleate syncytia. In the Drosophila embryo, this process occurs asymmetrically between founder cells that pattern the musculature and fusion-competent myoblasts (FCMs) that account for the bulk of the myoblasts. The present studies clarify and amplify current models of myoblast fusion in several important ways. We demonstrate that the non-conventional guanine nucleotide exchange factor (GEF) Mbc plays a fundamental role in the FCMs, where it functions to activate Rac1, but is not required in the founder cells for fusion. Mbc, active Rac1 and F-actin foci are highly enriched in the FCMs, where they localize to the Sns:Kirre junction. Furthermore, Mbc is crucial for the integrity of the F-actin foci and the FCM cytoskeleton, presumably via its activation of Rac1 in these cells. Finally, the local asymmetric distribution of these proteins at adhesion sites is reminiscent of invasive **podosomes** and, consistent with this model, they are enriched at sites of membrane deformation, where the FCM protrudes into the founder cell/myotube. These data are consistent with models promoting actin polymerization as the driving force for myoblast fusion.

### [Mm]igration

#### PubMed

1. CZH proteins: a new family of Rho-GEFs. The Rho family of small GTPases are important regulators of multiple cellular activities and, most notably, reorganization of the actin cytoskeleton. Dbl-homology (DH)-domain-containing proteins are the classical guanine nucleotide exchange factors (GEFs) responsible for activation of Rho GTPases. However, members of a newly discovered family can also act as Rho-GEFs. These CZH proteins include: CDM (Ced-5, Dock180 and Myoblast city) proteins, which activate Rac; and zizimin proteins, which activate Cdc42. The family contains 11 mammalian proteins and has members in many other eukaryotes. The GEF activity is carried out by a novel, DH-unrelated domain named the DOCKER, CZH2 or DHR2 domain. CZH proteins have been implicated in cell **migration**, phagocytosis of apoptotic cells, T-cell activation and neurite outgrowth, and probably arose relatively early in eukaryotic evolution.
2. Cortical actin dynamics facilitate early-stage centrosome separation. Proper centrosome separation is a prerequisite for positioning the bipolar spindle. Although studies demonstrate that microtubules (MTs) and their associated motors drive centrosome separation [1], the role of actin in centrosome separation remains less clear. Studies in tissue culture cells indicate that actin- and myosin-based cortical flow is primarily responsible for driving late centrosome separation [2], whereas other studies suggest that actin plays a more passive role by serving as an attachment site for astral MTs to pull centrosomes apart [3-6]. Here we demonstrate that prior to nuclear envelope breakdown (NEB) in Drosophila embryos, proper centrosome separation does not require myosin II but requires dynamic actin rearrangements at the growing edge of the interphase cap. Both Arp2/3- and Formin-mediated actin remodeling are required for separating the centrosome pairs before NEB. The Apc2-Armadillo complex appears to link cap expansion to centrosome separation. In contrast, the mechanisms driving centrosome separation after NEB are independent of the actin cytoskeleton and compensate for earlier separation defects. Our studies show that the dynamics of actin polymerization drive centrosome separation, and this has important implications for centrosome positioning during processes such as cell **migration** [7, 8], cell polarity maintenance [9, 10], and asymmetric cell division [11, 12].
3. The RhoGEF Zizimin-related acts in the Drosophila cellular immune response via the Rho GTPases Rac2 and Cdc42. Zizimin-related (Zir), a Rho guanine nucleotide exchange factor (RhoGEF) homologous to the mammalian Dock-C/Zizimin-related family, was identified in a screen to find new genes involved in the Drosophila melanogaster cellular immune response against eggs from the parasitoid wasp Leptopilina boulardi. RhoGEFs activate Rho-family GTPases, which are known to be central regulators of cell **migration**, spreading and polarity. When a parasitoid wasp is recognized as foreign, multiple layers of circulating immunosurveillance cells (haemocytes) should attach to the egg. In Zir mutants this process is disrupted and lamellocytes, a haemocyte subtype, fail to properly encapsulate the wasp egg. Furthermore, macrophage-like plasmatocytes exhibit a strong reduction in their ability to phagocytise Escherichia coli and Staphylococcus aureus bacteria. During encapsulation and phagocytosis Zir genetically interacts with two Rho-family GTPases, Rac2 and Cdc42. Finally, Zir is dispensable for the humoral immune response against bacteria. We propose that Zir is necessary to activate the Rho-family GTPases Rac2 and Cdc42 during the Drosophila cellular immune response.

### [Ii]nvasive

#### PubMed

1. Asymmetric Mbc, active Rac1 and F-actin foci in the fusion-competent myoblasts during myoblast fusion in Drosophila. Myoblast fusion is an intricate process that is initiated by cell recognition and adhesion, and culminates in cell membrane breakdown and formation of multinucleate syncytia. In the Drosophila embryo, this process occurs asymmetrically between founder cells that pattern the musculature and fusion-competent myoblasts (FCMs) that account for the bulk of the myoblasts. The present studies clarify and amplify current models of myoblast fusion in several important ways. We demonstrate that the non-conventional guanine nucleotide exchange factor (GEF) Mbc plays a fundamental role in the FCMs, where it functions to activate Rac1, but is not required in the founder cells for fusion. Mbc, active Rac1 and F-actin foci are highly enriched in the FCMs, where they localize to the Sns:Kirre junction. Furthermore, Mbc is crucial for the integrity of the F-actin foci and the FCM cytoskeleton, presumably via its activation of Rac1 in these cells. Finally, the local asymmetric distribution of these proteins at adhesion sites is reminiscent of **invasive** podosomes and, consistent with this model, they are enriched at sites of membrane deformation, where the FCM protrudes into the founder cell/myotube. These data are consistent with models promoting actin polymerization as the driving force for myoblast fusion.

---

## mv

- NCBI Gene
- GeneRIF
- Pubmed

| Search Term | Rank | Fields |
| --- | --- | --- |
| [Ii]nvasive | 10 | PubMed(1) |

  
hide/show details for mv

### [Ii]nvasive

#### PubMed

1. Serine protease MP2 activates prophenoloxidase in the melanization immune response of Drosophila melanogaster. In arthropods, melanization plays a major role in the innate immune response to encapsulate and kill the **invasive** organisms. It is mediated by a serine protease cascade and is regulated by serpins. The identification of the molecular components of melanization and the regulation of those components are still unclear in Drosophila melanogaster, although some genetic research on the activation of melanization has been reported. Here we report that Drosophila serine protease MP2 directly cleaves both recombinant and native prophenoloxidase-1. Overexpression or repression of MP2 in flies resulted in increased and decreased rates of cleavage, respectively, of prophenoloxidase-1. Moreover, serine protease inhibitor Spn27A formed SDS-stable complexes with MP2, both in vitro and in vivo. The amidase activity of MP2 was inhibited efficiently by Spn27A. Spn27A also prevented MP2 from cleaving prophenoloxidase-1. Taken together, these results indicate that under our experimental conditions MP2 functions as a prophenoloxidase-activating protease, and that this function is inhibited by Spn27A. MP2 and Spn27A thus constitute a regulatory unit in the prophenoloxidase activation cascade in Drosophila. The combination of genetic, molecular genetic and biochemical approaches should allow further advances in our understanding of the prophenoloxidase-activating cascade in insects and indirectly shed further light on protease-cascades in humans and other vertebrates.

---

## CG31694

- NCBI Gene
- GeneRIF
- Pubmed

| Search Term | Rank | Fields |
| --- | --- | --- |
| [Mm]igration | 6 | PubMed(1) |

  
hide/show details for CG31694

### [Mm]igration

#### PubMed

1. Regulation of Drosophila mesoderm **migration** by phosphoinositides and the PH domain of the Rho GTP exchange factor Pebble. The Drosophila RhoGEF Pebble (Pbl) is required for cytokinesis and **migration** of mesodermal cells. In a screen for genes that could suppress **migration** defects in pbl mutants we identified the phosphatidylinositol phosphate (PtdInsP) regulator pi5k59B. Genetic interaction tests with other PtdInsP regulators suggested that PtdIns(4,5)P2 levels are important for mesoderm **migration** when Pbl is depleted. Consistent with this, the leading front of migrating mesodermal cells was enriched for PtdIns(4,5)P2. Given that Pbl contains a Pleckstrin Homology (PH) domain, a known PtdInsP-binding motif, we examined PtdInsP-binding of Pbl and the importance of the PH domain for Pbl function. In vitro lipid blot assays showed that Pbl binds promiscuously to PtdInsPs, with binding strength associated with the degree of phosphorylation. Pbl was also able to bind lipid vesicles containing PtdIns(4,5)P2 but binding was strongly reduced upon deletion of the PH domain. Similarly, in vivo, loss of the PH domain prevented localisation of Pbl to the cell cortex and severely affected several aspects of early mesoderm development, including flattening of the invaginated tube onto the ectoderm, extension of protrusions, and dorsal **migration** to form a monolayer. Pbl lacking the PH domain could still localise to the cytokinetic furrow, however, and cytokinesis failure was reduced in pbl(ΔPH) mutants. Taken together, our results support a model in which interaction of the PH-domain of Pbl with PtdIns(4,5)P2 helps localise it to the plasma membrane which is important for mesoderm **migratio**n.

---

## CG45186

- NCBI Gene
- GeneRIF
- Pubmed

| Search Term | Rank | Fields |
| --- | --- | --- |
| [Mm]igration | 6 | PubMed(1) |

  
hide/show details for CG45186

### [Mm]igration

#### PubMed

1. Molecular requirements for actin-based lamella formation in Drosophila S2 cells. Cell **migration** occurs through the protrusion of the actin-enriched lamella. Here, we investigated the effects of RNAi depletion of approximately 90 proteins implicated in actin function on lamella formation in Drosophila S2 cells. Similar to in vitro reconstitution studies of actin-based Listeria movement, we find that lamellae formation requires a relatively small set of proteins that participate in actin nucleation (Arp2/3 and SCAR), barbed end capping (capping protein), filament depolymerization (cofilin and Aip1), and actin monomer binding (profilin and cyclase-associated protein). Lamellae are initiated by parallel and partially redundant signaling pathways involving Rac GTPases and the adaptor protein Nck, which stimulate SCAR, an Arp2/3 activator. We also show that RNAi of three proteins (kette, Abi, and Sra-1) known to copurify with and inhibit SCAR in vitro leads to SCAR degradation, revealing a novel function of this protein complex in SCAR stability. Our results have identified an essential set of proteins involved in actin dynamics during lamella formation in Drosophila S2 cells.

---

## Akt1

- NCBI Gene
- GeneRIF
- Pubmed

| Search Term | Rank | Fields |
| --- | --- | --- |
| [Ff]ilopodia | 1 | PubMed(1) |
| [Mm]igration | 6 | Process(1); PubMed(13) |
| [Ii]nvasive | 10 | PubMed(6) |
| [Mm]etastasis | 12 | PubMed(5) |
| [Cc]ell [Ii]nvasion | 13 | PubMed(2) |

  
hide/show details for Akt1

### [Ff]ilopodia

#### PubMed

1. Gene regulatory networks controlling hematopoietic progenitor niche cell production and differentiation in the Drosophila lymph gland. Hematopoiesis occurs in two phases in Drosophila, with the first completed during embryogenesis and the second accomplished during larval development. The lymph gland serves as the venue for the final hematopoietic program, with this larval tissue well-studied as to its cellular organization and genetic regulation. While the medullary zone contains stem-like hematopoietic progenitors, the posterior signaling center (PSC) functions as a niche microenvironment essential for controlling the decision between progenitor maintenance versus cellular differentiation. In this report, we utilize a PSC-specific GAL4 driver and UAS-gene RNAi strains, to selectively knockdown individual gene functions in PSC cells. We assessed the effect of abrogating the function of 820 genes as to their requirement for niche cell production and differentiation. 100 genes were shown to be essential for normal niche development, with various loci placed into sub-groups based on the functions of their encoded protein products and known genetic interactions. For members of three of these groups, we characterized loss- and gain-of-function phenotypes. Gene function knockdown of members of the BAP chromatin-remodeling complex resulted in niche cells that do not express the hedgehog (hh) gene and fail to differentiate **filopodia** believed important for Hh signaling from the niche to progenitors. Abrogating gene function of various members of the insulin-like growth factor and TOR signaling pathways resulted in anomalous PSC cell production, leading to a defective niche organization. Further analysis of the Pten, TSC1, and TSC2 tumor suppressor genes demonstrated their loss-of-function condition resulted in severely altered blood cell homeostasis, including the abundant production of lamellocytes, specialized hemocytes involved in innate immune responses. Together, this cell-specific RNAi knockdown survey and mutant phenotype analyses identified multiple genes and their regulatory networks required for the normal organization and function of the hematopoietic progenitor niche within the lymph gland.

### [Mm]igration

#### Process

1. epithelial cell **migration**, open tracheal system

#### PubMed

1. Drosophila tribbles antagonizes insulin signaling-mediated growth and metabolism via interactions with Akt kinase. Drosophila Tribbles (Trbl) is the founding member of the Trib family of kinase-like docking proteins that modulate cell signaling during proliferation, **migration** and growth. In a wing misexpression screen for Trbl interacting proteins, we identified the Ser/Thr protein kinase Akt1. Given the central role of Akt1 in insulin signaling, we tested the function of Trbl in larval fat body, a tissue where rapid increases in size are exquisitely sensitive to insulin/insulin-like growth factor levels. Consistent with a role in antagonizing insulin-mediated growth, trbl RNAi knockdown in the fat body increased cell size, advanced the timing of pupation and increased levels of circulating triglyceride. Complementarily, overexpression of Trbl reduced fat body cell size, decreased overall larval size, delayed maturation and lowered levels of triglycerides, while circulating glucose levels increased. The conserved Trbl kinase domain is required for function in vivo and for interaction with Akt in a yeast two-hybrid assay. Consistent with direct regulation of Akt, overexpression of Trbl in the fat body decreased levels of activated Akt (pSer505-Akt) while misexpression of trbl RNAi increased phospho-Akt levels, and neither treatment affected total Akt levels. Trbl misexpression effectively suppressed Akt-mediated wing and muscle cell size increases and reduced phosphorylation of the Akt target FoxO (pSer256-FoxO). Taken together, these data show that Drosophila Trbl has a conserved role to bind Akt and block Akt-mediated insulin signaling, and implicate Trib proteins as novel sites of signaling pathway integration that link nutrient availability with cell growth and proliferation.
2. A drosophila model for EGFR-Ras and PI3K-dependent human glioma. Gliomas, the most common malignant tumors of the nervous system, frequently harbor mutations that activate the epidermal growth factor receptor (EGFR) and phosphatidylinositol-3 kinase (PI3K) signaling pathways. To investigate the genetic basis of this disease, we developed a glioma model in Drosophila. We found that constitutive coactivation of EGFR-Ras and PI3K pathways in Drosophila glia and glial precursors gives rise to neoplastic, invasive glial cells that create transplantable tumor-like growths, mimicking human glioma. Our model represents a robust organotypic and cell-type-specific Drosophila cancer model in which malignant cells are created by mutations in signature genes and pathways thought to be driving forces in a homologous human cancer. Genetic analyses demonstrated that EGFR and PI3K initiate malignant neoplastic transformation via a combinatorial genetic network composed primarily of other pathways commonly mutated or activated in human glioma, including the Tor, Myc, G1 Cyclins-Cdks, and Rb-E2F pathways. This network acts synergistically to coordinately stimulate cell cycle entry and progression, protein translation, and inappropriate cellular growth and **migration**. In particular, we found that the fly orthologs of CyclinE, Cdc25, and Myc are key rate-limiting genes required for glial neoplasia. Moreover, orthologs of Sin1, Rictor, and Cdk4 are genes required only for abnormal neoplastic glial proliferation but not for glial development. These and other genes within this network may represent important therapeutic targets in human glioma.
3. Colorectal cancer progression: lessons from Drosophila? Human colorectal cancers arise as benign adenomas, tumours that retain their epithelial character, and then progress to malignant adenocarcinomas and carcinomas in which the epithelium becomes disrupted. Carcinomas often exhibit transcriptional downregulation of E-cadherin and other epithelial genes in an epithelial-to-mesenchymal transition (EMT), a mechanism first discovered in Drosophila to be mediated by the transcription factors Twist and Snail. In contrast, adenocarcinomas retain expression of E-cadherin and disruption of the epithelium occurs through formation of progressively smaller epithelial cysts with apical Crumbs/CRB3, Stardust/PALS1, and Bazooka/PAR3 localised to the inner lumen. Results from Drosophila show that morphologically similar cysts form upon induction of clonal heterogeneity in Wnt, Smad, or Ras signalling levels, which causes extrusion of epithelial cells at clonal boundaries. Thus, intratumour heterogeneity might also promote formation of adenocarcinomas in humans. Finally, epithelial cysts can collectively migrate, as in the case of Drosophila border cells, a potential model system for the invasive **migration** of adenocarcinoma cells.
4. Centrosomal kinase Nek2 cooperates with oncogenic pathways to promote metastasis. Centrosomal kinase Nek2 is overexpressed in different cancers, yet how it contributes toward tumorigenesis remains poorly understood. dNek2 overexpression in a Drosophila melanogaster model led to upregulation of Drosophila Wnt ortholog wingless (Wg), and alteration of cell **migration** markers-Rho1, Rac1 and E-cadherin (Ecad)-resulting in changes in cell shape and tissue morphogenesis. dNek2 overexpression cooperated with receptor tyrosine kinase and mitogen-activated protein kinase signaling to upregulate activated Akt, Diap1, Mmp1 and Wg protein to promote local invasion, distant seeding and metastasis. In tumor cell injection assays, dNek2 cooperated with Ras and Src signaling to promote aggressive colonization of tumors into different adult fly tissues. Inhibition of the PI3K pathway suppressed the cooperation of dNek2 with other growth pathways. Consistent with our fly studies, overexpression of human Nek2 in A549 lung adenocarcinoma and HEK293T cells led to activation of the Akt pathway and increase in β-catenin protein levels. Our computational approach identified a class of Nek2-inhibitory compounds and a novel drug-like pharmacophore that reversed the Nek2 overexpression phenotypes in flies and human cells. Our finding posits a novel role for Nek2 in promoting metastasis in addition to its currently defined role in promoting chromosomal instability. It provides a rationale for the selective advantage of centrosome amplification in cancer.
5. Increased centrosome amplification in aged stem cells of the Drosophila midgut. Age-related changes in long-lived tissue-resident stem cells may be tightly linked to aging and age-related diseases such as cancer. Centrosomes play key roles in cell proliferation, differentiation and **migration**. Supernumerary centrosomes are known to be an early event in tumorigenesis and senescence. However, the age-related changes of centrosome duplication in tissue-resident stem cells in vivo remain unknown. Here, using anti-γ-tubulin and anti-PH3, we analyzed mitotic intestinal stem cells with supernumerary centrosomes in the adult Drosophila midgut, which may be a versatile model system for stem cell biology. The results showed increased centrosome amplification in intestinal stem cells of aged and oxidatively stressed Drosophila midguts. Increased centrosome amplification was detected by overexpression of PVR, EGFR, and AKT in intestinal stem cells/enteroblasts, known to mimic age-related changes including hyperproliferation of intestinal stem cells and hyperplasia in the midgut. Our data show the first direct evidence for the age-related increase of centrosome amplification in intestinal stem cells and suggest that the Drosophila midgut is an excellent model for studying molecular mechanisms underlying centrosome amplification in aging adult stem cells in vivo.
6. Protein tyrosine phosphatase PTPN3 inhibits lung cancer cell proliferation and **migration** by promoting EGFR endocytic degradation. Epidermal growth factor receptor (EGFR) regulates multiple signaling cascades essential for cell proliferation, growth and differentiation. Using a genetic approach, we found that Drosophila FERM and PDZ domain-containing protein tyrosine phosphatase, dPtpmeg, negatively regulates border cell **migration** and inhibits the EGFR/Ras/mitogen-activated protein kinase signaling pathway during wing morphogenesis. We further identified EGFR pathway substrate 15 (Eps15) as a target of dPtpmeg and its human homolog PTPN3. Eps15 is a scaffolding adaptor protein known to be involved in EGFR endocytosis and trafficking. Interestingly, PTPN3-mediated tyrosine dephosphorylation of Eps15 promotes EGFR for lipid raft-mediated endocytosis and lysosomal degradation. PTPN3 and the Eps15 tyrosine phosphorylation-deficient mutant suppress non-small-cell lung cancer cell growth and **migration** in vitro and reduce lung tumor xenograft growth in vivo. Moreover, depletion of PTPN3 impairs the degradation of EGFR and enhances proliferation and tumorigenicity of lung cancer cells. Taken together, these results indicate that PTPN3 may act as a tumor suppressor in lung cancer through its modulation of EGFR signaling.
7. Regulation of Drosophila mesoderm **migration** by phosphoinositides and the PH domain of the Rho GTP exchange factor Pebble. The Drosophila RhoGEF Pebble (Pbl) is required for cytokinesis and **migration** of mesodermal cells. In a screen for genes that could suppress **migration** defects in pbl mutants we identified the phosphatidylinositol phosphate (PtdInsP) regulator pi5k59B. Genetic interaction tests with other PtdInsP regulators suggested that PtdIns(4,5)P2 levels are important for mesoderm **migration** when Pbl is depleted. Consistent with this, the leading front of migrating mesodermal cells was enriched for PtdIns(4,5)P2. Given that Pbl contains a Pleckstrin Homology (PH) domain, a known PtdInsP-binding motif, we examined PtdInsP-binding of Pbl and the importance of the PH domain for Pbl function. In vitro lipid blot assays showed that Pbl binds promiscuously to PtdInsPs, with binding strength associated with the degree of phosphorylation. Pbl was also able to bind lipid vesicles containing PtdIns(4,5)P2 but binding was strongly reduced upon deletion of the PH domain. Similarly, in vivo, loss of the PH domain prevented localisation of Pbl to the cell cortex and severely affected several aspects of early mesoderm development, including flattening of the invaginated tube onto the ectoderm, extension of protrusions, and dorsal **migration** to form a monolayer. Pbl lacking the PH domain could still localise to the cytokinetic furrow, however, and cytokinesis failure was reduced in pbl(ΔPH) mutants. Taken together, our results support a model in which interaction of the PH-domain of Pbl with PtdIns(4,5)P2 helps localise it to the plasma membrane which is important for mesoderm **migratio**n.
8. Branching morphogenesis of the Drosophila tracheal system. Many organs including the mammalian lung and vascular system consist of branched tubular networks that transport essential gases or fluids, but the genetic programs that control the development of these complex three-dimensional structures are not well understood. The Drosophila melanogaster tracheal (respiratory) system is a network of interconnected epithelial tubes that transports oxygen and other gases in the body and provides a paradigm of branching morphogenesis. It develops by sequential sprouting of primary, secondary, and terminal branches from an epithelial sac of approximately 80 cells in each body segment of the embryo. Mapping of the cell movements and shape changes during the sprouting process has revealed that distinct mechanisms of epithelial **migration** and tube formation are used at each stage of branching. Genetic dissection of the process has identified a general program in which a fibroblast growth factor (FGF) and fibroblast growth factor receptor (FGFR) are used repeatedly to control branch budding and outgrowth. At each stage of branching, the mechanisms controlling FGF expression and the downstream signal transduction pathway change, altering the pattern and structure of the branches that form. During terminal branching, FGF expression is regulated by hypoxia, ensuring that tracheal structure matches cellular oxygen need. A branch diversification program operates in parallel to the general budding program: Regional signals locally modify the general program, conferring specific structural features and other properties on individual branches, such as their substrate outgrowth preferences, differences in tube size and shape, and the ability to fuse to other branches to interconnect the network.
9. A functional RNAi screen for regulators of receptor tyrosine kinase and ERK signalling. Receptor tyrosine kinase (RTK) signalling through extracellular-signal-regulated kinases (ERKs) has pivotal roles during metazoan development, underlying processes as diverse as fate determination, differentiation, proliferation, survival, **migration** and growth. Abnormal RTK/ERK signalling has been extensively documented to contribute to developmental disorders and disease, most notably in oncogenic transformation by mutant RTKs or downstream pathway components such as Ras and Raf. Although the core RTK/ERK signalling cassette has been characterized by decades of research using mammalian cell culture and forward genetic screens in model organisms, signal propagation through this pathway is probably regulated by a larger network of moderate, context-specific proteins. The genes encoding these proteins may not have been discovered through traditional screens owing, in particular, to the requirement for visible phenotypes. To obtain a global view of RTK/ERK signalling, we performed an unbiased, RNA interference (RNAi), genome-wide, high-throughput screen in Drosophila cells using a novel, quantitative, cellular assay monitoring ERK activation. Here we show that ERK pathway output integrates a wide array of conserved cellular processes. Further analysis of selected components-in multiple cell types with different RTK ligands and oncogenic stimuli-validates and classifies 331 pathway regulators. The relevance of these genes is highlighted by our isolation of a Ste20-like kinase and a PPM-family phosphatase that seem to regulate RTK/ERK signalling in vivo and in mammalian cells. Novel regulators that modulate specific pathway outputs may be selective targets for drug discovery.
10. Drosophila tribbles antagonizes insulin signaling-mediated growth and metabolism via interactions with Akt kinase. Drosophila Tribbles (Trbl) is the founding member of the Trib family of kinase-like docking proteins that modulate cell signaling during proliferation, **migration** and growth. In a wing misexpression screen for Trbl interacting proteins, we identified the Ser/Thr protein kinase Akt1. Given the central role of Akt1 in insulin signaling, we tested the function of Trbl in larval fat body, a tissue where rapid increases in size are exquisitely sensitive to insulin/insulin-like growth factor levels. Consistent with a role in antagonizing insulin-mediated growth, trbl RNAi knockdown in the fat body increased cell size, advanced the timing of pupation and increased levels of circulating triglyceride. Complementarily, overexpression of Trbl reduced fat body cell size, decreased overall larval size, delayed maturation and lowered levels of triglycerides, while circulating glucose levels increased. The conserved Trbl kinase domain is required for function in vivo and for interaction with Akt in a yeast two-hybrid assay. Consistent with direct regulation of Akt, overexpression of Trbl in the fat body decreased levels of activated Akt (pSer505-Akt) while misexpression of trbl RNAi increased phospho-Akt levels, and neither treatment affected total Akt levels. Trbl misexpression effectively suppressed Akt-mediated wing and muscle cell size increases and reduced phosphorylation of the Akt target FoxO (pSer256-FoxO). Taken together, these data show that Drosophila Trbl has a conserved role to bind Akt and block Akt-mediated insulin signaling, and implicate Trib proteins as novel sites of signaling pathway integration that link nutrient availability with cell growth and proliferation.
11. A genetic screen in Drosophila for metastatic behavior. A genetic screen was designed in Drosophila to interrogate its genome for mutations sufficient to cause noninvasive tumors of the eye disc to invade neighboring or distant tissues. We found that cooperation between oncogenic RasV12 expression and inactivation of any one of a number of genes affecting cell polarity leads to metastatic behavior, including basement membrane degradation, loss of E-cadherin expression, **migration**, invasion, and secondary tumor formation. Inactivation of these cell polarity genes cannot drive metastatic behavior alone or in combination with other tumor-initiating alterations. These findings suggest that the oncogenic background of tissues makes a distinct contribution toward metastatic development.
12. Akt regulates centrosome **migration** and spindle orientation in the early Drosophila melanogaster embryo. Correct positioning and morphology of the mitotic spindle is achieved through regulating the interaction between microtubules (MTs) and cortical actin. Here we find that, in the Drosophila melanogaster early embryo, reduced levels of the protein kinase Akt result in incomplete centrosome **migration** around cortical nuclei, bent mitotic spindles, and loss of nuclei into the interior of the embryo. We show that Akt is enriched at the embryonic cortex and is required for phosphorylation of the glycogen synthase kinase-3beta homologue Zeste-white 3 kinase (Zw3) and for the cortical localizations of the adenomatosis polyposis coli (APC)-related protein APC2/E-APC and the MT + Tip protein EB1. We also show that reduced levels of Akt result in mislocalization of APC2 in postcellularized embryonic mitoses and misorientation of epithelial mitotic spindles. Together, our results suggest that Akt regulates a complex containing Zw3, Armadillo, APC2, and EB1 and that this complex has a role in stabilizing MT-cortex interactions, facilitating both centrosome separation and mitotic spindle orientation.
13. Regulation of Drosophila tracheal system development by protein kinase B. Protein kinase B (PKB, also termed Akt) is a phosphatidylinositol 3' kinase (PI3'K)-dependent enzyme implicated in survival signaling and human tumorigenesis. To identify potential targets of this protein kinase, we employed a genetic screen in Drosophila. Among several genes that genetically interacted with PKB was trachealess (trh), which encodes a bHLH-PAS domain transcription factor required for development of the trachea and other tubular organs. Trh activates expression of the fibroblast growth factor receptor Breathless, which, in turn, is required for directed **migration** of all tracheal branches. Using a combination of biochemical and transgenic approaches, we show that direct phosphorylation of Trh by PKB at serine 665 is essential for nuclear localization and functional activation of this regulator of branching morphogenesis.

### [Ii]nvasive

#### PubMed

1. A drosophila model for EGFR-Ras and PI3K-dependent human glioma. Gliomas, the most common malignant tumors of the nervous system, frequently harbor mutations that activate the epidermal growth factor receptor (EGFR) and phosphatidylinositol-3 kinase (PI3K) signaling pathways. To investigate the genetic basis of this disease, we developed a glioma model in Drosophila. We found that constitutive coactivation of EGFR-Ras and PI3K pathways in Drosophila glia and glial precursors gives rise to neoplastic, **invasive** glial cells that create transplantable tumor-like growths, mimicking human glioma. Our model represents a robust organotypic and cell-type-specific Drosophila cancer model in which malignant cells are created by mutations in signature genes and pathways thought to be driving forces in a homologous human cancer. Genetic analyses demonstrated that EGFR and PI3K initiate malignant neoplastic transformation via a combinatorial genetic network composed primarily of other pathways commonly mutated or activated in human glioma, including the Tor, Myc, G1 Cyclins-Cdks, and Rb-E2F pathways. This network acts synergistically to coordinately stimulate cell cycle entry and progression, protein translation, and inappropriate cellular growth and migration. In particular, we found that the fly orthologs of CyclinE, Cdc25, and Myc are key rate-limiting genes required for glial neoplasia. Moreover, orthologs of Sin1, Rictor, and Cdk4 are genes required only for abnormal neoplastic glial proliferation but not for glial development. These and other genes within this network may represent important therapeutic targets in human glioma.
2. Colorectal cancer progression: lessons from Drosophila? Human colorectal cancers arise as benign adenomas, tumours that retain their epithelial character, and then progress to malignant adenocarcinomas and carcinomas in which the epithelium becomes disrupted. Carcinomas often exhibit transcriptional downregulation of E-cadherin and other epithelial genes in an epithelial-to-mesenchymal transition (EMT), a mechanism first discovered in Drosophila to be mediated by the transcription factors Twist and Snail. In contrast, adenocarcinomas retain expression of E-cadherin and disruption of the epithelium occurs through formation of progressively smaller epithelial cysts with apical Crumbs/CRB3, Stardust/PALS1, and Bazooka/PAR3 localised to the inner lumen. Results from Drosophila show that morphologically similar cysts form upon induction of clonal heterogeneity in Wnt, Smad, or Ras signalling levels, which causes extrusion of epithelial cells at clonal boundaries. Thus, intratumour heterogeneity might also promote formation of adenocarcinomas in humans. Finally, epithelial cysts can collectively migrate, as in the case of Drosophila border cells, a potential model system for the **invasive** migration of adenocarcinoma cells.
3. Cell mixing induced by myc is required for competitive tissue invasion and destruction. Cell-cell intercalation is used in several developmental processes to shape the normal body plan. There is no clear evidence that intercalation is involved in pathologies. Here we use the proto-oncogene myc to study a process analogous to early phase of tumour expansion: myc-induced cell competition. Cell competition is a conserved mechanism driving the elimination of slow-proliferating cells (so-called 'losers') by faster-proliferating neighbours (so-called 'winners') through apoptosis and is important in preventing developmental malformations and maintain tissue fitness. Here we show, using long-term live imaging of myc-driven competition in the Drosophila pupal notum and in the wing imaginal disc, that the probability of elimination of loser cells correlates with the surface of contact shared with winners. As such, modifying loser-winner interface morphology can modulate the strength of competition. We further show that elimination of loser clones requires winner-loser cell mixing through cell-cell intercalation. Cell mixing is driven by differential growth and the high tension at winner-winner interfaces relative to winner-loser and loser-loser interfaces, which leads to a preferential stabilization of winner-loser contacts and reduction of clone compactness over time. Differences in tension are generated by a relative difference in F-actin levels between loser and winner junctions, induced by differential levels of the membrane lipid phosphatidylinositol (3,4,5)-trisphosphate. Our results establish the first link between cell-cell intercalation induced by a proto-oncogene and how it promotes **invasive**ness and destruction of healthy tissues.
4. Sin3a acts through a multi-gene module to regulate invasion in Drosophila and human tumors. Chromatin remodeling proteins regulate multiple aspects of cell homeostasis, making them ideal candidates for misregulation in transformed cells. Here, we explore Sin3A, a member of the Sin3 family of proteins linked to tumorigenesis that are thought to regulate gene expression through their role as histone deacetylases (HDACs). We identified Drosophila Sin3a as an important mediator of oncogenic Ret receptor in a fly model of Multiple Endocrine Neoplasia Type 2. Reducing Drosophila Sin3a activity led to metastasis-like behavior and, in the presence of Diap1, secondary tumors distant from the site of origin. Genetic and Chip-Seq analyses identified previously undescribed Sin3a targets including genes involved in cell motility and actin dynamics, as well as signaling pathways including Src, Jnk and Rho. A key Sin3a oncogenic target, PP1B, regulates stability of β-Catenin/Armadillo: the outcome is to oppose T-cell factor (TCF) function and Wg/Wnt pathway signaling in both fly and mammalian cancer cells. Reducing Sin3A strongly increased the **invasiv**e behavior of A549 human lung adenocarcinoma cells. We show that Sin3A is downregulated in a variety of human tumors and that Src, JNK, RhoA and PP1B/β-Catenin are regulated in a manner analogous to our Drosophila models. Our data suggest that Sin3A influences a specific step of tumorigenesis by regulating a module of genes involved in cell invasion. Tumor progression may commonly rely on such 'modules of invasion' under the control of broad transcriptional regulators.
5. APC loss-induced intestinal tumorigenesis in Drosophila: Roles of Ras in Wnt signaling activation and tumor progression. Adenomatous polyposis coli (APC) and K-ras are the two most frequently mutated genes found in human colorectal cancers. In human colorectal cancers, Wnt signaling activation after the loss of APC is hypothesized to be the key event for adenoma initiation, whereas additional mutations such as Ras activation are required for the progression from adenoma to carcinoma. However, accumulating data have led to conflicting views regarding the precise role of Ras in APC loss-induced tumorigenesis. Here, using Drosophila midgut as a model system, we show that in the absence of Ras, APC mutant epithelial cells cannot initiate hyperplasia, suggesting that Ras plays an essential role in tumor initiation. Conversely, activating Ras by expressing oncogenic Ras or Raf in APC-deficient cells led to a blockage of cell differentiation and to pre**invasive** tumor outgrowth, characteristics that are shared by advanced colorectal carcinoma in humans. Mechanistically, we find that Ras is not required for Wnt signaling activation after APC loss, although Ras hyperactivation is able to potentiate Wnt signaling by increasing the cytoplasmic and nuclear accumulation of Armadillo/β-catenin via mechanisms independent of JNK/Rac1 or PI3K-Akt signaling, partly owing to the downregulation of DE-cadherin. Together with the data from gene expression analyses, our results indicate that both parallel and cooperative mechanisms of Wnt and Ras signaling are responsible for the initiation and progression of intestinal tumorigenesis after APC loss.
6. A genetic screen in Drosophila for metastatic behavior. A genetic screen was designed in Drosophila to interrogate its genome for mutations sufficient to cause non**invasive** tumors of the eye disc to invade neighboring or distant tissues. We found that cooperation between oncogenic RasV12 expression and inactivation of any one of a number of genes affecting cell polarity leads to metastatic behavior, including basement membrane degradation, loss of E-cadherin expression, migration, invasion, and secondary tumor formation. Inactivation of these cell polarity genes cannot drive metastatic behavior alone or in combination with other tumor-initiating alterations. These findings suggest that the oncogenic background of tissues makes a distinct contribution toward metastatic development.

### [Mm]etastasis

#### PubMed

1. Centrosomal kinase Nek2 cooperates with oncogenic pathways to promote **metastasis**. Centrosomal kinase Nek2 is overexpressed in different cancers, yet how it contributes toward tumorigenesis remains poorly understood. dNek2 overexpression in a Drosophila melanogaster model led to upregulation of Drosophila Wnt ortholog wingless (Wg), and alteration of cell migration markers-Rho1, Rac1 and E-cadherin (Ecad)-resulting in changes in cell shape and tissue morphogenesis. dNek2 overexpression cooperated with receptor tyrosine kinase and mitogen-activated protein kinase signaling to upregulate activated Akt, Diap1, Mmp1 and Wg protein to promote local invasion, distant seeding and **metastasis**. In tumor cell injection assays, dNek2 cooperated with Ras and Src signaling to promote aggressive colonization of tumors into different adult fly tissues. Inhibition of the PI3K pathway suppressed the cooperation of dNek2 with other growth pathways. Consistent with our fly studies, overexpression of human Nek2 in A549 lung adenocarcinoma and HEK293T cells led to activation of the Akt pathway and increase in β-catenin protein levels. Our computational approach identified a class of Nek2-inhibitory compounds and a novel drug-like pharmacophore that reversed the Nek2 overexpression phenotypes in flies and human cells. Our finding posits a novel role for Nek2 in promoting **metastasi**s in addition to its currently defined role in promoting chromosomal instability. It provides a rationale for the selective advantage of centrosome amplification in cancer.
2. Loss of cell polarity drives tumor growth and invasion through JNK activation in Drosophila. Apparent defects in cell polarity are often seen in human cancer. However, the underlying mechanisms of how cell polarity disruption contributes to tumor progression are unknown. Here, using a Drosophila genetic model for Ras-induced tumor progression, we show a molecular link between loss of cell polarity and tumor malignancy. Mutation of different apicobasal polarity genes activates c-Jun N-terminal kinase (JNK) signaling and downregulates the E-cadherin/beta-catenin adhesion complex, both of which are necessary and sufficient to cause oncogenic Ras(V12)-induced benign tumors in the developing eye to exhibit metastatic behavior. Furthermore, activated JNK and Ras signaling cooperate in promoting tumor growth cell autonomously, as JNK signaling switches its proapoptotic role to a progrowth effect in the presence of oncogenic Ras. Our finding that such context-dependent alterations promote both tumor growth and metastatic behavior suggests that **metastasis**-promoting mutations may be selected for based primarily on their growth-promoting capabilities. Similar oncogenic cooperation mediated through these evolutionarily conserved signaling pathways could contribute to human cancer progression.
3. Oncogenic programmes and Notch activity: an 'organized crime'? The inappropriate Notch signalling can influence virtually all aspect of cancer, including tumour-cell growth, survival, apoptosis, angiogenesis, invasion and **metastasis**, although it does not do this alone. Hence, elucidating the partners of Notch that are active in cancer is now the focus of much intense research activity. The genetic toolkits available, coupled to the small size and short life of the fruit fly Drosophila melanogaster, makes this an inexpensive and effective animal model, suited to large-scale cancer gene discovery studies. The fly eye is not only a non-vital organ but its stereotyped size and disposition also means it is easy to screen for mutations that cause tumours and metastases and provides ample opportunities to test cancer theories and to unravel unanticipated nexus between Notch and other cancer genes, or to discover unforeseen Notch's partners in cancer. These studies suggest that Notch's oncogenic capacity is brought about not simply by increasing signal strength but through partnerships, whereby oncogenes gain more by cooperating than acting individually, as in a ring 'organized crime'.
4. Sin3a acts through a multi-gene module to regulate invasion in Drosophila and human tumors. Chromatin remodeling proteins regulate multiple aspects of cell homeostasis, making them ideal candidates for misregulation in transformed cells. Here, we explore Sin3A, a member of the Sin3 family of proteins linked to tumorigenesis that are thought to regulate gene expression through their role as histone deacetylases (HDACs). We identified Drosophila Sin3a as an important mediator of oncogenic Ret receptor in a fly model of Multiple Endocrine Neoplasia Type 2. Reducing Drosophila Sin3a activity led to **metastasis**-like behavior and, in the presence of Diap1, secondary tumors distant from the site of origin. Genetic and Chip-Seq analyses identified previously undescribed Sin3a targets including genes involved in cell motility and actin dynamics, as well as signaling pathways including Src, Jnk and Rho. A key Sin3a oncogenic target, PP1B, regulates stability of β-Catenin/Armadillo: the outcome is to oppose T-cell factor (TCF) function and Wg/Wnt pathway signaling in both fly and mammalian cancer cells. Reducing Sin3A strongly increased the invasive behavior of A549 human lung adenocarcinoma cells. We show that Sin3A is downregulated in a variety of human tumors and that Src, JNK, RhoA and PP1B/β-Catenin are regulated in a manner analogous to our Drosophila models. Our data suggest that Sin3A influences a specific step of tumorigenesis by regulating a module of genes involved in cell invasion. Tumor progression may commonly rely on such 'modules of invasion' under the control of broad transcriptional regulators.
5. Modeling tumor invasion and **metastasis** in Drosophila. Conservation of major signaling pathways between humans and flies has made Drosophila a useful model organism for cancer research. Our understanding of the mechanisms regulating cell growth, differentiation and development has been considerably advanced by studies in Drosophila. Several recent high profile studies have examined the processes constraining the metastatic growth of tumor cells in fruit fly models. Cell invasion can be studied in the context of an in vivo setting in flies, enabling the genetic requirements of the microenvironment of tumor cells undergoing **metastasis** to be analyzed. This Perspective discusses the strengths and limitations of Drosophila models of cancer invasion and the unique tools that have enabled these studies. It also highlights several recent reports that together make a strong case for Drosophila as a system with the potential for both testing novel concepts in tumor progression and cell invasion, and for uncovering players in **metastasis**.

### [Cc]ell [Ii]nvasion

#### PubMed

1. Sin3a acts through a multi-gene module to regulate invasion in Drosophila and human tumors. Chromatin remodeling proteins regulate multiple aspects of cell homeostasis, making them ideal candidates for misregulation in transformed cells. Here, we explore Sin3A, a member of the Sin3 family of proteins linked to tumorigenesis that are thought to regulate gene expression through their role as histone deacetylases (HDACs). We identified Drosophila Sin3a as an important mediator of oncogenic Ret receptor in a fly model of Multiple Endocrine Neoplasia Type 2. Reducing Drosophila Sin3a activity led to metastasis-like behavior and, in the presence of Diap1, secondary tumors distant from the site of origin. Genetic and Chip-Seq analyses identified previously undescribed Sin3a targets including genes involved in cell motility and actin dynamics, as well as signaling pathways including Src, Jnk and Rho. A key Sin3a oncogenic target, PP1B, regulates stability of β-Catenin/Armadillo: the outcome is to oppose T-cell factor (TCF) function and Wg/Wnt pathway signaling in both fly and mammalian cancer cells. Reducing Sin3A strongly increased the invasive behavior of A549 human lung adenocarcinoma cells. We show that Sin3A is downregulated in a variety of human tumors and that Src, JNK, RhoA and PP1B/β-Catenin are regulated in a manner analogous to our Drosophila models. Our data suggest that Sin3A influences a specific step of tumorigenesis by regulating a module of genes involved i**n cell invasi**on. Tumor progression may commonly rely on such 'modules of invasion' under the control of broad transcriptional regulators.
2. Modeling tumor invasion and metastasis in Drosophila. Conservation of major signaling pathways between humans and flies has made Drosophila a useful model organism for cancer research. Our understanding of the mechanisms regulating cell growth, differentiation and development has been considerably advanced by studies in Drosophila. Several recent high profile studies have examined the processes constraining the metastatic growth of tumor cells in fruit fly models. **Cell invasion** can be studied in the context of an in vivo setting in flies, enabling the genetic requirements of the microenvironment of tumor cells undergoing metastasis to be analyzed. This Perspective discusses the strengths and limitations of Drosophila models of cancer invasion and the unique tools that have enabled these studies. It also highlights several recent reports that together make a strong case for Drosophila as a system with the potential for both testing novel concepts in tumor progression and **cell invasion**, and for uncovering players in metastasis.

---

## Rab7

- NCBI Gene
- GeneRIF
- Pubmed

| Search Term | Rank | Fields |
| --- | --- | --- |
| [Mm]igration | 6 | Process(1); PubMed(7) |
| [Ee]xtracellular [Mm]atrix | 8 | PubMed(3) |
| [Ii]nvasive | 10 | PubMed(3) |
| [Mm]etastasis | 12 | PubMed(3) |
| [Cc]ell [Ii]nvasion | 13 | PubMed(1) |

  
hide/show details for Rab7

### [Mm]igration

#### Process

1. border follicle cell **migration**

#### PubMed

1. Direct detection of guidance receptor activity during border cell **migration**. Guidance receptor signaling is crucial for steering migrating cells. Despite this, we generally lack direct measurements of such signaling. Border cells in Drosophila migrate as a tightly associated group, but dynamically, with front and rear cells exchanging places. They use the receptor tyrosine kinase (RTK) PDGF/VEGF receptor (PVR) as a guidance receptor, perceiving the attractant Pvf1. Here we determine the spatial distribution of PVR signaling by generating an antibody that specifically detects activated PVR in situ. PVR activity is very low in migrating border cells, due to strong activity of cellular phosphatases. Measurements of signal at the cell cortex show variability but a strong bias for both total active PVR and specific activity of PVR to be elevated at the front versus side of the leading cell, often with several-fold difference in signal levels. This polarized active PVR signal requires the E3 ubiquitin ligase Cbl and the recycling regulator Rab11, indicating a dependency on receptor trafficking. The endogenous ligand gradient contributes to shaping of signaling by increasing the specific activity of PVR toward the source in front cells. Surprisingly, signaling is also elevated at the back versus the side of rear cells. This distally polarized distribution of active PVR is ligand independent. Thus the actual guidance signal transmitted in border cells appears to integrate perceived ligand distribution with cell polarity or cell orientation with respect to the cluster. A general implication is that both group configuration and extrinsic cues can directly modulate guidance receptor signaling during collective cell **migration**.
2. Dynamics of the basement membrane in invasive epithelial clusters in Drosophila. The basement membrane (BM) represents a barrier to cell **migration**, which has to be degraded to promote invasion. However, the role and behaviour of the BM during the development of pre-invasive cells is only poorly understood. Drosophila border cells (BCs) provide an attractive genetic model in which to study the cellular mechanisms underlying the **migration** of mixed cohorts of epithelial cells. BCs are made of two different epithelial cell types appearing sequentially during oogenesis: the polar cells and the outer BCs. Here, we show that the pre-invasive polar cells undergo an unusual and asymmetrical apical capping with major basement membrane proteins, including the two Drosophila Collagen IV alpha chains, Laminin A and Perlecan. Capping of polar cells proceeds through a novel, basal-to-apical transcytosis mechanism that involves the small GTPase Drab5. Apical capping is transient and is followed by rapid shedding prior to the initiation of BC **migration**, suggesting that the apical cap blocks **migration**. Consistently, non-migratory polar cells remain capped. We further show that JAK/STAT signalling and recruitment of outer BCs are required for correct shedding and **migration**. The dynamics of the BM represents a marker of migratory BC, revealing a novel developmentally regulated behaviour of BM coupled to epithelial cell invasiveness.
3. Spinster controls Dpp signaling during glial **migration** in the Drosophila eye. The development of multicellular organisms requires the well balanced and coordinated **migration** of many cell types. This is of particular importance within the developing nervous system, where glial cells often move long distances to reach their targets. The majority of glial cells in the peripheral nervous system of the Drosophila embryo is derived from the CNS and migrates along motor axons toward their targets. In the developing Drosophila eye, CNS-derived glial cells move outward toward the nascent photoreceptor cells, but the molecular mechanisms coupling the **migration** of glial cells with the growth of the eye imaginal disc are mostly unknown. Here, we used an enhancer trap approach to identify the gene spinster, which encodes a multipass transmembrane protein involved in endosome-lysosome trafficking, as being expressed in many glial cells. spinster mutants are characterized by glial over**migration**. Genetic experiments demonstrate that Spinster modulates the activity of several signaling cascades. Within the migrating perineurial glial cells, Spinster is required to downregulate Dpp (Decapentaplegic) signaling activity, which ceases migratory abilities. In addition, Spinster affects the growth of the carpet cell, which indirectly modulates glial **migration**.
4. The Bro1-domain-containing protein Myopic/HDPTP coordinates with Rab4 to regulate cell adhesion and **migration**. Protein tyrosine phosphatases (PTPs) are a group of tightly regulated enzymes that coordinate with protein tyrosine kinases to control protein phosphorylation during various cellular processes. Using genetic analysis in Drosophila non-transmembrane PTPs, we identified one role that Myopic (Mop), the Drosophila homolog of the human His domain phosphotyrosine phosphatase (HDPTP), plays in cell adhesion. Depletion of Mop results in aberrant integrin distribution and border cell dissociation during Drosophila oogenesis. Interestingly, Mop phosphatase activity is not required for its role in maintaining border cell cluster integrity. We further identified Rab4 GTPase as a Mop interactor in a yeast two-hybrid screen. Expression of the Rab4 dominant-negative mutant leads to border cell dissociation and suppression of Mop-induced wing-blade adhesion defects, suggesting a critical role of Rab4 in Mop-mediated signaling. In mammals, it has been shown that Rab4-dependent recycling of integrins is necessary for cell adhesion and **migration**. We found that human HDPTP regulates the spatial distribution of Rab4 and integrin trafficking. Depletion of HDPTP resulted in actin reorganization and increased cell motility. Together, our findings suggest an evolutionarily conserved function of HDPTP-Rab4 in the regulation of endocytic trafficking, cell adhesion and **migration**.
5. The Drosophila Dead end Arf-like3 GTPase controls vesicle trafficking during tracheal fusion cell morphogenesis. The Drosophila larval tracheal system consists of a highly branched tubular organ that becomes interconnected by **migration**-fusion events during embryonic development. Fusion cells at the tip of each branch guide **migration**, adhere, and then undergo extensive remodeling as the tracheal lumen extends between the two branches. The Drosophila dead end gene is expressed in fusion cells, and encodes an Arf-like3 GTPase. Analyses of dead end RNAi and mutant embryos reveal that the lumen fails to connect between the two branches. Expression of a constitutively active form of Dead end in S2 cells reveals that it influences the state of actin polymerization, and is present on particles that traffic along actin/microtubule-containing processes. Imaging experiments in vivo reveal that Dead end-containing vesicles are associated with recycling endosomes and the exocyst, and control exocyst localization in fusion cells. These results indicate that the Dead end GTPase plays an important role in trafficking membrane components involved in tracheal fusion cell morphogenesis and lumenal development.
6. Spatial restriction of receptor tyrosine kinase activity through a polarized endocytic cycle controls border cell **migration**. Border cell **migration** is a stereotyped **migration** occurring during the development of the Drosophila egg chamber. During this process, a cluster composed of six to eight follicle cells migrates between nurse cells toward the oocyte. Receptor tyrosine kinases (RTKs) are enriched at the leading edge of the follicle cells and establish the directionality of their **migration**. Endocytosis has been shown to play a role in the maintenance of this polarization; however, the mechanisms involved are largely unknown. In this study, we show that border cell **migration** requires the function of the small GTPases Rab5 and Rab11 that regulate trafficking through the early and the recycling endosome, respectively. Expression of a dominant negative form of rab11 induces a loss of the polarization of RTK activity, which correlates with a severe **migration** phenotype. In addition, we demonstrate that the exocyst component Sec15 is distributed in structures that are polarized during the **migration** process in a Rab11-dependent manner and that the down-regulation of different subunits of the exocyst also affects **migration**. Together, our data demonstrate a fundamental role for a plasma membrane-endosome trafficking cycle in the maintenance of active RTK at the leading edge of border cells during their **migration**.
7. Evi5 promotes collective cell **migration** through its Rab-GAP activity. Membrane trafficking has well-defined roles during cell **migration**. However, its regulation is poorly characterized. In this paper, we describe the first screen for putative Rab-GTPase-activating proteins (GAPs) during collective cell **migration** of Drosophila melanogaster border cells (BCs), identify the uncharacterized Drosophila protein Evi5 as an essential membrane trafficking regulator, and describe the molecular mechanism by which Evi5 regulates BC **migration**. Evi5 requires its Rab-GAP activity to fulfill its functions during **migration** and acts as a GAP protein for Rab11. Both loss and gain of Evi5 function blocked BC **migration** by disrupting the Rab11-dependent polarization of active guidance receptors. Altogether, our findings deepen our understanding of the molecular machinery regulating endocytosis and subsequently cell signaling during **migration**.

### [Ee]xtracellular [Mm]atrix

#### PubMed

1. The Gyc76C Receptor Guanylyl Cyclase and the Foraging cGMP-Dependent Kinase Regulate **Extracellular Matrix** Organization and BMP Signaling in the Developing Wing of Drosophila melanogaster. The developing crossveins of the wing of Drosophila melanogaster are specified by long-range BMP signaling and are especially sensitive to loss of extracellular modulators of BMP signaling such as the Chordin homolog Short gastrulation (Sog). However, the role of the **extracellular matrix** in BMP signaling and Sog activity in the crossveins has been poorly explored. Using a genetic mosaic screen for mutations that disrupt BMP signaling and posterior crossvein development, we identify Gyc76C, a member of the receptor guanylyl cyclase family that includes mammalian natriuretic peptide receptors. We show that Gyc76C and the soluble cGMP-dependent kinase Foraging, likely linked by cGMP, are necessary for normal refinement and maintenance of long-range BMP signaling in the posterior crossvein. This does not occur through cell-autonomous crosstalk between cGMP and BMP signal transduction, but likely through altered extracellular activity of Sog. We identify a novel pathway leading from Gyc76C to the organization of the wing **extracellular matrix** by matrix metalloproteinases, and show that both the **extracellular matrix** and BMP signaling effects are largely mediated by changes in the activity of matrix metalloproteinases. We discuss parallels and differences between this pathway and other examples of cGMP activity in both Drosophila melanogaster and mammalian cells and tissues.
2. Crag regulates epithelial architecture and polarized deposition of basement membrane proteins in Drosophila. The polarized architecture of epithelia relies on an interplay between the cytoskeleton, the trafficking machinery, and cell-cell and cell-matrix adhesion. Specifically, contact with the basement membrane (BM), an **extracellular matrix** underlying the basal side of epithelia, is important for cell polarity. However, little is known about how BM proteins themselves achieve a polarized distribution. In a genetic screen in the Drosophila follicular epithelium, we identified mutations in Crag, which encodes a conserved protein with domains implicated in membrane trafficking. Follicle cells mutant for Crag lose epithelial integrity and frequently become invasive. The loss of Crag leads to the anomalous accumulation of BM components on both sides of epithelial cells without directly affecting the distribution of apical or basolateral membrane proteins. This defect is not generally observed in mutants affecting epithelial integrity. We propose that Crag plays a unique role in organizing epithelial architecture by regulating the polarized secretion of BM proteins.
3. Rab9 and retromer regulate retrograde trafficking of luminal protein required for epithelial tube length control. Apical **extracellular matrix** filling the lumen controls the morphology and geometry of epithelial tubes during development, yet the regulation of luminal protein composition and its role in tube morphogenesis are not well understood. Here we show that an endosomal-retrieval machinery consisting of Rab9, retromer and actin nucleator WASH (Wiskott-Aldrich Syndrome Protein and SCAR Homolog) regulates selective recycling of the luminal protein Serpentine in the Drosophila trachea. Secreted Serpentine is endocytosed and sorted into the late endosome. Vps35, WASH and actin filaments differentially localize at the Rab9-enriched subdomains of the endosomal membrane, where Serpentine containing vesicles bud off. In Rab9, Vps35 and WASH mutants, Serpentine was secreted normally into the tracheal lumen, but the luminal quantities were depleted at later stages, resulting in excessively elongated tubes. In contrast, secretion of many luminal proteins was unaffected, suggesting that retrograde trafficking of a specific class of luminal proteins is a pivotal rate-limiting mechanism for continuous tube length regulation.

### [Ii]nvasive

#### PubMed

1. Dynamics of the basement membrane in **invasive** epithelial clusters in Drosophila. The basement membrane (BM) represents a barrier to cell migration, which has to be degraded to promote invasion. However, the role and behaviour of the BM during the development of pre-**invasive** cells is only poorly understood. Drosophila border cells (BCs) provide an attractive genetic model in which to study the cellular mechanisms underlying the migration of mixed cohorts of epithelial cells. BCs are made of two different epithelial cell types appearing sequentially during oogenesis: the polar cells and the outer BCs. Here, we show that the pre-**invasive** polar cells undergo an unusual and asymmetrical apical capping with major basement membrane proteins, including the two Drosophila Collagen IV alpha chains, Laminin A and Perlecan. Capping of polar cells proceeds through a novel, basal-to-apical transcytosis mechanism that involves the small GTPase Drab5. Apical capping is transient and is followed by rapid shedding prior to the initiation of BC migration, suggesting that the apical cap blocks migration. Consistently, non-migratory polar cells remain capped. We further show that JAK/STAT signalling and recruitment of outer BCs are required for correct shedding and migration. The dynamics of the BM represents a marker of migratory BC, revealing a novel developmentally regulated behaviour of BM coupled to epithelial cell **invasive**ness.
2. Elevated expression of the V-ATPase C subunit triggers JNK-dependent cell invasion and overgrowth in a Drosophila epithelium. The C subunit of the vacuolar H(+)-ATPase or V-ATPase regulates the activity and assembly of the proton pump at cellular membranes. It has been shown to be strongly upregulated in oral squamous cell carcinoma, a highly metastatic epithelial cancer. In addition, increased V-ATPase activity appears to correlate with **invasive**ness of cancer cells, but the underlying mechanism is largely unknown. Using the Drosophila wing imaginal epithelium as an in vivo model system, we demonstrate that overexpression of Vha44, the Drosophila orthologue of the C subunit, causes a tumor-like tissue transformation in cells of the wing epithelium. Overexpressing cells are excluded from the epithelium and acquire **invasive** properties while displaying high apoptotic rates. Blocking apoptosis in these cells unmasks a strong proliferation stimulus, leading to overgrowth. Furthermore, we show that excess Vha44 greatly increases acidification of endocytic compartments and interferes with endosomal trafficking. As a result, cargoes such as GFP-Lamp1 and Notch accumulate in highly acidified enlarged endolysosomal compartments. Consistent with previous reports on the endocytic activation of Eiger/JNK signaling, we find that V-ATPase stimulation by Vha44 causes JNK signaling activation whereas downmodulation of JNK signaling rescues the **invasive** phenotypes. In summary, our in vivo-findings demonstrate that increased levels of V-ATPase C subunit induce a Eiger/JNK-dependent cell transformation within an epithelial organ that recapitulates early carcinoma stages.
3. Crag regulates epithelial architecture and polarized deposition of basement membrane proteins in Drosophila. The polarized architecture of epithelia relies on an interplay between the cytoskeleton, the trafficking machinery, and cell-cell and cell-matrix adhesion. Specifically, contact with the basement membrane (BM), an extracellular matrix underlying the basal side of epithelia, is important for cell polarity. However, little is known about how BM proteins themselves achieve a polarized distribution. In a genetic screen in the Drosophila follicular epithelium, we identified mutations in Crag, which encodes a conserved protein with domains implicated in membrane trafficking. Follicle cells mutant for Crag lose epithelial integrity and frequently become **invasive**. The loss of Crag leads to the anomalous accumulation of BM components on both sides of epithelial cells without directly affecting the distribution of apical or basolateral membrane proteins. This defect is not generally observed in mutants affecting epithelial integrity. We propose that Crag plays a unique role in organizing epithelial architecture by regulating the polarized secretion of BM proteins.

### [Mm]etastasis

#### PubMed

1. Activated Cdc42 kinase regulates Dock localization in male germ cells during Drosophila spermatogenesis. Deregulation of the non-receptor tyrosine kinase ACK1 (Activated Cdc42-associated kinase) correlates with poor prognosis in cancers and has been implicated in promoting **metastasis**. To further understand its in vivo function, we have characterized the developmental defects of a null mutation in Drosophila Ack, which bears a high degree of sequence similarity to mammalian ACK1 but lacks a CRIB domain. We show that Ack, while not essential for viability, is critical for sperm formation. This function depends on Ack tyrosine kinase activity and is required cell autonomously in differentiating male germ cells at or after the spermatocyte stage. Ack associates predominantly with endocytic clathrin sites in spermatocytes, but disruption of Ack function has no apparent effect on clathrin localization and receptor-mediated internalization of Boss (Bride of sevenless) protein in eye discs. Instead, Ack is required for the subcellular distribution of Dock (dreadlocks), the Drosophila homolog of the SH2- and SH3-containing adaptor protein Nck. Moreover, Dock forms a complex with Ack, and the localization of Dock in male germ cells depends on its SH2 domain. Together, our results suggest that Ack-dependent tyrosine phosphorylation recruits Dock to promote sperm differentiation.
2. Rabaptin-5 and Rabex-5 are neoplastic tumour suppressor genes that interact to modulate Rab5 dynamics in Drosophila melanogaster. Endocytosis plays an important role in the regulation of tumour growth and **metastasis**. In Drosophila, a number of endocytic neoplastic tumour suppressor genes have been identified that when mutated cause epithelial disruption and over-proliferation. Here we characterise the Drosophila homologue of the Rab5 effector Rabaptin-5, and show that it is a novel neoplastic tumour suppressor. Its ability to bind Rab5 and modulate early endosomal dynamics is conserved in Drosophila, as is its interaction with the Rab5 GEF Rabex5, for which we also demonstrate neoplastic tumour suppressor characteristics. Surprisingly, we do not observe disruption of apico-basal polarity in Rabaptin-5 and Rabex-5 mutant tissues; instead the tumour phenotype is associated with upregulation of Jun N-terminal Kinase (JNK) and Janus Kinase (JAK)/Signal Transducer and Activator of Transcription (STAT) signalling.
3. Distinct functions for Rho1 in maintaining adherens junctions and apical tension in remodeling epithelia. Maintenance and remodeling of adherens junctions (AJs) and cell shape in epithelia are necessary for the development of functional epithelia and are commonly altered during cancer progression/**metastasis**. Although formation of nascent AJs has received much attention, whether shared mechanisms are responsible for the maintenance and remodeling of AJs in dynamic epithelia, particularly in vivo, is not clear. Using clonal analysis in the postmitotic Drosophila melanogaster pupal eye epithelium, we demonstrate that Rho1 is required to maintain AJ integrity independent of its role in sustaining apical cell tension. Rho1 depletion in a remodeling postmitotic epithelium disrupts AJs but only when depleted in adjacent cells. Surprisingly, neither of the Rho effectors, Rok or Dia, is necessary downstream of Rho1 to maintain AJs; instead, Rho1 maintains AJs by inhibiting Drosophila epithelial cadherin endocytosis in a Cdc42/Par6-dependent manner. In contrast, depletion of Rho1 in single cells decreases apical tension, and Rok and myosin are necessary, while Dia function also contributes, downstream of Rho1 to sustain apical cell tension.

### [Cc]ell [Ii]nvasion

#### PubMed

1. Elevated expression of the V-ATPase C subunit triggers JNK-dependent **cell invasion** and overgrowth in a Drosophila epithelium. The C subunit of the vacuolar H(+)-ATPase or V-ATPase regulates the activity and assembly of the proton pump at cellular membranes. It has been shown to be strongly upregulated in oral squamous cell carcinoma, a highly metastatic epithelial cancer. In addition, increased V-ATPase activity appears to correlate with invasiveness of cancer cells, but the underlying mechanism is largely unknown. Using the Drosophila wing imaginal epithelium as an in vivo model system, we demonstrate that overexpression of Vha44, the Drosophila orthologue of the C subunit, causes a tumor-like tissue transformation in cells of the wing epithelium. Overexpressing cells are excluded from the epithelium and acquire invasive properties while displaying high apoptotic rates. Blocking apoptosis in these cells unmasks a strong proliferation stimulus, leading to overgrowth. Furthermore, we show that excess Vha44 greatly increases acidification of endocytic compartments and interferes with endosomal trafficking. As a result, cargoes such as GFP-Lamp1 and Notch accumulate in highly acidified enlarged endolysosomal compartments. Consistent with previous reports on the endocytic activation of Eiger/JNK signaling, we find that V-ATPase stimulation by Vha44 causes JNK signaling activation whereas downmodulation of JNK signaling rescues the invasive phenotypes. In summary, our in vivo-findings demonstrate that increased levels of V-ATPase C subunit induce a Eiger/JNK-dependent cell transformation within an epithelial organ that recapitulates early carcinoma stages.

---

## l(2)gl

- NCBI Gene
- GeneRIF
- Pubmed

| Search Term | Rank | Fields |
| --- | --- | --- |
| [Ff]ilopodia | 1 | PubMed(1) |
| [Mm]igration | 6 | PubMed(22) |
| ECM | 7 | PubMed(1) |
| [Ee]xtracellular [Mm]atrix | 8 | PubMed(6); Component(1) |
| [Ii]nvasive | 10 | PubMed(18) |
| [Mm]etastasize | 11 | PubMed(2) |
| [Mm]etastasis | 12 | PubMed(16) |
| [Cc]ell [Ii]nvasion | 13 | PubMed(6) |

  
hide/show details for l(2)gl

### [Ff]ilopodia

#### PubMed

1. The leading edge during dorsal closure as a model for epithelial plasticity: Pak is required for recruitment of the Scribble complex and septate junction formation. Dorsal closure (DC) of the Drosophila embryo is a model for the study of wound healing and developmental epithelial fusions, and involves the sealing of a hole in the epidermis through the migration of the epidermal flanks over the tissue occupying the hole, the amnioserosa. During DC, the cells at the edge of the migrating epidermis extend Rac- and Cdc42-dependent actin-based lamellipodia and **filopodia** from their leading edge (LE), which exhibits a breakdown in apicobasal polarity as adhesions are severed with the neighbouring amnioserosa cells. Studies using mammalian cells have demonstrated that Scribble (Scrib), an important determinant of apicobasal polarity that functions in a protein complex, controls polarized cell migration through recruitment of Rac, Cdc42 and the serine/threonine kinase Pak, an effector for Rac and Cdc42, to the LE. We have used DC and the follicular epithelium to study the relationship between Pak and the Scrib complex at epithelial membranes undergoing changes in apicobasal polarity and adhesion during development. We propose that, during DC, the LE membrane undergoes an epithelial-to-mesenchymal-like transition to initiate epithelial sheet migration, followed by a mesenchymal-to-epithelial-like transition as the epithelial sheets meet up and restore cell-cell adhesion. This latter event requires integrin-localized Pak, which recruits the Scrib complex in septate junction formation. We conclude that there are bidirectional interactions between Pak and the Scrib complex modulating epithelial plasticity. Scrib can recruit Pak to the LE for polarized cell migration but, as migratory cells meet up, Pak can recruit the Scrib complex to restore apicobasal polarity and cell-cell adhesion.

### [Mm]igration

#### PubMed

1. Src42A modulates tumor invasion and cell death via Ben/dUev1a-mediated JNK activation in Drosophila. Loss of the cell polarity gene could cooperate with oncogenic Ras to drive tumor growth and invasion, which critically depends on the c-Jun N-terminal Kinase (JNK) signaling pathway in Drosophila. By performing a genetic screen, we have identified Src42A, the ortholog of mammalian Src, as a key modulator of both Ras(V12)/lgl(-/-) triggered tumor invasion and loss of cell polarity gene-induced cell **migration**. Our genetic study further demonstrated that the Bendless (Ben)/dUev1a ubiquitin E2 complex is an essential regulator of Src42A-induced, JNK-mediated cell **migration**. Furthermore, we showed that ectopic Ben/dUev1a expression induced invasive cell **migration** along with increased MMP1 production in wing disc epithelia. Moreover, Ben/dUev1a could cooperate with Ras(V12) to promote tumor overgrowth and invasion. In addition, we found that the Ben/dUev1a complex is required for ectopic Src42A-triggered cell death and endogenous Src42A-dependent thorax closure. Our data not only provide a mechanistic insight into the role of Src in development and disease but also propose a potential oncogenic function for Ubc13 and Uev1a, the mammalian homologs of Ben and dUev1a.
2. A Jnk-Rho-Actin remodeling positive feedback network directs Src-driven invasion. Current models of tumor cell invasion propose that oncogenic signaling converges upon key orchestrators of cytoskeletal dynamics, including c-Jun N-terminal kinase (Jnk) and RhoGTPase family members; these signals dynamically direct Actin remodeling proteins (ARPs) to catalyze the cytoskeletal changes required for **migration**. Src is a key driver of tumor aggression, metastasis and patient mortality. To clarify how Src regulates Actin dynamics to promote invasive **migration**, we performed a genetic modifier screen in a Drosophila model of invasion. Nine genes linked to Actin dynamics were identified that mediate invasion in situ. We found that ARPs were required for many oncogenic effects of Src including Mmp1 expression and initiation of apoptosis. Surprisingly, they were also regulators of Jnk pathway activity: both Src and the small GTPase Rho1 activated Jnk in a manner dependent on ARPs during invasion. Our results suggest that ARPs are not simply downstream executors of signal transduction pathways. Rather, they participate in a positive feedback network involving canonical oncogenic signaling pathways that promote tumor invasion.
3. Drosophila nonmuscle myosin II has multiple essential roles in imaginal disc and egg chamber morphogenesis. Morphogenesis is characterized by orchestrated changes in the shape and position of individual cells. Many of these movements are thought to be powered by motor proteins. However, in metazoans, it is often difficult to match specific motors with the movements they drive. The nonmuscle myosin II heavy chain (MHC encoded by zipper is required for cell sheet movements in Drosophila embryos. To determine if myosin II is required for other processes, we examined the phenotypes of strong and weak larval lethal mutations in spaghetti squash (sqh), which encodes the nonmuscle myosin II regulatory light chain (RLC). sqh mutants can be rescued to adulthood by daily induction of a sqh cDNA transgene driven by the hsp70 promoter. By transiently ceasing induction of the cDNA, we depleted RLC at specific times during development. When RLC is transiently depleted in larvae, the resulting adult phenotypes demonstrate that RLC is required in a stage-specific fashion for proper development of eye and leg imaginal discs. When RLC is depleted in adult females, oogenesis is reversibly disrupted. Without RLC induction, developing egg chambers display a succession of phenotypes that demonstrate roles for myosin II in morphogenesis of the interfollicular stalks, three morphologically and mechanistically distinct types of follicle cell **migration**, and completion of nurse cell cytoplasm transport (dumping). Finally, we show that in sqh mutant tissues, MHC is abnormally localized in punctate structures that do not contain appreciable amounts of filamentous actin or the myosin tail-binding protein p127. This suggests that sqh mutant phenotypes are chiefly caused by sequestration of myosin into inactive aggregates. These results show that myosin II is responsible for a surprisingly diverse array of cell shape changes throughout development.
4. Basolateral junctions utilize warts signaling to control epithelial-mesenchymal transition and proliferation crucial for **migration** and invasion of Drosophila ovarian epithelial cells. Fasciclin2 (Fas2) and Discslarge (Dlg) localize to the basolateral junction (BLJ) of Drosophila follicle epithelial cells and inhibit their proliferation and invasion. To identify a BLJ signaling pathway we completed a genomewide screen for mutants that enhance dlg tumorigenesis. We identified two genes that encode known BLJ scaffolding proteins, lethal giant larvae (lgl) and scribble (scrib), and several not previously associated with BLJ function, including warts (wts) and roughened eye (roe), which encode a serine-threonine kinase and a transcription factor, respectively. Like scrib, wts and roe also enhance Fas2 and lgl tumorigenesis. Further, scrib, wts, and roe block border cell **migration**, and cause noninvasive tumors that resemble dlg partial loss of function, suggesting that the BLJ utilizes Wts signaling to repress EMT and proliferation, but not motility. Apicolateral junction proteins Fat (Ft), Expanded (Ex), and Merlin (Mer) either are not involved in these processes, or have highly spatio-temporally restricted roles, diminishing their significance as upstream inputs to Wts in follicle cells. This is further indicated in that Wts targets, CyclinE and DIAP1, are elevated in Fas2, dlg, lgl, wts, and roe cells, but not Fat, ex, or mer cells. Thus, the BLJ appears to regulate epithelial polarity and dynamics not only as a localized scaffold, but also by communicating signals to the nucleus. Wts may be regulated by distinct junction inputs depending on developmental context.
5. Colorectal cancer progression: lessons from Drosophila? Human colorectal cancers arise as benign adenomas, tumours that retain their epithelial character, and then progress to malignant adenocarcinomas and carcinomas in which the epithelium becomes disrupted. Carcinomas often exhibit transcriptional downregulation of E-cadherin and other epithelial genes in an epithelial-to-mesenchymal transition (EMT), a mechanism first discovered in Drosophila to be mediated by the transcription factors Twist and Snail. In contrast, adenocarcinomas retain expression of E-cadherin and disruption of the epithelium occurs through formation of progressively smaller epithelial cysts with apical Crumbs/CRB3, Stardust/PALS1, and Bazooka/PAR3 localised to the inner lumen. Results from Drosophila show that morphologically similar cysts form upon induction of clonal heterogeneity in Wnt, Smad, or Ras signalling levels, which causes extrusion of epithelial cells at clonal boundaries. Thus, intratumour heterogeneity might also promote formation of adenocarcinomas in humans. Finally, epithelial cysts can collectively migrate, as in the case of Drosophila border cells, a potential model system for the invasive **migration** of adenocarcinoma cells.
6. The interdependence of the Rho GTPases and apicobasal cell polarity. Signaling via the Rho GTPases provides crucial regulation of numerous cell polarization events, including apicobasal (AB) polarity, polarized cell **migration**, polarized cell division and neuronal polarity. Here we review the relationships between the Rho family GTPases and epithelial AB polarization events, focusing on the 3 best-characterized members: Rho, Rac and Cdc42. We discuss a multitude of processes that are important for AB polarization, including lumen formation, apical membrane specification, cell-cell junction assembly and maintenance, as well as tissue polarity. Our discussions aim to highlight the immensely complex regulatory mechanisms that encompass Rho GTPase signaling during AB polarization. More specifically, in this review we discuss several emerging common themes, that include: 1) the need for Rho GTPase activities to be carefully balanced in both a spatial and temporal manner through a multitude of mechanisms; 2) the existence of signaling feedback loops and crosstalk to create robust cellular responses; and 3) the frequent multifunctionality that exists among AB polarity regulators. Regarding this latter theme, we provide further discussion of the potential plasticity of the cell polarity machinery and as a result the possible implications for human disease.
7. Requirements of Lgl in cell differentiation and motility during Drosophila ovarian follicular epithelium morphogenesis. The epithelial follicle cell layer over the egg chamber in Drosophila ovary undergoes patterning and morphogenesis at oogenesis. These developmental processes are essential for constructing the eggshell and establishing the body axes of the egg and resultant embryo, thereby being crucial for the egg development. We have previously shown that lethal(2)giant larvae (lgl), a Drosophila neoplastic tumor suppressor gene (nTSG) is required for the posterior follicle cell (PFC) fate induction during antero-posterior pattern formation of the follicular epithelium. In this report, we further characterize lgl in this epithelium patterning and the morphogenetic changes of specified border cells. Genetic interactions of lgl with discs large (dlg) and scribble (scrib), another two nTSGs in specifying the PFC fate reveal a cooperative role of this group of genes. Meanwhile, we find that loss of lgl function causes failure of follicle cells at the anterior to differentiate properly. The clonal analysis further indicates that lgl is necessary not only for the border cell differentiation, but also for control of the collective border cell **migration** via presumably modulating the apico-basal polarity and cell adhesion. Overall, we identify Lgl as an essential factor in regulating differentiation and morphogenetic movement of the ovarian epithelial follicle cells.
8. Molecular requirements for actin-based lamella formation in Drosophila S2 cells. Cell **migration** occurs through the protrusion of the actin-enriched lamella. Here, we investigated the effects of RNAi depletion of approximately 90 proteins implicated in actin function on lamella formation in Drosophila S2 cells. Similar to in vitro reconstitution studies of actin-based Listeria movement, we find that lamellae formation requires a relatively small set of proteins that participate in actin nucleation (Arp2/3 and SCAR), barbed end capping (capping protein), filament depolymerization (cofilin and Aip1), and actin monomer binding (profilin and cyclase-associated protein). Lamellae are initiated by parallel and partially redundant signaling pathways involving Rac GTPases and the adaptor protein Nck, which stimulate SCAR, an Arp2/3 activator. We also show that RNAi of three proteins (kette, Abi, and Sra-1) known to copurify with and inhibit SCAR in vitro leads to SCAR degradation, revealing a novel function of this protein complex in SCAR stability. Our results have identified an essential set of proteins involved in actin dynamics during lamella formation in Drosophila S2 cells.
9. Bendless modulates JNK-mediated cell death and **migration** in Drosophila. The TNF-JNK pathway is a highly conserved signaling pathway that regulates a wide spectrum of biological processes including cell death and **migration**. To further delineate this pathway, we carried out a genetic screen for dominant modifiers of the cell death phenotype triggered by ectopic expression of Eiger (Egr), the Drosophila TNF ortholog. Here we show that Bendless (Ben), an E2 ubiquitin-conjugating enzyme, modulates Egr-induced JNK activation and cell death through dTRAF2. Furthermore, Ben physically interacts with dTRAF2 and regulates Egr-induced dTRAF2 polyubiquitination. Finally, Ben is required for JNK-dependent tumor progression, cell **migration**, oxidative stress resistance and longevity. Our results indicate that Ben constitutes an essential component of the evolutionarily conserved TNF-JNK pathway that modulates cell death and invasion, tumor progression, stress response and lifespan in metazoans.
10. Planar cell polarity signaling in collective cell movements during morphogenesis and disease. Collective and directed cell movements are crucial for diverse developmental processes in the animal kingdom, but they are also involved in wound repair and disease. During these processes groups of cells are oriented within the tissue plane, which is referred to as planar cell polarity (PCP). This requires a tight regulation that is in part conducted by the PCP pathway. Although this pathway was initially characterized in flies, subsequent studies in vertebrates revealed a set of conserved core factors but also effector molecules and signal modulators, which build the fundamental PCP machinery. The PCP pathway in Drosophila regulates several developmental processes involving collective cell movements such as border cell **migration** during oogenesis, ommatidial rotation during eye development, and embryonic dorsal closure. During vertebrate embryogenesis, PCP signaling also controls collective and directed cell movements including convergent extension during gastrulation, neural tube closure, neural crest cell **migration**, or heart morphogenesis. Similarly, PCP signaling is linked to processes such as wound repair, and cancer invasion and metastasis in adults. As a consequence, disruption of PCP signaling leads to pathological conditions. In this review, we will summarize recent findings about the role of PCP signaling in collective cell movements in flies and vertebrates. In addition, we will focus on how studies in Drosophila have been relevant to our understanding of the PCP molecular machinery and will describe several developmental defects and human disorders in which PCP signaling is compromised. Therefore, new discoveries about the contribution of this pathway to collective cell movements could provide new potential diagnostic and therapeutic targets for these disorders.
11. Basolateral junctions are sufficient to suppress epithelial invasion during Drosophila oogenesis. Epithelial junctions play crucial roles during metazoan evolution and development by facilitating tissue formation, maintenance, and function. Little is known about the role of distinct types of junctions in controlling epithelial transformations leading to invasion of neighboring tissues. Discovering the key junction complexes that control these processes and how they function may also provide mechanistic insight into carcinoma cell invasion. Here, using the Drosophila ovary as a model, we show that four proteins of the basolateral junction (BLJ), Fasciclin-2, Neuroglian, Discs-large, and Lethal-giant-larvae, but not proteins of other epithelial junctions, directly suppress epithelial tumorigenesis and invasion. Remarkably, the expression pattern of Fasciclin-2 predicts which cells will invade. We compared the apicobasal polarity of BLJ tumor cells to border cells (BCs), an epithelium-derived cluster that normally migrates during mid-oogenesis. Both tumor cells and BCs differentiate a lateralized membrane pattern that is necessary but not sufficient for invasion. Independent of lateralization, derepression of motility pathways is also necessary, as indicated by a strong linear correlation between faster BC **migration** and an increased incidence of tumor invasion. However, without membrane lateralization, derepression of motility pathways is also not sufficient for invasion. Our results demonstrate that spatiotemporal patterns of basolateral junction activity directly suppress epithelial invasion by organizing the cooperative activity of distinct polarity and motility pathways.
12. Bazooka is a permissive factor for the invasive behavior of discs large tumor cells in Drosophila ovarian follicular epithelia. Drosophila Bazooka and atypical protein kinase C are essential for epithelial polarity and adhesion. We show here that wild-type bazooka function is required during cell invasion of epithelial follicle cells mutant for the tumor suppressor discs large. Clonal studies indicate that follicle cell Bazooka acts as a permissive factor during cell invasion, possibly by stabilizing adhesion between the invading somatic cells and their substratum, the germline cells. Genetic epistasis experiments demonstrate that bazooka acts downstream of discs large in tumor cell invasion. In contrast, during the **migration** of border cells, Bazooka function is dispensable for cell invasion and motility, but rather is required cell-autonomously in mediating cell adhesion within the migrating border cell cluster. Taken together, these studies reveal Bazooka functions distinctly in different types of invasive behaviors of epithelial follicle cells, potentially by regulating adhesion between follicle cells or between follicle cells and their germline substratum.
13. Rho1-Wnd signaling regulates loss-of-cell polarity-induced cell invasion in Drosophila. Both cell polarity and c-Jun N-terminal kinase (JNK) activity are essential to the maintenance of tissue homeostasis, and disruption of either is commonly seen in cancer progression. Despite the established connection between loss-of-cell polarity and JNK activation, much less is known about the molecular mechanism by which aberrant cell polarity induces JNK-mediated cell **migration** and tumor invasion. Here we show results from a genetic screen using an in vivo invasion model via knocking down cell polarity gene in Drosophila wing discs, and identify Rho1-Wnd signaling as an important molecular link that mediates loss-of-cell polarity-triggered JNK activation and cell invasion. We show that Wallenda (Wnd), a protein kinase of the mitogen-activated protein kinase kinase kinase family, by forming a complex with the GTPase Rho1, is both necessary and sufficient for Rho1-induced JNK-dependent cell invasion, MMP1 activation and epithelial-mesenchymal transition. Furthermore, Wnd promotes cell proliferation and tissue growth through wingless production when apoptosis is inhibited by p35. Finally, Wnd shows oncogenic cooperation with Ras(V12) to trigger tumor growth in eye discs and causes invasion into the ventral nerve cord. Together, our data not only provides a novel mechanistic insight on how cell polarity loss contributes to cell invasion, but also highlights the value of the Drosophila model system to explore human cancer biology.
14. Signaling pathways directing the movement and fusion of epithelial sheets: lessons from dorsal closure in Drosophila. Wound healing in embryos and various developmental events in metazoans require the spreading and fusion of epithelial sheets. The complex signaling pathways regulating these processes are being pieced together through genetic, cell biological, and biochemical approaches. At present, dorsal closure of the Drosophila embryo is the best-characterized example of epithelial sheet movement. Dorsal closure involves **migration** of the lateral epidermal flanks to close a hole in the dorsal epidermis occupied by an epithelium called the amnioserosa. Detailed genetic studies have revealed a network of interacting signaling molecules regulating this process. At the center of this network is a Jun N-terminal kinase cascade acting at the leading edge of the migrating epidermis that triggers signaling by the TGF-beta superfamily member Decapentaplegic and which interacts with the Wingless pathway. These signaling modules regulate the cytoskeletal reorganization and cell shape change necessary to drive dorsal closure. Activation of this network requires signals from the amnioserosa and input from a variety of proteins at cell-cell junctions. The Rho family of small GTPases is also instrumental, both in activation of signaling and regulation of the cytoskeleton. Many of the proteins regulating dorsal closure have been implicated in epithelial movement in other organisms, and dorsal closure has emerged as an ideal model system for the study of the **migration** and fusion of epithelial sheets.
15. [Dorsal closure in Drosophila. A genetic model for wound healing?]. Dorsal closure (DC) is a morphogenetic movement that establishes the dorsal ectoderm of the drosophila embryo. During this process, the two lateral epithelia stretch toward the dorsal midline, the suture line of the two leading edges. Cell **migration** during DC relies both on cell shape change controlled by the activity of the JNK pathway in the leading edge cells and modification of cell adhesiveness, probably dependent upon activation of the Dpp (TGF-beta) pathway. Coupling of the JNK and TGF-beta pathways is essential. The sequence of the cellular and molecular events of DC highlights interesting common features with wound healing in vertebrates. Like DC, wound healing relies on the **migration** of epithelia bordered by leading edges controlling the direction and speed of the movement. This review summarizes recent data concerning the control of epithelial morphogenesis during DC and the bases of wound healing. The molecular and cellular events that underlie these two analogous migratory processes are detailed, discussed and compared. We suggest that DC is a good genetic model for wound healing studying.
16. A Fasciclin 2 morphogenetic switch organizes epithelial cell cluster polarity and motility. Little is known about how intercellular communication is regulated in epithelial cell clusters to control delamination and **migration**. We investigate this problem using Drosophila border cells as a model. We find that just preceding cell cluster delamination, expression of transmembrane immunoglobulin superfamily member, Fasciclin 2, is lost in outer border cells, but not in inner polar cells of the cluster. Loss of Fasciclin 2 expression in outer border cells permits a switch in Fasciclin 2 polarity in the inner polar cells. This polarity switch, which is organized in collaboration with neoplastic tumor suppressors Discs large and Lethal-giant-larvae, directs cluster asymmetry essential for timing delamination from the epithelium. Fas2-mediated communication between polar and border cells maintains localization of Discs large and Lethal-giant-larvae in border cells to inhibit the rate of cluster **migration**. These findings are the first to show how a switch in cell adhesion molecule polarity regulates asymmetry and delamination of an epithelial cell cluster. The finding that Discs large and Lethal-giant-larvae inhibit the rate of normal cell cluster movement suggests that their loss in metastatic tumors may directly contribute to tumor motility. Furthermore, our results provide novel insight into the intimate link between epithelial polarity and acquisition of motile polarity that has important implications for development of invasive carcinomas.
17. The leading edge during dorsal closure as a model for epithelial plasticity: Pak is required for recruitment of the Scribble complex and septate junction formation. Dorsal closure (DC) of the Drosophila embryo is a model for the study of wound healing and developmental epithelial fusions, and involves the sealing of a hole in the epidermis through the **migration** of the epidermal flanks over the tissue occupying the hole, the amnioserosa. During DC, the cells at the edge of the migrating epidermis extend Rac- and Cdc42-dependent actin-based lamellipodia and filopodia from their leading edge (LE), which exhibits a breakdown in apicobasal polarity as adhesions are severed with the neighbouring amnioserosa cells. Studies using mammalian cells have demonstrated that Scribble (Scrib), an important determinant of apicobasal polarity that functions in a protein complex, controls polarized cell **migration** through recruitment of Rac, Cdc42 and the serine/threonine kinase Pak, an effector for Rac and Cdc42, to the LE. We have used DC and the follicular epithelium to study the relationship between Pak and the Scrib complex at epithelial membranes undergoing changes in apicobasal polarity and adhesion during development. We propose that, during DC, the LE membrane undergoes an epithelial-to-mesenchymal-like transition to initiate epithelial sheet **migration**, followed by a mesenchymal-to-epithelial-like transition as the epithelial sheets meet up and restore cell-cell adhesion. This latter event requires integrin-localized Pak, which recruits the Scrib complex in septate junction formation. We conclude that there are bidirectional interactions between Pak and the Scrib complex modulating epithelial plasticity. Scrib can recruit Pak to the LE for polarized cell **migration** but, as migratory cells meet up, Pak can recruit the Scrib complex to restore apicobasal polarity and cell-cell adhesion.
18. A genetic screen in Drosophila for metastatic behavior. A genetic screen was designed in Drosophila to interrogate its genome for mutations sufficient to cause noninvasive tumors of the eye disc to invade neighboring or distant tissues. We found that cooperation between oncogenic RasV12 expression and inactivation of any one of a number of genes affecting cell polarity leads to metastatic behavior, including basement membrane degradation, loss of E-cadherin expression, **migration**, invasion, and secondary tumor formation. Inactivation of these cell polarity genes cannot drive metastatic behavior alone or in combination with other tumor-initiating alterations. These findings suggest that the oncogenic background of tissues makes a distinct contribution toward metastatic development.
19. The polarising role of cell adhesion molecules in early development. Polarising a cell or an embryo is a crucial and recurrent event during development, as it is important for cell differentiation and **migration**. Cells can become polarised along their apical-basal axis and also within the plane of the tissue layer to which they belong. The embryo develops three axes: the anteroposterior, the dorsoventral and the left-right axis. Recent work indicates instructive roles for cell adhesion molecules in establishing not only apical-basal polarity but also planar cell polarity and, surprisingly, in the generation of left-right asymmetry in vertebrates. Signalling cascades that regulate polarity formation seem to be conserved among different organisms, thereby raising the intriguing question of whether this also holds true for the cell adhesion molecules.
20. The tumor suppressor Lgl1 regulates front-rear polarity of migrating cells. Cell **migration** is a highly integrated, multistep process that plays an important role in physiological and pathological processes. The migrating cell is highly polarized, with complex regulatory pathways that integrate its component processes spatially and temporally. The Drosophila tumor suppressor, Lethal (2) giant larvae (Lgl), regulates apical-basal polarity in epithelia and asymmetric cell division. But little is known about the role of Lgl in establishing cell polarity in migrating cells. Recently, we showed that the mammalian Lgl1 interacts directly with non-muscle myosin IIA (NMIIA), inhibiting its ability to assemble into filaments in vitro. Lgl1 also regulates the cellular localization of NMIIA, the maturation of focal adhesions, and cell **migration**. We further showed that phosphorylation of Lgl1 by aPKCζ prevents its interaction with NMIIA and is important for Lgl1 and acto-NMII cytoskeleton cellular organization. Lgl is a critical downstream target of the Par6-aPKC cell polarity complex; we showed that Lgl1 forms two distinct complexes in vivo, Lgl1-NMIIA and Lgl1-Par6-aPKCζ in different cellular compartments. We further showed that aPKCζ and NMIIA compete to bind directly to Lgl1 through the same domain. These data provide new insights into the role of Lgl1, NMIIA, and Par6-aPKCζ in establishing front-rear polarity in migrating cells. In this commentary, I discuss the role of Lgl1 in the regulation of the acto-NMII cytoskeleton and its regulation by the Par6-aPKCζ polarity complex, and how Lgl1 activity may contribute to the establishment of front-rear polarity in migrating cells.
21. Requirement of Drosophila I(2)gl function for survival of the germline cells and organization of the follicle cells in a columnar epithelium during oogenesis. The lethal(2)giant larvae gene, or 1(2)gl, encodes a widely expressed cytoskeletal protein which acts in numerous biological processes during embryogenesis and oogenesis, including cell proliferation, and morphogenetic movements. Having identified the nucleotide change occurring in the l(2)gl(ts3) sequence, we produced by site-directed mutagenesis the identical change leading to the substitution of a serine by a phenylalanine at position 311 of p127l(2)gl and introduced the modified l(2)glF311 gene into l(2)gl flies. The transgene can fully rescue the development of l(2)gl flies raised at 22 degrees C but causes drastic effects on their development at 29 degrees C confirming the temperature sensitivity of the phenylalanine substitution at position 311. Fertility of females, albeit not of males, was strongly affected. Temperature-shift experiments and microscopic examination of ovaries showed that the mutation blocked egg chamber development at the onset of vitellogenesis (stages 8-9) with growth arrest of the oocyte, incomplete follicle cell **migration** over the oocyte associated with abnormal organization of the follicular epithelium, and apoptosis of the germline cells, as measured by TUNEL assays. By comparison to wildtype, we found that p127F311 is already reduced in amount at 22 degrees C and delocalized from the cytoskeletal matrix, albeit without affecting the apical localization of myosin II, a major partner of p127. At 29 degrees C, the level of p127F311 is even more reduced and the distribution of myosin-II becomes markedly altered at the apices of the follicle cells. These data indicate that during oogenesis p127 plays a critical function at the onset of vitellogenesis and regulates growth of the oocyte, follicle cell **migration** over the oocyte and their organization in a palisadic epithelium, as well as viability of the germline cells.
22. The Scribble and Par complexes in polarity and **migration**: friends or foes? The Par complex [consisting of Bazooka (also called Par3), Par6 and aPKC] is a well-described regulator of cell polarity whose role in many aspects of cell morphogenesis is under intense investigation. Recently, another set of proteins known as the Scribble complex (consisting of Scribble, Discs large and Lethal giant larvae) has also been shown to be important in polarity regulation in several settings. Here, we describe the current status of Scribble in polarity and review evidence from various model systems that indicates an essential but context-dependent role for the Scribble and Par complexes in directed cell **migration**. Based on the known interactions of Scribble and Par complexes with each other and with other signalling pathways, we propose models by which Par and Scribble might interact to regulate cell **migration**.

### ECM

#### PubMed

1. Epithelial polarity proteins regulate Drosophila tracheal tube size in parallel to the luminal matrix pathway. Regulation of epithelial tube size is critical for organ function. However, the mechanisms of tube size control remain poorly understood. In the Drosophila trachea, tube dimensions are regulated by a luminal extracellular matrix (**ECM**). **ECM** organization requires apical (luminal) secretion of the protein Vermiform (Verm), which depends on the basolateral septate junction (SJ). Here, we show that apical and basolateral epithelial polarity proteins interact to control tracheal tube size independently of the Verm pathway. Mutations in yurt (yrt) and scribble (scrib), which encode SJ-associated polarity proteins, cause an expansion of tracheal tubes but do not disrupt Verm secretion. Reducing activity of the apical polarity protein Crumbs (Crb) suppresses the length defects in yrt but not scrib mutants, suggesting that Yrt acts by negatively regulating Crb. Conversely, Crb overexpression increases tracheal tube dimensions. Reducing crb dosage also rescues tracheal size defects caused by mutations in coracle (cora), which encodes an SJ-associated polarity protein. In addition, crb mutations suppress cora length defects without restoring Verm secretion. Together, these data indicate that Yrt, Cora, Crb, and Scrib operate independently of the Verm pathway. Our data support a model in which Cora and Yrt act through Crb to regulate epithelial tube size.

### [Ee]xtracellular [Mm]atrix

#### PubMed

1. Tissue design: how Drosophila tumors remodel their neighborhood. Drosophila genetics has long been appreciated as a powerful approach for discovering the normal functions of genes that act as oncogenes and tumor suppressors in human cancer. Recent studies have also highlighted its advantages for deciphering how such genes function during tumorigenesis itself. Here we detail studies relating to how tumors, generated in developing organs and adult stem cell-based tissues, remodel the tissue landscape to their benefit. Like mammalian tumors, insect tumors can dissolve **extracellular matrix**, recruit blood cells, migrate and invade other tissues. While much is known about how mammalian fibroblasts, immune cells and vasculature promote late tumorigenesis, less is understood about the very earliest stages of tumor development in mammals. Because Drosophila has fewer mitotic cells and a simpler tissue architecture, it affords easy detection and analysis of early clonal tumor growth. Drosophila studies have revealed both cooperative and competitive interactions between tumor and normal cells during early tumor growth. During development, these interactions typically occur with other proliferative progenitor cells, but in adult stem cell-based tissues, the stem cell niche can fuel tumor growth.
2. Epithelial polarity proteins regulate Drosophila tracheal tube size in parallel to the luminal matrix pathway. Regulation of epithelial tube size is critical for organ function. However, the mechanisms of tube size control remain poorly understood. In the Drosophila trachea, tube dimensions are regulated by a luminal **extracellular matrix** (ECM). ECM organization requires apical (luminal) secretion of the protein Vermiform (Verm), which depends on the basolateral septate junction (SJ). Here, we show that apical and basolateral epithelial polarity proteins interact to control tracheal tube size independently of the Verm pathway. Mutations in yurt (yrt) and scribble (scrib), which encode SJ-associated polarity proteins, cause an expansion of tracheal tubes but do not disrupt Verm secretion. Reducing activity of the apical polarity protein Crumbs (Crb) suppresses the length defects in yrt but not scrib mutants, suggesting that Yrt acts by negatively regulating Crb. Conversely, Crb overexpression increases tracheal tube dimensions. Reducing crb dosage also rescues tracheal size defects caused by mutations in coracle (cora), which encodes an SJ-associated polarity protein. In addition, crb mutations suppress cora length defects without restoring Verm secretion. Together, these data indicate that Yrt, Cora, Crb, and Scrib operate independently of the Verm pathway. Our data support a model in which Cora and Yrt act through Crb to regulate epithelial tube size.
3. Non-canonical roles for Yorkie and Drosophila Inhibitor of Apoptosis 1 in epithelial tube size control. Precise control of epithelial tube size is critical for organ function, yet the molecular mechanisms remain poorly understood. Here, we examine the roles of cell growth and a highly conserved organ growth regulatory pathway in controlling the dimensions of the Drosophila tracheal (airway) system, a well-characterized system for investigating epithelial tube morphogenesis. We find that tracheal tube-size is regulated in unexpected ways by the transcription factor Yorkie (Yki, homolog of mammalian YAP and TAZ) and the Salvador/Warts/Hippo (SWH) kinase pathway. Yki activity typically promotes cell division, inhibits apoptosis, and can promote cell growth. However, reducing Yki activity in developing embryos increases rather than decreases the length of the major tracheal tubes, the dorsal trunks (DTs). Similarly, reduction of Hippo pathway activity, which antagonizes Yki, shortens tracheal DTs. yki mutations do not alter DT cell volume or cell number, indicating that Yki and the Hippo pathway regulate cell shape and apical surface area, but not volume. Yki does not appear to act through known tracheal pathways of apical **extracellular matrix**, septate junctions (SJs), basolateral or tubular polarity. Instead, the Hippo pathway and Yki appear to act downstream or in parallel to SJs because a double mutant combination of an upstream Hippo pathway activator, kibra, and the SJ component sinu have the short tracheal phenotype of a kibra mutant. We demonstrate that the critical target of Yki in tube size control is Drosophila Inhibitor of Apoptosis 1 (DIAP1), which in turn antagonizes the Drosophila effector caspase, Ice. Strikingly, there is no change in tracheal cell number in DIAP1 or Ice mutants, thus epithelial tube size regulation defines new non-apoptotic roles for Yki, DIAP1 and Ice.
4. Basement membrane remodeling is essential for Drosophila disc eversion and tumor invasion. Organ and tissue integrity is often maintained in animals by a specialized **extracellular matrix** structure called the basement membrane (BM). Accumulated evidence indicates that BM remodeling occurs during development and tumor invasion. Although the BM organizes and functions at the organ level, most past studies have explored its biochemical and in vitro properties. In this study, we monitor the BM in vivo during developmental tissue invasion for disc eversion and tumor invasion in Drosophila and modulate BM integrity with genetic alterations affecting either the whole organism or the targeted discs or tumors. We observe that the degradation of BM by the discs or the tumors is an early event during invasion processes and that preventing BM degradation completely blocks both tissue and tumor invasion, indicating that modulation of BM is essential for developmental and tumor invasion. Furthermore, elements of the invasion machinery, including JNK-induced matrix metalloproteinase (MMP) expression, are shared by both disc eversion and tumor invasion processes. Moreover, we show that although expression of MMP inhibitor, TIMP, is sufficient to halt developmental invasion, inhibition of proteases by both TIMP and RECK are required to block tumor invasion. These data suggest that tumor cells have a more robust invasion mechanism and could acquire metastatic behavior by co-opting developmental invasion programs. This type of co-option may be a general feature contributing to the progression of tumors. Finally, although past efforts using MMP inhibitors have not yielded much success, our results strongly argue that BM modulation could be a critical target for cancer therapy.
5. Crag regulates epithelial architecture and polarized deposition of basement membrane proteins in Drosophila. The polarized architecture of epithelia relies on an interplay between the cytoskeleton, the trafficking machinery, and cell-cell and cell-matrix adhesion. Specifically, contact with the basement membrane (BM), an **extracellular matrix** underlying the basal side of epithelia, is important for cell polarity. However, little is known about how BM proteins themselves achieve a polarized distribution. In a genetic screen in the Drosophila follicular epithelium, we identified mutations in Crag, which encodes a conserved protein with domains implicated in membrane trafficking. Follicle cells mutant for Crag lose epithelial integrity and frequently become invasive. The loss of Crag leads to the anomalous accumulation of BM components on both sides of epithelial cells without directly affecting the distribution of apical or basolateral membrane proteins. This defect is not generally observed in mutants affecting epithelial integrity. We propose that Crag plays a unique role in organizing epithelial architecture by regulating the polarized secretion of BM proteins.
6. A junctional problem of apical proportions: epithelial tube-size control by septate junctions in the Drosophila tracheal system. The size of epithelial tubes is critical for the function of organs such as the lung, kidney and vascular system. However, the molecular mechanisms regulating tube size are largely unknown. Recent work in the Drosophila tracheal system reveals that septate junctions play a previously unsuspected role in tube-size control. Surprisingly, this tube-size function is distinct from the established diffusion barrier function of septate junctions, and involves regulation of cell shape rather than cell number. Possible tube-size functions of septate junctions include patterning of the apical **extracellular matrix** and regulation of conserved cell polarity genes such as Scribble and Discs Large.

#### Component

1. proteinaceous **extracellular matrix**

### [Ii]nvasive

#### PubMed

1. Src42A modulates tumor invasion and cell death via Ben/dUev1a-mediated JNK activation in Drosophila. Loss of the cell polarity gene could cooperate with oncogenic Ras to drive tumor growth and invasion, which critically depends on the c-Jun N-terminal Kinase (JNK) signaling pathway in Drosophila. By performing a genetic screen, we have identified Src42A, the ortholog of mammalian Src, as a key modulator of both Ras(V12)/lgl(-/-) triggered tumor invasion and loss of cell polarity gene-induced cell migration. Our genetic study further demonstrated that the Bendless (Ben)/dUev1a ubiquitin E2 complex is an essential regulator of Src42A-induced, JNK-mediated cell migration. Furthermore, we showed that ectopic Ben/dUev1a expression induced **invasive** cell migration along with increased MMP1 production in wing disc epithelia. Moreover, Ben/dUev1a could cooperate with Ras(V12) to promote tumor overgrowth and invasion. In addition, we found that the Ben/dUev1a complex is required for ectopic Src42A-triggered cell death and endogenous Src42A-dependent thorax closure. Our data not only provide a mechanistic insight into the role of Src in development and disease but also propose a potential oncogenic function for Ubc13 and Uev1a, the mammalian homologs of Ben and dUev1a.
2. A Jnk-Rho-Actin remodeling positive feedback network directs Src-driven invasion. Current models of tumor cell invasion propose that oncogenic signaling converges upon key orchestrators of cytoskeletal dynamics, including c-Jun N-terminal kinase (Jnk) and RhoGTPase family members; these signals dynamically direct Actin remodeling proteins (ARPs) to catalyze the cytoskeletal changes required for migration. Src is a key driver of tumor aggression, metastasis and patient mortality. To clarify how Src regulates Actin dynamics to promote **invasive** migration, we performed a genetic modifier screen in a Drosophila model of invasion. Nine genes linked to Actin dynamics were identified that mediate invasion in situ. We found that ARPs were required for many oncogenic effects of Src including Mmp1 expression and initiation of apoptosis. Surprisingly, they were also regulators of Jnk pathway activity: both Src and the small GTPase Rho1 activated Jnk in a manner dependent on ARPs during invasion. Our results suggest that ARPs are not simply downstream executors of signal transduction pathways. Rather, they participate in a positive feedback network involving canonical oncogenic signaling pathways that promote tumor invasion.
3. Basolateral junctions utilize warts signaling to control epithelial-mesenchymal transition and proliferation crucial for migration and invasion of Drosophila ovarian epithelial cells. Fasciclin2 (Fas2) and Discslarge (Dlg) localize to the basolateral junction (BLJ) of Drosophila follicle epithelial cells and inhibit their proliferation and invasion. To identify a BLJ signaling pathway we completed a genomewide screen for mutants that enhance dlg tumorigenesis. We identified two genes that encode known BLJ scaffolding proteins, lethal giant larvae (lgl) and scribble (scrib), and several not previously associated with BLJ function, including warts (wts) and roughened eye (roe), which encode a serine-threonine kinase and a transcription factor, respectively. Like scrib, wts and roe also enhance Fas2 and lgl tumorigenesis. Further, scrib, wts, and roe block border cell migration, and cause non**invasive** tumors that resemble dlg partial loss of function, suggesting that the BLJ utilizes Wts signaling to repress EMT and proliferation, but not motility. Apicolateral junction proteins Fat (Ft), Expanded (Ex), and Merlin (Mer) either are not involved in these processes, or have highly spatio-temporally restricted roles, diminishing their significance as upstream inputs to Wts in follicle cells. This is further indicated in that Wts targets, CyclinE and DIAP1, are elevated in Fas2, dlg, lgl, wts, and roe cells, but not Fat, ex, or mer cells. Thus, the BLJ appears to regulate epithelial polarity and dynamics not only as a localized scaffold, but also by communicating signals to the nucleus. Wts may be regulated by distinct junction inputs depending on developmental context.
4. Colorectal cancer progression: lessons from Drosophila? Human colorectal cancers arise as benign adenomas, tumours that retain their epithelial character, and then progress to malignant adenocarcinomas and carcinomas in which the epithelium becomes disrupted. Carcinomas often exhibit transcriptional downregulation of E-cadherin and other epithelial genes in an epithelial-to-mesenchymal transition (EMT), a mechanism first discovered in Drosophila to be mediated by the transcription factors Twist and Snail. In contrast, adenocarcinomas retain expression of E-cadherin and disruption of the epithelium occurs through formation of progressively smaller epithelial cysts with apical Crumbs/CRB3, Stardust/PALS1, and Bazooka/PAR3 localised to the inner lumen. Results from Drosophila show that morphologically similar cysts form upon induction of clonal heterogeneity in Wnt, Smad, or Ras signalling levels, which causes extrusion of epithelial cells at clonal boundaries. Thus, intratumour heterogeneity might also promote formation of adenocarcinomas in humans. Finally, epithelial cysts can collectively migrate, as in the case of Drosophila border cells, a potential model system for the **invasive** migration of adenocarcinoma cells.
5. The lethal giant larvae tumour suppressor mutation requires dMyc oncoprotein to promote clonal malignancy. Neoplastic overgrowth depends on the cooperation of several mutations ultimately leading to major rearrangements in cellular behaviour. Precancerous cells are often removed by cell death from normal tissues in the early steps of the tumourigenic process, but the molecules responsible for such a fundamental safeguard process remain in part elusive. With the aim to investigate the molecular crosstalk occurring between precancerous and normal cells in vivo, we took advantage of the clonal analysis methods that are available in Drosophila for studying the phenotypes due to lethal giant larvae (lgl) neoplastic mutation induced in different backgrounds and tissues.We observed that lgl mutant cells growing in wild-type imaginal wing discs show poor viability and are eliminated by Jun N-terminal Kinase (JNK)-dependent cell death. Furthermore, they express very low levels of dMyc oncoprotein compared with those found in the surrounding normal tissue. Evidence that this is a cause of lgl mutant cells elimination was obtained by increasing dMyc levels in lgl mutant clones: their overgrowth potential was indeed re-established, with mutant cells overwhelming the neighbouring tissue and forming tumourous masses displaying several cancer hallmarks. Moreover, when lgl mutant clones were induced in backgrounds of slow-dividing cells, they upregulated dMyc, lost apical-basal cell polarity and were able to overgrow. Those phenotypes were abolished by reducing dMyc levels in the mutant clones, thereby confirming its key role in lgl-induced tumourigenesis. Furthermore, we show that the eiger-dependent Intrinsic Tumour Suppressor pathway plays only a minor role in eliminating lgl mutant cells in the wing pouch; lgl-/- clonal death in this region is instead driven mainly by dMyc-induced Cell Competition.Our results provide the first evidence that dMyc oncoprotein is required in lgl tumour suppressor mutant tissue to promote **invasive** overgrowth in larval and adult epithelial tissues. Moreover, we show that dMyc abundance inside versus outside the mutant clones plays a key role in driving neoplastic overgrowth.
6. Aneuploidy-induced delaminating cells drive tumorigenesis in Drosophila epithelia. Genomic instability has been observed in essentially all sporadic carcinomas. Here we use Drosophila epithelial cells to address the role of chromosomal instability in cancer development as they have proved useful for elucidating the molecular mechanisms underlying tumorigenic growth. We first show that chromosomal instability leads to an apoptotic response. Interestingly, this response is p53 independent, as opposed to mammalian cells, and depends on the activation of the c-Jun N-terminal kinase (JNK) signaling cascade. When prevented from undergoing programmed cell death (PCD), chromosomal instability induces neoplasic overgrowth. These tumor-like tissues are able to grow extensively and metastasize when transplanted into the abdomen of adult hosts. Detailed analysis of the tumors allows us to identify a delaminating cell population as the critical one in driving tumorigenesis. Cells loose their apical-basal polarity, mislocalize DE-cadherin, and delaminate from the main epithelium. A JNK-dependent transcriptional program is activated specifically in delaminating cells and drives nonautonomous tissue overgrowth, basement membrane degradation, and **invasive**ness. These findings unravel a general and rapid tumorigenic potential of genomic instability, as opposed to its proposed role as a source of mutability to select specific tumor-prone aneuploid cells, and open unique avenues toward the understanding of the role of genomic instability in human cancer.
7. The expression of the Drosophila awd gene during normal development and in neoplastic brain tumors caused by lgl mutations. The abnormal wing discs (awd) gene of Drosophila is homologous to the nm23 gene of mammals, a gene whose expression is altered in metastatic tumors. Both awd and nm23 encode nucleoside diphosphate kinases (NDP kinases). We have examined the accumulation of AWD/NDP kinase during normal development by assaying enzyme activity in extracts. There is a nearly constant level of activity throughout larval and pupal development. We have examined the tissue-specific transcription of the awd gene by RNA in situ hybridization and by reporter gene expression. In imaginal discs and brains there is no detectable awd gene expression until the beginning of the third larval instar, despite the constant level of enzyme activity measured in extracts of larvae and pupae. The most intense awd gene expression in imaginal discs and brains occurs after the end of larval development. We have also examined awd gene expression in neoplastic brain tumors caused by mutations in the lethal giant larvae (lgl) gene. In lgl mutant brains, as in normal brains, awd gene expression begins during the third larval instar. No tumors form in brains from lgl-; awd- double mutant larva, so awd gene expression is required for tumor formation and/or proliferation. There is more accumulation of AWD/NDP kinase in lgl- mutant brains than there is in normal brains. Using an awd reporter gene, we show that this is a consequence of an increased proportion of awd gene-expressing cells in mutant brains. Using the same awd reporter gene as a marker of donor cells, we have confirmed the **invasive**ness of lgl-induced neuroblastomas.
8. Bazooka is a permissive factor for the **invasive** behavior of discs large tumor cells in Drosophila ovarian follicular epithelia. Drosophila Bazooka and atypical protein kinase C are essential for epithelial polarity and adhesion. We show here that wild-type bazooka function is required during cell invasion of epithelial follicle cells mutant for the tumor suppressor discs large. Clonal studies indicate that follicle cell Bazooka acts as a permissive factor during cell invasion, possibly by stabilizing adhesion between the invading somatic cells and their substratum, the germline cells. Genetic epistasis experiments demonstrate that bazooka acts downstream of discs large in tumor cell invasion. In contrast, during the migration of border cells, Bazooka function is dispensable for cell invasion and motility, but rather is required cell-autonomously in mediating cell adhesion within the migrating border cell cluster. Taken together, these studies reveal Bazooka functions distinctly in different types of **invasive** behaviors of epithelial follicle cells, potentially by regulating adhesion between follicle cells or between follicle cells and their germline substratum.
9. Crag regulates epithelial architecture and polarized deposition of basement membrane proteins in Drosophila. The polarized architecture of epithelia relies on an interplay between the cytoskeleton, the trafficking machinery, and cell-cell and cell-matrix adhesion. Specifically, contact with the basement membrane (BM), an extracellular matrix underlying the basal side of epithelia, is important for cell polarity. However, little is known about how BM proteins themselves achieve a polarized distribution. In a genetic screen in the Drosophila follicular epithelium, we identified mutations in Crag, which encodes a conserved protein with domains implicated in membrane trafficking. Follicle cells mutant for Crag lose epithelial integrity and frequently become **invasive**. The loss of Crag leads to the anomalous accumulation of BM components on both sides of epithelial cells without directly affecting the distribution of apical or basolateral membrane proteins. This defect is not generally observed in mutants affecting epithelial integrity. We propose that Crag plays a unique role in organizing epithelial architecture by regulating the polarized secretion of BM proteins.
10. A Fasciclin 2 morphogenetic switch organizes epithelial cell cluster polarity and motility. Little is known about how intercellular communication is regulated in epithelial cell clusters to control delamination and migration. We investigate this problem using Drosophila border cells as a model. We find that just preceding cell cluster delamination, expression of transmembrane immunoglobulin superfamily member, Fasciclin 2, is lost in outer border cells, but not in inner polar cells of the cluster. Loss of Fasciclin 2 expression in outer border cells permits a switch in Fasciclin 2 polarity in the inner polar cells. This polarity switch, which is organized in collaboration with neoplastic tumor suppressors Discs large and Lethal-giant-larvae, directs cluster asymmetry essential for timing delamination from the epithelium. Fas2-mediated communication between polar and border cells maintains localization of Discs large and Lethal-giant-larvae in border cells to inhibit the rate of cluster migration. These findings are the first to show how a switch in cell adhesion molecule polarity regulates asymmetry and delamination of an epithelial cell cluster. The finding that Discs large and Lethal-giant-larvae inhibit the rate of normal cell cluster movement suggests that their loss in metastatic tumors may directly contribute to tumor motility. Furthermore, our results provide novel insight into the intimate link between epithelial polarity and acquisition of motile polarity that has important implications for development of **invasive** carcinomas.
11. A genetic screen in Drosophila for metastatic behavior. A genetic screen was designed in Drosophila to interrogate its genome for mutations sufficient to cause non**invasive** tumors of the eye disc to invade neighboring or distant tissues. We found that cooperation between oncogenic RasV12 expression and inactivation of any one of a number of genes affecting cell polarity leads to metastatic behavior, including basement membrane degradation, loss of E-cadherin expression, migration, invasion, and secondary tumor formation. Inactivation of these cell polarity genes cannot drive metastatic behavior alone or in combination with other tumor-initiating alterations. These findings suggest that the oncogenic background of tissues makes a distinct contribution toward metastatic development.
12. Prospects of using Drosophila for insect neuroendocrine research. Classical and in vitro approaches for the analysis of the molecular components of neuroendocrine systems often disrupt their close interaction with other bodily systems, which is a crucial aspect of their function in vivo. "Genetic dissection" is an alternative, non**invasive** approach which involves the systematic generation of mutations in individual genes, followed by in vivo analysis of the phenotypic effects of altering a single protein at a time avoiding extraneous disruptions. Among insects Drosophila melanogaster is the most suitable model for this approach. This paper explores the application of genetic and molecular techniques available in Drosophila for studying its neuroendocrine system with special emphasis on the production of ecdysone and juvenile hormone. Strategies are described for the generation and identification of endocrine mutations, especially those affecting hormone synthesis and regulation. Once identified by a specific mutation, a gene in Drosophila can be cloned either by chromosomal microdissection and "chromosomal walk" or by transposon tagging. Methods for molecular analysis of the structure and function of a cloned gene and of the protein it encodes are available for further study. Alternatively, a gene can be cloned using heterologous DNA probes or oligonucleotides designed according to the amino acid sequence of a protein. Genes may also be cloned via their pattern of expression (using stage- or tissue-specific cDNA libraries or through transposon-mediated "enhancer detection." Anti-sense RNA, the replacement of the gene by in vitro manipulated versions, or mutagenesis of its endogenous copies can then be used for studying its function in vivo. Information about endocrine genes in Drosophila as well as material such as cloned genes and antibodies should be useful for the analysis of endocrine systems in other insects which are not amenable to genetic manipulations. Such information should be helpful in designing novel means for pest control based on the specific intervention with endocrine systems regulating insect development and reproduction.
13. Studying tumor growth in Drosophila using the tissue allograft method. This protocol describes a method to allograft Drosophila larval tissue into adult fly hosts that can be used to assay the tumorigenic potential of mutant tissues. The tissue of interest is dissected, loaded into a fine glass needle and implanted into a host. Upon implantation, nontransformed tissues do not overgrow beyond their normal size, but malignant tumors grow without limit, are **invasive** and kill the host. By using this method, Drosophila malignant tumors can be transplanted repeatedly, for years, and therefore they can be aged beyond the short life span of flies. Because several hosts can be implanted using different pieces from a single tumor, the method also allows the tumor mass to be increased to facilitate further studies that may require large amounts of tissue (i.e., genomics, proteomics and so on). This method also provides an operational definition of hyperplastic, benign and malignant growth. The injection procedure itself requires only ∼1 d. Tumor development can then be monitored until the death of the implanted hosts.
14. A protocol for genetic induction and visualization of benign and **invasive** tumors in cephalic complexes of Drosophila melanogaster. Drosophila has illuminated our understanding of the genetic basis of normal development and disease for the past several decades and today it continues to contribute immensely to our understanding of complex diseases (1-7). Progression of tumors from a benign to a metastatic state is a complex event (8) and has been modeled in Drosophila to help us better understand the genetic basis of this disease (9). Here I present a simple protocol to genetically induce, observe and then analyze the progression of tumors in Drosophila larvae. The tumor induction technique is based on the MARCM system (10) and exploits the cooperation between an activated oncogene, Ras(V12) and loss of cell polarity genes (scribbled, discs large and lethal giant larvae) to generate **invasive** tumors (9). I demonstrate how these tumors can be visualized in the intact larvae and then how these can be dissected out for further analysis. The simplified protocol presented here should make it possible for this technique to be utilized by investigators interested in understanding the role of a gene in tumor invasion.
15. Growth, metastasis, and **invasive**ness of Drosophila tumors caused by mutations in specific tumor suppressor genes. More than 50 genes have been identified in Drosophila by loss-of-function mutations that lead to overgrowth of specific tissues. Loss-of-function mutations in the lethal giant larvae, discs large, or brain tumor genes cause neoplastic overgrowth of larval brains and imaginal discs. In the present study, the growth and metastatic potential of tumors resulting from mutations in these genes were quantified. Overgrown brains and imaginal discs were transplanted into adults and beta-galactosidase accumulation was used as a marker to identify donor cells. Mutations in these three genes generated tumors with similar metastatic patterns. For brain tumors, the metastatic index (a measure we defined as the fraction of hosts that acquired secondary tumors normalized for the amount of primary tumor growth) of each of the three mutants was similar. Analysis of cell proliferation in mutant brains suggests that the tumors arise from a population of several hundred cells which represent only 1-2% of the cells in third instar larval brains. For imaginal disc tumors from lethal giant larvae and brain tumor mutants, it is shown for the first time that they can be metastatic and **invasive**. Primary imaginal disc tumors from lethal giant larvae and brain tumor mutants formed secondary tumors in 43 and 53% of the hosts, respectively, although the secondary tumors were, in general, smaller than the secondary tumors derived from primary brain tumors.
16. Neuroglian stabilizes epithelial structure during Drosophila oogenesis. The vertebrate L1 family of cell adhesion molecules (CAMs) and their fly homolog, Neuroglian, are members of the immunoglobulin (Ig) superfamily of CAMs. In general, Ig CAMs have been found to play critical roles in mediating axon guidance. One Ig CAM, NCAM, has also been implicated in maintaining epithelial integrity and suppressing metastatic dissemination of tumor cells. Other Ig CAMs, such as Nrg, are also expressed in epithelia. We thus tested the hypothesis that, like NCAM, Nrg might also be required for maintaining epithelial integrity and for inhibiting tumor invasion. We used the Drosophila follicular epithelium to determine the function of Nrg in vivo in maintaining epithelial structure, and in regulating the motility of migrating border cells and **invasive** tumorous follicle cells. Nrg(167) is expressed on the lateral membrane of follicle cells. Loss of Nrg(167) causes border cells to delay delamination and causes other follicle cells to delaminate inappropriately. The delaminated cells have aberrant epithelial polarity manifested as severe mislocalization of apical and basal membrane proteins, and uniform localization of lateral membrane proteins. Furthermore, loss of Nrg(167) dramatically enhances the **invasive** phenotype associated with loss of Discs Large, a neoplastic tumor suppressor. These results indicate that Nrg(167) stabilizes epithelial polarity by regulating junctional adhesion and function in normal and tumorous epithelia. Our data also suggest that Ig superfamily members have significant functional redundancy in maintaining epithelial polarity, with individual members playing subtle, unique roles during epithelial morphogenesis.
17. Increased type IV collagenase in lgl-induced **invasive** tumors of Drosophila. Loss of function mutations in the lethal giant larvae (lgl) gene causes neoplastic brain tumors in Drosophila. We have introduced a lacZ reporter gene into lgl mutant cells and used beta-galactosidase expression as a marker to monitor the growth of such tumors following transplantation into wild-type adult hosts. Whereas normal larval brains do not grow when transplanted, mutant brains can develop into enormous tumors that fill the entire abdominal cavity. To investigate whether these tumors are similar to mammalian tumors at the biochemical level, we examined the accumulation of a specific protein which is differentially expressed in mammalian metastatic tumors and is likely to be involved in the **invasive** and/or metastatic mechanism. Increased accumulation of a 72 kilodalton (kDa) type IV collagenase has been observed in several metastatic human tumors. Using antibodies directed against this human 72 kDa type IV collagenase, we show for the first time that Drosophila has a cross-reacting 49 kDa protein with gelatinase activity. In brains dissected from lgl mutant larvae, the accumulation of this 49 kDa gelatinase of Drosophila is increased compared to the level in brains dissected from wild-type larvae. In tumors derived from mutant brains, all of the cells express this protein. Moreover, the tumor cells that invade host organs express this protein. These data suggest that the metastasis of Drosophila tumor cells is similar to the metastasis of some human tumors at the biochemical level as well as at the cellular level.
18. Drosophila screening model for metastasis: Semaphorin 5c is required for l(2)gl cancer phenotype. Cancer metastasis is a complex process involving many genes and pathways. This complexity hinders the identification of molecules functionally required for this process. We have developed and used a Drosophila screening system to identify genes that are functionally important for tumorigenicity and metastasis. Deletion of Drosophila lethal giant larvae (l(2)gl) leads to highly **invasive** and widely metastatic tumors on transplantation into adult flies. Random homozygous P element insertions were screened for the ability to modulate the l(2)gl phenotype. Analysis of metastasis patterns of the lines containing P element insertions and lacking wild-type l(2)gl expression identified three homozygous mutations that dramatically alter tumorigenesis and/or metastasis. Semaphorin 5c (Sema 5c) is required for tumorigenicity, apontic overexpression suppresses metastasis but not tumorigenicity, and pointed up-regulation accelerates lethality of l(2)gl tumors. Furthermore, class 5 semaphorins are shown to be expressed in cancer cells and localized to the membrane. Drosophila Sema-5c and the mammalian homologs are transmembrane proteins with extracellular thrombospondin type I (TspI) repeats. TspI repeats are known in some proteins to bind and activate transforming growth factor (TGF)-beta ligand. Phospho-Mad and the downstream target gene vestigial were elevated in l(2)gl tumors, thus linking Drosophila neoplasia to the Dpp (TGF-beta-like) signal pathway. The activation of the Dpp pathway in l(2)gl tumors occurred only in the presence of Sema-5c. This study demonstrates that the power of Drosophila genetics can be applied to screen, identify, and characterize molecules that are functionally required for invasion and metastasis.

### [Mm]etastasize

#### PubMed

1. Aneuploidy-induced delaminating cells drive tumorigenesis in Drosophila epithelia. Genomic instability has been observed in essentially all sporadic carcinomas. Here we use Drosophila epithelial cells to address the role of chromosomal instability in cancer development as they have proved useful for elucidating the molecular mechanisms underlying tumorigenic growth. We first show that chromosomal instability leads to an apoptotic response. Interestingly, this response is p53 independent, as opposed to mammalian cells, and depends on the activation of the c-Jun N-terminal kinase (JNK) signaling cascade. When prevented from undergoing programmed cell death (PCD), chromosomal instability induces neoplasic overgrowth. These tumor-like tissues are able to grow extensively and **metastasize** when transplanted into the abdomen of adult hosts. Detailed analysis of the tumors allows us to identify a delaminating cell population as the critical one in driving tumorigenesis. Cells loose their apical-basal polarity, mislocalize DE-cadherin, and delaminate from the main epithelium. A JNK-dependent transcriptional program is activated specifically in delaminating cells and drives nonautonomous tissue overgrowth, basement membrane degradation, and invasiveness. These findings unravel a general and rapid tumorigenic potential of genomic instability, as opposed to its proposed role as a source of mutability to select specific tumor-prone aneuploid cells, and open unique avenues toward the understanding of the role of genomic instability in human cancer.
2. Metastatic ability of Drosophila tumors depends on MMP activity. We analyzed how cells from tumors caused by mutations in either lgl or brat use matrix metalloproteinases (MMPs) to facilitate metastasis in Drosophila. MMP1 accumulation is dramatically increased in lgl larval imaginal discs compared to both wild type and brat mutants. Removal of Mmp1 gene activity in lgl brain tumor cells reduced their frequency of ovarian micro-metastases after transplantation; whereas, removal of Mmp1 gene activity in brat tumor cells had no such effect. Host ovaries showed increased Mmp1 gene expression in response to transplantation of brat tumors but not of lgl tumors. Reduction of MMP activity in host ovaries by ectopic expression of TIMP significantly reduced both lgl and brat metastases in that organ. These results highlight the mechanisms that lgl and brat tumor cells use to **metastasize**. Our interpretation of these data is that secretion of MMP1 from lgl tumor cells facilitates their metastasis, while secretion of MMP1 from host ovaries facilitates brat tumor metastasis. This study is the first demonstration that Drosophila tumors utilize MMP activity to **metastasize**.

### [Mm]etastasis

#### PubMed

1. A Jnk-Rho-Actin remodeling positive feedback network directs Src-driven invasion. Current models of tumor cell invasion propose that oncogenic signaling converges upon key orchestrators of cytoskeletal dynamics, including c-Jun N-terminal kinase (Jnk) and RhoGTPase family members; these signals dynamically direct Actin remodeling proteins (ARPs) to catalyze the cytoskeletal changes required for migration. Src is a key driver of tumor aggression, **metastasis** and patient mortality. To clarify how Src regulates Actin dynamics to promote invasive migration, we performed a genetic modifier screen in a Drosophila model of invasion. Nine genes linked to Actin dynamics were identified that mediate invasion in situ. We found that ARPs were required for many oncogenic effects of Src including Mmp1 expression and initiation of apoptosis. Surprisingly, they were also regulators of Jnk pathway activity: both Src and the small GTPase Rho1 activated Jnk in a manner dependent on ARPs during invasion. Our results suggest that ARPs are not simply downstream executors of signal transduction pathways. Rather, they participate in a positive feedback network involving canonical oncogenic signaling pathways that promote tumor invasion.
2. Drosophila at the intersection of infection, inflammation, and cancer. Recent studies show that both cellular and humoral aspects of innate immunity play important roles during tumor progression. These interactions have traditionally been explored in vertebrate model systems. In recent years, Drosophila has emerged as a genetically tractable model system for studying key aspects of tumorigenesis including proliferation, invasion, and **metastasis**. The absence of adaptive immunity in Drosophila provides a unique opportunity to study the interactions between innate immune system and cancer in different genetic contexts. In this review, I discuss recent advances made by using Drosophila models of cancer to study the role of innate immune pathways Toll/Imd, JNK, and JAK-STAT, microbial infection and inflammation during tumor progression.
3. Drosophila as a model to study the role of blood cells in inflammation, innate immunity and cancer. Drosophila has a primitive yet effective blood system with three types of haemocytes which function throughout different developmental stages and environmental stimuli. Haemocytes play essential roles in tissue modeling during embryogenesis and morphogenesis, and also in innate immunity. The open circulatory system of Drosophila makes haemocytes ideal signal mediators to cells and tissues in response to events such as infection and wounding. The application of recently developed and sophisticated genetic tools to the relatively simple genome of Drosophila has made the fly a popular system for modeling human tumorigensis and **metastasis**. Drosophila is now used for screening and investigation of genes implicated in human leukemia and also in modeling development of solid tumors. This second line of research offers promising opportunities to determine the seemingly conflicting roles of blood cells in tumor progression and invasion. This review provides an overview of the signaling pathways conserved in Drosophila during haematopoiesis, haemostasis, innate immunity, wound healing and inflammation. We also review the most recent progress in the use of Drosophila as a cancer research model with an emphasis on the roles haemocytes can play in various cancer models and in the links between inflammation and cancer.
4. Loss of cell polarity drives tumor growth and invasion through JNK activation in Drosophila. Apparent defects in cell polarity are often seen in human cancer. However, the underlying mechanisms of how cell polarity disruption contributes to tumor progression are unknown. Here, using a Drosophila genetic model for Ras-induced tumor progression, we show a molecular link between loss of cell polarity and tumor malignancy. Mutation of different apicobasal polarity genes activates c-Jun N-terminal kinase (JNK) signaling and downregulates the E-cadherin/beta-catenin adhesion complex, both of which are necessary and sufficient to cause oncogenic Ras(V12)-induced benign tumors in the developing eye to exhibit metastatic behavior. Furthermore, activated JNK and Ras signaling cooperate in promoting tumor growth cell autonomously, as JNK signaling switches its proapoptotic role to a progrowth effect in the presence of oncogenic Ras. Our finding that such context-dependent alterations promote both tumor growth and metastatic behavior suggests that **metastasis**-promoting mutations may be selected for based primarily on their growth-promoting capabilities. Similar oncogenic cooperation mediated through these evolutionarily conserved signaling pathways could contribute to human cancer progression.
5. The Drosophila **metastasis** suppressor gene Nm23 homolog, awd, regulates epithelial integrity during oogenesis. The expression levels of the **metastasis** suppressor gene Nm23 have been shown to correlate positively or inversely with prognosis in different cancer cohorts. This indicates that Nm23 may be needed at different expression levels and may function differently in various tissues. Here we report a novel epithelial function of the Drosophila melanogaster homolog of human Nm23, abnormal wing discs (awd). We show a dynamic expression pattern of the Awd protein during morphogenesis of the Drosophila follicle cells during oogenesis. Loss-of-function awd mutant cells result in the accumulation and spreading of adherens junction components, such as Drosophila E-cadherin, beta-catenin/Armadillo, and alpha-spectrin, and the disruption of epithelial integrity, including breaking up of the epithelial sheet and piling up of follicle cells. In contrast, overexpression of awd diminishes adherens junction components and induces a mesenchymal-cell-like cell shape change. The gain-of-function phenotype is consistent with a potential oncogenic function of this **metastasis** suppressor gene. Interestingly, we demonstrate that the epithelial function of awd is mediated by Rab5 and show that the Rab5 expression level is downregulated in awd mutant cells. Therefore, awd modulates the level and localization of adherens junction components via endocytosis. This is the first demonstration of an in vivo function of Nm23 family genes in regulating epithelial morphogenesis.
6. Planar cell polarity signaling in collective cell movements during morphogenesis and disease. Collective and directed cell movements are crucial for diverse developmental processes in the animal kingdom, but they are also involved in wound repair and disease. During these processes groups of cells are oriented within the tissue plane, which is referred to as planar cell polarity (PCP). This requires a tight regulation that is in part conducted by the PCP pathway. Although this pathway was initially characterized in flies, subsequent studies in vertebrates revealed a set of conserved core factors but also effector molecules and signal modulators, which build the fundamental PCP machinery. The PCP pathway in Drosophila regulates several developmental processes involving collective cell movements such as border cell migration during oogenesis, ommatidial rotation during eye development, and embryonic dorsal closure. During vertebrate embryogenesis, PCP signaling also controls collective and directed cell movements including convergent extension during gastrulation, neural tube closure, neural crest cell migration, or heart morphogenesis. Similarly, PCP signaling is linked to processes such as wound repair, and cancer invasion and **metastasis** in adults. As a consequence, disruption of PCP signaling leads to pathological conditions. In this review, we will summarize recent findings about the role of PCP signaling in collective cell movements in flies and vertebrates. In addition, we will focus on how studies in Drosophila have been relevant to our understanding of the PCP molecular machinery and will describe several developmental defects and human disorders in which PCP signaling is compromised. Therefore, new discoveries about the contribution of this pathway to collective cell movements could provide new potential diagnostic and therapeutic targets for these disorders.
7. Transgenic expression of the Helicobacter pylori virulence factor CagA promotes apoptosis or tumorigenesis through JNK activation in Drosophila. Gastric cancer development is strongly correlated with infection by Helicobacter pylori possessing the effector protein CagA. Using a transgenic Drosophila melanogaster model, we show that CagA expression in the simple model epithelium of the larval wing imaginal disc causes dramatic tissue perturbations and apoptosis when CagA-expressing and non-expressing cells are juxtaposed. This cell death phenotype occurs through activation of JNK signaling and is enhanced by loss of the neoplastic tumor suppressors in CagA-expressing cells or loss of the TNF homolog Eiger in wild type neighboring cells. We further explored the effects of CagA-mediated JNK pathway activation on an epithelium in the context of oncogenic Ras activation, using a Drosophila model of **metastasis**. In this model, CagA expression in epithelial cells enhances the growth and invasion of tumors in a JNK-dependent manner. These data suggest a potential role for CagA-mediated JNK pathway activation in promoting gastric cancer progression.
8. Small is beautiful: what flies tell us about ERM protein function in development. Actin dynamics is recognized as being a determinant in many developmental processes and pathologies, such as cell polarity, morphogenesis and tumour **metastasis**. However, how actin interacts with the plasma membrane is poorly understood. Although numerous studies in cell culture point to the crucial role of Ezrin, Radixin and Moesin (ERM) proteins in the actin-membrane link, genetic approaches in mice have not yet revealed their activity during development. Drosophila has recently become an alternative and promising system for the genetic study of ERM protein function. This article focuses on advances made in flies, providing evidence for the evolutionary conservation of functional properties of ERM proteins, in addition to shedding new light on their importance for development.
9. Drosophila models for cancer research. Drosophila is a model system for cancer research. Investigation with fruit flies has facilitated a number of important recent discoveries in the field: the hippo signaling pathway, which coordinates cell proliferation and death to achieve normal tissue size; 'social' behaviors of cells, including cell competition and apoptosis-induced compensatory proliferation, that help ensure normal tissue size; and a growing understanding of how oncogenes and tumor suppressors cooperate to achieve tumor growth and **metastasis** in situ. In the future, Drosophila models can be extended beyond basic research in the search for human therapeutics.
10. Metastatic ability of Drosophila tumors depends on MMP activity. We analyzed how cells from tumors caused by mutations in either lgl or brat use matrix metalloproteinases (MMPs) to facilitate **metastasis** in Drosophila. MMP1 accumulation is dramatically increased in lgl larval imaginal discs compared to both wild type and brat mutants. Removal of Mmp1 gene activity in lgl brain tumor cells reduced their frequency of ovarian micro-metastases after transplantation; whereas, removal of Mmp1 gene activity in brat tumor cells had no such effect. Host ovaries showed increased Mmp1 gene expression in response to transplantation of brat tumors but not of lgl tumors. Reduction of MMP activity in host ovaries by ectopic expression of TIMP significantly reduced both lgl and brat metastases in that organ. These results highlight the mechanisms that lgl and brat tumor cells use to metastasize. Our interpretation of these data is that secretion of MMP1 from lgl tumor cells facilitates their **metastasis**, while secretion of MMP1 from host ovaries facilitates brat tumor **metastasis**. This study is the first demonstration that Drosophila tumors utilize MMP activity to metastasize.
11. Modeling tumor invasion and **metastasis** in Drosophila. Conservation of major signaling pathways between humans and flies has made Drosophila a useful model organism for cancer research. Our understanding of the mechanisms regulating cell growth, differentiation and development has been considerably advanced by studies in Drosophila. Several recent high profile studies have examined the processes constraining the metastatic growth of tumor cells in fruit fly models. Cell invasion can be studied in the context of an in vivo setting in flies, enabling the genetic requirements of the microenvironment of tumor cells undergoing **metastasis** to be analyzed. This Perspective discusses the strengths and limitations of Drosophila models of cancer invasion and the unique tools that have enabled these studies. It also highlights several recent reports that together make a strong case for Drosophila as a system with the potential for both testing novel concepts in tumor progression and cell invasion, and for uncovering players in **metastasis**.
12. Growth, **metastasis**, and invasiveness of Drosophila tumors caused by mutations in specific tumor suppressor genes. More than 50 genes have been identified in Drosophila by loss-of-function mutations that lead to overgrowth of specific tissues. Loss-of-function mutations in the lethal giant larvae, discs large, or brain tumor genes cause neoplastic overgrowth of larval brains and imaginal discs. In the present study, the growth and metastatic potential of tumors resulting from mutations in these genes were quantified. Overgrown brains and imaginal discs were transplanted into adults and beta-galactosidase accumulation was used as a marker to identify donor cells. Mutations in these three genes generated tumors with similar metastatic patterns. For brain tumors, the metastatic index (a measure we defined as the fraction of hosts that acquired secondary tumors normalized for the amount of primary tumor growth) of each of the three mutants was similar. Analysis of cell proliferation in mutant brains suggests that the tumors arise from a population of several hundred cells which represent only 1-2% of the cells in third instar larval brains. For imaginal disc tumors from lethal giant larvae and brain tumor mutants, it is shown for the first time that they can be metastatic and invasive. Primary imaginal disc tumors from lethal giant larvae and brain tumor mutants formed secondary tumors in 43 and 53% of the hosts, respectively, although the secondary tumors were, in general, smaller than the secondary tumors derived from primary brain tumors.
13. Drosophila brain tumor metastases express both neuronal and glial cell type markers. Loss of either lgl or brat gene activity in Drosophila larvae causes neoplastic brain tumors. Fragments of tumorous brains from either mutant transplanted into adult hosts over-proliferate, and kill their hosts within 2 weeks. We developed an in vivo assay for the metastatic potential of tumor cells by quantifying micro**metastasis** formation within the ovarioles of adult hosts after transplantation and determined that specific metastatic properties of lgl and brat tumor cells are different. We detected micrometastases in 15.8% of ovarioles from wild type host females 12 days after transplanting lgl tumor cells into their abdominal cavities. This frequency increased significantly with increased proliferation time. We detected micrometastases in 15% of ovarioles from wild type host females 10 days after transplanting brat tumor cells into their abdominal cavities. By contrast, this frequency did not change significantly with increased proliferation time. We found that nearly all lgl micrometastases co-express the neuronal cell marker, ELAV, and the glial cell marker, REPO. These markers are not co-expressed in normal brain cells nor in tumorous brain cells. This indicates deregulated gene expression in these metastatic cells. By contrast, most of the brat micrometastases expressed neither marker. While mutations in both lgl and brat cause neoplastic brain tumors, our results reveal that metastatic cells arising from these tumors have quite different properties. These data may have important implications for the treatment of tumor **metastasis**.
14. Increased type IV collagenase in lgl-induced invasive tumors of Drosophila. Loss of function mutations in the lethal giant larvae (lgl) gene causes neoplastic brain tumors in Drosophila. We have introduced a lacZ reporter gene into lgl mutant cells and used beta-galactosidase expression as a marker to monitor the growth of such tumors following transplantation into wild-type adult hosts. Whereas normal larval brains do not grow when transplanted, mutant brains can develop into enormous tumors that fill the entire abdominal cavity. To investigate whether these tumors are similar to mammalian tumors at the biochemical level, we examined the accumulation of a specific protein which is differentially expressed in mammalian metastatic tumors and is likely to be involved in the invasive and/or metastatic mechanism. Increased accumulation of a 72 kilodalton (kDa) type IV collagenase has been observed in several metastatic human tumors. Using antibodies directed against this human 72 kDa type IV collagenase, we show for the first time that Drosophila has a cross-reacting 49 kDa protein with gelatinase activity. In brains dissected from lgl mutant larvae, the accumulation of this 49 kDa gelatinase of Drosophila is increased compared to the level in brains dissected from wild-type larvae. In tumors derived from mutant brains, all of the cells express this protein. Moreover, the tumor cells that invade host organs express this protein. These data suggest that the **metastasis** of Drosophila tumor cells is similar to the **metastasis** of some human tumors at the biochemical level as well as at the cellular level.
15. Drosophila screening model for **metastasis**: Semaphorin 5c is required for l(2)gl cancer phenotype. Cancer **metastasis** is a complex process involving many genes and pathways. This complexity hinders the identification of molecules functionally required for this process. We have developed and used a Drosophila screening system to identify genes that are functionally important for tumorigenicity and **metastasis**. Deletion of Drosophila lethal giant larvae (l(2)gl) leads to highly invasive and widely metastatic tumors on transplantation into adult flies. Random homozygous P element insertions were screened for the ability to modulate the l(2)gl phenotype. Analysis of **metastasis** patterns of the lines containing P element insertions and lacking wild-type l(2)gl expression identified three homozygous mutations that dramatically alter tumorigenesis and/or **metastasis**. Semaphorin 5c (Sema 5c) is required for tumorigenicity, apontic overexpression suppresses **metastasis** but not tumorigenicity, and pointed up-regulation accelerates lethality of l(2)gl tumors. Furthermore, class 5 semaphorins are shown to be expressed in cancer cells and localized to the membrane. Drosophila Sema-5c and the mammalian homologs are transmembrane proteins with extracellular thrombospondin type I (TspI) repeats. TspI repeats are known in some proteins to bind and activate transforming growth factor (TGF)-beta ligand. Phospho-Mad and the downstream target gene vestigial were elevated in l(2)gl tumors, thus linking Drosophila neoplasia to the Dpp (TGF-beta-like) signal pathway. The activation of the Dpp pathway in l(2)gl tumors occurred only in the presence of Sema-5c. This study demonstrates that the power of Drosophila genetics can be applied to screen, identify, and characterize molecules that are functionally required for invasion and **metastasis**.
16. Differential regulation of the Hippo pathway by adherens junctions and apical-basal cell polarity modules. Adherens junctions (AJs) and cell polarity complexes are key players in the establishment and maintenance of apical-basal cell polarity. Loss of AJs or basolateral polarity components promotes tumor formation and **metastasis**. Recent studies in vertebrate models show that loss of AJs or loss of the basolateral component Scribble (Scrib) cause deregulation of the Hippo tumor suppressor pathway and hyperactivation of its downstream effectors Yes-associated protein (YAP) and Transcriptional coactivator with PDZ-binding motif (TAZ). However, whether AJs and Scrib act through the same or independent mechanisms to regulate Hippo pathway activity is not known. Here, we dissect how disruption of AJs or loss of basolateral components affect the activity of the Drosophila YAP homolog Yorkie (Yki) during imaginal disc development. Surprisingly, disruption of AJs and loss of basolateral proteins produced very different effects on Yki activity. Yki activity was cell-autonomously decreased but non-cell-autonomously elevated in tissues where the AJ components E-cadherin (E-cad) or α-catenin (α-cat) were knocked down. In contrast, scrib knockdown caused a predominantly cell-autonomous activation of Yki. Moreover, disruption of AJs or basolateral proteins had different effects on cell polarity and tissue size. Simultaneous knockdown of α-cat and scrib induced both cell-autonomous and non-cell-autonomous Yki activity. In mammalian cells, knockdown of E-cad or α-cat caused nuclear accumulation and activation of YAP without overt effects on Scrib localization and vice versa. Therefore, our results indicate the existence of multiple, genetically separable inputs from AJs and cell polarity complexes into Yki/YAP regulation.

### [Cc]ell [Ii]nvasion

#### PubMed

1. A Jnk-Rho-Actin remodeling positive feedback network directs Src-driven invasion. Current models of tumor **cell invasion** propose that oncogenic signaling converges upon key orchestrators of cytoskeletal dynamics, including c-Jun N-terminal kinase (Jnk) and RhoGTPase family members; these signals dynamically direct Actin remodeling proteins (ARPs) to catalyze the cytoskeletal changes required for migration. Src is a key driver of tumor aggression, metastasis and patient mortality. To clarify how Src regulates Actin dynamics to promote invasive migration, we performed a genetic modifier screen in a Drosophila model of invasion. Nine genes linked to Actin dynamics were identified that mediate invasion in situ. We found that ARPs were required for many oncogenic effects of Src including Mmp1 expression and initiation of apoptosis. Surprisingly, they were also regulators of Jnk pathway activity: both Src and the small GTPase Rho1 activated Jnk in a manner dependent on ARPs during invasion. Our results suggest that ARPs are not simply downstream executors of signal transduction pathways. Rather, they participate in a positive feedback network involving canonical oncogenic signaling pathways that promote tumor invasion.
2. dUev1a modulates TNF-JNK mediated tumor progression and cell death in Drosophila. Loss of cell polarity cooperates with oncogenic Ras to induce JNK-dependent tumor growth and invasion. To identify additional genes that modulate tumor progression, we have performed a genetic screen in Drosophila and found that loss of dUev1a, the ortholog of mammalian Uev1, suppressed lgl(-/-)/Ras(V12) induced JNK-mediated tumor growth and invasion. Furthermore, loss of dUev1a suppressed TNF ortholog Eiger-induced JNK-mediated **cell invasion** and cell death. Finally, dUev1a cooperated with Bendless to activate JNK signaling through dTRAF2. Together, our data indicate that dUev1a encodes an essential component of the evolutionary conserved TNF-JNK signaling pathway that modulates tumor progression and cell death in metazoan.
3. Basolateral junctions are sufficient to suppress epithelial invasion during Drosophila oogenesis. Epithelial junctions play crucial roles during metazoan evolution and development by facilitating tissue formation, maintenance, and function. Little is known about the role of distinct types of junctions in controlling epithelial transformations leading to invasion of neighboring tissues. Discovering the key junction complexes that control these processes and how they function may also provide mechanistic insight into carcinoma **cell invasion**. Here, using the Drosophila ovary as a model, we show that four proteins of the basolateral junction (BLJ), Fasciclin-2, Neuroglian, Discs-large, and Lethal-giant-larvae, but not proteins of other epithelial junctions, directly suppress epithelial tumorigenesis and invasion. Remarkably, the expression pattern of Fasciclin-2 predicts which cells will invade. We compared the apicobasal polarity of BLJ tumor cells to border cells (BCs), an epithelium-derived cluster that normally migrates during mid-oogenesis. Both tumor cells and BCs differentiate a lateralized membrane pattern that is necessary but not sufficient for invasion. Independent of lateralization, derepression of motility pathways is also necessary, as indicated by a strong linear correlation between faster BC migration and an increased incidence of tumor invasion. However, without membrane lateralization, derepression of motility pathways is also not sufficient for invasion. Our results demonstrate that spatiotemporal patterns of basolateral junction activity directly suppress epithelial invasion by organizing the cooperative activity of distinct polarity and motility pathways.
4. Bazooka is a permissive factor for the invasive behavior of discs large tumor cells in Drosophila ovarian follicular epithelia. Drosophila Bazooka and atypical protein kinase C are essential for epithelial polarity and adhesion. We show here that wild-type bazooka function is required during **cell invasion** of epithelial follicle cells mutant for the tumor suppressor discs large. Clonal studies indicate that follicle cell Bazooka acts as a permissive factor during **cell invasion**, possibly by stabilizing adhesion between the invading somatic cells and their substratum, the germline cells. Genetic epistasis experiments demonstrate that bazooka acts downstream of discs large in tumor **cell invasion**. In contrast, during the migration of border cells, Bazooka function is dispensable for **cell invasion** and motility, but rather is required cell-autonomously in mediating cell adhesion within the migrating border cell cluster. Taken together, these studies reveal Bazooka functions distinctly in different types of invasive behaviors of epithelial follicle cells, potentially by regulating adhesion between follicle cells or between follicle cells and their germline substratum.
5. Rho1-Wnd signaling regulates loss-of-cell polarity-induced **cell invasion** in Drosophila. Both cell polarity and c-Jun N-terminal kinase (JNK) activity are essential to the maintenance of tissue homeostasis, and disruption of either is commonly seen in cancer progression. Despite the established connection between loss-of-cell polarity and JNK activation, much less is known about the molecular mechanism by which aberrant cell polarity induces JNK-mediated cell migration and tumor invasion. Here we show results from a genetic screen using an in vivo invasion model via knocking down cell polarity gene in Drosophila wing discs, and identify Rho1-Wnd signaling as an important molecular link that mediates loss-of-cell polarity-triggered JNK activation and **cell invasion**. We show that Wallenda (Wnd), a protein kinase of the mitogen-activated protein kinase kinase kinase family, by forming a complex with the GTPase Rho1, is both necessary and sufficient for Rho1-induced JNK-dependent **cell invasion**, MMP1 activation and epithelial-mesenchymal transition. Furthermore, Wnd promotes cell proliferation and tissue growth through wingless production when apoptosis is inhibited by p35. Finally, Wnd shows oncogenic cooperation with Ras(V12) to trigger tumor growth in eye discs and causes invasion into the ventral nerve cord. Together, our data not only provides a novel mechanistic insight on how cell polarity loss contributes to **cell invasion**, but also highlights the value of the Drosophila model system to explore human cancer biology.
6. Modeling tumor invasion and metastasis in Drosophila. Conservation of major signaling pathways between humans and flies has made Drosophila a useful model organism for cancer research. Our understanding of the mechanisms regulating cell growth, differentiation and development has been considerably advanced by studies in Drosophila. Several recent high profile studies have examined the processes constraining the metastatic growth of tumor cells in fruit fly models. **Cell invasion** can be studied in the context of an in vivo setting in flies, enabling the genetic requirements of the microenvironment of tumor cells undergoing metastasis to be analyzed. This Perspective discusses the strengths and limitations of Drosophila models of cancer invasion and the unique tools that have enabled these studies. It also highlights several recent reports that together make a strong case for Drosophila as a system with the potential for both testing novel concepts in tumor progression and **cell invasion**, and for uncovering players in metastasis.

---

## heph

- NCBI Gene
- GeneRIF
- Pubmed

| Search Term | Rank | Fields |
| --- | --- | --- |
| [Mm]igration | 6 | PubMed(1) |
| [Ee]xtracellular [Mm]atrix | 8 | PubMed(1) |

  
hide/show details for heph

### [Mm]igration

#### PubMed

1. Conservation of the RNA Transport Machineries and Their Coupling to Translation Control across Eukaryotes. Restriction of proteins to discrete subcellular regions is a common mechanism to establish cellular asymmetries and depends on a coordinated program of mRNA localization and translation control. Many processes from the budding of a yeast to the establishment of metazoan embryonic axes and the **migration** of human neurons, depend on this type of cell polarization. How factors controlling transport and translation assemble to regulate at the same time the movement and translation of transported mRNAs, and whether these mechanisms are conserved across kingdoms is not yet entirely understood. In this review we will focus on some of the best characterized examples of mRNA transport machineries, the "yeast locasome" as an example of RNA transport and translation control in unicellular eukaryotes, and on the Drosophila Bic-D/Egl/Dyn RNA localization machinery as an example of RNA transport in higher eukaryotes. This focus is motivated by the relatively advanced knowledge about the proteins that connect the localizing mRNAs to the transport motors and the many well studied proteins involved in translational control of specific transcripts that are moved by these machineries. We will also discuss whether the core of these RNA transport machineries and factors regulating mRNA localization and translation are conserved across eukaryotes.

### [Ee]xtracellular [Mm]atrix

#### PubMed

1. Modifiers of notch transcriptional activity identified by genome-wide RNAi. The Notch signaling pathway regulates a diverse array of developmental processes, and aberrant Notch signaling can lead to diseases, including cancer. To obtain a more comprehensive understanding of the genetic network that integrates into Notch signaling, we performed a genome-wide RNAi screen in Drosophila cell culture to identify genes that modify Notch-dependent transcription.Employing complementary data analyses, we found 399 putative modifiers: 189 promoting and 210 antagonizing Notch activated transcription. These modifiers included several known Notch interactors, validating the robustness of the assay. Many novel modifiers were also identified, covering a range of cellular localizations from the **extracellular matrix** to the nucleus, as well as a large number of proteins with unknown function. Chromatin-modifying proteins represent a major class of genes identified, including histone deacetylase and demethylase complex components and other chromatin modifying, remodeling and replacement factors. A protein-protein interaction map of the Notch-dependent transcription modifiers revealed that a large number of the identified proteins interact physically with these core chromatin components.The genome-wide RNAi screen identified many genes that can modulate Notch transcriptional output. A protein interaction map of the identified genes highlighted a network of chromatin-modifying enzymes and remodelers that regulate Notch transcription. Our results open new avenues to explore the mechanisms of Notch signal regulation and the integration of this pathway into diverse cellular processes.

---

## kuz

- NCBI Gene
- GeneRIF
- Pubmed

| Search Term | Rank | Fields |
| --- | --- | --- |
| [Ff]ilopodia | 1 | PubMed(1) |
| [Mm]igration | 6 | Process(2); PubMed(7) |
| ECM | 7 | PubMed(1) |
| [Ee]xtracellular [Mm]atrix | 8 | PubMed(2) |
| [Mm]etastasis | 12 | PubMed(2) |

  
hide/show details for kuz

### [Ff]ilopodia

#### PubMed

1. Notch and Numb are required for normal migration of peripheral glia in Drosophila. A prominent feature of glial cells is their ability to migrate along axons to finally wrap and insulate them. In the embryonic Drosophila PNS, most glial cells are born in the CNS and have to migrate to reach their final destinations. To understand how migration of the peripheral glia is regulated, we have conducted a genetic screen looking for mutants that disrupt the normal glial pattern. Here we present an analysis of two of these mutants: Notch and numb. Complete loss of Notch function leads to an increase in the number of glial cells. Embryos hemizygous for the weak Notch(B-8X) allele display an irregular migration phenotype and mutant glial cells show an increased formation of **filopodia**-like structures. A similar phenotype occurs in embryos carrying the Notch(ts1) allele when shifted to the restrictive temperature during the glial cell migration phase, suggesting that Notch must be activated during glial migration. This is corroborated by the fact that cell-specific reduction of Notch activity in glial cells by directed numb expression also results in similar migration phenotypes. Since the glial migration phenotypes of Notch and numb mutants resemble each other, our data support a model where the precise temporal and quantitative regulation of Numb and Notch activity is not only required during fate decisions but also later during glial differentiation and migration.

### [Mm]igration

#### Process

1. border follicle cell **migration**
2. glial cell **migration**

#### PubMed

1. Spatially localized Kuzbanian required for specific activation of Notch during border cell **migration**. The transmembrane receptor Notch is used repeatedly during development for a variety of essential functions. During Drosophila oogenesis, Notch activity is required first to specify particular follicle cell fates, then to promote the differentiation of all follicle cell types, to promote border cell **migration**, and then to form dorsal appendages, raising the question as to how Notch activity is spatially and temporally regulated. Here we show the Notch activity pattern during oogenesis. Notch activation was found in many follicle cells at stage 6 but then at stage 9 was restricted to migrating border cells, despite uniform expression of Delta. Expression of Kuzbanian (KUZ), a metalloproteinase that can activate Notch as well as cleave other substrates, is enriched in border cells at stage 9; and dominant-negative KUZ caused a strong border cell **migration** defect, without affecting expression of markers of border cell fate or follicle cell differentiation. Constitutively active Notch rescued the **migration** defect due to dominant-negative KUZ, and conditional alleles of Delta and Notch also exhibited border cell **migration** defects. Expression of two different reporters of Notch activity was lost upon expression of dominant-negative KUZ. Taken together these results show that Notch activation and KUZ expression are restricted to border cells at stage 9 of oogenesis and are required for **migration**, but not differentiation, of these cells. This represents a previously unrecognized mechanism for achieving spatial restriction of Notch signaling.
2. TspanC8 tetraspanins regulate ADAM10/Kuzbanian trafficking and promote Notch activation in flies and mammals. The metalloprotease ADAM10/Kuzbanian catalyzes the ligand-dependent ectodomain shedding of Notch receptors and activates Notch. Here, we show that the human tetraspanins of the evolutionary conserved TspanC8 subfamily (Tspan5, Tspan10, Tspan14, Tspan15, Tspan17, and Tspan33) directly interact with ADAM10, regulate its exit from the endoplasmic reticulum, and that four of them regulate ADAM10 surface expression levels. In an independent RNAi screen in Drosophila, two TspanC8 genes were identified as Notch regulators. Functional analysis of the three Drosophila TspanC8 genes (Tsp3A, Tsp86D, and Tsp26D) indicated that these genes act redundantly to promote Notch signaling. During oogenesis, TspanC8 genes were up-regulated in border cells and regulated Kuzbanian distribution, Notch activity, and cell **migration**. Furthermore, the human TspanC8 tetraspanins Tspan5 and Tspan14 positively regulated ligand-induced ADAM10-dependent Notch1 signaling. We conclude that TspanC8 tetraspanins have a conserved function in the regulation of ADAM10 trafficking and activity, thereby positively regulating Notch receptor activation.
3. Spatially localized Kuzbanian required for specific activation of Notch during border cell **migration**. The transmembrane receptor Notch is used repeatedly during development for a variety of essential functions. During Drosophila oogenesis, Notch activity is required first to specify particular follicle cell fates, then to promote the differentiation of all follicle cell types, to promote border cell **migration**, and then to form dorsal appendages, raising the question as to how Notch activity is spatially and temporally regulated. Here we show the Notch activity pattern during oogenesis. Notch activation was found in many follicle cells at stage 6 but then at stage 9 was restricted to migrating border cells, despite uniform expression of Delta. Expression of Kuzbanian (KUZ), a metalloproteinase that can activate Notch as well as cleave other substrates, is enriched in border cells at stage 9; and dominant-negative KUZ caused a strong border cell **migration** defect, without affecting expression of markers of border cell fate or follicle cell differentiation. Constitutively active Notch rescued the **migration** defect due to dominant-negative KUZ, and conditional alleles of Delta and Notch also exhibited border cell **migration** defects. Expression of two different reporters of Notch activity was lost upon expression of dominant-negative KUZ. Taken together these results show that Notch activation and KUZ expression are restricted to border cells at stage 9 of oogenesis and are required for **migration**, but not differentiation, of these cells. This represents a previously unrecognized mechanism for achieving spatial restriction of Notch signaling.
4. Analysis of cell **migration** using whole-genome expression profiling of migratory cells in the Drosophila ovary. Cell **migration** contributes to normal development and homeostasis as well as to pathological processes such as inflammation and tumor metastasis. Previous genetic screens have revealed signaling pathways that govern follicle cell **migration**s in the Drosophila ovary, but few downstream targets of the critical transcriptional regulators have been identified. To characterize the gene expression profile of two migratory cell populations and identify Slbo targets, we purified border cells and centripetal cells expressing the mouse CD8 antigen and carried out whole-genome microarray analysis. Genes predicted to control actin dynamics and the endocytic and secretory pathways were overrepresented in the migratory cell transcriptome. Mutations in five genes, including ttk, failed to complement previously isolated mutations that cause cell **migration** defects in mosaic clones. Functional analysis revealed a role for the Notch-activating protease Kuzbanian in border cell **migration** and identified Tie as a guidance receptor for the border cells.
5. Cellular and molecular mechanisms of border cell **migration** analyzed using time-lapse live-cell imaging. Border cells in the Drosophila ovary originate within an epithelium, detach from it, invade neighboring nurse cells, and migrate as a coherent cluster. This **migration** has served as a useful genetic model for understanding epithelial cell motility. The prevailing model of growth factor-mediated chemotaxis in general, and of border cells in particular, posits that receptor activation promotes cellular protrusion at the leading edge. Here we report the time-lapse video imaging of border cell **migration**, allowing us to test this model. Reducing the activities of the guidance receptors EGFR and PVR did not result in the expected inhibition of protrusion, but instead resulted in protrusion in all directions. In contrast, reduction in Notch activity resulted in failure of the cells to detach from the epithelium without affecting direction sensing. These observations provide new insight into the cellular dynamics and molecular mechanisms of cell **migration** in vivo.
6. Notch and Numb are required for normal **migration** of peripheral glia in Drosophila. A prominent feature of glial cells is their ability to migrate along axons to finally wrap and insulate them. In the embryonic Drosophila PNS, most glial cells are born in the CNS and have to migrate to reach their final destinations. To understand how **migration** of the peripheral glia is regulated, we have conducted a genetic screen looking for mutants that disrupt the normal glial pattern. Here we present an analysis of two of these mutants: Notch and numb. Complete loss of Notch function leads to an increase in the number of glial cells. Embryos hemizygous for the weak Notch(B-8X) allele display an irregular **migration** phenotype and mutant glial cells show an increased formation of filopodia-like structures. A similar phenotype occurs in embryos carrying the Notch(ts1) allele when shifted to the restrictive temperature during the glial cell **migration** phase, suggesting that Notch must be activated during glial **migration**. This is corroborated by the fact that cell-specific reduction of Notch activity in glial cells by directed numb expression also results in similar **migration** phenotypes. Since the glial **migration** phenotypes of Notch and numb mutants resemble each other, our data support a model where the precise temporal and quantitative regulation of Numb and Notch activity is not only required during fate decisions but also later during glial differentiation and **migration**.
7. The function of leak and kuzbanian during growth cone and cell **migration**. Axonal growth cones require an evolutionary conserved repulsive guidance system to ensure proper crossing of the CNS midline. In Drosophila, the Slit protein is a repulsive signal secreted by the midline glial cells. It binds to the Roundabout receptors, which are expressed on CNS axons in the longitudinal tracts but not in the commissural tracts. Here we present an analysis of the genes leak and kuzbanian and show that both genes are involved in the repulsive guidance system operating at the CNS midline. Mutations in leak, which encodes the Roundabout-2 Slit receptor, were first recovered by Nüsslein-Volhard and co-workers based on defects in the larval cuticle. Analysis of the head phenotype suggests that slit may be able to act as an attractive guidance cue while directing the movements of the dorsal ectodermal cell sheath. kuzbanian also regulates midline crossing of CNS axons. It encodes a metalloprotease of the ADAM family and genetically interacts with slit. Expression of a dominant negative Kuzbanian protein in the CNS midline cells results in an abnormal midline crossing of axons and prevents the clearance of the Roundabout receptor from commissural axons. Our analyses support a model in which Kuzbanian mediates the proteolytic activation of the Slit/Roundabout receptor complex.

### ECM

#### PubMed

1. Glia **ECM** interactions are required to shape the Drosophila nervous system. Organs are characterized by a specific shape that is often remodeled during development. The dynamics of organ shape is in particular evident during the formation of the Drosophila nervous system. During embryonic stages the central nervous system compacts, whereas selective growth occurs during larval stages. The nervous system is covered by a layer of surface glial cells that form the blood brain barrier and a thick extracellular matrix called neural lamella. The size of the neural lamella is dynamically adjusted to the growing nervous system and we show here that perineurial glial cells secrete proteases to remodel this matrix. Moreover, an imbalance in proteolytic activity results in an abnormal shape of the nervous system. To identify further components controlling nervous system shape we performed an RNAi based screen and identified the gene nolo, which encodes an ADAMTS-like protein. We generated loss of function alleles and demonstrate a requirement in glial cells. Mutant nolo larvae, however, do not show an abnormal nervous system shape. The only predicted off-target of the nolo(dsRNA) is Oatp30B, which encodes an organic anion transporting protein characterized by an extracellular protease inhibitor domain. Loss of function mutants were generated and double mutant analyses demonstrate a genetic interaction between nolo and Oatp30B which prevented the generation of maternal zygotic mutant larvae.

### [Ee]xtracellular [Mm]atrix

#### PubMed

1. Glia ECM interactions are required to shape the Drosophila nervous system. Organs are characterized by a specific shape that is often remodeled during development. The dynamics of organ shape is in particular evident during the formation of the Drosophila nervous system. During embryonic stages the central nervous system compacts, whereas selective growth occurs during larval stages. The nervous system is covered by a layer of surface glial cells that form the blood brain barrier and a thick **extracellular matrix** called neural lamella. The size of the neural lamella is dynamically adjusted to the growing nervous system and we show here that perineurial glial cells secrete proteases to remodel this matrix. Moreover, an imbalance in proteolytic activity results in an abnormal shape of the nervous system. To identify further components controlling nervous system shape we performed an RNAi based screen and identified the gene nolo, which encodes an ADAMTS-like protein. We generated loss of function alleles and demonstrate a requirement in glial cells. Mutant nolo larvae, however, do not show an abnormal nervous system shape. The only predicted off-target of the nolo(dsRNA) is Oatp30B, which encodes an organic anion transporting protein characterized by an extracellular protease inhibitor domain. Loss of function mutants were generated and double mutant analyses demonstrate a genetic interaction between nolo and Oatp30B which prevented the generation of maternal zygotic mutant larvae.
2. Regulation of Notch signaling by Drosophila heparan sulfate 3-O sulfotransferase. Heparan sulfate (HS) regulates the activity of various ligands and is involved in molecular recognition events on the cell surface and in the **extracellular matrix**. Specific binding of HS to different ligand proteins depends on the sulfation pattern of HS. For example, the interaction between antithrombin and a particular 3-O sulfated HS motif is thought to modulate blood coagulation. However, a recent study of mice defective for this modification suggested that 3-O sulfation plays other biological roles. Here, we show that Drosophila melanogaster HS 3-O sulfotransferase-b (Hs3st-B), which catalyzes HS 3-O sulfation, is a novel component of the Notch pathway. Reduction of Hs3st-B function by transgenic RNA interference compromised Notch signaling, producing neurogenic phenotypes. We also show that levels of Notch protein on the cell surface were markedly decreased by loss of Hs3st-B. These findings suggest that Hs3st-B is involved in Notch signaling by affecting stability or intracellular trafficking of Notch protein.

### [Mm]etastasis

#### PubMed

1. Oncogenic programmes and Notch activity: an 'organized crime'? The inappropriate Notch signalling can influence virtually all aspect of cancer, including tumour-cell growth, survival, apoptosis, angiogenesis, invasion and **metastasis**, although it does not do this alone. Hence, elucidating the partners of Notch that are active in cancer is now the focus of much intense research activity. The genetic toolkits available, coupled to the small size and short life of the fruit fly Drosophila melanogaster, makes this an inexpensive and effective animal model, suited to large-scale cancer gene discovery studies. The fly eye is not only a non-vital organ but its stereotyped size and disposition also means it is easy to screen for mutations that cause tumours and metastases and provides ample opportunities to test cancer theories and to unravel unanticipated nexus between Notch and other cancer genes, or to discover unforeseen Notch's partners in cancer. These studies suggest that Notch's oncogenic capacity is brought about not simply by increasing signal strength but through partnerships, whereby oncogenes gain more by cooperating than acting individually, as in a ring 'organized crime'.
2. Analysis of cell migration using whole-genome expression profiling of migratory cells in the Drosophila ovary. Cell migration contributes to normal development and homeostasis as well as to pathological processes such as inflammation and tumor **metastasis**. Previous genetic screens have revealed signaling pathways that govern follicle cell migrations in the Drosophila ovary, but few downstream targets of the critical transcriptional regulators have been identified. To characterize the gene expression profile of two migratory cell populations and identify Slbo targets, we purified border cells and centripetal cells expressing the mouse CD8 antigen and carried out whole-genome microarray analysis. Genes predicted to control actin dynamics and the endocytic and secretory pathways were overrepresented in the migratory cell transcriptome. Mutations in five genes, including ttk, failed to complement previously isolated mutations that cause cell migration defects in mosaic clones. Functional analysis revealed a role for the Notch-activating protease Kuzbanian in border cell migration and identified Tie as a guidance receptor for the border cells.

---

## unc-4

- NCBI Gene
- GeneRIF
- Pubmed

| Search Term | Rank | Fields |
| --- | --- | --- |
| [Ii]nvasive | 10 | PubMed(1) |

  
hide/show details for unc-4

### [Ii]nvasive

#### PubMed

1. Genome-wide polycomb target gene prediction in Drosophila melanogaster. As key epigenetic regulators, polycomb group (PcG) proteins are responsible for the control of cell proliferation and differentiation as well as stem cell pluripotency and self-renewal. Aberrant epigenetic modification by PcG is strongly correlated with the severity and **invasive**ness of many types of cancers. Unfortunately, the molecular mechanism of PcG-mediated epigenetic regulation remained elusive, partly due to the extremely limited pool of experimentally confirmed PcG target genes. In order to facilitate experimental identification of PcG target genes, here we propose a novel computational method, EpiPredictor, that achieved significantly higher matching ratios with several recent chromatin immunoprecipitation studies than jPREdictor, an existing computational method. We further validated a subset of genes that were uniquely predicted by EpiPredictor by cross-referencing existing literature and by experimental means. Our data suggest that multiple transcription factor networking at the cis-regulatory elements is critical for PcG recruitment, while high GC content and high conservation level are also important features of PcG target genes. EpiPredictor should substantially expedite experimental discovery of PcG target genes by providing an effective initial screening tool. From a computational standpoint, our strategy of modelling transcription factor interaction with a non-linear kernel is original, effective and transferable to many other applications.

---

## Cont

- NCBI Gene
- GeneRIF
- Pubmed

| Search Term | Rank | Fields |
| --- | --- | --- |
| [Mm]igration | 6 | PubMed(1) |
| ECM | 7 | PubMed(1) |
| [Ee]xtracellular [Mm]atrix | 8 | PubMed(2) |
| [Ii]nvasive | 10 | PubMed(1) |
| [Mm]etastasis | 12 | PubMed(1) |

  
hide/show details for Cont

### [Mm]igration

#### PubMed

1. Analysis of cell **migration** using whole-genome expression profiling of migratory cells in the Drosophila ovary. Cell **migration** contributes to normal development and homeostasis as well as to pathological processes such as inflammation and tumor metastasis. Previous genetic screens have revealed signaling pathways that govern follicle cell **migration**s in the Drosophila ovary, but few downstream targets of the critical transcriptional regulators have been identified. To characterize the gene expression profile of two migratory cell populations and identify Slbo targets, we purified border cells and centripetal cells expressing the mouse CD8 antigen and carried out whole-genome microarray analysis. Genes predicted to control actin dynamics and the endocytic and secretory pathways were overrepresented in the migratory cell transcriptome. Mutations in five genes, including ttk, failed to complement previously isolated mutations that cause cell **migration** defects in mosaic clones. Functional analysis revealed a role for the Notch-activating protease Kuzbanian in border cell **migration** and identified Tie as a guidance receptor for the border cells.

### ECM

#### PubMed

1. Perlecan and Dystroglycan act at the basal side of the Drosophila follicular epithelium to maintain epithelial organization. Dystroglycan (Dg) is a widely expressed extracellular matrix (**ECM**) receptor required for muscle viability, synaptogenesis, basementmembrane formation and epithelial development. As an integral component of the Dystrophin-associated glycoprotein complex, Dg plays a central role in linking the **ECM** and the cytoskeleton. Disruption of this linkage in skeletal muscle leads to various types of muscular dystrophies. In epithelial cells, reduced expression of Dg is associated with increased invasiveness of cancer cells. We have previously shown that Dg is required for epithelial cell polarity in Drosophila, but the mechanisms of this polarizing activity and upstream/downstream components are largely unknown. Using the Drosophila follicle-cell epithelium (FCE) as a model system, we show that the **ECM** molecule Perlecan (Pcan) is required for maintenance of epithelial-cell polarity. Follicle cells that lack Pcan develop polarity defects similar to those of Dg mutant cells. Furthermore, Dg depends on Pcan but not on Laminin A for its localization in the basal-cell membrane, and the two proteins bind in vitro. Interestingly, the Dg form that interacts with Pcan in the FCE lacks the mucin-like domain, which is thought to be essential for Dg ligand binding activity. Finally, we describe two examples of how Dg promotes the differentiation of the basal membrane domain: (1) by recruiting/anchoring the cytoplasmic protein Dystrophin; and (2) by excluding the transmembrane protein Neurexin. We suggest that the interaction of Pcan and Dg at the basal side of the epithelium promotes basal membrane differentiation and is required for maintenance of cell polarity in the FCE.

### [Ee]xtracellular [Mm]atrix

#### PubMed

1. Perlecan and Dystroglycan act at the basal side of the Drosophila follicular epithelium to maintain epithelial organization. Dystroglycan (Dg) is a widely expressed **extracellular matrix** (ECM) receptor required for muscle viability, synaptogenesis, basementmembrane formation and epithelial development. As an integral component of the Dystrophin-associated glycoprotein complex, Dg plays a central role in linking the ECM and the cytoskeleton. Disruption of this linkage in skeletal muscle leads to various types of muscular dystrophies. In epithelial cells, reduced expression of Dg is associated with increased invasiveness of cancer cells. We have previously shown that Dg is required for epithelial cell polarity in Drosophila, but the mechanisms of this polarizing activity and upstream/downstream components are largely unknown. Using the Drosophila follicle-cell epithelium (FCE) as a model system, we show that the ECM molecule Perlecan (Pcan) is required for maintenance of epithelial-cell polarity. Follicle cells that lack Pcan develop polarity defects similar to those of Dg mutant cells. Furthermore, Dg depends on Pcan but not on Laminin A for its localization in the basal-cell membrane, and the two proteins bind in vitro. Interestingly, the Dg form that interacts with Pcan in the FCE lacks the mucin-like domain, which is thought to be essential for Dg ligand binding activity. Finally, we describe two examples of how Dg promotes the differentiation of the basal membrane domain: (1) by recruiting/anchoring the cytoplasmic protein Dystrophin; and (2) by excluding the transmembrane protein Neurexin. We suggest that the interaction of Pcan and Dg at the basal side of the epithelium promotes basal membrane differentiation and is required for maintenance of cell polarity in the FCE.
2. Organization and function of the blood-brain barrier in Drosophila. The function of a complex nervous system depends on an intricate interplay between neuronal and glial cell types. One of the many functions of glial cells is to provide an efficient insulation of the nervous system and thereby allowing a fine tuned homeostasis of ions and other small molecules. Here, we present a detailed cellular analysis of the glial cell complement constituting the blood-brain barrier in Drosophila. Using electron microscopic analysis and single cell-labeling experiments, we characterize different glial cell layers at the surface of the nervous system, the perineurial glial layer, the subperineurial glial layer, the wrapping glial cell layer, and a thick layer of **extracellular matrix**, the neural lamella. To test the functional roles of these sheaths we performed a series of dye penetration experiments in the nervous systems of wild-type and mutant embryos. Comparing the kinetics of uptake of different sized fluorescently labeled dyes in different mutants allowed to conclude that most of the barrier function is mediated by the septate junctions formed by the subperineurial cells, whereas the perineurial glial cell layer and the neural lamella contribute to barrier selectivity against much larger particles (i.e., the size of proteins). We further compare the requirements of different septate junction components for the integrity of the blood-brain barrier and provide evidence that two of the six Claudin-like proteins found in Drosophila are needed for normal blood-brain barrier function.

### [Ii]nvasive

#### PubMed

1. Perlecan and Dystroglycan act at the basal side of the Drosophila follicular epithelium to maintain epithelial organization. Dystroglycan (Dg) is a widely expressed extracellular matrix (ECM) receptor required for muscle viability, synaptogenesis, basementmembrane formation and epithelial development. As an integral component of the Dystrophin-associated glycoprotein complex, Dg plays a central role in linking the ECM and the cytoskeleton. Disruption of this linkage in skeletal muscle leads to various types of muscular dystrophies. In epithelial cells, reduced expression of Dg is associated with increased **invasive**ness of cancer cells. We have previously shown that Dg is required for epithelial cell polarity in Drosophila, but the mechanisms of this polarizing activity and upstream/downstream components are largely unknown. Using the Drosophila follicle-cell epithelium (FCE) as a model system, we show that the ECM molecule Perlecan (Pcan) is required for maintenance of epithelial-cell polarity. Follicle cells that lack Pcan develop polarity defects similar to those of Dg mutant cells. Furthermore, Dg depends on Pcan but not on Laminin A for its localization in the basal-cell membrane, and the two proteins bind in vitro. Interestingly, the Dg form that interacts with Pcan in the FCE lacks the mucin-like domain, which is thought to be essential for Dg ligand binding activity. Finally, we describe two examples of how Dg promotes the differentiation of the basal membrane domain: (1) by recruiting/anchoring the cytoplasmic protein Dystrophin; and (2) by excluding the transmembrane protein Neurexin. We suggest that the interaction of Pcan and Dg at the basal side of the epithelium promotes basal membrane differentiation and is required for maintenance of cell polarity in the FCE.

### [Mm]etastasis

#### PubMed

1. Analysis of cell migration using whole-genome expression profiling of migratory cells in the Drosophila ovary. Cell migration contributes to normal development and homeostasis as well as to pathological processes such as inflammation and tumor **metastasis**. Previous genetic screens have revealed signaling pathways that govern follicle cell migrations in the Drosophila ovary, but few downstream targets of the critical transcriptional regulators have been identified. To characterize the gene expression profile of two migratory cell populations and identify Slbo targets, we purified border cells and centripetal cells expressing the mouse CD8 antigen and carried out whole-genome microarray analysis. Genes predicted to control actin dynamics and the endocytic and secretory pathways were overrepresented in the migratory cell transcriptome. Mutations in five genes, including ttk, failed to complement previously isolated mutations that cause cell migration defects in mosaic clones. Functional analysis revealed a role for the Notch-activating protease Kuzbanian in border cell migration and identified Tie as a guidance receptor for the border cells.

---

## Sec24CD

- NCBI Gene
- GeneRIF
- Pubmed

| Search Term | Rank | Fields |
| --- | --- | --- |
| ECM | 7 | PubMed(1) |
| [Ee]xtracellular [Mm]atrix | 8 | Process(1); PubMed(1) |

  
hide/show details for Sec24CD

### ECM

#### PubMed

1. Trafficking through COPII stabilises cell polarity and drives secretion during Drosophila epidermal differentiation. The differentiation of an extracellular matrix (**ECM**) at the apical side of epithelial cells implies massive polarised secretion and membrane trafficking. An epithelial cell is hence engaged in coordinating secretion and cell polarity for a correct and efficient **ECM** formation.We are studying the molecular mechanisms that Drosophila tracheal and epidermal cells deploy to form their specific apical **ECM** during differentiation. In this work we demonstrate that the two genetically identified factors haunted and ghost are essential for polarity maintenance, membrane topology as well as for secretion of the tracheal luminal matrix and the cuticle. We show that they code for the Drosophila COPII vesicle-coating components Sec23 and Sec24, respectively, that organise vesicle transport from the ER to the Golgi apparatus.Taken together, epithelial differentiation during Drosophila embryogenesis is a concerted action of **ECM** formation, plasma membrane remodelling and maintenance of cell polarity that all three rely mainly, if not absolutely, on the canonical secretory pathway from the ER over the Golgi apparatus to the plasma membrane. Our results indicate that COPII vesicles constitute a central hub for these processes.

### [Ee]xtracellular [Mm]atrix

#### Process

1. positive regulation of **extracellular matrix** constituent secretion

#### PubMed

1. Trafficking through COPII stabilises cell polarity and drives secretion during Drosophila epidermal differentiation. The differentiation of an **extracellular matrix** (ECM) at the apical side of epithelial cells implies massive polarised secretion and membrane trafficking. An epithelial cell is hence engaged in coordinating secretion and cell polarity for a correct and efficient ECM formation.We are studying the molecular mechanisms that Drosophila tracheal and epidermal cells deploy to form their specific apical ECM during differentiation. In this work we demonstrate that the two genetically identified factors haunted and ghost are essential for polarity maintenance, membrane topology as well as for secretion of the tracheal luminal matrix and the cuticle. We show that they code for the Drosophila COPII vesicle-coating components Sec23 and Sec24, respectively, that organise vesicle transport from the ER to the Golgi apparatus.Taken together, epithelial differentiation during Drosophila embryogenesis is a concerted action of ECM formation, plasma membrane remodelling and maintenance of cell polarity that all three rely mainly, if not absolutely, on the canonical secretory pathway from the ER over the Golgi apparatus to the plasma membrane. Our results indicate that COPII vesicles constitute a central hub for these processes.

---

## S6k

- NCBI Gene
- GeneRIF
- Pubmed

| Search Term | Rank | Fields |
| --- | --- | --- |
| [Ff]ilopodia | 1 | PubMed(1) |
| [Mm]igration | 6 | PubMed(5) |
| [Ii]nvasive | 10 | PubMed(1) |
| [Mm]etastasis | 12 | PubMed(1) |

  
hide/show details for S6k

### [Ff]ilopodia

#### PubMed

1. Gene regulatory networks controlling hematopoietic progenitor niche cell production and differentiation in the Drosophila lymph gland. Hematopoiesis occurs in two phases in Drosophila, with the first completed during embryogenesis and the second accomplished during larval development. The lymph gland serves as the venue for the final hematopoietic program, with this larval tissue well-studied as to its cellular organization and genetic regulation. While the medullary zone contains stem-like hematopoietic progenitors, the posterior signaling center (PSC) functions as a niche microenvironment essential for controlling the decision between progenitor maintenance versus cellular differentiation. In this report, we utilize a PSC-specific GAL4 driver and UAS-gene RNAi strains, to selectively knockdown individual gene functions in PSC cells. We assessed the effect of abrogating the function of 820 genes as to their requirement for niche cell production and differentiation. 100 genes were shown to be essential for normal niche development, with various loci placed into sub-groups based on the functions of their encoded protein products and known genetic interactions. For members of three of these groups, we characterized loss- and gain-of-function phenotypes. Gene function knockdown of members of the BAP chromatin-remodeling complex resulted in niche cells that do not express the hedgehog (hh) gene and fail to differentiate **filopodia** believed important for Hh signaling from the niche to progenitors. Abrogating gene function of various members of the insulin-like growth factor and TOR signaling pathways resulted in anomalous PSC cell production, leading to a defective niche organization. Further analysis of the Pten, TSC1, and TSC2 tumor suppressor genes demonstrated their loss-of-function condition resulted in severely altered blood cell homeostasis, including the abundant production of lamellocytes, specialized hemocytes involved in innate immune responses. Together, this cell-specific RNAi knockdown survey and mutant phenotype analyses identified multiple genes and their regulatory networks required for the normal organization and function of the hematopoietic progenitor niche within the lymph gland.

### [Mm]igration

#### PubMed

1. A drosophila model for EGFR-Ras and PI3K-dependent human glioma. Gliomas, the most common malignant tumors of the nervous system, frequently harbor mutations that activate the epidermal growth factor receptor (EGFR) and phosphatidylinositol-3 kinase (PI3K) signaling pathways. To investigate the genetic basis of this disease, we developed a glioma model in Drosophila. We found that constitutive coactivation of EGFR-Ras and PI3K pathways in Drosophila glia and glial precursors gives rise to neoplastic, invasive glial cells that create transplantable tumor-like growths, mimicking human glioma. Our model represents a robust organotypic and cell-type-specific Drosophila cancer model in which malignant cells are created by mutations in signature genes and pathways thought to be driving forces in a homologous human cancer. Genetic analyses demonstrated that EGFR and PI3K initiate malignant neoplastic transformation via a combinatorial genetic network composed primarily of other pathways commonly mutated or activated in human glioma, including the Tor, Myc, G1 Cyclins-Cdks, and Rb-E2F pathways. This network acts synergistically to coordinately stimulate cell cycle entry and progression, protein translation, and inappropriate cellular growth and **migration**. In particular, we found that the fly orthologs of CyclinE, Cdc25, and Myc are key rate-limiting genes required for glial neoplasia. Moreover, orthologs of Sin1, Rictor, and Cdk4 are genes required only for abnormal neoplastic glial proliferation but not for glial development. These and other genes within this network may represent important therapeutic targets in human glioma.
2. A functional RNAi screen for regulators of receptor tyrosine kinase and ERK signalling. Receptor tyrosine kinase (RTK) signalling through extracellular-signal-regulated kinases (ERKs) has pivotal roles during metazoan development, underlying processes as diverse as fate determination, differentiation, proliferation, survival, **migration** and growth. Abnormal RTK/ERK signalling has been extensively documented to contribute to developmental disorders and disease, most notably in oncogenic transformation by mutant RTKs or downstream pathway components such as Ras and Raf. Although the core RTK/ERK signalling cassette has been characterized by decades of research using mammalian cell culture and forward genetic screens in model organisms, signal propagation through this pathway is probably regulated by a larger network of moderate, context-specific proteins. The genes encoding these proteins may not have been discovered through traditional screens owing, in particular, to the requirement for visible phenotypes. To obtain a global view of RTK/ERK signalling, we performed an unbiased, RNA interference (RNAi), genome-wide, high-throughput screen in Drosophila cells using a novel, quantitative, cellular assay monitoring ERK activation. Here we show that ERK pathway output integrates a wide array of conserved cellular processes. Further analysis of selected components-in multiple cell types with different RTK ligands and oncogenic stimuli-validates and classifies 331 pathway regulators. The relevance of these genes is highlighted by our isolation of a Ste20-like kinase and a PPM-family phosphatase that seem to regulate RTK/ERK signalling in vivo and in mammalian cells. Novel regulators that modulate specific pathway outputs may be selective targets for drug discovery.
3. Centrosomal kinase Nek2 cooperates with oncogenic pathways to promote metastasis. Centrosomal kinase Nek2 is overexpressed in different cancers, yet how it contributes toward tumorigenesis remains poorly understood. dNek2 overexpression in a Drosophila melanogaster model led to upregulation of Drosophila Wnt ortholog wingless (Wg), and alteration of cell **migration** markers-Rho1, Rac1 and E-cadherin (Ecad)-resulting in changes in cell shape and tissue morphogenesis. dNek2 overexpression cooperated with receptor tyrosine kinase and mitogen-activated protein kinase signaling to upregulate activated Akt, Diap1, Mmp1 and Wg protein to promote local invasion, distant seeding and metastasis. In tumor cell injection assays, dNek2 cooperated with Ras and Src signaling to promote aggressive colonization of tumors into different adult fly tissues. Inhibition of the PI3K pathway suppressed the cooperation of dNek2 with other growth pathways. Consistent with our fly studies, overexpression of human Nek2 in A549 lung adenocarcinoma and HEK293T cells led to activation of the Akt pathway and increase in β-catenin protein levels. Our computational approach identified a class of Nek2-inhibitory compounds and a novel drug-like pharmacophore that reversed the Nek2 overexpression phenotypes in flies and human cells. Our finding posits a novel role for Nek2 in promoting metastasis in addition to its currently defined role in promoting chromosomal instability. It provides a rationale for the selective advantage of centrosome amplification in cancer.
4. A core transcriptional network for early mesoderm development in Drosophila melanogaster. Embryogenesis is controlled by large gene-regulatory networks, which generate spatially and temporally refined patterns of gene expression. Here, we report the characteristics of the regulatory network orchestrating early mesodermal development in the fruitfly Drosophila, where the transcription factor Twist is both necessary and sufficient to drive development. Through the integration of chromatin immunoprecipitation followed by microarray analysis (ChIP-on-chip) experiments during discrete time periods with computational approaches, we identified >2000 Twist-bound cis-regulatory modules (CRMs) and almost 500 direct target genes. Unexpectedly, Twist regulates an almost complete cassette of genes required for cell proliferation in addition to genes essential for morophogenesis and cell **migration**. Twist targets almost 25% of all annotated Drosophila transcription factors, which may represent the entire set of regulators necessary for the early development of this system. By combining in vivo binding data from Twist, Mef2, Tinman, and Dorsal we have constructed an initial transcriptional network of early mesoderm development. The network topology reveals extensive combinatorial binding, feed-forward regulation, and complex logical outputs as prevalent features. In addition to binary activation and repression, we suggest that Twist binds to almost all mesodermal CRMs to provide the competence to integrate inputs from more specialized transcription factors.
5. Drosophila tribbles antagonizes insulin signaling-mediated growth and metabolism via interactions with Akt kinase. Drosophila Tribbles (Trbl) is the founding member of the Trib family of kinase-like docking proteins that modulate cell signaling during proliferation, **migration** and growth. In a wing misexpression screen for Trbl interacting proteins, we identified the Ser/Thr protein kinase Akt1. Given the central role of Akt1 in insulin signaling, we tested the function of Trbl in larval fat body, a tissue where rapid increases in size are exquisitely sensitive to insulin/insulin-like growth factor levels. Consistent with a role in antagonizing insulin-mediated growth, trbl RNAi knockdown in the fat body increased cell size, advanced the timing of pupation and increased levels of circulating triglyceride. Complementarily, overexpression of Trbl reduced fat body cell size, decreased overall larval size, delayed maturation and lowered levels of triglycerides, while circulating glucose levels increased. The conserved Trbl kinase domain is required for function in vivo and for interaction with Akt in a yeast two-hybrid assay. Consistent with direct regulation of Akt, overexpression of Trbl in the fat body decreased levels of activated Akt (pSer505-Akt) while misexpression of trbl RNAi increased phospho-Akt levels, and neither treatment affected total Akt levels. Trbl misexpression effectively suppressed Akt-mediated wing and muscle cell size increases and reduced phosphorylation of the Akt target FoxO (pSer256-FoxO). Taken together, these data show that Drosophila Trbl has a conserved role to bind Akt and block Akt-mediated insulin signaling, and implicate Trib proteins as novel sites of signaling pathway integration that link nutrient availability with cell growth and proliferation.

### [Ii]nvasive

#### PubMed

1. A drosophila model for EGFR-Ras and PI3K-dependent human glioma. Gliomas, the most common malignant tumors of the nervous system, frequently harbor mutations that activate the epidermal growth factor receptor (EGFR) and phosphatidylinositol-3 kinase (PI3K) signaling pathways. To investigate the genetic basis of this disease, we developed a glioma model in Drosophila. We found that constitutive coactivation of EGFR-Ras and PI3K pathways in Drosophila glia and glial precursors gives rise to neoplastic, **invasive** glial cells that create transplantable tumor-like growths, mimicking human glioma. Our model represents a robust organotypic and cell-type-specific Drosophila cancer model in which malignant cells are created by mutations in signature genes and pathways thought to be driving forces in a homologous human cancer. Genetic analyses demonstrated that EGFR and PI3K initiate malignant neoplastic transformation via a combinatorial genetic network composed primarily of other pathways commonly mutated or activated in human glioma, including the Tor, Myc, G1 Cyclins-Cdks, and Rb-E2F pathways. This network acts synergistically to coordinately stimulate cell cycle entry and progression, protein translation, and inappropriate cellular growth and migration. In particular, we found that the fly orthologs of CyclinE, Cdc25, and Myc are key rate-limiting genes required for glial neoplasia. Moreover, orthologs of Sin1, Rictor, and Cdk4 are genes required only for abnormal neoplastic glial proliferation but not for glial development. These and other genes within this network may represent important therapeutic targets in human glioma.

### [Mm]etastasis

#### PubMed

1. Centrosomal kinase Nek2 cooperates with oncogenic pathways to promote **metastasis**. Centrosomal kinase Nek2 is overexpressed in different cancers, yet how it contributes toward tumorigenesis remains poorly understood. dNek2 overexpression in a Drosophila melanogaster model led to upregulation of Drosophila Wnt ortholog wingless (Wg), and alteration of cell migration markers-Rho1, Rac1 and E-cadherin (Ecad)-resulting in changes in cell shape and tissue morphogenesis. dNek2 overexpression cooperated with receptor tyrosine kinase and mitogen-activated protein kinase signaling to upregulate activated Akt, Diap1, Mmp1 and Wg protein to promote local invasion, distant seeding and **metastasis**. In tumor cell injection assays, dNek2 cooperated with Ras and Src signaling to promote aggressive colonization of tumors into different adult fly tissues. Inhibition of the PI3K pathway suppressed the cooperation of dNek2 with other growth pathways. Consistent with our fly studies, overexpression of human Nek2 in A549 lung adenocarcinoma and HEK293T cells led to activation of the Akt pathway and increase in β-catenin protein levels. Our computational approach identified a class of Nek2-inhibitory compounds and a novel drug-like pharmacophore that reversed the Nek2 overexpression phenotypes in flies and human cells. Our finding posits a novel role for Nek2 in promoting **metastasi**s in addition to its currently defined role in promoting chromosomal instability. It provides a rationale for the selective advantage of centrosome amplification in cancer.

---

## fry

- NCBI Gene
- GeneRIF
- Pubmed

| Search Term | Rank | Fields |
| --- | --- | --- |
| ECM | 7 | PubMed(2) |
| [Ee]xtracellular [Mm]atrix | 8 | PubMed(2) |

  
hide/show details for fry

### ECM

#### PubMed

1. Epidermis-Derived Semaphorin Promotes Dendrite Self-Avoidance by Regulating Dendrite-Substrate Adhesion in Drosophila Sensory Neurons. Precise patterning of dendritic arbors is critical for the wiring and function of neural circuits. Dendrite-extracellular matrix (**ECM**) adhesion ensures that the dendrites of Drosophila dendritic arborization (da) sensory neurons are properly restricted in a 2D space, and thereby facilitates contact-mediated dendritic self-avoidance and tiling. However, the mechanisms regulating dendrite-**ECM** adhesion in vivo are poorly understood. Here, we show that mutations in the semaphorin ligand sema-2b lead to a dramatic increase in self-crossing of dendrites due to defects in dendrite-**ECM** adhesion, resulting in a failure to confine dendrites to a 2D plane. Furthermore, we find that Sema-2b is secreted from the epidermis and signals through the Plexin B receptor in neighboring neurons. Importantly, we find that Sema-2b/PlexB genetically and physically interacts with TORC2 complex, Tricornered (Trc) kinase, and integrins. These results reveal a novel role for semaphorins in dendrite patterning and illustrate how epidermal-derived cues regulate neural circuit assembly.
2. Integrins regulate repulsion-mediated dendritic patterning of drosophila sensory neurons by restricting dendrites in a 2D space. Dendrites of the same neuron usually avoid each other. Some neurons also repel similar neurons through dendrite-dendrite interaction to tile the receptive field. Nonoverlapping coverage based on such contact-dependent repulsion requires dendrites to compete for limited space. Here we show that Drosophila class IV dendritic arborization (da) neurons, which tile the larval body wall, grow their dendrites mainly in a 2D space on the extracellular matrix (**ECM**) secreted by the epidermis. Removing neuronal integrins or blocking epidermal laminin production causes dendrites to grow into the epidermis, suggesting that integrin-laminin interaction attaches dendrites to the **ECM**. We further show that some of the previously identified tiling mutants fail to confine dendrites in a 2D plane. Expansion of these mutant dendrites in three dimensions results in overlap of dendritic fields. Moreover, overexpression of integrins in these mutant neurons effectively reduces dendritic crossing and restores tiling, revealing an additional mechanism for tiling.

### [Ee]xtracellular [Mm]atrix

#### PubMed

1. Epidermis-Derived Semaphorin Promotes Dendrite Self-Avoidance by Regulating Dendrite-Substrate Adhesion in Drosophila Sensory Neurons. Precise patterning of dendritic arbors is critical for the wiring and function of neural circuits. Dendrite-**extracellular matrix** (ECM) adhesion ensures that the dendrites of Drosophila dendritic arborization (da) sensory neurons are properly restricted in a 2D space, and thereby facilitates contact-mediated dendritic self-avoidance and tiling. However, the mechanisms regulating dendrite-ECM adhesion in vivo are poorly understood. Here, we show that mutations in the semaphorin ligand sema-2b lead to a dramatic increase in self-crossing of dendrites due to defects in dendrite-ECM adhesion, resulting in a failure to confine dendrites to a 2D plane. Furthermore, we find that Sema-2b is secreted from the epidermis and signals through the Plexin B receptor in neighboring neurons. Importantly, we find that Sema-2b/PlexB genetically and physically interacts with TORC2 complex, Tricornered (Trc) kinase, and integrins. These results reveal a novel role for semaphorins in dendrite patterning and illustrate how epidermal-derived cues regulate neural circuit assembly.
2. Integrins regulate repulsion-mediated dendritic patterning of drosophila sensory neurons by restricting dendrites in a 2D space. Dendrites of the same neuron usually avoid each other. Some neurons also repel similar neurons through dendrite-dendrite interaction to tile the receptive field. Nonoverlapping coverage based on such contact-dependent repulsion requires dendrites to compete for limited space. Here we show that Drosophila class IV dendritic arborization (da) neurons, which tile the larval body wall, grow their dendrites mainly in a 2D space on the **extracellular matrix** (ECM) secreted by the epidermis. Removing neuronal integrins or blocking epidermal laminin production causes dendrites to grow into the epidermis, suggesting that integrin-laminin interaction attaches dendrites to the ECM. We further show that some of the previously identified tiling mutants fail to confine dendrites in a 2D plane. Expansion of these mutant dendrites in three dimensions results in overlap of dendritic fields. Moreover, overexpression of integrins in these mutant neurons effectively reduces dendritic crossing and restores tiling, revealing an additional mechanism for tiling.

---

## sowah

- NCBI Gene
- GeneRIF
- Pubmed

| Search Term | Rank | Fields |
| --- | --- | --- |
| [Mm]igration | 6 | PubMed(1) |
| ECM | 7 | PubMed(1) |
| [Ee]xtracellular [Mm]atrix | 8 | PubMed(1) |

  
hide/show details for sowah

### [Mm]igration

#### PubMed

1. Genes conserved in bilaterians but jointly lost with Myc during nematode evolution are enriched in cell proliferation and cell **migration** functions. Animals use a stereotypical set of developmental genes to build body architectures of varying sizes and organizational complexity. Some genes are critical to developmental patterning, while other genes are important to physiological control of growth. However, growth regulator genes may not be as important in small-bodied "micro-metazoans" such as nematodes. Nematodes use a simplified developmental strategy of lineage-based cell fate specifications to produce an adult bilaterian body composed of a few hundreds of cells. Nematodes also lost the MYC proto-oncogenic regulator of cell proliferation. To identify additional regulators of cell proliferation that were lost with MYC, we computationally screened and determined 839 high-confidence genes that are conserved in bilaterians/lost in nematodes (CIBLIN genes). We find that 30 % of all CIBLIN genes encode transcriptional regulators of cell proliferation, epithelial-to-mesenchyme transitions, and other processes. Over 50 % of CIBLIN genes are unnamed genes in Drosophila, suggesting that there are many understudied genes. Interestingly, CIBLIN genes include many Myc synthetic lethal (MycSL) hits from recent screens. CIBLIN genes include key regulators of heparan sulfate proteoglycan (HSPG) sulfation patterns, and lysyl oxidases involved in cross-linking and modification of the extracellular matrix (ECM). These genes and others suggest the CIBLIN repertoire services critical functions in ECM remodeling and cell **migration** in large-bodied bilaterians. Correspondingly, CIBLIN genes are co-expressed with Myc in cancer transcriptomes, and include a preponderance of known determinants of cancer progression and tumor aggression. We propose that CIBLIN gene research can improve our understanding of regulatory control of cellular growth in metazoans.

### ECM

#### PubMed

1. Genes conserved in bilaterians but jointly lost with Myc during nematode evolution are enriched in cell proliferation and cell migration functions. Animals use a stereotypical set of developmental genes to build body architectures of varying sizes and organizational complexity. Some genes are critical to developmental patterning, while other genes are important to physiological control of growth. However, growth regulator genes may not be as important in small-bodied "micro-metazoans" such as nematodes. Nematodes use a simplified developmental strategy of lineage-based cell fate specifications to produce an adult bilaterian body composed of a few hundreds of cells. Nematodes also lost the MYC proto-oncogenic regulator of cell proliferation. To identify additional regulators of cell proliferation that were lost with MYC, we computationally screened and determined 839 high-confidence genes that are conserved in bilaterians/lost in nematodes (CIBLIN genes). We find that 30 % of all CIBLIN genes encode transcriptional regulators of cell proliferation, epithelial-to-mesenchyme transitions, and other processes. Over 50 % of CIBLIN genes are unnamed genes in Drosophila, suggesting that there are many understudied genes. Interestingly, CIBLIN genes include many Myc synthetic lethal (MycSL) hits from recent screens. CIBLIN genes include key regulators of heparan sulfate proteoglycan (HSPG) sulfation patterns, and lysyl oxidases involved in cross-linking and modification of the extracellular matrix (**ECM**). These genes and others suggest the CIBLIN repertoire services critical functions in **ECM** remodeling and cell migration in large-bodied bilaterians. Correspondingly, CIBLIN genes are co-expressed with Myc in cancer transcriptomes, and include a preponderance of known determinants of cancer progression and tumor aggression. We propose that CIBLIN gene research can improve our understanding of regulatory control of cellular growth in metazoans.

### [Ee]xtracellular [Mm]atrix

#### PubMed

1. Genes conserved in bilaterians but jointly lost with Myc during nematode evolution are enriched in cell proliferation and cell migration functions. Animals use a stereotypical set of developmental genes to build body architectures of varying sizes and organizational complexity. Some genes are critical to developmental patterning, while other genes are important to physiological control of growth. However, growth regulator genes may not be as important in small-bodied "micro-metazoans" such as nematodes. Nematodes use a simplified developmental strategy of lineage-based cell fate specifications to produce an adult bilaterian body composed of a few hundreds of cells. Nematodes also lost the MYC proto-oncogenic regulator of cell proliferation. To identify additional regulators of cell proliferation that were lost with MYC, we computationally screened and determined 839 high-confidence genes that are conserved in bilaterians/lost in nematodes (CIBLIN genes). We find that 30 % of all CIBLIN genes encode transcriptional regulators of cell proliferation, epithelial-to-mesenchyme transitions, and other processes. Over 50 % of CIBLIN genes are unnamed genes in Drosophila, suggesting that there are many understudied genes. Interestingly, CIBLIN genes include many Myc synthetic lethal (MycSL) hits from recent screens. CIBLIN genes include key regulators of heparan sulfate proteoglycan (HSPG) sulfation patterns, and lysyl oxidases involved in cross-linking and modification of the **extracellular matrix** (ECM). These genes and others suggest the CIBLIN repertoire services critical functions in ECM remodeling and cell migration in large-bodied bilaterians. Correspondingly, CIBLIN genes are co-expressed with Myc in cancer transcriptomes, and include a preponderance of known determinants of cancer progression and tumor aggression. We propose that CIBLIN gene research can improve our understanding of regulatory control of cellular growth in metazoans.

---

## Ptp99A

- NCBI Gene
- GeneRIF
- Pubmed

| Search Term | Rank | Fields |
| --- | --- | --- |
| [Mm]igration | 6 | PubMed(2) |
| [Ee]xtracellular [Mm]atrix | 8 | PubMed(2) |

  
hide/show details for Ptp99A

### [Mm]igration

#### PubMed

1. Nemo regulates cell dynamics and represses the expression of miple, a midkine/pleiotrophin cytokine, during ommatidial rotation. Ommatidial rotation is one of the most important events for correct patterning of the Drosophila eye. Although several signaling pathways are involved in this process, few genes have been shown to specifically affect it. One of them is nemo (nmo), which encodes a MAP-like protein kinase that regulates the rate of rotation throughout the entire process, and serves as a link between core planar cell polarity (PCP) factors and the E-cadherin-β-catenin complex. To determine more precisely the role of nmo in ommatidial rotation, live-imaging analyses in nmo mutant and wild-type early pupal eye discs were performed. We demonstrate that ommatidial rotation is not a continuous process, and that rotating and non-rotating interommatidial cells are very dynamic. Our in vivo analyses also show that nmo regulates the speed of rotation and is required in cone cells for correct ommatidial rotation, and that these cells as well as interommatidial cells are less dynamic in nmo mutants. Furthermore, microarray analyses of nmo and wild-type larval eye discs led us to identify new genes and signaling pathways related to nmo function during this process. One of them, miple, encodes the Drosophila ortholog of the midkine/pleiotrophin secreted cytokines that are involved in cell **migratio**n processes. miple is highly up-regulated in nmo mutant discs. Indeed, phenotypic analyses reveal that miple overexpression leads to ommatidial rotation defects. Genetic interaction assays suggest that miple is signaling through Ptp99A, the Drosophila ortholog of the vertebrate midkine/pleiotrophin PTPζ receptor. Accordingly, we propose that one of the roles of Nmo during ommatial rotation is to repress miple expression, which may in turn affect the dynamics in E-cadherin-β-catenin complexes.
2. Ectopic expression screen identifies genes affecting Drosophila mesoderm development including the HSPG Trol. Gastrulation of the embryo involves coordinate cell movements likely supported by multiple signaling pathways, adhesion molecules, and extracellular matrix components. Fibroblast growth factors (FGFs) have a major role in Drosophila melanogaster mesoderm **migration**; however, few other inputs are known and the mechanism supporting cell movement is unclear. To provide insight, we performed an ectopic expression screen to identify secreted or membrane-associated molecules that act to support mesoderm **migration**. Twenty-four UAS insertions were identified that cause lethality when expressed in either the mesoderm (Twi-Gal4) or the ectoderm (69B-Gal4). The list was narrowed to a subset of 10 genes that were shown to exhibit loss-of-function mutant phenotypes specifically affecting mesoderm **migration**. These include the FGF ligand Pyramus, α-integrins, E-cadherin, Cueball, EGFR, JAK/STAT signaling components, as well as the heparan sulfate proteoglycan (HSPG) Terribly reduced optic lobes (Trol). Trol encodes the ortholog of mammalian HSPG Perlecan, a demonstrated FGF signaling cofactor. Here, we examine the role of Trol in Drosophila mesoderm **migratio**n and compare and contrast its role with that of Syndecan (Sdc), another HSPG previously implicated in this process. Embryos mutant for Trol or Sdc were obtained and analyzed. Our data support the view that both HSPGs function to support FGF-dependent processes in the early embryo as they share phenotypes with FGF mutants: Trol in terms of effects on mesoderm **migratio**n and caudal visceral mesoderm (CVM) **migratio**n and Sdc in terms of dorsal mesoderm specification. The differential roles uncovered for these two HSPGs suggest that HSPG cofactor choice may modify FGF-signaling outputs.

### [Ee]xtracellular [Mm]atrix

#### PubMed

1. Protein tyrosine phosphatases and neural development. During neural development, cells interact dynamically with each other and with the **extracellular matrix**, using cell signaling to control differentiation, axonogenesis, and survival. Enzymes that regulate protein tyrosine phosphorylation often lie at the core of such cell signaling. Protein tyrosine phosphatases (PTPases) are recognized as being of central importance here, and a growing family of PTPases are now known to be expressed in embryonic neurons and glia. Both receptor-like and cytoplasmic enzymes have been identified. The receptor family includes immunoglobulin superfamily members that influence cell-cell adhesion, proteoglycans that control neurite growth, and enzymes in Drosophila that regulate axon guidance and target cell recognition. Cytoplasmic PTPases are implicated in nerve cell commitment and potentially in the regulation of cell survival. This review outlines what we currently know about PTPases in the nervous system and presents concepts concerning their possible modes of action.
2. Ectopic expression screen identifies genes affecting Drosophila mesoderm development including the HSPG Trol. Gastrulation of the embryo involves coordinate cell movements likely supported by multiple signaling pathways, adhesion molecules, and **extracellular matrix** components. Fibroblast growth factors (FGFs) have a major role in Drosophila melanogaster mesoderm migration; however, few other inputs are known and the mechanism supporting cell movement is unclear. To provide insight, we performed an ectopic expression screen to identify secreted or membrane-associated molecules that act to support mesoderm migration. Twenty-four UAS insertions were identified that cause lethality when expressed in either the mesoderm (Twi-Gal4) or the ectoderm (69B-Gal4). The list was narrowed to a subset of 10 genes that were shown to exhibit loss-of-function mutant phenotypes specifically affecting mesoderm migration. These include the FGF ligand Pyramus, α-integrins, E-cadherin, Cueball, EGFR, JAK/STAT signaling components, as well as the heparan sulfate proteoglycan (HSPG) Terribly reduced optic lobes (Trol). Trol encodes the ortholog of mammalian HSPG Perlecan, a demonstrated FGF signaling cofactor. Here, we examine the role of Trol in Drosophila mesoderm migration and compare and contrast its role with that of Syndecan (Sdc), another HSPG previously implicated in this process. Embryos mutant for Trol or Sdc were obtained and analyzed. Our data support the view that both HSPGs function to support FGF-dependent processes in the early embryo as they share phenotypes with FGF mutants: Trol in terms of effects on mesoderm migration and caudal visceral mesoderm (CVM) migration and Sdc in terms of dorsal mesoderm specification. The differential roles uncovered for these two HSPGs suggest that HSPG cofactor choice may modify FGF-signaling outputs.

---

## CG12772

- NCBI Gene
- GeneRIF
- Pubmed

| Search Term | Rank | Fields |
| --- | --- | --- |
| [Mm]igration | 6 | PubMed(1) |

  
hide/show details for CG12772

### [Mm]igration

#### PubMed

1. Some novel intron positions in conserved Drosophila genes are caused by intron sliding or tandem duplication. Positions of spliceosomal introns are often conserved between remotely related genes. Introns that reside in non-conserved positions are either novel or remnants of frequent losses of introns in some evolutionary lineages. A recent gain of such introns is difficult to prove. However, introns verified as novel are needed to evaluate contemporary processes of intron gain.We identified 25 unambiguous cases of novel intron positions in 31 Drosophila genes that exhibit near intron pairs (NIPs). Here, a NIP consists of an ancient and a novel intron position that are separated by less than 32 nt. Within a single gene, such closely-spaced introns are very unlikely to have coexisted. In most cases, therefore, the ancient intron position must have disappeared in favour of the novel one. A survey for NIPs among 12 Drosophila genomes identifies intron sliding (**migration**) as one of the more frequent causes of novel intron positions. Other novel introns seem to have been gained by regional tandem duplications of coding sequences containing a proto-splice site.Recent intron gains sometimes appear to have arisen by duplication of exonic sequences and subsequent intronization of one of the copies. Intron **migration** and exon duplication together may account for a significant amount of novel intron positions in conserved coding sequences.

---

## mfrn

- NCBI Gene
- GeneRIF
- Pubmed

| Search Term | Rank | Fields |
| --- | --- | --- |
| [Mm]igration | 6 | PubMed(1) |

  
hide/show details for mfrn

### [Mm]igration

#### PubMed

1. Gain-of-function screen for genes that affect Drosophila muscle pattern formation. This article reports the production of an EP-element insertion library with more than 3,700 unique target sites within the Drosophila melanogaster genome and its use to systematically identify genes that affect embryonic muscle pattern formation. We designed a UAS/GAL4 system to drive GAL4-responsive expression of the EP-targeted genes in developing apodeme cells to which migrating myotubes finally attach and in an intrasegmental pattern of cells that serve myotubes as a **migration** substrate on their way towards the apodemes. The results suggest that misexpression of more than 1.5% of the Drosophila genes can interfere with proper myotube guidance and/or muscle attachment. In addition to factors already known to participate in these processes, we identified a number of enzymes that participate in the synthesis or modification of protein carbohydrate side chains and in Ubiquitin modifications and/or the Ubiquitin-dependent degradation of proteins, suggesting that these processes are relevant for muscle pattern formation.

---

## vvl

- NCBI Gene
- GeneRIF
- Pubmed

| Search Term | Rank | Fields |
| --- | --- | --- |
| [Mm]igration | 6 | PubMed(16) |
| [Ee]xtracellular [Mm]atrix | 8 | PubMed(1) |

  
hide/show details for vvl

### [Mm]igration

#### PubMed

1. EGF signalling regulates cell invagination as well as cell **migration** during formation of tracheal system in Drosophila. The Drosophila tracheal system is a network of epithelial tubes that arises from the tracheal placodes, lateral clusters of ectodermal cells in ten embryonic segments. The cells of each cluster invaginate and subsequent formation of the tracheal tree occurs by cell **migration** and fusion of tracheal branches, without cell division. The combined action of the Decapentaplegic (Dpp), Epidermal growth factor (EGF) and breathless/branchless pathways are thought to be responsible for the pattern of tracheal branches. We ask how these transduction pathways regulate cell **migration** and we analyse the consequences on cell behaviour of the Dpp and EGF pathways. We find that rhomboid (rho) mutant embryos display defects not only in tracheal cell **migration** but also in tracheal cell invagination unveiling a new role for EGF signalling in the formation of the tracheal system. These results indicate that the transduction pathways that control tracheal cell **migration** are active in different steps of tracheal formation, beginning at invagination. We discuss how the consecutive steps of tracheal morphogenesis might affect the final branching pattern.
2. Gene expression profiling of Drosophila tracheal fusion cells. The Drosophila trachea is a premier genetic system to investigate the fundamental mechanisms of tubular organ formation. Tracheal fusion cells lead the branch fusion process to form an interconnected tubular network. Therefore, fusion cells in the Drosophila trachea will be an excellent model to study branch fusion in mammalian tubular organs, such as kidneys and blood vessels. The fusion process is a dynamic cellular process involving cell **migration**, adhesion, vesicle trafficking, cytoskeleton rearrangement, and membrane fusion. To understand how these cellular events are coordinated, we initiated the critical step to assemble a gene expression profile of fusion cells. For this study, we analyzed the expression of 234 potential tracheal-expressed genes in fusion cells during fusion cell development. 143 Tracheal genes were found to encode transcription factors, signal proteins, cytoskeleton and matrix proteins, transporters, and proteins with unknown function. These genes were divided into four subgroups based on their levels of expression in fusion cells compared to neighboring non-fusion cells revealed by in situ hybridization: (1) genes that have relative high abundance in fusion cells, (2) genes that are dynamically expressed in fusion cells, (3) genes that have relative low abundance in fusion cells, and (4) genes that are expressed at similar levels in fusion cells and non-fusion tracheal cells. This study identifies the expression profile of fusion cells and hypothetically suggests genes which are necessary for the fusion process and which play roles in distinct stages of fusion, as indicated by the location and timing of expression. These data will provide the basis for a comprehensive understanding of the molecular and cellular mechanisms of branch fusion.
3. The genetics of cell **migration** in Drosophila melanogaster and Caenorhabditis elegans development. Cell **migration**s are found throughout the animal kingdom and are among the most dramatic and complex of cellular behaviors. Historically, the mechanics of cell **migration** have been studied primarily in vitro, where cells can be readily viewed and manipulated. However, genetic approaches in relatively simple model organisms are yielding additional insights into the molecular mechanisms underlying cell movements and their regulation during development. This review will focus on these simple model systems where we understand some of the signaling and receptor molecules that stimulate and guide cell movements. The chemotactic guidance factor encoded by the Caenorhabditis elegans unc-6 locus, whose mammalian homolog is Netrin, is perhaps the best known of the cell **migration** guidance factors. In addition, receptor tyrosine kinases (RTKs), and FGF receptors in particular, have emerged as key mediators of cell **migration** in vivo, confirming the importance of molecules that were initially identified and studied in cell culture. Somewhat surprisingly, screens for mutations that affect primordial germ cell **migration** in Drosophila have revealed that enzymes involved in lipid metabolism play a role in guiding cell **migration** in vivo, possibly by producing and/or degrading lipid chemoattractants or chemorepellents. Cell adhesion molecules, such as integrins, have been extensively characterized with respect to their contribution to cell **migration** in vitro and genetic evidence now supports a role for these receptors in certain instances in vivo as well. The role for non-muscle myosin in cell motility was controversial, but has now been demonstrated genetically, at least in some cell types. Currently the best characterized link between membrane receptor signaling and regulation of the actin cytoskeleton is that provided by the Rho family of small GTPases. Members of this family are clearly essential for the **migration**s of some cells; however, key questions remain concerning how chemoattractant and chemorepellent signals are integrated within the cell and transduced to the cytoskeleton to produce directed cell **migration**. New types of genetic screens promise to fill in some of these gaps in the near future.
4. Cell fate choices in Drosophila tracheal morphogenesis. The Drosophila tracheal system is a branched tubular structure that supplies air to target tissues. The elaborate tracheal morphology is shaped by two linked inductive processes, one involving the choice of cell fates, and the other a guided cell **migration**. We will describe the molecular basis for these processes, and the allocation of cell fate decisions to four temporal hierarchies. First, tracheal placodes are specified within the embryonic ectoderm. Subsequently, branch fates are allocated within the tracheal placodes, prior to **migration**. Localized presentation of the FGF ligand, Branchless, to tracheal cells that express the FGF receptor, Breathless, guides **migration**. Once cell **migration** is initiated, distinct cell fates are determined within each migrating branch. Finally, inhibitory feedback mechanisms ensure the correct assignment of these fates. Tracheal cell fate choices are determined by signaling cascades triggered by signals emanating from the tracheal cells, as well as by ligands produced by adjacent tissues.
5. ventral veinless, a POU domain transcription factor, regulates different transduction pathways required for tracheal branching in Drosophila. Cell **migration** is an important step in a variety of developmental processes in many multicellular organisms. A particularly appropriate model to address the study of cell **migration** is the tracheal system of Drosophila, whose formation occurs by **migration** and fusion from clusters of ectodermal cells specified in each side of ten embryonic segments. Morphogenesis of the tracheal tree requires the activity of many genes, among them breathless (btl) and ventral veinless (vvl) whose mutations abolish tracheal cell **migration**. Activation of the btl receptor by branchless (bnl), its putative ligand, exerts an instructive role in the process of guiding tracheal cell **migration**. vvl has been shown to be required for the maintenance of btl expression during tracheal tree formation. Here we show that, in addition, vvl is independently required for the specific expression in the tracheal cells of thick veins (tkv) and rhomboid (rho), two genes whose mutations disrupt only particular branches of the tracheal system. Indeed, we show that expression in the tracheal cells of an activated form of tkv, the putative decapentaplegic (dpp) receptor, is able to induce shifts in their **migration**, asserting the role of the dpp pathway in establishing the branching pattern of the tracheal tree. In addition, by ubiquitous expression of the btl and tkv genes in vvl mutant embryos we show that both genes contribute to vvl function. These results indicate that through activation of its target genes, vvl makes the tracheal cells competent to further signalling and suggest that the btl transduction pathway could collaborate with other transduction pathways also regulated by vvl to specify the tracheal branching pattern.
6. Branching morphogenesis of the Drosophila tracheal system. Many organs including the mammalian lung and vascular system consist of branched tubular networks that transport essential gases or fluids, but the genetic programs that control the development of these complex three-dimensional structures are not well understood. The Drosophila melanogaster tracheal (respiratory) system is a network of interconnected epithelial tubes that transports oxygen and other gases in the body and provides a paradigm of branching morphogenesis. It develops by sequential sprouting of primary, secondary, and terminal branches from an epithelial sac of approximately 80 cells in each body segment of the embryo. Mapping of the cell movements and shape changes during the sprouting process has revealed that distinct mechanisms of epithelial **migration** and tube formation are used at each stage of branching. Genetic dissection of the process has identified a general program in which a fibroblast growth factor (FGF) and fibroblast growth factor receptor (FGFR) are used repeatedly to control branch budding and outgrowth. At each stage of branching, the mechanisms controlling FGF expression and the downstream signal transduction pathway change, altering the pattern and structure of the branches that form. During terminal branching, FGF expression is regulated by hypoxia, ensuring that tracheal structure matches cellular oxygen need. A branch diversification program operates in parallel to the general budding program: Regional signals locally modify the general program, conferring specific structural features and other properties on individual branches, such as their substrate outgrowth preferences, differences in tube size and shape, and the ability to fuse to other branches to interconnect the network.
7. JAK-STAT pathway in Drosophila morphogenesis: From organ selector to cell behavior regulator. One of the main contributions of Drosophila to the JAK-STAT field is the study of morphogenesis. JAK-STAT signaling controls the formation of many different structures through surprisingly different morphogenetic behaviors that include induction of cell rearrangements, invagination, folding of tissues, modulation of cell shape, and **migration**. This variability may be explained by the many transcription factors and signaling molecules STAT regulates at early stages of development. But is STAT just acting as an upstream inducer of morphogenesis or does it have a more direct role in controlling cell behaviors? Here we review what is known about how the canonical phosphorylation of STAT contributes to shaping the embryonic and imaginal structures.
8. Dysfusion transcriptional control of Drosophila tracheal **migration**, adhesion, and fusion. The Drosophila dysfusion basic-helix-loop-helix-PAS transcription factor gene is expressed in specialized fusion cells that reside at the tips of migrating tracheal branches. dysfusion mutants were isolated, and genetic analysis of live embryos revealed that mutant tracheal branches migrate to close proximity but fail to recognize and adhere to each other. Misexpression of dysfusion throughout the trachea further indicated that dysfusion has the ability to both inhibit cell **migration** and promote ectopic tracheal fusion. Nineteen genes whose expression either increases or decreases in fusion cells during development were analyzed in dysfusion mutant embryos. dysfusion upregulates the levels of four genes, including the shotgun cell adhesion protein gene and the zona pellucida family transmembrane protein gene, CG13196. Misexpression experiments with CG13196 result in ectopic tracheal fusion events, suggesting that it also encodes a cell adhesion protein. Another target gene of dysfusion is members only, which inhibits protein nuclear export and influences tracheal fusion. dysfusion also indirectly downregulates protein levels of Trachealess, an important regulator of tracheal development. These results indicate that fusion cells undergo dynamic changes in gene expression as they switch from migratory to fusion modes and that dysfusion regulates a discrete, but important, set of these genes.
9. Regulation of cell **migration** during tracheal development in Drosophila melanogaster. Most of the knowledge concerning the intracellular mechanisms involved in cell locomotion have been obtained from in vitro studies of cells in culture. Many of the concepts derived from these studies have been partially confirmed in in vivo systems but numerous questions regarding the developmental control of cell **migration** remain to be addressed. Tracheal morphogenesis in Drosophila melanogaster embryos represents an in vivo model system to study the genetic control of cell **migration**. We review what is known about tracheal development and regulation of tracheal cell **migration**. We try to link these in vivo studies and the movement of cells over two dimensional substrates and elaborate on important questions which remain to be addressed in the future.
10. Function of the Drosophila POU domain transcription factor drifter as an upstream regulator of breathless receptor tyrosine kinase expression in developing trachea. Organogenesis of the Drosophila tracheal system involves extensive directed cell **migration**s leading to a stereotypic series of interconnected tubules. Although numerous gene products have been shown to be essential for tracheal morphogenesis, direct functional relationships between participants have not been previously established. Both the breathless gene, encoding a Drosophila fibroblast growth factor receptor tyrosine kinase homologue, and the POU-domain transcription factor gene, drifter, are expressed in all tracheal cells and are essential for directed cell **migration**s. We demonstrate here that ubiquitously expressed Breathless protein under control of a heterologous heat-shock promoter is able to rescue the severely disrupted tracheal phenotype associated with drifter loss-of-function mutations. In the absence of Drifter function, breathless expression is initiated normally but transcript levels fall drastically to undetectable levels as tracheal differentiation proceeds. In addition, breathless regulatory DNA contains seven high affinity Drifter binding sites similar to previously identified Drifter recognition elements. These results suggest that the Drifter protein, which maintains its own expression through a tracheal-specific autoregulatory enhancer, is not necessary for initiation of breathless expression but functions as a direct transcriptional regulator necessary for maintenance of breathless transcripts at high levels during tracheal cell **migration**. This example of a mechanism for maintenance of a committed cell fate offers a model for understanding how essential gene activities can be maintained throughout organogenesis.
11. Mutations affecting the pattern of the PNS in Drosophila reveal novel aspects of neuronal development. Through a systematic genetic screen, we have identified 55 mutations that affect the development of the PNS of Drosophila embryos. These mutations specify 13 novel and 5 previously characterized genes and define new phenotypes for 2 other known genes. Five classes of mutant phenotypes were identified in the screen: gain of neurons, loss of neurons, abnormal position of chordotonal neurons, aberrant neuronal trajectories, and abnormal morphology of neurons. Phenotypic analyses of mutations identified in this study revealed three novel aspects of PNS development. First, we have identified a novel gene that may be required to define glial versus neuronal cell identity. Second, our data indicate that neuronal **migration** plays an important role in pattern formation in the embryonic PNS. Third, we have identified mutations that cause a lack of sensory organs, but unlike mutations in proneural genes, do not affect the formation of sensory organ precursors. These genes may be required for key aspects of neuronal differentiation. Our studies suggest that approximately 70 essential genes are required for proper PNS development in Drosophila embryos.
12. ribbon encodes a novel BTB/POZ protein required for directed cell **migration** in Drosophila melanogaster. During development, directed cell **migration** is crucial for achieving proper shape and function of organs. One well-studied example is the embryonic development of the larval tracheal system of Drosophila, in which at least four signaling pathways coordinate cell **migration** to form an elaborate branched network essential for oxygen delivery throughout the larva. FGF signaling is required for guided **migration** of all tracheal branches, whereas the DPP, EGF receptor, and Wingless/WNT signaling pathways each mediate the formation of specific subsets of branches. Here, we characterize ribbon, which encodes a BTB/POZ-containing protein required for specific tracheal branch **migration**. In ribbon mutant tracheae, the dorsal trunk fails to form, and ventral branches are stunted; however, directed **migration**s of the dorsal and visceral branches are largely unaffected. The dorsal trunk also fails to form when FGF or Wingless/WNT signaling is lost, and we show that ribbon functions downstream of, or parallel to, these pathways to promote anterior-posterior **migration**. Directed cell **migration** of the salivary gland and dorsal epidermis are also affected in ribbon mutants, suggesting that conserved mechanisms may be employed to orient cell **migration**s in multiple tissues during development.
13. Drosophila Jing is part of the breathless fibroblast growth factor receptor positive feedback loop. In the developing Drosophila trachea, extensive cell **migration** lays the foundation for an elaborate network of tubules to form. This process is controlled by the Drosophila fibroblast growth factor receptor, known as Breathless (Btl), whose expression is activated by the Trachealess (Trh) and Tango (Tgo) basic helix-loop-helix (bHLH)-PAS transcription factors. We previously identified the jing zinc finger transcription factor as a gene sensitive to the dosage of bHLH-PAS transcriptional activity and showed that its mutations interact genetically with those of trh and btl. Here, we demonstrate that jing is required for btl expression in the branching trachea and dominantly interacts with known regulators of btl expression, including the ETS and POU transcription factors, pointed, and drifter/ventral veinless, respectively. Furthermore, the zinc finger-containing C-terminus of Jing associates with a btl tracheal enhancer in a Trh/Tgo-dependent manner in chromatin immunoprecipitation assays in vitro and interferes with btl in vitro and in vivo. Together, our results support a model by which Jing/Trh/Tgo complexes regulate btl transcript levels during primary tracheal branching.
14. Concentric zones, cell **migration** and neuronal circuits in the Drosophila visual center. The Drosophila optic lobe comprises a wide variety of neurons, which form laminar neuropiles with columnar units and topographic projections from the retina. The Drosophila optic lobe shares many structural characteristics with mammalian visual systems. However, little is known about the developmental mechanisms that produce neuronal diversity and organize the circuits in the primary region of the optic lobe, the medulla. Here, we describe the key features of the developing medulla and report novel phenomena that could accelerate our understanding of the Drosophila visual system. The identities of medulla neurons are pre-determined in the larval medulla primordium, which is subdivided into concentric zones characterized by the expression of four transcription factors: Drifter, Runt, Homothorax and Brain-specific homeobox (Bsh). The expression pattern of these factors correlates with the order of neuron production. Once the concentric zones are specified, the distribution of medulla neurons changes rapidly. Each type of medulla neuron exhibits an extensive but defined pattern of **migration** during pupal development. The results of clonal analysis suggest homothorax is required to specify the neuronal type by regulating various targets including Bsh and cell-adhesion molecules such as N-cadherin, while drifter regulates a subset of morphological features of Drifter-positive neurons. Thus, genes that show the concentric zones may form a genetic hierarchy to establish neuronal circuits in the medulla.
15. Hedgehog signaling patterns the tracheal branches. The elaborate branching pattern of the Drosophila tracheal system originates from ten tracheal placodes on both sides of the embryo, each consisting of about 80 cells. Simultaneous cell **migration** from each tracheal pit in six different directions gives rise to the stereotyped branching pattern. Each branch contains a fixed number of cells. Previous work has shown that in the dorsoventral axis, localized activation of the Dpp, Wnt and EGF receptor (DER) pathways, subdivides the tracheal pit into distinct domains. We present the role of the Hedgehog (Hh) signaling system in patterning the tracheal branches. Hh is expressed in segmental stripes abutting the anterior border of the tracheal placodes. Induction of patched expression, which results from activation by Hh, demonstrates that cells in the anterior half of the tracheal pit are activated. In hh-mutant embryos **migration** of all tracheal branches is absent or stalled. These defects arise from a direct effect of Hh on tracheal cells, rather than by indirect effects on patterning of the ectoderm. Tracheal cell **migration** could be rescued by expressing Hh only in the tracheal cells, without rescuing the ectodermal defects. Signaling by several pathways, including the Hh pathway, thus serves to subdivide the uniform population of tracheal cells into distinct cell types that will subsequently be recruited into the different branches.
16. drifter, a Drosophila POU-domain transcription factor, is required for correct differentiation and **migration** of tracheal cells and midline glia. The Drosophila drifter (dfr) gene, previously referred to as Cf1a, encodes a POU-domain DNA-binding protein implicated as a neuron-specific regulator in the developing central nervous system (CNS). We have isolated full-length dfr cDNA clones that encode a 46-kD protein containing the conserved POU-domain DNA-binding domain. The use of alternate polyadenylation sites produces two dfr mRNA transcripts that are first expressed in stage 10 embryos at 5- to 6-hr of development. A specific anti-dfr polyclonal antiserum generated against a dfr-glutathione S-transferase fusion protein recognizes a 46-kD protein on Western blots and has been used to analyze the cell-specific distribution of dfr protein during embryonic development. dfr protein is distributed in a complex expression pattern including the tracheal system, the middle pair of midline glia, and selected CNS neurons. We have carried out a genetic characterization of the dfr locus, previously localized to region 65D of the third chromosome, by generating a series of overlapping deficiencies between 65A and 65E1 that were used to isolate dfrE82, an EMS-induced lethal allele. Analysis of dfrE82 mutant embryos shows a disruption of the developing tracheal tree as well as commissural defects in the developing CNS. Based on an examination of a cell-specific marker for tracheal cells and midline glia, these defects appear to be caused by a failure of these cells to follow their characteristic routes of **migration**. The dfrE82 tracheal phenotype is rescued by a dfr minigene present as a P-element transposon expressing wild-type dfr protein in tracheal cells. These results suggest that the dfr protein plays a fundamental role in the differentiation of tracheal cells and midline glia possibly by regulating the expression of essential cell-surface proteins required for cell-cell interactions involved in directed cell **migration**s.

### [Ee]xtracellular [Mm]atrix

#### PubMed

1. Characterisation of Upd2, a Drosophila JAK/STAT pathway ligand. The characterisation of ligands that activate the JAK/STAT pathway has the potential to throw light onto a comparatively poorly understood aspect of this important signal transduction cascade. Here, we describe our analysis of the only invertebrate JAK/STAT pathway ligands identified to date, the Drosophila unpaired-like family. We show that upd2 is expressed in a pattern essentially identical to that of upd and demonstrate that the proteins encoded by this region activate JAK/STAT pathway signalling. Mutational analysis demonstrates a mutual semi-redundancy that can be visualised in multiple tissues known to require JAK/STAT signalling. In order to better characterise the in vivo function of these ligands, we developed a reporter based on a natural JAK/STAT pathway responsive enhancer and show that ectopic upd2 expression can effectively activate the JAK/STAT pathway. While both Upd and Upd2 are secreted JAK/STAT pathway agonists, tissue culture assays show that the signal-sequences of Upd and Upd2 confer distinct properties, with Upd associated primarily with the **extracellular matrix** and Upd2 secreted into the media. The differing biophysical characteristics identified for Upd-like molecules have implications for their function in vivo and adds another aspect to our understanding of cytokine signalling in Drosophila.

---

## CG11448

- NCBI Gene
- GeneRIF
- Pubmed

| Search Term | Rank | Fields |
| --- | --- | --- |
| [Mm]igration | 6 | PubMed(1) |

  
hide/show details for CG11448

### [Mm]igration

#### PubMed

1. A misexpression screen to identify regulators of Drosophila larval hemocyte development. In Drosophila, defense against foreign pathogens is mediated by an effective innate immune system, the cellular arm of which is composed of circulating hemocytes that engulf bacteria and encapsulate larger foreign particles. Three hemocyte types occur: plasmatocytes, crystal cells, and lamellocytes. The most abundant larval hemocyte type is the plasmatocyte, which is responsible for phagocytosis and is present either in circulation or in adherent sessile domains under the larval cuticle. The mechanisms controlling differentiation of plasmatocytes and their **migration** toward these sessile compartments are unclear. To address these questions we have conducted a misexpression screen using the plasmatocyte-expressed GAL4 driver Peroxidasin-GAL4 (Pxn-GAL4) and existing enhancer-promoter (EP) and EP yellow (EY) transposon libraries to systematically misexpress approximately 20% of Drosophila genes in larval hemocytes. The Pxn-GAL4 strain also contains a UAS-GFP reporter enabling hemocyte phenotypes to be visualized in the semitransparent larvae. Among 3412 insertions screened we uncovered 101 candidate hemocyte regulators. Some of these are known to control hemocyte development, but the majority either have no characterized function or are proteins of known function not previously implicated in hemocyte development. We have further analyzed three candidate genes for changes in hemocyte morphology, cell-cell adhesion properties, phagocytosis activity, and melanotic tumor formation.

---

## Apc

- NCBI Gene
- GeneRIF
- Pubmed

| Search Term | Rank | Fields |
| --- | --- | --- |
| [Mm]igration | 6 | PubMed(1) |
| ECM | 7 | PubMed(1) |
| [Ee]xtracellular [Mm]atrix | 8 | PubMed(2) |
| [Ii]nvasive | 10 | PubMed(5) |
| [Mm]etastasis | 12 | PubMed(1) |
| [Cc]ell [Ii]nvasion | 13 | PubMed(1) |

  
hide/show details for Apc

### [Mm]igration

#### PubMed

1. Colorectal cancer progression: lessons from Drosophila? Human colorectal cancers arise as benign adenomas, tumours that retain their epithelial character, and then progress to malignant adenocarcinomas and carcinomas in which the epithelium becomes disrupted. Carcinomas often exhibit transcriptional downregulation of E-cadherin and other epithelial genes in an epithelial-to-mesenchymal transition (EMT), a mechanism first discovered in Drosophila to be mediated by the transcription factors Twist and Snail. In contrast, adenocarcinomas retain expression of E-cadherin and disruption of the epithelium occurs through formation of progressively smaller epithelial cysts with apical Crumbs/CRB3, Stardust/PALS1, and Bazooka/PAR3 localised to the inner lumen. Results from Drosophila show that morphologically similar cysts form upon induction of clonal heterogeneity in Wnt, Smad, or Ras signalling levels, which causes extrusion of epithelial cells at clonal boundaries. Thus, intratumour heterogeneity might also promote formation of adenocarcinomas in humans. Finally, epithelial cysts can collectively migrate, as in the case of Drosophila border cells, a potential model system for the invasive **migration** of adenocarcinoma cells.

### ECM

#### PubMed

1. Integrin signaling is required for maintenance and proliferation of intestinal stem cells in Drosophila. Tissue-specific stem cells are maintained by both local secreted signals and cell adhesion molecules that position the stem cells in the niche microenvironment. In the Drosophila midgut, multipotent intestinal stem cells (ISCs) are located basally along a thin layer of basement membrane that composed of extracellular matrix (**ECM**), which separates ISCs from the surrounding visceral musculature: the muscle cells constitute a regulatory niche for ISCs by producing multiple secreted signals that directly regulate ISC maintenance and proliferation. Here we show that integrin-mediated cell adhesion, which connects the **ECM** and intracellular cytoskeleton, is required for ISC anchorage to the basement membrane. Specifically, the α-integrin subunits including αPS1 encoded by mew and αPS3 encoded by scb, and the β-integrin subunit encoded by mys are richly expressed in ISCs and are required for the maintenance, rather than their survival or multiple lineage differentiation. Furthermore, ISC maintenance also requires the intercellular and intracellular integrin signaling components including Talin, Integrin-linked kinase (Ilk), and the ligand, Laminin A. Notably, integrin mutant ISCs are also less proliferative, and genetic interaction studies suggest that proper integrin signaling is a pre-requisite for ISC proliferation in response to various proliferative signals and for the initiation of intestinal hyperplasia after loss of adenomatous polyposis coli (Apc). Our studies suggest that integrin not only functions to anchor ISCs to the basement membrane, but also serves as an essential element for ISC proliferation during normal homeostasis and in response to oncogenic mutations.

### [Ee]xtracellular [Mm]atrix

#### PubMed

1. Tissue design: how Drosophila tumors remodel their neighborhood. Drosophila genetics has long been appreciated as a powerful approach for discovering the normal functions of genes that act as oncogenes and tumor suppressors in human cancer. Recent studies have also highlighted its advantages for deciphering how such genes function during tumorigenesis itself. Here we detail studies relating to how tumors, generated in developing organs and adult stem cell-based tissues, remodel the tissue landscape to their benefit. Like mammalian tumors, insect tumors can dissolve **extracellular matrix**, recruit blood cells, migrate and invade other tissues. While much is known about how mammalian fibroblasts, immune cells and vasculature promote late tumorigenesis, less is understood about the very earliest stages of tumor development in mammals. Because Drosophila has fewer mitotic cells and a simpler tissue architecture, it affords easy detection and analysis of early clonal tumor growth. Drosophila studies have revealed both cooperative and competitive interactions between tumor and normal cells during early tumor growth. During development, these interactions typically occur with other proliferative progenitor cells, but in adult stem cell-based tissues, the stem cell niche can fuel tumor growth.
2. Integrin signaling is required for maintenance and proliferation of intestinal stem cells in Drosophila. Tissue-specific stem cells are maintained by both local secreted signals and cell adhesion molecules that position the stem cells in the niche microenvironment. In the Drosophila midgut, multipotent intestinal stem cells (ISCs) are located basally along a thin layer of basement membrane that composed of **extracellular matrix** (ECM), which separates ISCs from the surrounding visceral musculature: the muscle cells constitute a regulatory niche for ISCs by producing multiple secreted signals that directly regulate ISC maintenance and proliferation. Here we show that integrin-mediated cell adhesion, which connects the ECM and intracellular cytoskeleton, is required for ISC anchorage to the basement membrane. Specifically, the α-integrin subunits including αPS1 encoded by mew and αPS3 encoded by scb, and the β-integrin subunit encoded by mys are richly expressed in ISCs and are required for the maintenance, rather than their survival or multiple lineage differentiation. Furthermore, ISC maintenance also requires the intercellular and intracellular integrin signaling components including Talin, Integrin-linked kinase (Ilk), and the ligand, Laminin A. Notably, integrin mutant ISCs are also less proliferative, and genetic interaction studies suggest that proper integrin signaling is a pre-requisite for ISC proliferation in response to various proliferative signals and for the initiation of intestinal hyperplasia after loss of adenomatous polyposis coli (Apc). Our studies suggest that integrin not only functions to anchor ISCs to the basement membrane, but also serves as an essential element for ISC proliferation during normal homeostasis and in response to oncogenic mutations.

### [Ii]nvasive

#### PubMed

1. APC loss-induced intestinal tumorigenesis in Drosophila: Roles of Ras in Wnt signaling activation and tumor progression. Adenomatous polyposis coli (APC) and K-ras are the two most frequently mutated genes found in human colorectal cancers. In human colorectal cancers, Wnt signaling activation after the loss of APC is hypothesized to be the key event for adenoma initiation, whereas additional mutations such as Ras activation are required for the progression from adenoma to carcinoma. However, accumulating data have led to conflicting views regarding the precise role of Ras in APC loss-induced tumorigenesis. Here, using Drosophila midgut as a model system, we show that in the absence of Ras, APC mutant epithelial cells cannot initiate hyperplasia, suggesting that Ras plays an essential role in tumor initiation. Conversely, activating Ras by expressing oncogenic Ras or Raf in APC-deficient cells led to a blockage of cell differentiation and to pre**invasive** tumor outgrowth, characteristics that are shared by advanced colorectal carcinoma in humans. Mechanistically, we find that Ras is not required for Wnt signaling activation after APC loss, although Ras hyperactivation is able to potentiate Wnt signaling by increasing the cytoplasmic and nuclear accumulation of Armadillo/β-catenin via mechanisms independent of JNK/Rac1 or PI3K-Akt signaling, partly owing to the downregulation of DE-cadherin. Together with the data from gene expression analyses, our results indicate that both parallel and cooperative mechanisms of Wnt and Ras signaling are responsible for the initiation and progression of intestinal tumorigenesis after APC loss.
2. APC loss-induced intestinal tumorigenesis in Drosophila: Roles of Ras in Wnt signaling activation and tumor progression. Adenomatous polyposis coli (APC) and K-ras are the two most frequently mutated genes found in human colorectal cancers. In human colorectal cancers, Wnt signaling activation after the loss of APC is hypothesized to be the key event for adenoma initiation, whereas additional mutations such as Ras activation are required for the progression from adenoma to carcinoma. However, accumulating data have led to conflicting views regarding the precise role of Ras in APC loss-induced tumorigenesis. Here, using Drosophila midgut as a model system, we show that in the absence of Ras, APC mutant epithelial cells cannot initiate hyperplasia, suggesting that Ras plays an essential role in tumor initiation. Conversely, activating Ras by expressing oncogenic Ras or Raf in APC-deficient cells led to a blockage of cell differentiation and to pre**invasive** tumor outgrowth, characteristics that are shared by advanced colorectal carcinoma in humans. Mechanistically, we find that Ras is not required for Wnt signaling activation after APC loss, although Ras hyperactivation is able to potentiate Wnt signaling by increasing the cytoplasmic and nuclear accumulation of Armadillo/β-catenin via mechanisms independent of JNK/Rac1 or PI3K-Akt signaling, partly owing to the downregulation of DE-cadherin. Together with the data from gene expression analyses, our results indicate that both parallel and cooperative mechanisms of Wnt and Ras signaling are responsible for the initiation and progression of intestinal tumorigenesis after APC loss.
3. Colorectal cancer progression: lessons from Drosophila? Human colorectal cancers arise as benign adenomas, tumours that retain their epithelial character, and then progress to malignant adenocarcinomas and carcinomas in which the epithelium becomes disrupted. Carcinomas often exhibit transcriptional downregulation of E-cadherin and other epithelial genes in an epithelial-to-mesenchymal transition (EMT), a mechanism first discovered in Drosophila to be mediated by the transcription factors Twist and Snail. In contrast, adenocarcinomas retain expression of E-cadherin and disruption of the epithelium occurs through formation of progressively smaller epithelial cysts with apical Crumbs/CRB3, Stardust/PALS1, and Bazooka/PAR3 localised to the inner lumen. Results from Drosophila show that morphologically similar cysts form upon induction of clonal heterogeneity in Wnt, Smad, or Ras signalling levels, which causes extrusion of epithelial cells at clonal boundaries. Thus, intratumour heterogeneity might also promote formation of adenocarcinomas in humans. Finally, epithelial cysts can collectively migrate, as in the case of Drosophila border cells, a potential model system for the **invasive** migration of adenocarcinoma cells.
4. Sin3a acts through a multi-gene module to regulate invasion in Drosophila and human tumors. Chromatin remodeling proteins regulate multiple aspects of cell homeostasis, making them ideal candidates for misregulation in transformed cells. Here, we explore Sin3A, a member of the Sin3 family of proteins linked to tumorigenesis that are thought to regulate gene expression through their role as histone deacetylases (HDACs). We identified Drosophila Sin3a as an important mediator of oncogenic Ret receptor in a fly model of Multiple Endocrine Neoplasia Type 2. Reducing Drosophila Sin3a activity led to metastasis-like behavior and, in the presence of Diap1, secondary tumors distant from the site of origin. Genetic and Chip-Seq analyses identified previously undescribed Sin3a targets including genes involved in cell motility and actin dynamics, as well as signaling pathways including Src, Jnk and Rho. A key Sin3a oncogenic target, PP1B, regulates stability of β-Catenin/Armadillo: the outcome is to oppose T-cell factor (TCF) function and Wg/Wnt pathway signaling in both fly and mammalian cancer cells. Reducing Sin3A strongly increased the **invasiv**e behavior of A549 human lung adenocarcinoma cells. We show that Sin3A is downregulated in a variety of human tumors and that Src, JNK, RhoA and PP1B/β-Catenin are regulated in a manner analogous to our Drosophila models. Our data suggest that Sin3A influences a specific step of tumorigenesis by regulating a module of genes involved in cell invasion. Tumor progression may commonly rely on such 'modules of invasion' under the control of broad transcriptional regulators.
5. Drosophila apc regulates delamination of **invasive** epithelial clusters. Border Cells in the Drosophila ovaries are a useful genetic model for understanding the molecular events underlying epithelial cell motility. During stage 9 of egg chamber development they detach from neighboring stretched cells and migrate between the nurse cells to reach the oocyte. RNAi screening allowed us to identify the dapc1 gene as being critical in this process. Clonal and live analysis showed a requirement of dapc1 in both outer border cells and contacting stretched cells for delamination. This mutant phenotype was rescued by dapc1 or dapc2 expression. Loss of dapc1 function was associated with an abnormal lasting accumulation of β-catenin/Armadillo and E-cadherin at the boundary between migrating border and stretched cells. Moreover, β-catenin/armadillo or E-cadherin downregulation rescued the dapc1 loss of function phenotype. Altogether these results indicate that Drosophila Apc1 is required for dynamic remodeling of β-catenin/Armadillo and E-cadherin adhesive complexes between outer border cells and stretched cells regulating proper delamination and invasion of migrating epithelial clusters.

### [Mm]etastasis

#### PubMed

1. Sin3a acts through a multi-gene module to regulate invasion in Drosophila and human tumors. Chromatin remodeling proteins regulate multiple aspects of cell homeostasis, making them ideal candidates for misregulation in transformed cells. Here, we explore Sin3A, a member of the Sin3 family of proteins linked to tumorigenesis that are thought to regulate gene expression through their role as histone deacetylases (HDACs). We identified Drosophila Sin3a as an important mediator of oncogenic Ret receptor in a fly model of Multiple Endocrine Neoplasia Type 2. Reducing Drosophila Sin3a activity led to **metastasis**-like behavior and, in the presence of Diap1, secondary tumors distant from the site of origin. Genetic and Chip-Seq analyses identified previously undescribed Sin3a targets including genes involved in cell motility and actin dynamics, as well as signaling pathways including Src, Jnk and Rho. A key Sin3a oncogenic target, PP1B, regulates stability of β-Catenin/Armadillo: the outcome is to oppose T-cell factor (TCF) function and Wg/Wnt pathway signaling in both fly and mammalian cancer cells. Reducing Sin3A strongly increased the invasive behavior of A549 human lung adenocarcinoma cells. We show that Sin3A is downregulated in a variety of human tumors and that Src, JNK, RhoA and PP1B/β-Catenin are regulated in a manner analogous to our Drosophila models. Our data suggest that Sin3A influences a specific step of tumorigenesis by regulating a module of genes involved in cell invasion. Tumor progression may commonly rely on such 'modules of invasion' under the control of broad transcriptional regulators.

### [Cc]ell [Ii]nvasion

#### PubMed

1. Sin3a acts through a multi-gene module to regulate invasion in Drosophila and human tumors. Chromatin remodeling proteins regulate multiple aspects of cell homeostasis, making them ideal candidates for misregulation in transformed cells. Here, we explore Sin3A, a member of the Sin3 family of proteins linked to tumorigenesis that are thought to regulate gene expression through their role as histone deacetylases (HDACs). We identified Drosophila Sin3a as an important mediator of oncogenic Ret receptor in a fly model of Multiple Endocrine Neoplasia Type 2. Reducing Drosophila Sin3a activity led to metastasis-like behavior and, in the presence of Diap1, secondary tumors distant from the site of origin. Genetic and Chip-Seq analyses identified previously undescribed Sin3a targets including genes involved in cell motility and actin dynamics, as well as signaling pathways including Src, Jnk and Rho. A key Sin3a oncogenic target, PP1B, regulates stability of β-Catenin/Armadillo: the outcome is to oppose T-cell factor (TCF) function and Wg/Wnt pathway signaling in both fly and mammalian cancer cells. Reducing Sin3A strongly increased the invasive behavior of A549 human lung adenocarcinoma cells. We show that Sin3A is downregulated in a variety of human tumors and that Src, JNK, RhoA and PP1B/β-Catenin are regulated in a manner analogous to our Drosophila models. Our data suggest that Sin3A influences a specific step of tumorigenesis by regulating a module of genes involved i**n cell invasi**on. Tumor progression may commonly rely on such 'modules of invasion' under the control of broad transcriptional regulators.

---

## Stat92E

- NCBI Gene
- GeneRIF
- Pubmed

| Search Term | Rank | Fields |
| --- | --- | --- |
| [Ff]ilopodia | 1 | PubMed(3) |
| [Mm]igration | 6 | Process(2); PubMed(38); GeneRIFs(2) |
| ECM | 7 | PubMed(2) |
| [Ee]xtracellular [Mm]atrix | 8 | PubMed(7) |
| [Ii]nvasive | 10 | PubMed(13); GeneRIFs(1) |
| [Mm]etastasis | 12 | PubMed(10) |
| [Cc]ell [Ii]nvasion | 13 | PubMed(1) |

  
hide/show details for Stat92E

### [Ff]ilopodia

#### PubMed

1. JAK/STAT signalling in Drosophila controls cell motility during germ cell migration. The gonad is formed from two populations of cells originating at different locations: the primordial germ cells (PGCs), giving rise to either sperm or oocytes, and the somatic gonadal mesoderm precursors (SGPs), which support development of the gametes. Following the PGCs' migration during gastrulation, these two populations meet, forming the immature gonad. We present evidence that during embryonic development, the PGCs require the canonical JAK/STAT signalling cascade to migrate efficiently towards the SGPs. Loss of function for any element of the JAK/STAT pathway causes frequent germ cell mislocalisation. We have found that wild-type germ cells produce **filopodia** while they migrate through the mesoderm towards the gonad. Our observations suggest that PGCs use **filopodia** to migrate and to keep contact with each other. Interestingly, activation of the JAK/STAT pathway is required for these **filopodia** to form, and ectopic JAK/STAT activation enhances their formation.
2. JAK/STAT signalling in Drosophila controls cell motility during germ cell migration. The gonad is formed from two populations of cells originating at different locations: the primordial germ cells (PGCs), giving rise to either sperm or oocytes, and the somatic gonadal mesoderm precursors (SGPs), which support development of the gametes. Following the PGCs' migration during gastrulation, these two populations meet, forming the immature gonad. We present evidence that during embryonic development, the PGCs require the canonical JAK/STAT signalling cascade to migrate efficiently towards the SGPs. Loss of function for any element of the JAK/STAT pathway causes frequent germ cell mislocalisation. We have found that wild-type germ cells produce **filopodia** while they migrate through the mesoderm towards the gonad. Our observations suggest that PGCs use **filopodia** to migrate and to keep contact with each other. Interestingly, activation of the JAK/STAT pathway is required for these **filopodia** to form, and ectopic JAK/STAT activation enhances their formation.
3. Gα73B is a downstream effector of JAK/STAT signalling and a regulator of Rho1 in Drosophila haematopoiesis. JAK/STAT signalling regulates many essential developmental processes including cell proliferation and haematopoiesis, whereas its inappropriate activation is associated with the majority of myeloproliferative neoplasias and numerous cancers. Furthermore, high levels of JAK/STAT pathway signalling have also been associated with enhanced metastatic invasion by cancerous cells. Strikingly, gain-of-function mutations in the single Drosophila JAK homologue, Hopscotch, result in haemocyte neoplasia, inappropriate differentiation and the formation of melanised haemocyte-derived 'tumour' masses; phenotypes that are partly orthologous to human gain-of-function JAK2-associated pathologies. Here we show that Gα73B, a novel JAK/STAT pathway target gene, is necessary for JAK/STAT-mediated tumour formation in flies. In addition, although Gα73B does not affect haemocyte differentiation, it does regulate haemocyte morphology and motility under non-pathological conditions. We show that Gα73B is required for constitutive, but not injury-induced, activation of Rho1 and for the localisation of Rho1 i**nto filop**odia upon haemocyte activation. Consistent with these results, we also show that Rho1 interacts genetically with JAK/STAT signalling, and that wild-type levels of Rho1 are necessary for tumour formation. Our findings link JAK/STAT transcriptional outputs, Gα73B activity and Rho1-dependent cytoskeletal rearrangements and cell motility, therefore connecting a pathway associated with cancer with a marker indicative of invasiveness. As such, we suggest a mechanism by which JAK/STAT pathway signalling may promote metastasis.

### [Mm]igration

#### Process

1. border follicle cell **migration**
2. border follicle cell **migration**

#### PubMed

1. Paracrine signaling through the JAK/STAT pathway activates invasive behavior of ovarian epithelial cells in Drosophila. The JAK/STAT signaling pathway, renowned for its effects on cell proliferation and survival, is constitutively active in various human cancers, including ovarian. We have found that JAK and STAT are required to convert the border cells in the Drosophila ovary from stationary, epithelial cells to migratory, invasive cells. The ligand for this pathway, Unpaired (UPD), is expressed by two central cells within the migratory cell cluster. Mutations in upd or jak cause defects in **migration** and a reduction in the number of cells recruited to the cluster. Ectopic expression of either UPD or JAK is sufficient to induce extra epithelial cells to migrate. Thus, a localized signal activates the JAK/STAT pathway in neighboring epithelial cells, causing them to become invasive.
2. Requirement for JAK/STAT signaling throughout border cell **migration** in Drosophila. The evolutionarily conserved JAK/STAT signaling pathway is essential for the proliferation, survival and differentiation of many cells including cancer cells. Recent studies have implicated this transcriptional pathway in the process of cell **migration** in humans, mice, Drosophila and Dictyostelium. In the Drosophila ovary, JAK/STAT signaling is necessary and sufficient for the specification and **migration** of a group of cells called the border cells; however, it is not clear to what extent the requirement for cell fate is distinct from that for cell **migration**. We found that STAT protein is enriched in the migrating border cells throughout their **migration** and is an indicator of cells with highest JAK/STAT activity. In addition, stat(ts) mutants exhibited border cell **migration** defects after just 30 minutes at the non-permissive temperature, prior to any detectable change in the expression of cell fate markers. At later times, cell fate changes became evident, indicating that border cell fate is labile. JAK/STAT signaling was also required for organization of the border cell cluster. Finally, we show that both the accumulation of STAT protein and nuclear accumulation are positively regulated by JAK/STAT activity. The activity of the pathway is negatively regulated by overexpression of a SOCS protein and by blocking endocytosis. Together, our findings suggest that the requirement for STAT in border cells extends beyond the initial specification and delamination of cells from the epithelium.
3. JAK/STAT signalling in Drosophila controls cell motility during germ cell **migration**. The gonad is formed from two populations of cells originating at different locations: the primordial germ cells (PGCs), giving rise to either sperm or oocytes, and the somatic gonadal mesoderm precursors (SGPs), which support development of the gametes. Following the PGCs' **migration** during gastrulation, these two populations meet, forming the immature gonad. We present evidence that during embryonic development, the PGCs require the canonical JAK/STAT signalling cascade to migrate efficiently towards the SGPs. Loss of function for any element of the JAK/STAT pathway causes frequent germ cell mislocalisation. We have found that wild-type germ cells produce filopodia while they migrate through the mesoderm towards the gonad. Our observations suggest that PGCs use filopodia to migrate and to keep contact with each other. Interestingly, activation of the JAK/STAT pathway is required for these filopodia to form, and ectopic JAK/STAT activation enhances their formation.
4. Requirements of Lgl in cell differentiation and motility during Drosophila ovarian follicular epithelium morphogenesis. The epithelial follicle cell layer over the egg chamber in Drosophila ovary undergoes patterning and morphogenesis at oogenesis. These developmental processes are essential for constructing the eggshell and establishing the body axes of the egg and resultant embryo, thereby being crucial for the egg development. We have previously shown that lethal(2)giant larvae (lgl), a Drosophila neoplastic tumor suppressor gene (nTSG) is required for the posterior follicle cell (PFC) fate induction during antero-posterior pattern formation of the follicular epithelium. In this report, we further characterize lgl in this epithelium patterning and the morphogenetic changes of specified border cells. Genetic interactions of lgl with discs large (dlg) and scribble (scrib), another two nTSGs in specifying the PFC fate reveal a cooperative role of this group of genes. Meanwhile, we find that loss of lgl function causes failure of follicle cells at the anterior to differentiate properly. The clonal analysis further indicates that lgl is necessary not only for the border cell differentiation, but also for control of the collective border cell **migration** via presumably modulating the apico-basal polarity and cell adhesion. Overall, we identify Lgl as an essential factor in regulating differentiation and morphogenetic movement of the ovarian epithelial follicle cells.
5. Socs36E limits STAT signaling via Cullin2 and a SOCS-box independent mechanism in the Drosophila egg chamber. The Suppressor of Cytokine Signaling (SOCS) proteins are critical, highly conserved feedback inhibitors of signal transduction cascades. The family of SOCS proteins is divided into two groups: ancestral and vertebrate-specific SOCS proteins. Vertebrate-specific SOCS proteins have been heavily studied as a result of their strong mutant phenotypes. However, the ancestral clade remains less studied, a potential result of genetic redundancies in mammals. Use of the genetically tractable organism Drosophila melanogaster enables in vivo assessment of signaling components and mechanisms with less concern about the functional redundancy observed in mammals. In this study, we investigated how the SOCS family member Suppressor of Cytokine Signaling at 36E (Socs36E) attenuates Janus Kinase/Signal Transducer and Activator of Transcription (Jak/STAT) activation during specification of motile border cells in Drosophila oogenesis. We found that Socs36E genetically interacts with the Cullin2 (Cul2) scaffolding protein. Like Socs36E, Cul2 is required to limit the number of motile cells in egg chambers. We demonstrated that loss of Cul2 in the follicle cells significantly increased nuclear STAT protein levels, which resulted in additional cells acquiring invasive properties. Further, reduction of Cul2 suppressed border cell **migration** defects that occur in a Stat92E-sensitized genetic background. Our data incorporated Cul2 into a previously described Jak/STAT-directed genetic regulatory network that is required to generate a discrete boundary between cell fates. We also found that Socs36E is able to attenuate STAT activity in the egg chamber when it does not have a functional SOCS box. Collectively, this work contributes mechanistic insight to a Jak/STAT regulatory genetic circuit, and suggests that Socs36E regulates Jak/STAT signaling via a Cul2-dependent mechanism, as well as by a Cullin-independent manner, in vivo.
6. Socs36E attenuates STAT signaling to optimize motile cell specification in the Drosophila ovary. The Janus kinase/Signal transducers and activators of transcription (JAK/STAT) pathway determines cell fates by regulating gene expression. One example is the specification of the motile cells called border cells during Drosophila oogenesis. It has been established that too much or too little STAT activity disrupts follicle cell identity and cell motility, which suggests the signaling must be precisely regulated. Here, we find that Suppressor of cytokine signaling at 36E (Socs36E) is a necessary negative regulator of JAK/STAT signaling during border cell specification. We find when STAT signaling is too low to induce **migration** in the presumptive border cell population, nearby follicle cells uncharacteristically become invasive to enable efficient **migration** of the cluster. We generated a genetic null allele that reveals Socs36E is required in the anterior follicle cells to limit invasive behavior to an optimal number of cells. We further show Socs36E genetically interacts with the required STAT feedback inhibitor apontic (apt) and APT's downstream target, mir-279, and provide evidence that suggests APT directly regulates Socs36E transcriptionally. Our work shows Socs36E plays a critical role in a genetic circuit that establishes a boundary between the motile border cell cluster and its non-invasive epithelial neighbors through STAT attenuation.
7. PAR-1 kinase regulates epithelial detachment and directional protrusion of migrating border cells. Many cells that migrate during normal embryonic development or in metastatic cancer first detach from an epithelium. However, this step is often difficult to observe directly in vivo, and the mechanisms controlling the ability of cells to leave the epithelium are poorly understood. In addition, once cells detach, they must assume a migratory phenotype, involving changes in cytoskeletal and signaling dynamics. Drosophila border cells provide a model system in which a combination of forward genetics and live-cell imaging can allow researchers to investigate the cellular and molecular mechanisms of epithelial cell detachment and **migration** in vivo.We identified the Drosophila homolog of the serine/threonine kinase PAR-1 (MARK/Kin1) in a screen for mutations that disrupt border cell **migration**. Previous studies identified two proteins, Apontic and Notch, that indirectly affect border cell detachment by regulating transcription of downstream targets. In contrast, PAR-1 directly modulates apical-basal polarity between border cells and epithelial cells to promote detachment. Furthermore, PAR-1, but not the apical polarity complex protein PAR-3, promotes the directionality of transient cell protrusions, which border cells require for sensing the chemoattractant gradient.We conclude that PAR-1-dependent apical-basal polarity is required for proper detachment of migratory border cells from neighboring epithelial cells. Moreover, polarity controlled by PAR-1 influences the ability of migratory cells to sense direction, a critical feature of **migration**. Thus, this work reveals new insights into two distinct, but essential, steps of epithelial cell **migration**.
8. Colorectal cancer progression: lessons from Drosophila? Human colorectal cancers arise as benign adenomas, tumours that retain their epithelial character, and then progress to malignant adenocarcinomas and carcinomas in which the epithelium becomes disrupted. Carcinomas often exhibit transcriptional downregulation of E-cadherin and other epithelial genes in an epithelial-to-mesenchymal transition (EMT), a mechanism first discovered in Drosophila to be mediated by the transcription factors Twist and Snail. In contrast, adenocarcinomas retain expression of E-cadherin and disruption of the epithelium occurs through formation of progressively smaller epithelial cysts with apical Crumbs/CRB3, Stardust/PALS1, and Bazooka/PAR3 localised to the inner lumen. Results from Drosophila show that morphologically similar cysts form upon induction of clonal heterogeneity in Wnt, Smad, or Ras signalling levels, which causes extrusion of epithelial cells at clonal boundaries. Thus, intratumour heterogeneity might also promote formation of adenocarcinomas in humans. Finally, epithelial cysts can collectively migrate, as in the case of Drosophila border cells, a potential model system for the invasive **migration** of adenocarcinoma cells.
9. Initiating and guiding **migration**: lessons from border cells. Cell **migration** occurs in many different contexts. Amoebae and other isolated cells migrate in culture. In animals, 'professional' migratory cells of the immune system constantly survey the body for intruders, whereas other cell types perform specific developmentally regulated **migration**s. One simple model for the latter type of event is **migration** of border cells during Drosophila oogenesis. Recent findings have shed light on how border cell fate is induced and on how the **migration** is guided. This article discusses the implications of these studies and compares (invasive) **migration** through a tissue with what is known about cells crawling on a flat substratum.
10. The Drosophila cytokine receptor Domeless controls border cell **migration** and epithelial polarization during oogenesis. In mammals, the JAK/STAT (Janus Kinase/Signal Transducer and Activator of Transcription) signaling pathway is activated in response to cytokines and growth factors to control blood cell development, proliferation and cell determination. In Drosophila, a conserved JAK/STAT signaling pathway controls segmentation in embryos, as well as blood cell development and other processes in larvae and adults. During embryogenesis, transduction of the Unpaired [Upd; also known as Outstretched (Os)] ligand through the JAK/STAT pathway requires Domeless, a putative membrane protein with distant homology to vertebrate type I cytokine receptors. We have isolated domeless (dome) in a screen to identify genes essential in epithelial morphogenesis during oogenesis. The level of dome activity is critical for proper border cell **migration** and is controlled in part through a negative feedback loop. In addition to its essential role in border cells, we show that dome is required in the germarium for the polarization of follicle cells during encapsulation of germline cells. In this process, dome controls the expression of the apical determinant Crumbs. In contrast to the ligand Upd, whose expression is limited to a pair of polar cells at both ends of the egg chamber, dome is expressed in all germline and follicle cells. However, the Dome protein is specifically localized at apicolateral membranes and undergoes ligand-dependent internalization in the follicle cells. dome mutations interact genetically with JAK/STAT pathway genes in border cell **migration** and abolish the nuclear translocation of Stat92E in vivo. We also show that dome functions downstream of upd and that both the extracellular and intracellular domains of Dome are required for JAK/STAT signaling. Altogether, our data indicate that Dome is an essential receptor molecule for Upd and JAK/STAT signaling during oogenesis.
11. JAK/STAT signalling in Drosophila controls cell motility during germ cell **migration**. The gonad is formed from two populations of cells originating at different locations: the primordial germ cells (PGCs), giving rise to either sperm or oocytes, and the somatic gonadal mesoderm precursors (SGPs), which support development of the gametes. Following the PGCs' **migration** during gastrulation, these two populations meet, forming the immature gonad. We present evidence that during embryonic development, the PGCs require the canonical JAK/STAT signalling cascade to migrate efficiently towards the SGPs. Loss of function for any element of the JAK/STAT pathway causes frequent germ cell mislocalisation. We have found that wild-type germ cells produce filopodia while they migrate through the mesoderm towards the gonad. Our observations suggest that PGCs use filopodia to migrate and to keep contact with each other. Interestingly, activation of the JAK/STAT pathway is required for these filopodia to form, and ectopic JAK/STAT activation enhances their formation.
12. **Migration** of Drosophila intestinal stem cells across organ boundaries. All components of the Drosophila intestinal tract, including the endodermal midgut and ectodermal hindgut/Malpighian tubules, maintain populations of dividing stem cells. In the midgut and hindgut, these stem cells originate from within larger populations of intestinal progenitors that proliferate during the larval stage and form the adult intestine during metamorphosis. The origin of stem cells found in the excretory Malpighian tubules ('renal stem cells') has not been established. In this paper, we investigate the **migration** patterns of intestinal progenitors that take place during metamorphosis. Our data demonstrate that a subset of adult midgut progenitors (AMPs) move posteriorly to form the adult ureters and, consecutively, the renal stem cells. Inhibiting cell **migration** by AMP-directed expression of a dominant-negative form of Rac1 protein results in the absence of stem cells in the Malpighian tubules. As the majority of the hindgut progenitor cells migrate posteriorly and differentiate into hindgut enterocytes, a group of the progenitor cells, unexpectedly, invades anteriorly into the midgut territory. Consequently, these progenitor cells differentiate into midgut enterocytes. The midgut determinant GATAe is required for the differentiation of midgut enterocytes derived from hindgut progenitors. Wingless signaling acts to balance the proportion of hindgut progenitors that differentiate as midgut versus hindgut enterocytes. Our findings indicate that a stable boundary between midgut and hindgut/Malpighian tubules is not established during early embryonic development; instead, pluripotent progenitor populations cross in between these organs in both directions, and are able to adopt the fate of the organ in which they come to reside.
13. On the role of PDZ domain-encoding genes in Drosophila border cell **migration**. Cells often move as collective groups during normal embryonic development and wound healing, although the mechanisms governing this type of **migration** are poorly understood. The Drosophila melanogaster border cells migrate as a cluster during late oogenesis and serve as a powerful in vivo genetic model for collective cell **migration**. To discover new genes that participate in border cell **migration**, 64 out of 66 genes that encode PDZ domain-containing proteins were systematically targeted by in vivo RNAi knockdown. The PDZ domain is one of the largest families of protein-protein interaction domains found in eukaryotes. Proteins that contain PDZ domains participate in a variety of biological processes, including signal transduction and establishment of epithelial apical-basal polarity. Targeting PDZ proteins effectively assesses a larger number of genes via the protein complexes and pathways through which these proteins function. par-6, a known regulator of border cell **migration**, was a positive hit and thus validated the approach. Knockdown of 14 PDZ domain genes disrupted **migration** with multiple RNAi lines. The candidate genes have diverse predicted cellular functions and are anticipated to provide new insights into the mechanisms that control border cell movement. As a test of this concept, two genes that disrupted **migration** were characterized in more detail: big bang and the Dlg5 homolog CG6509. We present evidence that Big bang regulates JAK/STAT signaling, whereas Dlg5/CG6509 maintains cluster cohesion. Moreover, these results demonstrate that targeting a selected class of genes by RNAi can uncover novel regulators of collective cell **migration**.
14. Requirement for JAK/STAT signaling throughout border cell **migration** in Drosophila. The evolutionarily conserved JAK/STAT signaling pathway is essential for the proliferation, survival and differentiation of many cells including cancer cells. Recent studies have implicated this transcriptional pathway in the process of cell **migration** in humans, mice, Drosophila and Dictyostelium. In the Drosophila ovary, JAK/STAT signaling is necessary and sufficient for the specification and **migration** of a group of cells called the border cells; however, it is not clear to what extent the requirement for cell fate is distinct from that for cell **migration**. We found that STAT protein is enriched in the migrating border cells throughout their **migration** and is an indicator of cells with highest JAK/STAT activity. In addition, stat(ts) mutants exhibited border cell **migration** defects after just 30 minutes at the non-permissive temperature, prior to any detectable change in the expression of cell fate markers. At later times, cell fate changes became evident, indicating that border cell fate is labile. JAK/STAT signaling was also required for organization of the border cell cluster. Finally, we show that both the accumulation of STAT protein and nuclear accumulation are positively regulated by JAK/STAT activity. The activity of the pathway is negatively regulated by overexpression of a SOCS protein and by blocking endocytosis. Together, our findings suggest that the requirement for STAT in border cells extends beyond the initial specification and delamination of cells from the epithelium.
15. Border-cell **migration** requires integration of spatial and temporal signals by the BTB protein Abrupt. During development, elaborate patterns of cell differentiation and movement must occur in the correct locations and at the proper times. Developmental timing has been studied less than spatial pattern formation, and the mechanisms integrating the two are poorly understood. Border-cell **migration** in the Drosophila ovary occurs specifically at stage 9. Timing of the **migration** is regulated by the steroid hormone ecdysone, whereas spatial patterning of the migratory population requires localized activity of the JAK-STAT pathway. Ecdysone signalling is patterned spatially as well as temporally, although the mechanisms are not well understood. In stage 9 egg chambers, ecdysone signalling is highest in anterior follicle cells including the border cells. We identify the gene abrupt as a repressor of ecdysone signalling and border-cell **migration**. Abrupt protein is normally lost from border-cell nuclei during stage 9, in response to JAK-STAT activity. This contributes to the spatial pattern of the ecdysone response. Abrupt attenuates ecdysone signalling by means of a direct interaction with the basic helix-loop-helix (bHLH) domain of the P160 ecdysone receptor coactivator Taiman (Tai). Taken together, these findings provide a molecular mechanism by which spatial and temporal cues are integrated.
16. The social lives of migrating cells in Drosophila. Studies of cell **migration** in Drosophila are yielding insights into the complex interactions migrating cells have with each other and with the cells in their environment. Intriguing links between factors that promote cell **migration** and those that control cell survival have been reported recently. For example, migrating germ cells compete with the surrounding somatic tissue for the substrate of the lipid phosphate phosphatases encoded by the genes Wunen and Wunen2. Germ cells take up the dephosphorylated lipid and require it for their survival. In addition, the secreted growth factors called PVFs, previously thought to guide the **migration**s of hemocytes in the embryo, were found to function instead predominantly as survival factors. And in border cells, DIAP1 and Dronc, two proteins known mainly for their ability to regulate cell death, were found to control cell **migration**.
17. The Maf factor Traffic jam both enables and inhibits collective cell **migration** in Drosophila oogenesis. Border cell cluster (BCC) **migration** in the Drosophila ovary is an excellent system to study the gene regulatory network that enables collective cell **migration**. Here, we identify the large Maf transcription factor Traffic jam (Tj) as an important regulator of BCC **migration**. Tj has a multifaceted impact on the known core cascade that enables BCC motility, consisting of the Jak/Stat signaling pathway, the C/EBP factor Slow border cells (Slbo), and the downstream effector DE-cadherin (DEcad). The initiation of BCC **migration** coincides with a Slbo-dependent decrease in Tj expression. This reduction of Tj is required for normal BCC motility, as high Tj expression strongly impedes **migration**. At high concentration, Tj has a tripartite negative effect on the core pathway: a decrease in Slbo, an increase in the Jak/Stat inhibitor Socs36E, and a Slbo-independent reduction of DEcad. However, maintenance of a low expression level of Tj in the BCC during **migration** is equally important, as loss of tj function also results in a significant delay in **migration** concomitant with a reduction of Slbo and consequently of DEcad. Taken together, we conclude that the regulatory feedback loop between Tj and Slbo is necessary for achieving the correct activity levels of **migration**-regulating factors to ensure proper BCC motility.
18. JAK-STAT pathway in Drosophila morphogenesis: From organ selector to cell behavior regulator. One of the main contributions of Drosophila to the JAK-STAT field is the study of morphogenesis. JAK-STAT signaling controls the formation of many different structures through surprisingly different morphogenetic behaviors that include induction of cell rearrangements, invagination, folding of tissues, modulation of cell shape, and **migration**. This variability may be explained by the many transcription factors and signaling molecules STAT regulates at early stages of development. But is STAT just acting as an upstream inducer of morphogenesis or does it have a more direct role in controlling cell behaviors? Here we review what is known about how the canonical phosphorylation of STAT contributes to shaping the embryonic and imaginal structures.
19. Cellular and molecular mechanisms of single and collective cell **migration**s in Drosophila: themes and variations. The process of cell **migration** is essential throughout life, driving embryonic morphogenesis and ensuring homeostasis in adults. Defects in cell **migration** are a major cause of human disease, with excessive **migration** causing autoimmune diseases and cancer metastasis, whereas reduced capacity for **migration** leads to birth defects and immunodeficiencies. Myriad studies in vitro have established a consensus view that cell **migration**s require cell polarization, Rho GTPase-mediated cytoskeletal rearrangements, and myosin-mediated contractility. However, in vivo studies later revealed a more complex picture, including the discovery that cells migrate not only as single units but also as clusters, strands, and sheets. In particular, the role of E-Cadherin in cell motility appears to be more complex than previously appreciated. Here, we discuss recent advances achieved by combining the plethora of genetic tools available to the Drosophila geneticist with live imaging and biophysical techniques. Finally, we discuss the emerging themes such studies have revealed and ponder the puzzles that remain to be solved.
20. Genetic interaction screens identify a role for hedgehog signaling in Drosophila border cell **migration**. Cell motility is essential for embryonic development and physiological processes such as the immune response, but also contributes to pathological conditions such as tumor progression and inflammation. However, our understanding of the mechanisms underlying migratory processes is incomplete. Drosophila border cells provide a powerful genetic model to identify the roles of genes that contribute to cell **migration**.Members of the Hedgehog signaling pathway were uncovered in two independent screens for interactions with the small GTPase Rac and the polarity protein Par-1 in border cell **migration**. Consistent with a role in **migration**, multiple Hh signaling components were enriched in the migratory border cells. Interference with Hh signaling by several different methods resulted in incomplete cell **migration**. Moreover, the polarized distribution of E-Cadherin and a marker of tyrosine kinase activity were altered when Hh signaling was disrupted. Conservation of Hh-Rac and Hh-Par-1 signaling was illustrated in the wing, in which Hh-dependent phenotypes were enhanced by loss of Rac or par-1.We identified a pathway by which Hh signaling connects to Rac and Par-1 in cell **migration**. These results further highlight the importance of modifier screens in the identification of new genes that function in developmental pathways.
21. Hox transcription factors: modulators of cell-cell and cell-extracellular matrix adhesion. Hox genes encode homeodomain-containing transcription factors that determine cell and tissue identities in the embryo during development. Hox genes are also expressed in various adult tissues and cancer cells. In Drosophila, expression of cell adhesion molecules, cadherins and integrins, is regulated by Hox proteins operating in hierarchical molecular pathways and plays a crucial role in segment-specific organogenesis. A number of studies using mammalian cultured cells have revealed that cell adhesion molecules responsible for cell-cell and cell-extracellular matrix interactions are downstream targets of Hox proteins. However, whether Hox transcription factors regulate expression of cell adhesion molecules during vertebrate development is still not fully understood. In this review, the potential roles Hox proteins play in cell adhesion and **migration** during vertebrate body patterning are discussed.
22. The Jak/STAT pathway in model organisms: emerging roles in cell movement. The JAK/STAT pathway was originally identified in mammals. Studies of this pathway in the mouse have revealed that JAK/STAT signaling plays a central role during hematopoeisis and other developmental processes. The role of JAK/STAT signaling in blood appears to be conserved throughout evolution, as it is also required during fly hematopoeisis. Studies in Dictyostelium, Drosophila, and zebrafish have shown that the JAK/STAT pathway is also required in an unusually broad set of developmental decisions, including cell proliferation, cell fate determination, cell **migration**, planar polarity, convergent extension, and immunity. There is increasing evidence that the versatility of this pathway relies on its cooperation with other signal transduction pathways. In this review, we discuss the components of the JAK/STAT pathway in model organisms and what is known about its requirement in cellular and developmental processes. In particular, we emphasize recent insights into the role that this pathway plays in the control of cell movement.
23. Awd, the homolog of metastasis suppressor gene Nm23, regulates Drosophila epithelial cell invasion. Border cell **migration** during Drosophila melanogaster oogenesis is a highly pliable model for studying epithelial to mesenchymal transition and directional cell **migration**. The process involves delamination of a group of 6 to 10 follicle cells from the epithelium followed by guided **migration** and invasion through the nurse cell complex toward the oocyte. The guidance cue is mainly provided by the homolog of platelet-derived growth factor/vascular endothelial growth factor family of growth factor, or Pvf, emanating from the oocyte, although Drosophila epidermal growth factor receptor signaling also plays an auxiliary role. Earlier studies implicated a stringent control of the strength of Pvf-mediated signaling since both down-regulation of Pvf and overexpression of active Pvf receptor (Pvr) resulted in stalled border cell **migration**. Here we show that the metastasis suppressor gene homolog Nm23/awd is a negative regulator of border cell **migration**. Its down-regulation allows for optimal spatial signaling from two crucial pathways, Pvr and JAK/STAT. Its overexpression in the border cells results in stalled **migration** and can revert the phenotype of overexpressing constitutive Pvr or dominant-negative dynamin. This is a rare example demonstrating the relevance of a metastasis suppressor gene function utilized in a developmental process involving cell invasion.
24. Regulation of cell adhesion and collective cell **migration** by hindsight and its human homolog RREB1. Cell movements represent a major driving force in embryonic development, tissue repair, and tumor metastasis [1]. The **migration** of single cells has been well studied, predominantly in cell culture [2, 3]; however, in vivo, a greater variety of modes of cell movement occur, including the movements of cells in clusters, strands, sheets, and tubes, also known as collective cell **migration**s [4, 5]. In spite of the relevance of these types of movements in both normal and pathological conditions, the molecular mechanisms that control them remain predominantly unknown. Epithelial follicle cells of the Drosophila ovary undergo several dynamic morphological changes, providing a genetically tractable model [6]. We found that anterior follicle cells, including border cells, mutant for the gene hindsight (hnt) accumulated excess cell-cell adhesion molecules and failed to undergo their normal collective movements. In addition, HNT affected border cell cluster cohesion and motility via effects on the JNK and STAT pathways, respectively. Interestingly, reduction of expression of the mammalian homolog of HNT, RREB1, by siRNA inhibited collective cell **migration** in a scratch-wound healing assay of MCF10A mammary epithelial cells, suppressed surface activity, retarded cell spreading after plating, and led to the formation of immobile, tightly adherent cell colonies. We propose that HNT and RREB1 are essential to reduce cell-cell adhesion when epithelial cells within an interconnected group undergo dynamic changes in cell shape.
25. Genes that drive invasion and **migration** in Drosophila. Successful cell **migration** depends on the careful regulation of the timing of movement, the guidance of motile cells, and cytoskeletal and adhesive changes within the cells. This review focuses on genes that act cell-autonomously to promote these aspects of cell **migration** in Drosophila. We discuss recent advances in understanding the **migration** of the ovarian border cells, embryonic blood cells, primordial germ cells, somatic gonadal precursors, and tracheal cells. Comparison of genes that regulate these processes to those that promote tumorigenesis and metastasis in mammals demonstrates that studies in fruit flies are uncovering new genes highly relevant to cancer biology.
26. Branching morphogenesis of the Drosophila tracheal system. Many organs including the mammalian lung and vascular system consist of branched tubular networks that transport essential gases or fluids, but the genetic programs that control the development of these complex three-dimensional structures are not well understood. The Drosophila melanogaster tracheal (respiratory) system is a network of interconnected epithelial tubes that transports oxygen and other gases in the body and provides a paradigm of branching morphogenesis. It develops by sequential sprouting of primary, secondary, and terminal branches from an epithelial sac of approximately 80 cells in each body segment of the embryo. Mapping of the cell movements and shape changes during the sprouting process has revealed that distinct mechanisms of epithelial **migration** and tube formation are used at each stage of branching. Genetic dissection of the process has identified a general program in which a fibroblast growth factor (FGF) and fibroblast growth factor receptor (FGFR) are used repeatedly to control branch budding and outgrowth. At each stage of branching, the mechanisms controlling FGF expression and the downstream signal transduction pathway change, altering the pattern and structure of the branches that form. During terminal branching, FGF expression is regulated by hypoxia, ensuring that tracheal structure matches cellular oxygen need. A branch diversification program operates in parallel to the general budding program: Regional signals locally modify the general program, conferring specific structural features and other properties on individual branches, such as their substrate outgrowth preferences, differences in tube size and shape, and the ability to fuse to other branches to interconnect the network.
27. Function of the ETS transcription factor Yan in border cell **migration**. Invasive cell **migration** in both normal development and metastatic cancer is regulated by various signaling pathways, transcription factors and cell-adhesion molecules. The coordination between these activities in the context of cell **migration** is poorly understood. During Drosophila oogenesis, a small group of cells called border cells exit the follicular epithelium to perform a stereotypic, invasive **migration**. We find that the ETS transcription factor Yan is required for border cell **migration** and that Yan expression is spatiotemporally regulated as border cells migrate from the anterior pole of the egg chamber towards the nurse cell-oocyte boundary. Yan expression is dependent on inputs from the JAK/STAT, Notch and Receptor Tyrosine Kinase pathways in border cells. Mechanistically, Yan functions to modulate the turnover of DE-Cadherin-dependent adhesive complexes to facilitate border cell **migration**. Our results suggest that Yan acts as a pivotal link between signal transduction, cell adhesion and invasive cell **migration** in Drosophila border cells.
28. Systematic analysis of the transcriptional switch inducing **migration** of border cells. Cell **migration** within a natural context is tightly controlled, often by specific transcription factors. However, the switch from stationary to migratory behavior is poorly understood. Border cells perform a spatially and temporally controlled invasive **migration** during Drosophila oogenesis. Slbo, a C/EBP family transcriptional activator, is required for them to become migratory. We purified wild-type and slbo mutant border cells as well as nonmigratory follicle cells and performed comparative whole-genome expression profiling, followed by functional tests of the contributions of identified targets to **migration**. About 300 genes were significantly upregulated in border cells, many dependent on Slbo. Among these, the microtubule regulator Stathmin was strongly upregulated and was required for normal **migration**. Actin cytoskeleton regulators were also induced, including, surprisingly, a large cluster of "muscle-specific" genes. We conclude that Slbo induces multiple cytoskeletal effectors, and that each contributes to the behavioral changes in border cells.
29. Tousled-like kinase regulates cytokine-mediated communication between cooperating cell types during collective border cell **migration**. Collective cell **migration** is emerging as a major contributor to normal development and disease. Collective movement of border cells in the Drosophila ovary requires cooperation between two distinct cell types: four to six migratory cells surrounding two immotile cells called polar cells. Polar cells secrete a cytokine, Unpaired (Upd), which activates JAK/STAT signaling in neighboring cells, stimulating their motility. Without Upd, **migration** fails, causing sterility. Ectopic Upd expression is sufficient to stimulate motility in otherwise immobile cells. Thus regulation of Upd is key. Here we report a limited RNAi screen for nuclear proteins required for border cell **migration**, which revealed that the gene encoding Tousled-like kinase (Tlk) is required in polar cells for Upd expression without affecting polar cell fate. In the absence of Tlk, fewer border cells are recruited and motility is impaired, similar to inhibition of JAK/STAT signaling. We further show that Tlk in polar cells is required for JAK/STAT activation in border cells. Genetic interactions further confirmed Tlk as a new regulator of Upd/JAK/STAT signaling. These findings shed light on the molecular mechanisms regulating the cooperation of motile and nonmotile cells during collective invasion, a phenomenon that may also drive metastatic cancer.
30. Paracrine signaling through the JAK/STAT pathway activates invasive behavior of ovarian epithelial cells in Drosophila. The JAK/STAT signaling pathway, renowned for its effects on cell proliferation and survival, is constitutively active in various human cancers, including ovarian. We have found that JAK and STAT are required to convert the border cells in the Drosophila ovary from stationary, epithelial cells to migratory, invasive cells. The ligand for this pathway, Unpaired (UPD), is expressed by two central cells within the migratory cell cluster. Mutations in upd or jak cause defects in **migration** and a reduction in the number of cells recruited to the cluster. Ectopic expression of either UPD or JAK is sufficient to induce extra epithelial cells to migrate. Thus, a localized signal activates the JAK/STAT pathway in neighboring epithelial cells, causing them to become invasive.
31. Coactivation of STAT and Ras is required for germ cell proliferation and invasive **migration** in Drosophila. Primordial germ cells (PGCs) undergo proliferation, invasion, guided **migration**, and aggregation to form the gonad. Here we show that in Drosophila, the receptor tyrosine kinase Torso activates both STAT and Ras during the early phase of PGC development, and coactivation of STAT and Ras is required for PGC proliferation and invasive **migration**. Embryos mutant for stat92E or Ras1 have fewer PGCs, and these cells migrate slowly, errantly, and fail to coalesce. Conversely, overactivation of these molecules causes supernumerary PGCs, their premature transit through the gut epithelium, and ectopic colonization. A requirement for RTK in Drosophila PGC development is analogous to the mouse, in which the RTK c-kit is required, suggesting a conserved molecular mechanism governing PGC behavior in flies and mammals.
32. The planar polarity pathway promotes coordinated cell **migration** during Drosophila oogenesis. Cell **migration** is fundamental in both animal morphogenesis and disease. The **migration** of individual cells is relatively well-studied; however, in vivo, cells often remain joined by cell-cell junctions and migrate in cohesive groups. How such groups of cells coordinate their **migration** is poorly understood. The planar polarity pathway coordinates the polarity of non-migrating cells in epithelial sheets and is required for cell rearrangements during vertebrate morphogenesis. It is therefore a good candidate to play a role in the collective **migration** of groups of cells. Drosophila border cell **migration** is a well-characterised and genetically tractable model of collective cell **migration**, during which a group of about six to ten epithelial cells detaches from the anterior end of the developing egg chamber and migrates invasively towards the oocyte. We find that the planar polarity pathway promotes this invasive **migration**, acting both in the migrating cells themselves and in the non-migratory polar follicle cells that they carry along. Disruption of planar polarity signalling causes abnormalities in actin-rich processes on the cell surface and leads to less-efficient **migration**. This is apparently due, in part, to a loss of regulation of Rho GTPase activity by the planar polarity receptor Frizzled, which itself becomes localised to the migratory edge of the border cells. We conclude that, during collective cell **migration**, the planar polarity pathway can mediate communication between motile and non-motile cells, which enhances the efficiency of **migration** via the modulation of actin dynamics.
33. The Hippo pathway controls border cell **migration** through distinct mechanisms in outer border cells and polar cells of the Drosophila ovary. The Hippo pathway is a key signaling cascade in controlling organ size. The core components of this pathway are two kinases, Hippo (Hpo) and Warts (Wts), and a transcriptional coactivator, Yorkie (Yki). Yes-associated protein (YAP, a Yki homolog in mammals) promotes epithelial-mesenchymal transition and cell **migration** in vitro. Here, we use border cells in the Drosophila ovary as a model to study Hippo pathway functions in cell **migration** in vivo. During oogenesis, polar cells secrete Unpaired (Upd), which activates JAK/STAT signaling of neighboring cells and specifies them into outer border cells. The outer border cells form a cluster with polar cells and undergo **migration**. We find that hpo and wts are required for **migration** of the border cell cluster. In outer border cells, overexpression of hpo disrupts polarization of the actin cytoskeleton and attenuates **migration**. In polar cells, knockdown of hpo and wts or overexpression of yki impairs border cell induction and disrupts **migration**. These manipulations in polar cells reduce JAK/STAT activity in outer border cells. Expression of upd-lacZ is increased and decreased in yki and hpo mutant polar cells, respectively. Furthermore, forced expression of upd in polar cells rescues defects of border cell induction and **migration** caused by wts knockdown. These results suggest that Yki negatively regulates border cell induction by inhibiting JAK/STAT signaling. Together, our data elucidate two distinct mechanisms of the Hippo pathway in controlling border cell **migration**: (1) in outer border cells, it regulates polarized distribution of the actin cytoskeleton; (2) in polar cells, it regulates upd expression to control border cell induction and **migration**.
34. The JAK/STAT pathway is required for border cell **migration** during Drosophila oogenesis. During Drosophila oogenesis, border cells perform a stereotypic **migration**. Slbo, a C/EBP transcription factor, is required for this **migration**. Here we identify Drosophila Stat92E (signal transducer and activator of transcription) in a screen for gain-of-function suppressors of the slbo mutant phenotype. By clonal analysis for Stat92E and hop (Drosophila JAK) mutants we find that the JAK/STAT pathway is required in border cells for their **migration**. The activating ligand for the pathway, Unpaired, is expressed in polar cells. Polar cells are specialized cells which can induce border cell fate in anterior follicle cells. On its own, ectopic expression of Unpaired can induce ectopic expression of border cell markers including Slbo. However, Stat92E mutant cells still express normal levels of Slbo protein, thus Stat92E must regulate other targets critical for border cell **migration**.
35. The ballet of morphogenesis: unveiling the hidden choreographers. Several recent papers reveal new insights into the mechanisms by which cells turn their perceptions about fate into action, focusing on the role of Wnt signal transduction in cell polarization and **migration**.
36. Feedback inhibition of Jak/STAT signaling by apontic is required to limit an invasive cell population. In both normal development and in a variety of pathological conditions, epithelial cells can acquire migratory and invasive properties. Border cells in the Drosophila ovary provide a genetically tractable model for elucidating the mechanisms controlling such behaviors. Here we report the identification of a mutant, apontic (apt), in which the migratory population expanded and separation from the epithelium was impeded. This phenotype resembled gain-of-function of JAK/STAT activity. Gain-of-function of APT also mimicked loss of function of STAT and its key downstream target, SLBO. APT expression was induced by STAT, which bound directly to sites in the apt gene. The data suggest that a regulatory circuit between STAT, APT, and SLBO functions to convert an initially graded signal into an all-or-nothing activation of JAK/STAT and thus to proper cell specification and **migration**. These findings are supported by a mathematical model, which accurately simulates wild-type and mutant phenotypes.
37. Ecdysone signalling and ovarian development in insects: from stem cells to ovarian follicle formation. Although a great deal of information is available concerning the role of ecdysone in insect oogenesis, research has tended to focus on vitellogenesis and choriogenesis. As such, the study of oogenesis in a strict sense has received much less attention. This situation changed recently when a number of observations carried out in the meroistic polytrophic ovarioles of Drosophila melanogaster started to unravel the key roles played by ecdysone in different steps of oogenesis. Thus, in larval stages, a non-autonomous role of ecdysone, first in repression and later in activation, of stem cell niche and primordial germ cell differentiation has been reported. In the adult, ecdysone stimulates the proliferation of germline stem cells, plays a role in stem cell niche maintenance and is needed non-cell-autonomously for correct differentiation of germline stem cells. Moreover, in somatic cells ecdysone is required for 16-cell cyst formation and for ovarian follicle development. In the transition from stages 8 to 9 of oogenesis, ecdysone signalling is fundamental when deciding whether or not to go ahead with vitellogenesis depending on the nutritional status, as well as to start border cell **migration**. This article is part of a Special Issue entitled: Nuclear receptors in animal development.
38. JAK/STAT signaling regulates tissue outgrowth and male germline stem cell fate in Drosophila. In multicellular organisms, biological activities are regulated by cell signaling. The various signal transduction pathways regulate cell fate, proliferation, **migration**, and polarity. Miscoordination of the communicative signals will lead to disasters like cancer and other fatal diseases. The JAK/STAT signal transduction pathway is one of the pathways, which was first identified in vertebrates and is highly conserved throughout evolution. Studying the JAK/STAT signal transduction pathway in Drosophila provides an excellent opportunity to understand the molecular mechanism of the cell regulation during development and tumor formation. In this review, we discuss the general overview of JAK/STAT signaling in Drosophila with respect to its functions in the eye development and stem cell fate determination.

#### GeneRIFs

1. By clonal analysis for Stat92E and hop (Drosophila JAK) mutants we find that the JAK/STAT pathway is required in border cells for their **migration**
2. STAT signalling controls cell motility during germ cell **migration**.

### ECM

#### PubMed

1. Differential activities of the Drosophila JAK/STAT pathway ligands Upd, Upd2 and Upd3. JAK/STAT signalling in vertebrates is activated by multiple cytokines and growth factors. By contrast, the Drosophila genome encodes for only three related JAK/STAT ligands, Upd, Upd2 and Upd3. Identifying the differences between these three ligands will ultimately lead to a greater understanding of this disease-related signalling pathway and its roles in development. Here, we describe the analysis of the least well characterised of the Upd-like ligands, Upd3. We show that in tissue culture-based assays Upd3-GFP is secreted from cells and appears to interact with the extracellular matrix (**ECM**) in a similar manner to Upd, while still non-autonomously activating JAK/STAT signalling. Quantification of each of the Upd-like ligands in conditioned media has allowed us to determine the activity of equal amounts of each ligand on JAK/STAT ex vivo and reveals that Upd is the most potent ligand in this system. Finally, investigations into the effects of ectopic expression of Upd3 in vivo have confirmed its ability to activate pathway signalling at long-distance.
2. Drosophila perlecan regulates intestinal stem cell activity via cell-matrix attachment. Stem cells require specialized local microenvironments, termed niches, for normal retention, proliferation, and multipotency. Niches are composed of cells together with their associated extracellular matrix (**ECM**). Currently, the roles of **ECM** in regulating niche functions are poorly understood. Here, we demonstrate that Perlecan (Pcan), a highly conserved **ECM** component, controls intestinal stem cell (ISC) activities and ISC-**ECM** attachment in Drosophila adult posterior midgut. Loss of Pcan from ISCs, but not other surrounding cells, causes ISCs to detach from underlying **EC**M, lose their identity, and fail to proliferate. These defects are not a result of a loss of epidermal growth factor receptor (EGFR) or Janus kinase/signal transducer and activator of transcription (JAK/STAT) signaling activity but partially depend on integrin signaling activity. We propose that Pcan secreted by ISCs confers niche properties to the adjacen**t E**CM that is required for ISC maintenance of stem cell identity, activity, and anchorage to the niche.

### [Ee]xtracellular [Mm]atrix

#### PubMed

1. Differential activities of the Drosophila JAK/STAT pathway ligands Upd, Upd2 and Upd3. JAK/STAT signalling in vertebrates is activated by multiple cytokines and growth factors. By contrast, the Drosophila genome encodes for only three related JAK/STAT ligands, Upd, Upd2 and Upd3. Identifying the differences between these three ligands will ultimately lead to a greater understanding of this disease-related signalling pathway and its roles in development. Here, we describe the analysis of the least well characterised of the Upd-like ligands, Upd3. We show that in tissue culture-based assays Upd3-GFP is secreted from cells and appears to interact with the **extracellular matrix** (ECM) in a similar manner to Upd, while still non-autonomously activating JAK/STAT signalling. Quantification of each of the Upd-like ligands in conditioned media has allowed us to determine the activity of equal amounts of each ligand on JAK/STAT ex vivo and reveals that Upd is the most potent ligand in this system. Finally, investigations into the effects of ectopic expression of Upd3 in vivo have confirmed its ability to activate pathway signalling at long-distance.
2. Characterisation of Upd2, a Drosophila JAK/STAT pathway ligand. The characterisation of ligands that activate the JAK/STAT pathway has the potential to throw light onto a comparatively poorly understood aspect of this important signal transduction cascade. Here, we describe our analysis of the only invertebrate JAK/STAT pathway ligands identified to date, the Drosophila unpaired-like family. We show that upd2 is expressed in a pattern essentially identical to that of upd and demonstrate that the proteins encoded by this region activate JAK/STAT pathway signalling. Mutational analysis demonstrates a mutual semi-redundancy that can be visualised in multiple tissues known to require JAK/STAT signalling. In order to better characterise the in vivo function of these ligands, we developed a reporter based on a natural JAK/STAT pathway responsive enhancer and show that ectopic upd2 expression can effectively activate the JAK/STAT pathway. While both Upd and Upd2 are secreted JAK/STAT pathway agonists, tissue culture assays show that the signal-sequences of Upd and Upd2 confer distinct properties, with Upd associated primarily with the **extracellular matrix** and Upd2 secreted into the media. The differing biophysical characteristics identified for Upd-like molecules have implications for their function in vivo and adds another aspect to our understanding of cytokine signalling in Drosophila.
3. Tissue design: how Drosophila tumors remodel their neighborhood. Drosophila genetics has long been appreciated as a powerful approach for discovering the normal functions of genes that act as oncogenes and tumor suppressors in human cancer. Recent studies have also highlighted its advantages for deciphering how such genes function during tumorigenesis itself. Here we detail studies relating to how tumors, generated in developing organs and adult stem cell-based tissues, remodel the tissue landscape to their benefit. Like mammalian tumors, insect tumors can dissolve **extracellular matrix**, recruit blood cells, migrate and invade other tissues. While much is known about how mammalian fibroblasts, immune cells and vasculature promote late tumorigenesis, less is understood about the very earliest stages of tumor development in mammals. Because Drosophila has fewer mitotic cells and a simpler tissue architecture, it affords easy detection and analysis of early clonal tumor growth. Drosophila studies have revealed both cooperative and competitive interactions between tumor and normal cells during early tumor growth. During development, these interactions typically occur with other proliferative progenitor cells, but in adult stem cell-based tissues, the stem cell niche can fuel tumor growth.
4. Hox transcription factors: modulators of cell-cell and cell-**extracellular matrix** adhesion. Hox genes encode homeodomain-containing transcription factors that determine cell and tissue identities in the embryo during development. Hox genes are also expressed in various adult tissues and cancer cells. In Drosophila, expression of cell adhesion molecules, cadherins and integrins, is regulated by Hox proteins operating in hierarchical molecular pathways and plays a crucial role in segment-specific organogenesis. A number of studies using mammalian cultured cells have revealed that cell adhesion molecules responsible for cell-cell and cell-**extracellular matrix** interactions are downstream targets of Hox proteins. However, whether Hox transcription factors regulate expression of cell adhesion molecules during vertebrate development is still not fully understood. In this review, the potential roles Hox proteins play in cell adhesion and migration during vertebrate body patterning are discussed.
5. Drosophila perlecan regulates intestinal stem cell activity via cell-matrix attachment. Stem cells require specialized local microenvironments, termed niches, for normal retention, proliferation, and multipotency. Niches are composed of cells together with their associated **extracellular matrix** (ECM). Currently, the roles of ECM in regulating niche functions are poorly understood. Here, we demonstrate that Perlecan (Pcan), a highly conserved ECM component, controls intestinal stem cell (ISC) activities and ISC-ECM attachment in Drosophila adult posterior midgut. Loss of Pcan from ISCs, but not other surrounding cells, causes ISCs to detach from underlying ECM, lose their identity, and fail to proliferate. These defects are not a result of a loss of epidermal growth factor receptor (EGFR) or Janus kinase/signal transducer and activator of transcription (JAK/STAT) signaling activity but partially depend on integrin signaling activity. We propose that Pcan secreted by ISCs confers niche properties to the adjacent ECM that is required for ISC maintenance of stem cell identity, activity, and anchorage to the niche.
6. Drosophila unpaired encodes a secreted protein that activates the JAK signaling pathway. In vertebrates, many cytokines and growth factors have been identified as activators of the JAK/STAT signaling pathway. In Drosophila, JAK and STAT molecules have been isolated, but no ligands or receptors capable of activating the pathway have been described. We have characterized the unpaired (upd) gene, which displays the same distinctive embryonic mutant defects as mutations in the Drosophila JAK (hopscotch) and STAT (stat92E) genes. Upd is a secreted protein, associated with the **extracellular matrix**, that activates the JAK pathway. We propose that Upd is a ligand that relies on JAK signaling to stimulate transcription of pair-rule genes in a segmentally restricted manner in the early Drosophila embryo.
7. Hemocytes are essential for wing maturation in Drosophila melanogaster. Newly eclosed flies have wings that are highly folded and compact. Within an hour, each wing has expanded, the dorsal and ventral cuticular surfaces bonding to one another to form the mature wing. To initiate a dissection of this process, we present studies of two mutant phenotypes. First, the batone mutant blocks wing expansion, a behavior that is shown to have a mutant focus anterior to the wing in the embryonic fate map. Second, ectopic expression of protein kinase A catalytic subunit (PKAc) using certain GAL4 enhancer detector strains mimics the batone wing phenotype and also induces melanotic "tumors." Surprisingly, these GAL4 strains express GAL4 in cells, which seem to be hemocytes, found between the dorsal and ventral surfaces of newly opened wings. Ectopic expression of Ricin A in these cells reduces their number and prevents bonding of the wing surfaces without preventing wing expansion. We propose that hemocytes are present in the wing to phagocytose apoptotic epithelial cells and to synthesize an **extracellular matrix** that bonds the two wing surfaces together. Hemocytes are known to form melanotic tumors either as part of an innate immune response or under other abnormal conditions, including evidently ectopic PKAc expression. Ectopic expression of PKAc in the presence of the batone mutant causes dominant lethality, suggesting a functional relationship. We propose that batone is required for the release of a hormone necessary for wing expansion and tissue remodeling by hemocytes in the wing.

### [Ii]nvasive

#### PubMed

1. Paracrine signaling through the JAK/STAT pathway activates **invasive** behavior of ovarian epithelial cells in Drosophila. The JAK/STAT signaling pathway, renowned for its effects on cell proliferation and survival, is constitutively active in various human cancers, including ovarian. We have found that JAK and STAT are required to convert the border cells in the Drosophila ovary from stationary, epithelial cells to migratory, **invasive** cells. The ligand for this pathway, Unpaired (UPD), is expressed by two central cells within the migratory cell cluster. Mutations in upd or jak cause defects in migration and a reduction in the number of cells recruited to the cluster. Ectopic expression of either UPD or JAK is sufficient to induce extra epithelial cells to migrate. Thus, a localized signal activates the JAK/STAT pathway in neighboring epithelial cells, causing them to become **invasive**.
2. Socs36E limits STAT signaling via Cullin2 and a SOCS-box independent mechanism in the Drosophila egg chamber. The Suppressor of Cytokine Signaling (SOCS) proteins are critical, highly conserved feedback inhibitors of signal transduction cascades. The family of SOCS proteins is divided into two groups: ancestral and vertebrate-specific SOCS proteins. Vertebrate-specific SOCS proteins have been heavily studied as a result of their strong mutant phenotypes. However, the ancestral clade remains less studied, a potential result of genetic redundancies in mammals. Use of the genetically tractable organism Drosophila melanogaster enables in vivo assessment of signaling components and mechanisms with less concern about the functional redundancy observed in mammals. In this study, we investigated how the SOCS family member Suppressor of Cytokine Signaling at 36E (Socs36E) attenuates Janus Kinase/Signal Transducer and Activator of Transcription (Jak/STAT) activation during specification of motile border cells in Drosophila oogenesis. We found that Socs36E genetically interacts with the Cullin2 (Cul2) scaffolding protein. Like Socs36E, Cul2 is required to limit the number of motile cells in egg chambers. We demonstrated that loss of Cul2 in the follicle cells significantly increased nuclear STAT protein levels, which resulted in additional cells acquiring **invasive** properties. Further, reduction of Cul2 suppressed border cell migration defects that occur in a Stat92E-sensitized genetic background. Our data incorporated Cul2 into a previously described Jak/STAT-directed genetic regulatory network that is required to generate a discrete boundary between cell fates. We also found that Socs36E is able to attenuate STAT activity in the egg chamber when it does not have a functional SOCS box. Collectively, this work contributes mechanistic insight to a Jak/STAT regulatory genetic circuit, and suggests that Socs36E regulates Jak/STAT signaling via a Cul2-dependent mechanism, as well as by a Cullin-independent manner, in vivo.
3. Socs36E attenuates STAT signaling to optimize motile cell specification in the Drosophila ovary. The Janus kinase/Signal transducers and activators of transcription (JAK/STAT) pathway determines cell fates by regulating gene expression. One example is the specification of the motile cells called border cells during Drosophila oogenesis. It has been established that too much or too little STAT activity disrupts follicle cell identity and cell motility, which suggests the signaling must be precisely regulated. Here, we find that Suppressor of cytokine signaling at 36E (Socs36E) is a necessary negative regulator of JAK/STAT signaling during border cell specification. We find when STAT signaling is too low to induce migration in the presumptive border cell population, nearby follicle cells uncharacteristically become **invasive** to enable efficient migration of the cluster. We generated a genetic null allele that reveals Socs36E is required in the anterior follicle cells to limit **invasive** behavior to an optimal number of cells. We further show Socs36E genetically interacts with the required STAT feedback inhibitor apontic (apt) and APT's downstream target, mir-279, and provide evidence that suggests APT directly regulates Socs36E transcriptionally. Our work shows Socs36E plays a critical role in a genetic circuit that establishes a boundary between the motile border cell cluster and its non-**invasive** epithelial neighbors through STAT attenuation.
4. Colorectal cancer progression: lessons from Drosophila? Human colorectal cancers arise as benign adenomas, tumours that retain their epithelial character, and then progress to malignant adenocarcinomas and carcinomas in which the epithelium becomes disrupted. Carcinomas often exhibit transcriptional downregulation of E-cadherin and other epithelial genes in an epithelial-to-mesenchymal transition (EMT), a mechanism first discovered in Drosophila to be mediated by the transcription factors Twist and Snail. In contrast, adenocarcinomas retain expression of E-cadherin and disruption of the epithelium occurs through formation of progressively smaller epithelial cysts with apical Crumbs/CRB3, Stardust/PALS1, and Bazooka/PAR3 localised to the inner lumen. Results from Drosophila show that morphologically similar cysts form upon induction of clonal heterogeneity in Wnt, Smad, or Ras signalling levels, which causes extrusion of epithelial cells at clonal boundaries. Thus, intratumour heterogeneity might also promote formation of adenocarcinomas in humans. Finally, epithelial cysts can collectively migrate, as in the case of Drosophila border cells, a potential model system for the **invasive** migration of adenocarcinoma cells.
5. Initiating and guiding migration: lessons from border cells. Cell migration occurs in many different contexts. Amoebae and other isolated cells migrate in culture. In animals, 'professional' migratory cells of the immune system constantly survey the body for intruders, whereas other cell types perform specific developmentally regulated migrations. One simple model for the latter type of event is migration of border cells during Drosophila oogenesis. Recent findings have shed light on how border cell fate is induced and on how the migration is guided. This article discusses the implications of these studies and compares (**invasive**) migration through a tissue with what is known about cells crawling on a flat substratum.
6. **Invasive** and indigenous microbiota impact intestinal stem cell activity through multiple pathways in Drosophila. Gut homeostasis is controlled by both immune and developmental mechanisms, and its disruption can lead to inflammatory disorders or cancerous lesions of the intestine. While the impact of bacteria on the mucosal immune system is beginning to be precisely understood, little is known about the effects of bacteria on gut epithelium renewal. Here, we addressed how both infectious and indigenous bacteria modulate stem cell activity in Drosophila. We show that the increased epithelium renewal observed upon some bacterial infections is a consequence of the oxidative burst, a major defense of the Drosophila gut. Additionally, we provide evidence that the JAK-STAT (Janus kinase-signal transducers and activators of transcription) and JNK (c-Jun NH(2) terminal kinase) pathways are both required for bacteria-induced stem cell proliferation. Similarly, we demonstrate that indigenous gut microbiota activate the same, albeit reduced, program at basal levels. Altered control of gut microbiota in immune-deficient or aged flies correlates with increased epithelium renewal. Finally, we show that epithelium renewal is an essential component of Drosophila defense against oral bacterial infection. Altogether, these results indicate that gut homeostasis is achieved by a complex interregulation of the immune response, gut microbiota, and stem cell activity.
7. Gα73B is a downstream effector of JAK/STAT signalling and a regulator of Rho1 in Drosophila haematopoiesis. JAK/STAT signalling regulates many essential developmental processes including cell proliferation and haematopoiesis, whereas its inappropriate activation is associated with the majority of myeloproliferative neoplasias and numerous cancers. Furthermore, high levels of JAK/STAT pathway signalling have also been associated with enhanced metastatic invasion by cancerous cells. Strikingly, gain-of-function mutations in the single Drosophila JAK homologue, Hopscotch, result in haemocyte neoplasia, inappropriate differentiation and the formation of melanised haemocyte-derived 'tumour' masses; phenotypes that are partly orthologous to human gain-of-function JAK2-associated pathologies. Here we show that Gα73B, a novel JAK/STAT pathway target gene, is necessary for JAK/STAT-mediated tumour formation in flies. In addition, although Gα73B does not affect haemocyte differentiation, it does regulate haemocyte morphology and motility under non-pathological conditions. We show that Gα73B is required for constitutive, but not injury-induced, activation of Rho1 and for the localisation of Rho1 into filopodia upon haemocyte activation. Consistent with these results, we also show that Rho1 interacts genetically with JAK/STAT signalling, and that wild-type levels of Rho1 are necessary for tumour formation. Our findings link JAK/STAT transcriptional outputs, Gα73B activity and Rho1-dependent cytoskeletal rearrangements and cell motility, therefore connecting a pathway associated with cancer with a marker indicativ**e of inv**asiveness. As such, we suggest a mechanism by which JAK/STAT pathway signalling may promote metastasis.
8. Function of the ETS transcription factor Yan in border cell migration. **Invasive** cell migration in both normal development and metastatic cancer is regulated by various signaling pathways, transcription factors and cell-adhesion molecules. The coordination between these activities in the context of cell migration is poorly understood. During Drosophila oogenesis, a small group of cells called border cells exit the follicular epithelium to perform a stereotypic, **invasive** migration. We find that the ETS transcription factor Yan is required for border cell migration and that Yan expression is spatiotemporally regulated as border cells migrate from the anterior pole of the egg chamber towards the nurse cell-oocyte boundary. Yan expression is dependent on inputs from the JAK/STAT, Notch and Receptor Tyrosine Kinase pathways in border cells. Mechanistically, Yan functions to modulate the turnover of DE-Cadherin-dependent adhesive complexes to facilitate border cell migration. Our results suggest that Yan acts as a pivotal link between signal transduction, cell adhesion and **invasive** cell migration in Drosophila border cells.
9. Systematic analysis of the transcriptional switch inducing migration of border cells. Cell migration within a natural context is tightly controlled, often by specific transcription factors. However, the switch from stationary to migratory behavior is poorly understood. Border cells perform a spatially and temporally controlled **invasive** migration during Drosophila oogenesis. Slbo, a C/EBP family transcriptional activator, is required for them to become migratory. We purified wild-type and slbo mutant border cells as well as nonmigratory follicle cells and performed comparative whole-genome expression profiling, followed by functional tests of the contributions of identified targets to migration. About 300 genes were significantly upregulated in border cells, many dependent on Slbo. Among these, the microtubule regulator Stathmin was strongly upregulated and was required for normal migration. Actin cytoskeleton regulators were also induced, including, surprisingly, a large cluster of "muscle-specific" genes. We conclude that Slbo induces multiple cytoskeletal effectors, and that each contributes to the behavioral changes in border cells.
10. Paracrine signaling through the JAK/STAT pathway activates **invasive** behavior of ovarian epithelial cells in Drosophila. The JAK/STAT signaling pathway, renowned for its effects on cell proliferation and survival, is constitutively active in various human cancers, including ovarian. We have found that JAK and STAT are required to convert the border cells in the Drosophila ovary from stationary, epithelial cells to migratory, **invasive** cells. The ligand for this pathway, Unpaired (UPD), is expressed by two central cells within the migratory cell cluster. Mutations in upd or jak cause defects in migration and a reduction in the number of cells recruited to the cluster. Ectopic expression of either UPD or JAK is sufficient to induce extra epithelial cells to migrate. Thus, a localized signal activates the JAK/STAT pathway in neighboring epithelial cells, causing them to become **invasive**.
11. Coactivation of STAT and Ras is required for germ cell proliferation and **invasive** migration in Drosophila. Primordial germ cells (PGCs) undergo proliferation, invasion, guided migration, and aggregation to form the gonad. Here we show that in Drosophila, the receptor tyrosine kinase Torso activates both STAT and Ras during the early phase of PGC development, and coactivation of STAT and Ras is required for PGC proliferation and **invasive** migration. Embryos mutant for stat92E or Ras1 have fewer PGCs, and these cells migrate slowly, errantly, and fail to coalesce. Conversely, overactivation of these molecules causes supernumerary PGCs, their premature transit through the gut epithelium, and ectopic colonization. A requirement for RTK in Drosophila PGC development is analogous to the mouse, in which the RTK c-kit is required, suggesting a conserved molecular mechanism governing PGC behavior in flies and mammals.
12. The planar polarity pathway promotes coordinated cell migration during Drosophila oogenesis. Cell migration is fundamental in both animal morphogenesis and disease. The migration of individual cells is relatively well-studied; however, in vivo, cells often remain joined by cell-cell junctions and migrate in cohesive groups. How such groups of cells coordinate their migration is poorly understood. The planar polarity pathway coordinates the polarity of non-migrating cells in epithelial sheets and is required for cell rearrangements during vertebrate morphogenesis. It is therefore a good candidate to play a role in the collective migration of groups of cells. Drosophila border cell migration is a well-characterised and genetically tractable model of collective cell migration, during which a group of about six to ten epithelial cells detaches from the anterior end of the developing egg chamber and migrates **invasive**ly towards the oocyte. We find that the planar polarity pathway promotes this **invasive** migration, acting both in the migrating cells themselves and in the non-migratory polar follicle cells that they carry along. Disruption of planar polarity signalling causes abnormalities in actin-rich processes on the cell surface and leads to less-efficient migration. This is apparently due, in part, to a loss of regulation of Rho GTPase activity by the planar polarity receptor Frizzled, which itself becomes localised to the migratory edge of the border cells. We conclude that, during collective cell migration, the planar polarity pathway can mediate communication between motile and non-motile cells, which enhances the efficiency of migration via the modulation of actin dynamics.
13. Feedback inhibition of Jak/STAT signaling by apontic is required to limit an **invasive** cell population. In both normal development and in a variety of pathological conditions, epithelial cells can acquire migratory and **invasive** properties. Border cells in the Drosophila ovary provide a genetically tractable model for elucidating the mechanisms controlling such behaviors. Here we report the identification of a mutant, apontic (apt), in which the migratory population expanded and separation from the epithelium was impeded. This phenotype resembled gain-of-function of JAK/STAT activity. Gain-of-function of APT also mimicked loss of function of STAT and its key downstream target, SLBO. APT expression was induced by STAT, which bound directly to sites in the apt gene. The data suggest that a regulatory circuit between STAT, APT, and SLBO functions to convert an initially graded signal into an all-or-nothing activation of JAK/STAT and thus to proper cell specification and migration. These findings are supported by a mathematical model, which accurately simulates wild-type and mutant phenotypes.

#### GeneRIFs

1. signal activates the JAK/STAT92E pathway in neighboring epithelial cells, causing them to become **invasive**.

### [Mm]etastasis

#### PubMed

1. Drosophila at the intersection of infection, inflammation, and cancer. Recent studies show that both cellular and humoral aspects of innate immunity play important roles during tumor progression. These interactions have traditionally been explored in vertebrate model systems. In recent years, Drosophila has emerged as a genetically tractable model system for studying key aspects of tumorigenesis including proliferation, invasion, and **metastasis**. The absence of adaptive immunity in Drosophila provides a unique opportunity to study the interactions between innate immune system and cancer in different genetic contexts. In this review, I discuss recent advances made by using Drosophila models of cancer to study the role of innate immune pathways Toll/Imd, JNK, and JAK-STAT, microbial infection and inflammation during tumor progression.
2. Drosophila as a model to study the role of blood cells in inflammation, innate immunity and cancer. Drosophila has a primitive yet effective blood system with three types of haemocytes which function throughout different developmental stages and environmental stimuli. Haemocytes play essential roles in tissue modeling during embryogenesis and morphogenesis, and also in innate immunity. The open circulatory system of Drosophila makes haemocytes ideal signal mediators to cells and tissues in response to events such as infection and wounding. The application of recently developed and sophisticated genetic tools to the relatively simple genome of Drosophila has made the fly a popular system for modeling human tumorigensis and **metastasis**. Drosophila is now used for screening and investigation of genes implicated in human leukemia and also in modeling development of solid tumors. This second line of research offers promising opportunities to determine the seemingly conflicting roles of blood cells in tumor progression and invasion. This review provides an overview of the signaling pathways conserved in Drosophila during haematopoiesis, haemostasis, innate immunity, wound healing and inflammation. We also review the most recent progress in the use of Drosophila as a cancer research model with an emphasis on the roles haemocytes can play in various cancer models and in the links between inflammation and cancer.
3. Oncogenic programmes and Notch activity: an 'organized crime'? The inappropriate Notch signalling can influence virtually all aspect of cancer, including tumour-cell growth, survival, apoptosis, angiogenesis, invasion and **metastasis**, although it does not do this alone. Hence, elucidating the partners of Notch that are active in cancer is now the focus of much intense research activity. The genetic toolkits available, coupled to the small size and short life of the fruit fly Drosophila melanogaster, makes this an inexpensive and effective animal model, suited to large-scale cancer gene discovery studies. The fly eye is not only a non-vital organ but its stereotyped size and disposition also means it is easy to screen for mutations that cause tumours and metastases and provides ample opportunities to test cancer theories and to unravel unanticipated nexus between Notch and other cancer genes, or to discover unforeseen Notch's partners in cancer. These studies suggest that Notch's oncogenic capacity is brought about not simply by increasing signal strength but through partnerships, whereby oncogenes gain more by cooperating than acting individually, as in a ring 'organized crime'.
4. Cellular and molecular mechanisms of single and collective cell migrations in Drosophila: themes and variations. The process of cell migration is essential throughout life, driving embryonic morphogenesis and ensuring homeostasis in adults. Defects in cell migration are a major cause of human disease, with excessive migration causing autoimmune diseases and cancer **metastasis**, whereas reduced capacity for migration leads to birth defects and immunodeficiencies. Myriad studies in vitro have established a consensus view that cell migrations require cell polarization, Rho GTPase-mediated cytoskeletal rearrangements, and myosin-mediated contractility. However, in vivo studies later revealed a more complex picture, including the discovery that cells migrate not only as single units but also as clusters, strands, and sheets. In particular, the role of E-Cadherin in cell motility appears to be more complex than previously appreciated. Here, we discuss recent advances achieved by combining the plethora of genetic tools available to the Drosophila geneticist with live imaging and biophysical techniques. Finally, we discuss the emerging themes such studies have revealed and ponder the puzzles that remain to be solved.
5. Awd, the homolog of **metastasis** suppressor gene Nm23, regulates Drosophila epithelial cell invasion. Border cell migration during Drosophila melanogaster oogenesis is a highly pliable model for studying epithelial to mesenchymal transition and directional cell migration. The process involves delamination of a group of 6 to 10 follicle cells from the epithelium followed by guided migration and invasion through the nurse cell complex toward the oocyte. The guidance cue is mainly provided by the homolog of platelet-derived growth factor/vascular endothelial growth factor family of growth factor, or Pvf, emanating from the oocyte, although Drosophila epidermal growth factor receptor signaling also plays an auxiliary role. Earlier studies implicated a stringent control of the strength of Pvf-mediated signaling since both down-regulation of Pvf and overexpression of active Pvf receptor (Pvr) resulted in stalled border cell migration. Here we show that the **metastasis** suppressor gene homolog Nm23/awd is a negative regulator of border cell migration. Its down-regulation allows for optimal spatial signaling from two crucial pathways, Pvr and JAK/STAT. Its overexpression in the border cells results in stalled migration and can revert the phenotype of overexpressing constitutive Pvr or dominant-negative dynamin. This is a rare example demonstrating the relevance of a **metastasis** suppressor gene function utilized in a developmental process involving cell invasion.
6. Regulation of cell adhesion and collective cell migration by hindsight and its human homolog RREB1. Cell movements represent a major driving force in embryonic development, tissue repair, and tumor **metastasis** [1]. The migration of single cells has been well studied, predominantly in cell culture [2, 3]; however, in vivo, a greater variety of modes of cell movement occur, including the movements of cells in clusters, strands, sheets, and tubes, also known as collective cell migrations [4, 5]. In spite of the relevance of these types of movements in both normal and pathological conditions, the molecular mechanisms that control them remain predominantly unknown. Epithelial follicle cells of the Drosophila ovary undergo several dynamic morphological changes, providing a genetically tractable model [6]. We found that anterior follicle cells, including border cells, mutant for the gene hindsight (hnt) accumulated excess cell-cell adhesion molecules and failed to undergo their normal collective movements. In addition, HNT affected border cell cluster cohesion and motility via effects on the JNK and STAT pathways, respectively. Interestingly, reduction of expression of the mammalian homolog of HNT, RREB1, by siRNA inhibited collective cell migration in a scratch-wound healing assay of MCF10A mammary epithelial cells, suppressed surface activity, retarded cell spreading after plating, and led to the formation of immobile, tightly adherent cell colonies. We propose that HNT and RREB1 are essential to reduce cell-cell adhesion when epithelial cells within an interconnected group undergo dynamic changes in cell shape.
7. Genes that drive invasion and migration in Drosophila. Successful cell migration depends on the careful regulation of the timing of movement, the guidance of motile cells, and cytoskeletal and adhesive changes within the cells. This review focuses on genes that act cell-autonomously to promote these aspects of cell migration in Drosophila. We discuss recent advances in understanding the migration of the ovarian border cells, embryonic blood cells, primordial germ cells, somatic gonadal precursors, and tracheal cells. Comparison of genes that regulate these processes to those that promote tumorigenesis and **metastasis** in mammals demonstrates that studies in fruit flies are uncovering new genes highly relevant to cancer biology.
8. Gα73B is a downstream effector of JAK/STAT signalling and a regulator of Rho1 in Drosophila haematopoiesis. JAK/STAT signalling regulates many essential developmental processes including cell proliferation and haematopoiesis, whereas its inappropriate activation is associated with the majority of myeloproliferative neoplasias and numerous cancers. Furthermore, high levels of JAK/STAT pathway signalling have also been associated with enhanced metastatic invasion by cancerous cells. Strikingly, gain-of-function mutations in the single Drosophila JAK homologue, Hopscotch, result in haemocyte neoplasia, inappropriate differentiation and the formation of melanised haemocyte-derived 'tumour' masses; phenotypes that are partly orthologous to human gain-of-function JAK2-associated pathologies. Here we show that Gα73B, a novel JAK/STAT pathway target gene, is necessary for JAK/STAT-mediated tumour formation in flies. In addition, although Gα73B does not affect haemocyte differentiation, it does regulate haemocyte morphology and motility under non-pathological conditions. We show that Gα73B is required for constitutive, but not injury-induced, activation of Rho1 and for the localisation of Rho1 into filopodia upon haemocyte activation. Consistent with these results, we also show that Rho1 interacts genetically with JAK/STAT signalling, and that wild-type levels of Rho1 are necessary for tumour formation. Our findings link JAK/STAT transcriptional outputs, Gα73B activity and Rho1-dependent cytoskeletal rearrangements and cell motility, therefore connecting a pathway associated with cancer with a marker indicative of invasiveness. As such, we suggest a mechanism by which JAK/STAT pathway signalling may pro**mote metas**tasis.
9. Oncogenic cooperation between SOCS family proteins and EGFR identified using a Drosophila epithelial transformation model. MicroRNAs (miRNAs) are emerging as cooperating factors that promote the activity of oncogenes in tumor formation and disease progression. This poses the challenge of identifying the miRNA targets responsible for these interactions. In this study, we identify the growth regulatory miRNA bantam and its target, Socs36E, as cooperating factors in EGFR-driven tumorigenesis and **metastasis** in a Drosophila model of epithelial transformation. bantam promotes growth by limiting expression of Socs36E, which functions as a negative growth regulator. Socs36E has only a modest effect on growth on its own, but behaves as a tumor suppressor in combination with EGFR activation. The human ortholog of SOCS36E, SOCS5, behaves as a candidate tumor suppressor in cellular transformation in cooperation with EGFR/RAS pathway activation.
10. Rabaptin-5 and Rabex-5 are neoplastic tumour suppressor genes that interact to modulate Rab5 dynamics in Drosophila melanogaster. Endocytosis plays an important role in the regulation of tumour growth and **metastasis**. In Drosophila, a number of endocytic neoplastic tumour suppressor genes have been identified that when mutated cause epithelial disruption and over-proliferation. Here we characterise the Drosophila homologue of the Rab5 effector Rabaptin-5, and show that it is a novel neoplastic tumour suppressor. Its ability to bind Rab5 and modulate early endosomal dynamics is conserved in Drosophila, as is its interaction with the Rab5 GEF Rabex5, for which we also demonstrate neoplastic tumour suppressor characteristics. Surprisingly, we do not observe disruption of apico-basal polarity in Rabaptin-5 and Rabex-5 mutant tissues; instead the tumour phenotype is associated with upregulation of Jun N-terminal Kinase (JNK) and Janus Kinase (JAK)/Signal Transducer and Activator of Transcription (STAT) signalling.

### [Cc]ell [Ii]nvasion

#### PubMed

1. Awd, the homolog of metastasis suppressor gene Nm23, regulates Drosophila epithelial **cell invasion**. Border cell migration during Drosophila melanogaster oogenesis is a highly pliable model for studying epithelial to mesenchymal transition and directional cell migration. The process involves delamination of a group of 6 to 10 follicle cells from the epithelium followed by guided migration and invasion through the nurse cell complex toward the oocyte. The guidance cue is mainly provided by the homolog of platelet-derived growth factor/vascular endothelial growth factor family of growth factor, or Pvf, emanating from the oocyte, although Drosophila epidermal growth factor receptor signaling also plays an auxiliary role. Earlier studies implicated a stringent control of the strength of Pvf-mediated signaling since both down-regulation of Pvf and overexpression of active Pvf receptor (Pvr) resulted in stalled border cell migration. Here we show that the metastasis suppressor gene homolog Nm23/awd is a negative regulator of border cell migration. Its down-regulation allows for optimal spatial signaling from two crucial pathways, Pvr and JAK/STAT. Its overexpression in the border cells results in stalled migration and can revert the phenotype of overexpressing constitutive Pvr or dominant-negative dynamin. This is a rare example demonstrating the relevance of a metastasis suppressor gene function utilized in a developmental process involving **cell invasion**.

---
